# Serving organization goals by organizational information dissemination: An empirical study from the Communist Youth League of China

**DOI:** 10.1371/journal.pone.0280221

**Published:** 2023-01-20

**Authors:** Xueqing Zhou, Jielin Jing, Yushi Yin, Qing Liu

**Affiliations:** 1 Communist Youth League, Huainan Normal University, Huainan, China; 2 School of Management, Xi’an Jiaotong University, Xi’an, China; 3 School of Accounting, Shaanxi Technical College of Finance & Economics, Xianyang, China; 4 College of Tropical Crops, Yunnan Agricultural University, Puer, China; 5 College of Economics and Management, Huainan Normal University, Huainan, China; Sreenidhi Institute of Science and Technology, INDIA

## Abstract

From the perspective of news topic modeling, this paper investigated how the Communist Youth League of China (CYLC) uses organizational information communication to serve organizational goals—“Keep the Party Assured and the Youth Satisfied” (“让党放心, 让青年满意”). Using the Latent Dirichlet allocation (LDA) algorithm, we performed a topic analysis on 1898 news articles published on the CYLC website. We discovered that nearly all of the CYLC’s news centered on the achievement of its organizational goals, reflecting the characteristics of information dissemination that is highly supportive of organizational objectives. We discovered distinct differences in the dissemination of organizational information between the central, provincial, municipal, county, and school league committees through cluster analysis. The various league organizations have distinct positioning and distinguishing characteristics. In addition, correlation analysis reveals that higher-level league organizations prioritize the dissemination of “Keep the Party Assured” information. While lower-level organizations gradually implement “Keep the Youth Satisfied” initiatives. This paper fills a gap in research on mass organizations in the field of information dissemination and serves as a resource for other political organizations involved in public information dissemination.

## 1. Introduction

Numerous studies have documented how the dissemination of information by government and general organizations serves organizational objectives [1, 2]. However, few academics have focused on research pertaining to the dissemination of information about mass organizations. Particularly, there is a gap in the literature regarding the CYLC and information communication. Using news topic modeling in [3], this study examined how the CYLC uses information dissemination to achieve organizational objectives. This study assists individuals in perceiving and comprehending the CYLC, and is an essential resource for studying organizational communication and information dissemination.

Scholars have observed that organizational information dissemination has been beneficial for improving organizational effectiveness for a long time [4, 5]. [1] argued that not all information dissemination is effective and that quality information dissemination helps organizations manage their business processes, make decisions, and improve organizational performance. In addition, organizational information dissemination is significantly and positively related to organizational climate and organizational communication effectiveness [6]. Organizational information dissemination is also an important part of organizational communication that enhances individuals’ identification with organizational values. And the interaction between the individual’s identification with organizational values and the importance of the individual’s own work values determines the outcome of the socialization of organizational goals and values [7]. In short, effective information dissemination benefits the achievement of organizational goals.

It seems that the government’s dissemination of public information has received more attention from the academic community than the dissemination of organizational information in general. It is still controversial about what information should be published, what information should not be published, and to what extent. Although [8] states that “No matter what other differences there may be about public policy, there appears to be universal acceptance of dissemination.” However, this conclusion remains in disagreement. Some scholars conclude that reducing public signal precision or entirely withholding information may improve welfare [9]. However, other scholars believe that “public information should always be provided with maximum precision but, under certain conditions, not to all agents.” Restricting the degree of publicity is a bettersuited instrument for preventing the negative welfare effects of public announcements than restrictions on their precision are [10].” While there is disagreement about how the government should conduct public information dissemination, the conclusion that public information dissemination facilitates the achievement of organizational goals has remained well-established. Government public information dissemination is often motivated by three basic goals: increasing transparency, enhancing citizen engagement, and building collaboration [2, 11]. Public information dissemination can increase the legitimacy of the government and enable citizens to participate in public affairs, and public authorities can explain their actions to citizens [12]. In short, the government can achieve its goals through public information dissemination.

Our research relates to two aspects of expertise: organizational information dissemination and organizational goal achievement. Prior research on organizational information dissemination was focused on its effectiveness [13]. Although there are many studies on communication methods [14, 15], the evaluation criterion of these studies remains the effectiveness of communication [16]. Research on communication effectiveness is usually conducted in the form of surveys, questionnaires, or interviews. This approach has long been the standard for measuring information dissemination [16]. [17] refers to this approach as a "legacy approach" because, with the development of big data and information technology, the traditional standard evaluation methods have been challenged. Traditional data acquisition methods are expensive and difficult to collect compared to easily available big data [18]. In the era of big data, Social Network Analysis (SNA) is the fastest method of information dissemination analysis [16], which measures the conversations between users and then forms social networks based on the conversations [19].

According to [20], content analysis is "any technique for making inferences by objectively and systematically identifying defined properties of messages" (p. 14). Content analysis allows researchers to sift through large amounts of data in a systematic way with relative ease [21]. Thanks to the development of natural language processing, content analysis has also become an important approach to information communication research [22]. Based on [20]’s definition, current text mining methods in almost all disciplines fall under the category of content analysis [23, 24]. This approach de-mines information themes, sentiment classification, and sentiment temperature from a large number of texts [25–27], which provides a new reference for the study of organizational information dissemination.

Another central piece of knowledge relevant to our study is organizational goal attainment. Although many studies have shown that organizational information dissemination is related to organizational goal attainment [4, 5]. However, the mainstream research usually focuses on studying the association of organizational goal attainment with motivation Besser, 1995), effective decision making [28], organizational effectiveness [29], human resource management [30], and information technology strategy [31]. The relationship between organizational goal achievement and organizational information dissemination has rarely been studied separately. In terms of research methods, they focus on case studies [32], questionnaires [28, 29, 33], correlation analysis [30], or behavioral theoretical models such as goal-setting theory [34] or expectancy theory [35].

Although it has been pointed out that political parties communicate with citizens in various ways at different stages of their development, depending on the possibilities of technology [36–38]. However, the dissemination of information about mass organizations and political groups is still in the minority in comparison to the government and organizations in general. In particular, there is still a gap in the literature regarding research on the organizational communication of the CYLC. We linked organizational information dissemination and organizational goals and examined how the Communist Youth League of China used organizational information dissemination to serve organizational goal achievement. In terms of research methodology, we did not use the traditional standard methods of information dissemination research: theoretical models [7, 34, 39, 40] and data from surveys, questionnaires, or interviews [16]. Data acquisition for this type of approach is expensive and difficult to collect [18]. We used a lot of texts and used recent advances in machine learning and artificial intelligence to study the relationship between the CYLC’s goals and how it spreads information.

The CYLC is a group organization of advanced youth led by the Chinese Communist Party (CPC) [41]. Its widely known as the assistant and reserve army of the CPC [42]. Hu Jintao distilled the goal of the Communist Youth League of China as “让党放心, 让青年满意” [43, 44]. A popular and concise translation of this phrase is "Keep the Party Assured and the Youth Satisfied [45]." This paper investigated how the CYLC is using organizational information dissemination to serve organizational goal achievement through textual topic modeling of 1989 news items published on the CYLC website. The following are our main findings:

- The whole news of the CYLC can be divided into 2 themes: “Keep the Party Assured” and “Keep the Youth Satisfied”, with organizational information dissemination highly serving organizational goals.
- The distribution of information dissemination topics of the central committee, provincial committee, municipal committee, county committee, and school committee can be accurately categorized through cluster analysis. Organizations at all levels have distinctive features and clear goals, and they work independently according to their own positions.
- Higher-level league organizations pay more attention to the dissemination of information on “Keep the Party Assured”-related topics and effectively communicate these messages to lower-level league organizations. And the lower-level organizations gradually implement “Keep the Youth Satisfied” under the premise of implementing the spirit of the higher-level organizations.

We took advantage of the most recent advances in computer technology to conduct an empirical study utilizing a larger amount of data, and we focused on a field that has received little scholarly attention–the CYLC. This paper’s primary contribution is multifaceted. We present the CYLC from a novel perspective, which increases its visibility and comprehension. Our research serves as a guide for the organizational communication of other political groups and mass organizations. This research also adds a new point of view to the study of how organizational communication spreads.

**Fig 1:**
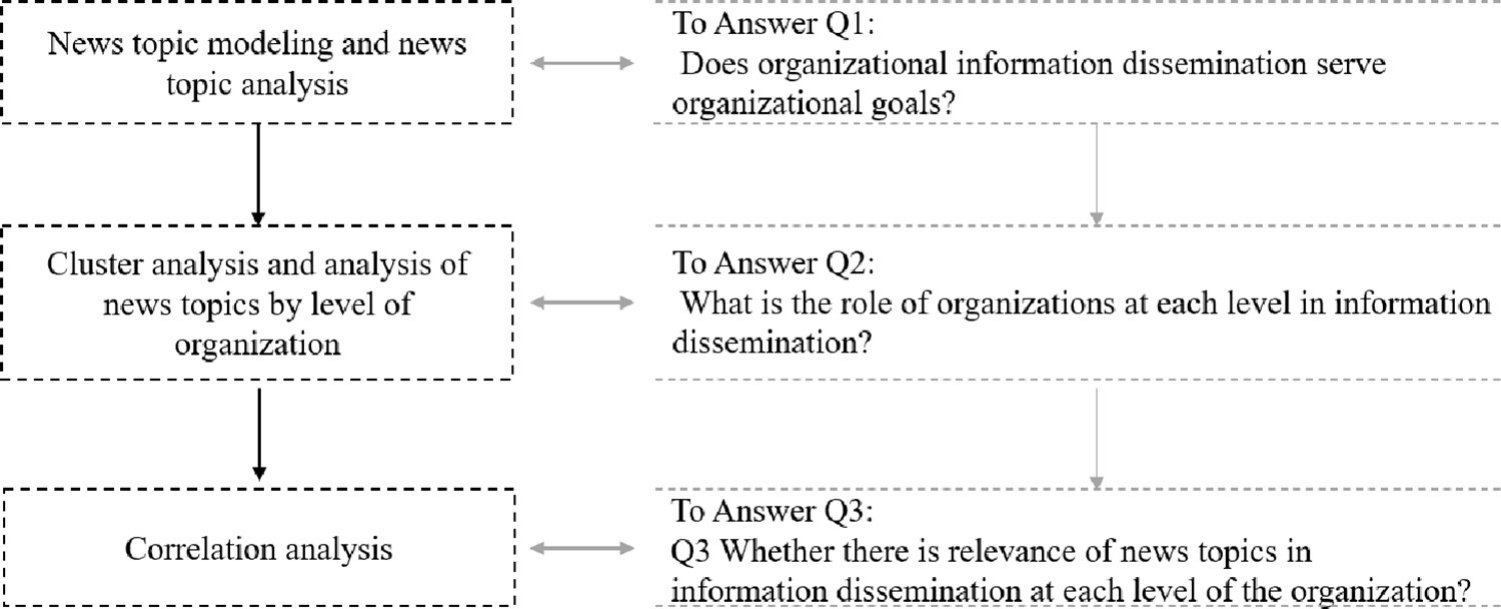
Flow of empirical analysis.

The rest of the paper is organized as follows: Section 2 presents the data and methodology. Section 3 is the empirical analysis section, as shown in Fig 1, in which we investigated the three core questions that are the focus of this paper. Section 4 is the conclusion.

## 2. Data and methodology

### 2.1 Data

The CYLC has an organizational structure similar to that of the CPC. As shown in Table 1, in this study, we focus on the first four levels of organizations of the CYLC and the school CYLC with special significance. In the subsequent part of this paper, the short names listed in Table 1 are used to refer to the organizations of the Communist Youth League of China.

**Table 1:** The organizational structure of focus in this study.

Level	Organization Names	Higher Level Organization	Short Name
1	Central Committee of the CYLC		Central -CYLC
2	Provincial Committee of the CYLC	Central-CYLC	Provincial-CYLC
3	Municipal Committee of the CYLC	Provincial-CYLC	Municipal-CYLC
4	County Committee of the CYLC	Municipal-CYLC	County-CYLC
Unclear	School CYLC	Varies by school jurisdiction	School-CYLC

Note: Central committee of the CYLC is the top management organization of the CYLC.

Since there are many types and levels of schools, such as universities, secondary schools, elementary schools, etc. Universities, in turn, contain several levels of universities, such as those directly managed by the Ministry of Education, provincial universities, and city universities. This leads to an uncertain hierarchy of school community organizations. In our case, we note that the news content is mainly about universities. Therefore, the data in our study is mainly about university league organizations. Most of the time, the provincial or municipal league committees are in charge of running these school leagues.

The research data were obtained from the CYLC’s official website (https://www.gqt.org.cn/). We used crawler technology to obtain all 1,898 news items that can be viewed in the five sections of “Main Messages of the Whole League,” “Provincial League News,” “Municipal League News,” “County League News.” and “School-CYLC News.” These five sections contain information from the Central-CYLC, Provincial-CYLC, Municipal-CYLC, County-CYLC, and School-CYLC. Table 2 displays the specifics of these news items.

**Table 2:** Statistics of the numbers of news for each category.

Categories	Number of News	Date Range
Important Messages of Central-CYLC	40	From 2021-05-18 to 2022-06-17
News of Provincial-CYLC	465	From 2021-08-29 to 2022-06-30
News of Municipal-CYLC	489	From 2021-08-31 to 2022-06-28
News of County-CYLC	471	From 2021-03-03 to 2022-06-29
News of School-CYLC	433	From 2020-09-28 to 2022-06-29
**Total**	**1898**	

The “Main Messages of the Whole League” are issued by the Central-CYLC in the form of documents, which are fewer in number and mainly publish some important news for the whole league. The other four columns are mainly in the form of news about the work carried out by the league organizations at various levels. The length of the news ranged from 112 to 5546 characters, with large differences, which are statistically described in Table 3.

**Table 3:** A statistical breakdown of the length of news content.

Statistical Indicators	Value
std	717.8
min	112.0
25%	480.0
50%	667.5
75%	1039.8
max	5546.0
mean	914.1

Note: The 25%, 50%, and 75% indicators are quartiles of the number of characters.

The number of characters is counted as Chinese characters.

**Fig 2:**
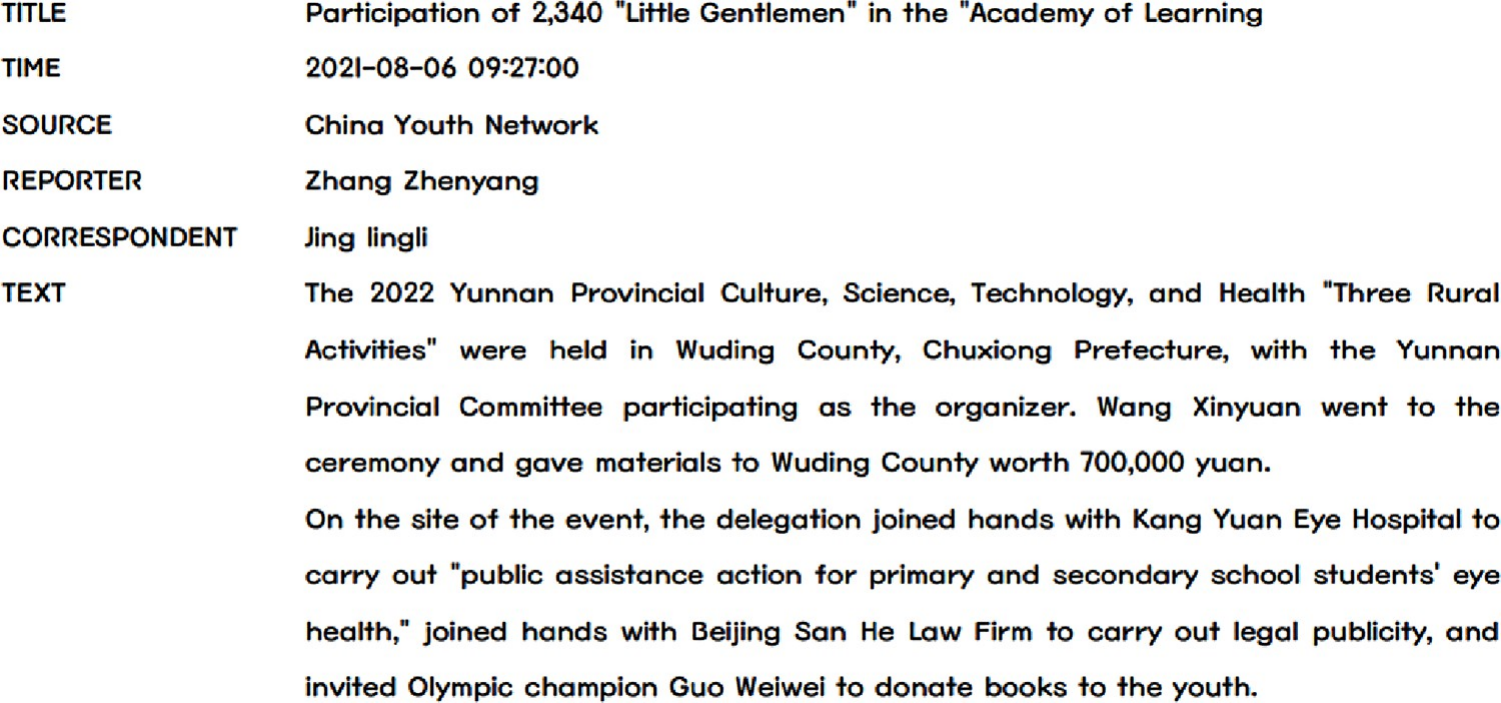
Information schematic of the news sample. **Note:** To meet the need for privacy protection, the names of persons and organizations in the information in the figure are all pseudonyms. It should be emphasized that the official website (https://www.gqt.org.cn/) of the Young Communist League of China (CYLC) is only available in Chinese. Therefore, the news content and terminology used in the manuscript are translations of the source content.

Fig 2 shows a sample of a news item. Since images do not contribute positively to the modeling of the news topics, we ignore the photos in the news when collecting the data. For further processing, we store all the data in a structured table indexed by news section and date for further processing, and the data style is shown in Table 4.

**Table 4:** A structured news list.

News Section	Date	Text
Provincial-CYLC	2021-08-29	Project Hope helps 60 freshmen pursue their dreams in Shaanxi …
Provincial-CYLC	2022-06-29	Helping teenagers heal “unhappy”, “Spiritual Growth Club” shifts the focus of mental …
Municipal-CYLC	2021-08-31	Liaocheng City held a special training course for good young pioneers in rural revitalization
School-CYLC	2022-06-06	Anhui Agricultural University held the fifth “Creators Salon” in 2022…

Note: This table only shows 4 of the 1898 data items.

### 2.2 News topic modeling

**Fig 3:**
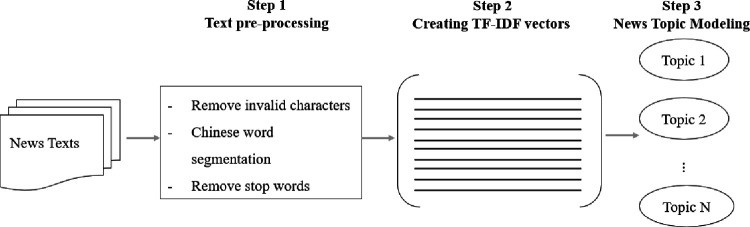
Flowchart of news topic mining.

In this study, there are 2 basic claims for news topic modeling. Firstly, we need to know the distribution of all 1898 news topics. Secondly, we need to know the news topic distribution at various organizational levels of the CYLC. To accomplish the above, 3 basic steps are required, the preprocessing of data, the term frequency–inverse document frequency (TF-IDF) word vector construction, and news topic modeling. Fig 3 presents this process.

#### Step 1 Data pre-processing

News texts that consist of multiple contents, except for the text, which will not contribute to the topic mining of the text, are referred to as noise in text processing [26]. In the data preprocessing process, we need to remove numbers, punctuation marks, extra spaces, and special characters that are difficult to understand, such as: (, [, {, &, etc.). And replace the web link in the text with the string “URL” and the username with the string “USERNAME”.

After removing invalid characters, the Chinese text must be divided into ordered words. Word segmentation is a necessary first step in processing Chinese language. In Chinese, however, sentences are represented as strings of Chinese characters or hanzi without similar natural delimiters, as opposed to English where sentences are sequences of words separated by white spaces. Consequently, the first step in a Chinese language processing task is to identify the word order in a sentence and mark appropriate boundary locations [46, 47].

In addition, there are some words that are repeated in the text but will not contribute to text classification or topic mining, and these words are called stop words [48]. Removing the stop words helps in the performance of natural language processing and is an important part of text data preprocessing [23]. A fundamental tool in text classification is a list of stop words that is used to identify frequent words that are unlikely to assist in classification and hence are deleted during preprocessing [49]. At the end of the data preprocessing, we use a list of stop words to improve the data quality.

#### Step 2 Building TF-IDF vectors

“Term frequency–inverse document frequency” (TF-IDF) is one of the most widely used term weighting schemes in modern information retrieval systems [50]. The “TF” in “TF-IDF” represents the frequency with which particular words appear in a document. Important words in a document are those with a high TF value. In contrast, the DF indicates how frequently a particular word appears in a set of documents. It determines how frequently the word appears in multiple documents, not just one. Because they occur so frequently in all documents, words with a high DF value are not significant. Therefore, the IDF, which is the inverse of the DF, is used to determine the importance of words in all documents. High IDF values indicate that words are uncommon in all documents, making them more significant t [51].

Using Christian et al. (2016) as a reference, the formula for TF (Term frequency) can be written as

$${TF}_{i,j}=\frac{n_{i,j}}{\sum_{k}n_{k,j}}.$$ (1)

In Formula (1), ***n***_***i*,*j***_ indicates the number of occurrences of word ***t***_***i***_ in document j. ***TF***_***i*,*j***_ indicates the frequency of word ***t***_***i***_ in document j. The formula for IDF (Inverse Document Frequency) can be described as

$${IDF}_{i}=log\frac{\left|D\right|}{1+|j:t_{i}\in d_{j}|}.$$ (2)

In Formula (2), |***D***| represents the number of all documents, |***j***: ***t***_***i***_∈***d***_***j***_| represents the number of documents containing the term, ***t***_***i***_. The denominator in the Formula (2) plus 1 is to avoid the situation where the denominator is 0. The TF-IDF is calculated as

TF−IDF=TF×IDF. (3)

#### Step 3 Topic modeling

Text mining is a subset of data mining that has the potential for greater business value than data mining because 80% of a company’s data is in text format [52]. Latent Dirichlet Allocation (LDA) is a probabilistic model that can model the topical information of text data. The LDA topic model can realize the dimensionality reduction representation of text in the semantic space, and it models the text with the probability of vocabulary, which alleviates the issue of data sparsity to some extent y [24, 27].

LDA is a three-level hierarchical Bayesian model, in which each item of a collection is modeled as a finite mixture over an underlying set of topics. Each topic is, in turn, modeled as an infinite mixture over an underlying set of topic probabilities [53]. In this paper, we use the LDA algorithm for news topic modeling to aggregate all news into 10 topics and assign a topic to each news item.

## 3. Empirical research

In this section, we use the analysis process depicted in Fig 1 to answer the question of how the CYLC uses organizational information dissemination to achieve organizational objectives.

### 3.1 Analysis of news topics

In this paper, we use the LDA algorithm to perform news topic modeling. How many topics to construct when modeling is a question that is worth exploring. Too small a number of topics will reduce the credibility of modeling, while too many topics will increase the difficulty of analysis. At this point, the lowest percentage of topics, accounting for only 0.4% of total news, may be of no practical significance. Topic modeling can divide all 1898 news items into 10 topics, and Table 5 shows the number and percentage of news items contained in those 10 topics.

**Table 5:** News topic popularity statistics.

Topic No.	News Count	Percentage
Topic 01	592	31.2%
Topic 02	263	13.9%
Topic 03	349	18.4%
Topic 04	386	20.3%
Topic 05	160	8.4%
Topic 06	86	4.5%
Topic 07	41	2.2%
Topic 08	7	0.4%
Topic 09	7	0.4%
Topic 10	7	0.4%

Table 6 shows the keywords and topic descriptions of the 10 news topics obtained by topic modeling, from which we can have a comprehensive understanding of the topic information obtained by topic modeling. Since the news contains some specialized vocabulary, in order to avoid ambiguities caused by translation, the Chinese expressions of key words and topic descriptions are noted in our table. The Keywords column in Table 6 shows only the 20 keywords with the highest relevance, and the more front-ranked keywords have the greatest impact on the topic.

**Table 6:** Keywords and descriptions of topics.

Topic No.	Top 20 Topic Keywords	Topic Description
Topic 01	Party history; red; theme; preaching; practice; spirit; students; academy; China; youth; century; era; story; history; organization; follow the party; school; culture; youth; university	Love the party; love the country; follow the party.
	In Chinese: 党史; 红色; 主题; 宣讲; 实践; 精神; 学生; 学院; 中国; 青春; 百年; 时代; 故事; 历史; 举办; 跟党走; 学校; 文化; 青少年; 大学	In Chinese:爱党; 爱国; 跟党走
Topic 02	construction; youth league organization; reform; promotion; grassroots; league cadres; society; secretary; advance; promotion; meeting; community; mechanism; politics; persistence; comprehensive; convening; establishment; youth league committee; requirements	Construction of the league organization; reform of the league organization; stringent requirements for league cadres.
	In Chinese: 建设; 团组织; 改革; 推动; 基层; 团干部; 社会; 书记; 推进; 提升; 会议; 社区; 机制; 政治; 坚持; 全面; 召开; 建立; 团委; 要求	In Chinese:团组织建设; 团组织改革; 从严要求团干部.
Topic 03	Entrepreneurship; Employment; Rural; Talent; College Students; Revitalization; Innovation; Enterprise; Project; Provide; University; Municipal Party Committee; Plan; Practice; City; Platform; Organize; Youth; Help; Policy	Assisting college students in innovating and launching their own businesses; assisting them in finding employment; encouraging the development of their hometowns.
	In Chinese: 创业; 就业; 乡村; 人才; 大学生; 振兴; 创新; 企业; 项目; 提供; 高校; 市委; 计划; 实践; 城市; 平台; 举办; 青春; 助力; 政策	In Chinese: 帮助大学生创新、创业; 帮助大学生就业; 大学生建设家乡
Topic 04	Volunteer; Volunteer; Community; Students; Classmates; Practice; School; Children; Knowledge; Society; Propaganda; Mass; College; Participation; Civilization; College Students; Anhui Province; Understanding; Classroom; Youth	Be a youth volunteer; teach in rural areas; use knowledge to repay the society.
	In Chinese: 志愿; 志愿者; 社区; 学生; 同学; 实践; 学校; 孩子; 知识; 社会; 宣传; 群众; 学院; 参与; 文明; 大学生; 安徽省; 了解; 课堂; 青春	In Chinese:做青年志愿者; 乡村支教; 知识回报社会
Topic 05	youth; love; children; hope; hut; caring; public welfare; project; action; children; left behind; plight; volunteer; hope project; assistance; municipal party committee; society; volunteers; family; difficulties	Caring for teenagers; caring for children; Project Hope.
	In Chinese:青少年; 爱心; 儿童; 希望; 小屋; 关爱; 公益; 项目; 行动; 孩子; 留守; 困境; 志愿; 希望工程; 帮扶; 市委; 社会; 志愿者; 家庭; 困难	In Chinese:关爱青少年; 关爱儿童; 希望工程
Topic 06	epidemic; volunteer; prevention and control; volunteer; frontline; epidemic prevention; material; commando; nucleic acid; participation; detection; group organization; community; new crown; anti-epidemic; recruitment; personnel; youth; pneumonia; all levels	Epidemic prevention and control; young volunteers.
	In Chinese: 疫情; 志愿者; 防控; 志愿; 一线; 防疫; 物资; 突击队; 核酸; 参与; 检测; 团组织; 社区; 新冠; 抗疫; 招募; 人员; 青春; 肺炎; 各级	In Chinese: 疫情防控; 青年志愿者
Topic 07	Youth; Representative; Ceremony; Fujian; Held; Fujian Province; Henan; Related; Participate; Henan Province; Launch; Social; Sichuan; Youth League; Planning; High School; Held; Training Class; Mental Health; Head	Conduct youth symposium; conduct youth training;
	In Chinese: 青少年; 代表; 仪式; 福建; 举行; 福建省; 河南; 相关; 参加; 河南省; 启动; 社会; 四川; 团省委; 规划; 高校; 举办; 培训班; 心理健康; 负责人	In Chinese: 开展青少年座谈会; 开展青少年培训
Topic 08	Young Pioneers; Young Pioneers; Working Committee; Red Scarf; Counselors; Primary School; Tianjin; Shaanxi Province; Shaanxi; Children; Practice; Xi’an; Excellent; Tianjin; Brigade; Shaanxi Provincial Party Committee; Juvenile; Show; Times; School	Young Pioneers; Young Pioneers.
	In Chinese: 少先队; 少先队员; 工委; 红领巾; 辅导员; 小学; 天津; 陕西省; 陕西; 少年儿童; 实践; 西安; 优秀; 天津市; 大队; 陕西省委; 少年; 展示; 时代; 学校	In Chinese: 少先队; 少先队员
Topic 09	County Committee; Guangdong; Guangdong; County Committee; Guangdong Provincial Committee; Volunteer; Xinfeng County; Changsha; Contribution; Xinfeng; Green; Eco; Environmental; Hunan; Hunan Province; Propaganda; Chengdu; Garbage; Shaoguan City; Learn from Lei Feng	Advocate green and environmental protection; carry out learning from Lei Feng activities.
	In Chinese:县委; 广东省; 广东; 团县委; 广东省委; 志愿; 新丰县; 长沙; 供图; 新丰; 绿色; 生态; 环保; 湖南; 湖南省; 宣传; 成都; 垃圾; 韶关市; 学雷锋	In Chinese: 倡导绿色、环保; 开展学雷锋活动
Topic 10	Safety; Training; Rescue; Creation; Skills; Emergency; Youth Civilization; Production; Ability; Collective; Site; Fire Fighting; Drill; Commando; Municipal Party Committee; Shandong Province; Flood Control; Promotion; Photo for; Workers	Let young people learn safety knowledge and learn to protect themselves.
	In Chinese: 安全; 培训; 救援; 创建; 技能; 应急; 青年文明; 生产; 能力; 集体; 现场; 消防; 演练; 突击队; 市委; 山东省; 防汛; 提升; 供图; 职工	In Chinese: 让青少年学习安全知识, 学会自我保护

**Fig 4:**
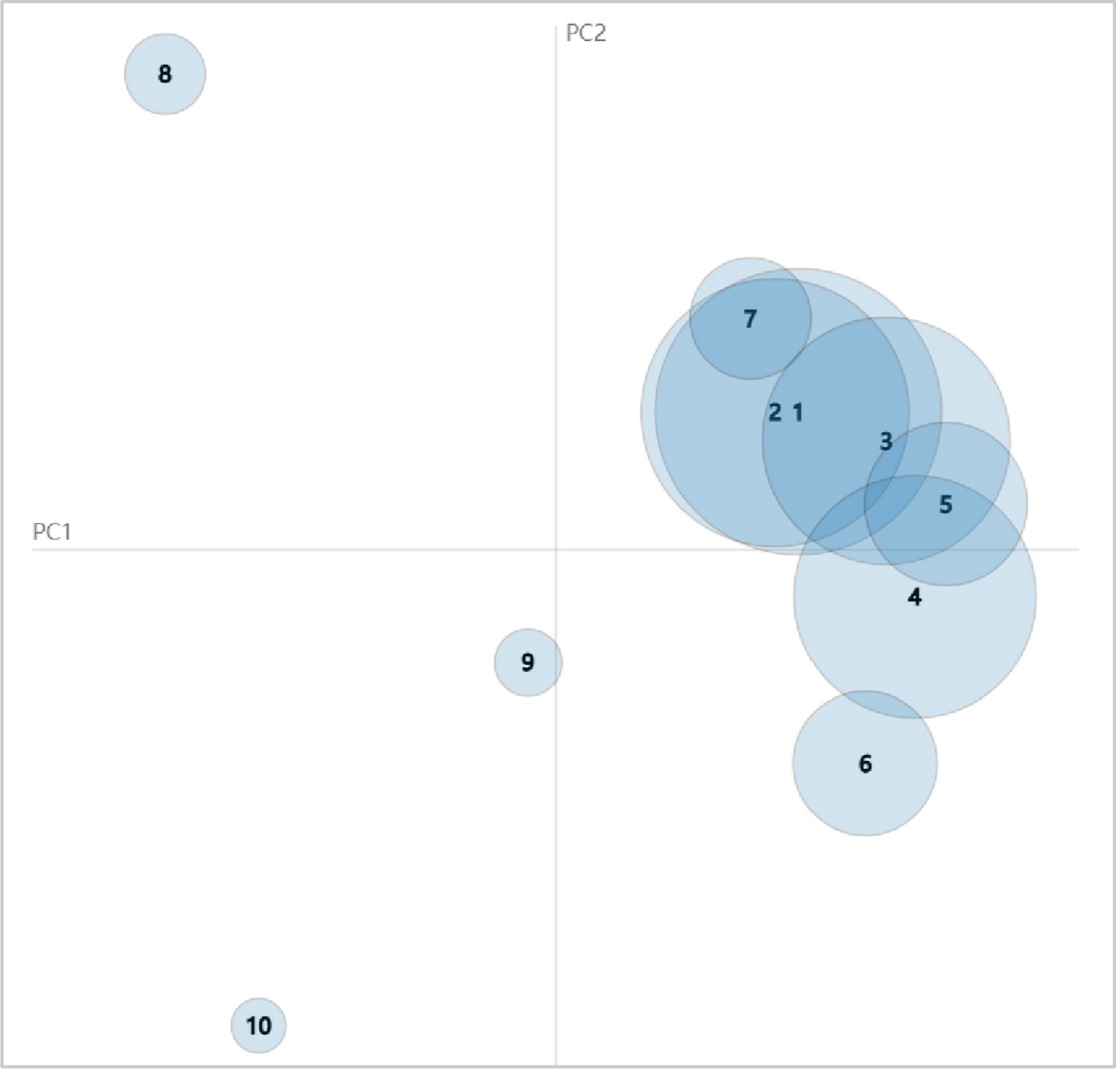
Inter-topic distance map.

To analyze the association between topics, as shown in Fig 4, we plot the topics as circles in the two-dimensional plane whose centers are determined by computing the distance between topics, and then by using multidimensional scaling to project the inter-topic distances onto two dimensions, as is done in [54]. The “PC” in the labels “PC1” and “PC2” on the horizontal and vertical axes in Fig 4 is an abbreviation for Principal Component. Principal component analysis (PCA) is one of the most important and powerful methods in the field of data analysis [55, 56]. We used this method for multidimensional scaling of the data to achieve the goal of presenting multiple topics in a two-dimensional image. The size of the circles represents the popularity of the topic.

**Fig 5:**
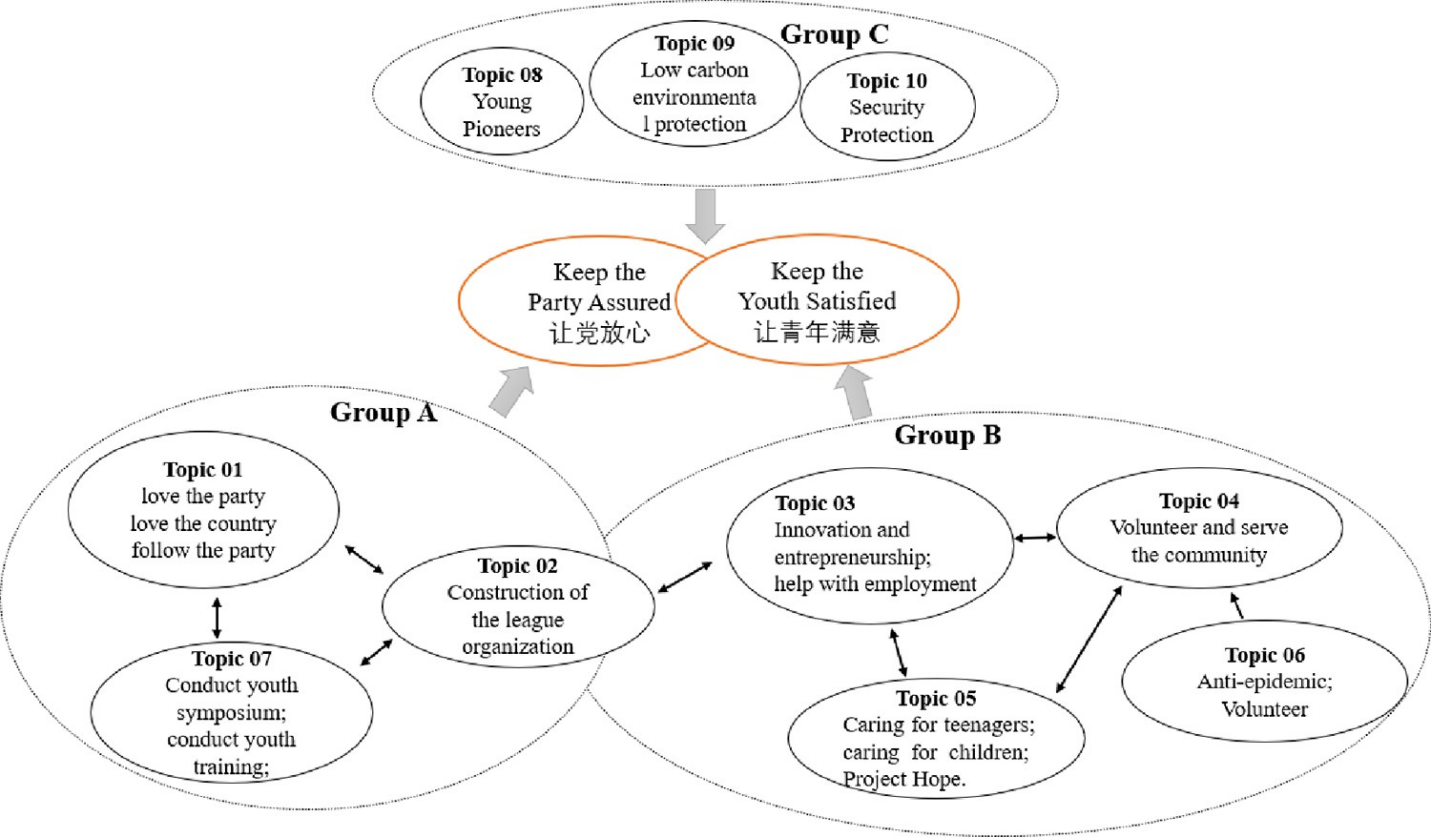
Topic grouping analysis.

As we can see in Fig 4, Topic 01 and Topic 02 are highly overlapping, and Topic 07 is also highly overlapping with Topics 01 and 02. These three topics are coming together to describe the same event. League organizations at various levels pursue the goal of “Keep the Party Assured” by conducting training, symposiums, league organization building, and education on love of the country and the party. The group A section of Fig 5 shows the above associations.

In addition, topics 03, 04, and 05 also overlap highly, especially topic 05, which is almost included by topics 03 and 04. The combination of topics 03, 04, and 05 illustrates the three themes of “innovation, entrepreneurship and employment”, “volunteers”, and “care for youth”. These topics clarify that the CYLC attaches great importance to the innovation, entrepreneurship, and employment of young people in the hopes that they will serve as volunteers and give back to society, in the hopes of achieving the goal of caring for young people through youth employment and volunteers, and in the hopes that young people will work where the motherland needs them. Although in Fig 4, the specialized vocabulary related to the epidemic caused Topic 06 to be more distant from Topic 04, we still confirm that Topic 06 is an extension of Topic 04 in a particular social context. Fig 4 also shows that there is a large overlap between topics 03 and 02, which shows that helping young people to establish their own businesses and employment is also an important part of the construction of league organizations and training of league organizations. In the group B part of Fig 5, topics 03, 04, 05, and 06 are shown to be important.

As shown in group C of Fig 5, the group of topics 07, 08, and 09 constitutes another combination of topics. The content of these topics is more independent and not related to each other, and they elaborate on other foci of the league organization’s work: the Young Pioneers (reserve league members), environmental protection, and safety education. The league organization cares about potential league members and wishes the youth to pay attention to environmental protection as well as their own safety.

As shown in Fig 5, we divided the ten topics into three topic groups. It is easy to see that topic group A highly serves the goal “Keep the Party Assured,” and topic group B serves the goal “Keep the Youth Satisfied.” While topic group C seems to be distant from group A and group B, however, they are the common concern of “the Party” and “the Youth”, and they serve the goal of “Keep the Party Assured and the Youth Satisfied”. In conclusion, the CYLC’s organizational information dissemination, which does a great job of helping the organization achieve its goals, is a great example of how mass organizations can use organizational information dissemination to achieve their goals.

### 3.2 The characteristics of information dissemination of league organizations at different levels

As reported in Section 2.1 of this paper, the 1898 news items used in this study were obtained from the website of the CYLC. All the news came from the five levels of the CYLC: the Central-CYLC, the Provincial-CYLC, the Municipal-CYLC, the County-CYLC, and the School-CYLC. Analyzing the characteristics of news dissemination at each level of the CYLC separately will help to understand the roles and differences in organizational information dissemination at each level of the CYLC. The website of the CYLC only displays 10 pages of news, and the information release frequency of the league organizations at different levels is not consistent, thus making the obtained news from different levels inconsistent in time range. We can see the detailed date ranges for each column in Table 2. For comparison purposes, for news data from all levels of the league, we consistently used data between 08/2021 and 06/2022. In total, 1,485 news items were published during that time, and Table 7 summarizes the distribution of news topics. The sub-tables of Panels A-E in Table 7 show the monthly distribution of topics for the five levels of organizations: the Central-CYLC, the Provincial-CYLC, the Municipal-CYLC, the County-CYLC, and the School-CYLC, respectively.

**Table 7:** News topic distribution of each level of league organization.

**Panel A Data of Central-CYLC**											
Month	Topic01	Topic02	Topic03	Topic04	Topic05	Topic06	Topic07	Topic08	Topic09	Topic10	Total
2021–08		2									2
2021–09		2									2
2021–10								1			1
2021–11		5	1								6
2021–12		6	2								8
2022–01	1	5									6
2022–02		2									2
2022–03		1									1
2022–04	2	2				1					5
2022–05											0
2022–06	1	3									4
Total	4	28	3	0	0	1	0	1	0	0	37
Percentage	10.8%	75.7%	8.1%	0.0%	0.0%	2.7%	0.0%	2.7%	0.0%	0.0%	100%
**Panel B Data of Provincial-CYLC**											
Month	Topic01	Topic02	Topic03	Topic04	Topic05	Topic06	Topic07	Topic08	Topic09	Topic10	Total
2021–08	2	1	3		3						9
2021–09	10	10	13	5	3	2	1				44
2021–10	22	5	11	5	1	8	1				53
2021–11	13	10	10	1	3	1	1				39
2021–12	14	13	6	8	4	3		1			49
2022–01	7	3	9	2	2		10	1			34
2022–02	1	14	3	2	4	4	3				31
2022–03	5	14	4	7		12	2		2		46
2022–04	15	17	9	5	4	7	1	1			59
2022–05	17	8	2	1	1	1	4	1			35
2022–06	20	12	18	4	5		5			2	66
Total	126	107	88	40	30	38	28	4	2	2	465
Percentage	27.1%	23.0%	18.9%	8.6%	6.5%	8.2%	6.0%	0.9%	0.4%	0.4%	100%
**Panel C Data of Municipal-CYLC**											
Month	Topic01	Topic02	Topic03	Topic04	Topic05	Topic06	Topic07	Topic08	Topic09	Topic10	Total
2021–08	2		1		2						5
2021–09	7	10	9	11	11	2	2		1		53
2021–10	11	3	10	7	2	2		1			36
2021–11	13	8	12	9	4	5	1		1	2	55
2021–12	16	7	22	8	9	2	1	1		1	67
2022–01	5	8	18	11	14	2	4				62
2022–02	1	5	17	9	3	2					37
2022–03	3	5	10	13	3	1					35
2022–04	7	4	10	3	2	6	1			1	34
2022–05	24	1	13	3						1	42
2022–06	14	8	19	12	9		1				63
Total	103	59	141	86	59	22	10	2	2	5	489
Percentage	22.0%	12.6%	30.1%	18.4%	12.6%	4.7%	2.1%	0.4%	0.4%	1.1%	100%
**Panel D Data of County-CYLC**											
Month	Topic01	Topic02	Topic03	Topic04	Topic05	Topic06	Topic07	Topic08	Topic09	Topic10	
2021–08	3	4	6	10	7	3				33	
2021–09	3	4	9	8	5	2				31	
2021–10	2	2	4	7	3	2				20	
2021–11	6	12	7	6	5	2	1			39	
2021–12	5	8	9	7	5		1		1	36	
2022–01	5	3	5	14	7					34	
2022–02	2	3	14	13	7	1			1	41	
2022–03	2	5	1	8	1	2				19	
2022–04	3	2	1	4	1	3			1	15	
2022–05	5	4	6	2	3	1				21	
2022–06	8	3	4	12	5	1				33	
Total	44	50	66	91	49	17	2	0	3	322	
Percentage	13.7%	15.5%	20.5%	28.3%	15.2%	5.3%	0.6%	0.0%	0.9%	100%	
**Panel E Data of School-CYLC**											
Month	Topic01	Topic02	Topic03	Topic04	Topic05	Topic06	Topic07	Topic08	Topic09	Topic10	
2021–08	10	1	2	7		1				21	
2021–09	11	2		4		1				18	
2021–10	7			1	1					9	
2021–11	11	1	1	7		1				21	
2021–12	16	2	1	3	1					23	
2022–01	7		2	6	1	1				17	
2022–02	1		5	3						9	
2022–03	4		6	4						14	
2022–04	5			3						8	
2022–05	13		1	4						18	
2022–06	8		3	3						14	
Total	93	6	21	45	3	4				172	
Percentage	54.1%	3.5%	12.2%	26.2%	1.7%	2.3%				100%	

To analyze the characteristics and differences of information dissemination of the league organizations at different levels, we first need to confirm whether there are identifiable and clear differences in the organizational information dissemination behavior of the league organizations at different levels. We have used cluster analysis to answer the above question. Clustering is one of the unsupervised learning algorithms. The k-means algorithm is a popular clustering algorithm based on error minimization [57]. In k-means clustering, N samples are assigned to k centroids by minimizing the mean square distance from the data points to the nearest centroid [58]. The k-means algorithm is based on the distance measure of spatial geometric distance, and the feature variables need to be normalized in order to avoid errors caused by too large a magnitude difference between the features [59].

We linked the five sub-table data of Panels A-E in Table 7 vertically. Each data sample contains 10 features from Topic 01 to Topic 10. Therefore, we obtain the matrix [55 x 10]. The topic feature matrix T is characterized as

$$T=\left\{ \begin{array}{c} {tc}_{0}^{0},{tc}_{1}^{0},\cdots {tc}_{9}^{0}\\ {tc}_{0}^{1},{tc}_{1}^{1},\cdots {tc}_{9}^{1}\\ \vdots \\ {tc}_{0}^{54},{tc}_{1}^{54},\cdots {tc}_{9}^{54} \end{array}\}.\right.$$

We use ***t c -i j***. to represent a month’s news topic feature of a certain level of the CYLC, ***i*** is the feature number and ***j*** is the line number. Since we are more interested in the distribution of topics for the dissemination of organizational information rather than the volume of news. Therefore, we divide each feature by the total value of the feature data in that row to convert the volume of news to a percentage. Finally, the data was normalized using the z-score method [60].

**Fig 6:**
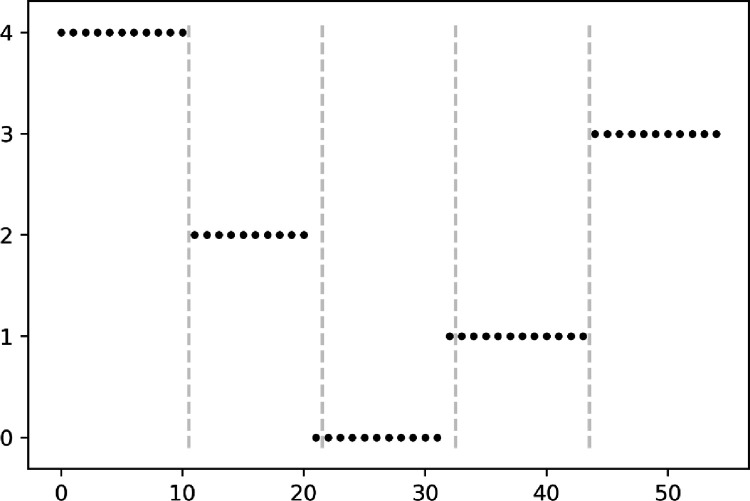
Scatterplot of news characteristics clustering analysis for each level of league organizations. Note: The order of the data in the figure is consistent with the order of the data samples in Table 6. The numbers 0,1,2,3,4 on the vertical axis of the image are only the classification markers obtained from clustering and have no other meaning.

We use the k-means algorithm to do cluster analysis on the matrix T after preprocessing the matrix. We set the number of clusters to be 5 and assume that k = 5.We can determine whether there are characteristic differences in organizational information dissemination at different levels of the CYLC by observing whether the clustering model can accurately separate the topic data of the five types of the CYLC. Fig 6 shows the results of the clustering analysis. The labels 0, 1, 2, 3 and 4 in the vertical axis of the figure represent the ordinal number of the classification, and this number simply represents the different categories and has no other meaning. The clustering results revealed that the Central-CYLC, School-CYLC, and County-CYLC sample data could be classified with 100 percent precision. Provincial-CYLC and Municipal-CYLC sample data were misclassified in a single instance each, but overall classification accuracy was 96.36 percent. Therefore, it is reasonable to assume that the organizational information dissemination characteristics of the different levels of the CYLC are distinct, and that there are clear differences between organizations.

**Fig 7:**
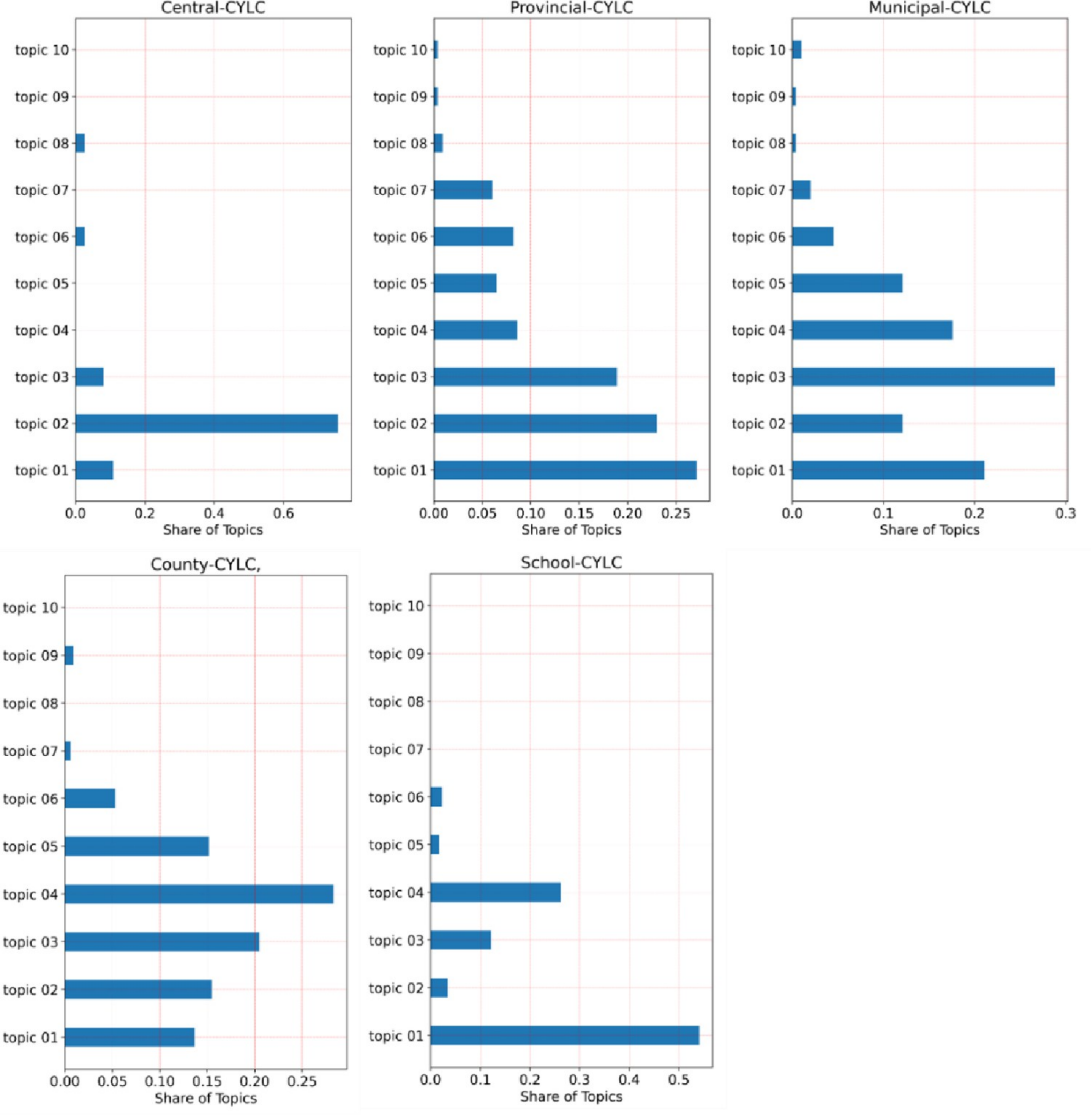
Distribution of news topics of league organizations at all levels.

**Fig 8:**
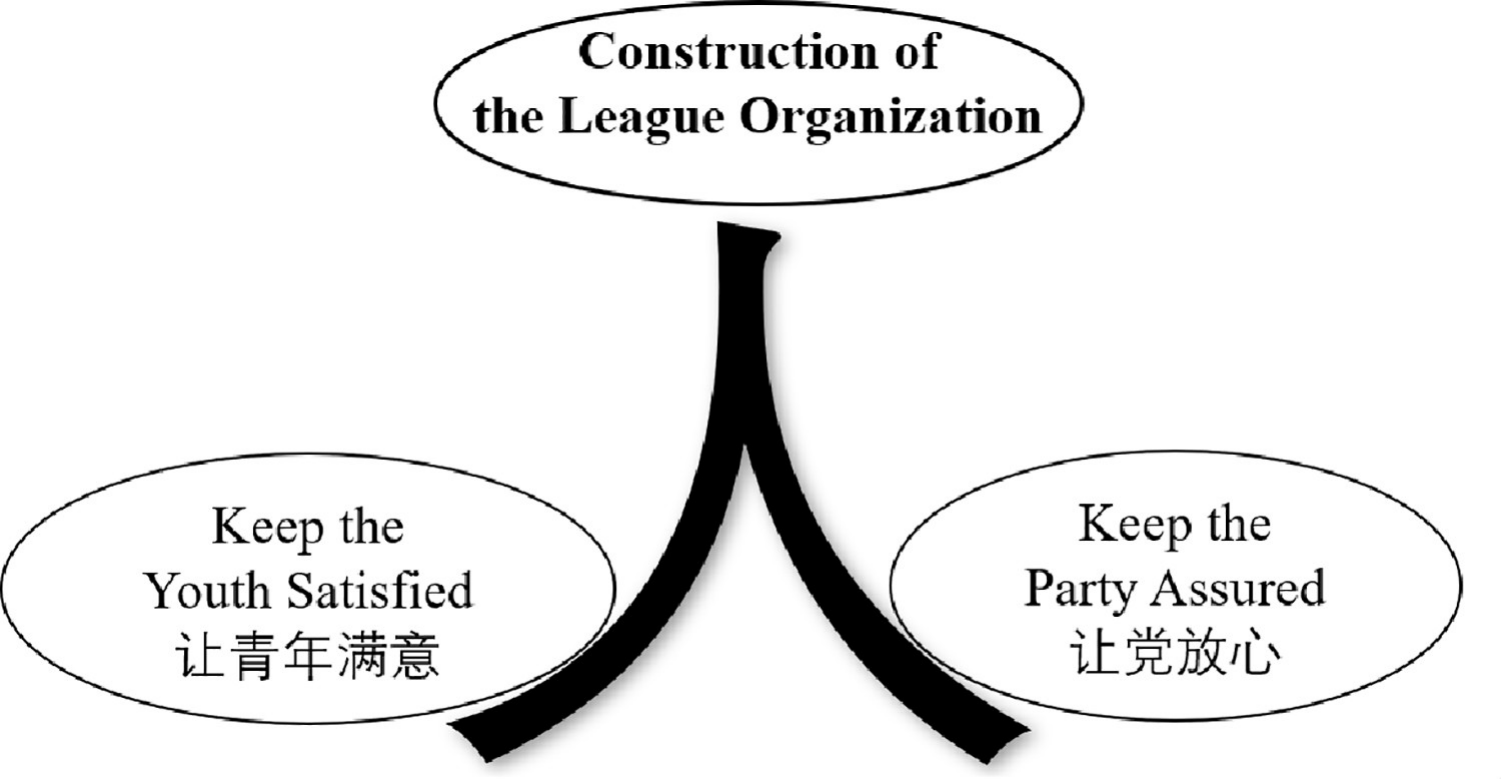
The central committee of the league drives organizational goals with organization construction.

Fig 7 shows the distribution of news topics for the league organizations at each level. Combining Fig 7 and Table 7, we can get a more in-depth understanding of the information dissemination characteristics of league organizations at different levels. About 94.6% of the news of the Central-CYLC focused on Topic 01, Topic 02, and Topic 03. In particular, the proportion of Topic 02 related to “construction of the league organization” was as high as 75.7%. It can be seen that, in the background of the strict governance of the Party [61], the CYLC attaches great importance to the construction of league organizations. In addition, Topic 01 and Topic 03 are the other two information dissemination focuses of the Central-CYLC. Topic 01 covers the theme of “love for the country and the party”, which serves the organizational goal of “Keep the Party Assured.” Topic 03 covers the theme of “helping young people to start their own businesses and employment”, which serves the organizational goal of “Keep the Youth Satisfied.” It seems that the Central-CYLC takes the construction of the league organization as a hand to promote the goals of “Keep the Party Assured” and “Keep the Youth Satisfied” to move forward. As shown in Fig 8, its information dissemination characteristics present the human shape of Chinese characters (the Chinese word for human is “人”).

Compared with the Central-CYLC, the information dissemination topics of the Provincial-CYLC are more dispersed. As shown in Fig 7, Topic 01 accounts for the highest proportion of news released by the Provincial-CYLC, followed by Topic 02 and Topic 03. It can be seen that the Provincial-CYLC attaches great importance to the information dissemination of “love for the party and the country.” It is highly concerned about the construction of the league organization and the employment and entrepreneurship of young people. In addition, Topic 04 and Topic 06, which are related to youth volunteers, are also the focus of the Provincial-CYLC. Finally, the Provincial-CYLC also takes into account the work of “caring for youth”. To sum up, the provincial committee is balancing the dissemination themes of “Keep the Party Assured” and “Keep the Youth Satisfied,” with “Keep the Party Assured” being the more important one.

As the subordinate organization of the Provincial-CYLC, the focus of the Municipal-CYLC began to migrate backward. The most important focus of the Municipal-CYLC is the “employment and entrepreneurship” work of young people, followed by “love for the party and the country”, “construction of the league organization”, “volunteers” and “caring for the youth”. The County-CYLC is the subordinate organization of the Municipal-CYLC, and its focus is further shifted back. The Municipal-CYLC is most concerned with “volunteer work”, followed by “youth entrepreneurship and employment”, then “league organization building”, “love for the party and patriotic” and “caring for youth” related topics. There is a hierarchical relationship from the top to the bottom among the provincial, municipal, and county league committees. As the organizational hierarchy moves down, the topic focus of their information dissemination also gradually moves backward. From the focus of “love for the party and the country” and “construction of the league organization”, the focus gradually shifted to “entrepreneurship and employment”, “youth volunteers”, “caring for youth”, “green, environmental protection”, “safety” and other relevant topics that are closer to reality. The topics of information dissemination are getting closer and closer to the lives of young people.

The School-CYLC is the league organization most closely associated with youth. Compared to the Provincial-CYLC, Municipal-CYLC, and County-CYLC, the School-CYLC is a special category of league organization. Secondary school league organizations are under the jurisdiction of the local league committee. In contrast, the college league committees are both influenced by the local league committee and subject to the jurisdiction of the Provincial-CYLC, and may also receive messages directly from the Central-CYLC. More than half of the news of the School-CYLC is related to Topic 01, accounting for 54.1% of all news. This shows that the School-CYLC takes “love for the party” and “love for the country” as the foundation of young people. 26.2% of the news topics of the School-CYLC are related to volunteers. The School-CYLC encourages young people to go to work where the motherland needs them and pay back society with knowledge. In addition to helping young people, “employment and entrepreneurship” and “construction of league organizations” are other concerns of the School-CYLC.

In summary, the organizational information dissemination of the Central-CYLC, the Provincial-CYLC, the Municipal-CYLC, the County-CYLC, and the School-CYLC each has its own characteristics, with clear features and clear goals. The Central-CYLC promotes organizational goals forward with league organization construction. The School-CYLC takes “love for the party” and “love for the country” as the foundation of young people. The Provincial-CYLC, the Municipal-CYLC, and the County-CYLC all place great emphasis on “love for the Party and the country” and “building the organization of the League”. However, the focus of work is becoming more and more specific, and organizations at all levels work flexibly according to where they are located, reflecting the maturity and initiative of the organization.

### 3.3 Topic relevance analysis of each level of CYLC

We used correlation analysis to investigate whether there is a correlation between information dissemination among the various levels of the CYLC. There are various methods to measure the correlation between two or more sets of serial data, such as regression analysis, Pearson correlation, Spearman Rank correlation, and Kendall’s Tau correlation [62]. These methods assess the correlation between data based on different data distribution assumptions. In our study, these methods do not affect the results of the analysis, and only slight differences in values exist. So, we used the easier and more accurate Pearson correlation to figure out if the same topics came up at different levels of the CYLC.

**Fig 9:**
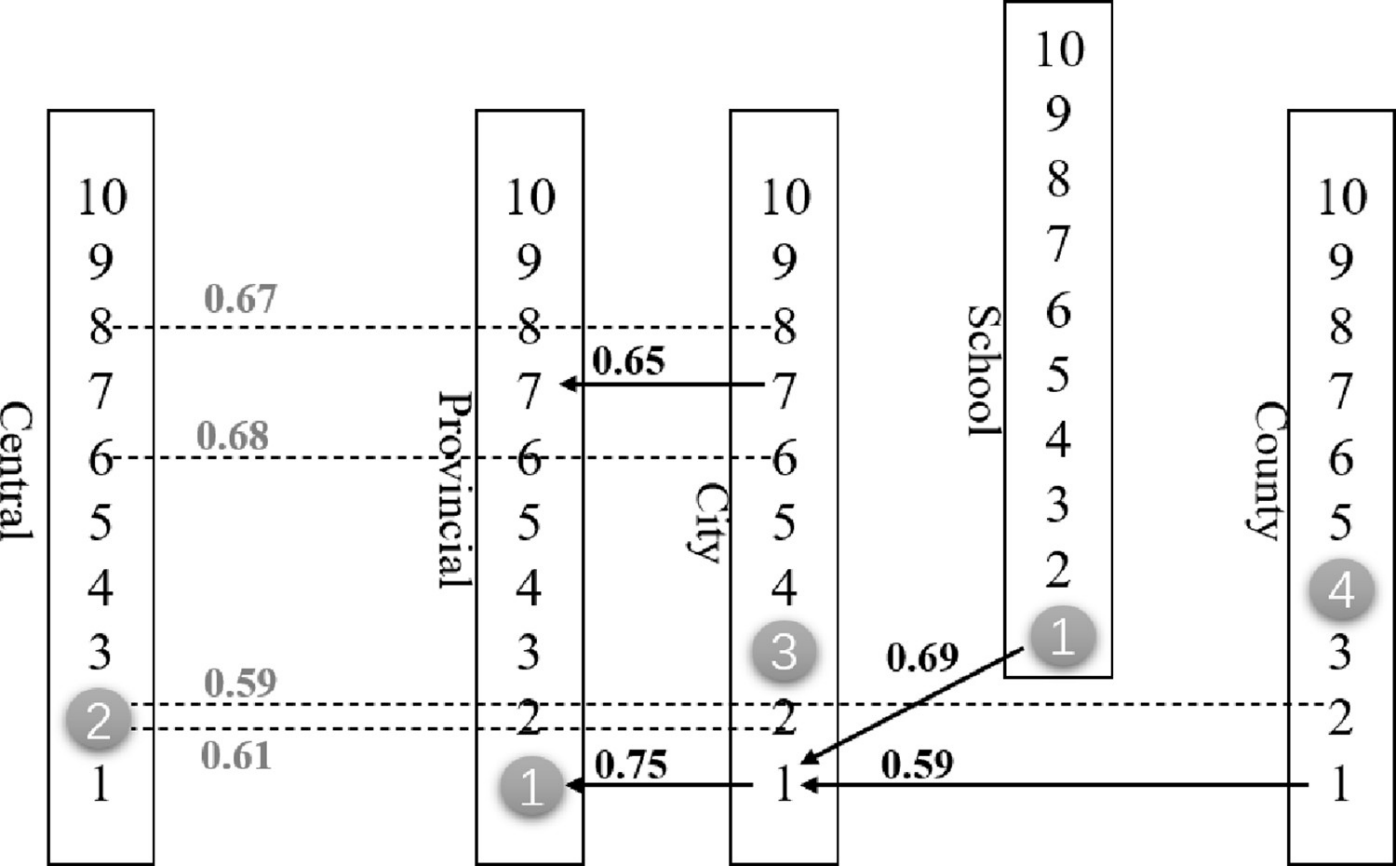
Graph of topic relevance between organizations. Note: The number in the figure represents the topic number. For example, the number 1 means Topic 01. The topics marked by white circles are the topics with the highest percentage of information published by the group at that level. Dashed lines indicate cross-level topic relevance.

Table 8 shows the results of the correlation analysis. To more clearly show the topic relevance between the various levels of the CYLC, we also drew a detailed relationship map. As depicted in Fig 9, there is a strong relationship between the Provincial-CYLC and the Municipal-CYLC, the County-CYLC and the Municipal-CYLC, and the School-CYLC and the Municipal-CYLC with regard to topic 01. The Topic 01 describes the core content of “love for the party and the country”, which highly serves the organizational goal of “Keep the Party Assured.” The relevance of Topic 01 shows that the lower-level league organizations will seriously study and disseminate the spirit of the higher-level league organizations. The above conclusion is supported by the fact that there is a pretty strong link between the Municipal-CYLC and the County-CYLC on topic 07, which is about training and conferences.

**Table 8:** Correlation analysis of news topics among committees.

	Central Committee	Provincial Committee	City Committee	County Committee	School Committee
Central Committee					
Provincial Committee					
City Committee	Topic02 0.60661 * Topic06 0.68056 ** Topic08 0.67082 **	Topic01 0.75448*** Topic07 0.65479**			
County Committee	Topic02 0.59433*		Topic01 0.59327*		
School Committee			Topic01 0.69489**		

Note: The table shows only the data with significant correlation analysis.

* represents significance level 0.05; ** represents significance level 0.02.

*** represents significance level 0.01.

There is no correlation between the information dissemination topics of the County-CYLC and the Provincial-CYLC as the superior and inferior levels. The high priority given to topic 02 by the County-CYLC (a ratio of 75.7%) and the high priority given to topic 01 by the Provincial-CYLC (a ratio of 27.1%) are perhaps the main reasons for the irrelevance of the communication topics between the two. This is a side effect of the high degree of autonomy of the Provincial-CYLC. However, both Topic 01 and Topic 02 highly serve the organizational goal of “Keep the Party Assured,” which shows that although the information dissemination of the Central-CYLC and the Provincial-CYLC serve the organizational goal from different perspectives, they have the same focus. Surprisingly, as the non-direct subordinate organizations of the Central-CYLC, the Municipal-CYLC, and the County-CYLC show a higher degree of correlation with the Central-CYLC on Topic 2. Considering the subordinate relationship between the Provincial-CYLC and the Municipal-CYLC, the Municipal-CYLC and the County-CYLC, as well as the cascading channels of information transmission. We believe that the Provincial-CYLC passes relevant information to the lower-level organizations: the Municipal-CYLC and the County-CYLC. Similarly, there is a high degree of correlation between the Central-CYLC and the Municipal-CYLC regarding topics 06 and 08, which are related to “anti-epidemic” and “young pioneers.” The League’s provincial committee played a role in relaying them.

Topic 03 is the communication focus of the Municipal-CYLC, while Topic 04 is the communication focus of the County-CYLC. These two topics serve the organizational goal of “Keep the Youth Satisfied.” We found that as the organizational level extends downward, the topics related to ’satisfying youth’ gradually become the most important concerns of grassroots organizations. The information about the topics of “Keep the Party Assured” can be transferred from higher to lower levels, which leads to the correlation between organizations at different levels regarding topic 01 and topic 02. In contrast, “Keep the Youth Satisfied” needs to be implemented by grassroots organizations, but grassroots organizations can not reverse this content to higher-level organizations for implementation, which leads to irrelevance between organizations at different levels regarding the topics of “Keep the Youth Satisfied.” As shown above, the higher-level league organizations pay more attention to the dissemination of the message about “Keep the Party Assured” and effectively convey these messages to the lower-level organizations. And the lower-level organizations gradually put the work of “Keep the Youth Satisfied” into practice under the premise of implementing the spirit of the higher levels.

This paper examines how the Communist Youth League of China (CYLC) uses organizational information dissemination to serve organizational goals. Although we use the Chinese Communist Youth League as the target of our study, essentially, our study centers around organizational information dissemination and organizational goals with content research. This is significantly different from the traditional research conducted on the Communist Youth League as a purely political group [63, 64].

## 4. Conclusion

Using 1,898 news items crawled from the website of the Communist Youth League of China as the research object, this paper presents a panoramic view of the information dissemination of the Communist Youth League of China using news topic modeling techniques and machine learning. We discovered that the CYLC’s dissemination of organizational information greatly supports the organization’s objectives. In addition, there is a distinct distinction between “information dissemination” at various levels of the CYLC, with each level operating independently according to its own characteristics and organizational goals, indicating a high level of organizational maturity. The Central-CYLC utilizes the construction of the League’s organization to simultaneously advance the goals “Keep the Party Assured” and “Keep the Youth Satisfied.” In addition, the higher-level league organizations prioritize the dissemination of the “Keep the Party Assured” message and effectively communicate it to the lower-level organizations. Under the premise of implementing the ’spirit’ of the higher levels, the work of “Keep the Youth Satisfied” is gradually implemented by the organizations at lower levels.

Although numerous theoretical studies confirm that organizational information dissemination can support organizational goals [1, 2, 65], there are comparatively few studies on large mass organizations. Our research provides a standard case study that fills a relevant gap in the literature. This study is both’methodologically’ and ’perspectively’ novel. We eschew purely theoretical analysis in favor of methods related to natural language processing and machine learning and examine a mass organization from the standpoint of organizational information dissemination. Our contribution has two components. First, the CYLC is the assistant and reserve army of the CCP, one of the most successful mass organizations, and relevant scholars will be interested in the organization itself. Furthermore, our study provides a new perspective to recognize and comprehend the CYLC. Second, we demonstrate how a successful mass organization uses organizational information communication to serve organizational goals, which is an essential reference for theorizing about organizational information dissemination [66].

Our study can be extended from three perspectives. The first is that social media has been widely adopted by league organizations at all levels, and compared to the information on official websites, the content on social media is richer and the amount of data is much larger than that on official websites. Based on the social media data, reproducing our study may lead to an enhanced version of the conclusion. Secondly, the data used in this paper are collected from the central website of the Communist Youth League, which helps to unify the data sources and reduce the difficulty of data acquisition. However, the provincial Communist Youth League websites also have richer information resources, and richer conclusions may be obtained based on the information disclosed by the Communist Youth League at various levels in multiple sources. Three, text sentiment mining is already a mature research method, and based on the existing research data and sentiment classification or sentiment temperature mining, it is also a meaningful problem to study the sentiment transmission between organizations at all levels.

## Supporting information

S1 Data (ZIP)
