# Supplementary material for: Serving organization goals by organizational information dissemination: An empirical study from the Communist Youth League of China
Source: PLoS One. 2023 Jan 20;18(1):e0280221. doi: 10.1371/journal.pone.0280221 (PMC9858461; doi:10.1371/journal.pone.0280221)
Supplement: S1 Data — (ZIP) [file pone.0280221.s001.zip › Supporting Data/Version of Chinese to English/Central Committee(English).docx]

2022-6-17 18 Editor's note: College students are valuable human resources, and the party and the government have always attached great importance to the cultivation of talents in colleges and universities and the employment of graduates. At present, under the interweaving influence of the century-old epidemic and the century-old changes, this generation of college students is experiencing employment difficulties that are different from the past. How to help students get more full and higher-quality employment is an important aspect for the Communist Youth League to serve the development of college students with heart and soul. Since 2014, the Central Committee of the Communist Youth League has further promoted the Sailing Plan·Internships for College Students in Central and State Organs, organizing college students to participate in government affairs internships in a planned, large-scale, and systematic manner, to experience government operations, workplace culture, and understanding of human sophistication to form a correct society. Cognition, effectively improve socialization ability. The relevant experience and practices are now sorted out and compiled for reference by all regions. —1—Yangfan Project Central and State Organ College Student Internship helps college students experience and grow in the social classroom. Since 2014, the Central Committee of the Communist Youth League and the Central Committee and State Organ Working Committee have continued to carry out the Sailing Project Central and State Organ College Student Internship ( Hereinafter referred to as the “Yangfan Project”, gradually explore and improve the implementation mechanism, actively play the role of practical education, continuously promote the in-depth development of the work, and effectively serve the growth of students. 1. Focus on educating people for the party, focus on tempering political quality, educating people for the party and educating talents for the country, so that college students can understand the basic logic of our party's governance of the country through the actual experience of working in institutions and participating in the operation of party and government departments. To deepen the understanding of the decisive significance of the two establishments, to continuously enhance the four consciousnesses, to strengthen the four self-confidences, to achieve two maintenances, and to be more firm in the belief in listening to the party's words and following the party's path of socialism with Chinese characteristics. important purpose of the plan. After years of exploration, a dual education model has gradually formed in which intern students not only participate in the political study organized by the party committees of ministries and agencies, the youth league committees of government agencies, and the party and youth league branches of various departments, but also participate in the study of temporary youth league branches for intern students. On the occasion of the 100th anniversary of the founding of the party in 2021, carry out the theme group day activities of learning a history, reading a book, and being worthy of a class, and organizing students to study in depth the important speech of General Secretary Xi Jinping at the celebration of the 100th anniversary of the founding of the Communist Party of China spirit, the spirit of the Sixth Plenary Session of the 19th CPC Central Committee, and books such as "Xi Jinping and Friends of College Students", deepen the understanding of the party's century-old struggle, enhance the ideological recognition and action follow of General Secretary Xi Jinping, and further strengthen the realization of the party's The second centenary goal and the confidence and determination to continue the struggle to realize the great rejuvenation of the Chinese nation. 2. Based on broadening horizons and actively cultivating practical ability, the Sailing Plan creates opportunities for college students to enter the central and state organs. When participating in daily work, the students personally experienced the efficient and rigorous working atmosphere of the central and state agencies, the intense and orderly work rhythm, and the good demeanor of the national civil servants who are politically minded, considerate of the overall situation, and dare to take responsibility. Especially since 2019, the Central Committee of the Youth League has coordinated various internship ministries and commissions to provide each student with a mentor to strengthen daily guidance. Through one-to-one teaching and guidance, students can broaden their horizons in participating in field research, organizing meetings, policy formulation and other business work, and further enhance their understanding of the operating mechanism of the national governance system and the working methods of state agencies; they are also sorting out materials , drafting manuscripts, asking for instructions and reporting, etc., to undergo training in daily work, and further improve socialization skills such as dealing with people, teamwork, communication and coordination, and language expression. 3. Improve the operating mechanism and continuously improve the management level After years of exploration and promotion, the operating mechanism of the Yangfan Project has been continuously improved and increasingly sound. In terms of participating ministries and commissions, from 5 ministries and 38 posts in 2014 to 47 ministries and 1,212 posts in 2021. In terms of job categories, it has gradually expanded from the simple government departments in the past to central media units and central financial institutions. In terms of time arrangement, the previous 1-month summer internship has been extended to a 3-month autumn internship. In terms of student sources, 156 domestic colleges and universities and 18 overseas colleges and universities have participated. Moreover, among domestic colleges and universities, there are 75 ordinary colleges and universities, accounting for 48.1%; there are 122 colleges and universities outside Beijing, accounting for 78.2%. In terms of selection procedures, highlight the recommendation checks of college league organizations, emphasize putting political quality first, formulate standards in terms of -3- academic level, social practice, etc., standardize registration, selection, publicity and other procedures, and ensure fairness and justice. In terms of training and management, a system including tutoring, daily management, logistics support, and comprehensive evaluation has been established to form a three-in-one mechanism of centralized training before the internship, collective learning during the internship, and exchange and sharing after the internship. In terms of service guarantee, we will purchase personal accident insurance for intern students, provide catering and transportation subsidies for college students in need, organize cultural and sports activities for college students outside Beijing who live in centralized accommodation, and establish a special mechanism for epidemic prevention and control. 4. Generate a linkage effect and promote the construction of the organization and regiment. Since the implementation of the Sailing Plan, the youth league committees of various ministries and commissions have actively cooperated, organized powerfully, and coordinated promotion. In the process of implementing projects and playing their roles, self-construction has been simultaneously strengthened. Highly recognized by the working committee and the party committees (party groups) of the internship ministries. The Youth League Committee of the National Development and Reform Commission summed up and formed a four-step working method of looking, listening, asking, and inquiring, with obvious effects and distinctive features. The Youth League Committee of the State Administration for Market Regulation set up a column "Sailing Plan" on the "Youth in the City" public account to promote learning exchanges and expand the effect of communication. The CPPCC National Committee, the Ministry of Culture and Tourism, and other organizations organize youth intern meetings and summary exchange meetings every year, so that the leaders of the ministries and commissions can deeply understand the work of the regiment in face-to-face exchanges. Through the implementation of the project, the work of various ministries and commissions not only provides opportunities for college students to participate in government internships, promotes talent development and service development; it also introduces young human resources for ministries and commissions to contribute wisdom and increase vitality. 5. Form an external publicity window and vividly tell Chinese stories. In 2021, Chinese-4-country students studying in universities in more than 10 countries including the United States, the United Kingdom, and Japan, and Hong Kong and Macao students studying in mainland universities will participate in the Sailing Project for the first time. Under the premise of abiding by the relevant regulations, many students recorded their internship life with text, pictures and videos, and made short videos, Vlogs, etc. and posted them on social media to show the true image of the Chinese party and government agencies from the first perspective. A new window for external publicity. By sharing what they have seen and heard, the students vividly told the stories of China and the story of the Communist Party of China's governance and administration to their classmates and friends abroad, so that the world can understand the code of China's development more comprehensively and objectively, and have a deeper understanding of the Chinese youth. A gene that is united with the party and walks with the times. —5—Shake Distribution: General Office of the Central Committee of the Communist Party of China, General Office of the State Council. All comrades in the Secretariat of the Central Committee of the Communist Youth League, the relevant ministries and commissions of the Central Committee, and the leaders of the provincial party committees. The main responsible comrades of the various departments and directly affiliated units of the Central Committee of the Youth League, and the main responsible comrades of the provincial Youth League committees. Shake The General Office of the Central Committee of the Communist Youth League Issued on June 17, 2022

2022-6-16 17 Congratulations to the 20th National Congress of the Communist Party of China, always follow the party, and forge ahead on a new journey. The fifth edition of the special issue of educational practice activities in Beijing, Tianjin, Jiangsu, and Guangdong. General Secretary Xi Jinping at the centennial celebration meeting The spirit of the important speech goes deep and solid in Beijing: focus on improving leadership, organization, and service, and give good practical ideological and political lessons to the young members of the league members in the capital. The spirit of the important speech at the conference, adhere to the first good standard, focus on consolidating the work pattern of "Three Forces, One Two Guarantees", organize and mobilize youths and league cadres in the capital to actively participate in the fight against the epidemic, so that the majority of young people can deepen their experience in the practice of great ideological and political lessons. Trust in the party, cultivate responsibility, improve quality and ability, and practice the youth vows of capital youth to rest assured of the party and strengthen the country with self-cultivation with practical actions. 1. Efforts should be made to enhance the leadership and always unite the young members of the league under the banner of the party. Issue guidelines for organizing learning and education to grassroots league organizations, and continuously strengthen the theoretical arming of young people from the four dimensions of experts speaking theory, league cadres giving lectures, youth role models telling deeds, and young pioneers telling stories. Give full play to the advantages of the new media matrix, set up relevant columns and topics on the all-media platform, produce and launch more than 30 new media products and more than 20 pictures and texts related to the spirit of General Secretary Xi Jinping's important speech, and do a good job in the interpretation of the party's innovative theory for youth, effectively promoting In the minds and hearts of teenagers. Strengthen the ideological and political guidance for young people, focus on creating high-quality cultural products, focus on learning speeches, Beijing Winter Olympics, and fighting the epidemic together, through themed youth songs, city roaming activities, H5, picture albums, books, etc., for the majority of young people I took a lively ideological and political lesson. 2. Efforts should be made to improve the organizational capacity and lead the young members of the league to fight against the epidemic. Resolutely implement the Party Central Committee's policy on epidemic prevention and control dynamic clearing, and in accordance with the deployment of the Secretariat of the Youth League Central Committee, set up a leading group of the Beijing Municipal Committee of the Communist Youth League to participate in the emergency response to the large-scale new crown virus epidemic, formulate and introduce emergency work plans, and establish and improve the city and district. The linkage mechanism of the group organizations at all levels, the main tasks in 8 aspects are determined, and a strong organizational system is built. Carried out backbone volunteer training in an orderly manner, organized league cadres, youth members, young volunteers, and college students to actively participate in community big data dispatch verification, nucleic acid testing, and material distribution, and provided voluntary services such as academic tutoring and language translation. Epidemic-2-more than 203,000 hours of practice. 3. Focus on improving service capabilities, and strive to be the strongest bridge between the party and the youth. Comprehensively survey the situation of young people in distress in the city, continuously improve the database of targeted assistance, and formulate and promulgate the "Opinions of the Capital Communist Youth League on Carrying out the Work of Handling Before Litigation". In-depth implementation of the "Beijing Youth Development Plan during the 14th Five-Year Period", regularly schedule the progress of tasks, implement detailed service projects for young people, carry out brand-themed exchange activities of Qingye brands such as Youth Civilization and Youth Commando, and guide young members of the league Based on the post, make contributions. Deepen the grass-roots mechanism, formulate the development plan for youth social organizations in Beijing, strengthen the construction of grass-roots organizations and positions such as community youth gatherings, and actively carry out activities such as epidemic prevention skills training, mental health counseling, and loving donations, and continuously enhance the sense of gain and belonging of young people. Tianjin: Pay close attention to study, publicity, dissemination, and practice to guide learning, publicity, and implementation of the spirit of important speeches. Focusing on the spirit of the important speech at the Centennial Celebration of the Founding of the League, we will continue to promote thematic education and practice activities through multiple forms, channels, and carriers, understand the original mission in the awareness of the great power of thought, gather the strength to forge ahead, and meet the party's second challenge with practical actions. Ten victories held. 1. Grasp the solid foundation of learning and smelting. The Tianjin Municipal Committee of the Communist Youth League immediately held a meeting (expanded) of the Secretary's Office to convey and study the important speech of General Secretary Xi Jinping, and study and deploy the study, publicity and implementation measures. A notice was issued to deploy the city to carry out a special action to study, publicize and implement General Secretary Xi Jinping's important speech. More than 42,000 grass-roots Youth League branches and more than 30,000 to 24,000 Young Pioneers squadrons in the city focused on learning, exchanging discussions, and talking freely about their experiences. The Municipal Youth Federation, Students' Federation, and Youth Working Committee organize youths in various fields to exchange and study experiences through symposiums, seminars, etc., and deeply understand the spirit of the speech. 2. Do a good job of preaching and strengthening leadership. Focus on the spirit of General Secretary Xi Jinping's important speech, the history of the Tianjin youth movement under the leadership of the party, etc., organize and carry out lectures, hold special lectures on studying and implementing General Secretary Xi Jinping's important speech, and plan and produce Tianjin Youth Celebration of the Centennial Theme Education Group (team) class , launched the themed educational practice demonstration group class and the red scarf themed cloud team class to welcome the 20th salute to the new era, and guide the members and young people to keep up with the new era of party building and forge ahead on a new journey with a high-spirited attitude. 3. Grasp strong communication and improve the effect. Actively innovate publicity methods, use a combination of newspapers, Internet, terminal, micro, and screen, carefully plan mainstream media publicity reports, set up a column for struggling youth, launch "Let's Talk Now" large-scale ideological and political interviews, and "New Era Youth Talk" A group of online cultural products popular among young people, such as youth speeches and "Invincible Youth" youth style recording, jointly spread the strong voice of the times that Tianjin youths please rest assured to the party and strengthen the country and build a strong network propaganda atmosphere. 4. Focus on excellence and practice to promote development. Adhere to multiple measures to deepen practical education, formulate and issue the "Notice on Continuously Deepening Haihe Youth Listening and Training Practice Activities", and build a four-listening, four-giving, and four-member service mechanism for serving young people; carry out centralized service activities for youth civilization, Through the combination of online and offline methods, guide the young members of the league to make contributions based on their posts; around the key negotiation issues of the CPPCC, organize the members of the Communist Youth League and the Youth Federation of the CPPCC to go to the Hexi District Elderly Care Center for investigation, guide the members of the CPPCC to innovate their work ideas, and actively Advise and advise the government to enhance effectiveness in deepening consultation work and writing proposals and suggestions. All localities have actively organized Qingma project trainees, youth commandos and other Qingye teams to participate in specific work such as epidemic prevention and control, household visits, social governance, etc., and actively play the role of young people in helping difficulties and emergency rescue. The role of the new force. Jiangsu: In line with the characteristics of young people, spread the important requirements of the Party Central Committee to the hearts of young people. The Jiangsu Provincial Committee of the Youth League adopts various methods such as organized learning, focused guidance, and full-coverage publicity. , solidly promoted the development of themed education and practice activities, quickly set off an upsurge of learning, publicizing and implementing the spirit of General Secretary Xi Jinping's important speech at the Centennial Celebration Conference of the League, continued to implement the whole process of career development, spread to the hearts of the majority of young people, and met the party's demands with practical actions. The 20th National Congress of the Communist Party of China was successfully held. 1. Strengthen the political leadership of young people and carry out solid study and education. Combining theme education and practice activities with learning, publicizing and implementing the spirit of General Secretary Xi Jinping's important speech, and connecting with party history learning and education, through various forms such as theoretical learning center group (expanded) meetings, symposiums, and report meetings, pay close attention to the organization and Demonstration of working backbone. Set up special classes for organized learning work, issue work reminders to promote organized learning, and mobilize cadresThe Ministry went to the young people to learn the spirit of General Secretary Xi Jinping's important speech in the form of a themed group class. Insist on connecting with reality to promote learning, organize youth members from all walks of life to contact their positions and professional characteristics, and carry out in-depth learning exchanges. Make the important speech "Jinjuye" into graphic posters and short videos, and publish them on Weibo, WeChat, Douyin, Kuaishou, etc. 2. Grasp the cognitive characteristics of young people and carefully organize youth presentations. Give full play to the brand advantages of group cadres on the podium, youth lecturer group, red scarf explainer, etc., carefully carry out a series of themed publicity activities, and focus on the youthful interpretation of the party's innovative theory. League cadres at all levels act in unison and concentrate on their actions, walk into the joint youth service stations they are in contact with, and take the lead to give speeches to the youth members of the league; the youth lecturers at all levels polish the blueprint of youthful speeches and write speeches for the first time Outline, carry out collective lesson preparation, and go to young people to carry out group-style and focused publicity; there are more than 80,000 Young Pioneers learning teams in the province, more than 1,200 hero squadrons, and 109,000 red scarf explainers. Work in shifts and go into the community , Nursing homes, red fronts and other venues to relay lectures; organize outstanding youth models to form a good youth sharing group, go to the youth to share their deeds, and lead the young members of the group to strive to be the five models. 3. Follow the law of youth growth, and extensively promote the practice of educating people. Give full play to the characteristics and advantages of practical education, combine with the youth education practice activities to welcome the 20th National Congress of the Communist Party of China, organize the ideological and political skills competition of hundreds of thousands of league cadres from colleges and universities across the province, and the grassroots league branch secretaries special competition. Give full play to the peer influence of the secretary of the Youth League branch to create classrooms for young people; organize young people to play the role of new force and commando in the fields of epidemic prevention and control, grassroots governance, and green environmental protection. Focus on college students' employment and entrepreneurship, psychological counseling during the epidemic, stress relief for high school and college entrance examinations, self-care for teenagers, etc., and provide targeted services for teenagers. 4. Keeping close to the actual needs of young people, continuously optimize the content supply. Focus on expanding the radiation effect of the new media matrix, continue to launch online themed group classes for youth learning, design and launch powerful audio and video interactive products in the '890 Ye Audio-Visual Museum, and carry out media interviews with the group flag and the party's song of youth. , In the province's 103 provincial-level youth learning associations, organize the learning cloud relay, continue to introduce online cultural products that young people love, make meaningful things interesting and effective, and continue to enhance the attractiveness and appeal of themed educational practice activities. —6—Guangdong: Focus on organization, youth flavor, and effectiveness, let the young members of the league learn the spirit of important speeches. Organize the study, publicity and implementation of the spiritual work of General Secretary Xi Jinping's important speech at the centennial celebration meeting of the founding of the Communist Youth League, and a rapid upsurge has emerged. 1. Highlight deep learning and detailed understanding, and promote organizational learning layer by layer. The Youth League and Provincial Committee immediately held a symposium on learning and implementing the spirit of General Secretary Xi Jinping's important speech, a special secretary meeting, a theoretical study center group study meeting, a special reading class, and held a youth evening school to quickly implement the first-in-class learning and demonstration learning of all government cadres. The cadres of the leading organs of the regiments at all levels in the prefectures and cities strengthen their personal self-study, actively write theoretical articles, and combine large-scale research, large-scale discussions, and large-scale lesson preparation to demonstrate and drive the cadres at all levels to carry out large-scale learning. Youth league schools at all levels and research forces of regiment affiliates research and develop special textbooks and courses, deepen the main training of regiment cadres, youth horse engineering classes at all levels and youth backbone training, carry out the delivery of classes to the grassroots, and promote the construction of Guangdong Youth Movement history teaching base under the leadership of the party and "A Brief History of the Guangdong Communist Youth League 1922-2022". Fully relying on the system mechanism of "Three Meetings, Two Systems and One Class" and the platform carrier of the Youth League, 271,300 grass-roots Youth League branches and Young Pioneers squadrons in the province have achieved full coverage of learning. 2. Highlight extensive coverage and innovatively carry out youth-flavored promotion. Relying on mechanisms such as League cadres giving lectures on the Party and Youth League, keeping in close contact with young people, one-on-one station and two-in-one cooperation, the secretaries of the League and Provincial Party Committees take the lead, and the cadres, representatives, and secretaries of the grassroots League (Working) Committees of the League at all levels go to the youth to carry out More than 500 keynote speeches were given. Continue to give full play to the role of various youth lecture groups such as the Youth Lecturer Group, actively and flexibly carry out lectures, and use green words and clear words to explain the spirit of General Secretary Xi Jinping's important speech. Nearly 100 lectures have been carried out, covering nearly 200,000 person-times. Give full play to the brand advantages of "Three Going to the Countryside", "Returning Home", "I Practice National Unity", "7-Youth Inheritance of Red Gene Volunteer Preaching" and other brand advantages, and make good use of various red revolutionary education bases such as the First Memorial Hall of the Tuanyi University, and the history of Chinese youth movements. Educational quality routes and other resources, organize young people to actively carry out research and publicity activities. Cooperate with mainstream news media to strengthen publicity and reports, give full play to the advantages of the group's new media matrix, carefully plan special columns, launch network cultural products and network theme activities, and continue to form a strong atmosphere. 3. Highlight the main business of educating people, and pay close attention to the effectiveness and implementation. Convened the Standing Committee (Expansion) Meeting of the Provincial Youth League Committee, the General Assembly of the Provincial Youth Federation, and the Plenary Session of the Provincial Youth Working Committee to systematically study and plan, and effectively implement the spirit of General Secretary Xi Jinping's important speech into the entire process of the Communist Youth League and youth work. Combined with Guangdong's actual situation, continue to deepen brand actions such as youth commandos, youth volunteers, and youth civilization, further promote the implementation of Guangdong's mid- and long-term youth development plans, and strive to solve youth problems of urgent difficulties and worries. Do a good job in employment support for key groups such as college graduates, and help the three projects of Cantonese Cuisine Master, Guangdong Technician, and Nanyue Family Economics, etc., continuously enhance the youth's sense of belonging to the League and their sense of job gain, and unite and lead the majority of young people in the province Join the new journey with a more high-spirited attitude, and welcome the victory of the 20th National Congress of the Communist Party of China with practical actions. Shake Distribution: General Office of the Central Committee of the Communist Party of China, General Office of the State Council. All comrades in the Secretariat of the Central Committee of the Communist Youth League, the relevant ministries and commissions of the Central Committee, and the leaders of the provincial party committees. The main responsible comrades of the various departments and directly affiliated units of the Central Committee of the Youth League, and the main responsible comrades of the provincial Youth League committees. Shake The General Office of the Central Committee of the Communist Youth League Issued on June 16, 2022—8—

2022-6-14 16 Congratulations to the 20th National Congress of the Communist Party of China, always follow the party, and forge ahead on a new journey. Important Speech Spirit Upsurge On May 10th, the 100th anniversary of the founding of the Communist Youth League of China was grandly held in the Great Hall of the People in Beijing. General Secretary Xi Jinping attended the meeting and delivered an important speech, comprehensively reviewing the glorious course of the Communist Youth League’s struggle against the Party, and profoundly clarifying The basic experience accumulated in the 100-year-1-journey of the Communist Youth League further points out the direction of the work of the Communist Youth League in the new era, and places ardent hopes on the healthy growth of the members and cadres of the Communist Youth League. Conscientiously studying, propagating and implementing the spirit of General Secretary Xi Jinping's important speech is the primary political task of the whole regiment at present and in the future. Youth league organizations at all levels acted quickly, taking learning the spirit of General Secretary Xi Jinping's important speech as the main content of the educational and practical activities on the theme of celebrating the 20th National Congress, always following the party, and forging ahead on a new journey, and launched a variety of learning activities covering a wide range. Organize the young members of the League to listen to the live broadcast of the conference through radio, television, and live webcasting, and exchange learning experiences through seminars, sharing lectures, writing experiences, etc., quickly setting off an upsurge of learning and publicity. 1. The leading organs and leading groups of the regiment take the lead in learning and in-depth study. The leading organs of the regiment at all levels act quickly, and invite members of the leading group of the party committee to watch the live broadcast of the conference together with the cadres, members of the league, and youth representatives from all walks of life, and listen to the important speech of General Secretary Xi Jinping; Organize and hold theoretical study center group study meetings and other special topics to convey the spirit of studying General Secretary Xi Jinping's important speeches, research and deploy relevant implementation measures; use important speeches as the core teaching materials for the education and training of regiment cadres, insist on combining them with planning work, and continuously promote the transformation of learning results. The Central Committee of the Communist Youth League, the All-China Youth Federation, the All-China Federation of Students, and the National Youth Working Committee held a symposium on the spirit of learning General Secretary Xi Jinping's important speech for the first time. The study contains great significance, profound connotations, and important requirements. The Federation of Students and the Young Pioneers organized the study, publicity and implementation work to make deployments, clearly requesting to deeply understand the ardent expectations of the General Secretary, to widely publicize the care and love of the General Secretary, to truly understand the important requirements of the General Secretary, and to fully implement the spirit of the General Secretary's important speech. The whole process of career development has spread to the hearts of young people. The Beijing Municipal Committee of the Communist Youth League invited the municipal party committee—2—mainly responsible comrades to discuss and communicate with young people from all walks of life in the capital, held a secretarial (expanded) meeting, deployed organizational learning, social publicity, centralized training, and implementation of key tasks, and made important speeches solidly Spiritual learning, propaganda and implementation. The Heilongjiang Provincial Committee of the Youth League held a secretary (expanded) meeting to convey the spirit of learning important speeches, formulate a learning and publicity implementation plan, and aim to implement the branch and cover all members of the league, deploy the province to carry out activities such as themed regiment days and social practices, and form a regiment organization A lively scene of concentrated action and active participation of young people. The Zhejiang Provincial Committee of the Youth League organized a symposium for young people, inviting 8 outstanding youth representatives in the fields of reform pioneers, scientific and technological workers, industrial workers, and volunteers to meet face-to-face with the Secretary of the Provincial Party Committee to exchange learning experiences, share youth stories, and express the spirit of the new era of meritorious service determination. The Anhui Provincial Committee of the Communist Youth League held a group day event on the theme of studying the spirit of General Secretary Xi Jinping's important speech. All the Standing Committee members of the Provincial Party Committee, the Provincial People's Congress, the Provincial Government, and the Provincial Political Consultative Conference responsible comrades attended to study and communicate with youth representatives from all walks of life, and set an example for the youth of the province's League members . The Jiangsu Provincial Youth Working Committee organized the national and provincial Qingma Project Young Pioneers worker class students to watch the live broadcast of the celebration conference, learn the spirit of the important speech in depth, and exchange learning experiences. The Hebei Provincial Committee of the Youth League held a symposium to celebrate the 100th anniversary of the establishment of the Youth League. The Provincial Party Committee, the Provincial People's Congress, the Provincial Government, the Provincial Political Consultative Conference team and the May 4th Medal Winners, Two Reds and Two Excellences, and youth representatives of the Youth League members exchanged learning experiences and grasped the core essentials. 2. The majority of regiment cadres and young backbones should learn and follow up in a timely manner. Relying on the mechanism of "one specific, one station, two alliances" in various regions, combined with the epidemic prevention and control arrangements, and through a combination of online and offline methods, organize representatives of the regiment, regiment cadres, Workers of the Young Pioneers and various young backbones focused on watching the live broadcast of the conference, giving full play to organizational advantages, forming a wide coverage, highlighting the leadership of representatives, earnestly studying and discussing, strengthening practice to promote learning, and improving learning effectiveness. The All-China Youth Federation mobilized members of the Youth Federation at all levels to watch the live broadcast of the conference, organized members to study and discuss the spirit of General Secretary Xi Jinping's important speech immediately, and edited and distributed members to study and discuss articles. Members of the Youth Federation at all levels adhere to the original study, in-depth systematic study, and practical study, and organize the "Strengthening the Country and Youth Talking" through a combination of online and offline forms --- the grassroots activities in the Youth Federation lecture hall, and organize the members of the Youth Federation to walk into the The spirit of the important speech was preached at grassroots level such as colleges and universities and communities. The students of Tsingma Engineering in various fields at all levels across the country watched the conference carefully, and held a symposium as soon as possible to learn the spirit of the important speech and have a heated discussion. The Shanghai Municipal Committee of the Communist Youth League organized 16 district league representative liaison stations to watch the live broadcast of the conference online, and held heated discussions on how to implement the important requirements put forward by General Secretary Xi Jinping for the league organization, young league members, and league cadres. The Hubei Provincial Committee of the Communist Youth League produced and launched the theme music group class of "Century Pursuit". Through situational interpretation + artistic presentation, cross-time and space dialogue, etc., the students and young pioneers of the Provincial Qingma Project were organized to study the spirit of important speeches and review the Communist Youth League and the Party. The history of struggle inspires the mental outlook of young people who are determined to strive for the first place. Beijing University of Science and Technology and China University of Petroleum (East China) jointly held a theme group class, through the organization of the celebration conference to share their feelings, the teachers of the ideological and political course interpreted the speech, the youth members of the group interacted and shared, reviewed the oath of joining the group, and signed the letter of determination, etc. Internalize the spirit of the conference into the spiritual motivation to study hard, practice hard, and aspire to serve the country. The Youth League Committee of Xi'an Jiaotong University held the 2022 themed group day event of the 100-year-old Youth League, the gift of youth, the 20th Daye, collectively learning the spirit of the important speech, and reviewing the school's Communist Youth League through four chapters: Baptism, Westward Journey, Innovation, and Endeavour. The journey of struggle under the leadership of the party. After the representative liaison station of the Xiangzhou League in Zhuhai, Guangdong, watched the conference collectively, they carried out a group class on the history of the party's youth movement, and combined with the history of the century-old youth movement, they deeply understood the ardent entrustment of General Secretary Xi Jinping. The Youth League Committee of CNOOC Flourishing Operations Company organized the young backbones of the offshore production line and various land departments to watch the live broadcast of the conference, and combined with a series of activities such as the commendation of "two reds and two excellent smelters", the flag presentation of young pioneers, and the establishment of projects by young people, they will learn the ideological results of important speeches It has been transformed into the actual effect of helping the high-quality development of Bohai Oilfield. The Jianhu County Party Committee in Yancheng, Jiangsu Province organized youth lecturers Tuan Ye and Red Scarf to give lectures, went to schools, enterprises, and villages, and carried out important speeches for young students, enterprise youths, and rural youths, covering more than 2,000 youth members. 3. The majority of league members and young people in various fields should study hard and learn deeply. League organizations at all levels insist on combining individual self-study and organizational learning, aiming at youth characteristics and local characteristics, making good use of three meetings, two systems and one lesson, red education resources, etc. Carrier of practice, carefully designed and solidly carried out learning and education activities. All levels of youth federations, students' unions and youth social organizations give full play to their advantages in extensive contact with young people, innovatively carry out various forms of learning activities, and strengthen the leadership of various youth groups. The Central Committee of the Communist Youth League organized Youth League members from all over the country to watch the live broadcast of the conference and listen to the important speech of General Secretary Xi Jinping. More than 10.52 million people watched the live broadcast of the conference through @共青团中央微博, WeChat video account, and Bilibili platform. The Youth League Committees of Inner Mongolia and Xinjiang Autonomous Regions distributed learning reminders by counties (cities, districts, and banners) and organized youth social organizations to watch the conference live. The Shanxi Provincial Party Committee of the Tuanshan made full use of the advantages of the Youth Home and the Youth Station, and organized League cadres at all levels, young League members, and Young Pioneers to exchange learning experiences face-to-face. The Jiangsu Provincial Committee of the Communist Youth League combined with the key publicity and coordination projects of the Central Committee of the Communist Youth League, organized young experts and scholars to actively write in-depth theoretical articles, and further made important speeches for the majority of young people. The Tuan Jiangxi Provincial Committee expanded the coverage of learning through new media, adopted the form of current affairs + Vlog, and filmed the provincial party committee's symposium on learning and implementing the spirit of important speeches on the spot. the interviewThe delegates at the meeting conducted an immersive record of the learning and implementation. The Fujian Provincial Committee of the League issued the "Work Tips for Learning and Education", improved the functional module of "Smart League Building", and mobilized League organizations at all levels in the province to carry out learning in a combination of online and offline. Up to now, a total of more than 69,000 regiment (headquarters) branches have carried out special studies such as studying the general secretary's speech at the centennial conference of founding the regiment, and carried out nearly 100,000 learning activities. The Henan Provincial Committee of the Communist Youth League, together with the mainstream media and the new media matrix of the Communist Youth League at all levels in the province, continued to launch the response to the conference, and set up a typical youth column of "Youth Shining Striving to Be a Pioneer", fully demonstrating the feelings and experience of the youth members of the province's youth learning important speeches and actively participating in modernization The high-spirited style and vivid practice of Henan construction. —6—(No text on this page)—7—Shake Distribution: General Office of the Central Committee of the Communist Party of China, General Office of the State Council. All comrades in the Secretariat of the Central Committee of the Communist Youth League, the relevant ministries and commissions of the Central Committee, and the leaders of the provincial party committees. The main responsible comrades of the various departments and directly affiliated units of the Central Committee of the Youth League, and the main responsible comrades of the provincial Youth League committees. Shake Issued by the General Office of the Central Committee of the Communist Youth League on June 14, 2022

2022-6-14 15 Congratulations to the 20th National Congress, always follow the party, and forge ahead on a new journey. On the occasion of the May 4th Youth Day, the theme education and practice activities of the Journey, the youth league organizations at all levels will conscientiously study, publicize and implement the spirit of General Secretary Xi Jinping's inspection of Renmin University of China's important speech and the spirit of the important reply to the youth team of China Aerospace Science and Technology Corporation's space station construction as a welcome The important content of the educational practice activities on the theme of the 20th National Congress of the Communist Party of China, always follow the party, and forge ahead on a new journey, taking the opportunity of celebrating the May 4th Youth Day and the centenary of the establishment of the regiment, and taking the theme regiment (team) day activities as the carrier, fully excavated Local characteristic resources guide young people to keep in mind the teachings of the Party, carry forward the spirit of the May Fourth Movement, aspire to national rejuvenation, and run the best results of contemporary youth on the track of youth. 1. Topical study and in-depth study, let the party's innovative theory enter the brain and heart, and the whole group deeply study Xi Jinping's new era of socialism with Chinese characteristics The glorious history, give full play to the advantages of organized learning, and form a strong learning atmosphere of classic theoretical topics and the latest spiritual follow-up learning. 1. Combination of various forms. All localities combine the study of General Secretary Xi Jinping's important speech and the spirit of replying to the letter with the implementation of General Secretary Xi Jinping's important thoughts on youth work, and the launch of the theme education and practice activities of celebrating the 20th National Congress, always following the party, and striving for a new journey. Through collective learning Organize youth league cadres and league members to study, systematically, and follow-up in the form of , theme discussions, exchange seminars, etc., to further strengthen the ideals and beliefs of fighting with the party, and to become a vanguard force in realizing the great rejuvenation of the Chinese nation. The Secretariat of the Central Committee of the Communist Youth League held several secretariat meetings, held study sessions for the theoretical study center group, conveyed and studied the spirit of General Secretary Xi Jinping's important speeches and replies in a timely manner, and researched, deployed and implemented measures. The Guangdong Provincial Committee of the Youth League focused on the spirit of General Secretary Xi Jinping's inspection of Renmin University of China's important speeches, combined with reading "Xi Jinping and College Students Friends" and "Chinese Youth in the New Era" white paper, etc., during the May 4th period. Discussion activities. Shanxi Provincial Party Committee organizes city (county) Youth League Committee Secretary, Youth Federation Chairman, Young Workers-2-Committee Director and College Youth League Committee Secretary Representatives to combine work exchanges and study the feelings and experience of General Secretary Xi Jinping's important speech, and express their steadfastness and hard work in their respective positions , Confidence and determination to shoulder heavy responsibilities. The Jiangxi Provincial Committee of the Communist Youth League organized a youth symposium on welcoming the 20th National Committee of the Chinese People's Political Consultative Conference. Youth League members, members of the Youth Federation, members of the Provincial Committee of the Chinese People's Political Consultative Conference, and young cadres of the Political Consultative Conference organs exchanged their experiences and experiences in learning Xi Jinping's socialist ideology with Chinese characteristics in the new era, combined with the performance of duties Practice, talk freely about feelings and perceptions, and improve the realm of thought. Youth League organizations in Hebei, Liaoning, Gansu, Anhui, Hainan and other places organized group members and young people from all walks of life to conduct in-depth exchanges and study the spirit of General Secretary Xi Jinping's important speech and reply letter by holding theoretical study center group study meetings, theoretical seminars, symposiums and other forms What they think and understand guides the majority of young people to establish a sense of family and country, and to aspire to continue to struggle and struggle forever. 2. Innovative formal practice. Relying on rich practical education resources, guide the majority of young people to learn the spirit of General Secretary Xi Jinping's important speech in practice, and learn the history of the Chinese youth movement led by the party. With the theme of Chinese youth's unremitting struggle for the great rejuvenation of the Chinese nation under the leadership of the party, the Central Committee of the Communist Youth League launched an exhibition on the history of the Chinese youth movement in the Central League School. , Listening to the Party’s Call to Fight the Reform Trend, and Young People’s Progress toward the Party in a New Era, exhibited more than 650 pictures and 230 precious cultural relics, documents, files, etc., comprehensively showing the century-old history of the Chinese youth movement under the leadership of the Party. With the theme of the establishment of the Chinese Socialist Youth League under the leadership of the Communist Party of China, the Guangdong Provincial Committee of the Youth League built a memorial hall for the first anniversary of the Youth League in the former site of Guangzhou Dongyuan. Keep in Mind the Mission: Four sections, exhibiting pictures, objects, sculptures, and —3—historical videos, etc., present a panoramic view of the birth of the Chinese Socialist Youth League under the leadership of the Communist Party of China a hundred years ago, and show that the Communist Youth League adhered to its original mission and worked hard. The historical responsibility of following the party and forging ahead on a new journey. The Gansu Provincial Committee of the League created the theme publicity and education positions of the Gansu Party Youth Movement History Exhibition and the Gansu Young Pioneers History Experience Hall, providing a richer learning and education base for the majority of young people, and providing a more influential activity carrier for the theme education practice . The Yunnan Provincial Committee of the Communist Youth League organizes a themed exhibition. Through three sub-exhibitions, including the Career Exhibition, the Art Exhibition, and the Immersion Exhibition, the youth time capsule is used as the main line and carrier, and the innovative real-scene interpretation and exhibition interaction allow young people to experience a century in the exhibition. The journey inspires the strength to forge ahead. The Zhejiang Provincial Committee of the Youth League established 1,073 youth preaching teams, organized youth league cadres, youth preachers, and members of the Red Scarf Tour Lecture Group to go to the grassroots and frontlines, and carried out more than 4,000 speeches on the spirit of General Secretary Xi Jinping's important speech, covering 500,000 young people. The Hubei Provincial Committee of the Communist Party of China organized a number of youth search teams for the new generation of the powerful country, and they went to places for youth ideological and political education such as revolutionary relics, new economic construction areas, and major engineering projects, and interacted online through the province's youth ideological and political education map , leading the majority of young people to strengthen their ideals and beliefs in immersive education. 3. Enrich the carrier extensively. Create and launch a series of learning products to continuously expand the coverage of learning. The Central Committee of the Communist Youth League released a series of heavy theoretical articles, aimed at young people to do a good job of explaining the important thoughts of General Secretary Xi Jinping on youth work and the history of the Chinese youth movement under the leadership of the party, and produced and launched the general secretary of the online themed group course of "Youth Great Learning" With young friends, the major achievements and historical experience of the Chinese Youth Movement led by the Party for a century, the special edition of the 100-year-old regiment-Exploring the Chinese Youth Movement-4-Historical Exhibition and other special content, mobilize the majority of League members to learn and follow up in time . Launched the Chinese Youth Sports History Learning Challenge mini-program under the leadership of the Party, through modules such as daily quiz, national challenge, friend battle, World War I to the end and other applications, such as self-study room, wrong question book, ranking list, etc., to continuously improve youth's ability Learning interest and learning effect. The Xinjiang Autonomous Region Committee of the Communist Youth League compiled and printed a series of working materials for the regiment, including the voice of the regiment, the speeches of the regiment, the courseware of the regiment, the norms of the regiment, and the deeds of the regiment, etc., to guide grassroots organizations to improve Combine the history of the study group well, and further promote the work of the Communist Youth League and youth. The Heilongjiang Provincial Committee of the Communist Youth League launched a 22-episode series of short video collections "Questions and Answers on Youth Learning", with the theme of General Secretary Xi Jinping's important thoughts on youth work and the development of the Communist Youth League, vividly showing the history of the Chinese youth movement under the leadership of the party. There is a boom in learning. The Shandong Zibo Municipal Committee of the Youth League adheres to the youth standpoint and the underlying logic, and organizes a knowledge contest on the history of the Youth League with the theme of "Forging ahead and keeping the original heart and youth presenting the 20th Daye". The competition platform of the four sections of the personal center creates a strong atmosphere for the majority of young people to learn about the history of the school and know the love of the group. 2. Inherit the spirit of the May 4th Movement, bravely undertake the mission of the times, and carry out extensive group and team activities. All regions make full use of important time points to innovate and carry out May 4th themed group (team) day activities with novel forms and rich content, so as to guide the majority of young people in their organizational life. Consolidate the theoretical foundation, inherit the spirit of the May Fourth Movement, and provide great ideological and political courses that combine with reality. 1. Give full play to the educational role of red resources, so that young people can feel the great power of the spirit of the ancestors in the ritual education, and enhance their advanced nature and sense of honor. The Jiangxi Provincial Committee of the League took advantage of the advantages of many old revolutionary areas, many party history events, and many revolutionary ancestors to organize and carry out centralized demonstration activities for new League member admission ceremonies in Gongqingcheng, Jinggangshan and other red education bases. More than 7,700 ceremonies were held, covering more than 1.6 million youth members. The Hunan Provincial Committee of the Communist Youth League organized a centralized demonstration activity for the entry ceremony of new league members. 1,000 representatives of new and old league members from Changsha, Zhuzhou, and Xiangtan gathered at Mao Zedong Square in Shaoshan to solemnly swear an oath under the flag of the league. The firm belief and mission of the state-owned company. The Jilin Provincial Party Committee of the League specially launched the online interactive display activity of "Showing My League Member ID" to review the update and iteration stories of the three generations of League Member ID cards. By generating exclusive electronic membership cards, young League members and old League members who have left the League over the age can rejoin the League Youthful memories of the times, sharing the glory of being a member. The Henan Provincial Committee of the Communist Youth League mobilized the league organizations of colleges and universities in the province to carry out a wide range of activities such as flag-raising ceremony, game experience, red movie viewing, orienteering, and a hundred red songs in praise of the century-old singing competition to guide the majority of league members and students to stay young forever. Enthusiasm, looking for direction and contributing strength from the new mission entrusted by the new era. The Working Committee of the Guangxi Autonomous Region Organs held the theme group day event of "Continuing the red blood to gather youth power". Youth representatives remembered the martyrs who devoted themselves to the revolutionary cause in activities such as party history story presentations, situational poetry recitations, original song singing, and knowledge quizzes. Understand the mission of young people in the new era. The Youth League Committee of Xiamen University held a group class to ask the party to rest assured and strengthen the country under the national flag. Representatives of the members lined up in a square formation to strengthen the country and rejuvenate the country. On the spot, the national flag was raised and the national anthem was held. Activities such as "Praise of the People's Republic of China" and singing group songs together provided a vivid education lesson on patriotism and ideals and beliefs for the youth of Xiamen University. 2. Create a red-seeking themed team (team) day event featuring "theoretical learning + visit practice" to guide young people to inherit the red gene and absorb the strength of struggle in immersive activities. The Tibet Autonomous Region Committee of the Communist Party of China held a practical education activity of "Red Education, Moisturizing Children's Minds" in the Border Youth Research Tour, and organized outstanding Young Pioneers and Young Pioneers counselors from border counties and districts in the region to participate in research and practice, allowing them to personally experience the great achievements Tibet has made under the leadership of the Party. General Secretary Xi Jinping and the Party Central Committee's affectionate feelings have further fostered the love of the Party, the country and socialism, and strengthened the consciousness of the Chinese nation's community. During the May 4th theme team day activity, the Chongqing Fuling District Committee invited the early builders of the 816 project to the project site of that year, organized the young pioneers to take the third-line metallurgical construction road again, and shared the heroes of the third-line metallurgical builders through micro-interviews The deeds lead the young pioneers to inherit and carry forward the third-line spirit and sow the seeds of serving the country. The Ankang Municipal Committee of Shaanxi Province carried out the theme group class activity of remembering the revolutionary martyrs and inheriting the spirit of the May Fourth Movement in the Revolutionary Memorial Park of Shiquan County. Typical stories of advanced youths, the young members of the league deepened their ideological understanding and received spiritual baptism while listening. 3. Combining voluntary service actions such as fighting the epidemic, community service, and employment assistance, integrate the group (team) day activities into the fervent practice of serving the society and the people. The Shanghai Municipal Committee of the Communist Youth League launched a cloud group class themed on May 4th with the theme of uniting and fighting against the epidemic, launched a screening of excellent works of youth guarding Shanghai, and invited typical young people who emerged in the fight against the epidemic to participate in cloud interviews, telling about their extraordinary stories in ordinary positions The story of the battle against the epidemic was simultaneously broadcast on the People's Internet-7-Sea Channel, Xinhua Finance and other platforms, with a total of more than 1.3 million views. The Guangxi Liuzhou Municipal Party Committee launched a special action to recruit talents for young people in Qingju Longcheng, creating brands such as Qingshijian, Qingyanxueyue, Qingnengli, Qingxuanjiao, etc., serving the growth and development of young people in Liuzhou, and promoting the return of college students to their hometowns and society Practice. 3. Extensive mobilization, comprehensive coverage, set off a wave of themed publicity During the May 4th period, various localities carried out extensive themed network activities to celebrate the 100th anniversary of the founding of the Chinese Communist Youth League, by launching original media products that are popular among young people, setting up special columns and online topic discussions, etc. , set off an upsurge of themed educational practice activities. 1. The league-affiliated media set up a column, issue a special issue celebrating the centenary of the founding of the regiment, publish important articles and the development of activities in various places. Through the People's Daily, Xinhua News Agency and other central media, the Central Committee of the Communist Youth League published "The Centennial Struggle and Historical Enlightenment of the Chinese Communist Youth League", "Singing the Song of Youth in the New Era——The Party Central Committee Caring for Youth with Comrade Xi Jinping at the Core and Youth Work Documentary" and other blockbuster articles, released the statistical bulletin of the Communist Youth League of China, and published more than 400 reports related to youth and youth work during the May 4th Movement. Youth league organizations in various places have extensively used newspapers, TV media, online new media, outdoor advertisements and other channels to carry out themed publicity reports with their own characteristics, various forms and rich content. 2. Carry out publicity around the theme of "Youth Heart to the Party and Contribute to the New Era". On May 4th, the Central Committee of the Communist Youth League broadcasted a special program on May 4th Youth Day, a special program on the May 4th Youth Day, an exhibition of excellent online cultural products of the Communist Youth League, and a century-old youth burning with me on 21 platforms including Learning Power, WeChat, Weibo, and Station B. Four Youth Day youth lectures and other content, watched by more than 14 million person-times; initiated online solicitation activities such as "Hundred Schools Relay Singing for a Hundred Years" and so on, more than 8-more than 20,000 people contributed articles, 10 topics were on the Weibo hot search list, and the reading volume exceeded 4.2 billion times; released the theme promotional film "Together Youth" celebrating the centenary of the founding of the regiment, the May 4th theme micro-film "Determination" and "Super Power" and other products, produced and builtThe group's centennial-themed posters, comics, wallpapers, etc., have accumulated more than 160 million views. League organizations around the world launched May 4th-themed webcasts, created themed promotional films, micro-movies, short videos, theme song MVs and other colorful online cultural products, and created interactive exhibitions such as original hand-painted H5, comics, and script killings. The network cultural products focus on showing the glorious history of the young members of the league under the leadership of the party in different historical periods who have continued to struggle and make contributions. In the cloud, they have inspired young people to follow the party closely and strive to serve the country. 3. Focusing on welcoming the successful convening of the 20th National Congress of the Communist Party of China, combined with the celebration of the centenary of the founding of the League, creating a more youthful and attractive atmosphere in various places. The Inner Mongolia Autonomous Region Committee of the Inner Mongolia Autonomous Region created the Youth Express Line, which set the city government station of Hohhot Metro Line 1 as the theme station, and the four exits were mainly colored in red, pink, blue, and orange, creating a young, bright, and refreshing feeling , to highlight the dedication, devotion and heart-warming of youth, with the combination of original hand-painting and graphic design, the 6 carriages are made into different themes such as thought leadership, national unity, innovation and entrepreneurship, volunteer service, marriage and friendship, and team leadership Train, to create a Communist Youth League that young people can always think of, find, and rely on. Group organizations in Guangdong, Shaanxi, and Chongqing put up themed posters on iconic buildings such as Canton Tower, Xi'an City Wall, Jiefangbei WFC, street bulletin boards, buses and subways, and large LED screens in business districts, displaying the celebration of the 20th National Congress, The theme slogans are always follow the party, forge ahead on a new journey, please rest assured the party, and strengthen the country. The Xi’an Municipal Committee of Shaanxi Province carried out a drone light show at the Daming Palace National Heritage Park on September 9, and used light changes to display the symbol of the new era of youthful heart and party building, to send festival blessings to young friends, and to inspire the majority of young people to take practical actions To welcome the victory of the 20th National Congress of the Communist Party of China. —01—(No text on this page)—11—Shake Distribution: General Office of the Central Committee of the Communist Party of China, General Office of the State Council. All comrades in the Secretariat of the Central Committee of the Communist Youth League, the relevant ministries and commissions of the Central Committee, and the leaders of the provincial party committees. The main responsible comrades of the various departments and directly affiliated units of the Central Committee of the Youth League, and the main responsible comrades of the provincial Youth League committees. Shake Issued by the General Office of the Central Committee of the Communist Youth League on June 14, 2022

2022-4-29 14 Editor's note: Since the "Deepening Reform Plan of the Central Committee of the Communist Youth League" was issued, the provincial Communist Youth League committees have taken the initiative to benchmark and check the table, earnestly assume the main responsibility for deepening the reform of the directly affiliated organs at the same level, and drive the deepening of the Communist Youth League organizations at the prefectural level Reform, guiding the pilot reform of grassroots organizations of the Communist Youth League in county areas. Among them, the Tuanshanxi Provincial Committee followed the deployment of the Provincial Committee and the League Central Committee, closely combined with its own reality, focused on focusing on the real, making practical moves, and seeking practical results, solidly promoting the deepening reform of directly affiliated agencies, and blazing a new path with distinctive characteristics. Its experiences and practices are now compiled and distributed for reference by other regions. —1—Tuanshanxi Provincial Party Committee has solidly promoted the deepening reform of directly affiliated organs. Central deployment, in accordance with the relevant requirements of the "Communist Youth League Central Committee Deepening Reform Plan", combined with its own reality, accurately grasp the three relationships, always adhere to the three orientations, focus on improving the three capabilities, and promote the deepening of the Communist Youth League and provincial committees with the spirit of nailing The reforms took effect. 1. Accurately grasp the three relationships, focus on issues and make precise efforts 1. Grasp the relationship between consolidation and development, and implement the reform and deployment to achieve a standard. The members of the team led all the cadres in the organization to earnestly study and understand the spirit of the series of important instructions issued by General Secretary Xi Jinping and the Party Central Committee on deepening the reform of the Communist Youth League. Deepen the deep mechanism problems that urgently need to be solved in the reform, anchor the reform goals of eliminating the persistent diseases of institutionalization, administrative reformation and root cause of institutional disorganization, focus on the main responsibilities and main businesses, and clearly propose that the Shanxi Communist Youth League will focus on all aspects of youth contribution and high quality The development goal requirements and the key work matrix of 7 Yi 3 smelters, the precise positioning of the general coordinates of deepening reforms, lay a solid foundation and make sufficient preparations for drafting the deepening reform plan of the Shanxi Communist Youth League and promoting the implementation of reform measures. 2. Grasp the relationship between comprehensiveness and focus, and improve the system and mechanism to achieve an effect. Focusing on the requirements of further improving the system and mechanism for strengthening the party's overall leadership and reforming the working mechanism of directly affiliated agencies, pay close attention to the shortcomings in daily management and operation, and -2- deepen the reform of agency system construction and operating mechanism. Adhere to the system concept and rule of law thinking to promote the improvement of governance efficiency, adhere to the simultaneous development of establishment, reform, and abolition, and improve 55 systems in various fields including strengthening the party's leadership, organization construction of the league, team management of the league cadres, team management of the league members, etc., among which Created 38 items, revised 17 items, and abolished 5 items to further clarify the specific functions of departments, standardize the work process, and straighten out the leadership and operation mechanism of the agency. Adhere to the idea of ​​making key breakthroughs, point to area, and overall advancement, focusing on establishing and improving the mechanism for implementing the important instructions of General Secretary Xi Jinping and implementing the decision-making arrangements of the Provincial Party Committee and the work requirements of the Central Committee of the Communist Youth League. The first topic of the Provincial Party Committee Secretary's "system" forms a closed-loop chain of learning and comprehension, implementation, supervision and handling, evaluation and questioning, and report feedback, leading the province's league cadres to continuously improve and strengthen the four consciousnesses, strengthen the four self-confidences, Be conscious of thinking and acting consciously in two ways. 3. Grasp the relationship between integrity and innovation, and strengthen theoretical research to highlight a word of deep smelting. Closely follow the requirements of deepening the research on the mechanism of political work and the law of youth work, strengthen theoretical research on institutions, draw spiritual strength from the century-old development of the Chinese youth movement under the leadership of the party, and actively explore and expand reform paths. To overcome the disadvantages of bureaucracy, break the boundaries of departments and divisions, set up a theoretical research project team for young cadres of the Youth League and Provincial Committee, select a full-time regiment cadre from each department to form a project working group, and innovate the operating mechanism of the general instructor as the general responsibility. Senior regiment cadres act as the general instructors, and organize members of the working group to participate in the drafting of important materials, compilation of important information, preparation of major meetings, and planning of major events through hands-on and mentoring methods, effectively improving the theoretical level and ability of young cadres in institutions , promote theoretical research to achieve new breakthroughs-three-breakthroughs, and provide a solid theoretical guarantee for the in-depth advancement of the Shanxi Communist Youth League reform. 2. Always adhere to the "three orientations" and deepen the measures for long-term success 1. Adhere to the political orientation and make practical achievements, and strictly implement the report on major issues. Implement the requirements of the dual leadership mechanism for the lower-level Youth League committees, and the provincial Youth League committees consciously and strictly abide by the "Regulations on Requesting and Reporting Major Issues of the Communist Youth League of China". 39. 28%, many work reports have been approved by the Communist Youth League Central Committee, Shanxi Provincial Party Committee, and provincial government leaders. At the same time, further standardize the requesting and reporting work of league organizations and organs at all levels in the province, formulate the "Provisions of the Shanxi Provincial Committee of the Communist Youth League on Requesting and Reporting on Major Issues", and "Working Rules for Requesting and Reporting on Major Events of the Provincial Committee of the Communist Youth League" to refine the requesting report Procedures and methods, clarifying the acceptance department, reply department and report time limit; Printing and distributing the "Report List for Requesting Instructions on Major Issues", clarifying the list of matters that municipal-level league organizations should ask, report, and file with the Youth League Provincial Party Committee and the list of matters that municipal-level committees need to submit to The list of materials for the report of the Provincial Committee of the Youth League; the introduction of the "Mistake Notification System", to register and report 17 types of errors such as incorrect format and content of the report, and copy them to the members of the Provincial Committee of the Communist Youth League to ensure the system of requesting instructions for major issues Implementation in Shanxi. 2. Adhere to the responsibility orientation and seek practical results, and continue to deepen the reform of directly affiliated institutions. Benchmarking further implements the requirements of the overall leadership responsibility for directly affiliated institutions, strengthens the leadership of party building and business work in institutions directly under the institutions, aims to provide professional and social support for the development of the Communist Youth League, and continues to deepen the reform of directly affiliated institutions. Establish Shanxi Provincial Youth League School, carry out education and training for Youth League cadres and young backbones in the province; reorganize Shanxi Provincial Youth Career Development Center, and carry out service work such as youth employment and entrepreneurship, protection of rights and interests, marriage and friendship, voluntary service, and poverty alleviation; The Shanxi Communist Youth League Media Center was newly established to carry out the propaganda, ideological and cultural work of the Communist Youth League. On the basis of reducing staff and downsizing in the early stage, reducing organization by 70%, establishment by 53.4%, and number of posts by 62.5%, further standardize the management of public institutions, and build a Establish a collaborative work system between government departments and directly affiliated public institutions, and do a good job in the second half of the article on the reform of public institutions. 3. Adhere to the problem-oriented and practical measures, and find the gaps to make up for the shortcomings based on the results of the benchmarking assessment. In-depth study and understanding of the results of the assessment and evaluation of the Shanxi Provincial Committee of the League by the Central Committee of the Communist Youth League. Shanxi Provincial Party Committee's Report on the Implementation of the 2021 Annual Assessment and Evaluation of the Central Committee of the Communist Youth League". In accordance with the working pattern of three forces, one guarantee and two guarantees of the Central Committee of the Communist Youth League, 14 aspects of work proposed in the "2021 Assessment and Evaluation Sub-item Opinions" were sorted out, and 21 specific improvement measures were sorted out, all of which were included in the key points of work in 2022, and promoted with a more rigorous style. All work has reached a new level. 3. Focus on improving the three kinds of capabilities, anchoring and forging the team 1. Go deep into the grassroots and integrate into the youth, and improve the ability to serve attentively and thoughtfully. Get rid of the thinking of institutionalization, insist on going to the grassroots level, and go to the youth to establish a clear orientation, and further promote the mechanism of close contact between government officials and youth. Formulate the "Communist Youth League Shanxi Provincial Party Committee Members Direct Contact Youth Youth System", strictly implement the requirements of the Shanxi Provincial Party Committee on provincial, city and county three-level Youth League committee members to go to the grassroots for 1/3, 1/2, and 2/3 of the time each year, and the team members take the lead Integrate into the youth to carry out the work, closely connect with the youth, and enhance the relationship with the youth. Print and distribute the "Notice on Doing a Good Job in the Work of Closely Contacting Youth with the Youth League and Provincial Party Committee Officials and Cadres", promote all professional and temporary cadres in the organization to preach in conjunction with the spirit of the Sixth Plenary Session of the Nineteenth Central Committee of the Party, and urge the county, township, and village three-level regiments Organize the re-election, guide the reform pilot work of the county-level Communist Youth League and the themed practical activities of "I do practical things for the youth", normalize and go deep into the grassroots league organizations, extensively contact young league members in schools, enterprises, communities, social organizations and other fields, and continue to promote the integration of government officials and youth. Improve the working skills of serving youth, and in 2021, the average person will directly contact the grassroots for more than 60 days. 2. Take multiple measures and share with multiple parties to improve the overall planning ability of cohesion and integration. Abandon administration and dependence, raise resources through social channels, allocate resources in accordance with the operating rules of social organizations, and strive to reduce administrative dependence to carry out work. Relying on the Shanxi Provincial Youth Foundation, and cooperating with units and institutions that are enthusiastic about public welfare, establish the Youth Civilization Public Welfare Fund, the Volunteer Service Fund, and the Youth Federation Public Welfare Fund, etc., to enhance the ability of the party's youth organizations to gather strength and integrate. In 2021, a total of Money and property amounted to 74.7122 million yuan, a year-on-year increase of 73.9%. Carry out the renovation of the Youth League History Exhibition Hall of the Office Building of the Provincial Committee of the Communist Youth League, deeply explore the endogenous resources of the party's youth organizations, strive for social support, and raise more than 1 million yuan in renovation funds. The new look of the education base is open to the society and shared with young people across the province. 3. Strict management and practical forging, enhance the pioneering ability of seeking improvement and thinking about change. Get rid of the idea of ​​official status, firmly establish the concept of "being a youth friend, not a youth's official father", and forge the vigor and forge ahead of the cadres. Benchmark the party's good cadre standards and General Secretary Xi Jinping's important requirements for young cadres, continue to strengthen the growth concept education of government officials, implement the "Six Regulations on Improving Political Position and Improving Work Style" of the Central Committee of the Communist Youth League, and formulate measures to overcome formalism and reduce the burden on the grassroots Five measures to improve the cadres' fault tolerance-6-correction mechanism, encourage cadres to want to do things, be able to do things, and do things well, and promote the overall improvement of the work style and work efficiency of the cadres through strict management. At the same time, in light of the new era, new requirements and new situations, revised and promulgated the "Shanxi Province Youth Civilization Activities Management Measures", "Shanxi Province May 4th Red Flag Youth League Committee (League Branch), Outstanding Communist Youth League Members, Excellent Communist Youth League Cadre Selection and Commendation Work Measures", Standardize the standards and procedures of appraisal and commendation, strengthen publicity and interpretation, enhance the participation and sense of gain of youth, and effectively enhance the value appeal and social reputation of the league organization among Shanxi youth. —7—Shake Distribution: General Office of the Central Committee of the Communist Party of China, General Office of the State Council. All comrades in the Secretariat of the Central Committee of the Communist Youth League, the relevant ministries and commissions of the Central Committee, and the leaders of the provincial party committees. The main responsible comrades of the various departments and directly affiliated units of the Central Committee of the Youth League, and the main responsible comrades of the provincial Youth League committees. Shake Issued by the General Office of the Central Committee of the Communist Youth League on April 29, 2022

2022-4-29 13 Congratulations to the 20th National Congress of the Communist Party of China, always follow the party, and strive for a new journey. The 20th National Congress, combined with the celebration of the 100th anniversary of the founding of the Youth League, insisted on organizing learning as the basic form, with the Youth League branch and the Young Pioneers Squadron as the basic unit, and extensively mobilized youth federations at all levels, student union organizations and youth associations affiliated to the Youth League. Learning, combined with mass-themed presentations, giving full play to the advantages of new media, has created a learning boom among leagues and team organizations at all levels and among young people. —1—1. Adhere to the combination of individual self-study and project learning, promote learning to be integrated into daily life, and focus on regularity 1. Adhere to hierarchical classification and mobilize young people to carry out self-study. Organize league members and young pioneers to study the history of the youth movement under the leadership of the party and the history of the league in a targeted manner. By participating in online themed group classes, reading related historical books, watching excellent film and television works, etc., learn from history. Get nourishment. The Central Committee of the Communist Youth League produced and launched the online themed group class of Youth Great Learning and the online themed team class of Red Scarf Love Learning to help young people learn more about the history of the Party’s youth movement and the great achievements of the new era, covering a total of 450 million league members and the Young Pioneers 230 million people. CompleteChina Railway adheres to the traditional + innovation model, compiles and distributes the "Railway Tuanxun" study magazine, sets up special columns such as Qingma micro-classes, organizes league cadres at all levels, Qingma backbones, and young lecturers to give special lectures, and uses online cloud platforms to organize epidemic control Youth league members in the district participate in learning, effectively improving the coverage and participation of learning. Heilongjiang integrates social resources, gives full play to the advantages of all parties, and cooperates with the "Chinese Communist Youth League" magazine to organize and carry out the Intensive Reading Contest of "Xi Jinping and College Students" to guide college students in the province to read important books, Feel the power of thought. 2. Focus on the theme of "The Centennial History of the Chinese Youth Movement under the Party's Leadership", and carry out the first special topic of learning and education. Deploy the whole regiment with the regiment branch as the unit, carry out the special study of the history of the party's youth movement, learn about major events, important meetings, and important figures in the history of the youth movement, understand the glorious history of the party's leadership of the Chinese youth movement, and lead the majority of league members to inherit the red gene, Take on the mission of youth bravely. As of April 22, the coverage rate of the Youth League branch reached -2-67.7%. The organs directly under the Central Committee of the Communist Youth League organize all party members and cadres to focus on the history of the party's youth movement and the history of the Communist Youth League. On March 30, the Central Committee of the Communist Youth League conducted a special study on "The Centennial History of the Chinese Youth Movement under the Party's Leadership". The Party Branches of various departments and the Party Committee Theory Center Group of the directly affiliated units all carried out at least one special study and seminar. All grassroots party branches and youth theoretical study groups compared the study of the single, and used the three meetings and one lesson, learning exchange meetings, etc. to carry out special learning. Xinjiang organizes college and middle school Youth League branches to uniformly carry out the theme group day activities of "The History of the Party's Youth Movement". Henan has built a networked learning position, conducting daily summaries, daily scheduling, and weekly reports on the learning situation of the Youth League branches, and conducting random inspections and supervision of the Youth League branches that have completed their studies. At present, 98,000 Youth League branches have carried out the study of the history of the Party’s youth movement , covering 2.404 million members. Relying on the path of regional group building, Tianjin Hebei District organizes the students of the Qingma Project in the region to check in at key points of the youth movement history education boutiques such as the Juewu Society, and leads the students to learn the history of the party's youth movement through immersive education. 3. Pay attention to ability improvement, and continuously strengthen the education and training of regiment cadres and youth work backbones. In the process of learning and education, the whole regiment has always insisted that the regiment cadres and young backbones learn one step first and learn one level deeper. League schools at all levels carefully arrange courses around the learning content, increase the proportion of learning content of the party's youth movement history and league history, organize special training, carry out special study and seminar activities, and use the content of youth movement history as an important part of youth Marxism at all levels and in various fields. In order to deepen the understanding of the party's innovation theory, we will carry out a series of trainings for young people in emerging fields. The Central Committee of the Communist Youth League held the closing ceremony of the 2020 class and the opening ceremony of the 2022 class of the National Youth-3-Year Marxist Training Project. College classes, state-owned enterprise classes, rural classes, social organization classes, and Young Pioneers worker classes have all completed centralized online training. Adhere to the "432" training system as the starting point, establish the "Information Officer" and "Preacher" system, set up the "Qingma Project" observation point, mobilize all levels of "Qingma Engineering" trainees to take joint actions, and continuously strengthen the effect of education and training. Shanxi built an online learning platform for youth, organized the main responsible comrades of the Provincial Committee of the Communist Youth League to record 12 online league classes, and opened them to grassroots league cadres, organized provincial league schools to design course content with high standards, and carried out 4 grassroots training activities . The Hebei Provincial Federation of Students studied in depth the important thoughts of General Secretary Xi Jinping on youth work, systematically studied the history of the party's youth movement, combined the learning activities with the spring training of staff organized by the student union, and achieved full coverage of 123 colleges and universities. Sinopec Group Corporation invited experts and scholars from Peking University to give lectures on the history of the party's youth movement to the Youth League branches. Through learning the history of the Youth League and understanding the situation of the Youth League, it will guide the youth of the Youth League to draw wisdom from history and show their responsibilities in the new journey. 2. Adhere to the combination of theoretical preaching and practical experience, promote face-to-face communication and see the effect 1. Highlight the party's innovative theory and integrate true feelings into preaching. The whole group actively mobilized various youth lecture groups such as the Youth Lecturer Group, the Red Scarf Tour Group, the Central Youth League School Promotion Group, the National Unity Promotion Light Cavalry, the Youth Federation Lecture Hall, etc., to go deep into the grassroots to carry out publicity, insisting on using friendly and relaxed language, wonderful With various stories, firm and persistent beliefs, true and sincere communication, and face-to-face interaction, it interprets the party's innovative theories for young people in a simple and simple way, tells the story of party history, youth movement history and the people who are determined to work hard under the party's training. Youth stories not only narrow the distance with young people, but also make the preaching activities more down-to-earth and popular. The organization directly under the Central Committee of the Communist Youth League held the "Theoretical Light Cavalry" Young Cadres Theoretical Publicity Competition. The contestants combined their learning experience and work practice to simulate the presentation to young people. The All-China Youth Federation and the Zhejiang Province Youth Federation organized the Youth Federation Lecture Hall and the All-China Youth Federation's Skilled Talent Sector to Enter the Grassroots Publicity Activities. The youthful dream story behind the country's most important weapon and high-tech technology inspires young people to take the road of serving the country with struggle and skills. Jiangxi continues to do a good job in five types of presentations, including demonstration lectures by regiment cadres, special lectures by theoretical scholars, group lectures by lecturers, mobile lectures at the grassroots level, and video lectures on online platforms, so that the party's innovative theories can reach young people most extensively. Henan explores and innovates, analyzes, lectures, and evaluates a new mode of theoretical lectures by the youth lecturer group, achieving precise interpretation with short and concise content, and normalizing the presentations through a combination of online and offline methods. 2. Relying on red resources, carry out small-scale and interactive presentations in various forms. Relying on the youth education bases at all levels of the regiment, the youth home, the youth study club and other regiment-affiliated positions, especially the red positions such as the party's youth movement history exhibition hall and memorial facilities, guide the majority of young people in immersive, experiential and interactive In the red atmosphere, subtly accept education and feel growth. Liaoning organized 18 college students' red theory propaganda groups with a total of 100 lecturers to carry out thematic lectures in stages. Before and after the Memorial of Learning from Lei Feng - 5-day, with the theme of Lei Feng spirit passed down from generation to generation, the theme of Lei Feng spirit was carried out for young students 25 field. Guangdong carried out a video exhibition and broadcasting activity for teenagers in love with Guangdong Hongye to inherit the red gene voluntarily, and launched a series of red background color metallurgy products in an economically powerful province, showing the youthful color of Guangdong's major engineering achievements, urban humanities and rural revitalization and development, and telling the story of the party leadership The history of the Chinese youth movement and the stories of Chinese youth in the new era. Zhuji, Zhejiang organically integrated more than 20 red resources in the city as offline teaching bases, and selected 30 small preachers in Ciwu, Fengqiao and other commemorative places to tell stories about party history and regiment history, and spread Fengqiao experience. The Youth League Committee of Hunan Normal University organized students to tell the story of Lei Feng to international students from 10 countries including Russia, India, Mali, Indonesia, Tajikistan, Congo (Brazzaville) and Syria in 5 languages ​​including English, Russian and French, guiding more college students Tell the story of Lei Feng to the world and spread the voice of China to the world. 3. Innovate the form of preaching and inject youthful vitality into the traditional preaching. In terms of publicity modes, all localities not only focus on tradition, but also focus on innovation; they not only pay attention to highlighting theoretical and ideological aspects, but also take into account vividness and practicality, forming a variety of publicity modes such as sharing sessions, report meetings, seminars and exchanges, and online presentations. Offline, take the form of sharing groups, squads, light cavalry, etc. to send lectures to factory workshops, campus lecture halls, army barracks, and farm yards; online make full use of modern technologies such as stages, sound effects, and scenery to create visually impactful and appealing scenes. The effect is to make the vivid stories come alive, make the preaching scene hot, and make the young audience on the scene ignite. Yunnan planned and produced a series of special programs on the theory dissemination of "Confident China Talk", and selected 20-year-old post-2000 young people as speakers of innovative theories, answering questions about China, the world, and Questions of the people and questions of the times show the responsibilities and responsibilities of young people with short documentary films shot during the search. Shaanxi has planned a series of special video programs such as Shaanxi Youth Talking about Ye. The main content is that outstanding young people narrate the great spirit of the Communist Party of China and share red historical stories. Hubei launched the "Always Follow the Party" and "Youth Volunteer Action" activities, selecting and training 100 outstanding volunteer preachers for the whole province, taking volunteer service stories as the main content and volunteer preachers as the main force, going deep into 100 community units, organizing and carrying out this Yu Volunteer Service Lecture Hall is a publicity activity, leading young volunteers to carry forward the spirit of voluntary service and contribute youth power to grassroots social governance. The Xilingol League in Inner Mongolia launched a publicity activity on ecological civilization for young people, celebrating the 20th National Congress, always following the party, and forging ahead on a new journey. Members of the ecological and environmental protection promotion group were invited to present to the youth through recitations on the theme of environmental protection, interactive games, and explanations of garbage classification knowledge. The vast number of teenagers will popularize science and environmental protection related knowledge, and improve the awareness of ecological civilization of the majority of teenagers. Xining, Qinghai organized the members of the city-level red scarf tour group to carry out an 8-day publicity and broadcasting of the city-level red scarf tour group around the general requirements of children's education and political lessons. Through stories, news, and examples , pictures, short videos, etc., to give lectures on topics such as "The Red Scarf Learns from the Two Sessions and Inherits the Red Gene", "Let Qinghai Bloom the Flower of National Unity", "New Development and New Concepts--Know the Five Must-Take Roads", etc. Set off a learning boom among the young pioneers in the city. -7-3. Adhere to the combination of productization and matrix, adding vividness and youthful flavor to education 1. Introduce integrated media products to provide learning content. According to the characteristics of teenagers' habits and the rules of new media platforms, give full play to the whole group coordination mechanism, produce and launch a batch of theoretical, ideological, and affinity media-integrated learning products, carry out cloud-based organizational learning, and continuously improve the learning effect. Shandong plans the column of Youth Heart to the Party, Learning History and Learning the Heart, relying on graphics, posters, short videos, animations, film and television dramas, audio and other carriers, to produce a batch of cultural products with clear themes, correct direction, rich content, and strong appeal , Empathize with young people with green words and green languages, and integrate learning and nourishing things into the daily life of young people silently. The Youth League Working Committee of Jiangsu Provincial Organs and the Youth League Committee of Jiangsu Provincial Radio and Television Corporation jointly launched the special program "Hot Blood Youth to the Future——2022 Qingming Yuhua Heroes and Cloud Sacrifice Sweeping", with more than 3.5 million viewers. The innovative organization launched the youth school online Joint education and joint construction will lead the youth of provincial institutions to forge ahead on a new journey and make contributions in a new era. Before the Ching Ming Festival in Anhui, an online martyr H5 with the theme of We Remember, They Are Still Alive was launched on the new media platform, vividly telling the touching stories of martyrs from Anhui, such as Chen Yannian and Chen Qiaonian, and leading young people to relive the glory of martyrs and heroes Deeds, continue to absorb the nutrients of growth from the value orientation and life pursuit of heroes. Jiayuguan, Gansu organizes members to learn about the history of the league through the Encyclopedia of League Affairs and the WeChat mini-program, creating an encyclopedia of history of the league that is at your fingertips and within reach of the history of the regiment, creates a strong learning atmosphere, and fully considers the learning habits of young people in the new era. Carry out group history study and education anytime, anywhere in the fragmented time. In Wuzhou, Guangxi, two feature films were filmed on Wuzhou’s early regiment history video "Revolutionary Firework Writing Youth" and "Passionate and Unforgettable Youth Song--Gongqing Hydropower Station Construction-8-Documentary" two feature films, encouraging young people to learn history, learn history, increase credit, Study history and respect morality, study history and practice. 2. Expand the scope of learning through the new media matrix. The whole group gives full play to the advantages of the new media matrix with 700 million fans, and continuously enhances the interactivity and sense of participation through topic interaction, online check-in, answering games and other forms, and forms a wide coverage of young people. The Central Committee of the Communist Youth League set up the topics of "Celebrating the 20th National Congress of the Communist Party of China, Forever Following the Party, and Forging A New Journey" for online interaction, and published articles on Party history, H5, posters, short videos, animations, etc. every day to guide young people to actively participate in discussions and interactions, and the reading volume reached 18.8 million. Carried out the live broadcast event with the theme of "Sacrificing Heroes and Martyrs during the Qingming Festival", with a total of more than 36 million views on 125 platforms including the "Learning to Strengthen the Country" platform, CCTV, and Zhihu, and 80 million person-times of flowers for the martyrs cloud. Sichuan has set up a series of columns on short video platforms such as Douyin and Kuaishou and the Great Chinese People series. By selecting the history of the party's youth movement and the deeds of outstanding youths that have emerged in the past century since the founding of the league, they are told in the form of short videos edited and edited. The heroic stories of generations of advanced Chinese youths are evocative and heroic, inheriting and carrying forward the revolutionary spirit. At present, 219 episodes of videos have been launched, with more than 900 million hits and nearly 50 million likes. Heilongjiang has fully grasped the needs of young people and gained insight into their interests, designed and produced original media products in the form of H5, further created an immersive and interactive experience, set up cloud check-ins in red places in the city, vividly displayed heroic deeds on the cloud, and described red history in detail. In-depth series of boutique exhibitions in various places. All-weather today, presenting flowers to the heroes and slowing down the live broadcast, taking advantage of major festivals, historical time nodes and other opportunities to help young people better relive the party's struggle and feel the power of faith. A total of 400,000 to 90,000 people participated in the activity, and the number of messages exceeded 300,000. Chamdo, Tibet set up the online learning column "History of the Centennial League Youth Talk", invited members of the youth lecturer group to lead the youth into the history of the century-old league through video explanations, and launched "The Creation Process of the Chinese Socialist Youth League", "Behind the League Songs" The Story of the People's Revolution", "Youth League Developing in the People's Revolutionary Movement", "Youth League Struggles in the Torrent of the Great Revolution" and other promotional videos. —01—(No text on this page)—11—Shake Distribution: General Office of the Central Committee of the Communist Party of China, General Office of the State Council. All comrades in the Secretariat of the Central Committee of the Communist Youth League, the relevant ministries and commissions of the Central Committee, and the leaders of the provincial party committees. All departments and units directly under the Central Committee of the Communist Youth LeagueThe main comrades in charge, and the provincial youth league committees are the main responsible comrades. Shake Issued by the General Office of the Central Committee of the Communist Youth League on April 29, 2022

2022-4-29 12 Editor's note: To meet and study, publicize and implement the 20th National Congress of the Communist Party of China is the main line of work throughout the year. Since the beginning of the year, the Communist Youth League has carried out educational and practical activities on the theme of welcoming the 20th National Congress, always following the party, and forging ahead on a new journey. Combined with the celebration of the 100th anniversary of the founding of the Communist Youth League, it deepened the ideological and political leadership of young people, and organized learning as the basic form, online and offline. Combining, extensively publicize the historic achievements and historical changes that have been made by the party and the country since the 18th National Congress of the Communist Party of China, publicize General Secretary Xi Jinping's care and concern for young people, learn from the glorious history of the Chinese youth movement under the leadership of the party, and actively guide the general public The league members and young people deeply understand the decisive significance of the "two establishments", strengthen the four consciousnesses, strengthen the four self-confidence, and achieve two maintenances, and welcome the victory of the 20th National Congress of the Party with practical actions. In order to guide the in-depth development of the promotion-1-moving theme education practice activities, a special issue is set up, and the experience and practices of various places are compiled and distributed in stages for the whole group to learn from. —2—Celebrate the 20th National Congress of the Communist Party of China, always follow the party, and forge ahead on a new journey. Since the whole regiment launched the themed education and practice activities of welcoming the 20th National Congress, always following the party, and striving for a new journey, the regiments and team organizations at all levels have insisted on taking organizational learning as the basic method, insisting on the combination of theoretical learning and practical experience, fully Give full play to the advantages of new media, through strengthening theoretical arming, organizing team activities, carrying out practical merit-making activities, strengthening service guidance, and doing a good job in publicity and cultural work, etc., unite and lead the majority of young people to firmly follow the party and make contributions in the new era, and meet the party's second with practical actions. Ten victories held. 1. Hierarchical and classified learning, strengthening theoretical arming. Through organized mobilization of league members and team members, learning in different levels and classifications according to local reality and the characteristics of young people groups, and at the same time mobilize youth federations at all levels, student unions of student unions and youth associations. Young people are widely involved in learning and education. The Central Committee of the Youth League conducts 4 special study sessions for the Youth League branch each year, develops an online monitoring system, and reports the progress every half a month. Relying on the mechanism of "one special, one station, two unions", organize members of the committees, representatives of the leagues, and students of the Qingma Project to carry out study and lectures, and clearly improve the history of the Chinese youth movement under the leadership of the party in the training courses of the league schools at all levels. Smelting related content ratio. Produced and launched "The General Secretary's Guide to the Vigorous Development of China's Youth Movement" and other 8 "Youth Great Learning" online theme group courses, covering more than 400-300 million young people. Through offline lectures, MOOCs, and the construction of physical lecture sites, Shanxi has built a brand of theoretical lectures on the theory of "Youth Lixiang Jin Xingshiye", and innovatively drawn the "Shanxi Youth Movement and Communist Youth League Historical Map". Guangdong gives full play to the educational role of revolutionary sites such as the Tuan Daye Memorial Hall, organizes an academic seminar on the historical value and mission of the Chinese Communist Youth League over the past century, conducts a province-wide competition on the history of the Communist Youth League for youth, and launches a hundred-year history Smelting series of microgroup courses. Inner Mongolia launched the "Welcome to the 20th National Congress, Always Follow the Party, Forge ahead on a New Journey" theme knowledge competition for young people in the region, and stimulate the enthusiasm of young people to learn in middle school and in school through methods such as points ranking. Tianjin launched three themed demonstration group classes, including Awakening the World——Zhang Tailei and Tianjin Youth Movement. Yunnan carried out the "Five One" learning activities, and combined with the characteristics of the frontier ethnic areas, carried out special lectures on ten thousand regiment cadres coming to the podium and being good thought leaders. Jiangsu included the study of the history of the Youth Movement into the learning list of the provincial youth learning community, organized activities such as "youth learning society gave lectures", normalized the organization of learning and exchanges on the history of the Youth Movement, and made good use of the ideas of the youth lecturer group to lead the brand, go deep into the grassroots, Go deep into the youth and tell the history of the youth movement under the leadership of the party. Hubei organizes colleges and universities to launch the Baisheng Lecture, with the theme of theoretical study——welcome the 20th National Congress, always follow the party, and forge ahead on a new journey Group classes, 100 provincial excellent speakers, and 100 provincial vitality league branches. Relying on Wangdao Youth College in Yiwu, Zhejiang, customized 10 special courses and 28 on-site teaching sites, established 43 propaganda groups including Wangdao Youth (Youth) Youth League, and carried out more than a thousand themed publicity activities at the grassroots level such as communities and enterprises . -4-2. Highlight the characteristics of practice and create high-quality activities. Give full play to the characteristics of the Communist Youth League's practical education in various places, seize important time nodes, combine local characteristics, and extensively tap educational resources, so that young people can strengthen their love for the party and enhance their theoretical identity in practice. . The Central Committee of the Communist Youth League organized the completion of the 17th Challenge Cup competition, issued five work guidelines of the Communist Youth League to promote the employment of college students, solidly promoted the community practice plan for college students, deepened the key project of the Student Union of the Union of Chemistry, I do something for my classmates, and launched the Youth Home Provide support for group learning and practical activities. Beijing organized the "Young Pioneers Gang Ye" activity in the capital, stood guard for the people's heroes in the name of the red scarf, carried out the "Pursuing Glorious Footprints, Inheriting the Red Gene" red theme research and education activities, and led the young pioneers in the capital to inherit the red gene in practical activities. Guangxi seized the time point of "March 3rd of the Zhuang Nationality" and carried out the "Pomegranate Flowers Blooming and Growing Road" to build a learning and practice activity for the awareness of the Chinese nation's community, sowing the seeds of national unity in the hearts of young people in Bagui. Hunan launched a million youths to learn from Lei Feng's voluntary service practice and education action, creating a 3354 special youth volunteer service work model that integrates the organization chain——position chain——project chain——cultural chain, metallurgical chain, and four chains. Ningxia organized educational activities on the theme of commemorating the revolutionary martyrs, inheriting the red gene, and offering sacrifices to heroes during the Qingming Festival, and recorded a series of promotional short videos on Zhengqingqingye, which reflected exemplary stories of youth groups such as returning home to start businesses and emerging fields who struggled with the party and made contributions to their posts. Jiangxi organized young pioneers to take the Red Scarf train, opened up a boutique route for the journey of rooting in the cradle of the red scarf, organized young pioneers to conduct a search for six social practice camps for children in Jiangxi, and guided the young pioneers to find great achievements, listen to struggle stories, and tell about the development around them . Shanxi has deepened the "Double Competitive and Double Prosperous" project, scientifically carried out skill training in agriculture and e-commerce, and released the -5-Digital Business Youth E-commerce cloud course, which has trained more than 10,000 rural youths. In Anhui, the Communist Youth League organized a series of non-profit job fairs to serve youth employment. At the same time, it set up a special topic of "Cloud Recruitment" on its website and WeChat official account, and contacted more than 10,000 companies to provide more than 21,000 jobs. Shandong continues to carry out the social practice activities of "returning to hometown", providing more than 17,000 social internship practice job needs, and organizing more than 500 activities such as special research, red search, and cloud practice. Qingyang, Gansu Province organized a spring tree planting and greening activity to celebrate the 20th National Congress of the Communist Party of China and jointly build youth Linye, to guide the majority of young people to take practical actions to improve their awareness of green environmental protection and practice the development concept of "Beautiful China". Tacheng, Xinjiang, centered on the practical activity brand of "Little Pomegranate Blossoms" for young people, launched a youth inheritance red gene stage play and a red sketch competition, covering all the children in the city. 3. Embody the main body of youth and strengthen media publicity All localities give full play to the leadership and influence of the new media matrix, aim at the ideological characteristics of young people, use green language and green language, produce and disseminate media products that young people like to hear and see, and let the voice of the party spread the most widely. Straight to youth. The Central Committee of the Communist Youth League organized the publication of "100 Years of the Chinese Youth Movement", and promoted the publication of key books such as "100 Years of the Chinese Communist Youth League" and related work related to the exhibition of the history of the Chinese Youth Movement. The media affiliated to the organization group set up a special column to comprehensively demonstrate the characteristic practices of the theme education practice activities in various places. Heilongjiang launched the first lesson of a special program on integrated media—towards the future together, and invited Winter Olympic champion Ren Ziwei and others to share their struggle stories and encourage young people to study hard and make achievements. . Guangdong Production launched a series of integrated media products such as the micro-documentary on the theme of "24 Hours in the Bay Area", "The Youthful Color of Super Engineering" Chinese style animation video - 6-channel, and the original song "Shining", to carry out major achievement education for young people. Jiangsu published the book "Hundred Archives of Youth Shine Hundred Years—Jiangsu Memories", which concentratedly displayed the important activities, meetings, events, etc. carried out by Jiangsu Youth League organizations at all levels and Jiangsu youths under the leadership of the Party over the past century through hundreds of archives. Sichuan launched the "Young People in Our Village" VLOG short video collection activity to attract young people from inside and outside the province to participate in rural revitalization, planning and carrying out the "Reassure the Party, Strengthening the Country, We" Answering Challenge, and developing a century-old group history. You must be familiar with this term and other new ideas Media products, strengthen media linkage publicity. Relying on the "Youth Search" online mini program, Henan sorted out the historical context of the youth's struggle with the party for a hundred years, and radiated the whole province to create 1 provincial-level core search line, 18 prefecture-level search lines and 100 red scarf search lines. Shaanxi filmed the special documentary "Anwu Youth Training Class" and the movie "Running", planned a series of video special programs such as "Youth 100" and "Shaanxi Youth Talking", and completed the editing work of "Shaanxi Communist Youth League Centennial History". Liaoning's original youth inspirational campus drama "Girls in Junior Years", the song "I Am in the Era of a Strong Country", the stage play "Guan Xiangying——His Youth", the theme group sitcom "Youth to the Party, Struggle to Strengthen the Country", etc. A batch of novel cultural products. In Tibet, the special column FM8848 on the spirit of the Sixth Plenary Session of the 19th Central Committee of the 19th Central Committee of the Tibetan-Chinese bilingual party was launched, and the online learning column "History of the Centennial Youth League Youth Talk" was set up. Taking the major events of the Communist Youth League as clues, it leads the youth into the history of the century-old Communist Youth League. The Youth League organizations in Shanxi, Jiangsu, Anhui, Shandong, Henan, Hubei, Guangdong, Guangxi, Sichuan and other places focused on the history of the Chinese youth movement under the leadership of the party, integrated resources from various places, launched exhibitions on the history of youth movements, and created a fine line of education on the history of youth movements. —7—Shake Distribution: General Office of the Central Committee of the Communist Party of China, General Office of the State Council. All comrades in the Secretariat of the Central Committee of the Communist Youth League, the relevant ministries and commissions of the Central Committee, and the leaders of the provincial party committees. The main responsible comrades of the various departments and directly affiliated units of the Central Committee of the Youth League, and the main responsible comrades of the provincial Youth League committees. Shake Issued by the General Office of the Central Committee of the Communist Youth League on April 29, 2022

2022-4-25 11 Editor's note: It is an important duty of the Communist Youth League as the party's assistant and reserve army to take the initiative to share the party's worries and take responsibility. In the past two months, local clusters of epidemics have broken out in many places, and the situation is very grim. League organizations at all levels should study and understand the important instructions of General Secretary Xi Jinping and the important decisions of the Party Central Committee, implement the specific deployment of the Secretariat of the League Central Committee, act quickly upon hearing orders, form youth commando teams and youth volunteer service teams, raise social resources, and do a good job Propaganda and guidance, leading the young members of the League to actively participate in the battle of epidemic prevention and control, and become a reliable force in the local epidemic prevention and control game. Standing up in the face of urgent, difficult, dangerous and heavy tasks, demonstrating organizational value with practical actions, is the political nature that the Communist Youth League should have in the new era. The relevant situation is now compiled and distributed for reference by all regions. —1—League organizations at all levels acted upon hearing the order to actively participate in the epidemic prevention and control battle. Recently, local clusters of epidemics across the country have shown the characteristics of multiple, wide-ranging, and frequent outbreaks, and the task of epidemic prevention and control is very arduous. To implement the important instructions of General Secretary Xi Jinping and the Party Central Committee, under the unified leadership of party committees and epidemic prevention and control headquarters at all levels, the Communist Youth League organizations at all levels moved after hearing the order, actively challenged the fight, and extensively mobilized young members of the League to devote themselves to the fight against the epidemic and contribute their strength. According to incomplete statistics, since the outbreak of this round of epidemic in early March, the Youth League organizations in relevant regions have formed more than 84,000 youth commandos and mobilized more than 2.6 million young commandos and young volunteers to participate in epidemic prevention and control. In the urgent, difficult and dangerous tasks, the responsibility of the young members of the league members in the new era is demonstrated. 1. Build a scientific mechanism and take the initiative to prepare for exams. On March 17, after the meeting of the Standing Committee of the Political Bureau of the Central Committee, the Central Committee of the Communist Youth League quickly held a meeting of the secretariat, a deployment meeting of directly affiliated organs, and a teleconference with the secretaries of the provincial Youth League committees to study Xi Jinping on a special topic. The important instructions of the General Secretary and the important requirements of the Party Central Committee have made special arrangements for the active participation of league organizations at all levels in epidemic prevention and control. The Youth League Committees of all provinces (autonomous regions and municipalities directly under the Central Government) implement the spirit of the Central Committee and implement the arrangements of the whole regiment. On the basis of normalized participation in epidemic prevention and control work, they improve the information transmission mechanism from the top to the bottom, and quickly invest in emergency response. Shanghai, Tianjin, Jilin and other provinces have launched emergency plans, coordinating the working forces and resources of league organizations at all levels to sink to the grassroots and provide guidance. Guangdong, Shandong, Sichuan and other provinces deployed the youth league organizations in the places where the local epidemic occurred to report to the party committee and headquarters as soon as possible, take the initiative to take on tasks, shoulder the burden, and integrate into the overall situation of epidemic prevention and control; guide the municipal youth league committees to establish a daily epidemic prevention and control system, To achieve effective scheduling and organic overall planning. Yunnan has established a group-2-organization linkage mechanism at all levels in the province to strengthen the linkage between organization, personnel, security, and publicity to form a joint force. 2. Carry out extensive mobilization and actively face the rapid development. Youth league organizations in various regions fully rely on the advantages of organizational mobilization, social mobilization experience and network mobilization characteristics, and give play to the leading role of grassroots league organizations, league members, league cadres and youth work backbones to drive Young people in various fields have enriched and strengthened the force of prevention and control work, and they can pull out and rush forward at critical moments. One is to form a youth commando team to charge forward. Polish up the work brand, give full play to the advantages of the youth commando team, such as fast pulling, institutionalization, and specialization, and become the basic carrier for organizing and mobilizing youths to charge forward. Various districts in Shanghai, universities, state-owned assets and other large-scale industries have formed 2,525 youth commando teams with 77,000 people to fight tough battles and carry out hard work on the front line of epidemic prevention. Tianjin has established more than 3,000 youth commandos, and more than 30,000 commandos are fighting in key fields such as medical rescue, transportation, material production, and project construction. Shenzhen, Guangdong Province mobilized municipal government agencies, resident central enterprises, and state-owned enterprises to form more than 1,300 commando teams with more than 36,000 people within a week. They were reorganized by the Municipal Committee of the Communist Youth League and entered the sealed-off and controlled areas in an orderly manner, which was fully recognized by the party committee and the government. . Square cabin hospitals and isolation points are being built all over the countryIn tasks such as construction, electric power, and communications, youth commando teams have been generally established, and temporary regiment branches have been set up, building the regiment's fighting fortress at the forefront of epidemic prevention and control. The second is to mobilize young volunteers to stick to the front line. Widely issue mobilization orders and proposals to promote the spirit of voluntary service and mobilize young people to participate in voluntary service nearby. Shanghai immediately issued a letter of proposal to the majority of league members and young people, issued guidelines to colleges and universities to further play the role of college league organizations, and issued a mutual assistance and caring initiative to community youth. The city's districts, college league organizations and youth voluntary service organizations have recruited more than 129,000 young volunteers, and a total of more than 419,000 people have been employed. In accordance with the principles of "accurate recruitment, nearby services, and reduced mobility", Jilin has extensively mobilized youth league members to report to the community on the spot and carry out voluntary services on the basis of counties, districts, and streets. A total of 36,000 people have been employed. Hohhot, Inner Mongolia has organized a total of 55,000 volunteers to participate in order guidance, community registration, household inspection and other work, covering more than 95% of the city's communities and Gacha villages. Shanghai, Shenzhen, Guangdong, Jiaxing, Zhejiang and other places have strengthened linkages with the Internet industry and business groups, taking advantage of the advantages of many young people in the industry, flexible working hours and locations, and wide influence of posts, and actively forming anti-epidemic youth volunteer service teams. , community flow adjustment and other aspects play an important role. The third is to guide the strength of the youth league work in colleges and universities to become the backbone. In view of the closed management of some colleges and universities and the normalization of nucleic acid testing, the Communist Youth League organizations in colleges and universities mobilized the work force of the Youth League to become the backbone of epidemic prevention and control on campus. The Youth League Committee of Fudan University organized 2,487 people to participate in voluntary services, of which 243 volunteers accompanied 276 children of front-line anti-epidemic workers at home online. Jilin University and other colleges and universities established 381 Qingma Commando Teams and Youth League Service Teams, with 47,000 key members participating, and guided the establishment of more than 1,000 temporary Youth League branches at isolation control points. Play an active role in appealing and soothing students' emotions. Yunnan organized student backbones to cooperate with the community to conduct phone checks on campus to publicize the latest epidemic prevention and control requirements; some medical colleges and universities were organized to give full play to their professional advantages and participate in the local epidemic prevention and control in an orderly manner. 3. Raise socialized resources, answer questions carefully and be more calm. All localities pay attention to the advantages of the youth organization system led by the Communist Youth League, relying on the strength of the Youth Federation, -4-Youth Enterprise Association, Youth Association, Youth Foundation, etc., to give full play to the strength of the Youth Home Taking the initiative to find out the anti-epidemic funds and material needs, and mobilize all sectors of society to raise resources through multiple channels. Shanghai launched the Youth War Epidemic, Hope to Warm Hearts and Helping Troubled Teenagers, surveyed and confirmed more than 3,200 families of minors in trouble, raised four-in-one love gift packs containing more than 20 materials, and formed a distribution commando team of more than 100 people Carry out a relay to get through the last 100 meters of assistance to ensure that love can be delivered to households and individuals in a timely and accurate manner. Mobilize the member units of the Youth Enterprise Association to give full play to the advantages of resource channels and donate about 1.5 million pieces of epidemic prevention and living materials such as protective clothing, medical masks, and drinking water. Guangdong launched an emergency operation to support Hong Kong's fight against the epidemic, raising materials worth more than 15 million yuan to help Hong Kong. Beijing, Tianjin, Shanxi, Liaoning, Jilin, Heilongjiang, Zhejiang, Fujian, Jiangxi, Hunan, Guizhou, Shaanxi and other places have launched special campaigns to fight the epidemic and hope to go together, raising medical supplies, urgently needed medicines, fast food and other funds totaling nearly billion. League organizations at all levels in Yunnan have raised more than 30 million yuan in total, collected more than 1.35 million pieces (sets) of medical supplies such as medical masks and protective gloves, more than 1.57 million boxes (pieces) of daily necessities such as instant noodles and mineral water, and allocated more than 4.2 million yuan in special group fees. In addition, all localities focus on online and offline targeted psychological and life assistance for young people. Shanghai adopts the model of "cloud companionship + interest courses + self-care counseling" to launch the "War Epidemic Cloud Nurturing Class" that cares about the children of frontline personnel in epidemic prevention and control. Jilin carried out 280 psychological counseling activities for isolation control points, covering 84,000 person-times. Tianjin announced that professional psychological counselors will provide direct services by telephone, and organized professional teachers to provide online art courses. Liaoning, Anhui, Hunan, Chongqing, Guizhou, Shaanxi, Gansu, Xinjiang and other places have made full use of the 12355 youth psychological assistance hotline, and launched epidemic prevention and control micro-lectures, anti-epidemic psychological tweets, etc., to help adolescents relieve their physical and mental pressure during the epidemic. 4. Carry out targeted guidance, inspire fighting spirit and become more determined. League organizations at all levels will take advantage of the new media matrix of the Communist Youth League, actively participate in online publicity work, strictly implement the responsibility system for ideological work, produce anti-epidemic publicity products, and timely and accurately release the epidemic situation in various places Progress and prevention and control measures, publicity and interpretation of policies and measures such as dynamic clearing and zeroing; actively clarify false information, counteract rumors and noises, and help everyone unify their thinking and understanding and strengthen their confidence in winning. Shanghai has launched special promotional topics such as "Youth Shou" "Shang Yeye", telling the stories of the anti-epidemic of grassroots frontline league organizations and youth members, and gathering positive energy online. Jilin strengthened its cooperation with the Propaganda Department of the Provincial Party Committee and the Cyberspace Administration of China, opened 14 columns including "Youth Fighting the Epidemic Power", and recorded nearly 7,000 anti-epidemic promotional videos. Jiangxi launched more than 130 short videos on anti-epidemic, planned original Weibo topics, and the reading volume exceeded 60 million, which was affirmed by the leaders of the provincial party committee. Shandong conducts in-depth research and judgment on the ideological signs reflected by some college students on the Internet, guides the youth league committees of colleges and universities and cooperates with relevant departments to effectively strengthen the ideological guidance and psychological counseling of young students, and effectively prevent and resolve potential risks. —6—(No text on this page)—7—Shake Distribution: General Office of the Central Committee of the Communist Party of China, General Office of the State Council. All comrades in the Secretariat of the Central Committee of the Communist Youth League, the relevant ministries and commissions of the Central Committee, and the leaders of the provincial party committees. The main responsible comrades of the various departments and directly affiliated units of the Central Committee of the Youth League, and the main responsible comrades of the provincial Youth League committees. Shake Issued by the General Office of the Central Committee of the Communist Youth League on April 25, 2022

2022-4-21 10 Editor's note: The grassroots is the foundation of all the work of the Communist Youth League, and deepening the reform of the Communist Youth League must be reflected in the overall activity of the grassroots organizations. Since the 18th National Congress of the Communist Youth League, the whole group has thoroughly implemented the important instructions of General Secretary Xi Jinping on establishing a clear orientation of grasping the grassroots. Taking the pilot reform of the organization of the Communist Youth League at the county level as the starting point, the grassroots organizations of the Communist Youth League have been promoted to strengthen political functions and restore social functions. Among them, the Youth League Jiangsu Provincial Committee focused on project-oriented work, socialized survival, flattened operations, diversified sources of cadres, diversified organizational methods, leading mobilization and network-based reform goals, insisted on integrated planning and deployment of reforms, insisted on innovative measures to promote reforms, and insisted on Deepen the reform and explore a road with Jiangsu characteristics for the pilot reform of the Communist Youth League organization in the county. Its experiences and practices are now compiled and distributed for reference by other regions. —1—Three Persistences of the Jiangsu Provincial Committee of the Youth League to deepen the pilot reform of the grassroots organization of the Communist Youth League at the county level. Focusing on the six specific goals of chemical and metallurgy, 25 pilot counties (cities, districts) have been driven to make great efforts to deploy reforms, promote reforms, and deepen reforms, so as to promote the pilot reform of grassroots organizations of the Communist Youth League at the county level to take root in the whole Jiangsu Province and achieve positive progress and results . 1. Adhere to the integrated planning and deployment of reforms. Establish a leading group and a special working class for the pilot reform of the grassroots organization of the Communist Youth League at the county level of the Communist Youth League and the provincial committee at the first time, and effectively assume the overall responsibility of the provincial Youth League committee. Anchor the basic goal of strengthening political functions and forming social functions at the county level, and aiming at the key and difficult issues of the pilot reform of the grassroots organization of the Communist Youth League in the county area, it lists the issues including party building, team building and team building included in the party committee inspection, county reform included in the reform supervision, and strengthening the Youth League education. The 18-item list, including cooperation, contacted with the Provincial Party Committee Organization Department, Provincial Party Committee Inspection Office, Provincial Party Committee Reform Office, Provincial Party Committee Education Work Committee and other departments. Held 8 special guidance meetings to review the introduction and implementation of reform plans in pilot counties (cities, districts) one by one. Members of the Youth League Committee Secretary Committee led teams to 25 pilot counties (cities, districts) for research, strengthened face-to-face communication with local party and government leaders, and promoted the establishment of all pilot counties (cities, districts) with the main responsible comrade of the party committee as the team leader The reform leading group in the country will formulate reform implementation plans and implement them after deliberation at the meeting of the standing committee of the party committee at the same level. 2. Insist on innovative measures to promote reform—2—1. New progress has been made in the diversification of organizational forms. The first is to promote the team building of the industry system. Taking the strengthening of team building in industry systems as an important path to promote active team building in the two new fields, the Jiangsu version of the guidelines for team building in industry systems has been detailed, and actively drawing on the strength of industry authorities and industry associations to focus on construction, transportation, express logistics, Internet and other industry fields, guiding the establishment of 22 county-level industry working committees. The second is to deepen the construction of youth homes. Clarify the construction standards and work requirements for grading and tiering, and divide into three categories: hub function type, contact service type, and window unit type, and gradually build an organization with ten types of service items, hundreds of flagship stores, and thousands of backbone administrators According to the pattern, 179 new youth homes were built in the pilot areas, accounting for 43% of the new increase in the province. The third is to promote the standardization of youth social organizations affiliated with the Youth League in the county. Introduced star rating management methods to improve the construction quality of youth social organizations affiliated to the League, and added 1,007 youth social organizations directly led, sponsored and directly contacted in pilot areas, driving a large number of social youth backbones to work directly with young people. The fourth is to vigorously build online youth communities. Pilot areas have generally established "tower-style" online youth communities to create online "Communist Youth Leagues" that are full of micro-power, wide-ranging influence, and accessible at your fingertips, and achieve global coverage and hierarchical mobilization of online youth communities. For example, the three-step walk in Gulou District, Nanjing established a three-level WeChat tower group for youth league members, covering more than 16,000 youth youth league members, and established a 5-minute direct access mechanism for emergency orders. 2. New breakthroughs have been made in the diversification of grassroots forces. One is to strengthen the regiment cadres and master the muscles and bones. Calculate the number of resident youths aged 6 to 35 served by each regiment cadre against the data of the Seventh Puye, and promote the increase of full-time regiment cadres in grassroots league organizations in pilot areas. Focus on outstanding young people in key fields such as the financial system, social organizations, and volunteer backbones, select and match strong temporary and part-time League cadres, and strive to achieve the number of part-time League cadres and full-time League cadres. The number of regiment cadres is 1: 1 requirement. The second is to identify the growth pole of strength. Guide the pilot areas to seek policy support and financial guarantees for youth affairs social workers and youth work officers, etc., carry out the community have my youth registration campaign, and promote 2068 backbone college student league members to report to the pilot area every week in a project-based way, and carry out various activities. Regular service and group work of less than 2 hours. The Nanjing Municipal Committee of the Communist Youth League strives for 2 million yuan in municipal financial funds every year to purchase social work services for young people. Through three-level funding guarantees at the city, district, and township levels, it is ensured that all 101 towns and streets in the city are equipped with full-time youth affairs social workers. The third is to make good use of the internal drive and incentive stick. Explore the establishment of a full-chain regiment cadre growth system of selection, education, management, and employment. 25 pilot counties (cities, districts) have established a hierarchical and classified post target responsibility system to realize the selection of regiment cadres. Construct a multi-dimensional incentive system of job incentives, salary incentives, job rank incentives, and development incentives, and care for the growth and development of the cadres of the Care Group. Xinyi City issued the "Implementation Plan for Comprehensively Strengthening the Management of the City's Communist Youth League Cadre Team", implemented a 2+1+X evaluation management system, and recommended 3 outstanding league cadres to the party committee organization department based on the evaluation situation and work results. 3. There is a new extension of the party and team integration education chain. Adhere to party building and team building, guide pilot areas to universally explore and establish a point-based quantitative recommendation mechanism, institutionalize promotion of excellent candidates to join the league, promote excellent candidates to join the party, and establish an integrated training model for party teams. Improve the working mechanism of promoting the best to join the party, actively seek the care and support of the organization departments of the party committees in various places, and focus on the key links such as the promotion of excellence standards, promotion indicators, joint cultivation, and standardization procedures. In the work system of the party organization to develop young party members, it truly assumes the political responsibility of delivering fresh blood to the party organization. Hai'an-4-City issued an implementation plan for the evaluation system of the advanced nature of the Communist Youth League members, implemented activities for members to compete for the first place, innovated the management methods of members, promoted the quantifiable, assessable, and testable progress of the members, and used the evaluation results as whether to be included in the recommendation An important basis for party candidates. Jiangyin City implemented the 345 smelter party team integration full-chain training model, organized 3 types of red scarf competition activities, grasped the 4 steps of points to join the league, and conducted comprehensive evaluations in 5 dimensions, effectively strengthening the party team connection. 3. Adhere to the implementation and deepen the reform 1. Support and guarantee are in place. First, policy support is in place. Promote the implementation of the three inclusions in the pilot areas (regiment building and team building are included in party building assessment, party building and team building are included in party committee inspections, party building and team building are included in education evaluation and education supervision) 100% implemented in place. Guided all localities to issue 127 supporting system documents such as the implementation opinions on strengthening the construction of the regiment cadre team, the inspection work list of the key content of the party building and the team building, and consolidated the reform results of the grassroots league organizations. The second is that the strength is in place. In the 25 pilot counties (cities, districts) Youth League Committees and their grassroots Youth League organizations, a total of 115 full-time League cadres, 382 temporary-job League cadres, 1,214 part-time League cadres, 2,185 youth affairs social workers, and public welfare post personnel were newly established. Institutions or specialized work institutions can effectively enrich the grassroots work force. The third is to guarantee the funds in place. Promote the pilot counties (cities, districts) to include 100% of the youth league building work funds into the overall plan of party building funds, and ensure youth work funds in the county-level annual budget. The average increase in work funds in pilot counties (cities, districts) in 2022 exceeds 40%. At the counties, districts, towns and streets, special funds for youth development work are generally set at the standard of 2 to 3 yuan per year for resident youths aged 6 to 35. Nanjing Jiangbei New District guarantees work funds according to the standard of no less than 3 yuan per year for resident youths at the district level, and no less than 150,000 yuan per year for each street. In 2022, the team building work funds will increase from 600,000 yuan to 2.64 million yuan Yuan. 2.The role is in place. One is to create a distinctive brand. Following the social mechanism to plan work projects, the province, city, and county implemented the "Dream Renovation + Care Plan" in an integrated manner, and built 5,543 "Dream Houses". The 71·61 Heart-warming Project organized and implemented by the Jiangning District Committee of Nanjing City was incorporated into the important project "I do practical things for the masses" by the district committee, and promoted the long-term pairing between 446 party members and cadres in the region and young people in trouble. The second is to continue to promote the socialization of resource allocation. The 25 pilot counties (cities, districts) will raise a total of more than 27 million yuan in social funds and more than 8 million yuan in social materials in 2021, a substantial increase over 2020. The third is to effectively increase the contribution to the overall situation. In the summer of 2021, after the outbreak of the new crown epidemic in Nanjing and Yangzhou, the province organized and mobilized more than 220,000 young league members to report to the community through organizational and social methods, set up 4,245 pioneer posts for league members, and formed 4,138 youth commandos. Contributing to youth at the forefront of epidemic prevention and control has been fully affirmed by the party, government and society. 3. Social awareness is in place. One is to ask young people to discuss the performance of their duties. Regularly carry out the work of "double statement and double evaluation" by the secretary of the Youth League branch, and select youth members of the youth league to conduct satisfaction surveys every month, which will be included in the comprehensive evaluation of counties (cities, districts) at the end of the year. The second is to invite young people to observe the progress of reform. Implement the youth observer project, focusing on reform pilot counties (cities, districts), organize youth representatives to go directly to the grassroots, participate in the activities of grassroots youth organizations as ordinary youths, and complete 2,550 observation reports and various questionnaires. An important basis for the evaluation of the implementation and operation status of grassroots reform projects. The third is the work - 6 - please comment on the results of the young people. Through questionnaires and third-party large-scale telephone spot checks, the young members of the group are collected to evaluate the work of the group organization, and no less than 10,000 people are interviewed by telephone one-on-one every month, so as to promote the grass-roots group organizations to take the satisfaction of young people as a barometer of their work. Ye. Fourth, let the society know the achievements of the reform. Make full use of the resources of mainstream media platforms at all levels and propaganda positions of regiment affiliates, and dig out typical experience and effective practices in the reform. Pilot counties (cities, districts) have released more than 620 reports on reform dynamics, including more than 110 reports from central media such as People's Daily, CNR, and China Youth Daily, comprehensively presenting the hot scene of the reform of the Communist Youth League in Jiangsu counties, and forming a good social environment. repercussions. —7—Shake Distribution: General Office of the Central Committee of the Communist Party of China, General Office of the State Council. All comrades in the Secretariat of the Central Committee of the Communist Youth League, the relevant ministries and commissions of the Central Committee, and the leaders of the provincial party committees. The main responsible comrades of the various departments and directly affiliated units of the Central Committee of the Youth League, and the main responsible comrades of the provincial Youth League committees. Shake Issued by the General Office of the Central Committee of the Communist Youth League on April 21, 2022

2022-3-1 9 Editor's note: Effectively controlling the incidence of adolescent mental health problems and improving the level of adolescent mental health counseling and services are important development goals proposed in the "Medium and Long-Term Youth Development Plan (2016-2025)". Since last year, local Youth League organizations have taken the opportunity of studying and educating the Party history and doing practical activities for the masses to coordinate and promote the construction and improvement of various departments to form a working mechanism of joint management, strengthen the construction of the 12355 youth service desk, and insist on policy advocacy and case assistance. Support and unify, promote the combination of online counseling and offline services, help young people solve their psychological confusion, and study, live and work more comfortably. Relevant experiences and practices are now compiled and distributed for reference by all regions. —1—Strengthen cooperation and linkage, increase information connectivity to help young people solve psychological confusion and promote healthy growth. In recent years, the mental health problems of Chinese young people have become increasingly prominent, and it has become an important aspect of the Communist Youth League to serve the growth and development of young people. Youth League organizations at all levels aim at the mental health needs of young people, use the 12355 Youth Service Desk as a front, build a collaborative linkage mechanism among various departments, use Internet information technology, integrate mental health experts, youth affairs social workers and volunteers, and effectively help young people solve their psychological confusion . 1. Take the initiative to strengthen interdepartmental cooperation and build a joint management system and mechanism. Local league organizations attach importance to policy advocacy and social advocacy, strengthen communication and coordination with education, health care, network information, public security and other departments, promote consensus and cohesion, and promote the formation of attention and support Institutional Mechanisms of Adolescent Mental Health. Relying on the Municipal Youth Work Joint Conference System, the Beijing Municipal Committee of the Communist Youth League will strengthen the mental health education and services for primary and middle school students into the "Beijing Youth Development Plan during the 14th Five-Year Plan"; launch 40 key projects of the Beijing Minors Protection Committee in 2021 Ye, including minors' mental health and family education. The Fujian Provincial Committee of the Communist Youth League established a special group for mental health education to build a team of full-time staff and volunteers to effectively play the role of youth affairs social workers affiliated to the Communist Youth League in various cities; in conjunction with Fujian Medical University, 91 youth affairs social workers affiliated to the Communist Youth League in the province To create and form a service network with 12355 Youth Service Desk as the center, branch hotlines in various cities, online and offline cooperation, and radiate the whole province. The Sichuan Provincial Committee of the Communist Youth League worked closely with the public security and education departments to form a psychological intervention team composed of psychological counselors and volunteers, and promptly intervened in the case of Lanjingye, Fighting Orphan, -2-suicide youth calling for help, and a certain elementary school student in Mianyang. Accidental death and other incident handling. 2. Actively use network technology to create a combined online and offline system. League organizations around the world attach importance to method innovation and means update, strengthen information construction and Internet technology application, promote the transformation and upgrading of 12355 youth service desk, and improve the ability and level of youth mental health services. The Guangdong Provincial Committee of the League strives for provincial financial support, and has invested a total of 17 million yuan in the past four years to build a "cloud seat" mechanism, play the role of the "Guangdong Provincial Affairs" platform, and effectively integrate the power of 12355 youth service desks in various cities. The Shanghai Municipal Committee of the Communist Youth League explores the quality monitoring and emergency response mechanism under the Internet psychological service model, cooperates deeply with influential Internet platforms in the society, and opens "warm-hearted" and "accompanying" psychological counseling services. The Yunnan Provincial Committee of the Communist Party of China developed a WeChat mini-program called Yunqinghu - a mobile phone to protect growth, and opened up activity areas, hot topics, crisis intervention and other sections to achieve multi-functional integration of psychological consultation, message Q&A, online assessment, and telephone inquiry . Combined with the intelligent construction of the Children's Palace, the Jiangsu Provincial Committee of the Youth League developed and perfected the Nanjing 12355 call platform to build a call center and business system; developed the 12355 service desk APP, covering modules such as online services, expert appointments, public welfare activities, volunteer services, and online classrooms . 3. Timely intervene in individual case assistance to help solve realistic psychological confusion. League organizations around the world attach importance to individual case assistance and crisis intervention, focusing on young people affected by the epidemic, addicted to online games, and involved in lawsuits and crimes. They design and launch corresponding service models, targeted Help teenagers solve psychological problems in a positive way. The Beijing Municipal Committee of the Communist Youth League targeted more than 55,000 students in 107 middle schools, using big data to carry out mental health examinations, established a psychological crisis identification and intervention mechanism, and actively intervened—3—and tracked students with extreme behavior tendencies; Provide psychological assistance to minors who are suing minors, and launch the Sunshine in Hope project to help teenagers involved in lawsuits and crimes heal their psychological trauma and improve their social adaptability. The Yunnan Provincial Committee of the Communist Youth League of China implemented a research project on the living conditions of youths in the express delivery industry. Relying on the 12355 youth service desk, it provided a special psychological hotline consultation for the courier brothers; continued to carry out the youth service month for the express delivery industry, online delivery consultation, and live broadcasts and online sessions on the theme of labor rights protection. Provide psychological counseling experience and other services to guide the courier boys to protect themselves and adjust their psychological pressure. Relying on the provincial 12355 youth service hotline, the Fujian Provincial Committee of the Youth League organized the compilation of the "Work Guide for Anti-epidemic Psychological Assistance Hotline", "Service Instructions for Fujian 12355 Epidemic Prevention and Control Psychological Crisis Intervention Service Team", etc., to widely publicize epidemic prevention and control policies and public health knowledge , Guide young people to respond to the epidemic with a positive attitude. The Gansu Provincial Committee of the Youth League provided 24-hour online professional answers and psychological counseling around the hot psychological issues that young people were concerned about during the epidemic prevention and control period. At the same time, it also focused on psychological counseling for medical staff and their underage children to help relieve tension and relieve mental pressure. Shake Distribution: General Office of the Central Committee of the Communist Party of China, General Office of the State Council. All comrades in the Secretariat of the Central Committee of the Communist Youth League, the relevant ministries and commissions of the Central Committee, and the leaders of the provincial party committees. The main responsible comrades of the various departments and directly affiliated units of the Central Committee of the Youth League, and the main responsible comrades of the provincial Youth League committees. Shake The General Office of the Central Committee of the Communist Youth League Issued on March 3, 2022—4—

2022-2-21 8 Comprehensively and strictly governing the Youth League Special Issue No. 5Editor's Note: In late January, the Sixth Plenary Session of the Eighteenth Central Committee of the Communist Youth League was held to implement the spirit of the Sixth Plenary Session of the Nineteenth Central Committee of the Party and to learn and understand the greatness of our Party The historical experience of self-revolution leading a great social revolution, closely centering on the fundamental plan of successors to the party's cause, has made special arrangements for implementing the requirements of comprehensive and strict party governance and promoting comprehensive and strict governance of the league, leading the whole league and mobilizing youth joy. Welcoming the 20th National Congress of the Communist Party of China, we will always follow the party and forge ahead on a new journey. At the meeting, Shanghai, Shandong, Henan, Hubei, Guangdong and other provincial youth league committees exchanged views, combined with their respective actual conditions, and introduced the experience and practices of promoting comprehensive and strict regiment management. These typical experiences are now excerpted for reference by other regions. —1——Shanghai, Shandong, Henan, Hubei, Guangdong and other places have taken multiple measures to promote the implementation of comprehensive and strict regiment management. Shanghai: Give full play to the functions of grassroots league organizations and highlight a strong character. The Shanghai Municipal Party Committee strictly implements The construction of grass-roots league organizations focuses on the weak links of urban community governance, and builds a working pattern in which the Communist Youth League participates in community governance personnel, projects, and mechanisms in coordination and linkage, so as to effectively enhance the political and social functions of the Communist Youth League. The first is to strengthen the construction of grassroots organizations. Innovate the organization form of the Youth League in the community, explore the dispatch of the Youth League Working Committee or establish a functional group organization in the community, and effectively improve the coverage of the organization. Optimize the organizational setting of the street and town league (work) committees, so that the staffing, functions, and operating mechanisms of the street and town youth (work) committees are more in line with the requirements of the party's grassroots work in the new era. Improve the platform mechanism of regional group building, promote the system of three checklists for regional group building, and promote the organizational sorting, institutional connection, and precise matching of group building resources. Strengthen the construction of district-level support platforms, give full play to the advantages of platforms such as the Youth Federation, the Youth Enterprise Association, the Youth Association, and the Youth Social Association, which are led by the Youth League Committee, to provide professional support for grassroots organizations. The second is to strengthen the strength of grassroots organizations. Improve the association system of youth industry committee members at the municipal and district levels to achieve full coverage at the district level. Implement the leading action of young talents in urban governance, deepen the district-school pairing and co-construction mechanism, strengthen the selection and training of young talents in community governance, and guide the four major groups of youth league backbones, young talents, young volunteers, and college teachers and students to enrich grassroots league organizations , to participate in community governance. The third is to enhance the ability of grassroots organizations. Carry out a number of key livelihood projects such as "Love Summer School" and "Youth Aid Enterprise Service Group". In 2021, there will be 2-543 love summer care classes, covering all streets and towns. More than 12,000 volunteers and more than 500 teachers will participate, and nearly 40,000 primary school students will benefit from it. The community-based youth center assisted Double Reduction, providing 4,905 sessions of public welfare services, covering more than 80,000 person-times. Shandong: Promoting the capacity building of regiment cadres, highlighting a hard-forged character Shake the Shandong Provincial Committee of the regiment to strengthen the capacity building of regiment cadres, promote the performance of duties, and strive to build a team of regiment cadres who are energetic, strong, and good at deeds. First, education and training measures are tough. Pay close attention to on-the-job training, establish a reminder and job adjustment reporting system for county-level Communist Youth League secretaries, rely on provincial league schools to organize training for new league cadres, and print and distribute the "Knowledge Handbook for Youth League Cadres". Pay attention to work guidance, implement the flat management of provinces focusing on counties, cities focusing on townships and towns, and comprehensively use methods such as compiling and distributing briefings, case guidance, observation and communication, etc., to achieve first-class benchmarking and table competition. Adhere to the principle of rewarding the excellent and punishing the inferior, implementing a one-vote affirmation for the secretary of the Youth League Committee of the city and county who performed well, and sending a commendation notice to the main responsible comrades of the party committee at the same level; Report criticism on formalism issues such as banners and posing for photos, and correct deviations in a timely manner. The second is to practice hard work style. Taking the opportunity of the Provincial Party Committee to implement General Secretary Xi Jinping's important instructions on Shandong's work, we launched ten actions to serve the ecological protection and high-quality development of the Yellow River Basin. Promote the group cadres to focus on the center and serve the overall situation, and hold the International Summit on Innovation and Development of Young Entrepreneurs for 4 consecutive years, resulting in 137 signed projects. Encourage cadres to practice front-line work methods, implement the Hope House child care project, conduct field visits to 151,000 families, raise 210 million yuan through organizational + socialization channels, and build houses and houses under construction 15,000 rooms. Promote cadres to overcome difficulties, focus on rural revitalization, and promote 14,000 to 3—good rural youths to join the village committees and committees, and issue "good rural youths" loans of 3.15 billion yuan. The third is that the cadre coordination mechanism is solid. Introduced standardized measures to strengthen the coordination and management of regiment cadres, and in recent years, put forward written suggestions on issues such as non-standard coordination procedures and unreasonable candidate structure for 10 units. Establish a regular communication mechanism with the party committee organization department, and promote the timely resolution of the problem of insufficient staffing of the city and county youth league committees. Henan: Deepen the construction and implementation of the system, highlighting a strict character. The Henan Provincial Party Committee takes the continuous improvement of the system list as the starting point, establishes and strengthens the party's leadership, shoulders the fundamental task of educating people for the party, and improves the service for youth. 95 items of system in 7 categories, including sexuality, effectiveness, etc., strictly grasp system construction and system implementation. The first is to improve the system construction, from serious to normal. Jointly dispatch the discipline inspection team, implement the quarterly self-inspection system for the internal departments and directly affiliated units of the Youth League Provincial Committee, and regularly report on typical cases of formalism and bureaucracy, show the progress of reforms in the province's reform forums, and promote the construction of wisdom groups Small-cut, large-scale and in-depth measures such as precise dispatching and interviews for key tasks such as , youth learning, and other key tasks, and the implementation of ideology, party building, party conduct and clean government. The second is to innovate the evaluation mechanism, from strict to everyone. The province's youth league cadres will be cross-level, project-based, and lead the work tasks, and the monthly defense competitions will be arranged, and the youth judging group will participate in the scoring and evaluation system.Combined with the plan, establish the KPI assessment rules of the Provincial Committee of the Communist Youth League and the provincial Communist Youth League's participation in the project work assessment index system to improve the scientific nature of the quantitative assessment of the strict governance of the League. The third is to establish a result-oriented approach and strictly implement it throughout the whole process. Improve and implement the supervision and accountability mechanism for the major deployment of the whole regiment, randomly visit the grassroots regiment organizations, and implement the deployment of superiors - 4 - to the point. Strictly implement the reporting system for instructions, clarify the list of reports before, during, and after the event, and compact the leading group of the Youth League Committee to comprehensively and strictly manage the main responsibility of the group. Establish a serious meeting style and discipline reporting mechanism, arrange closed-book examinations after the annual plenary meeting, and absentees participate in cram schools. Taking the opportunity of the comprehensive reform pilot project of the Communist Youth League in the county area, the "system list" was included in the training subjects of league members and cadres, and included in the compulsory content of grassroots league courses, and continued to strengthen the interpretation and publicity of the system. Hubei: Boost the work spirit of the Communist Youth League, highlight a lively character, and shake the regiment. The Hubei Provincial Committee further promotes the three constructions of clean and honest organs, hardworking and honest members, and youthful demeanor. The cadres and officers have weakened their entrepreneurial enthusiasm and lack of vitality in the organization of the regiment. One is to activate the directly affiliated agencies. Emphasis on mobilizing the power of youth and showing the passion of the cadres in the work, calling on the cadres of the provincial party committee to implement the six proposals for the construction of the spirit of the organization, to implement the original mission, to be more firm, to be more liberated in thinking and concepts, and to be more courageous in reforming and tackling difficulties One point, the work is promoted more openly and other four points" requirements, to create a healthy, orderly, high-spirited working atmosphere, and promote the efficient operation of the organization with vitality. The second is to use live regiment cadres. Combined with the law of growth of young cadres, promote the implementation of the regiment construction plan for cadres, actively build a platform for cadres to grow into talents, strengthen the education and training of growth concepts, form an orientation that focuses on hard work and responsibility, and cultivate good cadres who can be used and can take on heavy responsibilities. Adhere to the combination of strict management and deep love, implement the requirements of three distinctions, accurately regulate discipline and accountability, seriously investigate and deal with false accusations and frame-ups, and promptly clarify false complaints and reports, so that cadres can let go of their work within the scope of the system and rules. Work with confidence. The third is to bring alive the grassroots group-5-organization. Promote the extension of the three constructions of incorruptible organs, incorruptible cadres, and youthful demeanor to the leading organs of the city and county leagues, and take the three constructions as an important part of the work of comprehensively and strictly governing the regiment, and include them in the annual work report evaluation focus of the city and state youth league committees, and promote The leading organs of the League at all levels have established three constructions, a level-by-level supervision, follow-up and effectiveness mechanism. Guangdong: Improve the cadre assessment mechanism, highlighting a practical character. The Guangdong Provincial Committee of the Communist Youth League continues to improve the incentive system for cadres to perform their duties and conscientiously. Grasp the league organizations directly under it, and solve the outstanding problems of not being able to manage, not being able to manage, and not wanting to manage the cadres of the group organizations. The first is to strictly enforce political standards to ensure effective results. Persist in focusing on the center and serving the overall situation, and highlight the assessment of the implementation of the Party Central Committee's decision-making arrangements and General Secretary Xi Jinping's important instructions. The actual effectiveness of implementing the key work deployments of the Central Committee of the Communist Youth League and the Provincial Party Committee and promoting the high-quality development of the party's youth work in the new era is the main evaluation basis, and strengthen the guidance of evaluating heroes based on actual performance. Pay attention to the implementation of internal control and management systems, the standardization of the organization of documents and meetings, and the construction of style of work, and guide the cadres of the regiment to form a work habit of perfection and strictness. The second is to refine the assessment items and strive to verify the actual performance. Enhance the pertinence of assessment, and reasonably set measurable and comparable assessment indicators according to different assessment objects. In 2021, there are a total of 83 KPI indicators for various departments and directly affiliated units of the Youth League and Provincial Committee. Adhere to all-round, multi-angle and three-dimensional assessments, conduct a performance evaluation of all cadres every quarter, carry out cadres' work reports and honesty for 5 consecutive years, implement all-staff reports and overall evaluations, middle-level managers will go on stage to report their work every year and accept the annual test, and others Written reports by comrades will be scored and evaluated by all cadres. The third is to strengthen the combination of examination and application, and promote practical application. —6—Give full play to the basic role of assessment and evaluation in the selection and appointment of personnel, set the assessment indicators, determine the assessment plan and assessment results, and combine the assessment results with promotion, education management, supervision and discipline enforcement, heart-to-heart talks, etc. , effectively enhance the endogenous motivation for cadres to take on the role, and establish a working atmosphere for reform, innovation, and entrepreneurship. —7—Shake Distribution: General Office of the Central Committee of the Communist Party of China, General Office of the State Council. All comrades in the Secretariat of the Central Committee of the Communist Youth League, the relevant ministries and commissions of the Central Committee, and the leaders of the provincial party committees. The main responsible comrades of the various departments and directly affiliated units of the Central Committee of the Youth League, and the main responsible comrades of the provincial Youth League committees. Shake Issued by the General Office of the Central Committee of the Communist Youth League on February 22, 2022

2022-2-7 7 Editor's note: Organizing and mobilizing young people to receive education in the hot social practice is a major feature of the Communist Youth League's youth work for the party, and it is also an important method to grasp the fundamental plan of successors to the party's cause. Since 2021, league organizations at all levels have deepened the social practice of college students returning to their hometowns, further explored and summarized the new mechanism of normalized and long-term practical education, and guided college students to better understand national conditions, understand society, love their hometown, and serve the masses. Keep up with the party and take the path of success combined with the masses of the people. The relevant situation is now compiled and distributed for reference by all regions. —1—Leave organizations at all levels to deepen the social practice of college students returning to their hometowns and guide young people to know the national conditions, understand the society, love their hometown, and serve the masses. On this basis, further clarify the goals of educating people, reasonably expand the scale, give full play to the enthusiasm of the college and county youth league committees, deepen and promote the social practice of college students returning to their hometowns, and achieve good political and social effects. 1. Strengthen organizational leadership and form a work pattern for the whole regiment. The Secretariat of the Central Committee of the Communist Youth League attaches great importance to the mechanism and role of practical education, and conducts special research on the deployment of college students' social practice of "returning to their hometowns". After 2 years of exploration, the whole group conscientiously implemented the main responsibility, continued to optimize the working mechanism, connected the superior forces in the school with the needs of the grassroots outside the school, and adopted the methods of county and district implementation, college mobilization, local recruitment, and two-way selection to further clarify different levels and different levels. The work tasks of the league organizations in the field should be determined, so that the league organizations at all levels can play corresponding roles in the relevant links of the cooperation chain. By unifying work deployment, improving the working mechanism, formulating work plans, and setting up temporary league branches, etc., we will lay a solid organizational foundation for the normalization of the social practice of "returning home". Over the past year, more than 900 counties (cities, districts, and banners) have organized and carried out the social practice of "returning home". The Zhejiang Provincial Committee of the Youth League issued work guidelines based on the collection of typical cases, strengthened unified deployment, combined promotion with point-to-point areas, and promoted the coordinated implementation of all counties in the province through key counties, and paired with colleges and universities to form one county, one school, and one town The pairing mechanism of one hospital, one village and one class. A total of 11 cities divided into districts and 79 counties in the whole province carried out the social practice of "returning home", accounting for nearly 90%; nearly 35,000 college students participated. According to the actual situation of returning college students -2-, the Shaanxi Provincial Committee of the Communist Youth League established a temporary league organization and a volunteer service team in charge of the cadres of the Youth League and county committees to effectively promote the organization, standardization, and coordination of work operations. The Hebei Provincial Committee of the Communist Youth League mainly focused on key counties and districts, and led a total of 136 counties and districts in the province to carry out the social practice of "returning to their hometown", with a total of more than 12,000 college students participating. The Youth League Xinjiang District Committee organized 46 county-level Youth League committees to carry out the social practice of returning home, released more than 1,700 positions, and more than 2,500 college students signed up to participate. 2. Enrich the content of practice and continuously deepen the thinking. Lead the whole group to focus on the theme of celebrating the 100th anniversary of the founding of the party, actively expand practical projects, continuously enrich the practical content, focus on the strategy of rural revitalization, and combine "I do things for the youth" to enrich online + offline. Dual-path, organized and carried out nearly ten thousand activities such as field research, red search, cultural presentation, cloud practice, epidemic prevention and control voluntary service, and community report, guiding college students to deeply feel the changes in their hometown, deeply understand the major achievements of the party's century-old struggle, and strengthen A sense of responsibility and a sense of mission to serve the people and return home. The Shanghai Municipal Committee of the Communist Party of China organically combined party history learning and education with social practice by carrying out the spirit of General Secretary Xi Jinping’s important speech on July 1, concentrating on research, visiting the Centennial Exhibition of the Communist Party of China in Shanghai, walking into streets and towns to give lectures, and launching theme essay contests. . In response to the sudden outbreak in the province, the Jiangsu Provincial Committee of the Communist Youth League actively organized college students returning to their hometowns to participate in anti-epidemic voluntary services, assisting townships (streets) to do body temperature checks, information officers for registration and investigation, couriers for delivering materials, and popularization He is a propagandist of knowledge, leading the majority of young students to deeply understand the national conditions and cultivate patriotism while participating in the war against the epidemic. The Guangxi District Committee of the Youth League closely combined the community youth action, focusing on organizing no less than 10 college students to carry out social practice in 25 communities in Nanning City. Actively participate in community governance and service system construction. The Shandong Provincial Committee of the Communist Youth League organized the city and county Youth League committees to carry out activities such as hometown awareness training, hometown development cloud live broadcasting, and discussions and exchanges with local entrepreneurial youths in combination with local development characteristics, and led returning hometown college students to city exhibition halls, key projects, and pillar industries. All-round and multi-angle observation and research. The Sichuan Provincial Committee of the Communist Youth League built a cloud practice platform to unblock the connection channels between practice units and students, encourage students to use their spare time to work online after returning to school, and continue to improve college students' work ability and socialization skills. 3. Do a good job in service guarantee and gradually improve the students' sense of gain. The Central Committee of the League will play the role of the China Guanghua Science and Technology Foundation, select 400 teams to carry out practice in counties and districts, and give each team 2,000 yuan in activity funds, and at the same time do a good job in distributing volunteers Support guarantees such as a package for participants and free accidental injury insurance. The Guizhou Provincial Committee of the League requires all counties and districts to organize special training on relevant work skills, laws and regulations, and safety knowledge before carrying out activities, so as to improve the practical skills of college students and enhance their legal awareness and safety awareness. The Jiangxi Provincial Committee of the Youth League urges employers to sign temporary agreements with returning college students, provide financial guarantees, recruit qualified college students as part-time League cadres of the Youth League Committee of employers, and seek policy support from party and government departments. For example, the Organization Department of the Fuzhou Municipal Party Committee, the Municipal The Bureau of Education and Sports provides a living allowance of 2,000 yuan per month to college students, and the Bureau of Human Resources and Social Security of Yichun City and the Municipal State-owned Assets Supervision and Administration Commission purchase personal insurance for college students. The Chongqing Municipal Committee of the Communist Youth League established a WeChat group around the two groups of local college students abroad and foreign college students in Chongqing to keep track of the time when college students return to their hometowns, research and formulate a list of study and education, business training, skill practice, volunteer services, etc., and establish pre-job training and process management. ,-4-post-tracking and other whole-process management service modes. The Hubei Provincial Committee of the Communist Youth League actively sought all kinds of support, purchased personal accident insurance for college students who "returned to their hometowns" to participate in social practice, and uniformly distributed backpacks, T-shirts, epidemic prevention kits and other materials. 4. Innovate work measures, optimize and broaden the platform carrier. The Youth League Central Committee extensively solicits opinions and suggestions from organizations at all levels, and optimizes the post-docking system for college students to return to their hometowns to practice social practice. 29 provinces (autonomous regions, municipalities directly under the Central Government) and Xinjiang Production and Construction Corps have passed the Use this system to achieve accurate matching of people and jobs. Over the past year, a total of 1,172 units have released more than 27,000 projects, provided more than 45,000 jobs, and nearly 100,000 students from 2,377 colleges and universities have signed up to participate. The Guangdong Provincial Committee of the Youth League organically integrates the action of improving the employment and entrepreneurship ability of college students with the social practice of college students returning to their hometowns. Relying on the Yizhanyi platform and the social practice platform of returning to their hometowns, it realizes two-way precise docking of people and posts, improves job suitability, and develops practical posts accumulatively 97,000. The Jilin Provincial Committee of the League extended the chain of student source and local smelting, strengthened school-local cooperation, and jointly carried out the summer practice of Peking University and Tsinghua students in Jilin Xingye with the Organization Department of the Provincial Party Committee, and developed practical positions in provincial departments, universities, scientific research institutes, and high-tech enterprises. More than 80 Jilin college students from the two schools were mobilized to return to their hometowns to participate in post training. Relying on the syndicate cooperation + Ye, the Fujian Provincial Committee of the Mission, in conjunction with the Provincial Financial Bureau and the Provincial Rural Credit Cooperatives, promotes financial majors returning home to Ye Ye students to actively participate in the practice of rural revitalization. Group organizations in Beijing, Liaoning, Anhui, Hainan, Inner Mongolia and other regions carried out continuous publicity through multimedia platforms such as newspapers, microblogs, websites, and terminals, effectively expanding the spread and influence of activities. 5. Strengthen research and guidance, effectively improve the level of professionalism. The Central Committee of the Communist Youth League organizes investigations and research, continues to strengthen online and offline guidance, and holds -5-2021 national college students returning to their hometowns to practice social practice training courses for provincial and county-level youth league committees across the country. A total of 149 league cadres from the Youth League Committee and the Youth League Committee of colleges and universities provided special demonstration training; launched a series of 8 tweets on the "College Students Returning to Hometown Social Practice Training Cloud Classroom", and achieved full coverage of key county training through the form of online cloud classrooms; compiled and released " "College Students Returning to Hometown Social Practice Work Manual" to improve the work level of youth league committees at all levels; carry out online questionnaire surveys and online interviews with more than 200,000 college students, comrades in charge of social practice work of provincial youth league committees, and college youth league committees, and write "College students return to their hometowns" 2 research reports on smelting social practice, providing reference for relevant work decisions. The social practice of college students returning home has received great attention from party committees and governments across the country and positive responses from league organizations, forming a number of characteristic brand projects. The Henan Provincial Party Committee of the League has fully carried out the social practice of "Spring Geese Action", college students from Henan returning to their hometowns, combining the social practice of returning to their hometowns along the Yellow River with the cooperative action of millions of young people's ecological industry development along the Yellow River, to create a regional brand in Henan. The Shanxi Provincial Party Committee of the Tuan Shanxi organized a summer social practice for college students to return to their hometowns, specially designed the "Youth and Jin" logo, and customized uniform cultural shirts and badges to form a unified brand image. The Hunan Provincial Committee of the Tuanmen Group carried out the social practice of the third "Swallows Returning to the Nest and Returning Home". It was welcomed by the majority of college students and became one of the signature projects for serving youth. It was promoted 13 times by Guangming Daily, China Youth Daily, Changsha Evening News and other media. The whole society creates a strong atmosphere of loving hometown, returning home and building hometown. —6—(No text on this page)—7—Shake Distribution: General Office of the Central Committee of the Communist Party of China, General Office of the State Council. All comrades in the Secretariat of the Central Committee of the Communist Youth League, the relevant ministries and commissions of the Central Committee, and the leaders of the provincial party committees. The various departments and departments of the Central Committee of the Communist Youth LeagueThe directly affiliated units are mainly responsible comrades, and the provincial Youth League committees are mainly responsible comrades. Shake Issued by the General Office of the Central Committee of the Communist Youth League on February 7, 2022

2022-1-29 6 Editor's note: The All-China Youth Federation has thoroughly studied and understood the spirit of General Secretary Xi Jinping's important instructions on strengthening the education and training of young people in Hong Kong and Macao and promoting integration into the overall development of the country, and launched eight measures to benefit young people in Hong Kong and Macao, focusing on the strategy of the Guangdong-Hong Kong-Macao Greater Bay Area , to drive youth federations at all levels to innovate working mechanisms, enrich exchange projects, highlight ideological leadership, promote comprehensive exchanges, extensive exchanges, and in-depth integration between mainland and Hong Kong and Macao youths, promote Hong Kong and Macao youths to enhance their motherland awareness and patriotism, and enhance national identity, ethnic identity and culture agree. The relevant situation is now compiled and distributed for reference by all regions. —1—The youth federations at all levels carry out in-depth exchanges between the mainland, Hong Kong and Macao youths with rich and colorful highlights. In 2021, under the overall guidance of the All-China Youth Local realities, deepening the connotation of the activities, innovating the carrier, and focusing on overcoming the impact of the new crown pneumonia epidemic, held a total of 355 youth exchange activities between the Mainland and Hong Kong and Macao, covering a total of 22.34 million person-times online and offline. The influence, coverage and effectiveness of the activities continued to increase. It has achieved a new breakthrough in the youth exchange activities between the Mainland and Hong Kong and Macao. First, the rules are well-structured, and the layout of the article should be paid attention to. Strengthen the systematic and holistic design of youth exchange activities between the Mainland and Hong Kong and Macao, promote the advantages of provincial youth federations and coordinate their efforts, and strive to build a Hong Kong and Macao youth exchange system that is integrated and operates in a standardized manner. The All-China Youth Federation formulated and issued the "Implementation Specifications for Hong Kong and Macao Youth Exchange Activities in the Mainland (Trial)", which clarified the basic principles and practical orientation of Hong Kong and Macao youth exchange activities, and determined the three major sections of constitutional and basic law education, national conditions education, Chinese history and Chinese culture education9 This special project provides normative guidance for youth federations at all levels to carry out youth exchange activities between the Mainland and Hong Kong and Macao. The Guangdong Provincial Youth Federation benchmarked against the institutional documents of the National Youth Federation, gave full play to the role of the youth work mechanism in the Guangdong-Hong Kong-Macao Greater Bay Area, formulated the annual work plan for the Youth Concentric Circle in the Guangdong-Hong Kong-Macao Greater Bay Area, and mobilized 101 Guangdong-Hong Kong-Macau youth associations to jointly carry out exchanges Activity. The Youth Federation of Jiangsu Province amplifies the effect of the Youth Development Forum in Jiangsu, Hong Kong and Macao, and around themes such as culture, education, health, economy and trade, technology, etc., designs the cloud sharing activities for youth in Jiangsu, Hong Kong and Macao as a whole, and realizes the transformation from small-scale and long-term to high-frequency and wide-coverage. Shandong Province - 2 - Youth Federation integrates exchanges, internships, competitions, and practice in the four aspects of Shandong, Hong Kong and Macao youth exchange projects, designs and builds Shandong, Hong Kong and Macao youth exchange seasons, and better helps Hong Kong and Macao youth exchange projects to deepen and solidify through seven one-way methods . 2. Focus on the theme, closely related to the centenary of the founding of the party. Taking the opportunity of celebrating the 100th anniversary of the founding of the Communist Party of China, make good use of red resources such as revolutionary memorial sites and youth patriotic education bases, carry out the study and education of party history throughout the entire process of youth exchanges between the mainland and Hong Kong and Macao, and strengthen the ideological guidance of Hong Kong and Macao youths in a subtle way. The Youth Federation of Chongqing organized Hong Kong and Macao students to pursue the revolutionary footsteps and review the party's history. Revisit the stories of party history in the book, and understand the original aspiration and mission of the Chinese Communists in reviewing history. The Shanghai Youth Federation held a themed activity for college students from Hong Kong and Macao to grow under the national flag, and organized college students in Shanghai, Hong Kong and Macao to visit the Memorial Hall of the First Congress of the Communist Party of China and the Shanghai-Hong Kong Bank History Exhibition Hall, and listen to stories told by veterans who resisted US aggression and aid Korea, further enhancing the patriotic spirit of youth in Shanghai, Hong Kong and Macao. The Youth Federation of Shaanxi Province created the characteristic brand activity of "Youth Stories for the Party". From the perspective of Hong Kong and Macao youths, they vividly told the party's century-old struggle that they felt, and led Hong Kong and Macao youths to understand the heart of the century and cultivate the feelings of home and country. The Tianjin Youth Federation carried out the activity of Tianjin, Hong Kong and Macao youths raising the national flag and singing the national anthem together, organized youths in Tianjin, Hong Kong and Macao to participate in the flag raising ceremony, pay homage to the martyrs cemetery, and enhance the patriotic sentiment of Hong Kong and Macao youth through ritual education. 3. Implement policies according to categories, so as to be targeted. In response to the needs of Hong Kong and Macao youths in the Mainland for internships, employment, entrepreneurship, and life, hierarchical and classified exchange activities of different natures are designed to continuously enhance the experience and sense of acquisition of Hong Kong and Macao youths. Central and state agencies—3—Guan Qinglian recruited the first batch of more than 20 Hong Kong and Macao students from mainland universities to do internships in central and state agencies to help them understand and feel the national governance system at close range, and further deepen their understanding of the socialist system with Chinese characteristics. The Zhejiang Provincial Youth Federation established the Zhejiang Hong Kong and Macao Youth Talent Service Center, launched and implemented work projects such as dissemination of provincial conditions, talent reserves, internships and employment, and provided various services for young talents from Hong Kong and Macao to understand Zhejiang and stay in Zhejiang for development. The Youth Federation of Sichuan Province held the "Rong Piao Cup" High-level Talent Innovation and Entrepreneurship Competition, focusing on the three leading industries of aviation economy, electronic information, and biomedicine, providing a competition platform for Hong Kong and Macao youths to innovate and start businesses in Chengdu to display, communicate, and raise funds. The Youth Federation of Hainan Province launched an exchange activity for young people from Qiong'ao to talk about the Hainan Free Trade Port, building a platform for young people from Macao to study, study and work in Hainan. The Beijing Youth Federation, with Hong Kong and Macao youths studying, living and working in Beijing as the main body, initiated the establishment of the Hong Kong and Macao Youth Beijing Club, and strives to create a Beijing home for Hong Kong and Macao youths that Hong Kong and Macao youths in Beijing can think of, want to come, and be reliable. 4. Warm the heart and feelings, and increase the temperature of the activity. Pay attention to the emotional needs of young people in Hong Kong and Macao, and organize warm and affectionate exchanges at special time points such as the Mid-Autumn Festival, Spring Festival, and the beginning of school, so that they can feel the warmth of the motherland. The All-China Youth Federation is coordinating the youth federations at all levels, with youths from the Mainland, Hong Kong and Macao as the main body, carrying out five first-year New Year condolence activities. Through various methods such as Spring Festival group worship, visits and condolences, Spring Festival party, and experience of New Year customs, people who are affected by the epidemic cannot go home The young people in Hong Kong and Macao who celebrate the Chinese New Year are not alone. The Youth Federation of Anhui Province held the "Full Moon Mid-Autumn Festival · Youth with a Date" cloud Mid-Autumn Festival reunion event for youths from Anhui, Hong Kong and Macao. Using the WeChat group as the carrier, they spent the Mid-Autumn Festival together in the "cloud" and sang together the best wishes for a long life and a thousand miles. The Youth Federation of Fujian Province held the Wuyi Cultural Week, organized young students from Hong Kong and Macao from Fujian to visit Wuyi Mountain to visit their hometown stories, learn about Zhu-4-xi's life and Neo-Confucianism research, and stimulate the strong nostalgia of Hong Kong and Macao youth. The Youth Federation of Guangxi Autonomous Region held a welcome exchange meeting for students from Hong Kong, Macau and Guangxi to help students from Hong Kong, Macau and Guangxi adapt to life in mainland universities as soon as possible, and feel the enthusiasm and warmth from mainland compatriots from the beginning of enrollment. 5. Diversified communication, expanding the coverage of influence. Make full use of new Internet media methods, and continue to expand the breadth and depth of communication activities between the mainland and Hong Kong and Macao youths through various methods such as live broadcast of themes, secondary dissemination, and product production. The All-China Youth Federation coordinated the provincial youth federations in Beijing, Shanghai, Jiangsu, Zhejiang, Fujian, Guangdong, Sichuan and other provinces to host live broadcasts themed on "Qiqiao Folklore Festival", covering more than 5.5 million people at home and abroad. Hunan Province Youth Federation hosts My Internship Season Youth 'Vision Ye Jiejie' short video contest for Hong Kong and Macao students' internships. More than 110 students from Hong Kong and Macao participated in the competition, telling stories about the development of the mainland from the perspective of Hong Kong and Macao students. Douyin 2021 My Internship Season related topics The playback volume exceeds 5 million times. The Youth Federation of Hubei Province held the Yandi Cultural Season E-sports Carnival, specially organized Hong Kong and Macao youth special selection competitions, and carried out extensive mobilization and publicity through local auditions and online live broadcasts. It was reported by more than 30 mainstream media across the country, and the number of online topic readings exceeded 5,000 million people. The Youth Federation of Jiangxi Province held an online exchange event for compatriots in Jiangxi, Hong Kong and Macao to connect blood forever and build roots and dreams together. With the help of short video platforms such as Douyin and Kuaishou, the topic of 2021 Dragon’s Descendants was launched and punched in air, with a total of 77 views. 80,000 person-times, realizing the effective connection between activities and publicity chains. —5—Shake Distribution: General Office of the Central Committee of the Communist Party of China, General Office of the State Council. All comrades in the Secretariat of the Central Committee of the Communist Youth League, the relevant ministries and commissions of the Central Committee, and the leaders of the provincial party committees. The main responsible comrades of the various departments and directly affiliated units of the Central Committee of the Youth League, and the main responsible comrades of the provincial Youth League committees. Shake Issued by the General Office of the Central Committee of the Communist Youth League on January 29, 2022

2022-1-25 5 Editor's note: 2021 is the first year of the 14th Five-Year Plan, and it is also a link between the past and the future for the mid-term evaluation of the implementation of the "Medium and Long-term Youth Development Plan (2016-2025)" and the promotion of in-depth implementation year. League organizations at all levels actively report to the party committee and the government, actively assume the leading responsibility for promoting the implementation of the plan, rely on the joint meeting mechanism, combine local realities, and focus on youth needs, strengthen policy advocacy and social advocacy, and promote the introduction of more policy measures to promote youth development and practical projects, so that young people have a stronger sense of gain and a deeper experience of caring for the party. The typical experience and practices of Hebei, Guangdong, Shandong, Sichuan and other places are now being compiled and distributed for reference by other places. —1—Hebei, Guangdong, Shandong, Sichuan and other places promote the in-depth implementation of medium and long-term youth development plans in Hebei: deepening the construction of linkage mechanisms, cohesive efforts, joint management and joint efforts. Guided by the construction of a coordination and linkage mechanism, we will continue to consolidate and deepen the results of planning implementation, and promote the integration of youth development into the overall situation of reform, development and stability in the province. The first is to give full play to the role of the youth work joint meeting mechanism led by the party committee. Strive for the Standing Committee of the Provincial Party Committee to study and review planning implementation, joint meetings and other work matters for three consecutive years. On the basis of realizing the full coverage of the youth work joint meeting mechanism at the provincial, city, and county levels, promote the full coverage of plenary meetings and plan implementation. Into the provincial party congress report, driving all prefectures, cities and 119 counties and districts to write in the plenary session of the party committee at the same level or the government work report. The second is to promote the comprehensive and effective connection between youth development and the 14th Five-Year Plan. Take the lead in realizing the establishment of special chapters (sections) on youth development in all the 14th Five-Year Plans at the provincial, municipal and county levels, and set up a special section on promoting youth development and youth thought leadership and development for the first time in the provincial 14th Five-Year Plan , Shijiazhuang, the provincial capital, set up a special chapter and 4 special sections on building a youth-friendly city, and incorporated the provincial mid- and long-term youth development plan into the catalog list of the 14th Five-Year Special Plan. The third is to strengthen the coordination and supervision function of the joint meeting mechanism. Establish a briefing and reporting system, summarize the work progress of each member unit every quarter and report it to the provincial party committee and provincial government leaders; establish a task implementation reporting and supervision mechanism, and incorporate the planning implementation into the supervision of the provincial party committee system; General Secretary Xi Jinping's important work on youth work Incorporate ideas into the training courses of the Party School of the Provincial Party Committee, and—2—drive 7 cities and 74 counties to implement them as reference; strive for provincial financial support to include special funds for planning implementation in the annual budget, and drive 11 cities and 106 counties to include them in the local financial budget. Guangdong: Focusing on urgent, difficult, worrying and hopeful issues to improve the effectiveness of policy advocacy The Guangdong Provincial Party Committee adheres to the problem orientation, focuses on the housing security difficulties of fresh college graduates, continues to strengthen policy services under the framework of planning and implementation, and provides tangible help to young people. One is to improve the system design. In accordance with the spirit of policy documents such as the "Opinions of the General Office of the State Council on Accelerating the Development of Indemnificatory Rental Housing", the Ministry of Housing and Urban-Rural Development and the Provincial Department of Housing and Urban-Rural Development implemented the housing security service for fresh graduates of colleges and universities. 2022, it is clearly stated that by 2022, the proportion of newly added affordable rental housing in cities in the Pearl River Delta region will be no less than 20% for college graduates, and no less than 10% in cities outside the Pearl River Delta region. work goals. The second is to enrich the forms of protection. Coordinate the forces of all regions and units, explore the mechanism of serving the housing needs of college graduates through various forms such as physical guarantees + rental subsidies, encourage localities to focus on building public rental housing and talent housing, give priority to graduates to allocate rent, and support local governments to reduce or exempt college graduates Fresh graduates apply for public rental housing to purchase social insurance period conditions, and cooperate with social organizations to build youth post stations. At present, 28 public welfare youth post stations have been built in Shenzhen, Zhuhai, Dongguan, Huizhou and other places, serving more than 30,000 young people. The third is to unblock the information platform. Relying on the 12355 Voice of Youth platform to build an information query system for youth housing, to effectively solve the problem of information islands such as inaccurate housing supply and demand information, and inadequate policy publicity, and mobilize and guide—3—18 cities including Guangzhou, Shenzhen, and Foshan are on-line for There are 8,700 sets of public rental housing and talent housing released by youth. Shandong: Focusing on youth development, the city activates local endogenous power, and the Shandong Provincial Committee, with the support of the provincial party committee and the provincial government, seizes opportunities and makes serious plans. Taking the construction of a youth-friendly city as the main starting point, it vigorously expands Plan the path carrier for in-depth implementation. The first is to formulate and issue guidance documents. Focusing on the overall situation of the construction of a modern and powerful province in the new era and the concept of giving priority to youth development, the "Guiding Opinions on the Construction of Youth Development-Friendly Cities in Shandong Province" was issued in the name of the Provincial Youth Work Joint Conference, focusing on employment quality, entrepreneurial environment, 10 key areas such as housing security, old-age care and childcare, marriage, love and making friends, etc., clarify the basic principles, key content, and implementation subjects of youth development-friendly city construction, and take the lead in carrying out the construction of youth development-friendly cities on a provincial scale nationwide. The second is to study and formulate evaluation indicators. Focusing on the 10 key areas proposed in the "Guiding Opinions", research and formulate a youth development-friendly city index system, clarify 10 first-level indicators, 24 second-level indicators, 50 third-level indicators and the weights, acquisition paths, and responsible departments of each indicator , objectively evaluate the construction of youth development-friendly cities in various regions in a quantitative way, and use more social methods to mobilize the initiative and enthusiasm of local party committees and governments. The third is to vigorously strengthen publicity and guidance. The United Provincial Government Information Office held a press conference to publicly release the "Guiding Opinions" and evaluation indicators to the public, introducing the situation, background, objectives, principles, main content, organization and implementation of youth development-friendly cities, which aroused increased attention from all walks of life. focus on. The fourth is to establish and improve the working mechanism. Supporting the introduction of youth development-friendly-4-type urban construction operation guidelines and index interpretations, strive to build a scientific and operable work system, select Jinan and other areas with a good foundation and fast action to take the lead, and implement them in the 14th Five-Year Planning Outline, Both the government work report and the city's special planning clearly propose to build a youth development-friendly city, providing an experience template for the whole province. Sichuan: issuedThe Sichuan Provincial Party Committee attaches great importance to the implementation of the county-level pilot work of the medium and long-term youth development plan, and recommends Chenghua District of Chengdu City, Luojiang District of Deyang City, and Luxian County of Luzhou City as national pilot projects, and will be implemented in 21 provinces. 37 counties (districts) in 1 city (prefecture) were selected to carry out provincial-level pilot projects, and basically achieved full coverage of regional differences in regions, ethnic groups, resource endowments, and development levels. The first is to promote the integration of youth development into the overall development of the county. Promote the pilot counties (districts) to put forward youth development concepts corresponding to the local economic and social development goals in combination with the actual situation. Among them, 17 pilot counties (districts) propose to build youth-friendly (development) cities (districts), and use this as a guide to introduce and implement A series of policy programs serving youth development. The second is to improve the pilot work leadership mechanism. Each pilot county (district) has established a pilot work leading group headed by the secretary of the county (district) party committee. Incorporated into the county party committee and county government's annual target task assessment and party building report content, 18 counties (districts) increased their work force and established special work teams. The third is to strengthen the guarantee of pilot work resources. To promote the inclusion of pilot work funds into the financial budget at the same level, 37 provincial-level pilot counties (districts) have allocated a total of 5.855 million yuan of special funds for pilot work, of which Chenghua District of Chengdu City and Dongpo District of Meishan City have respectively allocated special funds of 500,000 yuan . The fourth is to improve the youth work joint-5-meeting mechanism. Explore a complete set of key promotion + supervision and assessment + debriefing and inquiry, overall planning, task book + checklist + responsibility system, implementation, monthly inventory + quarterly consultation + annual summary, effectiveness, policy + funding + establishment, guarantee, etc. Reproducible and scalable pilot working mechanism. —6—(No text on this page)—7—Shake Distribution: General Office of the Central Committee of the Communist Party of China, General Office of the State Council. Secretary of the Secretariat of the Central Committee of the Communist Youth League, relevant ministries and commissions of the Central Committee, and provincial party committees in charge of leadership. The heads of the various departments and directly affiliated units of the Central Committee of the Youth League, and the secretary of the Provincial Youth League Committee. Shake Issued by the General Office of the Central Committee of the Communist Youth League on January 25, 2022

2022-1-11 4 Comprehensively and strictly governing the League Special Issue 4铱 Grasping the management of the team of members of the League strictly and unswervingly listening to the party and following the party The fundamental task of the Communist Youth League at all levels is the party's educating people, and adhere to the construction of advanced nature In order to influence, continue to strictly enforce the development, education and management of members, explore the establishment of a bottom-up, segmented classification, and ladder promotion incentive mechanism for the new era of the Communist Youth League, focus on standardizing and strengthening education and management after joining the league, and cultivate people with faith, speech The team of members who are political, moral, pioneering, and disciplined strive to realize the political function of continuously delivering fresh blood to the party—1—. 1. It is better to be less than to be better. The team members have achieved phased results in controlling quantity and improving quality. Adhere to political standards as the primary standard for joining the group. In accordance with the requirements of strict standards, quality improvement, incremental control, and stock management Close the entrance of the members, improve the quality of the development of the members, and enhance the advanced nature of the members from the source. 1. Strict team member development standards and procedures. The whole group insists on including excellent ideological and political courses, passing 8 credit hours of group courses, and 20 hours of annual volunteer service hours as the prerequisites for joining the group. Implement the system of promoting the best into the group, joining the group with points, and evaluating the group, and achieve standardized procedures and complete procedures in all aspects of the group. The Jiangsu Provincial Committee of the Communist Youth League has fully implemented the "double accumulation and double evaluation" points into the league in junior high schools, quantified the development standards of league members in stages, promoted the combination of 2 points accumulation and 2 evaluations, and formed an integrated training chain from the Young Pioneers to the Communist Youth League. The Tibet Committee of the Communist Youth League has established a comprehensive evaluation mechanism for the whole process of joining the League by accumulating points before the League, carrying out evaluations of members, and signing a letter of commitment to join the League, and has regularly carried out verification and disposal of members who have violated regulations. The Jiangxi Provincial Committee of the Youth League carried out the verification and rectification of "young people" joining the league. In the 2020 province-wide "two reds and two excellent" selections, the grassroots youth league committees and their responsible persons who entered the league at a young age in 2019 were vetoed by one vote. 2. Strictly control the size and structure of the members. The whole regiment does a good job in regular member development, insists on the basis of county-level regulation and city-level regulation as a supplement, regulates the number of development, reduces the proportion of youth groups, and has now stabilized the ratio of junior and high school graduates to 30% and 60% %, and will be controlled at around 20% and 40% respectively by the end of 2022. Continue to optimize the structure, formulate development plans for league members in different fields, improve the -2-refined level of regulation in the school field, and increase efforts in the development of league members in the social field. The Xinjiang District Committee of the Communist Youth League has precisely adjusted and controlled the development of league members by field to the county level. In view of the fact that there are more young people in the rural areas of the four prefectures in southern Xinjiang, the proportion of league member development indicators in the social field has been increased to 10%. The development of league members is more scientific and reasonable, and the structure ratio Constantly optimize. 3. Strictly regulate the daily management of members. Formulate and implement the "Regulations on the Education and Management of Members of the Communist Youth League of China (Trial)", rely on the wisdom group building system, continue to straighten out the relationship between members and organizations, and write instructions and examples for filling out the "Volunteer Letter for Joining the League", focusing on new members. Establish electronic files for members, promote member files to enter student status files or personnel files, and take the initiative to do a good job in transferring the organization relationship of graduate student members in a timely manner. In the past three years, 43.02 million graduated student league members across the country have completed the transfer of organizational relations, and the average annual rate of connection between schools and societies has exceeded 90%. The whole regiment deploys a systematic inventory of criminal members, establishes a closed loop of discipline management, and fully completes the disciplinary punishment of historical stock criminal members, and more thoroughly solves the problem of being imprisoned with gang members. The Chongqing Municipal Committee of the Communist Youth League and the Municipal Education Commission jointly issued a document to include the files of student league members in the student files for unified management and transfer, opening up a two-way channel for organizations to find members and league members to organize. In 2019, 213,000 lost league members were recovered. The Hunan Provincial Committee of the Youth League has established a supervision and reporting mechanism for the construction of a "smart group construction" system, and paid close attention to the connection between schools and societies. In 2019 and 2020, the entry rate of new development group members reached 100%. 2. Learn party history, strengthen beliefs, and follow the party. The endogenous motivation of the league members to strive for excellence has been further stimulated and focused on the main responsibility of the party's education. Relying on the system of three meetings, two systems and one lesson, consolidate members at all levels To learn and educate the position, guide the majority of young people to listen to and follow the party, and improve the effectiveness of ideological and political leadership. —3—1. Organize the study and education of party history. Grasping the great opportunity of the 100th anniversary of the founding of the Communist Party of China, focusing on studying party history, strengthening beliefs, and following the party, with organized mobilization as the basic means, and the Youth League branch as the basic unit, carry out in-depth special study, themed group days, and organizational life. As of December 2021, 99.3% of the whole regiment's party history study and education branch coverage rate, 95.3% coverage rate of General Secretary Xi Jinping's important speech on July 1st, and 95.3% coverage rate, and the spiritual special topic study of the Sixth Plenary Session of the 19th CPC Central Committee The branch coverage rate is 71.8%. Organized and implemented the themed practical activities of "I am a leader in party history" and "I do things for my classmates", with more than 56 million participants and more than 4,000 typical service items. The Tianjin Municipal Committee of the Youth League and the Xinjiang District Committee of the Youth League have strengthened the supply of party history learning and education content, produced group lessons and theme group day courseware and audio and video materials by field and group, focused on creating a learning menu, and promoted grassroots group organizations to implement prescribed actions and self-selected actions special. The Liaoning Provincial Committee of the Communist Youth League and the Shandong Provincial Committee of the Communist Youth League seized the opportunity of the election of the village (community) league organization, held a special study on party history after the election of the league organization, took the study and education of party history as the first lesson of the new term, and cracked the organization of rural league members Develop difficult problems to learn. 2. Normalize daily learning and education. Focusing on strengthening the regular political education and training of league members, formulate and implement the regulations on the management of league member education, the guidance outline for middle school league class education, clarify the content of league member education, build a curriculum system for league class, build and promote the group entry ceremony, 14-year-old group birthday, 18-year-old Ceremony education system within the regiment, such as coming-of-age ceremony and overage leaving ceremony. The Beijing Municipal Committee of the Communist Youth League integrates the party's innovative theory propaganda and the study and education of the Four History into the preparation and development of major events, and will organize members to participate in the preparations for the 70th anniversary of the founding of New China and the 100th anniversary of the founding of the Communist Party of China, the Winter Olympics and Paralympics, etc. The preparation of major events, the guarantee of the Beijing Summit of the Belt and Road International Cooperation Forum, etc.—4—Major events serve as practical classrooms for ideological leadership. Focusing on 10 themes such as inheriting the red gene, science popularization and publicity, and cultural and artistic experience, the Tianjin Municipal Committee of the Youth League awarded the first batch of Tianjin youth practice education positions, set up a special area for party and team ceremony education, and displayed historical scenes in real scenes to enhance the experience and perception of young people . 3. Strengthen the construction of online and offline education platforms. Strengthen the construction of offline positions, formulate and implement the guidelines for the construction of middle school league schools, strengthen the construction of middle school league schools, and promote the construction rate of middle school league schools nationwide to 91%. Strengthen the supply of online products, launch a series of online theme group courses for young people, focusing on the youthful and popular interpretation of Xi Jinping's new era of socialism with Chinese characteristics , the cumulative number of students studying in the whole year exceeds 3 billion. The Henan Provincial Committee of the Communist Youth League vigorously strengthened the construction of youth league schools in middle schools, and carried out the work of "one construction, two evaluations and three promotions" of middle school youth league schools in the province, so as to promote the construction by evaluation, combine evaluation and construction, and promote the role play. Up to now, there are 4,283 middle school regiment schools in the whole province. The Anhui Provincial Committee of the Communist Youth League promoted the study and education of party history online and offline, launched a series of radio programs and online audio columns, and launched a series of radio programs and online audio columns. The Hunan Provincial Party Committee of the Tuan set up a special column on the Internet, and launched the short video "Chaoye Youth" on short video platforms such as Douyin and Kuaishou. The short video of red revolutionary figures in Hunan has accumulated more than 50 million likes, and launched "Those Red Revolutionaries Post-90s and 00s" , "History of the Party in Hands" and other cultural products attract young people to actively accept the study and education of party history. 3. I am a member of the league and I am proud. Positive progress has been made in the construction of practical carriers to enhance the advanced nature and sense of honor of the league members. The political consciousness of getting closer to the organization and the action consciousness of taking on the important task of national rejuvenation, those who have the greatness of the country in mind, play the role of new force and commando. 1. Carry out the evaluation of the advanced nature of the members and strengthen the positive incentives within the group. Improve the integrated education chain of the party and team, formulate and implement the guidance outline for the incentive mechanism of the Communist Youth League, build an incentive mechanism with the main methods of membership incentives, appraisal incentives, honor incentives, opportunity incentives, and development incentives, and carry out step-by-step progressive incentives. Formulate and implement the guidance outline for the evaluation of the advanced nature of league members. According to the characteristics of different age groups and groups in different fields, clarify the guiding standards of having faith, emphasizing politics, emphasizing conduct, striving for pioneers, and observing discipline. Quantifiable and evaluable. The Fujian Provincial Committee of the Youth League established a star rating system for league members, subdivided the basic obligations of league members into 1 to 5 stars and several secondary indicators, and used the evaluation results as the main reference for the evaluation of the first and the best within the group. The Hebei Provincial Committee of the Communist Youth League carried out the typical theme tree selection activity for outstanding young people in Hebei Qingzhixing, directly covering 17 industry fields and various key youth groups such as industrial manufacturing, agriculture and rural areas, poverty alleviation, innovation and entrepreneurship, so that outstanding models around them can play a good role model lead role. 2. Improve the training chain of the party and the league, and fulfill the political responsibility of promoting the best to join the party. In 2019, the Central Committee of the Communist Youth League formulated and promulgated the "Implementation Measures for the Work of the Communist Youth League to Promote Excellent Members to the Party (Trial)", which clarified the concept, working procedures, and responsibilities of the Communist Youth League in the work of "Excellent Promotion", and comprehensively standardized and strengthened the work of the Communist Youth League to promote excellent members to join the party. The Shanghai Municipal Committee of the Communist Youth League and the Party Working Committee of the Municipal Education Commission jointly promote the integration of high school and college students, and establish a training and development chain of continuous training, effective connection, and continuous development at the high school and college stages. The Shanxi Provincial Committee of the Tuan Shanxi Committee formulated the implementation measures for provincial enterprises-6-Communist Youth League members to recommend excellent members to join the party in light of the fact that Shanxi has a large number of heavy industry enterprises, and guided all localities and systems to give priority to recommending young people on the front line of anti-epidemic to join the party, so that Tui Youye can become a party organization to develop young party members main channel. The Hebei Provincial Committee of the Communist Youth League established the province-wide Youth League organization to promote and optimize the work process, and explored and formed the working idea of ​​3+1+1. 3 means that before the League members apply for joining the party, the Youth League branch must carry out an education on ideals and beliefs, hold a unified examination, and conduct a league member education review; the first 1 means that after the League members become active members of the party, the Youth League branch must Assist the party organization to conduct a centralized training; the second 1 means that after the league members are listed as the party's development targets, the league branch should assist the party organization to arrange for the league members to give a themed group class. 3. Serve the overall situation around the center and lead the members to make contributions in a new era. Institutionalize the development of voluntary services and social practice as an important carrier in the new era to highlight the advanced nature of members and innovate the organizational life of the group, polish the work brands of youth commandos and young volunteers, and deepen the "Three Going to the Countryside" and "Returning to Home" Report to the community (village) and the youth home and other mechanisms to play the function of the second classroom report card. Especially in 2020, the whole regiment mobilized more than 74,000 youth commandos and 1.63 million youths fought on the front line of the new crown epidemic prevention and control, which was widely praised by the party, the country and all walks of life; organized more than 5,100 youth commandos, More than 100,000 Youth League members participated in the flood prevention and relief work in Jiangsu, Zhejiang, Anhui, Jiangxi, Hubei, Hunan, Chongqing and other provinces and cities; organized more than 2,740 cadres stationed in front-line poverty alleviation regiments to fight against poverty, implemented rural revitalization and youth contribution actions, and participated in helping 10 impoverished counties and 2,446 impoverished villages; the majority of members of the league are widely active in the work of helping the youth together in the same boat, caring and helping actions, practicing thrift and opposing waste, and blooming youthful flowers. The Zhejiang Provincial Committee of the Communist Youth League actively created a carrier, strengthened organizational mobilization, and promoted league members to be able to see it at ordinary times, and to rush forward at critical moments. Since July 2020, it has promoted youth league members to go deep into the community to participate in voluntary services reaching 3.682 million person-times, 9. 80,000 league members joined the front line of epidemic prevention and control and flood control, and 1,821 league members submitted applications for joining the party. Combined with the community youth action, the Jiangsu Provincial Committee of the Communist Youth League carried out the work of reporting to the town, street and village associations for members of the student league in Jiangsu, and organized and mobilized the members of the student league in Jiangsu to participate in theGrassroots social governance and rural revitalization. The Sichuan Provincial Committee of the Youth League promotes all members of the League to become registered volunteers of "Volunteer Sichuan Ye", and organizes the majority of League members to actively participate in the guidance of online public opinion, promote the main theme, spread positive energy, and consciously safeguard national security. Shake Distribution: General Office of the Central Committee of the Communist Party of China, General Office of the State Council. Secretary of the Secretariat of the Central Committee of the Communist Youth League, relevant ministries and commissions of the Central Committee, and provincial party committees in charge of leadership. The heads of the various departments and directly affiliated units of the Central Committee of the Youth League, and the secretary of the Provincial Youth League Committee. Shake The General Office of the Central Committee of the Communist Youth League Issued on January 11, 2022—8—

2022-1-11 3 Comprehensively Strictly Govern the Youth League Special Issue 3铱Strengthen the management of the cadres in the cadres and strive to be loyal, clean and responsible Ideals and beliefs, caring for the vast number of young people, improving work ability, and tempering good work style are important requirements. Strict management of the regiment cadre team is an important task in promoting comprehensive and strict management of the regiment, so as to explore and improve the cadre work mechanism combining special appointment and combination As a starting point, strive to promote the construction of a team of cadres who are energetic, strong, and good at deeds. 1. The scientific nature of the selection and use of regiment cadres has been continuously improved—1—Adhering to the principle of party management of cadres, highlighting the standards of good cadres in the new era, following the "Regulations on the Selection and Appointment of Party and Government Leading Cadres", highlighting knowing young people, understanding young people, and loving young people It is an important requirement of the Communist Youth League to strictly control the selection and allocation of league cadres, and more and more outstanding talents in various fields who dare to take responsibility, have the courage to take responsibility, are good at deeds, and have outstanding performance are absorbed into the ranks of the Communist Youth League. 1. Highlight political standards and establish a clear career-oriented orientation. The organs directly under the Central Committee of the Communist Youth League held the first cadre and personnel work meeting, insisting on political quality as the primary criterion for selecting and employing personnel, highlighting political integrity and loyalty to the party, and strictly evaluating and inspecting cadres. At the same time, adhere to the cause first, focus on actual performance, vigorously implement the "five breakthroughs" requirements, and give priority to the promotion of candidates who have participated in Xinjiang, Tibet, poverty alleviation and other work with outstanding performance, forming a competitive situation of merit. The organs directly under the Shanghai Municipal Committee of the Communist Youth League have set up five orientations of "persistence" and "employment", and put the persistence in viewing whether cadres are responsible from the height of loyalty to the party as the first priority, so that more cadres who want to do things, are able to do things, and do things well stand out. The Shanxi Provincial Party Committee of Tuan sought the support of the Organization Department of the Provincial Party Committee to promote the simultaneous completion of the reelection of the county and village league organizations and party organizations. Establish a joint review mechanism for candidate qualifications during the reelection inspection, highlight political standards, and work with the organization and discipline inspection departments to do a good job in checking candidates. 2. Persist in all corners of the country and promote the diversification of cadre sources. The organs directly under the Central Committee of the Communist Youth League insist on broadening the sources, and the temporary cadres use the four methods of organizational selection, social selection, grass-roots selection within the League, and selection of directly affiliated units; the selection of part-time cadres focuses on party and government cadres, experts and scholars, and youth models. 4 types of groups, the backbone of the Youth League organization. Since the reform in 2016, a total of 202 temporary cadres and 89 part-time cadres have been selected and assigned. 31 provinces (autonomous regions, municipalities directly under the central government), 300 cities (prefectures, states, leagues), and 2102 counties (cities, districts, banners) across the country have been equipped with part-time cadres. Take multiple measures to enrich the work force of the grassroots Communist Youth League. On the basis of consolidating the traditional selection model, purchase services through the government, hire social workers for youth affairs, train and select youth committee members in villages (communities),—2—develop volunteer service projects, attract rebates ( In the form of part-time jobs for rural students, etc., multi-channel enrich the backbone. Since the reform and expansion of the county-level Communist Youth League’s grassroots organization pilots, 621 pilot counties (cities, districts, and banners) have added 207 administrative and career establishments, 593 more part-time workers, and 860 other workers. The national central enterprise headquarters, provincial enterprise headquarters, and university-level youth league committee secretaries have an average staffing rate of around 95%. 3. Improve the coordination and management mechanism and implement dual leadership responsibilities. The League organization assists the Party organization in managing the leading group and members of the group organization at the next level, which is a concrete manifestation of the regiment's dual leadership system in the management of the regiment cadres. The Central Committee of the Communist Youth League established and improved the system for the division of labor and adjustment of the leadership team of the provincial Youth League committee, and the daily reporting system for important matters of the team members, established a mechanism for sending members to attend the democratic life meeting of the leadership team of the lower-level Youth League committee, and actively cooperated with the provincial party committee to do a good job in the reelection and adjustment of the leadership team of the provincial Youth League committee. Equipped with inspection work. Coordinate and promote the overall allocation of cadres and leading teams of the leading organs and leading groups at the provincial, city and county levels, establish and improve the three normalized working mechanisms of sampling surveys, statistical notifications, and ledger management, build a closed loop of work, and promote the deployment rate of cadres and leading groups of the leading organs of the regiment , The on-the-job rate remains at a high level. All localities promote the implementation of responsibilities for coordinating work. The Guangxi District Committee of the Youth League has established a "reverse force" mechanism for the deployment of regimental cadres with "no one is exempted", no one is exempted, and both rewards and punishments are imposed. The Shandong Provincial Committee of the Youth League established a record system for the adjustment of team members of the county-level Youth League Committee, requiring the comrades in charge of the county-level Youth League committee to report to the Provincial Youth League Committee for the record. The Shaanxi Provincial Committee of the Communist Youth League formulated the "Methods for the Examination of Basic Knowledge of League Affairs for Proposed Youth League Cadres (Trial)", and conducted pre-employment examinations for proposed Youth League cadres. The Yunnan Provincial Committee of the Communist Youth League has strengthened the cooperative management of league cadres. In 2021, interviews will be held with the secretaries of the Youth League committees of 3 cities and 5 colleges and universities where the work is progressing slowly. 2. The ability of regiment cadres to perform their duties has been significantly enhanced—3—The education and training of regiment cadres is a leading, basic, and strategic project for the construction of regiment cadres. The Central Committee of the Communist Youth League studied and formulated the "Regulations on the Education and Training of Cadres of the Communist Youth League of China", which clearly established a training content system based on Xi Jinping's socialist ideology with Chinese characteristics in the new era. The Communist Youth League at all levels has taken multiple measures to strengthen the ideological refinement, political experience, practical training, and professional training of the cadres, leading the cadres to truly grow into experts in the work of the party's youth and the masses, and better serve the overall situation of the party and the country . 1. Focus on the improvement of the political capabilities of regiment cadres. The key settings include 3 course modules of political education, theoretical education and ability education, covering 19 course units and 73 specific courses of regiment cadre education and training course framework. In each training class, the study and implementation of Xi Jinping's socialist ideology with Chinese characteristics in the new era is always given priority, and the party spirit education runs through the training. The Central Committee of the Communist Youth League held a symposium on the education, training and theoretical research of the Communist Youth League cadres. In accordance with the idea of ​​doing academics and scientific research, two batches of 25 teams composed of personnel from the Central Committee of the Communist Youth League, the Central Youth League School and the Research Center were formed in a cross-professional and cross-post manner. A course teaching and research group, 119 people participated in the course development and teaching work. The Jiangxi Provincial Committee of the League organized and developed a batch of training courses for League cadres with Jiangxi characteristics such as "Good Style of Cadres in the Soviet Area and Healthy Growth of League Cadres". The Sichuan Provincial Committee of the Youth League selected more than 20 high-quality teachers at the provincial level and above to participate in the recording of high-quality courses, and to provide full coverage training for township (street) league cadres. 2. Construct a hierarchical and classified regiment cadre training system. The Central Committee of the Communist Youth League issued the "2020-2023 National Youth League Cadre Education and Training Plan" to improve the education and training system of regiment cadres in accordance with the method of hierarchical responsibility and overall planning. Work. Improve the reporting system and training notification system for regiment cadre training plans, incorporate 10 industry league (referring) committees into the training work system, and expand training coverage. Since the 18th National Congress of the Communist Youth League, the Organization Department of the Central Committee of the Communist Youth League has directly organized 114 main classes of education and training for cadres of each category, with a total of 10,873 participants, equivalent to 323,123 person-days of training. For the first time, the Heilongjiang Provincial Committee of the League’s Heilongjiang Provincial Committee’s special training course on promoting the ability of grassroots League cadres was included in the Provincial Party Committee’s Organization Department, the Provincial Party Committee’s Propaganda Department, and the Provincial Party School’s provincial-level education and training key shift plan and the Provincial Party School Party School’s special plan for student selection. 3. Strengthen the construction of training positions and the guarantee of teachers. Build a training position structure with the Central Youth League School and the Jinggangshan Base as the leader, and the Youth League schools at all levels and the education and training bases for regiment cadres complement each other and have a reasonable layout. In addition, in accordance with high-quality and professional requirements, build a shared pool of teachers for education and training of cadres, establish a working mechanism for cadres to go to the podium, and enrich the team of teachers for education and training of cadres through multiple channels. The Inner Mongolia District Committee of the Youth League deployed and carried out a skills competition for the leaders of the League on the podium throughout the region to improve the ability of the cadres to preach. The Anhui Provincial Committee of the Communist Youth League established the Research and Scheduling Center for the Key Work of Youth League Building in the Province to help the provincial league schools cultivate a team of teachers who can understand the grassroots, get on the podium, and write well. The Hebei Provincial Committee of the Communist Youth League held a competition, which was carried out in layers and categories for city and county Youth League committees and Young Pioneers, colleges and enterprises, and to improve capabilities during exchanges. The Sichuan Provincial Committee of the Youth League and the Chongqing Municipal Committee of the Youth League took the opportunity of the construction of the twin-city economic circle in the Chengdu-Chongqing region as a national strategy, and innovatively implemented the Sichuan-Chongqing Youth Training Camp, a cooperative innovation work project between the Youth League and the School, and jointly built the Sichuan-Chongqing Youth Worker Training Base. 3. The spirit of the League cadres' responsibilities further boosted the Youth League. After the 18th National Congress of the Communist Youth League, the Central Committee of the League immediately issued the "Six Regulations on Improving Political Position and Improving Work Style", declaring that it is consciously facing the new requirements of party building in the new era and comprehensively following the The firm determination to strictly govern the party and meet the high standards. The Communist Youth Leagues in various places implement the spirit of the eight central regulations and their implementation rules, strictly implement the six regulations within the league, get rid of the ideology and formalism and bureaucratic tendencies of the cadres and official standards of the league cadres, temper the rigorous work style, and build a good political ecology within the league. 1. Strengthen belief in deepening reform and overcoming difficulties. Under the direct -5- leadership of the Party Central Committee Secretariat, the Communist Youth League, the Youth Federation, the Students' Union of the Students' Federation, and the Young Pioneers focused on the reform goals of strengthening the three characteristics and eliminating the four modernizations, and conscientiously promoted the implementation of nine reform plans. The leadership of the party and the construction of the party It has been comprehensively strengthened, the leadership system, operating mechanism, working methods, and cadre structure have undergone profound changes, and the institutional setup, work layout, force allocation, and daily operation have all focused on the main responsibilities and main businesses. League cadres at all levels have further strengthened their ideals and beliefs in promoting the reform of the Communist Youth League, tempered their skills, effectively improved their ability to contact and serve young people, and changed their work to a new atmosphere and a new image of cadres. In October 2021, with the approval of the Secretariat of the Central Committee, the organs directly under the Communist Youth League Central Committee will focus on the deep-seated mechanism problems that urgently need to be solved in the reform of the Communist Youth League. Responsibilities, ensure that reforms are managed, responsibilities are compacted, and tasks are nailed down. The Guangdong Provincial Committee of the Communist Youth League promoted the introduction of the country's first provincial league school reform plan in the name of the provincial party committee and the provincial government, focusing on the main business of education and training of league cadres, and testing the reform results with the actual effectiveness of the improvement of the political capabilities of league cadres. 2. Changing styles in outreach to youth. The mechanism of close contact with youth is regarded as an important starting point for the cadres of the Central Committee of the Communist Youth League to improve their style of work, and it is organically integrated and promoted in an overall manner with the regiment cadres on the podium, the youth study, the special mechanism for contacting colleges and universities, and the working mechanism of joint films. It is clearly required that all professional and temporary cadres of the Central Committee of the Communist Youth League spend no less than 45 days per year in going to the grassroots level and contacting young people. In 2021, cadres of the Central Committee of the Communist Youth League will contact grassroots organizations for a total of 7,767 days, an average of 49.8 days per person; 638 theoretical lectures will be held, covering 103,900 person-times. The Jiangsu Provincial Committee of the Communist Youth League requires all league cadres at all levels to report to the Youth League (Working) Committee of the township (street) where they live or work, and to go down to the village (community) to carry out work in accordance with the principle of "proximity and convenience". The Anhui Provincial Committee of the Communist Youth League deployed the activities of "going to the grassroots, visiting youths, and delivering theories". The various departments of the government determined a number of research topics in combination with the annual key tasks, and formed written research results. 3. Strengthen the muscles and bones in the urgent, difficult and dangerous tasks. League cadres from all over the country carry forward the glorious tradition of "the party has a call, and the league has actions", and they are at the forefront and set an example in the work of epidemic prevention and control, flood prevention and disaster relief, poverty alleviation, and rural revitalization. Since the 18th National Congress of the Communist Youth League, 17 cadres have been selected and dispatched by the organs directly under the Central Committee of the Communist Youth League to participate in targeted poverty alleviation and rural revitalization work, and 5 cadres have been selected to participate in aiding Xinjiang and Tibet, and supporting the western regions and old revolutionary areas. The Beijing Municipal Committee of the Communist Youth League pays attention to training and training cadres in urgent, difficult and dangerous tasks. After receiving the task of epidemic prevention and control in Xinfadi, it organized the first batch of 49 cadres to arrive at the nucleic acid testing point for supervision within 2 hours. On the same day, it organized a team of 186 people to go to the scene . The Henan Provincial Committee of the Youth League, the Organization Department of the Provincial Party Committee, and the Provincial Poverty Alleviation Office carried out the work of the first secretary of the Communist Youth League in the whole province. In three years, more than 10,000 first secretary of the Communist Youth League were selected to join the front line of poverty alleviation and rural revitalization. The grassroots weathers the wind and rain, sees the world, and strengthens the bones. 4. The driving force of the cadres and officers of the regiment to start a business is effectively stimulated. Adhere to the combination of strict management and love, and pay equal attention to incentives and restraints. Young people are dedicated, loyal, clean and responsible backbone team. 1. Strengthen assessment and evaluation, and establish a clear orientation of rewarding the good and punishing the bad. The organs directly under the Central Committee of the Communist Youth League have established a performance appraisal system for the organs directly under the Central Committee of the Communist Youth League, which consists of key performance indicator assessment, party building element assessment, and comprehensive work assessment, and the performance evaluation results are used as an important basis for evaluating leading cadres. The Tianjin Municipal Committee of the Communist Youth League carried out the work of the youth league organization secretary's debriefing and evaluation work to the higher-level league organization. The youth league organization secretaries at all levels generally accepted the evaluation of the youth members of the league members and the comments of the higher-level league organizations, which stimulated the entrepreneurial enthusiasm of the league cadres and officers. The Chongqing Municipal Committee of the Communist Youth League deepened the evaluation system of whether the League members were satisfied with their work or not. The youths have the final say, and innovatively established a three-level report for the district and county Youth League committees to report to the Youth League and Municipal Committees, the grassroots Youth League committees to report to the higher-level Youth League organizations, and the Youth League branch's back-to-back satisfaction evaluation. Reviewing and evaluating the working mechanism. Since 2017, 73 Youth League branch secretaries have been dismissed due to dissatisfaction in back-to-back satisfaction evaluations. The Youth League Committee of the Xinjiang Production and Construction Corps has fully implemented the positive and negative list system for divisional and municipal Youth League secretaries, established a key-7-work month scheduling mechanism, publicly praised good work results, and resolutely notified poor implementation, and promoted the formation of an integrated promotion within the regiment. Good working situation of various key tasks. 2. Clarify the bottom line of discipline and strengthen supervision and accountability. The organs directly under the Central Committee of the Communist Youth League strengthen daily supervision, establish an internal inspection system, a system of disciplinary inspection proposals, and a working mechanism for disciplinary supervisors to effectively strengthen discipline constraints. The Guangdong Provincial Committee of the Communist Youth League issued the "Guidelines for Supervision and Discipline Enforcement within the Guangdong Communist Youth League (Trial)", which clarifies the responsibilities of the main bodies at all levels, and from the cluesDisposal, preliminary verification, result disposal, review and investigation, etc. regulate the supervision and discipline work process within the regiment. The Xinjiang District Committee of the Communist Youth League started with the existing system and norms, grasped the basics, grasped the daily routine, strictly enforced discipline and accountability, publicly notified typical cases on the network platform, and exerted the effectiveness of warning education. The Jiangxi Provincial Committee of the Communist Youth League conducted an in-depth investigation and rectification of fear, slowness, falsehood, mediocrity, and chronic illnesses. For those regiment cadres who failed to perform their duties, had a false style of work, and practiced fraud, they called out their surnames and reported criticisms, and strictly enforced discipline and accountability. The province notified 21 cadres of the criticism group. The Guizhou Provincial Committee of the Communist Youth League has continued to carry out the execution capacity building project in the province's Communist Youth League system in response to the lack of skills, panic and backward skills of the cadres, strictly implement the responsibility system for key tasks, and promote the work in a list-based and account-based way. Ineffective accountability and accountability, improve cadres' ability to perform their duties and work effectiveness. Shake Distribution: General Office of the Central Committee of the Communist Party of China, General Office of the State Council. Secretary of the Secretariat of the Central Committee of the Communist Youth League, relevant ministries and commissions of the Central Committee, and provincial party committees in charge of leadership. The heads of the various departments and directly affiliated units of the Central Committee of the Youth League, and the secretary of the Provincial Youth League Committee. Shake The General Office of the Central Committee of the Communist Youth League Issued on January 11, 2022—8—

2022-1-11 2 Comprehensively and strictly governing the regiment special issue 2铱Strengthening the organization of the regiment and making full use of the role of the party's assistants and reserve forces. Focus on activating the political and social functions of the Communist Youth League, continuously enhance the attractiveness and cohesion of the Communist Youth League, unite the young members of the Communist Youth League closely around the party, better play the role of the party's assistants and reserve forces, and provide a strong foundation for comprehensive and strict governance of the Communist Youth League. organization guarantee. -1-1. Strengthen the construction of the leading organs of the regiment, and effectively play its due political functions. The leading organs at all levels of the central steering group of the regiment adhere to the overall leadership of the party, standardize the reelection, and establish a working mechanism of one specific one station and two joint operations. Promote the group's congresses and committees at all levels to play political functions, achieve overall activity, and effectively perform the functions of leading organs. 1. Implement the party's overall leadership over the league organizations at all levels. The leading organs at all levels of the regiment adhere to and strengthen the overall leadership of the party, resolutely implement the spirit of General Secretary Xi Jinping's important instructions and instructions and the decision-making and deployment of the party central committee, and resolutely implement the requesting and reporting system for major issues. According to the "Regulations on the Work of the Party Group of the Communist Party of China", implement the requirement that the league organizations at all levels do not have a party group, and straighten out the decision-making mechanism of the league organization after the party group is abolished. Conscientiously implement the various systems and regulations of party building leading the league building, consolidate and deepen the county-level and below committee secretaries of the regiment to attend the meetings of the party committees and standing committees at the same level, and include the party building leading the league building work into the assessment content of the party building work of the leadership of the local party committee, etc. move. The Shandong Provincial Committee of the Communist Youth League and the Organization Department of the Provincial Party Committee jointly issued "Several Measures on Further Strengthening the Work of Grassroots Party Building and League Building under the New Situation", which clearly regards party building and League building as an important part of the responsibility system for party building work. The city and county party committees listen to at least 1 group work report. The Guangdong Provincial Committee of the Communist Youth League strictly implements the ten systems and mechanisms of "two maintenances" and formulates more than 200 specific detailed measures. It conducts supervision inspections and evaluation scores every quarter as an important basis for annual assessment. 2. Continue to improve the breadth of representatives of the leading body of the regiment. The Central Committee of the Communist Youth League formulated and promulgated the "Regulations on the Election of Local Organizations of the Communist Youth League of China", which clarified the whole process of the election of local organizations of the Communist Youth League. Efforts should be made to optimize the size and structure of the league's congresses and committees, especially to implement the requirements for the proportion of grassroots and frontline staff, and clarify that the proportions of grassroots and frontline staff in congresses, committees, and standing committees should not be less than 70%, 50%, and 25% respectively, and strengthen extensive and representative. Refine the qualifications and production procedures of candidates, comprehensively consider the willingness and ability of candidates to perform their duties, highlight political standards, broaden the selection horizon, improve the quality of candidates, and fully reflect the political, advanced, and mass requirements. In its reform plan, the Sichuan Provincial Committee of the Youth League clarified the requirements for the composition of delegates, committee members, and standing committee members of the three-level leagues at the provincial, city, and county levels. The proportions of grassroots frontline youths of the Hubei Provincial Committee of the Communist Youth League among the representatives of the Provincial Congress, the National Committee of the Provincial Committee of the Communist Youth League and the Standing Committee have increased to 75.5%, 58.6% and 46.2% respectively. 3. Promote representatives and members of the League at all levels to better perform their duties and responsibilities. Establish a working mechanism of "one dedicated, one station" and "two linkages", focusing on the construction of special committees at the city level and above, and the construction of liaison stations for delegation representatives in counties. For traction, build a work pattern that covers all fields horizontally, connects all levels vertically, and generally connects youth members. Up to now, 31 provincial-level Youth League committees and 280 municipal-level Youth League committees across the country have established special committees, and 2,671 county-level Youth League committees have established contact stations for county-level delegation representatives. , Member work participation. Relying on the special committees at all levels and the liaison stations of delegation representatives from all over the country, the "I do something for the youth" activity was carried out within the scope of the whole delegation. A total of more than 24,000 activities were carried out, and 385,000 delegation representatives and committee members participated. The Shanxi Provincial Party Committee of the Tuan Shanxi Province has established special committees at the provincial and municipal levels, and all 117 counties have established liaison stations for delegation representatives in county areas. The -3-level Youth League Committee will work together. During the period of May 4th and June 1st, the Hunan Provincial Committee of the Communist Youth League, combined with the practical activities of "I do things for the youth", guided the representatives and committee members of the Liaison Stations of League Representatives in various places to go deep into the grassroots to listen to opinions and understand needs, and strive to do practical things and do good things for young people . 2. Standardize the construction of grassroots organizations, continuously improve the cohesion and combat effectiveness of the league organizations, continuously consolidate and improve the grassroots organization system, extend to various youth social organizations, expand the effective coverage of youth league members, continue to promote the rectification of weak and lax league organizations, and make grassroots league organizations Organizations are built stronger and more dynamic. 1. Continue to expand the effective coverage of grassroots league organizations. The Communist Youth League at all levels insists on focusing on the grassroots, consolidating the construction of grassroots league organizations in traditional fields, continuing to expand the coverage of youth league organizations in emerging fields, and promoting the construction of all that meet the conditions for establishing a league. As of the end of October 2021, the number of league organizations across the country has increased by 764,000 or 27.1% compared with the end of 2018; the density of youth league organizations in the social field has reached 63/10,000 youths, compared with 32.9/10,000 youths at the end of 2018 The number of youth has nearly doubled; the number of non-public enterprise league organizations across the country has increased from less than 100,000 at the end of 2018 to 310,000. Taking industry system team building as a new growth point to enhance organizational strength, 329 new provincial and municipal levels have been established in taxation, health care, emergency rescue and other systems, construction, transportation, tourism and other industries, as well as the Internet, express delivery, logistics and other fields There are more than 1,500 league organizations in total, leading to the establishment of industry system league organizations nationwide covering 57% of the prefectures and cities, and building a vertically and horizontally intertwined organizational system that connects up and down. The Zhejiang Provincial Committee of the Youth League implemented the organization strength improvement trend project, focused on characteristic towns, non-public enterprises, social organizations, etc. to carry out special actions for group building, explored the establishment of groups in express delivery stations, community post stations, etc., and eliminated express delivery boys and Internet practitioners The group organization of groups such as the orthodox and the like covers blind spots. The 4th Committee of the Guizhou Provincial Youth League has stepped up efforts to build youth groups in ex situ poverty alleviation and relocation sites, established 743 youth groups, directly contacted 133,000 young people, and expanded the effective coverage of the group organizations. 2. In-depth implementation of the standardized construction of grassroots league organizations. The standardized construction of grassroots league organizations continued to advance, and the basic database of smart league building covering 3.65 million league organizations and more than 73 million league members was basically completed. Relying on the smart league building system, 85.9% of fresh graduate league members completed the organizational relationship transfer. Then, the previous phenomenon of leaving school and losing contact has been significantly improved. Formulate and promulgate the grassroots organization work regulations of government agencies, state-owned enterprises, and rural areas, revise and improve the work guidelines for the league (headquarters) branch to standardize and rank, and promote the gradual improvement of the grassroots work system of the regiment. The Henan Provincial Committee of the Youth League formulated the "Implementation Plan for the Standardized Construction of Basic-Level Youth League Organizations in the Province", and promoted the standardization and standardization of grass-roots Youth League organizations by compiling and distributing instruction manuals, drafting task lists, and setting key indicators. The bottom 20% of the Youth League branches will undergo key rectification. The Qinghai Provincial Committee of the Communist Youth League focused on rectifying the weak and lax League organizations. The Youth League branches of 4,641 villages (communities) in the province were reorganized according to the requirements. The Gansu Provincial Committee of the Communist Youth League focused on the construction of grassroots league organizations and the reform of grassroots organization of the county-level Communist Youth League to carry out supervision. Five supervision teams were established, led by members of the leadership team, to achieve full coverage of all prefecture-level and city-level youth league committees and 40 key county-level youth league committees. . 3. Vigorously promote the innovation of the group's grassroots organizational methods. The Communist Youth League at all levels combined with the grassroots organization reform of the Communist Youth League at the county level to promote the youth associations run by the Communist Youth League, and extensively established three types of youth social organizations led by the Communist Youth League, youth voluntary service, entrepreneurship and employment, art and sports. Up to now, there are more than 9,800 youth organizations at the county level, covering more than 50,000 to 20 million youths in various fields. In-depth implementation of the three-year action plan for the construction of youth homes, extensive construction of offline and online youth homes with effective coverage and stable functions, exploration of the operating mechanism of group organizations + youth homes + social organizations, and promotion of group organization, work, The positions are organically integrated at the grassroots level. For young people from new social classes such as non-public economic organizations and social organizations, a wide range of activities such as urban integration, employment and entrepreneurship, friendship and fellowship, and public service are carried out, and young people from new social classes are organized and mobilized to actively participate in grassroots social governance. In 2021, 3.881 million youth league members will participate in various urgent, difficult, dangerous, and heavy metallurgical tasks, and 903,000 college student league members will be active at the grassroots level in towns (streets) and villages (communities). The Jiangxi Provincial Party Committee of the League combined with the "New Era Jiangxi Rural Good Youth" selection and training plan, established Youth League clubs in 20% of the administrative villages in the province in 2021, further consolidating and expanding the Communist Youth League in rural areas. 3. Enrich the organization's operation and management mechanism, stimulate the internal effectiveness of the organization's work, take the deepening reform as the driving force, actively keep up with the pace of the times, walk in the forefront of the youth, enrich the organization's operation and management mechanism, and establish a flat working mechanism through the implementation of the project management model. Build a socialized work pattern and stimulate organizational work efficiency. 1. Implement project management mode. The organs directly under the Central Committee of the Communist Youth League implement the annual key project mechanism. In 2021, 11 annual key projects will be studied and determined to guide the organs directly under the Central Committee to focus on their main responsibilities and main businesses, and use limited resources and strength on the most important things. Based on the key project tasks they lead and participate in, the various departments of the agency research and determine the annual key performance indicators (KPI), establish scientific, standardized, and precise work orientation, and ensure that the work has goals, standards, and measurability. The Guangdong Provincial Committee of the Youth League takes the in-depth promotion of the "Lifeline Engineering" project as a command, promotes the various tasks of the regiment in stages and levels, strengthens the unified scheduling and guidance of the grassroots-6-construction work, and further consolidates the work pattern of "the whole regiment grasps the grassroots". The Tianjin Municipal Committee of the Communist Youth League formulated a project-based work list for village and community Youth League branches, and issued work manuals to accurately describe the work objectives, task contents, work processes, and assessment points of grassroots youth league organizations, promote the standardization of grassroots youth league affairs work systems, and better utilize grassroots youth league branches. Battle fortress role. 2. Establish a flat working mechanism. Explore the formation of an effective flat working mechanism, promote the work signals, resources, methods, and strength of the whole regiment to the grassroots, and ensure that the league organizations at all levels are in step, coordinated, and run smoothly. The Shandong Provincial Committee of the Communist Youth League implements a flat management mechanism that focuses on counties at the province and townships and towns at the city level, so as to achieve direct access to work signals and work pressure. In 2021, we will send good news to the main comrades in charge of the party committees of 7 cities, 47 counties, 27 enterprises, and 40 colleges and universities that have achieved outstanding results, and call and notify some lagging counties to effectively transmit pressure and stimulate motivation. The Hebei Provincial Committee of the Communist Youth League carried out research and guidance on the reform of the Communist Youth League at the prefectural and municipal levels and the pilot reform of the Communist Youth League at the county level, which integrated inspection, research, publicity, and interviews, and created a flat guidance and promotion mechanism to pass the work signal to the end. Relying on the comprehensive service platform, collaborative office platform and other working channels, the Ningxia District Committee of the Communist Youth League has established a system and mechanism for contacting the grassroots level, giving play to the role of the new media matrix of the regiment, and enhancing the timeliness of signal transmission and the close connection between the upper and lower levels of the league organizations at all levels in the region. sex. 3. Build a socialized work pattern. Improve organizational mobilization methods, get rid of the administrative way of thinking, focus on the central tasks of the party committee and the needs of young people, actively raise resources from the society, find helpers from young people, improve the resource allocation mechanism that is linked to the whole group, faces the society, and is open and shared. Volunteering, aiding students in poverty, employment and entrepreneurship, human-seven-talent training, practical education and other fields, form a project brand that serves the overall situation and serves young people well, and has stable political functions and social influence. In 2020, the League Central Committee at its own level will take the initiative to raise nearly 1.5 billion yuan in social funds for work, which is six times the annual financial project funds; foundations at all levels of the Youth League have extensively carried out socialized fundraising, and strive to stimulate the sense of ownership and awareness of young people. In the spirit of collectivism, all 580 million yuan of love funds raised were used transparently and efficiently to support the epidemic prevention and control work, greatly reducing the administrative dependence of the work. In accordance with the working mode of youth gathering + , the Youth League Beijing Municipal Committee has introduced bookstores, theaters, shopping malls and other social service units where youth groups gather as centers for youth practice education activities, and formed a youth gathering site + multiple The service model of "a normalized position" further extends the working arm of the Communist Youth League to serve youth. Shake Distribution: General Office of the Central Committee of the Communist Party of China, General Office of the State Council. Secretary of the Secretariat of the Central Committee of the Communist Youth League, relevant ministries and commissions of the Central Committee, and provincial party committees in charge of leadership. The heads of the various departments and directly affiliated units of the Central Committee of the Youth League, and the secretary of the Provincial Youth League Committee. Shake The General Office of the Central Committee of the Communist Youth League Issued on January 11, 2022—8—

2022-1-11 1 Shake Shake Editor’s Note: Strictly governing the league in an all-round way is an inevitable requirement for the Communist Youth League to follow the party, learn from the party, and continuously promote self-revolution; it is also an inevitable requirement for the party to be united and struggle, an important prerequisite for joining the great social revolution. Since the Eighteenth National Congress of the Communist Youth League, the Central Committee of the Communist Youth League has studied and understood the important requirements of General Secretary Xi Jinping on strict management of the League, adhered to the problem-oriented approach, guided by political construction, and strictly focused on the construction of the League organization, strict management of the League cadre team, and strict management of the League members. Strict management is the focus, and system construction is the guarantee, and efforts are made to build a work pattern that comprehensively and strictly manages the League. The whole group has effectively strengthened the ideological consciousness and action consciousness of strictly governing the group. The path has been continuously clarified and the effect has gradually appeared. Responsibility for striving for goals and tasks. Before -1-, when the whole party and the whole country are deeply studying and implementing the spirit of the Sixth Plenary Session of the Nineteenth Central Committee of the Party, the Sixth Plenary Session of the Eighteenth Central Committee of the League will be held soon, and important arrangements will be made for the fundamental plan of grasping successors, especially It is a special deployment to implement the requirements of comprehensively and strictly governing the party, promote the work related to comprehensively and strictly governing the league, unite and lead the youth of the youth league members, welcome the 20th National Congress with confidence, always follow the party, and forge ahead on a new journey. The Central Committee of the Communist Youth League has sorted out the relevant situation, and is now compiling and distributing special issues in 4 issues for learning from all over the world.One of the special issues on comprehensively and strictly governing the regiment.铱Take political construction as the command and always abide by what the party flag refers to. Through implementing Xi Jinping Thought on Socialism with Chinese Characteristics for a New Era, under the guidance of the party's political construction, we will continue to strengthen ideological forging and comprehensively improve political capabilities. After several years of hard work, the organizations at all levels of the Communist Youth League have significantly enhanced their awareness and effectiveness of putting a clear stand on politics, and the leadership and party building of the Communist Youth League organizations at all levels have been strengthened. 1. Always adhere to the overall leadership of the party, consciously achieve two maintenances, strictly abide by political discipline and political rules, continuously enhance the four consciousnesses, strengthen the four self-confidences, and achieve two maintenances, so as to ensure that the party's centralized and unified leadership runs through various business tasks of the group. —2—1. Resolutely implement the spirit of General Secretary Xi Jinping’s important instructions and the decision-making and deployment of the Party Central Committee. The whole regiment regards the implementation of the spirit of General Secretary Xi Jinping's important instructions and the decision-making and deployment of the Party Central Committee as the primary standard of politics. in the end. The organs directly under the Central Committee of the Communist Youth League have focused on a series of important instructions issued by General Secretary Xi Jinping on the work of youth and the Communist Youth League since the 18th National Congress of the Communist Party of China, improved the supervision and supervision mechanism, strengthened account management and process supervision, and established an evaluation mechanism for the handling of important matters to ensure that The requirements of the Party Central Committee have been implemented. The Guizhou Provincial Committee of the League regards studying, publicizing and implementing the spirit of General Secretary Xi Jinping's inspection of Guizhou's important speech as the primary political task and long-term strategic task, formulating a breakdown table of key tasks, improving the ledger of General Secretary Xi Jinping's important instructions, regular scheduling, and closed-loop management. The Hebei Provincial Committee of the Communist Youth League set up an inspection office, which included the spirit of General Secretary Xi Jinping's important instructions and the implementation of important requirements of the Party Central Committee into the supervision items. According to the specific deployment of the Provincial Party Committee and the Central Committee of the Communist Youth League, it adopted methods such as timely sub-handling, in-process supervision, and post-event reporting. , forming a complete supervision chain. 2. Solidly promote the inspection and rectification work of party committees at all levels to the group organizations. The leading organs and leading groups of regiments at all levels take it as a major political task to cooperate with inspections and inspections, and to carry out inspections, inspections, and rectifications as a major political task. The organs directly under the Central Committee of the Communist Youth League adhere to the idea of ​​unifying the solution of individual problems and the overall problem, and of treating the symptoms and the root causes. In response to the inspection feedback problem, they deeply analyze the common problems and general laws that arise, and strive to make up for the shortcomings of the system and mechanism. Up to now, 65 rules and regulations have been revised or newly formulated, 8 working mechanisms have been established, and 45 related plans and programs have been formed. At the same time, strengthen the work guidance for the provincial Youth League committees, put forward clear work requirements for the common problems found in the inspections of the provincial Youth League committees in recent years, and promote accelerated improvement. The Hubei Provincial Committee of the Youth League fully implemented account management to do a good job in the rectification work, unified leadership, precise policy implementation, and accountability in place to achieve results. 3. Strictly implement the reporting system for major matters. The whole regiment earnestly implements the "Regulations on Requesting and Reporting on Major Matters of the Communist Party of China" and the requesting and reporting system for major matters within the regiment, and promptly seeks instructions from the party committee and higher-level regiment organizations on the implementation of the Party Central Committee's decision-making and deployment, the important tasks of the regiment and the problems discovered during the promotion process According to the report, the party's centralized and unified leadership has been more resolutely and effectively implemented within the regiment. The Chongqing Municipal Committee of the Communist Youth League insists on a list management, sorting out and summarizing matters that need to be reported to the Chongqing Municipal Committee and the Central Committee of the Communist Youth League, clarifying the time limit for submission, regularly dispatching the implementation status, and using the implementation status as a reference for annual assessment and evaluation, and for each district and county The Youth League Committee's request for instructions and reports will be regulated. The Henan Provincial Committee of the Communist Youth League formulated a list of issues for requesting instructions and reports based on the annual work priorities and important time nodes, clarified the scope of matters that the lower-level Youth League organizations asked for instructions and reports to the Provincial Committee of the Communist Youth League, strictly submitted the procedures and methods, and included the implementation of the requesting and reporting system at the end of the year assessment. 2. Continue to deepen theoretical arming, constantly strengthen ideological forging, pay close attention to the study of political theory within the group, and consciously use Xi Jinping's new era of socialist ideology with Chinese characteristics to arm the mind, guide practice, and promote work. The consciousness of the whole group to learn theory and strengthen politics has been significantly enhanced . 1. Taking Xi Jinping's thought of socialism with Chinese characteristics in the new era as the primary political task of the whole regiment's theoretical arm. Strengthening the cultivation of political theory is the cognition of regiment cadres—4—the ability to settle down and live. Taking the study of the Central Committee of the Communist Youth League as an example to drive the regiment cadres at all levels, especially the leaders of the leading organs of the regiment, to read the original work carefully and earnestly The principle of true enlightenment. The Shandong Provincial Committee of the Communist Youth League strictly implements the system of the first topic, builds the learning brand of the Starlight Forum, invites experts, scholars, grassroots cadres, and youth models to go to the podium of the Provincial Committee of the Communist Youth League, formulates a theoretical learning system for youth in institutions, and sets up youth study groups in institutions. The practice has become typical of the provincial government. 2. Solidly carry out the study and education of the Communist Youth League to learn party history, strengthen beliefs, and follow the party. In carrying out the study and education of party history, pay attention to education and guide the whole regiment to feel the glory and responsibility from the party history, summarize the rules, and get inspiration. Further polish the original intention of unswervingly following the party; pay attention to the organic combination of learning history and educating people, always adhere to the fundamental task of educating people for the party, and strive to guide young members of the league to draw strength from party history and be more conscious and firm to the party. The party organizations moved closer together, continuously sending fresh blood to the party. As of the beginning of December 2021, 99% of the more than 3.1 million grassroots Youth League branches have organized party history study and education. In November, a survey on the study and education of party history by the whole regiment showed that 80% of the respondents said that their desire to join the party had become stronger and their motives had become purer; voice of the heart. Combined with the study and education of party history, the Tianjin Municipal Committee of the Youth League carried out work such as the youth lecturer group, the Youth Federation lecture hall, the red scarf tour group, and the youth group cadres on the podium, covering more than 2.69 million young people. 3. Go deep into the grassroots and go deep into the youth to carry out theoretical lectures. Comrades from the Secretariat of the Central Committee of the Communist Youth League took the lead in demonstrating and preaching, promoting the institutionalization and mechanism of regiment cadres on the podium, and regiment cadres going deep into the grassroots to preach has become a standard configuration for all levels of the regiment. A virtuous circle of better preaching is taking shape. Around the study and education of party history, the study of General Secretary Xi Jinping's important speech on July 1, and the study and implementation of the spirit of the Sixth Plenary Session of the Nineteenth Central Committee of the Party, etc., cadres at all levels combined the work mechanism of closely contacting youth work and linking film and hanging points to go deep into the grassroots and carry out extensive Preach. Provinces such as Liaoning, Guangdong, and Hainan have issued corresponding system documents for the work of the Youth League cadres on the podium, clarifying specific task requirements, and combining the work of the Youth League cadres on the podium with cadre assessment and incentives. The Sichuan Provincial Committee of the Youth League included the youth lecturer group into the Sichuan Provincial Party Committee's publicity group, which led to the establishment of young lecturer groups at the city and county levels. The Fujian Provincial Committee of the Communist Youth League opened youth lecture halls in league organizations at all levels to form a regular exchange and study mechanism for league cadres, and carried out 3 youth sharing sessions and at least 1 party class study every week. 3. In-depth practice of strict standards, comprehensively improve the quality of party building, constantly strengthen the awareness of political construction of the leadership team, and earnestly fulfill the main responsibility of grasping party building. Leadership is effectively implemented throughout the regiment. 1. Earnestly fulfill the responsibility of grasping the main body of party building. In 2021, the Secretariat of the Youth League Central Committee will study 29 issues suggested by the Party, promote the introduction of 14 institutional norms, and comprehensively consolidate the responsibility system for Party building. The organs directly under the Central Committee of the Communist Youth League explored the establishment of a list of responsibilities for comprehensively and strictly governing the party, clarified the basic elements of party building work, effectively strengthened the implementation of dual responsibilities for one post, innovated the party building liaison group mechanism, and strengthened the party leadership system of directly affiliated units. Inspection team to achieve full coverage of internal inspections of directly affiliated agencies. The Shanghai Municipal Committee of the Communist Youth League takes the regular inspection of the party building responsibility system as an entry point, and establishes 3 aspects of organizational life, discipline and style, innovation and excellence, and 13 index systems for the Communist Youth League Committee, and carries out normal inspections and -6- evaluations. The results of the evaluation and timely interviews with party organization secretaries who were assessed as unqualified to consolidate the main responsibility. 2. Strictly implement the responsibility system for ideological work. The whole regiment thoroughly implements the overall national security concept, adheres to the responsibility of guarding the territory, and fulfills the responsibility of guarding the territory, strictly implements the responsibility system for ideological work, conducts risk research, judgment and investigation in the field of youth work on a regular basis, strengthens positive publicity and education and ideological struggle, and strengthens the news of the regiment. The guidance and management of the publishing unit, strict supervision of the league-affiliated media, the establishment of a comprehensive review mechanism, conscientiously do a good job in the management and control of relevant public opinion, comprehensively find out and eliminate the blind spots of ideological work risks and the shortcomings of the system and mechanism in the league, adhere to the bottom line thinking, prevent and resolve Major risks have become the consensus of the whole group. The Guangdong Provincial Committee of the Communist Youth League has issued a survey and implementation method on the ideological dynamics and development status of young people. It conducts regular surveys and monitors the ideological and development status of various youth groups. Since 2020, it has completed the first large-scale online survey of more than 500,000 young people in China. Leaders at or above the level gave more than 40 instructions. The leading group of the Jiangsu Provincial Committee of the Youth League regularly conducts ideological research at the grassroots level, grasps new situations and trends in a timely manner, strengthens supervision and inspection, and promotes the establishment and improvement of ideological work organizations in the Youth League committees of all districts and cities. 3. Standardize and implement the coordination and management functions of lower-level league organizations. Improve the communication mechanism between the higher-level league organizations and the party organizations that directly lead the lower-level league organizations on major issues such as youth work. Improve the mechanism for fulfilling the responsibilities of coordinating and managing the leading groups of the Youth League Committee at lower levels in accordance with relevant regulations. The Liaoning Provincial Committee of the Youth League regularly communicates with the local party committees about the allocation of cadres and the training of young talents, so as to strive for the attention and support of the local party committees, and cooperate with the organization departments of the local party committees to do a good job in the follow-up training of the winners of the provincial May Fourth Youth Medal. The Jilin Provincial Party Committee of the Jilin Province - 7 - Implemented a seven-step progressive method, through the establishment of checks, providing policy basis, focusing on job transfer negotiations, organizing selection and recruitment, establishing a letter-to-business mechanism, strengthening communication with relevant departments of the party committee, and incorporating party building evaluations, Grasp the key steps of coordination and management, and continue to promote the improvement of the deployment rate of regiment cadres. Before attending the democratic life meeting, the Hunan Provincial Committee of the Communist Youth League collected and sorted out the highlights and shortcomings of the work of the Communist Youth League in various cities and prefectures, and formed relevant guidance for the democratic life meetings of the city and prefecture Youth League committees, and urged rectification after the meeting. Since 2020, the participation in the democratic life meeting will be realized Full coverage. Shake Distribution: General Office of the Central Committee of the Communist Party of China, General Office of the State Council. Secretary of the Secretariat of the Central Committee of the Communist Youth League, relevant ministries and commissions of the Central Committee, and provincial party committees in charge of leadership. The heads of the various departments and directly affiliated units of the Central Committee of the Youth League, and the secretary of the Provincial Youth League Committee. Shake The General Office of the Central Committee of the Communist Youth League Issued on January 11, 2022—8—

2021-12-2 30 Editor's note: In November 2019, General Secretary Xi Jinping sent an affectionate message to the 30th anniversary of the implementation of the Hope Project, requesting efforts to provide young people with new assistance and sow new hope. Over the past two years, the China Youth Development Foundation has studied and understood the spirit of the message, led local youth foundations to focus on the main business of the mission, anchored the goal of aiding students and educating people, continued to deepen reform and innovation, and strived to promote the high-quality development of the Hope Project in the new era. The relevant situation is now compiled and distributed for reference by all regions. —1—The main responsibilities of the focus group are anchoring the goal of aiding students and educating people to promote the high-quality development of the Hope Project in the new era. follow. In the past two years, the National Youth Foundation has systematically studied, publicized and implemented the spirit of the message, conscientiously implemented the deployment of the whole group, based on the new era, new mission, and new requirements, promoted work innovation with a strong sense of responsibility and mission, continued to deepen reforms, and strived to implement the spirit of the message Put it into practice, solidly provide young people with new support, sow new hope, and promote the high-quality development of the Hope Project in the new era. 1. Seriously study and understand the spirit of the message, and earnestly plan the blueprint for the development of the Hope Project in the new era 1. Improve your standing, pay close attention to learning, and deeply understand the essence of the spirit. The China Youth Development Foundation regards studying and implementing the spirit of the message as the primary political task. In accordance with the requirements of the Secretariat of the Youth League Central Committee, it continues to study and understand the important instructions and requirements of General Secretary Xi Jinping on the Hope Project, and accurately grasp the spiritual essence and practical requirements contained in them. Promoting the direction and fundamental follow-up of the Hope Project in the new era. According to the deployment of the whole group, the youth foundation associations in various places held study seminars immediately, focusing on the major issues such as what the youth foundation associations should do and how to do in the overall structure of the party's youth cause. At the same time, in-depth Rural areas, schools, and enterprises carried out lectures and research, broadly gathered social consensus, and actively promoted the transformation and upgrading of the Hope Project. Local party committees attach great importance to it, thoroughly study and implement the spirit of the message, and vigorously support the innovative development of the Hope Project in the new era. Provinces such as Jiangxi, Fujian, Henan, Guangdong, Heilongjiang, Yunnan, and Tibet successively held —2—meetings of the Standing Committee of the Party Committee to carry out study. Party committee leaders in Hebei, Shanxi, Hubei, Hunan, Shaanxi, Jilin, Qinghai, Tianjin and other provinces have issued instructions and put forward clear requirements for the completion of Project Hope. 2. Based on the overall situation, carefully plan, and formulate career plans from a high starting point. Always focus on the greatness of the country, closely follow the country 14The five metallurgical enterprises plan strategic goals and actively plan the Hope Project in the new era. One is to strengthen the top-level design in accordance with the requirements of the "National One Game of Chess". Implement the "Several Opinions of the Central Committee of the Communist Youth League on Vigorously Promoting the Development of the Project of Hope in the New Era", establish the Steering Committee for the System Planning of the Youth Foundation of the Communist Youth League, issue the "Guidelines for the Project of Hope in the New Era (2020-2025)", compile the "China Youth Development Fund Five-year Development Plan (2021-2025) of the Council. The second is to implement measures in light of actual conditions. Beijing, Hebei, Inner Mongolia, Sichuan, Guizhou, Xinjiang, Yunnan and other places issued provincial-level implementation plans, and actively promoted the inclusion of popular support projects by local governments to do practical things for the masses. Guangxi launched the Hope Project funding system of public welfare funding + government support + volunteer participation, and Guizhou proposed to implement an upgraded version of the Hope Project to help rural teachers and students improve their quality, which were written into the provincial 14th Five-Year Plan. The third is to promote the further optimization of the Hope Project system. A total of 14,511 hope huts have been built in Shandong, Hunan, Heilongjiang and other places to help children from families with poor living conditions realize their transformation from “hut rejuvenation” to “spiritual rejuvenation”. Hubei connects the Hope Project with the province's 14th Five-Year Plan, planning and launching a series of public welfare programs such as Hope with Feiye, Hope with Escort, and Hope with Homeland. 3. Strengthen integration, unity of knowledge and action, and incorporate the spirit of the message into the party building work. In the past two years, youth foundations at all levels have incorporated the spirit of learning messages as a key content in carrying out the theme education of "never forget your original intention and keep in mind your mission" and the study and education of party-3-history. The Theoretical Learning Center Group of the Party Committee of the China Youth Development Foundation has arranged several topics related to the spirit of learning the message, and strives to achieve a comprehensive understanding; in this year's party history study and education, the party members and cadres are organized to take the lead in pairing with the needy teenagers, and the ideological achievements of learning the spirit of the message Transform it into practical actions for young people to do practical things. The Anhui and Ningxia Youth Foundations compiled the spirit of the message into the political theory study manual of their units, and organized party members and cadres to continuously deepen their understanding by relying on offline and online learning activities such as "Morning Reading 90 Minutes" and "Youth School". The Heilongjiang Hope Project Party Branch has formed a youth commando team, which has been on duty at checkpoints in key communities for epidemic prevention and control since February 2020, and strives to practice and test the effectiveness of learning the spirit of the message in epidemic prevention and control. The Shanghai Youth Development Foundation set up a special course on party history for teachers who went to Shanghai to participate in the hope primary school teacher training class, and held the exhibition of the great man Mao Zedong, General Secretary Xi Jinping's entry into the Red Army primary school exhibition, Shanghai Hope Project Exhibition and other themed exhibitions to guide young people to learn more about The Chinese revolution, construction, reform and opening up, and the knowledge of the new era consciously inherit the red gene and continue the red blood. 2. Focus on the overall situation of the center and reform with determination to promote the high-quality development of the Hope Project in the new era. As of the end of 2020, the actual funding of 100,000+ yuan for academic studies has raised 638 million yuan, subsidized more than 246,000 students from impoverished families who have established files and registered cards, and turned the work deployment of the 18th regiment on three 100,000+ yuan into Visible and tangible political results. In February 2021, the China Youth Development Foundation won the honorary title of "National Advanced Collective for Poverty Alleviation". In the face of the sudden outbreak of new crown pneumonia, the National Youth Development Foundation system immediately launched the fight against the epidemic-4-epidemic, hoping to go together-the special action of the Hope Project, raising more than 581 million yuan in funds and 2.82 million in materials from all walks of life We have made positive contributions in supporting young volunteers, caring for young people in poverty due to the epidemic, and children of medical staff. The Ningxia Youth Foundation will start from the three aspects of homing enthusiasm, head-geese effect, and geese vitality to help young people returning to the countryside to start businesses, develop industries, and be able to find employment. Based on the goal of building a high-quality demonstration zone for common prosperity, Zhejiang Youth Development Foundation launched the five major plans of "Joining Wealth Together" to help students improve together, help the poor and accompany them, help doctors and protect them, help businesses set sail together, and help emergencies and grow together. 2. Focusing on three strengths and one degree, the field of work that takes responsibility has been newly expanded. Focusing on the main responsibilities and main businesses of the Communist Youth League, we will actively explore new ways for the Hope Project to play political and social functions in the new era, and continue to empower the grassroots construction of the Communist Youth League. Guizhou Youth Foundation will create a 12-year consistent system of the New Era Hope Experimental School, as the practice carrier of the upgraded version of the Guizhou Hope Project, and set up funds to implement the three plans of Xingxingye, Zhuangmiaoye, and Fertile Soilye, to promote the rural teachers in Guizhou, The comprehensive development of young people and the high-quality development of rural education. Chongqing Youth Development Foundation has innovatively implemented the health service of "teenagers' spiritual growth" and established four support systems of "platform hub, theoretical support, hardware foundation, and service support" to prevent, discover, and solve adolescent mental health problems in a timely manner. Focusing on the main line of building the awareness of the Chinese nation community, the Tibet Youth Development Foundation planned and implemented the Congenital Heart Disease Screening and Treatment Youth Dream Action and the Heart-Moisturizing Bacon Rescue Project, organized Tibetan children to go to the mainland for treatment, and participated in hands-on training with mainland teenagers. Hand in hand activities. The Youth Foundation of the Xinjiang Production and Construction Corps explored the construction of a Chinese culture experience hall, organized military and local youths to carry out the practice of "hand in hand and heart to party", and the Corps and more than 70,000 elementary and middle school students in the Mainland carried out online and offline communication activities on a regular basis. 3. Innovate the mobilization mechanism, achieve new breakthroughs in digital transformation and social mobilization models. The National Youth Foundation systematically implements the requirements of the Secretariat of the Youth League Central Committee, actively promotes the in-depth integration and integration of organizational mobilization and social mobilization, and extensively mobilizes the public, especially youth members of the Youth League, to participate in the Hope Project. During the fight against the new crown epidemic in 2020, relying on third-party online fundraising platforms and provincial-level online crowdfunding platforms such as Pro-Qing Fundraising, Jiqing Fundraising, Tuantuan Fundraising, Yunqing Fundraising, and Su Qingyi Fundraising, etc., mobilized more than one million person-times The amount of participation and donation exceeded 100 million yuan, and small donations from the public accounted for 54% of the total donations. The political function of practicing and educating people is realized in fundraising. In November 2021, the China Youth Development Foundation Pro-Youth Public Welfare Platform officially became one of the third batch of Internet public fundraising information platforms designated by the Ministry of Civil Affairs, and the National Youth Development Foundation system initially established its own information platform. In 2020, 224 projects will be launched on the digital platform of Zhejiang Youth Development Foundation, and more than 250,000 people will participate in donations, raising funds of 14.35 million yuan. The Yunnan Youth Foundation launched a mobile phone for public welfare——Yunqingchouye online crowdfunding platform. In the past two years, 156 crowdfunding projects have been released, with a total fundraising of more than 7.62 million yuan. Jiangxi Youth Foundation’s one-yuan donation project attracted 364,000 people to donate within five months, raising a total of 1.317 million yuan. Relying on the provincial-level digital government service platform for people's livelihood, Guangdong Youth Development Foundation innovates and promotes public welfare projects, and creates a large database of Hope Project research services. The Xinjiang Youth Foundation has built a digital platform centered on student funding, and carried out a series of crowdfunding projects in line with the healthy growth of Xinjiang teenagers, such as the Hope Peer Rescue and Care Program, the Communist Youth League Love Birthday Party, the Communist Youth League Love Tickets, and the Communist Youth League Love Football. 4. Promote transformation and upgrading, and hope that the project implementation organization will make new progress in its own construction. —6—Since 2019, the National Youth Development Foundation has followed the requirements of General Secretary Xi Jinping’s message, showing the spirit of self-revolution, insisting on problem orientation, and restricting the development of its own organization and personnel system, project management, brand building, process management, etc. With regard to the problem of the problem, drastic reforms have been made, and the overall appearance of the team has undergone significant changes. Shaanxi Youth Foundation initiated the Project Hope Public Welfare Research Institute, and built a large public welfare system consisting of public welfare research institute + public welfare competition + Qin Qingchou + public welfare micro-market + three-level database + five major plans of Hope Project, effectively solving the problems of project discovery, Landing, financing, supervision and other six difficult problems. With the goal of improving implementation performance, the Ningxia Youth Foundation has formulated work evaluation guidelines for the Hope Project and built a closed-loop management system from planning and design, organization and implementation to effect evaluation. The Hainan Youth Development Foundation has introduced a series of reform measures, focusing on solving the problem of aging cadres, deepening and upgrading the service platforms of the Hope Project at the city and county levels, and expanding the source of work forces in various forms. The Guangxi Youth Foundation has included Project Hope work into the annual work evaluation indicators of the Guangxi Communist Youth League, and included it in the key core tasks of performance evaluation. 3. Adhere to the correct direction of construction and fully integrate into the party's youth work system 1. Take the initiative to shoulder the important responsibility of doing youth work for the party. Adhere to the organic unity of leading ideas and providing services, strengthen ideological education in project design, guide young people to continuously strengthen their recognition of the party's leadership and the socialist system with Chinese characteristics, and promote the transformation of public welfare achievements into political achievements. Tianjin Youth Development Foundation implements the Jinlong Youth Accompanying Growth Program, carrying out a little love, writing a letter, making a video call, sending a gift, and making an appointment to visit five special activities. In the past two years, a total of 3,400 people have been organized - 7 - Tianjin teenagers participated in accompanying the growth and helped 2,050 poor students in Gansu Province. The Jiangsu Youth Development Foundation and relevant units established the Hope Project Alliance to promote the youth care project for troubled youths, raised 120 million yuan in two years, built more than 10,000 dream houses, and provided heart-warming services for de facto orphans . The Qinghai Youth Foundation launched the "Wings Plan" student aid project, focusing on solving the practical difficulties of secondary vocational school students, sending warmth to a large number of students from families with financial difficulties, and realizing the full coverage of Qinghai Hope Project aid for students at all stages. The Fujian Youth Development Foundation has set up the "Swan Goose Plan" for the growth of young talents to provide scholarships for outstanding students. With family and nostalgia as the link, it will guide and serve the "Double First-Class" from outside the province. It will build outstanding college students from Fujian to return to Fujian for employment and entrepreneurship. 2. Efforts should be made to find the correct position and play a unique role in the work structure of the Communist Youth League. Continue to play its role as a regular working force and an important support carrier in the field of youth development, concentrate working energy and resources on work that is closely related to the main responsibilities and main businesses of the regiment, and find synergy for the various work areas and functional departments of the regiment to play their roles point. The Beijing Youth Foundation uses the Hope Project Workstation as a carrier to focus on the growth concerns of young people, actively connect service projects, strengthen cooperation with youth federations, industry associations, and chambers of commerce, form complementary advantages, share experience, and encourage grassroots organizations to incubate third-party social organizations , to form a joint force of all walks of life to jointly implement the Hope Project. Henan Youth Development Foundation implements the Hope Primary School One School One Project aid construction system, seeks the support of the Provincial Department of Education, launches the Hope Primary School Demonstration School Construction Plan, carries out teacher training, demonstration course evaluation, and on-site demonstration teaching for Hope Primary Schools, and strives to improve the Hope Primary School. overall teaching level. The Shanxi Youth Development Foundation takes the children of poor families and left-behind children who have registered in rural areas as the main target, and provides targeted public welfare research services—8—Shanxi Red Scarf Hope Journey, to help rural students increase their knowledge and inspire their growth and success. Endogenous motivation. The Liaoning Youth Foundation, together with the Provincial Federation of Students and the Youth Working Committee, carried out the Youth Calligraphy and Painting Public Welfare Contest based on the spirit of General Secretary Xi Jinping's message, and the series of activities of calligraphy and painting appraisal + public welfare auction + 1 yuan for public welfare was the starting point, attracting more than 3,000 people from the whole province Teens get involved. 3. Adhere to the strategic positioning of building a scientific work system and cultivating a high-quality talent team. The China Youth Development Foundation initiated institutional reforms, restructured the organizational structure, streamlined the setting of departments and coordination agencies, and made the functional positioning of each department more professional and scientific. Continue to increase personnel training in key tasks and key positions, and strive to build a professional public welfare team that serves the main responsibilities of the Communist Youth League. Jilin Youth Foundation is committed to promoting the integrated development of fundraising, funding, service, and guidance. It cooperates with professional social work organizations to build a working model of "foundation assistance + social work case counseling" to effectively enhance the competitiveness of professional public welfare. Jiangxi Youth Foundation has adjusted its internal departments to adapt to the new situation, reformed the assessment and selection system, selected a group of outstanding professionals from non-profit charitable organizations to join the leadership team, and improved the organizational and combat effectiveness of the team. The Gansu Youth Foundation has included Project Hope talent training into the cadre education and training system of the whole regiment, focusing on selecting outstanding talents from youth work agencies and public welfare organizations, and exploring the construction of a high-quality professional public welfare talent team. 4. Continuously strengthen the awareness of the bottom line and effectively strengthen risk prevention and control. Adhere to the most stringent business standards and moral standards to restrain ourselves, earnestly grasp political and ideological risks, public opinion crisis risks, financial capital risks and other twelve major risk points, constantly improve the risk prevention mechanism and internal control mechanism, and conscientiously implement the risk management system. Prevention and control responsibility - 9 - responsibility. The Inner Mongolia Youth Development Foundation has established an overall planning and linkage mechanism to standardize and strengthen the management functions of levels, regions, and fields within the group, organically combine project configuration, implementation, and resource use, and unify them into the Inner Mongolia Hope Project brand system, and explore the implementation of Hope Project + new model of . Through measures such as improving rules and regulations, optimizing program standards, and standardizing work documents, the Railway Donation Center has built a standardized system with clear politics, clear concepts, standardized management, service standards, extensive brand influence, good effect evaluation, and strong supervision and guarantee. —01—(No text on this page)—11—Shake Distribution: General Office of the Central Committee of the Communist Party of China, General Office of the State Council. Secretary of the Secretariat of the Central Committee of the Communist Youth League, relevant ministries and commissions of the Central Committee, and provincial party committees in charge of leadership. The heads of the various departments and directly affiliated units of the Central Committee of the Youth League, and the secretary of the Provincial Youth League Committee. Shake Issued by the General Office of the Central Committee of the Communist Youth League on December 28, 2021

2021-12-2 29 Editor's note: Doing a good job in the work of emerging youth groups is an important political task assigned by General Secretary Xi Jinping and the Party Central Committee to the Communist Youth League. In recent years, the Beijing Municipal Committee of the Communist Youth League has insisted on researching first to find out the scale, type and characteristics of the capital's emerging youth groups, actively report to the party committee, actively seek support from various departments, and carry out work around contact coverage, leading ideas, serving growth, mobilizing meritorious service, etc. , forming a new mechanism and constructing a new pattern. Its experiences and practices are now compiled and distributed for reference by other regions. —1—Six Persistences of the Youth League Beijing Municipal Committee Promote the construction of a new pattern of work for emerging youth groups The scale, type and characteristics of emerging youth groups in the capital, actively report to the party committee,Actively strive for the support of various departments, and work together to promote the formation of a new pattern of work for emerging youth groups. 1. Adhere to research first and deeply analyze the characteristics of emerging youth Since 2020, the Beijing Municipal Committee of the Communist Youth League has conducted research on Beijing emerging youth aged 16-35 in the city, and formed the "Research Report on the Situation of Beijing Emerging Youth Groups", which calculated the Beijing emerging youth group. The base number of youth groups is between 1.8 million and 2.2 million people. On this basis, the preliminary scope is defined and divided into three types: Internet culture youth, independent literature and art youth, and new life format youth. Moreover, it summarizes the characteristics of the emerging youth group, such as the expansion of the number of young people, flexible employment forms, loose organizational dependence, diverse group needs, and social self-identity. Due to the lack of standardized supervision and other difficulties, the analysis shows that this group has five new influences on the economic and social development of the capital, namely, forming a new driving force for the capital's economic development, stimulating the new vitality of the capital's innovative development, meeting the new needs of citizens for a better life, and conserving the capital's culture. The new ecology of the capital has become a new subject of social governance in the capital. At the same time, it cooperated with more than 20 departments and units directly under the municipal government to gain an in-depth understanding of policies involving emerging youth groups, listen to relevant opinions and suggestions from all parties, and lay a good foundation for further strengthening the work of emerging youth groups. -2-2. Adhere to the leadership of the party committee and strive to be included in the overall layout of the party building. Take the lead in formulating "Several Measures on Strengthening the Work of Emerging Youth Groups in Beijing" (hereinafter referred to as the "Measures"), and proposed five aspects: strengthening top-level design, deepening ideological and political leadership, strengthening contact coverage, serving growth and development, and playing a positive role. 22 specific measures. The main leaders of the municipal party committee attached great importance to it, and instructed that the party building work of emerging youth groups be included in the pilot work system of party building work for new business forms and new employment groups, and educate and guide the majority of emerging youth groups to listen to and follow the party. The municipal party committee decided to include the "Measures" in the overall work arrangement of party building by the Office of the Municipal Party Committee's Party Construction Work Leading Group to issue the "Measures" to the party groups (party committees) of various departments (units) and district committees, which attracted the attention of party organizations at all levels and formed a general consensus. Various departments (units) launched policy measures around the research of emerging youth groups, and all district committees held district-level youth joint meetings to study and dispatch emerging youth groups. 3. Adhere to departmental coordination and promote a clear division of responsibilities. It is not the responsibility of the Communist Youth League to do a good job in the work of emerging youth groups. The Beijing Municipal Committee of the Communist Youth League adheres to the system concept, promotes the establishment of the idea of ​​a game of chess for the work of emerging youth groups, gives full play to the role of the youth work joint meeting mechanism, and actively cooperates with the Organization Department of the Municipal Party Committee, the Propaganda Department of the Municipal Party Committee, and the Municipal Public Security Bureau in the process of leading the formulation of the "Measures" ( Units), combine their respective responsibilities and work characteristics, conduct in-depth research on the career development, social security, life services and other issues of emerging youth groups in different regions and industries, and actively form corresponding measures, so as to explore the formation of party organizations and industry management departments Take the lead - 3 - A new work pattern in which the head office, all relevant departments and territories work together to manage together, and industry associations and platform companies participate together. The "Measures" focus on practicality and strong practicality. For example, the establishment of a new mechanism for handling complaints immediately, in conjunction with the Municipal Affairs Service Bureau, aimed at the concentrated appeals of emerging youth groups involved in the 12345 citizen hotline, and improved the working mechanism of handling complaints immediately and taking the initiative to govern. Another example is to launch new measures to promote development, and to launch a series of activities in conjunction with the Municipal Party Committee Propaganda Department, the Municipal Party Committee Cyberspace Affairs Commission, and the Municipal Science and Technology Commission. Key industries and important areas of economic and social development will play an active role. 4. Adhere to organizational coverage and expand work influence Organizational construction In order to further enhance the service for emerging youth groups and broaden the circle of friends of emerging youth groups, the Beijing Youth League Committee, the Municipal Party Committee Propaganda Department, the Cultural Resources Center, and more than 100 emerging youth groups in the city More concentrated cultural and creative parks and Internet companies have established working contacts, and piloted the construction of 15 dream-building space activities to serve emerging youth groups on a regular basis. In terms of network cultural youth, continue to expand the coverage of social organizations, cooperate with the Municipal Party Committee United Front Work Department, the Municipal Party Committee Internet Information Office, the Municipal Federation of Literary and Art Circles, the Municipal Writers Association and other departments (units), strengthen ties with emerging youth social organizations and communities, and cooperate with the municipal party committee social workers The Committee, the Civil Affairs Bureau and other departments actively promote the construction of social organizations affiliated with emerging youth groups in the fields of youth literature and new media. In terms of independent literary and artistic youth, guide the establishment of Beijing Design Youth Forum, hold the Youth Zhiye Biennale, and create a communication and display platform for them through project support, employment practice, holding salon competitions, etc., to promote independent literary and artistic youth in the lives of the masses , urban construction, and rural revitalization to play a greater role. In terms of young people in the new business format of life, organize working forces to go deep into places where delivery couriers are concentrated, —4—add WeChat and group chats with them, promote the establishment of 215 mobile league branches, and focus on promoting the completion of the establishment of leading companies in the express delivery industry headquartered in Beijing. Work with youth groups to practically expand the organizational coverage of emerging youth groups. 5. Adhere to service guidance, promote the growth and development of emerging youth, focus on career improvement, carry out the "New Youth School Rider Banye" project, and provide pre-examination counseling and scholarships for high-level and professional-level upgrades for takeaway boys who need to improve their academic qualifications. Invite deputies to the Municipal People's Congress, members of the Chinese People's Political Consultative Conference and emerging youth representatives to carry out face-to-face activities to understand the urgent, difficult and anxious issues in the work, life, growth and development of emerging youth, and call on all sectors of society to pay attention to emerging youth groups through suggestions and proposals. Lianhe Municipal Bureau of Human Resources and Social Security and Municipal Bureau of Justice provided more than 2,000 person-times of services in career development, social security, and legal consultation for emerging youth. The Human Resources and Social Security Bureau of Lianhe City further clarified the main responsibility of platform companies to protect labor rights and interests, standardized the employment management of new employment groups such as couriers, food delivery workers, and online car drivers, improved legal aid and dispute mediation mechanisms, and safeguarded the rights and interests of new employment forms of laborers. The Lianhe City Federation of Trade Unions and the Municipal Women's Federation have established more than 700 heart-warming post stations and Xiaoge gas stations in the city to provide voluntary services such as medical examinations, free clinics, and haircuts. Young practitioners address practical difficulties and needs in working life. 6. Adhere to play a role and help the overall construction and development of the capital The Beijing Municipal Committee actively plays the role of role models among emerging youth groups, does a good job in the selection of typical trees, organizes the takeaway boys and new literary youths of Beijing Youth Model Yezhong, and goes deep into emerging youth groups Gathering places and cultural and creative parks to carry out lectures. Make good use of the media matrix of the group, by recording everyone's great speeches, telling stories about the century-old party history, -5-upward youths, little brothers talking, and carrying out work such as small post big heroes video collection, telling emerging youth Positive energy stories, guiding them to love the Party, the country, and work hard. Jointly with the Propaganda Department of the Municipal Party Committee and the Municipal Federation of Literary and Art Circles to jointly hold the 5th China Internet Literature+Conference -- Youth Literature Talent Development Symposium, focusing on the high-quality creation of Internet literature, exploring and serving the growth of young Internet writers in the new era. Jointly with the Capital Greening Committee, the Municipal Urban Management Committee, the Municipal Public Security Traffic Management Bureau and other departments (units), around themes such as grassroots governance, community environment improvement, domestic waste classification, and block renovation, organize emerging youth groups to carry out professional creative design, providing high-quality services for the capital. Development injects youthful vitality. —6—(No text on this page)—7—Shake Distribution: General Office of the Central Committee of the Communist Party of China, General Office of the State Council. Secretary of the Secretariat of the Central Committee of the Communist Youth League, relevant ministries and commissions of the Central Committee, and provincial party committees in charge of leadership. The heads of the various departments and directly affiliated units of the Central Committee of the Youth League, and the secretary of the Provincial Youth League Committee. Shake Issued by the General Office of the Central Committee of the Communist Youth League on December 27, 2021

2021-12-2 28 Editor's note: Housing problems such as expensive housing and difficulty in renting are currently obstacles that restrict the high-quality development of young people. Relying on the youth development planning joint meeting mechanism, the Guangdong Provincial Committee of the Communist Party of China, in conjunction with relevant provincial departments, launched the youth housing plan and the housing security service action for fresh graduates of colleges and universities. From the two-way efforts of policy advocacy and practical projects, we found a good way to win praise from young people way. It can be seen that it is indeed difficult to solve the youth housing problem, but judging from the exploration of the Guangdong Provincial Committee of the Communist Youth League, the Communist Youth League is not impossible. Its experiences and practices are now compiled and distributed for reference by other regions. —1—The Guangdong Provincial Committee of the League explored the implementation of the youth housing plan and worked hard to solve the housing problem of college graduates. In view of the youth housing problem, the Guangdong Provincial Committee of the League and the relevant departments directly under the provincial government faced up to the difficulties and boldly explored, and launched the youth housing plan for college graduates. Graduate housing guarantee service action, and included it in the first batch of "Guangdong Youth People's Livelihood Practical Projects", centering on the positioning of insisting that houses are for living, not for speculation, in accordance with physical security + currency subsidies + social supplements + information Based on the idea of ​​modernization construction + industry standardization, promoting public rental housing and talent housing policies, launching preferential housing, youth post stations and other constructions, it really helps young people solve their worries and troubles. 1. Explore the path from policy advocacy, highlighting the youth people's livelihood attributes of housing. According to the spirit of the relevant documents of the State Council on strengthening the development of affordable rental housing, the Guangdong Provincial Committee, in conjunction with relevant provincial departments, launched a youth housing plan to guarantee housing for fresh graduates of colleges and universities. Service action, clearly stated that by 2022, the proportion of new supply of indemnificatory rental housing in cities in the Pearl River Delta region will be no less than 20% for fresh college graduates, and no less than 10% in non-Pearl River Delta cities. Target. In the name of the Youth Development Planning Joint Conference, coordinate the strength of relevant units in the province and cities, and seek housing benefits for fresh graduates of colleges and universities through multiple channels through the security method of currency + material. Encourage local governments to focus on building public rental housing and talent housing, giving priority to Fresh graduates of colleges and universities should allocate rent, and support localities to reduce or exempt college graduates from applying for public rental housing to purchase social insurance period conditions, etc., and provide a policy basis for solving the housing problem of college graduates. -2-2. Strengthen the coordination of information channels and break the island effect of information in the market Aiming at information island problems such as information asymmetry between supply and demand of housing sources and inadequate policy publicity, the Guangdong Provincial Party Committee and the Provincial Department of Housing and Urban-Rural Development rely on 12355 Voice of Youth The platform builds an information query system for youth housing, sets up housing information, policy interpretation, youth post, affordable rental housing and other sections, provides youth with the latest housing policies and information, and displays the existing public rental housing and talent housing that can be applied for , Youth Inn, and other types of low-income housing related housing information, providing instant free consultation services. So far, 18 cities including Guangzhou, Shenzhen, and Foshan have been mobilized and guided to launch 8,700 sets of public rental housing and talent housing for fresh college graduates to apply for in the youth housing information query system. Fresh college graduates can query housing information through the system45 million times. 3. Integrate resources from the perspective of housing supply and create an innovative model of security and subsidies. To solve problems such as housing difficulties, job hunting, and integration difficulties for young talents, the Guangdong Provincial Committee of the Youth League guides the Youth League committees of various cities to closely focus on physical security and rental subsidies. Explore and integrate social resources to build a youth station to provide up to 7 days of free accommodation for job-seeking college graduates, and to launch one-stop services such as job recommendation, employment counseling, workplace ability improvement, and policy consultation. Shenzhen, Zhuhai, Dongguan, Huizhou and other places have built 28 public welfare youth post stations, with 888 beds, serving more than 30,000 people in total. Yunfu City has launched 62 sets of housing for young people who can move in with their bags and have preferential rents for fresh college graduates. Those who meet certain conditions can also enjoy a rent-free policy. Up to now, 2,456 fresh college graduates in the province have solved housing problems through a combination of "physical security" and "rental subsidies". —3—4. Explore and innovate from the market mechanism, and mobilize the enthusiasm of multiple social subjects. The Guangdong Provincial Party Committee and the Housing Leasing Association mobilized housing leasing companies to launch preferential socialized leasing housing activities for college graduates, covering Guangzhou, Foshan, etc. In many cities, fresh graduates of colleges and universities can enjoy special discounts, no-reason check-out, one-for-one deposit, instant discount on payment and other preferential policies for renting houses; in cooperation with China Construction Bank Guangdong Branch, they launched the Youth Housing Bank worry-free graduation season campaign, in During the graduation season, exclusive preferential services are provided for fresh graduates of colleges and universities, and the greatest discount in all channels helps fresh graduates of colleges and universities solve their housing problems. The Guangzhou Municipal Committee of the Communist Youth League and relevant social organizations carried out the public welfare activities of the 2021 college graduates' graduation season "Harbour Care Plan", and launched a great gift package for employment and housing. The Tuanfoshan Municipal Party Committee and relevant leasing companies carry out housing preferential activities for fresh graduates of colleges and universities. Up to now, the province has launched a total of 9,500 sets of preferential socialized rental housing for college graduates. 5. Concentrate efforts on gathering areas to provide precise services for youth development. The Guangdong Provincial Committee of the Communist Party of China and the leasing company will promote the establishment of youth homes, community volunteer service stations and other service positions in youth apartments, youth communities and other housing rental venues. Youths are sent to Jiaye to provide various services such as cultural learning, physical fitness, leisure and entertainment, marriage and friendship, voluntary public welfare, entrepreneurship and employment. At the same time, all kinds of youth volunteer teams such as garbage sorting are established in the community to create a community atmosphere where they can live and work in peace and contentment, and enhance the youth's sense of gain, happiness, and security. The Nanhai District Committee of Foshan City built a Nanhai Youth Home camp in Guicheng Street, a commercial and residential community where young people gather, including a psychological consultation room, a volunteer center in Nanhai District, a youth activity training room, a party and group activity room, a college student activity base, and a green bar, etc. There are 6 function rooms, more than 70 activities have been carried out, and more than 2100 youths have been served in 4 years. It not only promotes the construction of grassroots league organizations, but also creates a warm and harmonious activity place for the majority of young people. In the next step, the Guangdong Provincial Committee of the Youth League will continue to work hard to further solve the problem of housing for fresh graduates of colleges and universities on the basis of previous work and exploration. One is to advocate the introduction of more youth housing policies. Continue to play the role of the Youth Development Planning Joint Conference, promote the inclusion of the housing security policy for college graduates into the province's affordable rental housing policy, and explore the formation of institutional achievements that can be replicated and promoted. The second is to continue to face colleges and universitiesHousing for fresh graduates launched practical projects for people's livelihood. Cooperate with the Provincial Department of Housing and Urban-Rural Development to formulate the 14th Five-Year Plan for housing development and the three-year action plan for the development of affordable rental housing, increase the supply of affordable rental housing, explore the provision of long-term rental apartments for college graduates, and strictly regulate the socialized housing rental market . The third is to provide more precise services for youth gathering community apartments. Continue to promote the establishment of youth homes in housing rental places such as youth apartments and youth communities, increase the efforts of the two new organizations to build groups, improve the youth organization system led by the Communist Youth League, and improve education, health, marriage, employment, etc. Youth care Focus on various policies, encourage young people to actively participate in public welfare activities, and ensure and support the integration of young people into the city in many ways. —5—Shake Distribution: General Office of the Central Committee of the Communist Party of China, General Office of the State Council. Secretary of the Secretariat of the Central Committee of the Communist Youth League, relevant ministries and commissions of the Central Committee, and provincial party committees in charge of leadership. The heads of the various departments and directly affiliated units of the Central Committee of the Youth League, and the secretary of the Provincial Youth League Committee. Shake Issued by the General Office of the Central Committee of the Communist Youth League on December 27, 2021

2021-12-2 27 Editor's note: Giving full play to the active role of group organizations in grassroots social governance is an important part of the modernization of the national governance system and governance capabilities. In recent years, based on the special advantages of Comrade Lei Feng's hometown, the head of the Sha Municipal Party Committee has launched a special action for the participation of young volunteers in Lei Feng Xingye to participate in social governance. A new way to organize and mobilize youth to participate in social governance. Its experiences and practices are now compiled and distributed for reference by other regions. —1—Tuan Chang Sha Municipal Party Committee Exploring the Five Joint Smelting Mechanisms to Organize and Mobilize Youth to Participate in Social Governance Wangcheng District, Changsha City, Hunan Province is the hometown of Comrade Lei Feng. Changsha has a fine tradition and solid foundation of learning from Lei Feng in voluntary service. In recent years, under the leadership of the Changsha Municipal Party Committee and the Hunan Provincial Party Committee, the Tuanchangsha Municipal Committee has insisted on taking youth as the main force and volunteer service as the main carrier to implement the special action of Lei Feng Xingye Changsha Youth Volunteers Participating in Social Governance. Work on the five aspects of football, platform, project and culture to actively explore new ways to organize and mobilize young people to participate in social governance. 1. Serving the overall situation of the center, the joint metallurgical construction team follows the work requirements of organizing youth to participate in social governance put forward by the Changsha Municipal Party Committee, focusing on organizing the majority of young volunteers more effectively, insisting on multi-party linkage, deepening ability training, and striving to build a strong Excellent work team. 1. Gather public welfare forces and expand the circle of friends. On the one hand, orderly recruited 100 group members and 243 individual members to join the Municipal Youth Volunteer Federation, and extensively gathered working forces such as national moral models, public welfare leaders, caring enterprises and college students. On the other hand, relying on the Municipal Youth Social Organization Incubation Center, 31 youth social organizations were incubated, and the number of youth social organizations affiliated to the municipal and district levels was increased to 50, which further enriched the organizational form of youth participation in social governance. 2. Strengthen classification training to improve combat effectiveness. Enlist the support of the Organization Department of the Municipal Party Committee to implement the Changsha Youth Leifeng Volunteer "Little Pepper" training project, and promote the formation of a team with teamwork and step-by-step efforts. The first level is aimed at about 150,000 college freshmen—2nd—1st grades in the city, and mainly provides general education training for volunteers; the second level is aimed at the backbone of volunteer teams in various fields, and is divided into 11 phases, with a total of more than 1,000 people trained; The three levels are aimed at industry leaders, and a total of 4 special training courses have been held. 3. Give full play to organizational advantages and build a professional team. Focusing on key areas of social governance such as AIDS prevention, ecological environment protection, and mental health counseling, 11 municipal professional volunteer service teams have been established in conjunction with relevant professional forces. Among them, the Mental Health Volunteer Service Team composed of experts from the Second Xiangya Institute of Mental Health continues to implement the Evergreen Nursing Youth Mental Health Escort Program. Up to now, more than 100 special lectures have been carried out, which has exerted a wide influence on young people. 2. Adhere to multi-party planning, Unicom and gather resources On the basis of making full use of party and government resources and utilizing resources within the league, actively explore and leverage social resources to promote the Unicom and integration of resources from all parties. 1. Make full use of party and government resources, the Dinghaishenzhen needle. In the major activities oriented to social forces carried out by the Municipal Party Committee and the Municipal Government, actively strive for seats and build a stage for youth social organizations to play their roles. Plannedly recommend leaders in the field of voluntary service to become members of the Chinese People's Political Consultative Conference, creating more opportunities for them to offer advice and suggestions on social governance. Actively coordinate with civil affairs and other departments to further smooth the channels for youth social organizations to undertake government purchases of public services. In 2020, a total of more than 3.2 million yuan of funds will be promoted. 2. Use the powerful leverage of resources within the living group. Strengthen the classified construction of youth social organizations, and for qualified youth social organizations, specify that the Communist Youth League Committee shall be the competent department of business, issue corresponding management measures in a timely manner, clarify three types of partnerships, and introduce a series of specific working mechanisms such as debriefing, reporting, and auditing . Set up emerging youth and social organization sectors in the Municipal Youth Federation Technical Secondary School-3, and orderly expand the effective coverage and political leadership of young people in emerging fields. 3. Leverage the infinite treasure house of social resources. Aiming at the urgent needs of young people, accurately design work projects, actively and extensively integrate social resources, and provide more sufficient material guarantee for project implementation. For example, the Happy Decibel project was designed for hearing-impaired children, and Changsha Bank was coordinated to support 400,000 yuan; the Caring for Longshan Baby project was implemented, and more than 360,000 yuan of social funds and more than 550,000 books were raised; Crowdfunding for metallurgical projects, raising a total of more than 1.87 million yuan for the purchase of protective materials, and condolences to more than 600 medical staff. 3. Highlight the standard of effectiveness, build a platform through joint operation and build a platform to implement the concept of "not seeking everything, but for what you want" and build a platform for young people to participate in social governance in the Internet and real space through joint construction and joint operation. 1. Build an online flat information platform. Vigorously promote the Volunteer Huiye APP, develop the Changsha plate Ye, the number of registered volunteers increased from more than 50,000 to more than 220,000 in one year, 1,713 registered volunteer organizations, and recorded a total of 1,151,000 hours of voluntary service Hour. During the epidemic, a total of 33,758 people participated in anti-epidemic voluntary services through the Changsha section of the Volunteer Huiye APP. 2. Offline regionalization to build a physical platform. Relying on the Youth Social Organization Incubation Center, it serves more than 110 youth social organizations on a regular basis, and provides comprehensive services such as office, conference, road show, exhibition, and communication. Based on the community, we have extensively carried out the construction of grassroots positions. A total of 114 Lei Feng volunteer workstations and 289 youth homes have been built, which are organically embedded in the 15-minute urban residents’ life circle. Those who have difficulty finding volunteers provide physical support for the goal. 4. Orient the quasi-needs and jointly promote the project Focus on the combination of the work of the party and government center and the actual needs of young people, carefully design work projects, and cooperate with youth social organizations in related fields to jointly implement, forming complementary advantages. 1. Insist on inheriting and carrying forward the spirit of Lei Feng. Taking Lei Feng spirit as the spiritual core for Changsha youths to carry out voluntary services and participate in social governance, take the initiative to shoulder the glorious mission of building a highland of Lei Feng spirit, and successively carried out themed activities such as Learning from Lei Feng in Lei Feng's Hometown and Presenting Lei Feng with Thousands of Flowers to promote Changsha's good people, The selection and search activities of Lei Feng around you, Changsha Good Boys in the New Era, and Changsha Upward Good Youths have broadly gathered the social consensus and public opinion atmosphere of City of Good People. 2. Persist in expanding and polishing brand projects. Focusing on helping to win the battle against pollution prevention and control, organized the Changsha Blue Youth Public Welfare Volunteer Action for three consecutive years, integrated funds of more than 1 million yuan, attracted 62 youth social organizations to participate, and carried out pairing construction with 170 primary and secondary schools and communities, compiled and published "21 Weeks Handbook for Cultivating the Habit of Household Garbage Sorting, 50,000 copies were distributed free of charge to primary and middle school students. Jointly with youth social organizations to organize annual visits to the Little Squadron of Environmental Protection and the Youth of Environmental Protection. Through project-oriented work ideas, life-oriented content design, socialized resource integration, diversified participants and organizational mobilization, through Young people drive families, influence communities, and implement the concept of green development into the daily lives of citizens. 3. Insist on solid and detailed basic projects. Focusing on leveraging the leverage of financial support and project evaluation, 50 high-quality projects were selected based on the college student volunteer service project competition, and a Lei Feng list that dynamically reflected youth participation was set up on the Volunteer Huiye APP. The number of people, the number of likes and other dimensions are comprehensively evaluated, and the real-time data of the platform is used to follow up and guide the development of the project. The city's youths have carried out a total of 407 activities through basic high-quality projects, with 16,000 participants and a total service time of 37,000 hours. 5. Demonstrate the function of educating people and coherently cultivate the culture. Always adhere to the fundamental task of educating people for the party, pay attention to strengthening the ideological and political leadership of young people in the whole process of work, vigorously inherit the red gene, and actively cultivate new people of the era. 1. Unify the logo and brighten the image. The name of the city's young volunteers is unified and clearly defined as Changsha Youth Lei Feng Volunteers, and a unified identification system including logos, flags, clothing, etc. is designed and launched, and efforts are made to create a distinctive brand of young volunteers. In the process of carrying out the Changsha Lanye project, we designed and produced themed mascots such as Lvbaoye and Jiewaye to help shape the public welfare image of the project. 2. In-depth exchanges to gather consensus. Hold the Changsha Youth Learning from Lei Feng Volunteering Cultural Forum, persist in carrying out various symposiums, thought salons and other activities for a long time, and flexibly and extensively organize public welfare figures, experts and scholars in various fields to conduct discussions and exchanges on learning from Lei Feng's voluntary service topics, and share experience in volunteer service projects , Actively provide advice and suggestions for young Lei Feng volunteers in Changsha to participate in social governance 3. Spread culture to boost morale. Mobilize the power of emerging youth groups, create and launch a series of excellent cultural products that reflect the spirit of volunteer service, such as "Love is like a star——Song of Changsha Youth Lei Feng Volunteers", and vividly demonstrate the youthful demeanor of writing Lei Feng stories in the new era with practical actions. The city's young volunteers had a positive impact. —6—(No text on this page)—7—Shake Distribution: General Office of the Central Committee of the Communist Party of China, General Office of the State Council. Secretary of the Secretariat of the Central Committee of the Communist Youth League, relevant ministries and commissions of the Central Committee, and provincial party committees in charge of leadership. The heads of the various departments and directly affiliated units of the Central Committee of the Youth League, and the secretary of the Provincial Youth League Committee. Shake Issued by the General Office of the Central Committee of the Communist Youth League on December 24, 2021

2021-12-2 26 Editor's Note: Youth Federation organizations at all levels conscientiously implement the important requirements of General Secretary Xi Jinping on doing a good job in the work of young people who have returned from studying abroad, hold high the great banner of patriotism and socialism, and actively contact, serve and guide returned students studying abroad For young people, help them understand the national conditions and integrate into the society, lead them to enhance their patriotism and ambition to serve the country, and promote their better achievements and all-round development. Recently, the Central Committee of the Communist Youth League sorted out this work, and the relevant situation is now compiled and distributed for reference by all regions. —1—Youth federations at all levels work together to unite and unite young people who have returned from studying abroad Returned young people who have studied abroad are an important part of the youth talent team and an important focus of youth united front work. Youth federations at all levels conscientiously implement the important requirements of General Secretary Xi Jinping on doing a good job in the work of young people who have returned from studying abroad, accurately grasp the group characteristics and interest demands of young people who have returned from studying abroad, build a special work system, and solidly carry out contact with young people who have returned from studying abroad , service and guidance have achieved positive results. 1. Strengthen organizational liaison, broadly unite and gather talents, give full play to the organizational advantages of the Youth Federation and its member groups, strengthen the organization and absorption of the backbone of young people who have returned from studying abroad, and guide qualified provincial-level Youth Federations to establish youth organizations for returned overseas students to effectively contact A group of youth associations with certain influence and organizational power have established a talent pool for young people who have returned from studying abroad, and strive to broaden the channels for contacting young people who have returned from studying abroad. The China Association of Young Scientists and Technologists took the mid-term appointment as an opportunity to recruit and add 23 young people who had returned from studying abroad as members and directors, and the provincial and municipal youth federations and youth science and technology associations organized the selection and absorption of 1,355 outstanding young people who had returned from studying abroad. The Guangdong Provincial Youth Federation has established a talent pool for overseas students in the Guangdong-Hong Kong-Macao Greater Bay Area. Through internal recommendations, organizational recommendations, and targeted invitations, 500 outstanding young people who have returned from overseas studies have been recruited to become the first batch of talents in the pool. The Youth Federation of Chongqing City and Yunnan Province has increased communication and coordination with the organization department, human resources service agencies and other units, established a wide-caliber talent pool for young people who have returned from studying abroad, and carried out dynamic updates. 2. Highlight the orientation of educating people and deepen ideological and political leadership. Taking the opportunity of celebrating the 100th anniversary of the founding of the Communist Party of China, through flexible and diverse forms such as study meetings, report-2-meetings, seminars, and webcast classes, promote the Youth Federation's Youth Unity and Forever Follow the Party to learn and educate young people who have returned from studying abroad to broadly cover the study and education of Party history, helping them understand the four histories. The ambition to serve the country. The youth federations of Tianjin and Jilin Province organize youth returning from studying abroad to carry out activities such as raising the national flag and singing the national anthem together. Through activities such as raising the flag, visiting party spirit education bases, and paying homage to heroes and martyrs, they increase their emotional identity and rationality for the party and the socialist system. agree. The Youth Federation of Shaanxi Province carried out the Red Culture Tour in Yan'an for young people who returned from studying abroad, organized the young people who returned from studying abroad to pay homage to the Yan'an Revolutionary Holy Land, feel the red feelings, and listen to the descendants of revolutionary martyrs tell about the indomitable and hard-working revolutionary spirit and nobility of the older generation of proletarian revolutionaries sentiment, and deepen their sense of responsibility to contribute to society and serve the country. The Youth Federation of Chongqing City and Guizhou Province invites young people who have returned from studying abroad to enter the countryside and experience farming. They use lectures, visits, experiences, and studies to witness the achievements of poverty alleviation and rural revitalization, and actively help to draw a grand blueprint for rural revitalization. Tell Chinese stories well and spread Chinese voices. The Youth Federation of Tibet Autonomous Region organizes young people returning from studying abroad to visit the Million Serfs Memorial Hall and the Lhasa Urban Planning Exhibition Hall to experience the all-round and historic achievements in economic and social development since the peaceful liberation of Tibet 70 years ago, and to strengthen their love for the Party, the country, and socialism Confidence and determination. 3. Take the initiative to set up a stage and actively mobilize to make contributions to give full play to the potential of young people who have returned from overseas studyBased on the intellectual advantages of talents, centering on major national strategies and local economic and social development tasks, etc., adopt a multi-frequency, localized, and small-scale approach to extensively carry out roadshows, project docking, seminars and exchanges, etc. Better development paves the way. The China Association of Young Scientists and Technologists has organized the China Youth Chip Science and Technology Innovation Competition and established the Zhong Nanshan Youth Science and Technology Innovation Award, etc., to guide returned youth to actively participate in the construction of major national scientific research projects and major scientific and technological platforms, and actively participate in frontier scientific research tasks in key fields. The Youth Federation of Shandong Province has held many high-level youth talent matchmaking and exchange meetings, inviting returned overseas youth and industry leaders to conduct in-depth cooperation and negotiations with relevant departments and enterprises through inspections, exchanges, etc., and some projects have reached cooperation intentions and signed contracts. The Sichuan Provincial Youth Federation held the 19th Haike Huiye Global Young Scholars Forum, inviting key enterprises and institutions to connect with young people who have returned from studying abroad, and building a platform for exchange and learning of young talents in key industries and disciplines. The Hunan Provincial Youth Federation held the 8th Overseas Returns Forum, and set up theme activities such as Opening Up and Prospering Hunan Overseas Returnees Talk, Young Overseas Returnees' Shared Development and Warm-hearted Companionship, Overseas Students Entrepreneurship Startup Support Program Project Selection Competition, Innovation and Entrepreneurship Special Exhibition, etc. To build a platform for innovation and entrepreneurship of young people who have returned from studying abroad. The Youth Federation of Hainan Province held the Overseas Chinese Youth Development Conference, carried out overseas Chinese youth investment project roadshows, closed-door meetings with representatives of overseas returnees and overseas Chinese organizations from various provinces, and issued the "2021 Hainan Free Trade Port Overseas Chinese Youth Development Conference Joint Declaration", actively Help the construction of Hainan Free Trade Port. Relying on Zhichuang City, an innovation and entrepreneurship base, the Shanxi Provincial Youth Federation promotes the effective connection of technologies, products, and projects brought by young people who have returned from studying abroad with domestic capital, and builds an important venture capital platform. Relying on the construction of Youth Federation Hope House, Henan Province Youth Federation organizes young people who have returned from studying abroad to go to the front line of the countryside and actively contribute to the strategy of rural revitalization. 4. Enthusiasm to solve problems and serve higher-quality development Focus on the pain points and difficulties of young people returning from studying abroad in terms of internships, employment, entrepreneurship, life, etc. - 4-points, combined with the study and education of party history, I do practical things for the masses We should pay attention to the work and life of young people, keep abreast of the practical difficulties and problems they face, and cooperate with relevant functional departments to provide better services for young people who have returned from studying abroad. The Beijing Youth Federation and relevant departments launched the first "Double Hundreds of International Youth Talent Matchmaking Meeting", which serves the introduction, recruitment, retention and employment of outstanding international young talents, and provides convenient services in terms of work permits, innovation and entrepreneurship, and integration in Beijing. The Shanghai Youth Federation launched a special action to gather talents from overseas, actively cooperated with outstanding foreign-funded enterprises to provide more than 100 internship positions, and cooperated with relevant departments to open the first acceptance service channel for youth entrepreneurship projects returned from overseas. The Guangdong Provincial Youth Federation launched services such as returnees' dreams, Chinese dreams, public welfare lectures, online one-on-one tutoring assistance, returnees' internship and employment plans, and established 58 WeChat groups based on overseas contact stations, focusing on the concerns of young people who have returned from studying abroad Topics, to provide them with practical information and experience sharing. The Tianjin Youth Federation held a symposium on "Haihe Qingting Listening" to enter the young people who have returned from studying abroad, listen to their needs and appeals, and open up the last mile of communication. The Youth Federation of Jilin Province launched an online survey on the development status of youth returning from studying abroad, and sorted out and formed a survey report. The Youth Federation of Yunnan Province created the work brand of "Yunjing Huiye" for returned youths who have studied abroad, and held service activities such as "Returnees·Government and Enterprise Special Sessions" and making friends. The Inner Mongolia Autonomous Region Youth Federation launched the "Swan Goose Returning Home" salon activity to establish a friendship and exchange platform for young people who have returned from studying abroad. —5—Shake Distribution: General Office of the Central Committee of the Communist Party of China, General Office of the State Council. Secretary of the Secretariat of the Central Committee of the Communist Youth League, relevant ministries and commissions of the Central Committee, and provincial party committees in charge of leadership. The heads of the various departments and directly affiliated units of the Central Committee of the Youth League, and the secretary of the Provincial Youth League Committee. Shake Issued by the General Office of the Central Committee of the Communist Youth League on December 24, 2021

2021-12-2 25 County-level Communist Youth League Grass-roots Organization Reform Special Issue No. 3 Editor's note: In order to thoroughly implement General Secretary Xi Jinping's important instructions on deepening the reform of the Communist Youth League, with the approval of the Party Central Committee Secretariat, in June 2021 The pilot reform of grassroots organizations has been expanded, covering 621 county-level Communist Youth League committees in 31 provinces (autonomous regions and municipalities directly under the central government) across the country. Active exploration and bold innovations have emerged in various places, and a series of distinctive approaches have emerged. Recently, the Central Committee of the Communist Youth League has selected some typical cases around reform goals such as diversification of sources of cadres, diversification of organizational methods, and socialization of survival. They are now compiled and distributed in three phases for reference by all regions. —1—Survival Socialization Shake Breaking the reliance on resource administration to find resources from the society and helpers from the youth should be the specialty of the Communist Youth League as a group organization. The pilot places actively explore, tap the internal resources of the organization, learn from the practices of excellent social organizations, adopt a socialized operation mode, mobilize the resources and power of the society and the youth, integrate the youth into work in a down-to-earth way, and strive to break the The administrative thinking and path dependence of how much money to give and how much to do. 1. Internet Crowdfunding Hope House in Laiwu District, Jinan City, Shandong Province 1. In-depth investigation of the actual needs of children and adolescents. For children aged 8 to 14 who do not have an independent living environment and a good learning environment, we are preparing for the Hope Hut public welfare project, relying on the original housing to create an independent learning and living space. The Youth League Committee took the initiative to connect with poverty alleviation, education, township (street) and other units, and determined more than 300 children of the right age who were in trouble, such as filing cards, subsistence allowances, disabilities, etc., focusing on single families, orphan families, and families who can take out a single room for renovation Re-screening, through on-site visits, and finally determine the service targets. 2. Internet + Public Welfare: Extensively raise social resources. Get rid of the administrative path dependence of relying solely on the party and government allocation and the superiors, and use project-based, product-based, and network-based methods to stimulate the powerful love power hidden in the general public and young people. Offline, relying on the District Charity Federation to apply for the establishment of the Laiwu District Committee of the Communist Youth League Yuanmengji-2-Jinye; strengthen project promotion and strive for charitable enterprises to donate 260,600 yuan; mobilize outstanding young entrepreneurs from Laiwu, members of the Youth Federation, etc. Donated 520,200 yuan through the Provincial Youth Foundation. Online, seizing the opportunities of 99 Public Welfare Day and 520 Public Welfare Day, etc., initiated the donation action of co-constructing the Laiwu Hope House. By mobilizing the Young Pioneers of the Communist Youth League at the grassroots level to organize the formation of an online team, we expanded the amount of matching donations and raised 998,500 yuan of funds , online crowdfunding has become the main source of construction funds. 3. Prioritize the construction of pilot projects and strictly control the quality. Formulate the promotion table of the construction plan of the whole district, reverse the construction period according to the time node, and promote the construction in batches and orderly. Insist on putting the quality and safety of Hope House in the first place, and hire a professional company to issue bidding announcements to ensure that the construction party's qualifications, construction, infrastructure and equipment quality meet the standards; strengthen the supervision and supervision of the entire project, and the supervisor will conduct a unified acceptance after the project is completed , implement environmental monitoring to ensure that every hut built can make children feel at ease and parents feel at ease. 4. Carry out "one-on-one" and pair-up assistance. Adhere to the combination of material assistance and spiritual assistance. For the 138 huts for children in distress that have been built, the Communist Youth League Committee, social organizations, and young volunteers carry out voluntary services at least once a month to promote the transformation from hut rejuvenation to spiritual rejuvenation. change. Establish the "Hope House Children's Care Project Volunteer Service Handbook", implement one household, one book, one person, and register in detail the personal and family conditions of the service objects, as well as the volunteer's visit time, service content, and service object appeals. Organize activities such as Children's Day cultural performances on June 1st, colorful holidays, dream-seeking growth summer camps, book rafting programs, and custom-made birthday cakes to serve children's growth from multiple perspectives. —3—II. Gathering Talents and Enabling Volunteer Huiye in Linhai City, Taizhou City, Zhejiang Province 1. Innovate the mechanism of voluntary service projects. Establish and improve the voluntary project acknowledgment mechanism, change assignment to acknowledgment, open up the main line of demand-release-claim-undertake-summary, attract and discover a group of positive and influential voluntary service leaders to become planners of voluntary service projects People, participants, lead volunteers and resources sink. Since 2021, 65 volunteer service projects have been accepted and undertaken. Promote the incubation of voluntary projects, hold the Linhai New Era Civilization Practice Volunteer Service Innovation Project Competition, and provide professional guidance and financial support for 31 high-quality voluntary service projects. 2. Focus on the construction of volunteer service teams. Using the Volunteer Huiye platform to vigorously promote the registration of volunteers, build a 1+4+N voluntary service framework, and under the leadership of the Linhai Volunteer Association, set up four major voluntary services: safe governance, theoretical propaganda, civilized practice, and convenience for the poor Organized and established 687 registered volunteer service teams to participate in social governance services on a regular basis. Promote the official registration of volunteer service teams as social organizations and improve professional capabilities. In 2021, a total of 6 volunteer service social organizations will be guided to complete registration with the civil affairs department. 3. Coordinate the deployment of volunteer service resources. Integrate various professional voluntary resources, and stimulate service potential enthusiasm through interactive participation. Regularly carry out team resource sharing meetings, communicate and coordinate volunteer resources needed in various fields, and focus on solving different volunteer needs in a certain area. Taking the "grid" as a unit, we integrated doctors, teachers, police and other forces within the jurisdiction to form more than 650 functional volunteer teams to achieve full coverage of emergency rescue volunteer services. Relying on community voluntary service sites, more than 2,300 voluntary services such as community housekeeping, psychological counseling, medical insurance-4-health, safety patrol and legal aid have been carried out. 3. The Shunde District, Foshan City, Guangdong Province promotes the implementation of the "Youth Innovation Action" 1. Extensively integrate and leverage social resources. With the help of the leading force of the party committee and the government, leverage the resources of all parties in the society, and promote the integration of resources by exploring the three modes of organizational role, position co-integration, and resource linkage. To create a high-quality public welfare brand serving young people around the direction of talent service, practical education, and youth care. 2. Venture philanthropy empowers grassroots organizations. Collect work projects from grassroots league organizations, youth social organizations, and volunteer service teams in the region, highlighting public welfare, innovation, and sustainability. Through project solicitation, project review, public announcement, project determination, project implementation, mid-term evaluation, organizational supervision and other links, an effective closed loop of work is formed. In 2021, a total of 23 Youth Yichuang Action Support Projects will be identified, covering themes such as youth ideological leadership, youth league positions, rural revitalization, cultural and creative public welfare, youth backbone cultivation, and voluntary services. 3. Strengthen the implementation of the revitalizing social function. Focusing on the construction of the rule of law at the grassroots level, implement the Moot Court into the Grassroots project of advocating the law and promoting the rule of law to be kind and moral, allowing young people to write and direct the rule of law dramas on the big stage of the rural party-mass service centers. Covering more than 4000 people. Focusing on the inheritance of traditional culture, implement the "Liyuan - Thousands of Charms and Baimei" project, effectively connect grass-roots front resources with the teachers of Quyi Club, and provide regular free Cantonese opera experience courses and training for children in the form of voluntary service. Focusing on enhancing the stickiness of the organization, relying on bookstores and other establishments to establish the Green Cafe Station Ye-5-Alliance, integrating a series of elements such as Q cute and cute Xiaofen Qingye IP, creative display, exclusive discounts for team members, etc., tailor-made various interest salon activities , conveying the full energy of the group, and enhancing the youth members' sense of participation and sense of gain. —6—(No text on this page)—7—Shake Distribution: General Office of the Central Committee of the Communist Party of China, General Office of the State Council. Secretary of the Secretariat of the Central Committee of the Communist Youth League, relevant ministries and commissions of the Central Committee, and provincial party committees in charge of leadership. The heads of the various departments and directly affiliated units of the Central Committee of the Youth League, and the secretary of the Provincial Youth League Committee. Shake Issued by the General Office of the Central Committee of the Communist Youth League on December 23, 2021

2021-12-2 24 County-level Communist Youth League Grass-roots Organization Reform Special Issue II铱Editor's Note: In order to thoroughly implement General Secretary Xi Jinping's important instructions on deepening the reform of the Communist Youth League, with the approval of the Party Central Committee Secretariat, in June 2021 The pilot reform of grassroots organizations has been expanded, covering 621 county-level Communist Youth League committees in 31 provinces (autonomous regions and municipalities directly under the central government) across the country. Active exploration and bold innovations have emerged in various places, and a series of distinctive approaches have emerged. Recently, the Central Committee of the Communist Youth League has selected some typical cases around reform goals such as diversification of sources of cadres, diversification of organizational methods, and socialization of survival. They are now compiled and distributed in three phases for reference by all regions. —1—Diversification of organizational methods to stimulate the vitality of grassroots organizations In the context of the weakening of the management and service functions of work units, the frequent flow of youth, and the diversification of interests and needs, the traditional organizational methods of building groups at work units are facing more and more challenges. During the reform, the pilot places started from the aspirational, professional, interesting and realistic needs of young people, promoted the establishment of youth associations by youth leagues, innovated the form of grassroots organizations, expanded the coverage of organizations, and enhanced organizational stickiness. 1. Set up a community-based practice team in Xuhui District, Shanghai 1. Systematic design to expand depth. The Communist Youth League Committee closely focuses on serving the people's city construction, regards post meritorious service and practical education as the main means, and regards cultivating the new form of grass-roots organization of the regiment as an important goal, and strives to unite and gather excellent young backbones who are professional counterparts, active in thinking and have potential . Focusing on the main responsibilities and main businesses, through scientific research and judgment, we have established 6 forms of young people's urban practice groups: young red commentators focus on telling red stories, strengthen ideal inspiration, and cultivate advanced models; youth career experience officials focus on service Urban construction, carrying out trainee training, and gathering young talents; young "Shuidiye" volunteers are focusing on promoting volunteerism, helping waterfront services, and showing a civilized image; young entrepreneurial partners are focusing on activating innovation engines, cultivating potential talents, and gathering entrepreneurship Talents; youth community builders focus on promoting community innovation, optimizing the community environment, and raising the temperature of the community; youth culture star promotion officials focus on continuing Xuhui culture, promoting Shanghai-style culture, and creating youth traffic. 2. Increase the breadth of social mobilization. Formulate the "Guidelines for the Work of the Xuhui Youth People's City Practice Group" and the "Recruitment Plan for the Xuhui Youth People's City Practice Group" to implement the management, operation, evaluation, incentive, and guarantee mechanisms of the Xiqing-2-year practice group. Grasp the basic structure of the League organization and mobilize the 33 district-affiliated League organizations in the whole regionRecommend young backbones extensively, conduct research and visits to some key industry league organizations, and dig out backbone candidates. Covering the strengths of young people in the two new areas, communicating with regional units and enterprises with large-scale park buildings, forming a publicity effect within the park, and giving priority to the inclusion of outstanding young people with certain professional reputation and Internet celebrity attributes. Lead the vitality of secondary vocational schools in middle schools, extend to 50 secondary vocational schools in the district, encourage young people to participate in the activities of the practice group, and focus on recruiting a group of "slash youths" and "professional talents" with special skills. Create a linkage platform for colleges and universities in the city. Relying on the regional endowment and the advantages of university talent resources, connect with the youth league committees of 13 colleges and universities such as Fudan University and East China University of Science and Technology, establish a district-school linkage platform, and promote two-way empowerment. At present, more than 300 backbone youth practice groups of various industries and age groups have been recruited, with a total of more than 4,500 members. 3. Community-based operations enhance brightness. In view of the characteristics of the stratification of youth social circles, through stratified community management, the communication space is open and orderly, and youth participation is facilitated. The first is to establish a "full-cycle" community management model. Clarify community operation rules, promote standardized construction, standardized operations, and project-based operations, realize unified identification, unified management, and unified services, and improve brand identity and youth experience satisfaction. Establish a partnership-style growth tracking training mechanism to strengthen the identity of members of the practice group. The second is to build a small but refined community operation team. Each practice group is equipped with a core group of 1+2+3+N (one group leader, two executives, three experts and N team leaders), strictly controls the size of the practice group, and regularly cleans up inactive members. The third is the delivery of high-matching community activities. Let the best regional resources approach young people with a human touch, online strategy-3-plan popularity gathering activities, offline organization interactive check-in tasks, continue to create community excitement, and transform location function advantages, cultural service advantages, and regional resource advantages for youth appeal. The fourth is "radiative" community linkage. Continue to expand the radius of activities, link up with youth gathering places such as script killing, paid self-study rooms, and dressing experience halls, and radiate to young people's work and life points such as long-term rental apartments for young people and gathering points for takeaway boys, so that the youth practice group will continue to go out. , continue to attract more young people to interact and join, and realize the two-way empowerment of regional resources supporting youth development and youth development helping urban construction. 2. Yangzhong City, Zhenjiang City, Jiangsu Province, builds the "Five Youths and Five Unions" community group building 1. Optimize the "Youth Home" and use it together. Relying on the community party-mass service center and the New Era Civilization Practice Institute to build the flagship store of the Youth House, set up the Youth House function store in the community, and achieve full coverage within a 15-minute walk. Organize activities in associations, invite young people to settle in, customize services based on the needs of community youth, enrich the circle of friends of young people, and build an eight-hour youth social field. 2. Advocate "Youth Officers" and team building. Establish a youth business committee, which is composed of members of the community league branch, director of the business committee, youth building directors and youth property representatives, youth league cadres, members of the Lianqing service station, and young property owners as members. Carry out public opinion collection, resident management, voluntary services and other related work to inject green power into the corridor autonomy of the community. 3. Planning Young people talk about things, and green governance joint discussions. Establish a Youth Round Table Youth Roundtable with 1+1+N members (1 party member instructor, 1 community cadre, and several members of the Youth League) to organize discussions online and offline at least every month 1 time, through visits, WeChat, etc. to collect work and life issues encountered by young people, - 4 - to deal with grassroots difficulties and troubles for the community residents. 4. Cultivate youth organizations and co-organize activities. Introduce various social organizations in the community, assign psychological counselors and social workers, and undertake various social practice and public service project activities. Relying on the owner's WeChat group, the community youth library was established, the activity recruitment order was issued, and the friendly club was established; the youth commando team was formed to actively participate in the urgent, difficult, dangerous, heavy and new tasks of the community; the youth professional service team was established to provide District grid check-in. 5. Improve the courtesy of young people and share services. Combined with the community public welfare bank, the "Youth Points Management Measures", "Youth Cards", and "Youth Points Manual" were launched, and the courtesy incentive policies and points redemption list were formulated by classification and classification, so that young members of the league can benefit from action points and points. Integrate into the community and participate in grassroots governance to realize self-worth. Up to now, the total number of points is more than 30,000 points, and the cumulative centralized exchange of more than 20,000 points. 3. The Youth League Organization of Dachuan District, Dazhou City, Sichuan Province Tentacles into the residential buildings 1. Innovative organizational settings. Adhere to the leadership of party building, rely on the community party branch, unite with youth social organizations such as the Rural Youth Enrichment Leaders Alliance, Youth Volunteer Association, and integrate social resources such as government agencies, enterprises, institutions, property owners, and young property owners in the jurisdiction to establish 1+4+N Union branch. According to the interests and hobbies of young people, 45 interest groups such as dance troupe, calligraphy club and yoga keep team have been established, a shared space has been established, and a shared tool room has been set up. 2. Build a platform for participation. Build a voluntary service platform, set up youth volunteer service teams in each community, set up youth volunteer service posts, establish a rotation work system, implement a volunteer service point exchange system, and form a new community governance pattern of youth participation, co-construction and sharing. —5—Build a grass-roots governance platform, establish two ledgers for members and youths, the leader of the group is responsible for contacting the youth in the building, mobilize the backbone of the members to serve as community grid members, information officers, and propagandists, and assist the community and the property company to develop residents Registration, policy promotion, conflict mediation and other work. 3. Rich contact carriers. Make good use of carriers such as Youth Lecturer Group and Youth University Learning, and build 9 carriers such as Sunshine House and Community Book Bar according to local conditions. A group representative contact point was established in the community, 35 group representatives settled in the group representative contact point, and 7 Youth League member receiving day activities were carried out. With the "Youth Home" as the main front, resources such as the Party-mass Service Center, Children's Companion Home, and Group Activity Center are integrated, and the working model of "League Cadres + Social Workers + Volunteers" is implemented to achieve close contact. —6—(No text on this page)—7—Shake Distribution: General Office of the Central Committee of the Communist Party of China, General Office of the State Council. Secretary of the Secretariat of the Central Committee of the Communist Youth League, relevant ministries and commissions of the Central Committee, and provincial party committees in charge of leadership. The heads of the various departments and directly affiliated units of the Central Committee of the Youth League, and the secretary of the Provincial Youth League Committee. Shake Issued by the General Office of the Central Committee of the Communist Youth League on December 23, 2021

2021-12-2 23 One of the special issues on grassroots organization reform of the county-level Communist Youth LeagueIridiumEditor's note: In order to thoroughly implement General Secretary Xi Jinping's important instructions on deepening the reform of the Communist Youth League, approved by the Secretariat of the Party Central Committee, in June 2021 The pilot reform of grassroots organizations has been expanded, covering 621 county-level Communist Youth League committees in 31 provinces (autonomous regions and municipalities directly under the central government) across the country. Active exploration and bold innovations have emerged in various places, and a series of distinctive approaches have emerged. Recently, the Central Committee of the Communist Youth League has selected some typical cases around reform goals such as diversification of sources of cadres, diversification of organizational methods, and socialization of survival. They are now compiled and distributed in three phases for reference by all regions. —1—Diversification of cadre sources shakes open the path of socialized selection. The key to reforming the grassroots organization of the Communist Youth League in the county is to reform the cadre selection mechanism. Under the leadership of the party committees, each pilot area adheres to all corners of the country, opens up the path of socialized selection, and strives to select outstanding young backbones with good political qualities, ambitions, and passion for youth work to the posts of the regiment, strengthen the grassroots work force, and realize multi-channel Selecting people, using multiple carriers, and motivating people in multiple ways. 1. Shapingba District, Chongqing City implements the "Hundreds of Millions" youth talent mechanism 1. Expand 100 young talents for grassroots governance. Adopt organization recommendation, youth recommendation, personal self-recommendation, etc., select 100 young talents to concurrently serve as deputy secretaries or group building instructors of 22 towns and streets, 16 departments, and 3 industrial park committees. Clearly organize and plan group day activities, promote the standardized construction of grassroots group organizations, guide the non-public enterprise group building work in the jurisdiction, contact and serve youth entrepreneurship and employment, and promote youth voluntary service projects, etc., to clarify the work objectives and tasks. 2. Select and send thousands of grassroots service youth talents. Based on the unique advantages of the concentration of colleges and universities in the jurisdiction, and give full play to the leading function of the organization, the first batch of more than 1,000 college students from 50 needy villages (communities) paired with 10 colleges and 50 grassroots league organizations (youth volunteer groups) were selected to form youth The work special class, with a cycle of one academic year, carries out three practical activities (promoting the implementation of a project, bringing a group of resources to the grassroots, and writing a senior government report) practical activities to build a school-local collaborative education and training system to give full play to the talents of young students. Party and League members empower the community and activate the role of the grassroots. 3. Recruit 10,000 grassroots public welfare youth talents. Firmly establish the concept that youth is not only the object of work, but also the resource of work, guide young people to go out of their homes and integrate into the community, and recruit more than 10,000 grassroots public welfare youth talents to form 1+22+N Town and street detachments, N industry detachments) backbone volunteer teams, implement fixed posts, responsibilities, and fixed lengths, open up the last mile of youth service to society, and continuously enhance the political and social functions of the Communist Youth League. 4. Optimize the growth path and stimulate endogenous motivation. Strengthen daily management and job incentives, implement "one person, one policy" mentoring, one person, one file (full record, one person, one post) classified assessment. Incorporate outstanding young talents in the annual assessment into the reserve candidate pool for full-time community workers in the region without examination. The region's youth work talent pool is managed dynamically, and at least 20 grassroots positions are allocated every year, and staff from government agencies, state-owned enterprises, and village (community) workers are recruited (recruited) in accordance with regulations and procedures. Establish a daily care, visit and condolence system such as organizing a physical examination every year, purchasing an insurance, and issuing a letter to enhance the sense of job acquisition for young talents. 2. Multi-level selection of cadres and regiment cadres in Pengshan District, Meishan City, Sichuan Province 1. Select excellent and strong government cadres. According to 1 official and 4 deputy (2 full-time, 1 temporary, 1 part-time) to complete the team of the Communist Youth League Committee, select 3 young cadres from the selected students to serve as secretary and deputy secretary, and select 1 outstanding cadre from the political and legal team to serve as secretary and deputy secretary. As a temporary deputy secretary, an outstanding young teacher from Jinjiang College of Sichuan University, a resident university, was selected as a part-time deputy secretary, and the team structure was significantly optimized. With the support of the district committee, one person was added to the staff of the Communist Youth League Committee, and one person was temporarily employed. Adopt a flexible method to increase the backbone, and rely on the school-site joint construction mechanism to select 4 college students from across the province to serve as youth officers in the Youth League Committee. —3—2. Strengthen the strength of the directly affiliated organization. A trainee base was established in the Youth Palace under the Youth League Committee, and 4 trainees were recruited to realize institutionalized appointments. Taking the adjustment of township administrative divisions as an opportunity, strengthen the secretaries of 6 towns (streets) league (industrial) committees, and select 6 outstanding young people from social organization leaders, western plan volunteers, village community cadres and other groups to concurrently serve as towns (streets) Deputy Secretary of the Youth League (Working) Committee. Strengthen the cooperation between the Youth League and Education, establish the Educational Youth League Work Committee, implement the dual-director system, and implement the middle-level treatment for the person in charge of the school league organization. The "Administrative Measures for League Cadres and Youth Officers" was issued to define work responsibilities, work objectives, rewards and punishments, and strengthen rigid constraints on work performance. 3. Weave a dense grassroots team network. Promoted 51 village (community) league organizations to fully complete the reelection, and the average age of league cadres dropped from 35 to 31.5 years old. Among them, 15 people, such as the leaders of rural youth getting rich and children with their mothers, also served as secretary of the league organization. Set up the college student alliance of Homecoming and Dream Project, reserve 411 college students from Pengshan, guarantee the normalized provision of voluntary services, and preliminarily realize that youth activities can be carried out at any time, and young volunteers can participate at any time. Cooperate with other group organizations to implement the Colorful Group, Love in Shou Township project, make good use of the power of social organizations, invest 220,000 yuan to build 13 service points in villages (communities) by purchasing services, and carry out normalized care for left-behind children and disadvantaged Group assistance, science popularization and other activities. 3. Gaochang District, Turpan City, Xinjiang Uygur Autonomous Region Breaks through two bottlenecks 1. Breaks down the bottleneck of weak strength. Unblock the growth channel, stimulate the vitality of the cadres of the government, actively recommend to the organization department of the district committee, and transfer 3 grassroots league cadres from the education system to the grassroots league cadres in the education system and 1 outstanding cadre selected from the township to the league district committee through internal selection methods. Select and assign one temporary deputy secretary and two part-time deputy secretaries, enrich and equip one young worker from the three one-four-support teams, increase the staffing rate of full-time regiment cadres from 57% to 100%, and the on-the-job rate is 100% % . Enrich grassroots strength, select and assign 128 members of the grassroots youth league (work) committees, and assign full-time, part-time, and temporary deputy secretaries; 22 middle school youth league organizations in the region have entered the middle school, and 91 village (community) youth league branch secretaries have all Enter the village (community) two-committee management team; the village (community) specially sets up young professional positions, and recruits 31 outstanding youth party and league members from the fields of outstanding western plan volunteers, returning college students, entrepreneurial youth, etc., and incorporates them into local projects Ed. 2. Break the bottleneck of ability and quality. One is the normalized delivery of nutrients. Carry out special training for new cadres, set the 15th of each month as the training day for cadres, and carry out study and training around the key tasks of the month, and 336 sessions have been carried out. The second is to strengthen guidance on a regular basis. Implement the cadres of the Youth League and district committees to lead the intervention mechanism; set up two special working committees, and implement the 84 league cadres and league representatives at the district and township levels. . The third is to normalize constraints and incentives. The Organization Department of the District Party Committee issued the "Implementation Opinions on Establishing the Management System of Youth League Cadres in Gaochang District" and "Implementation Plan for the Selection, Training, Management and Use of Youth League Cadres in Gaochang District". Settled two accounts every month (priority work list at the beginning of the month, feedback list at the end of the month), strengthened follow-up and inquiry through telephone surveys, on-site visits, etc., released 8 issues of red and black lists, and dealt with 19 league cadres who failed to perform their duties accountability. Implement grassrootsOrganize the secretary's debriefing evaluation mechanism. The evaluation results are linked to the evaluation, selection and appointment. A fund of 110,000 yuan is set up every year to reward outstanding grassroots organizations. 4. Constructing the Pyramid Smelting Model in Gaomi City, Shandong Province 1. Widen the perspective and consolidate the tower base smelting. Grasping the opportunity of the village (community) re-election,—5—relying on the two major network communities of the Youth League Committee—the person in charge of the alliance—the member units of the alliance and the Municipal Committee of the Youth League—the town (street) Youth League (work) committee—the youth community—community residents, Announcement on the general election was released to attract the participation of good young people in the countryside, experts in getting rich by young people, graduates from high-density colleges and technical secondary schools, etc. After the general election, all 910 villages (communities) achieved a League organization secretary, a team of Youth League branches, and a youth Committee members, 721 of whom joined the two committee members. 2. Cultivate and strengthen the body of the tower. Explore the implementation of open selection and competition for grassroots league cadres, select and transfer more outstanding young people to enrich the team of grassroots league cadres, and equip and strengthen the town (street) league (work) committee team. Start the Youth Project, attract high-density college students and youth affairs social workers to the town (street) league (work) committee for practice and exercise, and establish a reserve talent pool for young crops. Fulfill the responsibilities of the League cadres, establish a regular debriefing and evaluation mechanism, and form the KPI assessment results of the regiment cadres through the evaluation of party and government leaders + the debriefing of the regiment cadres + the evaluation of youth satisfaction. 3. Selection and use, forging tower tip metallurgy. The Communist Youth League Committee has 6 administrative establishments, and the number of subordinate institutions has increased to 5. Public institutions provide growth channels for grassroots backbone interventions. Part-time cadres, youth affairs social workers, etc. can enter the agency through written examination + interview + comprehensive ability assessment. One outstanding cadre with more than 5 years of work experience in grassroots organizations has been selected for entry career ed. For grassroots league cadres, town (street) youth cadres, rural good youths, outstanding youths in the education system and other groups, the selection and matching of part-time regiment cadres will be carried out, and 2 temporary posts and 2 part-time deputy secretaries will be selected. Every six months, a phase of "replacing training with cadres" is carried out, and 3 grassroots cadres are recruited to participate in the daily work and major activities of the Youth League Committee. —6—(No text on this page)—7—Shake Distribution: General Office of the Central Committee of the Communist Party of China, General Office of the State Council. Secretary of the Secretariat of the Central Committee of the Communist Youth League, relevant ministries and commissions of the Central Committee, and provincial party committees in charge of leadership. The heads of the various departments and directly affiliated units of the Central Committee of the Youth League, and the secretary of the Provincial Youth League Committee. Shake Issued by the General Office of the Central Committee of the Communist Youth League on December 23, 2021

2021-11-3 22 County Youth League Youth Social Organization Construction Special Issue No. 3 Editor's Note: League organizations at all levels implement the deployment of the League Central Secretariat, in accordance with the "County-Level Youth Social Organization Construction Work Plan" requirements, in order to prevent Focusing on the development of crazy growth, anti-shock, and anti-involution, focus on promoting the construction of three types of youth social organizations: volunteer service, entrepreneurship and employment, literature, art and sports, and strive to build a county with firm politics, standardized construction, outstanding functions, extensive coverage, and full of vitality The league belongs to the youth social organization system, which constantly stimulates the vitality of grassroots league organizations. Recently, the Central Committee of the Communist Youth League sorted out the relevant situation, and now it is compiling and distributing special issues in three issues for reference by all regions. —1—Promote Youth Social Organizations under Youth League to serve the overall situation and serve youth Youth League organizations at all levels focus on stimulating organizational vitality, actively explore effective ways to promote the active development of youth social organizations under Youth League in county areas, and guide the establishment of socialized operating mechanisms and diverse To promote the improvement of the ability to serve the overall situation and serve the youth. 1. Strengthen support and guarantee, and unblock resource support and supply channels. Based on the actual needs of the growth of youth social organizations affiliated to the Youth League in the county, fully explore and integrate project resources in various fields, promote the construction of institutionalized supply channels, and provide targeted personnel, positions, funds, etc. aspects of support. 1. Make full use of the resources in the group. Give full play to the coordination and allocation role of league organizations at all levels, promote the inclination of various resources in the league to youth social organizations affiliated to the county youth league, give priority to supporting them to undertake government procurement service projects carried out by the Communist Youth League, and provide multi-faceted guidance and guarantees for their growth and development. The Chongqing Municipal Committee of the Communist Youth League has upgraded and built a growth and development base for youth social organizations, promoted the "mentor system" and "pair assistance" and other models, carried out exchange and training activities on a regular basis, and provided diversified assistance to youth social organizations affiliated to the Youth League in the initial stage. The Jilin Provincial Committee of the Youth League established the incubation base of youth organizations in Jilin Province through the joint construction model of the province and district, and implemented the three-zero principle of zero threshold, zero fee, and zero closure, and provided free office and activity venues for youth social organizations affiliated to the county youth league. 2. Actively strive for government resources. Seize the opportunity of the reform of the social governance system and mechanism, strengthen cooperation with the departments of civil affairs, human resources, social security, development and reform, publicity, cultural tourism, sports, etc., and introduce relevant policies and measures, in the registration and filing of organizations, supply of venues, and incubation services—2— Intensify support in aspects such as business affairs and project undertaking and implementation. The Shanghai Municipal Committee of the Youth League cooperates with the Municipal Bureau of Human Resources and Social Security to provide youth social organizations with employment trainee positions in the form of government subsidies, which not only helps solve the bottleneck problem of human resources, but also opens up a new path for youth employment. The Communist Youth League Wuhan Municipal Party Committee and the Civil Affairs Bureau jointly held the "Outstanding Youth" venture philanthropy competition, allocating funds in the form of signing government purchase service agreements to support youth social organizations affiliated to the Youth League to carry out public welfare activities, and supported more than 80 public welfare projects in total. 3. Integrate and develop with Youth Home. Take the Youth Home as an important platform to support the construction and development of youth social organizations affiliated to the Youth League, guide youth social organizations affiliated to the Youth League to settle in the Youth Home through joint operations, entrusted operations, project cooperation, etc., establish and improve the youth social organizations affiliated to the Youth League and youth The co-construction and sharing operation mechanism of the Home of Youth forms an online and offline activity mechanism, which not only improves the operational capabilities of the Youth Home, but also provides a more convenient and efficient practice platform for youth social organizations affiliated to the League. According to incomplete statistics, as of now, more than 2,000 youth social organizations affiliated to the Youth League in counties have settled in Youth Home and other regiment-affiliated positions, showing a good situation of synergy, complementarity and integrated development. 2. Activate endogenous power, explore and establish a socialized resource raising model Based on the social attributes of youth social organizations, take the establishment of a socialized resource mobilization and integration mechanism as an important direction, extensively absorb various social resources, and continuously improve youth social organizations affiliated to the Communist Youth League Self-hemopoiesis and self-operating ability. 1. Strengthen social financing. Give full play to the role of various group-affiliated foundations and public welfare platforms, make full use of venture philanthropy, social donations, online crowdfunding and other methods, and broadly expand socialized fund-raising channels. Based on guiding youth social organizations to efficiently—3—dock the resources of party and government agencies, enterprises and institutions, the Shanghai Municipal Committee of the Communist Youth League explores and builds a bidding platform for youth social organizations. Up to now, a total of 58 biddings have been carried out, with a total project value of 30.24 million yuan. The Guangdong Provincial Committee of the Youth League launched the Yimiao Program to support the growth of voluntary service organizations, providing financial support for youth social organizations affiliated to the Youth League to carry out projects, and focusing on guiding them to carry out development around drug control and AIDS prevention, grassroots social governance, serving youth entrepreneurship and employment, and helping rural revitalization, etc. normalized activities. 2. Mobilize innovative social forces. With the help of resource platforms such as social organization incubators, universities, and enterprises, we should strengthen the cultivation and development of Youth League-affiliated youth social organizations through joint establishment of cultivation bases, establishment of expert committees, and counterpart support. The Jiangsu Provincial Committee of the Communist Youth League has established 48 training bases relying on social work departments and social work service agencies in colleges and universities to undertake the training tasks of the backbone of youth social organizations affiliated to the Youth League; it has established a youth community think tank and expert committee to establish a combination of full-time and part-time jobs, theoretical and The combination of practical and practical teaching staff forms a strong intellectual support. The Hebei Provincial Committee of the Youth League gives full play to the role of the hub platform of the Youth Federation, mobilizes each member of the Youth Federation of Entrepreneurs to connect with at least one youth social organization affiliated to the Youth League, and provides assistance in terms of financial donations, voluntary services, and intellectual services according to their own actual conditions . 3. Expand the selection of social talents. Based on the reality, explore and establish a socialized and multi-channel backbone talent selection mechanism, and plan to find, discover, train, and reserve outstanding young talents for youth social organizations affiliated to the Communist Youth League. On the basis of fully excavating outstanding youth models, the Henan Provincial Committee of the Youth League has built a regional youth talent pool on a county-by-county basis, making it an important source of backbone strength for youth social organizations affiliated to the Youth League. The Hainan Provincial Committee of the Youth League explored the activities of proactively looking for young people and youth into organizations, mobilizing and absorbing outstanding young people in rural communities to join—4—Youth social organizations affiliated to the Youth League, participating in various volunteers such as family companionship, academic guidance, self-care education, and loving donations Public welfare activities to realize the integrated promotion of the work of the county league and the construction of youth social organizations affiliated to the league. 3. Highlight functions, improve service capabilities and levels Highlight the service functions of youth social organizations affiliated to the Communist Youth League, design and carry out work closely in conjunction with the actual work of the Communist Youth League at the county level, and guide youth social organizations affiliated to the Communist Youth League to continuously improve their service capabilities in practice and effectively serve the grassroots function of the group organization. 1. Participate in grassroots social governance. Inspired by the community youth action, youth social organizations affiliated with the Youth League are taken as an important force in the organization system of the community youth league, and supported to undertake community youth service projects such as the 4:30 Classroom, Wisdom to Help the Elderly, Caring Action, etc., and guide them in Enhance functions, play a role, take root and grow stronger in community governance. The Shanghai Municipal Committee of the Communist Youth League established a key project team of youth social organizations "going to the community" to carry out a series of activities "going to the community", attracting the participation of more than 1,800 youth social organizations of various types, and realizing the close connection between youth social organizations and community needs. 2. Participate in rural revitalization actions. Promoting youth social organizations affiliated with the Communist Youth League to deeply participate in the rural revitalization and youth contribution action, continuing to implement the Youth Social Organization Partnership Program of the Communist Youth League, organizing 100 youth social organizations affiliated to the Communist Youth League in 22 provinces, and carrying out public welfare for 100 youths in relocation communities for poverty alleviation and resettlement Sexual service projects, providing regular services such as academic counseling, family companionship, and community integration. At the same time, extensively promote youth social organizations affiliated to the Communist Youth League to participate in key tasks such as the construction of the New Era Civilization Practice Center and Beautiful China Youth Action, and continue to play a role in publicizing and cultivating socialist core values, advocating rural civilization, and building beautiful villages. —5—3. Serving the growth and development of young people. Youth social organizations under the Guidance Group give full play to their advantages of being close to young people and providing professional services, and design and implement targeted service projects around the needs of young people in terms of physical and mental health, entrepreneurship and employment, marriage and dating, and protection of rights and interests, so as to effectively promote the growth and development of young people. Relying on the China Youth Entrepreneurship and Employment Foundation, the Central Committee of the Communist Youth League implements the Youth Entrepreneurship and Construction of Hong Kong Plan, and implements six measures for youth social organizations belonging to the Entrepreneurship and Employment League, focusing on standardizing construction, member recruitment, financial support, mentor allocation, event planning, Strengthen guidance and support in personnel training and other aspects. The Shandong Provincial Committee of the Communist Youth League led youth social organizations affiliated to the Communist Youth League to participate in child care projects such as Hope House, forming a new model of the Communist Youth League's work on caring for disadvantaged youth groups led by the Youth League organizations and deeply participated by youth social organizations. The Jiangxi Provincial Committee of the Youth League has established a hierarchical system of youth entrepreneurship organizations in the province, guiding the Youth Entrepreneurship social organizations affiliated to the Youth League to carry out in-depth incubation, training, publicity and other work, effectively expanding the coverage of the Youth League organizations serving grassroots entrepreneurial youth. —6—(No text on this page)—7—Shake Distribution: General Office of the Central Committee of the Communist Party of China, General Office of the State Council. Secretary of the Secretariat of the Central Committee of the Communist Youth League, relevant ministries and commissions of the Central Committee, and provincial party committees in charge of leadership. The heads of the various departments and directly affiliated units of the Central Committee of the Youth League, and the secretary of the Provincial Youth League Committee. Shake Issued by the General Office of the Central Committee of the Communist Youth League on November 4, 2021

2021-11-3 21 County Youth League Youth Social Organization Construction Special Issue No. 2 Editor's Note: League organizations at all levels implement the deployment of the League Central Secretariat, in accordance with the "County-Level Youth Social Organization Construction Work Plan" requirements, in order to prevent Focusing on the development of crazy growth, anti-shock, and anti-involution, focus on promoting the construction of three types of youth social organizations: volunteer service, entrepreneurship and employment, literature, art and sports, and strive to build a county with firm politics, standardized construction, outstanding functions, extensive coverage, and full of vitality The league belongs to the youth social organization system, which constantly stimulates the vitality of grassroots league organizations. Recently, the Central Committee of the Communist Youth League sorted out the relevant situation, and now it is compiling and distributing special issues in three issues for reference by all regions. —1—Enhance the ideological and political leading function of youth social organizations affiliated to the Communist Youth League Youth League organizations at all levels regard youth social organizations affiliated to the Communist Youth League in counties as an important practice platform for leading and cohesive youth, actively adapt to the group characteristics and operating laws of different types of organizations, and innovate the carrier, Extensive organization and mobilization, strengthening close ties, and striving to give full play to the ideological and political leading function of youth social organizations affiliated to the Communist Youth League. 1. Closely follow the main line of the theme, and carry out in-depth study and education of party history. The study and education of the Communist Youth League to learn party history, strengthen beliefs, and follow the party as an important carrier of ideological and political leadership of youth social organizations under the Communist Youth League, highlight the characteristics of youth in the social field, and innovate the learning mechanism and ways to continuously improve the appeal and effectiveness of work. 1. Based on group characteristics, optimize the learning mechanism. Closely follow the goals and tasks of party history learning and education, vigorously strengthen top-level design and planning and deployment, and actively establish a learning mechanism that adapts to the characteristics of youth in the social field. The Central Committee of the Communist Youth League incorporated the study and education of party history into the backbone training system of youth social organizations affiliated to the Communist Youth League and the quality evaluation system of organizational construction. The Jiangsu Provincial Committee of the Communist Youth League incorporated party history courses into the Youth Benefit Training Camp, and established a normalized learning mechanism for the backbone of youth social organizations through special training, social practice, and study and training. The Beijing Municipal Committee of the Communist Youth League carried out a series of seven activities including party history knowledge contests, interviews with young social workers, and capacity-enhancing lectures among youth social organizations, forming a systematic learning and education model of party history. 2. Highlight practical participation and innovative methods. Starting from the ideological and cognitive laws of members of youth social organizations, using the combination of online and offline methods, innovatively carry out a series of profound and fashionable novels such as youth-2-spring party classes, youth learning societies, party history learning salons, etc. learning activities. The Jiangsu Provincial Committee of the Communist Youth League planned and carried out a series of activities such as reading party history and understanding thoughts among youth social organizations, a series of activities such as reading and sharing activities on the theme of "reading party history and understanding thoughts", "like a hundred years" video relay challenge, and online check-in for party history learning, which has formed a wide range of youth in the social field. The Fujian Provincial Committee of the Communist Youth League held the "Red Dream Building Journey" learning and practice activities, organized the backbone of youth social organizations to conduct national conditions research, and used practical actions to trace the footprints of the martyrs and inherit the red gene. Shandong, Shaanxi, Jiangxi and other places organized youth social organizations to go to Jinggangshan, Yan'an and other revolutionary memorial sites to experience the party's history and development achievements on the spot, and guide them toIn the historical comparison, I deeply understand the irreplaceable and important role played by the Communist Party of China, and effectively strengthen the four self-confidences. 3. Combined with functional positioning, focus on practical results. Based on the professional advantages of youth social organizations in various fields, with various public welfare service projects as the starting point, organize youth social organizations to carry out extensive practical activities of "I do practical things for the masses", and promote the timely transformation of the effectiveness of party history learning and education into practical actions to participate in social governance . The Shanghai Municipal Committee of the Youth League organizes a series of activities throughout the city called "Learning Party History, Doing Practical Things, Youth Social Organizations in Action", and guides youth social organizations to conduct regular community micro-surveys, community salons and other activities to help solve difficult problems in community governance. The Hainan Provincial Committee of the Communist Youth League mobilized youth social organizations to participate in the rural revitalization · volunteer first voluntary service action in combination with the relevant deployment of party history study and education, and carried out activities such as policy presentations, education support, and health assistance at the grassroots level in rural areas on a regular basis to guide youth in various fields Accept ideological influence in the hot practice of rural revitalization. 2. Seize the key few and actively cultivate outstanding young backbones—3—Focus on discovering, selecting trees, and cultivating a group of outstanding young backbone talents with firm beliefs, superb business skills, bases at the grassroots level, and willingness to contribute in youth social organizations in various fields, and strengthen exemplary Incentives and political absorption guide it to play an exemplary and leading role in youth social organizations affiliated to the county league. 1. Focus on exemplary incentives. In the group's various selection and commendation activities at all levels, further increase the selection of backbones of youth social organizations. Since the beginning of the year, in the field of youth social organizations, a total of 2 people have won the China Youth May 4th Medal, 26 people have won the National Outstanding Communist Youth League Members, 6 people have won the National Excellent Communist Youth League Cadres, 9 collectives have won the May 4th Red Flag League Branch, 29 people Elected as a national progressive and kind youth. According to statistics, a total of more than 1,200 backbone youth social organizations across the country have been honored and commended by the League. At the same time, the Communist Youth League partner lectures have been widely carried out in various places, and outstanding youth social organization backbones have been organized to publicize their partner stories. Through the combination of online and offline methods, nearly 10 million young people have been affected. 2. Do a good job in backbone training. The Central Committee of the Communist Youth League regards the leaders of youth social organizations as an important training object, and cooperates with the Ministry of Civil Affairs to set up a social organization class for the Qingma Project, design and develop excellent courses such as "Theory and Practice of the Construction of Youth Social Organizations under the Youth League", and guide the establishment of normalized training mechanisms in various places . The Shanghai Municipal Committee of the Communist Youth League carried out regular training for youth public welfare backbones, formed a special course system such as "National Conditions Study Camp", and conducted more than 80 training sessions for more than 1,000 youth social organizations, covering more than 5,000 person-times. The Zhejiang Provincial Committee of the Youth League implemented the "Youth Pilot" series of training programs in depth, and organized the backbone of youth social organizations to receive professional counseling and participate in training exchanges. Youth league organizations in Guangdong, Fujian, Xinjiang, Heilongjiang, etc. generally use special training, salon discussions, practical teaching and other forms to achieve normalized training for the backbone of youth social organizations belonging to the 4th Youth League. 3. Strengthen political absorption. Through the establishment of an institutionalized selection mechanism, league organizations at all levels actively select and match junior and part-time league cadres from the backbone of outstanding youth social organizations, and build a platform for in-depth participation in the work of the grassroots Communist Youth League. For the backbone of qualified youth social organizations, actively recommend them to serve as party representatives at all levels, deputies to the National People's Congress, members of the Chinese People's Political Consultative Conference, representatives of the Youth League, members of the Youth Federation, etc., and guide them to make better use of their professional advantages and play an active role on a broader stage. The Jiangsu Provincial Committee of the Youth League explored the establishment of a special mechanism to recommend outstanding youth social organizations affiliated to the Youth League to become members of the Youth Federation, and recommended relevant backbones to serve as affiliated and part-time Youth League cadres at all levels. The Ningxia District Committee of the Youth League selected 9 deputy secretaries and part-time deputy secretaries from the youth social organizations affiliated to the Youth League, and absorbed 26 backbone members to serve as members of the Youth Federation. 3. Strengthen ideological leadership and maintain political security in a timely manner. Continue to pay attention to the ideological dynamics and development trends of youth social organizations, strengthen close contact, risk research and judgment, and ideological leadership to ensure political and ideological security in the field of youth social organizations belonging to the Youth League. 1. Do a good job of political checks. Always keep a firm grip on the political direction, constantly strengthen the political guidance to various youth social organizations affiliated with the Communist Youth League, keep abreast of the organization’s operating conditions and members’ ideological status, and deeply grasp the problems that may arise in terms of ideological understanding, member structure, activities, and foreign exchanges. and risks, to ensure that speech and behavior that deviate from the goals of the organization do not occur. The Shanghai Municipal Committee of the Communist Youth League insists on taking the development of youth social organizations affiliated to the youth league at the district level as the key content of ideological work, conducts regular surveys, strengthens risk investigation and judgment, and compiles "Observation on Shanghai Youth Social Organizations" every month to make sure that the results are clear and accurate. Surface combined. —5—2. Do a good job in daily monitoring. Guide youth league organizations at all levels to plan to strengthen regular contact with youth social organizations that have greater influence and coverage. Up to now, the group has closely contacted more than 8,300 related youth social organizations, doubling the number compared to last year. Through regular communication platforms such as open days and symposiums, various localities have an in-depth understanding of the development trend of various youth social organizations, actively help reflect urgent needs, solve practical difficulties, and promptly discover and promote the resolution of emerging problems. Relying on the dual-network interaction mechanism, the Zhejiang Provincial Committee of the Communist Youth League has a detailed understanding of the group size, distribution characteristics and ideological dynamics of youth social organizations in the grid, and actively strengthens contact services with organization leaders to achieve three-dimensional and information coverage. 3. Strengthen theoretical research. Give full play to the role of platforms such as the Communist Youth League and youth work high-end think tanks, the Communist Youth League community and social organization committees, and local youth league schools, and conduct in-depth investigations on related issues such as social organization building, youth social organization operation mode, and youth ideological characteristics in emerging fields Research, launch a series of research results. The Tianjin Municipal Committee of the Communist Youth League and Tianjin Normal University and other colleges and universities conducted a special survey to gain an in-depth understanding of the development status and needs of youth social organizations affiliated to the Youth League, and formed the research report "Research on the Optimization of Youth Social Organizations from the Perspective of Constructing a New Pattern of Grass-roots Social Governance" to promote county-level It provides a reference for the construction of youth social organizations affiliated to the Communist Youth League. —6—(No text on this page)—7—Shake Distribution: General Office of the Central Committee of the Communist Party of China, General Office of the State Council. Secretary of the Secretariat of the Central Committee of the Communist Youth League, relevant ministries and commissions of the Central Committee, and provincial party committees in charge of leadership. The heads of the various departments and directly affiliated units of the Central Committee of the Youth League, and the secretary of the Provincial Youth League Committee. Shake Issued by the General Office of the Central Committee of the Communist Youth League on November 4, 2021

2021-11-3 One of the special issues on the construction of youth social organizations belonging to the county-level leaguesIridiumEditor's note: League organizations at all levels implement the deployment of the Secretariat of the Central Committee of the Communist Youth League, in accordance with the requirements of the "Work Plan for the Construction of Youth Social Organizations at the County Level" to prevent Focusing on the development of crazy growth, anti-shock, and anti-involution, focus on promoting the construction of three types of youth social organizations: volunteer service, entrepreneurship and employment, literature, art and sports, and strive to build a county with firm politics, standardized construction, outstanding functions, extensive coverage, and full of vitality The league belongs to the youth social organization system, which constantly stimulates the vitality of grassroots league organizations. Recently, the Central Committee of the Communist Youth League sorted out the relevant situation, and now it is compiling and distributing special issues in three issues for reference by all regions. —1—Construct a youth social organization system that covers a wide range of youth leagues in the county. The league organizations at all levels closely focus on the reform goals of the Communist Youth League in the county, grasp the work direction, focus on adapting measures to local conditions, steadily expand the scope and improve quality, and promote the further expansion of organizational scale, organizational coverage and organizational functions Optimization, gradually forming a Youth League-owned youth social organization system in which grass-roots Youth League organizations and youth social organizations are organically coordinated in the county. 1. Highlight the quality of construction and continuously expand the coverage of organization construction Based on the reality of economic and social development, closely follow the differentiated characteristics and needs of young people, actively and orderly promote the construction of various youth social organizations affiliated to the Youth League, making them an important part of the grassroots organization system of the Youth League part. 1. Classification key promotion. Since the beginning of the year, the whole regiment has actively and steadily promoted the construction of youth social organizations belonging to cultural, art and sports groups on the basis of continuing to deepen the construction of youth social organizations belonging to voluntary service and entrepreneurship and employment groups. Up to now, the group has established more than 9,200 youth social organizations belonging to the group in three types of counties, with 6.85 million individual members and 548,000 group members. In terms of proportion, the coverage rate of districts and counties for voluntary services is 97.1%, that of entrepreneurship and employment is 92.2%, and that of arts and sports is 57.6%. 2. Focus on adapting measures to local conditions. For the central and western regions with a relatively weak foundation, establish a one-to-one smelting work promotion mechanism, and guide them to help grass-roots innovation and explore effective organizations through methods such as linking up points, grasping the next level, counterpart contact, and normalized research— 2—Construction mode. For areas with good foundations such as Beijing, Shanghai, Jiangsu, and Zhejiang, we will promote the organization to gradually extend its coverage to towns, streets, and urban and rural communities on the basis of active organization. The Jiangsu Provincial Committee of the Youth League insisted on self-increasing pressure, and proposed that on the basis of ensuring the realization of the three types of organization construction goals, within the year, more than 60% of the county-level Youth League committees in each districted city should build at least one new youth group organization under the Youth League. 3. Integrate into the organizational system. The general establishment and functioning of youth social organizations under the Youth League in county areas has effectively deepened the relationship between the grassroots Communist Youth League and youth social organizations, and has become a link for the Communist Youth League to contact, serve, and guide youth and other youth social organizations. The Chongqing Municipal Committee of the Communist Youth League established a joint meeting of youth social organizations, bringing 119 of the city's most influential youth social organizations into the meeting mechanism, and actively building and promoting youth social organizations through democratic discussions, capacity building, business guidance, industry self-discipline, and resource sharing. It is a platform for organizations to realize resource integration, exchange and mutual assistance, and collaborative development, and guide them to play a better role around the work goals proposed by the group organization. 2. Deepen guidance and evaluation, establish and improve a flat working mechanism to adapt to the characteristics and laws of youth social organizations, coordinate the efforts of the whole group to explore the establishment of a flat mechanism, continue to strengthen the influence and guidance of working forces at all levels and in various fields, and promote work signals, work Tasks and work resources go directly to the grassroots. 1. Establish a collaborative promotion mechanism. The various departments of the Central Committee of the Youth League and the units directly under it have established a working mechanism to coordinate the construction of youth social organizations under the Youth League, hold regular scheduling meetings, and promptly promote the resolution of problems encountered in the work. Provincial and municipal youth league committees generally establish a coordinated promotion mechanism to actively create conditions, build platforms, and —3—integrate resources for further advancement of related work. The Sichuan Provincial Committee of the Communist Youth League organized the cadres of the Provincial Committee of the Communist Youth League to directly pair up with 183 districts and counties, and took the effectiveness of the promotion work as an important reference for the annual assessment of cadres. According to the idea of establishing 1 supportive youth social organization + building 3 special youth social organizations + cultivating N youth social organizations, the Gansu Provincial Committee of the Youth League carried forward the work in a planned and step-by-step manner relying on relevant mechanisms. 2. Improve the level of information management. Relying on the "Reunion Partner" youth social organization information service platform, establish a national information database of youth social organizations affiliated to the Communist Youth League, grasp the construction situation of youth social organizations affiliated to the Communist Youth League at all levels and regions in real time, and strengthen the influence and guidance of grassroots youth social organizations in a targeted manner. Up to now, 11,069 Youth Social Organizations affiliated with Youth Leagues at the provincial, municipal, and county levels have registered and entered the platform to realize real-time grasp of the construction situation in various places and ensure that relevant progress is predictable, descriptive, and measurable. 3. Establish a scientific evaluation system. Establish a comprehensive evaluation mechanism based on the coverage, standardization, activity, and functioning of organizational construction, and strive to achieve a scientific assessment of the quality of various social organization constructions through on-site supervision and research, big data analysis, and questionnaire surveys. Evaluate and guide the league organizations at all levels to focus their work on improving the quality of construction. Hebei, Jiangsu and other provinces have explored the implementation of star rating and credibility assessment mechanisms for youth social organizations affiliated to the Communist Youth League, and effectively improved the quality of youth social organization construction through star ratings and quantitative assessments. 3. Focus on the direction of work and ensure the healthy and orderly development of various organizations. Strengthen the standardized management of youth social organizations affiliated to various groups, and strengthen work guidance in registration, member recruitment, resource integration, activity development, and external exchanges. — 4— guide to ensure that construction and development are always in the right direction. 1. Promote normative construction. All localities carry out organizational construction in accordance with the principles of having a charter system, having a backbone of work, having a stable team, and having a brand of activities, and promote the timely registration of youth social organizations belonging to the Youth League that meet the registration requirements with the civil affairs department. Up to now, 5,910 Youth Social Organizations affiliated to the Youth League have completed registration, accounting for 53.4% of the total number of Youth Social Organizations affiliated to the Youth League at the provincial, city, and county levels; There are 5412 companies with part-time league cadres, accounting for 48.9%; 6142 companies with fixed activity places, accounting for 55.5%; and 7138 companies with fixed office space, accounting for 64.5%. 2. Expand the coverage of team building. Promote the timely establishment of youth social organizations belonging to the Youth League that meet the conditions for forming a youth group. Up to now, 3,034 Youth League organizations have been established among the youth social organizations belonging to the Youth League, accounting for 27.4%. All localities have fully considered the looseness and mobility of members of youth social organizations affiliated to the Youth League, actively explored new work carriers, and further strengthened the effective coverage of young and middle-aged members of social organizations by establishing youth interest groups and youth working committees. The Sichuan Provincial Committee of the Communist Youth League and the Provincial Department of Civil Affairs jointly established a guidance and promotion committee for social organization industry league building work, issued work plans, issued guidance manuals, and strengthened advanced guidance. Hebei, Shandong, Gansu and other places give priority to working resources to youth social organizations that have established leagues, forming an incentive and guiding effect on other youth social organizations. 3. Strengthen daily management. Youth league organizations in various places regularly listen to the opinions of leaders and members of youth social organizations affiliated to the league, and continuously improve relevant mechanisms and work measures. Each county-level Youth League Committee conscientiously performs its supervisory duties, implements a review mechanism for annual work and major event reports, and urges youth social organizations affiliated to the Youth League that have been inactive for a long time and have not played an obvious role to rectify in a timely manner. Adjustment. The Fujian Provincial Committee of the Communist Youth League established and implemented the working mechanism of "three cleans, four guarantees, and five guarantees" to effectively guarantee the quality of construction of youth social organizations affiliated to the Youth League. —6—(No text on this page)—7—Shake Distribution: General Office of the Central Committee of the Communist Party of China, General Office of the State Council. Secretary of the Secretariat of the Central Committee of the Communist Youth League, relevant ministries and commissions of the Central Committee, and provincial party committees in charge of leadership. The heads of the various departments and directly affiliated units of the Central Committee of the Youth League, and the secretary of the Provincial Youth League Committee. Shake Issued by the General Office of the Central Committee of the Communist Youth League on November 4, 2021

2021-11-1 19 Editor's note: Tuan Shanxi Provincial Committee implementsThe whole regiment focused on grassroots deployment, anchored the construction of a team of grassroots regiment cadres with high political quality and strong professional ability, seized the favorable opportunity of the county and rural party organization change, and actively sought the support of the party committee and organization department, comprehensively Promote the simultaneous progress and completion of the reelection of the league organization, effectively consolidate and strengthen the grassroots organization construction of the regiment, and effectively enhance the organizational strength of the regiment. Its experience and practices are now compiled and distributed for the whole group to learn from. —1—The Shanxi Provincial Committee of the Tuan has four insistences to comprehensively promote the reelection of the county and rural league organizations and the party organization at the same time. The general requirements of the party's leadership, promoting grassroots democracy, strict employment standards, promoting reform and innovation, and comprehensively and strictly governing the regiments, promote the centralized reelection of county and village league organizations and the simultaneous reelection of county and township party and government leaders, and village (community) committees Advance, sync complete. As of the end of August, all 117 counties (cities, districts) and 1,061 townships in the province had held congresses of league members, elected a new leadership team of the league committees, and further strengthened grassroots work. 1. Adhere to the party building and the league building, firmly grasp the correct direction, and adhere to the party's overall leadership throughout the work of the county and rural league organizations, actively report to the provincial party committee, and actively seek support. The first is overall coordination across the province. Under the great attention of the Provincial Party Committee, we sought the support of the Organization Department of the Provincial Party Committee to establish a group leader with the Secretary of the Provincial Party Committee as the team leader and the Deputy Director of the Organization Department of the Provincial Party Committee as the deputy team leader. The head of the relevant divisions and offices of the Organization Department of the Committee is a leading group for the re-election work of the league organization, leading the whole process and advancing the re-election work at a unified pace. The second is the coordinated promotion of cities and counties. According to the overall arrangement for this reelection work, the reelection of the league organization and the reelection of the county and village party organizations will be deployed, promoted, and assessed at the same time. At the city and county levels, set up leading groups and institutions for the reelection work with responsible comrades from the party committee as the team leader, lead the formulation of the reelection plan for the county and township Youth League committees, preside over the mobilization and deployment meeting for the reelection work, lead and check the entire process of the reelection work, and coordinate and solve the reelection process specific issues in . The third is to ask for instructions and reports in a timely manner. On the basis of -2- conscientiously implementing various mechanisms for Party building and League building, the Shanxi Provincial Committee of the League, in accordance with the requirements of the reporting system within the League, has repeatedly asked and reported to the Central Committee of the League on issues such as the organizational procedures for the reelection and the requirements for candidates; Strictly implement the dual leadership system and the reporting system within the regiment, keep abreast of the deployment, training, and transfer of regiment cadres in counties (cities, districts) in real time, and actively play their due role in the transition work. 2. Adhere to the unity of the province's league organizations, actively strengthen the overall planning and guidance of the provincial and municipal youth league committees to study and implement the requirements of the provincial party committee and the league center for the re-election work, conscientiously implement the main responsibility, set up a special class for the re-election work, carefully organize and strengthen guidance, Vigorously promote the full implementation of the policy requirements for re-election. The first is to strengthen policy guidance. The Provincial Committee of the Communist Youth League and the Organization Department of the Provincial Committee of the Communist Party of China jointly issued the "Notice on Doing a Good Job in the Change of Youth League Organizations at the County, Township, and Village Levels", clarifying the requirements for guiding ideology, working principles, time arrangements, policy basis, and organizational leadership; The "Communist Youth League County (City, District) Committee Re-election Work Instruction Manual" and the "Communist Youth League Township Committee Re-election Work Instruction Manual" were compiled and issued to make detailed regulations on the re-election process, operation examples, candidate conditions, time nodes, etc. The second is in-depth research and guidance. Members of the Youth League Provincial Committee went to 11 cities and 26 counties (cities, districts) for research and guidance, discussed and communicated with 3 municipal party committee secretaries, 5 municipal party committee deputy secretaries, and 4 municipal party committee organization department ministers, and coordinated and promoted the youth league organization's reelection work; Held 18 symposiums at the three levels of the city, county and township to listen to opinions and suggestions, publicize policy requirements, improve the arrangements for the re-election, and promote the implementation of work. The third is to carry out regular scheduling. Establish a regular scheduling system for the reelection of the county and township Youth League committees, report the progress of the reelection of the county and township Youth League committees every week, issue work reminders for slow progress, guide the progress of the reelection work according to the node, and carry out question and answer questions about the reelection policy, and assist in solving various reelection problems. More than 120 Documents to ensure consistent work standards and unified actions for -3- reelection. The fourth is to promote reform simultaneously. The youth league committees at the city and county levels took the re-election as the starting point, actively innovated in organizational setting, cadre selection and matching, operating mechanism, and working methods, and promoted the implementation of reform measures. County and township Youth League committees generally establish a debriefing and evaluation system for the main person in charge and an incentive and restraint mechanism, and all township (town) Youth League committee secretaries are included in the management of the organization department of the county committee. At the same time, the league and county (city, district) committees actively seek the support of the party committees, and promote the implementation of the system of county and township youth league committee secretaries attending the same level of party committees and standing committees. The fifth is to broadly build consensus. In-depth transmission of work signals, closely follow the three-strength and one-step work pattern to plan the work for the next five years, and make comprehensive arrangements for the construction of the League organization, the team of League members, the team of League cadres, and the role of the League organization. In the process of drafting the work report, the Youth League committees at the county and township levels effectively condensed the consensus of doing a good job in the party's youth work through extensive solicitation of opinions. The province has held more than 1,300 symposiums and solicited more than 15,000 opinions from youth members. Sixth, adhere to the law and regulations. Adhere to the "Group Constitution", "Regulations on the Election of Local Organizations of the Chinese Communist Youth League", "Rules for the Election of the Chinese Communist Youth League's Grassroots Organizations", "Interim Regulations on the Work of the Chinese Communist Youth League's Rural Grassroots Organizations" Follow and guide all localities to carry out elections in strict accordance with laws and regulations, actively and steadily. 3. Adhere to the key minority, select a good team of strong youth league committees to implement the party's requirements for the reform of the Communist Youth League, based on the fundamental political positioning of the party's assistants and reserve forces, and take building a high-quality professional league cadre team as an important goal, combined with the overall The actual construction of the provincial league cadre team puts forward clear requirements for the number of posts in the committee and standing committee of the new middle league, the basic conditions for the secretary and deputy secretary of the county and township league committee, and the representative structure of the league congress. First, the Provincial Committee of the Youth League conscientiously implements the important requirements of General Secretary Xi Jinping on the young cadres-4-department and the League cadres, strictly implements the system of cooperative management of the League cadres, and assigns leadership comrades and 117 counties (cities, districts) to the municipal committees of 11 cities The secretary of the party committee sent a letter to support the allocation of cadres to further strengthen communication and cooperation in the coordination and management of cadres. The second is that the city and county Youth League committees adhere to the principle of "two ups and two downs", negotiate with the organization department of the party committee to nominate candidates, and actively recommend young cadres who love the cause of the Communist Youth League, have strong organizational skills, excellent personal qualities, and have prestige among young people as the secretary of the county and township Youth League committees. The third is to strengthen organizational checks and strict qualification review. It is recommended that relevant party committees cancel the nomination of candidates who do not meet the qualifications and have low youth voices, so as to ensure that the elected cadres are worthy of their positions and posts. During the reelection period, relevant party committees were urged to cancel 10 nominees for county-level youth league committee secretaries and 15 nominees for township youth league committee secretaries. 4. Adhere to the discipline and rules at the forefront, and strive to create a clean and positive atmosphere. Conscientiously implement the overall requirements for comprehensive and strict governance of the party, make solid plans to promote comprehensive and strict governance of the regiment, and always highlight discipline education and process supervision during the transition process, so as to achieve Education comes first, warning comes first, and prevention is in place. The first is to strictly examine the qualifications of candidates. Establish a joint review mechanism for candidate qualifications, highlight political standards, fully solicit opinions from organizations, discipline inspection departments and other departments on nominated candidates, accurately identify candidates, and publicize them within a certain range as required, effectively avoiding distortion and fraud. Those who have political problems, damage the interests of the country and the people, have misconduct, moral corruption, and serious violations of discipline and law will be resolutely rejected. The second is to unblock reporting channels. The Provincial Committee of the Youth League and the Youth League Committees of each city and county have opened special reporting telephones and mailboxes to promptly accept all kinds of reports related to the re-election of the Youth League organization, and resolutely correct and properly solve various problems discovered during the re-election process. The third is to carry out special supervision. Promote the discipline supervision of county and township youth league organizations as an important part of the supervision of the 5th leadership of the county and township leadership, and cooperate with the discipline inspection commissions and supervisory committees at all levels to focus on warning education, patrol inspections, on-site supervision, etc., to ensure that the transition process is smooth Upright and disciplined. Through the reelection, the structure of Shanxi Communist Youth League cadres has been further optimized, the age, education, work experience and other aspects have been greatly improved, the overall function of the county and township Youth League committees has been improved, and the organizational vitality has been enhanced. First, the political quality has been strengthened. Tuanxian (city, district) committee members are all members of the Communist Party of China, and 23.19% of them are from the front line of poverty alleviation and rural revitalization. CCP members account for 65% of the township youth league committees, and a group of young cadres who are full of vigor, excellent quality, rich practical experience, and high prestige among young people have taken up the grassroots work positions of the youth league. The second is to hang and fully complete. After the re-election, 117 counties (cities, districts) in the whole province have a total of 325 regimental county (city, district) party secretaries and full-time deputy secretaries, 123 temporary deputy secretaries, and 220 part-time deputy secretaries. 75. Increase from 21% to 100%; Township Youth League Committees are equipped with 1,061 Youth League secretaries, 1,924 deputy secretaries, and 6,218 propaganda committee members and organization committee members. The third is the age of education, one rise and one fall. After the re-election, among the members of the new team of the Tuanxian (city, district) committee, 635 have a bachelor's degree or above, accounting for 95.06%, an increase of 50.59% from the previous term; the average age is 29.97 years, a decrease from the previous term 0. 67 years old. Among the members of the new team of the township Youth League Committee, 2601 have a bachelor degree or above, accounting for 87.14%, an increase of 14.40% over the previous term; the average age is 28.2 years, a decrease of 1.82 years over the previous term. —6—(No text on this page)—7—Shake Distribution: General Office of the Central Committee of the Communist Party of China, General Office of the State Council. Secretary of the Secretariat of the Central Committee of the Communist Youth League, relevant ministries and commissions of the Central Committee, and provincial party committees in charge of leadership. The heads of the various departments and directly affiliated units of the Central Committee of the Youth League, and the secretary of the Provincial Youth League Committee. Shake Issued by the General Office of the Central Committee of the Communist Youth League on November 1, 2021

2021-11-1 18 Editor's note: Promoting high-quality employment of college graduates is a key area for the Communist Youth League to serve the youth and the overall situation. Since the beginning of the year, league organizations at all levels have resolutely implemented the deployment of the Fifth Plenary Session of the Eighteenth Central Committee of the Communist Youth League, taking the employment service action of college students from thousands of schools and colleges as a guide, focusing on graduates from poor families in local ordinary colleges and universities, and focusing on the 2021 graduates. The solid and accurate employment service has been praised by all walks of life, especially college graduates. The relevant situation is now compiled and distributed for reference. -1- League organizations at all levels solidly and accurately serve college graduates for employment. The total number of college graduates in the 2021 class of the country will reach 9.09 million, and the number of employed people will hit a new high. The Communist Youth League organizations at all levels resolutely implement the spirit of General Secretary Xi Jinping's important instructions on the employment of college graduates, follow the major decisions and deployments of the Party Central Committee and the State Council's "six stability" and "six guarantees", and focus on graduates from poor families in local ordinary colleges and universities , solidly promote the precise pairing and assistance of league cadres, continue to hold a series of recruitment activities for thousands of schools and ten thousand posts, carry out in-depth employment internships and other work, and fully serve the high-quality employment of college graduates. 1. Focus on key groups and more accurately help graduates from difficult families in colleges and universities. Graduates from poor families in local ordinary colleges and universities are the top priority in the employment of college graduates. On the basis of successfully completing three tasks of 100,000+ in recent years, the whole regiment continues to focus on serving the employment of graduates from poor families in local ordinary colleges and universities, and continues to carry out precise pairing assistance for league cadres. By finding out employment intentions and providing employment Provide guidance, recommend employment information, pay attention to employment progress, etc., and provide full-chain employment services for assistance targets. Up to now, a total of 28,925 league cadres have paired up to help 37,794 college graduates from difficult families find jobs. The Shandong Provincial Committee of the Communist Youth League launched the Jade Bird Project Employment Service Season, focusing on graduates of local ordinary undergraduate colleges and vocational colleges, and organized league cadres at all levels to provide full-process employment services for graduates from poor families. The Henan Provincial Committee of the League, together with 14 units such as education, human society, and the Disabled Persons' Federation, carried out the 2-Action of Public Welfare Support for Employment and Entrepreneurship. Relying on the intelligent employment service platform, more than 400 special job fairs were held to provide better education for college graduates from poor families. Targeted employment support. Inner Mongolia, Fujian, Hainan, Gansu, Qinghai, Ningxia, Xinjiang and other seven provincial league organizations carried out precise assistance work to achieve full coverage of colleges and universities in the region. 2. Give full play to organizational advantages and recruit jobs more widely Relying on the mobilization advantages of organization and socialization, league organizations at all levels fully coordinate and strive for resources from all aspects, and extensively recruit and connect job information. Relying on the job brand of Thousands of Schools, Thousands of Posts, Central Enterprises Cloud Recruitment, and 360 central enterprises and local state-owned enterprises, the Central Committee of the Communist Youth League provided more than 13,000 jobs, and mobilized members of Youth Federations and Youth Enterprise Associations at all levels to provide jobs 2.1 More than ten thousand. The Jilin Provincial Committee of the Youth League mobilized 71 enterprises in the Youth Entrepreneurship Park of the Youth League to provide 1,094 positions for the "Qingye" brand name and 1,046 positions for the development of "emerging business forms". The Heilongjiang Provincial Committee of the Mission coordinated the employment resources of 343 enterprises in the province, and provided 16,000 jobs to 80 colleges and universities in the province through pairing assistance. The Henan Provincial Committee of the Youth League held the New Youth OFFER Festival, mobilizing nearly a thousand companies to provide more than 80,000 jobs, and more than 190,000 college graduates participated in consultations and interviews. Chongqing and Sichuan Youth League organizations jointly carried out the "Shuangcheng Youth Talent Recruitment" campaign, recruiting more than 530,000 jobs from more than 11,000 companies. 3. Innovate the work carrier, and more efficiently promote the matchmaking between people and posts. To adapt to the urgent needs of online job hunting under the background of normalized epidemic prevention and control, the whole group actively explored online work carriers, and held 276 recruitment activities for thousands of schools and ten thousand jobs relying on the Internet. Organized 34,000 enterprises to provide a total of 1.224 million jobs, and more than 550,000-3-times of college graduates participated. The Central Committee of the Communist Youth League optimized and upgraded the youth employment service platform Tuantuan Micro-employment, attracting more than 10,000 companies to settle in, providing a total of more than 1 million jobs, and sending job information directly to the hands of college graduates. The Jiangsu Provincial Committee of the Youth League carried out 51 live broadcast recommendation activities in various cities, attracting 109,000 seniorsA total of 7,861 college graduates reached employment intentions by submitting their resumes and participating in interviews. Relying on the WeChat platform of Jiangxi Youth League, the Jiangxi Provincial Committee of the Tuan established a column for the employment and recruitment of college graduates with thousands of schools, ten thousand jobs, and jobs for you, and organized the league organizations of colleges and universities to release more than 3 million pieces of employment information. The Zhejiang Provincial Committee of the Communist Youth League mobilized employers to enter schools to send jobs, and held a total of 142 job fairs, attracting 241,000 college graduates to participate. The Tuan Tuan Inner Mongolia Autonomous Region Committee innovated the service model, created a series of short videos of “Tuan Tuan takes you to find a job” series, and recorded 30 public welfare live broadcast courses of Tuan Tuan Employment Help, online employment guidance, with a total of 630,000 people watching online. 4. Deepen employment practice and further help college students improve their socialization ability. The whole group will further promote the 2021 National College Student Internship Sail Plan, providing 438,000 government affairs and enterprise internship positions for college students, and some positions have passed the National College Student Internship Program. The service platform was released, and more than 150,000 resumes were received; 2,079 workplace experience activities were organized, and more than 170,000 college students participated. The Central Committee of the Communist Youth League, together with the Central Committee and the Work Committee of State Organs, continued to implement the summer internship program, organizing 770 college students (including international students) from 31 provinces (autonomous regions, municipalities) and the Xinjiang Production and Construction Corps, Hong Kong, Macao, and overseas to carry out summer internships in 47 ministries and commissions ; Cooperating with 10 enterprises including China Railway Group and -4- China Telecom to implement the central enterprise internship project, providing a total of 5,179 internship positions. The Sichuan Provincial Committee of the Communist Youth League, the Organization Department, the Education Department and other departments implemented the "Dream Chasing Plan", and recruited more than 20,000 various internship positions. The Fujian Provincial Committee of the Communist Youth League, the Department of Finance, the State-owned Assets Supervision and Administration Commission and other units have provided 15,000 internship positions for college students. The Tuanshanxi Provincial Party Committee organized the "Youth Prosperity Jin" internship practice activity, mobilizing enterprises and institutions across the province to provide 11,000 internship positions. The Hubei Provincial Committee of the Communist Youth League built an online service platform for college students' internship training. More than 5,000 college students signed up to participate, and the number of page views on the platform exceeded 1 million. V. Strengthen investigation and research to serve the employment needs of college students in a more targeted manner. The Central Committee of the Communist Youth League has established a direct contact system for the employment of the Youth League committees in 131 local colleges and universities across the country to keep abreast of the latest employment trends of graduates; On the basis of questionnaire surveys and field visits, the paper focuses on promoting the employment of graduates from poor families in local common colleges, strengthening the supply of job information, helping to improve socialization capabilities, and guiding college students to establish a correct outlook on employment. decision-making reference. Group organizations in Beijing, Jiangxi, Sichuan, Gansu and other regions have carried out in-depth surveys and research on the employment of college graduates in the region, dynamically grasped the front-line situation, and made timely research and put forward countermeasures and suggestions. In addition, local league organizations also combined the clues found in the survey to actively explore and solve employment-related "urgent, difficult, anxious and hopeful" problems, which were welcomed by college graduates. The Shandong Provincial Party Committee of the Tuanshan Group has built 223 youth post stations, providing one-stop services such as free temporary accommodation, policy interpretation, job promotion, business matching, and skill training for fresh college graduates. The Guangdong Provincial Committee of the Communist Party of China implemented the "Youth-5-Affordable Housing" plan, and mobilized enterprises to provide 3,685 sets of preferential and high-quality rental houses for the 2021 college graduates. —6—(No text on this page)—7—Shake Distribution: General Office of the Central Committee of the Communist Party of China, General Office of the State Council. Secretary of the Secretariat of the Central Committee of the Communist Youth League, relevant ministries and commissions of the Central Committee, and provincial party committees in charge of leadership. The heads of the various departments and directly affiliated units of the Central Committee of the Youth League, and the secretary of the Provincial Youth League Committee. Shake Issued by the General Office of the Central Committee of the Communist Youth League on November 1, 2021

2021-11-1 17 Editor's note: At present, more than 60% of the members of the Communist Youth League are students, and the activities of the Young Pioneers are basically in schools. Establishing a good cooperation mechanism between the Communist Youth League and the Young Pioneers is very important for the Communist Youth League and the Young Pioneers to perform their duties. The Communist Youth League (Young Pioneers) Work Steering Committee of the School was jointly established by the Chongqing Municipal Committee of the Communist Youth League and the Municipal Education Commission. It adheres to the joint construction mechanism, joint discussion on reforms, joint efforts in five educations, and joint promotion of services, so as to promote the improvement of the education chain of the party, the League, and the team. Better Play the function of educating people for the party. Its experiences and practices are now compiled and distributed for reference by other regions. —1—Chongqing Municipal Committee of the Communist Youth League established a cooperative mechanism for group education to promote the improvement of the integrated education chain of the party, league and team Committee), and jointly issued documents to clarify the functional positioning, main tasks, organizational form, and working mechanism. Both sides of the Youth League and Education Group in Chongqing use the Steering Committee as a collaboration platform, adhere to the joint construction mechanism, discuss reforms, focus on five educations, and promote services, explore the formation of a normalized and integrated coordination mechanism, and work together to promote the implementation of the "Central Committee of the Communist Party of China" by the Municipal Party Committee. Concerning the specific measures of "Opinions on Comprehensively Strengthening the Work of the Young Pioneers in the New Era", carry out the reform supervision of the Communist Youth League, the Students' Union, and the Young Pioneers every year, and make it clear that the score in the school's party building supervision and assessment shall not be less than 10%, and deepen the sampling evaluation of the student union reform The student satisfaction rate reached 99%, and the work of the Communist Youth League and Young Pioneers of the city's schools gradually achieved high-quality development. 1. Face-to-face group education, jointly build three mechanisms, and improve the work chain of the party, league, and team. First, jointly build an overall coordination mechanism. Under the Steering Committee, set up three special committees for colleges and universities, secondary vocational schools, and basic education, and establish communication and coordination mechanisms such as plenary meetings, special committee meetings, and special meetings. Every year, a plenary meeting is held with the main responsible comrades of both the Youth League and the Education Committee to formulate and issue annual work points to promote the organic connection of policies, effective linkage of work, timely sharing of information, and detailed implementation of tasks. Promote 97% of the counties (districts) to establish a working mechanism for the Secretary of the Educational Youth League Working Committee to serve as the (part-time) Deputy Secretary of the Youth League County (District) Committee. The second is to jointly build a research and supervision mechanism. Incorporate the Communist Youth League Committee into the Municipal Party Committee's Educational Work Leadership-2-Group and the member units of the Municipal Government Education Steering Committee, and incorporate the reform of college student league studies and association management into the investigation and supervision of party building and ideological and political work in public colleges and universities and the annual inspection of private colleges and universities. Accounting for 10%; Incorporate the work of the Young Pioneers into the supervision and assessment of party building in primary and secondary schools, and the score shall not be less than 10%. The third is to jointly build a mechanism for promoting the best to join the party. Implement the two general and two main requirements, and further improve the mechanism of promotion of excellent candidates to join the party, so that the promotion of excellent candidates becomes the main channel for the development of student party members in the school field, and the league members become the main source for the development of young student party members. The proportion of excellent party members reaches 100%. 2. League education, heart to heart, discussing four reforms to enhance the vitality of the league and team organization. First, coordinate the promotion of the reform of the Communist Youth League in schools. Focusing on the reform goals of strengthening the three characteristics and removing the four modernizations, the Youth League and Education jointly formulated "Several Measures for Deepening the Reform of the Communist Youth League in Schools" and a task list and reform evaluation form to dynamically grasp the reform of the Communist Youth League in various colleges and universities. The relevant responsible comrades give guidance , One school, one policy and one account to promote deepening reform. The second is to coordinate and promote the reform of the Young Pioneers. Incorporate the work evaluation of the Young Pioneers into the education evaluation of primary and secondary schools, and promote the realization of the four norms (regularly hold county and district youth congresses, standardize the establishment of school youth working committees, standardize the development of batches of teams, standardize the implementation of moral education directors and assign brigade counselors ). Jointly build a step-by-step growth incentive system for young pioneers, improve the incentive mechanism for joining the team, medals, honors, posts, practice, and promotion, and strive to enhance the sense of honor of young pioneers. The third is to coordinate and promote the reform of the student union. Jointly hold the student union reform work deployment meeting to achieve the leadership of the school party committee and the guidance of the youth league committee, the focus of the main responsibility and the main business, the streamlining of institutional personnel, the selection process of the best, and the strict governance of the committee. Grasping the "Student Congress" as a key point in deepening reform, we issued -3- "Guidelines for the Work of the Student Congress" to demonstrate and guide 100% of the city's colleges and universities to hold student congresses in a standardized manner. Jointly carried out a random sampling evaluation of the deepening reform of the student union, and the satisfaction of the interviewed students with the overall work of the student union and the staff reached 99%. Establish a system of debriefing and review meetings at the end of each semester to create a fresh and sunny organizational image. The fourth is to coordinate and promote the management of college student associations. Jointly forward the "Management Measures for the Construction of College Student Associations", incorporate the management of associations into the supervision of party building and ideological and political work in colleges and universities, incorporate the work of student associations into the overall pattern of ideological and political work and group work, and scientifically guide the youth league committees of colleges and universities to establish association management departments. Ensure that student associations are equipped with instructors and guide the healthy and orderly development of student associations in colleges and universities. 3. Group education should be practiced side by side, and the five educations should be carried out simultaneously to demonstrate the effectiveness of moral education. Jointly carry out activities such as learning and publicizing General Secretary Xi Jinping's important speech on July 1, party history learning and education, and the spirit of the Fifth Plenary Session of the 19th CPC Central Committee, designing and developing a small program of Q&A about party history knowledge, and holding party history learning and education theme exhibitions , built a four-in-one learning system of promoting learning, guiding learning, practicing learning, and supervising learning, and carried out various party history propaganda activities for more than 21,000 times, covering more than 852,000 young college students, and promoted party history learning and education. Deep and solid. Continue to carry out work such as "Youth Great Learning", "Tsing Ma Project", "Youth Hearts to the Party·Building Merits in a New Era", and guide young students to listen to and follow the party, and to be practitioners of "please the party at ease and strengthen the country". The second is to focus on intellectual education and solid foundation, and jointly grasp innovative practices. Based on the growth needs of young people, jointly organized the "Challenge Cup" selection competition, innovation training camp, small technological invention small production display, career role experience and other events, attracting more than 200,000 young students to participate. Mobilize Chongqing University, Southwest University and other 6-4-ring university innovation ecosystems to build youth entrepreneurship spaces, organize various youth entrepreneurship trainings on a regular basis, and guide young college students to actively participate in academic and technological innovation practices. The third is to focus on physical and aesthetic sports, and jointly grasp cultural and sports activities. For 40 consecutive years, a series of spring activities on the campus of college students have been held every year, integrating ideological and political education into cultural, artistic, and sports activities, and determining sub-items such as marathon competitions, singing contests, speech contests, and football contests by ordering online. Attract millions of college students to participate and pay attention. Innovate to carry out the cultivation and construction of excellent league school work brands, and continue to cultivate and build excellent league school work brands that can be demonstrated, promoted, and sustainable. The fourth is to focus on labor training and jointly grasp voluntary services. Promote the implementation of the "Opinions on Comprehensively Strengthening Labor Education in Universities, Middle Schools and Primary Schools in the New Era", jointly select and send young volunteers from Camellia to work in towns and villages, and organize volunteers from Smurfs to participate in the Wisdom Expo, Western Conference, and Talent Conference , Agricultural Expo and other competition services, and set up He Xiao Qing Ye and Jie Xiao Qing Ye college student volunteer service teams to carry out activities such as patrolling rivers and mooring rivers, planting and protecting greenery, and sorting garbage on a regular basis, educating and guiding young students to participate in voluntary services, In the process of public welfare activities, we can grow knowledge, increase talents, and make contributions. 4. Group education, hand in hand, jointly promote a series of services to promote the growth and development of young students. First, jointly carry out the work of caring for students in difficulties. Since 2020, we have persisted in carrying out the Winter Sunshine Warm You and Me Caring Action, opened Tencent’s online love system, and normalized point-to-point to help more than 3,000 left-behind children in deeply impoverished villages and towns realize their personalized New Year wishes; mobilized social forces to raise 4.41 million yuan , Send care to 31,000 left-behind children and teenagers from difficult families. Implemented public welfare projects such as Hope Project·Dream Action, -50,000-100,000+ student funding plan, and sponsored a total of 3,106 students from poor families. The second is to jointly safeguard the legitimate rights and interests of young students. Relying on the 12355 Youth Comprehensive Service Platform and the Youth Chongqing APP, etc., it provides young students with psychological assessment, online consultation and other public welfare services. At the same time, it provides 68 colleges and universities with a full coverage of the "Happiness Lecture" activity, and walks into 152 primary and secondary schools in remote areas for demonstration Carry out the "Growth Classroom" activities to provide online and offline dual protection for the mental health of young people. Actively do a good job in negotiating endorsements for the interests of young people, soliciting a total of 986 small proposals for red scarves and addressing 138 concerns of young pioneers. The third is to jointly carry out employment assistance for graduates from difficult families. Print and distribute the "Notice on Carrying out the Accurate Assistance Action for the Employment of College Graduates in Chongqing with Thousands of Schools and Jobs", and continue to carry out the employment service season activities of the Twin Cities Youth Talent Plan to provide a comprehensive job search and employment service for college graduates. Stand-by help. Since 2020, league organizations at all levels have been mobilized to jointly hold more than 4,000 online and offline job fairs, providing more than 860,000 jobs. League cadres at all levels have paired up to help nearly 10,000 graduates from poor families find employment. —6—(No text on this page)—7—Shake Distribution: General Office of the Central Committee of the Communist Party of China, General Office of the State Council. Secretary of the Secretariat of the Central Committee of the Communist Youth League, relevant ministries and commissions of the Central Committee, and provincial party committees in charge of leadership. The heads of the various departments and directly affiliated units of the Central Committee of the Youth League, and the secretary of the Provincial Youth League Committee. Shake Issued by the General Office of the Central Committee of the Communist Youth League on November 1, 2021

2021-9-9 16 Implementing the "Opinions of the Central Committee of the Communist Party of China on Comprehensively Strengthening the Work of the Young Pioneers in the New Era" Special Issue 3铱The Shanxi Provincial Youth Working Committee took action upon hearing the order and acted actively. In accordance with the deployment of the Youth League Central Committee and the National Youth Working Committee, it took the initiative to seek the support of the Provincial Party Committee and relevant departments, insisted on high-level promotion, insisted on coordinated efforts, insisted on problem orientation, and twisted key policies. In order to be practical and effective, promote the introduction of provincial implementation plans, with practical measures, many highlights, and good effects. Its experiences and practices are now compiled and distributed for reference by other regions. —1—Three Persistences of the Shanxi Provincial Party Committee to solidly promote the introduction of the implementation plan for the provincial Young Pioneers work. The work of the Young Pioneers in the new era has been fully deployed. The Shanxi Provincial Party Committee and the Shanxi Provincial Youth Working Committee organically combined the implementation of the spirit of the "Opinions" with deepening the reform of the Young Pioneers, focusing on solving the problems that restrict the reform and development of the Young Pioneers and the key needs of the grassroots. , work hard, and solidly promote the introduction of provincial implementation plans, for the whole groupImplement the "Opinions" and explore the Shanxi program. 1. Based on career development, three persistences to explore the path of Shanxi 1. Emphasize politics, plan early, and insist on high-level promotion. After the "Opinions" were issued, the Shanxi Provincial Party Committee and the Shanxi Provincial Youth Working Committee conscientiously studied and understood the spirit of the "Opinions", and actively reported the situation and suggestions to the Provincial Party Committee. The Education Working Committee of the Provincial Party Committee took the lead in meeting the requirements of the instructions, and jointly set up a special class with the Provincial Department of Education to draft the implementation plan, forming a joint force of group education cooperation. At the same time, according to the progress of the drafting work, timely report to the Provincial Party Committee and the Central Committee of the Communist Youth League for relevant matters, so as to ensure that the drafting work accurately implements the instructions and requirements of the Provincial Party Committee and the Central Committee of the Communist Youth League. 2. Emphasize strategy, strive for tenacity, and insist on synergistic efforts. The Shanxi Provincial Party Committee and the Shanxi Provincial Youth Working Committee are clearly aware that the "Opinions" have given them the greatest political confidence, but they lack the spirit to fight tough battles, and they cannot seize even the best political opportunities. From the very beginning, special forces were organized to systematically sort out relevant policy documents, and to learn about the situation and experience from brother provinces on difficult issues such as work funds, counselor allowances, and appraisal-2-recognition. Go deep into the grassroots to conduct research, extensively collect and refine fresh experience and practices from the front line, and provide practical support for policy formulation. In line with the tenacity of not withdrawing troops if the goal is not achieved, one step forward is one step forward, we have held more than 40 meetings with responsible comrades from the Organization Department of the Provincial Party Committee, the Provincial Organization Office, the Provincial Department of Human Resources and Social Security, and the Provincial Department of Finance to tackle key issues one by one , It has been a long time to achieve the goal and promote the smooth introduction of the implementation plan. 3. Emphasize practical results, focus on operations, and insist on problem orientation. In order to make full use of the major policy benefits of the "Opinions", the Shanxi Provincial Party Committee and the Shanxi Provincial Youth Working Committee focused on the obsessive issues that have long restricted the reform and development of the Young Pioneers and the key needs of the grassroots, exploring the use of task-based, project-based According to the working method of "construction", engineering, list, and plan, the "Opinions" were detailed one by one, the topic was established, and the implementation plan with 5 major projects and 26 measures as the main content was summarized, and the leading unit and cooperation were clarified item by item. Units, taking into account both policy systematization and practical operability. 2. Anchor first-class goals, nine bright spots. Hand over the Shanxi answer sheet 1. Focus on the shortcomings and weaknesses, so that the Young Pioneers organization covers a wider range. It is proposed to establish the Young Pioneers organization in an all-round way in the first grade of junior high school, guarantee the basic conditions for the organization of the Young Pioneers in rural areas, and promote the establishment of teams in all private primary and secondary schools through concentrated tackling and pairing guidance. Congress and other measures to strengthen the construction of the Young Pioneers organizational system in all areas. 2. Improve curriculum development and make the Young Pioneers activity courses more professional. It is proposed to increase the research and development and promotion of guidance books for activity courses such as the Red Scarf Book Department, and include them in student textbooks to ensure them, actively carry out activities such as micro-team class display, excellent case collection, inter-school observation-3-communication, and establish Young Pioneers activities in schools The course teaching and research group and other measures will consolidate the basic position of the Young Pioneers activity class throughout the process. 3. Relying on local resources, make the Young Pioneers more effective in practical education. It is proposed to use the rich resources of the Red Revolution, human history, and natural ecology to carry out the red scarf research and study activities, create a group of research routes and curriculum resources with Shanxi characteristics, and guide the Young Pioneers organizations at all levels in the province to continue the red blood. Further integrate with the strengthening of practical education, and focus on cultivating the emotional feelings of loving the party, the country, socialism and the hometown among the young pioneers. 4. Improve the education chain, so that the red genes of the party, regiment and team can be passed on more closely. Propose measures such as establishing a hierarchical and internally cohesive Young Pioneers ceremony education system, formally incorporate the red transmission activities into the province's Young Pioneers ceremony education system, and guide children to participate in passing party flags, national flags, military flags, regiment flags, team flags, etc. In the process of raising the red flag, we will deepen our understanding of the blood relationship between the party, regiment and team. 5. Make overall plans to solve problems, and make the team building of counselors more powerful. On the one hand, it is clearly stated that the honors, research results obtained, projects participated in, and experience exchange activities undertaken by the counselors in the Young Pioneers organization should be exchanged with the honors obtained by the education department, research results obtained, topics participated in, and experience undertaken activities are treated equally; on the other hand, four policy guarantees for inclusion are clearly proposed, namely: the Young Pioneers studios are included in the construction of subject studios of the same level of education department; , The winners of the counselor skills competition are included in the scope of the labor competition committee's merits, and the brigade counselor's allowance is included in the implementation scope of the head teacher's allowance to ensure that the counselors have the conditions to work, the officers have -4-platforms, the remuneration is guaranteed, and there is room for development. 6. Enrich the staffing and make the work functions of the grassroots working committees clearer. It is proposed that the city-level Youth League Committee should ensure that a dedicated department and a general counselor are dedicated to the work of the Young Pioneers, and the county-level Youth League Committee should ensure that one team member serves as the deputy director of the Youth Work Committee and one dedicated person is engaged in the work of the Young Pioneers. In terms of guaranteeing grass-roots small workers, there are people who are responsible for the work and who are responsible. 7. Implement quantitative standards to make the leadership and assessment mechanism of the Young Pioneers smoother. It is proposed that the education work leading groups of party committees at all levels include the work of the Young Pioneers in the main points of their annual work, and the joint youth work meeting will hear a special report from the functional departments once a year. Incorporate the work of the Young Pioneers into the supervision and evaluation of primary and secondary education and the standardized construction indicators of party building work, as a separate content in the party building assessment, and the score is not less than 10%. 8. Propose a list of responsibilities to make the group education cooperation mechanism clearer. On the one hand, it clearly puts forward four synergistic responsibilities of the education department, namely: incorporate the work of the Young Pioneers into the overall planning of improving the quality of compulsory education and promoting the reform of basic education, incorporate the construction of the Young Pioneers into the standardized construction of party building work in primary and secondary schools, and incorporate the work of the Young Pioneers into the The quality index system for running schools in primary and secondary schools includes the assessment of Young Pioneers counselors into the annual assessment of school teachers; on the other hand, it clearly puts forward three specific responsibilities for primary and secondary schools, namely: include the construction of young working committees in the scope of party building work, and include The performance of students in the Young Pioneers organization, such as red scarf medals, practical activities, and honor incentives, is included in the comprehensive quality evaluation system for students, and the selection of outstanding Young Pioneers collectives and individuals is included in the school's priority evaluation sequence. 9. Included in the financial budget to ensure more adequate funding for the work of the Young Pioneers. Propose—5—The working expenses of the provincial, municipal, and county-level youth working committees should be included in the financial budget at the same level. Taking 15%, 30%, and 55% of the proportion to be arranged and other measures, it is the first time in history to respond to and solve the problem that the small working committees at all levels have no special work funds. 3. Seize policy opportunities and make three efforts to form Shanxi characteristics 1. Focus on the main responsibility of the party's education and work hard on effectiveness. Focusing on the three contemporary issues of the Young Pioneers and the three outstanding issues of being unable to keep up, in terms of content, it focuses on the theme of inheriting the red gene and continuing the red blood, and refines the red scarf heart to the party into seven design plans for one activity , that is: tell a story of party history, walk into a base of party history, learn from a party member pioneer, search for new changes in a hometown, strive to be a red scarf explainer, carry out a research practice, carry out a ritual education, and use comprehensively By means of visits, role models, and immersion experiences, efforts should be made to use children's cultural language to carry out political education, so that party history education can be childlike and down-to-earth; in terms of form, we should pay attention to the complementary role of Young Pioneers' activity courses and school moral education work, Efforts were made to solve problems such as unprominent generalization of themes, random and unsystematic content, and adult-oriented carrier methods, and compiled and published three volumes of guidance books for activity courses "Red Scarf Books Series" divided into low, middle, and high school levels. Based on the theme of “Cultivating the Soul of People and Aspirations”, we provide segmented education and layered guidance to children and adolescents. 2. Improve the socialized work system, and work hard on "extensive". First, the work tasks of promoting the establishment of Young Pioneers organizations in Youth Palaces and Youth Homes affiliated to the Youth League will be clearly included in the annual key work evaluation system of the Youth League and municipal committees, and a clear work signal will be sent to the grassroots; Alternate members and others take the lead in serving as the Young Pioneers - 6 - off-campus counselors, and at the same time make clear and standardized requirements for the standards, appointments, training, etc. The foundation platform further integrates social resources, explores the establishment of small horse bookstores in primary and secondary schools across the province, and promotes the extension of Qingma Project to Shaoma Project; Fourth, in accordance with the general idea of marching towards society and entering online, systematically implement the red scarf Research and study activities, launched 100 red study check-in maps and 11 classic study routes online and offline, simultaneously compiled and published Shanxi's first "Red Journey" youth reader, and jointly launched "Red Scarf Research and Study Youth School" with the provincial TV station column etc. 3. Deepen the organizational reform of the Young Pioneers, and work hard on the "shencheye". The first is to cooperate with the Shanxi Provincial Party Committee Education Work Committee to issue 3 institutional documents, comprehensively promote the group entry into the team, finely deploy the red scarf medals and activities, and standardize the construction and operation of the school's young work committee; The construction of the Youth Work Committee’s double-clearing and zero-level action will promote the regular reelection of the county-level Youth Work Committee in the province; the third is to carry out the retrospect and self-inspection and rectification work on the deployment of the Young Pioneers’ counselors in the province, and further standardize the work of the Young Pioneers’ counselors at all levels. Selection, allocation, appointment and adjustment; the fourth is to formulate a three-level education and training plan for Young Pioneers counselors, and set up 69 red scarf tour groups at the provincial, city and county levels, and strive to create politically valuable, valuable work and a sense of social achievement. The fifth is to strive for the support of the Provincial Market Supervision Bureau, and incorporate the Young Pioneers entry ceremony into the province's quality standardization construction system. —7—Shake Distribution: General Office of the Central Committee of the Communist Party of China, General Office of the State Council. Secretary of the Secretariat of the Central Committee of the Communist Youth League, relevant ministries and commissions of the Central Committee, and provincial party committees in charge of leadership. The heads of the various departments and directly affiliated units of the Central Committee of the Youth League, and the secretary of the Provincial Youth League Committee. Shake The General Office of the Central Committee of the Communist Youth League Issued on September 9, 2021

2021-9-9 15 Implementing the "Opinions of the Central Committee of the Communist Party of China on Comprehensively Strengthening the Work of the Young Pioneers in the New Era" Special Issue 2铱Editor's Note: Since the publication of the "Opinions of the Central Committee of the Communist Party of China on Comprehensively Strengthening the Work of the Young Pioneers in the New Era" has been issued, the provincial Youth League committees have actively Strive for the support of the party committee and earnestly promote the implementation of the work. Up to now, 10 provinces including Beijing, Hebei, Shanxi, Jilin, Hubei, Hunan, Guangxi, Chongqing, Yunnan and Xinjiang have issued provincial implementation documents. The Central Committee of the Communist Youth League and the National Youth Working Committee sorted out the experience and practices of various places in promoting the introduction of implementation plans, improving relevant systems and mechanisms, refining key work measures, and strengthening resource support. This issue introduces relevant practices in detailing key measures and strengthening institutional support in various regions for learning and reference. —1—Refine key measures and strengthen institutional support to further consolidate the foundation for the work of the Young Pioneers in the new era. Beijing, Hebei, Shanxi, Jilin, Hubei, Hunan, Guangxi, Chongqing, Yunnan, Xinjiang and other places actively combined local actual details in the implementation plan The key measures proposed in the "Opinions" will focus on making detailed arrangements in terms of innovating the form of political leadership of the Young Pioneers, deepening the construction of the Young Pioneers' work policy system, and strengthening the guarantee of working conditions and resource support for the Young Pioneers. 1. Enrich the carrier of practice and enhance the sense of honor of the Young Pioneers Aiming at enhancing the sense of honor of the Young Pioneers, fully tap local resources, highlight local characteristics, innovate the form of political leadership of the Young Pioneers, enrich the carrier of the Young Pioneers' practical activities, and strive to improve the effectiveness of educating people. For example, the implementation plan of Beijing proposes to run the Youth Pioneer League School well, set up three-level capital youth pioneer gangs at the city, district, and school levels, and rely on the service guarantee mechanism for major events to organize and mobilize the Young Pioneers to participate in national events, international exchanges, and national ceremonies , Polish the red scarf and love the capital city practice education brand. The implementation plan of Hebei Province proposes to guide the young pioneers to learn and carry forward the spirit of Xibaipo, to carry out mutual assistance activities such as "Hand in Hand-to be a successor to communism", and to hold practical activities such as "juvenile military school review ceremony" and military summer camp. The implementation plan of Shanxi Province proposes to strengthen the publicity of outstanding leaders, revolutionary martyrs, reform pioneers, role models of the times, moral models and other advanced figures, and carry out labor practice education such as looking for craftsmen from a great country and walking into the harvest season with red scarves. The implementation plan of Jilin Province proposes to carry out activities such as current affairs and news classes, competition to be a red scarf explainer, etc., to create a social practice site for the Young Pioneers of Jilin Province—2—map. The implementation plan of Hubei Province proposes to deepen practical education brand projects such as "Hope Homeland", carry out practical experience activities such as "I am a small labor expert", "I am a small environmental protection envoy", and "Red Scarf Moot Court". The implementation plan of Hunan Province proposes to continue to carry out activities such as the story of the party, let me tell you the story of the party, 2035, where are you? Youth theme dialogues, etc., deepen the youth volunteer service action brand of Lei Feng's hometown to learn from Lei Feng, and carry out the campaign to wear the Lei Feng badge and strive to create Lei Feng style Small and medium teams, red scarf one yuan donation and other special activities. The implementation plan of Guangxi Autonomous Region proposes to vigorously carry out red scarf holiday activities such as public welfare and inclusive summer and winter camps and young pioneer training classes, continue to carry out activities such as pursuing red footprints and red scarf boundary monuments, and develop a friendly culture with children from ASEAN countries. Art exchange activities. The implementation plan of Chongqing City proposes to carry out in-depth activities such as "inheriting the spirit of Hongyan, striving to be a newcomer of the era" and "the voice of the red scarf", and deeply carry out the New Year care action for left-behind children in rural areas under "Winter Sunshine Warm You and Me". The implementation plan of Yunnan Province proposes to carry out comprehensive and in-depth education on building a sense of community of the Chinese nation, continue to carry out educational activities on the theme of "Pioneering from primary school to being a pioneer when you grow up", and strengthen exchanges with children in South Asia, Southeast Asia, and member states of the Shanghai Cooperation Organization. The implementation plan of the Xinjiang Autonomous Region proposes to carry out high-quality monthly unified Young Pioneers activity classes in the region, carry out in-depth pairing and hand-in-hand activities, ethnic unity and family-friendly summer (winter) camps, weekend camps, and You come to my house to eat Sanzi, I come to your house to eat moon cakes, I am in the city (countryside)There is a housekeeping activity and so on. 2. Strengthen the funding guarantee and optimize the working environment of the Young Pioneers Focusing on creating a better environment for the growth of children and adolescents, through various means to further strengthen the funding guarantee for the Young Pioneers' work, and make clear institutional arrangements. It is clearly stated in the implementation plans of various places that the red scarves, team badges, and red scarf medals worn by young pioneers for the first time will be uniformly distributed by primary and secondary schools. The implementation plan of Shanxi Province proposes that the working expenses of the provincial, municipal and county-level youth working committees be included in the financial budget at the same level. The proportions of 15%, 30%, and 55% will be arranged by the level finance; accelerate the construction of activity course content, increase the research and development and promotion of activity course guidance books such as the Red Scarf Book Series, and ensure that they are included in student textbooks. The implementation plan of Hunan, Yunnan and other places proposes to encourage all levels of finance to list the work budget of the Young Pioneers separately. If it fails to list it separately, in principle, it should list the work expenses of the Young Pioneers in the work budget of the Youth League Committee at the same level at a ratio of no less than 25%. The proportion of group organization fees supporting the work of the Young Pioneers is not less than 20%. The implementation plan of Hebei Province proposes that the proportion of league organizations at all levels to support the work of the Young Pioneers will be increased from 10% to 20%, and the squadrons will be united by schools to subscribe to team newspapers such as "China Youth Daily". The implementation plans of Jilin, Hubei, Hunan, Chongqing and other places propose that the public funds of all primary and secondary schools should include or list the working funds of the Young Pioneers. 3. Improve policy support and promote the construction of the counselor team Focus on enhancing the overall quality of the Young Pioneers counselor team and optimizing the member structure, focusing on further improving relevant policies and systems in terms of implementing political treatment, strengthening basic guarantees, deepening assessment and evaluation, and improving the incentive system. For example, the implementation plan of Chongqing City proposes that all districts and counties implement a system of double general counselors in the Youth League Committee and the education department, with formal cadres serving as counselors. The implementation plan of the Xinjiang Autonomous Region proposes to establish and improve the admission and exit mechanism of counselors, and the post of school counselors can be openly recruited to the public. The implementation plan of Beijing is put forward - 4 - to build a training system for Young Pioneers counselors at the city, district and school levels. The implementation plan of Hebei Province proposes to include the Young Pioneers counselors and the directors of the city and county youth working committees into the regiment cadre training, Qingma Project and other talent training systems. The implementation plans of Beijing, Yunnan and other places propose to increase support for counselors to participate in the evaluation of professional titles, and clearly set up the Young Pioneers-related evaluation subjects separately. The implementation plans of Beijing, Hebei and other places propose to carry out the evaluation of the titles of backbone counselors, counselor leaders, and special counselors, and implement equal treatment for disciplines. The implementation plan of Shanxi Province proposes that the winners of the vocational skills competition for counselors be included in the merits of the labor competition committee. The implementation plan of Hubei Province proposes to actively recommend outstanding Young Pioneers counselors to serve as candidates for the National People's Congress at all levels and CPPCC members, and to recommend outstanding players of the Young Pioneers counselors' professional quality exhibition activities to participate in the evaluation of Hubei Province Technical Experts and Hubei Province Youth Post Experts. The implementation plans of Shanxi, Guangxi and other places propose to set up counselor post allowances in accordance with the class teacher post allowance standards, and increase the total amount of school performance wages accordingly. —5—Shake Distribution: General Office of the Central Committee of the Communist Party of China, General Office of the State Council. Secretary of the Secretariat of the Central Committee of the Communist Youth League, relevant ministries and commissions of the Central Committee, and provincial party committees in charge of leadership. The heads of the various departments and directly affiliated units of the Central Committee of the Youth League, and the secretary of the Provincial Youth League Committee. Shake The General Office of the Central Committee of the Communist Youth League Issued on September 9, 2021

2021-9-9 14 Implement the implementation of the "Opinions of the Central Committee of the Communist Party of China on Comprehensively Strengthening the Work of the Young Pioneers in the New Era", one of the special issues铱Editor's Note: Since the publication of the "Opinions of the Central Committee of the Communist Party of China on Comprehensively Strengthening the Work of the Young Pioneers in the New Era", all provincial Youth League committees have actively Strive for the support of the party committee and earnestly promote the implementation of the work. Up to now, 10 provinces including Beijing, Hebei, Shanxi, Jilin, Hubei, Hunan, Guangxi, Chongqing, Yunnan and Xinjiang have issued provincial implementation documents. The Central Committee of the Communist Youth League and the National Youth Working Committee sorted out the experience and practices of various places in promoting the introduction of implementation plans, improving relevant systems and mechanisms, refining key work measures, and strengthening resource support. This issue introduces the relevant practices of promoting the promulgation of the provincial "Opinions" for reference. —1—Actively promote the introduction of the provincial-level "Opinions" implementation plan to strengthen the party's overall leadership over the work of the Young Pioneers in the New Era Actively seek the support of the party committee and deeply participate in the drafting of the implementation plan. Take this opportunity to promote the establishment of an implementation mechanism under the leadership of the party committee, further strengthen the party's overall leadership over the work of the Young Pioneers in the new era, and improve and implement the system of team leadership and team-education cooperation arrange. 1. Gain the support of the party committee and promote the research and introduction of the implementation plan. After the "Opinions" were issued, all provincial Youth League committees made special reports to the party committee at the first time, creating favorable conditions for the implementation of the work. Among them, the main comrades in charge of the party committees in Hebei, Shanxi, Guangxi, Yunnan, Xinjiang and other places issued specific instructions and instructions. , Hunan, Guangxi, Chongqing, Xinjiang and other places are included in the annual work points of the provincial education work leading group. The provincial Youth League committees in Beijing, Hebei, Shanxi, Jilin, Hubei, Hunan, Chongqing, Xinjiang and other places guided and promoted the city and county level Youth League committees to report to the party committees for support and achieve full coverage. Under the strong leadership and strong support of the party committees, the provincial youth league committees quickly set up special work teams to take the lead in drafting local implementation plans. During the drafting process, on the one hand, it focused on pooling the wisdom of all parties, and extensively listened to the opinions and suggestions of grassroots youth league committees, youth working committees, Young Pioneers counselors, Young Pioneers workers, educators, experts and scholars, etc. Put forward targeted solutions; on the other hand, focus on strengthening -2-departmental coordination, solicit opinions from relevant units such as the Education Department, Finance Department, Human Resources and Social Security Department, as well as relevant units at the city and county levels in multiple rounds, and carry out sufficient communication Negotiations, and strive to further implement the requirements of the "Opinions", improve the operability of work measures and the golden content of policies. After the drafting work is completed, the provincial youth league committees continue to strengthen the request for instructions and reports, promote the provincial party committees to conduct special research, and accelerate the progress of publishing documents. 2. Strengthen leadership responsibilities and promote departmental coordination to implement measures. The provincial Youth League committees will take the opportunity of introducing implementation plans to further strengthen the leadership responsibilities of party committees at all levels. The team educates people to connect. In the implementation plan that has been issued, all the responsible comrades of the local party committees at all levels are clearly required to serve as the honorary director of the Young Pioneers at the same level, and the governments at all levels specify a responsible comrade to contact the Young Pioneers for work. , Comprehensively strengthen the party's leadership over the work of the Young Pioneers. Among them, the implementation plan of Shanxi Province proposes that the youth work joint conference should listen to a special report from functional departments once a year; the implementation plan of Hubei Province proposes to establish an inter-departmental joint conference system for the work of the Young Pioneers. The implementation plans of Hebei, Shanxi, Jilin, Hunan, Guangxi, Yunnan and other places propose to incorporate the work of the Young Pioneers into the supervision and evaluation of primary and secondary education at all levels and the standardization construction indicators of party building work, as a separate content in the party building assessment, with a score of no less than 10 % . At the same time, the local implementation plans also make arrangements for the implementation responsibilities and working mechanisms of party committees and government functional departments, clarifying the main body of responsibility for each measure and the coordination mechanism in important fields and important projects. Jilin, Chongqing, Xinjiang and other places have also specified details. Division of labor arrangements and completion time limit requirements. 3. Improve the leadership of the whole group and promote the deepening of the group-education cooperation mechanism—3—In terms of deepening the leadership of the whole group, the implementation plans in various places have been clearly proposed, and the responsible comrades of the provincial and municipal Youth League committees, and the main responsible comrades of the county-level Youth League committees will serve as peers. The director of the Young Pioneers at the lower level will further strengthen the Communist Youth League’s leadership of the Young Pioneers in terms of politics, organization, team, and work, and earnestly fulfill the important political responsibility of leading the team. Among them, the implementation plan of Hebei Province proposes that the score and weight of the work of the Young Pioneers in the assessment indicators of the Communist Youth League at all levels should not be less than 30%. The implementation plans of Hebei and Shanxi provinces propose that the Young Pioneers counselors account for no less than 10% of outstanding youths and advanced workers commended by the Communist Youth League. The implementation plan of Shanxi Province proposes that youth league organizations at all levels should recruit outstanding Young Pioneers counselors as representatives of the congress, committee members, and standing members; Add a member of the team to serve as the deputy director of the Young Work Committee, and a dedicated person to work on the Young Pioneers; the number of Young Pioneers counselors in the training of regiment cadres at all levels should not be less than 20%. The implementation plan of Jilin Province proposes to incorporate the work of the Young Pioneers into the propaganda and ideological work pattern of the League, and strengthen the construction and publicity of the Young Pioneers on the Internet. The implementation plans of Hubei and Yunnan provinces propose that the main responsible comrades of the league organizations at all levels are the first responsible persons for the work of the Young Pioneers. The implementation plan of the Guangxi Autonomous Region proposes that no less than 20% of the group fees of the league organizations at all levels should be used to support the work of the Young Pioneers. In terms of deepening group-education cooperation, the implementation plans of various regions have clearly proposed to establish a system of dual directors of the provincial, municipal, and county-level youth working committees, specify that responsible comrades from the education department serve as directors of the youth working committees at the same level, and establish regular consultations on research work, major Mechanisms such as communication and coordination of matters, refinement of the education supervision list, mutual appointment of cadres and part-time jobs. The implementation plan of Hebei Province proposes that the Youth League committees and education departments at the county level and above should study the work of the Young Pioneers at least once every six months (semesters). The implementation plan of Shanxi Province proposes to incorporate the development of the work of the Young Pioneers into the quality index system of primary and secondary schools. The implementation plan of Chongqing proposes that the municipal and district levels hold at least one working meeting of the school Communist Youth League (Young Pioneers) Work Steering Committee every year, and hold at least one meeting of the directors of the Young Pioneers Committee every semester; Secretary, concurrently (suspended) as deputy secretary of the Youth League Committee at the same level. —5—Shake Distribution: General Office of the Central Committee of the Communist Party of China, General Office of the State Council. Secretary of the Secretariat of the Central Committee of the Communist Youth League, relevant ministries and commissions of the Central Committee, and provincial party committees in charge of leadership. The heads of the various departments and directly affiliated units of the Central Committee of the Youth League, and the secretary of the Provincial Youth League Committee. Shake The General Office of the Central Committee of the Communist Youth League Issued on September 9, 2021

2021-8-19 13 Editor’s note: Since 2000, the Chongqing Municipal Committee of the Communist Youth League has relied on the joint meeting mechanism of the city’s youth students’ off-campus education work, and has always adhered to the political, public welfare, professional, and practical aspects, actively building a matrix of out-of-school education under the Youth League , take the initiative to cooperate with school education to promote the innovative development of youth off-campus education. Its experiences and practices are now compiled and distributed for reference by other regions. —1—Four insistences and four strengthenings of Chongqing Municipal Committee of the Youth League to promote innovation and development of out-of-school education for young students. In the Youth League Committee. Over the past 21 years, the Chongqing Municipal Committee of the Youth League has insisted on integrating politics, public welfare, professionalism, and practicality throughout the entire work process, and promoted the establishment of an out-of-school education of the Youth League, including the Municipal Children's Palace + 38 district and county youth activity centers + 871 youth homes The field matrix covers a total area of about 210,000 square meters, offers more than 1,200 training programs, and serves more than 1.5 million youths annually. The third-party evaluation results show that the satisfaction of students and parents with related work is over 90%. 1. Adhere to political nature and strengthen ideological leadership. Always put the requirements of ideological and political leadership in the first place, and actively promote various tasks to focus on serving the political task of educating people for the party. The first is to strengthen political control. The joint meeting and the director's office meeting are held regularly, and the deployment and progress of various tasks are politically checked in a timely manner, and all parties are coordinated to promote the city's off-campus education work in an orderly and vigorous manner. Incorporate off-campus education work into the key issues of the Communist Youth League Committee Secretary Committee, and conduct political review of the implementation links such as activity content, educational projects, and personnel management. The second is to highlight political attributes. All off-campus activity venues are required to uniformly hang the party, regiment, and team badges, post and arrange elements such as the message from General Secretary Xi Jinping, the red scarf love learning column, the Chongqing Communist Youth League hot pot Hongmeiye IP image, etc., highlighting the cultivation of qualified builders and reliable To meet the political requirements of successors, create a strong atmosphere for educating people. The third is to innovate political education. Carefully designed in terms of theme setting, project declaration, course arrangement, etc., to promote elements such as ideals, beliefs, values, and moral concepts throughout various educational programs. In 2020, the Chongqing Youth Painting, Calligraphy and Film Competition will specially set creative themes such as "I and the Fourteenth Five-Year Lord", poverty alleviation, epidemic prevention and control, etc., to educate and guide young people to recognize the general trend of development and bravely shoulder the responsibilities of the times. Various off-campus activity venues focus on scientific and technological innovation, rural revitalization, ecological environmental protection and supporting the Chengdu-Chongqing economic circle, etc., organize young people to participate in scientific and technological innovation, conduct investigations and research, and participate in voluntary services. Metallurgical and other special projects. 2. Adhere to public welfare and strengthen social benefits Always follow the principle of public welfare, put social benefits in an important position, and strive to achieve the unity of social benefits, talent benefits and economic benefits. One is to maintain the stability of funding sources. Construct a fund guarantee system of central special lottery public welfare fund transfer payment + municipal lottery public welfare fund support + district and county fiscal funds matching, to provide stable financial support for infrastructure construction, equipment allocation, management and operation of various places. Among them, the city-level finance arranges 1.5% of the lottery public welfare fund every year, and has invested more than 1.3 billion yuan so far. The second is to ensure the inclusiveness of site operations. Adhere to free or low-paying services, deepen and expand service functions, make full use of leisure venues and time slots from Monday to Friday to open public welfare projects, and effectively respond to social concerns. The 4:30 classroom activities are held after class and during holidays, and public welfare services such as centralized nursing, academic guidance, cultural and sports activities are provided for students aged 6 to 12, and the practical difficulties of students and parents are solved in a targeted manner. The third is to highlight the inclination of service objects. Focus on young people from difficult families and special young people, as well as young people in remote areas, old revolutionary areas, and ethnic minority areas. Held the "Winter Sunshine Warm You and Me" public welfare activity for 9 consecutive years, and launched microYoung Gong Ye, Migrant Young Gong Ye. Invite representatives of groups such as children from needy families, youths from regions that aid Tibet, and families of anti-epidemic medical workers to participate in youth military summer camps. 3. Adhere to professionalism and strengthen service quality Always adhere to the direction of professional construction, and strive to promote the high-quality development of off-campus education for young students. One is to adhere to professional management. Issued more than 20 institutional documents such as "Chongqing City's Opinions on the Construction and Management of Off-School Activities Sites" and "Chongqing Interim Measures for the Fund Management of Youth Out-of-school Activities Sites" to standardize the construction, assessment, safe operation, and project declaration of the venues manage. Every year, a third party is entrusted to carry out special inspections and audits, satisfaction evaluations, evaluation and grading of each off-campus activity venue and the person in charge of the venue, and allocate funds and projects accordingly to promote professional development through system construction. The second is to create professional courses. Relying on the Municipal Children's Palace to build a central kitchen and cloud platform for youth off-campus education, and provide a unified product supply for all off-campus activity venues. Relying on local characteristic resources to create high-quality courses according to local conditions, realize the overall planning and compilation of course materials, unified distribution, online push of activities and projects, and offline promotion. During the COVID-19 epidemic in 2020, a combination of online and offline methods will be used to hold activities such as exhibitions of youth off-campus education achievements, and exhibitions and evaluations of research and practical courses. The third is to build professional teachers. Establish the city's juvenile students' off-campus education teacher pool, and on the basis of ensuring the quality of teachers in various places and the stability of teaching—4—, coordinate and dispatch education teachers to achieve intensive development. Coordinate and organize young lecturer group members, science and technology experts, non-genetic people, art and sports experts and other volunteers to support teaching in various off-campus activity venues. Persist in holding training courses for site managers, key teachers, and off-campus counselors every year. 4. Adhere to practicality and strengthen method innovation. Always adhere to the habits and lifestyles of young people, highlight experiential, participatory, and interactive features, and guide young people to experience growth and receive education in a natural and active state. On the one hand, focus on innovative practice methods. Online, push wallpapers, comics, games, videos and other themed online cultural products through the Cloud Children's Palace, carry out learning activities such as check-in challenges and answering games, and promote the core socialist values into the minds and hearts of young people in a way that young people like to hear and see. Offline, by holding activities such as special learning, theme discussions, publicity and exchanges, speech contests, calligraphy and painting, etc., we will focus on cultivating the deep feelings of the majority of young people who love the Party, the country and socialism. Since the beginning of this year, we have continued to deepen the youth learning action around learning party history, strengthening beliefs, and following the party. We have organized youth lecturer groups and red scarf tour group members to go to off-campus activity venues to focus on party stories and revolutionary ideas. Stories and hero stories carried out more than 600 themed lectures. On the other hand, focus on carrying out practical activities. Focus on making the major themes detailed, and focus on science and technology, arts and sports, national defense and military education, extensively carry out activities such as exchanges, performances, and competitions, and explore and carry out special education on safety, self-protection, and mental health. Organize youth national defense education theme demonstration activities, introduce military training skills demonstrations, and guide young people to improve their ideological understanding, temper their will, and enhance their overall quality through scenario simulation and interaction-5-practice. Focusing on the development of good activities, we have held talent competitions for elementary and middle school students for 16 consecutive years, held youth military summer camps for 9 consecutive years, held youth calligraphy and painting competitions for 8 consecutive years, and held Dream Classroom Nature Notes Competition for 6 consecutive years, with a total of more than 1 million teenagers benefit from it. —6—(No text on this page)—7—Shake Distribution: General Office of the Central Committee of the Communist Party of China, General Office of the State Council. Secretary of the Secretariat of the Central Committee of the Communist Youth League, relevant ministries and commissions of the Central Committee, and provincial party committees in charge of leadership. The heads of the various departments and directly affiliated units of the Central Committee of the Youth League, and the secretary of the Provincial Youth League Committee. Shake Issued by the General Office of the Central Committee of the Communist Youth League on August 20, 2021

2021-8-18 12 Editor's note: The Beijing Municipal Committee of the Communist Youth League takes the in-depth implementation of the "Medium and Long-term Youth Development Plan (2016-2025)" as the starting point, closely follows the positioning of Beijing, adheres to the first good standard, and highlights the speed, accuracy, and high practicality Requirements, actively promote the compilation of the "Beijing Youth Development Plan during the 14th Five-Year Plan" to provide a strong guarantee for the overall promotion of the high-quality development of the city's youth undertakings. The experiences and practices of Beijing are now compiled and distributed for reference by other regions. —1—The Youth League Beijing Committee quickly, accurately, and practically promoted the compilation of the 14th Five-Year Development Plan for Youth. planning"). In the process of promoting the preparation of the "Plan", the Beijing Municipal Committee of the Communist Youth League took active actions and took the lead in the preparation speed and quality of the plan. 1. Systematic planning and fast compilation 1. Reverse the construction period and advance the compilation work in advance. At the beginning of 2020, the Beijing Municipal Committee of the Communist Youth League began to compile the "Plan", determined the working principle of fast and tight under the premise of ensuring quality, formulated a systematic promotion plan, and started the preliminary research and demonstration and compilation work. In November, after the release of the "Central Committee of the Communist Party of China's Proposals on Formulating the 14th Five-Year Plan for National Economic and Social Development and the Long-term Goals for 2035", the Beijing Municipal Committee of the Communist Youth League studied and understood the relevant spirit, carefully studied the revision of the first draft, and published it on December 12. After reporting to the leaders in charge of the municipal party committee and the municipal government for review at the end of the month, it will be reported to the leading group of the municipal planning preparation work for a coherent review, and the opinions of relevant functional departments will be solicited at the same time. After the National Two Sessions in 2021, it will be further revised to form a deliberation draft in accordance with the relevant spirit of the "14th Five-Year Plan for National Economic and Social Development and Outline of Long-term Goals in 2035", which will be officially issued at the end of May. 2. Link up and down to form a joint force for planning and compilation. The Beijing Municipal Committee of the Youth League regards the compilation work as the top priority of the annual work, and established a leading group with the secretary as the team leader, members of the secretariat, and the main responsible comrades of the machine-2-official departments, and studied and formulated a detailed division of responsibilities and schedule schedule. At the key nodes of each stage of the preparation work, the leading group held 8 leading group meetings, listened to progress reports, conducted special research to solve problems, and effectively played the role of overall planning and overall promotion. Through scientific dispatching, the resources and forces of Beijing Communist Youth League agencies, directly affiliated units, and various aspects have been effectively integrated to form a joint work force of close coordination between the upper and lower levels, avoiding the phenomenon of fighting independently and duplicating labor. 3. Take the initiative to connect and build consensus among member units. Relying on the platform of the Municipal Youth Work Joint Conference System, the Beijing Municipal Committee of the Communist Youth League took the initiative to cooperate with the Propaganda Department of the Municipal Party Committee, the Municipal Development and Reform Commission, the Municipal Education Commission, the Municipal Human Resources and Social Security Bureau, and the Municipal Health and Health Commission, etc.54 Conduct in-depth communication with relevant member units of the country, straighten out the main responsible offices and specific docking personnel for key tasks, and achieve timely consultation and efficient linkage. During the preparation process, the opinions and suggestions of relevant member units were solicited in three rounds, and a consensus was reached through consultations one by one around the 123 feedback contents, laying a solid ideological and institutional foundation for determining the key tasks of the "Plan" and promoting the implementation. 2. Focus on the overall situation and be accurate 1. Implement the national medium and long-term youth development plan with the best standards. In-depth study and implementation of the important instructions and requirements of the Municipal Party Committee on speeding up the preparation of the five-year development plan for youth, planning and promoting the work of the Communist Youth League and youth in the capital with a higher position, a wider vision, and a larger pattern, and conscientiously implement the Central Committee of the Communist Youth League. Opinions on the In-depth Implementation of the Youth Development Plan (2016-2025), adhere to the high political position and the first good-3-standard, define the age range of teenagers as 6 to 35 years old, and demonstrate and propose a series of fourteen During the fifth metallurgical period, the youth development planning indicators that exceeded the national average level, especially in the fields of education, employment and entrepreneurship, and social security, designed a series of targeted youth development policy measures and practical projects. 2. Benchmark the overall situation of the city's 14th Five-Year Metallurgy to plan youth careers. Based on the functional positioning of the capital city and the overall situation of construction and development, combined with the important requirements of the Municipal Party Committee on strengthening the four centers' function construction, improving the four services' level, and realizing the six more prominent ones, benchmarking against the city's 14th Five-Year Plan Outline Clear key issues and major projects, actively respond to and fully connect with the content and key projects in the fields of young talents, education, employment and entrepreneurship, health, grassroots governance, and youth volunteer services in the "Plan", and actively cooperate with other relevant municipal-level special projects The specific arrangement of the plan should be fine-tuned. At the same time, the city’s 14th Five-Year Plan outline was written into the youth development column for the first time, clearly proposing to attract young people to rejuvenate the city, build an urban ecology suitable for youth development, and create employment innovations, improve career development capabilities, housing security, and create a living atmosphere. In terms of better serving the youth. 3. Relying on research and evaluation methods to conduct in-depth research and judgment on the needs of young people. Before the compilation work started, the Beijing Youth League Committee focused on various youth groups in Beijing and carried out more than 20 key researches. It persisted in regularly sorting out the data of the 12345 Citizen Hotline and the 12355 Youth Service Desk, and extensively collected opinions from all walks of life through online and offline channels. There are more than 17,000 suggestions for planning compilation, accumulating a large amount of first-hand materials on the growth and development needs of young people. After the preparation work started, the Beijing Municipal Committee of the Beijing Youth League, together with relevant parties, conducted a systematic evaluation of the implementation of the "Medium and Long-term Youth Development Plan (2016-2025)" and the city's 13th Five-Year Development Plan for Beijing-4-Beijing. The achievements in 38 aspects, 20 landmark policies and practical projects were sorted out, and the problems existing in the construction of the monitoring index system, the operation of the guarantee system, and the establishment of the work system were carefully analyzed, providing an important reference for the scientific preparation of the "Plan" . 3. Improve the mechanism and implement it. 1. Formulate the booklet project for the implementation of the planning task. In the process of compiling the "Plan", the opinions and suggestions of the member units of the joint meeting were absorbed, and the plan for the division of labor for the implementation of the plan was formulated in a timely manner, and the tasks specified in the "Plan" were refined into a sub-project including 130 specific tasks. The work tasks are defined by 1 to 2 main responsible units and several coordinating units, and at the same time, the main responsibilities and coordinating departments within the Communist Youth League Committee are clarified to ensure that the responsibilities of each task are clearly defined and implemented to the individual. 2. Give full play to the advantages of the Youth Work Joint Conference mechanism. Under the leadership of the Municipal Party Committee and the Municipal Government, the Youth Work Joint Conference has been promoted to continuously enrich the institutional connotation and improve the operating mechanism. A series of specific mechanisms have been formed in terms of meeting convening, topic collection, and supervision and implementation. At the same time, conscientiously perform the duties of the Joint Conference Office, insist on promoting the preparation and implementation of the "Plan", supervision and inspection, and effect evaluation, regularly summarize and organize the completion of key tasks of the plan by relevant units, promote the resolution of difficult problems in a timely manner, and strengthen guidance, supervision and implementation. 3. Vigorously excavate the guiding role of planning county-level pilot demonstrations. Adhere to high standards to promote national pilot and municipal pilot work, guide each pilot area to combine its own reality, highlight policy-5-policy orientation, carefully study and formulate pilot implementation plans, and continue to introduce iconic and affecting policies and practical projects. At the same time, focus on pilot projects to drive the general public, and use the annual work evaluation mechanism as a starting point to promote other districts and counties to strengthen the construction of the benchmarking county pilot "Liuyouye" standard, forming a competitive atmosphere of learning to catch up. Attachment: Catalog of Youth Career Development Planning during the 14th Five-Year Plan Period of Beijing—6—Appendix Catalog of Youth Career Development Planning During the 14th Five-Year Plan Period of Beijing 1. Guiding ideology 2. Main objectives 3. Key monitoring indicators Chapter 3. Development fields and development measures 1. Youth ideology and morality 1. Strengthen the education of ideals and beliefs for young people 2. Cultivate and practice socialist core values among young people in the city 3. Strengthen online thought leadership 4. Carry out ideological education and guidance for young people in different groups 2. Youth education 5. Improve the quality of school education 6. Strengthen scientific and technological innovation education 7. Strengthen social practice education Field youth talent team 3. Youth health 10. Improve youth physical health and sports participation 11. Strengthen youth mental health education and services 12. Strengthen youth health promotion work 4. Youth marriage and love 13. Strengthen youth marriage and family concept education and guidance 14. Take multiple measures to serve young people in marriage and making friends 15. Carry out youth sexual health education and healthy marriage and childbearing publicity and education 16. Guarantee the legal rights and interests enjoyed by young female workers during pregnancy, childbirth and breastfeeding 5. Youth employment and entrepreneurship 17. Promote improvement Promoting Youth Employment and Entrepreneurship Policy System 18. Strengthening Youth Employment Services 19. Promoting Youth to Participate in Entrepreneurship Practice 20. Strengthening the Protection of Youth Employment Rights and Interests VI. Youth Culture 21. Building Beijing Youth Cultural Boutiques 22. Enriching Youth Cultural Activities 23. Strengthening Youth Cultural Construction VII , Youth Social Integration and Social Participation—8—24. Improve the Beijing youth organization system led by the Communist Youth League under the leadership of the Party. Grassroots governance 27. Encourage young people to contribute to the new development of the capital 28. Enhance the national identity, national identity, and cultural identity of young people from Hong Kong, Macao and Taiwan. Improve the mechanism for the protection of the rights and interests of young people 32. Crack down on violations of the legitimate rights and interests of minors in accordance with the law 9. Prevent juvenile delinquency and crime Youth social security 36. Strengthen care, care and support for disabled youth 37. Improve the level of youth social assistance Chapter 4 Key Projects 1. Young Marxist Training Project 2. Youth Socialist Core Values Training Project 3. Beijing Youth Network Civilization Development Action-9-4, Beijing Youth Employment Promotion Plan V, Youth Innovation Beijing Smelting Project VI, Beijing Youth Cultural Quality Project VII, Beijing Youth Volunteer Service Brand Deepening Project 8. Youth from Beijing, Hong Kong, Macao and TaiwanYouth Exchange Plan IX. Contact and Guidance Service Plan for Emerging Youth Groups in Beijing Ten. Community Youth Meeting Construction Project Chapter V Organizational Implementation I. Give full play to the role of the municipal youth work joint meeting mechanism and strengthen the organization and leadership of the planning implementation II. Establish a sound city . District youth development planning system 3. Strengthen the construction of youth development promotion and service positions in the city 4. Guarantee youth development funds investment 5. Create a good social environment for planning implementation 6. Establish a monitoring, evaluation and supervision mechanism for planning implementation—01—( No text on this page)—11—Send to: General Office of the Central Committee of the Communist Party of China, General Office of the State Council. Secretary of the Secretariat of the Central Committee of the Communist Youth League, relevant ministries and commissions of the Central Committee, and provincial party committees in charge of leadership. The heads of the various departments and directly affiliated units of the Central Committee of the Youth League, and the secretary of the Provincial Youth League Committee. Shake Issued by the General Office of the Central Committee of the Communist Youth League on August 18, 2021

2021-6-28 11 Editor's note: The Henan Provincial Committee of the Youth League took the opportunity of learning party history, strengthening beliefs, and following the party to learn and educate, and actively promoted the "I do things for youth" campaign to be effective, focusing on college graduates from difficult families Provide employment assistance, work hard in job matching, concept guidance, mechanism guarantee, etc., and the work has achieved actual results. Its experiences and practices are now compiled and distributed for reference by other regions. —1—The Henan Provincial Committee of the Communist Youth League solidly carried out the targeted employment assistance work for graduates from difficult families in colleges and universities. Taking the opportunity of learning and education with the party, we will continue to deepen the employment service action for college students from thousands of schools and thousands of jobs, focusing on providing precise employment assistance to graduates from difficult families in colleges and universities, and actively promoting the "I do things for youth" campaign to achieve results. Since 2020, more than 5,000 league cadres across the province have paired up to help more than 13,000 college graduates from difficult families, and have helped 11,083 of them find employment. 1. Focus on job matching and build a broad platform with Zhiye 1. Collect and publish high-quality employment information, and strive to solve the problem of information asymmetry. Make full use of informatization and network means, rely on the online platform where graduates gather to set up special columns such as employment consultation, etc., collect and dynamically release information about civil servant recruitment, public institution recruitment, grassroots service projects, corporate recruitment positions, and special job fairs All kinds of information, such as information, are accurately pushed to the class of fresh graduates and previous graduates who have left school and are not employed. Since the beginning of this year, nearly 30,000 pieces of employment information have been released on various platforms, covering more than 790,000 graduates. 2. The province's joint efforts to provide jobs will focus on solving the problem of unsmooth channels. Coordinate the network platform and local talent markets, set up 1 provincial-level main venue and 18 municipal-level, N college-level sub-venues, forming a combined force of online, offline, and province-wide linkage services for youth employment. Among them, the New Youth OFFER Festival held the 2021 Henan Provincial College Graduates Spring-2-Air Double Election Meeting, which mobilized nearly 1,000 companies to provide more than 80,000 jobs, and more than 180,000 graduates participated in consultations and interviews; Henan Province's 2021 spring college graduates' double election will provide nearly 4,000 jobs for graduates. 3. Explore and carry out live broadcasting, and focus on solving the problem that the form is not novel. Take the initiative to get close to the life and communication habits of young students, learn from the current popular live broadcasting, design and carry out live broadcasting activities, and invite professional anchors and employer representatives to make professional comments on job-related information, and young graduates can interact online And submit your resume with one click. Up to now, it has successfully carried out 6 industry-specific live broadcasting activities including real estate construction, IT Internet, education and training, agriculture, forestry, animal husbandry and fishery, attracting a total of 200,000 online participation. 2. Focus on concept guidance and use Zhiye to guide the correct direction 1. Pay attention to publicity and guidance, and promote graduates to establish a correct outlook on employment. Focus on strengthening the guidance of employment concepts, focus on finding a group of outstanding graduates who have successfully employed themselves as youth employment boosters, and tell graduates face-to-face stories of struggle and growth through live interviews, campus interactions, recording public welfare videos, etc. Guide them to establish a concept of employment and success that can make meritorious deeds, can establish a career everywhere, struggle is the most beautiful, and labor is the most glorious. Since the beginning of this year, more than 3,000 activities of various types have been held, covering more than 430,000 college graduates. 2. Pay attention to practical education and promote graduates to participate in workplace experience in advance. Organize young students to extensively participate in employment internships and social practice, and guide them to consciously integrate career planning into the overall situation of national development. On the post side, 49 practice units with high-quality posts and good internship effects have been developed and constructed, which have been recognized by the Provincial Department of Human Resources and Social Security as Henan Youth Employment See-3-Practice Units. On the student side, social practice activities such as "Three Going to the Countryside" and "Returning to Hometown" were promoted in an orderly manner. In the summer of 2020, more than 550,000 college and technical secondary school students from 141 colleges and universities in the province formed a total of 6,332 practice teams, of which 18,491 students participated In order to return home to practice social practice activities. 3. Pay attention to entrepreneurial incentives and promote graduates to improve their enthusiasm for innovation and entrepreneurship. Strengthen the demonstration and leading function of the Innovation Youth Competition, hold a series of competitions such as the Henan Youth Innovation and Entrepreneurship Competition, the Challenge Cup Henan Youth Entrepreneurship Plan Competition, and the 2021 Challenge Cup Provincial Competition attracted 2,444 entries from 91 colleges and universities and 120,000 college students works to participate. Actively make good use of the Golden Seed Program for College Students Entrepreneurship, focusing on supporting college student entrepreneurship projects with certain operability and applicability, as well as good market potential, social value and development prospects, and continuously boosting the confidence of young student officers in entrepreneurship. 3. Focus on strengthening guarantees, and use manufacturing to gather strong synergy 1. Improve the resource integration mechanism and cooperate with multiple parties. 14 departments including education, human resources and social security, disabled people's federation, etc. launched a public welfare support action for college graduates in Henan Province in 2021 in a new era · new dream - employment and entrepreneurship. The member units of the Young Entrepreneurs Association provide jobs for key integration, and rely on the intelligent employment service platform to provide precise employment support for the employment of difficult graduates. Since the beginning of this year, the province's Communist Youth League system has promoted more than 2,800 online and offline recruitment activities, covering more than 700,000 college graduates, of which more than 400 activities focused on graduates from poor families. 2. Improve the tracking service mechanism and provide precise pairing assistance. Organize the province's league cadres to contact and serve 1 to 3 college and technical secondary school graduates, provide employment guidance, recommend employment-4-information, and follow up on employment progress, etc., and accurately promote the solution of each graduate's outstanding difficulties . For example, the Youth League Committee of Zhengzhou University of Light Industry created the three-color work method, which classifies and marks students according to their different employment status. Green indicates that they have been successfully employed or have entered a higher education; Cadres help each other in pairs. In this way, no one is missed in employment assistance, which has been widely praised by graduates. 3. Improve the linkage mechanism of work and advance in a criss-cross manner. Taking the youth development plan as the starting point, promote the member units of the provincial joint meeting to focus on launching a number of practical projects to serve youth employment, focusing on graduates from poor families. The Henan Provincial Committee of the League and the Provincial Department of Human Resources and Social Security signed the "Framework Agreement on the Joint Implementation of the Henan Youth Employment and Entrepreneurship Promotion Action (2020-2022)" to carry out in-depth cooperation around serving youth employment and entrepreneurship. A special group for youth employment and entrepreneurship work of the Communist Youth League and Provincial Committee was established, and special work meetings were held regularly to deploy and promote targeted employment assistance for graduates from difficult families. —5—Shake Distribution: General Office of the Central Committee of the Communist Party of China, General Office of the State Council. Secretary of the Secretariat of the Central Committee of the Communist Youth League, relevant ministries and commissions of the Central Committee, and provincial party committees in charge of leadership. The heads of the various departments and directly affiliated units of the Central Committee of the Youth League, and the secretary of the Provincial Youth League Committee. Shake Issued by the General Office of the Central Committee of the Communist Youth League on June 28, 2021

2021-6-17 10 Editor's note: The party constitution stipulates that the secretary of the county-level and below-county-level committees of the regiment, and the secretary of the league committee of enterprises and institutions, who are party members, can attend the meetings of the party committees and standing committees at the same level. "The Opinions of the Central Committee of the Communist Party of China on Strengthening and Improving the Work of the Party's Groups and Youth Leagues" clarifies that: the main person in charge of the Communist Youth League organization below the county level shall attend the relevant meetings of the party organization at the same level in accordance with the provisions of the party constitution. In recent years, the Anhui Provincial Committee of the League has focused on implementing this existing system and regulations. With the help of the Provincial Committee's Deep Reform Committee mechanism, it has insisted on coordinating high-level promotion with grass-roots coordination, insisted on combining party committee support with self-struggle, and insisted on pilot projects first and Coordinating with the general rollout, promoting the full implementation of 104 county-level administrative divisions in the province. Comprehending the existing system regulations and implementing them in combination with reality is talking about politics; studying the seemingly inconspicuous measures thoroughly, keeping pace with the times and making them concrete, is grasping reform. The practice and experience of Anhui is now compiled and distributed for reference by other regions. —2—The Anhui Provincial Committee of the Communist Youth League promotes the effective implementation of the attendance system of county-level Youth League secretaries The implementation of the attendance system of the secretary of the county-level Youth League Committee is the starting point, actively report to the party organization, adhere to the goal direction, strengthen guidance and supervision, extensively leverage on momentum, deepen the point-to-point, small-cut, large-depth promotion of relevant institutional arrangements to achieve results, and serve the whole country The provincial Communist Youth League deepened reforms to create favorable political conditions. After 5 years of continuous efforts, all the secretaries of the Communist Youth League committees of 104 counties (cities, districts) in the province have realized regular attendance at the standing committees of the party committees at the same level, and relevant work has achieved phased results. 1. Anchor specific goals, grasp key links, and take multiple measures to promote the effective implementation of the attendance system. 1. Set up benchmarks and strengthen pilots to lead the way. In response to the ideological concerns and wait-and-see attitudes of some counties (cities, districts) at the beginning of the reform, the Anhui Provincial Committee of the Youth League firmly seized the favorable opportunity of learning and education of "two learning and one doing" and took the solid study and application of the party constitution and party rules as the starting point. Taking Tongling City as a pilot, actively guide and promote the secretary of the Youth League Committee of the four counties (districts) in the city to attend the standing committee at the same level, and solidify it into an institutional arrangement. On this basis, the Tuan Tongling Municipal Party Committee further promoted the League Committee of 56 township (street) Youth League secretaries and 18 city- and county-directed enterprises and public institutions in the city by sending letters to county (city, district) party committees and conducting door-to-door coordination. All organization secretaries attend the party committee at the same level. Through the pilot project first, a clear demonstration effect has been formed across the province, and a good-3-good external atmosphere has been created for the overall advancement of the work. 2. Turn on the signal ‧, and keep pushing it at a high level. On the basis of summarizing the pilot experience, at the end of 2019, the Anhui Provincial Committee of the Communist Youth League took the initiative to ask for instructions and report to the Reform Office of the Provincial Party Committee and the Leading Group for the Reform of Provincial Party Committee Groups, and promote the inclusion of the "attendance system" in the work points of the 2020 Provincial Committee's Deep Reform Committee. Since 2020, the Anhui Provincial Committee of the League has actively communicated and coordinated with the party committees at the city and county levels, and promoted the party committees of 16 provincial cities and 104 counties (cities, districts) to include the attendance system as a key point of work for the deep reform committee at the same level. The leading group of the Anhui Provincial Committee of the Communist Youth League has always regarded the promotion of the implementation of the attendance system as an important reform matter. By holding secretarial meetings and reform work leading group meetings, etc., they insisted on listening to monthly progress reports, unifying their thinking, scheduling regularly, and reporting to the provincial party committee every month. The Reform Office reported the progress of the work. 3. Go to the front line, and go to the front for research and supervision. In May 2020, the Anhui Provincial Party Committee of the League and the Reform Office of the Provincial Party Committee went to Bengbu and Huainan to conduct a special survey on the implementation of the "attendance system". From October to November 2020, the Reform Office of the Anhui Provincial Committee of the Youth League went to Hefei, Tongling, Xuancheng, Huaibei and other places to carry out research and supervision, held 4 county (city, district) youth league committee secretary seminars, and went to 4 counties ( districts) to carry out on-the-spot research to further publicize policies and deliver reform signals. For the areas where the promotion work is relatively lagging behind, the Anhui Provincial Committee of the Youth League issued a special notice to guide the relevant Youth League and municipal committees to further improve their political positions and pay close attention to the implementation of the work, forming a good situation of grasping the implementation at each level and at all levels. 2. Focus on the main responsibility and main business, highlight political value, and continue to strengthen the attendance system to play its role 1. Calibrate the bull's-eye perspective, and help the work of the Communist Youth League to better integrate into the overall situation. The attendance system provides an important -4- platform for the league organization to better focus on the center and serve the overall situation according to the positioning of duties, which is conducive to the league organization to better find the entry point, combination point, and focus of the work. Hu Yima, then secretary of the Qingyang County Party Committee of the Tuan, said that after attending the standing committee of the county party committee to study the consumption poverty alleviation action plan in March 2020, the county party committee took the initiative to give full play to its organizational advantages and work characteristics, and timely connected with the county poverty alleviation and development bureau, agriculture and water bureau and other departments , organized a youth commando team to participate in the research and consignment of agricultural products, and achieved a total sales of more than 2 million yuan, which was fully affirmed by the secretary of the county party committee; , the Communist Youth League Committee was able to gain a more comprehensive understanding of the current situation and major problems of epidemic prevention and control in the region, and better mobilized young volunteers to go to the community to carry out voluntary services such as publicity, persuasion, temperature testing, and delivery of materials, which significantly improved the Communist Youth League's participation in epidemic prevention and control. Accuracy and effectiveness. 2. Provide learning opportunities to boost the political ability training of Communist Youth League cadres. By attending relevant meetings of the party committee at the same level, experiencing the decision-making process of important local work at close range, and learning knowledge in various fields of economy and society in real time, it plays an important role in improving the political judgment, political comprehension, and political execution of regiment cadres. Qian Yechao, Secretary of the Tuanzongyang County Party Committee, said that attending the Standing Committee of the County Party Committee will help to fully understand the work of the County Party Committee and the County Government Center, which greatly broadens the work horizon and improves the level of thinking. In the process, after a period of time, I obviously feel that my ability to observe, analyze and deal with problems has been enhanced, and my political position and political ability have been effectively improved. 3. Broaden the channels of voice and help the Communist Youth League to better perform their duties. The attendance system provides an effective way for league organizations at all levels to better play the role of a bridge and link between the party and youth, and helps grassroots league organizations to better upload the voice of youth to the party organization-5-organization. Peng Yanzang, secretary of the Youth League Committee of Guoneng Tongling Power Generation Co., Ltd., actively participated in discussions on youth work issues when attending the company's party committee, and promptly reportedIn view of the actual needs of young employees, a proposal was proposed that both the husband and wife of dual-career college students who have been married within five years of new employment should continue to enjoy a monthly housing subsidy of 1,500 yuan, which was adopted by the company's party committee to help the company further create a good atmosphere of keeping people in mind and win the company's young employees Generally praise Ye. At present, the Anhui Provincial Committee of the Youth League is organizing special forces to conduct a comprehensive inventory, in-depth analysis and systematic research on the experience and existing problems in the implementation of the attendance system. In the next step, the Anhui Provincial Committee of the Youth League will start by further optimizing the working mechanism, consolidating and improving work results, dig out and promote effective experience and practices in a timely manner, deepen the promotion of the attendance system and extend it to the township (street) first-level league organizations, and continue to promote grassroots organizations in the province. The construction of the group organization has reached a new level. —6—(No text on this page)—7—Shake Distribution: General Office of the Central Committee of the Communist Party of China, General Office of the State Council. Secretary of the Secretariat of the Central Committee of the Communist Youth League, relevant ministries and commissions of the Central Committee, and provincial party committees in charge of leadership. The heads of the various departments and directly affiliated units of the Central Committee of the Youth League, and the secretary of the Provincial Youth League Committee. Shake Issued by the General Office of the Central Committee of the Communist Youth League on June 17, 2021

2021-5-18 9 Editor's Note: The Fifth Plenary Session of the 18th Central Committee of the Youth League highlighted the actions of the whole regiment and paid close attention to implementation. Grasping the implementation depends on doing practical things. In recent years, the Communist Youth League Beijing Municipal Committee has centered on the work layout of three-dimensional one-time management, and has taken helping the poor and helping the poor and precise problem-solving as an important entry point for the construction of service capabilities. The basic duty of contacting service youth falls to the ground. —1—The Beijing Communist Youth League has made a multi-pronged approach to help the healthy growth of young people in trouble. In recent years, the Beijing Municipal Committee of the Communist Youth League has solidly promoted the implementation of the "Medium and Long-term Youth Development Plan (2016-2025)", and in accordance with the relevant deployment of the Central Committee of the Communist Youth League, adheres to the principle of dual support with wisdom and wisdom Based on the idea, build a six-in-one work system of education, life, medical care, legal, psychological and social support, precisely help teenagers in need, implement more than 40 demonstration projects at the municipal level, and drive 7,617 independent projects in all districts and townships of the city , directly helped more than 250,000 youths in need. 1. Strengthen guarantees and stabilize the mechanism-driven ballast metallurgy 1. Establish a policy framework and clarify the work targets. Jointly issued the "Implementation Opinions on Beijing Communist Youth League's Accurate Assistance Work for Adolescents from Families with Difficulties", which included youths under the age of 25 who enjoy the city's minimum living allowance, subsidy for living difficulties, and support for extremely poor people. With the deepening of the work, the objects of support will be further expanded to include youths with difficulties in life, disabled youths, left-behind children, youths who come to Beijing for work and their children, etc. 2. Improve organizational construction and strengthen work linkage. Establish and improve the city-district-street township-community four-level support service system to realize the unified scheduling and flexible allocation of work force and work resources. Organize front-line staff to participate in special training to improve the professional level of assistance work. Mobilize the city's league organizations at all levels, youth federations, -2-Sunshine Zone, social organizations, etc. to participate together, and explore the construction of special work teams such as "targeted assistance volunteers" and "little V bee volunteers". 3. Strictly implement responsibility and optimize work flow. The work of accurately helping young people in need is included in the annual work focus, as an important indicator for the annual assessment of district-level league organizations, and the progress of the work is tracked and inspected every month, forming a closed loop of work with plans at the beginning of the year, assessment at the end of the year, supervision during the process, and feedback on evaluation. All districts of the city take the safety construction assessment mechanism as the starting point, refine the three-level division of responsibility of district-street township-community, and promote the orderly development of various tasks. 2. Research and find out, and correct the compass of precise assistance 1. Jointly carry out investigations to find out the target base. Jointly with the civil affairs department, relying on the working force of the grassroots league and professional social work institutions, comprehensively and detailedly investigate the troubled teenagers in the city in the form of entering the village, entering the residence, and going to the household. Support the responsible person and form a database. Standardize the management of information statistics on new declarations, withdrawals and reductions, supervision records, etc., to ensure that information is updated in a timely manner and data is used accurately and flexibly. 2. Organize systematic research to identify outstanding needs. Organize professional forces to conduct empirical research on primary and secondary school students who face long-term or periodic difficulties in material life, spiritual life, and academic education, and organize a special survey on the psychological status and service needs of 18,000 primary and secondary school students in the city during the epidemic period. The "Investigation Report on Teenagers in Difficulties in Beijing", "Report on the Psychological Conditions and Service Demands of Primary and Secondary School Students in Beijing", etc., provide precise guidance and scientific basis for precise policy implementation. —3—3. Establish daily contact and follow up and promote in time. Under the premise of protecting the privacy of minors, the League organizations at all levels have established a daily contact mechanism with teenagers in trouble and their families, and followed up and understood the growth and development of the help targets through questionnaires, telephone communication, visits and condolences. The situation and feedback and evaluation of the assistance work, coordinate solutions or referrals to promote the resolution of difficulties and problems encountered in the assistance work, and timely analyze and report the ideological trends of the youth groups in difficulty. 3. Implement policies by category and create a booster for personalized help. For families with teenagers in need, the Lianhe Municipal Housing and Urban-rural Development Commission implemented a pilot project for direct special rent allocation of public rental housing. In 2018, a total of 49 eligible families met their daily needs. During the COVID-19 epidemic, a living allowance of 1,000 yuan was provided to 288 severely disabled young people living on minimum living allowances. Promote the normalization of special node assistance activities such as the two festivals to send warmth, and the city-level visits and condolences cover more than 1,000 youths on average every year. 2. Serve growth and development, and promote social integration. Launched 1,592 service projects such as "Helping you learn" and organized volunteers from universities such as Peking University, Tsinghua University, and Beijing Normal University to provide regular academic tutoring, covering a total of more than 19,000 person-times. To promote the employment and entrepreneurship of youth in difficulties, 536 employment assistance projects in the city have served 6,297 youths in total. Carry out activities such as "Passionate Ice and Snow, Meet the Winter Olympics" and "Grow Up with My Motherland" to guide the help targets to actively participate in community governance and enhance their sense of social integration and self-confidence. 3. Protect physical and mental health and protect legitimate rights and interests. Relying on the 12355 youth psychological and legal service hotline, it provides daily and professional consulting services. During the epidemic period, 3378 incoming calls were accepted, with a cumulative duration of 66419 minutes. Innovatively carried out the "Health Guard" campaign to provide version 2.0 "mental health services" for teenagers in trouble. In response to the psychological conditions and needs of primary and middle school students during the epidemic, 100 mental health lectures of "Starlight Self-care" were launched on campus, covering 135,500 person-times, with a satisfaction rate of 100%. 4. Precise assistance for individual cases and continuous deepening of services. While providing universal psychological counseling services, one-to-one case assistance is carried out in a planned way. Give full play to the role of the grass-roots service front for the rights and interests of the "Sunshine Belt", continue to pay attention to the urgent needs of teenagers in trouble, and carry out case services around the reconstruction of parent-child relationship, interpersonal communication, integration of peer groups, and correction of behavioral deviations. As of December 2020, a total of 1,369 teenagers have been served and 8,149 person-times of services have been provided. 4. Expand the path and dig deep into the reservoir of social support 1. Rely on the path of "fund +" to strengthen resource absorption. Relying on the Beijing Youth Development Foundation to carry out various donation activities such as the Hope Project's low-care, heavy-duty and disabled assistance activities, play the role of the Hope Project workstations in various districts, raise special love funds for troubled teenagers, and issue Hope Star 1+1 Scholarships, Student Sunshine Metallurgical grants, etc. Over the past five years, it has raised more than 20.26 million yuan and helped more than 11,000 people. 2. Consolidate the path of "volunteering + smelting" and improve service effectiveness. Fully mobilize the forces of colleges and universities, Youth Federation members, social organizations and other parties to vigorously cultivate youth service volunteer teams in various districts, implement long-term volunteer service projects such as loving companionship and growth, mobilize social resources to concentrate on troubled youth, and support the normalization of assistance work development. 3. Explore the path of "Internet +" and innovate the assistance mechanism. Give full play to the advantages of the Internet in social mobilization, and explore the efficient integration and precise allocation of social resources. For example, the Tuanfangshan District Committee opened a micro-wishing online store to guide social forces to help 370 teenagers fulfill their wishes; the Tuanmiyun District Committee developed a WeChat mini-program, which provided a convenient channel for teenagers in need to release their needs independently. —6—(No text on this page)—7—Shake Distribution: General Office of the Central Committee of the Communist Party of China, General Office of the State Council. Secretary of the Secretariat of the Central Committee of the Communist Youth League, relevant ministries and commissions of the Central Committee, and provincial party committees in charge of leadership. The heads of the various departments and directly affiliated units of the Central Committee of the Youth League, and the secretary of the Provincial Youth League Committee. Shake Issued by the General Office of the Central Committee of the Communist Youth League on May 18, 2021
