# Supplementary material for: Serving organization goals by organizational information dissemination: An empirical study from the Communist Youth League of China
Source: PLoS One. 2023 Jan 20;18(1):e0280221. doi: 10.1371/journal.pone.0280221 (PMC9858461; doi:10.1371/journal.pone.0280221)
Supplement: S1 Data — (ZIP) [file pone.0280221.s001.zip › Supporting Data/Version of Chinese to English/City Committee(English).docx]

2022-06-28 Jianwei) In 2022, the youth mental health service into the village (community) action was included in the people's provincial and municipal livelihood for the first time. As the main responsibility unit, the Luohe Municipal Party Committee of Henan Province has actively organized the youth League organizations at all levels of Luohe City, together with the relevant responsible units, to jointly do this practical work well and do the good things well. The Communist Youth League and Municipal Party Committee integrates and establishes a team of professional psychological services, which integrates social organizations, social work organizations and legal aid institutions. The project operation is implemented, the municipal level is responsible for unified training and guidance, and the specific docking activities of counties and villages. In the specific work, the Communist Youth League Municipal Party Committee has actively promoted the public welfare project of "Hope for You from the Heart" initiated by the Henan Provincial Youth Development Foundation, and guided the caring people from all walks of life to actively participate in it and jointly promote it. Build a team, improve service level of professional league to form more than 60 professional licensed personnel "psychological consultant + crisis intervention + psychological supervisor" ladder psychological volunteer service team, develop management system and work standards, design the training course system, combining with teenagers and parents' age, psychological characteristics, for adolescent self psychological adjustment, parent-child relationship, easy preparation, security, care, network addiction, frustration education, such as psychological science education, case counseling, psychological counseling and other targeted services. In the next step, the Communist Youth League Committee will prepare to establish the establishment of psychological professionals database in the city's Communist Youth League system to provide strong talent guarantee for adolescent mental health services. The Communist Youth League Municipal Committee will build six "Qingyi homes" in the city's high standard, as a normal mental health service work position, equipped with high-quality psychological counselors regularly on duty, to provide psychological counseling services. So far, case consultation files have been established for 52 teenagers. Designed and produced 300 copies of adolescent mental health action psychological case consultation files in villages (communities) in the city, with unified standards and unified storage, and protecting the rights and interests of teenagers in case consultation. Through various online and offline forms, walking into villages (communities), to carry out high-quality psychological lectures, group counseling and other service activities. At present, it has conducted 6 online parent-child courses, participating in more than 1,500 lectures; 12 offline activities, covering more than 10,000 teenagers. Combined with the professional knowledge of psychological counseling, the overall plan of annual specific activities has been formulated to fully ensure the practicality, coherence and professionalism of psychological counseling activities. In the next step, the League and Luohe Municipal Party Committee will continue to give full play to the advantages of the League organization, focus on the special youth groups such as left-behind and single parent, send the party's care to the teenagers in time, and escort the healthy growth of the city's teenagers.

2022-06-25 Group Gansu Qingyang municipal party committee to build "about" youth dating brand activities group Qingyang Gansu municipal party committee to build "youth" dating brand activists Ma Fuchun) in order to further promote the Gansu province long-term youth development plan (2018-2025) further implementation, youth, lead the function of youth condensed youth, youth, Gansu Qingyang municipal party committee innovation create "youth about" youth dating brand series activities, build youth platform for communication, communication, dating, active youth cultural life, guide the youth to set up the correct view of love, the outlook on life and values. Since February 28, the first phase of "youth" youth dating activities opened, group Qingyang municipal party committee adhere to the fixed issue a month, so far, has successfully held municipal special activities 4, county (area) level special activities 5 games, attract 272 list youth sign up, activity brand through online radiation accumulated more than 800000 people (times). Group Qingyang municipal party committee always adhere to the service normalized, for online registration and applicants identity information, dating information, classification built in youth information files, establish service tracking mechanism, early activity contact group, set up telephone hotline, and pay attention to authority and privacy protection, to safeguard the legitimate rights and interests of the youth. The relevant person in charge of the Youth League Qingyang Municipal Party Committee said that the activity followed the diversified characteristics of the post-80s and post-90s youth's personality, and made full use of the city libraries, green parks and other theme venues to organize and carry out various theme activities such as "reading friends" and "planting a friendship tree together". During the activity, we also provided dolls, cakes, fruits, snacks, gift boxes and other gifts for the participating youth to continuously improve the freshness and experience of the participating youth."The youth dating activity held by the CPC Communist Youth League has a distinct theme, which can combine reading and interpersonal communication, for our youth to build a noble, pure dating platform, let us read and meet in such a beautiful time, it is very meaningful."Said Xiao Li, a young man who participated in the event.

2022-06-25 Henan Pingdingshan returning college students to actively participate in the verification work of Pingdingshan, Henan: more than 200 returning college students to actively participate in member verification workers Pan Zhixian correspondent Zhao MengYan) in Henan province college students to the grassroots work to mobilize the deployment, the Pingdingshan municipal party committee organization to mobilize returning college students cadres to participate in the city in 2021 new development member verification work. In view of the actual situation of the large amount of basic league affairs and the insufficient work force, the Pingdingshan Municipal Party Committee of the Youth League immediately issued the Notice on Organizing Returning College Students to Participate in the Verification of the New Development League Members in 2021, and formulated a detailed work plan and made a work list. The youth League committee of each county (city, district) has conducted more than 30 verification and training sessions on the epidemic situation, online and offline activities. For the problems found in the verification, TuanPingdingshan Municipal Party Committee established a collection and summary mechanism for the verification problems, and implemented the "daily research and judgment, daily sales number". Up to now, Pingdingshan city has found more than 2306 categories of league file files, which has been reported to the grass-roots league branch rectification and implementation. Recently, the Youth League Pingdingshan Municipal Party Committee organized returning college students to participate in the 2021 development league member verification work training meeting. Recently, the cadres of returning college students from Pingdingshan City, Henan Province, participated in the on-site verification of the paper files of the development of league members in 2021."As a college student, I can use my holiday time to participate in the work of the Communist Youth League, which can not only make myself get practical exercise, but also deepen my understanding of the league organization. I think it is very meaningful!"Said Guan Zhiyuan, a college student volunteer from Zhengzhou Business School. During the summer vacation, college students returning from Pingdingshan City, Henan Province, actively participated in the online verification of electronic files for developing league members in 2021. Correspondent Zhao Mengyan / photo group Pingdingshan municipal party committee, said the next step, will be according to the group of Henan provincial party committee arrangement, to college students to the grassroots activities as an opportunity, combined with the actual practice, further innovation ideas, explore the carrier, effectively mobilize the home college students in practice to understand the national conditions, guide and help the young students combined with practice of "education", in the social classroom education, long ability, contribution.

2022-06-25 Anhui Maanshan city to build a professional, long-term youth volunteer service brand five member congress was held. Ma'Anshan Municipal Deputy Party Committee Secretary Zhang Quan attended and made a speech. The meeting elected 30 directors of the fifth council of the Ma'Anshan Youth Volunteers Association, and elected the fifth council, president, vice president, secretary general and deputy secretary general of the Municipal Youth Volunteers Association from the 30 people. Xu Lei, secretary of the Youth League Committee of the city's Guangzhou radio station, and Wang Jing, head of the city's 12355 Youth Comprehensive Service Center, shared their experiences as outstanding volunteers and excellent volunteer service organizations. Before the conference, the participants watched the propaganda video of the Municipal Youth Volunteers Association "Volunteer service is continuing to forge ahead in the new era". In the past four years, Ma'Anshan Youth Volunteers Association has continuously strengthened its own construction and improved its operation mechanism. By the end of 2021, county-level youth volunteers associations had been established in all the three counties and three districts of the city. At the same time, ma on shan youth league organization carefully designed service project, consolidate and deepen "our festival feeling warm childlike innocence", "benefit" charity "" warm winter "" green lemon "public" easy test for 12355 with you "" warm bee action " existing brand projects, strive to explore a batch of suitable for the grassroots actual, has guiding value and era characteristics of volunteer service excellent project. At the critical moment, the young volunteers also stepped forward. During the flood control period in 2020, the city set up 9 youth volunteer service teams and 109 youth commandos, and more than 3,928 young volunteers participated in the front line of flood control and flood fighting, carrying out volunteer services such as night dike patrol, danger screening and river barrier clearance. In the past four years, Maanshan youth volunteer service work has achieved fruitful results. "A Love Food to Warm a City" and "Little Umbrella" volunteer service team have won the gold medal and bronze award of the fifth China Youth Volunteer Service Project Competition respectively. Jiang Youming, a young volunteer, was awarded the national award of "Advanced Individual in Fighting COVID-19", and Wang Ruitong, a young volunteer, won the "12th China Young Volunteer Outstanding Individual Award". At the same time, the conference invited the Maanshan Municipal Party Committee Publicity Department, Municipal Civilization Office, Civil Affairs Bureau, Municipal Youth League Municipal Party Committee, Municipal Youth Entrepreneurs Association and other five units to recommend a volunteer service professionals as the honorary director of the fifth council of the Maanshan Youth Volunteers Association, and awarded the award certificate to the honorary director. At the scene, Zhang Quan, deputy secretary of the Maanshan Municipal Party Committee, said that in recent years, the city's youth league organizations at all levels and the majority of young volunteers adhere to the center, serving the overall situation, and carried out a series of effective volunteer services, which has effectively promoted the vigorous development of young volunteers. It is hoped that the Municipal Youth Volunteers Association will take this change as an opportunity, base itself on a new starting point and shoulder a new mission, and promote the city's volunteer service work to achieve new results.

2022-06-24 Wuhan) Recently, the final of the 2022 Wuhan "Outstanding Youth" public welfare venture capital Competition project was held. A jury composed of experts and scholars, representatives of the organizers, entrepreneurship mentors and media representatives selected 50 of the 139 projects for the preliminary trial, among which 30 projects will receive financial support from the competition. The entries of the competition cover five categories: youth ideological guidance, youth community service, focusing on innovation and entrepreneurship, boosting rural revitalization, and helping municipal social governance. Next, the organizers of the competition will carry out special training for the winning projects, optimize the project plan, guide the implementation of the projects, and guide the youth league members to show their talents and gain growth in serving the society with their youthful power and creativity. Wuhan "brilliant youth" public welfare venture capital competition sponsored by the Youth League Wuhan Municipal Party Committee and other units, this year has entered its fifth year. The first four sessions of the competition have funded 110 demonstration public welfare projects, serving more than 3.505 million young people online and offline. Source: China Youth Daily, June 24,2022, edition of the 02nd edition

2022-06-23 In Dezhou, Shandong province, we held the 2022 Annual Grassroots Youth League Cadres Special Seminar. Correspondent for the picture of China youth network Beijing, June 23 (reporter Zhang Jianwei, correspondent Yang Yanzhao) in order to effectively improve the grass-roots cadres business ability and work level, recently, Shandong Province Dezhou city grass-roots cadres special seminar held in the municipal Party school, more than 50 township (street) youth League committee secretary to attend. This year, the cadres training for the first time into the municipal party committee party school training class, relying on the municipal party committee party school to carry out the 5-day closed-end management teaching, training covers the ice development, spirit, green horse engineering, business ability, policy, education, honest education, mass work, red film and so on eight teaching plate, training effect get trainees consistent recognition and high praise.

2022-06-23 Fujian Ningde 16 departments power young students internship employment of Fujian Ningde 16 department power young students internship employment and many universities established partners Chen QiangTian HongHui) recently, the communist youth league of Fujian Ningde municipal party committee organization department, the municipal development and reform commission, municipal bureau of education and other 16 departments jointly issued the "about 2022 Ningde college students internship" sail plan "notice" (hereinafter referred to as the "notice")."Notice", this year in the party and government organs, institutions, state-owned enterprises and financial institutions, all kinds of scientific research institutions and large non-public enterprises and other units for no less than 3000 high quality internship, Ningde students and Ningde university students released and actively push, promote no less than 2000 college students to participate in summer internship, help college students in practice, establish the correct employment concept, the career development positioning, reserve work experience, improve employment core competitiveness, drive a group of provincial university Ningde students returning home employment. Members of the promotion meeting introduced the "Sailing Plan" and the main talent policy in Xiamen University. On June 17th, the "Sailing Plan" and the "Action of concentric Employment" went into Xiamen University, and attracted more than 100 students from all campuses of Xiamen University to participate. After the meeting, the Ningde Municipal Party Committee of the Youth League and the Xiamen University Youth League Committee had a discussion and exchange, and communicated on the 2022 Ningde University Students internship "Sailing Plan", student social practice cooperation in winter and summer vacation, Xiamen University Art Troupe benefiting the people performance, "Qingma Project" practice project, and reached the cooperation intention. In addition, in order to deepen the cooperation between the university, the Youth Ningde Municipal Party Committee also took the initiative to connect with Peking University, Tsinghua University, National People's Congress and other universities to promote the regular implementation of practice.

2022-06-29 Shandong Jining: more than 300 volunteers high quality complete Shandong tourism development conference service work Jining: more than 300 volunteers high quality complete Shandong tourism development conference service work JianWei correspondent BaiChen) on June 26 to 27,2022 tourism development conference successfully held in Jining, during the conference, more than 300 volunteers warm thoughtful, patient and meticulous service, post, serious and responsible attitude and things for first-class, the pursuit of excellence standard, become a tourism development conference a beautiful scenery line. The picture shows the volunteers of the 2022 Shandong Provincial Tourism Development Conference. Correspondent for the picture is to do a good job in the conference volunteer service work, the Communist Youth League Municipal Committee on the service, active as, accurate docking with Qufu Normal University, Jining University, Jining Vocational and Technical College, Shandong Vocational College of Technology and other related universities, after three rounds of selection and interview, to achieve the recruitment of more than 300 volunteers. To ensure the quality of volunteer service, pku made special volunteer training plan, hired five professional mentors site etiquette action, improve communication skills, highlight the "smile" service, batch organization to carry out centralized training 2 times, professional training 12 times, practice more than 30 times, effectively improve volunteers professional skills, knowledge reserve and actual combat ability. Volunteers are receiving etiquette training. After several rounds of selection and training and field drills, more than 300 volunteers were enthusiastically devoted to the service work of Shandong Provincial Tourism Development Conference, with active volunteers in the hotel reception, pickup station, car guidance, road guidance, point explanation, opening ceremony award, catering service and other work tasks. Volunteers are signing in for the guests. The picture for the correspondent is to strengthen the organization and leadership, the Communist Youth League municipal committee set up a special class for volunteer work, divided into 9 volunteer groups, established two ledger of volunteer personnel management and post responsibilities, detailed the responsibilities of each post, and defined the work tasks of each volunteer. The picture shows the Youth Jining Municipal Party Committee holding the volunteer service summary and exchange meeting of Shandong Provincial Tourism Development Conference. On the morning of June 28, the Communist Youth League Municipal Party Committee organized a volunteer service summary and exchange conference of the Shandong Provincial Tourism Development Conference, which systematically summarized the volunteer service work of the conference and praised the units and individuals with excellent performance. Next, the communist youth league of Jining municipal party committee will continue to carry forward the excellent tradition, sum up valuable experience, promote the construction of volunteer service system, study and formulate the management method for youth volunteer registration, volunteer service activity implementation rules, youth volunteer courtesy incentive measures, held youth volunteer service exchanges, promote and realize the youth volunteer service activities projects, high quality service center in the city and major festival activities.

2022-06-23 Hengshui, Hebei Province: The "Old Friends Plan" for the holiday, was held in Shuicheng District. Group Hengshui municipal party committee to carry out the "thousands of talents back home" to attract talent back to balance activities, to summer college students youth volunteers with autistic children, it is also a college students summer "a program under which officials" social practice one of a series of volunteer service activities, aims to expand through volunteer service activities, bring care for autistic children and families. During the activity, the teachers from Leqi Home showed the picture book "Leo and the Octopus" for the volunteers and the autistic children in the form of PPT. The story enabled the volunteers to have a preliminary understanding of the autism spectrum disorder. Subsequently, volunteers interact with autistic children through manual "octopus". The holiday "Old Friends Program" is a youth volunteer who graduated from high school and enters college and is in school. During the summer vacation, it is held weekly in the form of singing and dancing, charity sale, picture book sharing, handwork and so on, which has lasted for two weeks.

2022-06-23 Group of Jilin province Songyuan municipal party committee held the college entrance examination volunteer filling guidance lecture series Songyuan municipal party committee held the college entrance examination volunteer filling guidance lecture series pei-ilian wang), to help the college entrance examination students and parents understand volunteer filling policy, reasonable fill in the college entrance examination volunteer, Jilin Songyuan municipal party committee through the "youth" video "youth matsubara cloud classroom" college entrance examination volunteer filling guidance lecture series live activities. In the live broadcast, TuanSongyuan Municipal Party Committee invited teachers with many years of registration experience to analyze the advantages and disadvantages of colleges and universities in and outside Jilin Province in majors, regions and industries and fill in sections. College entrance examination volunteer instructors are broadcast live. At present, TuanSongyuan Municipal Party Committee has held three live public lectures on college entrance examination applications, with a total of more than 2,700 viewers.

2022-06-23 The Zhangjiakou Municipal Party Committee of the Communist Youth League has launched a volunteer service activity of "Respecting the elderly" in all counties (districts). During the activity, the Youth League Zhangjiakou Municipal Party Committee awarded the "Loving the Grass and Respect for the Elderly" municipal demonstration site in the Shengli North Road Convenience Service Center in Qiaodong District, mobilizing volunteers to help the elderly living at home to solve their daily life difficulties. Tuan Xuanhua District Committee organized volunteers to separate action, for each village "respect mutual canteen" to send fresh vegetables and fruits, and in Pangjiapu Town Hujialiang Village Fried oil cake, in Guo dumplings, send love and warmth for the elderly; TuanGuYuan County organized volunteers to carry out "warm sanitation workers heart lonely elderly" activities, send fruit, water, masks and other comfort, and take sanitation workers to the volunteer service station rest summer; Tuan Chicheng County organization volunteers, for the elderly voluntary blood pressure, explain the geriatric knowledge. Since the beginning of this year, Zhangjiakou city has carried out a total of 156 "inch grass love and respect for the elderly action", the total volunteer service time exceeded 42,800 hours, 7,082 volunteers participated, the average service time of 6.05 hours. In the next step, the Communist Youth League Zhangjiakou Municipal Party Committee will continue to give full play to the organizational advantages of the Communist Youth League, innovate the service forms, mobilize the youth league members and volunteers to join in the volunteer service of loving and respecting the elderly, and contribute their youth strength to the construction of a beautiful Zhangjiakou.

2022-06-23 The weaving Department and the Municipal Human Resources and Social Security Bureau launched the launching ceremony of the 2022 summer social practice activities of Qitaihe college students returning to their hometown in 2022. Since the beginning of the year, we have organized more than 1,000 college students to participate in the social practice of returning to their hometown. In this activity, we have organized more than 280 college students to return home to the grass-roots level and contribute to the construction of their hometown. At the ceremony, the Organization Department of Qitaihe Municipal Party Committee and the Municipal Human Resources and Social Security Bureau publicized and interpreted the talent introduction and employment internship policies, and the participating enterprises promoted the enterprises to show the city's youth development environment and enterprise development prospects to the students. Then, the returning students went into enterprises to carry out research activities, visited the short track speed skating champion hall, feel the innovation-driven development and the charm of the champion city.

2022-06-23 Held in Wuhan city, Hubei province in 2022 "brilliant youth" public venture capital contest held in Wuhan city 2022 "brilliant youth" public venture capital contest green net reporter LeiYu) recently, in 2022 Wuhan "brilliant youth" public venture capital contest project final, by experts and scholars, organizers, business mentors, media representatives of the jury from 139 projects selected 50 into the final, including 30 projects will be competition financial support. The entries of the competition cover five categories: youth ideological guidance, youth community service, focusing on innovation and entrepreneurship, boosting rural revitalization, and helping municipal social governance. Next, the organizers of the competition will carry out special training for the winning projects, optimize the project plan, guide the implementation of the projects, and guide the youth league members to show their talents and grow to serve the society with their youthful power and creativity. Wuhan "brilliant youth" public welfare venture capital competition sponsored by the Youth League Wuhan Municipal Party Committee and other units, this year has entered its fifth year. The first four sessions of the competition have funded 110 demonstration public welfare projects, serving more than 3.505 million young people online and offline. The competition has cultivated and supported a number of innovative public welfare projects, so that a group of young talents can grow up in practice. The 2045 Unmanned Driving Science Base took a group photo

2022-06-23 Group Heilongjiang gang committee for energy saving publicity week theme education activities group Hegang municipal party committee of energy saving publicity week theme education activities of Heilongjiang Hegang party committee, city, municipal government affairs service center respectively into the hope hut, to carry out the "green low carbon, energy saving first" energy saving week theme education activities, youth league committee, authority center and student representatives, a total of more than 200 people to participate in the activities. At the activity, participants watched the energy saving propaganda video, the student representatives read the energy saving proposal. Subsequently, the lecturers of the center focused the purpose, significance and practice of energy saving and low-carbon, and gave the students the theme course of "green and low-carbon, energy saving first". Finally, the members of the Youth Federation and the students live to make energy-saving publicity hand copies and carry out the selection of excellent works.

2022-06-22 The ninth "Youth Innovation Cup" Guangzhou Youth Innovation and Entrepreneurship Competition finals ended the ninth "Youth Innovation Cup" competition ended more than 100 mass innovation project cloud competition Lin Jie) recently, the ninth "Youth Innovation Cup" Guangzhou Youth Innovation and Entrepreneurship Competition finals ended. The final was carried out online, with 71 college students and 68 non-school youth entrepreneurship projects gathered in the cloud for the project road show and defense. The competition has set up 11 competition districts of Guangzhou, as well as Qiannan, Bijie and Anshun cities, and specially opened 18 competition areas, including Hong Kong and Macao competition, Taiwan competition, "Rural Revitalization" special competition and student competition, to create a hierarchical display and communication platform for young entrepreneurs in various fields. The competition are concentrated between the ages of 18 and 35. The competition has a special college student track, which has attracted nearly 300 college students from more than 31 universities. The organizer cooperated with relevant universities to launch the "Create youth" and "Challenge Cup" college students entrepreneurship project to participate, and dug out a number of outstanding college students entrepreneurs. According to statistics, the final project focuses on three new emerging pillar industries of new generation information technology, intelligent and new energy vehicles, five emerging advantages of intelligent equipment and robot, rail transit, new energy and energy conservation and environmental protection, new materials and fine chemical industry, digital creativity, accounting for more than 70%; covering rural revitalization, nanotechnology, cultural creativity and other fields, accounting for nearly 30%. Yu Wenhui, an expert judge of the competition and the founder of Thunderstorm Capital, said that the emerging pillar industries and competitive industries in Guangzhou are very popular among young entrepreneurs, and the quality and landing of the project have also been greatly improved, which is worth the attention of investors. The 9th "Youth Innovation Cup" Guangzhou Youth Innovation and Entrepreneurship Competition final evaluation scene. It is worth noting that the organizers have set up a special "rural revitalization" competition to mobilize young talents to participate in the rural revitalization work. Relying on Zengcheng District, the competition has specially opened the "Rural Revitalization" special competition, collecting 87 participating projects from the Greater Bay Area, involving rural cultural tourism, rural e-commerce, modern planting and breeding, prefabricated vegetables and other fields. The special competition also introduced the Guangzhou Rural revitalization fund for the first time. Guangzhou rural revitalization fund President, China rural cooperative economic management society, deputy director of rural construction committee said, will take the preparation "green gen cup" build talent gathering platform as an opportunity, for the depth of the rural CEO talent team, linkage related enterprises, jointly launched "rural (operation) CEO cultivate dragonfly plan", cultivate a group of willing to join country, understand the countryside, and can effectively docking city, docking market, and has good quality of rural youth leaders, continuous output for rural revitalization of talent. Recently, The State Council issued the Overall Plan for Deepening global Comprehensive Cooperation between Guangdong, Hong Kong and Macao in Nansha, Guangzhou. This year, the Guangzhou Municipal Party Committee, together with the United Front Work Department and the Municipal Foreign Affairs Office (the Municipal Hong Kong and Macao Office), set up the Hong Kong and Macao competition area based on Nansha, Guangzhou, attracting nearly 100 youth entrepreneurship projects, including 6 Hong Kong and Macao youth innovation projects advancing to the final, and two projects won awards and applied for the 2022 Guangzhou Excellent Youth Innovation and Entrepreneurship Talents. It is reported that the Guangzhou Municipal Party Committee of the Communist Youth League focuses on building platforms for transforming innovation achievements for college students, young people from Hong Kong, Macao, Taiwan, and "rural revitalization", providing "policy, talent, capital, technology, training, market" and other system docking. For the winning projects, it will be recommended to enter Guangzhou Hong Kong and Macao Youth Innovation and Entrepreneurship Service Center to provide rent-free venues and supporting services for eligible entrepreneurial projects.(Photo provided by the Communist Youth League and Guangzhou Municipal Party Committee)

2022-06-22 Henan Puyang to carry out the "youth about let love set sail" high-speed wedding activities in Henan Puyang organization to carry out the "youth about let love set sail" high-speed rail wedding activities in youth network reporter Pan Zhixian correspondent xiao-guang wang) " my lover is from shaanxi ankang municipality, for our love came to Puyang, before home need a whole day, Puyang high-speed opened today, greatly facilitate our later visit road. We sincerely wish the future of Puyang to develop as fast as its high-speed railway, and wish the people of Puyang a better and better life!"Wang Yongli, a young cadre from the Market Supervision Bureau of Puyang City, Henan Province, experienced the" Youth makes love set sail " - -the first ride Pu-Zhengzhou section of the Ji-Zhengzhou high-speed railway. On June 20th, the Pu-Zheng section of the Ji-Zhengzhou high-speed railway was opened to traffic. Henan Puyang Municipal Party Committee of the Communist Youth League organized 25 couples from all walks of life in the city to participate in the first ride experience activity of "Youth to Let Love Set sail". In this day, enough to go down in the annals of Puyang history, the new people have ten fingers linked, heart to heart, in Puyang East Station jointly opened their exclusive high-speed rail version of the sweet romantic group wedding. At 9:50 a. m., couples joined hands on the first G9205 train in Puyang, under the blessing of relatives and friends, toward the palace of happiness. A symbol of perfect, happy and happy 10 carriage is dressed up festive and romantic, red words, painted gold angea, for the new to create a unique warm environment. Yan Zhaomin, deputy secretary of the Puyang Municipal Party Committee, sent his best wishes to the newlyweds participating in the event, wishing their love and career to follow the development pace of Puyang high-speed railway era and move towards a better tomorrow. The opening of pu-Zheng section of ji-Zhengzhou high-speed railway is not only the end of Henan's "meter" shaped high-speed railway, but also the finishing touch of Puyang's traffic construction. With this as a sign, Puyang city has really broken through the traffic bottleneck constraints, entered the era of high-speed rail, joined the "circle of friends" of the high-speed rail city, and fully integrated into the new pattern of national development.

2022-06-22 Fuzhou departments into universities to attract talents Fuzhou departments into universities to recruit talents reporter Chen Tian HongHui) for nearly a month, the communist youth league of Fuzhou Fuzhou people club bureau, Fuzhou talent group department promotion group, walked into the Nanchang, Changsha, Wuhan, Nanjing, Hefei, Qingdao and other 18 cities, to carry out the "good years, gather Fuzhou" activities and college students' practice "sail plan" campus seminar. The move aims to enhance the willingness of college students to find jobs and start businesses in Fuzhou, and reserve talents for local economic and social development. According to Chen Hao, secretary of the Fuzhou Municipal Party Committee of the Communist Youth League, the "Good Years, gather in Fuzhou" activity is a platform for employment, internship, practice and tour for full-time college and college students, and is also a brand project of talent introduction work in Fuzhou. This project has been selected into the summer "three to the countryside" social practice project of the central Communist Youth League for two consecutive years, and won the brand project of the national "return to home" social practice activity. The seminar in "online + offline" way, four promotion group into Wuhan university, central south university, China university of petroleum, sun Yat-sen university, hehai university and other nine universities, to college students face to face promotion, detailed explanation content and supporting policies, and adopt the "cloud live" way to tsinghua university, Peking university, renmin university of China and other college students to promote online, invite college students to Fuzhou summer employment, internship, practice, tour. According to statistics, as of tonight, the number of "good time, gather in Fuzhou" talent introduction activity registered to 4313 people, 594 participating colleges and universities, the number of social practice applicants 2876 people. Source: China Youth Daily, June 22,2022, edition of the 02nd edition

2022-06-21 Wang Peilian) Recently, the Changchun Municipal Party Committee of the Communist Youth League has released more than 2,000 "returning to their hometown" social practice posts for college students from Changchun. The posts involve party and government organs at all levels, enterprises and institutions, towns, streets, communities, etc. Changchun college students' practice of "returning to their hometown" in social practice, with the purpose of "receiving education, developing talents and making contributions", requires a duration of no less than 40 days from the beginning of July to the end of August. Social practice is carried out in accordance with the principle of "independent application, voluntary registration, two-way selection, and selective selection". When the number of job applicants exceeds the number of job needs, the employer will select the best candidates for the internship. In recent years, the group of Changchun city party committee actively build outside Changchun students and home normalized practice bridge, guide students home practice, service in Changchun, talent city strategy, let Changchun, love Changchun, stay in Changchun, service, in the post feel hometown development and change, improve college students post practice ability.

2022-06-21 Jiangxi Pingxiang about "youth learning" about communication into the campus boom Jiangxi Pingxiang about "youth learning" about communication into the campus boom JianWei correspondent Chen cheng) to better play to the youth branch theory interpretation, demonstration leading "light cavalry", recently, the communist youth league Pingxiang in Jiangxi province municipal party committee launched the "youth learning" about communication into the campus theme activities. For days, the city's youth branch members at all levels to act quickly, into the campus, into the classroom, focus on meet, propaganda, implement the party's twenty big theme, listening, understand, remember with teenagers, simple, tangible, preach, xi jinping, general secretary in celebrating the 100th anniversary of the founding of the communist youth league of China's important speech spirit to teenagers, into the youth heart."Youth big learning" publicity and exchange into the campus activity site. In the process of the activity, the county (district), colleges and universities youth league organizations according to local conditions, individual conditions, play their own characteristics, invited members of the youth lecturers, to carry out extensive publicity. In Anyuan District, Long Chanyi, a member of the city's youth teaching group, encouraged the students to continue to maintain the passion of learning and learning, maintain the spirit of exploring, study hard and climb new heights. In luxi county, XuanJiangTuan members He Yue into the galaxy town middle school, to "firm system confidence reveal youth bear" as the theme, combined with the power of youth in all walks of life and the 2022 Beijing Olympics young athletes story, through the interaction, teachers and students reading, initiative the students with the power of example as the guide, forge ahead, bear as. The Youth League committee of the opening district invited members of the publicity group to visit the general school of Dengan Primary School and Jiangwan Branch School. Through sharing the deeds of Zhang Guimei, more than 300 teachers and students present knew the desire of children in mountainous areas for knowledge, and the important truth of changing their fate through education. In Pingxiang martial arts at the foot of the mountain wan longshan township HuaYun school, city youth branch member yi ni combined with exciting China space history, led the students to review the shenzhou 13 from launch to the complete process, from special can bear, especially can fight, special can research, special can offer four "special", learn to comprehend the profound connotation of the great manned space spirit."Youth big learning" publicity and exchange into the campus activity site. Zhang Huahai is not only a member of the city's youth teaching group, but also the deputy director of Anyuan Red Culture Research Center. He came to Pingxiang college, respectively from Anyuan road mine and workers 'early struggle, Anyuan road mine workers movement history, Anyuan spirit, realistic four aspects, tells the young students vivid history of Anyuan road miners movement, inspire contemporary youth to remember history, ambition, inspire faith, inspired, draw strength, from the communist youth league member's cutting edge exemplary role, in the realization of the great rejuvenation of the Chinese nation the Chinese dream of the new long march. Up to now, Pingxiang youth league organizations at all levels have carried out more than 20 publicity activities, covering 2,000 person-times. At the same time, online "Youth Pingxiang" and other new media platforms have opened up "Me and My Youth" micro stories, "Youth Leadership Group History Stories", "Red Scarf narrator tells Party History Stories" and other columns.

2022-06-21 Hunan Changde: Small youth organizations drive grass-roots big governance Hunan Changde: After the small youth organizations drive grass-roots big governance problem did not get the policy implementation and have the idea of revenge, the community grid member timely to Hunan Changde City Wuling District Changgeng Street political and legal committee member Hu Xiaoming reflected the situation. After coordinating with the petition departments to solve the problems left over from history, Wang Wei's mood has finally calmed down. Changgeng Street covers an area of 8.1 square kilometers, and has jurisdiction over 7 communities, 40 grids, and has a population of 35,800 people.in recent years, The street closely adheres to the goal of youth helping to "modernize community urban governance", Explore new ways for young people to participate in social governance in the new era; Building a five-level three-dimensional organization network of "street-community-grid-Party members-social forces", Taking the street and community two-level links as an opportunity, Select 56 outstanding young people under the age of 35 to enter the community "two committees" team, Young cadres account for more than 60%, Promote the street and community youth league cadres to sink the grid front line, More than 320 people have gathered in the grid terminals; Select a batch of pilot grids, Select outstanding young party member cadres as the first secretary of the grid Party branch, Each cadre is contracted with 10 households, Establish a "one to ten" assistance system. At the same time, the street based on the area in the unit, including Changde city drug rehabilitation center "woodpecker" youth police anti-drug team, Hunan arts and science college youth teachers, the city intermediate people's court youth judge litigation management team, often into the community and families, with rich and colorful activities to carry out the franco-prussian education. Changgeng Street has been rated for many years as the city, district peace street and the construction of no petitioning village (community) street. Before 2016, Changde city had an average of more than 20,000 petition cases per year. Since 2017, the municipal party committee municipal government in the city construction "no petition village (community)" work, implement "the party leading, grid integration, multiple mediation, give priority to with village", strengthen petition source prevention, rely on grass-roots organizations and social forces timely solve the masses, to ensure that "small not village, village street, petition in the county". At present, the city has established 14,000 wechat groups, with 2.6 million people enrolled, and has built 2,262 village-level mediation committees, covering 100%. In recent years, more than 95% of the conflicts and disputes in the city have been resolved within the county level, and the number of letters and visits above the city level has decreased by more than 20% annually in the past three years. In order to adhere to the demonstration and guidance and set up the work benchmark, the secretary of Changde Municipal Party Committee and the mayor personally led the case to visit and visit the masses to solve problems, held an on-site meeting to personally grasp the source management work, and demonstrated and led more than 13,000 cadres in the city to sink to the grass-roots level. District / county (city) secretaries and district / county (city) chiefs take the initiative to study and plan the construction of the county-level "terminal" work of public letters and visits, and take the lead in handling the backlog of letters and visits. Fifteen municipal leaders led the way to resolve 45 backlog letters and visits, and 460 leading officials at the county and department level solved more than 2,300 difficult letters and visits. China Youth Daily, June 21,2022, edition 03

2022-06-21 Shandong Jining held a "new era youth" large melting media preaching contest Jining "new era youth" large melting media preaching game Jianwei correspondent BaiChen) recently, the Shandong Jining municipal party committee municipal education bureau, city, city WeiJianWei, SASAC, municipal radio and television, the city's rural commercial Banks jointly organized "new era youth said" large melting media preaching activities held in Jining radio and television studio hall. The picture shows the contestants' speeches. Correspondent for figure this speech activity around "new era Jining youth said" theme, stick to the "youth struggle" "brave responsibility" power "I" "first-class, first, only", such as key words, about people, around things, express the good vision of the future life, love party patriotic love home feelings, etc., embodies the contemporary youth "is innovation, strives for seeking and enterprising" spirit and positive positive energy. Since the announcement of the solicitation announcement, the event has attracted wide attention and welcome from all walks of life, attracting active participation from state assets, education, public security, medical care, finance and other systems, as well as party and government organs, grass-roots party members, young students and other groups. After the review of the manuscripts and videos of the registered contestants by the organizing committee, 80 contestants entered the final competition. The picture shows the contestants' speeches. Contestants fully interpret the contestants around the theme of the speech. With lively language, rich and colorful form, from the people around, things, talk about youth ideal, youth responsibility, youth, show the contemporary youth positive, healthy, upward life attitude and fighting spirit, and called on young people to set up a strong era sense of mission, sense of responsibility and urgency, enthusiastically in hot reform and construction practice, not wasted Jing Jing time. Li Xian, from Jining detention center, told you how the girls protect the group of lost children with love; Wang Xiaodan, a physician from Jining municipal government hospital, shared with you how her young colleagues stick to the forefront of epidemic prevention and control, the first line, reverse, for the spread of the epidemic, ensure the safety of life, maintain the social stability, leaving the most beautiful retrograde war epidemic figure... after a day of fierce competition, finally selected the first prize 3, the second prize 6, third prize 10,30 excellence award.

2022-06-20 Henan Nanyang held "stamp age youth" exhibition Henan Nanyang held "stamp age youth" exhibition activities Pan Zhixian correspondent Zhang Ruoxuan) to further promote "welcome twenty big, always follow the party, new journey" theme education practice, on June 17, "stamp age youth-to celebrate the 100th anniversary of the founding of the Chinese communist youth league of China" photo exhibition offline tour launch ceremony was held in Nanyang city, Henan province. The photo exhibition is a special theme event planned by the Communist Youth League Nanyang Municipal Party Committee of Henan Province to celebrate the 100th anniversary of the founding of the Communist Youth League of the CPC, and was held both online and offline simultaneously. The activity was held by the Communist Youth League Nanyang Municipal Party Committee, Nanyang Municipal Propaganda Department, Henan Industrial Vocational and Technical College, Nanyang Municipal Network Information Office, Municipal Education Bureau, Municipal Bureau of Culture, Broadcasting and Tourism, etc. The photo exhibition is divided into 32 sections, tracing the 100 years from the square of stamps. More than 20 pieces of regiment history and cultural relics, including badges, MEDALS, MEDALS, league certificates and books, were also displayed from the 1950s. Launch ceremony scene, Nanyang Municipal Party Committee Deputy Minister of Propaganda Department Lei Hongbo said that holding the tour activities, is not only under the leadership of the Party of the Communist Youth League one hundred years of concentrated publicity, but also to educate and guide the majority of Nanyang city youth to inherit the red gene, carry forward the revolutionary spirit, condensed the strength of the practical action. Group Nanyang municipal party committee secretary Yang Wenjie, the next step, will be in Nanyang downtown city "three pavilion", liberation square, central square, Nanyang institute of technology, Henan industrial vocational and technical college, Nanyang medical college, Nanyang agricultural vocational and technical college and other colleges and universities, urban primary and secondary schools, and various counties continue to organize tour activities.

2022-06-20 Chengdu launched youth Volunteer Service Project Competition project implementation for no less than 2 years to participate in the excellence award of China Youth Network reporter Wang Xinxin) On June 18,2022 Chengdu Youth Volunteer Service Project Competition was launched. With the theme of "Youth Volunteer to Create a Civilization Model City in the New Era", the competition was jointly launched by 22 departments including Chengdu Municipal Party Committee, Chengdu Civilization Office, Chengdu Municipal Social Governance Committee, Chengdu Civil Affairs Bureau and Chengdu Branch of Agricultural Bank of China. The application projects cover 13 categories, including environmental protection, civilized practice, caring for children, services for the elderly, sunshine for helping the disabled, health care, emergency rescue and epidemic prevention and control, community governance and neighborhood watch, water saving and water protection, legal services and anti-drug education, covering all areas of urban governance. Contest poster. Adhering to the concept of "let the youth grow together with the city", the Communist Youth League Chengdu Municipal Party Committee will face the volunteer service projects in various fields of society that have been implemented for at least two years, select 60 excellent project awards, and promote them to the national and provincial youth volunteer service project competitions. At the same time, the competition will also collect the proposed volunteer service project ideas to be implemented, and set up the top ten creative projects awards. The organizer will build a platform and coordinate resources to support the incubation and implementation of creative projects. The organizer will set up an expert jury and youth jury, and open public evaluation channels to support real-time youth participation and evaluation. The competition will also support the youth volunteer service courtesy exchange salon, organize the young volunteers to carry out brainstorming, and expand the volunteer service skills. The competition has opened online declaration channels on the web end and mobile phones. All applicants can log on to the official website of the Chengdu Municipal Party Committee of the Communist Youth League from June 18th to June 30th (http: / / www.cdcyl.org. For cn /), enter the young volunteer "2022 Chengdu Youth Volunteer Service Project Competition Zone" for project application, and can also enter the young volunteer "Project Competition" through the wechat public account of "Qingjujin Guan City".

2022-06-17 China Youth Daily reporter Xing Ting) On the afternoon of June 14, Jinan Youth Work Joint Conference and the national youth development city construction pilot promotion meeting was held. The meeting further clarified the "Pilot Implementation Plan of Jinan Youth Development Friendly City Construction" and the decomposition of 12 key tasks, 36 small items and 141 specific work. Jinan city youth development friendly city construction leading group deputy leader, the Municipal Committee of the Standing Committee, the Municipal Federation of Trade Unions Party Secretary, chairman, the City District Party Committee Secretary Liu Ke attended the meeting and made a speech. Recently, the Inter-ministerial Joint Conference Office for the Implementation of medium-and long-term Youth Development Plan announced the list of pilot youth development cities and youth development counties, including 45 pilot cities (including municipalities directly under the Central Government) and 99 pilot counties, with a pilot period of 2 years. Jinan city was successfully selected among them. Liu Ke said in his speech that, From the four aspects of the central provinces and cities to this work, the rest of the country to youth development, Jinan's own development and its own responsibilities, Deeply understand why we should pay attention to the youth development of friendly city construction; To seriously study the "Jinan City Youth Development friendly City construction pilot implementation plan" and the key tasks decomposition of 12 major items, 36 small items, 141 specific work, Combined with the actual situation of the youth friendly city to grasp what; To clarify the "goals, problems, responsibilities, time limits", Plan to implement the youth development of a friendly city should be how to grasp; To make full use of the "scheduling, supervision, notification, assessment" way, To ensure that the city's youth development and friendly city construction work must be done well. Zhang Xi, director of the Office of Jinan Youth Development Friendly City Construction Leading Group and secretary of Jinan Municipal Party Committee of Jinan Youth League, introduced the progress of the construction of youth development friendly city and the Implementation Plan of Jinan Youth Development Friendly City Construction Pilot Project. Around the Jinan youth development friendly city construction pilot implementation plan work basis, expected goals, the main task, organize the implementation of four most, based on optimizing the urban environment and service urban development two dimensions, "seven optimization" and "five mobilization" and other 12 big plate for detailed interpretation, has been clear about the development of youth friendly city construction important direction and work path. The leaders of Lixia District, Huaiyin District and Municipal Bureau of Commerce, Municipal Culture and Tourism Bureau, Municipal Health Commission and Municipal Sports Bureau respectively reported the relevant situation of promoting the development of youth-friendly cities, and introduced their respective experiences and practices. Conference issued the Jinan youth development friendly city construction pilot key task decomposition table " (draft) and related materials, has been clear about the 141 important tasks, the next step will control task index list pays special attention to the implementation, adhere to excellence, strengthen scheduling, the whole domain as a whole, successfully complete the national pilot construction tasks, effectively promote the development of youth and urban high quality mutual promotion. Member units of Jinan Youth Development Friendly City Construction Leading Group, relevant units of the Municipal Youth Work Joint Conference, and leaders of all districts, counties and functional areas attended the meeting.

2022-06-17 Guangxi Nanning minors judicial social work service center unveiled Nanning minors judicial social work service center unveiled Nanning intermediate people's court, Nanning people's procuratorate, Nanning public security bureau and Nanning judicial bureau and other units, held in Nanning minors judicial social work service center opening ceremony and minors judicial social work service series of activities. Event, from the court, procuratorate, public security bureau, judicial bureau and the relevant person in charge of Nanning municipal committee for Nanning minors judicial social work service center, at the same time signed the Nanning Nanning intermediate people's court, the people's procuratorate public security bureau, Nanning Nanning justice bureau of Nanning league committee of Nanning minors judicial social work service center cooperation agreement. Then, the organizers held professional judicial social work agency service experience exchange, around the minors involved case intervention, family trial, tracking support, prevention of juvenile delinquency propaganda and family education work, such as the introduction of judicial social work force to carry out minors conditional not prosecution education, social investigation, build minors' mental health counseling system, carry out good family education, establish communication cooperation mechanism reached consensus. The establishment of Nanning Juvenile Judicial Social Service Center aims to strengthen the judicial protection of minors, innovate the social mobilization mode, participate in social governance, jointly boost the healthy growth of minors, and realize the organic connection between "professional case handling" and "socialized service".

2022-06-17 Guangdong Zhuhai: love power not to implement the employment to graduates Zhuhai: love power not to implement the employment to graduates correspondent Jin Xinling) on June 16, from Beijing normal university Zhuhai campus and branch, Beijing institute of technology Zhuhai college, Zunyi medical university Zhuhai campus, Zhuhai college of science and technology of the first 290 family difficulties and not implement the employment to students, received from the Zhuhai communist youth league to bank card of 666 yuan encourage gold and warm messages. In order to support the employment of college graduates in Zhuhai in 2022, the Communist Youth League system of Zhuhai, Guangdong Province recently launched to support the employment of college graduates in 2022. One of the key actions is the "key college students refueling Action". The Zhuhai Zhuhai municipal party committee of Zhuhai green league, Zhuhai green enterprise association, Zhuhai sea green synergy, raise 600000 yuan, will be 898 in pearl university family difficulties and has not implement the employment to graduates, take the way of direct student bank card, 666 yuan each refueling encourage gold, and send warm refueling encourage text messages. The students said that they received their blessings from the youth league organization. They felt warm and confident for a moment. They would adjust their employment mentality and positioning, and never give up actively. I believe that they could find a satisfactory job and take the first step in entering the "social university". Students who have received the encouragement gold and text messages have left messages on the background of the official WeChat official account of the Zhuhai Communist Youth League "Zhuhai Youth" - - " Come on together!”

2022-06-17 The donation ceremony and the "Pomegranate Flower Opening and Growth Road" - -the practice activity of forging the community consciousness of the Chinese nation were held in Liuzhou Youth Palace. Activities, group Liuzhou Liuzhou municipal party committee in recent years Liuzhou hope project work and new era of hope project development direction, mobilize Liuzhou communist youth league organizations at all levels and the social from all walks of life attach importance to and participate in the hope project, care about youth healthy growth, jointly promote the new era of Liuzhou hope project quality and efficiency. The activity aims to help left-behind children aged 6 to 16 and students from poor families in ethnic minority areas to solve small difficulties, realize small needs and light up small dreams. The Communist Youth League organizations, Youth Civilization, social organizations, caring enterprises and individuals, as well as teachers and student representatives participated in the activity. As of that day, the event has received donations of more than 500,000 yuan and planned to help 5,000 teenagers from poor families.

2022-06-17 The Harbin Municipal Committee of the Harbin Municipal Committee was held in Harbin Engineering University. During the activity, the Harbin Science and Technology Bureau interpreted the Harbin science and technology innovation support policies, and the Harbin Human Resources and Social Security Bureau introduced the Harbin employment and entrepreneurship policies and talent support policies of Harbin. Students of Harbin Engineering University in 2023 of Harbin Engineering University watched the promotional video.

2022-06-16 Xing Ting, the first young civil servant skills competition in Qingdao city, was officially launched recently. The competition is jointly organized by the Organization Department of Qingdao Municipal Party Committee, the Municipal Working Committee of Qingdao Municipal Party Committee, the Qingdao Municipal Communist Youth League Committee, and the Qingdao Special Class Office of Optimizing the Business Environment. It is understood that this is also the first Qingdao city for young civil servants held skills competition. According to the report, the competition aims to speed up the construction of high-quality professional civil servants, and further promote the "style and ability improvement year" activities. The competition is open to young civil servants at all levels of the city, to participate in the name of individuals and teams, and to create innovative and creative works around the key work and to submit the entries. Adopt the "1 + 3 + N" mode, namely "one theme, three types of works, and N forms"."One theme" is to "practice ability, strong ability, still practical work to change style" as the theme. Encourage the city's young civil servants based on the foothold official duty post, take the initiative to adapt to the requirements of the development of the new situation, practice good skills, temper "everything about politics, man, director heavy effect, achieving it top" style of ability, to be "things for first-class, only flag is to seize" youth "doer", guide the formation of advancement, than learning to catch up with, bear as, vigorously promotes the implementation of the thick atmosphere."Three types of works" are three types of business environment, policy popularization, and advanced deeds. Business environment works focus on the four "whole life cycles" of service enterprise, natural person, project construction, innovation and entrepreneurship; policy popularization works focus on epidemic prevention and control, education and health, medical security, urban management, market supervision, ecological environment, policy interpretation and knowledge popularization; advanced deeds works implement the spirit of the 13th Party Congress, focusing on urban construction and urban renewal, service and cadre construction, and promote the advanced deeds of individual and collective."N forms" means that the works can use text manuscripts, new media (micro video, PPT, H5, "one picture", etc.) and creative design and other forms. Recommended entries are accepted from now on, and the deadline is July 31,2022. In August, the host department of the competition exhibited its works and voted online on the official accounts of "Lide Gong", "Qingdao Organ Party Building" and "Qingdao Communist Youth League". In September, we collected online voting data and organized expert review. Publish the list of winners according to the selection results, and organize and carry out centralized exhibition activities. The competition will set up the first prize, second prize, third prize and several awards of excellence, issued in the city. Outstanding organization awards shall be awarded to the district, municipal and municipal units with outstanding organizational work performance. According to reports, the Qingdao Municipal Party Committee Organization Department and other relevant departments will take this competition as an opportunity to promote the organs at all levels to focus on key work and job responsibilities, to carry out professional training and skills training. We will actively build a platform for learning and exchange and interaction to encourage civil servants to update their knowledge, enhance their abilities and innovate their creativity. After the competition, we will continue to make good use of the results of the competition, take various forms to promote the publicity competition, play excellent works in the government service halls and service Windows at all levels, publicize policies, popularize knowledge, and display a good image of civil servants.

2022-06-16 The renovation project of 416 "dream cabins" and 206 "dreams cabins" has been fully completed. This means that in 2022, the municipal government's livelihood "dream hut" project in the problem of "one old and one small" has been completed. This year is the group of Jiangsu provincial party committee "dream transformation +" care plan in the second year, Zhenjiang for 206 without independent living and learning environment of 6 to 16 troubled children, on the basis of the original housing, provide "private" service, "three sides" (ground, metope, roof), with "six" (bed, wardrobe, bookcase, lamp, curtains, four pieces), create independent space, and purchase special life furniture, school supplies, etc., improve its living and learning environment, perfect the care support system. The reconstruction project of "Dream House" is the second consecutive year that the Zhenjiang Communist Youth League has independently undertaken the practical project of the livelihood of the municipal government, which is the concrete practice of the Communist Youth League to serve the overall situation around the center. In the past two years, with the joint efforts of the youth league organizations at all levels and all sectors of society in the city, the Zhenjiang Municipal Party Committee of the Communist Youth League has raised more than 7 million yuan of project funds and built 416 "dream cabins", exceeding the provincial construction task ahead of schedule. At present, the group of Zhenjiang municipal party committee is playing jointly issued with the civil affairs bureau of 2 + 2 pair support mechanism, and professional social organizations, in town university depth cooperation, overall cadres, township children supervisor, social organizations, university volunteers, complete the plight of teenagers key case tracking, care project, related assessment referral and other key work, promoting dilemma teenagers care support precision, specialization, brand, long-term.

2022-06-16 Fujian Longyan 14 departments jointly issued a document to carry out college students' internship "sail plan" Jianwei) recently, The Longyan Municipal Party Committee of Fujian Province, together with municipal Development and Reform Commission, Municipal Education Bureau, Municipal Bureau of Science and Technology, Municipal Industry and Information Technology Bureau and other 14 departments, jointly issued the "Notice on Carrying out the 2022 Longyan College Students Internship" Sailing Plan "", This year, in the city's units to collect no less than 2,700 public institutions, party and government organs, state-owned enterprises and financial institutions, all kinds of scientific research institutions and large private enterprises, For rock students and college students in the province, Promote no less than 1,400 college students to participate in summer internships. Up to now, Longyan city college students internship "sail plan" has mobilized 1,258 employers, providing 2,750 internship positions, attracting students to send 3,324 resumes.

2022-06-16 Jiangxi 7th Nanchang "Hongcheng Cup" Vocational Skills Competition held the 7th Nanchang "Hongcheng Cup" Vocational Skills Competition and the first Jiangxi Province Vocational Skills Competition Nanchang Selection E-commerce division Competition held Jianwei correspondent Cheng Hong, Sun Jianbo, Song Fei) June 13, Sponsored by Nanchang Municipal Human Resources and Social Security Bureau of Jiangxi Province, Nanchang Municipal Finance Bureau, Nanchang Municipal Federation of Trade Unions, Nanchang Municipal Party Committee of the Communist Youth League and Nanchang Municipal Women's Federation, The 7th Nanchang Municipal "Hongcheng Cup" Vocational Skills Competition and the first Jiangxi Provincial Vocational Skills Competition Nanchang Selection Competition E-commerce Division (Cross-border E-commerce) Vocational Skills Competition organized by Jiangxi Youth Vocational College was opened. Liu Anqing, vice president of Jiangxi Youth Vocational College, Xin jiagang, deputy director of Nanchang Employment and Entrepreneurship Service Center, Huang Hao, president of Transportation Management School of Jiangxi Transportation Vocational and Technical College, and Fang Xueliang, general manager of Xiamen Youyou Huilian Information Technology Co., Ltd. attended the opening ceremony and delivered a speech. More than 20 teams from other universities participated in the competition of theoretical knowledge and practical skills. Theoretical knowledge covers cross-border e-commerce industry and mainstream cross-border e-commerce platform knowledge, including industry knowledge and rules, commodity publishing, marketing promotion, customer service and other knowledge, assess the theoretical knowledge of cross-border business; design around the real cross-border e-commerce platform, contestants can experience Amazon platform operation process, simulation from store opening, product publishing, order processing, customer service, FBA delivery setting, marketing promotion, assess the comprehensive operation ability and innovation ability of cross-border e-commerce platform. In order to ensure that the event each work smoothly, Jiangxi youth vocational college established the Nanchang seventh "Hongcheng cup" vocational skills contest Jiangxi youth vocational college organization committee, the school dean Zheng Yanming as director of the organizing committee, and set up the team, contact group, security group, logistics group, monitoring group, technical support group, publicity group, activities, event evaluation group is responsible for the competition activities of organization and coordination, contact, security, system operation, publicity, etc. Before the competition, Zheng Yanming dispatched for many times to deploy each link of the competition, on-site inspection and guidance of related work. Competition provides a platform for learning and communication between colleges, effectively promote to promote learning, to promote evaluation, to build, to promote the development of new forms, new mode of foreign trade innovation, support the national cross-border e-commerce comprehensive experimental zone construction, cultivating innovation compound advanced cross-border electricity talent is of great significance.

2022-06-15 Bengbu Bengbu municipal party committee, Bengbu railway station youth experience red play kill Bengbu, Bengbu railway station party committee with youth experience red play to kill green net reporter hai-han wang) recently, Bengbu and Bengbu railway station party committee "youth learning-youth Young remember history, yong bear mission" theme TuanRi activities, through the script reading, scene simulation, scene, reproduction, personal expression, question answer, lead the youth experience "red play". The scripts "The Sun rises" Rising as usual "and" Based on the Lugou Bridge Incident and the Shuoxian Massacre (note: they are all public release scripts in the market), tells the growth and choice of the patriotic and progressive youth in the face of the current of The Times in 1937. In the more than four hours of immersive interpretation, with the advance of the plot, the youth members gradually integrate themselves into the historical "space dialogue", feeling the heroic and fearless journey and the original mission of the Communists."We are all from all over the world, coming together for a common revolutionary goal. When our comrades are in difficulties, our comrades must see their achievements, the light, and improve our courage."For the youth members, the review of the clues and the collective reading at the end of the play become a mirror of self-examination. Ding Aochen, a member of Bengbu Qingma Teaching Group and assistant duty officer of Bengbu Railway Station, said that "red script killing" is not only a "attack and defense" between characters, from costumes, character selection, script reading and then to text description, every scene of the script makes everyone feel the pursuit and dream of the revolutionary predecessors.

2022-06-15 Nanjing communist youth league to implement the "youth civilization pioneer" project power national civilization model city to create Nanjing communist youth league implementation "youth civilization pioneer" project power national civilization model city creator li chao) in 2022 is the seventh national civilized city to create cycle in the second year, Nanjing is the national civilization model city, the key to breakthrough years. In order to thoroughly implement the spirit of the Nanjing Spiritual Civilization Construction Steering Committee meeting and the National Civilized Model City creation and deployment Conference, Give full play to the role of the Communist Youth League in ideological education, practice education and service education, in the near future, The Communist Youth League Nanjing Municipal Party Committee combined with the actual work and the new characteristics and new requirements of the creation work, Formulated and issued the "Youth Civilization Pioneer" project- -Nanjing Communist Youth League to Help the National Civilization Model City Creation Work Plan ", For the characteristics of different groups of teenagers, Focus on strengthening youth ideal faith education, carry out "pioneer youth civilization" theme TuanRi activities, continue to deepen the communist youth league "unified action", "small pioneer" for civilization activities, extensive civilization create volunteer service, actively guide in ning college students to participate in creating activities, the implementation of the "civilization has my team listing" action, do plight teenagers care support, care to mobilize emerging youth group nine big initiatives, To guide the majority of youth league members and young pioneers around the relevant deployment of the Municipal Party committee and the municipal government, To contribute to the comprehensive construction of socialist modernization model city. After the announcement of the plan, the city's Communist Youth League at all levels and the Young Pioneers organizations responded quickly and acted immediately. On June 11, the Nanjing municipal party committee secretary RongFei into qinhuai district xinjiekou business block youth home, around "what is a civilized model city" "how to be a responsible civilization good citizen" "how to play the advantages of network distribution industry power civilized model city to create" "further care about service network about distribution industry youth group" about youth network about distribution theme, and listen to distribution staff to civilized model city to create ideas and Suggestions, mobilize them from yourself, combined with professional characteristics to create civilized model city. League Yuhuatai District Committee through the development of special publicity, issuing proposals, explain the creation of civilization attempts and other ways to spread the concept of civilization to the majority of youth, Create a strong creation atmosphere of "I know, I participate, I contribute"; Jiangbei New District Youth League Working Committee widely launched youth League cadres, young volunteers into the residential community, Orderly participation in garbage classification, small advertising eradication, corridor debris cleaning and other creative cultural practice work; The Youth League Gulou District Committee organized the youth league members to take to the streets, Participate in voluntary services such as civilized guidance, road environment improvement, and shared bike sorting, With practical actions to continue to set off the youth of helping to create a civilized model city upsurge; Jiangning Youth League District Committee established the city's first community "youth volunteer service station to help create the national civilized model city", To provide a learning and training platform and practice position for teenagers to participate in and help build the national civilized model city. Next, the Nanjing Communist Youth League will focus on the key areas and key links of civilization creation, do a good job of redeployment, mobilization, and deepening of activities, and strive to build a more characteristics of the Communist Youth League, more demonstration effect of the civilization creation work brand. At the same time, centering on the requirements of normal long-term help creation work, further consolidate the work responsibility, make unremitting efforts, will help the creation of work into the youth, into the daily life, so that the figure of youth active in every corner of the creation of civilization, and constantly promote the creation of civilized model city to achieve new results.

2022-06-14 Group Jilin Yanbian committee held "youth celebrate twenty" youth speech contest Jilin Yanbian committee held "youth celebrate twenty" youth speech contest Wang Peilian) recently, to celebrate the party's twenty victory, to celebrate the 70th anniversary of the founding of Yanbian Korean autonomous prefecture, Yanbian committee held a "youth to celebrate 20" youth speech contest. It is understood that more than 190 people have signed up for the competition. The competition will be divided into two rounds: preliminary rounds and final rounds. The Yanbian State Committee of the Communist Youth League hopes to select a group of civilized, heavy etiquette, good quality and good image contestants through the competition, and become the volunteers of the state celebration activities, publicize and promote Yanbian. Outstanding players in the activity will be included in the Yanbian Youth Image Ambassador talent pool. Competition players. Youth League Yanbian State Committee for the picture of the competition players. A few days ago, in the preliminary competition site, the contestants explained the changes of their hometown around the theme of the competition, showing the style and mission of the youth.

2022-06-14 Rizhao Municipal Youth League Committee and Shandong Normal University Youth League Committee held school-local cooperation symposium Rizhao Municipal Youth League Committee and Youth League Committee of Shandong Normal University held school-local cooperation symposium Jianwei correspondent Xiang Changhui) In order to accelerate the construction of a youth development friendly city, Deepening the cooperation and development of the school and local Communist Youth League, Enhance communication with students from Rizhao, Help the overall situation of the work of attracting talent and wisdom, On June 11th, The Shandong Province Rizhao Municipal Party Committee and the Youth League Committee of Shandong Normal University jointly held a school-local cooperation symposium, Zhao Wei, Secretary of the Communist Youth League Committee, and Zhang Meng, Deputy Secretary of Shandong Normal University, Li Wanying, deputy secretary of the Youth League Rizhao Municipal Party Committee, attended the symposium, More than 20 people from the Youth League Committee of School of Geography and Environment and School of Life Sciences of Shandong Normal University and representatives of Rizhao students attended the symposium. The Rizhao Municipal Party Committee and the Youth League Committee of Shandong Normal University jointly held a school-local cooperation symposium. Before the meeting, we watched the Rizhao city youth development friendly city theme propaganda video "Young Light Sunshine youth City". At the symposium, Zhao Wei, secretary of the Communist Youth League Committee of Shandong Normal University, introduced the overall situation of the Communist Youth League of Shandong Normal University, and raised his ardent expectations for the practical cooperation between the two sides."Green Bird Station" unveiling picture. The two around the "green bird project", in the service of college students social practice base, regular "return home" social practice activities, in the service of college students volunteer service platform, arrange students to Rizhao "three to the countryside" social practice volunteer service, to serve the growth and development of college students, beach art festival, innovation and entrepreneurship competition, to enrich the cultural life of young students, to add youth elements. At the same time, the League Rizhao Municipal Committee set up the "Green Bird Station" in Shandong Normal University to widely gather young students from Rizhao. For the "green bird station" stationmaster Liu Peiying issued a letter of appointment. Correspondent for figure finally, group Rizhao, deputy party secretary wan-ying li said that the school cooperation is group Rizhao municipal party committee and Shandong normal university youth corps committee hand in hand, help students grow up, boost the development of urban quality new starting point, the two sides will take the cooperation as an opportunity to give full play to the school to the youth, unite youth, condensed youth, fully absorb students good advice, promote more practical measures. The newly established "Bluebird Station" is the "heart bridge" for Rizhao Municipal Party Committee to contact and serve students from abroad in Shandong Normal University, the "new window" to promote the city, and the "outpost" for the Communist Youth League to attract talents. It will give full play to the important role of gathering students from Rizhao abroad. The stationmaster of "Green Bird Station" is the liaison officer of Rizhao students of Shandong Normal University. In the future work, he should pay attention to the power of youth, play the role of a bridge to publicize Rizhao and promote Rizhao, and become the publicity ambassador of his hometown.

2022-06-14 Jianwei, correspondent Chen Cheng, Pan Yujie) Recently, the first lecture of "Pingxiang Young Doctor Lecture Hall" hosted by the Pingxiang Municipal Party Committee of Jiangxi Province and undertaken by the Youth League Committee of Pingxiang College was held. The lecture was conducted in the form of "cloud live broadcast + offline centralized listening". Liu Yong, a professor and Party committee member of the School of Control Science and Engineering of Zhejiang University, was invited to give a special academic lecture. Tan Liqin, deputy secretary of the Youth League Pingxiang City Party Committee, presided over the lecture. Nearly 200 some college students and young people from various industries and fields participated in the study. Liu yong to "from artificial intelligence to intelligent robot", around the definition of the connotation and development of artificial intelligence, the typical application of artificial intelligence and robot technology and scenarios, as well as the future development direction, etc., combined with a large number of vivid specific cases for special academic lectures, elaborated the artificial intelligence to human society. During the exchange and interaction session, Liu yong discussed with his students online about how to view the popular "virtual idols", the relationship between digital images and artificial intelligence, and how to learn the basic subjects in the field of artificial intelligence well. Liu Yong, professor, doctoral supervisor and Party committee member of the School of Control Science and Engineering of Zhejiang University, has won the first prize of Natural Science of Zhejiang Province and the first prize of Science and Technology of Zhejiang Province. As the first author or corresponding author, he has published more than 100 papers in well-known journals and top robotics conferences, applied for more than 70 invention patents, and 33 patents have been authorized. It mainly studies autonomous robots and intelligent systems, robot autonomous planning and navigation control, visual recognition and mode recognition, etc. Young talent is the most active factors in urban development, "Dr. Pingxiang city youth lecture hall" activity is the pku "ping green homing" plan one of the series of activities, the purpose is to give full play to the talent demonstration leading role, promote Pingxiang nationality outside youth doctor contact with home, further build youth study hard, study hard, to promote, courageous strong learning atmosphere. In the future, more young experts and scholars from Pingxiang will be invited to return to their hometown to share knowledge, opening up a new window for more young people to grow up.

2022-06-13 Shandong Yantai held welcome twenty and celebrate building theme lesson activities in one hundred in Shandong Yantai "one hundred just elegance struggle is youth" celebrate 20 and celebrate building one hundred theme lesson activities green net reporter XingTing) on June 10, Yantai, Shandong province held "one hundred just elegance struggle is youth" welcome twenty and celebrate building theme lesson activities in one hundred. The activity is organized by the Organization Department of Yantai Municipal Party Committee and the Yantai Municipal Party Committee, and organized by Muping District Base Office and Youth League Muping District Party Committee. Youth League Yantai Municipal Party Committee secretary Xiang Fuliang, Muping District Committee Standing Committee, organization minister Ma Ji and other urban leaders attended, Muping district town streets, the units of the youth League committee secretary and young cadres, school members, young pioneers nearly more than 200 people attended. Activities are divided into "dedicated" "selfless dedication" "regret choice" three chapters, by Yantai municipal party committee organization department base teachers and base narrator, muping red scarf narrator, youth members, etc., by reading red letters, red stories, scene reading, singing red songs, let the young cadres deeply understand the communist party's mind and mission, strengthen the young cadres of patriotism education faith, let the red gene passed down from generation to generation. During the activity, the participating leaders were invited to the "May 4" youth post expert, "May 4" youth model, "May 4" youth pioneer collective awards."This vivid theme league class is very rich, allowing us Communist Youth League members to see the hard-won happy life today in a touching red letters and revolutionary stories, and will also encourage us league members to keep their original aspiration and dedicate their youth to the motherland."Zheng Wenyu, a youth league member of Shandong Traditional Chinese Medicine College, was quite touched. Next, group Yantai municipal party committee will organize to mobilize the members and youth from all walks of life in the era of realizing the great rejuvenation of the Chinese nation continue vigorous, brave, strive to write worthy of the party, the people, the era of The Times, with high morale and full state to meet the party's 20th victory.

2022-06-13 Guilin held "Chinese youth May 4th medal" "Guangxi youth May 4th medal" winners (collective) deeds held "Chinese youth May 4th medal" "Guangxi youth May 4th medal" winners (collective) deeds sharing Xie Yang) on June 10, sponsored by the Guangxi district party committee, Guangxi youth league, Guilin municipal committee, Guilin normal college youth corps committee to undertake "welcome 20 always follow the party new journey" - "Chinese youth May 4th medal" "Guangxi youth May 4th medal" winners (collective) deeds sharing held in Guilin normal college concert hall. The sharing meeting will be broadcast live online on the Internet simultaneously, and the young people in the whole district will watch it simultaneously through the Internet. The sharing meeting kicked off in the 26th China Youth May 4th Medal propaganda video "100 Years of Struggle Always Follow the Party"."Guangxi youth may 4th medal" winner and the team on behalf of Guilin public security bureau forensic brigade five brigade deputy battalion chief LeiLinZhen, Guilin qin pool experimental school vice principal Li Jiayuan, Guangxi fencing club director Dong Jiale, Guilin college of tourism students and Guilin bank rural revitalization work, respectively around xi general secretary in celebrating the 100th anniversary of the communist youth league of China's important speech spirit, tells the story of their youth struggle on the road. They interpret the thickness and breadth of their lives with their dedication, play the power of example in their respective posts, inspire and drive the majority of young people to practice their original aspiration, fulfill their original aspiration and fulfill their mission, and constantly write the youth chapter of "Please rest assured of the Party, a strong country has me". In the exchange and interaction session, the award-winning representatives gave answers to the confusion and problems encountered by young people on the growth path with their own experiences, and explained the mission of staying true to their original aspiration and having the courage to take responsibility, and the spirit of being pragmatic, dedicated, innovative and enterprising.

2022-06-11 Huo Tong Jianwei correspondent Su Jie Zhong Yiming) Recently, the "Red Scarf Tour Group" organized by the Youth League of Fujian Province Ningde Municipal Party Committee and the Municipal Youth Working Committee walked into Huotong Town to carry out learning and education publicity. Nearly 100 people including representatives of young Pioneers and young Pioneers counselors participated in the learning and experience activity. Pku, deputy secretary of "better" theory XuanJiangTuan members Liu Xiaobing to the young pioneers representatives propaganda xi general secretary in celebrating the 100th anniversary of the founding of the communist youth league of China's important speech spirit, at the same time, the young pioneers general counselor, "red scarf tour" members Ruan Guangqing to the young pioneers about the revolutionary predecessors, the red story."This learning and experience activity is full of harvest. I will bring back what I saw, heard and felt in the activity, light up the star torch with my own actions, and strive to be a good team member in the new era."Said Gong Haoqi, a representative of the Young Pioneers. It is reported that in recent years, the Youth League Ningde Municipal Party Committee by building a red education base, the establishment of the "red scarf tour group", the development of red courses, and the use of on-site teaching mode to give the children a good "walking party history class".

2022-06-11 Xing Ting, the first youth learning and practice space in Qingdao) On the morning of June 9th, Qingdao "Youth Teaching Group" municipal demonstration lecture and "youth Learning" publicity activity was held in the zero-carbon community energy exhibition hall of Olympic Center. Zhao Quanchao, deputy secretary of the Qingdao Municipal Party Committee of the Communist Youth League, and Zhang Lin, chairman of the trade Union of Qingdao Energy Group, attended the event and inaugurated the first Qingdao youth learning and practice space. It is understood that the "Qingdao Youth Learning and Practice Space" aims to build a communication platform for the city's youth learning and practice, and further improve the learning ability and practice level. The inaugurated zero-carbon community energy exhibition hall of Aofan Center is the first batch of zero-carbon community pilot projects created by Qingdao Energy Group in order to actively respond to the national dual-carbon strategy. Through visiting and learning, interactive games, we popularize the knowledge of "zero carbon" to the public and "zero carbon" and publicize the concept of "zero carbon". In order to deeply study, publicize and implement the important spirit of General Secretary Xi Jinping's speech at the conference celebrating the 100th anniversary of the founding of the Communist Youth League of China, and guide the youth league members of the city to make speeches, strengthen their style, improve their ability and work hard, the Qingdao Municipal Party Committee of the Communist Youth League organized a series of special publicity activities. At the scene of the preach, two municipal youth branch members, from Qingdao youth teachers Li Chunzhu and Qingdao energy group jiaozhou bay undersea gas pipeline youth commandos captain wei-bin zhang respectively to "when the pioneer, do model" and "young blood yong bear, gas into the sea dance dragon" to preach, combining the actual study and work, from the theory and practice two dimensions to share the learning experience. The representatives of the young commandos of Qingdao Energy Group took the youth oath, expressing the determination and confidence of all the energy youth to actively participate in the three years of urban renewal and urban construction, and implementing the spirit of the 13th Municipal Party Congress with practical actions. Next, the Qingdao municipal party committee will continue to deepen the youth branch team construction, organize youth lecturer into school, into the school, into the enterprise, into the community, to carry out the focus, interactive preaching, constantly promote learning propaganda boom, to put the city's youth ideological unity to xi general secretary's important speech spirit, continue to improve style ability, top, work rushed, with youth perspective li energetic spirit, with youth discourse power have my triumphalism.

2022-06-10 The Municipal Youth Volunteers Association carried out the "Warm Youth" in Jixi No.1 Middle School and Jixi Experimental Middle School. At 6 am, June 7, young volunteers in two test site near tents, carrying mineral water, put tables and chairs, etc., set up the "communist youth league love help test service station", to need students and parents free masks, disinfection wipes, 2B pencil, eraser, mineral water and other items, and provide candidates with mobile phones, watches and other contraband storage service. At the same time, the Youth League Jixi Municipal Party Committee invited the Jixi City Youth Mental Health Association psychological counselors, for the parents and candidates in need of psychological adjustment and counseling. In the early stage of the examination, the Communist Youth League committee also set up a love team, to need the candidates, one to one to provide love to send the test volunteer service.

2022-06-10 2022 "Good Time, gather Fuzhou" talent activity opened 2022 "Good time, gather Fuzhou" talent activity opening person Tian Hongwei) recently, 2022 "Good time, gather Fuzhou" talent activity opened, will provide the national full-time college students with employment, practice, practice, tour platform. This activity has two major themes: cold summer social practice and tourism. Tourism is divided into special line tour and independent tour. Social practice is divided into resident social practice and visiting social practice, and it is expected to provide 5,000 practical positions. The relevant person in charge of the Youth League Fuzhou Municipal Party Committee said that we welcome fresh and prospective graduates, local university students in Fuzhou, professional students in urgent need of economic and social development, and youth from Taiwan, Hong Kong and Macao to sign up. Students participating in the activity can enjoy accommodation, transportation and catering subsidies, free accommodation, transportation subsidies, green channel for medical treatment and free nucleic acid testing, personal accident insurance, electronic tickets and other discounts. Students participating in social practice activities will get the corresponding cold summer social practice certification; some students will be employed as young volunteers in Fuzhou campus. Up to now, the Fuzhou Municipal Party Committee of the Communist Youth League has carried out campus promotion activities to 12 colleges and universities in 8 cities of Fujian Province. In the future, we will also go to Wuhan, Changsha, Guangzhou and other places to carry out campus promotion meetings to promote more fresh graduates and prospective fresh graduates to develop in Rong. Students can follow the WeChat official account of "Youth Fuzhou" for more details of the activity and sign up.

2022-06-10 Group Longyan of Fujian province municipal party committee to carry out the troubled youth care activities Longyan municipal party committee to carry out the "green grain care-hope light micro wish" activities JianWei correspondent Tong Xiaodan) to implement teenagers to provide new power, sowing new hope, recently, group Longyan city of Fujian province for the city troubled youth "green grain care-hope light micro wish" activities, youth league organizations at all levels through issuing ceremony and household visits, the "micro wish" gift sent to the hands of the children's hands. This activity has been strongly supported by caring people and caring enterprises from all walks of life. The 203 "micro wishes" are collected by 43 caring people and caring enterprises, and the wish materials raised are worth more than 40,000 yuan. In the next step, TuanLongyan Municipal Party Committee will continue to carry out the care activities for teenagers in difficulties, launch the brand of "Qinghe Care- -Hope to Light up the Micro Wish", and contribute to the healthy and happy growth of more teenagers in difficulties.

2022-06-10 Group Ningxia Wuzhong city committee to carry out the "youth learning" preach Wuzhong city party committee to carry out the "youth learning" preaching figure China youth network Beijing on June 10 (xinhua zhang jianwei) on June 7, the Ningxia hui autonomous region Wuzhong city committee into Wuzhong city tax bureau member youth, through the way of cadres on the platform, xi jinping, general secretary of the important speech spirit. On the day of the activity, Wang Zhilan, deputy secretary of Wuzhong Municipal Party Committee and a member of the Communist Youth League Central Youth Lecture League Group, made a demonstration lecture entitled "youth heart to the Party, casting glory" in a hundred years. Subsequently, from the beginning of the Chinese youth movement and the birth of the Communist Youth League, the centenary journey of the Communist Youth League under the leadership of the Party, and the main experience and enlightenment of the Communist Youth League, based on the reality, the party further clarified the study and work priorities and thinking for the league cadres. The lecture was carried out through the combination of political height and practical significance, macro elaboration and micro interpretation, and theoretical communication and case analysis.

2022-06-10 Shandong Jining "green bird plan" cloud recruitment for youth jobs more than 13500 Jining "blue bird plan" cloud recruitment for youth jobs more than 13500 Jianwei correspondent BaiChen) recently, the Shandong Jining municipal party committee joint talent work leading group office, municipal human resources and social security bureau, the municipal people's government state-owned assets supervision and administration committee, Jining city holding group co., LTD., Jining talent development group co., LTD., to undertake "green bird plan" young talent cloud recruitment and 2022 summer college students practice launch activities successfully held. This activity, the Youth League Jining Municipal Party Committee combined with relevant units accurately excavated nearly 600 enterprises to provide more than 13,500 quality jobs, the number of positions increased by about 83.4% compared with the same period last year, achieving a new breakthrough, 440 organs, enterprises and institutions to provide more than 2,500 practice positions. The cloud recruitment category involves high-end equipment, high-end chemical industry, a new generation of information technology, new energy, new materials, medical health and other key industries, promote the college students and related enterprises docking talks, power young talent high quality employment entrepreneurship, college students practice, more than 560000 people through live watch simultaneously. For rich content, online live increased the talent policy, social security policy to preach, green bird station, young talent accelerator introduction and key enterprises, more frequency, multidimensional enterprise recruitment information, Shandong credit group co., LTD., Chen xin pharmaceutical co., LTD., lai electrical system (Jining) co., LTD., Shandong and big data co., LTD., Shandong is nuclear power technology co., LTD., head of the enterprise development situation and job demand, other key enterprises in the cloud recruitment site video promotion. This cloud recruitment activity is widely publicized through college Qingbird Station, student wechat group, QQ group and other platforms, unimpeded the channels of accurately push posts to students, and timely reply to students' concerns. In the future, the Youth League Jining Municipal Party Committee will continue to do a good job of follow-up visit, person-post matching, interview signing, workplace training, fellowship and other fellowship chain services. Next, group Jining municipal party committee will adhere to the young talents as an important strategic resources to promote the development of reform, with "youth vitality, youth charm, green power, youth gravity" to create a brand, cohesion to implement the service youth development "ten action", to build youth gathered "ji", "peace" ning " youth development friendly city, for the majority of young talents to employment entrepreneurship, create a good environment, build a broad stage.

2022-06-09 The Jining Municipal Party Committee has launched the first centralized training session for the volunteers of the 2022 Shandong Provincial Tourism Development Conference. Correspondent for figure China youth network Beijing on June 9 (reporter Ji'an-wei zhang, correspondent BaiChen) since 2022 tourism development conference in Shandong province volunteers registration start, from Jining culture system, qufu normal university, Jining vocational and technical college and Shandong technology vocational college volunteers actively register, the interview, selection, 276 recruit volunteers to stand out. Training KaiBanShi, group Jining, deputy secretary of dong-xu gao introduces the conference background and conference agenda, hope volunteers cherish service conference valuable opportunity, enhance active learning consciousness and bear dedication consciousness, to sign service standards and norms, to find their own shortcomings and short board, rapid growth into a good volunteer, with a sincere smile, professional enthusiasm service and selfless dedication, show Jining charm, youth. The training content covers the basic concept of volunteer service, volunteer service knowledge and skills of large-scale competitions, etiquette training and other courses. Vice President Qu Chengwei from Qingdao Youth League School has been engaged in the guidance and training of volunteers in large-scale games for a long time. He gave lectures on the concept and value of volunteer service in large-scale games, volunteer work analysis, basic service norms, volunteer management and other aspects. Photos show volunteers training on etiquette and codes of conduct. Correspondent for the picture " the conference volunteer appearance should be simple and generous, standing, sitting, walking, squatting, gestures have strict unified standards."At the training site, four volunteers from universities trained from the aspects of smile service, spiritual outlook and code of conduct training."We volunteers should maintain 100 percent enthusiasm, have a deep understanding of the culture and history of Shandong and Jining, better serve the guests at home and abroad, and truly become the youth and beautiful scenery of the conference."After a day of intense and orderly training, the volunteers still felt that they were not exhausted, and they were full of confidence in facing the important volunteer service tasks they would soon undertake. Early, group Jining municipal party committee issued the 2022 Shandong province tourism development conference volunteers should know should be manual, next, pku will continue to do a good job of volunteer dynamic management and selection, through concentrated teaching, scenario simulation, practice, experience sharing, strengthen the normalized training, organize young volunteers in etiquette training, Jining history and culture, foreign guests and oral communication, explaining skills, medical aid, mental health lectures, quality development and other training.

2022-06-08 The 2022 "Dream House" centralized delivery ceremony of "Dream House" was held in Nantong, Jiangsu province. At the ceremony, the participants watched the sitcom "Light up a Dream with Love" adapted from the true story of the Qidong City "Dream Transformation +" Care Plan. The Youth League Qidong Municipal Party Committee, the Youth League Tongzhou District Committee and the Youth League Haimen District Committee exchanged speeches as representatives of the county (city, district), providing practical experience for the cabin construction in the city, and exchanging work plans on the follow-up project care and service activities. Event held the award ceremony, the organizers for haian water group, the state administration of taxation, rugao county charity federation, qidong hui ping town chamber of commerce, longxin construction group co., LTD., Shanghai wan mu da furniture in Jiangsu cable company, Jiangsu guest electric power technology co., LTD., zhongtian cable and other eight love units and enterprises awarded love dedication award. In the process of Qidong counterpart assistance and cooperation, Qidong Youth Chamber of Commerce donated 10 "dream cabins" to Xixiang County, Shaanxi Province; Nantong Young Artists Association and Young Calligraphers Association presented calligraphy and painting works to the "dream hut". After the meeting, the participants went to Huilong Town, Qidong City to observe the "dream cabin" construction results. The relevant persons in charge of the Youth League Nantong Municipal Party Committee and the relevant persons in charge of the Qidong Municipal Party Committee attended the meeting. The secretaries of the Youth League Committee of each county (city, district), the persons in charge of the Hope office, the representatives of caring enterprises, students and other representatives attended the activity.

2022-06-08 In front of the Ling Campus, we will provide drinking water, umbrellas, emergency medicine, stationery and other love items for candidates and their parents. China youth daily client Nanning on June 7 (the youth newspaper reporter Xie Yang correspondent, Liang Yuwen, Huang Yifan Liu Xiaodan) today, 2022 the national college entrance examination, to deepen the "I do the practical work for youth" theme practice, help the students interpreta dream the college entrance examination, Nanning municipal party committee joint build eight bureau of Guangxi branch and other enterprises and social organizations, in 2022 Nanning youth volunteers "love help, help the future" volunteer service activities, "escort" for students. Nanning Municipal Party Committee of the Youth League and Nanning Radio and TV Station, called on and organized the majority of car friends and social vehicles to join the love to send the test "green ribbon" team, for the travel inconvenience, difficult candidates free test. In addition, the Nanning municipal party committee also united 13 love enterprises, recruited more than 300 young volunteers, in Nanning district 30 test site and Nanning subway line 1 to 5 near test subway station set up 57 exam volunteer station, for the examinee and parents with drinking water, umbrellas, emergency medicine, psychological counseling decompression, to route consulting services, and assist police and traffic police in the test site surrounding civilization guidance, order maintenance, etc. On the eve of the college entrance examination, the Nanning Municipal Party Committee of the Communist Youth League also launched the grassroots youth League Committee to carry out the "12355 with you", set up psychological counseling points, 12355 psychological assistance hotline and mini programs, and provide free pre-examination decompression and post-examination psychological assistance services. Psychological experts online to the examinee's psychological and emotional problems for stress relief counseling. According to statistics, the Nanning Municipal Party Committee of the Communist Youth League has carried out 20 college entrance examination decompression activities in 16 schools this year.

2022-06-08 Guangdong Qingyuan Municipal Party Committee held the theme activity of "Welcome 20, Strive to be a good member". Guangdong Qingyuan Municipal Party Committee held the theme activity of "Welcome 20, Strive to be a good member" theme activity, strive to be a good member " theme activity. The Communist Youth League Qingyuan Municipal Party Committee, Municipal Education Bureau and Municipal Civilization Office carried out the centralized demonstration activities in Fengxiang Primary School, New Beijiang Primary School and Boai Primary School. Youth League Qingyuan Municipal Party Committee secretary, the city young working Committee director Liu Gang delivered a speech. About 30,000 students from 309 primary schools joined the team during the June 1 holiday.

2022-06-08 Group of Anhui Bozhou municipal party committee around the college entrance examination students to carry out the series of theme activities Bozhou municipal party committee around the college entrance examination students to carry out the series of theme activities wang haihan) on June 7,2022 the college entrance examination, Bozhou of Anhui province municipal party committee unified high standard construction 12 youth college entrance examination comprehensive volunteer service station, according to the principle of the nearest, then, reasonable service area. Organized 43 youth social organizations, municipal organs, state-owned enterprises, non-public enterprises, community Party organizations and other volunteer service teams to provide careful, warm and warm examination services for 36,000 college entrance examination students in the city. According to the needs of students and parents, group Bozhou municipal party committee in the comprehensive service station set heat cooling, health care, hope engineering, employment, catering service desk, such as eight and free mineral water for students and parents, cooling medicine, Chinese medicine sachets, emergency stationery package, cool fan, etc., accurate classification to carry out the volunteer service. At the same time, volunteers set up parents' rest areas, inviting psychological counseling experts, young medical staff and emergency rescue personnel to be on duty in the service station, and provide special volunteer services such as psychological counseling and first aid treatment at any time. After the test began, the young volunteers sent sweet and delicious watermelon and mung bean soup to their waiting parents, and distributed leaflets on Project Hope and employment and entrepreneurship policies, explaining the possible problems and solutions during the college entrance exam. On the morning of July 7, Du Yan'an, secretary of Bozhou Municipal Party Committee, came to the scene to visit the volunteers, praised their dedication, and affirmed the standardized construction of volunteer service stations, optimizing the volunteer service process, and making great efforts to serve the college entrance examination students and their parents. After the college entrance examination, the Communist Youth League committee will also carry out a series of service projects and activities. For example, the "gaokao students see their hometown" activity, organize high school graduates into enterprises, projects, parks, communities, etc., to experience the development and changes of their hometown; youth 100 search card activity, visit Bozhou 100 most popular youth gathering places, attract college students inside and outside the city to practice in Bozhou, and contribute to the vivid practice of urban transformation and leapfrog development.

2022-06-08 Wang Peilian) Recently, the Jilin Municipal Party Committee of the Communist Youth League launched the "Beautiful China youth Action" in Jilin City, young volunteers to help create a civilized city volunteer service action. Jilin Youth League Municipal Party Committee launched young volunteers to help create a civilized city action.(Photo provided by the Jilin Municipal Party Committee) The action plan runs from early June to the end of October. The youth volunteer service team will use weekends to carry out environmental sanitation improvement on the north and south banks of the Songhua River, about 7.5 kilometers between Jilin Jiangwan Bridge and Linjiang Jiangmen Bridge, and all communities in Jilin City, especially the old residential areas. Volunteers clean up the garbage along the Songhua River.(Photo provided by CNPC Jilin Municipal Party Committee) On June 5th, more than 30 young volunteers from the "Gem Flower" volunteer service team of CNPC Jilin Petrochemical Company took the lead in action. Volunteers provided volunteer services such as cleaning debris, picking up garbage and cleaning guardrail near Binjiang West Road in Jilin City, helping Jilin City to build an ecological and livable city with practical actions.

2022-06-08 Shandong province dilemma school-age children student public welfare action held in Jining in Shandong province dilemma school-age children student public welfare action in Jining wei correspondent Bai Chen) recently, by the Shandong radio and television channel, agricultural channel joint Jining closes working committee, Jining municipal party committee, charity federation, city education bureau, Jining nine dragon education group departments, jointly launched the "light childhood-Shandong province dilemma school-age children student public welfare action" launch ceremony was held in Jining qufu. Zhang Weihua, deputy secretary of the Party Committee of Shandong Radio and Television Station, and Dong Bing, vice mayor of Jining City attended the event. The picture shows the launching ceremony of "Lighting Up Childhood- -The Public Welfare Action for School-aged Students in Shandong Province". In the activity, the student representatives of Qufu Nine Dragon School performed the gesture dance "Chinese Chinese characters". Representatives of the parents, students and representatives of outstanding graduates spoke. The students of Feicheng Jingxin School performed 5G connection. After the ceremony, the participants visited Qufu Nine Dragon School together. The picture shows the launching ceremony of "Lighting Up Childhood- -The Public Welfare Action for School-aged Students in Shandong Province". This "Light up Childhood- -Shandong Province School-age Children in Difficulties Public Welfare Action" will face the whole province, search, screening, rescue school-age children in need of help, through the "rescue, raising + education", free clothing, food, housing, learning and other comprehensive help. At the same time, combined with the "Hope House" child care project of the Jining Municipal Party Committee, it provides one-to-one care and assistance activities for children in difficulties, so that the children of this special group can have a warm, healthy and upward growth environment.

2022-06-08 Shandong Rizhao "small" volunteer training launched sunshine: "small" volunteer training officially launched for China youth network Beijing on June 8 (reporter Zhang Jianwei, correspondent XiangChanghui) to form a vigorous, trained volunteers, to ensure the provincial games volunteer service "efficient, professional, safe" operation, on June 4 to 5, Rizhao city in Shandong province committee volunteer work organization "small" volunteer general training, respectively held in Shandong sports institute, Jining medical college 2 universities, nearly 500 "small" took part in the training. At the training meeting, the responsible comrades of the volunteer department explained the work guarantee, the incentive measures and the emergency response measures of the volunteers. Therefore, the volunteers emphasized three points: first, do self-protection, strictly abide by the epidemic prevention and control requirements during the vacation; second, report the volunteer service, timely join the school volunteer group to obtain the return to school notice; third, obey the command and strictly observe the discipline of the event service. Battalion leader, squadron leader and captain leader should strictly perform their duties and implement the service task instructions. During the volunteer work department hired professional tutor for "small sea" taught health and epidemic prevention and safety knowledge, appearance training and grooming, basic etiquette reception training courses, through the "theory + practice" teaching means, the Shandong province games volunteer service required knowledge and skills to achieve mastery through a comprehensive study, make volunteers comprehensive quality and emergency ChuTu ability. In the next step, Rizhao Municipal Party Committee will continue to do a good job in volunteer training with universities stationed in Japan, and carry out special training activities with other departments of the Municipal Preparatory Committee to help the volunteers clarify their work responsibilities, effectively improve their volunteer service ability and adapt to the work pace of the provincial Games.

2022-06-08 Fujian Sanming held "green help trapped interpreta dream micro wish" concentrated demonstration Sanming Fujian held "green help trapped interpreta dream micro wish" concentrated demonstration wei correspondent officer yu flower Yu Lili) for the implementation of the Sanming left-behind children "weekend breakfast" spirit, according to the communist youth league Sanming committee "three hundred" action plan, recently, by the Fujian Sanming committee, Sanming youth federation, Sanming less committee Sanming "green help one trapped interpreta dream micro wish" concentrated demonstration activities held in sanyuan district fuhua community "young yan home". Pictures of the event site. At the scene of the activity, the young entrepreneurs and members of the Youth Federation claimed the micro wishes of 30 left-behind children, and gave them wish gifts, encouraging the children to study hard, enrich themselves with knowledge, and achieve life with knowledge. Young entrepreneurs, members of the Youth Federation and the children woven Dragon Boat Festival colorful rope, colorful rope, each other festival blessings. This activity is not only for the dream of left-behind children, but also a transmission of love, building a love platform for social caring people, and creating a good atmosphere of "great love of Sanming youth counterparts". Pictures of the event site. It is understood that the recent Youth League Sanming Municipal Party Committee issued the "" 300 action "to help" one district and six cities "construction implementation plan", called on the majority of youth league members to carry out the "300 action" as the implementation of "improve efficiency, improve efficiency, increase efficiency" an important starting point of action. Next, the city's communist youth league system will carry out comprehensive "best green help best sleepy interpreta dream micro wish" activities, collect left-behind children and other special groups, combined with the "youth league member grassroots" "young entrepreneurs old revolutionary base areas" and other activities, called for youth league members, young entrepreneurs, social love people claim micro wish and help achieve, continue to start "Sanming birth" brand, power "area six cities" construction.

2022-06-07 Shandong Jining young entrepreneurs, rural good youth into surishui Jining youth entrepreneurs, rural good youth into surishui wei correspondent Bai Chen) recently, to further study and implement the general secretary xi in celebrating the 100th anniversary of the communist youth league of China's important speech spirit, unity leading young entrepreneurs, rural good youth listen to the party, follow the party, new era, Shandong Jining bank organization youth entrepreneurs, rural good youth representatives to surishui technology innovation, rural revitalization, rural electricity, "hut" project, and share exchange activities. The picture shows young Jining entrepreneurs and good rural youth in Surabaya. Correspondent for figure youth representative field visited the surishui Ming hui electricity industrial park (city young talent accelerator), Shandong inter-city rail transit technology co., LTD. (Shandong youth innovation model unit), Shandong hope food co., LTD. (surishui green enterprise association unit), surishui micro public welfare association (Shandong youth innovation commandos), surishui sihu is Confucianism research base, Shandong leisure valley art granary, surishui deer demonstration area, such as the project unit, listened to the relevant situation, around enterprise transformation development, technological innovation, power rural revitalization, volunteer service to carry out the interactive learning. The picture shows the young representatives of Jining city visiting the high-quality enterprises in Sishui County. Correspondent for figure in Jining young entrepreneurs, rural good youth learning xi general secretary in celebrating the 100th anniversary of the founding of the communist youth league's important speech spirit sharing meeting, Shandong provincial rural good youth Zhou Qingqing, Jining youth entrepreneurs association, Jining may four outstanding youth zhang lu, city youth entrepreneurs association member, city returnees innovation and development association executive chairman Cao Sheng youth representatives, combined with personal development, Jining bank Surabaya branch President Li Yanping introduced the power rural revitalization, enterprise development and other financial policies. At the "Hope House" donation ceremony, Tuan Jining Municipal Committee donated 50,000 yuan for the construction of "Hope House". Han Yu, sincerely invited young entrepreneurs and young people to pay attention to children in their hometown, mobilize more caring people to participate in dedication and help more children in difficulties realize their dreams and grow up healthily.

2022-06-07 Group of Fujian Longyan municipal party committee to carry out the minor protection law theme campaign group Longyan municipal party committee to carry out the "youth since protect have you have me" theme rule of law campaign wei) in order to further promote the protection of minors, protect minors health, effectively prevent minors illegal crime, recently, the group of Fujian Longyan municipal party committee organization youth volunteers to the new era civilization practice red thou farmland base small square "youth since protect have you have me" theme of the rule of law campaign. Activities, young volunteers around the protection of minors law and the prevention of juvenile delinquency law to past masses legal knowledge, campus security, anti-drug leaflets, care manual such as promotional materials, for various cases of juvenile legal advice, and from the family protection, social protection, government protection propaganda obligation of the protection of minors, emphasized the guardian should perform guardianship duties in accordance with the law, the activity issued more than 200 copies of publicity materials. In recent years, the Communist Youth League Municipal Party Committee attaches great importance to the protection of minors' rights and interests. In view of the physical and mental development process and ideological and behavioral characteristics of minors, it actively carries out colorful publicity activities, such as sending the law into the campus, mental health counseling, safety and self-protection education, to escort the healthy growth of minors.

2022-06-06 Group of Anhui Wuhu municipal party committee to carry out the immersive reading sharing activities in Anhui Wuhu municipal party committee to carry out the "early summer city and young talents" immersive reading sharing activities the youth network Beijing on June 6 (reporter Zhang Jianwei, correspondent Yang Shanshan check WeiTing) recently, guided by the municipal committee of Wuhu in Anhui province, I TALK public class, Wuhu 365 tao room jointly planning "early summer city and young talents" immersive reading sharing activities in the red casting garden, nearly 30 youth from all walks of life to participate. At the event, the young people talked about Wuhu, the development and the future, shared their favorite books and experiences, and described the ideal future city with key words and stick figures. The atmosphere was relaxed and active. In the next step, the Youth League Wuhu Municipal Party Committee will continue to develop the "Qingsheng Reading Together" project, help the construction of "Book Wuhu", and build a thoughtful and interesting reading and exchange platform for more young people.

2022-06-06 Fujian Longyan in the national youth development city construction pilot city of Fujian Longyan in the national youth development city construction pilot city wei) according to the central propaganda department, the National Development and Reform Commission, the communist Youth league central 17 departments on the youth development city construction pilot documents, recently, long-term youth development planning implementation inter-ministerial joint conference office announced the national youth development city construction pilot and youth development county pilot list, among them, Longyan city of Fujian province in 45 pilot cities, shanghang county in 99 pilot counties. It is understood that the pilot cycle is 2 years. During the pilot process, The Interministerial Joint Conference Office will unite with the member units, Continue to strengthen the guidance to the pilot areas, Focusing on the Medium-and Long-term Youth Development Plan (2016-2025), Comprehensively and systematically implement the objectives and tasks specified in the Opinions on the Pilot Construction of Youth Development Cities, On the basis of clearly understanding the base number of youth development in the region, More from the policy and project level out of the real move, Efforts to solve the "anxiety" of young people, In terms of youth learning, living and working, Form a batch of replicable and popularizable practical experience, To better serve the growth and development of young people, Give full play to the innovation and creative potential of young people, We will strive to explore a new way for the high-quality development of cities and counties.

2022-06-02 Changsha, Hunan: with firm determination and practical measures to promote the county communist youth league reform effective Changsha, Hunan: with firm determination and practical measures to promote the county communist youth league reform effective wei correspondent Yu Xiaoling) since June 2021, the Changsha, Hunan province always to county reform pilot breakthrough as the global gripper, build the party leadership, government support, youth corps committee, the reform of the coordinated pattern, the communist youth league system "force once" improved, reveals the provincial capital of the communist youth league bear. Establish the "three" mechanism, strengthen the reform and organizational guarantee to establish the leadership and guarantee mechanism: the Changsha Municipal Party Committee of the Communist Youth League has repeatedly reported to the superior departments on the pilot work of the grassroots organization reform of the county Communist Youth League, and established the Changsha Communist Youth League system to promote the reform of the county Communist Youth League leading group, and defined the responsible person and the main responsibilities. Improve the work promotion mechanism: the work leading group regularly holds promotion meetings to follow up the reform progress. The group members respectively connect with one district, county (city) to guide the reform work. The group office makes a special report to the secretary meeting once a month. Strengthen the target assessment mechanism: combining the reality of Changsha county region, formulate the county communist youth league reform pilot division of responsibility, has six reform pilot work will advance, clear counties (city) each stage task, hierarchical classification work responsibilities, goals, rewards and punishments, enhance the work performance rigid constraints. Grasp the "three categories" key points, promote the basic indicators: Youth League committees at all levels adhere to the principle of the Party managing cadres, and actively seek the support of local Party committees, 9 county-level youth league committees have selected and allocated full-time, temporary and part-time deputy secretaries, standing committees and members. Key on core indicators: in terms of strengthening the working force of the league, the youth League committee of each district, county (city) supplemented the working force of more than 1,300 people through government employees, public welfare posts, temporary personnel, college students internship and other ways. Actively explore innovation index: group Changsha municipal party committee on the implementation of the "541" party team integration education work implementation opinions ", all districts and counties (city) youth corps committee (head) at the county level into the party's education leading institutions and education supervision institutions at the same level, the party with league building, team building into the education evaluation and education supervision. Adhere to the "three" outstanding, to ensure that the reform goal outstanding problem oriented: good education class evaluation, 8 hours class learning qualified, annual 20 hours volunteer service as a necessary condition for league, basic eliminate the grade, high school (secondary) class league branch cover blank phenomenon, put under the age of 14, no development number league, not according to the program violations, league development regulation to achieve scientific and standardized. Highlight the responsibility implementation: actively strive for the support of the party and government departments, promote the municipal Performance Office to include the "Party building with group construction" (including league construction, team building) into the "2022 District and County (City) Performance Assessment Measures", and make it clear that the score is 1 point. Highlight the results of the reform: the county-level region arranged the working funds of the youth League committee, the street league work and the following standards, and the nine districts and counties (cities) increased by an average of 179,400 yuan compared with the year before the reform. District, county (city) Party committees will incorporate the work of league building and team building into the inspection and supervision of county-level and municipal party committees and the annual performance assessment.

2022-06-01 Shandong Rizhao First Talent Salon was held Wei Correspondent Xiang Changhui) Further do a good job in the integration of industry and innovation, industry and talent integration, give play to the role of Rizhao high-level talent service station, recently, the first phase of "Love Rizhao Love Talents" Rizhao Talent Salon was successfully held. The activity by Shandong province Rizhao municipal party committee organization department, Rizhao people club bureau, Rizhao technology bureau, Rizhao municipal party committee, activities invite representatives from sunshine industry high-level talents in various fields, visit the urban development achievements, experience style characteristics, and around the city economic and social high quality development, optimize ecological talent development, and do a good job in talent service security in-depth discussion. In the next step, Rizhao will rely on various high-level talent activities to build a platform for communication between the city and talents, and promote the effective docking of "talent, industry and capital".

2022-06-01 Shenzhen city, Guangdong province issued "love minors ten practical" Shenzhen released "care for minors ten practical work" in the youth network reporter Wu Xin) in the 72nd "June 1" international children's day approaching, Shenzhen "bless children" poetry reading and 2022 annual "love minors ten practical" release activities held in Shenzhen hall. Excellent projects such as "Inheriting the Red Gene Youth Education Activity" and "Tutoring with Growth Action" were selected as this year's "Top Ten Practical Things for Caring for Minors". This activity is sponsored by the Publicity Department of Shenzhen Municipal Party Committee (Municipal Civilization Office), the Municipal Education Bureau, Shenzhen Youth League, the Municipal Women's Federation and the Municipal Working Committee. The inclusion of the top ten practical work are: "red gene" youth education activities, tutor with growth action, minors physical and mental health services, youth aesthetic education practice, children health action, youth kechuang theme activities, minors reading and viewing literacy promotion plan, primary and middle school students summer characteristic activities, the young pioneers culture position construction, children special care plan. At the scene of the event, the reciters affectionately recited a letter from revolutionary hero Xia Minghan to his mother "You will see the red flag we raised flying in the blue sky of the motherland" and "space teacher" Wang Yaping "a letter to primary and middle school students", encouraging the majority of children to establish great ambition, Ming great virtue, become great talent and bear great responsibility. The event also specially invited moral models, advanced figures, Olympic champions, infrastructure veterans and musicians to send holiday blessings to the children, encouraging the children to consciously integrate their life ideals into the great cause of national prosperity and national rejuvenation. Zhang Yingying, a national moral model, said, " Dreams do not shine. It is us who pursue our dreams. The road to pursuing the dream is full of hope and longing, which brings us unimaginable energy. May you strive for unremitting self-improvement and strive to be the dream pursuers of your great ideals!”

2022-06-01 Chengdu, Sichuan young pioneers "happiness Chengdu beautiful park city innocence to the party 20" children theme team day activities in Chengdu, Sichuan young pioneers "happiness Chengdu beautiful park city childlike innocence to the party 20" children theme team activities wei correspondent Zhao Qinghua) on May 31, the young pioneers of Chengdu, Sichuan province "happiness Chengdu beautiful park city childlike innocence to the party welcome 20" children theme team day activities held in Sichuan tianfu new district first primary school. Leaders from the Communist Youth League Sichuan Provincial Party Committee, Chengdu Municipal Party Committee, Chengdu Municipal People's Congress, Chengdu Municipal People's Political Consultative Conference, Chengdu Civilization Office, Chengdu Education Bureau and Tianfu New District Party Working Committee attended the activity. Activity site map. Correspondent for this activity by the Chengdu municipal party committee, Chengdu civilization office, Chengdu city bureau of education, tianfu new district, Chengdu less working committee, organized by the "party lead TuanJian, team building" work features, "welcome twenty big Chengdu development I growth" Chengdu eighth little will red scarf small proposal to promote achievement exhibition, Chengdu young pioneers "happy Chengdu beautiful park city childlike innocence to welcome the party twenty big" theme will three parts. Chengdu Young Pioneers primary and secondary school young Pioneers counselors, young Pioneers representatives at the scene to celebrate the "June 1". Activity site map. Correspondent for the activity scene, the young Pioneers and guests jointly visited the district (city) county, school young working committee in the promotion of the party building with the league building, team construction work of the characteristic highlights and work results. Representatives of the young Pioneers from the high-tech Zone, Jinjiang District, Qingyang District, Chenghua District, Longquanyi District and other districts (cities) and counties respectively showed the promotion and implementation of the outstanding proposals of "jointly building a red scarf college" and "setting up a red scarf volunteer service post" put forward by the young Pioneers at the 8th Municipal Youth Congress. We watched the example melodrama "Small hands holding big hands, build a happy City" jointly arranged by the counselors and young pioneers inside and outside the Tianfu New Area, as well as the three "I love beautiful Park City" red scarf original songs MV brought by the young pioneers of Tianfu New Area, Wuhou District and Jinniu District. Activity site map. Correspondent for figure activity in the end, held a "childlike innocence to the party" new team ceremony, the scene of the new players in the national red scarf speaker "please trust power have me" red children public class, under the young example led the oath and sign, officially became a young pioneers, out of becoming a good member, become a good party member of the first step. Joining the team, joining the league and joining the Party is the "life trilogy" for young people to pursue political progress, and joining the team is the first important thing in the children's "life trilogy". Chengdu Youth Working Committee earnestly implements general Secretary Xi Jinping's important discourse on the work of children and young Pioneers, systematically constructs a step of growth incentive system for young pioneers, gradually carries out batch team entry, segmented education, hierarchical incentive, and promotes the youth League, and strives to cultivate new people who can take on the great responsibility of national rejuvenation.

2022-05-31 Jianwei Correspondent Yu Xiaoling) " As a member of the Communist Youth League and a young engineer in the field of intelligent manufacturing, I feel that I was born at the right time and have a heavy responsibility on my shoulders."" 95 " after the new Changsha Guo ni said excitedly. Guo ni's team is a master's "detachment army" with an average age of less than 30 years old. The youth league organization has effectively brought young talents together with them. In more than three years, the company has overcome more than 100 technical problems and process bottlenecks, and created the first one (sets) in many industries. In recent years, the Changsha Municipal Committee of the Communist Youth League has promoted the overall reform deployment of the Central Committee of the city, fully participated in the first batch of pilot reform projects of the Communist Youth League, created the "four in one" education Changsha model, and greatly improved the "three forces". The Changsha Municipal Party Committee adheres to using the Party's innovative theories to cultivate people, set up youth teaching groups at the city and county levels, and conducted 282 lectures in government organs and schools, covering 14,700 youth league organizations and 296,000 youth league members. A total of 28,000 themed group and team daily activities were held, and a total of 4.88 million people participated in youth college studies, each with an average increase of 109.1%. Last year, with the theme of "youth heart to the party to build the new era" in the series of theme youth league day activities in the city, launched 10,984 grass-roots youth league organizations to carry out party history learning and education. Adhere to the cultural education, build the communist youth league "big school" in recent years, the Changsha municipal party committee cultivating "youth recommended officer" recommended 100 times, the young pioneers practice education base 120, push tree red small narrator 14036, writing huxiang revolutionary character story 100, to carry out the "seed growth camp", covering 153500 primary and middle school students. More than 500 typical young people, such as "Huxiang Young Talents" and "Youth May 4th Medal", were selected to tell the stories of living youth struggle and gather the strength to forge ahead. The Changsha Municipal Party Committee has always insisted on educating people in practice. We set up 300 youth commandos in the front line of the construction project, the "Young craftsmen" training program helped 166 young people to improve their academic qualifications free of charge, and the "evergreen Innovation" contribution campaign gathered 200 science and technology innovation talents. The joint conference on Changsha-Zhuzhou-Xiangtan integration was held to create ten key projects such as "Love Changsha-Zhuzhou-Xiangtan". We released a new three-year action plan to help rural revitalization, selected the "Top ten Leading geese" and included them in the city's rural industry talent funding project. We implemented the three-year action plan for one million teenagers, innovated the "Five Alliance" work method, promoted the community youth campaign, and provided volunteer services to more than 200,000 people. Adhere to the network education, build the communist youth League "family" league Changsha municipal party committee set up a new new media center, launched 708 original youth cultural products, create "Changsha youth in action" and other columns, the total number of fans 987000, the total broadcast (reading) 51.188 million times, youth home cloud platform entry activities 9330 times, a year-on-year increase of 105%. It is reported that since the reform, the Changsha Communist Youth League basic work force significantly enhanced, 9 county (city) administrative establishment increased by 21%, supplement 1312 people; coverage, 2705, increased by 43.2%, youth organizations increased by 283; social viability, the party committee attaches great importance to and listen to the special report, financial funding increased by 20.7%, raising social funds increased by 180.5%. By the end of 2021, the reform work was reported and recommended for more than 40 times by the provincial-level and above media, which promoted the in-depth development of the reform of the Communist Youth League at the county level.

2022-05-31 Haikou city, Hainan province held June 1 children's day performance in Haikou held 2022 June 1 children's day performance Ren Mingchao) on May 29, group Haikou, Hainan province and other units held "coconut city young with the party childlike innocence dream new journey" in 2022, the scene also launched the Haikou third "coconut green cup" youth cultural and sports activities. About 900 people, including youth representatives and their parents, attended the two-hour event. At the event, Li Yang, secretary of the Youth Haikou Municipal Committee, delivered a speech on behalf of the organizers, and 100 Young Pioneers sang "Team Song of Chinese Young Pioneers". The whole activity is divided into three chapters: "One Hundred years of inheritance, casting a red heart to the Party", "the light of the ideal reflects the Chinese youth", and "the stars set sail to shine the light of The Times". Among the 17 programs, there are both featured programs of the youth activity center, and excellent works selected by schools and other after-school training institutions, including dance, singing, recitation, sitcoms, model show, robot performance and other forms.

2022-05-30 ) In order to promote the construction of a youth-friendly city, Increase efforts to attract talents and talents, Expand the awareness of the young talent policy, Attract more young college students stationed in Japan to choose Rizhao and stay in Rizhao, recently, In Shandong Province, the Organization Department of Rizhao Municipal Party Committee, Municipal Education Bureau, Municipal Human Resources and Social Security Bureau, Municipal Communist Youth League Party Committee, and Municipal Talent Development Group jointly went to Shandong Water Conservancy Vocational College and Shandong Foreign Language Vocational and Technical University to carry out the " Growth together with Rizhao, "2022 Youth Development-Friendly City Activity Season and Young Talent Policy Information Conference, More than 600 teachers and students participated in the activity. Young talent policy briefing site. Around the construction of youth development friendly city, the Communist Youth League Municipal Committee introduced the construction of youth development friendly city in Rizhao from three aspects of construction background, promotion and goals, and told the young students about the "520" target measures related to youth development. We will focus on the "key small things" of young people in employment, education, housing, medical care, social security and other aspects, how to optimize the layout of urban functions, enrich the quality of life of young people, provide a broad stage, improve the policy system, and give young people more reasons to come to sunshine. At the same time, in order to promote the construction of the "city + university" community and improve the service ability of young students, the Communist Youth League Municipal Committee also introduced the progress of the "youth code" work for college students stationed in Japan, and collected opinions and suggestions on the spot. In the next step, the Communist Youth League Municipal Committee will cooperate with all units to continue to carry out a series of publicity activities in the good youth development friendly city activity season, and constantly improve the city "youth and power" index, so that the city is more friendly to young people and young people are more promising in the city, and attract more young college students stationed in Japan to choose Rizhao and stay in Rizhao.

2022-05-30 Ningde, Fujian: Theory propaganda into the grass-roots level gathers youth positive energy Ningde: Theory propaganda into the grass-roots level gathers youth positive energy Jianwei correspondent Su Jie) " Learning propaganda is inspiring, theory propaganda gathers energy. This face-to-face approach is more popular."Recently, organized by the Fujian Ningde Municipal Party Committee" Ning tuan "theory propaganda team into the community, enterprises, campus, to carry out the" mindong things the world new ideas I speak " publicity activities. In the Fu'an College Students Pioneer Park, the "Ning Small League" theory lecturer carries out face-to-face + point-to-point interactive communication with youth league members, young entrepreneurs and young entrepreneurs."As young entrepreneurs who are active at the grassroots level, we must thoroughly study and carefully publicize the spirit of General Secretary Xi Jinping's important speech, so that the voice of the Party can be deeply rooted in the hearts of the people."Said Li Yang, winner of the bronze award of the start-up agricultural and Rural" Youth " China Youth Innovation and Entrepreneurship Competition. At the Hubin Community Social Work Station in Dongqiao Development Zone, members of the publicity group spoke to the representatives of social workers in youth affairs, and exchanged discussions on how to promote social work work in youth affairs based on general Secretary Xi Jinping's important speech spirit. In the symposium, everyone listened carefully and actively discussed it, and expressed that they would bear in mind their original mission, firmly uphold their ideals and beliefs, practice the purposes of the Party, and do a good job for the young people wholeheartedly. For several days, The Communist Youth League and Ningde Municipal Party Committee insists on the learning, publicity and implementation of the spirit of General Secretary Xi Jinping's important speech as the main content of the education and practice activities with the theme of "Welcome the 20th Party Congress, Always follow the Party, and forge ahead in a new journey", Insist that the youth League cadres take the lead, Organizational learning, Innovation in the form of "menu-type points", "red scarf tour group", "small bench propaganda team" and so on, Study, publicize and implement the spirit of General Secretary Xi Jinping's important speech with vivid examples and down-to-earth language, For youth league members, young entrepreneurs, young entrepreneurs, young pioneers and other groups, Strengthen interactive guidance, By doing this with face-to-face preaching, Highlight the effective coverage, Promote publicity and publicity into universities, enterprises, and into the grass-roots level, Guide the general league members to keep pace with The Times, Show off the power of youth, Fully join the "Ningde chapter" hot practice, Take practical actions to welcome the party's 20th victory held. At present, the city's youth league organizations at all levels have carried out more than 200 theme publicity activities, with the audience of more than 10,000 people.

2022-05-30 Guangxi Hezhou Communist Youth League carries out Labor and Skills Competition Hezhou Communist Youth League carries out the theme of "Welcome 20 years" labor and skills competition and "Number 3" joint creation activities. Activities, the relevant person in charge of group Hezhou municipal party committee for cang zhao highway project 6 youth commandos flag and three youth home, and said, hope youth commandos firm ideal faith, take the initiative to undertake and conquer "daunting new" task, improve skills in practice, show youth in the project construction, make new achievements in the new era of work. The Cangzhao Expressway project takes the youth home as the carrier, combined with the characteristics of the youth home located on the construction site, to carry out a number of youth activities that can promote the project construction, promote the growth of the youth, and serve the local development. In the next step, the Communist Youth League and Hezhou Municipal Party Committee will continue to deepen the role of the Communist Youth League in contacting, serving, and condensing the youth, and lead the youth to shoulder a brilliant mission on the road to the exam in the new era.

2022-05-27 The first Zhenjiang Youth Talent Making Friends Culture Festival was launched in Zhenjiang, Jiangsu Province. At the opening ceremony, the relevant person in charge of Zhenjiang Municipal Party Committee issued the letter of appointment to 15 "Qingyuan Ambassador" representatives. Recently, Zhenjiang Talent Office and the Municipal Communist Youth League Committee launched the recruitment of "Qingyuan Ambassador" in the city. Through individual registration and organizational recommendation, nearly 100 enthusiastic people from all walks of life will serve as "Qingyuan Ambassador", and provide dating services and create development conditions for young talents in the future work and life. The theme promotional video of "Town" is Good to Meet You " was broadcast. The young talents of Zhenjiang enterprises were interviewed on the spot. 5 couples told the stories of meeting, accompanying and staying together in Zhenjiang and sent love messages to the young talents. Huang Han, a professor at the Party School of Jiangsu Provincial Committee and a doctor of social psychology, shared the knowledge of love psychology through a video. The youth talent dating cultural festival is divided into 1 opening ceremony and 8 theme activities. Organizer, co-organizers and other relevant person in charge, "jinshan prize", "dream creek award" winning enterprises, the city's financial institutions, health system, education system, in the town enterprise, in the town of young talents in universities to attend the opening ceremony, more than 500 young talents in eight jurisdiction city synchronization to carry out the "town" good meet you youth dating culture theme activities.

2022-05-26 The Shuangyashan Municipal Party Committee of the Communist Youth League "Easy to prepare for the exam 12355 with you" decompression activity, for more than 100 students. The lecture starts with a small experiment of faith, using humorous language and vivid cases to explain the importance of mentality, through psychological tests, small games, typical case analysis, reveal the source of stress to students, start from how to regulate emotions, relieve stress, combine case and interaction; teach students positive psychological hints to help students adjust cognition and drop the burden to create the best state before the test. The lecture was also broadcast live online, which students and their parents can watch online through the live broadcast platform.

2022-05-26 Guangxi Guilin youth volunteers into lingchuan teaching point to carry out the dissemination propaganda Guangxi Guilin youth volunteers into the franco-prussian propaganda month 26 (reporter Zhang Jianwei, reporter Jiang Bo) on May 24, the Guangxi zhuang autonomous region Guilin municipal united southern airlines engineering branch of Guangxi base and Guangxi construction company youth to Guangxi lingchuan yao township east teaching point, for students to send to "June 1" children's day gift, at the same time promote minors protection laws and regulations, enhance the minors of the rule of law concept, improve the awareness of self-protection of the minors. The Dongliang Teaching Point in Lantian Yao Nationality Township is a "miniature school" embedded deep in the mountains and built beside the terraced fields. It is a "field of hope" with only one teacher and six students. In Dongliang teaching point, there are six students in preschool, grade one and grade two, mixed in one class. The teacher needs to teach the preschool children first, and then teach the first and second grade students, so on. Activity pictures. Correspondent for figure at the event, young volunteers combined with the characteristics of young children, in order to avoid the boring course, activities in the form of "you, I will do", young volunteers from China southern airlines engineering technology branch, for the children with manual origami plane, and teach the principle of flight, come from Guangxi construction four construction company young volunteers for the children to teach you how to build buildings and other forms of interesting courses. During the activity, the young volunteer propagandists from the Guilin Municipal Committee of the Communist Youth League carried out publicity and education for the children to enhance the concept of the rule of law of minors and improve their awareness of protection. After class, the volunteers also to boldly speak the dream as the theme, and the children to talk about "a better tomorrow" exchange activities, and the school bags, skipping rope and other recreational and sports supplies as the "June 1" Children's Day gifts to the children.

2022-05-26 Guangxi Beihai Communist Youth League held the Youth Learning Knowledge Challenge Beihai Youth Learning Knowledge Challenge Beihai Youth Federation jointly held the "Welcome 20 Always follow the Party" - -2022 Beihai Youth Learning Knowledge Challenge and the special Competition of youth forging the Community consciousness of the Chinese nation. The competition was divided into two parts: written test and on-site answer. 36 contestants from 12 teams from Beihai counties (districts) and municipal league organizations participated in the competition. After two rounds of competition, the organizers were awarded the first, second and third prizes, among which the League Yinhai District Committee team won the first prize. Since the opening of the "Youth Learning" online theme group class in 2022, the Beihai Municipal Committee of the Communist Youth League has organized 720,000 youth league members to participate in the "Youth Learning" online theme group class learning activity, and continuously promote the normalization and systematization of theoretical learning.

2022-05-25 Zhuhai communist youth league fully support hengqin Guangdong Macao depth cooperation zone league team building work of Zhuhai communist youth league fully support hengqin Guangdong Macao depth cooperation zone league building team building work newspaper zhongqingwang reporter Wu Xin) Zhuhai hengqin Guangdong Macao depth cooperation zone provincial demonstration leave, joining ceremony was held in hengqin first middle school, cooperation zone more than 200 teachers and students and parents to participate in the activities. With the theme of "Welcome the 20th, Always Follow the Party, and forge ahead on a New journey", the activity was guided by Guangdong Provincial Youth League Working Committee, and jointly hosted by the Party Building Office of Hengqin Working Committee, Zhuhai Municipal Committee of the Youth League. It is the first gathering of team leaving, league joining ceremony and youth ceremony in the cooperation area. The event kicked off with the solemn national anthem. The 217 young pioneers who left the team were arranged neatly in front of the team flag, presented a lofty team salute, and bid farewell to the red scarf. The 16 new league members solemnly swore to the League flag to join the Chinese Communist Youth League. The Communist Youth League members and the young pioneers loudly shouted out "please rest assured, strong country have me" youth oath, affectionate singing song "I", full of enthusiasm and hope to the future to write a letter, solemnly into the youth mailbox, hand in hand across the door of youth. It is understood that since the establishment of the Hengqin Guangdong-Macao In-depth Cooperation Zone, the Zhuhai Municipal Party Committee and the Municipal Youth League Working Committee have fully supported the league construction and team construction of the service cooperation zone. The first school youth working committee in the cooperative zone was established in Hengqin Harolide School, and the first Young Pioneers' off-school practice education camp (base) was inaugurated in Hengqin Library. Relying on the Communist Youth League and the Young Pioneers, Qinao Youth carried out a series of open patriotism education classes and study and practice activities on a regular basis. Said the relevant person in charge of the Zhuhai municipal party committee, city less working committee, will continue to implement to promote the party, group, team education chain link, the practice requirements, continue to fully support service cooperation area group building, team building work, lead the qinao youth to the party, dream weavers, to continuously strengthen the leadership of the party and the party's construction to lay a solid organizational foundation.

2022-05-25 Gansu Gannan horseback youth XuanJiangDui grassland deep preach Gannan "horseback youth XuanJiangDui" through theory service youth "the last kilometer" JianWei communication Wang Cong) in order to further promote xi general secretary in celebrating the 100th anniversary of the founding of the communist youth league conference's important speech spirit heart into the brain, take root, Gansu Gannan Tibetan autonomous prefecture committee innovation preach form, organize high political consciousness, good theoretical level, preach experience of 15 grassroots young cadres and the masses formed the Gannan "horseback youth XuanJiangDui", and deep into the grassland recently carried out the first preaching activities."We will combine the reality of farming area, strengthen their own learning, enhance the political accomplishment, enhance the level of theory, adopt the way of farmers and herdsmen masses, xi general secretary in celebrating the 100th anniversary of the founding of the communist youth league of China's important speech spirit as XuanJiangDui current important preaching task, the first time to every corner of the grassland, to every herdsmen home."The captain said. Gannan "horseback youth XuanJiangDui" will normalize to carry out the party's policy, rural revitalization, social security, animal disease prevention and control, control drop out, such as school, laws and regulations publicity and education, and the practical problems existing in farmers and herdsmen youth life timely feedback to the township, government, become a bridge connecting government and farmers and herdsmen, through the theory service youth "the last kilometer". Next, Gannan league committee will innovation use the teenagers understand, remember, improve the propaganda about xi general secretary in celebrating the 100th anniversary of the founding of the communist youth league of China's important speech spirit of vividness and attraction, motivate a state of all nationalities youth to realize the great rejuvenation of the Chinese nation as own duty, in building "five Gannan", create "ten homes", realize rural revitalization, the Yellow River upstream ecological protection and high quality development party and government center work practice "please rest assured power have me" clank oath, take practical action to meet the party's 20 victory.

2022-05-24 Group of Jiangxi Pingxiang party committee to carry out the flood control and emergency rescue drill of Jiangxi Pingxiang party committee to carry out the flood control and emergency rescue drills improve water rescue "hard power" JianWei correspondent Chen cheng) on May 22, group of Pingxiang, Jiangxi province municipal party committee organization Pingxiang Jiangxi qing commandos, Pingxiang dawn rescue team in Pingxiang paradise lake reservoir to carry out the flood control and emergency rescue drill. The picture shows Pingxiang Ganqing commando captain Xiao Liu before the stressed requirements. Correspondent for figure "in view of the current situation of flood control situation, water rescue drill is necessary, but also test the emergency measures, equipment good time", Pingxiang ganqing commandos captain xiao liu said, carry out the practice, the purpose is to further enhance Pingxiang jiangqing commandos emergency rescue ChuTu ability, improve the emergency rescue organization, coordination, command system, to ensure that in the process of flood control and emergency rescue can master emergency rescue equipment operation technology, do rapid rescue, scientific rescue. The picture shows the female team members conducting a boat-turning and self-rescue drill. On the day of the drill, according to the group division of labor, tacit cooperation. In view of the characteristics of flood control emergencies, the water rescue drill has been carried out around the charge boat power disassembly and installation, rowing, turning self-rescue, O-type S-type driving technology and rescue of drowning people, achieving the expected purpose."Through this emergency rescue and flood control drill, I learned the wearing of emergency rescue equipment, equipment use and other related contents, and improved my understanding of flood control safety."Said Lu Jiaxin, a communist party committee cadre who participated in the drill. The picture shows a drill to rescue the drowning people. Correspondent for figure XiaoLiu introduction, every rescue face is precious life, the next step, they will continue to promote daily training, constantly improve personal rescue skills and teamwork emergency rescue ability, make preparations for water rescue, to ensure that in danger, can maximize the safety of the people's life and property.

2022-05-19 Youth heart to the party, the dream is Gansu Qingyang communist youth league series of activities to celebrate the 100th anniversary of the founding of the communist youth league of China youth heart to the party dream is Qingyang communist youth league series of activities to celebrate the 100th anniversary of the founding of the communist youth league of China jianwei) in late April to mid-may, Gansu Qingyang communist youth league organized a series of youth theme cultural activities, unity leading teenagers from all walks of life in the implementation of the "double wheel" drive, adhere to the "three" go hand in hand, strengthen the "four" development of "four" play a role of force and commandos in the new journey. On April 18, Qingyang municipal party committee held in huachi county nanliang town "inheritance nanliang spirit be new era" new members in Qingyang group demonstration activities, more than 140 new and old members through to the nanliang revolutionary martyrs monument flower basket, to flag solemn oath, visit nanliang revolutionary memorial hall, dun-Gansu revolutionary base development and the glorious struggle of the older generation of proletarian revolutionaries and the party's youth movement. The picture shows the new league members taking the league oath."Nanliang is the seat of the former Shaanxi-Gansu border Soviet government. The spirit of Nanliang, with 'facing the masses, adhering to faith, considering the overall situation, and being realistic and pioneering' as the main content, is our precious spiritual wealth."The person in charge of the League Qingyang Municipal Party Committee said that the centralized demonstration activities of joining the league is mainly to enable the youth members to further enhance the awareness of the members, firm ideals and beliefs, inherit the spirit of Nanliang, strive to be a new person of The Times, let the red gene, revolutionary fire from generation to generation. On May 4, group Qingyang municipal party committee organization to carry out the "youth counterparts, celebrate the May 4 th" emerging social organizations youth group visiting activities, through communication with youth representatives discussion, giving work life necessary supplies, to the city's four emerging youth social organizations nearly hundred youth representatives to send holiday greetings and blessings. Activities aimed to further enhance the communist youth league in Qingyang city youth group organization, leading force and service force, unity lead the youth stick to the ideal faith, have the courage to bear dedication, with their own practical action to practice "may 4th spirit", condensed the strength of youth, for the city's economic and social development of high quality youth strength. The picture shows the Youth League Qingyang Municipal Party Committee visiting the Blue Sky rescue Team. On May 10, the Communist Youth League, the Youth League, the Young Pioneers, and the Student Union organized youth members to watch the celebration of the 100th anniversary of the founding of the Communist Youth League of China, and carry out learning, publicity and implementation of the theme of the spirit of General Secretary Xi Jinping's important speech."General Secretary Xi Jinping's important speech is full of the CPC Central Committee's cordial care for the younger generation and its great importance to the cause of youth development."Youth League Qingyang Municipal Party Committee cadre Hou Yingying said, young people should have the courage to be the vanguard of a strong country, listen to the party to follow the party, practice excellent skills, bloom the flower of youth in the motherland most needed place. The picture shows Qingyang Vocational and Technical College watching the live broadcast of the celebration conference. Group Qingyang municipal party committee for days, Qingyang communist youth league city, county leading organs held a special meeting, study the deployment of the communist youth league study at all levels general secretary xi important speech spirit, 3464 grassroots league branch to carry out the special study, coverage reached 100%, the young pioneers brigade at all levels of synchronous learning. The city's young people have said that on the new journey, will bear in mind general Secretary Xi Jinping's earnest entrust, play the role of youth league force and commando, with the youth me, create a youth China, run the best results of the contemporary youth on the youth track. The picture shows the city's Communist Youth League representatives centralized commendation scene. Group Qingyang municipal party committee for figure on May 13, the CPC Qingyang municipal party committee propaganda department, Qingyang city education bureau, Qingyang municipal party committee jointly organized "celebrate 20, always follow the party, forge ahead new journey" to celebrate the 100th anniversary of the youth theme cultural activities, for the city's communist youth league "two red two excellent", the young pioneers "three outstanding" up "the good youth" "outstanding volunteers". Activities to review the history of the Chinese youth movement in one hundred as the main line, one hundred of youth, youth to the party, dream future, always follow the party, through the form of show, show the youth culture city youth members in the construction of Qingyang old, Qingyang take off, in the struggle, li progressive youth and spirit, firm the youth with the party, the new era of confidence and determination. The picture shows the youth league representatives of various industries reviewing the league membership oath. Tuan Qingyang Municipal Party Committee for the picture " to firm faith, aim high, and strive to become a firm belief in Marxism in the new era; study hard, good thinking, hard practice skills, and strive to become the cause of socialism with Chinese characteristics qualified builders; bravely shoulder the mission, forge ahead, and strive to become a unremitting struggle to achieve the great rejuvenation of the Chinese nation."Qingyang ShengYunfeng, deputy secretary of municipal party committee, said the youth to further study xi general secretary in celebrating the 100th anniversary of the founding of the communist youth league of China's important speech spirit, earnestly implement the Qingyang municipal party committee and municipal government decision deployment, with youth power and creativity stirred up the high quality development of old surging spring, with the wisdom of youth and sweat struggle out a more happy new Qingyang. The picture shows a youth-themed cultural activity performance celebrating the 100th anniversary of the founding of the Communist Youth League of China. Youth League Qingyang Municipal Party Committee for the map struggle is the most beautiful background color of youth, action is the most effective youth hone. In order to further enhance the organization force, leading force and service power of the city's Communist Youth League, Continue to thoroughly implement the spirit of General Secretary Xi Jinping's important speech at the conference celebrating the 100th anniversary of the founding of the Communist Youth League of China, Qingyang youth league organizations at all levels through the youth symposium, theme group (team) classes, discussion and exchange, expert publicity, cultural works creation and other forms, To lead the youth league members to learn and understand general Secretary Xi Jinping's hopes and requirements for the Communist Youth League organization, youth league members and cadres, Unswervingly listen to and follow the Party, To live up to the time, to The Times, to the people's sonorous oath, In Qingyang old area in the new journey of high-quality development contribution of youth strength.

2022-05-22 Group Jilin province Songyuan municipal party committee held welcome, the college entrance examination public live activities group Jilin Songyuan municipal party committee held welcome, the college entrance examination public welfare activities Wang Peilian) recently, in order to prepare for the college entrance examination students and their parents correctly cope with the psychological pressure, in good condition to meet the exam, group Jilin province Songyuan city party committee through "youth matsubara cloud classroom" video "clever points, easily" meet "test" public welfare live activities. Lu Hui, a national second-level psychological consultant and senior family education instructor, invited Lu Hui, a national second-level psychological consultant and senior family education instructor, to teach the lecture in the live broadcast (the picture for the photo). Parents and students interact with teachers online during the live broadcast. Students and parents watch the live broadcast (TuanSongyuan Municipal Committee) It is understood that in the next step, TuanSongyuan Municipal Committee will also carry out various online live broadcast activities around youth education, health, employment and entrepreneurship and other aspects, to create "Youth Songyuan Cloud Classroom".

2022-05-21 Recently, a video of Deyang League transforming houses for left-behind children has gone viral on the Internet, known as the Deyang version of the "dream transformation home". The video is presented in the communist youth league of Deyang municipal party committee to carry out the implementation of the "I and my hometown | dream transformation +" love is a part of the troubled children plan. Earlier this year, the communist Youth League Deyang municipal party committee launched a "I and my hometown" series of activities, around Deyang city red ruins, local traditional culture, urban development, rural revitalization, service youth and other directions to promote, intended to retain, attract young talents, help teenagers set up the construction of hometown, serve the motherland. The series of activities are divided into four single activities, including the "Looking for the most beautiful Deyang" network publicity activity, the "Deyang Study Tour" search activity, the "Dream Transformation +" care for children in distress plan and the "series of new media products" release plan."In the implementation process, we have continuously enriched the forms of activities and joined the 'village painting' campaign to help rural revitalization."The relevant person in charge of the Communist Youth League Deyang Municipal Party Committee introduced, the" Painting Village "action invited professional teams and young wall painting artists to enter, held the village transformation plan led by cultural and art wall painting, to achieve" one village, one characteristic "and" one wall, one landscape ", will last for one year.(Yuan Congjun, Lin Ling, Sichuan Daily all-media reporter Cao � @ Source)

2022-05-21 115 old photos tell the history of Liaoshen Youth Movement, and the theme exhibition of "the Communist Youth League and the Youth" was held in the memorial hall of the former site of the Communist Party of China Manchuria Provincial Committee. At about 10 a. m., the Communist Youth League was publicized through the online live broadcast, showing the youth power of the eventful years. The exhibition conveys the power of faith through the historical development of Shenyang Youth League through rich graphic boards. What is particularly noteworthy is that the 115 black and white old photos reproduce the red memory of the youth members of the League in Liaoshen carrying out the revolutionary struggle under the leadership of the Party. Of these precious old photos, some have been yellow, the picture is also a little fuzzy, some although washed by time, still clear as yesterday."Speaking of the establishment of the Communist Youth League in Liaoning province, we might as well turn our eyes to these old photos and look at these lovely young people. The photo is Guan Xiangying, the first member of the Communist Youth League in Liaoning, while working in Tailong Daily in Dalian. This photo shows a group photo of seven progressive students of Fengtian Medical College, who are Xiang Quanshen, Bai Xiqing, Yang Weijian, Zhou Dongjiao, Wu Zhizhong, Bi Tianmin and Wu Yingkai. In fact, they are the members of the earliest Youth League branch in Shenyang."Said the narrator, Wang Lu, pointing to the display board. These are two old photos full of youth. In Guan Xiangying's photo, he is wearing a long gown and a top hat, which is gentle and elegant, and his eyebrows show a heroic spirit. In another photo, seven young men in their prime, some in Zhongshan suits, with a pen in their left upper pocket, are also the fashion mark of that era, and from their firm eyes, they convey the power of faith. As for the background of the old photos, Li Shu, the writer of the exhibition, said that the Communist Party of China sent cadres to Shenyang to publicize Marxism, guide the workers' movement and establish the party and league organization. The first party organization and youth league organization in Shenyang area were simultaneously established in September 1925. In October 1927, the CPC Central Committee sent Chen to northeast China to establish the Manchuria Provincial Committee of the Communist Youth League in Shenyang. Zhao Shangzhi, the anti-japanese hero Zhao Shangzhi, was engaged in youth work in the Manchuria Provincial Committee of the Communist Youth League. The hot-blooded youth grease-print paper described on the old paper is already old, but the lettering above can still be analyzed. The documents displayed in the old photos highlight the national feelings of the hot-blooded youth in the turbulent years."This is on January 15,1931, the Mukden Municipal Committee of the Communist Party of China and the Mukden Municipal Committee of the Communist Youth League of China issued the Memorial Youth Message for Libuknesi, Luxembourg and Lenin. After the 918 incident in 1931, the communist party of Manchuria provincial party committee on September 19, the first declaration for the Japanese imperialist armed occupation Manchuria declaration, then, the communist youth league manchuria provincial party committee made 'northeast communist party members, patriots and soldiers immediately armed, join the anti-japanese national salvation' decision, published the letter of manchuria provincial party committee to China and South Korea. On September 20, the Manchuria Provincial Committee of the Communist Party of China and the Manchuria Provincial Committee of the Youth League issued a letter to the Korean Workers, Farmers, Students and the Working People for the Japanese imperialist military occupation of Manchuria."Wang Lu's explanation was sonorous and passionate. Li Shu introduced that after the September 18 Incident, the party and league organizations will be the central and provincial party committee documents printed into leaflets in the society, to publicize the anti-japanese propositions to the people. According to records, only the Communist Youth League Manchuria Provincial Party Committee issued more than 1,000 copies of all kinds of propaganda materials. In the live broadcast, Wang Lu's explanation and Li Shu's interpretation led the young audience to return to the historical scene, looking back at the growth of the youth power on the red fertile soil of Shenyang. The theme exhibition is divided into three parts: "Road of struggle", "Road of Revitalization" and "journey of the future". It shows the vivid practice of Shenyang youth league members striving hard on the youth track for the liberation, construction, reform and development of Shenyang under the leadership of the Party. At 11 o'clock, the online live broadcast ended, with more than 50,000 viewers. On the other end of the Internet, "onlookers" messages are still flooded."When I watched the live broadcast, I felt solemn and stirring, angry, feeling and proud. When I was a child, I learned a sentence that happy life was the blood of revolutionary martyrs. Today's exhibition made this sentence more specific in my mind."Zhao Yuting, 21, watched the live broadcast, saying that the group history class, which sowed the" red seeds ", has incorporated the red gene into the blood and immersed into the heart."Only by entering history and understanding history can we go further, and the power of youth is the future of society."Liu Qifeng, a college student, said he would pay tribute to the heroes with his struggling youth."Old photos are a 'family heirloom' to carry forward the revolutionary tradition and a vivid textbook to educate the young generation."Liu Xiuhua, director of the former memorial hall of the CPC Manchuria Provincial Committee, said, using the combination of offline and online exhibition, to break the barrier of time and space, can effectively improve the exhibition education communication effect and social influence, remember the revolutionary martyrs, ceaseless red blood. Original title: 115 old photos tell the history of Liaoshen youth movement story

2022-05-20 Guangdong Yangjiang City, Guangdong Province nearly 7,000 new league members into the league to join the league Jianwei correspondent Ruan Yizhen) recently, the Yangjiang Municipal Party Committee of Guangdong Province organized nearly 7,000 students in the city's middle schools to hold "celebrate the 20 will always follow the party to forge ahead new journey" young pioneers concentrated on the league ceremony. The young Pioneers joined the league in the centralized ceremony. At the ceremony, all the staff stood solemnly and sang the national anthem. The representatives of the league introducer read out the provisions on the obligations and rights of the league members in the Constitution of the Communist Youth League of China, read out the list of the new league members, and issued the league badges and certificates for the representatives of the new league members. Oath the picture. Under the leadership of the oath leader, the new league members of the middle school raised their right fist and solemnly swore to the league flag. The teenagers' uniform oath sound was bright and strong, and the old league members reviewed the oath of joining the league together, and once again firmly determined to forge ahead. Award pictures. At the scene of Yangjiang No.2 Middle School, TuanYangjiang Municipal Party Committee awarded the 2021 Yangjiang City "two red and two excellent" advanced individuals and collectives. In April this year, the Youth League Central Committee and the Guangdong Provincial Party Committee issued the 2021 "Two Red and Two Excellent" commendation decision, Yangjiang one person and one collective was awarded "National Outstanding Communist Youth League Member", "National May Fourth Red Flag Youth League Branch"; "Guangdong May Fourth Red Flag Youth League Committee", "Guangdong May Fourth Red Flag Youth League Branch Model", 11 individuals were awarded "Guangdong Excellent Communist Youth League Member", and youth members gave full play to the role of the party force and commando in all fronts. New members have said, must always remember the oath, with more full spirit to face life and study, struggle, play an exemplary role of members, practice the member's responsibility and bear, drive the students around positive enterprising, study hard ability, to grow up construction to create a good scientific foundation, to realize the great rejuvenation of the Chinese nation the Chinese dream. Yangjiang experimental middle school youth corps committee officials said, as a cadre and the people's teachers, should be based on teaching work, will always cultivate students grow up as the education goal, lead the league members seriously implement the xi general secretary's important speech spirit, inheritance and carry forward the spirit of "may 4 th", and the group work together, for the party to do the new era youth work, strive to cultivate batches of ideal, ability, bear a new generation of youth.

2022-05-20 Guangxi Guilin "youth learning" knowledge challenge held in 2022 Guilin "youth learning" knowledge challenge host Xie Yang) on May 18 in the afternoon, by Guilin Guangxi zhuang autonomous region, the state administration of taxation Guilin youth league committee jointly organized in 2022 Guilin "youth learning" knowledge challenge and youth cast in the community consciousness of the Chinese nation. Sixteen teams from the grass-roots league organizations in Guilin participated in the competition, which was divided into preliminary and final matches. After fierce competition, the League Lipu Municipal Party Committee team won the first prize, the League Lingchuan County Party Committee team, the League Resources County Party Committee team won the second prize, the League Yangshuo County Party Committee team, the League Quanzhou County Party Committee team, the Guilin Normal College Youth League Committee team won the third prize. The event aims to study and implement the spirit of General Secretary Xi Jinping's important speech at the celebration of the 100th anniversary of the founding of the Communist Youth League of China, focus on the main responsibility of educating the Party, deepen the achievements of party history learning and education, and unite and lead the majority of young people to welcome the 20th anniversary, always follow the Party, and forge ahead on a new journey.

2022-05-19 Hubei Shiyan around the "six new" booster talent gathering Shiyan municipal party committee around the "six new" booster talent gathering correspondent He Yulin Guangxin) for the further implementation of the Shiyan municipal party committee government "national entrepreneurship, youth first" and "college students (back) weir plan" decision deployment, group of Hubei Shiyan municipal party committee based on entrepreneurial youth and college students need, combined with the functions of the communist youth league, around the "six new", from learning, entrepreneurship, career, life to boost Shiyan talent gathering, for service innovation entrepreneurship and college students stay yan employment, boost Shiyan economic and social development. Shiyan City to commemorate the anniversary of the May 4th Movement theme group Day and Youth Innovation and Entrepreneurship 103 Carnival pictures. Correspondent for the picture of good "propaganda work", show the new environment of attracting talent to use the "youth Shiyan" all media matrix to open up the "college students to stay weir" column. Organize typical short videos of entrepreneurship and employment youth, push more than 30 stories of Shiyan college students, municipal social bureau and other departments, innovation and entrepreneurship incubation platform, realize the policy awareness and entrepreneurship policy coverage, and entrepreneurship incubator policy coverage, 62 periods, encourage and call on Shiyan, and hold the 103th anniversary of the May 4th Movement and Youth Innovation and Entrepreneurship Carnival. Original program "for love to stay" in the form of poetry and painting show four different types of young college students after graduation voluntary leave weir return weir construction hometown touching story, leave (back) weir entrepreneurship employment policy, and through live to the city youth "why struggle to go to the distance, home is a good place", 110000 people watch live online. The picture shows the release ceremony of the top ten stars of employment and entrepreneurship. Correspondent for the map to set up a good "example flag", stimulate the new vitality of the introduction of talent to push the evaluation of "college students to stay weir employment and entrepreneurship of the top ten stars". In conjunction with the city colleges and universities, from the local employment entrepreneurship outstanding graduates selected a batch of self-employment, take the lead to get rich, rooted in hometown, contribute to the society of the weir students advanced representative, sum up the successful experience, walk into the city colleges and universities to carry out the exchange discussion, about entrepreneurial employment experience, guide the weir students in Shiyan struggle, growth. At present, 3 campus activities, covering more than 400 people; shooting original green creation theme film "Let Flowers bloom". The film is based on Wang Taixiang, a young man from Zhushan County, Shiyan City, telling the struggle and touching story of his return home to establish the Tianying Left-behind Children Care Center after graduating from university. The premiere of Let the Flowers Bloom. Correspondent for the map to draw a good "talent map", inject new elements of talent retention organization to carry out the "campus talent ambassador" recruitment activities. Select outstanding students from Shiyan nationality as "Campus Talent Introduction Ambassador" to promote the talent policy and employment demand information of Shiyan, and carry out "Liuyan Employment Experience Day" activities. Twelve batches of more than 600 college students were organized to observe and exchange in key enterprises, key parks and major projects, so as to further understand the economic and social development, enterprise talent demand and employment service policies of Shiyan, so that college students can walk into Shiyan, feel Shiyan, and improve their willingness to find employment and entrepreneurship in Shiyan. The picture shows the issuance of qingchuang loans. Correspondent for pictures to build a good "service desk", add talent to retain new momentum has held Shiyan "youth creation salon" 5. Carry out entrepreneurship guarantee loan policy publicity and college students entrepreneurship support project application and guidance; continue to promote the "youth innovation loan" project. From January to April, Shiyan "Youth Innovation Loan" project lent 256 loans of 61.12 million yuan; the ninth Shiyan Youth Innovation and Entrepreneurship Competition of "I Choose Hubei Dream Car City" was launched. Select 15 high-quality projects, and the final is scheduled to be held in mid-June; actively strive to hold the 2022 "Create Youth" Hubei Youth Innovation and Entrepreneurship Competition (Science and Technology Innovation Special Project) in Shiyan. At present, the competition project application work has been started, and the final round is scheduled to be held in early June and the final before the end of June, and the application work of college students entrepreneurship support project in 2022. Up to now, a total of 43 projects in the city have submitted online application materials, and 5-50 yuan of free entrepreneurship support funds will be provided after the successful declaration. After active reporting and striving, the Hubei Provincial Committee of the Communist Youth League decided to implement the 2022 Hubei Province "College Students Volunteer Service Rural Revitalization Plan" in Shiyan City. Relying on the platform of the Central Committee of the Communist Youth League and the Provincial Communist Youth League Committee, 200 volunteers will be recruited from universities across the country and dispatched to service posts in counties (cities and districts). In principle, the service period is 1-3 years, and the relevant living allowances are fully guaranteed. On April 29, in the 2022 western Hubei Province plan recruitment and selection work mobilization and deployment video conference, introduced the situation of Shiyan city in detail, broadcast the Shiyan talent policy propaganda video, more than 100 universities in the province attended the conference, and the optimal policy strengthened the guarantee."Youth has a chance is you" youth talent fellowship activity pictures. Correspondent for woven "emotional network", broaden the new channels to stay to pay high attention to youth dating and marriage issues, actively advocate civilized dating new fashion, build a platform for young dating, actively call on foreign outstanding young talent for marriage in Shiyan, in 2021 organization "youth dating is you", "qin male chu" and other young talent fellowship activities, participate in nearly 2000 young people; joint "evening matchmaker" single men and women information database, hire matchmaker for single single youth dating "matchmaking", will form a long-term mechanism. In 2022, the Shiyan Municipal Party Committee of the Communist Youth League plans to carry out a youth talent association for young people of different ages, industries and fields, so as to retain people with "affection" and make the young people settle down to the maximum extent.

2022-05-19 "Dear young people, behind me is the National Rural Revitalization Model Village, where we can enjoy the rural beauty of the 'good place Yangzhou'."Xiao Zhao, a young man of new media creation, is participating in the "Beauty Search North Lake" TikTok challenge. HanJiang district in Yangzhou city, Jiangsu province fang lane town along the lake village, groups of youth and guest gathered here, relying on village youth league transition, they established youth rural youth league group, and as a "youth leader" as deputy secretary of village youth league organization, they built a home stay facility, open live, with "youth mode" propaganda of the rural humanistic beauty and beauty, attracted a group of young people return home business construction. In Zhuxi Street, the Hanjiang District Committee relies on the Wuyue business circle group building, to build the business circle of the youth league general branch, the red gene into the most prosperous economy, the most talent gathering, the most active information, the most diversified cultural business circle. Wuyue Square Bamboo West 31 youth position with "youth just red" "bamboo dream flying" "down-to-earth" "new youth style" "colorful youth" "original heart harbor" and other red punch points. In recent years, the group of Yangzhou municipal party committee continue to consolidate and expand the village (community) youth league organization transition and agricultural park group building "one hundred crucial" work achievements, on the basis of perfecting the traditional rural youth league organization system, take the initiative to adapt to the youth return and gathered to the industry trend, actively explore the rural economic organizations, industrial chain building group new model, strengthen the rural group building foundation. Keep up with the pace of township (street) party organization transition, according to the reform requirements of grassroots "three integration" in Yangzhou, strengthen the relevant requirements of the construction of grassroots youth league organization in the "second half of the" article, highlight the focus of serving rural revitalization, and establish and improve the work effect evaluation mechanism of the work. We will improve the system for returning college students to youth league organizations in towns (streets) where they live, and create a strong atmosphere for young people to devote themselves to rural revitalization. In Gaoyou City Tong Post and Telecommunications Business Park, the new phase of the Rural Revitalization Youth Innovation and Entrepreneurship Competition was launched, and 200 rural young people from all over the city participated in the competition. A total of 25 start-up groups were cultivated, and 13 outstanding rural revitalization projects were excavated. Bian Shengjie, chairman of the Posts and Telecommunications Business Park and a national pioneer of rural revitalization, said, " By cultivating local young rural revitalization talents in Gaoyou, more young people can love the countryside, take root in the rural areas and help the rural revitalization."In order to constantly play a typical role of youth, Yangzhou municipal party committee also organized" the national rural revitalization of youth pioneer pacesetter "green enjoy, promote the construction of Yangzhou young talent rural live base, set up youth electricity live college, cultivating HanJiang along the lake village" qiao fishing niang " rural local youth web celebrity team, normalized local talent for home, travel promotion, agricultural products live with goods activities, cultivating entrepreneurial youth, new youth professional farmers, rural youth rich leaders of rural young talents. The key to rural revitalization is in "people". Yangzhou league organizations at all levels through league position, league competition, guide more young talents rooted in rural, home innovation entrepreneurship, drive more rural surplus labor and the poor entrepreneurship to get rich, help rural build the Internet platform, cultivate culture, understand technology, good management, management of youth innovation entrepreneurial leaders. On the other hand, local township youth league committees actively organize training on modern agricultural science and technology and management skills, and train a number of modern young farmers and new young professional farmers to truly give youth energy to rural revitalization."Affected by the epidemic, many enterprises will be very difficult for them to survive this year, especially for our agriculture-related enterprises. Once their products are unsalable and their funds cannot be withdrawn, it will be difficult."A young farmer from Tianle Lake, Dayi Town, Yizheng City, said," Fortunately, this year's new farming elite is more preferential, and the application threshold is lowered, continuously simplified procedures, timely provided liquidity, and ensure the continuous development of innovative products."At the second "Green Painting Village" Youth Cultural Creative Design Competition, the young contestants not only described the beauty of the countryside by designing their painting works, but also applied for the rural cultural design and planning as a cultural project. Establish a "chief designer" training plan for outstanding young cultural and creative individuals, and allocate special funds to the enterprise where the "chief designer" works. The enterprise signed a training funding agreement with the "chief designer" and put it into practice, and recommended it to the Jiangsu Zijin Cultural Talents Training Plan simultaneously. It is understood that Yangzhou this year launched the implementation of the "rich people and strengthen the village youth contribution" action, the organization of young business enterprises "rural travel", accelerate the construction of supply and demand complementary, common development of the village enterprise consortium, at the same time, continue to deepen the "new rural elite" cultivation plan, expand and upgrade the Yangzhou "new rural elite" talent pool.

2022-05-17 Guangdong Huizhou: build "youth" into the youth heart of Guangdong Huizhou: build "youth" into the youth heart Jianwei correspondent Zou Lijuan) since "celebrate 20 always follow the party go new journey" theme education practice, group, Guangdong Huizhou municipal party committee based on party education, focus on youth perspective, youth demand, youth, youth league organizations in the city to the party, struggle for the " welcome 20 celebrate founding group in one hundred series of activities, unity led the teenagers firmly follow the party, the new era. The picture shows visiting the Huizhou Century-old Youth Games Exhibition."Did you leave home because you study?"" Studying is the happiest thing to ask."In the leadership of the Communist Party of China under the leadership of the exhibition, there is a dialogue across time and space. The picture shows the time dialogue booth in the exhibition. Correspondent for figure in addition, the group of Huizhou municipal party committee in more than 400 photo literature exhibition hall, set up virtual change, red phonograph, one hundred time axis, power knowledge challenge vivid experience device project, let the teenagers through "immersive preaching + experiential exhibition + interactive participation" learning mode, understanding under the leadership of the party for 100 years of Huizhou youth movement, attracted nearly 20000 teenagers to visit. Organize a novel theme group class theme group class picture. Correspondent for the figure " youth by hone and brilliant, life because of struggle and sublimation."Said Liu Siyu, a student from Guangdong Technician College who won the honorary title of" National Technical Expert " in 2021. Pku organization to carry out the "always follow the party, new journey" -Huizhou communist youth league may 4th theme group, in xinhua cloud platform, Huizhou TV show, with four generations of Huizhou youth youth struggle story as the main line, with recitation, flash, chorus, about one hundred heart, dialogue in Huizhou youth, youth Huizhou, the city millions of youth members gathered "cloud", fully show the new era of Huizhou youth in youth track to run, build welcome 20, youth achievements strong atmosphere. Launched a batch of "green language" products Huizhou youth league organizations at all levels relying on the new media matrix, combined with "youth branch" "party" action, launched "one hundred road Huizhou youth said" short video column, by 100 Huizhou youth with a unique perspective to share and tell 100 history story, with "green language" led the youth revisit youth heart to the party in one hundred, carefully launched the road of the light of youth shining revival " hello! Huiyang youth "youth, ideal when burning" "bo youth are doing" "cohesion rural revitalization with painting youth picture" "the definition of youth" theme video, series dynamic posters, party history online knowledge quiz "youth" network culture products, launched # youth heart to the new era # topic, nearly 100000 people participate in the topic interaction. Create a warm volunteer red Huizhou youth to participate in the "pick up running" volunteer service activities scene pictures. Correspondent for the picture to mobilize the city's youth league organizations at all levels, volunteer service organizations to carry out environmental protection, civilized practice, voluntary epidemic prevention, youth services, respect for the elderly and help the disabled, convenient services and a series of theme activities, In 12355, launched the "confident to welcome the examination of youth dream" - -Huizhou college graduates' psychological adjustment live class, public welfare online class counseling, More than 410,000 views alone; Youth League Huiyang District Committee organized the " cohesion of youth strength, On rural revitalization " public welfare run activities; Tuanboluo County Party Committee to carry out the "welcome the 20, always follow the party, forge ahead on the new journey" night running activities, Lead the majority of young people to "pick up and run" volunteer service to form a new fashion. During the May 4th Movement, the city has carried out more than 400 volunteer service activities, and nearly 10,000 people have participated. Huiyang District "gather youth strength, talk about rural revitalization" public welfare run pictures. Correspondent for the map " only foothold, can find a job, very grateful to the Communist Youth League committee to Huizhou fresh graduates to provide one-stop service."Lai Hui job young man WeNuo said. During the May 4th period, "Huizhou Young Talent Post" added 4 new stations, realizing the full coverage of the four districts. Through "1 terminal + N branch" construction way, with "talent station + community service + preferential housing" service mode, to meet graduates with employment, entrepreneurship, volunteer, rights, dating, urban integration, community services, so far, for nearly 4000 college graduates to provide all kinds of community services, help them quickly into Huizhou. Create a number of exclusive popular positions of wild Island youth cultural community "Youth Home" and "Huizhou youth culture and creative block" inaugurated. Correspondent for figure wild island "youth home" is the group Huizhou municipal party committee to explore a new demonstration offline comprehensive "store", relying on the "youth home" to explore new service mode, create wen gen incubation, art exchange, youth dating three "service desk", regularly carry out all kinds of cultural salon, theatrical performances, style, health lectures, exhibitions and other series of activities, become condensed youth, service youth "web celebrity". Up to now, the city has established 170 youth homes. Conduct a wonderful cultural feast in Huizhou "May 4 th" youth culture carnival, through the youth reading sharing, youth art exhibition, youth salon and other series of activities, in various forms, rich and colorful cultural activities to gather youth strength, perceive colorful youth culture, understand the new era of Huizhou youth. During the May 4th Movement, the city's youth league organizations at all levels organized more than 50 activities, such as youth painting and calligraphy photography exhibition and I with the regiment emblem flag. Huizhou youth with the charm of ink, the beauty of light and shadow, continued the magnificent chapter of youth, and celebrated the construction of the league for a hundred years.

2022-05-14 Beihai Municipal Party Committee held a themed League Day Activity "- -2022 Yinhai District Leaving the Team Joining Ceremony and" Please trust the Party to have me " theme League Day activity was held in Overseas Chinese Middle School in Beihai City, Guangxi Zhuang Autonomous Region. Activity aims to review the Chinese youth movement in one hundred years, strengthen the yinhai district middle school students to the communist youth league organization and the communist youth league member advanced understanding, further stimulate the district youth love party patriotic enthusiasm, enhance their sense of historical sense of mission, political responsibility and practice "please rest assured the party have me" oath action consciously. Next, group yinhai district party committee will be coagulation hearts meet force led the youth league organizations at all levels in the strengthening group's leading force up and down detail, on expanding the organization of the hard, in strengthening the service force and hard, make the communist youth league grassroots organization reform of the party and government period, development, youth hope, improve youth satisfaction and feeling. The activity was jointly organized by the Yinhai District Committee, Yinhai District Education Bureau, Yinhai District Working Committee for the Next Generation, Yinhai District Young Pioneers Education Working Committee, and Beihai City Overseas Chinese Middle School. More than 1,500 people from the Beihai Municipal Committee, Yinhai District Education Committee, Yinhai District, principals of various middle schools, secretaries of the Youth League Committee and young Pioneers counselors attended the activity.

2022-05-14 Gansu Longnan held "welcome the 20 youth ode to the Party" keynote speech contest Gansu Longnan held "welcome the 20 youth ode to the Party" keynote speech contest youth ode to the Party " keynote speech contest, the contest by the Youth League Longnan Municipal Party Committee, Longnan City Youth Federation hosted. With the theme of "Welcome the 200 and Always follow the Party to forge ahead with the new journey", the competition is divided into three stages: preliminary, semi-final and final. The preliminary competition was organized by the youth League committee of each county and district, and the final competition was organized by the Longnan Municipal Party Committee of the Communist Youth League. After the competition, 12 contestants entered the final. The game around the party's struggle major achievements in one hundred and historical experience, in one hundred happened in Longnan heroic deeds, touching story, express to the party, to the motherland, loyal love to home, show Longnan youth good style, guide the youth unswervingly follow the party, listen to the party, contribute to the construction of happiness new Longnan youth strength.

2022-05-13 Hong Kefei) recently, the Changsha Municipal Committee of the Party League and Hunan Radio jointly launched the "youth heart to the Party music ideological and political class". On the afternoon of May 11th, the first open class was opened in Hunan First Normal University. The picture shows the music, ideological and political class scene. Hunan Radio, Hunan Youth League, Changsha, Zhuzhou, Xiangtan, Hunan First Normal University, Hunan University of Technology and other 15 universities teachers and students; representatives of youth League organizations at all levels, outstanding youth representatives from all fronts in Changsha listened to the scene. At the scene, a hot-blooded original song "Fenghua", singing the youth melody of the first hundred years, but also opened the "youth heart to the party music ideological and political class" curtain. A hundred years ago, MAO Zedong led the establishment of the Changsha Socialist Youth League, and developed the earliest group of the Communist Youth League members in Changsha in Hunan First Normal University. Since then, the youth movement in Hunan has had its own core. The red ideal was conceived here, and the red prologue was written. Group after another of patriotic youth have practiced their original aspiration and continued to work hard to strengthen the country and serve the country."Youth heart to the party music ideological and political class" is planned to be divided into "preface-youth I" "establish ambition has ideal-we are all dreamers" "Ming Dade dare to bear-not the people" "can bear hardships into great talent-everyone is great" "willing to struggle to bear responsibility-roll up his sleeves to work hard" "end-youth to the party" six parts. Core value leading is xi jinping, general secretary in celebrating the 100th anniversary of the founding of the communist youth league remarks youth conference speech "have ideal, dare to bear, can bear, willing to struggle", this also with xi general secretary in tsinghua university last year hope young people "ambition, Ming virtue, into great, bear the requirements of the". The picture shows the students of Hunan First Division watching this special ideological and political class carefully. Hunan radio for figure music courses theory and immersive integration, in a series of classic music works, JiaoYuLu, Yuan Longping, ai patriotic example story, let ideological courses not only have depth, more temperature, guide the young students in the painting shuttle in the agitation years, inspiring the great spirit of national rejuvenation, thus more firm "youth, for the great bloom" ideal ambition. It is reported that the music, ideological and political courses will also go into a number of government organs, enterprises and schools in Hunan province to gather the youth power of implementing the strategy of "strengthening the provincial capital" and promoting the high-quality development of Hunan province.

2022-05-13 Anhui Huaibei city youth park opening Anhui Huaibei city youth park opening youth and more theme punch point in green net reporter wang haihan) recently, Anhui Huaibei city strong mountain group held in the city youth park "welcome 20 forever walk with the party new journey" strong mountain to celebrate the 100th anniversary of the group theme activities. The activity is divided into two stages: the unveiling ceremony and the theme group day activities. The theme league day activities were carried out simultaneously online and offline, divided into young Pioneers representative dedication, poetry recitation, youth representative sharing and other links. Huaibei Youth Park is located in the scenic area, Lishan Town, Lishan District, with youth square, youth trail, red corridor, sightseeing platform and other areas and facilities."Youth park is an important measure for us to explore and expand youth positions. Relying on the scenic area to build comprehensive, exemplary and public welfare youth service positions, youth park is positioned as a gathering place for youth, which can be used as an outdoor space for brainstorming, group building activities, youth friends and fitness walking."Wu Xiaomei, secretary of the Tuanolieshan District Committee, introduced that the Communist Youth League District Committee will make use of its position advantages to form an overall force to connect and guide the youth, and effectively integrate and accurately put resources. In the youth park theme square, with youth, struggle, future, interpreta dream as the theme design corresponding buildings and modules, into the square, greeted is "salute together youth" sculpture, the park also has "learning lei feng good example" new era of youth inspirational slogans, printed with party history, group history, team history of knowledge of "red education theme corridor", make young people favorite clock point. The park will also become the sixth young Pioneers after-school practice and education base in Lishan District. In recent years, the leishan area has formed the idea of "returning to the hometown for employment, investment and business development", and the youth league organization of the district will take the youth park opening as an opportunity to constantly promote the implementation of the theme activity of "dream building hometown Huai talent have you".

2022-05-11 Gu Jianwei, correspondent Fu Xiaomin) On the morning of May 10th, the establishment ceremony of Network Literature Youth Valley in Zibo City, Shandong Province was held in the Sky Orange Mass Entrepreneurship Art Space. Xu Qiang, secretary of Zibo Municipal Committee and chairman of Zibo Youth Federation, Liu Xiaoming, secretary and chairman of Zibo City of Literary and Art Circles Federation, and Tao Zhiyu, member of Party Group and director of Zibo Culture and Tourism Bureau attended the establishment ceremony. Presidium members of Zibo Network Writers Association, representatives of young network writers participated in the activity. Participants visited the construction of network literature youth Valley, and Mr.Guo Xiang, deputy secretary general of Zibo Network Writers Association and founder of Network Literature Youth Valley, introduced the construction, progress, effect and development direction, as well as the development and promotion of Zibo cultural industry. Sky Orange Holdings Co., Ltd. signed a cooperation agreement with Zibo Network Writers Association. Liu Xiaoming, chairman of the Municipal Federation of Literary and Art Affairs, and Tao Zhiyu, a party member of the Municipal Culture and Tourism Bureau, unveiled the network literature youth Valley. Youth League party secretary Xu Qiang for the network literature youth valley youth League branch unveiling. Some network literature writers sent a congratulatory video for the establishment of the network literature youth Valley. As the first in the province with hatch youth network literature creation base, network literature youth valley will improve Zibo city "modern atmosphere" of "modern temperament" and "vitality index" as the core, adhering to the "open, inclusive, interconnected, win-win" thinking concept, online to the sky city clouds as the carrier, offline based in the orange of the sky, online synchronous interaction, gather youth network literature writers, industry platform and related film and television culture industry, form the youth network literature IP complete industry chain.

2022-05-09 Xinjiang Korla City "Communist Youth League Cinema" officially launched, Xinjiang Korla City "Communist Youth League Cinema" officially launched, the first public welfare viewing! The opening ceremony and the first public welfare movie-watching activity were held in 103 Film Park. At the ceremony, the relevant person in charge of the Communist Youth League Korla Municipal Party Committee introduced the relevant situation of the "Communist Youth League Theater", and inaugurated the "Communist Youth League Cinema". At the same time, we held a Mother's Day public movie viewing event. At present, the "Communist Youth League Cinema" of Korla 103 Film Park, New Huijia Wheat Field Cinema and Tianbai Friendship International Cinema are listed simultaneously. When the youth Youth League members buy tickets at the "Communist Youth League Cinema", they can enjoy preferential treatment with the youth League member certificate. Learned, for the full performance of the communist youth league leading condensed youth, mobilize youth, contact service youth of the basic responsibilities, at the same time meet the korla youth growing spiritual and cultural demand, into a "youth ideological education position" "youth patriotism education base" and other functions as one of the integrated service platform, the communist youth league korla committee of Xinjiang grain source city culture investment co., LTD., jointly build the Xinjiang first youth theme cinema- "the communist youth league cinema". Tuan korla municipal party committee secretary Song Xin said: " we take the initiative as, innovative work way, strive to expand more conform to the actual needs of contemporary youth organization, efforts to build the communist youth league cinema members youth spiritual home, into youth ideological education positions, youth patriotism education base, volunteer service public welfare activities, carry out good youth story sharing, entrepreneurial youth exchanges, youth fraternity 'green' font size characteristic special activities, constantly inspire the youth firm ideal faith, determined to become, to serve the motherland, repay the society in the future."Next, the communist youth league korla committee will further strengthen the coordination service, strive to build the communist youth league studio for shaping youth correct world outlook, the outlook on life, values, strengthen the communist youth league member's sense of honor, sense of mission, sense of responsibility, motivate the city the youth members always follow the party, the new era, in the track of youth run out of the contemporary youth's best grades.(Correspondent Huang Wanli)

2022-05-09 Anhui Lu 'an League organization to deepen the "Rural Revitalization New Youth Plan" Anhui Lu' an League organizations at all levels to deepen the "rural revitalization new youth plan" China Youth network reporter Wang Haihan Wang Lei) " I hope that farming, entrepreneurship can be more and more handy, let the villagers' life better!"This is the Lu'an city young science and technology correspondent Xue Yejing has been a wish. Xue Yejing, who was born in a rural family in Huoqiu County, Lu'an City, has been trying to solve the pain point of a single traditional agricultural model and low benefits in her hometown. After graduating from graduate in 2016, Xue Yejing returned to her hometown to start her own business. He successively established Fenghao Aquaculture Cooperative and Fenghao Agricultural Science and Technology Co., Ltd., explored a scientific rice and shrimp planting and breeding system, and promoted more than 200 local households to achieve employment and increase industrial income. Xue Yejing is a participant and beneficiary of the New Youth Program of the Lu'an Communist Youth League. In recent years, Lu'an Communist Youth League has deepened the "New Youth Plan for Rural Revitalization", innovated and implemented five major actions, including youth innovation industry, youth education talents, youth culture, youth ecological protection and youth solid organization, and explored the rural revitalization work path that the party and government are satisfied with, what rural needs and young people can do. Thanks to the strong support of the youth league organizations at all levels in Lu'an and the superimposed support of various policies, the "First Xianhui" brand handmade noodles created by Li Xianhui, a post-85 young entrepreneur, has become the longevity brand product of Jinzhai County. The annual output value of the company has been nearly 100 million yuan, driving the cumulative income of the poor population of more than 3 million yuan. In 2021, Li Xianhui was awarded the "National Young Pioneer of Rural Revitalization". Lu 'an industrial development has outstanding resource endowment characteristics and obvious advantages. Lu' an melon piece, Huoshan Dendrobium, western Anhui big white goose and so on are all famous local characteristic agricultural products. How to "drain" the brand resources to the rural revitalization and development boom? Du Mingxing, deputy secretary of the Lu'an Municipal Party Committee, said that the Luan Communist Youth League has walked out of a unique way- -to build the brand of "Lu Xiaoqing", a public platform for youth innovation. Then, the professional team continues to optimize the brand management and operation, gather the industrial brand effect, deeply explore the needs of young and middle-aged people in rural revitalization in technology, industry and other aspects, and change the individual contact and fighting alone into efficient docking and coordinated operations. The Communist Youth League Municipal Committee also organized all counties and districts to build a number of "Lu Xiaoqing" brand villages around the "one village, one product". At the same time, the Lu'an Communist Youth League has carried out youth innovation and entrepreneurship competition and other activities, bringing together a number of high-quality youth innovation agricultural industry projects. Has hatched more than 10 rural leisure brigade, such as science and technology agriculture, electricity couplet of "green farm", choose tree and cultivate more than 40 industry demonstration leading rural youth entrepreneurial projects, and follow-up tracking services, for the rural industry "seed" docking government departments, financial institutions, provide policy, capital, technology and channel support. According to the data released by the Research group of "Youth and Talent-Friendly Cities Index Research in the Yangtze River Delta" of the Shanghai Academy of Social Sciences, the net outflow ratio of population in Lu'an in 2021 is about 25%, and the most outflow is young people. The core of rural revitalization is talent revitalization. Zhang Xiaoquan, head of the youth development department of the Lu 'an Municipal Committee, said that in order to solve the problem of rural revitalization in the old areas, the Lu' an Municipal Committee explored the implementation of the youth education action to attract, retain and cultivate talents."The Lu'an Youth League has improved the mechanism for youth league organizations and cadres at all levels to regularly contact the youth, and carried out activities for young entrepreneurs from abroad to participate in the return home week and summer practice."Zhang zhaoquan said the league dug deep into rural talents and allows young people to make suggestions for rural revitalization. By the end of 2021, more than 50 people were stored in the young leading talent pool of Lu'an. At the same time, the youth league at all levels in Lu 'an has organized practice bases, cultural venues and patriotic education bases with on-site teaching conditions, and built 19 rural teaching bases of Lu' an Youth College in the city. We will organize special training sessions on rural revitalization, and carry out special training activities around the knowledge required and expected of rural youth, including party and government policies, youth entrepreneurship, and youth health. With the "return" of groups of new youth, the Luan Municipal Committee of the Communist Youth League is constantly deepening the reform of grass-roots league organizations as a breakthrough point, strengthening the construction of township and village league organizations, and opening up the "last kilometer" of league organizations to serve rural revitalization. On the one hand, pay attention to give play to the advantages of grassroots league organization, actively select and train village reserve cadres, village reserve cadres into the village league organization leadership, select excellent village reserve cadres to township, county and municipal league organs hang (and) job training exercise; on the other hand, explore the city to establish the first league organization weak village, key villages, for rural youth labor education, social practice, volunteer service, to community (village), rural youth league vitality and rural youth social practice ability "double promotion". In recent years, the Lu'an Communist Youth League has also endowed the Hope Project, focusing on key groups such as rural left-behind children and poor teenagers from families, taking the construction of "youth home" as the starting point, and allocating working forces to build rural youth after-school activities."Relying on the New Era Civilization Practice Center (stations and stations), we will strengthen the construction of rural youth volunteer service teams, and carry out volunteer service activities such as policy publicity, civilized village customs, moral guidance, and literature and culture, so as to improve rural civilization."Zhang Zhu, party secretary of the Communist Youth League, said that in the future, we will continue to give full play to the organizational advantages of the Communist Youth League, to deepen the" new rural revitalization youth plan " as the whole, and constantly explore the new path of the old new youth areas to serve the rural revitalization.

2022-05-08 Sichuan Guangyuan Communist youth League) leveraged social capital nearly 10 million yuan, win provincial youth venture capital nearly 18.9 million yuan, support entrepreneurship youth 225, youth entrepreneurship park drive employment of more than 4,480 people... the Communist Youth League Sichuan Guangyuan Municipal Party Committee recently released data, recorded the achievements of the local youth league organizations at all levels service youth innovation and entrepreneurship. Since 2017, the Guangyuan Municipal Party Committee of the Communist Youth League has successively launched the "Youth Innovation Plan" and other projects to serve the needs of youth innovation and entrepreneurship. In the past two years, the Guangyuan Municipal Party Committee of the Communist Youth League has leveraged nearly 10 million yuan of social capital to consolidate and upgrade the Guangyuan Youth Mass Innovation Space. At present, 57 projects have settled in the Youth Innovation Park. The Communist Youth League Guangyuan Municipal Party Committee also strive for provincial youth entrepreneurship funds of nearly 18.9 million yuan, to support 225 young entrepreneurs. In 2021, the Guangyuan Municipal Party Committee of the Communist Youth League, while increasing its efforts to support youth innovation and entrepreneurship, focused on helping rural revitalization, and launched the first batch of youth venture funds, "Shuqing Revitalization Loan" and "Guangqing Post Loan", to mobilize and guide young volunteers to participate in rural revitalization. At the same time, the Communist Youth League Guangyuan Municipal Party Committee joined forces to launch the "farming cloud plan" to help rural revitalization, invested more than 1 billion flow resources, to support Guangyuan 100 rural "leading geese", 100 "rural electricity trademark soldiers", 100 more than 1 million fans of new farmers. The Communist Youth League Guangyuan Municipal Committee also cooperated with Shanghai Fudan University and Postal Bank Shanghai Branch and other universities and enterprises to assist returning young entrepreneurs to establish Guangyuan seedplanting and breeding professional cooperative. According to statistics, in the past five years, Guangyuan Youth Entrepreneurship Industrial Park has absorbed 278 youth entrepreneurship projects, incubated 220 enterprises in the park, and settled 57 projects in the Park, creating more than 4,480 people, contributing to the consolidation and expansion of the achievements of poverty alleviation and rural revitalization.

2022-05-01 Guangxi Hechi Communist Youth League held Open Day activities. On the day of the activity day, more than 20 young representatives from towns, enterprises, organs, schools, wechat and other netizens went to the Hechi Municipal Committee to communicate with all cadres of the Hechi Municipal Committee and understand the work functions of all departments. During the symposium, the secretary of Hechi Municipal Communist Youth League communicated with the youth representatives. The person in charge of Hechi Municipal Communist Youth League introduced the key work of Hechi Communist Youth League in 2022 to the youth representatives, and hoped that the youth representatives would make suggestions for the work of the Communist Youth League. Subsequently, the youth representatives went to the "National Youth Civilization" 110 police reception center of Jinchengjiang Branch of Hechi Municipal Public Security Bureau, and visited the youth activity room, cultural corridor and police reception center. Liu Yi, the director of the youth civilization number, introduced the situation and experience of the creation of the youth civilization number to the youth representatives. In the Young Pioneers off-campus activity base of Hechi City, the young representatives and the young Pioneers visited the Hechi Revolutionary Memorial Hall, and the young party members reviewed their vows of joining the Party.

2022-05-01 Shanxi Changzhi held the theme league day activities piano youth Daily Youth network reporter Hu Zhizhong) on the evening of April 29, the head of the party committee held the "welcome 20, always follow the party, forge ahead on a new journey- -my youth my group" theme league day activities. Twelve outstanding young people from different industries walked into the broadcast room to tell their own youth stories. The number of online viewers reached 388,000, and the online and offline viewers covered nearly one million teenagers in the city."One of the common characteristics we have chosen is that they are all working in the ordinary positions closest to the people's livelihood. They should study hard, work hard, abide by their duties and love their work."Zhang Zhaoxiang, secretary of the municipal party committee, said that these young people are all typical people around them, and their youth stories can be seen, touched and learned. Lei Wanli, the CNC lathe operator of the second branch factory of Shanxi Aerospace Tsinghua Equipment Co., LTD., is a typical representative of the struggling youth. He is only 27 years old for eight years. From the age of 19 to enter the enterprise, while learning while doing, not only proficient in a number of CNC system programming, but also continuous product technical innovation, to save a lot of costs for the enterprise. He also served as a number of key aerospace products, key parts precision processing tasks, the product qualified rate of 100%. Lei Wanli said that he likes to refine on his professional skills in the details and accumulate over a long period of time. The great craftsman spirit is the strongest driving force for him to constantly innovate and forge ahead. Maintenance and receiving tools, attendance registration, maintenance discipline, paying salary and labor insurance and welfare, and doing employee insurance... Cao Yilong, from the third Taihang Cleaning Team of Luzhou District Sanitation Department, has patiently and earnestly done the seemingly simple and small things in his work for 12 years."Only when people are safe can our work be done well. Although what we do is all insignificant things, our work is closely related to the urban civilization construction of Changzhi and the good life of the common people."Cao Yilong said that his teammates do "beauty" for the city, while he is responsible for the "city beautician" as a good logistics, as a good housekeeper, so that clean, clean and Changzhi citizens accompany every day. Guan Jiaxing, league branch secretary and deputy captain of Changzhi Blue Sky Rescue Team, has organized volunteers and volunteers to show love to poor areas for many times, taking the initiative to connect with sick poor children in hospital, carried out targeted assistance activities, donated money for charity sales for children with leukemia, and helped lost elderly go home. "Volunteer work is a happy thing"."If you have the time, you must come to the Taihang Memorial Hall of the Eighth Route Army in Wuxiang County, and listen to me tell you about the legend of the Eighth Route Army in the Anti-Japanese War."At the event site, from Wuxiang County primary school student Jia Beini is the youngest narrator. Jabini, who grew up listening to the stories of the Anti-Japanese War, became a small commentator at the Taihang Memorial Hall of the Eighth Route Army in Wuxiang County in 2020 and was rated as an outstanding student."I can not only tell the story of the Eighth Route Army to more people, but also learn a lot of knowledge outside the textbooks from practice."Ma Xiao, secretary of the Communist Youth League Committee of Changzhi No.5 Middle School, said that the students benefited a lot from watching the live broadcast and responded warmly, saying that they should conform to the example and the advanced, feel the power of example, and convey the spirit of example. Zhang Zhaoxiang introduced that the theme of "my youth and my League" is one of the important measures of the Changzhi Communist Youth League "welcome the 20th, always follow the Party, forge ahead on the new journey" theme education and practice activities. In the next step, the head of the municipal party committee will focus on the main responsibility and main business, to create the "one body, two wings and two drive" work system, successively launched the "Tuan Tuan blind box season", "Tuantuan grow together with you" theme cartoon and "100 youth talk changzhi" promotion film, with the strength to circle the majority of young people, with the sincere response to the majority of young people.

2022-04-29 Shi Jia) Near graduation, fresh graduate Liu Jian, a 2019 graduate majoring in new energy technology from Tongliao Vocational College, has been slow to find a suitable post. Liu Jian was a little worried. He said that due to the epidemic, " I have never been able to return to school, and all the campus job fairs have also been held online."The turnaround occurred in early April, when the school teacher gave Liu Jian a job list with the name of the company, the name of the job, and the proposed salary. Liu Jian looked at the form for more than 3,000 people, " the teacher also specially marked the key suitable position for me."Now Liu Jian enters Tongliao Seiko Sapphire Co., Ltd. to practice as an operation technician." I am satisfied with all the conditions, and I am ready to stay here and work after graduation."Behind this employment list, which covers the employment needs of 3,910 people, the Communist Youth League of Inner Mongolia Tongliao Municipal Party Committee has visited enterprises and schools for many times, and carried out seven "youth listening" activities with relevant units and universities in Tongliao City. In early March this year, TuanTongliao Municipal Party Committee came to Tongliao Kailu Industrial Park to visit research."Our company is now in the construction stage. Due to the epidemic, the difficulty of recruiting enterprises has become a difficult problem restricting our production."In the Kailu Biomedical Development Zone Jin Yufeng Biotechnology Co., Ltd. construction site, the head of the company is anxious to feedback their immediate problems. League Tongliao Municipal Party Committee to Tongliao City Kailu Industrial Park visit investigation. Pictures provided by respondents group tgo municipal party committee in visiting tgo vocational college, Tongliao city industrial vocational school and other colleges and universities in the process of many universities consistent feedback: affected by the outbreak, the graduates employment faces certain difficulties today, only with online interview or delivery resume, unit of choose and employ persons and students lack of face to face communication, or there is a certain gap. In view of the double difficulties of difficult recruitment and difficult employment, the main leaders of the Tongliao Municipal Party Committee and Tongliao Municipal Development and Reform Commission carried out the first "youth listening" activity to jointly study the solutions. The two sides spent a week collecting labor demand from enterprises in six industrial parks in Tongliao city one by one, and sorted out a labor list of 3,910 people. Lu Xianhong, director of the Youth Development and Rights Department of the Youth Tongliao Municipal Party Committee, said, " These positions cover front-line workers, management positions, professional and technical positions and other fields, and the educational requirements also involve various levels such as college and technical secondary schools."It is understood that" Youth Listening " is an important activity that the Inner Mongolia Autonomous Region Committee launched to normalize the youth contact, requiring the youth league organizations at all levels to go deep into the youth, listen to the demands of the youth, solve their urgent difficulties and worries, and enhance the feelings with the youth."'Green listening' is the process of constantly collecting and solving problems."Lu xianhong said. Youth League Tongliao Municipal Party Committee to carry out the "green listening" activities. It is only the first step, and how to achieve the effective matching between jobs and youth is the ultimate goal. With the employment list, the Youth League and Tongliao Municipal Party Committee held 7 consecutive "youth listening" activities continuously, visited and connected 7 relevant units and schools, and carried out work docking by industry and field, thus setting up a communication platform for enterprise employment and youth employment. Yin Haifeng, deputy secretary of the Communist Youth League Committee of Tongliao Vocational College, said: " The positions provided by the Communist Youth League Municipal Committee are all key enterprises in the city, with high gold content. It is really very timely."On April 7," youth Tongliao " public account online Tuan Tuan to help employment column. On April 8, the first cloud recruitment of "group to help employment" was launched, and more than 3,000 job information with the majority of young job seekers met in the "cloud"."Young people can find the latest employment and entrepreneurship positions here at any time and consult preferential policies to meet their needs for entrepreneurship and employment."Lu Xianhong introduced:" This is the Communist Youth League, the Municipal Bureau and the Municipal Human Resources and Social Security Center to create a regular youth and employment service platform."The employment list has also been widely released through the university youth league organizations in Inner Mongolia and the official employment information platform of Tongliao City. In this way, the League Tongliao Municipal Party Committee has built a bridge between youth and enterprises, which not only solves the urgent need of enterprises, but also meets the most urgent employment needs of the youth."After the epidemic situation improves, we will do a good job in epidemic prevention and control as well as in offline platforms."Lu Xianhong said:" The municipal Communist Youth League committee will organize enterprises to carry out double employment selection meetings in schools, and share jobs with the grass-roots level. The local Communist Youth League and employment departments will jointly organize recruitment activities according to local conditions to attract the local labor force for employment.”

2022-05-12 Gansu Longnan "celebrate 20 youth party" keynote speech contest in Gansu Longnan "celebrate twenty youth party" keynote speech contest Ma Fuchun) recently, to meet the party's 20, to celebrate the 100th anniversary of the Chinese communist youth league, unity lead the youth firmly follow the party, build new era, Longnan, Gansu province "celebrate twenty youth party" keynote speech contest, contest sponsored by the communist youth league Longnan municipal party committee, Longnan youth federation. With the theme of "Welcome the 200s and always follow the Party to forge ahead on the new journey", the competition is divided into three stages: preliminary, semi-final and final. The preliminary competition is organized by the youth League committee of each county and district, and the final is organized and carried out by the Communist Youth League committee. After the preliminary competition and the semi-final competition, the 12 contestants entered the final. On the scene of the competition, the contestants combined with their own learning, thoughts and understanding, personal experience and actual work practice, with vivid language, vivid examples and full of enthusiasm, expressed the deep praise to the Party and the motherland. The game around the party's one hundred struggle major achievements and historical experience, control xi jinping, general secretary of the new era of youth entrust, in Longnan heroic deeds, touching story, express to the party, to the motherland, loyalty to his hometown, fully shows the Longnan youth vigor, forge ahead, progressive good style, guide the member youth unswervingly follow the party, listen to the party, contribute to build happy new Longnan youth strength.

2022-05-09 Shandong Weihai: efforts to build youth development friendly city model Shandong Weihai: efforts to build youth development friendly city model in youth newspaper network reporter XingTing) not long ago, in Weihai innovation pioneer park China-eu membrane technology research institute, a national patent of water processing new materials-functional ceramic film has passed the pilot, the industrialization, and the key technology research and development is a group of young people who just graduated from Harbin (Weihai). On October 14,2021, Shandong proposed to promote the construction of "youth development friendly city" in the whole province, build a number of "young" youth development friendly cities, and introduce the first provincial "youth development friendly city" construction index system in China, providing strong guidance for the construction of youth development friendly city development in Weihai. In February this year, Weihai city issued the construction of youth development friendly city "mobilization", the youth priority development concept into urban development strategy and delicate city connotation, will "building youth development friendly city" has written the 16th party congress report and 2022 municipal government work report, in urban planning, construction, management process of youth elements, take care of the youth characteristics, inject youth vitality for delicate urban construction. Weihai port young talent apartment for youth housing security deployment, high positioning, coordination-Weihai immediately established the youth joint work conference innovation working mechanism, set up special class, deputy secretary of municipal party committee, government contact the communist youth league vice mayor as a double leader, 23 member units and eight districts, development zone linkage, wisdom, hit youth development friendly city construction combination, quickly build up youth development friendly "ecosystem". Building a youth-friendly city is a new "proposition" for most cities, and we need to "do your homework" before drawing. In the past three months, Weihai city has completed more than 20,000 valid questionnaires for asking questions and asking questions from young people. More than 30 reports on various industries were collected, and four special investigations and one comprehensive report were formed. This gives Weihai the "confidence" to build a youth-friendly city. Weihai college students to participate in robomaster machine a masters at the beginning of May, Weihai issued "building youth development friendly city implementation plan" (hereinafter referred to as the "plan"), around the "more friendly city to youth development, more youth for urban development" two dimensions, focus on youth in housing, education, employment, entrepreneurship, health, marriage, research launched a package of youth friendly policy package, a total of 12 aspects, 65 specific measures."Plan" in the top-level design, adhere to the overall layout, overall planning and coordination, integrated promotion. For example, considering the comprehensive consideration of youth innovation and entrepreneurship, housing projects, cultural experience, leisure and entertainment land guarantee, we will accelerate the digital and intelligent transformation of core business districts and blocks, reserve sufficient space for youth activities, and give top priority to youth in planning. In 2022 in the commercial complex to carry out the heart of "tiger" youth dating in the service, in addition to the "521" living subsidies, increase policy housing, talent apartment supply, pay more attention to the employment college students' examination service, free in the "youth station" bag in 5 days such as flexible care, from the fine service reflects the greatest sincerity. In terms of diversified supply, we have launched a series of activities and policies focusing on "eating", "playing" and "traveling" to meet the various needs of young people. According to the plan, Weihai city has clarified the roadmap and task book for building a youth-friendly city."By 2023, we will strive to strengthen the shortcomings of the policy, improve the policy system, and form the 2.0 version of the youth development-friendly city policy. In 2024, the dynamic monitoring of various youth development indicators will be more accurate, the youth friendliness and satisfaction will be further improved, and the newly introduced young talents will increase by 20%, making Weihai a model for a national youth development city."Tuan Tuan Weihai Municipal Party Committee responsible person said.

2022-05-01 The first batch of young Pioneers off-school practice education base in Liuzhou city, Guangxi Province was launched in Liuzhou Youth Palace (Liudong Palace). Liuzhou Municipal Party Committee and Liuzhou Youth Working Committee actively explore the socialized work mode of the Young Pioneers, linked with the Municipal Education Bureau and the Municipal Bureau of Culture, Broadcasting and Tourism to integrate resources to hold the "Welcome 20, Strive to be a good Team Member" in 2022 " Longcheng Youth, set out!"Liuzhou Young Pioneers practice education theme demonstration activity and young Pioneers after-school practice education base launch ceremony. At present, Liuzhou has established 7 autonomous region level, 10 municipal level and more than 50 county (district) level young Pioneers off-campus practice education bases, covering five categories, including revolutionary tradition, labor practice, science and technology and national defense, traditional culture, and ecological civilization, forming the "Longcheng Young Pioneers off-campus practice map" with Liuzhou characteristics. Base construction, has received more than 3000 young pioneers practice, covering the young pioneers more than 20 people, average every ten thousand young pioneers have more than 1.5 young pioneers outside practice education base, for the city 380000 young pioneers involved in nearby activities, support the city's 456 young pioneers brigade, more than 9000 young pioneers squadron to carry out the "red scarf narrator" "red scarf heart to the party" "red gene generations" theme education and research activities, play the young pioneers outside practice education base for children's education leading role.

2022-05-07 Sichuan Guangyuan: youth contribution write a new era struggle a new era of Sichuan Guangyuan: youth contribution new era struggle wei correspondent Cai Min) over the years, Sichuan Guangyuan municipal party committee always keep in mind that unswervingly follow the party, unremitting struggle for the cause of the party and the people and mission, solidarity led Guangyuan youth charge, bear as, in leading ideological and political construction youth, service youth entrepreneurship, win poverty engines, in rural revitalization, fighting COVID-19 youth sweat, with golden years writing youth contribution great chapter. For the 87,839 Communist Youth League members in Guangyuan, "seize the day and live up to the time" is their pronoun. It is their mission and responsibility to strive to write the chapter of youth without regret. Run, teenagers- -to love to help grow up in the sun, should have fire in the heart, eyes have light, warm and bright; should be riding a horse whip, dream distance, independent and strong. All these good things are inseparable from the guidance and infiltration of thought, morality and culture. For a long time, the Guangyuan municipal party committee adhere to do the party's loyal assistant, deepen the youth ideological and political lead, guide the youth tree prison "four" consciousness "four," four confidence "," two maintenance ", extensive" my youth my dream "" learning message spirit, show youth bear "" bloom war epidemic youth firm system confidence "" one hundred party history every day " and other theme activities more than 1400 games. Vigorously promote the "voice of the youth" and the key work of the league, "youth voice" construction work was "central league as" advanced typical league organization "; further promote" youth learning " online theme league class learning, online more than 8 million online learning, the study of the top in the province, around the party history learning activities, more than 200,000 teenagers online and offline party history learning education activities nearly 800 times. With the rapid development of economy and society, more and more people choose to work out for employment, and the problem of left-behind children becomes increasingly apparent. To this end, the Youth League Guangyuan Municipal Party Committee vigorously implemented the "Huiai Home" left-behind students home project, continue to carry out the "Child Partner Plan", "Happy School", "Toy Story" and other brand care projects, to achieve scientific and normal services for key youth groups. Up to now, the city has established 64 "children's companion homes", carrying out regular activities such as academic counseling, family companionship, and self-care education, and directly serving more than 2,000 left-behind children in rural areas, and playing a positive role in caring for and protecting left-behind children in rural areas and helping rural revitalization. In order to implement the municipal Party committee to build Guangyuan high-quality development talent engine, implement the talent gathering project, retain young people and take root in Guangyuan concept. Group Guangyuan municipal party committee adhere to youth demand oriented, focus on social integration, employment, dating, deepen development "community mutual aid plan", "green love plan", "dream plan" and other brand work, normalized community parent-child, elderly service activities, "gather talent edge is you" dating fellowship activities and "dream plan" "home" college students' social practice. In recent years, it has carried out more than 10 dating activities, served more than 3,000 young people of the appropriate age, and raised more than 7,000 internship positions for college students. The Cangxi Youth League County Party Committee was praised by the 2021 national college students' "return to their hometown" social practice activities. Li Jun, a young man born in the 1980s, gave up a high-paying job in the city and returned to his hometown to start his own business. He not only helped the villagers to get rich out of poverty, but also told the Chinese people about his experience in helping farmers as a deputy to the National People's Congress. All these, cannot be separated from the Youth League Guangyuan Municipal Party Committee behind the silent support and help. In 2017, Li Jun, as the leader of rural youth entrepreneurship and prosperity, won the "National Poverty Alleviation Award"; in 2021, Li Jun and Zhao Hailing won the title of "National Advanced Individual in Poverty Alleviation" at the National Poverty Alleviation Summary and Commendation Conference. Since 2017, the Youth Guangyuan Municipal Party Committee has successively launched the "Youth Innovation Plan" to serve the needs of youth innovation and entrepreneurship. In just two years, the Youth Guangyuan Municipal Party Committee has leveraged social capital of nearly 10 million yuan to consolidate and upgrade the Guangyuan Youth Mass Innovation Space. So far, 57 projects have settled in the Youth Innovation Park. Strive for the provincial youth venture capital of nearly 18.9 million yuan, to support 225 young entrepreneurs, the capital arrival rate ranked among the best in the province. Assisted returning entrepreneurial youth to establish Guangyuan seedplanting and breeding professional cooperative, and Shanghai Fudan University and other universities and postal bank Shanghai branch and other enterprises for cooperation, signed a purchase agreement of more than 3 million yuan. In 2021, while supporting the youth innovation and entrepreneurship, will focus on help rural revitalization, and quickly signed youth help rural revitalization "cloud plan", invested more than 1 billion flow resources, support Guangyuan hundred rural "leading goose", "rural electric trademark", more than 1 million fans new farmers; launched "Shu Qing revitalization loan", "post" first credit youth entrepreneurship fund 2.5 billion yuan, mobilize and guide the young volunteers to participate in rural revitalization. Stars do not ask the traveler, time pays off. In the past five years, Guangyuan Youth Entrepreneurship Industrial Park has absorbed 278 youth entrepreneurship projects, incubated 220 enterprises in the park, and created more than 4,480 people, contributing its youth strength to the consolidation and expansion of the achievements of poverty alleviation and rural revitalization."I am the most beautiful boy in this street and this street..." On April 30, Gu Ruining, a volunteer, led the children in the hottest dance at the children's companion home in Jiepai Village. The excited smiling faces of the children explained the love and trust of the "volunteers". At the age of 17, Gu Ruining is only a microcosm of the tens of thousands of young volunteers in Guangyuan city. Over the years, a total of 202,000 young volunteers have been registered in Guangyuan city. In Guangyuan, whether it is on the activity site or on the epidemic prevention front line, in the fields or in schools and enterprises, young volunteers can always be captured, and they have become the most brilliant presence among the crowd. They adhere to the concept of serving the overall work of the party and government center with voluntary service, To carry out the volunteer service in front of the Guangyuan Station of the West-Chengdu High-speed Railway and the preparatory service for the 13th Provincial Games; they actively participate in the epidemic prevention and control, Establish 80 "youth commandos", Scientific and orderly visits, epidemic prevention and publicity, temperature measurement and other epidemic prevention and control work; they actively help the resumption of work and production, Many times to the railway station for Guangyuan to go to Zhejiang Jiashan and other places for migrant workers to provide station services; they extensively organize and guide the league affiliated social organizations, youth volunteers to participate in the youth volunteer "love in the community", "beautiful in the countryside", the "youth volunteer", "love" in the journey " theme volunteer services;... up to now, Guangyuan city has registered 1,800 volunteer service teams through the "volunteer Sichuan", The cumulative volunteer service duration of 1.174 million hours, It has benefited more than 2.7 million people. Short steps, not a thousand miles; not small streams, not into rivers and seas. Next, group Guangyuan municipal party committee will adhere to the "grassroots", strengthen the construction of member team advanced nature, leading the Guangyuan youth and the communist youth league mass development, around the eighth party congress proposed "four big economy" "four big projects", in-depth implementation of youth work "difference" action, organization to mobilize the youth to speed up the construction of Sichuan modern youth in the modern youth sweat, reveal youth bear, with excellent performance to meet the victory of the twelfth party congress.

2022-05-07 Shandong: 8 city youth development friendly city construction plan officially released Shandong: 8 city youth development friendly city construction plan officially released network reporter XingTing on May 5, Shandong province youth work joint conference plenary meeting held, approved and officially released Jinan, Qingdao, Zibo, Jining, Weihai, Rizhao, Linyi, Liaocheng 8 city youth development friendly city construction plan, which marks the youth development friendly city construction work into a new stage of "further implementation". This is another "major action" after Shandong took the lead in launching the provincial youth development-friendly city construction in October 2021. The State Council Information Office recently held a press conference on the white paper "Chinese Youth in the New Era", which specifically mentioned the exploration and practice of building Shandong province into a youth-development province, laying the foundation for the national deployment of this work. Data show that the urbanization rate of China's young permanent urban residents has been significantly higher than that of the total permanent urban population, reaching 71.1%. Building a youth-friendly city plays an important role in promoting youth development, attracting young talents, stimulating urban vitality and enhancing the city's taste."Embrace urban renewal help youth development" in 2022 Qingdao urban renewal and urban construction youth BBS successfully held in Shandong will the work in a more important position to grasp, as the further implementation of xi general secretary about youth work important thought, promote economic and social development innovation, promote the development of high quality, centered on the people of the development of specific measures, is committed to promote the further implementation of long-term youth development planning. Since the beginning of the year, Shandong has made great efforts to promote the "ten innovations", put innovation at the core of the overall development position, and continuously enhance the innovation power of economic and social development. This not only provides a broader stage for young people, but also gives them greater responsibility and mission. As a new force for economic and social development and innovation, caring for and supporting youth development means to enhance the vitality of urban development and accumulate the sustainability of urban development. Jinan city organized the theme salon of "youth love China-fashion" youth-friendly city. In a certain sense, Shandong province to promote the construction of youth-friendly city is not only the result of urban development, but also the expectation of youth. At present, with the increasingly fierce social competition, young people are facing multiple pressures, such as graduation job hunting, housing, innovation and entrepreneurship, social integration, dating, support for the elderly, children's education, resulting in the youth "lie flat", "Buddha department" and other prominent problems. It is the purpose of solving the "urgent and anxious" problem that affects the growth and development of the youth and distributes the work energy, which is the meaning of building a youth development friendly city. Shandong tries to clear away the "obstacles" and "stumbling block" to the development of the youth, so that the city can gather more young talents, stimulate its innovation and competitiveness, make the city "young", and make the youth "more brilliant". It is a systematic project to build a youth-friendly city, which needs to unite all parties and form joint forces. The Shandong Provincial annual mobilization meeting held at the beginning of this year particularly stressed that the "construction of a whole-chain support mechanism for the growth of young people", and the concept of promoting youth priority development has become a social consensus. Centering on this focus, Shandong takes advantage of the trend and is systematically designed, responding to the new era with a new answer paper, and gathering a strong impetus for building a youth-friendly city. Weihai port area to build young talent apartment, further attract talents, retain talents by grabbing early city, county two levels of party congress and "two sessions" intensive favorable opportunity to promote Shandong youth friendly city construction around the party report and the government work report, has covered the province 16 cities, 102 counties (city, area)."Youth development" has become a high-frequency term for local Party congresses and people's congresses, and the working pattern of "Party committee leadership, government leadership, and all parties to jointly manage youth development affairs" has become clearer. At the same time, in order to further clarify the construction ideas, Shandong gave full play to the important role of the youth work joint conference, formulated the index system and operating guidelines, and standardized the working mechanism and working process. Since the launch of this work, it has received positive response from party committees and governments at all levels, paid unprecedented attention to the construction of youth development-friendly cities, and actively applied for innovation. Rizhao city in 2022 special youth recruitment and ten key industrial chain ZhaoCaiYinZhi cloud hire month activities launched, Jinan, Qingdao, Zibo, Jining, Weihai, Rizhao, Linyi, Liaocheng 8 city have established experts and scholars, research institutions, party and government functional departments, young masses, citizens, people's congress, CPPCC involved in special research group, the largest listen to opinions from all walks of life Suggestions. Local governments have strengthened policy advocacy and social advocacy, and launched a series of policy measures and practical projects tailored to young people. Up to now, all cities in Shandong have issued 109 policies related to youth development and 202 at the county level, effectively improving young people's sense of gain, belonging, happiness and security. Based on the reality, the economic foundation, resource endowment, population size and development characteristics of different cities are different, and the growth path and development needs of the young people themselves are also different. In the specific work, Shandong insists on the actual start, no "standard answer", no "fixed template". The Shandong Provincial Youth Work Joint Conference Office only provides reference indicators to guide all localities to determine construction goals and formulate construction plans and index systems according to local conditions, so that the concept of youth priority development can have more appropriate programs and richer connotations in different cities. Shandong takes policy service as the main direction and an important point of force, and aims to provide the most fundamental and effective service for youth development. We will accurately focus on the employment services, entrepreneurial environment, housing security, education and health and other key areas that are widely concerned by the young people in the province, and take real measures to deliver the real policy dividends to the young people.for instance, Jinan issued 640 million yuan of rental subsidies to low-and middle-income young people; Qingdao city has included the improvement of youth living conditions and transportation environment optimization into the government's practical livelihood projects; Zibo city has issued 25 policies to build a youth entrepreneurship-friendly city; Weihai city supported the construction of 920 youth innovation platforms; In Donggang District of Rizhao City, 17 urban studies and 740 young talent apartments were built and opened..., a practical and effective policy measures, An intimate and warm service action, We have helped young people solve many "urgent and anxious" problems, Let the concept of youth priority development to manifest, We will strengthen the foundation for building youth-friendly cities. In order to ensure the implementation of the work, Shandong has defined the "responsible persons" and planned the "road map", so that the development of youth friendly city construction is more reliable and dependent. In terms of working mechanism, the municipal Party committees and governments play the role of the construction subject, including the overall planning and promotion of the work, through the municipal joint committee, the municipal standing Committee and the provincial joint committee, the construction cycle is not less than two years, continue to promote the normal construction, the provincial joint meeting for scientific and effective evaluation. In the next step, Shandong will do a solid job in the construction of the first batch of youth development-friendly cities and counties (cities, districts), to form an effective demonstration and influence. Promote provinces and counties level 3 youth work joint conference mechanism more perfect, promote more systematic, universality youth development policy, make youth more active in urban development, comprehensive deepening long-term youth development planning effect, promote the youth and urban creativity mutual agitation, youth and urban quality development two-way promotion.

2022-05-07 Sichuan Guangyuan: youth contribution write a new era struggle a new era of Sichuan Guangyuan: youth contribution new era struggle wei correspondent Cai Min) over the years, Sichuan Guangyuan municipal party committee always keep in mind that unswervingly follow the party, unremitting struggle for the cause of the party and the people and mission, solidarity led Guangyuan youth charge, bear as, in leading ideological and political construction youth, service youth entrepreneurship, win poverty engines, in rural revitalization, fighting COVID-19 youth sweat, with golden years writing youth contribution great chapter. For the 87,839 Communist Youth League members in Guangyuan, "seize the day and live up to the time" is their pronoun. It is their mission and responsibility to strive to write the chapter of youth without regret. Run, teenagers- -to love to help grow up in the sun, should have fire in the heart, eyes have light, warm and bright; should be riding a horse whip, dream distance, independent and strong. All these good things are inseparable from the guidance and infiltration of thought, morality and culture. For a long time, the Guangyuan municipal party committee adhere to do the party's loyal assistant, deepen the youth ideological and political lead, guide the youth tree prison "four" consciousness "four," four confidence "," two maintenance ", extensive" my youth my dream "" learning message spirit, show youth bear "" bloom war epidemic youth firm system confidence "" one hundred party history every day " and other theme activities more than 1400 games. Vigorously promote the "voice of the youth" and the key work of the league, "youth voice" construction work was "central league recognition as" advanced typical league organization "; further promote" youth learning " online theme league class learning, online more than 8 million online learning, the study of the top in the province, around the party history of three hundred activities, more than 200,000 teenagers online and offline party history learning education activities nearly 800 times. With the rapid development of economy and society, more and more people choose to work out for employment, and the problem of left-behind children becomes increasingly apparent. To this end, the Youth League Guangyuan Municipal Party Committee vigorously implemented the "Huiai Home" left-behind students home project, continue to carry out the "Child Partner Plan", "Happy School", "Toy Story" and other brand care projects, to achieve scientific and normal services for key youth groups. Up to now, the city has established 64 "children's companion homes", carrying out regular activities such as academic counseling, family companionship, and self-care education, and directly serving more than 2,000 left-behind children in rural areas, and playing a positive role in caring for and protecting left-behind children in rural areas and helping rural revitalization. In order to implement the municipal Party committee to build Guangyuan high-quality development talent engine, implement the talent gathering project, retain young people and take root in Guangyuan concept. Group Guangyuan municipal party committee adhere to youth demand oriented, focus on social integration, employment, dating, deepen development "community mutual aid plan", "green love plan", "dream plan" and other brand work, normalized community parent-child, elderly service activities, "gather talent edge is you" dating fellowship activities and "dream plan" "home" college students' social practice. In recent years, it has carried out more than 10 dating activities, served more than 3,000 young people of the appropriate age, and raised more than 7,000 internship positions for college students. The Cangxi Youth League County Party Committee was praised by the 2021 national college students' "return to their hometown" social practice activities. Li Jun, a young man born in the 1980s, gave up a high-paying job in the city and returned to his hometown to start his own business. He not only helped the villagers to get rich out of poverty, but also told the Chinese people about his experience in helping farmers as a deputy to the National People's Congress. All these, cannot be separated from the Youth League Guangyuan Municipal Party Committee behind the silent support and help. In 2017, Li Jun, as the leader of rural youth entrepreneurship and prosperity, won the "National Poverty Alleviation Award"; in 2021, Li Jun and Zhao Hailing won the title of "National Advanced Individual in Poverty Alleviation" at the National Poverty Alleviation Summary and Commendation Conference. Since 2017, the Youth Guangyuan Municipal Party Committee has successively launched the "Youth Innovation Plan" to serve the needs of youth innovation and entrepreneurship. In just two years, the Youth Guangyuan Municipal Party Committee has leveraged social capital of nearly 10 million yuan to consolidate and upgrade the Guangyuan Youth Mass Innovation Space. So far, 57 projects have settled in the Youth Innovation Park. Strive for the provincial youth venture capital of nearly 18.9 million yuan, to support 225 young entrepreneurs, the capital arrival rate ranked among the best in the province. Assisted returning entrepreneurial youth to establish Guangyuan seedplanting and breeding professional cooperative, and Shanghai Fudan University and other universities and postal bank Shanghai branch and other enterprises for cooperation, signed a purchase agreement of more than 3 million yuan. In 2021, while supporting the youth innovation and entrepreneurship, will focus on help rural revitalization, and quickly signed youth help rural revitalization "cloud plan", invested more than 1 billion flow resources, support Guangyuan hundred rural "leading goose", "rural electric trademark", more than 1 million fans new farmers; launched "Shu Qing revitalization loan", "post" first credit youth entrepreneurship fund 2.5 billion yuan, mobilize and guide the young volunteers to participate in rural revitalization. Stars do not ask the traveler, time pays off. In the past five years, Guangyuan Youth Entrepreneurship Industrial Park has absorbed 278 youth entrepreneurship projects, incubated 220 enterprises in the park, and created more than 4,480 people, contributing its youth strength to the consolidation and expansion of the achievements of poverty alleviation and rural revitalization."I am the most beautiful boy in this street and this street..." On April 30, Gu Ruining, a volunteer, led the children in the hottest dance at the children's companion home in Jiepai Village. The excited smiling faces of the children explained the love and trust of the "volunteers". At the age of 17, Gu Ruining is only a microcosm of the tens of thousands of young volunteers in Guangyuan city. Over the years, a total of 202,000 young volunteers have been registered in Guangyuan city. In Guangyuan, whether it is on the activity site or on the epidemic prevention front line, in the fields or in schools and enterprises, young volunteers can always be captured, and they have become the most brilliant presence among the crowd. They adhere to the concept of serving the overall work of the party and government center with voluntary service, To carry out the volunteer service in front of the Guangyuan Station of the West-Chengdu High-speed Railway and the preparatory service for the 13th Provincial Games; they actively participate in the epidemic prevention and control, Establish 80 "youth commandos", Scientific and orderly visits, epidemic prevention and publicity, temperature measurement and other epidemic prevention and control work; they actively help the resumption of work and production, Many times to the railway station for Guangyuan to go to Zhejiang Jiashan and other places for migrant workers to provide station services; they extensively organize and guide the league affiliated social organizations, youth volunteers to participate in the youth volunteer "love in the community", "beautiful in the countryside", the "youth volunteer", "love" in the journey " theme volunteer services;... up to now, Guangyuan city has registered 1,800 volunteer service teams through the "volunteer Sichuan", The cumulative volunteer service duration of 1.174 million hours, It has benefited more than 2.7 million people. Short steps, not a thousand miles; not small streams, not into rivers and seas. Next, group Guangyuan municipal party committee will adhere to the "grassroots", strengthen the construction of member team advanced nature, leading the Guangyuan youth and the communist youth league mass development, around the eighth party congress proposed "four big economy" "four big projects", in-depth implementation of youth work "difference" action, organization to mobilize the youth to speed up the construction of Sichuan modern youth in the modern youth sweat, reveal youth bear, with excellent performance to meet the victory of the twelfth party congress.

2022-05-07 Shandong: 8 city youth development friendly city construction plan officially released Shandong: 8 city youth development friendly city construction plan officially released network reporter XingTing on May 5, Shandong province youth work joint conference plenary meeting held, approved and officially released Jinan, Qingdao, Zibo, Jining, Weihai, Rizhao, Linyi, Liaocheng 8 city youth development friendly city construction plan, which marks the youth development friendly city construction work into a new stage of "further implementation". This is another "major action" after Shandong took the lead in launching the provincial youth development-friendly city construction in October 2021. The State Council Information Office recently held a press conference on the white paper "Chinese Youth in the New Era", which specifically mentioned the exploration and practice of building Shandong province into a youth-development province, laying the foundation for the national deployment of this work. Data show that the urbanization rate of China's young permanent urban residents has been significantly higher than that of the total permanent urban population, reaching 71.1%. Building a youth-friendly city plays an important role in promoting youth development, attracting young talents, stimulating urban vitality and enhancing the city's taste."Embrace urban renewal help youth development" in 2022 Qingdao urban renewal and urban construction youth BBS successfully held in Shandong will the work in a more important position to grasp, as the further implementation of xi general secretary about youth work important thought, promote economic and social development innovation, promote the development of high quality, centered on the people of the development of specific measures, is committed to promote the further implementation of long-term youth development planning. Since the beginning of the year, Shandong has made great efforts to promote the "ten innovations", put innovation at the core of the overall development position, and continuously enhance the innovation power of economic and social development. This not only provides a broader stage for young people, but also gives them greater responsibility and mission. As a new force for economic and social development and innovation, caring for and supporting youth development means to enhance the vitality of urban development and accumulate the sustainability of urban development. Jinan city organized the theme salon of "youth love China-fashion" youth-friendly city. In a certain sense, Shandong province to promote the construction of youth-friendly city is not only the result of urban development, but also the expectation of youth. At present, with the increasingly fierce social competition, young people are facing multiple pressures, such as graduation job hunting, housing, innovation and entrepreneurship, social integration, dating, support for the elderly, children's education, resulting in the youth "lie flat", "Buddha department" and other prominent problems. It is the purpose of solving the "urgent and anxious" problem that affects the growth and development of the youth and distributes the work energy, which is the meaning of building a youth development friendly city. Shandong tries to clear away the "obstacles" and "stumbling block" to the development of the youth, so that the city can gather more young talents, stimulate its innovation and competitiveness, make the city "young", and make the youth "more brilliant". It is a systematic project to build a youth-friendly city, which needs to unite all parties and form joint forces. The Shandong Provincial annual mobilization meeting held at the beginning of this year particularly stressed that the "construction of a whole-chain support mechanism for the growth of young people", and the concept of promoting youth priority development has become a social consensus. Centering on this focus, Shandong takes advantage of the trend and is systematically designed, responding to the new era with a new answer paper, and gathering a strong impetus for building a youth-friendly city. Weihai port area to build young talent apartment, further attract talents, retain talents by grabbing early city, county two levels of party congress and "two sessions" intensive favorable opportunity to promote Shandong youth friendly city construction around the party report and the government work report, has covered the province 16 cities, 102 counties (city, area)."Youth development" has become a high-frequency term for local Party congresses and people's congresses, and the working pattern of "Party committee leadership, government leadership, and all parties to jointly manage youth development affairs" has become clearer. At the same time, in order to further clarify the construction ideas, Shandong gave full play to the important role of the youth work joint conference, formulated the index system and operating guidelines, and standardized the working mechanism and working process. Since the launch of this work, it has received positive response from party committees and governments at all levels, paid unprecedented attention to the construction of youth development-friendly cities, and actively applied for innovation. Rizhao city in 2022 special youth recruitment and ten key industrial chain ZhaoCaiYinZhi cloud hire month activities launched, Jinan, Qingdao, Zibo, Jining, Weihai, Rizhao, Linyi, Liaocheng 8 city have established experts and scholars, research institutions, party and government functional departments, young masses, citizens, people's congress, CPPCC involved in special research group, the largest listen to opinions from all walks of life Suggestions. Local governments have strengthened policy advocacy and social advocacy, and launched a series of policy measures and practical projects tailored to young people. Up to now, all cities in Shandong have issued 109 policies related to youth development and 202 at the county level, effectively improving young people's sense of gain, belonging, happiness and security. Based on the reality, the economic foundation, resource endowment, population size and development characteristics of different cities are different, and the growth path and development needs of the young people themselves are also different. In the specific work, Shandong insists on the actual start, no "standard answer", no "fixed template". The Shandong Provincial Youth Work Joint Conference Office only provides reference indicators to guide all localities to determine construction goals and formulate construction plans and index systems according to local conditions, so that the concept of youth priority development can have more appropriate programs and richer connotations in different cities. Shandong takes policy service as the main direction and an important point of force, and aims to provide the most fundamental and effective service for youth development. We will accurately focus on the employment services, entrepreneurial environment, housing security, education and health and other key areas that are widely concerned by the young people in the province, and take real measures to deliver the real policy dividends to the young people.for instance, Jinan issued 640 million yuan of rental subsidies to low-and middle-income young people; Qingdao city has included the improvement of youth living conditions and transportation environment optimization into the government's practical livelihood projects; Zibo city has issued 25 policies to build a youth entrepreneurship-friendly city; Weihai city supported the construction of 920 youth innovation platforms; In Donggang District of Rizhao City, 17 urban studies and 740 young talent apartments were built and opened..., a practical and effective policy measures, An intimate and warm service action, We have helped young people solve many "urgent and anxious" problems, Let the concept of youth priority development to manifest, We will strengthen the foundation for building youth-friendly cities. In order to ensure the implementation of the work, Shandong has defined the "responsible persons" and planned the "road map", so that the development of youth friendly city construction is more reliable and dependent. In terms of working mechanism, the municipal Party committees and governments play the role of the construction subject, including the overall planning and promotion of the work, through the municipal joint committee, the municipal standing Committee and the provincial joint committee, the construction cycle is not less than two years, continue to promote the normal construction, the provincial joint meeting for scientific and effective evaluation. In the next step, Shandong will do a solid job in the construction of the first batch of youth development-friendly cities and counties (cities, districts), to form an effective demonstration and influence. Promote provinces and counties level 3 youth work joint conference mechanism more perfect, promote more systematic, universality youth development policy, make youth more active in urban development, comprehensive deepening long-term youth development planning effect, promote the youth and urban creativity mutual agitation, youth and urban quality development two-way promotion.

2022-05-07 Chengdu, May 4) At 19:30 PM on May 4, a "Youth Dialogue" 2022 Chengdu Youth Theme song hosted by the Chengdu Communist Youth League Municipal Party Committee was held online. The song with the theme of "Youth Heart to the Party and Contribution to the New Era" revolves around three chapters: "Youth Dialogue Era", "Youth Dialogue world" and "Youth Dialogue to the Future". In Xinglong Lake Park, Dayun Village of Chengdu University, Dongan Lake Park, Kuanzhai Alley, Jiaozi Park, etc., more than 400 young teams and individuals sang more than 200 of their favorite songs. The song was broadcast live through more than 20 online platforms and Chengdu TV station, watched by more than 300,000 people, and 100,000 people participated in the interaction by sending bullet screens, forwarding updates and likes. The topic # Youth Dialogue Dialogue Youth attracted 2.2 million views. Live footage of the song club. The young people come from all walks of life. They integrate folk songs, rap, Acabella and other songs into the songs, using different forms of expression such as progressive style, scene style and immersion style. In the dialogue between singing and youth, the young people express their different selves. Leng Yuhang, the driver of the electric bus, said that we should more actively shoulder the mission and responsibility given to us by the "growing together between the youth and the city", and be a good guarantor of the urban rail transit operation. Huang Keyun, a young man in the new economy field, has strengthened the confidence of our young people in the technology industry by participating in the concert. By Xinglong Lake in Chengdu, Cao Yu, a graduate student of music major at Chengdu University, has a more intuitive feeling of "Park City": " Seeing Chengdu's openness and tolerance to us young people, especially' Rong Piao'youth, gives us the power to pursue our dreams in Chengdu. Xue Yuxin, singer of " 《's Most Beautiful Talent and head of the Music Treatment Society of Sichuan Conservatory of Music, said that as a young public welfare worker, he hopes that through this original song of caring for autistic people, more young people can pay attention to caring for vulnerable groups, participate in related public welfare undertakings together, and make the world full of love.

2022-05-04 Hubei Shiyan Memorial May 4th Theme Youth League Day and Youth Innovation and Entrepreneurship Carnival activities were held in Shiyan City to commemorate the 103rd anniversary of the May 4th Movement theme youth League Day and youth innovation and Entrepreneurship Carnival was held on month 4 (reporter Zhang Jianwei, correspondent He Yuguarong) recently, Sponsored by the Youth Shiyan Municipal Party Committee of Hubei Province, Wudang Mountain Airport Group Co., Ltd., Shiyan Radio and Television Station and other units co-organized the "Welcome the 20 always follow the Party to forge ahead new journey" Shiyan city to commemorate the 103rd anniversary of the May 4th Movement theme group day and youth innovation and entrepreneurship carnival held in the city media center, Standing Committee of Shiyan Municipal Committee and Minister of United Front Work, Chairman of the Municipal Federation of Trade Unions, Zhang Tao, Party Secretary of Zhang Bay District, attended the speech and announced the launch of the Youth Innovation and Entrepreneurship Carnival. Lu Xichang, deputy director of the Standing Committee of the Municipal People's Congress, Wang Xiao, deputy mayor of the municipal government, Li Xiang, vice chairman of the Municipal People's Political Consultative Conference, and nearly 200 leaders of related units and young representatives of various industries attended the meeting. Activity pictures. Group day with the "May fourth wave" historical scene began, the Chinese youth as a new social force on the stage of the history; then, the party flag leads the youth road first try the green screen synthesis technology, leading the city through the space tunnel of youth, with the May fourth movement youth students, new Chinese liberation army soldiers (communist youth league), the new era of grassroots youth league and contemporary youth identity time and space dialogue, review the Chinese youth movement under the leadership of the party; "Pass on Love" tells a touching story between the "Son of Shiyan," who has received financial support, and a female college student and the Project Hope.site of activity, Yunyang District single blood plasma collection station Co., Ltd. and other love enterprises donated 1 million yuan to the city hope office to subsidize the poor students in Shiyan, Continue to pass on the love; "Song of Youth Civilization" group image performance of Shiyan Dongfeng Motor, transportation, medical care, public security, electric power and other industries of love and dedication, hard work performance, Since the creation of Shiyan in 2001, There are 21 national youth civilization number, 183 provincial level, 612 municipal level; "The Banner of Young Volunteers floats up" with song and dance and video exhibition party members, youth, women and other 8 volunteer service corps, 702,000 registered volunteers in the creation of culture and health, poverty alleviation, helping the elderly and the young, large-scale competitions and other fields of dedication figure; "Leaving for Love" poems and pictures show four different types of young college students voluntarily return to build their hometown after graduation, Promote the retention (return) weir entrepreneurship and employment policy, And through the live broadcast to the city's youth issued a "struggle why go to the distance, hometown is a good place" strong call. Activity pictures. Correspondent for figure theme after the day, Shiyan youth innovation entrepreneurship carnival officially launched, "I choose Hubei interpreta dream car city" Shiyan ninth youth innovation entrepreneurship competition launch ceremony, Shiyan youth entrepreneurship pioneer training camp, Shiyan "green gen salon", college students leave yan employment entrepreneurship star campus action, college students "leave yan employment entrepreneurship experience day" activities held in the venue. The activity was broadcast live through the youth Hubei and Youth Shiyan video account, Shiyan Radio and Television, and Shiyan Daily client, and a total of 110,000 youth league members watched it online. Activity pictures. Correspondent for the picture

2022-05-03 Jincheng launched the 100th anniversary of the youth theme month activities, in the May 4th Youth Day approaching, the Jincheng Youth League Municipal Party Committee launched the youth theme month activities. It is reported that the youth theme month activities to "youth heart to the party, a hundred years of competitive youth" as the theme, including "lighthouse project" "strong foundation project" "model project" "pioneer project" four categories a total of 15 specific activities.among, The "Lighthouse Project" holds various thematic educational practice activities, celebrating the 100th anniversary of the league conference, and celebrating the 100th anniversary of the league symposium, Build the Communist Youth League history pavilion of Jincheng, release the theme song to welcome the 20th Party and celebrate the 100th anniversary of the founding of the Communist Youth League, "Youth and the Party set sail again", Constantly leading the thinking of the youth, Firm youth faith; "Strong Foundation Project" through the implementation of the young Marxist training project, to carry out the air youth League school activities, held the 2022 Jincheng Young Singer Competition, To fully serve the growth of young people, Raise the youth dreams; "Model project" to carry out the "new era of youth contribution" typical tree selection activities, Focus on displaying the spirit of Jincheng youth in the new era, And using the all-media platform, Multi-directional show the Jincheng Communist Youth League under the leadership of the Party to unite and lead the youth members to the new era of vivid practice; The "Pioneer Project" organized the theme activities of "Epidemic resistance war, the frontline regiment flag red" and the youth Civilization Open Week activities, Build a youth-themed bank, Carry out the youth contribution action and other activities, In leading, contacting and serving young people with more real measures, Make every effort to build a youth development policy system, Enhance the function of a youth development city, Establish a long-term mechanism to serve the youth growth, With practical actions, Promote urban development.(Reporter Zheng Lu)

2022-05-01 Chengdu, Sichuan province held the China Space Day Theme activity to Light up the Dream " theme activity was held in Chengdu, Sichuan Province. Event, Sichuan space system engineering institute narrator for build three innings southwest company subway line 27 project builders and build the second generation system in our country, starting from two satellite project gradually developed to nuclear bombs, missiles, rockets, earth satellite, manned space, deep space exploration and other major fields of development and achievements, fully embodies the China's space industry from scratch, from weak to strong development. The event site also has a space capsule immersive experience and VR space panoramic experience links. The four-day exhibition, with an average daily reception of about 1,200 people, is expected to receive more than 5,000 people. A total of more than 30 primary and secondary schools organize students to make reservations. The event was jointly organized by the Longquanyi District Committee of Chengdu City, Sichuan Province, the Party Committee of the Dongfang Sub-district Government and the Sichuan Aerospace System Engineering Research Institute, and undertaken by the Dongan Lake Sports Park Swimming Stadium Center, the Southwest Company of China Construction Third Bureau and Beijing Aixingke Aerospace Technology Co., LTD.

2022-04-30 Shandong Zibo communist youth league held league history knowledge contest Zibo communist youth league held league history knowledge contest XingTing) on April 19 solstice 25, Zibo municipal party committee joint public network to carry out the challenge "forge ahead keep beginner's mind youth gift 20" theme history knowledge contest, 7 days cumulative participation more than 1.26 million, wide coverage, high participation, cause good social response. The league history knowledge competition is carried out by using online answers. TuanZibo Municipal Party Committee has carefully designed the competition platform, including four sections: learning question bank, simulation practice, formal answer, and personal center, and set up a special team to compile the competition question bank. After the activity, all the participants will win the first, second and third prizes and the electronic certificate of excellence award according to the answer results. Group Zibo municipal party committee secretary xu qiang, said to carry out the group history knowledge contest, Zibo municipal party committee learning way innovation, platform development attentively, mobilization heart, launched the city's youth league organizations at all levels as the branch collective participation, concentrated competition, through all levels of new media platform and all kinds of media publicity, and by issuing daily reminder, real-time scheduling entry, regularly announced participation, strengthen the positive incentives. After the activity, the Communist Youth League Zibo Municipal Party Committee will commend a number of excellent organizations, and hold the offline challenge of "Youth League History Knowledge Learning Talent" according to the situation.

2022-04-28 Guangxi guests youth civilization collective field visit research activities in Guangxi guests communist youth league collective youth civilization to visit the group research activities, the Laibin city public security bureau traffic police detachment a brigade, xingbin district people's procuratorate office, Laibin city museum club department six units youth civilization collective, creating youth civilization collective to carry out the field visit investigation. The working group through field visits, listen to reports, access to stand books, discussion exchanges and other ways to in-depth understanding of the youth civilization collective creation work, to the performance of the collective affirmation, and visit the problems found in the process of on-site guidance. Working group said that all levels of the youth civilization collective, striving for youth civilization collective around the central work, find work fulcrum, play the role of youth groups, promote the systematization, standardization, standardization of youth civilization work, to further enhance the youth sense of belonging, honor, responsibility, give full play to the demonstration role, establish the civilization image, carry out quality service, build service brand, to the construction of a new era of socialism with Chinese characteristics magnificent Guangxi guests new chapter contribution youth strength.

2022-04-27 Xing Ting) On the morning of April 24th, the activity of "Building a Dream" - -I will grow together with entrepreneurs was launched. The activity was jointly organized by Qingdao Municipal Party Committee of the Communist Youth League, Qingdao Municipal Market Supervision Bureau, Qingdao Municipal Human Resources and Social Security Bureau, Qingdao Municipal Private Economy Bureau and Qingdao Municipal Private Individual Economy Association. At the launch ceremony, the representatives of young entrepreneurs issued the initiative of "forging ahead on a new journey to contribute to the new era" to the young people and all sectors of society in the city. Three representative representatives of young entrepreneurs made speeches on their typical deeds. The scene also held a "growth with the youth youth business, benefit the enterprise service volunteer group" flag ceremony. This activity launched 35 outstanding young entrepreneurs, entrepreneurs, from different industries and different fields, their typical entrepreneurial success stories are compiled under the title of "Striver". Next, Qingdao will release 17 measures to empower young entrepreneurs' entrepreneurial development, through close cooperation between departments, implement the "consultation" supervision, and create the "first lesson of entrepreneurship", and better promote the development of young entrepreneurs. At the same time, through the establishment of the "young entrepreneurs entrepreneurship exchange service platform", to carry out policy push, legal rights protection, talent exchange and other work.

2022-04-26 The Xingtai Municipal Party Committee of Hebei Province has launched a reading activity themed "Enjoy the youth and moisten the heart". In order to ensure the effect of the reading theme activity, the Xingtai Municipal Party Committee of the Communist Youth League launched a series of "cloud" activities such as "one million young people read a book together", "100 good books recommend youth" and "one million young people recommend good books" through the new media platform. Through these online activities, teenagers are encouraged to read selected chapters of classic books, "cloud relay" to share their reading experience, and recommend "100 good books" to teenagers, and encourage them to recommend a good book to carry out extensive reading. At the same time, the youth league organizations at all levels also rely on around the "city study" reading positions, build youth reading brand, establish "youth reading club", create new youth reading service mode, provide convenient, efficient, high quality diversified reading service platform, encourage youth league organizations at all levels according to the actual situation of reading positions, play to the characteristics of the communist youth league, carry out normalized reading activities, select "reading promoter" every month. Up to now, more than 190,000 people have participated in the "One Million Youth Reading", and the online activity of "One Million Young People Read a Book" has reached more than 110,000 people, with 3,567 messages. This activity aims to create an atmosphere of "love reading, reading good books and being good at reading" among teenagers, guide and encourage young people to develop reading habits, arouse repercussions among teenagers, and set off a reading boom among young people.

2022-04-26 Qingyang, April 26 (reporter Li Chuan) In order to further promote the "rural revitalization youth contribution" action, Fully implement the "youth xinglong" education project, Give full play to the advantages of the Communist Youth League in organizational, cultural, practical and service education, Deepening the results of the "I Do something for the Youth" practice activity, Strive to cultivate a team of outstanding young talents in the field of agriculture, rural areas and farmers, recently, The Youth League Qingyang Municipal Party Committee of Gansu Province and Qingyang Puji Health Consulting Service Co., Ltd. held the city's Communist Youth League "Build a Beautiful Home" youth action 2021 outstanding youth public welfare physical examination and 2022 launch ceremony, More than 30 representatives of the municipal "good youth" and the 2021 municipal "Clean and Civilized Star" demonstration households and young volunteers participated in the activity. Public welfare physical examination site. It is reported that the Qingyang City "Building a Beautiful Home" youth action is an effective carrier for the Qingyang Communist Youth League organization to lead the youth in all fields to participate in the revitalization of rural industries and the construction of rural customs and civilization. Since 2020, a total of 185 youth volunteer service activities (times), participate in youth 14896 people (time), normalized selection tree in the city "village have good youth" and the municipal "star" clean civilization " a model household 2000, got the social from all walks of life of aspiring youth and social organizations, build the whole society youth common concern, jointly help the rural revitalization of the good atmosphere. This public welfare physical examination sponsored 1,500 free outstanding rural youth for 1,000 yuan each. The activity was officially launched on April 19th and completed by the end of September.

2022-04-24 Hunan Xiangtan youth volunteers to the countryside to help spring farming fertile field surging youth power to seize the spring time Hunan Xiangtan youth volunteers to help spring ploughing Jianwei correspondent Zhang Heng Yang Jian) thank the young volunteers to help, this 'iron cow' can work in the field again."On April 20, Hunan XEMGroup motor test chief operator Luo Weiping and two colleagues came to Shebu Village, Shebu Town, Xiangtan County, free for the grain farmer Liu Shunliang maintenance rotary tiller. Find the fault, change parts, on the oil... less than an hour, after the maintenance of the rotary tiller again in the farm and happily in the run. The picture shows the maintenance machine. At the critical season for early rice transplanting, a group of young volunteers in red vests rolled up their trousers into the field to help farmers plant rice seedlings and throw rice seedlings. In another field, young farm operators skillfully operate farm machinery to plow the land. Youth League Xiangtan Municipal Party Committee related person in charge of the group introduced, more than 100 young volunteers to help the village to complete 300 mu of farmland land rotary tillage, transplanting, throwing seedlings operations. Help spring ploughing pictures. At the beginning of this year, the Xiangtan Municipal Party Committee of the Communist Youth League, together with the Xiangtan City Bureau of Agriculture and Rural Affairs, organized the establishment of the city, county, township "help spring ploughing preparation" youth volunteer service team, youth commandos. At present, the city has set up 681 youth volunteer service teams and youth commandos, with a total of 3,145 volunteers to participate in the policy delivery, science and technology delivery, agricultural materials delivery to the countryside, and services delivery to the countryside and other volunteer services.

2022-04-24 Rizhao Youth Development of Friendly City was held. Correspondent for figure China youth network Beijing on April 24 (reporter Ji'an-wei zhang, correspondent Xiang Changhui) recently, Rizhao city youth development friendly city to create work advance meeting, pku deputy secretary Li Wanying attended the meeting and speech, the county league (work) committee responsible for comrades, pku organs staff to attend the meeting. At the meeting, Donggang District made an exchange speech on building a friendly youth development city. Li Wanying arranged the next step of the city to create a youth-friendly city. First, to further improve the understanding of the work. The district and county youth league (work) committee should fully understand the necessity and importance of the construction work, take the initiative to report to the main leaders and leaders in charge of the district and county, and actively strive for the support and guidance of the party and the government. We will improve the system of youth joint meetings, timely adjust and supplement the member units, strengthen communication with the member units of the joint meetings, form joint efforts, and collectively study the policies and measures that benefit all aspects of youth work and life. Second, the further compaction of the work responsibility. Counties league (work) committee in further study of the communist youth league central 17 departments jointly issued on the opinions of the pilot, on the basis of the provincial "guidance" and the city upcoming related documents, as a whole regional economic and social development and youth group characteristics of the pilot, issued as soon as possible "implementation plan" as soon as possible. Third, we will further focus on our work priorities. Focus on urban employment quality, innovative entrepreneurial vitality, housing security level of youth "anxious sorrow" 10 aspects of key issues, focus on the characteristics of youth, youth demand, youth experience, with reference to the provincial index system, swallow policy as soon as possible, implement innovation projects, strive to improve the quality of urban function and youth fit, make the city more friendly to youth, more youth in the city. Next, the Communist Youth League Municipal Party Committee will further improve the working mechanism according to the relevant requirements of the Shandong Provincial Youth Work Joint Conference, prepare for the 2022 Municipal Youth Work Joint Conference, and promote the implementation of the creation of the work. Focusing on major events and serving the overall situation, we will promote the introduction of favorable policies and projects to serve the youth development, optimize the working, living and social environment of young people in cities, and make comprehensive efforts to attract talents, boost innovation and entrepreneurship, and stimulate urban vitality, so that more young people can choose Rizhao, start a business sunshine and take root in Rizhao.

2022-04-22 Jinan, Shandong Province, launched on April 20. The plan focuses on three key links to launch 10 hardcore measures to escort youth innovation and entrepreneurship in the face of the epidemic. According to the report, the plan was jointly launched by the Communist Youth League Jinan Municipal Taxation Committee, the State Administration of Taxation, the Jinan Municipal Young Entrepreneurs Association and the Jinan Municipal Student Federation. In terms of implementing tax benefits, according to the plan, Jinan will establish a two-way and accurate docking mechanism and the "grid" policy implementation mechanism; people will change "find policy" to "policy search", "grid workers" will use the golden Tax Phase III information system and the big data of the incubation center to accurately locate the entrepreneurs of the tax incentives; set up "double innovation tax service station" and through "cloud live" and "point to point" publicity, to ensure that the tax dividend will benefit the young entrepreneurs. In terms of solving practical problems, the plan focuses on deepening the cooperation between government, bank and enterprise, expanding the scope of tax credit loan application, solving the financing problems of small and micro enterprises; exploring the distribution service of "Qingchuang Bank", jointly launching the "Quancheng Green Business Inclusive Loan" with the Bank of Beijing, and increasing the loan support for start-ups under the epidemic situation. At the same time, tax big data as the medium to match start-ups and promote the connection between supply and demand in accordance with the market-oriented principles. We will support districts and counties in working with universities, research institutes, and well-known companies in building innovative buildings, entrepreneurial blocks, and maker Spaces, and provide at least 10,000 square meters of free space and 1,000 work stations for young entrepreneurs every year. In terms of expanding building platform, Jinan will set up "youth double gen service alliance", relying on the city's youth work joint conference mechanism, to provide one-stop, intensive "package service" for entrepreneurial youth, and integrate county resources, perfect a batch of incubators, accelerator, the space, entrepreneurship park and other youth double gen positions. In addition, it also includes the establishment of a municipal youth entrepreneurship mentor group of 200 people in different industries, strengthen the training of entrepreneurial youth, park leaders and entrepreneurial teams, expand the "training base", provide more entrepreneurship training projects for college students, and promote the transformation of innovation and entrepreneurship achievements.

2022-04-20 Shiyan communist youth league build service youth new system help employment entrepreneurship, warm Shiyan youth league service youth system jianwei correspondent He Yu) since this year, the Shiyan municipal implement the Hubei provincial party committee, Shiyan municipal party government on youth innovation entrepreneurship, leave weir weir work decision deployment, adhere to the power center work, wholeheartedly service youth as the starting point and the foothold of all work, strengthen service consciousness, strengthen the service function, rich service means, expand services, improve the service system, closely around the "three industries" build new service system, boost youth grow up. Around the youth growth career demand down-to-earth "help employment" vigorously carry out "thousands of schools" online recruitment activities, from more than 160 enterprises and institutions for all kinds of employment positions, employment 3633 people, through WeChat group, public push, field visits and other forms, 10 county (city, area), youth corps committee, youth league organizations, youth civilization unit, 1085 jobs targeted to inform the corresponding groups, including rural family youth labor, veterans, big (in) college students, etc. Organize the "take you into the enterprise" activity, organize college students to visit local quality enterprises by type, specialty and batch; invite relevant experts and "top ten employment and entrepreneurship stars" to publicize the economic and social development achievements, ecological civilization construction achievements and urban development prospects in Shiyan; organize the "visit home" experience activity to feel the hometown development and change; set up employment guidance team, youth career planning and entrepreneurship mentor group to publicize the employment and entrepreneurship policy in various universities. Around the youth realize self life value needs "help entrepreneurship" organization of Shiyan ninth youth innovation entrepreneurship competition series of activities, launched in Shiyan independent enterprises, individual management or engaged in agricultural cooperatives, and registered youth entrepreneurship projects and obtain industrial and commercial business license, finally select the national industrial policy, technical requirements, good market prospects, with employment ability 10 projects to give key support, provide entrepreneurship training, business incubation, project financing and policy consultation services; held Shiyan "green salon" series of activities. Each issue focuses on specific themes, with effective problem solving orientation, and regularly invites well-known investors, enterprise managers and young entrepreneurs to collide with ideas and inspire wisdom; holding "Shiyan Young Entrepreneurship Pioneer Training Camp". To recruit 50 young makers from the city, and invite senior lecturers in the industry to improve students' thinking and operation ability through experiential teaching such as team cohesion, outdoor expansion, sand table drill, investment and financing sharing and exchange meetings, and implement the "youth Innovation Loan" financial support project. By April 8,2022, a total of 340 loans, the amount of 71.91 million yuan, effectively solve the "financing difficulties" that entrepreneurs strongly reflect, was warmly welcomed by the majority of entrepreneurs and highly concerned by all sectors of society; promote the provincial "youth innovation park" pilot construction work. To strive for financial and policy support, invite relevant parties to work in the pilot youth innovation park, touch the facts, listen to ideas, make suggestions, help optimize the construction planning, solve the construction difficulties, and speed up the construction progress. Around the youth pursuit of happy family life needs coagulation hearts meet force "warm family" group Shiyan municipal party committee attaches great importance to the youth dating work, the youth dating activities as an important measure to care for youth growth, held online and offline public friendship activities more than 30 games, service youth nearly ten thousand people, help nearly hundred couples into the marriage hall. Timely collect and sort out policies on talents, taxation, supporting the elderly and children's education benefiting young people, publicize and push them to enjoy various preferential policies in time, relieve the pressure on the support of the elderly and children's education, and build a beautiful and happy family for young people. To promote college students to stay (back) weir work, actively strive for "college students in Hubei province volunteer service rural revitalization plan" in Shiyan, relying on the central, provincial committee platform to recruit 200 college students volunteers, sent to Shiyan township and the following service positions to carry out a 2-year volunteer service, to carry out youth public welfare activities as the integration of rural revitalization, for rural revitalization strategy training reserve young talents, boost young talent stay weir family employment. Next, pku will fully around the youth characteristics and interests, focus on strengthening youth ideological and political lead, service youth growth, pay attention to youth development rights and interests, create a good atmosphere for youth development pays special attention to the service youth work, the youth development system advantage into governance efficiency, in Shiyan construction in western Hubei province regional central city process better play to the role of youth powerhouse and commandos.

2022-04-25 Zaozhuang in Shandong province municipal party committee play "combination" power enterprises to return to work and production of Zaozhuang municipal party committee play "combination" power enterprises to return to work and production built wei) for the further implementation of the city overall new kou pneumonia epidemic prevention and control and economic and social development work conference spirit, Zaozhuang in Shandong province municipal party committee rapid response, proactive, to city youth chamber of commerce member enterprises and rural good youth visit work as the carrier, innovative methods, youth strength, to play power enterprises to return to work and production "combination"."Accurate" the word first, overall planning and linkage. After the lifting of the epidemic control instructions, cadres at all levels were immediately organized to visit key projects, member enterprises of youth chambers of commerce and business incubators. City, district (city) youth corps committee leadership in accordance with the principle of dependency, deep enterprise line, around the enterprise financing credit, hiring, logistics, staff auxiliary, volunteer service demand, find out base, establish the enterprise development problem parameter, inventory, targeted plan, to the communist youth league "six credit" financial map, "bird" plan "talent introduction," production season "match," volunteers "volunteer alliance" volunteer service, "date can" quality development, "green action" skills promotion business work as the breakthrough point, dig resources, accurate matching. Up to now, the city's youth league cadres at all levels have visited a total of 51 enterprises, sorted out 27 problems, 9 problems have been solved, and 18 problems are being solved in an orderly manner."Real" word for this, heavy quality and effect. The "Communist Youth League Zaozhuang Municipal Party Committee to carry out the" Great Study, Great Improvement and Great Implementation "action Plan" was issued, focusing on the ideological ice-breaking, effective implementation, work style construction, requiring the city's Communist Youth League cadres to resolutely be the party's assistant and reserve army with the blade inward and self-revolutionary attitude. In the face of the demands of enterprises' resumption of work and production, youth league organizations at all levels have taken real measures to actively solve problems and difficulties for enterprises. In view of the capital shortage of enterprises, vigorously promote "Lu Qing Hui enterprise loan", "Lu Qing farmers bear loan", "rural good youth loan" project, the cumulative low interest rate loan of 20.1 million yuan to 11 enterprises; during the epidemic prevention and control problem, timely assist the enterprise to purchase materials nearby, ensure the completion of the enterprise to attract talent problem, continue to develop " green bird plan? Date gather talent "recruitment activity, a total of more than 7000 positions in short supply, attracting more than 100,000 college students to watch online, more than 2000 college students into the enterprise online communication room," cloud " to see the development of the enterprise, employment intention reached 153 people, 169 people signed employment. In view of the problems of enterprise skills training, we have cooperated with Zhongzhong Education Zaozhuang Branch to carry out the 2022 public welfare training activities for enterprises in the whole city. Up to now, 301 registration information have been received."Fast" the word is to want, continue to send force. To fully mobilize the window unit youth in power enterprise to return to work and production when international vanguard, pku joint municipal straight committee, municipal administrative bureau, municipal examination and approval, municipal public security bureau, city revenue, silver circ, city power supply company to carry out the "I write youth" theme practice, around "green agent", service "project", promote "cloud service", training "strong skills" and other six practical measures, build "project, team specialization, service informatization" youth service team, to save enterprise time investment, improve for enterprise service efficiency. Youth organizations at all levels in the city have set up 89 young volunteer teams for epidemic prevention in enterprises to conduct comprehensive elimination of key enterprises, timely investigation of epidemic prevention and safety hazards, and help enterprises meet the requirements of resuming work and production as soon as possible. A mental health service team of 22 people was set up, relying on the rapid and practical flat working mechanism, to help young workers to quickly invest in the "acceleration mode" of enterprise resumption of work and production with an optimistic, firm and positive attitude. In the next step, on the premise of the epidemic prevention and control work, the Communist Youth League Municipal Committee will make every effort to serve all kinds of enterprises in the resumption of production and work, and to make concerted efforts to achieve both the epidemic prevention situation and stabilize the economy.

2022-04-19 The Chengde Intermediate People's Court of Chengde City and the Education Bureau of Chengde City held a press conference on the work of "one school, one Judge". Before the meeting, the Chengde Municipal Party Committee of the Communist Youth League, the Municipal Intermediate People's Court, the Municipal Education Bureau and the Municipal Youth Working Committee jointly issued the Notice on Promoting the Working Mechanism of "One School, One Judge", and fully implemented the "one school, one judge" system in colleges and secondary schools in Chengde. Chengde Municipal Party Committee selects professional team, integrate working force, innovate work carrier, clarify responsibilities, and develop the "task book", build the juvenile crime "firewall"; start the "training camp", innovate the work carrier, build the juvenile legal education "gas station", and put forward a series of detailed measures and clear requirements for promoting the working mechanism of "one school and one judge". Next, Tuan Chengde Municipal Committee will further strengthen the communication and cooperation with Chengde Intermediate People's Court and Chengde Education Bureau, and intensify efforts to create a new pattern of youth publicity and rights protection, expand the new path of "comprehensive guidance and classified guidance", and strive to build a new mode of youth publicity and rights protection with "two-way force and dual combat", striving to show greater responsibility, seek more achievements and create better achievements.

2022-04-19 Wuzhou Communist Youth League carried out team joint construction and co-education activities, and 30 Young Pioneers organized to carry out the pilot mechanism of joint construction and co-education. The types of league organizations include organs, enterprises, parks, secondary vocational schools, colleges and universities, etc. The Young Pioneers organizations take school brigades as the unit. In order to facilitate the development of the activities, the organizer took the nearest as the main principle, and finally determined the joint league organization and the young Pioneers organization, and combined with the characteristics of the league organization and the needs of the young Pioneers organization, jointly carry out the activities, to create the "one school, one product" young Pioneers work brand. Youth league organizations and young Pioneers organizations take the lead to carry out joint construction activities. The Youth League Committee of Wuzhou Branch of China Post Group and the Young Pioneers of Wuzhou Minzhu Road Primary School went to Wuzhou Special Education School together, Carry out the "carry forward Lei Feng spirit and strive to be a model in the new era" hand in hand activity; The Youth League Committee of CSGuijiang Shipbuilding Company and members of the Young Pioneers Brigade of Wuzhou Qianjian Primary School cleaned the campus; Wuzhou Maritime Safety Administration Committee of the Youth League explained water traffic safety and safety, drowning prevention knowledge to the young Pioneers of Xinxing Second Road Primary School, and took the young Pioneers to experience daily work; Wuzhou Circular Economy Industrial Park Youth League Working Committee and Wuzhou Longwei No.1 Experimental Primary School in Century Square, Longpolder District, The " charity sale, Happy sharing " vegetable charity sale activities; The Youth League Committee of Guangxi Industrial and Commercial Technician College and the Young Pioneers of Wuzhou Longwei Second Experimental Primary School, Jointly carry out the special children care activity with the theme of "team education together for the future".

2022-04-19 Group Zibo municipal party committee continue to carry out the "cloud recruitment" power enterprises to return to work and production of the communist youth league of Shandong Zibo municipal party committee continues to carry out the "cloud recruitment" power enterprises to return to work and production XingTing) recently, the communist youth league of Shandong Zibo municipal party committee actively play the advantages of the communist youth league organization, continue to carry out the "cloud recruitment" activities, to assist enterprises to solve the return to work and production stage of choose and employ persons tension, power enterprises to return to work and production. It is understood that the Tuanbo Zibo Municipal Party Committee solicited 750 high-quality positions (including 9,853 positions) from enterprises through visits, wechat, email and telephone inquiries. At the same time, through the "Green Bird Plan" small program, wechat public account and other forms of pushing the job demand plan to college students, attracting more than 50,000 college students to watch online, thus building the "cloud recruitment" supply and demand mechanism. At the same time, the group Zibo municipal party committee pay attention to perfect the "cloud recruitment" signing system, build "resume review-enterprise selection-online interview-online signing" four-step signing system, relying on the mic form, "green bird plan" platform collection resume, enterprise according to the resume online interview, has reached employment internship intention of more than 1300 people. In addition, TuanZibo Municipal Party Committee strives to optimize the follow-up service of "cloud recruitment", timely visits the employment and employment effect of students after "cloud recruitment", and tracks the problems at any time, so as to ensure the "whole-process" service. In view of the subsequent understanding of technical problems and the shortage of blue-collar technicians, Zibo municipal committee actively docking more than 20 universities, Shandong University, the Chinese Academy of Sciences space application center, national nano science center of more than 10 Zibo young talents, solve 25 micro and small enterprises 69 technical problems; contact Zibo job network docking city employment youth, recruit more than 800 blue-collar technicians for each enterprise. According to statistics, since this year, the Youth League Zibo Municipal Party Committee has held a total of 14 "cloud recruitment", reached more than 4,500 employment intentions, 587 employment contracts. In the next step, the TuanZibo Municipal Party Committee will continue to carry out the "cloud recruitment" activity. Through the "joint" mode of joint universities inside and outside the city, relevant departments and various recruitment websites, it will contribute to the city to attract talent and wisdom.

2022-04-18 The COVID-19 situation is gradually improving, and "Baymax" and "Red Vest" among the youth commandos are making their last resort. As a veteran who volunteered to support Wuhan in 2020 and Lanzhou Pulmonary Hospital in 2021, Zhang Ying, a young nurse, went to Lanzhou New Area, then the most severe city in Gansu Province, as the leader of the first medical support group of Lanzhou University First Hospital as early as March 8. Zhang Ying led the team members to complete the training and assessment, and deployed the division of work of the team members. She encouraged them with her rich experience and mental journey in fighting the epidemic. In the designated hospital, she even entered the isolation ward without hesitation, working hard, working day and night, until the successful completion of the tasks assigned by the hospital."As long as the country, our country needs it."Although she has elderly and sick in-laws and young children, and it is difficult to take care of her family because they are engaged in special industries, Zhang Ying believes that as a medical worker, they should go where patients need it most, which is the duty of a doctor. Since the outbreak of this round, Chai Wenbin, a worker of Lanzhou City Water Supply Group and a member of the Five Star Youth Volunteer Service Team, has not been idle. As soon as the epidemic prevention and control started, he put on his volunteer clothes and assisted the community to arrange the returning people to carry out the work of flow adjustment and bayonet guard. The Wuxingping Community of Xiyuan Street, where he lives, belongs to the Gaoping area, with a large number of residents living on the mountain. The medical staff walk to the nucleic acid testing points every day. In order to save the time and physical strength, Chai Wenbin used his private car to pick up the medical staff for free. Last year, when the epidemic occurred, Chai Wenbin led the charge, assisting in household nucleic acid sampling, material transportation, district inspection, and disinfection work in various residential buildings. In the face of a new round of epidemic this year, Chai Wenbin chose to set out without hesitation. In his opinion, he is not only a resident of the area, but also a Communist party member, and has the obligation to participate in the "war without smoke of gunpowder". Adhering to their posts, young people from all walks of life in Lanzhou write the story of the youth war "epidemic" with their responsibility and courage. Qilihe district triangle line community party secretary ma LAN and community party members and cadres fighting in the epidemic prevention and control line, not only the first time to build the community party branch, grid, grid, dean, party members, residents backbone linkage "six epidemic prevention" mode, also worry about the isolation personnel within its jurisdiction, for their distribution of the necessities of life, to solve all kinds of difficulties. One second still in reply to residents to resume work, the next second to provide psychological counseling for residents... eyes swollen, a hoarse voice, often disinfected hands also dry, these Malan do not care. She said that as the community secretary, the community is the position she firmly hold, must fight the spirit, overcome all difficulties."We're not afraid. we can do it! Although we cannot be at the forefront of epidemic prevention like medical personnel, we can protect the peace of the city in our own way."These days, Lanzhou Bus Group youth commando member Dai Haojun and colleagues often carry 20 kg of disinfection spray, uninterrupted disinfection around the station, inside and outside the compartment, armed waiting for medical workers, hotel control quarantine personnel; stick to the station, check the passenger health code, until the last passenger... in the face of the aggressive virus, galloping horse rental group Lei Feng team quickly organized the driver into epidemic security" attendant ", for the public travel, emergency car escort-24-7, point to point, on-call. A few days ago, some citizens expired after the quarantine period and lifted the sealing control. The group immediately sent 130 taxis to gather at the two isolation points to send the citizens to their homes safely, and reduced the fare of 3,175.6 yuan. To help the epidemic prevention and control, it requires the persistence of all walks of life. After receiving the task of cleaning the isolation point, Qilihe District Sanitation Service Center urgently dispatched more than 200 cleaning commandos, and it only took four days to complete the cleaning task of nearly 600 rooms in the six apartments unused for a long time."Epidemic" to spring warm, "clean" to return. After receiving the information from the disinfection unit, the Gansu Ark rescue volunteer service team set out for the first time, and launched an all-round disinfection operation in the roar of the fog machine. In many areas that are about to be unsealed, in recent days, there are busy elimination teams everywhere. The road is not lonely, why is the fear of wind and rain. During the current round of epidemic prevention and control period, there were 86 youth commandos and more than 3,400 young people in Lanzhou, who joined the battle against the "epidemic" and built a strong "protective wall" between the citizens and the virus. It was them who built the most beautiful scenery in Lanzhou this spring. China Youth Daily, April 18,2022, edition 02

2022-04-18 Guangdong Shenzhen Shenzhen first 20 list let volunteers everywhere by courtesy network reporter Wu Xin) recently, Shenzhen municipal party committee launched volunteer courtesy plan, and released the first 20 plan list, from health insurance, living consumption, public transportation, and other aspects for volunteers to provide more considerate, more thoughtful, more comprehensive service, let volunteers everywhere in Shenzhen by courtesy. In terms of insurance, Shenzhen Municipal Committee and Bank of China provide personal accident insurance for registered volunteers, Ping An Insurance, Tencent Micro insurance and other institutions to provide 80,000 free exclusive insurance quota for volunteers for epidemic prevention and control. In terms of credit investigation, volunteer service is listed as a personal credit score bonus point, and volunteers with good credit status are included in the credit "red list" to provide more convenient services for volunteers in administrative approval and other aspects. The Shenzhen Municipal Party Committee of the Communist Youth League also prepared performances for the volunteers, together with major theaters and concert halls in Shenzhen, to provide discount tickets, and hold a free special concert every year. In terms of transportation, Shenzhen Municipal Committee and Shenzhen Airport Group provide priority security channels for previous "100" volunteers, priority security and waiting service for previous "100" volunteers, Shenzhen Metro Group, 20 free bus cards for previous "100" volunteers, and Shenzhen Bus Group, RMB 50 for previous "100" volunteers. In terms of life, the Municipal Youth Development Foundation provides 200 hotel packages worth 1.0 million yuan, designs custom dresses for the "Best" volunteers, provides China Mobile, China Unicom and China Telecom, and gives a certain amount of phone fee. In terms of learning, the Shenzhen city joint Shenzhen book city, for successive "best" volunteers free 2000 total 100000 yuan worth of books, provide preferential benefits for volunteers, and the young volunteers into the "interpreta dream plan" funding, joint renmin university of China and other 21 domestic key universities, to provide young volunteers with high quality, low cost of continuing education. In order to better protect the health of volunteers, the Communist Youth League provides a batch of free health welfare packages (including 180,000 yuan) every year, providing free oral health check and exclusive special health welfare package, and free basic eye health examination for 500 volunteers. Not only is volunteers, volunteer children also have preferential treatment, the relevant controller of Shenzhen municipal party committee, the future will also hold "summer and summer vacation love guard" "winter camp" and other public welfare activities, for previous "best" volunteer children free escort and training services, free calligraphy education online courses, and provide volunteer children with "red scarf anchor group" special quota, organize volunteers to participate in spring outing, autumn, technology, art, red culture, traditional group building activities. As one of the cradles of volunteer service in mainland China, Shenzhen now has 2.8 million registered volunteers, who have become a beautiful scenery in the city's spiritual civilization construction and an important force in grassroots governance. Since the beginning of this year, a total of 126,500 volunteers in Shenzhen have participated in the epidemic prevention volunteer services, including 90,000 volunteer services, with 685,000 participants and more than 3.02 million hours. Volunteers responded overnight and went to the frontline, contributing to winning the battle against the epidemic.

2022-04-16 Shandong Liaocheng youth worker skills revitalization plan Shandong Liaocheng youth worker skills revitalization plan Cui Yan in the green newspaper network reporter XingTing) to continue to deepen the "youth achievements new Liaocheng" action, organize and mobilize the city's young workers based on post contribution, on April 14, Liaocheng city in Shandong province 2022 youth worker skills revitalization plan mobilization meeting and member youth commando training meeting. The on-site conference on the youth safety production skills promotion action, youth league members commandos to participate in the "urgent, difficult, dangerous and new" task, youth post skills competition, youth civilization creation and other work arrangements and deployment, I listened to the typical speeches of the 20th National Youth Civilization, 2019-2020 Shandong Province Youth Civilization, 2021 Shandong Province Youth Safety Production Demonstration Post, and 2021 Shandong Province Youth Safety Production Skills Improvement Action Demonstration county representatives, Honorary plaques were awarded to some outstanding youth collectively, Selected 157 professional emergency rescue backbone volunteers from the blue sky rescue, special combat rescue and frontier rescue as the township (street) league members youth commando instructors, And has carried out on-site training, Guo Yujuan, president of Emergency Management School of Liaocheng Vocational and Technical College, was invited to make a special report. It is understood that the youth workers skills revitalization plan is an important part of the Youth League Liaocheng Municipal Committee "youth achievements new Liaocheng" action. Tuan Liaocheng municipal party committee will play a youth civilization, youth post expert, youth production safety ShiFanGang, youth commandos "ShieGang team" outstanding youth collective and individual demonstration leading role, guide the city young worker improve pioneer consciousness and professional skills, for example in their respective positions, first-class, advanced, for the "six new Liaocheng" construction contribution youth wisdom and strength.

2022-04-16 Anhui Fuyang communist youth league mobilized youth from all walks of life in disease resistance line in Anhui Anhui Fuyang communist youth league to mobilize youth from all walks of life in disease resistance line reporter Wang Haihan Wang lei) since March this year, in the face of COVID-19 outbreak, Anhui Fuyang city youth league organizations at all levels, league association and young volunteers smell and move in resistance to disease a line, take practical actions to write youth bear. In early March, the Fuyang Municipal Party Committee of the Communist Youth League issued the Proposal to Youth League Organizations at all levels and Youth League Members. Youth League organizations at all levels in the city took immediate action to reserve and pre-recruit more than 4,000 young volunteers for epidemic prevention. After the outbreak of the epidemic, the Fuyang Municipal Party Committee of the Communist Youth League immediately issued the "Prevention and control of the epidemic, Start from Me" initiative, and nearly 40,000 Fuyang young people made the relay commitment. On April 6, the Fuyang Municipal Party Committee of the Communist Youth League called on the youth members over the age of 18 to report to the community. The local youth members responded to assist the community in nucleic acid sampling, order maintenance, site disinfection, temperature measurement, information input, epidemic prevention and publicity, and material distribution. At the same time, the Fuyang Municipal Committee of the Communist Youth League issued the Guidelines on Youth Volunteer Service for COVID-19 Prevention and Control and the Proposal of Fuyang Young Volunteers' Scientific and Orderly Participation in Epidemic Prevention and Volunteer Service, mobilizing youth league organizations and young volunteers at all levels to participate in the epidemic prevention and control work in a scientific and orderly manner. As of April 13, more than 8,000 young volunteers in the city have joined the fight against the epidemic, serving for more than 548,000 hours. On March 27, Fuyang city decided to build two makeshift hospitals in the Fuyang Sports Center and the Fuyang epidemic Convention and Exhibition Center. Fuyang Economic and Technological Development Zone Anhui New Road construction engineering Group after receiving the order, quickly assembled young commandos, rushed to the construction site. In the early morning of March 28, Fuyang sports center began construction, more than 50 young commandos scientific work, promote the progress of the project. In early April, in Yingshang County, the youth commandos of the North Anhui Branch of China Construction No.5 Bureau and Company 2 were divided into construction technology, material management, logistics support and other teams, to complete the construction of more than 2,000 isolation rooms in the county epidemic prevention and emergency project. At the same time, the Youth League Fuyang Municipal Party Committee quickly launched all walks of life to organize the formation of youth commandos. At the traffic epidemic prevention card point, young civilian auxiliary police work all night to maintain order, young medical workers at the community nucleic acid testing point, and express industry youth to register vehicle information. Young people in public transport, taxation, court, procuratorial, urban management and law enforcement, civil aviation, state grid power supply and other industries show their strength with practical actions. More than 190 youth commandos for epidemic prevention and control have been set up in the city. The Fuyang Municipal Party Committee of the Communist Youth League gave full play to the advantages of organization and socialized mobilization, and organized the fundraising work of epidemic prevention and control materials with the Municipal Youth Federation, the Municipal Youth Entrepreneurs Association, the Municipal Youth Entrepreneurs Association and the Municipal Youth Volunteers Association, with a total donation and materials equivalent to more than 970,000 yuan. The Fuyang Municipal Party Committee has also opened up channels for social donations. As of April 13, it has received nearly 100,000 social masks, including disinfectant, disinfection gel and alcohol, worth more than 200,000 yuan. Youth league organizations at all levels also connected with the first line in the first time to form a list of material needs. On April 5, Tuan Linquan County Party Committee issued the "Spring" city collection order, calling on people from all walks of life to donate money and materials, over the past week, in addition to epidemic prevention materials, received more than 40,000 yuan of donations from all walks of life. At present, Fuyang league organizations at all levels have raised donations and materials equivalent to about 700,000 yuan to ensure grass-roots league organizations to carry out epidemic prevention services. The Communist Youth League Fuyang Municipal Party Committee for the picture

2022-04-15 Zhongshan youth talent employment guidance online live classroom in Beijing to develop the communist youth league committee held in Beijing Zhongshan youth talent employment guidance online live class get youth attention, power Zhongshan economic and social development of high quality, on April 12, the communist youth league committee joint city in Beijing in Beijing to carry out the "young eagle nest" young talent theme activities- "rush young people" Zhongshan young talent employment guidance online live classroom. Xie Li, director of the Municipal Liaison Office in Beijing, visited the studio to promote Zhongshan City. Zhou Qiuhong, a teacher from the Employment and Entrepreneurship Guidance Center of the Student Affairs Office of Beijing University of Posts and Telecommunications, taught online. More than 2,000 young college students studying in the Beijing-Tianjin-Hebei region watched and learned through the online platform and interacted with experts and guests online. This activity is sponsored by Zhongshan Youth Social Work Guidance Center and Zhongshan Young Talent Work Promotion Association. The activity aims to promote the employment of college students, and takes the graduation season as an opportunity to form the double attention of urban publicity and youth growth. Before the event, xie, director of the invited part of Beijing Zhongshan young college students for discussion exchange, thorough understanding of young students demand, and live around Zhongshan industry development, urban innovation and development, young talent service, such as three aspects, the future development of Zhongshan and major platform to promote, at the same time, combined with "young eagle nest" project, encourage young talents to actively understand Zhongshan city, welcome to Zhongshan employment entrepreneurship, create their own better future in the development of Zhongshan city. Zhou Qiuhong teacher around the "career by my master" "resume production skills and interview minefield" and other content, in the job direction, job preparation, job skills, etc., give youth targeted guidance, detailed answer to young college students in the job will encounter practical problems, and give examples to the scene response analysis."What aspect do you think is more important in the analysis of career needs?"" What information can I paid attention to about the practice of college students in Zhongshan City?"And other questions, the guests answered one by one. Students have said on the bullet screen: " this guidance content is full of dry goods!"" Through this activity, I have a clearer understanding of the employment situation in Zhongshan."This activity to the Beijing-Tianjin-Hebei region as the origin, for a wide range of radiation. Online live classes broke the limitation of time and space and reduced the impact of the epidemic on offline recruitment activities. which not only attracted the attention of young college students in the Beijing-Tianjin-Hebei region, but also effectively spread based on the organizations of Zhongshan Young Talent Promotion Association and Zhongshan Town Federation, with more extensive attention. Next, the communist youth league of Zhongshan city committee will explore relying on the association for the promotion of young talents, young talent station organizations or positions, in Beijing-Tianjin-Hebei and other regions and cities will be "eagle nest" young talent theme activities normalized, effective innovation form, continue to respond to the concerns of young talent, service young talent demand, leading the young talent love township home hometown construction.

2022-04-15 Jincheng, Shanxi: "one county, one product" to stimulate the vitality of grass-roots youth League organizations now where? To achieve the 'last mile' of community youth services, we should not only rely on full-time youth league cadres, but also focus on mobilizing various forces."A few days ago, in the Youth League of Shanxi Province Jincheng Municipal Party Committee, a team members for the county (city, district) youth League committee secretary pilot work progress of the" soul torture " is ongoing: some work looks tall, the youth do not recognize? Can the young people in the trade city restaurant feel the warmth of the league organization? Emphasize several times that the work problem is not solved, who is the responsibility? Sharp questioning, so that some blush sweat, some to clear work ideas. Through on-site assessment, questions and sharing, let everyone see the gap, learn from each other, each has its own harvest. This is in recent months, the Communist Youth League Jincheng Municipal Party Committee in the promotion of the county Communist Youth League grassroots organization reform pilot work in one of the innovative work measures. As the only area in Shanxi Province to promote the pilot reform of the Communist Youth League, Jincheng Municipal Party Committee adhere to the leadership of the top leader, the standing Committee of the Municipal Party Committee studied the reform of the Communist Youth League, from the topic to "one county one product", Jincheng city is building a long-term mechanism for the pilot reform of the Communist Youth League. While exploring while reform " the whole city reform reform what? Where to change? How to change? In fact, we are also exploring while promoting, while carrying out while improving, after several rounds of supervision of the CPC Municipal Party Committee, under the continuous docking and guidance of the CPC Central Committee and the Provincial Party Committee of the Communist Youth League, our work path is gradually clear."Youth League Jincheng Municipal Party Committee related responsible person introduction. The municipal Communist Youth League Committee has improved the special class for the pilot reform of the Communist Youth League at the county level, determined the responsible leaders of the county (city, district) committee, defined the responsible persons of the pilot, and detailed the municipal package leaders, joint departments and the reform pilot instructors. On the basis of fully considering the resource endowment of each county (city, district), the working idea of "one county, one product" and "three steps" from the grass-roots level was finally established. The Communist Youth League Municipal Committee of the CPC adopted the "three up and three down" way to promote the reform: three times "please come up", in the initial launch stage, the secretary of the county party committee and team members were invited to the city for at least three times for work communication and arrangements. Three times "go down", by the secretary of the Communist Youth League, the deputy secretary, bao hang departments to the municipal county and the county party committee of the main leadership, in charge of the leadership, cadres for in-depth communication. The Communist Youth League Municipal Committee took the three-level league organization change as an opportunity to actively strive for the support of the Party committee, and fully matched the county and rural league cadres. According to statistics, Jincheng county cadres from the grass-roots cadres accounted for 42%, 25% higher than before the transition. In the team, full-time cadres of the youth league with bachelor degree or above accounts for 100%, graduate degree or above accounts for 10%, and CPC members account for 100%, which provides a strong talent and organizational guarantee for promoting the work of the grass-roots youth league. Recently, the circle of friends of many netizens in the southeast of Shanxi Province has been painted by the post-90s "little flower sister" Wu Meng � B.� B's TikTok number is called "little sister surname Wu". Different from the traditional "web celebrity", her duty is the secretary of the Youth League Committee of Yangcheng County. From hesitant to careful pilot, to popular become a young guest mentor, "little sister" with the support of the county, actively for hometown, promote Yangcheng brigade resources, promote the brand activities of the communist youth league, actively expand the communist youth league "friends", won the recognition of netizens, also become the popularity of the local youth endorsement. Driven by the "little sister" live broadcast, the Yangcheng Communist Youth League TikTok number "Youth Yangcheng" has nearly 40,000 fans."Seizing the online and offline gathering places of youth is one of the focal points of our reform. The pilot project is gradually rolled out to influence a group of people through one person, play a league flag on the Internet, speak for our hometown, and help rural revitalization."The purpose of Tuanyang County Party Committee is very clear, where the youth is, we will expand the service and position of the regiment. Facts have proved that this measure is playing a positive role, and the influence of the Yangcheng Group organization is expanding. Recently, an online survey launched by the Yangcheng Communist Youth League showed that more than 600 of the more than 4,000 young people participating in the survey were unemployed, and they expressed a strong interest in the e-commerce industry. The survey results provide a basis for the Yangcheng Youth League organization to serve the local youth. With the support of the county Science and Technology Bureau, Yangcheng County E-commerce Center Youth Home specially invited young entrepreneurship mentors and young entrepreneurs to carry out e-commerce training."The Communist Youth League County Party Committee will set up the training class classroom at the gate of the county bus station, returning young people can see as soon as the station, a total of more than 20 training sessions, very popular."TuanYangcheng County Party Committee introduced. Yangcheng for thousands of years of ceramic, sericulture industry culture unique charm, and the future of the core direction, is the cultural output, "as a grassroots cadres, we serve for the returning youth at the same time, also hope that through their development, spread Jincheng culture, boost local high quality agricultural products out of Jincheng, to the country"."Leading more young people to test the waters in the e-commerce field is also a task entrusted to us by the Party committee."Group yangcheng county controller introduces, yangcheng established led by the party committee head of the communist youth league organizations at the grass-roots level reform leading group, the county main leadership clearly proposed to promote more local and returning young people put short video, live and yangcheng development, with their own business development, play a leading role in the rural revitalization. Reform is "chemical reaction" is not the superposition of traditional work. Compared with other counties and districts, Jincheng urban area has prominent location advantages, the urban league organization takes the park and industry as an opportunity, integrates supporting youth home, social practice league school, talent station and other service positions, and gives full play to the linkage role of the position complex. Gaoping City of Jincheng has been established by the party secretary of the Communist Youth League and the deputy secretary of the Municipal Education Working Committee, and comprehensively established the youth league and education cooperation cross-office mechanism. At the same time, taking the village (community) organization change as an opportunity, combined with the municipal party committee "young eagle plan", the qualified young village cadres and college students nominated as the village (community) league branch secretary candidates, enrich the village level league organization talent force, nearly half of the village (community) has determined the change of the league organization intention candidates. Qinshui county by guide and promote enterprises and institutions and township youth league pair construction, revitalize the county league organization construction; Zezhou county lead the group work into the county annual work assessment system, cadres daily management and supervision by the party committee (group), league county, comprehensive and accurate evaluation of cadres of performance and work performance."Reform is not a superposition of traditional work, but a chemical reaction, first to find young people, to study the breakthrough of the work of grass-roots youth league organizations, and then to establish an assessment and incentive mechanism."In the view of the Communist Youth League Jincheng Municipal Party Committee, the above changes reflect the Jincheng Youth League organization through the reform, influence in the promotion, social functions in the play, the youth for the youth league organization service recognition is reflected," this is a qualitative change ". It is understood that in order to ensure the effectiveness of the reform, Jincheng city in addition to the league construction into the annual target responsibility assessment, but also introduced the Jincheng Doctor Association for a third-party evaluation, the reform of the league organization pilot work was twice reviewed, the review results were directly copied to the county (city, district) Party secretary. At the same time, the policy laboratory of the Communist Youth League- -Jincheng Communist Youth League Cadre Famous Teacher Studio was established to actively solve the reform difficulties encountered in all counties (cities, districts), and promote the experience of the whole city. In addition, the non-public economic organizations and social organizations, social organizations and unemployed college students have been set up at the municipal level. The core purpose is to attract, connect with and serve youth as much as possible. China Youth Daily, April 15,2022

2022-04-14 Inner Mongolia youth league celebrate twenty league theme activities: extensive "welcome twenty always follow the party go new journey" theme activities green network reporter Shi Jia) since this year, Inner Mongolia youth league organizations at all levels to meet the party's 20 as the main line, combined to celebrate the 100th anniversary of founding, phased organized learning, mass preaching, typical youth tree, new media culture products promotion "welcome twenty always with the party go new journey" theme education practice, attract youth active participation, armed with the party's theory, education to guide teenagers. For days, the group of Inner Mongolia Xingan league committee offline to carry out the "welcome twenty big, always follow the party, forge ahead new journey" theme learning salon series activities, through the cadres public class, the party history of the play to kill, learning discussion, knowledge contest, watching movies, reading red letters, let the young people deeply comprehend the party leadership of the glorious course of the Chinese youth movement. According to statistics, in 2022, the youth league organizations at all levels of Inner Mongolia Xingan League held 78 discussions and exchanges, 31 social practice activities, more than 50 special studies and 22 story sharing sessions. Online, the Inner Mongolia Xingan League Committee continued to organize 163 online themed group classes of "Youth Learning", covering 109,725 people, and 11 online series of "Red Scarf Love Learning", covering 41,656 people, focusing on improving the ideological, affinity and effectiveness of theoretical learning. The Communist Youth League of Inner Mongolia Xing'an League Committee to carry out the party history script killing activities. Pictures provided by respondents at the same time, the Inner Mongolia au to preach "youth branch" as the carrier, to play the role of "youth love", "build" six types of speaker "propaganda matrix, youth cadres, first secretary of residency, returning entrepreneurial youth, outstanding volunteers, college students practice, red scarf narrator, in rural areas, communities, enterprises, schools and other grassroots line, focus, normalized theory policy, the" truth "into" sharing ", the good voice extends to the deep at the grassroots level. According to statistics, more than 60 publicity activities were held in 2022, covering more than 2,400 young people. Members of the Inner Mongolia Xingan League Youth Lecture League are preaching. In addition, the youth league organizations at all levels of Inner Mongolia Hinggan League carried out a series of activities such as "League Youth Party History and League History Knowledge Competition", "Casting the Community Consciousness of the Chinese nation Theme Speech Competition" and "Sing the Future" red songs singing. Recorded "one hundred years", "reunion youth song", "please rest assured that the party strong country has me" MV, "prairie fire square square" gesture dance, the original song "youth to the future". The youth League branches and young Pioneers of all schools in Inner Mongolia Xingan League held various team day activities, such as learning discussion, story sharing, essay soliciting and speech, visiting and search, and volunteer service, covering more than 100,000 people.

2022-04-14 "Chengde May 4th youth said" online deeds sharing held "Chengde May 4th youth said" deeds sharing held "Chengde May 4th youth said" online deeds sharing activities, synchronous announced Chengde youth May 4th medal (model) selected recommended results, 10 comrades won the "Chengde youth May 4th medal pacesetter" honorary title, 40 comrades won the "Chengde youth May 4th medal" honorary title. Since the launch of Chengde Youth May 4th Medal (Model) selection and recommendation activity in January this year, more than 200 young people from all walks of life in the city have participated in the activity by organizing and reporting and media recommendation. The organizing committee finally determined 50 formal candidates through the preliminary qualification examination, online publicity, and deeds review, to participate in the "Chengde May 4th Youth Talk" story sharing activity. During this event, 50 official candidates of the Chengde Youth May 4th Medal exchanged their achievements in rural revitalization, epidemic prevention and control, flood fighting, helping the Winter Olympics, innovation and entrepreneurship, and other grass-roots frontline work through online video display, so as to create an atmosphere of catching up and striving to be the first. Participate in the activities of youth said, as a new era of Chengde youth, to bear era responsibility in the pursuit of progress, to realize the value of life in the permanent struggle, let the youth in comprehensive deepen the "three implants", promoting "four achievements", "second" entrepreneurship "with brilliant brilliance, to speed up the construction of the development of high quality" ecological city, charm of Chengde " to make new contributions.

2022-04-13 Quanzhou city to carry out "our festival qingming" online theme activities in 2022 "our festival qingming" online theme activities JianWei correspondent, Lu Yaokun Wu Junwen) group of Fujian Quanzhou municipal party committee, city student federation, city less working committee jointly develop rich content, various forms of "our festival qingming" online theme activities, guide the student members, young pioneers to carry forward the traditional virtues, remembering sages martyrs, set up the civilized fashion, power epidemic prevention and control. Recently, due to the need of COVID-19 prevention and control, primary and secondary schools in Quanzhou have suspended all offline teaching and activities, but "classes are not suspended". The Communist Youth League Committee, the Municipal Students' Federation and the Municipal Youth Working Committee have extensively carried out online memorial activities for heroes based on the online platform. During the qingming day, pku launched "online offering heroes" special activities, with online bow to sages martyrs flowers, express speech message, browse martyrs deeds, let the student members, young pioneers learning and remember the feats of revolutionary heroes, with online flowers, network message express respect for revolutionary martyrs, in the pursuit, remembering martyrs inherit the revolutionary spirit, carry forward the fine tradition, perception happy life is hard-won. Up to now, 396,167 people have participated in the online "Online Heroes Festival" online activity, with 152,951 messages left."Cloud" group (team) to long under the unified deployment of pku, various counties (city, area) team organization to "our festival qingming" as the theme, through the "cloud", "cloud" team class, the theme "cloud classroom" activities, combined with the current national resistance "disease", overcome difficulties, open online flag-raising, qingming festival, salute heroes, red scarf for chapter activities. According to statistics, during the qingming festival, the city county (city, area) team organization conducted more than 400 games "cloud" class, "cloud" team class, and through innovation "epidemic prevention" characteristic chapter, carry out more than 300 cloud red scarf activities, guide the student members, the young pioneers to review the party history, understand qingming festival, master epidemic prevention knowledge, in the epidemic prevention hero strength, enhance love party, patriotism, love the socialist emotion. This year's Qingming Festival coincides with the epidemic, League (teams) at all levels in the city organized and timely carry out the theme activities of "hand in hand red scarf civilization inheritance", Advocate the majority of student league members, young pioneers to lead the family and friends to establish the concept of civilized worship, inherit the new fashion of civilization, With online memorial, flower memorial, family memorial and other civilized and low-carbon memorial methods, To express the memory of our ancestors, While strictly observing the requirements of the prevention and control work, Cultivate and establish the civilized and green concept of student league members and young pioneers, To actively become an advocate and disseminator of the wind of civilization, So as to drive more citizens to practice civilized sacrifice, Form a civilized and healthy new social fashion. Propaganda creation help epidemic prevention during the qingming festival, Quanzhou student members, young pioneers in learning at the home at the same time, also combined with Quanzhou sea silk culture, minnan culture, and family create propaganda works, with poetry, painting, express high respect for the revolutionary pioneer, disease resistance hero, with calligraphy, copy, show the profound connotation of traditional festivals, with music, dance, inheriting civilization, spread civilization. These various forms of literary and artistic works not only publicize the knowledge of epidemic prevention, but also express their most sincere respect and blessing to the workers on the frontline, and express their support to the whole people in the fight against the epidemic.

2022-04-12 Hanzhong, Shaanxi: Let Red become the youth background color Hanzhong, Shaanxi: Let Red become the youth background color member " practice experience activity. Youth League Hanzhong Municipal Party Committee for the picture of China youth network Beijing on April 12 (reporter Zhang Jianwei) in the recent report to the seventh plenary meeting of the 13th Committee of the Shaanxi Province of the Communist Youth League, "theme group class", "seek sympathy", "based on the grassroots", "public welfare volunteer" has become liu Yang's high-frequency words. These words seem to be independent but actually connected. From the washing of thought to the transformation of practice, exploring their root causes, ultimately thanks to the "red" power."Party history study and education is the main line."Liu Yang introduced, in 2021, the Hanzhong Communist Youth League classified policy, for the youth, 189 youth theory study groups to set up the party history study boom, for the grassroots cadres, more than 30 training courses to enhance the" party spirit " cognition, for the youth group, more than 7000 theme visits, veteran party visits, theme team day activities, with immersive experience, red gene planted nearly 700,000 young heart."Recall the eventful years to spread the red gene" old party members search for sympathy activity pictures. Youth League Hanzhong Municipal Party Committee for a wider range of learning also needs to innovate more forms, with more strength. According to Liu Yang, a total of 1.991 million young people in Hanzhong in 2021 will have participated in online learning through the online platform of "Youth Big Learning". In addition, the Hanzhong Communist Youth League also added 10 outstanding young people to the municipal youth lecturers' group, and carried out 103 theme publicity activities such as "Party history learning and education" and "celebrating the 100th anniversary of the founding of the Party", effectively realizing the deep learning of young people in a wider range of areas. The study and education of party history should not only closely follow the important history, but also combine the present, and should seek both outside and inside."In fact, we are constantly performing vivid 'party history lessons' around us."Liu Yang said that Hanzhong fully strengthens the effect of the typical Communist Youth League in tree selection, and continues to take" excellent Communist Youth League members "," Hanzhong Good Youth "and other brand activities as the traction, to stimulate the endogenous learning motivation of the majority of young people," because The Times are more compatible, the incentive effect of people and things around them may be stronger."Hanzhong city in the 2021 Spring Festival travel rush "warm winter action" service time ranked the third in China. In many cases, the results of ideological learning are difficult to explain, and action has become the most direct expression. Under the repeated situation of COVID-19 epidemic, communities and epidemic prevention checkpoints are "volunteer red"; 1800 competition cities and social volunteers are active inside and outside the arena, protecting the green waters and mountains. More than 200 environmental volunteer teams carry out volunteer service activities such as Qinba ecological environment protection and Hanjiang River basin protection, creating three work characteristics of "quality, construction and love" in Shaanxi Province... Hanzhong "Protect Mother River and Care for Great Qinling Mountains" tree planting activities. The Communist Youth League Hanzhong Municipal Party Committee for the party history learning and education is talking about the history of the Party, read is the party situation, focus on the future, guide is the present. From ideological guidance to institutional guarantee, from characteristic practice to brand building, from organizational mobilization to the spontaneous action of the youth, the "red practice" highlighting the youth characteristics coordinated by the Hanzhong Communist Youth League has achieved phased results, and will continue to make contributions around the central work of the Party and government."In 2022, we will focus to meet and study propaganda implement the party's 20, combined to celebrate the 100th anniversary of the league, widely carry out' celebrate the 20, always follow the party, forge ahead new journey 'theme education practice, unremitting armed with xi Jinping thought of socialism with Chinese characteristics youth mind, promote the youth party history learning education normalized, with the great party spirit, deepen the learning education achievements, continue to' I do practical things for the youth'practice, unite to unswervingly listen to the party, follow the party, the new journey, the new era."Liu Yang said.

2022-04-12 Anhui Chuzhou "five platform" build young talent gathering "strong field" group of Chuzhou, Anhui province municipal party committee to build "five platform" to build young talent gathering "strong field" jianwei correspondent Wang Rui Zha WeiTing) since 2022, the group of Chuzhou, Anhui province municipal party committee adhere to the inline, actively build "five platform", build gathering young talents "strong field", high standard and high quality to promote the development of young talents, efforts to the city ZhaoCaiYinZhi "quality breakthrough year" to make due contributions. Build a practice training platform, be a good innovation carrier of "incubator" for party and government reserve talents, select Chuzhou college students as grass-roots league cadres, vigorously carry out the social practice of "returning home", and enhance the sense of belonging and identity of college students to their hometown. The selection of 280 Chuzhou college students to serve as deputy secretary of the township (sub-district) youth League (work) Committee, to further broaden the source channels of grass-roots youth league cadres, so that young students can train and grow at the grass-roots line. Focus on the survey of "985", "211" and "double first-class" university of Chuzhou registered students, establish the information database of outstanding college students from Chuzhou, and establish the institutionalized channel for students from outside and their hometowns. At present, 1 have been 442 outstanding college students, including 133 postgraduate students and 10 doctoral students. We have implemented the special campaign for youth employment services, and organized more than 50 enterprises to visit the Longpan Street and Three towns and Chahe Town of Lai'an County to "send posts to the countryside", so that young people can achieve "find employment at home". During the winter vacation, we carried out the practice activity of "Zhihui Chuzhou" chain "at home" into the "eight industrial chains" for college students, organized more than 210 college students to study and practice in more than 20 enterprises, understand the employment environment of enterprises, and meet the job needs on site. We will improve the youth employment service chain, and select 85 young post experts with the Municipal Human Resources and Social Security Bureau, not only helping unemployed young people achieve employment, but also pay attention to promoting the growth of young workers, and providing high-quality young talents for high-quality development. Build innovation entrepreneurship platform, lit youth achievement dream "catalyst" play the advantages of the city youth league, city green enterprise association of contact, regularly organize Chuzhou young entrepreneurs and outstanding young entrepreneurs in the Yangtze river delta region interaction, held "condensed youth strength, optimize the environment of business," youth entrepreneurs salon, young entrepreneurs hand in hand to grow up together. The training class of "Start Your Enterprise" was held in Chuzhou City Vocational College to guide college students to enhance their entrepreneurial awareness, enhance their entrepreneurial ability and devote themselves to their entrepreneurial practice. In the middle of March, the "Elite wanderers Return to Chuzhou" College Students Returning for Entrepreneurship Competition was held, and 8 demonstration bases (parks) of college students returning for entrepreneurship were selected to encourage young people to realize their life dreams in rural revitalization and inject fresh blood into the development of their hometown. Build overseas contact platform, gathering booster Chuzhou development "new force" research and formulate "Chuzhou foreign young talent workstation operation method (try out)", actively promote the construction of foreign young talent workstation, strive to build workstation discovery, introduction, service the global outstanding young talents "outpost" and "post", let young talents timely understand Chuzhou talent New Deal, gather elite, the introduction of quality projects, promote economic and social development of Chuzhou. At present, it has established two young talent workstations in Shanghai and Beijing, and established regular contacts with nearly 40 outstanding young people, including assistant professor and doctor Wu Wen of Peking University, and contacted more outstanding young talents through them. By 2022, no less than five overseas youth workstations will be built. We will build a friendship platform to help young talents expand the "circle of friends", hierarchical classification and implement precise policies, customize different activities focusing on the needs of different youth groups, plan and implement a series of "Youth Joy +" youth talent exchange activities, and retain young people with solid services. Since 2022, has successively carried out the "eight industrial chain" special young worker dating fellowship activities, "reading book has hui" reading communication, "yue swim line chunhui" obligation tree planting and a series of theme dating fellowship activities, attract more than 240 agencies, enterprises and institutions youth involved, by helping them to create opportunities to meet, find a life partner, promote their home in chuhe.

2022-04-12 Guangxi north sea "sea silk road green gen cup" youth entrepreneurial innovation competition in Guangxi north sea "sea silk road green gen cup" youth entrepreneurial innovation competition opener Xie Yang correspondent Li Qibao) "sea silk road green gen cup" the north sea sixth youth entrepreneurial innovation competition preliminaries held, contest to "struggle in one hundred road entrepreneurship is youth" as the theme, aims to improve youth entrepreneurial quality, attract more young entrepreneurial innovation talents to investment in the north sea. The competition will set up entrepreneurship and innovation group and creative innovation group. The preliminary competition will be carried out in the form of "5 + 4" (namely 5 minutes road show, 4 minutes question and answer). The judges will score the projects from five aspects: project function positioning, market evaluation, financial operation, team situation and social benefits. The top 30 projects will be promoted to the final competition. After the preliminary competition, the organizing committee will organize pre-final training, semi-final, pre-final training and final, and provide a full range of services for the entries to settle in Beihai. Since its launch on December 17,2021, the competition has attracted students from 21 domestic and foreign universities, including Tsinghua University, Peking University, Fudan University and Manchester University in the UK. Through the preliminary screening and evaluation, 187 projects were selected from the 853 entries to enter the preliminary competition, including 60 entrepreneurship and innovation groups and 127 creative and innovation groups. These entries have 504 independent intellectual property rights such as patents and soft works. The competition is jointly organized by Beihai Municipal Talent Office, Beihai Municipal Party Committee, Beihai Industry and Information Technology Bureau, Beihai Science and Technology Bureau, Beihai Human Resources and Social Security Bureau, Beihai Industrial Park Management Committee, Beihai High-tech Zone Management Committee, Beihai Marine Industrial Park Management Committee and other departments.

2022-04-11 Gansu Qingyang 2022 public art training class officially support 100 difficult families children Gansu Qingyang 2022 public art training class class Li Chuan) to promote the "double minus" opinions ground work, make "service efficiency" authority, play to the positive role of municipal youth palace in after-school service, on April 9, Gansu Qingyang 2022 public art training class opening ceremony and "longdong college volunteers outside social practice base" opening ceremony held in Qingyang city youth palace. Huang Jing, secretary of Qingyang Municipal Youth League Committee, and Sun Bo, Secretary of the Youth League Committee of Longdong College attended the ceremony. More than 100 representatives of Qingyang Youth Palace staff, all teachers and students and parents attended the ceremony. Song Zhenhui, president of Qingyang Youth Federation, presided over the ceremony. Qingyang Municipal Party Committee and the Youth League Committee of Longdong College jointly set up a college student volunteer social practice base in Qingyang Youth Palace, employing Longdong College with excellent college students with professional ability and certain teaching experience as teachers, and opening a total of 5 classes of art, dance and electronic piano. Adhering to the principle of "pure public welfare, zero charge", the training recruits 100 children from poor families in Xifeng Urban District, Qingyang City. The course is conducted by weekend in-semester teaching and concentrated summer teaching, and will end at the end of December this year. Huang Jing said that the establishment of Longdong College student volunteer social practice base and the opening of this public welfare art training class is not only a measure of the deep integration of Qingyang City Communist Youth League and Longdong College Youth League Committee, but also a breakthrough to create a new situation of the city's youth after-school education work. It is reported, through the college students power public welfare training mode, Qingyang city communist youth league to better play to the role of college students volunteer teacher youth model, practice volunteer spirit, reflect educational value, using professional director, pragmatic "I do the practical work for the masses" "I do something for youth" theme practice, strong guarantee the healthy growth of difficult family children, the full implementation of the communist youth league "cultivating era, education, lead the children do the communist cause successor" the fundamental task, reveal the new era of college students' spirit and responsibility.

2022-04-07 Sichuan Liangshan group clever solution fire prevention propaganda problem with a lollipop in lighter Sichuan Liangshan group clever solution fire prevention propaganda problem Wang Xinxin) to improve the masses of forest grassland fire prevention consciousness, qingming festival holiday, the communist youth league in Sichuan Liangshan yi autonomous prefecture committee in xide county, DeChang county organization carried out the "youth volunteer protect life" as the theme of volunteer service activities and forest grassland fire prevention propaganda and education action. During the activity, the volunteers gained their understanding and support for fire prevention work in the form of "use my one lollipop for you to smoke less smoke". According to the feedback of the daily forest and grassland fire prevention team, during the supervision and inspection of the fire prevention card points, the confiscated lighters are prone to produce resistance, which is not conducive to the development of fire prevention work. Volunteers are promoting fire prevention. Therefore, the volunteers set up a "love forest box" containing a lollipop in the forest and grassland fire prevention standard card point. At the same time, the staff gave the lighter a lollipop, and explained that collecting the lighter was not the purpose, just to better carry out the forest and grassland fire prevention and fighting work. I hope you can understand and support it. In Dechang and Xide counties, the local youth league organizations have set up 65 "love forest boxes"."Love the forest box" to a certain extent to ease the contradiction between the masses and the card prevention point personnel, to ease the mood of the confiscated personnel. At present, the situation of Liangshan forest and grassland fire prevention is grim. According to the relevant deployment requirements, Liangshan Municipal Committee of Forest and Grassland Fire Prevention has organized cadres and staff to sink to serve as "safety officers" and "propagandists" in Huili City, Dechang County and Yide County, and strictly investigate the risk of fire sources entering the mountain. Local youth league organizations also carried out "youth volunteer to protect life" forest and grassland fire prevention training sessions in primary and secondary schools to strengthen students' ideological understanding, and promote the forest and grassland fire prevention propaganda from their families to the community, so as to realize small hands holding big hands.

2022-04-07 Group Shijiazhuang municipal party committee held emerging youth representatives symposium Shijiazhuang youth representatives symposium green reporter Fan Jiangtao) recently, Shijiazhuang municipal party committee, Shijiazhuang Internet industry group organized a "trillion city achievements" emerging city youth field youth representatives exchange symposium, further understand the emerging youth group characteristics, ideological dynamic and interests, encourage the emerging areas of youth do "trillion city youth achievements" activities of propagandists and practitioners. Symposium site. Group Shijiazhuang municipal party committee for the meeting, new media from youth, e-sports industry entrepreneurship youth, fast electricity business senior operation experts speak freely with their own reality, share their learning experience, entrepreneurial story, working experience and target ideal, and for their own ideological confusion and development bottleneck, expressed the hope of the communist youth league organization. The staff of the Shijiazhuang Municipal Party Committee of the Communist Youth League listened carefully to the speeches of each representative, and said that they would carefully sort out, summarize, and give timely feedback on the ideas, consensus and reasonable demands of the young people attending the meeting. At the same time, the Shijiazhuang Municipal Party Committee of the Communist Youth League will also provide more communication platforms for the development of young people in emerging areas, give policy support, and effectively make the Communist Youth League organizations become their "bosom friends" that they can remember, find, rely on and trust.

2022-04-06 Shandong Rizhao city to carry out the "inherit martyrs legacy to be young" activities Rizhao "inherit martyrs legacy to be young" qingming festival theme team day activities wei correspondent XiangChanghui) to guide the youth of revolutionary martyrs, red gene, carry forward the Chinese excellent traditional culture and revolutionary spirit, further strengthen patriotism education, group Shandong Rizhao municipal party committee organization at all levels under the premise of strictly implement the relevant provisions of COVID-19 epidemic prevention and control, "inherit martyrs legacy to be young" qingming festival theme team day activities. Rizhao Experimental Primary School organized the young Pioneers to express their high respect to the revolutionary heroes through the theme team meeting. Rizhao No.4 Experimental Primary School carried out the theme of "Under the National flag feeling Qingming Festival" education activities. Correspondent for the picture of Jinan Road Primary School to carry out the "Tomb Sweeping Festival classical reading" sharing activities. Correspondent for the picture

2022-04-04 Jinan communist youth league: condensed youth power win epidemic war Jinan communist youth league: youth power win outbreak war XingTing) on April 2 morning, group Jinan municipal party committee held the city communist youth league system to participate in the epidemic prevention and control work video, further implement xi general secretary of epidemic prevention and control work important instructions, earnestly implement the provincial and municipal party committee government and group Shandong provincial party committee relevant requirements, around fully condensed the city youth strength block the spread of the epidemic chain, win the epidemic war work mobilization and deployment. According to the unified arrangement of the whole city, the Jinan Municipal Party Committee of the Communist Youth League participated in the work of the community control group, and, together with other relevant municipal departments, carried out the investigation of the personnel base in the "sealed control area", "control area" and "prevention area" to provide detailed data support for the implementation of special personnel and management. It is understood that in the current emergency state of epidemic prevention and control, Jinan joint headquarters at all levels, special teams, streets and communities all need a large number of manpower, need to transfer personnel from all levels of departments, the Jinan Communist Youth League will actively participate in, obey the arrangement, and resolutely implement the tasks. As of April 1, the Jinan Municipal Party Committee of the Communist Youth League has dispatched 52 personnel from government organs or affiliated public institutions to join various special groups or sinking communities to directly participate in the epidemic prevention and control work. According to the requirements of the meeting, the Jinan Municipal Party Committee should guide the city's teenagers to strictly abide by the epidemic prevention requirements, continue to carry out health education, and enhance their personal awareness of self-protection. Under the leadership of the university Party committee, the youth League committee of the university should do a good job of ideological and psychological guidance for students, life, study and care. At the same time, relying on the youth league organizations at all levels, youth committee, youth federation, youth associations, set up the epidemic prevention and control youth commandos, fully consider the area, unit or industry of the actual demand of epidemic prevention and control, under the guidance of the party organization, around the community control, flow traceability, nucleic acid detection, isolation point project construction work, to play the leading role of youth pioneer. Up to now, there are more than 150 youth commandos for epidemic prevention and control in Jinan. In addition, a special campaign was launched for epidemic prevention and control for young volunteers. Youth league organizations at all levels should do a good job in the recruitment, training, scheduling and guarantee of young volunteers for epidemic prevention and control in combination with the actual situation of their respective jurisdictions, industries or units. Up to now, the city has recruited 3,315 reserve young volunteers for epidemic prevention and control reserve, among which 1,001 young volunteers have participated in the frontline prevention and control work. The Jinan Communist Youth League will also call on the city's all levels of youth civilization to play a role of example. At present, there are 1,121 youth civilization collectives above the municipal level in the city. Each civilization collective will continue to play a leading role, based on their own job responsibilities, to inject youth momentum and warm heart strength into the effective epidemic prevention and control work. Relying on the youth league organizations at all levels, youth working committee, youth association association and youth Entrepreneurs Association, Jinan Communist Youth League provides effective efforts to fight the frontline workers and their families, and to care for the disabled or elderly living alone, left-behind elderly and left-behind children, scattered orphans, troubled children, disabled people, pregnant women, special disadvantaged groups and low-income families.

2022-04-03	Guiyang: stick to the "epidemic" line warm people stick to the "epidemic" line warm people-Guiyang volunteers active in the epidemic prevention and control work of the city at all levels of epidemic prevention and control reserve volunteer recruitment order, so far, a total of 996 young people signed up in the epidemic prevention line, including students, workers, waiters... they use their own actions for Guiyang fuel, warm the masses."We have already arranged it here, and 11 quarantined personnel can come here."Deng Ruihan called while making the record. Born in 1998, after graduating from university in 2020, he became a volunteer of the Western China Project and came to work in the Yousqueeze Sub-district office of Nanming District. On March 30, The Fuyuan Bin Hyatt Hotel in Guiyang became the isolation point. As Deng Ruihan had worked as a volunteer in the Yardir Hotel before and was familiar with the procedures, he applied to become a volunteer again and came to work at the isolation site of the Fuyuan Bin Hyatt Hotel in Guiyang. After entering the isolation point, Deng Ruihan quickly got familiar with the layout of the rooms in the isolation point, and worked with other staff and volunteers to eliminate the elevators and rooms, so that he could arrange the quarantine personnel to move in as soon as possible. On the evening of March 30, more than 50 people were placed in the hotel. Wearing protective clothing, delivering food and water, organizing nucleic acid sampling... this is Deng Ruihan's daily routine. Most of the volunteers working in the isolation sites are in their early twenties, like Deng. In the eyes of their parents, they are still children, but when the epidemic has hit, they have become "soldiers" against the epidemic."Although the work is hard, it is worth contributing to the prevention and control of the epidemic."Deng Ruihan said. In order to ensure the life safety and health of the broad masses, Wangjiawan District organized regional nucleic acid testing in Qianling Town, Yunyan District at 22:00 on March 29. After learning the news, Gao Jie, a volunteer of the Western Project working in Qianling Town, immediately rushed to the Wangjiawan epidemic prevention site and became one of the epidemic prevention volunteer team."I am a volunteer and I should do something that I can to contribute to the epidemic prevention and control."Said Gao Jie. Gao Jie is mainly responsible for assisting residents in nucleic acid testing and maintaining order at the site. Gao put on her protective clothing and never removed it until the first round of nucleic acid sampling ended at 3 am the next morning. At this point, Gao's protective mask is full of water vapor. Gao Jie dared not drink water or go to the toilet, afraid of wasting protective clothing. She walked through the crowd and repeatedly reminded everyone: " Keep your distance."It is reported that in the Wangjiawan area, the first round and the second round of regional nucleic acid test results are negative."It was not easy for everyone, and everyone was very happy to hear about the negative test results."Said Gao Jie."Please keep a safe distance of one meter."For several hours, Wu Conghas been standing at the entrance of a nucleic acid testing point in Guanshan Lake, guiding people to queue up for nucleic acid testing. This week, two new imported cases of COVID-19 were confirmed in Guiyang, making the epidemic prevention and control situation grim. Wu Congling is one of the 68 employees of China Construction Technology Group who joined the volunteer team under the call of the youth league organization. On April 2nd, Wu Congling came to the testing site early, and did his own disinfection work, and then began a day of volunteer work. Wu Congling is mainly responsible for maintaining the order at the scene and assisting the masses in nucleic acid testing."To contribute to the epidemic prevention and control is a valuable experience in our life."Wu Congling said. Like Wu Congling, 68 volunteers from China Construction Technology Group were assigned to each epidemic prevention point according to the needs of each community. They gave up spending time and rest with their families and stuck to the epidemic prevention front and contributed to the city's epidemic prevention and control work.(Wei Xin, reporter of Guiyang Daily Rong Media)

2022-04-02	Weifang Municipal Party Committee: Five Volunteer Service Modules: Xing Ting, reporter of the Epidemic Prevention and Control Youth Daily) Since the outbreak of this round of epidemic, TuanWeifang Municipal Committee has innovated and improved the modular volunteer service system for COVID-19 since 2020, In accordance with the principle of "nearby recruitment, standardized participation in services, and help not to make trouble", Set up five volunteer service modules, including "front-line environmental disinfection, flow adjustment and testing organization, love materials fundraising, material errands and delivery, and dedication for devotees", With 14,000 young volunteers in the city, Organized 461 volunteer teams, Serving communities with 529, We will fully devote ourselves to the battle against the epidemic. For a line xiaosha prevention and control, group Weifang municipal party committee to establish "environment xiaosha module" youth volunteers, kite volunteers, spring rain volunteers, thunder rescue, Jinyang volunteers youth social organizations professional forces, quickly docking epidemic prevention and control command, the first accurate sinking to sanitizers demand of community, village and other public places, the efficient xiaosha work, cumulative environment xiaosha covers an area of 56 million square meters, and actively guide home sanitizers small video, guide residents home disinfection. In view of the flow testing, Weifang Municipal Committee established a young volunteer team of "personnel flow and nucleic acid testing organization module", organized 20001,000 volunteers and young students to participate in the local personnel flow work, effectively alleviate the problem of grassroots cadres take the lead, 6000 young volunteers into the line, deep into the community and village, cooperate with the local party committee, government and professional forces, participate in the nucleic acid testing site organization and order maintenance. At the same time, the group Weifang municipal party committee to establish "love supplies raise delivery" module youth volunteers, through the youth league, green enterprise association and county, town, different levels of organization, raise N95 masks, medical protective clothing, goggles, alcohol, disinfectant, such as instant noodles, biscuits, mineral water and other living materials worth more than 256 ten thousand yuan, continuously for disease resistance line transport disease resistance supplies. We will actively mobilize relevant enterprises of the Youth Federation, the Youth Enterprise Association and 16,000 rural young people to give full play to their strengths, and participate in material operation and ensuring stable supply and demand, so as to ensure both epidemic prevention and control and ensure supply and production. Focusing on the practical problems such as the shortage of living materials for residents in the sealed and control area and the control area, the Weifang Municipal Party Committee of the Communist Youth League established the "errand delivery module" youth volunteer module to carry out targeted assistance for key groups. In the work, we made full use of "Youth Community" and platforms such as "Express Industry Youth League Working Committee", actively guided the leading role of express boys, young social workers and other groups contacted by youth league organizations, and organized more than 4,000 young volunteers to actively participate in the work to provide convenience for the control of residents. In addition, the group Weifang municipal party committee to deepen the "dedication for devotees" action, form a "dedicated for devotees module" youth volunteer team, take the initiative to contact with frontline doctors, public security, volunteers and other epidemic prevention and control personnel, understand their life needs, help their family children run errands agent, tutoring company, compulsory haircut services, and to help keep war disease rear.

2022-04-02	On March 15, Linyi, Shandong province, reported a new confirmed case of COVID-19, marking the battle against the city. Linyi Municipal Party Committee of the Youth League immediately united with the Municipal Youth Federation, Municipal Students' Federation, Municipal Youth Enterprise Association, municipal Youth Volunteers Association issued an initiative to organize and mobilize youth league members, youth Federation members, youth enterprise association members, youth volunteers to act, start from themselves, listen to the command, do first, let the youth bloom in the front line of epidemic prevention and control. Mengyin county was the first confirmed case in the city. As soon as the epidemic appeared, Guo Jing, secretary of the Communist Youth League Working Committee of Mengyin Street, turned from a breastfeeding Bao mother to a "soldier against the epidemic" and never returned home again. Guo Jing, who is also the director of the western district of the urban community, is also responsible for the nucleic acid testing of more than 1,500 people in 11 old residential areas and family hospitals within her jurisdiction. She organized personnel, household survey, registration information, data collection, and guaranteed medical supplies... She stayed on the front line of epidemic prevention and control, and was deeply imprinted on her face."In this 'critical juncture', I can't just think that my children need to take care of, there are more community people waiting for my colleagues and me to guard, I have to charge ahead."Guo Jing said. Since the outbreak, the communist youth league extensive resources force, actively organize the evaluated integratedly young cadres and distant love alliance, learn lei feng love association, micro dream association and other social love groups in epidemic prevention and control volunteer service, to carry out the nucleic acid testing personnel registration, household screening, policy propaganda, order guidance, etc. Despite the epidemic, young people should take action. Young Party members of the Youth League and Linyi Municipal Party Committee took the initiative to sign up for community epidemic prevention volunteers, and assisted the community to carry out voluntary activities such as temperature measurement and code testing, isolation and control, door-to-door service, and order maintenance. Whether for the "war" disease postponed the wedding after the first secretary of gao, after 90 young teachers rock, or holding infants to three feet platform after 90 teachers LuNa, stick to nucleic acid detection line after 95 XingRuilin, Yang Shuyu, or "hate not have three head six arms" grassroots cadres Liu Hanqing, stick to the outbreak of a line interview reporter Chu Feifei, Song Tianjian, they are the epitome of Linyi league youth fighting epidemic prevention and control line. The frontline of epidemic prevention and control is also a strong test of the organizational strength of the Communist Youth League at all levels. City, county, township three-level youth league organization linkage, Lanshan District, Luozhuang District, Hedong District, Tancheng County and other county district youth League committee set up hundreds of youth commandos. The township streets green committee based on their own functions, actively launched youth members at all levels in the epidemic prevention and control of a line, lanshan district YiTang town, luozhuang street, junan county ping town nearly hundred evaluated integratedly, launched youth volunteers, village young grid team, youth members, the party activists, college students volunteers to carry out the sampling point order maintenance, foreign personnel screening, epidemic prevention site on duty, volunteer support registration, etc. Youth civilization at all levels has also sounded the clarion call to fight the epidemic. Linyi 120 Emergency Command Center set up four levels + personnel echelon and emergency consulting echelon, and the members petitioned to join the alarm line; Hedong District Fire and Rescue Brigade actively organized emergency response drills during the epidemic period, and the substation transportation inspection team of State Grid Linyi Power Supply Company carried out power maintenance of key power protection fields to ensure power supply. The youth volunteer teams are in action: the Communist Youth League Tancheng County Committee quickly established the youth commandos to carry out the epidemic investigation and registration volunteer service activities; the youth volunteer team of Mengyin County Tax Bureau to assist in the nucleic acid testing task of more than 1500 people in a community distributed throughout the country. They are born after the "1990 s" and the "2000 s", and even after the "hard work" and "hard struggle". They use their practical actions to make their youth bloom on the front line of epidemic prevention and control.

2022-03-31	Jiangsu su Suzhou: gaining paving escort Jiangsu Suzhou: gaining paving escort function advantages, focus on cohesion "two in" two with "new action and" I do the practical work for the masses "practice, the implementation of the" six stability "and" six protect "task," stick to "cloud employment, stable employment, promote employment" of the main line, to ensure that college students employment work steadily. In 2021, the number of registered college graduates in Suzhou was 28,821. By the end of December 2021, the employment rate of registered college graduates in Suzhou had reached 98.49%.1. Make a good advance, for college graduates to accumulate momentum. One is to light up the ability point of college graduates. Cooperate with various departments and municipal colleges and universities to carry out extensive employment counseling, innovation and entrepreneurship, volunteer service and other activities, such as the "U + Spread Your Wings" employment and entrepreneurship ability improvement plan for college students, and the social practice of "three to the countryside" and "returning to home", so as to guide college students to strive to be knowledge-based, innovative and skilled talents. Integrating the resources of youth Innovation and Entrepreneurship Consulting Service Center, youth Innovation and entrepreneurship organization service group, youth Innovation and Entrepreneurship mentor Group and other resources, relying on various youth innovation incubation bases, providing free entrepreneurship training and free accurate entrepreneurship guidance for young entrepreneurs in Suzhou. Second, to improve the practical ability of college graduates. The three-year 10,000 youth internship plan has been steadily promoted. Since 2019, the youth employment internship work in Suzhou has achieved remarkable results, and the number of internship positions and the number of participants in the employment internship have shown a rapid growth trend. At present, there are 907 employment internship bases in the city. From January 2019 to December 2021, the city provided 29,740 employment internship positions, and organized 11,460 people to participate in the employment internship. Third, to stimulate the initiative of college graduates. A total of 515 vanguard posts for the league members have been set up in the city, calling on college students to actively report to the local village and community youth league organizations and actively participate in the front line of epidemic prevention and control. Jointly with the Municipal Development and Reform Commission and other units issued the "Implementation Opinions on the Implementation of the" Youth Contribution Action " in the construction of major projects in the city, calling on the majority of young people to play the role of a new force and a commando in the construction of the city's major projects, and strive to make contributions and create results. We will thoroughly implement the "new rural Elite" cultivation and development plan, provide employment internship positions in agricultural production, sales, logistics, and e-commerce, guide college graduates to make contributions where the motherland needs it most, shine at the grass-roots level, and practice the youth oath of "please rest assured that the Party has my country" with practical actions.2. Show a combination of fist, pave the way for college graduates. First, we will tap the potential of employment and entrepreneurship. The 2021 China Yangtze River Delta Youth Business Summit Forum was held. More than 400 outstanding young entrepreneurs from the three provinces and one city of the Yangtze River Delta gathered in Suzhou and signed 18 youth business cooperation projects, with a total investment of nearly 6 billion yuan. To organize young entrepreneurs to actively participate in activities such as "city and county trips" of Yangtze River Delta Youth Business, G60 Yangtze River Delta Science and Technology Innovation Corridor, "Dialogue Youth Business Help Development" Young Entrepreneurs Forum and other activities, and to build communication channels for talent introduction, talent cultivation, platform construction and service integration. Jointly with Suzhou Mass Entrepreneurship and Innovation Center, we carried out 5 "Let China See You" youth Innovation and Entrepreneurship Project road show (Suzhou Station) biomedicine and equipment manufacturing special activities, and promoted 150 industrial projects. Second, we will expand employment channels. Highlight the location advantages of Suzhou, and accurately connect the talent needs of enterprises and university talent resources. Actively "go out". Integration to promote the "campus Suzhou Day", into the domestic double first-class universities, united with Suzhou leading enterprises, leading talent enterprises, well-known foreign enterprises, scientific research institutes and other 898 units, for college students to provide more than 40,000 jobs, received more than 3,800 resumes, reached 31 university-local cooperation, industry-university-research cooperation. Fully carry out the Suzhou campus talent introduction plan, the whole year is divided into 5 lines, organized 857 enterprises to 23 universities, attracted more than 5,000 graduates to apply for jobs, collected 4,300 resumes, and recruited outstanding young talents for the high-quality development of Suzhou. Warm "please come in". The Suzhou campus wisdom introduction activity was carried out, and 80 master and doctoral students from famous universities made technical breakthroughs on 36 projects from 21 enterprises. Held in 2021 "elite hui-school masters bo Suzhou line" series of activities, from tsinghua university, Peking University and other 18 "double top" universities 182 graduate students gathered in Suzhou, through negotiation, understand Suzhou innovation entrepreneurial environment, cultural history, strengthen the docking with unit of choose and employ persons, to attract more outstanding young talents to Suzhou innovation entrepreneurship. Third, we will enable employment and development. For 13 consecutive years, "organizing and providing 100,000 jobs for fresh college graduates" has been included in the practical project of Suzhou municipal government. Offline and recruitment will be promoted simultaneously on the cloud, and college graduates can help find full employment by holding cloud double selection meeting, "Waiting for you in Suzhou" live broadcast with posts, offline high-quality characteristic recruitment, and daily public welfare market for college graduates. In 2021, more than 18,000 employers in the city provided more than 241,000 jobs for college graduates, up 15.4% year on year, effectively alleviating and balancing the structural problems of difficult employment for enterprises in Suzhou and job seekers.3. Build a solid guarantee line, and provide the escort for college graduates. First, we will provide fine services. Relying on the "Su Qinghui" youth service cloud platform, provide characteristic for college graduates to guide, employment entrepreneurship, life services, style, family care, social integration package of services, through the "gen" Chinese youth innovation youth entrepreneurship comprehensive service platform can assign county youth entrepreneurship organization, for more ordinary graduates entrepreneurship mentor services, online training, investment and financing docking and other comprehensive services. Second, to provide reliable financial credit guarantee. Develop the exclusive "Huiqing Loan" online application green channel to provide financial credit opportunities for young entrepreneurs in Suzhou. At present, it provides financial credit to 1413 young entrepreneurial enterprises with a credit line of RMB 360 million, and helps rural youth to innovate and start, issuing "new Rural Elite loan" of RMB 135.86458 million yuan. Third, we will provide multiple policy guarantees. Promote "on strengthening the Suzhou talent rental housing security work several opinions" gusu key industry shortage talent plan rules for the implementation of the gusu talent "youth" plan " and other 13 youth development policy documents, effectively optimize Suzhou youth development policy system and working mechanism construction, from the source to eliminate entrepreneurial employment youth trouble back at home.(China Youth Daily, China Youth Network reporter Yang Baoguang) Jiang Yan, Deputy Secretary of the CPC Suzhou Municipal Party Committee, Source: China Youth Daily, March 31,2022, edition 05

2022-03-31	Changchun Communist Youth League led the cadres and young volunteers to fight the battle. As of 24:00 on March 27, the total number of cases reported in Changchun has exceeded 13,000. In the face of the sudden outbreak, the Changchun Municipal Committee of the Communist Youth League responded quickly and moved quickly, recruiting volunteers in batches, raising and distributing epidemic prevention materials, and the youth league cadres rushed to the front line of fighting the epidemic.......... Changchun Communist Youth League led the youth league cadres and young volunteers to fight the epidemic to the end. On March 12, Changchun city began to implement the closed management. Young volunteers began to be busy every day, and they stuck to the front line of epidemic prevention with responsibility and responsibility. This time, the Changchun Municipal Committee of the Communist Youth League implemented socialized and precise volunteer recruitment, according to the district, street (township) and community (village) recruitment of volunteers, orderly arranged volunteers to achieve the nearby post. Up to now, the Changchun Municipal Committee of the Communist Youth League has recruited four groups of epidemic prevention volunteers, and a large number of young people have responded positively once a recruitment notice was issued. Now, the youth league organizations at all levels in Changchun have established 113 volunteer service WeChat working groups, with more than 15,000 volunteers and more than 10,000 volunteers reserve. In order to meet this round of the epidemic, the Changchun Municipal Committee of the Communist Youth League put forward the working idea of "focusing on one goal, building six teams, and implementing four links". Focusing on one goal is to give full play to the advantages of the league organization, mobilize all available resources and forces, and fully cooperate with the key tasks of epidemic prevention and control in Changchun. Six teams have established six types of special service teams to support community prevention and control, raising and deployment of epidemic prevention materials, supporting key prevention and control areas, supporting key work tasks, and ensuring the living needs of residents. Do real four links, that is, do real social recruitment of volunteers, staff pre-job training, post personnel safety protection, tracking management four links. Recently, every morning at 6:30, volunteer Li Jinsong reports to Yangpu Community in the Jingkai District. He has been joining the "community frontline prevention and control volunteer service team" for more than 10 days, and has maintained the order of the nucleic acid testing site, input information, door-to-door nucleic acid testing for the elderly with mobility difficulties, and distributed antigen testing kits door to door. In his service position, Li insisted on not drinking water or going to the bathroom for a day. When he returned home in the evening, he felt a backache and took off his protective clothing. The clothes inside were full of sweat. As he became more skilled in volunteer work, Li has more tasks in training new volunteers. With his help, 18 volunteers quickly took charge. In a 25-member "team to support key prevention and control areas", the average age is only 24. They marched overnight from Nong'an County in Changchun city to Jiutai District to join the nucleic acid testing team. Zhang Chunlai, 25, a former armed police soldier, always volunteered to arrive at the nucleic acid testing site in Yingcheng Street, Jiutai District, when he came to collect nucleic acid for close contacts. He said, " I am a soldier. I have to work first."Tong Lijun and Sun Jinshi are a young couple, running a moving company, they are members of the" epidemic prevention materials deployment team. ". As the epidemic hit, the couple were busy carrying quarantine supplies, both drivers and stevedores. Now, both the couple keep their phone on 24 hours a day and on call. In Changchun community epidemic prevention front line, there is a professional public welfare disinfection team. They were originally the Changchun Volunteer Rescue Team, composed of volunteer heroes and volunteers. After the outbreak of the epidemic, they quickly assembled again, in the nucleic acid testing interval, carrying 35 kilograms of the fog disinfection machine, the area of the nucleic acid testing point for no dead corners of elimination. In order to improve the delivery time of living materials, the Changchun Youth League has also recruited a "living materials distribution team" composed of 62 T3 travel drivers to be responsible for delivering goods for the citizens purchased online. At present, the fleet has delivered more than 7,000 orders for the citizens. At the same time, Changchun also has 32 youth commandos, a total of 1,019 young people, fighting in all areas to ensure the normal operation of the city. Without moving the troops and horses, food and grass go first. The Changchun Youth Development Foundation provides material support for the anti-epidemic forces, including volunteers. This is a team with epidemic prevention experience to supplies supplies and transportation in advance in response to the epidemic that may come at any time. At the beginning of the current outbreak, members of the Changchun Youth Federation and members of the Youth Enterprise Association under the call of the Changchun Youth Development Foundation gave generously. At present, the Changchun Youth Development Foundation has raised 460,000 N95 masks, 1 million disposable surgical masks, 68,000 protective suits, 50,000 medical gloves, 32,000 isolation masks, and more than 5.5 million yuan worth of disinfection and insulation supplies, all of which have been delivered to the frontline of epidemic prevention. In order to ensure that the epidemic prevention materials are collected and delivered immediately and improve the transmission efficiency, the Changchun Youth Development Foundation has made precise deployment according to the epidemic situation in all counties and districts. At the same time, the Changchun Youth Federation also transferred more than 50 vehicles to set up a Changchun Youth Federation epidemic prevention supplies."I sign up!"On March 9, the Changchun Municipal Committee Working Committee issued an emergency notice to support the community front line, the Youth League Changchun Municipal Committee cadres have invited to fight. At present, while organizing volunteer services and raising materials, all the cadres of the Changchun Municipal Party Committee have fallen to the front line of epidemic prevention, and formed a team of cadres fighting the epidemic, which has been fighting for 20 consecutive days. Some government league cadres are transferred to nucleic acid testing agencies for coordination and supervision; some are dispatched to the streets to serve the epidemic; others are on duty at the checkpoints. At various epidemic prevention posts, youth League cadres constantly switch between the roles of card-duty volunteers, nucleic acid testing and code scanner, and material transfer labor force, and are on standby 24 hours a day to act bravely. The original youth face was blocked in the foggy protective mask after, the tender hands become red and wrinkled.... Changchun youth and the regiment cadres are racing against time, and the epidemic battle. During the rest, they will encourage each other in the WeChat work group and the circle of friends, " live up to the youth, live up to the time, and will fight the 'epidemic' to the end!"China Youth Daily, China Youth Network, reporter Wang Peilian, source: China Youth Daily, March 31,2022, the 02nd edition

2022-03-30	Ningbo yinzhou: with youth to field "two-way to" date long-term youth development planning county pilot Ningbo yinzhou: with youth to field "two-way to" date after overseas returnees postdoctoral HongYu � Xing jing � he is very glad to return first choice yinzhou, and become the first "bo and yinzhou" plan of young talents, has developed the best domestic technology of bulletproof ceramic materials. In addition to Hong Yu � � there are three young postdocs in the school to work in the school, in their strengths in their respective fields. To win the youth is to win the future. Ningbo yinzhou district of Zhejiang province has always attached great importance to youth development, become the national long-term youth development planning after the county pilot, has launched a series of big measures: youth development work was included in the government work report and area "difference" general rules outline, in the name of the district government issued the yinzhou district youth development "difference" plan, to carry out the seven youth projects, 62 youth related proposals submitted to yinzhou district two sessions, issued 30 youth development policies. From entrepreneurship and employment to platform support, to dating, schooling and medical treatment, culture and entertainment, good policies, good services and good atmosphere have attracted more and more young people to stay in Yinzhou. Youth and Yinzhou are together, two-way rush, grow together. In October 2021, the third "Yin Li" conference sponsored by the district government, launched the first young talent special policy: for entrepreneurial young talents, give the team maximum 100 million yuan, to the newly introduced young talents, leaders, top talents, senior talents, world top 200 overseas universities, graduates and young high skilled talents will be given one-time subsidies, up to 1 million yuan. At the conference, 90 after Shandong youth Zhao Lin also appeared on the stage. He is a doctor from Tsinghua University. He was just admitted to the Publicity Department of Yinzhou District Committee and is showing his talents in a new field."Yinzhou is a vibrant city and is very concerned about young people. I wanted to make a break here when I heard that doctoral students could enjoy a subsidy of 350,000 yuan."This year, like Zhao Lin, 17 graduates, into Yinzhou district-level departments. In addition to the financial subsidies, Yinzhou also has many supporting platforms to serve the youth. Young Talent Station provides 7 days of free accommodation for college graduates in Yin. Youth in the same time, but also can enjoy policy guidance, job recommendation, training exchange and other one-stop services; district college students (youth) pioneer park for startup youth "escort", build a "park + grassroots + university + enterprise" global incubation chain, form a large enterprise, research institutes, university innovation subject innovation ecological network, 205 mentors, service youth more than 30000 young, provide jobs 10000,100 youth entrepreneurship activities every year. Thanks to the support of the youth entrepreneurship and innovation platform, Ye Chenglong, a young man born after 1995, founded Yima Dang Fresh Trading Company. With a group of young people, he built the first smart agriculture enabling base in the province; the "Yima adoption- -Smart adoption Digital Agriculture Service Platform" project won the national "Creative Youth" Youth Innovation and Entrepreneurship Competition bronze award; and he was selected into the "Pan-Entrepreneurship Yinzhou Elite Leading Program" talent project, and received the youth entrepreneurship subsidy of 1 million yuan. In Yinzhou, youth gravity is growing. More and more young people choose Yinzhou. Since last year, 31,400 new college graduates have been in the district, ranking among the top of the city, introducing 1116 masters and top 100 universities in the world, setting a record high."Direct hit the pain point" let Yinzhou more youth attractive youth attracted into, but also to stay. In order to better meet the needs of young people, in this year's Yinzhou top ten practical people's livelihood projects, two major young people's livelihood projects were included, respectively for the construction of primary and secondary schools, kindergartens and the "the most beautiful way to school" comprehensive transformation. Up to now, the district has started the construction of two new schools and six completed schools, and carefully customized six "the most beautiful roads to school", with a total investment of 1.424 billion yuan. Young people gather because of the city, and the city prospers because of the youth."Ground stall economy" fire all over the big cities, "go out stall" become the popular word of Yinzhou youth. Tuan Yinzhou District Committee launched the "Yin Ling Youth Night Market", which packaged the products, services, public welfare and activities to the southern Commercial Water Street, Li West Corridor, future Community and other places. A total of 22 night markets were carried out throughout the year, attracting 682 young people to participate in the stall, and 280,000 young people watching."The youth night market gave us a stage to show off our talents. I was so happy to see so many people applaud me."Lu Qi, a student from Nottingham University of Ningbo, performed an original song at the night market and met a group of friends. Now, "Yin Ling Youth Night Market" has become a youth culture IP and characteristic night economy brand. For Yinzhou, half city and half township, youth cultural activities are also held in the countryside. In Tiantong Street, Soochow Town, a live broadcast of hornet's nest head anchor "Miss Guo", attracted 63,000 young people online and offline, in Jiangshan, more than 3,500 young warriors participate in the Gohe Sparta warrior competition, different colors of youth PK, in Dongqian Lake, the bonfire festival, 100,000 young people. In the rich and colorful youth cultural activities, youth landmark places also more and more, happy coast ecological sports park, crane youth sports park fitness places increasing, "box" and "may 4 blocks" youth creative blocks, 67 "youth home", 117 "yong love e home" to provide intimate comprehensive services for youth. These youth elements are making Yinzhou even more attractive. Whether it is the top-level design, or the key projects, Yinzhou takes "creating favorable conditions for the youth" as the premise, pay more attention to the interests of the youth and the practical needs to start, pay more attention to the spiritual communication and emotional resonance with the youth. After the Tokyo Olympic Games, Yang Qian, the first gold medal winner, joined the "Yin Ling" post-00s publicity group in her hometown and was hired as the "Yinzhou Image Promotion Ambassador". She said: " As an athlete, I am very proud to celebrate the 100th anniversary of the founding of the new era; as a native of Yinzhou, I am very happy to show the world the spirit of Chinese youth."In the youth example, many after 80,90,00 youth preacher, into the fields, factory workshop, office buildings, rural community, to carry out the" Yin bell literary micro preach "" yinzhou youth blacksmith said "" young Dr Young said " and other forms of preaching activities, through the youth vision, youth thinking, youth discourse, good yinzhou story, China story. No struggle, no youth. In Yinzhou, such "two-way go" is staged all the time. Typhoon hit, more than 600 young people volunteered to participate in flood and typhoon overnight, within 10 days to help affected farmers to sell 100,000 jin of grapes; this outbreak, youth social organization blue sky emergency rescue team iron shoulder, 40 young members for 14 consecutive days, 24 hours uninterrupted nucleic acid test samples, is still in... now, the district registered young volunteers have reached 109,000 people, widely involved in social governance. In Jiangshan, Yunlong, Qiuai and other towns where the floating population gathers, volunteer services also greatly enhance the sense of urban identity and social integration. They achieve integral settlement through volunteer services and transform them into a stable registered population. China Youth Daily China Youth Network reporter Jiang Yutong source: China Youth Daily

2022-03-28	Lei Yu) On March 25, in the exhibition hall of the Wuhan Youth League, Wuhan celebrated the 100th anniversary of the founding of the Chinese Communist Youth League exhibition exhibits donation ceremony was held. The special exhibition will tour the government offices, campuses, enterprises, communities and rural areas, so that more youth members can review the centennial league history. When built in one hundred, collectors, shaanxi construction machinery co., LTD. tiancheng built machine deputy general manager jiang xiaoping to Wuhan municipal committee of free donated 18 pieces of Wuhan youth movement history physical data, including different historical period, group fee card, volunteer book, etc., and free borrow exhibition 28 (38 pieces) across the country youth movement history physical data, in Wuhan to celebrate the 100th anniversary of the founding of the communist youth league of China special exhibition activities. The special exhibition, jointly launched by the Wuhan Municipal Party Committee of the Communist Youth League, Wuhan Culture and Tourism Bureau, Wuhan Revolution Museum and Wuhan Youth League School, will be officially held in the memorial halls of the five major meeting sites of the Communist Party of China around the May 4th Youth Day this year. The exhibition will take important historical events and advanced typical figures in the centennial history of the Chinese Communist Youth League as clues, and show the glorious course of the Wuhan Communist Youth League uniting and leading generations of young people to struggle with the Party under the leadership of the Party. Source: China Youth Daily

2022-03-24	Sichuan Guangyuan City Lizhou District "small housekeeper" on-line Sichuan Guangyuan City Lizhou District "small housekeeper" on-line, this is the group Guangyuan City Lizhou District Committee to realize the "home school community" tripartite linkage, guide the young pioneers to report, participate in the community governance of a new measure. Community housekeeper build mobile book bar in the community space, they bring their idle books, toys to the bar, provide residents with a free reading, teaching salon, entertainment and leisure integrated reading space; facing the severe epidemic situation, the community housekeeper called on the residents to strengthen daily exercise, enhance resistance, win the epidemic battle. At the event, Mr.He Juan of the Community Committee of Ju Road answered interactive questions about family education, school education, social education, subject education and reading habit formation. This will further improve the community group building and the young pioneers organization construction, "off-campus +" resource integration, team mobilization, position effective use, improve the young pioneers social work system, realize community flexible governance, build state area "small housekeeper" young pioneers characteristic practice and grassroots governance brand.

2022-03-21	The first film league school in Nanning, Guangxi was inaugurated. Film league school to "welcome 20 always go with the party new journey" as the theme, open red movie, theme class, film league school "card signature", review the league oath courses, and the sheng world film international cinema (jiangnan shop) 5 cinema renamed "ideal hall", as the film league school exclusive hall. At the same time, the organizers used the Palace Museum to make the Study Manual of the Film League School and 12 scene stamps. The youth League organizations at all levels (youth league branches) and the young Pioneers can cover the stamps of the study manual to exchange or carry out the theme activities. The establishment of Nanning Film League School by Nanning Youth League Municipal Committee aims to continuously deepen the party history learning and education, strengthen the ideological and political construction of young people, and consolidate the construction of the league organization activities. In the next step, the Nanning Municipal Party Committee of the Communist Youth League will take the film youth League school as the main position to promote the regular development of the "four histories" learning and education in the city, and organize a series of themed league day and team day activities in a way that is popular with teenagers.

2022-03-21	In Zhenjiang, Jiangsu college students concentrated cloud attracting activities in Zhenjiang national college students concentrated cloud attract activities reporter li chao) on March 18, Zhenjiang held 2022 hundred college students concentrated cloud attracting activities, by promoting Zhenjiang talent policy, organize thousands of enterprises, hundreds of colleges and universities, thousands of college students to carry out online communication, build talent and enterprise docking "magpie bridge", blowing more young talents to join Zhenjiang assembly. The event was guided by the guidance of Jiangsu Provincial Department of Human Resources and Social Security, hosted by Zhenjiang Municipal Party Committee and Municipal Government, and organized by Zhenjiang Talent Office and Zhenjiang Human Resources and Social Security Bureau. It attracted 110 universities from Jiangsu, Anhui, Shandong, Henan, Hubei and other provinces to attend the event, including 70 universities outside the province. On the same day, the organizer released the talent demand for 13,684 college graduates collected from 1,058 enterprises and public institutions in Zhenjiang."Last year, we introduced 38,600 college graduates, including 19,100 with a bachelor's degree or above, up 6 percent year on year. The number of registered residents from outside the city increased by 12.8 percent year on year. The' Chinese City talent attraction ranking'ranked 14th to 41st in China."Zeng Wenbing, director of Zhenjiang City Human Resources and Social Security Bureau, said that this year, Zhenjiang city human resources and social security department will base on the new positioning of" building 40,000 college students ", planning to introduce 40,000 college students and 20,000 skilled talents in the whole year. Source: China Youth Daily, March 21,2022, edition of the 02nd edition

2022-03-18	Guangdong Chaozhou hundred youth theory about "cavalry" ready to go Guangdong Chaozhou hundred youth theory about "cavalry" ready to build wei correspondent Liao Yukun) recently, the group of Chaozhou municipal party committee of Guangdong province held a "lighthouse project" -Chaozhou communist youth league "welcome twenty ten, always follow the party, the new journey" theme information and "Chaozhou youth branch" collective lesson preparation. Members of Chaozhou youth teaching group, city and county (district) level youth League committee organs comrades attended the meeting. Collective lesson preparation meeting is on-site. Correspondent for figure in 2021, Chaozhou 65 "Chaozhou youth branch" members actively take part in Chaozhou communist youth league "learning party history strong faith with the party" series of theme preaching activities, grassroots, into the campus, enterprise, combined with the party history learning education, based on the actual post, spread the party's voice, preach the party's policy, annual cumulative about 358 times, online covering 368000 people. Conference, group Chaozhou municipal party committee decided to award outstanding performance xiu-jie deng, zhenrong Yang, shen become warped, Guo Jiaqiong, zhuang, Zheng Jiong, Zhong Xin, Guo Xiangyu, Wei Chuan, Chen Qin 10 comrades "outstanding young lecturer" title, further stimulate the young lecturer to preach enthusiasm, solid promote youth theory to preach work in-depth development. This year, in order to meet, study, publicize and implement the Party's 20th Congress, let the Party's voice the most extensive to young people. Group Chaozhou municipal party committee through directional hiring, organization recommendation, personal recommendation, selection from various fields industry high theoretical accomplishment, preaching ability of outstanding youth adjustment to enrich the "Chaozhou youth branch" team, including party and government cadres, theoretical experts, party history experts, young teachers, media workers, entrepreneurial youth, etc. At present, the "Chaozhou Youth Lecture Group" has 119 members. The letter of appointment in 2022. Correspondent for figure at the scene also held in 2022 the first collective lesson preparation, invited han shan normal college, vice President of the school of history and culture, Chaozhou municipal party committee XuanJiangTuan members, part-time Wen Jianqin to " don't forget the beginner's mind always follow the party: one hundred communist youth league endeavour preach theme, around the glorious history of the Chinese youth movement, with a lot of historical materials and rigorous argument, expounds the relationship between the history and the party. Next, we will organize the teaching group groups to carry out research and make courseware, create high-quality shared courseware, put it on the theme group class network platform, and lay a solid foundation for the young lecturers to better carry out the theme of "Welcome 20 and Always follow the Party on a new journey". Theme lecture collective lesson preparation. Correspondent for figure youth branch members and cadres have said, will combine their respective jobs, the theory of the spirit, policy, the party's history and the history of the leadership of the youth, youth movement language and youth feelings, the "truth" into "little story", the theory logic into life logic, let youth theory armed work more ground gas, more temperature.

2022-03-17	Tuan Songyuan municipal Party committee: To provide a "spiritual feast" for the isolated college students, Wang Peilian) "Teacher Xu, I was the student who called you yesterday, after talking with you, I am not so anxious..." on the morning of March 14, psychological counseling volunteer Xu Hui received a feedback call from the isolated college students in Songyuan. At present, 350 students from Jilin University of Agricultural Science and Technology are under quarantine in Songyuan. In order to ease the students' possible tension, anxiety and loss, TuanSongyuan Municipal Committee opened a "spiritual oxygen bar" hotline for the youth service platform. Fifty-two psychological counseling volunteers were recruited to provide online 24-hour online counseling services. Xu Hui said that every morning, the psychological counseling volunteers would send some text, pictures and videos to interact with the quarantined students to guide them to actively face the epidemic and eliminate anxiety. It is understood that the Songyuan Municipal Committee of the Youth League through the "Youth League cadres + volunteers + students" work mode, timely understand the needs of isolated students, to guarantee services. In order to enrich the students' lives during the quarantine period, the TuanSongyuan Municipal Party Committee also organized a short video competition of "Drinking two cities together". Soon after the event began, many entries were received from the students being quarantined. Not only that, the Youth League Songyuan Municipal Committee also launched a succulent adoption activity, giving each college student in isolation a pot of succulent plants, making the isolation room full of vitality. In addition, the Songyuan Municipal Committee of the Communist Youth League has also set up six temporary youth league branches in the Songyuan isolation point of Jilin University of Agricultural Science and Technology, calling on the youth league members to maintain a good attitude and actively cooperate with the isolation. At the same time, we also contacted 6 high school class youth league branches to pair up with the temporary youth league branch, established a wechat group, and carried out various forms of online activities.

2022-03-15	Li Yan, a young man from Northeast China who moved to alcohol live streaming, set up a sales volume of 470,000 yuan in a single live broadcast not long ago. Behind this amount, cannot leave the league organization to help him."Not only sales, the number of fans, the number of groups, per capita stay time are all indicators to measure the effect of a live broadcast. Professional live streaming teams should be able to clearly know the demands of each live broadcast and set the theme of each live broadcast... "In 2021, by participating in a training activity called" Rocket Live streaming Training Camp ", Li Yan and his live streaming delivery team learned the relevant knowledge of live streaming comprehensively and systematically for the first time. The 8-day training activity included a one-day big class and a 7-day tutor to "run with you", including job segmentation, live broadcast skills and operation ideas. Benefiting from the "one-to-one" guidance of its professional tutors, this young team has not only improved its business, but also made many like-minded friends. Located in the first flow economic industrial base in Jiangxi Province, at the intersection of Dongsheng North Road and Shunwai Road, Jingdong Town, Qingshan Lake District, Nanchang City, has attracted a large number of new young people like Li Yan to work, start businesses and pursue their dreams in 2021. Emerging young people are full of personality and large mobility, and the work of the park was once "difficult to organize coverage" and "difficult to play a role". Base league committee secretary Huang Plateau introduction, in order to better cohesion and service youth, flow economic industrial park after the establishment, joint ByteDance monthly "LIVE salon" "spark city conference" live "rocket" camp "anchor" trainee "real estate agent training" "selected alliance with goods king offline selection will" online training exchange activities, improve the youth league member level and professional skills. Based on the 2021 when Nanchang city was identified as the pilot city of the industry system in the league construction, the Communist Youth League seized the opportunity to study and deploy the league construction work of the industry system, issued the Communist Youth League Nanchang Municipal Committee on Further Strengthening the Work Plan of the Industry System, and established a special class of the industry system in the league construction. According to the actual economic and social development of Nanchang, all departments of the Communist Youth League Municipal Committee comprehensively sorted out the stock resources and carried out thorough research in the subdivided fields. Due to the need, the Communist Youth League Working Committee, namely, Hongcheng Street Art, Flow economy industry and postal and express delivery, were successively built. Nanchang Youth League organization establishes the industry working committee among the emerging youth people, extends the "working arm" of the group, makes friends with the young people, and creates a "warm nest" for the emerging young people."Where the youth league member is, we will build where the youth League working committee is."League Nanchang Municipal Party Committee of the relevant responsible person introduced, according to the industry management characteristics and regional distribution, three industry league working committees have a number of league branches, the city's busker group, flow economy industry, express logistics industry youth members all into the league organization management. In addition, in order to promote the smooth development of the newly established industry League working committee work, the League Nanchang Municipal Committee will guide the establishment and improvement of the league system, select and match all levels of the league organization members. To carry out job training for the new secretaries of the youth League working Committee to improve the professional ability of the youth League cadres. Today, Jiangxi has a short shuttle to the base and subway station in 20 minutes a day, providing convenience for young employees; in order to solve the problem of youth dining, the park opened a canteen; less than 1 year, 20 youth members of the base submitted the application. After the establishment of the youth League organization, the enthusiasm of emerging young people to participate in social governance was further enhanced. In 2021, with the recommendation of the Nanchang Municipal Party Committee of the Communist Youth League and the Qingshan Lake District Party Committee of the Communist Youth League, five people in the park were elected members of the Municipal and District Youth League and members of the District CPPCC. In the future, the Communist Youth League Nanchang Municipal Party Committee in the annual "two red and two excellent", "Nanchang Youth May 4th Medal", "youth post expert" and other selection and commendation, will be inclined to the industry system youth league cadres, youth league members. Nanchang Post Express Industry Working Committee of the Youth League organized youth league members to send care and warmth to delivery men throughout the city, combined with the party building "red Post" and "youth home" and other service positions, to provide intimate services such as comfortable rest, endurance and supply for delivery men, and the centripetal force of the league organization has been constantly enhanced. In order to standardize and promote the Nanchang street culture and art performance market, the Publicity Department of the Nanchang Municipal Party Committee, the Nanchang Municipal Communist Youth League and other relevant departments jointly launched the "Hongcheng Street Art". Since the establishment of Hongcheng Street Art and Youth League Working Committee, it has continuously planned a number of street concerts, street culture and art festival activities. To celebrate the 100th anniversary of the founding of the Communist Party of China, the Communist Youth League Working Committee organized young musicians to hold a red song tour in many landmark venues in Nanchang for one consecutive week. Organized youth members to visit and study in Ruijin, the revolutionary holy land, and jointly launched the original song "My name is the Soviet", as a tribute to the 90th anniversary of the founding of the Soviet Republic of China and paying tribute to the revolutionary martyrs. Regular public welfare classes are held, and industry professionals are invited to give open classes to young artists, teach vocal music skills, and improve their professional quality. In the future, group Nanchang municipal party committee will seize the industry system league organization construction work pilot opportunity, intensify fishing expeditions in the field of other emerging industries, combined with the actual bold exploration building path built group, innovation group model, summarizes the industry system built group useful experience, strive for in areas such as VR industry built more with the characteristics of Nanchang industry system league organization. Source: China Youth Daily, March 15,2022, edition of the 01st edition

2022-03-17	Shandong Linyi cadres help package contact children volunteer service activities in Shandong Linyi cadres help package contact children volunteer service activities jianwei correspondent Guo Jingjing) to the "hope cabin" volunteer service do real fine, establish cadres long-term support mechanism, better service cabin host healthy growth, the Shandong Linyi municipal party committee combined with "learn lei feng volunteer month" work arrangement, lead the city cadres at all levels to help package contact "hope cabin" children "learn lei feng" volunteer service activities. The cadres of the towns and streets of Luozhuang District helped the children in the Hope House with homework, made beds, talked with the parents, told stories to the children, publicized the knowledge of safe electricity use and prevention of school violence, and sent rice and noodles and other living materials to the families in need. The Youth League Committee of Yandi Town of Yinan County organized volunteers to help the children of "Hope House" and taught them to make dumplings; the Youth League Committee organized cadres to carry out the "reading with growth" activity to enrich the extracurricular life of Xinji Town League cadres to check the use of the hut and do the room health maintenance work. Tancheng county youth league cadre and youth volunteers walk into "hope small hut" in children's home, carefully check the construction of small hut and use a circumstance, check whether volunteer help activity is implemented in place. The Communist Youth League officials, volunteers and the children play games together, clean up their rooms, and publicize safety knowledge such as preventing carbon monoxide poisoning. The cadres and young volunteers sent badminton, rope skipping, basketball, gobang, chess, notebook, neutral pen and other sports and school supplies, encouraging the children to develop their interests and hobbies and maintain a strong body. They also clean up and tidy up their rooms together with the children to help them develop the good habit of practicing hygiene. Next, the group of Linyi municipal party committee will continue to focus on the "hope hut" children's actual needs, organize the city cadres at all levels joint youth volunteers to carry out the family companionship, psychological counseling, academic counseling, feeling city, wish to achieve volunteer service activities, help children to develop good habits of learning and living, realize from "cabin refresh" to "color" spiritual growth.

2022-03-17	Guizhou guyang, Guizhou patrol forest volunteer service propaganda demonstration activities "xiao Lin" patrol Lin forest volunteer service propaganda demonstration activities JianWei correspondent Chen Kai) recently, by the group of Guiyang municipal party committee, Guiyang forestry bureau, Guiyang forest office, Guiyang youth federation and other units held "xiao Lin" patrol forest volunteer service propaganda demonstration activities in deer guan provincial forest park, Guiyang expensive district (city, county) "xiao Lin" volunteer representatives, Guiyang industry member youth representatives, rural two levels of government Lin representative more than 30 people. The demonstration activity mainly includes the Guiyang Luchongguan provincial forest park patrol, the park biodiversity status survey, the implementation and publicity of the "Guizhou Province Volunteer Service Measures" and other contents. Activities, the forest park management office staff to introduce the basic situation of the park, development change, qian kernel ecological staff lead you through interactive participation in the park biodiversity survey activities, enjoy the deer chong forest park beauty also learned a lot of plant science knowledge, learn the volunteers tour content, methods, matters needing attention. Since May 2021, group Guiyang municipal party committee, municipal forestry bureau, city forest long system office jointly launched the Guiyang your Ann "xiao Lin long" volunteer service action, facing the society to recruit citizens as a "xiao Lin long" volunteers, nearby, then patrol forest protection, fire prevention propaganda, garbage cleaning, uncivilized behavior to dissuade, forestry protection propaganda and other volunteer service. The activity aims to organize and mobilize the majority of young people and other social forces to respond to the call of the central, provincial and municipal Party committees with practical actions, promote the participation of the whole people, and guide the young people to serve to "strengthen the ecology" and help "strengthen the provincial capital". In this demonstration activity, the Communist Youth League Municipal Party Committee and the Municipal Forestry Bureau awarded the forest park with the first "Guiyang Gongqing Forest- - 'Xiaolin Long' ecological civilization education and practice base" plaque. In the next step, we will cooperate with 18 Guiyang municipal forest chief responsibility areas to further clarify the scope of "Xiaolin Chief" volunteer service action, standardize the content of volunteer service, and continue to carry out "Xiaolin Chief", "Qinghe Qinghe" and other green brand projects to ensure the effectiveness of the activities and protect the clear water and green mountains.

2022-03-12	Xie Yang, on the eve of the 44th National Arbor Day), the Hechi Municipal Party Committee of the Communist Youth League and the Hechi Forestry Bureau jointly carried out the "Lin Xiaoqing" Arbor Day volunteer service activity in Hechi City. Tree planting activities begin, the relevant person in charge of group from Hechi city silver protects inspect branch, Hechi city Jincheng district party committee, jinchengjiang district township, Hechi city social work service center and 29 departments of youth volunteers on a "carry forward the lei feng's spirit, be era pioneer" theme lesson, called on young volunteers to actively participate in volunteer service, improve youth volunteer service ability. During the tree planting activity, more than 120 "Lin Xiaoqing" volunteers came to the tree planting activity site of Hechi Airport. Under the guidance of the technicians, they carried out various forms of volunteer activities, such as planting flowers and trees, maintenance operations, disease and insect control, and facility maintenance.

2022-03-12	Anhui Tianchang: create "home" young pioneers help "double reduction" Anhui Tianchang: create "home" young pioneers help "double reduction" Wang Haihan, correspondent, Qian Yonglai Yang Jiali) " a faucet 15 minutes dripping 200 mg, if there are a lot of faucet dripping every day, how much water to waste a year ah! I must remind my parents to close the tap when I go home!"Recently, Anhui Province Tianchang city first primary school six (14) squadron young pioneers Wang Mengling in the Tianbao community young working committee organized" beautiful day I am a small actor " environmental knowledge lecture said. In order to give full play to the advantages of young Pioneers education organization and practical education characteristics, and cooperate with the "double reduction" work, the Youth League Tianchang Municipal Party Committee and the Municipal Young Working Committee to create "young Pioneers at home", using the winter vacation, weekend to organize young pioneers to report to the community young working committee, to participate in red education, vocational experience and other activities."Where the needs of teenagers are, the services of the community young working committee will follow everywhere. Encourage children not to indulge in video games, and build a bridge between community young working committees in schools, families and communities."Tianbao community young working committee member Wang Jiamei said, in order to enrich the youth after school life, the community young working committee combined with the characteristics of the youth growth stage, regularly arrange them to participate in social practice, community governance, held a" colorful holiday "," I am a small speaker " and other activities."The Tianchang Municipal Committee of the Communist Youth League will combine the deepening of the reform of the grass-roots organizations in the county level, relying on the 'youth home' and other positions, to create the young Pioneers practice education map, and enhance the service and attraction of the young pioneers within the community."Youth League Tianchang Municipal Party Committee related responsible comrade said. Photo taken by Xu Xianzhe, Guangling Street, Tianchang City

2022-03-11	Tangshan, Hebei: Youth Volunteer Service Month Activity Launch to the Future " Youth Volunteer Service Month activity. The organizer selected three demonstration activities of sunshine assistance, community assistance, respect and volunteer encouragement: in the school for deaf children, volunteers and children play table hockey, do free diagnosis and rehabilitation guidance; in the basic community, organize the elderly people to celebrate the collective birthday, make fruit paintings and read a letter from young children to their parents; in the transportation hub volunteer service station, visit young volunteers, send heating equipment, service equipment and souvenirs. This volunteer service month activity will focus on six aspects: publicity to help the Winter Olympics, Promote the popularization of ice and snow sports, Promoting the Winter Olympics culture, Serve and guarantee the families of frontline personnel for the Winter Olympics; inch grass love respect the elderly action, For the needs of the elderly, To carry out voluntary services in humanistic care, material assistance and other aspects in communities, rural areas and welfare institutions; We will promote the development of demonstration volunteer service sites for young people who love and respect the elderly, Caring for special groups, With the empty-nesters, left-behind children and the disabled, To carry out emotional care, academic counseling, legal aid and other care and assistance volunteer services; Green and environmental protection services, Around Arbor Day, March 12th, Carry out practice and publicity volunteer service activities in green planting, green travel, bird loving and bird protection; The Better Life Volunteer Service, Organize young volunteers in rural areas to carry out volunteer services such as science popularization, literature and art to the countryside, skills training, help, education and assistance; Civilized life advocacy, We will resist old rules and bad habits, promote the virtues of thrift, and promote civilized customs.

2022-03-15	Jining: reunion love 2022 the first stop youth dating activities in Jining: reunion love 2022 the first stop youth dating activities held group Jining municipal party committee for figure China youth network Beijing on March 15 (reporter Zhang Jianwei, correspondent jingbo), group of Shandong Jining municipal party committee in the city first people's hospital successfully held in 2022 "reunion love" the first stop youth dating activities, from the city's enterprises and institutions of more than 60 single outstanding youth to participate in. The picture shows the first stop of the Communist Youth League Jining Municipal Party Committee reunion love 2022 youth dating activity site dating activity through interactive games, eight-minute dating and other young people willing to accept the forms, both ice-breaking team cooperation links, and sweet game interaction, a number of young men and women found the right object, successfully hand in hand. In the next step, the Communist Youth League Municipal Party Committee will continue to deepen the brand of "reunion and love" youth dating activities, carry out various themed activities on a regular basis, and build a platform for single young people to develop friendship, harvest love and grow up.

2022-03-16	Tongren to carry out "I donate a tree concentric to build chunhui forest" activities Tongren in 2022 "I donate a tree concentric to build chunhui forest" activities on March 16 (reporter zhang, correspondent Chen Kai) recently, Guizhou including 2022 "I donate a tree concentric to build chunhui forest" municipal demonstration activities held in the mountains Dan street flag tun village. The Standing Committee of Tongren Municipal People's Congress, Tongren Municipal Party Committee of the Communist Youth League, Tongren Forestry Bureau, Municipal Federation of Trade Unions, Municipal Women's Federation, and Wanshan District participated in the demonstration activities.site of activity. After the launch ceremony, under the guidance of the technicians, we shovel the soil to participate in the tree planting activities. In recent years, led by Tongren municipal forestry bureau, municipal federation of trade unions, municipal women's federation, further implement the new era of socialism with Chinese characteristics and jinping general secretary visited Guizhou important speech spirit, by giving full play to chunhui action social mobilization mechanism, cling to the affection, nostalgia, friendship "emotional line", township scene, xiangyin, nostalgia of "homesickness line" and filial, gratitude, feedback, return "line of virtue", gather more social forces power high quality "ecological city" "industrial city" project. Since the launch of 2018, the activity has attracted more than 1.5 million people to participate, integrated and raised funds of more than 10 million yuan, and driven the construction of 14 Chunhui Industrial parks. Since this year, Tongren city has raised more than 900,000 yuan. All districts and counties also adopted the "online + offline" crowdfunding method to raise public welfare funds for the construction of Chunhui Forest, and simultaneously carried out the activity of "I donated trees for my hometown to jointly build a Chunhui Forest".

2022-03-14	Jining youth power the Yellow River basin ecological protection and high quality development volunteer service action "the Yellow River shore is home" Jining youth power the Yellow River basin ecological protection and high quality development volunteer service action Jianwei correspondent BaiChen), by the Shandong Jining municipal party committee, municipal ecological environment bureau and Jining city departments jointly sponsored by "the Yellow River shore is home" Jining youth power the Yellow River basin ecological protection and high quality development volunteer service action activities held in Liangshan county. Activities, deputy secretary of pku Gao Dongxu is introduced in the river along the Yellow River river patrol river protection, water quality monitoring, afforestation, the Yellow River volunteer service activities such as good results, issued the "green force green beach" ecological promotion, "Yellow River power" red continuously and "happy home" power beach people live three rich Jining power of the Yellow River basin ecological protection and high quality development of volunteer service projects. The picture shows the leaders attending the meeting concentrated unveiling for the youth community activities positions. The leaders of Jining Municipal Party Committee attended the meeting opened the Jining Young Pioneers off-school practice education base- - "Yellow River Memory Hall", "River Xiaoqing" Yellow River basin ecological protection science hut, "sapling" community mental health counseling station and "Qingchuang Jining" community youth entrepreneurship service station and other youth community activity positions. Jining Ecological and Environmental Protection Volunteer Association and Shandong Jiayuan Testing Technology Service Co., Ltd. donated popular science items and rapid testing service projects to the "He Xiaoqing" popular science cabin. Industrial Bank Jining Branch, Xinwo Construction Group Co., LTD., Liangshan County Rural Good Youth League to the province to "strengthen the construction of rural grass-roots Party organizations" task team Liangshan County general team, the provincial first secretary of Liangshan County temporary Party branch donated green seedlings. In the community service center and cultural square, 15 youth volunteer team for beach area people provide free health examination, health consultation, free hair shaving, fire safety knowledge, worker rights and interests protection consultation, employment and entrepreneurship policy propaganda, anti-drug dissemination education, youth mental health education, agricultural products quality and soil safety quality testing and other convenient services, let beach area people really experience "sincere service, convenient home" one-stop convenient, beneficial service, get the masses consistent high praise. The picture shows carrying out volunteer service activities in the community party-mass service center and the cultural square. Group Jining municipal party committee for figure group will serve the Yellow River beach relocation community measures into the "I for youth" work list, focus for the children of the Yellow River beach and entrepreneurial youth do the practical work, solving, do good, scientific design, elaborate "Yellow River memory hall" young pioneers practice education base, "river" the Yellow River ecological protection science hut, "saplings" community mental health counseling station and "green" community youth community service activities, for the beach residents construction youth happy home. Next, Jining pku will be "youth help the ecological protection of the Yellow River basin and high quality development volunteer service action" is suggested, adhere to the upper and lower linkage, prominent regional characteristics, based on work effect, continue to carry out the brand, serialized volunteer service projects, build can promote work brand, with youth, dedication and bear good "Jining in the Yellow River story".

2022-03-11	Fuzhou for young talents to carry out the electricity training power rural revitalization of Fuzhou for young talents to carry out the electricity training power rural revitalization of acquisition reporter Tian Hongwei) on March 9, the communist youth league of Fuzhou municipal party committee and China post group co., LTD., Fuzhou branch and other units, in Fujian live ecological garden jointly organized the "green village time" rural revitalization of youth with goods host training class. The training lasted for 3 days, focusing on theoretical training in case analysis, short video production, live broadcast room construction, product selection and speech techniques and other practical applications, and practical training based on the hardware platform of Fujian Live streaming Ecological Park. The trainees are mainly aimed at fresh graduates in Rong universities, members of Fuzhou Youth Federation, returning youth entrepreneurs, youth cadres in the village, college students and village officials, rural revitalization demonstration village backbone and youth League branch secretaries, etc. Huang Ting, a student majoring in fashion design at Minjiang University, participated in the activity through the introduction of the school teachers. She was deeply impressed by the lecturer's introduction of how to locate the short video account, find the target audience, how to shoot the shots, create scripts and other content. Xie Jingguo, a post-90s student and the youth league branch secretary of Pengxie Village, Hunan Town, Changle District, Fuzhou, is also interested in the course. He had been working out of the field. Last year's village election, he chose to go home to join the rural revitalization work. Xie Jingguo hopes to learn the relevant knowledge of online e-commerce through training, and exchange experience with other friends who work in rural areas, so as to explore a way out and find opportunities for the development of his hometown. Wang Chuanshu, who combines the Internet with rural revitalization in Yongtai County, Fuzhou, runs wechat official accounts and short video accounts with more than 110,000 followers. He hopes to improve his knowledge system in short video and online e-commerce through training. As the secretary general of Yongtai County Youth Innovation Association, he also hopes to introduce good training content and mode to Yongtai County to serve more young people returning home to be entrepreneurs. Chen Guoxing, the village branch committee of Guanzhong Village, Jingxi Town, Minhou County, Fuzhou, has a lot of feelings about the "first e-commerce village- - 'Daxi Village' model sharing" brought by Huang Changhui, an expert in the rural revitalization industry. His village has a certain scale of goldfish industry, which has many differences from the Xinjiang Daxi village shared by the lecturer. Huang Changhui's sharing inspired Chen Guoxing to think more. It is understood that students with excellent results in this training can obtain later commercial cooperation and employment opportunities through the internal incubation system of Fujian Live streaming Ecological Park. China Post Group Co., Ltd. Fuzhou Branch, relying on postal e-commerce channels, will provide high-quality agricultural products, supporting expert guidance, information consultation, practical training platform, start-up loans, customized delivery and logistics, and other services for young talents in youth entrepreneurship projects and e-commerce live broadcasting industry.

2022-03-10	Anhui Bengbu start "happiness Bengbu youth action" volunteer service activities Bengbu learn lei feng volunteer service month in 2022 and "happiness Bengbu youth action" volunteer service activities China youth network Beijing on March 10 (reporter Zhang Jianwei correspondent Zhang Zheng check WeiTing) recently, the Bengbu in Anhui province municipal party committee joint straight authority committee, civil affairs bureau of Bengbu learn lei feng volunteer service month in 2022 and "happy Bengbu youth action" volunteer service activities launch ceremony was held in the huaihe river culture square. The Municipal people's Congress, the Municipal People's Political Consultative Conference and the organizers attended the launch ceremony. The city's outstanding youth volunteers, outstanding youth service organizations and project representatives participated in the activity. At the launch ceremony, Han Jian, Party Secretary of the Communist Youth League, introduced the implementation of the "Happy Bengbu Youth Action" and read out the commendation list of outstanding individuals, outstanding organizations and outstanding projects in 2021. Leaders presented the certificates of honor to the award-winning volunteers and volunteer project representatives, and presented flags to the outstanding organization representatives. Anhui youth volunteers outstanding individual, taihu world culture BBS sixth annual meeting of outstanding youth volunteers representative Du Hongyang speak, he said volunteer service career is a noble cause, is an important carrier of spiritual civilization construction, is the spirit of contemporary young people love and accept the fashion, where need, where there are our youth volunteers, at the same time, he called on the whole city youth fully carry forward the lei feng spirit, do lei feng, lei feng internalization, externalized in line, let the lei feng spirit in Bengbu generations. Volunteer service is an important symbol of social civilization and progress, and learning from Lei Feng Volunteer Service Month is an important carrier of volunteer service. In order to fully display the good image of Bengbu youth volunteer service organization, 20 outstanding youth volunteer service projects were displayed on the activity site. After the launch ceremony, the young volunteers also carefully prepared at the scene for hair, home appliance maintenance, youth rights consulting, electricity water gas propaganda, fraud propaganda, tax consultation, health consultation, business employment policy and other convenient volunteer service, more than 40 young volunteers to participate in voluntary blood donation, contribute 7700 ml of fresh blood. In the next step, Bengbu city will take this activity as an opportunity to lead the youth league members to actively participate in the volunteer service, practice the "Lei Feng Spirit" with practical actions, and build a happy Bengbu together.

2022-03-09	Group Chuzhou municipal party committee to carry out the voluntary tree planting and communication fellowship activities of Chuzhou municipal party committee to carry out the "yue swim line chunhui" voluntary tree planting and communication fellowship activities wei correspondent Wang RuZha WeiTing) recently, group Chuzhou, Chuzhou, Chuzhou youth volunteer association, young entrepreneurs association jointly in south qiaoqiao zhang wide town to "with a tree to build youth forest" as the theme of the voluntary tree planting and dating fellowship activities, to protect green, fellowship friendship and volunteer service, is the practice of "green water castle peak is the jinshan yinshan" concept of action, It is also a good platform for promoting the exchange of young talents and serving their growth and development. On the morning of the same day, more than 100 Communist Youth League members and young volunteers from the municipal organs and institutions and the municipal Youth Enterprise Association members cooperated well, digging pits, supporting trees, planting, planting soil, sowing the seeds of green to the green water and green mountains. This "Yue Tour Line Chunhui" voluntary tree planting is the third phase of Chuzhou Communist Youth League of Chuzhou "Youth Yue +" youth talent exchange series of activities. In 2022, Youth League Chuzhou Municipal Party Committee around the city's "double recruitment and double introduction" work mobilization conference deployment, Based on the reality, exploit the advantages to the full, Carefully planned the young talent "youth Yue +" series of activities, We will continue to carry out a series of young talent exchange activities, such as "Yue Enjoy Green Cafe", "Yue Reading Book You Hui", "Yue Tour Chunhui", "Yue Heart Warm Yi Hui", "Yue Watch Movie Watching", "Yue Chat Tea Si Hui", "Yue Listening Concert", "Yue Dynamic Fitness Hui", By inviting leaders of relevant department leaders, experts and scholars, members of "Chuying Zhihui +" youth think tank and other role models to share with the young people face to face, Organize young people to carry out cultural, sports activities and volunteer services such as reading, sports and watching movies, Help young people to expand their "circle of friends", Gather the "new synergy" for development, For Chuzhou high-quality development of a solid talent cornerstone.

2022-03-09	Shandong Rizhao youth innovation and development conference held Rizhao youth innovation and development conference held month 9 (reporter Zhang Jianwei, correspondent phase chang hui) recently, Shandong sunshine youth innovation and development conference, pku relevant person in charge to attend the meeting, counties, functional areas, straight group (work) committee principal, municipal green league committee representative, city green enterprise association secretariat, pku authority staff to attend the meeting. The meeting conveyed and learned the spirit of the ninth Shandong Provincial Communist Youth League, the first plenary session of the 14th Rizhao Municipal Committee of the Communist Party of China, the first session of the 19th Rizhao Municipal People's Congress, the first session of the 11th Municipal CPPCC, and the "innovation to revitalize the city" mobilization conference, Read out the main responsible comrades of the CPC Municipal Committee in the Rizhao Communist Youth League 2021 annual assessment good report instructions and requirements, The work of the Communist Youth League, the work of the Town and Street Youth Working Committee, the "youth home", the rural good youth, the "Green Bird plan", the young Pioneers work, the youth volunteer service work, and the work of the youth entrepreneur Association of the outstanding collectives and individuals were notified and praised. At the meeting, we summarized and analyzed the work experience of the past year: "Party building leads the league building" is a strong political guarantee, around the center, serving the youth is a clear direction of work, the upper and lower linkage, multi-party coordination are important organizational advantages, at the same time, with the "six deficiencies" to accurately judge the existing gaps and deficiencies. Juxian Youth League County Party Committee, Lanshan District Andongwei Street Youth League Working Committee, Rizhao Bank Youth League Committee and Rizhao Vocational and Technical College Youth League Committee, respectively, around the national county Communist Youth League reform pilot work, town and street youth working Committee work, enterprise youth work and university youth work to make an exchange speech. The departments and departments of the Communist Youth League released the innovation of key work in 2022. Xu Guannan, the research and development team of Rizhao Port "Xinxin" automated container terminal, and Chang Yanlei, the national young pioneer of rural revitalization, respectively delivered the theme of "Youth Innovation Power in Rizhao Wonderful" Butterfly Change "" and "Let the Flowers of Youth bloom in the vast rural world".

2022-03-09	Gannan, Gansu province to carry out the "student dream cast people" public welfare activities Ma Fuchun) On March 4, "light up the light of the dream, help youth set sail, build" ten homes "Gannan, Gansu Province" student, dream, cast people " public welfare student aid conference was held. Yang Jun, deputy secretary of Gannan State Party Committee, attended the meeting and delivered a speech. Yang Guanglong, a member of the state government party group and deputy governor, presided over the meeting. Yang Jun fully affirmed the outstanding contribution of the prefectural Communist Youth League organization to the public welfare education cause in Gannan, hoping that the aided students are self-reliant and unswervingly study hard; caring people for a long time to do a deep and practical public welfare cause; organizations at all levels take the initiative to give full play to the role of a bridge. At the meeting, Yang Xuefeng, deputy director of the Standing Committee of Gannan People's Congress, introduced the donations and donations for public welfare in 2021, and Wang Haiqing, secretary of Gannan Party Committee of the Communist Youth League, reported the relevant work. Representatives of the caring units distributed grants to the representatives of the subsidized students and signed a "one-to-one" funding agreement. According to reports, last year, the Youth League Gannan State Committee, the state Youth Foundation widely docking all kinds of social love resources, a total of 7.24 million yuan, material value of more than 952,000 yuan, funded the state college and technical secondary school students more than 640 students, more than 2,500 primary and secondary school students. Since the launch of the "charity education campaign", Gannan Prefecture has been strongly supported by many caring units and caring people inside and outside the province, and has raised a total of 550,000 yuan, supporting a total of 188 college students and 12 primary and middle school students.

2022-03-08	Group Chuzhou municipal party committee to learn lei feng love volunteer service into the community activities Chuzhou municipal party committee to learn lei feng care volunteer service into the community activities the youth network Beijing on March 8 (reporter Zhang Jianwei, reporter Wang Jiecha WeiTing) on March 4, Anhui Chuzhou Chuzhou CPPCC office, Chuzhou, Chuzhou city vocational institute and Anhui Chuzhou ning highway development co., LTD. Further Xia Gang community "carry forward the new era of lei feng spirit pavilion city youth positive energy" learn lei feng love volunteer service into the community activities. At the event site, carried out love haircut, love free diagnosis, anti-fraud publicity and other volunteer service activities. Volunteers from Chuzhou City Vocational College half root grass volunteer service team have carried out volunteer services such as TCM massage and blood pressure measurement for the masses, which have been highly praised. The Communist Youth League Municipal Committee, the Municipal Red Cross Society and Anhui Chuning Expressway Development Co., Ltd. also carried out care and sympathy activities among disabled families, empty-nesters and poor families, and sent them rice oil and other comfort products.

2022-03-04	Jinan, Shandong Province: Juli to build a youth development-friendly city demonstration model Xing Ting) On March 1st, The Communist Youth League of the 17th Jinan Fourth National Committee (expanded) meeting was held, According to the meeting deployment, In 2022, Jinan Communist Youth League will grasp the four key words of "innovation, pioneering, hard work and responsibility", Firmly grasp the " ideological guidance, Serving the overall situation, serving the youth " three major work directions, Anchor "walk in the forefront, comprehensive creation" "three walk in the front" always follow, general positioning, total navigation, Unite and lead the youth league members to actively participate in the construction of a modern and strong provincial capital in the new era, We will build a model for youth-friendly cities. In summarizing the work of the past year, the Communist Youth League Jinan Party Secretary Zhang Xi used four "tight" and four "more" to explain: guide the youth to listen to the party more effectively, follow the party and the government, service center work more accurate; closely contact the youth, youth growth and development environment more optimized, focus on the strict governing league, the league construction and reform deeper. In deepening the thought lead, in 2022, Jinan communist youth league will be to meet and study propaganda implement the party's 20, combined to celebrate founding in one hundred, to "celebrate 20, always follow the party, forge ahead new journey" theme education practice is suggested, to carry out the "challenge to keep beginner's mind youth tribute 20" theme activities, do a good job in "the glorious course of the party leadership of Jinan youth movement exhibition". Further explore the brand connotation of "post number team player", and make good use of "youth learning", "youth publicity group", "Qingma Project", "theme team Day" and other carriers. At the same time, it will comprehensively promote the construction of a youth development-friendly city, study and formulate the "Implementation Plan" and "Index System" to create a youth development-friendly city in Jinan, and guide and promote districts and counties to create youth development-friendly urban areas. We will give full play to the function of the joint conference on youth work, launch a special campaign of "Youth Development of Friendly Cities", and coordinate and promote all member units to introduce a number of high-value guarantee measures to meet the general needs of young people. Jinan communist youth league will also strive to build the first fusion young talent pilot area, promote the cooperation, promote talent chain and industry chain, innovation chain depth fusion, the implementation of springs youth industry talent gathering action, combined with the "green bird plan" brand work, normalized "line" "school season" "graduation season" "employment service season" "practice season" and other theme services. In 2022, the Jinan communist youth league will continue to launch "I do the practical work for teenagers" list, continue to promote "rural good youth can project", deep "" hope hut "dilemma children love project", launched "action" warm bee "express little brother care service project", deepen the "" youth community volunteer service project ", continue to carry out" youth after class delay service "and" love help the public welfare action ", etc. To power the new era of modern provincial capital construction, Jinan communist youth league will implement the "youth work" action, actively carry out production integration project docking negotiations, signing show work, continue to stimulate green league, youth enterprise association work vitality, high quality to undertake the sixth Chinese youth volunteer service project competition and volunteer service in Jinan exchanges in 2022.

2022-03-04	Baoding, Hebei province first family education service station in community Baoding first family education service station in community wei correspondent Sun Ning) home school collaborative education is the highlight of education reform this year, the family education promotion law after formally implemented on January 1,2022, local education departments actively launched a variety of home-school collaborative education new measures, build up a variety of innovation education new model. The picture shows the League Baoding Municipal Party Committee donating books to the family education service station. Correspondent for the figure on March 4, Baoding first "family education service station" officially settled in xinghua garden community, xinghua garden family education service station is by Baoding city education bureau, youth corps committee, women's federation, closes working committee, Baoding family education expert committee, Baoding normal affiliated school, xinghua garden community formed together, condensed social forces, together will implement collaborative education. Ge Xin, Party secretary of Baoding, said, "Home-school collaborative education into the community is the cohesion of social warmth. Such warmth will be transmitted to every teenager, and fully stimulate children's enthusiasm for learning with visible warmth, and such learning enthusiasm will become a positive and continuous motivation for children."

2022-03-04	Hefei: Wang Haihan and Wang Lei) Besides her continuous work, Gao Jing, a young entrepreneur born in the 1990s, always takes time to lead her team of 10 friends to learn theoretical knowledge together. Even if she is busy with her work, she will share the learning links of the party history, the relevant policies of non-public enterprises, and China's economic development knowledge in the group on time. Gao Jing is the head of the Straw Hat Image Company in Chaohu City, Anhui Province. She also has another identity- -the "graduate" of the non-public enterprise class of the "Yangma Project" in Hefei city in 2021. Last October, in the non-public enterprise class, she not only communicated with students in the same industry, such as new media and live broadcasting, but also learned about the work content of e-commerce, social organizations and other industries. At the end of the five days of class, she felt "reluctant to part". What impressed her most was that she could not fully answer some knowledge of the history of China's Party and China's economic development."Why is my theoretical knowledge so weak? Usually too busy with the work, neglected to study."When she went back, she threw these questions to her team friends and found that they were scratching their heads. Since then, regular learning of theoretical knowledge has become a required course for the team. The Hefei city "Qingma Project" has been carried out for 13 years. In 2020, Hefei was designated as the pilot city of the "Qingma Project" by the CPC Provincial Committee of Anhui Province. On the basis of college students backbone class, group Hefei municipal party committee joint organization, publicity, education, state, civil affairs, agriculture, party school and other departments and units, add state-owned enterprises, non-public enterprise class, social organizations, rural class, college youth teachers class, the young pioneers worker class and other six shifts, training students about 300 people a year. The study of "Qingma Project" not only enriched Gao Jing's theoretical knowledge, but also changed her business thinking. Before, after she received the shooting task, she would come to the text materials, then go to see the scene, and then start to shoot."At that time, it was more 'shooting for the sake'. Now, I told the team to understand the policy, know what rural revitalization is doing, and make the film more profound."Gao Jing said. After coming back from the "Qingma Project", she asked the team to go to the site as soon as the party A received the information, and then collect information online and do the homework in front. When filming a party building propaganda video in The town, Gao Jing named the protagonist as Chen Yuan Dao, who died at the age of 32. The team went to the former residence several times to discuss the scripting ideas with the narrator and the town publicity committee members, and strive to restore the history and reflect the new local development and changes. During that time, the team members kept brainstorming, and finally, "a letter home" in the form of "time dialogue" with the martyrs, by the town of the generation told Chen Yuan hometown education, environment, infrastructure and other aspects of the improvement. A cadre feedback: " Your films are very deep, let people are willing to watch."Encouraged by Gao Jing, who tried to shoot more works that are from the perspective of the people and easy to understand."Nearly 100 films were made last year, and the theme of rural revitalization accounted for more than half."Gao Jing felt that her work thinking had changed- -no longer just to publicize the performance of the unit, but to convey the party's thought, policy line and development concept, so that more people can understand the new changes in her hometown. The picture shows that Gao Jing (right) also wants to find an opportunity to take college students into the fields to carry out the "nest small group broadcast room", introducing the food, the scenery, the agriculture and the tourism projects of Chaohu Lake from the perspective of the returning students. Chen Tao, 36, has maintained the habit of visiting agricultural products at the grassroots level every month. From the farmers' home handmade bacon, sausage, salted fish, sesame oil, peanuts, to the town collective economy characteristic industry, he will understand on the spot. It was qingma training that ignited his entrepreneurship. Previously, he worked for a rural tourism company. Although he had a dream of starting a business, he could not find the right product and direction. In 2019, he participated in the first rural class of Qingma Project in Anhui Province, and served as the temporary youth League branch secretary of the rural class, and became classmates with the head of the village collective cooperative, farmers, entrepreneurs and other groups. He learned that the common pain point-agricultural products information asymmetry, poor sales. After class, he learned poverty alleviation cases and knowledge of e-commerce, and determined the entrepreneurial idea of building an upward platform for agricultural products. In the second half of 2019, Anhui Dream E-commerce Company came into being. At first, Chen Tao found a tomato product at a production base, which was sold on the shelves, but the packaging was damaged. Later, he tried packaging them in foam boxes, but the cost was too high and he sold little. He sank down to visit households, trying to find suitable for transportation, high added value of characteristic products. He is installed in his heart a pair of homemade agricultural products e-commerce "map" - -Chaohu City has what agricultural bases in the city, in what season, what products mature, he is clear. Every time he finds a good product, he will surf the Internet to compare prices, cost and packaging. Over the years, he has moved the rice and shrimp from the poverty alleviation factory in Zhegao Town to the Internet, and sold the clean water lotus root and quail eggs from a company to restaurants... In 2020, the company's sales volume exceeded 10 million yuan. During the outbreak, Chen tao also helped a student from Qingma Class and a village resident in Huangshan city to sell unsalable tea. When participating in the training of "Qingma Project", the teacher said that "cultivating young political talents with loyal political character" has always encouraged Liu Xiaowei, secretary of the Youth League Committee of Zhegao Town, Chaohu City, to take root at the grass-roots level."The spirit of Qingma is invisible, but it is reflected in the words and deeds of his daily work."She was the secretary of the Communist Youth League Committee for four years. On the one hand, she actively carried out quality development activities such as revisiting the revolutionary road; on the other hand, she helped the young people in great detail and enhanced their sense of belonging. In the cold winter, when volunteers stand guard, Liu Xiaowei always provides timely logistics support, even a pot of boiling water, a box of hot rice, a greeting; on weekdays, she wants to provide policy advice, small loans and other services, help to functional departments through procedures and seal; contact the Hefei Municipal Party Committee and town party and government departments to help subsidize and comfort the troubled youth... she felt that "Qingma Project" also gives people a sense of social responsibility, forming the mission consciousness of "go wherever you need". During the 2020 flood season, all the local "qingma students" will start the flood control work. During the epidemic prevention period, those on duty at the nucleic acid testing sites were mostly youth league members. She also serves the knowledge learned by the Qingma Project to rural revitalization. Last summer, the town Youth League Committee and the College of Business Administration of Chaohu University launched a simulation marketing competition to let local students see the changes in their hometown. Some students have specially designed cartoon images for the local unique "� X Mountain Yang" traditional Chinese medicine museum. At present, Liu Xiaowei has set up a volunteer service library composed of more than 100 young people. At ordinary times, many young village cadres volunteered to participate in volunteer services. It is understood that in Hefei at all levels "green horse students" training period, the youth league organizations and the industry departments fully involved in student education, assessment, feedback, and to carry out theoretical study, practice, communication, situational simulation, knowledge competition, such as tracking training activities, organize students to emerging industries, rural revitalization, community governance a line. The relevant person in charge of the Hefei Municipal Committee of the Communist Youth League said that the next step will explore relying on the Hefei "Qingma Project" Research Institute, focus on improving the teaching quality, broaden the talent recommendation channel, further enhance the attraction and influence of the "Qingma Project", and polish the golden signboard of the Communist Youth League for the Party to educate people.

2022-03-04	Group Shenyang municipal party committee started "the youth service warm neighborhood" theme practice group Shenyang municipal party committee started "the youth service warm neighborhood" theme practice report wang reporter wang Chen) to inherit lei feng spirit, carry forward the volunteer culture, youth and Shenyang youth volunteer association launched today "the youth service warm neighborhood" theme practice, the city has professional skills of youth volunteers into more than 30 community (village) to provide volunteer service. In the community, young volunteers according to the needs of residents, young volunteers take the combination of residents "order", "volunteer service organizations" to carry out volunteer services. The volunteer service teams of the Municipal Health Commission, China Construction Second Bureau and other enterprises and public institutions and the youth through the social registration have set up professional youth volunteer teams to provide more than 20 volunteer service projects such as legal and regulatory consultation and lectures, housing maintenance and free medical and health consultation. North from second bureau north youth volunteers hao-dong liu said: " I am honored to participate in the volunteer service, I am mainly responsible for the security temple community residents on old house common problems and maintenance, put their professional knowledge to serve the people, I hope to contribute to youth strength, do more practical work for the society, show the style of the youth volunteers."According to the introduction, the early recruitment of volunteers is hot, only a week has more than 300 young volunteers with professional qualifications actively signed up. In the next step, the Shenyang Municipal Party Committee of the Communist Youth League will continue to practice the concept of "two neighbors" as the main line, and actively guide young volunteers to extensively participate in the community construction. In the early stage, it will go deep into 500 communities in the city to carry out regular volunteer services.

2022-03-03	Heze communist youth league three work was written into the municipal government work report Heze communist youth league three work was written into the municipal government work report wei correspondent Cui Lu) recently, at the first meeting of the 20th Heze City People's Congress, the mayor on behalf of the municipal government to the congress government work report: " to promote the development of youth-friendly city construction; building hope hut 1000,100 small lotus school."Among them, hope hut and small lotus school work was included in Heze City in 2022 ten livelihood matters. Photo of the first session of the 20th Heze Municipal People's Congress. In recent years, Heze Municipal Party Committee and Heze Municipal Government have always attached great importance to the work of the Communist Youth League and the youth, and actively created opportunities and build a stage for the majority of young people to display their talents. It is of great significance to build a youth-friendly city, attract more young talents to stay in Heze, and constantly improve the vitality and competitiveness of the urban development of Heze to promote the high-quality development of Heze and create a new situation of backward development. Pku around creating youth development friendly city related work, youth work joint meeting, coordinate various departments at all levels, practical measures, build development platform, held a series of activities, maximum solve the importance of youth difficult, continue to improve the youth development environment, efforts to promote the development of Heze youth yearning city, friendly city, the city of youth. Youth Station provides one-stop services for young people who apply for jobs. Youth League Heze Municipal Party Committee for the map to do a good "hope cabin", "small lotus school" two key projects of people's livelihood. In the work report of the municipal government, it was clearly stated that 1,000 hope cabins and 100 small lotus schools should be built. The work of Hope Hut and Xiaohe School has been included in the 10 livelihood matters in Heze City in 2022. Since the launch of the project in 2020, the Heze Communist Youth League at all levels has raised 15.52 million yuan through various online and offline channels to build 2,676 hope cabins, and more than 3,000 children in distress have moved into the cabins. The number of hope hut construction and the number of funds raised are among the best in the province. At present, the city's youth league cadres at all levels are carrying out a comprehensive and detailed survey work. In 2022, they will complete the established goal of building 4,000 cabins in three years on the existing basis. Hope Cabin Pictures. The "Xiaohe School" Rural Left-behind Children Care project is a rural left-behind children project initiated by the Heze Municipal Committee. The project organizes college student volunteers to carry out one-month volunteer service activities such as family companionship, interest care expansion, self-care education and civilized etiquette during the summer vacation. In the past four years since the launch of the project, the city has built 366 "Xiaohe Schools", serving more than 20,000 left-behind children and serving 810,000 hours. Xiaohe School has become a practical project of people's livelihood recognized by the party and government at all levels and praised by the masses. Many media have publicized and reported, and was awarded the bronze prize of the fifth China Youth Volunteer Service Project Competition. In the summer vacation of 2022, the CPC Communist Youth League Municipal Committee will focus on the Yellow River Beach area and other key areas, and open at least 100 small lotus schools to provide more intimate summer voluntary childcare services for left-behind children in rural areas. Photo of Xiao He School. In the next step, the Youth League Heze Municipal Party Committee will thoroughly study and implement the spirit and work deployment of the municipal Party Congress and the people's Congress, focus on the two key projects of "Hope House" and "Little lotus School" with our heart, and do the people's livelihood well.

2022-03-03	Youth small mayor out of the "rural new life office" youth small mayor out of the "rural new village youth town" flash shop "New Year teahouse" exploded into the circle, the average daily reception of more than 600 people, often from Chengdu, Hangzhou, Wenzhou and other places to punch in here. Why is a "New Year teahouse" located in the village so popular? It turned out that this is the shared space of the youth town "entrepreneurial team partner" - -rural New Life Firm. During the Spring Festival, the "different pattern" was transformed into a "New Year teahouse", operating for 45 days from the first to the fifteenth day of the first lunar month, and then resumed its original function. The staff told reporters: " During the Spring Festival, it was transformed into a pop-up shop 'New Year teahouse', just to meet the ritual sense of the city youth, to recall the childhood taste of the New Year, but also to attract and increase the popularity."Henghe Chen Village is only 4 kilometers away from the city center, three facing water, is an ancient water village, with a history of more than 700 years. Mainly in agricultural production, location, natural and cultural resources have advantages, which has become the first choice for the Youth League Jiaojiang District Committee to build a youth town. After selected yokoghe chen cun, group jiaojiang district party committee and jiaojiang district agricultural water bureau, Chen street offices and other units to establish normalized contact, for 200000 yuan youth town project special funds, through the bidding form successfully introduced a creative enterprise for youth town "partner" entrepreneurial team ", and set up the youth town working group-" town housekeeper ", guide the village collective established yokogawa Chen tourism development co., LTD. In early 2021, a group of young "beautiful boys" came to Chen Village, whose "small town residents" established "rural newborn offices" in a hut at the head of the village. Here, the water tank, door panels have changed, most of the objects in the house are manually transformed, big to sofa, small to ornaments, and even the field of green Onions, radish, vegetables are pulled out as bonsai, rural collision of literature and art, old objects collision new ideas, here was made into a real version of the "yearning life". Chen Ting is the head of the rural Xinsheng office. She was once a youth league cadre. Because of her love for the countryside, she closely followed the boom of rural revitalization, quit to start her own business, and focused on serving the rural design and development. She told reporters: " As long as you have an idea to come to the village, the rural freshmen office can help them solve the problem."Chen Ting still remembers Qiu Mengying's first visit to the village. At that time, Qiu Mengying, a young literary man, came to the Youth Town of Henghe Chen Village with great enthusiasm and wanted to land in a rural slow mail shop. " We very welcome such young people with ideas, but considering the low income of the slow mail shop, I suggest that she add an affiliated business form, such as beverage shop and cultural and creative shop."" Rural Xinsheng Office not only gives me reasonable advice, but also helps to connect drinks, cultural and creative resources, and store design for me to choose."Qiu Mengying said. As the leader of the "entrepreneurial team partner" of the youth town, Chen Ting's first thing was to join the Youth League Jiaojiang District Committee to effectively integrate the idle resources under the framework of the overall planning. Chen Ting said: " Every summer, people from the surrounding areas come to Henghe Chen Village to enjoy the lotus flowers, but there is no other commercial ecology in the village, and tourists can not stay, let alone the chance to spend money. There are 131 old street buildings of the Ming and Qing Dynasties in "" Henghe Chen Village, which are quite distinctive and condense many stories and legends. T-word Ming Old street, century-old turret, seaside ancient library... culture and turmoil once parallel here. And this old street is historically a commercial street, why can't it be reopened?"With this idea, Chen Ting and the team planned a series of recruitment, investment activities. Youth Town adopts the business model of "one-yuan rent recognition", focusing on cultural and creative organizations, bookstores, cafes, live broadcast teams, design teams, agricultural products exhibition and sales, art exhibition exhibition and other categories, and simultaneously formulate and improve the business examination and award subsidy mechanism, to reward instead of rent. Jiang Yuejiao stood out from the 23 registered youth teams. Growing up in Taizhou city, she has been yearning for the original ecological life in the countryside."I didn't dare to go to the countryside because I couldn't find a suitable job in the countryside."Taizhou Jiaojiang District Committee to create the youth town, let Jiang Yuejiao's rural yearning has the opportunity to realize."The first time I came to the Youth Town of Henghe Chen Village, I was attracted by the antique scenery here. After understanding, I found that here for us to think of rural entrepreneurship youth to provide a variety of intimate services, let me move."Then, Jiang Yuejiao chose a small house in the youth town, invested 100,000 yuan to design and decoration, opened a named" rural house "cafe, finally found their own" heaven and earth " in the countryside. Now, there are 15 young entrepreneurs like Jiang Yuejiao officially moved into the horizontal river Chen Village youth Town. The agricultural output value of Henghe Chen Village accounts for a high proportion of the village collective assets, and the collective land mainly grows vegetables, rice and lotus. These agricultural products still present the characteristics of extensive and scattered type, technical content is not high, lack of brand effect, low added value. To break the deadlock, group jiaojiang district party committee and Chen Ting operation team in rural new firm convened a group of town farmers, town activists, village enterprise cooperation, agreed to build "two insect field" green farm, construction of fruit and vegetable planting sales, farming labor education, CSA (community support farming), farming experience, camping base, such as the integration of small comprehensive sharing leisure farm."Jku will yokohawa chencun into youth town, built the youth and rural 'heart bridge', attract more and more youth to open our shop here, let us the ancient village renewed youthful vitality, with the help of people, the villagers through the communist youth league to provide rich cart, began to sell lotus seed cake, lotus leaf chicken specialty, started business at home."Said Xu Xigen, the village party secretary of Henghe Chen Village. With more and more young people gathered, the commercial ecology of Henghe Chen Village has been gradually improved. Chen Ting, who has experience in youth league cadres, began to strengthen the construction of grass-roots youth league organizations and enhance the internal momentum of helping common prosperity. Under the guidance of the jiaojiang district party committee, through the rural new office discussion, determine the youth town youth league branch, composed of 34 people members covers entrepreneurship team, youth merchants, yokogawa chen village and surrounding farmers and the youth, and invited youth (college students) business mentor, district water experts as town life mentor, continue to explore the common rich practice path, to "rich ten path" build town life "rich ten scene". At the same time, with the help of "double hundred double service" syndicated cooperation advantage, the town is equipped with a can provide ground services, financial services, legal services and volunteer service considerate youth youth team, not regularly in youth town civilization persuasion, garbage classification, five water treatment, planting green, epidemic prevention and control, order maintenance, tourist consultation guide youth volunteer service, add warm background for the town. With the "youth town", the Youth League Jiaojiang District Committee's dating activities have a new venue. Yokohama Chen Village has set up a number of "pro-friendly" activity bases such as "Flower Sea in May" and "Love you 105 degrees". In 2021, a total of 5 youth dating activities will be carried out, serving more than 300 young farmers and talents. Rural new life office is not only a window for young people to enter the countryside, but also a source to further stimulate young people to participate in rural development. Staff told reporters, "during the Spring Festival, the youth town 'entrepreneurial team partner' tide set around street cultural tourism festival, extract yokohogawa chencun rich water system and local resources, the lotus seed soup, plum blossom cake specialty snacks, clay, cutting rice and other traditional customs, 'New Year flag wall' New Year's" teahouse "' rich cart" web celebrity elements fusion, these attract youth play, are we in rural new office imagination collision."Yu Feng, secretary of Taizhou Jiaojiang District Party Committee, introduced that through the construction of youth town, it has cultivated a group of young talents to participate in rural governance and common prosperity, improved the skills and quality of young people participating in rural construction, and implanted" youth power " into the beautiful countryside. At the same time, Yu Feng also said frankly, " At present, the youth town has built a bridge for young people and the countryside, but there is still a long way from realizing the real demonstration of effective rural revitalization and common prosperity. After all, the strength of the Communist Youth League is limited. The development of Yokohe Chen Village also needs to gather all kinds of resources. How to coordinate the support and participation of the party, government, enterprises and social resources, we are still exploring new paths and opening up new points."China Youth Daily, March 03,2022,05 edition

2022-03-03	Shandong Heze employment service season and 2022 cloud recruitment activities held "green bird plan selected Heze" employment service season and 2022 cloud recruitment activities held in China net reporter XingTing) on March 1, "blue bird plan selected Heze" employment service season and 2022 cloud recruitment live activities, the activity sponsored by Heze talent work leading group office, Heze municipal party committee organization department, Heze, Heze people club bureau, Heze sasac, live watch 150000 people. According to the introduction, the activity was held to promote the development of Heze youth friendly city creation, help the high-quality development of the Yellow River basin, continue to deepen the "green bird plan" work project, to serve the whole city to attract talent and wisdom work overall situation. Lin Yongjing, party secretary of Tuan Heze, said that Heze is now at a new historical starting point, with obvious location advantages, Peony Airport and Lunan high-speed railway, successively into the "aviation era" and "high-speed railway era"; the industrial drive continues to strengthen, "231" characteristic industry; the policy superposition effect is more prominent. He sincerely invited all the young talents to choose Heze and display their talents. The head of the department promoted the brand work of "Green Bird Project", "Youth Post", "Heze Green Post", "Xiaohe School" and "Rural Revitalization Partner"; the organization Department of Heze Municipal Party Committee explained the preferential policies for introducing college graduates and talent housing security, and the bureau introduced the "three branches and one support" plan and "enterprise introducing college graduates subsidy". In addition, eight employers, including Tongsheng Investment and Development Group, Fangming Chemical, Danhong Pharmaceutical, and Chenxi School in the High-tech Zone, introduced 730 positions through a live webcast."The 'Green Bird Project' platform has opened up a new recruitment channel for enterprises. Through zero-distance communication with college students, it has built a bridge and link of effective communication between employers and college students."Transportation investment and development group related responsible person said. It is understood that the next step, group Heze municipal party committee will take this activity as an opportunity to give full play to the "green bird plan" platform carrier role, accurate mining high quality jobs, accurate promotion matching jobs, in key universities as a whole built with good "green bird station", planning to carry out special in key universities recruitment, practice, perception and communication series of activities.

2022-03-03	"Bird return sail Yantai" 2022 spring breeze send post cloud recruitment "bird return sail Yantai" 2022 spring breeze send post cloud recruitment hot wei correspondent Zhao Jing) to help talent attract, service college students employment, February 28 morning, by the Shandong province Yantai municipal party committee organization department, group Yantai municipal party committee, Yantai human resources and social security bureau, the teng human resources group co., LTD. "bird return sail Yantai" 2022 spring breeze send post cloud recruitment activities successfully held. More than 900,000 people were attracted to watch it online through the live webcast. The job fair began with two short films of Yantai, which systematically and introduced the cultural, economic and social development of Yantai and nature. Event invited to the teng human resources group co., LTD., Yantai constant state chemical additives co., LTD., run group co., LTD., smoke building group co., LTD., Yantai wang meat co., LTD., Shandong nuclear power equipment manufacturing co., LTD., mountain han han xin technology co., LTD., Shandong peng xiang automobile co., LTD., Yantai zhenhua commercial group co., LTD., 9 key enterprises to promote quality job demand. At present, more than 11,000 jobs have been collected, laying a solid foundation for helping young people start businesses and find jobs. In addition, this activity also builds a variety of communication bridge between job seekers and enterprises through Tencent conference room, wechat group and other ways, and makes every effort to improve the effectiveness of employment through cloud interaction. In the early stage of the activity, the Communist Youth League Municipal Committee made full use of WeChat, Weibo and other platforms to publicize preheating and interpret policies through long pictures, posters and short videos, and actively create a strong atmosphere to attract young people to smoke and stay in smoke for employment and entrepreneurship. The "green bird plan" employment service season and "green bird return sail Yantai" 2022 spring breeze send cloud recruitment activities, to Yantai students in Yantai, Yantai, Yantai students and solve Yantai enterprise of choose and employ persons as the starting point, the organizers together, local, universities, enterprise linkage, designed to build young students, service ZhaoCaiYinZhi window, pooling platform of intelligence resources. Next, the Youth League Yantai Municipal Party Committee will continue to follow up the interview, signing progress, comprehensively do a good job of young talent contact services, continue to serve the talent recruitment and wisdom and college students' employment and entrepreneurship.

2022-03-03	Hefei: "youth volunteer community" focus on grassroots governance "the last kilometer" Hefei: "youth volunteer community" focus on grassroots governance "the last kilometer" green net reporter wang haihan wang lei) lives in Hefei yao area small Du Gang Huang Wenjing is Anhui medical university biology students, in addition to the winter vacation, every Wednesday afternoon and weekend, she participated in the community and nearby community volunteer service on time."Under the leadership of the community party and league organizations, I have tried to visit the empty-nesters and participate in the epidemic prevention and control."Huang Wenjing said. Since the beginning of this year, the Hefei Municipal Party Committee and the Municipal Youth Volunteers Association have focused on the "last kilometer" problem of grassroots governance, and unified the deployment of "youth volunteer community" activities to help the community epidemic prevention and control, environmental remediation, service for the elderly, the civilization creation, and care for the youth. Wang Zimeng, who lives in the dormitory community of Sanxiaokou Museum, Hefei, is a member of the "Lu Xiaozhi" volunteer service team in Luyang District. This winter vacation, she participated in the community young working committee organized by the home cleaning action, winter warm sun sympathy and other activities. The picture is Wang Meng to participate in sympathy activities since January this year, Hefei municipal party committee, city youth volunteers association touch city area 2738 village empty-nesters, disabled teenagers, key teenagers group actual demand, summary of nearly ten thousand college students volunteers and enterprises and institutions, social organizations, social work institutions and other young volunteers' professional expertise, free time, form the volunteer service supply and demand list."The community has signed an agreement with Anhui Forestry Vocational and Technical College for mutual assistance, to carry out environmental protection theme publicity in the district community every month, and will carry out environmental protection fashion show activities in March."Hefei High-tech Zone Shufengwan community secretary Wang Xian said, the community also coordinated the college and the district community property company cooperation, using the advantages of the garden discipline, and strive to organize students into the community to carry out" green protection, love green " activities, improve the environmental quality. The Youth League Committee of Anhui University of Traditional Chinese Medicine paired up with the Modian Community of Hefei New Station to carry out special volunteer services such as free traditional Chinese medicine appropriate technology diagnosis, first aid skills teaching, traditional Chinese medicine specimen display, traditional Chinese medicine health sachets making, fitness method teaching and so on. Ye Lanlan, secretary of the Communist Youth League Committee of the university, said, " We also pair up with all the campuses and the communities near the affiliated hospitals to carry out volunteer services to improve the people's health literacy."On the basis of collecting the opinions of the Hefei Communist Youth League and the Municipal Youth Volunteers Association from the youth league leaders of colleges and universities in each district and district, the street youth league organization sorted out the service list to realize the effective connection between the development needs of the community, residents and the professional supply of young volunteers. In the process of carrying out the community volunteer service, the Hefei Municipal Party Committee of the Communist Youth League and the Municipal Youth Volunteer Association explore and cultivate a number of characteristic community volunteer service projects. There are three party members active in the Sunac Hefei branch of The Property Service Group Co., Ltd. in the Hefei Economic Development Zone, two of whom are born in the post-1990s generation. These days, they take the community residents and old party members to carry out cleaning floor mat, screen window, air conditioning filter net and other convenient services. Most of the old communities around Wuhu Road Street in Baohe District, Hefei, where some elderly people and empty nesters live. Li Xianglian, the team leader of the community Blue Lotus Volunteer Service Center, understood the situation, and paired up with many old communities, and took volunteers to help the elderly cook, clean up, dry the quilts, and talked with the elderly every day. On March 1st, volunteers from Hefei Water Supply Group and Yicheng Street Youth League Working Committee also came to explain the knowledge of water use, with listening leakage equipment to test whether water leakage and water quality. The graph is volunteers come to detect water quality in addition, the Hefei municipal party committee to explore building volunteer service data platform, to upgrade to Hefei App volunteer service module, volunteer online registration, activities, time certification, points, insurance and community masses "order", "platform" order ", volunteers" order " and other functions.(All the pictures are provided by the volunteers Department of Hefei Municipal Party Committee)

2022-03-03	Nanchang: industry system building group, build a "warm nest" Nanchang: industry system building group, make a "warm nest" Chen Zhuoqiong) for content planning, sold chili sauce, wine fish, now to live wine northeast youth li yan in not long ago set a single live sales of 470000 yuan. Behind this number, without the league organization to help him."Not only the sales, sales, fans, per capita stay time are the indicators of a live broadcast; professional live broadcast team should clearly know the demands of each live broadcast, set the theme of each broadcast..." In 2021, by participating in a training activity called "Rocket Live broadcast training camp", Li Yan and his live broadcast team learned the relevant knowledge of live broadcast for the first time. The eight-day training session included a one-day big class and a seven-day mentor to "run with you", including job segmentation, live broadcast skills and operation ideas. Benefiting from the "one-to-one" guidance of its professional mentors, this young team has not only improved its business, but also made many like-minded friends. Located in the first flow economic industrial base in Jiangxi Province, at the intersection of Dongsheng North Road and Shunwai Road, Jingdong Town, Qingshan Lake District, Nanchang City, has attracted a large number of new young people like Li Yan to work, start businesses and pursue their dreams in 2021. The new professional youth are full of personality and large mobility, and the work of the park was once faced with "difficult organizational coverage" and "difficult to play a role". Base league committee secretary Huang Plateau introduction, in order to better cohesion and service youth, flow economic industrial park after the establishment, joint ByteDance monthly "LIVE salon" "spark city conference" live "rocket" camp "anchor" trainee "property agent training" "selected alliance with goods king offline selection will" online training exchange activities, improve the youth league working level and professional skills. In 2021, Nanchang city was identified as a pilot city of the league construction of the industry system. Nanchang Municipal Committee of the Youth League seized the opportunity to study and deploy the league construction work of the industry city, issued the work plan of strengthening the league construction of the industry system, and set up a special team of the industry system. According to the actual economic and social development of Nanchang, all departments of the Municipal Communist Youth League Committee comprehensively sorted out the stock resources, and carried out thorough research in the subdivided fields. According to the needs and conditions, they successively established three industrial working committees, namely, Hongcheng Street Art, flow economy industry and postal and express delivery. Nanchang Youth League organization establishes the industry working committee among the emerging youth people, extends the "working arm" of the group, makes friends with the young people, and creates a "warm nest" for the emerging young people."Where the youth league member is, we will build where the youth League working committee is."League Nanchang Municipal Party Committee of the relevant responsible person introduced, according to the industry management characteristics and regional distribution, three industry league working committees have a number of league branches, the city's busker group, flow economy industry, express logistics industry youth members all into the league organization management. In addition, in order to promote the smooth development of the newly established industry League construction work, the League Nanchang Municipal Committee guided the establishment and improvement of the league system, the selection and allocation of the league organization members at all levels. To carry out job training for the new secretaries of the youth League working Committee to improve the professional ability of the youth League cadres. Today, Jiangxi flow economy industrial base has a short shuttle bus to the base and the subway station in an average of 20 minutes every day, providing convenience for young employees. In order to solve the problem of youth dining, the park has opened a canteen. In less than a year, 20 youth league members of the base have submitted their applications for party membership. After the establishment of the youth League organization, the enthusiasm of emerging young people to participate in social governance was further enhanced. In 2021, with the recommendation of the Nanchang Municipal Party Committee of the Communist Youth League and the Qingshan Lake District Party Committee of the Communist Youth League, five people in the park were elected members of the Municipal and District Youth League and members of the District CPPCC. In the future, the Communist Youth League Nanchang Municipal Party Committee in the annual "two red and two excellent", "Nanchang Youth May 4th Medal", "youth post expert" and other selection and commendation, will be inclined to the industry system youth league cadres, youth league members. These industry system groups are also in action. Nanchang Post Express Industry Working Committee of the Youth League organized youth league members to send care and warmth to delivery men throughout the city, combined with the party building "red Post" and "youth home" and other service positions, to provide intimate services such as comfortable rest, endurance and supply for delivery men, and the centripetal force of the league organization has been constantly enhanced. In order to standardize and promote the Nanchang street culture and art performance market, the Publicity Department of the Nanchang Municipal Party Committee, the Nanchang Municipal Communist Youth League and other relevant departments jointly launched the "Hongcheng Street Art". Since the establishment of Hongcheng Street Art and Youth League Working Committee, it has continuously planned a number of street concerts, street culture and art festival activities. Organize youth members to visit the revolutionary holy land of Ruijin to learn the original song "My name is the Soviet", to pay tribute to the 90th anniversary of the founding of the Soviet Republic of China and pay tribute to the revolutionary martyrs. Regular public welfare classes are held, and industry professionals are invited to give open classes to young artists, teach vocal music skills, and improve their professional quality. To celebrate the 100th anniversary of the founding of the Communist Party, the Communist Youth League Working Committee organized young musicians to hold a red song tour in many landmark venues in Nanchang for one consecutive week. In the future, group Nanchang municipal party committee will seize the industry system league organization construction work pilot, intensify fishing expeditions in the field of other emerging industries, combined with the actual bold exploration building path built group, innovation group model, summarizes the industry system built group useful experience, for in areas such as VR industry built more with the characteristics of Nanchang industry system league organization.

2022-03-01	Group Jiangxi Yingtan committee: "three poly" play the role of committee "three poly" play the role of Jiangxi Yingtan committee started potential, from the organization propaganda, youth development and service overall three aspects actively play a role of staff assistant, to deepen the reform of the communist youth league, effectively fulfill the mission of "three responsibilities" provides the intellectual support and decision-making reference. In accordance with the principle of "work-related, similar regions and similar interests", all committee members and alternate committee members will be incorporated into the special committee. We will improve the working mechanism of the special committees, establish a system of reporting on the performance of their duties and year-end reporting, and clarify the work requirements of "one annual research report, two plenary meetings, and three grassroots investigations". The Standing Committee of the Communist Youth League regularly hears the work report of the committee, praises and rewards outstanding members, and reminds those who perform poorly and participate in few activities; meanwhile, employs outstanding NPC deputies and CPPCC members as instructors to incubate the committee "think tank" through "one-to-one" guidance, and guides more experts, scholars and professionals to participate in the work of the committee. Over the past two years, we have made more than 20 work achievements, presenting 11 proposals, suggestions and public opinions to the Municipal People's Congress and the Municipal People's Political Consultative Conference. The special committee carries out investigations and research every year, focusing on one key work in this field. For example, in view of the weak construction of grass-roots youth league organizations in towns and rural areas, we organized committee members to carry out the "Spring Festival grassroots" activity, obtain first-hand information, plan in advance and carefully promote the change of village (community) youth league organizations. After the transition, the average age of the village (community) youth league branch secretaries decreased by 8.8 years compared with the previous one, and the number of team members entering the "two committees" in the village (community) increased by 73.1% than before the transition. Rural revitalization of the problem of the lack of young talent, committee hang point contact 18 township (street) youth corps committee, thorough schiscosomiasis understand the actual situation, joint municipal party committee organization department and other eight departments issued a "new era of Jiangxi province rural good youth" selected culture plan, annual breeding township "good youth" 580 people, the municipal "good youth" 56 people, all into the rural "green horse project" training object. In view of the imbalance of the implementation of the medium-and long-term youth development plan, the committee members are organized to carry out special supervision, evaluation and research in different fields. Up to now, more than 140 key tasks have been basically completed, and the "9 key projects" have been included in the city's "14th Five-Year Plan" column. We will focus on the role of young people, guide the committee members to participate in democratic consultation with special committees in an orderly manner, and constantly promote the effective transformation of the policy of building a youth-and talent-friendly city. We carried out the "Six One" study and discussion research activity, collected more than 1,000 opinions and suggestions from more than 3,600 young people, focused on their "urgent and anxious" problems, carried out the "five actions to serve the youth", and completed 13 "practical work" projects, and the satisfaction rate of young people reached 96 percent. Organized the members of the special committee to participate in the activities such as "I offer a policy for the 'two sessions' ", "The Communist Youth League and the deputies to the NPC and CPPCC members" and "Youth simulation proposal", and timely refined the research results into policy suggestions. Among them, the proposal of "Promoting Youth Innovation and Entrepreneurship and Helping Rural Revitalization and Development" was included in the supervision of the provincial and municipal CPPCC committees. Giving full play to the role of members of the special committee, we promoted the establishment of three overseas youth league working committees in Beijing, Shanghai and Shandong province, selected 78 liaison officers for college students in 48 cities across the country, and regularly carried out the "green Eagle returning home" college students internship activities. In the whole year, 19 "college student internship bases" were built, and 287 internship positions were connected with 51 units, which further strengthened the contact between the youth league organization and Yingtan students and young entrepreneurs. Source: China Youth Daily, March 01,2022, edition 03

2022-02-28	The new journey of the opening ceremony of the youth Red Culture and Education Base in Qingyuan City, Guangdong Province, was held in the city's youth activity center. The relevant person in charge of the Youth League Qingyuan Municipal Party Committee said that Qingyuan City youth red culture and education base is the red position of the youth to learn the history of the party, the history of the team, the city's primary and secondary schools, the city youth activity center to play a position role. The base by "figure said the party history" "socialist core values" propaganda corridor, city youth red culture education VR digital exhibition and safety education exhibition hall, city youth series works exhibition three room, covers an area of about 230 square meters, configuration of Qingyuan city red education VR learning system and patriotism series, etc. Next, the venue will be open to teachers, students, teenagers and parents of primary and secondary schools in the city. The activity by the Qingyuan City Civilization Office, the City Education Bureau of guidance, the Youth League Qingyuan Municipal Party Committee, the city youth activity center hosted. The fourth-level researcher of Qingyuan Education Bureau, the person in charge of the Coordination Department of Qingyuan Civilization Office, and the person in charge of the Municipal Youth Activity Center attended the opening ceremony, and experienced the VR learning chair, VR sightseeing desk and other equipment.

2022-02-28	Jining young students cloud recruitment second live activity held "green bird plan to help enterprises climb" 2022 Jining young students cloud recruitment second live activity held year vice minister Li Ping introduced green bird station. Group Jining municipal party committee for China youth network Beijing on February 28 (reporter Zhang Jianwei, correspondent BaiChen) by the group of Shandong Jining municipal party committee, municipal talents office, municipal technology bureau, the municipal human resources and social security bureau, sasac departments, China unicom Jining branch of Jining "green bird plan to help companies climb" 2022 young students cloud recruitment activity was held smoothly on the morning of February 24. During the period, 993 recruitment positions in various enterprises and institutions, 240 employers, and more than 7,100 people were needed, covering the new generation of information technology, high-end equipment, medical care and health, financial securities, high-end chemical industry and other industries."Green Bird plan to help enterprises climb" cloud recruitment broadcast room, the major enterprises HR introduced the job demand in detail, a barrage kept jumping in the broadcast room. The event was broadcast through China Unicom APP, with a popularity of 300,000 people. The picture shows Gao Cong, deputy secretary of the Youth League Jining Municipal Party Committee, explaining the policy of introducing young talents. Youth League Jining Municipal Party Committee for the live broadcast set up a total of young talent introduction policy promotion, green bird station introduction, young talent accelerator introduction, key enterprises and units promotion, live lottery and other links, to the majority of young talents to sincerely invite entrepreneurship and employment. China unicom Jining city branch, Shandong lutai holding group co., LTD., Jining architectural design and research institute, lai commercial bank co., LTD., Jining branch, Shandong is nuclear electric power technology co., LTD., Shandong Intel force optical communication development co., LTD., HR, head of the enterprise to promote the unit and job information. Six key enterprises conducted real-time interaction with young talents in the broadcast room to timely answer their job consultation. Next, group Jining municipal party committee will continue to follow up, matching, interview signing, workplace training, fellowship communication and the whole chain service, continue to consolidate cloud recruitment experience, deepen the "green bird station" "young talent accelerator" construction, condensed more outstanding young students, widely touch service industrial park, actively help "double recruit double lead" work.

2022-02-25	Henan Luoyang "youth station" to provide free accommodation for young talents "youth station" for young talents to provide free accommodation in youth network reporter pan zhixian) is located in Henan Luoyang zhongzhou road maple leaf hotel west work area "youth station", a few days ago welcomed the first guest-from Nanyang job youth wen-dong zhang. During the winter vacation, Zhang Wendong got an interview notice from an optical technology enterprise in Xigong District. He came to Luoyang from his hometown Nanyang for the interview and needed to live in Luoyang for a few days. He learned that the "youth Station" in Luoyang was available for young people to move in for free, and he immediately submitted the application materials on his WeChat official account, and it was approved the next day. For a fresh college graduate, the Luoyang city "youth Station" offers a 1-5 days, three times a year of free admission opportunities, greatly reducing the economic burden of job seekers. In order to make job youth in Luoyang first "home" warm and convenient, in January, Luoyang began construction "youth station", mainly close to the business circle, park, build a one-stop youth talent service platform, for Luoyang employment youth talents to provide free accommodation, employment guidance, social integration and other series of services, help youth faster and better into Luoyang, take root in Luoyang. On February 22, he Wei, member of the Standing Committee of Luoyang Municipal Committee and minister of Organization, inaugurated the municipal demonstration "youth Station" in Dongshan Huayue Hotel, located in Luolong District, Luoyang City, Henan Province. The door is posted with the "youth station" logo, the room is fully equipped with slippers, towels and other daily necessities, the overall decoration style is simple and fashionable, warm and convenient. The person in charge of the hotel said that the hotel allocated a special floor to build a "youth post station", and equipped with public rest areas, to the young people who come to live in a rest, communication platform. According to the "Luoyang City Building a Youth-friendly City Action Plan", during the "14th Five-Year Plan" period, Luoyang city will focus on the implementation of the youth housing and security project, the construction and operation of the youth post stations. Luoyang youth post project by the CPC Luoyang municipal party committee, Luoyang people's government construction, by the communist youth league Luoyang, Luoyang talent office, Luoyang civilization office, Luoyang bureau of finance and counties, focus in Luoyang city build one-stop youth talent service platform, to luo, back to young talents to provide free accommodation, employment guidance, social services, guide them to choose faster and better in Luoyang, Luoyang, Luoyang, grow together with Luoyang, win-win future. The recent meeting of the second plenary (expanded) meeting of the 12th Luoyang Municipal Party Committee and the economic work conference of the Luoyang Municipal Party Committee clearly pointed out that a number of "youth post stations" should be built with high standards. The communist youth league of Luoyang party secretary Li Huamin, Luoyang is now in luolong area, west work area, jianxi, mengjin district, old city, yanshi, � e river area, and yi bin area and longmen park youth station 21, more than 1500 beds, to luo, back to young talents to provide free accommodation, employment guidance, social integration and other services, build a one-stop young talent service platform. Li Huamin said that the youth station has the conditions of position, logo, service and security, to provide high-quality occupancy experience for young people and relieve their job-hunting pressure. Li Huamin said that the Luoyang Municipal Committee of the Communist Youth League also arranged youth homes in the youth station, relying on the youth home to timely organize and carry out relevant activities, to provide a platform for young talents to communicate and exchange. At the same time, the youth station will also provide employment and life services for the young talents, equipped with special personnel to be responsible for the timely receiving and processing of occupancy applications, providing them with one-stop services, further promote the construction of a youth-friendly city, and attract the youth strength to revitalize the glory of Luoyang. According to the Implementation Plan of the Construction of Youth Post Stations in Luoyang City in 2022 (Trial), the free accommodation time of youth post stations is essentially one day to 5 days for one person, who can apply for 3 times a year, and the annual cumulative free accommodation time is less than 15 days. Apply to stay at the Youth Post Station, Should meet the following conditions: full-time graduates of ordinary institutions of higher learning or above (the year of graduation, one year before graduation and one year after) can come to luo enterprises to apply for a job or under 35 years old, Senior workers with secondary vocational or technical secondary school education or above (they must provide the certificate of academic qualifications and the vocational qualification certificate or skill level certificate recognized by the state); Both the universities and the registered residence are outside Luoyang City and have no own housing in Luoyang City District (fresh graduates in Luoyang University after graduation, Applicants still applying during the period of employment); in good condition, No major diseases or infectious diseases; Abide by the relevant regulations of the youth post station. According to the introduction, the young people who meet the conditions need to be 3 days to 5 days in advance, log in the Luoyang network wechat public account or Luoyang youth WeChat public account of the Luoyang youth station online service system, according to the nearest principle of choosing to enter the site, fill in the relevant information, complete the registration application. Qualification notification will be received within 48 hours. After receiving the successful SMS message, you can check in at the youth station according to the hotel occupancy procedures on the application check-in day. Those who are unable to check in on time for some reason should apply for adjustment online more than one day in advance on the application date. Those who do not check in without any reason will be disqualified from the current year."The facilities are complete and the service attitude is very good, too. The stay experience is great!"Cui Xiao, 24, is full of praise for staying in the" youth Station " in Luoyang a few days ago. Cui Xiao family lives in Song County, is a graduate student of social work major of Gansu University of Political Science and Law. Just after the Chinese New Year, he received an interview notice from a unit in Luolong District, Luoyang City."It took nearly two hours from my home to Luolong District, and it's not economical to stay in a hotel."Cui Xiao said that he was hesitant not to go to the interview."I saw in Luoyang Daily that the whole city was building a youth post station, so I tried to apply and passed the examination the next day."Cui Xiao said," I have a place to live, I can go to the interview!"Cui Xiao checked in the youth station in the city convenient hotel (Luoyang city government store), the station environment is clean and comfortable, the staff service is also very considerate."They also encouraged me to adjust my mind and wish me a successful interview. It felt very warm."Cui Xiao said. Talking about his career planning, Cui Xiao is very firm: " The construction of Luoyang youth-friendly city has given us so many opportunities and guarantees for our young people. I will seize the opportunity and strive to work in Luoyang!"He Wei, member of the Standing Committee of Luoyang Municipal Party Committee and minister of organization, said that building a number of" youth post stations " is an important deployment for Luoyang Municipal Party Committee to build a youth-friendly city and an important measure for Luoyang to attract and retain talents. Luoyang city will always adhere to the problem-oriented and demand-oriented city, timely understand and accurately grasp the actual needs of young talents, and gradually build and improve the youth post station with full functions, good service and many characteristics.

2022-02-25	The Ningxia Yinchuan Municipal Party Committee held the "Hope Desk" distribution ceremony in Yinguang Village, Jingui Town, County. At the ceremony, the leadership of the Youth Yinchuan Municipal Party Committee donated multi-functional learning books and smart eye protection lamps worth more than 210,000 yuan to 127 student representatives from 6 counties (cities) in Yinchuan. After the ceremony, the Yinchuan municipal party committee team members visited part of the donated difficult youth families, will "hope desk" and eye lamp to students' home, and charged the counties (city) district youth corps committee head to "hope desk" one by one to the aided students, and details about the aided students support needs, continue to do a good job of follow up. In the next step, the Yinchuan Municipal Party Committee of the Communist Youth League will mobilize the city's Communist Youth League system and other public welfare social organizations to take the "Hope Project" project as the carrier to steadily promote the "Hope Desk" care project around the growth needs of teenagers. More than 30 people attended the ceremony, including members of the Yinchuan municipal Party committee, Jingui Town, leaders of the county and district (city) youth league committees and representatives of the aided students.

2022-02-25	Xie Yang) After the Spring Festival, the Liuzhou Communist Youth League of Guangxi actively coordinated all resources to organize all counties and districts to carry out the special car action of "returning to work". The action was supported by the Bank of Guilin Liuzhou Sub-branch, the Agricultural Bank of China Liuzhou Branch, and the Liuzhou Pharmaceutical Co., LTD. The Liuzhou Communist Youth League "return to Work" service special car action was carried out in Liucheng County, Luzhai County, Rongan County and Rongshui County at the same time. Young volunteers distributed "Tuan Tuan epidemic prevention love bags" equipped with medical masks and disinfectant wipes to migrant workers, which also contained dry food and drinking water such as brewing snail noodle and bread, reducing the risk of migrant workers being exposed to novel coronavirus during their trips. In order to ensure the safety of the rural migrant workers, the young volunteers took the temperature of every migrant worker before getting on the bus, and went all out to build a strong defense line against the epidemic. The counties and districts organized the special car operation, sending nearly 900 young workers to Guangdong. Next, Liuzhou Communist Youth League will concentrate on recommending and delivering workers for local key industrial enterprises to ensure the employment needs of enterprises. Combined with the development of online and offline skills training, they will have rural youth with unique skills to stay in Liuzhou to jointly help the high-quality industrial development of Liuzhou city.

2022-02-25	Guangxi Beihai Communist Youth League visited in-depth visits and representatives of Beihai Youth Entrepreneurs Association visited the member enterprises of the Association. During the visit, the person in charge of the TuanBeihai Municipal Party Committee said that the enterprise should pay attention to the construction of corporate culture, strengthen innovative ideas, adhere to the concept of "people first, life first", ensure safe production, and lead the high-quality development of enterprises. During the visit and exchange, the young entrepreneurs discussed and communicated with the visiting group members on how to further realize the resource integration of the members, broaden the cooperation channels, and achieve win-win group development, saying that they should make good use of the platform of the association, integrate resources, develop in common, and make contributions to the economy of Beihai city. After understanding the development of the enterprise and the implementation of the production safety work, the visiting group had in-depth discussions on the development needs of the enterprise and the enterprise management mode, solved the problems for the development of the enterprise, and made suggestions and suggestions. In the next step, the Communist Youth League Beihai Municipal Party Committee will continue to focus on the main duties and main business, lead, organize and mobilize, contact and serve young entrepreneurs and makers, carry out regular enterprise visits, understand the needs of enterprises, and play the role of the Communist Youth League as a bridge.

2022-02-25	Qingdao 2022 first youth recruitment "cloud" open Qingdao 2022 first youth recruitment "cloud" open to provide more than 5000 jobs XingTing) on February 22 in the afternoon, "green bird plan only Qingdao" 2022 Qingdao youth talent "cloud recruitment" launch ceremony and the first activities, activities by Qingdao municipal party committee organization department, Qingdao human resources and social security bureau, the communist youth league Qingdao municipal party committee, provide more than 230 quality enterprises for college graduates more than 5000 jobs, just two hours live attracted 1.2 million people online watch. Wu Xuexin, deputy director of the Organization Department of Qingdao Municipal Committee, Zhang Haijun, Secretary of Qingdao Municipal Committee of the Communist Youth League, Dong Haibo, director of Qingdao Public Employment and Talent Service Center, and Zhao Quanchao, deputy secretary of Qingdao Municipal Committee of the Communist Youth League attended the event. It is understood that the first activity around the computer hardware and software, communication services, e-commerce, mechanical equipment, bio-medical and other industries. Scan qr code can read "cloud fair" job summary table "green bird plan" WeChat small program according to introducing, the Qingdao municipal solid "green bird plan" series of activities, through the construction of jobs database, organize special fairs, promote "Qingdao young talent station" form, actively build post accurate intelligent docking platform, young talents and local development of supply and demand docking platform, contact youth platform, attract young students and talents to green entrepreneurship, promote the "phoenix also nest". Since 2020, the Qingdao Municipal Party Committee of the Communist Youth League, the Organization Department of the Qingdao Municipal Party Committee, the Qingdao Municipal Human Resources and Social Security Bureau, the Qingdao SasAC and other relevant units have held 9 " Green Bird Plan? Only in Qingdao "Qingdao young talents" cloud job fair ", and at the same time, it guided the district and municipal youth League committee to carry out more than 60 various" cloud job fair " activities.

2022-02-24	Green bird in 2022 plan cloud recruitment event successfully held "blue bird dream sunshine" in 2022 bluebird plan cloud recruitment event successfully held in Beijing on February 24 (reporter Zhang Jianwei, correspondent XiangChanghui) to help youth development friendly city, promoting ZhaoCaiYinZhi, further service college students employment, on February 22, by the CPC Rizhao municipal organization department, Rizhao human resources and social security bureau, Rizhao municipal party committee, Rizhao talent development group jointly hosted "green bird dream sunshine" green bird plan cloud recruitment event successfully held in 2022. This activity set up the municipal main venue, in Donggang District, Lanshan District, Juxian County, Wulian County set up a sub-venue, through a number of new media platforms on the whole network synchronous live broadcast, link to the province and even the whole country, overseas job-hunting talents. This cloud recruitment relies on the "Green Bird Project" small program, and provides more than 8,000 quality jobs for job seekers to choose. Before the cloud recruitment began, the "Youth Sunshine" and "Rizhao Talent Introduction Policy" were played. More than 40 HR people went into the live broadcast room to release their posts, introducing the development of enterprises, job demand and salary, and answered questions from netizens on the spot. During the live broadcast of the event, the whole Internet was watched more than 1.61 million times. Next pku will do cloud recruitment in the second half of the article, continue to push employment information, organization of enterprises and institutions online answering questions, interview, actively carry out policy interpretation, skills training, entrepreneurship counseling, practice and other personalized employment services, better realize job information and talent information accurate matching, promote the employment of college graduates entrepreneurship.

2022-02-23	Youth League Bozhou Municipal Party Committee: the first day of school good civilized traffic first class Youth League Bozhou Municipal Party Committee: the first day of school good civilized traffic first lesson Jianwei correspondent Zhang Zhenzhen Zha Wei Ting) " Do you know what small details will affect traffic safety?"" How can we travel in a civilized way?"...... recently, in the lecture hall of Bozhou No.9 Middle School, the Youth League Bozhou Municipal Party Committee of Anhui Province invited Zhao Fei, a police officer from the traffic police detachment of the Municipal Public Security Bureau, to teach the students the first lesson of" civilized traffic you and me together ". Zhao Fei in more than 20 years as a traffic police, around the "small details big safety", "I am a civilized small traffic police", "traffic safety publicity caravan" and other themes of large-scale publicity activities, the first to compile traffic regulations into textbooks, free into the classroom. Event, Zhao Fei through the combination of graphic, video, in case, introduces the students do not wear a seat belt, red lights, such as wearing a helmet may not obey the traffic rules, focus on the traffic safety precautions, and the scene for the students demonstration error and the correct way to wear a helmet, guide the students to firmly establish a "safe travel, civilized travel" consciousness, small widening hand, create a civilized city."I know it's very dangerous to drive the doors at random."" Cross the road must look at the signal lights, do not look at your mobile phone."" Through today's class, I have learned more about the traffic regulations."" I go home to tell grandma this knowledge, let her also pay attention to traffic safety. In the first lesson of "......, the students actively participated in the interaction and said that they benefited a lot. It is reported that "civilized traffic you and me together" the first lesson is the combination of the civilized organization to carry out one of a series of activities. In addition to invite the traffic police field teaching, strengthen the traffic consciousness of primary and middle school students of civilization, pku also joint education department, using her small green, her small group two group animation image, made a vivid activity "civilized traffic you and me" animation video, then will rely on the school group, the young pioneers organization in delay class, class, etc. At the same time, the Youth League Bozhou Municipal Party Committee will also hold the "strive for civilized guard to create a civilized city" theme team class competition, carry out relevant essay activities, "civilized practice", with "children's words" to explain the knowledge of civilization creation and a better life.

2022-02-23	Yantai: build youth development friendly city of Yantai: strive to build youth development friendly city make beautiful harbour city more "young" Yantai municipal party committee for China youth network Beijing on February 23 (xinhua Zhang Jianwei) on February 18 morning, the 14th congress of the communist party of China Yantai, vice governor, party secretary of the thirteenth committee of the communist party of China to the conference entitled "swagger new era vigorous shape new advantages in the socialist modernization new journey take the lead in the first" work report. The report pointed out that we should build new advantages in social programs that improve people's wellbeing, and take the lead in joint contribution and shared benefits. We will steadily promote income increase and prosperity. Adhere to the employment priority, maintain zero employment family dynamic zero; support entrepreneurship to promote employment, improve the "one-stop" entrepreneurship services, build a youth development friendly city, for the city's youth work pointed out the direction forward, for the majority of young people to smoke employment and entrepreneurship to provide a strong guarantee. The municipal Party committee and the municipal government attach great importance to the youth work, and put the youth work in a very important position to plan, deploy and promote. In 2021, the 14th Five-Year Plan Youth Development Plan of Yantai City (hereinafter referred to as the "Plan") was compiled and promulgated, issued and implemented in the name of the Municipal Party Committee and the Municipal Government. This is the first youth development plan in Yantai City. According to the plan, we should adhere to the basic principles of the party managing youth, youth first, serving the overall situation, and build a youth-friendly city that is "cities are more friendly to youth development and youth are more promising for urban development". Under the care and support of the Municipal Party Committee and the municipal government, the Communist Youth League Municipal Party Committee led the establishment and improvement of the "youth Work Joint Conference" mechanism, which is responsible for the implementation of the Plan and the coordinated and promotion of the construction of a youth development-friendly city. Since the establishment of the joint conference mechanism, all levels and departments of the city have accurately issued a series of youth inclusive policies, launched a number of youth livelihood projects, and actively create a good environment for young people to smoke for employment and entrepreneurship, so that young talents will become a bright sign of Yantai. Focus on youth employment. Implementation of the "green bird return sail Yantai" lead to stay plan, to carry out a series of cloud recruitment activities, 32000 jobs, establish "the" green bird station "37," youth station "30, hiring" campus ambassador "55 people," smoke employment experience day "," Yantai students see home " and other activities, organize more than one thousand of students back to smoke practice every year. Focus on innovation and entrepreneurship among young people. Green entrepreneurs across the BBS, Peking University entrepreneurship camp, college students entrepreneurship competition, young talent electricity training activities, set up overseas young talent alliance, lending LuQing benchmark, lu green farmers bear loan 515.3 million yuan, built "youth employment entrepreneurship line laboratory" 22, fu can assign young entrepreneurs, entrepreneurial youth growth and development. Focus on youth cultural services. Give full play to the educational function of Jiaodong red resources, carry out a series of activities of "learn the history of the Party, strengthen faith, follow the Party", implement the "Dream building plan" for youth in emerging fields, undertake the "Dynamic Youth Dance Port" Shandong Province Youth hip-hop Competition, and hold the first Yantai Youth Culture and Art Festival, which greatly enrich the cultural life of youth. Focus on youth social integration. Young volunteers were organized to participate in the frontline of environmental remediation, civilized transportation and grid governance, and 18,000 young volunteers were recruited to participate in COVID-19 prevention and control. We carried out "Express Youth Service Month" and "Warm Bee Action" care activities in the city, built 72 "youth homes", 22 "youth communities" and 20 youth friendship activities in 2021, involving more than 4,000 young people. We will focus on safeguarding the rights and interests of young people. The Communist Youth League launched a campaign of "face to face with deputies to people's congresses and members of CPPCC committees" to open channels for young people to express their interests. The "Hope House" child care project was implemented, raising a total of more than 8 million yuan and 476 houses built. Give full play to the advantages of the city's main position outside the youth palace, and provide high-quality after-school education for 30,000 young people every year. In the next step, the Communist Youth League Municipal Committee will thoroughly implement the spirit of the 14th Party Congress of the city, firmly establish the working concept of "striving for the first class, only the flag is to seize", to implement the "Yantai City" Fourteenth Five-Year "Youth Development Plan" as the guidance, focus on building a youth development friendly city, to attract more young people to smoke for employment and entrepreneurship.

2022-02-22	Handan, Hebei: Live with "post" to serve youth accurate employment with "post", Handan Municipal Party Committee Cyberspace Office and Handan Employment Bureau jointly carried out the "Spring breeze Action" live with post recruitment and employment and entrepreneurship policy publicity series activities. This activity adopts the mode of "live broadcast + online recruitment", mainly for college graduates, returning migrant workers, rural labor force who are willing to transfer to employment, rural youth from poor families and other groups. During the activity, the head of the human resources department of many enterprises in Handan City became the "talent introduction anchor", introduced the enterprise situation online, immediately answered the job-hunting doubts, and pushed the jobs in management, marketing, personnel, specialized skills and general workers. Up to now, special activities have been held in Congtai District, Feixiang District, Economic Development Zone and Feixiang District, a total of 61 enterprises have provided online explanations, more than 120 enterprises have provided more than 20,000 jobs, attracting more than 1.07 million interactive views, delivering 8,425 resumes, and reached the initial employment intention of more than 3,520 people. In the next step, the organizer will also carry out the special network job fairs and other employment assistance activities, and provide more services through college students' "returning to their home" social practice, youth post internship plan, the countryside into the community, youth innovation and entrepreneurship competition and other platforms and carriers, to help enterprise employment and serve more youth employment and entrepreneurship.

2022-02-22	Group Jinan municipal party committee held young talent air recruitment group Jinan municipal party committee held "green bird helps fly dream springs" young talent air recruitment jianwei correspondent Peng Chong) recently, the Jinan municipal party committee in Shandong province jointly multisectoral units successfully held "green bird helps fly dream springs" 2022 high quality development young talent air recruitment activities. The cloud recruitment activities will be held in Jinan daily newspaper group media center, activities take "2 + 7 + N" mode, namely the launch ceremony and 2 jobs focus (four pillar industries, new industry new forms new ground enterprise), synchronous opened seven online interview studio, follow up held N cloud enterprise Open Day activities, netizen questions from the host, enterprises, head of the scene, each enterprise also synchronous meeting studio, will promote, and the intention of employment college students interview activities. We will open up the green channel between young talents and enterprises in the four pillar industries in Jinan, serve young talents for employment and entrepreneurship, and create a new highland for young talents gathering in the four pillar industries."Green Bird Help Fly Dream Spring City" 2022 Yellow River Basin high-quality development of young talent air recruitment activities will further expand the "cloud employment" new mode, increase the "cloud" job new channels, for higher quality and more full employment for college students to set up a "cloud" ladder.

2022-02-22	Guizhou Tongren college students "development with my hometown" symposium held "chunhui action kite plan" Tongren young college students "with my hometown development" symposium jianwei correspondent Chen Kai) recently, "chunhui action kite plan" Guizhou Tongren young college students "with my hometown development" symposium held in van net academy, the relevant person in charge of the municipal party committee organization department, the straight committee attended the meeting. At the meeting place, the young students spoke enthusiastically. From Peking University, tsinghua university and other "985", "211" engineering university Tongren students, previous graduate employment students and the city college graduates in 202214 representatives, combined with professional, growth experience, around the rural revitalization, "dual core", tourism development, urban construction about the understanding and feelings, said will play their own advantages and professional expertise, as a good hometown development of propagandist, participants, contributors. Tongren municipal party committee secretary, municipal party committee talent work leading group leader Chen Changxu stressed that the relevant departments at all levels should earnestly implement young college students back to the plan, the introduction of a group of outstanding young talents to copper business employment, promote "talent" drive "resources", to speed up the realization of "area five" goals to provide strong talent support. Before the meeting, 52 representatives of young college students visited Tongren Rural Revitalization Industrial Belt and the Zhongnan Gate Historical and Cultural Tourism Area. At the meeting, played the "One Belt dual Core" and "Chunhui Action Kite Plan" propaganda video, the Municipal Human Resources and Social Security Bureau, the Communist Youth League Municipal Party Committee leaders respectively introduced the Tongren city related talent policy, "Chunhui Action Kite Plan" related content. Responsible comrades of the municipal departments concerned attended the meeting.

2022-02-21	Fan Jiangtao, Zhu Hongyuan) During the Beijing 2022 Winter Olympics and Paralympics in Tangshan, Tangshan, Hebei Province, dispatched professionals to participate in the service guarantee work of Zhangjiakou Competition area. In view of the professional tutoring needs of such left-behind children, the Tangshan Municipal Committee of the Communist Youth League and Tangshan Normal College jointly carried out one-to-one youth volunteer service activities such as "Your small home has me in the Winter Olympics". Before the Spring Festival, the Tangshan Municipal Party Committee of the Communist Youth League took the initiative to coordinate with the situation of the public security, health and other relevant departments, and counted a total of 120 class tutoring needs of 35 service objects. Immediately, Tangshan Normal University organized volunteers to establish contact with the service object families one by one, detailed understanding of the children's winter vacation homework progress, schoolwork weaknesses and other information, to "life one policy, one teacher, more teaching, pure public welfare" as the principle, free schoolwork tutoring program. During the Spring Festival, 118 young college student volunteers used the nail App online course service, which not only realized "face to face", but also recorded and saved the teaching process for children to review. Each class plan is compiled by young volunteers according to the psychological characteristics, preferences and personal characteristics of primary and secondary school students. Source: China Youth Daily, February 21,2022, edition of 03

2022-02-18	Anhui Huaibei municipal party committee to see home youth students hometown Huaibei municipal party committee to see young students see home home correspondent Wang KaiWeiTing) to guide more Huaibei college students understand the hometown of industry, culture, urban development and change, inspire love hometown, promote hometown, serve hometown enthusiasm, attract more young talents to huai employment entrepreneurship, recently, the Huaibei city of Anhui province municipal party committee held "huai just spring line build dream new era" young students to see home activities. The picture shows the students visiting the city exhibition hall. In the process of the activity, the correspondent first visited the city exhibition hall, so that the students could feel the development and change process of their hometown and the future development plan in the exhibition hall, and further cultivate the hometown feelings of the young students. Afterwards, the young students visited the food expo museum and some high-tech enterprises. In the enterprise exhibition hall, the students learned about the manufacturing process of integrated circuit and electronic circuit board, which enriched their knowledge and horizon. The picture shows the students visiting the high-tech enterprises. After the activity, everyone said that the development of his hometown changes with each passing day, through this activity has a deeper understanding of Huaibei in the urban development planning, industrial development status and other aspects of the situation, he also want to contribute to the development of his hometown after graduation.

2022-02-17	Nanjing, Jiangsu Province: Full cycle of youth employment and entrepreneurship services, for college students, college graduates, workplace youth, young talents and other youth growth of different stages, tailored to the full cycle of high-quality service mode, effectively guarantee the majority of young people growth, entrepreneurship and employment. We divide the youth growth into four stages. The first stage is the college students just entered the university, the so-called junior college students stage, the second stage is the student graduation looking for a job, just into the social stage; the third stage is the graduate has worked, namely the workplace youth stage, the fourth stage is some workplace youth do well, become a certain leading talent, we call the young talent stage. Different stages have different needs, so we carry out the corresponding services around different needs. Around college students, we focus on innovative form carriers to guide them to perceive and love Nanjing. One is to send out the youth gift package to guide college students to perceive Nanjing. For four consecutive years, the opening ceremony of college freshmen in Nanjing, successively sent out the "six service plans", "six service plans", "three posts", "eight youth action" and other youth gift package, open "my Nanjing" App page, to show the college students in Nanjing Nanjing affinity; second, to carry out wonderful activities to guide college students to understand Nanjing. Continue to promote the "college students see Nanjing" plan, continue to carry out the Nanjing College Students Double Innovation Festival activities, promote college students to appreciate the ancient capital, feel the innovative vitality, touch the pulse of development; third, organize practice, guide college students to integrate into Nanjing. We have continued to promote internships in "entering government offices, parks, enterprises, communities, villages and campuses", and encourage Party and government organs at all levels, public institutions, various enterprises and social organizations to provide support for college students to participate in social practice. In the past four years, about 34,000 college students have worked on the job. We have increased our support for college graduates to help them stay in Nanjing. First, targeted measures to improve the degree of human post. We will optimize and improve the policies and measures of the "Ningju Plan" for young college students, and accurately pass on the practical measures of Nanjing for being sincere in treating, loving and caring for college students online through new media such as government microblog, wechat public account, and the university portal website of Nanjing. Offline innovation has carried out the graduation season activities for college students, promoting the employment assistance plan of "thousands of schools and enterprises and thousands of posts". In the past four years, about 1,800 special job fairs of "Ningju Plan" have been held, providing about 1.41 million jobs (times), and more than 1.5 million new insured college students; second, reflecting the temperature of Nanjing with actual support. We coordinated the introduction of housing rental subsidies, employment subsidies, social insurance subsidies, and entrepreneurship support policies, as well as public employment services, and increased support for graduates to start businesses and find jobs in Nanjing. Rental subsidies for college graduates exceeded 260,000 people, and subsidies exceeded 2.8 billion yuan. Characteristic launched the "one-time interview subsidy" policy, benefiting nearly 100,000 college graduates in Nanjing to interview, issued subsidies of about 98 million yuan; third, to simplify the process to improve the processing speed. Implement smart network business, in the university portal page and "my Nanjing" App edition set up zone, provide "ning gather plan" landing "one-stop" point-to-point consulting services, realize "one window acceptance, one window service, one window" function; cancel college graduates in ning employment agreement verification, employment adjustment, employment registration and other enterprises employment demand, realize quality job information input, whole network, let "data more run, youth run errands". For the youth who have been on the post, we create good conditions to stimulate the working youth to make contributions to Nanjing. First, we will use platforms to promote young people in entrepreneurship and employment. Actively cultivate science and technology enterprises, incubators, mass innovation space, university innovation port, urban "silicon lane", and high-tech park to provide a good environment for youth entrepreneurship and employment; second, to promote youth job level improvement. Further promote the action of "One line, one team out". In recent years, 174 municipal youth skills competitions have been held in subdivided industries, and 52,000 young skills talents have improved their post skills through skill competitions and post training; third, promote the growth of youth innovation projects through resource docking services. Continue to hold "win in Nanjing", "the door will" "youth" and other brand entrepreneurship competition, orderly youth and training camp, "let you see you" innovation entrepreneurship project roadshow resources docking activities, accumulated 1054 green project in Nanjing young college students outstanding entrepreneurial project selection, has 17 projects to "foster unicorn enterprise". The Municipal Innovation Investment Group was inaugurated, and set up a guide fund of up to 5 billion yuan for start-up projects, providing strong support for the development of youth innovation projects. College students who have become young talents are sincere enough to attract young talents to gather in Nanjing. First, we will strengthen the policy traction for young talents. Taking the "Purple Mountain Talents Plan" as the whole, introduce the "Peak, Pioneer, Ningju, Elite" series plan, build a policy system of talent introduction and service; encourage policy implementation is inclined to young talents, among which the proportion of municipal plan to support young talents is not less than 40%; second, strengthen the housing security of young talents. For young talents such as overseas returnees and postdocs, we will build a young talent post station and an international talent block integrating "living + office + social networking" functions to effectively solve the transitional needs of young talents. Implement the talent housing plan for young talents, give priority to the supply of talent apartments and affordable rental housing, adopt the "order type" way to raise high-quality commercial housing for targeted supply to young talents, and help the development of young talents; third, strengthen the service support for young talents. Offline launched "purple mountain talent card", online build "purple mountain talent wisdom cloud platform", covering business office, policy cash, housing, education, financial support and other total factor services, dedicated to create a quality and convenient service environment for young talents; young talents "green ying fu" project, through "I think", "returnees", "research", stimulate young talents cross-border cooperation potential, to support young talents, when the protagonist. Rong Fei, Deputy Secretary of Nanjing Communist Youth League, China Youth Daily, February 17,2022

2022-02-17	Qingdao eight youth action Qingdao municipal party committee implement eight youth actors XingTing) in 2022, Qingdao municipal party committee will propaganda to meet and study to implement the party's 20 for the main line, combined to celebrate the 100th anniversary of the 20th, extensive "welcome 20, always follow the party, new journey" theme practice, "in" focus on the party center work, service youth reality needs, the communist youth league for " three dimensions to find the breakthrough point and power, closely around the center of the city work, vigorously implement the eight youth action. On February 15, the seventh (expanded) meeting of the 17th Qingdao Communist Youth League was held. At the meeting, it conveyed the spirit of the Sixth Plenary Session of the 18th CPC Communist Youth League and the spirit of the ninth Full Committee of the 14th Shandong Province; deliberated and approved the Confirmation Case of the 7th Plenary Session of the 17th Qingdao Communist Youth League of Qingdao Municipal Committee; summarized the work of the members of the CPC Communist Youth League in 2021, and arranged the work tasks in 2022. The secretary committee of the Qingdao Municipal Committee, the seventeenth members of the Communist Youth League Qingdao Municipal Committee, not the members of the district Youth League Committee, the youth League working Committee, the municipal units, the head of the Youth League Qingdao Municipal Committee and the head of the Youth League Committee. According to the introduction, the eight youth actions include the youth pilot action, youth talent gathering youth action, youth creation pioneer action, rural revitalization youth contribution action, youth volunteer action, social governance youth force action, youth development friendship action, grassroots organization force promotion action. According to the work arrangement, in 2022, the Qingdao Municipal Party Committee of the Youth League will vigorously promote the comprehensive and strict governance of the league, pay attention to the political construction, organizational construction, system construction, the management of the league cadres and league members, with the work style of "strict, true, fine, solid, fast", to promote the comprehensive improvement of the work, and help the high-quality of the city's economic and social development.

2022-02-17	Tuanzhao municipal party committee to carry out member enterprises visit research activities Rizhao municipal party committee to carry out member enterprise visit research activities Jianwei correspondent phase chang hui) recently, group Rizhao, deputy secretary of Shandong province Li Wanying led to part of the city green enterprise association unit, the development situation, problem demand, work Suggestions to carry out the visit research, send policy to service for the enterprise, promote the development of high quality. The Communist Youth League Municipal Party Committee, Guoji Medical Management Co., Ltd., part of the municipal Youth enterprise Association, and the secretariat of the Municipal Youth Enterprise Association participated in the investigation. The district and county youth League committee and the Youth League Working Committee of functional areas respectively organized and investigated the activities of member enterprises in the region. Research, pku, city green enterprise association research group for the first time to Rizhao city people's government about Rizhao in 2022 "seek improvement in stability" high quality development policy list notice and Rizhao city people's government office about the first quarter of 2022 steady growth measures of ten documents to the member enterprises, help enterprises to timely understand policy, enjoy the relevant policy support. In the next step, the Communist Youth League Municipal Party Committee and the Municipal Youth Enterprise Association will unite and lead the member enterprises to focus on the center and serve the overall situation, focus on the effective role of the "Young Entrepreneur Growth Plan", and stimulate the enthusiasm of young entrepreneurs for innovation and entrepreneurship. Organize and carry out economic and trade investigation activities around the high-quality development of enterprises, accurately and actively understand the needs of enterprises, and deliver policies and services for enterprises. Organize industry dialogue, outside exchange and training activities around enterprise exchanges and cooperation and entrepreneurs' ability improvement. We will continue to enhance the sense of honor, belonging and gain of young entrepreneurs.

2022-02-16	Shanxi Jincheng "335" mode to promote the communist youth league grassroots organization reform "335" mode to promote county communist youth league grassroots organization reform pragmatic Jianwei correspondent Fan Yao) reform pilot work started, Shanxi Jincheng as the whole city to promote the county communist youth league grassroots organization reform, one of the three pilot cities, Jincheng municipal committee closely around the "six" target, solid exploration "335" grassroots organization reform mode, by adhering to the "three", maintain "triple linkage", achieve "five changes", comprehensively promote the county communist youth league grassroots organization reform in Jincheng go deep. Adhere to the "three combination", clear what the reform of the grass-roots league organizations on the basis of in-depth research, the reform of the grass-roots league organizations and the characteristics of the county development, the league situation, the youth situation closely together, take measures according to local conditions, fast and steadily to promote the implementation of the reform. Combined with the development of counties: the economic and social conditions of Jincheng county (city, district) are different, and the development level is not the same. Based on the advantages of the downtown area, the city has expanded the coverage of the two new organizations and the emerging youth groups as the focus of reform. Zezhou has a rich cultural tourism and health care industry, and actively makes breakthroughs in the development of cultural tourism and health care and rural revitalization construction. Gaoping and Yangcheng non-public economy and social organizations are more developed, efforts to innovate in the setting and operation mechanism of the youth league organization; Qinshui and Lingchuan have a large and small population, relatively weak economy, clear the "joint village-level youth League working committee" model, consolidate the rural grassroots foundation. Combined with grassroots league: jin cheng municipal committee to the city county rural tertiary youth league transition as the gripper, with strong grassroots cadres strength, 450 youth into the cadre team, Jincheng county cadres average age reduced before 1 year old, team full-time cadres of bachelor's degree or above accounted for 100%, the communist party members accounted for 100%. After the change, the youth League Jincheng Municipal Party Committee immediately organized a special training meeting, combined with the county league cadres, league members, the league pragmatic classification measures, to guide the county (city, district) youth league committee to clear the reform goals. Combined with the characteristics of green situation: urban youth are concentrated, Take the grass-roots organization form of the innovation group as the main direction to drive the comprehensive promotion of the reform; A high proportion of young students in Gaoping, Explore the establishment of league and education cooperation and cross-part-time working mechanism; Many rural youth in Zezhou and the distribution is not concentrated, Focus on through the "list report" system to force the township youth League committee work of vitality; Yangcheng gathers the strength of young people in the field of e-commerce, Through the "three-network fusion", Create a new position of the online regiment; Qinshui will encourage the enterprise youth strength as the first choice, Actively explore the working methods of "one construction, one concurrently", "two-way empowerment" and "three support", Promote mutual construction of local enterprises and groups; Lingchuan youth to the county, outside the city flow more, Explore the working mechanism of "one station and two links", Further expanded the "radius" of the Communist Youth League. The committee makes full use of the linkage advantages of the youth league organizations at the provincial, city and county levels, and works closely together and makes concerted efforts to extend the reform to the grassroots level from top to bottom. The provincial Communist Youth League Committee adheres to a "guide" word: establish and improve the package piece connecting point, counterpart guidance, work scheduling mechanism, and give support to the municipal and county youth league organizations in the direction guidance, method guidance and daily supervision. League committee to strengthen a "unified" word: Jincheng party committee and organization, propaganda, people and other departments jointly set up a special team, promote the pilot reform of counties (cities, districts); introduce joint meeting and information reporting system, make the county communist Youth League grassroots organization reform pilot work flow chart, Jincheng County Communist Youth League grassroots organization reform pilot evaluation guidance schedule, introduce the third party social organizations for evaluation, comprehensive combing, accurate evaluation, promote the reform to achieve practical results. The Communist Youth League County Party Committee highlights a "creation" word: focus on work innovation, widely carry out the league construction instructors, league construction inspectors, league construction demonstration members "three members to help the project", effectively improve the activity and standardization of the grass-roots youth league organizations. Build a new matrix of all-media publicity, publicize the progress and results of the pilot reform work in an all-round and multi-channel way, and create a strong atmosphere for the pilot reform of the county Communist Youth League grassroots organizations in the whole society. Realize the "five change", decoding the reform of grassroots organizations to overcome the problem of institutionalization, administrative, order, project work: the project of 22 new projects, created a "youth", "youth lecturer", "youth painting club", "youth drama club" and a series of youth popular advantage project. Get rid of the dependence of administrative resources, gather various resources through "purchase + integration": on the one hand, actively strive for government funds to purchase projects, strive for the government to buy 3.5 million yuan, further improve the youth social work service mechanism; on the other hand, fully integrate social resources. The "Jincheng Youth Development and Growth Public Welfare Fund" was set up for the society to serve the young people in need. Solve the disadvantages of the hierarchical system, actively explore the "continuous, horizontal and vertical" flat work mode: continuous and horizontal, namely the pilot youth league organization actively study, through the horizontal addition of the youth League committee and a variety of youth professional institutions, increase the basic force of the county youth League committee, but also reduce the work signal layer by layer transmission layer of weak situation. It means to strengthen the management through the regional industry integration of the weakened league organization. Break the conventional thinking, broaden the channels of league cadres: break the identity, occupation, level restrictions, attract outstanding young backbone to join the league organization. Yangcheng, through the "1234" working method, one election, two selection, three ways and four modes; Lingchuan actively join the "domicile" to establish the "enclave" league organization, timely spread the party policies and ideas to the youth members, and realize the cohesion and guidance of the youth. Activate "peripheral nerve", gather youth: set up the youth league committee, youth league working committee, youth league association, youth home, youth station, achieve full coverage of county youth, expand the two new organizations, emerging youth group organization coverage, strengthen the platform construction, Zezhou, to provide opportunities for returning youth entrepreneurship; Lingchuan exploration innovation group representative liaison station model, establish the "liaison station-special group-league representative" liaison service system.

2022-02-16	Sunshine "blue bird plan students homing" patriotic viewing activities remember history salute the most lovely people in Rizhao "blue bird plan students homing" college students patriotism theme activities Beijing on February 16 (reporter Zhang Jianwei, reporter XiangChangHui) recently, Shandong sunshine municipal party committee organization department, the city people club bureau, municipal education bureau, pku, city talent development group jointly carry out "green bird plan students homing" college students patriotism theme activities, from Beijing university, Shandong university, Jilin university, ocean university of China and other universities more than 150 Rizhao college students to attend. During the activity, the students watched the films such as "Sniper", "Shuimen Bridge of Changjin Lake" and so on, feeling the great spirit of resisting the United States and aiding the DPRK, always remember the history, always cherish the patriotic heart, and strengthening the will to serve the country. During this period, in order to strengthen the contact between the local Communist Youth League, and better play the role of outside Rizhao college students in promoting their hometown, the students who participated in the activity were hired as the stationmaster of the school "Green Bird Station". Patriotic movie watching to strengthen the feelings of home, home perception of the cohesion of nostalgia. In the next step, the "Green Bird Project Students Return home" series of activities will continue to organize young students to have social discussions and interactions, so that more young students can feel the changes in their hometown and the talent development environment, and stimulate their enthusiasm for their hometown to start businesses and find employment. Pku will continue to respond to the youth needs, strive to make the youth and sunshine growth, common development together, promote the development of youth friendly city, help enhance the city of youth attraction, cohesion, bearing capacity, expand the youth participation in urban development, innovation, contribution, with youth power booster sunshine wonderful butterfly change.

2022-02-15	Rizhao communist youth league: holiday open volunteer exhibition as the communist youth league: holiday open volunteer exhibition bear JianWei correspondent XiangChanghui) during the year of the tiger, group of Shandong sunshine committee to mobilize volunteers and volunteer service organizations in completes their own protection at the same time, active in civilization practice, with action practice of volunteer spirit, reveal the youth bear. During the whole holiday, young cultural volunteers were also active in the urban study rooms of Rizhao, providing services such as assisting in epidemic prevention, book sorting, civilized guidance, and answering questions and doubts. Correspondent for the graph to vigorously carry forward the "dedication, love, mutual assistance, progress" volunteer spirit, and expand the government service work social participation and public awareness, pku joint Rizhao government affairs service center, the new government service hall enabled as an opportunity to create "zheng" youth government affairs volunteer service brand, normalized to the public to recruit "government service" young volunteers, the new era of government service civilization practice volunteer activities, in the hall to provide business consulting guidance, civilized behavior advocacy, for the elderly and provide deputy, with do and other services. In the municipal government service hall of Rizhao, Bluebird Post and Rizhao college students returning home to practice actively carry out volunteer service activities such as information consultation, civilized persuasion and order maintenance. Volunteers carry out epidemic prevention services in the city study. Correspondent for figure in recent years, pku to study, citizens, volunteers tripartite demand oriented, follow the "study is suitable, citizens welcome, volunteers can" principle, efforts to build city study youth culture volunteer service brand, on weekends and holidays, joint city study release youth culture volunteers recruitment, recruit volunteers 433 people, service time 6495 hours, help delicate city construction, guide more citizens into the study, enjoy high quality public reading service.

2022-02-14	Hebei Baoding: build cultural confidence to help youth development in Hebei Baoding: build cultural confidence to help youth development jianwei correspondent Zhao Bin) on February 11, Baoding, Hebei province municipal party committee staff into Baoding cultural industry development group co., LTD. (hereinafter referred to as the "wen hair group"), and wen hair group company leadership, youth representatives to build "youth friendly city" work on the discussion. The picture shows the relevant situation at the construction site of China Ancient Animal Museum. Correspondent for figure group is responsible for comrade, is the construction of Chinese ancient museum project phase is expected to open to the public on October 1,2022, the museum with ancient fossils as the carrier, show the origin of ancient humans and vertebrate, evolution, development process, is a modern ancient digital museum for the world, after the completion of the project will become the largest natural museum in Asia. Group Baoding municipal party committee secretary GeXin said, the culture lead, is the fertile soil for the healthy growth of teenagers, cultural confidence, is the ideal cornerstone of youth go up, the communist youth league Baoding municipal party committee and hair group culture hand in hand, is an important progress symbol of "youth friendly city", but also the depth of the municipal party committee strategic deployment. During this discussion and exchange, Baoding Municipal Committee and Chengfa Group reached consensus on the cultural construction of "youth friendly city". In the future, the Youth Baoding Municipal Party Committee and Chengfa Group will work together to build a new cultural stage for young people, start a new cultural journey, and provide an effective cultural guarantee for their talents to grow up.

2022-02-14	Beijing, February 14 (reporter Zhang Jianwei, correspondent Xiang Changhui) At the beginning of the New Year, the Communist Youth League Rizhao Municipal Party Committee of Shandong Province quickly started the "first war" of the New Year. To convey the spirit of learning from provincial and municipal meetings. Quickly convey learning the province in 2022 work mobilization meeting, the provincial communist youth league focus on video, provincial party committee 14 nine times (expand) meeting and municipal party committee spirit, around the "ten innovation" work requirements, in-depth study of the communist youth league work, the breakthrough point, anchor "leading, comprehensive create" "three walk before" always follow, general positioning, total beacon, adhere to the "things for first-class, only flag is", quickly into the work state, strictly implement the provincial, city relevant work deployment, promote Rizhao communist youth league work comprehensive breakthrough, innovation. Collective wisdom and strength of innovative planning work. In accordance with the deployment requirements of the Rizhao Municipal Party Committee to realize the wonderful "butterfly change" year, to meet, learn, publicize and implement the party's 20th year as the main line, combined with the celebration of the 100th anniversary of the founding of the league, focusing on the realization of "five have five promotion", the implementation of five "youth innovation actions". Play the role of "one competition, one, four brands" driving engine, through the youth development chain, provincial games volunteer recruitment, training, management, use, help "one meeting, invigorate a city", implement "young entrepreneurs growth plan", stimulate the enthusiasm of young entrepreneurs, with "green brand" innovation practice to help high-quality economic development; consolidate and deepen "I do practical things for youth", strive to create a youth development friendly city. We will continue to change our work style. To grasp the style of change, grasp the ability to improve, grasp the innovation implementation, promote innovation and excellence "three grasp and one promote" as the core, strengthen the construction of the organization, form everyone grasp learning, strong quality, improve ability, team exhibition image, common progress, can achieve the work pattern. Focusing on the change of work style, we will implement the work requirements of "strict, true, fine, solid and fast", strengthen the construction of executive force, advocate the practical and practical guidance, and adhere to the truth-seeking and pragmatic work style. Centering on the improvement of the ability, with the "green fan" school, youth culture corridor, youth reading gathering as the carrier, through the "comparison, learning, evaluation, exposure", to improve the entrepreneurial ability and level of cadres, officials and officials. Focusing on the implementation of innovation, we will focus on the practices of domestic and provincial advanced cities, identify the innovation point of the overall central work, and carry out activities such as "top sharing" and "brainstorming". We will establish systems for supervising key work and special work, and work hard to implement the work. Centering on creating the first to compete for excellence, taking the "flowing red flag" as the starting point, establish the "competition" system of monthly evaluation and quarterly evaluation of district and county departments, and activate the cadre team innovation and excellence "a pool of living water".

2022-02-14	Hangzhou Fuyang: The "Youth Students Alliance", to the emergence of the COVID-19 epidemic, has become a new position for the Communist Youth League to organize young students to "list" to participate in volunteer services."I am now at home, I can participate in the service," "I can go in need" and "I also sign up"... "Concentric Volunteer peer- -Actively participate in epidemic prevention Volunteer Service Proposal" released just two hours, 86 returning college students signed up to participate in the epidemic prevention and control volunteer service. A person in charge of the Hangzhou Fuyang District Committee said that due to the overall arrangement of the district epidemic prevention and control headquarters and the safety of the volunteers themselves, the registered students were arranged to report to the village committees and communities where they live, and cooperate with the territory to carry out some epidemic prevention work within their capacity. He Yuting, a Fuyang student studying in Wenzhou Medical University, is mainly responsible for assisting community organizations in nucleic acid testing. As the nucleic acid testing started very early, she changed her late sleep habit and set four or five alarm clocks for herself every morning."As a probationary party member and a medical student, I am very happy to have such a volunteer service experience."In the nucleic acid testing site, there are enthusiastic small citizens gave her a hand-warming treasure, a" sister, hard work " let her feel the happiness of volunteer service. In front of the village committee building of Xinsha Village, Dongzhou Street, Fuyang District, many old people were full of praise for the "red vest", "thanks to the little girl, otherwise the mobile phone operation really can not come," "the little girl is pretty fast, and she can get it in half a minute." "She helps me every day"... the old girl, a junior student of Nanchang Institute of Technology Xu Jiayi, during the COVID-19 outbreak, she and her classmates volunteered to volunteer service in the village, help the village elderly online quickly complete the nucleic acid test appointment, reduce the waiting time in the cold wind. She said: " I feel very sad to see me 'sick' in my hometown. I feel very honored to participate in the epidemic prevention services."Zhou Aonan, a freshman at Zhejiang Wanli University, started a family of three together with her parents. They get up at 4:50 every morning and put on two pairs of socks and two pairs of woolen pants to keep themselves warm. After arriving at the nucleic acid detection point, put on the protective clothing, goggles, gloves and other protective equipment, and start the post service at 6 o'clock on time. Mother is responsible for checking the health code, my father is responsible for assisting the appointment, Zhou Aonan is responsible for maintaining the order, " this job is actually very hard, midway I feel almost can not stick to it, but to see my father, mother and so many people are working hard, I also stick to it, I point a thumbs up for myself."Song Xiangyi, a senior two student who is also studying in Fuyang Middle School, has participated in various public welfare activities with her mother since she was a child. During the epidemic, he became a "Courier", purchasing supplies for residents in the epidemic prevention area. At first, he learned about the needs of residents through the circle of friends and Weibo. With the expansion of the prevention area, the demand for materials was also increasing. Song Xiangyi built a new mutual aid document for the epidemic situation, and residents can fill in the needed materials through this document to facilitate volunteers to make statistics and purchase. During this period, he and his companions ran errands more than 100 times together, providing daigou services for more than 150 families. Since the Hangzhou COVID-19 outbreak, Hangzhou Fuyang district party committee to give full play to the "youth tower" youth "youth" and "youth league alliance" organization system advantages, effectively mobilize grassroots TuanGan, youth members, returning college students, youth volunteers, a total of 26000 people involved in epidemic prevention and control volunteer service, the 1265 grassroots, 32 social public welfare organizations rushed, with practical action to practice "please rest assured, power have me" youth oath, test in the big test reform, reveal the youth bear. Source: China Youth Daily, February 14,2022

2022-02-11	Chenzhou "full journey warm winter action" volunteer service in Hunan Chenzhou 2022 "full journey warm winter action" travel volunteer service activities build wei correspondent, QinZisong Li Jia) in response to the communist youth league about the 2022 Spring Festival "lei feng hometown learn lei feng" youth volunteer action initiative, Chenzhou youth league member, Chenzhou youth volunteer federation association of volunteers, wearing red vest, shuttle in Chenzhou car hub station, highway intersection, high-speed railway station, provide warm heart help for past passengers. At Chenzhou High-speed Railway Station, Chenzhou Railway Station, Chenzhou Bus Station, Beijing-Zhuhai Expressway and other places, volunteers took their temperature, scanned the QR code, publicized COVID-19 epidemic prevention instructions, carried luggage and helped buy tickets, and sent holiday blessings to passengers. It is understood that in the 2022 "Love Full Journey Warm Winter Action" Spring Festival travel volunteer service activities, Chenzhou city has nearly 60 young volunteers every day, not affected by the cold weather and the Spring Festival holiday, uninterrupted to carry out volunteer service activities, the activities will continue until the end of the Spring Festival travel rush. During the activity, the volunteers said that they would make their personal efforts to contribute to the prevention and control of the epidemic, the safe Spring Festival and the safe Chenzhou.

2022-02-11	Anhui Hefei youth League members actively participate in snow removal and freezing resistance.) The snow drops sharply, wake up overnight outside the window is covered in snow. In the face of low temperature and snow weather, the Hefei Municipal Party Committee of Anhui Province and the Hefei Youth Volunteers Association immediately issued proposals to the youth league organizations at all levels, youth league members, youth volunteer service organizations and young volunteers to actively participate in the fight against snow and ice volunteer service for young volunteers. The city's youth league organizations at all levels and the majority of young volunteers heard the order, quickly listed, actively participate in the first line of snow removal and frost resistance. In the ice and snow, a red vest and a dough flag have become the warmest bright color in Hefei. Early in the morning of February 7, the female volunteers from the "little A-Sao Help group" in the Wulimiao community were busy. Wearing red vests, they braved the wind and rain to clean up the snow to ensure the safety of the people. Deng Lu is one of the "little sister-in-law help group", she said, at 8 o'clock in the morning, eight small sister-in-law all gathered in place, considering the first day of work after the holiday, the masses travel volume is relatively large, they took shovels and other snow removal tools focus on the investigation of farmers' markets, shops, pharmacies, bus stations, community roads and other areas."After four hours of continuous operation, the snow in key areas has been cleared."Deng Lu said that the snow is still under way, and the snow-clearing work can not stop. Today and tomorrow, for the area of easy to slide and frozen location, they will also send people to patrol and clean up, to ensure traffic safety. On the first day of the Year of the Tiger, Zhang Dongli, a young volunteer from Yinhe Community, Wuhu Road Street, Baohe District, was particularly busy. Early in the morning, to see the WeChat group constantly flashing snow sweeping initiative, Zhang Dongrui resolutely signed up."Our main snow-clearing team includes female volunteers, parents and students from the nearby No.48 Middle School."Zhang Dongmei said that after a morning of fighting, the snow on the main road of the community has been cleared."Several young students worked in full swing, driving the drive of the whole snow-clearing team."Zhang Dongmei said that the snow-clearing team has gathered more than 50 volunteers to carry out snow-sweeping activities in the main streets, back streets, and in public places. At the same time, to persuade and mobilize the units stationed in the district, businesses along the street to take the initiative to carry out snow clearing and deicing activities."As long as the snow doesn't stop, we have to continue it."Zhang said the snow-clearing team is fully ready. In the face of the rain and snow weather, Furong community gathered more than 100 volunteers to set up more than 30 snow sweeping emergency teams, mainly on the area of farmers' markets, residential areas, unit entrances, branch roads and other areas to carry out snow shoveling and deicing work. After several hours of fighting, the snow on the entrances was finally cleared from the main road and the residential area, providing convenience for residents to travel."This is convenient, volunteers cleared the road early in the morning."Living in Furong community Crescent neighborhood committee Rongxiang Garden community, Ms.Li said. The enthusiasm of the community volunteers also led many residents in the area. Early in the morning, two high school students, Chen Jingjing and Wei Minxuan, from Bihu Lanting and ZhongHuancheng Yundi, came to the community and volunteered to ask to join the snow clearing team. It is reported that on the first day of snow removal, the city's youth league organizations at all levels launched more than 2,000 people to participate in the snow shoveling and deicing action, in the urban streets, fields, community campus, organs, everywhere are young volunteers busy figure, for the masses to clean out a "safe road" and "warm road".

2022-02-10	League Wuzhong city "three lead" to strengthen youth ideological and political education group Wuzhong city "three lead" to strengthen youth ideological and political education jianwei) since 2021, the Ningxia hui autonomous region Wuzhong city party committee focus on the party successor the fundamental plan, with "three lead" strengthen youth ideological and political education, to lead condensed youth, mobilize youth, contact service youth basic responsibility, unity lead the youth members always follow the party, new journey, with practical action to meet the party's 20th victory. To commemorate the 102nd anniversary of the May 4th Movement. Wuzhong Municipal Committee for the map to strengthen ideological guidance, education of young people to listen to the party, follow the party. Adhere to the theme of education as the carrier, to carry out "panoramic" learning and teaching, through the "13344" work method, guide the city's 126 league (work) committee, 2420 league (general) branches to carry out party history learning education full coverage. For the purpose of consolidating the foundation of faith, we carried out "guidance" lectures, "five laws and four lectures", and 120 lectures on General Secretary Xi Jinping's July 1 important speech and the spirit of the Sixth Plenary Session of the 19th CPC Central Committee, attracting more than 13,000 people. Xu Jingjing, the counselor of Qingtongxia Young Pioneers, was recommended to be selected into the national "Red Scarf Publicity Group" and the "Yangma Project" lecture group. Adhere to the red gene as the core, to carry out the "immersive" activities, organize teenagers to carry out the red studies, red landmarks, youth, be red small narrator, "orchid spirit" inheritance, may youth poetry more than 170 games, and broadcast "the Yellow River young said" short video more than 60, lead the youth in the practice of immersion, experience, strengthen the patriotic emotion, inheriting red genes, continuously red blood. The spirit of the Sixth Plenary Session of the 19th CPC Central Committee. Wuzhong Municipal Committee to strengthen practice guidance, and promote young people to consciously practice the socialist core values. Focus on COVID-19 epidemic prevention and control battlefield, started "youth war" disease ", build" personnel recruit, material donations, psychological mutual assistance "three closed-loop working mechanism, recruit young volunteers formed 61 service, in 285 epidemic prevention sites for volunteer service, set up volunteer material collection, distributing epidemic prevention materials, to form a" love team ", open psychological assistance hotline. Focus on the big stage of rural revitalization, leading the "new countryside and new youth", and relying on the "green" brand farms, employment bases and innovation studios. We mobilized 2,008 college students from Wu Zhongji to participate in the social practice of "returning to their hometown", injecting "green vitality" and "green momentum" into rural revitalization. Focusing on the main window of volunteer service, we implemented the "Youth Volunteer Tour", carried out youth volunteer services on a regular basis, supported five youth social organizations such as the "Yellow River Guard" and five volunteer service projects such as the "Strive to be the River Xiaoqing", and carried out practical activities such as the "Protect Mother River" beach purification action. We will strengthen policy guidance to serve the prominent problems of "urgency, difficulties and hope". Through the practice of "I do practical things for the masses", we will promote closer contact with young people and serve young people more accurately. We will deepen the connotation of the "Project Hope" project, and extend the traditional single donation to improve the quality of basic education, youth health escort, scientific and technological innovation, and cultural training. We implemented 61 projects, including "Promoting education and education", "community services for poverty alleviation from inhospitable areas" and "youth aviation model science into campus", and raised 6.14 million yuan of hope materials, benefiting 3,386 young people. We expanded the coverage of the "Communist Youth League around us", promoted the construction of all municipal league committees and liaison stations for county-level league representatives, established 142 "youth homes", carried out 820 activities, focused on young people in emerging areas, built five small bee post stations, and cultivated 30 youth social organizations and 90 backbone members of youth social organizations. We will expand the service extension of "youth warm heart", and promote the long-term regular activities such as "12355 youth escort", "Youth Love" and "Youth Creation theory", to serve the real needs of youth in employment, marriage, dating, social integration, and rights and interests protection.

2022-02-11	Hebei Zhangjiakou: with the games "volunteer blue" illuminate the city "warm red" group Zhangjiakou municipal party committee: with the games "volunteer blue" illuminate the city "warm red" Liu Haowen Wu Yufan) Beijing Olympics games in full swing........., dressed in blue and white uniforms of the games Zhangjiakou division city volunteers in service post youth enthusiasm. The Youth League Zhangjiakou Municipal Party Committee actively explores the meeting point of urban volunteer service and urban operation, closely adheres to the needs of urban residents, and carries out characteristic urban volunteer service. In Guyuan County, city volunteers took off their time to provide family companionship, interest training and learning difficulties guidance to the families and children of the frontline staff participating in the Winter Olympics in Chongli. City volunteers gave full play to their professional expertise, guided the children to make handcrafts, exercise their hands-on ability, and patiently answered the difficult questions left in class and winter vacation homework. While accompanying and taking care of the children, the staff serving in Chongli can reduce their care and devote themselves to serving the Winter Olympics. In district, under the boom of "a pier is hard to find", rotational rest city volunteers with children, using the color mud at hand making Beijing Olympics mascot "ice pier", "snow", in the process of production for children about "ice pier" "snow" and behind the Chinese culture, and will make the finished product, presented to the children, let them realize the "pier freedom". At the peak period of the Spring Festival holiday, the headquarters of all counties (districts) in Zhangjiakou predicted in advance, and timely increased the service frequency and sent more volunteers at the urban volunteer service stations of the high-speed railway stations and long-distance bus stations to ensure the smooth and safe travel of passengers during the Spring Festival travel rush. City volunteers actively provide bus guide services, maintain bus order, and help passengers with mobility difficulties to carry large luggage, and the majority of passengers have praised the city volunteers' thoughtful service one after another. Many Zhangjiakou competition area city volunteers also spontaneously organized, came to the downtown blood station located in the counties (district) mobile blood collection vehicles to donate blood. Up to now, urban volunteers in Zhangjiakou competition have donated 25,000 milliliters of blood. Group Zhangjiakou municipal party secretary RongHaiGuang said, " to serve the winter games as an opportunity, we built the 'N +' city volunteer service content, to comprehensively promote volunteer service normalized, project, gradually improve the volunteer service system, accumulated experience, exercise team, running mechanism at the same time, let volunteers become a beautiful beautiful scenery line, make more deeply rooted in the hearts of the volunteer service, let the city more civilized, more tender.”

2022-02-10	Jining Municipal Committee to carry out children "warm winter action" the Communist Youth League Jining Municipal Committee to carry out children "warm winter action" for China Youth network Beijing February 10 (reporter Zhang Jianwei, correspondent Bai Chen) recently, In order to help rural children in difficulties have a safe, civilized and warm holiday, The Communist Youth League at all levels in Jining, Shandong Province focuses on the "Hope House", Linkage pair volunteers, returning college students, young social organizations, construction support units and other loving forces, To fully promote the "Warm Winter Action", To provide family companionship, psychological counseling, academic counseling, wish fulfillment, self-care education and other services. Warm bag distribute pictures of the event. In the drawing supply activity of the Youth League Jining Municipal Party Committee, The Communist Youth League Municipal Party Committee raised more than 100,000 yuan, Warm packs to 1,039 children with 1,015 cottages in the city, It mainly includes packing boxes, scarves, hats, gloves and daily necessities, Help the children through the cold winter; "Hope hut 100 yuan thousand dream" dream micro wish activity, Made a letter to the hope hut children and a micro-wish collection card; Free 2022-2023 Love insurance package for all the Hope House assisted children, The total insurance amount reached 643 million yuan; Carry out self-protection education centering on preventing accidental injury and personal injury, transportation, electricity safety and winter sports safety, Enhance teenagers' awareness of self-protection, Take practical measures to enhance the care for the assisted children. Volunteers from Liangshan County sent popular knowledge and warm bags to the children. Recently, the youth League organizations at all levels combined with the work of warm package distribution work, simultaneously to carry out visits, schoolwork counseling, psychological counseling, safety publicity and other activities, to help them safely and warm through the Spring Festival and winter vacation.

2022-02-09	"Youth Meeting Room" enables youth. Is there a way to know more outstanding young people in industries?"Holding the microphone, it was Li Zhichun, director of the Youth Working Committee of the Qingdao Hip-hop Dance League, and several colleagues, who were the leading roles of the dialogue. On the other side are leaders from relevant departments of the Qingdao Municipal Youth League, Qingdao Culture and Tourism Bureau and Qingdao Federation of Literary and Art. This is a vivid scene of the "Youth Meeting Room" activity held by the Licang District Committee of Qingdao City. The theme is to respond to the demands of young people and answer their puzzles. Open communication has achieved good results: street-hop alliance plans to promote hip-hop public welfare classes in the community, let street-hop into the masses, transfer the positive energy of youth; their learning and friends demands have also been promised by the relevant departments. How to ensure that the services and resources sink to the grass-roots level, and avoid the problems such as dislocation and poor connection with the actual needs of the youth, has become an urgent problem for the Communist Youth League at the grass-roots level to solve. Combined with the history of the party learning education, group licCang district party committee focus on the actual needs of grassroots youth, to organization, contact, service, leading the youth work guidance, set up youth work service platform, accurate docking grassroots and youth demand, promote work, service, resources to sink to the grassroots level, for grassroots youth league organizations can assign, coagulation hearts meet force service youth members at the grassroots level. In practical work, Licang district party committee set up the platform thinking, innovative ideas, using WeChat small program build online youth work service platform, the "order" service, "project", "network" operation "and" dynamic " management mode, collect grassroots and the actual demand at the same time, pay attention to integrate work resources, the activities, volunteer service online, according to the demand to match resources, provide services. At present, the platform dynamically provides 13 various service projects and activities. Activities such as "Youth Meeting room" and Love Library have been carried out regularly at the grass-roots level, and more than 50 other activities have been carried out offline. In this way, the grassroots youth league organization through the youth work service platform, for the youth and service youth league members ability and level has been effectively improved. It is worth mentioning that the "youth Meeting room" activity initiated by the grass-roots youth League committee has now been included in the youth work map of the whole city. According to the project, institutionalization, platform, the brand ideas, the Qingdao municipal party committee is trying to build "youth meeting room" 2.0 upgrade version, establish to listen to youth voice, youth problems, help youth development of long-term mechanism, constantly enlarge its service, communication, cohesion, lead the comprehensive function and brand effect, power youth friendly city construction. On the basis of building the platform, the "demand-oriented" working mode can be implemented smoothly- -streets and communities as the "demand side", select projects through the platform, send them to WeChat group for soliciting opinions, and decide whether to carry out activities according to the actual needs of youth league members; the project demand is summarized by the Youth League District Committee and pushed to the "supply side" of youth social organizations and volunteer service teams, so as to realize the effective connection between supply and demand. After the completion of the project will be by the "demand side", "supply side" and "league committee" tripartite evaluation, evaluation results through the platform data precipitation, realize dynamic, accurate data statistical analysis, show the development of different projects, hatch truly meet the needs of youth members, and promote the normalized development of youth work. The Initial Heart Social Work Service Center positioned as the community public space operation (hereinafter referred to as the "Initial Heart Social Worker") is a youth social organization successfully incubated through the youth work service platform."Five years ago, the original heart social worker was just founded. The biggest problem is the lack of ability to go out. To put it bluntly, they do not have the ability to be self-hematopoietic, and the scope of activities is only limited to one or two communities."Pang Xiao, head of the original social work group, recalled. In 2019, Chu Xinxin social workers officially entered the youth work service platform in Licang District, putting mature projects on the platform for more communities in the district to choose from, in order to meet more needs. To Pang Xiao's surprise, after the platform promotion, the echo plan for the mental health of teenagers in distress, and the new Era parent salon for parents under the "double reduction" policy were very popular in the community, and even became popular projects. At the same time, the financial support provided by the platform enables the original social workers to improve the quality of their activities more easily, and the more social organization resources linked by the platform also greatly expand the "circle of friends" of the original social workers. Up to now, through the youth work service platform, five youth social organizations have regularly entered the platform to carry out activities and provide services, pooling their youth strength and serving grassroots governance while serving the youth. In the platform operation, the Youth League Licang District Committee innovates the operation mode and fully releases the vitality of the platform. In the early stage, with the Youth League Licang District Committee as the leading group, raising special funds for financial support. After the platform running smoothly, to achieve the expected results, timely to the youth social organization self operation management as the leading, is given priority to with community party construction funds and social financing, youth corps committee special funds for auxiliary operation mode, help more youth social organizations into community governance, civilization, volunteer service, form sustainable with hematopoietic ability of self operation mode. In the specific work, the Licang District Committee of the Communist Youth League signed open contracts with all the volunteer service teams or social organizations settled on the platform, and approved the service content, support funding standard and evaluation method of each activity in advance, and settled at zero cost. According to the actual needs of the grass-roots level and the youth league members, the special fund support will be given by the number of activities and the comprehensive evaluation. According to the feedback of different projects from the grassroots, Licang District Committee of the Communist Youth League gave different levels and levels to the projects, which is integrated into the market mechanism of resource allocation. Projects that have entered the platform for 3 months will exit the platform, with 5-6 such projects."To build a youth service platform for youth work is an attempt to promote the construction of the grassroots youth league and serve the youth work mode. To actively explore the 'lever solution' to promote the work in the way of the platform gathering strength, integrate and leverage more resources, serve the needs of the grassroots and the youth, and help the grassroots and empower the youth."League Licang District Party Committee Secretary Liu Dianshuai introduced."The Qingdao Communist Youth League will continue to focus on the needs and expectations of the youth, innovate its work ideas, actively build a platform, and widely gather resources, so that the Communist Youth League serves the youth with depth, temperature, and strength, and effectively improve the organizational strength, leadership strength, service strength and overall contribution."Said Zhang Haijun, party secretary of the Communist Youth League in Qingdao. China Youth Daily, February 09,2022

2022-02-08	Hunan Xiangtan more than 2000 young volunteers to carry out the Spring Festival Hunan Xiangtan organization more than 2000 young volunteers Spring Festival volunteer service activities wei correspondent, FuDingChen week) in 2022 Spring Festival, the Xiangtan city of Hunan province party committee and various counties (city) area, Xiangtan by the youth league committee of more than two thousand young volunteers, on the premise of strictly implement the epidemic prevention and control requirements, during the Spring Festival for passengers with high quality volunteer service. Wang Jiayu, a young volunteer from the Xiangtan Liancheng Volunteer Association, is a Xiangtan native, studying in Hunan Urban Construction Vocational and Technical College. She chose to spend her holiday as a volunteer, the second year she participated in the Spring Festival travel rush. According to the arrangement, she will volunteer at Xiangtan Railway Station and Bantang City Railway Station this year."It is very hard to volunteer in winter, but I feel worth it to see the heartfelt smiles of the passengers, and I am very happy."Wang Jiayu said. At Xiangtan North Railway Station, Zuo Wenqiu, Ning Suyun, Xiao Qiulian and other volunteers of Jiuhua Volunteer Association, were busy guiding passengers, answering passengers' inquiries, and making ginger tea to warm them up for the epidemic prevention. The bright red vest became a beautiful scenery in the station. Xie Yibo, president of the Jiuhua Volunteer Association, told us that during the Spring Festival travel rush, the association will arrange 12 people every day in two groups to provide volunteer services for epidemic prevention and control, guidance and consultation, order maintenance, convenience and benefit, key passengers, traffic safety persuasion and emergency rescue. At the same time, the association also with minmetals real estate (Xiangtan) development co., LTD. Party branch hand in hand, set up a love team with 6 cars, waiting in the north station exit or underground garage, once there are disabled, the elderly travel inconvenience of special passengers, in the city can provide free shuttle services, directly to the door. This year's "Full of Journey and Warm Winter Action" Spring Festival travel volunteer service activities are carried out in accordance with the principle of "pre-recruitment and reserve, moving due to needs, adjusting measures to local conditions, focusing on large stations, streamlining posts, optimizing services, strengthening training and safety first". Before the Spring Festival travel rush, the Communist Youth League and Xiangtan Municipal Party Committee and other departments and units took the initiative to meet the needs of the transportation departments and various stations, reasonably arranged the allocation of volunteer service personnel, and trained the volunteers. During the Spring Festival travel rush, volunteers went to the front line to steadily carry out volunteer services according to the actual needs of epidemic prevention and control publicity and reminders, consultation and guidance, and ticket assistance.

2022-02-07	Ningxia Wuzhong to carry out the "" wu "warm winter action" series of activities "wu" little to warm winter "series of youth volunteer service activities wei correspondent Qiu Xiao) since winter, the Ningxia hui autonomous region Wuzhong city committee fully arouse the enthusiasm of youth, organize youth volunteers to participate in" "wu" warm winter "action, take concrete actions in the economic and social development line practice" action is better than words ". College students "return to their hometown" social practice activities. Start in 2022 Wuzhong city students winter vacation "home" social practice, recruit Wu Zhongji "home" college students 368, and send it to authority, township (town), village (community) involved in grassroots social governance, civilization, guide and organize college students based on practice, participate in hometown construction, through rural health knowledge propaganda, grassroots zone spreading activities, the health and care to the countryside, farmers, the fields, the fields. The "Warm Winter Action" volunteer service work. Offline to recruit more than 100 young volunteers in the bus station travel volunteer service activities, through check in entrance health code, travel code, guide passengers in ticket hall, ticket places to help security and baggage handling, to meet the Spring Festival passenger security, for cold winter travel passengers to warm and love, line listed county two levels of the communist youth league new media matrix frequency resonance, open up "good epidemic prevention festival" column, regularly published epidemic prevention and control policy and popular science knowledge, remind citizens friends to celebrate the Spring Festival, don't forget security."Warm and warm childlike innocence" Spring Festival sympathy activity. Through field research, household visits and other forms, special groups such as helping villages and building communities were visited during the Spring Festival, concerned about their learning and living conditions, and understood their difficulties and needs. In this activity, 225,000 yuan worth of condolence materials were distributed, covering more than 450 young people.

2022-02-04	Shandong Dezhou New Year sent warm into the first secretary of the Communist Youth League dispatched to help the village Dezhou Youth League, the Municipal Youth League, the municipal Youth enterprise Association sent warm into the first secretary of the Communist Youth League dispatched to help the village correspondent for the picture China youth network Beijing February 4 (reporter Zhang Jianwei, correspondent Fan Yichao) recently, Shandong Dezhou Municipal Party Committee, Municipal Youth Federation and Municipal Youth Enterprise Association sent warmth to the first secretary of the Communist Youth League Municipal Party Committee dispatched to help the village- -Liu Qinxin Village, Zhongding Township, Qingyun County, ceremonially, The Communist Youth League Municipal Committee, the Municipal Youth Federation, Dezhou High-tech Entrepreneurship Service Center, Dezhou Trading Logistics Development Co., Ltd., Dezhou Finance Investment Holding Group, Ltd. presented 300 sets of scarves, gloves and hats to all the elderly over 60 years old in Liu Qinxin Village, Value of RMB 10,000, Give 50 sets of children's cotton clothes for left-behind children in Liu Qinxin Village, Value of 6,000 yuan; Municipal Youth Enterprise Association, Shandong Bohai Spring Wine Co., Ltd. presented 150 new spring red lanterns to Liu Qinxin Village, Value of 4,000 yuan. At the event site, the attending leaders tried on cotton clothes for the representatives of left-behind children, wore scarves, hats and gloves for the elderly representatives, and gave them the blessing of the New Year. Then, the participating leaders visited the representatives of the old Party members.

2022-02-02	Guangdong Xinyi: Want a big bag, want an exhaust fan, want a basketball... the Year of the Tiger Spring Festival is coming, in Maoming Xinyi city, Guangdong province, a wall full of pink wishes. These community residents, after the community grid consultation, there will be party members to claim. Xinyi City, secretary of the general Party general branch of the second community, said that everyone has a voice from the bottom of my heart, but sometimes always buried in the bottom of my heart, seize the residents "open mouth" needs, set up a "wish wall", to solve the wish needs of the community people. Approaching the end of the year, walking in the streets and lanes of Xinyi City, you can be seen the "red vest" figure everywhere, they are either sending Spring Festival couplets, or visiting, or in the city renovation of the appearance. With the theme activity of "Our Festival", Xinyi City has carried out cultural activities such as sending Spring Festival couplets to enhance the awareness rate, participation rate and satisfaction of "Building a Civilized City in Guangdong Province" among the residents."The written Spring Festival couplets are more interesting than the printed Spring Festival couplets."Liang Minghong, secretary of the General Party Branch of Xinyi Tangmian Community, said that Party members led the Communist Youth League members and literary volunteers in women's groups to the community site to write Spring Festival couplets and" fu " for the residents, sending the blessing to the hearts of the residents. In Xinyi City, 23 volunteers are maintaining order, guiding the public and calling their numbers. Li Ming, who is in his 80s, gave a thumbs-up to the volunteers: " My legs are bad, and I need to queue up to the hospital. Now I can go to the community to help us old people."It is reported, appropriate city give full play to the role of evaluated integratedly party organization axis, improve the community party construction coordination mechanism, organization guide jurisdiction unit working party members actively participate in party members claim micro wish, theoretical policy, fire safety propaganda, vaccination volunteer service community, public welfare activities, to further enhance the warm and service level of grassroots governance, completes the" last kilometer " of the masses.

2022-02-02	Group Yantai municipal party committee to carry out the "public welfare farmers" the communist youth league Yantai municipal party committee to support village to carry out the "public farmers" the youth network reporter XingTing) near the Spring Festival, to continue to do a good job of Bao Village support, recently, Shandong group Yantai municipal party committee in Yantai qixia temple town government held a "public farmers" dividend ceremony, for mengjiagou villagers bonus funds more than 24000 yuan. Xiang Fuliang, secretary of Yantai Municipal Committee, and relevant personnel, Gu Fanglei, vice mayor of Qixia Municipal Government, some members of Qixia CPPCC, members of Qixia Youth Federation, young entrepreneurs of the city and representatives of villagers of Mengjiagou Village attended the distribution ceremony. Since the launch of the village work, the leadership of the Youth League Yantai Municipal Party Committee led the team to the village in the first time, through discussions and exchanges, field visits to grasp the village situation and the people, carefully comb the development of the village, carefully set ideas, planning and goals. To effectively solve the villagers of apple unsalable, Shandong the teng human resources group co., LTD., general manager of wang teng, Yantai kai CNC technology co., LTD., chairman Lv Chunzhe, Shandong wide source group co., LTD., general manager GuanJun, Shandong environmental technology co., LTD., general manager Feng Changfeng young entrepreneurs have extended a helping hand, through the village party branch led cooperatives to help villagers sell apple more than 15000 jin. After the ceremony, the relevant comrades of the Youth League Yantai Municipal Party Committee and the young entrepreneurs visited the poor families in Mengjiagou Village, learned more about the physical conditions and living conditions of the 6 old party members and the people in need, sent them condolence gifts to the festival, told them to take good care of their health, and encouraged the poor people to face life actively.

2022-02-01	Zhangjiakou area city volunteers ready for the games volunteers | Zhangjiakou area city volunteers ready learned from Zhangjiakou pku, Zhangjiakou area city volunteers training, selection and people post matching work has been completed. A total of 53,435 city volunteers in Zhangjiakou are ready to provide all-round and multi-angle city volunteer services for the Beijing Winter Olympics and Paralympic Games. In accordance with the principle of "dividing by region, task and group", 53,435 urban volunteers were organized in Zhangjiakou through extensive launching, selecting the best, including 2,735 urban volunteers in key areas, 50,700 environmental security cities, and participated in volunteer services in key areas and environmental security cities during the competition. In terms of urban volunteers in key areas, 98 urban volunteer service points will be set up, and 2735 city volunteers will provide information consultation, civilization guidance, language translation, cultural promotion, emergency rescue and other volunteer services, to highlight the civilized image of the host city and tell China story well. In terms of environmental security, city volunteers will provide volunteer services to ensure the smooth and orderly city operation and clean urban environment. Zhangjiakou conducted 111 general volunteer training through online and offline; taking advantage of test activities and test competition opportunities, accurately formulated daily operation plan, organized 814 urban volunteers to carry out whole process and actual combat test, accumulated service time of 20084.5 hours, trained backbone, trained team, and accumulated experience for urban volunteers.(Reporter Yang Mingjing)

2022-01-30	Ningxia Guyuan Municipal Party Committee to carry out the Spring Festival sympathy and research activities Ningxia League Guyuan Municipal Party Committee to carry out the "sympathy and research activities of the heart of the youth" sympathy and research activities. All the 5 groups of the Youth Guyuan Municipal Party Committee went deep into four counties and one district, visited 80 young families in extreme poverty, and sent 500 yuan to each poor youth. Combined with the research activities of the Tuan Guyuan Municipal Party Committee at the beginning of the year and the research work requirements of the Tuan Ningxia Hui Autonomous Region Party Committee, Guyuan Municipal Committee formulated and issued the Notice of Guyuan Municipal Committee on the 2022 Spring Festival sympathy Activities and research work, Focusing on the themes of the change of township youth League committees, the construction of grass-roots organizations, youth employment and entrepreneurship, and the growth of young people, Group goes deep into urban and rural communities, enterprise organizations and other grass-roots frontline, In accordance with the principle of "communication, investigation and research, business supervision, and work promotion", To solicit opinions and suggestions from the youth league members on the work of the Communist Youth League and Guyuan Municipal Party Committee, Listen carefully to what the grassroots needs and wants, Timely sorting out of the research situation list, Scientific planning for key work in 2022, Ensure that the stationing point research achieves practical results.

2022-02-09	Ningxia Wuzhong study and implement the party's spirit of the sixth plenary session of the 19th learning class class Wuzhong city committee study and implement the party's spirit of the sixth plenary session of the 19 special class Beijing February 9 (reporter Zhang Jianwei) to thoroughly study and implement the party's sixth plenary session of the 19th and the central provincial main leading cadres study and implement the party's spirit of the sixth plenary session of the special seminar spirit, on February 7, the Ningxia hui autonomous region Wuzhong city committee study and implement the spirit of the sixth plenary session of the 19th special class, pku secretary zhang chaired class, all the staff to attend. Zhang Yuan stressed that all the cadres should closely around the study and implement the party's spirit of the sixth plenary session of the 19th and the central provincial main leading cadres to study and implement the party's spirit of the sixth plenary session of the 19 seminar spirit, adhere to deep study fine enlightenment, study the original, learn spirit essence, grasp the essence essence, unswervingly with xi jinping new era of socialism with Chinese characteristics armed mind, to guide practice, promote work. We will study and implement the guiding principles of the Plenum, coordinate all tasks and measures in the Communist Youth League system, and take concrete actions to implement the spirit of the session. This special study class will last for two days from February 7th, through intensive learning, personal self-study, communication and mutual learning, and warning to promote learning.

2022-01-30	Shaoxing, Zhejiang to nearly ten thousand in Shaoxing youth send "gift package" to nearly ten thousand in Shaoxing youth send "gift package" Li Jianping) recently, Shaoxing, Zhejiang municipal party committee, China mobile, China, China telecom Shaoxing branch launched "Shaoxing youth New Year traffic call blessing package", youth welfare; joint city post "warm bee action" for the delivery industry youth send shao gift package. This is one of the five major theme activities of "We will stay in Shaoxing together" initiated by the Shaoxing Municipal Party Committee and the Shaoxing Municipal Government to promote the resumption of work and production and the regular epidemic prevention and control work. Focusing on young migrant workers, young children and their children, we have carried out public welfare care winter camps for children of enterprise employees and children of young migrant workers in communities, and launched more than 10 public welfare courses and activities, such as manual labor skills, quality development, and painting and calligraphy training. In Xinchang County, the Communist Youth League Committee actively arranged the "micro wish" of key teenagers, sending Spring Festival gift package and blessing for 43 youth to the winter vacation hosting class and Spring Festival traditional culture theme activities- "traditional" to solve the worries of young migrant parents; to visit the ancient village, enjoy the flavor, to enjoy the ancient village culture and natural scenery and experience the Spring Festival flavor. In addition, Shaoxing Municipal Committee also launched an online recruitment activity of " 'more" and "no' ", providing nearly 10,000 jobs for young people; municipal and county youth palaces launched preferential policies for reducing spring course training fees for young children, and lighting the "micro wish" of 1000 children of migrant workers and teenagers in difficulties.

2022-01-30	Inner Mongolia Chifeng youth ice and snow festival teenagers "pattern" play snow Shi Jia) on January 26, "celebrate the future Winter Olympics youth" youth festival, Inner Mongolia, in the "Great Wall ice and snow ribbon" tourism route on the Meilin valley ski resort main venue launched. On the ice-covered football field, 10 players from the football team, professional ski teams from Shanxi, Beijing and other places and friends from all over the country competed; on the ski resort, more than 30 single participated in fun interactive games; at the launch ceremony, the first batch of 101 "Ice and snow child painting" paintings were exhibited."Ice and Snow children painting" Chifeng City youth painting Exhibition. Image was provided by the respondents. It is understood that the Chifeng Youth Ice and Snow Festival has carefully designed three categories of "cultural and tourism publicity", "sports competition" and "interactive experience" around the boutique tourism route promotion, friendship, ice sports, and youth practical education. The main venue of Meilin Valley Ski Resort in Karqin Banner will launch projects such as ice and snow and snow child painting, Frozen, ice and snow young players, ice and snow hero meeting. The festival will last for a month from January 23 to February 20. Players of the Chifeng Hongshan County Primary School football team play ice football. Image was provided by the respondents. During the activity, 2022 Chifeng city teenagers of all nationalities from all walks of life, will be around the "Great Wall ice and snow ribbon" boutique line, in Chifeng valley nanshan ski resort, WengNiuTeQi Yulong sand lake, wulam cloth scenic area of different ice and snow playground and scenic area to share, and shoot "clock ice ribbon pass the games dream" theme publicity photos and video, promote culture of ice and snow sports, spread the games culture, make Chifeng ice and snow sports label. Hold the "ice and snow young players" Chifeng city youth winter camp. For young pioneers aged 10 to 13 and student league members aged 14 to 16 to carry out public welfare winter camp, theme research and other activities. Participate in the "snow and ice young player" Chifeng city youth winter camp teenagers experience skiing. Image was provided by the respondents. The Chifeng Youth Ice and Snow Festival is organized by the Youth Chifeng Municipal Party Committee, Chifeng Culture and Tourism Bureau, Chifeng Sports Bureau, Chifeng Education Bureau and Chifeng Youth Development Foundation.

2022-01-30	Jinan "warm bee" warm the heart of the Courier brother Jinan "warm bee" warm the heart of the Courier brother Jianwei correspondent Peng Chong Xu Jiaying Chen Ziyang) " your package will be delivered soon!"A simple word, to protect the people's livelihood, to serve the people's lives. Especially during the epidemic, the "Courier boy" was praised as a retrograde warrior. In order to help build a youth-friendly city, explore new ideas for youth work in emerging fields, and protect the legitimate rights and interests of "express delivery boys", Shandong Province and Jinan city care for express (takeout) industry youth "Warm Bee Action" activity was held in Tianqiao District.site of activity. Correspondent for the scene of the opening of the provincial express industry youth League working Committee, Tianqiao District Express (takeout) industry youth work Committee. The provincial and municipal youth League committees and the districts and counties at all levels of Jinan city have raised a total of more than 410,000 yuan of love materials and distributed them to the young people in the express delivery (takeout) industry. Service new youth growth, Jinan "warm bees" in action to better service condensed express (take-out) industry from youth, to promote care Courier, take-out member "warm bee action" continue to carry out, improve express (take-out) from youth sense of belonging, pku city federation of trade unions to build "outdoor workers post" for express little elder brother sanitation workers to carry out volunteer service.site of activity. In addition, the Communist Youth League Municipal Committee also united with the city library to create a "Courier brother reading club" in the Quancheng study for the Courier brother presented electronic book card, regularly carry out reading club, little brother parent-child interaction salon and other activities to enrich the little brothers' amateur life and spiritual food. City books, Jinan Rural Commercial Bank and other relevant units set up a little brother home, to provide the "delivery boy" and "delivery boy" with rest, rest, drinking water, charging, microwave heating, leisure reading and other basic services, so that the "little brothers" feel warm. In the next step, the Communist Youth League Municipal Party Committee will continue to promote the in-depth development of activities such as "Express Industry Love Station", "Courier boy" reading station, "motorcycle driving test Green Channel" and so on, so as to effectively improve the quality of young people in new fields and stimulate the development vitality of young people in new fields.

2022-01-29	Correspondent Li Li) On January 28, the staff of Baoding Municipal Committee of Baoding, Hebei Province visited China Telecom Baoding Company and had a discussion and exchange with the youth representatives on the work of building a "youth friendly city". At the symposium, Ge Xin, secretary of the CPC Baoding Municipal Committee, said that Baoding Telecom practices the responsibility of a central enterprise, and continues to give full play to its industry advantages in communication services and youth employment, so as to provide effective support for attracting young talents to stay in Baoding. Zhang Baodong, secretary of the Party Committee of China Telecom Baoding Company, proposed that it would combine the daily production work, increase the publicity elements of the youth friendly city, form the service window effect of the business hall, light up the "youth friendly city", cultivate a strong young pioneer team, and constantly enhance the vitality of the youth, serve the youth and lead the youth. In this discussion and exchange, Tuan Baoding Municipal Committee and China Telecom Baoding Company reached a consensus on building a "youth friendly city". In the future, the Baoding Municipal Party Committee and Baoding Telecom Company will work together to establish a one-stop service platform for young people and provide more warm life service support for young people.

2022-01-29	Sunshine the communist youth league "warm action" care express little brother sunshine communist youth league for "warm action" care express little brother Jianwei correspondent XiangChanghui) recently, the Shandong Rizhao municipal postal administration organization care sympathy express little brother "warm action", to express business season line express personnel concentrated sympathy and discussion, city postal administration chief Ma ShouDong, deputy secretary of pku Li Wanying, city postal administration deputy director, tertiary researcher Fan Cheng, deputy secretary (temporary) wang xigang, some express company, express little brother representative of more than 20 people to participate in the activities.site of activity. In the activity, the correspondent inspected the working environment of the provincial youth civilization express customer service department, understood the daily work and life of the customer service staff, and talked with the main responsible person in charge of the express company, the head of the customer service department of the provincial youth civilization express, and the representative of the Courier boy. Vice secretary wan-ying li stressed that in recent years, pku attaches great importance to the service condensed express industry youth, lay solid express industry youth organization, cities and counties two levels of express industry youth organization construction to achieve full coverage, timely attention to youth demands, in the rights and interests maintenance, dating, security since the deep care work, efforts to do the practical work for them, problem solving, to ensure that the youth organizations in youth difficulties can afford, find, reliable.

2022-01-30	Heilongjiang Yichun Municipal Party Committee and Municipal Youth League visited the activity of "Youth League members send warmth" to visit students in distress, sending New Year wishes and holiday wishes to 16 students and families in the friendly area. During the visit, representatives of Yichun Municipal Committee and Municipal Youth Federation sent students more than 7,000 yuan worth of stationery, books, drawing boards, basketball, New Year gifts such as rice, noodles, oil, and 1,000 yuan. On the eve of the activity, Tuan Yichun Municipal Committee released a short video of "Dream Micro Wish" to care for students in distress through the TikTok platform. Loving people from all walks of life responded positively, and the child's "micro wish" was soon all claimed.

2022-01-28	Group of Quanzhou, Fujian province municipal party committee held a "campus to ambassador" of Quanzhou, Fujian province municipal party committee "campus to ambassador" bank jianwei correspondent Wu Ruiyuan) " brick reveals a kind breath, let I believe that hometown Quanzhou is our eternal home, the future there will be more spring students return to the land, written love spell dare to win the story."Participating in the World Heritage Quanzhou gave Xu Jintao to Zhengzhou University a full sense of belonging. On January 24, Quanzhou Municipal Party Committee of the Youth League of Fujian Province opened the world heritage tour of "Campus Talent Ambassador". By enhancing students' sense of belonging, identity and pride in their hometown, it helped "Campus Talent Ambassador" to be the "spokesperson" of Quanzhou talent work. During the activity, "Campus Talent Ambassador" visited Luoyang Bridge, enjoyed the Quanzhou Sea silk heritage and charm, visited West Street, listened to the new generation of youth combining modern culture with Minnan traditional culture to create an urban composite space, and experienced the charm of traditional culture, listened to Nanyin music, and experienced the visual, auditory and tactile senses. During the discussion, the Communist Youth League Municipal Committee introduced in detail the industrial, economic, social development and future planning direction of Quanzhou in recent years, and had a sincere dialogue on further playing the influence, appeal and motivation of "campus talent ambassador" to attract more outstanding college graduates to spring for employment and entrepreneurship and "campus talent ambassador". Xie Yanling study in Fujian engineering institute, said activities make her a comprehensive understanding of Quanzhou good innovation entrepreneurial environment and excellent employment development environment, enhance her identity of the city of Quanzhou, " the bank let me see the current Quanzhou is the key to accelerate the development of high quality, more than ever need talent strong support, strengthened my home employment confidence, I will also send Quanzhou youth, Quanzhou youth image, to promote the talent supply and demand accurate docking, gather more outstanding students to spring director contribute a youth power."The relevant person in charge of Quanzhou municipal party committee, said the next step, pku will continue to deep talent" harbor plan ", combining cultural heritage influence and urban development opportunities, attract more students become" campus ambassador ", multi-channel, multi-way propaganda Quanzhou world culture, talent policy, policy of choose and employ persons needs, let more students know Quanzhou, Quanzhou, take root in Quanzhou.

2022-01-28	Wuzhong City "three to the countryside" and "I do practical things for the masses" and "I do practical things for the masses" activities officially launched Jianwei) On January 25,2022 Wuzhong City cultural science, technology and health "three to the countryside" and "I do practical things for the masses" activities in Litong District Jinji Town Street Square officially launched. The Communist Youth League committee organized young volunteers to carry out various forms of volunteer service activities. College students "return to their hometown" social practice activities. Start 2022 Wuzhong city students winter vacation "home" social practice, cities and counties two levels to recruit 368 "home" college students, and send it to the township (town), village (community) to participate in grassroots social governance, guide and organize students based on practice, participate in hometown construction, through the rural health knowledge propaganda, grassroots zone spreading activities, the health and care to the countryside, farmers, the fields, the fields. The "Warm Winter Action" volunteer service work. Offline recruit more than 100 volunteers in the bus station to travel volunteer service activities, through check in entrance health code, travel code, guide passengers in ticket hall, in security places to help security and baggage handling, to meet the Spring Festival passenger security, online Wuzhong communist youth league cities and counties two levels of new media matrix with frequency resonance, open up "good epidemic prevention section" column, regular new epidemic prevention and control policy and popular science knowledge."Warm and warm childlike innocence" Spring Festival sympathy activity. Through field research, household visits to support village, to build community and other special groups of youth during the Spring Festival sympathy, concerned about youth learning, living conditions, understand their difficulties and needs, and to send them holiday blessings, the cities and counties in two levels issued 225000 yuan worth of sympathy supplies, covering more than 450 teenagers.

2022-01-27	Jiangsu Huai 'an youth station party history learning education "C" Jiangsu Huai' an youth station party history learning education "C" youth network reporter Li Runwen) " in the process of explanation, I seem to be immersive in that era!"Speaking of the glorious deeds of the Xin 'an tour group, Zhu Enyi, the red scarf narrator of Xin' an Primary School, Jiangsu Province, sighed. Zhu Enyi is a fourth-grade student and one of the young interpreters of the Xin'an Tour Group History Memorial Hall. The experience of explaining the history of the new journey to teachers, students and guests has made this little girl well-trained and generous, and become a unique scenery in the museum. Last summer vacation, she also participated in the red search activity of "Little Hero revisiting the New Travel Road" organized by Xin'an Primary School, where she approached the glorious history of the new brigade during the study tour and practiced the spirit of independence of the new brigade. Since 2021, the Huai 'an Municipal Party Committee of the Communist Youth League has promoted the party history learning and education in an all-round way, served young talents, and contributed pioneers to the high-quality development of Huai' an through the action of "youth staying in Huai". Group Huai 'an municipal party committee secretary he � Xing jing � group Huai' an municipal party committee of the history of the party learning education as a major political task, relying on Huai 'an red resources, focusing on the youth group thought lead the main business, innovative learning methods, rich learning carrier, comprehensively promote the party history learning education deep walk, guide the city's youth learning party history, strong faith, follow the party. Teenagers become the party history learning speaker on the eve of last children's day, xi jinping, general secretary to "xinan tour" Alma mater, xinan elementary school of the young pioneers reply, I wish them a happy holiday and the children, and encourage them to combine the actual growth to learn party history, following the example of the hero model character, firmly listen to the party, follow the party's determination, efforts to achieve all-round development. Huaian less working committee, deputy director of the young pioneers general counselor chun-chun xu said: " we organize the city team organizations at all levels in-depth study and implement xi general secretary to xinan elementary school five (8) squadron young pioneers important reply spirit, mining new brigade glorious history rich connotation and era significance, actively use the red scarf to preach, search, studies, guide the youth party's history, learning red story, inheriting red genes."" young friends, we are now located in Xuyi County Huanghuatang Town of the New Fourth Army military memorial hall......"Yin Xiao spoke of the history of the New Fourth Army as well off. She is a student of the "Iron Army Road" youth learning Club in Xuyi County. By changing military uniforms, playing leggings and other ways, everyone is immersed in experiencing the spirit of the Iron Army. The Huai'an Communist Youth League Municipal Committee organized the "post-8090" teaching group to launch "micro courses" for party history publicity with small cuts and small examples, using offline activities and online live broadcast to transfer the party history learning and education courses to the "basic units" and "nerve endings" of the youth league organization and the youth league members."The Huaian District Committee will be the great spirit of the Party and the lofty spirit of Zhou Enlai integrated inheritance, combined with Huaian local culture, red history, Tao Xingzhi education thought, with the youth perspective, youth language design launched the Xin 'an Tour History Memorial Hall, Zhou Enlai's former residence of the youth learning club lines, become a favorite place for teenagers to punch in."Tuan Huaian District Party Committee Secretary Zheng Shan introduced."As a college student in Huai 'an University, I deeply feel the temperature and depth of the Huai' an Communist Youth League Municipal Party Committee to our young students."Yi Yuxuan, a 2018 student from Huaiyin Institute of Technology, is the executive chairman of Huai'an College Students Public Welfare Alliance. She has rich experience from the class volunteer service activities to the fourth China (Huaian) International Food Expo. In the summer of 2021, the Huai 'an Municipal Party Committee of the Communist Youth League carried out the summer internship activities for Huaian college students, organizing 5,000 college students to "see Huai' an", and let them understand and fall in love with Huai'an. Yi Yuxuan is also one of the 5,000 students. She explained that the "help Feng spread the wings" plan, the collective opening ceremony and the collective graduation ceremony made her feel that "my heart is safe in Huai'an", which is the "temperature". College students' summer internship, volunteer service, support for entrepreneurship and employment in the Huai River will help college students to accumulate work experience and improve their comprehensive ability, which is "depth". Through many practical experiences in the university, she has also established her own career goals."Huai 'an has become my second hometown. After graduation, I hope to stay in Huai' an to display my ambition, pursue, dream, and create a better future with Huai'an!"Yi Yuxuan's experience is the effective embodiment of the Huai' an Youth League Committee service" phoenix "and" wild goose " project. Around the "stay in phoenix" project, the Youth League Huaian Municipal Party Committee actively undertook the first "collective graduation ceremony" and "collective opening ceremony" of Huai'an college students, around the "college students stay in huai employment", "college students entrepreneurship support" and other six aspects, issued the college students "leave in phoenix" project comprehensive service gift package. In the summer of 2021, Xia Mingjun, a student from Jiangsu Electronic Information Vocational and Technical College, was selected as the "Tour of Huaian Youth Experience Officer" to visit huai'an planning venues, high-tech enterprises, cultural and tourism projects, and entrepreneurial bases...... He felt the vigorous development and cultural history of the city. In 2020, Li Liyuan graduated with a PhD from Northwestern Polytechnical University. With the wish to contribute to the development of his hometown, he returned to Huaian and taught at Huaiyin Institute of Technology. In December of the same year, he became attached with Yu Luhua, a woman led by the Communist Youth League of Huai 'an City, a female doctor from the First People's Hospital. "My wife is from Shandong, and she wanted to go home, but I stayed in Huai'an."In 2021, Li Liyuan and his wife also participated in the "920 Youth Talent Group Wedding" organized by the Municipal Communist Youth League Committee, and spent their whole life under the witness of their relatives and friends. Group Huai 'an municipal united front work department minister Zhu Yiting, around the "wild goose" project, pku also in Beijing, Shanghai, Nanjing, xi' an and other universities gathered city, innovation to set up a "wild goose to xinghuai" college youth talent contact station, around the "1234" service system to carry out policy propaganda, emotional contact, service deputy activities, set up Huai'an native students and hometown development with frequency resonance platform. Youth bear achievements in the new period of Huai'an municipal party committee organization department director yan said: " in early 2021, we determine the ten key projects, distributing department basic work order, evaluated integratedly, authority, schools and industry league work guidelines, around six major themes help grassroots solve practical difficulties, regular grassroots, wide research, listening, service, improve the coordinated development of youth league efficiency, improve the scientific and effective service."In the summer of 2021, Hongze District issued an orange rainstorm alert and an epidemic notice on the same day. The Hongze District Committee of the Communist Youth League has urgently set up 15 youth commandos and high-quality volunteer service teams of more than 500 people, becoming the main force on the front line of epidemic prevention and control."The Communist Youth League Committee through the development of the first line, the team'theme activities, guide us to make contributions in the front line."Hongze District youth commando director Chu Zhiguo said."This is the first gift I received this year. I have the delicious food and the motivation to learn."Xiao Ke, who lives in Huaiyin District, said happily that this is the daily care and visit to the factual orphans carried out by the Communist Youth League District Committee, and to send the poor factual orphans grants and comfort items. It is reported that in 2021, more than 1,200 books and 160 puzzle toys were distributed to 162 de facto orphans in Huaiyin District, lighting up 159 "micro wishes" of de facto orphans. TuanLianshui County Party Committee carried out the "dream transformation +" 10 yuan donation activity, Lianshui County 163 dream cabins in 50 were built by teenagers "ten yuan" love donation. It is reported that in 2021, the Huai 'an Communist Youth League focused on the "dream hut" project of the provincial people's livelihood, raising a total of 9.95 million yuan of construction funds and built 710 "dream cabins", which effectively improved the original simple learning and living environment of the actual orphans and provided a "world of dream building" for children to grow up."Looking at the children's clear eyes, I can feel that they know that my legal knowledge can protect them."Young lawyer Huang Keyi, a young lawyer, said about the" Youth Journey to the West class ". The Huai'an Municipal Party Committee of the Communist Youth League has included "protecting youth by law" into the key work of preventing juvenile crimes throughout the year, continuously enhancing the concept of rule of law among teenagers, especially those in weak rural areas, and constantly promoting the comprehensive development of their physical and mental health. CAI Zhenying, head of the rights and interests Department of the Youth League Huaian Municipal Party Committee, said that so far, the "Law Protection Youth West Journey Classroom" has entered 44 schools in Huai'an, and the city has held 113 activities such as open classes on the rule of law, covering 120,000 teenagers.

2022-01-25	Changzhou: Let young talents and urban development with the same frequency resonance and become "new Changzhou people". In 2019, Wu Fan, who has studied and worked in the United States for eight and a half years, gave up his job at Harvard University and his green card in the United States and chose to return to China to serve his motherland with the advanced technology and knowledge he has learned. With the strong support of the Yangtze River Delta Physics Research Center and the Tianmu Lake Advanced Energy Storage Technology Research Institute, Wu Fan has set up a scientist's studio to carry out research in the field of sulfide all-solid-state battery technology and solid-state electrolyte materials. In his work and life, Wu Fan gradually fell in love with Changzhou, a city carrying his dream of scientific research. Over the years, focusing on the city positioning of "international intelligent manufacturing city and central axis hub of the Yangtze River Delta", Changzhou is eager for talent and innovation. Taking this opportunity, the Changzhou Youth League organization also has sufficient conditions and opportunities to achieve talents and undertakings. Wu Zhenyu, party secretary of the Changzhou Communist Youth League, said that they will further help to build an ecology of comprehensive development of talents, use more efforts to introduce young talents, more concrete measures to cultivate young talents, better services to retain young talents, and promote the development of young talents and urban development with the same frequency resonance. On December 17,2021, the German special live broadcast activity of "Overseas Students watching Changzhou" and the opening ceremony of Changzhou Overseas Youth Innovation and Entrepreneurship Friendship Station were held through the "cloud video". The bilingual live broadcast attracted 28,700 online views at home and abroad. The Changzhou Municipal Party Committee of the Communist Youth League has successfully held three such activities, leading overseas young people to visit Changzhou and approach Changzhou with their cameras. He Zheng, a young man from Changzhou, is a staff member of the German station. After graduating from Jiangsu Institute of Technology, He Zheng went to the University of Duisburg-Essen in Germany. After graduation, he stayed in Germany for many years. After the establishment of the friendship station, He Zheng made the Changzhou students feel the warmth of their home by explaining the epidemic prevention knowledge online, the local epidemic prevention policies in Germany, and issuing the "epidemic prevention gift packages" offline. During the epidemic, more than 1,000 Changzhou students benefited from Germany alone. In order to strengthen the service "front" of overseas young talents and promote the intellectual return of overseas young talents, the Changzhou Municipal Party Committee of the Communist Youth League uses the Internet thinking to actively create a "strong magnetic field" to attract talents. Relying on the 14 local returnee youth talent service stations listed in the Sino-German Industrial Innovation and Cooperation Center and other parks, and establishing three overseas youth friendship stations based on the German Rheinruhr Student Association, they have initially formed the working mode of online "cloud tour", "cloud recruitment", offline "friendship station" and "service station". Group Changzhou, vice secretary of municipal party committee, vice President of youth federation, they will continue to promote fellowship station and service station construction work, break the information, time and space and cultural barriers, under the background of normalized epidemic prevention and control innovation service in often students group, speed up and the multi-level, multi-theme, multi-channel communication between overseas youth. In October 2021, Changzhou launched "famous school cooperation line innovation entrepreneurship to win the future" activities, the city four sets of team leaders led into Chengdu, Wuhan, Shanghai, Dalian and other places, with the universities, research institutes, business depth of cooperation, invite young talents to often innovation entrepreneurship, issued "not stick to one style drop talent" the strongest voice. Through active linkage with the local Student League and the University Youth League Committee, the Changzhou Municipal Party Committee jointly built a liaison service station for Changzhou college students. Changzhou college students serve as liaison officers and urban publicity ambassadors to carry out Changzhou policy promotion, youth liaison service, employment and entrepreneurship information release, and other work, to attract more young students to develop regularly. Changzhou, with the Yangtze River Delta Physics Research Center and the Tianmu Lake Advanced Energy Storage Technology Research Institute, is the most ideal place for Wu Fan to realize his dream. After he came to Changzhou, the local youth league cadres came up to give Wu Fan a "one-to-one" service, so that he could focus on research and development work. Working for nearly 3 years, Wu Fan adheres to the road of independent research and development, so that the all-solid-state battery technology has reached the world's leading level. Currently, they have achieved success in the laboratory. With 22 post-90s doctoral students and even post-00s doctoral students gathered here, he encouraged these young people to take root in Changzhou and serve Changzhou. Coincidentally, in the New Power Entrepreneurship Center of Changzhou Tianning Economic Development Zone, the technical team of Changzhou Middeck Optoelectronic Technology Co., Ltd. is exploring the next 0.1 mm breakthrough. In 2014, Middeck entered the New Power Green Innovation Space. Under the escort of the whole chain service of the Youth League Committee and the park, Midk focuses on technological innovation, and the high-precision laser products are 10 meters away from ± 1.5 mm, reaching the international leading level. Within six years, Middeck grew from a start-up to a company listed company on the Youth Innovation Board. At present, Changzhou green chuang space more than 20. Jiayidu Youth Innovation Workshop, the first youth innovation space in the province, took the lead in launching the "Jiayidu Youth Entrepreneurship Plan", recruiting 30 high-quality youth entrepreneurship projects for the whole city every year, and giving 2.5 million yuan of free funding and an angel investment fund of 5 million yuan. In the past 7 years, Jiayidu has incubated more than 400 enterprises, including 1 enterprise listed on the New Third Board, 19 Longcheng Talent Project projects, 142 jiayidu Youth Entrepreneurship Plan, and created more than 1,800 jobs. Starting from the support of site, capital, policy and other factors, the Changzhou Municipal Party Committee of the Communist Youth League has built a "hardcore" guarantee for the youth innovation project. By cooperating with local resources, they have built three youth entrepreneurship and innovation project roadshow bases, Jiayu Xindu, New View and Xinxin Courtyard, providing a one-stop service platform for project display, policy consultation, investment and financing docking for entrepreneurial teams. In recent years, the Changzhou Municipal Party Committee of the Communist Youth League has helped to apply for more than 300 high-quality youth innovation projects to participate in the "Creative Youth" national and provincial competitions. The number of project awards and the number of promoted projects have always ranked first in the province. Zheng Yiqin, 33, is the director of the West Taihu Lake International Talent Community in Wujin District, Changzhou. The international talent community has 50 "talent apartments", and 70% of the residents are young science and technology innovation talents from all over the world. How to provide them with more convenient services and make them feel warm in a foreign land is the focus of Zheng Yiqin's daily work. Zheng said the community is equipped with more than 10 "bilingual butlers", who can communicate smoothly with foreign youth in English and other languages. Last year, a British man told Zheng that he wanted to get a driver's license in Changzhou to facilitate travel. Zheng Yiqin and his colleagues took turns to accompany the young man to fill in the form of application, examination and physical examination. After several months of hard work, the young man finally got his driver's license. Garden-style community, log color decoration style, modern supporting facilities, just put into operation of the Oriental young talent community let young people call heart. As the first high-standard young talent apartment complex in Changzhou, the first phase of the Oriental Youth Talent Community Project covers an area of 36 mu and can provide 952 talent apartments. At present, Changzhou is comprehensively promoting the project of "youth retention and talent excellence". It plans to build 100,000 talent apartments that meet the needs of young people within five years. The construction of 16,000 talent apartments and 4,500 apartments have been completed. Let the young people can struggle and live, let Changzhou become the "first choice" in the hearts of the youth become the goal and power of the work of Changzhou Municipal Youth League. China Youth Daily, January 25,2022

2022-01-25	Wuhu in Anhui province young talent workstation (Shanghai station) held in Anhui Wuhu young talent workstation (Shanghai station) founding ceremony and Wuhu nationality in Shanghai outstanding young talent exchange symposium Beijing on January 25 (reporter Zhang Jianwei, correspondent YuWen check WeiTing), Wuhu in Anhui province young talent workstation station (Shanghai) establishment ceremony and Wuhu nationality in Shanghai outstanding young talent exchange symposium successfully held in Shanghai. Yang Lei, Secretary of Wuhu Municipal Party Committee, and Wang Lei, Deputy Secretary of Wuhu City attended the meeting. More than 30 representatives of the second group of Wuhu City "100-day Training" and representatives of outstanding young talents from Wuhu in Shanghai attended the meeting. Event, read out the Wuhu young talent workstation (Shanghai) established file, Wuhu party secretary Yang lei, deputy secretary of Wang Lei respectively for webmaster Wu Junfeng, deputy stationmaster Fu Zhian, Qian Bang, Xu Zhaohui, Liu Huan, and Wang � t Ming certificate, and jointly for Wuhu young talent workstation (Shanghai). In the discussion and exchange session, the outstanding young talents from Wuhu made an exchange speech based on their personal growth experience in Wuhu, their learning and working feelings in Shanghai, and their suggestions and expectations on the economic development of their hometown and the work of young talents. Finally, Yang Lei introduced the recent development and change and future development goals of Wuhu from three aspects: Wuhu-great, Wuhu-great potential, Wuhu-and future potential. He pointed out that the main purpose of the youth talent workstation is to build a service platform of information, emotional interconnection and mutual elements for Wuhu young talents in Shanghai, and to all Wuhu young people abroad: hometown has charm, endorsement, potential, nostalgia, collection, sincerity, and participation. Before the event, the delegation from the Wuhu Municipal Party Committee of Anhui Province visited the G60 Industrial Internet Innovation and Application Experience Center, AVIC Huadong Optoelectronics (Shanghai) Co., LTD., Shanghai Inventory Food Co., Ltd., LTD.

2022-01-29	Jining express industry in Jining city in Shandong province Jining city express industry group committee founding meeting and care for express industry working youth "warm bee action" sympathy activities held jianwei correspondent Bai Chen) on January 25, Jining city, Shandong province express industry group committee founding meeting and care for express industry working youth "warm bee action" sympathy activities successfully held. Sheng Zhenkai, deputy secretary of Jining Municipal Party Committee of the Communist Youth League, and Pang Lei, member of the Party group of Jining Postal Administration attended the event. Members of the Youth League Working Committee of the express delivery industry and representatives of youth League members attended the event. The picture shows the Jining Express Industry Youth League Working Committee establishment meeting activity site. Correspondent for the picture meeting read out the "on the establishment of Jining Express Industry Youth League Working Committee approval" document, and for the establishment of the city express industry Youth League working Committee, the newly elected secretary of the industry Youth League Working Committee and express industry youth representatives respectively made a statement. After the meeting, the "Warm Bee Action" condolence activity was held for the youth of the express industry. The Communist Youth League Committee sent life materials and warm bags for the care of the young people, and sent the New Year to the blessings of the express industry. The picture shows the establishment of the Jining Express Industry Youth League Working Committee. In recent years, The Youth League Jining Municipal Party Committee and the Municipal Postal Administration jointly carried out the "Express Industry Youth Service Month" activity for successive years, Purchase gloves and masks and other protective and epidemic prevention materials and visit express delivery enterprises; Make and distribute express delivery youth public welfare consultation service cards, Relying on the 12355 Youth service desk, Contact a professional law firm, Provide public welfare consulting services for young express delivery workers; Relying on the "youth home", learn from Lei Feng volunteer service station and other positions and some love units to set up "warm heart station", Provide free drinking water, rest, charging, inflation, emergency medicine and other outdoor workers, And mobilize the love of enterprises, social organizations to participate in various care services.

2022-01-28	Group Jining municipal party committee to carry out the public opinion "5" to listen and Spring Festival blessing activities group Jining municipal party committee to listen to public opinion "5" and "our festival" in 2022 Spring Festival blessing theme activities JianWei correspondent Bai Chen) recently, the Shandong Jining municipal party committee to east red temple community xintiandi garden community to carry out public opinion "5" and "our festival" 2022 Spring Festival blessing theme activities, at the same time the party branch and village party branch to carry out the joint thematic party day activities. Yu Lingdong, party secretary of the Communist Youth League, and Sheng Zhenkai, deputy secretary of the CPC, led the "people secretary" service team members and party members of the organization to visit and send holiday wishes and sympathy to the community residents and senior party members. The picture shows the youth League Municipal Committee in the community calligraphy activities site. The Communist Youth League Municipal Committee invited young calligraphers from Jining Young Calligraphers Association to write Spring Festival couplets and fu characters for the community residents. Red paper, brush, ink, inkstone lined up, young calligraphers brush brush ink, a variety of books show their own charm. A piece of ink fragrance overflowing Spring Festival couplets and "fu" word spread all over the desk. Although the Spring Festival couplets are small, the friendship is strong, which not only has the beautiful vision of "bidding farewell to the old year and welcoming the New Year", but also has the love feelings of "praising the Party's grace and feeling the Party sentiment". Party members of the branch led the community residents to send out more than 200 Spring Festival couplets and "fu" words, and the activity site was filled with a cheerful and strong festive atmosphere. The picture shows young calligraphers writing Spring Festival couplets at the scene. Correspondent for the picture then, the "people secretary" service team came to the home of the old party members, to them to extend the New Year wishes and send condolence items. Yu Lingdong had a cordial conversation with the senior party members, talked about their family affairs and temperature, and told them to take good care of the epidemic prevention and control measures. He also sincerely hoped that the senior party members and the residents of the community could actively make suggestions and suggestions to create a better life. The interviewed veteran party members said that the relevant activities have made them truly feel the care and warmth of the Party, and will continue to play the waste heat to contribute to the construction of a harmonious and beautiful home. Through this activity, the interaction and communication of the joint construction branch was further promoted, and the link between the "people secretary" team of the Communist Youth League Municipal Committee and the residents was tightened. While carrying forward the traditional folk culture, it also sends the blessing, warmth and festival to the residents, which is warmly praised by the residents of the community.

2022-01-26	Sunshine "home" winter vacation social practice first interns in place sunshine "home" winter vacation social practice first interns have in place JianWei correspondent XiangChangHui) recently, Shandong Rizhao municipal party committee to participate in the "green bird plan students homing" series of activities in 2022 Rizhao college students "home" winter vacation social practice, got the unit of choose and employ persons positive response and the enthusiasm of college students to participate in. After the early post push, student registration, department review and unit screening, the winter vacation social practice activities finally ushered in the first batch of lovely little green birds. The picture shows the meeting of the "Green Bird Project" in Donggang District. Due to the COVID-19 outbreak, the number of jobs has decreased, but the enthusiasm of returning students. Among the 554 posts already issued by various districts, counties and municipal departments, a total of 9,646 resumes have been collected, and 526 students have reached the internship intention. This social practice covers the whole city and covers a wide range of industries. This winter vacation social practice activities cover 112 units in all districts and counties of the city, including government organs, administrative institutions, state-owned enterprises, large private enterprises, etc., involving banking, finance, Internet, transportation, machinery manufacturing and other industries. The number of positions, good quality, rich variety, to meet the diverse needs of Bluebird students. Practice recruitment procedures are standardized, and the job position is highly attractive. The release of internship positions and the recruitment of interns are strictly in accordance with the requirements, and the relevant regulations on epidemic prevention and control are strictly complied with. They can work only after meeting the standard conditions. Meeting will be held before work, and pre-job training such as short video, photography knowledge training and official etiquette course-based training. The number of post-2000 interns is wide, and the role of the main force is more prominent. In this winter vacation social practice activities, the post-2000s accounted for 76% of the total number of post deliveries, becoming the backbone force of the internship work, and further highlighting the role of the main force in the new era. The activities are rich and diverse in forms, and the students' sense of gain is significantly improved. In addition to work practice, all districts and counties lead bluebird students to carry out a variety of activities, so that they can feel the changes in their hometown in the process of social practice, and improve their sense of work gain and happiness.

2022-01-24	2022 Jining young students "cloud recruitment" activity held "Green Bird plan to help enterprises climb" 2022 Jining young students "cloud recruitment" activity held in the year of young talent introduction and rural revitalization of youth partners and other preferential policies. Correspondent for the picture of China youth network Beijing on January 24 (reporter Zhang Jianwei, correspondent Bai Chen) on January 22, Jining City, Shandong Province, "green bird plan to help enterprises climb" 2022 young students cloud recruitment activities were held. Jining Communist Youth League, together with the Municipal Talent Office, Municipal Science and Technology Bureau, Municipal Human Resources and Social Security Bureau, Municipal SasAC and other departments, has accurately identified 870 high-quality recruitment positions and 6,035 demand plans, covering high-end equipment, medical care and health, financial securities, high-end chemical industry and other industries. About 153,000 people watched live streaming through multiple platforms. The picture shows the scene of the "cloud recruitment" activity of "2022 Jining Green Bird Plan to Help Enterprises climb". This live broadcast has set up a "cloud promotion", "cloud interview", "cloud sharing" and other three scenes. At the first scene, Gao Cong, deputy secretary of the Communist Youth League Municipal Committee, introduced the preferential policies such as the introduction of young talents and the rural revitalization of youth partners issued in Jining city in recent years, interpreted the incentives from undergraduate students to doctors, and issued a sincere invitation to the majority of young talents to start businesses and find employment. The heads of 12 heavyweight enterprises and institutions and cadres "stationed in the enterprise climbing" promoted the unit situation and recruitment position information on the spot. More than 20 key enterprises in the "cloud interview" site through the "cloud interview room" and young talent real-time interactive interview, interview negotiation, the city's more than 170 employers simultaneously online, timely answer the young talent job consultation. In recent years, the Jining municipal party committee, Jining municipal party committee talent office jointly built 12 "young talent accelerator", for young talents entrepreneurship mentor training, practical technology training, entrepreneurial project operation, scientific research incubation and sharing technology, dating fellowship services, formed the "scientific research institutes + industrial park + industry enterprise" three-layer layout, "talent promotion + enterprise incubation" good pattern of two-wheel drive. The picture shows the scene of the "cloud recruitment" activity of "2022 Jining Green Bird Plan to Help Enterprises climb". Correspondent for the live activities, pku vice minister li Ping introduced the youth station policy, invited five new bird station master through video and young students, nankai university Jining bird station master Zhang Bolin classmates and Shanghai jiaotong university Jining bird station master Wei Jiru students walked into the third studio, to share their study and work. This activity through the university green bird station, students WeChat group, QQ group, etc., smooth the channel of accurately push posts to students, timely reply to students' concerns. At the same time, the activity has also established the enterprise HR communication group, timely convey the progress and requirements of cloud recruitment work, carefully create an online job application cloud platform, to help enterprises climb and forge ahead. In the next step, the Communist Youth League Municipal Party Committee will continue to do a good job of the whole chain of services, such as tracking and return visit, job matching, interview and signing, workplace training, friendship and exchange, continue to consolidate the cloud recruitment experience, deepen the construction of "Green Bird Station" and "young talent accelerator", gather more outstanding young students, and widely touch the service industrial park.

2022-01-24	Dali, Yunnan: The contradiction between taking care of your children and your own heavy work has become a headache for many young parents. Don't worry, this holiday, to help you take care of your children!"" tuantuan in order to accompany the children to spend a happy winter vacation is also hard! Come on quickly and spend your first holiday after the 'double reduction' happily with Tuantuan."On January 15, the first batch of winter vacation activities were launched on the official WeChat Dali Municipal account" Youth Dali " in Yunnan Province. By the Dali state committee, the Dali municipal party committee, Dali city government, city education sports bureau, municipal party committee organization department, Dali erhai popular science education center, Dali service center, Dali university, youth community, art and youth development center and other units to provide support for these activities, not only welcomed with parents, also received the attention of the people from all walks of life, have claim class, as a volunteer teacher."Erhai Protection Little Guide" is a brand project carried out by the Dali Municipal Committee every winter and summer vacation. This year is the 8th issue. This project, with the theme of "exercising language expression ability, learning ecological and environmental protection knowledge, and cultivating the spirit of volunteer service", was rated as the national "four hundred" advanced model in 2018. There are currently 157 primary and middle school students serving as interpreters, the oldest of 14 and the youngest 6 years old."You and secure work, the child to 'small green companion'!"The most touching parents, do not charge any fees" Xiaoqing companion " love care project, 5 hours a day, divided into morning classes and afternoon classes, the course content including sports exercise, reading appreciation, puzzle games, popular science education, homework guidance, etc. The project emphasized: " Medical workers, grass-line workers, children of migrant workers and left-behind children are preferred."The Youth League Committee of Dali University recruited 120 college students to pair up with Xiaguan Third Primary School and Beixin MoExperimental Primary School to conduct online activities such as" Sports Together to Welcome the Winter Olympics "and" Cloud Reading Together "; Dali Aier Eye Hospital prepared" Little Dream chaser " student professional experience activities for the children."We are continuing to plan projects to help parents solve the problem of having children during the holidays."Said Qian Li, part-time deputy secretary of Dali. How to enrich the extracurricular life of teenagers after the "double reduction" is an important topic of the Dali State Party Committee and the Dali Municipal Party Committee of the Communist Youth League since 2021. To this end, they excavate and integrate social resources, promote the construction of "off-school youth working committee" and "youth home", build a socialized youth work platform, and expand the youth position outside the school. On December 16,2021, Dali City Taihe Street Wanhua Community Youth Working Committee was established, which is the first community youth working committee in Dali Prefecture. Among the more than 30,000 people permanently living in the Wanhua Community, there are more than 10 ethnic minority populations such as Hui, Tibetan, Yi, Dai, Bai and Mongolia. The community was named National National Unity and Progress in 2016."Communities are the basic cells of the city, with the most contact and the closest approach to teenagers."Wang Sihan, secretary of the Dali Municipal Committee of the Communist Youth League, said that the Community Youth Working Committee relies on the community and works through the working platform of" the Communist Youth League + youth social organizations + the Community Youth Working Committee " to complement and organically link the school, so as to enhance the social participation and integration of the Young Pioneers. In fact, the first after-school youth working committee in Dali Prefecture, Feiyang Dance School, has shown the vitality of the young Pioneers since its establishment in 2018. Feiyang Dance School Youth Working Committee has more than 50 off-school counselors and 3,000 young Pioneers. Children combined with their own characteristic innovation team day, team, such activities, let the young pioneers outside can participate in the young pioneers activities, such as the annual October as patriotic education month, love the party, motherland, hometown, art theme education activities; Dali intangible cultural heritage, Bai music, Bai overlord whip, Bai string four Bai "intangible" project as long-term teaching courses, built bai "intangible" inheritance classroom, into more than 60 primary schools, kindergartens, carry out "intangible into campus" public after-school service, training more than 40,000 students. In July 2021, the Flying Dance School's External Youth Working Committee performed a large-scale music and dance sitcom marking the 100th anniversary of the founding of the Communist Party of China. More than 50 off-campus counselors and more than 300 young Pioneers presented a series of vivid character stories, such as Erhai Lake protection administrators, poverty alleviation team members, anti-epidemic medical staff, non-genetic inheritors, and volunteer teachers, on the stage in means of music, dance, drama and folk art, which caused a great response in the society. The off-school Youth Working Committee not only brings happiness to urban children, but also benefits left-behind children in mountain villages in public welfare volunteer teaching. Wang Xue, a young teacher from Feiyang Dance School, first went to the volunteer school in Dali Weishan County Democratic Revolution Primary School. Most of the students there are left-behind children, and the children and teachers are very eager to dance. On the day of the teaching, a teacher said to Wang Xue, " Don't give up on us. We want to dance!"" At that moment, I strengthened my determination to continue to volunteer teaching."Wang Xue said. Over the past year, more and more young people have joined the "Youth Help 'Reduction' " volunteer service team set up by the Dali State Party Committee and the Dali Municipal Party Committee of the Communist Youth League. In the second half of 2021, Dali gathered the young people from the hip-hop dance training schools to participate in the "hip-hop dance into the Campus Activity" organized by the Communist Youth League State Committee, and led the teachers and students to dance the dynamic hip-hop dance in many primary schools, and "hi" turned over the campus. Dali Nuoyi Biotechnology Co., Ltd. held insect science exhibitions in many primary schools, which opened students' eyes and marveled: " I have never seen so many insects, so there are so many attention."While selecting areas and units with good foundation for Party construction, many district units and strong youth work force to set up off-campus working committees", the Youth League Dali Municipal Party Committee also relies on the production and business sites of Wutong Academy, Dafang Bookstore, Enlightenment K stack and other enterprises to build "youth home", continuously expanding the position of Dali youth activities. For some time, the Wutong Academy Youth Home in Dali has become popular. Young people can exchange the "Youth Study" with coffee coupons and receive the Dali Communist Youth League. The learning club activity of "Send you the first cup of coffee in winter" held by Wutong College Youth Home has attracted many league members and young people. Under the system of encouraging and guiding the youth homes to carry out innovative activities, the youth home characteristic activities, such as the singing of Wanhua Community and the "Little Judge" professional experience of Mayang Law Firm, have become more and more influential among young people. League-run projects such as "Protecting Erhai Lake Volunteer service and keeping homesickness", "one-hour public welfare", "growth compass" and "Yan returning home social practice" are also becoming more and more popular among young people. Qian Li introduced that the expansion of youth positions outside the school not only helps to "reduce the burden and improve the quality", and creates a good "ecosystem" of students' comprehensive quality education, but also makes many young people benefit from this "circle of friends". After the establishment of Dali Xizhou Blue Xu Tie-dye Studio, the staff participated in the Dali Prefecture "Entrepreneurship and Poverty Alleviation Good Youth" training, and went to Shanghai Jiao Tong University for exchange and study. Yang Donger, a young Bai man in Dali after 1995, returned to Dali from the United States and Australia and set up a creative team of "Wilderness Animation", which will show the scenery of Erhai Lake and Bai folk culture in Dali in the form of local original cartoons and animation. The first comic volume of Cangshan Booklet, published by Yunnan People's Publishing House, has been read more than 10 million times on various major cartoon platforms, and was selected into the "motivation" Chinese original animation publishing support plan of the Central Propaganda Department in 2020."Wilderness Animation" has participated in the Creative Yunnan Cultural Industry Expo twice. "Cangshan Brochure" and its surrounding products have attracted many readers. The cartoon "Dali Family Style" created by the team has become the poster of Dali Chuangwei City. China Youth Daily China Youth Network reporter Zhang Wenling source: China Youth Daily

2022-01-23	Zhengzhou, Henan province: "green" brand collective in action "green" brand collective in action to make the ground particularly slippery, to the city traffic has brought a lot of safety risks. The Zhengzhou Municipal Party Committee of the Communist Youth League issued a timely notice, calling on the city's youth commandos and youth civilization number and other "green" brand collective to immediately carry out snow removal and de-icing action, and go all out to ensure smooth traffic. The city's "green" brand collective responded quickly, 47 different industries of youth commandos and "youth civilization" in their respective posts to carry out snow deicing, vehicle emergency repair, order maintenance, line inspection and other activities, for the safety of the public travel escort. Early in the morning, at the bus station of Kaixuan Road, Jianshe Road, Zhengzhou city, Zhengzhou Bus First Operation Company 203 Road "Youth Civilization" members braved the cold, cleared the departure yard and the surrounding snow, and threw snow melting agent on the icy road. Bus B5 Road "Youth Civilization" party and league members volunteer service team volunteers also quickly gathered, to clear the snow on the road, to ensure that the line is smooth flow. At 10 o'clock in the morning, Hengtian Heavy Industry youth commandos gathered on the main road of the company, shovel by shovel to remove the road snow, to ensure the smooth production. In order to facilitate the residents to the hospital, Zhongmou County fire rescue brigade youth commandos came to the county people's hospital near, cleaning the gate and trails on both sides of the snow. Zhengzhou MIXC Youth League branch youth commandos gathered early in the morning that day, boil cold ginger tea, clean up the snow in front of the mall. After the shopping mall opened at 10 a. m., they delivered hot ginger tea to the delivery boys, couriers and passing citizens at the delivery channel, so that everyone could feel the warmth and care in the cold winter. Reporter He Ke, our correspondent Zhou Man

2022-01-20	Yanji to build a "youth vitality" city medium-and long-term youth development planning county-level pilot youth league organization to boost the youth high-quality development of Yanji to build a "youth vitality" city well-paid work, back to his hometown Yanji City, Jilin Province, to start a business. Three of his fellow villagers who returned to Yanji from first-tier cities became his entrepreneurial partners. In their opinion, Yanji not only has a strong Korean regional characteristics, but also constantly releases its youthful vitality, which is the main reason to attract them. In 2021, Yanji city was designated as the pilot county (city) for the implementation of the National Medium-and long-term Youth Development Plan. Yanji to "boost youth high quality development, building youth dynamic city" as the goal, by building youth service position, focus on youth development work, the youth thought lead, employment entrepreneurship support, talent training, mental health, safeguard youth legitimate rights and interests, expand youth social integration and participation, deepening, create a livable, appropriate learning, appropriate industry of yenji "youth dynamic city" characteristic card. When he first returned to his hometown, Wu Zhenyu did not know much about the specific entrepreneurship policies of his hometown because he had already left home for many years. After becoming attached to the Yanji Youth League organization, Wu Zhenyu and his entrepreneurial partners took many detours. For returning home to start businesses and employment talents, the league organization takes the initiative to send a variety of affordable "service gift packages". The youth league organization invited Wu Zhenyu to attend the industry exchange meeting, helped him understand the national and local support policies to start businesses, and also provided him with a free office area for the Youth Pioneer Park. Not only that, from business registration, legal advice to apply for small and micro business loans, the youth league organizations have taken the initiative to help Wu Zhenyu."When we see the need, the league organization can always help us easily. Problems that can not be solved temporarily, they will also help to coordinate for many times."Wu Zhenyu said. The "service gift package" organized by the youth league was also sent to the rural fields. After graduating from university, Zhang Guozhen returned to his hometown of Chaoyang Chuan Town, Yanji City, and set up Gu Xiangrui Planting Farm. In the early days, Zhang Guozhen was short of funds. During the visit to the countryside, the cadres of the Communist Youth League of the League Yanji Municipal Party Committee learned about Zhang Guozhen's difficulties and took the initiative to help him coordinate the application for a special loan of 250,000 yuan. Zhang Guozhen used loans to buy forklifts, plant protection machines and other farm tools. Now, as a leader in returning home to start businesses, Zhang Guozhen has not only created a high-quality rice brand, but also led more than 30 villagers to increase their income and become rich together. In June 2021, in order to increase the support for young people returning home to start businesses, the Communist Youth League Yanji Municipal Party Committee cooperated with Yanbian Rural Commercial Bank Co., Ltd. to invest 2 million yuan to set up the municipal risk guarantee fund, and launched a special loan policy of 20 million yuan to start businesses. The interest of the first year of the loan was fully discounted by the municipal government finance. Up to now, 16 returning business loans have been issued to Yanji city, with a total loan amount of 6.55 million yuan. At the same time, relying on the Yanji Youth Pioneer Park and entrepreneurship sharing space, Yanji provides 4,000 square meters of comprehensive office operation and logistics storage space for young people, and provides free "one-stop" entrepreneurship practice services such as registration, incubation site and loan financing. Since the youth home was established in Yuanyue community, junior high school student Park Yu (pseudonym) has spent the winter and summer vacation here. His parents work abroad all the years, and Park's extracurricular life with his grandparents is boring. But at the youth's home, Park attended a weekend holiday class where college student volunteers helped him with his homework. Not only that, he can also attend interest classes such as painting, paper cutting, calligraphy, Lego and taekwondo. Jin Ye, secretary of the Youth League branch of anyue Community, said that there are still many left-behind children like Park Yu in the district. Because their parents are not at home for years, the community youth home takes the responsibility of helping their children broaden their horizons and enrich their extracurricular life. At present, anyue Community Youth Home has set up six service stations, including Sunshine Station, Service Station and Rights Protection Station, to provide menu services for vulnerable groups including special children, left-behind women, disabled people, unemployed and other vulnerable groups within the jurisdiction. In addition to the weekend and holiday classes, the youth home staff and volunteers also organize the children to visit playgrounds, natatorium, ski resorts, or take them to experience rural life. In 2021, relying on the platform of Youth Home, Xinyue Community carried out more than 20 practical activities of "doing practical things to the Party and me for the masses". Through safety knowledge publicity, epidemic prevention and control, and helping the community to carry out the creation of a civilized city, we strive to create a found and trusted youth service position for the youth in the community. It is understood that there are 6 streets in Yanji city, each street has one such a youth home, these youth homes have become an important carrier to serve the youth and a comprehensive platform for all kinds of young people in the area to participate in social practice. After finishing college in Changchun, Cao Rui, a 25-year-old girl from Yanji, joined the Yanbian Light Love Association and started her public welfare entrepreneurship. Now she is the secretary of the Youth League committee of the association. In 2021, Cao Rui participated in the Light Love Association's Safe Class and de facto Orphan Relief projects. She introduced that the docking of the project school, information collection, funds, resources and site docking are all due to the coordination of the Communist Youth League Yanji Municipal Party Committee. In recent years, in order to deepen the youth help rural revitalization action, Yanji has also supported a number of young e-commerce anchors to carry out such as "anchor + bring goods" and "anchor + tourism". At the same time, it also regularly organizes young e-commerce anchors and township youth wealth leaders to participate in agricultural products exhibitions in Beijing, Shanghai, Shenzhen and other places, to improve the popularity of special agricultural products in Yanji city, and to connect more resources and platforms for young anchors. At present, in Yanji city, there are 152 young social organizations, private non-public enterprises and social organizations, such as the Light Love Association and e-commerce anchor organizations. Supporting the healthy and orderly development of young social organizations, serving the needs of young people and leading them to participate in social governance is another top priority of the Communist Youth League Yanji Municipal Party Committee. The cleanliness and tidiness of Yanji city is inseparable from the daily efforts of young volunteers. To gather the vanguard strength of young volunteer service, deepen volunteer service and consolidate the achievements of city creation, the Yanji Municipal Party Committee of the Communist Youth League regularly carried out brand volunteer activities such as "protecting mother River, helping the river chief system" and "Road chief system" inspection. At the same time, in terms of enhancing youth social participation, the Yanji Municipal Party Committee of the Communist Youth League also regularly carries out educational and public service volunteer service activities to attract young people to participate, so that the services of the Communist Youth League organization will benefit more young people. In the next step, Yanji city will continue to build a youth service position and promote the field of youth service, so as to make Yanji more youthful vitality. Source: China Youth Daily, January 20,2022, edition of the 01st edition

2022-01-20	The 2022 Guangxi Youth Exchange Committee of Guangxi Youth League, vice chairman of Guangxi Youth Federation, and young people from Guangxi and Hong Kong visited the Guangxi Ethnic Museum. China Youth Daily client news (He Rilin China Youth Daily China Youth Network reporter Xie Yang) "To promote harmony and brave innovation" 2022 Guangxi and Hong Kong youth exchange activity was held in Nanning, Guangxi recently, more than 90 young people from Guangxi and Hong Kong participated in the event. On the day of the event, the young people of the two places visited Guangxi Museum for Nationalities and Tencent Short Video Live Nanning ASEAN Operation Center, which not only deepened the understanding of Hong Kong youth on mainland culture and experienced the spirit of ethnic unity in Guangxi, but also enabled the young people of the two places to feel the power of new media by participating in digital content production. The event was jointly hosted by the Liaison Office of the Hong Kong Special Administrative Region Government in Guangxi, the United Front Work Department of the Party Committee of the Guangxi Zhuang Autonomous Region, the Guangxi District Committee of the Communist Youth League and the Guangxi Youth Federation. In the next step, the Guangxi District Committee of the Communist Youth League and the Guangxi Youth Federation will continue to carry out a series of youth exchange activities, such as the good Guangxi-Hong Kong youth exchange and the summer internship program for college students from Hong Kong, Macao and Taiwan. In the context of the normalization of epidemic prevention and control, we will actively explore a new model of practical cooperation between the young people of Guangxi and Hong Kong, and further promote the friendly exchanges between the young people of Guangxi and Hong Kong.

2022-01-21	Jinan to carry out the "Hope cabin winter warm" series of volunteer sympathy activities, Jinan to carry out the "Hope cabin winter warm" series of volunteer sympathy activities, Jianwei correspondent Peng Chong, Xu Jiaying) to promote the "I do practical things for teenagers" activity, Realize from "new" to "spiritual color", Help the healthy growth of teenagers and children in difficulties, recently, Shandong Province Jinan Municipal Party Committee, the Municipal Youth Federation organized all levels of the city's youth League organizations, youth League members, love volunteers, love units and enterprises to carry out the "hope hut winter warm" volunteer sympathy series of activities, Sent to the "New Year growth gift package" for the children, Do a good job of children care "in the second half" article. Changqing District Youth Volunteers Association joined hands with 56 young volunteers to jointly carry out the "Warm delivery" Hope House public welfare activity. Teams of 17 volunteers, to 10 street town, 82 villages, more than 100 hope hut, before the cold wave, more than 100 bed warm quilts to Changqing each hope hut children, one of them is qian (not a real name) children, when the volunteers are coming out of the house, quietly put a letter into the hands of volunteers, one wrote, " your love makes us feel the warmth of society and pride in a great country!"Young notes on the paper left the children to all walks of life Thanksgiving, but also revealed the love of the motherland. Jinan Municipal Party Committee and Jinan Youth Federation have carried out the "Spring New Spring Art" public welfare class, sending cultural courses such as street dance public welfare teaching and calligraphy guidance to the children in the Hope House, among which the hip-hop public welfare courses have accumulated nearly 100 classes, covering hundreds of young people. Not only to enhance children's healthy body, to explore their talent potential, but also to let children keep sensitive thinking, cultivate self-confidence, self-improvement, perseverance, bold character, to maintain a healthy and positive mental state. Under the explanation of the narrator of Shandong Science and Technology Museum, the children visited all kinds of interesting scientific experiments one by one: tilt hut, mirror world, static hair generator, etc. The children operate the experimental model and observe the scientific principles behind the life phenomenon found everywhere in the game. By experiencing popular science games and watching popular science videos, the children's enthusiasm and interest in active exploration and active thinking were aroused, and their understanding of science and their thirst for knowledge were deepened in a relaxed and pleasant atmosphere. Next, pku will continue to take pair help, accurate help children care services and communication docking, continue to carry out family companionship, academic counseling, wish, psychological counseling and other volunteer service activities, such as the need to improve the level of youth growth care services, to carry out various forms of practical and effective volunteer service activities.

2022-01-20	Haikou communist youth league open 2022 youth "warm winter action" Haikou communist youth league open 2022 youth "warm winter action" Ren Mingchao) "warm way home, to the future", on January 16, the eve of Spring Festival, service security Haikou civil aviation line personnel and youth volunteer representatives in Haikou meilan international airport (hereinafter referred to as the "meilan airport") issued to guarantee 2022 travel service declaration. They also sent their New Year wishes to the returnees through flash MOBS. This year, the group of Haikou municipal party committee in accordance with the "advance reserve, need, adjust measures to local conditions, station, streamline jobs, optimize services, strengthen training, safety first" principle, continuous service travel "warm winter action", organization called for youth from all walks of life, volunteers in meilan airport, Haikou bullet train station, train station eight main sites provide station guidance, security assistance, epidemic prevention propaganda, order maintenance, information consulting services. It is understood that the youth "Warm Winter Action" has launched more than 70,000 young people and volunteers, and served more than 500,000 people of various groups.

2022-01-19	Shenyang Youth Volunteers Warm Winter Action) sponsored by the Communist Youth League and Shenyang Youth Volunteers Association, the warm winter action was recently launched in Shenyang. For the first time, the campaign has cooperated with youth volunteer organizations in the airport, railway station, passenger transport, subway and taxi industries. The Shenyang Railway Station Youth Volunteers Association recruited 130 college student youth volunteers to carry out volunteer services in Shenyang Railway Station, Shenyang North Railway Station and Shenyang South Railway Station. The Shenyang Metro Volunteer Service team recruited more than 80 young volunteers to carry out volunteer services in the Olympic Sports Center subway station and other Spring Festival travel concentrated stations. Shenyang Rongqiang Lei Feng volunteer team recruited "Lei Feng Love Team", "Party Member Team" and operated more than 2,000 taxis to become mobile volunteer service stations, with the goal of improving the service quality, to provide travel consultation, luggage transportation, help the old and the young and other volunteer services. The youth volunteer service team of Shenyang Passenger Transport Group Co., Ltd. recruited more than 80 young volunteers to carry out volunteer service activities in key points such as South Square of Shenyang North Railway Station and West Square of Shenyang Railway Station. Wang Chenyang, head of the Youth League Committee of the No.4 Company of China Railway No.9 Bureau, believes that the young volunteers in Shenyang who stick to the busy Spring Festival travel rush provide help to returning students and passengers who need care, and improve the travel efficiency of passengers. They have warmed the city with the spirit of volunteering, and volunteer red has become the most beautiful scenery in Shenyang.

2022-01-18	Nearly 30 children celebrate the festival, accompanied by their mothers and safe volunteer teachers. Most of the children in the "childlike innocence harbor" are left-behind children. Compared with ordinary children in ordinary families, parents pick up to school, tutoring homework, play games, go out to learn knowledge and so on are an extravagant hope. To further care about the rural left-behind children, Ganzhou municipal rural revitalization of residency teams combined with unit functions and rural revitalization work requirements, introducing the landing of "childlike innocence harbor" project province "difference" key support village-warehouse village, aims to build with rural left-behind children care service system construction, normalized service of rural left-behind children of the communist youth league work.

2022-01-18	Qinhuangdao youth civilization "I do practical things for the masses" practice activity launched the group Hebei Qinhuangdao Municipal Party Committee launched the youth civilization "I do practical things for the masses" activity Zhuang Town Dashi Primary School held in 2022 (Spring Festival) Qinhuangdao youth civilization "I do practical things for the masses" practice activity launch ceremony. At the scene of the ceremony, Qinhuangdao Heating Co., Ltd. and its subordinate youth civilization collective presented love gifts of 10,000 yuan value to the rural left-behind children in Lulong County. During the Spring Festival, the city youth civilization collective will be around the rural left-behind children, life difficult lonely old man, epidemic prevention and control line staff, during the Spring Festival line worker, in the Spring Festival local migrant workers and Courier service object such as actual need, organization to carry out care sympathy, psychological counseling, legal escort, convenient service activities.

2022-01-18	Guangxi Beihai Communist Youth League carried out the "roundtable talk" activity, putting forward specific measures, planning the activity content and showing innovative ideas around the key work of the CPC Beihai Municipal Party Committee in 2022. On the activity, the cadres and staff for how to do a good job of youth thought guidance, promote the reform of the county Communist Youth League and the young Pioneers reform, the implementation of the youth, deepen the Communist Youth League brand project construction four key work to speak, a total of nearly 180 suggestions. In the next step, the TuanBeihai Municipal Party Committee will review the suggestions collected by the "round table" activity, select the best, and actively plan to promote the 2022 work highlights and results.

2022-01-16	Wuhu communist youth league "reading list" light plight children scholarly dream Wuhu communist youth league "reading list" light plight children scholarly dream green net reporter wang haihan wang lei) on January 14 morning, the Wuhu communist youth league power people's urban construction building "reading sheet" interpreta dream future activities concentrated donation ceremony held in mirror lake village street horse factory village. A group of 535 left-behind rural children and urban migrant children have received their favorite books. In the early stage, the Wuhu Municipal Party Committee of the Youth League raised money for the whole city. More than 300 people, including youth league cadres, enterprises and institutions, young entrepreneurs, youth League members, and caring people, as well as Wuhu Xinhua Bookstore, Three squirrels, Zhishan Culture and other caring enterprises, responded positively, donating materials equivalent to 135,000 yuan. Among them, Wuhu Xinhua Bookstore directed donation of fangcun Street, Machang Village rural library, Tianmin school library worth 10,000 yuan of love books. Three squirrels provided 80,000 yuan worth of warm gift bags to 535 children. Subsequently, the League Wuhu Municipal Party Committee selected 50 books involving inspirational growth, red classics, popular science knowledge, science fiction stories and other fields to form a "total reading list". Each child chooses 2 books in the book list as his micro wish, and the Communist Youth League Municipal Party Committee will donate a party history learning and education book according to the primary school, middle school, high school and other different school age levels. A few ordinary tile-roofed houses, a few simple tables and chairs, this is xiao Chen (pseudonym) home. Xiao Chen, 14, is already a junior high school student and is interested in books. As his parents died, he had to live with his elderly grandparents, and reading extracurricular books became a "luxury". In class, Xiao Chen developed a strong interest in the Forbidden City as described by the teacher. This activity, Xiao Chen immediately selected the dream of "Six hundred Years of the Forbidden City" and "Shining Red Star". Yanyan (pseudonym), a 7-year-old girl in Puxi Village, Yijiang Town, has only a mottled walls, a bumpy concrete floor and a few pieces of old furniture. Because his grandfather was disabled and bedridden for a long time, he could only rely on his father to support a big family of six people. In the face of the difficult and difficult life, sensible she never complained, "The Ordinary World" and "Red Star over China" is her choice of the wish book list. Xiao Lei (pseudonym), a 13-year-old boy from Xinba Village, Nanling County, is about to enter junior high school. He is in a poor family due to illness. He overcomes difficulties and studies hard, and has certificates posted on the walls of his home. Xiao Lei loves science passionately. " I want to know what is behind the stars."This time, he did not hesitate to choose the Three-Body Problem and Stephen Hawking: My Universe."We hope to bring happiness and plant hope to our children through the activities. We will jointly organize the Communist Youth League to carry out the 'Little Migratory Bird' winter camp in Jiangcheng, so that more teenagers in difficulties can have a happy childhood."Said Zhang Qian, administrative director of Three Squirrels Co., Ltd."The campaign will effectively convey the care of the Party, the government and all sectors of society to key youth groups. The wish is small, but significant. Youth league organizations, young entrepreneurs, caring people from all walks of life to build the children's dream of books."The relevant responsible comrade of the Communist Youth League Wuhu Municipal Party Committee said that in the future, it will continue to give full play to the organization and mobilization advantages of the Communist Youth League, and include caring and helping teenagers in difficulties in the key work content.

2022-01-16	Quanzhou youth painting and calligraphy photography contest winning works exhibition in the form of art to guide teenagers healthy up Quanzhou held successive youth painting and calligraphy photography contest effective wei correspondent, Wu Junwen Huang Jingya) recently, "always to the party calligraphy exhibition" Quanzhou third youth painting and calligraphy photography contest award-winning works exhibition was held successfully, in the contest, Quanzhou teenagers with calligraphy, art, photography and other different art forms, review the red history, describe one hundred struggle, praise the prosperity of the motherland and hometown development. This is the third event jointly organized by Quanzhou Municipal Party Committee, Quanzhou Municipal Civilization Office, Quanzhou Education Bureau, Quanzhou Federation of Literary and Art circles and other units. In the previous competitions, a total of nearly 30,000 calligraphy, art and photography (including short video) entries have been received. Contest held around the era theme " held the contest, to celebrate the 100th anniversary of the founding of the communist party of China, promote the party history learning education go deep, acura under the leadership of the party Quanzhou economic and social development achievements, combined with spiritual civilization education, further guide the youth know Dang, listen to the party, follow the party, advocating civilized healthy green lifestyle, cultivate the correct aesthetic concept and good artistic accomplishment."Youth League party secretary Xu Yue in the third session of the Quanzhou City youth painting and calligraphy photography competition award works exhibition opening ceremony speech said. According to introducing, Quanzhou third youth painting and calligraphy photography contest to "celebrate the 100th anniversary of the founding of the communist party of China" as the theme, since its launch last September, get Quanzhou relevant units, schools and society attaches great importance to and strong support, attract the youth calligraphy and painting lovers and the social active participation of all walks of life, received 11297 entries, contributions are the highest number. Since the launch of Quanzhou Youth Calligraphy and Painting Photography Competition, the theme of each session is related to the background of The Times and the current hot spots of Quanzhou. Quanzhou coincided with 2020, the key period to create the national civilized city, and heritage, to meet the world is also Quanzhou citizens concerned about events, therefore, the second event to "create civilized city power ShenShi heritage to meet the world movement" as the theme, contestants closely around the theme, created a batch of content health, unique style, novel ideas, elegant style of excellent works. Professional judges to ensure scientific justice to adhere to the principle of openness, fairness and justice, to ensure that scientific review, standard review, academic review, democratic review, each event, the organizers have hired a number of well-known artists and university art college professor review committee for review, most of the judges for the Chinese calligrapher's association, China artists association, China photographers' association members, has considerable professional level and artistic attainments. Before the review, the organizer will hold a review preparatory meeting to emphasize the review principles, review plan and relevant regulations. Since the competition was held for three years, a total of 1,698 award-winning works have been selected, including 519 calligraphy works, 775 art works and 404 photographic works."The competition is aimed at Quanzhou teenagers, first of all, in the creation to reflect the theme of the session, in addition, in the calligraphy skills, there are also relatively high requirements. Specifically speaking, it is to see its taking the method, should take the ancients, the way to be positive, at the same time, but also need to have a certain originality, can reflect their own appearance."Said calligraphy group judge Wang Jianqiang. Art group judge Dai Yiqiang said: " The art review in the work of the shape, creativity has professional standards at the same time, but also to see whether it is around the theme of the activity creation."Works of both ideological and artistic" these award-winning works integrate ideological and artistic as one, express the Quanzhou youth love the party, love the motherland, love the hometown feelings, expressed the time to listen to the party, always follow the party's firm faith."The third competition review committee introduced in the review. It is reported that the previous award-winning works are both sports, rich types, smooth and unrestrained calligraphy poems, colorful magnificent pictures, but also magnificent moments of light and shadow, they are closely related to the theme and have a considerable artistic level, representing the overall level of Quanzhou youth painting and calligraphy photography art creation. In addition, each event, the organizers are in the city organization to carry out excellent award-winning works tour, and editing excellent works, further display activities, lead the teenagers in the beauty, beauty, appreciation, create beauty in the process of improving aesthetic and humanistic accomplishment, help youth ideological guidance, civilization, heritage, etc.

2022-01-14	Putian "dawn love action" build family communication "cloud" bridge Putian "dawn love action" build family communication "cloud" bridge Jianwei correspondent, Xu Yongpan, Li Jianghua Tang Fei) "thank you, thank you, 13 years, I finally saw my son......" Aunt Huang while wiping tears, while holding the sunshine social worker's hand for a long time. Because Huang is disabled and can't speak Mandarin, she has never seen her since his son was in prison. In order to realize the family dream of the minor children and their families, at the beginning of 2022, the "Morning Care Action" - -child "Cloud" meeting activity was held by the Putian Municipal Committee of Fujian Province and Putian Prison of Fujian Province. A team of cadres of the Youth League Committee, prison police and Sunshine social workers went to the families of the prisoners in advance to debug the video connection equipment in advance. A total of More than 80 prisoners and their families participated in the meeting. Since 2013, the Putian Municipal Committee of the Communist Youth League and Fujian Provincial prisons have jointly held 11 parent-child meetings for minor children of prisoners. In particular, under the normal prevention and control of the epidemic in prisons, Putian Municipal Party Committee of the Communist Youth League, Putian Prison and Putian Sunshine Youth Affairs Service Center have overcome many difficulties and opened a "sunshine special line" to build a bridge of family communication between prisoners and their children through video connection meetings. Let the prisoners feel the warmth of the family, feel the care of the prison and the care from the society, so as to promote the prisoners to confess and repent, actively reform, and establish the confidence and determination to turn over a new life. At the same time, let the children of the prisoners find the "missing love", promote the emotional communication between the prisoners and their children, and help them to tide over the difficulties and grow up healthily. It is reported that over the years, the Putian Municipal Committee of the Youth League, Putian Prison and other units have helped more than 1,500 families of prisoners, visited more than 8,000 families times, coordinated and solved more than 300 problems such as household registration, enrollment and parent-child relationship of minor children of prisoners, winning the trust of teenagers in distress with solid work results.

2022-01-14	Anhui Huainan network writer association youth league branch established Anhui Huainan network writer association youth league branch established Jianwei correspondent, Xu Xiaoxia check Weiting) recently, Anhui Huainan network writer association in Huainan economic and technological development zone party and mass activity service center held the youth league branch founding meeting. Tuan Tang Bin, deputy secretary of Huainan municipal party committee, Huainan writers association chairman jin fung, Huainan municipal party committee four researcher Zeng Wei, Huainan municipal authority league committee secretary Ren Yi, deputy secretary Lu Shawei, Huainan city network writers association chairman Jiang Kai (pen name: green) and Huainan network writers association council representatives, members to attend the meeting. By voting, the congress elected Pan Yijing, Liang Miaomiao (pen name: Jiushuihui), Cheng Jiale and Dai Yujie were elected as members of branch Committee of Huainan Network Writers Association of the Communist Youth League; Pan Yijing as branch secretary of Huainan Network Writers Association Committee of the Communist Youth League, Liang Miaomiao (pen name: Jiudishui) as deputy Secretary, and Cheng Jiale and Dai Yujie as members. Founding meeting, deputy secretary of Huainan municipal party committee Tang Bin delegation municipal party committee of Huainan network writers association branch committee congratulations, hope the establishment of youth league branch can give full play to the bridge and link, timely understand and reflect the Huainan network writers association member development needs and desire, grasp the thoughts of young writers, being a good party organization's right-hand man. Combined with the actual situation, we will carry out a variety of activities, improve the comprehensive quality and professional ability of young network writers, create a growth platform, according to the situation and characteristics of young network writers, constantly innovate service means, so that young network writers can create more excellent works with social value and promote positive energy. After the meeting, Huainan City network writers Association held the "Huainan City new era youth theme essay contest" press conference.

2022-01-19	Jining people's congress representative CPPCC members face to face birth policy supporting measures in 2022 Jining "communist youth league and NPC, CPPCC face to face" activities JianWei correspondent BaiChen) recently, the Shandong Jining municipal committee in 2022 "the communist youth league and NPC, CPPCC members face to face" symposium, part of the NPC, CPPCC members, city WeiJianWei, city family planning association responsible comrades, marriage and family psychology, parenting services related social organizations, enterprise responsible for comrades to participate in the symposium. The picture shows the scene of the "Communist Youth League and NPC deputies and CPPCC members face to face" activity in Jining City. This year's "face to face" activity to "improve the youth birth policy supporting measures" as the theme. At the symposium, the municipal Health Commission, the Municipal Family Planning Association, the marriage and family psychological field, parenting service field related social organizations, enterprise responsible comrades combined with the actual situation, to exchange the impact of youth fertility psychological concepts, parenting methods, parenting costs and other factors. The deputies to the Municipal People's Congress and members of the Municipal People's Political Consultative Conference combined with the Jining Urban and Rural Youth Birth tility Research Report and the speeches of the relevant work field, responded to the relevant issues, and put forward their own views and suggestions. Sheng Zhenkai, deputy secretary of the Communist Youth League Jining Municipal Party Committee, introduced the preliminary investigation situation and relevant suggestions on the theme of "improving the youth birth policy and supporting measures", He pointed out that youth birth protection rights and interests is an important part of youth development rights and interests, It is hoped that through the "face to face" activity can expand the attention of deputies to the municipal people's Congress and members of the municipal CPPCC on the rights and interests of youth development, We sincerely request the deputies to the Municipal People's Congress and the members of the Municipal CPPCC to reach a consensus with the Communist Youth League, Combined with the construction of Jining city for youth development and friendly city, Propropositions, countermeasures and suggestions conducive to youth development, Through various channels, and in various ways, Actively reflect and publicly appeal to the party and government departments, To play a positive role in promoting youth development. In the next step, the Communist Youth League Municipal Committee will play an important role of "face to face" activities. Through "face to face" activities, representatives and reflect the general demands of young groups through institutionalized channels like the NPC and the CPPCC, they will provide the basis for government decision-making, and promote the introduction of policies and measures conducive to youth development, including protecting the rights and interests of young people.

2022-01-14	Zhejiang Shaoxing "green farmers Market" to help new farmers entrepreneurship (China Youth Daily, China Youth network reporter Li Jianping) today, Shaoxing City, Zhejiang Province, nearly 100 "new farmers" signed up for the "green farmers market cloud on the Spring Festival". The activity to the flavor of the New Year, the year customs, excellent products as the theme, will be through the "green farmers excellent products" WeChat small program, to help the sales demand of the "new farmers" free online, selling agricultural products. According to reports, affected by the epidemic, some "new farmers" products are facing difficulties to sell on the market. The Shaoxing Municipal Party Committee of the Communist Youth League and the Municipal Rural Youth Entrepreneurship Federation jointly launched the "Green Farmers Market Cloud Spring Festival Festival", which helps the "new farmers" to open up sales, and provides convenient channels for consumers from inside and outside the city to buy high-quality, green and healthy agricultural products in Shaoxing. Local Internet celebrity anchors will also open a live broadcast with goods, with agricultural products purchased online being mailed within 48 hours. Li Yiqing, president of Shaoxing Rural Youth Entrepreneurship Federation and deputy general manager of Zhejiang Yijing Dairy Co., LTD., said, " These days, we are selecting goods, distributing high-quality tea, Torreya grandis, rice and other products to the Qingfarmers Market Spring Festival Festival platform."" Spring Festival is coming, relatives and friends in Shandong want to buy Xinchang Giant Buddha Longjing tea and Shengzhou squeezed noodles."Guo Huifang, a 65-year-old Shaoxing citizen, immediately told her distant relatives and friends to attend the Qingnong Market. Xiao Liu, a graduate student from Tongji University, has just started working in Shaoxing. He also wants to buy some Shaoxing special New Year goods and send them to his parents in Zhangjiajie, Hunan province. The Communist Youth League Shaoxing Municipal Party Committee for the picture

2022-01-13	Anhui Wuhu: promote the basic communist youth league cooperation in Anhui Wuhu: promote the basic communist youth league cooperation wang lei) recently, Anhui Wuhu municipal party committee and Anhui engineering university committee signed a comprehensive strategic cooperation framework agreement, the two sides will be around the thought lead, volunteer service, innovative entrepreneurship, practice, group belongs to new media, grassroots organization construction, etc. Heads of 14 grass-roots youth league organizations in Wuhu city and heads of youth League committees of 14 secondary colleges of Anhui Engineering University also signed cooperation framework agreements respectively. The cooperation includes the work exchanges of "youth Marxist training project" and "youth lecturers", cultivate volunteer public welfare brands around civilized city construction, ecological protection, poverty alleviation and social publicity, social investigation, volunteer teaching, youth culture and art exchange on weekends and summer vacation, and recommend outstanding college students to participate in government practice, industrial practice, etc. Liu Ning, secretary of the Party Committee of Anhui Engineering University, said that the university has high compatible with the economic and social development of Wuhu city in terms of talent training majors. The university adheres to comprehensive, multi-level and multi-point cooperation, strives for support and development through contribution, and strives to achieve win-win, win-win and multi-win. Source: China Youth Daily, January 13,2022, edition of 05

2022-01-13	Suzhou youth new media alliance for the first time plenary meeting and youth Suzhou e purchase launch ceremony successfully held youth new power e to the future correspondent Gu Qiufeng) on January 10, by the Suzhou municipal party committee net letter office, Suzhou business bureau, Suzhou municipal party committee of Suzhou youth new media alliance first plenary meeting and youth Suzhou e purchase launch ceremony was held in Suzhou China shipping wealth building. Leaders and leaders of Jiangsu Youth League Committee, Suzhou Municipal Committee, Cyberspace Administration, Municipal Bureau of Commerce and Youth League Municipal Committee attended the event. This activity aims to mobilize the strength of the alliance member units, give full play to the unique social role of the youth new media, and record and spread the youth scene of the Suzhou youth to strive for the new journey and contribute to the new era.site of activity. Group Suzhou municipal party committee for the figure for better docking resources, break the barriers, this activity of Suzhou youth new media alliance in the municipal party committee net letter office, pku under the guidance of network, released a "youth Suzhou said", "youth not YOUNG" and so on 54 key projects in 2022, and set up three youth new media alliance workstation, strive to project operation to stimulate creative power. The first video of "Youth Suzhou said" "We are in Suzhou, Together to the future" was also released, which launched the "Youth Suzhou e-purchase" project and the "Youth Suzhou" IP image collection activity, and three young new media representatives exchanged their experiences and feelings.site of activity. The "Youth Suzhou e-start purchase" project will design and release the youth exclusive "youth blessing bag" in the major commercial carriers, and promote consumption through the "youth Suzhou" joint name, youth characteristic design, youth exclusive preferential treatment and other ways. The IP image of "Youth Suzhou" will further enhance the perception and recognition of Suzhou Communist Youth League among the youth league members, and better promote the interaction of various youth innovation subjects and produce the aggregation effect, and play the good role of "youth Suzhou" brand in the wave of digital economy. The relevant person in charge of the CPC Suzhou Municipal Committee introduced that in the New Year, the CPC Communist Youth League Committee and the youth New Media Alliance will focus on the new chapter of the "strong, rich, beautiful and high" new Suzhou modernization construction, and tell the stories of Suzhou and youth stories with more and more novel works.

2022-01-12	Jiangxi Gao 'an: League representative liaison station gather youth strength Jiangxi Gao' an: League representative liaison station gather youth strength Zuo correspondent Xi Chen) to actively build a "performance platform" and a "contact platform" for ordinary youth league members, further play the leading role of league representatives. On January 7th, the opening ceremony of gao'an Youth League representative liaison station was held. Xiong Wu of the Youth League, the establishment of the establishment of Gao 'an League representative contact station is the Communist Youth League Gao' an Municipal Committee to further contact the youth of a new measure, but also at all levels of league representatives and members to do a good job of the new channel pioneer model. Gao'an city regiment representative liaison station opening ceremony. China youth network correspondent Xi Chen for figure after the opening ceremony, the first "national rural revitalization of youth pioneer" winner, the communist youth league yi chun city fourth congress, the communist youth league third congress Ding Dan made the first preach, more than 30 social entrepreneurship youth and returning college students sign up to listen to the preaching and around the "new farmers" "help rural revitalization" youth "entrepreneurship" home " and other topics to carry out interactive discussion. Liu Xingchen, secretary of the Communist Youth League Haian Municipal Party Committee, said that in the next step, the Communist Youth League Haian Municipal Party Committee will rely on the youth League representative liaison station to carry out various youth activities such as reading sharing meeting, such as "Gao'an Youth Talk" and "Weizhou Talent Lecture", to build a platform for young people to find employment, entrepreneurship, marriage and dating. Into the youth, understand the youth, really do practical things for the youth to do, do good things.

2022-01-12	Hefei, Anhui province, "Lu Xiaozhi" volunteers into the police camp on the professional experience class: "Lu Xiaozhi" volunteers into the police camp on a professional experience class China youth network reporter Wang Haihan) " a sight of the police uncle and aunts, there is a sense of security, today with them to watch the police flag raising ceremony, feel particularly shocked."Said Huang Zhiwei, a volunteer from Class 6 (3), No.3 Primary School, Anqing Road, Hefei city. On January 10 morning, coincides with the Chinese people's police day, Hefei luyang district three xiao mouth street XiPing door community less committee organization jurisdiction Anqing road third primary school "LuXiaoZhi" volunteers students into the Anhui province public security department to carry out the "police professional experience" practice, the communist youth league luyang district party committee, sanxiao mouth street, Anqing road the third primary school head to participate in the activities. The picture shows a group photo at the event site. Pictures for the Tuan Tuan Luyang District Committee to provide the activity site, "Lu Xiaozhi" people lined up to watch the police flag-raising ceremony, all the team ceremony. Li Jianzhong, vice governor of Anhui Province, party secretary and director of the Provincial Public Security Department, led all the police to solemnly swear. After the flag-raising ceremony, under the guidance of the narrator, the "Lu Xiaozhi" visited the Anhui Provincial People's Public Security History Exhibition Hall, and jointly learned the development process of the Anhui Provincial public security cause, as well as the efforts of the police in serving the masses, cracking down on crimes, maintaining stability and other work. The picture shows the students listening to the explanation. Photo provided by Tuan Luyang District Committee

2022-01-12	Group Yichun municipal party committee to carry out the "youth warm care sanitation workers" activities group Yichun municipal party committee to carry out the "youth warm care sanitation workers" activities jianwei correspondent BaiChen) recently, Heilongjiang Yichun encounter snow freezing extreme weather brings inconvenience to the production and living at the same time, more let work in outdoor sanitation workers face a severe test. In order to express their respect and care, recently, the League Yichun Municipal Party Committee organized young volunteers to carry out the "activity" of Youth Warm Current Care for sanitation Workers, so as to pay tribute to the urban beauticians who stick to their posts in the cold winter, and convey warm and positive energy. At the event, the sanitation workers sent 100 electric heat treasures, 1,180 hot posts and milk and gloves. The sanitation workers said that although the weather was cold, everyone was very warm. In recent years, the Communist Youth League Municipal Committee has carried out many theme activities such as "Care for the sanitation workers to warm the cup action" and "Salute to the city beautician", while sending the warmth of the youth league organization to the sanitation workers, but also created a good social atmosphere of more respect and understanding of the sanitation work, and more care and support for the sanitation workers.

2022-01-12	Reporter Li Jianping) Today, Ningbo City, Zhejiang Province, was officially launched. This activity will focus on precise assistance and assistance, and focus on the work of caring for young people in difficulties. It is divided into four sections: lighting up the micro-wish, hand in hand in Daliang Mountain, love care class, and the city warm heart action. League Ningbo municipal party committee requirements, counties and the cadres according to the actual situation to form a dream team, in towns, streets, unit, completes all kinds of difficult family youth contact, visit, field touch work, especially for low family minor children, disabled teenagers, inmates teach minor children, seriously ill children, orphans, left-behind children and other groups, key visit the touch, by the social caring people claim help light micro wish. In combination with the east and west cooperation plan between Ningbo city and Liangshan Yi Autonomous Prefecture of Sichuan Province, various organizations in Ningbo have continuously strengthened mutual visits and learning, and carried out the "Ningbo-Liangshan" children hand in hand summer camp and the "Warm Winter Care for Ten thousand People to realize their dreams" micro-wish activity, so as to send warmth to left-behind children and children with special difficulties in Liangshan. Since the beginning of the year, Ningbo has sent 591 sets of winter clothes to teenagers in Liangshan, increased support and guidance to young entrepreneurs, funded students, donated sports equipment to Butuo County, Liangshan Yi Autonomous Prefecture, and implemented the "Happy Playground" project. In addition, this year Ningbo new love care class and city warm heart activities, will make full use of the league volunteer service social mobilization ability and the advantages of teachers, explore the long-term mechanism, solve the summer child care problems; to provide labor rights, workplace adaptation, mental health, and create an atmosphere of understanding, respect and care in the whole society. Source: China Youth Daily, January 12,2022, edition of the 02nd edition

2022-01-12	Jining city e-commerce industry vocational skills competition successfully held the "youth e-commerce" Jining city e-commerce industry vocational skills competition successfully held the skills competition opening. Correspondent for figure China youth network Beijing on January 12 (reporter Ji'an-wei zhang, correspondent BaiChen) recently, by Jining city of Shandong province, human resources and social security bureau, Jining municipal party committee and China post group co., LTD. Jining branch guidance, Jining electricity chamber of commerce hosted "youth electricity" 2021 the city electricity industry vocational skills competition successfully held in Shandong vocational institute of technology. Nearly 100 players from Jining manufacturing and processing industry, e-commerce industry, e-commerce industry, vocational colleges and other fields participated in the competition. The picture shows the scene of "Youth E-commerce" 2021 The city's e-commerce industry vocational Skills Competition. According to the different professional categories of the contestants, this competition is divided into two groups: enterprise workers group and college teachers group. It is carried out in two directions of domestic trade e-commerce and cross-border e-commerce. The competition is carried out by systematic sand table practice simulation and systematic theoretical knowledge examination. Through the fierce competition of visual marketing design, online transaction management, online customer service, e-commerce data analysis and application, and network promotion and other projects, 16 outstanding individual awards were finally produced. The competition also invited e-commerce enterprises to do cross-border e-commerce and new media e-commerce wonderful sharing, for Jining e-commerce enterprises and young e-commerce talents to bring the latest domestic trade and cross-border e-commerce information. Next, pku will adhere to build power Jining electricity industry innovation and development platform, electricity enterprise exchanges and cooperation and group development platform, "alliance, project +, + base + talent" integration development chain "two platform a chain" working train of thought, constantly optimize the youth electricity alliance activity brand, further enhance the level of youth electricity industry development.

2022-01-11	The Yangzhou Youth League Organization creates "Dream Transformation +" care matrix ornaments and personalized furniture for the factual orphans' dream building "Dream Sea". There are neat extracurricular books on the shelves, which is the room transformed for Xiaoying by the Yangzhou Municipal Party Committee of the Yangzhou Youth League in Jiangsu Province. It is understood that since 2021, the Yangzhou Municipal Party Committee of the Communist Youth League has taken the transformation of the living space and pairing care of practical orphans aged 6-16 as the focus of the practical project of youth livelihood, which has cared for 251 practical orphans and completed the transformation of 208 "dream cabins". Xiao Ying (pseudonym), 12 years old, lives in Chengnan New District, Gaoyou City. Her father died three years ago. Xiao Ying's mother has to do odd jobs and receive a subsistence allowance, and her family is heavily in debt. Xiao Ying's bedroom is small, damp, dark, and covered with old furniture. After learning about the situation, the young volunteers took the initiative to pair up with Xiaoying and actively applied for the "Dream House" renovation plan. After learning that Xiaoying liked the ocean, the volunteers selected the ocean-themed beds, bookcases, tables and chairs for Xiaoying."I like the vastness of the sea. When I grow up, I want to be a guard of the navy to protect the motherland."Xiaoying shared her dream with the volunteers. Xiaogang (pseudonym), also a factual orphan, admired astronaut Nie Haisheng the most, and volunteers decorated space-themed wallpaper and star-shaped desk lamps in his transformed hut."Dreaming flying in the sky in the new room."Xiaogang's joy to overflow. When the Communist Youth League found that due to the death of their parents' disability, divorce, abandonment and missing contact, the orphans not only need the transformation of their living environment, but also need emotional care such as family care, daily care and confidence remodeling. For continuous care fact orphans, Yangzhou municipal party committee make full use of social resources, overall integration project carrier, the Yangzhou characteristic volunteer service project "hongzhi action" and "dream hut" transformation plan, explore building "dream transformation +" care matrix, form the plight of Yangzhou characteristic youth care service brand, for more difficult teenagers build dream space. Through the "Hongzhi action" volunteer service professional team organization and training, Yangzhou Hanjiang District Huaiisi Town Linqiao Village young volunteer Sun Qian pair of the facts of the village orphan Jia Yu (pseudonym). Due to the death of her parents, Jiayu has been living with her grandmother, living in the dilapidated old house. Without the care of her parents and relying on her in life, she gradually lost confidence in her study. After Sun Qian learned about Jiayu's situation, she not only helped her apply for the "dream cabin" renovation plan, but also encouraged Jiayu not to be discouraged because of the setbacks in life. Sun Qian not only made regular monthly field visits for schoolwork counseling and psychological counseling, but also signed up for the local "Red scarf publicity group", encouraging her to go to the platform to become a small "teacher" and tell her students the story of the history of the Party. In helping Jiayu again and again in the "lesson preparation" and "speech", Sun Qian found that Jiayu became more and more serious, more and more cheerful."Every child has a dream, and the orphans also have their own stars. We volunteers can not only do daily care, but also need to help them form a good habit of striving for their dreams."Sun Qian wrote on the care record chart. It is reported that in 2021, Yangzhou county-level youth League organizations raised nearly 4 million yuan for the construction of the "dream cabin", distributed more than 1 million yuan of grants and gifts, and carried out more than 2,800 visits and more than 30 care activities. Next, group Yangzhou municipal party committee will continue to "dream cabin" as the gripper, in accordance with the "advanced service, systematic planning, project implementation" working train of thought, before the Spring Festival plan to start the second batch of "dream cabin" construction, solidly put the practical work, do well, for Yangzhou more troubled teenagers provide continuous service, help the children light dream, dream set sail. Source: China Youth Daily, January 11,2022

2022-01-11	Henan luohe communist youth league more power service youth development of Henan luohe communist youth league more power service youth development jianwei correspondent Liu Yong) since this year, the Henan luohe city party committee to promote long-term youth development planning landing effect is suggested, practice building mechanism, build a platform, strong team, tree brand, cultivating work concept, more power, more measures simultaneously, service youth development. We will establish mechanisms to provide long-term guarantees for promoting the implementation of the plan. The Communist Youth League Municipal Party Committee has improved the joint conference system for youth work at the city and county levels, laying a solid foundation for better promoting the implementation of youth development plans. The Rules of Procedure of the Joint Conference on Youth Work was issued to continuously improve the policy system and working mechanism of youth development in Luohe. The youth development plan will be incorporated into the 14th Five-Year Plan of Luohe City to strengthen the top-level design of youth development. We will build a platform to make solid progress in entrepreneurship and innovation among young people. The Communist Youth League Committee held the Youth Entrepreneurship and Innovation Competition for three consecutive years, with a total bonus of 1.12 million yuan and more than 300 entrepreneurial projects. The youth entrepreneurship and innovation project database and talent mentor database have been established, and 1 person is recommended to join the China Youth Entrepreneurship mentor Group and 6 people to join the Henan Provincial Youth Entrepreneurship mentor Group, and the municipal youth entrepreneurship mentor group has been established to provide precise assistance to young entrepreneurs. Internship bases have been established in the Youth Entrepreneurs Association and the "Youth Civilization" units to provide 367 young people. Four units in Luohe were identified as youth employment internship units in Henan Province, and carried out the "Spring Goose Action" - -the "social practice activity" of Luohe college students, providing 336 positions, so that students from Luohe can further understand their hometown and serve their hometown. Strong team, extensive development of youth workers skills revitalization project. Relying on the youth entrepreneurship and employment internship bases, the youth League committees carry out classified training for young people. Further carry out the activity of "mentor and apprentice", and constantly improve the professional skills of young workers and their ability to deal with problems through mentoring. It has hosted the Henan Selection Competition of "Zhenxing Cup" National Youth Vocational Skills Competition for five consecutive years, and was awarded the Provincial Excellent Organization Award. Thirteen young people from Luohe have successively won the first place in the project, 18 young people participated in the "Revitalization Cup" National Youth Vocational Skills Competition on behalf of Henan Province, and 3 Luohe youth successively won the bronze MEDALS in the National Youth Vocational Skills Competition. In the city excavated a number of moral noble, skilled grassroots young workers typical, into the enterprise and campus propaganda 13 times, to guide the majority of young workers and college students to establish a correct outlook on life and values. Tree brand, strengthen the "green" word team brand construction. Pku in the city "green" font size collective to "post achievements in the new era of one hundred exhibition" as the theme, to the party history learning education help optimize the business environment, "I do something for the youth" theme practice as the main content of nearly hundred games, in the city's economic development of high quality highlights the youth power. With project construction as the center, the joint Municipal Development and Reform Commission has set up 58 youth commandos in key areas such as manufacturing and modern service industry, giving full play to the role of youth commandos in the main battlefield of economic and social development, especially in serving the key work of the Party and government, and promoting the smooth completion of key construction project tasks. Jointly with the Municipal Emergency Bureau, we held the safety publicity and consultation day centralized publicity and emergency drill and observation activities, and awarded the title of "Youth Safety Production Demonstration Post" to 15 collectives. Jointly with the Municipal Human Resources and Social Security Bureau, we carried out the selection work of "Youth Civilization" in 2021, and named 40 collectives as "Youth Civilization". We will nurture it and lead young people to rural revitalization. The CPC Communist Youth League Municipal Committee, together with the Organization Department of the CPC Municipal Committee, the Municipal Bureau of Agriculture and Rural Affairs, and the Municipal Bureau of Rural Revitalization, implemented the selection and training program of "Good Youth in every Village" to inject youth momentum into rural revitalization. At present, it has established a database of rural outstanding young talents, absorbing 1,253 outstanding young people, and elected 130 young people as the first "Rural Good Youth". We have carried out 11 professional and technical training for outstanding young people and 21 face-to-face technical guidance for outstanding young people, organized 13 live online broadcasts, and recommended 35 people to join the "leader Goose" training program for rural youth leaders to get rich, helping the "head goose" to improve their ability, growth and development.

2022-01-11	Linyi Communist Youth League: 644 million "loan" moving youth to create the future Xing Ting) Since 2021, Linyi Communist Youth League innovation work measures, carry out youth credit support plan, effectively solve the financing difficulties of young entrepreneurs, vigorously promote all kinds of youth groups entrepreneurship and employment. Linyi Communist Youth League supported a total of 1822 young people, support funds reached more than 644 million yuan. Among them, credit supports 1,331 rural youth and college students, issued 276 million yuan of business loans, 429 rural guarantees, 275 million business loans, labor-intensive small businesses, 62 enterprises founded by young people, and issued 93 million business loans. In the specific work, Linyi Communist Youth League highlights the cooperation and joint construction, and strives to build a credit support plan work system under the guidance of the Communist Youth League, the supervision by the People's Bank of China and handled by financial institutions. Regularly hold work coordination meetings, joint meetings, etc., to clarify the work objectives, unify the loan conditions, simplify the project process, and actively coordinate and solve the practical problems and difficulties in the work. We will extensively mobilize financial institutions to participate in the work of start-up loans, establish a reward and subsidy mechanism for start-up guaranteed loans, and further stimulate the enthusiasm of their work. At the same time, around the key groups, special youth loan services, play the communist youth league extensive advantages, relying on WeChat, weibo and other new media and newspapers, television, traditional media radio media coverage, improve project awareness and attention, and with the help of handling bank branches, wide coverage and familiar with jurisdiction personnel characteristics, the implementation of "the village whole club" work, for the first time to provide financing services and entrepreneurial support. In addition, Linyi Communist Youth League also played a "combination of fist", launched the rural good youth loan, benchmark interest rate loan, agricultural bear loan, college students entrepreneurship loan combination package, to serve many groups, focus on reducing the threshold of loan guarantee. For rural youth, individuals and small and micro enterprises with good credit, positive projects and positive repayment, credit loans; to entrepreneurs or entrepreneurial teams recommended by the city and county Communist Youth League, according to the situation, to apply for special financial discount support. Next, Linyi communist youth league will continue to optimize the youth credit support plan, improve the financial product portfolio, reduce business guarantee threshold, increase financial support, provide fast and convenient service, "loan" the youth go up, forge ahead, to accelerate the city "from big to strong, from beautiful to rich, from new to fine" strategic transformation contribution youth strength.

2022-01-11	The Yangzhou Youth League organization builds dreams for the factual orphans, the "Star Sea" ornaments, personalized furniture, and extracurricular books are neatly placed on the shelves, which is the room transformed by the Yangzhou Municipal Party Committee of Jiangsu Province. It is understood that since 2021, the Yangzhou Municipal Party Committee of the Communist Youth League has taken the transformation of the living space and pairing care of practical orphans aged 6-16 as the focus of the practical project of youth livelihood, which has cared for 251 practical orphans and completed the transformation of 208 "dream cabins". Xiao Ying (pseudonym), 12 years old, lives in Chengnan New District, Gaoyou City. Her father died three years ago. Xiao Ying's mother has to do odd jobs and receive a subsistence allowance, and her family is heavily in debt. Xiao Ying's bedroom is small, damp, dark, and covered with old furniture. After learning about the situation, the young volunteers took the initiative to pair up with Xiaoying and actively applied for the "Dream House" renovation plan. After learning that Xiaoying liked the ocean, the volunteers selected the ocean-themed beds, bookcases, tables and chairs for Xiaoying."I like the vastness of the sea. When I grow up, I want to be a guard of the navy to protect the motherland."Xiaoying shared her dream with the volunteers. Xiaogang (pseudonym), also a factual orphan, admired astronaut Nie Haisheng the most, and volunteers decorated space-themed wallpaper and star-shaped desk lamps in his transformed hut."Dreaming flying in the sky in the new room."Xiaogang's joy to overflow. When the Communist Youth League found that due to the death of their parents' disability, divorce, abandonment and missing contact, the orphans not only need the transformation of their living environment, but also need emotional care such as family care, daily care and confidence remodeling. For continuous care fact orphans, Yangzhou municipal party committee make full use of social resources, overall integration project carrier, the Yangzhou characteristic volunteer service project "hongzhi action" and "dream hut" transformation plan, explore building "dream transformation +" care matrix, form the plight of Yangzhou characteristic youth care service brand, for more difficult teenagers build dream space. Through the "Hongzhi action" volunteer service professional team organization and training, Yangzhou Hanjiang District Huaiisi Town Linqiao Village young volunteer Sun Qian pair of the facts of the village orphan Jia Yu (pseudonym). Due to the death of her parents, Jiayu has been living with her grandmother, living in the dilapidated old house. Without the care of her parents and relying on her in life, she gradually lost confidence in her study. After Sun Qian learned about Jiayu's situation, she not only helped her apply for the "dream cabin" renovation plan, but also encouraged Jiayu not to be discouraged because of the setbacks in life. Sun Qian not only made regular monthly field visits for schoolwork counseling and psychological counseling, but also signed up for the local "Red scarf publicity group", encouraging her to go to the platform to become a small "teacher" and tell her students the story of the history of the Party. In helping Jiayu again and again in the "lesson preparation" and "speech", Sun Qian found that Jiayu became more and more serious, more and more cheerful."Every child has a dream, and the orphans also have their own stars. We volunteers can not only do daily care, but also need to help them form a good habit of striving for their dreams."Sun Qian wrote on the care record chart. It is reported that in 2021, Yangzhou county-level youth League organizations raised nearly 4 million yuan for the construction of the "dream cabin", distributed more than 1 million yuan of grants and gifts, and carried out more than 2,800 visits and more than 30 care activities. Next, group Yangzhou municipal party committee will continue to "dream cabin" as the gripper, in accordance with the "advanced service, systematic planning, project implementation" working train of thought, before the Spring Festival plan to start the second batch of "dream cabin" construction, solidly put the practical work, do well, for Yangzhou more troubled teenagers provide continuous service, help the children light dream, dream set sail. Liu Feng China Youth Daily China Youth Network reporter Li Runwen source: China Youth Daily

2022-01-10	Jiangsu Taizhou 753 troubled families received New Year gift Jiangsu Taizhou 753 troubled families received New Year gift reporter li chao) on January 7,2022, in Jiangsu Taizhou pharmaceutical 2021 (Gaogang) "dream transformation +" care plan appreciation ceremony and 2022 construction launch ceremony, a batch of troubled children received a special gift, from zhongjian installation group co., LTD. Don100 copies of the China youth daily. It is reported that since the launch of the "Dream Transformation +" care plan, the Youth League Taizhou Youth League Municipal Party Committee has focused on building the "333" working mechanism, actively explored the "material help + spiritual care" help system, and scientifically and orderly transformed 453 "dream cabins". Since 2021, the Taizhou municipal party committee in the normal support work, continuous implementation of psychological counseling, schoolwork counseling, family company, interest training special services, on the basis of focusing on the difficult youth spiritual needs, for 753 dream cabin building object (453 already built and 300 proposed) to subscribe to the China youth daily and other newspapers and magazines, let the children by reading open vision, increase knowledge."Thank you, uncle and aunt, I will study hard and be a useful person to the society in the future."Holding the stationery she received, the little girl from Qintong town brook said."The cabin carries the dream of children to sail away. The change of hardware conditions is the first step to realize the dream, and the growth change from 'cabin renewal' to 'spiritual renewal' is the ultimate goal we hope to achieve."Youth League Taizhou Municipal Party Committee related responsible comrade said.

2022-01-09	Liaocheng group is youth palace socialized operation to explore into the right track Shandong Liaocheng group is youth palace social operation mode exploration into the right track in the youth network reporter XingTing) on January 6, Liaocheng youth comprehensive service center (Liaocheng city youth palace) and Shandong care health service group signed a cooperation framework agreement, which marks the city group of youth palace social operation mode of exploration on track. Xu Baoliang, Party Secretary of Tuan Liaocheng, Liu Juan, the second-level researcher of Liaocheng SASAC, Zhai Jian, member of the Party Leadership Group and deputy director of Liaocheng Education and Sports Bureau, Li Qian, Deputy Secretary of the Communist Youth League and Liaocheng Municipal Party Committee, Wei Taojun, deputy Secretary of the Party Committee of Shandong Nursing Health Service Group, and Wang Hongbing, assistant general manager, attended the signing ceremony, earlier stage, In accordance with the relevant arrangements for the reform of the Communist Youth League and the Young Pioneers and the relevant requirements for the reform of municipal public institutions, According to the Opinions of the Central Committee of the Communist Youth League and the National Youth Working Committee on Further Strengthening and Standardizing the Management of the Youth Palace of the Youth League, " we should perform the duties of standard customization, code of conduct, resource integration and service provision, With open thinking, socialization mode, professional means to vigorously promote the youth practical education, social education " related requirements, Youth League Liaocheng Municipal Party Committee, Liaocheng Youth Working Committee, Liaocheng Youth Comprehensive Service Center and Shandong Nursing Health Service Group held several rounds of consultations, A cooperation intention was reached. Learned, Liaocheng youth comprehensive service center as the city youth outside activities exclusive place, youth social education important position, minors ideological and moral construction and promote quality education, the construction of socialist spiritual civilization, in the education to guide teenagers set up the ideal faith, cultivate the rule of law consciousness, develop interests, promote physical health, enhance innovation spirit, improve scientific quality and practice ability, temper moral quality, practice social responsibility, realize all-round development plays an important role. The signing of the agreement is not only an important exploration to promote the quality and efficiency of youth off-campus education and improve the supply-side structural reform of education, but also a strong support for promoting the comprehensive and healthy growth of teenagers and building a new education ecosystem centered on education. In the next step, Liaocheng Youth Comprehensive Service Center will actively promote the success of cooperation projects, lead the more healthy development of youth-related industries, promote the formation of more standardized industry standards, meet the more diversified needs of the city's youth, and effectively promote the comprehensive improvement of the comprehensive quality of the city's youth. Leaders of relevant departments of Shandong Health Health Service Group, relevant departments of Liaocheng Municipal Committee, Liaocheng Youth League Working Committee, Liaocheng Education League Working Committee, representatives of Youth League organizations of Dongchangfu District, Development Zone, High-tech Zone and Young Pioneers organization, and all the staff of Liaocheng Youth Comprehensive Service Center attended the signing ceremony.

2022-01-09	Shandong Yantai first Shandong youth development planning officially released Shandong Yantai first youth development planning officially publisher XingTing) recently, Shandong Yantai municipal party committee, municipal government issued the "Yantai" difference "youth development plan" (hereinafter referred to as the "planning"), marks the first based on Yantai youth reality, rich in Yantai regional characteristics of youth development planning officially released, opened the new era of youth work. It is understood that the "plan" is divided into "overall requirements", "main tasks", "key projects", "organization and implementation" four parts. In the "general requirements" part, "planning", adhere to the party tube youth, youth, service, overall situation, overall basic principle, to build "city more friendly to youth development, youth more for urban development" youth development friendly city, and communities in by 2025, form the characteristics of Yantai youth development policy system and working mechanism, the city's youth ideological and political quality and comprehensive development level significantly, in the new era of modern city construction force and commandos. In the "main task" part, "the" plan " has been clear about the youth ideological and moral construction, youth education career development, youth employment entrepreneurship, youth health protection, youth cultural services, youth social integration, youth rights and interests maintenance, the prevention of juvenile crime and other eight key areas, and targeted formulated article 25 specific development measures. In the "key project" part, the "planning" put forward the youth thought lead promotion engineering, young talent to attract agglomeration engineering, youth innovation entrepreneurship can assign project, youth music engineering, youth culture and art project, youth society to promote the project, youth healthy growth escort project and so on seven key projects, and has been clear about the corresponding measures. In the "organization and implementation" part, "planning", city and county party committee and government to improve the planning implementation of the youth work joint conference mechanism, pay attention to strengthen the planning and local economic and social development planning and related special planning, the plan implementation funds into the budget at all levels, establish the youth development index monitoring system, formulate and adjust policies and measures to promote youth development, guide the youth to participate in planning implementation and supervision, to ensure that the plan landing. According to introducing, as an important top design of Yantai youth development, the implementation of the "planning" will effectively meet the youth in health, employment, entrepreneurship, housing, education, marriage development in the field of demand, build for youth growth broad development platform, in promoting the development of youth itself at the same time, add vitality for the city, gather talent, improve quality.

2022-01-09	Nanjing released young talents "Qingying" project reporter Li Chao) January 7, Nanjing young talents "Qingying" project press conference and the first "Qingying Wu Hui" theme activity was held in Fanji Bookstore, 28 outstanding young representatives from various industries attended. On January 4th, Nanjing Municipal Party Committee held a talent work conference and a leading national innovative city construction conference. Centering on the requirements of "striving to build a national important talent highland and making talents become the most distinctive sign of the high-quality development of Nanjing", the Nanjing Municipal Party Committee and the Municipal Youth League actively implemented the requirements of the meeting, focused on the work of young talents, and took multiple measures to establish a multi-level, field and three-dimensional young talent service system. It is understood that in 2022 will be fully launched Nanjing young talents "fu" project, strive to gather a group of deep patriotism, has a certain vision of young entrepreneurs, a group of innovative vitality, development potential of young professionals and a number of high level of knowledge, fit Nanjing industry demand returnees young talents, to carry the service of Nanjing talents and innovation work youth bear. According to the introduction, Nanjing young talents "Qingying empowerment" project includes "Qingying wu thinking", "Qingying overseas returnees camp", "Qingying achievements", "Qingying high research class" four sections."Qingying Wu Hui" will invite young talents from all walks of life to exchange, discuss and cooperate according to different themes of urban development. On Friday, at 5 pm, Fixed time, fixed process, Forming a brand memory; "Qingying Overseas Returnees Camp" takes young returnees as service objects, To build the first stop of short-term "training" for international students returning to China, By means of teaching, discrimination, experience and study, Carry out an in-depth investigation of the city conditions, To guide overseas returnees to understand the situation of the Party, national conditions and social conditions; "Qingying Achievements" will focus on the development of key industries in each sector, Organized about 500 members of the Youth Federation and members of the Youth Chamber of Commerce to conduct research and discussions, brainstorming, discussion and exchange, Promote the implementation of cooperation projects; The "Qingying High Research Class" is for young entrepreneurs, In 2022,8 batches of research and exchange activities will be held. There are not only centralized training and interactive exchanges within the city area, but also visits and special studies in advanced areas and well-known universities, as well as national conditions visits and exchange visits in the supporting areas. The theme activity of "WuSi Hui" held on January 7th is not only a salon sharing meeting, but also a platform for exchange, including "collision of ideas, creation and sharing, going to the distance". Meanwhile, it is also the function of link, which makes everyone want to work together and go to the distance together."I think" at the same time homophonic "May 4", representing the power of vigorous youth. The theme of the first "I Si Hui" is "near to the yue, far to come- -cohesion to serve young talents". Rong Fei, deputy secretary of the Nanjing Municipal Committee of the Communist Youth League and vice chairman of the Nanjing Youth Federation, exchanged and shared on the theme of "How to do youth work well". He stressed that youth work is essentially a cohesive work for the people, and the mental outlook and working status of the youth can reflect the construction status and development prospects of an enterprise. In the sharing, he summed up the "eight-one" working method of the non-public youth league construction, which inspired and promoted the young entrepreneurs attending the conference to help the enterprises gather the young people through the construction of the youth league organization. Subsequently, the participants were divided into four groups for group discussions around the theme of the activity. After the group discussion, each group representative conducted a group review and shared the discussion results with everyone. According to the introduction, in the future, the brand activities will be held around the theme of young talents.

2022-01-09	Wuhan Youth Volunteers Association, Wuhan Youth Volunteers Association, Wuhan Youth Volunteers Association held the fourth general meeting of Wuhan Youth Volunteers Association. At the meeting, 15 people, including Wang Yong, manager of Jianghan Branch of Hubei SF Express Co., Ltd., and Hua Yuchen, a teacher of Ganghua Primary School in Qingshan District, Wuhan city, were employed as lecturers of "Zhi Qingchun" Wuhan Youth Volunteer Service Lecture Group. It is reported that Wuhan Youth Volunteer Service Teaching Group is committed to comprehensively strengthening the construction of the association's volunteer service training system, to create a professional team of trainers. More than 200 members attended the meeting by combining "online + offline". Lei Jing, deputy secretary of Wuhan Municipal Party Committee of the Communist Youth League, He Maolin, head of Hubei Youth Volunteer Action Promotion Center and secretary general of Hubei Provincial Volunteer Association, head of publicity Department of Wuhan Municipal Party Committee, Civil Affairs Bureau and other relevant departments attended the conference. Bai ϟ a-zhi, president of the third council of the Association and general manager of Hubei Tianqin Weiye Enterprise Management Co., Ltd., donated 100,000 yuan to Wuhan Youth Development Foundation to set up a special fund of the association. At the conference site, the special fund donated the first support fund to donate 200 copies of personal accident insurance for the Wuhan Yangtze River Rescue Volunteer Team. The meeting elected a new leading body of the association. Bai � zhi was elected president of Wuhan Youth Volunteers Association; Wang Yong, Wang Yuchen, Liu Pei, president of Wuhan Xiaoyi Brothers Social Work Service Center, Dong Ming, head of Dong Ming Volunteer Team of Wuhan Software Engineering Vocational College, He Mingzhe, general manager of Hubei Zhonglixun Human Resources Technology Co., Ltd. was elected vice president; Wang Fan of Wuhan Youth League School was elected chief supervisor; He Mingzhe was elected secretary general.

2022-01-08	Wang Xinxin, founder of Sichuan Guang 'an Youth New Media Alliance) Sichuan Guang' an Youth New Media Alliance was recently established and held its first member conference in Guang'an, Sichuan province. The Guang 'an Youth New Media Alliance is jointly initiated by the Cyberspace Office of Guang' an Municipal Committee and the Guang 'an Municipal Committee of the Communist Youth League. It is composed of Party and government organs, new media enterprises, institutions and employees related to Guang' an Youth work. It is a non-profit alliance organization. The first member meeting of the Alliance deliberated and passed the Constitution of Guang 'an Youth New Media Alliance, and elected the first secretary general, deputy secretary general and standing director members of Guang' an Youth New Media Alliance. The meeting issued the "Care for Youth Mental Health Initiative", and launched the youth volunteer service projects of "Clear Action" and "Care for Mental Health Action". The relevant person in charge of the municipal party committee said, the founding of cross city youth new media alliance, to further standardize the order of the network, to purify the network environment, promote the healthy development of new media, condensed youth ideological consensus, promote the construction of socialist modernization is of great significance, will promote cross into Chongqing metropolitan area, speed up the completion of Chongqing cooperation demonstration zone.

2022-01-08	Yidao Youdao and netease Cloud Classroom donated 1,000 sets of Youdao logic English learning materials worth 150,000 yuan to some schools in four counties (districts) of Yinchuan city. Next, group Yinchuan municipal party committee will continue to support and assist the public welfare activities, and called on people from all walks of life to join Yinchuan public welfare activities, provide better education resources for children, help basic education, cultivate a group of ideal, bear, grateful heart of socialist successors.

2022-01-08	In Tangshan, Hebei, the conference was held in North China University of Science and Technology. At the meeting, the relevant officials of Tangshan Municipal Party Committee and the leaders of North China University of Science and Technology watched the volunteers of "Burning Snow" MV, listened to the preparation report of the volunteers, and distributed materials and awarded flags to the volunteer group; the volunteer teachers and student representatives spoke. The Tangshan Municipal Committee of the Communist Youth League organized the selection and recommendation, training and promotion, testing and drills. 81 Winter Olympics volunteers participated in the training, which significantly improved their service awareness and professional quality.

2022-01-07	Jinan communist youth league "eight formula" to promote the construction of youth political talents Jinan communist youth league "eight formula" promote youth political talent builders XingTing) in recent years, the communist youth league of Jinan municipal party committee in integration of youth talent pilot area under the core framework, focus on cadres forging, green horse project extension, pushing the party work linkage, further optimize the political discrimination, platform, talent, career, youth success work path, through the "bat, optimization, refinement, education, use" the "eight formula", to promote the construction of youth political talents. In terms of "bat", will strictly from solid pays special attention to the cadre team construction, according to the "clean shell league, adjust lax league, for outstanding league, promote most of the youth league" thinking, both league "thin body and muscle", "bone and can assign", according to the industry layout, organization type, group distribution, adjust measures to local conditions to promote the standardized construction of the league. Adhere to the problem-oriented, in the city to carry out the style of cadres in the action, to solve the problem of "no sense", "no post", no state "," no cadres. In addition, adhere to the classified management. For full-time cadres, make dynamic updates, take the political assessment as the primary reference for the adjustment of the cadres, and smooth the communication channels to the organization department; For temporary and part-time cadres, standardize the construction of the league organization, strengthen the training, clarify the job responsibilities, strengthen the management functions, shorten the adaptation period, extend the working period and enhance the sense of identity. In terms of "selection", the overall consideration for the implementation of the promotion of excellent party membership. Pay attention to risk prevention, establish a three-level linkage system of "Party committee leadership, Youth League committee leadership and branch implementation", with "integral standard" as the quantitative ruler and "integral file" as the process guide. At the same time, it adhered to the selection selection, designed the Points Standard for Active Party Members, defined 20 basic indicators, and set seven additional points from the three aspects of work contribution, evaluation and volunteer service. Before joining the Party, they have passed theoretical examinations and democratic evaluation of party members and the masses. After joining the Party, new party members and young active applicants will be organized to "guarantee" and check the "short links" according to the "points standards", forming a closed-loop management system of "striving for excellence, reflecting and improving". In the aspect of "intensive education", classified implementation to promote the green horse project. Pay special attention to the districts and counties, schools and various fields green horse engineering teaching base construction, improve the student selection management, curriculum system, cultural products training mechanism, to achieve "classification training, hierarchical implementation, comprehensive coverage" training effect, add online courses, micro group, the annual new "green horse engineering" rural class, social organization class. At the same time, we will focus on the national strategies of ecological protection and high-quality development of the Yellow River Basin, strengthen the research and development of courses within the league, and comprehensively improve the pertinancy and effectiveness of qingma Project training. In terms of "making good use of it", the integration of industry and education provides a broad open platform, builds a youth innovation platform for schools, enterprises and places, builds a pilot zone for the integration of industry and education, and effectively opens up a new path for the connection between schools and enterprises. The "Innovation Youth" youth innovation and Entrepreneurship Competition will be transformed from driving to traction, adhere to the three steps of mining, cultivation and support, realize nearly 200 outstanding youth innovation projects, and successively promote the establishment of a youth innovation investment fund with a total scale of 1 billion yuan. Innovate the new mode of "service team + industry team + school-enterprise and local liaison", promote the precise docking between domestic well-known universities, universities and provincial ordinary colleges, vocational colleges and enterprises stationed in Jinan, and attract and gather more outstanding young talents.

2022-01-07	Guangxi Guilin college students "early experience" social practice activities for college students "early experience" social practice in green network reporter Xie Yang) on January 5, Guangxi Guilin municipal party committee held in Guilin college students "early experience" social practice mobilization meeting. At the meeting, school representatives, employer representatives and student representatives signed a tripartite agreement on social practice. After more than two months of planning, through the collection of professional characteristics and personal specialties of Guilin direct units, urban areas, directly affiliated enterprises and institutions, matching the college graduates of 2022 and 2022 graduates, finally determined that more than 130 students reached a preliminary intention with relevant units. The activity is sail landscape dream between Guilin city-Guilin "youth development friendly city" one of the ten projects, the purpose is to organize Guilin college students in Guilin winter vacation enterprises and institutions to participate in social practice, experience in Guilin life, for willing in Guilin job entry or voluntary social practice exercise students provide platform and opportunities, attract more young people know Guilin, into Guilin, stay in Guilin.

2022-01-06	Shaanxi Xianyang: study the history of the party, strong faith, Xianyang to follow the party: study party history, strong faith, go with the party, the city youth league organizations at all levels, order, guide the city's youth deep learning, fine enlightenment, certainty, learn from the history of the party in one hundred, the learning education achievements into the powerful power of youth. Learn to understand combined, firmly continue to struggle "youth faith". We should adhere to the principle of "one key opens one lock", and carry out party history learning and education in the way of "classification, hierarchical classification and mass classification". Among the young Pioneers, Make telling the story of the Party the main content, "I will tell the story of the Party, Strive to be a red scarf narrator, "" childlike innocence to the party, Red songs flying "and" Red scarf heart to the Party " and other theme activities, Cultivate the feelings of loving the Party and loving socialism since childhood; Among the student league members, Guide them to prison their ideals and beliefs, Deploy to carry out the history of the Party, strong faith, follow the Party " theme League day activities and the party history theme League class, Let the knowledge of party history into the brain into the heart, Stimulate the power of advance; Among the young cadres, Highlight the situation and policy education, Promote special reading classes, "Youth League cadres on the platform", Conduct a keynote speech contest, Build a complete knowledge system and form a profound and rational cognition. At the same time, through the Youth Federation, the Students' Federation, the youth association affiliated to the youth League, the wide organization of "reading red classics", "Party history youth said" and other activities, the youth learning atmosphere of youth is constantly strong. Figure | "tell the party history story, promote high start" theme speech contest learn xuan simultaneously, keep in mind that always go with the party "youth mission". The party history education "implement to the end of the organization", with the youth league branch as the basic unit to carry out the party history study, organization life, theme youth league day and other activities, give full play to the role of "youth teaching group", face to face to tell the story of the Party, promote learning and education into the daily, grasp often. Party history education "runs through the Internet", launched a series of online group courses on "Youth Study", Weibo and wechat "Party History Study every Day" column, launched online and offline "Qingming Festival Heroes" online and offline, and launched online activities such as "Why to join the Party" and "May 4th Spirit Inheritance with Me". The party history education "through to the group history education", combing group with the unity of the party, with the glorious course of the struggle, dig wei wild revolutionary martyrs started May 4th, sowing fire integrating ecology revolutionary course and system arrangement, the city linkage held the 102nd anniversary of the "May 4th movement" activities, take practical actions to interpret "flag refers to is the flag". Figure | "study party history, strong faith, follow the party" Xianyang to commemorate the 102nd anniversary of "the" May 4th movement "commendation congress practice, singing high start" the song of youth "."Responsibility in place" strong responsibility. Focus on deepening reform, strictly governing the league, strengthen the organization of grass-roots league organizations, relying on the "smart league building" system, real-time grasp the grass-roots branch to carry out party history learning and education dynamics, really let the party history education "live" up, let the study "hot" up, let the branch "move" up."Service in place" strong contribution. Solid development "I do something for the youth" practice education, post merit theme activities, through the implementation of the "youth have me" project, provide meet youth growth, employment, physical and mental health, social integration, dating and other urgent needs of service, the city's youth to group identity and the sense of belonging is more intense."Implementation in place" strong action. Focus on the implementation of "youth" action, hold Chinese young entrepreneurs Xianyang activities, to business, to enterprise, to talent selection; "ten outstanding youth" "outstanding youth" and other typical youth, launched "green 100000 +" entrepreneurship training, unity and lead the youth in Xianyang new era all the way to the "Yang".

2022-01-06	Shanxi Jincheng Communist Youth League: The "chemical reaction" in the reform with full-time personnel our role of full-time is reflected? To open up the last mile of community service for the youth, we should not only rely on full-time youth league cadres, but also focus on mobilizing various forces."A few days ago, in the Youth League Jincheng Municipal Party Committee, a team members for the county (city, district) youth League committee secretary pilot work progress of the" soul test " is ongoing: some work looks tall, the youth do not recognize? Can the young people in the trade city restaurant feel the warmth of the league organization? Emphasize several times that the work problem is not solved, who is the responsibility? Sharp questioning, so that some reporters blush and sweat, some to clarify the work thinking. Through on-site assessment, asking questions and sharing, let everyone see the gap, learn from each other, each has its own harvest. This is in recent months, the Communist Youth League Jincheng Municipal Party Committee in the promotion of the county Communist Youth League grassroots organization reform pilot work in one of the innovative work measures. As the only area in the province to promote the pilot work of the reform of the Communist Youth League, Jincheng Municipal Party Committee adhere to the leadership of the top leader, the standing Committee of the Municipal Committee of the reform of the Communist Youth League, from the topic to one county one product, Jincheng City is building a long-term mechanism for the pilot reform of the Communist Youth League. While exploring while reform " the whole city reform reform what? Where to change? How to change? In fact, we are also exploring while promoting, while carrying out while improvement, after several rounds of supervision of the CPC Municipal Party Committee, under the continuous docking and guidance of the CPC Central Committee and the Provincial Party Committee of the Communist Youth League, our work path is gradually clear."Youth League Jincheng Municipal Party Committee deputy department level cadre Wei Bo introduced. The municipal Communist Youth League Committee has improved the special class for the pilot reform of the Communist Youth League at the county level, determined the responsible leaders of the county (city, district) committee, defined the responsible persons of the pilot, and detailed the municipal package leaders, joint departments and the reform pilot instructors. On the basis of fully considering the resource endowment of each county (city, district), the working idea of "one county, one product" and "three steps" from the grass-roots level was finally established. The Communist Youth League Municipal Committee of the CPC adopted the "three up and three down" way to promote the reform: three times "please come up", in the initial launch stage, the secretary of the county party committee and team members were invited to the city for at least three times for work communication and arrangements. Three times "go down", by the secretary of the Communist Youth League, the deputy secretary, bao hang departments to the municipal county and the county party committee of the main leadership, in charge of the leadership, cadres for in-depth communication. The Communist Youth League Municipal Committee took the three-level league organization change as an opportunity to actively strive for the support of the Party committee, and fully matched the county and rural league cadres. According to statistics, Jincheng county cadres from the grass-roots cadres accounted for 42%, 25% higher than before the transition. In the team, full-time cadres of the youth league with bachelor degree or above accounts for 100%, graduate degree or above accounts for 10%, and CPC members account for 100%, which provides a strong talent and organizational guarantee for promoting the work of the grass-roots youth league. Recently, the circle of friends of many netizens in the southeast of Shanxi Province has been a post-90s "net red" and "little flower sister" Wu Meng � B brush screen.� B's TikTok number is called "little sister surname Wu". Different from the traditional "web celebrity", her duty is the secretary of the Youth League Committee of Yangcheng County. From hesitant to careful pilot, to popular become a young guest mentor, little sister with the support of the county, actively for hometown, promote Yangcheng brigade resources, promote the brand activities of the communist youth league, actively expand the communist youth league "friends", won the recognition of netizens, also become the popularity of local youth endorsement. Driven by the "little sister" live broadcast, the Yangcheng Communist Youth League TikTok number "Youth Yangcheng" has close to 40,000 fans."Seizing the online and offline gathering places of youth is one of the focal points of our reform. The pilot project is gradually rolled out to influence a group of people through one person, play a league flag on the Internet, speak for our hometown, and help rural revitalization."Shi Hui's purpose is very clear, where the youth is, she will expand the service and position of the regiment. Facts have proved that this measure is playing a positive role, and the influence of the Yangcheng Group organization is expanding. A recent survey of an online survey launched by the Yangcheng Communist Youth League showed that more than 600 of the more than 4,000 young people participating in the survey were unemployed, and they showed strong interest in the e-commerce industry. The survey results provide a basis for the Yangcheng Youth League organization to serve the local youth. With the support of the county Science and Technology Bureau, the Youth Home of Yangcheng County E-commerce Center specially invited young entrepreneurship mentors and young entrepreneurs to carry out special e-commerce training for everyone."The Communist Youth League County Party Committee will set up the training class classroom at the gate of the county bus station, returning young people can see as soon as the station, a total of more than 20 training sessions, very popular."Shi Hui introduction, yangcheng for thousands of years of ceramic, sericulture industry culture unique charm, and the future yangcheng youth electricity development core direction, is cultural output," as a grassroots cadres, we serve for returning youth at the same time, also hope that through their development, spread Jincheng culture, boost local high quality agricultural products out of Jincheng, to the whole country."" lead more young people in the field of electricity water is the task of our party committee " Shi Hui introduction, yangcheng established party head led by the communist youth league grassroots organization reform leading group, the county main leadership to promote more local and returning young people put short video, live and yangcheng development, with their own business development, play a leading role in the rural revitalization. Reform is "chemical reaction" is not the superposition of traditional work. Compared with other counties and districts, Jincheng urban location advantages are prominent, the urban Youth League committee takes the park, industry as an opportunity, integrate the supporting youth home, social practice league school, talent station and other service positions, give full play to the linkage role of the position complex. Gaoping City of Jincheng has been established by the party secretary of the Communist Youth League and the deputy secretary of the Municipal Education Working Committee, and comprehensively established the youth league and education cooperation cross-office mechanism. At the same time, taking the village (community) organization change as an opportunity, combined with the municipal party committee "young eagle plan", the qualified young village cadres and college students nominated as the village (community) league branch secretary candidates, enrich the village level league organization talent force, nearly half of the village (community) has determined the change of the league organization intention candidates. Qinshui county by guide and promote enterprises and institutions and township youth league pair construction, revitalize the county league organization construction; Zezhou county lead the group work into the county annual work assessment system, cadres daily management and supervision by the party committee (group), league county, comprehensive and accurate evaluation of cadres of performance and work performance."Reform is not a superposition of traditional work, but a chemical reaction, first to find young people, to study the breakthrough of the work of grass-roots youth league organizations, and then to establish an assessment and incentive mechanism."In Wei Bo's view, the above changes reflect the influence of Jincheng League organization through reform, social functions are playing, and the youth recognition of the league organization service is reflected." This is a qualitative change ". It is understood that in order to ensure the effectiveness of the reform, Jincheng city in addition to the league construction into the annual target responsibility assessment, but also introduced the Jincheng Doctor Association for a third-party evaluation, the reform of the league organization pilot work was twice reviewed, the review results were directly copied to the county (city, district) Party secretary. At the same time, the policy laboratory of the Communist Youth League- -Jincheng Communist Youth League Cadre Famous Teacher Studio was established to actively solve the reform difficulties encountered in all counties (cities, districts), and promote the experience of the whole city. Not only that, but also at the municipal level, a non-public and social organizations covering the three modules of non-public economic organizations, social organizations, and unemployed college students were established. The core purpose is to attract, contact and serve young people as possible.

2022-01-04	Zhejiang Shaoxing youth league officially launched special online fairs Zhejiang Shaoxing youth league officially launched online fairs Li Jianping) recently, by the group of Shaoxing, Zhejiang province municipal party committee launched "with you" more "have" to " online recruitment activities officially launched, key services affected by the epidemic prevention and control of employment enterprises and youth employment needs. To return to work and production and epidemic prevention and control under the normalized youth employment service work, the Shaoxing municipal party committee positive response, according to the central, Zhejiang provincial party committee about "one thousand school post" employment support special action deployment, through hierarchical organization to raise and socialization, the first time after the outbreak of Shaoxing enterprise production labor demand, collected from more than 300 companies, a total of more than 10000 jobs. Among the recruitment enterprises, Zhongcheng Holdings, Xilin Men Group, Seiko Group, Wanfeng Art Group, Baoye Group and other well-known enterprises at home and abroad, such as Wolong Group, and the job categories cover manufacturing, Internet, social services and other fields. This special online recruitment activity relies on the "pro-youth Innovation" platform for one month. The majority of young people can directly send resumes with one click and cloud "peer-to-point" matching. At the same time, Shaoxing also provides employment policy for employees to Shaoxing, up to 500 yuan per person; recruit workers before March 31,2022, more than one year and participate in social insurance for more than 3 months, not less than RMB 250 yuan / person. The Communist Youth League Shaoxing Municipal Party Committee for the picture

2022-01-04	Group of Anhui Wuhu municipal party committee actively help city social governance in Anhui Wuhu municipal party committee actively power city social governance wei correspondent Wang LuZha wei ting) to youth work "difference", improve the overall situation contribution, power Wuhu economic and social development, group of Wuhu in Anhui province municipal party committee through the "four green" construction positive power city social governance."Green" escort. Relying on the "Jiuzi Safe Escort Group" of Wuhu City, focusing on key youth groups such as urban floating children and minors involved in crimes, cultivating and incubating 6 key projects, and innovatively set up the "Young Bird Station" care work station, "Green Miao Mu Xin" service station, to create a new brand to help social governance in the city."Green" reading. A series of activities, such as the youth reading program, such as the youth lecture hall, and the "total reading list", were implemented to realize the people's future and cultivate their interest in reading."Green" collective. Deepen the creation of "green" brand project, carry out the theme practice activities of "post bugle player team together listed youth achievements I first", a total of more than 200 youth civilization number collective participation. One youth collective was awarded national youth civilization, and nine youth collectives were awarded provincial youth civilization."Youth" volunteer. We will comprehensively promote the construction of county-level associations for youth volunteers. Deepen the brand connotation of "Lei Lifeng Travel", hold the second Youth Volunteer Service Project Competition, build a display, communication and resource service platform, and carry out more than 10,000 volunteer service activities around the "35" time node. We will deepen the "Protect Mother River" campaign, and carry out a series of themed activities such as "Green Movement, Youth and Beautiful Wuhu". One person won the 10th "Mother River Award" - -Green Guard Award.

2022-01-07	Hefei held the communist youth league and people's congress, CPPCC members face to face GongTuan Hefei municipal committee held "the communist youth league and people's congress, CPPCC members face to face" symposium Wei correspondent Fang Ding Zha WeiTing) recently, Hefei, Anhui province in 2022 "the communist youth league and people's congress, CPPCC members face to face" (hereinafter referred to as "face to face") symposium. The theme of the symposium is "Improving the mental health level of youth services". The deputies of Hefei Municipal People's Congress, members of the CPPCC, leaders of Hefei Municipal People's Congress, Municipal People's Congress, Municipal CPPCC, Municipal Education Bureau, Municipal Health Commission, Municipal Fourth People's Hospital and other relevant departments, as well as experts from the "face to face" research group of the Municipal Communist Youth League Committee, and representatives of social organizations attended the meeting. Zhang Leiyong, deputy secretary of the Communist Youth League Hefei Municipal Party Committee, attended the meeting. At the symposium, the responsible comrade of the Rights and interests Department of Hefei Municipal Youth League introduced the "face to face" activities in detail. Team leader, associate professor of Anhui architecture university Zheng Min detailed interpretation "face to face" research results- "Hefei adolescent mental health service research report", head of the youth service social organizations, combined with the work practice, around the discussion theme and research results report, to Hefei adolescent mental health service related work made the communication speech. Conference, the party and government organs of Hefei youth service policies and government work to carry out the interpretation, National People's Congress, CPPCC members of the current adolescent mental health services in Hefei, for the research report and youth social organizations on behalf of the present situation, difficulties and fully exchanged views, targeted put forward Suggestions, consensus. In summary, Zhang Leiyong proposed that we should pay attention to the mental health of teenagers, and promote the party and government, schools, mass organizations and society to make joint efforts to enhance the supply capacity of psychological services. Take high-quality proposals as the carrier, promote the development of social psychology institutions, and improve the mental health service system. To participate in the "face to face" activities of National People's Congress, CPPCC members have said, will be around the "improve adolescent mental health services" theme, actively to 2022 provinces, proposals, proposals, and Suggestions, good duties, and the communist youth league organization close collaboration, strengthen psychological science, deepen work collaboration, increase policy care, to do a good job of adolescent mental health services, the construction of Hefei characteristics of youth mental health service system. In the next step, the Hefei Municipal Party Committee of the Communist Youth League will further improve the research report according to the spirit of the symposium, and track the representatives and members, so as to provide strong support for the centralized voice of the "two sessions".

2022-01-07	Rizhao: volunteer youth power day blessing ceremony Rizhao: volunteer youth power day blessing ceremony wei correspondent XiangChanghui) recently, 2022 "hospitable Shandong" will launch ceremony and early light according to China (sunshine) New Year's day blessing ceremony held in the scenic spot, the activity, Rizhao joint qufu normal university, Rizhao blue sky rescue team and Rizhao ark rescue team recruited 120 volunteers, participate in related activities of preparation and field volunteer service. At 5 a. m., when the weather was cold, 50 volunteers from Rizhao Blue Sky Rescue Team and Rizhao Ark Rescue Team had already arrived at the blessing ceremony of the Performing Arts Square in Wankou Scenic Spot. They did their respective duties, did a good job in temperature measurement, "health code" inspection and other epidemic prevention and control work, and maintained the order of the ceremony site. In the crowd, one figure is busy. At 7 a. m., 70 volunteers from Qu Normal University had already arrived at the school gate and took a bus to the Olympic Water Sports Park to pray for the welcome and run around. On the road, the person in charge reminded the matters needing attention to ensure the safety and smooth progress of the work. Arriving at the site of the welcome run, the volunteers immediately put themselves to work, actively served the athletes, prepared for the event, kept the athletes' belongings, and assisted in maintaining the order of the event site. Volunteers' logistics service work has become a beautiful scenery at the activity site.

2022-01-04	Wei correspondent Xu Yuling) Recently, Fuzhou City, Fujian Province, Fuzhou Young Pioneers cartoon image release ceremony was held in Fuzhou Youth Activity Center. In order to show the healthy, sunny and positive spirit of Fuzhou Young Pioneers, To create a cultural "business card" highlighting Fuzhou characteristics, To create a brand image in line with the characteristics of the children group, Carry out education and guidance in a way that children like, Guide the majority of children to love their hometown and strive to be good team members in the new era, On November 20th, Under the guidance of the Fujian Provincial Youth Working Committee, Fuzhou Youth League Municipal Party Committee and Fuzhou Youth Working Committee launched the "Fuzhou Young Pioneers cartoon image and cartoon name collection" activity, Has received wide social attention, Get a positive response across the country, The entries include both computer graphics by professional designers, There are also children's hand paintings. As of November 30, a total of more than 400 excellent works reflecting the regional characteristics of Fuzhou and in line with the physical and mental characteristics of children were received. After the primary election, a total of 20 cartoon characters and 10 character names were selected. Combined with the moral of the entries, the expert review committee finally selected the cartoon characters and names of Fuzhou Young Pioneers. Fuzhou Young Pioneers cartoon image, the boy named "Fu Fu", the girl named "Zhouzhou state", the name together, is "Fuzhou", with distinct regional characteristics. Cartoon image is simple and lively, full of the flavor of The Times and a high regional recognition degree, the design of the Q version is more in line with the current children's preferences. The red scarf worn on the chest represents the specific identity of the young Pioneers."Fu" clothing from the Fuzhou famous scenic spots three fang seven lane, the middle of the belt is the seal character, shoes from the elements of Fuzhou banyan trees; "state" skirt from the Fuzhou Sanbao oil paper umbrella, wearing Fuzhou jasmine hair ornaments, jasmine petals shawl, foot auspicious cloud shoes, holding the young pioneers torch."This meaning is very good, they are two very lovely, very in line with the image of our young pioneers sunshine."Cartoon characters" Fu "" State " attracted the attention of many young pioneers. On the scene of the event, for the first time, the cartoon cartoon "Fuzhou Young Pioneers was Born in Fuzhou State" was released for the first time, and a rich and colorful artistic performances were also performed, and the "Top Ten Red Scarf Small Proposal" was awarded. In the future, the images of "Fuzhou" and "Fuzhou" will be used for the application of printing and animation derivatives. As measures to encourage the young pioneers and spread the knowledge of the young pioneers, they will become a close partner of the 620,000 young pioneers in Fuzhou and grow up with them.

2022-01-03	The police of "Yunjing Studio" found in the case that the lack of family education is an important reason for children to commit crimes. The hospital has specially invited marriage and family instructors to provide a thought-provoking parental education for negligent parents. In the class, the teacher pointed out the role positioning and role of family members in the family through multiple dimensions, such as family environment, family system structure and family communication mode, providing parents with family education mode and coexistence experience, so as to build a harmonious and healthy family model. In recent years, the Lu'an Communist Youth League has strengthened education and guidance, highlighted the source governance, strengthened key prevention, integrated all forces to carry out the seedling protection action, and constantly optimized the growth environment for young people. Among them, the Lu'an Youth League organization strives to build a mental health barrier. Relying on the municipal 12355 youth comprehensive service desk, a volunteer team of 15 young psychological counselors has been set up, and the youth psychological counseling services have been carried out on a regular basis, serving 550 people. During the epidemic prevention and control period, 8 psychological counseling volunteer service points were set up in key closed isolation areas; a 24-hour service hotline was opened, and 52 professional psychological counselors were publicly recruited to provide psychological counseling volunteer services. In the fourth quarter of this year, the Communist Youth League Municipal Committee carried out mental health services into the campus activities. The city completed a total of psychological questionnaire survey of 11,460 primary and middle school students, held 28 mental health lectures, and 346 youth self-care education theme team activities. At the same time, the city has focused on building care and help barriers. We carried out the activity of "Youth Warm Winter Tour Volunteer Manchu Gao City", helped 220 children from difficult groups, such as left-behind children and minor children of prisoners, and invested 310,000 yuan. The Communist Youth League Municipal Party Committee has also explored and implemented the projects of "Rural Revitalization Love Classroom" and "Mountain Doll Strong Growth Plan", raising an investment of 116,000 yuan. Tuan Tuan Lu'an Municipal Party Committee strives to build a self-protection education barrier. A special class for youth safety self-care education was set up, with more than 2,000 cadres and more than 2,000 youth volunteers, and more than 534 volunteer service teams, carrying out summer safety self-care education in 140 towns (streets) and 1,811 villages in stages and groups. In addition, the Lu'an Communist Youth League strives to build a judicial protection barrier to prevent and reduce juvenile delinquency. Procuratorates at all levels in Lu'an city have piloted the brand of juvenile criminal procuratorial work, and relied on social services such as the 12355 youth service desk.

2022-01-02	Inner Mongolia Baotou city to build regional league to build the first pilot unit of Inner Mongolia Baotou city to build regional league building the first pilot unit stone jia) in order to improve the attraction and cohesion, expand effective coverage of the communist youth league work, Inner Mongolia Baotou city qingshan district party committee further promote regional league building work, build regional league to build the first pilot unit. On December 29, Baotou City Qingshan District Xianfeng Road street youth work conference and regional youth league building joint meeting of the first plenary meeting was held. The meeting deliberated and approved the "List of member units of Xianfeng Road Street Youth Work Committee" and "Rules of Xianfeng Road Street Youth Work Committee", and released the first batch of Xianfeng Road Street youth work resource sharing and demand list. Delegates from Baotou Fourth Hospital and Baotou Youth Development Center made speeches on the resources and needs of the unit. Baotou City Qingshan District regional group construction joint meeting of the first plenary meeting site. Image was provided by the respondents. Meeting also examined and approved the pioneer road street regional group building joint meeting member unit list "and" pioneer road street regional league building joint meeting system ", Baotou qingshan district pioneer street party committee vice secretary dong-bo wang combined with qingshan district to carry out the youth long-term development planning six pilot projects, introduces the pioneer street in 2022 plan 20 youth development practical projects. At the event site, Baotou City "Red Scarf Classroom" youth community service project received 100 sets of love books. As Baotou regional league building first pilot unit, qingshan district pioneer street will fully integrate the communist youth league and youth work resources, build the streets as the core, community organizations, jurisdiction at all levels of youth league organizations and youth members, young pioneers to participate in the regional party building + league building new pattern, solid promote the communist youth league and the young pioneers work heart and "new".

2021-12-31	Hebei Province Handan Municipal Party Committee held the "National Youth Civilization" awarding ceremony Zhaohe Brigade, held the "National Youth Civilization" awarding ceremony and youth civilization observation and exchange activities. At the scene of the event, Handan Municipal Committee for the "National Youth Civilization" collective-city traffic patrol detachment Zhuhe brigade women's squadron awarded. Participants focused on watching the women's squadron gesture exercises and motorcycle training results drill, visited the party and league activity room, police camp culture, command center, etc., to understand the detailed understanding of the women's squadron to create a national youth civilization work content and results. In recent years, in 26 create youth civilization activities of the organizing committee member unit care and support, Handan municipal committee often create new, by strengthening ideological management, process management, the main management, organization "youth civilization" create collective at all levels in optimizing the business environment, convenient huimin service, civilized city, normalized epidemic prevention and control work play a pioneer demonstration role, further polish new era youth civilization brand, produce good economic, social and talent benefits. In 2021, six youth collectives in Handan won the 20th "National Youth Civilization", and 17 youth collectives were awarded the "Hebei Province Youth Civilization". About 70 people participated in the activity, including the heads of some members of the organizing committee of Handan City, and the representatives of the collective heads of the provincial and municipal youth Civilization activities from 2021 to 2022.

2021-12-31	"Harbin Bank Cup" Harbin Youth Innovation and Entrepreneurship Competition held "Harbin Bank Cup" Harbin Youth Innovation and Entrepreneurship Competition held "Harbin Bank Cup" Harbin Youth Innovation and Entrepreneurship Competition final. After the selection of the audition, preliminary competition and semi-final competition, the 16 events shortlisted in the final competition competed on the same stage. In the final, five experts in technology, venture capital and incubation were specially invited as judges. After the competition, the two groups will win the cash prize of the first prize 50,000 yuan, the second prize 30,000 yuan, and the third prize 10,000 yuan respectively. The Harbin Municipal Party Committee of the Communist Youth League will focus on business incubation and guidance, media publicity and display, investment and financing docking and other related aspects, to provide support and services for all kinds of outstanding youth entrepreneurship projects participating in the competition to take root and better develop in Harbin.

2021-12-31	Heze "three points force" to create "temperature" community less working committee Heze "three points force" to create "temperature" community less working committee Zhang Jianwei correspondent Cui Lu) "we are the communist successors, inherit the glorious tradition of revolutionary ancestors, love the motherland, love the people..." recently, in jia and the community party service center, the Chinese young pioneers Chengwu County Wenting street jia and community work committee founding meeting held here. Youth League Chengwu County Party Committee, Wenting Street Youth League Working Committee, Jia He community and school responsible comrades and representatives of the young Pioneers attended the launch ceremony. In order to further implement the central "opinions" and provincial "several measures" file spirit, group Heze municipal party committee, city less working committee attaches great importance to community construction, as the implementation of the party building, team building, promote the reform of the young pioneers, strengthen the young pioneers work mechanism construction of important measures, grasp the time, find support, integrated project point "three", has built 14 temperature community committee, build up "campus + campus" young pioneers organization mode. Grasp the "time point", concentrate on making efforts. In order to solve the problem of the establishment of the community young working committee, Heze City young working Committee used during the summer vacation, relying on the "small lotus school" project point to set up the community young working committee. Through the unified issuance of the "Activity Plan on the Establishment of Community (Village) Young Working Committee during the Summer vacation", standardize the process of the establishment of community young working Committee, and urge counties and districts to establish work ledger, clear time nodes, establishment location, and record activity projects. And the municipal young working committee, as the guiding unit, has held many meetings to schedule the construction progress, set up a benchmark, grasp the first to promote after breaking, one by one, and 14 community young working committees have been established. The young working committee by the community party branch secretary as the director of the young working committee, select outstanding community cadres, "five old" personnel, enthusiastic parents, on-the-job teachers or college students volunteers and other groups as the community young pioneers counselors, and issued appointment letters for them. Find the right "support point", strengthen the work guarantee. Smooth blocking point, actively strive for the support of the local township party committees and governments, by the director of the township youth working committee led the communication, the township youth League committee secretary specific docking guidance of the site selection and staffing. Through visiting and investigation, select the community to support and cooperate, learning tables and chairs, venues, and community preparation close to the school; establish rules and regulations according to the "five one" standard, that is, an operation team, a pair of schools, a brand practice, a "red scarf medal" features; brand linkage, make full use of the existing community brand projects, realize departmental linkage and resource sharing."Little lotus school" play to the advantages of college students volunteer service left-behind children, huacheng volunteers in peony district shuangjing community set up "little tadpoles energy station" summer counseling, chengwu jia river community "dream classroom" public hosting "green" signboard project, dongming horse set community planning "green volunteer" "green classroom" and other activities are the community less service effective carrier of the young pioneers. Integrate the "project points" to enhance the community vitality. After the establishment of the community youth working Committee, it released the activity "menu" for the community, sending all kinds of young Pioneers activities to the community in the form of "order", so that the community becomes the position for the young Pioneers to strengthen ideological and political education and an important base to expand social practice. Carry out party history publicity activities. Members of the city red scarf tour group respectively went into the community to carry out 23 party history education lectures with the theme of "youth to the Party to struggle for the strong country", covering more than 1,600 team members. The Nancao Community Youth Working Committee of Juye County has set up the "Red Scarf Radio Station", with five columns, such as the hundred years of the Party history and the spring breeze idioms. The red scarf lecturers regularly publicize the Party's principles and policies and its history through the radio, and create a strong atmosphere of learning party history and education. Carry out public welfare classroom activities."Little Tadpole Energy Station" in Shuangjing Community, Mudan District provides summer guidance for more than 350 young pioneers in the area, offers colorful classes such as "legal education and training, skilled craftsman handicraft class, traditional culture class accompanied by gifts, and carries out summer camp activities such as" Poor Children Swan Project ". Yuncheng pan crossing town pan west village, Guo Tun town Shao set village less committee organization young pioneers to carry out the "small hand" ban burning straw, civilized village I create, fraud propaganda volunteer service activities, the young pioneers in the nearby area to carry out labor practice, volunteer service activities, to participate in community governance, rich amateur life, also injected a fresh blood for the community.

2021-12-30	Weihai Youth League Organization: Be a good cooperation bridge between the local Communist Youth League with a complete set of underwater intelligent operation equipment, which is expected to be marketed next summer."Yu Changli, associate professor of shipbuilding and Marine engineering at Harbin Institute of Technology (Weihai), said that the underwater intelligent robots produced and developed in China only have detection functions and rely on imports, and the research and development results will break this dilemma. In just a few months, why can a team of professors and graduate students "spark" in the cooperation with a local enterprise in Wendeng District, Weihai City, Shandong Province? This starts with the "gathering in Weihai" action. In March this year, the Weihai Municipal Party Committee of the Communist Youth League played the role of the Communist Youth League as a "bridge" and a "link" between the schools, and launched the action. Through in-depth investigation of 151 industry-university-research needs of 79 enterprises, more than 170 Taishan scholars, master and doctoral supervisors, and graduate students from many universities were invited into the enterprises."Our team has been doing the research and development of Marine intelligent equipment, and the industrialization achievements are rarely transformed. And power tools mainly engaged in manual production of tools, low level and technology added value."Yu Changli said. In the third phase of high-level young talents "gathering in Weihai" into Wendeng District, Yu Changli led the team to visit the power tools, and finally reached a cooperation agreement. In the next step, the two sides also plan to carry out cooperative research and development of underwater unwinding intelligent equipment for ship propellers. The "Gathering Weihai" action has set up a high-level communication platform and closely combining talents, science and technology with enterprise development. Up to now, a total of 30 industry-university-research projects have reached cooperation intentions and 9 projects have been implemented. Around the three-year action of "talent development", Weihai Municipal Party Committee also united with Shandong University (Weihai) and other universities, Set up "Qingling Weihai" ideological and political education, "Qingjun Weihai" innovation and entrepreneurship, "Qingrong Weihai" school culture, "Qingzhi Weihai" public service four-class studios, Led the holding of more than 650 theme activities such as "100 schools and 1000 posts" precision employment internship action for college students, To stimulate the new drivers of the integrated development of the local Communist Youth League in an all-round way, The total signing of more than 60 school-enterprise cooperation agreements, To promote 14,000 quality jobs for college students, Signed by more than 4,800 college students, To promote the proportion of college students to increase substantially for two consecutive years. Correspondent, Chang Xiaohua, China Youth Daily, China Youth Network reporter Xing Ting source: China, China Youth Daily

2021-12-30	Guizhou including: party committee cadre team construction reform "20" communist youth league grassroots reform focus on "selection, culture, tube, examination, with" Guizhou including: party committee cadre team construction reform "20" recently, by the Guizhou including municipal party committee comprehensive deepening reform committee, including deepening the reform of the communist youth league cadres team construction implementation opinions "(hereinafter referred to as the" implementation opinions ") issued. This is the first special document on deepening the reform of the construction of cadres of the county Communist Youth League since the pilot reform was expanded, which is of symbolic significance. Tongren city is located in the eastern part of Guizhou Province, with a total population of 4.4803 million, of which more than 1.2 million are young people. In June this year, two districts and 8 counties under the jurisdiction of Tongren were identified as the pilot areas of the county Communist Youth League. Since the pilot reform, the Tongren Municipal Committee of the Youth League to carry out in-depth research, firmly grasp the league cadre reform "nose" targeted force, the remedy to the case of the drafting of the "implementation opinions" and the organization, human resources, education and other departments of communication and consultation, finally approved by the Municipal Party Committee of the reform."Implementation opinions" focus on the lack of mass cadre management characteristics, the source channel single, constraint incentive mechanism such as insufficient problems, to city and county two level youth league authority, especially county youth league team, on the basis of grassroots cadres, adhere to ShiCe classification, hierarchical management, through the reform of cadre selection, training, management, assessment, use mechanism, remove "official standard" thought, overcome the "administrative" thinking, strive to build a high-quality professional cadres, continuously for the party training youth work backbone, to achieve high quality development. This has a total of 20 specific measures of the "implementation opinions" not only "full of dry goods", but also has a lot of traction, innovative, breakthrough specific measures."Document scope jumped out of the traditional sense within the system of 'cadres' category, both applicable to cities and counties of youth corps committee organs (including internal institutions, subordinate units) cadres, schools, organs, institutions, state-owned enterprises, villages and towns (street), village (community) field cadres, also applies to the park, non-public enterprises, social organizations and other emerging areas of cadres, and other work in the communist youth league or assume the corresponding responsibilities of the backbone."Youth League Tongren Municipal Party Committee concerned responsible comrade said. The Implementation Opinions propose that, Vigorously select outstanding young cadres who love young work into the city and county youth League committee organs, Municipal and county Youth League committee team members appear vacant, In principle, complete the allocation within 3 months; Establish a regular matching mechanism for hanging part-time cadres in the municipal and county youth League committees, Working hours are usually two years; It can take open recruitment, targeted recruitment, comparative selection and transfer, open selection and other ways, Select and assign part-time cadres of municipal and county Youth League committees; Release of external conditions such as region, identity, occupation, age, level, household registration and so on, Expand the source channels of grass-roots youth league cadres; Establish an open competition mechanism for school youth league cadres at the city and county levels; Explore the project-based employment mechanism, Municipal and county youth League committees can develop "youth work project commissioner" positions. In terms of training, establish the implementation of cadres talk system, the main responsible party organizations at all levels should strictly implement the "five one" (hear a report, attend a meeting, make a instruction, research, solve a problem) requirements; cadres training into the city and county cadres education training plan, the annual arrangement, the municipal and county level. In terms of management, To uphold the leadership of the Party, Strictly implement the provisions of the dual management system of cadres, The appointment and removal of secretaries and deputy secretaries of youth league organizations (including temporary and part-time cadres) at all levels, Party organizations at the same level shall solicit the opinions of the league organizations at higher levels in advance and go through written procedures; Adhere to the "retain the backbone, with capital", To keep the ranks of full-time youth league cadres relatively stable, In principle, for one full term, Generally not transferred during the term of office, Avoid the group's post as a springboard of the "traveler" mentality, keen on the surface of vigorous and other short-term behavior; Full-time cadres of the municipal and county Youth League committees should work in the youth League for no less than 3 years, The secretaries and deputy secretaries of other grass-roots youth league organizations shall generally serve for no less than two years. In terms of assessment, establish and implement the assessment system for the ability of the cadres of the league, and take the assessment results as an important basis for the annual assessment; strictly implement the assessment system of the league secretary, and the higher league organizations shall report to the corresponding Party organization and organization department. In terms of use, perfect the outstanding cadres and outstanding cadres in other areas of the normal two-way communication mechanism, explore building cities and counties organs and grassroots cadres engaged in youth work career growth development system, the outstanding grassroots cadres as a source of cadres important "reservoir", according to the classification of job performance for post incentive, salary incentive, job rank incentive, development incentive, etc. In addition, in view of the county youth league leadership the "key minority", Tongren municipal party committee organization department, pku supporting issued by the including city (county) youth corps committee leadership and team members started the evaluation implementation method (try out) ", to" what, who to test, how to test "make specific provisions, the county youth corps committee team and secretary do well or do badly to" have a statement ", implement rewards. Source: China Youth Daily

2021-12-29	Recently, Zhang Ruhu, a young rural entrepreneur who lives in Qugou Town, Xiangshan District, is busy carrying goods. The 31-year-old has long run a soy products processing plant, but has little money to expand. In April this year, he submitted the application materials for financial discount interest to the League Huaibei Municipal Party Committee of Anhui Province, and in less than a month, the funds arrived. Zhang Ruhu regrets that the real money type to help for him in the initial stage of entrepreneurship, let him have the confidence to continue to start a business. This year, Huaibei, like Zhang, enjoyed 47 young grass-roots entrepreneurs with discount funds. The Huaibei Municipal Party Committee of the Communist Youth League, together with the financial and financial departments, has carried out the evaluation of the financial discount interest projects for youth entrepreneurship loans, focusing on supporting the youth entrepreneurship projects with good market prospects, strong development momentum, strong industrial drive, safe, green and environmental protection. In the past three years, it has provided 1.8158 million yuan to 155 eligible projects. Real money and silver financial support, butler service escort, point-to-point business guidance... since this year, the Communist Youth League Huaibei Municipal Party Committee from the policy, capital, platform, environment and other aspects for the youth innovation and entrepreneurship to create a good environment. Zhang Kaiqiang, the head of the celery base in Sipu Town of Suixi County and a young returning entrepreneur, was rated as the young pioneer of rural revitalization in Anhui Province this year. His success is also inseparable from the strong support of the youth league organization. This year, Tuansui County Party Committee set up a celery planting youth club, which went to the base to understand the production and operation situation and invited agricultural experts to give on-site guidance. This year, the Huaibei Municipal Party Committee of the Youth League launched grass-roots youth League organizations and cadres, in the city's brand research, the establishment of Huaibei city entrepreneurial youth talent pool, regional assigned grass-roots league cadres as a good entrepreneurial youth waiter. In addition, the Youth League Huaibei Municipal Party Committee, the Municipal Talent Office and other units also rely on the youth apartment to build the Huaibei youth talent station. Li Yandong, secretary of the CPC Huaibei Municipal Committee, said that the talent station will provide one-stop services such as temporary accommodation, employment guidance, innovation and entrepreneurship exchange, and urban integration services for young people who come to Huai for employment and entrepreneurship, and make the talent station a warm first stop for young talents to Huai.

2021-12-29	Let quantum mechanics into teenagers Jinan create a new technology exploration platform for quantum mechanics into teenagers Jinan create a new technology exploration platform XingTing) on December 26, a new youth technology exploration platform in Jinan, Shandong province, this means that represented by quantum mechanics of frontier science and technology will walk into more teenagers, help hatch the children's dream of science and technology. On the same day, Jinan Communist Youth League and Jinan Municipal Party Committee signed the signing ceremony of Jinan Quantum Technology Research Institute and the first 2021 Jinan Youth Palace Science and Technology Carnival opened in Jinan Youth Palace. With the theme of "Dream building, Innovation and growth", this science and technology carnival builds a platform for teenagers and children to explore science and technology, stimulates children's creativity and hands-on ability, and plants the seeds of science and technology in children's hearts. Jinan youth palace of science and technology small people brought wonderful science experiment show at the opening ceremony, in addition to the wonderful science drama show, science "air" show, and the quantum knowledge, robot model experience, technology model airplane handmade experience, wei to hut, lego building experience, ClicBot cell robot, intelligent calligraphy robot, robot dance performance, etc., shows the power of science and technology and cool. Quantum popular science knowledge exhibition, rockets and beidou satellite exhibition, intelligent calligraphy robot colorful activities of this activity is to further implement the national scientific quality action plan spirit and the opinions of the central committee of the CPC on strengthening the new era of young pioneers work requirements, group Jinan municipal committee actively seek quality popular science education resources, take the lead with Jinan quantum technology institute reached strategic cooperation, the latter long as our quantum science research and applied basic research frontier and popular science education base in Shandong province. It is understood that the Jinan municipal committee and Jinan quantum technology research institute will focus on the formation of springs future talent training popular science experts, springs future talent science and technology creative competition, the implementation of springs future talent optimal training plan, common construction of science positions, joint research and development of science resources package plate cooperation, to quancheng youth promote public science knowledge.

2021-12-29	Gannan communist youth league start national unity youth action communist youth league start national unity youth actor Ma Fuchun correspondent He Xuezhong) to continue to deepen education of national unity and progress, casting the Chinese nation community consciousness, further organize and mobilize the youth actively in beautiful Gannan construction, recently, the Gansu Gannan committee joint zhuo county united front work department united front work department, the communist youth league zhuo county "national unity youth action-blowing" five no Gannan "new horn" ten homes "new journey: the communist youth league in action" series of activities started. At the launch ceremony, Wang Haiqing, secretary of the Youth League Gannan Prefecture Party Committee, earnestly hoped that the young people should strengthen their ideals and beliefs, strive to be the practitioner and defender of a harmonious home, play the role of example, promoting and promoting national unity; and take the initiative to cultivate the new people who build up the sense of community of the Chinese nation. CAI, Deputy Secretary of Zhuoni County Party Committee, put forward specific requirements for carrying out this activity: highlight the theme, sing the theme of national unity and progress; focus on the key points, promote the implementation of a series of activities; strengthen the guarantee to ensure the safe and smooth development of this activity. During the activity, the youth representatives went to Yang Tusi Memorial Hall of Zhuoni County, Gensha Village, The Demonstration Village of Zhuoni County, and the well-off Village in Larluhe Village, Kangduo Township, Zhuoni County, to carry out research exchanges, visits and study of the Red Revolution.

2021-12-29	Xi'an young volunteer: " My city, I come to guard!"Xi'an young volunteers built the epidemic defense line in the snow:" My city, I will guard!"" " kid, do you have an ID card? At "...... in Xi'an in Shaanxi Province, the center of COVID-19, the nucleic acid testing site in the cold and snow of volunteers made people in the closed community feel warm. A total of 811 local confirmed cases have been reported in Xi'an since December 9, as of 24:00 on December 27."jump into action! Build a defense against the epidemic- -the youth league members should adhere to scientific epidemic prevention and strive to be a model of self-discipline and epidemic prevention. At the same time, actively report to the community, to carry out the relevant volunteer service work!"At the critical moment of epidemic prevention and control, the Xi 'an Municipal Committee of the Youth League, Xi' an Youth Federation and Xi'an Volunteers Association, immediately issued a proposal."Before one had hung up, the other phone call came in again!"As soon as Xie Jiajia, a cadre of the Yanliang District Committee of the Communist Youth League, the phone's phone call was released. At that time, Xie Jiajia, who received more than 300 phone calls a day, had already formed a conditioned reflex. "I can guess what he wanted to ask before speaking.". Some volunteers who have previously participated in the epidemic prevention and control have voluntarily participated in the prevention and control work. In less than two days, Xie Jiajia has set up two volunteer groups, each with 500 people, both of which are a daily reserve of volunteers. Li Zhengxi, a former soldier, is a "senior" epidemic prevention and control volunteer. Since 2020, he has been on the front line of prevention and control for every epidemic outbreak in Xi'an. The daughter once asked Li Zhengxi, " Dad, what is a volunteer?"He thought and replied," He who is willing to do something, not in return."For several days, Li Zhengxi, as the leader of the volunteer team, has claimed the task at 5 am every day and was busy until 10 PM. When he returned home late at night, he felt pain in his waist and calf muscles, but he had to leave a day's message on wechat and plan for the next day. He shut himself alone in his small room, afraid to approach his wife and daughter. In order to facilitate his work and ensure the safety of his family, Li has stayed in the office of the epidemic prevention and control command center in recent days. In Yanta District, Liu Panfeng, secretary of the Communist Youth League Committee of the Dayanta Sub-district Office, has been working continuously for more than half a month. "At the busiest time, he slept for more than four hours in two days.""I need masks," "I want protective clothing," " We lack foot covers... Every day, Liu receives calls from various nucleic acid testing points in his area. He must find ways to put the epidemic prevention materials in place in the first time. His big Wild Goose Pagoda sub-district office has 22 communities and about 70,000 residents. According to a press conference on COVID-19 prevention and control in Xi 'an, some of the three major transmission chains of Xi' an, the most serious family hospitals of Chang'an University, are within the jurisdiction of the Big Wild Goose Pagoda Street Office. The day before each round of nucleic acid tests, Liu was busiest and needed to be equipped with all supplies. After several days of continuous work, Liu and the volunteers felt tired, but also constantly refreshed their work efficiency. Before, the nucleic acid test of 70,000 to 0080,000 people could be completed within two days. " Now, it takes only seven hours!"" My city, I'll keep it!"Liu Panfeng, who sent this sentence on wechat, has not been home for more than half a month. The seven-year-old son will kiss his father across the screen when he makes a video phone. Hearing the child said, "Dad, I miss you," Liu Panfeng's heart is warm, "I hope the epidemic can end as soon as possible, go home to accompany the child."Yang Zhi is the head of an education and training company in Xi'an. As a native of Hubei province, he deeply felt the warmth of people from all over the country when the COVID-19 outbreak broke out in 2020. This time, he led his employees to join the Yanta district volunteer service activity, "want to do something for the city."The most tiring time, Yang, zhi persisted in the snow for more than 8 hours, wechat steps recorded more than 20,000 steps. It was also on this day that he really felt the "warm" of Xi'an people. As a volunteer to maintain order at the community nucleic acid testing sites, Yang zhi and his colleagues have only done basic protection to save money on epidemic prevention materials. It was getting dark because of the large number of people, and the nucleic acid testing was not over yet, so they had to move the testing table to the lighted place to continue their service."We wanted to carry the test table away, but several female nurses refused to help when they saw us without protective clothing and were worried about the risk of infection."Looking at several female nurses carefully lifted the table, Yang Zhi was warm" blow ". In addition to maintaining the order of the nucleic acid testing points, Yang Zhi has to make streaming phone calls every day."As long as everyone actively cooperates with the epidemic prevention work, the city can tide over the difficulties as soon as possible."Yang Zhi said. December 23 is the volunteer Qu Jinjin's birthday. On her birthday, she received an unexpected surprise- -Zhang Yiren, the party secretary of Tuan Yanliang District, came with a cake to celebrate her birthday."As of 18:00 on December 28, a total of 21,432 young volunteers in the Communist Youth League system have been organized to serve them, with a total service time of 208,983 hours."The relevant person in charge of the Youth League Xi 'an Municipal Party Committee told reporters that these volunteers are active in the city's streets, villages, communities, in the centralized nucleic acid detection, handling of protective materials, personnel control and investigation, closed community material support, and psychological counseling play a huge role. China Youth Daily, China Youth network trainee reporter Han Rong, reporter Shi Jia, Sun Haihua, source: China Youth Daily

2021-12-29	The 6th United Nations China Youth Environmental Forum was held in Yangzhou, Jiangsu Province, on December 26,2008. Wang Qian, Director of the Project Planning Program of the United Nations Environment Programme Representative Office in China, Han Hua, Deputy Secretary of the Yangzhou Municipal Party Committee, and Chen Chao, Director of the Shanghai International Cultural Exchange Center, delivered speeches respectively. Yuta Uchiyama, Professor of the Graduate School of Human Development and Environment, and Tim Christophersen, Director of the Ecology Division of the United Nations Environment Programme, delivered keynote speeches. The forum also invited representatives from the United Nations, the World Canal Historical and Cultural City Cooperation Organization and domestic universities to have a roundtable dialogue. Well-known scholars in the field of environment, representatives of universities and scientific research institutions, and representatives of Yangzhou youth attended the activity. It is reported that in recent years, the Yangzhou Municipal Communist Youth League has deeply implemented the "Action of gathering youth strength to help the Beautiful Yangzhou", and built a volunteer team of "River Xiaoqing" with more than 10,000 people within three years. Mobilize the city's youth to extensively carry out ecological civilization education and practice activities such as green planting and green protection, low-carbon travel and garbage classification, and guide the young people to extensively spread the concept of green environmental protection and advocate a green lifestyle. At the same time, relying on relevant resources, the Grand Canal Ecological Protection Youth Learning Society is built, and the comprehensive use of sitcoms, micro lectures, scanning code listening, and interesting answers are open to young people on a regular basis, so that the majority of young people can fully feel and participate in the practice of ecological protection in Yangzhou. The forum is hosted by the United Nations Environment Programme's Representative Office in China, supported by the World Canal Historical and Cultural City Cooperation Organization, Yangzhou People's Association for Friendship with Foreign Countries, and the Sino-Foreign Cultural Exchange Center of the Ministry of Education, and organized by Yangzhou Ecological Science and Technology New City, Yangzhou Municipal Party Committee and Shanghai Cultural Exchange Center.

2021-12-29	Tuanrizhao municipal party committee and Beijing university youth league committee deepening cooperation agreement with Beijing university municipal party committee deepening the cooperation agreement in Beijing on December 29 (reporter Zhang Jianwei, correspondent XiangChanghui) to further promote the communist youth league service youth employment entrepreneurship, power "ZhaoCaiYinZhi" work, on December 26, group of Shandong Rizhao municipal party committee to Beijing to participate in "just gather sunshine-through school" Beijing university special recruitment activities, organize students to recruitment, and the Beijing university youth corps committee signed deepening the cooperation agreement, strive to promote the complementary advantages, resource sharing, and build. Hu Guodong, Secretary of the Youth League Committee of Peking University, and Li Wanying, Deputy Secretary of the Municipal Youth League of Rizhao attended the signing ceremony. The Signing ceremony is on-site. Correspondent for the figure the signing is the group sunshine municipal party committee and Beijing university youth corps committee to carry out the major decision of strategic cooperation, the two sides will take the cooperation as an opportunity to give full play to the youth, youth, unite youth, youth, to build the "three bases" -graduate student employment trainee base, rural revitalization of youth volunteer pioneer base, Beijing university doctoral lecturers preach station base, achieve "two promotion" -promote science and education integration and production collaboration level, to achieve "one goal" -talent sharing, mutual benefit and win-win. In the special recruitment activity of "Caiju Rizhao-Direct to Famous Schools" in Beijing universities, the Communist Youth League Committee also promoted Rizhao Youth Station. At the recruitment activity site, the convenient services such as free accommodation, employment and entrepreneurship policies, life and travel to the young students by means of yapo display and publicity leaflets.

2021-12-28	Gansu Linxia start youth national unity progress education activities Gansu Linxia start youth national unity progress education activists Ma Fuchun) for education to guide the youth cast up the Chinese nation community consciousness, on December 24, Gansu province Linxia committee joint Linxia bureau of education, ethnic committee held "river young pomegranate hand in hand new era" Linxia youth national unity progress education theme activities launch ceremony. Zhang Lequn, deputy secretary of Gansu Provincial Committee, attended the ceremony and delivered a speech. Ma Bin, member of the Standing Committee of Linxia State Committee and Minister of United Front Work Department, attended the ceremony and delivered a speech. Zhang Lequn stressed in his speech, the youth national unity publicity and education work to adhere to from the reality of ethnic areas, promoting organization youth, guide youth, service youth, safeguard the legitimate rights and interests of the work, make them firmly establish a "five identity", "three cannot" thought, promote ethnic areas youth in promoting scientific development, promote social harmony, consolidate the healthy growth in the national unity, achievements. State league organizations should thoroughly study and implement the important thoughts of General Secretary Xi Jinping on strengthening and improving ethnic work, adhere to the unity of loving party and loving socialism, strengthen the community consciousness of young Chinese nation, vigorously carry out the activities of "pomegranate seeds and one family" series of activities, strive to promote the high-quality development of the publicity and education of youth ethnic unity, and actively carry out various communication activities. Ma Bin pointed out in his speech that in recent years, Linxia state attaches great importance to youth national unity progress propaganda and education, the Chinese nation community consciousness as an important standard of all kinds of school schools, khalid ents, as a compulsory course of school education, promote the Chinese nation community consciousness education into the campus, into the teaching material, into the classroom, into classroom teaching, theme activities, social practice, make the Chinese nation community consciousness infiltration and rooted in the youth of all nationalities. According to introducing, "Hezhou youth pomegranate flowers hand in hand new era" Linxia youth national unity progress propaganda education theme series activities for nearly half a year, will through organizing youth cultural exchange studies, painting and calligraphy exhibition, national unity progress tour, national unity, short video creation contest, excellent theme team selection show according to the characteristics of teenagers, close to the needs of teenagers series of activities, education guide the state of youth cast the Chinese nation community consciousness, be a new era Linxia national unity progress "pomegranate seeds". Linxia Deputy director of the Standing Committee of the People's Congress Chen Hong, vice chairman of the CPPCC, chairman of the state Federation of Trade Unions Gao Hua attended the ceremony. He � B, Party Secretary of the Youth League of Linxia Prefecture, presided over the ceremony. Linxia State Education Bureau, the Ethnic Affairs Committee, the Bureau of Culture and Tourism, the Federation of Literature and Art, and more than 800 youth representatives of all ethnic groups attended the ceremony.

2021-12-27	Group of Anhui Province Huangshan Municipal Party Committee held a rural revitalization theme salon activity group of Huangshan Municipal Party Committee held the "creative Huangshan Youth Young- -township about growth" rural revitalization theme salon activities Zhang Jianwei correspondent, Cai Hongyun Zha Wei Ting) recently, Sponsored by the Talent Office of Huangshan City, Anhui Province and the Huangshan Municipal Party Committee of Anhui Province, The "Creative Huangshan Youth Young- -Rural About Growth" rural revitalization theme salon activity organized by the Youth League Huizhou District Committee and Huangshan Youth Entrepreneurs Association was successfully held in the hydroponic base of Huizhou District Flower Zhiyun Flower Technology Co., LTD., Youth representatives of Huangshan Municipal Party Committee and relevant municipal departments in the field of rural revitalization attended the meeting. At the salon site, the young representatives combined with the development situation of rural revitalization in Huangshan city in recent years, and exchanged views on the characteristic industries of rural revitalization, the "new wind mouth" of rural tourism, and the revitalization of idle rural land. Promote rural revitalization is thoroughly study and implement the spirit of the spirit of the sixth plenary session of the 19th, since this year, Huangshan city focus on "strong industry", focus on "quality", relying on resource endowment and industrial base, accelerate the characteristic industry mass efficiency enhance rural revitalization of endogenous power, around power rural revitalization development, municipalities departments have said, will revolve around rural tourism, digital economy, live electricity, and other fields, build all-round, multi-level and wide public service platform. At the same time, we will strengthen the development of talent teams, create a better environment for entrepreneurship and employment, improve incentives for young people to return to their hometowns to start their own businesses, and support the development of talents in rural revitalization. Wang Kun, deputy secretary of the Youth League Huangshan Municipal Party Committee, pointed out in the activity that the youth salon should be more informal, without the venue and the scale. The focus is to open their hearts, speak freely and express their opinions. Through the salon, we will take the initiative to approach the youth, listen to their "aspirations", pay attention to their development needs, understand the difficulties and actively promote the solution of problems, effectively enhance the effectiveness of serving the youth, and make the Communist Youth League become more friends of the young people. Next, the Huangshan municipal party committee will be "creative Huangshan youth Young" theme season activities as the lead, stick to the main line, normalized youth salon activities, help youth entrepreneurial enterprise development and youth group growth, with creative light Huangshan, inspire youth in creative innovation, double recruit double lead, rural revitalization, release more youth energy in the industry development.

2021-12-25	Youth volunteers for the Beijing Winter Olympics in universities have prepared "warm-heart gift packages" worth more than 2,000 yuan for each volunteer, and held a symposium. To complete the games volunteer team organization work, the Tangshan party committee, north China university of science and technology youth corps committee detailed planning, strictly implement the Beijing winter Olympics organizing committee and Hebei provincial party committee related arrangements, play to the role of territorial youth league organization, deep volunteer group organization communication, research activities, understand the volunteers thought dynamic, physical state, training effect, etc., timely solve the difficulties found, the overall state of volunteers to achieve dynamic control. Before the volunteers left for the Winter Olympics, the Tangshan Municipal Committee invited local experts to conduct special training on Tangshan's history and culture, Dazhao spirit and earthquake spirit, encouraging the volunteers to bring the glorious image of Tangshan as a "heroic city" to Chinese and foreign friends. Since the launch of volunteer work in Zhangjiakou in Beijing 2022 Winter Olympic and Paralympic Games, the Hebei Provincial Committee of the Communist Youth League has coordinated supply and demand docking, stratified training and drills, and sent management teams to the Genting venue group to provide humanized management and services. According to the arrangement and deployment of the superior, the Tangshan Municipal Committee ensured and helped volunteers to participate in volunteer services in an orderly manner, and helped the successful holding of the Beijing Winter Olympics and Paralympic Games.

2021-12-23	Yunnan Mengzi: Implant OKR Assessment Endogenic Entrepreneurship momentum Little Bee " volunteer publicity team has recently become the focus of media attention. The team, composed of more than 500 young pioneers from 13 ethnic groups, won the honorary title of the 2021 National Grassroots Theory preaching Advanced Collective on November 19. It is one of the 42 national theory preaching advanced collectives commended by the Propaganda Department of the CPC Central Committee. In the Mongolian red scenic area, patriotism education base, schools, communities, nursing homes, parks, "a red scarf, a microphone, a yellow vest", the average age of 10 "little bees", with vivid, simple, plain, popular words, party stories, stories, become a fresh force in the Mongolian party history learning education. The publicity team promoted the C position of the party history publicity, which is one of the achievements made after the implementation of the OKR assessment method of the Mengzi Municipal Party Committee of the Communist Youth League. This year, the OKR (Objectives and Key Results) assessment method, which is widely used by many enterprises, has been applied to the cadre management of the Communist Youth League and Mengzi Municipal Party Committee."OKR, as a set of management tools that activate employee potential, encourages people to create everyday unimaginable goals and key results. In the process of reaching their goals, both teams and individuals will make breakthroughs."Said Zhang Ruihang, party secretary of Tuan Mengzi. In Mengzi, the Standing Committee of the CPC Municipal Committee studied and discussed them in the name of the Municipal Office, Mengzi Municipal Committee formulated 17 supporting measures and work manuals, and explored the OKR assessment method with "work objective" and "key achievement" as the core evaluation, established the "double assessment and three evaluation" mechanism, and strengthened the management of cadres. Zhang Ruihang introduction, draw lessons from enterprise management methods of OKR assessment method of specific approach is: to the central committee of the communist youth league about the county basic youth league reform target requirements, every quarter, issued by the county youth corps committee to the youth league organizations at all levels, everyone according to their own working methods, combined with the weak aspects for yourself to formulate and complete the quarterly goals."Double assessment", namely, peacetime assessment of league cadres + OKR work performance assessment; "three evaluations", namely, evaluation of superior league organization + evaluation of party organizations at the same level + evaluation of service objects (youth league members), with quarterly assessment cycle. After the trial operation in the municipal Communist Youth League organs, now, the OKR assessment method has been implemented to all the grass-roots youth league organizations in Mengzi city. The Communist Youth League Municipal Party Committee conducts quarterly supervision over the grass-roots youth league cadres, and the township (sub-district) youth league (working) committee and the municipal directly affiliated youth league organizations regularly feedback the assessment results. Take the quarterly assessment cycle, set specific and measurable work objectives and measures, so that each work process becomes intuitive and transparent OKR assessment method, make the work of the league cadres more purposeful and planned, the work measures more clear and feasible, and the work performance can be quantified. In 2021 loans and entrepreneurial loan work, pku responsible for the work of the cadres of the task number set up for monthly, every quarter should be completed, set up propaganda, deal with, submit the trinity working group, every day before work and every Friday, monthly report, summarize the work, and submit the bank, task expires, for XiaoHao. Two months later, the data showed that before the adoption of the OKR assessment method, the completion rate of loan exemption and start-up loan was only 12.5% from January to August; after the adoption, the task completion rate increased to 110% in two months from August to September. In the development of league members in 2021, the grass-roots cadres were decomposed according to the tasks issued by the Communist Youth League Municipal Committee, combined with the situation of various fields and industries in various fields, and set the development tasks into monthly and quarterly targets, and carried out according to the time node. By October, 1,052 tasks had been completed, and the completion rate had increased from 73.86% to 85.39%. At the same time, from October to December this year, the Communist Youth League Committee also completed the city's "society connection" rate of more than 90%, the new development of the league members electronic files 10 0% establishment target. To this end, the Communist Youth League committee and each township (street) youth League committee have assigned special personnel responsible for the "social connection" work, the league organization relationship connection with problems, to deal with the existing problems every day, strengthen the communication and communication with the youth League committee of other cities, to solve the difficulties for the league members. Strengthen the league, condensed extensive work force using daily assessment and OKR work performance appraisal, not only can real-time tracking cadres work results, let everyone around the main business overcome difficult, at the same time, also stimulated the enthusiasm of grassroots cadres, over the past year, grassroots organizations combined with their own advantages, create different characteristics of the work window. In late October, in the ancient pomegranate garden of Xiaohongzhai Village, Xin'ansuo Town, Mengzi, Zhang Xian, president of Mengsheng Pomegranate Production and Marketing Professional Cooperative, shuttled among the luxuriant and large canopy ancient pomegranate trees, showing the audience the red pomegranate that had just been picked from the trees and peeled off like crystal clear gems. Mengzi has a history of more than 800 years and has the title of "the hometown of pomegranate in China". In the ancient pomegranate garden of more than 200 mu, the pomegranate is on average 135 years old, and the largest one is 197 years old. Born in 1992, Zhang Xian is a new farmer working in the pomegranate garden. This year, the Mengsheng Pomegranate Production and Marketing Professional Cooperative has planted 12,000 mu of pomegranate trees, with an output of nearly 4 tons per mu. In order to help fruit farmers explore the market, the TuanMengzi Municipal Party Committee held the promotion meeting of "E-commerce helps farmers, I speak for Mengzi pomegranate" in the ancient pomegranate garden, inviting a number of cooperatives and other e-commerce platforms to conduct live broadcast."This event has solved our urgent need."Zhang Xian said."Where the youth is, the league organizations are built, and the services of the league organizations extend there."Zhang Ruihang introduction, this year, the group since the youth gathered industry, field, region, established a social committee, build the" youth league built in the industrial chain "" league organizations built in the volunteer service "" league organizations built in the pioneer park "," league organizations built in practice base "" league organizations built in the business circle "five league alliance brand, youth report to the organization mode into youth league organizations built in the youth" home "working method, let" organization to find youth ". This new model has enriched the life of young people from all walks of life in Mengzi."KO" hip-hop youth league branch held "hip-hop flash mob" in many business districts in the city; Mengma wedding youth league branch held "Muye Street (gai) sub" youth exhibition; Tuesday cafe youth league branch organized "Youth Reading Club" and "youth entrepreneurship salon"; youth entrepreneurship park, e-commerce industry association youth league branch organized youth employment and entrepreneurship training activities, also came to the youth together with the establishment of the youth league branch. Up to now, through the group affiliated platform, "E-commerce to help farmers I speak for Mengzi pomegranate" to help Mengzi rural youth sell more than 10,000 tons of pomegranate, apple for youth dating "Nanhu, happiness" for 5, promote the success of youth; 12 youth decoration festival ", the total sales exceeded 120 million yuan; to" team + project + grassroots league building ", more than 100 internship students are assigned to specific projects according to their specialties and expertise, to ensure that each college student has a team, each team project, and each project is organized."Through the league building alliance, the working force of the league, the youth activities position and the working methods of the league have been greatly expanded, and the win-win working method for the welfare of the youth has been explored."Mengzi city social field youth League working Committee secretary Yang Feng said. Data show that this year, Mengzi has built 59 new youth league organizations in the social sector, with a growth rate of 137%. Youth League organizations basically cover key enterprises in emerging fields such as industry, agriculture, service industry and e-commerce."After a year of practice, it has been proved that the OKR assessment method has given full play to the internal motivation of the league cadres and enhanced the new momentum of entrepreneurship."Zhang Ruihang said. China Youth Daily, December 23,2021, edition 06

2021-12-22	Changchun international youth urban ice and ice fashion week activity kicks off Changchun international youth urban ice and ice fashion week activity launch Wang Peilian) recently, by the Changchun city, Changchun culture, radio and television and tourism bureau, Changchun youth federation jointly hosted the 25th Changchun ice and snow fashion series activities- "urban ice and snow youth rong" Changchun international youth urban ice and ice fashion week activities. The activity was actively responded to by more than 50 universities in and outside Jilin Province, and nearly 3,000 young college students participated. Snow and ice fashion activity week will be held by college students snow and ice and snow clothing design competition, the selection of college students urban snow recommendation officer, campus ice sculpture art design, youth ice and ice art exhibition series of activities, and play the role of youth consumption experience and promotion, show Changchun youth fashion innovative thinking and urban ice culture, power Changchun ice and snow economic high quality development. At the opening ceremony, the famous calligrapher Yao Junqing presented his calligraphy works to express his best wishes for Changchun's ice and snow culture. Representatives of foreign students studying and living in Changchun performed poetry recitation. Young people in the emerging fields used a wonderful Hanfu show to perfectly integrate the traditional clothing culture with snow and ice fashion, showing the good spirit of the young people in Changchun in the new era.

2021-12-28	Xing Ting) On December 25, Shandong Heze Municipal Party Committee and Heze Youth Federation jointly held the 2021 Heze "Hope House" distress children care project report performance. Heze Municipal Party Committee Secretary Zhang Xinwen attended and delivered a speech, and the Communist Youth League Shandong Provincial Party Committee Secretary Liu Tiandong delivered a speech. Heze city leaders Yang Xiaoling, Zhang Qianshan and part of the love units, love enterprises, responsible for love individuals and assisted children attended."Hope hut" dilemma children care project report performance to "sowing hope light dream" as the theme, the love unit, enterprises, individuals and difficult children through songs, recitation, chorus, dance, and other forms, witness "hope hut" children care project achievements, feel the peony warm heart positive energy. Zhang Xinwen said in his speech that last year, the Shandong Provincial Party Committee of the Communist Youth League launched the "Hope House" project in Heze City, and successively allocated 16.41 million yuan of support funds to Heze City, injecting warm love into the "Hope House" project and adding strong momentum to Heze City. Since the launch of the project, a total of 2,676 "hope cabins" have been built in the city, the living environment of more than 3,000 children has been improved, and more than 6,000 volunteers have paired up with children in distress. A large number of children in distress have realized a profound change from material to thought, from "cabin renewal" to "spiritual renewal". In the next step, we should pay more attention to the needs of children in difficulties, work together, and persistently adhere to the construction of the "Hope House" project. It is hoped that social organizations, caring enterprises and people from all walks of life will actively participate in and mobilize extensively to add more warmth and contribute more strength to the healthy and happy growth of children in difficulties. Liu Tiandong fully affirmed the Heze city "hope cabin" construction achievements. He said the "Hope House" project has been included in the province's Communist Youth League to serve the ecological protection and high-quality development of the Yellow River Basin. It is hoped that the youth League organizations at all levels in Heze City will take "Hope House" as a practical measure to serve the overall situation of the party and government, build a model project to care for the growth of children, become a public welfare brand condensing social love, and truly manage and use "Hope House" well, so as to make this public welfare brand condensing social love better and stronger. Zhang Xinwen and Liu Tiandong respectively presented charity contribution MEDALS to caring units, enterprises and individual representatives. Yang Xiaoling and Zhang Qianshan extended growth gift bags to the representatives of the assisted children. Lin Yongjing, party secretary of the Communist Youth League of Heze City, reported the implementation of the "Hope House" work in Heze city in 2021, and announced the launch of the construction of the "Hope House" in 2022.

2021-12-25	In Fuyang city, Anhui province to carry out the "warm winter" public welfare action in Anhui Fuyang city to carry out the "warm winter" public welfare action me YaoTai han sea charity special fund, the communist youth league Fuyang city heart public social work service center branch in Fuyang YaoTai ocean park jointly launched "left-behind Eva smiled" -Fuyang city "warm winter" public welfare action. During the activity, 50 left-behind children from the Sanyi nine-year school in Xingliu Town, Yingquan District, received a "warm winter love gift package" donated by people from all walks of life. After the activity, the children watched the sea lion performance and the dolphin performance under the leadership of the volunteers and the staff. After the launch of the action, Yaoshan Ocean Park also carried out the public welfare activity of "scarf mother winter needle love" for the society. Loving people and volunteers can get free "love knitting bags" and personally weave scarves, hats and other items for left-behind children. At the scene, leaders of Yingquan District Civilization Office, Fuyang Heart Public Welfare Social Work Service Center, TuanYingquan District Committee and other relevant departments, as well as "Chinese Good Man" Wang Xia and 50 left-behind children participated in the activity.

2021-12-24	The Qingdao Communist Youth League has felt the warmth of the city from a place called "youth talent station". At present, Qingdao city has built 18 "young talent post stations". For the majority of foreign students who come to Qingdao to find jobs, the young talent station is like the "first stop of warm heart": providing one-stop services of free accommodation, policy consultation, job promotion and other services within 7 days, so as to solve the problem of "transition period" for young talents to Qingdao. In Liao's view, this is "very intimate to students who have just graduated and saves a lot of money."Since the development of party history learning and education, Qingdao Communist Youth League has accurately focused on the needs and expectations of the young people, steadily carried out "I do practical things for the youth", serves the young people in an all-round and multi-angle way, promotes the transformation of learning and education achievements into the vivid practice of serving the young people, and effectively improves their sense of gain and happiness. To achieve smooth employment and successful entrepreneurship is the key to the growth of young people and realize their dreams. Qingdao Communist Youth League aimed at the needs of the youth, to help the youth employment and entrepreneurship. Since this year, Qingdao Communist Youth League has held 10 cloud recruitment fairs for Qingdao young talents and special job fairs for universities, realized "online employment service, online recruitment", organized and carried out "Green Bird Project Skills Improvement Season", and accurately meet students' needs through campus job fairs and talent policy publicity in Qingdao. Lu Chuan (not his real name), who has participated in the summer internship, said, " This is a good combination of theory and practice. I cherish this opportunity and hope to return to continue to work here after graduation."Aiming at the pain points and difficulties of youth entrepreneurship, the Qingdao Communist Youth League has opened up the" capital chain "to empower youth innovation and entrepreneurship by deepening the youth innovation and entrepreneurship pioneer action, and built a" service chain " to help youth innovation and entrepreneurship. Among them, the "Youth Innovation Pioneer Legal Escort" action organized 60 legal service stations and 230 young lawyers to provide legal services for more than 430 times; connected with Qingdao Rural Commercial Bank and other financial units, and provided low-interest loans for Qingdao outstanding youth innovation and entrepreneurship. More service lists are launched: the first 100 young entrepreneurship mentors to carry out entrepreneurship training, entrepreneurship guidance, pair assistance, etc.; Youth Innovation Pioneer Civilization Post customized "one person to run the service chain" for young entrepreneurs; establish a comprehensive youth innovation and entrepreneurship service platform and capital enabling platform, etc. For rural talents, Qingdao Communist Youth League has steadily promoted the "Rural Good Youth Enabling Action", adhering to both "expanding the vision of tree selection" and "strict audit", and actively building a stage, providing opportunities and creating conditions for young people to serve in rural grass-roots services. To this end, Qingdao Communist Youth League implemented the rural revitalization youth contribution action, launched five youth campaigns of "Rural industry transformation and upgrading" and "Rural talent cultivation", held 17 "Rural Revitalization youth Lecture Hall", selected more than 2000 rural good youth, organized 800 youth league organizations and youth social organizations to help 2000 rural youth entrepreneurship leaders, and actively build various communication platforms to help youth development. The "Love Qingdao, Make Qingdao Better" youth parlor activity held in the city's youth league organizations at all levels is another major measure. The meeting room focuses on the areas of concern to young people, listening to the demands of young people, linking to the various resources, and doing practical things for young people to solve their problems. Up to now, the meeting room has invited more than 20 business departments, more than 50 experts in related fields, for more than 300 young people in different industries to carry out more than 40 various activities. The Qingdao Communist Youth League has also actively set up a volunteer service platform. Ten volunteer service online courses, including "Volunteer Recruitment" and "Understanding Volunteer Service for Large-scale Games", have been launched, providing a volunteer service practice platform for large-scale competitions. It has organized nearly 10,000 young volunteers to participate in volunteer service during the 2021 Qingdao Marathon and the Boao Forum for Asia Global Health Forum Second Conference. To mobilize social forces to help teenagers living in difficulties, the Qingdao Communist Youth League gives full play to its organizational advantages, coordinates social resources, and promotes the healthy growth of teenagers with practical actions. In the process of promoting the construction of the "Hope House" child care project, Qingdao Communist Youth League expanded fundraising channels, actively explored the new mode of "Internet + public welfare", and extensively mobilized youth league members to donate money. In 2021, a total of more than 5.2 million yuan was raised for the construction of "Hope House" and 112 "hope houses" were built. The Qingdao Communist Youth League also helps the assisted children to achieve the "spiritual rejuvenation" through the "cabin rejuvenation" by carrying out volunteer services and pair assistance. In addition, Qingdao Communist Youth League also strives to mobilize social forces to help the children in difficulties realize their wishes; cooperate with related enterprises to provide pair assistance and summer care for the children of migrant workers. In the next step, the Qingdao Municipal Party Committee of the Youth League will closely follow the difficulties, pain points and obstacles in the growth of youth, establish and consolidate the long-term mechanism of "I do practical things for youth", centering on other actual needs of youth employment and entrepreneurship, volunteer service, friendship, social integration, and rights and interests protection, and help the growth and development of youth. China Youth Daily China Youth network reporter Xing Ting correspondent Wang Li Zhao Xiaochen

2021-12-23	Fujian Fujian: tree 100 "crucial 120 days" double 'youth meritorious action "advanced collective Fuzhou: choose tree 100" crucial 120 days' double 'youth meritorious action "advanced collective Ji' an-wei zhang correspondent Xu Jingxian) on December 22 morning, practice new ideas merit new Fuzhou" crucial 12 0 days' double for "youth meritorious action" style display activities held in taijiang district "cangxia family" life hall. The activity selected the tree "attack 120 days of 'double struggle' youth contribution action" 100 advanced collective. Lin Jian, deputy secretary of Fuzhou Municipal Committee, Li Teng, deputy secretary of Fujian Provincial Committee of the Communist Youth League, and Huang Jianxiong, member of the Party group of Fuzhou Municipal Government attended the event and presented plaques to the advanced collective representatives. The activity was guided by Fujian Provincial Party Committee of the Communist Youth League, and jointly organized by Fuzhou Municipal Party Committee, Municipal Development and Reform Commission, Municipal Industry and Information Technology Bureau, Municipal Self-regulation Bureau, Municipal Commerce Bureau, Municipal State-owned Assets Supervision and Administration Commission and other units."The 120 days of 'double competition' youth contribution action" is the Youth League Fuzhou Municipal Party Committee around the center to serve the overall situation, implement the spirit of the 19th CPC Central Committee and the spirit of the provincial and municipal Party Congress. Activities combined with the municipal committee of the "build six cities, start five international brands, the implementation of nine special action" deployment requirements, in the investment, relocation, technical expansion, speed up production and other fields to strive for the youth civilization, encourage the majority of young people to actively participate in the construction of modern international city. In the style exhibition activity, the young people showed their achievements through group mouth allegro, music and sand painting, poetry recitation, chorus and other forms, and expressed their responsibility and strength to participate in the "double competition".

2021-12-23	The Youth League Zibo Municipal Party Committee took multiple measures to create the "youth community". Correspondent for figure China youth network Beijing on December 23 (xinhua Zhang Jianwei, correspondent li xiang) since this year, Zibo committee earnestly implement the communist youth league of Shandong provincial party committee urban and rural community youth into action work deployment and "I does the practical work for youth" work requirements, in the process of building "youth community" to explore the practice of "youth cultivation, popular science knowledge, culture service" into the community and N characteristic project "3 + N" mode, inject youthful vitality for grassroots social governance. Recently, the Shandong Provincial Party Committee of the Communist Youth League determined that 8 communities such as Jijiacun Community, Fangxian Town, Zhangdian District, Zibo City were included in the first 100 "youth communities" in the province, and the number of communities ranked the third in the province."Youth popular science" into the community. To focus on the needs of young people and promote the creation of precision and efficiency. We carried out special research on the needs of community youth, and solicited and collected their opinions and suggestions in an all-round way. All municipal, district, county, town and community league cadres participated, and visited more than 2,800 young people. Formulate the "Implementation Plan on Promoting the creation of" Youth Community "", hold the city's "youth community" creation work promotion meeting, establish the pilot "youth community" work ledger, clarify the list of community service needs and the list of league organization work projects, highlighting the participation and role of teenagers."Youth popular science" into the community. Correspondent for the map to build a resource platform, link to long-term service projects. The city's "youth community" creation training and resource docking exchange meeting was held, 16 social organizations in various fields, project leaders and 21 pilot community party committee (branch) secretaries and leaders of the league organization service project matching and docking, reached a number of landing intentions. Link city youth science and technology education association, city youth artists association and other social organizations in the community landing "youth science" "art school", "wisdom" "etiquette training" and other public service projects, districts and counties simultaneously carried out the "group hosting", "wheat ta" "green classroom" "like Yang partners" "TikTok station" has the characteristics of the communist youth league. Shandong University of Technology practice group into the "youth community". Correspondent for pictures to carry out "youth cultivation", stimulate the endogenous power of the community. Through the introduction of centralized training by professional social work organizations and on-the-spot interviews, the secretaries of the pilot community party committees (branches) will be guided to "personally grasp", and the heads of youth league organizations, social workers and grid members will "work together". We will actively integrate the talent advantages of the community local youth working committee, young talent association, college students, youth in emerging fields, and "rural good youth" to serve the actual needs of community residents differently. Relying on the science and technology counselor team of the Municipal Youth Science and Technology Education Association, the application of science popularization facilities for community workers to improve the utilization efficiency of science popularization positions. Contact professional social organizations to enter the "youth community" dispatched team, regular support community youth work to promote promotion.

2021-12-22	Fujian Quanzhou held the first state-owned enterprise youth talent training in Quanzhou the first state-owned enterprise youth talent training (green horse class) successfully held on December 22 (reporter Zhang Jianwei, reporter Guo Yang bin) recently, Fujian Quanzhou municipal party committee, its Quanzhou power supply company jointly organized the first state-owned enterprise youth talent training (green horse class), the city state-owned enterprises at all levels, a total of 42 students trainees and complete the training course completion. The training to young marxist training project as the core, invited the party history learning education provincial party committee, municipal party committee XuanJiangTuan members, overseas Chinese university doctoral supervisor, professor Lin Huaisheng, Quanzhou evening news deputy editor, senior reporter wang, Quanzhou municipal party committee party school professor Yang, associate professor Feng Weiwei famous mentor, around the spirit of the sixth plenary session of the 19th party spirit of Fujian province, Quanzhou 13th party spirit, the current economic situation and public opinion crisis public relations theme, for all the students. It is understood that the 42 students in this training course are selected in accordance with the strong political quality, good theoretical foundation, strong willingness to study, high work performance recognition standards, from the city's all levels of state-owned enterprises, outstanding young people. Students who have a particularly excellent performance after the training will also be included in the city's "Qingma Project" talent information database, and they will focus on the recommendation. In the next step, the Quanzhou Municipal Party Committee of the Communist Youth League will regularly hold the "Yangma Project" training course in various fields to guide the majority of the youth of overseas Chinese hometown to firmly listen to the Party and follow the Party, and strive to grow into qualified builders and reliable successors of the cause of socialism with Chinese characteristics.

2021-12-21	The 12th Hebei Province Handan City Youth Innovation and Entrepreneurship Competition held the rematch of the Dancheng City Youth Innovation and Entrepreneurship Competition. The second round will be held both online and offline, divided into project roadshow and judges' questions and comments. The competition jury is composed of university professors, cultural and creative experts, and leaders of design enterprises, which conducts a comprehensive evaluation on the theme, innovation, and practicality of the project works. After a two-day competition, 22 entries will emerge from 75 final entries and will compete for the highest award in the final. The competition with "creative youth enjoy Handan" as the theme of youth and design, aims to dig Handan profound cultural heritage, launched a batch of characteristics of Handan products and urban gifts, cultivate a batch of youth and talent, build a entity enterprise and creative team negotiation communication platform, power Handan regional cultural tourism center construction, further enhance Handan "idiom, tai chi" city brand influence. Since the launch of the competition in early October 2021, a total of 313 teams and individuals from inside and outside the province have registered, and received more than 630 project works, covering Handan characteristic gifts, intangible cultural and creative products, local specialty packaging, etc. In the near future, the competition organizing committee will hold the final, and carry out a series of supporting activities such as network display, project promotion, training and guidance, negotiation and exchange. The activity is jointly organized by the Communist Youth League Handan Municipal Party Committee and Handan Bank.

2021-12-21	Weifang: reform and innovation stimulate rural revitalization of "green power" to explore party hope, country, the communist youth league can work path of Weifang: reform and innovation inspire rural revitalization of "green power" active bear, positive, give full play to their advantages, to implement rural revitalization of "green power" is suggested, explore a party hope, rural needs, the communist youth league can work way, for Weifang innovation improve "three mode" (namely xi general secretary has praised "model" "Weifang model" Weifang model "shouguang mode"), to speed up the building rural revitalization of qilu model zone accumulated surging youth energy. In the field of Moujia Town, Gaomi, Weifang, Wang Weili, 32, is busy harvesting spinach, which is the first order of procurement cooperation between a well-known domestic e-commerce platform and a ten-mu family farm. As a good rural youth elected by Gaomi, Wang Weili explored the creation of the "three societies in one" farmer interest connection model, which increased the village collective income by 150,000 yuan annually, and the average annual income of farmers by 100,000 yuan. The core of implementing the rural revitalization strategy lies in people, and youth strength is the key. Through the multi-dimensional working mechanism of "selection, training and education", Weifang Communist Youth League cultivates "leading geese", drives a large area, guides young people to grow up in the rural areas and empower the revitalization. Through the innovative implementation of the selection and cultivation plan of "Good youth in every Village", the CPC Weifang Municipal Party Committee has explored the "political chain" and "industrial chain" double-chain cultivation mode, which has cultivated a large number of effective forces who firmly follow the Party and are responsible for rural revitalization. In the "political chain", Weifang Communist Youth League brought the rural good youth into the talent work system of the organization department, set up the young talent party branch in the towns and streets, and promoted the rural good youth into the training vision of the organization department. This year, there are 1,225 rural good youth into the village "two committees" team, 99 rural good youth as the village party branch secretary. On the industrial chain, explore the "the communist youth league organization personnel, training school arrangement teachers, vocational college to provide security" training mode, 14 training in rotation more than 1000 rural good youth, rural good youth alliance, and actively promote "rural good youth loan", "rural revitalization of good youth loan", etc., for rural entrepreneurial youth loans of more than 300 million yuan. Rural revitalization should not only be driven by good young people, but also involve tens of millions of young people. Weifang Communist Youth League takes multiple measures, around the ideological and political guidance, implement the youth teaching group "four advance" action into rural, community, enterprises and schools, make the new ideas become the beacon of the rural youth, actively organize the rural youth marketing, agricultural planting and other training, around the rural customs and customs, green and environmental sacrifice activities, make the new fashion become the consensus of the rural youth. Today, the city's 16,000 rural good youth active in the fields, the grass-roots line. Gao Yuanliang is a post-90s entrepreneurial youth. Thanks to the support of the local Youth League committee and the follow-up support of Weifang Youth Innovation and Entrepreneurship Competition, in recent years, his enterprise has introduced perfect imported chain technology and equipment, and built the first foie liver demonstration base in line with international standards. After the completion of the base, it will greatly improve the output and quality of products, and will also drive more than 600 local farmers to increase production and income. The higher education here is rich in resources and strong strength, especially agriculture, in the forefront of vocational and technical education. How to bring this advantage into the hot wave of rural revitalization? Weifang Communist Youth League has walked out of a unique way- -the implementation of "school-local cooperation" to help rural revitalization action, through the two focal points of local and university youth league organization, set up a "high-speed channel" of resource introduction, demand handed out, and "youth power" surging. Centering on the connection between the two resources of the university, the Weifang Municipal Party Committee of the Communist Youth League organized the Communist Youth League committee of all counties, cities and municipal universities to pair up through "one-to-one" and "many-to-many" ways. County and urban youth League committee actively docking relevant local government departments and rural entrepreneurial youth, deeply mining the technical and industrial needs in rural revitalization; university youth League committee will extensively contact the resources of universities, especially vocational colleges, accurate matching and coordination with local needs, change individual contact, fight alone for efficient docking and coordinated operation. At the same time, the Weifang communist youth league will "project, chain, platform, systematic" concept through every aspect of the work, through the youth innovation entrepreneurship competition, gathering high quality from all over the country and agricultural industry project, and through the whole chain tracking, service, support, foster youth power rural revitalization of the industry "army". The "incubator" built through various youth innovation competitions has unique advantages in promoting long-term development and providing soft services well. Relying on the follow-up tracking service mechanism of the competition, Weifang Communist Youth League actively matches the "seed players" in these rural industries, and won credit products such as "rural good youth loan" and "Luqing benchmark loan" to support the horse and send a ride."The rooms before were empty, dark and damp, and now the rooms are bright and orderly, fashionable and elegant, has really undergone earth-shaking changes!"Xiao Ying, 14, of Weifang Changle, described it as his new" hope hut " in his composition. Weifang Communist Youth League deeply combined with the practical activity requirements of "I do practical things for the masses", focusing on doing practical things and good things for the teenagers, so that the rural youth groups can share a happy life and soak up the sunshine and rain together. By further promoting the "Hope House" child care project, the Weifang Communist Youth League has built a warm shelter for the vulnerable teenagers in rural areas, and helped the children achieve the "spiritual renewal". In financing, Weifang Communist Youth League focuses on the use of social mobilization and network fundraising channels to raise 16 million yuan for the whole society; in the construction process, encourage cadres and young volunteers to participate in the planning, and formulate supporting policies to award and subsidize the county Youth League committee. For each hope hut built at the grassroots level, the municipal Youth League Committee helps to obtain 3600 yuan for the subsequent cottage construction. Up to now, 829 cabins have been built in the city, benefiting 854 children in distress, and more than 350,000 people have participated in the cabin fundraising construction and volunteer services. To consolidate and expand the achievements of poverty alleviation with the comprehensive promotion of rural revitalization, the education of rural youth is always the key, but also the concern of the Weifang Communist Youth League. In recent years, Weifang Communist Youth League has continued to carry out the "one-to-one love student aid" activity. Through the Hope Project platform and the online public welfare platform, donors can choose the recipients and regularly donate grants to students from poor families. Up to now, the city has subsidized 274 students from poor families, and granted 646,200 yuan in grants. The Weifang Communist Youth League has also found a new breakthrough to help rural revitalization through the integration of urban and rural community youth. We will carry out the centralized action of jointly building youth communities, select 34 urban and rural connecting communities as examples, coordinate youth volunteer service forces into the community, and fully integrate community resources such as community activity room and new Era civilization practice center, to create an upgraded version of "Youth Home". Zhao Zhonghua, Party secretary of Weifang Communist Youth League, said, " We will continue to give full play to the organizational advantages of the Communist Youth League, take the 'youth power' of rural revitalization, encourage more young people to participate in the practice of rural revitalization, make our countryside more beautiful, livable, happy and civilized, and make new and greater contributions to building a model of rural revitalization in Qilu."China Youth Daily, China Youth Network, reporter Xing Ting, source: China Youth Daily, December 21,2021, edition of the 01st edition

2021-12-22	Jining City to study and implement the spirit of the sixth Plenary session of the 19th CPC Central Committee special seminar held Jining City to study and implement the spirit of the sixth Plenary session of the 19th CPC Central Committee special seminar held the site. Correspondent for the picture of China youth network Beijing on December 22 (reporter Zhang Jianwei, correspondent Bai Chen) recently, sponsored by the Youth League of Shandong Province Jining Municipal Party Committee, Jining vocational and technical College undertook the Jining City youth study and implement the spirit of the sixth plenary session of the 19th CPC Central Committee special seminar was held. Yu Lingdong, a member of the municipal publicity group and secretary of the municipal Communist Youth League, made a comprehensive interpretation and in-depth interpretation of the spirit of the sixth Plenary session of the 19th CPC Central Committee. More than 100 teachers and students representatives attended the activity. At the special seminar, three member representatives of Jining City "Youth Publicity Group" from different industries and different positions, combined with their own work and learning reality, to share the vivid practice of transforming their learning results into their work enthusiasm and work results. Zhang Lili, a teacher of the School of Marxism of Jining Vocational and Technical College and the third prize winner of the Jining Micro Party Class Competition, with the title of "Drawing the wisdom and strength from the hundred years of the Party history of the Party", Shared the four historical periods and great achievements of the century of struggle; Ma Dengyue, member of Jining Youth Federation, Jining May Fourth Excellent Youth, secretary of the Party branch of Shandong Zhibo owei Education Group, with the title of "contributing youth strength in the new journey", I shared my thoughts, thoughts and insights in the process of growing up, In the green language to tell a vivid example; Jiang Yuying, a grid member of Xianying Street, Rencheng District, and the third Jining Red Story Explanation Competition, took the title of "Learning the spirit of the Sixth Plenary Session of the 19th CPC Central Committee to be the successor of the new era", I shared my work at the grass-roots level. Next, pku will organize cadres at all levels, youth branch members, youth league members, etc., to the topic as the breakthrough point, in the form of combining online, regions, batches into the campus, into the community, into the enterprise, into the "two new" organization, to carry out universality, focus, interactive theme.

2021-12-21	Jining youth moot court contest performance held Jining youth moot court contest performance and youth constitution publicity and education activities held on February 21 (reporter Zhang Jianwei, correspondent Bai Chen) recently, "rainbow umbrella method with youth" Jining youth moot court competition performance and youth constitution publicity and education activities held in Shandong Jining city high-tech zone court. The Municipal Working Committee, the Municipal Public Security Bureau Party Committee, the Municipal Youth League Bureau, the Municipal Education Bureau, the Seventh Procuratorial Bureau of Justice, the Municipal Procuratorate, the Municipal Intermediate People's Court and the high-tech Zone attended the event. The county and district youth League committees, excellent organizational units, excellent instructor representatives and participating students representatives participated in the activity.site of activity. Correspondent for figure activity review the moot court contest moments, won the first prize in the contest of four schools of Jining Confucius international school, Jining HuoJia street primary school, Jining experimental junior high school, Jining haida line know school for the field performance, and for the competition excellent instructor representative, excellent organization units, the award-winning schools. At the beginning of the year, the "Rainbow umbrella method with youth" Jining Youth moot court competition activities, more than 70 schools in the city, nearly 3,000 primary and middle school students participated. The video of the event will be broadcast through new media platforms such as wechat public account.

2021-12-20	Wuzhong city held long-term youth development planning association Wuzhong city long-term youth development planning association meeting and organize youth in modern beautiful new Wuzhong construction kick-off zhang) for in-depth study and implement general secretary xi important discourse about youth work, summarizes the city to carry out the Ningxia hui autonomous region, medium-and long-term youth development plan (2019-2025) ", recently, Wuzhong city held long-term youth development planning association and organize youth in modern beautiful new Wuzhong construction kick-off. Gao Jianbo, member of the Standing Committee of the CPC Municipal Party Committee and minister of the Propaganda Department attended the meeting and spoke. Peng Yong, deputy secretary general of the CPC Municipal Party Committee presided over the meeting, the leaders of the members of the fourth Committee of the Fourth Committee of the Communist Youth League and all the staff of the Communist Youth League Committee and more than 30 people attended the meeting. Conference conveyed the study party's sixth plenary session of the 19th, the autonomous region party committee 14th plenary session and the sixth party congress spirit, studied the Wuzhong city the implementation <about strengthening and improving the new era party building work condensed youth in zone construction opinion> implementation plan "the implementation of <district youth in" nine key industries "" ten "ten projects" four promotion action " ten measures> task division plan and solicit the opinions of the member unit Suggestions. Municipal Education Bureau, Human Resources and Social Security Bureau, Agriculture and Rural Affairs Bureau, health Commission four member units exchange speech. The meeting required that, standing at a new starting point towards the second centenary goal, all members should further mobilize the enthusiasm, initiative and creativity of the young people. To earnestly study General Secretary Xi Jinping's important remarks on youth work, to guide youth to bear the mission of The Times in the journey of building a modern and beautiful new Wuzhong; to strengthen the theoretical arm, to arm youth with Xi Jinping Thought on Socialism with Chinese Characteristics for a new Era, to uphold and innovate, to lead them; to focus on accurate service to ensure their better growth. The meeting stressed that youth is the fundamental force to ensure that the cause of the Party is passed on from generation to generation. All member units should be deeply aware of the great responsibility and glorious mission of youth work in the new era, always bear in mind the original mission, improve the standing position pattern, bravely shoulder the responsibilities, lead and mobilize the young people to work hard in building a modern and beautiful new Wu Zhong, and jointly write a new chapter in the development of the city's youth cause. Next, pku will take the meeting as an opportunity to further implement xi general secretary xi important thoughts about youth work, implement the city youth work joint conference office overall coordination role, under the support of various departments, strive to optimize the environment of youth development, strengthen youth policy integration, accurate focus on youth concern of key areas, coordinate relevant departments units launched more good youth development policies and measures, services, platform carrier, effectively build youth development ecosystem.

2021-12-19	Fuzhou green horse students in primary and secondary schools about the spirit of the sixth plenary session of the 19th hundred "green horse" students into primary and secondary schools about the spirit of the sixth plenary session of the nineteenth youth newspaper youth network trainee reporter Tian Hongwei) from Jiaxing red boat to two bombs, China's first liberation car story, on December 15 in the afternoon, a by college students to pupils to carry out the young pioneers theme team class held in Fuzhou. This team class, displayed by the members of the Qingma class of Minjiang University, is the first demonstration class of the spirit theme publicity activity of the sixth Plenary Session of the 1900 Qingma Central Committee of the "100 classes" of the 19th CPC Central Committee in Fuzhou. The launching ceremony was held in Golden Harbor Experimental School of Cangshan District, Fuzhou city. The activity was hosted by Fuzhou Municipal Party Committee of the Communist Youth League and Fuzhou Student Federation, and organized by the Youth League Committee of Minjiang University and Cangshan District Party Committee of the Communist Youth League. In the next three months, 100 college students from Fuzhou "Qingma Project" will go into the city's primary and secondary schools to carry out 100 group classes and team classes to preach the spirit of the sixth Plenary session of the 19th CPC Central Committee. The activity will invite relevant party and government leaders, experts and scholars, and advanced figures as "Qingma mentors" to improve the theoretical level of the students and provide theoretical support for the activity. First demonstration class, XuanJiangYuan around the sixth plenary session of the 19th "why we can succeed" "future how can we continue to succeed" and other topics, with small story interpretation, with small incision reflect big theme, explain the significance of the sixth plenary session of the 19th and two one hundred goals historical intersection of youth bear. Zhu strontium yuan, a junior student of Minjiang University. And she introduced that for the theme team class, the members of the publicity group studied the spirit of the Sixth Plenary Session of the 19th CPC Central Committee for many times, and discussed the class design and presentation."I transformed the theoretical study of the Sixth Plenary Session of the 19th CPC Central Committee into classroom practice, from learner to speaker, I gained a lot of knowledge in this process, which is also very helpful to my future teaching work."Shangguan Haolong, deputy secretary of the Fuzhou Municipal Party Committee, said that through the selection of college students with strong politics, firm faith and comprehensive quality to primary and secondary schools to preach the spirit of the sixth Plenary session of the 19th CPC Central Committee, this mode of "the Party leads the league and the league", is an exploration to guide the construction of ideological education for young people.

2021-12-19	Ningxia evalution push rural children "happy house" care project group Ningxia natural Guyuan municipal committee launched rural children "happy house" care project learning education "know" three "practice, focusing on" four promotion action ", based on the hope project quality upgrade, raise 150000 yuan innovation" happy house "care project, the natural Guyuan 30 rural children living room redesign decorate, equipped with the necessary furniture and learning articles, built" happy house ". After the launch of the project, the Youth League Guyuan Municipal Party Committee organized the Communist Youth League cadres to carry out household surveys, collect the basic family information, housing situation, transformation willingness and other information, form a "one household, one file" data sheet, select and determine 30 care and help objects and publicize them. According to the working process of "field measurement, planning and design, construction transformation, equipment, and completion acceptance", through socialization, standardization and project operation, according to 5000 yuan / home standard, and organize the special team to accept the cabin one by one, and the quality standard is not discounted, clear and manage, manage and use well. The Youth League Guyuan Municipal Party Committee has also established a "1 + 1" pair assistance mechanism, organized volunteers to regularly carry out family companionship, academic counseling, psychological counseling, wish fulfillment and other services, and strive to promote the transformation of the assisted children from "cabin renewal" and "life renewal" to "spiritual renewal".

2021-12-17	Group Quanzhou city committee push dream weavers plan gather emerging youth league Quanzhou city committee push dream weaver plan gather emerging young youth network trainee reporter Tian Hongwei) Fujian Quanzhou is the communist youth league central emerging youth group "dream weavers plan" contact one of the key cities, how to provide resources and platform for their growth and development, is one of the focus of the communist youth league Quanzhou municipal party committee. With goods anchor Zhang Qiuli has been suffering from no channel system to learn live with goods knowledge. Recently, she participated in the youth e-commerce live public welfare training class held by Quanzhou Municipal Party Committee."Different from the scattered content that we have contacted before, the live broadcast training held by the Communist Youth League Municipal Party Committee is more systematic and directional," Zhang said. " The training has gathered a group of aspiring young people to learn together and make progress together. The course content is rich and practical, and the teachers are also very professional. Friends are taking notes and taking photos on their mobile phones, for fear of missing important knowledge points."Kang Zhiwei, a training service provider, said that there were 20 modules, involving the construction and use of live broadcast rooms, live broadcast operation skills, fan traffic promotion and promotion, short video shooting and editing, Internet legal knowledge, etc. Up to now, four training courses have been held, inviting lecturers with rich experience in live broadcasting to give lectures through "theory + practice, case + analysis, demand + answer". More than 300 local young anchors and e-commerce practitioners have participated. To guide the emerging youth into the rural revitalization of work, the Quanzhou municipal party committee organization and coordinate five emerging youth community, led the revitalization of rural services, integration of youth league, territorial youth league, community organization resources, and territorial rural pairing, technical support, rural planning, industry consulting, electricity sales, talent training and other activities. In order to guide the emerging young people to participate in social governance and serve the poor people, the Quanzhou Municipal Party Committee of the Youth League and Fujian hip YAYA hip-hop association held several "Hip-hop Love Public Welfare performance" activities. All the proceeds from the performance will be used for students in need and art training for rural children. Under the background of the success of Quanzhou's World Heritage Site this year, the performance activities also combined Quanzhou intangible cultural heritage skills such as Nanyin and puppets with fashionable elements such as rap, graffiti and skateboarding to create a program with local characteristics. According to the head of the Fujian hip-hop YAYA hip-hop club, hip-hop is not only synonymous with fashion and fashion, but also a new way to express love and mutual assistance.

2021-12-16	Fuzhou launched "one hundred green horse hundred classes" theme publicity activities Fuzhou launched "one hundred green horse hundred classes" the 19th CPC Sixth Plenary session of the spirit of the theme publicity activities February 16 (reporter Zhang Jianwei, correspondent Fang Chen Wang Congcong) December 15 afternoon, The launch ceremony of the sixth Plenary Session of the 19th CPC Central Committee on the spirit theme publicity activity was held in Jinwan Experimental School, Cangshan District, Fuzhou City, Fujian Province, The activity was sponsored by the Communist Youth League Fuzhou Municipal Party Committee and the Fuzhou Municipal Student Federation, Minjiang University is organized by the Youth League Committee, Cangshan Youth League District Committee, Cangshan District Education Bureau, Cangshan District Youth Working Committee, About 150 people from Fuzhou universities, municipal "qingma project" college students, teachers and young pioneers attended the launch ceremony. The participants presented flags to the "ma Project" students from 8 Fuzhou universities and launched the theme publicity activities. The 100 college students of the Fuzhou Young Marxist Training Project will go to Fuzhou middle schools and primary schools to carry out 100 group classes and team classes, publicize the spirit of the sixth Plenary session of the 19th CPC Central Committee and the provincial and municipal Party congresses, and carry out diversified and characteristic publicity.site of activity. Correspondent for figure "hundreds of green horse hundred class" preach the party's spirit of the sixth plenary session of the first demonstration class, by minjiang college green horse class "youth salary classroom" XuanJiangTuan for CangShan gold harbor experimental school six (4) squadron of the young pioneers to follow the general secretary, struggle of youth set sail again theme class, college students preach with small story interpretation big truth, with small incision reflect big theme, to explain the significance of the sixth plenary session of the 19th and two one hundred goals history of youth bear. Lin Yuxiang, chief counselor of the Young Pioneers in Cangshan District, said that the college students are close to those of primary school students. They know what kind of class content and forms they like, which is easy to stimulate students' interest and enthusiasm in learning. The youthful vitality of college students 'lecturers has deeply infected the young pioneers, and this kind of college students' preaching to primary school students has produced a special chemical reaction between them.site of activity. Correspondent for figure group Fuzhou, deputy secretary of shangguan HaoLong said by selecting political quality, firm faith, comprehensive quality of college students to primary and secondary schools to preach the spirit of the sixth plenary session of the 19th, college students use understandable, lively simple language to speak, said the content of the students like, said the words have a strong affinity and appeal. Facing the characteristics of young people, facing the connotation of The Times, constantly innovate ways, through the mode of "the Party leads the league, the league leads the team", to explore new ways and new practices in guiding the ideological education construction of young people. The activity will also continue in Fuzhou primary and secondary schools 100 games, for three months, subsequent will be set up in Fuzhou "green horse mentor library", hire party and government leaders, experts and scholars, advanced figures and other teachers from all walks of life as a tutor, improve green horse class students overall theoretical level, for activities to provide strong theoretical support.

2021-12-16	Group Huludao municipal party committee to build mock court help youth growth middle school students judge Huludao municipal party committee to build mock court help youth growth wang) recently, Huludao municipal municipal court, Liaoning province, municipal judicial bureau and other units in Huludao city teachers' secondary school affiliated jointly carried out the "constitutional knowledge into the campus and teenagers moot court trial view activities", cultivate teenagers set up constitutional consciousness, create a good atmosphere of the rule of law education. With a clear sound of the hammer, a "juvenile version" moot court officially opened, this moot court activity is a mock trial with the theme of private lending. At the event, the students demonstrated from the announcement of the court discipline, checking the identity of the original and the defendant, to the court investigation, the court debate, the final statement, until the sentencing of the court, which enhanced the students' awe for the law. Lu Qinfang, deputy secretary of the Huludao Municipal Party Committee, said the mock court gave a special lesson to more than 800 teachers and students in the school. The mock court helps to innovatively carry out legal education for minors, which is conducive to strengthening the legal education for teenagers, enhancing their awareness of the rule of law, helping them to gradually develop the thinking habits and behavior habits of consciously abiding by the law, finding the law when problems arise, and solving problems by the law, and guiding them to participate in the practice of the rule of law. Since the reform of the grass-roots organization of the Communist Youth League at the county level, the Huludao Municipal Committee of the Communist Youth League has focused on the main responsibility and main business, gathered young people, protected the growth of young people, helped the modernization of urban social governance, and contributed to the construction of a higher level of rule of law and a safe Huludao.

2021-12-16	Tuanrizhao municipal party committee held a national symbol of day theme "spark reading" Tuanrizhao municipal party committee held a national symbol of day theme "spark reading" years net Beijing on December 16 (reporter Zhang Jianwei, correspondent XiangChang Hui) in order to further carry forward the spirit of patriotism, guide the young pioneers to remember history, do not forget the national humiliation, work hard, recently, the Shandong Rizhao municipal party committee held in the city study national symbol of day theme "spark reading". This reading club invited Li Fei, a member of the Rizhao Red Scarf Tour Group and the outstanding Young Pioneers counselor of Rizhao City, to lead the young Pioneers to truly understand the Nanjing Massacre with the title of "Inheriting the China" and remember the history of blood and tears, suffering and humiliation of the Chinese nation. Reading club activity site. Correspondent for the picture, Mr.Li teacher to "red letter" as the entry point, told the representative of the Communist hero story, and "take the baton, run the relay race" this image metaphor, encourage the team members with practical action to take over the hero of the baton. After the reading club, the young pioneers walked into the city study "Red Scarf Reading Corner", enjoy wandering in the sea of red books, to learn more knowledge of the party history, revolutionary history, example and pioneer stories.

2021-12-16	Tangshan Municipal Committee of Hebei held the key work of the Communist Youth League. Meeting convey learning about the implementation of the young pioneers counselor political quality promotion plan notice spirit, report around 2021 new development member input, social cohesion, middle school construction, prevent youth crime, information submitted assessment indicators completed, Tangshan counties (city, area), development zone (area) is mainly responsible for comrades report around related work communication, the Tangshan comrade is mainly responsible for the city of the communist youth league how to better work specific requirements. The meeting also conducted on-site training on the statistical work in the league in 2021. The leading group of the Tangshan Municipal Party Committee, the heads of all departments, counties (cities, districts), development zones (administrative areas) attended the meeting.

2021-12-16	Guangxi Hezhou "12355" help youth self-care education Guangxi Hezhou "12355" help youth self-care education 2355 youth service desk, to the Babu District Longshan Primary School to carry out the theme of "12355" into the campus and youth self-care education activities. By playing educational videos, distributing brochures, case introduction and other ways, the activity publicized sex education knowledge to students, and interspersed with the rule of law, drug control and AIDS prevention knowledge contest, so as to enhance students' self-protection awareness. In recent years, Hezhou 12355 youth service desk to "I do the practical work for the masses" as the gripper, "four haves" standard "namely" fixed place and necessary facilities, a unified name and logo, professional service team, fixed service project "to carry out the specific construction, explore the" TuanGan + social workers + volunteers "mode, normalized" 12355 " rule of law education, psychological counseling since the youth offline service activities. At the same time, the League Hezhou Municipal Party Committee focuses on training the "12355" volunteers to participate in offline activities, through the "12355" volunteers and "Guangxi volunteer" system contact channel, established the Hezhou 12355 youth volunteer service team, the "12355" volunteers into the "Guangxi volunteer" system management, issued the "Hezhou 12355 Youth Service desk Volunteer Management System", improve the assessment and supervision ability and improve the access mechanism, make the "12355" volunteers play a role, improve the ability of the Communist Youth League to serve teenagers.

2021-12-14	Group Rizhao municipal party committee to attend special recruitment and project promotion youth station Rizhao municipal party committee organized students to participate in Wuhan, Nanjing recruitment and special promotion Rizhao youth station Zhang Jianwei correspondent XiangChanghui), group of Shandong Rizhao municipal party committee to Wuhan, Nanjing in 2021 "sunshine-through schools" colleges and universities special recruitment activities, organize students to participate in the recruitment, and promote Rizhao youth station. Recruitment site. The recruitment activity is sponsored by the Organization Department of the Municipal Party Committee and the Municipal Human Resources and Social Security Bureau. During the activity, the city introduced the economic and social development of Rizhao, and introduced the talent policy of Rizhao in detail. More than 60 key employers have participated in the recruitment, providing more than 1,300 recruitment positions, including 38 public institutions and more than 300 career establishment positions, attracting college students from Wuhan University, Huazhong University of Science and Technology, Nanjing University, Nanjing Normal University and many other famous universities to consult and apply for employment. According to statistics, Wuhan and Nanjing two stations received 2223 resumes, including 19 doctors, 1888 master's degrees, and 719 people reached intentions. Public institutions provide a total of 315 recruitment plans, with 1,214 applicants. During the activity, the CPC Communist Youth League Municipal Committee gave full play to the organizational advantages of the Communist Youth League to undertake the organization and launch work of some students. The municipal and county youth League committees have actively contacted students from more than 20 schools, including Wuhan University, Central China Normal University, Nanjing University and Southeast University, to participate in the recruitment activities.site of activity. In the activity, the Communist Youth League Committee also promoted the Rizhao Youth Station. At the recruitment activity site, the convenient services such as free accommodation, employment and entrepreneurship policies, life and travel to the young students by means of yapo display and publicity leaflets. Rizhao youth station since operation, has built up "four" service system, through special publicity, experience, such as a variety of ways, youth station construction efficiency continues to improve, the cumulative service young students more than 360 people, contribute to more than 70 people to find the right job, become young students to Rizhao job employment warm heart first stop.

2021-12-14	Tongchuan: deep public service in the communist youth league "three promotion" Tongchuan: in the youth train, deep public service in the communist youth league "three promotion" zhang) informatization, intelligent age has brought the social environment, living habits and concept of profound change, how to make the new era of the communist youth league public welfare activities more youth needs of personal value, social integration and interest, shaanxi Tongchuan municipal committee with a four years of "youth train" public welfare activities give the answer. Implementing the goals of project, socialized and flat reform of the Communist Youth League, Accurately crack the relatively limitations of social resource mobilization ability of League organizations, The short board of vague awareness of efficiency and efficiency, Through the direct launch of the youth league organization and the efficient cooperation of the functional departments, Social organizations proactively follow up, Extensive participation of public welfare forces, Organize special youth public welfare campaigns to remote mountainous schools, deeply poor villages, happy communities, and rural revitalization and happy life, Effectively stimulate and orderly attract public welfare organizations and the dispersed public welfare organizations and forces contained in the society, Gradually established a 24 well-known public welfare teams covering the city, Tuan Tuan Tuan, with more than 1,800 people, It has realized the improvement of league organization and organization and service. The implementation of branding boosts the leading force. Pku cohesion will be "youth train" as the communist youth league to participate in social governance, in poverty completed important public welfare project brand, in normalized, precision, every year after in-depth research, listening face to face to determine the service direction, around a specific youth group, fine realize every wish, let every young volunteers, public welfare harvest friendship in the activities, the honor, awarded to "group public group" league organization, youth honor chapter. In order to improve the restriction bottleneck of the irregular development of youth social organizations and the lack of endogenous motivation, The Communist Youth League Municipal Committee won the support of the Civil Affairs Bureau and the Municipal Rural Revitalization Bureau, Start the implementation of the "benefit the youth and a total of well-off" Tongchuan city youth public welfare venture capital partner plan, And social organizations to help fight poverty action, Set up the project capital allocation fund of 200,000 yuan, Focus on supporting ten categories of projects, including poverty alleviation assistance, health assistance, psychological rehabilitation, and skills training, To serve the poor and vulnerable youth groups by means of project venture capital competition and project financial support, Focus on supporting and cultivating 15 outstanding public welfare projects, Extend the establishment of youth social organizations project pool, capital pool, talent pool, talent pool, It has realized the deep integration and improvement of the "social governance" work force, means of participation and service objects, Attract social organizations and public welfare forces to follow the Communist Youth League, To provide rich social resources for the continuous development of the "youth special train". Project-based construction to boost organizational strength. Before the Spring Festival in 2018, the first trip "to depth funding youth train", pku select seven depth is funding, collecting left-behind poor children micro wish 307, fully to clear activities in the implementation of resource investment, expected goals and social assessment, take online recruiting social accurate claim, reflect love put forward each micro wish no more than 365 yuan, public claim, public delivery, the influence of youth train firmly in the activities of 290 volunteers, 2019 micro wish coverage to 507 people, to further expand brand influence. In 2020, pku to carry out the consumption for poverty alleviation requirements, organization sales enterprises into poverty alleviation factory, poor home, preferred appropriate jun bitter buckwheat vinegar, corn dragon beard tea, walnut chili sauce, such as poverty alleviation products, to create 169000 yuan worth of "copper xiaoqing consumption for poverty alleviation package" mountain gift, in each package claim, will not less than 10% of the income as love public welfare fund, free to urban community difficult people, sanitation workers, migrant workers, public welfare jobs, send, realize the poor income, public love power, and the masses happiness promotion path closed loop, effect multiplication. In 2021, the Communist Youth League Committee, together with the Yancheng Communist Youth League Committee, launched the youth train for rural revitalization and happy life, and arranged the micro-wishes of 1,000 left-behind children in the city to gather the public love power of Yancheng to Tongchuan and realize the diversity of the youth train. Since 2020, the Yancheng Municipal Party Committee of the Youth League has funded the establishment of two youth "science and technology innovation dream classrooms" for the migrant relocation and resettlement communities in Tongchuan city, and the professional guidance team of "Tongren Technology" provides talent guarantee, and carries out artificial intelligence training such as data programming and 3D printing. Social mobilization boosts the improvement of service power. In the "youth train" public welfare activity organization, the whole chain of the Communist Youth League committee adopts the socialized launch, organization, implementation, summary and evaluation. In the fight against COVID-19, the Communist Youth League Municipal Committee quickly set up a "one and four wings" work structure with the Communist Youth League as the core and the Youth Federation, the Youth Entrepreneurs Association and the Youth Volunteers Association as the main force, and quickly set up 131 youth commandos to fight on the front line of the epidemic. In July 2021, Shangluo rainstorm flood disaster, pku WenXun move, the first time to the city's youth initiative, youth entrepreneurs, young volunteers, social love donations, in contact with Shangluo Red Cross docking, precision shortage supplies, city youth volunteers association, copper green home, N + 1 love student association active response, widely mobilize propaganda love enterprises, public welfare organizations donated 300000 yuan worth of bedding, flashlight, raincoat, rain boots, shovels, disinfectant and emergency medicine relief supplies. At the inaugural meeting of Tongchuan Youth Volunteers Association in August 2021, members of the Charity group became the mainstay of the first members. In key actions such as poverty alleviation, rural revitalization, and cultural protection, the Communist Youth League will continue to fully release its organizational vitality in social mobilization and resource integration. Pku key support of "sound for love" yellow ribbon public action, "copper Xiaoqing" home rural child protection system project, "mountain children see city" public summer camp and teenagers "hope" mental health science sand table activities cultivate a group of professional youth social workers, explore formed the "government funded authorization, social work team to undertake, the communist youth league coordination management" of personalized, customized service mode, make public social work team government service orderly powerful supplement.

2021-12-14	Guangxi Nanning held 2021 youth volunteer service project exchange fair Guangxi Nanning held 2021 youth volunteer service project exchange fair, city hope engineering office and city association jointly organized in 2021 Nanning youth volunteer service project exchange fair (hereinafter referred to as the youth rendezvous) was held in Nanning youth activity center. At the launch ceremony, the relevant person in charge of the Guangxi District Committee of the Communist Youth League pointed out that the youth Chronicles Fair is a very meaningful exploration. Young volunteers and youth volunteer service organizations should earnestly implement General Secretary Xi Jinping's series of important instructions on the cause of young volunteers, devote themselves to building a magnificent Guangxi of socialism with Chinese characteristics in the new era, and constantly promote the high-quality development of volunteer service. It is understood that the Youth Volunteers Fair is under the working mechanism of Nanning Municipal Committee Youth Volunteer Service Collaborative Innovation Center (hereinafter referred to as the Center), extensively integrating social resources, building a platform for communication activities, cultivating volunteer service organizations, and boosting the project, normalized and socialized development of the youth volunteer service work in Nanning. The youth volunteers Fair highlights outstanding, warm response. First, the overall coverage of the participating projects is wide, and the quality is higher than in previous years. A total of 29 youth volunteer service projects have been selected and competed for annual excellent projects. The projects have a wide coverage and higher quality than in previous years, covering rural revitalization, public welfare and environmental protection, student assistance, youth creation and employment support, assistance for vulnerable groups and other fields. Second, the function of resource integration of the center is highlighted. Since its operation in 2020, the center has raised 6 million yuan of funds and materials, and supported the smooth implementation of 29 volunteer service projects. A total of 13 projects between caring enterprises and youth volunteer service organizations reached a preliminary cooperation intention and signed a letter of intent to jointly implement the project, and the first batch of signing amount reached 330,000 yuan. Third, the participants are multiple. Both the traditional social volunteer service organization, volunteer service team in colleges and universities, also from institutions volunteer service organizations, in Nanning state-owned enterprises volunteer service team, private enterprises volunteer service and love enterprises and foundation, fully promote the parties in the project design, operation experience exchange, team building and extensive in-depth exchanges. In the next step, Nanning Municipal Party Committee of the Communist Youth League will continue to take the Youth Volunteers Fair as an opportunity to give full play to the "bridge link" function of the Communist Youth League organization, innovate social mobilization methods, explore and build the brand of youth volunteer service projects, and promote the high-quality development of youth volunteer service in Nanning.

2021-12-13	Group Taiyuan municipal party committee held "youth branch" first preaching Tuan Taiyuan municipal party committee held "youth branch" first preaching zhang correspondent wang) recently, Taiyuan, Shanxi province "youth branch" learning propaganda and implement the party of the sixth plenary session of the 19 and province the 12th party congress spirit first preaching activities held in the city library lecture hall. Yang Jiji, member of the Standing Committee of the CPC Municipal Committee and minister of the Publicity Department, Zhao Jing, deputy secretary of the Provincial Communist Youth League Committee and other leaders attended the event, and issued letters of appointment and certificates to the members of the youth teaching group and the good youth representatives of Taiyuan City. Municipal party history learning education eighth tour steering group responsible person on the scene to guide. Representatives of Taiyuan Taiyuan Youth Lecture Group and youth representatives from all walks of life attended the activity. Among the first five young lecturers, there is a three generations of cultural career, resolutely water the flower of cultural prosperity, and the youth pledge of "trust the party" into the new era in the green journey of high-quality development, the dedication, dedicated and dedicated to the children's hard gardener, and the young fighters in the anti-fraud line, and the "love power" to lead the new trend of youth learning model. The lecturers focused on the spirit of the sixth Plenary Session of the 19th CPC Central Committee and the 12th Provincial Party Congress to carry out publicity, and the party's innovative theory and the decision and deployment of the provincial Party Committee were popular and vivid explained. After the first lecture, the Communist Youth League Municipal Committee will continue to organize young lecturers to send them to the grass-roots level and to the front line.

2021-12-13	Qinhuangdao held rural revitalization of innovative entrepreneurship "cloud classroom" Qinhuangdao rural revitalization of innovative entrepreneurship "cloud classroom" in the green newspaper network reporter FanJiangtao) on December 7 solstice 8, group of Qinhuangdao municipal party committee, municipal bureau of finance jointly held in 2021 Qinhuangdao rural youth innovation entrepreneurship and "green horse project" rural revitalization, entrepreneurial youth backbone training courses. More than 200 students from the city's rural youth becoming rich leaders, rural entrepreneurial youth, rural e-commerce business youth, new agricultural business entities (youth) and youth staff from grass-roots township governments and two village committees participated in the training. According to introducing, the training class invite colleges and universities, party school and experts and scholars in related fields, around the party history learning education, dissemination education, rural revitalization, rural electricity, etc., respectively to the communist party of China in 100 "open the new era of law of China <civil code> spirit interpretation" the youth innovation entrepreneurship help rural revitalization "" short video, live with agricultural products marketing ", use in the form of network video teaching. After listening to the class, the students said that through the training, it has strengthened the systematic learning of all aspects of knowledge, which is of great help to enhance their own thinking, the awareness of the rule of law, innovation, entrepreneurship, and work. It is understood that the Qinhuangdao municipal party committee, municipal finance bureau has for many years rural youth innovation entrepreneurship project training, and training and "green horse project" rural revitalization, entrepreneurial youth backbone training, in-depth research understand students group actual needs, elaborate training teachers and course content, expanding the coverage of youth backbone, has achieved good results. In the next step, the organizer will continue to innovate the work carrier, enrich the work content, integrate social resources, and gather and lead the majority of young people to contribute their youth strength to the city's rural revitalization and economic and social development.

2021-12-13	Wang Xinxin) On December 10th, the Chengdu Municipal Party Committee of the Communist Youth League held the 2021 college student club style exhibition activity at Chengdu University. The event attracted about 6,000 associations from 65 universities in Chengdu. Through the combination of online and offline methods, the organizers selected 100 top 100 student associations and 30 best "happy Life associations" in urban construction, economic and social development, voluntary public welfare, culture and education, Tianfu culture and other categories. In the process of organizing the activity, the Chengdu Municipal Party Committee of the Communist Youth League actively guided the university associations to combine their professional advantages, contribute to the development of the city, and boost the creation of a civilized model city. Chengdu University hip-hop Dance Club, Chengdu Jincheng College Wushu Association, Chengdu Polytechnic University Electronic Robot Association and other societies show the perfect combination of Tianfu culture and modern technology through the integration of traditional martial arts and technological robots. Xihua University Knife Painting Association and other student associations depict the print of "Park City under the Snow Mountain" through the knife painting skills, expressing the college students' beautiful vision and confidence in the development of Chengdu. Tang Lu, president of Hanlin Yuxiu Traditional Culture Exchange Association of Southwest Petroleum University, said that the activity identified the key points of the concerns of schools and the needs of students, set up a stage for college associations to communicate, and unblocked the channels for college students to integrate into urban development and serve the society. League of Chengdu municipal party committee was also held here in the city university youth corps committee work exchange, in-depth study and implement the spirit of the sixth plenary session of the 19, around how to further pay special attention to the successor the fundamental plan to the university youth league work, strengthen the young college students' political thought lead, guide college students in national construction, realize self growth, etc., in the communication discussion, put forward the specific work measures.

2021-12-12	Wang Haihan, from Shushan District, Hefei City, Anhui Province), was established on December 9. So far, the 14 middle schools, 35 primary schools and 1 nine-year system school in Shushan District of Hefei city have all established a young working committee and carry out their work. This marks the further improvement of the organization construction of the Young Pioneers in the area, the further improvement of the institutionalization, specialization and systematization level of the work of the Young Pioneers, and the continuous expansion of the attraction, cohesion and influence of the organization of the Young Pioneers. In the afternoon, in the neat and clear team singing, the founding meeting of Jinhu Primary School Youth Working Committee was held. The Shushan District Committee and relevant responsible members of the school; Meanwhile, the Youth Working Committee held a book donation ceremony to donate learning and popular science books to left-behind children in remote township schools; all the young pioneers watched the first live broadcast of "Tiangong Classroom" together. The school young working committee responsible comrade introduced, after the establishment of the young working committee, will regularly continue to carry out lei Feng volunteer service, young eagle holiday team, theme research, party history learning education and other series of theme activities, to further build the young Pioneers work brand. For a long time, the Youth League Shushan District Committee has implemented the work requirements of the "Young Pioneers Reform Plan", and jointly cooperated with the education and sports departments to explore the young Pioneers organization construction, team construction and position construction. In the next step, the youth league Shushan District Committee will also rely on the position of the good school youth working committee, to perform the work responsibility of "leading the whole youth league", to realize the ideological guidance and growth of the young pioneers.

2021-12-12	Yantai, Shandong Province "Smoke employment Experience Day" activity reporter Xing Ting) December 9-10, "choose Yantai youth without free" college students "smoke employment experience day" and municipal organs "learning, practice" young party members forum was successfully held. The activity is organized by the Talent Office of the Organization Department of the Shandong Yantai Municipal Party Committee, the Yantai Municipal Party Committee, the Municipal Working Committee of the Yantai Municipal Party Committee, the Youth League Committee of China Ocean University, and co-organized by the Yantai Public Employment and Talent Service Center and the Party and Mass Work Department of the High-tech Zone. On December 9, the ocean university of China teachers and students have visited wanhua chemical group co., LTD., in Yantai environmental protection dredging co., LTD., Shandong green leaf pharmaceutical co., LTD., through field visits, listen, and enterprise executives, human resources manager, sea outstanding alumni face to face communication, understand the three enterprise development, corporate culture, future planning, salary, and so on and so forth. On the evening of December 9th, the teachers and students of Ocean University of China moved into the youth station. The full range of services and comfortable accommodation environment made the teachers and students of Ocean University really feel the "first stop" of the warm smoke. In their spare time, the young students read the youth post station and the young talent policy manual, and understood the talent policy of Yantai city in detail. On December 10th, "Choose Yantai youth without worry" college students "Stay in smoke and employment Experience Day" and municipal organs "learn, think and practice" young Party members forum was held in Chengyue Hotel, High-tech Zone. Group Yantai, deputy party secretary of zhang said, Yantai Marine development planning and industrial structure and the ocean university of China professional setting matching degree is very high, sea university teachers and students personally into Yantai key park, enterprises, project site view communication, can better understand Yantai economic and social development, enterprise talent demand, employment service policy, strengthen the identity of Yantai enterprise identity, city identity, cultural identity. He sincerely invited more young talents to join Yantai and share the development opportunities. Wang Yuhao, chief of the Development Planning Section of Yantai Development and Reform Commission, introduced in detail the two sectors of new Marine momentum and double-carbon new opportunities, and six advantageous industries such as high-end chemical industry, bio-medicine, food processing and electronic information. Yantai city public employment and talent service center talent treatment section deputy chief Cui Ni introduced in detail the introduction of talent living subsidies and housing subsidies and other policies as well as the application process of various subsidies. China ocean university youth corps committee practice secretary xing-yu zhou said the sea general will take this activity as an opportunity, around the "green bird plan", comprehensively strengthen cooperation with Yantai, form the enterprise to tripartite young talents foster win-win sharing new mode, for Yantai more high quality talents, power Yantai economic development of high quality.

2021-12-10	"Zhejiang" has gathered the youth forces against the epidemic and gathered the youth strength leg service, to send them vegetables, rice and other daily security supplies."Wang Pengfei, a Courier from Shangyu District, Shaoxing City, Zhejiang Province, is one of the volunteers who voluntarily contacted the league organization and joined the fight against the epidemic. After checking the travel code and health code, Wang Pengfei and his teammates followed the first group of volunteers to the Xihe Community in Shangyu District to carry out relevant volunteer services. Since December 5, COVID-19 outbreaks have occurred in Ningbo, Shaoxing and Hangzhou, Zhejiang province. The provincial Communist Youth League has acted quickly to join the front line of fighting the epidemic. The Shangyu District Committee of the Communist Youth League and the Organization Department of the Communist District Party Committee launched the call of "Party members of the list, I will contribute to the fight against the epidemic", and called together more than 3,500 party members of the anti-epidemic commandos. At about 11 o'clock on December 8, the "youth Shangyu" public number issued a volunteer recruitment order to the society, and more than 200 people signed up within an hour. In addition, the Shangyu District Committee of the Communist Youth League contacted the local Logistics Industry Working Committee of the Communist Youth League, organized more than 200 express delivery and delivery boys to participate in the epidemic prevention and control work, to participate in door-to-door investigation, point order maintenance, telephone contact personnel and other work. From 2 o'clock on December 7, Zhenhai District of Ningbo implemented closed management, in principle, "only in, not out". The Youth Zhenhai District Committee immediately organized and mobilized more than 2,000 people to participate in volunteer services for epidemic prevention and control."I will", "always be ready", "say where you need, and immediately be in place"... In the young volunteer group in Zhenhai District, we will respond to the front line of epidemic prevention and control. At 4 o'clock on December 7th, Fei Kejin, the head of Haigang Community Health Station of Longsai Medical Group, Ningbo Zhenhai District, received a notice and was assigned as the head of the nucleic acid sampling point in the playground of Guisi Primary School. She was on the scene just after an hour later. At that time, it was dark, she was not familiar with the terrain when she observed the situation of the sampling point and stepped on the empty, her right foot sprain and swollen into "steamed bread". Despite the pain, Fei worked at the nucleic acid sampling site and walked more than 10,000 steps on crutches. Jin Min, 29, is a thoracic surgery nurse at Li Huili Hospital. As one of the hospital's first medical staff to reinforce the Zhenhai district, Jin Min called her boyfriend before leaving, and the two decided to delay their wedding date. In order to ensure the supply of people's livelihood, "Keao Agriculture" of Zhenhai District Qingchuang Farm, with the help of young cadres in Jiulong Lake Town, rushed to harvest fresh vegetables to ensure people's livelihood during the epidemic. On the evening of December 6, the Linjiang community in Zhenhai prepared multiple trucks of food to ensure the daily life of the people in isolation, but the food distribution became a big problem. Therefore, all the staff of "Xiao 6 Buy vegetables" joined the separate vegetable packaging team, packed more than 1,000 pieces of food overnight, and sent them to the epidemic prevention line. At 9 o'clock on December 7,11 materials delivery vehicles, 35 people formed a delivery team, breakfast and instant noodles, Babao porridge, mineral water and other materials sent to Zhenhai � potential screen � auditorium and other nucleic acid testing sampling points."This is an overnight procurement, to the on-site medical staff, volunteers, you have worked hard!"Li Yubin, the donor of the supplies, said that he contacted volunteers from the Tianlu volunteer team overnight to provide a hot breakfast for doctors and volunteers on the front line. Nucleic acid testing, door-to-door mapping, point order maintenance, and close contact with the telephone contact of personnel... As of press time, more than 6,000 people have participated in the epidemic prevention and control volunteer service work in Zhenhai. On the morning of December 8, Zhejiang Youth Volunteers Association issued a written proposal, calling on volunteer associations and young volunteers at all levels in the province to actively and participate in the epidemic prevention and control in an orderly manner, and another group of youth forces are gathering. Hangzhou, December 9, China Youth Daily, China Youth Network reporter Jiang Yutong, correspondent Cong Yang source: China Youth Daily, December 10,2021, edition 03

2021-12-10	Anhui Bengbu: volunteers as the city "youth spokesman" group to build "field" volunteer service system of Bengbu: volunteers when the city "youth spokesman" after the annual meeting concluded, BBS chairman Yan Zhaozhu saw a group of young people dressed in red clothes, recognize them as accompany you 4 days of young volunteers, he took the initiative to take photos with volunteers. Press the shutter down, leaving a precious commemorative photo. The sixth annual meeting of the Taihu Lake World Cultural Forum is a feast of civilization and culture. During the activity, 131 young volunteers from Bengbu became the beautiful scenery. They were carefully selected and trained by 613 registered youth from the Communist Youth League Bengbu Municipal Party Committee, including 81 students and 50 social people who have already worked. In fact, there are all young volunteers at the International New Materials Industry Conference, the Anhui Provincial Games, and the Bengbu Music and poetry Festival celebrating the 100th anniversary of the founding of the Communist Party of China. Under the call of the youth League organization, the young people from universities, organs, enterprises, Youth Volunteers Association, Youth Enterprises Association, Youth Federation and other organizations showed their style in the activities. The process of training young volunteers is not accomplished overnight. The Communist Youth League and Bengbu Municipal Party Committee has been thinking about how to build a standardized volunteer service system of "no longer leave the field", and build a platform for young people to grow up and communicate. During the Taihu Lake Forum, the volunteers provided high-quality services to nearly 500 Chinese and foreign guests from more than 30 countries and regions in different positions."It is a rare opportunity to understand the cultures of different countries."Liu Chang, an English volunteer from the Bengbu Tax Bureau, has gained growth. Liu Chang, 28, is a native of studying abroad for her first participation in a large-scale volunteer event. On the bus to the hotel at the station, she adjusted her mood and took the initiative to introduce the local customs and characteristic industries of Bengbu to the South Korean mission. Unexpectedly, the other side successively interactive questions: " What is the professional setting of the university? What is the legend behind the name of Longzhu Lake? In "", from staying in the hotel to arranging the travel schedule, to checking and coordinating the daily schedule and translation, we are always present at the time, and every night, the volunteers will meet to review the work of the day."Liu Chang said," Many foreign guests said they want to know more about Bengbu. Volunteers should regard themselves as their hosts. We represent not only individuals and units, but also young people in Bengbu."" in the network literature sub-forum, I understand the experts' views on the new forms of literature and art."Du Hongyang, a post-2000 student from Bengbu Medical College, who is interested in traditional culture and has broadened his vision during the volunteer service process. Get up at 5 am every morning for breakfast and go to the venue to distribute conference materials. Guests have come one after another, and Du Hongyang wants to maintain order and accept inquiries."It's some tired, but every day is very full."Du Hongyang regrets that the volunteers are also the city image ambassadors. Through narration and etiquette expression, they provide information for foreign guests and build a bridge to convey the culture. Although Du Hongyang is young, he is already a "veteran" of volunteer service. In 2018, at the 14th Anhui Provincial Games, he signed up as a volunteer at the 14th Anhui Provincial Games in Bengbu. In the past three years, he has participated in more than 380 volunteer service activities, such as the International New Materials Industry Conference, free medical treatment in mountain villages, epidemic prevention and control card points and other occasions."Volunteers are first and foremost a devotee, and volunteering is not really easy."But he felt that through the training of volunteer service, he had developed the quality of facing difficulties and perseverance. In Bengbu, there are many enthusiastic young volunteers like Du Hongyang, but how to reasonably allocate and manage them to exert their talents has become a test question in front of the league organization. According to the staff of the Publicity Department of Bengbu Municipal Party Committee of the Communist Youth League, in the past, the youth volunteer service activities had some phenomena, such as scattered themes and unclear service scope. The youth league organizations of the city did "their own work", which cannot form a unified youth volunteer service brand, which weakened the attraction of volunteer service to young people. At the beginning of the year, the Communist Youth League Bengbu Municipal Committee formulated the annual volunteer service plan of "Happy Bengbu Youth Action" around the central work of the Municipal Party Committee, and determined the nine main action lines of "Offering the Party, Clean rivers and lakes, Clean villages, health and health, civilized New style, Clear network, caring for tomorrow, Sunshine Helping the Disabled, and Warm Winter Action". In the "Happy Bengbu Care for Tomorrow" action, more than 300 volunteers provided volunteer services such as handmade, academic guidance, family companionship and self-care education for left-behind children and poor children in groups. Song Haoyu, a student from Bengbu Technician College, is one of the volunteers. In the children's homes in the communities and streets, Song Haoyu has led the children to learn picture books, clay production skills, and vocal music and dance skills for many times. At the end of each activity, there will always be children asking Song Haoyu and others, " brothers and sisters, when do you come again next time? In the "Happy Bengbu Warm Winter Action" activity, the League Bengbu Municipal Party Committee, together with the Youth League Committee of Bengbu Railway Station, organized "clam Xiaoqing" and "Little Sweet Orange" to participate in volunteer services in transportation, Spring Festival transportation and epidemic prevention. Many young people have been affected and have signed up as volunteers. At present, Bengbu Youth League has organized more than 300 volunteer activities around the nine major actions, with a total of more than 20,000 volunteers. For a long time, the Youth Bengbu Municipal Party Committee has also explored the operation and brand building of youth volunteer service projects, and Wuhe County's "Xiaohe umbrella" public poverty alleviation volunteer service project is one of them. One day in the winter vacation of 2016, Wan Gongda, a teacher from Toupu Middle School in Toupu Town, Wuhe County, drove to the school. When he passed a crop field covered with snow, he found that there were no obvious safety signs beside the canal. Five primary students playing by the water were at risk of falling down at any time."What about your parents?"He quickly pulled over and asked about the children. The oldest child said, " My parents haven't returned home for the Spring Festival yet!"At that time, Wan Gong was shocked and wanted to do something for the left-behind children in his hometown. Later, he learned that the Communist Youth League Wuhe County Party Committee to carry out the "small lotus umbrella" volunteer service project, he took the initiative to exchange ideas to the Communist Youth League County Party Committee. Since then, every winter and summer vacation, the Communist Youth League County Party Committee will make the "anti-drowning" billboards, placed in the township remote ponds, and to the township schools to carry out anti-drowning propaganda. Since 2016, TuanWuhe County Party Committee has promoted the construction of 6 "Xiaohe umbrella" volunteer service centers and 19 "Xiaohe umbrella" volunteer service stations at the village level, and carried out activities such as youth self-care education and "star picking class". This year, as a pilot county of the national county Communist Youth League reform, the Communist Youth League Wuhe County Party Committee continues to integrate social forces, and the "Xiaohe Umbrella" project has gathered more than 300 volunteers from various industries."We mobilize the youth league organizations at all levels, youth civilization, youth social organizations, youth organizations to participate in 'happiness Bengbu youth action activities, improve the incentive mechanism, the cohesion brand, make volunteer service cohesion, service youth platform, let volunteers show the Bengbu city image' youth spokesperson, for happiness of Bengbu."Tuanbengbu municipal party committee director said. China Youth Daily, December 10,2021, edition 01

2021-12-09	A teacher was sentenced to life in prison for assaulting female students. This case can be successfully filed, but also from a "mailbox" start. In 2020, the Zhangzhou Municipal People's Procuratorate, the Communist Youth League and the Zhangzhou Municipal Party Committee, and the Zhangzhou Municipal Education Bureau jointly launched the "youth escort mailbox" mechanism combining online and offline. Offline is in Zhangzhou schools set up physical mailbox, responsible for management. Receiving local reports for help online, such as wechat mini program, email, hotline, etc. Shen Zhaojin, director of the seventh procuratorial department of the Zhangzhou Municipal People's Procuratorate, said that after the girl's parents learned that their child was sexually assaulted by a teacher, they left a message through the online "youth escort mailbox", and the procuratorate immediately supervised it. After filing the case, considering the child's future study and privacy, the Procuratorate, together with the Education Bureau and the Zhangzhou Municipal Party Committee of the Communist Youth League, handled the transfer for him and started a new study and life. Shen pointed out that many parents have a misconception that only when their children have obvious scars can they report to the police. By opening the "youth escort mailbox" in schools, is equal to parents and minors opened a convenient channel for demand, at any time, can be delivered at any time, help to protect the rights and interests of minors of the network woven dense and wide. In order to make the mailbox really play a role, 3 units together formulated the "campus" youth escort mailbox "cooperation memorandum". Among them, the physical mailbox selection point to take into account the convenience and privacy, convenient for students to go to school or recess activities when the delivery, and will not cause attention because of the delivery action. The height of the mailbox should be built according to the actual situation of the height of minors in school, and the help hotline and the QR code should be marked outside the mailbox. The memorandum also stipulates that the mailbox responsible for the delivery of the letter to the day, according to the specific situation of the event to contact the handling of the relevant departments. If the responsible commissioner delays in performing his duties or intentionally conceals clues, causing serious consequences, it shall be dealt with by the competent administrative department or unit according to the cadre administrative authority; if it constitutes a crime, the criminal responsibility shall be investigated according to law; if the relevant unit or supervisor prevents the staff from reporting the case, he shall be given a heavier punishment. Chen Luojia, head of the publicity and rights department of the Youth Zhangzhou Municipal Committee, said that he hoped he would never receive help or reports about the damage of minors, but from a practical point of view, the mailbox does have the value and needs. Since its establishment more than a year ago, the city's "youth escort mailbox" at all levels has received a total of 21 cases for help, involving minors being assaulted, school bullying, psychological counseling and other aspects. This summer, set up in the Zhangzhou Youth League social work agency Lu Binghua youth social work service center of the youth escort mailbox, received a number of minors by the training institutions teacher indecent report. According to Ding Rulan, deputy director of the center, the informant did not want to disclose his identity information or have direct contact with the judicial organs, only providing clues and evidence through the "youth escort mailbox" set up in the social work service center. This shows that the "flexible" work of the Communist Youth League can help the "rigid" work of the judicial organs under some special circumstances. After close cooperation from all parties, the teacher of the training institution was quickly brought to justice. Recently, the highest inspection and the central jointly issued " about the national juvenile procuratorial work social support system demonstration construction notice, clear from November 2021 to October 2022,80 regions across the country to carry out a one-year juvenile procuratorial social support system demonstration construction work, Zhangzhou selected as a pilot area. In fact, as early as 2019, Zhangzhou became the first batch of pilot areas for the Central Committee of the Communist Youth League and the Supreme Procuratorate to carry out the construction of the social support system for uninspected work. In order to promote the protection of the rights and interests of minors, Zhangzhou People's Procuratorate, Zhangzhou Municipal Committee and other departments have jointly established three teams of youth social workers, retired cadres, spring bud safety officers, and provided ideological guidance, accompanying support, psychological counseling and difficult assistance services; and entrusted the social work agencies to conduct psychological adjustment, deviation behavior correction and other services, and organized hundreds of adolescent mental health mentors in various rural primary and secondary schools. The Zhangzhou Municipal Party Committee of the Communist Youth League has also established a regular contact mechanism for NPC deputies, CPPCC members and the youth to build a platform for teenagers to express their interests and demands. At the fourth session of the 13th National People's Congress, the deputies of the Zhangzhou Municipal People's Congress made suggestions to the Ministry of Education on exploring and innovating the campus "youth escort mailbox" mechanism and severely cracking down on the sexual assault cases of minors. Source: China Youth Daily, December 09,2021

2021-12-09	Dingxi held to study and implement the spirit of the sixth Plenary session of the 19th CPC Central Committee symposium Gansu Gansu youth study and implementation of the spirit of the sixth plenary session of the 19th CPC Central Committee symposium Ma Fuchun) recently, Gansu Dingxi city held youth from all walks of life to study and implement the spirit of the sixth plenary session of the 19th CPC Central Committee symposium. Dingxi Municipal Party Committee Deputy Secretary Wen Weidong attended the meeting and made a speech. Wen Weidong stressed that, as a new era of Dingxi youth, to often jump down, sink to the line, and the masses want to work together, with the value of the gain and happiness of the masses; to innovation, enterprising spirit, take the lead to break the thought ice, innovative ideas, entrepreneurial consciousness and practical spirit, with the action to create real achievements, make due contribution to the high-quality development of my hometown. Zhang Jiang, deputy secretary of the Municipal Party Committee of TuanDingxi, always raised the banner of "revolutionary ideal is above the day" in the spirit of "the spirit of the day". Young representatives from many industries exchanged views and made speeches, and talked freely about their learning gains, experiences and work plans. Song Yun, the principal of Jiaoshan Primary School in Lintao County, said that the Young Pioneers are the main force to achieve the second centenary goal, and the Chinese dream eventually becomes a reality in their hands. She will turn the expectation of "centennial dream round" into a firm faith in the cause of the red scarf. As an industry leader, Cao Baiping, the head of Lintao County Xingping Peony Industry Company, is very proud of the great development and changes of the motherland, full of confidence in the future of his hometown, and more confident of his decision to return home to do business."We will continue to work hard to cultivate and attract more young people to join the flower industry, so that the countryside becomes a fascinating world, agriculture a desirable industry, and farmers an enviable profession."

2021-12-09	Taiyuan group backbone training camp party history learning education into the campus successful ChengJiangTuan Taiyuan municipal party committee to build "youth learning" group backbone training camp to party history learning education "voice" into the heart wei correspondent Wang Yishu) since the party history learning education, group Taiyuan party held "party history, strong faith, follow the party" - "youth learning" in 2021, the party history into the campus form, joint city league school invited provincial, municipal party committee party school, party history education related teachers, deep straight schools group backbone about the party history of the team. The training camp focuses on the history of understanding, credit enhancement, history of morality, history and practice, focusing on the study of the Party history, covering the new democratic revolution, socialist revolution, reform and opening up construction, and socialism with Chinese characteristics. Activities aimed at education guide Taiyuan city group backbone to understand the party's glorious history, feeling the party's beginner's mind mission, grasp the party's innovation theory, recognize the party's spiritual pedigree, inheriting the party's red gene, more consciously armed with xi new era of socialism with Chinese characteristics, better unite and condensed the youth members listen to the party, follow the party. In pku, municipal league, municipal league, all the school TuanGan organization, "learn party history, strong faith, follow the party" - "youth learning" in 2021 Taiyuan league backbone training camp party history learning education into campus training activities in recent successful packaged, the city's 78 schools to carry out concentrated training, the schools to carry out the independent training 277 games, covering 17469 group learning backbone.

2021-12-09	When the reform of the Communist Youth League, the Hedong District of Linyi, Shandong Province, grasped the construction of the youth league cadre team "ox nose" to open the "blocking point" to release the vitality of the grass-roots youth League organization and gained high popularity. As early as a year ago, the Wuyue Community, Hedong District, Linyi City, Shandong Province, was faced with a shortage of teenagers due to the majority of residents aged 20-40. And in the Hedong District grass-roots league organization, a similar situation is not uncommon. In order to supplement the work force, Hedong District launched the first batch of full-time youth affairs social worker recruitment examination in 2020. At 33, Wu Xiangling was successfully selected and served in Wuyue Community. In the past year, she innovatively carried out the building "micro-autonomy", cultivated the characteristic building culture of "I Yue Wu Jia Lou Yue Lou", created a wechat public account, and organized various "youth community" cultural activities on a regular basis. With the support of the special work funds of the Communist Youth League Hedong District Committee, the community music festival of spending 50,000 yuan has changed from "unthinkable" to reality."Echelon" with strong work force is mostly small, miscellaneous, difficult, but each thing connects the trust and recognition of the youth league organization, testing the work ability and faith of the youth league cadres to serve the young people. In fact, the grass-roots youth league organizations "no work, no money, will not do, do not want to do" the situation has a long history. In order to effectively solve this problem, Hedong District closely grasped the construction of the league cadres, strengthened the top-level design and support from the party and government level, thus further strengthening the political and social functions of the Communist Youth League, and revitalizing the vitality of the grass-roots league organization. Since the official launch of the reform of the county Communist Youth League at the beginning of 2021, the "echelon type" matching with strong working force has become the first measure taken by Hedong District. At the district level, it timely added 4 working forces of public institutions for the Communist Youth League District Committee, and was equipped with two temporary deputy secretaries and 5 part-time deputy secretaries. The cadre allocation rate and post rate within the establishment of the Communist Youth League District Committee reached 100% and were maintained normally. At the same time, in the township (street), by the secretary of the town and street party (work) committee as the director of the youth work committee, with 11 town and street youth league (work) committee secretary at the same time, for each town and street to recruit a full-time youth affairs social worker and as a full-time deputy secretary of the Youth League committee. In the village (community), 21 full-time youth affairs social workers were publicly recruited as the deputy secretary of the Youth League Committee. Liu Gen, secretary of the Party branch and Youth League Committee of Wuyue Community, described Wu Xiangling's role as "grounded" and "sticky". "With 8 years of rich street work experience, she not only serves as a bridge between residents, property management, streets and district departments, but also coordinates more resources to sink the community to directly serve the residents."The problem of solving no money, everywhere "begging for alms" dilemma was solved, the problem of money surfaced again. In the actual work, no money to handle affairs, everywhere "begging" is often the grass-roots cadres have to face the embarrassment. Take the Hedong District Committee as an example, the previous annual work expenditure was less than 200,000 yuan, while each rent for the local Grand Theater was 100,000 yuan. The Hedong District Committee had to try every means to save expenses and reduce the scale and number of activities. After full investigation, the party and government departments supported by the red document to the League Hedong District Committee full work confidence. The Hedong District Party Committee Office and the District Government Office jointly issued the Measures for the Management and Use of Youth Work Funds in Hedong District, which clearly included the youth work funds into the district-level financial budget. According to the above documents, according to the total area of 14-35 youth resident population, according to the standard of 8 yuan per person, set up 1.04 million yuan of youth work security funds, for position construction, learning exchange training, youth characteristic service, youth friendly city construction, etc., which for the grassroots communist youth league leverage more social resources provides the basic guarantee. After the establishment of the special work funds, the Hedong District Youth League organization for the number of practical work for teenagers quadrupled. In particular, we will organize a number of groups of rural young people to carry out e-commerce training in Shanghai and Hangzhou, so that rural e-commerce will blossom everywhere and give new momentum to the development of characteristic industries such as local hardware manufacturing and deep processing of agricultural and sideline products. Not only that, Liu Kehan, secretary of the Youth League Hedong District Party Committee, observed that more and more township (street) youth League committee secretaries had only completed the "prescribed actions" before, and instead began to take the initiative to consider the "voluntary actions" in line with the local reality. In order to improve the working ability of grassroots cadres, the Hedong District Committee specially issued the Opinions on the Implementation Plan of Promoting the Growth of League Cadres, which incorporated the training of League cadres training into the overall plan of cadre education and training in the district, and carried out ability training on a regular basis. Every year, we will recommend 30 youth league cadres with good comprehensive quality, excellent work and great potential to the organization department. Through temporary posts, exchanges and job rotation, we will send them to rural revitalization, petition and stability maintenance, demolition and construction for training, and become the backbone of promoting key work. In the Phoenix Ridge street project demolition site, a youth commando composed of 76 cadres have been fighting for several days. Most of them had no experience in demolition work and first went to people's homes and were kicked out in less than 20 minutes. With the promotion of the experienced old league cadres the new people, these members gradually learn how to bring the policy to the hearts of the people."Each group is a battle fortress, and these young team members dare to take responsibility, overcome difficulties, and have played a vanguard role in the demolition line."Phoenix Ridge street office director Sun Chengwei evaluation. Get the front-line experience of the group stem, in the service of the youth post burst out of energy. In just one and a half years, the cadres of the Communist Youth League in Hedong District raised 6.2 million yuan of social funds to build 300 hope cabins, which made the children in difficulties feel warm. Through the "actual performance", in addition to the centralized evaluation on the performance of cadres at the end of each year, and serving as an important basis such as excellent evaluation and post transfer and promotion, it also innovates to open up the rising channels of cadres outside the system. These include the part-time cadres and youth affairs social workers outside the system through targeted examination and talent introduction; and determining the full-time village (community) youth league secretary and youth affairs social workers with a reward of 300 yuan per month."No one, no money, no way to do it, and where is the way out?" - -Hedong District's reform is targeted and interlinked, forming a systematic, closed and virtuous cycle."It has effectively reversed the situation that the grass-roots cadres of the youth League are small, weak, and their role is not obvious. It has not only enriched the working force of the grass-roots league, but also realized the ability to go up and down, strong constraints and effective incentives, and effectively promoted the grass-roots league organizations to play a role."Liu Kehan regrets. With the advancement of the reform, Hedong District also issued policy documents such as the Implementation Opinions on Strengthening the Construction of Grass-roots League Organizations by Classification and Classification, especially paying attention to solving the problem of effective coverage of league organizations in the two new areas, and further activating the work of the Communist Youth League at the county level. As an Internet + cultural and creative enterprise, The annual sales of Jia Painting Culture and Creative ranks first in the same industry in China, and the average age of nearly 500 employees is only 28 years old. In 2019, after the establishment of the Youth League Committee, the company set up a demonstration post for league members, built a youth activity room, and opened up a learning conference room for league members. Young employees also spontaneously organized the "Anning Band" and held regular birthday parties every month. The rich league building activities and perfect learning system gave the warm family of talents from all over the country. According to statistics, since the reform, the number of Hedong District cadres from 179 to 410, including 92 cadres outside the system, full-time cadres rate 100%, hang, part-time cadres increased by 200%; organize 136 youth commandos, youth commandos 2200; 15 outstanding township (street), district league (work) committee secretary were promoted reuse, the grassroots cadres are exciting."Young people are the most active and dynamic force among the whole social force. Only when young people have vigor and confidence can the region have vigor and development."In hedong district party committee secretary Juan shinyo view, reform must cling to the" people " the key factor, must be around the cadre growth blocking point difficulty suit the remedy to the case, only the enthusiasm of young cadres initiative, truly solid foundation security, the work situation will open, can effectively drive more young people in regional development. China Youth Daily, December 09,2021, edition 03

2021-12-07	Jiangsu Taizhou, xinghua both volunteers to help "warm winter action" Jiangsu Taizhou, xinghua volunteers to help "warm winter action" morning), in Taizhou pku, jiangyan TuanOuWei, XingHua TuanOuWei guidance, xinghua dandelion volunteer association, jiangyan district pioneer public development promotion association, dainan town youth corps committee, dainan town veterans service station jointly held a "rural revitalization of warm winter action" public welfare activities launch ceremony, for XingHua DanNa town veterans, Ganzi Tibetan autonomous prefecture, Sichuan low income Tibetan, jiangyan district skin disease rehabilitation disabled elderly vulnerable people to send warm. On the same day, the activity distributed 400 yuan to each of the 31 veterans, purchased 395 sets of high-quality cotton clothes for 395 troubled teenagers and low-income Tibetans, and donated 50,000 yuan of warm winter donation of "Dream Transformation + Care Plan" to the Taizhou Communist Youth League Municipal Committee. In the afternoon, more than 30 volunteers from Xinghua, Jiangyan with winter quilts, bedding and other materials came to the Jiangyan District dermatology prevention and treatment of the Qinhu rehabilitation area to visit the disabled elderly. It is reported that the activity was initiated by Jiangyan Pioneer Public Welfare Development Promotion Association and jointly donated by Xinghua Dandelion Volunteer Association. It is a public welfare practice of volunteers from both places to jointly care for teenagers, veterans, disabled elderly and other special groups in Tibetan areas and other places. Shen Yan, deputy secretary of Taizhou Youth League (temporary post) and secretary of Xinghua Youth League, said that "Warm Winter Action" is an important public welfare brand implemented by the Taizhou Communist Youth League at the beginning of the year, and is a livelihood project to effectively consolidate and expand the achievements of poverty alleviation and rural revitalization. The annual "Warm Winter Action" has been fully launched. This year, the Taizhou Communist Youth League will focus on the "Dream transformation +" care and service objects, and carry out the action at the municipal level around the "three batches".

2021-12-07	Fujian Sanming: Fujian enterprise linkage write a new chapter for Tibetan students Fujian Sanming: care Tibetan students enterprise linkage write a new chapter on February 7 (reporter Zhang Jianwei, correspondent, Xiao Weijie Yu uli) recently, the Fujian Sanming municipal organization its Sanming power supply company power grid volunteers, Sanming college pomegranate seed studio volunteers to east middle school Tibet class "youth protect young volunteer counterparts" 125 international volunteer day activities.site of activity. Deng Ran, a teacher from Sanming School of Marxism, preached the spirit of the sixth Plenary Session of the 19th CPC Central Committee of the Tibetan class of Sanming School, encouraging students to feel the party, listen to the Party, follow the Party, inherit the red gene, and gather the strength to forge ahead.site of activity. A year ago, 9 Power Grid volunteers of State Grid Sanming Power Supply Company successfully paired up with 9 Tibetan students from Liedong Middle School. From unfamiliar to familiar, they enhanced the distance between each other and made the flower of Sino-Tibetan friendship bloom warmly in Sanming. A year later, nine multi-ethnic college students of Sanming University will continue their love and pair up with a new group of Tibetan students. In the future, they will communicate and interact with the power grid volunteers and the Tibetan children to help the Tibetan children grow up happily.site of activity. Correspondent for figure volunteers and Tibetan students in the form of new and old pairs, to the urban green road and light station to promote the party's spirit of the sixth plenary session of the 19, garbage sorting, help strange greenway demand user download registration online its APP and other volunteer service activities, and in the post to celebrate "youth protect young interpreta dream hidden Eva" activities, jointly make "unity patriotic with the party, dedication and mutual assistance and progress" wish.site of activity. It is understood that Liedong Middle School Tibet class was founded in 1995, which is a key project of education aid to Tibet in Fujian Province, and it is also the only mainland Tibetan class junior high school in Fujian Province. Over the past 25 years, more than 1,200 students have been admitted to various institutions of higher learning, and more than 900 students have graduated from universities and returned to Tibet to work, becoming the backbone or backbone of all walks of life in Tibet. Since running the class, the Xizang Class of Liedong Middle School has been awarded the honors of "National Advanced Collective in Education and Aid to Tibet" and "National Demonstration School for Ethnic Unity and Progress Creation Activities".

2021-12-07	The opening ceremony of the "Book Reading" to the "Reading Good Books" reading room " was held in Pingling Primary School, Fengtang Town, Lingshan County. This year, the whole district has obtained 15 "Hope Reading" approved schools and 19 "Guangxi Green Space". Pingling Primary School is the only approved school in Qinzhou City. The project provides hardware and books, sets up reading rooms, reading corridors and reading corners, carries out public reading services, sinks reading resources to rural primary schools, and unblocks the "last kilometer" of reading in rural primary schools. In order to thoroughly study and implement General Secretary Xi Jinping's important instructions on promoting reading for all and building a scholarly society, To implement the important arrangements of the Party and the government for nationwide reading, Focusing on the hope project is the fundamental task of the Party to educate people, In April, 2021, Guangxi Youth League District Committee and Guangxi Youth Development Foundation launched the "Hope Reading" public welfare project, By increasing the guidance of reading content, innovating the "hope reading" methods, and establishing a reading service system, Highlight the educational function of Project Hope, It has provided reading services for more than 80,000 rural teenagers in the region, Effectively empower grassroots youth league organizations, Extend the Communist Youth League working arm, To provide new help to the rural youth, Sow a new hope. Increase reading content lead, innovation multiple reading way to celebrate the 100th anniversary of the founding of the communist party of China, "hope reading" to "elegant eight Guangxi reading DangEn" as the theme, widely organize teenagers to carry out the history of the party learning education, key reading xi new era of socialism with Chinese characteristics and the history of the communist party of China theme publications. Combined with party history learning education requirements and youth reading characteristics, recommend "xi seven educated youth years" xi and college students friends "" party history for youth "" red legend "red letter" the xiangjiang river 1934 to die and other books, encourage teenagers in reading red gene, firm ideal faith, draw strength, let the students understand the party's glorious history, feeling the party's mission, efforts to grow into the socialist builders and successors. The "Hope Reading" public welfare project has widely attracted teenagers to participate in reading through "reading partners", "" books "sending hope", "pleasant reading and sharing", "happy reading journey" and "smart reading". For hundred hope primary school and rural primary school to carry out the "hope students to the party, red gene" theme publicity and education, make "reading cloud" WeChat small program, through online participation channels, through the cloud walk long march (Guangxi), cloud view red memorial, red classic, cloud, red stories, red spirit "hope reading cloud" series of activities, enrich the party history learning form, improve reading experience, inheriting red gene, continuously red blood, for teenagers buried the seeds of ideal and faith, attract nearly 26000 students to participate in. Play the advantages of youth league organizations to build "reading" service system deeply grasp the rural youth from "books" to "read good books" demand change, in April project launched, Guangxi youth foundation to mobilize outstanding young entrepreneurs to hope project donations, set up 10 million yuan "swallow hope fund", for rural teenagers to carry out the "reading" to provide financial support. At the same time, through organized mobilization methods, the league members and young pioneers are encouraged to actively participate in it, expand the amount of donations and the coverage of the projects, and enhance the influence of "Hope Reading"."Hope to read" public welfare project give full play to the advantages of youth league organization, focus on the key support in rural revitalization of resettlement for poverty alleviation "Guangxi green space", to carry out the red education, the implementation of psychological classroom, micro wish, popular science reading, fun sports, difficult support normalized care project, promote teenagers to develop good reading habits, boost rural talent revitalization. One is to provide psychological assistance services for teenagers. In combination with the 12355 platform, it provides mental health reading services and education for rural ethnic minority teenagers, left-behind children and orphans, timely prevent, discover and solve their mental health problems, and promote their harmonious physical and mental development. Second, to popularize scientific and technological knowledge to young people. We will carry out scientific and technological activities focusing on popular science reading, science and technology production, and scientific experiments to cultivate young people's interest in science, improve their creative ability to create science and technology, and expand their scientific horizons. Third, to carry out youth sports training. Through table tennis, badminton, basketball and other knowledge lectures and skill training, we can increase the interest and experience of youth sports, and help rural teenagers to enjoy fun in physical exercise, enhance their physical fitness, improve their personality, and temper their will. Fourth, we will pool our strength to help young people in need. We will integrate social resources to raise grants to reduce the financial burden of poor students and help them successfully complete their studies. County-level Communist Youth League organizations send "micro-wish" items to the hands of teenagers through centralized distribution and household visits, so that they can deeply feel the love and warmth of the big socialist family. School schools are a place for education and a place to cultivate people. The "Hope Reading" public welfare project has set up a guarantee mechanism in Hope Primary School, improved the construction of facilities, coordinated the organization and construction, established the reading system, and played a good "combination of fist" of the construction of "scholarly campus". Improve the facilities and equipment of the school library of the project, create a "Hope reading room", class book corner, and campus reading corridor, create a warm reading atmosphere, build a reading display platform, and post reading excerpts, reading experience, and good book recommendation for teachers and students. The project has set up a leading group with the participation of the secretary of the township (town) Youth League Committee, the school principal and the director of the youth working Committee. It is responsible for designing, planning and arranging the reading activities of teachers and students, clarifying the reading tasks of teachers and students, arranging 20 minutes of reading every day, and arranging one reading class every week to ensure the normal reading of teachers and students. The Project also establishes a book borrowing, open reading room, reading and lecture system, implements the funding guarantee system, updates books timely, subscribes to the newspapers and periodicals needed by teachers and students; establishes a reading evaluation system for teachers and students, sets up a reading record sheet, reading practice activities, special reading test, etc. The establishment of rural youth reading education "hope reading" public welfare project in the school, "Guangxi Green space" attracted western plan volunteers, research team members, invited celebrities, outstanding entrepreneurs and other caring people from all walks of life to form an enthusiastic support mentor team, regularly provide reading counseling volunteer services for teenagers. Through scientific guidance, children can learn to read and love reading, nourish the vitality of youth reading, help rural education with the power of reading, and light up the light of education. At the same time, we will expand social participation, mobilize and organize civil servants, press and publication workers, and college students to join the team of "Hope Reading" promoters, to spread reading concepts, carry out reading promotion, and provide reading guidance. Reading is an important way to help young people acquire knowledge and increase their wisdom, and an important way to inherit civilization and improve their quality. Guangxi youth development foundation will continuously improve the service ability and public efficiency, strive to read "reading" public welfare project as a distinct theme, extensive, influential, characteristic of Guangxi public brand, bring new reading cognition and experience, realize rural teenagers from "read books" to "read" profound change, let more rural teenagers benefit. China Youth Daily, December 07,2021, edition 202

2021-12-06	Group of Fujian Quanzhou municipal party committee of constitutional garden theme activities Quanzhou municipal party committee of constitutional garden theme activities jianwei correspondent Wu Ruiyuan) on December 4,2021 is the eighth national constitution day, to strengthen the constitution learning propaganda, on December 3, in the afternoon of Fujian Quanzhou city people's procuratorate, Quanzhou municipal party committee in okanagan valley new village rule of law park joint constitutional garden activities, for Quanzhou two real elementary students, community residents sent to a rich rule of law "feast".site of activity. Group of Fujian provincial party committee for figure activities through constitutional knowledge puzzle, law puzzle, the rule of law elements paper-cutting art, drawing postcards, I pose with "constitution", the constitution knowledge to the residents, guide teenagers to use legal weapons to protect their legitimate rights and interests, improve the youth research method, law, law-abiding, usage enthusiasm, at the same time, the youth affairs social workers also patience for teenagers legal questions.site of activity. Group of Fujian provincial party committee for figure group the relevant person in charge of Quanzhou municipal party committee said, activities aims to further implement the sixth plenary session of the spirit, promoting the construction of community rule of law, strengthen the legal consciousness of the masses, the next step, pku will to the franco-prussian propaganda into community activities, further carry forward the spirit of the constitution, set up the constitutional authority, spread the constitutional culture.

2021-12-06	"Mountain green flow children's palace" into Jining jiexiang county primary school "mountain green flow children's palace" into Jining jiexiang county Liang Bao temple town canal garden primary school on February 6 (reporter Zhang Jianwei, correspondent Bai Chen) recently, "party history know DangEn red gene generations" and "mountain green flow children's palace" into Jiaxiang county Liang Bao temple town canal garden primary school activities were successfully held. The province sent "to strengthen the construction of rural grass-roots Party organizations" task force in Jining City general liaison officer, the provincial youth palace related leaders and Jining City, Jiaxiang County Youth League Committee responsible person to participate in the activity. At the launch ceremony, Zhang Wenming, a member of the director office of Shandong Youth Palace, donated materials to the school and awarded the provincial "Mobile Children's Palace" palace flag. The teachers of Shandong Youth Palace signed a relevant agreement with the team of sound, sports and beauty science teachers in Liangbaosi Town to establish a long-term cooperative relationship. Teachers from Shandong Provincial Youth Palace have created a set of "characteristic courses" for the young Pioneers, and doctors from the Affiliated Eye Hospital of Qianfoshan Hospital conducted a vision examination for the children.site of activity. Correspondent for figure from the canal garden primary school, canal primary school, Liang Bao temple town left-behind children school more than 400 students, under the organization of the music, painting, performance, kechuang popular science, fun go boutique public welfare class, the children benefit from this activity, a "I am more interested in learning", is the heartfelt joy of the children. In the next step, the Communist Youth League Municipal Committee will continue to improve the quality education of teenagers as the focus, focus on caring for rural left-behind children, build a volunteer service platform, and do a good job of resource docking link.

2021-12-05	On the occasion of the 36th International Volunteer Day, the latest data disclosed by the Chengdu Municipal Committee of the Communist Youth League shows that the number of young volunteers registered in Chengdu has exceeded 1 million. According to the "Qingju Jincheng City Volunteer Service" platform, since this year, Chengdu has added 350,000 new registered young volunteers, 952 new volunteer service organizations, 23,000 new volunteer service projects, and 1.22 million hours of new volunteer service."Qingjujin Guancheng City Volunteer Service" platform page. The 31st Summer Universiade ("Chengdu Universiade") to be held in Chengdu has boosted the enthusiasm of young people in Chengdu to participate in volunteer service. Since the launch of the Universiade, all the young volunteers in Chengdu have been mobilized to actively participate in the service of the Universiade. In 2018, Chengdu won the right to host the Universiade. The Chengdu Municipal Party Committee of the Universiade, seizing the opportunity of upholding the concept of "running a good meeting, invigorating a city", launched the "Qingju Jin Guancheng City Volunteer Service Platform" platform in that year to explore and promote the innovative development of youth volunteer service platform, projects and teams. The "Qingju Jin Guancheng City Volunteer Service" platform integrates volunteer registration, demand collection, project release and other functions. As a youth volunteer service supply and demand docking platform and Chengdu youth volunteers big data system, "green jin city city volunteer service" platform set up with the communist youth league central "volunteer", Sichuan province "volunteer", Chengdu civilization volunteer network data collaborative mechanism, and the platform access to tianfu citizens cloud government service platform, tianfu tong (APP) citizens life platform, realize the volunteer service demand real-time "transformation", youth with "can participate in". In order to make the youth volunteer service better fit urban development needs, the Chengdu municipal party committee relying on the above platform to establish the "masses point-platform sent single-single volunteer orders-mass evaluation" service mode, guide the youth volunteers into community volunteer service demand collection, developed the concentric war disease, colorful dream, green warm sunset, feeling full journey 10 categories of 133 small categories of volunteer service projects. Group Chengdu municipal party committee also established with the city community, library, museums and other public cultural services, tax, social security and other comprehensive service hall, Chengdu east bus, Shiyang bus transportation hub, park green road and other public activities of volunteer service demand normalized collection work mechanism, promote volunteer service coverage in the city. In order to meet the diversified and personalized needs of the citizens, the Chengdu Municipal Party Committee conducts classified team building: forming the professional volunteer service team by "recruiting" the industry leaders and backbone members; cultivating the independent planning and independent "veteran" team around the low threshold and everyone participation. Up to now, Chengdu has set up more than 100 various youth volunteer service teams.

2021-12-05	Tell the people story walk new descend tell the people story walk good new descend road Zhang Wenling) on December 2, group of Kunming, Yunnan province in spring city people preach demonstration site, chenggong district new era civilization practice center, the people to preach to study the results of the spirit of the sixth plenary session of the 19th into clerkstarts, become see, touch the work effect. Feng Xianru, the lecturer of the Kunming Municipal Party School, with the title of "explaining the spirit of the plenary session"; Kunming Haikou forest farm narrator Chen Mona told, since 1956, under the leadership of the party, several generations of forest farm workers hard struggle, selfless dedication, greening barren hills, achieved an ordinary and glorious cause. Simple words, emotional speech, touching stories won the scene bursts of applause. Wang Yanyan, secretary of the Kunming Communist Party Committee, said that at present, the municipal Communist Youth League at all levels has set off a wave of multi-channel publicity and learning of the spirit of the plenary session, taking the implementation of the spirit of the plenary session as a mission and strength, and transforming their ideals and beliefs into practical actions to fulfill their duties.

2021-12-05	Jiamusi city to speed up the development of youth electricity theme group Heilongjiang Jiamusi municipal committee, city youth league organization "to speed up the development of youth electricity boost good city economic revitalization" theme activities woven business, press and publication, culture and art and sports more than 20 Jiamusi youth electricity federation, to carry out "speed up the development of youth electricity business boost good city economic revitalization" theme activities. Members of the Youth Federation came to the Jiamusi Youth E-commerce Federation Base and communicated with e-commerce enterprises on the development prospects and policy needs of the e-commerce industry.

2021-12-05	The second "Youth 'Benefit' " Tangshan Public welfare project Competition final held the public welfare project and volunteer service project competition final was held in the multi-function hall of Tangshan Youth Palace, Hebei Province. Thirteen projects from all kinds of youth social organizations and college student associations in the city were selected and entered the final. The competition was launched in July, and covers helping the growth and development of young people, charity assistance, volunteer services, social services and other fields. After qualification examination, project first evaluation, public welfare salon, road show final evaluation and other links, 1 first prize, 4 second prizes and 8 third prizes were selected. In the next step, the Tangshan Municipal Party Committee will continue to expand the coverage of the Communist Youth League organization, cultivate and gather more young social organizations to participate in the development of public welfare undertakings and social governance innovation, and contribute its youth strength to the new situation of "three efforts to build" in the new era. The public welfare project competition, jointly organized by the Tangshan Municipal Committee and the Tangshan Bank, aims to promote the incubation and cultivation of public welfare projects, guide young social organizations to actively participate in social governance, and promote the project operation, socialized mobilization and institutionalized development of the city's youth service work.

2021-12-05	Bozhou: innovation mode for youth dating service precision, normalized group Bozhou municipal party committee: innovation mode for youth dating service precision, normalized network reporter Wang Haihan) on December 2, the youth network reporter came to Bozhou city landmark in Anhui province-Lin medicine Lin hai scenic spot, central area, Bozhou city party committee, city brigade group, city women's federation to build youth communication place "Bozhou outstanding youth communication base" (corner) striking. On the wooden building beside the trail, the blue "male god card" and the pink "goddess card" are neatly arranged. Single young people who have been registered and reviewed into the dating database can scan the QR code for the detailed information of young men and women. The picture is an aerial photo of the mountain corner. The activity area is loving, which has become a favorite point for young people and trusted by their parents. This year's "Qixi" day, hundreds of single young people went here, according to statistics, a total of more than 850 single youth information inquiry invitation. Single youth information card and information database is the epitome of the Youth League Bozhou Municipal Party Committee's service of youth marriage and dating work. In recent years, TuanBozhou Municipal Party Committee has innovated the three combination ideas of "online + offline", "platform + activity" and "service + guidance" to realize the precision and normalization of youth dating services. Bozhou Municipal Committee staff introduced, in the past, the league organization to carry out less similar activities, the activity matching degree is not high. Some single young people in their units call to say that they need to make friends, but they are introverted and concerned, and they are unwilling to give their names and information. To this end, the League Bozhou Municipal Party Committee called party and government organs, enterprises, institutions, schools and other units of single youth discussion, to understand the single youth dating needs, mate criteria and open information list, develop scientific and detailed single youth information statistics, and through technical means, clear query information open, confidential items, maximize the image of youth image, protect youth privacy, let youth rest assured. Offline, the Bozhou Municipal Party Committee of the Communist Youth League analyzed the work industry, region, educational background and mate selection needs of single young people in the information database, and regularly carried out regular "matchmaking" activities. In 2021, in the city's power supply company, cultural and tourism group, traffic control group and other units of the special dating fraternity, 11 pairs of youth successfully took off the single. This made the "blind date party" organized by the league become "hot property", and the enterprises called "urging": " When do you give us a special fraternity for our company?"In recent days, the Youth League Bozhou Municipal Party Committee has once again summarized and combed the information of high-quality single youth, building an online communication and dating service platform of" Love is you in Bozhou Yuan ", and the city, counties (districts) can realize information exchange and activity linkage. The platform has put more than 1,000 people in the library, and it is planned to be launched in the near future. It is equipped with information retrieval, private message reminder and other functions to facilitate single young people to find their "favorite objects" at any time.(Photo provided by Bozhou Municipal Committee; video shot and edited by Wang Haihan, China Youth Network, China Youth Network)

2021-12-03	Zhejiang Province Taizhou Municipal Party Committee to carry out the "one thousand youth mobility" activity to learn the spirit of the sixth Plenary session of the 19th CPC Central Committee Taizhou, Zhejiang Province to carry out the "one thousand youth mobility lecture" activity member for the picture of China youth network Beijing on December 3 (reporter Li Chuan correspondent Cai Xiaoxi) recently, The Youth League Taizhou Municipal Party Committee of Zhejiang Province carried out the "one thousand youth mobile lecture" publicity activities in 9 counties (cities, districts) and Taizhou Bay New Area in full swing, Hundred youth speakers with cadres at all levels, young cadres of Party and government organs and young models as the main members, Into the organs, into the enterprises, into the community, into the countryside, into the campus, into the network to carry out the spirit of the sixth Plenary session of the 19th CPC Central Committee. Huangyan district public security bureau youth corps committee carried out "the party spirit of the sixth plenary session of the 19 th" preaching activities, focusing on the agenda and significance of the sixth plenary session of the 19, from the development of the party, the party's historical experience of the resolution of the central committee of the communist party of China about the party's struggle in one hundred major achievements and historical experience of the main content of the resolution. Spark young publicity group young lecturer came to the district knowledge association to carry out the "concentric theme day" and study and implement the spirit of the sixth plenary session of the 19th CPC Central Committee publicity activities, spark young publicity group leading mentor Ren Yin walked into Taizhou Luqiao District Hailong sanitary ware Co., LTD. There are more than 20 ethnic minority employees in the enterprise. Ren Yin, combined with the changes around him, preached the spirit of the sixth Plenary Session of the 19th CPC Central Committee with the theme of "love gathering pomegranate seeds, singing the song of common prosperity"."Thousands of youth mobile talk" publicity activities into the enterprise. Correspondent for figure wenling city ZeGuo town youth corps committee joint propaganda, trade union do line, carefully as a whole, organization cadres, member youth XuanJiangTuan into the enterprise, into the workshop to carry out the spirit of the sixth plenary session of the 19th preaching activities, guide the teenagers to the thought and action unity to the plenary session spirit, further build consensus, firm confidence, enhance morale and energy. Youth XuanJiangYuan with "theory + explanation", to the enterprise worker of the sixth plenary session of the 19th significance, main spirit, rich connotation, and combining the reality and the grassroots changes, talk about feelings, experience, vision, guide everyone to draw wisdom and strength from the plenary spirit, further stimulate staff motivation aggressive, the spirit of the sixth plenary session of the 19th into the new journey. The Youth League Committee of Ganjiang Town, Yuhuan City, led the young volunteers to go deep into the fields, to carry out publicity activities, and to thoroughly study and implement the spirit of the sixth Plenary Session of the 19th CPC Central Committee. At the scene, the young volunteers used lively dialects, combined with the local characteristics of Ganjiang, to publicize the spirit of the sixth Plenary Session of the 19th CPC Central Committee of the farmers."Thousands of youth mobile talk" propaganda activities into the rural areas. Correspondent for the picture "one hundred years of perseverance, major achievements table a table, historical experience can not be forgotten, ten adhere to the party leadership: the first, if there is no Communist Party, there is no new China, where are we happy to find..." Huangyan �� township central primary school playground, students and teachers sit together, attentively listen to a wonderful "allegro preach". The speakers publicized the spirit of the Sixth Plenary Session of the 19th CPC Central Committee in the way of allegro, and spoke colloquial and down-to-earth on the great achievements and great life changes since the founding of the Party, so that the spirit of the Party's policy could enter the people with the "sound" of allegro. Chen Shijun, the village ambassador of Shanghai �� Township and the creator of the program, is also planning to create the allegro "Six lectures and six doing" in Zhejiang ", hoping to attract more people to pay attention to and learn from the spirit of the combination of the Sixth Plenary Session of the 19th CPC Central Committee.

2021-12-03	The Working Committee of Non-Public Enterprises established the inaugural meeting of the Working Committee of Non-Public Enterprises. At the meeting, the Handan City Non-Public Enterprise Youth League Working Committee (Youth Working Committee) personnel composition list was read out, and passed the "Communist Youth League Handan City Non-Public Enterprise Working Committee working Rules (interim)". Two Handan city non-public enterprise youth league working committee member representatives spoke. Conference pointed out that the establishment of Handan city non-public enterprise league committee, is the non-public economic organizations youth bridge and the link, is also the new era of the communist youth league work effective organization form, is an important measure to strengthen the work of the communist youth league, and Handan municipal party committee to further lead condensed youth, mobilize youth, contact service youth powerful exploration. Next, Handan city non-public enterprise league committee will take the initiative to play to the industry advantage, organize learning training, skills, innovation, dating and other activities, in the service enterprise reform and development and youth growth play a bigger role, unity led the non-public enterprise league youth to speed up the construction of modern regional central city youth strength.

2021-12-03	Liuzhou, Guangxi, Xi reporter Yin Xining, 2021) Smoke is billowing in front, how to evacuate primary school students? What can teenagers help when they encounter passers-by who fall to the ground? On December 1, more than 2,000 teachers and students at a primary school in Liuzhou city, South China's Guangxi Province. On the same day, by the communist youth league of Liuzhou municipal party committee, Liuzhou federation of trade unions, Liuzhou women's federation, Liuzhou education bureau, Liuzhou fire rescue team, the young pioneers of Liuzhou working committee jointly sponsored by Liuzhou "love life improve survival ability" theme education work-2021 Liuzhou youth survival ability training theme team demonstration activities in keelung development zone primary school. Holding a fire extinguisher and friends to put out the fire, cover the nose out of the smoke to the playground collection, close look at the medical staff to implement CPR... activity site, teachers and students jointly carry out fire emergency evacuation drill, fire knowledge and fire emergency rescue knowledge learning, fire equipment use training and other links, learn to deal with the emergency response and self-rescue mutual rescue knowledge, enhance fire safety awareness. According to the introduction, Since the theme of "Caring for life and improving viability" in Liuzhou, Guangxi was officially launched in October this year, The CPC Communist Youth League Municipal Committee, together with the Municipal Federation of Trade Unions, the Municipal Women's Federation and other 11 departments, issued the "Liuzhou City" Care for Life and Improve Surviability "theme Education Work Plan", Lead the establishment of a theme education expert team and a survivability training teacher team, Focus on the majority of primary and secondary school students, women and children, the elderly and the disabled and other weak self-rescue ability in the face of disasters, More than 200 viability training sessions, To constantly improve the quality of the people's survival ability, Truly protect the safety of people's lives and property.2021 Liuzhou Youth viability training theme team day demonstration activity site.(Photo provided by Liuzhou Municipal Party Committee of the Communist Youth League) Primary school students came on stage to watch medical staff demonstrate CPR methods.(Photo provided by Liuzhou Municipal Party Committee of the Communist Youth League) Teachers and students participated in the fire evacuation drill together.(Photo provided by Liuzhou Municipal Party Committee of the Communist Youth League)

2021-12-02	Shaoxing: "red script to kill" take-away little elder brother immersive party history of Shaoxing, Zhejiang: "red script to kill" with take-away little elder brother immersive party history Jiang Yutong) on November 30 afternoon, Shaoxing, Zhejiang province KeQiao district party committee organization take-out little elder brother launched a party history theme "red script to kill" about the spirit of the sixth plenary session of the nineteenth, through the role play, lead you immersive to understand the revolutionary spirit, draw strength in reviewing the history of the party."Red script kill" has injected "young state" into the study of party history. With innovative forms and role playing, it builds an immersive party history learning and education platform, allowing the youth and their ancestors to have a "space dialogue" and "brainstorming" with a sense of history, and understand the century-old history of the Communist Party of China. Back to the War of Liberation, although the different characters have a different historical destiny, but the justice and the truth will not change because of the different role plays. This lasted nearly 5 hours of "red script kill", let the little brothers feeling quite deep. Deng Jinwei said, " I seem to have experienced the turbulent history and experienced the revolutionary passion behind that history. Through this innovative learning and education way to review the history of the Party and learn the spirit of the Sixth Plenary Session of the 19th CPC Central Committee, I can not only learn the history of the Party, but also exercise my logical thinking ability. Wang Feng, the regional manager of "" Ele. me ", said that the" red script kill "activity was very popular with the delivery boys, and everyone registered very enthusiastically." To convey the spirit of the Sixth Plenary Session in this form, which is more close to the reality of the delivery boys, and can also better realize the significance of publicity and education."Photo provided by the League Shaoxing Municipal Party Committee

2021-12-02	Shandong Heze: more than 6000 loans to help youth innovation and entrepreneurship in Shandong Heze: more than 6000 loans to help young innovative entrepreneurs XingTing) to further help youth innovation and entrepreneurship, strive to solve the youth capital bottleneck problem in the process of entrepreneurship, Heze group joint Heze city people's bank to jointly carry out the "LuQing benchmark loan" project. Since this year, the Youth League Heze Municipal Party Committee has reviewed a total of 64 loan applications, the loan amount of 78.41 million yuan. Among them, 53 young entrepreneurs have received 61.02 million yuan of loans that have been issued in place, saving more than 1.8 million yuan of interest for young entrepreneurs. It is understood that the main body of the "Luqing benchmark loan" project is small and micro enterprises founded by young people under the age of 45. Enterprises applying for loan shall have clear property rights, sound system, legal operation, pay taxes according to regulations, good credit, and be in urgent need of financial support. The production and operation projects engaged in by the enterprise are low-carbon environmental protection, high scientific and technological content, with strong growth, in line with the national industrial policy. For the "four new" enterprises and youth entrepreneurial talent projects, priority will be given to the youth entrepreneurship competition winners, young veterans, and the "good youth" at the village level. Every time a young person applies for a loan, the youth league organization of the county and district will cooperate with relevant banks to have a detailed understanding of his family background, personal personality, interests and other aspects. After the preliminary screening, the recommendation will be issued, and then the bank will further professional review. Such cooperation ensures that there is no bad record of youth entrepreneurship loans. In recent years, Heze City Youth League organizations at all levels through various new media platforms, to have entrepreneurial ideas of policy introduction and other forms to introduce this preferential measures, focusing on returning entrepreneurial youth, college graduates, youth e-commerce, rural good youth and other groups to promote. Heze Municipal Committee also established a youth loan WeChat group for young entrepreneurs to answer questions and exchange entrepreneurial experiences. Zhou Guangwei, a young entrepreneur in Juancheng County, is the beneficiary of the Luhua benchmark loan. After graduating from college, Zhou returned to his hometown of Juancheng County with a dream of starting a business and founded a mechanical equipment company with two friends. But the startup process has not been smooth sailing. Do machinery and equipment industry, the early need to invest a lot of money, but the return cycle is longer. Lack of sufficient funds, the enterprise development has encountered difficulties. It happened that the Tuan Juancheng County Party Committee and the Juancheng Rural Commercial Bank to carry out the "youth entrepreneurship finance night school" publicity activities, the activity mentioned the "Lu Qing benchmark loan" to help young people on the road of entrepreneurship case. Zhou Guangwei immediately submitted the loan application, Tuan Juancheng County Party Committee and the first bank field research, timely issued for him a 1 million yuan business loan, to solve his urgent need. In the view of the relevant person in charge of Heze Rural Commercial Bank, the issuance of youth entrepreneurship loans also makes the bank find a lot of high-quality customers who can cooperate for a long term in the future, which is a win-win thing. The relevant person in charge of Heze Municipal Party Committee said that it would combine the combination of the "Luhua benchmark loan" product policy, continue to deepen the cooperation between League Bank, strengthen the financial support for youth entrepreneurship, continue to provide financial support services for the majority of youth in Heze, especially rural youth to start innovation and entrepreneurship, and help young entrepreneurs realize their dreams.

2021-12-01	Huangshi Municipal Party Committee of Hubei Province has set up a service platform for a practical training platform for youth innovation and entrepreneurship to serve youth innovation and entrepreneurship. Youth League Huangshi Municipal Party Committee organized 44 young people to step into the Huangshi youth science and technology live network red base, Carry out vocational skills training for e-commerce live-streaming talents; Ma Jie, a distinguished lecturer of the School of Economics and Management of Tsinghua University and a distinguished Chinese youth entrepreneurship tutor of the Central Communist Youth League, was invited to bring the "first live class" to the students; Promote the implementation of Hubei "Qingchuangdai" financial support project in 2021, Loan made to 18 enterprises, Total of 7.35 million yuan; Selected 5 youth innovation enterprises to participate in the 2021 "Innovation Youth" Hubei Youth Innovation and Entrepreneurship Competition; Helped 18 youth creative enterprises to obtain a total of 360,000 yuan for college student entrepreneurship support project in Hubei Province in 2021; Organize the Huangshi City Youth Enterprise Association, " To Challenge yourself, Win in the future " exchange and learning activities; Organized representatives of young entrepreneurs to donate 100,000 yuan to four primary and secondary schools in Qusong County, Xizang Province, Tibet. The Youth Huangshi Municipal Party Committee has also built a "12355" youth service desk in Huangshi City to provide regular online psychological counseling services for its teenagers; by the middle of November, it has received more than 700 consultation calls. During the gaokao, the CPC conducted 8 "decompression" actions, the special program "Voice of Youth- -psychological counseling for college entrance examination" online, and the online listening to more than 50,000 people, and the "12,355 easy test preparation with you" in 6 middle schools, covering more than 3,000 students with the Municipal Procuratorate, Municipal Education Bureau, and 67 times covering more than 20,000 people.

2021-12-01	Wuxi Communist Youth League: To volunteer service for youth "partners without shares" in the Internet of Things Expo, I feel the strong strength of Wuxi's scientific and technological innovation."Han Tian, a student of Wuxi University, praised the experience of volunteering for the conference together with more than 420 college students. Recently, because she served as the chairman of the Wuxi Student Federation and had an excellent performance, she was in the internship of a science and technology innovation enterprise in Wuxi as recommended by the Wuxi Municipal Party Committee of the Communist Youth League. Han Tian's experience is a vivid epitome of the Communist Youth League Wuxi Municipal Party Committee's innovation and entrepreneurship services for young people. Since this year, the Wuxi Municipal Party Committee of the Communist Youth League has visited the youth and served the youth, focusing on youth innovation and entrepreneurship, providing diversified services for all kinds of employment and entrepreneurship youth in Wuxi, and helping to build a local science and technology innovation highland. Group Wuxi municipal party committee deputy secretary xue said, pku will continue to implement the whole chain youth talent "tin" plan and "tin green I gen" youth innovation entrepreneurship, normalized provide meticulous and thoughtful service, help youth double gen talent agglomeration, service support, ecological can assign, truly become youth innovation entrepreneurship "no partner" on the road. Chang'an Shenghui, co., Ltd., is a "new group leader". In May last year, he as Wuxi "taihu lake talent plan" top talent team a member of the team, Wuxi municipal party committee of the youth talent pool, and Wuxi municipal party committee recommended to the Jiangsu provincial party committee united front work department for a 1-year credentials the exercise, created a scientific research frontline workers in Jiangsu province to the communist youth league system of the first case. In the past year, An Shenghui has actively given full play to his professional advantages and actively connected with Jiangsu Institute of Industry and Research and universities. The "Youth Scientific and Technological Achievements Through train" is guided by the technological needs of enterprises, and is carrying young scientific and technological talents and a large number of achievements to hundreds of high-tech enterprises in Wuxi."Thanks to the Wuxi Municipal Party Committee of the Communist Youth League for timely collecting, sorting out and reporting the difficulties of enterprises, it is the scientific and technological achievements through train to help us get through the demand end of enterprises and the innovation end of research and development institutions."Said Qi Yu, co., director of Wuxi Zhongke Integrated Circuit Co., LTD. Under the coordination of the youth league organization, Lin Jun, an associate professor of Nanjing University, has completed the supply and demand case docking with the enterprise on the chip manufacturing "bottleneck" technology, and solved the technical problems for it. At the just-concluded 2021 Wuxi Taihu Talent Summit, in order to bring the numerous fruits of young scientific and technological innovation to the majority of high-tech enterprises, the Wuxi Municipal Committee of the CPC Youth Group led the "Science and Technology Innovation Jiangnan" Taihu Bay University Youth Maker Summit Forum. During this period, 5 academicians and young talents from more than 20 universities came to Wuxi with 260 projects; 24 projects reached landing intention with many sectors in Wuxi and signed letters of intent for cooperation, with the total amount of projects exceeding 10 billion yuan."When I was working in Wuxi, my position was very compatible with my major, which gave me an opportunity to have a deep perception of Wuxi."Recently, Li � N, a student at Lomonosov National University in Moscow, Russia, is working in Wuxi based on the" New gravity " overseas student Jiangsu employment internship program. This year, the Wuxi Municipal Committee of the Communist Youth League and the Wuxi Youth League have further extended their talent introduction to overseas countries, actively coordinating more than 2,400 quality employment internship positions, and widely publicized them to universities in North America, Europe, Australia and other places, covering tens of thousands of overseas students. At present, a large number of overseas young talents like Li � N are being "attracted" to Wuxi to start a business through post internship, city situation investigation, social situation experience, communication and sharing. Zou Hao, founder of Qingying Medical Technology, visiting professor of Stanford University, a professor of Tsinghua University and a young man born after 1985, is one of them. In 2016, after the Wuxi Municipal Party Committee of the Communist Youth League identified it as the training object after investigation, it has been connected with the latest local entrepreneurial policies and resources every quarter for five consecutive years. In April this year, when we learned that Zou Hao's recent research direction had shifted to intelligent technology, the Communist Youth League Municipal Committee immediately invited him to Wuxi to investigate related industries and connect with the resources of the park. During this year's Taihu Talent Summit, Zou Hao's entrepreneurial project "Wuxi Intelligent Technology Research Institute" was finally launched in Wuxi Liyuan Economic Development Zone. Zou hao's team of entrepreneurial partners to Wuxi includes both scientific research academicians from China and the United States, artificial intelligence experts, and a number of doctoral students. Zou Hao from the "attracted" talent, to become the ambassador to help Wuxi to attract talents. Zhou Yuyang, who graduated from King's College London, came to Wuxi to work this year. With the help of the Tuan Xishan District Committee, she carried her bag into the Dongcheng International Youth Talent Community. Zhou Yuyang said: " The international young talent community provides a good living environment for overseas returnees and a platform for us to communicate and interact."The community currently has 260 fashion apartments, which are now almost full. The community front desk regularly pushes information about courban youth activities to young talents. It is understood that the, Youth League Wuxi Municipal Party Committee this year in the city to implement the "tips to build tin hope" returnee youth to celebrate the 100th anniversary of the founding of the Party theme series of activities, Jointly with the Organization Department of the CPC Municipal Committee and the United Front Work Department of the CPC Municipal Committee, we launched the demonstration training course of "patriotic struggle and dedication" spirit education and party history learning education for high-level overseas returnees in Wuxi, And together with Wuxi Talent Office to create a "heart to heart to tin" Wuxi young returnees talent friendship brand activities, The city will be more than 100 "youth homes", cinemas, theaters and so on, Integrated and set up as theme reading areas, public welfare training places, youth literature and art classes and other learning service positions around young returnees, We will further provide an environment for young returnees to respect talents, encourage innovation and live a carefree life."Entrepreneurship" partner: youth interpreta dream base spectrum "tin hui" new movement in 2021 China Wuxi "taihu cup" international elite innovation entrepreneurship competition, wuqing, Wuhan city, focus on youth innovation entrepreneurship quality project 283, and through the policy promotion, project guidance, "tin ambassador" employment, attention, motivation and support of youth entrepreneurship, let more young friends see Wuxi, understand Wuxi, and choose Wuxi. How to continue to provide services for innovative and entrepreneurial youth after they come to Wuxi? Based on the "Xiqing I Innovation" innovation and entrepreneurship plan, Wuxi Municipal Youth League has launched the "I Innovation 8" service youth innovation and entrepreneurship measures, including the construction of I innovation community and planning I innovation network. These measures, from making investment connections to recommending entrepreneurial teams to participate in the competition, are really helping the young talents to set sail and realize their dreams. Through the linkage of city, county (city) and district league organizations, competition, Wuxi introduced youth entrepreneurship and innovation projects to all sectors. In Wuxi Xinxiang Information Technology Co., LTD., founded by Xu Peipei, its semiconductor core system project has realized three domestic technological innovations and broken the monopoly of foreign technology. After understanding the needs of Xu Peipei's team, the Xinwu District Talent Office and the Youth League Xinwu District Committee gave the start-up capital, financing support, living security and other supporting policies to help the project to implement smoothly. Ma Tianquan, a young entrepreneur, has enjoyed the policy benefits of the "Xiqing I Innovation" innovation and entrepreneurship plan. This year, he led his team to participate in the second China Yixing Taihu Bay International Youth Elite Science and Technology Innovation Challenge, with the second prize in the city competition, and finally landed in Yicheng Street, and Wuxi Yiguan Intelligent Technology Co., Ltd. was established. Talking about the implementation of the project, Ma Tianquan expressed his voice with two "full people": " I am full of sincerity in attracting young talents here. I am full of confidence in the future in this city."Jiao Peng China Youth Daily China Youth Network reporter Li Chao Jiao Minlong source: China Youth Daily (December 01,2021, edition 01)

2021-11-30	Group Wuzhong city party committee organization to carry out the 2021 constitution special study group Wuzhong city party committee organization to carry out the 2021 constitution special learning youth network Beijing on November 30 (reporter Zhang Jianwei) to thoroughly study and implement the party's spirit of the sixth plenary session of the 19, earnestly implement the central committee about in-depth study propaganda and implement the constitutional decision deployment, in accordance with the requirements of relevant documents, on November 29, the Ningxia hui autonomous region Wuzhong city committee held the franco-prussian education special study. Conference, deputy secretary of pku Zhou Jing led everyone to learn the communique of the sixth plenary session of the 19, xi jinping, the basic spirit of the rule of law thought and the core principles and the 18th party congress comprehensively advancing the rule of law new achievements review, improve league cadres of general secretary of the rule of law thought and the understanding of the spirit of the sixth plenary session of the 19th. At the same time, I focused on studying the relevant chapters of the Constitution of the People's Republic of China and the general provisions of the Party Constitution of the Communist Party of China, so as to promote the party and league members to enhance their awareness of the Constitution and the law, and perform their duties in accordance with the Constitution, the law and regulations. The Constitution special topic study site. Group Wuzhong city committee for figure meeting to learn the Wuzhong city committee in accordance with the city committee office on the "124 in 2021" national constitution day and "constitution publicity week" publicity week notice, and issued by the pku about organization to carry out the "12.4" national constitution day and "constitution publicity week" activities work plan ", arrange the related work deployment. Finally, the participants watched the small video, titled "The Rule of Law is together", and conducted a special test of the Constitution to test everyone's understanding of the Constitution. The special study of law, improve the youth League cadres and workers to study law, understand the law, law and use the ability.

2021-11-29	Chuzhou held "new era good young" advanced deeds conference in Chuzhou city, Anhui province, "new era good young" advanced deeds conference Ji 'an-wei zhang correspondent, Wang Xinyi check WeiTing), by Chuzhou, Anhui province, Chuzhou party committee, city education bureau, city women's federation, city closes working committee jointly sponsored "childlike innocence to praise the party in one hundred" in the second and third quarter of 2021 Chuzhou "new era good young" advanced deeds conference held in clear primary school of Chuzhou. The activity kicked off in the wonderful opening dance "The Star Torch is brighter". Conference scene, we know the firm, unyielding optimistic girl Yang Xin, pass red culture "red scarf preacher" stingy, with paper to describe a better life, with voice spread civilization "confident girl" in the small 18 new era good young, watched the zhao minjia, Li Boyang two new era good young winner advanced deeds promotional clips. Later, Jin Xiaoyang and Zou Peng brought the ethereal drum poetry recitation of "childlike Innocence to the Party" and the poetry recitation of the martial arts series of "Young China said". In addition, the young pioneers of Qingliu Primary School also brought a wonderful dance program "The Field Mountain Flower is Another Red" for the press conference. At the press conference, each one of the infected people fully demonstrated the patriotism of the contemporary youth of Chuzhou, the dare to venture and the pursuit of excellence."The future of the motherland is in our hands, striving to be a good youth in the new era!"In the solemn oath of the good youth, the activity came to an end. By holding the advanced deeds of "New Era Good Youth", we will constantly explore and publicize the advanced models around them, guide the majority of children to actively cultivate and practice the core socialist values, and help them to button the first button of life, so that they can learn by example, set a model and set goals.

2021-12-01	The West Youth League, Hezhou Youth League and Tuan Fuchuan County Party Committee held the first "Youth League Hope House" unveiling ceremony and public welfare education activities in Tangbei Complete Primary School in the Ancient Town of Fuchuan City. Youth League Hezhou Municipal Party Committee related responsible person delivered a speech. Hezhou city love enterprises donated books, sports equipment and sports supplies to the school. In the next step, TuanHezhou Municipal Party Committee will continue to integrate social resources to jointly build the "Hope House of the Youth Federation" to protect the healthy growth of young people. At the same time, it also calls on caring people from all walks of life to pay attention to rural school education and care for young people in underdeveloped areas, so as to help the revitalization of rural talents.

2021-11-29	Qingdao Municipal Party Committee held the First Youth Innovation and Creative Festival Zhang Jianwei) Recently, the "Youth Innovation and Creativity and Wisdom to Lead the Future" Qingdao first Youth Innovation and Creative Festival achievements exhibition and Youth Innovation and Creativity Forum was held in the Qingdao artificial intelligence living room. The activity site for the city's youth issued the initiative to actively carry out "innovative, creative and creative activities", and carried out innovative thinking, creative design, AI intelligence as the theme of youth innovation and creative and creative forum. To this year, the group of Shandong Qingdao municipal party committee, city working committee for the city youth "youth wisdom future" Qingdao first youth innovation festival, aims to through innovative and creative works for selection improve Qingdao youth scientific innovation quality, creative level, creative ability, to create a good atmosphere for entrepreneurial city construction. Since its launch in July, the activity has attracted the active participation of young people from 10 districts of the city, and received more than 300 kinds of innovative and creative works. After careful evaluation by professional judges, a total of 140 works such as "Future Star Little Maker", "Future Star Designer" and "Future Star Little Academician" were selected, and 8 Qingdao youth innovation and creative practice bases have been set up.

2021-11-29	In 2021 (Longyan) youth song held on both sides of the concentric sing youth in 2021 (Longyan) youth song held Ji'an-wei zhang correspondent Zeng Qiuzhao, Zhang Yuhong) on November 27, sponsored by the group of Fujian Longyan municipal party committee, Longyan city, youth federation, minxi vocational and technical college, Longyan youth art association "concentric sing youth" in 2021 across the strait (Longyan) youth song in minxi vocational and technical college. In the evening, several young singers from both sides of the Taiwan Straits sang, and a young and lively song "To youth" opened the party. Young people from both sides of the Straits sing to share their hometown. The whole song is rich and colorful, including solo, guitar, piano, band and so on. It is reported that the "cross-strait concentric singing youth" 2021 cross-strait youth song is the first time in Longyan city held cross-strait youth exchange song, the evening of more than 300 young people gathered together, taste the essence of the culture, mutual love of compatriots on both sides of the Straits.

2021-11-28	Guangxi Liuzhou promote the reform of the county communist youth league Guangxi Liuzhou promote the county communist youth league reform xi reporter Yin Xining) "build the foundation of reform and development, expand the surface of reform and development, strengthen the effect of reform and development", recently, Guangxi Guangxi Liuzhou held long-term youth development plan (2018-2025) the joint meeting and the county communist youth league organization reform work will advance. This year, Liuzhou Committee of the Communist Youth League proposed to explore the construction of youth development friendly city, focusing on 10 key areas such as youth ideology and morality, youth health and marriage, to build the foundation of reform and development; through the support of Guangxi Longcheng Youth Development Foundation and Liuzhou Youth Development Center platform building, expand the reform and development; focus on promoting "ju Longcheng" to attract talents, guided by Rongshui and Liunan, drive all non-pilot counties and districts to promote reform and enhance the effect of reform and development with local characteristics. According to the introduction, under the background of the Communist Youth League further promoting the reform of the county grass-roots organizations, Liuzhou city has become the only city in the whole city to promote the reform of the county Communist Youth League, with the spirit of driving a nail in the reform, let the reform dividend benefit the grass-roots youth. In the implementation of the county reform, all 10 counties and districts promoted the introduction of the implementation plan for the reform of the county grass-roots organizations of the Communist Youth League at the same level. The relevant responsible departments received tasks, requirements and responsibilities, and worked together to promote the reform to go solid and deeper, and achieved initial results.

2021-11-26	Qiqihar Municipal Party Committee of Heilongjiang Province organized young volunteers to carry out snow clearing activities, youth league members and young volunteers to participate in the volunteer service action of snow clearing activities, and cooperated with relevant departments to carry out snow clearing work in an organized and planned way. Since winter, to cope with the heavy snow weather, the city's youth league organizations at all levels under the unified arrangement, youth volunteer recruitment work, so far the city 16 county (city) district party committee have established TuanShu youth volunteer service, recruiting 1241 young volunteers, to snow, ready to participate in winter snow work, for the city to cope with heavy snow weather, improve management ability to reserve youth strength.

2021-11-26	Hefei city "civilized city beautify the city" volunteer service activities in Hefei city "civilized city beautify the city" volunteer service activities, young volunteers according to the nearby principle, under the guidance of the territorial youth league organization, go deep into the community, take to the streets, to participate in environmental health management. In Xiaoyaojin Street, Luyang District, Hefei City, Young volunteers clean up the white garbage in the community green belt, cigarette butts on the road, psoriasis on the wall; In Chaohu City District, Middle school student volunteers use their spare time, Participate in the campus surrounding city appearance and order management along with the urban management law enforcement team members and sanitation workers; In Xinji Community, Daxing Town, Yaohai District, Young volunteers to the area of the oil road, Carry out the volunteer service activities of "beautifying homes, greening and pruning"; In Yinghuai Community, Yicheng Street, Baohe District, Young volunteers go to bus stops, road lampposts, bulletin boards, urban squares, back streets and other places to clean up small advertisements; On Tianxiaquan Road, Xiaomiao Town, Shushan District, Volunteers on both sides of the road and the surrounding health dead corners for the "carpet" cleaning, At the same time, to promote the knowledge of environmental protection to the residents. Since August this year, the Hefei Municipal Party Committee of the Communist Youth League has carried out a campaign to create centralized volunteer services, calling on the youth of the city to carry out a series of activities such as city creation publicity, city beautification, civilized travel, care services, and city creation knowledge survey.

2021-11-26	Tangshan Municipal Committee started 12355 grassroots contact point psychological testing work group Tangshan Municipal Committee started 12355 grassroots contact point psychological testing work grassroots contact point psychological testing work online training. This psychological general test work is relying on Tangshan 12355 youth service desk, for the city's 14 counties (city, districts) 12355 middle school grass-roots contact point students to carry out free psychological test. The organizer uses the Internet analysis technology of the way to process and analyze the data, so as to provide a complete and effective psychological evaluation report. The activity aims to guide students to pay attention to their mental health, improve their ability to understand themselves, develop themselves and improve themselves, and provide guidance for schools to carry out mental health education and develop students' psychological potential. More than 30 people in charge of the youth League committee, teachers and mental health guardians of the 12355 grassroots contact point schools participated in the training.

2021-11-25	Fujian green federation ecological service group Ningde station activity launched Fujian green federation ecological service group Ningde station activity launched Zhang Jianwei correspondent Xu Jingxian) on November 24, Fujian green league ecological service group "youth concentric protect clear water and blue sky" Ningde station protection Huo Tongxi series of activities in Ningde city banana city town xi pond village Taiji square launched. Li Teng, deputy secretary of Fujian Provincial Committee, Li Yan, member of the Standing Committee of Ningde Municipal Committee and minister of Publicity Department, Chen Yi, vice mayor of Ningde Municipal Government and river chief of Huo Tongxi, members of provincial and municipal youth Federation and nearly 100 people attended the event. Pictures of the event site. Correspondent for figure event, vice mayor Chen Yi announced protection HuoTongXi series of activities started, li teng, deputy secretary of the leadership guest to provincial green league member ecological volunteer service, Ningde normal college green environmental protection volunteer service volunteer service flag, volunteer representatives read the youth concentric protect clear water blue sky initiative, provincial green league ecological service head party to preach ecological environmental protection knowledge. Subsequently, the leaders and guests attending the activity carried out the river patrol work of Xizhi Village and participated in the proliferation and release activities. Volunteers scientifically released 100,000 bighead fish fry and soil dace seedlings supported by the Scientific Release Special Fund of Beijing Entrepreneurs Environmental Protection Foundation. Pictures of the event site. Since the 18th CPC National Congress, the CPC Central Committee has made unprecedented efforts to promote ecological progress, and has made major strides in building a beautiful China. Recently, the Youth Federation of Fujian Province deeply studied, publicized and implemented the spirit of the Sixth Plenary Session of the 19th CPC Central Committee, Xi Jinping's ecological civilization thought and the spirit of General Secretary Xi Jinping's important speech during his visit to Fujian, and guided the ecological service groups of the province to carry out a series of activities. At present, in the upper, middle and lower reaches of Huotong Creek, the Youth League service group and the youth environmental protection volunteer service team have successively held the "Protecting the clear water and blue sky" activities. All the local units have also actively participated in various forms of outdoor publicity, river patrol and river protection, encouraging more people to participate in the river and lake protection work. Pictures of the event site. Since the beginning of this year, the Youth Federation at all levels in Fujian Province have thoroughly implemented the "Youth Federation Organizing and Serving Thousands of Villages" plan and the "Hope House" project, and actively explored the working mechanism of the Youth Federation organization to help rural revitalization and participate in social governance innovation. The province has established science and technology, beautiful rural planning and construction, industry revitalization, electricity to help agriculture four types of youth league members service 53, mobilize more than 500, and professional areas and rural technical support, rural planning and industry revitalization, e-commerce sales, talent training and other service activities nearly 200; docking remote rural school preparation youth league "hope hut" more than 130, mobilize youth league members through donations, software support, teacher training, effectively make up for the rural schools in the "second classroom" education deficiencies.

2021-11-24	Liuzhou: Four aspects to do a good job of the county Communist Youth League grassroots organization reform Guangxi Liuzhou: the quasi "machine, bureau, potential" under the good county Communist Youth League grass-roots organization reform "a chess game" Zhang Jianwei correspondent, Liu Tingting Wei Zhuli) since June this year, Liuzhou city is the first and only Guangxi city to promote the grass-roots reform of the Communist Youth League at the county level, Go all out to promote the reform of the county Communist Youth League grass-roots organizations, Reform and breakthroughs in four aspects: expanding the selection and management of cadres of the Communist Youth League, improving the organizational setup and operation, standardizing the development and education of the Communist Youth League, strengthening the Party's leadership mechanism and support and guarantee, Under the spirit of nail the good county communist Youth League grass-roots organization reform work "a game of chess". Take the opportunity of reform to quasi "star plate", forming a "party committee attaches importance to + department support + implementation of the Youth League committee" reform pattern. Party and government leaders attach great importance to strengthening the reform guarantee. Liuzhou Municipal Party Committee and Liuzhou Municipal Government took the initiative to grasp the correct direction of reform, open up the reform ideas, broaden the reform channels, and take the lead to steadily promote the development of the grassroots Communist Youth League organization work at the county level. Multi-sectoral support and pooling synergy for reform. The Organization Department of the Municipal Party Committee, the Municipal Organization Office, the Reform Office of the Municipal Party Committee, the Municipal Party Committee of the Liuzhou Municipal Party Committee, the Municipal Education Bureau and other departments communicated repeatedly to jointly seek policy breakthroughs in the selection and appointment channels, institutional establishment, performance incentive, and strengthening management, and finally establish a coordination mechanism of multi-department joint management. We will clarify the responsibilities of local leaders and promote the implementation of the reform. At the municipal level, we went to conduct in-depth research and guidance for 22 times, communicated with the main leaders and responsible leaders of the county and district Party committees, and won preferential funds, personnel and resources for the county and district Communist Youth League. To guide all counties and districts in the city to introduce the implementation plan for the reform of the local Communist Youth League county grassroots organizations, and to establish the main leaders or responsible leaders of the county and district Party committees as the convenor, and the relevant responsible departments to lead the tasks, clear requirements, clear responsibilities, and jointly promote the reform to go solid and deep. We will scientifically "draw chess charts", optimize reform and design arrangements, and focus on "key areas" for reform and innovation. The Liuzhou Municipal Party Committee of the Communist Youth League has repeatedly communicated about the reform matters with the relevant municipal departments and the municipal counties and districts, extensively solicited the reform opinions, and had discussions, discussions and individual visits with the grass-roots youth league members to gather the wisdom and scientific reform. In terms of reforming and innovating the cadre selection and management mode of cadres of the Communist Youth League, we explored "nine paths", such as open examination, open selection, open employment, temporary and part-time jobs, targeted recruitment, selective transfer, purchase of services, selection and guidance, and candidate recommendation. For example, enrich the cadres from the non-full-time cadres outside the Communist Youth League system, so that the proportion of temporary posts and part-time jobs in the city, county (district) youth League committee is not less than 50%; explore new ways to encourage cadres' professional title and post; and promote the volunteers of the Western Plan to apply for township institutions from written examination, etc. In terms of reforming the league organization setup and operation, relying on the party and mass service center, enterprise activity center, the embedded layout of the youth home, affects the construction of "party building + league building + unit" hierarchical WeChat tower group, form 5 minutes youth information reach working mechanism; promote "communist youth league + social organization" working method, establish "youth order, youth league committee, social organization order" service mode, implement the system of the youth and the league viscosity. In terms of reforming the development and education management mechanism, the league member implements the points system to join the league, clarify the assessment conditions of volunteer service and league class study; promote the process and standardized construction of league member file management, enter the league member files into the paper student files, and enter the personnel files after entering school or participating in work. We will promote the establishment of a whole-process training and assessment system for league members to reserve talents for the Party's cause. In terms of improving the leadership mechanism of the Party and support and guarantee, the league building and team building will be included in the random supervision and research contents of the grass-roots Party building work; and promote the reform of the grass-roots organizations of the county Communist Youth League into the deployment of comprehensively deepening the reform and the task list of 2022. Strong reform of strong "fall chess pieces", share experience, to create a replicable, can promote the group building brand. On the one hand, the pilot counties and districts are good practice to drive. Holding a seminar on the reform of the county Communist Youth League grassroots organizations, sharing the reform experience in Rongshui and Liunan, and driving all the non-pilot counties and districts to combine the local characteristics. The Youth League Rongshui County Party Committee took the lead in setting up the youth development center, and increased the establishment of two people. Liunan District effectively integrates the resources of famous teachers, builds the city's first county-level and district-level young pioneers' work famous teacher center group, and promotes the professionalization and professionalization of the young pioneers' work in urban areas. On the other hand, non-pilot county reform has their own characteristics. Counties and districts to serve the overall situation of the party and government center and to serve the young people, In the reform combined with their own characteristics: Rongan County broke through the establishment restrictions, Set up a Youth Development Center, Prepare 3 fully funded enterprises; Liucheng County focuses on strengthening the construction of the youth league school, Established the only Guzhai Mulam Ethnic Township Youth League School, To help cadres of ethnic minority youth grow up; Luzhai County Innovation Group organization contact information, Relying on the "Youth Home" to build a maker space and other service platforms popular among young people, Improve the service function of the regiment-affiliated position; Liujiang District to broaden the selection channels of youth League cadres, Establish a talent pool of "good rural youth", For the grass-roots league cadres selection do good reserve.

2021-11-29	Jining young pioneers practice education theme team day activities held "red scarf heart to the party" Jining young pioneers practice education theme team day activities held Zhang Jianwei correspondent Bai Chen) on November 25 afternoon, group Jining municipal party committee, Jining joint closes working committee, city women's federation, city culture and tourism bureau in Jining li bai memorial hall "red scarf heart to the party" Jining city young pioneers practice education theme team day activities. Jining City Working Committee deputy director of the Working Committee Li Chunxing, the City Culture and Tourism Bureau four-level researcher Liu Fenglai, deputy secretary of the Communist Youth League Municipal Committee Gao Cong and other relevant leaders attended. Municipal Cultural Relics Protection Center, Rencheng District Customs Working Committee, Rencheng District Youth League District Committee, Rencheng District Women's Federation, Rencheng District Education and Sports Bureau, Rencheng District Culture and Tourism Bureau and other relevant leaders, Rencheng District Young Pioneers counselors, Young Pioneers and other representatives participated in the activities. The picture shows the scene of Jining Young Pioneers off-school practice education theme team Day activity. Correspondent for the picture activity in the young Pioneers "flag song" opened, the team members in front of the Li Bai Memorial Hall singing "we are the successors of communism", the activity for the Jining Li Bai Memorial Hall "Jining Young Pioneers outside the school practice education base" unveiled, and for the red scarf volunteer commentator awarded a letter of appointment. Jining Red scarf tour group members carried out the theme of the "liberation of Jining battle hero" party history theme publicity, excellent red scarf narrator shared the "Taibai Tower and the Red General" keynote speech. Subsequently, the representative of the Young Pioneers brought the traditional youth culture program "Youyou Deer Ming". The picture shows the scene of Jining Young Pioneers off-school practice education theme team Day activity. After the ceremony, the Jining Li Bai Memorial Hall was officially opened. The participants visited the Li Bai Memorial Hall together, the red scarf narrator carried out the voluntary explanation activities, and the team members carried out the traditional culture experience activities. Taibai Tower is a famous Chinese historical and cultural building, a cultural relic protection unit in Shandong Province, and a patriotism education base in Shandong Province. It is an important building to witness Li Bai's residence and travel in Jining. In recent years, the city has deeply excavated the rich Li Bai cultural heritage, vigorously promoted and inherited the excellent traditional culture, and strive to provide high-quality public cultural services for the general public. Taibai Building has become an important cultural meeting room and a beautiful city name card of Jining District.

2021-11-22	Anhui Suzhou youth constitution, civil code legal knowledge contest group Suzhou municipal party committee held a "better life civil code accompany" youth constitution, civil code theme legal knowledge contest on November 22 (reporter Zhang Jianwei, reporter Zhang Can check WeiTing) recently, group Suzhou, Anhui province in Suzhou city second middle school "better life civil code accompany" youth constitution, civil code theme legal knowledge contest. After fierce competition, the municipal team won the first prize, Sixian team and Xiaoxian team won the second prize, �� qiao District team, Dangshan County team and Lingbi County team won the third prize respectively. This knowledge competition, after the preliminary selection, six teams from the city to participate in the competition. The competition focuses on the legal knowledge closely related to the study and life of teenagers in the Constitution and the civil Code, and is divided into three parts: required questions, rush questions and risk questions. In order to stimulate the enthusiasm of the students to participate, the competition also set up an interactive answer link, the municipal Bureau of Justice law popularization volunteers gave the civil code books and small gifts for the students who answered the correct questions, the audience rushed to answer. The purpose of this knowledge competition is to further deepen the publicity and education of the rule of law among teenagers, strengthen their awareness of the rule of law, enhance their practical ability of the rule of law, cultivate their legal thinking of respecting, studying, abiding by and using the law, and develop the good habit of finding the law and relying on the law for solving problems. During the whole competition, all the team members were full of spirit, close cooperation, witty answer to answer, and presented a wonderful legal knowledge "feast" for the audience.

2021-11-20	Yunnan Lijiang solid party history learning education of Lijiang solid party history learning education-rich form of the party history sincerely for the practical goal task, combined with consolidate deepening "don't forget the beginner's mind, keep in mind that the mission" the theme education achievements and celebrate the 100th anniversary, elaborate organization, vigorously promotes the implementation, promote the city party history learning education high standard high quality orderly, by the party history learning education of the central seventh steering group. Recently, party members of the Huaping County Commission for Discipline Inspection and Supervision attended a vivid Party history lesson at the Party spirit education base of Huaping Girls' High School. Under the guidance of Zhang Guimei, a national outstanding Communist Party member and the winner of the July 1 Medal Medal, the party members of Huaping County Commission for Discipline Inspection read the Seven Law Long March, reviewed the oath of joining the Party, and watched the performances of "Red Mei Zan" and "Wan Jiang"."I have gained a lot from this party history lesson."Party member Luo Yanjuan said with deep feeling, in the future to take Zhang Guimei teacher as a benchmark, to find the gap, clear direction, gather power, with practical actions to interpret the original mission of the party members. This is the scene of Lijiang City in the party history learning and education in the in-depth study of Zhang Guimei's spirit of "learning advanced, creating first-class" theme practice activities. Since the launch of party history study and education, Lijiang City has combined the study and education of Party history with the spirit of Zhang Guimei, given full play to the power of example, and told historical stories by example through grass-roots propaganda, party spirit education and other forms, so that the century-old history of the Party came into the hearts of the people. Up to now, organized 263 lectures of Zhang Guimei's advanced deeds, started the construction of Zhang Guimei Party spirit education base; 5 counties and 87 counties (districts) and municipal departments of the city, carried out concentrated study and exchange discussion, and held 4625 special organizational meetings. Draw spiritual strength, the city's party members and cadres burst out of the struggle "fourteenth five", forge ahead in the new journey of powerful power. A number of Zhang Guimei advanced workers have emerged successively, including Chen Yaowu, national outstanding Communist Party member and national advanced individual in fighting COVID-19, Mu Runhua, national March 8 Red Flag bearer in fighting COVID-19, Peng Ping, winner of the National May Day Labor Medal, Yang Shunqiong, national advanced individual in Food safety, and provincial model worker. With good live red resources innovation form further study party history in the ancient city open south institute, Lijiang municipal party school teachers in the open south institute monument, visited the exhibition hall, around the study of party history, feeling "open south spirit", combined with the actual work and personal feelings, and the day in "into the red base to carry out the party history education" party members for warm communication. This is the concrete practice of Lijiang City to deeply excavate the red resources in the study and education of the party history, build the party spirit education base, and guide the party members and cadres to pursue the red footprints and feel the lofty spirit through the on-site objects, vivid explanation and interactive experience. Since the development of party history learning and education, Lijiang city has made good use of red resources, built learning platforms, innovated learning forms, and closely combined the requirements of the central government with local characteristics to learn Party history. Ancient city comprehensive service company when a day sanitation workers experiential learning education way, establish the ancient city party spirit education base, mobilize 2864 party members and cadres to attend when a day sanitation, when a green, when a day parking, when a star toilet cleaner experience activities, the party spirit education classroom from indoor to work, the content of the party spirit education from books to practice. Focusing on the youth group, the Lijiang Municipal Committee of the Communist Youth League released online theme group (team) courses of "Youth Learning" and "Red Scarf Love Learning" through WeChat and Weibo, learning columns of "Learn the History of the Party and follow the Party" and "Read the Party History every Hundred Years", and mobilized 829,000 young people to participate in online learning. We will design 8 research routes for teenagers, and carry out online and offline and rich theme publicity activities, such as "Red Heart to the Party" summer research, so as to ignite the enthusiasm of young people to learn the history of the Party in a way that is popular with young people. In early September, 1,405 students from the No.2 Middle School in Ninglang Yi Autonomous County attended the school as scheduled. This is a epitome of Ninglang County's combination of the study and education of party history with the promotion of the central work of the county Party committee and the county government to solve the problem of the children of the people and the children of the migrant workers relocated to inhospitable areas. It is understood that with the joint efforts of all levels and departments in Ninglang County, not only the construction project of Ninglang No.2 Middle School has been fully completed, but also the expansion project of Ninglang County No.3 Kindergarten and Bell Middle School has also been completed as scheduled. 51.5% of the students in the county have studied in the county seat. Party history learning education, Lijiang municipal party committee put forward to develop a batch of agricultural industry, consolidate a batch of poverty achievements, improve a batch of infrastructure, solve a batch of ecological problems, optimize a batch of business environment, strengthen a batch of energy security, upgrade a batch of wisdom service, improve a batch of governance results, resolve a batch of disputes, do a batch of people's livelihood security "ten" emphasizing "I do the practical work for the masses" practice, strengthen the masses' sense of happiness security. Lijiang Municipal Government Affairs Service Administration Bureau innovated the clear policy book of benefiting farmers made by Jinshan Street, Gucheng District, established the service guide "know on the code", provided 1,734 qr code of 60 departments and enterprises in the window and bulletin board, built a convenient service platform, and set up a bridge connecting convenient services. Lijiang city tax department through retired soldiers data information and tax cloud platform natural person database, corporate library data comparison, analysis of retired soldiers enterprises can enjoy the preferential policies, to accurately carry out the preferential tax policies, help veterans at ease employment as the goal, to settle retired soldiers 117 enterprises telephone communication and household visits, make veterans should enjoy the state support policies.(Our reporter He Qian)

2021-11-20	The communist youth league of Yantai municipal party committee light children "light of hope" benefit more than 690 children communist youth league Yantai municipal party committee light children "light of hope" in the green newspaper reporter XingTing) from June 2020 the communist youth league of Shandong Yantai municipal party committee launched the "hope hut" child care project, so far, the city online raise money more than 800 ten thousand yuan, built hope hut 483, built Jining, Texas and Chongqing wushan 210, benefit more than 690 troubled children. Yantai youth league organizations at all levels focus on the original document tent card family, disabled families, disabled teenagers, orphans, orphans, key children, low family children of 8 to 14, through the docking department, household visits, establish parameter, details about children living space, housing safety status, guardian will and other relevant information, grasp the difficult teenagers real situation. After accurate investigation, the city needs to build 476 hope cabins and 483 rooms from 2020 to 2020.21, exceeding the construction task. At the same time, according to the deployment of the Shandong Provincial Party Committee of the Communist Youth League, 150 cabins in Dezhou and Jining were implemented in three years, and each city built 50 cabins each year. At the same time, the project into the city's party history learning education "I do the practical work for the masses" focus on the people's livelihood, "hope hut light city landmark" activities, invited three Yantai Olympic champion for the project, in the city more than 5000 elevator advertising screen, more than 3000 taxi electronic screen on the posters and slogans, effectively expand the influence. Through the organization of the Youth Federation, the Youth Enterprise Association, the Youth Civilization number, the rural good youth to hold a project symposium, the Youth League Yantai Municipal Party Committee gathered various forces to build a cabin. In addition, the Youth League Yantai Municipal Party Committee raised multiple funds, accurately launched Yantai Xiwang Group, Blue and White catering, Zhenhua Group and other enterprises subordinate more than 1,800 stores all into the "consumption donation" system, a total of 4.76 million donations, accounting for more than 55% of the province's flow, driving the province's hope house "consumption donation" more than 2.66 million yuan. In 2020, Tencent "99 Charity Day" raised 1.45 million yuan, in 2020, Tencent "520 Public welfare season" raised more than 1.38 million yuan, and in 2021, "99 Charity Day" raised more than 1.25 million yuan. In the process of construction, the Youth League Yantai Municipal Party Committee adhere to the "one household one policy, one house one gear", strict construction acceptance, normal tracking services. Organize cadres of the Communist Youth League at all levels and members of the Youth League to regularly "look back" on the built cabins, focusing on the construction quality and environmental sanitation of the cabins.innovatively carry out the "star" cabin evaluation work, recruit volunteers to establish a pair support mechanism with the assisted children, carry out volunteer services such as ideological guidance, psychological counseling and habit formation, and organize the assisted children to participate in activities such as feeling city, red scarf research, skills experience summer camp.

2021-11-19	Party history learning education new model played Shiyan youth work strongest "433" party history learning education new model played Shiyan youth work strongest voice Zhang Jianwei correspondent he yu) since the party history learning education, the communist youth league in Hubei Shiyan youth league advantages, comprehensive mobilization, classified guidance, careful planning, promote the city's youth party history learning education, learning, into the brain, in the heart, to ensure that the party history learning education has achieved solid results. The "four-type linkage" learning system forms a joint educational force. Adhere to classified guidance, grasp the focus of teenagers of different ages and different structures of party history learning and education, formulate a learning and education system with its own emphasis, and promote the differentiation and specific learning and education among teenagers."Panoramic" self-study, "hand in hand" guidance. Make and distribute the city's youth party history learning education program, starting from the direction of youth learning interest, hobby characteristics, with personal self-study, special discussion, tour publicity, ceremony education and other forms, to attract students to look up. Up to now, the city's 5,765 youth league branches have all launched the party history learning and education activities, covering 1.08 million teenagers."Tutoring type" preaching, face to face delivery. A youth party history education publicity group and red scarf lecturers covering outstanding young theory experts and young backbone from all walks of life were established. 61 lectures were held for primary and middle school students and young people in all fields of society. Nearly 30,000 young people participated in offline learning, strengthening the simple feelings of teenagers to love the Party, their country and their hometown."Side type" class, heart to heart to witness. To carry out the "100 stories", "youth heart to the party singing new journey", "one hundred years of the party history youth said", "party history today" cultural product production, innovative use of telling red stories, singing red songs, create red culture works and other youth popular learning carrier, constantly enhance the appeal and appeal of the party history learning education."Fun-style" activities, push them side by side. More than 90 interactive activities such as "Youth Learning Party History together", "Learning Party History with Tuan" and "Party History Story script Kill" were held. Through the interpretation of Party history, interesting answers and ranking competition, the interest of party history learning was effectively enhanced, and more than 350,000 young people enthusiastically participated. The "Trinity" activity mode firmly implements the law of quasi-youth to implement the important requirements of General Secretary Xi Jinping on the learning and education of the Party history for teenagers, and strives to build an activity mode with "visiting, Myanmar and new" as the main line, singing the main theme of "learn the history of the Party, strengthen faith, and follow the Party". Search for the red memory, ceaseless spiritual blood."Take you to clock in" "red scarf heart to party-red gene, pursue revolutionary footprints" theme activities, offline led youth, young pioneers for red landmark, dig home heroes, advanced figures, red history, to teenagers interest, understand, remember the story of party history education, let the teenagers through the clock, viewing form draw home "red nutrient". Remembering the revolutionary martyrs, review the party oath. During Tomb-sweeping Day, martyrs Day, the theme group (team) day activities such as "party", more than 38,000 teenagers online sacrifice heroes, patriotism; offline theme group (team) class 876, to remember the heroes, memorial martyrs, inherit and carry forward the fine tradition of revolutionary martyrs; in the "May 4", "61" and other important time nodes, to standardized, serious ceremony education, leading the party, league, team closely linked red chain. Innovate the education carrier, play and live the revolutionary spirit. Carry out "youth to the party" situational theme class demonstration activities, activities with stage, poetry recitation, song and dance performances and other forms of the development of the party, the city more than 90,000 teenagers through the live pursuit of red footprint, guide the youth to learn the glorious history of the party, feel the party; fusion new media form, widely collect the city at all levels of the "youth gift" series of short video, enthusiastically eulogize the brilliant achievements of the party. Focus on what the teenagers want to think, formulate a "I do something for the youth" program, determine 20 specific tasks, and effectively open up the aorta of the youth growth needs. Focus on long-term volunteer service. Focusing on the creation of culture and people's livelihood, Implementing the Community Plan " Initiative, Nearly 3,000 young volunteers have been organized to carry out more than 900 volunteer service activities, such as cultural creation, environmental protection, assistance in difficulties, and care for children, To create a good social atmosphere and a beautiful urban environment for cultural creation; To do our best in epidemic prevention and control, A total of nearly 300 youth commandos and youth volunteer service teams have been organized, Participated in serving more than 7,000 youth commandos and youth volunteers, More than 20,000 copies of anti-epidemic propaganda materials were distributed, Various medical and daily epidemic prevention materials worth more than 500,000 yuan, Build a strong youth barrier against the Delta virus. Anchor the employment and entrepreneurship to sincerely promote development. Implementing the "Qingchuang loan" project, A total of 31.47 million yuan of discount interest loans were issued, The loan amount ranks among the top in the province; Support rural youth entrepreneurship "rural well-off" 45 projects; Report 23 college students entrepreneurship support projects in Hubei Province, Strive for support funds of 720,000 yuan; Carry out the "live broadcast with the post" online job fair, Provide more than 1,200 jobs; Holding the 2021 Shiyan Young Entrepreneurship Pioneer Training Camp; Invite the famous domestic business mentors, For 50 people such as young entrepreneurial talents in various industries, heads of start-up enterprises, rural youth leaders, returning entrepreneurial youth; Select young entrepreneurship models and young entrepreneurs' representatives to set up a youth entrepreneurship mentor group, More than 10 activities such as "Youth Entrepreneurship Lecture Hall", "Entrepreneurship Elite online sharing meeting" and "thousands of schools" have been carried out in colleges and universities. Focus on the hot spots and difficult points to do practical things sincerely. For 20 consecutive years, the Project Hope Campaign to Realize a Dream has been launched. From January to October, we raised 13,042,400 yuan of public welfare funds for Project Hope students, Help for 6,785 students from poor families, New project approved 6 hope primary schools; Strive for the support of the "Yao Fund" project, Organize professional volunteers to volunteer teaching in 32 rural primary schools in Yunxi County and Danjiangkou City, Regular "hope and micro-wish" activities to care for left-behind children, A total of "micro wish" materials worth more than 500,000 yuan, More than 5,000 micro wishes of left-behind children; Deepen the work of pre-youth and uninsured, We launched 603 "Safe Action", legal education and mental health themed activities, Audience of 130,000; Polish the "12355" work brand, Together with the Legal Aid Center of the Municipal Bureau of Justice and the Mental Health Association of the Municipal Hospital of Traditional Chinese Medicine to establish the municipal "12355 Youth Psychological Consultation Base", Accumulated service of more than 1,000 person-times; Shiyan Taihe Hospital Mental Health Promotion Center; Held "youth appointment is you" youth talent fraternity 11 times, Build an interactive dating platform for single young people.

2021-11-19	Zaozhuang municipal party committee in the party history learning education "heart" and "new" new word pledge movable type to Zaozhuang municipal party committee in party history learning education "heart" and "new" Zhang Jianwei) party history learning education, group of Shandong Zaozhuang municipal party committee closely learning education objectives, to carry out the "learn the party history strong faith with the party" overall arrangement, innovative learning carrier, pay attention to practice, response to youth demands, the party history learning education and inspire youth patriotism and bear, guide the city youth to learn party history of the "compulsory course". Zaozhuang Communist Youth League explores a new mode of learning party history, and circles the fans of the majority of young people. Innovation party history learning education activities, for the youth in the city, party history theme to preach, red songs, revisit the party, league, team oath, such as activities, search for Zaozhuang martyrs cemetery, hug calves gu 115 division martyrs monument, railway guerrillas memorial, taierzhuang war memorial red base and martyrs cemetery, a total of 4425 team organization in the concentrated learning activities, covering 350000 young pioneers. Strengthen the history of the party learning education activity viscosity, pku with young people popular way lit youth party history learning enthusiasm, online "red gene green inheritance" "green listen to the history of the party" column, the original MV "my party is a little sweet", actively participate in the # 1921 # weibo topic interaction, released more than 1000 new media products, viewed nearly 150 million people, simple about the party's extraordinary years in one hundred. Good party history education activity courses, mobilize the majority of youth members to actively participate in the "youth study together" series of online theme league classes, under the leadership of well-known anchors, network V review the history of the Party, a total of more than 1.1 million youth members to participate in the study, so that the youth in touch of the history of the Party. The Communist Youth League and Municipal Party Committee set up a party history learning and education youth publicity group, to carry out experiential publicity around the party history story, and to guide the youth to learn the Party history into the mind and heart. Typical propaganda enhanced persuasion, the Communist Youth League committee selected a group of ideological and political political literacy, high theoretical level, strong expression ability teachers, young typical, outstanding cadres to set up young teaching groups, select outstanding young pioneers counselors to set up a red scarf tour group, each member of the history of the Party according to their own experience. Grassroots preaching to enhance penetration, organization to preach "four" activities, XuanJiangTuan into schools, communities, villages, enterprises to focus, interactive preaching, vivid for youth, the history of the party, the historical logic clearly, the system of confidence thoroughly, the city's youth league organizations at all levels to preach 130 times, more than 30000 member youth to accept the baptism of the party."Immersive" preach to enhance the appeal, pku organization to preach team situational preaching, lead teenagers to visit the revolutionary soldiers, red scene, drama performance, from a touching story, a mottled objects to revisit history, to preach more approachable, lively, attract young people "every speak will listen to". To create a meaningful and interesting practice position of the Youth League Party Committee to build the youth party history learning education characteristic position, continue to enhance the attraction of party history education. Make full use of basic youth league organizations, the basic organization as the basic unit, to branch meeting, theme, theme TuanRi, special training form to carry out the history of the party learning education, organize youth in the "youth home" about party history knowledge, watch red film, organization salon discussion, the history of the party learning grassroots education, covering 5802 branch in the city. Broaden the channels of youth communication, pku, city less working committee give full play to the role in the flow of children's palace, public summer camp, "hand care" feeling city activities, organize youth clock red base, watch red movies, learn to sing red songs, in the form of the silent red seeds implanted youth heart. To do a good job of both needed and effective service platform, the Communist Youth League Municipal Committee to help the youth to solve problems as the center of the circle, constantly expand the forms, enrich the carrier, outline the biggest "concentric circles" to serve the youth. For the overall situation of the service center, it promoted the work of youth work safety, carried out the promotion action of youth work safety skills, jointly cooperated with the Municipal Emergency Management Bureau to launch the establishment of youth work safety demonstration posts, sorted out the key positions and weak links of 121 key enterprises, and organized the docking training of 1,290 registered safety engineers. Effectively help the work of attracting talent and wisdom, build 9 "youth post stations", realize the full coverage of Zaozhuang District (city), and provide "one-stop" services such as free accommodation, employment guidance and urban integration for young people in jujube. Carry out summer college students practice activities, 621 college students in 318 units to practice, organized 3 batches of more than 200 key university students to participate in the "outside students home travel" activities, and constantly improve the enthusiasm of students outside Zaozhuang to know home, love home, and stay in their hometown. Enabling youth development, to take the village (community) "two committees" as an opportunity to promote 491? Forty good young people into the village (community) "two committees" team, 390 good youth as the village-level league organization secretary. We cooperated with financial institutions to launch the "Good Youth Loan for Rural Revitalization and Rural Revitalization", helping 201 good young people with 85.74 million yuan, covering the last mile of serving good young people in rural areas. We have promoted the school-local cooperation of the "Green Bird Project", and held activities such as "Zaozhuang, Shandong-College Talent Through Train" and special job fairs for fresh graduates. More than 300 enterprises have provided more than 8,000 positions, attracting 12,000 college students. We will safeguard the rights and interests of children, accelerate the implementation of practical benefits to the people in 2021, vigorously promote the construction of "Hope cabins", raise 3.6 million yuan from various sources, and start the construction of 278 new cabins, with 56% of the annual task completed. Focus on doing solid swimming volunteer service, build daily publicity, special training, hidden trouble investigation and key supervision "four in one" working mechanism, establish the attention of the key personnel ledger, install 159 swimming marks, compiled a comprehensive, three-dimensional youth life safety net. We will provide pair services, carry out regular volunteer services such as family companionship and academic counseling, and organize 820 left-behind children to participate in activities such as feeling the city and achieving micro-wishes.

2021-11-19	Yangzhou Communist Youth League: Learn the History of the Party for the Youth. Tell the story of the history of the Party well, spread the history of the Party in a way popular with the youth, and widely use the "online + offline" way to study and implement the spirit of party history learning and education."This form is very novel, I before play script kill" "impressive, experience is too strong"... in guangling development zone, community league general branch is organizing youth to participate in the party history special red script killing activities, youth randomly selected role, fighting in the front line officers, hidden in the enemy camp underground party members, suffering people, play traitors, bandits, mysterious atmosphere instantly spread. After reading the script, young people use language to express their opinions and positions, or conceal and fabricate information to mislead their opponents. After rounds of evidence search and public chat, the smoky revolutionary history, gradually revealed in the fog... as the plot page by page opened, the youth slowly into the heart of the characters, gradually regard himself as a key member of the current of The Times, put himself into the historical sense of the "space dialogue" and "brainstorming". Youth League organizations at all levels in Yangzhou are committed to making the century-old Party history study alive in colorful and lively forms. They adopt offline learning methods such as red theme scripts and immersive drama performances, so that abstract theories can be seen, felt and learned, and promote the study and education of Party history to set off a boom and achieve results. Hanjiang district huai see town youth corps committee secretary Sun Qian said: " the marxist youth and youth said party history activities we invited the HanJiang district party committee party school youth member representatives and national youth representative for good good party history project education, like such activities we held once a month this year, strive to make youth members can attend, we also invite youth branch into the enterprise to carry out the enterprise."Innovation learning form, let the youth branch" on the platform, said party history, exhibition ", arouse the interest of youth learning history of the party, the party history learning education theme into a story, with small environment analysis of the big situation, with things reflect development, with sweet speak good truth, let the party history in one hundred" home " into ordinary people, leading the teenagers Ming party history, feeling."Hello, young friends, welcome to the last Gaoyou youth learning club line, I am the leader Xiaoqing today, today I will take you to review the last battle situation, understand the battle story, feel the spirit of the battle, now please follow me into today's study."In Gaoyou, every weekend, youth league members will come to the Youth Learning Club to understand the historical stories and feel the spirit of the Anti-Japanese War. There are also four red lines in Yangzhou, which are committed to reviewing the history of the Red Party and experience the red culture through immersive learning. Yangzhou is an important part of the central Soviet anti-Japanese base area and the Huainan anti-Japanese base area. At present, more than 150 revolutionary sites and patriotic sites have been excavated. Youth league organizations at all levels made full use of the spirit of the Iron Army of the New Fourth Army, the Huaihai Campaign spirit and other revolutionary spirit resources, deeply explored the deeds of the older generation of revolutionaries and revolutionary heroes, and used the red sites and memorial venues in the city to build five red youth learning club lines in the city. In the form that is popular with the masses, we will make the red resources hot and study the history of the Party alive, and effectively guide and mobilize young people to study history clearly, increase credit, study history Chongde, and study history effectively. On the one hand, using party history stories and red positions to infect teenagers. Baoying County used the Zhou Enlai Youth Reading Office to carry out the league joining ceremony, Gaoyou City used the "last battle" of the Anti-Japanese War of Resistance to carry out the theme league day activities, and Jiangdu District used the "Guo Village Defense War" to create a mobile propaganda platform and classroom for party history education. Youth league organizations at all levels conform to the thoughts and expectations of young people, seek new activities in form, and constantly extend them in content, making the learning and education work in their hearts, so that young people can have deep empathy with historical figures, historical stories and historical spirit. On the other hand, use historic achievements and beautiful blueprints to inspire young people. Youth league organizations at all levels make use of the glorious history of Yangzhou for three times to encourage young people to be the first and open the "Yangzhou Historical Achievements Classroom" to help young people understand the great achievements in the history of Yangzhou. Yangzhou Municipal Party Committee combines the party history learning and education with solving practical problems, highlights the deep understanding, adheres to the problem-oriented approach, transforms the learning results into the power and ability of the masses to do practical things and solve problems, and promotes serving the people to solve problems with practical results."The child's father died, and the mother was also seriously ill. Seeing the child growing up, now he can only sleep in the same room with his grandfather. Thanks to the village and the enthusiastic enterprise for building us the dream hut, and the volunteers who have been helping us. Thank you."In Ganquan Street, Xiao Qi (pseudonym)'s grandmother told everyone about the changes in the family. Under the help of hongzhi action, Xiao Qi has his own bedroom. With the help of school teachers, cadres of the village league general branch and social work volunteers, Xiaoqi, a previously autistic girl, gradually walked out of the haze and grew up in the sun. The Yangzhou Municipal Communist Youth League has vigorously implemented the key projects of youth care. Relying on the "Hope Village School", the in-depth implementation of practical work for the youth- - "Hongzhi Action", "Warm Winter Action" and other projects. We will implement the "Dream Transformation +" care plan, adhere to building dreams with love, by constantly expand the channels of assistance, actively strive for resources, give full play to the role of "dream hut" position and hub, to carry out regular services for "de facto orphans". A total of 3.382,500 yuan of project funds were raised, and 186 cabins were built. Establish municipal youth affairs social work professional committee, in the city to build six comprehensive service platform "the space", 20 service window "workshop" and other youth affairs cultivation base, school of social development in Yangzhou university, the city of all kinds of social organization service platform, large social work service institutions set up five training base.

2021-11-25	Rizhao city new 200 "hope cabin" dream set sail, Rizhao 200 new "hope hut" Zhang Jianwei correspondent XiangChang Hui) since this year, according to the Shandong provincial party committee "about release" I do the practical work for youth "work list notice requirements, sunshine municipal party committee combined with the actual, the" dream, "in the communist youth league" I work for the youth "work list, promoting cabin construction, cling to" cabin construction "and" volunteer service " two articles, ensure the cabin often warm, hope often bright. This year, with the focus on disabled people, teenagers from disabled families and teenagers from poor families, household survey, field investigation and analysis have set the target of 200 new cabins. By the end of October, construction of all 200 "hope cabins" had started. At the same time, looking back at the "Hope cabin" built in 2020, a third party will pay a return visit to the quality and use of the built cabin, and immediately require the builder to repair and rectify the cabin with quality problems. A new round of "Hope House" construction was launched in the first time. On March 11, the Municipal Poverty Alleviation Office, the Civil Affairs Bureau and the Municipal Disabled Persons' Federation held the 2021 "Hope House" work site promotion meeting to strengthen the linkage and joint construction of departments. Cities and counties through wall chart operations, establish ledger, log records, the work to promote the situation of clear, clear base number, high efficiency. According to the "Implementation Manual" and "Implementation Standard", the construction clear paper of "Hope House" was combed and issued to ensure the standardization and standardization of "Hope House" construction. Adhere to the problem-oriented, on-site office, clear "district and county to tackle key problems, the municipal bottom line, adapt measures to local conditions, seek truth from facts, take the lead to complete, to ensure the quality of the" work requirements. The implementation of "daily promotion, weekly scheduling, monthly summary", strict project process, safety clearance, acceptance clearance. Formulate "one household, one plan", fully discuss and determine the plan, drawings, budget and other details. Extensive launch, raising funds adhere to the project leading, "online + offline" combination, multi-channels to raise construction funds. Offline, actively strive for straight related departments support, focus on key time launched more loving people, in the office area, large business super, university town crowded area set up offline fundraising point, 1860 young volunteers face to face to explain, point to point project, the key promotion friends donation mode, the city's party and government organs, enterprises and institutions, social organizations, colleges and universities, youth, green association and loving people from all walks of life to participate in, emotional devotion, full support, common love matrix. Online, new media and other platforms are used to widely promote the "Hope House" project to the society, and expand the brand influence and financing scale of "Hope House". Actively encourage young people to participate in online donation activities. During the 520 "Tencent Public Charity Season", the total number of funds raised by the city's four districts and counties ranked among the top 50 in the province, and was awarded the honorary title of "' Tencent Public Charity Season'Hope House Child Care Project" by the Provincial Communist Youth League Committee. During the "99 Public Welfare Day", the total fundraising volume of Rizhao city also ranked among the top in the province. Three-level acceptance, to ensure the quality of the Youth League committee repeatedly put forward to adhere to the practical principles, avoid blindly improve the construction standards, at the same time to always tighten the safety string, the existing problems timely discovery, timely solution, timely rectification, to ensure that the project construction level gradually improved. After the completion of the cabin, the implementation of the township and street youth League committee, district and county youth League committee, youth League committee three-level acceptance policy. Township street youth League committee preliminary acceptance, the whole process and help to coordinate the decoration scheme and engineering materials. The district and county youth League committee shall conduct re-inspection, organize the staff to check whether the daily necessities of the "Hope House" are fully equipped, and check whether the service object information data sheet, information collection disclaimer, project service agreement and other materials are complete. The Communist Youth League and Municipal Party Committee organized professionals to carry out the final acceptance, combined with the Municipal Engineering Testing and Consulting Group, conducted standardized management of the project testing and acceptance, conducted strict testing according to the national standards, and carried out spot inspection and reinspection throughout the city. Volunteer service, continuous security to deepen "hope" service efficiency, completes the "second half" volunteer service, youth league organizations at all levels, universities, industry departments, social volunteer organizations actively innovation "hope" volunteer recruitment way, according to the characteristics of volunteers to form college students "grass partner", love mother service, industry departments, service team, improve service ability and coverage, volunteer service activities form "week" mechanism, to ensure that to help the children in the home every week. More than 12,000 volunteer services, such as family companionship, schoolwork counseling, and habit formation, were provided in the form of pairing, covering more than 400 children in the "Hope House" project. Districts and counties organized 600 volunteers to send school supplies, warm bags and other supplies to the "Hope House" and "Hand in Hand Care Action" assisted children, and provided free physical examination for more than 100 assisted children. We have carried out 15 "Feel the City" activities in Rizhao Ocean Park, Science and Technology Museum, Museum, City Study and other places, so that the children assisted in the hut can feel the change and development of the city, appreciate the charm of the modern city, and feel the warmth and care of the society. Establish a cabin comprehensive evaluation mechanism, formulate and unified Posting health management system, regularly organize health evaluation, cabin works collection, "cabin star" evaluation and other activities, through encouraging selection and praise, education and guide teenagers to develop good living habits, cultivate children's self-reliance, optimistic attitude towards life.

2021-11-22	The Qingdao Municipal Committee held the spirit of the sixth Plenary Session of the 19th CPC Central Committee of the spirit of the 19th CPC Central Committee Zhang Jianwei) Recently, the Qingdao Municipal Committee of the CPC Central Committee to study the spirit of the sixth Plenary session of the 19th CPC Central Committee. All the staff of the Communist Youth League and municipal Party committee organs and the responsible comrades of the affiliated units attended the meeting. The first topic was set at the meeting, with Zhang Haijun, Party Secretary of the Communist Youth League, leading the keynote speech of General Secretary Xi Jinping at the Opening Ceremony of the Fourth China International Import Expo. Subsequently, all the participants studied the full text of the communique of the Sixth Plenary Session of the 19th CPC Central Committee carefully. We have said, the plenary session comprehensively summarizes the party's struggle in one hundred major achievements and historical experience, called on the whole party and all the Chinese people of all ethnic groups to unite more closely around the party central committee with comrade xi as the core, fully implement xi new era the ideas of socialism with Chinese characteristics, vigorously carry forward the great founding spirit, not forget the suffering yesterday, worthy of today's mission, not the great dream tomorrow, to achieve the second goal in one hundred years, realize the great rejuvenation of the Chinese nation of the Chinese dream and unremitting struggle. The communique of the plenary session has profound thought, rich connotation and inspiring. In the next step, the Communist Youth League Municipal Committee will thoroughly study and implement the spirit of the plenary session, set off a learning upsurge among the vast number of youth league members of the city, lead the majority of young people based on their posts, work hard, pioneering and innovative, and contribute their youth strength to the construction of a modern national metropolis.

2021-11-18	Group Haikou municipal party committee to communicate learning the spirit of the sixth plenary session of the 19th Haikou municipal party committee to communicate learning the spirit of the sixth plenary session of the 19th Zhang Jianwei correspondent, Liu Yanzhao Zhou Yutong) on November 15 morning, group Haikou municipal party committee of Hainan province held theoretical study group (expand) learning, communicate learning the spirit of the sixth plenary session of the 19th. Communist Youth League committee, all cadres and staff and heads of subordinate units to attend the meeting. Members of the leading group made a speech on the study of the spirit of the Sixth Plenary Session of the 19th CPC Central Committee. The meeting stressed that the Sixth Plenary Session of the 19th CPC Central Committee is a meeting of great historical significance held at a major historical juncture. The plenum stressed that the "two establishment" is of decisive significance to the development of the cause of the Party and the country in the new era and to advancing the historical process of the great rejuvenation of the Chinese nation. Plenary session of the central committee of the communist party of China on the party's one hundred struggle major achievements and historical experience resolution, a new era of xi the ideas of socialism with Chinese characteristics, with "ten insist" summarizes the valuable historical experience of one hundred struggle, is a marxist programmatic document, is a new era of the communist party of China to keep in mind the mission, uphold and develop socialism with Chinese characteristics of political declaration, is the history as a mirror, create the future, action guide to realize the great rejuvenation of the Chinese nation. Group Haikou party secretary Li Yang requirements, the city at all levels of the communist youth league, youth league, union, young pioneers organization to study well, good publicity, implement the spirit of the sixth plenary session of the 19 as the current and future a period of primary political task, quickly learning propaganda to upsurge, strengthen the consciousness of "four", firm "four confidence", "two maintenance". We should improve our political standing and truly learn and understand it. To learn what it really is, Through mastery, Combine the study and implementation of the spirit of the plenary session with the study and implementation of the spirit of the 18th National Congress of the Communist Youth League and the second, third, fourth and fifth Plenary sessions of the 18th CPC Central Committee, Combined with the study and education of party history, To fully understand the great achievements, great significance, and great experience of our Party's century-old struggle, To guide the city's young people in the "study of the history of the Party, strong faith, follow the Party" learning and education, more firm ideals and beliefs, Strengthen our mission responsibility, Pay more attention to drawing on wisdom and strength, Constantly enhance the rational identification of the Party, So as to be more consciously a firm believer in and faithful practitioner of Xi Jinping Thought on Socialism with Chinese Characteristics for a New Era, Resolutely do " the Party has a call on, The regiment took action. ". To enrich the carrier of activities, improve the effectiveness of publicity. To rely on the new media platform, Adhere to the mass dissemination, Use good practical products, Give full play to the role of the "youth teaching group", Innovative development to carry out simple, tangible and effective publicity, discussion and other interpretation activities, With the league member meeting, league branch committee, league group meeting, group class, young Pioneers squadron meeting as the main organization way to carry out the theme team day activities, hold learning exchange meeting, organize essay and speech, etc., To promote the spirit of the plenum into schools, rural areas, communities, and enterprises, To preach and spread the spirit of the plenary session in the language of teenagers, Drive the city's youth and the public to learn about the spirit of the central government, Constantly consolidate institutional confidence, We will unswervingly listen to the Party and follow it on the path of socialism with Chinese characteristics. We should implement the spirit of the plenary session in light of the actual work. To be guided by the spirit of the Sixth Plenary Session of the 19th CPC Central Committee, In light of the reality of Hainan comprehensively deepening reform and opening up and building a free trade port, Adhere to the central task of serving the overall situation, To fulfill the duties and missions of leading and gathering youth, organizing and mobilizing youth, connecting and connecting and serving youth, Comprehensively strengthen the building of the Communist Youth League in the new era, We will focus on planning the comprehensive and strict governance of the Communist Youth League, To further promote the deepening of the reform of the Communist Youth League, Help China (Hainan Free Trade Port) youth Dream Avenue construction, Effectively enhance the leading force, organizational, service and contribution degree of Haikou Communist Youth League, Unite and lead the city's youth to make contributions based on their own duties, To welcome the 100th anniversary of the league with excellent results, In the construction of Hainan Free Trade Port, resolutely shoulder the responsibility of Haikou youth, Be a pioneer, take the lead, and set an example, Let, " Please reassure the party, Strong country has me " has become the common aspiration of the new era.

2021-11-18	Henan Puyang "take-away little brother" help the national civilized city to create Puyang "take-away little brother" power national civilized city to create Ji'an-wei zhang) recently, "do civilized knight and civilized city" - "take-away little brother" power national civilized city to create activity launch ceremony held in Puyang city center square, from (Puyang) operation center of more than 100 "take-away little brother" for the solemn oath. Puyang Municipal Party Committee Propaganda Department, Youth League Puyang Municipal Party Committee, Municipal Public Security Bureau traffic police detachment responsible person to participate in the activity. In her speech, Li Yali, vice minister of the Publicity Department of Puyang Municipal Party Committee, thanked the "little brother" for doing his best, without remuneration, serving the society and making the contribution to the creation of Puyang city. Under the requirements of the whole city to promote the centralized registration of electric bicycles and wear safety helmets, She hopes that, "Takeout boy" strives to be the propagandist and guide of civilized traffic cycling, Standardize cycling, wearing helmets, wearing work clothes, riding civilized, not jaywalking actively supervise and persuade uncivilized cycling behavior, Become the vanguard force of Puyang civilized traffic cycling; She hopes that, The general public friends do civilized people, To be a participant in civilized traffic cycling, Do not speed, do not go the wrong way, do not violate manned, not on the motor vehicle lane, To transfer the positive energy of civilized traffic, With practical actions to drive more people around to join the upsurge of creating the city. At the scene of the launch ceremony, Chang Hongbo, a brigade leader of Puyang City Traffic Police Detachment, read out the civilized traffic cycling proposal, and the participating leaders distributed masks and warm heart bags for the "food delivery boys". After the launch ceremony, Chang Hongbo carried out traffic safety knowledge training for the "delivery boy"."Civilized knights" will distribute civilized traffic publicity pages for citizens in the future food delivery process. As a new era of emerging youth, "take-away little brother", "delivery brother" like small bees shuttle in the streets of Puyang fields, give full play to the "little brother" "directly to the home, wide coverage" unique work advantages, and the center in the city, key work perfect fit, in the national civilized city, the national health city to create activities, gather the vanguard of emerging youth groups.

2021-11-18	Anhui Xuancheng held "yi" fluent English "volunteer activities promote the first phase of" yi "from" fluent English "volunteer English horn activities successfully held China youth network Beijing on November 18 (reporter Zhang Jianwei, correspondent, trail good beauty check WeiTing) on November 14, Anhui Xuancheng municipal party committee, xuan city youth volunteers association joint Hefei university of technology (Xuancheng campus) English in aofeng park east square successfully held the first phase of" yi "from fluent English" volunteer English horn activities. On the day of the activity, the volunteer teachers and the students came to the park square. Under the guidance of the volunteer teachers, the students introduced themselves, communicated and interacted with them in English, learned English tips and common phrases, and sang the English song "Friendship lasts forever" together. In the final oral practice, the students played in groups of fun games and English scene dialogue. The students were enthusiastic, and the atmosphere was very active and active. The communication activity lasted for more than two hours. In the laughter of the volunteers and the students, the first phase of the "' Yi Qi" from Fluent Speaking English " volunteer English corner activity was successfully concluded. This small world in Aofeng Park has built a platform for the students to communicate in English, and also built a platform for the students of the University of Technology to spread their love and knowledge. This activity not only exercised students 'oral English expression ability, but also stimulated students' enthusiasm for learning English. Students said that the activity was novel in form and rich in content, which made people harvest full and hoped that it could be held more in the future."Benefit" fluent English "volunteer English Angle project is group Xuancheng municipal party committee, city green association and union (Xuancheng campus) youth corps committee cooperation of the" youth volunteer " project, in the future, group Xuancheng municipal party committee will further strengthen the cooperation with colleges and universities, innovative volunteer form, expanding volunteer service new ideas, youth love, popular volunteer service activities, let the volunteer service, participate in civilized practice has become a new social fashion.

2021-11-16	Fuzhou to carry out the "hope project lesson" prevent bullying Fuzhou to carry out the "hope project lesson" prevent bullying reporter Chen Qiang) in order to effectively prevent bullying, create a friendly, happy, warm, harmonious campus environment, the recent Fuzhou group organization joint public welfare organizations to carry out the "hope project lesson" activities. Tuanlianjiang County Party Committee, together with the county Procuratorate and the Big windmill Social Work Center, carried out the theme activity of "One Class in the Hope Project, Zero Bullying in a Safe School". In class, social workers around "what is bullying" "away from bullying" "bullying" and "to help each other, love others" four aspects, and the students learn to prevent bullying related knowledge, to the students introduced the kinds of bullying, performance, and the students discussed the bullying coping methods. Social workers also handed out bullying prevention manuals to students, hoping for them to use them to spread bullying prevention knowledge on campus. Under the guidance of the group horsetail district party committee, to farce youth affairs service center organization social workers into horsetail town tingjiang central primary school, launched the "hope project lesson of prevent bullying" public welfare classroom activities, let the students to have a basic understanding of bullying, in the bottom of bullying seeds took root at the same time, help build a good atmosphere of friendly campus.

2021-11-13	Guiyang to strengthen garbage classification propaganda mobilization Guiyang to strengthen garbage classification propaganda mobilization work leading group office, to comprehensively promote Guiyang living garbage classification work, build the city mobilization, full participation, a good public opinion atmosphere and social environment, Guiyang will fully strengthen the garbage classification propaganda mobilization work, a variety of forms of garbage classification related content. Social publicity was all rolled out throughout. Make full use of the city's large LED screen, taxi, bus car LED display screen and organs, enterprises, institutions, the community publicity column to carry out garbage classification publicity. At the same time, we will increase airports, railway stations, bus stations and other crowded places of garbage classification publicity and advertising efforts, so that garbage classification is deeply rooted in the hearts of the people. Household mobilization covers all households. By the municipal comprehensive administrative law enforcement bureau, pku, district neighborhood and social organizations jointly formed more than 30000 people garbage classification volunteer team, strongly promote garbage classification knowledge household propaganda and garbage classification on the guidance, ensure to December 31, garbage classification propaganda covers every family in the city, everyone understand the garbage classification knowledge. At the same time, the industry departments will carry out garbage classification publicity for the management and service objects to ensure the full coverage of garbage classification publicity in units, enterprises, schools and farmers' markets. Media coverage is comprehensive. Traditional media and new media work together to popularize garbage classification knowledge through MG animation videos, illustrations, posters, H5 and other ways. According to this year's garbage classification questionnaire, more than one-third of the participating citizens learned about garbage classification through online media.(Reporter Tang Li)

2021-11-17	Jining young entrepreneurs training excellent traditional culture activities held Jining young entrepreneurs training excellent traditional culture activities held. Correspondent for figure China youth network Beijing on November 17 (xinhua Ji'an-wei zhang, correspondent Bai Chen) on the morning of November 12, sponsored by the Jining municipal party committee, city green enterprise association, build international investment (Shandong) co., LTD. To undertake the "ancient new sound, study mountain" young entrepreneurs study excellent traditional culture activities held in qufu mountain. Responsible members of the Communist Youth League Committee and representatives of the Municipal Youth Enterprise Association participated in the study and discussion and exchange. During the activity, the members held a discussion on the cultural salon "The Value of Confucianism in the New Era", visited the project of ploughing and studying academies, and felt the unique charm of Nishan, a traditional cultural holy land. Young entrepreneurs said that in the birthplace of Confucius and the birthplace of Confucian culture, by discussing "Confucianism", promoting the extensive and profound nature of traditional Chinese culture, combined with the development needs of modern enterprises, refining Chinese management ideas from Confucian classics, to empower the growth and development of enterprises. In the next step, the Communist Youth League Municipal Party Committee and the Municipal Youth Enterprise Association will further integrate resources, actively carry out colorful salon activities starting from the actual needs of the member enterprises, constantly improve the exchange and learning platform for young entrepreneurs, and promote win-win cooperation.

2021-11-12	Haikou city in 2021 Haikou young pioneers counselor training held in 2021 young pioneers counselor training organizers Ren Mingchao) November 8 solstice 10, group Haikou municipal party committee, Haikou city bureau of education and other units jointly organized in 2021, city, district young pioneers general counselors and the young pioneers brigade counselors in the city, a total of 206 people to participate in the training. Li Yang, secretary of Tuan Haikou Municipal Party Committee, attended the opening ceremony and delivered a speech. She hoped that the counselors who participated in the training would cherish the learning opportunity, strengthen communication, really learn something, and constantly improve their professional ability and work level. This training course lasts for three days, the course is compact and rich in content, which not only makes the students 'young Pioneers' work have a clear direction and new ideas, but also strengthens the students' belief to be an excellent young Pioneers counselor.

2021-11-18	Zhang Jianwei) On November 11, the sixth Plenary Session of the 19th CPC Central Committee concluded successfully and issued a meeting communique. Plenary summarized the party's struggle in one hundred major achievements and historical experience, called on the whole party and army the Chinese people of all ethnic groups to unite more closely around the party central committee with comrade xi as the core, fully implement the xi new era, the ideas of socialism with Chinese characteristics, vigorously carry forward the great founding spirit, not forget the suffering of yesterday's brilliant, worthy of bear today's mission, not the great dream of tomorrow, to achieve the second goal in one hundred, realize the great rejuvenation of the Chinese nation of the Chinese dream and unremitting struggle. As soon as the communique of the plenary session was announced, it aroused a warm response among the youth of Qingdao. They all expressed that they should thoroughly study and implement the spirit of the plenary session, firmly uphold their ideals and beliefs, keep in mind their original mission, work hard and move forward bravely, and contribute their youth strength to the high-quality development of Qingdao. Zhang Haijun, Secretary of the Communist Youth League, has profound thought and rich connotation, and summed up the major achievements and historical experience of the Party's centenary struggle and struggle. The century-old history of the Party is a glorious history in which young people unswervingly listen to the Party and follow the Party. As a cadre of the Communist Youth League, I deeply feel the glorious mission and the great responsibility. Next, pku will thoroughly study and implement the spirit, in the form of youth happy to promote the party's struggle in one hundred major achievements and historical experience, in the city's youth learning boom, lead the youth do not forget the suffering of yesterday brilliant, worthy of today's mission to bear, not tomorrow's great dream. Unity led the city general youth members, strengthen the consciousness of "four", "four" confidence "," two maintenance ", mind, innovation, hard work, yong yi, personal future with the national fate, the youth struggle into the cause of the party and the people, into the city's overall economic and social development, based on post, growth, contribution to youth strength, compose the youth. Xiao Yuqing, secretary of the Youth League Working Committee of Tieshan Street, West Coast New Area, Qingdao, is as strong as iron, and now he is stepping over from scratch. As a cadre of the Communist Youth League in the new era, I feel honored and proud after learning the communique of the Sixth Plenary Session of the 19th CPC Central Committee, and at the same time, I feel a great responsibility and a mission on my shoulder. I will learn to grasp the spirit of the sixth plenary session of the middle of the 19th, at the same time completes the propaganda work, to guide the youth firm ideal faith, and physically, do the practical work for youth, solving problems, for our new good quality development in the city, build socialist modernization demonstration leading area condensed youth wisdom, contribution pioneer power, take practical actions to practice "please rest assured, power have me" youth oath. Yan Zhen, president of Qingdao Student Federation, is an important historical juncture at the intersection of the two hundred years. The plenary session summed up the achievements and experience of the past century from a strategic perspective and pointed out the direction of future development. As young students, we should deeply understand the spirit of the plenary session, fully understand the party's brilliant achievements in one hundred years, not only to "understand the past", but also to "understand the future". We will integrate our personal growth into China's development, unswervingly follow the Party, and fulfill our mission and responsibility as a youth. The future belongs to the youth, and the hope is placed on the youth. The past hundred years, is a hundred years of brilliant achievements, the next hundred years, is our young people. Young students should be firm in their ideals and beliefs, cultivate patriotism, develop excellent skills, and actively integrate into the society, serve society and contribute to the society. Their youth should inherit a century of glory, and run us well in the new journey of realizing the second centenary Goal. The Sixth Plenary Session of the 19th CPC Central Committee is a meeting of great historical significance held at an important historical juncture. Learning the content of the communique of the plenary session, but also made me more clear about my mission as a grass-roots young pioneers workers and responsibility. I will further bear in mind my original mission, firmly uphold my ideals and beliefs, practice the purposes of the Party, inherit the red gene, train new people of The Times, pursue reform and innovation, and unite and lead the young pioneers to listen to the Party and follow the Party. Through carrying out a variety of colorful young Pioneers activities, let the team members deeply feel the timeliness and appeal of the young Pioneers activities, and truly integrate into the activities. At the same time, we will also internalize the inheritance of the red gene and learning from the pioneer example into the actions around us, and clarify our responsibilities and responsibilities. To make comprehensive preparations for building a great modern socialist country and realizing the Chinese dream of the great rejuvenation of the Chinese nation, and let the "please rest assured of the Party, a strong country has me" become a practical action for every member of the team. National youth federation vice chairman, Shandong youth federation agricultural sector director of Qingdao youth federation KaiSheng Haofeng agriculture co., LTD., general manager of the sixth plenary session of the party's 19th struggle major achievements and historical experience, review our party adhere to the basic principle of marxism combined with Chinese concrete reality to solve the main tasks facing different historical period, lead the Chinese people to create a better life. As a member of the Youth Federation, I will firmly support the leadership of the Communist Party of China, stick to my duties, and actively participate in the work and activities of the Youth Federation, and actively promote the rural revitalization strategy, change through technology and innovation, and promote the digital and intelligent upgrading of the whole industrial chain of modern agriculture. Live up to the mission, live up to the great dream. Song Yunpeng, deputy director of Qingdao Emergency Center, listened to the important speech of General Secretary Xi Jinping. As a member of the Youth Federation, I was particularly excited. The Resolution of the CPC Central Committee on the Major Achievements and Historical Experience of the Party's Struggle in One Hundred Years is a summary of the Party's experience in the new historical period. Under this guidance, I will closely focus on General Secretary Xi Jinping's requirement to give priority to people's health in development, devote myself to the fight against COVID-19 and improve people's health literacy, and provide all-round and full-cycle health services for the people. At the same time, we should give full play to the role of members of the Youth Federation, unite and lead the young medical workers around them to firm up their ideals and beliefs, innovate, increase their talents, display their talents, sweat and inspire their youth, and do our best to realize the great rejuvenation of the Chinese nation. On behalf of Qingdao Young Entrepreneurs Association, the general manager of Qingdao Huatong Financial Holding Co., LTD., the Sixth Plenary Session of the 19th CPC Central Committee is an important meeting held at the critical juncture of the historical convergence of the "Two Centenary Goals". For one hundred years, the party leads the people in the great struggle, has accumulated valuable historical experience, this is: adhere to the leadership of the party, adhere to the people first, adhere to the theoretical innovation, adhere to the independence, adhere to the Chinese road, adhere to the mind, adhere to the innovation, dare to struggle, adhere to the united front, adhere to the self revolution. The meeting sounded the horn for young entrepreneurs to forge ahead on a new journey. Industry and diligence, young entrepreneurs have unified their thoughts and actions into the spirit of the Sixth Plenary Session of the 19th CPC Central Committee. The second "centenary" is approaching, and the "Chinese dream" of national rejuvenation is coming ahead. In the face of the challenges of enterprise development, young entrepreneurs need to work hard, and lead enterprises forward step by step with a pragmatic style, practical attitude and innovative thinking. In this process, every young entrepreneur will find a stage of life, take his ambition as the direction, take his diligence as the driving force, and strive forward together with the enterprises, the state and the private capital. Xie Bin, deputy director of the Operation Department of the Family Entertainment Division of Hisense Jumei Beautiful Company, looking back on the past hundred years of struggle, the Party united and led the people to endure hardships, overcome difficulties and create great achievements. When youth prospers, the country prospers. When youth is strong, the country is strong. As a new force in the cause of building socialism with Chinese characteristics, young people should not forget their revolutionary ancestors, their ideals and dreams, and be down-to-earth and pragmatic to realize the road to a strong country in the process of overcoming difficulties. I as a member of the hisense beautiful company youth powerhouse, also as a member of the hisense group youth pacesetter, when led by group party committee, youth corps committee, further study the spirit of the sixth plenary session of the 19th, closely around the "is committed to scientific and technological innovation, leading to intelligent as the core of advanced manufacturing, with high quality products and services happy billions of families" enterprise mission, down-to-earth hard work, contribute youth for national development. From November 8 to 11, the sixth Plenary session of the 19th CPC Central Committee was held in Beijing, marking a new step forward in the great rejuvenation of the Chinese nation. In the whole party set foot on the second centenary goal of the road, as a young party member, was born in a great era is our lucky, to contribute to the great cause is our responsibility! In the post of a bus attendant, I carry a ticket bag shuttle in the streets every day, witnessing the prosperity of the motherland, feeling the happiness of life, but also in the process of service constantly harvest touched and warmth. In the future work, I will not forget the beginner's mind mission, always keep consistent with the party on thought, action, the people's yearning for good travel as a goal, based on jobs to provide high quality car service, history in power construction, steadfast struggle, give full play to the responsibility of the young party members, do the second one hundred goal and striver Qingdao cieme, director of the cieme intangible chunhui liu troupe Shan chao comprehension of the party's struggle, in one hundred. I will study deeply, understand the spirit of the sixth plenary session of the 19th, adhere to history practice, practice, continue into the campus, into the community, understanding, listen to the ideas and needs of the youth, further strengthen cultural confidence, write a good story, good works, in the song of a new era dream, a dream, also let willow cavity the traditional culture generations development, inheritance and prosperity. Qingdao metro operation branch of youth civilization director Wu Zheng hundred years of struggle, forge ahead at the time. Over the past hundred years, the Party has led the people in their great struggle and accumulated valuable historical experience with the Ten Principles. In the new descend, we will unite to lead the subway youth, thoroughly study and implement the spirit of the sixth plenary session of the 19th, vigorously carry forward the great founding spirit, focusing on the city to optimize the business environment, the national civilized model city to create strategic tasks, comprehensive construction, operation, development, management, capital operation "five contest", with higher stance, with more solid measures to livelihood, with stronger bear exhibition, to match urban development strategy, help improve city quality level, contribute to speed up the construction of modern international metropolis metro youth power. In the next step, the Qingdao Municipal Communist Youth League Committee will thoroughly study and implement the spirit of the Sixth Plenary Session of the 19th CPC Central Committee, set off a learning upsurge among the youth league members of the city, and lead the young people to base themselves on their posts, work hard, pioneering and innovative, and contribute their youth strength to the construction of a modern international metropolis.

2021-11-14	Zhang Jianwei) Youth people are the main force of innovation and entrepreneurship. Improving the ecology of youth innovation and entrepreneurship, and enabling all kinds of enterprises and young talents to attract, stay, and develop well, is a necessary requirement for stimulating the vitality of urban innovation and entrepreneurship. Group Qingdao municipal party committee according to the Shandong provincial party committee "green and qilu" work "promote influence, consolidate position construction, strengthen financial support, accurate professional fu assign, innovation policy integration" requirements, focus on youth innovation in key areas, around the bottleneck restricting youth innovation entrepreneurship, innovative launch including ten plate "green pioneer action", with "project, institutionalization, platform, the brand" work ideas to build youth innovation entrepreneurship "service chain" and "ecosystem", help build youth development friendly city. Focus on optimization of service, pioneer to build "post" "cloud" to create pioneer "post". Actively in straight departments, large enterprises and window units set up more than hundred "green gen pioneer civilization ShiFanGang", build "one do a good service chain", bright identity, commitment, practice skills, practical effect, actively organize open week, efforts to be good "the bartender", through bright sun process list, "one-stop" matters, handheld APP online business measures, simplify the process, with high quality and efficient service polished youth civilization "green word brand", guarantee youth innovation entrepreneurship. The Youth team of Qingdao 12345 Government Affairs Service Hotline, the Jimo Branch office of the Municipal Bureau of Ecology and Environment, and other youth teams were awarded the national Youth Civilization number. Qingdao Rural Commercial Bank has established a comprehensive "Youth Innovation Pioneer Civilization Demonstration Post" in all branches of the bank, opened green service channels, provided consulting services, business management, wealth management and value-added services for youth innovation and entrepreneurship, and gradually built a whole-process, comprehensive, convenient and standardized single-window service system. Construction of youth innovation pioneer "cloud". Qingdao will jointly build the Industrial Internet youth public service platform, and comprehensively serve the youth innovation and entrepreneurship, internship, employment and employment around the ecological industrial Internet platform. Relying on the "convenient" Qingdao "citizens comprehensive service APP development" green e "youth comprehensive service cloud platform, provide youth employment services, entrepreneurship support, rights and interests maintenance, dating, volunteer service, policy consultation, learning, communication, psychological counseling, financial services, social integration services, fusion through qin island, tunnel e, guosen through online service resources, build youth affordable, find, use the" one-stop " integrated service cloud platform. Optimize and upgrade Qingdao 12355 Youth Service Desk, integrate and design 8 service functions such as online consultation, psychological evaluation, legal inquiry and activity release, and link professional legal and psychological service institutions such as Deheng Law Firm, Shenzhen Mental Health Center and Shenzhen Heart Talk psychological service to escort the healthy growth of teenagers in an all-round way. Focusing on professional support, qingchuang pioneer set up a "station" to open a "class" to establish a legal escort "station". Firmly establish a "the rule of law is the best business environment" concept, joint city justice bureau, city lawyers association, integration of de heng, qin island, kang, strategy and other well-known law firms, comprehensive launch "green and pioneer legal escort" action, released for the first time, the Qingdao youth innovation entrepreneurship legal service manual, select the city of more than 230 outstanding young lawyers formed "green and legal services", set up within the city set up 60 "green and legal services", for the social service hotline, timely online for entrepreneurial youth provide tangible, normalized legal services. Carry out the youth innovation pioneer "class". Joint venture capital institutions, legal institutions, industry associations and other "Youth Pioneer Lecture Hall" and "Rural Revitalization Youth Lecture Hall", inviting experts and scholars, professionals, entrepreneurship examples to explain policies and regulations, entrepreneurship knowledge, entrepreneurship cases for entrepreneurial youth through the combination of online and offline ways, to provide all-round entrepreneurship guidance. We have implemented the "Rainbow Umbrella" publicity and taught the rule of law for teenagers, held moot court competitions for teenagers, and held more than 200 youth rule of law lectures, both online and offline, covering nearly 1 million teenagers. We will build a platform, regular and institutionalized "Youth Meeting Room 2.0", listen to the aspirations of young entrepreneurs, connect with relevant government departments, and help solve the problem of youth innovation and entrepreneurship. Focusing on the platform construction, youth pioneer to help "loan" to build "park" group "group" to launch youth pioneer "loan". Strive to resolve young entrepreneurs financing difficulties, lack of mortgage, high cost, program numerous pain points difficult problems, innovative financial support, rich financial products matrix, joint Qingdao agribusiness bank innovation launched green and pioneer basic loans, e, talent, discount loans, farmers bear loan 5 types of products, establish "online and offline" "the" urban + rural "" discount + guarantee "" product matrix + convenient service "comprehensive service architecture, through youth innovation entrepreneurship" capital chain "the last kilometer," credit " aspiring youth innovation entrepreneurship. Since the beginning of this year, a total of 460 million yuan of youth innovation pioneer loans have been issued to support more than 2,880 young urban entrepreneurs, young entrepreneurs, good rural young people and young people returning to their hometowns. Among them, qingchuang Pioneer e-loan adopts online processing, unsecured, pure credit, low interest rate, one-stop convenient services and other modes, and after the product was launched in less than three months, the loan scale reached 220 million yuan, which is widely favored by young entrepreneurs. Construction of youth innovation pioneer "garden". Adhere to factor drive, coupling linkage, effective integration of district youth innovation business incubation platform resources, joint Shanghai youth entrepreneurship international incubator, haier card, tianan digital city platform enterprises, strive to build youth innovation entrepreneurship demonstration service carrier and incubation space, built in the city of Qingdao youth innovation entrepreneurship base, rural revitalization of youth entrepreneurship base more than 50, to provide entrepreneurial youth skills training, project incubation, financial support and market development and other comprehensive services. Joint spring in industrial capital group, capital venture capital institutions, build youth innovation venture capital can assign platform, using the "ecological chain VC + city partners" model for start-ups from angel round to IPO full cycle capital services and the whole process of solutions, to provide new track, make new plans, create new space, promote new breakthroughs. Set up the youth innovation pioneer "group". In the city to select 100 in enterprise management, industry operation, business incubation, rural revitalization, financial investment, legal consulting has rich experience of experts of "green gen pioneer mentor group", around the advanced manufacturing, digital economy, modern service industry, rural revitalization, urban operation and other areas such as all-round fu can power youth innovation entrepreneurship. We implemented the "Rural Revitalization and Youth Achievements" campaign, organized and mobilized 800 youth league organizations, youth organizations and social organizations in the city to help 2,000 rural young people at all levels, young people returning to their hometowns and rural entrepreneurship leaders, and actively participate in rural revitalization, innovation and entrepreneurship. Taking the opportunity of the change of the municipal Youth Entrepreneurs Association, more than 10 young entrepreneurs from Shenzhen and Shanghai with investment and cooperation relations with Qingdao have been innovatively recruited to further strengthen the communication and cooperation between young entrepreneurs in Qingdao, Shenzhen and Shanghai. Focusing on the environment, the pioneer set up "home" to promote "line", "competition" to create the pioneer "home". In the city 10 district construction "Qingdao youth station" 18, through the standardization construction, convenient application, for foreign students to green employment entrepreneurship within 7 days free accommodation, policy consultation, job promotion, entrepreneurial guidance, urban into the one-stop service, relieve to green employment entrepreneurship students accommodation cost, employment confusion, not familiar, from the personalized demand to solve "key things", build young talents stay green warm heart first stop. Up to now, he has served more than 300 young talents, among whom more than 60 people have worked in the youth. Carry out the youth innovation pioneer "line". Joint in universities, youth entrepreneurship association, rural good youth organizations to carry out the "green and pioneer park" and other activities more than 10 times, organize nearly 600 people college students, entrepreneurial youth, rural good youth into the incubator, entrepreneurial base, industrial park, excellent enterprises, etc., through the policy explanation, discussion communication and understanding practice, help young entrepreneurs understand Qingdao entrepreneurial environment, improve youth entrepreneurial vision, find the suitable entrepreneurial carrier, effective docking entrepreneurial resources. Hold the youth innovation pioneer "competition". Qingdao "Future Innovation � q to Youth" Youth Innovation and Entrepreneurship Competition was held, striving to build a platform for achievement display, learning exchange, resource docking and project incubation for the majority of innovation and entrepreneurship youth, so as to promote learning, research, innovation and production through competition. In the 10th Shandong Youth Innovation and Entrepreneurship Competition " in 2021, a number of projects from Qingdao with high science and technology content, great market potential, good social benefits and high investment value stood out, winning 2 first, second and third prizes of the competition, and 6 excellence award projects.

2021-11-12	Jining Jining municipal party committee of youth production safety ShiFanGang create unit site verification work Jining municipal party committee to carry out the youth safety production ShiFanGang create unit site verification work Zhang Jian correspondent BaiChen) to promote youth safety skills promotion action, promote youth production safety ShiFanGang process, normalized, better test daily production safety management, recently, the Shandong Jining party committee to "four not two straight" way to apply to create 2021 youth production safety ShiFanGang related enterprises, team to carry out the on-site verification. The picture shows the on-site verification of the establishment unit of the youth safety production demonstration post. The inspection team of the CPC Municipal Party Committee randomly selected the second class of tooth manufacturing workshop of No.1 Main Engine Machinery Co., LTD., and the construction project department of Xintiandi International Business Center of Jining City Investment Holding Group, and went directly into the production site to understand the daily production status of the relevant enterprise teams. The person in charge of the enterprise introduced the safety production and self-inspection, daily safety management practices and related highlights of the practice. Verification team carefully check the hidden risk screening, operation scheduling, duty record parameter, such as detailed understanding of the enterprise young worker work condition, safe production operation, site management, safety responsibility, major hazard control, production safety training, etc., and for "green AnGang" create work puts forward the guidance promotion Suggestions.

2021-11-11	Jiang Yutong, the founder of the first Internet industry Youth League Working Committee in Taizhou, Zhejiang Province) On November 4th, Taizhou, Zhejiang Province, Linhai City Internet Industry Youth League Working Committee and Youth New Media Alliance was established. Tuanlinhai city committee to promote the national county communist youth league organization reform pilot work as an opportunity to fill the gap in the new field, strengthen the contact, emerging youth group after Taizhou first express industry in August, after three months of baseline research, discussion, organization planning, layer upon layer elected the Internet industry league committee secretary and members, a total of seven people, covering the more than 200 Internet companies in the city. Newly established linhai city Internet industry league committee will stimulate the Internet industry youth work initiative and enthusiasm as the focus, play a guiding role as a whole, continue to promote the conditions of the Internet industry enterprises to establish youth league organization, in the Internet industry party organizations, enterprises and build a communication bridge between youth, to strengthen the Internet industry own development as the key point, efforts to sing good "youth theme". To deepen the service Internet industry from youth brand construction, linhai municipal party committee organization department two new branch, linhai network letter office, linhai city bureau, linhai city public security bureau, business bureau, linhai city market supervision bureau, linhai city big data bureau will be around the "double wings" group to build a mechanism, with the Internet industry to promote the communist youth league and youth work "resource sharing, project, youth to discuss". After the meeting, linhai city green law league secretary-general, linhai city XuanJiangTuan director into a masterpiece the Internet industry legal risk prevention theme, for the Internet industry in the process of operation may face labor disputes, illegal advertising, unfair competition, trade secrets and other legal risks, and infringement of citizens' personal information, illegal intrusion computer information system crime common criminal risk, and share how to correctly prevent the risk.

2021-11-11	Bengbu: make "field", the standardization of volunteer service system of Anhui Bengbu: make "field", standardization of volunteer service system hai-han wang haihan wang lei correspondent Xu Zi) on October 13 afternoon, taihu lake world culture BBS sixth conference after the closing, this happened: BBS chairman YanZhaozhu saw a group of young people dressed in red clothes, recognize them as accompany you 4 days of young volunteers, he immediately offered to photo with volunteers. The shutter was pressed, leaving behind a precious commemorative photo. The sixth annual meeting of the Taihu Lake World Cultural Forum, with the theme of "Mutual Learning between civilizations: Building a Community with a Shared Future for mankind", is a feast of civilization and culture. During the activity, 131 Bengbu young volunteers (including 30 English volunteers) became the bright scenery. They were carefully selected and trained by 613 registered youth from Bengbu Municipal Committee of Anhui Province. To complete the task successfully in large-scale activities and show the youth style of the city is a test of the ability of volunteers, but also a test of the ability of the organizational unit. In fact, young volunteers were present at the International New Materials Industry Conference, the Conference on Enhancing the High-quality Development Drivers of the Less Developed Regions of the Yangtze River Delta, and the Music and poetry Singing celebrating the 100th anniversary of the founding of the Communist Party of China. Under the call of the Bengbu Municipal Committee of the Youth League, the young people from universities, organs, enterprises, Youth Volunteers Association, Youth Enterprises Association, Youth Federation and other organizations showed their style in the activities. The cultivation and cohesion of young volunteers is not an overnight process. How to build a "non-end" and standardized volunteer service system, and build a platform for young people to grow, communicate and make progress? The reporter came to Bengbu for an interview. During the Taihu Forum, the volunteers worked hard on the arrival, guest registration, guest registration, venue guidance, and conference assistance, providing quality services to nearly 500 Chinese and foreign guests from more than 30 countries and regions. Statistics from the Communist Youth League Bengbu Municipal Party Committee show that 81 students were among the volunteers and 50 social people who were already working."It is a rare opportunity to learn about different cultures and contact foreign affairs."From tension to adaptation, Liu Chang, an English volunteer from the Bengbu Tax Bureau, has gained growth. The 28-year-old is a native studying abroad, her first large-scale volunteer service. She admitted that when she first received the whole mission with the Korean mission, she was a little afraid that the other side was not satisfied. On the bus to the hotel at the station, she adjusted her mood and took the initiative to introduce the local customs, characteristic enterprises and industries of Bengbu. Unexpectedly, the other side of the things interested in, a series of interactive questions: " how is the professional setting of the university? Is there any legend and origin of the name of Longzhu Lake?"" was present from hotel stay to scheduling, to checking, coordinating daily schedule requirements, and translators."Liu noted that the Korean mission has high requirements for some etiquette, dining and occupancy details, but the mission is amiable and takes the initiative to greet itself every morning. They will also fully understand the temporary schedule changes. Every night, the volunteers will have a meeting to resume the work on the same day and sort out the plan for the next day."The few days of experience made me feel a lot. Many foreign guests said they wanted to know more about Bengbu. We volunteers should regard themselves as the host country. We represent not only the individuals, not only the units, but also the young people in Bengbu."Liu Chang also has a new understanding of volunteer service." What we do is to work behind the scenes for others, but only by doing our best to do the details first, can every link be carried out in an orderly manner. While "" serves the network art sub-forum, I have learned a lot of experts' views on the new forms of literature and art."Du Hongyang, a post-2000 student at Bengbu Medical College, is interested in traditional culture and has broadened his vision during his volunteering process. Get up at 5 o'clock every day, have breakfast as soon as possible, and then go to the venue to assemble and distribute the meeting materials. Guests come one after another, and Du Hongyang wants to maintain order, guide the guests, and accept their inquiries."I was a little tired, but I gained all the money, and every day was full."" When I pick up foreign guests from Azerbaijan, I can feel their enthusiasm. They want to take photos with the volunteers and say goodbye to us in English."Du Hongyang regrets that the volunteers are also the city image publicity ambassadors. Through telling and etiquette expression, they provide the guests around the world with first-hand and fresh information, and build a bridge to display and convey the culture. Although Du Hongyang is young, he is already a "veteran" of volunteer service ". In October 2018, at the 14th Anhui Provincial Games, he first signed up as a volunteer, responsible for placing tables and chairs, maintaining order and guiding the athletes. He appeared in the International New Materials Industry Conference, volunteer services for free medical treatment in mountain villages, and epidemic prevention and control card points. From freshman year to senior year, he has participated in more than 380 volunteer service activities in Bengbu."Volunteers are first and foremost a devotee who develop and progress with the city. Volunteering is not easy, some of the tasks are complicated."But he felt that through the training of volunteer service, he has developed the quality of facing difficulties, perseverance, in life and study encountered problems, will consider more details. In Bengbu, there are many enthusiastic young volunteers like Du Hongyang, but how to reasonably allocate and effectively manage them to exert their talents has become a test question in front of the league organization. Group Bengbu municipal party committee propaganda department staff, in the past, youth volunteer service activities exist scattered theme, service scope is not clear, the city's youth league organization "each", some project service people, and some areas, unable to form a unified youth volunteer service brand, weakened the appeal of volunteer service to the youth. At the beginning of this year, the Communist Municipal Committee of Bengbu formulated an annual volunteer service plan around the central work of the Municipal Party Committee. With the theme of "Happy Bengbu Youth Action", the nine unified activities of "Hundred Party, Clean Rivers and Lakes, Clean Village, Health, New Style of Civilization, Clear Network, Care for Tomorrow, Sunshine Disabled, Warm Winter Action" as the main line began, and the youth league organizations at all levels and youth volunteer associations in the city participated in it regularly. Taking the "Happy Bengbu Care for Tomorrow" action as an example, more than 300 volunteers provided volunteer services such as handmade, academic guidance, family companionship, and self-care education for left-behind children and poor children in groups. Song Haoyu, a 2020 student from Bengbu Technician College, is one of the volunteers. In the children's homes in the communities and streets, Song Haoyu became the "little teacher" of the children. He used his major to help the children in difficulties in the community develop their interests and skills. In April, he travelled to the Diaoyutai Street Children's Home twice, taking them to learn about picture books and teach them to make terracotta warriors. In May, he offered some more clay-making lessons, and also taught vocal music and dance."It taught them the dance moves of the song" You Smile So Good, " and they felt a great sense of accomplishment."At the end of each activity, before Song Haoyu and others left, there will always be children asked:" Brother and sister, when do you come back again next time? At present, the Youth League organizations at all levels in Bengbu city have carried out more than 300 volunteer service activities around the nine major actions, with more than 20,000 volunteers, who are active in the streets and alleys. In the "Happy Bengbu Warm Winter Action" activity, the League Bengbu Municipal Party Committee, together with the Youth League Committee of Bengbu Railway Station, organized "clam Xiaoqing" and "Little Sweet Orange" to participate in volunteer services in transportation, Spring Festival transportation and epidemic prevention. Many young people were affected and "attracted" to sign up to become volunteers. For a long time, the Youth Bengbu Municipal Party Committee has also explored the operation of youth volunteer service projects and brand building, and created a number of excellent volunteer projects. Wuhe County's "Xiaohe umbrella" public welfare poverty alleviation volunteer service project is one of them. One day in the winter vacation of 2016, Wan Gongda, a teacher from Toupu Middle School in Toupu Town, Wuhe County, drove to the school. After a crop field covered with snow, he suddenly found that five pupils were playing by the water beside the canal, at risk of falling down at any time. And there is no obvious safety sign beside the pond."What about your parents?"He quickly pulled over and asked about the children. The oldest child said carefully, " My parents haven't returned home for the Spring Festival yet!"At that time, Wan Gong was so shocked that he felt that he would do something for the left-behind children in his hometown."There are many left-behind children in my class. They look insecure in their eyes and smile rarely."Later, he learned from his colleagues that the Communist Youth League Wuhe County Party Committee innovatively carried out the" Xiaohe umbrella " volunteer service project for left-behind children, and he took the initiative to exchange ideas to the Communist Youth League County Party Committee. Since then, every summer and summer vacation, the Communist Youth League County Party Committee will make "drowning prevention" billboards, placed in each township remote pond; before the holiday, the joint county blue sky rescue team to each township schools to carry out drowning prevention safety publicity."I also became a volunteer of the 'Little Lotus Umbrella', using my free time to do more meaningful things. The children's innocent and grateful eyes were my constant motivation."Wan Gong said. Since 2016, TuanWuhe County Party Committee has gathered social forces and continued to care for the growth of young people in difficulties. At present, it has promoted the construction of 6 township "small lotus umbrella" volunteer service centers and 19 village-level "small lotus umbrella" volunteer service stations, carried out self-protection education activities such as fire prevention, electricity prevention and drowning prevention; held "small lotus umbrella" public welfare run according to the needs of young people; organized professional volunteers to hold "star picking small class" class tutoring activities in the county welfare home. As a pilot county of the reform of the National County Communist Youth League, with the deepening of the reform, the Communist Youth League Wuhe County Party Committee insists on the integration of social forces. The "Xiaohe umbrella" project gathers more than 300 young volunteers, covering the members of the county youth Federation, members of the youth innovation association, five old volunteers, psychological counselors and other groups. Volunteers for juvenile delinquency prevention are also the Bengbu Communist Youth League brand project. In recent years, the city's Longzihu District Youth League Committee has set up a special volunteer service team of 20 people with a "suitable adults" system to provide temporary guardianship of the minors involved, conduct psychological counseling and psychological evaluation for the infringed teenagers, visit the minors involved, and write social investigation reports."We maximize the integration of resources, to mobilize the youth league organizations at all levels, youth civilization, youth social organizations, communities to participate in the happiness of Bengbu youth action activities, improve the incentive mechanism, brand, cohesion, make volunteer service cohesion, service youth platform, let volunteers show the image of Bengbu city 'youth spokesman', for happiness of Bengbu."Said Han Jian, party secretary of Tuan Bengbu.

2021-11-11	Shandong Yantai: make young students to smoke employment warm heart first stop in Shandong Yantai: make young students to smoke employment warm heart first stop in the first stop in the youth network reporter XingTing) to effectively help the city "ZhaoCaiYinZhi" work overall situation, deepening the development of youth friendly city construction, in April this year, Yantai municipal party committee organization department, the communist youth league committee launched the "youth station" project, relieve to smoke youth accommodation cost high, job pressure problems, build young students to Yantai employment warm heart first stop. Now, the project has been included in the "Yantai City three-year Action Plan to accelerate the gathering of young talents to smoke employment and entrepreneurship", and included in the key matters of Yantai Party history learning and education "I do practical things for the masses". In terms of specific construction, the Youth League Yantai Municipal Party Committee adhere to standardization, city and county linkage, to the point with the surface. At the municipal level, the logo of youth post stations is designed uniformly, issuing work guidelines, clarifying the internal design, online application, service content, etc., and highlighting the youth elements and local characteristics. County-level joint brand chain hotels for cooperation, various post stations are located in convenient transportation, complete supporting facilities, complete indoor hardware. At present, the city has built 30 post stations, basically realizing the full coverage of the district and the city. In order to make it convenient for young talents to apply for occupancy, the Yantai Municipal Party Committee of the Communist Youth League jointly developed and launched the WeChat mini program of "Yantai Youth Post Station" together with technical forces, bringing 30 post stations in the city into online management, and clarifying the occupancy conditions and conventions of the post stations. By logging in the small program, upload the interview notice, resume, graduation certificate and other supporting materials, you can complete the online application. The staff of the post station can complete the audit within 24 hours and check in after passing the examination. The mode of application without meeting and approval within a deadline is implemented. At the same time, the "youth station" has achieved accurate employment services. Before moving in, the station service specialist took the initiative to connect with job-seeking young people, understand the needs of employment and entrepreneurship, and help connect with relevant government departments and enterprises. During the check-in period, the post service specialist provides job seekers with supporting services such as post occupancy, policy consultation, employment guidance, urban integration and legal aid. The "Youth Station" was included in the "Green Bird Return Sailing Yantai" plan, which released more than 11,000 quality posts with Yantai Municipal Organization Department and Yantai Municipal Human Resources and Social Security Bureau of 410 high-quality enterprises, and organized more than 900 young students from colleges and universities inside and outside the province to carry out internships in enterprises, public institutions, towns and streets in the summer vacation. After the interview, the service specialist follows up the progress of their job hunting and recommends more suitable positions for the young people who have not successfully entered the job. In addition, in the process of building a "youth station", the Youth League Yantai Municipal Party Committee pays attention to the diversification of publicity and promotion. Take the initiative to connect with human resources and social security, education and other departments, with the help of the special recruitment activities of "Yantai Famous Enterprises and Famous Enterprises" and "Industry + Talent", promote the project, talent policy and post information of the station, and improve the awareness of the station. Participate in the city's talent policy publicity group, and publicize the youth post station policy "face to face", so that more young students can know about the post station. Make the propaganda video of Yantai Youth Station, and increase the "exposure" of the station through the mainstream media and the new media of the league.

2021-11-10	Reporter Zhu Juanjuan) The reporter learned from the Youth League Yichang Municipal Party Committee, today, the city's first 10 young talent station officially opened. College students who come to Yichang for jobs can enjoy up to 15 days. In addition, the young talent station will also provide supporting services such as employment guidance, policy push, job release and social integration. The 10 young talent station distribution in the city is convenient transportation, good comprehensive conditions near the business circle, respectively are: convenient city Yichang train station, li maple hotel Yichang train station, li maple hotel Yichang wanda plaza store, green hao tai Yichang wanda riverside store, ester hotel tourist center, beautiful chain fashion hotel, orange hotel white longgang shop, Yichang international trade hotel, Yichang orange hotel pedestrian street store, Yichang people's livelihood hotel. According to the introduction, all graduation within two years outside the city (household registration and the school are outside Yichang city) bachelor degree and above, can apply for check-in. To create a "one-stop platform" for serving young people to apply for jobs, and to solve the worries of college students living in the post station. The Yichang Municipal Party Committee of the Communist Youth League integrated the county and municipal level of the youth league cadres and the youth league association strength, and specially equipped each youth talent station with a "chief service officer"."Strive to be an ideological 'guide' of college students, a 'good helper' on the way to job hunting, a 'heart-warming person' to solve problems, and be a charming 'promoter' of Yichang."Chief service officer representative, regiment point military area committee secretary Li Wei promised at the event site. Source: China Youth Daily, November 10,2021, edition 04

2021-11-10	Fujian Sanming League organization for single youth matchmaking Fujian Sanming League organization for single youth matchmaking network reporter Chen Qiang) sponsored by the Fujian Sanming League organization "group about the bar" youth dating activities recently held in Sanming City Sanyuan District cultural and creative park. With the theme of "Rush that Year", the activity set up interactive links such as "You draw, I guess" and "listening to songs", which attracted 20 young people from enterprises and institutions in Sanming City to participate. Through cultural and entertainment projects, they enhanced the understanding of the two sides and successfully helped 3 young men and women. In order to solve the problems of narrow circle, busy work and difficult making friends, which are generally faced by young people in the system, the Sanming Municipal Committee adhered to the city and county synchronization, and innovatively launched an integrated online and offline youth dating platform. In the past year, a total of 91 various activities have been carried out, covering 2,130 single young people, successfully matching 152 pairs, and obtaining 28 pairs of marriage certificates. According to the introduction, the Youth League Sanming Municipal Party Committee established a general database on the official WeChat platform online, set up two platforms offline in the north and south areas of the city, regularly organized small gatherings within 30 people to communicate and interact, according to the "equal conditions, complementary advantages" to accurately select and match the participants. This year, six special friendship activities for young teachers were held in municipal schools alone, covering 128 single young teachers. At present, Sanming city has established 15 "Tuan Tuan about bar" positions. The relevant person in charge of the Youth League Sanming Municipal Committee said that in the future, it will continue to improve the mechanism construction, innovate the form of activities, highlight the linkage and interaction, through the regular expansion of the scope, regular database construction and regular tracking return visit, the launch of the "Tuan Tuan Date Bar" brand, truly serve the youth, help and gather the youth. Source: China Youth Daily, November 10,2021, edition 04

2021-11-10	Jinan Communist Youth League issued Zhang Jianwei correspondent Peng Chong for the snow and snow cold wave weather recently, Jinan city fell heavy snow, local snow, snow is rare for many years. To prevent the snow weather to the traffic travel and production and living, the Jinan municipal party committee, Jinan youth federation issued a mobilization order, called for the city youth league organizations at all levels, the youth organizations, youth members, youth volunteers and other social youth from all walks of life to play a leading role, take concrete action to participate in dealing with the snow and ice in the cold weather. Shovel ice and remove snow, youth first. Youth league organizations at all levels and the youth working committees should organize the youth league members of their own units and industries in an orderly manner, and actively participate in the removal of snow and ice on the premise of ensuring their own safety. Municipal young Entrepreneurs Association and other youth organizations should actively respond to the call of the municipal party committee and the municipal government, first of all, to "sweep the snow in front of the door". Under the premise of ensuring their own safety, the city's volunteer service organizations and volunteers actively participated in the snow removal and deicing action, and took the lead in the nearby buildings, hospitals, streets, schools, hospitals, bus stations, bus stations, market towns and other public areas to eliminate the inconvenience and safety risks caused by low temperature and blizzard to people's travel. Warm feeling to send warm, the youth comes first. The city's youth league organizations at all levels, the youth working committee to organize young volunteers to the "hope cabin" children and "Jin Hui to help the elderly" elderly home visit, ask about the warm winter situation, help check travel safety, heating safety and electricity safety, timely investigation of safety risks, to ensure that the "one old and one small" safe and warm winter. Members of the Youth Federation and members of the Youth Enterprise Association should give full play to the vanguard role of young groups and try to help the surrounding affected enterprises to resume production. Civilized transportation, youth comes first. Youth league members should travel in a civilized way, abide by the traffic rules, do not rush the road, occupy the road, yield to pedestrians, keep a safe car distance, create a good travel order, and reduce the burden of urban traffic. At the same time, a wide release of publicity of rain and snow weather safety tips, to remind travel citizens, especially the elderly, children, pregnant women and other people to do a good job of protection, to prevent slippery road fall injuries. Safety publicity, youth first. The majority of youth league members should actively publicize the concept of cold prevention and safety, publicize the concept of safety in response to low temperature blizzard to neighbors and relatives, improve the people's awareness of prevention and response, and enhance the self-protection ability of the masses. Youth moves up, the spring city has power. Let us take the initiative to extend our hands, work together, care for home, to the cold blizzard prevention contribute youth strength.

2021-11-10	Fujian Sanming group to build "group" brand service single youth Fujian Sanming group building "group" brand service single youth newspaper in the youth reporter Chen qiang) recently, sponsored by the Fujian Sanming municipal party committee "group" youth dating activities in Sanming sanyuan red mountain 1 cultural creative park, from the enterprises and institutions of 20 single young men and women. The activity took the theme of "Rush that Year", set up "you draw I guess", "listen to songs", "DIY food" and other popular links that young people like, to enhance the understanding of both sides through cultural entertainment, and three pairs of young men and women successfully matched. To solve the system of youth generally face narrow circle, busy, dating, Sanming municipal party committee adhere to the cities and counties synchronization, innovation launched "group about" online integration youth dating platform, nearly a year 91 activities, covering single youth 2130 people, successful 152 pairs, marriage for 28 pairs. According to the introduction, the Youth League Sanming Municipal Party Committee established a general database on the official micro line, set up two platforms offline in the north and south areas of the city, regularly organized a small party within 30 people to communicate and interact, according to the "equal conditions, complementary advantages" way to accurately select matching participants. This year, six special social activities for young teachers were held in municipal schools alone, covering 128 single young teachers. At present, the city has set up 15 "Tuan Tuan Date Bar" positions, and wechat groups attract nearly 100 single young people from schools, airports, government organs and enterprises to join them. The relevant person in charge of the Youth League Sanming Municipal Party Committee said that in the future, it will continue to improve the mechanism construction, innovate the form of activities, highlight the linkage and interaction, through the regular expansion of points, regular database construction, regular tracking visit, the launch of the "Tuan Tuan Date Bar" brand, truly serve, help and gather the youth.

2021-11-10	Yichang, Hubei province, the first 10 young talent station in Hubei Yichang first young talent post for college students longest free accommodation 15 days net ping green network reporter Zhu Juanjuan) the reporter learns from the communist youth league in Hubei Yichang committee, today, the city's first 10 young talent station, to Yichang college students can enjoy the longest 15 days free accommodation, and supporting employment guidance, policy push, post release, social integration and other services. Shang Liang, vice director of the Organization Department of Yichang Municipal Party Committee, and Fan Xuzhong, secretary of the Communist Youth League Yichang, jointly unveiled the first batch of young talent stations.(Yichang municipal party committee for figure) the 10 young talent station distribution in the city convenient transportation, good comprehensive conditions near the business circle, respectively are: convenient city Yichang train station, maple hotel Yichang train station store, �� maple hotel Yichang wanda plaza store, green hao tai Yichang wanda riverside store, yester hotel tourist center store, beautiful fashion hotel, orange hotel bai longgang shop, Yichang international trade hotel, Yichang orange hotel pedestrian street store, Yichang people's livelihood hotel. Those who have a bachelor degree or above outside the city (household registration and the school are outside Yichang city) within 2 years of graduation can apply to check-in. Aiming to create a "one-stop platform" for young people to apply for jobs, in order to effectively solve the worries of college students living in the post station, the Yichang Municipal Party Committee of the Communist Youth League integrated the county and city level cadres and the youth league association strength, and specially equipped each youth talent station with a "chief service officer"."Strive to be a 'guide' for college students, a 'good helper' on the way to job hunting, a 'heart-warming person' to solve problems, and a 'promoter' for charming Yichang."At the event site, the chief service officer representative, regiment point military district Committee secretary Li Wei commitment.

2021-11-09	Guangxi Nanning City "Red Scarf Growth Experience Camp" series of activity launch camp " and the minor protection volunteer service activity launch ceremony, was held in Nanning Youth Activity Center, Guangxi Zhuang Autonomous Region. On the day of the event, the organizer held the flag awarding ceremony of "Red Scarf Growth Experience Camp" and the opening ceremony of "Nanning Public Security Volunteer Service Workstation". Relevant person in charge of Nanning Municipal Committee said, since 2020 and Nanning Public Security bureau established "youth escort" minors protection professional volunteer team, anti-bullying, riot terrorism, psychological crisis intervention, drug prevention and other safety education activities more than 200, covering 25,000 teenagers and their parents; "care for minors care for the motherland flowers" unprotected publicity month activities 36, benefiting more than 11,600 people. The activity aims to let all departments cooperate with each other and work together, strengthen the protection of minors in a more rich and diverse, solid and effective way, and help children to enhance their self-protection awareness, master self-protection methods, and establish a correct concept of self-protection of the rule of law. In the next two months, "red scarf growth camp" and minors protection volunteer service series activities will integrate youth activity center, Nanning public security volunteer service workstation and other professional resources, for 7 to 12 teenagers safety education, art, language performance, physical exercise, life skills learning and other comprehensive quality promotion activities, extend minors protection service content, promote the growth and development of minors. The activity was jointly sponsored by Nanning Municipal Party Committee, Nanning Municipal Public Security Bureau and Nanning Municipal Youth Working Committee. About 100 people from Nanning Municipal Party Committee, Nanning Municipal Public Security Bureau, Nanning Municipal Youth Activity Center, young students and young volunteers attended the event.

2021-11-09	In Mudanjiang City, Heilongjiang Province, 712 young volunteers have officially raised young volunteers to participate in scientific wearing masks. Up to now, 712 young volunteers have been successfully stored through online registration, youth league organizations at all levels and offline youth volunteer organizations convening. On November 5, the staff of the Mudanjiang Municipal Party Committee of the Communist Youth League selected 16 key areas and more than 20 key routes according to the personnel flow, organized young volunteers to divide into four routes to persuade people who did not wear masks and distribute new masks, and gave detailed explanations to pedestrians who did not wear masks correctly. At the same time, the Mudanjiang Municipal Party Committee also organized youth volunteer service teams of organs, enterprises and institutions to carry out regular persuasion work around their stations, advocating people not to gather to chat, keep a safe distance, and not to participate in various mass activities during the epidemic prevention and control period, so as to further weave a tight "publicity network" for epidemic prevention and control.

2021-11-09	Jining Municipal Party Committee to carry out the work style rectification of colleges and universities to promote the reform and implementation of Jining City to carry out the work style rectification and promotion and the key work of the Communist Youth League mutual inspection and mutual learning action Jianwei correspondent Bo Chen) November 4 to 5, In order to further implement the key work deployment of the Central League Committee and Shandong Provincial Committee of the Youth League, Promote the implementation of the reform of the Communist Youth League in colleges and universities, Jining Municipal Committee of the Youth League carried out the rectification and improvement of college and league cadres and the mutual study of the key work of the Communist Youth League, Responsible comrade of the Communist Youth League Municipal Party Committee, Jining Vocational and Technical College, Shandong Vocational College of Technology, Jining Technician College, Qufu Far East Vocational and Technical College Youth League Committee responsible comrades attended. The picture shows the work site of the rectification and promotion of colleges and universities. Correspondent for the action to field view, visit, view information, combined with the early stage of the college style of rectification project supervision research and the colleges and universities problem rectification, public commitment, does the practical work to the ground, for this year college cadres style rectification improve work results and since 2021 the reform of the communist youth league key task to promote mutual learning mutual inspection. At the same time, the paper discussed the implementation of key tasks such as party history learning education, green bird plan, hope hut and so on, and exchanged and shared, and interviewed the evaluation of the style change and job satisfaction of the league and study cadres. This mutual inspection and mutual learning is an important carrier for the Communist Youth League Committee to implement the reform and evaluation of the Communist Youth League in universities. Through mutual inspection and mutual learning among schools, the university Youth League Committee is further promoted to standard advanced, find gaps, improve, and promote the implementation of the reform of the Communist Youth League in the observation, learning, evaluation and comparison.

2021-11-09	Zhenjiang City, Jiangsu Province held the Government Service Skills Competition Bureau and the Youth League Zhenjiang Municipal Party Committee jointly held the 2021 Municipal Government Service Skills Competition and the Government Service Youth Civilization Quality Service Special Competition Final. Ten teams from government service, tax, fire protection, public security, market supervision and other industry systems participated in the finals. The final is carried out in the form of on-site competition, a total of required answer, answer, scenario analysis 3 links, around the party history learning education, administrative examination and approval service business and youth civilization related knowledge, focus on policy understanding, problem solving, communication service and other aspects of the comprehensive ability. After the competition, Yangzhong Municipal Bureau of Administrative Examination and Approval won the first prize, and the other participating teams won two second prizes, three third prizes and four winning prizes respectively. Next, Zhenjiang government affairs office, municipal civil service bureau and group Zhenjiang municipal party committee will lead the youth civilization collective, government affairs civil servants service system, business experts yong bear "to be a model, demonstration, walk in the forefront" Zhenjiang mission, normalized "town" special service activities, do "top three strong three than run", contribute to the construction of modern new Zhenjiang youth strength.

2021-11-08	Youth League Qidong Municipal Party Committee to carry out the "sunflower" care action Youth League Qidong Municipal Party Committee to carry out the "sunflower" care action to carry out the "sunflower" six build integrated care action. Youth League Qidong Municipal Party Committee according to local conditions, to carry out the "dream cabin" renovation project according to the person, raising a total of 600,000 yuan, 30 "dream cabins" have been transformed. Qidong Municipal Party Committee also held "hand holding hands" and "dream transformation +" care youth activities, "inheritance", "reading", establish "many to one" academic tutoring mechanism for school teachers, cadres, village cadres, cadres tutoring more than 40 games, open summer college students "cloud classroom" 6 games, held 10 times. In addition, the Youth Qidong Municipal Party Committee also cooperated with the Qidong Maternal and Child Health Care Hospital to carry out the public welfare activity of "dream transformation + health care", carrying out blood examination, vision screening, height and weight measurement, internal and surgical examination for teenagers. Source: China Youth Daily, November 08,2021, edition 02

2021-11-06	Group Huaibei municipal party committee of the dissemination into the night market campaign of huaihuai "jianghuai dissemination line" of the "dissemination night market" concentrated campaign JianWei correspondent Cao Qi WeiTing) in order to further enhance the youth of the rule of law consciousness, set up the rights concept, recently, the group of Anhui Huaibei municipal party committee organization "escort youth" volunteer service members in huaihuai "jianghuai dissemination line" of the "dissemination night market" campaign, through publicity materials, giving balloons, interactive games to teenagers popularize legal knowledge, the dissemination propaganda work and the night market economy organic integration, let the dissemination really have "fireworks".site of activity, Members of the "Escort Youth" volunteer service team made balloons with the legal logo, Attract teenagers to stop and watch, By issuing balloons, your questions and my answers, legal knowledge contests and other forms, Guide young people to actively read law books, Stimulate the enthusiasm of teenagers to understand the relevant legal knowledge; Issuing "Huan Huan statement", "Interpretation by Case" and other legal materials, Tote bags, legal mouse pads and other legal items, More than 1,500 copies of laws and regulations such as the Constitution, the Civil Code and the Law on the Protection of Minors, Help teenagers to learn and master legal knowledge from books at any time; Through the face-to-face communication, Understand the problems of teenagers in detail, Seriously solve the legal doubts for young people, At the same time, increase the legal knowledge publicity for parents, Work together with their parents to help their children establish the concept of legal rights protection. Members of the "Escort Youth" volunteer service team have said that the form of the night market law popularization activity is more novel and interesting, and there are more people coming and going, which can make the public feel the legal atmosphere while playing, and have a better learning effect. They hope to participate in more such activities in the future to escort the healthy and happy growth of teenagers in Huaibei together.

2021-11-05	Group Shiyan municipal party committee: build youth "craftsman" create "five" authority group Shiyan municipal party committee: build youth "craftsman" create "five" authority Jianwei correspondent Jin An) to enhance the level of cadre specialization, refinement, strengthen precision execution, Hubei Shiyan municipal party committee to "five" authority to create activities as the gripper, outstanding post "training", strive to build a to meet the needs of high quality development youth "craftsman". Focus on ability improvement, to create a learning organization. Adopt the spirit of theoretical central group study, the series of important speeches of the provincial Party Committee and the municipal Party Committee, and the spirit of the series of plenary sessions, branch themed Party day activities, "youth study" and reading classes, and accurately grasp the pulse of high-quality development. At present, the organization has organized and carried out 13 central group study, 10 branch theme party day activities, the city's "youth" study for 12 seasons, opened 2 reading classes, organized 5 special seminars, and effectively consolidated the ideological foundation of "carrying forward the spirit of craftsman post training". Focus on ideological emancipation, to create a high-efficiency organs. With "thought ice-breaking lead development breakthrough" as the theme, adhere to the combination of "introduction" and "going out", carry out "learn advanced, standard, find the gap, reason, direction" big discussion, big research activities, invite experts and scholars to carry out "young cadres how to speak politics", xi jinping, general secretary "July 1" important speech spirit and CPC five eleventh plenary session spirit, "my secret" special counseling, led the communist youth league system focus view tour and red base to visit education activities, guide the party members and cadres to improve the political stance, unified thought, problem solving, promote work. We will make good use of the active government OA and the "smart group building" system, build a "double micro" platform, improve government efficiency, reduce government costs, and ensure government security. Focus on the problem investigation, to create a model organization. Combined with "Shiyan civil servants online online assessment system", control the party branch secretary, the leading cadre and party member responsibility, control responsibilities and "ten star" to create commitment content, adhere to the weekly report, record, season evaluation system, the party responsibility implementation with the annual party member democratic appraisal and civil servant assessment results, guide the party members and cadres model duties, form the party construction, the party affairs, everyone role together. Focus on the post training, to create a pragmatic organization. To implement the spirit of the fifth eleventh plenary session as the theme, continue to deepen I do the practical work for youth, "double report, double report" activity mechanism, such as active planning construction "Shiyan digital economy incubator" plan ", the implementation of" weir plan "," power "volunteer city", "Beijing weir youth kecan alliance" set up, four key work, carry out "three training", "three practice one fine" activities, constantly improve the level of party members and cadres post takes office and the ability to serve for the people. Focus on the rectification of work style, to create a clean type of organs. A leading group for the construction of clean organs of the Communist Youth League and the Municipal Party Committee was established, which was led by the office to carry out three special rectification efforts, including poor service quality and efficiency, weak discipline constraints, and lack of responsibility. Through the implementation of the work system of first inquiry and responsibility, weekly clearance, and repeated deduction, we will focus on building a clean organ with "good political ecology, positive employment orientation, solid work style of cadres, and excellent development environment", and promote the comprehensive and strict governance of the Party to develop in depth and cover all officials and workers.

2021-11-04	Yangzhou communist youth league: learn the party history for the practical work of youth: Yangzhou communist youth league for youth since this year, Yangzhou city, Jiangsu province communist youth league according to the "the party history in one hundred, build the red position, for the people to implement the" requirements, further contact youth, listen to the youth voice, healthy growth of the party history as the member of the most vivid textbooks, drive youth to learn party history of the "compulsory course". The Yangzhou Municipal Party Committee insists on telling the story of the history of the Party with youth language, spreading the history of the Party in a way that is popular with young people, and extensively using the "online + offline" way to study and implement the spirit of party history learning and education."This form is very novel, before I play every week in script kill" "impressive, experience is too strong" in straight development zone, community group is organizing youth to attend the party history project red script kill activities, youth randomly selected role, fighting in a line of officers, hidden in the underground party members, the people, cunning traitors, vicious bandits, etc., mysterious atmosphere instantly. In the process of role-playing, the smoky revolutionary history is gradually revealed in the heavy fog...... As the plot unfolds, the young people slowly walk into the hearts of the characters, and gradually regard themselves as a key member of the current of The Times. Hanjiang district huai see town youth corps committee secretary Sun Qian said: " we invited HanJiang district party committee party school of youth member and youth and the national up for good youth, bring party history project education, activities like this we held once a month this year, strive to make youth members can attend, we will invite youth branch into the enterprise to carry out the special."Yangzhou youth League organizations at all levels innovate the form of learning and publicity, let the youth teaching group" on the platform, say the history of the Party, show style ", stimulate the interest of young people in learning the history of the Party, the big theme of the party history learning and education into small stories around, so that the century-old history of the Party" fly into the ordinary people's homes "."Hello, young friends, welcome to the last Gaoyou youth learning club line, I am the leader Xiaoqing today, today I will take you to review the last battle situation, understand the battle story, feel the spirit of the battle, now please follow me into today's study."In Gaoyou, every weekend, youth league members will come to the Youth Learning Club to understand the historical stories and feel the spirit of the Anti-Japanese War. There are also four red lines in Yangzhou, which are committed to reviewing the history of the Red Party and experience the red culture through immersive learning. Yangzhou is an important part of the central Soviet anti-Japanese base area and the Huainan anti-Japanese base area. At present, more than 150 revolutionary sites and patriotic sites have been excavated. Youth league organizations at all levels made full use of the revolutionary spiritual resources such as the Iron Army spirit of the New Fourth Army, the Huaihai Campaign and other spirit, deeply explored the deeds of the older generation of revolutionaries and revolutionary heroes, and built five red youth learning club lines in the city with the red sites and memorial venues in the city. On the one hand, using party history stories and red positions to infect teenagers. Baoying County used the Zhou Enlai Youth Reading Office to carry out the league joining ceremony, Gaoyou City used the "last battle" of the Anti-Japanese War of Resistance to carry out the theme league day activities, and Jiangdu District used the "Guo Village Defense War" to create a mobile publicity platform and classroom for party history education. On the other hand, use historic achievements and beautiful blueprints to inspire young people. Youth league organizations at all levels took advantage of the three glorious history of Yangzhou to encourage the young people to be the first and open the "Yangzhou Historical Achievements Classroom" to help the young people understand the great achievements in the history of Yangzhou. Yangzhou Municipal Party Committee combines the party history learning and education with solving practical problems, highlights the deep understanding, adheres to the problem orientation, transforms the learning results into the power and ability of the masses to do practical things and solve problems, and promotes serving the people to solve problems with "practical results"."There is no way, the father died, and the mother was also seriously ill. Seeing the child grow up, now he can only sleep in the same room with his grandfather, thank the village and the enthusiastic enterprise for helping us to build the dream hut, and the volunteers who have been helping us, thank you."In Ganquan Street, Xiao Qi (pseudonym)'s grandmother told everyone about the changes in the family. Before Xiaoqi, no room, even no room to learn, under the help of Hongzhi action, Xiaoqi has his own bedroom. School teachers, cadres of the village League branch and social work volunteers helped Xiao Qi get out of the haze and grow up in the sun. The Yangzhou Municipal Communist Youth League has vigorously implemented the key projects of youth care. Relying on the "Hope Village School", the in-depth implementation of practical work for the youth- - "Hongzhi Action", "Warm Winter Action" and other projects. The implementation of the "Dream Transformation +" care plan was implemented, adhering to building dreams with love. By constantly expanding assistance channels and actively striving for resources, giving full play to the role of the "dream hut" position and hub, and carrying out regular services for the "factual orphans". A total of 3.382,500 million yuan of project funds were raised and 186 cabins were built. Establish municipal youth affairs social work professional committee, in the city to build six comprehensive service platform "the space", 20 service window "workshop" and other youth affairs cultivation base, school of social development in Yangzhou university, the city of all kinds of social organization service platform, large social work service institutions set up five training base.

2021-11-04	Beijing Chaoyang District Committee: Expand the organizational form and the youth "one button Touch" Spring Chaoyang "WeChat public account registered for the" Red Haitang " red theme script killing activity is a new experience."In the past, I always looked at the profile of the characters whenever I like. Red Begonia needs to answer the questions first to match the characters in the show."Mention this detail, the demon demon was impressed."Red Begonia" is a script killing product based on the martyr Ma Jun independently developed by the Chaoyang District Committee of the Beijing Youth League. The young people involved can feel the original story of the revolutionary ancestors for over half a century ago through role-playing. A young man who played Red Begonia with the demon said: " The script allows us born in peace to 'travel back to that era and become the man we admire'. Facing the difficulties and tests he faces, it also makes us think, what choices will we make when we are in that dangerous environment?"The script to kill" Red Begonia " is in recent years, the Youth League Chaoyang District Committee for young people to develop an immersive series of party history learning education products of a microcosm. Group chaoyang district party committee secretary Sun Boyang said, from youth reading group, "one hundred elegance is youth" -new era youth red street dance theme contest, to "let faith not confused" youth blind box, to "learn July 1 speech spirit-mind map" learning courses, group chaoyang district committee with "high quality youth thought guide product service platform" do traction, developed a series of can reach young people's thought leading products. In fact, "touch" is also the key word of the Chaoyang District Committee to work directly to the youth. In the reform process of the grassroots organization of the Communist Youth League, in addition to the translator of advanced ideas, the Chaoyang District Committee of the Communist Youth League takes the community youth gathering and the industry youth League working committee as the starting point to realize the "one-key touch" of the youth. Diversified youth structure means diverse needs. In order to produce thought leading products that can really let young people into the ears, into the brain and into the heart, the Chaoyang District Committee of the Communist Youth League has built a "central kitchen" for the research and development of products, actively improving their own product research and development capabilities and expanding product types. The original script to kill "Red Begonia" is a beneficial attempt to crack the "thought-leading products and young people love two skins", especially with the help of social resources, to provide more professional support for the products. Zhang Xinxin, the screenwriter of Red Begonia, is a senior young screenwriter. He has rich experience in creating drama and secret room stories before. As the "external brain" introduced by the Chaoyang District Committee of the Communist Youth League, he is also very willing to contribute his experience and wisdom to high-quality youth thought leading products. To "break" thought lead product and professional script kill "wall", "red begonia" beta stage also invited a number of professional script kill practitioners to participate in the content and form of modification, for example, the story began before increasing character test link, let players wear blind, mutual hand shoulder into the room, etc., are virtually improve the script play. Sun Boyang said that the Chaoyang District Committee of the Communist Youth League holds the concept of "all staff are product managers" in the research and development of young ideas, leading products. Every league cadre should have the whole chain working ability from "market due diligence, research and development, output, publicity, channel" to the final "data precipitation, private domain traffic construction". With continuous research and development of high-quality products as the core, since 2021 alone, the Chaoyang District Committee has raised more than 4 million yuan from various projects from society, enterprises and government purchase. Chaoyang District extends from the East Second Ring Road in Beijing to the east and North Fifth Ring Road. It not only covers a large area, but also contains rich various economic forms. There are a large number of young people in emerging industries. Young people from various industries bring vitality to the great development of the region, but their professional backgrounds and interests vary greatly. How to effectively touch them has become a required answer for the Chaoyang District Committee of the Communist Youth League. Has 68 covering chaoyang district all street township community youth, is the traditional advantage of chaoyang district committee, but they did not stick to the traditional, but dynamic optimization of youth hui store layout, where there are youth gathered, where to promote youth construction, around the rent apartment, film industry, art blocks, voluntary public welfare, street dance industry, different areas of venture capital, science and technology, as a whole to promote the district characteristic theme store construction. In September, the "22 Courtyard Street Community Youth Club" adjacent to the "Today Art Museum" opened for business."Today Art Museum" gathers a large number of young artists, including many studios of young artists and related resources of universities in many surrounding areas. The Chaoyang District Committee of the Communist Youth League will connect with the resources of expert think tanks, universities, public welfare organizations and enterprises, and regularly open up art salons there to create a good cultural atmosphere for the youth in the area."22 courtyard street community youth hui" and the traditional street township youth hui, is in territorial Shuangjing street party working attaches great importance to support, ZhuQu enterprises, colleges and universities under the cooperative participation resources social raising products, mainly focus on service "art, public welfare, volunteer" three youth pioneer group, hope in the future to become "culture and art, organization incubation, community governance" three big youth gathering place. In September this year, the "Free Yu Youth Hui" was officially inaugurated in Beijing Jianguomen Free Yu, which is also the first youth Club located in a long-rent apartment in Chaoyang District, aiming to contact the young people directly in the way of "living". The Chaoyang District Committee hopes that the youth exchange located in the long-rent apartment can form a "double-cycle service mechanism", on the one hand, to provide a platform for social support and social participation, to promote the reconstruction of the youth group relationship; on the other hand, to explore the systematic "research-design-service-feedback-lead" model, to improve the precision of the Communist Youth League service."Two new" organizations and emerging youth groups are the important youth forces in the development of Chaoyang region. In the form of work coverage, it has established Beijing Street Dance Industry, Chaoyang Catering Industry League Committee, Chaoyang Lawyers Association Youth League Committee, and Chaoyang Lianjia, 360,58 City and Blue City Brothers. Through the "service menu" mode, customized project service packages in two new fields to open up the chain of work coverage, backbone cultivation and organization coverage, so as to mature and develop one. Ma Heng has been in charge of the youth gathering since the construction of the first youth home in Chaoyang District in 2010. As the secretary-general of the District Youth Social Workers Association, he also has another identity- -the part-time deputy secretary of the Communist Youth League District Committee. In Chaoyang District, youth League cadres are not only cadres in the traditional system, but also youth League organizations open the door to more outstanding industries who are willing to serve the youth. In June this year, taking advantage of the reform of the Communist Youth League, the Chaoyang District Committee of the Communist Youth League added a temporary deputy secretary and a part-time deputy secretary, respectively from LiulStreet and the Bar Association. Since then, the Chaoyang Communist Youth League has basically formed a full-time organizational structure to adapt to the characteristics of Chaoyang youth, including one full-time secretary, two full-time deputy secretary, one temporary deputy secretary, and three part-time deputy secretary. In the view of the Chaoyang District Committee of the Communist Youth League, the bar association is the advantage of Chaoyang. Now the secretary of the Communist Youth League Committee of the Bar Association comes as a part-time deputy secretary, which virtually increases the resources to provide legal guarantee for the young people from all walks of life in the region. In addition, the temporary deputy secretary will directly face 43 townships in the district, play their previous grassroots work experience, enhance the communication between the Youth League Committee and the townships, in order to carry out the work more accurately; can directly connect with about 180 education system units in Chaoyang District, consolidate the basic secretary of the District Youth Social Workers Association, will help the construction of future youth home. Zhang Shuai, secretary of the Communist Youth League Committee of the Bar Association and part-time deputy secretary of the Communist Youth League District Committee, mentioned that many young lawyers have enthusiasm and professionalism, but they lack platforms and opportunities to display and exercise. In the early days of COVID-19, they set up lawyers to provide public welfare legal services to more than 70 small and medium-sized enterprises affected by the epidemic, and spent more than 40 million yuan involved in resolving the dispute."This provides a platform for young lawyers to exercise both their personal abilities and reflect their social values."From the service object to the work force change, so that the work backbone of the Communist Youth League constantly enriched. Chang Kai is one of the first delivery boys to join the "Beautiful Chaoyang Knight" team. When walking from street to street every day, he will upload the social uncivilized behavior to the wechat mini program of "Chaoyang People manage the City". And the "beautiful Chaoyang housekeeper" and "beautiful Chaoyang Knight" composed of real estate agents and food delivery boys have become an important force in Chaoyang community governance. It is understood that more than 1,000 registered "beautiful Chaoyang Knight" by taking photos and uploading, reflecting more than 1,500 community problems, has become a social district of Chaoyang District to achieve "before action" participation path. Source: China Youth Daily (November 04,2021, edition 01)

2021-11-09	Heze Municipal Party Committee to carry out youth safety production skills promotion action to the "key post" into "safety post"! Group Heze municipal party committee to carry out the youth safety production skills promotion action youth network reporter XingTing) since this year, group Heze municipal party committee joint Heze emergency management solid youth production safety skills promotion action, guide young worker production safety consciousness, improve safety production skills, participate in production safety management, the key industry in key areas of key enterprises "key post" into "safety", take concrete actions to contribute to the city's production safety work youth strength. According to the list of enterprises under the supervision of production safety in Shandong Province, the youth league organizations at the city and county levels actively connected with the emergency management departments, visited the key supervision enterprises in the region, sorted out the key positions and weak links, and started the establishment of "youth production safety demonstration posts". It to 15 provincial demonstration posts and 48 municipal demonstration posts. Youth at all levels organize and guide the creation of innovative work carriers of safety responsibility, and generally carry out activities such as warning education, post training and operation method promotion around safety responsibility, safety system and safety skills. Tuandan County Party Committee summarized the basic experience and practices, promoted the practice of "four four" in Class B of Shandong Shangshun Chemical Co., LTD. To promote the creation of youth safety production demonstration post; TuanChengwu County Party Committee and County Emergency Management Bureau organized Qingangang founders to carry out safety knowledge and rules and regulations examination, carried out "hand copy safety responsibility list" activity for young workers; the production team of Heze Jindou Feed Co., Ltd. carried out the "hidden trouble photo-feedback reward" activity, set up safety hazard information group, established hidden trouble search, feedback and rectification mechanism to promote safety production. At the same time, focusing on the skills of young workers, the Youth League Heze Municipal Party Committee integrated resources to carry out skills training activities. On the one hand, the Municipal Emergency Management Bureau jointly held a special training class of "Youth Safety Production Skills Improvement Action" in Heze City, and effectively strengthened the "four capabilities" of young workers' risk control, safety operation, hidden trouble investigation and emergency treatment. At the same time, the training institutions will give full play to the faculty and professional advantages of the training institutions to provide free teaching materials and course guidance. The municipal Communist Youth League has set up 6 online learning platforms for exam preparation and learning wechat groups for more than 800 young workers who prepare for registered safety engineers, and invited 12 instructors to answer questions in the group. In addition, the Youth League Heze Municipal Party Committee seized the opportunity of the "safety production month", organized the "youth safety said" skills lecture hall, "green force to build peace" volunteer services, to mobilize young workers to participate in the safety production publicity.

2021-11-08	Group Qingdao municipal party committee launched "rural revitalization of youth achievements" action Qingdao municipal party committee launched "rural youth achievements" action jianwei correspondent Liu Jie) to seriously implement the Qingdao rural revitalization work conference spirit, closely around the municipal party committee on speeding up the construction of rural revitalization of qilu model zone to promote common prosperity and other file requirements, group of Shandong Qingdao municipal party committee launched the "rural revitalization of youth achievements" action, launched the "five youth campaign" help rural revitalization. We will fight the youth campaign of "rural industrial transformation and upgrading". In agriculture-related areas (cities), we will extensively carry out "Rural Revitalization youth lecture halls", establish "rural revitalization youth mentor group", establish rural revitalization youth entrepreneurship bases, and hold youth innovation and entrepreneurship competitions of "Creating the future to youth", so as to build a youth enabling matrix for industrial development and help revitalize rural industries. We will fight the "rural talent cultivation" youth campaign. We will integrate "I do practical things for youth" with helping rural revitalization. Focusing on serving, gathering and cultivating young talents, we will organize and implement projects such as "cultivating young seedlings", "Yan returning to the nest" and "building post stations", cultivate local talents to revitalize townships, serve foreign talents to return home, mobilize social talents to the countryside, and help the revitalization of rural talents. Fight the youth battle of "prosperity of rural culture". We will carry out regular social practice activities such as "New Era civilization practice", "Three to the countryside", "returning to home" and "Weekend youth volunteer action" and other regular youth volunteer service activities to lead youth league members to contribute to the revitalization of rural culture. We will fight the youth battle of "livable rural ecology". In accordance with the requirements of the "Beautiful China Youth Action", we will make solid progress in the construction of ecological and environmental protection youth volunteer teams and ecological civilization education and practice base, organize a variety of ecological and environmental protection theme practice activities, and guide youth league members to become participants and practitioners of ecological civilization construction, and contribute to rural ecological revitalization. Play a good battle of "strong foundation enabling" youth. Firmly establish a "big grasp the" grassroots work orientation, solid grass-roots organization construction crucial action, strengthen the league organization, cadres and subordinate position construction, continue to promote the communist youth league strong foundation, style improvement, position consolidation, power rural organization revitalization, unity led the city's youth members to build rural revitalization of qilu model contribution to youth wisdom and strength.

2021-11-02	Wuhu creates a platform for the returning youth: Hui youth momentum helps to enrich the rural revitalization. Whenever she is free, she stays in the family farm orchard to prune the vines and think about it's picking business plan next year. In addition, she also won awards in the selection activities of the rural revitalization demonstration youth innovation project jointly organized by the Communist Youth League and Wuhu Municipal Party Committee, the Municipal Rural Revitalization Bureau and other units. Chen Yuanyuan admitted that the competition has refreshed her development ideas- -on the one hand, relying on the power of science and technology to improve the function of the smart farm, introducing more leisure, sightseeing and fishing elements; on the other hand, stepping up the establishment of e-commerce brand, to build an e-commerce sales platform, to help the high-quality agricultural products in my hometown out of the countryside. Since this year, in order to implement the deployment of the Central Committee of the Communist Youth League, the Anhui Provincial Committee of the Communist Youth League and the Wuhu Municipal Government, the Wuhu Municipal Committee of the Communist Youth League has mobilized more young people to participate in rural revitalization and modern agricultural construction by selecting youth demonstration models and excavating high-quality projects. Chen Yuanyuan, the leader of industrial revitalization, is the head of Legrape tao Family Farm, Liulang Town, � b District, Wuhu Bay. As the "second generation of farmers", she knows the hardships of her parents taking root in the fruit planting and wholesale industries for more than 30 years. When he came home for a holiday, Chen saw his parents bending over in the field and felt very distressed."Dad, mom, I will come back and do farming with you!"She immediately said that the future study of agricultural intelligence, mechanization knowledge, to help parents to reduce the burden. In 2017, Chen Yuanyuan graduated from university and returned home with seven like-minded friends to set up the grape Peach Family Farm, exploring planting standards while promoting product channels. Lock the direction of smart agriculture, Chen Yuanyuan went to Beijing, Shanghai, Nanjing and other places to learn from the knowledge of the agricultural Internet of Things, using detectors, collectors, digital sensors to collect the soil temperature, PH and other values, and accurately control the amount of water and fertilizer. In 2020, her family farm officially introduced a smart system to establish a complete QR code traceability mechanism for agricultural products. Managers can remotely shut down the shed, water, temperature measurement, fertilization and other operations on their mobile phones. The farm receives 70,000 people a year. Chen Yuanyuan also took the lead in popularizing the standardized planting technology of smart agriculture, absorbing 12 enterprises to set up the Wuhu Sunshine Fruit Tree Consortium project, to provide unified technical service guidance and employment positions for farmers. At present, the consortium has organic yellow peach, more than 30 popular picking varieties such as peach, grape, raisins, sunshine rose grape has become a "burst", a mu of land output value of more than 40,000 yuan."By increasing fruit quality and sales, the pace of technology will be faster."She said with emotion. Now, Chen Yuanyuan has also joined the � b District Young Entrepreneurs Association and regularly participate in exchange activities. She hopes to use the platform of the league organization to attract more young people with ideas to work together. In recent years, the Youth League Wuhu Municipal Party Committee has actively promoted and selected demonstration models of rural revitalization, helped build demonstration bases (parks) for college students returning to their hometowns, held more targeted youth innovation competitions such as agriculture and tourism integration, characteristic e-commerce and cultural revitalization, and provided a platform for intelligent and sentimental college students returning home to make contributions. In recent years, a group of post-80s and post-90s "pilots" (plant protection drone operators) have been active in the fields of Nanling County, Wuhu City. They come from an agricultural science and technology company founded by Wan Zewen, a young returning man. The company provides plant protection and flight prevention services, UAV special agents, and complete plant protection solutions. At first, Wan started his own business in Nanjing. When communicating with relatives in his hometown, he learned that someone had tried to spray agents with drones, which was very effective and efficient. But the current situation is that many farmers can't afford drones. With the mentality of trying, Wan Zewen returned to his hometown in 2018 to buy more than 80 professional drones and stepped into the agriculture field that he had never set foot in before. He adopts the business model of "company + base + peasant household" to provide affordable UAV plant protection services for retail investors, enterprises, cooperatives, family farms and other business entities. In addition, with the assistance of TuanNanling County Party Committee, Wan Zewen invited plant protection experts to teach the team the prevention experience of diseases, insects and insect pests, and explain the standard operation, maintenance, and relevant legal knowledge of agricultural machinery. At present, his entrepreneurial team has 56 people, with an average age of 31. Under the recommendation of the youth League organization, they have participated in the youth entrepreneurship competitions for many times, and constantly polished their business ideas. Over the past two years, the company has trained 264 plant protection drone pilots for free, serving an area of 1.6 million mu last year."Popular science education for young pilots is very important. For example, when sowing herbicides, we should learn to observe the surrounding terrain and cash crops, and should not bring losses to the people."He believes that on the road to help rural revitalization, we should not only cultivate" small white pilots ", but also cultivate young new farmers who can identify diseases, insects and insect pests, and provide targeted advice to farmers. The efforts of the young "pilots" also made Wan Zewen gratified. During the busy farming season, they live a life of wind and sun. With two boxes of mineral water in the trunk of the car, it is normal to start before dawn. In the slack season, Wan Zewen and the league organization together to find ways to teach "flying hand" agricultural machinery sales, balers, logistics and freight and other skills and knowledge, so that them flexible employment. Starting from 2021, the Wuhu Municipal Party Committee of the Communist Youth League has specially held a rural class for the young Marxist training project, with young leaders engaged in agricultural science and technology services like Wan Zewen, to inject scientific and technological strength into the development of rural grass-roots industries. The key to rural revitalization lies in people. The growth of rural children needs to be paid great attention to. Over the years, the Wuhu Communist Youth League has gathered the love resources from all walks of life to accurately help the young people in need and light up the light of hope in their hearts. A bright desk lamp, a clean desk, a neat bookcase, a quiet learning world... this year, the Wuhu Municipal Committee and the Civil Affairs Bureau to create "dream space" dream troubled children special action, for the city's rural 100 left-behind children, migrant children, no raised children tailored learning space, to provide each child with bookcases, desks, study chairs, eye lamps and books, and fundraising activities in the city. On May 19th this year, as soon as the donation channel was opened, the Youth League committee of Wuhu Xinxing Casting and Pipe Company and the youth League branches of the factory area immediately forwarded it, and the party and league members of the company started online donations. Within a week, youth league organizations at all levels, enterprises and institutions, caring people and members of the Youth Federation raised nearly 180,000 yuan to provide financial guarantee for the construction of "building a dream building space". Xiao Yue (pseudonym), who lives in Xingang Town, Fanchang District, lives on his father, with visual disability. The family can only rely on their father for odd jobs and subsistence allowance subsidies, TV sets, electric fans and refrigerators are the only electrical appliances in the home, the only decoration is pasted on the whole wall of awards. Tuan Wuhu Municipal Party Committee tailored for Xiao Yue adjustable seat, sent to the smooth and smooth desk, bookcase, let Xiao Yue love can not put it down. Xiao Yue, 13, hopes to become a teacher in the future and change the fate of more people with her knowledge."We selected two families with children in difficulties in Fanchang District to carry out the pilot 'dream building' space, and built 100 'dream building Spaces' in the city within one month."League Wuhu Municipal Committee rights and interests department a staff introduction. Group Wuhu party secretary Yang lei said the next step, the communist youth league in Wuhu around the rural revitalization strategy, combining the actual needs of the villages and towns, the agricultural project, set up 10 covers art design, marketing, public service and other types of rural revitalization of youth volunteer service, open youth to the countryside channel, improve the long-term mechanism of guiding youth service rural revitalization. China Youth Daily China Youth Network reporter Wang Haihan Wang Lei correspondent, Zhang Lingzhi source: China Youth Daily

2021-11-02	Nanning city hope project public day and public student plan start Nanning hope project public day and "hibiscus flower" public student plan initiator Xie Yang) on October 30, the 32nd anniversary of the implementation of the hope project, Nanning hope project public day and "hibiscus flower" -Nanning hope project 10 74 love trunk public student plan launch ceremony was held in Guangxi Nanning China grand theatre square. At the launch ceremony, the participating leaders jointly awarded the honorary plaque and certificate of the Hope Project to the caring enterprises and individuals who care about and support the public welfare undertakings of Nanning Hope Project. The Young Pioneers performed programs such as "Young China" and "Listen to me to Say Thank You". Yin Jiangyue, a traffic music radio reporter of Nanning Radio and Television Station, gave a public welfare speech on "practicing the original mission on the road to poverty alleviation and witnessing the great changes in the countryside on the road to public welfare", calling on the public to contribute their love. In the applause, the leading guests jointly launched the "hibiscus flowers open" - -Nanning Hope Project 1074 love trunk public welfare education program. This activity also innovative public welfare mode, set up love blind box area, all the sales money into the "hibiscus flower" public welfare fund pool; and open hope market, set up charity stalls, rural revitalization of agricultural products, love handicrafts, hope primary school students paintings site charity sale, charity sale money donated to Nanning Hope Project Office as the original fund of Nanning Youth Development Foundation. This activity is hosted by Nanning Municipal Party Committee of the Communist Youth League and Nanning Radio and TELEVISION Station, and organized by Nanning Hope Project Office, Nanning 1074 Transportation Station, Nanning Youth Activity Center, Nanning Youth Entrepreneurs Association and Nanning Youth Volunteers Association.

2021-11-02	"One hundred red boat is youth" national youth preaching contest final held in Jiaxing "one hundred red boat is youth" national youth preaching contest finals held in Jiaxing south lake in red boat 4 anniversary, by the Jiaxing municipal party committee of the communist youth league, China communist youth league magazine, youth abstract magazine, Jiaxing city investment development group co., LTD., Jiaxing bank to undertake "one hundred red boat is youth" national youth preaching contest final in jia guang group melting media center building studio held in the form of network broadcast. PI Jun, Party Secretary and chairman of China Youth Publishing Association Co., LTD., and Wang Huilin, vice mayor of Jiaxing city, delivered a speech online. Then the competition officially began. In the final, the contestants preached the spirit of the Red Boat, the theory of the Party and policies. Some told stories at the grass-roots level, some told the past of South Lake with deep feeling, and some carefully combed the spiritual inheritance of revolution, construction, reform and different historical periods in the new era, expressing the passionate feelings of loving the Party, the country and socialism. After the competition, Hu Xianzhong, the main judge of the competition and the curator of the Youth Sports History Archives of the Central Communist Youth League, commented on the contestants from the source, content and form of the contestants. In the end, Zhu Chenglong, Bai Haotian, Li Hengwei and Sun Linlin group, Jiang Shuo, Nie Ziqing won the first prize. The above six contestants were recommended by the State Grid Youth League Committee, China Railway Construction Youth League Committee and Tongxiang Media Center Youth League Committee. The group, Mao Xiaoting, Zheng Xiaojuan, Yuan Hang, Yang � Diana, Yanqing, Qin Yuchen, Zhu Ying, Zhan Xiaoxuan, Wang Hanyu and Zhang Ruipeng, Qi Yijun, Zu Jialing, Jiang Xiao, Li Changzhou, Zhang Jing and Cui Ying won the second prize. This competition aims to promote the youth theory propaganda work to keep pace with The Times, improve and innovate, and really let the new ideas in the youth group internalized in the heart, externalized in the practice. At the same time, the competition also uses A number of new technologies such as AR augmented reality to empower the contestants, so as to further enhance the appeal and appeal of the lecture. It is reported that the "Centennial Red Boat is youth" national youth publicity competition lasted more than half a year, has been through the league organization selection, expert evaluation, online voting and final and other procedures, in more than 1200 works selected 170 winning works, among which the first 20 entered the final. The competition was warmly welcomed by the young people. During the week of online voting, the total number reached 4.22 million votes and more than 5.12 million voting page views. In addition, in the process of holding the final of the competition, Jiaguang Group fully provided technical support and personnel support to ensure the smooth operation of the competition live broadcast.

2021-11-03	Jinan: Invite young people to share the future. Fresh graduates will become the leading role of the city. On the evening of June 18th, "The graduation ceremony of 2021 Jinan College students started in Jinan" was held. In the name of a city, a grand graduation ceremony for college students is a first time in Jinan, with only one purpose: to invite more young people to grow up together with the city. Event, young students not only received the Shandong provincial party committee standing committee, Jinan municipal party committee secretary Sun Licheng solemnly promise- "the city will always' gentle, waiting for you, waiting for flowers", "also received the real" package ": open restrictions," double gen " policy is enjoy, the implementation of the housing, rental and transportation subsidies, etc."The millennium of ancient city is young, lakes and mountains are just young."Now, Jinan ushered in a rare development opportunity, to promote the construction of" youth development friendly city " has reached a consensus in the city."Call you with the dream of a city" - -in Jinan is not true. The construction of a "youth development-friendly city" has been clearly written into the "Jinan Yellow River Basin Ecological Protection and High-quality Development Plan", which is also reflected in the "14th Five-Year Plan" of Jinan City and this year's work report of Jinan Municipal Government. The construction of "youth development friendly city" has the characteristics of overall, systematic and realistic, which requires not only the "clairvoyant eye" of the overall situation, but also the meticulous "embroidery work". Jinan unimpeded channels to participation in the deliberation and administration of state affairs, ask ordinary young people, to ensure that the work of young people. Recently, a 2021 youth simulation CPPCC proposal solicitation activity focusing on the construction of a "youth development-friendly city" is under way. The activity was held by the Jinan Municipal Party Committee of the Communist Youth League, the General Office of the Jinan CPPCC and the Jinan Municipal Students' Federation. It conducted extensive research on economic development, social governance, quality of life, innovation and entrepreneurship from the perspective of young people. At present, the activity has collected 562 simulation proposals, are being under guidance and evaluation by experts, the next will evaluate the best and recommended to participate in the urban two sessions. Building a youth-friendly city is also a special topic of the Jinan CPPCC this year. Group Jinan municipal party committee and Jinan CPPCC club health and legal system committee jointly undertake many "building youth development friendly city project" discuss "symposium and" the communist youth league and NPC, CPPCC members face to face " activities, according to the development of the youth demands and specific problems, invited different youth representatives and education, people club, building, business, brigade, administrative examination and approval departments, head of face to face consultation, timely solve or transfer related problems. The discussion found that the quality of urban life needs to improve the problems for the attention of many young people. To this end, Jinan Municipal Party Committee of the Communist Youth League took the initiative to connect with business, cultural tourism and other departments, plan and implement a series of activities such as "Youth Quality Life Festival" and "New Youth Day", and carefully create quality consumption landmark scenes popular with fashion shopping, entertainment, play and experience in Impression Jinan, Huayi Film Town and other places. Facts have proved that these activities are very popular among young people, and the passenger flow and consumption of the corresponding landmark places generally increase by more than 30%, thus achieving a positive interaction between boosting the consumption level and improving the quality of life. In order to encourage college students and young people from all walks of life to stay and develop, Jinan city gave full play to the discussion and coordination role of the Youth Work Joint Conference, and played a set of "combined fist" of youth work. For college graduates, Jinan Education Bureau and Jinan Human Resources and Social Security Bureau have jointly implemented the "Choose Jinan for a Win-win Future" employment action for college graduates, and launched policies such as "Quancheng Scholarship", employment and internship subsidy, "talent retention award" and "talent price fund" in Jinan colleges and universities. Labor and employment departments specifically implement the graduate recruitment, registration and real-name registration, adjustment and change, file transfer and other work. In response to the housing problem, the employment, housing, human resources and social security departments have jointly issued new policies, stipulating that eligible college graduates can apply for a rental subsidy of 500-1,500 yuan per month for three consecutive years. Currently, the policy has benefited more than 20,000 people. Qualified high-level talents can also get housing subsidy, 100,000 yuan for master, 150,000 yuan for doctor, BCD talents, 1 million yuan, 700,000 yuan and 400,000 yuan for subsidies respectively, A talents implement "one person one policy, one case one discussion". So far, 6,160 people have received a housing subsidy of 700 million yuan. In addition, Jinan has launched 6,838 "apartments for young talent" to give priority to the needs of young talents. The Jinan Municipal Party Committee of the Communist Youth League, in collaboration with education, civil affairs, health, sports, cultural tourism and other departments, has introduced a series of measures in children's schooling, after-school care, elderly care services, and public culture to solve the development of young people to the maximum extent. In July this year, the Yellow River Basin high-quality development of the school-enterprise youth innovation platform was officially established. The platform is jointly built by the Jinan Municipal Party Committee of the Communist Youth League and the relevant municipal departments. Up to now, more than 10 universities, such as Tsinghua University, Xi'an Jiaotong University and Shandong University, have accurately connected with Shandong Advanced Technology Research Institute, Qilu Pharmaceutical Group and Inspur Group to build an integrated mode of "government, industry, university, research and financial consumption" in an orderly manner. In the specific process of promoting the "youth development friendly city", Jinan does not do one size fits all, fully consider the reality at the grass-roots level, adopt the strategy of pilot first and promoting in batches, and some districts and counties took the lead in exploring and solving problems and accumulating experience. Huaiyin District is located in the west of Jinan city, one of the earliest planned five main cities in the city center. Jinan West Railway Station, one of the five hub stations of Beijing-Shanghai high-speed railway, has 387 high-speed trains directly to 297 cities in China. Huaiyin District, fully considering the characteristics of young talent gathering and high mobility, took the lead in Shandong Province to launch the creation of a "youth development friendly city" in the city, led by the "Huai Young" brand, and the district committee secretary and the district chief personally promoted the creation of the work. In the process of specific promotion, the district has identified Greenland Qilu Gate-Beijing-Shanghai meeting room as the core area of "youth development friendly city", and accelerated the construction of youth development service center. Signed strategic cooperation agreements with 10 universities in Jinan under "One School, One Project", encouraged grassroots creative work in accordance with "One street, One brand", and created a "youth development friendly street". In addition, also the establishment of Huaiyin talent enabling port-young talent venture capital service base. As one of the first trials of provincial youth development planning, Shizhong District has created the social scene of "Luneng Fan Community" for youth, and created the youth park model of Jinan International Innovation and Design Industrial Park. Lixia District strives to build an international first-class youth friendly community represented by the Central Business District and Shunjing International Community. Today, the pulse of the city's youth is felt by more and more young people. Sun Lulu, who is engaged in the cultivation and incubation of public welfare organizations in Jinan, is almost synchronized with the urban growth. Since 2017, she has covered 51 professional social organizations, helped more than 1,400 people get employed, and raised more than 50 million yuan, becoming a benchmark for local public welfare services."Jinan is a city with temperature. Her tolerance gives young people the chance to try and error. As long as we stick to it, it is sooner or later to succeed. We are full of confidence in ourselves."Sun Lulu regrets. Group Jinan municipal party committee secretary zhang xi, the next step, Jinan municipal party committee will give full play to its functional advantages, under the leadership of the party committee government urged functional departments and the social coordination from all walks of life, fully support the youth society into participation, improve youth quality of life, efforts to get through the policy "the last kilometer", leading the youth in the economic construction and social development to play the role of powerforce and commandos. China Youth Daily, China Youth Network reporter Xing Ting source: China Youth Daily

2021-11-05	Zibo most beautiful youth model sharing held "please party trust power have me" Zibo best youth model sharing held jianwei), "please trust party power have me" Zibo best youth model sharing held in Zibo vocational college, Zibo municipal party committee propaganda department, pku, Zibo daily, Zibo radio and television related leaders to attend the sharing and won the title of "Zibo most beautiful youth" outstanding youth representatives. More than 400 young representatives from all walks of life attended the sharing meeting, and 350,000 teenagers watched the live broadcast online. Award prizes to the most beautiful youth winners in Zibo. Zibo Municipal Committee for the picture sharing meeting in Zibo Youth Snail Choir children beautiful melody opened. Nine award-winning representatives from various fields and industries shared them on the scene, and 11 award-winning representatives shared them online, respectively telling their youth struggle stories based on their posts respectively. Example sharing meeting site. The "Zibo most beautiful Youth" tree selection activity selected a total of 20 "Zibo most beautiful youth", 50 "Zibo upward to the good good youth". Choose tree activities focus on the city from all walks of life and grassroots line, find, elected a batch of new era model practice of socialist core values, take the lead to the spread of positive energy around good youth, inspire the youth misfortune, chong DE for good, actively participate in the construction of practical open, quality vitality, ecological harmonious modern group of vivid practice of big cities.

2021-11-02	A patient with a positive nucleic acid test was diagnosed as COVID-19 in Wulian County, Rizhao City, Shandong Province, on the evening of May 25. With COVID-19 (common type) after consultation on the early morning of The 26th. As of 24:00 on October 31, the county seat, with 510,000 people, had accumulated 10 confirmed cases and nine asymptomatic infected people. The epidemic is an order. Tuanrizhao municipal party committee act quickly, according to the Rizhao municipal party committee municipal government epidemic prevention and control work unified arrangement, the first time to the city youth league organizations at all levels, cadres and the youth initiative, youth commandos and form, volunteer service actively participate in epidemic prevention and control work, contribute to win the epidemic prevention and control war of youth strength."Under the gathering point of the street lamp I saw and I hurried to wear various volunteer clothes, I carefully distinguish the words on their clothes: ark rescue team, blue sky rescue team...." 26 morning, as a member of the first echelon of epidemic prevention youth volunteers, the Rizhao municipal party committee after 90 cadres Teng Yu sat on the bus to Wulian County, then wearing "sunshine youth volunteers" blue vest Teng Yu did not expect, the next 40 hours, this vest has not been off. Outbreak occurred within a few hours, the first by Rizhao municipal party committee cadres, the ark rescue team, blue sky rescue team, strong-arm reaction team, woodpecker volunteers with rich experience in epidemic prevention and control of the first echelon, young volunteers quickly into war "disease" line, to wulian to assist nucleic acid detection guide, disinfection kill, order maintenance, convenient service volunteer work, with a sense of urgency, sense of responsibility and mission, youth strength, blowing the outbreak "charge"."Our first echelon has dispatched five vehicles and 13 people. In addition to seven fog machines, we have also brought 15 sets of protective suits and other personal protective equipment and daily necessities. While fighting the epidemic, we will also protect ourselves. Please don't worry."Blue sky rescue team member Lin Xi said. On October 26, in the dormitory of No.1 Middle School in Wulian County, several young volunteer teams, including Ark Rescue Team and Blue Sky Rescue Team, actively assisted the epidemic prevention and control department to set up prevention and control testing points. After the nucleic acid testing task was completed, the nucleic acid testing points were sterilized. Volunteers, dressed in protective suits, protective masks, and professional disinfection equipment such as fog machines and drone control drones, sterilized nucleic acid testing sites according to the unified command, fighting against the epidemic against time to contain the spread of the epidemic. Together with the community workers, they have built a solid epidemic firewall for the residents."In the critical period of the city's epidemic prevention and control work, the Ark Rescue Team, as an emergency rescue force, will do its best to take the epidemic prevention and control as the most important work at present, and unite as one to shoulder its due social responsibility for winning the battle against the epidemic."Ark rescue captain Ma Xianchao said."Please line up, wear a mask and wait patiently."" Please line on the yellow line!"" Please get your ID card, complete the information input and nucleic acid sample collection according to the instructions!"In the county many nucleic acid testing site, many wearing blue vest grassroots cadres shuttle in the crowd. The young volunteers of the Youth League Wulian County Party Committee immediately contacted with the Baolian community, quickly assisted the community to complete the layout and construction of the nucleic acid testing site, planning the diversion channel, order maintenance, registration and sampling, and were on duty in the community 24 hours a day to carry out personnel registration and epidemic prevention publicity. At the same time, youth league organizations at all levels and youth league affiliated social organizations in Rizhao have actively organized and mobilized young volunteers to participate in the epidemic prevention and control. The youth Working Committee has organized young volunteers to actively participate in epidemic prevention and control volunteer service activities, publicize epidemic prevention and control knowledge, distribute masks, and take multiple measures for epidemic prevention and control."Advance registration work is far more complicated than I imagined, I am responsible for the community of the masses is given priority to with the elderly and children, qr code scanning, perfect personal information, minor children do these easy for youth is helpless for the old man, I can only guide over and over again, some simply directly agent."Teng Yu recalled."Please don't worry, keep the distance apart, if you encounter any problems in scanning the code or testing, please directly find our volunteers, wearing a blue vest, with this logo."Volunteer Peng Zhaohui pointed to the regiment emblem and guided the people who came for nucleic acid tests. The epidemic will not withdraw, and the volunteers will not withdraw."In order to further provide volunteer services for epidemic prevention and control, the second echelon of volunteer services for epidemic prevention and control, composed of cadres of the Municipal Communist Youth League and volunteers from many volunteer service organizations, such as the Municipal Safety Volunteer Association, the Fire Volunteer Association and the Shancheng Volunteer Team, has been on standby."Youth League Rizhao Municipal Party Secretary Mou Yaowen introduced. Shining group emblem, blue vest, red figure, followed by a more colorful volunteer team. These figures have formed a solid and powerful emergency response force in Rizhao. They have fulfilled the original mission in the epidemic prevention and control work, demonstrated the youth background of the contemporary youth with their responsibilities, built a strong security line, and gone forward bravely. China Youth Daily China Youth network reporter Xing Ting correspondent Zhuang Yan source: China Youth Daily

2021-10-30	Lanzhou Youth: I organizations and young volunteers to fight against the epidemic and contribute to the COVID-19 prevention and control work. In just 72 hours, there are 870 volunteer service organizations, 5338 volunteers, 76 volunteers entering high risk areas, 99.4% and post-00s volunteers, 47.43%, over 310 vehicles and 105 grassroots service points, serving more than 1.6 million people. In response to the initiative, Lanzhou Youth Federation members launched WeChat love fundraising solitaire, only 48 hours, 43 Youth Federation members solitaire, donated a total value of 56,800 yuan. Lanzhou Youth Volunteers Association timely grasped the information of the third nucleic acid testing point in Lanzhou, and immediately sent 7,500 bottles of drinks worth more than 60,000 yuan donated by caring enterprises to 10 epidemic prevention checkpoints in Lanzhou. According to the needs of the testing points, the Tuanchengguan District Committee organized volunteers and uniformly distributed them with materials, and carried out 23 disinfection operations, with a total area of 162,000 square meters. The Tuan Qilihe District Committee organized 63 volunteers to assist in the third round of nucleic acid testing and sampling in various street outlets, and donated more than 2,000 materials. As of Monday, the city has set up 138 youth commandos, dispatched 6,700 young volunteers, prevented and killed 614 key sites, covering an area of 320,000 square meters, built 262 nucleic acid testing sites, delivered 521 epidemic prevention materials, and spent 163,000 hours of volunteer service. The group organized to receive supplies worth 870,000 yuan, including masks, protective clothing, goggles, instant noodles, mineral water, beds and bedding.

2021-10-30	Held in Hefei, Anhui province the first rule of law micro video contest in Hefei, Anhui province held the first rule of law micro video contest in Anhui traffic radio, Hefei thermopower group, the city green jointly sponsored by Hefei power "happy growth" the first rule of law micro video contest sharing and new product exhibition. This activity also promoted the youth rights theme script kill, the distribution of youth happy growth self-service manual, online youth happy growth service platform, through more youth rights work new, inspire more caring people to consciously invest in the cause of youth rights and interests protection and crime prevention. Next, group Hefei municipal party committee will take this activity as an opportunity to continue to seriously explore "youth rights and interests work innovation", and relying on the city youth association, city 12355 service desk, youth law, psychology, protect propaganda guide education, efforts to improve the youth rule of law consciousness, safeguard the legitimate rights and interests of teenagers, to prevent and reduce juvenile crime.

2021-10-28	Dezhou green league with Xinjiang pair primary school activities "letter hand in hand" Dezhou green league with Xinjiang head tun river farm primary school "letter hand in hand" activity on October 28 (reporter zhang, reporter Fan Yichao) recently, Shandong Dezhou city youth federation joint group LingCheng district party committee, ling city teachers study school affiliated primary school through the form of letters, and Xinjiang 12 division head tun river farm primary school partners pair, jointly "letter hand in hand" activities.site of activity. In the activity, the students will turn their sincere hearts into words to convey each other's blessings. A letter full of deep feeling, the two hearts close, not only conveyed the true feelings and hope, but also let the students understand each other's study, life, build a bridge of friendship between the two of the two places, hand in hand happy growth. In the letter, the students not only introduced themselves and their study and life in their class, but also introduced the local customs of their hometown. Many students also attached small cards and small gifts carefully prepared by themselves, and some students also invited them to their friends in Xinjiang to study and play in Dezhou.site of activity. Dezhou Youth Federation has actively organized the school young Pioneers of the whole city to carry out in-depth "hand in hand" and "pair up" activities with primary and middle school students in Xinjiang. So far, more than 1,200 students in Dezhou have become friends with students from Toutunhe Farm Primary School in Xinjiang. Through exchange letters, further enhance the young pioneers' understanding of various ethnic culture, create a good atmosphere of national unity, at the same time let the Chinese nation unity and mutual assistance, mutual respect and love of the traditional virtue to inheritance, further enhance the young pioneers national unity consciousness, the concept of national unity members rooted in the heart.

2021-10-26	Quanzhou municipal committee hierarchical classification accurate support service youth employment entrepreneurship group Quanzhou municipal committee hierarchical classification accurate support service youth employment entrepreneurship Zhang Jianwei correspondent Wu Ruiyuan) for a period of time, the group of Fujian Quanzhou municipal party committee close contact youth, wholeheartedly help youth, to the new era of youth growth demand, with hierarchical classification accurate training as the breakthrough point, integrate resources, create conditions for youth development, building platform, and youth development, progress, youth group contact coverage, powerful help youth improve skills and understanding level, help youth employment entrepreneurship. In order to better attract outstanding college graduates to return to spring and provide solid talent support for Quanzhou to comprehensively promote high-quality development and go beyond, the Quanzhou Municipal Party Committee of the Communist Youth League continues to carry out the social practice activities of "returning home" for college students. This summer vacation, group Quanzhou municipal party committee to implement the "sail plan", for party and government organs, institutions, research institutions, state-owned enterprises, financial institutions and other units for 2790 jobs, attract from Beijing normal university, Nanjing university, Wuhan university and Quanzhou local universities 1873 to spring outstanding students in social practice change, listen to policy, talk about development, firm a stay spring employment entrepreneurship determination and confidence. Support entrepreneurship has "school" for small and medium-sized enterprise entrepreneurial youth in the Internet, big data, artificial intelligence, impact, face management innovation, thinking innovation, technology innovation, channel innovation, market innovation, group Quanzhou municipal party committee implementation "Quanzhou new generation entrepreneurs quality promotion training plan", founded "green college", held "gen" super public class "," rural revitalization of rural youth anchor " special training activities, help different industries entrepreneurial youth exchange learning, cooperative innovation. Since its launch in 2018, the "Super Open Course" has been held for 8 periods, and well-known experts and scholars from home and abroad have been invited to Quan to exchange and discuss with more than 3,000 outstanding young entrepreneurs, effectively helping them with new knowledge and ideas, improve their standing, expand their horizons, arm their minds, and start a scientific business. Support green business have "action" to foster industry innovation development "leader", help young entrepreneurs comprehensively improve management ability, social responsibility, culture, group Quanzhou municipal party committee all-round, multi-level, wide field build learning promotion, research cooperation platform, for "create the second generation" growth development to provide quality service and effective power. Together with high-quality resources, we have innovated to build a first-class learning carrier, carefully selected courses, organized teachers and inspection routes, and systematically trained more than 200 young entrepreneurs and "creative second generation". Group of Quanzhou municipal party committee of the relevant departments, said the next step, Quanzhou municipal party committee will continue to pay attention to, accurately identify different youth group demand, actively explore, summary, innovation service mode, effective integration of the social resources from all walks of life, build a "learning + practice" closed loop, hierarchical classification make entrepreneurial youth cognitive breakthrough platform, skills promotion platform, healthy growth platform, communication and cooperation platform.

2021-10-26	Group Texas municipal party committee for "express little brother" open psychological counseling to listen to relieve pressure group Texas municipal party committee for "express little brother" psychological counseling zhang correspondent Fan Yichao) recently, in order to help express little elder brother relieve work pressure, group of Shandong Texas municipal party committee, Dezhou postal administration jointly carried out the peak season service security and Courier psychological counseling activities, for a line express personnel opened "counseling", help them properly release physical and mental pressure, more healthy work and life, from 10 express enterprise employees a total of more than 20 people participated in the activity. Han Mingfang was invited, a national second-level psychological consultant and the first top ten psychological consultant in Dezhou, to carry out face-to-face and one-to-one psychological counseling for enterprise customer service personnel and front-line Courier. Activities, through the rich case, vivid explanation, to solve employees emotional management, dispute resolution, improve customer satisfaction, social recognition, correct understanding of family relations in life and the staff concern, relieve their psychological pressure, for the "double a" "double" business season to send a decompression "timely rain". In the next step, the Communist Youth League Municipal Committee will give full play to the role of the Communist Youth League Working Committee of the express delivery industry, further find the right service entry point in urban integration, employment and entrepreneurship, psychological counseling, social security, legal aid and other aspects, and actively create a good working and living environment for the express workers to help them solve practical difficulties.

2021-10-26	Guizhou), the opening ceremony of Lizi Village, Qixingguan Town, Bijie City and the "Chunhui Action Kite Plan" seminar was held in Lizi Village, Jichang Town, on October 21. This is the first Chunhui Society established after the Communist Youth League Guizhou Provincial Party Committee launched the "Chunhui Action Kite Plan"."Chunhui Action" is a golden signboard of the Communist Youth League of Guizhou province. For decades, it has been advocating "carrying forward Chinese civilization and feeding back their native relatives", actively playing the emotional bond role of "family affection, nostalgia and friendship", inspiring travelers to return to their hometown, and jointly promoting the economic and cultural development of their hometown. In September this year, Lan Shaomin, deputy secretary of the Guizhou Provincial Party Committee, called for "establishing a mechanism to bring workers back, so that kites can fly high and high-quality workers are outside the province. As long as Guizhou has the opportunity and needs, he can be summoned and come back."The "Chunhui Action Kite Plan" was born. The plan around the "find, get tight, critical moment drew back" working train of thought, focus on mobilizing migrant workers, social talents, college students three types of people, cling to the affection, affection, friendship, scenery, accent, nostalgia homesickness line and filial piety, gratitude, feedback, return virtue line, inspire heart in Guizhou, holding home native feelings, to ensure that the "kite" constant line. An official in charge of the Guizhou Provincial Committee of the Communist Youth League said, " The Chunhui Action Kite Plan will strive to become a new platform to serve outstanding social talents, migrant workers, and college students to return to Guizhou to work, start businesses, invest and provide public welfare services."In this activity, Guizhou Province Chunhui Action Development Center Director Wang Chaoqian, Youth League Bijie Party Secretary Tang Yu attended and awarded the license. This year is the first year of rural revitalization. Tang Yu said that he expects the "Chunhui Action Kite Plan" to explore and solve the current problems facing the rural industry, promote the good development of the Chunhui industry, and truly guide the local people to devote themselves to rural revitalization.

2021-11-01	Wuzhong city, county two levels of youth corps committee issued epidemic prevention and control reserve volunteers recruitment to youth war "disease" power I Wuzhong youth actively be war epidemic volunteers let youth shine in "epidemic" line built wei), Ningxia hui autonomous region Wuzhong city, county two levels of epidemic prevention and control reserve volunteer recruitment, Wuzhong city youth from all walks of life, only 12 hours, 780 youth through online participation in epidemic prevention volunteer service."Material transportation, street community can contact me" Yu Xiufang saw the Communist Youth League Municipal Party Committee volunteer recruitment order, the first time plus wechat to register into the "Wuzhong City Youth Commando". Knowing that the nucleic acid sampling site was in short supply, Liu Xinyu, a college student majoring in clinical medicine, volunteered to volunteer to maintain the order of the team at the sampling site and help people scan the code to register. She said, " Each generation has its own historical mission. When the city we live in is facing the test of the epidemic, it is my responsibility and obligation to fulfill my social responsibility and contribute my youth strength. I am willing to fulfill the youth pledge of 'Please rest assured that the strong country has me' with practical actions!"" Snow Spring Dairy first donated 1,000 boxes, if not enough, you can add."Man Xuefeng, a member of Wuzhong Youth Enterprises Association, took the initiative of donating 1,000 boxes of milk to donate 1,000 boxes of milk to visit frontline volunteers and contribute to the prevention and control of the epidemic. In accordance with the latest requirements of Wuzhong COVID-19 Prevention and Control Headquarters, On the basis of summarizing the experience of epidemic prevention, On October 22, the Wuzhong Municipal Committee of the Communist Youth League issued the notice on giving full play to the role of the municipal youth League organizations at all levels and the general Communist Youth League members to resolutely win the battle against the epidemic, In the "1 + 5 + X" mode (1 Wuzhong City epidemic prevention and control youth volunteer commandos, 5 county-level epidemic prevention and control youth volunteer commandos, X grassroots youth volunteer commandos), Set up the command center, comprehensive coordination group, publicity and guidance group, psychological counseling group, logistics support group, We mobilized 125 young volunteers who had been vaccinated and tested negative for nucleic acid to carry out volunteer services. At the same time established the cities and counties, professional support team, a total of 12 WeChat group, arrangement is responsible for real-time update release epidemic prevention and control situation, protective guidelines and epidemic prevention policy announcement, volunteer service demand, volunteer service team, to ensure that youth volunteer service force can know oneself and know yourself, smell, science, to implement the municipal party committee and government all policy decisions.

2021-10-30	Gansu Zhangye: young volunteers to resist disease line exhibition as Gansu Zhangye: young volunteers fight disease line exhibition after released the epidemic prevention and control youth volunteers reserve recruitment order "epidemic prevention and control health youth volunteers reserve recruitment order, issued" Zhangye city disease resistance youth volunteers management measures "Zhangye city youth volunteer organizations at all levels recruit reserve epidemic resistance responsibilities" Zhangye city disease resistance youth volunteers work code of conduct, to the city's epidemic prevention and control youth volunteers work guidelines, for a line of volunteers issued volunteers epidemic prevention safety manual. On the front line of epidemic prevention and control, young volunteers participated in medical rescue, convenient services, order maintenance, psychological assistance, publicity and guidance, care and assistance, transportation, environmental governance, and logistics support in the area where they lived nearby. Up to now, the youth League committee of Zhangye City, county (district) level has selected and recruited 2,232 reserve young volunteers from the applicants, and 1,660 people have worked.

2021-10-25	Anhui Huaibei: 8 "escort youth" education service group "post" Anhui Huaibei: 8 "escort youth" education service group "post" youth newspaper network reporter Wang Haihan) recently, Anhui Huaibei municipal party committee joint relevant departments, set up Huaibei city "escort youth" rule of law publicity and education service team, set up 8 service group. At the launch ceremony, Hu Jing, deputy secretary of Anhui Provincial Committee of the Communist Youth League, put forward clear requirements on how to effectively improve the pertinancy and timeliness of legal publicity and education from three aspects: accurately grasping the relationship between the law and the state, the law and the youth, and the object of law popularization. On the same day, the "escort youth" service team representatives combined with their own work to put forward a law popularization plan. From late October 2021 to December 2021, eight legal publicity and education service groups will use the after-school time in primary and secondary schools to provide services around legal publicity, net net, drug control, traffic safety, anti-electricity fraud, illegal campus lending, mental health and other services. Among them, the city's intermediate people's Court legal publicity and education service group mainly guides primary and middle school students to carry out mock court activities, guide students to play the role of judges, parties, lawyers, witnesses and other witnesses, to provide evidence, cross-examination and other court examination procedures, so that primary and middle school students feel the judicial justice and legal majesty. The rule of law Publicity and Education Service Group of the Municipal Education Bureau has made a blackboard newspaper, handwritten newspaper, opened a special reading party to read the Law on the Protection of Minors, and encouraged associations to make law-based newspapers and other forms, so that primary and middle school students can better understand the legal knowledge through personal participation. In addition, the Municipal People's Procuratorate legal publicity education service through virtual juvenile crime cases, guide primary and middle school students to study the law; the municipal network letter method propaganda education service will simulate the network security protection situation, propaganda against malware, network telecom fraud, personal information protection, phishing mail content, enhance students' network security awareness.

2021-10-24	The 15th Hefei International Cultural Expo opened in Hefei on October 22 in East China's Anhui Province. The first time in the "Maker Space" exhibition area organized by the Hefei Municipal Committee of the Communist Youth League launched the cultural and creative products- -the blind box "Heroes" of Hefei key industrial chain. The word "little chivalry", which originated from the Hefei dialect, means "youth" and "children". At the same time, the Hefei Municipal Party Committee of the Communist Youth League uses the blind box as a fashion toy to personification the image of the industry, so as to promote the key industries of Hefei in a form popular with young people, implying that the 12 key industrial chains in Hefei are like thriving young people, riding the wind and waves with unlimited prospects. It is understood that this is pku is six consecutive years to participate in wenbo exhibition work, exhibition to service and growth as the main line, divided into small guest, college students and guest, youth and the three plates, including artificial intelligence, culture and education industries, field content, covering the Yangtze river delta G60 kechuang corridor youth innovation and development alliance, youth innovation entrepreneurship competition comprehensive exchange innovation elements, for the national youth and thought, temperature, effective "circle of friends". Photo provided by the League Hefei Municipal Party Committee

2021-10-22	Youth League Taiyuan Municipal Party Committee to carry out lighting up a thousand micro wish activities Youth League Taiyuan Municipal Party Committee to carry out the "study of the Party history and practice the original aspiration" to light up a thousand micro wish activities Li Chen correspondent Wang Yishu) in order to further promote the party history learning education, To steadily carry out the "I do practical things for the masses" practical activities, Small incision to solve the problem of "urgent worries" for young people, To continuously enhance the youth people's sense of gain and happiness, Encourage young people to listen to the Party, feel the Party's kindness, and follow the Party, Shanxi Taiyuan Municipal Party Committee and Municipal Youth Working Committee jointly carried out the "study the Party history and practice the original aspiration" to light up thousands of micro wishes, On October 20, the "micro wish" lighting ceremony was held in Taiyuan School for the deaf. Municipal party history learning education tour guidance of the eighth group deputy leader Xue Yujun, secretary of the Communist Youth League Zhao Zhiyuan, director of the city young working committee Xu Chao attended the event. The lighting ceremony kicked off in the children's grateful sign language song "Let Love Pass out". The traditional martial arts performance showed the combination of effort and beauty, and the modern fancy rope skipping perfectly presented the superb skills and wonderful cooperation. The program was full of vitality and wonderful, which won the applause of the students. At the activity ceremony, the participating leaders issued certificates for the representatives of the caring units. In his speech, the aided students' representative expressed their gratitude to the Party, the government and all sectors of the society. He said that he would be grateful, study hard, become talents as soon as possible, and repay the society. The activity ended with the chorus of "Glorious, the Communist Youth League of China". Since the launch of the "micro wish" activity, the Communist Youth League Taiyuan Municipal Party Committee and the Municipal Youth League Working Committee have collected 1,296 "micro wishes" through online solicitation and offline visits. As soon as the "youth Taiyuan" public account was released, the city's youth league organizations at all levels, all front of the youth members and love units responded quickly, actively claimed, lasted for a month, 1296 gifts carrying deep warmth have all been sent to the children, small "micro wish" by a little fire of love all "light up". To meet the small wish of thousands of teenagers in difficulties is also the big wish of the Communist Youth League to serve their healthy growth. Next, Taiyuan pku, city working committee will take this activity as a new starting point, continue to promote "I do the practical work for the masses" activities, the party history learning education achievements into service the vivid practice of teenagers, strengthen teenagers feeling, happiness, to strive to write a comprehensive construction of socialist modern country in Taiyuan, fully reproduced "splendid Taiyuan city" cheng jing contribution youth strength.

2021-10-28	High-level young talents "gather in Weihai" into Nanhai New Area, Shandong Province. China youth network Beijing on October 28 (reporter Zhang Jianwei, correspondent Liu Kejia) recently, the fourth phase of high-level young talents "gathering in Weihai" successfully held. More than 80 Taishan scholars, master and doctoral supervisors and graduate students of Harbin Institute of Technology (Weihai) have conducted industry-university-research docking with key enterprises in Nanhai New Area. A group of high-level young talents learned about the enterprise culture, cutting-edge technology, industry-university-research needs of key enterprises in Weihai, and personally felt the charm of project agglomeration and innovation-driven park. In the subsequent school-local cooperation discussion and exchange meeting, the participants watched the talent propaganda video of Weihai City and the Nanhai New Area City propaganda video together. The Youth League Committee of Harbin Institute of Technology (Weihai) awarded the plaque of "College Students' Social Practice Base" to three enterprises, including Mingchuan Automobile, Nanhai New Energy and Zhendi Technology. The activity was jointly organized by the Organization Department of the CPC Municipal Party Committee, the CPC Communist Youth League Committee, the Municipal Human Resources and Social Security Bureau, and the Youth League Committee of Harbin Institute of Technology (Weihai), and organized by the Party and mass and the Human Resources Department of Nanhai New Area, aiming to do a good job in the article of "introducing, using, cultivating and retaining" of young talents, and promote the deeply integrated development of the university, government and enterprises. At present, the high-level young talents "Gathering in Weihai" activity has been successfully held for 4 periods, with more than 250 Taishan scholars, master and doctoral supervisors, and graduate students participating. Through joint efforts, universities, enterprises and parks have reached 16 industry-university-research cooperation intentions, and 3 projects have been implemented.

2021-10-27	Jining city first youth palace young pioneers working committee established Jining city first young pioneers youth palace working committee established China youth network Beijing on October 27 (reporter Zhang Jianwei, correspondent Bai Chen) recently, Jining city in Shandong province held the first youth palace young pioneers working committee-zoucheng youth palace less working committee establishment ceremony. The Youth League Jining Municipal Party Committee, the Standing Committee of the Zoucheng People's Congress, the Youth League Zoucheng Party Committee, the Municipal Bureau of Education and Sports related officials, social caring people, young Pioneers and young Pioneers counselors representatives participated in the activity. During the activity, some of the "Jining Young Pioneers Social Practice Base" and "Zoucheng Red Scarf College" and "Zou Lu Spark Young Pioneers Studio" were officially inaugurated, and the League Zoucheng Municipal Committee appointed members of the "Red Scarf Lecture Group", and awarded certificates for Zoucheng outstanding Young Pioneers counselors and outstanding Young Pioneers (collective) representatives. Social love enterprises held a donation ceremony of the "Happy Bud" activity. In order to further promote the study and education of youth party history, the event held "Youth dialogue" Sister Jiang "", "youth dialogue" Qu Qiubai "," pay tribute to the smallest martyr in the Republic " and other party history propaganda.

2021-10-24	Shandong Linyi city in Shandong province Guangdong youth working committee founded in Guangdong youth working committee founded XingTing) to build Linyi nationality in Guangdong youth resource sharing, mutual benefit and win-win, means of development carrier platform, strengthen communication, contact the power "double recruit double lead" in the city, on October 19, Shandong Linyi city in Guangdong youth working committee founding meeting held in Shenzhen treasure party-mass service center. Du Yifang, deputy secretary general of Linyi Municipal Government and temporary Party branch secretary of the third batch of Research and practice Team to Shenzhen, attended and delivered a speech. Xu Wenming, secretary of the Communist Youth League, attended and delivered a speech. The meeting was presided over by Zhang Xiao, Party Secretary of the Mobile Party Member of Linyi City in Guangdong and director of the Liaison Office of the Municipal Government in Guangdong. According to statistics, there are a large number of young people of Linyi nationality who are active in Guangdong universities, scientific research institutes, government institutions and social organizations, and a considerable number of young Linyi entrepreneurs are developing and growing in this fertile land of innovation and entrepreneurship. The team members of the Linyi Municipal Working Committee in Guangdong Youth are composed of 1 director, 6 deputy directors, 15 members and 1 secretary general. They work under the leadership of the Linyi Municipal Party Committee of the Communist Youth League and the Mobile Party Committee of the Linyi Municipal Party Members in Guangdong. More than 60 members of the floating party members of Linyi counties in Guangdong, outstanding young representatives from all walks of life in Guangdong, and some members of the third batch of research and practice team in Shenzhen attended the meeting. It is understood that in the promotion work, Linyi Municipal Working Committee in Yueqing will grasp the correct political direction, give full play to the role of a bridge, identify the entry point in project planning, business negotiation, capital operation and other aspects, and promote the coordinated development, resource sharing, mutual benefit and win-win situation among young people. At the same time, it actively serves the needs of Linyi young people in Guangdong in innovation and entrepreneurship, marriage, dating, and rights and interests protection, attracts and drives more entrepreneurs to return to their hometown to invest and do business, and creates a distinctive work brand.

2021-10-22	Group Bozhou municipal party committee held emerging youth group communication activities Bozhou municipal party committee held "recall the red boat spirit Shared good times" emerging youth group communication activities Zhang Jianwei correspondent, chun-hui liu check WeiTing) recently, "recall the red boat spirit Shared good times" emerging youth group communication activities held in Anhui Bozhou vortex river river red boat. More than 30 people, including representatives of emerging youth groups in Bozhou area, attended the activity. More than 30 emerging young people from all walks of life in the city watched the Bozhou cultural tourism propaganda video. In the form of story sharing and talk show, they shared their love and good at experience in network broadcast, work creation, publicity and operation, and exchanged their entrepreneurial journey. Since this year, the Bozhou municipal party committee of the full implementation of emerging youth group "web celebrity change net" red " action, through based on effective coverage, build participation in the carrier, weave dense contact network, gradually build by the communist youth league emerging youth group work service system, lead, condensed emerging youth, help them grow up, innovation and development.

2021-10-21	The Youth Working Committee jointly held the outstanding Young Pioneers counselors in 2021. After the preliminary selection, 19 young Pioneers counselors participated in the young Pioneers activity display and evaluation. The competition conducts a comprehensive evaluation of the young Pioneers 'basic knowledge written test, the young Pioneers' knowledge situation defense and the young Pioneers 'activity counseling skills display, and examines the working ability and comprehensive quality of the young Pioneers' counselors. After the competition, 8 contestants entered the Young Pioneers activity counseling skills display competition, among which Cui Yongchen, the Young Pioneers team counselor from Luhe Primary School in Zengdu District, won the first prize. The top 6 contestants in this comprehensive performance will also represent Suizhou to participate in the Hubei Provincial Young Pioneers Counselor Professional Quality Competition.

2021-10-21	Shijiazhuang "Create and Win Youth" Youth Employment and Entrepreneurship Training Camp and Youth Employment and Entrepreneurship Training Base awarding Ceremony of Shijiazhuang Youth Employment and Entrepreneurship Training Camp Activity Year. Representatives of entrepreneurial entrepreneurs from Luquan District spoke, telling about their entrepreneurial experience. Excellent youth entrepreneurship representatives from Beijing and Tianjin will share on the road show, build a platform for youth entrepreneurship and exchange, promote the convergence of entrepreneurial elements in Beijing, Tianjin and Hebei, and realize the perfect docking of entrepreneurial resources in Beijing, Tianjin and Hebei. In recent years, the Shijiazhuang Municipal Party Committee of the Communist Youth League has held more than 50 activities such as "Create Win Youth" Youth Entrepreneurship Training camp, entrepreneurship lecture hall and entrepreneurship online round table, covering more than 30,000 young people, providing professional entrepreneurship guidance for the young entrepreneurs in the city. For three consecutive years, the Shijiazhuang Youth Innovation and Entrepreneurship Policy Compilation has been issued, so that the city's innovation and entrepreneurship policies will benefit young entrepreneurs. At the meeting, the Shijiazhuang Municipal Party Committee of the Communist Youth League also awarded 50 youth employment and entrepreneurship internship bases. Build youth employment entrepreneurship trainee base is the Shijiazhuang municipal party committee service youth employment entrepreneurship is an important measure, aims to mobilize social resources, to provide a powerful platform for youth to participate in social practice, relieve the pressure of employment, create a strong atmosphere of concern and help youth employment entrepreneurship, help youth work experience, improve the ability of employment entrepreneurship work, lead the youth active in innovative entrepreneurship practice. The event was hosted by Shijiazhuang Municipal Party Committee and organized by TuanYuhua District Party Committee, Peking University Incubator, Tianjin Peking University Science and Technology Park and Shijiazhuang Peking University Science and Technology Park. Nearly 100 young entrepreneurs participated in the event.

2021-10-21	Changsha Municipal Party Committee of the Communist Youth League: "Qinghe Power" promotes the gathering experience of talents, etc., to attract young talents to enter Changsha, understand Changsha and choose Changsha."The person in charge of the Changsha Municipal Party Committee said that the youth League organization, gathering and serving the outstanding youth is one of the responsibilities of the youth League organization, and it is also an attempt of the youth League organization to strengthen the fine management of young talents and customize the exclusive services for young talents under the new situation. Liu Guangjian, a ―――――――――― graduate of Peking University majoring in software engineering, is from Qianjiang city, Hubei Province. In 2021, he left his first stop on campus in Changsha, Hunan province. Liu Guojian, who was born in April 1997, has had many plans for the future, considering multiple areas of work in various cities at home and abroad. Changsha, with low housing prices, good educational resources, profound red heritage and high quality of life, has become an important alternative for him earlier. In July 2021, Liu Gangjian participated in the "College Students' Changsha Tour" activity jointly organized by the Organization Department of Changsha Municipal Party Committee (Talent Office) and Changsha Municipal Party Committee of the Communist Youth League. Finally, he chose to work in Changsha and join Hunan Caixin Financial Holding Group. In the first half of this year, Wu Guiying, member of the Standing Committee of Hunan Provincial Party Committee and secretary of Changsha Municipal Party Committee, led a team to many well-known universities and research institutes in Beijing to attract talents and talents. Thereafter in Changsha municipal party committee under the guidance of talent office, Changsha municipal party committee set up special work organization, set up activity organization, contact with group, publicity materials, comprehensive support four groups, transferred more than 20 young cadres to participate in service, launched the national college students in Changsha experience exchange activities, in early June to Beijing, Shanghai field docking nine colleges and universities. In July, 70 students from the first batch of 7 universities, including Peking University, Tsinghua University, Renmin University of China, Beijing University of Aeronautics and Astronautics, Beijing Institute of Technology, Beijing Normal University and China University of Political Science and Law, arrived in Changsha. In August, the second batch of 57 outstanding students from "double first-class" universities participated in the "National College Students' Changsha Experience and Exchange Activity" to feel the urban vitality of Changsha."Through field industrial investigation, policy publicity and urban experience, we will attract young talents to visit Changsha, understand Changsha and choose Changsha."The person in charge of the Changsha Municipal Party Committee said that the youth League organization, gathering and serving the outstanding youth is one of the responsibilities of the youth League organization, and it is also an attempt of the youth League organization to strengthen the fine management of young talents and customize the exclusive services for young talents under the new situation. It is reported that the background of this activity is that Changsha to promote the implementation of the "young talent dream building project". Changsha municipal party committee municipal government leadership believes that Changsha to create national center city to realize basic modernization, must further implement the strategy of innovation leading, open rise, always adhere to the talent priority development, promote all kinds of talent team construction, cohesion to build innovative entrepreneurial talent highland, fully stimulate social creativity, and proposed to build a more open and effective young talent policy system, in the next five years to attract reserve 1 million young talents in Changsha employment entrepreneurship goals. The introduction of talents to domestic well-known universities has become one of the important tasks. Zhang Linyuan, who studied mineralogy at Peking University, expressed his feelings directly during a week-long visit: " I fell in love with this city!"A large number of college students positioned Changsha as a" vibrant city that can ignite the enthusiasm of youth " after the visit. They believe that the scientific research spirit of making hybrid rice from the experimental field to the world is actively moving towards the advanced manufacturing industry of Industry 4.0, and the cultural industry represented by "broadcasting Xiang Army" and "publishing Hunan Army" leads the trend for a long time... such Changsha is an entrepreneurial stage that can arouse the passion of struggle, and can become the "priority volunteer" for their future employment and settlement. External inline warmth extension "service area" group Changsha municipal party committee also introduced the xiangjiang river district league introduced 15 Beijing university, Wuhan university graduate students to Changsha, for 1-2 months summer job internship, and actively participate in the Internet yuelu summit, Hunan port contact weeks work, and tongji university, Shanghai jiaotong university youth corps committee established close ties, enhance the school friendship, promote the talent introduction pass forward. In the spring of 2018, Feng Junkai, who works in Shanghai, received a "call order" from his parents to return to Changsha- -they saw the "22 Changsha New Talent Policies" forwarded by their friends, and they quickly contacted Feng Junkai, who graduated from Tongji University. Feng Junkai had the idea of going back to his hometown to work. At this time, he happened to see an article in the group of his Alma mater entitled " Zhonglian, China Railway, Sany, 124 enterprises to recruit 3,627 people, the highest annual salary of 600,000 yuan, do you come?! The WeChat tweet. The tweet, released by universities across the country Changsha Municipal Committee on April 23, said that the "gold content" of the policy is a "magnetic field" to attract talents, and Changsha has become the choice of more and more young talents. On May 3, the Communist Youth League Committee held a precise job fair to connect with high-quality enterprises and face national outstanding talents. Previously, the Changsha Municipal Party Committee of the Communist Youth League widely released the recruitment information to colleges and universities across the country through various influential WeChat and microblogs. We welcome colleges and universities across the country to register and come to Changsha together to "find a job" for the job students. Not only did Feng Junkai immediately decide to book tickets to Changsha to attend the "Youth Industry Talent Recruitment matchmaking Meeting" in May, which also attracted Tang Zhiyu, deputy secretary of the Youth League Committee of Tongji University, to the scene to observe. He had a strong interest in the new talent policy and the detailed organization implementation in Changsha. Zhou Kun, head of the Changsha Municipal Communist Youth League, said that since 2017, cadres of the Changsha Municipal Communist Youth League have carried out research activities to directly contact and serve the youth. All cadres and staff went to 61 universities to promote "Changsha Talent Deal 22", carried out special research with 798 students and had discussions with some student representatives; visited Xiangjiang New District, Jingkai District, High-tech Zone, Yuelu Technology Industrial Park, investigated the talent service window, and some entrepreneurial enterprise representatives. They found that in addition to the first-tier cities of well-known college graduates, local enterprises also recruit more local quality graduates, but suffering from local college students often robbed by large domestic enterprises, or because the information contact is not timely, to local enterprises, often lead to local high quality students in the autumn and spring in advance in the process of "collective" dig and established the employment intention. Therefore, the talents cultivated and cultivated by local colleges and universities are far from enough to fill the job gap of local enterprises. Poor employment is not able to open up the "last kilometer" of local college talents to stay in Changsha. At present, there are 57 colleges and universities in Changsha, with an average annual number of nearly 600,000 graduates. As one of the most dynamic and potential subjects in the talent market, how to effectively connect with enterprises has become a major problem in the job market. Accordingly, the Communist Youth League Municipal Party Committee took the initiative to cooperate with universities to build a bridge for key industrial enterprises to introduce talents, combined with the comprehensive investigation on the needs of enterprises in the early stage, and contacted students from 37 universities such as Tongji University, Hunan University, Central South University, Hunan Normal University and other 37 universities inside and outside the province. In addition, more than 2,000 job-hunting students participated in the docking negotiations with 72 key industrial enterprises. The nest can attract the phoenix. League of Changsha municipal party committee in the municipal party committee, municipal government attaches great importance to and under the guidance of the municipal talent office, respectively in furong district, Changsha high-tech zone, Changsha, by the open area, liuyang city, liuyang by the open area built six Changsha young talent post municipal demonstration site, a total of 219 beds, for more than 4600 people to Changsha young talents to provide quality service. Talent Station releases the latest post information in the city to young talents every day, accurately recommends posts with high matching degree with the help of big data platform, dynamically updates the "Talent Employment Honor List", and also holds industry observation activities to enhance the sense of identity and belonging of young talents to Changsha. In recent three years, the Changsha municipal party committee also carried out the "love changzhutan" "fate house" youth public friendship activities such as 11, direct service single youth more than 3000 people, and through the city youth league, city green enterprise association, city farmers and other organizations, widely condensed youth, build learning training, entrepreneurial support, social activities, such as functional platform, promote youth exchanges in different areas."New citizens" growth channel talent "attract" is the foundation, and realize the dream is the key to "stay". Through the government purchase of services in Changsha, the city coordinates about 5,000 grassroots public welfare jobs to recruit ordinary college graduates every year. Entrepreneurs among college graduates will be given entrepreneurship training subsidies, one-time start-up fee subsidies and business site rent subsidies, and 100 outstanding youth entrepreneurship projects will be selected every year, with a maximum of 500,000 yuan free subsidy according to 50% of their actual effective investment. At the same time, Changsha takes industrial development as the main direction, has identified 22 industrial emerging and advantageous industrial chains, and introduced preferential policies such as "22 Changsha Talent New Policies". The Changsha Municipal Party Committee of the Communist Youth League carried out the action plan of "youth contribution Industrial chain" in accordance with the government's main direction of industrial development, to stimulate the development potential of youth "mass entrepreneurship and innovation". Zhou Bowen, the head of Hunan Ruitu Intelligent Technology, is a member of his hometown to start his own business. Ruitu Intelligence was founded in 2016, led by Academician Wang Yaonan of the Chinese Academy of Engineering. It is a national high-tech enterprise with machine vision as the core technology. Most of its core team members have worked in first-tier cities in Beijing, Shanghai, Guangzhou and Shenzhen."Although first-tier cities have their charm, Changsha is obviously more suitable for hard work and life."Zhou Bowen said that after returning to Changsha, the economic environment of emerging industries in Changsha has provided good conditions for their development- -more than 100 billion-level industrial clusters for entrepreneurial enterprises' innovative technology industry landing, providing a good application scenarios and industry resources guarantee. More importantly, government departments and mass organizations have set a platform, creating many opportunities for cross-border communication for entrepreneurs. Group Changsha municipal party committee under the leadership of Changsha young entrepreneurs association, has emerged a large number of excellent enterprises, such as tea YanYue color, and friends, fruit ah, ShengXiangTing, black classic, etc., become a Changsha web celebrity business card, pku organization of various communication activities, let more enterprises to realize the cross-border cooperation, especially like their technology enterprises and traditional industry has a communication platform between big enterprises. It is reported that most of the company's employees have bought cars and settled down in Changsha, with none of the core members actively lost. Youth League Changsha Municipal Party Committee also conducted in-depth research on young talents, found, reserve more than 100 outstanding young scientific and technology talents, through extensive launch, open roadshow, strict review, selected 23 people named "Hunan young talents"; continuous young post expert professional quality promotion class, covering more than 530 job experts, Changsha "ten line of top craftsmen", criminal technology, rail transit industry, fire industry, selected a number of professional youth model. Data from the seventh census released recently showed that the population of Changsha has exceeded 10 million for the first time, 3 million more than in 2010, showing a population agglomeration effect. The total number of talents in Changsha has increased from 1.1 million in 2017 to more than 2.8 million in 2020, gathering a strong intellectual momentum for the city's high-quality development. October 21,2021, Version 06

2021-10-22	Xuzhou, Jiangsu province held the 72nd anniversary of the founding of the Young Pioneers Jiangsu city held the 72nd anniversary of the Young Pioneers I " Xuzhou Young Pioneers to celebrate the 72nd anniversary of the young Pioneers held in Xuzhou Beijing Road Primary School. During the activity, the counselors of the Xuzhou Red Scarf Tour group told the example stories of "the people's victory of the people", "the alley premier" and "a special roll name". The young Pioneers of Beijing Road Primary School also interviewed Meng Qingxi, former Party secretary of Jiawang Mazhuang Village, Ma Jing, representative of the 19th CPC National Congress and party branch secretary of the fourth class of sewer, Huang Long, monitor of Wang Jie class, and Luo Shuai, one of the top ten outstanding youth in Xuzhou. Before the event, the relevant comrades view tongshan real small education group flag escort display, attend tongshan real small education group "team education class" and for the new appointment of small counselor representatives, understand "red scarf heart to the party" party history learning practice, and players in the "huaihai campaign spirit" theme party history learning education class. League of Jiangsu provincial party committee, group, Xuzhou, Xuzhou municipal bureau of education, Xuzhou committee, city young pioneers work society, tongshan district education bureau, tongshan district, tongshan small education group, Xuzhou sunflower practice experience base, from troops, fire control, sewer four, jia wang ma zhuang village counselors, and tongshan small education group young pioneers, a total of more than 360 people to participate in the activities. The activity is organized by Xuzhou Municipal Party Committee, Xuzhou Education Bureau and Xuzhou Young Working Committee, organized by Tongshan District Education Bureau and Tongshan District Young Working Committee, and co-organized by Xuzhou Sunflower Practice Experience Base and Tongshan Real Small Education Group.

2021-10-20	Huangshan municipal party committee to carry out the network security publicity week "youth day" group Huangshan municipal party committee to carry out the network security publicity week "youth day" activities Zhang Jianwei correspondent Sun Ke Zha WeiTing) to popularize knowledge of network security, enhance network security consciousness, create a clear network environment, recently, by the group of Anhui Huangshan municipal party committee, new city experimental school, city public security bureau, city telecom branch jointly organized network security publicity week "youth day" home activities held in Huangshan city new experimental school. Activities through the development of "strive to be a good young netizen" signature, visit the network security theme painting exhibition, watch the network security propaganda video, holding "5G" network science and network security special lectures and other forms, to further strengthen the youth network security knowledge reserve, enhance the ability to prevent network security risks. The activity also issued a "network security proposal", mobilize the majority of youth league members to build a network security steel Great Wall, further in the city's youth tree prison "network security for the people, network security by the people" concept. This activity has played a positive role in promoting the safe and civilized Internet access of teenagers in Huangshan city. Everyone said that in the future, they will effectively cultivate good network behavior habits, enhance safety awareness and protection skills, strive to be the disseminator of network positive energy, and strive to be the "Huangshan young good Internet users".

2021-10-17	Xi 'an to commemorate the 72nd anniversary of the founding of the Young Pioneers theme demonstration activity held Xi' an to commemorate the 72nd anniversary of the Young Pioneers founding theme demonstration activity held Sun Haihua) "Please rest assured, strong country has me" Xi'an to commemorate the 72nd anniversary of the young Pioneers founding theme team day activity was held recently. On the scene of the activity, thousands of young pioneers through hope, growth, care three chapters, 8 links of the performance, the interpretation of the new era young pioneers "learn the history of the Party, feel the party, listen to the party, follow the party, the party, please rest assured, the strong country has me" spirit of The Times. The activity was hosted by the Xi 'an Municipal Party Committee of the Communist Youth League and the Xi' an Youth Working Committee, and organized by the Communist Youth League Baqiao District Party Committee, the Baqiao District Education Bureau, and the Baqiao District Youth Working Committee. The activity in the young Pioneers flag, salute, singing team song. In the performance of "Review the team history, remember the party grace" chapter, the team members reviewed the glorious history of the Chinese Young Pioneers; the youth committee representatives told the young pioneers to "pioneer from primary school and grow up"; a thousand young pioneers to the Communist Party of China and the Young Pioneers. The activity also held a grand entry ceremony for the new team members, held the red education base, primary school students 100 meters painting exhibition and other observation activities."The development of the Team Building Day activities will encourage and educate the young pioneers to learn the history of the Party, strengthen their faith, and firm their determination to listen to the Party, feel the Party's grace, and follow the Party."Xu Hui, party secretary of the Communist Youth League, expressed the hope that the young pioneers would carry forward the glorious tradition of revolutionary martyrs, take over the" torch "of struggle, mind ideals, love the party and the country, set the vanguard example, and strive to be the" red children " in the new era.

2021-10-15	Group Changchun city committee to commemorate the 72nd anniversary of the young pioneers founding theme day group Changchun city committee held to commemorate the 72th anniversary of the Chinese young pioneers founding theme day activities Wang Peilian) recently, the Jilin Changchun city committee joint Changchun city party committee propaganda department, Changchun city bureau of education bureau and other departments jointly carried out the "red scarf heart to the party always follow the party" to commemorate the 72nd anniversary of the Chinese young pioneers founding theme day activities. The activity was held in the Gaoxin Huigu School of Changchun New District, where more than 4,000 children celebrated the "113 th" Young Pioneers' founding Day together. At the beginning of the activity, the red scarf small guide introduced the colorful young pioneers activities such as "red scarf small bookworm", "red scarf small maker", "red scarf small Ji 'an general", "red scarf small lark" and "red scarf small master" to the guests in turn, describing the happy life of the young pioneers in the new era in children's words. In the exhibition area of "Red Scarf grows up with the motherland and strive to be a good player in the new era", the leaders of the activity awarded MEDALS to the outstanding Young Pioneers of Changchun Samsung Red scarf Medal, and enjoyed the dance "Red Scarf Heart to the Party", poetry recitation "Please trust the Party to have me" and other programs. Then accompanied by music, the central venue entry ceremony officially began, the team flag, singing team songs, the small team members solemnly swear under the team flag. After the ceremony, Song Baoyin, the ideological and political mentor of Changchun youth, the winner of "Learn from Lei Feng Gold Medal" and the former political commissar of Jinzhou Military Division, told a vivid party history education lesson for the young members, encouraging the young pioneers to inherit the red gene and carry forward the red spirit.

2021-10-15	Jining City to celebrate the 100th anniversary of the founding of the Communist Party of China young students keynote speech recitation performance was successfully held on October 15 (reporter Zhang Jianwei, correspondent Bai Chen) on October 13, To celebrate the 100th anniversary of the founding of the Communist Party of China and further promote the study and education of Party history, Guide the city's young students to listen to the Party from childhood, strong faith, follow the party, On the occasion of the "1013" Young Pioneers Founding Festival approaching, By Jining municipal party committee, Jining municipal committee concerned about the next generation of working committee, Jining municipal party committee network letter, Jining education bureau, Jining less working committee jointly organized "thousands of people recite the party history of the school-Jining to celebrate the 100th anniversary of the founding of the communist party of China young students keynote speech performance" held in Jining sea line school. Jining Municipal Working Committee, the Communist Youth League Committee and the Municipal Network Information Office, the Municipal Education Bureau, Jining High-tech Zone and other relevant leaders attended the representatives, the winning works, counties (city, district) youth League committee, young pioneers and young pioneers counselors representatives, Jining Haida Xingzhi school teachers and students at the scene to watch. Jining city youth online red speaker appointment ceremony. Correspondent for the figure this performance is divided into "a single spark can start a prairie fire", "the red army is not afraid of expedition difficult", "red gene generations" and so on three chapters, focusing on the "thousands of people recite the history of the party" activities some outstanding works, activities held the "thousands of people recite the history of the party" award-winning works on behalf of the award ceremony, the first batch of Jining youth online red XuanJiangYuan appointment ceremony."Thousands of people recite the history of the Party" is an important measure of the party history learning and education of young students in Jining city. Since the launch of the activity, more than 1,400 schools and more than 800,000 young students have participated in the activity. A large number of excellent speech and recitation works were selected through the university-level preliminary contest, a county-level semi-final and a municipal final.

2021-10-14	Zhenjiang City, Jiangsu Province, "Five One" promotes the construction of a youth development-friendly city layout meeting. China Youth Network Beijing, October 14 (reporter Zhang Jianwei, correspondent Miao Jinjie) recently, Jiangsu Province Zhenjiang Municipal People's Government Information Office, Zhenjiang Municipal Youth Work Joint Conference Office jointly held the "14th Five-year" youth development plan press conference. As the city's first youth development plan, the biggest feature is that in the overall goal, it has formally proposed to create a youth-development-friendly city. Next, the Youth League Zhenjiang Municipal Party Committee will promote the construction of "youth development friendly city" through the "five one" measures, so that the city is more friendly to youth development and the young people are more promising to urban development. Clear about a goal. To promote the "youth development friendly" city construction as the overall goal of youth work, uphold the "city to develop, youth must first to develop" the important concept, constantly explore the actual law of scientific evaluation index, constantly build the characteristics of Zhenjiang youth development system, constantly improve the working mechanism in the leading enterprises in the province. Deepen a path. Focus on youth work joint conference mechanism of effective operation, establish the group departments counterpart contact, coordination, supervise the joint conference members of the working mechanism, set up youth development research center, hire college scholars, follow villager, entrepreneurs, forming youth development think-tank, pay attention to information sharing, project consultation, comprehensive supervision, joint research youth issues, coordinate youth policy, to carry out the youth project. Study a system. Under the guidance of statistical departments, a set of youth development index monitoring system and an effective evaluation system should be improved. Build "two single tables", sort out a list of considerable and tangible needs for Zhenjiang youth, a list of projects for young and middle-aged urban development, organically combine the demand list with the list of projects, and draw a matching table for youth development-friendly cities. Form a series of reports. Relying on the youth development research center, gradually formed the Zhenjiang youth development research report, the Zhenjiang youth development data analysis report, the Zhenjiang youth development index system construction report, according to a series of basic reports, annually formed the Zhenjiang youth development annual report, further to the perspective of youth, the youth needs into the urban development planning and system. Introduce a package of policies (projects). Focusing on the youth's worries and troubles in graduation job hunting, innovation and entrepreneurship, social integration, dating, elderly support, children's education and other aspects, we actively promote the party and government at all levels in the city to introduce a series of landmark support policies and implement a series of universal practical projects.

2021-10-13	Sanya held "academician lecture hall" special activities of young talent of Sanya municipal party committee held "academician lecture hall" young talent special activities zhang correspondent Wu Yuhua) recently, Sanya of Hainan province municipal party committee, Hainan tropical ocean institute and Sanya federation jointly held "academician lecture hall" young talent special activities, specially invited to Chinese Academy of Sciences Zhao Zhao screen of Hainan tropical Marine college 200 teachers and students entitled "don't forget the beginner's mind, must always-to life science life" lecture. Hainan Tropical Ocean College, relevant leaders of Sanya Municipal Party Committee, Municipal Development and Reform Commission and Communist Youth League Municipal Party Committee attended the activity. At the lecture, Academician Zhao Guoping, academician of the Chinese Academy of Sciences, told the life course of building, seeking, pursuing and realizing his dream based on the original mission of "the centenary of the Founding of the Party". From the hesitation in the scholarly family to inspirational and cultivation of education, from the rural classroom farmer teachers learn to be a responsible person to the university hall to learn in practice, even if the journey is difficult, also never forget to love their family, patriotism, adhere to the original intention of scientific research. When talking about the problem of how the youth grow in the new era, Academician Zhao Guoping proposed four points: always adhere to be ambitious, have to see the world, live and transform the world, always adhere to see the stars, be thoughtful, logical, dialectical and innovative life pattern, and always adhere to the life values of hard work, excellence and serve the people. Finally, the teachers and students of Hainan Tropical Ocean College asked questions, and Academician Zhao Guoping answered them one by one. MAO Yunxiang, vice president of Hainan Tropical Ocean College, made a summary. The Sanya Municipal Party Committee of the Communist Youth League will combine the party history learning and education work, give full play to the organizational advantages, actively mobilize the city's youth League cadres, and strive to provide more and better services for the young people.

2021-10-13	Ma on shan college students entrepreneurship employment salon series activities held 2021 China Maanshan "ten thousand enterprises" college students entrepreneurship employment salon series activities successfully held zhang correspondent wang jun check WeiTing) by ma on shan talent work leading group, city high-level talent association, city talent office, city people club bureau, city bureau, bureau of education, pku to undertake 2021 China ma on Anshan "ten thousand students into one thousand enterprises" college students entrepreneurship employment salon activities recently held in Anhui university of technology, cool cube, the gen space. At the Youth Home of Anhui University of Technology, more than 30 in-service college students and entrepreneurial youth representatives from the university participated in the activity. The participants also publicized and answered the questions of their concern on employment and entrepreneurship policies, employment situation and employment concept. In the Cool Cube Maker Space, More than 50 people from Maanshan Normal College, Ma'Anshan Vocational and Technical College, Maanshan Technician College, Wanjiang Institute of Technology in-service college students backbone and young entrepreneurship representatives of Cool Cube maker space participated in the salon activities, Sharing at the meeting, Cheng Lei, an outstanding entrepreneurial youth, closely combines college students' employment and entrepreneurship ideas and their own entrepreneurial learning experience, I shared with you my entrepreneurial insights and mental journey, Encourage college students to devote themselves to innovation and entrepreneurship practice, Select Ma on Shan, Stay in Ma on Shan, Rooted in Ma'Anshan, Encourage everyone to dare to pursue their dreams, Diligent in realizing your dreams, Bear the historical responsibilities entrusted by the new era, Live up to the youth of the time. Liu Shaozhu, deputy chief of the entrepreneurship service Department of the Municipal Human Resources Management Service Center, preached the latest entrepreneurship service policy for the participants, and guided everyone on how to make good use of the policy welfare and establish a correct view of career selection. During the activity, the young people also exchanged and interacted with the sharing guests. The atmosphere was warm and the interaction was frequent. Next, pku will be around the startup city, service "double recruit double lead" goal, adhere to the "youth priority development" concept, to further promote the ma on Anshan <long-term youth development plan (2018-2025)> implementation plan ", the construction of Yangtze river delta youth sister city," one thousand enterprises "activities for the gripper, combined with" I do something for youth " theme practice, extensive employment policy, entrepreneurship pioneer into campus, fairs and other brand activities.

2021-10-12	Taizhou, Jiangsu: "Phoenix return nest" nine years warm returning students said, put forward the need to extend the practice time, which makes the organization of the "Phoenix return nest" summer practice of the Jiangsu Taizhou Municipal Party Committee into the "happy trouble". Taizhou alias "Fengcheng"."Phoenix Return to the Nest" summer practice for college students, which is a brand activity jointly sponsored by Taizhou Talent Office and "Fengcheng Youth Talent Gathering Plan" of Taizhou Youth League Municipal Committee. It has been held for 9 consecutive periods and has been welcomed and sought after by Taizhou college students. Entering the ninth year, the "Phoenix return nest" is further upgraded. This year, the Taizhou municipal party committee launched the city, county (area) two levels, respectively from the party and government organs, financial units, enterprises, assets management, public welfare undertakings, grassroots services, scientific research platform, medical institutions, legal services in more than nine thousand units matching more than 3000 jobs, successfully attracted 1605 college students to internship, refresh the number of jobs and records again. This year's phoenix nest " is different from previous years. COVID-19 has occurred in Nanjing and Yangzhou, and the epidemic prevention and control situation in Taizhou suddenly became serious."We are young, let's go up first!"The 623 college students located in the village and community posts have applied to the local youth league organizations to go to the front line of epidemic prevention and control, and to enrich the fight against the epidemic at the grass-roots level. In Shagou Town, Xinghua City, Hu � h, a student from Changzhou University, works to assist towns and villages in the initial screening of flow."At the peak of the task, several of us had to undertake more than 1,200 people a day, calling all night and confirming repeatedly confirming.""This holiday is not the most comfortable, but the most fulfilling heart" "the biggest harvest is the sense of responsibility"... the students grow in the responsibility."I came to Taizhou once before, and I thought it was very beautiful. I hope to experience a round of substantial practice here."On the afternoon of July 6, Li Wenlu, a graduate student from Southeast University, appealed on the phone. Li Wenlu is neither a Taizhou native nor has any internship experience in Taizhou. She was attracted by the city through the official microblog of the school's Youth League committee. According to statistics, a total of 58 foreign students came to Taizhou to participate in social practice. Hot summer days, sudden showers, typhoons landing and epidemics coming... bring great challenges to our daily work."In just six weeks, the students showed strong adaptability and excel in the post internship and 'seven one' practice activities."Zhai Wenzhou, Party secretary of the Youth League Taizhou, said," To deepen the summer practice of college students, in recent years, we have been exploring new practices and striving for a strong guarantee. This year, we not only formulated the "Credit Management Rules" to strengthen the student management, but also carefully produced the students' address book and the "Zhixiang Printing Collection", so that the students can punch in as a souvenir, and leave a good memory."Yuan Jiayue, a student majoring in agriculture and forestry economic management at Yangzhou University, said that through the summer practice of" The Phoenix returns to the nest ", she has made a big step forward on the road of realizing her entrepreneurial dream and helping the rural revitalization. During her internship in Baimi Town, Jiangyan District, she encountered difficulties, and the farmers' loofah became unmarketable. After investigation, Yuan Jiayue found that the loofah has a unique load-bearing structure, which is a special material with excellent buffer performance and environment friendly, which can be applied to packaging, sound elimination, filtration, thermal insulation, shock absorption and other fields, and has great commercial value and sales market. Therefore, she consulted the teachers of the school and used her professional knowledge to lead the planning of dianhe Village loofah industry extension project. At present, the project has entered the processing link, and has successfully promoted the employment of 56 people. The key to rural revitalization lies in the people. This year, the League Taizhou Municipal Party Committee took rural revitalization as an important direction for organizing young students to carry out activities."Speak for hometown flavor, live with goods training" special help farmers live, "cloud push" Taizhou's most popular food strategy; launch a study tour rural action, organize the visit of modern agricultural industrial base; carry out "red footprint big search", "a wonderful plan to Taizhou" discussion... Qingyi public summer care class is the youth League Taizhou Municipal Party Committee, Taizhou Civil Affairs Bureau and other units to recruit young volunteers, for 7 consecutive years for left-behind children, de facto orphans and other teenagers to provide public welfare care services. This year, the class has covered all the towns and villages in the city, and the "Phoenix returns the nest" college students have become good teachers and helpful friends for the children's summer vacation."On the first day of the task assigned in the afternoon, the students could take out the lesson preparation notes at 9 PM. On the next morning, they rushed to the village to teach the lower grade students. Moreover, the class atmosphere was very good, and the children liked it very much that the villagers rushed to sign up."Township youth league cadres to" phoenix return nest " college students point a big praise."Under the background of 'double reduction', the number of service objects of summer care classes has increased significantly this year, and the management pressure has increased significantly. Fortunately, a group of college students have actively participated in the whole work is not only orderly, parents are also full of praise."Sun Lei, deputy director of Taizhou Civil Affairs Bureau, said," Watching the countryside and dedicating love is our original intention. Children will become college students, to realize their life dreams, and will also bring this love back. Six years ago, Deng Liuliu participated in the summer practice of "Phoenix Return to the Nest" for the first time, practicing in Lengjia Village, Zhaoyang Town, Xinghua City, responsible for the construction of "rural library". In 2016, she signed up again to exercise in the Xinghua Municipal Human Resources and Social Security Bureau to assist in the receipt and management of college graduates' files. From the village head to the desk, from book management to file management, the summer practice experience in her heart buried the seeds of serving her hometown. In 2018, Deng graduated from university and chose to work in Taizhou. In colleges and universities, there are many Taizhou college students like Deng who want to go home to work in their hometown. Taizhou trains and sends tens of thousands of university freshmen every year. They are distributed in major cities across the country. Many of them are eager to return to them and build their hometowns. According to the current situation and needs of young students from Taizhou, Taizhou Municipal Committee of the Communist Youth League set up a youth student fraternity with cities as a unit to more accurately contact and serve the majority of young students from abroad, and further build a platform for two-way communication between their hometown and students."In the past year, the Municipal Talent Office and the Communist Youth League Committee have piloted student associations in Beijing, Xi'an and Harbin, and achieved remarkable results."Ju Linhong, deputy director of the Organization Department of Taizhou Municipal Party Committee and secretary of the Working Committee of Non-public Enterprises and Social Organizations, said," This summer, we set out a student association in nine cities, including Nanjing, Changsha, Shanghai, Wuhan, Chongqing and Tianjin, and on this basis, we established the Taizhou Student Association. According to statistics, the existing 12 student associations can cover more than 26,000 college students from Taizhou. In the new "" school year, we will organize students in Beijing to carry out an ice-breaking fellowship, a party history competition, a research and exchange, and a hometown lecture, and enhance their friendship through four activities. At the same time, we will also provide you with a series of conveniences such as city guide, postgraduate entrance examination sharing, academic mutual assistance, and growth strategy."Said Song Jiacheng, a student of Tsinghua University and president of the Taizhou Students (Beijing) Association. At the founding meeting of the Taizhou Student Association on August 25th, all 12 associations released their activities and service menus for the next school year. This year, the Taizhou Municipal Party Committee of the Communist Youth League has selected 104 outstanding college students as the backbone of the local associations, who will be active in the university campus and speak for their hometown of Taizhou. The Communist Youth League Taizhou Municipal Party Committee also strengthened communication and docking with the local Taizhou Chamber of Commerce and enterprises, and reached cooperation agreements to strive for more rich resources for the fraternity. Li Huanjun, chairman of Jinchen Group, said, " Homesickness is the strongest. Wherever the Taizhou Student Association is built, our local branches will provide office offices and activity venues to spare no effort to help them carry out their activities."China Youth Daily China Youth network reporter Li Runwen, correspondent, Qiu Junyi Jiang Lin source: China Youth Daily

2021-10-10	Yichun to carry out the "please rest assured that the strong country has me" theme league day activities at all levels to carry out the "please rest assured that the party has me" theme of the party to have me " as the theme, with the league branch as the unit, to carry out a variety of forms of theme league day activities. Centering on the theme of "Please reassure the Party that the strong country has me", the activity was carried out mainly online and combining online and offline. Online picture and micro-video collection activities, such as "I and the National flag" and "confession to the motherland"; offline, the youth League branch conducts commemorative activities. The First Youth League Branch of the Fifth People's Hospital sang the national anthem and league songs; the Youth League branch of Class 1,2020, Medical Technical College of Yichun Vocational College; the Yichun Taxation Bureau of the State Administration of Taxation and the Youth League Committee; and they organized the Youth League Committee to conduct class meetings and exhibitions to express their best wishes for the great motherland. The city's 934 youth league branches have carried out a series of 1,776 themed youth league day activities, including "Please trust the Party to have me", covering more than 20,000 youth league members.

2021-10-10	The Youth League Committee of the Municipal Hospital of Traditional Chinese Medicine carried out the theme activity of "I Do Practical Work for the Youth" and "Traditional Chinese Medicine into the Government- -Health Lecture". Activities invited the national youth federation committee, the passengers hospital of traditional Chinese medicine, deputy party secretary, dean Liu Jianhang around nine health problems, from the great contribution of Chinese medicine culture, Chinese medicine in disease resistance, combined with the root of the traditional Chinese culture and contact, and lead the medical team to site yizhen, for authority youth to solve health problems. The activity aims to guide young people to pay attention to health, strengthen self-health care, consciously develop good life habits, with the best body, sufficient energy into the busy work and life, and open the golden key to health.

2021-10-09	Sunshine: spark reading pilot young pioneers red holiday sunshine: spark reading pilot young pioneers red holiday China youth network Beijing on October 9 (xinhua Zhang Jianwei, reporter XiangChanghui) recently, the group of Shandong Rizhao municipal committee in the city study cherry garden library to celebrate the 72nd anniversary of the founding of the People's Republic of China "spark reading". This reading club invited Su Huicong, a member of Shandong Province Red Scarf Tour Group and the excellent Young Pioneers counselor of Rizhao City, to have a special party history learning trip with the young Pioneers with the title of "72 Years, So the Coming of New China". By displaying rich historical materials and pictures and telling example stories, the team members were guided to understand the heroic spirit of the Chinese people in resisting aggression under the leadership of the Party and the great achievements made in building a socialist country. In order to deepen the young Pioneers' party history learning and education activities, Enrich the children's Party history learning reading materials, In April, 2021, The Communist Youth League Municipal Committee created the "red scarf reading Corner" in the city study cherry garden hall, Carefully selected more than 500 red books, party history story picture books, comic books and so on, Build a platform for children's party history learning and reading, And combined with Rizhao TV station invited the party history research Institute President Yin Dewen, front-line counselors and red scarf narrator, Around how the young Pioneers learn the history of the Party, Recorded and broadcast the young Pioneers "Learn the History of the Party" TV interview program, At the Cherry Garden in the City Study on the first Saturday of the month, Invite the members of the provincial and municipal red scarf tour groups and the winners of the red scarf commentator competition, Conduct public lectures and reading and sharing activities to study Party history. Up to now, the "Spark Reading Club" has been successfully held for 4 periods, the "red Scarf Reading Corner" red books borrowed 3,000 people, the education and guidance of more than 10,000 young pioneers.

2021-10-08	Yinzhou: To strengthen the education and management of the league members to celebrate the centenary of the new era; as a native of Yinzhou, I am very happy to show the world the spirit of Chinese youth."Recently, Yang Qian, the first gold winner of the Tokyo Olympic Games and the post-00s from YangJialong Village, Yinzhou District, Ningbo City, Zhejiang Province, opened a special lecture for the" Yin Ling " post-00s publicity group through VCR. The publicity group is jointly built by the Publicity Department of Yinzhou District Committee and Yinzhou District Committee of the Communist Youth League, which is also yinzhou's further efforts in strengthening the education and management of the youth League members after becoming the comprehensive pilot of the grass-roots organization reform of the Communist Youth League. On September 1, Yinzhou Youth Service Center broadcast "Generation 1 Is Here" the first lesson, which attracted more than 100,000 views. It was Zhu Chunyan, who entered the service center last year, who was in charge of the event. The service center is a subordinate institution of the Youth League Yinzhou District Committee."Targeted recruitment, is yinzhou Communist Youth League grass-roots organization reform of the biggest measure."Xin Chunyi, secretary of Yinzhou District Party Committee, said that what kind of person to choose and how to choose the right person to enter the position of the league is the first step in the whole process of management of the league cadres, but also a very critical step. Yinzhou selects cadres through open examination, open recruitment, open recruitment, open selection and directional recruitment. In November 2019, the Yinzhou District Committee of the Communist Youth League Bureau publicly admitted five youth League social workers through the Civil Affairs Bureau, who stood out from 700 applicants and are now sending five of the youth League working committees. According to the relevant requirements of superiors at that time, the status of social workers can only be used in the community. The Yinzhou District Committee of the Communist Youth League and the civil affairs and financial departments negotiated for many times, and finally attached these social workers to the social organization innovation park under the Civil affairs Committee. The salaries were paid by the Civil Affairs Committee, and the performance assessment was determined by the Communist Youth League District Committee. All the missing social workers could be made up in time to ensure the formation of a long-term mechanism. In yinzhou dragon village, there are more than 7000 migrant youth, the local youth league branch by setting up "581" youth volunteer service team (581 homophonic "I help you", means that all over the world, acting together, reporters), absorbed a lot of foreign youth backbone, and one of the fellow will liu hired as deputy secretary of the league branch, focus on volunteer service activities. This has not only stimulated the enthusiasm of foreign youth to participate in grassroots governance, but also promoted more foreign youth through the backbone of foreign youth. Local league organizations may give certain remuneration to the appointed league cadres according to their workload. He Libin, deputy secretary of the Zhejiang Provincial Party Committee of the Communist Youth League, said: " The cause of the Communist Youth League mainly relies on the promotion of the youth league cadres. The key to the reform of grass-roots organizations is to promote the leading cadres of the youth League at all levels to remove the 'official standard' and carry out the 'de-administrative' work. Grassroots league organizations should target a wider range of youth groups and train more young political backbone for Party organizations."Before the reform, Yinzhou only district village three level 288 cadres, to the system cadres, village community cadres, part-time cadres only the youth League district committee and part of the towns and streets. In the face of the problems of the poor channels of absorbing social talents in the selection and management of youth League cadres, the weak business ability, the insufficient overall planning of the league work force, and the insufficient scientific management, the Organization Department of Yinzhou District Committee led by the people and social security, the organization office and the Youth League Committee jointly formulated the Regulations on the Whole Process Management of Yinzhou District Youth League Cadres."Management" from all over the world, the overall linkage of "education", can be the "management", performance oriented "use", humanistic care "love". Carry out chain management from five aspects of "selection, education, management, management, use and love", so as to make the selection mechanism more reasonable, more accurate education and training, more strict daily management, more effective assessment and evaluation, and more perfect guarantee mechanism. After becoming the expanded reform pilot project, Yinzhou insists on "no channel without change, no strength", continuing to consolidate the first part of the institutionalized reform, improving the party leadership mechanism and support; unimpeded the selection of young backbone; reconstructing the basic organization construction and operation of the group; and strengthening the education and management of league members. The Communist Youth League Yinzhou District Committee also actively sought for the support of the party and the government. Chu Yinliang, member of the Standing Committee of Ningbo Municipal Party Committee and secretary of Yinzhou District Party Committee, specifically instructed that "further promoting the reform pilot project is trust and great trust, and we must do a good job and become a first-class". In addition, in accordance with the work requirements of "four synchronization of the Party and the Youth League", in the first half of 2021, Yinzhou District Committee of the Communist Youth League will cooperate with the district Committee inspection team to carry out league construction inspections in 6 towns and streets (departments), effectively promoting further consolidating the work of the Communist Youth League at the grass-roots level. Under the guidance of Ningbo Municipal Party Committee of the Communist Youth League, the "Spark Chain" youth league cadre management project at the urban level has successfully "taken charge" of the digital reform and application project of Zhejiang Communist Youth League. Yinzhou District Committee, to select the new round of part-time deputy secretary and temporary cadres, adhere to the joint training of the youth backbone, and the organization Department of the District Committee to carry out a one-month "smith special training camp", focusing on the village, the young cadres in the "challenge". In terms of reconstructing the construction and operation mode of grass-roots organizations of the league, Yinzhou District Committee of the Youth League further sorted out the organizational forms of various youth gathering, comprehensively rolled out the "ten pooling modes of league construction", carried out a new round of league construction actions in non-public and two new fields, and added 230 enterprise league organizations. Xin Chunyi, secretary of Yinzhou District Committee, said that he had the honor to become the pilot area of the grassroots organization reform of the National Communist Youth League twice. Yinzhou will continue to make great efforts to take the "four whole regions to build a strong" + "seven panoramic demonstration" as the core, integrate into the digital reform wave of Zhejiang Province, and do a good job in the second half of the reform! China Youth Daily China Youth Network reporter Jiang Yutong source: China Youth Daily

2021-10-08	Dezhou youth civilization collective to revolutionary martyrs cemetery Dezhou youth civilization collective to revolutionary martyrs cemetery Beijing October 8 (reporter Zhang Jianwei, correspondent Fan Yichao) in order to carry out ideal and faith education and revolutionary tradition education, Cultivating and practicing core socialist values, Leading the city's vast number of professional youth to remember the revolutionary martyrs, Remember the glorious deeds of the revolutionary martyrs, Cherish today's happy life, a few days ago, Shandong Dezhou Municipal Party Committee organized a total of 30 collective representatives of 13 youth Civilization number, including State Grid Dezhou Power Supply Company Center Business Hall and Decheng Power Supply Center, to go to Dezhou Revolutionary Martyrs Cemetery to carry out martyrs' Day activities. Pay tribute to the monument and memorial hall of the people's heroes, and listen to the general situation of the memorial hall and the deeds of the revolutionary martyrs. Correspondent for the picture of the youth have said that the spirit of the revolutionary martyrs through time and space, constantly inspire us, to participate in this activity is a washing of the soul. As a youth representative of the new era, we must stick to their posts, fully fulfill our duties, do every ordinary thing well down-to-earth, and let the revolutionary spirit be passed on from generation to generation.

2021-10-04	The 13th China air show volunteer service over 80000 hours the 13th China air show volunteer service over 80000 hours Wu Xin) the 13th China air show on September 28 solstice on October 3rd in Zhuhai, 955 young volunteers full-service air show, volunteer service over 80000 hours, "blue sky event volunteer red" become a beautiful beautiful youth scenery line. It is understood that 955 volunteers from the Zhuhai campus of Zunyi medical university, Guangdong vocational college of science and technology, Beijing Institute of Technology Zhuhai college, Zhuhai city vocational college of art, divided into peripheral inspection post, logistics support post, core regional post, the average age is only 19 years old. During the air show, they were dressed in "red vests", relying on the five "Blue Sky cabin" volunteer service stations and various service stations, providing a full range of volunteer services such as air show publicity, air show information inquiry, traffic guidance and emergency rescue. Tao Baolong, from the School of Aviation, Zhuhai University of Beijing Institute of Technology, served as the leader of the catering team in the volunteer service, mainly responsible for the temperature detection of customers entering the restaurant and maintaining the order of the restaurant. WeChat records show that he takes 30,000 steps per day."Classroom learning can make us gain knowledge, and volunteering in large-scale activities can not only exercise our ability, but also feel the joy of helping others. Yu Yu � j from Zhuhai art vocational college said, can in the 100th anniversary of the founding of the communist party of China and the 72nd anniversary of the founding of new China to participate in air show volunteer service is a glorious thing, " only a strong country, have personal safety, happy life, we have'please rest assured, power have me bear mission, to do everything, for the national development, urban construction."Hou Wentao, secretary of the Zhuhai Municipal Committee of the Communist Youth League, said that the warm, thoughtful, meticulous, professional and efficient service of the young volunteers was praised by the citizens, tourists and exhibitors, showing the spirit of the youth of the Special Zone, and polishing the name card of Zhuhai" Youth City and Capital of Vitality " with practical actions.

2021-10-02	Hefei, Anhui: More than 500 young people sing the song of youth tribute to the founding of new China reporter Wang Haihan Wang Lei) Every era, there are their songs; behind each song, there is a struggle of youth time. At 8 o'clock this morning, the Youth League Anhui Provincial Committee, Youth League Hefei Committee and Youth League Shushan District Committee jointly produced MV " Listen! Youth is singing! It has been launched on the new media platform of Anhui Province and some provincial media and provincial departments and bureaus. In the video of......... for over 5 minutes of young Pioneers, Communist Youth League members, university teachers and students, scientific research team of the Chinese Academy of Sciences, fire officers, new energy vehicle practitioners and environmental industry practitioners, More than 500 young people from 16 units appeared on, They came to Chang'an automobile new energy automobile welding automation workshop, China university of science and technology Qian Xuesen statue, Anhui Mao Zedong statue of Anhui medical university, Anhui agricultural university auditorium, defense university of technology electronic confrontation institute, China environmental valley, science island big science device, of shushan district fire rescue brigade scenario, Singing songs like "My Motherland", "The East is Red" and "Chinese Military Soul" with the imprint of The Times. According to the introduction, the video design script, thinking grinding, shooting and editing lasted two weeks, aiming to express the contemporary youth do not forget the road, firm original belief, dedicated to the youth of the party, determined to serve the country spirit of style, show the vivid practice of all fronts to help the national social development.

2021-10-02	Xing Ting, Qingdao Youth who won good results in the Shandong Province Youth "Mass Entrepreneurship and Innovation" Competition) On September 28, the 10th " Shandong Youth Innovation and Entrepreneurship Competition award ceremony was held in Jinan. After fierce competition and wonderful display in each link, Qingdao youth has achieved excellent results in this competition: a total of 12 projects in Qingdao have won prizes. Among them, 2 projects won the first, second and third prizes of the competition, and 6 excellence award projects. Among them, the dual-carbon integrated platform project from Qingdao city and the industrialization platform project of high value-added natural metabolites won the first prize in the competition. The Qingdao Municipal Party Committee of the Communist Youth League won the Competition Excellent Organization Award. It is understood that the competition to "youth create Qilu win in the future" as the theme, a total of more than 1,100 projects to participate in the number of projects for the most ever, the participating projects focus on the "top ten" industries, generally represent the new technology, new forms of business, new mode of development direction. Next, the Qingdao municipal party committee will take the youth innovation entrepreneurship competition as an opportunity to cohesion service "project", "youth" I do the practical work "as the point, to help youth innovation entrepreneurship as the goal, according to the" project, platform, institutionalization, the brand work, "by opening" green and pioneer class, pioneer, green and pioneer, green and pioneer garden, green and pioneer, pioneer post, green and pioneer loan, pioneer, pioneer cloud, green and pioneer list " ten section activities, promote green and pioneer action. At the same time, more platforms will be built to form more results and comprehensively help Qingdao's high-quality development.

2021-09-30	Meishan, Sichuan: "green and plan" relief youth entrepreneurship "pain points" to 142 entrepreneurial youth issued "unsecured, no guarantee, zero interest" business loans 12.33 million yuan in Sichuan Meishan: "green and plan" rescue youth entrepreneurship "pain points" green and plan ', I got a lot of money and business support, which added my entrepreneurial confidence."Fan Finland, the founder of a media studio in Meishan, Sichuan province, from running business with a SLR camera to an annual turnover of more than 300,000 yuan, said that the" Youth Innovation Plan " is a guide to her own business."Youth Innovation Plan" is the short term of Sichuan Youth Entrepreneurship Promotion Plan, which aims to help young entrepreneurs get a ride through venture capital support and entrepreneurship project guidance. In recent years, the Meishan Municipal Party Committee of the Communist Youth League has provided tangible services and support to young entrepreneurs through the "Youth Innovation Plan" and other work projects. In 2013, Fan Finland ended his "north drift" life and returned to his hometown Meishan to try the wedding camera work. After mastering the shooting and editing skills, she had the idea of starting her own studio. Buying a SLR camera, Fan Finland started a business alone."When the studio was first founded, I could only take some orders for a few hundred yuan. Without equipment, in the media industry."Fan Finland said that the purchase of new equipment requires a lot of capital investment, which is a big problem for her new business. At a loss, a friend recommended the "Youth Innovation Plan" to Fan Finland. If the project is successfully selected, he can not only get an unsecured, guarantee-free and zero-interest loan support of 30,000 yuan to 100,000 yuan. However, he can also get "one-to-one" help from a business mentor. With a try mentality, Fan Finland began to provide the registration materials as required."From the beginning of the registration, the staff of the Meishan Communist Youth League patiently and carefully guided me to make the business plan and fill in the cash flow statement, which gave me a more reasonable and practical improvement of my entrepreneurial project."Fan Finland said. In addition to understanding the urgent loan support, Fan Finland was also surprised by the entrepreneurial mentor's help and support."My mentors not only provided me with professional and technical support, but also introduced me to the right customers, helped me to expand my business, and gradually opened up the market."Like Fan Finland, ex-soldier Fang Cong was once suffering from a shortage of start-up capital and a lack of entrepreneurial experience. After returning to his hometown Meishan, Fang Cong's first business failed."When I first started my business, I always felt that I could do everything, neither I chose the right direction, nor I had the experience in starting a business."In 2017, he chose to start with his familiar field of agricultural materials and embark on the road of starting business again."When the company was first founded, it was short of capital, sales and publicity, and its development still faced many problems. The 'Youth Innovation Plan' not only enabled me to get a business loan of 100,000 yuan, which solved the company's urgent need for starting the business, but also provided the free help and guidance from my business mentors, which also increased my confidence on my way to starting a business."After the entrepreneurs applied for the" Youth Innovation Plan ", the Meishan Municipal Committee of the Youth League organized the entrepreneurship mentors to conduct a field investigation of the application project, and conducted further research and guidance on the applicants' entrepreneurial environment, the viability and market prospects of the entrepreneurial project. In Fang Cong's company, his business mentor told him that he could not always sit in the store and wait for the business, but to take the initiative to go out and communicate with consumers more. The entrepreneurial mentor's words suddenly woke up Fang Cong. He found that while helping farmers solve problems in production scenarios, his products have also gained trust step by step. Now, Fang Cong has grown from the former entrepreneurial "small white" to Meishan city rural youth to get rich leader. As the business grew bigger, he began to figure about how to give back to others. In addition to participating in various social welfare activities, veterans, disabled and five guarantees became his focus when recruiting employees. Employee Chen Mingjie watched his boss's career grow stronger."Since I went to the company in 2018, he has changed two cars."Chen Mingjie was disabled due to injuries while working outside. After his introduction, he came to the agricultural materials company founded by Fang Cong and became one of his first employees. Ran Liyuan, a college student and college student entrepreneur, was one of the first beneficiaries of the program. The start-up loan and the help of his entrepreneurial mentor provided by the "Youth Innovation Plan" once gave him the opportunity to restructure his team, adjust the direction and successfully overcome the entrepreneurial starting difficulties when facing the dilemma of not even paying his employees' salaries. After the company got on the right track, Ran Liyuan found that at that time, Meishan city had no business incubator base for entrepreneurs, and more young people who wanted to start businesses and were starting businesses like him lacked a platform to obtain information and learn entrepreneurial knowledge. With the help of his business mentor, he invested and established the Pengshan District Business Incubation Park in Meishan City, providing entrepreneurs with free entrepreneurship training, office space, market information allocation, industrial and commercial registration and other services."As a beneficiary of the Youth Innovation Program, I hope to benefit more entrepreneurs from the experience and lessons of my own entrepreneurship."Ran said. It is understood that the Meishan Municipal Party Committee of the Youth League focuses on the most difficult and prominent problems of youth entrepreneurship, tries every effort to integrate resources to make practical measures and new measures, takes practical measures to get through the policy blockage, relieve the pain points of entrepreneurship, break down the difficulties of development, and help young people innovation and entrepreneurship. Since its launch in March 2015, Meishan "Youth Innovation Plan" has issued a total of 12.33 million yuan of business loans to 142 young entrepreneurs, with the capital amount and repayment rate ranking among the top in Sichuan Province. China Youth Daily, China Youth Network reporter Wang Xinxin, intern, Ma Xiaoqing source: China Youth Daily

2021-10-09	League Jining Municipal Party Committee to carry out the "advocate tree civilization new wind resist marriage customs" special action League Jining Municipal Party Committee to carry out the "advocate tree civilization new wind resist marriage customs" special action Jianwei correspondent Bai Chen) recently, In order to further promote the new trend of The Times, To guide the vast majority of young people to abandon the old rules and bad habits, Establish a correct view of love and marriage, outlook on life and values, Youth League Jining Municipal Party Committee firmly seize the "National Day" wedding peak favorable opportunity, Fully integrate civil affairs, commerce, public security, cultural tourism, market supervision and other resources, Clear division of labor, lock in responsibilities, collaboration, co-cohesion, In the city-wide " advocating a new civilization, Resycomarriage evil " special action. At the source of the mouth to eliminate the picture for the newlyweds signed "refuse vulgar marriage, do civilized couple" commitment. Together with the civil affairs department, in the city to promote the "wedding certification" service to the traditional wedding culture into the marriage registration ceremony, around the theme of civilized wedding and resist marriage activities, marriage education guidance; mobilize more than 150 couples to sign "refuse vulgar marriage, do civilized couple" commitment, basically achieve "every registration commitment", constantly establish "promote, push, teach, sign" long-term mechanism, to prevent and curb marriage practices at the source of marriage. Before the marriage of education to remind the picture shows the hotel person in charge in the signing of the "advocate against" agreement. The correspondent, the business and tourism departments, signed the "promotion" agreement with more than 220 hotels in the city to undertake wedding events, built the "advocate new civilization" publicity corner in the hotel, hung slogans and billboards; reminded the newlyweds to ensure that there is no vulgar marriage behavior during the wedding banquet. Together with the market supervision department, and the city's more than 500 wedding companies signed agreements, the wedding practitioners for education, training and signed a letter of commitment, strictly check the wedding link, and further strengthen the publicity, education and guidance before the marriage. In the process of strengthening the supervision of Li ge town village level red and white council civilized wedding training meeting. Give full play to the role of the youth working committee, village (community), grid and youth league branch, education and training for more than 120 township and village-level red and white council staff, included in the work of the council members, commitment to dissuade the new young people in the wedding process to track and supervise, prevent the resistance of marriage work in the whole process of marriage. The picture shows volunteers promoting the "initiative" agreement. Correspondent for the picture of the city sent to the office, to create a "help cadres in the village + village 'two committees' members + young volunteers" mode, the implementation of grid guarantee system, layer upon layer compaction responsibility, do a good job in the masses into the household publicity, guidance work. During the visit, to the masses to publicize the relevant requirements of changing customs and the bad influence of bad customs and marriage, vigorously break the trend of ostentation, important face, blind comparison, vulgar "marriage" and other bad customs, so that the concept of civilized new marriage customs is well-known, deeply rooted in the hearts of the people. Together with the urban management department, pay attention to the wedding outdoor activities, found that the marriage trouble vulgar behavior timely disposal. Cooperate with the public security departments to form a supervision and punishment mechanism for marriage disturbance and vulgar behavior. For the vulgar wedding parties who have a bad influence, the relevant responsible persons shall be investigated for legal responsibility in accordance with the Criminal Law, the Public Security Administration and Punishment Law and other relevant laws and regulations according to their circumstances. In the atmosphere, volunteers publicize the "initiative" agreement in the Marriage Registration Office of the Civil Affairs Bureau. Correspondent for pictures to increase media publicity and public opinion supervision efforts, vigorously carry out the theme of the promotion of civilized new wind, resist the vulgar marriage noisy publicity and volunteer service activities. Make " boycott the marriage make the communist youth league to recruit propaganda, print propaganda color page, National Day before and after the large hotel super, wedding studio, wedding supplies and youth gathered cultural center, library cultural center, using electronic display loop, distribute leaflets to youth in a variety of ways, such as the civilization fresh air propaganda and education work in place, create a civilized and noble wedding atmosphere.

2021-09-30	Jining build "young talent accelerator" help youth accelerate growth Jining build "young talent accelerator" help youth accelerate growth jianwei correspondent Bai Chen) to link key enterprises and institutions talent demand and young talent demand supply, focus on industrial park, research institutes, key enterprise quality development talent reserve support and talent promotion service, Shandong Jining party committee, municipal talent office elaborate planning, widely, jointly build "Jining young talent accelerator", to promote the young talent back innovation entrepreneurship, power double recruit double lead set up a new platform. Since 2020,3 A-level accelerators, 4 B-level accelerators and 5 C-level accelerators have been identified, forming a good pattern of three-level layout of "scientific research institutes + industrial parks + industrial enterprises" and "talent promotion + enterprise incubation". Actively strive for the support of the Municipal Party Committee and the municipal government, take out real money to protect funds to actively strive to write the construction of the "young talent accelerator" related policies into the municipal government "on the implementation of the" Zhihui Jining to draw the holy City "project to accelerate the gathering of innovative and entrepreneurial talents in the new era" document. For the identified young talent accelerator, according to the preliminary investment, construction plan, expected goals and other factors, A, B and C are determined. The municipal finance will grant 300,000,200,000,000 and 100,000 yuan, 000 respectively, specially for the young talent accelerator work, and cast the youth talent acceleration platform with "real money". Clear standards, establish rules and regulations, relying on the system construction direction pku, municipal party committee talent office jointly formulated the interim measures for Jining youth talent accelerator management service, clear young talent accelerator declaration subject and conditions, selection procedures, support and supervision and management policy, provide policy support for the creation of the accelerator. At the beginning of the application for the establishment, it is necessary to support the formulation of their own units "Young Talent Accelerator Construction Plan" and "Young Talent Accelerator Management Measures" and other systems, and set up a special team to carry out the establishment work, to provide strong support for the effective operation of the young talent accelerator. Extensive launch, strict investigation, adhere to the high standard selection of tree quality to protect the extensive launch of the county (city, district) youth League committee, talent office, the industrial parks, scientific research institutes, industrial enterprises and other units to actively declare, seriously carry out the creation of the work. The Communist Youth League and Municipal Party Committee worked with relevant departments to carry out field visits, focusing on the hardware conditions, service capabilities, incubation achievements, development prospects, and creation intentions, so as to ensure the stable accelerator organization team, efficient service, outstanding ability, and excellent quality. Shandong Intelligent Robot Application Technology Research Institute focuses on service manufacturing enterprises, connects with nearly 100 enterprises such as Chenxin Pharmaceutical, Taishan Glass Fiber and Xinyi Group, has solved 30 technical problems in the industry, trained more than 300 industrial talents, promoted more than 100 applied technologies, and realized the project contract turnover of more than 30 million. Shandong north university industrial park management co., LTD. As the first double first-class universities and the government at the county level, private capital investment in the university science and technology park, service youth entrepreneurial enterprises (team) 22 (a), accumulative total service college students more than 800 people, build the "gen space-incubator-accelerator-industrial park" the whole life cycle incubation chain. Jining High-tech Zone Talent Development Group Co., Ltd., Shandong Business Power Network Technology Co., Ltd. and other accelerator institutions have also focused on the development of young talents, and have carried out effective incubation and cultivation services. Regular scheduling, careful guidance, continuous tracking supervision guarantee effect to ensure the quality of young talent accelerator, pku, municipal party committee talent office to establish joint management supervision mechanism, clear personnel accelerator management service work special class, establish management supervision parameter, regular scheduling accelerator to create management, system implementation, talent cultivation, technology transformation and enterprise incubation, and so on and so forth. In the next step, the Communist Youth League Committee and the Municipal Talent Office will conduct in-depth research, analyze, coordinate and solve the problems encountered in the operation process of the accelerator, so as to continuously improve the operation level of the accelerator for young talents and attract more young talents to facilitate entrepreneurship and innovation.

2021-10-02	Weifang communist youth league to carry out the "youth to the party struggle power" theme learning education activity Weifang communist youth league to carry out the "youth to the party struggle power" theme learning education activity green net reporter, XingTing) recently, Weifang the communist youth league to "youth to the party struggle power" as the theme, actively carry out special learning education activities, raising the youth party history learning education new upsurge. Weifang Communist Youth League gave full play to the demonstration and driving role of learning in the first step, held a special publicity and report meeting on learning and implementing the spirit of General Secretary Xi Jinping's "July 1" important speech in the first time, and invited members of the publicity group of Weifang Municipal Party Committee to give professional interpretation and lectures. At the same time, the propaganda into the school, into the enterprise, into the community, into the countryside of the propaganda "four" action, "youth teaching group" members scattered to every corner of the grass-roots level, spread with the lively "green language" to the Party to the hearts of the youth. Weifang Communist Youth League also organized school teams at all levels to organize and carry out various activities with the theme of "please trust the party, a strong country has me", with the heart and love on the "first lesson". Weifang diesel city experimental primary school to "please rest assured, power have me" as the theme to carry out the red education activities, for low middle grade respectively good three lessons: red team education before the first lesson, red revolutionary spirit comprehension, red inheritance aspire to class, requires all the young pioneers inheritance red gene, do successors to the communist cause. In the process of carrying out the party history learning and education for teenagers, Weifang Communist Youth League also pays attention to the quasi-connotation and essence, and closely combines learning the spirit of General Secretary Xi Jinping's "July 1" important speech with the youth ideological and political guidance and main business. Actively optimize the construction of "double database" of ideological and political youth, raising 118 excellent ideological and political courses and 100 lecturers; give full play to the online learning platform of "youth learning", covering 6 million youth league members. Next, Weifang youth league organizations at all levels will continue to further study xi new era the ideas of socialism with Chinese characteristics, around the "youth to the party struggle power" theme, design the theme of characteristic education, to build people satisfied with modern quality city is suggested, grasp the youth thought guide the main business, unity lead the teenagers don't forget to beginner's mind, self-motivated, to achieve the second goal in one hundred contribution youth strength.

2021-09-29	Haikou Communist Youth League to promote grass-roots organization reform Ren Mingchao) On September 27, Haikou held the county Communist Youth League grass-roots organization construction pilot work meeting, to promote the reform of the city's grass-roots Communist Youth League. The meeting made a specific explanation of the implementation plan of implementing the Guiding Opinions of the Central Committee of the Communist Youth League on expanding the pilot reform of the grassroots organizations of the county Communist Youth League in Haikou City. The district committees and municipal units of Haikou city deliberated on the implementation plan and the task list, and put forward suggestions on the reform of the grass-roots organizations of the Communist Youth League at the county level. Lin Haining, member of the Standing Committee of Haikou Municipal Party Committee and secretary general of Haikou, asked all grass-roots units to combine the actual situation of the region and the department, with extraordinary determination, extraordinary measures, extraordinary, extraordinary strength, to give the maximum support from human, financial, material, system and other aspects, and truly implement the task requirements of the pilot reform. A system of joint meetings, a system of monthly report work and a system of supervision and reminder should be established, so as to supervise, notify, interview and hold units and persons in whom the reform progress is slow and the work is not paid attention to accountable.

2021-09-29	Anhui Bengbu youth league organizations at all levels for different industries youth friendship Bengbu: railway, cities and counties youth fellowship for different industries youth network reporter wang haihan) recently, China railway Shanghai bureau group co., LTD., youth corps committee of Bengbu municipal party committee in Anhui province, held "edge in high-speed rail, confession about" youth fellowship friendship activities. More than 60 outstanding single league members from railway units and enterprises and institutions in Bengbu participated. Among the participants, there are both railway drivers who witness the leapfrog development of high-speed railway speed and technical personnel who escort the safe transportation of the motherland, "angels in white" who heal the wounded and rescue the dying, and teachers who deeply cultivate the platform and contribute silently. In order to enable young people in different industries to share good times in a romantic and warm environment. The organizer specially has a net red punch points, dessert drinks area, romantic dating area and other areas, and intimate preparation of activity souvenirs. That day in the morning, after random grouping, the fellowship activities officially began. The novel "ice-breaking game" situation alleviates the tension and embarrassment of young men and women. Through "treasure hunting marriage, a line", "moon walk", "dry land curling", and "ice-skin moon cake DIY" and other interesting games, the distance between each other is narrowed. In the "Bring love home" session, single young men summon up the courage to offer roses to express their love. In the end, seven young men and women joined hands successfully."I don't do much manual activities, so I was impressed by the mooncake process. It was done by two people together. Although there was a mold, my original mooncakes made were very rough. Thanks to the patience of the girls in the group who helped me a little bit. In the process of making, we talked a lot, were curious about each other's industry, and unconsciously found a lot of common ground."Wang Tao, 26, is an assistant duty officer at Bengbu Railway Station. He is introverted and never fell in love. He was originally driven by curiosity to participate in activities, but it gave him new feelings about life and happiness. This activity is the 10th anniversary of the "special edition" of the "Yuan in High-speed Railway" brand series of the Railway Shanghai Bureau Group Co., Ltd. This featured brand work, which was first held in 2011, has been held more than 700 times, covering more than 25,000 single young people. A total of 140 single young people have met their own fate through the event. Wu Peng, secretary of the youth league committee of the railway Shanghai bureau group co., LTD., said, in recent years, the railway cause develops rapidly, the railway young worker work rhythm and pressure increase ceaselessly, often ignored, missed their own fate. This activity builds a platform for young friends to communicate, show themselves and pursue happiness, and guides young people to establish a healthy and upward mental outlook and an optimistic and positive attitude towards life.

2021-09-27	Handan city six youth collective won the title of "national youth civilization" Handan city six youth collective won the 20th title of "national youth civilization" litigation service center), Handan city real estate transaction management center, wuan supply and marketing cooperatives blue sky hotel catering, Handan city public security bureau traffic patrol police detachment zhu he brigade women squadron, Handan power supply branch substation operations room qi village operations class, bank of communications Handan branch business department was awarded the national youth civilization collective. Combined with the practical activity of "I do practical things for the masses", Centering on the activities of "triple, four innovation and five optimization" in Hebei Province and Handan City of "transforming style, improving efficiency, comparing responsibility and making contributions", The Communist Youth League Handan Municipal Party Committee organized and launched 161 national, provincial and municipal "youth civilization" to create a collective, Combining it with the characteristics of the industry, Based on their own posts, Carry out theoretical learning, volunteer service, skill competition, service process optimization and other creative activities, In the fields of government services, window positions, commercial services, social services, front-line units, and service platforms to display the civilized style of the youth in the new era, To speed up the construction of a modern regional central city that is prosperous, strong, civilized and beautiful.

2021-09-26	Tian Hongwei, trainee reporter of China Youth Daily) " Hello, have you ever been to Xiamen recently? What is the specific round-trip time?"In Fengze community, Quanzhou, Fujian province, the Guangming City youth affairs social workers repeated the conversation on the phone. Since Putian, Quanzhou, Xiamen and other outbreaks, the communist youth league of Fujian province Quanzhou municipal party committee launched various counties (city, area) league social work agencies, organize youth affairs social workers take "online + offline" service mode, logistics, psychological counseling, personnel screening, policy interpretation, epidemic prevention propaganda and assist the community personnel nucleic acid detection, form "youth affairs social workers + psychological counseling expert volunteers" team, opened Quanzhou youth psychological counseling service line "22212355", for teenagers. Up to now, a total of 116 youth affairs social workers from 12 league-affiliated social work organizations in Quanzhou have participated in the epidemic prevention and control, serving more than 720,000 people. A team of 121 psychological counseling experts, 394 wiring services, a total service time of 125 hours. Quanzhou youth affairs social workers actively cooperate with the community workers to do a good job of telephone investigation of personnel in medium-high risk areas, and file registration. To assist all counties (cities, districts) to unload, store and distribute the love materials as soon as possible, so as to ensure that air defense materials are sent to all epidemic prevention lines in the first time, and make every effort to ensure epidemic prevention and control materials. We actively carried out nucleic acid testing for all staff, effectively ensuring the high-quality and efficient progress of nucleic acid testing. Youth league organizations at all levels in Quanzhou extensively popularize the knowledge of epidemic prevention and control through new media channels such as wechat, Weibo, TikTok and video account, guide young people and the people to view the epidemic correctly and rationally, and enhance their self-prevention awareness and protection ability. At the same time, we will cooperate with the community to do offline publicity work and guide residents to reduce unnecessary outings during the epidemic period. Since the outbreak, a total of 896 wechat tweets have been posted, reading by 1,792,000 people.

2021-09-24	Guangxi Hechi Communist Youth League carried out the "Youth League organization service thousands of villages" activity Guangxi Hechi Communist Youth League carried out the "Youth League organization service activity of" thousands of villages "Chi City Youth League and other departments, to hold the" Youth League organization service thousands of villages " in Liuwei Town, Jinchengjiang District. During the activity, the Youth League Hechi Municipal Party Committee and the relevant officials of Hechi Youth Federation awarded the plaque of "Youth Federation Hope House" to Kenyan Primary School in Liuwei Town, and donated reading stationery, books, sports goods and other materials. At the same time, the organizer hired three off-campus counselors for the "Hope House", aiming to encourage the teachers and students to actively create a scholarly campus, firm ideals and beliefs, study hard, and strive for a better tomorrow of the motherland. In the activity of "Young Scientists on campus", the counselors outside the school led the students into the hall of science through humanoid robots and deformed robots to display, and through questions and answers of popular science knowledge."12355" Growing up without trouble? "" psychological counseling activities, red scarf propaganda activities, "I take a picture with the national flag" and other activities actively guide children to calmly face the troubles in life, stimulate students' patriotic enthusiasm. The person in charge of the Youth League Hechi Municipal Party Committee said that the school should strengthen the management of education and teaching, constantly improve the quality of education and teaching, strengthen the management of the "Hope House", and truly make the "Hope House" a "love home" welcomed by students and trusted by parents.

2021-09-30	Group Jining municipal party committee successfully completed 2021 China (qufu) international Confucius culture festival the 7th nishan world civilization BBS volunteer service corps Jining municipal party committee successfully completed 2021 China (qufu) international Confucius culture festival the 7th nishan world civilization BBS volunteer service work JianWei correspondent Bai Chen) on September 27 to 28,2021 China (qufu) international Confucius culture festival, the 7th nishan world civilization BBS held in Jining qufu. With their professional knowledge, full enthusiasm and smile, 158 young volunteers provided high-quality, efficient, enthusiastic and thoughtful all-round volunteer services for the successful convening and experts and scholars attending the conference. A group photo shows volunteers at the closing ceremony of the 2021 China (Qufu) International Confucius Culture Festival and the 7th Nishan World Civilization Forum. After layers of selection, Zhang Jingya, a post-2000s college student, became a "one-to-one" young volunteer in the expert group. She said: " It is a great honor to be a 'one-to-one' volunteer for experts and scholars, and to learn closely from so many respected experts and scholars. Although the two days of work is very hard, but the bitter there is sweet, let me exercise themselves."To achieve the festival" precision to the heart, delicate warm heart, etiquette run heart " requirements, pku adhere to the best, from qufu normal university, Shandong vocational institute of technology selected the high service level, strong discipline concept, good image temperament of 158 young volunteers, and through the common training and field practice dual-track mode of volunteers to volunteer basic knowledge, Confucian culture, first aid knowledge, etiquette manners group training. The picture shows the volunteers answering the questions for the participating guests. Volunteer service comes from the heart, but also in the line. Volunteers participated in training and practical combat drills for many times, and learned about the meeting schedule, venue distribution, and practiced smiling expressions and standing posture in advance. On the day of the activity, volunteers were dressed in uniform and shuttled inside and outside the venue, and they could be seen in almost every corner of the venue. The picture shows the volunteers in charge of the etiquette work. Correspondent for figure group Jining municipal committee invited Jining vocational and technical college art and design department of traditional clothing innovative design studio is responsible for the teacher design and made the unique volunteers clothing, clothing will Confucian culture, regional characteristics, festival will visual system integration, clothing pattern to mountain as the center, the Confucian p 5 culture of "righteousness" reasonable integration, fully embodies the characteristics and volunteers youth vitality, expressed the "with walking culture good Chinese story" design concept.

2021-09-24	Youth League Chizhou Municipal Party Committee held the 2021 youth civilization work training meeting youth League Chizhou Municipal Party Committee held the 2021 youth civilization work training meeting Jianwei correspondent, Cao Longyun Zha Wei Ting) recently, the Youth League of Anhui Province Chizhou Municipal Party Committee held the 2021 youth civilization work training meeting. More than 70 people from the county (district) Youth League Committee, the municipal Youth League working Committee, the Jiuhua Mountain Scenic Area and the development zone, and the provincial and municipal youth civilization creation group attended the training. Zhang Guodong, vice minister of the Youth Development and Rights and Interests Protection Department of Anhui Provincial Committee, combined with the publicity video of youth civilization, taught about the five aspects of historical evolution, basic concepts, requirements for creation, and how to manage and selection of youth civilization. At the meeting, the youth League branch of the EPC General Contracting Department of the collective Chizhou Jiangjingyuan Construction project, the second ward of the Third People's Hospital, and the leaders of the municipal youth Civilization collective Xiangyang Kindergarten respectively made exchange speeches. Chizhou, deputy secretary of municipal party committee Zheng Biao stressed that each create collective to improve ideological understanding, firm ideal faith, adhere to create activities and center work organic integration, to the young staff thought and action unity to the center, guide the youth in epidemic prevention and control, civilization, rural revitalization, high quality economic development work show new bear, new as. To optimize the service, expand the influence, accurately grasp the creation process, create the standard, as the standard to achieve the "five first-class" goals, strict selection procedures, adhere to the dynamic management, explore the review mechanism, and strive to create a new situation of "youth civilization" work. To strengthen management, combine, continue to carry out "I do something for the youth" "youth civilization open week" activities, promoting the party history learning education, post work action, education guide professional youth continuously red blood, bear era responsibility, to create results for the construction of the new stage of modernization "three optimal Chizhou" contribution youth strength.

2021-09-23	Shiyan: Learn the history of the Party to do practical things Shiyan: Learn the history of the Party to do practical work correspondent Jin An Wang Yidan) Recently, Hubei Province Shiyan Municipal Party Committee and the Municipal Women's Federation went to Xiping Village to carry out the September branch theme Party day and "I do practical things for the masses" activities. Communist Youth League Committee, Municipal Women's Federation, Xiping Village all Party cadres and part of the masses representatives attended the meeting. Theme Party Day activities. Correspondent for figure meeting, Hubei medical college professor Chen Xinxin to "glorious history in one hundred, struggle new journey", from the learning objectives and meaning, main framework and content, the learning method and requirements of three aspects, simple to xi general secretary "July 1" important speech spirit made special counseling, guide everyone to understand and accurately grasp the general secretary xi "July 1" important speech spirit and it contains new ideas, new ideas, new judgment, to further learn the party's history in one hundred, strengthen the ideal faith, for the private good practical work. Sign the cooperation agreement. Correspondent for figure around the party history learning education "I do the practical work for the masses", pku actively for, in xiping village start "hope to fly" care of migrant workers trailing children public service projects, from September 2021 to February 2022, the introduction of social professional public welfare institutions for xiping village 40 rural migrant workers trailing children provide spiritual and developmental care support, to promote the healthy growth of specific minors. At the meeting, the main person in charge of the Communist Youth League Committee signed a cooperation agreement with the person in charge of Shiyan Yimin Social Work Service Center, the "Hope to Fly" project organization, donated 50 "Hope Micro Wish" gift packages to the project recipients, and visited some young people in difficulties. Donation of the "Hope Micro Wish" gift package. Correspondent for the picture meeting asked all cadres and workers and the village "two committees" party members, to listen to the party. The key to implementing the policies formulated by the CPC Central Committee is to rely on officials. Party members are the core of officials. We must listen to the Party wholeheartedly, follow the Party in firm faith, and deepen our consciousness of thinking and action in studying the history of the Party and doing practical things. We must do the Party's work well. Party members and cadres should base themselves on their own posts, carry forward the "screw" spirit, play the vanguard and exemplary role of party members, and always maintain the advanced nature of party members. Is the strict self-requirements. Party members and cadres should be honest and self-disciplined, clean, respect the power in their hands, and build "family style"; always keep the people in mind, keep the safety of the people, pass the party's voice and policies to the people; constantly improve their ability and level, accurately grasp the development situation of The Times and the people yearn for a better life, do well for the people, and serve the people well.

2021-09-21	Lanzhou: "Children of the Stars" are too diverse to celebrate the Mid-Autumn Festival ". The picture shows volunteers teaching autistic children to dance movements. Lanzhou, Sept. 19 (Xinhua) - -Min Min (pseudonym) and Ning Ning (pseudonym) are autistic brothers who usually hide behind their mother Xu Yan. Only when they see what they are interested in can they reach out their hands and show a shy smile on their faces. As the Mid-Autumn Festival approaches, the brothers guess lantern riddles, sing and play instruments with a group of "stars" and volunteers... the "little stars" radiate their own light. Autism, also known as autism, often has language but is difficult to communicate. It is immersed in its own world and is known as the "child of the stars". The Lanzhou Municipal Party Committee of the Communist Youth League held a special care activity for the children of "Our Festival Mid-Autumn Festival" to embrace the stars on August 18. Volunteers sang songs to tell the origin and legends of the Mid-Autumn Festival, so that the children could understand the customs of traditional festivals. What makes the children most happy is that when the music sounded, this special group of children under the encouragement of their parents, bravely stood in the middle of the stage, seriously doing every dance movement. Xu Yan was pleased to see her son's rhythmic performance on the stage."Usually I don't have much opportunity to contact the outside world. I hope my children will participate in more such activities and better integrate into social activities."According to Lanzhou city youth volunteers association, vice President of Ma Xiaofei, the Mid-Autumn festival approaching, through songs, singing, African drum playing, dance, children enhance interaction, enhance its optimism and confidence, with love to embrace the children from the stars, let autistic children and families feel more social love. Ma Xiaofei said that the activity is one of the series of the Communist Youth League Lanzhou Municipal Committee caring for special children. Now, every weekend, special education teachers, college student volunteers and volunteer teachers will be invited to teach special children about their specialties, including free singing, painting, handwork, African drum and electronic organ. Up to now, 310 volunteers and 4,126 special children have participated in the special children education training class.(intact)

2021-09-24	Qingdao: to grassroots transition as an opportunity to choose good youth "leader" Qingdao municipal party committee to grassroots transition as an opportunity to choose good grassroots youth "leader" JianWei correspondent Liu Jie) since this year, the Qingdao municipal party committee according to the city's "transition" work overall arrangement, comprehensive start grassroots youth league transition, selection with strong league team, the city's 1928 village (community) complete transition, 1459 "two committees" as league secretary, 181 rural good youth as league secretary. Focus on "one leader", strengthen the party's overall leadership over the transition work. Always adhere to the leadership of party building, to lead the league building, to promote party building, strengthen the party's overall leadership over the transition work, the whole process, implement the main responsibility, grasp the organization and implementation, to ensure that the party building leadership is the most solid. First, to integrate into the overall planning. Actively strive for party committee policy and work support, the league centralized transition into the village (community) "two committees" transition unified deployment, "to support qualified village (community) league secretary and excellent" rural good youth team "into the" two committees ", in principle, each village (community)" two committees "team at least one young cadres under the age of 35, improve the communist youth league organization" requirements into the city's village (community) "two committees" transition work opinion, achieve high standard planning, high quality. Second, we need to optimize the process design. Formulate and release the Reference Manual for the Change of Village Youth League Organization (2021 edition), guide the district (community) Youth League Committee to produce the guidance manual for the transition of the Youth League organization, clarify the key points, reference flow chart, common documents and other standards, and ensure that the thinking of the transition is clear, standardized, rigorous and orderly. Third, we will strengthen our sense of responsibility. Set up a special work class, establish a four-level linkage and coordination mechanism of "the Communist Youth League Municipal Party Committee- -district (city) - -town (street) - -village (community)", and tighten the responsibility chain. The team members of the Communist Youth League Municipal Party Committee, the head of the grass-roots organization construction special class took the lead, and three supervision groups were set up to carry out field visits, heart-to-heart talks, research and supervision, focus on the guidance, and effectively promote the high-quality implementation of the transition work. Adhere to the "two first", grasp the key links of the transition work. In the work of the transition, we should accurately grasp the requirements, steps, procedures and timing of the transition, focus on the key points, and steadily and orderly advance all the work. On the one hand, adhere to the bottom first, scientific analysis and judgment. As a whole "online", "offline" two resources, in good "wisdom building" system, on the basis of the rural youth database, grassroots organizations strengthen field visits, online contact, positive and census, public security household registration data analysis, comprehensive MoPai registered league members, strive to maximize eliminate organization cover blind spots. Adhere to the pass front, do "three clear", that is, find out the current operation of the league organization, find out the situation of the district communist Youth League members, find out the rural good youth and outstanding youth under the age of 35 in various fields, one by one analysis and judgment, establish a work ledger, for scientific decision-making, accurate identification of people to provide "first-hand information". On the other hand, adhere to the pilot first, to the point with the surface spread out. To guide the Youth League Committee of the West Coast New District to take the first trial, and strive to create a model for the league organization change, and lead the point and demonstration. Fully stimulated the enthusiasm and creativity of each district (city) youth League committee to take measures according to local conditions and take the initiative. Shibei Youth League District Committee drew the transition process map and wall chart operation, and Jiaozhou Youth League Municipal Party Committee implemented the "three sections, five rings and six points" work method, which effectively promoted the implementation of the work. By timely summarizing the experience and practices of the grassroots in optimizing the convening of league members and standardizing the qualification examination, it has played a role of standard guidance and demonstration and driving. Implement the "three in place", to ensure that the good team, change the spirit. Adhere to the goal-oriented, problem-oriented, combined with the target and task requirements of the transition, integrate the resources of all aspects of all aspects, to ensure the smooth work of the transition. The publicity is launched in place. For members of youth mobility, active thinking, and network fit higher characteristics, make full use of the "three micro end" media, form online propaganda matrix, improve the awareness of youth members and participate in the transition of political enthusiasm, guide organization youth members fully exercise their democratic rights, earnestly fulfill their obligations, in order to participate in the transition in accordance with the law. Strict discipline is in place. We will strictly control the entrance control, implement the joint qualification examination system at the district (city) and town (street) two-level levels, strictly compare the qualification conditions and the "negative list", cooperate with the public security, organization and discipline inspection departments, and prescreen the key candidates. We will strengthen control over the process, and ensure that legal procedures are not flexible, prescribed steps are not reduced, and democratic rights are not withheld. Open an open telephone to timely answer, accept and reflect the problem, with a rigorous attitude, strict procedures, strict operation to create a clean and upright transition environment. Select excellent and match strong in place. We will strengthen the mission of cultivating continuous strength for the Party and conducting political recruitment, broaden the vision of selection and employment, and truly select outstanding young talents with excellent political quality, significant head goose effect, dedication and love of youth work into the youth league organization team, so as to inject fresh vitality into the high-quality development of the village (community). After the village (community) "two committees" change, basically realize all the village (community) "two committees" team has at least 1 young cadres under 35 years old; 2859 youth under 35 years old into the "two committees" team, including 289 rural good youth; 132 youth under 35 years old as the party organization secretary, including 19 rural good youth. We should combine the "four combinations" and effectively improve the leadership, organization, service and overall contribution of the league organization. Standing in the overall situation of the city, thinking and planning for the transition work, through a series of effective measures, to promote the youth league organization work into the city's "a game of chess". One is to combine with the party history study and education. The whole process of the general election will be taken as an important opportunity for the youth league members to participate in the party history learning and education and an effective carrier to test the learning results. Through concentrated learning, communication, exchange and discussion, duty commitment, and "I do practical things for the youth", the youth league members will be educated and guided to understand the original mission and inherit the red gene. Second, with the town and street youth working committee construction combined. Expand the town (street) youth working committee members, the new village (community) youth league organization secretary incentive measures, explore the backbone force into the street youth league working committee team, further enhance the town (street) youth working committee members sense of mission and identity, enhance the work activity and youth recognition. Third, it is combined with the training of good rural youth. During the transition period, we will give full play to the head goose effect of "good rural youth", start the pairing of rural revitalization youth, set up 10 rural revitalization youth volunteer service teams, and set up projects such as industry drive, hand in hand care, ecological protection and emergency response, so as to unite and gather more aspiring young people to participate in rural revitalization. Fourth, it is combined with the reform of the county Communist Youth League grass-roots organization. The change as an important entry point to strengthen the construction of the grass-roots league, through the selection of excellent and strong team, improve the system, consolidate the grass-roots foundation, for the central county of the Youth League grass-roots organization reform to expand the pilot work to lay a good foundation for the work, to achieve "seamless" work.

2021-09-26	Wuzhong city cadres learning xi general secretary "July 1" important speech spirit project reading class successful graduation Wuzhong city cadres learning xi general secretary "July 1" important speech spirit project reading class successful graduation jianwei), Ningxia hui autonomous region cadres learning xi general secretary "July 1" important speech spirit project reading class in Qingtongxia, from Wuzhong city straight authority, non-public enterprises, social organizations, schools and other fields of 30 cadres attend reading class. This reading class lasts for five days from September 14 to 18. Reading class to "follow the party, please let the party power have me" as the theme, set up experts and scholars special counseling, interview national "July 1" medal winner Wang Lanhua advanced deeds, group seminars, visit red education base, etc, to thoroughly study and apply the general secretary xi "July 1" important speech and in 2021 the central party school (national school of administration) young cadres training KaiBanShi important speech spirit, focus on improving young cadres political ability and professional ability, promote party history learning education continue to go deep. During the study period, everyone devoted themselves to thinking and actively discussed it. The participants said that they would "stay true to their original aspiration and keep their mission in mind" in their future work, further strengthen their faith, and show new achievements in their work practice. Next, Wuzhong city will pay special attention to the cadres at all levels of partial stratified education training work, to improve Wuzhong city cadres at all levels of political ability and business level, education guide cadres at all levels when the political "wise", "more action of" vanguard ", unity led the city's youth in the construction of modern beautiful new Wuzhong new journey practice" please rest assured, power have me " youth oath.

2021-09-24	Jianwei correspondent Bo Chen) "Select Jining" - -the 2021 summer vacation "Return to home" social practice activity in 2021 was successfully concluded recently. Group Jining municipal party committee combined with "I for the youth" "green bird plan practice season" activities, joint city human resources social security bureau and other units, actively help the city ZhaoCaiYinZhi, service college students' employment, strengthen contact and outside Jining students docking, service outside the students perception of Jining, Jining development change, inspire students home employment entrepreneurship enthusiasm, solid promoting activities. From July to August this year, the Communist Youth League Municipal Committee actively mobilized college students to participate in the "summer social practice activities" in 2021, with a total number of 8,350 people. For college students' social practice experience, insufficient understanding of home, lack of personal career planning, insist from set out actually, based on the college students need and the communist youth league, focus on young talent gathering and young students employment guidance service, from post solicitation, volunteer recruitment, mid tracking, late summary, and other aspects, to participate in the summer social practice of college students to provide the whole process of intimate service. Social practice research. On August 13th, the social practice group of "Red Dream Building for Rural Revitalization" of University of Science and Technology Beijing came to Jining to carry out rural revitalization research activities, encouraging and guiding "green bird students" to devote themselves to grass-roots development, and contribute their youth strength to rural revitalization. On August 20, Qingdao university academy of fine arts youth corps committee secretary XiaoYuling teacher led "youth power, diffuse painting surSurabaya" rural revitalization practice into the Jining surishui county China village slope village, focusing on the rural education backward, assist the local education department to carry out the wall painting, art teaching, visiting veterans, do their best to improve rural basic education status, promote play slope village rural civilization construction. Volunteer services. Correspondent for the picture is fully protect people's health and safety, to further do a good job in Jining new crown vaccination, pku according to the requirements of epidemic prevention work, widely recruited by the youth league members, youth social organizations, youth civilization, summer returning college students, the young pioneers counselors of "home youth volunteers", actively participate in vaccination volunteer service. Up to now, more than 1,800 volunteers have invested in vaccination volunteer services in all counties (cities, districts).

2021-09-20	The Youth Heihe Municipal Party Committee held the 2021 "Golden Autumn Student Aid" Hope Project "Dream Realization Action" grant distribution ceremony. Sixty-eight freshmen from poor families and excellent character and poor students received financial aid. At the ceremony, the organizer issued a total of 85,000 yuan of grants to the poor students, and issued certificates to the representatives of caring units, caring enterprises and caring people. Since July this year, the Heihe Municipal Party Committee of the Communist Youth League has launched the 2021 "Golden Autumn Students" hope Project "Dream Realization Action" within the city. Since the launch of the event, the city's youth league organizations at all levels have mobilized social forces from all walks of life, raising a total of 362,800 yuan of grants, which is expected to help 280 poor teenagers. Since the launch of poverty alleviation work in 2016, youth league organizations at all levels have raised a total of 3.5537 million yuan in grants, and carried out 74 activities to help poor students, helping nearly 3,300 poor students.

2021-09-19	Jiangsu Taizhou: "phoenix also nest" nine years feeling warm home students in Jiangsu Taizhou: "phoenix also nest" nine years feeling warm home students reporter li runwen summer practice is not over, there are many parents call, put forward the request to extend the practice of time, which makes the "phoenix also nest" summer practice organizers-group of Jiangsu Taizhou municipal party committee into the "happiness". Taizhou alias "Fengcheng". Summer practice of "Phoenix Return to Nest" is a brand activity jointly sponsored by Taizhou Talent Office and "Fengcheng Youth Talent Gathering Plan" of the Communist Youth League Committee. It has been held for 9 consecutive periods and has been welcomed and sought after by Taizhou college students. Entering the ninth year, the "Phoenix return nest" is further upgraded. This year, the Taizhou municipal party committee launched the city, city (area) two levels, respectively from the party and government organs, financial units, enterprises, assets management, public welfare undertakings, grassroots services, scientific research platform, medical institutions, legal services and nine categories thousand units matching more than 3000 jobs, successfully attracted 1605 college students to internship, again refresh the post and the number of most. This year's "phoenix return nest" is different from previous years. Nanjing and Yangzhou have been outbreaks, and the situation in Taizhou suddenly became severe."We are young, let's go up first!"The 623 college students located in the village and community posts have applied to the local youth league organizations to go to the front line of epidemic prevention and control, and to enrich the fight against the epidemic at the grass-roots level. In Shagou Town, Xinghua City, Hu � h, a student from Changzhou University, works to assist towns and villages in the initial screening of flow."At the peak of the task, several of us had to do more than 1,200 people a day, calling us all night and checking it repeatedly.""This holiday is not the most comfortable, but it is the most comfortable heart", "the biggest harvest is the sense of responsibility"... the students grow in the responsibility."I came to Taizhou before and I thought it was very beautiful. I hope I can experience a round of substantial practice here without accommodation."On the afternoon of July 6, Li Wenlu, a graduate student from Southeast University, made her claim on the phone. Among the many students who have signed up for the summer practice of "Phoenix Return to the Nest", Li Wenlu is somewhat different. She is neither a native of Taizhou, nor has any internship experience in Taizhou. Through the official micro information of the school Youth League Committee, she was attracted by the city. According to statistics, a total of 58 foreign students came to Taizhou to participate in social practice. Hot summer days, sudden showers, typhoons landing and epidemics coming... bring great challenges to your daily work. The students are nervous and busy, although hard but never complain."In just six weeks, the students showed strong adaptability and excel in the post internship and 'seven one' practice activities."Zhai Wenzhou, party secretary of the Communist Youth League in Taizhou, said," To deepen the summer practice of college students, we have been exploring new practices and striving for a strong guarantee. This year, we not only formulated the "Credit Management Rules" to strengthen the student management, but also carefully made the students' address book and the "Zhixiang Printing Collection", so that the students can punch in as a souvenir, and leave a good memory."Yuan Jiayue, a student majoring in agriculture and forestry economic management at Yangzhou University, said that through the summer practice of" The Phoenix returns to the nest ", she has made a big step forward on the road of realizing her entrepreneurial dream and helping the rural revitalization. During her internship in Baimi Town, Jiangyan District, she encountered difficulties, and the farmers' loofah became unmarketable. With questions, feelings and dreams, Yuan began her story with "Phoenix returns its nest". After investigation, Yuan Jiayue found that the loofah has a unique load-bearing structure, which is a special material with excellent buffer performance and environment friendly, which can be applied to packaging, sound elimination, filtration, thermal insulation, shock absorption and other fields, and has great commercial value and sales market. Therefore, she consulted the teachers of the school and used her professional knowledge to lead the planning of dianhe Village loofah industry extension project. At present, the project has entered the processing link, and has successfully driven 56 people to find employment, creating a "small miracle". The key to rural revitalization lies in the people. This year, the League Taizhou Municipal Party Committee took rural revitalization as an important direction for organizing young students to carry out activities."Taste of hometown, live with goods training" special live broadcast to help farmers, "cloud push" Taizhou the most popular food strategy; launch a study tour rural action, organize a visit to modern agricultural industrial base; carry out "red footprint search", launch "a wonderful plan to Taizhou" discussion... organize more than 40 wonderful activities during practice. Qingyi Public Welfare Summer Care Class is a practical project for the people of Taizhou Municipal Party Committee and Taizhou Municipal Civil Affairs Bureau for seven consecutive years. It recruits young volunteers during the summer vacation to provide public welfare care services for left-behind children, factual orphans and other young groups in distress. This year, the Qingzai charity summer care class has covered all the towns and towns in the city, and the "phoenix return nest" college students have played an important role in the children's summer good teachers and helpful friends."On the task assigned in the first afternoon, the students could take out the lesson preparation notes at 9 PM. On the next morning, they rushed to the village to teach the lower grade children. Moreover, the whole class atmosphere was very good, and the children liked it very much that the villagers rushed to sign up."Township youth league cadres to" phoenix return nest " college students point a big praise."Under the background of 'double reduction', the number of service objects of summer care classes has increased significantly, and the management pressure has increased significantly. Fortunately, a group of college students have actively participated in the work, which makes the whole work not only orderly, but also the parents are also full of praise."Sun Lei, deputy director of Taizhou Civil Affairs Bureau, said," Watching the countryside and dedicating love is our original intention. The children will go out from here, and later become a college students, to realize their life dreams, and will certainly bring back this love again. Six years ago, Deng Liuliu participated in the summer practice of "Phoenix Return to the Nest" for the first time, practicing in Lengjia Village, Zhaoyang Town, Xinghua City, responsible for the construction of "rural library". In 2016, she signed up again to exercise in the Xinghua Municipal Human Resources and Social Security Bureau to assist in the receipt and management of college graduates' files. From the village head to the desk, from book management to archives management, in her heart buried the seeds of service hometown. Because of these two summer experiences, Deng returned to Taizhou to work in Taizhou after graduating from University in 2018. In colleges and universities, there are many Taizhou college students like Deng who want to go home to work in their hometown. Taizhou trains and sends tens of thousands of university freshmen every year, who are distributed in major cities across the country, eager for a good platform to return to and build their hometowns. According to the current situation and needs of young students from Taizhou, Taizhou Municipal Committee of the Communist Youth League set up a youth student fraternity with cities as a unit to more accurately contact and serve the majority of young students from abroad, and further build a platform for two-way communication between hometown and students."In the past year, the Municipal Talent Office and the Communist Youth League Municipal Party Committee have been building pilot student associations in Beijing, Xi'an and Harbin, and have achieved remarkable results."Ju Linhong, deputy director of the Organization Department of Taizhou Municipal Party Committee and secretary of the Working Committee of Non-public Enterprises and Social Organizations, said," This summer vacation, we are in Nanjing, Changsha, Shanghai, Wuhan, Chongqing, Tianjin, Guangzhou, Chengdu, Hangzhou, on this basis of the Taizhou Student Association. According to statistics, the existing 12 student associations can cover more than 26,000 Taizhou college students. In the new school year of "", we will organize students to break in Beijing to carry out a fellowship, a party history competition, a research and exchange, a hometown lecture, and enhance their friendship through four activities. At the same time, we will also provide you with a series of convenience such as city guide, postgraduate entrance examination sharing, academic mutual assistance, growth strategy and life services."Said Song Jiacheng, a student of Tsinghua University and president of the Taizhou Students (Beijing) Association. At the founding meeting of the Taizhou Student Association on August 25th, all 12 associations released their activities and service menus for the next school year. This year, the Taizhou Municipal Party Committee of the Communist Youth League has selected 104 outstanding college students as the backbone of the local association, who will be active in the university campus and speak for their hometown of Taizhou. The Communist Youth League Committee also strengthened communication and docking with the local Taizhou Chamber of Commerce and enterprises, and reached cooperation agreements to strive for more rich resources for the fraternity. Jinchen Group is a well-known enterprise in Taizhou, with branches in many cities across the country. Li Huanjun, chairman of the Group, said affectionately, " Homesickness is the strongest thing. Wherever the Taizhou Student Association is built, our local branches will provide office offices and activity venues, and spare no effort to help them with their activities.”

2021-09-19	On September 17, Danzhou city, the central city of western Hainan Province, held a donation ceremony in 2021.20 freshmen from poor universities were funded. This year, 637 college graduates have received applications, raising more than 2.43 million yuan since June. Since the implementation of the "Hope Project Dream Action" in 2011, Danzhou has traveled to 16 towns and townships in the city, raising a total of more than 15 million yuan and helping more than 3,000 students realize their dreams in university. Danzhou has won the "Hope Project Dream Action Organization Award" and "Advanced Collective Award" of Hainan Province for 10 consecutive years.

2021-09-18	Xuancheng league social organizations held "first aid" training xuan city in Anhui province league social organizations organized "first aid" training jianwei correspondent, Ding Wenjing check WeiTing) in order to further enhance the xuan city youth volunteers first aid consciousness, improve the youth volunteers in the emergency state, communal skills, recently, led by the group of Xuancheng in Anhui province municipal party committee, city QingZhiXie, xuan city medical emergency center jointly develop xuan city league social organizations "first aid" training. The training session was divided into 4 batches, with 50 volunteers participating. During the training, the teacher explained the CPR emergency method to the volunteers in detail, and taught the volunteers how to judge consciousness, artificial respiration, chest compressions, etc. During the on-site practice, the teacher also used a mannequin for the volunteers to simulate the CPR mannequin.

2021-09-18	Group Taiyuan municipal party committee establish students homing workstation power Taiyuan municipal party committee establish students homing workstation power provincial school cooperation correspondent Wang Yishu) recently, the Shanxi Taiyuan municipal party committee around the province, city to deepen provincial school cooperation decision deployment, explore to gather new forms, in the national 42 first-class university construction in Shanxi students build workstation, attract more outstanding youth to and employment entrepreneurship. Gather the "window" of the countryside and show the new style of Taiyuan. Since its establishment, under the workstation's close cooperation with the Municipal Committee of the Communist Youth League and the Talent Office of the Municipal Party Committee, relying on the platform of each workstation, it has reprinted and publicized various kinds of information about Taiyuan's economy and society, science, technology and culture, innovation and development, ecological environment, urban style and talent introduction, to help young students feel the changes in their hometown and listen to the warm local dialect. Strengthen guidance, and build an information transmission "platform". Through the integration of online and offline services, the workstation publicizes and promotes the latest provincial and municipal talent introduction policies, student selection, civil servant recruitment, and recruitment information of enterprises and public institutions. At present, each workstation has released a total of 160 provincial and municipal talent policies, 3 Taiyuan city propaganda videos, 3 talent policy propaganda videos, and 520 kinds of employment and recruitment information, involving more than 25,000 positions. Gather talents and build a "think tank" for young talents. Workstation pay attention to find outstanding young talents, collect students in school, professional, employment intention and other information, establish 1169 outstanding young talent database, into the provincial and municipal youth talent pool, recommended to the provincial departments of young talents, while relying on the youth talent station, to face and entrepreneurial employment of young talents to provide online comprehensive services. Warm-hearted service, dredge the policy transmission "the last kilometer". Workstation pay attention to understand the actual needs of young students, timely collect students feedback talent policy questions and advice, master young talents and common demands and policy problems existing in the process of landing, through talent policy propaganda and implementation of the implementation of the "the last kilometer", real-time follow-up student demand and timely feedback to the relevant departments, has collected answer students outside all kinds of talent policy questions 120. Matching, build talent "bridge". During the period, we cooperated with the transfer policy meeting in 18 universities, organized and mobilized 670 outstanding students; During the cooperation between the provincial and municipal organizations, we mobilized more than 90 teachers and students from 5 double first-class universities in Shanghai to participate in the provincial-university cooperation symposium. Reprinted and recommended more than 60 pieces of policies and job demand information related to the "12 bases" of provincial and university cooperation, and mobilized 124 students from 9 universities including Peking University and Tongji University to carry out summer practice and practical practice. At present, "Taiyuan Student Home Workstation" has radiated more than 5,000 Shanxi students.

2021-09-17	Youth League Handan Municipal Party Committee held a seminar on youth mental health seminar on youth service desk work deployment and youth mental health seminar. Conference, 12355 youth service desk director introduced Handan city 12355 youth service desk hotline answering and operation, experts around 12355 hotline and network consulting small program concentration of teenagers weariness, adolescent rebellion, addicted computer phones, sensitive parent-child relationship and other psychological problems in-depth analysis, discussion and communication, and starting from their own professional field for Handan 12355 make recommendations. Experts attending the meeting said that to strengthen adolescent mental health education and comprehensively improve their mental health quality, we should pay attention to the influence of parents and teachers, and build an adolescent mental health prevention and control system integrating family, school and society. Next, group Handan municipal party committee will continue to improve Handan 12355 professional service level, increase basic research on mental health problems, explore "3:30 after-school service", "school social workers" 12355 into the campus form, accurate object, accurate ShiCe, using group of media platform, regular psychological knowledge sharing, 12355 heart live activities, let more teenagers and parents know psychological service hotline, truly 12355 the brand makes teenagers feel, benefit teenagers "intimate hotline".

2021-09-22	TuanRizhao municipal party committee continue to promote the youth committee construction group sunshine youth committee construction JianWei correspondent phase Changhui) for the implementation of the party building requirements, further strengthen the villages and towns (street) group work force, group of Shandong sunshine municipal party committee active action, elaborate organization, continue to promote youth committee construction, evaluated integratedly green committee around the summer volunteer service carried out a variety of colorful activities. The picture shows the Shijiu Street Youth Working Committee organizing volunteers into the community to bring AI robots to the children. Centering on the theme of "energy saving and carbon reduction and green development", the youth working committees of Donggang District will give full play to the youth organization and social appeal, change the object of work into the working force, and carry out a series of publicity and education activities in different groups and fields. For example, relying on the resource advantages of building enterprises, the Shijiu Street Youth Working Committee organizes volunteers into the community to bring independent AI robots to the community children, expand the knowledge of garbage classification, and enhance the children's environmental awareness through teaching and fun. The Houcun Youth Working Committee gathered young volunteers, put up energy saving posters in the canteens and office buildings, and issued the "2021 Energy Conservation Publicity Week and Low Carbon Day Proposal" along the streets, which went to the streets to clean up and create a green living environment with practical actions. Lanshan District Lanshan each town street green working committee around the grass-roots organization construction, green bird plan, hope cabin, volunteer service and other aspects to carry out 24 activities. Among them, the Youth Working Committee of Dongwei Sub-district has made solid progress in epidemic prevention and control work, organized young volunteers to publicize COVID-19 prevention and control in shopping malls and markets within the jurisdiction, distribute masks, and do a good job in household publicity, so as to improve the public's awareness of prevention. Lou town youth working committee organization college students volunteers to GeZi ditch village hope hut, to help children with schoolwork counseling, safety education and other services, learn drowning knowledge, to help children guardian for drowning supervision responsibility to remind, improve guardian safety awareness, pays special attention to the family education, implement the supervision care responsibility. Juxian Juxian Town Street Youth Working Committee to play the organizational advantages, actively mobilize green bird students, rural good youth, young volunteers and other backbone forces to participate in the "hope cabin", epidemic prevention and control and other volunteer service activities. Liu Guanzhuang Town Youth Working Committee organized the youth home to report to college students to the "Hope House" within the area to pair up to help, to provide professional guidance for the pair of children. Qiao Shan Town Youth Working Committee launched college students in the jurisdiction to carry out a pair of volunteer service activities in the "hope cabin" children's home, to communicate with the pair of children, and to provide tutoring, housework and other volunteer services. The Youth Working Committee of Xiaodian Town organized resident college students, resident good rural youth and young volunteers of Lei Feng Rescue Team to actively carry out voluntary epidemic prevention and control activities such as epidemic prevention publicity and mask distribution. The towns and streets of Wulian County in Wulian County have actively organized members of the Youth Working Committee to help with rural revitalization, typhoon and flood control, and epidemic prevention and control. Zhaohe Town Youth Working Committee implements the work requirements of the Town and Street Youth Working Committee, innovates the "three and three" mode, builds a platform for the central work of the Party and government and the actual work of youth, for young people to grow up, and organizes and mobilizes the majority of young people to make contributions to the rural revitalization work. In order to thoroughly implement the children-based education concept, pay attention to children's interests, give full play to children's subjectivity, and let children participate in the activities, we carry out the "I grow, I am happy" theme education activities with the community and the joint construction kindergarten.

2021-09-17	Wu Zhong: promote the youth "four history" learning education into the brain into the heart Wuzhong: promote youth "four" learning education into the brain into the Chinese youth network Beijing on September 17 (xinhua Zhang Jianwei) to further promote youth "four" learning education, Ningxia hui autonomous region youth league, cadres and communist youth league linkage, good teenagers love "four classes", guide the teenagers learn party history, strong faith, follow the party. Take the lead by example, on a good "demonstration class". City and county youth corps committee for demonstration, to theoretical study group learning, detailed learning system, thoroughly study and implement the general secretary xi "July 1" important speech spirit, the study and study the new Chinese history, reform and opening up, socialist development, learning, tutoring, personal, communication, project, "five" way, online watching video, knowledge contest, offline party class guidance, party spirit education, closed test form, drive the city youth talk about comprehension, comments. Carry out the "youth study", covering the city, county, township, village four youth league organizations, to the "May 4", "61" as an opportunity, the city and county youth league team members went to the grass-roots line, into the youth side, in-depth branch, for the youth party lessons, speak the history of the party. Under the leadership of the Communist Youth League municipal committee, the city's 138 youth League (work) committee, 2312 youth league (general) branches carried out the party history learning education, the coverage rate of 100%. Integrate resources, speak good "lecture class". We will strive to build the "8090", "9000", "Red scarf Lecture Group" and "Yellow River Youth" youth publicity brands, and pay attention to the exemplary and leading role of the "May Fourth Youth Medal", "Excellent Communist Youth League Cadres" and "Good and Good Youth". At the same time in the primary and secondary schools preferred different ages of "red small narrator", and set up "small narrator training class" and "Chinese small lecturer training class", to carry out regular, targeted professional training, and strive to improve the business ability of small narrator. Various counties (city) branch to organs, enterprises, rural areas, schools, the new era civilization practice station, carry out "learning party" "learning'July 1 important speech spirit, practice socialism is a great call" "youth to the party power" preaching activities, using yanchi li tableland bank revolutionary site, revolutionary history memorial park, concentric red army west levy memorial hall "home" revolutionary sites, memorial and other red resources, make it "characteristic textbook", let the red local materials into the campus, let the red story into the classroom, let teenagers into the revolutionary memorial hall. The whole group leads the team to create a "faith class". The county (city) teams organized the theme activity of "Red Scarf Heart to the Party", to drive the school young pioneers organization to constantly enrich the history of the Party learning and education. The city's 324 schools less committee, 3312 young pioneers squadron, 131000 young pioneers active action, to carry out the "red scarf heart to party" "red scarf love learning", "I tell the party's story", painting competition, invited the young pioneers with a "red scarf heart to party" theme class, the young pioneers themselves about "fighting new crown youth deeds" "heroic deeds of defending national territorial sovereignty", the border in "recite, sing, create, write, draw" inspire the city's young pioneers love party patriotic enthusiasm, lit the young pioneers "faith lesson". Learn, think and practice, and enrich the "practice class". In the "May 4 th" Youth Day, "June 1" Children's Day, "July 1" Party Day and other important time nodes to carry out the "youth to the Party" concentrated joining ceremony 80 times."I and the emblem / flag, flag / flag", "study, party history, strong faith, follow the party" the "four" knowledge contest, celebrate the 100th anniversary of the founding of the communist party of China and commemorate the 102nd anniversary of the "May 4th movement" youth poetry, "study party history party En go" red viewing activities, party history, strong faith, follow the party "" inheriting the party's glorious history of the Chinese nation community consciousness " theme TuanRi activities 178 times, Covering 60,000 teenagers, Make the majority of teenagers in the scene experience practice exercise more firm faith to follow the party, Strive to make the "four history" learning education "move" up, "live" up, Comprehensively enhance the "temperature" of party history learning and education in the hearts of young people.

2021-09-17	Sunshine "green sail" after class youth volunteer service and add new project sunshine "green sail" after-class youth volunteer service and add new project JianWei correspondent XiangChanghui) in order to further enrich the "green sail" after class youth volunteer service content, improve the quality of service afternoon, on September 15, one fund children's safe small classroom sunshine project launch ceremony held in Rizhao new era civilization practice guidance center hall. Launching ceremony of One Foundation Children's Safe Little Class Sunshine Project. Correspondent for figure activity requirements, the "green sail" after class youth volunteer service to strengthen cooperation with the cooperation of school teachers, seriously participate in the "safe small classroom" safety education lesson preparation, master the teaching content and methods, design an interactive, high participation, vivid and interesting courses, let the children in the classroom can subtly feel the influence of safety knowledge."Qingfan" after-school youth volunteer service. Correspondent for figure since march group sunshine joint municipal bureau of education launched the "green sail" youth volunteer service after class, the service into school after class delay class, with sports, national defense education, safety education, environmental protection, science popularization project into campus after-school service courses, covering 18 urban and rural primary school, more than 2500 students, volunteers 110 people. In the next step, the Communist Youth League Municipal Party Committee will continue to adhere to the demand-oriented approach, dig deep into the professional resources of the "Qingfan" youth volunteer service team, create highlights of after-school service, and polish the "Qingfan" volunteer service brand.

2021-09-16	Bozhou group Bozhou municipal party committee "four force" to promote nonpublic league building work group "four force" promote non-public group building work JianWei correspondent, chun-hui liu check WeiTing) since this year, Anhui Bozhou city party committee to promote non-public enterprise organization, organization construction and enterprise development depth fusion as the goal, combine the group building and grasp business, vigorously implement the group in the city to build "climbing" plan, through "force by" force ", strong foundation, short board, ideas, grasp innovation, activation, comprehensively promote nonpublic group building work quality continues to improve. With the party building with the league building as the leadership, enhance the work "resultant force". Actively connected with the Organization Department of the CPC Municipal Committee and the Non-public Working Committee of the CPC Municipal Committee, comprehensively investigate the current situation of non-public enterprises and social organizations and the construction of the city, so that "the base situation is clear". Make systematic data synchronization between "intelligent group building" and "non-public economic and social organizations", jointly issue the Implementation Opinions on Comprehensively Promoting "Establishing mass organizations and Expanding the coverage of Party Work" in non-public Enterprises and Social Organizations in the city, and further clarify and refine the non-public league building work. Since this year, qiaocheng District non-public enterprises and social field youth league working committee, Mengcheng Anhui new cultural industry investment Co., Ltd. Youth League branch of 118 enterprises and youth league organizations have been established. With practical research as the cut-in, enhance the construction of the "power". Combined with quarterly view analysis, "January a lesson a practice" thematic party day activity, non-public party instructor visit, focus on "service youth entrepreneurship employment, strengthen non-public enterprise group building" theme, around the "team construction, organization construction, system construction, position construction, activity organization" five aspects, such as research, to promote communication to promote development, actively explore the effective way of non-public enterprises and social organizations. In the second quarter of the communist youth league work site view analysis and thematic party day activities to Anhui youth pond health industry co., LTD., that bo pharmaceutical co., LTD., HuaXuan hall pharmaceutical co., LTD., raising mansion health industry co., LTD. And other non-public enterprises to study, by listening to introduction, see information, around the construction of youth organization construction, youth employment, youth home construction work for field research and communication. Focus on scientific management, and enhance the organization's "ability". We will improve the youth league organization work system of non-public enterprises, improve the working mechanism, issue the Work Manual on Youth League Construction of Non-Public Enterprises and Social Organizations, improve the work standardization and construction capacity of non-public enterprises, give full play to the substantive role of non-public enterprise youth league organizations, and prevent them from becoming "showy". In the city's grass-roots youth league branches, we innovatively carried out the activity of "one lesson, one practice and one enlightenment" a month, and a micro group class lecture, a social practice, a learning and perception exchange, effectively promoting the normalization and standardization of the league organization life of non-public enterprises. Anhui province idle film and television technology co., LTD., youth league branch held "read red classic strength" micro group lecture hall, and to the dabie mountain diamond red education base learning activities, through the activities we have said to actively study the history of the party, from the power of the party faith, the spirit of red, enhance red memory, follow the footsteps of the party, for the realization of the next goal in one hundred. Take the activities as the starting point, enhance the team "vitality". According to the characteristics of non-public enterprises, we regularly organize the league construction leaders of non-public enterprises to have discussions, centralized teaching, training and exchanges, build a learning platform, and improve the work level and business ability. The activities such as Bozhou Enterprise Youth Elite Key Training Class, Youth Entrepreneurship Sailing Action- -Bozhou Youth E-commerce Entrepreneurship Talent Symposium, and Maanshan Copper area economic and trade investigation were held to promote the communication between non-public enterprises in Bozhou city and enterprises in the Yangtze River Delta region.

2021-09-16	Hefei: Let the people "push the door" and have books to read and sit in the same position in Hefei Xinghua Academy every day to review their lessons. He told the China Youth Daily reporter that he would pick up a book, relax and read for a while, looking out of the window, the lake of the park is sparkling."Coriko" and "scholarly and culture" are now often used to describe the city spirit of science and humanities in Hefei. Now in Hefei, whether it is the park lake, the green space center, or the "city core" area, the best location and scenery is always "give way" in the reading space, and the reading time has also become better because of the beautiful "background". Since the introduction of the Implementation Plan for The Construction of Urban Reading Space in Hefei city in 2017, the city and county governments have invested more than 300 million yuan to build 110 open-government-led urban reading Spaces, taking the lead in forming a "15-minute reading circle" in provincial capitals in China. In 2020, the urban reading space will be open to 6.92 million readers, 1.37 million books are borrowed, and 7,013 various activities will be held."The specific practice of building reading space in Hefei can be summarized as six aspects, namely, integration of library and store, integration of two industries, multiple functions, two-wheel drive, construction of network system, convenience and benefit to the people."Chen Jiangli, deputy director of the Hefei Municipal Culture and Tourism Bureau, concluded. The sustainable development of reading space cannot be separated from mechanism innovation. The path explored by Hefei is the "two-wheel drive" of government dominance and market allocation power. For government investment in construction, the required funds shall be shared by the municipal and district finance in a ratio of 1 ∶ 1. After completion, zero cost will be given to the enterprise for operation, and the operating expenses such as rental fees and property fees shall be borne by each district. In addition, the book purchase fee and the unified distribution fee of the municipal library shall be borne by the municipal finance."Enterprises can provide personalized services for a fee, and all the service personnel come from enterprises."Chen Jiangli introduced that at present, the city has a total of 15 market entities for operation, with more than 600 employees. Among them, 10 urban reading Spaces, such as Half Mu Fangtang, � Street Yue Study, Shiguang Library, etc., reached 50,000 to 100,000,000 yuan, basically reaching the break-even and starting to step into a virtuous cycle track. In addition, the relevant departments arrange a special fund of 6 million yuan for the cultural industry every year to implement awards for the operating units with a "good" annual management and service performance assessment."The emergence of urban reading space has expanded the public reading services and made up for the lack of public libraries in terms of coverage, convenience and affinity."In the opinion of Professor Lu Hejian of Anhui University, Hefei was able to build such a scale of reading space in a short time because of the strong financial support, and it is also the government's responsibility to strengthen the city's public cultural services."Integrating libraries and stores" is another feature of the urban reading space. Lu Mei, a staff member of Xinghua Academy in Hefei, said that the "library + bookstore" makes the reading space have a strong book source to support it, and the citizens can enjoy the "trinity" convenient service of borrowing books, buying books and reading books just at home. According to the planning requirements, the urban reading space should also have the "4 + X" function. Located in Zhuxi Community, Changqing Street, Hefei city, Fangtang is the largest reading space in the city, with four standard service functions: reading, activity, display and leisure, and is compatible with characteristic service functions such as mass entrepreneurship space, small public theater and 4:30 school. This summer vacation, the Zhuxi community opened a free summer care class based on the reading space. Zhang Siyu from Chaohu University worked together with other college student volunteers to provide professional tutoring and quality education services to 30 primary school students in the community."In addition to the summer vacation, during the future weekends and winter holidays, we will create a long-term trusteeship mechanism to link more university resources, attract more college student volunteers to participate in it, and truly do practical things for the people."Said Wang Zhenglin, deputy secretary of the Zhuxi Community Party Committee. The layout and construction of the 110 reading Spaces are also forcing the innovation of the library management system to ensure that the circulation of books keeps up with the pace of the reform. Within the city, the online service platform and library management software are used to vertically realize the borrowing management and effective distribution of public books in all urban reading space by the city library vertically, and horizontally realize the resource sharing of all operating units with complementary advantages. In May this year, Baohe District further promoted the practice activity of "I do practical things for the masses", and launched the pilot library "general and branch library system"."The book business management is concentrated in the general library. Through the management platform, we urge the branch library and the grassroots service points to open the borrowing, and organize and carry out the book borrowing and reading promotion activities throughout the district."Yu Jiaping, director of the Baohe District Library, said that people can borrow books and return books at home, and have books to read at the door. In order to promote the nationwide reading work, the "reading bank" developed by Baohe District Library relying on the book cloud system was officially launched in June this year. Readers can get the corresponding knowledge coins by borrowing, participating in activities and donating books, and exchange the prizes in the points mall."The establishment of a reader reading encouragement and moderate feedback system is to create a good atmosphere for reading more books, reading good books, and constantly guide readers to enhance their interest in reading, develop reading habits and improve their reading ability."Yu said. China Youth Daily China Youth Network reporter Wang Lei source: China Youth Daily

2021-09-15	Wuhu Wuhu municipal party committee focus on youth growth boost people's urban construction in Anhui province group focus on youth growth boost people's urban construction JianWei correspondent, Chen Zixin check WeiTing) in order to further implement the Anhui Wuhu municipal party committee about people's urban construction work deployment, earnestly concern and protect troubled children, group Wuhu municipal party committee to carry out a series of activities focus on youth growth, boost people's urban construction. Focus on children in difficulties and create a learning space. Wuhu city has carried out a special action to help rural revitalization and create a "dream building space" to realize the dream of children in distress, accurately identified 100 children in distress in the city, and issued a pledge to the city, raising a total of 170,800.00 yuan, tailored learning area for each child, equipped with learning tools. Focus on freshmen in need, love for students to realize their dreams. The Hope Project 2021 "Love Dream University" campaign was launched to help students. As of August 30, municipal youth league organizations had raised 996,571.00 yuan to provide one-time grants to college students from poor families. Focus on key young people to escort their safe growth. Give full play to the advantages of "Jiuzi Peace Escort" youth rights service group, integrate the city resources of youth service, incubate 6 characteristic project brands, and innovatively set up two offline service workstations to guide and promote the orderly participation of social forces in social governance, and escort the healthy growth of key youth groups.

2021-09-13	Fuyang Youth Innovation and Entrepreneurship Competition Beijing Competition successfully held "Smart Future Youth Leader" 2021 Fuyang Youth Innovation and Entrepreneurship Competition Beijing Competition successfully held Jianwei correspondent, Cao JieZhen Wei Ting) recently, Sponsored by the Fuyang Municipal Committee of the Communist Youth League of Anhui Province, Fuyang Human Resources and Social Security Bureau, Municipal Investment Promotion Center and Municipal Talent Office, The 2021 Fuyang Youth Innovation and Entrepreneurship Competition co-organized by Fuyang Modern Rural Industrial Park, Northwest Anhui Internet Headquarters Base and Shuangqing Courtyard, and Fuyang Yingquan Rural Commercial Bank- -Beijing Competition Area was held in Fuyang Municipal Human Resources and Social Security Bureau, Adopt the "online + offline" approach. A total of 20 projects have been registered in Beijing, including 4 energy conservation and environmental protection projects, new materials projects 3,3 Internet projects, 2 advanced manufacturing projects, 5 information technology projects, 1 advanced agricultural project, 1 new energy project and 1 biomedical project. There are 15 registered project entrepreneurship group projects and 5 creative group projects, including 7 doctoral teams and 4 master's teams. Most of the projects come from entrepreneurial companies and university entrepreneurial teams. After the online evaluation of the competition judges, a total of 11 teams' innovation and entrepreneurship projects were displayed online. According to the order of the lottery, each contestant went on the road show online, and the judges scored them. In the competition, each project showed the good spirit of the entrepreneurial youth. After fierce competition, the spirit eagle long sky project, α amylase inhibitor project and "home tao" living space project won the first, second and third prizes in this competition respectively. Winin the division will be recommended to participate in the rematch and final. The final and final competition will be held in Fuyang in October, with gold, silver, bronze, excellence awards and other award categories, with a maximum bonus of 100,000 yuan. The participating projects will also enjoy preferential entrepreneurship support policies and high-quality entrepreneurship incubation services in Fuyang Hefei Modern Industrial Park and Northwest Anhui Internet Headquarters Base.

2021-09-15	Hope cabin construction site will be held in Jining hope cabin construction site will be held in Jining JianWei correspondent Bai Chen) recently, hope the cabin construction site will be held in Jining, the Shandong provincial party committee relevant departments responsible for comrades, 16 city youth corps committee is mainly responsible for comrades, various counties (city, area) youth corps committee is mainly responsible for comrades to attend the meeting. The conference was held in the form of video conference, with the main venue located in Jining, and the other 15 cities set up sub-venues. The picture shows the site of the Hope cabin construction promotion site. At the meeting, the secretaries of the Youth League Committee of Zaozhuang, Jining, Tai'an, Linyi, Liaocheng and Heze, 6 cities in southwest Shandong, made an exchange speech. The responsible comrades of the rights and interests Department of the Provincial Youth League Committee and the relevant responsible comrades of the Sishui County Micro public welfare Association gave the training and the specific implementation of the hope hut project in the province. The day before the meeting, Liu Tiandong, secretary of the Shandong Provincial Party Committee of the Communist Youth League, and his delegation went to Zaozhuang and Jining to carry out the "four no and two straight" investigation, and carried out a sampling inspection on the already built "hope hut". In the next step, the Youth League Jining Municipal Party Committee will take this meeting as a new starting point to further promote the implementation of the project, to ensure the accurate coverage of every child in difficulties, make good use of every love donation, and build every "hope hut".

2021-09-15	Wuzhong city "elegance" youth XuanJiangTuan into the community theme Wuzhong city in Ningxia "elegance" youth XuanJiangTuan into the community theme preach network Beijing on September 15 (reporter Zhang Jianwei) to thoroughly study and implement the general secretary xi "July 1" important speech spirit, recently, Ningxia hui autonomous region Wuzhong city "elegance" youth XuanJiangTuan into yu people street east community "learning July 1 important speech spirit practice socialism is a great call" theme preaching activities, more than 30 members youth to listen to preach. Community presentation site. Zhang Tongtong, Xu Jingjing and Ding Yue, members of the Qingtongxia "Fenghua" youth publicity group, preached at the scene, calling on the majority of young people to practice the youth oath of "please trust the Party and the strong country has me" with practical actions. After the lecture, Xu Yana, deputy secretary of the East Community Party Committee of the East, said that community cadres should settle down and often "go from street to street" to listen to the voice of the masses, solicit the opinions of the masses, know more about the troubles of the masses, worry, try to solve practical difficulties for them, do more affordable things to satisfy the masses. Community presentation site. Group Wuzhong city committee for the next step, pku will learn "four history" with sum up experience, observe reality, promote work, to improve organization, leading force, service and overall situation contribution as the goal, to carry out "I do the practical work for the masses" "I do something for teenagers" practice, the learning results into work, continuously improve the sense of teenagers, happiness and security.

2021-09-16	Group Zibo city committee to "city township" power grassroots youth league vitality promotion Zibo committee to "city villages and towns" power grassroots youth league vitality boost JianWei) to fully implement the general secretary of the important thoughts of youth work, deepening the county communist youth league organization at the grassroots level reform, Zibo in Shandong province municipal party committee to establish and improve the "city grasp the villages and towns" work implementation mechanism, improve town (street) the organization vitality of the communist youth league. Visit the "Hope hut" children. Youth League Zibo Municipal Party Committee for the map to clear responsibility, the implementation of the "city to grasp the township" main body. The team members take the lead in catching it. Each person contacted two towns (streets) and 10 villages (communities), emphasizing the two tasks of youth league organization construction and youth development, consolidating the three foundation of the grassroots, the whole league leadership and the Communist Youth League online, and combining online and offline contact and guidance, to help coordinate and solve 27 problems. Organ department room directional grasp. Establish a contact mechanism, each department at least once to contact more than 2 towns (streets) every month for research, find out and solve problems. District and county youth League committee normal grasp. Establish the dynamic management ledger of districts and towns (sub-districts), implement the "4 + 1" mechanism for the cadres of the district and county Youth League committees, and work in the organs for four days and at the grass-roots level for one day a week, so as to promote the cadres to truly enter the grass-roots level, approach the youth, and directly contact, serve and guide the youth. Zibo Youth Rural Revitalization Innovation and Entrepreneurship Competition. Youth League Zibo Municipal Party Committee for the map precision force, the prison "city grasp the township" focus. Grasp the organization construction, consolidate the grass-roots foundation. Youth working committees and young talent associations were set up in 88 towns (sub-districts) of the city, a list of basic responsibilities was formulated, and four mechanisms, including assessment and evaluation, and regular research work, were strictly implemented. Grasp the village (community) "two committees" transition opportunity, in the city's 2,405 villages, 522 communities, to simultaneously carry out the youth league organization transition. Grasp the team management, enhance the organizational vitality. We paid attention to the representativeness of members of the Youth Working Committee and added 126 people over the past year. Organized 110 training courses for town (street) league (working) committee secretaries, rural outstanding youth backbone courses and youth pioneer forums at all levels. The Zibo Youth Rural Revitalization, Innovation and Entrepreneurship Competition was held, recommending 529 good young people to become active members of the Party, 180 people joining the Party, 1,025 people as members of the "two committees" of the village (community), and 239 people as party branch secretaries of the village (community), and issuing the "Good Youth Loan" of 60.86 million yuan. Grasp the role to play, to form the social function. To guide the town (street) to steadily promote the "hope cabin", "youth community", "I do practical things for the youth" and other key work of the provincial Communist Youth League, strong supervision, to seek practical results. The project of "one town (street) and one product" was carried out, and 88 key characteristic projects, including "Qidu Spring Rain", achieved initial results. To the community research, to understand the needs of the youth. League Zibo Municipal Party Committee for the map rewards and punishments at the same time, in order to "city grasp the township" actual effect. Mutual learning and mutual comparison are promoted together. Hold 5 on-site observation and comment meetings (promotion meetings) to create a strong atmosphere of comparing with learning and catching up. Establish the "town (street) Youth Working Committee backbone work group", and create an online platform for work signal transmission and youth league work communication. The Forward guide award is advanced. For the overall or special work performance of outstanding performance of the town (street), timely through sending good news, written praise and other forms of incentive, and in the typical report above the provincial level, the main shift of students transfer training priority. The municipal finance has a regular annual budget of 100,000 yuan to support outstanding league construction projects. Reverse restraint promotes backward development. Innovate the form of reporting and evaluation, through the questionnaire survey, telephone spot check, on-site evaluation of the town (street) group (work) committee evaluation. The secretary of the youth League (work) committee, whose work style is not true, ineffective performance of duties, and low satisfaction of the youth, has instructed the district and county youth League committee to timely remind the interview and supervise the rectification. In the next step, the Youth League Zibo Municipal Party Committee will take the reform of the county Communist Youth League to further improve the implementation mechanism of the "city grasping the township" work, and constantly improve the organization, leading force and service power of the town (street) Communist Youth League through the sinking of resources and service.

2021-09-14	Zhang Yueyue, a summer college student in Jin'an District, Anhui Province, and three other friends ended a one-month volunteer service activity. In the Jiuligou community in the district, they have experienced epidemic prevention and control, telephone notification, information registration, and maintaining the order of vaccination successively. It is reported that the Jiuligou community youth league branch has 1 secretary, more than 200 registered youth members, after a bottom of the bottom, the area has 269 minors. The community youth League branch focuses on the needs of helping vulnerable youth groups, helping filial piety for the elderly, and developing civilized living habits, and mobilizes young volunteers to take the initiative to report to the community. This summer, the community also connected with the Youth League Committee of the Department of Mechanical and Electrical Engineering of West Anhui College, and selected college student volunteers to carry out voluntary publicity activities against COVID-19 vaccine. Since this year, the Youth League Jinjin-An District Committee has promoted the close integration of youth work and social governance through the "four-in-one" working mode of strengthening the organization, building positions, creating projects and finding resources. In addition to providing voluntary services, building a youth position is also an important part of the work. In recent days, the league Jinan District Committee on the area of two youth community pilot-Jinyu community and Jiuligou community youth positions to transform and upgrade, to achieve office space, activities, places to learn, improve the service level of community league organization. This summer, nine li ditch community branch joint area property units to carry out community flea market activities, to the area of youth and residents of the public awareness of frugality and environmental awareness. Jinyu community integrates the resources of social organizations, and innovatively establishes a platform of "site sharing, precise ordering, integration and pairing". In summer vacation, the community organization built "Green Kwai Paradise" to "light up children's heart lamp, build every heart house" and fill every space for children, and built "bud" and "rainbow" volunteer service, providing psychological counseling, epidemic prevention and control, respect and love support. In the next step, the Communist Youth League Jin'an District Committee will improve the organization system of the community youth league, improve the community youth work project, and mobilize the youth league members to actively participate in community governance around the most urgent needs of the community people.

2021-09-13	Jinan young pioneers to carry out the "first lesson" theme education activities Jinan young pioneers organization "first lesson" theme education activities China youth network Beijing on September 13 (reporter Zhang Jianwei, correspondent Sun Han Peng Chong) recently, the Jinan municipal party committee, city less committee organization young pioneers at all levels in the city to "please trust power have me" as the theme, the focus of the party history education, focus on the fall of 2021 the first lesson theme education activities, guide the city the youth to love party, patriotism, love socialism, with the strongest open a new learning life. The first lesson of the school theme education activities. Group Jinan municipal party committee for the figure through the opening ceremony, the flag speech, theme squadron and revisit in the league to celebrate the 100th anniversary of the congress of the founding of the communist party of China and the young pioneers on behalf of the activities, comprehensive, stereo, multi-level, multi-dimensional cultivate patriotism, constantly guide the young pioneers in the city's young pioneers firm ideal faith, take practical actions to practice "please rest assured power have me" clank oath.

2021-09-13	Group Wuzhong city party committee to carry out the health special knowledge lecture group Wuzhong city party committee to carry out the "healthy life love" health knowledge lecture jianwei) to fully implement the spirit of the fifth plenary session of the 19 and the 2021 national health work conference spirit, further implement the opinions of the State Council on the implementation of healthy China action "and" healthy Ningxia action (2019-2030) " requirements. Recently, the Youth League of Ningxia Hui Autonomous Region Wuzhong Municipal Party Committee invited the safety and health education center propaganda and education department staff to carry out a "healthy life love health counterparts" special knowledge lecture. In the lecture, the teacher focused on the knowledge of CPR site emergency rescue, the prevention and improvement of common occupational diseases and the protection knowledge of novel coronavirus. Through professional knowledge, rich content and interesting language, we can vividly tell the correct method of cardiac resuscitation, the prevention and treatment of common occupational diseases (periarthritis of shoulder, cervical spondylosis, lumbar spine disease), the transmission route of novel coronavirus and the Delta strain. Lecture using quiz interaction, case sharing and demonstration teaching way, a relaxed and lively atmosphere, serious, in entertaining atmosphere to the youth about safety and health knowledge, guide everyone to set up the correct health view, further improve the ability to prevent disease prevention, constantly promote the "national health level promotion action", to boost the construction of "Wuzhong" health.

2021-09-12	Wang Haihan, correspondent Bao Yuqiao) On the afternoon of September 10, Hefei, more than 400 volunteer service teams and 85,000 volunteers in Baohe District, Hefei, had a unified management brand and cultural symbols. The district's "Sister Bao" volunteer service team was officially established, and will carry out 3,000 various services throughout the year. The establishment of the "Sister Bao" volunteer service team is a concrete measure to implement the deployment of the Hefei civilization creation work, adapt to the new goal of building a national civilized model city in Hefei, and also the due meaning of promoting the practical activities of "I do practical things for the masses". After the establishment of the brand, it will help create a volunteer service atmosphere in which all the people participate, build and share. It is reported that the "Bao elder sister" volunteer service team will be the area "Bao Meili" female volunteer service team, small a sister help group, psychological counseling volunteer group, community purchasing volunteer service team and other functions of different volunteer service organizations to integrate resources, unified management, scientific deployment, to break the management barriers. It also includes the "Rainbow Appointment Relay" volunteer service team established to implement the "double reduction" policy. The team will build a three-way linkage volunteer service mode of "home, school and community" with teaching staff as the main body and community and parent volunteers as the supplement. Volunteer service has always been the brand work of Baohe District, Hefei. At ordinary times, the district direct departments and the streets and towns pay attention to cultivating and reserve volunteer service forces around social governance, and have experience in building and managing emergency volunteer service teams. During the epidemic period last year, many community youth league organizations in Baohe District relied on the community-community-building group three-level network circle, and mobilized young volunteers in the district to form a "war'epidemic" team " in a short period of time to help with joint prevention and control. Photo taken by Xie Wenjun

2021-09-12	On ┙� Day, the Youth League Committee held the ideological and moral construction of minors and a public welfare donation ceremony for the opening season. At the event, Kaide Group gave 100 Kaide love bags and learning necessities to the teenagers. It is understood that the Youth League Daxing District Committee, relying on the sunshine zone community youth gathering in the area, starting from the youth preferences of teenagers, customized the "sunshine zone healthy growth card" for 100 teenagers, providing professional psychological sand table experience, calligraphy and painting training, growth planning and other teaching services. And lasted 4 days of minors ideological and moral construction propaganda pop-up shop, is to further promote the implementation of minors ideological and moral construction of important measures, through publicity display, card commitment, activity experience, psychological counseling, legal aid and other diversified activities, the propaganda and education really into the daily life of teenagers. At the same time, the teenagers who benefited from the activity also spontaneously became the link to expand the influence of the activity, leading more teenagers into the 41 community youth groups in the district. Here, the confusion and problems they encountered in the process of learning and growth can be solved in time. This has also become one of the examples of Daxing District to continuously strengthen the ideological and moral construction of minors and provide normal services for the protection of teenagers' rights and interests. In addition, the Youth League Daxing District Committee also supports and helps young people to grow up through the Hope Project Education Action and the "Xingqing" series of legal popularization and self-protection activities.

2021-09-12	The second Guangzhou Volunteer service contest final held the second Guangzhou Volunteer service contest final held Youth Daily reporter Lin Jie) today, the second Guangzhou volunteer service contest final held in Guangzhou Youth League School. The 10 contestants focused on the themes of "Lei Feng Spirit in the New Era", "Fighting COVID-19", "grassroots social governance", "rural revitalization", "emergency volunteer service", "college students" and "volunteer service for helping the disabled", and based on their own volunteer service experience. Since the launch of the competition in July this year, more than 100 volunteers from the Communist Youth League organizations, volunteer service organizations, universities, communities, enterprises and public institutions in Guangzhou have actively signed up to participate. After layers of selection, 35 outstanding players entered the semifinal, and the final 10 players entered the final. In order to improve the finalists volunteer service micro course design and development, teaching of actual combat ability, Guangzhou group school specially held a special training course, invited experts and scholars in various fields, from the course design, implementation process to present skills, etc., to enter the semi players for all-round, multi-level, point-to-point training guidance and practice. On the scene, the post-00s college students use the Internet to carry out "cloud volunteer teaching" and "cloud company" to help rural volunteer service; vaccination site volunteers share the pre-job training of "elderly vaccination blood pressure measurement", professional volunteers' epidemic prevention knowledge; sign language teachers let volunteers experience the living world of hearing disabled through interactive experiential training, and build the information exchange between hearing disabled and healthy people. In the end, Huang Xiaoshu from Guangzhou Metro operation headquarters and Zhang Yirong from South China Agricultural University won the first prize in the competition, and Chen Minshan from Guangzhou Baiyunshan Hutchison Whampoa Traditional Chinese Medicine Co., Ltd. won the "Internet Popularity Award". As a judge, Ye Zhihai, deputy dean of the School of Art and Pearl River Film Academy, said that the contestants were very careful in the design of teaching content and the application of teaching methods, and also used a variety of teaching methods and rich and meaningful cases. Their personal style and language expression fully showed the style of the volunteer lecturers. I hope they will become the "dandelion" to spread the spirit of volunteer service and sow the "seeds" of volunteer service in the new era. Tu Minxia, president of Guangzhou Youth League School (Guangzhou Volunteer College), said that she hoped that through such an open and inclusive platform, she would carry forward the volunteer service spirit of "dedication, friendship, mutual assistance, assistance and progress" in the whole society, and cultivate a team of young volunteer lecturers who are firm in faith, responsible, good and useful.

2021-09-12	Chengdu youth league 13th six standing committee of Chengdu youth league 13th sixth standing committee held xinxin wang) on September 10, Chengdu youth federation of 13th sixth standing committee held in Sichuan tianfu new district, meeting learning xi general secretary in celebrating the 100th anniversary of the founding of the communist party of China's important speech spirit and the 11th CPC Sichuan party committee nine times, Chengdu municipal party committee of the 13th nine plenary session spirit, from all walks of life youth committee representatives around organizational function, role, work summarizes the communication. Li Jialin, secretary of Chengdu Party Committee, attended the meeting and delivered a speech. Ren Na, deputy secretary of Chengdu Party Committee and chairman of Chengdu Youth Federation, presided over the meeting. For a long time, the members of the Chengdu Youth Federation have made remarkable achievements in uniting and leading the youth, helping the rural revitalization, serving innovation and entrepreneurship, caring for the vulnerable and groups, and participating in the epidemic prevention and control. Li Jialin, Party secretary of the Chengdu Communist Youth League, discussed and communicated with members of the members of the Youth League on how to help the high-quality economic and social development of Chengdu. He said that the Chengdu Communist Youth League should be a good navigator, postman, waiter and propagandist of the members of the Youth Federation, to provide more opportunities and platforms for the growth and development of the youth league members, and to promote the common development of the youth league members and the city. Li Jialin hope the youth league members always keep in mind the political honor, more youthful, more ground, more popular, based on the industry reality, aiming at key areas, leading and driving the youth for Chengdu construction practice new development concept of park city demonstration area, make important driving the development of the high quality growth pole and new power source. Members of the participating Youth Federation said that as a witness and participant of the rapid development of Chengdu, it is a great honor to make progress with the city. We should unite and lead the young people of all ethnic groups and all walks of life in the city to further contribute their youth strength to the economic and social development of Chengdu. The meeting also deliberated and adopted the Work System of Direct Contact with Youth by Members of Chengdu Youth Federation and the Implementation Rules for the Proposals and Suggestions of Chengdu Youth Federation. Representatives of the committee members from all walks of life put forward suggestions on the work of the Municipal Youth Federation.

2021-09-11	Ningbo Volunteer oath Service 14th National Games Sailing Competition 14th National Games Sailing Competition reporter Li Jianping) today, the 14th National Games Sailing Competition (Ningbo competition area) young volunteer oath conference held in Ningbo University of Finance and Economics Xiangshan Campus. The 153 young volunteers who participated in the competition signed a letter of commitment and took a collective oath. Jin Xin, Secretary of Ningbo Municipal Communist Youth League Committee, Zhou Bo, Party Secretary of Ningbo University of Finance and Economics, as well as relevant guests from Ningbo Municipal Sports Bureau, Municipal Education Bureau, Municipal Red Cross Society, Municipal Communist Youth League Committee and Xiangshan County Government attended the meeting. It is reported that this is the 14th National Games (referred to as the National Games) sailing competition, Hangzhou 2022 the 19th Asian Games test competition for the first time settled in Ningbo, is also the first time for Zhejiang Province to host the final stage of the National Games official competition. In this competition, teams from 22 provinces and regions will compete for gold MEDALS in 8 events. To safeguard the event, Ningbo municipal party committee, city volunteer association after early layers of selection and strict audit, from Ningbo university, Ningbo university of finance and economics, Ningbo Nottingham university and other three universities and social volunteers selected 153 young volunteers, participate in the event of volunteer service work, they will provide the comprehensive management of venues, news, publicity, reception guidance, logistics and other volunteer services. Shi Hanxiao for the picture

2021-09-10	Guizhou Qingzhen: Multiple measures to strengthen the end of the Communist Youth League organization [the Communist Youth League grassroots reform] Guizhou Qingzhen: multiple measures to strengthen the end of the youth league organization settled, nearly 140,000 young teachers and students. How to play the vocational education youth? Taking advantage of the opportunity of the second batch of pilot reform of the national county Communist Youth League, the Youth Municipal Party Committee proposed the joint construction and sharing of campus and campus to promote the "five district linkage" between campus and campus, campus and block, campus and park, campus and community, campus and mountainous area. Taking the linkage between campus and community as an example, TuanTown Municipal Party Committee has carried out the "Community Youth Action". Up to now, 10 vocational education colleges have participated in more than 2,000 community volunteer services with youth volunteer associations and student federations as the main body. In order to effectively promote the integration of school and local, the Municipal Party Committee of TuanTown has jointly created an exclusive "youth card" due to various needs, providing high-quality and convenient services for the youth of vocational colleges and social youth to experience research, social practice and travel transportation. At present, all the preparatory work of the "youth card" has come to an end, and it is expected to be launched through the small program at the end of September, with the first batch of audit quota of 10,000 people. This is just a microcosm of the reform of the local Communist Youth League. In the reform process of the grassroots organization of the Communist Youth League, the Youth Municipal Party Committee actively explored and launched a series of reform measures to strengthen the end of the league organization from the aspects of working force, position construction and serving the youth. Two days ago, Hu Juan, who lives in Baini Village, Hongfenghu Town, Qingzhen City, Guizhou Province, was recommended as the part-time deputy secretary of the Youth League branch of the village. Before this, as a young party member in the village, although Hu Juan is working outside, but the village large and small things, she has been very concerned about. In Qingzhen city, each village not only has a full-time youth league branch secretary, but also is equipped with two part-time deputy secretaries of the youth league branch. The organizational system of contacting the youth and serving the overall situation is constantly improved. This should benefit from the Youth Town Municipal Party Committee in the Communist Youth League grass-roots organization reform in the process, to promote the establishment of the ladder "special hanging" cadre team. By strengthening the strength of cadres layer by layer, we will consolidate the foundation for the youth league organization and service, provide more opportunities for the youth to exercise and improve, and stabilize the "basic plate" of the organization team construction."The Municipal Party Committee of TuanTown has promoted the three-level full-time, part-time and temporary cadres in counties and villages, and established a talent system covering party and government organs, enterprises and institutions, and non-public economic organizations."According to Gao Lei, deputy party secretary of TuanTown, optimize the" 1 + 2 + 2 + 2 "model, including one full-time secretary, 2 full-time deputy secretaries, 2 full-time deputy secretaries, 1 full-time secretary, 2 temporary deputy secretary and 2 deputy secretary, and one full-time branch secretary in the village (residence) in the" 1 + 2 " mode. On the basis of the full coverage of the vertical three-level Communist Youth League cadres, the Communist Youth League Town Municipal Party Committee actively promotes the horizontal promotion from four aspects, and constantly improves the organizational system of contacting the youth and serving the overall situation. Volunteers of the "Three Support and one Support" and "Western Plan": Under the guidance of the Communist Youth League, young people have devoted their youth to the front line of rural revitalization and epidemic prevention and control. Up to now, more than 180 volunteers have been sent to grassroots youth league construction and comprehensive civil affairs administration. Horizontal establishment of youth commandos covering all fields: in Qingzhen city, 28 different youth commandos, rushed to the grass-roots urgent, difficult and dangerous heavy task first line. Promoting the construction of volunteer teams for civilized practice in the new era: We have set up 105 volunteer service teams, 225 teams, more than 290 registered volunteer service organizations, more than 79,000 real-name certified volunteers, and carried out more than 7,700 volunteer activities. Horizontal establishment of social organizations that gather youth to serve youth: On the basis of social organizations such as Youth Volunteer Leaders Association and Rural Youth Leaders Association, TuanMunicipal Party Committee has also explored the establishment of literary and artistic social organizations to build a platform and create conditions for "literary and artistic youth" to better play their role in the new era. The scattered "1 + 12 + N" youth home is not far from the Qingzhen city center, and a youth park integrating sports culture, sports and leisure culture, and ecological environment culture has become a good place for local youth to punch in recently, attracting an average of 500-800 people every day. Into the park, there are both boxing, badminton and other sports places, as well as go-kart, sightseeing small train, barbecue, tea restaurant and other leisure places. In a pavilion in the center of the park, an open shared library is under construction. The person in charge of the park introduced that the following here will not only display all kinds of books loved by the young people, but also will carry out book rafting, hold reading sharing and other activities. Where the youth is there, the service of the youth league organization extends there. As the park became functional, many young people found that the familiar "youth home" has been moved in. In fact, "Youth Home" has not only become a place where more and more young people gather, but also a platform for cultural exchanges."I didn't expect that out of the school gate, I could also see the league organization, giving people a very cordial and warm feeling."Students of Guizhou Industrial and Commercial Vocational and Technical College were both surprised and happy when they saw the four words" Youth Home "in the" Guizhou Intangible Cultural Heritage Creation " in Wuyue Square, Qingzhen City. In Qingzhen city, "Youth Home" as the Communist Youth League to contact and serve the youth "stores", throughout the area of "Star Cinema", "Geometry Bookstore" and other nearly 30 popular places by the young people. In the past, the "Youth Home" of "Dexin Bookstore" regularly held reading sharing with middle school students and vocational education college students, which attracted many students to participate. The "Youth Home", established in Fengyu Community, Zhanjie Town, carries out interesting activities such as pottery classes for children. Since the reform of the grassroots organization of the Communist Youth League, the "1 + 12 + N" youth home station model covering 12 townships has been formed on the basis of the original 12 townships (towns and streets). The "N" "youth homes" scattered in all regions and industries of the city have made youth and social organizations find a sense of belonging and honor, and also enhance the emotional identity of youth groups to youth league organizations. Fengyu Community, the street town of the Communist Youth League, is the largest resettlement site for the relocation of poverty alleviation in Qingzhen city. There are 1,966 people from 483 households from 8 towns and townships here, and the left-behind children and the elderly account for more than half. How to make them adapt to the new life as soon as possible, Tuanqing Town Municipal Party Committee as the initiative, from the details, to the community people to send warmth. Zhou Changjiang, a volunteer of the Western Project, is a part-time deputy secretary of the Tuanzhan Street Town Committee, and has been sinking in Fengyu Community for a long time, and the people have already become their families here. He still remembers that one family whose grandfather and father died soon after they moved over, and the two children lived with their grandmother. Children's hygiene habits and study conditions are not optimistic. During the household visit, Zhou Changjiang found that the child had been relatively autistic and unwilling to communicate with others. Therefore, Zhou Changjiang, a social worker major, visited many times and took the initiative to communicate with his children, gradually teaching them to get familiar with community life, and to take them to and from school."Now both children have become much more cheerful. They often help the community with garbage sorting, and actively participate in the daily activities organized by the community."Zhou Changjiang said. This is just one of the many incidents that need to be guided and dealt with. In order to enable more professional forces to intervene and better help the community residents to integrate into the urban life, the Municipal Party Committee of Tuanqing Town, together with Qixin Social Work Service, a social work organization affiliated to the Communist Youth League, arranged two social workers to stay in the community for a long time through the "New Citizens Dream Bridge" action in the way of government purchase. On September 4, two social workers had officially reported to the community."The majority of left-behind elderly and children in the relocated communities, there are certain safety risks in daily life, and there are also widespread problems such as academic difficulties and parent-child conflicts."Qixin Social Work Service supervisor Luo Yunnan is a social worker teacher in Guiyang Kindergarten Higher Normal School. She introduced that they had done some regular services in the community before, and the subsequent local labor union continued to go deep into the community to evaluate the needs of children in the community, and to investigate the public environment, safety management, daily monitoring, family education and other aspects of the community. Precise intervention should be classified for special intervention, and also focus on gratitude education and confidence education."In June, Guizhou launched the pilot work of expanding the reform of grassroots organizations of the county Communist Youth League, and identified 20 counties (cities and districts) including Yunyan District of Guiyang city as the pilot counties of grassroots organization reform of the youth League."The Guizhou Provincial Party Committee of the relevant person in charge introduced, the scale of this round of reform pilot work expanded 10 times than the previous year 10 times, among which Tongren city 10 counties as this round of reform pilot counties, open the whole city outside the municipality to promote the pilot. The Guizhou Provincial Party Committee of the Communist Youth League took the work of Tongren as the focus of this round of reform, and strives to solidify the effective practices of strengthening the construction of the grass-roots league organization, expand the influence of the reform, and issue the good voice of Guizhou. Source: China Youth Daily (September 10,2021, edition 01)

2021-09-09	Jingdezhen municipal party committee to carry out the 2021 funding difficult college students in Jiangxi Jingdezhen municipal party committee in 2021 "study party history does the practical work-hope project interpreta dream action" funding difficult college students activities Chen Zhuoqiong) recently, organized by the Jingdezhen municipal party committee in 2021 "study the party history does the practical work-hope project interpreta dream action" funding difficult college students' activities began. The 43 national, provincial and municipal youth civilization (creation) of the collective actively responded to the call of the Communist Youth League, and the league cadres at all levels together into the counties (city, district) villages (communities), to the poor families to carry out the work of pairing students. It is reported that this year, the Jingdezhen Municipal Party Committee hope project aid activities a total of 165,000 yuan, for superior project funds 60,000 yuan, a total of 225,000 yuan, funding 45 freshmen from poor families (including registered poor families). Source: China Youth Daily

2021-09-06	Dezhou city "hope project interpreta dream action" grants in 2021 Dezhou "hope project interpreta dream action" grants in network Beijing on September 6 (reporter Zhang Jianwei, correspondent Fan Yichao) group of Shandong Texas municipal party committee implementation 2021 hope project "interpreta dream action", lasted two months since July, after the school and township (street) youth corps committee screening, the final audit visit verification, group support object, launched several love unit to raise funds 330000 yuan, funding 64 difficult university freshmen.

2021-09-06	Haikou City launched "Xiao Er" Youth Entrepreneurship Volunteer Service Project Wei Correspondent Zhou Yutong) In order to help optimize the business environment of Haikou city, To effectively meet the entrepreneurial needs of young groups such as young entrepreneurs, local college students, local first-time entrepreneurs and so on, The Youth League Haikou Municipal Committee, the Municipal Human Resources and Social Security Bureau, the Municipal Entrepreneurship Small Loan Guarantee Center, and the Municipal Youth Federation, jointly launched the Haikou City "Youth Innovation store small two" youth entrepreneurship volunteer service project, Taking the opportunity of college students returning to their hometown in summer vacation, From July to August, the volunteer service window was opened in the commercial registration area of Haikou government affairs Center and four district government affairs halls, Provide the "one-stop" policy consultation volunteer service in the mode of "offline publicity-online consultation-offline cash". Volunteer Service Project Service "Menu". Correspondent for figure for Haikou city foreign and local micro, small and medium enterprises young entrepreneurs, combing integration at all levels entrepreneurial resources and people club department pratt & whitney loan subsidies services, and through the advantage of youth social organizations and social service, the entrepreneurs gathered city, district government affairs center to provide normalized consulting services, broaden the entrepreneurs to understand pratt & whitney policy and service channels, help entrepreneurs through policy to cash "last link". At the same time, it also forms a good complementary support with the functions and services of the government service hall, further enriching the humanized service content. Group Haikou municipal party committee and city youth entrepreneurship association formulated the "Haikou city youth entrepreneurship volunteer service menu", including "policy package" "service package" "project package" three major service package a total of 14 services, to the broad entrepreneurial youth entrepreneurship microfinance discount policy, one-time business subsidies, free business training and enterprise worker training subsidies, and launched the policy lecture hall, entrepreneurship seminar, business market and youth business incubators in youth entrepreneurship brand services and activities. In our daily work, we will actively build a entrepreneurial volunteer service system with clear process, substantial content and various forms through online and offline ways, such as distributing publicity materials, explaining supporting policies, guiding young people to join the service wechat groups and so on. Policy lecture hall activities. Since the launch of the activity in late May, it has gradually realized the synchronous development in the four districts and has been welcomed by small, medium and micro entrepreneurs and government halls at all levels. By the end of August, it has served more than 1100 young entrepreneurs, established 5 WeChat groups (1 at municipal level and 4 at district level), benefiting 300 young entrepreneurs; effectively responded to more than 320 online and offline consultations and 40 policy interpretation tweets by WeChat public account; provided 2; 12 young entrepreneurs registered in Haikou; recommended 1032 employees of 42 enterprises to apply for employee training subsidy and 300,000 yuan.

2021-09-04	Chengdu green low carbon series practice Chengdu green low carbon series practice fast to be "green low carbon AI small talent" when "green low carbon AI little talent" in the fall of 2021, by the communist youth league of Chengdu, Chengdu science and technology association, Chengdu less working committee of the new semester "red scarf first lesson" green low carbon series practice activities officially launched yesterday. In order to further strengthen the young pioneers of green low carbon concept and the cultivation of environmental protection consciousness, this activity combined with the Chengdu living garbage management regulations promotion, aims to through small widening hand drive family, schools and society to participate in creating the development of green low carbon social atmosphere, in order to speed up the construction of practice the new development concept of park city demonstration area. In order to convey the concept of "carbon neutrality", improve the awareness of ecological civilization, and create a new fashion of green and low-carbon life, this activity will take the daily life of the Young Pioneers as the starting point, aiming to comprehensively enhance the practice of low-carbon environmental protection concept through the dissemination of life and interest. Based on the "Chengdu Red Scarf" and "Popular Panda" wechat public account platforms, online activities such as low-carbon life tips and knowledge sharing challenges, and offline activities carry out practical education activities around green travel and garbage classification."Online + offline" four big activities, will set up for the young pioneers green low carbon environmental protection concept- "green low carbon life" cloud launch campaign, will rely on the "Chengdu red scarf" WeChat public activity announcement, the young pioneers through WeChat small program activity interface point sponsorship, propaganda to citizens advocate green environmental protection, low carbon life. At the same time, the school young Pioneers university (middle school) team combined with the first lesson of the new semester red scarf, organized the young pioneers to carry out green and low-carbon theme learning activities, guide the young pioneers to learn carbon peak, carbon neutral basic knowledge, establish the concept of green and low-carbon environmental protection."Carbon peak, carbon and knowledge science" online experience activities, relying on the "Chengdu red scarf" WeChat public release carbon peak, carbon and knowledge interesting animation micro team class, the young pioneers learn carbon peak, carbon neutral related science knowledge, improve the understanding of green low carbon development, develop a good habit of garbage classification, care for the environment. The "Green and low-carbon AI Little Talent" fun challenge activity is an online fun challenge task with a green and low-carbon concept, taking urban space, energy, transportation as the theme design, and integrating carbon peak, carbon neutral and popular science knowledge, etc. The majority of young pioneers can participate in it. This activity will select 100 "green and low-carbon AI small talent"."Red scarf carbon plan small proposal" solicitation, the young pioneers, the young pioneers collective around green travel, garbage classification, energy conservation and emission reduction, low carbon cycle, etc., through small investigation, research, form a "red scarf carbon plan small proposal", guide the young pioneers firmly establish a consciousness of energy conservation and emissions reduction, develop green low carbon way of life. Finally, 10 "Red Scarf Carbon Plan outstanding small proposals" were selected, which will be displayed during the eighth Municipal Youth Congress in Chengdu. It is understood that the young Pioneers brigade in Chengdu school squadron as a unit, using the young Pioneers activity class weekly class, with micro team class, micro speech and other children easy to accept, sow the "seed" of green low-carbon environmental protection concept. It is understood that the one-month new semester "red scarf first lesson" green and low-carbon series of practice activities, will cover more than 20,000 young pioneers squadrons and one million young pioneers in Chengdu.(Reporter Zhao Zijun)

2021-09-04	Anhui Lu 'an) On September 1, the Publicity Department of the Communist Party of China Lu' an Municipal Party Committee and the Communist Youth League jointly held the "Centennial Party History Youth Talk" - -Party history learning micro publicity contest. More than 100 people from the city's counties, municipal units and schools watched the event, and more than 750,000 people watched the show live online. The contestants are the post-80s and post-90s youth people selected from various counties, districts, municipal organs and school youth league organizations. In the competition, they based on their own work, combined with the knowledge of party history, introduced ordinary people around, moving things around, with vivid expression of the glorious history of the Communist Party of China, and the Communist mission, show the spirit of Lu'an youth enterprising, brave mission. On the field, they actively participate in the cause of youth theory propaganda, enhance the attraction and dissemination of propaganda. Next, they will go to villages, communities, enterprises, schools and party and government organs. A total of 5 first prizes, 8 second prizes and 10 third prizes were awarded.

2021-09-03	22 Courtyard Street Community youth gathering in Beijing Chaoyang Shuangjing Street inaugurated 22 Courtyard Street community youth gathering completed! Or will become a young pioneer new card jun) on September 2,22 courtyard Street community youth hui in Beijing Chaoyang Shuangjing Street inaugurated, or will become chaoyang literature and art, public welfare, community governance youth pioneer new card.22 Courtyard Street Community Youth Club focuses on serving the three young pioneer groups of "art, public welfare, volunteer", committed to building the three youth gathering places of "culture and art, organization incubation, community governance", focusing on improving the three mechanism guarantee of "Party building support, district-level platform, and territorial resources", and carrying out 14 high-quality project products. First, centering on "building a gathering place for young literature and art pioneers", and relying on the art resources of 22 Art Institute Street and Today Art Museum, we will introduce college students in the district to set up a pavilion volunteer service team. Regular art salons are opened to create a good cultural atmosphere for the youth in the area. Second, centering on "building a pioneer gathering place for youth public welfare organizations", inject the "Sunshine Partner" plan of the Chaoyang Communist Youth League, divert the resources of public welfare organizations and social organizations to the community youth gathering, and through the establishment of incubation, cultivation and communication platform, improve the ability of social organizations to participate in community governance. Third, centering on "building a pioneer gathering place for youth community governance", establish a contact mechanism with the Kowloon community, implement the pilot deployment of the youth community action of the Central Communist Youth League, and guide young groups to participate in community governance. Wang Hongtao, deputy secretary of pku, said the communist youth league to rely on youth position platform to expand youth ideas lead the work channel in the new era, around the characteristics and needs of chaoyang youth, focusing on youth thought lead, youth participation in community governance, emerging youth groups contact services, workplace youth growth, intensify the design of youth activities, enhance the vitality of youth league, promote the construction of the youth service system. Under the guidance of the party building in the youth Hui construction, the local Party committee should attach importance to the socialized resource raising mechanism under the support and multi-party coordination, and explore the formation of a "replicable and popularizable Shuangjing Youth Hui store model room".

2021-09-03	Rizhao: "hand in hand zebra crossing" love care warm heart guard sunshine: "hand in hand zebra crossing" love care warm heart guard wei correspondent Xiang Changhui) recently, Rizhao volunteer workers federation, Rizhao hand in hand volunteers association volunteers to assist the traffic police to maintain the campus surrounding road traffic order, to ensure the safety of students into the campus. The nursing teams of 12 schools of Rizhao Compulsory Workers Federation have all started to start their posts, sending more than 400 volunteers. Rizhao city hand in hand volunteer association "hand in hand zebra crossing" action in the city of 16 schools, Lanshan District 5 schools to carry out nursing activities, dispatched more than 450 volunteers. Since its launch, volunteers have been working on the road of nursing posts, regardless of wind, rain, cold and heat. With their fist and love, they support a safe sky for the children, and practice the volunteer service spirit of "dedication, friendship, mutual assistance and progress" with practical actions.

2021-09-02	Fuzhou to port forward to attract four thousand students to Fuzhou to attract four thousand students study reporter Tian Hongwei reporter Chen Qiang correspondent Xie YuShu) on August 31, to attend the "good years in Fuzhou" study activities, Anhui university students from east China university of science and technology dragon is leaving Fuzhou, return to Shanghai, began a sophomore study life. He said: " This activity made me realize the unique location advantages of Fuzhou and feel the sincerity of Fuzhou thirst for talents."By the communist youth league of Fuzhou municipal party committee joint departments to carry out the" good years in Fuzhou " study activities, in local social practice, visiting social practice, line travel, provide 7646 social practice jobs and more than 20 visit route, set up more than 50 research theme, from 506 of colleges and universities at home and abroad of 4213 young students to participate in the practice. On June 25, Lin Baojin, member of the Standing Committee of Fujian Provincial Party Committee and secretary of Fuzhou Municipal Party Committee, presented the flag to the event, and issued a sincere invitation to the young students: " If you are ideal, Fuzhou will achieve you attentively. If you choose to take root, Fuzhou will not live up to you!"Ding Ye, a doctoral student from Tongji University from Fuzhou, took an internship in Fuzhou Municipal Committee of the Youth League this summer and participated in the specific implementation of the study tour. She said that in order to run the activity well, the leaders of the Communist Youth League Municipal Committee specially solicited opinions from the interns who participated in the preparation of the activity, so that everyone could think about how to provide a better study tour experience for the students and meet the practical needs of the students."Fuzhou is not only beautiful, but also more enthusiastic."Cheng Yuheng, a master's student from Renmin University of China in Jiangxi province, was full of praise for the trip." The organizer provided free dormitory sharing and transportation subsidies in the university town, and prepared bags with epidemic prevention materials and Fuzhou cultural and creative study tour. During the investigation, I took the 'Good Time' theme subway and watched the 'Sail for Good Time' theme light show. These study tour theme elements made people feel the enthusiasm of Fuzhou from the bottom of their hearts."In order to enhance the communication between young students, deepen the understanding of Fuzhou, Fuzhou municipal party committee also planned" sail good time "," teacher say good time "," tea "," enjoy "and other 16 supporting activities, invited" July 1 " medal winner Lin Dan old model, veteran cadres, the whole party and young students communicate with. The person in charge of Fuzhou Municipal Party Committee said that 227 study tour logs and 299 research reports were collected. The selected excellent research report will be submitted to the leaders of Fuzhou city for reference and submitted to the relevant departments to promote the transformation of practical results. The Communist Youth League Municipal Committee also deeply integrated the activity with the "Furong 500" and college student internship "Sailing Plan", covering the study tour groups to the prospective of college students who graduated from high school, included the interns from Fuzhou into the young talent information database, and established a tracking service mechanism to do a good job in the reserve of young talent resources.

2021-09-07	Linyi city hope project interpreta dream action grant ceremony held in Linyi city 2021 hope project interpreta dream action grant ceremony held wei correspondent Guo Jingjing) recently, Linyi, Shandong province in 2021 hope project interpreta dream action grant ceremony held in east four conference room, Linyi government, municipal party committee propaganda department, straight committee, city bureau of education, pku, Linyi silver protects inspect branch and donate love unit representative, counties youth corps committee, high-tech industry development zone committee, straight committee, interpreta dream action student representatives, about more than 80 people to attend the meeting. At the ceremony, the participating leaders gave 4,000 yuan of grants to each of the 8 student representatives, awarded honorary plaques to the representatives of the caring units, and the representatives of the caring units and the representatives of the aided students made speeches respectively. In this year's dream realization action, the county and district youth league organizations and joint volunteers to visit students' families, widely launched caring enterprises, caring people to actively participate in the donation, forming the city of a game of work force, the city raised a total of 2.651 million yuan of grants, 642 students to realize the dream university.

2021-09-03	Haikou city "coconut green" rural youth entrepreneurship through train into xiuying district Haikou city "coconut green" rural youth entrepreneurship through train activities into xiuying district long stream, west show, sea show three town correspondent Zhou Yutong) on August 30 solstice on September 1,2021, Haikou city "coconut green" rural youth entrepreneurship through train into xiuying district long flow town, west show town, sea show town. Nearly 200 people attended the activity, including members of the Municipal Youth Federation, members of the city leaders of rural revitalization work in Zuntan Town, young entrepreneurs, entrepreneurship mentors and agricultural experts. Towns held "face-to-face" exchange meetings with Allies of the Rural Revitalization Youth Alliance. Correspondent for figure activities in the form of "Allies face to face" meeting, the scene around the towns of the local youth entrepreneurship plugging and difficult problems, focusing on entrepreneurial thinking is not open, narrow sales channels, insufficient promotion, and the scene to collect local entrepreneurial youth in entrepreneurial needs, hope, think, for the next step as a whole township resources power rural revitalization of the work preparation. The event also awarded the Changliu Town, Xixiu Town and Haixiu Town Youth League. He was awarded prizes to the "Changliu Town Rural Revitalization Youth League", "Xixiu Town Rural Revitalization Youth League" and "Haixiu Town Rural Revitalization Youth League". At the end of the activity, agricultural technology experts and other professional mentors and volunteers also went to Changliu Town, Xixiu Town and Haixiu Town to examine their industrial development, provide technical guidance, industrial financing, sales resources docking and other entrepreneurial services, to solve the practical problems encountered in the production and operation of rural youth. At the same time, invite the relevant planning team to conduct packaging planning for its product publicity. Through train team visited Changliu Town, Xixiu Town, Haixiu Town project base. Since this year, Haikou City "Coconut Qing" rural youth entrepreneurship through train activities have carried out 13 times, into 10 towns, services covering the public and rural entrepreneurship youth more than 700 people, field investigation of 17 industrial projects, effectively serve the rural entrepreneurship youth. The activity is jointly organized by Haikou Municipal Committee of the Communist Youth League, Haikou Municipal Office of Cyberspace and Municipal Youth Federation, and organized by Haikou Rural Youth Rich Leaders Association.

2021-09-03	Dongying evaluated integratedly youth committee than show communication Dongying city held "youth challenge" town street youth committee than show communication Beijing on September 3 (reporter zhang, correspondent Yang Ze) to further implement the new situation of grassroots party construction work requirements, earnestly implement the "city evaluated integratedly" deployment arrangement, fully stimulate grassroots youth work vitality, recently, the group of Dongying city committee in Dongying city youth home youth committee than show communication activities. The city's 39 town and street youth working committee through the form of PPT road show, from the youth working committee self-construction, the implementation of key work, the town and street brand construction and work innovation, introduced the work situation since its establishment in July last year. Wang Haiming, deputy secretary of the Communist Youth League Municipal Committee, summarized and commented on the work of the youth working committee of each town and street. After a day of challenge competition, 17 town and street youth working committees were praised as "2020-2021 Excellent Town and street Youth Working Committee". The meeting stressed the need to focus on resource integration. All county and district youth league committees should take the reform of the grass-roots organizations of the county Communist Youth League as an opportunity to innovate the integration and allocation of the league resources, further extend all kinds of resources to the town and street youth league organizations, and realize organizational joint construction, resource sharing, position sharing and work linkage. We should pay close attention to institutional building. Through the establishment and improvement of the assessment and evaluation mechanism, regular research mechanism, dynamic adjustment mechanism of members, and contact mechanism of members, the work responsibility of the town and street Youth Working Committee is further compacted to strengthen the role of the Youth Working Committee and brand creation. We must effectively give full play to our role. Outstanding lead condensed youth, service center, strengthen the function of its own construction, give play to the youth committee organization advantage, resource advantage and strength advantage, clear "resources list", clear youth "demand list", consultation form "project list", strive to solve the problems facing the youth and the demand, make the youth work into the center, the service general situation, to the youth committee with good use. Next, pku will earnestly play a good role of street youth committee platform and mechanism, accelerate the grassroots youth work organization diversification, survival socialization, work project, flat, running backbone diversification, realize the reform measures of the communist youth league, in the grassroots ground work, further strengthen the basic foundation, efforts to create a new situation.

2021-09-02	Rizhao more measures and ensure that "hope" construction service double promotion Rizhao more measures and to ensure that "hope" construction service double promotion wei) this year, group of Shandong Rizhao committee will "hope hut" children care project as "I do the practical work for the masses" concrete gripper, promoting "hope" children care project, baseline, strict, optimal service, to the "hut" built in the most need children heart, realize the construction service double promotion. The youth league (work) committees, focusing on disabled people, disabled families and poor families, have scientifically identified 200 new cabins through three steps: household survey, field investigation, analysis and judgment. Construction of cities and counties through wall chart operations, the establishment of ledger, log records, the work to promote a clear situation, clear base, high efficiency. Organize and distribute the "hope cabin" construction paper to ensure the standardization of "hope cabin" construction and standardization. The implementation of "daily promotion, weekly scheduling, monthly summary", strict project process, safety clearance, acceptance clearance. Formulate "one household, one plan", fully discuss and determine the plan, drawings, budget and other details. Pictured is the 'House of Hope' photo. After the completion of the three-level acceptance policy, the township Youth League Committee, the district and county youth League (work) committee and the Communist Youth League Committee are implemented to ensure safety and quality clearance. Fund raising offline, and actively strive for the relevant municipal departments to support funds of 300,000 yuan. Promote enterprises and institutions, caring enterprises and people to raise money or claim cabins. To promote and promote the "Hope House" project in large local supermarkets, and reached 16 "consumption donation" cooperation, with 83 participating merchants, including 2 rural youth e-commerce households. Actively carried out the "Red Scarf to Build the Hope House" charity sale, donating a total of 273,000 yuan. Online, the new media matrix is used to do a good job in publicity and promotion, and relying on the Internet to actively carry out fundraising activities. The amount of funds raised by the city's four districts and counties all ranks among the top 50 in the province. The picture shows the volunteer service team providing services to the cabin children. Tuanrizhao municipal party committee for figure volunteer service hut comprehensive evaluation mechanism, formulate and unified post health management system, regular organization health appraisal, hut works, "hut star" appraisal activities, by encouraging praise, education guide teenagers to develop good habits, cultivate children self-reliance, optimistic attitude towards life. According to the characteristics of the volunteers, many volunteer service teams, such as "grass partners", caring mothers and industry departments, were set up to provide tracking services, such as schoolwork guidance, artistic enlightenment, urban and rural teenagers hand in hand, and love care, to meet the personalized needs of the assisted children.

2021-09-02	Liaocheng city to deepen the reform of the county communist youth league grassroots organization work "pioneer" for deepening the county communist youth league grassroots organization reform "pioneer" wei correspondent Han Xiao) group Shandong Liaocheng municipal party committee thoroughly implement the central guidance on the expanding of the county communist youth league grassroots organization reform and the Shandong provincial party committee about work requirements, with "three cattle spirit" as the guide, actively explore building reform "four dimensional stage", to sing the county communist youth league grassroots organization reform comprehensive "highlight" to lay the first foundation. We will continue to share the signals of all-round reform. According to the characteristics and needs of regional youth, guide the reform pilot Dongchangfu District to formulate detailed reform measures, list special list, locate the responsibility subject, set up special class to promote, ensure the effectiveness of the Communist Youth League reform, guide the counties (cities, districts) to participate in and seek breakthroughs through WeChat group sharing, telephone communication and field research. We will build a support system for supporting the Party and the Communist Youth League. Joint municipal party committee organization department issued on the implementation of the provincial party committee organization department provincial party committee <about further strengthening the grassroots party building work under the new situation several measures> implementation plan ", the content into the comprehensive governing party assessment system and county party committee supervision content, and various counties (city, area) party comprehensive communication, the grassroots party with league building work assessment index into the assessment content of party organizations at all levels, points accounted for not less than 5%. Establish a mechanism for young cadres to participate in politics from a wide perspective. The party committees of counties (city and district) always insist to study the work of the Communist Youth League regularly, listen to the work report of the head of the league organization at least once a year, study and solve outstanding problems; attend the head of the head of the league at the city and county level, and improve the participation and political quality of the city and county level. We will broaden the growth path of grassroots youth league cadres. Municipal party committee organization issued a notice, from grassroots selected eight "90" outstanding young cadres to pku authority credentials the exercise, adhere to the "equal treatment with cadres standard, daily assessment" of "double" principle, start "1 + 334" cadres echelon project, further improve the quality of the credentials the cadre matching, effectively strengthen the power, broaden the source of cadres.

2021-09-01	Jining city held the city's youth anti-drug knowledge contest Jining held youth anti-drug knowledge competition for figure China youth network Beijing on September 1 (reporter Zhang Jianwei, correspondent BaiChen) recently, in order to further strengthen the youth drug prevention publicity and education work, select outstanding students in the province youth anti-drug knowledge contest, Jining drug office, city bureau of education, pku jointly organized by "healthy life, green non-toxic" as the theme of the city's youth anti-drug knowledge contest. Executive deputy director of the Municipal Narcotics Control Office, the Municipal Public Security Bureau anti-drug control detachment leader Zhou Zhonghua attended the event and delivered a speech. The competition is divided into two rounds of promotion and finals. A total of 15 teams from 14 counties and municipal schools are divided into three groups according to the order of drawing lots, and the top two teams of each group a total of 6 teams entered the final. The contents of the competition include China's anti-drug policies, anti-drug laws and regulations, anti-drug history, drug knowledge, drug harm and drug prevention knowledge. The three answer links of "each show", "first attack", "and" final sound " were tense and interesting, and the wonderful performance of the contestants won the applause of the audience. The picture shows the contestants answering the questions in the anti-drug knowledge competition. After four rounds of fierce competition, the economic development district team finally won the first prize, Wenshang, Liangshan team won the second prize, Jinxiang, Qufu, municipal team won the third prize. By Jining city drug control office, city education bureau, municipal Youth League committee, Jining children's vocal music association of 6 experts composed of judges, from the competition selected 6 outstanding seed players, after training will represent Jining city to participate in the province's youth anti-drug knowledge competition.

2021-08-31	Sunshine "the communist youth league grass school" love service deeply rooted in the hearts of the people sunshine "the communist youth league grass school" love service deeply rooted in figure China youth network Beijing on August 31 (xinhua Zhang Jianwei) "the communist youth league grass school" is by Shandong Rizhao group in 2011, innovation implementation of care for rural left-behind children, help poverty of crucial public welfare projects. Through school-local cooperation, college students are recruited to carry out free "colorful courses" such as civilized etiquette, safety and self-protection, red theme education, traditional culture, sports, handicraft, and young Pioneers class every summer. The project has won the gold medal of the first China Youth Volunteer Service Project Competition, the national "Four 100" best volunteer service projects, the National Youth Volunteer Service Excellent Project, the first outstanding volunteer service projects in poverty alleviation and other honors. In the past 10 years, the "Little Grass School" has grown from 0 to 1, and then now blossomed everywhere, which has condensed the painstaking efforts of youth league organizations at all levels and groups of volunteers. By 2021, the city has built 403 "Communist Youth League Grass schools", covering 61 towns (streets) and more than 400 villages, serving more than 22,000 children, and more than 3,000 college student volunteers writing stories about youth and love. In 2009, the Youth League Committee of Jufeng Town, Lanshan District, explored the establishment of a "love classroom" to care and help the left-behind children during the holidays. After the municipal and district youth League committees found this model, they timely summarized and improved it, standardized the establishment of the "small grass school", and gradually replicated and promoted it in the whole city. With the care mode of "youth League organization + volunteers + rural left-behind children", the Communist Youth League explored a new path for the Communist Youth League to undertake the government youth affairs and serve the young people's livelihood. In 2021, relying on the New Era Civilization Practice Center, the city will build 30 "Communist Youth League Little Grass Schools" and carry out the "Little Grass School" summer student care service, recruiting 160 volunteers and more than 800 students. Moving forward steadily, in helping the rural revitalization play the role of the powerhouse and commandos dilemma children in the school found the warmth of campus, home warmth, the heart got care and comfort, college students volunteer through teaching, the national conditions, people, society have a deeper understanding, thus more determined in the grassroots line after graduation, meritorious rural revitalization of the courage and faith. The league has organized "one-to-one" pair assistance to the school through the "Hope Project", "Dream Realization Action", "Hand in Hand Care Action" and other projects. Youth league organizations at all levels have also taken schools as the carrier to strengthen the coordination of work resources of grass-roots Party and league organizations, and built poverty alleviation projects such as "youth homes" and "good home". Every year, the volunteers use their own practical actions to encourage, guide and lead more people to join the volunteer team, and extensively participate in large-scale competitions, civilized guidance, poverty alleviation and disability assistance, environmental protection and other volunteer services. As a "grass school" volunteer said, " flowers bloom a season, but leave me all my life. I will go on on the volunteer road with such joy and emotion."At present, the municipal and county-level youth League committees have established fixed contacts with Qufu Normal University, Shandong University of Science and Technology and other universities through university-local cooperation. In addition, also attracted the Shandong normal university, Shandong university, ocean university of China, southwest university, and other provincial universities volunteer service team involved in summer social practice, volunteer recruitment, selection, training and organization, at the same time, to absorb public inspection department, health, fire and other industries volunteers and youth affairs social workers, all kinds of social volunteer organizations.beginner's mind remains the same, the old tree flowers in the name of the grass in the road of love this year, pku joint municipal government, education department, will "the communist youth league grass school" and summer student hosting service work, help alleviate summer pupils "care" problem, a total of 30 "the communist youth league grass school" to carry out the summer student hosting service. Before the opening of the school, I inspected the hardware environment and enrollment situation of "Little Grass School", and polished the golden brand of "Little Grass School" that reassured parents, volunteers and children.

2021-08-31	Liaocheng rural revitalization of good youth pioneer special training in Liaocheng city rural revitalization of good youth pioneer special training wei correspondent Han Xiao) on August 27, by the group of Shandong Liaocheng municipal party committee, Liaocheng agriculture and rural bureau of Liaocheng city in 2021 high-quality farmers advanced training agricultural products electricity class (rural revitalization of good youth pioneer workshops) in Liaocheng rural practical talent training college, pku, city agriculture and chiping district relevant units responsible for the comrades to attend the opening ceremony. More than 90 rural good youth under 45 years old, rural entrepreneurial youth and high-quality new farmers from Dongchangfu District and Linqing City have carried out a 7-day training. This training course is carried out in the mode of training camp, focusing on the live sales of rural e-commerce, cultivating a number of key rural youth e-commerce by means of teachers' teaching and guidance, practical operation practice, field visits and exchanges, and promote and expand the channels for farmers to start and increase their income.

2021-08-31	Linyi: let the red gene into youth blood Linyi: let the red gene into youth blood jianwei correspondent Du Erqi) in November 2013, xi jinping visited Shandong Linyi pointed out that the river bank Mongolia spirit and Yan'an spirit, jinggangshan spirit, xibaipo spirit, is the precious spiritual wealth of the party and the country, to carry forward the new era conditions. In recent years, the League Linyi Municipal Party Committee has thoroughly implemented the important instructions of General Secretary Xi Jinping, seized the opportunity of carrying out party history learning and education, guided the youth to draw strength in inheriting the red gene and carrying forward the Yimeng spirit, and unswervingly listen to the Party and follow the Party. Let the red landmark become the youth "immersive" classroom Menglianggu Campaign Memorial Hall, Daqingshan Victory Breakthrough Memorial Hall, East China Field Army Memorial Hall and other "Red Landmarks" have witnessed the difficulties and turns of the revolutionary ancestors, indomitable search process, is the tangible position for young people to carry out patriotic education. From the three dimensions of "intuitive, situational and experiential", TuanLinyi Municipal Party Committee carries out the concrete and warm immersive education of Yimeng spirit in the way that teenagers can understand, see and experience. Intuitive onsite teaching. More than 200,000 school team cadres and young pioneers have been organized to visit and study in the party spirit education bases, patriotism and revolutionary tradition education bases. Carry out the experience activities of "telling red stories" and "telling Yimeng together with small hands", and organize "little red narrator" to carry out volunteer publicity activities at the old site of the East China Field Army on weekends, winter and summer vacations, so as to attract parents to participate in it. A total of 9 seasons of summer research activities of "taking the university with the history of my hometown" have been carried out, so that prospective college students can understand their hometown in an all-round way and bring Yimeng spirit to all over the world. Scenario-type interactive participation. More than 600 practical activities with the theme of "Youth heart contributes to the Party in the New Era" and "Growing up under the National flag" were carried out. Through micro-group classes and red stories, young people can receive the baptism of Yimeng spirit. Nearly 1,000 "red scarf guides" were selected and trained to perform red melodramas such as "Wang Er Primary School" and "Hero Chen Ruoke" from time to time, and educated 300,000 young pioneers. On March 8 this year, the city's 1600 primary and secondary school young pioneers to carry out the "salute to the heroes, learn from the example" new semester concentrated flag-raising ceremony, 1.4 million young pioneers, 30,000 young pioneers counselors, with a flag, sing a song. Experiential depth perception. Relying on the party spirit education base, red tourist attractions, etc., continue to promote red education field experience, organize teenagers to participate in rolling rice, grinding, flipping pancakes, insoles, transport rations experience before support labor, by retrace road, fire bridge comprehension river bank Mongolia spirit, let teenagers "line know" "play" middle school "do grind", let the red gene, revolutionary generations. Let red preach teenagers "diversified" teaching material in recent years, group Linyi city close to different youth group characteristics, adhere to the "online" and "offline" combination, "go out" and "please come in", with distinctive forms of preaching, release red preach the biggest transmission effect, let the river bank Mongolia spirit infiltration young mind. Stratified focus, to build a hierarchical and integrated youth education system. For example, strengthen the systematic training of young students, relying on the "green horse project learning pioneer" training program, for the young pioneers, through "learning, listening, speaking, competition", established the red scarf tour group 7, covering 560,000 young pioneers, nearly 10 young pioneers to participate in the "study party history to Yimeng", "red Yimeng heart to the party" red propaganda contest, etc. Cloud online talk, to create an online and offline youth thought to lead the "concentric circles". Give full play to the advantages of new media, integrating and connecting the use of resources such as wechat and Weibo, carry out the "Yimeng Little commentator" style display activity, attracting 263,000 young pioneers to participate online, and the total video clicks reached more than 40 million. Set up "My Hometown Yimeng Mountain" and "FM Sound Red Story" columns, organized more than 13 million youth league members to participate in more than 50 "Youth Learning" online theme group classes. Outside the city extension, promote Yimeng spirit to go out. Adopt the mode of "red publicity + talent and wisdom + achievement transformation", and carry out "Yimeng spirit into universities". More than 400 "Yimeng Spirit 100 Schools volunteer publicity groups" have been established, which has visited more than 70 cities in more than 10 provinces, and has conducted more than 400 lectures in more than 300 universities such as Nankai University and Fudan University, covering nearly 100,000 young students. Advanced model speak, give play the leading effect of advanced youth typical. Insist on educating the people around us, select a group of young people who inherit the Yimeng spirit and practice the socialist core values every year, carry out face-to-face, small-scale and interactive communication with vivid examples, and guide the young people to take the example as the benchmark and consciously inherit the Yimeng spirit. Let the red gene group green work "engine" kinetic energy as the bridge and the link of the youth, Linyi youth league organizations at all levels to lead the river bank Mongolia youth always holding the party banner, consciously into Linyi development, in high quality development, social construction of the first line, service the masses forefront created outstanding youth performance. We will make new progress in integrating it into rural revitalization. With "the village has good youth" selected culture for the gripper, build "optimization, cultivation, development" chain selected culture mechanism of country, the country good youth training plan into the city's key project training theme shift, set up and relying on the municipal good youth league accumulated view, training activities, "river bank Mongolia country good youth credit support plan" has been 2004 good youth loans of 450 million yuan. High-quality service development when the pioneer. Give full play to the unique advantages of active thought, new knowledge and strong creativity, show their wisdom and wisdom in industrial cultivation, scientific and technological creation, and system innovation, and carry out innovative youth work safety activities of "check hidden dangers, help training, help rectification, help promotion and help development". The "2021 Registered Safety Engineer Public Welfare Training" covers nearly 1,000 young people, and 18 provincial youth civilization collectives and 5 national youth civilization collectives have emerged. Based on the contribution to the job responsibilities. To promote the work of "Green Bird Plan only gathered in Yimeng", the cities and counties have jointly collected more than 1,500 winter and summer vacation internships, served more than 2,000 college students, and built 18 "youth post stations" with complete hardware facilities, water, electricity and heating supply, and convenient transportation.

2021-08-31	Zibo City Youth Federation "three efforts" to carry out the party history learning and education meeting site. Beijing, August 31 (reporter Zhang Jianwei, correspondent, Liu Wencai Zhou Hongliang) launched 12 "youth learning society-members on the Party history" online micro party class; held "green reading" reading sharing meeting; organized youth league members to participate in the "hope cabin children care action"... Zibo City, Shandong Province closely around the history theory, history credit, history Chongde, theory and practice education, leading the city members of the party history learning education "three efforts", continue to promote the party history learning education go deep and solid. The picture shows the members of the Zibo City Youth Federation visiting the Party History Hall. Correspondent for the map focus on form innovation, promote the party history study "speak up". Give full play to the role of youth league members to promote learning, launched 12 "youth learning club-members speak party history" online micro party class, relying on WeChat public number show different youth league members youth, share party history learning comprehension; six members go grassroots party history activities, schools, rural, community, and other grassroots units, through opera performances, poetry recitation, music, drama tour, drive the youth to understand the glorious history of the party, enhance the feelings and trust of the party. Focus on extensive participation, to promote the study of party history "move". Adopt the mode of "theoretical learning + practical teaching", highlight the vividness and flexibility, and create a "classroom around" for party history education. Hold "green reading" party history reading sharing meeting; organize visit Zibo history exhibition hall, "hundred light-party leadership China (Zibo) industry century" theme pavilion, Beihai Bank underground money printing office and other red revolution venues, immersive party history learning education; relying on the city green book association, green art association, green photo association collected nearly 300 works for young art lovers, held "dream new era of youth" calligraphy art photography network exhibition 6. The picture shows a collection of calligraphy and photography of "Dream of youth in the new era". Correspondent for the map to focus on working for the people, to promote the party history study and education "real". The City Youth Federation carefully planned, multi-point force, comprehensive promotion, The "I do practical things for the youth" practical activities as an important content of the party history study and education, Organize the members of the Youth Federation to participate in the "Hope House Child Care Action", Actively helping teenagers and disadvantaged and vulnerable groups; Implementing the "Youth Federation Serving a Thousand Villages" plan, Help to revitalize rural industries, It has set up rural rural professional service teams such as "science and technology", "beautiful countryside", "rural planning and construction", "and e-commerce to help agriculture", A total of 23 youth League members were paired up with eight villages, Provide agricultural technical guidance, rural beautification and greening, e-commerce business training and other services; Carry out "rural good youth" pair to help activities, Members of the Youth Federation in promoting agriculture, finance, commerce, law and other related fields, Regular face-to-face communication activities with good rural youth, Provide effective support in policy consultation, industrial cooperation, technical guidance, learning and training, To continuously deepen the mechanism for maintaining close ties with the youth, Help young people to solve the urgent and anxious practical things.

2021-08-31	Hunan Liuyang more than 70 "hope hut" delivered a table a chair hope more than 70 "hope hut" delivery before school construction) wall renovation, wooden bed wardrobe, textbooks are stacked neatly, through the sun through the blue-gray curtain gap pair of college students volunteers donated handmade gifts... four months ago, here was a dim and debris room, has been transformed into "hope hut", for liuyang, 10 years old small trace town, xin (pseudonym), " is like in a dream, Never thought I could have a room that beautiful ". Xiao Xin, who was remarried by her mother and was huddled in a messy room with her grandmother and brother, is the recipient of the 2021 "Hope House" youth care project of the Liuyang Municipal Party Committee in Hunan Province. On August 31, at the beginning of the semester this autumn, caring people sent furniture, books and other materials to install, which means that the "Hope House" was officially built, and also marks the successful completion of the more than 70 "Hope House" youth care project in 2021. In recent years, with the improvement of poverty alleviation policies, family conditions like Xiaoxin have improved, but their desire to have an independent learning and living space is difficult to realize. Therefore, group, liuyang liuyang civilization do in 2020 in the city launched "hope hut" care project, built 30 hut, in 2020 in 2021, improve the difficult reading teenagers living and learning environment, let them live with dignity, learning confidence, the future hope, this year to "hope hut" project into "I do the practical work for the youth" brand project. Xiao Ping (pseudonym), 12 years old, is ill and her mother is leaving home in Puji Town, Liuyang City. Her two children live in a house without any interval with their grandparents respectively. Having a separate room is the little girl's mind."I have a bed of my own, and a very big desk, I am very grateful to help my uncle and aunt, I will study hard, live up to their expectations..." Xiao Ping wrote in her diary. From 30 households last year, more than 70 households were renovated this year. Behind the doubling of the data reflects the original mission of "doing practical things for the youth", and also tests the wisdom of the overall arrangement of the Communist Youth League committee, the spirit of the youth league cadres to fight again and the temperature of this loving city. In April this year, the "hope cabin" project was just arranged, the village (community) youth league organization immediately found out the situation, truthfully declared. In June this year, the Communist Youth League and volunteers visited field visits. In the face of the pressure of transformation funds, the method of the Communist Youth League committee is to concentrate on doing great things. The Communist Youth League Municipal Committee is responsible for collecting soft decoration materials such as love furniture and home textiles; the Municipal Civilization Office is responsible for collecting schoolbags, stationery, tutoring books; the township and street youth league (work) committee is responsible for the hardware renovation and construction supervision of the local hut."A set of furniture is worth 6,000 yuan, and the Liuyang Furniture Industry Chamber of Commerce raised 45 sets of furniture online."" Xinhua Bookstore provides 70 children with charity books such as "The Four Great Classics" and "100,000 Reasons"."" alarm clock, desk lamp and other children to read the necessary supplies, the city civilization office contributed to the whole process. The "" cabin acceptance is qualified, rest assured to move in."The municipal Housing Authority staff obligation to carry out the cabin renovation quality and environmental testing. During the four months of......, many caring units and people in Liuyang have donated generously, and more than 70 "hope cabins" have taken on a new look. During this summer vacation, a dozen children in Liuyang village prevent drowning charity lessons. In Yongan "half mu square pond" activity room, the town Youth League committee organized 14 left-behind students to carry out the "childlike innocence to the Party" paper-cutting activity... this is a summer collective support activity organized by local volunteers."The 'Hope House' project is a public welfare brand of Liuyang that cares for teenagers and gathers love. It is a demonstration project from 'single' student aid to 'comprehensive development' and from 'universal benefit' to 'precision'. In the past two years, it has achieved remarkable results and won a lot of attention."Zhang Yuqi, party secretary of Liuyang, said," We will continue to write the 'second half of the' article 'of accompanying and helping, so as to realize the magnificent transformation from' cabin renewal 'to' spiritual renewal '."According to the principle of territoriality, the Communist Youth League Municipal Committee guided Liuyang Volunteer Federation and other public welfare organizations to provide" one to one "volunteers nearby, combined with the summer social practice activities of" Yan Return to Home " college students, to carry out services such as family companionship, academic guidance, micro-wish achievement and so on, so as to ensure the tracking and management in the later stage."For the child's situation, what the child needs, and what kind of services we provide. Can not clean, we will come to teach hand in hand; need learning guidance, we will jointly return home college students to carry out activities."City volunteers union volunteer sister Zhang said."Good rain knows the season, when spring happens."In the" Hope House ", each recipient child received a calligraphy gift from a pair of" Yan Return to the Home " college students volunteers, encouraging them to be hopeful and grow up. It is reported that the "Hope House" project is a continuous livelihood project, which plans to improve the existing family learning environment of poor teenagers in Liuyang over three years. It is expected that by 2022,150 cabins will be built, benefiting more than 240 young people. Volunteer follow-up service will grasp as the second half of the project, realize teenagers "1 + 5 + X" support measures, namely for difficult teenagers to build a high quality learning, living environment, provide warm heart company, academic counseling, psychological counseling, habits, micro wish to achieve five aspects of tracking service, meet the needs of each child's several personalized, make long-term support system to the ground.
