# Supplementary material for: Serving organization goals by organizational information dissemination: An empirical study from the Communist Youth League of China
Source: PLoS One. 2023 Jan 20;18(1):e0280221. doi: 10.1371/journal.pone.0280221 (PMC9858461; doi:10.1371/journal.pone.0280221)
Supplement: S1 Data — (ZIP) [file pone.0280221.s001.zip › Supporting Data/Version of Chinese to English/County Committee(English).docx]

2022-06-29 Group of Hunan Yongzhou ningyuan county to carry out the village household against fraud propaganda group in Hunan ningyuan county to carry out the village household against fraud propaganda jianwei correspondent Tao Shiqi) for the current telecom network fraud cases, June 27 morning, group of Hunan Yongzhou ningyuan county staff and volunteers into the village household face-to-face communication with the masses, popularize knowledge of preventing telecom network fraud. The picture shows entering the village to publicize the knowledge of preventing telecom network fraud. In the process of household publicity, the community staff to the villagers to publicize the national anti-fraud App, issued anti-fraud leaflets, to take the form of a case statement to remind the residents to remain vigilant, improve the awareness of fraud prevention. The activity aims to enhance the awareness and level of the villagers to actively prevent telecom network fraud through the household anti-fraud propaganda. The Communist Youth League County Party Committee will continue to go deep into the masses, do a good job of publicity, let the knowledge of fraud prevention deeply rooted in the hearts of the people.

2022-06-27 Li Jianping) On June 25, the friendship exchange activity between Chinese and Tibetan youth in Derong County, Sichuan Province and Tonglu County, Zhejiang Province was launched in the Youth Palace of Tonglu County. The exchange activity lasts for one week. Deze Renma, party secretary of TuanDerong County, said that Tonglu County and Derong County became attached due to the construction assistance, which is the second time for the county to come to Tonglu to carry out exchange activities. The young representatives of Derong will visit the Tonglu County Urban Planning Center, Ye Qianyu Memorial Hall, the Equestrian Center and other venues to carry out research activities, and experience the intangible cultural heritage paper cutting and seal cutting art activities."We hope that through these activities, the Derong young people can fully feel the cultural history and traditional culture of Tonglu, and appreciate the charm of Jiangnan water towns, so as to further strengthen the friendship between Derong and Tonglu youth, and lay a solid foundation for the cooperation, exchange, joint construction and sharing of the Communist Youth League of the two places."Said Xu Yuanjun, Party secretary of Tuan Tonglu County. Derong County of Sichuan Province and Tonglu County of Zhejiang Province carried out friendship exchange activities between Chinese and Tibetan teenagers. Photo is provided by the Publicity Department of Tonglu County Party Committee

2022-06-25 Anhui linquan: dream college volunteers to carry out calligraphy public class Anhui linquan: "dream college" into the primary school volunteers to carry out the hard pen calligraphy public class Wang Haihan) on June 23, Anhui linquan new era civilization practice center, the communist youth league linquan county party committee joint jingu primary school to carry out the "welcome twenty big, calligraphy ode DangEn" calligraphy writing activities. Qin Xiang, the initiator of the "Dream College" project and a volunteer in rural calligraphy teaching for 26 years, explained the requirements and rules for writing the lyrics of "No Communist Party, No New China". The 39 young Pioneers wrote the Chinese characters with one stroke, and after 30 minutes, they handed in the clean and standard pen calligraphy works to the judges. In the following brief evaluation, awards and experience sharing, the team members spoke freely. Chai Xinyue, a student of the school four (1) Squadron, said, " I learned to sing this song when I was a child. I feel very proud when I write the lyrics today! It is reported that the "Dream College" is a volunteer service provided by the youth league organizations at all levels in Fuyang City, Anhui Province, for rural schools, left-behind children and other groups. The picture shows a group photo of teachers and students from linquan County Party Committee

2022-06-25 The Wuzhong District of Suzhou, Jiangsu Province, held a story sharing meeting for the winners of the May 4th Medal. Conference, the first "Suzhou youth May 4th medal" nomination winner, Jiangsu MaiXinLin aviation science and technology co., LTD., party secretary, chairman, district part-time vice secretary zhang around the "entrepreneurship with the party" theme, about his lead team struggle, make the enterprise in the Shanghai stock exchange kechuang board listed, growth into the Suzhou aerospace industry first struggle. At the event, Wuzhong District Committee also held the opening ceremony of 360 degree Youth Home and youth Learning Club. By further focusing on the three functions of "leading youth gathering, organizing and mobilizing youth, connecting and serving youth", further optimizing "sincere, intimate, intimate, warm, comfortable and secure", truly realize zero distance from youth, expand "youth home" coverage and recognition, improve the communist Youth League service contribution.

2022-06-24 Li Jianping) On June 22, Xinchang County and Quzhou City Qujiang District was held in Xinchang County. Four lecturers of Xinchang County "Tianmu New Fire" youth Group vividly preached the deeds of the rich model to the audience. Yu Xinru, the "8090" speaker of Qujiang District, told the good story of the countryside through the cloud connection. As the two areas of Zhejiang Shanhai Cooperation, TuanXinchang County Committee and Qujiang District Committee have actively deployed a series of interactive communication activities such as theoretical study, connected publicity and practical research around the publicity work of the common rich youth, so that the young people of Shanhai can collide with more sparks of wisdom in the exploration and practice of common prosperity. It is reported that Xinchang County "Tianmu new fire" youth publicity group is jointly built by the publicity Department of the County Party Committee, the Youth League County Party Committee, mainly to "8090" and 00 after the youth, has selected and cultivated the backbone of the young speakers 56,7 senior mentors, more than 200 lecturers in various industries. Xinchang County "Tianmu new fire" youth publicity group member Shi Yulu preach "education together rich we are in action". Photo by Wang Haowei

2022-06-22 Xindu district in Chengdu, Sichuan province "sweet" meet intern activities start Chengdu, Sichuan province xindu district "sweet" meet interns start jianwei correspondent Zhao Qinghua) recently, Chengdu, Chengdu district party committee organization department, xindu district party committee club committee, xindu district talent development service center, xindu district committee jointly organized rong floating talent new hui "sweet" meet intern activities, and take this opportunity to establish a "chain in enterprise" -xindu industry youth alliance. Forty-three outstanding college students from 13 universities across the country walked out of the ivory tower and gathered in the city meeting room of Xindu District, Chengdu city, Sichuan Province, to attend the launch ceremony, opening the first half of their career. Photo of the launch ceremony of "Xiang" meeting interns. Correspondent for figure xindu district talent development service center relevant controller introduces, xindu district has all kinds of talents of 338300 people, has from the "talent depression" to "talent highland", is formed with new characteristic talent 2.0, xindu district to speed up the high level technology innovation platform, realize the "five chain fusion", to promote industry circle strong chain to provide solid talent guarantee, let talent in fragrant city, let "rong float" soul in place. At the event site, the three employers signed an agreement with the intern representatives and sent "Talent Card-YOUNG card"; Xindu District Committee and Xindu District Committee of the Communist Youth League signed a contract with Sichuan Sound School to start a new situation; the Organization Department of Xindu District Committee, Chengdu Xindu District Committee, China Gas Turbine Research Institute, Chengdu Engine Co., Ltd., CRRC, China Railway Investment and Construction Company, Ltd., and the Xindu District Industrial Youth Alliance was officially established. Next, TuanOuWei will continue to deepen the sweet meet interns project, build college students and youth league normalized contact platform, continue to linkage in universities and internship units, to help more young college students came to the city, taste sweet city beauty, feeling new, with practical action to the construction of Chengdu metropolitan circle north center to provide solid talent support.

2022-06-20 Kunming Jinning District Committee of Yunnan Province recently issued a work plan to further help small, medium and micro enterprises rescue development. In order to implement the special action of tax reduction and fee reduction, on June 17, the Youth League Kunming Jinning District Committee set up a tax volunteer service team of 76 people, to optimize the business environment, to small, medium and micro enterprises to preach, to help enterprises rescue. In the Implementation Plan of the Ten Special Actions of Yunnan Province to Further Help Small, Small and Medium-sized Enterprises, the special action to cut taxes and fees is to highlight the implementation of the large-scale tax rebate policy, and to reduce or reduce the payment of some taxes and fees, administrative fees and government-managed funds. To this end, volunteer service teams send classes to their homes, provide tailored policy guidance for taxpayers with different types and different needs, accurately release the dividends of tax refund and tax reduction policies, and help taxpayer payers should fully know and enjoy the new combined tax support policy gift package. At present, it has publicized the tax rebate and tax reduction policy for 301 times for the enterprises within the jurisdiction."After the service team came to the enterprise to do publicity, we submitted the materials, the tax was reduced."Said a small and micro business official. At the same time, the volunteer service team also carried out the "cloud problem solving" activity, providing cloud tax-related consultation for taxpayers through the Internet, wechat, telephone, email, etc., timely answering tax-related questions, unimpeded online handling channels, and reducing the number of taxpayers entering the hall.

2022-06-29 Group Maanshan city in Anhui province and county to carry out the "transforming customs ten years old" theme team activities in Anhui county county to carry out the "transforming customs ten years old" theme team activities for China youth network Beijing, June 29 (reporter Zhang Jianwei, reporter Dai Qing check WeiTing) to promote changing customs work, foster local custom civilization, power rural revitalization, recently, the group of Anhui ma Anshan county county central primary school brigade less "transforming customs ten years old" theme team activities."Speak civilization, tree new wind, do not compare, no trouble."At the team meeting, the young Pioneers counselors and teachers combined with the examples around them, briefly introduced the importance of changing customs and the harm brought to the society and the burden of the family, advocating the young pioneers to lead by example, dare to be the first, and strive to be the small pioneer of breaking the old customs. Subsequently, the student representative came to the stage to read out the "change customs ten years old" proposal, calling on everyone to start from themselves, do not do "ten years old", inherit and carry forward the Chinese traditional virtues of thrift, hard work, consciously refuse to blindly compare and extravagance and waste. After the team meeting, the young pioneers have expressed that they should actively publicize and mobilize the friends and elders around them, do not do "ten years old", and do the propagandists and practitioners of changing customs. Next, the county and the county will change social traditions into the campus activities as an opportunity to in the county primary and secondary schools continue to change social traditions propaganda activities, with classic reading, red scarf, essay painting and calligraphy competition forms, guide children to develop thrift, civilization, small widening, with children to influence their parents, to advocate fresh air, "ten years" do not change social traditions concept into the family, into the society.

2022-06-14 Ningxia red temple fort district care for difficult teenagers first micro wish issued Ningxia red temple fort district first "light micro wish" distribution ceremony wei) to further implement the xi general secretary about youth work important thoughts and important discourse about children and young pioneers work, continue to deepen the "I do the practical work for youth" practice, constantly improve the plight children care system, continue to focus on the healthy growth of rural children. On the morning of June 11, the Ningxia Wuzhong city HongSiBao district party committee in the red temple fort town people's government on the second floor conference room care difficult teenagers first "light micro wish" distribution ceremony, ceremony, TuanOuWei propaganda director high dream sweet first to teenagers and parents preach solution "youth since protect drowning prevention" safety education knowledge, from the first aid method, drowning swimming precautions, drowning should be how to save themselves in three aspects, with typical examples warning students, education everyone to cherish life, away from dangerous water. The activity for a total of 16 teenagers on the scene of the "micro wish", due to traffic safety and epidemic prevention and control work and other reasons, the Communist Youth League District Committee will be the "dream micro wish" activity happily extended, the remaining 34 "micro wish" by the township, the love materials to the donated youth who could not be present. Next, TuanOuWei will continue to increase the intensity of resource integration, play to the grassroots of the cohesion and appeal, continue to promote the communist youth league power poverty crucial results, a variety of ways, all-round, multi-channel to promote "light micro wish" activities solid and effective, with love, heart for the growth of teenagers.

2022-06-29 The regiment, Guangdong Shixing county committee held to study and implement xi secretary in the celebration of the 100th anniversary of the founding of the Communist Youth League of China important speech spirit special training class Guangdong Shixing county committee held to study and implement xi secretary in the 100th anniversary of the founding of the Communist Youth League of China at the important speech spirit, Special training course Jianwei correspondent Chen Rui) On June 27th, The Shixing County Party Committee of Shaoguan City, Guangdong Province held a special training course on learning and implementing the important speech spirit of General Secretary Xi Jinping at the 100th anniversary of the founding of the Communist Youth League of China, By Lu Yuqun, vice principal of the Party School of Shixing County Committee of the CPC; Secretary of the Youth League Committee of all townships, organs, enterprises and institutions, and middle schools, More than 80 counselors of the young Pioneers, provincial, municipal and county-level outstanding league cadres and league members attended the training. Scene pictures of the special training class. During the lecture, Lu Yukun from the "brief introduction of the Communist Youth League of China, the historical task changes and contributions of the Communist Youth League in the past hundred years, the basic experience of the Communist Youth League youth movement in the past hundred years, and the youth heart to the party contribution in the new era" four aspects, Comprehensively reviewing the glorious course of the Communist Youth League unswervingly following the Party and striving for the cause of the Party and the people, Reviewing general Secretary Xi Jinping's hopes and requirements for youth league organizations, youth league cadres and youth league members at the conference celebrating the 100th anniversary of the founding of the Communist Youth League of China, To encourage the young people to keep in mind the general Secretary's instructions, Draw wisdom and add strength from history. Scene pictures of the special training class. Correspondent for the next step, TuanCounty ShiXing Wei will continue to study publicity and implement general secretary xi a series of important speech spirit, organize youth members, industry youth outstanding youth typical deep ShiXing county schools, enterprises, communities, grassroots line, object, focus, interactive preach, through learning and the party concentric, listen to the party order, with the party struggle of youth, inspire leading the youth members based on post, be a model, to meet the party's 20th victory with honors.

2022-06-27 Group ningyuan county of Hunan province, bureau of education to carry out anti-drug propaganda into campus activities of Hunan ningyuan county, bureau of education to carry out anti-drug propaganda into campus activities JianWei correspondent Tao Shiqi) in order to further create a strong atmosphere of drug control, recently, the Yongzhou of Hunan province ningyuan county united county education bureau organized more than 10 volunteers, in the county Confucian temple square, county twelve small anti-drug propaganda activities.site of activity. In the county Confucian Temple Square, volunteers explain the harm of drugs to the passing people, guide them to actively promote anti-drug knowledge to their family and friends; in the county 12 primary school, by distributing anti-drug publicity materials, guide the students to watch anti-drug knowledge boards, visit the drug base, "refuse drugs and cherish life" signature activities.site of activity. This activity issued a total of more than 1,100 anti-drug publicity materials, next, will continue to mobilize the county youth to actively participate in the anti-drug publicity and education work, to create a "healthy life green non-toxic" good life.

2022-06-23 Ningxia red temple fort area to carry out the "river" I protect mother river clean beach volunteer service Ningxia red temple fort area to carry out the "river" I protect mother river clean beach volunteer service built wei) recently, the Ningxia hui autonomous region Wuzhong city red temple fort district party committee organization 20 western plan volunteers in clear cloud lake park "river" in 2022 I protect the mother river clean beach volunteer service activities. Activities, the young volunteers dressed in red vest, with plastic gloves, holding garbage bags and other tools are divided into two teams, respectively along the clear cloud lake and plastic runway pick up cigarette butts, clean up the lake, lawn, white garbage, plastic bottles, after 2 hours of labor, clean up the clear cloud lake around more than 1000 meters, the lake environment has improved significantly. In this activity, the young volunteers fulfilled the solemn promise of caring for wetlands with practical actions, further improved the ecological environment of Qingyun Lake, promoted the concept of green environmental protection, and enhanced the awareness of ecological and environmental protection. The clean beach cleaning action is not only to pick up garbage, but also to call on everyone to act together to be the advocate, defender and practitioner of the mother river protection.

2022-06-22 Hainan's first party team integration training center in lingshui li autonomous county in Hainan first party team integration training center opening Ren Mingchao) recently, lingshui the communist youth league party team integration training center opening ceremony was held in the county youth palace red scarf practice square, the center is the first party team integration training center in Hainan. It is understood that the Lingshui Communist Youth League Party Team Integrated Training Center pays attention to the establishment of "two lines and one cycle" through the growth process of youth ladder incentive system, to achieve the combination of online and offline "full-staff, whole-process, all-round" education mode. Online, the training center has built a platform of "Red Scarf Dream Post" to solve the problem of the party team connection fault, record the growth track of the team members and league members in detail, take points as the incentive carrier, and visualize the growth track, so as to provide reference for joining the team in batches, promoting the league and promoting the excellent to join the Party. Since the platform was launched in May this year, more than 13,000 parents and more than 12,000 Young Pioneers have registered. In May and June, more than 13 offline activities were held, with more than 30,000 clicks. Offline, with the party team integration training center as the position, the school connects and connects with the school "Red scarf Medal" competition results through the variety of immersive, experiential and interactive evaluation results. The two lines are connected, forming a whole chain and closed-loop ideological and political education system from young Pioneers, league members to party members.

2022-06-21 Changsha county, Hunan province, 184 volunteers become "hope hut" host "heart" friends in Changsha county, Hunan province: 184 volunteers become "hope hut" host "heart" hope friend China network reporter HongKeFei) on June 19, Changsha normal college campus, 184 young volunteers and "hope hut" owners into the university, red, opened a trip to care for."Hope cabin" host in the company of youth volunteers into college Changsha county provide activities on the same day, "hope hut" owners in the company of college students volunteers, into the preschool education institute of all kinds of training room, immersive experience psychological group auxiliary, picture books, sand games, music games and creative manual activities. They also walked into the Xu Teli Memorial Hall, in the small game designed by the narrator and volunteers, a comprehensive understanding of the life of the red famous teacher Xu Teli, to receive the red education. It is reported that the Changsha County Party Committee of the Communist Youth League accurately focuses on the needs and expectations of teenagers. Through the extensive gathering of social forces, it has built a "hope hut" for 50 teenagers without independent learning and living environment. It plans to build 100 rooms this year. After the completion of the "Hope House" provided by the Changsha County Party Committee, how to better help the young people in difficulties and realize the "new hut replacement" to "spiritual renewal" has become a concern of the local government and the society. The School of Preschool Education of Changsha Normal University cooperated with the Changsha County Committee of the Communist Youth League to implement the "Heart Care of Hope House" to help teenagers. According to 10 villages and towns 50 "hut" layout, according to "a teacher docking a town" docking "a team docking" principle, guided by teachers party members, student party members, party activists, league members participate in 50 support team, a total of 184 college youth volunteers, focus on "mental health", "aesthetic education growth" and "academic support" theme, provide professional company, design growth scheme, let young volunteers do children grow on the road "bosom sister" and "bosom brother".

2022-06-17 Gansu dangchang first "chang" grassroots theory preach contest successfully ended the first "chang" grassroots theory preach contest successfully ended jianwei correspondent mother Xiaoyan) recently, sponsored by Longnan county committee of Gansu province, dangchang county people's government, the CPC down county party committee propaganda department, down chang style, radio, television, and tourism, down county education bureau, downchang county committee, downchang county women's association of the first "ring down chang" grassroots theory preach contest final held in time square. Twelve contestants from all walks of life in the county brought various forms and rich theme lectures."Li chang" grassroots theory propaganda contest final scene picture. After the fierce competition of the preliminary round and the final round, a total of 12 contestants entered the final. At the final scene, the contestants combined with the red cultural resources of Dangchang County, their own job responsibilities, advanced typical stories around them and the masses to preach."Li chang" grassroots theory propaganda contest final scene picture. Through fierce competition, 1 first prize, 2 second prizes, 3 third prizes and 6 excellence awards were finally selected. In the process of preaching, there were many art performances with strong national characteristics, and the programs took various forms and won the unanimous praise of nearly 10,000 audiences."Li chang" grassroots theory propaganda contest final scene picture. Correspondent for figure the preach contest focus on "celebrate the party's 20 victory held" the main line, is to xi jinping, the ideas of socialism with Chinese characteristics as guidance, thoroughly study and implement xi general secretary about propaganda and ideological work important performance, is to carry forward the great founding spirit, adhere to seek improvement in stability, innovation, dare to struggle, solid, focus on solid, short, forging board, lift flag, morale, education, culture, exhibition image mission task, is to further discover and cultivate the county grassroots theory about talents, Effectively promote Dangchang county grass-roots theory propaganda work to go deep and solid a grand meeting.

2022-06-17 Guizhou kaili "one more than three strict" promote college students "home" social practice results in Guizhou kaili municipal party committee "three strict" promote college students "home" social practice results JianWei correspondent Chen Kai) in recent years, Guizhou qiandongnan kaili municipal party committee organize young college students using winter and summer vacation period to carry out government internship and volunteer service activities, to "one more than three strict" promote college students "home" social practice quantity expansion, quality improvement. Multi-channel development position. The social practice of college students "returning to their home" will be incorporated into the medium-term and long-term youth development plan of Kaili City and the training work of serving young college students and local talents, Effectively integrate the resources of administrative organs, enterprises and social organizations through superior communication and horizontal coordination, Ensure the universality of the "return to hometown" internship units and the quality of the internship positions; This year, Kaili city successfully launched the towns (streets), municipal units, enterprises to release more than 270 positions on the "return home" internship system, The job category covers multiple areas, At the same time, employers will strive to buy personal accident insurance for college students who participate in social practice; Actively coordinate with various organizations at all levels to encourage returning college students to participate in the work of effectively linking the achievements of poverty alleviation with the rural revitalization strategy, Constantly improve the socialization skills of returning college students, Establish institutionalized channels for college students and their hometowns, We will further promote college students to return home for employment. Strict dispatch and training work. The employer identified a leader in charge of the "homecoming" social practice activities, ensure high level promotion, efficient scheduling, high quality completion, strictly implement the interns, ensure good office and living conditions; analyze and analyze the employment intention of the students, to ensure that the interns effectively go to the "homecoming" social practice training and pledge meeting, etc, and improve the overall quality of interns. Strict safety and health management system. During the epidemic period, we actively cooperated with Kaili First People's Hospital to provide free nucleic acid testing for interns, strictly check the "double code" according to the management system, collect personal epidemic declaration form, COVID-19 prevention and control, and sign the "return home" social practice agreement with interns and employers; do the holiday safety and health management before holidays, open the interns for social practice, and be responsible for each intern. Strictly carry out the assessment. According to the relevant management measures, interns and volunteers are divided into three grades: "unqualified", "qualified" and "excellent"; shared through the new media platform, and attract more returning college students to participate. The appraisal form of social practice activities records the learning experience and achievements of young people in detail, and issues certificates to interns who complete their work. In the social practice activity of "Returning to Home", more Kaili students return to their hometown, go to the society, go deep into the community and enter the masses, increase their personal skills in practice, help the development of their hometown with practical actions, show the responsibility and responsibility of the youth in the new era, and contribute their youth strength to the "Big Kaili".

2022-06-16 Group xinfeng county "six big change" stimulate the communist youth league reform xinfeng county to promote the "six big change" stimulate the county communist youth league reform jianwei correspondent Liang Zuer) since Guangdong Shaoguan municipal party committee to carry out the "reform crucial standard governance", combined to promote the national county communist youth league organization reform pilot work, xinfeng county around strengthening the political function, form social function, to promote the "six big change", actively explore ideas for the county communist youth league reform. Since the reform, Xinfeng Communist Youth League has continued to take the lead in various work, and has been awarded the national summer "Three to the countryside" social practice activity excellent unit for two consecutive years. The league brand project "My Hometown I Construction" has become the only national excellent project in the province in 2022. The source of the League cadres has changed from "single" to "diversified", and explores to solve the problem of socialized selection path is not smooth. The League Xinfeng County Party Committee adheres to the "two-wheel drive" of new training and excellent, and constantly strengthens the construction of the League cadre team. On the one hand, we break the inertia of thinking, attract talents, and attract outstanding young backbone to join the league organization. Through the "Youth Forum" and "Longma Project", we explore the development force from the service objects, absorbing 121 returning college students, rural revitalization volunteers, and the cadres of towns and villages. On the other hand, improve the appraisal mechanism, issued the new venture party joint training recommended talent work plan " the new venture town (street) group (work) secretary of the term performance and started evaluation method, establish the county cadre database, comprehensive evaluation of cadres' daily performance, style construction, and so on, stimulate the cadre motivation, vitality. The organization mode has changed from "simplification" to "diversification", and explored ways to solve the problems of not excellent organizational form and weak vitality. The Xinfeng County Committee promotes the league to achieve "vertical and horizontal interaction" with the framework of "combination of blocks and vertical and horizontal interaction". Launch youth gathered more assets group, takeout industry, Internet industry, education and training industry, beauty industry set up more than 70 new social organizations, new "youth reading association" "youth troupe" group association, jointly undertake all kinds of social practice, public service project activities, promote pku "tangible coverage". Relying on the "Learn from Lei Feng" volunteer service station, village (community) party and mass service center, grace study, to build 17 "youth homes", absorb social organizations to enter, form an atmosphere of "youth order, youth home order, social organizations order", and promote the "effective service" of youth league organizations. Jointly with 100 caring businesses in the county, the "Yunjin Volunteer Benefit" volunteer welfare plan was launched to realize the "two-way empowerment" of enterprises and land, promote common development, and promote the "beneficial radiation" of the youth league organization. Group work by "temporary task" into "order project", explore to solve the long-standing and recurring group work situation actively explore establish project to promote working mechanism, launched social organizations to undertake cultivating the "new", "cloud" "youth" club "cloud" miao "" love camp "," love "a batch of teenagers thumb up with the characteristics of the" green size " brand project. Such as actively promote school-local cooperation projects, China normal university and university to carry out "cloud seedlings" and "my hometown I construction" projects, provides a platform for returning college students feedback home, service home; Dongcheng feng counterpart headquarters implementation "east new collaboration youth dream" love student three years action plan, raise 150,000 yuan for poor students situation verification, file records and subsequent return visit, become an important force to promote the innovation of the local Communist Youth League work. Working resources have changed from "relying on administration" to "multi-party integration", exploring and trying to solve the problem of lack of funds. On the one hand, and vigorously seeking financial support, the working funds of the Communist Youth League have been doubled than before the reform for two consecutive years. At the same time, actively integrate the party and government resources, the county youth palace by the county education bureau subordinate institutions allocated to the county youth League committee subordinate institutions, to achieve the county youth league management of the youth activity position "0 breakthrough". On the other hand, the Communist Youth League Xinfeng County Party Committee actively explores the operation mechanism of youth work under the guidance of the Communist Youth League and the operation of social organizations, and guides the youth league organizations at all levels to fully integrate social resources. Since the reform, the total value of social resources has exceeded 700,000 yuan. Management mode has changed from "multi-layer management" to "continuous, horizontal and vertical", Explore and solve the problem of distortion, Lian is through the horizontal establishment of the Youth League Committee or the Youth League working Committee and a variety of youth professional institutions, Increase the force of the basic plate, Reduce the attenuation of working signals, Such as promoting the county state-owned assets group to establish the youth League working committee, Set up five youth league branches by relying on its subsidiaries, Explore the establishment of the two new Communist Youth League working committees, Direct management services in the county "two new" youth league organizations, Direct contact with more than 1,000 league members and youth, We have pushed the league building work in various fields into a flat, scientific and standardized track. It means to strengthen the management through the regional industry integration of the weakened league organization. For example, to explore the establishment of a "big youth league building" pattern with township youth league committees as the core, township industrial park enterprises, public institutions and youth league organizations jointly build by the two new social organizations, directly contact more than 6,000 youth league members and young people, and strengthen the management and implementation of grass-roots youth league organizations. Youth way from "traditional interview" into "multiple platform network", explore to solve the problem of network covering youth does not reach the designated position xinfeng county based on youth keen network gathered new characteristics, one is a smooth vertical work channel, league as the "backbone", the township (street) (league (work) committee, league, "two new" league for "branches", villages (communities) for the "branches" of the communist youth league organization tree system, establish 371, normalized online youth community service members, youth more than 18000 people, will contact service youth positions to the network. At the same time, focusing on the new network media, leading young people to participate in the youth entrepreneurship e-commerce youth league organization base and youth exchange base, improve the organizational power of grass-roots youth league organizations, help township youth to recommend hometown specialties, and publicize the achievements of local rural revitalization culture.

2022-06-14 Inner Mongolia ordos Wu flag: condensed youth power inspire mission in Inner Mongolia Wu flag: condensed youth power inspire mission bear JianWei correspondent Wang Chen) in 2022, Inner Mongolia ordos flag to xi general secretary in celebrating the 100th anniversary of the founding of the communist youth league of China's important speech spirit as guidance, establish the "five heart, the party" distinct guidance, to the advanced, pursued beyond, lead the youth stick to "center, beginner's mind, determination, confidence, perseverance", in the clerkstarts bear as, achievements in sheer regret life. The Communist Youth League should always educate people for the Party and always become a political school leading the ideological progress of the Chinese youth. Wu Shen flag adhere to the youth to the party's "center", to "welcome 20, always follow the party, new journey" as the main line, thoroughly study and implement xi general secretary in celebrating the 100th anniversary of the founding of the communist youth league of China's important speech spirit, extensive "youth learning", mobilize "youth branch" learning "grassland light cavalry" into the fields, into the home, into the people, the party's voice to each youth, spread to more grassroots youth, help the youth firm ideal faith, ambition, far. The picture shows the special study of the spirit of General Secretary Xi Jinping's important speech at the celebration of the 100th anniversary of the founding of the Communist Youth League of China. The Communist Youth League Wushen Banner Committee serves to keep the "original aspiration", As a good bridge between the Party and the youth Wushen Banner to play the league organizations throughout the grass-roots line, deep youth around the greatest advantage, Adhering to the "original aspiration" of serving the people, To create a national youth development county pilot project as the entry point, Practice the concept of youth priority in development, Further carry out "green listening", "green" enjoy "," return to home " and other brand projects, Fully relying on the resources and channels entrusted by the Party, Providing tangible help to the youth, To solve the concerns and troubles of young people in innovation and entrepreneurship, social integration, dating, children's education, and support for the elderly, Make Wushen more youth-friendly, Let the youth be more effective in Wushen, We will earnestly fulfill our political responsibility of consolidating and expanding the young mass foundation of the Party's governance. As of June 2022, the flag now has 247 grass-roots youth league branches, 12 large state-owned enterprises, and 27,870 youth league members. The youth league organizations cover all fields of the flag. The picture shows the Wushen Banner organization to carry out the "deepening the warning education to build a solid ideological defense line" league cadres clean government education. Only when the Communist Youth League has the courage to make a self-revolution can the Communist Youth League keep pace with The Times, youth development, and practice and innovation. The Wushen Banner has set up the "determination" of reform and innovation, Continue to break the conventional thinking with the courage of the blade inward, Carry out extensive work projects needed by the Party and the government and expected by the young people, Consolidate the achievements of the work of cadres, At the same time, combined with the Gacha village (community) "two committees" change, Excellent selection and strong grass-roots youth League branch secretaries; Formulate a list on rural revitalization, returning to their hometown to start businesses and caring for children, Effectively enhance the sense of gain of children and youth; The party building with the league construction, team construction work into the flag level evaluation and assessment, Promote the implementation of the reform requirements of the Communist Youth League in government organs, communities, schools and enterprises, In the all-round, high standard forging coruscate the Communist Youth League high spirited style of The Times. The picture shows Wushen Banner organizing the "Welcome the 20th Youth in Action". As the Communist Youth League, it should consciously fulfill its responsibility and always become the vanguard force in organizing the permanent struggle of the Chinese youth. At present, the Wushen Banner to the construction of high-quality green Wushen as the leadership, strive to promote the "five Wushen" and "three districts and two places" construction, to provide the majority of young people with a great stage and opportunity to show their skills. Wu Shen flag set up the "confidence", actively play the role of youth powerhouse and commandos, unity led the youth to the grassroots, to science and technology, to difficulties, constantly enrich "youth civilization" "youth volunteers" "youth commandos" "green font" brand connotation, in economic quality development, normalized epidemic prevention and control, ecological civilization construction, casting the Chinese nation community consciousness for the key areas, combining and focus, in reform and development to show new bear, seek new as, improve the organization, leading force, service force and the overall situation contribution. The picture shows Wushen Banner carrying out the theme activity of "Welcome the 20th Strive to be a good" team member. The communist youth league committee for figure "perseverance", to the governing party comprehensive governing group firm governing group "perseverance", to "the flag is the flag to" for all the work of the communist youth league guide, for each member cadres action consciousness, through vigorously promotes the theoretical study, work implementation, system implementation, cast prison cadres to be loyal political character, cultivate bear work style, make its mood clear, seriously started and responsible. We will fulfill the political responsibility of leading the whole League and the team, work hard to move forward at the pass, standardize and strengthen the working mechanism for joining the League and joining the Party, further play the function of the education chain connecting the Party, the League and the team, and actively recommend and transfer new forces to Party organizations. The general requirements of strict management will be implemented in the work and construction of the Youth Enterprise Association, the Young Pioneers and the Youth Federation, so that the Party's youth organizations and youth social organizations will be more dynamic and powerful. Youth heart to the party, a hundred years to set sail again. Standing at a new historical starting point, Wushen Banner will know heavy weight, self-reflection and self-encouragement, gather wisdom, strive to move forward, constantly enhance the leadership, organization and service force of the league organization, unite and lead the majority of young people to struggle for a youthful wisdom and sweat with a better and more magnificent future.

2022-06-13 Henan jiaxian county "four" steadily push forward youth development work of jiaxian youth county "four" steadily promote youth development work wei strong correspondent Zhao Xiaolin) to promote the development of the party's youth work high quality, Henan Pingdingshan jiaxian county has always adhere to unity led youth league organizations at all levels, cadres and the masses of youth members listen to the party, follow the party, earnestly implement the central, provincial party committee, municipal party committee, the party of series of policy decisions, to "ability style construction year" as an opportunity to create a new chapter of service jiaxian youth growth development. Adhere to the example of the demonstration. Carry out the first "return home to start a business good youth" selection activity. The notice on the 2021 "Good Youth Returning Home to Start a Business" Selection Activity in Jiaxian County was issued, clarifying the selection conditions, strict selection criteria, and selected and commended 10 typical young people who have made outstanding contributions in Jiaxian economic work. Offline exchange experience, sharing experience, online building communication platform, expand the propaganda influence, give full play to the advantages of youth leading the first of social atmosphere, to create a good atmosphere of leading youth innovation and entrepreneurship, make "returning entrepreneurial good youth" spirit motivation, drive the jiaxian youth involved in their hometown development, the construction of beautiful countryside. Adhere to the appeal-oriented approach. We will carry out extensive research and research activities on the needs of youth development. To organize and formulate youth questionnaires to lay a solid foundation for the next range of research activities at different levels and fields and investigate the needs of young people. We will establish a system of joint youth meetings, cooperate with all member units to participate together, hold regular working meetings, accurately focus on key areas of concern to young people, and jointly study and launch more policies, measures and service projects conducive to youth development. Adhere to practical improvement. Carry out "return to home" college students in 2022. Combined with the provincial party committee about 2022 "spring goose action" -yu college students "home" social practice work requirements, play to the county "students" advantage, using jiaxian, jiaxian communist youth league and other public platform release practice information, clear content, including government practice, enterprise practice, public service, community service, rural revitalization, part-time exercise and cultural propaganda, organize college students return to their home to participate in social practice, establish college students and hometown contact institutionalized channels, help young students improve the social ability. Stick to your skills. To carry out the "everyone with certified youth first" skills training activities. Mobilize youth to actively participate in, organize fire emergency, mental health counseling training activities, such as joint jiaxian public education special training teacher qualification examination, joint Pingdingshan technician college held public training courses, to further promote the county youth skills training, skills employment, skills income, skills, let more youth realize skills, a certificate in hand. At present, it has carried out four skill training sessions of "everyone certified youth first", covering more than 500 people.

2022-06-13 Mother Xiaoyan) In order to help relieve pressure and easily prepare for entrance examination for secondary school or college, Jiuquan City, Gansu Province, has carried out a series of decompression activities for entrance examination for secondary school or college in May this year. By inviting experts to give centralized teaching, the school youth League committee organized by itself, it provides good suggestions for the candidates in the life attitude, emotional stability, easy preparation and other aspects of the exam, so as to reduce the psychological pressure of the students before the exam, and enhance the confidence of preparing for the exam. Jiuquan Xinyuan School psychological counseling teachers have carefully designed a colorful and interesting group psychological development activities for the ninth grade teachers and students. Through letting all the teachers and students experience muscle relaxation, passion rhythm and other ice-breaking activities, so that the students put down the pressure and trouble in the laughter. Relying on the 12355 youth service desk, Subei County Middle School has carried out the theme class meeting of "Easy preparation for the examination 12355 Walk with you" for college entrance examination decompression and "actively adjust the attitude and calmly face the high school entrance examination" to guide the students to maintain a calm and calm mind, eliminate interference and overcome anxiety. The warm-hearted measure of "a letter home, a hotline, a video and a blessing" in Jiuquan Prison has injected "heart" motivation into the children who are about to take the college entrance examination and cheer them on. Dongfeng Middle School organizes career experience activities, which is conducive to strengthening students 'professional awareness, improving students' sense of responsibility and initiative, and laying a solid foundation for the future volunteer professional application and career planning, which has received a positive response from students.

2022-06-13 Anhui first city group on behalf of the contact station to carry out the "lu xun and youth" lecture in Anhui Fuyang city group representative liaison station to carry out the "lu xun and youth" theme lecture JianWei correspondent, Wang Jianing check WeiTing) recently, Anhui Fuyang city group contact station invited young scholars, author, China lu xun research society member Yu Zhongda, to carry out the "lu xun and youth" as the theme lecture, lecture in simple, easy to lead the youth into the wild and rational spiritual world. More than 60 league members and young people participated. Lecture from "do today's youth why like lu xun", "lu xun like close to what kind of youth", "lu xun is always permeated with rich' youth '", "youth theme has always been an important content of lu xun's works", "lu xun always with youth", "lu xun's hope in youth" and "four Suggestions to contemporary youth" seven parts. Yu Zhongda introduced "Lu Xun and youth", and also made suggestions to modern youth: first, people should learn an elephant, to refine the heart in the society; second, to transform themselves, then transform the society and transform the world; third, to observe, wait, stick to and work; fourth, to break the servility and construct an independent personality.

2022-06-12 Jiangxi Dongxiang: children with mother build "childlike innocence harbor" Jiangxi dongxiang: children with mother build "childlike innocence harbor" rao taken Nanchang on June 10, June 8, in Fuzhou city, Jiangxi province Dongxiang tiger wei township fang village committee, children with mothers are carried out with left-behind children fun games, practice calligraphy, handmade and other activities. The Dongxiang District Committee of the Communist Youth League has set up 13 "Childlike Heart Harbor" public welfare projects for left-behind children in all towns and streets of the district, equipped with "children with mothers", organized various characteristic activities to provide intimate and warm services for left-behind children, and cared for their healthy and happy growth with maternal love.(Shi Yu, Rao Fangqi) Children and their mothers are practicing calligraphy with left-behind children. Rao Fang its photo

2022-06-11 Group of Guizhou Tongren wanshan district party committee to carry out anti-drug campaign in Guizhou wan district to carry out the "anti-drug propaganda into m-don't let drugs into my home" theme activity on 11 (reporter zhang, reporter zi-xuan Yang) recently, the Guizhou Tongren wanshan district party committee joint bijiang district CDC, including source and social work service center to carry out the "anti-drug propaganda into m-don't let drugs into my home" theme activities. Activity pictures. Correspondent for figure activities, by setting up anti-drug propaganda banners, anti-drug volunteers anti-drug publicity materials, explain the harm of drugs and drug prevention knowledge, with anti-drug knowledge further propaganda to the masses about the laws and regulations of poppy, encourage everyone to find drug-related illegal timely report to the relevant departments, advocating residents to expand their "circle of friends", do anti-drug propagandist, actively participate in anti-drug propaganda work, guide home age youth careful friends, not involved in complex places, do not accept strangers' food and beverage. In this publicity activity, more than 300 copies of anti-drug publicity materials and anti-drug souvenirs were distributed, and more than 600 people participated.

2022-06-10 Anhui Chizhou Qingyang procuratorial Open Day activities of Anhui county joint mu child miao studio "hand in hand to implement the" two 'to protect the future "procuratorial Open Day activities JianWei correspondent, HongLiLi check WeiTing) recently, the Chizhou Anhui county joint Qingyang county people's procuratorate, Qingyang county experimental primary school departments held by" hand in hand to implement the "two" to protect the future " as the theme of the procuratorial Open Day activities. The procuratorial Open Day activity invited more than 40 experimental primary school teachers and students and parents to visit the county procuratorate inspection work area, the procuratorate police explained the functions and deployment of the procuratorial organs on the spot. After the visit, mu tong protect miao studio members to the teachers and students, parents on a vivid rule of law education lesson, content around the law on the protection of minors "and" family education promotion law " two aspects, not only from the perspective of minors to children the franco-prussian education, also from the perspective of family education to parents friends inspired, also had the prize after class. Rich activities are like colorful childhood. The children from the rule of law class painted the theme of "Protecting the you of the youth in the law" in the name of the law, and selected the excellent works and awarded the awards.

2022-06-10 Jianwei Correspondent Zuo Yi) This year, 6,483 students from Chaohu city went to the college entrance examination room to set sail for their dreams. In order to provide the examinee with convenient high quality and efficient volunteer service, during the college entrance examination, chaohu municipal party committee in Anhui province, the city urban management bureau organization youth volunteer in five college entrance examination "love help test volunteer service", provide students with love stationery, free water, epidemic prevention supplies, emergency medicine, temporary rest, bus guide and other warm heart service, to assist the examinee exam, provide intimate guarantee for the examinee and parents. The picture shows the charity help examination volunteer service station. Correspondent for figure "our exam service station, have for the examinee disinfection bandage wound, provide stationery for candidates, shade for parents summer, although things are small, but hope to do their best, for them call" chaohu city yi qi love social work service center Feng Liyong first response group chaohu municipal party committee called, to join the youth volunteer team, in seven college entrance examination to provide examination service. The picture shows a group photo with his family. Correspondent for the figure to ease the pressure of examinee, the "love to test volunteer service" volunteer volunteers holding "koi possessed" college entrance examination steady win "" riding the examinee ollie to "slogans, encourage students to calmly, June 8 afternoon, the university entrance exam ended, candidates from the examination room, have stopped, in the" group to help gold exam title photo area " photo."Thank you for your water, we will cheer up, keep our love, go to the mountains and seas", out of the examination room of Wang Yuqing finger photo version, high-spirited. In the next step, the League Chaohu Municipal Party Committee will continue to carry forward the volunteer service spirit of "dedication, friendship, mutual assistance and progress", and gather the majority of young people to carry out various forms of volunteer service activities.

2022-06-09 The Jinghai District Committee of Tianjin Municipality has moved the "publicity platform" to the first line to preach. Beijing, June 9, China Youth Network (reporter Zhang Jianwei, correspondent Wang Shumin) " Young friends, We must implement the spirit of General Secretary Xi Jinping's important speech at the conference celebrating the 100th anniversary of the founding of the Communist Youth League of China, To be a model of hard work and selfless dedication, To be a brave and good at fighting, Constantly hone my skills on the post, Constantly enhancing capabilities, Be a screw that will never rust... " In the production workshop of China Railway Construction Bridge Engineering Bureau Group Construction Assembly Technology Co., Ltd., Liu Hongli, secretary of Tianjin Jinghai District Committee and member of the youth publicity group, issued a call to the young workers. In order to promote the important speech spirit of General Secretary Xi Jinping at the celebration of the 100th anniversary of the founding of the Communist Youth League of China to take root at the grass-roots level, the Jinghai District Committee of Tianjin and the grass-roots youth League organizations continue to innovate the carrier form, moving the "propaganda platform" to the front line and to the youth. The picture shows Wang Wenbin, the youth representative of China Railway Construction and Assembly Technology Co., Ltd., China Railway Construction Bridge Engineering Bureau Group, sharing his experience. On the scene, the members of the youth publicity group combined the innovation and entrepreneurship stories of outstanding young people during the centenary of the group to preach the young workers in plain language. At the same time, they visited and learned from the workshop to understand the corporate culture and the production process of prefabricated buildings, and had in-depth communication with the young workers. The picture shows the base league members reviewing the oath of joining the league."I volunteer to join the Communist Youth League of China, firmly support the leadership of the Communist Party of China, abide by the constitution of the league, implement the resolutions of the league, fulfill the obligations of the league members, strictly observe the discipline of the league, study hard, work actively, bear hardships first, enjoy in the back, and struggle for the cause of communism."The presence of the Communist Youth League members to review the league oath, clang oath echoed in the factory, become to encourage everyone to do a good job in the new generation of outstanding youth lead street lamp. Liu Hongli said: " Members of the publicity group will extensively visit government organs, rural areas, communities, enterprises, schools and other places to carry out on-site discussions, household visits, artistic performances, calligraphy and photography exhibitions, ideological and political courses and other forms of publicity activities, and comprehensively and accurately interpret the spirit of General Secretary Xi Jinping's important speech.”

2022-06-09 Group ningyuan county of Hunan province to carry out "welcome twenty love help the college entrance examination" activities group ningyuan county of Hunan province "welcome twenty love help the college entrance examination" activities wei correspondent Zheng Hao Tao Shiqi) after the college entrance examination, senior students say goodbye to the tense learning atmosphere, vigilance gradually relax, with relatives and friends and in and out of the KTV and other entertainment places, easy for social curiosity, thus affected by drugs. During the college entrance examination, the Ningyuan County Party Committee of Yongzhou City, Hunan Province organized young volunteers to come to the test site to carry out the activity of "Welcome the 20th Love to Help the college Entrance Examination". At the same time, they distributed anti-drug small fans, and other publicity materials to the students and their parents, and carried out anti-drug publicity and education activities while sending blessings to the students. Volunteers to the past parents and students explain the common drugs and the current easily appear in the entertainment places "fairy water", "laughing" gas "," ecstasy "," jump candy " and other new types of drugs, harm and criminals used induction means, guide the examinee cherish life, away from drugs, have a happy and healthy summer.

2022-06-07 Sichuan pengzhou rong Hong Kong, Macao and Taiwan youth exchange activities Chengdu pengzhou rong Hong Kong, Macao and Taiwan youth exchange activities network reporter wang xin) recently, sponsored by the communist youth league Chengdu municipal party committee, the communist youth league pengzhou municipal party committee, pengzhou to promote foreign affairs bureau to jointly undertake "concentric to rong build a dream of Chengdu" rong Hong Kong, Macao and Taiwan pengzhou perception line activities held in Chengdu pengzhou. Group pengzhou municipal party committee for figure activities, Hong Kong, Macao and Taiwan youth visited the pengzhou international sister city pavilion, pengzhou city planning hall, understand pengzhou, foreign cultural exchange, future urban planning and construction, in osmanthus town Jincheng community research volunteer service, community culture construction, the development of the home stay facility, also in pear flower reading rural library discussion, around the "new Chengdu, new youth, new as" communication discussion, and participated in the dragon Boat festival folk culture experience activities. It is reported that the Communist Youth League Pengzhou Municipal Party Committee will continue to do a good job in building the youth platform, deepen the youth exchange activities, do excellent "with Peng You line" brand activities, promote the youth and Rong Piao youth, Hong Kong, Macao and Taiwan youth, college students and other groups of exchanges, exchanges, to attract more young talents to pay attention to Pengzhou.

2022-06-06 Yunnan longchuan county "magic student plan" centralized ceremony in Yunnan longchuan county "hope project 1 + 1-magic student plan" centralized ceremony held wei correspondent Cao Yanhong) in order to consolidate longchuan poverty engines, make poverty foundation more stable, more sustainable, continue to promote youth care support work, recently, Yunnan Dehong dai jingpo autonomous prefecture longchuan "hope project 1 + 1-magic student plan" centralized ceremony held in prince tree township nine years school. Thirty representatives of the Communist Youth League Party Committee, the county Party committee, the student Union of the county Education and Sports Bureau, the Education and Sports Bureau, the relevant leaders of the nine-year school of Wang Zishu Township and some teachers and students attended the distribution ceremony."Hope Project 1 + 1- -Fantasy Student Aid Plan" centralized distribution ceremony site. The "China Youth Development Project" won "Hope Project 1 + 1" from Ningbo Fantasy Investment Management Co., Ltd. and China Youth Development Foundation. We funded 175 originally registered girls from poor families in grade one primary school in Longchuan County, aiming to provide study and living allowances to help them better complete their studies and grow up healthily and happily. The subsidy standard is RMB 1,000 / person, and the total subsidy is RMB 175,000; The subsidy is distributed twice, with RMB 500 / person, with a total of RMB 87,500. Zhao Weigang, deputy secretary of the Communist Youth League county Party Committee. To introduce the students of the project implementation background and source, I hope the students to bear in mind general Secretary Xi Jinping's earnest entrust, in the future study and life, determined to childhood, have dreams, love learning, love labor, love the motherland, love the people; to start from small, start from the side, and strive to be a good member of the new era. The representative of the aided students speaks. The representative of the students said: Thank you very much for your caring people and donations. I will redouble my efforts to study in the future and be a good team member. In the next step, the Communist Youth League County Committee of the Communist Youth League will continue to promote the work of youth assistance, strengthen the cooperation between the Communist Youth League education and schools, strive for more Hope projects in Longchuan, and provide scholarship and grant policy assistance for more students from low-income families.

2022-06-05 Beijing fengtai 800 college students in flow service fengtai 800 college students in flow service prevention and control, fengtai district party committee recently mobilized Beijing institute of electronic science and technology, capital university of economics and trade, capital medical university established a composed of 800 college students "college students flow screening online volunteer service", to assist in big data screening, flow, etc. On May 17, students from Beijing Institute of Electronic Science and Technology formed a volunteer service team of 427 people to assist Fengtai District to carry out the big data investigation work. As of June 1, the volunteers had investigated 29,525 people, and the service lasted 1,000 hours. On May 20, the Capital University of Economics and Business gathered 260 college student volunteers to carry out big data screening volunteer services. After receiving the training, the students immediately went on duty, became "investigators" to carefully verify the situation of the risk personnel, and fill in the information. In just a few days, the volunteers were skilled in their work and became more and more efficient. As of June 1, the volunteers had investigated 10,739 people, and the service time lasted for 600 hours. On May 22, Capital Medical University received the task of supporting the district CDC for Disease Control and Prevention, which gathered 113 student volunteers to assist in the flow adjustment work, and carefully summarized and classified the common problems, and sorted out the "reference guide for the flow adjustment language". As of June 1,4,114 volunteers had served for 600 hours."The flow transfer work is a gear that fits into each other," said Li Hui from Capital Medical University. Just two hours after receiving the recruitment notice, a volunteer team of more than 100 people at the university gathered together. As the backbone of the students involved in the recruitment work, I watched the growing data of the background registration and heard the firm words of the volunteers, and I felt proud to devote myself to the front-line work of epidemic prevention and control. Li Hui said that in the process of conducting the survey work, the volunteers never retreated in both the surge of tasks and the encounter of complicated parties. Actively claim the flow adjustment task, timely feedback to solve the problems encountered, and summarize the review after the end of the work every day. Everyone is like a gear, biting and turning together, promoting the rapid completion of the flow adjustment work. It is understood that since this round of the epidemic, the Fengtai Youth League District Committee has fully integrated resources from universities to help the epidemic prevention and control. In conducting data flow of screening volunteer service at the same time, also accurate docking central university for nationalities, Beijing forestry university and other universities volunteers, start the "cloud accompany war" disease "online care action, for disease resistance line staff children to provide online company, cloud counseling services, until the end of the summer, effectively solve the worries of frontline medical workers and community workers, continue to resist disease line of" logistics ".(intact)

2022-06-01 Guangdong Shaoguan Xinfeng children's day theme activities into the rural primary school in Guangdong province Xinfeng June children's day theme activities into the rural primary school Beijing June 1 (reporter Zhang Jianwei, correspondent Liang Zuer) on May 31, Shaoguan Xinfeng county, Guangdong, Dongguan dongcheng Xinfeng counterpart support headquarters "east new collaboration childlike innocence dream" June 1 children's day theme activities into the dragon town to stone village primary school, in the form of collective garden, caring sympathy, holiday greetings to the children, host related leaders and teachers and students a total of more than 100 people participated in the activities. At the scene, the activity opened in the national anthem and the team singing began, arranged "Grandpa Xi taught to remember the heart", "team history I know", "sapling confidence" and other forms of prize question and answer garden activities. It is understood that the, in recent years, To serve the healthy growth of teenagers, TuanXinfeng County Party Committee continued to promote the establishment of a comprehensive social protection network for the rights and interests of teenagers, Linkage with Dongguan Dongcheng Xinfeng Counterpart Assistance Headquarters to carry out the three-year action plan of "Dongxin Cooperative Dream Realization" for Huilong Town, Constantly improve the discovery, reporting and response mechanism of troubled teenagers in the county, Dynamic tracking of the status quo of troubled teenagers, Timely coordinate and help them solve practical and difficult problems, And play the role of a bridge between service, To call on social forces to jointly care for the young people in distress, next step, Tuan Xinfeng County Party Committee will continue to strive for support from all social parties, Mobilize social forces to participate in children's care and service activities.

2022-06-01 Lintao County, Dingxi City, Gansu Province, young Pioneers "unlock" social classroom experience intangible cultural heritage, search for punch card, when the narrator... Gansu Lintao Young Pioneers "unlock" social classroom Li Chuan correspondent, Wang Qingxiang Fu Ying Qi) video primary, field training, star rating, award letters of appointment... recently, Lintao County, Gansu Province, a group of red scarf docters will be officially appointed as the Longyou Revolution memorial Hall, Lintao Museum, intangible cultural heritage exhibition center and other venues "red scarf narrator". This is the vivid epitome of the socialization reform of the young pioneers in Lintao County. Since the county communist youth league grassroots organization reform pilot, according to the implementation opinions on the construction of new era social work system spirit, group Lin county, lintao county committee to promote the young pioneers social reform as an opportunity to actively cooperate with "double minus" work, from the counselor team enrich, the young pioneers activity design, campus practice base construction, promote red scarf "hand" social resources, make the new era of the young pioneers more active, full of vitality. Lintao County Young Pioneers school counselor Yao Hui is telling the children about the history of the Party eclectic talent school counselor "promising" " wearing the red scarf again feeling kind and novel, to the team back to the moment, I felt the heavy responsibility."In 2021, lintao county people's procuratorate prosecutors Du Yan was lintao county little committee hired as counselors, become one of the first batch of the county counselors, with her to accept the offer, and TV host, youth electricity, lawyers, revolutionary venues narrator, psychological counselors, the genetic bearing, retired cadres and other 11 outstanding representatives from all walks of life. After the county-level "young Pioneers outside the school counselors", through the young Pioneers big, squadron "appointment order", the county young working committee "overall order" way was invited to organize the young Pioneers activities, a total of 72 activities, covering more than 2,100 young pioneers. Lintao county young pioneers organizations at all levels also have action, in accordance with the "every two squadron not less than one outside counselor" equipped with requirements, from students' parents, district police, whole hired outside counselors, to carry out the "campus security with me" around me "my communist party member" "I am a little dentist" "red scarf little reporter" and so on more diverse practice education. At present, Lintao County has more than 380 off-campus counselors at all levels, and uses these off-campus counselors as the media to leverage all kinds of resources, striving to achieve the seamless connection inside and outside, and jointly open a new chapter of the young Pioneers' work with the vigorous and diverse social forces. Since 2016, TuanLintao County Party Committee and Lintao County Education Bureau have vigorously promoted the "Young Pioneers activities into the class schedule" to strictly ensure the young Pioneers activities every week. After several years of exploration and precipitation, the young Pioneers etiquette gradually standardized, the young Pioneers activities gradually diversified, to do the "interesting, meaningful" both. In 2021, Lintao County "red scarf for chapter" implementation rules issued, young Pioneers organizations at all levels will be "red scarf medal" evaluation and incentive system into the whole process of young Pioneers activities, "red scarf medal" become the growth of the growth of the shining beacon. Lintao County Jiaoshan Primary school squadron instructor Yu Lan found the class of students "star" into the wind, Design " Salute to Hero, Aspire to since childhood " young Pioneers activities, Guide the team members to visit and find the true heroes, And to the search activities deeply touched the team members awarded "Xiangyang chapter"; Some school young working committee for all members and reserve members issued with the school-based characteristics of the "red scarf chapter manual", Beca a's of of for for children... Also, Lintao County young working Committee timely for the counselors, teachers and young pioneers to build a growth and display platform, Organized and carried out a series of county-level activities, such as "Young Pioneers Counselor Style Competition", "Young Pioneers Activity Quality Class Selection", "I speak for my hometown" short video contest, "Red Scarf Talk on Party History" speech contest, "Red Scarf Radio Station", And with the county education bureau jointly issued the "Young Pioneers counselors assessment rules", With the rich and colorful young Pioneers activities to enhance the vitality of the young Pioneers organization, Improve the performance level of the young Pioneers counselors and stimulate the young pioneers' sense of honor, Strive to cultivate new people of the worthy of national rejuvenation. Recently, Lintao County Intangible Cultural Heritage Protection Center was approved as one of the first young Pioneers after-campus practice bases in Dingxi City ", leather carving, clay sculpture, traditional murals, paper cutting, shadow play... after-campus practice base, these are not only in the need of protection of intangible cultural heritage skills, but also an important channel for the team to explore the mystery of traditional culture, exhibition center by setting up intangible heritage experience courses, make this research practice become the second classroom for children. The heat of the practice base is not reduced, almost every day, the young pioneers come to carry out research. In order to better carry out the young pioneers activities, practice base hired the genetic bearing as the young pioneers counselor, under the guidance of the instructor teacher, the players of the county intangible culture after a preliminary understanding, according to their interests to choose characteristic experience course, by the inheritance teacher on-site teaching, intuitive feeling the charm of intangible culture. Lintao county museum into the campus series of activities lintao county museum is also the players outside practice "clock resort", combined with "519 museum day", the museum is not only open to visiting studies, more actively the museum "move" into the campus, held the power of the museum "museum" keynote speech contest and a series of colorful activities, the practice education base for the young pioneers organization and the young pioneers nearby off-campus research activities building platform, promote the comprehensive growth of the young pioneers. The socialized development of the young Pioneers focuses on the long flow. League Lintao County Party Committee, Lintao County young Working Committee will continue to firmly grasp the new era of young pioneers work socialization development direction, with open thinking, socialization mode, professional means, in the construction of the new era of young pioneers socialization work system exploration and practice, carefully plow the young pioneers work "responsibility field".

2022-06-01 Group of Anhui Wuhu bay � b district party committee to carry out love fold wing angel viewing activities of Anhui bay � b district party committee to carry out the "feeling warm June 1 love fold wing angel" viewing activities wei correspondent, TaoHui Fang check WeiTing) on May 29, Anhui Wuhu bay � b district party committee joint area disabled persons' federation, district cultural tourism sports bureau, bay � b area sunflower volunteer association "feeling warm June 1 love fold wing angel" viewing activities, invited 20 disabled children family parent-child time. On the day of the event, 20 groups of families came to the cinema to sign in and scan the code in an orderly manner. The cinema specially provided a viewing hall for 80 people, and carefully prepared the interesting movie "Spirit Hotel 4: A Great Adventure" for the children. In the next step, Tuanwan � b District Committee will continue to pay attention to the growth of various special groups of teenagers, introduce more social resources, mobilize more young volunteers to continuously carry forward and practice the Lei Feng spirit, to care for disabled children and protect the healthy growth of teenagers.

2022-05-31 Group of Anhui Wuhu yijiang district party committee to carry out yizhen volunteer service activities of Anhui yijiang district party committee to carry out the "meet with home doctor and health" volunteer service activities JianWei correspondent, Tu Guangxing check WeiTing) recently, the group of Anhui Wuhu yijiang district party committee organization to carry out the "meet with home doctor and health" as the theme of yizhen volunteer service activities. Activities, the medical staff to the residents on the family common disease prevention and health knowledge, around the propaganda theme, combined with the actual field signing, yizhen consultation, health science, focus on volunteer service activities, at the same time with banners, folding, poster boards, health literacy prize questions and a variety of channels to strengthen basic public health services and health knowledge propaganda, popularize family doctor signing service related preferential policies. This activity for more than 80 residents for free free clinic, a total of more than 100 copies of publicity materials, has been widely recognized by the area residents. In the next step, the Communist Youth League Yijiang District Committee will continue to carry out volunteer service activities on the front line, create the youth league-affiliated brand projects, further play the role of the youth league organization and service in leading the youth, and enhance the influence of the Communist Communist Youth League.

2022-05-31 Shandong ling city communist youth league: be a good dilemma teenagers "close friend" Shandong ling city communist youth league: be a good dilemma teenagers "close friend" jianwei correspondent Li Wei) on May 28, in Shandong province Dezhou city ling town party history education cultural center, filled with thick love and laughter, young volunteers with more than 30 children launched a vivid and interesting "no love children ling distance" theme activities, led the children and hope hut children visit ling city party history hall, museum, science and technology museum. The picture shows volunteers and children in distress playing group building games together in the Qizhen Party History Museum. In recent years, TuanDistrict Committee has accurately focused on the needs and expectations of teenagers, earnestly implemented the important instructions of General Secretary Xi Jinping on youth work, created a strong social atmosphere of caring for the healthy growth of teenagers, and effectively improved their sense of gain and happiness. Make overall plans and accurately mobilize the TuanLingling District Committee to connect with education, civil affairs, disabled Persons' Federation, medical insurance and other departments, and fully grasp the basic information of poor families, disabled families, subsistence allowance families, orphans, disabled teenagers, disabled teenagers and other groups in Lingcheng District. We have made comprehensive use of policies and measures such as social assistance, social welfare and security guarantee to integrate and expand security resources, increased security efforts, formulated the Implementation Plan of the Social Work Service Project for Children and Adolescents in Difficulties in the "Hope House", and made overall planning and precise launch. The picture shows children in distress visiting a water conservancy and power generation model in Qizhen Science and Technology Museum. Correspondent for figure widely MoPai YanXuan object established TuanOuWei + social work organization + township (street) youth corps committee MoPai group, four roads, for the eligible children door-to-door visits, research, door to door, according to the "comprehensive MoPai, key screening, comprehensive priority, special" principle, multidimensional control, find out difficult children's family situation, to ensure that rescue object choose real, and unified establish support work parameter. Tuanling City District Committee focuses on the needs and expectations of young people in difficulties, and makes precise efforts to create a good environment for the healthy growth of young people in difficulties. In the process of promoting the construction of the "Hope House" difficult child care project, the Communist Youth League District Committee expanded fundraising channels, actively explored the "Internet + socialization" mode, extensively launched donations, raised more than 1.1 million yuan for the construction of "Hope House", and built 93 "hope houses". The picture shows children in distress experiencing Ai glasses in Mizhen Science and Technology Museum. Correspondent for figure positive docking of civil affairs, education, disabled persons 'federation, women's federation and other departments, establish and family, community, school information communication, coordination mechanism, district civil affairs bureau led "ling city minors protection center", TuanLing city district party committee regulation established "sun sunshine youth service center", optimize the resources, strengthen force, form the civil investment as a whole, TuanLing city district party committee link regulation, the third party professional service mode, is committed to create "not love children line ling distance" boutique service brand. Tuanling City District Committee standardizes the volunteer service system, promotes the project, normalization and diversification of volunteer services, and establishes the "1 + 3 + N" volunteer service standards for children in difficulties. Multi-group participation, namely "professional social workers pair", "Party and government organs love volunteer pair", "targeted professional love volunteer help" as the help of the volunteer teams, give full play to the advanced personal knowledge resources and artistic resources of professional social workers and professional love volunteers, to improve and improve the life skills of children in difficulties. Diversified service content: Children in distress have their own characteristics, and everyone's needs are different. Volunteer service for every child in distress is "privately customized". Multiple service dimensions: the combination of "poverty alleviation", "heart support" and "technology assistance", not the previous single material assistance as the main method, and the multi-in-one perfect assistance system, truly realize the volunteer service activities to benefit children in difficulties. Improve the volunteer management mechanism. Relying on the "Volunteer Hui" and the Lingcheng District Volunteer Association, we will strengthen the daily management and activity approval of more than 600 pair volunteers. Conduct pre-job training for volunteers, and provide whole-process guidance. We will improve the reward and punishment mechanism for volunteers, formulate a volunteer service evaluation system around the effectiveness of assistance, and issue certificates of honor to volunteers with excellent performance to increase their sense of identity and gain. In the next step, Tuanling City District Committee will closely focus on the difficulties, pain points and blockage points in the growth of teenagers, and establish a long-term mechanism of consolidating "close friends" to help the growth and development of teenagers through volunteer service, social integration, and rights and interests protection.

2022-05-27 Anhui Chuzhou langya district build youth branch "flow micro class" work brand Anhui Chuzhou Langya district build youth branch "flow micro class" work brand wang reporter wang haihan) recently, Chuzhou city, Anhui province langya district youth branch members, clear street league committee secretary li ran to jurisdiction non-public enterprises-wheat elevator co., LTD., around xi jinping, general secretary to celebrate the 100th anniversary of the founding of the communist youth league of China's important speech spirit of micro preaching activities. Li Ran used the people and things around him to guide the youth league members to make contributions based on their posts, and communicated with everyone face to face."I started at a grass-roots level. No matter how busy and tired I work every day, I have to spare from half an hour to an hour to study books and learn new technologies. I hope to carry out more similar theoretical publicity activities in the future."The enterprise youth league branch secretary, Chuzhou City May 1 labor medal winner Kong Xiaowei said. In recent days, Langya District Youth League organization has made full use of the publicity force of the youth teaching group work, and has successively organized 10 young lecturers to the district youth league representatives liaison stations, communities, non-public enterprises, schools and other places to carry out mobile micro-league class theme publicity activities, covering more than 300 youth league members. In order to further improve the effectiveness of the lecture, the youth lecturers' group tailored the speech manuscripts and publicity methods for different publicity objects. For the youth members of enterprises and students, we can quickly shorten the distance and attract attention; for the youth members of government organs and institutions, especially grassroots cadres, mainly adopt "lecturer interpretation + communication discussion", pay attention to deep theoretical interpretation and work guidance. Wang Qixia, deputy head of Langya District Youth Lecture Group and a psychological teacher of Chuzhou No.4 Middle School, led the youth league members to learn theoretical knowledge through PPT display and short video broadcast, so as to stimulate the interest of the enterprise youth in learning. Zhang Lirong, secretary of the Youth League General Branch of Yuanqi Forest Beverage Company, said, " The production task of the youth league members is heavy. It is very popular to carry out the publicity in this accurate and fast way."In addition, Ning Renyi, a member of the Langya District Youth Lecture Group and the head of the Fengshan Sub-district Youth League Working Committee, encouraged the league representatives to actively discuss and solve the problems when proposing and solving the publicity based on the current key work of the Communist Youth League. The person in charge of TuanLangya District Committee said that in the future, it will expand the coverage of publicity, enrich the carrier of publicity activities, and build the mobile micro group class into a brand work serving the growth of youth. Photo provided by Tuanlong Langya District Committee

2022-05-27 Li Xinyi, a student, and Wang Xinyi, reporter of China Youth Daily) On May 26, the Chengdu-Chongqing Youth Collaborative Innovation Center was held in Chengdu and Chongqing through video link. The promotion meeting is jointly hosted by Chengdu Chenghua District Committee and Chongqing Shapingba District Committee of the Youth League, aiming to further strengthen the coordination, exchange and sharing of young talents between Chengdu and Chongqing, and organize and mobilize the youth of Chengdu and Chongqing to contribute to the construction of Shuangcheng economic circle. The meeting site. In recent years, the youth league organizations at all levels in Chongqing and Sichuan have actively seized the major strategic opportunity of the construction of the Chengdu-Chongqing twin cities economic circle, and taken the opportunity of participating in the national county Communist Youth League grassroots organization pilot reform to explore a series of innovative experiences and practices. Group of Chengdu Chenghua district party committee to social organizations as the main body, qing green gen enterprise as the main force, coalition of Chongqing shapba district party committee, explore the integration platform sharing, resource sharing, innovation, project construction, economy, employment, service sharing, youth ring Chengdu-Chongqing youth collaborative innovation center, build formed the latest achievements of the youth organization service center, for Chengdu-Chongqing youth interactive growth. Ring Chengdu-Chongqing youth collaborative innovation center depth into the national county communist youth league organization reform pilot, focus on Chongqing green gen resource elements integration channel, linkage Chengdu-Chongqing technology managers service platform, released the project amount of 36.4 million yuan opportunity list, the project covers wisdom city construction, big data application, industrial Internet innovation application and digital brigade, etc. The meeting also launched the first release of Chengyu youth youth innovation platform. With wechat mini program as the carrier, the platform opens many functions such as information exchange, resource docking, project release and talent recommendation.

2022-05-27 Guangxi: The Communist Youth League of Cangwu County has launched the opening ceremony of the pilot "Youth Employment Port" to open up the "last kilometer" of serving youth employment. In the activity, the participants visited the exhibition hall of youth entrepreneurship achievements, and investigated the development of young entrepreneurship representatives, including Wangfu hawthorn flower tea, Lion sea green plum salted duck eggs, Shiqiao sweet potato press, pear port beancurd, Liubao tea, wooden double rice wine and other featured agricultural products and sales methods. Cangwu county through in the form of point with surface realize the full coverage of the villages and towns "youth employment port", nine villages and towns in accordance with the unification, standardization of employment information release, employment entrepreneurship youth skills training and entrepreneurial youth propaganda studio, and other functional areas, to improve the county township youth employment entrepreneurship ability, for Wuzhou continue to carry out "youth employment port" construction provides new ideas.

2022-05-27 Hubei province, the county committee to promote "green loan" group, the county committee to promote "green loan", "green loan" support projects, through multi-channel publicity, optimize the approval process, establish transport mechanism, to provide financial guarantee for youth innovation and entrepreneurship; relying on the "youth city" WeChat public release "green and loan" financial support project information, loan conditions, quota, interest rates and deadline, application process and related key information, strengthen the contact with market supervision and management department, with SMS point to point propaganda "green loan", let the policy accurate coverage entrepreneurial youth. Tongcheng county party committee at the same time to establish "green gen loan reception room", assigned someone is responsible for the preliminary examination work, strict audit standards, prepare paper loan application form template, data list, flow, provide entrepreneurial youth consulting, contact services, timely to meet the requirements of the loan and have actual needs of entrepreneurial youth for examination and approval work. The Youth League County Committee, Tongcheng County Branch of People's Bank of China, Public Employment Bureau, Finance Bureau and Rural Commercial Bank, hold special meetings, timely grasp the loan issuance information, and promote the orderly issuance of loans; As of May 13,2022,183 million yuan, 933 loans; the number and amount of loans ranked first in Hubei Province.

2022-05-20 Shandong mountain: "hope hut" light lake children light of hope of Shandong mountain: "hope hut" light lake children light of hope for China youth network Beijing on May 20 (reporter Li Chuan correspondent, Wang Mengjie) Yin Jipeng recently, Shandong weishan mountain town Zhang Lou lake fishing village of 11 small (a pseudonym) has a wedding, she finally has the independent learning and living space. Clean room, clean desk, bright desk lamp, warm bed cabinet, these originally thought that the remote scene did not expect to realize all in just a few days time. A "hope hut" is also a space for children to pursue their dreams."Hope hut" children care project start immediately two anniversary, it is understood that since the project implementation, group weishan county party committee in strict accordance with the provincial and municipal youth league work deployment, adhere to the "high positioning, system planning, strengthen execution, pragmatic", the working train of thought, around accurate MoPai, social fundraising, control construction quality, follow-up tracking services and other key links, to promote the "hope hut" child care project. Up to now, the county has raised more than 1 million yuan to build 82 "hope cabins", sending love and hope to 86 children in distress. At the same time, in order to help like minimized living on the island, the ship children can also have their own a "hope", group county party committee to overcome transportation inconvenience, high transportation cost, wet natural environment, complex hull construction difficulties, adhere to the principle of never leave a child, comprehensive MoPai construction, let the light of hope illuminate every plight children. At present, Weishan County has built 2 boat cabins, cabins on the island 17. With the completion of the "hope cabins", the seeds of love also slowly take root and sprout in the hearts of the assisted children, along with them to thrive. At the same time, on the basis of helping children in difficulties improve their living and learning environment, TuanWeishan County Committee takes care service as an extremely important "second half" of the "Hope House" project, implements the "1 + 3 + X" volunteer service system, and continuously deepens the service efficiency of "Hope House". On the basis of the monthly fixed household visits, organized the "lotus sweet reading" public books rafting, "youth guardian, love New Year", "love in hope, gratitude have you," and "plant hope tree, light youth dream", "dream, dream" hope hut children feel city, "warm childlike innocence, green birds," and "green lotus cloud mail" cloud hand and other volunteer service activities, promote the implementation from "cabin refresh" to "spirit"."Youth breeds infinite hope, and youth create a better tomorrow...", General Secretary Xi Jinping's important speech at the celebration of the 100th anniversary of the founding of the Communist Youth League of China is full of deep feeling, reflecting the CPC Central Committee of the concern and love for the youth. In the next step, Tuanweishan County Party Committee will further strengthen its mission responsibility, consolidate and expand the effectiveness of the "Hope House" child care project, and help more children in difficulties to light up the light of adversity and build the dream of a beautiful youth.

2022-05-24 Group of Guangdong shixing county to carry out the "youth mock court" activities of Guangdong shixing county to carry out the "youth moot court" activities Jianwei correspondent Chen Rui) recently, the group of Shaoguan city of Guangdong shixing county united county procuratorate, the county court, county judicial bureau, county public security bureau and county education bureau held a unique "youth moot court" activities. This activity was held in Shixing County Danfeng Primary School legal education base, more than 50 primary and middle school students to participate in the activity. Activity pictures.mock court " is a normal activity to prevent juvenile crime work of Shixing County, through typical, vivid cases to increase the publicity effect of law popularization, to educate the majority of teenagers to enhance self-protection awareness and ability, consciously resist and prevent injury, prevent juvenile crime has far-reaching significance. Activity pictures. Correspondent for figure in the activity, the students dress code, look solemn, from the court discipline, check the original identity, to the court investigation, court debate, the final statement, sentenced in court, completely in accordance with the standardized trial procedure, highly restore a real trial scene, let the scene participation and attend students experience the whole process of the trial, and accepted a vivid rule of law education. Students have said that the mock court gave them a more intuitive understanding of the court trial process, the harm brought by illegal crimes, and the fair authority of the law. Activity pictures. The "immersive" rule of law practice activities, rigorous procedures, standard, relaxation and orderly, let the students in the relaxed atmosphere to understand the legal knowledge, guide teenagers to take the initiative to study the law, law, law usage, usage "code" bright heart, let the rule of law in the heart of every youth bud. At the successful end of the trial was the sound of the final hammer. Activity pictures. Correspondent for figure after the trial, also added the franco-prussian corridor visit learning, prize knowledge, drug oath, emphasis on the popularization of the constitution, the civil code, criminal law and the prevention of juvenile delinquency, the minors protection law knowledge, and issued a batch of publicity materials, the activity a warm atmosphere, inspired the students' enthusiasm. Activity pictures. Correspondent for figure the next step, the county will unite relevant departments to prevent juvenile delinquency series of activities, the youth rule of law education as an important content of service teenagers, let the county teenagers have a deeper understanding of legal knowledge, greater fear of law, in learning life to do an active research method, usage, respect law, law-abiding small citizens.

2022-05-21 Chongqing Qijiang: river patrol river chief " in the action of the garbage. The communist youth league in the qijiang district party committee for figure Chongqing on May 20,19, the qijiang district CPPCC, the communist youth league in the qijiang district party committee, district women's federation, longsheng town, build second bureau of southwest branch "Xiaoqing" volunteers and militia inspectors in the qijiang district longsheng town double arch village of five cloth river patrol river protection, protect blue sky river environment regulation. It is understood that the Wubao River is a tributary of the Chongqing section of the Yangtze River, originating from the Qijiang River, flowing through Wansheng, Banan, is a level of water source protection area in Chongqing. Since this year, in order to implement the spirit of Chongqing municipal general river chief conference, Qijiang district recruited part of the district CPPCC members as "member river chief", participate in the river (section) water environment protection publicity, supervision and other work, boost the "river chief system" work fully implemented. At the Shuanggong Village hydropower station dam, Xie Linna, secretary of the Qijiang District Party Committee of the Communist Youth League, inspected the ecological flow and water quality of the Wubao River basin, and learned about the daily management and protection of the river and the environmental improvement along the coast. Then, together with volunteers, militia inspectors into the river environmental remediation, and jointly maintain the ecological environment of the Wubao River."We will strengthen our sense of responsibility, strictly perform the management duties of 'committee members and river chiefs', be good inspectors, propagandists and demonstrators of river management, and continue to intensify our efforts to patrol and protect rivers."Said Xie Linna. At the same time, we should continue to give full play to the role of the Communist Youth League and the Young Pioneers, encourage members of the Communist Youth League, young Pioneers and young volunteers to participate in the standardization, institutionalization and normalization of river management and protection, and create an ecological environment of the Wubao River with smooth rivers, clear water, green banks and beautiful scenery.(Hu Hong, Zhou Yin)

2022-05-20 Jiangsu Province Qidong Municipal Party Committee recommended more than 300 posts live broadcast recommended more than 300 posts of provincial college students employment assistance "send post through train" live broadcast post recommendation activity (Qidong special session), held in Qidong City, Nantong City, Jiangsu Province. More than 10 enterprises participating in the activity provided more than 300 jobs for college students, attracting more than 2,000 college students to watch online. At the scene, Zhu Fengxiang, secretary of the Qidong Municipal Committee, invited college students to visit, study, exchange and display their talents. During the period, the relevant person in charge of the enterprise walked into the broadcast room, publicize from the working environment, employee salary and welfare, the company's development status, employee promotion channel and other aspects, and promote jobs online. The live recommendation is the innovative exploration of the Youth Qidong Municipal Party Committee to promote the gathering of young talents. In the next stage, the Communist Youth League Qidong Municipal Party Committee of the Communist Youth League will continue to implement the " Youth Talent employment plan, connect with local enterprises, extensively contact Qidong college students abroad, one to one help and guidance services, and strive to attract more young talents to return to employment and entrepreneurship.

2022-05-18 Group of Nantong, Jiangsu province rudong county continue to build 30 "dream house" group rudong county continue to build 30 "dream house" face transformation six things in place "construction standard, between 35" dream house "last year, on the basis of the county charity federation fundraising, encourage town (area, street) self-raised, called for more people in love enterprises to join the donation team, continue to build 30" dream house "."Dream transformation +" care plan, group east county to promote the establishment of "1 + 3 + 6" care service system, linkage at the county level, town (area, street), village (community) tertiary organization, organization town (area, street) part-time youth affairs workers, county youth charity supermarket, fresh air public service center, etc, as volunteers to participate in all kinds of care service. This year, Rudong County, as the national county Communist Youth League grass-roots organization reform to expand the pilot project, from the grid members, joint defense members and other township government purchase service personnel to hire part-time youth affairs workers in town (district, street) workers, innovation into them into the "dream transformation +" care service team. In addition, group east county also explore with natural resources bureau "youth volunteers" team, revenue "love mother" team, power supply company communist party team, green chamber of commerce love entrepreneurs cooperation, will be more volunteer service organizations and "dream transformation +", make care service become more rich, real, warm heart.

2022-05-18 Group lianshui county of Jiangsu province: with youth bear polish "volunteer red" lianshui county of Jiangsu province: with youth bear polish "volunteer red" youth network Beijing on May 18 (reporter Zhang Jianwei, correspondent Zhang Lei), group Huai'an of Jiangsu lianshui county issued epidemic prevention and control volunteer service call, in just 2 days, the county has recruited reserve youth volunteers 490 people, formed more than 20 epidemic prevention and control youth commandos to the epidemic line."Inadvertently saw the county in recruiting epidemic prevention and control volunteers, I signed up", young party member volunteers xiao li, is a member of the youth committee youth commandos, "the outbreak, as a party member, as a once protected in the" SARS " period after ninety, I should stand up, stand out for the society, for my hometown."Help the elderly to apply for a" double code ". Correspondent for map group county committee all cadres all sink to the pair community, assist the community to carry out "knocking", "gatekeeper", door to door preach epidemic prevention and control policy, issued "inform", assist the community platoon to ripple back ripple personnel; carry out epidemic prevention screening, to enter the personnel into the community do double code inspection, information registration, temperature measurement and other volunteer services. Since the epidemic prevention and control, the Communist Youth League County Party Committee has not only completed visits, epidemic prevention and publicity, and guard duty, but also provided assistance to the special groups of the elderly, young, sick, disabled and pregnant. For the elderly who "play" smart phones, help them apply for health code and travel code; take the initiative to help special groups lift heavy objects and deliver materials on the premise of "gatekeeper"."The epidemic is heartless and people have love. We young volunteers will polish the party emblem with our actions and interpret the youth mission with dedication."Said Li Liangliang, party secretary of the Communist Youth League.

2022-05-16 Group Chizhou in Anhui province Qingyang county to carry out the party's youth movement "four" group the party's youth movement in Anhui province to carry out the party's youth network in Beijing on May 16 (reporter Zhang Jianwei, correspondent liu ting check WeiTing) recently, the Chizhou in Anhui province Qingyang county organization carried out the party's youth movement history exhibition into organs, into the campus, into the villages and towns, into the community activities, create a strong atmosphere of learning group history. The graphic exhibition is collected by TuanCounty Party Committee, and made into posters, exhibited in organs, towns, villages (communities) and schools. Text exhibition is divided into glorious Chinese communist youth league, the new democratic revolution period, socialist revolution and construction period, reform and opening up and socialist modernization construction, the new era of socialism with Chinese characteristics and so on five chapters, through the precious historical pictures and vivid text description, shows the communist youth league of China from the birth of the day, always unswervingly listen to the party, follow the party, in our revolution, construction, reform, various historical period, politics, economy, culture, social and other fields, for the party for the youth, gathering youth power to make a positive contribution, play an important role. Enter the government organs- -strengthen the responsibility of the youth league cadres. The youth League county committee took the lead in arranging the history of the Party's youth movement, the purpose is to guide the league to not forget the instructions of their ancestors, bear in mind the original mission, bravely choose the responsibility of The Times, conscientiously perform their duties, inherit the fine tradition, give full play to the role of a bridge connecting the youth, and strive to unite the young people closely around the Party. Into the campus- -to deepen the ideological understanding of teenagers. Primary and secondary schools in the county in the form of theme league day, team day and other on, the Communist Youth League members, young pioneers to learn the history of the party's youth movement. By understanding the history of the league in advance, the young pioneers laid a solid ideological foundation and action consciousness; through reviewing the history of the students, strengthened the socialist ideal and faith, enhance the sense of belonging and identity to the Communist Youth League organization, and said to be a qualified Communist Youth League member. Into the township- -enhance the grassroots youth dedication awareness. The history of the Party's youth movement was arranged and exhibited in 11 towns and townships of the county, encouraging the young people at the grassroots level to actively participate in the vivid practice of rural revitalization and epidemic prevention and control, to display their talents and youth in the vast rural land, and to become a young people of the new era who dare to bear hardships and are willing to contribute. Into the community- -to stimulate the social youth to forge ahead strength. In furong community youth home layout exhibition of the party's youth movement, namely give full play to the position of the youth home, but also let more social youth struggle and the communist youth league in one hundred, feel the beginner's mind mission, draw strength, in the ordinary jobs, efforts to do a new era of talent, as a social youth. Next, group Qingyang county will continue around "celebrate 20 always follow the party forge ahead new journey" theme to carry out a series of activities, guide the county member youth to forge ahead, yong when pioneer, with bear and faith polished youth background, with dedication and struggle writing era, to meet the party's 20 victory with honors.

2022-05-13 Shandong Feicheng: promote the reform of the county communist youth league grassroots organization with new kinetic energy Shandong Feicheng: promote the reform of the county communist youth league grassroots organization with new momentum XingTing) in Shandong fat city to promote the reform of the county communist youth league grassroots organization, unity lead the youth based on jobs, achievements, a large number of potential youth rapidly grow into business backbone, innovation, youth on all fronts of economic and social development. Through the three-step linkage of the party and the league, now, Feicheng has formed a good pattern with the party leading the league and the league connecting. The first step is to build organizations together. Relying on the 10 municipal Party working committees, the system youth working committee was established, and 144 first secretaries served as dispatched village league construction instructors at the same time. Among the non-public enterprises and social organizations that have established Party organizations, 129 new youth league organizations were established. The second step, mind synchronization. We will carry out education and education on learning the history of the Party, strengthen faith, and follow the Party, and on the theme of "Welcome the 20th Plan, Always follow the Party, and forge ahead on a new journey" to strengthen the ideological foundation for young people to listen to the Party and follow the Party. The third step is to test the performance together. The league building work will be included in the overall planning and annual assessment of the party building work, and the "league building is unqualified and the Party building is not excellent". The party building with the league building work into the scope of the municipal party committee inspection and supervision, clear "three check seven see" inspection content. At the same time, the Feicheng Communist Youth League has formulated four incentive measures to stimulate the enthusiasm of young officials. Carry out the "two red and two excellent", innovative and effective youth collective individual and other typical tree selection activities, Put outstanding performance into the category of evaluation at all levels, Give honorary incentives; Increase the publicity and promotion of typical young people, With the power of example to encourage the majority of young people to forge ahead, forge ahead, Give spiritual incentives; Continue to increase the guarantee of policy system, practical projects, public culture, health care and education to give development incentives; Implement the preferential promotion system, Priority to recommend good political quality, strong professional ability to join the party and league organizations, Those who have made remarkable achievements in rural revitalization, grass-roots governance and other fields will be included in the training sequence of village (community) reserve cadres, Give growth incentives. In the specific reform, feicheng Communist Youth League focuses on five contents. Serve "industrial strong city", promote the healthy development of young entrepreneurs association, carry out integration communication, training empowerment, help and package drive series of measures; regularly carry out "green bird program to peach township" college students return to hometown social practice, build the interactive platform between university and enterprise talents; deepen the "youth safety production skills promotion" action, guide youth to lead to build the safety bottom line. Service "rural revitalization", with rural revitalization of the youth work action is suggested, to carry out "looking for rural revitalization partner" action, "township green" industry docking activities, build project promotion, supply and demand docking, industry cooperation platform, successively released quality project 13, article 27 supply and demand information, 3 times industry matchmaking meeting, on the fat peach industry chain formed "green peach revitalization club", guide the young talent power rural revitalization. Serve the "urban promotion", carry out the "youth report to the community" activities in the organs, participate in the community epidemic prevention and control, science popularization, environmental protection, etc.; implement the "community youth vitality promotion" action in the urban and rural communities, build the work brand of "community youth school", and expand the "circle of friends" of the community youth. Serve the "cultural leading", launch the working brand of "Qingyan Peach Capital", create excellent cultural works, innovate publicity forms, and help urban promotion and cultural communication. During the outbreak, the MV "So Many People" recorded by young people in emerging fields effectively boosted the morale of the epidemic. Serving "common prosperity", launched the volunteer service project of "Hope House Color Action", 172 young volunteers pair up to help 75 "Hope House" assisted children, carried out a total of 81 volunteer services, and presented 291 pieces of assistance materials (sets). The "Qingqing School" left-behind children care project, built 8 volunteer teaching centers, recruited more than 30 college students volunteers to provide services, covering more than 200 children.

2022-05-12 Anhui Fuyang city for young cadres clean education theme salon in Anhui province municipal party committee to carry out the "green said" young cadres honest education theme salon JianWei correspondent, dan-dan sun check WeiTing) recently, the Fuyang city in Anhui province joint municipal party committee organization department, city municipal discipline inspection committee supervisor committee and other units to carry out the "green said" young cadres clean education theme salon, municipal party committee, secretary of the municipal discipline inspection commission, city committee director Chen JianLi to attend activities and preach honest party class, in recent years, new promoted appointed some young party member leading cadres to participate in activities. At the scene of the activity, we watched the warning education feature film "young cadres violation of discipline and law warning record", issued the "button good clean politics" the first button "- -young cadres discipline and law education pocket book", carried out the "manual cut clean" activity, some young cadres representatives exchanged the feelings and feelings of clean politics. The meeting called for improving the political standing and fully understanding the significance of strengthening the clean education of young cadres. To thoroughly study and apply xi general secretary on strengthening the supervision and management of young cadres education important, the implementation of the party central committee on strengthening the construction of the new era of clean culture deployment requirements, more consciously the comprehensive governing party requirements into daily work practice, the good growth of the "steering wheel", fasten clean "seat belt". The meeting pointed out that to strengthen the case warning, with negative examples as a mirror to tighten the string of discipline rules. For a few young cadres have "vitality, less concentration, less maturity, less education, less experience, too little suffering; too self, less public interest, personal interests first, high expectations, great contrast, heavy burden, heavy use, light supervision, management loopholes", the responsibility, adhere to the responsibility, strengthen the education management supervision of new party, new entry, newly promoted young cadres, and guide young cadres from the negative typical cases, learn experience and lessons. The meeting stressed the need to forge strong skills and strive to be loyal, clean and responsible cadres in the new journey of the new era. To strictly observe the political, power, communication, life, family, and adhere to the party, meticulous study and hard to take the work, responsible for the people, take the mass line, a healthy bottom line, clean and honest mirror, strive to grow into a firm faith, serve the people, diligent, responsible and honest cadres.

2022-05-10 The Liangxi District and the launching ceremony of the third "Xi" Youth Culture Festival was held in Wuxi. Ten thousand young talents participated in the online launch ceremony through the live broadcast. The "Youth Talent Light Chasing Plan" was launched on the site. The Youth League Liangxi District Committee signed the Strategic Cooperation Agreement on Youth Development Central City Construction (Young Talent Field) with Zhihu, Zhaopin, and Taihu Talent Development Center, and held the unveiling of the Guangdong-Hong Kong-Macao Taihu International Youth Talent Community and the occupancy ceremony of talent representatives. The organizer also appointed "Wuxi Youth Observer" and "Liangxi District Youth Question Officer" to release the List of Youth Observation Projects. Young talents participated in social governance at a deeper level through online questioning and offline observation, and put forward youth plans for the development of Wuxi and Liangxi. For the first time, Zhihu launched the " Youth, Know Liangxi?"Series of activities; In May, we will leave the first" Zhihu Liangxi Youth "subway train in the Yangtze River Delta, build a city flash mob exhibition space" Wow " museum, release a subway vlog creative competition, and hold 100 youth round table Zhihu sharing salon. At the same time, "Liangxi Communist Youth League" has entered Zhihu, which will attract more than 1 million young intellectual friends online, create a professional community of more than 10,000 young talents gathered, focus on 1,000 young talents in various fields, and cooperate with 10 Zhihu brother cities to jointly help the development of young talents. Next, liangxi district will implement a more powerful liangxi talent plan, issued "about promoting the construction of the new era of talent strong area opinions", continue to carry out talent service month activities, issued a white paper on talent development, participate in the Yangtze river delta integration center city alliance, expand ZhaoCaiYinZhi "friends", build education, close to new heights of talent development.

2022-05-07 Group of Anhui dingyuan county to carry out "welcome 20 always follow the party forge ahead new journey" series of theme activities in Anhui dingyuan county to carry out "welcome 20 always go with the party endeavour new journey" series of theme activities wei correspondent wu � S) during the "May 4 th" youth day, group of Chuzhou, Anhui province dingyuan county organization to carry out "welcome 20, always follow the party, endeavour new journey" series of theme activities, unity lead the county youth not young, not times, not people, strive to run on the race track of youth."Youth" force organization, held the theme of the group day practice activities. Around the theme of "salute one hundred glory youth challenge to the party" the theme, on April 24, the county organization "welcome 20" practice, in "yuan lang baking" group representative liaison station, invited to the outstanding communist youth league, youth CPPCC, young entrepreneurs, state-owned enterprise youth, youth volunteers, Courier little elder brother, epidemic prevention and control of frontline workers, home employment students to attend. During the activity, the professional baking teacher demonstrated the cake making process for the youth members, and they experienced the baking making process together. In the end, the young volunteers sent the cake to the frontline volunteers, the families of the frontline medical workers, the hard-working delivery boys and others."Green" heart gather strength, the organization of centralized demonstration joining ceremony. On April 29, the youth League county Committee to carry out the "youth show the motherland always follow the party" as the theme of the new league members to join the league ceremony demonstration activities, in the new Dingyuan county campus held the "youth show the motherland, always follow the party" new league oath ceremony, more than 20 new development of the league members participated in the activity. Youth league comrade is responsible for the history of the league, introduces the significance of the ceremony, the scene issued chapter, member card, wearing badge, led all the members in the face of the bright flag of brief, solemn, warm oath ceremony, let new members have experienced a baptism of the heart, accepted a serious and serious education, for the county communist youth league organization injected new vitality."Youth" talk heart, to carry out the returning youth dialogue discussion. On April 30, the county bureau in the bridge town of the third meeting site, organize more than 20 home old and new member youth in the league under the solemn oath, chorus "glory, China communist youth league", then, the county is responsible for comrades and returning youth representatives face to face dialogue, combined with returning youth thought, thought, needed, to further improve the youth organization work, better condensed youth, service youth."Youth" order to promote, help to create a civilized city work."May 4" youth Day, the Communist Youth League County Committee organized young volunteers to the community to carry out health cleaning activities, the community each floor of the residents in front of the "psoriasis" centralized eradication, the corridor of the garbage for a comprehensive cleaning. At the same time, the young volunteers also guide the community residents to take the initiative to participate in the volunteer service work of the community nucleic acid testing, patiently and actively providing services for the elderly who participate in the nucleic acid testing. The Communist Youth League County Party Committee will carry out the environmental sanitation management work of guaranteeing the guaranteed community grid point on a regular basis, create a beautiful home that satisfies the residents, and help the county to strive to become a provincial civilized city.

2022-05-07 Group of Inner Mongolia castle peak district party committee held "youth appearance" theme team day group of Inner Mongolia Baotou castle peak district party committee held "youth looks like" theme team day activists Shi Jia) to celebrate the 100th anniversary of the founding of the communist youth league of China, recently, the Inner Mongolia Baotou castle peak district party committee held a "welcome 20 always follow the party forge ahead new journey" - "youth appearance" online theme team day activities. The event was broadcast live online, consisting of five chapters: "Youth faith", "the cutest person", "our youth role model", "countermarch people" and "Castle Peak Youth Talk". The picture of two generations of young people applying to join the league kicked off the live broadcast. Veteran party member Chen Wenxue traces the story of his youth and the party, the new era youth to the party, listen to the party, follow the party; "Inner Mongolia Youth May 4th Medal" winner, Winter Olympics performance volunteers, Inner Mongolia autonomous region outstanding cadres share their stories of struggle; Baotou city youth vows to the camera. The "Appearance of Youth" online themed team day activity has been viewed 35,000 times online. Wang Youxuan, a student from Baotou city, Inner Mongolia, visited Chen Wenxue, a veteran party member.

2022-05-06 Group of Guangdong shixing county to carry out talent salon group Guangdong county party committee "party lead the youth entrepreneurship, power shixing economic leapfrog growth talent" theme salon activities wei correspondent Chen Rui) recently, group of Shaoguan city of Guangdong province shixing county party committee to "party building, group to promote the party building", organized held "the party leading youth entrepreneurship, power economic leapfrog growth talent" theme salon activities. The organization department of the county Party Committee, the county people and social bureau, the county agriculture and rural bureau, the county bureau, the county, Shixing Rural Commercial Bank and other units in charge of leaders and nearly 20 young entrepreneurs participated in the activity. On the day of the activity, the Communist Youth League County Party Committee organized relevant units to first visit enterprises to have an in-depth understanding of the epidemic prevention and control situation, business status, development strategy and needs of enterprises, and encourage enterprises to strengthen their confidence, seize policy opportunities, and unswervingly take the road of innovation and development. After the investigation, the Communist Youth League Committee and the leading group Office of the County Party Committee held a symposium for young entrepreneurs in the Gutang station of the talent station. At the meeting, young entrepreneurs combined their own reality, around the reform and innovation, introduction and training of talents, implementation of policy help, help hometown construction and other exchanges; relevant departments around the youth entrepreneurship, employment assistance policies, Shixing cultural revitalization, rural revitalization and other conditions. The activity also invited outstanding entrepreneur Mr.He Zhudong to share his entrepreneurial experience and impart entrepreneurial experience for young entrepreneurs, and called on young entrepreneurs to keep up with the pace of The Times, and constantly innovate their ideas and abilities around the work center of the Party committee and the government. At the meeting, Shixing Rural Commercial Bank also introduced how to solve the financial difficulties for young people entrepreneurship. In the next step, the Communist Youth League County Party Committee will shoulder the youth responsibility of "youth heart to the party, contribute to the new era", actively explore the new mode of "Party building +" and "league building +", the party building, league building work with enterprise development, cultural construction, to accelerate the construction of a new stage of modernization and contribute youth strength.

2022-05-06 Shandong ling city set up "love alliance" to explore new models of community governance in Shandong ling city "love alliance" to explore the communist youth league participation in community governance network Beijing on May 6 (reporter Zhang Jianwei, correspondent gao lili) to explore the communist youth league to participate in community governance model, recently, Shandong Dezhou ling city set up "love alliance", district party committee organization department, TuanOuWei, qi streets, the streets and other units responsible for comrades, love ambassador and outstanding volunteers represent more than 20 people to participate in the activities. Issue letters of appointment to the representatives of the "Love Ambassador". The "Love Alliance" is a non-governmental, non-profit social charity organization led by the Organization Department of the Lingcheng District Committee and the Communist Youth League District Committee, The alliance adheres to the working concept of "voluntary and dedication to society", With 12 urban community party and mass service centers as the main position, Gather the strength of enterprises, social organizations, individual industries and businesses and other social caring people, Provide volunteer service-related resources based on actual conditions, Giving love to the society through public welfare feedback, financial assistance, volunteer service and other forms, By mobilizing emerging youth groups to participate in the "Love Alliance" work, Let youth forces play a direct role in the last mile of community-level governance. We will build a platform for young people, social organizations and other youth forces to work together to solve the concerns and troubles of the residents, so that more young people will become participants, practitioners and leaders in grassroots social governance. Issuing certificates of honor to the representatives of "outstanding volunteers". Correspondent for figure group district party committee will continue to explore "youth league organization, to community volunteer service as the main body, with various group participation as the main form" the new mode, "love alliance" into the communist youth league organizations involved in community governance work new carrier, improve the regional "organization, resource sharing, position sharing, linkage" work pattern, guide condensed youth group return to community, community, community, to realize the community governance of new situation, speed up the construction of international first-class center contribution youth strength, show the bear era.

2022-05-06 Dalian Dalian district to carry out the "cloud class" talk youth as Dalian plulan district: "cloud class" talk youth youth reporter wang Chen) "review one hundred years history, youth struggle story, watch the epidemic resistance film touching moment..." the national May fourth red flag youth corps committee-Dalian district committee today in Dalian plulan district to launch "welcome 20 always follow the party with the new journey" theme "cloud class", guide the youth rooted patriotism, carry forward the spirit of may 4th, bear in mind the mission. In the "cloud class", Sun Si, secretary of Pulandian District, led the young people to review the history of the league. Four front-line young representatives of rural revitalization and scientific and technological innovation shared their stories of struggle and youth dreams through video link. The short film "Youth Against the Light" brings you to feel the responsibility and responsibility of young people during the epidemic prevention and control period. At the same time, the Communist Youth League District Committee also carried out the online activity of 100 new league members to join the league, and thousands of old league members reviewing the league oath, leading the district youth league members to learn from the youth example, keep in mind the original aspiration of joining the league, and practice the youth oath. The youth league members have expressed that they will shoulder the historical mission, unswervingly listen to the Party, feel the Party's grace, follow the Party, work hard, and do not idle. According to reports, the League Dalian Pulandian District Committee will also carry out "a hundred years of youth ambition, new journey" youth typical selection tree publicity, held "youth heart to the party to the new era" Pulandian District youth from all walks of life to celebrate the 100th anniversary of the founding of the Chinese Communist Youth League theme symposium and a series of activities.

2022-04-26 The Communist Youth League Tianjin Peace District Committee organized the centralized service month activities to collectively carry out the centralized service month activities in their respective units. The young notaries of Heping District Notary Office received visitors in the reception hall, distributed publicity materials such as the Civil Code, Notary Law, Youth Civilization Service Card and other publicity materials, and answered common notarization questions about houses such as housing transfer, inheritance and entrustment and agency. The young police officers from the Second Civil Trial Court of the People's Court of Heping District answered the questions of the visiting parties and guided the parties to resolve the disputes through pre-litigation mediation. The First Procuratorate of the People's Procuratorate of Heping District brought information remotely during the epidemic period, used procuratorial technical support to crack down on crimes and timely and effectively safeguard social security; conducted online publicity of law popularization, resolved social contradictions and spread positive energy of the rule of law. Youth league members of Heping District Hospital of Traditional Chinese Medicine carry out voluntary service activities such as voluntary consultation, diagnosis and treatment, and blood pressure measurement for patients, strengthen pre-examination and triage, and improve the service level. The Youth Civilization number of Chronic Disease Department of Nanshi Street Health Service Center of Heping District collectively carried out medical consultation activities to introduce family doctor signing services for the elderly and free physical examination services for the elderly. The Obstetrics and Gynecology Department of Heping District Obstetrics and Gynecology Hospital carries out convenient services such as medical guidance and health consultation, and takes the initiative to "show its identity, image and commitment". The young firefighters from the Fire and rescue station of the Heping District Fire and Rescue Detachment conducted demonstration activities to explain and display the performance, technical parameters and operating procedures of the equipment. Xi'an Road Fire and Rescue Station of Heping District Fire and Rescue Detachment held a business competition activity, to improve its professional skills and level through the business competition, and to show a good mental outlook and superb business ability.

2022-04-26 Shuangliu district, Chengdu, Sichuan: more than hundreds of youth hired part-time cadres of Chengdu shuangliu district: hundreds of youth hired part-time cadres xin wang) on April 22, Chengdu, Sichuan province shuangliu held the communist youth league grass-roots organization reform pilot advance and cadres "mix" -shuangliu district town (street), village (community) part-time cadres hiring work. At the meeting, more than 100 young people from enterprises and universities were hired as part-time league cadres at all levels in Shuangliu District. At the meeting, the Shuangliu District Committee of the Youth League to 9 young entrepreneurs, outstanding young enterprises issued the town (street) youth league (work) committee part-time deputy secretary of the letter of appointment, and signed the part-time youth league cadre management responsibility letter. Youth League Shuangliu District Committee also awarded 10 college students to the school members of the part-time village (community) youth league branch deputy secretary letter of appointment, and signed a part-time youth league cadre management responsibility letter. It is understood that a total of 118 college students will be employed as part-time deputy secretaries of the village (community) youth league branch in Shuangliu District. So far, Shuangliu District has formed a district-level, town (street), village (community) three-level part-time youth league cadre system. The meeting also made arrangements for the key work of the Communist Youth League in Shuangliu District in 2022, requiring the league organizations and cadres at all levels to focus on the center, serve the overall situation, and go all out to complete various tasks.

2022-04-25 The Volunteer Incentive Plan in Xinfeng County, Shaoguan City, Guangdong Province- -the "Yunjin Volunteer Benefit" was officially launched. Correspondent for figure China youth network Beijing on April 25 (xinhua Ji'an-wei zhang, correspondent li) to promote new venture to create the national civilized city work, normalized volunteer service activities, create strong volunteer service social atmosphere, recently, Shaoguan county propaganda department, county civilization office, the county jointly promote new venture volunteer incentive plan- "cloud bun volunteer hui" officially launched and launched. At the ceremony, the relevant person in charge of the county party committee propaganda department for the love business representatives issued a cloud bun volunteer hui love business logo. Launch ceremony site. Correspondent for figure meeting, all the love business representatives signed the "cloud bun volunteer" pledge, the first in "cloud bun volunteer" love businesses include travel agencies, hotels, catering, entertainment, wedding, wedding, supermarkets, hairdressing, fitness, training industry, a total of 110, the implementation of the volunteers in cultural tourism, entertainment, enjoy the people's livelihood concerns related preferential or preferential treatment. In addition to providing incentive services for volunteers, the volunteer service incentive program will also invigorate the market after the epidemic. In the next step, the Communist Youth League County Committee will continue to make efforts, combined with the reform pilot work, linkage more social resources, mobilize more businesses to join the "bun volunteer benefit", seek benefits for more volunteers, and steadily promote the work of building a national civilized city in Xinfeng County.

2022-04-20 Anhui Chuzhou langya district held young pioneers civilized etiquette display activities in Anhui langya district held "please rest assured power have me" young pioneers civilization etiquette show wei correspondent, wang pei-pei check WeiTing) to meet the party's twenty big, thoroughly study and apply xi new era of socialism with Chinese characteristics, further advancing the reform of the young pioneers, standardize the young pioneers organization culture construction, reveal the young pioneers cultural appeal, strengthen the young pioneers of glory. Recently, the Youth League of Chuzhou City, Anhui Province, Langya District Committee, Langya District Education and Sports Bureau, Langya District Civilization Office, Langya District Young Working Committee jointly held the "please rest assured that the party strong country has me" Langya District, the third Young Pioneers civilized etiquette style exhibition activities. The young Pioneers etiquette style exhibition activity adopts online display, with a total of 15 teams from the whole district to participate in the exhibition. The activity is divided into two links: young Pioneers basic etiquette display and dynamic squadron style display. In the young Pioneers etiquette display link, the young Pioneers through the red scarf and the team cadre logo wearing, formation arrangement, three-level report, flag out, team salute, singing team song, call sign, flag withdrawal and other etiquette, to show the young Pioneers' unique organizational culture, showing their sense of honor and the sense of organizational belonging. Dynamic squadron style display link, each team closely around the hope and entrust of General Secretary Xi Jinping, combined with the help to create a civilized city of Chuzhou city, for a wonderful micro team display. The young Pioneers and the counselors reported the sound setting of the squadron and the development of the "five small schools" activities through colorful forms such as storytelling, poetry recitation, singing and dancing, painting, and manual court exercises. From the team members to tell the story of the squadron, show the wonderful moment, shout out the squadron inscription and other forms to show the school "dynamic squadron" to create activities distinctive, lively, full participation, normal, the team members are confident and self-improvement, innovation and creation, give full play to the role of collectivism spirit and small master role. After the careful selection of the evaluation group, Chuzhou second primary school eagle squadron, Chuzhou Qingliu primary school Jingya squadron won the special prize. The remaining 13 teams won the first and second prizes respectively.

2022-04-20 In Guangxi industrial county to carry out the "speak for help" series of practice in Guangxi industrial county "speak for help" series of practice Xie Yang) since this year, the Guangxi industrial county party committee to meet the party's twenty as the main line, combined to celebrate the 100th anniversary of the county youth league organizations at all levels, organizations of the young pioneers to carry out the hundred league history, one thousand players for pioneer, youth help revitalization as the main content of the "speak for help" series of practical activities. Xingye County organized the establishment of a league history publicity group composed of 100 league secretaries and cadres, going deep into villages (communities), communities, workshops, construction sites, campus and other production and life lines to carry out 240 league history publicity demonstration classes, building a solid ideological foundation for the league members of the county. The county elected thousands of young pioneers to be the "small pioneer", village (community) "little Lei Feng", folk culture "small inheritors", walked into the Xingye County literature and history hall, Chenghuang Town Zhensheng building and other off-school practice bases, to carry out the "red map" xing "research", "red scarf search for the party's centennial footprints" 80 times. Carry out the theme team class to learn the "Zhuang March 3" folk culture, organize the young pioneers to participate in more than 200 times, ceaselessly red gene. Societe generale county organization of young cadres, teachers and members, the young pioneers "youth" volunteer service, deep into the fields, to carry out the "spring service I first, youth pioneer example" activities, organize agricultural skills training 26 times, be a good spring "charge", "technical group", "field", help the masses win the food battle.

2022-04-18 Tianjin Jinnan District held youth civilization centralized service activities Implementing the spirit of the Sixth Plenary Session of the 19th CPC Central Committee, Further carry forward the spiritual connotation of the youth civilization of "dedication, cooperation, excellence and dedication", Tianjin Jinnan District Committee took the opportunity to celebrate the 100th anniversary of the league, Launched the "Welcome 20 always follow the Party to forge ahead on a new journey" youth civilization centralized service month activity, Unite and lead the youth civilization to create a collective and the majority of young people to establish the work concept of serving the people, love and dedication, Actively do practical things for the masses, solve difficult problems, To meet the party's 20th victory held with outstanding results. Jinnan district 15 kindergarten, Tianjin macro shun joint property management service group co., LTD., build river west town mud sell village league branch, jinnan tenth kindergarten league branch youth civilization create collective into the community, through posters, brochures, to popularize the residents of green environmental protection, epidemic prevention and control knowledge. The Youth Civilization Department of the Small Station of Jinnan District Justice Bureau created a collective innovative form, carried out cycling law popularization publicity activities, and distributed leaflets and publicity materials of the Constitution, Civil Code, Criminal Law and other laws and regulations to the passing masses, to answer the legal questions about civil infringement and marriage inheritance raised by the masses. The Youth Civilization of the Youth League Branch of Jinnan Economic Development Zone and the District Human Resources and Social Security Bureau held the theme of "Help enterprises to develop, and attract talents" live broadcast activity with posts, and organized enterprises to recruit college graduates through the network live broadcast. Pilot the future (Tianjin) business management co., LTD., league branch youth civilization to create a collective joint south economic development zone management committee, sailing future (Tianjin) productivity co., LTD. For development zone enterprise "specialization, new" policy online briefing, invited nankai university science Dr Yan Hong teacher introduced "specialization, new" small and medium-sized enterprises gradient cultivation policies. Jinnan District Government Service Office assistant lead team youth Civilization creation collective in the district government service center to carry out the "volunteer service" as the main business assistant activities. The activity will set up publicity points at the entrance and service desk, arrange special personnel in the self-service area to provide guidance to the application online staff, and provide assistant agents for the elderly, the sick, the disabled and pregnant and other special groups, so that the visiting staff more warm, comfortable and gratified.

2022-04-15 Tianjin Communist Youth League: Take social organizations as the fulcrum to leverage the biggest force of serving teenagers Hu Chunyan) social worker Han �, � a "cloud classroom" with the theme of youth safety and self-protection has be so popular. A few days ago, the event quickly flooded into the official account of "Chenqing e-color" of the Beichen District Committee of Tianjin Municipality. " Because there were too many people, we had to divide them into two phases."This hot scene let Han � hurried feeding core Chang Jin Jin � she knows, most of them are" old fans "and" iron fans "," the children like us very much, recently are at home online classes, looking forward to having activities to play together."This is one of the youth Tianjin Beichen District Committee relying on youth social organizations. To get through the county communist youth league organization reform at the grassroots level "the last kilometer", in recent years, the youth league organizations to develop youth social organizations as the fulcrum, to explore the group "buy vegetables", "cooking", evaluated integratedly village "order", village "teenagers" taste ", the social from all walks of life" review " social full closed operation mode, move up the whole society service youth biggest force. Cultivate the soil suitable for the growth of social organizations to do grass-roots labor coordinator, as a social worker, Han � rough � ya first ten years, are concerned about the people's family affairs. When she quit her stable job in the neighborhood committee and settled down to run the Xingyao Future Social Work Service Center, most of her family and friends did not understand. But she hopes to "realize her ideals while she is young", and "like to deal with people and be willing to serve people" is her original intention to establish a youth social organization. She has been serving the community residents. She not only understands the rules and procedures of grassroots government operation, but also knows the most concerned issues of the common people and the troubles of people of different ages. She developed her ability at coordination and communication, and launched a series of volunteer service projects to meet the needs of different residents. In the early days of its establishment, both the site and the personnel were difficult problems. The Beichen District Youth League organization helped her to coordinate about 1,000 square meters in the community Party and mass service center as a fixed place for office work and activities. More importantly, through government purchase, the brand project of the social work service center was included in the series of projects of the "Weekend Practice Camp" of the Communist Youth League District Committee, and included in the Young Pioneers series of "competition for chapter" activities. Nowadays, the towns and villages in the whole district can "order" according to the characteristics and preferences of teenagers in the district, and teenagers can "taste" a variety of colorful activities and "big meals" at home. League beichen district party committee deputy secretary XingHongDan said, while the youth group growing diversity, personalized service demand, one side is the number of grassroots cadres is very limited, develop more and better youth social organizations, has become an important way of the communist youth league contact service guide youth, " the development of social organizations both help to promote the reform of the communist youth league, is also beneficial to extend the youth work force."In recent years, the Communist Youth League Beichen District Committee makes full use of the Communist Youth League resources and talent advantages, under the great strength to cultivate a good social soil suitable for the growth of professional institutions of youth work. At present, it has guided the establishment of 10 youth social organizations and recruited more than 100 full-time social workers. On the one hand, relying on the "youth home" and other league positions to coordinate the town and streets, provide space and funding support for social organizations; on the other hand, the community social work service youth incentive system, youth social work project points system, establish youth social work rotation mechanism, two-way communication mechanism, public venture capital project incentive mechanism, forming the complement of "municipal full-time social workers + social organization + community social workers". Let professional people do professional things is like the seed of fertile soil. With the support and endorsement of youth league organizations, a number of rapidly growing youth social organizations also increasingly show the unique professionalism, flexibility and youthful vitality of social forces. Qing set sail social work service center is beichen the communist youth league guidance established the first "green" social work service center, in accordance with the "social work work independently, social participation" ideas, the social work service center to explore "social work + social organizations + community + volunteers + community enterprise" five club linkage working mode, become the communist youth league directly facing and service youth comprehensive window and platform. In 2017, the social organization received the first public welfare project of the youth league organization to "Pay the bill" - -the "Careful Communication and Love Walk together" youth crime prevention program. It is a special service for teenagers with behavioral bias or bad behavior tendency. At that time, a boy was very nervous with his parents, he was depressed for a long time, and his sleep was very poor. His mother insisted on taking him to participate in the activities. While giving professional counseling, the psychological consultant encouraged him to participate in various volunteer service activities. Gradually, the boy found his confidence in serving others, and his sunny and confident smile reappeared on his face. After years of deep work in the prevention of juvenile delinquency, the social work service center has also become the first socialized judicial and social service center for minors in Tianjin. Zhang Kun, the head of the center, said that the community where the center is located has a large floating population, and the family economic conditions of the residents vary greatly, and there are many "marginal" teenagers. In the survey, they learned that, especially after the implementation of the "double reduction" policy, many migrant workers children did nothing to do on weekends, and their parents were unable to discipline them. In response to this phenomenon, Zhang Kun and his colleagues contacted the community and opened a young eagle class for these children. It was also during the group activities in the Young Eagle class that the social workers found two troubled teenagers. One of the children suffered great psychological trauma from the death of his mother. Zhang noticed that the child was always cautious and did not like to communicate with others. The social worker immediately communicated with the community in time. With the consent of his father, the social worker repeatedly went into the child's home for psychological counseling, encouraging and helping the child to get out of the haze slowly. XingHongDan said, in recent years, the youth league organizations actively explore social operation mode, combining youth interests integrate youth social organization resources, gradually expand the service scope to the rule of law education, psychological counseling, security, quality development, traditional culture, schoolwork counseling, and other aspects, such as government purchase services to support excellent social organization growth. Let more teenagers have a sense of gain in recent years, the Youth League Beichen District Committee carefully polished out a number of hands, the masses recognized, targeted strong service brand. More and more young people are also taking the initiative to volunteer services and participate in community governance. Since the outbreak of this year, beichen district youth league organizations quickly condensed more than 4000 young volunteers, formed 16 youth commandos, 243 teams in epidemic prevention and control, " social organizations in organization scheduling volunteers has played a very important role, some social organizations led volunteers actively contracted part of the nucleic acid detection point of the service task."Said Xing Hongdan. By Han � Chuntun �, the "small public welfare experience officer" activity, founded by the � people, is to let the children understand the big truth in practice. For example, in a recent educational activity with the theme of "civilized dog raising", social workers encouraged children to go into the community residents to conduct research and listen to different views; independently refer to various materials, think about the relationship between man and animals and the harmony between man and nature, and finally show their views through research reports and debates. Safety self-care education class is not a simple scripted lesson, young social workers designed safety knowledge "everyone to find fault" activities, as well as safety knowledge questions and answer contests, teaching and fun."A lot of kids just run over to our office."Han � onion ψ Pan Dan � and many children's parents have also volunteered to volunteer in recent years. Fang Wei, minister of Tianjin Municipal Committee, said that the process of leading and helping social organizations promotes the self-innovation, and that the growing strength of youth social organizations also extends the tentacles and strength of youth league organizations, making the service more considerate and closer to the youth.

2022-04-14 Meizhou Wuhua: The first county-level permanent youth volunteer commando Meizhou Wuhua: The flood control drill is very "hardcore" in the Meijiang River waters, more than 40 young people emergency gathered, under an order, all the staff quickly and orderly busy. On the water, six young people drove two charge boats quickly driving, after a rescue, a "drowning people" safely arrived at the shore, and then rushed to the next rescue point. On the shore, three young people monitored the "drowning people" of the rescue... and the tense atmosphere made everyone on the scene hold their breath."That's the end of today's exercise!"League Wuhua County Party Secretary Li Yifeng's words, let the people on the scene realize that this was a flood control drill. The flood control drill team is five county youth volunteer commandos, was founded on November 27,2021, is guided by wuhua county civilization office, five county director, five county volunteer federation, five county emergency rescue association, 995 emergency rescue brigade of Guangdong province squadron, five county club jointly launched youth organizations, is a set of road rescue, water rescue, communication rescue, emergency rescue, jungle rescue, medical rescue, comprehensive, multi-functional, professional social emergency rescue and relief team. Xue Wentao, a young man born after 1985, is the team leader of this team. With more than 10 years of volunteer service experience, he told reporters modestly, " I am a temporary team leader. I will have more professional and capable people in the future, and let them lead this team."Xue Wentao has participated in volunteer services since 2010 and is one of the first volunteers in Wuhua County. He set up the first love team volunteer service team in Meizhou city and served as the leader. This love team brought together a group of caring young people, and insisted on carrying out the "Red Ribbon" volunteer service activities every year. For more than 10 years, Xue Wentao has led the charity team to 16 towns in the county, carrying out charity actions such as poverty alleviation, poverty assistance, education, care for left-behind children, and lonely elderly people. He is the first person in the county to bring goods to rural farmers, and helped more than 200 farmers sell agricultural products. In order to help improve the quality of Wuhua agricultural products to expand sales, he also opened a food processing factory to create the brand of Hakka characteristic products "Ping Yuan" brand, continuously and effectively help local farmers get rich."After so many years of volunteering, I also want to upgrade my volunteering and help people in need with a more professional strength."Talking about why willing to participate in the five county youth volunteer commandos, wen-tao xue said:" more and more volunteers, but the original form of volunteer service can no longer stimulate my satisfaction, now more and more natural disasters, professional rescue can save lives, life first, this is what I should do."In addition to work, Xue Wentao talks to friends about volunteer service. He introduced, " Wuhua County has a strong volunteer service atmosphere, which is the first county in Meizhou city to carry out volunteer service work. Thanks to the guidance and guidance of the villagers, I also joined the volunteer service under his drive and found the interest in life of helping others."Hu Donghui, one of Xue Wentao villagers, Wuhua County, lives in Shenzhen. Dong-hui hu told reporters that he went to Shenzhen entrepreneurship after little success joined the baoan district volunteers federation, then slowly have a little achievement, think should do something for my hometown, joint Shenzhen qin jiang culture research villagers donated money launched the five China county volunteer federation, drive more people to help their hometown development."I am a beneficiary of the reform and opening up, and volunteer service is a symbol of modern civilization. Although my hometown Wuhua is slightly behind that in Guangdong, our spiritual civilization should not lag behind. I just want to bring the advanced practice of volunteer service in Shenzhen to my hometown."Now, Hu Donghui, who is already the executive vice president of the Guangdong Provincial Social Work and Volunteer Cooperation Promotion Association, has more opportunities to mobilize more resources to promote the development of spiritual civilization in his hometown. Under his promotion, people outside Wuhua County also established a mechanism to help their hometown regularly and repay the support for their hometown. Wuhua county youth volunteer commandos is also established under the promotion of Hu Donghui, he as commandos general coordinator, responsible for the integration of various resources, " our five county youth volunteer commandos rescue equipment, is the most advanced in the communist youth league organization, some time ago, adjacent xin city have rescue mission, Meizhou city rescue association emergency dispatch our equipment and team to participate in the rescue work."Hu Donghui also said:" Wuhua County Youth volunteer commandos and other social rescue forces are different, we are the head of the Communist Youth League county Committee, will gather more aspiring young people professional strength, is an effective supplement to the government rescue force."At present, wuhua youth volunteer commandos organization propaganda, grassroots governance, medical aid, water rescue, jungle search, supervision, vehicle rescue, communication support, training, logistics, the main task is to popularize knowledge of all kinds of emergency rescue, improve social response ability, to participate in various rescue activities, to assist the government in emergency rescue work, encourage youth in the service society, help others in the selfless pursuit of life, reflect the youth bear. Li Yifeng, party secretary of the Youth Wuhua County, told reporters: " In the process of epidemic prevention and control, the youth commandos are the backbone of the epidemic prevention."It is understood that in January 2022, the commandos sent more than 30 vehicles, serving 60 nucleic acid sampling sites, and transporting 360 samples. Li Yifeng introduces: " the water rescue drills to improve the wuhua youth volunteer commandos flood control and emergency team comprehensive quality and emergency disposal ability, make the commandos can orderly, quickly and efficient flood control and emergency relief work, earnestly implement the wartime state, wartime practice, wartime discipline of flood control and disaster relief work requirements, early preparation, early prevention, create a call up, to fight, war win team, efforts to wuhua county flood control and disaster relief, emergency rescue, forest fire prevention and control, maintenance and stability work play a positive role."Through drills, commando team not only master charge boat driving technology, strengthen the adaptability of the water rescue, also accumulated certain driving experience and rescue experience, for solid wuhua county water rescue work, give full play to the role of youth force and commandos, help people to solve practical difficulties, maximum protect people's lives and property safety laid a strong foundation.[Expert comments] Wuhua County youth volunteer commandos have three characteristics: the party committee support, the youth League county committee guidance, to ensure the political orientation of the commandos; local elites support, professional guidance, to ensure the operation guarantee and professional strength of the commandos; the youth of the whole society actively respond, to ensure the mass character and driving force of the commandos. It is hoped that the youth volunteer commandos of Wuhua County will overcome difficulties and serve the masses from the four major areas of rural revitalization, rural governance, rural mutual assistance and rural ecology, find the right entry point, explore deeply, build consensus, and continue to form a new brand of Wuhua volunteer service.- -Tan Jianguang, a research and training expert of China Volunteer Service Federation and president of Guangdong Social Work and Volunteer Cooperation Promotion Association, and a reporter of China Youth Network of China Youth Daily

2022-04-11 Meizhou city, Guangdong province, the first county-level youth flood control volunteer commandos set up the flood control drill is very "hardcore" the first county permanent youth volunteer commandos severe, Meijiang water rescue site let people stop to watch, this is the Guangdong Meizhou city Wuhua county youth league committee established Wuhua county youth volunteer commandos flood control drill. On the afternoon of March 31, more than 40 young people gathered in the Meijiang waters of Wuhua County, Meizhou City, Guangdong Province. Under the order, all the people were busy quickly and orderly. On the water, six young people drove two charge boats quickly driving, after a rescue, a "drowning people" safely arrived at the shore, and then rushed to the next rescue point. The shore, 3 young people to rescue the drowning people for the body index monitoring....... The tense atmosphere made everyone at the scene hold their breath."That's the end of today's exercise!"Li Yifeng, party secretary, made the people on the scene realize that this was a flood control drill. The flood control drill team is five county youth volunteer commandos, was founded on November 27,2021, is guided by wuhua county government, five county volunteer federation, five county emergency rescue association, 995 emergency rescue brigade of Guangdong province squadron, five county club jointly launched youth organizations, is a set of road rescue, water rescue, communication rescue, emergency rescue, jungle rescue, medical rescue, comprehensive, multi-functional, professional social emergency rescue disaster relief team. Xue Wentao, a young man born after 85, is the captain of this team. With more than 10 years of volunteer service experience, he modestly told reporters: " I am a temporary captain, and I will have more professional and capable people in the future, so let them lead this team."Xue Wentao has participated in volunteer services since 2010 and is the first group of volunteers in Wuhua County. He set up the first love team volunteer service team in Meizhou city and served as the team leader. This love team gathered a group of caring young people with car owners, insisting on the annual "red Ribbon" love care test for the Wuhua County college entrance examination volunteer service activities. For more than a decade, Xue Wentao has led the team of volunteers to visit 16 towns and townships in the county to carry out poverty alleviation, poverty aid, education, care for left-behind children, and lonely elderly people. He was the first person in the county to bring goods to rural farmers, helping more than 200 farmers sell agricultural products. In order to help improve the quality of Wuhua's agricultural products and expand sales, he also started a food processing factory, to create the Hakka characteristic product "Pingan Yuan" brand, to continue to effectively help local farmers get rich."After so many years of volunteer service, I also want to upgrade my volunteer service and help people in need with a more professional strength."Referring to why willing to participate in the five county youth volunteer commandos, wen-tao xue said:" more and more volunteers, the original volunteer service form no longer can inspire me, now more and more natural disasters, professional rescue can save lives, life first, this is what I should do."Xue Wentao likes drinking tea and studies cultural and creative products. In addition to work, what he talks about most with his friends is volunteer service. He introduced, " Wuhua County has a strong volunteer service atmosphere, which is the first county in Meizhou city to carry out volunteer service work. Thanks to the guidance and guidance of the villagers, I also joined the volunteer service under his drive and found the interest in life of helping others."Hu Donghui, one of Xue Wentao villagers, Wuhua County, lives in Shenzhen.02 Commando run from follow villager support Hu Donghui tells reporters that he went to Shenzhen entrepreneurship after little success joined the baoan district volunteers federation, then slowly have a little achievement, feel that should do something for his hometown, joint Shenzhen qinjiang culture research folks donated money launched the five county volunteers federation, drive more people to help their hometown development."I am a beneficiary of the reform and opening up, and volunteer service is a symbol of modern civilization. Although my hometown Wuhua is slightly behind that in Guangdong, our spiritual civilization should not lag behind. I just want to bring the advanced practice of volunteer service in Shenzhen to my hometown."Now, Hu Donghui, who is already the executive vice president of the Guangdong Provincial Social Work and Volunteer Cooperation Promotion Association, has more opportunities to mobilize more resources to promote the development of spiritual civilization in his hometown. Under his promotion, people outside Wuhua County also established a mechanism of regular assistance with their hometown to return to and support their hometown. Wuhua county youth volunteer commandos is also established under the promotion of Hu Donghui, he as commandos general coordinator, responsible for the integration of resources, " our five county youth volunteer commandos rescue equipment, is the Meizhou city communist youth league organization, some time ago, next door xin city have rescue mission, Meizhou city rescue association emergency dispatch our equipment and team to participate in the rescue work."Hu Donghui said. Hu Donghui added: " Wuhua County Youth Volunteer Commando and other social rescue forces, which are different. We are set up under the guidance of the county Youth League Committee, and will gather more professional forces of aspiring young people, which is an effective supplement to the government rescue force."At present, wuhua youth volunteer commandos organized propaganda, epidemic prevention and control, medical aid, water rescue, jungle search, vehicle rescue, communication support, training supervision, logistics, a total of nine phalanx, the main task is to popularize knowledge of all kinds of emergency rescue, improve social response ability, to participate in various rescue activities, to assist the government in emergency rescue work, encourage youth in the service society, help others selfless dedication of practice life pursuit, reflect the youth bear. Li Yifeng, secretary of the Wuhua County Communist Youth League Committee, told reporters: " The youth commandos are the backbone of the process of epidemic prevention and control."It is understood that in January 2022, the commandos sent more than 30 vehicles to serve 60 nucleic acid sampling sites and transport 360 specimens. Li Yifeng introduces: " the water rescue drill aims to improve the five China youth commandos flood control and rescue team comprehensive quality and emergency disposal ability, make five China youth commandos can orderly, rapid and efficient flood control and emergency relief and relief work, earnestly implement the wartime state, wartime practice, wartime discipline of flood control and disaster relief work requirements, early preparation, early prevention, create a call up, to fight, win team, efforts to wuhua county flood control and disaster relief, emergency rescue, forest fire prevention and control, maintenance and stability work play a positive role."Through drills, five China youth volunteer commandos players not only master charge boat driving technology, strengthen the adaptability of the water rescue, also accumulated certain driving experience and rescue experience, for a solid five hua county water rescue work, give full play to the role of youth force and commandos, help people to solve practical difficulties, maximum protect people's lives and property safety laid a strong foundation, ready to contribute to the county flood control work youth power at any time.[Expert comments] The establishment of Wuhua County Youth Volunteer Commando is an inheritance in the first hundred years of the regiment. From the commandos to the present volunteers, and then to the youth volunteer commandos, this is the continuation of the inheritance of a young pioneer role, and also the process of the professional development of volunteer service. This youth volunteer commando has three characteristics: the party committee support, the youth League county committee guidance, to ensure the political orientation of the commandos; local support, professional guidance, to ensure the commandos operation guarantee and professional strength; the whole society youth actively respond to ensure the mass and driving force of the commandos.- -Tan Jianguang, a research and training expert of China Volunteer Service Federation and president of Guangdong Social Work and Volunteer Cooperation Promotion Association

2022-04-11 Anhui yijiang district organization to mobilize young people from all walks of life involved in epidemic prevention volunteer corps yijiang district party committee organization to mobilize young people from all walks of life in epidemic prevention volunteer service China youth network Beijing on April 11 (reporter Zhang Jianwei, correspondent, Li Lei super check WeiTing) " now is 10 PM at night, I in high speed bayonet pass you the current vehicle access, the volunteers also hard."Recently, Cheng Jian, a volunteer of Lei Feng Love Team in Yijiang District, Wuhu City, Anhui Province, recorded a simple video to share in the volunteer group, and prompted passing drivers to prepare health codes and travel codes, and conduct temperature measurement, registration and nucleic acid testing according to the guiding order."The expressway intersection is the first pass to prevent the importation of the epidemic. Our volunteer service starts at 6 am, working three shifts a day, and assisting at the expressway intersection 24 hours a day."He said that the main task of the young volunteers at the highway intersection is to conduct double codes and double checks, epidemic prevention and health registration, and to assist professional medical workers in nucleic acid tests. Cao Yanzhen, the post-1990 generation, is a volunteer veteran and signs up to participate in various public welfare activities whenever she is free. She has been involved in assisting the volunteer work of the high-speed bayonet for more than a week. " coincides with the Qingming holiday, the flow of the high-speed bayonet has increased compared with usual. I have been relatively skilled in these work, and I am very satisfied to contribute a little contribution to protect my own city. The "" Expressway intersection requires volunteer assistance, and you can register if you meet the requirements."Before the Qingming Festival, the staff of the Yijiang District Committee of Wuhu City completed the recruitment of the expressway young volunteers within half a day. During the 3-day holiday, a total of 50 young volunteers served at two expressway intersections within the jurisdiction of Yijiang District. This volunteer contact group has been particularly "lively" since March 15."I want to attend, Saturday and Sunday for all day."" Lei Feng love team to sign up, please arrange the time."On March 15, the group Yijiang District Committee organized the first time mobilization and social recruitment of volunteers, every day, the group appointment registration solitaire news constantly refresh. After two rounds of recruitment, 712 young volunteers from all walks of life have signed up. At present, more than 160 people have participated in the construction of nucleic acid testing shed, guiding signs and the maintenance of order maintenance at the testing points under the arrangement of the street league. At the same time, the youth league organizations at all levels in Yijiang District encourage volunteers with volunteer service experience in epidemic prevention and control, medical psychology and other disciplines to participate in the assistance work. Zhang Shanshan is a college graduate of the class of 2021. After preparing for a job at home, she immediately contacted the local community after seeing the recruitment information of volunteers, and then worked on duty at the gate of the community, and experienced the hard work of nucleic acid testing."Although I only did some small things and the work content was repeated, I communicated patiently and explained with the residents coming in and out, which improved my ability to deal with problems."She sighed. The Jiuzi Oral Youth League Branch of a non-public enterprise in Yijiang District of Wuhu city also responded to the call and actively arranged 23 medical staff to go to the isolation points and nucleic acid collection points for support. Soon after the league branch secretary Fu Hongrui got married, she learned that the quarantine site was in urgent need of medical staff. She has voluntarily signed up for the 14-day volunteer service twice, and she is still working in the quarantine site. The workload of the isolation site is very heavy, and Fu Hongrui often loses a mouthful of water a day because he wears isolation clothes for a long time. After the day of work, I was soaked with sweat."There is no insurmountable hurdle for one heart."Fu Hongrui said as he sent WeChat greetings to his wife. The Yijiang District youth volunteer service team is still growing. Every day, the volunteers of Yijiang District Social Organization Federation carefully arrange the scheduling, docking and guarantee of each volunteer post; Yuan Xin, a middle school teacher who has participated in the epidemic prevention work in 2020, volunteered to take up again to assist the community and the community in nucleic acid testing."The reason why we can extensively mobilize young volunteers in a short time and gain the trust of the object of service is that we usually pay attention to the establishment of a long-term working mechanism, strengthen the construction of grass-roots youth league organizations, maximize the cohesion of youth, and change the object of service into the service force."Tuan Yijiang District committee responsible comrade said.

2022-04-09 Linquan, Anhui province: Young people on the frontline of epidemic prevention and control. Linquan County of Fuyang City, Anhui Province, combined with the reform of the national county Communist Youth League, further integrated the resources of the league members, gave full play to the role of the youth league members as a new force, and demonstrated the youth responsibility on the front line of epidemic prevention and control. Youth members, please, the county party committee organization department issued by the organization agatanoatae unit party members and cadres actively involved in the epidemic prevention and control work line notice, the county immediately transferred personnel selected work, youth members actively practice "the party has called for action" oath, have recommended into the epidemic prevention and control line work."Please go here to cooperate with the temperature measurement. Have you left Linquan County recently? Have you been going to other places recently?"" Hello, please wear a mask, and show your health code and travel card with the inspection data."This is when Zhu Wenlei works on the front line of epidemic prevention. As a community staff member of TuanLinquan County Party Committee, he has been involved in epidemic prevention and control for more than 10 days. In the reform of the county Communist Youth League, Linquan County set up a youth development comprehensive service center, recruiting four staff members."Dai Jie, Li Xue and Wang Meiting respectively cooperated with young volunteers at the east gate, west gate and south gate of the county government to carry out the guidance and joint inspection of the peak post."According to the relevant person in charge," this work has been carried out for five consecutive days, the center staff and young volunteers are never late and absent, sometimes in order to work, at 7 am in the morning, politely guide into the gate staff to scan the code to measure the temperature."According to statistics, a total of 2,456 youth league members in the county participated in the epidemic prevention and control work, including 375 people in the youth league association and 2,081 people in towns and streets."We publicized the advanced deeds of the young people through the 'youth Linquan' platform, and called on the young volunteers in the county to devote themselves to the epidemic prevention and control work, to do the strong wind and grass, when the fire is really gold."Tuan Linquan County Party Committee related responsible person said. Youth home, I reported to " this spring is destined to be unforgettable, my friends and I feel the meaning of youth, more feel that today's happy life is hard-won."Yang Qi, a young volunteer, has said this. Recently, he went to the "Youth Home" in Chengnan Street to assist the community in the epidemic prevention and control work."Not as soon as necessary."" The owners of the community, scan the code can enter, thank you for your cooperation."" Hello, please show me your pass."Yang Qi in the new town community Hua an City royal Chen garden site intense work, far can hear his voice. Since March 2022, more than 120 youth league members have taken the initiative to report to the "youth home" of villages (communities), actively carrying out "personnel survey outside the province", "household verification information" and "love helping the elderly", to coordinate and deal with various situations and timely report work. Since last year, the Youth League Linquan County Party Committee has issued the Notice on the Construction of the materialized "Youth Home" (Interim), which standardizes the basic positioning, establishment process, construction content and related requirements of the materialized "youth Home". At present, Linquan County has established a county-level youth home, and the construction of youth homes in each township (street) has achieved full coverage. It has innovatively created a materialized youth home platform, and opened up the "last kilometer" for the Communist Youth League to directly contact and serve the youth."Youth home", the Communist Youth League around the youth."Young people's participation in volunteer service activities such as epidemic prevention and control has further enhanced their sense of social responsibility and opened up channels for their growth."According to the relevant person in charge of the league," the township (street) league organization every year to report to the youth league members for a comprehensive evaluation, volunteer service length, the number of activities as an evaluation reference, the outstanding performance of the youth league members to commend."Up to now, the" Youth Home " platform has carried out more than 30 service activities, benefiting a total of more than 2,000 people. In the fight against the epidemic, I relay "smile, put on a mask, the 'epidemic army' retreat...." recently, the youth League members organized by the Youth League Linquan County Party Committee recorded the gesture dance is popular on the Internet, they use the song of youth for the fight against the epidemic to cheer up. Early in the morning, Linquan No.5 Middle School, Linquan County New Huiying Middle School playground, students are rehearsing the program."Through online and offline methods, we will actively guide the young pioneers to participate in an orderly manner, do their contribution to the fight against the epidemic, and spread positive social energy."The county youth working committee related responsible person said. At present, a total of 12 primary and secondary schools, more than 20,000 young pioneers participated in the epidemic prevention program recording, to continue the fight against the epidemic, to create a good social atmosphere."Forward, forward, forward! Our team to the sun.............. "" the blue sky rescue team, the day Wolf rescue team members singing red songs, in the streets of the city for disinfection operations. It is understood that the League Linquan County Party Committee organized 15 league associations to form the "youth commandos", they went all over the community, the building, measurement with their feet, mouth propaganda, with the spirit of selfless dedication of volunteers to practice their duties and mission. One of the team members, Peng Zian, once won the honorary title of "The Most Beautiful Linquan Person". When the epidemic broke out at the end of 2019, he and his son Peng Jack formed a father-son team, risking the risk of the epidemic for two consecutive months. Since March 28 this year, Peng Zi'an and his wife Chen Liqi, his wife is responsible for driving, he is responsible for spraying, formed an anti-epidemic disinfection husband and wife file, has been shuttling between the local primary and secondary schools, kindergartens, continuous fighting, spraying disinfection."What about the lack of protective suits, medical masks, disinfectants and alcohol? Let's relay!"At the request of the association, the League Linquan County Committee issued the" Quan " collection order on April 5, to collect epidemic prevention materials from young committee members, young entrepreneurs, young entrepreneurs, volunteers and social caring people. Dai Hao, a young entrepreneur, has been busy traveling between young entrepreneurs with epidemic prevention supplies for several days. He said, " As young entrepreneurs in the new era, we should have a sense of social responsibility, take the initiative to take responsibility and be meticulous, and demonstrate the exemplary role of young people with practical actions."" 2,000 medical masks, 180 boxes of 84 disinfectant, 200 bottles of alcohol.............. " The whistling collection, microlight convergence.9 Hours!"Spring" city collection under the order of the spring speed is exciting. The Times need responsibility, responsibility to achieve the future."Participating in epidemic prevention and control and maintaining people's safety is not only an effective way for the youth members to serve the society, but also an bounden responsibility and responsibility. The youth in the new era should strive to become talents who can be used and shoulder heavy responsibilities, and live up to their youth and youth."Tuan Linquan County Party Committee related responsible person said.(Li Dongdong)

2022-04-09 Nearly 300 Hainan ChengMai youth volunteers in epidemic prevention line nearly 300 ChengMai youth volunteers in epidemic prevention frontline committee, at present, our province cities and counties have been sporadic or clustering COVID-19 outbreak confirmed case report, ChengMai epidemic prevention situation, to do the county epidemic prevention and control work, build the epidemic prevention and control line, so far, the communist youth league Chengmai county has recruited through multiple channels reserve nearly 300 young volunteers in various industries. It is reported, according to the provinces and counties two levels of epidemic prevention and control headquarters epidemic bulletin, the communist youth league Chengmai county joint county youth volunteer association, the first epidemic prevention and control volunteer service emergency work plan and mechanism, at the same time, the communist youth league Chengmai county joint several public welfare association, organization to carry out the duty during the epidemic prevention and control system, quickly in the epidemic prevention and control of the first line, ensure do on-the-job, at any time, at any time. In chengmai each nucleic acid detection sampling point, the communist youth league, young volunteers wearing "blue waistcoat", assist government staff to do a good job of returnees information registration collection, cooperate with medical staff layout sampling site, maintain sampling order, guide to nucleic acid sampling masses scan health code, etc., to provide necessary help for the masses.(Reporter Gao Yi)

2022-04-07 Group of Sichuan Qingchuan county to carry out the "qingming festival heroes" series of activities of qingchuan county of Sichuan province "qingming festival heroes" series of activities wei correspondent Yao Tianxiang) recently, Guangyuan city, Sichuan province qingchuan county organization county primary and secondary schools to carry out the "qingming festival heroes" special activities, with the county teenagers remember history, memorial heroes, inheritance "red gene". Fangshi town central primary school tomb-sweeping day martyrs tomb activities pictures. On April 1, the Fangshi town central primary school organized all the school teachers and students, with the mood of reverence, came to the Fangshi martyrs cemetery held the Tomb-sweeping Day martyrs' tomb activities. All the teachers and students stood in awe, played and sang the national anthem, to the revolutionary martyrs laid a wreath, flowers basket, deeply remembered the martyrs who died in the revolutionary war, all the young Pioneers to review the team oath, express the young pioneers to inherit the revolutionary martyrs will, study hard, work hard, struggle for the cause of communism lifelong strong determination. Jianfeng Town central Primary School Qingming Festival activity picture. Correspondent for the map of Jianfeng town central primary school to carry out the " remembering the revolutionary martyrs, Promote the red tradition " theme activities, Holding the qingming Festival "in memory of revolutionary martyrs" theme national flag raising ceremony, The secretary of the school Youth League Committee introduced the origin and significance of the Qingming Festival, Teach the students to remember the history, Remembering the revolutionary martyrs, Carry forward the red tradition, Representative of the young Pioneers spoke, To the heroic sacrifice of the revolutionary martyrs to express the high respect, infinite memory, And called on the young pioneers to inherit the revolutionary martyrs' legacy, Promote the revolutionary tradition, Keeping in mind the teachings of the Party, Listen to the party, Follow the party, Cherish today's hard-won good life, Establish lofty ideals, Honest and brave, Self-discipline and self-improvement, Strive to love the motherland, Good youth with lofty ideals; Conduct themed class meetings, Tell the red story; Watch the film of revolutionary history, Educate and guide all the young pioneers and communist Youth League members to remember the achievements of the martyrs forever, Cherish today's good life. Guanyindian Township Central Primary school theme activity picture. Guanyin shop township central primary school to carry out the "Qingming festival martyrs, hiking, grinding will" theme activities, 15 km journey, the students do not say bitter or tired, help each other, together; after the red song chorus activities, recall the war years, remember the fearless dedication of the revolutionary predecessors and the achievements of the Chinese revolutionary cause, the school brigade counselor speech, good to find the children to the revolutionary martyrs to pay high respect, encourage the children to learn from the revolutionary martyrs, miss them to remember them, inherit their red genes. Guanzhuang Middle School Qingming Festival activities. Correspondent for figure GuanZhuang middle school to carry out the "backbone bearing one thousand jins, love cast teacher soul" qingming festival activities, school youth corps committee secretary organized students to donghekou earthquake ruins park qingming festival martyrs activities, in he qingfang martyrs tomb about he qingfang teacher in the earthquake to save trapped students give heroic deeds and fearless dedication, organize students cleaning cemetery, respects memorial wall, silent and chrysanthemum, express the heart of the reverence of the martyrs and deep condolences to the victims.

2022-04-07 Shandong Ningjin County "three in place" to ensure a good "visit" work group Shandong Ningjin County Party Committee tamping work "three in place" to ensure that the good youth "visit" quality Xiaowei correspondent, Wu Haoran Cui Yihao) in order to further deepen the Ningjin "rural (community) good youth" selection and training work, Promote the practice of "I do practical things for the youth", Actively serve the youth development, Help to create youth-friendly cities, According to the deployment requirements of the Provincial Communist Youth League Committee and the Municipal Communist Youth League Committee, Through the implementation of the "three in place" measures to carry out the rural good youth "visit" work, Find out the difficult blocking points of the selection and training work, We will better "attract talents" and "empower" young people in rural areas (communities)."County-level + villages and towns" horizontal to the edge, to ensure that the visit in place. The Communist Youth League County Party Committee adopts the three-level linkage method of "the Communist Youth League County Party Committee + the town and street youth working Committee + the village (community) youth League organization secretary" to realize the full coverage of "the good rural youth" visit activities. At the county level, according to the principle of "adjacent regions, easy to organize, similar personnel and easy to visit", the Communist Youth League County Committee divided two teams to visit 47 good young people at the provincial and municipal levels.evaluated integratedly level, to the county 12 villages and towns (street) youth committee issued "about rural good youth" visit " work notice, give full play to the evaluated integratedly (work) committee, green committee functions, widely launched more than 100 township (street) youth committee and 561 village (community) youth league secretary work force, for the nearly three years of the county, evaluated integratedly, village level 865 rural (community) good youth industry, classification don't visit activities."Online + offline" vertical to the end, to ensure that the epidemic prevention is in place. In combination with the provincial and municipal notice requirements and the form of COVID-19 prevention and control, the Communist Youth League County Party Committee carried out the visit activities in various ways, not only "key to key", but also "face to face", so that the visits were warm and hot. Offline visits, the county according to the standard of not missing one person, establish "provincial, municipal, county, evaluated integratedly, village (community)" five MoPai parameter, in early march part evaluated integratedly in batches held 31 field symposium with 296 good youth discussion, field household into enterprise 136 visit good youth 167, widely collect demands and Suggestions. Online research, after mid-march adhere to the epidemic prevention and control of the bottom line, 12 evaluated integratedly youth committee actively innovative service way, let rural good youth visit activities continuously, not dropped, take "video link", "telephone visit", "WeChat research", visit good youth 283, further clarify the development measures, strengthen the visit work."Demand asking + solve difficulties" is promoted simultaneously to ensure that the effect is in place. The Communist Youth League County Party Committee visited various activities to have a detailed understanding of the difficulties and pain points of young people in production, life, employment and entrepreneurship. Through the visits, it sorted out 239 demands and suggestions and established a summary ledger, so as to effectively solve the development and urgent expectations of the youth industry according to the needs. The county will look for "rural revitalization partner" action combined with visiting activities, actively contact the county organization department, county club bureau and other seven departments, combing the relevant policy documents, form covers "housing", "green enterprise" credit "youth station" and more than 10 policy of the ningjin youth talent policy compilation, further find work point and focus, to provide targeted good youth "order" service, only 2021 for "ningjin rural good youth alliance" centralized credit 200 million yuan, truly realize "financial fu, youth take-off". Young people, return mulberry catalpa. During the epidemic period, the "Ningjin County Rural Good Youth Alliance" actively organized 302 good young people to participate in the front line of epidemic prevention and control, participated in various volunteer services such as village residence duty, nucleic acid testing, and "two look and one test", and donated a total value of isolation clothing, disinfectant and other epidemic prevention materials worth more than 50,000 yuan. In the next step, the Communist Youth League county Party Committee will further increase the rural good youth "visit" work efforts, to take more practical measures, to provide more practical services.

2022-04-03 Ten years for children with autism hold up umbrella ten years for children hold up umbrella for autism youth net reporter Hu Chunyan) on April 2, in the 15th "world autism care day" approaching, the communist youth league of Tianjin ninghe district committee of the UN network Tianjin ninghe company "sunflower" youth volunteer service to autistic children home "send home" education " activities. During the activity, the young volunteers donated paintbrushes, chess boards, building blocks, and other love materials that can improve their hand and eye coordination ability, and communicated with the children through interactive games and painting. One of the autistic children was twins and twins. In the third grade of primary school, the two siblings successively suffered from "regressive autism". The originally lively and cheerful children gradually lost the ability to express themselves and take care of themselves."In recent years, it is the volunteers who have given us help and encouragement that has made me full of hope for life!"In the face of the" Sunflower " youth volunteer service team for years of care, the child's mother was very strong and moved. Since 2012, the TuanNingHe district party committee organization, its Tianjin NingHe company to carry out the corporate responsibility, around the special children such as autism, carried out the care children grow volunteer service project, help more than 30 special children, in special education school donated a "sunflower" happy classroom, establish family, school, service "trinity" normal communication mechanism, real-time follow up special children growth, help relieve parents anxiety. A rehabilitation knowledge learning and practice group was set up to assist the teaching assistants to carry out daily courses. Through color painting, interactive games and program performances, a "heart bridge" between volunteers and special children was set up, so as to help the children get out of their own space and feel the care from the society. Ten years of company, "sunflower" for the "children of the stars" to hold up the protection umbrella. The "Sunflower" Youth Volunteer Service Team of State Grid Tianjin Ninghe Company will continue to pay attention to autistic children, continue to carry out the "sending education to households" activity, continue to expand the service content of youth volunteer service projects, give full play to the brand influence of "Sunflower", and create a good atmosphere of caring for the vulnerable groups in society.

2022-03-22 Xianghe youth in Hebei Province, who protect the epidemic with enthusiasm. They stick on the front line of fighting the epidemic and protect the safety of thousands of families. As one united, we will surely win!(China Youth Daily China Youth Network reporter Shi Weiqiang video: provided by the Communist Youth League Xianghe County Party Committee)

2022-03-21 "Point line", promote jia xiang students "home", "line" combination, promote jia xiang students "home" jianwei) to lead the broad students home internship entrepreneurship, help rural revitalization, participate in social governance, Shandong Jining jia xiang county in accordance with the national "home" county work requirements, adhere to the "line" combination of working ideas, will return college students social practice grasp often grasp, change "home" for "dedication", inject "green" vitality for rural revitalization. Precise grasp the focus "point", issued youth "assembly order". A total of 436 "green bird plan" internship positions, through the "Jiaxiang county" WeChat public number and WeChat group release "green bird assembly", attracted more than 1100 people through the "green bird plan" small program, according to the "two-way choice, reasonable deployment, nearby" principle for 436 college students assigned internship positions. More than 3,300 college student volunteers in the county reported to their villages (communities) during their return home, to carry out volunteer services such as "young eagle classroom" and living environment improvement. More than 60 returning college students have joined the activity of "recruiting new talents", appointed 7 students to become the county-level "Green Bird Station" stationmaster, and built a "heart bridge" connecting and serving students abroad. Actively extend the work "line", blow the youth "action number". We will guide returning students to contribute their youth to the development of their hometowns, help with the epidemic prevention and control lines, and be good "epidemic prevention workers". Help the safety production line, be a good "supervisor". Thirty-nine college students volunteers followed the staff to the production site of the town and streets of the enterprise to carry out electricity and fire inspection, and investigate potential safety risks. Help the national anti-fraud line, be a good "promoter". Carry out anti-electric fraud centralized publicity activities, and promote the "National Anti-fraud Center" APP in supermarket stores, streets and alleys. Help ban burning line, be a good "propagandist". Adopt the combination of "online + offline" method for three-dimensional publicity. Continue to expand the service "surface", play the youth "dream song". Make solid progress in "looking for rural revitalization partner" action, combing summary entrepreneurial talent support, young talent support related policies, to carry out "auspicious students feel home" activities, organize home college students to ZhengDe education base, youth community, youth talent accelerator, key enterprises, close perception home new changes, strengthen the students of home identity, sense of belonging. The first batch of 30 outstanding college students to hire back to the village (community) part-time training, for the county economic and social development to cultivate a high comprehensive quality, rich experience at serving the masses, and good at the youth reserve talent team.

2022-03-20 Group Ningxia qingtongxia municipal committee to meet 20 red scarf search activities qingtongxia municipal committee to meet 20 red scarf search activities Jianwei correspondent Zhang Tongtong), Ningxia hui autonomous region qingtongxia municipal party committee to carry out "welcome 20, be players" red scarf search activities, unity leading the city teenagers firmly follow the party, new journey. Qingtongxia city red scarf search team with the school shaoxian brigade as the support, Set up a red scarf search team in each first brigade, Xu Jingjing, member of the "National Red Scarf Tour", He Lingling and Wu Lijuan, outstanding Young Pioneers counselors of Qingtongxia City, were hired as the leaders, Relying on the red resources, Combined with the rich historical and cultural resources of Qingtongxia, Carry out investigation, search, visit and practical experience activities, Looking for the party's footprints, looking for red footprints, looking for advanced models, Through a series of searches, Integrate red genes into the blood, soak them into your heart, Guide teenagers to establish a correct world outlook, outlook on life and values, Bear the responsibility and mission of socialist builders and successors. Since the red scarf search activities, qingtongxia city 15 red scarf search team more than 160 small players, successively along the footsteps of xi grandpa xi jinping visited the Yellow River river bay, "July 1" medal winner Wang Lanhua, also went to the Korean veterans Cao Xinli grandpa listen to his story of revolution, search for the young pioneers theme team class 3, through different forms of glorious revolutionary history, feel the great achievements of reform and opening up, feel characteristic regional culture, inspire the emotion of love hometown. Next, qingtongxia red scarf search team in the spring semester and summer vacation to lead the members around the party footprint, "I see hometown new changes" "search around my youth model", snow maple brigade patriotism education base and other themes to find the great achievements, struggle story, tell the development around; autumn semester to "grandpa xi instruction in mind", "dialogue party 20 representatives" as the main content, lead the young pioneers pay attention to the party 20, understand the party's 20th.

2022-03-19 Group of Jiangsu built lake county: in the "disease" line bloom youth Yancheng built lake group county: in the "disease" line bloom youth under the grim situation of Yancheng built lake youth county as the initiative, organize the group, youth volunteers and all kinds of youth social organizations actively and orderly into COVID-19 epidemic prevention and control work, in the epidemic prevention and control of the first line show build lake youth power."Hello, let's register your relevant information on epidemic prevention and control." " How many people are there? Are they all vaccinated yet? How many needles? Family recently to go to other people... " Liu Liu is a northern Jiangsu program volunteer, is also a member of the county youth commandos. Since March 14, when the county launched a "knock on the door action", her workplace has been moved from the office computer to the doorstep of the residents in the community."These days the throat is really more painful" Liu Liu said, he usually do not talk, now knock on the door, every home to keep understanding the situation, try to use the shortest time to grasp the most complete information."Sometimes, when you have to climb three buildings in one night, and you knock from door to door, and you often miss people, so you need to visit people for a second or third time, until everyone's situation is verified."Liu Liu, who usually loves sports, always carries a sports kettle with him. He is filled with water in the morning, but he is too busy that he often drinks back at night. In the party and mass service center of Xihu Street Town Community, 24-year-old Ni Qinghu is busy recording the information of the community staff. Ni Qinghu, a freelancer, immediately signed up when he saw the recruitment notice for the epidemic prevention and control reserve on the wechat official account of the Jianhu Communist Youth League. A few weeks later, when the staff of the Communist Youth League county Committee posted the demand for volunteer positions in the group, he immediately contacted him and said that he was mobile during working hours, and that young people could use computers and could work immediately. In the community, Ni Qinghu is responsible for the information input of more than 1,000 households in the three areas. In addition to normal data entry, Ni qinghu also often receives unexpected tasks. In the early morning of March 16, Ni received a notice that the national nucleic acid test 81 sampling site lacked information registrars. Ni has worked in the wind for 17 hours since 4 am on March 16. Xia Zhengting, 29, who is a young Pioneers brigade counselor at Xiufu Primary School, has helped weave a tight protective net in the rear of the epidemic. The severe situation of epidemic prevention and control has broken the pace of people's life. Xia Zhengting realized that for the Young Pioneers, what is happening right now is a vivid and profound textbook. She planned and carried out the theme activity of "Small Hands, Big Hands, Childlike innocence, Fighting the epidemic together" to guide the young Pioneers to deeply understand the knowledge of epidemic prevention and control, strengthen their self-protection awareness, and improve their self-protection ability. The young pioneers are encouraged to become "small guards against the epidemic", with the pen as the "fight" and the paper as the "rong", and to pay tribute to the epidemic prevention workers through children's paintings, hand-copied newspapers and short videos. Counselor propaganda scientific epidemic prevention knowledge (photo) do strong wind grass, when the fire really gold. Youth league members are the link to highlight the organizational function and strengthen the connection between the Party and the league. In this great battle against the epidemic, the youth league members of Jianhu moved forward bravely and took the initiative to shoulder the mission entrusted to them by The Times.(Jian Qingxuan)

2022-03-17 Group ningyuan county to carry out the youth forest volunteer service action of Hunan ningyuan county to carry out the "add new green seedlings" young Lin volunteer service action Jianwei correspondent plate ling bead) in march, warbler grass long, Yongzhou, Hunan province ningyuan county county 57 youth league, 29 young pioneers, more than 3700 people, carried out the "add new green seedlings" planting youth forest volunteer service action, planting camphor trees, pine, cypress, camellia trees and so on more than 25000 plants. We roll up our sleeves, carry hoes, shovels, dig pits, help the seedlings, fill the soil, step on the solid, water... in our efforts, a vibrant green landscape jumped before us. The small group children and the small red scarves in Ningyuan have spread the ecological concept of "clear water and green mountains are golden mountains and silver mountains" with practical actions, created a good atmosphere of planting, protecting and loving green, and further enhanced the responsibility and mission of children and youth league members to be "green practitioners".

2022-03-16 Tuan Lingcheng District Committee has steadily promoted the "Hope House" child care project Jianwei correspondent Gao Lili) " My brother is very professional, very careful, and has a good attitude. Finish the hair feeling the whole person is much more beautiful! Thank you, the volunteer brothers and sisters, who are so concerned about us."In a hope hut in Zhengjiazhai, Lingcheng District, Dezhou City, Shandong Province, young volunteers patiently listened to their children's requirements for their hair style and trimmed them with a" energetic " hairstyle. Volunteer service pictures. Recently, the Youth League Lingcheng District Committee of Dezhou City, Shandong Province organized young volunteers into the "hope cabin" to carry out public welfare hair cutting, love free clinic volunteer service activities, to provide free haircut services, oral examination, vision examination. Helping the work is not just about renovating rooms. Building the "hope cabin" is the "first half", and the follow-up care is the "second half". Integrate security resources, for the hut continued "escort" group ling city district party committee will volunteer work as a "hope hut" project important "second half", efforts to advantage, continue to hut "escort", coordinate ling city resources power project operation, carry out the implementation of the "hope hut" assisted children specific care measures, do real normalized, project volunteer service. Jointly with the Civil Affairs Bureau to build a project of government purchase of youth social work services for children with difficult "Hope House" as a pilot, further promote the government's purchase of social work services for teenagers, and improve the operation of the "Hope House" child assistance project. Adopt the mode of "youth league organization + professional social workers + caring volunteers", and reach in-depth cooperation with four professional social work organizations. Enrich the volunteer support team and carry out volunteer training, effectively improve the service ability and quality level of the volunteers, and make the volunteer service more intimate and more professional. Volunteer service pictures. Correspondent for figure together love power, for the hut continued "warming" love gathering strength, love build hope, in 2021, ling city will "hope hut" pairing work into ling city "party history learning education" practical claim activities, the party and government organs, enterprises and institutions, under part of the party members to participate in the claim in the district "hope hut" plight children. According to the different characteristics of each child, we should pay attention to the professional characteristics of education, medical care, psychology, legal and other industries, and attract professional psychological counselors, doctors and teachers as professional love volunteers. At the same time, the follow-up assistance work of children in "Hope House" will be included into the juvenile security work system. So far, ling city has built 93 households complete pair claim, and continue to carry out the "wish" reached, colorful holiday feeling city, volunteer family, academic counseling, psychological counseling, for "hope hut" children provide more intimate help, efforts to make hope hut "warming" link more perfect. Volunteer service pictures. Correspondent for map innovative service mode, Continuously "protect" the innovative service mode for the cabin, Establish the "1 + 3 + N" Hope House Volunteer Service work standard, "1" is household once a month, "3" is the three types of groups, namely "professional social workers pair", "Party and government organs love volunteer pair", "targeted professional love volunteer help", "N" is a number of service sections, Carry out volunteer service activities, including family companionship, psychological counseling, self-care education, academic counseling, wish fulfillment, feeling the city, warm winter action and other aspects, Using learning from Lei Feng Memorial Day, International Family Day, National Day to Help the Disabled, June 1 " Children's Day, Mid-Autumn Festival, International Volunteer Day, New Year's Day, Spring Festival and other important time nodes, Focus on planning and carrying out service activities close to the needs of children in the "Hope House", Improve the social attention and influence of the children in the difficulties of the "Hope hut". Since the "hope hut" children care project launch, ling city volunteer services around 93 have built hut has carried out the "wish", "youth warm line" visit sympathy, love, family company, homework counseling, colorful holiday feeling city and other forms, rich content of characteristic service activities more than 60 times, giving support materials accumulated more than 40000 yuan.

2022-03-16 Guangdong Shaoguan new venture launched "double product double review" integral league system held middle school communist youth league "double product double evaluation" integral league system propulsion will month 16 (reporter Zhang Jianwei, correspondent Li Joyo) to strengthen the national county communist youth league organization reform, promote integral league implementation, build the whole process training evaluation mechanism, promote the county middle school league standard quantifiable, evaluable, inspection, 2022 Shaoguan city middle school communist youth league "double product double evaluation" integral league work propulsion will be held in new venture first middle school. Youth League county committee responsible person, the county middle and high school, secondary vocational school youth League committee responsible person attended the meeting. Conference, the xinfeng county, head of the comprehensive analysis of the implementation of the "double product double evaluation" integral into the work to promote the importance of the work of the county communist youth league and the young pioneers, emphasized the "double product double evaluation" integral into the overall requirements, under the new situation to promote the communist youth league and the young pioneers reform, promote fully active team work pointed out the path. Then, the municipal middle school communist youth league teacher studio host of new venture middle school communist youth league "double product double evaluation" integral into the detailed interpretation, he said, "double product double evaluation" integral mainly focus on "league activists" and "league development object" how to produce two links, and combined with the school team building to share the integral in the league work experience and practice. At the meeting, the leaders of the youth League committee also exchanged views and shared their experience based on the actual situation of the school. Since comprehensively promoting the reform of the grass-roots organizations of the county Communist Youth League, the youth league organizations at all levels in Xinfeng County have made a lot of beneficial explorations, and obtained some experience and practices worth popularizing. In the future, Xinfeng County will explore an important way to join the league with "double accumulation and double evaluation" points, form a team work system and personal growth record in the middle school, improve the contribution of the team work in the overall situation of the school work, and provide reference for the reform of the school Communist Youth League and the Young Pioneers.

2022-03-15 Anhui Wuhu Bay � b district youth social organization rhythm � b jin art association established Anhui Province Wuhu Bay � b district youth social organization rhythm � b jin art association established Jianwei correspondent, Ni Linlin Zha Weiting) recently, Anhui Wuhu Bay � b District rhythm � b Tianjin Art Association held the inaugural meeting, Cui Qiang, Party Secretary of Tuanwan � b District, He Xiangdong, vice chairman of the District Literary Federation and director of the Cultural Center, District literary federation vice chairman and secretary general Zhu Xingfu attended the meeting, About 50 people in charge of the brother association and members of � b-Tianjin Art Association attended the conference, Witness the establishment of � b jin Art Association. Meeting chaired by group bay � b district party committee deputy secretary cui � D, written learning xi general secretary in China federation 11 big, Chinese writers association top ten speech at the opening ceremony, discuss through and read out the bay � b area rhythm � b Tianjin literary association articles of association, director, elected association, supervisors, vice President, supervisors, and President, will read out the association secretariat appointment list and statement. Finally, Secretary Cui Qiang's delegation of � b District Committee extended warm congratulations to the establishment of � b Tianjin Art Association of Wuhu City, and put forward the three requirements of how to adhere to the construction of the association, highlight the characteristics, and grasp the team construction of the association. Rhythm � b-Tianjin Art Association was established spontaneously by the people who love literature and art in the area. Before that, people communicated and contacted through the "Colorful Voice" WeChat group. In recent years, they have been invited to participate in the 2022 Bay � b District Federation of Literature and Art Gala, "All People Sing and Sing Bay � b" to celebrate the Mid-Autumn Festival and welcome the National Day. Their member Yang Yingfa also wrote the single "I Sing for Bay � b". In order to standardize group activities and help the development of members, under the call of the � b District Committee, the � b District Art Association was officially registered and established during the Spring Festival of 2022. In the future, the � b District Committee will also contact more related youth talents through the � b Tianjin Art Association, so as to enrich the spiritual and cultural life of the people in � b area and enhance the cultural soft power of � b Area.

2022-03-15 Group of Hunan ningyuan county "four new" measures stimulate reform of Hunan ningyuan county "four new" measures stimulate the communist youth league organization reform JianWei correspondent BaiJing) as one of the county communist youth league organization reform pilot area, Yongzhou of Hunan province ningyuan county to deepen the reform of the communist youth league, with party building led to lead, in the youth, leading youth, service youth mining young talent, opened up a new league path, for the communist youth league promotion "three force once" new kinetic energy. Create a "new path" for the growth of youth league cadres. Adhere to the selection of excellent and strong youth League county committee team, Continue to strengthen the team building, In 2019, there were two temporary deputy secretaries and one part-time deputy secretary; In 2020, two subordinate institutions were established: county youth Network Culture Development Center and county youth Volunteer Guidance Center, And through the public institution examination to recruit 2 staff; In 2021, to complete the centralized election of the youth league organization in 422 villages (communities) in the county, Village (community) youth league organization team members of 863 people; Further recruit 90 college students as deputy secretary of the township (street) Youth League Committee, Strengthen the township (street) youth league organization work force, The vitality and combat effectiveness of grass-roots youth league organizations have been consolidated and enhanced. We will improve the "new mechanism" for developing youth league organizations. Continue to improve the organization system of the county league, Relying on the 18th county Youth League Congress, Gradually build the "liaison station-special group-group representative" liaison service system; In 2021, taking the opportunity of the centralized transition of the youth league organization, A total of 442 exchange groups for counties, towns and villages, In the county 20 towns (streets) covering the establishment of a materialized "youth home" and centralized licensing; Deepening the construction of the online Communist Youth League, Open "Dream Ningyuan" video number in the whole year released 57 videos, Total of 1.679 million page views +, The centennial video of youth tribute to the founding of the Party "Youth determined to build the heart of the Party youth dedication new journey" was adopted and forwarded by "Youth Hunan" and other heavyweight platforms; Carry out the "Hope Project one yuan donation" and other love education activities, Raised more than 1 million yuan to subsidize more than 1,000 poor students in the county. Since 2018, the Ningyuan Youth Volunteer Service Team has provided 158 volunteer services, covering up to 49,000 people. Grasp the real growth of the league members "new cultivation". Pay attention to standardize the pre-league education, grasp the quality of league education, organize the county league organizations at all levels to carry out the party history learning education, "three meetings and one lesson", and input the "smart league building" system, the first time reached 100%; for 45 middle schools and 100% in the county, the league school building rate reached 100%; in 2021 reached 100%; the "social connection rate" remained above 98%. Extensive development of Ningyuan County "green horse project" training, "double red and double excellent", "youth post expert" and other typical tree selection activities, in the Lotus Primary school held "childlike innocence to the party to welcome the birthday to be a good youth of The Times" concentrated into the team demonstration activities, effectively enhance the young pioneers sense of honor and the sense of belonging to the organization. In 2021,52 youth league members were trained, and more than 180 outstanding young league members and young pioneers, and 15 advanced organizations were encouraged and commended. Lead the party team to jointly build a "new fashion". Strengthen the mechanism of Party building and team building, bring the pilot work of the county Communist Youth League into one of the key work of comprehensively deepening the reform; bring the Party building and team building into the inspection and supervision of the county Party Committee, the school education evaluation and education supervision and no less than 10% of the total score; bring League building and team building into the Party building work deployment and annual assessment, and the funds included in the Party building fund plan. Establish county education league committee, by the county education bureau outstanding youth as the youth development minister; middle school (secondary vocational) league secretary into the unit middle level and above cadres treatment, in principle, not exceed half of the school teachers, the school, the secretary of the superior opinions, and to the higher league organization and education administrative department at the same time.

2022-03-10 Zhejiang Ninghai, Zhejiang Ninghai released volunteer service courtesy incentive method Li Jianping) recently, Zhejiang Province, Ningbo City, Ninghai County Spiritual Civilization Construction Committee Office, the county Civil Affairs Bureau, the Communist Youth League county Party Committee, the county volunteer association jointly issued the "volunteer service courtesy incentive measures" (trial). Thirteen farmers, including Wang Zhenghong, were identified as one of the first "five-star volunteers" in Ninghai County. Ninghai county registered volunteers star rating points to five stars, in Ningbo WE volunteer platform volunteer service accumulated credit for 100 hours as "one star volunteer", 300 hours as "two star volunteers", 600 hours as "samsung volunteers", 1000 hours as "four star volunteers", 1500 hours as "five-star volunteer". For 2,500,3,500,000,5,000 hours, bronze, silver and gold medals respectively, and 10,000 hours. According to the Ninghai County volunteer service courtesy incentive method, in the recommendation and selection of the most beautiful civil servants, outstanding Communist party members, moral models, the most beautiful people, excellent teachers, the most beautiful angel, model workers advanced, the May 4th youth medal, outstanding Communist Youth League member, 38 red flag bearer and other honors, give priority to outstanding volunteers under the same conditions. Representatives of the awarded outstanding volunteers can be invited to participate in the relevant festivals, celebrations and other important activities in the county. Migrant workers can get the corresponding quantitative points according to the credit service time on the Ningbo WE volunteer platform. Star volunteers can enjoy the corresponding financial preferential services such as credit loan line and interest rate fluctuation in the designated banks with the rated star certificate. A mechanism of points exchange and volunteer mutual assistance should be established. Excellent volunteers can exchange an equal amount of points on the basis of volunteer service time, and the points can be exchanged for corresponding items or equivalent length of volunteer service feedback. According to reports, in recent years, Ninghai County has shown a good development trend, the volunteer service force continues to grow, more than 140,000 registered volunteers, more than 2,000 volunteer service teams. Since 2021, the county has carried out a total of 20,048 various volunteer service activities, and 175,660 people have participated in the volunteer service activities. Jiang Pan Wu Wuchuan

2022-03-08 Group Jiangsu yizheng municipal party committee to build "xiaoqing" characteristic volunteer service brand group Jiangsu yizheng municipal party committee to build "instrument" xiaoqing characteristic volunteer service brand green net reporter Li Runwen) on March 4, in Yangzhou Yangzhou Yangtze river park the bund, young volunteers wearing red vest, will be scattered on the Yangtze river beach of plastic bags, dead branches, plastic bottles of garbage picked up. While cleaning up the garbage, the volunteers also publicized the common sense of banning fishing in the Yangtze River to jointly maintain the good ecological environment of the Yangtze River. According to reports, Yizheng city has 27.3 kilometers of the Yangtze River coastline and 231,000 mu of water area, which is not only valuable resources, but also the focus of environmental protection work. Youth Yizheng Municipal Party Committee led the joint agricultural, maritime, environmental protection and other departments to launch the "Yi" to protect the Yangtze River, "Yi Xiaoqing" to protect the river environmental protection public welfare activities, with practical actions to protect the hometown mother river."The Yangtze River is our mother river. As a youth league member, we should shoulder the responsibility, � ν � heart, charge to the front line, and do the propagandist of the great protection, participants and practitioners of the Yangtze River."He Zijun, secretary of the Communist Youth League Committee, said that this is the epitome of the Communist Youth League solid development of youth volunteer work. Yizheng Youth Volunteers Association was established in July 2021, with 66 members, 22 directors and 2,274 registered volunteers. With the theme of "Yi Xiaoqing" volunteer service, the association has established 13 youth volunteer service teams with different characteristics, such as "True young Youth Commando Team", to provide volunteer services such as environmental protection, safety construction, production safety, rural revitalization and group care, showing the new image of Yizheng youth. During the Yangzhou International Horticultural Expo, the volunteers with green shirts and white hats were one of the most youthful and beautiful scenery of the Expo. Flow diversion, order maintenance, ticket assistant, tourist service, consultation and question answering... More than 3,600 young volunteers' high-quality, efficient, civilized and intimate service has won unanimous praise from tourists at home and abroad. In the room of Yizheng City horse town plight teenagers-a pair of twin sisters, warm breath: white walls, clean floor tiles, warm cots, pink and blue bedding, and pink curtains, and a beautiful bookcase neatly placed all kinds of books... Yizheng city youth volunteers association director, secretary of Hong Lizhu, the twin sisters reported as the Yizheng City "dream cabin" first service object. Well designed and carefully transformed, the twin sisters' dream hut has taken on a new look. It is reported that in 2021, the Youth Yizheng Municipal Party Committee raised more than 400,000 yuan to complete the construction of 33 "dream cabins". As of March 2022, the second batch of 35 "dream cabins" has also started construction.

2022-03-04 Recently, the Organization Department of the CPC Guangxi Donglan County Committee and the CPC Donglan County Committee selected 165 outstanding college students to take part-time training in the grassroots league organization. In accordance with the principle of "suitable people and posts, both integrity and ability, and making the best use of their talents", Donglan County adopts the way of "organization recommendation + individual recommendation" to create a vertical expansion post setting mode of "county-township-village", and actively introduces excellent college students from Donglan. Among the 165 outstanding college students selected, there are 1 doctor, 3 master's degree, 137 bachelor's degree, 24 junior college students, 22 CPC members and 143 members of the Communist Youth League. Part-time positions include deputy secretary of the Communist Youth League County Party Committee, part-time deputy secretary of the Communist Youth League Committee of each township (town) and part-time first secretary of the Communist Youth League branch of each village (community). Donglan County adopts the "centralized + decentralized" and "offline + online" mode to carry out the work. Let college students in the summer and cold holidays, implement "offline" field work, implement "online" network work, "cloud" to assist the grassroots youth league organizations to do related work, and establish the township (town), village (community) party organization "help management system", clearly accept the dual management of the youth League and county party committee and service units. In addition, the county issued the donglan county selected outstanding college students to grassroots organizations part-time exercise plan, for the part-time cadres tailored the "four responsibilities" and seven tasks, through strengthening grass-roots organizations, form a youth team, promote hometown culture, to carry out volunteer service, form a boost rural revitalization of work "resource list".

2022-03-04 Group kaiyang county to learn lei feng volunteer service activities in Guizhou kaiyang county "learn lei feng when pioneer behavior" volunteer service activities on March 4 (reporter Zhang Jianwei, correspondent Chen Kai) spring march, recovery, in the 60th "learn lei feng" anniversary arrived, in order to further learn lei feng's glorious deeds, practice lei feng "unity, friendship, mutual assistance, dedication" volunteer service spirit, on March 2, Guizhou Guiyang kaiyang in county easy to carry out "learn lei feng when pioneer behavior" volunteer service activities. Fire and rescue workers enter households to publicize fire safety. In the activity, the volunteers measure blood pressure, blood sugar, Chinese medicine moxibustion therapy and the elderly basic diseases, and promote the knowledge of the people, fire rescue workers explain in detail fire alarm, escape and rescue methods, family fire hazards and how to correctly call 119 alarm fire safety knowledge, guide the people to safely use fire, electricity, enhance the awareness of disaster prevention and mitigation and emergency avoidance and self-rescue and mutual rescue ability. Xu Daiwen, a veteran party member, presented commemorative badges to young volunteers, fire and rescue workers, and young pioneers. In addition, young volunteers, female volunteers, firefighters, young pioneers also went deep into the community old party members home, to help clean up, clean up debris, listen to the old people tell red stories."This is the commemorative medal of the 50 years of glory in the Party issued by the centenary of the Party. It is the Party's education and trained me. All my life, I have to listen to the Party and follow the Party."Xu Daiwen, 76, an old party member, said excitedly. Medical staff are doing for the old man of traditional Chinese medicine moxibustion physiotherapy correspondent for figure next step, the group KaiYang county will be "35" learn lei feng day as an opportunity to continue to conduct online "better life volunteer qian" series of theme activities, the upsurge of learn lei feng spirit in the county, guide the youth active in volunteer service activities, practice the new era of lei feng spirit, promote learn lei feng spirit volunteer service normalized.

2022-03-03 Group in Guizhou longli county "four" to strengthen volunteer management in Guizhou longli county "four" to strengthen volunteer management training wei correspondent Zhao Jing) in recent years, Guizhou buyi miao autonomous prefecture longli county for college students volunteer service plan volunteer team construction, to "four insist" as the gripper, constantly strengthen the management, volunteers to further guide encourage college students west plan volunteers at the grass-roots level, grass-roots, service, dedication, grassroots, provide talent support for longli county economic and social development. Deepen learning effect, cultivate politically loyal volunteer team to strengthen training, strengthen quality training: Longli County 57 western plan to recruit volunteers for pre-job training, ideological and political, professional quality, county conditions and national customs and other relevant knowledge of systematic training, let it do work dare to rush dare to fight ideas. Carry out ideological and working skills training from time to time, and consolidate and improve their political consciousness, working ability, professional level and work style, so that they can be able to take responsibility in their work. Insist on rotation exercise, strengthen learning depth: every year more than 80% of the volunteers arranged in various towns (street) a line jobs, and divided into two phase three months rotation, through YaDanZi, task, give opportunities, let volunteers in practice improve quality, enhance ability, more familiar with the youth corps committee business, booster group standardization, standardization work to a new level. Detailed system reform, training director on the volunteer team perfect organization system, strengthen the supervision and supervision: formulate and sign the western college students volunteer service plan safety and health responsibility and the 2021 college students volunteer service plan service agreement, clear project office, service units, responsibilities and obligations between volunteers, ensure that volunteers and the specific head of the service unit information flow, smooth communication. At the same time, to realize the whole process of supervision from the beginning to the end of volunteer service, adhere to the management of promoting the work, to promote the development of service to strengthen the management of volunteers, dynamically grasp the work and life of volunteers, and timely formulate rectification measures and solutions to the existing problems. Establish incentive system, strengthen the proactive: introduced "longli county college students volunteer service plan volunteer assessment method (try out)", combined with the actual situation and not regular and regular appraisal, through assessment, evaluation, reporting on activities to carry out three years transfer policy, let a batch of high political quality, professional level, writing skills, strong communication ability, overall ability good outstanding volunteers to enter institutions to participate in the youth corps committee construction work. Establish regular visit system, strengthen communication: the county, head of the active volunteer service unit, through the service unit, volunteers talk about ideological quality, working methods, business level, timely grasp the volunteers on-the-job started situation, understand the difficulties encountered in the process of work, adjust management ideas and work mode, to ensure that the volunteers thought not wrong, work no trouble, style on the leak. In 2021, Longli County Party Committee held five symposiums and visited towns (streets for more than 20 times). Strengthen the mechanism guarantee, cultivate excellent volunteer team to improve the guarantee mechanism, encourage responsibility: in order to better gather the strength of youth league members, stimulate the entrepreneurial enthusiasm of volunteers, give full play to the vanguard role of volunteers, guide the majority of volunteers to better take root at the grass-roots level and serve the grass-roots level. The Communist Youth League County Party Committee strengthened the organization and leadership, improved the working mechanism, implemented the financial support, and provided the logistics support and support for the volunteers to carry out the service work, so that the volunteers could devote themselves to the volunteer service work. Obey double management, disciplined rules: the county called volunteers and service units meeting, clear volunteers to strictly abide by the rules and regulations of each service unit, the service units in accordance with the "who of choose and employ persons, who benefits, who is responsible for" and "training and use" principle, good education, management, planning, care about volunteers, let volunteers consciously do Ming discipline, understand the rules, discipline, rules. Build platform, encourage clerkstarts: according to the Guizhou provincial party committee of "chunhui action kite plan", the county focus on volunteers, establish talent pool, for volunteers to build entrepreneurship training, public training platform, for expired volunteers root grassroots to create favorable conditions, provide employment information, and provide support for work in all kinds of selection and help. Optimization project depth, cultivate dedicated volunteers focus on project participation, condensed service strength: to youth poverty alleviation, grass-roots roots, service society, caring left-behind children for the main line, combined with thought lead and care service, concentrated learning and long-term radiation as the focus, to carry out a series of fruitful volunteer service activities, build the "I for all, all for me" thick atmosphere. In 2021, actively coordinate volunteers to participate in longli county large-scale activities of volunteer service, participate in Longli county to create a national civilized city mobilization conference, the second "virtue volunteer qian" volunteer service project competition, 2021 elderly tennis exchanges in Guizhou province more than 10 times, the total number of participants reached 120 people. Focus on project innovation, transfer service behavior: the county in endeavour community founded the youth dreamworks, youth volunteers and rejuvenating the country night school activities, launched the "4:30 class" "colorful holiday class" "childlike innocence guardian plan" "technology drops GO" financial aid for poverty alleviation volunteer service activities, such as the audience of 50000 people. At the same time longli wash ma town "draw new life" volunteer service project in provincial project competition won the gold medal, "art build childlike innocence circle" -cultural feedback endogenous dynamic community development training plan, "qian yue paper QianZhi now song" love left-behind children inheritance intangible cultural love action and "pay" full " employment volunteer service won the silver award. Focus on the project lead, help rural revitalization: carry forward the "party has called for group action" fine tradition, organize western plan volunteers around the "four new" main "four", according to the deployment of the county, the county government, around the industrial development, infrastructure consolidation, rural custom civilization inheritance, rural fusion in rural revitalization of related volunteer service activities, drive the masses, with the service spirit drive people actively active, make fresh civilization born in rural revitalization work.

2022-03-03 Fujian anxi for senior students held 18 adult Fujian anxi for senior students held 18 adult ceremony Chen Qiang trainee reporter Tian Hongwei) " the students just now in the witness of teachers and parents, solemn oath, from nestled under the knee of children into an independent shoulder adult shoulder, and will then embark on a new journey, hope the students cherish time, not youth, efforts to grow into'ambition, Ming teacher, into talent, bear the new era of tea township talent."On February 27, Liu Yongqiang, head of Anxi County, cheered up 720 senior three students at the 2022 18th-year-old adult ceremony ceremony held in Anxi No.1 Middle School in Fujian Province. According to Lin Shuieng, the party secretary of the county, this is the first time the county magistrate has attended the event and made a speech since the 18-year-old ceremony was held. Yong-qiang liu encouraged students in his speech "adult, success, success", he said, " eighteen is the most precious years, the most moving years, the best journey, hope the students high, down-to-earth, as soon as possible become the pillars of the country and society, always do not forget parents put up, meticulous care, teacher preaching, tireless teachings, chang huai heart, gratitude, feedback, piety, awe to walk every step of life."The event kicked off with the solemn national anthem. 720 senior three students solemnly took an oath to the national flag. Guests and their parents wore 18-year-old adult badges, presented constitutional readings, the principal's greeting cards and a book focusing on the 40 years of development of Anxi."Wearing the adult badge and walking through the 'adult door', I understand that there is a new journey and responsibilities on my shoulders."Senior three student Chen Ziyang said after the end of the activity, as Liu Yongqiang county magistrate said in his speech, 18 these two words together, it just is a" wood " word, wood can be towering, can block the wind and rain, can do pillars. It is reported that since 2021, the Youth League Anxi County Party Committee to implement the central Committee of the Youth League "national middle school students 18 years old adult ceremony norms (trial)" as the starting point, launched the "Anxi young home" brand work, promote the county 14 middle schools to carry out a series of 18 years old adult activities, help 5233 students better toward the adult stage. Source: China Youth Daily, March 03,2022, edition 03

2022-03-02 Inner Mongolia Xingan league families right front flag 207 troubled teenagers benefit "cloud teaching" Inner Mongolia Xingan league division right front flag: college students to carry out the "cloud teaching" 207 troubled teenagers beneficiaries Shi Jia) " I teach three sixth grade children, from the beginning does not adapt to, to then eager to speak, after class, their transformation let me feel deeply."Recently, at the" Cloud Volunteer teaching "sharing meeting, Zhang Lantian, a student in Inner Mongolia Agricultural University, shared his experience of participating in the" cloud volunteer teaching " volunteer service activities in the winter vacation. Zhang Lantian, through the "cloud volunteer teaching" volunteer service activity, together with 60 college student volunteers from Inner Mongolia, Heilongjiang, Shanxi, Gansu and other places, became the "little teacher" of 207 young people in Keyouqian Banner of Inner Mongolia Xingan League. While spreading knowledge, he also sent to them warmth and hope. According to reports, the "cloud volunteer teaching" volunteer service activity was jointly initiated by the Inner Mongolia Xingan League Front Banner Committee, Xingan League Xiangyang Environmental Protection Youth Volunteers Association, Inner Mongolia Xingan League youBanner Youth Volunteers Association, and has been held for three consecutive sessions. A total of 221 college students have participated in the volunteer teaching, benefiting 470 teenagers. On February 18th, the "cloud volunteer teaching" sharing meeting held."We carefully combed the information of teenagers in our daily work, and recommended 270 teenagers, single parent and no support. They really need more care and help from all sectors of society."Inner Mongolia Hingan League Branch Civil Affairs Bureau child welfare unit director Guo Shuyun said. Finally, after the telephone communication one by one and soliciting the opinions of the teenagers and their guardians, 207 students were identified as the service objects of this "cloud volunteer teaching"."Most of the 61 volunteers who participated in the cloud volunteer teaching program are college students registered in Keyouqian Banner. They also want to repay their hometown in this way."Hinggan League Xiangyang Environmental Protection Youth Volunteers Association president Du Tieyi introduced. During the "Cloud Volunteer teaching" activity, college student volunteers speak English to the students. Pictures provided by the respondents, the Inner Mongolia league right front banner "cloud teaching" activities, volunteer accumulative total volunteer service for more than 3000 hours, 61 college students volunteers patiently understand the actual needs of teaching object, according to the psychological characteristics, personality characteristics, learning situation, etc., establish "one person one policy" teaching work plan."The" cloud teaching " volunteer service activities around the right flag single parent, left-behind teenagers, to carry out academic counseling, psychological counseling, growth services, by organizing outstanding youth league pair rural left-behind children, provide 'one to one' or'one to one many service, the advantages of the communist youth league resources to troubled teenagers, to block poor intergenerational transmission to make an effort."Regiment Inner Mongolia Xingan League branch right front banner Committee secretary Bao Tianjiao said.

2022-03-02 Group Zhangjiakou, Hebei province Chicheng county learn lei feng love blood donation activities Zhangjiakou, Hebei province held "learn lei feng love youth blood and love with love" love blood donation activities, winter (residue) Olympic city volunteers, "learn lei feng love youth blood and love" love blood donation activities, cheer with love for athletes, with practical action to pass the warmth of love. Under the initiative of the Communist Youth League Chicheng County Party Committee, the majority of youth league members and volunteers enthusiastically signed up to participate. At the scene of the activity, the blood donation comrades queued up for registration, measured blood pressure, and tested for blood collection in an orderly manner, showing the good spirit of the Akagi youth. The cool breeze outside the car bursts, and the car inside is warm. In just a few hours, 44 young people have participated in the blood donation team and donated 171,100 m l of blood to the Zhangjiakou Central Blood Station. When we got their own blood donation "certificate", we felt very happy and proud.

2022-03-02 Anhui susong county "send method into the campus" "spring visit enterprises" series of activities "Anhui su county" send method into the campus "" spring visit enterprises "series of activities wei correspondent Zhu Lei check WeiTing) to promote the province to improve the work style for the people for the enterprise optimal environment conference spirit, recently, the group of Anhui province susong county organization county people's court league branch, county youth corps committee to long shop town" the first lesson law with you "and" spring visit enterprises, help solving problem " series of activities, the county, the county people's court and the county revenue youth corps committee more than ten people to participate in the activities. On The same day of the event, Chen Shijian, deputy chief judge of 90% Court of Susong County People's Court, with the theme of "study the law and know the law, escort the growth", Start with the small things and legal stories around you, It briefly explained the Law of the People's Republic of China on the Protection of Minors, the Law of the People's Republic of China on the Prevention of Juvenile Crime, the Compulsory Education Law of the People's Republic of China and other laws and regulations that are closely related to teenagers, Call on young people to study the law, understand the law, usage, abide by the law, Take the lead in advocating, abiding by and defending the socialist rule of law, Young tax officials preach personal income tax and other tax law knowledge to the school teachers, This paper mainly introduces how to declare the six special additional deductions of personal income tax, how to operate the mobile APP and so on, Interaction and communication with the teachers, And remind everyone to pay attention to personal information security when using the personal income tax APP, risk prevention. Susong county people's court and Susong county tax bureau volunteers further long shop town jurisdiction enterprises to carry out the "New Year visit all of enterprises, help solve problems" activities, to "into the enterprise door, enterprise trapped, help enterprises, promote the enterprise into" as the theme, through the door-to-door visit, policy list, about laws and regulations, etc., to enterprise policy, service, ask demand, solving problems. In the factory workshop of Anhui Jiangda Environmental Sanitation Equipment Co., LTD., Dai Shiqing had an in-depth communication with the workers working hard, understood the operation situation of the enterprise, listened to opinions and suggestions face to face, popularized legal knowledge, and publicized the policies benefiting enterprises. The two sides also had in-depth exchanges with the enterprises on the practical issues such as contract disputes, labor disputes, and tax-related issues of new and high-tech enterprises frequently encountered in the business activities. In the face of the opinions and suggestions made by the enterprise representatives, the site personnel or supplemented, or recorded, and presented the relevant policy publicity materials to the enterprise. In Changpu town good Jixin clothing factory, we understand the employment situation to the person in charge, and explained the key content of the civil code to the workshop staff, in view of the workshop staff are mostly female this characteristic, but also focus on the publicity of anti-fraud propaganda, remind them to cover their money bags. This series of law popularization activities is one of the measures taken by the Youth League branch of Susong County People's Court and the Youth League Committee of Susong County Tax Bureau to actively implement the cooperation requirements of "Youth League construction". The two sides will create a win-win youth league activity platform on the basis of joint construction.

2022-03-01 Sishui County Party Committee of Shandong Province will the needs and provide practical services, promote the standardized construction of youth League representative liaison stations, and open up the "last kilometer" of serving young people. With ideological guidance as the fulcrum, and the young people heart to strengthen the theoretical arm, temper the ability to perform their duties. We regularly organized contact meetings and special studies and discussions on youth league deputies, conducted solid study and education on Party history, deeply studied the spirit of General Secretary Xi Jinping's important speech on July 1 and the spirit of the Sixth Plenary Session of the 19th CPC Central Committee, strengthened the theoretical arm of deputies, and organized more than 20 online and offline training sessions. We will pay attention to the performance of our duties and lead the cohesion of young people. Representatives at all levels played an exemplary and leading role, and continued to drive the youth league members to participate in the "Youth Learning" online theme Youth League class, covering 240,000 youth league members, absorbing more than 30 league representatives to set up the Sishui County "Youth Publicity Group", and organized a series of activities of youth Party history learning and education activities. Guided by clear needs, we build a face-to-face performance platform with young people to collect social conditions and public opinions. Online, through the "Surabaya Communist Youth League" WeChat public platform "Public Opinion Express" mini-program to open the door to accept advice, regular job rotation to solve the youth confusion, and offline monthly centralized discussion and communication, to "understand the youth needs face to face". We will fully implement the working mechanism of "monthly scheduling and quarterly summary", establish a work ledger for the deputies to contact their youth, and encourage the youth league deputies to fulfill their duties. We will improve the liaison mechanism to cover more teenagers. Establish a "1 + 10 + 1 + N" liaison mechanism, that is, one youth league representative should contact at least 10 youth league members, contact one youth league branch, and carry out N activities based on special teams. Representatives at all levels visited communities, rural areas, schools and enterprises to collect social conditions and public opinions, carry out policy publicity, and help the youth league organizations to give suggestions. Set up a special team to accurately identify diverse needs. According to the occupation and nature of the work of the league representatives, four special groups were set up for youth volunteer service, youth innovation and entrepreneurship, youth ideological guidance, and care for young people. Since 2020, it has carried out more than 200 volunteer service activities, contacted 1,165 rural good young people, strive for more than 36 million yuan of "rural good youth loan", and held five youth talent fellowship activities to provide convenience for youth members' innovation and entrepreneurship, marriage and marriage, and friends. To do real service for the purpose, and the youth hand in hand service league members "zero distance". On behalf of the group contact station for the gripper, comprehensive focus on member demand, build online business platform, explore the group representative, members, guide the grassroots organizations to carry out the league fee collection, youth league relations and other related work, collect youth difficult requirements, let contact station really become close contact youth service station and reception station. Over the past this year, it has served 12,000 league members and solved more than 300 problems. Do practical things for teenagers. The liaison station played the role of town and street group representatives and other groups, and cooperated with the Sishui Micro Public Welfare Association to promote the upgrade of the Surabaya "Hope House" project. So far, a total of 219 cabins have been built. The implementation of the group representatives, young volunteers and "hope hut" assisted children pair support activities, regular concentrated visits, in the province's "hope cabin" construction work as an advanced model, publicity and promotion. We will integrate it into community-level social governance. We will promote the construction of "youth communities" in communities on pilot projects, open up "community micro-governance" youth discussion halls, and guide league members and league representatives to make suggestions and suggestions on community-level social governance. The league representatives actively responded to the work call of the center, recruited more than 600 league members and youth volunteers, set up volunteer service posts in the personnel gathering areas, and demonstrated their youth responsibility on the front line of COVID-19 prevention and control. Source: China Youth Daily, March 01,2022, edition 04

2022-02-28 Guizhou Tongren Shiqian County "four modernizations and four promotion" deepening the reform of grass-roots organizations Guizhou Shiqian County "four modernizations and four promotion" deepening the reform of the county Communist Youth League grass-roots organization Jianwei Correspondent Li Tefang) Since the reform of the county Communist Youth League grass-roots organization, Shiqian County Party Committee of Tongren City, Guizhou Province firmly grasps the working pattern of the Communist Youth League "three forces once" in the new era, Based on the responsibilities and missions, serving the overall situation, Committed to transforming service objects into service forces, Promote the branding, institutionalization, specialization and normalization of volunteer services, Constantly improve the Communist Youth League's leadership, organization, service and contribution to the overall situation, Unite and lead the youth league members to contribute their youth wisdom and strength to the social and economic development of Shiqian."I have had your hair cut and got ready for the Chinese New Year. This kind of real service is very good, thank you."Grandpa Wang, an old man who lives alone in Pingyang community, said with a smile to the volunteers who cut his hair. On January 23, the "youth love action vitality spring capital" charity righteousness scissors activity in Pingyang community is in full swing. Since the "green love action vitality spring" series of activities officially launched, TuanShiQian county around red culture, green ecology, rural revitalization, change of poverty alleviation, new era civilization practice, social governance, etc., formed more than 20 green love action volunteer service team, carry out love, clinic, public help, warm winter action, environmental regulation, the rule of law about theme series activities nearly 60 games, participate in the volunteers of more than 500 people. In recent years, shiqian county combined with the actual, built the "intangible youth said" "run small green" "chunhui love supermarket" "tiangong bookstore" and so on a batch of representative volunteer service brand, through brand project demonstration, guide, drive the youth in the form of volunteer service actively participate in social governance, transfer love, carry forward the positive energy, further condensed youth strength, play the role of youth in the vital force in the rural revitalization. Seeing the length of his volunteer service increasing rapidly, Li Donghai smiled with satisfaction. Li Donghai, Shi Qian college students federation, the winter vacation has been stuck to COVID-19 epidemic prevention and control volunteer service jobs, to participate in volunteer service activities is a normal Li Donghai holiday open way, winter and summer vacation has not yet begun, has been early start planning home volunteer service activities, what, how to do, to achieve what effect. In May 2020, Li Donghai officially registered as a volunteer, and successively took the lead in carrying out a series of volunteer service activities such as Love in Shiqian, Shuangyang Project, Warm Winter Action, ecological and environmental protection. In one and a half years, the service time has reached more than 500 hours. With the help of the volunteer service information system, we unify the content, format and record method, timely, complete and accurately record the volunteer service situation, and provide the basis for the volunteer commendation and encouragement, the number of volunteer services has been effectively expanded. Up to now, more than 40,000 volunteers have been registered in Shiqian County, with a total service time of about 550,000 hours."You must memorize the COVID-19 Prevention and Control Measures in Shiqian County during the Spring Festival in 2022, so that you can guide the people coming to Qian in the first time and save time. And we must do a good job of prevention and control when doing information registration, to ensure their own safety."He Tingyong, a person in charge of the Communist Youth League County Party Committee, is training the volunteers who will go to the epidemic prevention and control card post. This is his fourth pre-job training on epidemic prevention and control before the Spring Festival. Only by letting the volunteers know the work content, can we better play the role of volunteer service. Before organizing each volunteer service activity, TuanShiqian County Party Committee will conduct relevant skills and knowledge training for the volunteers to promote the professionalization of the volunteer service team and improve the service ability. Since 2021, volunteer service capability training for 4 times, with more than 140 trainees, 2 volunteer service backbone training sessions, more than 40 trainees, epidemic prevention and control training for 6 times, and more than 200 trainees, combined with the city's "five batches" work, organized 9 special training sessions with more than 320 participants."Grandma, medical insurance is a guarantee to see a doctor and buy medicine, because your legs are not convenient, today we want to use the most convenient way to pay the urban and rural residents medical insurance, you see this is..." county tax bureau young volunteers for Shiqian Huaxia community for the legs of more than 30 urban and rural residents pension and urban and rural residents medical insurance."Hello, please show your health code and travel track, and take your temperature here and register. Thank you."Has become the mantra of Shiqian expressway exit epidemic prevention and control card point volunteers. During the Spring Festival, some returning college students and volunteers from the Western China Project went to the front line of epidemic prevention and control, transforming from being guarded to guarding others. In addition, volunteers have also appeared at other high-speed outlets and epidemic prevention and control service points in townships (streets). Care for the elderly and children left behind, ecological and environmental protection, publicity of laws and regulations, poverty alleviation and disabled assistance, civilized persuasion, entrance examination for secondary school or college education, intangible cultural heritage class..., where there is a need, there are volunteer services. Since 2021, Shiqian County has carried out more than 200 volunteer service activities, with more than 1,000 people participating in the volunteer service and more than 4,000 people benefiting from it.

2022-02-28 Shandong muping district party committee continue to build "hope hut" love public welfare brand group muping district party committee continue to build "hope house" love public welfare brand jianwei correspondent Liu Jian) group of Shandong Yantai muping district party committee launched the "hope hut" love public welfare activities, has been for 19 troubled children built independent and comfortable learning living space, for the children brought the hope of love. Recently, Tuan Muping District Committee organized volunteers to walk into the two "Hope House" homes built in Guanshui Town, Muping District, Yantai City, Shandong Province, to continue to promote the follow-up volunteer care activities to understand the children's living conditions. Nini, who lives in Jia Village, Guanshui Town, Muping District, Yantai City, died and her mother ran away from home and was raised by her grandparents, who was paralyzed in bed due to bone hyperplasia. Her 68-year-old grandmother carried the burden of the whole family. Nini sensible and stable, study hard, the performance has been very excellent. After learning about the relevant situation, the Tuan Muping District Committee immediately contacted the relevant government departments to help Nini apply for practical orphans subsidies, and organized volunteers to go home for many times to send daily necessities, help her academic homework, and communicate and give guidance on her mental health problems. Han Han, who lives in Hao Gezhuang, Guanshui Town, Muping District, Yantai City, had her parents divorced and her father lost contact. Her mother was in prison, she was raised by her grandmother, who had to rely on crutches because of cerebral thrombosis. After the completion of the "Hope House", Han Han's learning and living environment was greatly improved, and the whole family also rekindled the hope of life. The volunteers had a detailed understanding of Han Han's recent situation and the difficulties in her life. As the school kicked off, they encouraged Han Han to set up lofty aspirations and study hard in the new semester. Since the launch of the "Hope House" charity public welfare project, many caring people and volunteers in the district have been driven to pay attention to the children in distress. By organizing the Muping District Committee of volunteers to form directional assistance pairs with children in difficulties in the whole district, we continue to organize and guide more local volunteer services to contribute love and gather great love, and strive to build "Hope House" into a model project of caring for children's growth and a public welfare brand of condensing social love.

2022-02-27 Tonglu, Zhejiang: The Youth Daily of China Youth Network reporter Jiang Yutong, correspondent Xu Yuanjun) "Look, this is our Jinxiao base, at the beginning of the team only more than 100 people, but developed to the full four brigades of more than 1800 people". Rao Yangyang, a "youth power" young speaker from the Xinhe Township Youth League Committee, is explaining the origin of Jin Xiao's spirit to the young pioneers. The "Youth power" youth speaker introduced the spirit of Jin Xiao to the young pioneers.(Photo provided by Tonglu County Party Committee) In order to celebrate the 100th anniversary of the founding of the Chinese Communist Youth League, continuous red blood, on the morning of February 25, the Youth League Committee of Xinhe Township, Tonglu County, Zhejiang Province and Xinhe Primary School jointly carried out the "welcome the centenary of the red gene" education practice activities. Seventy young pioneers from Xinhe Primary School rewalked the revolutionary road and visited the memorial hall of the Jinxiao Detachment of the People's Liberation Army in eastern Zhejiang. Five red scarf speakers told the heroic deeds of the soldiers to all the teachers and students based on their own studies. In recent years, tonglu county around "tell the story of the party, inheriting red blood", has carried out teenagers red search, red youth party history, youth revolution, red education activities, since the party history learning education accumulated more than 1000 teenagers came to the old revolutionary base areas to receive red education. The Young Pioneers of Xinhe Primary School visited the digital interactive wall of red cultural relics in the Jinxiao Detachment Memorial Hall.(Photo provided by Tuan Tonglu County Party Committee)

2022-02-25 Group of Hubei Huangshi city, Hubei province, municipal party committee organization home college students see home group Hubei daye city organization home students see home, city people club bureau organization to carry out the "feeling is big big swan return" home college students see home activities, attract from tsinghua university, Beijing university, Hunan university, south China technology and other universities of 28 daye students gathered together. Representatives of returning college students visited Huangshi Dongbei Motor Co., Ltd., Proge Core Technology Co., Jin Co., Ltd., etc. Employment Department of Daye Social Bureau and Municipal Talent Office answered questions for college students, and publicized the employment and entrepreneurship situation and talent policy, and encouraged young talents to return to their hometown for development. In this activity, the "Daye College Students Alliance" was also established to enhance the contact between Daye students, publicize hometown policies, understand the development situation of their hometown, further enhance the feelings of Daye students to "know their hometown and love their hometown", and guide them to help the construction of their hometown through practice and training, employment and entrepreneurship.

2022-02-25 Ningyuan Yongzhou, Hunan province anti-drug fraud first lesson preaching Hunan ningyuan: anti-drug fraud propaganda good school first lesson net Beijing, February 25 (reporter Zhang Jianwei, reporter Tao Shiqi) recently, group of Hunan Yongzhou ningyuan county, county less working committee in mountain primary school 10 schools to carry out the "anti-drug first lesson" and "fraud first lesson" preaching, with easy content to analyze the harm of drugs and means of telecom network fraud, warned students in daily life away from drugs, telecom fraud. Yinshan Primary School publicity site. Through the first lesson of the semester, for the students to build a "refuse drugs, prevent fraud" firewall, the students said that they will actively participate in the anti-drug anti-fraud propaganda, strive to be the anti-drug anti-fraud propaganda.

2022-02-24 The first red scarf volunteer service team in Xinfeng County, Shaoguan City, Guangdong Province, was established on December 24 (reporter Zhang Jianwei, correspondent Li Excellence) In order to thoroughly study and implement General Secretary Xi Jinping's important discussion on the work of children and young Pioneers, Give full play to the advantages of the young Pioneers in organizational education, independent education and practical education, Further guide the young Pioneers to carry out practical activities, Serving the healthy growth of young children and young children, Promote the construction of Xinfeng County young Pioneers socialized work system, Inheriting the Spirit of Lei Feng, On February 21st, The first red scarf volunteer service team in Xinfeng County, Shaoguan City, Guangdong Province, was established in Xinfeng Experimental Primary School. At the establishment ceremony, the school played the ceremony of singing the national anthem, issuing the flag and taking the oath. The young pioneers of the Red Scarf volunteer service team said that they would practice the volunteer service spirit of "dedication, friendship, mutual assistance and progress" with practical actions, and actively participate in more volunteer activities to help more people. Let the hand of mutual assistance will be closely linked, the heart of love will be heart linked, learn to care for others, let love in mutual aid transmission, let love in dedication together. Live pictures. The school red scarf volunteer service team is mainly responsible for the teacher introduction, the establishment of the red scarf volunteer service team can give full play to the young pioneers' organizational education advantages and practical education characteristics. The service team will, in accordance with the plan, the service team will carry out the red scarf volunteers "Together to the future" Lei Feng month theme social practice, "big hand holding hands" theme social practice, young Pioneers public welfare social practice education and other theme activities, leading the healthy growth and all-round development of the young Pioneers. Next, the county will take this opportunity to, combined with the national county communist youth league organization reform pilot task, joint county committee and red scarf teacher studio, promote the county comprehensive establish red scarf volunteer service, volunteer station and feng feng, red scarf practice base, youth palace youth activities normalized volunteer service activities, guide children to learn to carry forward the lei feng's spirit and "dedication, love, mutual aid, progress" volunteer service spirit.

2022-02-24 Shandong Qingdao cieme district: build youth "stay" talent station Qingdao cieme: build youth "stay" talent station cieme district party committee with the support of the district party committee organization department, the construction operation young talent station, has served nearly hundred jobs, entrepreneurial youth, provide warm heart habitat, become cieme love to cherish, open and inclusive city image display window. Nesting to attract phoenix, flowers to meet butterflies. After preliminary research demonstration, group cieme district party committee to further broaden the goal, anchor young talent diversification, diversified employment demand, and its Qingdao cieme power supply company and other enterprises and institutions cooperation intention, through the integration of resources, building platform, expand work dimension, strive to promote young talent post service to upgrade. Play the unique advantages of all walks of life, the post promotion, training discussion, high quality enterprise visit and dating fellowship in the service menu, constantly assign for youth, create suitable for youth development environment, affects to attract more outstanding young talents to cieme stay in cieme, for one thousand is cieme provides the rapid development of talent reserve and intellectual support. Source: China Youth Daily

2022-02-23 Group of Shandong Weihai, Shandong province party committee to carry out the Olympic theme painting activities painting pier pier Olympic group in Shandong province party committee to welcome the Olympic theme painting activities Beijing on February 23 (reporter Zhang Jianwei, correspondent Zhao Yang) to celebrate the Olympic Games, to encourage Chinese athletes, recently, Weihai, Shandong province party committee to carry out the second phase of Rushan youth activities- "draw the games together to the future" theme painting activities. During the activity, the staff of the Communist Youth League committee first explained the painting knowledge, the design concept and related knowledge of the Winter Olympic mascot "Bingpier" and "Snow" of the Winter Paralympic Games. Pictures of the event site. Under the careful guidance of the teachers of the Communist Youth League Committee, the young friends and young volunteers who participated in the activity played their creativity and portrayed the mascot "Bingdun dun" and "Snow Rong", expressing their love for the Olympic Games in a unique way."I am very happy to participate in the painting activity organized by the Communist Youth League Municipal Committee. We can help with the Beijing Winter Olympics together. I hope the athletes can get good results in the Olympic Games."Said a participating partner. The "draw the games to the future" theme painting activity is pku building "Rushan youth hui" brand series of activities, "Rushan youth" to "I for youth" as the foothold, build "youth home", "youth center", organize youth training, volunteer service, rural revitalization activities, effectively meet the needs of youth learning, entertainment, participation, growth, really through the "last kilometer" service youth.

2022-02-23 Anhui Huaibei mountain area: focus on rural revitalization of field research in Anhui shan area: focus on rural revitalization of the field research JianWei correspondent, Li Xiaoning check WeiTing) in recent years, Anhui Huaibei mountain district earnestly implement the development of the "people-centered" development thought, adhere to the farmers in the rural revitalization of the subject status, to safeguard the fundamental interests of farmers, promote farmers common prosperity as a starting point and the foothold, respect farmers will, give full play to the masses, unite and lead the broad masses of farmers, promote rural comprehensive revitalization. In order to understand the understanding of people from different villages and different ages of rural revitalization strategies and policies and the relevant contents of living environment improvement, the Youth League Xiangshan District Committee has actively planned and scientifically designed, and carried out extensive field research work. Five college student volunteers were recruited through the WeChat official account online, and six research groups were formed with the District Agriculture and Water Bureau and the Qugou Town government to carry out a two-day questionnaire survey on rural revitalization and living environment improvement in Qugou Town. In the form of a questionnaire survey, the household survey was carried out on the majority of farmers, and the villagers 'understanding of rural revitalization and the feelings of living environment improvement were collected, and the survey results were analyzed and summarized, so as to better understand the villagers' awareness of the rural revitalization strategy. The survey was mainly for six villages, Lulou Village, Zhanglou Village, Xuji Village, Zhonglou Village, Mengzhuang Village and Liulou Village, and a total of more than 300 questionnaires were collected. The questionnaire mainly involves issues about road infrastructure and public facilities, sewage treatment, household toilets, irrigation and water conservancy, living environment improvement, and rural revitalization. Vigorously implementing the rural revitalization strategy and building a beautiful and harmonious countryside is an inevitable requirement for realizing the Chinese dream, and also a necessary move to ensure the victory of the battle against poverty. The development of this research activity is conducive to promoting the steady and long-term implementation of the rural revitalization strategy, and organically linking the coordinated promotion of rural revitalization strategy with winning the battle against poverty.

2022-02-22 Jiangxi Shangrao City Guangfeng District Committee held a young talent symposium "young talent symposium, more than 80" back to hometown " college students participated in the activity. At the symposium, college students exchanged speeches about their own study experience, career planning and the social practice experience; the relevant leaders of Tuan Guangfeng District Committee promoted the economic and social development, young talent work and talent policy to young college students. Next, group guangfeng district party committee will continue to play a good role of bridge and link, continue to deepen the "home" social practice project, youth talent association, training practice and a series of activities, increase the output of "nostalgia" of the young talents, for college students and young talents to provide opportunities and platform, build talent gathering "new heights".

2022-02-22 The Haimen District Committee of Nantong City, Jiangsu Province, held a single youth fellowship activity- - "Towards the Sea to the Future" Zhongtian Iron and Steel special youth friendship event in Haimen District. At the event site, the relevant officials of Haimen District Committee presented commemorative certificates and gifts to the successful youth representatives; the Talent Office of Haimen District Committee and Zhongtian Iron and Steel Group signed a strategic cooperation agreement for youth dating talents. This fellowship is one of the series of activities mainly created by the youth dating charity brand "Yuan Dongzhou Wish" in Haimen District. The next stage, the haimen district party committee will continue to play under group political, advanced and mass advantages, through dynamic maintenance in the sea single youth database, with good "spring post" and a number of youth dating places, normalized youth building fellowship activities, continue to extend the private custom service chain package of practical measures, continue to serve in the sea single youth, boost youth and talent friendly city construction.

2022-02-22 Group of Anhui Wuhu fanchang district party committee wall draw volunteer service activities in Anhui Fanchang district party committee to carry out the "boost rural revitalization painted beautiful homes" wall painting volunteer service activities Jianwei correspondent Wang Yijia WeiTing) recently, Wuhu in Anhui province fanchang district party committee, hills joint Wuhu mechanical engineering school organization volunteers in hills bay shop village "boost rural revitalization painted beautiful homes" as the theme of the wall painting volunteer service activities. Through communication and interaction, visits and research, and on-the-spot inspection, the local youth league organization and the young volunteers have determined the specific location of the wall painting, on the basis of respecting the public opinions of the villagers and soliciting the opinions of the village committee. Volunteers carefully designed the drafts and drew them carefully with their deep art skills. When the lifelike and beautiful characters and colorful picture scenes are displayed on the wall, they attracted the passing residents to stop and watch, and attracted bursts of praise. The vivid and colorful hand-painted walls not only beautify the environment, but also further boost the publicity of new social civilization, adding a trace of vitality and vitality to the construction of beautiful countryside. In the next step, Tuan Fanchang District Committee will actively organize volunteers to carry out rural revitalization wall painting volunteer activities, and further beautify the rural environment through the form of rural hand-painted walls, improve the quality of rural culture, enrich the connotation of rural publicity and education, and lead the majority of young people to contribute to the construction of a beautiful Fanchang.

2022-02-21 Chenzhou city of Hunan province zixing member youth power thousands of volunteers net lake lake, Hunan zixing youth members in action network Beijing on February 21 (reporter Zhang Jianwei, correspondent Guo Yuxiang) recently, the Chenzhou city of Hunan province zixing municipal party committee organized a composed of hundreds of party members, youth commandos, power "beautiful dongjiang, build and share" thousands of volunteers net lake lake activities. Clean lake lake protection activity pictures. The launch ceremony of the activity was held in the wharf of Bailang Town, Zixing City, Hunan Province, and in Xingning, Bamianshan, Dongjiang, Qingjiang, Huangcao, Chukou and other towns around the lake, the township youth League committee organized young volunteers to carry out activities simultaneously. Clean lake lake protection activity pictures. All the volunteers dressed in uniform, holding tongs, trash cans (bags) to clean up the garbage along the Dongjiang River. Some volunteers also went to the market town shops, homestay hotels, farmhouses and other places in the towns around the lake, to publicize the purpose, significance, measures and policies of environmental protection and governance by hanging banners, posting slogans and issuing proposals, so as to drive the masses to consciously participate in and actively cooperate with the renovation work. Clean lake lake protection activity pictures. It is reported that the net lake protection activity is a strong Zixing city to promote the east river lake basin environmental protection and governance "spring thunder action" another vivid embodiment.

2022-02-21 Baohe District Committee of Anhui Province jointly carried out the "opening week" series of seedling protection action. Jianwei correspondent Zou FangZha Wei Ting) February 15, The first day of the new semester in Hefei, Anhui Province, The Youth League Working Committee of Wuhu Road, Baohe District, Hefei City, Anhui Province, planned with the sub-district "anti-pornography" grassroots station and grid service center in advance, Launch the "4 + X" joint law enforcement force, Centralized public security, traffic police, market supervision, urban management, young grid member volunteers to carry out the "light up the school season station good guard post" of the "school week" series of seedling protection action, Fully open the "nursing mode", For the majority of teachers and students to travel "escort". The comprehensive law enforcement joint nursing action will continue to be carried out in Qingnian Road Primary School and Tunxi Road Primary School for a week. Escort safe open to school road traffic safety is an important part of the children's safety, river traffic police brigade combined with daily JieChuJing and patrol prevention and control work, according to the "person, create positions, responsibility, timing" principle, adjust measures to local conditions route, in the campus around important road off patrol or stationed work specification guide motor vehicle parked, timely guidance, maximize vehicle traffic efficiency in front of the school. Arrange police on duty during school and school hours, arrive 30 minutes in advance and leave 30 minutes after school, cooperate with school security personnel to maintain school and school order and vehicle guidance to ensure the safety of teachers and students; start nursing warning for bad weather and bad road conditions, and open up a "green channel" for students to be the campus "patron saint". Traffic police are patrolling. In order to ensure the food safety of students in the opening season, Wuhu Road street market supervision and law enforcement personnel launched a comprehensive inspection of the school canteen, including a variety of canteen rules and regulations, canteen staff health certificate, full-time food safety management personnel, disinfection and sterilization and other projects. Focus on the food safety responsibility, raw materials and food procurement, certificate request, on the operation room, storage room, dining place, various hardware facilities and equipment for the inspection, to ensure the safety of the "bite" of teachers and students. In addition to the campus food safety inspection, the "opening week" series of seedling protection action will also expand the inspection scope to supermarkets and cultural supplies around the campus. In the inspection process, law enforcement officers also publicized the relevant provisions to the business and urged operators to consciously abide by the relevant laws and regulations, requiring businesses in the purchase link to good commodity safety, shall not sell children's toys harmful to the physical and mental health of minor students's toys. Law enforcement officers conduct inspections. The Urban Management Department of Wuhu Road Sub-district has strengthened the governance of the city appearance and environment around the campus, and carried out a special rectification in the opening season. By strengthening the school around the road occupation operation, mobile vendors, distributing small advertisements, non-motor vehicles disorderly parking and other disorderly rectification, advocate the surrounding business stores standard operation. At the same time, the law enforcement officers also publicize the laws and regulations to assume the scope of responsibility of "three guarantees before the door", keep the ground clean; arrange the law enforcement officers to go on school and school peak hours, guide and persuade the mobile vendors, according to the laws and regulations, to provide students with a clean, beautiful and safe campus environment. Build a campus security control network Wuhu road street police station focuses on campus surrounding security problems, carried out the rectification, docking with the police ahead of school, scientific deployment of police, around the campus and campus internal clinics, monitoring room and other key parts of the safety inspection, the problems found in the inspection timely rectification, for safety problems anxiety school and student parents to eat a reassuring. Police officers maintain safety around the campus. Correspondent for the picture warm heart escort, help the young volunteers and the street "anti-pornography and anti-illegal" grass-roots workstation grid members also stood on the front line of nursing care, for the teachers and students of the school escort. At 7:30 in the morning, the volunteers maintained the order around the campus at the school gate. At the same time, the volunteers also told the students to "wear masks well, keep a distance, do not gather together, and wash their hands frequently...", and helped to guide the students to walk into the school more than one meter apart, queuing for disinfection and temperature measurement in an orderly manner. The "4 + X" law enforcement linkage "opening week" series miao action through joint traffic police, public security, market supervision, urban management, youth volunteers and other forces timely eliminate the potential campus security risks, Wuhu road street after continue to carry out "opening week" series miao action, the "4 + X" law enforcement linkage center as a normalized work, as an important link of youth action, community governance, will I really do something for youth.

2022-02-20 Anhui nanling county "young chicken about, help village" theme TuanRi activities group nanling in Anhui province county held "ChuFeng about, power country" theme TuanRi activities JianWei correspondent Li Xuan check WeiTing) recently, sponsored by the group of Wuhu in Anhui province nanling county "ChuFeng about, power country" theme TuanRi activities in nanling xu town town smoothly. Eleven college students from Nanling who participated in the winter vacation social practice participated in the activity. The students first came to Chihu Village, Xuzhen Town, Nanling County. Under the leadership of the local staff, they successively visited various functional rooms and farming cultural centers to understand the construction of local basic cultural facilities and farming culture. Later, we came to the Yu Changjun Martyrs Memorial Hall to learn the story of the life of the revolutionary martyrs who established the first red peasant movement regime in Anhui province."If I know I will die, I hope to comfort my father", "blue blood today spread the enemy courage, the ancient people"... to see these poems left by martyr Yu Changjun, all people shed tears, grief, more determined the students to cherish the present life, and strive to serve the country confidence. At the second stop, the students arrived at the Dapu Modern Agricultural Research Institute. Under the guidance of the narrator, the college students learned about the development of Dapu in recent years, and visited the greenhouse breeding, soilless cultivation and plant domestication. At the botanical garden, you can feel the different looks of plants from tropical rain forests and deserts. Take a sightseeing bus, all around Dapu, and enjoy the development style of the industrial park from different perspectives. Dapu rural residence, understand the local development plan, visit the "new" rural construction style, feel the charm of the rural courtyard in the new era. This "young phoenix appointment, help the countryside" theme group day activity, mainly in the way of visiting and learning, leading all college students friends, review the history, looking forward to the future.

2022-02-20 Jianwei) Recently, the youth league organizations at all levels of Liupanshui City, Guizhou Province, have actively carried out voluntary tree-planting activities to add some green to the beautiful Panzhou. Liuguan Street Youth League Working Committee organized volunteers to participate in the voluntary tree planting activities, volunteers actively allocated roxburgh rose saplings, according to the pit, put saplings, return soil, pouring root water, compaction steps of tree planting activities, with practical action called on the masses of cadres and the masses to actively participate in the construction of ecological civilization. Pictures of the tree-planting site. The Youth League Committee of Tuan Tuan Panzhou Municipal Party Committee organized more than 230 young volunteers and young cadres to carry out voluntary tree-planting activities. We began to shovel, shovel soil, support seedlings, fill pits, watering, plant saplings, after a morning of hard work, a total of 1166 osmanthus seedlings planted, planting area reached 10.05 mu. Pictures of the tree-planting site. On the first day of the Spring Festival, the Youth League Committee of Jichangping Town actively organized young volunteers to participate in voluntary tree planting activities. We worked together to plant 1,600 Mawei pine plants, which added a new green to the chicken farm ping, and also started a beautiful journey in the New Year. Pictures of the tree-planting site. The Youth League Committee of Xiangshui Town organized young volunteers to carry out the voluntary tree-planting activity of "afforestation boosts rural revitalization". Volunteers helped each other and planted more than 200 roxburgh rose seedlings to contribute to boosting rural revitalization and protecting the ecological environment. Pictures of the tree-planting site. The Youth League Committee of Baotian Town organized more than 110 people from young volunteers at the town and village levels to carry out voluntary tree-planting activities, and planted more than 150 osmanthus trees in front of the Zhongyi Government Affairs Center. The Youth League Committee of Gujiaying Township organized young volunteers to carry out a voluntary tree-planting activity of "afforestation and greening and boosting rural revitalization", and a total of 1,600 Mawei pine saplings were planted. The Youth League Committee of silt Township organized volunteers to participate in the voluntary tree planting activity of "carrying out afforestation and boosting rural revitalization". According to the pre-planned planting area, we work together, closely cooperate together, dig pits, hold seedlings, help trees, cultivate soil, step on the solid.... After a morning of busy, rows of newly planted trees on both sides of the new village bayberry garden road stand upright. Pictures of the tree-planting site. Tuanpan State Municipal Party Committee for the map

2022-02-16 Group linquan county in Anhui province party committee to carry out the love cold camp theme activities of Anhui linquan county party committee organization to carry out the "love cold camp happy with me" theme activities JianWei correspondent, Li Dongdong check WeiTing) for the further implementation of the "double minus" work spirit, rich youth students business cultural life, promote the practice of education key project implementation, recently, the group of Fuyang city in Anhui province linquan county party committee, south street in youth home "love cold to camp happy with me" as the theme of the volunteer service activities. During the activity, the volunteer teachers gave wonderful lectures to the students. From theory to practice, it opened the door for the students to have a new world to explore their extracurricular life, let the students understand a lot of knowledge outside the textbooks, enrich their horizons and increase their knowledge. Finally, toys, Spring Festival couplets and other items were also distributed to the students who participated in the activity. Yao Quanheng said in his speech, to carry out the "love cold to camp happy with me" theme volunteer service activities, mainly in order to further implement the "double minus" work spirit, promote the youth actively participate in social governance, enrich young students amateur cultural life, strive to solve the problem of young children holiday "care difficult"."Double reduction" is not simply to reduce the burden, but to let schools and parents reasonably arrange their study and life, to complete the learning with a higher quality, to achieve better learning results. In the next step, Tuanlinquan County Party Committee will continue to promote the childcare service work for left-behind children, create a learning and living environment where parents can rest assured and their children are happy during the winter vacation, and better meet the practical needs of different parents. At the same time, it provides a platform for returning college students to show themselves, effectively do practical things for the youth, and further use the living youth home, to ensure the vitality index of the youth home.

2022-02-18 Group of Hunan Zhuzhou city tianyuan district party committee held the games send warm series of Hunan tianyuan district party committee held the games send warm series of theme activities in Beijing on February 18 (reporter Zhang Jianwei, correspondent Chen Zixuan) recently, the group of Hunan Zhuzhou city tianyuan district party committee linkage tianyuan district civil affairs bureau, mount tai road street garden community, hong yi social workers, innovation to "cadres + young volunteers + young interns", in the province, the first community youth action pilot community held "celebrate the games he yuanxiao" send warm series of theme activities. Send warm activity site. In the early stage of the activity, Tianyuan young volunteers sat around to prepare yuanxiao and angelica eggs, and sent the hot yuanxiao and angelica eggs to the elderly living alone, the elderly, sanitation workers, to express their holiday blessings to them. At the scene of the activity, the district invited teenagers and the first group of youth organization interns of the "Sailing Plan" to bring you wonderful programs, such as hip-hop dance show, pop singing, dragon lantern dance and other youth popular activities. Send warm activity site. In addition, there are interesting games, contests with prizes, guessing lantern riddles and other popular activities. Before the end of the activity, young volunteers distributed a letter to Tianyuan District Residents and anti-drug publicity materials to popularize anti-drug knowledge to teenagers. Send warm activity site. According to the understanding, Since the launch of the Community Youth Initiative, The Communist Youth League Tianyuan District Committee earnestly implements the relevant work deployment of the Working Committee, the District Committee and the Communist Youth League Municipal Committee, Organized the Hunan emerging youth community volunteer style SHOW Changzhutan special activity, With the dream building program and community youth action, Multi-dimensional and multi-channel contact and service for emerging young groups, Mobilize emerging young groups to actively participate in community volunteer service activities, next step, The Communist Youth League District Committee will further deepen the community youth action, Integrate and enhance the existing brand work with the community youth action, Strive to build a group of party and government support, social influence, youth have a sense of gain of work brands, Guide more young people to combine what they have learned, Conduct voluntary services at the community level and participate in community governance, Explore a new path of "community youth action".

2022-02-17 Group Liupanshui city in Guizhou province pan state anti-drug campaign group Guizhou pan state municipal party committee anti-drug campaign jianwei) in order to further strengthen drug prevention education propaganda work, Guizhou Liupanshui city pan state municipal party committee during the Spring Festival, returning staff characteristics, joint countryside (town, street) group (work) committee in carrying out anti-drug campaign, drug control knowledge to the masses in the first time, opened the "go out home New Year anti-drug education" theme of anti-drug campaign. Double phoenix town youth volunteers to the masses and the farmers' market workers issued anti-drug brochures, anti-drug leaflets, and introduce to the masses the types of new synthetic drugs, harm, guide the masses pay attention to the drug problem, strengthen self prevention, consciously resist drugs, activities issued more than 2000 promotional materials, covering more than 2000 people. The picture shows the scene of the anti-drug publicity activities. Tuan Panzhou Municipal Party Committee for the picture is Baotian town market day, Baotian Town Youth League Committee, together with the anti-drug office, justice office, comprehensive management office, trade union, women's Federation science and technology and cultural service center and other units, in the town of justice to carry out anti-drug publicity and education and the anti-drug publicity and education of the migrant home activities. More than 200 anti-drug brochures, more than 120 drug propaganda bags and more than 100 anti-drug propaganda aprons were distributed, covering more than 300 people. The picture shows the scene of the anti-drug publicity activities. Youth League Panzhou Municipal Party Committee for the old camp township youth League committee, joint justice, comprehensive treatment office, drug control office, trade unions, women's federation and other units as well as "back to home" practice of college students, in the old camp township government building door for centralized publicity. The activity mainly focuses on the publicity of anti-drug knowledge, insists on guiding the majority of residents to establish the awareness of "refusing drugs and cherish life", and comprehensively enhances the public awareness of anti-virus. The activity covered more than 500 people, distributed more than 500 copies of publicity materials such as anti-fraud brochures and anti-drug prevention brochures, and more than 300 anti-drug publicity handbags. The picture shows the scene of the anti-drug publicity activities. Tuanpan State Municipal Party Committee for the picture of the Zhuhai Town Youth League Committee and the town drug control office, the old factory neighborhood committee village cadres to use the market day to carry out the "Zhuhai Town during the Spring Festival anti-drug publicity and education activities". At the event site, from what is drugs, the harm of drugs to individuals, families and society, drug-related violations and other aspects of the publicity. Through reading and learning publicity materials and listening to the on-site explanation of anti-drug youth volunteers, the activity covered more than 600 people, distributed more than 600 copies of anti-drug publicity materials, received 50 people's consultation from the masses, and entered more than 20 street shops for publicity. In the next step, the Tuan Panzhou Municipal Party Committee will further integrate resources to carry out a series of anti-drug publicity and education activities, improve the people's awareness of drug control, drug resistance, anti-drug, enhance the ability to resist drugs, vigorously create a good social atmosphere of national drug control, so that the flower of drug control spread in every corner.

2022-02-16 Guangdong Shaoguan new venture to carry out the "weekend new green edge" dating activities in Guangdong new venture to carry out the "our festival yuanxiao" festival theme dating activities JianWei correspondent Li Excellence) on February 13, Guangdong Shaoguan xinfeng county, new venture youth dating guidance center jointly held a "weekend new green edge" single youth dating activities, service youth dating needs, set up a relaxed and healthy dating platform, activities attracted more than 20 single people to participate in. At the scene of dating activities, flowers embellished a romantic and warm atmosphere. With the progress of the game, the male and female guests gradually broke the ice, deepening their understanding of each other and experiencing different feelings. Rich interactive games and full communication, to build a good platform for everyone to make friends, the young people who participated in the activity have said: this activity is relaxed and warm, can know more young friends, creating opportunities for them to communicate. It is reported that in order to effectively promote the county Communist Youth League grass-roots organization reform work is effective, the county Communist Youth League Xinfeng County Party Committee linkage social resources, is committed to creating a "new Qingyuan" youth dating brand activities, give full play to the organizational advantages, for young friends to expand the "circle of friends", better contact youth, service youth. Next, the county will give full play to the county youth dating guidance center platform, strengthen the service of youth, to "new green" youth dating project as the gripper, multiple linkage, for young friends from all walks of life build communication and development of friendship platform, rich youth amateur cultural life, guide set up the correct dating, help solve youth dating problem, make xinfeng all kinds of young talent more feeling, in xinfeng, peace, safety, to speed up the development of xinfeng make greater contributions.

2022-02-16 The founding meeting of Guizhou Yanhe Tujia Autonomous County College Student Chunhui Service Club was successfully held. China youth network Beijing, February 16 (reporter Zhang Jianwei, correspondent Yang Jie) in order to effectively promote the "Chunhui action" upgraded version of the work to go deep and solid, focus on the "Chunhui action kite plan" contact three groups of people, gather strength in the new era. Recently, the founding meeting of Chunhui Service Club from Yanhe Tujia Autonomous County, Tongren City, Guizhou Province was successfully held. Relevant responsible comrades of the Communist Youth League County Party Committee and more than 40 college students from Yanhe nationality attended the meeting. At the meeting, the relevant responsible comrades of the Youth League Committee read out the "college student Chunhui service club establishment approval document", the university student Chunhui service council members of the office documents; the Youth League committee members of the river university students Chunhui service club card, and for the new office of the university student Chunhui service council 5 members issued a letter of appointment. Subsequently, Zhang Feng, the part-time deputy secretary of the Communist Youth League County Party Committee, interpreted the spirit of the Guizhou Province "Chunhui Action Kite Plan" document; Cui Tianyi, the president of the University Student Chunhui Service Club, made a speech. Issue the letter of appointment to the members of the university student Chunhui Service Club Council. At the end of the meeting, Yu Peng, secretary of the Communist Youth League county Committee, hoped to attend the college students: to do his career full of enthusiasm. To realize the importance of implementing the "Chunhui Action Kite Plan", unify the thought and action, further enhance the sense of responsibility and mission of the youth, and more actively contribute to the work of Chunhui Service Club; everywhere to find resources. Give full play to their own subjective initiative, make good use of their own interpersonal resources and social resources, vigorously publicize the "filial piety, gratitude, feedback, return" Chunhui concept, for the implementation of the "Chunhui Action kite plan" to open up the way; focus on the project. To adhere to the innovation and development, broaden the thinking, borrow the reform of grass-roots youth league organizations to promote, the college students Chunhui service club into a high-quality brand public welfare organization, better raise social resources; honest and pragmatic gather energy. For the people, pragmatic, clean, fundamentally in the pragmatic. Young officials should work hard, dare to explore, have the courage to take on responsibilities, avoid formalism, and shoulder the important responsibilities of The Times with hard work.

2022-02-16 Anhui tianchang city of Anhui launched the youth revitalization project condensed talents tianchang city launched the youth revitalization of the youth project condensed talents hai-han wang, correspondent li-li Yang) recently, Peking University PhD student, tianchang straight street part-time league committee deputy secretary of Xia Shiyong feel feeling "roof", after pre-job training, he became a grassroots cadres. Under the organization of the Youth League Tianchang Municipal Party Committee, Xia Shiyong returned to his Alma mater, Tianchang Middle School, and shared his study knowledge with his classmates, and called on them to return to their hometown after graduation. Recently, Tianchang city held the "Tianji Talents" youth revitalization project and tianchang registered college students concurrently serve as the deputy secretary of the grass-roots youth League organization work launch ceremony. In 2022, the city appointed 82 college students as part-time deputy secretaries of the youth league organization in 16 towns (streets) and 52 villages (communities)."First-line rural areas need more young talents. These part-time teams come from universities from all over the country. They have knowledge, vision and ambition, and they master advanced science and technology. They will go to the grassroots level to help rural revitalization."Tianchang municipal party committee organization minister Wang Lin said. Luo Qinqin, a 2021 student from Huazhong University of Science and Technology, is a part-time deputy secretary of the Qianqiu Sub-district Youth League Working Committee. During the winter vacation of 2022, she participated in activities such as helping teenagers in distress, volunteers' "Warm Winter Action", and "Youth Secretary said". It is reported that the Tianchang City youth revitalization project also includes the "reunion of young talents" one-stop youth talent service platform, and has organized a summer camp for graduates, college students' summer social practice, TED salon and other activities. As the new semester of 2022 just begins, the Youth League Committee of Tianchang Middle School and the Youth League Committee of Binghui Middle School are vigorously preparing for the "Graduate Summer Camp" activity. Xu Jiahui, secretary of the Youth League Committee of Tianchang Middle School, said, " What we are most looking forward to is that these outstanding young students can achieve something in learning and return to their hometown."Tianchang city college students' summer social practice has been held for 10 consecutive sessions since 2012, which is a" time-honored brand " group-run quality project. This year, the summer social practice activities will be included in the youth revitalization project unified planning. Li Song, the part-time deputy secretary of the Youth League Branch of Changting Village, is a junior student of Zhuhai College of Jilin University. He has participated in the summer social practice of college students in Tianchang City twice. This summer vacation, he can directly go back to his hometown and work at the grassroots level to participate in the practice, which is a good opportunity for him and other students who are about to face employment. Behind the high-quality economic development of Tianchang City, Anhui province, comes from the injection of talent vitality. The relevant person in charge of the Youth Tianchang Municipal Party Committee said that the greatest significance of the youth revitalization project is to build a bridge for students who go out to study to participate in the construction of their hometown.

2022-02-15 "Gongshu Zhaohua" east and west cooperation love donation ceremony held Shanhai hand in hand, love warm thousands of miles "Gongshu Zhaohua" east and west cooperation love donation ceremony held Jianwei correspondent Li Qi) recently, "Gongshu Zhaohua" east and west cooperation love donation ceremony held in Zhaohua District on the second floor video conference room. The ceremony was presided over by Liu Liang, the head of the Communist Youth League District Committee. The donated left-behind children, children in need and some parents attended the ceremony. The picture shows the charity donation ceremony scene. Correspondent for figure donation ceremony, group Hangzhou gongshu district party committee secretary LuYun by video speech, she said " Guangyuan city and Hangzhou gongshu district is the east-west cooperation pairing area, both youth corps committee has been closely linked, gongshu youth league district committee the purchasing a batch of study, life, sports goods to support zhaohua youth learning life, hope through our contribution for zhaohua youth do something can."The picture shows the charity donation ceremony scene. The Gongshu District Committee donated the cloud to the Zhaohua Youth League District Committee, and donated 17,500 yuan worth of school supplies, daily necessities and sports goods to the 15 poor left-behind children being funded. Liu Liang, head of the Youth League Zhaohua District Committee, said that we will be good "masters", actively seize the opportunity and take the initiative, so that the cooperation between the eastern and western regions of the Communist Youth League of the two places has been further deepened. At the same time, will be a good close friend, set up a good image of the new era of the communist youth league of organizing youth, guide youth, service youth, maintenance of youth responsibility, strengthen the dedication and service consciousness, build a broad platform for growth, provide more development opportunities for exercise, let the youth dare to have a dream, have the courage to pursue, diligently interpreta dream. In the next step, the League Zhaohua District Committee will conscientiously do a good job of cooperation with the League Gongshu District Committee, constantly enrich the content of cooperation, and push the cooperation work to the in-depth.

2022-02-15 Lujiang county, Hefei city, Anhui province project to promote the construction of "youth" lujiang county project to promote the construction of "youth" JianWei correspondent, Wu Yunmin check WeiTing) Hefei, Anhui province adhere to lead the "youth" condensed youth, mobilize youth, contact service youth, pay attention to the integration of party and government, social, market resources, for a certain area of youth league effective coverage, work to provide a solid organization and position. According to the "Five Have" construction standard of the Three-year Action Plan for Deepening the Construction of "Youth Home" (2020-2022) of the Central Communist Youth League Committee, the "Youth Home" in Lujiang County insist on making more prominent organizational functions, more perfect service functions and more active project activities. The Youth Home Study Society conducts organized study, and studies party history on four topics: the new democratic revolution, the socialist revolution and construction, reform and opening up, and the new era of socialism with Chinese characteristics. We will carry out special lectures to tell vivid stories about the Party history, clear historical logic, thorough confidence in the system, and clear political pursuits, so as to promote the study and education of the Party history among young people. Carry out the "red scarf love learning" activity, strengthen the study and understanding of the red story, invite the old Red Army to tell about the extraordinary years for the young pioneers, tell about the revolutionary struggle process, guide the children to be grateful to the martyrs, Thanksgiving to the Party, and establish a correct outlook on life and values. Youth volunteers have carried out regular COVID-19 prevention and control and pre-registration of nucleic acid testing information at high-speed rail stations, helping to check health codes and remind people to wear masks and get their luggage. Organize young volunteers to carry out the "Spring Festival, clear five waste" activities to clean the rural road garbage, pond dead branches and telephone pole small advertisements, with hard work for the rural "clean beauty". With the snow as the order, the young volunteers took the initiative to shovel snow and break the ice in the depth of the main and secondary roads and streets to ensure smooth traffic, and to ensure the safety of the people. Home of youth red scarf school, entertaining to help "double minus" college students summer "a program under which officials" actively report to the "youth" home, by opening self-cognition, ability breakthrough, music appreciation, party history learning, science experiment courses, let the children in the process of learning direction, guide the children to set up the ideal of life."Youth Home" organized the children to do handwork, paper cutting, clay sculpture, etc., to introduce them to the safety knowledge, folk culture and Chinese traditional culture knowledge, while enriching their winter vacation life, but also make them learn more knowledge and skills. The "youth Home" in Lujiang County pay attention to the integration of all kinds of social resources, give full play to the characteristic functions of the position, widely publicize the activities and services of the "Youth Home" to the teenagers, enhance the attraction of the "youth Home" position to the teenagers, and help promote the various service projects of the league to realize tangible and daily around the teenagers.

2022-02-14 Gansu Linze youth help live with goods to empower the county industry revitalization of Gansu Linze youth help live with goods to empower the county industry revitalization month 14 (reporter Zhang Jianwei, correspondent Wang Mengting) in recent days, The Linze County Party Committee of Zhangye City, Gansu Province, focused on exploring a new model of "Internet celebrity economy", Deeply involved in the work of helping farmers to increase their income, Organize college students returning to their hometown in winter vacation to set up a youth live broadcast volunteer service team, By mining and cultivating Internet celebrities, building a communication platform, organizing live broadcasting with goods and other ways, Juli to polish the "youth with goods hi buy Linze" activity brand, Expand the sales channels of "Lin" brand products, To lead young people to contribute their youth to the main battlefield of rural revitalization, Help linze to achieve high-quality economic development. Aggregation of "green resources" to boost the integration of agriculture and commerce. With the Internet interaction mode set up "Linze new youth" to help farmers "antenna". Focusing on the organic integration of "what the market wants", "what is Linze" and "what we can provide", accurately linking local agricultural products resources to play a combination of offline sales and online live broadcast, and improve the integration and exposure of agricultural products resources. Relying on the youth live broadcast volunteer service team to create a distinctive local youth web celebrity live broadcast team, and actively explore the use of new media to promote home rural agricultural products and tourism and cultural products. Live broadcast scene. Correspondent provided pictures to explore the "building mechanism" to broaden the coverage of live broadcast. With the help of the county held major festival and time node, explore the use of "group, engine drive, resource leverage, linkage" work, "will live online + offline industry", will set in studio on agricultural origin, through origin field experience, live, live interaction, let the Internet users intuitive feel linze good mountain good water good products, promote live with goods, green and brand youth fashion into the country, into the base, for rural revitalization can assign "green power", to help solve the specific problems, promoting cultural tourism, on the basis of the concept of innovation. Cultivate the "leading goose" enabling industry to build new achievements. Give full play to the organizational advantages of the Communist Youth League, Organize the recruitment of returning college students, returning entrepreneurs returning to the countryside, rural youth becoming rich leaders, members of professional farmers' cooperatives, entrepreneurial bosses, e-commerce practitioners, Internet live broadcasting industry practitioners and other young entrepreneurs, According to their different needs, Focusing on entrepreneurial theoretical knowledge, live streaming e-commerce, short video production, community e-commerce and other new business forms and cutting-edge theories and related laws and regulations, live streaming tool application, personnel and product packaging, live streaming positioning, live streaming copywriting planning, product shooting and editing and data promotion and other content training, Cultivate and build a group of e-commerce live broadcasting talents who love their hometown, have strong professional ability and high theoretical quality, With the "leader goose" to drive the "big team", Effectively expand the visibility and influence of special agricultural products in Linze County, Leading young people to show their talents in rural revitalization, New achievements. At present, it has held 3 special training courses on "Hi Shopping Linze", contacted 12 youth network celebrities, driven 160 returning students, agricultural cadres and youth league members to participate in 18 public welfare live broadcasts, and sold more than 250 kinds of temporary products such as agricultural special, cultural and creative products.

2022-02-11 Jianwei Correspondent Liu Nan) In order to guide young students to participate in the publicity and education of the "Four histories" in the form of social practice, Organize college students to devote themselves to the grassroots front-line to participate in the rural construction work, Establish normalized and institutionalized college students and contact channels with hometown league organizations, To attract more young students to return to their hometowns, employment and entrepreneurship, The Malipo County Party Committee of Wenshan Zhuang and Miao Autonomous Prefecture of Yunnan Province has actively carried out the social practice activities of "college students returning to their nest" and returning to their hometown in 2022, The county offers eight positions in total, Nine college students signed up for the event. Through college students' professional direction and job demand of two-way matching, group malipo county party committee organization college students into the party and government organs, institutions, the township government and the village committee line post, undertake specific work, multi-channel to participate in grassroots governance, policy propaganda, epidemic prevention and control work, on February 8, to participate in the social practice of college students have all mount guard. During the activity, Lu Dengmei, a college student serving the Yidu Village Committee of Yangwan Township, said: This is the second time for me to participate in the social practice activities. This time, I chose to serve the towns and villages to help the COVID-19 prevention and control work. On the first day, I went to the 345 card point together with the village committee staff to participate in the border patrol activities. Serving malipo county people's hospital of college students li-qiu huang said: "the paper end jue shallow, must know it to practice", as a medical student, we should have enough theoretical knowledge reserves, more through practical experience, to let oneself further understand the society, in practice, exercise their ability, cultivate their toughness, find out their own shortcomings and gap, next, I will use the opportunity of social practice, give full play to their professional knowledge, with action practice beginner's mind, with dedication interpretation responsibility. Organize college students to actively participate in hometown social practice activities, on the one hand, let college students to apply their professional knowledge to social practice, through continuous practice and reflection, find a favorable career path, to better employment and entrepreneurship to accumulate more experience in the future; on the other hand, to deeply understand the construction of their hometown, perceive the vitality of hometown, enhance the confidence in hometown development, encourage them to actively participate in the construction of hometown.

2022-02-15 Group of Guangdong shixing county to carry out the "childlike innocence harbor" caring left-behind children series activities of Guangdong shixing county to carry out the "childlike innocence harbor" care left-behind children series activities built wei) on February 11, Guangdong Shaoguan shixing county joint luo dam town youth corps committee in shixing luo dam town flood village to carry out the "childlike innocence harbor" care left-behind children series activities, for the children to send caffeine arts and New Year wishes. It is reported that Tuanxing County Party Committee set up a "childlike innocence harbor" project site for rural left-behind children in Dashui Village, Luoba Town, Shixing County in 2020. In order to actively play the role of the Communist Youth League as a "childlike innocence harbor" and do a solid job of caring for rural left-behind children, the Communist Youth League Guangdong Shixing County Party Committee has actively mobilized and organized caring enterprises and people to carry out a series of activities in the project point. At the same time, keep close contact with the "child companion mother", regular home visits, in-depth understanding of the children's family life, learning situation. In the next step, the Youth Guangdong Shixing County Party Committee will actively mobilize more caring enterprises and people to send care and warmth to the left-behind children, and create a good atmosphere for their physical and mental health growth.

2022-02-14 Anhui Hefei luyang district "five to" systematic to build the door of the young pioneers luyang district "five to" systematic building home of the young pioneers jianwei correspondent Zhang � D check WeiTing) to fully implement the opinions of the CPC central committee on strengthening the young pioneers work spirit, comprehensively deepen the reform of the county communist youth league effect, promote the young pioneers reform task, Hefei in Anhui luyang district party committee by perfecting the system and mechanism, strengthen team construction, integrate jurisdiction resources, building platform between the school and the community, primary and secondary school community less 100% full coverage. The extensive establishment of the community Youth Working Committee has effectively responded to the "double reduction" policy, and has also further strengthened the emotional relationship between adolescent families and the community. The young pioneers organization to home group LuYang district party committee attaches great importance to the young pioneers work, deepen the collaboration, set up district bureau, district committee in charge of the "double" system less committee, as a whole in the young pioneers work, absorbed the education, culture, finance, publicity, politics and law, civil affairs, women's federation, science and technology association, disabled persons' federation and closes working committee departments become less committee, realize effective docking resources. TuanOuWei, district bureau, district less committee jointly issued the LuYang young pioneers grassroots organization innovation work pilot plan, according to the district less committee guidance, township streets as a whole, community (village), the implementation of comprehensive three rounds, building area-street-level three of the young pioneers outside the organization system, for 11 township streets, 28 village (community) less committee provides organizational guarantee. The young pioneers positions to the home to adhere to the overall integration, co-construction and sharing, relying on the district level service center between the masses, the construction of representatives, youth volunteer service center, youth science and technology innovation laboratory, youth ground in all kinds of youth service positions 31, released luyang district youth activity position "picture", let jurisdiction all kinds of service youth resources within reach. Actively build the district-street-residence three-level young pioneers practice education position system, and build a team room team corner in the community. Give full play to the local advantages of the district, continue to innovate to build the "Lu Xiaozhi" social practice base, weave a dense young Pioneers practice base network, and organize the whole regional community young working committee to carry out various cross-regional communication and practice punching activities on a regular basis. On the basis of the scientific establishment of the young Pioneers organization, build a strong working team of the young Pioneers outside the school. According to the "outstanding political standards, to ensure having both ability and political integrity" principle, to concentrate on the young pioneers work team construction, absorb jurisdiction social organizations, enterprise resources, play a moral model, community "five old" and public security, fire control, medical treatment, health supervision, mental health and other fields of talents into the community working committee member team. At present, more than 100 industry elites such as traffic police, public security, entrepreneurs and public welfare sponsors in the area have become after-school counselors of the community, carrying out voluntary lectures and organizing practice activities on a regular basis to escort the healthy growth of young pioneers in the community. The young pioneers gathered at home through a variety of community practice activities, to attract the young pioneers out of the school, into the community. In community team, holiday team, volunteer team, and other forms, normalized civilization persuasion, empty nest sympathy, YongJunYong, environmental protection and other social practice, in the community activities at the same time launched "LuXiaoZhi" brand, "build" a community a characteristic project "," small propagandist "," small epidemic prevention guards "" little calligrapher "" small environmental protection guards " LuXiaoZhi in action activities such as traction brand project. Push volunteer service project to the door according to the party leading, party team integration construction, integration of all kinds of community resources, promote the volunteer force into the community, relying on the community in the surrounding pilot community to explore "LuXiaoZhi" service, further launched community children and parents to participate in, normalized to carry out all kinds of volunteer service activities, to guide the young pioneers in the community activities, service, growth, power community governance, positive service center. Next, group LuYang district party committee will further integrate resources, continue to explore new young pioneers to participate in community management and social services, condensed school, family, community tripartite consensus, strengthen institutional guarantee, solving practice problems, form work force, coagulation hearts meet force to activate the young pioneers outside work source, promote the stable and healthy development of the young pioneers career.

2022-02-14 Guangdong new county 50 returning college students gathered home new changes Guangdong new venture "youth heart to the new era" returning college students symposium Jianwei correspondent Zhu Qianqian) on February 10, Shaoguan xinfeng county organization department, xinfeng county, new feng talent do jointly carry out "youth heart to the new era" xinfeng returning college students symposium, attracted nearly 50 returning college students gathered together, common perception new feng new changes, development goals, and the career planning, hometown development describe beautiful blueprint. The picture shows the meeting site. Correspondent for the picture meeting, the county people and social bureau, the county party committee respectively on the new talent policy, employment situation and employment demand and economic and social development were introduced, encourage the majority of returning college students to deeply understand their hometown, pay attention to the development of their hometown, called on the majority of returning college students to contribute to the construction of their hometown. While talking about their personal learning and growth experience and the confusion and problems encountered in their future personal development plans, they also actively made suggestions and suggestions to Xinfeng County in external publicity, urban infrastructure construction, cultural inheritance and innovation, and vocational education development. The picture shows the person in charge of Xinfeng County Talent Office introducing the relevant talent policies of Xinfeng County in detail. In the next step, the tuan Xinfeng County Party Committee will continue to cooperate with all relevant departments to actively explore new modes of cultivating young talents, carry out various forms of high-quality talent introduction activities, better gather and serve the young people, help Xinfeng students to return back to their hometown for employment and entrepreneurship, and inject new youth vitality into the high-quality development of Xinfeng.

2022-02-11 Zhejiang jiaojiang "youth town life +" rich rural revitalization of rich connotation Zhejiang jiaojiang "youth town life +" rich rural revitalization of rich connotation JianWei correspondent, Cai Xiaoxi Yang Shasha) in recent months, more than seven hundred years old henghe chencun, since Taizhou, Zhejiang province municipal party committee started the "youth town" work, jiaojiang district party committee relying on henghe chencun location advantages and natural, human resources advantages, on the basis of beautiful rural hardware construction, introduce rural tourism, encourage rural community, leading the villagers to accompany long-term construction, former quiet ancient village was injected with a lot of fresh elements. At the beginning of the New Year of 2022, the "New Year teahouse", a pop-up shop in Yokoghe Chen Village, has received an average daily reception of more than 600 people. The picture shows the young people in the New Year teahouse in the youth town. Correspondent for figure we have learned, relying on the "one yuan to rent" business model, focusing on wen gen, bookstore, cafe, live team, design team, agricultural products exhibition, art curator, such as category, attract 23 youth team, has nine officially entered, synchronous development improve the business awards complement mechanism, to reward the rent. Among them, the "New Year Teahouse" Outlines the "spiritual sharing space" of the state youth with its original and simple design, warm and cordial experience, which drives the dual recovery of rural culture and economy. The renewed youthful energy of the old town calls, Among them, mainly fully relying on the young Literary and Art Workers Alliance, Youth Enterprises Association, Youth (agricultural) Entrepreneurs Association, Youth Volunteers Association and other organizations, The Youth League Jiaojiang District Committee selected and set up a "small town life home" rural revitalization young talent team, Among them, 33 people are included in five major youth groups, including small town artists, activists, farmers, volunteer families and big housekeepers, The town is equipped with a close youth league team that can provide landing services, financial services, legal services and volunteer services, The league members include entrepreneurial teams, young businesses, small town travelers, performers and other more interesting rural youth, 18 people, Jointly participate in the planning of new development and new business forms of traditional villages, such as town construction, line development, product development, cultural and tourism activities, Rich youth town connotation. During the Spring Festival, the joint youth town "entrepreneurial team partner" held "catch tide set around the street" cultural tourism festival, extract yokohawa chencun rich water system and local resources, the lotus seed soup, plum blossom cake specialty snacks, clay, rice traditional traditions, "New Year flag wall" "New Year teahouse" "rich cart" web celebrity elements blend with each other. We will further energize rural revitalization.

2022-02-11 Anhui Dangtu: 83 college students part-time youth league to help rural governance reporter Wang Haihan) "science counterparts" rural science, "warm winter little migratory birds" holiday with reading, "youth micro energy" series of preaching... from January 17, in the Maanshan City, Anhui Province, the county returning college students came to the township "youth home" to carry out the "warm winter colorful holiday" activities, for rural revitalization, grassroots governance power. On the morning of February 10th, the winter vacation activity came to an end. Qi Hao, the part-time deputy secretary of Tangnan Town, is a doctoral student of China University of Petroleum. During the part-time job, he not only learned a lot of skills, but also learned the basic tasks of industrial development in the village under the current background of rural revitalization, and learned how the village committee can play a role to help the villagers solve practical problems. Wang Yafei, from Maanshan Vocational and Technical College, is a part-time member in Dalong Town, Dangtu County. He organized young people to carry out red classic reading activities and led primary and middle school students to visit the red landmarks."I feel that the grassroots is another 'school', where I directly talk with the people, and I really feel that 'serving the people' is not a slogan."In September last year, the Youth League Dangtu County Party Committee through online registration, offline to local universities collection of" double channels ", for the county 10 towns recruited part-time youth League secretary, part-time Communist Youth League secretary and village (residence) part-time league branch secretary a total of 83, including 1 doctor, master 5,1 retired college students. They came to the assigned towns to report for duty in the winter vacation, to carry out poverty alleviation, volunteer services, industrial assistance, national conditions research and other activities, and to have a face-to-face communication with local young cadres, teachers, doctors, farmers, workers and entrepreneurs. In recent years, the Youth League Dangtu County Party Committee and the Talent Office of the County Party Committee jointly carried out the "Top Ten Youth Entrepreneurship Stars in Dangtu County" selection activity, including the winners in the Dangtu County talent pool. At the same time, Dangtu County selects about 10 outstanding young entrepreneurial talents (teams) every year, and gives them an annual fund subsidy of 30,000 yuan for three consecutive years. The picture shows returning college students' part-time league cadres to carry out activities in Dangtu County Party Committee

2022-02-10 Group of Shaoyang city of Hunan province SuiNing county condolences a line staff of Hunan Suining county condolences Spring Festival line staff JianWei correspondent Chen Qianling) recently, group of Shaoyang city of Hunan province SuiNing county joint green forest pharmacy, oasis huikang development co., LTD., tea restaurant, sympathy and visit fighting in the Spring line staff, send them caffeine arts and New Year wishes. Volunteers first visited the Suining County traffic police brigade to visit the traffic police on the front line of the Spring Festival travel rush, and sent them ginger tea, bread, warm baby, masks, hand sanitizer and other comfort products. Then, the group Suining county led the volunteers have came to the Suining car station, bus stops, Suining bus terminal, condolences the bus drivers, bus drivers and station staff, and visited the stick in the bus station "warm winter action" young volunteers, understand their service content, service difficulties and COVID-19 epidemic prevention and control, and so on and so forth. More than 200 copies of the condoling items were distributed. Since the Spring Festival travel rush started on January 17, the Youth Suining County Party Committee has organized more than 50 youth volunteer service teams to enter the station to serve passengers. In the next step, the Communist Youth League County Party Committee will continue to strengthen the construction of the volunteer service team, organize and call on the young people to participate in the volunteer service and epidemic prevention and control work of the Spring Festival travel rush, to ensure the safe travel of the people of the county during the Spring Festival.

2022-02-10 Guangdong: nearly thousands of xinfeng college students home development in Guangdong: nearly thousands of xinfeng students lit youth "torch", hometown development committee for figure China youth network Beijing on February 10 (reporter zhang, correspondent Qiu ChuYing) since January 2022, Guangdong Shaoguan xinfeng county widely called xinfeng college students to participate in home practice, active in hometown career development, get warm response, attracted nearly thousands of xinfeng college students active participation in rural revitalization, warm journey, love, love and sympathy, ecological environmental protection propaganda and other volunteer service. College students returning to their hometowns go from street to street to publicize epidemic prevention and control, fire prevention and electricity safety. League Xinfeng County Party Committee for the youth responsibility, light up the "revitalization" of Xinfeng towns and streets grassroots training college students at the desk seriously study, while practice as. In Fengcheng Street, college students promote COVID-19 prevention knowledge, in Matou Town, they enter every door to promote forest fire prevention and frost prevention; in Meikeng Town, college students organize wall painting renovation to enhance the rural spirit; in Shatin Town, college students go to the streets, and try to promote party building... returning college students provide free ginger tea. Group xfeng county for figure youth dedication, light up the fire of "warmth" in wide high-speed xinfeng service area, xinfeng bus terminal, return home college students volunteers become a "calligrapher" "waiter" "guide", for xfeng passengers provide free ginger tea food, Spring Festival hanging, voluntary spring, xinfeng tourism publicity materials and other services, won the passengers thumb up. Returning college students carry luggage for passengers at Xinfeng passenger station. Group xfeng county for figure youth in the afternoon, light the fire of "public" xfeng county set up feng lei feng volunteer service station, launched youth become public power, for pedestrians travel inquiries, lost and found, difficult for help, mobile phone charging and other convenient and beneficial service, and relying on volunteer station warm heart sympathy, volunteer service theme, some theme activities, provide the most intimate practical public service at the same time, reveals the spirit of xfeng youth.

2022-02-09 Suzhou, susu Wuzhong district actively build young talents "strong magnetic field" Suzhou Wuzhong district actively build young talents "strong magnetic field" green net reporter li chao) recently, Suzhou Wuzhong district held "people to Suzhou for" special young talent symposium, listen to the opinions of innovative entrepreneurial environment, promote the talent policy measures to further optimize, accelerate the pace of modernization of Wuzhong talent development advice. According to the introduction, the quarterly "young talent symposium" held once is one of the many work measures of Suzhou Wuzhong District Committee Organization Department and Suzhou Wuzhong District Committee to contact and service young talents. In recent years, Wuzhong District focuses on the gathering and training of young talents, adheres to improving the working mechanism, strengthening the development platform, optimizing the development ecology, improving the development support system of young talents, and promoting the continuous improvement of the development level of young talents. By improving the talent policy system and issuing the "Implementation Opinions on Further Strengthening Youth Innovation and Entrepreneurship", a total of 53 Soochow young Soochow science and technology entrepreneurs were selected and gave them the right to recommend high-level talents at the district level. They have innovatively "introduced talent through competition", and have held young maker competitions for 11 consecutive years, collecting more than 1,800 innovation and entrepreneurship projects, and nearly 200 projects have taken root in Wuzhong. Adhere to the "financial help", the creation of "youth loan", the newly created Soochow innovation and entrepreneurship guide fund, "Soochow loan" and other financial products to provide financing support to young entrepreneurial talents, a total of 200 start-up enterprises to provide more than 45 million yuan loan support, support discount of more than 5 million yuan. Pay attention to "campus talent introduction", carried out activities such as "excellent student Wu Zhongxing", college students from key universities to Wu internship, carried out "100 school talents air seminar" for 100 universities, more than 200,000 students watched online, and received more than 2,000 resumes. In addition, Wuzhong District serves as an "incubator" for precision education cultivation, establishing more than 40 municipal mass innovation Spaces, talent incubators and science and technology innovation industrial parks, providing professional incubation and pilot experiments, and cultivating more than 40 young leading talent enterprises. Build a "meeting room", build the first "Yangtze River Delta Youth Craftsmen Development Base" in the province, hold "Yangtze River Delta exchange activities such as Yangtze River Delta Young Craftsmen Development Conference", and organize young craftsmen to hold exhibitions in 14 cities around the world. Soochow Youth Business School was established to carry out the "Young Entrepreneurs Rbaton Project" training program to provide regular training to young entrepreneurs. We have built the "golden Platform" of Huicai, focused on the "3 + 3 + 3" industrial cluster, established 24 postdoctoral workstations above the provincial level, set up contact platforms such as "Zhihui Station" for overseas doctor Wuzhong, and gathered 206 young overseas returnees."Peace of mind" makes talent feel at ease. Wuzhong District has implemented the "Youth Residence" plan, created "free 7-day free rental" and "free intermediary fee rental" services, introduced YOU + and other youth apartment brands, and provided rental subsidies for more than 1,800 young talents. In the past three years, the accumulated housing subsidies of more than 30 million yuan."Learning at ease" makes talent rest assured, implement the education guarantee plan for talent children, establish the "Soochow High-level Talent Children Base School", regularly hold a summer camp for talent children, and organize Wu culture experience activities."Future" adds wisdom to talents. It has launched "Qinghui" mobile service platform, sets up "Future" service module, links employment and entrepreneurship policies, industrial distribution and enterprise recruitment information, and provides full-scene innovation and entrepreneurship consultation."Wuzhong is definitely a good place to live and work."Said Zhan Junfeng, a member of Suzhou Thomas Robot Group Alliance and chairman of Suzhou Thomas Robot Group Co., LTD. As a scientific and technological entrepreneurship talent recommended by the Organization Department of Wuzhong District Committee and the Communist Youth League District Committee to participate in the evaluation with "not only academic qualifications", Zhan Junfeng deeply felt the pragmatism and inclusiveness of the local talent policy. For the future, he is full of confidence, and is ready to actively embrace the wave of digital economy, and add luster to the "The most beautiful Wuzhong in Paradise in Suzhou" with real performance!”

2022-02-09 Wang Haihan, in Suixi County, and intern Cai Hailong, who are here in Huaibei City, Anhui Province.) " Finally, I can play basketball with my classmates."Ren Ziyi, Grade 7 of Suixi County Concentration School in Huaibei City, Anhui Province, said excitedly. On the same day, Suixi County Party Committee, together with the County Education Bureau and Anhui Youth Expo magazine, went to Suixi County School and TiBuddha Central School to carry out "micro wish" activities, sending New Year gifts to 50 left-behind children. At the event site, a little girl's micro wish is a bucket of cooking oil. When asked about the reason, the child said wisely: " Because grandpa was ill recently, in poor health, the home is running out of oil, so that grandpa doesn't have to go out to buy it. Since January this year, the Youth and Suixi County Party Committee has collected "micro wishes" from teenagers in the county through the "Youth Hui" service alliance platform and the WeChat tower group to help poor teenagers in Suixi County "realize their dreams". Up to now, Sui Xi County Youth Entrepreneurs Association, Sui Xi Project Youth League Branch of China Construction Third Construction Bureau, and Sui Xi County Youth Volunteers Association have claimed and helped complete 245 "micro wishes". Tuan Sui Xi County Party Committee also took the opportunity of teenagers returning to their hometown in winter vacation to organize youth League organizations at all levels to promote the return visits of more than 600 "Rain and Dew Plan" for assisted young people in the county. Since January 11, the heads of the town and village (community) youth league organizations have successively visited the villages to check the implementation of the funds and listen to the aspirations of the aided students. TuanSuixi County Party Committee, together with Suixi County Education Bureau and Baishan Town Government, held a "warm winter package" distribution ceremony in Liushi Primary School in Baishan Town, delivering scarves, hats, gloves, thermos cups, warm water bags and children's masks to 30 poor teenagers as winter daily necessities. During the event, some recipients become helpers. Recently, sui Xi County Party Committee organized a volunteer service team for children and young pioneers, including helpful students. During the Spring Festival, the children sent their own dumplings to the streets to share with the traffic police, sanitation workers, bus drivers and other groups.

2022-02-08 Jiangsu Huai 'an lianshui county start troubled youth care action in Jiangsu lianshui start "youth cohesion heart zhiyuan" care action wei correspondent, JuMengYa well) recently, Huai' an, Jiangsu province green league technology sector joint Tuanlianshui county to Huai'an lianshui county to carry out the "youth cohesion heart zhiyuan" care action, visit condolences troubled youth families, and held a symposium to visit entrepreneurial youth representatives. Visit teenagers in distress. Ma Rong, head of the science and technology group of Huai'an Youth Federation and deputy head of Lianshui County Government, Li Liangliang, secretary of Lianshui County Party Committee, and representatives of the Municipal Youth Federation went together to visit the families of teenagers in distress, and presented extracurricular books, toys and other extracurricular supplies and daily necessities for the children. Subsequently, the science and technology sector group of Huai'an Youth Federation visited the Gold Rush and Innovation Valley Entrepreneurship Incubation Base of Lianshui County Economic Development Zone to visit the entrepreneurial enterprises and held a symposium. At the symposium, young representatives of entrepreneurs shared their feelings around their own work and entrepreneurial experiences, and actively offered suggestions on local development. Watch the Startup Companies. Correspondent for the national outstanding youth league member, entrepreneurial youth representative, Jiangsu dream packaging new material technology co., LTD., head wang said, " as a entrepreneurial youth committed to environmental science and technology career, I will cherish the good time every day, focus on their own business, friendly and equal to everyone everything, is the so-called: don't forget to beginner's mind, seize every day, not young."In recent years, Tuanlianshui County Party Committee has continuously strengthened the care and assistance for factual orphans, left-behind children and other youth groups in difficulties, and built 163" dream cabins ", helping to consolidate the effective connection between poverty alleviation achievements and rural revitalization. At the same time, it has held youth entrepreneurship competitions for many times, giving support to policies, bonuses and other parties.

2022-02-08 Xi Reporter Han Rong) In Jidong County, Jixi City, Heilongjiang Province, a young man named Gao Xincheng did not expect a chance this year to make him become a boss and realize his dream of starting a business. After graduation from university, Gao Xincheng has been working outside, in June this year, he heard that the Youth Jidong County Committee is supporting the county aspiring youth employment, entrepreneurship, they returned to his hometown to find the Jidong County Committee, under the guidance of he successfully joined the Jidong County Youth Employment and Entrepreneurship Association. After a period of observation, Gao Zhencheng found that with the improvement of living standards, many women pay more attention to beauty and health preservation, so he had the idea of opening a beauty shop. He received support after exchanging his ideas with the local league cadres. With the help of the Communist Youth League, Gao Zhencheng not only successfully handled the business license, chose the right address to negotiate the rent, but also applied for a small loan of 50,000 yuan, within 3 years of interest-free, his Shunyi beauty and health pavilion was successfully opened. In jidong youth employment entrepreneurship association, and gao have more than 50 people, they in jidong youth employment entrepreneurship training center by learning beauty salon, chef pastry, live with goods, web production, a practical skills course employment, entrepreneurship changed their life, the communist youth league work from the past not familiar to frequent thumb up. In fact, behind the youth work of the Youth Jidong County Party Committee is in full swing, a group of cadres with ideas and energy are inseparable. In May 2021, after the Central Committee of the Communist Youth League issued the Guiding Opinions on Expanding the Pilot Reform of Grassroots Organizations of the Communist Youth League at the County level, the Heilongjiang Provincial Party Committee of the Communist Youth League sounded the "charge call" of the reform in the province, and the Jidong County Party Committee of the Communist Youth League was one of them. Select cadres and allocate teams, open supermarkets in the market, enter enterprises to build organizations, help the poor, volunteer services... Since the pilot reform, Jidong County Party Secretary, Jixi City, Heilongjiang Province, Lei Sheng has witnessed the unprecedented changes of the Jidong Communist Youth League since the reform. Lei Sheng recalled that at the beginning of the reform of the grass-roots league organization, the league Jidong County Party Committee had only 3 cadres. On the basis of completing the work assigned by the Jidong Municipal Party Committee of the Communist Youth League, it will also undertake the central work of the county Party committee and the county government, such as poverty alleviation, epidemic prevention and control, and the creation of a national civilized city."There is no one and no money to carry out the work within the regiment, insufficient and they is inadequate."Said Lei Sheng. At the same time, due to the older age of some grass-roots league cadres, the level of specialization is not high, the lack of enthusiasm for entrepreneurship, the management and incentive mechanism of the league cadres is not perfect, seriously restricting the rapid development of various undertakings of the county Communist Youth League. In order to break the "no one has no money, development depends on alms" embarrassing situation, group Jidong county think of ideas, find ways, strive for party committee and government support, to the social helper, find resources... through public recruitment, competition way from the reform only 3 cadres, equipped with 6 part-time deputy secretary, through government purchase recruitment 3 public welfare post college students, really match the team, with a strong team. At the same time, the Youth League Jidong County Party Committee also further optimized the age structure of grass-roots youth league cadres, adjusted the school, township over-age youth league cadres 7, to inject new vitality into the grass-roots youth league organizations. In order to completely break the embarrassing situation of "no one has no money, the development of career depends on begging", TuanJidong County Party Committee also actively applied to the county government for the special fund of 200,000 yuan, which has been approved."Grass-roots organizations are the basis of all the combat effectiveness of the League. How to play the 'central nerve' role of the leading organs of the county league and fully stimulate the" cell " vitality of each grass-roots league organization is the topic that the Heilongjiang Provincial Party Committee of the League has been deepening the exploration."Said Xu Changyong, party secretary of Heilongjiang Province. With the continuous enrichment of our work force, more reform measures have been implemented. Study and distribute the "three meetings and two systems and one lesson" standard record book; formulate and issue the "Jidong County Development League Member Working Measures"; strictly implement the "ten-step method", actively implement the points system method... the Communist Youth League Jidong County League School and 19 middle schools league schools, means that the league standard is more strict, the league education is more scientific, effectively strengthen the ideological and political education of middle school students in Jidong County, improve the education and management of league members. Zhang Guilin, a part-time deputy secretary of the Tuan Jidong County Party Committee, found that most small and micro businesses in Jidong County have been hit hard by the COVID-19 outbreak, but small supermarkets have not been affected and achieved considerable profits. After selecting addresses, talking about rent, decorating, placing shelves, running to the market, entering goods, advocating charity sale... Tuan Jidong County Party Committee has established the first offline entity love supermarket initiated by the Communist Youth League and operated by the majority of youth volunteers- -Jidong "Tuan Xiaoqing" love supermarket."The profits of the supermarket will all be used as the Jidong County charity fund for the poor students, used to help poor families, excellent character and learning students and social welfare love undertakings."Zhang Guilin said. At the same time, the group Jidong county innovation also carried out the "love pair of one" poor student aid action, three county standing committee, 18 are section leaders, 26 deputy section leaders and social caring people to the county more than 80 students "one to one" pair support, greatly enhance the influence of the communist Youth League and presence in society. Jidong County Youth Service Center is also a highlight of the Youth League Jidong County Party Committee to deepen the county grassroots reform of the Communist Youth League. Through socialized recruitment, three public welfare personnel will be recruited to specialize in youth affairs, and they will regularly organize activities such as red education, literary and artistic creation, competitive competitions and legal lectures that are popular with teenagers. In addition to vigorously consolidating the basic plate of education, township, community grass-roots organizations on the basis of the Youth League Jidong County Party Committee also focused on trying to expand the coverage of the two new organizations, joint party and government organs, public institutions, enterprises to carry out volunteer services, research, discussion and other activities, to continuously enhance the influence of the Communist Youth League. It is understood that the League Jidong County Party Committee has repeatedly organized Jidong County people's Congress representatives, Jidong County party representatives and a total of more than 30 people in Jixi city many enterprises, to visit and discuss and exchange, and has in 15 "two new" enterprises set up the league branch, awarded the "youth commando" flag. At the same time, it also actively communicated and coordinated with the six qualified organs and public institutions and the party organization leaders of Jidong County Rural Commercial Bank. In accordance with the recommendation of the Party organization and the approval of the county Youth League committee, the league branch was established and equipped with the league branch secretary and members."Big and bold, exploration and innovation, is the biggest feature of the county Communist Youth League grass-roots organization reform."Lei Sheng admitted that the next step will continue to play the positive role of the youth League organization in gathering the youth and serving the youth, consolidate and expand the achievements of the reform of the grassroots organizations of the Communist Youth League, sing the voice of the reform for the 100th anniversary of the founding of the Communist Youth League in 2022, and hand in a perfect answer.

2022-02-07 Chongqing Fengjie: winter holiday public hosting class care children liberation parents Chongqing fengjie: winter holiday public hosting class care children liberation parents wei) to actively implement the "double minus" policy, promote "I do the practical work for the masses" practice to the further development, the Chongqing Fengjie county party committee to youth palace, xiangshan community youth home, bamboo community youth home and football association, provide love for county pupils winter vacation hosting service, with practical action to solve the problem of "winter vacation no one to take care of their children". On the first day of the winter vacation charity care class, the children had already obediently sat in the classroom and took out their winter vacation homework to write down. The hosting class service is free, but the quality of the course can not be paid at any discount. Not only do volunteer teachers help the children with their homework, but they also carry out ideological and interesting ideological and political education, mental health, physical exercise and puzzle games according to the premise of their age and growth rules. According to the relevant controller introduces, winter holiday hosting service focus for fengjie county outbreak line medical workers children, model worker children, difficult family students, urban migrant workers trailing children, left-behind children of 6 to 12 years of age, by the students for free, parents of voluntary to help the worker family to solve the holiday children care problem. In the next step, the Communist Youth League County Party Committee will further integrate social resources, play a role in driving radiation, use winter and summer vacations and weekends, with public welfare trusteeship projects as the core, and expand and create practical experience activities with research as the main body.

2022-02-04 Chongqing Fengjie: reunion peace township warm heart dumplings "New Year" Chongqing Fengjie: reunion peace township warm heart dumplings "New Year" Beijing on February 4 (reporter Zhang Jianwei, correspondent YueQing), the group of Chongqing Fengjie county further docking support village-peace village to the village to carry out the "winter sunshine warm you and me" left-behind children New Year care activities. In the activity, we make dumplings together, play games, words reunion, happy. The children said that the dumplings they made were very delicious. Activity pictures. The leaders of the Communist Youth League County Party Committee gave a New Year gift package to more than 50 children, asked them about their study and life in detail, and encouraged them to be strong and brave, and study hard. Through intensive visits, more left-behind children whose parents did not go home could spend a happy and peaceful Spring Festival. Activity pictures. Tuan Fengjie County Party Committee for the map

2022-02-04 Fujian get ping: get a Taiwan youth New Year of cross-strait feelings of Fujian get ping: get a Taiwan youth New Year of love Chen Qiang trainee reporter Tian Hongwei) Spring Year, the communist youth league of Fujian province, get a flat rural business bank, get a platform and park management committee organized Taiwan youth and local returning students a total of more than 40 people to carry out "youth across the Taiwan straits get a friendship" "five one" spring series of activities. The young people visited xingxingxiang agricultural ecological agriculture, agricultural ecological agriculture, Donghu ecological agriculture, Fujiude Farm and other Taiwanese entrepreneurship bases and Beiliao Village Tea Museum, to learn about the entrepreneurial stories of young entrepreneurs in Taiwan. Young people from both sides of the Taiwan Straits experienced and interacted, had a deep understanding of Zhangping Narcissus tea, ecological agricultural planting technology and the standard production technology of alpine oolong tea, and learned the "Internet + agriculture" e-commerce sales model. After the visit, the young people also participated in the symposium to welcome the New Year, shared the New Year's Eve dinner, and carried out reading congratulatory letters.

2022-02-01 Linquan, Anhui Province: More than 100 college students volunteer to help students sweep their health codes. College student volunteers help passengers get out of the station. Photo by Guo Jinshan by busy college student volunteers. Guo Jinshan photo to help passengers out of the station. The Spring Festival is approaching, as "the most populous county in Anhui province" and a national well-known labor export county, Linquan County of Fuyang City ushered in the peak of passenger flow returning home. In recent days, in the Linquan County high-speed railway station, passenger station, the Communist Youth League Linquan County Party Committee recruited 139 college students volunteers, wearing red vests to join the "warm winter action", with their own warm service, warm the way home."Uncle, please wear a mask."" Dear passenger friends, scan the code to enter, thank you for your cooperation."" Sorry, please show your vaccination certificate."At the Linquan high-speed railway station, Li Sizhen, a sophomore at Chizhou University, gets busy whenever the high-speed train arrives. Two days ago, a blind couple got out of the station. She and two other volunteers took them to the nucleic acid test site. After completing the test, they also helped them to the taxi stop, waiting for the taxi to briefly explain the driver, put the couple's luggage in the trunk, and told them to be careful when they got off. During the volunteer service period, Chai Rong, a senior student from Anhui Normal University, worked to assist the staff at the entrance and waiting hall of Linquan Passenger Station. At the entrance, she will tell passengers how to buy tickets, tickets and enter the station, so that they can have a pleasant ride experience. Encounter some vulnerable groups, she will also guide them to use Alipay and scan Ankang code."Some elderly people, whose children are working in other places, do not have mobile phones or ID cards on them. We have to help to call to verify the information and register, and we also send them to the bus home as needed."Chai said. Xie Mengyu, a junior from North University of China, was the first to participate in the "Warm Winter Action", mainly helping passengers check and install the health code."An old woman with her granddaughter was in a hurry to drive home. Because she could not drive the health code, the staff asked her to ask the volunteers for help."The old man did not have Alipay on his mobile phone, and he did not know how to apply for the health code. While Xie Mengyu asked other volunteers to help coax the children, he installed it step by step, and explained the process of punching in for the elderly man with the health code. Although in a hurry to catch the bus, the old man listened patiently."She said that now everywhere to health code, after learning, it is convenient to go out, but also expressed gratitude to thank us, said that the Chinese New Year is hard here."The recognition of the passengers made Xie feel guilty of being needed and made her realize the meaning of volunteer activities.(Han Zhenzhen, Li Dongdong)

2022-01-29 Hunan Tianjin Municipal Party Committee to carry out Spring Festival sympathy activities for Left-behind Children Jianwei Correspondent Huang Peng) to call on the whole society to care for left-behind children, and to guide the relatives of left-behind children to return home to work, to attract more talents for the development of Tianjin. On January 27, the Communist Youth League Tianjin Municipal Party Committee of Changde City, Hunan Province, launched a Spring Festival condolence activity with the theme of "Left-behind children are heart to heart, and have a family reunion dinner together". Activities invited nine left-behind children and their guardians to attend, family reunion dinner, Tianjin municipal party committee, organization department minister chun-hua ye talked with left-behind children and their guardians, invited their relatives home to work, and for the children on the consolation money and school supplies gifts, encourage the children to live a healthy life, happy learning.

2022-01-29 She in Anhui province: "green heart warm" into left-behind children heart nest in Anhui county: "green heart warm" into left-behind children heart nest south-south university for nationalities "small students" winter vacation practice team youth volunteers came to changxi township chang xi village left-behind children's home to carry out the "green heart warm" volunteer service activities, and left-behind children to celebrate the Spring Festival in advance. Although the weather is some cold, but did not stop the volunteers to send warm footsteps, we walked into the village of left-behind children's home, for left-behind children prepared New Year "gift package (school supplies, sports goods, extracurricular books)", read with them, and talk with the children, detailed understanding of their study, life, encourage them to be grateful, knowledge and enterprising, grow up to realize dreams, give back to society, serve the motherland, the warmth in the cold winter of the warmth."Our books are collected from the whole society. We encourage donors to write messages to children on their books and send blessings to children in mountainous areas on the occasion of the Spring Festival is approaching."Said Zhang Ying, a young volunteer from South-Central University for Nationalities. As the Spring Festival approaches, due to the need of epidemic prevention and control, some parents of left-behind children working outside China have responded to the call to consciously reduce their mobility and choose the Spring Festival in other places. Although their parents are "absent" from this year's Spring Festival, their love and warmth are never late. Activities also invited Anhui provincial intangible cultural heritage "Huizhou paper-cut" representative inheritance Wu Xiaomei teach children paper-cutting, under the guidance of Huizhou paper-cut not genetic bearing, volunteers with the children to make new window, feel the Huizhou flavor, different modelling of paper-cut for every child's good wishes for the New Year."This year my father can't come back to accompany me for the New Year, I will give my cut good window flowers to my father, I hope my father in other places for a good New Year, happy."Han Zixuan, a left-behind child in Changxi Village, Changxi Township, said to the volunteers with a smile, with his newly cut" fu " window flower. It is reported that since 2022, rapidly county give full play to the communist youth league to participate in the new era civilization practice initiative, relying on the new era of civilization practice, rural "Renaissance" and children-behind children home position resources, "green heart warm" volunteer service activities 20 times, for more than 200 disabled children, left-behind children, floating children provide life care, academic counseling, family company services, let the children feel the warmth of the society.

2022-01-29 Guangdong Shaoguan new county to carry out college students "home" social practice in Guangdong province in 2022 winter vacation college students "home" social practice wei correspondent, Qianqian Huang Qinyi) to further expand the coverage of the party league work and unity lead the league youth active in home construction, group of Shaoguan, Guangdong xinfeng county joint xinfeng county talent station actively carry out winter vacation in 2022 college students "home" social practice activities. Recently, Xinfeng County returned to the hometown of social practice activities forum was held in the conference room of the Youth League Committee, the county party Committee organization department talent room, the Youth League Committee responsible comrades and 23 Xinfeng college students returned to attend. Conference, head of the group organization home college students learned the sixth plenary session of the 19th and cities and counties, from new venture "home" the background and significance of social practice, previous activity review, 2022 winter vacation arrangement, related requirements and security to carry out general training, and according to the government affairs practice, enterprise practice and volunteer service type, classification to carry out the deployment and special training. County party organization department of the talent room responsible comrade interpretation of the new era of Shaoguan city "thousands of thousands of talent" talent project and Xinfeng County talent introduction related policies and role. The two representatives of returning college students expressed their hope to further deepen their understanding of their hometown through this practice, and they will always pay attention to and actively publicize the development of their hometown in the future. The other participating college students introduced themselves and said that they would actively devote themselves to social practice, actively fulfill the obligations of the young people, apply their professional knowledge and existing personal ability to life, and make a contribution to their hometown. It is reported that the winter vacation college students "return to home" social practice activities, attracted a total of 121 returning college students to sign up. A total of 103 college students were recruited through recruitment and screening, telephone interview and job adjustment procedures. Among them, 47 college students were assigned to various towns (streets) and other units to carry out the theme of rural revitalization government practice; 56 college students participated in organizing colorful volunteer service activities.

2022-01-28 Anhui Hefei luyang district rider little brother get exclusive New Year package Hefei, Anhui: youth league organization for takeaway little elder brother send New Year gift package youth network reporter wang haihan wang lei) this morning, under the guidance of Hefei, Anhui province, Hefei, Hefei (Hefei) youth corps committee to carry out the "volunteer haircut to send warm handwritten Spring Festival couplets wishes" care leave fat rider little elder brother activities. In the Sanxiaokou Street Party and Mass Service Center of Luyang District, more than 30 riders received their exclusive Chinese New Year gift package. At 8 am, young volunteers in Sanxiaokou Street began preparing volunteer services such as voluntary haircuts and handwritten Spring Festival couplets. At the event, the "flavor of the New Year" thick, New Year, free haircuts, handwritten Spring Festival couplets and other projects lively. Rider little brothers in the work gap came, their own "order", feel the warmth of volunteer service. In addition, young volunteers in Luyang District also carried out activities such as warm Spring Festival travel rush, epidemic prevention and control, comfort for the elderly and help the poor, and neighborhood watch.

2022-01-27 Zhejiang Yuhuan Municipal Party Committee to carry out "Tuan Tuan invite you to take a family photo" activity Yuhuan Municipal Party Committee to carry out "Tuan Tuan invite you to take a family photo" activity month 27 (reporter Zhang Jianwei, correspondent, Wu Yiran, Chen Qianwei Liang Yuzhe) " come, look at the lens, one, two and three, eggplant."Click click click, with the crisp sound of the shutter, in the Yuhuan City Red Gate photography studio, a family left jade New Year new Yuhuan people's family photo frozen in the camera. To give jade new jade more from yuhuan "second home" warmth and love, on January 4, Taizhou city, Zhejiang province yuhuan municipal party committee in yuhuan new youth WeChat public release "invite you to take family photo" activities, invite jade new jade family, add thick happiness flavor for them. On January 12, "group, please take family photo" activities, from Yibin, Sichuan province ChenSi discussion family is the first family, as an ordinary take-out man, he riding electric car shuttle in yuhuan gate streets, running between restaurants and customers, when you see "group, ask you to take family photo" activities, immediately signed up. On the same day, he took his wife Liu Yanjun and a pair of lovely twin daughters to the Yuhuan Red Gate Photography Studio, and changed into festive Chinese costumes to take a family photo. When he saw the final film, Chen Siyan said excitedly: " This year we are going to take the baby in Yuhuan for the Spring Festival, this family photo records the happy moment of our family in Yuhuan, very happy!"site of activity. At the same time, a blue uniform large group family photo is particularly attracting attention, they are from the Yuhuan fire rescue brigade left jade New Year fire rescue workers. Young fire and rescue workers took Chinese knots, couplets, big red envelopes, ice-sugar gourd and other accessories, with their most familiar training ground and fire trucks as the family photo background, a group to a family photo, a class to a family photo, all the staff to a collective big family photo. This year, Yuhuan fire rescue brigade a total of 33 fire rescue personnel to stay jade for the New Year, stick to their posts. Zhang Zhenyuan, from Zhumadian, Henan province, said that this year is his sixth year in Yuhuan. At the family reunion moment, he will work with his teammates to protect the safety of the people of Yuhuan."Today, when taking a family photo, we recorded the good memories through the lens, and felt the strong flavor of the New Year from the" second hometown ". I am very happy."Hu Dongxi, the driver of the fire and rescue team, who came from Tongling, Anhui province, said. By January 24, the Communist Youth League committee had taken free family photos of 100 new Yuhuan people from different occupations and different places. Some are ordinary workers, doctors and nurses, and police and firefighters; they come from all over the country, including Sichuan, Hubei and Anhui provinces. Jin Binjie, party secretary of the Communist Youth League, said: " A family photo, freeze the happy year. We hope that through the family photo activity, let the new jade jade for the New Year people really feel our care and blessing, have a happy Spring Festival in Yuhuan!.”

2022-01-27 Group of Kunming, Yunnan province wuhua youth lawyers branch cover lawyers industry Kunming wuhua district party committee set up young lawyers branch wen-ling zhang) on January 25, the communist youth league of Kunming wuhua district party committee, wuhua judicial bureau relying on wuhua lawyers industry league committee, set up wuhua youth lawyers branch and juvenile crime prevention youth lawyers, provide legal services for teenagers. Wuhua District is one of the central urban areas of Kunming, with 82 law firms. On November 18,2021, the Youth League Wuhua District Lawyers Industry Working Committee was established in Taihe Tai (Kunming) Law Firm, which is the first district-level lawyer industry working committee in Yunnan province. Li Xin, secretary of the Wuhua District Party Committee, introduced that relying on the teaching group and service group established by the Youth League working Committee, it is a supplement to the establishment of young lawyer talent pool in Wuhua District, the construction of industrial technical talent pool in Wuhua District, and the construction of Wuhua science and education innovation pilot zone. At the same time, it will promote the comprehensive activity of the grass-roots league organizations in the lawyer industry, and gather the youth with the effective coverage of the organization. The teachers and service group will cooperate with the schools to organize the legal knowledge lecture hall, promote the prevention of school violence and teach the connotation of legal culture, and carry out the community and rural volunteer service activities, so as to realize the "zero distance" contact between legal service and community residents. Participated in the activities such as "Young Lawyers' Action to prevent Youth Crimes" initiated by the Lawyers Industry Youth League Working Committee of Wuhua District, provided legal aid to minors, opened green channels, gave priority to acceptance and assignment, and selected lawyers with a strong sense of responsibility, enthusiastic about the protection of the rights and interests of minors, and rich experience in handling cases. The service team will empower them in various ways to help young lawyers to participate in this work, and continue to create a good legal environment for the healthy growth of young people with professional strength.

2022-01-26 Shanghai changning: teenagers at home with public opinion through train Shanghai changning: teenagers on home with public opinion through train in the youth network reporter Wei Qi � sole ┙ yu yu rescue � "open public opinion intersection practice the whole process of people's democracy" -the Shanghai communist youth league "the new youth learning club" the third phase of the release ceremony and build the whole process of people's democracy best grassroots practice youth BBS held in Shanghai changning district ancient north citizen center, The "Youth Shanghai" Changning site was opened, Changning League representative liaison station, Changning Youth Industry Committee meeting hall unveiled, The youth representatives shared their feelings of participating in building democracy. Liu Meiyue, secretary of the Youth League General Branch of the Middle School affiliated to East China University of Political Science and Law, said that as a middle school with the rule of law, the school has formed independent choice, no school uniform days and campus mobile phone management regulations in recent years, and students have participated in the whole process of its formulation. For example, the "external food into the campus of several regulations" was formulated from a student with Kanto boiled into the classroom, and accidentally spilled the incident. After the incident caused disputes between the teachers and students, under the guidance of the school Youth League Committee, the Student Affairs Center issued questionnaires to all the teachers, students and parents, and the school organized debate competitions, campus hearings and other activities. Finally, the congress democratically formed the campus regulations. Liu said that the school encourages students to pay attention to the difficulties and pain points in campus life and social governance, and put forward their own most real ideas, hoping that every student can participate in the campus management through democratic participation, democratic consultation and democratic decision-making. Changning District Hongqiao riverside garden industry authority deputy director Zhang Ye eshi said that before their industry authority, due to the poor property service caused residents resentment, industry authority want to hold the owners 'meeting to change the property, but because did not invite residents to participate widely, listen to the residents' opinions, resulting in rumors and conflict. Change after industry authority lessons, formulated the property hiring method, by each building elected two residents representatives, and industry authority property hiring team, separately visit should mark property company in the project, the photos, video to the owner group let residents score, eventually the subject industry is the highest scoring property, its service has been recognized by the residents. Zhang Yeshi said: " The whole process of people's democracy, let us find the greatest common divisor in the small things in the community, so that the residents can better achieve a better life through extensive consultation, joint discussion, joint construction, joint governance and shared benefits."At the event, Wu Bin, deputy secretary of the CPC Youth League, said that, relying on the first local advantages, the Changning Communist Youth League has explored a new path, I hope to further strengthen the political guidance, resource integration, mechanism guarantee, continue to promote the youth integration into the urban governance system and governance capacity modernization, in the whole process of people's democracy construction.

2022-01-26 Group of Shenyang, Liaoning province Dadong district party committee held immersive theme exhibition of Shenyang Dadong district party committee held "pioneer youth youth is red" immersive theme exhibition Wang Chen) recently, the group of Liaoning Shenyang Dandong district party committee held in Shenyang yatt city "pioneer youth youth is red" immersive theme exhibition, theme exhibition set up "young China said" and "seeking the truth" and so on six theme scene. In order to enhance the sense of participation, interaction and experience of the youth, the Communist Youth League District Committee has provided its exclusive seal in each scene. As long as all the seals are collected on the "Pioneer Youth Participation Card", you can get the "Pioneer Youth Certificate". At the same time, the online "Youth Big East" public account platform is also synchronized with the "youth oath" collection activities, so that the majority of young people to express their best wishes to the motherland and as a pioneer youth clang oath. Visited the theme exhibition of zhongjian bureau Shenyang north company manager group general secretary in a fan thinks, theme exhibition let the youth in the clock experience feeling "young is strong China strong" ideological lead, in one hundred from all walks of life patriotic youth, revolutionary youth, progressive youth era miniature guide new era youth don't forget to beginner's mind, take the initiative to bear.

2022-01-24 Chongqing fengjie to carry out the "youth to help farmers gather force forward" volunteer service activities in Chongqing fengjie to carry out the "youth to help farmers gather force forward" volunteer service activities jianwei correspondent Zhang Yuehua) recently, Chongqing fengjie county to carry out the "youth to help farmers gather force forward" navel orange picking + live with goods volunteer service activities, in the name of the youth power societe generale xingcheng, youth and navel orange. Activity pictures. In the activity provided by the Fengjie County Party Committee, the Communist Youth League County Party Committee organized volunteers of college students in the Western Plan, together with the youth League branch of the "Three carrying poles" e-commerce company and Chongqing young spicy star Donald Duck team, opened a live broadcast in Jiangnan Village, Yongle Town, Fengjie County, Chongqing, to help farmers increase their income. In addition, 48 volunteers shuttled through the dense fruit trees to help farmers harvest and transport navel oranges. The event helped farmers pick and sell more than 5,000 pieces of navel oranges. Activity pictures. TuanFengjie County Party Committee for the relevant responsible person in charge said that the main purpose of this activity is to gather and lead the majority of young people in the rural revitalization to show the youth style, to strive to create a " Industrial Xingcheng? Strong county to enrich the people " a new situation, the construction of beautiful fengjie face to contribute to the youth strength.

2022-01-26	Zhejiang Yuhuan Municipal Party Committee to carry out the "send you gift package" activity "Send out you gift package" activity Jianwei correspondent, Zhang Sihan, Luo Kevin Wu Yiran) " This is our heart, I hope you can have a happy year in Yuhuan."Recently, the League Taizhou City, Zhejiang Province Yuhuan Municipal Party Committee and the city Volunteer Association into Yucheng Street �� Qing community to carry out the" group to send you New Year gift package " activity, for some of the jade Spring Festival people to send rice, cooking oil, Spring Festival couplets, masks and other sympathy supplies.site of activity."Thank you!"That afternoon, the sanitation worker, who was cleaning up the garbage by the roadside, took over the heavy gift bag and said gratefully. Dai Changjian, a native of New Yuhuan from Fuyang, Anhui province, has not returned to his hometown for two years because of the COVID-19 outbreak. In response to his family members, he chose to stay in Yuhuan this year in response to the government's call."It was very good to spend the Chinese New Year in Yuhuan. After all, the epidemic is not stable now, and the government is also very concerned about us, and it sent us a gift package, which made me feel very warm."Dai Changjian said with a smile. Yuan Limei and Yuan Yuan, from Sichuan province, who have worked in Yuhuan for ten years, return to their hometown almost every year for the Spring Festival, but this year they discussed with their families and finally decided to stay in Yuhuan for the Spring Festival."As long as the family is together, it is the same wherever the Spring Festival. And Yuhuan is also our second hometown, here the New Year is as happy, warm."Yuan Limei said. At the same time, the city's township (street) youth League committee organized young volunteers to act, the "group of Spring Festival goods gift package" to the delivery boys, enterprise employees, medical staff and other 300 new jade ring families left jade for the Spring Festival, and with them to clean the room, paste Spring Festival couplets, hang the word "fu", to welcome the upcoming Spring Festival. Learned, to actively implement the epidemic prevention and control requirements, let more new yuhuan people stay in yuhuan New Year, build warm "second home", on January 4, pku officially launched "I celebrate in yuhuan group love express" series of activities, including "please take family", "group send you goods package" "group you send love home" "help you find a job" group "help you realize micro wish", let more new yuhuan people feel in yuhuan New Year happiness and belonging.

2022-01-24	Group of Huaibei city of Anhui province district winter vacation safety education series activities in Anhui province winter vacation safety education series activities JianWei correspondent, Zhao Yingying check WeiTing) in order to ensure that all teachers and students spent a safe, happy, full winter vacation, strengthen the safety consciousness of teachers and students and self-protection consciousness, and prevent and prevent safety accidents, group of Huaibei city of Anhui province, district less district bureau of education launched school winter vacation safety education series of activities. Keep your precautions in mind. Community police were invited into the campus and gave the children a holiday "safe and nutritious" meal. In the course, they use vivid examples, colorful propaganda pages, to tell children about how to prevent telecom fraud during the holidays, with some drugs, to educate students away from drugs; the booming Chinese Year, in knowledge questions and answers, students realize the importance of banning burning and release. Traffic safety is kept in mind. The police officer of the traffic police detachment taught the students about traffic safety during the holiday. The police officer uncle told the statistics of traffic safety accidents for primary and middle school students, and asked the students to abide by the relevant traffic rules and laws during the holiday to protect their own safety. Through such a traffic safety education class, let the children master a lot of safety knowledge and skills, and can basically overcome the bad habits in daily traffic life, to avoid danger. Theme class meeting together mobilization. Class to carry out the theme of the holiday safety education squadron class, emphatically from the students during the holiday epidemic prevention and control, fire safety, traffic safety, food safety, personal safety made detailed education, and suggest the students make good study plan during the holiday, into the social big classroom to participate in the practice.

2022-01-24	Group Shaoguan city of Guangdong xinfeng county to carry out difficult groups Spring Festival sympathy activities in Guangdong xinfeng county to carry out the "love xinfeng warm spring" Spring Festival sympathy volunteer service activities jianwei) in the Spring Festival approaching, Guangdong Shaoguan xinfeng county joint individual private enterprise volunteer association organized nearly hundreds of volunteers to carry out the "big love xinfeng warm spring" Spring Festival sympathy activities, for difficult groups to care and holiday wishes. Volunteers expressed their condolences. The activity strictly complied with the epidemic prevention and control requirements, and sent volunteers into groups to 90 poor people in the county. While sending milk, noodles, towels and other comfort items and comfort money, they publicized the popularization of water, electricity and gas safety, the epidemic prevention and control situation and measures. Volunteers expressed their condolences. It is understood that this year has been the eighth year of Xinfeng County private Volunteers Association to carry out the Spring Festival sympathy activities, this year to the poor families and Spring Festival materials a total of 57,527.1 yuan, a total of donations of more than 650,000 yuan. Next, TuanXinfeng County Party Committee will continue to publicize and launch, calling on more caring people to participate in supporting volunteer service, so that volunteer service can play a greater role in economic and social development, Gongyi Wei creative culture, rural revitalization and other aspects of the work.

2022-01-17	Group of Hunan Yongzhou ningyuan county to carry out the youth care education activities of Hunan Ningyuan county to carry out the "youth since protect peace Spring Festival" youth care education activities Jianwei correspondent He Shuxuan) to enhance youth self protection consciousness and ability, peace celebrate the Spring Festival holiday, recently, the group of Hunan Yongzhou ningyuan county organized more than 20 young volunteers in jiuyi mountain west bay village with "youth since protect safe Spring Festival" as the theme of youth care education activities. Through household visits, young volunteers around accident injury protection, traffic safety, home safety, fraud prevention prevent electric fraud and other content, from the importance and protection methods to the children of the west bay village of the detailed explanation, let the children fully realize the importance of safety since the education, can do he has rules, you have a way, to improve the youth safety consciousness and safety ability to deal with emergencies.

2022-01-16	13,000 Letters To Exchange Letters to Kunshan and Atushi Young Pioneers, reporter Li Runwen) " Write back! Friend Atosh wrote back!"On the afternoon of January 13, the children of the 5 (6) Squadron of Lujia Central Primary School in Kunshan City, Jiangsu Province, were excited to send letters and bookmarks sent thousands of miles away. Recently, under the care and support of the Kunshan Aid to Xinjiang Working Group, 13,000 successful pairs of successful young pioneers from Kunming and Afghanistan introduced their beautiful hometown and shared a happy life through letters."He sent me a letter, three postcards, and a picture of how we played together."Get a letter from the atushi friend, Lujia Central Primary School five (6) Squadron of Pan Yucheng wiped his hand, carefully opened the envelope."Welcome to my home. There are many fruit trees in my hometown, Dagaliang Township, Atushi City. I hope you can like them..." Pan Yucheng While reading the letter, he can't wait to share this joy with his friends."The letter hand in hand activity is very meaningful. I am very glad to get to know my friends of Atushi Nluk Middle School. He introduced his hometown and sent me blessings, and I also wrote my wishes to him in the letter."Pan said," I know he is in Xinjiang, far away from us. I hope he can come to Kunshan one day."Pan Yucheng also specially marked Atush's position on the map, because there was a friend who he had never met before. In the letter, the Young Pioneers of Atushi not only introduced the local conditions and customs of their hometown, and wrote down their dreams and wishes, but also issued warm invitations to their distant friends on behalf of their hometown. At the same time, the unique culture of Atushi has also broadened the vision of the Kunshan Young Pioneers and made them fascinated by the unique local conditions and customs of Xinjiang."Letters close the hearts of children, although they are far away, their hearts are close together."Chen Yan, a counselor of the Fifth (6) Squadron of Lujia Central Primary School, told reporters that since the pair have written letters, students have often asked her about Atushi, and students' enthusiasm for writing has been stimulated."The exchange of letters has further enhanced the understanding of the cultures of all ethnic groups and created a good atmosphere for ethnic unity."Group kunshan municipal party committee group Xuanminister, less, deputy director of the week said, group kunshan municipal party committee, city less working committee will continue by letter hand in hand, love package, team class, open both red scarf communication channels, let love my Chinese seeds rooted in every youth heart, guide the young pioneers set up ambition, work hard, hand in hand together to serve the motherland.

2022-01-17	Group of Anhui bay � b district party committee held "jin wutong" weekend talent salon in Anhui bay � b district party committee held "jin wutong" weekend talent salon figure China youth network Beijing on January 17 (reporter Zhang Jianwei, correspondent, tao HuiFang check WeiTing) in order to further promote Anhui province Wuhu bay � b area talent team construction, power bay � b area of high quality development, realize bay � b vertical rise, recently, Anhui Wuhu bay � b area "jin wutong" talent salon weekend. Representatives of 25 outstanding entrepreneurs in Bay � b district and leaders of relevant universities in the district discussed how to retain talents and cultivate local talents. The theme is "Fengqi Wutong, Caijuwan � b". On the same day, Yin Qiong, secretary of the Bay � b District Party Committee, delivered a speech for the event and announced the official launch of the "Tianjin Wutong" weekend talent salon. The salon communicated on talent introduction and strengthening cooperation through theme sharing, dialogue and exchange, and on-site chat, and the universities in the region put forward effective suggestions on strengthening school-enterprise cooperation. In the next step, Tuanwan � b District Committee will closely focus on the work deployment of Wuhu "Ziyun Ying" talent plan, combined with the actual talent work in Bay � b District, adhere to the reform and innovation, do fine and solid "Qingyun Plan" and other young talent brands, and play the role of young talents, and contribute to the vertical rise of Bay � b.

2022-01-25	Chongqing Fengjie: Winter: Jianwei Correspondent Zhang Yuehua) Recently, the "Winter Sunshine warms you and me" care action held by the Chongqing Fengjie County Party Committee of the Communist Youth League went into some mountain schools to send winter warmth to the children. The staff of the Youth League Fengjie County Party Committee came to the Yunwu Primary School in Fengjie County, which is more than 1,200 meters above sea level, and distributed their school uniforms, scarves, gloves and hats to the children. At the event, the caring people also personally put on new clothes, hats and scarves for the children. And cordial communication with the children, a detailed understanding of their study, life, encourage them to aspire to, study hard, return to the society. Put on a new school uniform, wearing warm gloves, hat, a young face filled with a happy smile, the children are also with their most sincere and simple way, to express their gratitude. According to the introduction, the "winter sunshine warms you and me" care action, is mainly aimed at the rural left-behind children, children from poor families, by meeting the children a small wishes, to convey the warmth of the society."To carry out the caring action is not only to give children material help, but also to sow the seeds of children's young hearts, mutual assistance and gratitude with love action, the next step, the Youth League County Party Committee will continue to strengthen publicity, collect more love from the society, let them feel the warmth of the social family."Tuan Fengjie County Party Committee comprehensive department staff member Song Qiang said.

2022-01-20	Wang Xinxin, a young pioneer of Changshou District, Chongqing municipality, has launched a theme activity of sending proverbs greeting cards to the garrison guards. At the launch ceremony, 30 representatives of the young Pioneers wore bright red scarves, saluted and flags, reviewed vows, and proposed to send greeting cards to the border guards. The participants also watched the film and television works of "Head of the Country and Guard the Border Heroes" Qi Fubao "," Tian'anmen National Flag Coming to the Karakoram " and other films. The children will select a greeting card printed with the characteristic scenery of the longevity area, write what they want to say to the border uncle, and then send it from the campus post office of our school. The border soldiers will interact with the children in letters. The person in charge of the Chongqing Longevity District Committee of the Communist Youth League said that the immersive and interactive theme activity aims to make the young pioneers more deeply understand the border guards, educate and guide the young pioneers to enhance their awareness of national defense, and cultivate the feelings of home and country. Under the guidance of Chongqing Youth Working Committee and hosted by the Changshou District People's Armed Forces Department, the Education Committee and the Youth League Committee, more than 9,000 greeting cards bearing the blessing of the young pioneers will be delivered to the border guards before this year's Spring Festival.

2022-01-15	Tianjin pku to carry out the "respect help trapped" volunteer service special sympathy activities Tianjin youth community youth action "to help trapped" volunteer service special sympathy activity dragon) on January 12, Hunan jin pku joint Xiangyang street street, Xiangyang street community community youth action "respect help trapped" volunteer service special sympathy activities, for Xiangyang street community five difficult old man sent the warmth and care. During the activity, the young volunteers sent pork, rice, cooking oil and other living materials to every elderly person in need, talked with them cordially, understood their recent health and family conditions, and timely grasped their current practical difficulties. For the elderly with physical difficulties, community volunteers take the initiative to help pickle pork. The Communist Youth League Committee, the streets and the community visited the elderly with warmth to the home. A cordial greetings from the young volunteers also added to the lively home, making them feel the company like relatives. The "respect to help trapped" volunteer service special sympathy activities, kicked off the 2022 Tianjin city community youth action, to lead more youth into community volunteer service, power community volunteer service activities normalized, institutionalization, continuously consolidate community organization construction, dynamic community group work, improve group service Tianjin city community construction ability and level is of great significance. Next, the Communist Youth League Committee will continue to closely follow the central work of the Municipal Party Committee and the key work of the Changde Communist Youth League Committee, combined with the actual Tianjin community, to better implement the community youth action series of special activities

2022-01-21	Jianxing County, Shaoguan City, Guangdong Province, has launched a new round of "Love 1 + 1" student aid campaign. Jianwei) has launched a new round of "Love 1 + 1" student aid campaign to help students solve their difficulties in study and life. Recently, the group of Shaoguan city, Guangdong province shixing county united county volunteer association to carry out the "love 1 + 1" student household visit a baseline survey work, during the visit, the volunteers understand the difficult students family situation, learning life, and so on and so forth, at the same time, actively promote the support policy in our country education and encourage difficult students to study hard, with knowledge to change fate, return society with love. At present, we have contacted 31 businesses and enterprises to subsidize (continue to subsidize), and the next children from poor families will be eligible to help them, so as to provide a strong guarantee for the poor students to successfully complete their studies. In recent years, TuanShiXing county to give full play to their own advantages, gradually improve the various functional institutions and working mechanism, actively participate in social public welfare and charity activities, build a good platform for difficult students interpreta dream university, to carry out four years "love 1 + 1" student action, a love business enterprise 110 households, funding 110 difficult students, funding 132000 yuan.

2022-01-17	New venture in Shaoguan, Guangdong province love student three years action officially launched in Guangdong xinfeng "east new collaboration youth interpreta dream" love student three years action officially launched China youth network Beijing on January 17 (reporter Zhang Jianwei, reporter Li Excellence) in positive response to implement the county county government "good love", help difficult families students solve growth problems, recently, the joint Dongguan dongcheng xinfeng counterpart support headquarters to dragon town pond village village, new village, ancient pond village "east new collaboration youth interpreta dream" love student visit activities. In accordance with the requirements of COVID-19 prevention and control, more than 20 volunteer teams of "Xinfeng College Students + Dongguan Loving Enterprises" were set up to set up 44 subsidized students in "one-to-one" groups and send gifts. It is understood that by dongcheng xinfeng counterpart support headquarters, xinfeng county jointly launched "east new collaboration youth interpreta dream" return long town love student action for three years, will return long town pond village village, new village, ancient pond village left-behind teenagers provide long-term support services, establish "one to one" student plan, help teenagers grow up better. In the next step, the Communist Youth League County Party Committee will continue to integrate social resources, multi-party linkage, carry out more student aid activities, further build a platform for social forces to participate in the love donation, and promote social organizations, volunteers, caring enterprises and other social forces to actively participate in the love action, and benefit more young people in need.

2022-01-13	Fujian wuyi mountain group to carry out laba care activities in Fujian wuyi mountain group to carry out laba care activities in reporter Chen qiang) group Fujian wuyi mountain party committee recently joint pleasure social work center, catering association and other youth social organizations to carry out the "feeling warm wuyi local Chinese New Year" laba care activities, for migrant workers, frontline workers and community residents to more than 1000 steaming laba porridge. On the morning of January 10, more than 40 community youth action volunteers served Laba porridge to delivery boys, delivery workers, sanitation workers, traffic police and community residents. Volunteers also packed laba porridge, visited forest police and poor families, and sent festive greetings and blessings. Volunteers also issued "Youth" force, "Youth" as the "cover" and "Civilized Transportation Travel" proposals to participants, advocating migrant workers and citizens to actively cooperate with COVID-19 prevention and control work, pay attention to travel safety during the Spring Festival travel rush and the New Year, and try to stay in their work places for the Spring Festival.

2022-01-13	The Langya District Party Committee of Chuzhou City, Anhui Province, has implemented the system of integrating points into the league. Correspondent for figure China youth network Beijing on January 13 (xinhua Zhang Jianwei, correspondent, Zhu Rui check WeiTing) "heard that new members are pay attention to academic performance and the teacher's opinion", " now is different, heard that the school began to integral system, mainly examine the comprehensive performance in school, I think this way is the opportunity to our own hands, the higher the integral, the more actively participate in the school communist youth league practice, can be priority into the communist youth league member!"Chuzhou City, Anhui Province, Baoshan school, two students are having a heated discussion. Since 2021, the Langya District Committee of Chuzhou City, Anhui Province has focused on the comprehensive and strict governance of the League, from the source of "education and promotion", to provide institutional guarantee for the selection of good people and the right people. At present, Chuzhou Baoshan School is being taken as the pilot to vigorously promote the integral league system, and has achieved certain results, attracting the youth League committee of all brother counties of the city to come to observe and study. From August 2021, Baoshan School began to formulate the "Youth League Management Measures for Joining the League". The implementation object is the non-league member students in the first grade of the school, and to design the young Pioneers' integral entry manual, and the league member study manual according to the moral, intellectual, physical, aesthetic, labor and social practice. From the ideological and political evaluation, volunteer service, group class learning, honor and commendation and other diversified aspects of the assessment. First according to the integral situation by the young Pioneers squadron "presumption", then sent to the brigade "review", and finally by the school youth League committee "determination", layer upon layer of check, finally determine the excellent personnel as the league training object, become an active member of the league. Through team cooperation, from the source of the check, standardize the league recording procedures. Strengthen ideological guidance, focus on training education, standardize the construction of middle school and league schools, formulate league class learning courses, and guide the league activists to participate in league class learning, league affairs test, volunteer service and other practical learning, to deepen students' understanding and mastery of league knowledge. In addition, students are required to use holidays to enter the street communities to serve the society. The volunteer service time should not be less than 20 hours, and the theme group class study should not be less than 8 class hours. Finally, for the qualified assessment and meet the standard objects, according to the points established as their development objects. Xu Lisha, deputy secretary of the Youth League Committee of Chuzhou Baoshan School, said, " The school has been actively exploring the work of the league school, thinking about how to use students' easy to understand way to learn theoretical and pragmatic action. Strengthen the ideological guidance of the league members, and promote the ideological growth of the league activists."Langya district since the implementation of the league integral system, in the clear development member work objectives to break the past" see the league " mode, on the basis of scientific evaluation and democratic appraisal, layer upon layer, specification league formalities, from the source guarantee the quality of the development, student enthusiasm improved, one than learn to catch up with. It further enhances the students' sense of sanctity and pride in joining the league. In the next step, the League Langya District Committee will also timely adjust it according to the actual situation, so that the evaluation content is more perfect, the evaluation standard is more scientific, adhere to the strict entry of the league, and constantly improve the quality of new developed league members, so as to strengthen the construction of the overall advanced league members of the district.

2022-01-10	Volunteers in Fenghua District, Ningbo, visited Ningbo Li'an Technology Co., Ltd., Zhejiang Province, where volunteers from the Fenghua District Volunteer Association sent Laba porridge to migrant workers at 11:00 today."This is my first time to drink 'Laba porridge'. It tastes sweet and delicious to feel the warmth of 'home'."Said a migrant worker from Ganluo County, Liangshan Yi Autonomous Prefecture, Sichuan Province. Chen Li, deputy secretary of the Fenghua District Committee of the Communist Youth League, said that outdoor front-line workers, new employment groups and migrant workers are the important forces in the economic and social development of Fenghua District in the new era. On the eve of the lunar laba festival, fenghua district party committee organization department, youth league party committee, district youth volunteers association, district youth association to "yong love e home" as the carrier, launched 1 + N laba warm heart activities, namely through a bowl of "laba porridge", let the express little elder brother, delivery little elder brother pass love, make work in fenghua outdoor line workers, stay staff feel the warmth and care of the city. At present, 30 "Yongai e Home" stations in Fenghua District have been open to the outdoor frontline workers such as express delivery boys, food delivery riders, online ride-hailing drivers and sanitation workers. During the two-day warm festival, volunteers sent more than 600 bowls of Laba porridge to outdoor frontline workers, quarantine workers, migrant workers and other groups. Volunteers from Fenghua District of Ningbo city deliver warm Laba porridge to outdoor workers. Shi Hanxiao for the picture

2022-01-07	Shixing County, Guangdong Province, set up the first Young Pioneers after-campus practice education camp in Beijing, January 7 (reporter Zhang Jianwei) in order to thoroughly implement the "Opinions of the CPC Central Committee on comprehensively strengthening the Work of the Young Pioneers in the New Era", Further integrate red resources and educational resources, Smooth operation mechanism, Escort for the healthy growth of children in the whole county, League Shaoguan City, Guangdong Province, Shixing County Party Committee united county Education Bureau, county young working Committee to fully tap all fields of social resources, Selected the local off-campus practice base suitable for the young Pioneers, It provides an important platform for the young pioneers to carry out after-school practice, patriotism education and all kinds of knowledge popularization. On the afternoon of January 4th, the opening ceremony of the first "Young Pioneers after-school practice education camp" in Shixing County was held in Shixing Hongwei (the former site of Guangdong Provincial Party Committee). A total of 40 people from the Communist Youth League Committee, county education Bureau, counselors of Shenzhou Primary School attended the opening ceremony. The young pioneers wearing bright red scarves took the oath. During the activity, the Communist Youth League County Party Committee, the county education bureau, Shen Suo town responsible comrades jointly unveiled the first young Pioneers outside the school practice education base of Shixing County- -Shixing County Hongwei. The young pioneers listened to the red narrator of Hong Wai to explain the revolutionary deeds of Shi Hongxing Wai, understood the arduous history of the red revolution, and reviewed the years of hard struggle of the revolutionary ancestors.visit and learn. TuanShi xing county for figure then, the young pioneers came to shixing county red culture education base-the Guangdong provincial party committee underground traffic station revolutionary history exhibition hall visit study, understand the red revolution from the bud to the course of the development of the national unity, shows the China of the communist party have the courage to explore, adversity survival, strive to develop the spirit of the red. The young pioneers also said: " I really like to participate in practical activities in such bases outside the school, which let us learn more revolutionary stories outside the classroom."visit and learn. It is reported that the Communist Youth League County Party Committee gives full play to the educational role of the "red education base", this group of three positions as the county's first red education theme of the young Pioneers outside the school education practice camp, launched the young pioneers to approach the revolutionary history, inherit the revolutionary fire. Next, the county will be on this basis to build the extension of the traditional culture base, patriotism education base, anti-drug education base, youth home, volunteer service base, science and technology education base and other types of base into the young pioneers campus practice base, form the campus benign complementary system, promote the construction of campus "the home of the young pioneers".

2022-01-07	Hunan ningyuan county youth branch members to preach into the campus activities group ningyuan county to carry out the party and the sixth plenary session of the 19th party spirit micro preach youth network Beijing on January 7 (reporter Zhang Jianwei, correspondent plate beads) to thoroughly study and implement the spirit of the sixth plenary session of the 19th and the 12th congress of Hunan province, guide the county broad youth members to learn to promote line, to practice, unswervingly listen to the party, party, follow the party, in the new era new journey writing more beautiful youth answer. Recently, the Ningyuan County Party Committee of Yongzhou City, Hunan Province, organized Ouyang Yingying and Ouyang Lingdong, members of the county youth teaching group, to carry out the spirit of the sixth Plenary Session of the 19th CPC Central Committee and the 12th Party Congress of the province in Ningyuan County Experimental Middle School and Ningyuan County Aihua School. Carry out a micro lecture in Aihua School. Correspondent for figure briefing, the students watched the preaching theme film "" you "story", young lecturer from the party's struggle in one hundred major achievements, historical experience and historical significance of six aspects expounds the party's sixth plenary session of the 19th congress of Hunan province significance and profound connotation, motivate students to learn from the party's one hundred struggle glorious history and great achievements, firmly listen to the party, follow the party's determination, to achieve the second goal in one hundred, actively engaged in modern new Hunan.

2022-01-06	Fengxiang, Shaanxi: During the grassroots reform of the Communist Youth League, I served as the leader of the reform leading group to promote the work."The words of the Party secretary of Fengxiang District, Baoji City, Shaanxi Province are resounding. We need to gather strength and focus on the key of" the Party leads to the leadership of the Communist Youth League, and the Communist Youth League better adheres to the leadership of the Party ", to improve various systems and mechanisms. At the district Party Congress held in September 2021, the pilot reform work of grassroots organizations of the Communist Youth League was written into the "work report" of the Standing Committee of the District Committee. As a continuous in the national, the provincial county communist youth league grass-roots organization reform pilot counties, more than a year, fengxiang youth league organizations at all levels under the strong leadership and support of the grassroots common mechanism of operation, insufficient resources, main positioning is not allowed, constantly try to innovation, cohesion party bring out good building, make the communist youth league cause with vigor and vitality. Assessment "baton" compaction responsibility in Fengxiang, "league building, team building is not qualified, the party building is not excellent" has become the consensus of grass-roots Party organizations."Assessment is the 'baton'. With this 'baton' to consolidate the responsibility of party building and youth league building, the importance of primary-level Party organizations has significantly increased, and promoted the reform of community-level youth league organizations to deepen and strengthen."Fengxiang District Party Committee Organization Department Executive Vice Minister Lu Haitao told reporters. According to the 5% proportion, the league building and team building assessment in Fengxiang is included in the party building score, and serves as the basis for the party building work deployment content and the annual target responsibility system assessment. Not only that, the party building with the league building, team building work implementation situation has also been included in the party committee (party group) secretary to grasp the party building annual report review content and the party committee inspection and supervision content. In the view of Zhang Jianfeng, party secretary of Biao jiao Town, "matching the secretary of the village League general branch is the primary issue to further enhance the vitality of the rural League organization". In the town, the village two committees is taken as an important opportunity, "what constitutes the situation", "how the number of youth members", "league organization activities are held regularly" and other issues are clear, and on the basis of a comprehensive grasp of the rural youth ideological dynamic, to ensure that will want to be, can be as the youth absorption into the league cadres. At present, among the 20 village-level league organizations that have completed the transition, the new secretary of the village League general Branch are all members of the village committees, with college degrees or above and under 35 years old, realizing the goal of younger and high education of the league organization team. The role of the "sword" has also been fully played in Fengxiang."Through political inspections, we will pay more attention to primary-level Party organizations and devote more energy to the work of the Communist Youth League and the youth."District committee inspection office responsible person introduction. In several rounds of inspection by the district committee, a total of 5 district direct departments, 7 towns, 23 villages have put forward inspection feedback opinions. After a series of reforms, the league construction work has been included in the assessment of the regional target responsibility system and the key points of the organizational work, and regular supervision is carried out. All-round, three-dimensional and closed-loop inspection and rectification supervision system has been initially constructed in Fengxiang. In the recent demonstration activity of joining the Young Pioneers in Fengxiang District, the drum team composed of 32 young pioneers of the First Primary School was particularly eye-catching."This year, the Communist Youth League District Committee and the District Youth League Working Committee raised more than 30,000 yuan to give support to them, and our drum team took on a new look."Competitive save a small young pioneers counselor Li Jianting told reporters. Since the implementation of the pilot reform of the youth League grassroots organization, "vigorously supporting the reform and innovative development of the Communist Youth League" has become the consensus of the successive district committee leaders of Fengxiang District. With cadres, give resources, strong positions.... the district Committee to give the all-round policy tilt, for the smooth promotion of the pilot reform escort. In Fengxiang, the district finance included the work funds of the Communist Youth League and the Young Pioneers in the budget of the Communist Youth League District Committee, which was fully guaranteed according to the standard of 1 yuan per person. At the same time, the organization department allocated part of the party fee, as the party building with the league construction special work funds. Multiple departments jointly issued management measures, coordinated the overall implementation of the work funds of 12 town youth League committees, and took the lead in the city to pay remuneration to the village (community) youth league branch secretaries, which effectively guaranteed the operation of grass-roots organizations and stimulated the vitality of the work of the league. At the same time, "expanding the coverage" has become an important starting point to consolidate and upgrade the grass-roots youth league organizations. In Fengxiang, 27 grass-roots youth league organizations and 12 Youth Working Committee have been adjusted, and the district direct organ youth League Working Committee and Education Youth League Working Committee have been set up to create a number of positions for party building and league building of "two new" organizations."The establishment of the Enterprise Youth Working Committee has attracted more and more young people to join us, which has greatly improved the innovation and development ability of our enterprises."As a witness of the reform of the Communist Youth League, after 85 returning entrepreneurs," Boya clothing " youth working committee director Lou Xiaojiao said. Education "touchstone" promotion effect to effectively improve the quality of young party member development, fengxiang district party committee organization department, OuZhi authority, TuanOuWei jointly issued by "the communist youth league push the party work guidelines (trial)", formed the "define a scope, clear two conditions, distinguish between three paths" of "123" push the party work method, effectively solve the grassroots youth league in push the party often encountered "push what, how to push, who to push" problem."At present, the grassroots youth League organization 'promotion' has gradually become the main channel for party organizations to develop young Party members."Sun Ruiqing, secretary of the Fengxiang District Party Committee of the Communist Youth League, said that everyone who is striving to get close to the Party organization actively has also become the new consensus and new fashion of Fengxiang youth. In recent years, Fengxiang District Committee has made great play to the advantages of the Communist Youth League's political competence and talent gathering, and is committed to building a team of high-quality young talents. The secretary and deputy secretary of the Communist Youth League District Committee, the general counselor of the District Young Pioneers and other cadres have successively went out and sank to the southeast coast for temporary study and training at the grass-roots level, so that their ability and quality have been effectively improved. Zhang Jinli, who was transferred from the secretary of the Communist Youth League District Committee to the mayor of Changqing Town, once went to Kunshan, Jiangsu province, which was ranked at the top of the "top 100 counties in China"."My experience in Kunshan Huaqiao Economic Development Zone has not only broadened my horizon, but also been refreshed by the working ideas and methods of advanced areas."Said Zhang Jinli. Since 2020,10 grass-roots cadres of the Communist Youth League have come to the youth League district Committee for rotation. Such learning and training is expected to achieve the "full coverage" of the cadres of the town and directly affiliated schools in three years. In the transition of the village committees in 2021, the Communist Youth League District Committee made every effort to promote the village Party branch members under the age of 35 to serve as village League branch secretaries and support excellent village League branch secretaries to enter the village committees, which effectively solved the problem of weak reserve force of grass-roots Party organizations and made a good talent reserve for the implementation of rural revitalization."By suppressing responsibility, providing resources and educating new people, the political and social functions of the Fengxiang Communist Youth League organization are gradually being restored and established, and the Party building has brought out a good league building."Sun Ruiqing said that in the next step, Fengxiang will continue to uphold the concept of" reform and start again ", build a mechanism, strong function, increase effectiveness, and constantly explore a new path of grassroots organization reform of the Communist Youth League. China Youth Daily, reporter of China Youth Network, Sun Haihua, source: China Youth Daily

2022-01-05	Yunnan malipo communist youth league seven "double hundred" project will work in practice malipo communist youth league seven "double hundred" project will work in practice wei correspondent knife nan) in 2021, for grassroots youth thought lead less, the effect is not obvious, Yunnan Wenshan zhuang and miao autonomous prefecture malipo communist youth league implemented seven "double hundred" project, vigorously carry forward the "old mountain spirit" and "west spirit" of the new era, targeted crucial breakthrough, improve the contribution of the communist youth league organization, leading force, cohesion and service situation, earnestly will work dry in practice, walk in the forefront. We will carry out the "Double Hundred" project of ideological and political guidance for young people. Form hundreds of youth party history XuanJiangTuan: in the county schools foster "little red XuanJiangYuan", deep red education base as good party history volunteer narrator, organization watch "red scarf love learning", "youth learning" online series theme class, team, to carry out the "red scarf love learning young pioneers party history red story 100", in the youth heart strengthen love party patriotic love socialism feelings, efforts to grow into builders and successors to the cause of socialism with Chinese characteristics. To carry out the best project to preach down: malipo "youth branch" "red scarf branch" and "five old XuanJiangTuan", so far, the XuanJiangTuan members to preach 206 games, covering teenagers 53592 people, promote the communist youth league organization become the youth in practice learning socialism with Chinese characteristics and communist schools. The Youth League Malipo County Party Committee was awarded the "Yunnan Province 'Yunling People's Publicity'Demonstration Site" by the Publicity Department of the CPC Yunnan Provincial Committee and the Party History Learning and Education Leading Group Office of the CPC Yunnan Provincial Committee. We will carry out the "double hundred" project to standardize the construction of grass-roots youth league organizations. Hold hundreds of grassroots cadres, young pioneers counselor training: the county cadres, the young pioneers counselors started and ability promotion training, the spirit of the fifth, "July 1" important speech spirit, party history education, rural revitalization strategy, state the tenth party congress spirit, grassroots affairs into training, improve grassroots cadres' ideological and political construction and comprehensive ability, cultivate young marxist 282. Carry out 100 league branches key sorting and rectification work: successfully completed the county village (community) league organization change, relying on the "wisdom league construction system" to the county 105 villages (communities) elected 470 new members, completed the information update, and the county 524 league organizations for annual education evaluation. We will implement the "double hundred" project to serve youth development. Hundreds of youth entrepreneurship employment training: organize 100 rural young entrepreneurs to participate in "power rural revitalization, electricity talent public training plan", joint market supervision and administration, disabled persons' federation to the township market to carry out "you business I support, you loan I discount" as the theme of business guarantee loan policy campaign, provide consulting platform for youth employment, help them to better entrepreneurship, reduce the burden of entrepreneurship and entrepreneurship costs, benefit entrepreneurial employment youth more than 500 people, accept the bid "loan free help fill" and small business guarantee loans 106, cumulative 46 successful 7.38 million yuan. Organize hundreds of youth friendship activities: implement the "1 + 100" league cadres directly contact youth system, establish regional, industry, organizations and other emerging youth contact channels, carry out the "home yan nest to help growth" returning college students social practice activities, so that young students in understanding the county situation by education, long ability and contribution. In light of the actual situation of epidemic prevention and control, online and offline youth dating activities are held to make friends, focusing on serving the needs of public security, border defense, hospitals, school teachers and other groups. We will carry out the "double hundred" project to safeguard the rights and interests of young people. Carry out 100 young people to participate in the "mock trial into the campus" activity: to the students around things as cases, carefully selected cases, write scripts, by the students role play to perceive the majesty of the law, in the township (town) middle schools, urban secondary vocational middle schools fully spread out the mock trial into the campus activities, covering more than 8,000 students. Carry out 100 publicity activities for the prevention of juvenile crimes: joint schools to carry out juvenile legal education, AIDS prevention education, anti-drug education publicity activities and safety lectures, covering 81,500 teachers and students. We will carry out the "double hundred" project to jointly govern and jointly build urban projects in cities. Organize hundreds of youth volunteers to participate in volunteer service: use the national volunteer service platform, volunteer platform to form laoshan youth volunteer service, absorb 106 registered volunteers around malipo play "four big campaign" goal, effectively launch and lead the youth vigor, spirit, talent into the grassroots community governance and service, comprehensive youth participation in community, community governance work, total service time 2700 hours. Carry out 100 volunteer service activities for the masses: closely combined the caring for youth volunteer service project with the practice activity of "I do practical things for the masses", released 17 volunteer service activities on the Youth Home cloud platform, and carried out youth volunteer service on a regular basis, and 1,589 people participated in the activities. Based on the Communist Youth League participating in rural revitalization, epidemic prevention and control, and social governance, they have organized more than 1,000 volunteer services and dispatched more than 50,000 volunteers. Implementation of the young Pioneers construction "double hundred" project. Hundreds of young pioneers pro martyrs activities: in order to avoid entrance problems caused work cohesion, in 2020, based on malipo national middle school, iron works township central school, fierce adit township central school, Dong Gan town central school, new 123 pairing young pioneers, the communist youth league member of malipo county unknown martyrs or long relatives martyrs 55 martyrs, ensure martyrs work continuously, responsibility is not absent. To carry out the centenary of the founding of the Party, organize more than 100 drum teams in the county to carry out the "border drum horn ringing for the centenary of the Party" activities, so that the "sound of the drum horn" rang through the Malipo border area. Implement the sunshine growth plan "double hundred" project. Help hundreds of troubled children: relying on social organizations volunteers to carry out the "hoe grain plan", "colorful" holiday "," feeling warm childlike innocence "," red ribbon "and other activities, encourage and guide the volunteers and left-behind children to establish" one to one, one help one " pair service, normalized academic counseling, family company, since the care education, love interpreta dream and other care activities. In combination with public welfare projects, a total of 54,300 yuan of charity funds was raised to subsidize 25 poor students. More than 200,000 yuan of stationery, computers, drum equipment, winter shoes, quilts and cotton clothes were raised to help 1,585 children in distress realize their "micro wishes". Seven cases of free child diagnosis were contacted, and one child with congenital heart disease was organized to the Southern Theater Command General Hospital for surgery, effectively preventing the families from returning to poverty due to illness."Hundreds of youth example growth" activities: to carry out the "two red two optimal" selection activities, selection for a batch of outstanding typical, in each unit, school organization hundreds of industry youth example in various fields, jobs, innovation, entrepreneurship, patriotic dedication, self-improvement, charity positive incentive story, with excellent spirit infiltration youth thought, with typical deeds inspire youth growth. Recommended to the central, provincial, state, a total of 208 outstanding youth by the central, provincial, state, county recognition.

2022-01-14	Group of Guangdong province shixing county to carry out the road traffic safety propaganda volunteer service activities of Guangdong province shixing county to carry out the "youth feeling warm safe travel" road traffic safety propaganda volunteer service activities built wei) for the implementation of the CPC Central Committee, province, city, county on road traffic safety work major decision decisions, further strengthen shixing county the masses road traffic safety consciousness, create a more stable and orderly reason traffic environment. Recently, the Youth League of Shaoguan City, Guangdong Province and Shixing County Volunteer Association in Taiping Town community, Zhongfu Village, Chengnan Town Xincun Village and Shensuo Town Nanfang Village and other places to carry out the "youth warm safety travel" road traffic safety publicity volunteer service activities.site of activity. TuanShiXing county for figure activity scene, volunteers, "a helmet", "road traffic safety initiative" and other publicity materials, sent to the relevant area of the general residents, and for the residents explained the drunk driving drunk driving, agricultural vehicles illegal manned, no seat belt, do not wear a safety helmet, the serious harm of traffic violations, advocate people abandon traffic habits, safe driving, remind everyone to always abide by traffic safety regulations, develop good civilized traffic habits, let the masses understand the profound connotation of safety and great significance of travel. Next, TuanShixing County Party Committee said that it will continue to carry out good road traffic safety education work, effectively improve the awareness of the residents of Shixing County, and strive to create a strong atmosphere of law-abiding, civilized travel, consciously abide by the traffic rules.

2022-01-12	Group of Hefei in Anhui province lujiang county sympathy difficult student activities to carry out the "winter warm sun with love counterparts" sympathy difficult student activities jianwei correspondent Zhang Yu Zha WeiTing) to carry out the new era of civilization practice to help the needy volunteer service, help difficult family students solve practical difficulties, let them feel the TuanTuan care and warm, group of Hefei, Anhui province lujiang county in recently carried out the "winter warm sun with love counterparts" sympathy difficult youth activities. Tuan Lujiang County Party Committee through careful investigation, later review, determine the list, statistics of 115 poor students. The Party secretary and deputy secretary of Tuan Lujiang County respectively led teams to various towns (parks) to visit some students in need, and visited 35 students in need. Visit process, and difficult students face to face cordial conversation, detailed understanding of their family condition and physical condition, and encourage them to face the difficulties at home not depressed, to strong face life, set up the correct ideal faith, study hard, with a grateful heart, become determined to return society, the construction of hometown useful talent, and sent to them to the grant. According to statistics, a total of 50,000 yuan was distributed in the condolence activity, making the families of poor students feel the warmth of the league organization, and create a strong atmosphere of mutual help, warmth and harmony. In the New Year, TuanLujiang County Party Committee will continue to integrate social love resources to do a good job of pairing up funding and helping the students in need, and continue to promote the warm current of love in Lujiang County.

2022-01-12	League of Anhui province tianchang committee "three fusion" build youth around youth youth league "three fusion" build youth around the communist youth league JianWei correspondent Wang ShuoWeiTing) group of Chuzhou, Anhui province is committed to using market, emotion, interests, such as social mechanism, using the youth like communication, communication, gathering and contact, strengthen the mass, social hierarchy of network media construction."The Communist Youth League + 'net red' new media", brand operation. The Youth League Tianchang Municipal Party Committee now runs 1 WeChat public account platform "Communist Youth League Tianchang Municipal Party Committee", maintaining nearly 30,000 young users, and runs 8 online "Youth Home" cloud platforms, conducting activities in the form of youth "ordering". This year, 131 activities have been released, attracting 3,847 youth members to participate. A youth League organization was established in Tianchang Municipal Financial Media, and FM104.8 host Beibei was specially invited as the convener of the "Voice of Youth" activity, and online youth radio programs have been launched from time to time. Since the reform, online radio programs have been launched by young volunteers, covering more than 20,000 young people."The Communist Youth League + Network service Home", platform service. We will explore and build a one-stop youth service network platform for "gathering young talents". In young people customary WeChat platform to build small programs, from the big new students from the eve "understanding", college reading "participation", "return to" three stage work deployment, enhance the understanding of the new era of young people, help them understand the development status of everlasting, enhance their feelings and hometown stickiness, and effectively to the city's talent introduction strategy propaganda, let young people return with enthusiasm, have way, hope."The Communist Youth League + online information publicity", promote positive energy. Tuan Tianchang Municipal Party Committee is committed to the traditional young pioneer selection and commendation become more full of vitality, more nature of The Times. On the basis of the past "conference recommendation-government publicity-document commendation", the process of "network new media style display" was added. Whether it is "two red and two excellent", "youth pioneer" or "young role models around" in some aspects have strengths and contributions, all use the network new media all aspects, multi-angle to show the vanguard strength, shaping the model model. Let the young role model pioneer "more down to earth", let all the young people actively strive to be a typical, play a role model role.

2022-01-05	Hubei xianxian young pioneers campus practice demonstration and farming culture festival ended Hubei xianxian young pioneers campus practice demonstration activities and the first farming culture festival successfully ended wei correspondent to qing huang day tang ziwei) recently, the group of Hubei enshi tujia and miao autonomous prefecture xianfeng county, county town people's government organization xianxian young pioneers brigade counselor, outstanding young pioneers represent more than 90 people in zhongbao town in 2022 xianxian young pioneers campus practice demonstration activities and the first farming culture festival. The activity was presided over by Wang Chengxi, the general counselor of the young Pioneers of Xianfeng County. At the same time, Xianfeng County youth farming research base was established in Zhongbao Maherd tun, county concerned about the next generation working committee responsible comrades attended the activities.site of activity. Correspondent for figure this activity combines the party history learning education, the young pioneers brigade counselors and young pioneers less by visiting loyal fort martyrs cemetery, to the revolutionary martyrs wreath, to listen to the revolutionary hero story, listen to the hero story of revolutionary martyrs heroic deeds, learning the lofty spirit of the hero model, inheritance red gene, the continuation of the red blood.site of activity. This activity took the farming culture festival as an opportunity, the young pioneers participated in vegetable planting, glutinous rice cake production and other agricultural experience, in the local labor, enhance the young pioneers actively practice the Chinese traditional virtues of thrift consciousness. In the next step, the Communist Youth League County Party Committee will give full play to the advantages of organizational education, independent education and practical education of the young Pioneers by combining the rural revitalization strategy and the "double reduction" policy, broaden the practical activities and carriers, and enrich the after-school life of the young Pioneers.

2022-01-04	Guangdong Shenzhen longgang district communist youth league coagulation hearts meet force to promote the reform of grass-roots organizations longgang communist youth league adhere to the party lead, coagulation hearts meet force to promote the reform of grass-roots organizations about the party's struggle in one hundred major achievements and historical experience resolution, fully show the world the one hundred big party is youth vigor and vitality, the great attitude. As a mass League organization of advanced youth under the leadership of the Party, the Communist Youth League must take the in-depth study and implementation of the Resolution as the primary political task at present and for a period to come. Always adhering to the leadership of the Party is the most fundamental and important historical inspiration and the most fundamental guiding principle for guiding the reform and development of the Communist Youth League in the new era. All along, Longgang Communist Youth League stands in the overall height of the cause of the Party, grasps the party building and the league building team building work, and effectively promotes the organization construction of the League. In order to improve the "steering wheel" for the party construction of the new round of pilot reform of the CPC, Longgang District Committee adheres to the "upper and lower" linkage: improve the top design, actively win the support and leadership of the Party Committee, broaden the scope of the CPC, and vigorously improve the organization, leading force and service power of the CPC. We will improve the work of the Communist Youth League and the Communist Youth League."Since May 2021, Longgang District was listed by the Central Committee of the Communist Youth League as a pilot unit to expand the reform of the county Communist Youth League grassroots, Longgang District took out great energy to grasp the Party building with the league building work, the league building work into the overall pattern of the Party building, with the planning and deployment. First, give full play to the role of the party building assessment as a "baton", promote the work of league building and team building into the party building work assessment, and put forward clear scores. Second, the party building led the league construction, team construction work into the party committee inspection and supervision, forming a mechanism and inspection list. Party organizations at all levels have comprehensively attached more importance to the work of the Communist Youth League, forming a reform work pattern featuring the leadership of Party committees, government support, the focus of the Communist Youth League, and the coordination of all departments, and effectively driving the implementation of other reform measures. Longgang district seize the county communist youth league organization reform pilot opportunity, firmly establish the basic idea of "party building", strengthen party integration, multiple form expansion quality, form "resource integration, team aggregation, work fusion" youth work party and government joint conference system, the party's youth work system and youth development city construction, formulate effective service overall situation, promoting long-term youth development planning policy measures and work projects, strengthen the grassroots youth feeling, promote youth development urban construction depth practice."I am a grass-roots youth league leader, not long ago became a probationary party member, on the way to become a party member, the youth League organization has taught me a lot of things."Said a youth league cadre from the street. There are many other examples of developing from a Communist Youth League member to a party member in Longgang. Starting in 2021, TuanOuWei by perfecting the youth political talent training mechanism, in-depth implementation of the "green horse project", joint district party committee party school longgang district first youth marxist training project and "push to join the party" training class, from OuZhi authority, streets, school organizations and the young pioneers of 40 outstanding young people, the cadres is one of the students. Up to now, 29 students have submitted their applications for party membership, and 11 have become active members of the Party. Among the young people in Longgang, they have trained a number of young Marxists with both political firmness, comprehensive quality and both political integrity and ability. At the same time of "excellence", Longgang Youth League District Committee also pays attention to the combination of "excellence", "training" and "training", and attaches great importance to the development navigation of "optimization" objects. Longgang TuanOuWei adhere to the implementation of the party tube youth principle, comprehensively push the party work, the "push" into the party member development work plan, joint organization department formulated "on the development of the youth league push the party work guidance", the implementation of the "two general, two main, the" requirements, push the best party as an important political function of the youth league, improve the party, the youth league joint training education mechanism, after the league specification procedure of the party. Up to now, 81 recommendation forms have been received for youth League organizations, of which 3 are designated as probationary party members, 19 are designated as development targets, and 46 are designated as active party applicants. At the same time, the Longgang Youth League District Committee focuses on the implementation of the system of promoting excellent Party membership, the standardized construction of the team, strictly selects and manages the League cadres without sticking to one style, standardizing and strengthening the joint management work of the League cadres, and promotes the Party building and the League building work to create a new situation."In the reform work in 2021, the Longgang Youth League District Committee has comprehensively promoted the three-level full-time, part-time and temporary league cadre staffing of the district and street social organizations, and established a talent system covering party and government organs, enterprise parks and other fields."Longgang Communist Youth League District Party Committee Secretary Lu Xiqun introduced. At the district level, the government follows the "1 + 1 + 2" mode to optimize the youth league district committee leadership structure, that is, one full-time secretary, one full-time deputy secretary, and two part-time deputy secretaries. At the street level, the 11 street youth League working committees in the district are all equipped with 1 full-time secretary of the Youth League Committee and 1-3 part-time deputy secretaries. At the same time, the organization structure of the community youth League was optimized. 100% of the community youth League committee secretaries were also members of the community "two committees". During the centralized transition in March 2021,676 new members of the youth League Committee were produced in 111 communities in the district. In this reform, the Longgang Youth League District Committee in addition to the selection of the youth League cadres, in other youth work is also equipped with a strong working force. For example, in the work of the Young Pioneers, Longgang District promotes the honorary director of the District Committee, the Secretary of the Youth League Committee and the Director of the Education Bureau as the "double director" of the Youth League Committee, promotes the cooperation between the District Committee and the District Education Bureau, enters the seat system, and constantly strengthens the direct guidance of the work of the school. Broaden the party team widely established youth activities "main position" in recent years, longgang district committee constantly broaden the coverage of the communist youth league, in addition to achieve full coverage in the traditional field, in the Internet, social organizations, non-public enterprises and other emerging areas of party organizations set up a batch of "two new" youth corps cell, mission built with the party pace, constantly improve the league organization system, improve the quality of construction."It is actually difficult to find like-minded friends in a fast-paced city like Shenzhen. After the Communist Youth League District Committee established the Communist Youth League Working Committee of the Internet industry, it brought us 'retail investors' together, so that I can also have a circle of my own in this rapidly developing city."Said an Internet youth. It is understood that the Longgang District Internet Industry Party Committee and the Internet Industry Youth League Working Committee were inaugurated in the Longgang District Party and Mass Service Center on September 14,2021. The Youth League Working Committee of the Internet industry has 9 members, including 1 secretary, 2 deputy secretaries, 6 other members, and 7 youth league organizations. At the end of the opening ceremony, on the same day, the members have said: " the party flag is the youth League flag direction, will actively play the role of the Internet youth League working Committee members, do a good job in the Internet enterprises of the Communist Youth League and the young people under the new situation."At the same time, longgang TuanOuWei combined with longgang district" a brand "" a characteristic " the characteristics of the community between the community service center, enterprise park youth homes, community less committee, constantly play the role of party building lead TuanJianDui build demonstration, bring all kinds of youth favorite activities to the community, let the youth can find their own circle at home. On the basis of the high-standard operation of social organizations such as Longgang District Volunteer Federation and Longgang District Zhihui Youth Innovation Public Service Center, Longgang Youth League District Committee has also established literary and sports social organizations to build a platform and create conditions for "literary and artistic youth" to play a better role in the new era. Youth League organizations should send the Party's care to the young people, do everything possible to solve their problems, and become the force that young people can remember, find and rely on when they encounter difficulties, so that they can enhance their sense of belonging, identity and gain in the reform of grassroots organizations of the Communist Youth League at the county level. Next, Longgang Youth League District Committee will continue to promote the Party building with the league building as the focus of the reform, and strive to explore the effective practices to strengthen the construction of grass-roots league organizations, form institutional results solidified down, constantly expand the effective coverage of the work of the Party in the youth and the Communist Youth League, and promote the Party building with the League building work to create a new situation.

2022-01-02	Zhejiang and Sichuan League cadres as the representative of the host to help the east and west cooperation development Li Jianping) " Hello! Welcome to'Shan Hai Hand? Rich'Xiangshan Leibo agricultural products live special show!"Recently, in the" Chao Xiangshan Sea " broadcast room in Xiangshan County, Ningbo City, Zhejiang Province, Shen Yi, a cadre of the Youth League Xiangshan County Party Committee, and Xiangshan youth network anchor brought goods to sell agricultural products in Leibo County, Sichuan Province. Leibo County is located in the southwest of Sichuan Province, the east of Liangshan Yi Autonomous Prefecture and the north bank of the lower Jinsha River, with navel orange, tea, mountain sunflower, ahu water shield, Gastrodia elata and other characteristic agricultural products. In June 2021, according to the national cooperation arrangement between the east and the west, Xiangshan County and Leibo County signed the "Framework Agreement on Cooperation between the East and the west", formally forming a pair relationship. Shen Yi, who was the anchor in the broadcast room, also served as the deputy secretary of the Communist Youth League Leibo County Party Committee, and was sent to Xiangshan County for exchange and study."When I first became the anchor, I spoke for the special agricultural products of my hometown."Tuan Xiangshan County Party Committee deputy secretary Lu Lu also as a guest guest broadcast room with goods. The live broadcast attracted nearly 10,000 people, 52,000 likes, and the total sales of agricultural products worth 36,000 yuan. In the next step, Xiangshan will bring more project assistance to the youth of Leibo, and write a youth movement for "continuing the feelings of mountains and seas, taking the road of common prosperity". Cold xiao for the picture

2021-12-31	Luyang district in Hefei, Anhui province "Lu Xiaozhi" volunteer service into the community of Hefei, Anhui province Luyang district "Lu Xiaozhi" volunteer service into the community zhang correspondent Zhang � D check WeiTing) "aunt, your car on the awning is not safe, the traffic police uncle told us a lot of traffic accident cases, you can go to the opposite help demolition, my mother's hood is in the", from Hefei, Anhui province third primary school class 4 young pioneers Chen oak to remove hood owners, Chen oak is also a member of the "Lu Xiaozhi" volunteer service. Launch the young pioneers into the community, to participate in the demolition of electric awning propaganda persuasion work, help eliminate road traffic safety hidden trouble, play the young pioneers cutting edge exemplary role, radiation influence around family and friends, jointly enhance the level of urban civilization, this is XiPingMen less committee after the establishment of the first "LuXiaoZhi" series of volunteer service activities. Recently, luyang district, Hefei city, Anhui province, group LuYang district party committee, west Pingmen less committee hired jurisdiction district of Anqing road, Anqing road police station, parents, residents party members and other seven members of community young pioneers counselor team, and in the west Pingmen community service center built the first community young pioneers Angle, further enrich the young pioneers off-campus activities. Event, from Hefei Anqing road third primary school four (4) squadron of the young pioneers representative Chen spoke to speak, expressed into the community to participate in extracurricular activities, said to take an active part in the beneficial development of physical and mental activities, enthusiastic public welfare undertakings, actively participate in the volunteer activities, become a glorious "Lu Xiaozhi". Chen Qingya, a parent of the students who live in the dormitory of the Public Security Department, also promised that he would actively cooperate with the work of the school young working committee and the community young working committee in the future to contribute his own strength to the work of the community young pioneers. During the activity, Luyang traffic police brigade a squadron of three-level police chief Cheng Qiao explained the safety risks of electric bicycle awning and related road traffic safety laws to the "Lu Xiaozhi" volunteers, and led the "Lu Xiaozhi" volunteers into the provincial public security department dormitory community to carry out publicity and persuasion work.

2021-12-28	Guizhou Wanshan District has set up a temporary committee site of "College Students Chunhui Service Club". Correspondent for the picture of China youth network Beijing on December 28 (reporter Zhang Jianwei, correspondent, Chen Mengyao Yang Zixuan) recently, Guizhou Province Tongren City Wanshan District college students Chunhui service club temporary committee in the "Tongren youth home" officially established and started the service club members and management personnel recruitment work. At the establishment ceremony, Wu Fei, deputy secretary of the Communist Youth League District Committee, introduced the basic situation of Wanshan District College Students Chunhui Service Club, arranged and deployed the next work, and also set up the member recruitment team of the service club on the spot."College Students Chunhui Service Club" temporary committee establishment ceremony site. It is reported that Wanshan District "Young College Students Chunhui Service Club" is a social organization directly under the Communist Youth League District Committee, mainly to undertake, cultivate and participate in the youth League District Committee initiated and organization and operation of the youth management work, service projects, public welfare undertakings. At present, the recruitment of members and the service club management personnel is under way.

2021-12-26	Guizhou Honghuagang District Committee: Care for the elderly love the elderly warm winter "volunteer spirit, guide the youth to respect the elderly, do practical things for the elderly, solve difficulties, on December 20, sponsored by the Communist Youth League Honghuagang District Committee" warm winter care " for the elderly winter theme activity was launched. At the event site, the professional volunteer team provided the elderly with traditional Chinese medicine pulse pulse, blood pressure measurement, health knowledge popularization, anti-fraud knowledge publicity and other services, and sent love eggs to the elderly. The old people who love literature and art dressed in gorgeous national costumes performed wonderful dance programs, showing the positive spirit of the elderly in Honghuagang District. According to the introduction, this activity will last for one month, will hold various cultural activities and volunteer service activities, enrich the spiritual and cultural life of the elderly, enhance the sense of gain, happiness and security, and promote the creation of "respect for the elderly, worship virtue" social atmosphere.(Sheila Chan)

2021-12-25	Renqiu City, Cangzhou, Hebei Province, held the joining Ceremony Year Center for New Social Development League Members, and organized the joining ceremony of new social development League members in the social field in 2021. The organizer read out the list of the new members, and issued the league member card and league emblem to the new members. The new members wore the league emblem, and the representatives of the new members spoke and solemnly swore to the league flag. After the ceremony, the new league members visited the youth Center, the Innovation Building Youth Entrepreneurship Base and the Innovation Building Youth League Branch. At the ceremony, the Tuanqiu Municipal Committee spoke. More than 40 people attended the ceremony, including the leaders of the League cadres, and members of the new development in the social field.

2021-12-24	Group Chengdu, Sichuan province jianyang warm winter sympathy activities Sichuan jianyang municipal party committee to carry out warm winter sympathy activities) to further promote the traditional virtues of the Chinese nation filial piety old love kiss, power happiness jianyang, recently, the communist youth league of Sichuan jianyang municipal party committee love enterprises in jianyang city 25 nursing homes to carry out the "green heart love around is warm winter" sympathy activities, for nursing homes nearly 800 elderly people bring the social from all walks of life care and warmth. On the day of the activity, the staff of the Communist Youth League and the enterprise youth volunteers sent winter clothes, milk, rice and other gifts to the elderly in the nursing home, and talked with the elderly to understand their living conditions. Grandpa Zhang, 78, of Hefeng Town Nursing Home, wore a new hat and a new scarf and said, " The new hat is very warm, much warmer than wearing my own one. I am really so happy!"Love enterprise youth volunteer representative said that the family is an old, if there is a treasure. Every family has an old age, and it is everyone's responsibility to do a heart for every old man. Passing on this love and warmth is the responsibility of Jianyang enterprises to give back to the society and the people in their hometown. Youth League Jianyang Municipal Party Committee staff introduced that this series of sympathy activities have attracted a lot of young volunteers to participate in, they have played an important role in the organization and development of the activity process, reflecting the demonstration role of the "youth commando". We hope to encourage more young people to participate in it and contribute to the cause of respecting and loving the elderly in Jianyang. It is reported that the Communist Youth League Jianyang Municipal Party Committee will combine the "I do practical things for the masses" practice activities, combined with caring people from all walks of life, continue to carry out a variety of special group care activities, so that the special groups can feel the warmth of the society in the activities.

2021-12-23	Shaanxi Lantian: Play the trilogy to break the game "selection and training" is a new process, is the dare to break the circle, diligently strive for all kinds of resources process."Said Guo Jinghua, party secretary of Lantian County, Shaanxi Province. Since the pilot reform, the Youth League Lantian County Party Committee in the grass-roots league organization of the work force equipped to break the bureau, in enriching the grass-roots work force to find ways, efforts, the vitality of the grass-roots league organization was further stimulated."We go back to our hometown is to change our hometown, develop our hometown, and contribute our college students to the implementation of the rural revitalization strategy."In Lantian County Nancun Town Xinhua village party league square, village youth league branch part-time deputy secretary Wang Chenyu affectionate speech. Her "micro-group class" brought tears to the eyes of many people present. In order to strengthen the grass-roots work force of the League, the League Lantian County Party Committee has recruited and selected Lantian college students to return to their hometown as grass-roots cadres of the League. A total of 267 graduate students and undergraduate students from 19 universities including Shanghai Tongji University and Jilin University actively registered, and 48 college students were employed as grassroots part-time league cadres for one year after passing the qualification examination, interview and publicity. Wang Chenyu, from Northwest A & F University, is one of them. To the grassroots people, how to play their effectiveness is the top priority. The Lantian County Party Committee and 48 college students signed the target responsibility letter for part-time youth League cadres in Lantian County, requiring that during the term of office, they must carry out a theme of party history learning and education publicity, participate in the promotion of a volunteer service project, and write a research report in combination with the economic and social development of Lantian. College students, who served as part-time cadres of the Communist Youth League, were enthusiastic and entered the role of work ahead of time. They planned a theme activity of "Enjoy watching the changes of their hometown and sharing the rural revitalization" during the National Day period. The planning, organization and implementation, and later publicity and reporting of the activity are all completed by college students independently. These part-time league cadres are not only enthusiastic about their work, but also build a communication bridge between school and local cooperation. Zhang Ziyi, part-time deputy secretary of the Youth League Committee of Sanli Town, introduced school resources into Lantian, and put more college student volunteers into the Lantian summer practice of "three to the countryside", care for left-behind children and help rural revitalization activities. As the winter vacation of 2021 approaches, the part-time youth league cadres of college students from all over the country will successively return to Lantian to start their own "grassroots Communist Youth League experience" during the winter vacation. The Lantian County Party Committee of the League has focused on reforming the source and selection of league cadres. Since the pilot reform, 409 new grassroots cadres at the county, town, and village levels have been added, effectively enriching the grass-roots work force of the League. The County Party Committee allocated 4 organizations to the Youth League Committee to establish the first youth development service center in Shaanxi Province; with 27 part-time deputy secretaries, 19 secondary school League secretaries with high political quality, strong professional ability and strong sense of responsibility were selected for the first 19 rural revitalization demonstration villages in the county, and 351 villages (community). To solve the problem of lack of establishment and personnel, funding guarantee is the premise of the work of the Communist Youth League. The Lantian County Party Committee and the county government included the reform funds of the Communist Youth League into the financial budget, giving priority to the full amount. A total of 770,000 yuan was added since the pilot project. With the standard of 2 yuan per person, the county is 354,000 yuan (177,000 youth base aged 6-35); the young Pioneers is 36,000 yuan; the Town and Street Youth League Committee is 380,000 yuan. Young people are scattered in various industries and fields, and they have diverse needs. How to contact them and how to serve them well? On the basis of cultivating youth social organizations belonging to the Youth League, Lantian County Party Committee issued the Measures for the Administration of Youth Social Organizations in Lantian County (Trial), dispatched the Youth League Working Committee of Youth Social Organizations, cultivated 5 youth social organizations belonging to the Youth League, and contacted 15 youth social organizations. In order to strengthen the management of youth social organizations affiliated to the Youth League and attract more young people around the organization, the Youth League Lantian County Party Committee has successively established four major Youth League alliances: Lantian County New Youth League, Lantian Literary and Art Youth League, Lantian Sports Youth League and Lantian Youth Public Welfare Alliance, attracting 2,700 young people. Cultivate backbone, singing league collaboration "chorus" lantian county took the lead in education league committee, elected seven members of the division of responsibility system, respectively responsible for the league organization construction, league school construction, the young pioneers, team organization volunteer service, group learning activity planning and youth learning tasks, to ensure that the school field, team can be responsible for each task."Schools are an important position for the work of the Communist Youth League. We should give full play to the functions of the Education Communist Youth League Working Committee and promote the work of the Communist Youth League and the Young Pioneers to achieve greater achievements."Lantian County education youth League working Committee secretary Yang Ziyou said. In the practice of reform, in terms of top-level design, the seat system of the head of the county Youth League committee has entered the educational leading institutions and supervision institutions at the county level, which truly enhances the voice of the Communist Youth League in the school field. The secretary of the Communist Youth League County Party Committee is also the deputy secretary of the Party Committee of the Education and Science Bureau, and the secretary of the county Education Communist Youth League Working Committee is also the deputy secretary of the Communist Youth League County Party Committee. The substantive cross-serving and deep integration realize the common discussion of important issues, important issues and difficult issues. Joint county teaching bureau "on further strengthening and improving the county opinions of the communist youth league work" lantian county to strengthen primary and secondary school communist youth league cadres and the young pioneers counselor team construction implementation opinions ", clear middle school youth corps committee (branch) secretary according to the middle level at cadres with management, nine-year school youth league committee (branch) secretary of the young pioneers brigade counselors according to the moral education director level and included in the deputy field reserve cadre training sequence. When the team cadres participate in the professional title evaluation and calculate the workload, they should convert the work of the league into the corresponding workload and other contents, which forms a specific and feasible institutional arrangement for the work of the Communist Youth League and the Young Pioneers in the school field. Lantian County has 125 young Pioneers brigade. Before the reform, the Youth League Lantian County Party Committee, the county young working committee directly facing the grass-roots schools, teaching points to grasp the work, the work area is wide, the effect is not good. In order to ensure that the work of the young Pioneers at the town and street level is implemented and responsible, the Communist Youth League County Committee and the Party Committee of the Education and Science Bureau innovatively appointed 19 town and street chief counselors in the city, defined the post setting and responsibilities, and implemented the treatment of the deputy school level. This measure rationalizes the young Pioneers 'county-level, town-street and school three-level team system, provides promotion space for grass-roots young Pioneers counselors, and fully activates the "one river of spring water" of grass-roots young pioneers' work."The theme of our group class today is" Please rest assured that a strong country has me "."Lantian County Chengguan Middle School Youth League Committee Secretary Yang Yuan is in a group class. The Youth League School of Lantian County Chengguan Middle School has incorporated the league school training into the whole process of league member development, forming five categories of curriculum system: basic education, ideal and faith education, socialist core values education, situation and policy education, social practice and volunteer service practice education. From students applying to join the league to become an activist, to league member training and league cadres training, three characteristic training courses of "league activists, league members and league cadres" are set up, and different courses are set up for different classes. The youth League school has set up four departments, namely teaching and research, training, practice and art, with six systems in teacher management and student management. Since its establishment in May 2020, it has carried out six training courses for league activists, training courses for league members and league cadres, training and educating 926 league members and 198 league cadres. In December 2020, Chengguan Middle School Youth League School was rated as one of the first middle school model Youth League schools in Xi'an city. It is an important task for the Communist Youth League to participate in the innovation and boost of community-level social governance and promote left-behind children in rural areas. The "Kanglei Plan" project of caring for rural children implemented by Shaanxi Zero Two Nine Public Service Center has been implemented in Lantian County for more than six years. Tuan Lantian County Party Committee and Shaanxi Zero Ijiu Public Service Center have built 4 Lantian County childlike innocence bays through the mode of position, joint building and sharing of public use. Fifteen social organizations, including Shaanxi Zero Ijiu Public Service Center and Country Garden Lantian County Targeted Poverty Alleviation and Rural Revitalization Project Department, have made regular services, covering more than 7,200 children. In the normalized care for left-behind children work, the lantian county groping out of the "communist youth league + social organizations + volunteers" work mode, reform pilot six months, a total of 2.3519 million yuan, formed by the group position point, group county sent single, social organizations order normalized care for rural children work mechanism. China Youth Daily, China Youth Network, reporter Sun Haihua, correspondent, Guo Jinghua, source: China Youth Daily, December 23,2021, edition of page 06

2022-01-03	Suixi County, Anhui Province) " Come and take a look, have a look, charity sale, fresh vegetables!"Recently, the Chrysanthemum Town, Xinzhuang Town, Shipu Town, Suixi County, Huaibei City, Anhui Province, echoed with the shouts of young volunteers. This is a special" canteen " - -the Youth League Suixi County Party Committee, the Siping Town Youth League Committee and the Suixi County Celery Planting Youth Club jointly carried out a charity sale. Many pedestrians joined the "show love happy shopping", less than half a day, the stall vegetables sold out. Vegetables are provided free of charge by sui Xi County Celery Planting Youth Club, a youth club affiliated to Suixi County. The charity sale lasted for three days, raising a total of more than 1,000 yuan for poor teenagers to realize their "micro wishes". To further promote the county communist youth league organization reform expand pilot work, further innovation of grassroots organization form, extend service youth tentacles, group SuiXi county for the county towns (park), enterprises and institutions, social organizations, establish SuiXi county celery planting youth club, "bud" seed research and development youth club, SuiXi county live talent, SuiXi county development zone "aluminum way" youth club, the Xi Lin Qing reading club, "cart" youth volunteer service and so on more than a dozen forms of TuanBan youth club. The associations are built, planning projects to advance. Sui Xi Youth Association has created characteristic "green brand" brand projects combined with the actual situation, and carried out more than 30 service activities such as volunteer service, employment and entrepreneurship, art and sports. Among them, Suixi County Celery Planting Youth Club promotes the project of "charity sale to light up micro wish", realizing the dream of "micro wish", "budding" seed development youth association, "aluminum Way" youth association for more than 10 youth entrepreneurship exchange activities; "cart" youth volunteer service club encourages youth to participate in epidemic prevention and control, straw burning ban; and Nanping Sunshine Sports Colorful Club to carry out related activities. The county will also include the leaders of the youth associations into the training objects of Suixi County Young Marxist training project, absorb the backbone of the youth associations as the cadres of the village (community) youth league organization, open up the communication channels of the youth league, and ensure that the outstanding youth representatives from all walks of life have an "identity" in the youth league organization. The picture shows the members of the "little cart" youth volunteer service club walking into the villagers' homes to help dry the corn. Members of Sui Xi Live Talent Youth Club participate in the county e-commerce training

2021-12-29	Anhui Huaibei Leishan District Youth League organization with youth "painting" rural revitalization picture scroll Anhui Huaibei Leishan District Youth League organization with youth "painting" rural revitalization picture scroll Wang Haihan, correspondent, Rao Yanqin Mulin) " Help me take the white paint, on the color of the wings, this picture will be completed!"In recent days, in Anhui Province Huaibei City Lie Mountain District Songtuan town and village, a group of young people in a piece of courtyard wall back and forth busy. They are the young volunteers who participated in the "Beautiful Lie Mountain Painting Youth" painted village volunteer action. Through their brushes, a series of national style painting appeared on the wall. Since this year, in order to help the rural revitalization strategy, Tuanlie Mountain District Committee has organized the youth to carry out the voluntary action of painting the painted countryside. So far, 70 wall paintings have been painted, totaling 1,600 square meters, attracting 10,000 people to visit and punch in. Tuanlie Mountain District Committee excavates the red education resources in Lieshan District, enhanced the understanding of the party history and the rural revitalization strategy, trained new rural young talents, encourage and encourage and guide rural young people to take root at the grass-roots level, innovation and entrepreneurship, and participate in the deliberation and administration of state affairs. Recently, the affiliated school of Huaibei Normal University launched a "red landmark punch card" activity, and 60 primary school students listened carefully to the young speaker Song Wulong's affectionate explanation. This year, the Tuanlie Mountain District Committee selected seven red landmarks, such as the well Red Education Base and the Pomegranate Museum, to open classroom columns such as "red search", "labor picking" and "volunteer practice". The Communist Youth League District Committee also led the establishment of the "Lishan Help Group" volunteer service team, to carry out the youth to help farmers, comprehensive environmental improvement, garbage classification, and river protection actions.

2021-12-23	Feixi county in Anhui province held the fourth secretary of the youth corps committee lecture contest held the fourth secretary of Anhui Feixi county lecture contest lesson zhang correspondent, Xie Changhao check WeiTing) recently, by Hefei feixi teaching bureau in Anhui province, feixi county party committee of the fourth youth corps committee secretary of the lecture contest in Hefei eight Ming high school to a successful conclusion. The contest for feixi each school youth corps committee secretary, youth corps committee member, class league building instructor cadres, new era around xi jinping, the ideas of socialism with Chinese characteristics and the spirit of the sixth plenary session of the 19, the 100th anniversary of the founding of the communist party of China, the 72nd anniversary of the founding of new China, the 102nd anniversary of the May 4th movement theme innovation design league class content and form. The jury is composed of the school department of the Hefei Municipal Party Committee, the Hefei Municipal Education Bureau, the Youth League Committee of the Youth League Feixi County Party Committee, the Feixi County Education and Sports Bureau and other relevant officials. In the free speech session, the contestants explained their understanding, perception and experience of the positions of the league cadres or the league construction instructors around the league member education and daily work. In the lecture presentation session, the contestants took the party history study, national security, national unity, virtue cultivation, youth dream and other contents as the main line, and made reasonable use of physical objects, games, songs, audio and video and other methods, from the activity objectives, activity preparation, activity process, guidance points, and evaluation methods. After fierce competition and competition, Hu Jiangqin from Feixi Hongtu Middle School won the special prize, and another two contestants won the first prize, four contestants won the second prize, and eight contestants won the third prize. After the game, Hefei municipal party committee school vice minister Bi Xiaojian summary review, from the ideological content, content, practicality, innovative to the performance of the players, and from the theme orientation, curriculum, display way put forward targeted guidance and Suggestions, encourage the players to use in the future work, efforts to improve. The secretary of the Communist Youth League Committee said that the class competition is the youth league Feixi County Party Committee to implement the reform task of the county Communist Youth League and strengthen the construction of the middle school Communist Youth League cadres. The competition has set up a learning and exchange platform, promoted the self-education and mutual education of the league cadres, created a new mode of ideological guidance, and stimulated the new vitality of the grass-roots league.

2021-12-21	Nanning "hope classroom" remote support help rural revitalization of Nanning "hope classroom" remote support help rural revitalization in the youth newspaper reporter Xie Yang) on December 17, by Nanning hope engineering office, national wealth life insurance co., LTD., Shanghai dandelion education development foundation, the party hosted the party with the party-2021 intellectual rich "hope classroom" launch ceremony and national wealth starlight "hope house" donation activities held in Guangxi Nanning longan pingshan township central primary school. At the event site, Nanning Hope Engineering Office, Guofu Life Insurance Co., Ltd., and Shanghai Dandelion Education Development Foundation signed three-year cooperation agreements with Pingshan Township Central Primary School of Long'an County and Yanjiang Town Junior High School respectively to open the Zhifu "Hope Classroom" project. The project provides one-to-many, one-to-one, remote and interactive online teaching methods, sending art, music and English interactive courses to the two schools every week. By integrating high-quality teacher resources from Fudan University, China Academy of Art and other universities, recruiting college students and volunteers from professional associations in developed areas to form volunteer teaching teams, through the Internet + education way, make volunteer teaching services more flexible, and realize cross-regional integration and sharing of high-quality education resources. On the same day, Guofu Life Insurance Co., Ltd. donated 39,000 yuan worth of tablet computers to Yanjiang Town Primary Middle School to build Guofu Xingguang "Hope Library" for Pingshan Township Central Primary School in Long'an County, donating tablet computers and books worth 26,500 yuan.

2021-12-20	Guangdong rescue aid xinfeng team set up grassroots reform to promote group social organization diversified Guangdong rescue aid xinfeng team established youth network Beijing on December 20 (reporter Zhang Jianwei, reporter Li Excellence) to promote the county communist youth league reform work effectively, combining local enterprise development actual demand, recently, in Guangdong rescue aid 28 social emergency rescue basic training class graduation ceremony, composed of more than 20 volunteers of Guangdong rescue aid xinfeng team in xinfeng lingnan red world was formally established. After 13 days of theoretical knowledge study, volunteers from Guangdong Rescue Assistance Team passed the assessment and professional practice study of emergency rescue theory, and successfully graduated. At the graduation ceremony, the volunteers also conducted a practical graduation combat drill, showing off the professional emergency rescue knowledge they had learned during the training.site of activity. Correspondent for figure understands, Guangdong rescue auxiliary is Guangdong youth development foundation (hereinafter referred to as Guangdong youth foundation) to create volunteer organizations, is composed of the society enthusiastic people from all walks of life volunteer to provide emergency, rescue, rescue auxiliary service volunteer service, is a uniform, disciplined auxiliary emergency team. Guangdong rescue aid Xinfeng team, is the establishment of the county, county emergency administration jointly promote the main to assist the government in disaster relief, rescue, maintain the order of various large-scale activities, is new venture to expand the influence of social organizations and promote the volunteer service professional, diversification, project important exploration. When completed, the team will also provide training activities and professional advisory services for the safety and rescue of large-scale activities, and provide discipline training and skills training for teenagers aged between 12 and 17 to help cultivate their all-round development.

2021-12-20	Group in Changsha city of Hunan province tianxin district party committee in cohesion and service youth in Hunan Changsha tianxin district party committee: in cohesion and service youth youth reporter HongKeFei) in Changsha city, Hunan province the communist youth league organization since the pilot reform, actively build youth "gas station", youth "cornucopia", to get through the youth, condensed youth, service youth "the last kilometer". After the reform, the league Tianxin District Committee staff increased to 7. With the support of the local Party committee, the youth working committee was set up in the whole district organs and units, and the Youth Working Committee worked together. Recrurecruited staff from web celebrity enterprises, deputies to the NPC and CPPCC members to complete the election of 98 district-level league organizations, of which 80 were born in the post-1990s. Tianxin Youth Commando team actively participated in the "youth war epidemic" operation. In August this year, the Communist Youth League Committee led more than 3,000 young volunteers to actively join the front line of epidemic prevention and control in Tianxin District. During this period, a news link of "Volunteer women volunteer volunteer to fight the epidemic" went viral on wechat. The former female soldier is Zhou Yingxiang, the 24-year-old secretary-general of the Tianxin District Youth Volunteers Association. In the face of the outbreak, she immediately launched young volunteers in the area to go to the front line of epidemic prevention for services. Through the government purchase services and appointment of youth work officers, after the reform, Tianxin District youth social organizations, increased 15 teams to 16 colleges and institutes, 23 university-level community branch, forming "1 + 16 + 23 + N" social matching, project employment mode; plan development Tianxin new net red joint youth League committee, 191 new field "two new"; 90 "green" typical model, organize and mobilize them to participate in project construction, investment promotion and civilization creation center work.

2021-12-19	Jiangsu Yizheng held youth talent exchange activities Jiangsu Yizheng held youth talent exchange activities you road peer " Yizheng young talent exchange activities. At the scene, the activity through breaking the ice interaction, listening to songs, love trap, creative puzzle and other links, to the young friends in Yizheng to build a communication platform. Activities aimed to implement the "Yangzhou" difference " youth development plan (2021-2025) file spirit, strengthen the youth love, family education and guide, attract more high-level young talents to stay in yizheng, love in yizheng, in yizheng, the activity by yizheng city talent work leading group office, yizheng municipal party committee, yizheng city federation of trade unions, women's federation, Jiangsu optimal chemical co., LTD.

2021-12-25	Tianjin Beichen District Committee carries out "National Road Traffic Safety Day" publicity activities Tianjin Beichen District Committee carries out "National Road Traffic Safety Day" publicity activities Bureau Beichen Branch, Transportation Management Bureau and other units to carry out "National Road Traffic Safety Day" publicity activities. The staff of the Youth League Beichen District Committee set up points in crowded places to distribute road traffic safety publicity materials, guide the masses to learn publicity materials, improve traffic safety awareness, strictly observe traffic safety laws and regulations, enhance traffic safety prevention ability, pay attention to safe travel, civilized driving, and avoid the injury of traffic accidents. At the same time, the group beichen district party committee staff and beichen team police officer with publicity materials into residents in the traffic safety knowledge propaganda, chat to the residents and the harmfulness of violation of road traffic laws and regulations analysis, persuade people consciously resist traffic habits and dangerous driving behavior, not take the overcrowding, speeding, overloading, overload, put an end to drinking, unlicensed, fatigue, speeding and running a red light violations of traffic laws and regulations. The activity also invited traffic safety volunteers, delivery boys, transportation enterprise representatives and other important participants of road traffic safety to participate together.

2021-12-24	Create brand Yang advantage Shandong Pingyuan County "youth community" created in action create brand Yang advantage Shandong Pingyuan County "youth community" created in action Zhang Jianwei correspondent Li Yuzhe) since the Youth League Shandong Provincial Party Committee issued the "on the implementation of community youth action to carry out youth friendly community to create a pilot work plan", Tuanping County Party Committee combined with its own reality, Through the three steps of "fighting, learning and doing", Create a brand and promote advantage, Innovative breakthrough in the building of "youth community", Build youth service stations in urban communities by relying on party and mass service centers, Build a "one station, four links and two-way service" mode of "organization integration, position integration and group integration". The Communist Youth League County Party Committee has built two youth communities in Lianchi Community and Nancheng Community of Longmen Street, and established youth homes with high standards. The rest of the communities have realized resource sharing and facility sharing. Among them, Longmen Street Lotus Pond Community was identified as one of the first youth communities in the province. In addition, relying on the party and mass service center, the establishment of community youth service station, contact with the community youth talents, youth league members, young pioneers, young social organizations these four groups. At the same time, the work of the Communist Youth League, the Youth Home to jointly gather the youth strength of community governance. Since the "youth community" activities, the county, street youth corps committee based on reality, combining the community situation, the introduction of social organizations into the community, to carry out "you I describe dream, sharing happy time" painting activities, community "parent-child games", college students summer children service practice and other more than 20 community activities, guide teenagers widely participate in community governance, make community more friendly to youth, more promising youth to the community.

2021-12-24	Group of Huangshan city, Anhui province Huangshan district party committee to carry out care sanitation workers public welfare party committee of Anhui Huangshan district party committee to carry out the "warm a" orange "beautiful a city" care sanitation workers Ji'an-wei zhang correspondent huang �� check WeiTing) recently, the Huangshan city, Huangshan city, Anhui province Huangshan district party committee organization Huangshan district youth volunteer association and youth members to carry out the "a warm" orange "beautiful a city" care sanitation workers public welfare action. At the event site, the organizer distributed hand-warming gifts such as frostbite cream and hand cream to the sanitation workers, and also sent winter greetings to them."Although the gift is small, but we really feel that the party committee and the government and all sectors of society are full of care and attention to us, let us not only warm hands, warm hearts, but also warm."Said a sanitation worker who received the gift excitedly. Next, the Youth League Huangshan District Committee will continue to gather the Huangshan District Youth Volunteers Association and other youth social organizations to carry out the "I do practical things for the masses" and "I do something for the youth" practice activities, calling on the majority of young party members, youth league members, young pioneers to take the lead in respecting the sanitation workers, pay attention to civilized etiquette, care for environmental health.

2021-12-17	Xuyi, Jiangsu: Diversified youth service brand reform pilot county. Xuyi County Party Committee construction of "44513 + X" the Communist Youth League cadres work team training mode, select the best with strong league work backbone; 13 towns (street) youth affairs social workers have been allocated to 10, the town (street) youth League committee has also formed a positive and three deputy league organization leadership pattern, clear full-time, temporary, part-time deputy secretary. Xuyi has more than 40 bank insurance and other financial institutions, 10 county according to the "where the youth, youth league is built where" work concept, active docking county people's bank, after MoPai, the county financial institutions of more than 300 people under the age of 35,10 county united county people's bank established the communist youth league 10 county financial industry working committee, strengthen the contact with youth. The Xuyi County Party Committee has newly established "Xuyi County Rural Revitalization Youth Entrepreneurs Alliance", "Xuyi County Sports Association Youth Home" and "Lixin Red Scarf Mutual Aid Association", including the liaison stations, the United Youth Service Station and the Youth Learning Club; established the working mechanism of the community youth action, including personnel, projects, positions and resources.

2021-12-22	Group Anhui tianchang party committee to build one-stop league school education situation in practice Anhui tianchang party committee education reporter hai-han wang) Hu Guangyang is Anhui tianchang second middle school new development members, speaking of the school league school curriculum, he praised: " before the group class, is mostly the teacher about teaching material, now not only have video teaching, the teacher will take us to the outdoor development, to participate in volunteer activities, very interesting."Since this year, under the guidance of the school Youth League Committee, he has fully participated in the school courses, and got full marks for graduation. Tianchang City is one of the pilot areas for the organization reform of the primary Communist Youth League at the county level. The city takes strengthening the political education and practical education mechanism of the league members as the starting point to further standardize the construction of the league school from the institutional level. As of October this year, the city's 37 middle schools have all established youth league schools. With the teaching position, how to let the theoretical knowledge of the party into the brain into the heart? How to attract students in the youth league school setting and the youth league class teaching? The Municipal Youth League Committee formulated the "Tianchang City Middle School League School Construction Management System", issued the "Tianchang City League Class Textbook" and "School League School Students Record book", encouraging the city's middle schools to broaden their ideas and make bold innovations."One-stop situational group class education came into being."Youth League Tianchang Municipal Party Committee responsible comrade explained, under this mechanism, the group class teaching in addition to the routine curriculum, but also includes labor education, social practice, volunteer service and other activities, to achieve the school and off-school teaching situation through."Participating in the group class is equivalent to participating in the ideological and political class, moral and law class, outdoor practice class at the same time. Teachers of the Youth League school will lead students to the red education base, to participate in the community for grassroots governance, or to the streets to help the construction of urban civilization."Tianchang two middle school, deputy secretary of the Youth League Committee Huang Kun introduced. This year, the green mountains and green mountains are the gold and silver mountains labor practice group class, the students carry out afforestation action; summer, the teachers and students came to the Guangling street Tianbao community is the community assistant activity; in December, the school held the national memorial day... Huang Kun said, through the situational teaching, let the league school to become a platform to improve young students' political identity literacy."Team education used to be relatively serious, but now we interpret the truth brilliantly."Huang Dawei, deputy secretary of the Communist Youth League Committee of Tianchang No.2 Middle School, said that the school's youth league class teachers also often compete fiercely to ensure the quality of the youth league classes. In Tianchang Middle School, an old school in the city, the league-run associations have become the carrier of league class teaching."Since the implementation of one-stop league class education, the school Youth League Committee has linked team education with community activities to stimulate students' interest in learning the Party and league knowledge."Xu Jiahui, secretary of the Day Middle Youth League Committee, introduced that the school put the party and league history melodrama interpretation into the league class content, in the club activity festival performance, interaction, performance and watch the students are warmly responded.

2021-12-19	Anhui Tianchang City established the non-public enterprise Youth League Working Committee inaugural meeting. The relevant person in charge of the CPC Municipal Committee read out the establishment documents of the non-public working committee and the list of committee members. Through the establishment of the non-public youth League working Committee, Tianchang City will further play the role of party building and leading the youth league building, take "gathering youth, serving enterprises and society" as a breakthrough point, to further unite, condensing and lead the young employees of non-public enterprises to promote Tianchang high-quality development and dedication of youth strength. Up to now, Tianchang city has established 772 youth league organizations (including youth league branches) in non-public enterprises. Since this year, as one of the pilot counties of the county Youth League reform, the Youth League Municipal Committee guides the non-public enterprises to join the Sunflower "green" publicity group, promote the momentum, continue to understand the basic information of young workers, grasp the actual needs and ideological dynamics of the youth, improve the standardization of the league organization, gather the youth strength, participate in the "youth learning", strengthen the ideological and political guidance to the youth members.

2021-12-16	Fujian Zhenghe: "Mobile Youth Palace" to the campus Fujian Zhenghe: "Mobile Youth Palace" to the campus committee and the county youth Palace jointly held the "mobile youth Palace" sent teams to the countryside activity, the activity for the first time into the Zhenghe County Dongping Town Dongping Central Primary School. Dongping town is a famous old revolutionary base area in northern Fujian. So far, 38 red revolutionary heritage (old) sites have been kept intact. This activity has the theme of "Red gene is passed down from generation to generation to strive to be a good youth in the new era". Through singing revolutionary songs, listening to revolutionary stories, simulating war scenes and other contents, the red gene and revolutionary fire are passed on from generation to generation. In the class, Cao Maochang, a 63-year-old party member, told stories about his heroic deeds of his revolutionary ancestors. In 2021, the "Mobile Children's Palace" activity to the countryside has been organized for four sessions, covering the four towns of the county, participating in more than 1,000 young pioneers. Next, Zhenghe County will continue to play the brand effect of "mobile Children's Palace", successively to the rural grassroots primary schools to send team classes, send services, send love, further expand the party building with league building, league building with team building radiation effect, solid development of "I do practical things for teenagers", with practical actions to help the healthy growth of children.

2021-12-15	Zhou Runqing donated 150,000 yuan to build two hope cabins, Maocun Town Central Primary School and Guangfeng District Youth Activity Center. We hope that the hut mainly serves the left-behind children in rural areas in the area, provides places for reading after class and book resources, and cultivates children's good reading habits. Tuan Guangfeng District Committee equipped the school with computers, printers, bookcases and 1,000 children's books, and customized a set of anti-drowning and ancient poetry books; combined with the red scarf activities, regular reading sharing, and customized notebooks to encourage young pioneers to develop good reading habits. Guangfeng District Youth Activity Center Hope House is also in construction in an orderly manner, will be put into use in the near future.

2021-12-14	Group of Shandong Zaozhuang taierzhuang district party committee more measures and build college students "home" social practice platform to participate in social practice help hometown development of the communist youth league taierzhuang district party committee more measures and build college students "home" social practice platform branch, young college students to carry out the summer teaching social practice activities, for drowning prevention propaganda and education. Correspondent for figure China youth network Beijing on December 14 (reporter Li Chuan correspondent Xue Jie) " winter vacation is coming, we adults want to go to work, work is very busy, busy to take care of children, children at home is not play mobile phone is watching TV, I heard that you organize youth volunteers back home social practice, is really too good, solve our mind a big difficult."Recently, Ms.Li, Canglang Temple Village, P Zhuang Town, said."Hello, this winter vacation we want to continue to go to Taierzhuang to participate in the 'return home' social practice activities. We benefited a lot from the summer vacation activities, not only to exercise the ability, but also for the children to do real things."Zhang Chen, a student from Qingdao University of Science and Technology, also called the Taierzhuang District Committee of Zaozhuang City, Shandong Province. Winter vacation is coming, a phone call from Taierzhuang district college students outside to the Youth League Taierzhuang District Committee office, they actively signed up for the Communist Youth League Taierzhuang District Committee organized the "return to home" social practice activities, inspirational to participate in the rural revitalization strategy, do practical things for the people. College students work hard in their practical posts. Correspondent for figure party history learning education, group taierzhuang district party committee to boost rural revitalization as the starting point, to solve the problem of holiday "care" as the breakthrough point, combined with "I do the practical work for youth", "I do the practical work for children" practice, organize "home" social practice, guide college students to actively participate in rural revitalization strategy, help college students improve social skills, establish outside college students and hometown contact institutionalized channels."The water of Canglang xi, can wash my tassels; Canglang water muddy, can wash my feet."Canglangmiao Village of Zaozhuang City is the first choice for social practice pilot projects. There are more than 20 left-behind children here, and the problem of" difficult care " during holidays is particularly prominent. Group taierzhuang district party committee joint area closes working committee, district bureau, district civil affairs bureau, district women's federation and other units relying on canglang temple village cloud stream children service station, canglang temple village committee, "Canglang family" water home stay facility resource advantage to establish college students return home social practice base, group taierzhuang district party committee, evaluated integratedly, the village-level youth league trinity service guarantee. During the summer vacation, young college students carried out summer volunteer teaching social practice activities in Canglangmiao Village, Peizhuang Town, Taierzhuang District. This summer vacation, Qingdao University of Science and Technology "three to the countryside" social practice team came to Canglangmiao Village, Peizhuang Town, Taierzhuang District to carry out a 10-day college students "three to the countryside" summer social practice activities. Volunteers carry out theme courses such as "handicraft making", "English reading", "homework tutoring", "origami craft making", "music" and subsequent series of interesting courses for students. Adhering to the principle of "edutainment", they encourage students to actively participate in classroom interaction by means of explanation and demonstration, scene experience and fun games. The arrival of volunteers has greatly solved the problem of "difficult discipline" for rural students in the summer vacation, and reduced the burden for parents."Hello, I am Liu Yifan, I saw the return of my hometown social practice post collection order, I think of the youth League committee internship", "I am Taierzhuang college students volunteer service group Li Dian, I want to participate in the epidemic prevention volunteer service"... an email, a phone call, a WeChat, all contains the college students want to contribute to their hometown, improve their social skills. Return to my hometown for social practice activities, discussion and exchange meeting. In this regard, the Youth League Taierzhuang District Committee acted quickly to build a bridge of social practice. On the one hand, it organized students to go deep into the frontline positions of party and government organs and public institutions, to undertake specific work, especially in the party history study and education, epidemic prevention and control and other aspects. On the other hand, students are organized to carry out poverty alleviation, helping the disabled, academic guidance and service to the masses in the countryside and communities, youth homes and 4:30 classes, so as to promote the volunteer spirit of "dedication, friendship, mutual assistance and progress".

2021-12-14	Guangdong Shenzhen longgang district: provide youth "home" warm heart service in Shenzhen longgang district: provide youth "home" warm heart service reporter wu xin) since 2021, the Shenzhen longgang district committee actively implement the long-term youth development plan, to the county communist youth league organization reform comprehensive pilot as an opportunity, around leading condensed youth, mobilize youth, contact service youth basic responsibilities, established 23 youth home entity position. With the "youth home" as the starting point, we will stimulate the vitality and efficiency of the youth league organizations at all levels to gather the youth and serve the youth, promote the organizational form innovation of the youth league, and make the tangible and regular service projects."No.54 Mobile Class" is a characteristic "store" of "Youth Home" in Longgang District. It takes the form of youth "order and order", and carries out regular activities popular with young people, such as reading sharing meeting, film sharing meeting, music roadshow, photography teaching, food sharing, painting experience, outdoor sports and interest teaching. While integrating the resources within the league, it also actively use the strength of youth social organizations to customize practical and effective services for the majority of young people, which are welcomed by the young people. The Shenzhen-Hong Kong-Macao Youth Innovation and Entrepreneurship Service Center and the DCC Cultural and Creative Park are also the two unique stores in the "Youth Home" system of Longgang. Joint jurisdiction industrial park, enterprises, businesses, etc, design theme, deep Hong Kong and Macao youth innovation service center main "professional" design, built "dream book", "youth room" youth "green shed" and other entity positions, have launched the "CEO noon share" "small round table" "building horse" "green yue read" and other brand activities, to provide more professional services for youth. DCC Cultural and Creative Park "Youth Home" has established an integrated center with "legal affairs + finance + psychological consultation" as the main body, with maker space, fitness room and 24-hour self-service area as the carrier, providing young people with a full range of services such as learning, leisure, innovation and entrepreneurship exchange. The person in charge of the Youth League Longgang District Committee introduced that the 23 youth homes in the district are directly contacted by the district, street or community youth league organizations, and special personnel are stationed to timely understand the ideological trends and actual needs of the youth, and provide personalized and all-round services. Some stores have specially set up an "echo wall" functional area, which has become a "tree hole" for young people to talk about their needs, and has created a warm home in line with the characteristics and preferences of young people in the new era. Some selected youth representatives from different fields to set up youth meetings to encourage young people to speak out in their "home". At the same time, the league organization will constantly optimize the service projects according to the "Voice of Youth", break through the traditional one-way transportation service mode, and support and guide the youth associations to independently launch activities, so that "home" becomes the activity position of youth self-management and self-service, realizing the organic combination of serving youth and gathering youth. Referring to the experience of 24-hour convenience stores, the construction of district-level "youth home" flagship store, each street "youth home" franchise store, the community "youth home" shi many stores. At present, it has built 2 district-level youth homes, 8 street-level youth homes, 8 community-level youth homes and 5 youth homes in enterprise parks, realizing the "youth homes" with direct construction or contact guidance. Through the dot matrix effect, we will continue to enhance the vitality of the "Youth Home" and improve the frequency and coverage of youth service activities. Next, the Longgang District Committee of the Communist Youth League will continue to promote the construction of more "youth homes" in the youth gathering places of all over the district. At the same time, with the construction of "youth homes" as the influence, it will contact youth organizations more extensively, integrate social resources more intensively, and further improve the service function. We will continue to optimize the "one-stop" comprehensive platform with ideological guidance as the core, youth-oriented service, and goal at achieving political and social functions, so as to provide the youth with "doorstep" warm heart services.

2021-12-13	On December 11, Li Chao), the MOTOTO coffee shop, located in Shiquan Street, Gusu District, Suzhou city, Jiangsu Province, and many youth members are learning and communicating. Originally, here is holding a safe to "the first cup of coffee in the winter" of the theme, league Gusu District Committee, district level organs youth, street youth league working committee, community league branch, "two new" league branch and other industry league members a total of more than 30 people attended. It is understood that this activity is guided by the Youth League Suzhou Municipal Party Committee, sponsored by the Youth League Gusu District Party Committee, Gusu District Shuangta Street Youth League Working Committee, MOTOTO Coffee and Suzhou Gusu District Small Youth League Public Service Center jointly organized. The activity is divided into six links: offline space awarding of youth learning club, party history knowledge micro competition, coffee culture experience, heart-warming coffee and rafting coffee. Through the theme party class, competition interaction, coffee rafting and other forms, the upsurge of party history learning and education and in-depth practice service has been set off among the youth league members. In the theme activity, Lu Yuanyuan, a member of the "Youth Lecture Group" in Gusu District, gave the theme of "learning, publicizing and implementing the spirit of the Sixth Plenary Session of the 19th CPC Central Committee" for the youth league members present. Her emotional explanation aroused strong resonance with the audience. During the warm-hearted coffee session, the young people handed the on-site warm-hearted coffee to the social workers and medical workers in Jinfan Road Community and Jinfan Road Health Service Center to thank them for their hard work on the front line of epidemic prevention. The last link- -rafting coffee, set off the climax of the whole activity. Youth league members from all walks of life jointly raised funds and bought 10 cups of coffee coupons, so that the sanitation workers, traffic police, urban management, couriers and other outdoor people could enter the store to exchange for free tasting. It is understood that the event location is the first "youth learning" offline promotion space in Gusu District, the activity will last until January 12,2022. In the future, the Gusu District Committee will set up a number of youth gathering places, Internet celebrity card point "youth learning" space, the youth learning club position to the youth, the party's theoretical knowledge to the youth heart, with "green language" to lead the youth, call on the youth, the youth learning into the youth daily.

2021-12-13	Wuhu in Anhui province first people's city practice base in Anhui Jiujiang district first people's city "youth partner" practice base Ji 'an-wei zhang correspondent Yang JieZha WeiTing) recently, the Wuhu of Anhui province Jiujiang district party committee, bay street party working and Anhui engineering university mechanical engineering institute (school of artificial intelligence), material science and engineering institute in fengming lake community signed the people's city "youth partner" to build an agreement. Fengming Lake community, as the first district people's city "youth partner" practice base, was officially listed. According to the "Youth Partner" agreement of the People's City, the two sides will carry out all-round and in-depth cooperation in school and local co-construction, resource sharing, talent training, grass-roots services, entrepreneurship and employment, and establish a long-term mechanism for cooperation, innovation and common development. Fengming Lake Community, the first phase of the activity, will focus on building a "smiling flower" love classroom, organize more than 800 books from inside and outside the region, organize students to donate more than 800 books, and carry out fixed teaching activities on Saturday and Sunday."Cities are more friendly to youth development, and young people are more promising to urban development."In the next step, Tuanjiujiang District Committee will accurately connect the current situation and needs of the towns, streets and communities within the district with the people's and city partner units of the district to carry out joint construction. Build a broader stage for young people to practice and innovate, create more favorable conditions for young people to make contributions, and mobilize the young people in the whole region to be the "youth partners" in people's city construction with practical actions.

2021-12-13	Hunan Yongzhou ningyuan county group linkage when "matchmaker" Hunan Yongzhou ningyuan county group linkage when "matchmaker" Zhang Jianwei correspondent, Zheng Minghui Fang Guotao) to build young talent communication platform, broaden the channels of young talent dating, show young talent sincere, optimistic, upward spirit, recently, Hunan ningyuan county focus on the city's annual "talent theme day", the county united county federation of trade unions, county women's federation issued a notice, held dating fraternity, together when the "matchmaker", power young talent "off" single ". Sodality to "youth meet, fate jiuyi" as the theme, for the single young talents of enterprises and institutions. County organs, enterprises and institutions, central, provincial, municipal units in Ning under the age of 35, and women over 20 years old, male over 22 years of age of single youth can register for the participation, without any charge. To ensure the quality of the event, the first 200 people are limited to participate in the on-site event. At the same time, the activity time is arranged in the middle of December of the "Talent Theme Day", and the site is selected in the Dafeng Village of Xiaguan Tourism Area. It is reported that Ningyuan County will hold similar dating activities every year, in the past five years, more than 1,100 young people have participated in the activities, a total of 226 people hand in hand to "take off the single". Because the selected this year is full of attraction, the interactive links are interesting and fashionable, which attracted the majority of young talents to sign up.

2021-12-11	New venture county, Shaoguan city, Guangdong province "learn lei feng" volunteer service station in Shaoguan city, Guangdong province opened the first "learn lei feng" volunteer service station officially opened on February 11 (reporter Zhang Jianwei, reporter li zhuo hao) in order to further enhance the new venture volunteer service work, make volunteer service activities more convenient and beneficial, to create a national civilized city to create a good environment. Recently, the first "Learn from Lei Feng" volunteer service station in Xinfeng County, Shaoguan City, Guangdong Province was held in Fujiang Qiaotou Fitness Park, Xinfeng County. Xinfeng County publicity Department, county housing and construction Bureau, county public security Bureau, youth league Xinfeng County Party Committee and other relevant leaders and county volunteer service teams, school representatives attended the opening ceremony. At the event site, the leaders and guests attending the event jointly inaugurated the first "Learn from Lei Feng" volunteer service station in Xinfeng County. After the opening ceremony, the county standing committee, propaganda department minister Zhong WeiSu to new venture first "learn lei feng" volunteer service successfully built a warm congratulations, and hope the county departments, schools, and social organizations, volunteer service to "learn from lei feng" volunteer service built as a new starting point, vigorously inheriting lei feng spirit, actively build "close to the masses, practical" learn lei feng volunteer service platform, carry forward the "dedication, love, mutual assistance, progress" of volunteerism. Volunteers on duty at the post station provide drinking water for the passing people. Correspondent for figure understand, xinfeng "learn lei feng" volunteer service station daily management maintenance by the xinfeng county, on duty time is mainly weeknight, holidays will increase open during the day according to the actual demand, can provide volunteers registration, books, newspapers, mobile phone charging, emergency medicine, drinking water and other convenient services. In addition, the volunteer service station will also carry out special volunteer service projects such as voluntary clinic, charity cutting, civilized traffic publicity, environmental cleaning and more on, and radiate to communities, schools and scenic spots to extend the volunteer service projects. The volunteer station was officially opened. Correspondent for figure next group xinfeng county will be united county units, schools and social organizations to further improve the volunteer service station service content, expand service areas, innovative service form, let learn lei feng volunteer service activities diversification, normalized, the volunteer service station into a new venture convenient and beneficial service platform, show the forefront of volunteer service window, volunteer service of emerging carrier, to provide more convenient volunteer service and broader participation platform.

2021-12-09	Jiangsu Changzhou wujin district first 22 "dream hut" built Jiangsu Changzhou wujin district first 22 "dream hut" built green reporter li chao) renovated metope, fresh wall painting, plain roof, elegant cot, comfortable four sets, special desk... winter sun through the blue curtains, "slip" into the 10 square meters of "dream cabin", gently is quiet reading small Lin (pseudonym) face. Xiao Lin, 14, lives in Xueyan Town, Wujin District, Changzhou. When he was a child, his mother died of illness and his father was in prison. Xiao Lin has lived with his elderly grandparents since childhood, living on the meager subsistence allowance. Because the house was not completely built, the family is crowded in a small room to sleep. Lying on the cabinet to learn the small Lin, often looking forward to have their own small space. Since this year, group jin district party committee to implement "dream transformation +" care plan, the "dream hut" as the construction of "I do the practical work for teenagers" key projects, focus on 6 to 16 years old "orphans" group, through socialization, standardization, project operation, explore group led by depth participation, social organizations, full integration of social resources, volunteer service accurate plight of youth group care work new concept, new mode, high quality hut construction work to the ground. Xiao Lin is one of the first 22 people to care for him. After the transformation, he finally has his own "dream cabin". One table, one chair, one bed, one cabinet, one lamp and one curtain, are the seemingly ordinary objects, which are full of the deep feeling and care of the youth league organizations at all levels and all sectors of society to the young people in difficulties, especially the "de facto orphans"."On the road to common prosperity, every child should be seen, every dream deserves caring, and every smile is passed on."Jiang Yuchen, deputy secretary of the Youth League Wujin District Party Committee, said that so far, Wujin district has built 22" dream huts " to help teenagers in difficulties pick up their confidence and pursue their dreams."The '+' in the 'Dream Transformation +' is an important extension of the dream plan and the breakthrough direction of the transformation and upgrading."Jiang Yuchen said that building a" dream cabin "is only the first step, and the" second half of the article "is the" touchstone " to test the effectiveness of the care plan. Next, the Wujin Youth League District Committee will continue to weave a dense "organization network", "resource network" and "service network", through material help and spiritual care, to provide precise chain, continuous and intimate services, to help them realize the transformation from "renewal cabin", "renewal life" to "renewal spirit".

2021-12-08	Wuxuan County, Guangxi, build a system to build a platform, China Youth Daily reporter of China Youth Network Xie Yang) since July this year, The Wuxuan County Party Committee of Laibin City, Guangxi Province, and EGO Wuxuan Internet + Mass Entrepreneurship and Innovation Base, Actively explore the "1 + N" model, By building "a system", Building "a platform", Carry out the "one cultivation", Providing "N services" according to the actual needs of young entrepreneurs, Focus on solving the difficult problems faced by youth entrepreneurship, Guide and stimulate the innovative thinking of entrepreneurial youth, Provide the policy guidance for them, Improve the success rate of entrepreneurship. With the construction of the youth league organization in two new areas as the starting point and the incubation base as the basis, the Communist Youth League Wuxuan County Party Committee has built a three-level organization system of "The Communist Youth League County Party Committee- -The Wuxuan Town Youth League Committee- -EGO Wuxuan Internet + The Youth League branch of the mass entrepreneurship and innovation Base". EGO Wu Xuan Internet + double gen base integration Wu Xuan location, policy and high-tech industry aggregation advantages, to build a set of Internet electricity, Wu Xuan preferred experience center, e-commerce talent training base functions such as one of the big data entrepreneurial innovation industry platform, and for enterprise managers, enterprise youth, youth to get rich leader held regular electricity professional knowledge training, business salon, industry exchanges. At present, a total of 21 enterprises have successfully settled in EGO Wuxuan Internet Mass Entrepreneurship and Innovation Base, involving young entrepreneurial teams, college students' entrepreneurial online businesses, Internet creative companies, third-party service enterprises, intelligent technology enterprises and other fields. Since 2021, relying on Wuxuan County Youth Entrepreneurship and Employment Demonstration Base, held 41 skills training, entrepreneurship competition, 1,350 young people and more than 200 people, "Our Chinese Dream-culture into the intangible New Year", "March 3", with the highest popularity of 200,000 people. In addition, the base also held a total of 3 industry fellowship activities for young entrepreneurs, benefiting 150 young people.

2021-12-08	Group of Anhui province bay � b district party committee service enterprise development big visit activities in Anhui province bay � b district party committee to carry out the "double contact, into one thousand enterprises, four send a suit" big visit activities JianWei correspondent, TaoHui Fang check WeiTing) in order to further service for the enterprise, to implement the "enterprise call, department report" service enterprise development tertiary response mechanism, recently, the Anhui province Wuhu bay � b district party committee to contact enterprise Wuhu Australia ran ma auto parts co., LTD., Wuhu rich, sports goods co., LTD., Wuhu and vehicle equipment co., LTD., visit activities. Group bay � b district party committee is mainly responsible for comrades led into enterprise visit, to contact enterprises in the indoor visible location installation bay � b area "four" packages to public sign, to the enterprise propaganda the spirit of the sixth plenary session of the 19 and 11th congress in Anhui province, issued the 11th congress in Anhui province report key brochures publicity materials, such as detailed understanding of enterprise production and operation situation, future development planning and the current difficulties, and consult the three contact enterprise to "double contact, into one thousand enterprises, four send a suit to promote development" work opinions and Suggestions. In the future, Tuanwan � b District Committee will further strengthen its service efforts, enhance the awareness of active service, and help enterprises solve problems.

2021-12-07	Guangyuan city, Sichuan province, youth talent friendship activities held a "green" heart "creek" accompany young talent dating fellowship activities on February 7 (reporter Zhang Jianwei, correspondent, Xu Chuanliang Yao Tianxiang) recently, sponsored by Sichuan Guangyuan Qingchuan county organization department, county talent office, county to undertake "see" green "heart" stream "accompany" young talent dating fellowship activities in Guangyuan city, Sichuan province 4A scenic area qingxi ancient city, 100 from party and government organs, enterprises and institutions, outstanding youth representatives to participate in the activities.site of activity. The activity was opened in Jinsheng Yingxiang, with more than 100 participants wearing Hanfu, starting from learning the traditional etiquette of Han culture. A boy and a girl formed a team to participate in a series of interesting interactive games such as "wearing Hanfu, doing Han rites", "spreading love", "shooting", "watching morality", "flying flowers", "Elegant pop pot", "Chinese style," Chinese charm "," appreciating tea art "and" Tea Music Qingxi ".site of activity."The ancient city of Qingxi has a history of more than 1,700 years, in such an ancient city to learn the Han culture, to participate in the friendship, I think it is a very interesting thing, as if the dream back to the Tang Dynasty, meet after the evening romantic."As a hanfu lover, Yang Yuyan has been looking forward to this friendship event, and she is very satisfied with today's event schedule.site of activity. It is understood that Qingchuan County youth talent friendship activities have been successfully held for five consecutive years, is an important platform for Qingchuan to strengthen talent exchanges, enhance the feelings of youth. Tuanqingchuan County Party Committee will continue to take talent construction as the goal, build a good "magpie bridge" to help young men and women meet together, and regularly hold relevant activities to solve the practical problems of young men and women in the county.

2021-12-07	Group of Shandong Rizhao lanshan district party committee to carry out the franco-prussian propaganda volunteer service activities of Shandong Rizhao lanshan district party committee to carry out the franco-prussian propaganda volunteer service activities network Beijing on December 7 (reporter Zhang Jianwei, correspondent XiangChangHui) recently, group Rizhao in Shandong province lanshan district party committee joint district justice bureau, public security bureau in Anton county to carry out the national constitution day the franco-prussian propaganda volunteer service activities. Activities, the volunteers for past residents issued the constitution brochures, constitution pages, civil code manual, the protection of minors and other publicity materials, to explain the origin of the national constitution day and the constitution knowledge, and through listing legal cases in residents life, strengthen the legal and legal consciousness, consciously study law, law-abiding, usage, when their rights and interests are violated, learn to use legal weapons to protect their legitimate rights and interests.

2021-12-05	Handan City, Hebei Province promoted the establishment of the off-campus red scarf park Shexian County booster village (community) young working committee set up the off-campus red scarf park community) youth working committee established the off-campus red scarf park. Group county county for the school resources limited rural, relying on the village cultural activity center, rural culture, such as existing resources, close to rural reality, around the "beautiful countryside, rural revitalization, get rich guide" cultivate the goal of reserve forces, focusing on red inheritance education, national defense military education, Chinese traditional culture, local folk culture, exquisite arts and crafts, honest farming culture carrier, let the children grow outside school activities. Village (community) less committee in statutory holidays, weekend holidays and winter vacation normalized activities, so far, has established the campus youth practice base 10, a total of more than 60 times, provide services for more than 1200 teenagers, gradually formed the young pioneers of after-school education and after-school service system, build "young home" of primary and middle school students.

2021-12-03	Jianwei Correspondent Zhang Yuehua) In order to further deepen the cooperation between Shandong and Chongqing, build a young talent highland, and help to introduce talent and attract investment. Recently, the first Shandong-Chongqing cooperative youth talent station in Fengjie County, Chongqing city was officially opened in Kuizhou Hotel. Shandong Binzhou City Poverty Alleviation and Development Leading Group Office, members of Fengjie County People's Government, county Human Resources and Social Security Bureau, Communist Youth League County Party Committee and Chijia Group attended the opening ceremony. Under the strong support of the east and west cooperation funds in Binzhou City, Shandong Province, the Communist Youth League County Committee, the Organization Department of the County Party Committee and the Human Resources and Social Security Bureau of the county. It has a total investment of 500,000 yuan, with 20 rooms, 40 beds and a small meeting room that can accommodate 30 people. Through standardization construction, convenient application, considerate service, to apply for the fengjie youth, Shandong to offer investment, business negotiation of enterprise staff provide free accommodation within 3 days, policy consultation, investment negotiation and other one-stop service, at present, online application channel has been opened, through the "youth" WeChat public choose home youth-young talent station can apply for check-in.

2021-12-02	Group of Anhui yu an district party committee in Anhui province selected 80 village (house) league branch in Anhui province regarding Lu 'an yu an district party committee selected 80 village (house) league branch first book reporter Wang Haihan) " when you receive the letter, congratulations on your residency selected career with a youth beautiful background, you more than a' first secretary of the league branch position, welcome to join the family!"Recently, Anhui Province, Yu 'an District, Xikou Township landing Gang village village team deputy leader Cheng Xianpeng received a group of Yu' an District Committee responsible person sent a letter. Yu'an district is a former state-level poverty-stricken county. After poverty alleviation, it continued to send rural revitalization teams to 80 villages this year. In order to do a good job in the pilot reform of the grass-roots organizations of the Communist Youth League at the county level, the Youth League District Committee strengthened the construction of the village (community) grassroots league, and selected young people from the 80 villages to serve as the first secretary of the league branch of each village. League Yu'an District Committee responsible comrade introduced, these people are young, clear thinking, work passion, passion for the cause of the league, and the term of office is generally three years, relatively long, the work responsibilities of the task force and the league branch work responsibilities have overlapping, there is a basis for combination. Cheng Xianpeng is a staff member of the Joint Department of the Lu'an Municipal Party Committee. In June 2021, as the eighth batch of Anhui selected cadres came to Xikou Township as the deputy leader of the village team. He found that village-level cadres are generally young people, lack of voice in the village cadres, it is difficult to promote the work of the league."The characteristic of youth league cadres is a sense of innovation and open thinking."Cheng Xianpeng introduced that in order to help the development of rural industries, they plan to prepare for a bamboo board factory according to local conditions on the basis of full research. After site selection, site coordination, and multi-party contact, the bamboo board factory is about to start construction, and it is currently recruiting employees. In view of the "first secretary of the branch of the league" this new post, Cheng Xianpeng said that he would adhere to the party building with the league building, do small things, do a good job, and "attract talents", "cultivate talents", "retain talents" and "secure talents" for rural revitalization. Chengnan town rural revitalization workstation deputy stationmaster Chen Min and the Communist Youth League also have an indissoluble bond. After graduating from college in 2019 and attending work, she insisted on participating in various activities organized by the Yu'an District Committee of the Communist Youth League. In October 2021, she was cadres to the Party branch of Taowan Village, Chengnan Town. Subsequently, after the Youth League Yu'an District Committee and Chengnan Town Party Committee research, served as the first secretary of the Taowan Village Youth League branch. After nearly two years of poverty alleviation work experience, Chen Min paid special attention to the teenagers in need. Through visits, she understood the living conditions and academic conditions of the teenagers, took the initiative to contact the caring enterprises in the area, and sent condolence money and materials to the teenagers in need."In just over more than a month, I feel that grassroots work needs to be integrated into young forces. From grassroots Party building to rural revitalization, young comrades need to bend down and think down, and turn theories and policies into practical things for the people."Chen Min said. In the next step, the Youth League Yu'an District Committee will adhere to the party building with the league building and promote the league building, and constantly improve the organization, leading force, service force and the overall contribution of the Communist Youth League.

2021-12-01	Tengzhou City, Shandong Province to "five forces driven" to promote the reform work implementation effective Tengzhou City "five forces driven" to promote the county Communist Youth League grass-roots organization reform implementation effective Jianwei correspondent Xiang Changhui) since the county Communist Youth League grass-roots organization reform pilot work started, Tengzhou City, Zaozhuang City, Shandong Province, earnestly implemented the "Guiding Opinions on expanding the Pilot Reform of the County Communist Youth League" requirements, Focusing on the four reform objectives of the selection of league cadres, the establishment and operation of the league organization, the education and management of league members, and the leadership guarantee, Improve the station position, systematic planning, overall promotion, reform and innovation, Identify the target nodes, Refine the 40 specific measures, Gather multiple forces, We will effectively promote the reform work to take root and go deep and solid. We will strengthen the matching mechanism of "two-wheel drive" reform. We will continue to strengthen the ranks of cadres of the Communist Youth League and improve the assessment and training mechanism, so as to pool the strength of young people for the implementation of the reform work. We will innovate the mechanism for selecting and selecting youth League cadres. According to the number of posts, full-time and part-time cadres, and strong "professional and concurrently hanging" cadre team. Implement "interpreta dream plan" quality expansion, by collecting youth, youth home service needs, pku actively strive for funds to buy professional youth affairs social work service project in youth home normalized activities, and for the society to recruit youth volunteers to participate in the organization and development, innovation "1 + 2 + N" mode with strong ZhenJieTuan (work) committee work force. Innovation cadre appraisal cultivation mechanism, tengzhou combining the local reality, explore characteristic path, focus on "fu can", "issued" on further strengthening the city cadres tenure target management of the implementation opinions ", implement classification hierarchical management, improve the" 3 + x " term target management system, establish term commitment, performance evaluation, selection and appointment, results application mechanism. Formulate the Reporting and Evaluation System for Youth League Cadres, carry out the annual reporting and evaluation work step by step, and incorporate the work of Party building and youth league building into the reporting and evaluation content of Party committees at all levels. We will strengthen the activation force of the "one body and two wings" to optimize the operation mechanism. By rationalizing the league league organizations in traditional fields, expanding the coverage of the league organizations in emerging fields, and extending the service tentacles of youth groups, a new pattern of "one body and two wings" will be constructed in an all-round way. Up to now, 7 youth league organizations have been established in the municipal Youth League Working Committee, the Education Youth League Working Committee, and the Youth League Committee of the Health and Planning Bureau, with total training more than 1,000 young entrepreneurs and good rural youth. Adhere to the youth league-run youth associations, set up youth volunteer associations, young artists association and other youth associations, absorbed more than 600 young volunteers, and carried out more than 900 activities such as volunteer service, social practice, epidemic prevention publicity, public welfare classes, and youth painting and calligraphy exhibitions. More than 50 various communities, such as town and street youth league organizations, Bluebird students, rural good youth, young entrepreneurs, young volunteers and so on, have been built. Strengthen the "internal and external repair" to grasp the real education mechanism to increase the internal force. Adhere to the principle of combining inside and outside, ideological guidance and social practice, "both inside and outside" to grasp the education and cultivation of league members. Strict member education management, formulate "tengzhou school field" double product double evaluation "integral into the league work implementation opinions" on further strengthening the construction of the city's middle school league plan and other files, the member development, member advanced evaluation, league school construction, campus practice base work deployment arrangement. Improve the practice education mechanism, relying on the local a tower six pavilion, defense science and technology education base, Shandong youth rule of law education base, nursery ecological civilization education practice base of all kinds of education resources, build "1 center + N base" youth social practice base, guide the youth members out of the school to carry out labor education, social practice, volunteer service, community registration and other kinds of activities. At present, it has organized youth league members to carry out more than 20 social practice and education activities. We will strengthen the effectiveness of the "three lists". Integrate social resources. Relying on the party and the mass positions, we integrated social organizations, organs, schools, property management and other forces to build more than 40 "youth homes" positions in the city, realizing the overall coverage and normal activities. Build a service platform. We will focus on the needs of young people and the people, implement the supply-side reform of youth services, and carry out voluntary services on a regular basis. Polish the event brand. With the "green" brand project as the guide, constantly deepen the service content of "Party building leading youth", enhance the service effectiveness. We will strengthen the five-sphere integrated system and improve the guarantee mechanism to add impetus. Strengthen the effectiveness of the party building and league construction work, bring the league construction and team construction work into the party building work deployment and annual assessment, and bring the school league construction and team construction work assessment into the year-end comprehensive supervision and evaluation of education. Establish the inspection work list of the key contents of the Party construction and group construction, and bring the party building and group construction and team construction work into the inspection and supervision content of the municipal Party Committee, and apply it in the inspection work in the second half of the year. We will deepen the integrated training mechanism of the Party and the League, combine the first secretary and the Party building instructors at all levels, strengthen the guiding force of the league organization work, and take the "Xiangyang Plan" project as the starting point to deepen the mechanism of promoting the Party membership. Up to now, 186 league members have become Party members through the league organization. At the same time, the Communist Youth League Municipal Committee and the Municipal Bureau of Education and Sports have promoted the secretary of the Communist Youth League as a member of the municipal education leading group, and the secretary of the Communist Youth League Working Committee also serves as the deputy secretary of the Communist Youth League, to strengthen the cooperation and interaction of the Youth League and education.

2021-12-01	Group of Anhui Jiujiang district party committee to carry out the "painting piano heart" activities of Anhui Jiujiang district party committee to carry out the "painting in the heart" volunteer activities JianWei correspondent Yang Jie check WeiTing) to carry forward the spirit of patriotism, further inheriting Chinese traditional culture, recently, Wuhu city of Anhui province clear water street Qingyuan community youth league branch invited jurisdiction difficulties, floating children "painting piano heart" activities. Volunteer Teacher Zeng, with the title of "childlike innocence to the Party, Build the Chinese Dream together", introduced the history of the young Party propaganda. The heroic story of "four crossing the Chishui River" inspired the ideals and beliefs of the youth patriotism. The children used their imagination to put their patriotic feelings on the paper, and expressed their sincere love for the Party with calligraphy and painting. At the end of the activity, the clank and simple music sounded leisurely, from the "General Order" momentum, to the "prosperous National music", "I and my motherland" gorgeous euphemism, as if to tell the Communist Party of China take the bull by the horns, the battlefield, leading the Chinese people to the new era of happy life glorious course.

2021-11-30	Hunan Yongzhou ningyuan youth work joint meeting of the second plenary meeting ningyuan youth work joint meeting of the second plenary meeting Ji'an-wei zhang correspondent Pan Lingzhu) recently, Hunan Yongzhou ningyuan youth work joint meeting of the second plenary meeting, the youth party secretary, county youth work joint meeting convenor comrade Fang guotao chaired the meeting and summary speech, county youth work joint meeting member unit responsible for comrades to attend the meeting. At the meeting, the organization department of the county Party Committee, the county people and social security bureau, the county education bureau and other youth work joint conference member units based on their own duties, on the implementation of the "CPC Central Committee, The State Council issued <medium and long-term youth development plan (2016-2025)>" situation for exchange speech. Finally, on how to make better play to the role of youth work joint conference system, promote the development of youth work, youth party secretary, county youth work joint meeting convener fang guotao put forward three opinions: to deeply understand the importance of implementing development planning, youth xing country, youth strong, strong country, establish a system of youth work joint conference, is an important measure to carry out the national planning. We need to ensure that all tasks of the plan are effective, provide targeted guidance to the characteristics of their diverse ideological and cultural personalities, and unite and lead young people to "feel the Party's kindness, listen to the Party, and follow the Party's lead."To further form a joint force to promote the implementation of the plan, each member unit should compare their respective responsibilities and functions, and work together to do a good job in youth development. In the next step, the Communist Youth League County Party Committee will effectively play the role of overall coordination, and actively do a good job of communication and coordination between the member units, orderly and effectively complete the key work. Effectively transform the youth struggle enthusiasm into a strong driving force to promote the high-quality economic and social development of the county, for the implementation of the "three high and four new" strategy, the implementation of the "five districts and two cities" work deployment, the construction of a modern new Ningyuan to make new and greater contributions.

2021-11-30	Linquan county in Anhui province to build urban reading space crack youth reading problem linquan county party committee to build urban reading space crack youth reading problem Ji'an-wei zhang correspondent, dongdong check WeiTing) since the county communist youth league reform, Fuyang city in Anhui province linquan county party committee on youth demand problems, crack urban space, reading leisure problems, create beautiful environment, happiness full of urban reading space, let the young people in the community, can enjoy the full service such as reading, leisure. In the newly opened library of Daizhuang Community Youth Home in Chengnan Street, Linquan County, Huang Xiaoli came here to read his favorite book whenever he was free. As early as a few months ago, it was just an idle sales department, under the planning of the local party committee and government departments, it became the place of the community youth home. She said, " I have always wanted to have a place to read books. In just a few months, the community helped us build a library and a very good reading platform. The community did a great good thing for us."The community youth home has not only raised more than 10,000 books, but also realized information exchange with the county library, thus borrowing and returning the library books at the youth home. At the same time, the layout in the library, hire professional service personnel, so that young people enjoy the beauty of reading. It is understood that the space to benefit the people, Linquan County in view of the space limitations of the city for many years, the community service function is not comprehensive and other problems, the newly built community public service housing recovery, by the community to use. The person in charge of the community youth home said: " Young people need places for activities. We follow a service concept, giving all these Spaces to the masses."Dai Zhuang community is to seize this opportunity, more than 1,800 square meters of urban space out of 1,500 square meters, not only built a community library, but also built a science museum, self-study room, the elderly activity center and other places, to meet the spiritual and cultural needs of the masses."We combine the study of the reform of the Communist Youth League in the county level, in the process of integrating the construction of the city's reading space and the construction of the urban community, take more measures to reflect the value and characteristics of the youth, and significantly enhance the adsorption force and influence of the community."Tuan linquan county party committee said. Linquan County takes the building of urban reading space as an important part of people's livelihood construction. According to the characteristics of the community and the needs of the young people, while meeting the spiritual and cultural needs of the young people, it further improves the service level and ability of the community, and explores the "Linquan mode" of community governance.

2021-11-30	Shandong Linyi "I do the practical work for the masses" preaching activities into the community Linyi county "speak the practical work, enlightenment thought, party, go with the party" - "I do the practical work for the masses" preaching activities into the community zhang) recently, the group of Shandong Dezhou Linyi county "speak the practical work, thought, party, follow the party" - "I do the practical work for the masses" preaching in constant street xin garden community, the county, constant eight yuan street community, xin garden community and grassroots youth community representatives, on behalf of more than 40 people to attend. To earnestly study propaganda and implement xi jinping, the ideas of socialism with Chinese characteristics, give full play to the youth grassroots party secretary about demonstration leading role, the county in the county as secretary of the country good youth, youth typical youth group elected 10 outstanding youth "county communist youth league system at the grass-roots party secretary XuanJiangTuan". At the scene of the activity, the deputy secretary of the Youth League Party Committee Wang Yanxue read out the county Communist Youth League system grass-roots party organization secretary publicity group members list. XuanJiangTuan members hengyuan street Zhang MiaoLian village party branch secretary Hu Shuai around "do the practical work for the masses forever'not closed" theme, combining their own work practice, made a wonderful preach, how to let the masses really feel the young cadres to do the practical work "temperature", requires us in the process of doing the practical work for the masses, conscientious, only practical thought, style, action never close, can really do the practical things to the heart. Activities, combined with the communist youth league system to carry out the "I do the practical work for the masses" to preach requirements, speak good Linyi communist youth league "I for youth" the new era of youth story, and the county "I do the practical work for the masses" announced six service content, vice secretary yan-xue wang about youth volunteer service development to preach, youth around the county, county youth volunteer association organization guide the youth, volunteers in economic and social development, service guide youth to participate in community governance system in detail. Next, the county will combine the county communist youth league cadres training, cadres direct contact with the masses to continue to preach, organize XuanJiangTuan members combining work practice, in understandable, happy way good learning understanding of the spirit of the sixth plenary session of the 19, speak good why can, why marxism, why socialism with Chinese characteristics, speak good party history learning education since the party committees and governments, party members and cadres for the masses to guide the masses from all walks of life, especially teenagers firmly listen to the party, feeling, follow the party's faith.

2021-11-27	Wang Haihan, reporter of China Youth Network of the Group Youth Daily) " We old people do not leave the community, but we can often listen to young people tell red stories and the latest policies, and our life after retirement is not so boring."In November this year, Hefei Baohe District Feihe Town" red ring " publicity group was established. District Jia Da ying community grandma Huang mentioned the publicity group, can not help but praise a few words."Spread the party's innovative theories, principles and policies, can better gather the strength of the cadres and the masses."Baohe District Feihe Town publicity office director Shi Qin introduced, the town specially set up 18 people" red ring " publicity group. Among the members are veteran Party members who have witnessed urban development, grassroots workers and young volunteers who stick to the front line of epidemic prevention and control. The three generations of old, middle and young speakers often consult each other and carry out preaching in plain language and new media means. Recently, around the spirit of the sixth Plenary Session of the 19th CPC Central Committee, 18 speakers have achieved all coverage of all village residents."To be trusted and needed by the villagers is my greatest happiness."Xu Wenwen, a young volunteer, has long been working on the front line of epidemic prevention and control, expropriation and relocation security, and people's livelihood services. As soon as the" Red Ring " publicity group was prepared, she signed up. She will be the things encountered in the work into the publicity materials, to the villagers about the Party constitution and rules, guide everyone to participate in the area services. Active member of the Party Zhou Tingting, post-90s propaganda front staff Wang Jing, ecological protection worker Geng Xudong..... In Feihe Town, we often can see young speakers active in the relocation line, project site, residential areas and other places.

2021-11-26	Longquan city, Zhejiang province "position + brand" service tangible soul "position + brand" service tangible soul longquan party committee organization department of municipal party committee service center for position, integration of volunteer service force, 20 volunteer service team in longquan city service center, more than 450 "small spring" party member volunteer service team play the important role of cohesion, the heart and hearts."Xiao Qingquan" is the brand of party and mass volunteer service in Longquan. Under the organization and launch of the Organization Department of Longquan Municipal Party Committee, the Publicity Department of Longquan Municipal Party Committee, the Municipal Youth League Committee and other departments, the volunteer service cause has developed rapidly. At present, more than many volunteers have registered for 72,000. Since the beginning of this year, the total service participants have exceeded 200,000, and the service time is 635,000 hours. Excellent non-governmental volunteer service organizations such as Ant tribe and Blue Sky Rescue Team have emerged. In the municipal "small spring", longquan municipal party committee organization department actively build "1 + 13 + N" global matrix brand pattern, formed the dragon town "immediately", west street street "west street" and sword street water community "five micro", north township river village "something you talk" township, village party brand, for the service at all levels. Source: China Youth Daily

2021-11-24	Gansu Linze County: love "three left-behind" volunteer service warm heart: love care "three left-behind" volunteer service warm heart January 24 (reporter Zhang Jianwei, correspondent Li Ying) in order to further implement the Gansu Province rural "three left-behind" personnel and extremely poor people care service action, Combined with the practical activities of "post training, business competition" and "I do practical things for the masses", Linze County Party Committee of Zhangye City, Gansu Province gave full play to the role of league organizations at all levels in the care and service action, Leading and driving young league organizations at all levels, Continue to carry out youth volunteer service activities such as "four help and four send" with the main content of filial piety, the elderly, the young and the poor, Truly the rural "three left-behind" people and extremely poor people care services to in-depth, Do fine and solid. Care for the elderly tree new wind love righteousness cut, cut out filial piety old new normal. The organization and mobilization of the town youth league organization to carry out the "green warm sunset" care from the "head" warm-warming activities, normalized into the elderly living alone in each town to solve the problem of hair difficulties, haircut, and contribute youth strength to the rural revitalization."drying quilt than filial piety", drying a new fashion of filial piety. A selection working group composed of grass-roots organizations and youth representatives was set up, and to actively carry out family civilization creation activities with the theme of "drying quilts than filial piety". Towns live alone over 65 years old home, take "two look, two touch, a smell", the old man quilt "review", and chat with the old man, understand the old man daily life, by voting, selected filial piety old love family, in the moral red black list of the public, vigorously carry forward the respect, love, pension, filial piety, attract the masses consciously in family civilization, promote the formation of socialist family civilization.volunteering. Correspondent for the traditional festival to send warmth, send filial piety old new fashion. Make full use of the Mid-Autumn festival, chongyang and other traditional festivals, organize towns and townships, youth volunteers, caring people, etc., to nursing homes, living alone home, to carry out "love old filial piety to respect" as the theme of activities, including dumplings, eat long table banquet, visit condolences, health clinic, love cut, health regulation to help activities, for the old people to send holiday greetings and sincere wishes. Care for children has a temperature of "bosom sister" and "bosom brother" care is normalized. Continue to carry out the "hand to hand youth walk together" hand in hand, the county 208 "bosom brother" "bosom sister" and left-behind children formed a "1 + 1" pair support, young cadres, young volunteers incarnation "bosom sister" "bosom brother", normalized academic support, psychological counseling, humanistic care, material sympathy, etc., constantly optimize the learning life environment, make their family care, academic teaching, heart have embellish. Join hands to care for children, and help the happy growth. Together with caring enterprises, more than 106,000 yuan of stationery, books, clothes and other materials to left-behind children to encourage them to study hard and make progress every day. Family care, study, teaching, mind, moist, safe and guaranteed. Seize the "June 1" children's day, winter and summer vacation and other important nodes, organize young cadres, youth volunteers, relying on the youth home, colorful hut and other places, increase the rule of law publicity, extensive "green build dream studies camp" "colorful classroom" and summer security, anti cult, minors the franco-prussian propaganda, love care and other volunteer service activities, the escort for the healthy growth of minors.

2021-11-16	Guangxi Guigang port south group representative contact station set up green "bridge" Guangxi port port south: representative of the sinking township street set green "bridge" in the youth newspaper network reporter Xie Yang) since the county communist youth league reform pilot work, the Guangxi Guigang port south committee through the innovation contact station work, improve the group representative takes the quantitative evaluation system, improve the towns (street) group representative station started rate, strengthen the communication and contact between grassroots cadres and youth. On October 26, the liaison station of Zhanjiang Town delegation in Gangnan District launched the activity of "gathering youth strength and helping rural revitalization". In July this year, the South District of Guigang City introduced reform measures to build the brand of the town level league representative liaison station, and required the national, autonomous region, city, district, town (street) league representatives, in addition to the school students, all into the town (street) league representative liaison station. The person in charge of the Youth League Port South District Committee said that building the work brand of the town league representative liaison station is to enable more than 80% of the city, district and town league representatives to perform their duties more conveniently and contact the youth league members more frequently. Before the group representatives want to enter the "station" activities must be from the villages and towns to district, now the group representative contact station sink to the town level, on behalf of the group into the "station" activities become more convenient, especially the city, district level of the group representative directly into the village, household activities, grassroots close contact members, youth, started ability and space greatly improved. By the end of October, Gangnan District had invested more than 200,000 yuan to build nine town-level league representative liaison stations. Gangnan District has formed a grid performance platform with the district league representative liaison station as the general branch and the town (street) league representative liaison station as the extension. Port also develop "about further improve the league committee contact group, group representative contact youth member" two "working mechanism", "port league representative started quantitative evaluation method" and a series of documents, clear group representative annual activity theme, activities, work content and process, and the activities, activity time, contact members and youth league branch number of specific requirements, at the same time to strengthen the assessment constraints. According to carry out a study training, a research visit, a volunteer service activities "three one" requirements, nine town (street) group on behalf of 29 special team to organize representatives more than 100 activities, "station" more than 500 city, district, town group representatives to participate in the "station" activities contact youth member more than 10000 people, organize research visits, volunteer service for more than 3000 hours.

2021-11-15	Shandong Rizhao j electricity express industry group committee established Rizhao j electricity express industry league committee established Ji'an-wei zhang correspondent phase Changhui) to implement the party with the party building, to promote the party working mechanism and the county communist youth league grassroots organization reform pilot requirements, promote Rizhao j electricity express industry quality development, recently, the Rizhao city in Shandong province j electricity express industry working committee founding meeting, the county, county business service center is responsible for comrades and electricity express industry league committee members to attend the meeting. Since the reform of the grassroots organizations of the county Communist Youth League, Rizhao Juxian Youth League County Party Committee and the county business and trade service center in close cooperation, Establish the whole county e-commerce, express delivery industry ledger, Understand the distribution of youth; Promote the establishment of Juxian e-commerce Promotion Association (Juxian Youth E-commerce Industry Alliance), To absorb 16 e-commerce enterprises (incubation platforms) and 6 express delivery enterprises to join in, The average express delivery fee was reduced by 1.10 yuan per order, Express delivery order transaction volume increased by nearly 20%, To achieve mutual benefit and win-win results in the e-commerce express delivery industry; Relying on the Juxian E-commerce public service Center, Yanzhuang Street e-commerce incubation base, postal express and other e-commerce express distribution center to establish a service organization, Realize the grasp point with the surface, Contact covering 43 e-commerce enterprises and 6 express delivery companies, Expand the coverage of youth contacts.

2021-11-15	The first "Red Scarf" master studio in Xinfeng County, Guangdong Province, Zhang Jianwei, correspondent, Liang Zuer, Li Excellence) In order to thoroughly implement the "Opinions of the CPC Central Committee on Comprehensively Strengthening the Work of the Young Pioneers in the New Era" document spirit, Comprehensively promote the reform and innovation of the Young Pioneers, Improve the construction of the young Pioneers' working team system, Give play to the leading role of young Pioneers, recently, The opening ceremony of the first "red scarf" master studio in Xinfeng County, Shaoguan City, Guangdong Province was successfully held in Xinfeng County No.1 Primary School, Tuan Xinfeng County Party Committee, Xinfeng County Education Bureau, Xinfeng County No.1 Primary School and other relevant principals and members of the famous teacher studio attended the ceremony. Members of the famous teacher's studio to carry out the young Pioneers training activities. At the ceremony, the leaders attending the activity jointly inaugurated the teacher studio and issued the appointment certificate for the members of the teacher studio. Members of the studio have expressed that they will seize the opportunity, make pragmatic innovation, take serving the growth of students as the starting point of work, innovate working methods, deepen and optimize the brand work carrier of the young Pioneers, and help teenagers to take the first step in life. Subsequently, the members of the famous teacher studio carried out the first exchange and research activity, and deployed the implementation, management and division of work of the studio. Members of the famous teacher's studio to carry out the young Pioneers training activities. According to the introduction, Xinfeng County "red scarf" teacher studio is composed of many local provincial and county level excellent brigade counselors, the establishment of the studio is another important measure to comprehensively deepen the reform of the Communist Youth League and the Young Pioneers and Xinfeng County. Next, the Communist Youth League County Committee and the county Youth Working Committee will take this opportunity to enhance the demonstration and leading role of the young Pioneers teachers, create a group of highly, distinctive, influential, fruitful young Pioneers brand, enhance the organizational vitality of the young Pioneers, standardize the system construction of the young Pioneers work team.

2021-11-14	The Pingnan County Party Committee helps the young entrepreneurs to stand up: " It is not the e-commerce business, but the high rent."If the store rent is removed, can mobilize the enthusiasm of young people entrepreneurship and improve the success rate? A pilot project in Pingnan County, Guigang City, Guangxi Province, may give people some inspiration. In October 2019, the Tuanpingnan County Party Committee conducted a questionnaire survey on the entrepreneurial status of more than 110 local young people. What impressed Li Xiaoli, party secretary of Tuanfeng County, most was that all the "lack of funds" when asked about what was the "lack of funds" thing."At that time, it was a critical period of the battle against poverty. We hoped to help young people start a business and reduce their costs through the power of the youth league organization, so that some young people who have just been lifted themselves out of poverty or have returned to their hometowns to their hometowns could dare to start businesses and relax."Li Xiaoli said. After various contacts, TuanPingnan County Party Committee reached a cooperation with a local real estate development company to jointly build a Qingchuang Street in Pingnan County. By the real estate development company in Pingnan County Yujiang Dijing Community to provide a number of entrepreneurial youth area of 56-127 square meters of shops, the implementation of rent-free 3 years plus two years of preferential rent policy, TuanPingnan County Party Committee selected and recommended poor village entrepreneurs and returning entrepreneurial young people to enter and operate. On November 26,2019, of the first batch of eight young people who signed contracts, two were young entrepreneurs from poor villages and five had returned to their hometown to start businesses. Wu Jinyu, of Sanhe Village, Danzhu Town, Pingnan County, is one of the first young entrepreneurs to enter. A few years ago, Wu Jinyu's family was identified as a registered poor family. In order to increase his income, he entered a factory, an apprentice in a restaurant and a driver. Over the years, he saved tens of thousands of yuan in savings and borrowed money from friends, so he decided to open a snail noodle shop."I saw that the store was in the street and near the community. The location was very good, and there was no other snail noodle shop in the nearby area."Wu Jinyu said that what impressed him most was the rent-free policy, which is much less risky for young people like him. Now, after more than two years of operation, the snail noodle shop opened by Wu Jinyu not only attracts the surrounding residents to consume, but also often visits the citizens across the Xun River. In addition to attracting customers, he has also opened up the food delivery business. The daily turnover of the store can maintain at around 1,000 yuan. Yao Yanzhuang, 33, is also one of the first young entrepreneurs to enter, running a kitchen appliance shop. In 2019, he saw that the Communist Youth League County Party Committee launched the policy of Qingchuang Street, and felt that he met the conditions, so he signed up with the mentality of having a try. When it first settled in, the business in the block near Qingchuang Street was still in its infancy, and the surrounding residents were used to shopping in the old town center opposite the Xun River."At that time, there were not many people passing by at the door every day, but fortunately, the rent-free policy helped us relieve a lot of business pressure."Yao said, he believes that as long as the number of businesses increases, Qingchuang Street will gradually become a market, and customers' consumption habits will naturally change. Since last year, some residents have delayed their renovation plans due to the COVID-19 outbreak due to reduced income. But Yao Yanzhuang relies on service and reputation, gradually opened the market."Water heater, range hood as daily necessities every household can use, and the old use is broken also have the need for new."Yao said that although e-commerce is cheaper, brick-and-mortar stores have better after-sales advantages. Yao Yanzhuang calculated an account, he rented the 127 square meters of shop surface, according to the market price monthly rent to 2,500 yuan, peak season rent can account for about 30% of the operating costs, off-season, rent accounted for about 50% of the operating costs. The rent-free policy makes him not flustered in the off-season. He can focus on using the three-year rent-free period to reduce costs, do a good job in service and attract customers. After more than two years of careful management, the business in his store gradually on the right track, in the off-season, the monthly turnover of more than 20,000 yuan, and the monthly turnover to 70,000 or 80,000 yuan."None of the 13 green innovation enterprises that have settled in right now have closed down or changed hands."Pingnan royal river emperor scenery industry service co., LTD. Management He Wei machine told the green newspaper network reporter, at the beginning, the company is willing to cooperate with the county to build green and a street, on the one hand is in response to the national support youth entrepreneurship policy, on the other hand is also hope to through the entrepreneurial youth, pull sentiment, speed up the cultivation village business ecology, drive other property sales, improve the occupancy rate of village residents. In his opinion, the high success rate of Qingchuang Street is that the rent-free policy has greatly reduced the financial pressure of operators. In addition, young entrepreneurs should also investigate and evaluate their entrepreneurial projects before entering in, so as to avoid homogeneous competition and entrepreneurial projects that do not meet the needs of community residents. In order to help the settled young entrepreneurs to better operate, the property management company will regularly hold regular publicity activities to inform the owners of the community about which businesses are nearby and which services can be provided. Tuanpingnan County Party Committee also regularly organizes experts to provide door-to-door guidance, and holds training activities on youth entrepreneurship related policies, such as youth entrepreneurship loan, to provide as much support as possible in terms of technology and capital. Wu Yu, who runs a cake shop in Qingchuang Street, has deep feelings about the operating difficulties of physical stores in the past two years. After she opened the shop, she knew that, in addition to the early decoration, equipment investment, every day as long as the shop opened the water and electricity costs will spend more than 60 yuan, as well as labor, raw materials and other costs. Free rent policy, so that the first business to enjoy a great operating advantages."If you have to pay the rent, you have to sell at least two more birthday cakes a day to earn them back."The daily customer volume of the cake shop is not stable, and the profit mainly depends on the birthday cake. The profit of selling cakes and bread is very thin, in order to accumulate contacts and establish customer loyalty and consumption habits. Wu Yu also often sends products and preferential activities through WeChat Moments and social media. Now, in addition to the residents of the surrounding community, there are many residents of the old town on the other side of the Xun River."I am quite confident about continuing to make the store bigger and stronger."Wu Yu said that after a year or two of hard work, her perception of the market direction, the ability to control consumer demand is stronger, coupled with the support of the government and relevant departments, the operation of the store is increasingly prosperous."Even if I collect the rent in the future, I will continue to open the store here, because the surrounding facilities are better, and the occupancy rate of the surrounding community is getting higher and higher, and then our revenue capacity will be enough to offset the pressure of the rent."She said. China Youth Daily China Youth Network reporter Xie Yang trainee reporter Yin Xining source: China Youth Daily

2021-11-14	Zhang Jianwei) The opening ceremony of Yanhe Tujia Autonomous County in Guizhou Province was held at the Party School of the County Party Committee. Zhang Lang, deputy secretary of the county Party Committee, and Cui Yuqin, vice chairman of the county CPPCC, attended the unveiling ceremony and inaugurated the youth League school. County directly related departments, each township (street) in charge of responsible comrade or youth league (work) committee secretary, county straight middle school, county secondary vocational school youth League committee responsible comrade to attend the meeting. Zhang Lang pointed out that the Communist Youth League, as an advanced youth organization led by the Party, bears the glorious responsibility of uniting, educating and serving the youth. The establishment of the county youth league school is of great significance for strengthening the standardization and institutionalization of the education and training of the Communist Youth League, further playing the role of the party's assistant and reserve army, and promoting the reform of the grass-roots organizations of the Communist Youth League. Zhang Lang stressed that the youth league school should develop the education and training function, do a good job in ideological guidance, give full play to the role of youth cohesion, do a good job in young talent reserve work, and do a good job in the construction of the youth league school itself. We must firmly uphold our ideals and convictions, uphold the leadership of the Party, educate and guide league members to have the confidence and determination to always follow the Party, and strive to cultivate a contingent of league cadres who are "politically firm, have in style, reassure the Party and satisfy the young people."We should seize the favorable opportunity, adhere to the organic integration of ideological guidance and education and training, vigorously carry out the education and training of youth league cadres and youth league members, be the "thought pool" and "gas station" of the majority of youth and youth league cadres, and effectively play the bridge role of training and condensing young people. We should play the role of radiation, closely combine the actual situation of Yanhe County and the needs of the young people, innovate and carry out new practical courses such as skills training and business incubation, build a platform for the young people to improve their abilities, and continuously deliver fresh blood for the economic and social development of the river. To focus on the overall situation of the county, education and guide the county youth to thoroughly implement the spirit of the 14th Party Congress, the wisdom of the county youth members of the county together, along the river high quality development model, set an example, make new achievements, condensed the strong force to promote the construction of modernization.

2021-11-25	Shaanxi fengxiang group representative contact station at the 19th session of shaanxi group representatives at the sixth plenary session of the 19th project learning zhang) for in-depth study and implement the spirit of the sixth plenary session of the 19, grasp xi general secretary of the important thought of youth work, solid promote the county communist youth league grassroots organization reform work, recently, Baoji, shaanxi fengxiang group held the spirit of the sixth plenary session of the nineteenth special learning, in the form of online group representative contact station some comrades to participate in learning. Meeting learning to convey the sixth plenary session of the nineteenth central committee of the CPC communique the central committee of the communist party's major achievements and historical experience and xi resolution, general secretary of the central committee of the CPC about the party's major achievements and historical experience resolution, understand the party's mission, comprehensively deepen the party's struggle in one hundred great achievements and the understanding of historical experience. The meeting pointed out that the Sixth Plenary Session of the 19th CPC Central Committee is an epoch-making meeting held at the important moment of the Party's centenary birthday. The Resolution of the CPC Central Committee on the Major Achievements and Historical Experience of the Party's centenary struggle, deliberated and adopted at the plenum, on the basis of a comprehensive review and summarizing the course and major achievements of the Party, summarizes the historical significance of the Party's centenary struggle from a more extensive perspective. The summary of these five aspects, namely, based on the land of China and looking to the future of mankind, reflects the relationship between the CPC and Marxism, world socialism and human society development, and connects the historical, theoretical logic and practical logic of the CPC's century-old struggle. The meeting called on youth league organizations at all levels and youth league representatives in the district to bear in mind the glorious responsibility of the Party's assistants and reserve forces, and faithfully implement the specific requirements of educating people for the Party put forward in the resolution. We should study and implement the spirit of the Sixth Plenary Session of the 19th CPC Central Committee as the primary political task of the Communist Youth League organizations at all levels in the current region. Learn, think, and practice, To unify the thought and action into the spirit of the plenary session, Efforts to study on a deeper level, on a higher understanding, practice on the first step; To try to fight hard, Combined with the actual situation of Fengxiang, Bold innovation, fast and steady to promote the reform of the county Communist Youth League grassroots organizations pilot tasks, Constantly improve the leading force, organization force, service force and the overall contribution of the regional Communist Youth League; Keep your mission in mind, According to the deployment requirements of the District Committee and the District Communist Youth League Committee, To study the spirit of the plenary session with the implementation of the current key work closely combined, We will coordinate the study and education of Party history, I do practical things for the people, youth ideological and political guidance, rights and interests protection, youth volunteer services and other work, At the end of the year to hand over a satisfactory "youth answer paper".

2021-11-14	Jiangsu lianshui: with good youth learning line to carry forward the patriotic spirit of Jiangsu lianshui: with good youth learning line to carry forward the patriotic spirit zhang correspondent well Chen xu) in order to further promote the history of the party learning education into the brain into the heart, strengthen youth ideological and political lead, guide teenagers listen to the party with the party, recently, group Huai'an lianshui county "youth to struggle to the party power" series of activities, led the youth members and activists representatives into the youth learning club line, strengthen the patriotism, improve khalid ents results."North Jiangsu Xiao Yan'an" youth learning club line site. Correspondent for figure "northern Jiangsu small Yan'an" youth learning club line through each point scene reduction, multimedia technology immersive experience and cultural relics exhibition, the northern Jiangsu people in the war of resistance of indomitable spirit show incisively and vividly, lead the youth to revisit revolutionary struggle, inheritance to carry forward the spirit of patriotism. Youth league members visited the "North Jiangsu Xiaoyan" an Memorial Hall. Correspondent for figure since this year, lianshui county through joint lianshui county propaganda department, relying on the "fire" patriotism education base alliance, fully tap lianshui old revolutionary base red gene, series red star victory square, the memorial, school and military museum built representative youth learning club line, at the same time as a new development league ceremony designated point and member youth party history education designated point, launched a "one-stop" youth learning line. Since the establishment of the line, it has received more than 2,000 youth league members, young Pioneers, primary and middle school students, and extensively carried out the theme activities of "Please rest assured that the Party, the strong country has me", "One Hundred Years of the Party history and Youth said", "Youth struggle to the Party to strengthen the country" theme youth League Day, the league joining ceremony and other activities. It is understood that TuanLian water county to build strong "youth learning club" line work force, with full-time, temporary, part-time cadres as the main body, recruiting social workers, youth volunteers and other groups as auxiliary force, and strengthen the training to the team, the "youth learning club" learning line grinding more smooth, improve the atmosphere of the line, the course richness, youth's sense of harvest.

2021-11-22	Chongqing fengjie county "in the name of youth" explore youth development planning path Chongqing fengjie county "in the name of the youth" to explore the three gorges reservoir youth development planning path Zhang Jianwei) northeast Chongqing Fengjie county is located in the three gorges reservoir area, in December 2020, by the central as the national long-term youth development plan (2016-2025), one of the pilot counties. Under the guidance of the Central Committee of the Communist Youth League, Fengjie County earnestly implements the requirements of the Party management of youth work, reexamines, reconsiders and repositions the key issues concerning youth development, actively builds the youth development policy system and working mechanism, gathers the youth development force, and Fengjie County youth development planning pilot work has achieved remarkable results. Based on the overall situation, grasp the "three propositions", form a work pattern focusing on the youth, adhere to the principle of the Party's management of the youth, put the youth development in an important position, to provide a strong organizational leadership and institutional guarantee for the implementation of the Plan. First, high station planning, improve the working mechanism. On April 14, the pilot work of the plan was held, and on April 20 and May 31, the standing Committee meeting of the county Party Committee and the government executive meeting were held respectively to study the youth development work and deploy the pilot tasks. Timely adjustment and enrichment of the county youth work joint meeting, by the county party secretary as the general convenor, will be held in late this month. To further strengthen the standardization of the joint meeting system operation, establish weekly meeting, month, quarterly deployment and annual assessment mechanism, integration of demand, resources, project "three list" and nine key projects, efforts to form a "horizontal joint, vertical linkage, hierarchical responsible, field integration" pattern of youth development. The Chongqing Municipal Party Committee of the Communist Youth League went to Fengxian for many times for field research and guidance. In October, the "Planning" work promotion meeting was held in Fengxian. Leaders of 10 district and county youth League committees went to Fengxian to exchange and learn the pilot work experience. Second, high-standard design, strengthen institutional support. In accordance with 44 measures in the ten areas of the Plan, 71 youth opinions were collected, 27 major tasks in nine categories, including youth navigation, youth contribution and youth care, were defined, and 30 responsible departments were implemented. In the county "14th Five-year plan" to set up the "promote the all-round development of youth" special festival, "Fengjie Youth Development Center, the south bank of the Yangtze River youth research and practice base" two projects included in the Fengjie "14th Five-year plan" construction project database. Third, we promoted it efficiently and made precise arrangements. Focus on the youth needs, to carry out the "kui youth learning party history", "the party's story" "new youth yao youth" learning activities, such as "green orange a guest" and "orange" volunteer "return orange" service brand, such as county finance matching funds of 1 million yuan construction talents, reading, marriage, warm bee "youth station", really help young aggravation. Focusing on the demand side, focusing on the "six major actions", building a security system for serving the youth is closely related to the objectives and tasks of the Plan, focusing on the needs of the youth, together with the member units to promote the introduction of inclusive policies, to do a good job in the young people's livelihood. First, we will implement political-leading action. Focus on "founding in one hundred" theme, the youth party history in the county party history learning education overall plan, the youth branch ability promotion into the party school dry teaching training plan, organize more than 230 green horse engineering students to the red flag canal, jinggangshan, deng xiaoping cadre institute to accept the party spirit exercise, guide the youth party history, strong faith, follow the party. Second, we will implement a campaign to live and work in peace and contentment. Together with the Organization Department of the County Party Committee and the Human Resources and Social Security Bureau of the county, we issued ten Measures for Training and Encouraging Kuizhou Talents, to provide preferential policies for young talents in terms of housing, salary and children's education. We have formulated the 8090 " cadre training plan to meet the development needs of key industries such as navel orange, glasses, and cultural tourism, as well as social programs such as education, medical care, and science and technology, so that young officials can have a platform and space for growth. The "Youth Home" station project has been implemented, and the pleasant reading station, talent station and youth meeting rooms have been built, providing one-stop temporary accommodation, career guidance, policy publicity and other services for young people applying for jobs, covering more than 3,000 people. Third, we will implement innovation and entrepreneurship initiatives. We have set up a leading group to support returning to their hometowns to start businesses, promoted county government offices to issue the Thirteen Policies and Measures for Supporting Returning for Entrepreneurship and Promoting Rural Revitalization (Trial), and established a point-to-point service model of "enterprise + bank" to stimulate the innovation, entrepreneurship and creativity of young people in all fields, and promote rural revitalization. Up to now, it has attracted nearly 2,000 young people returning to their hometowns, lent nearly 5 million yuan, and 23 enterprises have increased their income by more than 2 million yuan. Fourth, the implementation of life care action. Promote the county government office to issue the Implementation Plan for the Pilot Reform of Marriage Customs in Fengjie County, explore the establishment of a "1 + N + N" county and rural work linkage mechanism, strengthen the construction of marriage and family counseling centers (stations, rooms) and other positions, and strive to create a "new generation of new fashion" civilized marriage customs. Carry out the "Green Orange love" and "full orange love" friendship activities, serving more than 1,100 single young people throughout the year, and organize 12 couples to attend the group wedding. Pre-marital check-up service centers have been set up to provide free pre-marital medical examinations for more than 2,300 young people. Lecture halls on youth marriage and childbearing have been held, and maternal and child health care experts have been regularly invited to teach 6,000 young people of the appropriate age to serve the marriage and childbearing needs of young people. Fifth, we will implement the action to protect our rights and interests. Cooperate with the county government office to set up a leading group for the protection of minors in Fengjie County, with the deputy county head as the group leader and the leaders of 34 responsible departments as the members, to timely study and solve the uninsured related problems and protect the legitimate rights and interests of minors. We have held 50 activities, such as 12,355 services, youth public welfare reading, and family education lectures, serving more than 6,000 young people. Focusing on the "double reduction" policy, with "I do something for the youth", we have expanded the "4-30 classroom" after-class services, and provided summer childcare services for more than 3,000 young people. We built a four camp of high-quality traditional culture research, carried out 46 social practice activities for young people and served 3,200 young people. Exploring the mode of "the Communist Youth League + enterprise", entering pingan Township, Yong'an Middle School and other places to carry out the Spring Festival "send warmth" activities, visiting more than 3,000 young people in difficulties, supporting 160 poor students, and caring for more than 3,000 left-behind children. Sixth, we will implement the action to reduce the burden on education. Strive for the county financial investment of 1.398 billion yuan to improve the conditions of 199 schools, to ensure that each central field town has a public kindergarten 1 so, the new urban public kindergarten 5, new 2,000 degrees, public kindergartens in the kindergarten children accounted for 60.29%, the universal benefit rate of preschool education reached 98.66%. We vigorously implemented the action plan to "support the elderly", upgraded 16 township nursing homes in the county, built 16 nursing centers and 74 community service stations, with 22 beds per 1,000 elderly people, and reducing the burden of young people taking care of the elderly. Focus on the integration point, highlight the "three leading", create a broad stage for youth, youth development work into the overall reform and development of the county, and strive to give play to the advantages of youth, highlight the pilot demonstration and guidance. First, humanistic guidance, so that the atmosphere of "poetry taste long" is more and more strong. Relying on the lecture hall and Baidi poetry Society, we will educate and guide young people to develop good study habits of "three and one hour" every day. We continued to carry out the "Six Advanced Poetry Progress" activity, organized more than 1,100 young people to participate in the "Talent and Beauty" Poetry Challenge, and selected 8 contestants from the "Chinese Poetry Conference". Relying on the "Return to the Three Gorges", we have cooperated with 108 universities including Beijing Normal University to build a national poetry culture research base and cultivate the feelings of young poetry and books. Second, the typical guidance, so that the spirit of "serving the festival, unremitting self-improvement" deeply rooted in the hearts of the people. Select trees to commend the "Kuizhou Youth Model", guide them to inherit and carry forward the Fengjie spirit of "serving the festival and unremitting self-improvement", and play a leading role in the central work of the Party and government. Shi Shenglan, "National May Day Labor Medal", Zhou Xiaomeng, "Municipal Advanced Individual in fighting COVID-19", Li Yuhong, "Good youth for Good in the City", and Yu Hongmei, "the most beautiful village official of the 1990 s", have emerged in the county. Third, innovation leads, so that the trend of "rushing out of Kuimen world wide" continues to appear. Taking the opportunity of creating the city's only national innovative county as one, it has carried out industry-university-research cooperation with 40 units such as China Rural Center, Chongqing University and Chongqing Institute of Electronic Engineering, built 21 platforms such as mass innovation space and Kui Business Pioneer Park, expanded the county Youth Chamber of Commerce and Youth Promotion Association, and cultivated more than 300 innovative young entrepreneurs. In May this year, the youth creative enterprise "Kui Feng Wine Industry" appeared at the Silk Road International Expo. The "Kui Think Tank" was established to pool the wisdom and the strength of the youth, fully flooding the innovation and entrepreneurship vitality of the county.

2021-11-13	Jimusar County Second Middle School to carry out the "Communist Youth League Love birthday party" activity Jimusar County No.2 Middle School to carry out the "Communist Youth League love birthday party" activity body care and help work, focus on deepening the "Communist Youth League love birthday party" work. On the afternoon of November 11th, the Communist Youth League Committee of Jimsar County No.2 Middle School organized a "love birthday party" for the special birthday boys. At the birthday party, the students performed a carefully prepared hip-hop dance, guitar playing and singing, national instrumental music ensemble, piano performance and other performances to bring the activities to a climax. Then the principal of the county No.2 Middle School cut the birthday cake for the children, and the secretary of the school Youth League committee wore the birthday crown for the children. With the warm and cheerful "birthday song" melody, the students have closed their eyes, clenched their small fists and made their own birthday wishes. The whole birthday party is full of warmth and happiness, and every child's face is filled with a happy smile. Finally, the county No.2 Middle School principal also sent a love gift package to the children. Through this "Communist Youth League love birthday party", let the children harvest the cake and blessing, felt the deep love of the Communist Youth League, further enhance the cohesion of the league organization, firm the young pioneers feel the party, to listen to the party, follow the party confidence and determination.

2021-11-15	Mingxi: The first "Youth Innovation Festival" series of activities of the first "Youth Innovation Festival" series of activities were launched. Correspondent for figure China youth network Beijing on November 15 (reporter Zhang Jianwei, correspondent Xu Jingxian), group, Sanming committee, Sanming youth federation guidance, group mingxi county joint county talent office departments start "dream weavers and Ming creek" the first "green gen festival" activities, November 2 solstice 4, invited the Wenzhou, Zhejiang province short video live center head to Ming three days of "new media concentrated camp" with actual combat training skills, more than 80 entrepreneurial youth, secondary vocational colleges teachers and students training, nearly 5000 people through live to watch simultaneously. On November 4, the first live broadcast of "Yue See Mingxi Youth I Speak" was held. More than 900 people were watched in the broadcast room, and the sales amount of dried meat and kiwi fruit was more than 9,600 yuan. Live with goods. Next, Mingxi County will also hold the "Youth Dream Star" selection, "Happy See Mingxi Bright Future" short video shooting competition, and the county financial media center to build the "Youth Foreign Exchange" broadcast room, and implement the "Youth Dream loan" financial project, open a start-up business ability improvement class and other series of activities.

2021-11-15	Anhui province regarding Lu 'an yu an middle school (secondary) league school construction to achieve full coverage of 51 league school completed in Anhui Lu' an yu an middle school (secondary) league school construction to achieve full coverage of Zhang Jianwei correspondent, Jiang Yulong check WeiTing) for promoting the county communist youth league organization reform task, play to the advantages of middle league in middle school ideological and political education, constantly consolidate the middle school league building foundation, league of Anhui Lu'an yu an district party committee adhere to the "software" system and "the" hardware " position with deployment, by the end of October, yu an 51 middle school (secondary vocational) league school construction complete comprehensive listing. Combing the "software", establish rules and regulations to highlight the league school norms. In middle school league construction work to promote, group yu Ann district party committee clear specialist is responsible for, detailed schedule, one is to strengthen work communication, establish middle school league work group, organize all middle school league, head to strengthen communication, exchange experience, try first middle school take the lead to share working methods, for part of the new office league head "mentoring". Second, to strengthen training guidance, has twice organized the middle school league organization training, from the middle school construction significance, goals, construction content and methods to give theoretical guidance, require the middle school actively strive for party support, promote the league school construction and middle school league development, education management foundation group work mutual integration, promote each other. Three is to strengthen the construction of specification, group yu an district party committee formulated the work manual, from the school constitution, organizational structure, training curriculum system, teachers construction and teaching management, training management system, student assessment method, etc., comprehensively combing the relevant rules and regulations, form a reference assembly, to ensure that the middle school league construction rules-based and standard. Perfect the "hardware", intensive and practical prominent position characteristics. Standard position construction is one of the five important goals of middle school construction, group yu an district party committee through "Thursday" to the middle school group visiting survey, for yu an middle school number is more, and most of the villages and towns, rural middle school, group yu an district party committee in the construction of the middle school base base, school resources, adhere to the school conditions, classification guidance. On the one hand, the schools are required to be solemn, standardized, simple and practical as the principle, highlighting the party, the league logo, in line with the political requirements. Guide all schools to check the league flag, league emblem, league song, league oath, timely update the organization structure, league school management system, and student training system on the wall, adhere to the principle of intensive economy, take the league and team activity room as the daily work position, make full use of multi-functional classroom, library and other resources, combined with the school youth League committee studio and league member activity room, guarantee the fixed activity site. At the same time, encourage qualified middle schools to fully explore and integrate educational resources, and actively explore and expand the positions outside the school. Currently yu an district 51 middle school (secondary vocational) have been set up, the next group yu an district party committee will continue to strengthen visit contact, control demonstration school construction standard to strengthen guidance, set up a typical, play a demonstration effect, focus on the role of league school, let the middle school really become the grassroots of youth league reform a "blade".

2021-11-13	TuanPingnan County Party Committee helps young entrepreneurs stand: " What breaks the collapse of physical stores is not e-commerce, but high rent."If the store rent is removed, can mobilize the enthusiasm of young people entrepreneurship and improve the success rate? A pilot project in Pingnan County, Guigang City, Guangxi Province, may give people some inspiration. In October 2019, the Tuanpingnan County Party Committee conducted a questionnaire survey on the entrepreneurial status of more than 110 local young people. What impressed Li Xiaoli, party secretary of Tuanfeng County, most was that all the "lack of funds" when asked about what was the "lack of funds" thing."At that time, it was a critical period of the battle against poverty. We hoped to help young people start a business and reduce their costs through the power of the youth League organization, so that some young people who have just been lifted themselves out of poverty or returned to their hometowns could dare to start businesses and relax."Li Xiaoli said. After various contacts, TuanPingnan County Party Committee reached a cooperation with a local real estate development company to jointly build a Qingchuang Street in Pingnan County. By the real estate development company in Pingnan County Yujiang Dijing Community to provide a number of entrepreneurial youth area of 56-127 square meters of shops, the implementation of rent-free 3 years plus two years of preferential rent policy, TuanPingnan County Party Committee selected and recommended poor village entrepreneurs and returning entrepreneurial young people to enter and operate. On November 26,2019, of the first batch of eight young people who signed contracts, two were young entrepreneurs from poor villages and five had returned to their hometown to start businesses. Wu Jinyu, of Sanhe Village, Danzhu Town, Pingnan County, is one of the first young entrepreneurs to enter. A few years ago, Wu Jinyu's family was identified as a registered poor family. In order to increase his income, he entered a factory, an apprentice in a restaurant and a driver. Over the years, he saved tens of thousands of yuan in savings and borrowed money from friends, so he decided to open a snail noodle shop."I saw that the store was in the street and near the community. The location was very good, and there was no other snail noodle shop in the nearby area."Wu Jinyu said that what impressed him most was the rent-free policy, which is much less risky for young people like him. Now, after more than two years of operation, the snail noodle shop opened by Wu Jinyu not only attracts the surrounding residents to consume, but also often visits the citizens across the Xun River. In addition to attracting customers, he has also opened up the food delivery business. The daily turnover of the store can maintain at around 1,000 yuan. Yao Yanzhuang, 33, is also one of the first young entrepreneurs to enter, running a kitchen appliance shop. In 2019, he saw that the Communist Youth League County Party Committee launched the policy of Qingchuang Street, and felt that he met the conditions, so he signed up with the mentality of having a try. When it first settled in, the business in the block near Qingchuang Street was still in its infancy, and the surrounding residents were used to shopping in the old town center opposite the Xun River."At that time, there were not many people passing by at the door every day, but fortunately, the rent-free policy helped us relieve a lot of business pressure."Yao said, he believes that as long as the number of businesses increases, Qingchuang Street will gradually become a market, and customers' consumption habits will naturally change. Since last year, some residents have delayed their renovation plans due to the COVID-19 outbreak due to reduced income. But Yao Yanzhuang relies on service and reputation, gradually opened the market."Water heater, range hood as daily necessities every household can use, and the old use is broken also have the need for new."Yao said that although e-commerce is cheaper, brick-and-mortar stores have better after-sales advantages. Yao Yanzhuang calculated an account, he rented the 127 square meters of shop surface, according to the market price monthly rent to 2,500 yuan, peak season rent can account for about 30% of the operating costs, off-season, rent accounted for about 50% of the operating costs. The rent-free policy makes him not flustered in the off-season. He can focus on using the three-year rent-free period to reduce costs, do a good job in service and attract customers. After more than two years of careful management, the business in his store gradually got on the right track, in the off-season, the monthly turnover of more than 20,000 yuan, and the monthly turnover to 70,000 or 80,000 yuan."None of the 13 green innovation enterprises that have settled in right now have closed down or changed hands."Pingnan royal river emperor scenery industry service co., LTD. Management He Wei machine told the popular newspaper network reporter, at the beginning, the company is willing to cooperate with the county to build green and a street, on the one hand is a response to respond to the national support youth entrepreneurship policy, on the other hand is also hope to through the entrepreneurial youth, pull sentiment, speed up the cultivation village business ecology, drive other property sales, improve the occupancy rate of village residents. In his opinion, the high success rate of Qingchuang Street is that the rent-free policy has greatly reduced the financial pressure of operators. In addition, young entrepreneurs should also investigate and evaluate their entrepreneurial projects before entering in, so as to avoid homogeneous competition and entrepreneurial projects that do not meet the needs of community residents. In order to help the settled young entrepreneurs to better operate, the property management company will regularly hold regular publicity activities to inform the owners of the community about which businesses are nearby and which services can be provided. Tuanpingnan County Party Committee also regularly organizes experts to provide door-to-door guidance, and holds training activities on youth entrepreneurship related policies, such as youth entrepreneurship loan, to provide as much support as possible in terms of technology and capital. Wu Yu, who runs a cake shop in Qingchuang Street, has deep feelings about the operating difficulties of physical stores in the past two years. After she opened the shop, she knew that, in addition to the early decoration, equipment investment, every day as long as the shop opened the water and electricity costs will spend more than 60 yuan, as well as labor, raw materials and other costs. Free rent policy, so that the first business to enjoy a great operating advantages."If you have to pay the rent, you have to sell at least two more birthday cakes a day to earn them back."The daily customer volume of the cake shop is not stable, and the profit mainly depends on the birthday cake. The profit of selling cakes and bread is very thin, in order to accumulate contacts and establish customer loyalty and consumption habits. Wu Yu also often sends products and preferential activities through WeChat Moments and social media. Now, in addition to the residents of the surrounding community, there are many residents of the old town on the other side of the Xun River."I am quite confident about continuing to make the store bigger and stronger."Wu Yu said that after a year or two of hard work, her perception of the market direction, the ability to control consumer demand is stronger, coupled with the support of the government and relevant departments, the operation of the store is increasingly prosperous."Even if I collect the rent in the future, I will continue to open the store here, because the surrounding facilities are better, and the occupancy rate of the surrounding community is getting higher and higher, and then our revenue capacity will be enough to offset the pressure of the rent."She said. Source: China Youth Daily, November 12,2021, edition of page 08

2021-11-12	Linze county, Gansu province to "volunteer red" become the most warm civilization background group linze county let "volunteer red" become the most warm civilization background Zhang Jianwei correspondent Ma Yuanyuan) to promote linze county national civilized city, give full play to the communist youth force and commandos, corps of Gansu Zhangye linze county focus, to develop urban and rural environmental health regulation, rural "three left-behind" and most needy people care action, guide the young volunteers to participate in, from "want me to do" to "I want to do", "volunteer red" become flowing scenery line, gathered into civilization to create majestic power."I often see volunteers wearing red vests picking up garbage and cleaning up the debris in the corridor. I also want to sign up as a volunteer to participate in the volunteer service activities in the community and contribute to the construction of the community."County vocational education middle school league member Yang classmate said. Cities are our homes, and everyone is a participant and a beneficiary of building a national civilized city. The Communist Youth League County Party Committee actively guides the youth league members and the community residents to devote themselves to the work of building a national civilized city as masters, and forms an atmosphere of "everyone participates, everyone observes, everyone contributes and everyone shares". Young volunteers and community staff, party members and cadres together on the area of health dead corners, flower beds, weeds, piles to thoroughly removed, young volunteers shuttle in the area courtyard streets, become a different landscape in the community. We thoroughly clean up the scattered garbage and weeds in the green belt on both sides of the road under the jurisdiction, clean up the small advertisements, "psoriasis" and other corridor, for the community and stores individual parked vehicles to communicate, and guide the owners to standard parking. After everyone's hard work, the garbage accumulated on the corridor road disappeared, the travel road became clean and bright, disorderly parked vehicles orderly, the area has taken on a new look. More than 100 volunteers participated in this volunteer activity, cleaning up more than 50 disorderly piles, more than 40 small advertisements, and more than 70 garbage and weeds. League members, youth together, clean the community ground health, cleaning the size of the publicity board, at the same time set up the national civilized city to create publicity points, improve the awareness rate of the work, let the new wind of civilization "blow up", residents' life "beautiful up". Love relay for left-behind personnel "warm" in order to further implement the rural "three left" personnel and most needy people care service action, combined with rural revitalization of "post big training, business big tournament", "I do the practical work for the masses" practice, actively mobilize the youth league organizations, youth league MoPai, clear base, establish parameter, organization county-rural youth cadres, village youth corps branch secretary, young volunteers household visit "three left" personnel and the most needy, find out base, do relatives to, health, major difficulties, demand hope "four clear". Combined with the practical activity of "I do practical things for the masses", In line with the principle of "moving the true feelings, thinking of practical measures, and doing practical things", Starting from the urgent needs of the "three left-behind" people and the extremely poor people, Combined with the traditional Double Ninth Festival, Together with the county Women's Federation and the county emin community held the urban and rural care for the elderly with the theme of "caring for the elderly and accompanying the Double Ninth Festival", We visited more than 100 old people, And for the elderly people present to provide a love righteousness scissors service, Talk to the old man, Combined with the "post training and business competition" activities of rural revitalization, Young cadres enter villages and households to publicize care policies and implement various assistance, Through pre-holiday sympathy, temporary assistance, subsistence allowance and other means, To ensure that the "three left-behind" people and the people in extreme poverty eat and wear warm clothes, Focus on solving their practical difficulties, Take concrete actions to enhance the people's sense of gain, happiness and security, To cultivate young cadres' professional quality and their ability to serve the masses, . Youth power for the epidemic prevention and control "points" in the face of the sudden outbreak of new champions league, linze county for the county youth league organizations at all levels and the general youth initiative, at that time, the youth league organizations at all levels, the field youth response to mobilize and call, form a youth commandos, recruit youth volunteers, actively raise supplies, publicity devoted to the outbreak on a line. The Communist Youth League County Party Committee issued three Linze County youth epidemic prevention and control proposal, youth volunteer reserve recruitment order and health volunteer reserve recruitment order through its wechat official account, and more than 300 volunteer applications were received in just three days. One by one, the "red vest" in the personnel investigation, information entry, bayonet on duty, to maintain order, social publicity and other important posts shine and fever.

2021-11-09	Group in Gansu province linze county epidemic prevention and control do not relax civilization and city momentum foot group in Gansu province linze county epidemic prevention and control do not relax civilization and city momentum foot JianWei correspondent Zhang Yuehua) since the epidemic prevention and control work, around the civilized city create goal, Gansu province Zhangye linze county adhere to the outbreak and the city two line war, should, frequency resonance, through the theme education, thought lead, volunteer service. Publicity and guidance and other ways, called on the majority of young people for the county civilized city to create work into a bright youth color. Overall planning, compaction of the responsibility line. We should give full play to the role of the Communist Youth League as a bridge, and organize and mobilize all grass-roots youth League organizations and young Pioneers organizations to vigorously build a civilized city in the fight against the epidemic. The grassroots youth corps committee, non-public and social organizations and less brigade, common responsibility, to implement epidemic prevention and control and civilized city to create work task, build up the "linkage, block, full" working situation, form everyone involved in epidemic prevention and control, everyone participate in the civilized city to create good atmosphere, promote the epidemic prevention and control and civilized city to create "to" "hands hard". All the staff to launch, to build a total line of civilization. We will actively mobilize and extensively publicize the construction of spiritual civilization, timely push information about the knowledge and meeting of civilized cities, and organize youth league members to conduct civilized persuasion, civilized patrol streets, "volunteer cleaning", and help pair communities to carry out environmental remediation and other volunteer service activities in environmental remediation and epidemic prevention and control. During the epidemic period, more than 100 youth members took the lead in the promotion of civilized city, the socialist core values, civic convention, city knowledge and knowledge to families; while on duty to help residents clean the building, dump garbage, buy and send, built a "love civilization line" of mutual help and clean their homes. Extend services to light up the "fight against the epidemic" line. Focus on the main responsibilities and main businesses, Make full use of online platforms such as "Youth Learning", "Tuan Tuan Micro Class" and "Red Scarf Love learning", We will improve the ideological and political guidance of young people, at the same time, Using online themed team lessons, QQ group and other online platforms, Combine scientific epidemic prevention class and city creation knowledge with online ideological and political class learning, Guide the young Pioneers and youth league members to actively create a series of paintings, essays and video works, such as epidemic prevention and control, the creation of a civilized city, ecological and environmental protection, and network security, Carry out the " small hand, big hand, Civilization goes together "," Strive to be the small guard against the epidemic ", Effectively encourage the whole society to actively participate in epidemic prevention and control and create a good atmosphere of civilized city creation.

2021-11-12	Jiu jiang district of Anhui province "youth home" held "save around" training in Anhui province jiu jiang district "youth home" held "save around" fire safety and facilities control training Ji'an-wei zhang correspondent, Yang Xiaohui check WeiTing) in order to further strengthen fire propaganda and education work, enhance the community volunteer fire safety disposal ability, recently, Anhui Wuhu Jiujiang district "youth home" held "save around" fire safety and facilities control training. Training meeting, young volunteers combined with reality, for the family fire basic knowledge, building rules, daily fire safety inspection, found rectification fire hazard, how to save escape, organize evacuation knowledge focused and training, and the demonstration of the use of all kinds of fire control equipment, let every attending volunteers and micro grid member can master fire prevention skills and escape skills such as common sense, consciously attaches great importance to fire safety, keep on high alert. Through the fire knowledge training, participants have a deeper understanding of the importance of fire safety, further enhance the district residents and volunteers fire safety awareness, improve the fire prevention ability in real life and fire escape self-protection ability, for peace civilization Wuhu created a good atmosphere.

2021-11-11	Fenghua district of Ningbo, Zhejiang province appointed national moral model for youth XuanJiangTuan branch of Ningbo fenghua district party committee appointed national moral model for youth XuanJiangTuan lecturer- (zhongqing net in the popular newspaper reporter li) recently, the communist youth league of Ningbo fenghua district party committee held youth theory member appointment ceremony, officially hired the national moral model Cui Yi for fenghua district "source youth branch" invited lecturer."This letter of appointment is both an honor and a mission. I want to play a role of good moral model, drive more people around to join the preaching team, preach the party's line, principles, policies, efforts to speak good Chinese story, Zhejiang story, fenghua story, with common ideal faith solidarity, inspiring, inspiring, lead more youth dedicated youth, compose life."Cui said. Cui is a native of Fenghua District, studying at Guilin University of Electronic Technology. She tried eight times to protect her classmates. In November 2021, she was named the 8th National Moral Model. In the recording of the micro group class "youth responsibility", she called on: " the youth members, let us live up to The Times, live up to the time, with the responsibility to fill the picture scroll of youth, to create a beautiful and brilliant life!"According to statistics, up to now, the Youth League Fenghua District Committee has more than" source "," piece feather " and other more than 200 young speakers, a total of online + offline, TV + network and other forms of publicity activities, the audience of nearly 100,000 people, all-round to promote the popularization of youth theory publicity, improve the coverage and influence of communication.

2021-11-11	Lanzhou Xigu District, Ni Jiayi, a young pioneer of Class 4, Grade 6, in the Third Primary School of Welfare Road, Xigu District, Lanzhou city, participated in the election and was successfully elected as the new Young Pioneers team leader."I will be strict with myself, try my best to serve the students, and strive to be a good bridge and link between teachers and students."In her election speech, Ni Jiayi said confidently. Shortly after the beginning of this semester, Welfare Road No.3 Primary School issued a notice for the whole school, Ni Jiayi saw that the opportunity is rare."This is a good opportunity to show myself and serve my classmates. I will try my best for it."With the encouragement of her parents and teachers, Ni Jiayi immediately signed up and began to actively prepare for the campaign. Design your own campaign posters, talent posters, show their talents, interests, hobbies, use the holidays to write a campaign speech, in addition, and actively learn from the young pioneers, deepen the understanding of the young pioneers organization... since the campaign, Ni Jiayi go all out. On the day of the campaign, Ni Jiayi successfully completed the written test and the interview, and also sang a song called "The New Era of Good Youth", which won the applause of the Young Pioneers' representatives. In the final voting session, she was recognized by the young Pioneers representatives of the whole school and was successfully elected."In recent years, with the continuous strengthening of the integration of league and education, the specialization of the young Pioneers 'activities has been continuously strengthened. By strengthening the sense of ceremony of the young Pioneers' activities, the sense of honor of primary school students and the sense of belonging to the young Pioneers have been effectively enhanced."Welfare three small brigade counselor Shi Xin said. Before that, the leaders of the three primary schools were appointed by teachers. Due to the lack of sense of ceremony, the leaders's sense of honor is not strong, and the recognition of students is not enough, which affects the healthy development of the young Pioneers organization. Since 2019, Xigu District has vigorously strengthened the cooperation between the youth league and education, and established the Education Youth League Working Committee, which is specially responsible for the team work of the education system. At the same time, a targeted and operational league and education cooperation mechanism has been established to promote the standardization and professional construction of the Communist Youth League and the Young Pioneers in the school. Before Li Yanlin, the secretary of the Xigu District Education Youth League Working Committee, he served as the party secretary of a middle school for many years."Since the promotion of league education cooperation work, the school league, team work has been more solid, the level of professional work is also higher, young students have a personal sense of gain."Before the district held the communist Youth League organization related meeting, are the school youth League committee secretary attended, now are in charge of the principal; before the school and the young pioneers work and no effective supervision mechanism, now, by the league organization and education administrative department regularly supervise the school work, the school communist Youth league work also into the party building assessment... after the formation of the education cooperation mechanism, from the education bureau to the school, the work of the school, these Li Yanlin see in the eyes, happy in the heart."Due to the lack of an effective working mechanism, the Communist Youth League did not deal with the education department before, and some schools did not have a good understanding of the league and team activities, believing that it would affect the normal education and teaching, and the league and team work and school education have two skins."In the view of He Lifeng, secretary of the Xigu District Party Committee of the Communist Youth League, the effective operation of these mechanisms has deeply integrated the organization work of the Communist Youth League and the Young Pioneers into the whole process of school education and teaching, and played an important role in the work of moral education. Riding the east wind of the county Communist Youth League reform, Xigu District to further strengthen the system construction, now, the education system league, team work by the education League working committee of the specific responsibility, the district Communist Youth League and young Pioneers work deployment, by the youth League district committee arranged to the education League working committee, education League working committee for all levels of schools to grasp the implementation. At the end of each year, the Communist Youth League Xigu District Committee and the Xigu District Education Bureau form a special class to assess the work of the education Communist Youth League working Committee and the school youth League and team, and make comments and evaluation. Since the establishment of the league and education cooperation mechanism, some positive effects are emerging. In promoting the implementation of the theme league day and team day, Li Yanlin included "youth learning" into the league and team theme activities every Friday. The teacher led the students to carry out learning together, which not only ensured the practical development of learning, but also expanded the coverage and improved the learning effect."Since the mechanism guarantee, the previous coverage rate has increased from only about half to 80 or 90%, and the learning effect is much better."Now, the school system" youth learning " has become the vanguard of this work in Xigu District. In addition, the assessment and supervision has also formed a complete set of mechanism. The League Xigu District Committee attaches great importance to the information exchange of daily work. Through regular distribution of work letters and grassroots highlights on the new media platform, it builds a platform for mutual learning and mutual learning, so that everyone can improve their work ability and promote the overall level of the work of the school league and the team. In Liuquan Middle School of Xigu District, with the deepening of the youth league education and cooperation, the work of the Communist Youth League and education and teaching complement each other, and the education work of the school presents a new atmosphere. In this year's concentrated party history learning education, Liuquan Middle School Youth League Committee carefully organized a set of "red stories", through audio, video, comics and other multimedia means presented, with the form of storytelling, the knowledge of party history interspersed, deeply students like."We took the initiative to change the traditional way of teaching party history knowledge through lectures, and explored the form of learning party history popular with young students, which has achieved very good results."Yao Xingxing, secretary of the Youth League Committee of Liuquan Middle School, said that this was inspired by the party history learning experience of other youth League organizations. In the work brief issued by the Tuan Xigu District Committee, she noticed that some schools explored this model well, so specially learned from the implementation in our school."League and education cooperation is a systematic project. We focus on the overall goal of moral education, combined with the current goals and requirements of the county Communist Youth League reform, strengthen the top-level design, pay attention to grass-roots exploration, practice has proved that this cooperation mechanism is conducive to playing the advantages of league and education, and help to jointly cultivate young people in the new era."He Lifeng said.

2021-11-10	Shaoguan county, Guangdong province to carry out rural youth electricity training in Shaoguan county, Guangdong new youth electricity training rural youth correspondent Ji'an-wei li) recently, Shaoguan county, Guangdong xinfeng county in the county party committee party school in 2021 new venture "youth power rural revitalization" rural youth electricity training, the activity attracted the county township more than 50 returning youth, grassroots cadres to attend. Training course site. Correspondent for figure training meeting, Guangdong rural e-commerce association teachers around electricity development, agricultural marketing skills, electricity field, etc, at the same time, hope that through electricity knowledge training, broaden the horizons of the masses, promote new venture electricity industry and agricultural products sales basic industry, broaden the sales channels of agricultural products, help xinfeng agricultural products through electricity channels "going out". In addition, the class also expanded the TikTok shooting skills and the mode and method of realization. Training course site. This training has deepened the students' understanding and understanding of the development of rural e-commerce, cultivated and expanded the rural e-commerce talent team, expanded the new ideas of the development of rural e-commerce, and provided a strong talent guarantee for the sound and rapid development of e-commerce in Xinfeng County.

2021-11-08	Chongqing Fengjie County "Youth Home" Yue reading station was officially established "Youth Home" Yue reading Post station. Correspondent for figure China youth network Beijing on November 8 (xinhua Zhang Jianwei, correspondent Zhang Yuehua) in accordance with the "where the youth, youth league is where" requirements, actively adapt to the change of the youth gathering, distribution and communication, vigorously promote the communist youth league organization form, working force, service around youth realize tangible, daily, recently, Chongqing fengjie county committee for excellence academy- "youth home" reading post licensing, post was formally established. Excellence Academy "Youth Home" Yue Reading Station position collection of youth public welfare reading, family education lectures, mental health counseling, friendship and other activities, the environment is spacious and open, comfortable and leisurely. Fengjie county is committed to the "home of youth," reading station into the communist youth league to actively adapt to the new era of the distribution of the youth gathering characteristics and the needs of the diversified form of youth work organization, online service services, "youth" youth home "youth" position layout, build the youth around the communist youth league "store". It is understood that the "youth home", as a grass-roots position to contact and serve the youth, and constantly play its role in serving the youth groups, and has achieved certain results. The establishment of the Excellence Academy- "Youth Home" Yue reading station, for Fengjie County youth work to open a new window, but also for the majority of young people life to create a new platform. The relevant person in charge of the Youth League Fengjie County Party Committee said that it will further promote the Fengjie medium-and long-term youth development plan, the county Communist Youth League grass-roots organization reform double pilot work. And will rely on group representative contact station, business circle, community, park, schools and other places and space to build services- "youth home" reading post, talent station, happiness station, the station, red scarf station, etc., make Fengjie youth home "post" brand, provide more meet the needs of contemporary youth cultural products, meet the needs of Fengjie youth reading and personal development, on the basis of forming national reading normalized learning, discussion and communication atmosphere, through the communist youth league organization service, contact youth "the last kilometer".

2021-11-08	Jianwei correspondent, Liang Zuer, Li Excellence) On November 5, the 2021 "Qingma Project" Youth League cadre training class was held by the Shaoguan Xinfeng County Party Committee in the Party School of the County Party Committee. Xinfeng County each town (street) youth league (work) committee, village youth league cadres, county straight youth league organization, middle school youth league committee, "two new" youth league organizations, a total of more than 60 youth league cadres participated in the training. Relevant leaders of the Communist Youth League Municipal Party Committee were present to guide. Training course site. Correspondent for figure during training, head of the party conveyed the new venture 14th party spirit, around the responsibilities of the communist youth league mission, infrastructure, work ideas, and comprehensively introduces the communist youth league reform pilot work deployment progress, focus on strengthening the basic team basic principles, working methods, organizational procedures and practical experience, designed to help grassroots cadres skilled business, improve ability, master skills, become the communist youth league work as soon as possible. Training site. The training also invited the relevant person in charge of the youth League municipal committee group publicity department on the village (residence) league branch construction, three meetings a lesson system, youth home construction and operation, smart league building system operation and other aspects of business training, to further enhance the comprehensive ability of league cadres. The students have said that the training schedule is compact, the learning content is rich and comprehensive, while learning new knowledge and improving the theoretical level, it also deepened the work exchange between the grass-roots league organizations, truly achieving the "ideological charging, action refueling". In the next step, the Communist Youth League County Party Committee will take this training class as an opportunity to continue to improve the professional quality and comprehensive ability of the county grass-roots youth league cadres, and vigorously promote the pilot reform of the national county Communist Youth League grass-roots organizations.

2021-11-06	Group muping district party committee to deepen the "partner plan" construction "youth community" group muping district party committee to deepen the "partner plan" construction "youth community" JianWei correspondent Li Jin) since this year, the group of Shandong Yantai muping district party committee continue to deepen the youth social organization "partner plan", by strengthening the introduction, cultivate organization leaders, build resources docking platform, promote social organizations to participate in the "youth community" pilot to create, actively into the community grid governance. I participated in the hygiene cleanup. Together with the Muping District Firefly Love Volunteer Service Team to carry out environmental sanitation clean-up and remediation activities in the old community, focusing on the investigation and rectification of health dead corners and health weak spots, effectively improve the health conditions of the old community, and create a beautiful and clean living environment. Care for children and I contribute. Joint muping seven color light minors rehabilitation center implementation "light star light and love" care children growth project, organization love volunteers for special teenagers to study articles for daily use, a youth volunteers pair support troubled children, in the material and spiritual level to give dual care, transfer love, FuZhi FuZhi. I care for the elderly first. Organize young volunteers to carry out warm activities for the lonely elderly in Muping District Tonghai Road community, to help the lonely elderly living in difficulties to clean up, chat with the elderly, do a rich delicious food, to send the elderly to the warmth of family. I help with environmental remediation. All volunteer service teams were launched to visit the Qinshui River Park to carry out environmental protection volunteer service activities, focusing on cleaning up the sanitary dead corners, road leaves and garbage in the green belt. At the same time to the past masses of civilized publicity, advocating environmental health, for the general public to create a clean environment.

2021-11-04	Guangxi Hechi city donglan county: make points into the league become "tide" up Guangxi Hechi city donglan county: make points into the league become "tide" green net reporter Xie Yang) since this year, the Guangxi donglan county combined with the national county communist youth league organization reform, in the county national middle school set integral system into the league work pilot, promote the league process is quantifiable, evaluation, inspection, make points into the league become "tide". Up to now, 92 students have joined the league through the points system. The pilot work establishes the principle of scientific evaluation by strengthening the "top-level design". Focus around league rules members shall have the basic conditions and the basic obligations, according to the basic performance of "politics, character, pioneer, discipline", positive incentive bonus "happy dedication, comprehensive evaluation", reverse deduction score rules for quantification, strict positive incentive bonus and reverse supervision deduction standard, the reality of the league activists into concrete operational score "hard index". The "points system" implements the principle of points accumulation, consisting of basic score, positive incentive plus points and reverse supervision deduction points, namely "total score = basic score + positive incentive plus points", points accumulated to 80 points is given to "red star", less than 60 points is bright "yellow star". The setting of the evaluation standard increases the proportion and weight of the "league class", "red classroom", "youth learning" and "volunteer service" set up by Donglan County National Patriotism Education Demonstration Base, strengthen the ideological and political education of activists in the League, and realize the differentiation of common standards and individual rules. Up to now, the pilot has accumulated 1,248 class hours of league classes, and the average completion rate of youth learning has reached 393%. More than 200 student league members and league activists have participated in off-campus practice volunteer service activities such as epidemic prevention and control, building a civilized city, and learning from Lei Feng. The "point system" implements the strict reverse supervision of the "one-vote veto" system for the league students who have problems with their ideals and beliefs and do not abide by the law. And adhere to the principle of "spiritual reward first", strengthen the positive incentive, give full play to the "points system" of the "baton" role, effectively encourage the league members to catch up with, strive to be a pioneer. The pilot combined with integral system into the league of the evaluation results, select typical tree, selected advanced star, civilization star, progress star, discipline, contribution, excellent star field star member, and as an important reference of the "May 4 th" recommendation, let members always keep enterprising, bear as, were the first to fight for their superior state of mind, continue to enhance the member's advanced nature and sense of glory.

2021-11-05	"Wan shan youth care plan second station" heart troubled children "wanshan youth care plan second station" heart troubled children, warm heart network Beijing on November 5 (reporter Zhang Jianwei, correspondent liu � Z) to earnestly "wanshan youth care plan" follow-up work, the party committee and government care and the social from all walks of life love to the heart, recently, group of Guizhou wanshan district joint Guizhou high-speed group operating division Tongren center love team into the home, Dan Yuan, dragon, dragon, south yi relocation community visit condolences activities. During the visit, the leading team members of the Communist Youth League District Committee and the enterprise love team were divided into five groups to go into the five resettlement sites to understand their life and learning situation with the troubled children and their families in detail, and sent the children school bags, books, colored pens and other school supplies. The accompanying volunteers paired up with the children on the spot, and publicized the youth psychological counseling hotline 12355 to the children. The condolence group also talked with the left-behind children as a "bosom sister", encouraging the children to move forward bravely and strive to be the pillars of the all-round development of morality, intelligence, physical education, beauty and labor. Accompanied by community volunteers, enterprise love team unanimously said: " looked at the children get the new bag happy appearance, feel your efforts is worth it, then I will be more careful investigation a baseline, understand the needs of the children, pay attention to the children's mental health, the family conditions difficult left-behind children to register one by one, to ensure that the party and the government care to bring more difficult children, let them feel the warmth and hope."In the next step, the Wanshan District Committee will continue to do a good job of youth care work, strengthen volunteer training, develop and expand the volunteer team, promote the" youth care plan " in Wanshan District to further develop, go deep and solid.

2021-11-11	Group of Anhui jin district party committee to help youth solve the problem of "urgent sorrow" in Anhui jin district held about "youth is you" youth 2021 Ji 'an-wei zhang correspondent Zhang check WeiTing) recently, the league of Anhui Lu' an jin an district party committee joint district straight committee, district women's federation held "youth about edge is you" youth fellowship activities, from OuZhi units and township street, a total of 40 outstanding single youth to attend activities, straight committee, TuanOuWei, district women's federation head to attend the activities. Activities with "youth edge is you" as the theme, to "relaxed, romantic, warm" as the tone, by holding "flour", "you draw," I guess "," you five cents, together is a " rich variety of forms of game, help the guests quickly break the ice, and began to know each other, recognize, familiar, open a pleasant, warm romantic journey, in this harvest friendship, germination of love. This activity is rich "I do the practical work for youth" practice of concrete measures, is the implementation of Jinan long-term youth development planning effective path, aims to further condensed youth, service youth, do a good job of youth related, build a platform power show youth good spirit, guide the youth set up a positive marriage view. In the next step, the Communist Youth League District Committee will continue to carry out various forms and rich contents of dating activities based on the party history learning and education, effectively help solve the problem of "urgent difficulties and sorrow" of young people, and constantly improve the happiness and belonging of young friends.

2021-11-10	Du district in Anhui province youth rule of law publicity and education activities "escort youth" double minus "method" Huaibei city, Anhui province du district youth rule of law publicity and education activities launch ceremony Zhang Jianwei correspondent, Ding Jianan check WeiTing) recently, "escort youth" double minus "method" youth rule of law publicity and education activities launch ceremony held in du huaihai east road school, OuZhi each relevant units responsible for comrades, "escort youth" volunteer service representatives, some school staff representatives, student representatives, a total of more than 90 people to participate in the activity. During the activity, the person in charge of the Duji District Public Security Sub-bureau read out the "Duji District" escort youth "double reduction" and "law" "legal publicity and education activities implementation plan". Leaders attending the meeting presented flags to the "escort youth" legal publicity and education volunteer service team. The participants observed the demonstration lesson of the rule of law brought by the volunteer service group of the three traffic police brigades. This "escort youth 'double reduction' has 'law" "law publicity and education activity is not only an important measure to implement Xi Jinping's thought on the rule of law, and build a safe Du ji, but also a powerful carrier to carry out legal education for teenagers under the" double reduction " policy, and guide the healthy growth of teenagers. Next, TuanOuWei will take the rule of law publicity and education activities as an opportunity, combined with the "I do something for the youth" theme practice, relying on the nine rule of law publicity and education services, in the primary and secondary schools around the legal propaganda, net, drug control, traffic safety, electric fraud, campus illegal lending, bullying, school violence, mental health and other innovation to carry out the youth rule of law publicity and education work.

2021-11-09	Shixing county, Guangdong province to carry out environmental popularization and minors ideological and moral construction campaign Shixing county, Guangdong province to carry out the "carbon peak, carbon neutral" environmental popularization and minors ideological and moral construction campaign launch ceremony jianwei correspondent Zhang Yuehua) recently, Shaoguan city, Guangdong province Shixing county "carbon peak, carbon neutral" environmental protection popularization and minors ideological and moral construction campaign launch ceremony was held in danfeng primary school. The Communist Youth League Municipal Party Committee, the County Party Committee of the Publicity Department, the Communist Youth League County Party Committee and the Education Bureau jointly opened the "carbon peak, carbon neutral" environmental protection popularization and minors' ideological and moral construction publicity activities launch ceremony. The picture shows the launching ceremony of "carbon peak, carbon neutral" environmental protection popularization and ideological and moral construction of minors. TuanShiXing county for figure launch ceremony, the county party committee, propaganda department minister jun-feng li said that the green economy is the trend of human development, is also to maintain the economic sustainable and healthy development of the latest power and energy, to borrow the flow campaign as an opportunity to actively guide students and the masses firmly establish a low carbon consciousness, actively participate in shixing county green development strategy. We should dare to be the propagandist of environmental protection popularization, encourage and mobilize people around us to actively participate in the practice of carbon peak and carbon neutral, and make new and greater contributions to the high-quality development of Shixing. The picture shows the launching ceremony of "carbon peak, carbon neutral" environmental protection popularization and ideological and moral construction of minors. Tuan Shixing County Party Committee for the map, as it is understood, The "carbon peak, carbon neutral" environmental protection popularization and minors' ideological and moral construction publicity activities, Sponsored by the Office of Shixing County Spiritual Civilization Construction Committee and the Shixing County New Era Civilization Practice Volunteer Service Corps, Organized by Tuan Shixing County Committee, Co-organized by Shixing County Education Bureau, For the county primary and secondary schools, county central communities, related enterprises, The activity cycle is from November 2021 to March 2022, Aaims to promote the popularization of "carbon peak, carbon neutral" environmental knowledge in the jurisdiction, Let the environmental protection knowledge and skills as well as the ideological and moral construction of minors related policies and knowledge into schools, into communities, into enterprises, Form a good situation in which everyone knows, everyone participates and everyone is responsible.

2021-11-06	Fengjie County, Chongqing, has held a practice and experience activity to experience the charm of "Kui Embroidery" and inherit the "intangible Cultural Heritage" culture. Correspondent for figure China youth network Beijing on November 6 (reporter Ji'an-wei zhang, correspondent yue-hua zhang) recently, the Chongqing fengjie county organized 19 young pioneers to Chongqing arts and crafts master, kuizhou embroidery inheritance Shi Shenglan studio, develop the feeling "kui embroidery" charm heritage "intangible" cultural public welfare practice experience. Young pioneers came to Shi Shenglan kui embroidery studio, walls hung around all kinds of kui embroidery works, sitting in a wheelchair Shi Shenglan began to tell the story of her study hard kui embroidery, countless times, countless times want to put down, but constantly forward in countless times, Shi Shenglan from drawings, line, embroidery, mounting to the young pioneers explained the birth of kui embroidery. Through the practice of Shi Sheng kui embroidery studio, the young pioneers to further deepen the interest in kui embroidery culture, let the children closely appreciate the "intangible" culture kui embroidery works, unveiled the mystery of the kui embroidery skills, to further enhance the youth of excellent traditional culture pride, let kui embroidery culture better carry forward and inheritance.

2021-11-05	Fengjie in Chongqing held "green orange love love TA" dating fellowship activities in Chongqing fengjie "green orange love love TA" friendship activities jianwei correspondent Zhang Yuehua) to effectively serve the youth realistic demand in dating, guide the youth set up the correct dating, recently, the Chongqing fengjie county, fengjie county women's federation jointly held the fourth "green orange love love TA" dating fellowship activities. More than 60 single young people from various units in the county gathered together to talk about their youth and friendship. This activity set up three chapters of "orange love first encounter", "orange love first acquaintance" and "orange love true confession", many male and female guests through "hand in hand and love", "hug" and other interactive games to open their hearts and close the distance. The atmosphere continued to heat up in the interesting and lively game, and in the end, a total of 5 male and female guests were successfully selected. It is understood that this friendship activity aims to help young groups to enhance communication, and build a platform to open their hearts, show themselves, and look for a good relationship. In the future, TuanFengjie County Committee and Fengjie County Women's Association will continue to innovate the form of activities, carefully plan fellowship activities, accurately provide fellowship and dating services, and promote the formation of a multi-level youth "circle of friends" with diversified participation, joint construction, co-governance and sharing.

2021-11-04	Youth Daily reporter Li Chao) Today, Qidong City, Jiangsu Province held a new generation of entrepreneurs symposium. The new generation of 12 entrepreneurs in Qidong combined their own realities, exchanged views on innovation and entrepreneurship, enterprise management ideas, and difficulties in development, and put forward suggestions on business environment, talent introduction, trade services, agricultural development and other aspects. Qidong Gong Xinming, deputy secretary of municipal party committee, pointed out that the organization held "from generation to generation qi qi qi" qidong new generation entrepreneurs symposium, the purpose is to release a clear support the healthy growth of new generation entrepreneurs strong signal, inspire the city new generation entrepreneurs vigorously carry forward the entrepreneurial spirit, in the modern new journey to innovation, vigor, and first-class. Learned, to vigorously cultivate "zhang type" entrepreneurs, continue to strengthen the new generation entrepreneurs education guide, group qidong municipal party committee launched "qi qi and share" new generation entrepreneurs training project, plan to face in qidong innovation entrepreneurship outstanding new generation entrepreneurs, returned young talents, new young professional farmers, etc., 1 annual entrepreneur summit BBS, 2 concentrated theoretical study, 3 times out project, four discussion chat activities.

2021-11-04	Beijing, Nov. 4 (Anhui) - -Zhang Jianwei, correspondent Guo Chao, Wei Ting) " Take a look, have a look, charity sale, fresh vegetables! Don't miss it when passing by!"The Chrysanthemum Town, Xinzhuang Village, Sipu Town, Suixi County, Huaibei City, Anhui Province, echoed with the shouts of young volunteers, and passers-by involuntarily looked at the stalls. It turned out that this is a special "grocery store" at the Sipu Chrysanthemum Festival- -recently, Suixi County Party Committee, Huaibei City, Siping Town Youth League Committee and Suixi County Celery Planting Youth Club jointly carried out a charity sale. Early in the morning, the charity sale was held in the temporary booth of the chrysanthemum Festival agricultural products exhibition area. Members of the youth club and young volunteers held vegetables and enthusiastically shouted through the street, becoming a beautiful scenery of the chrysanthemum Festival. Many pedestrians joined the "show love and happy shopping", and the purchase scene was extremely hot, and in less than half a day, the vegetables at the charity sale booth were sold out. Youth associations affiliated to the Communist Youth League are a vivid practice of innovating the grass-roots organization form of the Communist Youth League, which provides an organizational support for enhancing the ability of the Communist Youth League to lead and gather the youth and contact and serve the youth. It is reported that the vegetables in the charity sale were provided free of charge by sui Xi County Celery Planting Youth Club, a youth club affiliated to the Suixi County Youth League. The charity sale lasts for 3 days, and a total of more than 1,000 yuan is raised, which will realize the dream of the "micro wish" for the poor teenagers in Sipu Town, so that the love can be continued. The charity sale has received attention and support from all walks of life. Many people also "introduced" people around them to support the charity sale after buying vegetables. The people said that the activity is very meaningful, which not only bought fresh vegetables, but also gave their love. In the next step, Sui Xi County Party Committee will continue to promote the pilot reform of grassroots organizations of the Communist Youth League at the county level, take practical measures, emphasize practical results, do practical things, actively integrate social resources, innovate the form of serving young people, transfer the positive energy of youth, and promote the activity and role of youth social organizations affiliated to the Youth League.

2021-11-02	"Jinhui elders" volunteer service into the Anhui Wuhu Jiujiang community "Jinhui elders" volunteer service activities into the Anhui Wuhu Jiujiang community jianwei correspondent Yang Jie check WeiTing) in order to further carry forward the Chinese nation filial piety to respect traditional virtues, recently, the group Wuhu of Anhui province Jiujiang district party committee joint civil affairs bureau, youth volunteer association held in spring community medical center "Jinhui elders" volunteer service series of activities. Medical volunteers from Wuhu First People's Hospital and Maternal and Child Health Care and Family Planning Service Center carried out free medical consultations for the elderly in the community. Set up an inquiry desk to popularize the knowledge of drug safety to the elderly, patiently answer the questions raised by the elderly, help the elderly to understand their own health status, and receive and serve more than 100 elderly people on the spot. Volunteers from working in the bank introduced the common types and common criminal methods of telecom fraud to the elderly, combined with the typical fraud cases, targeted to publicize telecom anti-fraud knowledge to the elderly, improve the elderly awareness of fraud and fraud. In order to strengthen the publicity of health knowledge and health services for the elderly, health services and preferential policies, young volunteers distributed leaflets to the elderly, read the policies to the elderly, and sent the policies, knowledge and services to their families and to the elderly. The respect month series of activities from the care for the old man life daily, put the "I do the practical work for the masses" concept into practice, with breadth have temperature volunteer service activities increase respect for the elderly love, help the elderly propaganda, really do good for the elderly, for the formation of the "love, respect, help the elderly" good atmosphere.

2021-11-03	Chuzhou city, Anhui Dingyuan green league "hope" in Anhui Dingyuan green league "hope" built wei correspondent wu � S check WeiTing) recently, the group of Chuzhou, Anhui province municipal party committee organization part youth league members, young entrepreneurs, etc., to Dingyuan bridge town bridge primary school, carry out the city's first green league "hope" listing and donation activities, for rural schools, four difficult families to send to love supplies and consolation money. Youth league "hope hut" is initiated by the Anhui provincial party committee, provincial youth league, group Chuzhou municipal party committee, city youth league widely launched youth league members from all walks of life, social caring people and enterprises to participate in, through donations, software support, teacher training, in the remote rural construction includes talent learning, scientific practice, sports training "hope hut", make up for the shortage of rural schools in the "second class" education. In this activity, the League Dingyuan County Party Committee held the bridge town "hope cabin" construction symposium and the Youth Federation "hope cabin" opening ceremony. At the ceremony, the Youth League of Chuzhou Municipal Party Committee, Bridge Town Bridge Primary School, the Youth League members entrepreneurs, Bridge Town, the Youth League Party Committee jointly unveiled the Youth League "Hope House", and visited the Bridge Town Hope House service position.

2021-11-03	Guangdong Shaoguan city new county to carry out the young pioneers red research activities in Guangdong province new venture to carry out the "learning party history inheritance red gene" young pioneers red research activities Jianwei correspondent Li Excellence) recently, the Shaoguan city xinfeng county united county education organization horse town central primary school, juntun primary school more than 30 young pioneers representatives to the new venture of the communist party first party branch site-juntun xiumu primary school exhibition hall to carry out the young pioneers "learning party history inheritance red gene" red research activities. Under the guidance of the narrator and teacher, the Young Pioneers visited and studied in the exhibition hall of Juntun Xiumu Primary School. Before the beginning of the activity for the Communist Youth League Xinfeng County Party Committee, the head of the Communist Youth League County Party Committee and the Education Bureau awarded the exhibition hall "Xinfeng County Young Pioneers off-school practice education Base" for the unveiling. Under the guidance of the young party members, the young pioneers began to study the red tour, a detailed understanding of the "first party branch" and the red story of the local revolutionary martyrs. Through the "Red Scarf Love learning" red q & A interactive game, to further deepen the understanding and understanding of Xinfeng red story. After the visit, the team members reviewed their entry oath and sang the team song together under the team flag. Organize the young pioneers to carry out the red knowledge question and answer game in the study room of the exhibition hall. It is reported that in October this year is the first "Xinfeng red scarf culture month", the county young pioneers organizations at all levels relying on the school, the young pioneers outside the school practice education base and other young pioneers positions inside and outside the school, organized the red scarf narrator, red scarf chapter and other series of young pioneers cultural activities. Next, the Youth League Xinfeng County Party Committee will further play the role of the young Pioneers off-school practice education base, build a "young Pioneers home", relying on the base to carry out study card, visit learning, practical experience and other activities, guide the young Pioneers to learn the history of the Party, feel the party grace, so that the red gene and revolutionary spark passed on from generation to generation.

2021-10-27	Mingguang city in Anhui province to carry out minor traffic safety campaign in Anhui province minors traffic safety campaign zhang correspondent Yang Qun check WeiTing) recently, Chuzhou, Anhui province organization youth volunteers and young pioneers on behalf of the mingguang minors traffic safety civilization campaign, better response to the call to create the national civilized city, to build a better home. On the day of the activity, the little "red scarves" came to the intersection early to publicize the importance of wearing helmets, remind them to wear good helmets, advocate civilized travel, and strive to be civilized people. Young volunteers, students' parents and students also distributed the leaflet of "Civilized Traffic, You and Me Walk together" together, calling on more citizens to work together to create a safe, smooth, harmonious and orderly road traffic environment. Finally, the traffic police also issued an initiative, hope that the general public riding electric vehicles to consciously abide by the traffic safety laws and regulations, so that safety with us.

2021-10-26	Fengyang County, Anhui province launched the "group take you visit the sea" series of activities launched the "group take you visit the sea" series of activities zhang Jianwei) "I want to buy bookshelves and many books" "I want to repair the third floor floor" "I want to buy a lot of flowers and plants dress up the campus, once into the school can smell the flowers"... in a classroom, "group take you visit the sea" theme activities, students scrambling to tell their good wishes for campus construction. Recently, the fengyang county to build 1 group-the county bureau of youth league branch, banqiao town youth league branch, county data resources bureau youth league branch, jujube lane town youth league branch to banqiao town two shop primary school "tour you" theme activities, the theme activity aims to gradually deepen the communication between volunteers and township left-behind children, cultivate teenagers reading habits and thinking way, improve logical thinking ability, let left-behind children in the emotional sustenance and comfort. More than 60 students and 13 volunteers participated in the activity, and distributed more than 100 classic books of Chinese children's literature books, and more than 200 bookmarks, notebooks, markers and other small gifts."Tuan Tuan takes you to visit the sea" series of activities is the Tuan Tuan Fengyang County Party Committee planning, by the Communist Youth League County Party Committee organized a group to implement the implementation of the theme activities, the choice of Banqiao Primary School as the first stop of the activity, there will be a series of related activities in the future. Bring books into the campus, let the students develop reading habits.

2021-10-26	The opening ceremony of the first batch of young Pioneers campus practice education bases was established in Xinfeng County, Guangdong Province. Correspondent for figure China youth network Beijing on October 26 (xinhua Zhang Jianwei, correspondent, Liang Zuer Li Excellence) to comprehensively strengthen the new era of the young pioneers, foster the young pioneers social practice carrier, actively cooperate with the national "double reduction" policy goal, Guangdong Shaoguan xinfeng county united county education bureau, county committee fully tap social resources in various fields, jointly selected the first local suitable for the young pioneers campus practice base, for the young pioneers labor practice, patriotism education, popular science education practice platform. Recently, the opening ceremony of the first young Pioneers outside the school practice education base was held in Xinfeng County Museum, and 30 young pioneers attended the ceremony. Red scarf narrator explains the red story of Xinfeng revolutionary martyr Li Renyu. During the activity, the leaders of the Communist Youth League Party Committee, the county education bureau and the county museum jointly inaugurated the first young Pioneers after-school practice education base- -Xinfeng County Museum. Review after the team oath, wearing a bright red scarf of the young pioneers successively to li renyu square and county revolutionary history exhibition hall to visit learning, listen to the "red scarf narrator" on new revolutionary martyrs li renyu deeds, in the "red scarf party history" interactive quiz session, the young pioneers have incarnation little teacher, actively raise your hand to answer, tells the history of the inspiring story."Red Scarf Study the history of the Party" interactive question and answer session. It is reported that the first batch of young pioneers off-campus practice education base has a total of 5. Taking the lead in building the after-school practice education base of the Young Pioneers is one of the important measures to promote the pilot reform of the grass-roots organizations of the National Communist Youth League. Next, will fully combine the new era of civilization practice center, patriotism education base, youth rule of law education base, popular science base, youth home and other kinds of public venues, continue to dig the construction of more young pioneers outside practice education base, explore the school complement and organic linkage of the young pioneers practice education system, set up the "home of the young pioneers". And organize the young Pioneers to the education base to practice the "punch card", to carry out more different characteristics, rich content of the young Pioneers activities.

2021-10-24	Anhui Wuhu Women group dry live broadcast 8 hours live broadcast Wang Haihan Wang Lei) " This is my exclusive product- -FengDan, is the national geographical indication protection products, its root skin has analgesia, antipyretic, anti-allergy, anti-inflammatory, immunity and other medicinal purposes."On the morning of the 20th, Guo Lin pointed to rows of packaged specialties in Nanling County, Wuhu City, Anhui Province. Guo Lin, born in 1990, is the deputy secretary of the Youth League Committee of Hewan Town, Nanling County, Wuhu City, the deputy secretary of the General Party Branch of Lvling Village, and a veritable "Internet celebrity" cadre in Nanling County and even Wuhu area. Going out of the small mountain village and then returning to her hometown to struggle, she planted the live broadcast sales model in the hearts of the villagers. Growing up in the green Ridge village head, she has a deep feelings for this land. Since graduation, she has been working in Shanghai, earning over 10,000 yuan a month. In 2015, Lvling village recruited village cadres, and Guo Lin applied back to the village, becoming the first female college student to return to work. She went deep into the villagers' homes to understand the causes of poverty, and strive for help policies and funds. In 2018, Lvling village committee election, she entered the village committee with a high number of votes. She knew that her efforts were recognized by the villagers. Her story with Hewan Town e-commerce starts with the epidemic in 2020. Hewan town is located in the west of Nanling County, is a distribution center of agricultural and sideline products, rich in agricultural products resources, local chicken, rice, rare Chinese medicinal materials are famous. During a visit, Guo found a large backlog of eggs, and she wanted to help them through e-commerce. She was the first time in Huangshan village when the cargo master broadcast, with a mobile phone into the farmers' home live to explain why the poor households in Bay village sold more than 60,000 stranded eggs, more than 2,000 hens. This also let Guo Lin taste sweet, then, she tried to build Taobao stores and Alibaba wholesale network, and at the same time to build wechat small programs, Pinduoduo and other online platforms, specially selling Hewan Town agricultural and sideline products, to help 42 farmers and large households who need to open online stores to open online stores. Guo Lin Guolin has also established a small program of "rural small shop agricultural and sideline products sales platform", and opened online stores for each village, so that the interested poor households can learn the e-commerce knowledge for free, the whole process of tracking and help the poor households to sell agricultural and sideline products."Help them solve their urgent needs and promote high-quality agricultural products by using e-commerce live streaming."Guo Lin said. At work, Guo came into contact with Zhang Xianhong, who has ALS with only two fingers to move. When Guo Lin went to his home for the first time, he found that the backlog of agricultural products was serious and in urgent need of help. She has opened special live broadcasts for many times. In 2020, Zhang Xianhong's agricultural and sideline products sales amount reached more than 100,000 yuan. Zhang Xianhong also sent a long letter of thanks to Guo Lin. Now, Zhang Xianhong learned to shoot videos and open live broadcasts, he also told Guo Lin: " Girl, thanks to you, my chicken is not worried about selling now! I also helped others in the village sell a lot of eggs."In April 2020, Guo Lin set up a multi-functional farmhouse shop applet. The local government has built a new hewan Town e-commerce public service center next to the long-distance passenger station in Hewan Town to facilitate agricultural products to "go out of the village and into the city". In May of the same year, under the guidance and suggestion of TuanNanling County Party Committee, Guo Lin led the establishment of e-commerce broadcast room and e-commerce volunteer studio, launched the "Farm Small Shop" e-commerce volunteer service project, and recruited more e-commerce volunteers to participate. In 2021,50 live-streaming activities have been held. Volunteer service activities have visited all the poverty alleviation bases in Hewan Town, helping a large number of disabled people and lonely elderly people to sell their home agricultural products."Incubation and teaching skills are far more important than going alone."Guo and his volunteers walk into the fields every month or guide them to open live stores. Brother township pitaya unsalable, Guo Lin will be their live broadcast skills, she also live cultural tourism products. What impressed her most was to help an old woman living alone in Lvling village sell her eggs. The old man raised more than 30 chickens, on weekdays, an egg are reluctant to eat, put in the refrigerator, waiting for people to come to buy. Guo quickly sold her eggs through live streaming. Now, there are more than 100 products on various platforms of "rural Shop", creating an e-commerce pattern of "one town one center, one village one online shop". Guo Lin has a habit that before putting new products on the shelves, she leads volunteers to check the quality of agricultural products and help farmers connect with businesses, so that they can feel at ease. Since 2020, Hewan Town has built an online and offline agricultural products exhibition and sales service center and an e-commerce enterprise incubation base integrating product display, e-commerce training, supporting services and warehousing and logistics. The town's 18 village collective cooperatives have all set up online shops. Guo Lin personally demonstrated, taught the villagers to pick up their mobile phones, open the studio, to help the town to form a new situation of "everyone can bring goods, every product is easy to sell". In recent years, other counties, cities have have agricultural business entities to contact with Guo Lin live with goods matters. She also shared rural policies and tourism scenery in her personal video account, speaking for her hometown. The longest live broadcast, Guo Lin stood for eight hours, no rest in the middle, after the end of the feet raw pain."It is gratifying that many people know me and LvRidge Village through short videos. If I can drive the elderly and villagers in my hometown to increase their income through e-commerce, I think I have realized my life value."She said.

2021-10-21	Shaanxi Wushu: Cultivating young "elites" to contribute to rural revitalization is a rare opportunity for me to experience myself."Said Li Shanshan, a cadre of tax" elite talent ", at the recent opening ceremony of the Rural Revitalization Tax Service Center in Wugong County, Shaanxi Province. In July this year, to improve the overall quality of young cadres, wugong county revenue launched the "star youth elite" plan, through innovation full set of young cadre training system, selection with strong residency players, set up the rural revitalization service post, set up "rural revitalization service youth pioneers" measures, for young cadres in the rural revitalization, service key enterprises to provide a broader practice training platform. So far, 25 young officials have signed up for the plan. Wugong County, located in the hinterland of the Guanzhong Plain, is one of the birthplaces of Chinese agricultural civilization, known as the "Agricultural Capital of China". In recent years, Wugong County has established the e-commerce model of "buying the northwest and selling the whole country", and the industrial brand matrix effect is prominent, and has gained the reputation of "the first county of e-commerce in northwest China", providing a broad growth space for the "elite" youth. The establishment of the rural revitalization tax service center will become another major channel to serve the growth of the "elite" youth. It is understood that the rural revitalization of the tax service center aims to give play to the function of tax, to help rural revitalization enterprises timely urgent difficult to solve tax problems, through the implementation of policies, service, talents, training, farmers, tax bank, data "six one" service measures, do a good job in the key to the service for the people. Relying on the center, wugong county will be the county 522 households involved in agriculture, forestry and fishing enterprises establish power rural revitalization management parameter, for the key agricultural enterprises, led by the bureau and listed in the "elite" training plan of young cadres "one to one" package, do service early intervention, policy early informed, collection and management early cohesion, help enterprises bigger and stronger. The county also set up composed of 18 tax young cadres "rural revitalization service youth pioneers", the country to promote rural revitalization of tax preferential policy subdivided into support rural infrastructure construction, promote the development of agricultural enterprises, promote rural consumption, promote rural basic public services, stimulate rural entrepreneurship employment eight aspects, 26 categories, compiled into the support of rural revitalization strategy tax preferential policy compilation. Young tax officials, cadres walked into the 2021 Northwest Golden Fruit Harvest Festival, Xianyang City "celebrate the harvest sense of party grace" and featured agricultural products live broadcast with goods and other activities, the "support for rural revitalization strategy tax preferential policy compilation" sent to enterprises. Young officials also visited 58 specialized agricultural cooperatives and held policy guidance seminars in batches to solve tax-related demands for enterprises. As the cadres of the "elite talent" training plan, Li Shanshan and Li Jing were rotated to the working team of Waziyao Village, Sufang Town, to practice in the front line of rural revitalization. Under the leadership of the working team in the village, the village has determined two industrial revitalization projects of dairy goat breeding and developing characteristic planting, established a professional cooperative, introduced nearly one million yuan, raised more than 150 dairy goats, planted 90 mu of Onions and 30 mu of honey seeds and melon, and is expected to achieve an annual income of 1.5 million yuan. At present, all the 25 young people included in the "elite talent" training program have established a "one-to-one" pair assistance relationship with their helping families. Yang Shuo, Chen Yanyan, China Youth Daily, China Youth Network reporter Sun Haihua source: China Youth Daily

2021-10-21	Gansu Zhangye, linze county "wish" for children light dream in Gansu Zhangye, linze county "wish" for children light dream Ji'an-wei zhang) since "I do the practical work for the masses" practice, group Zhangye, Gansu province linze county positive response, to help rural left-behind children and poor students realize "wish" in the heart, the county, county closes working committee, county bureau of education jointly carry out "hundreds of young interpreta dream line" youth guardian action, mobilize social forces care, care for difficult teenagers. Up to now, caring people from all sides of the society have sent shoes, clothes, books, basketball, schoolbags and other 21 "micro wish" gifts, and the Communist Youth League County Party Committee will centrally publicize the micro wish claim situation, and conduct centralized distribution in various schools. Since April this year, the county, the county less working committee organization to mobilize the grassroots organizations, schools to carry out the "wish" solicitation, after early solicitation, careful screening, finally within the scope of the county to determine 133 children "wish", the WeChat public, weibo and other media propaganda, the social love people from all walks of life actively claim, light children's dream. This "micro wish" dream realization activity aims to let the children left behind, special, difficult families feel the care of the party and the country and the care of all walks of life, so as to draw on the positive spiritual strength to thrive. At the same time, it also provides a "love direct" channel for social caring people. During the activity, the Communist Youth League County Party Committee, the county youth working committee and some caring people visited the campus under the leadership of the school teachers, to understand the young Pioneers construction and education and teaching situation, and on the "please rest assured that the party, the strong country have me" activity held a symposium.

2021-10-21	Guizhou Bijiang: Chunhui Action into the source of youth when the Communist Youth League grassroots reform to help the youth solution policy, find resources, to support Guizhou Bijiang: Chunhui action into the source of youth "life", how to do? Young entrepreneurs want to use local materials at home to develop industrial economy, lack of technology, do not understand the policy, how to do? What should the children in the inhospitable areas want a more after-school life? In Bijiang District, Tongren City, Guizhou Province, the Communist Youth League has actively helped young people solve their policies, find resources and give them support, with these problems being solved. Initiated chunhui action, condensed youth strength in the Bijiang District Bahuang Town wooden lane village, many villagers eat on the "industrial rice". The Strawberry industrial base and the Guanyu loquat industrial base not only increase the income of the villagers' income, but also attract many young people who were originally employed in other places to return to their hometowns to start their own businesses. In addition to the preferential policies of the Tongren Municipal government to encourage migrant workers to return to their hometowns to start their own businesses, it also benefited from the injection of the Communist Youth League at the grass-roots level. A few years ago, Yang Zaizhang, a project manager at a company in Shanghai, wanted to bring the experience he had learned abroad back to his hometown of Bijiang District to help it develop. "I planned to return to my hometown early, but I didn't know what he could do or how to do it," he said. In May 2017, under the guidance of Bijiang District Committee of Tongren City, Chunhui Society was established in Mulong Village, Bahuang Town. Yang Zaizhang not only became the president of Chunhui Society of Mulong Village, but also was elected as the deputy director of the village committee and the secretary of the village Youth League branch. Under the leadership of the "two committees" of Mulong Village, we gave full play to the advantages of members' funds, resources and qualifications, transferred more than 800 mu of land, planned to build Mulong Chunhui Industrial Park, and built 100 mu of Guanyu loquat industrial base and 100 mu of strawberry industrial base, so that the people in my hometown could eat industrial food. In order to avoid the "competition" of the industrial development of the same village, Yang Zaizhang took advantage of muong village has provincial road through the village to open a farmhouse. While his career is on the right track, Yang Zaizhang drives everyone to start businesses and find jobs nearby, and share their experience with the aspiring young people in the village. Led by Yang, several young people in the village returned to their hometown. They not only bought cars, but also bought houses in downtown Tongren city. Yang Zaifa, a member of Chunhui Society, has an annual net income of about 200,000 yuan, and Guo Qiyu, a supervisor of Chunhui Society, has about 300,000 yuan."At present, 800 mu of more than Chunhui Industrial Park has formed an industrial loop of the park, and the slope and ladder project has completed 50% of the planning progress."With the early good development, Yang Zaizhang is full of confidence in the future. In Bahuang Town, actively promoting the integration construction of "party and league society" has become a work characteristic."Among the 15 league branch secretaries in the town, there are 9 like Yang Zaizhang who are the president of the Chunhui Society, and 45 league cadres from 15 villages (communities) in the town, including 19 members of the 'two committees', to further promote the integration of the 'party and society'."Dai Rui, head of the Tuanba Huang Town Committee, said. As one of the first group of young entrepreneurs to come back in the village, Yang Zaizhang has formed an indissoluble bond with the local youth league organization. He admitted that the establishment of Chunhui Society was not smooth. At that time, the Youth League Committee of Bahuang Town organized the youth league cadres to publicize the concept of the Chunhui Society, and later, a villager took the initiative to provide the empty ancestral house to the Chunhui Society as a position."The Youth League Committee launched the Chunhui fund raising activities every year, to give a certain financial support for our industrial development, but also to the relevant departments to coordinate, the industrial route has also been opened up."When it comes to the league organization in the establishment and development of Chunhui Society, Yang Zaizhang suddenly listed a lot. Help the horse, and send a ride. In the development process of Chunhui society, the advantages of the integration of the construction continue to play, the village party branch actively support the leadership of the League branch and Chunhui society work, the League branch, Chunhui club major matters are reported to the branch in advance, the branch strictly check. With "kinship, blood relationship and industry relationship" as the emotional bond, Chunhui Society organized an online and offline symposium on "I develop together with my hometown" to continuously expand its team. At present, 77 people from Mulong village alone have joined the Chunhui Society. Invite influential, pay attention to the development of his hometown, keen on public welfare undertakings for the Chunhui emissary, elected capable, dedicated, willing to work as the backbone of the Chunhui Society, hire entrepreneurs, teachers, retired cadres, college students as the Chunhui emissary, has become the "routine operation" to enrich the Chunhui talent team. After the establishment of Chunhui Society, capital, policies, technology and other problems have been settled, and internal resources have been effectively revitalized. In the past, a few people in the village mastered certain planting techniques, but kept each other. With the platform of Chunhui Society, Mulong Strawberry Association was established, which is generally responsible for the coordination of technology, sales and publicity and other work. Mulong Strawberry develops together and its popularity is continuously improved. It is worth mentioning that the Chunhui Society has also attracted talent resources. Yang Zaizhang gave an example. In the process of developing the Guanyu loquat planting industry, those who are willing to join them may not return to the village immediately. They can buy shares and pay regular dividends through the company's production and operation. The power of the Communist Youth League is not only in the village, but also not only to help the development of rural industries. In Bijiang District, there are eight relocation sites for poverty alleviation from inhospitable areas. The Youth League Bijiang District Committee helped the "New Citizens Dream Bridge" project, achieved full coverage of eight grass-roots league organizations, and established Chunhui Society, Youth Home, youth volunteer poverty alleviation night school. In Tongren, the first community red scarf growth station was built in Dajiaochong community."We recruited 18 college students volunteers from the Western China Plan to serve as cadres in the resettlement site, enriched the working force of the league, set up more than 50 youth volunteer service teams, developed 42 Chunhui members, and hired 65 Chunhui emissaries."Bijiang District Committee Secretary Hou Yemin introduced, Bijiang District Committee focused on the ideological guidance of the resettlement youth, the community league branch, young working committee regularly held the theme day, theme team day activities and the normal" youth teaching group into the resettlement " activities, guide the majority of young people to feel the party grace, listen to the party, follow the party. The integration construction of "Party and Youth League Society" not only plays a role in the village, but also in the community, Chunhui power is adding luster to the "New Citizens Dream Bridge" project. In the community, most children choose to come to the youth home after school. There is a book rafting station, a hope class to meet different interests, and college students and teachers to help help homework. Liu Qinqin, a student from Tongren Kindergarten Normal College, volunteered to teach her children to dance in the community when she was in school. After graduation last year, she chose to stay here and work. Liu Qinqin felt that since the beginning of the class, none of the 25 children who learn to dance with her have ever been left behind, which is a sense of achievement. In the dwarf tun community, the appeal of Chunhui action has attracted 14 teams from industries, universities and social organizations to form a volunteer service alliance, to promote the construction of Chunhui public welfare supermarket, Chunhui Thanksgiving cultural position, and extensively carried out Chunhui student aid, Chunhui Heart line and other activities. TuanBijiang District Committee set up an online "youth group" three circle of friends, combined with the offline youth home to carry out service work, the returning college students, aspiring youth as Chunhui messengers, actively guide them to participate in comprehensive community governance, help community development. During the summer vacation this year, college students from Cape Chong and Zhengguang communities returned home to actively participate in the activity of "College Students pair up to Help Chunhui Society", contributing to the construction and development of the new community. The relevant controller of Guizhou provincial party committee, including the communist youth league in the county to promote the communist youth league organization reform pilot work, with chunhui club construction as the breakthrough point, promote the socialization of resource allocation way, let chunhui power become the important force of the county communist youth league reform, enrich grassroots work strength, expand the party's ruling mass base, make obvious achievements for rural revitalization and local governance. October 21,2021, Version 01

2021-10-21	The Sui Xi County Party Committee of Huaibei City, Anhui Province, jointly held a collective birthday party to celebrate the Double Ninth Festival for empty nesters. During the activity, the young volunteers put on birthday hats for the birthday man, watched the art programs brought by the volunteers with the elderly, sang the song "Happy Birthday" and ate the birthday cake together. In the next step, the Youth League Sui Xi County Party Committee will continue to carry out activities such as "Grandparents' Big Class" and "Youth Escort" to further strengthen the awareness of youth league members in volunteering.

2021-10-19	Jiangxi Yichun gaoan: organize left-behind children "village" tender study in Jiangxi Yichun gaoan: organize left-behind children "village" warmth study left orange correspondent XiChen) on October 17, by the communist youth league Yichun municipal committee, Yichun qing, yi chun city youth activity center, the communist youth league gao an municipal committee to undertake "love household baby village-left-behind children care line" gao an special activities, gao an pku organized 21 left-behind children out of the countryside, led the children into the museum, red education base to study. At the first stop of the activity, the children came to the Pavluo pastoral complex tourist attraction in Gao'an City. On the big lawn, the volunteers of Gao'an Youth Volunteers Association took the children to play quality development games, and then the children experienced many outdoor projects. The Communist Youth League Yichun Municipal Party Committee prepared a set of four famous works for each child. Next, the children walked into Wuxun Science and Education Hall in Gao'an City. 8 red scarf interpreters from all primary schools in the city brought relevant explanations. Children stare big eyes to listen to the scientists from home out of the life story: Wu Youxun is a famous physicist, educator, member of the Chinese Academy of Sciences, is one of the pioneers and founders of Chinese modern physics research, known as the "founder" of Chinese physics research, the sanqiang qian, Qian Weichang, Yang, Deng Jiaxian, li and other scholars were Wu Youxun students. The last stop of the trip is the Yuan's Blue and White Museum of Gao' an City. While appreciating the exquisite Yuan blue and white porcelain, the children can intuitively feel and understand the history of Gao'an and the Yuan blue and white culture. Liu Xingchen, deputy secretary of the Gao Communist Youth League, said that these experiences can broaden the children's horizons, increase their knowledge, guide them to set up the ideal of striving to serve the motherland, and give their young hearts into a bunch of warmth.

2021-10-19	Group ningyuan county to carry out the "anti-drug fraud" on campus "ningyuan county to carry out the" anti-drug anti-fraud into the campus "preaching Ji'an-wei zhang correspondent Tao Shiqi) since September, the group of Hunan Yongzhou ningyuan county joint anti-drug anti-fraud XuanJiangYuan successively in ningyuan county eighth middle school, ningyuan county third middle school and mingde xiangnan school" drug anti-fraud into the campus " preaching activities. Lecture site. In the activity, the young speaker first explained the serious consequences of drugs, drugs, combined with the actual cases, improve the students' network drug vigilance; then to "prevent fraud, you and me", through the analysis of telecom network fraud data and a series of students involved in network fraud of typical cases investigated by the public security organs, let the students fully understand the means of telecom fraud, improve the ability to detect fraud, prevent being cheated. Young volunteers distributed anti-drug and anti-fraud brochures. The young volunteers distributed the anti-drug and anti-fraud brochures to the students, so that the students can have a comprehensive and detailed understanding of the anti-drug and anti-fraud knowledge, and effectively improve the youth's ability of poison to identify, resist virus and anti-virus. Oath the picture. At the end of the activity, the speaker led the students to mobilize the enthusiasm of the students to participate in the anti-drug struggle, and to create a strong atmosphere of anti-drug publicity.

2021-10-17	Sichuan set up the first county-level youth volunteer service guidance center financial full guarantee Sichuan established the first county-level youth volunteer service guidance center green network reporter xin-xin wang) on October 14, Sichuan province, Guangyuan city lizhou district youth volunteer service guidance center was inaugurated, this is the first county-level youth volunteer service guidance center in Sichuan province. The Lizhou District Youth Volunteer Service and Guidance Center is managed by the Lizhou District Committee of the Communist Youth League. It is a public welfare institution with two approved institutions, and the required funds implement the budget management method of "unified management of revenue and expenditure and full guarantee". Lizhou District Youth Volunteer Service Guidance Center founding conference. The functions of the center include: unite various youth volunteer organizations and youth volunteers to provide volunteer service, guide work, support and cultivate youth volunteer organizations, establish youth volunteer organization network, establish and perfect youth volunteer service project system, promote the development, implementation and evaluation of youth volunteer service projects; and carry out the communication and cooperation of youth volunteer work. Qiao Lu, secretary of the Communist Youth League Lizhou District Committee, said that the establishment of the Youth Volunteer Service Guidance Center is a specific result of Lizhou District to promote the reform of the Communist Youth League county grassroots organization and improve the level of youth volunteer service, and is a powerful measure to improve the volunteer service organization system and promote the sustainable development of volunteer service. Lizhou District has a permanent resident population of more than 600,000, and the demand for volunteer services is large, and the volunteer service activities are relatively active. At present, Lizhou District has 856 volunteer service teams registered on the "Volunteer Sichuan" platform, with about 33,000 volunteers. Among them, the Lizhou Communist Youth League, the 25 volunteer service teams directly contacted by the system, carried out 405 volunteer service activities from March 2020 to October 2021. The volunteer service duration was more than 160,000 hours, and more than 14,000 people participated. It is understood that, as the urban location of Guangyuan City, Lizhou District is facing a greater pressure of enrollment, medical treatment, employment and other pressures, financial resources is not rich, the establishment of public institutions is especially tight. Chang Zongming, member of the Standing Committee of Lizhou District Committee of Guangyuan City and the Department of the Organization Department, said that it is not easy to squeeze out financial funds and the establishment of public institutions to invest in youth volunteer services, which reflects the firm determination of Lizhou District Committee and district government to care about and support the work of the Communist Youth League. AnZongMing requirements, youth volunteer service guidance center focus on the overall economic and social development, the youth volunteer service work and the communist youth league work sink into the youth masses, let the group move, hot up, live, let the youth members for example, actively seek service opportunities, mining service content, characteristic service, for the masses, for social share, for harmony.

2021-10-08	Jiangsu Lianshui County Party Committee implements the "Dream Transformation +" care plan Jianwei Correspondent Pan Man Jingqi) A warm and bright cabin, with a warm little bed and a clean bookcase. This small world, although small, is fully functional, quiet and comfortable... At the end of September, the two sisters of Qingqing (pseudonym), Lianshui County, Huai'an City, Jiangsu Province, saw a different room at home, once old furniture, damaged doors and Windows, dim learning environment have taken a new look."Thank you very much for helping your uncles and aunts and giving us a room of our own. The room is very beautiful and the learning environment is much better, and I like it."Qingqing said gratefully, facing the new room, she looked at the decoration on the wall and the wardrobe. She was so happy that the children benefited from the" dream transformation + " care plan of Lianshui County Party Committee, Jiangsu Province, and the Qingqing sisters had a better living space. Due to various reasons, the green sisters have been living with their grandparents, grandma paralyzed in bed, grandpa is also old, the family has no financial source, can only rely on his grandfather farming and monthly subsistence allowance for a living, the green sisters also became a "de facto orphan".monotonous old narrow bed and shabby wardrobe, table has been the four people live for many years, to have a beautiful home is a long time dream of the sisters."We focus on the fact of unattended children aged 6 to 16, taking into account orphans living in distress. With the construction of 'dream house' as the new button and the normal pair care, we build a series of care project matrix, and build a '1 + 3 + 6' care service system to protect the healthy and happy growth of teenagers in distress."At the scene, Zhang Lei, deputy secretary of TuanLianshui County Party Committee, introduced that in view of the poor living conditions and lack of living facilities," Dream House " relying on the original living rooms for planning, design, decoration, and equipped with necessary furniture and daily necessities."In the process of transformation, we focus on protecting children's self-esteem from causing a pressure on them."Zhang Lei told reporters that in the early stage, TuanLianshui County Party Committee organized volunteers, village cadres went deep into the Qingqing home, many times to solicit the Qingqing wish, and combined with the actual situation of the cabin, decided to adjust the white system, the Qingqing room to rebuild. On the first day of moving into her new room, Qingqing said she was so excited that she was not falling asleep all night."Their help will always be in mind, and I want to repay them with excellent results. When I grow up, I also want to be a caring person like them."" Our tuupanshui County Party Committee plans to raise 3 million yuan in two years to build 263 'dream cabins' and help 263 children build their dreams together. At present, we have completed the construction of 168 'dream cabins'."Said Li Liangliang, party secretary of TuanCounty."In the next step, TuanCounty Party Committee will further strengthen its work, continue to play a good role as a bridge, and unite with caring organizations from all walks of life to help the construction of 'dream cabin'. At the same time, caring enterprises and people will be mobilized to provide long-term pair assistance. Through family companionship and psychological counseling activities, precise chain and continuous intimate services will be sent to the 'dream cabin', so as to truly light up the lamp of hope for the growth of children in difficulties.”

2021-10-19	Anhui chaohu youth volunteers "weekend about" release civilization energy in Anhui chaohu youth volunteers yong to create a pioneer "weekend about" release civilization energy zhang correspondent left yi check WeiTing) " before we come, nervous, afraid of residents ignore our persuasion, this twice to the nanyuan xincun, persuade everyone to home private fly line charging line, after the charging pile for electric vehicles, uncle aunt very understand, have support the charging pile charging, maintain the safety of the electricity area."From Hefei vocational and technical college level 20 clinical professional class 3 zhi-qiang hu said, on October 13 and October 17, zhi-qiang hu twice consecutive attended by the chaohu lake in Anhui province municipal party committee called" civilization you I weekend about " volunteer service, and he further community to carry out civilized volunteer service other young volunteers also have the same feelings. In order to further consolidate the civilized city to create achievements, since October 2021, led by chaohu city in Hefei, Anhui province, Hefei vocational and technical college, chaohu city youth volunteer association, chaohu city yi qi love social work service center of youth volunteer service team into the street community, nursing homes continue to carry out the "civilization you and I over the weekend about" volunteer service activities. Young volunteers in colleges and universities as the main body, actively launched the city street youth volunteer force, in the streets to rectify disorderly parking place vehicles, community fly line charging stop, civilization dog persuasion, love who movement propaganda as the key content of volunteer service, at the same time to participate in community psoriasis cleaning, corridor cleaning miscellaneous, etc. Up to now, the "Weekend Date" volunteer service activity has gone into 8 communities, participated in more than 200 young volunteers, distributed more than 1,000 publicity materials, and persuaded residents to recover more than 50 flying line charging lines.it is reported, Continuing the previous "weekend appointment" campaign, The Youth Chaohu Municipal Party Committee not only uses weekends to organize volunteer services, Usually will also be according to the needs of the street community, Combined with the time for college young volunteers to carry out volunteer services, In the form of "order-order-delivery service", Collect volunteer service needs for community civilization creation, After the Communist Youth League Municipal Committee received the order, Docking with youth volunteer service organizations and arrange task division, Then deliver the service, Related municipal unit youth league organizations, youth volunteer organizations, After the university volunteer service team receives the volunteer service demand of the Communist Youth League and the Municipal Party Committee, Develop a volunteer service plan, Get the task, We will carry out voluntary services in building civilized cities.

2021-10-18	Yu an district in Anhui province red scarf "small horn" narrator contest Anhui district red scarf "small horn" narrator preliminaries completed Zhang Jianwei correspondent, Jiang Yulong check WeiTing) recently, regarding Lu 'an of Anhui Lu' an yu an district party committee lecturers, district education bureau, district civilization create guidance center, area less committee held "please rest assured, power have me" yu an district red scarf "small horn" narrator game. The competition is divided into two stages: preliminary competition and final competition. The preliminary competition is conducted in the form of video selection. The Communist Youth League District Committee was widely launched, and all township and urban schools actively participated in the competition. After multiple recommendation, more than 50 video works were received in the preliminary competition, covering various themes and fields such as revolutionary history memorial facilities, museums, major engineering projects, poverty alleviation and rural revitalization. After discussing the evaluation criteria, the judges watched all the videos of all the red scarf "small trumpet" guides one by one, and finally determined 20 groups of contestants to participate in the final. It is reported that yu'an District "red scarf" small speaker commentator final will be held in the middle of October, when 20 groups of contestants will be on the stage to show the young pioneers practice "please rest assured, strong country have me" oath of "red scarf" style.

2021-10-12	Hainan xiuying youth commandos to port for stranded vehicle passengers to coconut city care Haikou xiuying youth commandos to port for stranded vehicles passengers to coconut city care Zhang Jianwei correspondent Zhou Yutong) recently, affected by the typhoon weather, the qiongzhou channel, xiuying port, new port, south port, cause ferry vehicles, passengers, to solve the problem of driver passenger drinking food, calm stranded people, group of Haikou city of Hainan province, xiuying district league organization more than 50 people youth commandos to the scene to love aid. Due to the bad weather stopping the port, a long queue lined up at Xiuying Port and the new port. Young assault volunteers comforted passengers again and again, and guided drivers and passengers to scan the code and follow the official account of the new port to ensure that they had timely access to weather and sailing information. For the convenience of stranded people get drinking water and edible food, xiuying district emergency called youth commandos and evaluated integratedly volunteers in xiuying port, new port, south port and railway station to carry out emergency security volunteer service activities, and set up two flow volunteer service point, provide emergency items to receive point and free distribution of mineral water and other materials.

2021-10-15	Jiange county, Sichuan province held youth talent collective wedding jiange county, Sichuan province held a "double sword guan Chen" young talent collective wedding on October 15 (reporter Zhang Jianwei, correspondent Li Yuanyuan) recently, "double sword guan Chen" jiange county young talent collective wedding, held in Guangyuan city, Sichuan province Ji'an men pass. As at the wedding scene, 100 newlyweds from the Jiange county grass-roots front under the guidance of the MC, wearing Hanfu, Han ceremony, according to the traditional wedding process, four worship, meals with prison, drink, knot hair, book mandarin duck, hand in hand towards a new stage of life, open a new chapter of life. After the ceremony, 100 new people went to the millennium pass downstairs three offer wine and blessing: one pray for the love is sweet, two pray for happiness and health, three pray for life and death, and Jianshan Pass building together with the heart. After blessing, the new people boarded the door to make vows of love, open a better future.site of activity. Correspondent for the completion of the traditional wedding ceremony in Jianmen Pass, the newlyweds from the "Han style national rhyme" to the "contemporary beauty village", in Jiange County double flag beauty village taste tofu, Jianmen Pass native chicken and other to the local intangible cultural heritage food characteristics of the mountain flavor theme wedding banquet, with fireworks in the original township achievement of the new case. Tuan Jian Ge county party committee to contact youth, service youth as the starting point, continue to deepen the "I do the practical work for youth" practice, in addition to actively organizing and planning the collective wedding, also developed "meet" sword " dating WeChat small program, to effectively solve the problem of good sword young talent marriage, realize emotional retention provides a guarantee. Dong Hongjie, a young man from Yixing Town, said, " As an 'outsider', my first impression of Jiange is its beautiful mountains and clear waters and its beautiful environment. In 2019, I met my lover here. She is lively, beautiful and generous, and she is the person that I want to accompany me all my life. Today, as a member of the 'new Jiange people', I am very grateful to the county party committee and the county government for our care and care, as well as the organization Department and the Communist Youth League County Party Committee to provide the communication platform, which is also a kind of encouragement and support for us to take root in Jiange and serve in Jiange.”

2021-10-14	Anhui Province DushDistrict help civilized tourism let "civilization" visible Anhui Province DushDistrict volunteer service special group help civilized tourism let "civilization" see Zhang Jianwei correspondent, Ding Jianan Zha Weiting) civilized tourism I first, volunteer service into the scenic area. Recently, Huaibei City, Anhui Province, Duji District group representative liaison station volunteer service special group organized part of the league representatives and youth members to carry out civilized tourism volunteer service activities in the double building flower sea, to the majority of tourists to promote the concept of civilized tourism, to volunteer service to drive civilized tourism. During the activity, the volunteers wore red vests and were divided into two teams. One team assisted the staff to measure the temperature at the entrance and exit of the scenic area, and guided tourists to queue up to buy tickets into the park in accordance with the epidemic prevention requirements of the scenic area; the other team entered the scenic area to maintain order in areas with large flow of people and provide route guidance and other services for tourists."Hello, please queue up at the entrance, measure the temperature and enter in an orderly manner."Volunteers enthusiastically guided tourists to get tickets and line up into the park."Where the scenic spot tickets are sold" "How to visit" for the various questions raised by tourists, everyone patiently answered. At the same time, the volunteers also actively publicize the knowledge of civilized tourism and environmental protection to the tourists, calling on the tourists to strive to be ambassadors of civilization, maintain the scenic spot environment, follow the order of the scenic spot, and jointly maintain a good atmosphere of the scenic area. In the next step, the Duji District League representative liaison station will give full play to the functions of each special group, carry out colorful youth activities around the central work of the district Committee and district government, and build the league representative liaison station into a warm harbor and gas station for the youth league members.

2021-10-09	New venture, Guangdong province measures to promote team organization construction in Guangdong group collaboration work advance will promote team organization construction JianWei correspondent Li Excellence) as the national county communist youth league organization reform pilot, Guangdong Shaoguan xinfeng county always around the fundamental task of khalent ents and party and government work center, actively adapt to the communist youth league deepening reform of new forms, education comprehensive reform and contemporary youth characteristics of new development, efforts to promote team construction, create mechanism, reform, activities of group collaboration new pattern. A few days ago, the Youth League Xinfeng County Party Committee, Xinfeng County Education Bureau jointly organized the county primary and secondary school youth League committee secretary, the young Pioneers brigade counselors held the Xinfeng County Youth League education cooperation work promotion meeting and team work training meeting. At the meeting, the relevant person in charge of the Communist Youth League County Party Committee conveyed the spirit of the "July 1" important speech to the county youth League committee secretaries and the counselors of general Secretary Xi Jinping's "July 1" important speech. Subsequently, Xinfeng County Middle School Communist Youth League master studio host and young Pioneers master studio host has to the middle school Communist Youth League and primary and secondary school young Pioneers work deployment. Subsequently, the county middle school Communist Youth League master studio host and the young Pioneers master studio host respectively to the county middle school youth League committee secretary and primary and secondary school young Pioneers counselors for the basic league affairs, team affairs knowledge training. During the training, the participating youth league cadres and counselors discussed the construction of the middle school youth league school, points into the youth league, red scarf competition, team work training mechanism and other work.

2021-10-08	Group of Hunan Lanshan county to carry out anti-drug anti-fraud into campus public welfare publicity activities Lanshan county to carry out anti-drug fraud into campus public publicity activities jianwei correspondent Chen Mengyao) since September 10, group of Yongzhou of Hunan province Lanshan county continue to carry out anti-drug anti-fraud into the campus public publicity activities, has accumulated in the county four schools, a total of more than 1200 students. During the activity, the newly developed anti-drug lecturers of the Communist Youth League County Party Committee in 2021 publicized the harm of drugs, and comprehensively summarized the anti-drug publicity work of the Communist Youth League system of Lanshan County some time ago. The activity also distributed anti-drug publicity materials on the scene, calling on the youth representatives to actively participate in the anti-drug publicity work, it is reported that before this, the Communist Youth League County Party Committee held in the Lanshan County vocational secondary school anti-drug knowledge special lecture, and organized more than 300 young people to participate in the anti-drug knowledge answer activities.

2021-09-30	The Farmers' harvest Festival was held in the First Mountain Historical and Cultural District of Xuyi County. The theme of this activity is "celebrate the harvest and common prosperity sense of party". At the scene, the relevant person in charge of the Jiangsu Provincial Committee of the Xuyi County awarded the central Committee "Rural revitalization youth contribution action key county" plaque, Xuyi County Party Committee received the card. Leaders of Jiangsu Provincial Committee of the Communist Youth League, Jiangsu Provincial Supply and Marketing Cooperative Association and other relevant departments jointly launched the "Rural Revitalization Network Live broadcast" competition in Jiangsu Province. The event site set up the Xuyi new agricultural elite exhibition products appreciation area, "new agricultural elite exhibition" brand products are the Jiangsu Communist Youth League for the province's rural youth tailored agricultural products brand, aimed to help rural young entrepreneurs to improve the brand influence of agricultural products and the added value of products. The Xuyi new farming elite exhibition area by Jiangsu Longcheng Agricultural Development Co., Ltd., Jiangsu Red fat fat lobster industry Group, Huaian Huai-da Agriculture Co., Ltd. and other 13 enterprises, selected sunshine rose grape, lobster, rock sugar pear and other 26 featured agricultural products.

2021-10-07	Anxi: coagulation hearts resist disease does the practical work for the people service bear anxi: coagulation hearts resist disease does the practical work for the people service bear learning education and solve practical problems, focusing on the epidemic prevention and control of the masses "anxious sorrow" problem, a vivid "I do the practical work for the masses" practice flourish, the party history learning education results into service to the masses, built a strong fortress guard tea township people's life safety. At the beginning of the epidemic, the Leading Group of Party History Learning and Education of the CPC Fujian Provincial Committee called on the whole province to further promote the practice of "I do practical things for the people" in resolutely winning the battle of epidemic prevention and control. Anxi heard the order and responded quickly, mobilizing party members, cadres and volunteers to go to the front line of the fight against the epidemic to test the effectiveness of the "I do practical things for the masses" practice on the battlefield of epidemic prevention and control."About 300 people will be needed at the nucleic acid test site tomorrow!"Late at night, a phone call cut through the peace of the night. What if there don't enough manpower, not enough supplies?...... " I am a party member, I take the lead!"" I have supported Wuhan in the fight against the epidemic. I have the experience!"" If the epidemic does not retreat, we will not retreat!"...... a sound of party members and cadres from different industries in the tea township with action, to interpret the most beautiful voice of fighting the epidemic. Party members and cadres risked their lives and rushed to the front line of epidemic prevention and control."I'll go first!"Grassroots medical staff to the responsibility of party members, rushed to the front line of epidemic prevention. On September 19, in the morning, anxi hospital party committee, anxi hospital party committee assembled 271 medical staff 35 nucleic acid sampling team and 2 tributaries, with the stars to dragon township, overnight nucleic acid detection, medical staff sleep on the ground, after a short rest, they continue to work, with responsibility and bear woven dense epidemic prevention and control "safety net". A pair of sweat blisters white hair wrinkled hands, a sweat-soaked clothes, practicing the original aspiration of a party member. Whether in hospitals at the forefront of epidemic prevention and treatment, in road stations with traffic, or in villages and communities, party members and cadres can be busy everywhere. Their selfless dedication protects our lives with responsibility and responsibility, and cares for the city of Tea Township. The little red Riding Hood and red vest that can be seen everywhere have also become a beautiful scenery in the tea village. Where there are difficulties and where there is a need, there are volunteers. In order to further do a good job of the county's "external import prevention, internal rebound prevention" work, effectively improve the people's awareness of prevention and control, do a good job of personal protection, to ensure the stability and stability of the county's production and life order, in recent days, Anxi County has organized a centralized epidemic prevention and control persuasion volunteer service activities. In urban areas, 126 volunteer service teams for epidemic prevention and control and persuasion are deployed to provide volunteer services every day. In spite of the virus, they walked from street to street to publicize the knowledge of epidemic prevention and control to the public, persuade businesses, shop operators and employees along the streets to consciously wear masks consciously, and implement prevention and control measures such as "three-code joint inspection" and temperature measurement. At the critical moment of epidemic prevention and control in the county, the Civilization Office of Anxi County Party Committee, together with the Communist Youth League Committee of Anxi County Party Committee, issued an emergency recruitment order to the young people of the county. On September 16,320 volunteers were recruited to 63 nucleic acid testing sites in Fengcheng, Chengxiang and nei to assist in the first round of nucleic acid testing; 43 volunteers were recruited to provide epidemic prevention and control materials; On September 21,300 volunteers were recruited to complete the second round of nucleic acid testing in three towns; On September 22,11 volunteers were recruited to assist in the distribution of epidemic prevention and control materials. All towns and townships carry out volunteer service activities for epidemic prevention and control in light of their actual conditions. According to statistics, 24 towns and townships in the county invest 174 volunteer service teams and 2,150 people every day. On September 15, Anxi County decided to start a nucleic acid test for a large population. The county party committee and government held a series of epidemic prevention and control meetings to evaluate the current epidemic prevention and control situation and deploy relevant work. On the same day, the county teachers and students, staff completed the second round of large crowd nucleic acid testing. Twenty-six off-campus epidemic prevention and control inspection teams have carried out all-round inspections and persuasion work. At the same time, 13 convenient nucleic acid collection points were set up and successively put into use. On September 17, Anxi County added 126 new nucleic acid testing convenience service points, and put into the intense and orderly work. To reduce personnel gathered, block the spread of the epidemic, better guarantee the safety of people lives, and orderly meet the needs of the enterprise and the masses, anxi county administrative service center advocates "delay, booking, online, handheld, mail office" not meet approval, built an invisible line in front of the outbreak, efficient power epidemic prevention and control normalized, realize "isolation virus, not isolation service". Anxi is also working hard to ensure the smooth flow of epidemic prevention materials, industrial chain and supply chain, timely issuing documents and providing policy services. Within the jurisdiction of Anxi County, all freight vehicles leaving Anxi can leave Anxi with their health code green code, negative nucleic acid test and normal temperature test. Freight vehicle passengers who intend to leave Anxi should pay attention to the schedule, actively advance to the nearest convenient nucleic acid sampling point and strictly implement relevant epidemic prevention and control measures. There are 13 convenient nucleic acid sampling points in Anxi from 8:00 to 18:00 every day. Seal control area of living materials special distribution, the masses have thumb up. On the basis of mapping the supply capacity of large supermarkets and vegetable basket bases in the early stage, Anxi County Industrial Informatization and Commerce Bureau timely connected with Longjuan Township government. In principle, it was delivered every three days, and at the same time according to the actual demand, implementing precise point to point, to achieve every household. The supplies on the special car are moved to the cold chain car, transported to Longjuan Township, and then by the township government for the second deployment. The township organize a refrigerated truck, five pickup truck, the supplies to five containment area village department, then by the third deployment in the village, each village according to the size, organize five to seven volunteers, and 3 to five village cadres, each household in accordance with the number of the supplies to the door, truly containment area villagers never leave home can get the life supplies."The epidemic is merciless, with continuous supply."Anxi has focused on epidemic prevention and control and ensured stable supply and prices. It has conducted daily price monitoring and timely grasped market sales and inventory changes to ensure that market prices are stable and people's lives are not affected. At the same time, it has signed emergency contracts with some relevant institutions, suppliers and associations within and outside the province to ensure timely transportation in emergency situations and ensure market supply. At present, Anxi Tieguanyin autumn tea into the production period. How to coordinate the production and sales of autumn tea while doing epidemic prevention and control has become the top priority. On September 27, the Guidelines on Epidemic Prevention and Control during the Autumn Tea Production period were issued to standardize health management measures for tea miners and tea workers related to safety. On September 29, the autumn tea production and marketing docking conference was held, and 5,199 tons of Anxi autumn tea were ordered on the spot, and the unit price was slightly higher compared with last year. It is worth mentioning that in order to ensure the outbreak during the county autumn tea production and sales orderly, anxi issued "on further promoting the outbreak of autumn tea production and marketing work opinion", including: support tea farmers home, accurately solve labor problem, take the initiative to strengthen technical services, expand sales channels, actively broaden online sales, comprehensively boost consumer confidence, to smooth logistics channels, strengthen financial support, strengthen organizational security. Behind the measures, it reflects the original intention of Anxi to carry out the practical activity of "I do practical things for the masses", so that the broad masses can feel the effectiveness of party history learning and education in the epidemic prevention and control work. Timely and effective psychological counseling is an important part of comprehensively winning the battle against the epidemic. From September 25 to 27, the Publicity Department of Anxi County Party Committee and Anxi County Rong Media Center held a live broadcast with the theme of "Epidemic prevention cloud classroom health, I will help". The three live broadcasts attracted 52,600 views, answered 97 questions online, and received 235,600 praise. In the "Cloud Classroom", photo), doctors and psychological counselors introduced epidemic prevention and mental health knowledge respectively, and answered questions raised by the people in the control area, control area and isolation point, and shared typical cases. In the live broadcast, doctors carefully explained the characteristics, incubation period, source of infection, transmission route and personal protection of novel Coronavirus, and explained the key points of home disinfection and protection; advocating the public to develop good habits such as more ventilation, hand washing frequently and keeping clean. In addition, Anxi quickly set up a psychological counseling volunteer service team, opened a 24-hour psychological assistance hotline to provide psychological protection services, help frontline cadres and staff of epidemic prevention and control to relieve and relieve, and guide front-line cadres and staff to combine work and rest to maintain mental health. In order to reassure frontline workers, Anxi County has also issued ten heart-warming measures to care for frontline workers in epidemic prevention and control, and encourage them to unite to win the battle of epidemic prevention and control. The ten measures include cash to implement the subsidy policy, increase a line identification use cadre talent, open title appraisal and personnel recruitment green channel, improve the annual appraisal excellent order proportion, inductrial injury treatment and insurance guarantee, to ensure the physical and mental health, frontline personnel rescue melancholy, pay attention to found in a line and test the party activists, to reduce the burden of the grassroots, increase advanced typical awards and propaganda. Special party fees escort the epidemic prevention and control work, allocating 600,000 yuan from the managed party fees to visit medical workers and grassroots Party members and cadres on the front line of the epidemic prevention and control struggle; support grassroots Party organizations in the epidemic prevention and control work, including the purchase of epidemic prevention and control drugs and materials; and subsidize Party members and masses who have encountered difficulties due to COVID-19. For the benefit of the people, silk hair will prosper. Looking at the land of tea township, the practice of "I do practical things for the masses" is in full swing, truly convenient and benefiting the people of the people, Anxi people's sense of gain, happiness and security is constantly enhanced.(Zhang Jinchuan)

2021-09-28	Ziyuan County of Guangxi held the Guangdong and Guangxi Cooperative Rural Youth Innovation and Entrepreneurship Training Course 2021 Guangdong and Guangxi Cooperative Rural Youth Innovation and Entrepreneurship Training Course Jianwei Correspondent Wang Lisu and Tan Xinyu) to thoroughly study and implement General Secretary Xi Jinping's important thoughts on youth work and the important speech spirit when inspecting Guangxi and Guilin. Recently, the Guilin City Resources County Committee of the Communist Youth League of Guangxi Zhuang Autonomous Region received financial support from the Guangdong-Guangxi Cooperation Committee, and successfully held the 2021 Guangdong-Guangxi Cooperation Rural Youth Innovation and Entrepreneurship Training course in Ziyuan County. In order to consolidate the achievements of poverty alleviation, we have led and led young people to actively participate in the rural revitalization strategy, trained a number of innovation and entrepreneurship talents for rural revitalization, and actively promoted the talent training project of "thousands of thousands of villages". The Opening ceremony of the training class was on the site. The trainees of this training class include grassroots cadres from 7 townships in Ziyuan County and entrepreneurial youth from 47 poverty-stricken villages and the cultivation objects of "thousands of townships and good youth" in Ziyuan County. Osmanthus commune commentator for you to introduce the osmanthus series of products. This training course set up special courses are mainly include "agricultural products live with goods and short video marketing", "family farm management", "deep processing and packaging sales of agricultural products" and "village home stay building and tourism product development", in line with the needs of resource county rural youth entrepreneurship needs. Training class completion site. Correspondent for figure during the training, all the students combined with theoretical knowledge to carry out field teaching, visit Guilin QianYe group agricultural products co., LTD., luohan fruit town and Guilin xiufeng district osmanthus commune, not only close visit the arhat fruit industry from raw material processing to product packaging sales of the whole industry chain, also understand the osmanthus as a Guilin city, in Guilin thick culture is how intertwined with historical development, learn excellent enterprises bigger and stronger advantage industry, vigorously develop regional advantages of agricultural products successful experience, for the county rural young cadres innovation entrepreneurial ideas.

2021-09-28	Anhui Province Taihu County Party Committee to carry out a series of veterans employment and entrepreneurship activities Anhui Province Taihu County Party Committee to help veterans employment and entrepreneurship series of activities Jianwei correspondent, Yin Cheng Jincha Wei Ting) in order to further implement the General Secretary Xi Jinping at the Party history learning and education mobilization conference important speech spirit, Take "I do practical things for the masses" as the starting point of the work, Further expand the channels for the employment and entrepreneurship of ex-servicemen, Help taihu Shuangyong model county, a few days ago, Youth League Taihu County Party Committee of Anqing City, Anhui Province, together with county Economic Development Zone, county People's Armed Forces Department, County Veterans Affairs Bureau, county Youth Entrepreneurs Association, county Youth Volunteers Association, We will organize a series of activities of "Welcome Home" in 2021 to help veterans find jobs and start businesses in 2021. Activities on the same day, present more than 20 veterans, under the guidance of the enterprise narrator into each company campus, dining area, office area and production workshop, detailed understanding of the product production process, enterprise management concept and development situation, as well as employment demand, salary and post development space, enterprise advanced production equipment, automated production line and good enterprise culture left a deep impression on veterans. Visit all the way, feeling, taihu lake membrane industry development trend rapidly, part of the activities of the veterans hope to join the enterprise construction as soon as possible personal dream, expressed the personal employment intention and left the contact information, then enterprises will be one by one telephone visit, according to its personal ability specialty and enterprise employment demand, tailored in jobs and development prospects, an interview invitation. In the subsequent employment and entrepreneurship forum, the enterprise respectively on the salary, living security, promotion prospects and other aspects of the introduction, to issue the "olive branch" to the veterans who participated in the activity. Two outstanding veterans of previous years shared their own entrepreneurial stories with this year's new veterans, telling them to strengthen themselves and walk out of their own new world. A new veteran made an exchange speech on the feelings of visiting the enterprise, and made a statement that he will change his role as soon as possible, strive to improve his skills, cherish the work opportunities, and always maintain the true character of a hard-working soldier.

2021-09-26	Xianyou County, Fujian Province established the "12345 + Youth Volunteers" service mechanism. Fujian Xianyou County Party Committee innovatively established the "12345 + Youth Volunteers" service mechanism to solve the problem of public medical treatment and drug use. Jianwei Correspondent Zheng Xuemei) " There are no heroes falling from the sky, only ordinary people who stand up."Facing the sudden attack on the Delta strain, they broke through once again and stepped forward. From the city to the countryside, from the streets to the alleys, from the Internet to the offline, the "red vest" figure can be seen everywhere. They either collected and input at nucleic acid testing sites, or provided support at logistics materials, or went from street to street. They appeared in every place where they were needed, with a bright "volunteer red" to build epidemic prevention and control "iron wall" for prevention and control. With the upgrading of epidemic prevention and control measures, Xianyou County, Putian City, Fujian Province, has implemented traffic control, Fengting Town, and other towns have gradually begun to control it. Many patients have stopped taking medicine, and can not go out to buy medicine, so the situation is very urgent. How to solve the problem of medication when special groups such as chronic patients and lonely elderly people solve the problem? The party has a call, and the league takes action. Epidemic prevention and control, volunteer first. In order to effectively do a good job in epidemic prevention and control, and help people effectively solve urgent problems such as emergency drug use and emergency medical treatment during the epidemic prevention and control period. Xianyou county and Putian city 12345 platform jointly established "12345 + young volunteers" for the practical service mechanism, lead the integration of xianyou volunteer association, xianyou love help group, Xianyou sponge group and other social organizations set up the first xianyou youth love help trapped commandos, 72 young volunteers, nine love vehicles active "out", "in the epidemic prevention and control line yong when" relay "," relay "through" love relay ", multi-channel for the masses during the epidemic prevention and control of emergency medication, the epidemic prevention and control of the county made a positive contribution. As of September 24, the commando team has undertaken the city's "12345" platform sent 127 cases of public help, drugs have been delivered 118 cases, help medical treatment 1 case, involving 15 towns in the county. Since many drugs cannot be purchased in the county, they need to be purchased from Putian, Fuzhou and other places, under the linkage of Putian 12345 platform and the Youth League Committee, Xianyou has opened three green channels: the first is the youth League Committee and the county team and the county. In order to improve work efficiency, people who have urgent need to buy drugs can register for help through the "12345" telephone. The help information will be uniformly submitted to the Youth League county Committee, and the Youth League county Committee will coordinate it to volunteers for drug distribution, forming a baton after another love transmission."It is just errands, but some drugs are 'life-saving drugs' for residents, and we have to race against time to deliver them."Said Xiao Xiongyao, head of the Xianyou Volunteer Association. Volunteers are on standby to send the medicine as soon as they receive information for help. For example, the Putian volunteers sent the drugs to the Xianyou bayonet point, and then the Xianyou volunteers delivered the drugs to the patients' homes by a relay way. In order to prevent cross-infection, the distribution sites generally choose the intersection or the community on duty point. Volunteers disinfect the outer packaging of the drugs. In the process of love relay, all the non-contact relay deliveries are adopted. In order to facilitate the delivery, the Communist Youth League County Committee also made uniform handbags, and presented a bag of masks and an epidemic prevention love card to each person to send warmth to the people, and guided the people to improve their awareness and ability of epidemic prevention, and strengthen their confidence and determination to overcome the epidemic.

2021-09-20	Sichuan jianyang in 2021 the second double gen dating activities in Sichuan jianyang in 2021 the second double gen dating successful ending he xin) on September 16, Sichuan jianyang committee of Chongqing tongliang district party committee, Deyang zhongjiang county, Meishan county, group, group to the county, Ziyang anyue county held "origin east red male state" jianyang in 2021 the second double gen dating activities in 2021. The Communist Youth League of the seven regions signed the Framework Agreement on Action Cooperation in Helping Chengdu-Chongqing Economic Circle and the Framework Agreement for Action Cooperation in Helping ChengdemCapital. According to the cooperation framework agreement, the seven communist youth league organizations, the grassroots youth league, volunteer service, outstanding youth exchanges, youth innovation and power rural areas such as strengthening exchanges and cooperation, jointly promote build around youth communication platform, build youth innovation project brand, youth work cooperation win-win mechanism, unity led the league youth active in, speed up the construction of Chengdu-Chongqing shuangcheng economic circle and the city development of the new journey. The event attracted nearly 200 young men and women from the local area and surrounding areas to participate, and launched a picture cloud live broadcast online simultaneously, with more than 20,000 viewers. The scene of 6 young men and women successfully hand in hand, and intentionally connected to develop nearly 20 people. The event site also set up the Jianyang, Tongliang, Zhongjiang, Renshou, Yanjiang, Lezhi, Anyue regional youth innovation market exhibition area, to widely promote the urban characteristics and youth entrepreneurship products to the young people present, to help the youth entrepreneurship, rural revitalization and urban development. According to group Jane Yang related responsible comrade, in recent years, in the context of the construction of Chengdu-Chongqing shuangcheng economic circle, Jane Yang continue combined with Chengdu "eastward" strategy, to unity, guide, service youth for work foothold, attract youth hui rong poly Jane, guide young talents root JianYang, construction JianYang, speed up the formation of all kinds of high-level talents continuously together. To carry out this mass entrepreneurship and innovation dating activities, mainly based on the needs of young people, to build a strong public welfare, high integrity and positive youth dating channels and innovation and entrepreneurship exchange platform, to enrich the spiritual and cultural life of single young workers.

2021-09-18	Baisha isha of Hainan province 170 difficult students successfully receive grants in Hainan baisha 170 difficult students successfully receive "love student interpreta dream action" grant wei correspondent Wu Yuhua) on September 15,2021 baisha "love student interpreta dream action" grant distribution ceremony held in Hainan baisha government complex building sixth floor conference room, 170 difficult students from baisha each successfully received 5000 yuan, including 45 for hope project student project pledge, 125 funded by baisha. County Committee member of the Standing Committee, the county "love student realization dream action" activity leading group leader He Jinsong attended the activity and awarded the plaque for the representative of the love enterprise. It is reported that since 2008, Baisha has carried out the "love for students to realize the dream action" activity for 14 consecutive years, a total of 9.702,886 million yuan raised, a total of 1,894 students in need. In 2021,660,698 million yuan was collected, among which 36 private enterprises, including Hainan Jiayuan Ecological Agriculture Comprehensive Development Co., LTD., LTD., Baisha Nanhong Concrete Co., LTD., Baisha Fu'an Real Estate Co., Ltd., and Hainan Spring Real Estate Development Co., LTD., donated 583,900 yuan. Baisha County Party Committee propaganda department, county rural revitalization bureau, county education bureau, county civil Affairs bureau, county ethnic affairs bureau, county federation of Trade unions, youth League committee, county women's federation, county federation of industry and commerce and other counties "love student dream action" activity leading group member units mainly responsible person, love enterprises, students, parents to participate.

2021-09-13	Group of Hunan Ningyuan county to support youth entrepreneurship employment power rural revitalization of Hunan Ningyuan county support youth entrepreneurial employment help rural revitalization of JianWei correspondent BaiJing) Hunan Yongzhou Ningyuan county as a national county communist youth league organization reform pilot area, group ningyuan county always adhere to play to the grassroots demonstration role, promote the construction of youth league organizations and "support youth entrepreneurship employment, help rural revitalization" to achieve interactive win-win. Since 2015, TuanNingyuan County Party Committee has built Ningyuan County Youth Employment and Entrepreneurship Training Base through in-depth cooperation with Ningyuan County Business Incubation Base (national mass entrepreneurship space), focusing on youth entrepreneurship and employment, and helping rural revitalization. It has opened up a new path for aspiring young people to start businesses and find employment in Ningyuan County, and has become a new driving force to stimulate the Ningyuan Communist Youth League to promote the "three forces and once". Over the years, the Youth League Ningyuan County Party Committee has attached great importance to the youth entrepreneurship and employment work, and strengthened the youth entrepreneurship and employment services through system construction. First of all, the establishment of a special work class, to strengthen the youth entrepreneurship and employment services work special force. Tuan Ningyuan County Party Committee set up the Youth Development Department (Rights and Interests Department), and at the same time set up a special class for youth entrepreneurship and employment, forming a working system of "special class promotion and department implementation". Secondly, carry out special research to provide the reference basis for youth entrepreneurship and employment services. Tuan Ningyuan County Party Committee carried out special research and organized a symposium on the service of returning young people for entrepreneurship and employment in Ningyuan County. At the meeting, 11 returning young people for entrepreneurship representatives exchanged entrepreneurial experience with each other. After the meeting, Tuan Ningyuan County Party Committee wrote a research report, put forward reasonable suggestions, effectively guide the scientific development of entrepreneurship and employment work, and further improve the service work system for youth entrepreneurship and employment. Finally, strengthen organizational leadership. A leading group for youth entrepreneurship and employment of the Communist Youth League and County Party Committee has been established to clarify the key work of youth entrepreneurship and employment, and all departments will jointly manage it to form a joint force to promote youth entrepreneurship and employment in Ningyuan County. We will focus on practical projects to provide effective assistance for young entrepreneurs and deepen the youth entrepreneurship and innovation competition. In 2016, the joint space ningyuan the first "ningyuan wing" youth entrepreneurial innovation competition, and for five consecutive years to undertake ningyuan "ningyuan wing", "the China" "China" and wing "and youth" youth innovation entrepreneurship competition, to undertake and participate in all kinds of innovation entrepreneurship competition, project roadshow, 30,20 entries, in the national competition have achieved excellent results, the entrepreneurial wave, has become a support and attract excellent entrepreneurial project normalized activities. To help young people improve their innovation and entrepreneurship skills. Six consecutive SIYB business training class, held "ningyuan network entrepreneurship training" "ningyuan enterprise tax knowledge training" "ningyuan business training follow-up service activities" "electricity poverty alleviation training" series of training activities, further cultivate youth innovation entrepreneurial innovation spirit and practice ability, to "stable employment, strong foundation, rich hometown" theme as the gripper, carry out ningyuan employment service platform field training activities, 40 enterprise human resources to participate in the training. We will actively support young e-commerce people in their innovation and entrepreneurship. Jointly organized a series of online and offline e-commerce training public welfare classes and "village broadcasting with goods" activities, held seven training sessions of "village broadcasting with goods" training class, built a talent chain of "village broadcasting with goods", and cultivated a total of 500 students, among which 100 outstanding students signed a live broadcast base. In 2020, the company held 80 training sessions, training more than 4000 people; invited Ou Jinping, deputy county magistrate, to become "super cargo", and carried out large-scale live delivery activities with village broadcast excellent students, attracting more than 50,000 netizens, selling 5939 agricultural products, the total order amount exceeds 160,000 yuan, and driving the Hunan agricultural products sales of nearly 530,000 yuan. Build a platform to provide all-round services for young entrepreneurs to promote the construction of Ningyuan County youth employment and entrepreneurship internship base. Set up "youth employment and entrepreneurship trainee base", for 2 years of rent reduction and electricity, computer, network; entrepreneurship guidance and training, organized by 18 contracted mentors free one-to-one professional guidance to provide the base, relieve youth employment pressure, help them accumulate work experience, build a platform for enterprises, further create a good social atmosphere of care for youth entrepreneurship employment, in 2019 Yongzhou innovation entrepreneurship drive employment demonstration base. The Ningyuan County Youth Entrepreneurship Chamber of Commerce was established. In order to create a good atmosphere of "mass entrepreneurship, youth innovation" and build a platform for entrepreneurial youth skills promotion, the CPC guides Ningyuan to hold the training course for the executive promotion of enterprise team to teach, more than 100 young people to attend the training session, and more than 140 young business managers. We will carry out employment and recruitment work on a regular basis. The Communist Youth League County Party Committee, together with the county Human Resources and Social Security Bureau and the county Science and Technology and Industry Bureau, launched the "Spring Breeze Action" live job fair online and offline, providing public employment services such as job introduction, skills training and employment policies, and building a platform for private enterprises and workers' employment. A total of 48 online and offline job fairs were held, with 37 enterprises participating in the job fairs, providing 11,560 jobs. A total of 11,560 online and offline jobs were registered, and 4,057 jobs were employed. We will actively promote the spirit of innovation and entrepreneurship, and support both ambition and wisdom. Adhering to the idea of "bringing entrepreneurship to employment and promoting poverty alleviation through employment", we provide free e-commerce poverty alleviation and other entrepreneurship courses, and provide high-quality entrepreneurship services and entrepreneurship guarantee loans to help the poor labor force achieve employment. In recent years, the county actively to the central, provincial, youth youth to participate in the selection of "huxiang youth talents", "Yongzhou youth talents" "youth civilization", "youth" young post expert "awards, including county the space and settled enterprises Hunan Kant jia forestry technology co., LTD., awarded" Yongzhou youth civilization "honor, county the space general manager zhi-ying tang named" youth post expert "," Yongzhou forestry technology co., LTD. Chairman OuYangYaLi named "Huxiang youth talents" "Yongzhou youth post expert" and other honors.

2021-09-10	Shandong ling city in Shandong province "hope" public fair activities in Shandong ling city "hope" public fair activities the youth network Beijing on September 10 (reporter zhang, reporter gao lili) on September 7 solstice 8, Shandong province Dezhou city lingcheng district party committee, ling city volunteer association joint fraternity social workers, good social worker, hong de social worker and love unit organization in Shandong ling city "hope" public fair activities, for "hope" children care project to raise money. Event with "love ring", "love blind box" "charity" and other areas, potted plants, flowers, articles for daily use, handicrafts and other kinds of sale, ling city teachers study school affiliated primary school red scarf volunteers took to the streets to the bazaar, for ling city "hope hut" construction to raise money. People heard that public welfare, love, one after another, each area in front of bustling people."Do my best, everyone public welfare", the activity site is most touched by the parents who take their children to do public welfare, the businesses who provide free goods for the volunteers, the stall owners in the night market, the people who have prizes but not, the free milk tea shops... every participant is paying attention to and helping the children in trouble with practical actions. This activity, taking the "Hope House" public welfare market in Lingcheng District, Shandong Province, as a platform, gathered public welfare resources from inside and outside the area, and narrowed the distance between social organizations, caring enterprises and caring people, and integrated social forces to actively participate in public service projects. Further expand the social awareness and brand influence of the "Hope House" project, extensively stimulate the psychological resonance of all sectors of society, and let the love "benefit" up.

2021-09-27	Group of Anhui province solid town county to carry out the traditional culture into the campus activities of Anhui province solid town county to carry out the traditional culture into the campus activities built wei correspondent Yu Dan check WeiTing) recently, Bengbu, Anhui province solid town county invited solid town paper-cut art association, county intangible paper-cut project heritage ms hua-fei liu into the solid town second primary school, led the young pioneers to experience the fun of traditional culture paper-cut art. The picture shows Ms.Liu Huafei, a professor of paper-cutting. President Liu taught the young Pioneers how to cut paper from the steps of paper selection, drawing and depiction, and even the male students who love to play began to learn paper cutting. While teaching paper-cutting skills, President Liu also explained the history and significance of paper-cutting with the young pioneers, so that the students can learn the traditional craft of paper-cutting and also understand the connotation of paper-cutting culture. The picture shows the students showing the paper-cutting results. Through this activity, the children not only understood the art of paper-cutting, but also realized the responsibility of serving as small citizens in the new era- -we have the responsibility to inherit and carry forward the long national treasure of the Chinese nation.

2021-09-26	Anhui su county "four intentions" to strengthen the online construction of the communist youth league in Anhui su county "four intentions" to strengthen the construction of the communist youth league online jianwei correspondent, Zhang Meifang check WeiTing) when the vast majority of the youth in brush TikTok, chat WeChat, strengthen the coverage of youth groups and lead, you must aim at cyberspace, strive to develop new platform new positions."Where the youth is, the construction and work of the league will be extended," has become the "online declaration" of the Communist Youth League. In recent years, the Susong County Party Committee of Anqing City, Anhui Province has carefully cultivated youth network publicity talents, established online youth community, operated online service platform, held online theme activities, vigorously promoted the construction of the online Communist Youth League, narrowed the distance between the Communist Youth League and youth league members and young Pioneers, and further expanded the network coverage of the Communist Youth League. Carefully cultivate a group of youth network xuan talent in 2018, TuanSuSong county in the county grassroots youth league organizations, the young pioneers organization selected talents in various field, selected 108 high political stance, theoretical foundation, strong writing ability as the communist youth league system network propagandist, covering cadres, the young pioneers counselors, young volunteers, college students, etc, mainly responsible for writing and promoting local youth and the communist youth league work, network public opinion monitoring and public opinion guidance, participate in online theme TuanRi activities, etc. At the same time, the Communist Youth League county Party Committee often holds the network publicity staff business work training, and regularly commend a number of outstanding network propagandists, to give certain material and spiritual rewards. Heart to create a group of online youth community the county set up propaganda and network work, in addition to the minister fixed for the authority staff, the rest are trainee college students, teachers, the department in addition to responsible for material collection, layout design, manuscript proofreading, manuscripts and video release, is also responsible for direct contact group online youth community. To better service youth, condensed youth, good youth work, the county according to the contact personnel industry, identity, demand, to contact work object and service object classification, respectively with the county young pioneers work group, directly under the middle school, township communist youth league system work group 14 (WeChat group 12, QQ group 2), eight, college students funding service group of college students power SuSong league network propaganda group of three, all kinds of volunteer service group, direct contact member youth of more than 2000. The establishment of various youth communities also better promotes the communication and learning among the youth league members, and facilitates the development of activities and the docking of resources. In order to reflect the voice of young online service platforms, respond to their demands, and safeguard their rights and interests, Tuanusong County Committee relies on the "smart group building" system, Actively develop online service platforms, Built and long operated the "Voice of Susong Youth" wechat public account, There are already more than 94,000 followers, Timely push the work trends of the Communist Youth League every day, Regular posting of web "benefits", Including the Susong Communist Youth League to help the youth public examination public welfare training class, Susong youth summer public welfare training class, Susong nationality college students "return to home" summer government affairs practice post and other activities, To constantly address the practical needs of various youth groups, Become the county youth widely praised, the circle of friends in the most attention to the WeChat public number. At the same time, the Youth League Susong County Party Committee also created the official TikTok number of "The Voice of Susong Youth", regularly pushing all kinds of current political hot news and videos showing the achievements of youth style and grass-roots team organization work, held the first TikTok live youth dating activity, and built a platform for more than 40 single young people to make friends. To alleviate the visual fatigue of young netizens, group susong county relying on college students power the communist youth league propaganda work team, produced by college students take turns WeChat public graphic, through the "triple system", daily push exquisite graphic and video, on the one hand, can make the release of the public and TikTok is more youth love, on the other hand also alleviate the office staff less, understaffed, and strengthen the working force of the group. Organize a series of online theme activities to enrich the forms and carriers of theme group and day activities, Group susong county has organized to carry out the "march learn lei feng", "qingming festival heroes", "the most beautiful green worker", "the most beautiful war epidemic youth" selection, "may 4th spirit have I", "less to meet the congress, be players", "flying youth dream new journey to study and implement the spirit of the fifth plenary session of the 19 th" micro preach video selection, " please rest assured, Strong country has me "theme micro heart language video collection," red scarf narrator " selection and other online theme activities, Educating teenagers through the glorious course of the Party, Encourinspiring youth with tremendous changes in the country, To inspire young people with General Secretary Xi Jinping's important speech, We will further strengthen ideological and political guidance on the Internet. TuanSuSong county rely on "online communist youth league", in the ideological lead, volunteer service, poverty engines, flood fighting and rescue work has achieved good results, the next step will continue to innovate work form, expand the scope of service, enhance the cohesion of the communist youth league of youth, organization, appeal, promote the county communist youth league organization reform to truly implement the ground.

2021-09-10	Beijing, Sept. 10 (Xinhua) - -By Zhang Jianwei, "I want a new bag," "I want a plush doll," and "I want a basketball."... These small wishes may be insignificant for many people, but they are an expectation for some children from poor families. Group Yongzhou of Hunan province Ningyuan county collected the rural revitalization of residency support point of paradise town fragrant flower bridge village 11 families difficult left-behind children micro wish, take widely launched love people online claim "micro wish" way, to mobilize caring people from all walks of life extend warm hands, 11 children "micro wish" all reached. On September 8, Tuan Ningyuan County Party Committee sent the "micro wish" gifts to the children one by one. Love to help the child Zhang Chenxi said: " I finally have a big plush doll, after I can hold it to sleep at night, will never be afraid again.”

2021-09-23	Anhui su: let every township youth corps committee have "skills" when the communist youth league reform deepening "a township (town) a" Anhui su: let every township youth corps committee have "skills" township secretary He Liang some shy, basic is to shout, mind practice many lines are useless. With the small audience, the planned interactive session was cancelled. This year, he Liang, who has been working at the grass-roots level for three years, stepped on the post of the Communist Youth League for the first time. He wondered how to make morels, black fungus, dried bamboo shoots, trichosanthis seeds, organic tea and other featured agricultural products on the e-commerce express train, so he tested the water to carry out the "hometown has good gifts, waiting for you" public welfare agricultural assistance project. The live broadcast room is funded by the township, and the live broadcast platform and technical support are provided by the local young entrepreneur- -Manxiang E-commerce Company."Talking about advantages, word of mouth, stories and feelings, I learned words from other anchors and tried my best to impress netizens."After many brewing, in the middle of August, He Liang summoned up the courage to appear in the studio again:" Hello! I am He Liang, secretary of the Youth League Committee of Chen Han Township. Welcome to the group League studio!"Opening with the story of General Chen Wu from the Three Kingdoms Period and General Chen Han in the late Yuan and early Ming dynasties, he told about the origin of the place name of Chen Han Township."Netizens, do you want to 'taste' after all? We are a remote mountainous town, the ingredients are pure natural green pollution-free is a major feature. Diaoyutai Reservoir is the drinking water source protection area in Susong County. Surrounded by mountains on three sides, the water quality is clear and sweet, and the fish is delicious."He Liang then went into the topic and introduced wild fish growing in Diaoyutai Reservoir in Chenhan Township. A few minutes later, the sound of the order came, two hours of live transaction a total of 52 orders, He Liang confidence doubled. Chenhan Township to build the brand of "help farmers live broadcast", is part of the work of "one township (town) one product" of Tuanusong County Party Committee. Since this year, the League Susong County Party Committee has focused on the main responsibility and main business, stimulated the vitality of the grass-roots league organization, promoted the sinking of high-quality resources, gathered efforts to build the grass-roots Communist Youth League characteristic work brand, to make up for the vitality, attraction and insufficient branding of the league, so that the young people have a real sense of participation and gain. This year, Zhang Xueyu, born in 1994, took office as secretary of the Communist Youth League Committee of Qianling Township. During the summer vacation, she led 24 volunteers to tutor nearly 150 left-behind children in rural areas."During the visit, I saw the children's eager eyes and pure smiling faces, and I deeply felt that the left-behind children need more care. By volunteering, let the kids know that, despite their absence, many parents are still caring."Zhang Xueyu regrets that only by going deep into the grassroots to face the masses, can we more clearly understand the needs of the masses. Volunteers found that children needed family companionship, learning places, and expanding their knowledge. To this end, the township youth League committee will be each village (residence) of the rural library to use up, to create a "feeling warm childlike innocence with love growth" care for rural left-behind children activity project. At each activity, the volunteers will guide the children to share the books they have read, or read the good words and sentences in a short piece of books. Then, the volunteers helped the children with their homework, explaining legal knowledge and drowning prevention knowledge. In the early days of the semester, volunteers also found that many left-behind children had signed up with their grandparents, but many elderly people were not well-educated and unable to move. So, they are divided into several teams, someone offered to help the children queue, sign up, someone to carry the children's luggage."At the grassroots level, only by providing precise volunteer service can we maximize the power of volunteer service."According to Zhang, they will use the holidays and weekends to provide regular companionship services to help the children develop good habits and enrich their spiritual and cultural life. The Youth League Committee of Fuyu Town carried out the project of "Good Helper for Small Grid Members", recruited 25 returning college students, sent them to each grid for training and division of labor, and regularly organized "grid members" into the community to carry out volunteer services such as health renovation, disability care, policy publicity, and help vaccination."When we came to explain the vaccination policy, some villagers said they had toothache and did not dare to get vaccinated. We explained the contraindications; someone was not convenient to go out with children, and they offered to help take care of the children."Chen Jianqiu, a returning college student, said that participating in the activity has deepened her understanding of the Communist Youth League. I used to think that the Communist Youth League is an organization responsible for the development of the league members, but now I realize that the Communist Youth League is "where it is needed". In addition, the Youth League Committee of Zuba Township carried out the project of "Inheriting Cultural Youth Tour", and organized local youth league members to publicize and promote local culture such as Wenci and fish noodles. In August, Hong Miaomiao, secretary of the Youth League Committee of the township, organized youth league members to systematically sort out the development of nan Ci through on-site investigation, watching programs and collecting words and images, and made a micro video of "Tuan Tuan Speaking Intangible Cultural Heritage" and spread it online. BeiTownship Youth League Committee implemented the "Youth Red Dream Publicity Project", Huikou Town Youth League Committee carried out the "Safe Construction, Youth Volunteer Travel" project, and Liuping Township Youth League Committee implemented the "Innovative grassroots governance, promote rural revitalization" project... in Susong County, the Communist Youth League activities are more and more distinct, and their coverage is more and more wide. In June this year, the Youth League Susong County Party Committee held a rural revitalization "one township (town) one product" creation work promotion meeting, the township communist Youth League brand creation of detailed analysis and comments, encourage the township youth League committee based on the local reality, boost the rural revitalization. The youth League county Party Committee will also conduct a dynamic evaluation of the activities of the grass-roots youth league organization in the form of "substituting awards instead of subsidies", and give rewards and support at the end of the year. He Liang felt that the live broadcast also brought great inspiration to the work of the Communist Youth League in the village- -the youth League cadres should follow the trend of The Times, learn new skills and new skills, continue to promote the excellent league members and agricultural youth to walk into the broadcast room, and bring goods for the agricultural products in their hometown with their skills and specialties."In the future, the township Youth League committee will give full play to the advantages of organization and resources, absorb the excellent local e-commerce enterprises and rural innovation youth, carry out rural live broadcast training and entrepreneurship training activities, and effectively serve the youth employment and entrepreneurship."" In the past, the grass-roots youth league organizations are used to the 'foot on the watermelon skin' type of work mode, to what time node, or what theme to carry out activities. Now, we encourage the grass-roots youth league organizations to incubate a number of high-quality and normal activities, so that the youth league members can make a difference, so as to enhance the contribution and presence of the grass-roots youth league organizations in serving the overall situation of the party and the government."Said Yu Ling, party secretary of Tuansusong County. China Youth Daily reporter Wang Haihan Wang Lei source: China Youth Daily

2021-09-23	Anhui province territory promoting key youth group management service corps in Anhui province territory party committee promoting key youth group management service wei correspondent Jiang Yuecha WeiTing) since 2021, the territory of Chuzhou in Anhui province district party committee unified deployment and pku leadership, attaches great importance to the key youth group management service, by creating projects, build positions, legal propaganda, solid key youth group management service work. Create the "Guardian Angel". In order to further implement the legal education and protection and care for young people, and promote the all-round development and healthy growth of young people, the "Guardian Angel" brand public welfare project was launched by the Chinese Communist Youth League Committee, together with the Chuzhou Beautiful Home Social Work Service Center and the Chuzhou Organizing Committee. Project for the adolescent parents of parents to carry out the classroom, group counseling, psychological counseling activities, form the government, school, family tripartite linkage, implement prevention, intervention, to adolescent mental health service and guidance, actively build conducive to adolescent mental health environment, accumulated more than 6000 students and parents to participate in, the parents, schools, society, consistent high praise. The news and information of "Guardian Angel solves Problems and Protect Miao Action to Help Growth" has been followed by People's Daily, Xinhuanet and other central mainstream media. In order to more effectively do minors psychological counseling, especially the left-behind children, pku in honeysuckle streets, streets, dragon pan street set up a "guardian angel" project psychological counseling workstation, hire several counselors with professional qualified psychological counseling for parents free counseling services, guide to help parents to carry out the prevention, intervention, comprehensively protect the healthy growth of minors. Build characteristic positions. TuanOuWei joint happy streets in the joy of street new era civilization practice has set up the south territory "youth", enrich the spiritual and cultural life of teenagers, with library, group representative reception room, psychological counseling room, youth activity room four functional areas, can effectively radiation surrounding three streets, eight villages and towns, for the majority of teenagers set up learning, communication, promotion, mutual platform, enrich the spiritual and cultural life of teenagers. We will publicize the legal system. With three "national double zero demonstration community" as the gripper, actively carry out legal propaganda, psychological counseling and prevention of juvenile delinquency volunteer service activities, earnestly especially the rights and interests of teenagers and crime prevention work, printed the newly revised protection of minors interpretation manual 1500 copies, further community school propaganda, actively create a healthy, safe and harmonious growth environment for teenagers. At the same time, give full play to the role of principals of the rule of law, seriously combine the actual work, with the theme of "stay away from danger, stay away from crime" in various primary and secondary schools regularly hold lectures on the rule of law, analyze the typical cases of juvenile crime, and further enhance their self-awareness of protection. Care for key groups. Together with the "Voice of Youth 12355 Rights Protection and Service Alliance", relevant experts and volunteers were invited to the school and organized more than 10 public welfare activities such as mock court, safety knowledge and drowning prevention publicity for the protection and growth of youth rights and interests. Help vulnerable groups, and carried out many youth volunteer activities to help the disabled. For the special group of left-behind children in rural areas, the "Spark Plan" has been formulated, which carried out a series of activities such as "Yitong Spark" growth camp, dream library, psychological counseling public welfare class, and micro wish claim.

2021-09-22	Anhui tianchang city to carry out electric fraud propaganda volunteer service activities in Anhui province to carry out electric fraud propaganda volunteer service activities jianwei correspondent, Hua Yufeng check WeiTing) in order to further enhance the image of the city, Chuzhou city in Anhui province tianchang municipal party committee joint propaganda department and police station departments actively organize volunteers to prevent electric fraud propaganda action, booster tianchang city national civilized city to create work. During the event, volunteers popularized common fraud methods to the public through household visits and posted posters. Promote the application by explaining the installation process of the "National Anti-Fraud Center" APP, and help the public to install it. Up to now, volunteers have promoted and installed the APP for more than 3,200 people. The development of this activity has not only further enhanced the vigilance and attention of the masses to prevent telecom fraud, but also created a good social atmosphere for the whole people to prevent fraud, and further enhanced the residents' awareness of prevention. Youth League Tianchang Municipal Party Committee will continue to carry out anti-fraud propaganda and "national anti-fraud center" APP promotion of volunteer service activities, for Tianchang city to create a civilized city to contribute to the youth glory.

2021-09-13	Xiangshan district in Anhui province less committee organization salute teachers 'day activities in Anhui province less committee organized "respect teachers, feeling teacher" salute teachers' day activities JianWei correspondent QuWan check WeiTing) on September 10,2021 is the 37th teachers 'day, in order to express respect to teachers, further enhance the consciousness of youth respect teacher, set a new example, create strong teacher's day atmosphere, Huaibei city in Anhui province xiangshan less committee organize "respect teachers, feeling teacher" salute teacher's day. Carry out themed Thanksgiving activities. The school working committee organized a series of recitation activities, such as "warm blessing", "reciting Thanksgiving for students" and "children's heart". Lang lang reading voice tells the deep feelings between teachers and students, and the beautiful words praise the love for teachers. At the same time, through painting blackboard newspaper, singing a series of songs for Thanksgiving teachers, making handmade cards, recording short videos and other ways to express their love and respect for teachers, strengthen the communication between teachers and students, home-school communication, enhance the feelings of teachers, students and parents. Carry out thematic education activities. This year marks the centenary of the founding of the Party and the first year of the 14th Five-Year Plan. The Xiangshan District Youth Working Committee and the District Education Bureau continue to carry out the "four histories" learning education and the special education of teachers' ethics, earnestly publicize and implement the policy of strengthening teachers and benefiting teachers, organize teachers to carry out exchange activities, strengthen teachers, and improve the ability level of teachers. Extensive online publicity. Xiangshan District Young Working Committee through the Xiangshan Communist Youth League WeChat public platform widely publicized teachers 'Day activities within the jurisdiction, popularize teachers' Day related knowledge, advocate green environmental protection, thrift Thanksgiving Teachers' Day, strengthen the construction of youth honesty and self-discipline, enhance the awareness of hard work and plain living through online activities to promote the district strong festival atmosphere. Through the "courtesy of teachers, feeling of teachers" salute teachers' Day series activities in the district online and offline hot development. Further close the friendship between teachers and students, stimulated the majority of young people to respect teachers, love teachers, respect teachers' enthusiasm, so that the friendship between teachers and students is more consolidated, further enhance the feelings of teenagers to love teachers, to create a strong atmosphere of caring about teachers and love teachers in the district.

2021-09-15	Group of Anhui shou county county issued north youth entrepreneurship loans financial discount funds of Anhui shou county county issued north youth entrepreneurship loans financial discount funds JianWei correspondent Zhang Yan check WeiTing) recently, the Anhui Huainan shou county county held 2021 Anhui youth entrepreneurship loan financial discount distribution ceremony and young entrepreneurs symposium, activities presided over by the TuanShou county principal, get discount entrepreneurial youth, a total of more than 20 people to attend the meeting. Distribution ceremony, group county county principal introduces the 2021 north Anhui youth entrepreneurship loan financial discount project overview, to get discount entrepreneurship youth financial discount funds, young entrepreneurs everyone opinions, share their own exchange of entrepreneurial course, entrepreneurial experience and the difficulties and problems encountered in entrepreneurship, the atmosphere is warm. The financial discount interest work of youth entrepreneurship loan in northern Anhui province is an important measure launched by the Communist Youth League to better help and support youth entrepreneurship and employment, and to promote youth entrepreneurship. Since 2013, has accumulated for the county 399 young entrepreneurs, declare the Anhui youth entrepreneurship loan financial discount funds of 3.4269 million yuan, which involves breeding, agricultural processing industry, labor intensive or technology intensive industry and commerce, services, and other fields, effectively alleviate the young entrepreneurs early capital problems.

2021-09-18	Group of Anhui province du district party committee to carry out the Mid-Autumn festival creative fan DIY activities in Anhui province du district party committee to carry out the "our festival" creative fan DIY activities JianWei correspondent, Ding Jianan check WeiTing) Mid-Autumn Festival will come, to vigorously carry forward the excellent traditional culture of the Chinese nation, on September 14, Huaibei city of Anhui province, du set district party committee joint du set district civilization office in du district huaihai east road school carried out "our festival Mid-Autumn festival" creative fan DIY activities. The picture shows the students showing their results. At the beginning of the activity, the teachers of Huaihai East Road School introduced the origin of the Mid-Autumn Festival, customs and the significance of the group fan through interactive questions and answers, so that the children could better understand the cultural connotation of the Mid-Autumn Festival. The picture shows the volunteers guiding the students as depicted. Then, the volunteers distributed excellent group fans, colorful paint, and a full variety of painting tools to the students. A pure white fan and colorful painting paint caused great interest, the students have picked up the brush, serious conception, imagination, combined with flowers, animals, cartoon characters to draw a unique fan, with childlike innocence will be a plain fan painted a novel and pure Mid-Autumn festival picture scroll. Through this activity, let the children experience the fun brought by hands-on participation, further understand the unique charm of traditional culture in the joy, but also let them more cherish Chinese traditional culture more, consciously become the disseminator and promoter of Chinese traditional culture.

2021-09-04	Guangxi Mashan: singing the rural revitalization of the song of youth in Guangxi Mashan: singing the song of rural revitalization of the youth tuan Mashan county committee to carry out a series of activities, make full use of this summer, organize 2021 college students summer "home" and primary and middle school students in their hometown to participate in social practice, make the party history learning education "I do the practical work for the masses" activities to the ground work. Since March, the Communist Youth League Mashan County Committee organized 19 league (work) committee and 119 young first brigade leaders to carry out the party history learning education, radiation led 312 league (total) branch more than 8000 members and 1305 young pioneers more than 37,000 young pioneers. To express the memory of the martyrs through the patriotism education base in the district of the county, to help the young students to understand the heroic deeds of the red story speech contest, watch the red revolutionary songs and watch the "first lesson", and actively guide the majority of young people to establish patriotic ideas, cultivate love home, patriotism and love socialism. Build a good party building with a "platform", to ensure that the brave tide have the responsibility to guide and help the majority of young students on the good and reality of the "big ideological and political class", in the social classroom education, long ability, make contributions. The communist youth league masan county committee through "masan youth circle", the county media center and the villages and towns and units public platform to the whole society called for more than 100 (time), publicly recruited to the "2021 masan county students" home "summer social practice" 93 horse college student volunteers sign up for "into the grassroots, service" activities. At the same time, the ancient village village ancient tang village, zhou deer town altar sand village, village party leading village youth league as active, strengthen communication, rely on residency first secretary support unit, respectively, organized the Guangxi art college students, Guangxi university for nationalities green home environmental protection association summer "a program under which officials" in the village with art joint performance, condolences, whole, painting wall, rural ecological environmental protection propaganda theme activities. Qiaoli, Bailongtan and other township youth League committees to increase publicity efforts, and has organized more than 10,000 young students to participate in the summer social practice activities in this village nearby. The Mashan County Committee of the Communist Youth League takes policies to fully mobilize the enthusiasm of young students, use the summer vacation of 2021 to return to the countryside or in the countryside, and consciously participate in the work of the local Party committee and government. Assist in the daily work. Mainly in the township poverty alleviation workstations, party and government offices, civil affairs offices, rural construction comprehensive service centers and other departments to assist in the collection and sorting of all kinds of archives. According to preliminary statistics, as of August 28, college and technical school volunteers have received 76 people and mediated disputes; collected more than 10,000 documents; checked 528 party members; verified projects for poverty alleviation and 219 people; assisted 83 village collective economic work; and prepared 37 NPC meetings. Participate in the epidemic prevention and control work. Proactively assisted all townships and departments in the epidemic prevention and control work. During this period, college and technical secondary school student volunteers participated in the volunteer service activities of vaccination sites, assisted more than 660 people to mobilize vaccination and maintain the order of the vaccination site, and posted 1,327 "two-code joint inspection" posters, and more than 5,630 copies of publicity materials were distributed. Participate in rural revitalization work. Participate in the villages and towns and units to create the national civilized city propaganda activities 526 people, household to distribute more than 5600 publicity materials, simulation questionnaire survey 441 people, 362 civilized parking persuasion, persuasion market uncivilized stalls 232 people, visit condolences to special groups 80 people, to carry out the "protect the mother river" cleaning activities 154 people. Assisted in the construction of 617 "three micro", 124 villages, "2,600" sanqing "," 1,900 "three demolition", 143 painted walls, to help to improve the rural revitalization style work.(Huang Qiheng, Wei Zhenlong, Du Zhe)

2021-09-10	Anhui Jinan youth community officially listed Anhui jin youth community built wei correspondent, Du QingHe check WeiTing) according to the requirements of community youth work, in order to better serve for district residents, create a convenient environment, broaden the service function, group of Anhui regarding Lu'an Jinan district party committee adhere to the "hardware", "software" together, to the two pilot community (jinyu community, nine ditch community) youth positions to upgrade, and recently listed, implements the office space, activities, learning place, to enhance the community youth group construction service level. Innovation service position, service function "full" up. In accordance with the requirements of reasonable layout, beautiful and tidy, distinctive characteristics and strong atmosphere, we will set up propaganda positions to widely publicize the positive energy content such as filial piety, love and relatives, civilization creation and volunteer service. Jinyu community has built the "Green Kwai Park", with the concept of "lighting up every heart lamp belonging to children, building every heart house belonging to children, and filling in children's every space belonging to children". The two pilot communities have set up "Bud" volunteer service teams and "Rainbow" volunteer service teams to provide volunteer services such as psychological counseling, epidemic prevention and control, respect and love care, and love support for residents in the area. The Party building leads the league building, the red atmosphere "thick" up. In accordance with the principle of position sharing and multi-use and time-sharing use, a multi-functional activity room has been built, equipped with perfect facilities, and the "red story corner" has been built. Through regular activities such as "Theme Party Day", "Theme team Day", "red reading club", "drowning prevention summer forum" and "parent school". The implementation of cultural and sports venues, activity venues "use" up. The youth community has been built into a youth activity room and open to the public free of charge, which is divided into reading room, dance room, fitness room and popular science room, equipped with TV, audio, chess, table tennis table and other equipment, and the teenagers in the area can sing, dance, practice calligraphy in the youth activity room. Jin'an district youth community position construction to improve the community residents happiness index as the ultimate goal, do the standard in place, function in place, personnel in place, service in place, to maximize the service efficiency for the people, to further enhance the overall service quality of the community, for better community youth action work laid a good foundation.

2021-09-04	Beijing dongcheng district: with the youth example told the story of the youth, the party story with young example fan not morning correspondent Hou Xudong) recently, the Beijing dongcheng district party committee to mobilize 1274 youth league branch, more than 40000 members to participate in the "please rest assured power have me" theme activities, explore a both pay attention to "know", and emphasize the effectiveness of "line" guidance, service youth road. The Dongcheng District Committee of Beijing has set up the Dongcheng Communist Youth League Youth Publicity Group to encourage young examples to tell the stories of the Party, revolution and heroes well. This is to preach as the carrier of the red blood line, ceaseless inheritance to today. The publicity group innovates the form of publicity, and carries out the differentiated, differentiated and personalized publicity for the student league members, the youth team members and the league members who have entered the work positions, so that the young people can talk to the youth. At the same time, relying on the "Dongcheng Friends" wechat public account, TikTok, Kuaishou, B station and other social media platforms, the network language is integrated into the propaganda content, and it stimulates the feelings of knowing history, love the Party and patriotism in a way popular with young people. Relying on the rich red resources of Dongcheng District, the publicity group also spoke in red landmarks such as the old site of the editorial department of New Youth, CAI Yuanpei's former residence and the memorial place of the March 18 Massacre, so that the young people could on the scene, measure history with their steps, and promote the study and education of the Party history to show confidence and confidence. In addition, the league dongcheng district party committee widely launched youth to participate in the celebration of one hundred service and volunteer service, for Henan, flood raising donations and culture, medical system, group organization, "capital" hope, "play" childlike, "medical" health, "group" help growth theme practice, for accurate help teenagers provide online schoolwork counseling, visiting condolences "star of hope" teenagers, for 30 excellent students to apply for grants, organize troubled teenagers to watch red drama. According to the introduction, The majority of league members and youth volunteers actively participate in the urban volunteer service task of the 100th anniversary of the founding of the Party, The jurisdiction has 20 urban service stations and 19 key community sites, Extensive carry out voluntary services such as directions, civilized traffic, order maintenance, cultural publicity, environmental beautification, neighborhood watch and other volunteer service activities, The cumulative time is 17,000 hours, The service group is about over 200,000 person-times; Primary school and middle school rich and colorful party history knowledge competition, patriotism education base visit and party history learning theme practice activities are attracting more and more youth league members, young pioneers to join in, Will " please rest assured that the party, The slogan of strengthening China " is transformed into a deep recognition of the unity of knowledge and action.

2021-09-13	Wuzhishan city of Hainan province "listen to the party, feeling party, follow the party" about cultural activities and "hope project interpreta dream action" donation ceremony wuzhishan "listen to the party, feeling party, follow the party" about cultural activities and "hope project interpreta dream action" donation ceremony Jianwei correspondent Zhou Yutong) recently, wuzhishan "listen to the party, feeling party, follow the party" about cultural activities and "hope project interpreta dream action" donation ceremony held in wuzhishan city south holy town spring village culture square. Wuzhishan city towns, the relevant departments of the main leaders, representatives of the publicity groups, love enterprises, love units and the villagers participated in the activity. Another highlight of this activity is the literature and art and "hope project interpreta dream action" merger, wuzhishan city has long insisted to carry out hope project interpreta dream action, now the project has been carried out for 19 years, help poor students round the university dream, take concrete actions to respond to a call by the municipal party committee municipal government, in power rural revitalization, promote the development of education, service youth growth play a role. The activity also prepared the "prosperous times Wuzhishan", "never forget the original mind", "Xinjiang", "China in the lights", "the power of Renaissance" and other artistic performance programs, the program also interspersed with the party building three rural knowledge questions and answers, in the form of the audience learned the 100th anniversary of the founding of the party, Hainan free trade port construction, rural revitalization and other policies. The final online and offline donations will be used to help poor students realize their dream of college. The event was organized by the Office of the Leading Group of Rural Affairs of the CPC Hainan Provincial Committee, the Hainan Provincial Department of Agriculture and Rural Affairs, and the Hainan Provincial Rural Revitalization Bureau. Sponsored by the CPC Wuzhishan Municipal Committee, Wuzhishan Municipal People's Government and Hainan Radio and Television Group (Group). Wuzhishan municipal committee of the rural work leading group office, the CPC Wuzhishan bureau of agriculture and rural areas, Wuzhishan rural revitalization bureau, the communist youth league committee, Wuzhishan tourism and culture of radio, film and television sports bureau, wuzhishan south holy town people's government, Wuzhishan media center, Hainan radio and television green agricultural science and technology co., LTD.

2021-08-31	Group xinfeng county of Guangdong province to carry out love student volunteer activities love student dream set sail in Guangdong xinfeng volunteers to action for figure, China youth network Beijing on August 31 (reporter Ji'an-wei zhang, correspondent Li Excellence) recently, the Shaoguan xinfeng county organization xfeng individual private enterprise volunteer association to carry out the "student Ming, plastic dream cast" love student volunteer activities, the social people warm and caring care to the hearts of teenagers. This year's program focuses on the rural revitalization and consolidation and development of poverty alleviation, sending about 160,000 yuan to 49 students. It is worth noting that, according to the requirements of COVID-19 prevention and control, the activity was changed from the previous concentrated activities to point-to-point visits. Volunteers went from street to street to each student's home to talk about their life and study, give them encouragement, and personally sent the "student gift package". It is understood that since 2016, Xinfeng County charity education charity project has been carried out for six years, up to now, a total of 208 local high school students, college students, a total of 850,000 yuan of grants.

2021-09-09	Hedong dong district of Shandong province cadres "chain" management system of Linyi hedong district cadres "chain" management system promoting the county communist youth league organization reform pilot work wei correspondent Zhou Zhipeng) Shandong Linyi hedong district focus on the county communist youth league organization reform pilot requirements, improve the cadres "selection, education, tube, with" chain management system, "change" the cadres team new vitality. In terms of selection and allocation, a "3 + 1" cadre team system has been established. In the organs of the Communist Youth League District Committee, the allocation and post rate of cadres in the establishment reached 100% and kept maintained. According to the principle of "supplementary", the Standing Committee of the District Committee assigned two temporary deputy secretaries and five part-time deputy secretaries. In towns (streets), the secretary of the Party (work) Committee serves as the director of the Youth Working Committee. Before the end of the year, the Communist Youth League District Committee and the human resources and social security department will recruit a full-time youth affairs social worker for each town and street. In the village (community), absorb young veterans, rural good youth and other outstanding youth as youth league cadres. In the field of non-public enterprises, we will promote the establishment of youth working committees in large building business districts, e-commerce, logistics and other industries. In education and training, we should grasp the three key links of education and training, practice and pair help. The Hedong District level has issued the Measures for the Management and Use of Youth Work Funds in Hedong District. According to the annual standard of 8 yuan per person for permanent residents aged 14 to 35,1.04 million yuan is guaranteed annually, from which 200,000 yuan is listed as the training funds. Every year, 30 cadres to the organization department to the frontline training. The implementation of party organization leaders and youth league cadres "one to one" pair of help. In the management assessment, we should improve the three mechanisms of term commitment, performance record, and retreat. Organize the league cadres to sign the annual target responsibility letter, establish the "four-in-one" evaluation mechanism of party and government evaluation, league cadre report, inner-league competition and youth examination, to provide an important basis for promotion and use. Establish the mechanism that the league cadres can enter and exit, define the exit "4 situations", and maintain the advanced nature and purity of the team. In terms of guarantee incentive, the "four incentives" are development incentive, honor incentive, promotion incentive and salary incentive. Part-time cadres and youth social workers outside the system can enter district institutions through means of targeted examination and talent introduction. According to the proportion of 20%, the district determines the full-time village (community) youth league secretaries and youth affairs social workers with outstanding performance, and each person will be awarded 300 yuan per month.

2021-09-07	Anhui chaohu college students "home" social practice MSchaohu in 2021 college students "home" social practice successful MSC wei correspondent Liu Chaohui) chaohu joint chaohu people club bureau, chaohu talent office, combined with the current party history learning education "I do the practical work for the masses" practice, organize the 2021 summer college students "home" social practice, to attract the young talents home, contribute to the construction of the hometown youth strength. Activities since its launch in June, pku for the city's party and government organs, institutions, village (community), enterprises to collect college students "home" social practice job demand, collecting practice 118, through online form independent registration, combined with the needs of unit of choose and employ persons and students learn professional, practice experience, such as practical situation, finally selected the 93 college students to each unit in the city to participate in social practice. Before the official post, the Communist Youth League Committee also combined with the Chaohu Red Cross Association held a "return to home" social practice college students emergency rescue staff training class, a total of 40 practical college students to participate in the training, training aimed to further improve the emergency rescue ability of social practice college students back to their hometown. It is reported that Chaohu City, as the only pilot county and city of the social practice of returning to Hefei hometown, has four teams rated as the key practice team of college students' rural revitalization in 2021. Through the social practice of returning to their hometown, college students are organized to participate in the rural governance, party history publicity, government services and other central work.

2021-09-08	Anhui si county implementation village "choose green push" project Anhui si county implementation of village "choose green push" project training rural young talent wei correspondent, LuoJia super check WeiTing) with the development of economy and society, more and more rural youth migrant workers, rural cadres "two committees", "green" party member problem is still prominent, for rural youth difficult, difficult to leave, education, Suzhou city in Anhui province to implement "choose green push" project as the gripper, continuously training village reserve cadres, youth backbone."Choose green push" project mainly through the normal village-level reserve cadre recruitment, young talent recommended, for the village youth league secretary with broaden horizons, for the village "two committees" team with the personnel, village-level party member development reserve young talents, and gradually solve the problem of village-level young talent shortage, continue to the rural grassroots injected "source". Two ways layer upon layer screening and selection of talent open recruitment "choose youth". Every year led by the county party committee organization department, joint group county, county people club bureau and other units, open recruitment under the age of 35, college degree or above village reserve cadres, through the "public ability test + structured interview + qualification" way, select outstanding youth to the village, has three consecutive times for the public recruitment 556 under 35 years of college degree village (community) reserve cadres. Party organizations feel the platoon "push excellence". In town (development zone) as the unit, pay attention to from the poverty alleviation team leader, rural rich expert, veterans, returning employment students, social organizations recommended reserve cadres, according to the village party organization recommended, personal recommendation, party organization, township process can director, want to director to absorb into the village-level reserve cadres. At the same time, Sixian county will open the door and strict examination combined, all "select youth and push excellent" reserve cadres must go through the organization, discipline inspection, the court and other 16 departments, to ensure that the political clearance, excellent quality, no violation of law and discipline. Up to now, the county 187 villages (communities), each village normal to maintain more than 3 reserve cadres, and at least one village identified a reserve cadres concurrently deputy secretary of the league organization or to assist the village league organization work. Five systems front-line training personnel record management system. Sixian County issued eight Opinions on Further Standardizing the Use and Management of Reserve Cadres at the Village Level. According to the requirements of "one person, one file, one township, one library, one county", we established a ledger person by person and implemented dynamic adjustment, which further standardized the whole process of the selection, training and appointment of reserve cadres. Centralized training system. According to the principle of "what, what", the basic party construction, group, collective economic development as a training focus, each year at least 2 annual reserve cadre training class, organize a group of outstanding reserve cadre to Suzhou cadre institute, Zhejiang university and other developed areas to participate in the training, improve political theory and practical ability. Tracking and training system. It is clear that the village team leader, the village cadres, and the village secretary will be "help" to track and train the village-level reserve cadres to ensure the sustainability of the training. Townships (development zones) regularly hold reserve cadres "challenge competition" to build a stage for reserve cadres to show their ability. Temporary post exercise system. Taking towns and townships as units, reserve cadres from various villages (communities) are regularly transferred to the township Communist Youth League, organization office, rural revitalization office and other departments for temporary post training, so as to broaden their working ideas and improve their comprehensive ability. First-line training system. Guide the reserve cadres in the village as the initiative, the front-line exercise, the implementation of the "package piece package group", practical burden, pave the way, around the rural revitalization, environmental remediation, civilized city and other central work, the implementation of big training, big exercise, for the village "two committees" cadre reserve to lay a solid foundation. The four major measures were taken to improve the retention of talents and strengthen the basic treatment. We will improve the mechanism for increasing the basic remuneration of reserve cadres year by year, pay the basic salary of the deputy village "two committees" and increase it year by year. At the same time, we will purchase "three insurance" of endowment insurance, medical insurance and personal accident commercial insurance for urban and rural residents for village-level reserve cadres, and community reserve cadres will enjoy "five social insurance and one housing fund". Strengthen assessment management. The star rating of the reserve cadres system, by the organization department of the county Party Committee and the Youth League county Party Committee of the reserve cadres on the work of the reserve cadres at the end of each year, directly linked to the performance reward, and to issue 4,000 to 14,000 yuan according to one star to five star. We will strengthen policy support. We will actively provide reserve cadres with project resources, entrepreneurship policy support, loan discount interest and other services, encourage and guide reserve cadres to run professional cooperatives, take the lead in getting rich and leading getting rich. Strengthen political incentives. Pay attention to political incentives, for outstanding performance, county-rural generally recognized reserve cadres, priority recommended to join the party, give honor, priority selection into the village "two committees" team, as of mid-August, the county has a total of 98 reserve cadres development for the communist party member, 61 into the village "two committees", 46 outstanding reserve cadres temporary village, deputy secretary of the party.

2021-09-07	Anhui Qingyang County "Lei Feng supermarket" ushered in a new batch of college students Anhui Qingyang County "Lei Feng supermarket" ushered in a new batch of college students Wei correspondent, Qiu Zhefeng check Weiting) recently, Anhui Qingyang County Lei Feng supermarket ushered in a batch of college students, and at the scene to get Lei Feng passbook, points exchange. In order to build a practical platform for college students returning to their hometown, guide and encourage outstanding college students to dedicate their youth at the grass-roots level, and spend a meaningful and fulfilling summer vacation, Qingyang County Party Committee of Anhui Province recruited college student volunteers from the society to build a civilized city and help with COVID-19 prevention and control, which was actively corresponding. During the summer vacation, according to the arrangement of the Communist Youth League county Party Committee, college students actively participated in the volunteer "three-yard joint inspection", conducted random investigations and inquiries on the vaccination situation in the whole county, and cooperated with the community to carry out vaccination work. We enthusiastically devoted ourselves to the creation of a civilized city, issued the National Questionnaire on the Creation of Civilized Cities at the County Level, participated in activities such as random photographing of uncivilized behaviors and He Xiaoqing, and jointly protected the achievements of the creation of a civilized city. This summer volunteer service activity for college students can be registered by points system, and exchange their favorite daily necessities in the Lei Feng supermarket. After the exchange of Lei Feng supermarket, the 2021 summer practice exchange forum was held in the conference room on the second floor of Furong Community. At the meeting, the college students spoke freely, talking about their experience, feelings, experience and shortcomings, which greatly aroused the enthusiasm and enthusiasm of the college students to participate in the volunteer service, and all said that they had spent a substantial and unforgettable summer vacation. In the next step, the Youth League Qingyang County Party Committee will adhere to the center, serve the overall situation, innovate ways and methods, enrich the activity carrier, organize and guide more youth league members to participate in the volunteer service cause, and contribute youth wisdom and strength to the new stage and modern "Jiuhua holy environment, beautiful Qingyang" construction.

2021-09-07	Yunan district in Anhui province comprehensively promote the county communist youth league organization reform pilot work in Anhui province regarding Lu 'an yu district comprehensively promoting the county reform of the communist youth league organizations at the grass-roots level pilot work wei correspondent Yang RuiZha WeiTing) in June 2021, Anhui province regarding Lu' an yu district in the national county communist youth league organizations at the grass-roots level reform pilot areas, regarding Lu'an is the only included in the county. Since the reform pilot work, yu an district party committee government and group Lu'an municipal party committee attaches great importance to, care, group yu an district party committee carefully planning, made solid progress, earnestly implement the requirements of the national county communist youth league organization reform, through elaborate deployment, improve the work system, stick to the key link, detailed target task, clear time limit, vigorously promotes the work implementation, reform work is solid and orderly. High-level promotion, strengthen the guarantee, the good "rudder" and "sail" communication and report frequently. The main leaders and leaders in charge of the Yu'an District Party Committee and district government listened to the reform work report for many times. At the same time, the Communist Youth League District Committee made two special reports to the Communist Youth League Municipal Party Committee. The Communist Youth League District Committee adheres to the accuracy of absorbing, improving and building consensus, the accuracy of every word and sentence, based on the reality and the strength of integrity and innovation, and formulated the Yunan version of the pilot task list of the county Communist Youth League around the four aspects of the reform content, which is specific for 40 reform matters. The reform atmosphere is strong. Lu 'an Municipal People's Government, Lu 'an Group, Yu' an release, Yu 'an District People's Government and other mainstream official micro voice reproduced, many youth League cadres also actively forwarded the pilot information, which caused great attention of the majority of the number of cadres and the masses. The WeChat official account of the Communist Youth League District Committee has set up special topics such as "reform dynamics" and "grass-roots youth League construction" to create a good working atmosphere in the whole district. Up to now, the Communist Youth League Anhui Provincial Committee "Wanqing Pioneer" wechat public account 6 times published the Yu'an District reform pilot work information. High organizational specifications. On August 15,2021, the district party committee held the 35th standing committee meeting, listen to and study the county communist youth league organization reform pilot work and grassroots youth league organizations "3 + 1" team construction work, set up by the district party committee secretary, deputy group leader, district party committee organization department minister and the district government deputy team leader, related 13 OuZhi units principal for members of the national county communist youth league organization reform pilot work leading group members. Strong factor guarantee. Make it clear that the township (street) at the beginning of the year budget to arrange the Communist Youth League work funds are not less than 20,000 yuan, to ensure that the special funds are dedicated. According to the number of posts, the full-time cadres of the youth League district committee, township (street) and high-tech zone of the youth league should be allocated, and timely adjust and supplement within three months of relevant personnel changes. This year, the district government continues to increase the support for the village (community) work of the Communist Youth League, the district Committee Standing Committee to study to determine the village (community) every year not less than 5000 yuan of the Communist Youth League work funds into the financial budget, and combined with the centralized transition, with a strong village-level league organization team. Scientific planning, optimize the mode, improve the "quality" and "efficiency" to grasp the real system design. System summarizes the development of grassroots league building work, the objective analysis of the present stage achievements, specifically points out the weak links and deficiencies, accurate to establish rules and regulations, in the party, pushing optimal recommendation, the party with league building (team building) inspector, member development management, grassroots league team construction system file. Improve the working mechanism. A two-way part-time education system has been established. The main responsible members of the District Communist Youth League Committee enter the leading Party's education institutions at the same level, and the responsible members in charge of the District Communist Youth League Committee enter the Party's education supervision institutions at the same level. We will strictly implement the dual management system for league cadres and the system for requesting instructions and reporting within the league. Further implement the working mechanism of "one specialty, one station and two links", and the liaison stations of district league representatives regularly carry out work for young people. Strengthen contact and guidance. The implementation of the "Thursday" action is carried out, and every Thursday, a member of the district Communist Youth League Committee leads the team to carry out investigations and visits for the league organization in the traditional field to improve the construction quality of the league. TuanOuWei cadres contact grassroots league organization system, is divided into four areas, each area clear one or more league party committee cadres contact guidance, the area of the league organization construction, league organization activities, league position operation, member development management and cadres style construction work to guide the supervision. Strengthen the branch construction, give full play to the role of the league construction instructor, to the traditional field of the league organization construction, the "two new" organization field of the league organization construction, two hands hard, to realize the new construction. The cancellation of the cancellation, the integration of the integration, the strong construction, improve the standardized construction level of the youth League branch. Adhere to the meeting must speak business, everyone strive to learn business, change the practice of an annual training for a quarter of the meeting model, to learn to promote business, to increase efficiency. Grasp the theme of the group day. The implementation of the theme league day "month recommendation" system, with the theme league day "ten action" as the starting point, to promote the implementation of organizational life, constantly in the form, content, the effect of continuous force, to carry out colorful league day activities, more and more grass-roots league organizations and league members like such a league organization life. Through the establishment of "Yu 'an District Youth Development Center of Lu' an City", to enhance the youth work force of Yu'an District. In-depth visits to youth, youth order, league organization orders. Through in-depth visits and investigations at the grass-roots level, the cadres of the Communist Youth League at all levels actively carry out the theme of "I do something for the youth" practice activities, to solve the needs and expectations of the youth. The "Xingzhi School" held by Taizi Temple Club in Gulou Street, the "4:30 Class" and "public welfare class" set up by Jinma Community in Pingqiao Township, and the Rong Love Rainbow Home built by Mahe Village in Xin'an Town have all well solved the practical problems such as summer vacation safety and schoolwork guidance for teenagers in the area, and have been praised by the residents. Close contact with the youth, the youth league organization call, the youth Daily to. To "around the center, the service general situation" as the main line, unity led the youth members for the new era good yu an construction youth strength, vaccination, prevention and control propaganda "red" ma 3 jia 3 "became a beautiful beautiful scenery line," yu "see youth power and city" college students summer social practice, attract more than 40 yu an college students to sign up, in the main district to create civilization, etc. Fully rely on youth, youth league organization needs, youth action. To "yu" see youth volunteer service station, youth home, group representative contact station, actively docking regarding Lu 'an public welfare volunteer service center, regarding Lu' an youth entrepreneurs association, regarding Lu'an youth association social work service center, district volunteers association organizations to raise social resources, promote in volunteer service, needy student, employment, talent training, practice education and other fields to form more, more high quality "green" font size project brand.

2021-09-06	Linquan, Anhui, Fuyang, Anhui, draw the private enterprise development "concentric circle" Wei correspondent, Gu Kaiyun check Wei Ting) " China's economic development can create the Chinese miracle, the private economy."Recently, the CPC Youth Linquan County Party Committee of Fuyang City, Anhui Province, held a training course on innovation and entrepreneurship for private entrepreneurs to charge up young entrepreneurs and empower innovation projects. The training closely focused on the writing of the business plan, the skills of the road show, innovative thinking, innovative marketing under the new market environment four aspects, in an interactive way to mobilize the enthusiasm of the students, with a large number of vivid cases, detailed data, the latest theoretical results, for a simple, authoritative and insightful explanation. Group of Fuyang city in Anhui province linquan county party secretary Lv Yehong said in class mobilization: " training is to further optimize the business environment, stimulate private enterprise entrepreneurial enthusiasm, the young talents together to better independent business, help students to improve the new market environment of innovative marketing understanding and grasp, system under the background of entrepreneurial policy and entrepreneurial skills."Linquan youth entrepreneurs association, new generation association and part of the villages and towns (street) youth entrepreneurs to discuss each other in learning, complement each other, students DaiHao said the training content is rich, further understand the new stage of county economic development trend, again clear the enterprise development goals to be closely related to urban development, common progress, to contribute to the county economic development youth strength. It is reported that the group linquan county party committee to the county communist youth league organization reform pilot as an opportunity to build youth around the indispensable communist youth league as the goal, further play to the role of the communist youth league condensed youth, service youth, lead the youth, unity led the county entrepreneurial youth active in economic and social development, condensed more youth for building beautiful hometown with youth blood, draw the county economic development "concentric circles".

2021-09-03	Anhui Susong County Party Committee held the 2021 "Yuan Ju Susong Youth Date" single youth online friendship activities Wei correspondent, Zhang Meifang Zha Weiting) In order to implement the "Anhui Province Medium-and long-term Youth Development Plan (2018-2025)", Help single young people in Susong County, Anqing City, Anhui Province to build a dating platform, Expand the communication channels, a few days ago, Tuan Susong County Party Committee representative liaison station through the "Susong youth voice" official TikTok number held the 2021 "Yuan Ju Susong youth about" the county single youth dating network live broadcast activities, Live streaming time is 2 hours, It attracted 5,000 people views. Due to the requirements of COVID-19 prevention and control, in order to adapt to the development and changes of young people in the new era and meet the needs of single young people, the contact station of Tuan Susong County Party Committee played the role of new media, opened the first live Douyin, and attracted the majority of single young people to actively participate by shooting video trailers and inviting professional hosts. During the live broadcast, the single young people in various organs and public institutions actively connected with the host to introduce the nature of their work, hobbies, mate selection criteria and other aspects, which attracted the attention of many young people. For the busy grass-roots staff, this kind of activity participation form is convenient, but also very relaxed and happy, which not only protects everyone's information security, but also lets everyone express themselves bravely. During the event, the staff of the liaison station of the Youth League representative of the Susong County Party Committee also conducted publicity and mobilization on the epidemic prevention and control work.

2021-09-02	The franco-prussian propaganda into the shitai county clove town library village mu child dot "escort growth" care left-behind children rule of law the franco-prussian propaganda into Anhui county clove town library village wei correspondent, later check WeiTing) in order to further strengthen the left-behind children rule of law education, improve the self-protection consciousness of left-behind children, care for the healthy growth of left-behind children. Recently, the Shitai County Party Committee of Chizhou City, Anhui Province, relying on the "Mu Tong dot stone" studio, together with the county procuratorate, the county women's Federation to Kushan Village, Dingxiang Town to carry out the "care for left-behind children rule of law escort growth" publicity activities. Event, group stone county a line in library village conference room to left-behind children "teenagers since the care manual" COVID-19 health epidemic prevention manual "assault prevention manual and love contact card, procuratorate personnel in vivid language and case to introduce left-behind children" conflict with classmates "," how to wash your hands, wear masks, "," know and protect our body " legal and self-protection knowledge, and the left-behind children personal and family basic situation made a simple investigation, late for special difficulties will continue to follow. TuanShitai County Party Committee delegation also to 2 left-behind children at home condolences, to them sent bags, notebooks, colored pens and other school supplies, encourage them to study hard, self-reliance and self-improvement, healthy growth, do ideal and ambitious children in the new era. This activity improves the left-behind children's rule of law awareness and self protection skills, has achieved good effect, the next step, the county will continue to rely on "mu child stone" studio, and unite more units and social caring people care youth series activities, continue to left-behind children material and ideological double care, continue to deepen the youth rule of law education, since the education, build safeguard the legitimate rights and interests of teenagers, promote the healthy growth of teenagers good social atmosphere.

2021-09-01	Lanshan county of Hunan province volunteers with youth to protect the health of the masses "green" Hunan Lanshan county volunteers with youth to protect the health of the masses "green" wei correspondent Wang Yujue) for days, Yongzhou of Hunan province, Lanshan county party committee to party history learning education as an opportunity, to party leading group building as the breakthrough point, positive response to recruit more than 20 young volunteers in county central hospital, central hospital vaccination nucleic acid testing center to carry out volunteer service activities, help the county will be coronavirus vaccination work orderly, smooth and efficient. At the vaccination site of the north hospital of the county central hospital, the young volunteers wearing green vests maintained order at the vaccination site, providing publicity and guidance, information registration and other services for the people who came to be vaccinated. The green vest visible everywhere has become a beautiful scenery of the vaccination site. Every morning, Li Wei, a junior at Xiangtan Institute of Technology, arrives at the vaccination site to coordinate and solve sudden problems at the scene, answer questions for people with doubts, and help them better receive the COVID-19 vaccine. Although it was very hard every day, Li Wei always had no complaint. The original aspiration remains unchanged, the epidemic prevention and control, youth in the front. There are many other young volunteers like Li Wei. They fully carry forward the spirit of volunteer service, contribute their youth to the regular epidemic prevention and control with their own actions, and protect the "green" health of the people with their youth. In the next step, Tuan Lanshan County Party Committee will continue to lead the youth of the county to practice the spirit of volunteer service, and implement the party history learning and education "I do practical things for the masses, I do practical things for the youth" practice activities.

2021-09-01	In July 2021, Chaohu City, Hefei City, Anhui Province, launched the "Love Dream University" to help college students in difficulties, Under the mode of "led by the Party and government, led by the Youth League Committee, linked by departments and social assistance", After two months of work and progress, The procedures of preliminary examination, review, household verification and publicity have been completed, According to the degree of difficulty, 328 freshmen will be assisted, Thirty-four college students enjoy tracking student assistance (including 13 from the Hope Project, Dream realization plan of 21 people). In addition, 9 college students enjoy continuous and targeted enterprise assistance, and 61 freshmen in line with the Rain and Dew Plan project are expected to register at the end of September. This year, under the leadership of the Chaohu Municipal Party Committee and the Chaohu Municipal Government, Chaohu City to assist the difficult college students work leading group member units closely cooperate, A total of 1.476 million yuan of various educational funds were integrated and raised, Among them, TuanChaohu Municipal Committee Hope Project Grant 275,000 yuan, Hefei Youth Development Foundation grant of 172,000 yuan; Chaohu Municipal Civil Affairs Bureau Lucky lottery public welfare fund of 189,000 yuan, Hefei Civil Affairs Bureau lucky lottery to wish you a college grant of 56,000 yuan; Chaohu Municipal Health Commission Population Fund grant of 120,000 yuan; Chaohu City Federation of Trade Unions golden autumn grant (undergraduate part) of 95,100 yuan, dream realization plan grant of 135,900 yuan; Chaohu City Women's Federation Spring Bud Program grant of 69,000 yuan, Hefei Women's Federation Spring Bud Program Grant of 15,000 yuan; Chaohu Municipal Working Committee Care grant of 30,000 yuan, Hefei Municipal Working Committee student realization dream realization grant of 30,000 yuan; Chaohu Charity Association Dream Realization Plan Grant of 106,000 yuan; Chaohu City Rural Revitalization Bureau is expected to help 61 freshmen, The estimated amount of assistance is 183,000 yuan. It is reported that in the past nine years, the "Love Realizing a Dream University" has helped a total of 4,117 college students in need, and distributed 14.265,700 yuan in grants. For six consecutive years, the "tracking student aid" registration platform has been opened on the wechat official account of the "Chaohu Communist Youth League", which is convenient for college students who had particularly poor families to register online. At the same time, combined with the student work to further consolidate poverty crucial achievements, to ensure that the city eligible for declare by inputting tent card poverty monitoring family children student a leak, a many, 2021 student 139 inputting tent card poverty monitoring children to attend the college entrance examination, 119 people determine the entrance guarantee support, another 20 people for application situation, reread, employment does not meet the conditions for assistance. In addition, since 2016, the Communist Youth League Municipal Committee has launched the "six youth projects" of "youth assistance" and "youth education" to explore new ideas for youth work, actively connect with caring enterprises and relevant municipal units and towns and streets, and build a platform for summer work-study programs for college students with difficulties, and has recommended more than 400 college students to carry out work-study programs. Next, group chaohu municipal party committee will try to help difficult college students work to the depth and breadth, but also will further standardize capital management, innovative working mechanism, improve the system of tracking support, incentive guide healthy success, make student activities to mobilize social forces, carry forward the era of new air "love project", become a service society, service youth, service students aspire to become, serve the "talent project" of the motherland.

2021-08-31	Bozhou city qiaocheng district established Chinese medicine industry Bozhou city qiaocheng district of Chinese medicine industry committee JianWei correspondent, He Jiaxing check WeiTing) in order to further adapt to the youth employment distribution, effective configuration work force, expand the scope of service, recently, the group of Anhui Bozhou city qiaocheng district committee organization representative enterprises in the Chinese medicine industry symposium, study the establishment of the communist youth league Bozhou city qiaocheng traditional Chinese medicine industry working committee. According to the agenda, the persons in charge of each TCM enterprise respectively made detailed reports on the number and proportion of the youth league members of the enterprise, and put forward suggestions on the establishment of the Communist Youth League Working Committee. Subsequently, the Youth League District Committee decided to establish the Working Committee of Qiaocheng District of Bozhou City, and defined the main responsibilities: the leadership of the industry, develop the development plan, the annual plan and the relevant work system; the second is to strengthen the registration, the registration and the daily management of the industry; and actively assist the relevant tasks. In the next step, the Communist Youth League District Committee will further increase the investigation of the areas and industries where the youth population is concentrated, continue to promote the establishment of the dispatched Communist Youth League Working Committee, further expand the coverage and influence of the Communist Youth League organization, and give full play to the effectiveness of the work.

2021-08-30	Luyang District Committee of Hefei City, Anhui Province aims to build a "store" for emerging youth Jianwei Correspondent Zhang � D Zha Weiting) In order to thoroughly study and implement the important instructions of General Secretary Xi Jinping on deepening the reform of the group during the 14th Five-Year Plan period, We will fully implement the pilot reform of the primary-level youth League organizations of the Communist Youth League, The Luyang District Committee of Hefei City, Anhui Province played the radiating role of the street regional league construction working mechanism, Broaden the construction of the "youth home" positions, To promote the joint construction and sharing of "youth homes" with street party and mass service centers, two new organization positions, emerging youth positions and other youth gathering places, Establish a "store" -style "youth home" that can directly contact young people and bring them within reach."In the future, when we wait for the order, we finally have a place to stay, here can charge and blow air conditioning, good!"Yao Big Brother, a food delivery man, is located in the Luyang District Fortune Square Party and mass Service Center, the" Little Brother supply Station ", while charging his mobile phone and other orders, while resting. On August 17, luyang district Bozhou road street first "little brother supply station" officially opened, wealth square building youth corps committee, joint in enterprise zheshang bank, combined with the party history learning education "I do the practical work for youth", adjust measures to local conditions will be convenient service to delivery rider, delivery little elder brother, sanitation workers and other outdoor workers, also make depot a characteristic building "youth home". The picture shows volunteers helping the delivery boy. Correspondent for the picture " I am mainly responsible for the wealth square around the distribution task, usually the meal peak time is busy is ok, idle down there is no place to go, sometimes on the roadside electric car sitting, encounter high temperature and rainy days, are very inconvenient."Fortune Square, dense buildings, many white-collar workers. Couboys, delivery boys and sanitation workers are busy shuttling between business buildings every day. They are the "beauticians", "ferryman" and "little bees", and also the "most familiar strangers". They make Luyang more beautiful with their hands and hope to get respect and care from the society. In order to express their care for them and solve their problems such as "difficult eating, drinking water and resting", the first "little supply station" in Bozhou Road Street was officially "opened in the hall on the first floor" of Building A, Fortune Square. Meituan delivery boy Xiao Zhang, after delivering the delivery order, quickly walked into the "little brother supply station" to rest."Later, come here. I just saw, this 'supply station' in everything, very thoughtful, the last time I broke off the mask, into the building, send the order late deducted money, later encountered such a situation, I will come here."The open" Little Brother Supply Station " is not only equipped with leisure facilities such as sofas, tables and chairs, but also with drinking water, charging equipment, wireless networks, books and emergency medicine. In addition, considering the characteristics of outdoor work and climate change, daily necessities such as umbrellas and masks were also added."Sometimes it's not that I don't want to read, but I don't have a chance. One is expensive to buy books, two is usually very busy, also have no time to go to the library, here you can share to read red books, take time to recharge their own... "" Brother supply station "is not only the" rest "of the little brothers, but also the" base " for young people to learn. Fortune Square building Youth League committee, will send the service to the little brother side at the same time, but also hope to send learning to their hearts."This year, everyone said to study the history of the Party. I didn't know what to learn before. Today, I saw so many red books in the Little Supply Station. I set a small plan for myself, and strive to read the "a brief History of the Communist Party of China" in this month, just the party and mass service center volunteers also told me that there are regular lecture courses, next time I also listen to!"Danbird express delivery officer Xiao Liu from the school has the dream of joining the party, but usually busy work, delayed the pace of learning, in the" little brother supply station ", the former dream was awakened."Sometimes people are a little bit biased against our profession. In fact, many of our colleagues also love reading very much. Due to the particularity of ordinary outdoor work, we have no conditions to learn at any time. Recently, our peers in Jiangsu were admitted to the civil servant news, very encouraging us, just the conditions here are suitable, I will often come to read the book!"Xiao Liu's view is not unique, wealth square building party and mass service center in the little brother needs visit research, in the service at the same time, will learn sent to the side. The "little brother supply station" is not only equipped with a 24-hour book borrowing cabinet, but also set up a shared "party history learning corner", to meet the different reading needs of the little brother. In addition to reading paper books, the "Little Brother Supply Station" has also set up a real-time interactive screen of "Learning Power", complete with policy theories, learning content, and red film and television. Extend reading, gain the power of knowledge, encourage more boys to fall in love with learning, improve themselves, harvest growth. The picture shows the delivery workers showing a COVID-19 epidemic prevention and control warning card. Correspondent for the picture " Hello, I am 'hungry' takeaway, this is your order. In addition, this is a COVID-19 epidemic prevention and control warning card, which not only has the location and telephone information of the vaccination sites in the Bozhou Road street, but also has some prevention tips, so you can have a look!"Food delivery staff Wang elder sister, these days in the delivery, the incarnation of the epidemic prevention and control propagandist. In the building Party and mass service center in Fortune Square, the "little brother supply station" has placed the "epidemic prevention and control warning card". After the rest, the delivery men mostly take the initiative to take some, in the delivery of express delivery, delivery, to the community people, building white-collar workers for epidemic prevention publicity."With the pressure on epidemic prevention and control, we enjoy care while we hope to contribute to the epidemic prevention and control. How is that saying, 'All for one, I for all'!"Express, delivery boys large number, strong mobility. According to this characteristic, the Fortune Square Building Youth League Committee relies on the party and mass service center position, and actively mobilize them to become the "epidemic prevention propagandists" around the masses. When delivering express delivery and takeout, the knowledge of epidemic prevention will be sent to the door to open up the "last meter" of epidemic prevention propaganda, and effectively build a strong dam for epidemic prevention and control.

2021-08-28	New venture county, Guangdong province volunteers love student again action in Guangdong province new venture volunteers love student again action morning correspondent Li Excellence) recently, the xinfeng county party committee organization new venture individual private enterprise volunteer association to carry out the "student zhi, plastic dream cast" love student volunteer activities, the social caring people warm and caring care to the hearts of teenagers. This year's program sent about 160,000 items and money to 49 students. According to the requirements of epidemic prevention and control, the charity activity was changed from the previous concentrated activities to point-to-point visits. Volunteers went from street to street to each student's home to talk about their life and study, give them encouragement, and personally sent the "student gift package". It is understood that since 2016, Xinfeng County charity education charity project has been carried out for six years, up to now, a total of 208 local high school students, college students, a total of 850,000 yuan of grants.

2021-08-27	Guangxi Shanglin organized 80 volunteers to carry out the educational practice activities for returning college students who "make suggestions and help development". By visiting the red revolution positions, entering villages and enterprises, returning college students can experience the vigorous development momentum of rural industrial revitalization closely. At the awarding ceremony of the forum and the symposium, the Party leaders of Bawei Town shared "eight Points" with the volunteers to encourage the young students to continue to struggle. The relevant leaders of the Youth League Committee of Dafeng Town, Baiji Town, Sanli Town and Xiaoxian Town and the volunteer representatives of "Returning Home" respectively elaborated on the changes they saw and made suggestions on the development of Shanghai Lin. The activity aims to help and guide college students to feel the changes in their hometown, remember the struggle of the Party, and enhance the sense of responsibility and mission of serving the people and returning to their hometown.

2021-08-26	The Huaibei Lieshan District Committee organized young volunteers to help the vaccination group organization and young volunteers to participate in the vaccination work. The Tuanlie Mountain District Committee mobilized returning college students and young volunteers to form five youth commandos for epidemic prevention and control, and more than 200 people went to various vaccination sites to participate in volunteer services. Volunteers provide services such as vaccination registration, publicity and guidance, and order maintenance for the vaccinated masses. To promote the epidemic prevention and control, TuanLie mountain committee also launched cadres at all levels, youth league members, youth typical vaccination pioneer model, adhere to the "should meet, should be as early as possible" principle, take the initiative to apply for vaccination, orderly participate in vaccination work, mobilize the relatives and colleagues to participate in vaccination, to ensure that the vaccination work obtains the actual effect.

2021-08-25	Anhui xiao county youth league organization to build 15 minutes epidemic prevention volunteer service work in Anhui xiao county youth league organization to build 15 minutes epidemic prevention volunteer service work hai-han wang, correspondent, longitudinal ZhaoQin Yan Xin) recently, a "xiao xinxian county hospital vaccination point need volunteer support" WeChat to Anhui xiao county WeiJianWei youth corps committee, head of the mobile phone, WeiJianWei youth corps committee contact group xiao county county immediately. Received a request for help, Tuanxiao County County Party Committee immediately contacted the area of the Longcheng Town Youth League Committee and social organization Xiaoxian Day Wolf rescue brigade responsible person, timely organized 11 young volunteers to the vaccination site to carry out activities. Xiaoxian county is located at the junction of four provinces, including Jiangsu and Henan provinces, which poses challenges to the epidemic prevention and control work. To this end, in accordance with the requirements of the epidemic prevention and control volunteer scheduling exercise simulated in the early stage, TuanCounty Party Committee specially formulated a "15-minute working circle" mechanism for volunteers' epidemic prevention and control. When receiving tasks and needs, volunteers can be arranged to enter the epidemic prevention and control work site within 15 minutes. On August 17, the Zulou Town Health Center in Xiaoxian County contacted the Communist Youth League County Party Committee and said that volunteers were needed to participate in the epidemic prevention and control publicity. Tuanxiao County County Party Committee immediately contacted with the head of the Zulu Town Youth League Committee, within 10 minutes organized the town straight unit 6 volunteers to arrive. In recent days, volunteers assist the staff in distributing, guiding, consulting and maintaining order, providing one-to-one service for the disabled and disabled, and in the main entrances and exits and main roads and shops along the streets, and timely understand the isolation of returning people in the risk areas. Isolates only need to send their needs through mobile wechat, and volunteers will deliver their living supplies to their homes in the first time.

2021-08-24	Congjiang County "Sailing Plan": Provide practice platform for college students "Sailing Plan": to provide practice platform for college students, at the beginning of a lot of things do not understand, but my brothers and sisters, sisters, uncles and aunts are very patient, again and again to teach me, let me improve my ability, exercise the courage."August 20, is Pan Mengyun in Congjiang County Agriculture and Rural Bureau internship expired day. She said: " In the past month's internship, I have learned a lot and accumulated valuable experience for adapting to and integrating into the society in the future."Pan Mengmeng is a freshman at Guizhou Normal University. At the end of last semester, she listened to her classmates talk about the internship, and also had the idea of going to practice. Later, she saw the 2021 Congjiang County "sail plan" registration announcement. On July 20, Pan began a one-month internship at the Congjiang County Agriculture and Rural Affairs Bureau. During the internship, under the guidance of the teachers, Pan got familiar with the work process of related business and department affairs, and also learned a lot from the teachers. Among them, what impressed her most is that everyone often works overtime late into the night, the selfless dedication of the spirit and perseverance."I am very glad to have such an internship experience. Seeing everyone being busy makes me realize what is the mission and responsibility given by The Times. As a young man in the new era, I should also be firm in my ideals and beliefs, practice my skills down-to-earth, and do my part for the development of the country in the future."Said Pan Mengyun."Sailing plan" is the communist youth League congjiang county to thoroughly implement the Guizhou medium-and long-term youth development plan (2019-2025), implement the relevant requirements of "six stability and six protection", guide college students to actively participate in internship, help them better establish the correct employment concept, reserve work experience, enhance the competitiveness of employment of the important measures. At the same time, through practice, the young people will continue to mature and rational, and make their ideals and beliefs more firm. They will be guided to actively participate in the three strategic actions of "rural revitalization, big data and big ecology", so as to provide strong and sustained youth wisdom and impetus for striving to create a new future of rich people and colorful ecological beauty. After the Communist Youth League issued the "Sailing Plan" registration announcement, it received the county positive response and strong support from all levels of government institutions and enterprises, providing a total of 42 internship positions. After the active registration, screening and recommendation process and two-way selection between employers, 27 college students finally went to all levels of the county organs and enterprises for a one-month internship life."Since the development of party history learning and education, we have combined with the practical activities of 'I do practical things for the masses' to actively guide college students to practice in social practice, hoping that they can improve their professional level and practical ability through practice. We will continue to do a good job in the 'Sailing Plan' to provide a broader platform for more college students to enter, integrate into the society and serve the society."Said Yang Zhou, deputy secretary of the Communist Youth League Committee of Congjiang County.(Xia Hua, reporter of Guizhou Daily Tianyan News)

2021-08-23	Jiangsu yizheng: 1870 young volunteers build disease "youth defense line" Jiangsu yizheng: 1870 young volunteers build disease "defense line" youth prevention and control of youth volunteer recruitment, the city authority young cadres, grassroots cadres, returning college students, high school graduates, summer interns and other youth actively sign up, together to fight disease. A total of 1,870 young volunteers contributed their youth in information registration, order maintenance, material distribution, prevention and control publicity, and nucleic acid testing. More than 800 college students returning to their hometowns became "combatants", "propagandists" and "platoon soldiers" at the grassroots level to publicize the epidemic prevention requirements.

2021-08-19	Group of Shandong Liaocheng gaotang county to fund college students household survey visit group Shandong Liaocheng gaotang county to fund college students household survey visit morning) to fund part of family difficult college students "interpreta dream university", combined with "I do the practical work for the youth" requirements, on August 17, gaotang county to "2021 hope project interpreta dream action large public welfare student activities", the county "yucai scholarship" student project to fund seven college students carried out household survey visit, further understand to fund college students' study, life, dynamic thought. During the visit, the relevant staff verified the information of the college students to be rescued, carefully checked the identity documents, admission notice, admission major and tuition amount, etc., recorded the valid information, and formed a standing book. During the activity, the visitors communicated with each college entrance examination freshmen and their families, learned about the living status, physical health status and income sources of their family members in detail, and encouraged the college students to maintain a positive attitude, enhance their confidence in study, set up lofty ideals, study hard, and realize their life value. In the next step, Tuan Gaotang County Party Committee will continue to follow up the situation of planned funded college students to help students realize their dreams in college. The picture shows the household investigation and visit

2021-08-16	Guangdong new county youth BBS (sixth) successfully held Guangdong province new county youth BBS (sixth) successfully held morning correspondent Zhu Qianqian) on August 13, Guangdong xinfeng county united county returning college students social practice temporary youth league branch, to carry out the "please rest assured, power have me" as the theme of the youth BBS. During the activity, the relevant person in charge of the Communist Youth League County Party Committee learned to convey the spirit of General Secretary Xi Jinping's "July 1" important speech to more than 20 returning college students present. After watching the video of the dedication of the 100th anniversary of the founding of the Communist Party of China, the returning students who participated in the activity actively shared their impressions, university learning experience and work impressions of participating in the social practice of their hometown, enhanced mutual understanding of each other, and encouraged each other by giving youth message postcards to each other. Everyone said that such a forum form is very novel, the activity is also very meaningful. In the future study and work, I will continue to give full play to what I have learned, go deep into the grass-roots level, and strive to be a new youth of the new era to reassure the Party and satisfy the people. It is reported, xinfeng youth BBS initiated by the county, since 2019, each period with different theme, for xinfeng county different industries, different academic background of young people provide communication, interactive platform, aims to build for xinfeng youth interaction, growth platform, guide the youth to set up the correct beginner's mind mission and dream, actively set youth, the wisdom of youth, youth force, with the action of youth service home, the construction of hometown.

2021-08-13	Group Hunan ningyuan county to carry out summer anti-drug propaganda activities Hunan ningyuan county to carry out the "green non-toxic youth since" summer anti-drug propaganda activities Jianwei correspondent Zheng Hao) recently, the Yongzhou of Hunan province ningyuan county organization "home" college students volunteers to the villages (communities) to carry out the "green non-toxic youth since protect" summer anti-drug propaganda activities. In the propaganda and education activities, the volunteers explained "what is drugs", "the harm of drugs", "Drug-drug history in China" and "how to prevent drugs" in the form of PPT courseware and video. At the same time, in order to further expand the effect of the propaganda and education activities, the volunteers also went to the homes of teenagers who could not attend the activities to distribute anti-drug brochures. Through the summer anti-drug propaganda and education activities, the anti-drug knowledge will be sent to the door to deepen the teenagers' cognition of the harm of drugs, enhance their vigilance against the temptation of drugs, and improve the consciousness of staying away from drug infringement.

2021-08-13	Jianwei correspondent Zhang Zhuo) When the youth meets the childlike innocence, when the dedication lights up the dream, "the warm childlike innocence colorful holiday" makes the children's summer vacation no longer lonely. Rich and colorful theme activities, patient and meticulous companionship and guidance, this summer children's warm smile, young volunteers to protect. Set up the "chess" overall situation thought arrangement deployment fast since the summer, the Ningxia hui autonomous region Guyuan jingyuan county actively organize summer hometown social practice college students volunteers and young volunteers play their own advantages, in jingyuan county seven administrative villages carried out a 20-day "colorful holiday" volunteer service activities, make unified manual, unified flag, uniform clothing, continuous care for rural left-behind children, to participate in more than 400 students. The picture shows the volunteer service activity site. TuanJingYuan county for figure to build "package" characteristic course design details adhere to the "individualized, learning, characteristic", college students volunteers according to students 'age, knowledge structure, interests, and combined with their own expertise, carefully prepared the tai chi, electronic organ, calligraphy, paper cutting, such as more than 70 characteristic courses, extended sports, literature, patriotism, gratitude, legal, promote faith, cultivate the effect of interest, the seeds of love into the children's heart, make "colorful holiday" children dream leap place. The picture shows a volunteer service work manual. Jingyuan county for figure cohesion "a rope" social force resources integration wide deep optimal colorful holiday "widely gathering social forces, will Jingyuan college students" home "social practice, China building materials group" good "colorful holiday, China university of geological community activities and Beijing university graduate teaching activities effectively combined, more than 80 college volunteers to participate in" colorful holiday "play a role of characteristic platform, the youth home," 4:30 class "and rural school children's palace into" colorful holiday " main position. With the new media platform, use the wechat public account to publicize the "colorful holiday". We pool various resources to send basketball, teaching AIDS such as badminton, watercolor pens as well as puzzle toys for left-behind children. The 20-day "Colorful Holiday" volunteer service activities, the playground and the classroom echoed with the children's loud reading and laughter, and the rich and interesting courses accompanied them through an unforgettable holiday time. The picture shows a group photo of the volunteer service activities. Group jingyuan county for figure, teaching gain li practice, learn with growth group jingyuan county "colorful holiday" volunteer service activities, not only for left-behind children brought care and knowledge, also provides volunteers with valuable teaching practice, the next step of work, the county will continue to and area outstanding student volunteers to strengthen contact, active docking, gather more excellent resources, further enhance zhi double help power, broaden children's field of vision.

2021-08-12	Group of Shandong gaotang county to carry out online "hope house" teaching activities, group of Shandong gaotang county to carry out online "hope house" teaching activities for China youth network Beijing on August 12 (xinhua zhang) to do "hope hut" follow-up support activities, group gaotang county of Liaocheng city in Shandong province and Shandong university "angel in action" volunteer group joint online "hope house" teaching activities. In the first few weeks before the activity began, all the members of the volunteer teaching group of Shandong University had actively contacted with Gao Tang's "Hope House" and carefully prepared all the tutoring courses. Considering the particularity of the children in the "Hope House" and their different learning levels, the members of the volunteer teaching group choose to contact the children one to one. After clarifying their respective needs, the targeted tutoring content covers almost all grades from primary school to junior high school. At the same time in order to ensure the safety of the children in the summer, enrich everyone's summer life, volunteers also cooperated with Shandong university first aid association, invited the first aid association of ying-mei miao students for the children against drowning safety education, help "hope hut" the children spend a more healthy, colorful and safe summer vacation. In the process of anti-drowning safety education, the children have turned on the cameras and microphones, and actively interacted with the keynote speaker, Miao Yingmai.

2021-08-12	Shandong Linyi county fu can rural "good youth" build "new engine" new youth [new hometown] Shandong Linyi county "rural good youth fu can action" build "new" engine JianWei correspondent Sun YuNa) in recent years, the group of Shandong Dezhou Linyi county focus on party and government needs, youth hope, continue to deepen the "rural good youth fu can action", do "choose, culture, help, couplet", foster "leader", for rural youth "policy", lead the youth active in rural revitalization of the main battlefield. On the basis of "selection" to enrich the rural youth reserve talent pool to explore the establishment of "regional + field double channel" promotion procedure, each township (street) youth league committee to establish normal training contact mechanism, "good youth" base cultivation; youth league committee through daily visits, posters, broadcast, public promotion, broaden channels, broaden horizons, mobilize various forces to find around "good youth", while fully grasp the rural revitalization industry projects, actively absorb rural talent back policy. Through the three-year selection and training program, more than 2,600 young people have been cultivated, and 147 "good youth" at the city and county level have been elected. With "training" as the focus to provide effective support to strengthen the connection with organizational departments, town and street party committees, do a good job in visiting and recommending work, and deepen the political training of "good youth". Since 2019,72 "good youth" have been recommended to join the Party and become 95 as active members; Considering the village "two committees" in 2021,151 "good youth" have entered the village (community) "two committees" according to the procedures. In accordance with the principle of equal emphasis on selection and training, we will highlight industrial empowerment, provide employment and entrepreneurship skills training for "good youth", carry out professional training on financial policy interpretation and modern agricultural production and operation, and provide classified, batch and personalized training content. Combined with the theme education activities of "Learning the Party history, strengthening the faith and following the Party", the "good youth" was included in the key training objects of the party history study, and the "Youth praises the Party in its youth" was carried out. With "help" as the carrier to help industrial extension and joint development of the "good youth" selection work included in the "I do practical things for the youth" matters, carry out visiting activities, field investigation and research "good youth" innovation and entrepreneurship projects, detailed understanding of the difficulties and needs in the process of innovation and entrepreneurship, master the industrial development and future planning. Provide special preferential services, invite financial institutions and members of the county youth enterprise associations to become "good youth" development consultants, and provide professional and technical guidance, policy support, legal advice and other support. Strengthen bank cooperation, build a financing platform, and establish a green channel for loan application. Visited 26 "good youth" people; helped 7 "good youth" to successfully apply for entrepreneurial preferential loan of 3.83 million yuan, recruited 6 entrepreneurial "good youth" to join the county Youth Enterprise Association; conducted 3 centralized training sessions, 1 outward bound training session, and 3 exchange discussions. On August 11,2020, Dezhou Rural "Good Youth" promotion site meeting was held in Qianyang Village, and awarded to its "Rural 'Good Youth' research and practice Base". On April 23,2021, the "Rural Good Youth Alliance" was established. The establishment of the platform promotes the communication between the "good youth", and promotes the cross-township and cross-field allocation and use of resources. The alliance establishes WeChat group, strengthens the daily contact of members, holds various communication and learning activities, and realizes resource sharing and group development. We will actively build connectivity channels for alliance members and social organizations, include qualified "good youth" into the Youth Enterprise Association, invite association members to form development pairs with "good youth", and expand contact channels in enterprise development, production safety, legal guarantee and other aspects. Tuan Linyi county in the "rural good youth" selected culture plan, according to the selection is the foundation, culture is the key principle, to promote the advanced typical drive youth, to build contact platform condensed youth, with various resource integration service youth, to enhance political literacy, outstanding industry can promote youth growth, strengthen the good youth identity sense, feeling.

2021-08-12	Anhui SuiXi county young pioneers counselors vocational skills contest in Anhui province Suixi county 2021 correspondent feeds China youth Beijing on August 12 (reporter Zhang Jianwei, correspondent Guo Chao check WeiTing) recently, Huaibei city of Anhui province Suixi county, Suixi county bureau of education, SuiXi county committee jointly held in 2021 SuiXi county young pioneers counselors vocational skills contest, the layers of selection recommendation, from the county 21 young pioneers counselors participated in the competition. Youth League committee, county education bureau party group responsible person attended the activity, Youth League committee, county education bureau related responsible comrades to participate in the activity. The competition combined with the activity of "celebrating the 100th anniversary of the founding of the Communist Party of China", with the theme of "red scarf heart to the Party", through the comprehensive knowledge test, activity design and lecture, professional skills display and other links. In the end, a total of 3 counselors won the special prize, 6 counselors won the first prize, 12 counselors won the second prize, and 3 counselors won the single prize. The competition highlights the practice of competition, training, learning and construction through competition, which plays a leading role of excellent counselors, and builds a platform for the young Pioneers counselors in Suixi County to share, communicate and learn.

2021-08-11	Hai'an City of Jiangsu Province set up a "understand" secretary studio to promote the non-public field league organization construction, manage well, live up the enterprise league organization secretary to carry out league construction business training. Correspondent for figure China youth network Beijing on August 11 (xinhua Ji'an-wei zhang, correspondent Wang Zhichao) this year, Nantong, Jiangsu province haian municipal party committee with party building, from the park party instructor team selected young party members as group instructor, form a group of around in the non-public youth "understand", secretary of the think tank. The secretary of "understanding" consists of a group of league construction instructors who understand the party building, technology, business and youth, integrating "management, service, education, activities" and other responsibilities."Understand" The secretary is not surnamed Dong but knows everything. Up to now, Party Secretary Understand has received and answered 981 questions, solved 118 problems together, and promoted 401 new league organizations in the social field. The participation of non-public youth people in league activities has increased by 80%, and the satisfaction rate has increased to 98%. The "soft power" has spawned the "hard power" in the non-public sector."There are many young employees in our shopping mall. In order to gather young strength and boost the development of enterprises, we often cooperate with nearby village league organizations to carry out league building activities, which is very popular among young employees."stagnation Xinghu 001 commercial square" understand " secretary Tan Xing introduced. The picture shows Haian Huji street "understand" secretary visited the area of enterprises. Correspondent for figure group haian municipal party committee in line with "where the youth, youth league organization is built" principle, according to the non-public league building work needs, in the youth intensive business circle, park, industry establish "understand" secretary studio, explore established 1 + 4 + N chain positions, for each "understand" secretary studio with a full-time "understand" secretary, four part-time "understand" secretary, leading non-public enterprise league organization and village league group building, realize "regional as a whole, group management". We will further deepen the work of regional league construction, improve the quality of the league construction, and work with the Party organizations to innovate the working methods of joint organization construction, joint activities, and resource sharing, so as to realize the precise docking and concentrated development of the co-construction units. The picture shows the young workers in Hai'an chemical fiber industry holding a reading party in the "understand" secretary studio near the enterprise. In addition to the daily resident studio, "understand" the secretary is more often "ambush" around the youth in the non-public field. Wang Sai, party secretary of Daxin Group, is one of them. As the manager and chief quality officer of the technical quality department of the enterprise, Wang Sai led the team to develop the secondary structure T-shaped wall fixture system to help the enterprise overcome the technical difficulties. As the deputy secretary of the street Youth League Working Committee, Wang Sai organizes the street youth activities, and leads the enterprise youth to participate in the youth activities such as love and public welfare, friendship, learning and discussion. He is the "understanding" secretary in the eyes of the group colleagues and the street youth. Youth League Hai'an Municipal Party Committee focuses on the organs, enterprises and institutions personnel, social organizations, university party members, typical youth figures, village (community) cadres, college graduates and other groups of outstanding party members and league members, equipped with a strong "understand" secretary think tank. So far, the city has been equipped with 15 full-time "understand" secretaries and 60 part-time "understand" secretaries. The "Understand" secretary studio covers 10 industrial parks and 5 business districts, covering 15 districts, towns and streets, and contacting and serving more than 20,000 young people in non-public fields. The picture shows the "understanding" secretary stationed in Hai'an Fashion Nylon Industrial Park patiently answering questions from young people."Our enterprise has a lot of migrant workers, large mobility, 'understand' secretary organized a number of friendship activities, became several pairs of marriage, so that the migrant workers here steadfast home."Said Yang Yang, head of Shuanghong Textile Co., LTD., said," The fellowship activity not only solved the personal problems of young employees, but also helped the company retain foreign talents. The secretary understands us too well."In order to fully meet the needs of non-public enterprises, the" Understand "secretary studio set up three list system, namely the resource list, demand list, annual list, respectively showing the resources provided by the" understand " secretary, the needs of enterprises and young workers, as well as the list of projects generated by the collision between the two sides."Understand" secretary of the studio for the enterprise elaborate learning materials, coordinate training and activities, a youth branch members, youth post expert, outstanding young entrepreneurs and youth social organizations, such as resources, into the park, into the enterprise, to the youth side, in the first half of this year learning training 25 games, youth style 20 games. We coordinated 23 economic disputes and labor disputes among enterprises, and carried out 10 publicity and consultation activities on employment and social security policies.

2021-08-11	Shandong jiaxiang county "four new" to create a new model of youth community in Shandong jiaxiang county "four new" to create a new model of youth community JianJianwei correspondent BaiChen) Jining city of Shandong province jiaxiang county adhere to the "organization, platform construction, service optimization, brand lighting" work goal, stick to "I do the practical work for youth" requirements, pilot build jiaxiang county Jiaxiang street community 2 youth community, adhere to strengthen the community youth, with youth, youth service, let the power of youth in the community, let the dedication of youth shine in the community. Relying on the "youth community" brand creation activities, the community aims to build the "youth HUI" youth work brand. It has successively guided the establishment of youth social organizations such as "Easy Thinking Reading Club", "Jiaxiang County Young Calligraphers Association" and caring for the scissors and other youth community organizations and volunteer service organizations. The picture shows students reading at the Easy Reading Club. The youth community elected the young backbone under 45 years old to join the community industry committee. In the change of the community resident committee, nearly 50% of the 72 elected resident representatives were under 45 years old; in the construction of the community building group, the community Party committee equipped 2 "youth officers" for each community, so as to combine the experience and energy advantages of traditional governance with the professional and resource advantages of youth governance. The picture shows the blue Sky Rescue team teaching residents about cardiopulmonary resuscitation, Heimlick first aid method, drowning prevention and other first aid measures. Around the working mechanism of community "youth HUI", we explored the "YUE" series activities, including "YUE" enjoy reading time, let the youth find the best through reading; "YUE" enjoy and happy life, into the community to carry out safety knowledge publicity, teach cardiopulmonary resuscitation, Heimlick method, drowning prevention methods, "YUE" enjoy wonderful life, organize "our Chinese Dream-culture into the community", "summer vacation-childhood different", "our festival" and other activities.

2021-08-11	Dingyuan county in Anhui province, hundreds of village cadres "nest" solution people sorrow "Quyang goose" project to return hundreds of village cadres "nest" solution area staff, just graduated from university this year he hasn't had time to adapt to the change of identity, has experienced several "event" in the town, burning straw, flood control, epidemic prevention and control, every experience accelerated his growth. In May, Luqiao town comprehensive ban burning straw, Zhang Bin and two colleagues on duty every day; in July, grade 10 wind and heavy rainfall hit, Zhang Bin and the community cadres immediately transferred residents, arranged them to stay in the hotel temporarily, and declare the disaster subsidies for everyone. As the director of the homestead reform office of Luqiao Town, Zhang Bin also carefully and systematically studied the policy materials issued by the county, and became the "homestead reform expert" combined with the practical problems encountered in the work. Colleagues, villagers have what questions are chasing after Zhang Bin asked, he are carefully answered. Zhang Bin said frankly: " Since my work, I have gained a new understanding of rural work, and the grass-roots work is complicated. When recognized and praised by the masses, I feel a lot of hard work is worth it!"In recent years, in view of the village (community)" two committees "cadres are older and other phenomena, Dingyuan County implemented the" Quyang wild goose return " project, from the village rich leaders, outside businessmen, college graduates, veterans, young reserve cadres to introduce and cultivate a number of young village cadres. This year, the Youth League Dingyuan County Party Committee innovative ideas, learn the history of the Party, do practical things, open a new bureau, from the "Quyang wild goose return" talent pool to absorb college students to the village level league cadres team, to solve the village level league organization work force lack of strength, cadres old problems. So far, 153 village-level cadres have been taken office. In September 2020, Yu Xue, head of Zhongtang Village Youth League Branch of Erlong Hui Township, Dingyuan County, became a member of the "Quyang Yan Return" project. She started from sorting out basic affairs such as documents, and has participated in civilized publicity, rural construction and other work. From road renovation and house demolition, to marital quarrels and neighborhood disputes, village committees help solve them. Yu Xue felt that, with these grass-roots work experience, do the work of the league is also handy, " I often remind myself, when the league cadres, to be patient and meticulous, more to do practical things, do real things for the youth."Shan Natural, 22, was elected as the deputy secretary of the Youth League branch of The Village, Xishi Store Town. She took the lead in strengthening theoretical study and urged and guided the young people in the village to carry out online themed group classes of" Youth Learning " every week. In combination with the work of the village center, she organized college students returning (in) to carry out COVID-19 vaccination and epidemic prevention and control."When the villagers don't cooperate very well, I will talk to the villagers about their health and talk about family affairs."She realized that in the work practice, we should learn to grasp the psychology of the masses, and deal with the masses in a soft and flexible way." In the process of doing practical things for the villagers, we can make a contribution to develop our hometown, and the sense of gain is still very strong."Liu Qiong, who was born in 1992, has left the town for 10 years since she went to university. This year, she passed the "Quyang goose" exam officially "home", become a youth member of the community neighborhood committee. From the workplace "small white" to "expert", Liu Qiong did not less self-pressure."Read more, listen more, ask more and run more, these eight words are my grassroots work guide."Recently, Liu Qiong has been going to publicize epidemic prevention knowledge. Every time, she would explain to the villagers that "the vaccine is a scientific means of epidemic prevention" and other relevant knowledge. Slowly, the villagers became proactive towards the vaccination. Liu Qiong's hard work and meticulous working attitude has won the recognition of the village cadres. The predecessor of the village committee told Liu Qiong: " Your arrival has solved the problem that people are not familiar with the computer business."Liu Qiong will jokingly respond:" As a qualified community 'network manager', I will continue to 'fill up' and 'speed up' for your work."In the next step, the Youth League Dingyuan County Party Committee will enrich the working force of the youth league organizations at all levels in the county through multiple channels by training and selecting village (community) youth committee members and absorbing college students returning (in) townships, and smooth the promotion channels of grass-roots youth league cadres. China Youth Daily China Youth network reporter Wang Haihan Wang Lei correspondent, Luo Chengye

2021-08-11	Longzi lake district in Anhui province to carry out the minors protection law preaching activities in Anhui longzi lake the franco-prussian into the community guardian "young you" jianwei correspondent, Lu Weifeng check wei ting) to protect the legitimate rights and interests of minors, popularize the protection of minors law, to create a healthy social atmosphere, recently, the youth Bengbu longzi lake district joint Anhui university of finance legal culture XuanJiangTuan to carry out the basic protection of minors preaching activities. During the activity, the members of the publicity group explained the relevant legal knowledge of the Law on the Protection of Minors by popularizing legal knowledge and explaining typical cases, and the publicity contents covered the campus safety, personal safety, legal culture, psychological education and other contents of minors. According to the diversified characteristics of the audience, the use of simple language, vivid typical cases, with image intuitive courseware, legal knowledge and safety knowledge into the life scene, let the guardian build a safety legal defense line in the heart. In the interactive session, the publicity group interacted with the parents by asking questions to understand the parents' feelings about the current "minor protection" environment and the sources and suggestions of the infringement. Finally, the members of the publicity group also distributed leaflets to parents to popularize the legitimate rights and interests of minors in home protection, school protection and social protection.

2021-08-10	Anhui Xuanzhou District "5 + 10 + N" system to improve the Communist Youth League service quality and efficiency of Anhui Xuanzhou District Committee "5 + 10 + N" system to improve the Communist Youth League service quality and efficiency of Jianwei correspondent Feng Xiang Zha Weiting) Anhui Xuancheng Xuanzhou District Committee actively seize the national Communist Youth League grass-roots organization reform pilot opportunities, We will make solid progress in reforming the organizational setup and operating mechanism, Constantly improve the organization system of the county league, According to the phrase " Where is the youth, The principle that the league organization is built where ", Build xuanzhou District Youth Home (flagship store), Driven by the construction of a "youth home", Effectively contact youth organizations, Scientific integration of social resources, Optimize and improve service quality and efficiency, We will effectively reach the "last mile" of contacting and serving the youth. Xuanzhou District Youth Home flagship store officially opened on August 3. Leaders at all levels of the Communist Youth League, Hefei Normal University, Communist Youth League, Xuanzhou District Committee and other levels attended the opening and unveiling ceremony. After comprehensive investigation and in-depth planning, the "Communist Youth League Store" grasps the requirements of "innovative organization, aggregate resources, effective guarantee and function formation", innovates to establish the "5 + 10 + N" system, and gives full play to the functions of the Communist Youth League.5:5 stations: "Xingzhi School" Xiangxiangqiao Village Station, minors protection work station, group representative liaison station, children service station, Xinhua Bookstore youth reading space station; 10 includes 10 functional areas including children's growth park, youth talent community and youth dating space; N includes legal aid, conflict mediation and psychological counseling. The store will take the pilot reform of the grassroots organization of the Communist Youth League as an opportunity to continuously integrate the elements of the Communist Youth League, highlight the needs of the youth, pay attention to the role played, and strive to build a model of the Communist Youth League in Xuanzhou of "leading and condensing the youth, organizing and mobilizing the youth, and contacting and serving the youth".

2021-08-10	New venture county, Guangdong province youth innovation "cloud teaching" speak good red story of Guangdong new venture county youth innovation "cloud teaching" speak good red story map China youth network Beijing on August 10 (reporter Zhang Jianwei, correspondent Li Excellence) recently, in COVID-19 epidemic prevention and control on the basis of the normalized situation, group of Shaoguan city, Guangdong province xinfeng county organization to carry out a series of summer back home social practice work. Among them, the "Good Heart" social practice team of Guangdong Vocational College of Foreign Language Arts, with the theme of "Love the Party, love the country and love the hometown", launched a 6-day free online characteristic course of "Youth Cloud Volunteer Teaching" for the students of Xinfeng County Experimental Primary School, Shaoguan City. The picture shows a screenshot of the "Youth Cloud Volunteer Teaching" class. In order to improve the interest of online courses and improve the teaching effect, the practice team carefully made a number of paper-cut animated videos and micro-class videos with local red stories for the students to help teenagers to enter the history of the Party and understand the history of the Party in a vivid and vivid way. In addition, the "cloud volunteer teaching" course also includes "we sing to you" music theme class, "we enter the military life" sharing theme class, "We draw the hero in our heart" art theme class, "cloud tour" red education practice class and so on. The picture shows the "cloud tour" red education practice class scene. It is reported that in recent years, the Youth League Xinfeng County Party Committee actively launched Xinfeng college students to carry out "return to hometown" social practice activities, aimed at calling on the majority of young students to use winter and summer vacation time in their hometown to carry out practice, effectively guide college students to understand their hometown, love hometown, master the policy, service hometown.

2021-08-09	Guangdong new venture to carry out the "road Xiaoqing" summer social practice in Guangdong new venture to carry out the "road Xiaoqing" youth summer social practice activities wei correspondent Pan Siyu) to better play to the role of young volunteers in creating civilized city, rich youth summer social practice, since August, group Shaoguan xinfeng county joint relevant departments to carry out new venture "road Xiaoqing" youth summer social practice. Before the activity, the traffic police brigade of Xinfeng County Public Security Bureau was invited to carry out professional training, and explained the main responsibilities, work objects, work objectives, duty norms and other work of the "Lu Xiaoqing" traffic volunteers in detail, and demonstrated the station position, command and language norms on the spot. After the training, "Road Xiaoqing" young volunteers immediately went to work, to carry out civilized traffic volunteer service activities, pedestrians, non-motor vehicles running red lights and other uncivilized behaviors to persuade, and distribute traffic safety brochures to pedestrians. For the elderly with physical difficulties, the volunteers took the initiative to provide help, showing the volunteer service spirit of "dedication, friendship, mutual assistance and progress", and passing on the love with the heart, which won the unanimous praise from the public. It is understood that this "Lu Xiaoqing" volunteer service activity is one of the 2021 Xinfeng County youth summer social practice series of volunteer service activities. The activity will last for a month, mainly set up in four major traffic intersections in the county seat to carry out civilized traffic persuasion. More than 60 young volunteers have volunteered for 200 hours.

2021-08-09	Gaotang county in Shandong province to carry out the "small" love visit activities in Shandong province to carry out the "small" love visit activities wei) recently, group Liaocheng Gaotang county joint Liaocheng city is good social work service center, Gaotang youth volunteer association organization youth volunteers and returning college students, middle school students for lonely old man send warmth, for troubled children send grants "small" love visit activities. Young volunteers, divided into three teams, went to six towns and streets to distribute love materials, subsidies and grants to nine elderly people and nine children. Volunteers for each old man sent to the summer cool bag (summer cool, electric fan, mosquito nets, emergency flashlight) and 500 yuan living subsidies, detailed understanding of the old man's life, health, remind the old people should pay attention to heat cooling, for the children issued a total of 8200 yuan grants and learning package, detailed understanding of their learning and thoughts, encourage them to work hard, inspire the courage out of trouble and endogenous strength, feedback family, in the future to society.

2021-08-05	) Henan province suffered extreme weather, serious urban waterlogging disasters occurred in Zhengzhou and other places, causing heavy casualties and property losses. In order to improve the self-rescue ability of youth self-protection ability, Xiaoxian County Party Committee of Suzhou City, Anhui Province and Xiaoxian County fire rescue brigade recently held the urban waterlogging youth self-protection and self-rescue knowledge training, young volunteer representatives from Xiaoxian Longcheng Town, Shengquan Town and some primary and middle school representatives about 60 students attended the training knowledge lecture. Event, fire commanders for detail when urban waterlogging occurs, how to self-help, and by watching video, live demonstration explained the sudden flood how to protect life safety and emergency common sense, as well as emergency response procedures and how to save themselves, let teenagers fully realize the importance of emergency self-help knowledge and skills. Special emphasis on the younger students, when the waterlogging disaster at the same time found someone encountered accident danger, can not take the decision to rescue, should immediately call for help and timely police. After the training, the fire officers led the teenagers to visit the rescue equipment, showed them the use of rescue equipment in the process of wading and disaster avoidance, and guided the teenagers to experience the use of rescue equipment. Through this activity, let the majority of teenagers have a full-round understanding of waterlogging drowning self-rescue knowledge, further enhance the safety awareness of teenagers, enhance the self-protection and self-rescue ability of teenagers, for the majority of teenagers to further build a solid safety "dam".

2021-08-04	Shandong gaotang started the second batch of "hope hut" MoPai construction work Shandong gaotang started the second batch of 10 "hope hut" MoPai construction work wei) for solid in 2021 the second batch of "hope hut" early baseline screening work, recently, Shandong Liaocheng gaotang county into evaluated integratedly, to select the lonely children comprehensive MoPai visits, carefully planning deployment, for the subsequent "hope hut" child care project to lay a solid foundation. Since the launch of the "Hope Cabin" child care project, the Communist Youth League County Party Committee has thoroughly implemented the deployment requirements of the superiors, strengthened its responsibility, and made every effort to promote the work of the "Hope Cabin" project. Construction has now started on the second batch of 10 "hope cabins" in 2021. In the next step, Tuan Gaotang County Party Committee will focus on children in difficulties, continue to deepen the "Hope House" child care project, high standards, strict requirements, strong service follow-up project, do a good job in fund raising, construction and volunteer assistance work, effectively improve the living and learning environment of the assisted children, to ensure that no child is left behind. It is reported that the "Hope House" project is aimed at children aged 8 to 14 without an independent living and learning environment, mainly for registered or actually poor families, relying on the original housing to create an independent space, planning, design, decoration, equipped with necessary furniture and daily necessities, to improve the living and learning environment. After the completion of the cabin, caring volunteers will be provided to help solve the difficult problems in growth and learning, and promote the formation of good habits and self-reliance ability.

2021-08-04	Anhui SuiXi county launched "grandparents big class" seminar "grandparents" in Anhui SuiXi county speak wei correspondent, Chen check WeiTing) recently, the group Huaibei city of Anhui province SuiXi county, county less working committee jointly sponsored "intergenerational parenting-grandparents big class" (the fourth issue) into SuiXi county town Cui Lou village between the service center of the party and the masses. More than 100 parents of left-behind children attended the activity. SuiXi county youth volunteers association, vice President of Guo Ziwen combined with real cases in daily life, popularize emergency rescue knowledge for all, to simulate the scene, demonstrate trauma dressing skills and children drowning, shock, scald treatment method, help grandparents learn to deal with all kinds of accidents, save the lives of the family in the crisis. The lecture also invited to Huaibei city "top ten" new era civilization practice XuanJiangYuan, SuiXi county Lin huan town people's mediation committee mediator Chen, combined with the case around, to popularize the civil code and other legal knowledge, help grandparents to master solve common legal problems in daily life, so as to promote family harmony and the healthy growth of teenagers and children. TuanSuixi County Party Committee and Suixi County Youth Working Committee jointly launched a series of lectures on "Grandparents' Big Class". The theme of "Family Class" aims to help the grandparents of left-behind children in rural areas master the scientific methods of intergenerational parenting, so that young people can grow up healthily and happily.

2021-08-04	Shandong gaotang county of veterans and troubled children to visit condolences to Shandong gaotang county of veterans and troubled children visits condolences activities wei) in order to further consolidate and enhance the poverty crucial effect, combined with "I do the practical work for the masses" "I do the practical work for youth" requirements, recently, the gaotang Shandong Liaocheng county for veterans and "hope the old cabin" troubled children to carry out the "big visit, big screen, big sympathy" visit activities. In the home of the children in difficulties in the "Hope House", the youth League cadres and volunteers asked about the "little master"'s study and living conditions in detail, and carried out characteristic assistance activities. At each visit, the volunteers cleaned up the veterans, asked them about their physical conditions and their lives, and listened to them tell them their own battle stories and the war years. In this "big visit, big investigation, big sympathy" activity, the league cadres and volunteers went from home to house, detailed understanding of the veterans, "hope hut" children in difficulties, clear the problems existing in the process of helping and solve them in time, effectively improving the satisfaction of the masses.

2021-08-03	Shandong lixia district build grassroots work for the people of the "youth sample" of Shandong lixia district party committee to build grassroots work for the people "youth sample" wei correspondent Sun Wencai) since this year, the group of Shandong Jinan lixia district party committee focus on the party needs and youth, explore the "345" work mode, build young people, social organizations and other youth power participation platform, together to solve the residents worry about things, worry, let more youth become grassroots social governance participants, practitioners and leaders. Create the "youth butler" brand, to solve the problem of "who will do it", "let the community residents save worry, let the youth take the initiative to participate in" is the original intention of the youth community creation. In order to provide one-stop "butler" service for community residents, The Lixia District Committee created the brand of "youth butler", and led the establishment of the three-level "youth butler" work structure of "district-street-community", which fundamentally solved the problem of "who will do it". At the district level, set up youth community creation leading group, serve as the "think tank", "supervisor", "waiter", integrate district resources, model construction and promotion of "youth community"; street establish "youth community" project team, promote the "youth public welfare" project, cultivate youth organization and youth autonomous force; community establish "youth community club", build resource linkage platform for "community-society", mobilize youth forces to participate in community governance. Relying on the "youth public welfare" project, solving the problem of "what to do", "let professional people do professional things, let more people become professional people" is the key to the creation of the youth community. TuanOuWei to youth social organizations will as the starting point, by project operation, to mobilize, cultivate youth strength as the goal, the universities, social organizations and professional strength "link" to the community, set up "youth public welfare" project, through organizational management and operation, solve the problem of "what", gradually form "party lead community governance and service project" "youth community public venture capital project" "youth social organizations cultivating development project" "youth social organization ability promotion project" four categories project 198, provide service 1300 activities, cumulative service residents more than 30000 people."Build a display platform and realize the social value" is the goal of creating a youth community. TuanOuWei in the community established "enjoy youth" multiple supply and demand platform, with "youth community" as the center, fully link quality resources, "cooperation", form "community + social organizations + university alliance + emerging youth" five linkage of "enjoy youth" platform, so far, has carried out more than 140 theme activities, volunteers more than 3200 people, service community residents more than 12000 people. Next, the league district party committee will continue to further implement the general secretary xi the important thoughts about youth work, relying on the "345" work mode, cling to the community governance difficulties, youth concern, residents demand focus, according to the different actual needs of different communities, will be more youth power "link" to the community, guide the condensed youth group return to the community, service community.

2021-08-03	Guangdong Xinfeng County set up return home college students social practice temporary youth League branch Guangdong Xinfeng County set up return home college students social practice team temporary youth League branch Wei correspondent, Pan Siyu Zhu Qianqian) in order to further strengthen the education and management of college students to participate in the Xinfeng County "return home" social practice activities in Shaoguan City, Enhance the organization of the regiment, Promote Xinfeng outstanding college students to actively participate in the rural revitalization strategy, On July 31st, In Xinfeng County, Shaoguan City, Guangdong Province, the 2021 annual "return to home" college students' social practice team temporary Youth League branch committee was held in the conference room of the Communist Youth League County Party Committee, Relevant person in charge of the Youth League county Committee and temporary youth League branch members attended the meeting. Conference, the county related comrades responsible for the specific explanation of the practice precautions and related requirements, establish branch committee and the evaluated integratedly returning college students "one to one" contact mechanism, and requires temporary youth league branch to give full play to the exemplary role, assist good unit to complete the task, set up a good image of "home" social practice of college students. It is understood that this is the first new venture back home social practice college students temporary youth league branch, by play to xinfeng returning college students love township enthusiasm, professional expertise, organization volunteer service, service, rural youth BBS, county research activities, expand the appeal and influence, let returning college students contact society, understand society, service society, strengthen for the hometown construction to do the practical work, do good deeds of social responsibility.

2021-08-03	Chaotian district, Guangyuan city, Sichuan province to establish the first "rural revitalization studio" in Guangyuan city, Sichuan province Chaotian district to establish the first "country revitalization studio" figure China youth network Beijing on August 3 (reporter Zhang Jianwei, correspondent Xiong Fei Qiao Lan) recently, Guangyuan city, Sichuan province Chaotian district li town organized a Shanghai university of engineering technology college of mechanical and automotive engineering, li town to build rural revitalization of the studio signing and opening ceremony meeting. Leaders of the Party Committee of School of Mechanical and Automotive Engineering of Shanghai University of Engineering, Guangyuan Municipal Party Committee, relevant leaders of District Rural Revitalization Bureau, Chaotian District Committee, Party Committee and government of Lijia Town, branch secretaries of villages (communities), and some student representatives of Shanghai University of Engineering Technology attended the meeting.at the meeting, The parties had in-depth exchanges on the school-local cooperation to promote the implementation of the rural revitalization strategy, Discuss the development direction of Lijia Town and the way to get rich together, Find the best combination for school-local win-win cooperation, Entrepreneurial youth representatives to Jian, District Rural Revitalization Bureau, the Youth League District Committee respectively made exchange speeches, Wang Dongyang, deputy Party secretary of the School of Mechanical and Automotive Engineering, Shanghai University of Engineering and Technology, Will seriously do a good job of the "rural revitalization studio" work, Give full play to the advantages of school disciplines, make use of superior social resources, and constantly introduce social forces to participate in the rural revitalization work, Strive to build this studio into a whole district, the whole city and even the whole province can copy and usable cooperation practice model. Next, TuanOuWei will continue to do a good job of contact youth, youth service, through the rural revitalization studio the platform, attract and drive the masses of young talents, experts and scholars to participate in the rural revitalization work, and do a good job of talent training, entrepreneurship employment guidance, constantly promote the depth of fusion, university to achieve win-win cooperation.

2021-08-02	Anhui Dingyuan county more than 100 college students for the village youth league construction vitality Anhui Dingyuan county: more than 100 "Quyang goose to" college students for the village youth league construction Wang Haihan Wang Lei correspondent, Huang Zhengnan Luo ChengYe) Anhui Dingyuan county furnace bridge town seven community after 95 staff zhang just graduated this year, has not had time to adapt to the identity change, he has experienced several "events" in the village, from straw burning to flood control risk, every experience accelerated his growth. In May, Luqiao town into the burning state, Zhang Bin and the other two colleagues on duty every day, there is no illegal straw burning phenomenon; in July, Luqiao town occurred large-scale flood disaster, class 10 wind and short-term heavy rainfall hit, Zhang Bin and the village committee cadres together in the first time to transfer the villagers. Zhang Bin said frankly: " Since my work, I have gained a new understanding of rural work, and the grass-roots work is complex and heavy. When I am recognized and praised by the masses, I feel very proud!"In recent years, in view of the village (community)" two committees "cadres age older phenomenon, Anhui dingyuan county innovation implementation" Quyang goose " project, from the village rich leader, outside business, full-time college graduates, veterans, young reserve cadres introduced, cultivate a batch of ability quality of young village cadres. This year, the Youth League Dingyuan County Party Committee from the "Quyang wild goose return" talent pool to absorb more than 100 college students to the village level league cadres team, strengthen the village level league organization strength. Yu Xue, head of the Youth League Branch of Zhongtang Village, Erlong Hui Township, Dingyuan County, became a member of the "Quyang Wild Goose Return" project in September 2020. She started from the basic affairs such as sorting out documents, and has been skilled and deeply involved in civilization publicity, rural construction and other work. In June, she became a grassroots league member and worked part-time on other affairs. She regrets that the big to road renovation, house demolition, small to husband and wife quarrel, neighborhood disputes, all need the village committee to solve, with these grass-roots work experience, do the work of the group is also handy. She often told herself that to become a cadre of the Communist Youth League, it is more patient and meticulous to deal with everything at the grass-roots level, " less empty vacation, less empty talk, less summary, more work, do practical things, do real things."Shan Natural, 22, was selected as the deputy secretary of the league branch of Xisdian Village, Xisisdian Town. She took the initiative to learn the league business knowledge, took the lead in strengthening theoretical study, and urged and guided the youth members in the village to carry out the" Youth Learning " online theme league class every week. In combination with the work of the village center, she organized COVID-19 vaccination and epidemic prevention and control for college students returning (in). Born in 1992, Liu Qiong has been leaving Jiepai Town for 10 years since she went to university. In 2021, she passed the "Quyang Goose Return" exam to "returned" and became the youth member of Jiepai Community Neighborhood Committee. From the work of the "small white" to the policy and business memorized in the heart of the "expert", Liu Qiong did not have less extra class. In recent days, she learned business knowledge, while mapping the information of the village to get rich leaders, in driving the village collective income and employment and entrepreneurship contribution. In the next step, the youth League Dingyuan County Party Committee will enrich the working force of the county youth league at all levels through multiple channels by training and selecting village (community) youth committee members and absorbing college students returning (in) townships, and further smooth the promotion channels of grass-roots youth league cadres.

2021-08-02	Leling city in Shandong province Bluebird students hometown tour Shandong Leling municipal party committee to carry out the "students hometown see revitalization hand in hand-2021 Leling students hometown activities" wei) to stimulate Leling home college students love party patriotic home enthusiasm, to experience home social and economic development achieved fruitful results, great changes, recently, the league in Shandong Dezhou city Leling municipal committee "students hometown line to see the revitalization-Bluebird students hometown" activities, Leling home college students a total of more than 40 people. The activity visited four livelihood projects, including the central Park project, elderly care service center, smart medical care service platform, Shandong Zhonggu Starch Sugar Industry Co., Ltd., Hebei and Shandong Border Region Revolutionary Memorial Park, and key enterprises and scenic spots."I don't have time to visit my hometown at school. Today, through this activity, I feel that the construction of my hometown is getting better and better. After graduation, I hope to return to my hometown. I also want to introduce my hometown to my classmates from other places and bring them to visit the beautiful Leling."Said Wang Zhenze, from the Beijing University of Chemical Technology. Through the field visit, the college students personally felt the new development, new changes and new image of their hometown. It is reported that the Communist Youth League Municipal Party Committee will take the Green Bird Plan and college students returning home as an opportunity to continue to build the "Le Ju Le Chuang" youth entrepreneurship and employment brand and the "Le Ju Le Chuang" Le Ju Le Chuang " to the music youth talent service brand, to form the effect of introducing talents, developing talents and retaining talents.

2021-07-31	Group of Ningbo, Zhejiang fenghua district party committee held common rich youth said Zhejiang Ningbo fenghua district party committee said common rich youth activities in youth network reporter Ji'an-ping li) 30 afternoon, Zhejiang Ningbo fenghua district party committee "I write youth in the window" common rich fenghua youth said activities, in the national common prosperity demonstration area village model village."I am a young village party secretary. I want to ask you how to better integrate into the countryside, promote rural revitalization, and do a good job in rural work."Fenghua District Jiangkou Street Qianheng Village after 85 village secretary Zhang Libo to Tengtou Village Party Secretary Fu Pingan. National outstanding communist party member, fenghua district teng village party secretary Fu average share "rural revitalization and common prosperity" experience, said to adhere to the party construction and the right direction, innovation digital development resource integration, deepen the "rich" with "rich" mode, give full play to the development of the party joint "means", adhere to the green ecological development path, focusing on young talent team construction, the more youth become love rural, love farmers, love rural revitalization of a new generation of "three rural" entrepreneurial innovation army. Chen Lei, deputy secretary of the Youth League Committee of Fenghua Rural Commercial Bank, said that the youth are duty-bound to help achieve common prosperity. Financial youth will firmly serve "agriculture, rural areas, farmers, small and micro businesses", actively help digital transformation, rural revitalization, rural youth entrepreneurship and other work, and improve the work quality of inclusive finance through financial innovation and digital empowerment. Fenghua district level green chuang farm "farmers" shared their entrepreneurial experience, said that young rural entrepreneurs can drive agricultural development, promote rural economic prosperity and achieve common prosperity from the aspects of intensive land, increasing production and efficiency, green production, innovative technology and so on. Ying Yingying, chairman of Ningbo Miss Honey Food Co., Ltd., as a young farmer maker, she called on more people to pay attention to food safety, plant conscientious agricultural products, so that more high-quality agricultural products can go to the city, make life better, and the countryside makes urban people more yearn for it. More than 20 young delegates visited Tengtou Village to learn about the development process of Tengtou in detail. Li Li, secretary of the Communist Youth League District Committee, said that the youth representatives should be firm in the belief of "common prosperity", make the determination of "innovation and entrepreneurship", maintain the perseverance of "solidarity and mutual assistance", gather youth consensus among various industries and groups, lead the youth to build together, and make youth contributions to boosting the construction of the demonstration area of common prosperity.(Photo provided by Cold Xiao)

2021-07-28	Anhui chaohu city for college students to return home social practice post group chaohu municipal party committee to carry out the "return home" social practice college students' emergency ambulance training JianWei correspondent, liu chaohui check WeiTing) recently, for the implementation of the Anhui provincial party committee organization college students to return home social practice requirements, group chaohu municipal party committee after collecting post, student registration, two-way selection, a total of more than 120 college students to return home social practice jobs. Before the official registration, the League Chaohu Municipal Party Committee organized some college students returning to their hometown to participate in the training class of emergency rescue workers, to improve the emergency rescue ability of college students returning to their hometown. The training activity includes the development of the Red Cross, the new concept of medical rescue, cardiopulmonary resuscitation, trauma rescue, and the disposal of common emergencies and accidental injuries. The training was carried out by a combination of theoretical explanation and demonstration operation. The participants participated in the on-site drill after listening to the class. Through the training, I deeply learned and understood the basic theoretical knowledge of emergency rescue, and initially mastered the basic essentials and skills of emergency rescue, such as cardiopulmonary resuscitation and on-site first aid methods for airway foreign body infarction. In the next step, the League Chaohu Municipal Party Committee will continue to carry out relevant professional training, popularize emergency rescue knowledge, and expand the emergency rescue team of young volunteers.

2021-07-26	Shandong Linyi county to carry out the "looking for red footprints feel revolutionary feelings" studies activities of Shandong Linyi county to carry out the "looking for red footprints, feel revolutionary feelings" studies activities youth network Beijing on July 26 (reporter Zhang jianwei, correspondent lu qi) recently, the group of Shandong province Dezhou Linyi county organization "green micro lecture hall" into the NingJin studies base, "hope hut" eight assisted children to ningjin red revolution education base research activities, two "green bird plan" summer college students volunteers to participate in the activities. Activities, the children enthusiasm, actively participate in, the "live" up the party history activities, to solve the problem of learning revolutionary history only in books, the children in the way in the process of listen, see, swim, deeply understand the glorious history of the communist party, listen to the heroic deeds of revolutionary ancestors, firm study hard, the faith to serve the motherland. The picture shows a group photo of the red study activity. Correspondent for figure the studies activities into the ningjin county, Shandong bohai military region teaching brigade revolutionary traditional education base, cricket happy valley, integrated use of field teaching, classroom teaching, discussion, communication and other training methods, largely reduce the students for historical knowledge acceptance degree of boring, enhance the interest on the learning process of interest. This flexible teaching method makes students have fun in play, gain knowledge in fun, and experience red culture.

2021-07-20	Shandong gaotang county launched "I do the practical work for youth" ten action Shandong gaotang county launched "I do the practical work for youth" ten action jianwei) according to the Liaocheng and gaotang county related requirements, for the party history learning education achievements constantly into the practical work, solving, open new action, promoting "I do the practical work for youth" practice, gaotang county focused on the party's party and government needs, youth hope precision, focus on the following "ten action", help youth development. Country good youth can assign action to actively play a role of youth in the rural revitalization and commandos, combined with the actual practice, the implementation of rural "good youth" foster "service method" six steps "(i. e., widely, specification, cultivating, strong, convincing, pragmatic, propaganda), build rural" good youth "" six "double new" (namely "," new media new vision "," new farmers "," new ideas, new thinking "," new era of new life "," new civilization "," new momentum new forms ") mode, industry to establish good youth alliance. Through the youth League branch, the youth talent association and other organizations, we will gather and integrate the youth strength, and concentrate our efforts to boost rural revitalization and development. We will keep close contact with county and town (sub-district) financial institutions, increase policy support for "good youth" business loans, and solve the problems of insufficient funds and resources for "good youth" in the process of entrepreneurship. We will build youth innovation service stations in the whole county to provide financial support, training services and e-commerce services for rural "good youth", and effectively serve the growth and development of rural "good youth". We will organize the "Financial Assistance Matching Meeting for Rural Good Youth Development", and invite rural "good youth" to discuss cooperation projects with financial institutions and provide financial support, so as to boost the development of rural "Good youth" projects. The urban and rural communities initiative has upgraded three "youth homes" in urban and rural communities to jointly build "youth communities".? Explore suitable for the creation of the community, innovative youth salon, entrepreneurship guidance, employment training, interest exchange services, extensive youth fellowship activities, let more youth with community feelings, professional knowledge to participate in the community governance team, truly become of community autonomy participants, community active drivers, community disseminator of positive energy. Youth XunXunTuan "six into" activities from the grassroots cadres, youth cadres, party and government organs, youth league members, youth teachers, youth theory experts, youth media workers, social organizations, youth backbone, "two new" organization youth backbone, college students village official, community youth party members, typical youth in such areas of outstanding youth recommend gaotang youth lecturer, set up "gaotang youth tour". We will focus on studying and implementing Xi Jinping Thought on Socialism with Chinese Characteristics for a New Era and the Party's glorious history in a century, and publicize the Party's advanced theories, Party history and the Youth League, national conditions, situations and policies, and stories of youth examples. Focusing on the activities to celebrate the 100th anniversary of the founding of the Communist Party of China and the study and education practice of "learning the Party history and strengthening the faith of following the Party", we will promote publicity into government organs, schools, rural areas, enterprises, communities, and networks to open up the "last mile" of youth theoretical armed work. Implement youth production safety skills promotion action to actively carry out "youth production safety ShiFanGang" to create work, joint emergency management widely mobilize grassroots production safety unit, engaged in safety production inspection collective actively participate in creating, in visiting line production workshop, team, project and other grassroots safety production unit, promote green AnGang process, normalized, explore typical, find, inspire green AnGang collective create enthusiasm at all levels. In order to help the development of the e-commerce industry, improve the training and incubation system of young e-commerce talents, and attract representatives of key e-commerce enterprises, financial institutions and training schools to jointly form the "Youth E-commerce Alliance". In rural youth entrepreneurship to get rich "leader" training plan, rural youth electricity cultivation project as the gripper, common young entrepreneurs organization, through the mentor pairing, entrepreneurship counseling, project cultivation, financial support, elements of docking services, integration of various education training resources, to carry out industry training courses, planned, targeted to carry out base training, practice.? High tang "youth station" project focus for graduating college students and unemployed young solve high tang young talent accommodation, job docking problems, founded "youth station", for college graduates and unemployed youth equipped with complete hardware facilities, water and electricity equipment, build warm heart retention environment, strengthen the follow-up service, to enhance the young students' sense of belonging and gain."Hope cabin" dilemma children care project continue to deepen the "hope hut" dilemma children care project, through online "520" "99 public day" and offline funds, strictly implement the "secretary of the youth corps committee grasp quality", high standards to promote hope cabin construction, timely to build cabin "look back" supervision investigations, to ensure that the cabin construction withstand inspection. Do a solid job in the "Hope cabin" two-to-one volunteer pairing assistance, to realize the growth and transformation from "cabin renewal" to "spiritual renewal color". Vigorously implement the "green birds to nest wisdom hui Gaotang" action to continue to hold the "green birds to nest wisdom hui Gaotang" 2021 Gaotang county college students in winter and summer vacation to return home practice activities, organize the county government institutions, communities, village level to actively connect with Gaotang college students. The visit and exchange activities and symposiums of "Green birds returning to visit gao Tang" were deeply carried out to enhance college students' understanding of their hometown and lay a good emotional foundation for them to return to their hometown for employment and entrepreneurship after graduation. Increase the exchange of youth painting and calligraphy relying on gaotang County "the hometown of Chinese painting and calligraphy art" gold signboard and painting and calligraphy town characteristic projects, through the county youth calligraphy association and other two youth teams, external exchange, internal service, promote the development of painting, calligraphy and painting industry, form a pulling effect, actively promote and promote Gaotang. Youth volunteer service action to strengthen the construction of youth volunteer team, widely launched youth groups to participate in volunteer service, organize skills training, improve professional level, extensive "drowning" "love nursing hand zebra crossing" "escort college entrance examination" "small hand widening" "poor" "care" yellow rose "" emergency rescue "" good reading "volunteer service projects, build" green warm Jincheng " youth volunteer service brand, increasing influence and popularity. League Gaotang County Party Committee will take "I do practical things for the youth" practical activities as "learn the history of the Party, strong faith, follow the party" of the party history learning education, accurately focus on the "ten practical things", press the compaction work responsibility, jointly promote the practical implementation, effectively enhance the sense of gain and satisfaction of teenagers.

2021-07-26	Li Jianping, the Communist Youth League of Chun'an, Zhejiang Province, has created a platform of "Innovation College" recently to help the rural revitalization of Qiandao Lake. Chun'an County is the practice and innovation base of "clear water and green mountains are gold and silver mountains". At present, Chun 'an County "Qingchun Innovation College" of Chun' an County integrates the "Qingpu Maker Competition" into the "One College Student in One Village" cultivation program, attracting young college students and young people returning to their hometown to start businesses and find jobs to develop modern agriculture, ecotourism, health tourism and other industries. The communist youth league chunan county officials said, "green and college" platform to service youth entrepreneurial employment in the countryside for the purpose, for entrepreneurial youth innovation entrepreneurship exchange platform, build interactive, closely linked township and youth talent community and youth innovation entrepreneurship hub organization, absorb outstanding entrepreneurs, experts, scholars, outstanding entrepreneurial youth become "green and mentor", for innovative entrepreneurial youth and enterprises to provide guidance support, promote chunan county rural industry upgrading. Fang smiled for the picture

2021-07-19	Shandong gaotang the second batch of summer college internship release Shandong gaotang the second batch of summer college internship jianwei) to further help ZhaoCaiYinZhi, service college students employment, promote lead and employment mark, help young students perception gaotang, understand society, improve skills, group gaotang Liaocheng county launched in early June in 2021 "bird nest gaotang" college students' summer social practice activities. The first batch of registered college students have employed 106 people after their intention of registration and examination. In order to further expand the coverage and better meet the needs of all units and returning college students, the second batch of college students returning to practice recruitment work. The activity time will be from July to August 2021, and the internship time will not be less than one month. The details will be determined by each practice unit according to the actual situation, from the date when the students report to the post. Recruitment objects are college students enrolled in or graduated within one year of school; actively participate in party organization activities, CPC members, outstanding Communist Youth League members and outstanding student cadres are prioritized in the recruitment objects; have good ideological and political quality and strong comprehensive ability, able to complete the summer public welfare internship plan according to the schedule. It is reported that the selection and recruitment of the Youth League county committee is responsible for, in the centralized summary, unified notice of the students through the audit.

2021-07-19	Anhui Qimen County youth volunteers to carry out the river chief system publicity activities group of Anhui Province Qimen County Party Committee organized young volunteers to carry out the river chief system publicity activities Jianwei correspondent, Xie Zhihong Zha Wei Ting) recently, the group of Anhui Province Huangshan City Qimen County Party Committee organized young volunteers to carry out the river chief system related publicity activities. During the activity, the township youth League committee organized young volunteers to set up a publicity and consultation desk in the central street section of the town, distribute publicity materials of the river chief system along the street, publicize the work of the river chief system to the villagers, and publicize the knowledge of banning illegal sand mining and electric fish that destroyed the ecological security of the river. Explain the importance of protecting river courses in detail, and crack down on the behaviors that destroy river courses' safety. The past masses have expressed praise to the river chief system propaganda action and strong support, they stopped to listen in detail and asked one by one, the staff are patiently answered one by one. It is reported that in recent years, TuanQiMen county actively to "practice'long river", protect the mother river " as the theme to carry out various forms of activities, mainly activities as the carrier, enhance the awareness of protecting the ecological environment, improve the enthusiasm and initiative of people to participate in the long river system work, with practical action to protect their beautiful homes.

2021-07-19	Anhui Lingbi county "micro wish" interpreta dream and basketball friendly held in Anhui province Lingbi county "micro wish" interpreta dream distribution ceremony and basketball friendly held jianwei correspondent, Xie Mengmeng check WeiTing) recently, Suzhou city, Anhui province Lingbi county "micro wish" interpreta dream distribution ceremony (county 9 middle school station) and basketball friendly was held smoothly, Xie Mengmeng, deputy secretary of county 9 middle school vice principal Zhu Tong and county 9 middle school all the teachers and students to participate in the activities. At the event site, the leaders attending the meeting distributed sports shoes, sportswear, basketball, table tennis rackets, and other "micro-wish" materials, and encouraged them to study hard, have dreams, and always maintain a positive heart. After the distribution ceremony, the county no. 2 middle school basketball team and the county no. 9 middle school basketball team held a friendly basketball match. With a clear whistle, the high throw of the basketball lit up the passion of the game, the two teams quickly launched a fierce attack, jump shot, breakthrough, three points, strong play, attracted the sidelines audience applause, repeatedly applauded. The smooth holding of the basketball game made the teenagers feel the strength and warmth of the group, strengthen the sense of unity and cooperation, show the youth style of the teenagers striving to make progress and never give up, and promote the close communication between the school. Tuan Lingbi County Party Committee actively connected with the county No.9 Middle School to collect the "micro wishes" of teenagers, including the spiritual level, such as a family companionship, learning guidance, story sharing, etc., but also including the material level, such as school supplies, daily necessities, extracurricular books, etc. Combined with the specific reality, to meet the small wish of every youth, with the heart and affection to do practical things, do good things, do things around them, to send the warmth to the hearts of the youth, so that the youth really feel the effectiveness of the party history learning and education.

2021-07-17	Yuexiu district of Guangzhou city, Guangdong province for Guangdong youth entrepreneurship platform yuexiu district for Guangdong youth entrepreneurship platform Lin Jie) on July 15, a large bay area of Guangdong youth innovation entrepreneurship base (yuexiu), a large bay area youth international cultural exchange center (yuexiu) in Guangzhou, will provide a set of teenagers for cultural exchanges, entrepreneurial employment, public welfare practice is equal to the integration of the comprehensive service platform. Tsim Sha Tsui, Central, Macau Tower, the archway... this exchange center jointly built by the United Front Work Department of Yuexiu District Committee of Guangzhou and Yuexiu District Committee of Guangzhou, has a strong flavor of Hong Kong and Macao flavor. In the office area, some enterprises are consulting the policies and operation methods, some enterprises have set up the office cards, and some enterprises are applying for the stationed office. In the activity room, young people from Guangdong, Hong Kong and Macao are warmly exchanging ideas and talking freely. A large bay area of Guangdong youth innovation entrepreneurship base (yuexiu) with a total area of 1035.32 square meters, set with Hong Kong and Macao youth business incubation multi-function room, project roadshow center, Shared office area, large bay area youth salon area, conference room, form a complete set of personal broadcast room, sharing coffee room space, provide about 40 open station, can accommodate 150 people activities, office communication leisure function. Deng Jinggan, the head of the center, said that more than 20 entrepreneurial teams from Hong Kong and Macao are interested in trying to settle in the base, covering business services, new-generation information technology, international education, cultural creativity and biomedical industries. Since its trial operation, it has attracted 2,000 students from all over the country and from Hong Kong and Macao, and attracted more than 100 Hong Kong and Macao schools and famous schools at home and abroad to participate in the activities."Strengthening the construction of youth innovation bases is an important starting point for the implementation of youth innovation and entrepreneurship services."The relevant person in charge of Guangzhou Yuexiu District Committee said, based on the youth development, take the initiative to build a platform, combined with the work requirements of" I do something for the youth ", with the youth innovation base as the position, to solve the most concerned, the most direct, the most practical difficulties and problems. The exchange center is jointly built in the mode of "government site provision, institutional financing and construction, professional team operation, and public welfare services" to promote extensive exchanges, comprehensive exchanges and deep integration among young people in Guangdong, Hong Kong and Macao, and enhance its centripetal force towards the motherland. In the future, the Exchange Center will also work with Hong Kong Youth Federation, Hong Kong Youth Association and Macao Yuexiu Youth Federation to organize and implement youth exchanges and cooperation projects between Hong Kong and Macao to build a platform for the young people of the three places. In addition, the "Yueqing Cup" 2021 Yuexiu District Young Maker Xinxing Entrepreneurship Skills Competition was awarded today. After multiple link examination selection, palm test information, hui core technology, virtual pictures and other high-quality enterprise project stand out, the final selection of eight entrepreneurial youth identified will win the title of Guangzhou yuexiu district young guest nova, up to 100000 yuan reward and enjoy the yuexiu district in housing security, children, health care and other aspects of talent treatment.

2021-07-15	Shandong muping district party committee held in 2021 in the grassroots cadres training league mouping district party committee held in 2021 in the China youth network Beijing on July 15 (reporter Zhang Jianwei, correspondent li) in order to further consolidate the village (community) youth league transition, combined with the history of the party learning education, improve grassroots political quality and business ability, promote the communist youth league each work to the ground. Recently, the Muping District Committee of Yantai City, Shandong Province held the 2021 district grassroots cadres training class, towns, departments, enterprises, schools, social organizations, rural (community) areas of more than 50 people participated in the training. Sun Fuhai, secretary of Tuan Muping District Party Committee, introduced the significance and course characteristics of the training class in the opening speech, exchanged his own work experience and training methods, and put forward the hope and work discipline requirements to the students. It is hoped that through concentrated learning and training, the participants can help them to better understand and master the basic knowledge and methods of the party history and the grass-roots youth league building work. The training lasts for 4 days, covering special lectures, business training, on-site observation, exchange and sharing. Training, invited MouPing district party committee party school zhang vice President, Yantai municipal party committee party school professor zhang, Yantai public network director zhang respectively, the history of the party, enlightenment beginner's mind, the history of the CPC from MAO zedong poetry "new media operation" and other content of special teaching, help trainees to further enhance the party spirit cultivation, improve the ability to work. Participants also visited the kunyu mountain revolutionary memorial hall, by retracing the road of "faith" experience, the students deeply feel the communist party of China, in leading jiaodong army and civilians for national independence and people liberation, made a magnificent, inspiring jiaodong revolutionary spirit, more strengthened the grassroots group build confidence and determination. In order to quickly shorten the distance between the students, a temporary league branch was established on the opening day and a new branch committee members were elected, forming a temporary organization and management mode with the league branch members as the group leader responsibility system and the organization of students for teaching and daily management. And use the evening time planned and held a theme of "new era youth said" publicity activities, all the students around the grassroots cadres "how to effectively organize the youth, serve the youth, cohesion youth" shared their feelings and talked about the harvest plan.

2021-07-15	Anhui jingxian county "children service station public small class" class in Anhui jingxian county "children service station public small class" class JianWei correspondent Zhang Xin check WeiTing) in Anhui xuan cheng jingxian county Jing Chuan town Jing Jing learning children service station, children under the guidance of the volunteer teacher picked up colorful brush on the paper fan, a lovely flag, the emblem, the words "100" on paper, the children to express the centenary of the pure blessing. In the Taohuantan town children's service station, the children sit up, a child is trying to introduce themselves to the friends, it turned out that the volunteer teacher guided the children to carry out the small host class learning, we laugh together, feel the fun of learning. The "children service station public small classroom" is sponsored by jingxian county county, the township youth corps committee to undertake a public welfare volunteer service project, and this year jingxian county communist youth league system "does the practical work for the masses" one of the practice point, mainly for primary school mobile left-behind children and double worker family children, using the community (village) both positions, organize volunteers, social workers open public small classroom, help teenagers healthy growth. This year, a total of 12 stations will be opened, including 8 municipal stations and 4 county-level stations. Each station is equipped with a full-time webmaster, responsible for the daily operation of the site. Each site in the semester and winter and summer vacation classes, courses to ideological guidance, schoolwork guidance, psychological counseling, supplemented by safety self-protection, science education, culture and sports, law publicity, ecological environmental protection and other courses, and combined with the characteristics of the township, to build a "3 + N + 1" public welfare curriculum system. During the history of the party learning education, the county closely with "I do the practical work for the masses" theme practice, to "children service station public small class" project as the carrier, in the county widely to the masses, priority to select the masses demand strong, build station class basic conditions good community (village), preparing children service station, open "public small class". So far, two stations have been open, and another 10 will open by mid-July.

2021-07-14	Shandong Gaotang County held drowning prevention knowledge training activities Shandong Gaotang County held "cherish life to prevent drowning" knowledge training activities network Beijing on July 14 (reporter Zhang Jianwei) summer is coming, the temperature rises, students drowning accidents into the high incidence period. In order to educate students cherish life, prevent drowning, safe summer, since April this year, gaotang group Liaocheng county will prevent drowning work as the current and future a period of time youth safety priorities, do early mobilization, early deployment, early action, implement, actively launch gaotang love public welfare association and other social organizations to carry out drowning prevention knowledge training activities. The picture shows the training activity site. During the presentation, the volunteers explained the "six no" against drowning: no swimming without permission; no swimming without parents or teachers; no swimming in unfamiliar waters; no swimming without safety facilities or rescue personnel; and no students without water knowledge. Knowledge of drowning self-rescue and mutual rescue: remind, dissuade and report danger; do not be nervous, relax, hold breath and breaststroke legs on water, how to save plastic bags and drinking water bottles in water, use bamboo poles, throwing items, throwing rope, etc. The activity aims to enhance the safety awareness of teenagers, so that teenagers understand the knowledge of drowning emergency rescue, and more importantly, let the important understanding of life is deeply rooted in the hearts of teenagers, to lay a solid foundation for the life safety of teenagers.

2021-07-14	Anhui duji district to celebrate the 100th anniversary of the party scene type party class to celebrate the 100th anniversary of the founding of the communist party party class Jianwei correspondent, Liang Beibei check WeiTing) "sing a folk song to the party, I put the party than mother..." recently, in the melodious prelude, Anhui Huaibei duji district "struggle a hundred years road set sail new journey" to celebrate the 100th anniversary of the founding of the communist party of China party type party class in the east lake happy world began. Huaibei municipal party committee of the party history learning education second circuit steering group leader, city women's federation work office director xiu-li sun, Huaibei normal university music college party secretary of zhang, du set district party committee secretary Jiang Ying, deputy secretary of district Sun Lulu, district Liang Longji, district CPPCC chairman chestnut four team leadership, OuZhi departments party branch secretary, outstanding communist party member, outstanding party workers, advanced grassroots party organizations, and the local masses more than 400 people to listen to the unique party class. Sun Lulu read about the CPC du district party committee of the outstanding communist party member, outstanding party workers and advanced grass-roots party organization decision, Jiang Ying for "glory in 50 years" commemorative medal winner Guo Honghui, Zhang Yujin, Zhang Yanzhao issued commemorative medal, for "two excellent first" awards, district party committee, organization minister Wang Shengchen led the new party representative of the oath. In the party class, Guo Honghui, a veteran party member of "Glory in the Party for 50 years", recalled the eventful years and reviewed the history of struggle; Niu Suping, Ge Zhiguo, Zhang Benqiang and Zhang Hongyun, four "good people of China", conveyed positive energy to everyone. Their simple language, sincere emotion, moving, more encourage the scene of the party cadres to draw the strength of example, firm ideals and beliefs, strive to take on as. The whole situational party class will art and party history learning education organic integration, through the instrumental music playing "sing a folk song to the party", song dance "south lake red boat", dance the red army is not afraid of expedition difficult program, such as, the party's glorious course, great spirit through the way of "change" people to preach, build "the party festival people's festival" atmosphere. The party class not only let the audience feel the charm of art, but also experienced the vivid party history learning and education, but also highlights the party members, cadres and the masses to gather together, inherit and carry forward the glorious tradition and fine style of the Party, to realize the modern and beautiful Duji in the new stage. The situational party class by the communist party committee, the district people's government, the district party committee party history learning education leading group, the communist party party committee organization department, the district party committee propaganda department, the CPC party committee, du district culture, tourism and sports bureau, the communist youth league du ji district party committee to undertake, du district public cultural service center, the district cultural center specific execution.

2021-07-13	Shandong gaotang county to carry out the poor elderly, troubled children visit sympathy activities gaotang county to carry out the "Jin Hui", "help old" hope "hut big screen big visit big sympathy activities jianwei) combined with" I do the practical work for the masses "" I do the practical work for the youth "requirements, since May this year, the gaotang county party committee in Liaocheng city, Shandong province, township youth corps committee to" Jin Hui help old "poor old man," hope hut "difficult children to carry out" big visit, big screening, big sympathy " visit activities. The Communist Youth League county party committee organs, the town and street youth League committee came to the "Jinhui to help the elderly" poor elderly home, detailed inquiries about the recent living situation, volunteer help, combined with the "five one" volunteer help requirements, to carry out help. During the period, the youth league, the volunteers to carry out the "golden" big sympathy activities, for the old man sent a portable flashlight, summer cool, crutches, edible oil and other supplies worth 500 yuan, also for each old man sent 500 yuan in cash, and for the old man framed painting and calligraphy hanging home, in material, spiritual and other aspects to help the old man. In the home of the children in difficulties in the "Hope House", the youth League cadres and volunteers asked about the "little master"'s study and living conditions in detail, and carried out characteristic assistance activities. The "big visit, big investigation, big sympathy" activity, the league cadres go from house to house, a detailed understanding of the "Jinhui to help the old" poor old people, "hope hut" difficult children production and living conditions, and the problems existing in the process of helping to be solved in time.

2021-07-09	Guizhou wan mountains "classroom" promote party history "heating" group of Guizhou wanshan district party committee "classroom" promote party history learning education "heating" youth network Beijing on July 9 (reporter zhang, correspondent Yang han) party history learning education, group of Guizhou Tongren wanshan district party committee combined with the characteristics of youth group, close to the work practice, innovative methods, with red classroom scenario studies, mobile classroom door learning, cloud classroom fingertips guidance for the gripper, education and guide teenagers know history love party, patriotic, promote party history learning education "heating". The picture shows the scene research site. Correspondent for the picture scene study, "red classroom" strong experience firmly grasp the "red classroom" function positioning, focus on the local red "resources". Inheriting the red gene through field objects, interactive experience, vivid explanation and other ways, and continuously enhancing the attraction and appeal of red activities. The secretary of the Youth League Wanshan District Committee took the lead to visit the Wanshan New Era Patriotism Education Demonstration Base and study, paying respects to the great achievements of history and listening to the patriotic stories. Young volunteers and youth league cadres were organized to visit cinnabar Ancient Town to carry out Party history learning and education by reciting classics and sharing red stories. At an important time point, the party day activities with the theme of "sacrificing the martyrs, learning the history of the Party, and realizing the thought" were taken as an opportunity to carry out activities such as remembering the revolutionary martyrs and reviewing the oath of joining the Party. At present, more than 50 series of activities have been carried out, including "Reviewing the Party membership oath", "Remembering the revolutionary martyrs" and "Red Story Sharing Meeting". The picture shows the scene of "sending school" and "mobile classroom". Correspondent for pictures to send school, "mobile classroom" wide publicity to take flexible and diverse ways, the party's knowledge into the grass-roots party members, cadres and the masses, let the glorious course of the Party deeply rooted in the hearts of everyone, and further promote the party history learning education to go deep and solid. The Wanshan District Committee carried out extensive learning and education of "learning the history of the Party, strengthening faith and following the Party". Together with the District Women's Federation carried out the activity of "Tell the Red Story of Wanshan" at the grass-roots level in Gaoping Youth Lake Village, giving a vivid party lesson for the local Party members and cadres, villagers' representatives and women's representatives. Organized the community volunteers to carry out the special activity of "I will tell the Party's story" based on the 4:30 class and the youth rural revitalization night school carrier. Mobilize the party cadres with high ideological understanding, strong comprehensive ability, cadres, western plan volunteers, Chunhui emissary to set up "youth lecturers", to send education to the door, on-site training and other ways to carry out the party history propaganda into the community, into the classroom, into the family and other activities. At present, the Communist Youth League District Committee has organized more than youth teaching groups and volunteers to carry out more than 100 lectures, benefiting more than 3,000 people and teenagers. The picture shows the "cloud classroom" learning. Correspondent for figure fingertips, "cloud classroom" guide "cloud classroom" to break the traditional learning mode, make learning education out of the limitation of space, space, distance, let the youth can make full use of fragmented time, anytime, everywhere, and through online browsing, online interaction, deepen the understanding of party history, improve the coverage of party history learning education. Tuan Wanshan District Party Secretary took the lead in shooting a video of party lessons. Innovate the form of reading party history and sharing story learning, and organize volunteers of the Western Plan to share and read a party history story on the WeChat platform of "Wanshan Youth". In the WeChat public account of "Wanshan Youth", we set up "Party history for a hundred years to read every day", "Party stories I me" and other party history learning and education columns. Through the online Communist Youth League platform to carry out "youth learning", online answer interaction and other activities. At present, the Wanshan District Committee of the Communist Youth League has sent more than 120 articles related to the party history learning and education through the WeChat platform of "Wanshan Youth", and mobilized more than 6,000 young people to participate in the party history learning and education.

2021-07-09	Shandong Pingyin County construction vitality "youth community" to help improve grassroots governance Wei correspondent, Zhang Zhichao, Liu Yang) in order to implement the league central Committee "community youth action" requirements, Improve the overall contribution degree, The Communist Youth League Jinan Pingyin County Committee in Shandong Province has issued "Implementation Opinions on Building a Party Building Leading Youth Grid Service Management System to Further Improve the Level of Grassroots Social Governance", "Notice on the Creation of Youth Communities to Promote Improving the Level of Grassroots Governance" and other documents, Clear about the creation of Pingyin County "youth community" of 8 categories of 22 project indicators, Explore the implementation of the youth community "3127" construction ideas, We will help improve community-level governance capacity."3" refers to the "three types of organizations", that is, the sound community youth working committee, youth League organization and youth working committee. Adhere to the party building with the league building, in the county city community level extension to the party organization secretary as the director, the league organization secretary as the executive deputy director, the area of the "youth home" stationmaster, property person in charge and other members of the youth working committee. We continued to lay a solid foundation for the league, and took the opportunity of changing the term of community committees to help 128 young cadres into 29 community committees, and elected 71 young people to join the league organizations, with an average age of only 28. Adhere to the whole league led the team, in the community to explore the establishment of the league organization secretary as the director, the young Pioneers work director as the deputy director of the young working committee, effectively strengthen the "youth community" construction organization and leadership, enhance the work motivation."1" refers to "a position", that is, to build a good "youth home". Relying on the community new era civilization practice station, service center and other existing site, according to a fixed place, someone responsible, marked, have "youth exhibition" photo wall, have normal "five" requirements activities, invite youth recommendations, rich innovation carrier, build "home" youth volunteers and youth friendship bridge, with revitalize the home of the "youth", build the party to build a demonstration site."2" refers to the "two teams", that is, cultivating a good youth grid (building leader) assistant team and the youth win-win post volunteer team. We selected 101 young grid assistants and 924 young building chief assistants for each community, with a rate of 93.5% and 87.2% respectively, basically forming a team of grid (building chief) assistants with a perennial stay at home, sufficient number and obvious effect. Focus on contacting and serving art, sports and volunteer service youth groups, hire people as art and design consultants, part-time youth grid assistant and other "youth win-win posts", and form a "part-time" youth volunteer team that serves online in school and community service during holidays."7" refers to the "seven projects", namely, seven volunteer service projects, such as focusing on city creation inspection and promotion, household waste classification, youth war "epidemic" action, community youth classroom, community governance improvement, youth action to help the elderly, and volunteer quality improvement. Centering on the central work of the county, focusing on the construction requirements of "youth community", the key content and implementation methods of 7 projects are designed with great efforts, to guide the community "youth" path is clear and have rules to follow. In the 2021 youth war campaign, 954 young volunteers have been mobilized to serve for an average of more than 100 hours, making a strong contribution to the fight against the epidemic. In the next step, the Communist Youth League County Party Committee will continue to make good use of the "3127" work ideas, focus on the needs of the residents, the youth needs, and the community needs to accurately gather the strength of the youth, coordinate all kinds of social resources, constantly broaden the working ideas, and strive to create a youth community "Pingyin mode" with Pingyin characteristics, youth co-construction and sharing.

2021-07-08	Xinfeng County, Guangdong Province, held a series of activities to celebrate the centenary of the founding of the Party, Guangdong Province, held a series of activities of "Please rest assured that the Party power has me" to celebrate the centenary of the centenary of the founding of the Party. Correspondent for figure China youth network Beijing on July 8 (xinhua zhang, correspondent Cheng Xin Chen CaiYing) recently, group of Shaoguan, Guangdong province xinfeng county launched comprehensive youth members, youth pioneers, group staff earnestly study and apply xi general secretary in celebrating the 100th anniversary of the founding of the communist party of China conference speech spirit, combined with the party history study to carry out the "please party power have me" series of activities to celebrate the founding in one hundred years. The Communist Youth League County Committee guided the Fengcheng Street Youth League Working Committee and the Party Building Office to organize the theme of "Please trust the Party, the strong country has me". Through the closed-book test, 1 special prize, 2 first prizes, 4 second prizes, 6 third prizes and 8 excellence awards were selected. Xinfeng various party members and cadres, youth members and volunteers spontaneously mobilize practice "please rest assured, power have me" youth oath, "sing a folk song to the party" the great party, I want to say to you "" please rest assured, power have me "and so on more than 30 issues produced by the micro video, only" July 1 " on the day of the clicks reached 100000 more times.

2021-07-08	Shandong boxing "four-wheel drive" promote youth party history learning education in Shandong boxing "four-wheel drive" promote youth party history learning education deep solid wei correspondent Li Lian Zhao Shuyue) party history learning education, Shandong Binzhou city boxing county according to the superior arrangement, highlight the characteristics of teenagers, through the "creative" "competition", "experience" "pragmatic" - "four-wheel drive" mode, promote all fronts youth active learning, enhance faith, unswervingly follow the party. Highlight the "creative style", with vivid, close to the youth members to carry out learning. Through the "youth study together to learn the history of the Party" series of online theme group class, party history study online answer and other ways, organize the county youth to study the history of the Party seriously, online theme class each group class participants are about 15,000 people. The activity of "Reading classics and Promoting the Chinese Dream of righteous Youth" Party history learning and education reading sharing meeting has been carried out. Up to now, works such as "Boxing Youth", "May 4th Spirit Inheritance With Me" and hand-drawn power supply youth cartoons have been produced successively. With the "childlike innocence to the party" as the theme, the organization of "I salute to the party flag", "sing a folk song to the party", "the party story I will tell" and other activities, so far, the county has carried out more than 150 practical activities, participating in the young people in about 30,000 people. Highlight the "competition", to learn to catch up, fierce way to carry out learning. To "learn party history strong faith with the party" the party history reading activities as important gripper, organize youth league organizations at all levels to carry out reading red classics, red songs, red stories, party history knowledge contest of various forms of "competition" learning education, let the general youth in than learn to catch up with learning competition deep enlightenment, into the brain into the heart. Up to now, the county at all levels of the youth league organization held "competition type" party history learning education 45 times. Highlight the "experience", to the immersive, intuitive grounded way to carry out learning. The county to carry out the theme team, more than 850 games, cao wang town youth corps committee, county fire brigade youth corps branch, the third primary school 20 youth to the revolutionary history memorial hall, river bank Mongolia red education base, Meng Lianggu battle memorial hall to carry out the party history learning education, in the scene learning experience to deepen the understanding and understanding of the history of the party. Highlight the "practical type", "I do practical things for the youth", promote the party history learning education to go deep and solid. To carry out free movie viewing activities, public training lectures, college road shows, employment service season, internship season, special recruitment season and other theme services, to provide a full range of services for young talents. At present, 42 "Hope House" child care projects have been completed and 23 are under construction; 5 Hope primary schools and more than 50 children in distress, so that the poor youth can feel the warmth from the league organization. We held the signing ceremony of "Helping Rural Revitalization and powering Rural Good Youth" and Party Construction with Rural Commercial Bank, and issued credit loan cards to good rural youth to solve the financial problems restricting industrial upgrading. Next, the Communist Youth League county Party Committee will take the party history learning education as an opportunity, to "I do practical things for the youth" list as the starting point, strengthen the management by objectives, grasp the implementation of the work, around the center, serve the overall situation, the party history learning results into the practice of promoting the work, help teenagers grow up.

2021-07-07	Beijing, July 7 (reporter Zhang Jianwei, correspondent Wu Lei) in order to further promote the party history learning education deeply rooted in the people's hearts, a few days ago, The Yongning County Party Committee of Yinchuan City, Ningxia Hui Autonomous Region and the Yongning County Civilization Office carried out the red film screening activity on campus with the theme of "Youth Micro Cinema Red Cinema Exhibition", By watching the excellent red classic films, let the majority of teachers and students understand the glorious course of the Party, Remembering the revolutionary martyrs, Inherit and carry forward the glorious tradition, Strive to grow up as a successor to the socialist cause. The picture shows the screening scene of Ningxia New Oriental Technical School. Youth League Yongning County Party Committee for the picture "youth micro cinema red film screening" activity to give full play to the campus cinema line close to the needs of the youth group, youth needs, through carrying out red theme film screening activities in colleges and universities, to guide the majority of young students to love the party, love the country, love socialism emotion. Next, the Youth League Yongning County Party Committee will actively guide the youth league members to accept the influence of red culture, so that the red gene, revolutionary fire passed down from generation to generation.

2021-07-07	Anhui jin district to carry out the history of the party study and celebrate the one hundred theme group Anhui jin district party committee of party to carry out the history learning education and celebrate the 100th anniversary of the theme activities wei correspondent, Du Qinghe check WeiTing) to celebrate the 100th anniversary of the founding of the communist party of China, further solid party history learning education, recently, group of Anhui Lu'an jin district party committee organs all cadres to jinzhai "pursue red footprints celebrate in one hundred" party history education and celebrate the 100th anniversary of the theme activities. In Dawan Village, we learned the special film of General Secretary Xi Jinping's inspection of Jinzhai, visited the photo exhibition of "Dawan Village on the Road to Chasing a Dream", the site of the Soviet Revolution in 14 townships of Sixth District of Lu' an, and the Dabie Mountain Farming Folk Culture Exhibition Hall. In the Dabie Mountain Farming Folk Culture Exhibition Hall, the outstanding Party members in 2020 were commended. Deputy Secretary of the Communist Youth League District Committee, Party branch Secretary (presided over the work) Liu Yi for everyone on a "learn the history of the Party and Ming early mind to know the mission" special party class. City youth speaker, deputy secretary of the Youth League District Committee Du Qinghe to "recall the red history, continue" as the theme to carry out the party history of micro propaganda, around the three sacrifices of Jinzhai, tell the solemn and stirring red history. With simple language and a "down-to-earth" way, the speaker moved the party history class into the old red site, and the scene to confirm the original aspiration, let the red story and the brilliance of the Party deeply rooted in the hearts of the people. Later, all the members went to the Red Army Square in Jinzhai County and the Jinzhai County Revolution Museum to receive red education, remember the martyrs, carry out flower baskets and review the party membership vows.

2021-07-06	Group of Shandong province plain county to carry out the "youth, youth-hundred meters scroll party childlike innocence is the Chinese dream" activities of Shandong plain county to carry out the "youth party-one hundred meters picture scroll party innocence on the Chinese dream" theme activities wei correspondent) wei correspondent Bai Chen, Shandong Dezhou plain county to carry out the "youth is youth party-one hundred meters scroll party childlike innocence on the Chinese dream" theme activities. The picture shows the chorus of young league members and young pioneers. The activity began in the red song chorus "Without the Communist Party, no new China", "singing a folk song to the Party", the young Pioneers representatives wearing red scarves, with full spirit and young voice to express the new era of children's love for the party and the country. The picture shows the representatives of youth league members and young pioneers holding their hands and drawing a 100-meter picture scroll. The representatives of the youth league members and the young Pioneers drew the party history on the 100 meters picture scroll, and drew the 100 meters picture scroll. At the end of the activity, representatives of the youth league members and the young Pioneers took photos with the party flag. Through this activity, let the red seeds take root and sprout in the hearts of the children, and establish the children's feelings of home and country. Let the young people in the activities of strong faith, education, so that the red culture directly to the hearts of the people.

2021-07-06	Guizhou wan mountain area to carry out the "summer heat, safe class" activities in Guizhou wan mountain area sites to carry out the "summer heat, safety class" theme activities wei correspondent xiao-qing wu) to enhance the residents summer heat cooling emergency knowledge, recently, the group of Guizhou Tongren wanshan district party committee organization volunteers in the relocation community to carry out the "summer heat, safe small class" theme activities. The picture shows the scene of the heatstroke safety small class activity. The community community volunteers first explained the main symptoms of heatstroke, common treatment measures and effective prevention methods to the students, and then told the students what measures should be taken for these symptoms, if they still felt uncomfortable to go to the hospital for treatment immediately. Finally, remind the students to often pay attention to the weather forecast to understand the weather changes to do a good job of prevention, but also let the students go home can let their parents cook "coix seed lotus seed soup", "summer mung bean soup" and other soup to relieve the heat. The picture shows the scene of the heatstroke safety small class activity. Correspondent for Tuwang home community volunteers to the children targeted to explain the main symptoms of heatstroke, common treatment measures and effective prevention methods, and reminded them to often pay attention to the temperature change to do a good job of prevention, the most important thing when summer comes is not to swim in the river privately. In addition, it is also recommended to the children in the summer suitable for food, such as banana, hawthorn, semen coicis lotus seed soup, mung bean soup and other food. The picture shows the scene of the heatstroke safety small class activity. Correspondent for the picture is to enhance the community residents' awareness of heat prevention and summer vacation, improve their emergency knowledge of heat prevention and cooling, Chengnan Yi community volunteers carried out household safety education and publicity activities. Heat prevention and cooling is an important task of community safety management. While doing a good job in daily publicity and education, we pay comfort the subsistence allowances and entitled groups, and take multiple measures to carry out care activities to effectively prevent heatstroke and other safety accidents, and ensure the safety of community residents through the hot summer. At the same time, relying on the community propaganda board, radio and other propaganda positions to publicize the knowledge and cooling prevention and slogans, popular science summer hot weather correct summer methods and skills. The picture shows the scene of the heatstroke safety small class activity. The correspondent of the Longsheng community volunteer teachers, combined with the characteristics of the students, explained to the children the symptoms of heatstroke, the basic knowledge of heatstroke prevention and how to deal with the heatstroke situation. Teach students to understand the wind oil essence, Huoxiang Zhengqi water and other commonly used heatstroke medicine. At the same time, the "4:30" class also do a good job of heat prevention and cooling measures, inspection and maintenance of indoor air conditioning, equipped with the corresponding heat prevention medicine. The picture shows the scene of the heatstroke safety small class activity. The volunteers explained to the children the relevant knowledge of heatstroke prevention, heatstroke prevention and first aid. Tell the children to keep enough sleep, which is conducive to maintain physical and mental health and good learning state, but also by supplement vitamins, eat fresh vegetables and fruits to enhance resistance. The picture shows the scene of the heatstroke safety small class activity. Volunteers from Maoping community explained to us the students the main symptoms of heatstroke, common treatment measures and effective prevention methods, and reminded the students to pay attention to the weather forecast and do a good job of prevention. Go out in summer to do a good job of heat prevention work, pay attention to drink more water, can not exercise violently under the sun. Through carrying out this activity, let the residents of the relocated community understand and improve the common sense of heat prevention and cooling, popularize the knowledge of cooling science, guide them to scientific heat prevention and cooling, further enhance the students' awareness of summer heat prevention, improve the life concept of health and safety in summer, so that the residents also have a deeper understanding of heat prevention and cooling.

2021-07-02	Anhui shucheng county to listen to the whole party history story theme activities in Anhui province shucheng county organization to listen to the whole party history story theme activities China youth network Beijing on July 2 (reporter Zhang Jianwei, correspondent, Xiong ShuTing check WeiTing) on the eve of July 1, Anhui province regarding Lu'an shucheng county three "childlike innocence harbor" focus on "red scarf heart to the party, listen to the whole party history story" theme activities. In the process of the activity, the three "childlike innocence harbor" respectively invited five local old party members to the children side, combined with their own personal experience, for the children told a touching story of the party history. The children said that they could listen to the stories face to face and understand the history of the hard struggle led by the Party, which made us understand that the present happy life has not come easily. At the end of the activity, the young pioneers tied a bright red scarf for the old party members, and solemnly saluted to the old party members. It is understood that the activity by the group ShuCheng county and country garden ShuCheng county precision rural revitalization projects joint organization for poverty alleviation, aims to organize the young pioneers around the whole experience about the party history, strengthen the youth learning the history of the party "sense", guide them to a whole example, firmly establish a party history, ode DangEn, follow the party's determination, strive to become a qualified successor to socialism.

2021-07-02	Group xinfeng county various activities to celebrate one hundred xinfeng county various activities to celebrate the one hundred wei correspondent Cheng Xin Pan Yanhui) on June 30, group Shaoguan city xinfeng county in the county style square "show youth style Yang volunteer behavior" activities, activities in flash, rap, collective modelling, industry party members, members, youth volunteers, a total of 180 people to attend. The picture shows a flash dance for the centenary of the founding of the Party. Correspondent for the picture youth League county committee organized "Jane" dance art center 3 dance teachers, in the county sports square to carry out the "I dance for the founding of the party" flash mob activity, with cheerful dance to show the vitality of the new era of young people, with dance jump out of the blessing of the party's birthday wishes and love for the great motherland. In the interview activity of "I want to say my truth to the Party" for the youth league members of various industries, the youth league members of the party, government, police, military and civilians rushed to express their centennial birthday wishes to the Party in front of the interview lens. A medical league member said, "the current vaccination task is still grim. In the centenary of the founding of the Party, I will stick to my post, based on my own duty, and try to celebrate the birthday of the Party with hard work."The picture shows the digital "100" figure presented by the league members and youth volunteers in various industries. Correspondent for figure activities, more than 100 industry members, youth volunteers in the face of the camera, put the digital "100" and heart pattern, each with a flag and a flag, chorus "no communist party, no new China", with song sing the love of the motherland, love of the party, the love of a happy life.

2021-07-01	Shandong sishui county "youth community" project power grassroots social governance work Shandong surishui county to carry out I do the practical work for the youth, "youth community" run out of grassroots social governance "acceleration" activities wei) since this year, Shandong Jining sishui county innovation implementation of "youth community" project construction, adhere to the pilot first, strengthen organizational security, attract condensed youth, focus on action lead, explore youth through the "micro governance", new ways to participate in social governance, run out of the grassroots social governance "acceleration". TuanSishui County Party Committee issued the Implementation Opinions on the Construction of "Youth Community" to Help Grassroots Social Governance, and identified Jinquan Community of Sihe Street and Shengquan Community of Jihe Street as the pilot communities of "youth community" in the county. The launching ceremony of the "Youth Community" project in Sishui County and the symposium on "Gathering youth power to help grassroots social governance" were held in Shengquan Community of Jihe Street. Three actions were released: youth volunteers to serve the community, "Building youth from generation to generation" and youth, "youth helps safe community". Promote the association of Sishui County Youth Volunteers Association to intervene in the community, and promote the youth projects of co-governance related units to settle in the community. The pilot communities formulated work implementation plans and promotion plans, implemented wall chart operations, and made every effort to promote the pilot work. Make the use of the village (community) "two committees" to change, further select and strengthen the urban community youth league organizations, promote the young grid members to enter the community youth league organizations according to the procedures, and guide the youth league members to participate in grass-roots democratic consultation. Relying on the party building and the league building, establish the league organization as soon as possible for the conditions of the league property service enterprises as soon as possible, and strengthen the guidance, training and support for the league work of the property service enterprises. Relying on the community new era civilization practice station, the party and mass service center and other positions, to build the community "youth home", and relying on the position to carry out regular youth service activities. We will carry out volunteer service activities such as learning from Lei Feng and reporting to the community, establish and improve the ledger of college students' volunteers, and guide and mobilize league members to participate in community governance by relying on community youth league organizations, "youth homes" and youth volunteer organizations. The "community micro-governance" youth council hall has been set up to stimulate the innovative vitality of young groups and empower community governance. Excavate and cultivate outstanding youth league backbone in property enterprises, contact and gather a group of outstanding young residents community party secretaries and young reserve cadres in residential areas, and expand the "reservoir" of youth participating in community governance. The Sishui County Youth Volunteers Association was established. Carry out the "Green Bird Plan Zhihui Sishui" college students to the countryside volunteer service activities. We have launched a series of activities to celebrate the 100th anniversary of the founding of the Party, study youth, and share the history of the Party, so as to foster and practice core socialist values. Focusing on the prevention of juvenile delinquency and juvenile self-care education, we have been socialized to mobilize young people to strengthen their sense of social responsibility and dedication, actively participate in the publicity of volunteer services, and help the construction of safe communities. We will carry out a special campaign to select and train "Community Youth Pioneers", actively select young people who have made contributions to community governance, and promote outstanding young talents to help urban social governance at the grassroots level. Next, the county will continue to adhere to the party building, focus on grassroots social governance ability, focus on group building focus, community affairs difficulties, youth concern, residents focus, guide the youth play the role of powerforce and commandos, realize community work sharing, form has the characteristics of siyouth backbone new mode to participate in grassroots social governance.

2021-07-01	Hubei xianfeng first "youth cinema" was officially established the first "youth cinema" officially established figure China youth network Beijing on July 1 (reporter Zhang Jianwei), Hubei enshi tujia and miao autonomous prefecture xianxian county committee established the first "youth cinema", and carry out "learn party history, feeling DangEn, follow the party" red activities, the county more than 70 members of all walks of life youth watched the red film "above the cliff". The picture shows the youth league members watching the movie carefully. Tuan Xianfeng County Party Committee for the picture of the film heroes and the enemy battle of wits and courage, showing the communist party members selfless dedication, brave sacrifice, unyielding revolutionary spirit. After watching the film, the youth members felt a lot, and said that in the future work and life, they will take the revolutionary ancestors as an example, learn from the heroes, remember the heroes, carry forward the revolutionary spirit, draw hard strength, with the struggling youth to pay tribute to the centenary of the Party.

2021-06-30	Group of Anhui du district party committee to carry out the social organization "research" activities of Anhui du district party committee to carry out social organizations "research" activities JianWei correspondent, Jiang Yan check WeiTing) in order to further expand the coverage of work, enhance cohesion and influence, on June 25, Huaibei city of Anhui province, du district party committee joint district women's federation, district WeiJianWei to jurisdiction social organizations field visit research, strive to promote qualified social organizations to establish a league organization. Research group mainly visited the six private hospitals, and talk with the relevant responsible comrade face to face, the basic situation such as detailed baseline, and around the social organization construction, how to guide the youth members play a vanguard exemplary role, improve youth theoretical accomplishment and political quality, etc., to communicate with the relevant person in charge. Youth league members are an important part of social organizations. The establishment and improvement of social organization and youth league organizations is a powerful starting point to steadily promote the work of "Party building leads league building and promotes Party building". The Youth League Duji District Committee will take this investigation as an opportunity to further strengthen the contact with social organizations, play an exemplary and leading role, lead the youth of social organizations to strive for excellence, inject impetus into the development of social organizations, and constantly expand the organizational coverage and work coverage of the Youth League.

2021-06-29	Guangdong Xinfeng county "321" work method to improve volunteer service management Guangdong Xinfeng county "321" work method to promote volunteer service management steps Jianwei correspondent Cheng Xin) group Shaoguan Xinfeng county always to volunteer team service management as a political task in grasp, constantly explore established three strengthening, two promotion, a improve the "321" method of work, further standardize the volunteer team service management, promote the volunteer team high standard, high quality, high efficiency development, also in the volunteer service activities won the recognition and affirmation of the masses. Strengthen training and improve service ability. The Communist Youth League County Party Committee publicized the party's policies and regulations and the latest current politics through the "Xinfeng Communist Youth League" WeChat public account, Guide youth league member volunteers to actively participate in "youth learning", Further enhance the political literacy of the volunteer team, The determination to listen to the Party and follow the Party; Early stage of each volunteer service activity, All organized volunteers to carry out "crash" training, Emphasize the matters needing attention, service content, responsibilities of each post, Ensure that the volunteer service is standardized and colorful; For volunteers who have employment needs, The Communist Youth League County Party Committee gives full play to the advantages of contacting with relevant units, More than 20 training sessions on "Chinese pastry + Western baking" skills promotion and employment promotion, Collect volunteers to repay the society and help the rural revitalization of the love. Strengthen management, enhance the awareness of self-discipline. The volunteer associations shall carry out the general elections in accordance with the regulations, Several presidents and several directors are elected, The president and the director shall, as the volunteer daily service and management personnel, Regular meeting meetings, In view of the problems in the service management of the volunteer team, Study and formulate measures, Form a normal and long-term management mechanism; Before the volunteer service activities, The staff of the Communist Youth League County Committee and a director of the volunteer association are the initiator and person in charge of the volunteer service activity, Full-process tracking service, docking management, To ensure that all volunteer services are foolproof, absolutely safe; Encourage volunteers to establish a mentoring relationship, Strengthen communication during activities and in daily life, "Hand in hand" teaches volunteering experience, Managing possible mispractices of new volunteers, Enhance the self-discipline consciousness of self-management and self-improvement of the volunteers. Strengthen the audit, improve the quality of the team. When recruiting social personnel to participate in volunteer activities and register as volunteers, listen to the political audit opinions of public inspection and law department and community (village committee), resolutely prevent members such as "impure motives" and introduce new members, review the life circle and friends circle, prevent "gang gang" circle; volunteer service activities, the county committee actively assist and inform "who is responsible" and "who is responsible" regulations, jointly maintain the overall image of the volunteer team.

2021-06-29	Group regarding Lu 'an in Anhui province shu county solid social cohesion league in Anhui Lu' an shucheng county solid social cohesion work JianWei correspondent, Xiong ShuTing check WeiTing) to expand the effective coverage of youth league, solve the problem of the school student member "lost" after graduation, league in Anhui province regarding Lu'an shu county more measures simultaneously, solid social cohesion work. Do a good job in the league member files inventory and sorting work. For early, high school graduates of the archives and the material is not complete, deployed within the scope of the county deployment inventory and sorting work, the paper file lost, wrong, official seal or leakage, fuzzy, "wisdom group building" system is not recorded, unified information error to fill and record work, to ensure that the member organization relations before the material integrity. Combing and refining the transfer process of group organization relationship. According to the work requirements of the Anhui provincial party committee, further comb and refine the league organization relationship transfer work process, for junior middle school, high school and university type of league organization relationship transfer steps flow chart, through the county WeChat public platform widely released, facilitate different learning stage members can clear as soon as possible into the organization, understand into method, strengthen the social cohesion work foundation in the county. Further optimize the league organization relationship transfer program. Seriously summarize cohesion work experience in recent years, for member paper files and organization relationship separation, further optimize the transfer program, in the county "members carry files and introduction to the league-into the league review materials-into the league through the" wisdom "system into application-out of the league with introduction receipt information agreed or refused to member out" transfer process, do member himself, paper materials and online league relationship "trinity". Set up a communication platform between the society and the league members. Timely adjust the smart league construction organization tree, establish wechat communication groups in towns and townships annually, and release the group QR code through the public account platform, so that university graduates and league members can join the corresponding township communication group, facilitate the graduate league members to consult and communicate with the league organization transfer, and greatly reduce the transfer error rate. At the same time also enhanced the communication between the township youth League committee and the graduate members, so that the members graduate not "lost contact". Up to now, the county league member file inventory and information registration work has been basically completed. In the next step, the Communist Youth League County Party Committee will take the work of "student and social connection" as an opportunity to continuously strengthen the construction of grass-roots youth league organizations and further consolidate the foundation of the work of the Communist Youth League.

2021-06-26	Gansu Tianshui Qinzhou District Committee to promote the Party history learning educator Ma Fuchun Wang Hao) Since the Party history learning and education of the Party history, the Communist Youth League Gansu Tianshui Qinzhou District Committee, early planning, detailed arrangements, high standards to promote the Party history learning and education work. The League Qinzhou District Committee held a mobilization meeting to refine the division of responsibilities and formulate the implementation plan. Open a reading class with the theme of "one book, one book, one story, learn every day, everyone speaks"; relying on the local red resources General Deng Baoshan Memorial hall, planning and shooting the red micro party class "Adhere to the original ambition perseverance", which was identified as one of the 100 outstanding league classes by the Gansu Provincial Committee. Comprehensively carry out on-the-job party members into the community activities, carry out more than 10 party branch pair construction activities. For different groups of young people, red publicity activities were carried out, and the Qinzhou Group Group Century-old Party History column was opened on the wechat public account of "Qinzhou Youth" to create an education and publicity base for extensive young people to participate in and in-depth learning. Through the theme class team meeting, theme team day, theme publicity and other forms, the Communist Youth League system of Qinzhou District organized with the theme of "learning the history of the Party, strong faith, follow the Party" learning education. Among them, the Youth League Qinzhou District Committee hosted the concentrated league joining ceremony, batch into the team demonstration activities, thousands of youth members singing red songs, "I send the blessing to the Party" handwritten newspaper competition and other activities, has won the attention and praise from all walks of life. We will make solid progress in the "Rural Revitalization and Youth Development" Initiative, and make a list of the "I do something for the youth" initiative to help them solve their urgent and anxious problems. Up to now, contact Shenzhen Jize Water Purification Equipment Co., Ltd. to donate 320 water purifiers for free to Qinzhou villages and communities; carry out visits and report more than 30 teenagers with special difficulties, and conduct a "youth friendship activity in May". To help young people grow up, to carry out "Tuan Tuan help employment", and to provide more than 500 various internship jobs. A series of practical things, such as "summer cool" to help secondary school and college entrance examination, and "golden autumn aid" to donate money for poor college students.

2021-06-25	Group of Shandong plain county measures and to prevent drowning safety education group of Shandong plain county measures and to prevent drowning safety education in Beijing on June 25 (reporter Zhang Jianwei, correspondent Li Yuzhe) with the rising temperature and the holiday, teenagers wading, play with water, swimming, etc, drowning accidents into the multiple period, prone period. Recently, the group of Dezhou in Shandong province plain the county to "I do the practical work for the masses" as the gripper, through "online + offline" all kinds of channels and platforms, measures simultaneously, linkage, through the theme team, set up warning signs, emergency drills, volunteer service and other forms, deepening drowning propaganda and education, enhancing teenagers drowning safety net, gradually improve the safety awareness of teenagers and parents. The picture shows the blue sky rescue team against drowning safety education into the campus. Correspondent for pictures to carry out team education, increase publicity efforts. Launch the county's primary and secondary school league, team organization to actively carry out drowning theme team class, improve the prevention of drowning safety awareness. Strengthen education and training, learn self-help knowledge. Jointly with Pingyuan County Yiqi Public Welfare Development Service Center, Blue Sky Rescue Team and other social organizations to carry out "cherish life to prevent drowning" safety education into the campus, into the community and emergency drills. Set up safety slogans to prevent drowning accidents. Together with the river Chief's Office and the Water Resources Bureau, we set up warning signs in dangerous waters and set up swimming resistance teams to carry out river patrols and lakes. Increase network publicity, enhance the awareness of drowning. Relying on the "youth Plain" WeChat public platform to prevent drowning safety publicity.

2021-06-25	Guangdong Xinfeng County "three grasp three promotion" to promote "youth learning" participation rate group Guangdong Xinfeng County Party Committee to take "three grasp three promotion" to promote "youth learning" participation rate Jianwei correspondent, Liang Zuer Cheng Xin Pan Yanhui) since this year, Combined with the party history learning and education, Xinfeng County Party Committee of Shaoguan City, Guangdong Province innovation "three grasp three promotion" work method, To improve the mechanism, innovation carrier, rich connotation, By exerting the magnet gravity effect of the "youth learning" platform, Constantly lead the county youth league members to take the initiative to learn, thinking of learning, happy to learn "youth learning" online group class, The participation rate has long been the first place in the city, Further enhanced the cohesion and combat effectiveness of the grass-roots youth league organizations. Focus on the "key minority" to promote our work. To grasp the leaders of youth league organizations at all levels, counselors and teachers, youth members with certain influence, play its key minority, extensive publicity, leading model, training guidance, jointly promote the "youth learning" work go deep; establish the county party secretary as the group leader, deputy secretary as deputy leader, members of the organization of "youth learning" leading group, clear responsibilities and tasks, form the industry, regional league organization "youth learning" contact mechanism, to leading group this "youth learning" to form a boom. Focus on the "concentrated attack", promote the promotion of learning. We should focus on the key league organizations with many league members' outflow and weak league construction work, and focus on the league organizations in schools, enterprises and public institutions with relatively concentrated league members, and carry out targeted and centralized efforts. For the scattered grass-roots league organizations, we encourage the establishment of 122 WeChat contact groups, urging group administrators, group administrators with "one to one" and "one to many" package tracking group members to complete the task of "youth learning", forcing them to improve the participation rate of "youth learning". For members of authority, school league organization, every Monday, Friday afternoon and weekend regular notification, timely remind boarding students with good rest days "debris" time to listen to watch "youth learning" online class, has issued "youth learning" task 14 period, school members "youth learning" reference rate rose 6% from the same period last month. Grasp "than learn to catch up", promote habit formation. We should create an atmosphere of mutual learning and catch-up between league organizations, and grasp the habit of teenagers' active learning and love of learning. Establish and improve the "youth great study" assessment, reward and punishment system, According to the affiliation relationship of the industry, system and regional youth league organizations, Establish a weekly evaluation and notification mechanism, Linked to the evaluation of the first evaluation, Including it in the individual annual work assessment; The Youth League County Party Committee commented on the participation rate and progress rate of each league organization weekly, To form a competitive atmosphere of comparing, learning and catching up among the youth league organizations; Using the weekly class meeting and evening self-study time for school students, Increase the "youth big study" exchange and discussion topics, Let the participating students have something to say, Let the students who did not participate in learning consciously want to "make up for the lessons"; Mobilize the majority of students' parents to participate in the "youth learning" supervision and supervision, Form a learning situation of joint management inside and outside the class, Promote the league members to develop the habit of consciously learning "youth learning". Xinfeng County Party Committee will continue to improve the effectiveness of the learning system of "guide learning, teaching, learning, and the online learning of" comparing learning, practice learning and supervision ".

2021-06-24	Anhui huai ning: I do something for the youth, skills training into the heart of Anhui huai ning: I do something for the youth, skills training into the heart built wei correspondent, Cheng Minqin check WeiTing) recently, the Anqing city in Anhui province huai ning county joint blueberry center in solo show blueberry heights planting base held the blueberry summer cultivation management technology training site, yellow pier town 40 blueberry planting base technology responsible for comrades to attend the attendant. The on-site activity guided and trained the pruning and management of young blueberry gardens, the pruning and fertilization of early-maturing varieties, and the key technical points of the prevention and control of diseases and insect pests in summer. Zheng Wei, a young entrepreneur, said, " Through this activity, I have a more comprehensive understanding of the green prevention and control of blueberry diseases, insect pests and diseases. When I went back, I immediately conducted a health examination of the planted blueberry seedlings."It is understood that the group huai ning county visited research entrepreneurial youth, after understanding the entrepreneurial youth demand skills training, immediately play organizational advantage, to develop" the party history, strong faith, follow the party "learning education activities as an opportunity to carry out" I do something for youth " theme practice, really help youth solve practical difficulties, solve the problem of anxiety, enhance its cohesion and combat effectiveness.

2021-06-24	Shandong Linyi county "youth, youth party" speech contest successfully held Shandong Linyi "youth party" keynote speech contest was successfully held jianwei correspondent Sun YuNa) on June 21 in the afternoon, the history of the party, in one hundred, heart to the party- "youth party" keynote speech contest final in Shandong Dezhou Linyi city lecture hall. Linyi County Party Committee, Shandong Xinhua Bookstore Linyi branch and the Youth League County Party Committee related departments responsible for comrades to participate in the activity. The picture shows the final scene of the speech contest. To warmly celebrate the 100th anniversary of the founding of the Communist Party of China, Looking back on the magnificent centenary journey of the Communist Party of China, To witness and record the vicissitudes and changes of the Chinese nation together, Enhance the sense of identity and mission of party members, cadres and outstanding youth, To encourage and guide party members and cadres to seek their original aspiration and fulfill their mission from the extraordinary history of the Party, In late May, Under the careful guidance of the Linyi County Commission for Discipline Inspection, the Organization Department of the County Party Committee, the Publicity Department of the County Party Committee, and the United Front Work Department of the County Party Committee, The Communist Youth League Linyi County Party Committee united with the county Federation of Trade Unions, the county party committee and the county direct organs working committee, the county women's Federation to study the history of the Party, celebrate a hundred years, the heart to the party- - "in the prime of youth ode to the Party" theme speech contest. The picture shows the final scene of the speech contest. Correspondent for figure activities by the county administrative examination and approval service, xinhua bookstore Linyi branch, county media center, county retired veteran cadres bureau support, extensive attention of various departments at all levels, from various units, township (street), health care, education, judicial and other industries in various fields of more than 40 players to sign up for the game, after intense preliminary, final, finally decided the first prize 2, the second prize 4, the third prize 6. In the final scene, the contestants either review the history of the Communist Party of China, review the glorious tradition of revolutionary ancestors, or tell the advanced deeds of the excellent Communists around them, advocate the majority of party members to be the vanguard model; or combined with their own work, express the party, the people, the motherland... with the speech reviewed the glorious history of the Party, determined the Communists to seek happiness for the Chinese people, the rejuvenation of the Chinese nation's original aspiration and mission.

2021-06-23	Anhui Province Langxi County Party Committee "three attention" to carry out drowning knowledge publicity group Anhui Province Langxi County Party Committee "three attention" to carry out drowning knowledge publicity Jianwei correspondent, Ma Shuhui check Wei Ting) every June to August is the high incidence of drowning accidents, strengthen the safety education and management of children is particularly important. In order to effectively prevent students from drowning accidents and effectively protect the safety of students' lives, the Langxi County Party Committee of the Youth League, Xuancheng City, Anhui Province, integrate all forces, highlight the "three attention", and effectively pay attention to the prevention of teenagers and children from drowning prevention work. Pay attention to strengthening the construction of campus positions. Organize the primary and secondary school young working committee to carry out the anti-drowning theme team meeting, a speech under the national flag, a letter to parents, watch the warning education propaganda video, anti-drowning handwritten newspaper competition, anti-drowning knowledge competition and other activities, to further enhance the safety awareness of teenagers, guide the young pioneers firmly establish anti-drowning awareness. We will give full play to the role of community-level organizations. The grass-roots league organizations of each towns extensively mobilized youth league members and volunteers to go into villages (communities) to carry out volunteer service activities such as warning board maintenance, safety investigation, banner hidden dangers, publicity and education on campus in each waters. Give full play to the role of grassroots "public welfare small classroom children service station" and "youth home", while doing a good job of youth schoolwork services, interest training, do a good job of drowning prevention knowledge popularization. Focus on improving students' self-help ability. Since late May, TuanLangXi county party committee joint education bureau, organize blue sky rescue team in the county primary and secondary schools, kindergartens to carry out "cherish life prevent drowning" theme education publicity and education, from the professor wear life jacket, cardiopulmonary resuscitation operation steps, heim, first aid method, further improve the students' self-protection ability and safety awareness. Up to now, 36 activities have been held in 25 schools, benefiting 7,053 people.

2021-06-22	Feidong county, Hefei, Anhui province "communist youth league cinema" officially listed "Anhui feidong county" communist youth league cinema "awarding ceremony and red education viewing activities jianwei correspondent wang check wei ting) recently, Hefei, Anhui province feidong county" communist youth league cinema " awarding ceremony and red education viewing activities held in our yue square star anecdote studios. City, county related department staff, the county middle school youth league organization leader and this year's college entrance examination of more than 100 graduates participated in this activity. Zhang Leiyong, deputy secretary of the Hefei Municipal Committee, first delegation the Hefei Municipal Committee to express warm congratulations on the official opening of the "Communist Youth League Cinema". He called on the youth league organizations at all levels to continue to focus on the requirements of "condensing the youth, serving the overall situation, being a good bridge, and strictly governing the youth league", expand the organizational position, extend the work tentacles, and further condense the youth. Centering on the central task of the reform and innovation of the Communist Youth League, we should take the initiative and constantly innovate our working methods. It is hoped that the "Communist Youth League Cinema" can play a good role and become a comprehensive service platform integrating the functions of "ideological education position for teenagers", "patriotism education base for teenagers", "volunteer service position for public welfare activities" and other functions. And encourage the majority of youth league members to firm ideals and beliefs, determined to become talented, with excellent results and outstanding performance in the future, to serve the motherland, return to the society, the construction of hometown. In the activity, we watched the "youth, Hello" legal education propaganda video, and then watched the "Red Sea action", to the students present to enter the university campus for a legal education and patriotism education baptism. In the future, the "Communist Youth League Cinema" in Feidong County will continue to guide the majority of young people to learn history, history history, history and history through excellent red films. After the activity, Zhang Leiyong and his delegation came to the revitalization community of izhen Town, Feidong County to investigate the creation of "Double Zero Community".

2021-06-22	Guizhou river tujia autonomous county "red scarf XuanJiangTuan" began one hundred I tell Guizhou river tujia autonomous county "red scarf XuanJiangTuan" began to China youth network Beijing on June 22 (xinhua Zhang Jianwei) to celebrate the 100th anniversary of the founding of the communist party of China, to further strengthen the youth patriotism education, ideal faith education and revolutionary tradition education, inspire the enthusiasm of children patriotic, love home, cultivate ideas, faith, sentiment of socialist successors. Recently, the "Red Scarf Publicity Group" in Yanhe Tujia Autonomous County, Tongren City, Guizhou Province, walked into Ganxi Town Central Complete Primary School, Qitan Town Central Complete Primary School and Yanhe Experimental Primary School respectively to carry out party history learning and education publicity activities. On the three school grounds, Members of the red scarf publicity group took turns to preach, Yang Yitong took "Learn History and Practice red ts to the Party" as the theme, It tells the story of China from the birth of the Communist Party of China to a hundred years of journey, A century-old party history, from revolutionary leaders to heroes, from the Red Boat spirit to the spirit of fighting the epidemic; CAI Chang, to find the footprints of our ancestors, Inheriting the Red Culture, He Long and Guan Xiangying led the red army advancing along the river, The story of the famous "Qi Tan battle" that happened on the red hot land of Qitan town; Song Shengrui preached the "little hero Li Aimin" anti-Japanese story; Zhang Yifan preached "Forever 9 years old", the head of the red scarf publicity group Zhang Zixin preached "the motherland will not forget". Several red scarf XuanJiangYuan with red historical figures and economic and social development of revolutionary martyrs, heroes, advanced models, in simple language to express with certainty, to return the motherland determination, and from the primary school pioneer, grow up pioneer, unswervingly listen to the party, follow the party, to be a new era of good players talk. It also makes all the teachers and students of all schools have a more profound understanding of the revolutionary spirit of the Communists' brave difficulties and firm faith in the difficult years, and deepens the ideal and faith of knowing the history, loving the Party, knowing the history and loving the country, firmly learning the history of the Party, strengthening the faith, and following the Party.it is reported, Yanhe Tujia Autonomous County "Red Scarf Publicity Group" was formed in positive response to the requirements of the county party committee and government on promoting the requirements of party history learning, education and publicity, This group of red scarf publicity group by Yanhe County experimental primary school vice principal Du Gaofei, Shaoxian brigade brigade counselor Yang Xueqin, experimental primary school teacher Zhang Yanli, Shaoxian brigade team leader Zhang Zixin, young Pioneers Zhang Yifan, Song Shengrui; Qi Tan center, deputy secretary of the Party branch Cai Ying, less first brigade brigade counselor Zhang Hai, Qi Tan finished primary school teacher Luo Jiao, young Pioneers Cai Chang; Deputy secretary of the Party branch Yang Shengting, Shaoxian brigade brigade counselor Yang Xiuchong, Ganxi finished primary school teacher Tian Xiaoli, young pioneers Yang Yitong 14 people composed of, This activity covers more than 4,800 school teachers and students. Next, autonomous county 14 red scarf XuanJiangTuan members will walk into the county of the rest of the township school to carry out the "flag leads my growth" series of activities theme tour, focus on tell the story of the party, the revolutionary story, the story of the hero, leading the masses of the county children understand the party's glorious history, feeling the party's original mission, strengthen the love party, patriotism, love socialism emotion, let red gene, revolutionary generations.

2021-06-23	Henan fu ditch county to carry out drowning prevention into the campus preach live Henan fu ditch county to carry out the "cherish life, prevent drowning" into the campus preach wei) recently, the communist youth league city of Henan Zhoukou fu ditch county joint county blue sky rescue team into the town of junior middle school to carry out the "cherish life, prevent drowning" preach education. At the event site, the Communist Youth League County Party Committee and the Blue Sky Rescue Team combined with previous cases to teach the teachers and students the knowledge of drowning prevention, outdoor swimming precautions, the use of rescue tools, cardiopulmonary resuscitation and other knowledge, and demonstrated the correct steps and methods of drowning rescue. And requires students to do "six forbidden": 1, no private swimming; 2, no unauthorized swimming with others; 3, no swimming without parents or teachers; 4, not swimming to unfamiliar waters; 5, not to swim without safety facilities, no rescue personnel; 6, do not know water students unauthorized rescue. At the end of the activity, the young volunteers of the Youth League County Committee issued the proposal of "Cherish life, prevent drowning" to the students.

2021-06-22	Shandong district "immersive" learn party history activation carrier open new "immersive" party history activation carrier open new bureau in Shandong district party history learning education both "new" and "heart" correspondent Ma Junting) as the birthday of the communist party of China, party history learning education is in full swing, the communist youth league of Shandong Zaozhuang district committee actively innovate party history learning methods, explore immersive, interactive learning education mode, promote the party history learning education results into the brain into the heart to see actual effect. Sing the party history study education propaganda "chorus", deeply study the party history, learn, think and practice. The Communist Youth League District Committee has earnestly implemented the relevant requirements of the provincial, municipal and district committee on Party history learning and education, and promoted the Party history learning and education with high standards and high quality through the "five studies" mechanism. The main responsible comrades take the lead in learning: formulate the implementation plan of the Communist Youth League District Committee on the Study and Education of Party History, clarify the learning content and work arrangements, and use the "three meetings and one lesson" and the theme Party day activities to promote the study of Party history. Systeatics of theory Central Group: After the mobilization meeting on April 8th, I actively participated in the special study class of the district theory center group, took the century-old history of the Communist Party of China as the basic time clue, and promoted the study in a comprehensive and systematic way."Classroom in the Air" tutoring: We set up a column of "Party History Learning" on the WeChat official account of the Municipal Communist Youth League, and forwarded a series of columns of "One Hundred Years of Original Heart- -Today in the Party History" every day. Effectively combined with the platform of "Youth Learning University", the online teaching of classroom in the air has achieved remarkable results."Smart League building" follow-up learning: Relying on the "smart League building" system, the league organizations at all levels in the district are organized to carry out special study of party history education, timely update the learning data of the league organization in the district, and regularly supervise the learning situation of the league branch. 293 league branches and more than 10,000 league members have participated in the study. Feeling experience exchange: invite the party school teacher to the district cadres for "adhere to the overall leadership of the party, strive to realize socialist modernization-to celebrate the 100th anniversary of the seminar, and the grassroots visit research, understand young needed, hope, to help young people solve problems of work and life, continue to enhance about appeal and appeal, singing party history to preach" chorus ". To build an immersive "realistic school" of party history learning and education, to learn the history of the Party and enter the mind and heart."Reality a": during the qingming festival, with the help of surrounding red resources, through the "qingming festival heroes", the listening to the old hero, remembering the revolutionary martyrs, indoctrination education for interactive experience, through listening, discussion, understanding, and other forms, let red knowledge silently into the classroom, implanted student heart."Reality 2": the primary school with "red scarf heart to the party" as the theme organization perform all kinds of red melodrama more than 60, a touching party history story on the stage, with "to speak, to play as the soul", the way of "party history content and stage" clever fusion, teacher teaching middle school, students in enlightenment, in the audio and visual feast, deep enlightenment red classic, absorb red nutrition and strength, has benefited more than 70000 young pioneers."Solid three": joint area youth volunteer association to carry out "the history of the party, does the practical work, green volunteer, into the community" theme activities, based on volunteer service, among the masses to open the walking "party class", through guessing song, sing red songs and "party history" people popular form, in the red song "listen to the party", in the prize contest "review", the knowledge, interesting, to sing to promote learning, to use. At present, it has gone into 6 communities, serving more than 5,000 people. Dedicated to the study of party history education public welfare for the people "great practice", study the history of the Party, to promote action. As the history of the party learning education gradually, to improve the effectiveness of the party history learning education, TuanOuWei in the party history education "learning" at the same time, seriously pays special attention to the "do" article, focusing on the party and government needs and the youth hope, formulate the city communist youth league "I work for the youth" list, will implement party history learning education."Hope hut" ignites hope: to create a "hope hut" for children aged 8-14 without independent living and learning environment, equipped with necessary furniture and learning and daily necessities, and recruit love pair volunteers to provide companionship services. So far, 45 "hope cabins" have been built."Green school" power growth: through socialization, project to raise funds, joint Zaozhuang college faculty resources, in rural schools to build exclusive music classroom, equipped with music equipment, free music courses, has pilot tax guo town central primary school "green music school", weekly organization students volunteers for seven students in music classes, so far teaching 42, benefit more than 400 students, plan four new "green school"."Youth post" dream weavers: in order to further optimize the talent environment, attract outstanding youth to city employment entrepreneurship, through contact coordination, has built the first lu family hotel and Yiyuan villa 2 youth post, for outstanding youth provide free accommodation, policy interpretation, skills training, job promotion, youth fellowship function in one of the "one-stop" service, for job students to build to the city job employment "warm heart home". The next step of the youth League district committee will continue to deepen the party history learning education, in the majority of youth league members set up the party history learning education new classroom, set off a new upsurge of learning the history of the party, everywhere to tell the history of the party, the learning effect into practical action, with "embroidery kung fu" to do a good job of service youth big article.

2021-06-21	Group in Anhui province county county organization compulsory haircut volunteer service activities in Anhui province county county organization compulsory haircut volunteer service activities China youth network Beijing on June 21 (reporter Zhang Jianwei, correspondent Zhang Ji check WeiTing) recently, group ma on shan in Anhui province and county county organization mechanical and electrical engineering school class 27 volunteers into the taiping le ling nursing homes volunteer haircut voluntary service activities, take concrete action to send the old man to warm heart service. In the nursing home, volunteers for more than 20 elderly people for voluntary haircuts, for the elderly "spirit", each old man's mental outlook took on a new look, meticulous service let the elderly faces are filled with a happy smile and praised their skills and spiritual beauty. In the next step, the Communist Youth League County Party Committee will continue to carry out volunteer service activities such as caring for the elderly, constantly improve the happiness index of the elderly life, and effectively take the practical activities as an important content of the party history learning education, consciously do practical things for the people as the eternal theme.

2021-06-20	One hundred volunteers of the Shucheng County Party Committee continued to carry out volunteer service activities to help the examination. In the early stage, Tuan Shucheng County Party Committee publicly raised materials and recruited volunteers through WeChat public account, and nearly 100 young volunteers signed up to participate. On the morning of June 14, volunteers set up volunteer service points outside the five test centers in the county under the high temperature, providing mineral water, masks, erasers, 2B pencils, test pads and other materials, to create a good waiting environment for candidates and their parents. The volunteer service lasts until the end of the senior high school entrance examination on The 16th. In addition, during the college entrance examination on June 7 and 8 this year, Tuanshu City County Party Committee set up love entrance examination volunteer service points and organized volunteer services. More than 9,600 bottles of mineral water, 1,000 disposable medical masks, nearly 1,000 sets of test tools, and several other heat prevention and cooling materials were distributed to examinees and their parents during the activity. More than 90 volunteers participated in the volunteer service.

2021-06-18	The Xinfeng County of Guangdong Province organized the "red vest" to participate in the epidemic prevention and control volunteer service campaign. Correspondent for figure China youth network Beijing on June 18 (xinhua Zhang Jianwei, correspondent Cheng Xin) recently, Guangdong COVID-19 outbreak affects the hearts of the whole people, xinfeng county actively organize youth volunteer service, active in epidemic prevention and control work, in the flow screen line, in the epidemic prevention and control knowledge propaganda line, in the new crown vaccination line, test organizations, youth volunteer service party history learning education results. In combination with the actual vaccination work, Xinfeng County Party Committee strictly set volunteer recruitment conditions, and thanked and declined the young volunteers who returned to high-risk areas, had not been vaccinated, and were unwell recently. The Red Vest volunteers use zongzi to celebrate a birthday on the Dragon Boat Festival. Correspondent for figure new venture county youth volunteers take the initiative to bear, to "stand out, rushed, withstand, play win" hardship, willing to bear spirit, into the hometown new epidemic prevention and control work, with practical action service hometown folks vaccination, with sweat and hard work to prove the new era of young people's social responsibility. The picture shows Pan � x, a volunteer of "Red Vest", assisting the vaccinated people to collect face information. On the second day after the college entrance examination, Pan � x, a young girl who had just graduated from high school, but an old volunteer, put on a "red vest" to participate in the vaccination volunteer service team. She showed everyone that "young people have responsibility and the country has strength"."Although I am young, I can afford the task assigned by the youth league organization," she said. "Although I am still a student, I am obliged to contribute to the prevention and control of the epidemic in my hometown and to the prosperity of the country."The picture shows the volunteer service team of Xinfeng County veterans reporting to the COVID-19 vaccination site. The volunteer service team of veterans in Xinfeng County took the initiative to connect with the Youth League County Committee. In the epidemic prevention and control, the military continued to carry forward the style of "retired not fade, retired not quit, leaving the army without the Party", and practiced the original mission with practical actions.

2021-06-20	Yan Liang group organization to help farmers sell melon 5 million jin Yan Liang group organization to help farmers sell melon 5 million jin of Huang Bo) " Yan Liang Communist Youth League, Yan Liang Qinglian can help me a lot this time, since signed the underwriting agreement with Shaanxi Fruit Trade Group, our melon sales increased several times this year."Zhang Xing, general manager of Yanliangke Nongmelon and Vegetable Professional Cooperative in Yanliang District, Xi'an city, Shaanxi Province, said happily, while picking crisp and sweet melons. At the Yanliang Melon Industry Culture Festival at the end of April this year, Shaanxi Fruit Industry Trade Group Supply Chain Management Co., Ltd. signed a melon underwriting agreement with Yanliang Agricultural Professional Cooperative, underselling the melon at the purchase price always higher than the market price of 5 cents / jin. The signing of the agreement was completed by the Yanliang Communist Youth League with the support of the United Front Work Department of the Communist Youth League. Yan Liang is a famous hometown of melons and fruits, famous for producing melons in Shaanxi. In recent years, the Yanliang District Committee of the Communist Youth League and the Yan Liang Youth League have actively participated in the local leading industries, steadily carried out the practice activities of "I do practical things for the youth", and tried to expand the sales channels of Yanliang melon. As early as in March of this year, the communist youth league YanLiang district party committee secretary zhang repeatedly contact shaanxi provincial party committee, shaanxi province green league, with the support of shaanxi provincial party committee united front minister Li Zong, successful contact shaanxi youth electricity federation Chen Bingxian, shaanxi fruit industry trade group supply chain management co., LTD., manager jia, deputy manager wang Ji'an, etc., and invited them to yan. After many times of matchmaking, contact, investigation and negotiation, Shaanxi Fruit Trade Group Supply Chain Management Co., Ltd. and Yanliang Agricultural professional cooperative successfully signed a melon underwriting agreement. Yan the organization, the company also reached a sales agreement with 300 retail farmers, estimated that only one can help retail households stable income of more than 20,000 yuan.

2021-06-18	Shandong ling city: build hope, love unit interpreta dream "wish" in Shandong ling city: with build hope, love unit interpreta dream "wish" for China youth network Beijing on June 18 (reporter Zhang Jianwei, reporter gao lili), the communist youth league in Shandong Dezhou city lingcheng district party committee, its Shandong electric power company Dezhou city power supply company a line, visit Dezhou ling city town village "hut" children, for them to the enterprise donation "micro wish" gift, let them feel the holiday blessing and warmth. After the launch of the "Hope Lodge" construction project was launched, the State Grid Shandong Electric Power Company Dezhou Lingcheng District Power Supply Company actively contacted and pledged two hope lodges, thus realizing the hut with a new look. The picture shows the representatives of caring enterprises giving their new clothes and stationery to their children. Correspondent for figure every to a child home, volunteers and children and their guardian cordial conversation, thorough understanding of the children's learning life, and the "wish" love gifts, stationery to children, encourage children to filial piety, study hard, be a good comprehensive development of students, as soon as possible, the construction of hometown, give back to society. At the same time, the love enterprise volunteers to the hope cabin circuit maintenance. From the power line to the power equipment, switches, are careful and detailed inspection, to ensure the safety of the hope cabin. The picture shows the volunteers of the circuit maintenance of the hope cabin. In the next step, the Communist Youth League Lingcheng District Committee will continue to pay attention to the growth of "Hope House" children, improve the service level of caring for children, actively select volunteers to form a pair with children, regularly carry out love help activities, and strive to do a good job in the "second half" of hope cabin.

2021-06-17	Ningxia jingyuan county singing youth party history learning education "quintet" Ningxia jingyuan "youth heart to party long march road" party history learning education and theme TuanRi network Beijing on June 17 (reporter Zhang Jianwei, correspondent Wu Lei), Ningxia hui autonomous region, the communist youth league jingyuan county joint education sports bureau, organize some cadres, young teachers, young volunteers and outstanding communist youth league and young pioneers on behalf of more than 100 people, to Liupanshan "youth heart to party to the long march" party history learning education and theme TuanRi activities, fully singing youth party history learning education "quartet". Retrace the footprints of the Long March, recall the history to know the party. All the staff along the "red army path" walk 2.5 kilometers to Liupan mountain red army long march memorial hall, by retrace the long march road experience of party history of education, guide the youth learning the glorious history, feel the party's struggle, cultivate youth historical responsibility, the sense of mission and sense of honor, in their hearts to sow a "party, respect the party, love party" seeds, motivate the county youth inheritance red genes, continuously red power, let the revolution. Remember the story of the Long March, inherit the history of the party genetics. Cadres, volunteers, the communist youth league, the young pioneers tells the story in the red army trail the red army long march in the river, bloody xiangjiang river, wujiang river fifteen historical stories and heroic deeds, make everyone experience revolutionary predecessors firm political belief, remember the revolutionary feats, carry forward the hard struggle, courage, unity and cooperation of long march spirit, further firm faith in socialism with Chinese characteristics, strengthening the consciousness of "four", "four confidence", "two maintenance". The picture shows the event site. Tuan Jingyuan county Party Committee for resinging red songs, firm faith sense of party grace. A red song is a period of history, everyone waving the red flag and flag singing "no communist party there would be no new China" "glorious, the Chinese communist youth league" childlike innocence to the party " and other red songs, with beautiful passionate melody, affectionate moving voice, emotional performance expressed the deep memory of revolutionary martyrs, shows the new era of youth uplifting spirit. Review the oath of joining the Party, stick to the original aspiration and listen to the Party. In front of the Liupanshan Red Army Long March Memorial Hall, all the staff reviewed the oath of joining the Party, joining the League and joining the team. The solemn oath expresses our determination to carry forward the revolutionary tradition, firm our ideals and beliefs, and our confidence to take the Long March in the new era, and always reminds ourselves of their sacred mission and historical responsibilities. Study the knowledge of the party history again, determined to struggle to follow the Party. All the staff presented flower baskets for the revolutionary martyrs, visited the Liupanshan Red Army Long March Memorial Hall, and recalled the magnificent revolutionary course together, deeply remembered the great achievements of the revolutionary martyrs, and felt the connotation of The Times of the red culture. Through listening to the characteristic party class, let everyone understand the history, temper the spirit, enhance the consciousness, brave to do walk in the forefront of The Times of progress, pioneers, devotees.

2021-06-16	Anhui Qingyang county "red textbook play" let party history learning education thorough childlike innocence Anhui Qingyang county "red textbook play" let party history learning education thorough childlike innocence jianwei correspondent, Qiu Zhefeng check WeiTing) in order to further promote the party history learning education thorough childlike innocence, group Chizhou, Anhui province Qingyang county innovation form, in Qingyang county fourth primary school pilot held "childlike innocence to the party happy growth" red textbook play. From the school nine classes of young pioneers with the most passionate patriotism, the most pure feelings, with young TongYin child performance one by one about young zhou enlai "reading for the rise of China", Zhu De pole, golden hook, batman zhang ga, little hero rain, draft do not forget to dig well, monument, red story on the textbook. The young actors on the stage are full of deep feeling, freely, fully expressed their love for the party and the motherland, thinking about history and the pursuit of ideals. The red textbook play, combining educational orientation and innovation form, let the students feel and experience in the activity in a revolutionary story, a red classic, not only to the children an unforgettable party history lesson, ideological class, more let the children at the beginning of the red culture of thought and emotion, help students set up the correct world outlook, the outlook on life, values, be "don't forget the beginner's mind, keep in mind that the mission" inheritance and love party patriotic successors.

2021-06-21	Group of Anhui jin district party committee joint tax bureau "tax, praise one hundred" sympathy group in Anhui jin an district party committee joint tax bureau "tax innocence, praise one hundred" sympathy activities jianwei correspondent Liu Yi check WeiTing) recently, the league of Anhui Lu'an jin an district party committee joint jin district tax bureau in jin an wood town hope primary school joint "tax innocence, praise one hundred" sympathy activities, district, jin district party committee, wood factory town party committee related responsible for comrades and other activities. Activities, the young pioneers to the leaders of the red scarf, deputy director of the district tax bureau office, youth tax school students Wang Qian to the young pioneers emotional about the red tax story in the western Anhui revolutionary base area, encourage students to continue along the footsteps of the revolutionary predecessors, strengthen the "listen to the party with the party" red feelings, the red jiangshan passed down from generation to generation. At the event site, the district tax bureau and the Communist Youth League District Committee donated school supplies to the representatives of the poor students, and delivered the love funds spontaneously donated by the youth league members of the youth tax school to Muchang Hope Primary School. Zhang Cheng, the representative of the Young Pioneers, solemnly promised to study scientific and cultural knowledge hard, listen to the Party and follow the Party, repay the Party and the motherland with excellent results, and never live up to the care and expectations of all sectors of society.

2021-06-09	Sichuan Chaotian District to carry out the party history study one hundred propaganda into the campus activities to learn a hundred years of party history to do the era of new people Sichuan Chaotian District to carry out the party history study one hundred propaganda into the campus activities map China youth network Beijing, June 9 (reporter Zhang Jianwei, correspondent Xiong Fei Qiaolan) recently, Sichuan Province, a member of the Youth Publicity Group- -Qiao LAN, secretary of the Communist Youth League District Committee, and Xiong Fei, deputy secretary of the Communist Youth League District Committee, respectively visited the Neutron Experimental School and Lianghekou Town Primary School to carry out the "Party History Learning Education- -100 lectures into the campus activities", They made the theme of "Learning the History of the Party to be a New Person of The Times" and "Learning the Red Gene of the Hundred Years of Party History". The party history publicity activities mainly from the youth why to learn the history of the Party, how to learn the history of the Party and how to do after learning the party history and other three aspects, deeply elaborated the great significance of carrying out the party history learning and education activities. In teenagers should how to learn the party history, XuanJiangTuan teacher focuses on the Chinese young vanguard history, the Chinese communist youth league history, and through learning the party constitution, the regiment chapter chapters, make the teenagers further deeply understand the party, the tertiary, team, in a unique way lit the patriotic enthusiasm of the students, guide the teachers and students from learning faith and strength, do "history, history, chong DE, history practice" era pioneer, yong do have ideal, ability, bear new era. The picture shows the party history study one hundred propaganda into the campus activities. In the Neutron Experimental School, Qiao Lan, secretary of the Communist Youth League District Committee, explained the glorious history of the Communist Party of China with detailed historical materials by playing videos, showing pictures and telling red stories. Principal Wang Guoyong made a concluding speech, he asked young students to seriously study the history of the Party, read the original work, the theory with practice, to seriously listen to the Party, follow the party, study hard, love the party and the country. In Lianghekou Primary School, Xiong Fei, deputy secretary of the Communist Youth League District Committee, combined the party history learning education with the students' learning and life, learned the past and learned, cleverly explained the importance of youth learning the party history, told a series of real events in the revolutionary years, and inspirational stories in real life. More than 200 people from the District Education and Science Bureau, the whole cadres of the Communist Youth League District Committee, and the teachers and students of the two schools participated in the activity.

2021-06-08	Promote the history of the party learning education into the heart into the history of the party learning education into the masses heart Wuzhong District Mudu town to celebrate the 100th anniversary of the founding as the main line, through the classic reading, red singing, story, painting and calligraphy hanging, exhibition, in the form of popular for the history of the party learning education into the masses heart. According to the introduction, Mudu town through holding to sing red songs as the theme of the competition, review the red classics, guide the masses of party members to sing the theme of The Times, gather the positive energy of The Times, to create a "always follow the party, faith from generation to generation" good atmosphere, so that the party history learning education into the eye into the ear more into the heart. At the same time, the town also widely collect reflect the town development of new journey, social new fashion, the new style, hometown new change excellent original literary works, covering literature, painting and calligraphy, photography, folk art, quyi, etc., break the past party history learning stereotypes, through all kinds of exhibition activities to further show the town struggle and outstanding achievements.

2021-06-04	Ding'an County, Hainan Province, customized Party history learning education "special meal" Wei correspondent Zhou Yutong) Since the development of Party history learning education, Hainan Dingan County Party Committee, United County Education Bureau and other departments customized party history learning education "special meals" for teenagers, Using the green language language, Open up the online and offline integration and communication path of teenagers to learn party history, By organizing a series of "Eight-One" theme activities, such as "looking, reading, speaking, painting, talking, sounding, writing, and moving", With the "young way" ignited the enthusiasm of young people to learn the party history, Leading young people to inherit the red gene from the party's century-old struggle history, Button up the first button of your life. It seems that- -around the theme of "Remembering revolutionary martyrs, searching for red landmarks", more than 12,000 teachers and students from 18 primary and secondary schools in the county were organized to visit revolutionary venues such as Huangzhu Martyrs Cemetery and Mruishan Revolutionary Education Base to take the red route to remember the revolutionary martyrs. Organized more than 10,000 young people to watch "I and my motherland", "I and my hometown", "the founding of the Party" and other red films, education and guide the county youth to carry forward the red tradition, inherit the red gene, continuously continue the communist spirit blood. Read-around the "read red classics, praise in one hundred chapter" this theme, combined with "reading childhood" party history red story, "praise party history" scholarly parent-child reading activities, primary and secondary school theme reading contest activities, carry out more than 30 red classic publications and red classic, poetry reading activities, about 6000 teenagers, education guide the county teenagers deeply understand why "can", "marxism why" line ", why" good ", unswervingly listen to the party, follow the party. To tell it- -around the theme of "learning the history of the Party and cultivating patriotism and love for the Party", the first province's "red scarf" volunteer service team was set up in Mother Mountain. On weekends, they took turns during winter and summer vacations to explain the red story of Mother Mountain to the tourists and research teams in the revolutionary base of Mother Mountain. Through the role model of the "Red Scarf" commentator volunteer service team, we will continue to spread the red voice more actively, and cultivate the feelings of love for the Party, patriotism and socialism, so that the red gene and revolution can be passed on from generation to generation. Draw up-around the "the history of the party I know" this theme, the organization of the county primary and middle school students to participate in the hand copy newspaper activities, received a total of 119 hand copy newspaper works, more than 19,000 people watch the hand copy newspaper works and vote, further expand the coverage of the party history learning coverage, education and guide them to understand the glorious course of the party. Talk about-around the "inheritance red gene gathering youth power" this theme, youth league organizations at all levels during the "May 4 th" organization 330 theme TuanRi activities, invite outstanding party member, youth May 4th medal winner, outstanding cadres, outstanding members to share dedication story, communication growth experience, feeling with the party beginner's mind, keep in mind that the party's mission, education guide member youth par example, learning advanced, struggle with the party. Sounds-around the "youth heart to the party free trade port" this theme, with the history of the party education as the main content, invited youth branch and "red scarf" XuanJiangTuan members, experts and scholars, hero models, party members and cadres into the county schools to preach, education to guide their firm ideal faith, in the comprehensive construction of the socialist modernization country in the new journey of flying youth dream. Write-around the "new era good young hearts to the party" theme essay competition, organize the county youth in the form of prose, poetry, review the party's glorious history, acura the party's great achievements, imagine a better future, education guide them draw confidence from the progress of the party's glorious history, set up the lofty ideal, firm forward power. At present, the activity has received 458 youth manuscripts. Move-around the "revisit the red story, red spirit" activity theme, organize young students to actively participate in online party history knowledge interactive learning, through "youth knowledge" online knowledge contest, "youth learning" online group, "red scarf love learning" online team to build party history learning education "cloud classroom", by sharing activities, exchange feelings, enhance the youth party history learning interactivity and effectiveness of education. Various volunteer service activities such as "I do practical things for the youth" and learning from Lei Feng are carried out offline.

2021-06-02	Guizhou river autonomous county XuanJiangTuan into the grassroots to carry out the party history learning education Guizhou river autonomous county party history learning education theory XuanJiangTuan preach report into the cultural and educational health system and the street wei), Guizhou Tongren river autonomous county party history learning education county XuanJiangTuan preach report respectively in the county bureau of education on the third floor conference room and peace street. Wang Xi, a member of the county party committee and secretary of the Communist Youth League Yanhe Autonomous County, made a speech report, and more than 200 cadres and workers of the county education and health system and Heping Street attended the report. At the report, Wang Xi to "review a hundred years of the Party history to remember the original mission" as the theme, the party history of the key points of education for in-depth interpretation, for the party cadres on a systematic, vivid party history education class, ideological and political class, party spirit cultivation class. The report pointed out that the history of the Party is the most touching chapter in the history of China in modern times. It is the best textbook and nutrient, and we should fully understand the significance of carrying out the study and education of the Party history. First, we need to integrate and understand ideas. Second, we need to take overall account. Third, we need to create an atmosphere. After listening to the report, the party members and cadres have said to preach the report as an opportunity to strengthen the party history learning education, comprehensive system study the history of the party, learning practice enlightenment firm faith, learn from the strength of the party in one hundred, further understanding, credit, chong DE, practice, truly learn the history of the party, enlightenment thought, does the practical work, open a new bureau.

2021-06-16	Guangdong Province Xinfeng County Party Committee to carry out youth civilization to create work exchange activities Guangdong Province Xinfeng County Party Committee to carry out "celebrate the founding of the party to gather youth strength" youth civilization to create work exchange activities to take photos. Correspondent for figure China youth network Beijing on June 16 (reporter Zhang Jianwei, correspondent Cheng Xin) in order to further strengthen the youth team ideological and moral and cultural construction, promote industries, units service people, social civilization image, recently, group Shaoguan xinfeng county actively coordinate, organization new venture fire rescue brigade and wide high-speed xinfeng toll station to carry out the "founding one hundred gather youth strength" youth civilization to create work communication activities, through field visits, discussion, watching short video, help to promote mutual progress, for excellent and advanced to the founding in one hundred. The picture shows the fire and rescue personnel showing the fire and rescue equipment for the visiting students. Correspondent for figure field visit learning link, xinfeng fire rescue brigade for publicity on the aspects of "safe production" knowledge, wide high-speed xinfeng toll station a staff regrets "for the first time into the fire rescue brigade, the first time to see all kinds of fire rescue equipment, the first comprehensive understanding of safety knowledge of fire and electricity, deeply by fire rescue workers civilization image, profound knowledge, excellent style, they are my mind male god". The picture shows all the representatives exchanging fiercely at the symposium. Correspondent for figure discussion communication link, big wide high-speed xinfeng toll station representative rushed to xinfeng fire rescue brigade "scriptures" youth civilization create work experience, xinfeng fire rescue brigade Chen Wei said "the party in the heart, there are the people in the heart, steadfast in their respective positions, everyone's post is youth Wen Minggang, every unit can be called the youth civilization". Next, the Youth League Xinfeng County Party Committee will combine the party history "I do practical things for the masses" activities, in the civilized law enforcement supervision, civilized service to the masses and other aspects, continue to encourage advanced, spur backward, with high standards, strict requirements, real style to guide each unit to create "youth civilization".

2021-06-17	Xie Jiaji in Anhui province youth volunteer association to carry out the Dragon Boat Festival to send warm volunteer service activities reed the Dragon Boat Festival filial piety the world Xie Jiaji in Anhui province youth volunteer association to carry out the Dragon Boat Festival to send warm volunteer service activities jianwei correspondent Fang Yuan check WeiTing) to inherit and carry forward the traditional excellent culture of the Chinese nation, in the "our festival Dragon Boat Festival" is coming. A few days ago, Anhui Huainan Xiejiaji district youth volunteer association organized the star of hope education technology Co., LTD., Bank of Communications Huainan branch, Xinhua hospital, Jinde industry, coal field geological exploration team volunteers came to gudui township nursing home to carry out a warm series of activities for the elderly. Let them feel the festival atmosphere and care from the society, spend a strong love of the Dragon Boat Festival. During the volunteer service activities, the volunteers and the elderly people sat together to make zongzi, chat, and send them comfort items. More than 40 volunteers from all walks of life gave full play to their professional expertise. Volunteers from Xinhua Hospital provided blood pressure measurement and other free medical services for the elderly. The young pioneers of the school brought artistic performances to the elderly, with continuous applause and wonderful applause. Love is the Dragon Boat Festival, love in the nursing home. While carrying forward the spirit of volunteer service by Xie Jiaji District Volunteer Association, it passes on love to more people in need of help and gives them warmth and care.

2021-06-10	Yanchi County, Ningxia, has carried out the practical experience activity of "I will tell the story of the Party- -the red scarf narrator". Correspondent for figure China youth network Beijing on June 10 (xinhua Ji 'an-wei zhang, correspondent wu lei), Wuzhong city, Ningxia hui autonomous region in Ningxia party history education visit-Yanchi county revolutionary history memorial park to carry out "the party's story me-red scarf narrator" practice experience activities, more than 100 young pioneers to participate in the activities. The picture shows "the story of the party I will tell- -red scarf narrator" scene. Correspondent for figure under the guidance of four red scarf narrator, the young pioneers visited the salt pool revolution memorial hall, the historical pictures and old objects let the young pioneers seem to return to the war s, more truly feeling today's happy life comes from the correct leadership of the party, from the revolutionary martyrs, from the people's hard struggle, they will inherit and carry forward the red spirit, in inheritance continuously red blood, be new times in the forward. The picture shows "the story of the party I will tell- -red scarf narrator" scene. If I tell the story of the Party, the revolutionary martyrs and the heroic deeds written with their blood and life will always inspire the young pioneers to work hard and work hard. Study the knowledge of the Party, carry forward the deeds of the Party, combine the history of the Party with practice, with plain words and colorful practical activities to the school, family, society issued the "red scarf" the strongest voice, the young pioneers with practical actions to "learn the history of the Party, strong faith, follow the Party", pay tribute to the founding of the Party.

2021-06-16	Lingbi county, Anhui province party history learning education youth XuanJiangTuan into the enterprise the party history of learning education youth XuanJiangTuan into the enterprise jianwei correspondent Wang XueZha WeiTing) recently, Huaibei county, Huaibei city, Anhui province, the party history XuanJiangTuan learning education youth XuanJiangTuan members Li ZhiYuan to Lingbi county youth entrepreneurs association for the history of the party learning education, entrepreneurs association members on behalf of a total of more than 40 people to participate in learning. Li Zhiyuan to "draw strength from the party history education" as the theme, combined with the history, the party's revolutionary road and the party's construction, from the direction, choose, support, market, the whole style, the patriotic six aspects indicate the direction for the enterprise development path, guide the young entrepreneurs from the party's development process for economic development direction, accurately grasp the new development stage, new development concept, new discovery pattern of profound connotation, no distractions, solid hard work, for the construction of the new stage of modern beautiful lingbi youth power."Through today's party history study and education, I deeply feel that the development process of the Party is not easy."The students said that they will take the party history learning and education as a good opportunity to strengthen the theoretical arm and improve their entrepreneurial skills, bear in mind their original mission, consciously fulfill their responsibilities and obligations, follow the laws of the market, and constantly work hard to accelerate the pace of enterprise reform and innovation. Tuan lingbi county party committee to party history learning education as an opportunity to lead the youth firmly grasp the "history, history, chong DE, history" goal requirements, party history, enlightenment thought, does the practical work, open new bureau, led the county youth singing "party history learning education" strongest voice, to forge ahead new stage of modern lingbi construction new journey, in order to celebrate the 100th anniversary of the founding of the communist party of China with honors.

2021-06-16	Group in Anhui province to carry out the party history learning education demonstration preaching and "youth branch" lesson preparation training group in Anhui province party history learning education demonstration preaching and "youth branch" lesson preparation training JianWei correspondent, Jiang Yan check WeiTing) for the solid education, the party history, guide the general youth members from learning faith, wisdom and strength, recently, the group Huaibei city of Anhui province du set district party committee to carry out "learn the history of the party, strong faith, follow the party" party history learning education and "youth branch" lesson preparation training. Huang oda, director of the study room of the Huaibei Municipal Party Committee, member of the provincial lecturers expert pool, member of the municipal Party history learning and education special publicity group, was invited to make a party history demonstration lecture entitled "continuous red blood practice the original mission". Liang Beibei, secretary of the Youth League District Committee, presided over the meeting. More than 50 people who were in charge of the league (working) committee of the development zone, members of the youth teaching group, representatives of the "two red and two excellent" and some youth league members attended the meeting. At the training meeting, Huang oda used "Qingyan" to comprehensively and systematically explain the significance of carrying out party history learning and education, focusing on four historical periods about the original mission and historical responsibility of the Communist Party of China in four historical periods, and shared his own thoughts on how to carry out publicity work among young people. Next, the youth branch will be in accordance with the established arrangement, around the "Yang youth celebrate one hundred birthday" theme, to carry out the history of the party learning education to preach into the countryside, into the community, into the campus, into the enterprise, into the authority, into the network "six into" activities, build a strong atmosphere of the history of the party, guide the teenagers listen to the party, feeling DangEn, follow the party.

2021-06-16	Xinfeng County, Guangdong Province, took a photo at the opening ceremony of the "Chinese pastry + Western baking" skills promotion training class in Xinfeng County, Guangdong Province. Correspondent for figure China youth network Beijing on June 16 (xinhua Zhang Jianwei, correspondent Cheng Xin) to further consolidate improve poverty crucial achievements, promote poverty engines and rural revitalization effective cohesion, practice the party history "I do the practical work for the masses" requirements, recently, Shaoguan, Guangdong new county people club bureau, xinfeng county, Dongguan dongcheng street counterpart support xinfeng headquarters joint Dongguan dongcheng street club training branch, Dongguan east for new venture youth carried out a "Chinese pastry + western baking" skills to promote employment training class. In the early stage of the training, Xinfeng County Human Resources and Social Security Bureau and Tuan Xinfeng County Party Committee comprehensively investigated the employment wishes of young people, fully communicated with the Xinfeng headquarters of Dongguan Dongcheng Street, actively connected with the Human Resources and Social Security Sub-bureau of Dongguan Dongcheng Street, and finally determined the training content. The picture shows the training teacher explaining the pastry and baking skills to the students. Correspondent for the picture training class, the teacher site demonstration, one detail explanation precautions, one student to guide the practical operation, to ensure the training effect, to achieve the "quick" training purpose. The training teacher said, "During the training in Xinfeng County, I feel the importance that the county party committee and government leaders attach to the work of youth groups. I will definitely teach them all what I have learned, so that they can add bricks and tiles to the rural revitalization of beautiful Xinfeng."The picture shows the students discussing their learning experience. After the training, the students sat together to exchange their learning experience and "pastry + baking" skills. A student said, "This training, set up my entrepreneurial confidence, but also let me master a certain pastry and baking skills, I will make a big cake for the motherland on the day of the founding, to share with poor primary school students, let them more feel the party grace, follow the party."Xinfeng county people social bureau, the county plan in the second half of 2021, in stages, targeted skills training courses, organization post promotion, through the xinfeng poor youth entrepreneurship to promote employment, employment to promote the development of benign interaction, realize the good effect of entrepreneurship drive employment multiplication, better serve xinfeng economic and social development.

2021-06-12	Huangshan district in Anhui province first community less founded in north community I do something for youth to build "home young pioneers" -Anhui Huangshan district first community less committee in north community JianWei correspondent Ye Qingcha WeiTing) to celebrate the 100th anniversary of the founding of the communist party of China, promote youth party history learning education, recently, Huangshan city in Anhui province Huangshan north community less committee founding ceremony held in the community new era of civilization practice station. The establishment of the community working committee will give full play to the role of the community working committee in moral education, encourage the young pioneers to report to the living community, participate in the community garbage classification, environmental protection publicity, volunteer service and other public welfare volunteer activities, do a good job in the community "little master", and constantly enhance the sense of honor and belonging of the young pioneers. Activities on the same day, the community working committee combining party history learning education "I do the practical work for the masses" practice activities, held the dragon north community four clover public class seminar, invited yu-juan liu teacher on a vivid parent-child family education class, tells the story of parents and children communication skills, analyze children's psychological and physiological characteristics, let parents learn to understand the children, listen to children, through effective communication to build a good family parent-child relationship. Next, longbei community young working committee will take the establishment of the district's first community young working committee as an opportunity, combined with the party history learning education, actively explore, innovate the management mode of children, guide the realization of children to firmly listen to the party, follow the party's determination.

2021-06-01	Beijing, June 1 (reporter Zhang Jianwei) in order to celebrate the 100th anniversary of the founding of the Party, Thoroughly study and study the party history of Zaozhuang City, especially in Xuecheng District, Make full use of the rich red resources of the whole city and the whole district, Shandong Province Zaozhuang City Xuecheng District Committee, together with the District Education and Sports Bureau, District Poverty Alleviation Office, District Veterans Affairs Bureau for the majority of the district to carry out the "learn a hundred years of Party history sing a hundred red songs" party history learning education red publicity into the campus activities, The Communist Youth League District Committee hopes to preach the magnificent history of the Party and sing the enduring red songs, Educate the next generation to know history love the Party, know history patriotic, know history love city love district, Let the red gene pass on from generation to generation. TuanOuWei invited love local party history of poverty office, deputy director of the Lv on May 19,20,21 in north city middle school, XiZhong middle school and longtan experimental school to preach activities, lu will happen in the red story and Zaozhuang communist struggle history, in the party in one hundred struggle history to young students, plastic students patriotic love party love hometown feelings, and into Chinese and history textbook knowledge, let students learn the cultural knowledge, and accepted the ideological and moral education baptism, received good effect, three days a total of more than two thousand member youth scene listened to the preach. North near the city middle school learning about the title of the history of the red film of Zaozhuang party, combined with four red films related to Zaozhuang, panoramic review of the party in the process of the new democratic revolution hard exploration and struggle, shows the communist party for the cause of national liberation, especially the outstanding contribution of zhu daonan, Yang Shide story moved many students to tears. Xi Zhong middle school party history learning about the title is "Xue Cheng double heroes, with a division", through Zaozhuang "Dong Cunrui" Chen Jinhe martyrs complete the crucial task and "desperately" Hu Chengyi martyrs fought heroic deeds, reviewed the party first crucial forces of Shandong military area 8 in this life, the students sincerely for the composed of south army iron male proud. Longtan experimental school party history learning about the title is "the exotic, not the motherland", through the xue city participated in the Korean war veterans, led the students to revisit the fierce battle of the republic, volunteers ice and snow changjin lake, bitter ganling classic example let the children respect for war veterans.

2021-06-07	Group of Anhui province suNing county held "wish" volunteer service activities in Anhui province county "light micro wish interpreta dream with me" volunteer service activities Wei correspondent Cheng Yuan WeiTing) to help rural left-behind children and poor students realize "wish" heart "little dream", recently, Anhui province suNing county invited caring people to SuNing county huang Ji'an primary school to carry out the "light micro wish interpreta dream with me" volunteer service activities, send special gifts for the children. In the "micro wish" dream realization ceremony, the county and township youth League committee staff and caring people together for Huangjian township 15 poor students personally sent school bags, picture albums, books, reread machine and other 15 "micro wish" gifts, and encouraged them to aspire to study hard, strive to be a good teenager in the new era. The children who received the gifts have expressed their sincere thanks to the caring people, and all showed a happy smile. Since April this year, the youth League county party committee, the county young working committee organized and mobilized the grass-roots youth league organizations, schools to carry out the "micro wish" collection activities, through the door-to-door understanding, directional consultation, personal submission and other ways to collect a total of "micro wish" 94, after the network release, love claim and other processes have been all lit up. This week, these "micro wishes" gifts will be sent by the township cadres to the children needed throughout the county. This "micro wish" series of activities aims to let the left-behind, special, difficult families in the children really feel the care of the party and the care of people from all walks of life, so as to draw a positive spiritual strength to thrive. The activity also provides a channel for caring people to "reach" the hands of children. During the activity, the county and caring people a line of people in the township staff and school teachers visited the school library and classroom, detailed understanding of the school young pioneers construction and education teaching situation, and strengthen the rural primary school campus management, deepen rural left-behind children contact and improve the education teaching quality related to the discussion.

2021-06-01	Zhaohua district of Sichuan province to carry out the party history learning education knowledge contest in Sichuan Zhaohua district to carry out the "party history strong faith follow the party" party history knowledge contest Beijing on June 1 (reporter Zhang Jianwei, correspondent peng wang), group of Guangyuan city, Sichuan province Zhaohua district party committee joint Guangyuan city Zhaohua district party committee propaganda department held the "party history strong faith follow the party" party history knowledge contest. The game, zhaohua district formed seven teams, 21 players, players are from zhaohua district youth, focus on the brief history of the communist party of China "the 100 years of the communist party of China" zhaohua district old revolutionary base areas history " the communist of the constitution of China party history learning education and history know JiMing law related learning content. The picture shows the competition site. Correspondent for the picture, the competition sets up individual required answer, group required answer, group rush answer, risk questions, additional questions, audience interaction and other links, novel form, rich content. Must answer link players all confident, answer like flow, rush answer link, everyone eye disease quick hand, calm response. The players on the field showed their skills, and the audience applauded constantly under the field. The contestants competed fiercely in the catch-up competition. Each team's solid theoretical foundation and excellent psychological quality showed incisively and vividly in this competition. The picture shows the competition site. After a fierce competition, the results of the competition were finally announced, the agency team won the first prize, the first secretary, the state-owned enterprise system team won the second prize, the education system, health system, return home entrepreneurship team won the third prize, the town team won the award of excellence.

2021-06-09	Group of Anhui xuanzhou district party committee to carry out the party history learning education and rural revitalization of Anhui xuanzhou district young entrepreneurs association to carry out the party history learning education and "six crucial, three years climbing" rural revitalization investigation research activities wei correspondent, TaoZixuan check WeiTing) recently, the group of Anhui province xuanzhou district party committee association of young entrepreneurs to 20 youth entrepreneurs BiQiao town, Zhu Qiao township, xuanzhou economic and technological development zone, party history study education and rural revitalization, TuanOuWei secretary wang xiaochuan to participate in research activities. The youth enterprise association first visited the town Jiangshan Village New Fourth Army two detachment headquarters site. By watching the display of photos, the deeds of the brief introduction, heroes list, let everyone understand the New Fourth Army of the second detachment in the leopard bridge to carry out the revolutionary heroic deeds, feel the revolutionary martyrs are loyal to the party, brave hardships and dangers, the spirit of selfless dedication. Subsequently, the entrepreneurs went to Xuanzhou Economic and Technological Development Zone, went deep into the production workshop and storage line, and visited Jinbo packaging, Huateng latex, Qunxing magnetic material, Jinbowei Home furnishing and other 4 enterprises. Association members came to Zhu Qiao township nine bao village screw pepper base, Zhu Qiao beishan industrial agglomeration area (ceramsite industrial park and calcium carbonate industry) after the site visit, Zhu Qiao township townships Jiang Jingbo held a symposium with young entrepreneurs, young entrepreneurs around Zhu Qiao township industry development planning, industrial development, rural cultural tourism theme in-depth exchanges. In the next step, the Tuanxuanzhou District Committee will closely focus on the "3 + 5" "action, and actively guide young entrepreneurs to focus on the" 3 + 5 " modern industrial system.

2021-06-09	Ningxia pengyang county to carry out the "clock red footprint-red scarf small reporter search" group Ningxia pengyang county to carry out the "clock red footprint-red scarf small reporter search" activities wei correspondent Wu Lei) recently, the group of Ningxia hui autonomous region evalution pengyang county organization to carry out the "clock red footprint-red scarf little reporter search" activities, recall one hundred glory, to celebrate the 100th anniversary of the founding. Representatives of counselors from 5 primary schools in the county seat and 50 red scarf little reporters attended the activity. The picture shows the "punch in red footprint- -red scarf little reporter search" scene picture. Correspondent for the past extraordinary years-in Pengyang County museum, small reporters follow the narrator to understand the profound history, visit MAO zedong, Peng Dehuai and other proletarian revolutionaries of the glorious legacy, relics, and the precious revolutionary relics, revisit the glorious history; visit the work since the establishment of the county, understand under the leadership of the Chinese county government led Pengyang people carry forward the "Peng Yang spirit" and "three bitter style" has made a series of achievements. I can personally experience the struggle of the Party over the past 100 years, and think about why the Communist Party is "able" and why socialism with Chinese characteristics is "good" in the search and practice. The picture shows the red scarf little reporter studying the history of the Party in Pengyang County Museum. Correspondent for figure memorial revolutionary martyrs, and martyrs cemetery, young pioneers one step by step, came to the revolutionary martyrs monument flower basket, respect, and fighting video image, as if heard 70 years ago, in revolutionary martyrs, in their certainty, resolute eyes, as if saw they rushed the fire beacon, for the victory of the revolution, brave forward."History must not be forgotten. Heroes need to remember it. We are always ready to be a qualified socialist successor."Red scarf small reporter said. The picture shows the red scarf reporter in the RenRiver Martyrs Cemetery oath. Correspondent for figure read hard in one hundred-ended Ren Shan martyrs cemetery memorial visit, red scarf small reporter came to small fork township party history, Qiao canal MAO zedong long march camp, under the guidance of the narrator, concrete image understanding of the party led the people to build new China, building socialism, leading the reform and opening up red footprint, inspire the hometown of red culture identity and affinity. The picture shows the red scarf small reporter "listen to" the party history. The red gene is passed down from generation to generation- -after the activity, the young reporters have published their feelings, thoughts, thoughts, and gains- - " the story around them is the most infectious! This search activity, can let us better know the party history story on the land of Pengyang, so as to better cherish the present, look to the future... "" Living in the revolutionary martyrs with blood and life for the peaceful environment, I should be more firm to study hard, perseverance, make himself a more good youth..... "" a promising nation can not be without hero, a promising country can not be without pioneer ". The comrade in charge of the activity said, " Peng Yang is a city with a glorious revolutionary tradition. In the past hundred years, this red hot land on the bank of the Ru River has left many touching red stories. The red Footprint activity is to make full use of local red resources for vivid ideological and political education courses, so that more young people can become visitors and sharing of the red history in their hometown, encourage people to stay true to their original aspiration and keep their mission in mind, and let the red gene be passed down from generation to generation.”

2021-05-31	Zhejiang Xiangshan County held the history of the Party youth labor education practice activities Zhejiang Xiangshan held "learn the Party history know farming" youth labor education practice activities correspondent Shi Han Xiao Lu Lu China Youth Daily youth network reporter Wang Shanshan) barefoot in the wet rice fields, Holding the tender green seedlings in his hand, Bunthe seedlings in the soil, Experience a different idyllic scenery with your family, friends, and children, Experience a different farming culture and customs... On May 29th, In order to guide the young people to learn the history of the Party, devote themselves to labor, and taste the harvest, Xiangshan County Party Committee of Ningbo City, Zhejiang Province carried out the youth labor education practice activity of "learning the Party history and knowing farming" in the Communist Youth League farm. At the scene, Xiangshan County youth Labor education practice base was inaugurated, the young Pioneers representatives initiated the food saving initiative, while the party history learning education and farming experience activities combined, to carry out the "red classroom on the ridge of the field" party history theme learning education. More than 120 young people experienced the hardships of labor and farming culture. They went barefoot, rolled up their trousers, and walked down to the field with great interest to plant the rice seedlings taught by agricultural experts on site."Good fun, good fresh, before always know the scene of farmers farming in the book, now finally can personally experience once, although hard, but endless fun, let me know how to cherish the food more."The young pioneers bent down while planting rice seedlings and said.

2021-05-31	Let every child by the Xiangjiang River can tell the story at the door let every child by the Xiangjiang River can tell the door story at home, located in the Guangxi Zhuang Autonomous Region, Quanzhou County, the Red Army Long March Xiangjiang Campaign Memorial Park was opened, become the main position for party members and cadres and people from all walks of life to carry out the theme of education. Also in the summer vacation of this year, the Quanzhou County Education Bureau and the Youth League Quanzhou County Party Committee, the Quanzhou County young Working Committee jointly held the "red scarf narrator" training class opened, the Quanzhou County youth activity center director, the young Pioneers general counselor Zhou Kangli is responsible for the training of students and teachers. In the first phase of the training, not many children were signed up in the county. Although there were only more than 10 students in the class, Zhou Kangli thought that as long as these students were well trained, people to visit the Red Army Long March Xiangjiang Campaign Memorial Park people to listen to the children's explanation, infected, see their growth and progress, will spontaneously spread the party history learning and education model for teenagers. Zhou remembers that when they first took over the students, some were introverted and not expressive; some were delicate, and their parents said they wanted lunch. And reviewing the arduous history of the Xiangjiang Campaign is the best education for the children."It is impossible for children to understand this history and feel the spirit of the Red Army by rote."Zhou Kangli said that every red scarf commentator's first class is to retrace the Long March road. Quanzhou County, Xing'an County and Guanyang County in northern Guangxi were the places where the Xiangjiang Campaign took place. When Zhou Kangli took the children to ohtsubo, pingshan, phoenix mouth, first four ferry, came to irrigation Yang new wei, Xingan guanghua shop, the state foot mountain shop three resistance point, in the north to the xiangjiang river scene to listen to the red army "victory, breakthrough, sacrifice" revolutionary deeds, the children to the growth of the land, have a deeper perception and understanding, also understand the "red army blood xiangjiang river crossing, river bottom is loyal" solemn and stirring and the hard-won happy life today. Zhou Kangli has been in a habit for several years to conducting professional training on the history of the Party every Saturday, and organizing children to enter the museum on Sunday. At the beginning, some students because of the tension, some explain is not fluent, was visited the memorial hall of the public criticism; and some children, because of the continuous explanation, show a state of exhaustion. Zhou Kangli will always stand by and encourage her children to stick to it. " As General Secretary Xi Jinping said, no matter how difficult it is, think about the Long March of the Red Army and the bloody battle of the Xiangjiang River. Stick to it in the most difficult times, so that you can keep achieving miraculous victories."The Red Army Long March Xiangjiang Campaign Memorial Hall in every booth, every sculpture has a touching story. In the study and explanation over and over again, the students will continue to have new insights and growth. Reporter interview, many students have mentioned the "rob ferry protect radio" story, when the red army from the first ferry crossing the river, in order to avoid generator, transmitter immersion, the soldiers head against the enemy's fire, at the foot of the cold xiangjiang river water, even if he was injured must protect the machine, a person fell, another horse. People were deeply affected by the team spirit of the Red Army soldiers. Jiang Mingzhong, an 11-year-old commentator on the red scarf, said that he realized that in a team, the sense of cooperation and responsibility can ensure the realization of the goal. Jiang Zhou Jinlin, a fifth-grade student from Quanzhou County Chengguan Complete Primary School, has attended the red scarf explanation group for more than two years. During the May Day holiday this year, many people came to visit the memorial hall. Because the explanation group is a team cooperation, each red scarf narrator is responsible for a position, relay commentary. From 10 a. m., she kept explaining, and when she was really thirsty, she would sip a little saliva, afraid of drinking too much to go to the toilet, causing the visitors to miss her responsible explanation part. At the lunch break, the friends do not talk and laugh as usual, have finished eating to seize the rest, because we know, the afternoon will still be a sea of people, they must hold on."We always remember what the counselor said- -always deserve the red scarf on our chest."Jiang Zhou Jinlin said. It is understood that since the National Day on October 1,2019, the Quanzhou County red scarf commentator has carried out 63 explanations, the average commentary duration of each child has been more than 200 hours. With more and more students coming here, Quanzhou County red scarf guide training class has more than 200 students. Through learning and training, they give a wheelchair veterans grandpa, to Korea with the medal of the old hero, to uniform party building soldiers, police, judges, prosecutors, doctors, teachers, to colleges and college students and children of the same age, also to Hong Kong, Macao and Taiwan compatriots, foreign friends."Whenever I see visitors stop to listen, be moved to tears, and thumbs up because of the children's stories, I will deeply feel the value and significance of this work."Zhou Kangli said that she is willing to stay as the chief counselor of the Young Pioneers all her life, so that every child by the Xiangjiang River can tell stories at home. China Youth Daily, China Youth Network reporter Xie Yang source: China Youth Daily

2021-06-03	Chengmai County Party Committee of Hainan Province boosts Party History learning and Education Group with "1 + 2 + 100" with "1 + 2 + 100". Correspondent Zhou Yutong) In recent days, The Communist Youth League of Hainan Province Chengmai County Party Committee adhere to the "1 + 2 + 100" ("1" is to unswervingly follow the party in a basic direction; "2" is the two musts, Be sure to deeply understand and think carefully, Be sure to apply what you learn, to learn to promote the use of the two principles; "100" is to carry out "I do practical activities for the masses" 100 activities) principle, By expanding coverage, grasping characteristics and solving difficult problems, To carry out the party history learning and education, Promote the "I do practical things for the masses" and 100 characteristic activities landing effective, Set off the county at all levels of league organizations, league cadres, youth league members, young pioneers learn to practice the history of the party upsurge, Unite and lead the majority of young people in promoting the high-quality development of beautiful Chengmai achievements. Youth League Chengmai County Party Committee adhere to the guidance of the party building, with activities as the carrier, embedded in the party history elements. Through the "party building + volunteer", "party building + research", "party building + red culture" and other modes, repeatedly organize youth into the county site pond north red interactive education base and other red positions to carry out the party day activities; guide the league organizations at all levels to help the "two sessions", holiday scenic spots volunteer service, help "expo" series volunteer service more than 20 games, 7 research on entrepreneurship and employment, league transition and other work. At the same time, the coordination mechanism should be improved, and the "1 + 2 + X" Party building alliance should be established, and the publicity Department of the county tourism and culture bureau and the schools to create "explanation groups", "tour groups" and "publicity groups", gather the strength of people of different ages and different regions, and enter the red bases, cultural centers, museums, libraries and other areas to carry out the explanation work. Youth League Chengmai County Party Committee in the party history learning and education, Firmly grasp the core of actually doing practical things for the people, Focus on the main positions and responsibilities, Working around the center, Formulate the "I do practical things for the masses", Continue to promote the "Learn foreign language ABC to build Hainan FTP" English classroom into towns, townships, schools, enterprises and institutions, Through the foreign teachers' characteristic teaching, Create a good atmosphere for people to learn foreign languages; The Youth League county Committee is also committed to improving the village cultural activity room "activity", Guided all towns and townships to carry out 12 weekend public welfare classes, Organize volunteers and social workers to use youth homes, youth league organizations and other positions to gather young people in the area to learn drowning prevention, drug control, spoken English, pinyin practice and other content, We will strive to make young people grow up happily in the care of the Party and the government."How to ignite the 'young way' of the youth party history learning enthusiasm, is the county at all levels of the youth league organizations, cadres need to master the basic skills."Youth League Chengmai County Party Secretary Cao Yujin introduced, the Youth League County Party Committee adhere to the purpose of serving the people wholeheartedly, the use of" Internet + " to help learn the history of the Party enthusiasm. Using WeChat working group, WeChat public account, party building garden, LED electronic screen and other positions, push, post a series of policies, meeting spirit, leadership speech spirit, knowledge points and other content, actively participate in online knowledge contest, create a multi-dimensional learning atmosphere; open a new four history learning column, push including "today in the party history", "historical moment" and "century pioneer" and other pictures. According to statistics, the column has pushed nearly 100 articles, the total reading of more than 1,000 times."We will organize and guide the youth league organizations at all levels to promote 'red' learning, publicity, activities content orderly, continuously promote the party history learning education achievements into vivid service youth practice, especially on June 1, officially launched in cooperation with the south China sea network red scarf about the history of the party 12 project, let red gene, revolution fire from generation to generation."Cao Yujin said, the next step, the Youth League Chengmai County Party Committee will adhere to the combination of" online + offline ", continue to organize the youth into the red base to carry out red education, consolidate the red gene; play the county youth entrepreneurship association party branch, the county financial system party organization and other positions, increase the linkage efforts, expand the coverage of different levels of groups.

2021-05-28	Huangshan district in Anhui province to prevent telecom fraud theme XuanJiangTuan Huangshan district party committee to carry out the "prevent telecom fraud financial knowledge into the campus" theme preach JianWei correspondent Ye Qing Cai Kaihao) to actively prevent telecom network new crime concentrated propaganda month activities, promote youth financial literacy education, recently, the Huangshan city in Anhui province joint public security bureau branch, bank of China taiping branch, Anhui Huangshan jinqiao village bank, dragon north community in Huangshan tourism management school "prevent telecom fraud financial knowledge into the campus" theme preach. The picture shows the scene of the lecture session. Correspondent for figure briefing, the police, bank staff respectively from the concept of money laundering, the process of money laundering and harm, financial institutions main anti-money laundering measures and other eight aspects, introduced to teenagers anti-money laundering knowledge and in the telecom fraud emergency measures, by sharing the latest telecom fraud news, remind everyone to improve security awareness. The development of the seminar has improved the financial knowledge level and financial risk prevention awareness of teenagers. Students have said that they would publicize their knowledge to more people in the future. The picture shows the school teachers and students listening to the lecture content carefully. Correspondent for figure since this year, the group Huangshan district party committee to "I do the practical work for the masses" "I do something for the youth" theme practice as an important content of party history education, through joint relevant departments, further "drowning" "telecom fraud" prevention "prevent bullying" theme to preach, propaganda way, innovation, to enhance youth awareness, efforts to create a good social environment, promote social harmonious development.

2021-05-27	Malipo county, Yunnan province "sunshine assistive" volunteer service into the special-education class special love to you-malipo county, Yunnan province "sunshine assistive" volunteer service into the special-education banpo county for China youth network Beijing May 27 (xinhua Zhang Jianwei) in order to further promote the traditional virtues of the Chinese nation, gradually form the whole society to understand, care, help the disabled good atmosphere, recently, Yunnan Wenshan zhuang and miao autonomous prefecture malipo county party committee in Laoshan (malipo) youth public service center volunteers into malipo county first primary school special-education class, into the special-education class children world. The picture shows the scene of the "Sunshine Helping the Disabled" volunteer service activity. In order to better integrate into the special education children, the volunteers carefully designed an interactive game suitable for the children of the special education class. In the activity, according to the strengths of different children, the volunteers led the children to carry out handmade paper cutting and clay creation, so as to exercise their practical ability and encourage them to play their strengths. With the help of the volunteers, the children cut out the flowers, pinched their favorite animals, and played their favorite games... Although the children's movements are slightly clumsy, but the smiles are warm and bright, maybe the volunteers and these special children can not have a normal language communication, but the smiles and zero distance interaction have become the best "language". The Communist Youth League County Party Committee will continue to encourage volunteers to pay attention to special children, so that great love can spread and the sunshine of care from all walks of life can warm the hearts of every disabled person.

2021-06-07	Group of Anhui Huangshan district party committee to carry out the love to send test volunteer service in Anhui Huangshan district party committee to carry out the "I do something for youth love escort help success" love to send test volunteer service correspondent Ye Qing check WeiTing) for the students and parents, group Huangshan Huangshan district party committee, district transportation bureau recently in Huangshan "I do something love escort to help success" love to send test volunteer service launch ceremony. The picture shows the Anda taxi love motorcade. Correspondent feeds activities on the same day, from anda taxi team love of nearly 20 taxi orderly parked in the gate of Huangshan mountain, anda taxi company, head of the party branch and "love driver" representative completes the epidemic prevention and control measures, abide by the traffic laws and regulations, create a good ride environment, for candidates to provide priority quality service commitment. The Youth League District Committee, District Transport Bureau and Huangshan Middle school leaders respectively for the love to send the car hanging green ribbon, and on the front of the "love to send the test team" logo. The picture shows the launch ceremony of the volunteer service. In 2021, more than 1,000 students in Huangshan District will take the entrance examination for secondary school or college. On the exam day, candidates and their parents can take the initiative to show the middle and high school certificates and stop immediately, and the vehicle will not charge any fees. If some candidates need vehicles to pick them up, they can also call the team volunteers. There are also two love cars outside the test site. Candidates, in case of emergency, they can ask the love car for help. It is reported that this year is the 16th year of charity volunteer activities in Huangshan District. In the past 16 years, the Youth League Huangshan District Committee has actively organized the Party member volunteers and young volunteers of "love to send two examinations" to stick to the original aspiration, rain or shine, the epidemic situation, and adhere to the "escort" of the numerous students in Huangshan. At present, the Youth League Huangshan District Committee actively combines the party history learning and education work, seriously carry out the theme of "I do something for the youth" practice activities, to do a visible and tangible practical work for the youth around within its capacity.

2021-05-31	Guizhou wan mountain sites to carry out drowning prevention safety education activities in Guizhou wan mountain sites to carry out "cherish life, prevent drowning" safety publicity and education activities JianWei correspondent Yang Han) as the summer approaching, the temperature is rising, prevent drowning publicity and education become an important task of community safety education, in order to enhance the community parents and students safety awareness, improve the safety ability, avoid drowning accidents. Recently, the Wanshan District Committee of Tongren City, Guizhou Province organized volunteers to carry out the safety publicity and education activities of "cherish life, prevent drowning" in various resettlement sites, so that the community parents and students can firmly establish the idea of "safety first". The picture shows household visits to distribute safety knowledge of drowning prevention safety. Correspondent for figure dragon community TuanOuWei volunteers joint dragon auspicious social workers, sunshine volunteers association volunteers combining the actual situation of the community, household visits at the same time issued safety drowning prevention knowledge manual, told the measures to prevent drowning and encounter drowning self-rescue and communal safety knowledge, and emphasizes the seriousness of the drowning accident, requires parents to attach great importance to the child safety problems and education guide. The picture shows the volunteers explaining the anti-drowning safety publicity and education to the children. Correspondent for figure your garden community volunteers first organized the "cherish life to prevent drowning" safety education propaganda, mobilize the children to actively participate in the drowning safety publicity and education activities, and then watch drowning safety education, image directly about the harm of drowning and drowning prevention method, if not alone to the pond, river or field ditch to play water, play, not alone or and companions to the river, and when hair drowning danger accident, how to save yourself, how to timely to nearby adults for help, not to organize rescue, etc. The picture shows the volunteers explaining the safety precautions against drowning to the students. In the du community activity, the volunteers explained to the students the safety precautions against drowning, especially on how to protect themselves when drowning, away from dangerous waters. And with 6 key safety guidelines to remind children to pay attention to safety, enhance safety awareness, improve self-protection ability.1. No diving, often due to shallow water, causing cervical injury and lifelong paralysis; 2. Do not push people into the water, not to hit others or hit the pool side injured; 3. Water, do not put others into the water, to avoid choking water suffocation; 4. When water activities, feel cold or cramps, should immediately shore to rest; 5. No swim in the water without safety facilities, no rescue personnel; no water rescue 6. The picture shows the volunteers explaining the relevant safety knowledge about drowning prevention to the residents in detail. Correspondent for figure wang home community wang home community volunteers to the residents to prevent drowning related safety knowledge and how to take first aid methods correctly, don't let the child casually or water alone to swim, play, prevent drowning events, let parents alert, fully realize the dangers of drowning safety accident, let's share the responsibility of care for children together, for the teenagers safe and healthy growth to create a good environment. The picture shows the tea shop community volunteers explaining the safety knowledge to the students. In the tea shop community activities, the tea shop community volunteers explained to the students what is drowning, the cause of drowning accidents and the abnormal characteristics of the body when drowning, the measures to prevent drowning and the safety knowledge of self-rescue and mutual rescue when encountering drowning events. Volunteers asked students to write down and read aloud "No drowning prevention": not swimming; no swimming without permission with others; no swimming without parents or teachers; no swimming without safety facilities, no rescuers; no swimming in unfamiliar water; and no swimming for students without water knowledge. Help students to firmly establish a sense of safety awareness. In order to ensure the safety of the community residents and promote the community residents to firmly establish safety awareness, the community carries out a series of work to prevent drowning. One is to carry out "cherish life, prevent drowning" safety education propaganda, prevent drowning safety publicity and education activities; the second is to use 4:30 class to let students watch drowning safety education, let students more intuitively understand the harm of playing water and pay attention to safety matters: third, through household visit publicity and education, teach community residents some self-help knowledge, deepen drowning safety education, make residents master the knowledge of self-help ability. Fourth, strengthen publicity and education, through the form of leaflets to let residents understand the relevant self-help knowledge, improve residents' awareness of safety. Through the development of "cherish life, prevent drowning" safety publicity and education activities, let parents and students realize the harm of drowning, realize that life is only once. Make the concept of drowning prevention deep into the hearts of students 'minds, further improve the students' awareness of drowning prevention, expand the knowledge of students' drowning prevention, to prevent the occurrence of drowning accidents.

2021-05-24	Ling city in Shandong province "red scarf to build hope hut" activities in Shandong province red city "red scarf to build hope house" activities youth network Beijing on May 24 (reporter Zhang Jianwei, reporter gao lili), group of Shandong Dezhou ling city district party committee, district closes working committee, district education sports bureau, district less working committee joint in Shandong ling city fourth experimental primary school "hand in hand, light hope" - "red scarf to build hope hut" activities. The picture shows the scene of the charity sale. The young Pioneers will sell their unused books, stationery, toys and personal daily necessities, hoping to raise more funds for the "Hope cabin", and build the dream of building bricks and tiles as soon as possible. The picture shows the scene of the charity sale. The charity sale raised a total of 8,459 yuan, which will be donated to the Shandong Provincial Youth Development Foundation and used for the construction of the "Hope House" in 2021. Next, the next step of the youth League district committee, district young working committee will continue to make efforts, calling on the district young pioneers organization, young pioneers to actively participate in the construction of "hope cabin" action. We will gather a little love into a river to contribute the "red scarf" to comprehensively promoting rural revitalization.

2021-05-19	The Lianhua County Party Committee of Jiangxi Province went to QiMountain to carry out the party history learning education of "carrying forward the revolutionary spirit and gathering the strength". All the staff came to chessboard Mountain, under the leadership of the village secretary, visited the old site on the ridge and the village history hall. The old objects in the hall and the deeds of the New Fourth Army made everyone realize the spirit of three years of guerrilla war between Hunan and Jiangxi, "firm faith, brave and brave". After the activity, the youth members of the Lotus League expressed that they would unswervingly follow the Party, turn the youth heart to the Party, learn the "four histories", praise the party grace, and strive to be a good youth in the new era.

2021-05-18	A county youth league organizations "resource link methodology" medium-and long-term youth development planning at the county level in Hubei fan city shall break the barriers to youth work a youth league organizations at the county level "resource link methodology" for four years, li had a headache involved minors rescue and education work, now has a new presentation. In November last year, the Fancheng Youth League organized 11 departments, including the Political and Legal Commission, the public security, the procuratorate, the court and the Communist Youth League Committee, to jointly issue documents to build a social support system for the judicial protection of minors in Fancheng District. Not long ago, Li Xuejun's youth mentor group held an online online micro class on the rule of law, which attracted 300,000 views. Why can a county-level youth league organization promote the formation of 11 functional departments to join forces? Recently, a reporter from China Youth Network of China Youth Daily walked into Xiangyang to explore the transformation of youth work in Fancheng."In most parts of the central and western regions, there are only two or three county-level youth League committees, short of money and resources, and it is difficult to coordinate with other departments."During the visit, a local old youth league cadre told reporters that the difficulty of work," in the youth League county committee, to do achievements, only toss their own."With the increasing siphon effect of regional central cities, more and more young people flow to big cities, and practical difficulties such as lack of youth work force in county areas, lack of vitality of grass-roots league organizations, limited league work funds, and limited coverage of emerging professional youth begin to appear. In October 2019, the Central Committee of the Communist Youth League carried out the county-level pilot work of youth development planning in seven counties (cities and districts), including Shanghai Xuhui, Shenzhen Baoan and Xiangyang Fancheng, and explored and improved the youth work operation mechanism of "Party committee leadership, government primary responsibility, the Communist Youth League coordination, and all parties jointly manage youth affairs". In November, fan district committee, district government office to establish youth work joint conference system, by the district party committee, district government in charge of the leadership as a convener, OuZhi 27 units responsible for comrades as members, break the previous policy scattered departments of youth work barriers, leverage each department resources, every year to solve 1-2 youth development problems. Change for the youth, change from passive to active. The Fancheng District Committee promoted the Publicity Department of the District Committee, District Political and Legal Committee, Human Resources and Social Security Bureau, Education Bureau and Civil Affairs Bureau to set up five special working groups within the joint meeting, and built a communication platform through the implementation of the "double group leader system". The Communist Youth League District Committee took the initiative to visit the entry point of the youth work of each functional department. In the Fancheng District People's Procuratorate, the problem long troubling Li Xuejun began to be solved. The police officer of the minor inspection office was also the deputy secretary of the Communist Youth League District Committee, and jointly implemented the "Green Shield Plan" to protect the youth rights and interests. She set up a mentor group with professional social workers and psychological teachers to regularly communicate on the prevention of juvenile crimes and publicize the rule of law. A 14-year-old junior high school boy has a antagonistic tension between father and son. To attract the attention of his parents, he deliberately grabbed his classmate's mobile phone, was caught by the police, and walked to the verge of being sentenced. To save the child, prosecutors and professional social workers intervened to educate him for a long time. Under the guidance of prosecutors and social workers, the child's personality gradually changed, and the relationship between father and son was improved. According to relevant laws and regulations, and referring to the social worker observation report, prosecutors made a decision not to prosecute the child."Later he took the high school entrance examination and now he is in high school."Li said that thanks to the platform built by the Youth League committee, they were able to help and educate their children. Since the launch of the pilot, Shao Chenchen, secretary of the Fancheng District Party Committee of the Communist Youth League, is that "youth work is more justified and no longer the Communist Youth League alone". She introduced that the district party secretary now participates in at least five league activities every year. Compared with big cities, the population of young people in counties is small, and relatively few resources can be integrated. Tuan FanCheng committee from build way 21 creative park transformation opportunity, cooperation with the park, called more than 50 youth social organizations and young entrepreneurs, youth resources, youth organization belonging, recreational life, growth and development of three demand, build FanCheng youth center, regular youth activities, extended group work "arm"."We are diligent in running government departments and social institutions, linking both sides to mobilize resources and build a bridge with young people. The Communist Youth League spends small amounts of money to do big things."This is a pilot since, Shao Chenchen summed up a set of resource link methodology. During the COVID-19 outbreak, Guo Kaiyan, head of Xiangyang Xinqiao Psychological Career Development Center, led more than 150 psychological consultant volunteers to contact the 12,355 youth service platform of Xiangyang Municipal Party Committee, answered more than 200 phone calls, and successfully resolved 15 extreme incidents. Focusing on the psychological counseling agency, which rose during the epidemic period, the Communist Youth League District Committee implemented the "Green Joy Plan" for youth physical and mental health, provided psychological counseling for rural left-behind children and middle and senior high school students, and created online micro-courses for parent-child education. In the process of psychological consultation and research jointly with the Communist Youth League District Committee, the brand influence and radiation population of the organization were gradually expanded, and its business scope was expanded. At present, the agency has investigated the psychological counseling needs for left-behind children in 10 counties (cities and districts) in Xiangyang, and provides free psychological counseling to 16 schools. In the view of Song Haipeng, member of the Standing Committee of Fancheng District Committee and minister of organization, youth work is related to various government departments and has a great responsibility. The work of the Communist Youth League at the county level is like a "small horse and a big cart". The implementation not only depends on the youth league organization, but also needs the support of the government departments in the mechanism. In the past two years, the pool of deepening reform, activated by the pilot program, is quietly spreading in the local area. The effect of "resource link" is increasingly emerging in Fancheng, and the influence of the Communist Youth League in the youth groups is increasing. On April 9th this year, the Fancheng District Human Resources and Social Security Bureau took the initiative to contact the Communist Youth League District Committee when holding the 2021 "Private Enterprise Recruitment Month" and "retired fishermen" in Fancheng District, asking them to jointly undertake the expanded publicity. Under the launch of the District Communist Youth League Committee, more than 80 young entrepreneurs have signed up to attend the conference, providing nearly 800 youth jobs. Tuan Xiangyang party secretary Zhang Xingrui said, the communist youth league to rely on the deepening reform of dongfeng, have the courage to break through self, take the initiative to plan, to determine the long-term youth development plan of youth work joint conference mechanism, gathering youth work related government departments and the social from all walks of life, link more rich, higher quality resources to provide considerate service for youth, truly become the youth can afford, find, reliable power. In the recent "14th Five-Year Plan" of Xiangyang City, youth development was included in the special chapter, making long-term plans from the aspects of youth ideological guidance, youth education, youth healthy growth, youth employment and entrepreneurship, youth social integration, youth legitimate rights and interests, and youth social security. Hu Lin China Youth Daily China Youth Network reporter Lei Yu source: China Youth Daily

2021-05-17	Guizhou wan district primary school to carry out the party history of the education activities into the campus: red scarf heart to the party history education into the campus JianWei correspondent Shi Sisi) learn party history, follow the party, forever, Guizhou Tongren district primary school through a variety of party history learning education into the campus activities, strengthen the patriotic feelings, inheriting red genes, with practical action to the 100th anniversary of the gift.site of activity. The teachers and students of the sixth grade of Tongren Fourth Primary School set foot on the spring research activity with the theme of "Praise the centenary of the Party and Strive to be the New People of the Times", in order to present a gift to the 100th anniversary of the Party. Nearly 500 teachers and students walked into the New Era patriotism education base in Wanshan District to explore the history of mercury Capital and feel the care of the Party. This research activity enables students to understand and learn the "four histories" through practice, practice the core socialist values, let the red gene be implanted in the hearts of students, and let the red culture become the source of moral education.site of activity. Tongren No.22 Primary School launched a column titled "Party History Study", and the principal Wu Yujun organized all the teachers to study "Xi Jinping: The Governance of China". This activity, aims to let teachers learn history Ming, learn history increase credit, learn history Chongde, learn history practice; let the students listen, follow the party, inherit the red gene, strive to be a new person of The Times ideal ambition.site of activity."We are the successors of communism, inherit the glorious tradition of revolutionary ancestors, love the motherland, love the people, the bright red scarf fluttering in the chest..." early in the morning, into Tongren city 24 primary school, the students passionately sing the classic red song "we are the successors of communism", the students are using the morning reading time to carry out red song singing, reciting classic red stories and other daily party history activities, these activities make the students in singing and reciting while understand the red culture, the history of the brain into the heart.site of activity. The teachers and students of Tongren 29 Primary School face the morning light, with the warm breeze, and the school football field is ready to start a unique study tour. In the study, the tutor led the children to sing red songs, sing the national anthem, understand the story of the national anthem, receive patriotism education, remember the sweet, called on the children to study hard and make their own contributions to the prosperity of the country.site of activity. In order to stimulate all the teachers and students of the school to learn the history of the Party, listen to the party, feel the party grace, follow the party to go with the determination and enthusiasm. Through the party history study and education, carry forward the red tradition, inherit the red gene, and let the spirit of patriotism firmly rooted in the hearts of teachers and students. To this end, Tongren city 34 primary school red scarf radio station "party history run childlike innocence" came into being."Whenever I heard the school radio telling stories about the history of the Party, I would listen carefully. Gradually, I learned a lot about the history of the Chinese revolution and understood the hardships of the War of the Anti-Japanese War. We should study more seriously, " said the students who listen to the party history.site of activity. Correspondent for the picture of Daping ethnic primary school to carry out to " tell a good pioneer story. Inheritance red gene "as the theme of reading, story games, through the theme activities, greatly improve the whole school teachers and students learn party history, praise, follow the party's enthusiasm, for students set up the correct aesthetic concept, cultivate noble sentiment, set up the correct outlook on life has played a great guiding role in childhood, motivate everyone consciously with action practice" lei feng " spirit, to be an excellent young pioneers.site of activity. The primary school of Renshan School held a story-telling competition with the theme of "Red story for me, strive to be a good boy with red gene". By organizing children to tell the stories of the Party, revolution, heroes, and the stories of outstanding Communist party members around them, we can feel the original mission of the Party, enhance the love for the Party, and strengthen the belief of children to be the successors of communism.site of activity. Correspondent for the picture of the fish pond primary school to carry out the "childlike innocence to the party story I will tell" competition activities, the activity site, the students vividly told the red classic story learned."Create the People's Army", "Pan Dongzi", "Rain", "Liu Hulan", "Zhang Gaga" and other stories through the children's national self-esteem, self-confidence, pride; inspired the students' patriotic enthusiasm, determined the children love the party patriotic ideal and faith.

2021-05-26	Cangxi county, Sichuan province to carry out young talents dating fellowship activities in cangxi county, Sichuan province to carry out young talents dating fellowship activities on May 26 (reporter Zhang Jianwei, correspondent Yang Xuanping), group of Guangyuan city, Sichuan province Cangxi county joint organization department of the county party committee, the county federation of trade unions and other units in cangxi nan Yang hui open "cangxi 520 romantic" -in 2021 Cangxi young talents dating fellowship activities. More than 200 single young people from the county party and government organs and state-owned enterprises and institutions came to the scene, looking for good luck. Like the sky, the blue and white balloons and table flowers are not only full of a warm and romantic atmosphere, but also accompanied by a trace of cool summer. The male and female guests entered the venue in an orderly manner and sat opposite each other, and introduced themselves and met each other. Driven by the enthusiasm of the host, the guests entered the game, and the guests held talent competitions such as singing and dancing, as well as interesting interactive games such as "what does chewing gum stick" and "poker pass table tennis". The youth fellowship activities, to further implement the Sichuan province long-term youth development plan (2017-2025) file spirit, promote the county youth better growth, faster development, but also promote the county party history learning education "I do the practical work for the masses" series of activities to further, help youth marriage development, attract more outstanding young talent rooted in Cangxi, the construction of cangxi.

2021-05-27	Group of Anhui Dingyuan county to carry out the history of the party learning education knowledge test group in the party history learning education knowledge test jianwei correspondent, Yang Wanying Cai Kaihao) recently, to promote the youth party history learning education, further test learning results, strong learning atmosphere, to do theoretical learning harvest, ideology, Anhui group of the county party organizations and the party history learning education knowledge test, the county grassroots cadres and the young pioneers counselors. The test content covers the important time node of the communist party of China and the Chinese communist youth league, important meetings, important activities, as well as the basic party affairs, league knowledge, comprehensively test the history of the party learning education, to promote learning, to promote the purpose of the unity of knowledge, to promote the county party history learning education plays an important role. Dingyuan county grassroots cadres and young Pioneers counselors have said that the test content is comprehensive, most of the questions have a certain difficulty, need to carefully read the original work carefully, learn the original text, understand the principle to get the answer. Through answering questions, enhanced the mastery of party history knowledge. Since the development of party history learning education, the Communist Youth League County Party Committee has taken "online + offline", "collective learning + self-study", "go out + invite in" and other ways, constantly internalize the party history learning in the heart, externalized in practice, forming a good learning atmosphere in the whole county.

2021-05-14	Group of Henan fugou county to prevent network fraud into campus activities of Henan fugou county to carry out the "prevent telecom network fraud" into the campus activities) in order to further enhance the youth security awareness of the new network fraud, on May 12, Henan fugou county county education bureau, public security bureau, procuratorate to carry out the "prevent telecom network fraud into campus" activities into fugou county JiHongChang middle school, to prevent telecom network fraud theme education. At the lecture, the volunteer speakers popularized the knowledge of anti-fraud, shared practical cases, and analyzed the common mode and means of fraud. The "prevent telecom network fraud" publicity into the campus activities, to the students widely popularized the common knowledge to prevent telecom network fraud and encounter fraud processing methods, to always maintain security awareness, protect personal privacy, away from bad information, calm analysis, to prevent telecom fraud.

2021-05-25	Group si county in Anhui province county party committee to carry out the "dandelion lecture hall" into the campus activity group of Anhui si county county party committee to carry out the "dandelion lecture hall" send method into the campus activities JianWei correspondent FanYanCai Kaihao) recently, the group of Suzhou city, Anhui province see county county joint see county people's procuratorate to yucai school to carry out the "dandelion lecture hall" send method into the campus activities, nearly 2000 students to participate in the activities. Dou Wenhui, a young prosecutor, delivered a legal report on the theme of "distinguish roles and refuse to play school violence". The picture shows the scene of the lecture activity."Class, have you ever seen the cartoon film Doraemon?"" to take a look at!"In order to quickly lead everyone into the campus violence situation, the prosecutor chose to use a classic animation work to introduce the propaganda theme, quickly found the" common language "with the students, opened everyone's" chatterbox "."You know who Nobita is always being bullied?"" Fat tiger!"Next, the prosecutor still asked the students questions about the experiences of the leading characters in the animation, leading them to gradually learn the classification, role, harm and how to avoid school violence step by step."Calm, brave, clever deal, abandon money, out of danger... timely report."At the end of the activity, the prosecutor led all the students to read the" 40-character anti-bullying Law "together, to consolidate the propaganda results again, education and guide everyone to resolutely say" no " to the school violence. The picture shows the volunteers distributing leaflets on youth safety and self-care to the participating students. Before the meeting, the volunteers distributed more than 2,000 youth safety self-care leaflets to the students, and introduced the key points of fire prevention, anti-virus prevention, anti-drowning, anti-violence prevention, anti-abduction and anti-fraud knowledge, etc. In the next step, Tusi County Party Committee will continue to work with Sixian Procuratorate into more schools, "childlike innocence harbor" and "youth home", carry out the "Dandelion Lecture Hall" legal publicity project, and promote the practice of youth safety self-protection and crime prevention work.

2021-05-08	Kaili held "red hearts to praise the party in one hundred" young pioneers work watch Kaili held "hearts to praise the party in one hundred" young pioneers work watch work watch and "red scarf medal" activity launch ceremony China youth network Beijing on May 8 (reporter Qin Liang correspondent Pan Shuhong) to celebrate the 100th anniversary of the founding of the communist party of China, the 102nd anniversary of the May 4th movement, while promoting the young pioneers reform, on May 6, Guizhou kaili "red to praise the party in one hundred" young pioneers work watch and "red scarf medal" activity launch ceremony. More than 140 people participated in the activity, including relevant leaders of Qiandongnan Autonomous Prefecture Party Committee, members of Kaili Education Youth League Working Committee, and counselors of all primary and secondary school brigades in the city. In Kaili No.4 Middle School, the delegation listened to the reform work of the Young Pioneers in Kaili City, observed the theme squadron meeting of "Celebrating the centenary of the Founding of the Party", participated in the selection activity of "I am a red scarf speaker- -my Party History story", and voted for the small speaker. The middle school has been strengthening the party building leading the league building leading the team building, adhere to do a good job of team connection, standardize the team building, leaving the team ceremony, held the theme team meeting and other work. In Kaili No.4 Primary School, the delegation visited the school red education base and the young Pioneers' room, and listened to the experience introduction and implementation of Kaili No.4 Primary School in "Batch Joining the School". Through learning advanced and typical methods, the young Pioneers work observation will have effectively strengthened the construction of the young Pioneers counselors, further enhanced the cohesion, combat effectiveness and appeal of the young Pioneers organization, and comprehensively promoted the integration of the Party, the Youth League and the team. Subsequently, kaili city "Red scarf medal" medal activity launch ceremony was held in Kaili No.4 Primary School. As a new starting point for the work of the Young Pioneers in Kaili City, the launching ceremony of the medal competition activity will boost the reform of the evaluation mechanism of the Young Pioneers in Kaili City, and enhance the sense of honor of the young Pioneers.

2021-05-25	Group in Anhui province district party committee leave league theme TuanRi group in Anhui province district party committee leave league theme TuanRi activities youth network Beijing on May 25 (reporter Zhang Jianwei, correspondent, pan dan-dan pan CAI Kaihao) recently, group in Fuyang city, Anhui province district party committee in Fuyang city 18 middle school successfully held "remember one hundred youth dream in the future" -to celebrate the 100th anniversary of the departure league theme TuanRi activities. Lu Yunlong, secretary of Youth Yingzhou District Committee, Yin Shu, part-time deputy secretary of Communist Youth League District Committee, secretary of District Education Youth League Working Committee and chief of District Education Bureau, Shen Dawei, secretary of the General Party Branch and principal of Fuyang No.18 Middle School, and more than 400 students from 18 Middle School participated in the activity. The picture shows the participating leaders presenting the league member certificate and wearing the league emblem to the new league members. The ceremony is divided into three chapters: "bidding farewell to childhood and setting sail", "inspiring youth, flying dreams" and "a hundred years of brilliant red heart to the party". In the dance "We under the Begonia Tree", the ceremony began. After playing the national anthem, Shen Dawei, secretary of the Party of Fuyang 18 Middle School, delivered a warm speech. Subsequently, through the team flag, call sign, treasure red scarf, withdrawal flag and other ceremonies, the young pioneers over the age of 14 left the team, officially bid farewell to the golden childhood, and entered their youth. During the joining ceremony, Hou Yan, the youth League secretary of the school, announced the list of new members. The leaders attending the meeting issued the league member certificate and wore the league emblem to the new league members. The new league members took the oath to the league flag under the leadership of the secretary of the school Youth League Committee. 50 outstanding students joined the Chinese Communist Youth League with honor.

2021-05-24	Group in Anhui province Fengtai county micro public book rafting activities in Anhui province county to carry out the "knowledge inheritance love" micro public book rafting activities in Beijing on May 24 (reporter Zhang Jianwei, correspondent, Hu Yuxian Cai Kaihao), by Huainan city, Anhui province fengtai county joint fengtai county bureau of education, Fengtai young entrepreneurs chamber of commerce, fengtai small fish net and other units of "knowledge inheritance love" micro public books rafting activities into the central school, donated books, students milk and learning package, etc., pass love and warmth, Hold up a clear sky of knowledge for rural students. The picture shows a group photo of the donated children and the caring people. At the donation ceremony, Fengtai Xiaofish net love netizens, excellent training and excellent counseling center, Antai company, love active taekwondo, sunshine baby children's photography, Huainan Canhang trade and other love enterprises donated more than 700 books, 100 puzzle toys, 20 mosquito lights and 20 boxes of milk for students. Representatives of caring enterprises presented books to the children and guide them to read. The relevant responsible comrade of the Communist Youth League County Party Committee said that in the future, more such love activities will be carried out in order to help the rural poor students realize more wishes, so that the children can feel the warmth and care of all walks of life. I hope the children can learn more knowledge from the books.

2021-05-21	Ling city in Shandong province middle school students leave the demonstration ceremony in Shandong ling city "learning party history, strong faith, follow the party" leave the league demonstration ceremony in Beijing on May 21 (reporter Zhang Jianwei, reporter gao lili) recently, Shandong province Dezhou city ling city middle school students leave the demonstration ceremony held in ling city experimental middle school. The Communist Youth League District Committee, the relevant responsible members of the District Education and Sports Bureau and the principal of the experimental middle school, the secretary of the Communist Youth League Committee, the new league members and more than 600 teachers and students of the experimental middle school witnessed this solemn moment. The picture shows the new league members taking off and collecting the red scarves. The ceremony opened the ceremony in the national anthem, then the team flag, the young Pioneers representatives led the team members to recall the happy time accompanied by the red scarf, presented the lofty team ceremony, singing the most beautiful team song, treasure the red scarf fluttering on the chest, quit the team flag. The young pioneers over the age of 14 left the team and officially entered their youth. The picture shows the new league members taking a solemn oath. The leaders attending the meeting issued the league seal and league member certificate for the new members, the old members wore the medal of honor and responsibility for the new members- -the league emblem, 33 new members solemnly swore to the league flag, with practical actions to show that they are worthy of the glorious title of the Communist Youth League. With "Glory, The Communist Youth League of China", the demonstration ceremony of "learning the history of the Party, strengthening faith and following the Party, leaving the Party" and joining the League came to an end. After the ceremony, students from the experimental Middle School brought a poem reciting "Ode to China" and singing two programs called "Follow the Communist Party" to present a gift to the 100th anniversary of the founding of the Party. During the activity, Zheng Wenjie, a member of the Lingcheng District publicity group and a member of the District Human Resources and Social Security Bureau, was invited to preach on the theme of "learn the history of the Party, strengthen faith, and follow the Party" to all the students.

2021-05-21	Group in Anhui provincial party committee held party history of youth reading sharing group in Anhui province party committee held "read the history of the party, keep the beginner's mind" mirror lake youth reading sharing jianwei correspondent Wang Yue Cai Kaihao) recently, group of Anhui province Wuhu mirror lake straight authority working committee held in Wang Jiaxiang memorial park "read the history of the party, keep beginner's mind" mirror lake youth reading sharing (second), OuZhi authority youth party members and members participated in the activities. The reading club, with the theme of "Youth to the Party and power", includes visiting and learning, online group classes, feeling sharing and sending messages to the youth. At the beginning of the activity, the representatives of the party members and league members visited the Wang Jiaxiang Memorial Park, reviewed the glorious struggle course of the revolutionary ancestors, and reviewed the oath of joining the Party and joining the Youth League under the leadership of Xu Longbin, the secretary of the working Committee of the organ. Afterwards, the youth representatives watched the online theme group class of "Seeking Faith in the Historical Footprints" taught by Teacher Gao Yanjie from Anhui Normal University. By understanding the story of the revolution in Jinghu, they thought deeply about the great power of faith in depth. Eight young representatives selected part of the original content to lead everyone to learn, and talked about the reading experience combined with the actual work, which aroused the strong ideological resonance of the young people present.

2021-05-20	Shandong city "red scarf" volunteer family "hope hut" in Shandong city "red scarf" volunteer family "hope hut" network Beijing on May 20 (reporter Zhang Jianwei, correspondent gao lili) to further implement the party history learning education requirements, to carry out the "I do the practical work for the youth" practice. After preliminary recruitment, the Lingcheng District Committee of Dezhou City, Shandong Province, recently organized eight "red scarf" volunteer families to join hands with the "Hope House", contributing to the "red scarf" to help more children in difficulties improve their learning and living environment. This visit aims to lay a foundation for further volunteer activities and achieve specific assistance to each family in combination with the current situation. The picture shows visiting children in distress. Correspondent for figure "red scarf" volunteer family has been built, proposed "hope" help children visit, for "hope" family to fruit, milk and other daily necessities, to troubled children on school supplies, close interaction with them, detailed understanding of the children's daily life, learning, listen carefully and do their best to help them wish. At the same time, the "Red Scarf" young volunteers also play games together with the children to accompany the troubled children in the "Hope House". Next, ling city district party committee will continue to strengthen the tracking maintenance of "hope hut" maintenance, promote "peer light hope" in hand "-" red scarf to build hope hut "project, continuous gathering social love power, recruit more volunteer family, regularly to children care company, learning counseling, psychological counseling support activities, cultivate children self-reliance, optimistic attitude towards life, from" cabin refresh "to" spirit refresh ".

2021-05-20	In Anhui province held the paintings in one hundred young pioneers theme activities in Anhui province held "childlike innocence to the party painting in one hundred" young pioneers theme activity figure of China youth network Beijing on May 20 (reporter Zhang Jianwei, reporter correspondent Wang Xuan Cai Kaihao) to celebrate the 100th anniversary of the founding, strengthen the consciousness of patriotic love party, inheriting the excellent traditional culture of the Chinese nation. Further create the activity atmosphere, strengthen the sense of honor of the young pioneers to be "good members in the new era". Recently, 100 young pioneers from all schools in Bagongshan District gathered on the playground of Bagongshan District No.5 Middle School, together to participate in the "childlike innocence to the Party, one hundred paintings to welcome a hundred years" site 100-meter painting and calligraphy activities. This activity is jointly organized by the Communist Youth League Bagong Mountain District Committee, Bagong Mountain District Education and Sports Bureau, and Bagong Mountain District Women's Federation. This painting and calligraphy activity aims to inherit and carry forward the traditional Chinese painting and calligraphy art through the development of children's painting and calligraphy creation, show the cultural characteristics of their hometown in the eyes of the children, stimulate primary and middle school students to love the motherland, love their hometown emotion, for the 100th anniversary of the founding of the Communist Party of China. The activity not only provides a stage for the students who love painting and calligraphy to show their talents and show themselves, but also promotes the construction of a harmonious and civilized campus in Bagongshan District. Event, the students around the "to celebrate the 100th anniversary of the founding of the communist party of China" this theme, or her, express, describe the warmth of the family scene, show the beautiful picture of home, also praise love and dedication, with childlike innocence of painting and calligraphy works, a realistic picture, close to life.

2021-05-20	Pingyuan county in Shandong province each middle school organization entrance examination decompression activities high school organizations in Shandong province Dezhou plain entrance examination for secondary school decompression activities JianWei correspondent, Feng Yuanyuan Li Yuzhe) entrance examination for secondary school or college approaches, recently, to help the students relieve the pressure before the exam, Shandong province Dezhou Pingyuan county each middle school youth corps committee carried out various forms of entrance examination for secondary school or college decompression activities, help students to adjust the psychological state, morale. The picture shows the students carrying out the activities in the psychological counseling room. Correspondent for the first middle school through the mobilization meeting, open reception student counseling room, to carry out pre-examination psychological counseling activities. Guide students to carry out physical relaxation training, help students release pressure, let students face the study and life with a sunny attitude. The picture shows the plain county second middle school sprint examination mobilization meeting. Correspondent for the picture of the ninth grade of all the teachers and students of the second middle school held in the school gymnasium "I can do what I take action I succeed-gather force forward sprint high school entrance examination mobilization meeting". Psychological counseling teachers through experience, games and other ways of psychological counseling, stimulate students' initiative and enthusiasm, experience and perception in the game, cultivate the spirit of teamwork and perseverance, to help students to meet the test easily and confidently. The picture shows the psychological decompression counseling scene. In the next step, the Communist Youth League County Party Committee will continue to serve the youth mental health education, innovative publicity and education forms, with the help of "youth Plain" and other new media platforms, actively push decompression and other skills and knowledge, to help the majority of candidates to calm the mind to meet the exam.

2021-05-20	Group in Anhui province east to the county to carry out hope bag distribution and spiritual growth public class group Chizhou city in Anhui province east to the county to hope project hope bag distribution activities and youth spiritual growth public class Jianwei correspondent Su Rui Cai Kaihao), Chizhou city in Anhui province east to the county united county hope public service center to carry out the "student education celebrate founding one hundred green love peer send hope bag" 2021 hope project hope bag distribution activities and youth spiritual growth public class. TuanDongzhi County Party Committee, Dongzhi County hope public service center, love enterprise Anhui Zhushiling Health consulting Service Co., LTD., and 3 volunteers participated in the activity. During the activity, the staff is divided into two groups simultaneously. To each school, to the children briefly introduced the hope project "hope bag" project and content, and encourage the children to study hard, aspire to become; cultivate morality, cultivate character; learn to behave from childhood, know how to be grateful, good exercise and strong body; wish all the children healthy and happy growth. Subsequently, the volunteers gave the children a public welfare class for youth spiritual growth, or for the children participating in the activity on a simple, vivid and interesting mental health education open class, or led the children to carry out a lively and happy quality development extracurricular activity class. During the teaching process, the volunteers interacted with the children on the spot, and guided the children to solve their own psychological defects with a positive and cheerful attitude, so as to help the children establish healthy psychological values. During the activity, the two teams visited 11 schools in 9 towns and townships, distributed 867 hope school bags, and carried out 11 public welfare classes for spiritual growth.

2021-05-18	Pingyuan county, Shandong province cadres to carry out the party history learning education Shandong Pingyuan county cadres to xibaipo, positive ding county yuanzhuang village party history learning education China youth network Beijing on May 18 (reporter Zhang Jianwei, correspondent, feng Li Yuzhe), Shandong Dezhou Pingyuan county organization county part of the communist youth league cadres for one hundred history, to Chinese revolution xibaipo, positive definite county yuanzhuang village party history learning education, guide the communist youth league cadres learning revolutionary spirit, firm ideal faith. On the same day, the cadres came to Tayuanzhuang Village of Zhengding County to visit and learn the "Tongfu Smart Farm" and the construction of the new countryside. Through the study, the cadres learned about the great changes in Tayuanzhuang Village in recent years. During the study, the league cadres visited the Xibaipo Memorial Hall, the site of the second Plenary session of the seventh Central Committee and other important revolutionary historical sites, in-depth understanding of the glorious history of Xibaipo and the advanced deeds of the older generation of revolutionary martyrs, review a wonderful and gorgeous, magnificent historical picture scroll.

2021-05-17	Group of Anhui Huangshan district party committee to carry out the youth since protecting education theme activities of Anhui Huangshan district party committee to carry out the "youth" youth since protecting education theme activities JianWei correspondent Ye Qing Cai Kaihao) to further promote the construction of peace school, harmonious campus, popularize knowledge of earthquake disaster reduction, improve the emergency response ability, ensure the life safety of all the teachers and students. Recently, the Youth League Huangshan District Committee of Huangshan City, Anhui Province, organized the Huangshan No.1 Middle School and Tangkou Central School to carry out an emergency evacuation drill of "earthquake prevention and disaster reduction". The picture shows the orderly evacuation of teachers and students during the emergency drill. Correspondent for figure at 9:40 points on the day, in soup central school, the bell just rang, a rapid alarm, the students immediately in the corner of the classroom, table head squat down emergency hedge, 20 seconds after danger alarm, the students under the command of the teacher quickly left the classroom, according to the class columns, head, low chest, cat waist respectively from both sides along the corridor quickly and orderly evacuated to the school playground. The whole evacuation drill process only took 1 minute and 08 seconds, and all the teachers and students were tense, fast, and orderly, and successfully completed the task of the emergency evacuation drill. The picture shows the staff distributing the emergency knowledge publicity materials to the students. In the process of the activity, the scenic area fire brigade staff also used vivid language and practical cases to popularize the knowledge of earthquake prevention, emergency, rescue and other aspects, and distributed fire safety knowledge brochures, mountain flood disaster prevention knowledge, home fire manuals and other more than 300 copies.

2021-05-14	Linyi city luozhuang district youth clock red landmark Linyi LuoZhuang district youth clock red landmark morning correspondent Tengpiety) Linyi city luozhuang district party committee to further implement the "party history, strong faith, follow the party" learning education requirements, to carry out the "red landmark" "qingming festival heroes" "the party's story" I " theme outstanding, distinctive, various forms of education activities, guide teenagers to listen to the party, feeling DangEn, go with the party. Linyi No.5 experimental primary school brigade committee organized the young pioneers to the Menglianggu campaign memorial hall to remember the revolutionary martyrs, more than 200 young pioneers to play red songs to pay tribute to the martyrs, review the team oath to let the young pioneers remember the martyrs legacy. More than 300 young pioneers from Linyi No.40 Middle School walked into Yimeng Red landmark- -Fei Daqingshan Red Education Base to explore red resources and feel the revolutionary spirit at close range. Linyi seventh experimental primary school is formed with the young pioneers as members of the red studies practice team, around the "explore red footprint" and "Thanksgiving martyrs" action ", clock in the east China revolutionary martyrs cemetery and Linyi new fourth army headquarters, in the" party history, strong faith, follow the party " red studies further comprehend the party's revolutionary spirit, the young pioneers planted red seeds in the heart. Since April, when the learning education of Luozhuang District of Linyi City launched "learning the history of the Party, strengthening faith and following the Party", it has carried out more than 50 activities such as finding red footprints, watching red movies and reading revolutionary poems, which have enriched the red knowledge reserve of teenagers and made them feel the power of red faith.

2021-05-14	Group including Guizhou wanshan district party committee to carry out youth sharing salon activities group wanshan district party committee to carry out the "new youth, new fashion, the history of the party, go with the party" youth sharing salon activities zhang correspondent Yang han) recently, group including Guizhou including wanshan district party committee to carry out the "new youth, new fashion, study, party history, go with the party" youth sharing salon activities. More than 30 people, including youth representatives, urban school youth League leaders, young Pioneers counselors, and volunteers from the west, attended the event. In the activity, the old party members first described their own stories to describe the history from the brilliant decline to the policy closure of the mercury mine to the rise of the "two transformations", showing the spirit of the cadres and the masses of Wanshan to take responsibility and contribute. Subsequently, seven young representatives told you the red story of the revolutionary ancestors. Everyone in the scene sang "Glory, The Chinese Communist Youth League", and compiled the Chinese knot together.

2021-05-09	Sichuan Pengshan: Young Party members team to grab agricultural time Sichuan Pengshan: Young Party members team to grab agricultural time youth network reporter Wang Xinxin) " Thank you young doll to help me insert seedlings, otherwise this year can miss the farming time."On May 7, the season of" double robbery ", a group of young helpers visited the farmland of Wang Jincheng, a veteran Party member in Lijia Street, Pengshan District, Meishan District, Meishan City, Sichuan Province. On the same day, more than 20 young Party members from the Communist Youth League Pengshan District Committee and Lishan Village General Party Branch of XieStreet rolled up their trousers, rolled up their sleeves, pulling seedlings, transport seedlings, seedlings, seedlings... young Party members full of energy, the scene is busy. The picture shows the young party members planting rice seedlings in Wang Jincheng's field. Wang Jincheng, more than 60 years old, is a veteran Party member in Lishan Village, his father and wife are ill, and his son work outside all the year round. The family only has more than 4 mu of farmland, Seeing the "rush to plant" season is almost over. The local youth league organization learned of this situation, and immediately united with the Lishan Village General Party Branch, Xiejia Street, organized more than 20 young party members to come to Wang Jincheng field to help them plant rice seedlings. In just three hours, Wang Jincheng's paddy field was covered with green "new clothes".

2021-05-08	Group of Shandong plain county safety into the campus, into the community activities of Shandong plain county safety into the campus, into the community activities correspondent, Feng Yuanyuan Li Yuzhe) to further enhance the safety awareness of children, improve children for drowning, fire accident prevention and control ability, recently, the group of Shandong province plain county joint county yi Qi public welfare service center to carry out the "safety into the campus, into the community" activities, organize volunteers against drowning and fire safety propaganda and education. In class, volunteers played videos of drowning to understand drowning behaviors and where. In the process of telling, volunteers invited students to participate in the closed breath challenge and feel the feeling of suffocation during drowning. Volunteers came to Longmen Street Jubao community to carry out fire propaganda into the community activities, to popularize fire prevention knowledge for the children. During the activity, the volunteers systematically explained the fire safety knowledge to the children through "theoretical explanation + classroom practice", including the fire characteristics and hazards, fire response and prevention. The lecturers guided the children to wear gas masks, and the children actively participated in the classroom interaction. The fire publicity into the community activities, to the children widely popularized the home fire prevention knowledge and escape and self-rescue skills, improve their fire safety awareness and the ability to deal with emergencies.

2021-05-07	Group of Huaibei city of Anhui province Suixi county held rural youth talent exchange activities in Huaibei city of Anhui province, sui xi county held rural youth talent exchange activities correspondent Hou Dong Cai Kaihao) on May 2, group of Huaibei city of Anhui province sui xi county, four shop town government in sui xi county four shop town sui garden organization to carry out the "fire inheritance youth dream" suixi county rural young talent exchange activities in 2021. Tuan Sui Xi County Party Committee, Siping Town and more than 60 rural young people from Siping Town attended the activity. Activities to read play "red iron" opening, for three four shop town home young entrepreneurs interviewed, returning entrepreneurial youth to share their own entrepreneurial experience, they are active in rural revitalization, promote economic development, for the hometown people provides more nearby employment jobs, become four shop town rural revitalization of the youth "leader". Subsequently, all the participants held a collective initiative, advocate the change of customs, promote the new style of civilization, highlight the new concept of civilization, create a positive energy of youth, and jointly build a civilized hometown. During the activity, the young people participated in the activities such as "shaking hands", beating the drum and passing flowers, group hug, youth dare to "grab", to complete the team tasks together, close the distance between each other, open their hearts and show themselves. In the next step, the Youth League Suixi County Party Committee will continue to do a good job in young rural talents, expand the channels for young people to find employment and entrepreneurship and make friends, guide them to establish a healthy and progressive view of career and marriage, and unite and gather young people to actively help rural revitalization.

2021-05-06	In Heping Primary School in Yaohai District, Hefei city, the Young Pioneers' environmental protection wish card holds to protect the environment and protect the Earth around the theme poster of Earth Day. Recently, in the online red scarf micro class, accompanied by video narration and graphic introduction, the young pioneers of the district had a vivid party history education class. Recently, the committee, with revolutionary events and heroic stories, has invited external counselors, college student volunteers and reading club social organizations to record micro-team lessons, radiating thousands of young pioneers inside and outside the area. In 2019, the Youth League Working Committee of Hefei Economic Development Zone established the community Youth Working Committee with Furong Community as a pilot, set up the working mode of "guidance of the community Party and league leaders + participation of the whole process of the young Pioneers counselors in the school", and built a practical education platform for the team members. Furong community young working committee deputy director Hao Cheng introduced, the community has created a "five-color youth heart to the party" brand project, including the red pioneer group, green environmental protection group, orange care group, blue management group, purple culture group and other projects. More than more than 80 activities, such as "I Draw the National Flag for the Motherland" and "Strive to be a small guard of environmental protection", with a total of more than 9,000 participants. Ding Xinyue, a student from Hefei 168 Rose Garden School, participated in the "Civilization with me" project. He compiled the knowledge of garbage classification into a small ballad, and set up a publicity team with his team members to publicize garbage classification knowledge in the community."Community is an important activity position for teenagers besides school and family. We should break the boundary between school and age, and innovate a new mode of community activities suitable for the growth characteristics of young pioneers."Anhui province young working committee staff, in recent years, Anhui to expand the young pioneers organization and activities channels, in the community, rural, youth palace and other places to establish a brigade or squadron, promote the young pioneers activities from the school to the community. At the beginning of this year, Anhui Xuancheng Dongqiao Community Youth Working Committee was established, relying on the city's children service station project and site, actively carried out the "red scarf medal" work and "four history" learning and education activities, to build the young Pioneers after-school work system. At the end of April this year, Hefei Lu 'an Road Primary School Rongcheng Garden Branch School Youth Working Committee and the fourth bureau of the first company Youth League Committee to Chuning intercity railway project department, listen to the youth about the history of China's railway construction. The young Pioneers personally sent their own workers-themed cartoons to the site builders. Since last year, after the establishment of Tianjing Lake Community, Tongguan District, Tongling City, the working mode of "the community takes the initiative to build the platform, educational resources out of the campus, young pioneers and parents out of the house", and carries out young pioneers activities suitable for the characteristics of children after school. Among them, the Tianjing Lake Community Youth Working Committee opened a "colorful summer growth hand in hand" children's summer camp. The summer camp offers professional tutoring in the morning, and in the afternoon, it offers five interest classes: children's calligraphy, children's go, small host, fun English, and children's taekwondo. The Happy Community Youth Working Committee invited senior experts in the radio industry to teach the children the professional knowledge of radio and related red stories, organically combining patriotism education with science popularization, leisure and educational elements. Huang Xiaoli, a member of the Tianjing Lake Community, said that the establishment of the after-school young working committee can help teachers to do a better job in training children, so that the young pioneers can effectively participate in, integrate into the community work and community services, and learn life skills. May 06,2021 Version 04

2021-04-28	Dafang county held to commemorate the "May 4 th" poetry recitation contest generous held the "party history" and commemorate the "May 4 th" poetry recitation contest morning) on April 25, the communist youth league generous county held "party history, strong faith, follow the party" and commemorate the 102nd anniversary of the May 4th movement poetry recitation contest, entered the final of 20 outstanding teams to participate in the competition. Competition, the team topic selection originality is unique, the players chanting passionate, forceful, through the use of creative video, accompanying dance, scene props, the vivid interpretation of red classics, the work behind the emotional experience and cultural memory better passed out, show a generous youth inheritance red genes, open the era of youth journey. After more than two hours of wonderful competition, dafang siyuan experimental school "youth China" won the first prize, dafang county experimental high school "never disappear", Guiyang one golden school "red flag fluttering" won the second prize, three yuan middle school "party history, feeling, with the party have red boat spirit", dafang county first middle school "strong faith, xu party soul", Evergrande national middle school "one hundred journey, don't forget to beginner's mind" won the third prize, dafang county third middle school and other four team works won the excellence award.

2021-04-28	Ren Chen, secretary of the State Street Youth League Working Committee, came to the Wulou village committee to communicate with the village cadres. Since August 2019, Ren Chen has been the responsible person for Hu Hongying's help and her daughter. When she first walked into Hu Hongying's home, her home was chaotic, and Ren Chen was greatly impacted. Ren Chen actively connected with the Poverty Alleviation Office and the civil affairs department to help Hu Hongying apply for a severe disability certificate and enjoy the rural pension benefits five years in advance. Hu Hongying's daughter Wang Caixiang was identified as a secondary mental disability, who was included in the subsistence allowance and enjoyed the disability subsidy. Usually fruit, New Year's Eve dumplings... Ren Chen to "small" care, let the mother and daughter feel "big" warmth. At present, China has comprehensively succeeded in fighting poverty, and rural revitalization is a major task to realize the great rejuvenation of the Chinese nation. Jiuzhou Sub-district will shift the poverty alleviation work system to rural revitalization, and take concrete actions to make the foundation of poverty alleviation more stable and the results more sustainable.

2021-04-27	"Youth night school" for rural talents to make up lessons under a tired, a busy day of young people with notebooks from all directions came to class. Among them are the young entrepreneurs who are busy in the fields, and some township cadres who go from street to street in rural work. In Tianliu Town, Weifang, Shandong Province, a local "youth night school" is set up for the growth of these rural talents. It is called "local" because the "youth night school" targets the young people who are rooted in the countryside and are willing to struggle in the countryside. The students include not only town cadres under the age of 40 years old and members of the two village committees, but also young people who have returned to their hometowns in the fields of cultivation, breeding, education and training."The advantages of young talents are active thinking, have a wide range of knowledge, passion, and vitality, and have a strong willingness to participate in the management of village-level affairs. But weaknesses are also obvious, such as lack of experience in rural work and the ability to deal with complex things."Said Dong Yongliang, Party secretary of Tianliu Town. He will not deal with the common people, does not know how to deal with the transition of the village two committees and the comprehensive improvement of rural areas, and understands the latest rural policies... the lack of skills of these young talents worries Dong Yongliang, who has been working in rural areas for 16 years. In Dong Yongliang's view, the original intention of opening the "night school for youth" is to "let these young people make up for the lesson of growth and reserve their youth strength for rural revitalization". In this "youth night school", most of the courses are set around rural work, which is also a strong "local" flavor. The teaching content includes more than 20 courses, such as "Some Thoughts on the Change of the Two Rural Committees", "Dealing with Rural Work with the Rule of Law" and "Land Business Knowledge". Considering the needs of the development and improvement of young talents, we also specially arranged the written examination and interview skills for civil servants and public institutions. On March 1 this year, "youth night school" officially opened, once a week, the course has been scheduled until August. To tell the truth- -from the very beginning, the "youth night school" set this unwritten rule. On the platform is a rich rural old party work experience cadres, small to the tone of talking to the people, big to the urgent, difficult and dangerous work, vivid cases at hand, greatly improve the enthusiasm of the students. Yuan Qing, a 29-year-old township official, had the opportunity to apply what he had learned. For the first time to participate in the transition and take charge of the voter identity check work, Yuan Qing believes that the "youth night school" let me "have confidence in learning.""We looked forward to the night school early before Monday."Cui Murray, 33, who has been assistant to the branch secretary of Cuijia Village for two years, admitted that he was himself as a primary school student. Tian liu town mayor Jin Shengkai introduced, now, the town has established the young talent party branch and young talent alliance, further smooth outstanding rural youth to join the party channel, for the rural grass-roots party organizations to transport young blood.

2021-04-27	The "4:30 Class" of Hui Hui received a nickname of "Xiao Hui Hui" last year. The cordial name was given to him by a group of pupils in their teens. Every day after school, these children, carrying their bags, walk into the "4:30 class" that Liu Hui is responsible for on time, take out their books, and spend the time before going home here. In 2019, the government of Shilou County relocated 174 natural villages to agou Village, Lingquan Town, and 860 families went out of the small mountain village and lived in buildings, becoming new citizens of the "Longshan Waterfront" community. Among the 3,400 new citizens, more than 120 pupils' parents mostly work in farmers. Every afternoon, some fail to leave their homes and have to "herd sheep" outside. Some are left-behind children who live with their grandparents for years. How to help their children with their study has become a worry for many parents. Children's living conditions are better, but how can they meet their spiritual needs? In order to integrate the children relocated from inhospitable areas into the new environment and improve their academic performance, in May last year, the TuanShilou County Party Committee and the Shilou County Youth Volunteers Association opened a "4:30 class" in the "Longshan Waterfront" community to provide public space for the children to do their homework after school and during holidays. This is the first "4:30 class" in the relocation community of Shilou County. Liu Hui is the head of the 4:30 class. A few years ago, when he formed a volunteer team with several young people to visit poor families in some villages, he saw that many children could only do their homework at a small dinner table. Some children lived with their grandparents and didn't know who to explain when they did not understand the problems. These children have a common problem- -the weak foundation of cultural courses. Some children go to primary school, but even 3 + 2=5 such a simple arithmetic problem is difficult to understand. Many families used to be in poverty and could not afford to pay for remedial classes for their children."4:30 Class" is called "Youth Community Space", with more than 100 square meters of room in a library, psychological counseling area and activity space. Some of the volunteers are from local volunteer teaching teams, who give lectures during the day and go to the "4:30 class" after class to help them with their homework and carry out sports and sports activities. Liu Wenwen (not her real name), 11, is one of the first students to come here. She has been living with her grandparents, who were at average grades and couldn't help solve problems, and sometimes couldn't finish her homework. After coming to the 4:30 class, two volunteers helped her explain her homework every night. She doesn't have to bring home her unfinished homework. Within a year, her academic performance rose to 15th place in her grade. The biggest change is the child's character Xie Yifan lives in Shilou County in Changzhi College normal major freshman. This winter vacation, she volunteered at the 4:30 class class. At first, she thought the "4:30 class" was like a nursery class, where the children came from school for a while, the volunteers watch it and not let the children happen. But when she went, her mind changed. On the 15th day of the first lunar month this year, volunteers in the 4:30 class led their children to cook dumplings together. Liu Wenwen handed the first dumplings in her bowl to Xie Yifan. She was instantly touched."There are a lot of touching things moving."One noon during the National Day holiday last year, Liu Hui went out to do business, it was very hot, out of the community not far, there were two children behind him, while calling" little Hui Hui ", while handed him a popsicles. Liu Hui was moved to tears. Liu hui felt that the "4:30 class" not only improved the children's grades, but also changed their personalities. Some children used to live a loose life in the village, although their parents were sent to the "4:30 class", but they ignored the volunteers; some children did not have good living habits, and even go to the toilet directly in the corridor and yard. To this end, Liu Hui and the volunteers helped the children develop good habits by rewarding the points. As time passed, they took the initiative to maintain the hygiene here and clean up their activity area when they left. The children have found their friends here. One day, a student quietly reminded Liu Hui that "his class has a birthday". Later, with the help of Liu Hui, the children prepared a cake and sang the birthday song in the 4:30 class class."We focus on celebrating our birthdays that month."Liu Hui said. In less than a year, more than 6,000 people have signed in at the gate of the 4:30 class. Now, there is no need for the staff to open the door, the children will consciously come together when after school time, find out the key hanging in a specific place to open the door to study activities, some children stay home until 10 p. m. Xie found that these children were more flexible and quick than they were young. They would not only ask "why", but also ask if there were other ways to solve problems ". Outside of schoolwork, children gradually opened up and share their thoughts with volunteers. Xie Yifan is often asked "what the college life is like". Because reading is a normal major, Xie Yifan's ideal is to be a teacher. This volunteer experience made her feel the difficulty of education in advance. For example, meeting naughty children needed ways to communicate."Communication requires skill, or it will be counterproductive."These were something the school had not yet taught her yet. Students who expect more professionals to do not attend the 4:30 class are the target of Liu and the volunteer program. Liu Hui plans to visit to understand the real reason why they did not participate, hoping to help more children in need with the "4:30 class"."Of course, our volunteers also need to be more professional."Liu Hui said that the small" 4:30 class "needs not only volunteers who can help their children with homework, but also volunteers who are good at psychological counseling, so that more children can come out of the" hidden corners " and embrace more care. Liu Hui often thinks about the difference between rural children and urban children, and believes that cultivating extracurricular interests is the key. He expects "4:30 class" kids to be as versatile as town kids. To this end, in the last year, "4:30 class" opened cultural activities on weekends, including art, calligraphy, music and so on. Liu found that the courses were more interesting to children, "more people than usually come to do their homework". Liu Hui believes that some children have a weak academic foundation. If they can have a good skill, even if their academic performance is not ideal in the future, there may be other paths for growth and development. He hopes the 4:30 class will inspire children's imaginations, stimulate their potential and nurture talented teenagers. The changes and smiling faces of the children prompted Xie to decide to continue to volunteer this summer. She hopes to take her children to see more movies and share more feelings about growing up. China Youth Daily China Youth Network reporter Ning Di source: China Youth Daily

2021-04-23	Shanghai Xuhui: China Youth Daily (April 23,2021) 01 More than half of young people in Xuhui District do not know what the agglomeration industry of Xuhui Binjiang Media Port, Wisdom Valley, City, Creative Warehouse, Fenglin Bay; 43.24% of young people say "good to live and work in Xuhui District, but worry about working and living here"; more than 40% suggest Xuhui District "provide richer career planning guidance and more adequate internship opportunities". In October 2019, Xuhui District of Shanghai was listed as one of the pilot areas for the implementation of the National Medium-term and long-term Youth Development Plan by the Central Committee of the Communist Youth League. Before making the planning, the Xuhui District Committee of the Communist Youth League designed the questionnaire of "The New Development of Xuhui and Xuhui Youth" on the basis of field visits and symposiums, and then "read the youth" with the research first. Xuhui District has both large riverside industrial agglomeration belt and small and beautiful international buildings, artificial intelligence industrial agglomeration area, and esports game industry development space, both Shanghai primary and secondary schools, and several well-known Grade A hospitals. In this suitable for working and living area, there are 373,000 permanent young residents, accounting for 34.4 percent of the total population. How are the young people doing here? How do they want to develop in the next five years? Group xuhui district committee given planning-follow the youth "priority" and "positive development" concept, efforts to create a bigger stage for youth youth, develop wider channels for youth to participate in urban governance, to provide more possible for youth enjoy quality life, for youth feel city temperature add better power, for the youth have identity gather stronger consensus. Housing, employment, life are the "three major" " coordinate Xuhui, Sunday to declare the Guangqi plan, I hope you pass by can introduce the next process."This is a question initiated by a netizen on Zhihu. The full name of the "Guangqi Plan" is "Xuhui District Guangqi Talent Action Plan", which is initiated by the Organization Department of Xuhui District Committee, aiming to attract all kinds of talents to innovate and start in Xuhui. The plan directly "hit" young people settling in and working in Xuhui. For example, for outstanding management talents or teams in enterprises promoting industrial innovation and transformation and upgrading industrial development level, no more than RMB 500,000 shall solve the periodic housing demand for innovative talents, service talents, management talents and professional talents, and apply for the rental subsidy of the previous year. According to the investigation of the Xuhui District Committee of the Communist Youth League, "housing, employment and life" are the "three major issues" that young people are most concerned about. Take housing, for example. " If the government is going to introduce employment-related policies or projects right now, what are your ideas and suggestions?"66.45 percent of young people believe that" providing affordable houses can retain people ". It is worth noting that this group of young people are mainly registered students in Xuhui District; the employed young people are mainly employed by Internet companies, with a monthly income of about 10,000 yuan. In addition, they are particularly concerned about whether the government can "do the job fair well", "strengthen career planning guidance" and "provide sufficient internship opportunities". After getting these opinions and suggestions of the young people, the youth league organization also took active action. The reporter noted that in January 2020, Xuhui District issued the "Xuhui District Promoting Employment Policy Implementation Opinions", a special policies to encourage employment, encourage entrepreneurship and youth probation for young people. In November last year, Xuhui District issued a talent recruitment order, providing the first batch of more than 4,000 job gift packages, attracting 1 million people to watch the "Gathering the Future" Xuhui District Outstanding Youth Talent Cloud Selection Meeting online. In March 2020, in order to better customize the "14th Five-Year Plan" development "small plan" for the youth, the Xuhui District Committee of the Communist Youth League also conducted an analysis and research on the big data of Xuhui District youth. According to the report, the Xuhui district has attracted many white-collar workers, college students and teachers. Interestingly, the whole Xuhui District exudes a "single wang" atmosphere, and the proportion of unmarried young people in the district is as high as 73.64%. In terms of the male-female ratio, the number of men were more unmarried, accounting for 60.62%. It is also rich in programmers, with nearly 80 percent of men. To this end, the Youth League Xuhui District Committee actively organizes the "Youth Benefit Friends +" community development plan to create suitable dating opportunities for young people; on the other hand, it tries to build the youth league organization, plan the Xuhui District Regional Youth Work Construction Committee, and regularly serve these "two new" organizations. Shanghai Mihayou Network Technology Co., Ltd. is one of the first alliance members of the Xuhui District Online Game Enterprise Alliance. Recently, Kuang � h, the head of the Miha youth league construction group, has made a general review of the situation of the league members of the company, and set up a preparatory group for the youth league construction. The next step is to start the establishment of the youth League committee of the company under the guidance of the superior youth league organization. One of the characteristics of Shanghai Xuhui District is that young people have high education, high income and high consumption level. After the material guarantee, they have a higher demand for the spiritual sense of participation and gain. The Xuhui Youth big data analysis report shows that nearly 50% of Xuhui young people have a high consumption level, compared with only 27.61% accounting for them nationwide. According to the big data, the group has an obvious preference for chat, social networking, shopping, video, audio, and news reading, and the penetration rate of literary App is relatively high. The young people also like to punch in at the West Bund Art Museum, Yu Deyao Art Museum, Shanghai Drama Art Center and Shanghai Museum of Contemporary Art Museum in Xuhui District, with an average frequency of 2.3 days per month. At the same time, they also have a higher political learning consciousness and initiative."Young, high income, literary and artistic, with a sense of political participation" - -this is a typical portrait of Xuhui youth."We have been thinking about what platform such a group of quality young people can build for them to have a sense of participation and gain."Group xuhui district committee, deputy secretary (work) lu � Z kun, at the end of last year, for the full implementation of the long-term youth development plan (2016-2025), xuhui youth group formed" xuhui youth people's city practice group ", began to recruit youth red narrator, youth career experience officer, youth" water " volunteers, youth entrepreneurship partners, youth community building division, youth culture star push officer six categories of volunteers. The appearance of these people, make Xuhui more "youth force"."difference" opening year, according to the diverse needs of xuhui youth, xuhui departments joint force formed "xuhui district youth development" difference "planning index system (draft)", the core index involves 10 areas, 27 indicators, through big data youth dynamic data acquisition, hierarchical classification analysis, personalized customization, work for youth development policy to provide a more detailed, more scientific basis. On April 23,2021, Version 01

2021-04-22	Qingchuan County held "star torch with growth" distribution ceremony Qingchuan County held "star torch with growth" distribution ceremony morning correspondent Xu Chuanliang) On April 16, the Communist Youth League Qingchuan County Party Committee, Qingchuan County young working Committee in Qingchuan County Qiaozhuang town central primary school held "star torch with growth" distribution ceremony. It is reported that the "star torch with growth" is the original book of the Communist Youth League Qingchuan County Party Committee, Qingchuan County Youth Working Committee, is to further explore a new way and a new carrier to serve children, to promote the healthy growth of children. The Qingchuan County Party Committee of the Communist Youth League and the Young Youth Working Committee of Qingchuan County established the editorial board of "Star Torch with Growth". The book "Star Torch with Growth" has a total of 10 chapters, which explain life knowledge, safety knowledge, young Pioneers' knowledge and the form of young people, so as to cultivate the sense of honor and organizational sense of belonging from the source.

2021-04-22	Huichuan, Guizhou: We will take multiple measures to communicate and seek more cooperation opportunities. Young people who want to start a business can also come to the community if they have no place to operate. If it is feasible, they do not need to pay any fees. With a legal business place, you can do a business license."When it comes to the community mass innovation center, Song Tingting, a young entrepreneur in Zimalu Street, Huichuan District, Zunyi City, Guizhou Province, started talking. At the beginning of the business, the high office rents and decoration costs had deterred Song Tingting."Office space, policy docking... are all difficult problems for us. With the community maker space as a platform, it is much more convenient."Through the entrepreneurial support of the community mass innovation center, Song Tingting's company is now on the right track. Now, she has also become one of the community youth entrepreneurship instructors, providing help for more young people entrepreneurship. In recent years, wash the road street committee attaches great importance to youth entrepreneurship work, promote business loans, rent subsidies and other preferential policies, establish one-stop service positions in the community, provide all kinds of agency services, provide high quality, efficient and convenient service for youth entrepreneurship, regular organization youth entrepreneurship training, as a whole on-the-job party members, non-public enterprise party organization resources, for intention to entrepreneurial youth one to one, more-to-one contact guidance. Up to now, the street has carried out all kinds of publicity, training more than 30 times, training more than 1,000 people.

2021-04-19	Hubei Xianfeng County Party Committee carried out the volunteer service activity of "Learn the history of the Party, strong faith, offer blood, follow the Party", correspondent Qin Qi) " The first time to donate blood, I am still a little nervous. But the thought of the revolutionary hero for the country shed his head, shed blood, are not afraid of death, I offer some blood and afraid of what......"On the morning of April 17th, the Xianfeng County Committee of the Communist Youth League and the Xianfeng County Volunteer Association carried out the volunteer service activity of" learning the party history of the Party, strengthening faith, offering blood, and following the Party ". On the same day, 23 volunteers donated 7,900 milliliters of whole blood to the enshi Prefecture central blood bank. Since the launch of the Party history education activities, the Communist Youth League County Party Committee has combined the study and education of Party history with voluntary service and solving the practical difficulties of the masses, constantly enhancing their sense of responsibility and mission, and serving the people and the society with practical actions.

2021-04-15	The Communist Youth League Xianfeng County Committee to carry out the party history theme education activities of the Communist Youth League Xianfeng County Committee to carry out the "learn the history of the Communist Party, strong faith, follow the Party" theme education activities morning correspondent Qin Qi) in order to further inherit the red gene, promote the party history learning education to go deep and solid. On April 12, the Xianfeng County Committee of the Communist Youth League organized all cadres and workers and volunteers from the western part of the county plan to go to the old revolutionary base areas to carry out the "study the history of the Party, strong faith, follow the Party" red education base visit activities. On the day of the activity, all the members came to the big village meeting site of the CPC Hubei Central Branch, the Red Army Cave and the Weijia courtyard. They listened carefully to the explanation of the narrator's explanation, read the pictures and read the materials, and deeply felt the revolutionary martyrs' revolutionary style of hardship and simplicity, firm revolutionary faith and fearless revolutionary spirit. Before the old site of the meeting, Hu Daocheng, party secretary of the Communist Youth League Party Committee, gave us a vivid party lesson with the title of "Remember the history and bear the mission". Hu Daocheng reminded everyone to remember the history, to be firm ideal, to be ideal, the pursuit of the youth, to study and practice, to be knowledge, ability, to innovate and create, to be responsible, to lead the civilized fashion, to be moral, cultured of the new youth."Although a great man is gone, his character still exists. Through the red education base visit activities, deepen the understanding of the revolutionary history of the communist party of China, also let me deeply feel the hard-won peace, in the future study, will continue to strengthen the study of the history of the party, achieve history, history, chong DE, history of the goal, contribute to the construction of xianfeng."Said Meng Enze, a Western Project volunteer.

2021-04-14	Zhejiang jiaojiang: "silver green pair" party history of Zhejiang jiaojiang: "silver green pair" party history reporter Dong Qingshui) recently, Taizhou jiaojiang six young party members, members to the 91-year-old whole Chen Shuqin home, sit around, listen to Chen tells a jiangshan island liberation, fighting experience, and brought him a red song, a bundle of books, party history audio and sound warm greetings. This is the party history learning "Yinhua Alliance" created in Jiaojiang District, Taizhou City, Zhejiang Province, so that the youth Party league members and retired cadres can study and exchange together. In Jiaojiang, there are many similar elderly party members with similar legs and Chen Shuqin, in view of their inconvenient travel, activities difficult to contact characteristics, Jiaojiang District Committee Organization Department, veteran cadres Bureau and League Jiaojiang District Committee together to guide the young party members, league members "silver green pair", send customized party lessons for senior party members. In addition to the paper red books, young volunteers also guide retired party members to use the "Learning Power" platform, wechat public accounts, small programs, etc., to broaden the party history learning channels from traditional books to "fingertips", so that retired party members can review the history of the Party from an all-round and multi-angle, recall their original aspiration to join the Party. In order to further excavate the red cadre system of rich mine, Jiaojiang also set up "silver green forum". Combined with the theme party day, cadre honorary retirement, theoretical study meeting, etc., invited retired cadres advanced typical to work with young party members, league members to "study the history of the Party, read the classics together, talk about the future". Jiaojiang also encourages retired cadres and party members and young party members, league members to serve as the "community party building grid members", from sanitation to order maintenance, from conflict mediation to industry authority change, together to serve for the community residents. Chen Chang, vice director of the organization department of Jiaojiang District Committee, said that the "silver-green pair" can not only help solve some learning difficulties of retired senior cadres, but also let young people grow up quickly through the old comrades 'lighting lights'. It is reported that up to now, the district has more than 240 youth party league members and retired veteran party members pair up, to carry out door-to-door learning, pair to help learning. Under the leadership of the senior party members, 50 youth Party members participated in the governance of the community.

2021-04-12	Anhui Huangshan district: fire knowledge into the campus escort youth safety growth group Huangshan district party committee: fire knowledge into the campus, escort youth safe growth JianWei correspondent, wang leqing Zhou Huanhuan) to do the safety education of fire prevention, let the young pioneers and young pioneers counselors more understanding of fire safety knowledge, strengthen self-protection awareness and self-help ability, recently, Anhui Huangshan Huangshan district party committee, area less committee, sweet elementary school less committee organization invited Hefei help fire center Shi Feng instructor, for the young pioneers on a vivid lecture on fire safety knowledge. Stone instructor from the fire control knowledge "four understand four will", around the major fire events, for example, in the form of video, pictures to show the serious consequences of fire, and around how to prevent fire in life the young pioneers of the hot issues and fire extinguishers, safety hammer and other fire equipment for vivid and detailed explanation, let the young pioneers to the use of fire equipment of a deeper understanding. The fire safety knowledge lecture is a good popularization of fire safety knowledge, further enhance the awareness of fire safety awareness, so that the young pioneers fully understand the harm of fire, improve fire self-rescue ability, escort the safe and healthy growth of teenagers.

2021-04-09	Ningxia Pengyang County school team organized the Qingming Festival martyrs activities to the party to remember the martyrs, praise the century of Ningxia Pengyang County school team organized the Qingming festival heroes activities to follow the party- -search for red prints, inherit the red flame " red study travel activities. Beijing, April 9 (reporter Zhang Jianwei, correspondent Ma Rong) under the guidance of team organizations at all levels, Ningxia Hui Autonomous Region Pengyang County youth in different forms, remember and mourn the martyrs, carry forward the national spirit, carry forward the martyrs' legacy, ceaseless red gene, firm ideals and beliefs. Remember a hundred years of old history under the guidance of the organization. School league organizations and young Pioneers support and lead the organization, independent education and education of practical education with the theme of "remembering the heroes", speak the politics of "children", and enhance their sense of honor for the Communist Youth League members and young pioneers and their simple feelings for the Party. The picture shows Pengyang County No.4 Middle School "remembering the revolutionary martyrs, inherit the red gene" learning the party history strong faith with the party theme education activities. Inherit the red gene in ritual education. Holding the national flag raising ceremony, keynote speech activities to express the memory and respect of the martyrs, holding the mausoleum ceremony, offering flowers and flowers, silence, revisit the league, team oath, sing the national anthem, group song, red song, participate in the online memorial activities, remember the history, remember the martyrs; promising, vowed to be pillars. The picture shows Pengyang County No.4 Primary School "red scarf heart to the party" practice activities- - "learn the history of the Party and recall the martyrs feel the party grace" theme class team meeting. Correspondent for the picture in the class team class learning role model pioneer. Guide teenagers in listening to a specific vivid story, watching a touching video feel revolutionary collectivism, revolutionary optimism, revolutionary heroism, cherish the happy life, set up the broad ideal, with the martyrs not the footprints, become a new era of useful to the nation to the country. The picture shows a series of activities of "learning the history of the Party and honoring the heroes", "remembering and remembering the revolutionary martyrs and carrying forward the national spirit". Correspondent for pictures in the search for the practice of firm ideal and faith. Lead the youth to visit the revolutionary Ren River martyrs memorial hall, Xiaochagou Mao Zedong Long March camp, Liupanshan Red Army Long March memorial hall and other red holy places, listen to the old Red Army long March story, understand the Pengyang area magnificent revolutionary struggle history; lead the members to revisit the Long March, walk several kilometers to visit the martyrs cemetery; arrange drawing board newspaper pictorial, writing experiences and insights. It is reported that this activity has covered more than 10,000 school team organizations.

2021-04-09	Green bird plan-Dezhou (Linyi) school industry talent double selection meeting held green bird plan-Dezhou (Linyi) school industry talent double selection meeting held Beijing on April 9 (reporter Zhang Jianwei) in order to thoroughly implement the green bird plan school cooperation, Do a solid job in party history learning and education "I do practical things for the masses- -do something for the youth" practice activities, To further improve the employment rate and employment quality of college graduates, Build a two-way choice platform for college graduates and employers, On the morning of April 7th, The Youth League of Linyi County Party Committee of Dezhou City, Shandong Province organized 7 enterprises to go to Shandong Jianzhu University Bluebird Plan- -Dezhou (Linyi) school-based and local industry talent double selection meeting was successfully held in Shandong Jianzhu University. The double selection meeting was held in the form of combining on-site job fair and enterprise briefing. Seven high-quality enterprises, including Sutong Development Co., Ltd., Shandong Aofu Environmental Protection Technology Co., Ltd., Shandong Sen and Construction Engineering Co., Ltd., participated in the on-site job fair, providing nearly 200 professional positions for graduates to choose. In order to make students have a deeper understanding of Linyi County and related enterprises, Wang Yanxue, deputy secretary of the Communist Youth League County Party Committee, introduced the basic situation, industrial development and talent policies of Linyi County through the briefing. The four enterprises also took the battle to introduce the advantages, development prospects and talent training. Double selection meeting site. Group Linyi county for the job fair is the implementation of the "green bird plan" college students employment accurate support action of concrete practice, the Linyi county industry development, enterprise talent planning and Shandong construction university discipline advantage organic combination, the integration of civil construction, material engineering, energy and chemical industry of three concentrated jobs. In order to provide students with more accurate services, more opportunities and greater choice space, in the early stage of the activity, the Communist Youth League County Party Committee conducted a thorough investigation, learning and enterprise communication, and the "Bluebird Station" stationmaster preheating publicity. Through the publicity, discussion and other precise docking and communication, the students have a full understanding of Linyi and enterprises, and reached a preliminary employment intention. On April 17th, the Communist Youth League County Party Committee will continue to organize enterprises from six major industries, including machinery industry, electronic information industry, chemical industry, sales industry, teachers class and comprehensive industry, to hold a special job fair in Dezhou College. Next, the pku will take more accurate measures, more thoughtful service continue to carry out the "green bird plan" series of activities, help the county "ZhaoCaiYinZhi" key work, between the enterprise and outstanding young talents set up the service bridge and link, to further enhance the attraction and cohesion of youth, support more young people home employment entrepreneurship, realize their youth dream.

2021-04-08	On the map of the state and province, Guiding County is not particularly striking. A comprehensive pilot reform of grassroots organizations of the Communist Youth League that began in October 2019 brought attention to the 300,000 county; the reform also caused waves in this peaceful western town. The influence of the reform of the Communist Youth League system also began to be "out of the circle". Zhang Lifang, party secretary of Guiding County Committee, interviewed by the China Youth Network of China Youth Daily, mentioned several times that the comprehensive pilot work of the Communist Youth League grassroots organization reform promoted the work of the central government. This is reflected in almost every aspect of the reform. In June 2020, a once-in-a-century rainstorm flooded a large field in Yunwu Town, Guiding County, flooding a single floor in deep places. Yunwu Town has a rare dam of 10,000 mu in the mountainous province of Guizhou. It is a high-quality producing area of vegetables, Yunwu gong tea and golden chrysanthemum. The vegetable output is about 42,000 tons, and the annual sales volume is about 100 million yuan. Seeing pepper, beans and other vegetables are about to harvest, but all were blisters, originally can be sold to two or three yuan a jin of vegetables, is likely to only rot in the ground. When the farmers in Yunwu Town are worried about this, a news came: the town's Jiuwang agricultural development company is willing to buy water-soaked vegetables. At that time, the agricultural company, initiated by three young people, was founded for more than half a year, but has not been able to open the market in Yunwu Town. In December 2019, Yang Peng, as the representative of the Youth League Committee of Yunwu Town, jointly established Jiuwang Company with two other large vegetable companies to serve in the connection of vegetable production and marketing. Yang Peng has a deep understanding of the small and scattered planting state of farmers in Yunwu Town: although the scale of vegetable planting in the town is not small, everyone has been lack of bargaining power in the market; if the market is not good, there will be a large number of vegetables can not be sold in the field. Yang Peng remembered that the vegetables were not soaked for a long time, but there was just some sand on the surface, but the vegetable vendors did not want to accept such dishes, Jiuwang company bid to buy, at least to let the farmers return their money. Despite the acquisition, Jiuwang company lost some money, but won the trust of local farmers, and people began to sell the vegetables to Jiuwang Company. In July 2020, Yang Peng became a member of the Yunwu Town Party Committee due to his outstanding work in the town Youth League Committee, and his work was even more busy. Yang Peng is not an individual case. Since the reform of the Communist Youth League was launched in Guiding County in October 2019,7 outstanding league organization leaders have also served as members of the Party organization at the same level, and 53 village (neighborhood) league branch secretaries have entered the two village committees- -which also reserves young talents for the future development of Guiding County. Ma Long, the party secretary of Tuan Guiding County, is also one of them. He was transferred to the old city reconstruction headquarters of Guiding County to directly participate in the front-line work with the most contradictions and the most complicated disputes. Since serving as the secretary of the Communist Youth League Committee of Yunwu Town, Yang Peng has also participated in the construction of the United Village Communist Youth League Working Committee. This is a great initiative of the reform of the Communist Youth League in Guiding County, namely, "the youth league organization is built on the industrial chain", and Jiuwang Company is an important link in it. Yunwu town of vegetables, Yunwu gong tea, crayfish breeding three industries, nine villages have their own focus. Since 2019, three villages with similar industrial structure have built the youth league working committees of vegetable industry villages, and the other six villages have built the youth league working committees of gong tea industry villages and ecological fishery villages respectively. Yang Peng recalled that at the beginning of the construction of the united Village Youth League Working Committee, he first "started" from his familiar large industrial households and friends, and gradually developed to the scale of 100 people. The joint village youth League working committee was initially completed, but the villagers did not buy it. The job turnaround came at the end of March 2020. The industry in Yunwu Town is mostly seasonal labor, late March and early April is the tea picking season, in the past there is no shortage of labor force, but during the epidemic in 2020, a confirmed case appeared in Yunwu Town center, even if the return to work and production, people are not dare to go out. Yang Peng called on his members to pick tea in the WeChat group of the joint village youth league working committee. The young people soon gathered up a picking team of more than 100 people. This solves the urgent need of tea farmers, the appeal of the joint League working Committee also makes the villagers sit up and take notice. With the young people of the working committee leading the demonstration, people have successively walked out of their homes and resumed production. Now, Guiding County has set up 8 joint village youth League working committees, basically forming a working mode of "local agricultural companies contact the sales market, the youth League working committee organizes labor force, village-level cooperatives or large households organize picking". Yang Peng said that with the help of the east-west poverty alleviation cooperation platform, Jiuwang company sold large vegetables to Nansha District of Guangzhou, making vegetable prices more stable for vegetable farmers. If you eat in restaurants in Guiding County, you will probably see the "help help" advertisement on the wall; walking on the road, it is not difficult to see the "help" riders- -this is the local errand service brand in Guiding County. Since 2019, more than 20,000 registered volunteers in Guiding County have joined another "Help Gang" team- -the "Youth Help Gang" Volunteer Association. Since the launch of the comprehensive pilot reform of the Guiding County, County Party Secretary Zhang Lifang has named the brand for the county's volunteer service team. Su Jiajia, deputy secretary of the Tuan Guiding County Party Committee, witnessed the growth of the volunteer service brand. At the beginning of 2020,10 confirmed cases of COVID-19 were found in Guiding County, a small county of more than 30 0,000 people, panicked. But Su Jiajia saw that many young people volunteered to be on duty at the epidemic prevention and control card point, while the young people who rested on shifts used their precious meat vegetables and food to deliver food to the card point. According to statistics, at that time, nearly 2,000 youth and league members participated in the work of 271 epidemic prevention and control card points on duty. Yang Peng remembers that during the COVID-19 outbreak, young people from the "Youth Help Gang" volunteer service team drove their own trucks to provide free food for residents in resettlement sites from inhospitable areas. Some village league branch secretaries contribute to the pickup trucks, several volunteers every day in the cold wind standing in the carriage, holding loudspeakers to shout, telling everyone not to visit, "Red Xi delay, Bai Xi simply do."Reform over the past year, your county of 137 "youth help help" volunteer service, distributed in the county departments, town (street), village (house), schools and non-public enterprises, by the masses, organization, full orders, the young people to carry out volunteer activities has more than 5000 times: your county health system "youth help" volunteer service according to the characteristics of the community to the elderly, the disabled, normalized health clinic; The "Youth Help Gang" volunteer service team in Baoshan Street, together with the teachers and students of Qiannan Youth College School, carried out the "Hope Project to Accompany Action"...... in the relocation site of poverty alleviation from inhospitable areasAt the end of October 2020, the Central Committee of the Communist Youth League commended the advanced individuals and advanced collectives in youth volunteer service in the fight against COVID-19, among which was the "Youth Help Gang" volunteer service team of Guiding County. In the Guiding County Financial Media Center, the proportion of young people under the age of 35 accounts for 65%. On the wall of the financial media center are the photos, names and positions of the youth league members- - "Bright identity" of the league members, which is an important part of the reform of Guiding County. Show their identity and let the league members have a more sense of honor and responsibility. During the epidemic, young people in the Guiding County Financial Media Center were also active on the front line. People panicked about the unknown new virus, and the relative lag of information exacerbated the problem. The young people took to the street checkpoints and entered the local hospital infection department to report the latest information about the outbreak in the county. Wu Lihua, director of Guiding County Financial Media Center, felt that the comprehensive pilot work of the grassroots organization reform of the Communist Youth League united the youth members more closely and promoted the business promotion. In addition to their own work, they also undertook volunteer services in Putonghua training and other work. The normal youth league organization life listens to the voices of ordinary workers. Zhan Ke, the secretary of the youth league branch of Shengyuan Pipeline Company, talked with the youth league members. He learned that many colleagues' children are studying in nearby primary schools. The temperature is high in summer, and some schools do not have any refrigerators, and the food ingredients are easy to deteriorate. After reporting to the company leaders, he launched a joint donation, and several enterprises in Guizhou Changming Economic Development Zone jointly donated more than 20 refrigerators to nearby schools. Your county party secretary Ma Long mentioned, since the reform, the county rural areas, organs, enterprises, schools in various fields of youth league coverage has reached 100%, in addition, also according to the member identity nature, organization league cadres, school members, government institutions, enterprises, community, rural members, two new social areas of six types, "six type" three than "activities," strengthen the identity of young workers and sense of responsibility, let youth active in service economic and social development, achievements ". China Youth Daily, China Youth Network reporter Li Yajuan source: China Youth Daily

2021-04-07	Ningxia qingtongxia "red scarf branch" speak good party history story Ningxia qingtongxia "red scarf branch" speak good party history story Beijing on April 7 (reporter zhang, correspondent tongtong zhang), the Ningxia hui autonomous region qingtongxia city bureau of education, qingxia "red scarf branch" to ceremony and party history preaching activities, qingtongxia "red scarf branch" all the lecturer and red scarf narrator, qingtongxia fifth middle school students to participate in the activity. The "Red Scarf Lecture Group" lecturer Xu Jingjing, the red scarf narrator Wang Ni demonstrated the lecture "Tell the red Gene of the Party history", and the story of the red scarf narrator "resist the United States and aid Korea". The picture shows the event site. Correspondent for figure to preach, the students have issued a speech, " just xu teacher preach let me tears purpose but also benefit my benefit, she in the form of vivid to show us how to make a good preaching, as a red scarf, as a red scarf narrator to preach, I feel very glorious, I must seriously study party history, with you like good party history, lead everyone to understand party history, the study of the spirit of the party, firm faith with the party."Red scarf narrator Tian Yulin said excitedly. After the exchange, the students filled in the card "I want to tell you the centenary of the founding of the Party", " This year is the centenary of the founding of the Party, and I hereby wish the motherland prosperity, peaceful country and peaceful people, better and better!'Without the Communist Party, there is no new China', without the Communist Party, there is no our happy life now, as middle school students, we want to thank the party to thank the motherland!"Student Bao Wenjing wrote. The picture shows the event site. The purpose of this activity is to encourage the members of the teaching group to tell the party history stories with the most beautiful voice, vivid stories, affectionate narration and passionate speeches, lead the children around to understand the history of the Party, learn the spirit of the history of the Party, firm faith to follow the party, draw from the history of the Party to the strength to pursue their dreams. In the future, the qingtongxia municipal party committee, qingtongxia bureau of education, qingtongxia less committee to "red scarf branch" as the gripper, continue in the city's primary and secondary schools, community committee, patriotic education practice base of the young pioneers activities to carry out the history of party education propaganda activities, in the form of the children like the party history, vivid, into the heart, further study the history of the party, guide the city children learn party history, strong faith, follow the party, the new journey, meet the centenary with excellent results.

2021-03-29	Guizhou Province Wanshan District Committee promotes "four senses" to build "four an Home" Wanshan District, Guizhou Province: "four grasp" enhances "four senses" to build "four an Home" Jianwei correspondent Wang Guiren) Since the implementation of the "New Citizens Dream Bridge" project, The Wanshan District Committee of Tongren City, Guizhou Province, gave full play to the volunteers, Integrate all resources, Linkage of departments at all levels, To explore a series of service activities in the relocated community, To answer questions for the relocated people, Serve the relocated people well with your heart and affection, We will make every effort to build a bridge between the Party and the hearts of the people, farmers and citizens, poor and rich, Actively create a "four'an Home", Constantly improve the sense of security, gain, belonging and happiness of new citizens. In order to further enhance the safety awareness and self-protection awareness of the relocated people, the Communist Youth League District Committee has built a strong community "safety net" in an online and offline way. Online, the "12355" youth service platform has been opened to provide telephone services such as emotional counseling, psychological counseling and legal knowledge popularization for the resettlement site teenagers through the telephone hotline. At present, I has received more than 2,300 telephone inquiries from parents and teenagers. At the same time, in wanshan youth WeChat public network platform push youth crime knowledge, entrance examination for secondary school or college decompression, psychological counseling and other comprehensive service information more than 130; offline, joint justice, public security, politics, education and other departments set up community legal rights protection volunteer service, provide legal aid, laws and regulations publicity, such as public legal lectures and publicity activities more than 110 times, household visit more than 240 publicity. At the same time, with the "4:30 classroom" as the carrier, more than 60 themed activities such as production safety, epidemic prevention and control, fire prevention and theft prevention were carried out, creating a harmonious and stable good atmosphere in the community, and effectively enhancing the "sense of security" of the people. Grasp employment services to promote industry safety, enhance the people's sense of gain should not only let the relocated people move, but also to ensure that the relocated people are stable, can gradually get rich, and fully eliminate the worries at home. The Communist Youth League District Committee focuses on the long-term livelihood of the relocated people and living and working in peace and contentment. It has actively cooperated with trade unions and women's federations and other departments to provide free vocational skills training such as auto repair, housekeeping, cooks and excavators to the relocated people in various resettlement sites, so as to realize "learning skills in hand and not worry about employment" and help the relocated people find jobs and start their own businesses. At the same time, the training class of young anchors to help farmers was held in the youth lecture hall of Wanshan District Industrial Park, teaching practical and effective "short video + live broadcast" courses for more than 30 relocated young people, helping Guizhou goods out of the mountain, helping farmers to increase their income and become rich, and constantly improving the self-income generation and employment ability of the relocated young people. In addition, it has organized various entrepreneurship policy consulting services such as "Youth Help Loan", distributed more than 10,000 copies of entrepreneurship policy publicity materials, jointly carried out more than 10 job fairs and other related activities, and provided more than 2,000 jobs. Up to now, through publicity, training, recommendation and other ways, it has helped more than 40 relocated people to open supermarkets, restaurants, barbershops, etc., realizing the dream of entrepreneurship, and driving the employment of more than 180 people. Through the implementation of various employment services, the relocated people have realized the nearby employment, and enhanced the "sense of gain" under the intimate service of the majority of volunteers. In order to make the relocated people have a sense of belonging to the community, the Communist Youth League District Committee further promotes the integrated construction of the "Party and Youth League Society" (Party branch, Youth League branch, and Chunhui Society) in accordance with the working idea of "Party building guidance, League building cohesion, and Chunhui help". In the organizational equipment, In each resettlement point in the district to establish the youth league branch 8,1 non-public enterprise youth league branch, Equipped with 28 full-time youth league cadres, Set up 8 volunteer service teams of the "New Citizens Dream Bridge" project, More than 140 registered volunteers, Held more than 80 various volunteer service activities, Serving more than 10,000 people; In the position construction, In order to let the nearby workers can work in peace of mind, "Project Hope Accompany Action" project, "4:30 Class", "Children's Paradise", "Community Dream Center" and other classes, More than 10,000 class hours have been opened, Long-term services for more than 500 relocated teenagers to provide homework counseling, interest training and other services; In terms of capital investment, A total investment of more than 100,000 yuan, Improve the positions and service functions of the Communist Youth League in all resettlement sites in the region; In the study, Organize night classes twice a week at the relocation site, To carry out various teaching activities, such as literacy and word reading, sports, literature and art, Enrich the daily cultural life of the masses, It meet their spiritual needs. Through the continuous improvement of the public service system, we ensure that the community residents live in a comfortable and comfortable place, and find a real "sense of belonging" in the resettlement site. Grasp the humanistic care to promote peace of mind, improve the happiness to effectively solve the resettlement part of the elderly and study children no care at home, academic tutoring, TuanOuWei actively explore "scud", "three agents" volunteer service brand, by recruiting community "scud", for the community people purchasing materials, send warm, help solve 52, at the same time to recruit "three agents" volunteers more than 60, deliver classes, door to door to send warm volunteer service activities, a total of more than 300 times. In addition, the working committee directly under the district carried out the activity of "hand holding hand for a happy summer vacation" to enrich the holiday life of the children in the relocated community. In addition, in order to better solve the living needs of special groups, care for the mental health of special groups, and improve the quality of life of the disabled, volunteers from the hairdressing and medical industries are irregularly invited to carry out charity cutting and free clinic activities, and provide free hairdressing and medical services for special groups in the community. Through this series of care measures, the relocated people have obtained a full range of care and care, and effectively improve the happiness of the relocated people. Next, TuanOuWei will continue to work hard to make volunteer service fit move the emotional and life needs, the love warm into resettlement homes, to help move people quickly into the new life to provide strong guarantee, to create "four homes", let move people feel full of security, sense of gain, sense of belonging and happiness.

2021-03-24	Li Jianping, the reporter, today is the "World Water Day" and the 29th and the 34th "China Water Week". This year's theme is "Cherish water, love water". The League Xiangshan County Party Committee of Zhejiang Province has organized 38,000 young people to participate in different forms of water conservation public welfare publicity activities, starting from me, starting from the bit by bit. Affected by the drought, from February 26 this year, Xiangshan County launched urban and rural water supply drought level emergency response. In order to enhance the awareness of "understanding water, protecting water and saving water", TuanXiangshan County Party Committee released the online "saving water" punch card help action, designed water-saving H5 small program, and launched teenagers to forward water-saving messages for relay activities. In Xiangshan County, 33 young Pioneers formed a like-like "Xiangba Red scarf" water-saving publicity team, which has conducted 172 online and offline publicity sessions in campuses, communities and enterprises, involving more than 120,000 students and citizens. Sheng Zihan, a representative of the Excellent Young Pioneers of Zhejiang Province, shot a water-saving video and proposed the county "red scarf" to save water and protect water with practical actions. The "Yunfan Squadron" of Dancheng No.4 Primary School in Xiangshan County set up a parent-child water-saving volunteer service team, and carried out 56 water-saving publicity and water supervision volunteer service activities in rural areas and islands on weekends and holidays. The county teenagers can also light up the Xiangshan water-saving map by completing 50 water-saving answers, and get the "water saving red scarf"."Last winter to this spring, Xiangshan was really dry. Today, I came all the way that the water level of the reservoir along the way is very low. I hope these activities will make more people realize the importance of saving water."Said Zhang Ying, a young man who participated in the" Yidong Youth Leading Water saving " public welfare activity in Xiangshan County. Teenagers from Xiangshan County, Zhejiang Province participated in the public welfare activities of water saving and water protection.(Photo provided by Shi Hanxiao)

2021-04-08	Group mengcheng county in Anhui province to carry out the youth party history education practice group mengcheng county to carry out the "sacrifice martyrs learn party history bear mission" youth party history education practice on 8 (reporter Zhang Jianwei, correspondent Zhang Lu Cai Kaihao) recently, group Bozhou city, Anhui province mengcheng county organization to carry out the "offering martyrs learn the party history bear mission" youth party history education practice. More than 90 people participated in the meeting, including all the staff of the Education Youth League Working Committee, leaders of the Youth League Committee, representatives of young Party members, representatives of the young Pioneers of the primary school affiliated to Bozhou Teachers College. In the solemn atmosphere, the representatives of the Young Pioneers presented a flower basket to the martyrs 'monument, and spoke to the martyrs, the cry is firm and confident, inspiring the young people to remember the revolutionary history, inherit the martyrs' patriotism.site of activity. After the ceremony, all the staff held flowers, paid their respects to the martyrs 'monument, and presented flowers to the martyrs' tomb. Under the guidance of the narrator, the representatives of the league members and the young Pioneers visited the martyrs memorial hall. Finally, we spontaneously swept the ground leaves of the martyrs cemetery, carefully wiped the martyrs tombstone, with their own practical actions to express their respect for the revolutionary martyrs. This activity, let the young party members, league members, young pioneers received a profound party spirit education, further understand the revolutionary history of the Chinese nation and the national spirit of unremitting self-improvement, clear history entrusted to the new generation of successors of the sacred mission, enhance their sense of historical mission and national responsibility.

2021-03-19	Shandong pingyin youth win-win post volunteers help home development pingyin county hiring 341 youth win-win post volunteers normalized power home development JianWei correspondent Zhang Zhichao) for the youth power normalized condensed to the county social governance, rural revitalization center, in February 2021 Shandong Jinan pingyin county issued the system on deepening the youth win-win post work notice, successfully selected 341 outstanding youth as youth win-win post volunteers, the average age of 20, including China agricultural university, China university of petroleum and other college students 311, accounted for more than 91.2%. To give full play to the advantages of youth, youth street town green committee science set up volunteer jobs, including "the online service at school, holiday village registration" youth grid (village) assistant (part-time) hiring 236, accounting for 69.6%, "when a good county school bridge" green bird station master hiring 33, industrial design, media art and other professional 23 college students were selected as propaganda design consultant, mainly engaged in logo design, video production services. By holding appointment ceremony, issuing letters of appointment, praise and encouragement, the sense of belonging and honor for volunteer service positions was effectively enhanced. Young people all expressed that they would cherish this platform, constantly temper their skills in practice, and strive to contribute their youth strength to the development of their hometown, and finally achieve the goal of "win-win".

2021-04-02	Shaanxi district held "the founding of one hundred heroes red gene generation" activities group district party committee held "one hundred heroes red gene generation" theme activities wei correspondent HuLei) to remember the revolutionary martyrs, to commemorate the 100th anniversary of the founding, the red gene, recently, the group of shaanxi Shangluo district committee in Shangluo revolutionary martyrs cemetery held a "founding one hundred heroes red gene generation" theme activities. Business district party committee propaganda department, district party committee office, district party committee DuZhaShi, district women's federation, district, district federation, district association for science and technology, veterans affairs, district bureau of statistics, district fire brigade 12 OuZhi departments and shaanxi transportation construction group company business branch, district city company, district rural credit cooperative association, district middle school, volunteer federation and other six schools, enterprises, social organizations participated in the activity. Shangluo Revolutionary Martyrs Cemetery buried 27 revolutionary martyrs during the agrarian revolutionary war and the liberation war, among which the famous martyrs were Tang Shu, former commander of the Northwest Workers and Peasants Revolutionary Army, and Gong Defang, former commander of the second division of the Henan, Hubei and Shaanxi Military Region. In the cemetery, the pines and cypresses and green trees are hidden, and the monument of revolutionary martyrs is solemn and towering. In front of the memorial hall hangs a banner of "Red gene for the heroes of the centenary of the founding of the Party". The atmosphere is solemn and respectable. On the day of the event, more than 200 people sang the National Anthem of the People's Republic of China. The national anthem was loud and clear throughout China, stirring everyone's heart. After playing and singing the national anthem, the heads of all departments wore white flowers, presented flower baskets to the revolutionary martyrs, and bowed three times to the revolutionary martyrs with the most solemn and respectful attitude. After laying the flower basket, all the staff stood upright, to the revolutionary martyrs for 1 minute, 60 seconds, to express infinite grief, to show the will to work. Subsequently, Li Changli, deputy director of the Shangzhou District Bureau of Statistics, member of the municipal youth teaching group and winner of the first "Shangzhou District May 4th Youth Medal", taught the micro group class with the theme of commemorating the 100th anniversary of the founding of the Party. Xu Pengjia, an outstanding representative of the Communist Youth League and a soldier of Beixin Street Fire Station, made a speech around the delegation to inherit and carry forward the spirit of the May Fourth Movement and strive to bear the great responsibility of national rejuvenation. Through this activity, the majority of young people, cadres and the masses received a profound ideological education and soul baptism. We all agreed that we should follow the example of the martyrs, inspire the great achievements, inherit the red culture, carry forward the spirit of patriotism, never forget the original aspiration, remember the mission, have the courage to take responsibility, work hard, strive to create a new situation of high-quality development of Shangzhou, and contribute our wisdom and strength to write a new chapter of catching up and surpassing in Shangluo.

2021-04-07	Dangshan county in Anhui province: the history of the party to preach into the "youth" dangshan county in Anhui province: the history of the party to preach into the "youth home" Beijing on April 7 (reporter Zhang Jianwei, correspondent Zhou Huanhuan) recently, Suzhou city in Anhui province dangshan county in dangshan city "youth learning club" in the history of the party project preaching activities, deputy secretary of Anhui provincial party committee Hu Jing to attend the report. County league, team cadres and young pioneers representatives a total of more than 100 people listen to the lecture. At the report, Hu Jing taught in detail the history of the century-old struggle of the Communist Party of China with the title of "The Red Code of the Hundred-old Party History". He had a distinct theme, rich connotation and was easy to understand, which has both theoretical height and practical significance. He first expounds the significance of learning the history of the party and further study the party history methodology, and then focus on the major events of the communist party of China in one hundred, important meetings and important figures, analyzes the history of the ten period, from wake up, stand up, rich, strong four dimension analysis explained the history of the party, with "true, good, beautiful" answered "why marxism to" "the communist party of China can" "socialism why good" three representative questions. Later, Hu Jing and the representatives of the Young Pioneers talked interactively, encouraging them to learn the history of the Party well, inherit the red gene, develop high skills, and aspire to serve the motherland. Next, the county youth league organizations at all levels and the young Pioneers organization will further seize the favorable opportunity, using the youth popular form of the youth, guide the county youth league members to fully understand the history, deepen rational identification, not afraid of risks and challenges, brave to shoulder the mission of a strong country. We will maximize the initiative and enthusiasm of young people to participate, actively participate in the great practice of socialism with Chinese characteristics, and contribute our youth to the realization of the Chinese dream of national rejuvenation.

2021-03-24	Guizhou: "qing qinghe" river patrol volunteer service into the wan mountain wood fir river Guizhou: "qing qinghe" river patrol volunteer service into the wan mountain wood fir river wei correspondent Song Zishun) to strengthen river environmental health protection, the implementation of the "green water castle peak is the jinshan yinshan" development concept, further play a role of volunteers in power long river work, on March 22nd afternoon, group of Guizhou including wanshan district party committee joint auspicious social workers in Dan street social work station, in the "qing qinghe" river patrol volunteer service activities. More than 10 volunteers and community residents participated in the activity. Volunteers and community residents put on their coats and armbands, and entered the river patrol to carry out garbage collection, river protection and publicity work. Along the river bank, we cleaned up the people on the bank, the white garbage, plastic, cigarette butts and other garbage in the grass, and at the same time, registered and recorded the ecological problems of the river, and publicized the concept of environmental protection to passers-by and fishing enthusiasts. The activity won the unanimous praise of the masses, and some people even took the initiative to join the river patrol team. Volunteers said: " This activity made us all realize the importance of protecting water resources, and protecting the environment should start from ourselves, starting from around."This activity, accumulated a total of 6 bags of plastic, cigarette butts and other kinds of garbage, accurately recorded the environmental conditions along the river, at the same time to carry out environmental protection concept publicity and civilized play persuasion, a total of more than 80 people. Through the activities, the volunteers with practical action to improve the quality of river ecological environment, realize the cedar river "smooth river, clear water, green shore, jingmei" contributed to the ecological goal, at the same time further called for the masses to cherish water resources, love water resources, jointly maintain beautiful ecological environment, create the concept of "everyone involved in" social atmosphere.

2021-04-02	Longde County, Ningxia young Pioneers, the Communist Youth League members set off the climax of the party history study Wei correspondent Ma Rong) This year is the 100th anniversary of the founding of the Communist Party of China. Learning Party history is a key to understand where the Communist Party of China comes from and where it is going. In order to welcome the centenary birthday of the Party, the young Pioneers and the Communist Youth League members of Longde County, Guyuan City, Ningxia Hui Autonomous Region, actively carry out various forms of activities to study the history of the Party in patterns, walking "new" and more "heart". Study the history of the party. The class counselors of Longde County No.2 Primary School organized all the team members to watch the red movie in the ideological and political class classroom and by the deputy school (school) director of the Party School of the CPC Central Committee (National School of Governance), A special lecture on CPC History given by Professor Xie Chuntao, Also use the reading class to learn red books; Longde County third primary school squadron counselors in the "from the primary school party history always follow the party" ideological and political class, Organize the team members to watch the "Red Scarf Love learning season 2" and " Inheriting the red gene, "The special program" The Communist Party of China Starting From Here ", Let the team members deeply understand the birth of the Chinese Communist Party in the white terror of the bloody wind process; The ideological, political and history subject teachers of Longde County No.4 Middle School, with the help of the subject characteristics, In the party history learning and education into the classroom activities, Explain the historical development of our party in detail, And held the party history knowledge competition activities, Promote the majority of teachers and students to further study the knowledge of party history. Know the party grace. Longde county school young pioneers, youth corps committee through organizing theme class meeting, team, hand copy newspaper, blackboard, "learn from lei feng" volunteer activities, and students essay share hero story, let all the young pioneers, the communist youth league members review the communist party of China led the Chinese people through the extraordinary years, and since the reform and opening up led the Chinese people to prosperity, democracy, civilization, happiness, to create a brilliant achievement, inspire the teenagers to let others live a better life, put the limited life into infinite serve the people to beginner's mind and mission. Ode to the party. Longde County No.4 Middle School, Longde County No.3 Primary School, Guanzhuang Township Central Primary School, Wenbao Township Central Primary School and other schools held the theme flag-raising ceremony. Teachers and students representatives called on the young pioneers, the Communist Youth League members from the primary school party history, do a promising good young man, unswervingly listen to the party, do the party good children since childhood. Start from small things, start from now, be the best, contribute to the construction of socialism with Chinese characteristics. Sing the party song. Sing the classic red songs, review the red history with the songs, and play the youth movement with the melody. Teachers and students of Longde No.4 Middle School carried out the "red song loud" activity, singing "No Communist Party, no New China" to express their love for the country and national pride; Longde No.3 Primary School sang the glorious course of the Party, praised the great achievements of the party and thanked their warm care; the radio members popularized the background and connotation of the song "No New China without the Communist Party", and played the song, creating a good environment for learning the history of the Party. Through various forms, rich content of "learning history" education activities, motivate guide the young pioneers and the communist youth league members understand the communist party of China in one hundred beginner's mind mission, deep understanding of the communist party of the communist party of China in one hundred struggle, profound perception of the communist party of China in one century, seriously grasp the communist party of China in one hundred theoretical innovation, cherish the communist party of China, vigorously promote the revolutionary spirit of revolution, cultivate and practice the socialist core values, efforts to become moral, intellectual and physical comprehensive development of socialist builders and successors.

2021-04-06	East to county in Anhui province to carry out youth party history education and qingming festival offering heroes activities in Anhui province to carry out youth "learn party history, strong faith, follow the party" party history education and tomb-sweeping day memory of revolutionary martyrs wei correspondent Liu Jingzhou huan) recently, Anhui Chizhou east to county civilization office, the county organization youth "learn party history, strong faith, follow the party" party history education and qingming festival memory of revolutionary martyrs activities. County civilization office and the youth League county committee organized more than 20 young party members, 50 league members, 50 young pioneers to participate in the activity. In front of the solemn monument, all the staff stood to the revolutionary martyrs, played and sang the "National anthem of the People's Republic of China", the Civilization office and the Youth League county committee party member representatives presented a flower basket to the martyrs, all the staff observed a minute of silence, expressing their infinite grief for the revolutionary martyrs. The representatives of the young Pioneers of Zhengde Primary School read the red poem "" Red Scarf "to Salute to the Dear Party", offering infinite respect to the martyrs with their affectionate poetry recitation, and expressing the faith of the young pioneers in unswervingly inheriting the revolutionary spirit. Party members, league members and the young Pioneers reviewed the party membership oath, the league membership oath and the team membership oath in turn. In the solemn oath ceremony, we deeply remembered the revolutionary martyrs, learn and inherit the lofty spirit of the revolutionary martyrs, encourage the young people to remember the revolutionary history, inherit the legacy, inherit the national spirit, and stimulate their patriotic enthusiasm. After the oath, all the staff around the monument, pay respects to the martyrs monument, to express their remembrance and reverence for the revolutionary martyrs. Then, came to the revolutionary martyrs memorial hall, review the east to this red land, countless revolutionary martyrs arduous struggle process.

2021-03-29	Group in Anhui province Taihe county to carry out the rule of law peace construction volunteer publicity group of Fuyang city in Anhui province Taihe county to carry out the rule of law peace construction volunteer publicity activities JianWei correspondent, Yu Jinpeng Zhou Huanhuan) to further promote the construction of peace, further expand the construction of peace propaganda coverage, improve people's safety awareness and self prevention consciousness, recently, group of Fuyang city in Anhui province Taihe county organization youth volunteers in taihe temple square to carry out the "rule of law and peace home" peace construction volunteer publicity activities. Activities, the young volunteers to past the masses on the protection of minors, prevent telecom network fraud prevention, safe production, and other kinds of closely related to the daily life propaganda color page, the patience to answer the masses, explain all kinds of high form of fraud, and remind the masses to improve self awareness, prevent all kinds of illegal and criminal behavior, protect the life and property safety of themselves and his family. Peace construction has only a starting point, but no end point. By organizing peace construction youth volunteer propaganda activities, help residents to raise the public awareness of peace and civilization consciousness, guide the masses concerned about minors, prevent telecom network fraud, build peace construction everyone to participate in, social peace everyone share thick atmosphere, for the construction of civilized and harmonious, safe and orderly peace and contribution youth strength.

2021-03-26	Dangshan county in Anhui province ancient city "home of the youth club" classes of dangshan county in Anhui province "youth club" classes youth home club " classes, the public welfare class around civil servants written test content, invited the industry senior teacher on the focus of the civil service exam training, and help students analysis, ease the test pressure, for young examinee learning opportunities, learning club more than 40 students to participate in concentrated teaching. For the further implementation of the long-term youth development plan (2016-2025), strengthen the position of youth home, TuanDangShan county relying on the ancient city of DangShan academy "youth" home "establish" youth home ", integrate teachers, form a branch, through the" green read, green, enjoy, green gen "form, build youth around the communist youth league" stores ". In the next step, the Dangshan County Party Committee will continue to rely on the Youth Home, pay attention to their needs, listen to their thoughts, grasp the pulse of youth growth, build a service platform for youth growth, and escort the development of youth.

2021-03-23	Ningxia Jinfeng district: micro pavilion party history for the new era good young Ningxia Jinfeng district: "the road to beginner's mind" micro pavilion for the new era good young wei correspondent Ma Rong), the Ningxia hui autonomous region Yinchuan jinfeng district party committee, jinfeng district city north street rainbow bay community in the "road" micro pavilion "party history, praise, follow the party, be a good young" theme activities, Yinchuan six education group 13 at 20 students into the "beginner's mind" micro pavilion to learn. Rainbow Bay community Party committee specially invited the area old party member Wu Yun to explain for the children, " children, do you know the origin of the party flag?"In grandpa Wu Yun's question, the children began to study that day. The "Road of the original aspiration" micro-exhibition hall is composed of three chapters: "remembering the original aspiration", "keeping the original aspiration" and "practicing the original aspiration". Grandpa Wu Yun led the students to review the history of the Party, recall the original aspiration, and understand the glorious course since the founding of the Communist Party of China. Students have expressed that they want to learn from the heroes, start from the small things around them, start from their own, inherit and carry forward the fine tradition of the revolutionary martyrs, study hard. After the visit, the students came to the Youth Home of the "Original Heart Road" micro exhibition hall to hold a symposium on exchange and learning, and review their vows of joining the League together. At the symposium, the students rushed to speak, " today I understand the birth of the party, felt the greatness of the Communist Party of China, through grandpa's explanation, let me more firm as a sense of mission, as the party's reserve army, I must study hard, do a useful person to the society."Student Li Nize said excitedly. Study the history of the Party, praise the party grace, follow the Party, this activity not only inspired the students' patriotic feelings, learn the spirit of the hard struggle of the Communist Party of China, but also make everyone realize the hard-won good life now, encourage everyone to follow the footsteps of the ancestors, do not forget the original aspiration to follow the Party, strive to be a good youth in the new era.

2021-03-25	Group Anhui Jiujiang district party committee held party history knowledge learning preach Anhui Jiujiang district party committee held 8090 branch party history knowledge learning preaching JianWei correspondent Yang JieZhou Huan huan) on March 23 afternoon, the group of Anhui Wuhu Jiujiang district party committee held "party history, strong faith, follow the party" party history knowledge learning preaching, invited Jiujiang district 8090 branch Sun teacher made entitled "party history enlightenment thought to promote development" special counseling, Jiujiang district group representatives, authority members and youth volunteers, a total of more than 40 people participated in the preaching. Sun Yongdong is the party branch secretary and principal of Tanggou Central School in Jiujiang District. He is mainly engaged in history teaching, with rich teaching experience and in-depth research on the party history. He was awarded as a senior teacher, and has won the title of Wuhu Key Teacher, Wuhu Advanced Individual of Teacher Ethics, Wuhu Outstanding Youth, Jiujiang Good Teacher and so on. At the beginning of this year, he took the initiative to register for the recruitment of Jiujiang District 8090 teaching group, and undertook the research and teaching of the party history section. Sun Yongdong teacher with profound theory, vivid historical facts, full of passion for the communist party of China 100 years of hard struggle, the glorious history of new China and so on the thorough and detailed explanation, through the important events of the communist party of China struggle, young revolutionary leader story lead you to review the communist party of China led the people in the arduous struggle. The lecture is rich in content, which is both theoretical and easy to understand, and has a strong theoretical guiding significance for all the youth league members to study the history of the Party. After the lecture, the scene also carried out the party history knowledge question and answer, the atmosphere reached a climax. After preaching, the youth feel deeply, said to cherish the "party history, strong faith, follow the party" the opportunity to learn education activities, further understand the history of the party, do history, history, chong DE, history, constantly inspire for the party started and responsible, bear as the vigor, to celebrate the 100th anniversary of the founding with honors.

2021-03-11	Anhui Province Huangshan District Committee to carry out the "Civil Code" knowledge lecture activities Anhui Province Huangshan District Committee to carry out the "" code "with the youth and law" knowledge lecture activities Jianwei correspondent, Wang Leqing Cai Kaihao) 2021 is the "eighth five" law popularization of the year. For the full implementation of the youth rule of law education outline), improve the youth rule of law literacy, to prevent juvenile illegal crime, promoting teaching, legal, recently, the Huangshan city in Anhui province Huangshan district party committee, district committee, soup center school organization to "" code "with young and law" as the theme of the "civil code" knowledge lectures. Soup mouth center school education teacher through vivid examples of the civil code involving minors large network reward spending should return, minors' family rights, the temptation of network bad information, network violence, network fraud and other related content, called on students to consciously research method usage, law-abiding, jointly create and maintain a good environment of the rule of law. By conducting the knowledge lecture activities, effectively popularize the knowledge of the civil code of teenagers, strengthen the consciousness of the rule of law, cultivate the youth of the rule of law belief, carry forward the spirit of the rule of law, build the district publicity learning thick atmosphere of the civil code, in efforts to build a higher level of peace Huangshan district, the rule of law to lay the foundation.

2021-03-18	Group in Anhui province county held the first micro group class group in Anhui province county held the first "don't forget to beginner's mind mission contribution youth strength" micro group game jianwei correspondent, He Chen Zhou Huan) to celebrate the 100th anniversary of the founding, encourage guide youth study history, history, publicity and implement xi new era of socialism with Chinese characteristics, on March 15, group ma on shan in Anhui province county in the county party construction service center held the first "don't forget the youth don't forget to beginner's mind mission contribution strength" micro group game. The competition attracted the county youth to sign up. After screening, a total of 21 from different positions in the county and youth members to participate in the on-site selection. Game, the players around the "don't forget the beginner's mind mission contribution youth strength" theme, in a lively, thorough and effective way about the party's history, advanced theory, system advantage, etc., at the same time, the players also combine the theme of innovation, according to their own interests, combined with current politics, news, teaching, highlights the youth vitality, youth positive energy, shows the determination of youth mission and faith. Finally, after a fierce competition, Li Xue from the county Data Resources Administration Bureau taught the micro group class "Capital Youth" won the first prize with a high score of 95.6. Held the theme micro group class aims to break through the traditional teaching way of teaching, let people around the things around, trying to speak really, speak vivid, feeling, with excellent works to inspire the member youth growth, encourage mountain youth show attitude, "young people" voice, to create a new stage of modern happiness in the construction of the majestic youth power.

2021-03-22	Ningxia hui autonomous region jinfeng district held red story sharing meeting Jianwei correspondent Ma Rong) "today I want to tell the story of lei feng, uncle lei feng wherever, do the good..." recently, a unique "childlike innocence to the party-hero story I will tell" red story sharing meeting in Ningxia hui autonomous region Yinchuan Jinfeng district Shanghai west road street read sea home community began, attracted many teenagers and parents stop to watch. At the event site, the contestants shared the stories of the revolutionary heroes they knew and loved."Today I am telling the story of Wang Erxiao, the hero of the anti-Japanese War. Although he is young, he is very brave in facing the enemy and is an example for us to learn from."Contestant Chen Xingbo said that he was very happy to participate in today's event, and he also heard many stories of other heroes and martyrs. He will study hard and be a qualified socialist successor. After the story-sharing meeting, the community staff presented the carefully prepared red books to the contestants."This activity is one of the series of activities we held to 'pay tribute to the Party', and also the beginning of the party history learning and education among the youth league members and young pioneers. In the future, we will closely focus on the theme of 'study THE history of the Party, strengthen our faith, and follow the Party', continue to use colorful study and education activities on Party history, deepen the ideological and political guidance of young people, and strengthen our ideals and beliefs."Tuan Jinfeng District Party Secretary Wang Li said. It is reported that the activity is jointly organized by Yinchuan Civilization Office, Jinfeng District Committee, Jinfeng District Shanghai West Road Street Yuehai Wanjia Community, aiming to deepen youth patriotism and patriotism education and revolutionary tradition education, inspire them to remember the history, cherish the present and look forward to the future.

2021-03-22	Group in Anhui province county cohesion "three promotion" strengthen the new era of lei feng spirit in Anhui province east to county cohesion "three promotion" strengthen the new era of lei feng spirit jianwei correspondent Liu Jing Cai Kaihao) in the 58th lei feng day, group Chizhou in Anhui province east to deepen the county of the new era of lei feng spirit connotation, led the county youth league organizations at all levels based on the new era, show new as, with more effective action to boost learn lei feng volunteer service activities flourish. Find the right focus, improve the pertinences. The county's grassroots youth league organizations will integrate volunteer service activities into all aspects of the economy and society, actively participate in the construction of beautiful countryside, rural living environment improvement, protection of "mother River" action, love and respect for the elderly and other key work, a total of 17 activities, participating in more than 200 young volunteers. Effectively for the civilized county to create a new fashion. Strengthen the cohesion, enhance the long-term effectiveness. To deepen learn lei feng volunteer service activities, the pku give full play to the role of condensed youth, form east to county youth volunteer federation, the social organizations, youth volunteer service youth work force, break the existing constraints, for east to county youth to participate in learn lei feng volunteer service plan out a new path. Highlight the action force, enhance the pragmatism. The Communist Youth League County Party Committee actively explored the means to improve the efficiency of volunteer service, set up a research group to Qingyang County to study and exchange the construction of Lei Feng Supermarket, studied and promoted the establishment of Dongjun Volunteer Supermarket, built a volunteer action supply platform, improved the interactivity and transparency of volunteer service, and stimulated the new vitality of volunteer service activities.

2021-03-19	Anhui Province Chaohu Municipal Party Committee to carry out jointly planting youth forest theme league day activity Chaohu Municipal Party Committee to carry out "protect mother river" jointly planting youth forest voluntary tree planting theme league day activity Jianwei correspondent Zhou Yu) the most spring benefits, planting trees add green is right. Recently, chaohu committee joint chaohu forestry and garden construction service center and Anhui chaohu peninsula national wetland park management center, organization evaluated integratedly and emerging areas, in the nest enterprise, social governance scheduling center, social organizations and other youth representatives of more than 60 people, to chaohu zhe gao river wetland park tree planting point, to carry out the "green line to build ecological civilization of chaohu-" protect mother river "plant youth forest tree planting activities" theme TuanRi activities. Walking from Guishan Park to Zhogao River Wetland Park, along the way, the "Protect Mother River in Action" activity was held to pick up garbage and promote the concept of environmental protection. Arriving at the voluntary tree planting site, the staff of the Linyuan Center introduced the overall situation of the wetland around the lake and the Zhegao River wetland project, and then carried out the voluntary tree planting activity. In The face of the spring rain, the young people, rolled up their sleeves to work hard, carrying saplings, digging pits, helping seedlings, filling the soil, watering and other series of work with an orderly, tacit understanding. Young people brave when the guardians of ecological civilization, with their own practical actions to plant chaohu youth forest. The theme youth league day activity made the youth league cadres further clarify their own responsibilities, and also made the volunteers further establish the concept of nature protection, and enhance the environmental awareness and social responsibility awareness of all staff. In recent years, the chaohu municipal committee adhere to the concept of green water peak is the jinshan yinshan, unswervingly ecological priority, green development, has carried out the tree planting green, "small hand", protect the mother river and a series of activities, to encourage and guide the youth and young pioneers for ecological civilization, green home builders, environmental protection practitioners, for the city's teenagers set up the "love green water castle peak starts from me" ecological concept. It has created a good atmosphere for everyone to care for the environment and contributed their youth to building a beautiful and livable Chaohu Lake.

2021-03-19	Ningxia yongning county held the history of the party learning education seminar activities Ningxia yongning county party history learning education seminar activities Jianwei correspondent Ma Rong) to celebrate the overall victory in poverty battle, to unite youth, condensed youth, with the power of youth to lead more youth to join the rural revitalization of the work, into the rural revitalization of the development. Recently, by the Ningxia hui autonomous region committee, the CPC Yinchuan municipal party committee propaganda department, Yinchuan committee, the communist party committee propaganda department, the yongning county committee, yongning county federation of literature and art, group Minning town committee of the "youth to the party drive mountain love youth said" keynote speech contest in Minning town FuNing community conference room on the second floor. Zheng Jinxuan, Minister of the United Front Work Department of the Standing Committee, Duan Yan, Secretary of the Youth League Committee of Ningxia Vocational and Technical College, and Wang Yanju, Deputy Secretary of the Yinchuan Youth League Yinchuan Committee attended the competition. More than 120 people, including representatives of grassroots youth league organizations, youth representatives and student representatives of Ningxia Vocational and Technical College, attended the speech contest. The speech contest was also broadcast live on the "Youth Yinchuan" TikTok platform. Before the competition, the contestants and the student representatives of Ningxia Vocational and Technical College came to Linan winery, Zhao Hong Farmer Culture Courtyard and Minning Town History Hall to visit the development history of Minning Town. The person in charge's wonderful explanation made everyone have a deeper feeling about how Minning changed from the former "dry beach" to the current "golden beach". At the beginning of the competition, the 13 contestants were full of spirit and passion. They expressed the most beautiful youthful attitude of coming to Minning Town with the majority of youth. They felt under the leadership of the Party and the government, the east and west cooperation fruitful poverty alleviation results, and felt the deep friendship of the people of Fujian and Ningxia across the mountains and seas. Wang Dayu, secretary of the Youth League Committee of Yinchuan Senior High School, and other seven judge teachers scored and commented on the speech program from four aspects of speech content, language expression, body language and field effect. Finally, they selected 1 first prize, 2 second prize, 3 third prize, 7 excellence awards, and 1 of the most popular award. Tong Hailong, a grade 18 student from Northern University for Nationalities, won the first prize for his speech titled "Towards the Mountain", and Deng Wenxia's speech by a grade 20 student from Ningxia University, won the most popular award on TikTok live broadcast platform. Near the end of the game, Ningxia vocational and technical college youth corps committee secretary Duan Yan and MinNing town youth corps committee secretary Zhao Rui respectively on behalf of Ningxia vocational and technical college and Minning town pair to build the site signing ceremony, Minning town will serve as the Ningxia vocational and technical college students' social practice base, to carry out a variety of forms of pair construction activities. This speech contest brings us not only the enjoyment of hearing, but also the edification of the soul. Left behind is not only moved, but also encouraged. It has inspired the passion and responsibility of the young people in the new era, have the courage to strive to be the backbone of China, stay true to our original aspiration, keep our mission firmly in mind, and forge ahead with hope.

2021-03-19	Chengdu jianyang, Sichuan province to carry out anti-drug education into the campus theme activities don't let drugs into the campus good school first lesson of Sichuan jianyang city to carry out anti-drug education into campus theme activities He Xin) to meet the 31st international drug day, continue to pay special attention to the youth drug prevention education work, on March 15, the Sichuan jianyang municipal party committee joint city bureau of education, city, DDc to carry out "don't let the drugs speak good school first lesson" anti-drug education into the campus theme activities. Activities, jianyang youth anti-drug social workers with humorous vivid, plain language, by watching anti-drug case video, live interactive questions, drug prevention knowledge, drug knowledge contest, for the event and through the live webcast of the primary and middle school students in the city to the importance of drug control, the harm of drug use and teenagers should be how to prevent drug resistance, and to the city's students initiative not to let drugs into the campus. Jian Yang, vice mayor, municipal public security bureau chief, inspector general Zhong Shibin in sharing with the students stressed that teenagers is the future of the motherland and the hope of the nation, more alert to drugs, to understand the drug knowledge, know the harm of drugs, consciously away from drugs, actively report drug illegal crime, resolutely fight against drug illegal and criminal behavior. Tuan Jianyang Municipal Party Committee related responsible comrade introduced, teenagers are in the physical and psychological development period, heavy curiosity but the ability to distinguish between right and wrong is weak, the lack of understanding of the harm of drugs, easy to be subject to the infringement of drugs. This activity hopes to make full use of the favorable opportunity of students in the new semester, constantly enhance the ability of students to identify, prevent, and resist drugs, and guide the majority of students to establish the concept of "healthy life, green and non-toxic" in their study and life. More than 200 teachers and students participated in the event. More than 60,000 primary and middle school students in the city listened to a vivid anti-drug publicity class through the online cloud live broadcast.

2021-03-18	Yingquan District, Fuyang City, Anhui Province to carry out the "River Xiaoqing" Clean Beach Action to Learn from Lei Feng volunteer service activity Zhang Jianwei, correspondent Huang Qian, Zhou Huan Huan) in order to carry forward the spirit of Lei Feng, enhance environmental awareness. Recently, the Yingquan District Committee of Fuyang City, Anhui Province, together with the "River Xiaoqing" volunteer service team of the District Audit Bureau and the Youth Volunteer service team of the District Tax Bureau, carried out the 2021 "River Xiaoqing" Clean Beach Action in Yingquan District. Environmental protection is a kind of consciousness, but also an action. Activities, young volunteers in the river scenic zone to carry out the beach action, on the bank of the rubbish, along the river of plastic bags, waste paper, glass bottles, cans and other garbage debris sorting cleaning, to the past people promote water resources protection, care for the environment and other ecological environmental protection concept, young volunteers take concrete actions to practice environmental protection concept propaganda lei feng's spirit. Participate in the activities of young volunteers have said, later will continue to participate in clean beach, starts from oneself, be a good ecological civilization protection propagandist and practitioners, with practical action to practice "green water castle peak is the jinshan yinshan" environmental protection concept, to protect the ecological environment become a new social fashion, contribute to the city's ecological civilization construction."River Xiaoqing" is the general term of the young people who participate in the protection of the mother river, help the river chief system and practice the ecological civilization. It is the assistant of the river chief and the participant and supporter of the implementation of the river chief system, the advocate and practitioner to protect the health of rivers and lakes, and the main force to promote the volunteer service of ecological civilization. In the future, the Communist Youth League District Committee will regularly hold river patrol and river protection activities, organize and mobilize more youth league members to participate in the vivid practice of "River Xiaoqing" volunteer service, enhance the awareness of water protection and love of water protection, and jointly protect the clear water and green mountains.

2021-03-18	Fujian Nanping City Wuyi Mountain "1314 color run" activity held Fujian Wuyi Mountain City "1314 color run" activity held yishan City Wufu Town tourist service center square, to participate in the "look up brilliant, open the new journey" rice fragrant cup "1314 color run" activity. At the event, more than 600 contestants, with the help of young volunteers, dressed themselves up with "rainbow powder", wig, headband, hair cards and stickers. Starting from the Wufu Town tourist service, they passed 500 mu of colorful rape flowers and ran along the colorful ring town road. The 5.2 km "Love supply station" and 10.1 km "Youth Singing station" of the TuanWuyishan Municipal Committee attracted many contestants to stop and interact."Qingyuan" dating activities, romantic sketches and "520" landmarks attract contestants."1314 color run" activity is wuyi city to meet the 100th anniversary, the implementation of the party history learning education related spirit, deepen the Fujian provincial party committee "learning, research, and implement" activities, promote consumption, promote tourism, and promote the development of wuyi city tourism with high quality, to speed up the body brigade fusion, the development of rural tourism, global tourism of a attempt.

2021-03-12	Group of Shandong Province Dezhou City Linyi County Party Committee to carry out the post to the countryside tour job fair group of Shandong Province Linyi County Party Committee to carry out the recruitment caravan to send the post to the countryside tour job fair theme volunteer service activities Jianwei correspondent Zhang Mingrui) in order to carry forward the spirit of Lei Feng, Advocating the volunteer spirit of "dedication, friendship, mutual assistance and progress", a few days ago, Group of Linyi County Party Committee, Shandong Province, Dezhou County, united county Human Resources and Social Security Bureau, county industry and Information Technology Bureau and other organizations of more than 20 enterprises in Deping town to carry out the "Linyi County recruitment caravan to send posts to the countryside tour job fair", To promote the orderly development of the job fair, Volunteers will simultaneously carry out themed volunteer service activities, Officially opened the 2021 learning from Lei Feng Volunteer Service month prelude. At the site of the activity, the young volunteers provided the public with post docking, policy consultation, safety publicity and other aspects of the publicity color pages through the display panel publicity. The activity distributed more than 300 publicity pages. The Communist Youth League County Party Committee proposed that the students outside Linyi County take the initiative to understand their hometown, understand their hometown enterprises, to join the construction of the hometown team, with knowledge and talent for the economic and social development of Linyi with passion and sweat, achievements, to achieve their own life ideals. Group Linyi county, Linyi county youth volunteer association called on the county member youth inheritance lei feng spirit, participate in volunteer service, emotional service for the people, transfer social positive energy, cultivate and practice the socialist core values, in the county to learn lei feng volunteer service activities of the new climax, for the construction of the county strong people rich political tong and new Linyi contribute their youth strength.

2021-03-18	Group dangtu county in Anhui province to carry out the franco-prussian campaign in Anhui province dangtu county to carry out the franco-prussian campaign JianWei correspondent, TongMeng wan Zhou Huanhuan) in order to further strengthen the rule of law propaganda work, correctly guide consumers to safeguard their legitimate rights and interests, recently, group of ma on shan in Anhui province dangtu county joint yellow pool town youth corps committee, judicial offices, market supervision and management departments organize youth volunteers in shops more concentrated pedestrian street area for 3.15 consumer rights, the franco-prussian propaganda activities. Event, the young volunteers issued to the masses "the consumer rights protection law" food safety law " and other publicity materials, to consult the masses on consumer rights and interests protection laws and regulations knowledge, and combined with the real case around to guide to safeguard their legitimate rights and interests, advocating quality consumption concept, improve safety awareness and self-protection ability. This activity distributed more than 200 copies of publicity materials, and received more than 20 on-site consultation.

2021-03-18	Anhui Huaibei City, Xiangshan District West Street Youth League Working Committee to carry out the memory of the Party history, Anhui Province, Xiangshan District West Street Youth League Working Committee to carry out the memory of the Party history of the Party class activities Jianwei correspondent, Hu Wenjing, Zhou Huanhuan) in order to effectively carry out the party history learning theme education activities, Constantly create a new situation in the work of the Communist Youth League, Grasp the characteristics of teenagers, Close to the needs of teenagers, We will tell the stories of the Party, revolution, and heroes well, Guide them to listen to and follow the Party, a few days ago, The Youth League Working Committee of West Street, Xiangshan District, Huaibei City, Anhui Province, organized outstanding representatives of young Pioneers, party members and league members to come to the home of revolutionary veteran Li Wenxiang, Listen to the old man tell the story of the battle. The 98-year-old Li Wenxiang, told us about the War of Resistance against Japanese aggression, the War of Liberation and other countless extraordinary years, and gave you a vivid party lesson with his personal experience. The old man's story brought us back to the past years, the children lamented that without the revolutionary martyrs of the happy life of today. Teenagers are the hope of the motherland and the future of the nation. The youth stage is also a key period for the formation of the outlook on life, world outlook and values. To carry out the study and education of Party history among young people is not only an important part of the CPC Central Committee's plan for the study and education of Party history in the whole Party, but also an urgent requirement to promote the healthy growth of young people and train the builders and reliable successors of the socialist cause in China. In the future, group mountain committee will continue to speak good red story, red gene, absorb the strength and confidence of struggle forward, build the history of the party, understand the history of the strong learning atmosphere, the history of the experience and wisdom into practical measures to study and work, inspire everyone's historical sense of mission and sense of responsibility.

2021-03-16	Group of Anhui province huaining county to learn lei feng volunteer tree planting activities in Anhui province huai ning county to carry out the "youth heart to the party volunteer you I" learn lei feng volunteer tree planting activities JianWei correspondent, Liu Xiaoli Zhou Huanhuan) to inherit and carry forward the lei feng's spirit, practice of green ecological concept, recently, the Anqing city in Anhui province huaining county joint county forestry bureau, public ridge town organization more than 40 young volunteers to public ridge town three shop village stone ridge "youth heart to the party volunteer you" learn lei feng volunteer tree planting activities. During the activity, the young volunteers were enthusiastic and energetic, and the tree planting site showed an atmosphere in full swing. We division of labor and cooperation, shovel digging, fill the soil planting trees, each link of the orderly connection, tacit understanding, conscientiously completed each step of the planting of trees, hits the newly planted saplings stand on the land, for the early spring added vitality. Subsequently, the volunteers came to Gongling Central School to carry out the forest chief system, forest fire prevention into the campus publicity activities, the volunteers actively publicize the forest chief system and forest fire prevention knowledge to students, parents issued forest fire prevention card and other publicity materials, to further enhance everyone's awareness of forest fire prevention, establish the concept of love and forest protection. More than 300 saplings were planted, and more than 1,000 leaflets were distributed. In the next step, the county Party Committee will carry out a series of volunteer service activities in combination with the "Learn from Lei Feng Activity Month", guide the league organizations and youth league members at all levels to actively participate in it, strive to be the inheritor, practitioner and promoter of Lei Feng spirit, and contribute its youth strength to promoting the construction of "two districts and two centers" in Huaining.

2021-03-16	Group of Anhui province Qianshan municipal party committee to learn from lei feng theme volunteer service activities of Anhui province Qianshan municipal party committee to carry out the "learn from lei feng" theme volunteer service activities JianWei correspondent Xie Qian Zhou Huanhuan) to further carry forward lei feng's spirit, advocating "dedication, love, mutual assistance, progress" volunteer spirit, show contemporary Qianshan youth good spirit, recently, group Anqing city in Anhui province Qianshan municipal party committee joint tianzhu mountain tourism school youth corps committee, qian mountain power supply company youth corps committee to carry out the "youth to learn from lei feng to build civilized city" theme volunteer service activities. Sixty volunteers organized by TuanQianshan Municipal Party Committee and the Youth League Committee of Tianzhu Mountain Tourism School came to Erqiao Park to carry out the volunteer service activity of "picking up garbage and cleaning the environment". Young volunteers are not afraid of hardship and dirty, and constantly pick up garbage in the green belt and grass of the park and purify the environment. During the activity, the volunteers practiced the plastic restriction order, used waste newspapers to make homemade garbage collection bags in advance, and collected the collected garbage collected and put them into the trash can. At the same time, the volunteers also actively urged the people walking in the park, calling on everyone to regulate their own behavior, improve environmental awareness, and build a better home. Subsequently, the volunteers of TuanQianshan Municipal Party Committee and Qianshan Power Supply Company came to Qianshan Special Education School together. The volunteers gave a vivid and vivid lesson on electricity safety education to the students and parents of the special education school through the form of cartoon display and case explanation. The students listened carefully and enthusiastically raised their hands to answer the questions. The volunteers also gave small gifts to the students who answered correctly. Although the course is simple, it effectively deepens students 'knowledge of electricity safety and improves students' ability to take care of themselves. After the course, the volunteers walked into the class, introduced Lei Feng to the students, interpreted Lei Feng's spirit in a simple way, and taught the students to advocate truth, goodness and beauty, and do good things within their ability. With the help of the volunteers, the students painted the portrait of "Lei Feng" according to their own imagination of Comrade Lei Feng, and held up their own finished portrait. The students' faces showed a sincere and sweet smile. At the end of the activity, the volunteers presented schoolbags, colored pens, notebooks and other learning stationery to the students, and encouraged them to build up confidence, face life bravely, show themselves boldly, and live a healthy and happy life every day. March 2021 is the volunteer service activity month of Qianshan City. Qianshan Municipal Party Committee issued a call to "learn from Lei Feng and build the spirit of a civilized city". In the next step, TuanQianshan Municipal Party Committee will carry out all kinds of volunteer service activities.

2021-03-16	Group Anhui province district party committee to carry out the finless porpoise protection volunteer tour group Anhui province district party committee organization volunteer finless porpoise protection volunteer tour activities jianwei correspondent, Li Tianyi Zhou Huanhuan) to earnestly study and implement the Yangtze river protection law of the People's Republic of China, in mid-march, group Anqing city of Anhui province district party committee, bureau of agriculture and rural areas organization more than 20 volunteers to Anqing city xijiang Yangtze finless porpoise protection base for volunteer patrol activities. During the activity, the Youth League Daguan District Committee organized the youth league members and youth volunteers to clean up around the Xijiang finless porpoise protection base. Thanks to their active efforts, protecting the plastic bags, old fishing nets and other garbage around the base were quickly cleaned up, creating a safe and clean living environment for the finless porpoises. Then, the Daguan District Committee of the regiment and the young volunteers, under the leadership of the captain of the finless porpoise protection base, boarded the patrol speedboat, to patrol the waters of the reserve, and observed the living conditions of the finless porpoises. The volunteer patrol activity aims to encourage the public and all sectors of society to participate in the protection of the Yangtze finless porpoise and the ecological environment restoration of the Yangtze River waters with the practical actions of the youth league members and youth volunteers, so as to jointly create a green and beautiful new grand view.

2021-03-15	Taihe county in Anhui province to carry out the "I do the practical work for the masses" practice group of Fuyang city in Anhui province to do the practical work for the masses, listen to public opinion, does the practical work "activities build wei correspondent Wu Qian Cai Kaihao) to promote the party history learning education, seeks deep seeks real" I do the practical work for the masses "practice, practice service for the people, promote party history study education, recently, group of Fuyang city in Anhui province county organization" I do the practical work for the masses, listen to public opinion, does the practical work " visit research activities. Yu He, party secretary of Taihe County, led a team to caiiao Town, Piusun Town and Guxian Town and other package service units to carry out research. The research team arrived at Cailiao Town, Picsun Town and Jiuxian Town to carry out research. Through exchanges and discussions, they learned more about the local safety construction, the effective connection between poverty alleviation and rural revitalization, and the protection of minors. Carry out face-to-face communication with the town and village cadres and the masses, communicate with the practical issues of education, health, travel, employment and other issues strongly reflected by the masses, and collect the hot and difficult issues in production and life that the masses strongly reflect and urgently need to solve on the spot. Research group also field investigation of the local youth entrepreneurship, listened to the youth enterprise operations, face to face understand the difficulties encountered in the process of youth enterprise development and entrepreneurship, the recent youth entrepreneurship loans small discount project site interpretation, encourage young entrepreneurs to listen to the voice of the party, the party's policy, do more good beneficial to the development of enterprises and hometown, and the county how to further service youth entrepreneurship employment work for advice."I do practical things for the masses- -visit the people, listen to the public opinion, do practical things" activity is a concrete measure to promote the study and education of party history, practice the purpose of serving the people, and promote the learning and education at that time. Youth League Taihe County Party Committee, in strict accordance with the requirements of the activity notice, will be this activity and the development of heart-to-heart talk, the annual democratic life to investigate the problem combined, multi-angle, multi-level, multi-aspect to solicit opinions and suggestions from the masses. Next, will be in accordance with the requirements of the work, to the opinions of the masses proposed suggestion sorting, registration by inputting, and can solve the problem quickly, to report to report problems, work together to concentrate, guide, serve the masses development, for poverty engines and rural revitalization effective cohesion provide due help, with honors to the 100th anniversary of the gift.

2021-03-12	Anhui Tongling teenagers protect the Yangtze river volunteer service activities teenagers protect the Yangtze river volunteer service bureau, Tongling middle school youth corps committee in binjiang ecological park "protect the Yangtze river mother river, protect the blue sky dream" theme education volunteer service activities, guide Tongling high school students in policy to understand the significance of the Yangtze river protection, rooted in volunteer service ecological environmental protection concept. During the activity, the staff of the Public Legal Service Management Department of Tongling Justice Bureau explained the legislative background and significance of the Yangtze River Protection Law carefully. Geography teachers of Tongling No.3 Middle School led students to conduct practical exploration, and inspired students to think about the whole ecosystem of the Yangtze River by exploring the fish population changes in the Yangtze River. After the study, the students became small volunteers and came to the river to clean up the garbage. In recent years, Tongling city has guided young students to learn a set of environmental protection teaching materials, visit an environmental protection education base, and participate in an environmental protection volunteer service in primary and secondary schools, making the concept of environmental protection, thrift, low-carbon consumption and green development become a norm.

2021-03-12	Sichuan Qingchuan county: learning traffic safety knowledge to be guide and propagandist in Sichuan Qingchuan county: learning traffic safety knowledge to be guide and propagandist Zhang Jianwei correspondent Xu Chuanliang) on the arrival of "lei feng day", Sichuan province qingchuan county traffic police brigade in Qiao Zhuang town center primary school conducted traffic safety knowledge lectures and civilized youth volunteer service activities, learning traffic safety knowledge when traffic safety guide and propagandist. Traffic police brigade police to "abide by the traffic safety law harmony and peace 100%" as the theme to carry out the traffic safety knowledge lecture, explained in detail how to walk safely, ride, ride and the ways to avoid traffic accidents. Through the way of lectures, to better help students to understand the traffic safety knowledge, improve the self-protection ability and traffic safety awareness, can effectively avoid the occurrence of traffic accidents. After the lecture, the police also led the students to Binhe Road Street, to assist in dredging traffic, maintain traffic order, dissuade uncivilized traffic behavior volunteer service, remind drivers to fasten their seat belts, and strive to be traffic safety guides and propagandists. Students can participate in social life through volunteer service, carry forward the Lei Feng spirit, enjoy the happiness brought by dedication, deepen the students' understanding of the Lei Feng spirit, form a good atmosphere of learning from Lei Feng, and strive to be a little Lei Feng in the new era. The development of this activity, not only let the students know the importance of traffic safety, improve the awareness of traffic safety, so that the students develop good traffic habits from childhood, put an end to jaywalking, running red lights and other unsafe behaviors. And through the way of practice, also let the students more profound understanding of helping others, willing to contribute to the Lei Feng spirit.

2021-03-10	Group Bazhong, Sichuan province en Yang district party committee to create a new situation the young pioneers cause Bazhong, Sichuan province en Yang district party committee: comprehensively strengthen the political leading efforts to create a new situation the young pioneers career jianwei correspondent Xu Hong fang haiyan) recently, group Bazhong, Sichuan province en Yang district party committee held the study and implement the opinions of the central party of China on comprehensively strengthening the new era of the young pioneers work "(hereinafter referred to as the" opinions ") workshops. At the special meeting, the original text of the Opinions was conveyed and interpreted, discussed and exchanged, and made arrangements for the implementation of the current Opinions. Conference pointed out that the "opinion" as the first in the history in the name of the central committee issued to strengthen the young pioneers work file, with xi comrade as the core of the CPC Central Committee for children and the young pioneers work important, profoundly illustrates the nature of the young pioneers organization orientation, responsibility function, further answer, clear declared "young pioneers are what kind of organization" "young pioneers cultivate what kind of people" and other basic, source problem, pointed out the direction of the young pioneers for the new era, outline the blueprint. Meeting requirements, All cadres of the Communist Youth League district Committee should adhere to the leadership of the Party, Led the youth league organizations at all levels in the district to conscientiously study the spirit of the "Opinions", In various forms of extensive publicity and interpretation, Mobilizing all parties to participate, Strive to form a good situation in which the whole society jointly cares about and supports the work of the Young Pioneers; To prepare for the second Enyang District, With the working force of the Communist Youth Working Committee, The close combination of the organization construction of the young Pioneers and the connection work of the Party and the league, Adhere to the working principle of "Party building leads the league building and promotes the team building", Strive to form a good situation in which the Party, the Communist Youth League and the team promote mutual progress and common development; Make full use of the "June 1", "113" team building day and other young Pioneers major festival activities to carry out a good young Pioneers entry ceremony, "Red scarf medal" two-star chapter selection and other series of activities, To build the young Pioneers honor incentive system, Strive to create a new situation in the cause of the Young Pioneers.

2021-03-09	Guizhou province Zunyi renhuai for youth financial service platform for Guizhou zhunyi renhuai for youth financial service platform jianwei correspondent Chen Kai) " thanks to pku and maotai agri-businesses bank for our youth entrepreneurship build such a good financial service platform, we must use a good platform, efforts to entrepreneurship, give back to society."After the" Rural Youth Financial Entrepreneurship Service Station " awarding ceremony, several youth innovation enterprises on behalf of the legal person and Moutai Rural Commercial Bank site held a credit granting ceremony. Recently, Zunyi City, Guizhou Province, Renhuai City "syndicate" linkage efforts, TuanRenhuai Municipal Party Committee held the "Rural Youth Financial Entrepreneurship Service Station" awarding ceremony with Maotai Rural Commercial Bank. After the awarding ceremony, Moutai Rural Commercial Bank became a new member of the "Rural Youth Financial Entrepreneurship Service Station". Award ceremony, pku, set up in banking institutions "rural youth financial entrepreneurship service station", aims to further promote renhuai youth especially rural youth "double gen" financial services, for want to, can director of "double gen" youth financial information consulting, financial products recommendation, credit financing services, financial knowledge training, etc., to encourage more youth positive innovation entrepreneurship. In the next step, the Communist Youth League Municipal Committee and Moutai Rural Commercial Bank will give full play to the resource advantages of "bank" and the organizational advantages of "youth league", strengthen resource integration, policy coordination, linkage and coordination and information sharing, and build a good entrepreneurship platform for "mass entrepreneurship" youth. Strengthen the establishment of "green" brand financial products, encourage and support the development of young people entrepreneurship products, make youth entrepreneurship product and service manuals, etc., tailored to young entrepreneurs, and enhance the credibility of "green" brand products. Do a good job in the "youth" service "articles", strengthen two-way communication, take the initiative to sink the service, strengthen financial knowledge training, vigorously carry out financial knowledge publicity, focus on research and solving prominent problems, improve the ability of young people to deal with risks, and provide convenient services for youth entrepreneurship. The Renhuai supervision group of Zunyi Banking Regulatory Sub-bureau said that all banking institutions should attach great importance to the financial services of young people, especially rural youth, in entrepreneurship, actively build entrepreneurship service platforms, introduce a series of preferential policies, effectively help solve the practical difficulties of youth entrepreneurship, and vigorously support young people, especially rural youth, in innovation and entrepreneurship. After the awarding ceremony, the person in charge of the credit management department of Moutai Rural Commercial Bank explained in detail the inclusive financial policies related to youth entrepreneurship and development. The representatives of Qingchuang invited to attend the ceremony said that they would respond positively, actively convey the good policies of financial services in the young Chamber of Commerce, and guide the 168 enterprises of the Youth Chamber of Commerce to make good use of all kinds of "green" financial service products. It is understood that up to now, TuanRenhuai Municipal Party Committee has completed the joint construction of "Rural Youth Financial Entrepreneurship Service Station" with two banking institutions, Guizhou Bank Renhuai Branch and Moutai Rural Commercial Bank. In the next step, the Renhuai Municipal Party Committee will give full play to the role of a bridge, strengthen the contact with the banking institutions within the jurisdiction, and further strengthen the joint construction and joint creation with the banking league organizations on the basis of serving the youth innovation and entrepreneurship, so as to gather more strength for the city's economic development and rural revitalization strategy.

2021-03-09	Anhui Province Maanshan Dangtu County Party Committee group of Anhui Province Dangtu County Party Committee to carry out "love in Dangtu" dream micro wish activities Wei correspondent, Tong Meng Wan Cai Kaihao) recently, "love in Dangtu" dream micro wish activities into Dangtu County, Anhui Province. Huang Yueyi, deputy secretary of the Dangtu County Party Committee, representatives of Dangtu County Young Entrepreneurs Alliance, representatives of social caring people and donated students attended the ceremony. Activities by visiting MoPai, group dangtu county to protect the river center school collected 50 "wish", and through WeChat public number to the public love information, got the people from all walks of life of positive response, dangtu county young entrepreneurs alliance and a number of social enthusiasts have claim micro wish, for "micro wish" interpreta dream. At the event site, the volunteers distributed wish gifts to the micro-wish student representatives, transmitting the warmth and love of the society to the hearts of the children, and encouraged them to study hard, cultivate good moral character and civilized habits, and strive to be good teenagers in the new era. In 2021, the Youth League Dangtu County Party Committee will continue to organize and mobilize volunteers and caring forces from all walks of life to promote regular material assistance and spiritual care activities, and implement the work of caring for teenagers.

2021-03-05	Anhui Dingyuan county home students new era civilization practice of Chuzhou city, Anhui province Dingyuan county organization returning college students new era civilization practice wei correspondent Wu � S He Xiangyu Zhou Huanhuan) to lead young students do brave bear national rejuvenation era, in the process of social practice by education, long ability, contribution, recently, Dingyuan county of Chuzhou city, Anhui province, county jointly organized the "students hometown line together for revitalization of" new era of civilization practice, 18 Dingyuan key college students were invited to attend. The first stop of this activity is Anhui Chuzhou national agricultural science and technology park, the event held the "dingyuan county new era civilization practice agricultural science base" opening ceremony, then the young students have visited the characteristic agricultural products exhibition hall and agricultural industry planning hall, Anhui the fungus industry technology co., LTD., jianghuai watershed test station, and experience the fun of picking mushrooms."This is the first time that I have seen mechanized agricultural production and seen a new look of agriculture in Dingyuan."Said Yang Chengxin, a student at the University of Science and Technology of China. Through a series of visits, experience activities, to further lead the college students to approach their hometown, into the grass-roots level. At the symposium held after the visit, Chen Jiahong, the executive deputy stationmaster of the Jianghuai Branch Ridge Experimental Station, first shared the advanced deeds of Zhang Zijun, growing from a sheep boy in northwest China to a sheep expert who cultivated new varieties, and from a college science and technology worker to a worker involved in the front line of rural revitalization. Everyone has said that to follow the example, to walk with the model, to pay tribute to the faith."As a rural person, I think the biggest change in my hometown is that the network environment has become better. I can never make video phone calls from before, but now I can really use 4G."Said Cao Yong, a student at the Beijing Technology Institute of Technology."It is in line with public opinion to restrict the discharge of fireworks and firecrackers during the Spring Festival, but the ancient city should increase the promotion of Dingyuan culture and strengthen health and environmental management."Said Dai Hongwei, a student at Xiamen University."My hometown is faced with the shortage of talents. We should not only rely on our feelings to retain people, but also attract talents with policies and treatment."Said Huang Chengchen, a student of Jinan University. At the symposium, young students offered suggestions and suggestions for the development of their hometowns, highlighting the image of Dingyuan youth in the new era with practical actions. After the symposium, the Communist Youth League County Committee presented the book "Xi Jinping and College Students Friends" to young students, encouraging them to firm their ideals and beliefs, cultivate the people's feelings of the people, work hard, highlight the strength of youth, realize their personal value, and let the light of youth illuminate the way forward of the great rejuvenation dream. Through this activity, the young students saw the great changes in their hometown, felt the warmth of their hometown, learned about the policies and measures of returning to their hometown to start businesses and find employment, and won the unanimous praise of the young students. Everyone said that in the future study and life, to release the youthful passion of youth, to pursue the ideal of youth, to constantly improve their comprehensive quality, to contribute to the new stage of modernization and beautiful distant construction of youth strength.

2021-03-05	Group of Huaibei city in Anhui province district for the party history reading sharing league in Anhui province district party "know the history of the party, party history, party history reading sharing wei correspondent, Lv Weixu Cai Kaihao) for the further study and apply xi new era, the ideas of socialism with Chinese characteristics, strengthen the party spirit education, firm ideal faith, further enhance youth historical sense of mission and sense of responsibility, encourage members play an exemplary vanguard role, Huaibei city in Anhui province district service center for" know the party history, party history, party history " reading sharing. At the sharing meeting, the youth members reviewed the birth, setbacks and suffering of the Communist Party of China, and saw the continuous growth of the Party with a single spark and a prairie fire to start. Looking back on history, we can clearly see that whether in the new democratic revolution and socialist revolution, or in the great new revolution of socialist construction and reform and opening up, there are both ups and downs, waves, and the difficulties of hesitation and trapped in the mire. Through the study of the party history reading club, the youth league members reviewed the great course of our party to overcome all difficulties and continuous development, strengthened the ideals and beliefs, strengthened the party spirit cultivation, and enhanced the sense of responsibility and mission. In the future, we will continue to study the history of the Party and the country, so as to sum up the historical experience, grasp the laws of history, enhance the courage and strength to explore and advance, and be a pioneer in the new era with full enthusiasm and high morale.

2021-03-04	Guizhou three sui county grassroots youth league organizations to carry out the New Year voluntary tree planting activities in Guizhou three sui county grassroots youth league organizations to carry out the New Year voluntary tree planting activities wei correspondent Chen Kai) recently, Guizhou Qiandongnan three sui county each township (street) youth corps committee organization plan volunteers, youth members to carry out voluntary tree planting activities, take concrete action for three sui county to add new green. Changji Town, Tonglin Town, Bong Town, Wubi Street Youth League Committee and other volunteers and youth league members to carry out voluntary tree planting activities carried out. At the tree planting site, we waved a hoe to dig pits, planting seedlings, soil watering, to contribute to the construction of ecological beautiful countryside. According to statistics, the total planting of osmanthus, camphor, rowpa, yew and other seedlings more than 1200 plants. Through this tree-planting activity, we can further improve the youth volunteers and youth league members' awareness of environmental protection, strengthen the sense of responsibility of green home, and play the role of youth for the ecological civilization construction of Sansui County.

2021-03-03	Ningxia Lingwu City held a "spring breeze action" large-scale job fair correspondent Ma Rong) in order to further do a solid job of "six stability" work, Fully implement the task of "six guarantees", To deal with the problem of enterprise spring recruitment and employment, On the morning of February 25, Yinchuan Lingwu Municipal Party Committee of Ningxia Hui Autonomous Region, 2021 Lingwu Municipal Human Resources and Social Security Bureau, Veterans Affairs Bureau, the Federation of Trade Unions and other units held the 2021 Lingwu Municipal "Spring Breeze Action" large-scale job fair, Provide job seekers with recruitment and job registration, exchange and negotiation, policy consultation and other comprehensive services, To promote the employment and entrepreneurship of young people in the city. This activity focuses on urban and rural surplus labor force, veterans, college graduates and other employment difficulties groups. More than 40 enterprises, such as Ningxia Yimei Biological Co., Ltd., Meituan Delivery Company, Ningxia Victoria Construction Engineering Co., Ltd., have provided more than 400 technical and service-oriented jobs for young job seekers, distributed more than 330 publicity materials, attracted more than 1,000 people to browse and consult, and initially reached the employment intention of 169 people.

2021-03-03	Longyan city of Fujian province to get flat outstanding students return view practice Fujian get flat to carry out the "double first-class university" outstanding students return view practice committee organization from Beijing university, Zhejiang university, Shanghai jiaotong university, Xiamen university and other 25 schools more than 40 "double first-class university" get flat outstanding students, return to see the practice activities. Two days of view practice, the students have visited dragon car revolution memorial hall, cherry blossom tea garden, hong ding farm development co., LTD., for town west mountain village young college students electricity sales platform, get a flat "city" fusion development exhibition hall, Fujian dragon steel new materials co., LTD., the Mars research base, etc. Through the on-site observation, the students accepted the baptism of the party spirit, appreciated the modern industrial civilization, shared the achievements of rural transformation and youth entrepreneurship, and witnessed the all-round rapid development and amazing changes of their hometown. The organizer also held a symposium for outstanding students, where Chen Tiancai, Member of the Standing Committee of Zhangping Municipal Party Committee and Minister of the United Front Work Department, Vice Mayor Zhang Muzhi, and Li Wenjing, Vice Minister of the Organization Department of the Zhangping Municipal Party Committee attended the meeting. At the symposium, the student representatives talked about their feelings, experiences, suggestions and development in terms of study and life, career development planning and their thoughts on local development. In addition, the students also watched the publicity video of Zhangping city construction, and received the gift of "Xi Jinping and College Students' Friends" and the talent policy compilation manual. Through the on-site observation and practice and symposium, we can further understand the good development environment of Zhangping, the policies related to talent introduction and the development and change of my hometown, and enhance the sense of identity of my hometown and the confidence in serving my hometown well. In the next step, TuanZhangping Municipal Party Committee will also hold various forms and rich content activities to mobilize outstanding students to take the initiative to integrate into the overall economic and social development of the city, and effectively enhance the sense of responsibility of serving their hometown.
