# Supplementary material for: Serving organization goals by organizational information dissemination: An empirical study from the Communist Youth League of China
Source: PLoS One. 2023 Jan 20;18(1):e0280221. doi: 10.1371/journal.pone.0280221 (PMC9858461; doi:10.1371/journal.pone.0280221)
Supplement: S1 Data — (ZIP) [file pone.0280221.s001.zip › Supporting Data/Version of Chinese to English/Province Committee(English).docx]

2022-06-30 Why is the topic of Hainan Dream Avenue hot on Weibo. This is the "Hainan Dream Building Action- -Dream Building Avenue Youth Collection Order" activity jointly initiated by the Hainan Provincial Party Committee of the Communist Youth League, CNOOC and Sina Hainan on the Weibo platform. Activities will be "happy dream", "ecological dream", "ocean dream", "technology dream", "world dream", "dream" space dream "six dreams to" sun "," deep blue "," plain green "," lemon yellow "," dynamic orange "," ivory "six" background "youth", called on netizens to participate in weibo topic discussion, the six big "youth background" as the theme to share personal declaration or story, for Hainan free trade port construction condensed youth strength. After the launch of the event, netizens actively discussed and participated, and the total topic was read more than 20 million times. Why can this microblog topic initiated by the Hainan Provincial Party Committee of the Communist Youth League attract wide attention? This cannot be separated from the importance of youth development from the state to the local level in the activity process, and is also closely related to the great development of the construction of Hainan Free Trade Port in recent years."I actively participate in the school community held environmental protection lecturers, 'Blue Star Guard' ocean guard, old clothes donation, volunteer teaching, epidemic prevention and control volunteers, etc. I firmly believe that fighting youth is the most beautiful background, the construction of the free trade port needs each of us to contribute, come on!"@ Hao Kaixin refueling to join the topic interaction, and share his vivid story of his participation in the construction of Hainan Free Trade Port. Yang Jianguo said that as a new Wenchang person, choosing Wenchang is the vigorous high-quality development of Wenchang, the key node of the core leading area of the free trade port, and that Wenchang is becoming a commercial space highland in China and even the world. Bin, deputy secretary of Hainan provincial party committee, in recent years, Hainan provincial party committee and government attaches great importance to youth work, in the free trade port construction of national strategy with the support of many favorable policies, both the introduction of young talents and local young talents, is the key to support, for youth in Hainan free trade port entrepreneurship provides a high quality environment. In this big environment, the youth from different fields also take struggle as the most beautiful background color of youth, some inherit red genes and interpret their original intention and mission in different battlefields; some take green youth as the guardians of nature; others go to refresh the depth of the sea depth and breadth; others take root in the fields, always hold Chinese jobs firmly in their own hands... youth have become the construction of Hainan Free Trade Port. Young people set sail in the fertile soil of Hainan, and Hainan also lives up to their expectations. The report of the 8th Party Congress of Hainan Province clearly pointed out that " youth are the new force in the construction of Hainan Free trade Port. We should care for and love our young people and make Hainan a fertile ground for more young people to achieve their dreams."In June this year, the national list of youth development city construction and youth development county pilot was announced, and Haikou, Sanya Jiyang District, Qionghai and Qiongzhong Li and Miao autonomous counties were selected. Xu Changbin introduced that the four places will promote the construction of a youth development demonstration city of Hainan Free Trade Port, and pilot innovation to build three "youth blocks" in Haikou, actively create a policy environment and social environment to meet the diversified and multi-level development needs of the youth, and actively promote the implementation of youth services and development projects. Zhu, which is trending on Weibo, is not done in a day. Dream avenue since its founding, always with big ideological thought leading platform as the core, to serve Hainan free trade port construction as the center of gravity, coordinate the party and government and social resources, extensive contact national youth organizations and outstanding youth talents, efforts to develop dream avenue circle of friends and service, elaborate youth work national brand project, actively promote the work more close to the youth, more into the youth, more deeply rooted in the youth. In June last year, the Hainan Provincial Committee of the Communist Youth League and the Department of Education and Youth Work of the Liaison Office of the Central Committee of Macao established contacts to deepen the activity of Qiongao Youth on Hainan Free Trade Port and signed a strategic cooperation agreement, providing a broader space for cooperation between the youth exchanges and industrial upgrading of the two places. This year, the project was awarded as a key cultural and tourism exchange project between the mainland and Hong Kong and Macao in China in 2022 by the General Office of the Ministry of Culture and Tourism. In may this year, the Hainan provincial party committee also cooperate with the communist party committee of network letter to build Hainan youth network public communication base, relying on the dream avenue platform advantage, further carry out good Hainan youth netizens, youth network anchor skills camp, practice camp and other activities, to attract, cultivate and accept more high-quality youth network talent, service in Hainan free trade port talent development. In addition, the Hainan Provincial Committee of the Communist Youth League also established the Hainan Youth New Media Alliance to further strengthen the contact, communication, close cooperation and cooperation of the new media industry of the free trade port and form joint forces, so as to continuously contribute its youth strength to promoting the online public welfare communication of Hainan youth. From April 10 to 13 this year, General Secretary Xi Jinping visited Sanya, Wuzhishan and Danzhou, Hainan province, for investigation and guidance, and delivered an important speech. League of Hainan provincial party committee carried out "Chinese youth dream season" activities, to Hainan youth demonstration practice education camp (base), is the dream avenue national youth "big politics" practice education base as the platform, innovation and development of "dream avenue island train-power mark" series of youth theme education practice, lead a group of teenagers pursue general secretary, further guide the youth fully understand the significance of free trade port construction in Hainan."Young people should actively participate in the construction of the Free trade Port of Hainan, and practice the pledge of 'Please rest assured of the Party, and a strong country has me'."In Maona Village, a theme group class told by the village cadres let Haikou Haikou School Youth League Committee secretary Xu Guoren feel the feelings of the general secretary, love the people, the construction of Hainan Free Trade Port, but also felt the hardships of the cadres in the village. Following in the footsteps of General Secretary Xi Jinping, wang Yingmiao, a graduate student of Hainan University, for the first time fully learned about the customs of clothing, food, living and transportation of the Li people. Through the real display of cultural relics, he got into close contact with the Li culture. A graduate student of Hainan University, felt excited."Research is a mobile classroom with educational and practical significance. We look forward to continuing the study to better inherit the local excellent culture for young people from all over the country.""This practical education activity around the island gives the answer to how to realize the Chinese dream and build a free trade port."Xu said. Source: China Youth Daily, June 30,2022

2022-06-30 Fujian: Welcome the 20th theme education practice activities held in Fujian, "Welcome the 20th theme, always follow the Party, forge ahead on a new journey" theme education practice activities held in the network, Beijing, June 30 (reporter Zhang Jianwei), June 28, The Communist Youth League Fujian Provincial Party Committee and China Mobile Fujian Company jointly held the theme of "Welcome 20, Always follow the Party, forge ahead on a New Journey" in Longyan Changting, Li Teng, Deputy Secretary of the Fujian Provincial Party Committee of the Communist Youth League, Luan Xiaowei, Party Secretary, Chairman and General Manager of China Mobile Fujian Company, attended the event and inaugurated the first "youth red education base" of China Mobile- - "The former site of the Communist Youth League Fujian Provincial Party Committee", Longyan Municipal Committee of the Standing Committee, Minister of the Publicity Department Yang Yi attended the event and delivered a speech, China Mobile Fujian Company Party Committee member, deputy general manager Qiu Baohua presided over the event. More than 50 youth representatives of provincial and county level mobile companies, youth representatives of Longyan and Changting league members attended the on-site activities. The picture shows the release of the "youth red education research boutique route". Fujian Provincial Committee, The two sides simultaneously released the first Fujian Communist Youth League & Fujian Mobile "Youth Red Education Research Quality Route", Held the provincial, city, county Communist Youth League, mobile companies "three-level joint innovation" strategic cooperation agreement signing ceremony, By launching the "Thousand Screen Plan", Strengthening ideological and political education among young people; Build a red education base together, To build a new position of youth ideological education; Implementing the "Youth Cloud Leadership Program", Carry out the digital Fujian youth contribution action; Carry out the "Fujian Communist Youth League & Fujian Mobile Digital Intelligence Rural Ambassador Plan", Promoting rural modernization and development; Develop the "Fujian Communist Youth League & Fujian Mobile and Miao Student Aid Program", Jointly protect the growth of young people; Implement the "Fujian Communist Youth League & Fujian Mobile to help rural revitalization youth contribution action", To help rural young people find jobs and start their own businesses; To build the "5G + Fujian Provincial College Students internship 'Sail Plan' " project, Improve the level of work digitalization, information technology and other seven aspects, Further strengthen the three-level co-construction and league and enterprise cooperation, We will deepen the implementation of more carrier projects to guide and serve the growth of young people, To empower the growth of Fujian youth in the new era. The picture shows the signing ceremony of the "three-level joint innovation" strategic cooperation agreement. Photo provided by the Communist Youth League and Fujian Provincial Party Committee

2022-06-30 Xinjiang: Xinjiang Autonomous Region Committee held the 2022 culture, science, technology and health "three to the countryside" demonstration activity launch ceremony Jianwei correspondent Adili) According to the relevant requirements of the Publicity Department of the Party Committee of Xinjiang Uygur Autonomous Region, In order to thoroughly study, publicize and implement Xi Jinping Thought on Socialism with Chinese Characteristics for a New Era and the spirit of the Sixth Plenary Session of the 19th CPC Central Committee, Learn and publicize the spirit of General Secretary Xi Jinping's important speech at the celebration of the 100th anniversary of the founding of the Communist Youth League of China, To implement the spirit of the third Plenary Session of the 10th Party Committee of the Autonomous Region, Focusing on the main line of "Welcome the 20th Plan, always follow the Party, and forge ahead on a new journey", To guide and help young students to "receive education, grow talents and make contributions" in social classes, The Youth Xinjiang Uygur Autonomous Region Committee held the 2022 culture, science, technology and health demonstration activity launch ceremony in Changji Prefecture on June 27. Communist Youth League District Party Secretary Maimaiti Jiang Davuti attended the ceremony and made a speech. The picture shows a group photo of the participants. At the launch ceremony, Zuo Lahasen, member of the Standing Committee of Changji Prefecture Party Committee, evaluated the positive impact of the cultural, technology and health "three to the countryside" on the economic and social development, the training of youth members, and the care for the masses of the cultural life. Chen Zixuan, a student of the "Red Scarf Small Class" college student volunteer service team, made a speech. He said that in the "three to the countryside" social practice activity, the volunteers will play their professional expertise, take the initiative to learn, with a serious and responsible attitude, in the practice activities to live up to the youth and The Times. District secretary to buy jiang I, and Changji state party committee committee for "youth branch" about service, "grassroots" send literature and art youth literary and art workers service, "fire" science and technology youth science and technology workers service, "young marxist training project" college class service, college students "a program under which officials" volunteer service, "red scarf small classroom" college students volunteer service, "health clinic" youth medical workers service. The picture shows the secretary of the Communist Youth League District Committee, awarding the flag to the volunteer service team. Jiang Dawu, Party Secretary of the Communist Youth League District Committee, made a speech on the active practice of General Secretary Xi Jinping's ardent expectations for the youth and the general goal of serving Xinjiang's social stability and long-term peace and stability in the practice of "three to the countryside". He proposed to put the personal ideal into the national and national cause, closely around the overall goal of social stability and stability in Xinjiang, efforts to make oneself to serve the motherland, the construction of hometown useful talent, pillars, and to participate in "a program under which officials" social practice and all kinds of volunteer service young friends put forward three hope. We must unswervingly feel the Party's kindness, listen to the Party, and follow the Party, and firmly establish a firm ideal of sincere dedication to the motherland and the people. To make persistent progress in learning, in the training of perseverance to grow up, through the active participation in the "three to the countryside" series of activities, in the broad grassroots world to find the value of life, to achieve lofty ideals, with the struggle in the grassroots to write a magnificent chapter of life. To the broad grassroots heaven and earth, into the simple grassroots masses, make all ethnic friends, sincerely for the masses, sincere for the people, in the service, the service of the hot practice deep understanding national area sentiment, further anchor life value and struggle direction, in the service to the motherland, benefit the society, serve the people grassroots practice to meet the party's 20th victory. More than 50 people from the Xinjiang Uygur Autonomous Region Party Committee, the Xinjiang Uygur Autonomous Region Party Committee and the prefectural people's Government, and members of the "three to the countryside" volunteer service teams participated in the activity. After the launch ceremony, the "three to the countryside" volunteer service teams will successively carry out various forms of volunteer service activities.

2022-06-29 In 2022 Henan hope project interpreta dream action start 2022 hope project interpreta dream action start jianwei correspondent liu yong) to help 2022 difficult young students interpreta dream university, from now on to the end of September, the Henan provincial party committee, Henan province youth development foundation jointly launched "welcome twenty big interpreta dream new era-2022 Henan hope project interpreta dream action". Every 2022 the college entrance examination in Henan province, and was admitted to full-time ordinary colleges and universities of difficult family college freshmen (military academy, normal and commissioned and other kinds of free students and private colleges), through the group county (district) committee to apply for "hope project interpreta dream action" funding, after audit, can get more than 4000 yuan of grants."Henan hope project interpreta dream action" is initiated by the Henan provincial party committee, Henan province youth development foundation in 2004 in the country first a funding poor college students' public welfare activities, the activity in the social support from all walks of life and enthusiastic participation, by 2021 to raise 302 million yuan, help 91817 poor students to complete their studies."Hope" is never someone's business, it comes in from so many people, and eventually it passes on to so many more people! In 2022, Henan Hope Project Dream Realization action, I look forward to your participation.1. Network donation: scan the qr code below for online donation 2. Bank donation: (please indicate the dream action) name: Henan youth development foundation bank: Zhengzhou bank co., LTD. Road branch account: 999156000400001585 3. Site donation: Zhengzhou Jinshui district, 17 green building room 1712 Henan province youth development foundation (Henan hope engineering office) donation hotline: 0371-65902382

2022-06-29 Not long ago, Xiao Xin, a student at Jingyi Ethnic Primary School in Yuzhong District, Chongqing, was unhappy as soon as he arrived at school. But she quickly adjusted her mood. The 10-year-old told herself she wore it to crush broken hair, not to look bad. She tried to be as happy as possible, not to let her classmates' words affect her mood. That's what the psychologist has taught her. Since last fall semester, two experts at the 12355 Youth service desk in Chongqing have opened a "Spiritual Growth Club" in Jingyi Minzu Primary School to teach children how to overcome "unhappiness". Now, as a public welfare project of adolescent mental health initiated by the Chongqing Municipal Committee of the Communist Youth League, the experiment of "Spiritual Growth Society" is being implemented in more schools, communities, youth activity centers and other places in Chongqing. This "small lever" has leveraged the "big pattern" of adolescent mental health. The children learned to cure the "unhappy" Jingyi ethnic primary school around the old communities, densely populated. Liu Wei, a teacher in charge of mental health education at the school, said that the children's family conditions and growing environment vary greatly, among which the mental health status of the children from low-income families and single-parent families is not optimistic."When I greet my children at the school gate in the morning, they often cry."Liu said he hopes to help the children cure" unhappy things."At the school gate to see the children crying to school, she always pulled the child aside to chat, chat about the child laughed. But she also felt that one man's power was too limited. Last September, Li Lihua and Zhao Shu, psychologists at the Chongqing 12355 Youth service desk, joined the school, providing great support to the school. They have opened a "spiritual growth club" in the school to help their children deal with their "unhappiness". Through painting, handwork, music, drama and other forms, mixed metaphorical stories and games, children's emotions that can not be express in words are expressed in another form, and experts walk into the children's inner world, and provide support to them. During the activity, Xiao Xin felt that the teachers were "like not teachers, but friends". She learned a lot of ways to cure her unhappiness, such as playing dolls and write their worries on notes and then tear them off. Xiao Xin admitted that in the past encountered injustice, some things belong to their own privacy, not convenient to tell his mother, can only endure. But after doing it according to the teacher's method, "the heart is very refreshing, the trouble is gone". After a semester of the pilot, Liu Wei saw changes in both the individual case and the overall situation. In the artistic expression section, several children's early paintings are dark and messy in lines. Now, Liu Wei sees more and more bright colors, and the children can better accept themselves and their classmates. To some extent, the pilot project of the "Spiritual Growth Society" in Jingyi Minzu Primary School responds to the common problems reflected in a survey organized by the Chongqing Municipal Party Committee of the Communist Youth League and the Department of Psychology of Southwest University. The survey covered more than 80,000 teenagers in 36 districts (counties) in Chongqing. The survey found that the initial mental health problems of primary and middle school students are mainly manifested as sensitivity, weariness, loss, mania, etc., which are easy to be mistaken as the ubiquitous rebellious stage of teenagers. Li Lihua said that the children in life and study, the "unhappy", if not intervene in time, will form self-attack, turn around to hurt themselves, over time, will evolve into a mental health problem. To this end, the above survey suggests that through the 12355 youth service hotline and other platforms, relying on the expert team, timely find the signs of adolescent psychological problems, to provide psychological counseling, pressure relief and other services for teenagers, to prevent and resolve their mental health risks. In Jingyi Minzu Primary School, experts and teachers specially chose the fourth grade as a pilot. They believe that this stage is a turning point for the physical and mental development of primary school students. If they are not dealing with their daily "unhappiness", they may evolve into mental health problems, and if they can give timely guidance, it will have a positive impact on the future. At present, the "Spiritual Growth Club" has become an important part of the Chongqing Communist Youth League "Heart Hope Project" youth mental health public welfare project. The Chongqing Youth Development Foundation has obtained 1.3 million yuan of project funds from the China Youth Development Foundation to build 17 spiritual growth stations and carry out spiritual growth activities including the "Spiritual Growth Society" project. With the support of the project funds, Chongqing Experimental Foreign Language School has upgraded the psychological counseling room of the school, and built the spiritual growth station of the Project School, with music relaxation chairs, hug dolls, evaluation system, etc. The whole space is arranged warm and natural."This is where the students can relax."Chen Wenjing, director of the school's psychological teaching and research section, said that students will take the initiative to go to the spiritual growth station, to seek help from teachers, three times a week of psychological counseling in a full state for a long time. Prepare for a rainy day, subtly affect adolescent mental health, in March this year, the Chongqing municipal party committee issued "about the" heart hope "to further safeguard the legitimate rights and interests of teenagers implementation plan, in the traditional hope project to provide material security, adhere to the" watering flower root, education heart ", focus on youth mind cultivation, mental education, mental health, etc."Heart Hope Project" proposes to build a "practice park", establish a "relatives and friends group", build a "growth club", draw an "electrocardiogram" and other work plans, to move the focus of adolescent mental health care work forward, to explore the new concept and new carrier of care for adolescent groups. At present, 14 schools in 6 districts of Chongqing have launched the "Heart Growth Society" project, and 7 municipal demonstration "practice parks" have been identified in 4 districts. The pilot work of "Heart Hope Project" has been fully rolled out in Chongqing. Lin Sen, head of Chongqing 12355 youth service desk, said that the traditional psychological consultation room is not enough to meet the changing psychological characteristics and needs of teenagers. If flexible and diverse forms are used, it will help teenagers to open their hearts in the state of natural relaxation. Chongqing web celebrity clock in "18 ladder" SiShu travel space, parent-child communication, psychological counseling activities and "18 ladder" good fruit lane good culture fusion, designed the "18 ladder good" practice, by professional social organizations "social workers" operation, with immersive role-playing, learning experience to attract the participation of teenagers. Wang Lijuan, the head of the "appendix search social worker", said that in view of the psychological problems caused by family and parent-child conflicts to teenagers, they have carried out a series of "parent-child electrocardiogram" activities to create opportunities for parents and children to open their hearts and listen, so that the cracks in the relationship caused by the lack of communication and companionship are healed. In the Chongqing Children's Palace, the "practice garden" exists in another form. The insect agents teach children to make animal specimens, learn to make kimchi in the "Treasure Garden", and experience sewing beautiful clothes in the workshop... Mental health education is integrated into these seemingly unrelated activities and courses."If you get the fabric you don't like, accept it and make the clothes you like."In Qianqiu Hua Workshop, psychological expert Mei Ying said that the course seems to be a manual class, in fact, to let the children know" what to want- -what- -what ". Lin Sen said that the "Heart Hope Project" is a systematic project. On the one hand, internal integration to promote the "practice garden", "growth club", "electrocardiogram", "friends and friends"; on the other hand, external linkage to establish the communist Youth League leading, multiple participation and win-win cooperation, so that the quality service can benefit more teenagers. Source: China Youth Daily, June 29,2022, edition of the 201st edition

2022-06-28 Group Beijing municipal party committee to carry out the anti-drug theme works collection group Beijing municipal party committee to carry out the "the franco-prussian education future research method to help growth" anti-drug theme work collection JianWei correspondent Sun Shihao) recently, the Beijing municipal party committee rights department joint Beijing youth law and psychological counseling service center of Beijing school students at all levels for the "the franco-prussian education future research method to help growth" anti-drug theme work solicitation. The picture shows the excellent work "Never be a partner with poison". Correspondent for figure activities since June 1, received a total of in the city 13 district 649 works, including video creation works 68,581 art design works, 87, a total of universities submitted works, 277,132 works, 153 elementary school works, implements the colleges and universities, primary and secondary schools, secondary schools, technical schools and other full coverage of schools at all levels. The students expressed their understanding of the harm of drugs through words and brushes, which further showed the publicity effect of the anti-drug work in the capital for 30 years. The awareness of recognizing, recognizing and preventing drugs was gradually deeply rooted in the hearts of teenagers. The picture shows the excellent work "refuse drugs, embrace life". On June 24th, the expert review meeting of anti-drug theme works collection activity was held in Beijing Youth Palace. Five experts from drug control, art design, education and other fields, after careful selection and discussion, finally reviewed 60 excellent works, including 10 first prizes, 20 second prizes and 30 third prizes. Green and non-toxic, healthy growth, the next step, the Beijing Communist Youth League will grasp the school, the key position of anti-drug propaganda, for the healthy growth of a non-toxic blue sky for the growth of teenagers in the capital.

2022-06-28 "Fall in love with Guangdong red" video show in 2022 "fall in love with Guangdong red" video show in 2022 JianWei correspondent YueQing) to meet the party's 20 victory, the Guangdong party committee propaganda department, Guangdong province, Guangdong education department, Guangdong culture and tourism hall, Guangdong province, Guangdong, Guangdong province volunteer federation deepen Guangdong youth inheritance red gene volunteer preaching action, joint "fall in love with Guangdong red" video show activities in 2022. Recently, the event led young people to "visit" Jiangmen, an agricultural city in the Guangdong-Hong Kong-Macao Greater Bay Area, to listen to the stories of young people from Hong Kong and overseas Chinese hometown using modern agricultural technology to help rural revitalization. The picture shows the publicity video exhibition of "Falling in Love with Guangdong Red" walking into Jiangmen, the hometown of overseas Chinese. In the Greater Bay Area, Hong Kong youth Robert Luo told them his entrepreneurial story in the Greater Bay Area. In 2016, he and his team partner graduated from the Chinese University of Hong Kong. He was attracted by the Greater Bay Area settlement policy and decided to start his own business in Jiangmen. After overcoming various difficulties such as scientific research and technology and Typhoon Hato in 2017, his team won the gold medal of the 2019 China Youth Innovation and Entrepreneurship Competition, and was established in 2020 as a vegetable planting base with 10,000 people... In recent years, he has actively participated in youth exchange activities between Guangdong, Hong Kong and Macao, and led over 8,000 Hong Kong schools and associations to Guangdong and more than 600,000 Hong Kong youth online."Benefit from The Times, give back to The Times."Luo Wei Te's words in the speech touched many teenagers who watched the live broadcast. The host introduces the Impression hall of Wuyi Characteristic Agricultural products to the young people. In Taishan China Agricultural Park, the representatives of the new young farmers in Jiangmen have introduced the modern agricultural technology to the young people through the propaganda videos, and talked about their own journey to returning home. Graduated in the national "rural revitalization" development strategy, became the young farming leader leading the farmers around; Huang Zhaopei led the young team under the average age of 34, to build a modern agricultural service management platform to realize "air" agricultural management analysis, South China agricultural University graduate Chen using water quality deployment, tracking testing, unmanned patrol, to create an important green crab supply base in south China, Li Jijin using design skills "old market activation", the 80s and 90s old rural buildings into a modern living park, attracting more than 100,000 passengers. Through their stories, teenagers feel the new impetus that the rural revitalization strategy brings to the Greater Bay Area. An official from the Guangdong Provincial Committee of the Communist Youth League said, " This year marks the 25th anniversary of Hong Kong's return to the motherland, and the exchanges between young people of Guangdong, Hong Kong and Macao will be more frequent and closer in the future."It is reported that this year, the Communist Youth League Guangdong Provincial Party Committee and a number of units to carry out video exhibition activities throughout the year, aiming at leading the majority of teenagers online punch card to witness the development of the Bay Area, rural revitalization, social governance, cultural strong province, scientific and technological innovation, beautiful Guangdong and other achievements. In the next phase, we will continue to lead young people to visit Zhuhai, Guangdong province, and listen to the stories of the Greater Bay Area behind the Hong Kong-Zhuhai-Macao Bridge.

2022-06-28 The Ningxia District Committee makes every effort to create a vibrant "youth community", The Communist Youth League Ningxia District Committee attaches great importance to, High-level push, To coordinate the inclusion of community youth actions in the pilot party committee reform list of Ningxia Hui Autonomous Region, Focusing on community-level social governance plans, Focusing on the main responsibilities and main business of the Communist Youth League, Actively explore the "3343" working mode, Promote young people to actively participate in the construction of community governance and service systems, Focus on building the organization, resources, project, personnel "four in one" working mechanism, Further active in the organization and work of the community groups, It has effectively promoted the "three forces once" in the field of social governance. Centering on improving the organizational system of community groups, we should focus on the construction of "three carriers". First, we will strengthen the construction of youth social organizations affiliated to the youth League. We incubated and cultivated 18 youth social organizations, and implemented 16 youth service projects on a regular basis. We have organized 4,450 volunteers to participate in more than 500 various service activities, covering 8,257 teenagers and 16,049 service objects. Second, we will deepen the construction of community "youth homes". Relying on the community "Youth Home" and other positions, we gathered the strength of the youth and carried out 246 volunteer services, covering 1,575 young people, so that the young people can grow together with the community. Third, improve the work network of the community group. Relying on the community grid work service mode, we will build online and offline "two networks" to serve and lead teenagers, so that young people who are keen on public welfare undertakings and actively participate in community governance can quickly find and integrate into the organization, and have the space and stage to display their talents. Centering on enriching the work force of the community group, we grasp the construction of "three teams". First, we will play the leading role of organizational forces. Each community shall have a volunteer of the Western Plan as the full-time deputy secretary of the league branch, specifically responsible for the work of the community youth action; and hire the part-time deputy secretary of outstanding college students and social organizations to enhance the working force of the community league. Give vertical play to the institutional advantages of party building and team building, form a "party team" linkage and joint construction pattern, gather 26 units to build together, and build a "large league building" pattern of "community + school + volunteers + district units". Second, give full play to the basic role of professional forces. We will further promote the construction of community "youth homes", introduce 18 volunteer service teams such as "Cuncao Heart" through projects, and provide public welfare services such as green environmental protection, intelligent assistance for the elderly, and caring for vulnerable youth groups, so that youth social organizations can be continuously incubated. Third, we will leverage the synergistic role of social forces. We have cooperated with civil affairs departments and social organizations to carry out 4,775 hours of volunteer service activities, including legal education for teenagers, mental health counseling, and family classes. Carry out the work of "league members report to the community", and a total of 70 league members report. Docking with 7 universities, including Northern University for Nationalities and Ningxia Vocational and Technical College of Finance and Economics, we carried out the "four links" activities of joint construction, joint organization, joint discussion and brand joint creation, so as to enrich the work force of the community league. Focusing on improving the community youth work project, we will focus on the construction of "four types of communities". First, to provide public services for teenagers and build warm communities. The community after-school youth working committee has been set up, and volunteer service activities such as "4:30 classroom" and "love library" have been implemented, with a total service time of 2,858 hours, covering 3,455 people. Second, we will innovate the public participation of young people and build dynamic communities. We implemented the project of "school-local and assisted community governance", collected 30 projects for young Marxists in preaching and caring for teenagers, connected with volunteer teams from nine universities including Ning University, and innovated the mechanism of youth participation. Third, to participate in the construction of a public environment and build beautiful communities. The "Civilized City Construction" project was implemented, and volunteer service activities such as garbage sorting and community environmental improvement were carried out, with a total service time of 3,158 hours, covering 7,033 people. Fourth, pay attention to the vulnerable youth groups, and build a harmonious community. We have implemented the "Caring for vulnerable Teenagers Project", carried out the activity of "Light up micro wishes to be dream ambassadors", established a list of vulnerable teenagers in communities, and established " one-to-one assistance with enterprises and social organizations under our jurisdiction, covering 2,982 people. Focus on innovating the operation mechanism of the community Communist Youth League, and focus on the construction of the "three mechanisms". First, we will improve the socialized operation mechanism. Form a joint construction and resource sharing mechanism with enterprises, universities and social organizations under the jurisdiction, and promote college student league members to be part-time deputy and secretary of community youth League branch. Second, the establishment of a project-based management mechanism. In combination with the actual situation of the community, we will integrate social resources to launch youth service projects, connect with social organizations and university volunteer teams to carry out normal services, and form a long-term cooperation mechanism for service projects. Third, we will improve the work information sharing mechanism. We will give full play to the role of "Smart Youth League Building" and "Youth Home Cloud platform", release information on the needs of youth activities, do a good job in publicity and mobilization, make good use of the new media platform belonging to the Youth League, and strengthen the publicity of the pilot work experience. Next, the communist youth league of Ningxia district party committee will be in accordance with the central unified deployment, comprehensively promote the implementation of the community youth action, build the group participation, should, carry out the effective work situation, form the communist youth league social characteristics of era organization system, working mode and operation mechanism, for the comprehensive construction of economic prosperity beautiful national unity environment beautiful people's rich socialist modernization beautiful new Ningxia youth.

2022-06-28 Jianwei correspondent Ma Xiaolong) Recently, the 2022 Henan Province medium-and long-term youth development planning work promotion meeting and training class opening ceremony was held in the provincial Youth League school. Wang Anlin, deputy secretary of the Provincial Communist Youth League Committee, attended the meeting and delivered a speech. More than 100 people attended the meeting, including the relevant departments of the Provincial Communist Youth League Committee, the departments of the planning committees of the provincial cities, the departments of some counties (cities, districts), and the Youth League cadres of Hami, Xinjiang. The meeting stressed the need to earnestly fulfill the mission and responsibility of serving young people in combination with the solid progress of people's livelihood. We should take strengthening the ideological guidance of young people as the main line and guide them to strengthen their original aspiration. We should take helping young people to solve their practical difficulties as the bottom line, and solve their worries and troubles in their graduation, job-hunting, dating, and children's education. We need to make helping young people to make contributions a high line, and organize and mobilize young people to highlight their youth in helping to promote high-quality development. We need to make safeguarding the legitimate rights and interests of young people the red line, deepen education on the rule of law, strengthen mental health services, establish a mechanism to respond to and intervene in hot rights and interests, and effectively safeguard the legitimate rights and interests of young people. The meeting called for the in-depth implementation of the plan to constantly create a good atmosphere for the whole society to care for the development of youth. We need to improve the effectiveness of the joint conference mechanism and consolidate the achievements of the full coverage of the joint youth work mechanism at the city and county levels. We need to strengthen the application and implementation of youth policies, and establish and improve relevant policies on youth education, culture, housing, and employment. We need to do a good job in the pilot construction of youth development-oriented cities and counties, and develop a number of replicable and popularizable practical experience that young people are suitable for learning, living and working. We should intensify research and publicity on youth development, promote special topics of youth development, and tell the stories of high-quality and all-round development of youth in Henan. It is reported that the training course will last for 5 days, which will focus on learning the important speech spirit of General Secretary Xi Jinping in celebrating the 100th anniversary of the founding of the Communist Youth League of China, focusing on the development of Henan youth in the new era to carry out training, and constantly improve the policy service level of the Communist Youth League. Training class invited the central maintenance youth rights, China youth research center, provincial development and reform commission, provincial radio and television stations and other units of experts on youth comprehensive development policy and practice, the new era of Chinese youth white paper and the new era of youth development, people-centered new urbanization strategy, all media era of traditional culture youth innovation expression, etc. At the opening ceremony, the Youth League Luoyang Municipal Committee, Luohe Municipal Committee of the Youth League and the Youth League Committee of Kaifeng Gulou District exchanged advanced experience in local planning. After the opening ceremony, Wang Anlin, deputy secretary of the Provincial Communist Youth League Committee, taught the first lesson of "Learning the Spirit of the General Secretary's Important Speech and Serving the Youth- -Promoting the In-depth Implementation of the Medium-and long-term Youth Development Plan in Henan Province". During the training, the participants will also focus on " 'How to empower the youth by anchoring the' two guarantees 'and implementing the" ten strategies'. " 'How' '"'?"" How to optimize the environment for youth development and boost them to grow up?"And other content for the seminar and exchange.

2022-06-28 Hainan college students' business plan competition provincial final and award ceremony ended in 2022 "challenge cup" college students in Hainan province provincial final and award ceremony ended JianWei correspondent Wu Yuhua) recently, sponsored by the Hainan provincial party committee, Hainan province student federation in 2022 "challenge cup" college students in Hainan province provincial final and award ceremony was held in Hainan university. Relevant officials from Hainan Provincial Party Committee of the Communist Youth League, Party Committee of Hainan University, Hainan Association for Science and Technology, and Hainan Provincial Education Department attended the award ceremony. Some leaders of the University Youth League Committee, members of the participating project team, and representatives of young teachers and students participated in the activity. The event around power Hainan free trade port construction, complete, accurate, fully implement the innovation, coordination, green, open, sharing five development concept, relying on the building dream road brand, focusing on scientific and technological innovation and future industry, rural revitalization and rural modernization, social governance and public services, ecological environmental protection and sustainable development, cultural creativity and regional cooperation and so on five areas, in order to build innovative entrepreneurship platform for more young talents, accelerate the transformation of scientific and technological achievements. Since its official launch in May this year, the competition of the provincial colleges and universities, according to the 13th "challenge cup" Chinese college students business plan competition evaluation rules, after field selection and provincial primary two stages, from more than 1000 entries selected 107 outstanding works into the final final, including 47 works took part in the final field defense. After the intense competition, a total of 12 gold medal works, 30 silver medal works of "4.6um, 8.5um high-power dual-wavelength quantum cascade laser" and 65 bronze award works were selected. Hainan University, Hainan Normal University, Qiongtai Normal University, Haikou School of Economics, Hainan Economic and Trade Vocational and Technical College were awarded the Excellent Organization Award.

2022-06-28 Guizhou communist youth league learning xi general secretary in celebrating the conference of the 100th anniversary of the founding of the communist youth league of China's important speech spirit special training held the communist youth league learning in Guizhou general secretary xi in celebrating the 100th anniversary of the founding of the communist youth league of China's important speech spirit special training held JianWei correspondent Chen Kai) recently, Guizhou Communist Youth League learning from the important speech spirit of General Secretary Xi Jinping at the celebration of the 100th anniversary of the founding of the Communist Youth League of China was held in Guiyang, Secretary of the Youth League Committee of each city (prefecture), responsible comrade of the Youth League Committee of enterprises, organs and public institutions, Members of the county (city, district) Youth League Committee, A total of 123 people, including all departments and units directly under the Provincial Communist Youth League Party Committee, attended the training. Live pictures. Correspondent for figure training class, provincial party secretary Shi Qilin publicity around Guizhou communist youth league general secretary xi in celebrating the 100th anniversary of the founding of the communist youth league of China's important speech spirit and the 13th party congress in Guizhou spirit, made the title "follow the great party casting youth glory unity lead youth in colorful Guizhou modernization flying youth dream new journey to realize life" project presentation report. Li Jian, deputy secretary of the Provincial Communist Youth League Committee, and Deng Shoucheng, the second-level inspector, respectively gave special guidance on deepening the reform of the Communist Youth League, comprehensively and strictly governing the Communist Youth League and the Red Scarf Pioneer project. The training course also specially invited well-known experts on the history of the youth Games and leaders of the Central Youth Games History Archives of the Communist Youth League Committee to give special guidance to the participants. The relevant departments of the Provincial Communist Youth League Committee and directly affiliated units gave practical lectures on the "five actions" of youth achievements and the propaganda ideological and cultural work of the Communist Youth League. At the same time, the students were organized to visit the Guizhou Youth Movement theme exhibition Chen under the leadership of the Party, Guizhou Youth Home Yihaodian, Guiyang Network Audio-visual Industry Youth League Working Committee, and Guiyang Young Talents Dream Building Station. Participants have expressed that the training is rich in content, highly professional, and highly theoretical. Through this training, they will have made new improvements in their theoretical accomplishment, new progress in action implementation, and new breakthroughs in team spirit. Group of Guizhou provincial party committee will hold three provincial "learning xi general secretary in celebrating the 100th anniversary of the founding of the communist youth league conference's important speech spirit project training", demonstration drive cities and counties, colleges and universities hierarchical special training, cadres of all types at all levels, the young pioneers counselors, social organizations training as a whole, the leading organs at all levels, universities, enterprises and institutions cadres and the young pioneers counselors, youth league, emerging youth groups into the training.

2022-06-27 Group Henan provincial party committee to carry out the normalized sinking group Henan provincial party committee "strong ability, forging style, grasp the implementation of the" sink grassroots work JianWei correspondent Han Ding a) recently, Henan provincial party committee secretary team and cadres continue to carry out the "strong ability, forging style, grasp the" normalized sink work, demonstration drive the provincial cadres at all levels really sink, heart with love for accurate and effective service, hard work solid promote key work, with the youth in the process of ground gas, experience, style. In Gongyi City, Wang Dubo, Deputy Secretary of Henan Provincial Party Committee, preached the spirit of General Secretary Xi Jinping's important speech to grassroots league cadres, And walk into the youth innovation and entrepreneurship base, towns, enterprises, Check the development of college students "report to the grassroots" practice activities, the construction of Qingyi home, In-depth exchanges with young entrepreneurs, youth psychological volunteers, the first Youth League branch secretary, college students, and representatives of youth League cadres, Encourage the youth league members to bear in mind General Secretary Xi Jinping's earnest entrust, Study hard and work hard, Consciously integrate "ego" into "ego", Create a wonderful life in the struggle, To contribute the youth wisdom and strength to the construction of modern Henan. In Shangcai County, Wang Anlin, deputy secretary of Henan Provincial Party Committee of the Communist Youth League, came to Wolong Street Mairen Community, Shangcai Jiu Primary School, Shangcai Sixth Middle School and other places to visit the development of community adolescent mental health services and the construction of Qingyi Home, and supervise the promotion of adolescent mental health services into the village (community). In Wu Zhixian, Wang Qiang, deputy secretary of the Henan Provincial Party Committee, had in-depth exchanges with Henan Hanxiang Ecological Agriculture Professional Cooperative in Qiaomiao Town, to understand the situation of youth innovation and entrepreneurship, and supervised and served the employment and entrepreneurship of college graduates. In Junxian County, Wang Jiwei, deputy secretary of Henan Provincial Party Committee of the Communist Youth League, came to visit to Xinzhen Town to check the application and promotion of the platform of "College students reporting to the grassroots", and to have an in-depth understanding of the experience summary and effect improvement after the final evaluation of the pilot reform of the Communist Youth League at the county level. In hui county, deputy secretary of Henan provincial party committee hai-ping wang into the south village town, JiTun town, and deputy secretary of township college students part-time one-on-one interview, focus on learning propaganda xi general secretary in celebrating the 100th anniversary of the founding of the communist youth league of China's important speech spirit, the county communist youth league grassroots organization reform work situation. In Jiyuan city, Wang Xin, the second-level inspector of Henan Provincial Committee of the Communist Youth League, went to schools, villages and state-owned enterprises to learn more about the construction of "Qingyi Home", enterprise youth league construction, and youth innovation and entrepreneurship. In Yuzhou City, Han Bing, the second-level inspector of the Henan Provincial Party Committee of the Communist Youth League, came to Damuchang Village, Fuliang Town, to learn and exchange the spirit of General Secretary Xi Jinping's important speech together with the youth league members. Henan Provincial Youth League Party Committee organ cadres group went to Xinzheng City, Xin'an County, Pingdingshan City Zhanhe District, Xiuwu County, Mianchi County, Shenqiu County, Guangshan County and other 31 county Communist Youth League grassroots organization reform pilot counties (city, district), Focus on promoting the implementation of General Secretary Xi Jinping in the celebration of the 100th anniversary of the founding of the Communist Youth League of China at the spirit of the study and publicity work, the county Communist Youth League grass-roots organization reform work, "youth mental health services into the village (community) action" people's livelihood and other annual key tasks of the Henan Communist Youth League, Learn together with grassroots youth league cadres, want to be together, and work together, And the county level youth League committee comrades seriously summed up the county Communist Youth League reform final evaluation experience, Further analyze the problems existing in the work, For the next step of the reform work to find a practical improvement path; Help the county-level youth League committees to actively strive for party and government support, Effectively promote the establishment and improvement of the party building, leading the league building and team construction mechanism, Continuously expand the source channels of youth league cadres; Comprehensively coordinate the injection of resources from all aspects of society, To realize the university youth league committee and the county youth League committee pair and joint construction, resource sharing, Promote the implementation of the "Qingyi Home" and "everyone holds a certificate, youth comes first" skills training programs at the grass-roots level; Planning together with the county-level Communist Youth League committee members, Innovate and build the upgrade group is a new media platform, Build a strong integrated education chain of the party team; Working together with the grass-roots youth league cadres, Promote the construction of youth associations, Attract and gather young volunteers to serve the overall situation, For the county Communist Youth League organization to enhance the "three forces once" increase power, With more high spirit and more outstanding results to meet the party's 20 victory.

2022-06-27 Group Guizhou provincial party committee held the international day theme campaign group Guizhou provincial party committee held the international day theme campaign green reporter Li Yajuan) to meet the 35th "626" international drug day, June 24 morning, Guizhou provincial party committee held "safe Guizhou youth counterparts" in Guizhou youth "6.26" international day theme campaign. The students of Weining County No.9 Middle School visited the anti-drug education base of Weining County, watched the anti-drug knowledge publicity board, understood the simulation of drugs, learned the "mask" of the new drug camouflage, and also watched the anti-drug propaganda video "The Disguiser". Guiyang Caiai Sunshine Public Welfare Center gave a lecture entitled "" healthy life green and non-toxic "teenagers away from action", and introduced the concept, types of drugs and the impact on the body, about the harm of drugs to families, society, the country, as well as the importance of drug control and anti-drug. The staff of the center also organized the students to carry out interactive activities such as anti-drug knowledge answers, and distributed the anti-drug manuals, the "Juvenile Protection Law", the "Prevention of Juvenile Crime Law" and other legal books. In Fukangxin District, Xiongshan Street, in Weining County, the Youth League Weining County Party Committee organized a youth anti-drug volunteer service team to publicize anti-drug knowledge for the people in the resettlement sites, and distributed more than 1,000 copies of anti-drug brochures. The Guizhou Communist Youth League and the Young Pioneers took the opportunity of the 626 International Day against Drug Abuse to carry out more than 100 anti-drug legal publicity activities. The Guizhou Provincial Committee of the Communist Youth League also produced a short interactive video titled "Small theater for law popularization, Deep Drug Trap, Avoid in workplace". In the next step, the Guizhou Provincial Committee of the Communist Youth League will continue to expand the team of young drug volunteers and carry out extensive publicity of drug law among teenagers.

2022-06-27 Fujian communist youth league to promote college students 'employment advance Fujian communist youth league to promote college students' employment advance will build wei) on June 24 morning, the Fujian provincial party committee organized Fujian communist youth league to promote college students 'employment video advance, to deepen the implementation of the "Fujian communist youth league to promote college students' employment action" to mobilize and deploy. Shao Mingsong, deputy secretary of the Provincial Communist Youth League Committee, attended the meeting and delivered a speech. The relevant departments and units of the Provincial Communist Youth League Committee attended the meeting. The deputy secretaries of the Youth League Committee, the provincial Youth League Working Committee of the Youth Development Department, the responsible members of the school department, and the Youth League Committee of the directly affiliated universities attended the meeting online. Conference pointed out that the current employment situation is very serious, the youth league organizations at all levels should deeply understand pay special attention to the extreme importance of "the communist youth league to promote college students' employment action", seize the key node, play employment battle, to improve the political stance, carry political responsibility, to do a good job of Fujian college graduates employment in a more prominent position, grasp the real work, for Fujian "employment" "employment" work to make positive contributions. Meeting requirements, Youth league organizations at all levels should take the "Fujian Communist Youth League to promote the employment of college students" as the whole, Further clarify the objectives and tasks, Compaction work responsibility, Go ahead, without a discount, Complete the annual target with quality and quantity guaranteed; Strengthen coordination, Innovation and linkage mechanism, The youth League committees of all colleges and universities should cooperate with the city and county-level youth League committees, Strengthen communication and linkage, Ensure seamless and continuous work; Strengthen policy interpretation, Do a good job of publicity and guidance, Help college students to establish a correct view of employment, career selection view, Further improve the awareness rate of college students on the employment policy; Strict work style, Strengthen work discipline, No formalism, no superficial work, Serious, solid efforts to help the measures into practice, With the practical action to promote the employment of college students to meet the party's twenty great victory held. It is reported, since the "Fujian communist youth league to promote college students 'employment action" launched in April this year, the general college low-income family students employment support plan, college students' rural entrepreneurship support plan, college students volunteer service "two plans", college students practice "sail plan", college students' community practice plan is steadily promoted, periodic progress. Up to now, the province has mobilized 3011 employers, provide 31409 internship positions, 1263 cadres and 3495 college graduates, has successfully helped 1698 difficult students to achieve employment; college students rural entrepreneurship assistance plan, 85 projects, is currently selecting entrepreneurial projects; "two plans" volunteer service, has completed registration, a total of 7644 people for less development plan, 2441 people for the western plan.

2022-06-27 Beijing, June 27 (reporter Zhang Jianwei, correspondent Sun Shihao) This year is the 30th anniversary of the anti-drug work in Beijing, On the occasion of the 35th International Day against Drug Abuse, Under the guidance of Beijing Municipal Drug Control Office and Beijing Communist Youth League Municipal Party Committee, Sponsored by Beijing Drug Control Education Base Management Center, The "Healthy Life Operation Co., Green and Non-toxic", Beijing Anti-drug Education Association, Beijing Family Construction Promotion Association, Beijing Drug Control Volunteer Association, Beijing Youth Law and Psychological Counseling Service Center- -anti-drug publicity theme activity into the Youth Road of Metro Line 6 Station, Relevant comrades from Beijing Drug Control Office, Beijing Municipal Party Committee, Beijing Drug Control Education Base, Beijing Metro Operation Co., Ltd. and anti-drug volunteers participated in the activity. Anti-drug propaganda scene pictures. This activity through the visit of anti-drug publicity boards, explain the simulation of drug models, distribute publicity leaflets and other forms for the masses, especially the majority of teenagers to popularize anti-drug knowledge, further improve the awareness and consciously resist drugs, so that the "healthy life green non-toxic" concept deeply rooted in the hearts of the people. On the morning of the activity, the relevant comrades of all units and anti-drug volunteers distributed more than 1,500 copies of publicity materials. The relevant comrades of Beijing anti-drug education base and anti-drug volunteers introduced the content of the display board and the simulation drug model in detail for the past masses, to publicize the anti-drug knowledge. Anti-drug propaganda scene pictures. Correspondent for figure with the deepening of anti-drug propaganda and education work, the Beijing communist youth league innovation anti-drug propaganda and education method, with good online two modes, always adhere to the masses especially teenagers to participate in anti-drug propaganda and education work, as a whole resources, with the help of all kinds of propaganda platform, has received a good prevention effect. In the next step, the Beijing Communist Youth League will continue to thoroughly implement the important instructions of General Secretary Xi Jinping on drug control work, adhere to the guidance of specialization, socialization and public welfare, and build a brand of anti-drug publicity and education work with the capital characteristics of the city.

2022-06-27 Recently, reporter Lei Yu), the opening ceremony of Hubei College Students' Innovation and Entrepreneurship Intellectual Property Rights Protection Assistance Center was held in Hubei Youth Innovation Park (Optical Valley) Demonstration Park. Zhou Senfeng, secretary of Hubei Provincial Party Committee of the Communist Youth League, Cheng Hao, deputy director of Hubei Intellectual Property Bureau, and Liu Renshan, vice president of Zhongnan University of Economics and Law, inaugurated the work service station. The service station is the first service station for college students in Hubei province. At the event site, the organizer issued letters of appointment to the members of the second batch of expert service group from the industry and academic circles, and set up a volunteer service team for innovation and entrepreneurship IP rights protection assistance in Hubei Youth Innovation Park. The volunteer service team is established by outstanding master and doctoral students and senior undergraduates from the Law School and School of Zhongnan University of Intellectual Property. It will provide legal advice and services for the youth innovation park and young entrepreneurs in need of the province under the guidance of the Rights Protection Assistance Center."Like a large convenience supermarket, service stations provide a clear way for entrepreneurs to help them avoid losses and improve efficiency."Said Xiong Penghang, founder and CEO of Wuhan Haocun Technology Co., Ltd., an enterprise settled in Hubei Green Innovation Park (Optical Valley) Demonstration Park. Hubei College Students Innovation and Entrepreneurship Intellectual Property Rights Protection Assistance Center is the first "mass entrepreneurship and innovation" intellectual property rights protection institution in China established in Zhongnan University of Economics and Law under the series of our reports in March 2018. Since its establishment, the center has given full play to the organizational advantages of the Communist Youth League, the functions of the Intellectual Property Office and the disciplines of universities, and jointly carried out research, consultation, legal rights protection and training on the "mass entrepreneurship and innovation", and actively promoted college students' innovation and entrepreneurship, and helped their growth. According to introducing, the next step, the Hubei provincial party committee will give full play to the rights aid center and optical valley demonstration park work service station, work integration, coordination, resource integration, relying on multiple advantages, improve the provincial youth innovation entrepreneurship comprehensive service system, is committed to stay to Hubei entrepreneurship youth youth priority policy supply, youth friendly service experience, youth legal aid, to speed up the construction of a new development pattern area contribution youth strength. Report link: 1. "College students entrepreneurs patent rights protection where to go" on June 21,2017,05 edition <ϟ 1>cyol.com/html/2017-06/21/nw. D110000zgqnb_20170621_1-05.htm 2. The State Intellectual Property Office launched July 13,2017 D110000zgqnb_20170621_1-05.htmcyol.com/html/2017-07/13/nw. D110000zgqnb_20170713_1-05.htm 3. "Intellectual Property Protection should keep up with the footsteps of" Mass entrepreneurship and Innovation "", " D110000zgqnb_20170713_1-05.htmcyol.com/html/2017-08/07/nw. D110000zgqnb_20170807_4-01.htm 4. "" Mass entrepreneurship and Innovation "education calls for Intellectual Property Protection Course", August 18,2017, version 01, D110000zgqnb_20170807_4-01.htmcyol.com/html/2017-08/18/nw. D110000zgqnb_20170818_6-01.htm 5. The first National Survey Report on Intellectual Property Protection of College Students for Mass Entrepreneurship and Innovation was released on September 26,2017cyol.com/html/2017-09/26/nw. D110000zgqnb_20170926_1-09.htm 6. "Hubei Youth Innovation Center issued the first lesson of" Mass entrepreneurship and Innovation "", December 26,2017cyol.com/html/2017-12/26/nw. D110000zgqnb_20171226_4-01.htm 7. "China's first College Students" Mass entrepreneurship and Innovation "intellectual Property Rights Protection Agency was established" on March 30,2018, version 01, http: / / zqb.cyol.com/html/2018-03/30/nw. D110000zgqnb_20180330_5-01.htm (Photo provided by Hubei Provincial Party Committee of the Opening Group of the Work Service Station) (Photo provided by Hubei Provincial Party Committee of the Innovation and Entrepreneurship Assistance Group of Hubei Youth Innovation Park)

2022-06-27 Group Jiangxi provincial party committee actively completes college graduates employment service group Jiangxi provincial party committee actively do a good job of college graduates employment service JianWei correspondent Huang Zhangyue) since this year, group Jiangxi provincial party committee for college graduates employment problem, actively looking for solutions, play to the advantages of youth league work, actively do a good job in employment service. Jiangxi Provincial Committee of the Communist Youth League introduced work measures to promote 1,398 teachers and cadres from 98 universities in the province to help more than 3,360 students, to give more than 500 office supplies "gift packages", more than 200 employment resumes and workplace etiquette course gift packages, to help graduates quickly improve their employability and enter the employment rhythm. Through the implementation of the "sail hand in hand" plan, for 500 low-income family students job skills promotion, first month "three risks" subsidies, let low-income family students from worries; the "challenge cup" contest attract 117 universities 375 entrepreneurial teams, more than 2200 youth, create a good entrepreneurial atmosphere, improve youth entrepreneurial confidence. More than 302,000 internships, etc., recruited 486 college volunteers to volunteer service in Tibet and Xinjiang, held 6 double selection meetings, providing 9744 jobs, covering 106 universities, helping 7145 students, providing real help for college graduates.

2022-06-24 Jiangxi: innovation form to preach go "new" and "heart" in Jiangxi: innovative form to preach go "new" and "heart" Jianwei correspondent Xiao Shudan) for days, the Jiangxi provincial party committee organization extensive youth learning "youth" about exchange theme activities, will xi general secretary in celebrating the 100th anniversary of the founding of the communist youth league of China's important speech spirit spread to the hearts of every youth. Up to now, youth league organizations at all levels in the province have held more than 1,200 online and offline publicity and communication activities, such as study and discussion, discussion and exchange, drama performance, cloud oath, and practical education, covering more than 800,000 people, and watched more than 100 million online live broadcasts. A still photo from the drama " Red Rock."Although I grew up listening to the stories of the revolutionary martyrs, I have never felt their stories as I are today."" I must cherish the good life now, and be a responsible and responsible youth of the new era."Recently, nearly 14,000 teachers and students of Jiangxi University of Science and Technology watched the red drama with the theme of" Welcome 20 and Always Follow the Party "and" Micro-group class " performance online, and expressed their feelings in the interactive comment area. Nanchang New Fourth Army former site of the propaganda section chief Cui Yiwen to the young pioneers to explain the story of the site of the Nanchang New Fourth Army. Correspondent for figure in yu zhang elementary school, Nanchang new fourth army headquarters propaganda section chief Cui Yiwen told the young pioneers about the Nanchang new fourth army under the condition of extremely hard, with infinite loyalty to the party's cause and clinging to the revolutionary ideal, for three years of guerrilla war, let the present teachers and students deeply realize to inherit and carry forward the revolutionary spirit and fighting spirit, bear the historical task. Liu Zhongxing, secretary of the Nanchang Municipal Party Committee of the Communist Youth League, used six key words: crossing the "inner volume", refusing to "lying down", fighting for "retrograde", never "Buddha", accelerating "running" and being "lonely and brave", and combined with the actual development of Nanchang and the ideological dynamics of young college students. Students from Nanchang Aviation University recited "Lovely China". Correspondent for figure Nanchang aviation university students read "lovely China", full chorus "I and my motherland", east China jiaotong university through "preach + practice" mode thoroughly study and implement xi general secretary of the important speech spirit, civil architecture institute to carry out the "youth heart to the party, the new era" new league "cloud oath" and synchronization to preach, god bless college lead youth through sickle rush in the harvest rape labor education practice understanding "struggle is the most beautiful youth background, action is the most effective hone" youth. Next, the Jiangxi provincial party committee will continue to innovation rich "youth big learning" offline activities, widely in the youth about face to face, interactive, experiential, with teenagers understand, remember the xi general secretary of the important speech spirit, speak, live, let the youth accept education in the emotional resonance.

2022-06-24 Fujian province "thousand post dream grassroots" action plan launched in Fujian province action plan start JianWei) on June 22, the Fujian provincial party committee of Fujian provincial province, Fujian provincial department of education, human resources and social security hall, the China strait talent market in the provincial party committee authority in Fujian province "thousand post dream" grassroots " action plan launch ceremony and the first advanced typical preaching activities. Preaching activities, the province's rural revitalization of key county unit of choose and employ persons, sanxiang new materials co., LTD., general manager Xia Ruiqi, 2018 volunteer service plan in underdeveloped areas students volunteer representatives, Ningde municipal party history of propaganda education Huang Jingyi, Fujian jin aviation agriculture co., LTD., general manager HongLiangBin, Sanming sea ford chemical co., LTD., Fujian qingshan paper co., LTD., welder zhang, China strait talent market senior economist, senior career instructor, Fujian people club hall business mentor Chen Xinjia came to share respectively. League provincial party committee deputy secretary li teng around the "thousands of schools post dream grassroots" action plan for deployment, stressed to seize the graduate job window period, sprint period, to further strengthen the ideological guidance, in-depth implementation project, strengthen the coordination, guide good service young college students to grassroots employment entrepreneurship, strengthen the construction of grassroots talent team, contribution to comprehensively promote the development of high quality beyond strength. Li Teng, deputy secretary of the Provincial Communist Youth League Committee, Li Xuan, member of the Party Group and deputy director of the Provincial Department of Education, Liu Tao, the second-level inspector of the Provincial Department of Human Resources and Social Security, and Wang Wen, member of the Party Group and deputy general manager of the China Strait Talent Market, came to the stage to launch the action plan of "thousands of schools and thousands of posts building dream grassroots". The activity also watched the "thousands of schools and thousands of posts" propaganda video. Leaders of about 40 people from the Provincial Communist Youth League Committee, the Provincial Department of Education, the Provincial Department of Human Resources and Social Security, the China Strait Talent Market and other relevant departments, representatives of employers and college graduates in key rural revitalization counties and other counties (cities and districts) with large demand for employment attended the launch ceremony. On June 23, the first online and offline recruitment activity of the action plan of "Building a Dream for a grassroots level" in Fujian Province was launched at the same time. The first offline recruitment activity was held in Minjiang University Rongqiao Cultural and Sports Center. One hundred and 100 employers from all over the province provide 2,791 jobs for graduates, covering the Internet economy, green economy, cultural and tourism economy, Marine economy, rural revitalization and other industries. It is reported that the action will be carried out regularly throughout the year, to carry out special job fairs in districts and cities or counties (cities, districts) with large job demand, and timely to carry out 2-3 offline special job fairs according to the epidemic situation, planned to organize 1,000 enterprises, to provide more than 10,000 jobs.

2022-06-23 Jianwei Correspondent Chen Kai) Recently, the final final of the 13th "Challenge Cup" Guizhou College Students Business Plan Competition was held in Guizhou Transportation Vocational and Technical College. After university-level promotion and provincial evaluation, 280 works from 48 universities in the province entered the final examination, and the participants conducted on-site defense. Pictures of the event site. Correspondent for the competition by the Guizhou provincial party committee, Guizhou education department, Guizhou human resources and social security hall, Guizhou association for science and technology, Guizhou association, set up science and technology innovation and future industry, rural revitalization of agriculture and rural modernization and social governance and public services, ecological environmental protection and sustainable development, cultural creativity and regional cooperation and so on five track, aims to guide and motivate college students will learn knowledge and economic and social development, stimulate innovation spirit, cultivate entrepreneurial consciousness, promote social ability. Picture of the defense scene. Since the launch of the competition in January this year, more than 100,000 young teachers and students from 71 universities in the province have actively participated, with a total of 9,006 entries. The number of entries increased by 31.5% and 545.6% respectively compared with the previous competition, and the scale of entries and the number of entries reached a record high. Group, deputy secretary of Guizhou provincial party committee li Ji'an, "challenge cup" Guizhou province college students business plan competition is an influential and authoritative young students innovation entrepreneurship, is also an important carrier of the communist youth league service function, to guide the college young students around the "four new" main "four", in the construction of "four areas a highland" plays an important role in promoting, also for next year "challenge cup" countries created a strong innovative entrepreneurial atmosphere. The first, second and third prizes will be selected in this competition, and some excellent works will be selected to participate in the national final held in the second half of the year. In addition, it will also build a platform for college students to innovate and start, provide strong support, actively promote the transformation of scientific and technological innovation achievements, and better boost the high-quality economic and social development of Guizhou.

2022-06-23 Youth of Henan provincial party committee held "youth club school" youth backbone online training group "youth club school" and youth social organization backbone, emerging areas youth backbone online training JianWei correspondent Zhang Heng) recently, the Henan provincial party committee held in 2022 "youth club school" and youth social organization backbone, emerging youth backbone online training, from across the province more than 400 youth social organization backbone, emerging youth backbone, young volunteers backbone to participate in the training. The training course for youth social organizations, emerging areas of youth demand, elaborate design course content, invited the central school affairs director wei ping wei depth interpretation xi general secretary in the 100th anniversary of the congress's important speech spirit, training first innovation in small class teaching, focusing on young network writers, the media writers, express little brother, network about car driver work life actual needs set up professional courses. During the training, invited Zhengzhou university professor Jiang Meihua, professor Liu Jiayao, Henan university lecturer xiao-dong han respectively is cultural confidence and building cultural power, the new era of social work in community governance, the communist youth league history and reality made special counseling, contact provincial post administration personnel Li Xiaokang, provincial road transport association legal minister, provincial writers association network writer Su YueXi career development policy guidance and professional promotion service, enrich students' knowledge structure, improve the ideological quality, professional ability. This online training conscientiously implements the training discipline, records the viewing time of each student through the background of the system, and carries out wechat group learning and communication activities after class. The students have expressed that they will serve the society based on their own duties, cherish the post achievements, and strive to run the best results of the youth on the youth track, and meet the party's 20th victory with more excellent results. It is reported that the Henan Provincial Party Committee of the Communist Youth League specially included the emerging youth groups, youth social organization backbone into the province's Communist Youth League training plan, and continue to build the "youth society school" work brand. Since 2017, the provincial level has directly held five series of training courses for young people, youth affairs and the backbone of youth social organizations, serving nearly 1,000 emerging young people, effectively expanding the "circle of friends" of the Communist Youth League.

2022-06-21 Jianwei) Recently, the Yellow River Basin in Shandong Province was held in Yanggu County, Liaocheng City. With the theme of "the bank of the Yellow River is my hometown, I take disaster prevention and mitigation", the activity aims to improve the level of emergency rescue youth volunteer services in the province and help the ecological protection and high-quality development of the Yellow River Basin. Pictures of the event site. Group of Shandong provincial party committee for figure, Shandong provincial party committee, Shandong water conservancy department, emergency management department, Shandong Yellow River bureau, Liaocheng city, yanggu county relevant leading comrades to attend the closing ceremony, the scene watched the provincial emergency rescue volunteers backbone water rescue drills of the Yellow River, Liaocheng Yellow River flood control power and the Yellow River culture display. More than 70 young emergency rescue volunteers and volunteer service workers from 16 cities in the province participated in the training camp. Pictures of the event site. At the ceremony of the Shandong Provincial Committee of Liaocheng Municipal People's Congress, chairman of the Municipal Federation of Trade Unions, delivered a speech, Yanggu County, emergency rescue young volunteers representative made a statement. Cui Cunyong, member of the Party Leadership Group, deputy director of Shandong Yellow River Bureau and secretary of the Party Committee of the directly affiliated institutions, and Gao Xixin, the second-level inspector of Shandong Provincial Water Resources Department, respectively awarded MEDALS and certificates of honor to the winning teams and individual representatives of the skill competition in the training camp. Pictures of the event site. League of Shandong provincial party committee for figure group deputy secretary Zhang Shouheng for Shandong emergency rescue youth volunteer service team professional volunteer team and social volunteer team flag, and for the provincial emergency rescue youth volunteer service work deployment, requires the provincial youth league organizations at all levels and emergency rescue youth volunteer service to strengthen ability building, highlight the actual combat training, extensive training drills, skills and other activities, to improve the emergency rescue ability and level. Next, the provincial Communist Youth League Committee will continue to improve the service system, strengthen the capacity building, take the initiative to "take the lead" and "be the vanguard" in the NATIONAL strategy of the Yellow River, continue to mobilize the youth League organizations at all levels and the majority of youth volunteers with their youth dedication, protect the peace of the river, and welcome the party twenty victory with outstanding achievements.

2022-06-22 Gansu province to carry out the "communist youth league and people's congress, CPPCC members face to face" theme activities in Gansu "communist youth league and people's congress, CPPCC members face to face" theme activities JianWei correspondent Li Jing) recently, the Gansu provincial party committee joint province people club hall, provincial postal administration, in Lanzhou conducted the "post road" care express little elder brother-Gansu province "communist youth league and the people's congress, CPPCC members face to face" theme activities. Group of Gansu provincial party committee, provincial postal administration, provincial, CPPCC, provincial party committee, provincial people club hall, provincial public security department traffic administration and other related units office responsible for the activities, part of the provincial, CPPCC members, young entrepreneurs, the Courier on behalf of a total of more than 40 people attended the activity. Pictures of the event site. Correspondent for the picture to hold this theme activity, aimed to implement complementary advantages, resource integration, strengthen the express industry organization construction, talent team construction, enterprise culture construction, effectively protect the legitimate rights and interests of the Courier group, promote the sustainable and healthy development of the express industry, with practical actions to meet the party's 20th victory. The delegates visited the Gansu Communist Party Youth Movement History Exhibition Hall. The theme activity is divided into three stages. In the first stage of the donation ceremony, Gansu province postal service party secretary, deputy director li Jie speech, Lanzhou big health health management co., LTD., general manager Ms.Zhang Mengqiu to the provincial party committee donated care express little brother free health examination card, the leaders to the express little brother representative issued sympathy package and stable employment policy brochures, Gansu province 12355 youth service desk expert zhang li care express little brother psychological counseling and legal aid work is introduced. In the second stage, the participants were organized to visit the Gansu Party Youth Sports History Exhibition Hall, Gansu Young Pioneers History Experience Hall and Gansu Youth Home. It reviewed the glorious course of the Communist Youth League under the leadership of the Party. In the third stage, the Communist Youth League face to face symposium of the CPPCC, focusing on the theme of "How to better represent and safeguard the rights and interests of the Courier", enthusiastically spoke, listened to their aspirations, had interaction and communication, and answered questions on the spot. Pictures of the event site. Correspondent for the next step, the Gansu provincial party committee will give full play to the provincial youth joint conference office, to "planning" implementation is suggested, collaborative relevant departments, guide "express little brother" listen to the party with the party, in post work, improve service quality, ensure security and stability, further enhance the sense of honor, sense of belonging, feeling, make "express little brother" become a new era of laborer model, become good youth, for the implementation of rural revitalization strategy, speed up the construction of happiness new Gansu, constantly create a prosperous Gansu contribution youth strength.

2022-06-22 Shaanxi province youth volunteers association four session four member congress held shaanxi youth volunteer association four times member congress held on June 22 (reporter Ji'an-wei zhang, correspondent zhang wen-hao) recently, "always follow the party youth volunteer line" shaanxi youth volunteer association four session four member congress held in China oil changqing oilfield company. Xu Yongsheng, secretary of the Communist Youth League of Shaanxi Province and chairman of the Provincial Youth Federation, and Yu Haojie, Deputy Secretary of the Party Committee and chairman of CNPC Changqing Oilfield Company attended the meeting. Miao Fu, president of Shaanxi Youth Volunteers Association, and some representatives of members, caring enterprises and think tank experts attended the meeting online and offline. The meeting heard from Miao Fu, president of Shaanxi Youth Volunteers Association, on the annual work report in 2021, made a systematic summary of the work of the past year, and made detailed arrangements for this year's work. And for shaanxi province youth volunteers association 2021 outstanding individual, organizations, project representatives, for the fourteen games and special Olympics volunteer service work outstanding individual, organizations, project representatives, for the shaanxi province youth volunteers association experts, to get the 2021 "volunteer public love enterprise" award, added part of the association members, the leaders for the association of volunteer service base. After the meeting, the first volunteer service backbone training class was also held.

2022-06-21 The 8th Congress of the Chinese Young Pioneers of Shaanxi Province has concluded in Xi'an. The meeting deliberated and adopted a resolution on the work report of the Seventh Shaanxi Provincial Working Committee of the Chinese Young Pioneers, and elected the eighth Shaanxi Provincial Working Committee of the Chinese Young Pioneers, composed of 137 members. The conference stressed that the province's young pioneers work to deepen political guidance, a clear banner of training communist successors. Strengthen the training and incentive, and continue to enhance the sense of honor of the young pioneers. Consolidate the grass-roots foundation, and comprehensively enhance the vitality of the young Pioneers organization. Highlight practical education and promote the socialized development of young pioneers in the new era. Strict political standards, strengthen the construction of young Pioneers team counselors. Strengthen the policy guarantee, improve the system and mechanism of the young Pioneers' work. After the closing of the conference, the first plenary session of the eighth Shaanxi Provincial Youth Working Committee was held. The meeting pointed out that the new provincial young working committee should further enhance the sense of historical mission and political responsibility. We should firmly grasp the working mechanism, implement the Opinions of the Central Committee of the CPC Central Committee on Comprehensively Strengthening the Work of the Young Pioneers in the New Era, and promote the construction of the young Pioneers working system under the leadership of the Party Committee. We will continue to improve the operation mechanism of "the whole regiment leads the team", and strengthen the cooperative working pattern of the league and the team. Consolidate the practical achievements of "league teaching as one family", continue to play the positive role of committee members, and build an efficient working mechanism of young pioneers in the new era. Source: China Youth Daily, June 21,2022, edition of the 02nd edition

2022-06-21 Fujian: The first offline job fair of the first offline job fair was held in Fuzhou Liming Vocational and Technical College. On the same day, 114 employers were organized to provide 3,510 jobs for vocational college graduates, involving construction engineering, manufacturing, information technology, art and design, medicine, economic management, business, hotel management and other industries, attracting more than 1,300 graduates to participate in the on-site application, and initially reached 515 employment intentions. There are 50 higher vocational colleges similar to Fuzhou Liming Vocational and Technical College in Fujian Province. In recent years, with the increasing enrollment scale of vocational colleges, there are more than 60,000 graduates every year. In order to gather the resource and strength of private vocational education and private enterprises, To help solve the problem of "difficult employment and difficult employment" under the new situation, The United Front Work Department of the Provincial Party Committee, the Provincial Communist Youth League Committee, the Provincial Federation of Industry and Commerce, and the Provincial China Vocational Education Association jointly carried out the "hundreds of schools of concentric employment" action, In addition to the offline on-site job fair, In the early stage, also relying on the Fujian Province graduates employment and entrepreneurship public service network cloud recruitment system, For The province's vocational college graduates and all kinds of enterprises to provide all-weather, continuous line of accurate online job hunting and recruitment docking services, Focus on building a characteristic brand to help industry and education integration and deepen school-enterprise cooperation, Focus on providing services to build a modern economic system. Up to now, the campaign of more than 12,000 vocational college graduates have been organized by 100 schools for recruitment, and 718 enterprises have provided more than 17,000 jobs. Nine districts and cities in the province have also launched the action of "one hundred schools and ten thousand posts of concentric employment" respectively, organizing vocational colleges to connect the employment supply and demand with related enterprises.

2022-06-21 Group of Tianjin municipal party committee held "guard" food "heart not" food "light" practice group of Tianjin municipal party committee held "guard" food "heart not" food "light" youth theme practice education activities in the youth network reporter chun-yan hu) on June 19, group of Tianjin municipal party committee in Tianjin baodi district party committee held "guard" food "heart not" food "light" youth theme practice education activities. Through " Inheriting the Red Spirit, Ceaseless red blood, "" spring planting a grain of millet, "" Who knows the meal on the plate, Everything is hard. "" A porridge, a meal, When thinking about the hard-won " and other links, Organize young people through the Young Pioneers micro-ceremony, Enhance the sense of honor and organizational sense of belonging of the young Pioneers; Led the young Pioneers and qingma students to go deep into the field to carry out an immersive food saving and environmental protection class, Invite agricultural experts to tell about the rice and wheat production process; Lead the teenagers to experience the field work, Guide the young people to feel the working people, " enough to steam the summer heat and the earth gas, Back burning inflammation sky light " hardships; Led by youth affairs social workers to carry out interesting knowledge games related to food saving and environmental protection, Cultivate youth food saving habits, Especially in the current global epidemic environment, Guide young people to understand the "big people's livelihood" through the "small rice grains". Together (the young pioneers and young horse students experience in the field farming group Tianjin municipal committee for figure) (youth food environmental protection theme manual activities group Tianjin municipal committee for figure), the relevant person in charge of Tianjin municipal party committee, said the city's communist youth league organization will continue to guide youth actively implement xi general secretary important requirements, "resolutely stop food waste behavior, to cultivate the habit of saving", proud of advocating saving, said no to food waste behavior.

2022-06-20 Yunnan communist youth league held "one thousand school employment one to" graduates double selection will provide nearly 60000 Yunnan communist youth league held "one thousand school employment one to" graduates double selection Zhang Wenling) recently, the communist youth league of the Yunnan provincial party committee in Yunnan university, Kunming college and other universities held "one thousand school employment one to" graduates double selection, provide job information, open channels, help them to employment smoothly. In Yunnan University, 126 units from Beijing, Shanghai, Zhejiang, Fujian, Sichuan, Chongqing, Yunnan, Inner Mongolia and other provinces, including state-owned enterprises, institutions and private enterprises, provided 4330 jobs; Kunming University invited 126 enterprises and institutions, providing 2996 jobs for graduates, involving medicine, news media, social services, IT, construction, agriculture, finance, education and other industries. The armed forces department of Yunnan University also set up the recruitment consultation point in the job fair site, and many students came to consult. According to the introduction, college students joining the army can enjoy tuition compensation, postgraduate extra points, employment incentives and other preferential policies. Lin Bowen, a 2020 pre-school education graduate from Kunming University, has reached a preliminary employment agreement after communicating with a kindergarten in Jianshui County, Yunnan Province. She believes that the standard will be very standardized, give students more choices, the school for the students docking unit, and the students 'major combination, the unit to recruit is more in line with the students' expectations, the employer to provide the salary, working environment, after the training expressed satisfaction. The relevant person in charge of the Youth League Committee of Kunming University introduced that the youth League cadres will be organized to complete the employment assistance work of graduates from poor families, give full play to their own advantages, provide more, better and higher quality employment channels for graduates, and do a solid job in the service of youth employment. It is reported that since this year, the Yunnan Provincial Party Committee of the Communist Youth League has organized 54 recruitment activities of "thousands of schools" in 29 universities, with more than 2,000 units providing nearly 60,000 positions.(Photo provided by: Kunming University Youth League Committee)

2022-06-20 The 10th "challenge cup" Guangxi college students business plan competition final held the 10th "challenge cup" Guangxi college students business plan competition final held in the youth newspaper reporter Xie Yang) the 10th "challenge cup" Guangxi college students business plan competition final, held on June 17 solstice 18 in Guangxi university acacia lake campus. Since the launch of the competition, a total of 12,037 works from 78 universities in Guangxi were received, and the number of entries and the number of final works have hit a record high. The entries covered scientific and technological innovation, rural revitalization, social governance, public services, ecological and environmental protection, cultural creativity and regional cooperation. After qualification examination and judge evaluation, 227 works from 52 universities entered the final defense, and their works were displayed in the library of Guangxi University for Nationalities on The 18th. Through the final, 28 works will be represented by Guangxi to participate in the national competition. Group of Guangxi district party committee signed with relevant units "challenge cup" business incubation special cooperation agreement, for the winning excellent entrepreneurial team business consulting, office space, skills training, support funds, promotion and other related public welfare services, strive to hatch every year a batch of high technology content, strong application, characteristic of Guangxi college students excellent entrepreneurial projects. The competition is co-sponsored by the Communist Youth League Guangxi District Committee, the Education Department of the Autonomous Region, the Autonomous Region Association and the Guangxi Students' Federation.

2022-06-20 Sichuan "dream plan" college students 'social practice launch ceremony held in 2022 in Sichuan province "dream plan" college students' social practice launch ceremony held jianwei correspondent Zhao Qinghua) on June 17 afternoon, to "welcome twenty big, always follow the party, forge ahead new journey" as the theme of 2022 in Sichuan province "dream plan" college students' social practice launch ceremony held in Sichuan light chemical university. Wang Honghui, secretary of the Party Committee of Sichuan Light and Chemical University, delivered a speech, and relevant responsible comrades of Zigong Municipal Talent Office made talent promotion. In 2021, outstanding intern representatives of "Dream Chasing Plan", outstanding internship base representatives, Zigong Municipal Party Committee and Provincial Financial Youth League Working Committee spoke respectively. The launch ceremony of the 2022 "Dream Chasing Plan"."General Secretary Xi Jinping in visiting Sichuan provides direction guidance and fundamental guidance for us to do a good job in the employment of college graduates."The Sichuan provincial party committee is responsible for comrade, said the Sichuan province organizations at all levels will put xi general secretary of the important instructions and earnest entrust into the concrete practice of college graduates, relying on the" dream plan " for college students to participate in social practice to create more opportunities, provide better conditions, help them in the practice rich life experience, temper excellent skills, through the real job exercise set up the correct concept of employment, to lay a solid foundation for the future graduation employment, to realize their life ideal. The launch ceremony of the 2022 "Dream Chasing Plan". Correspondent for figure according to introducing, this year's "dream plan" focus on scientific and technological innovation, rural revitalization, social governance, and other fields, internship throughout the province 21 city (state) party and government organs, enterprises, public welfare organizations, research institutes and other units, will raise and release 40000 internship, help not less than 20000 college students to internship. At the same time, this year also set up "rural revitalization internship" special, released 100 "summer rural revitalization post"; around the regional talent attraction to carry out the "provincial strategic cooperation", Sichuan provincial talent office and strategic cooperation universities outside the province to organize internship, through professional experience to help college students to improve employability and vocational skills, help local party and government, employers and employment work. The "Dream Plan" Project is carried out on the "Youth Hui" platform "Dream Plan" section of "Tianfu New Youth" in the official wechat account of Sichuan Youth League Committee. All posts are announced through the "Dream Plan" platform, and students and employers will select the positions and objects in both directions. The platform is open to operation throughout the year, and the winter vacation and non-concentrated internships are carried out by employers and students according to the actual situation. After the successful double selection, students should fulfill their relevant commitments and internship obligations. The employer will also provide necessary work and living security for the internship students, and conditions will also give certain internship allowance or accommodation and transportation allowance. More than 150 people attended the launching ceremony, including youth League Working Committee of Sichuan Provincial Government, Provincial Enterprise League Working Committee, Provincial Financial League Working Committee, organization Department of Zigong Municipal Party Committee, Zigong Municipal Committee, Sichuan Light Chemical University and other units, representatives of employers, representatives of interns and students. Background data: "dream plan" is a organization of full-time college students into organs, into enterprises, into financial institutions, research institutes, into social organizations, into the grassroots, into the rural post internship as the main content of social practice, aims to guide students to participate in social practice and professional experience to enhance their employment entrepreneurship ability. Since its launch in 2014, it has raised more than 160,000 jobs and helped more than 100,000 college students take internships.

2022-06-20 Hunan: "millions of youth meritorious rural revitalization strategy practice education action" start the communist youth league of Hunan provincial party committee joint provincial agriculture and rural areas hall launched "millions of youth meritorious rural revitalization strategy practice education action" jianwei correspondent wen-long sun) for the implementation of the Hunan provincial party committee rural work conference and Hunan provincial party committee rural revitalization of the leading group meeting spirit, To implement the key tasks of the two gangs ", According to the work requirements of Hunan Provincial Party Committee on "making a good table of Hunan rice", Mobilizing young people to join in the rural revitalization strategy, recently, Hunan Provincial Party Committee of the Communist Youth League and Hunan Provincial Department of Agriculture and Rural Affairs, We jointly launched the "Millions of Young People Giving Thanks to the Rural Revitalization Strategy"."Millions of youth meritorious rural revitalization strategy practice education action" to "Chinese food Hunan rice youth flavor" as the theme, highlight the characteristics of the communist youth league work, focus on "industry", for "talent" service, through the organization, work, team and the project, form the political construction as the main line, comprehensively promote the five plans, deepen the three guarantee mechanism of "153" mode. By strengthening ideological and political guidance, strengthening the network of grass-roots organizations, strengthening the grass-roots backbone team, and consolidating the working position of the league, we will guide the young people to integrate their personal ideals into the cause of the Party and the country, and make contributions to the front line of rural revitalization. Focus on implementing youth talent cultivation plan, young entrepreneurs achievement plan, youth volunteer service plan, youth employment entrepreneurship support plan, youth care support plan, through five years, the provincial youth league organizations at all levels training rural revitalization talent 10000, help 10000 rural orphans, disabled, poor students and low-income students to complete their studies, help 10000 rural youth entrepreneurship employment, organize 100000 youth to participate in rural social construction, mobilize 1 million youth in rural revitalization strategy. It is understood that in order to ensure the work to carry out the ground, the communist youth league of Hunan provincial party committee in the work scheduling, resource security, evaluation incentive constantly strengthen and perfect system and mechanism construction, give full play to the provincial rural revitalization work leading group, strengthen work coordination and work guidance, actively integrate internal and external resources, project, social work, efforts to build rural revitalization of youth meritorious action of Hunan model.

2022-06-20 Shanxi communist youth league: all-round cohesion youth new work practice "please rest assured, power have me" oath Shanxi communist youth league: all cohesion youth new work this theme, Shanxi communist youth league adhere to xi jinping new era the ideas of socialism with Chinese characteristics as guidance, lead the provincial youth listen to the party, follow the party, consciously practice "please rest assured, power have me" youth oath. As an important part of the study and life of the youth league members and the young pioneers, the theme team activities have become increasingly rich in forms and achieved remarkable leading results. During the May 4th Movement, 54,000 league organizations in Shanxi Province and 2.030 million youth members watched the live broadcast celebrating the 100th anniversary of the founding of the Communist Youth League of China; and conducted extensive ceremony education, learning discussion, knowledge contest, theme presentation, red poetry recitation, art performance, where youth members and young pioneers receive the baptism of thoughts. During the Qingming Festival, the province widely carried out online worship, learning discussion, theme recitation and other forms of "Qingming Festival heroes" activities. Since the whole youth league has carried out the education and practice activities with the theme of "Welcome the 20th Plan, Always follow the Party, and forge ahead on the new journey", the youth league organizations at all levels in Shanxi Province have actively created a strong atmosphere of high spirits, unity and progress. First, adhere to the leading organs to set an example. The study and implementation of the important speeches, the important instructions and instructions made by General Secretary Xi Jinping, and the important statements put forward were taken as the "first topic" of the theoretical learning center group, and the theoretical research project team for young cadres was set up. Second, we have made solid progress in organized learning.75.17% of the league branches carried out thematic learning activities, covering 1.113 million members, and the network knowledge contest of "Welcome 20, Always follow the Party and forge new Journey", with more than 1.828 million participants. Third, to create the "youth sound jin line" propaganda brand. Organized from the three dimensions of province, city and school, made online "China (Shanxi) Youth May 4th Medal" winners deeds sharing meeting and "Youth to the Party does not live up to the People- -Youth striver said" network sharing meeting, with more than 1.4 million views; more than 1,200 offline publicity activities, covering 236,000 youth league members. Fourth, we will strengthen theoretical research and educational training. We compiled the Annals of the Communist Youth League of Shanxi Province, innovatively drew the Historical Map of the Shanxi Youth Movement and the Communist Youth League, edited and published the Ideological and Political Guidance of the Young People (the second volume), and presented 1,000 copies to the grass-roots youth league organizations. Fifth, to improve the effectiveness of publicity. Coordinate TV, radio, major newspapers and websites, organize the league media matrix to extensively publicize and report the important speech of General Secretary Xi Jinping and the warm response caused among the teenagers in the province. We continued to promote online topics such as # Youth and Jin Struggle with I # and # I speak for core values #, which were read by 390 million times. Bear responsible, leading the youth in the new era of drum struggle in April this year, Shanxi while more input COVID-19 outbreak, the provincial communist youth league organization under the unified command of party committees and governments at all levels and epidemic prevention departments, a total of 235 volunteer organizations, 17775 youth in disease resistance line, to carry out 1002 volunteer service activities, the cumulative volunteer service time 177217 hours. The provincial Communist Youth League has extensively organized and mobilized the youth to forge ahead and contribute, and become a new force to promote economic and social development. First, we will help deepen the rural revitalization and revitalization project.trained 10,3700 rural youth e-commerce talents, recommended 8 outstanding rural youth to win the title of "National Rural Revitalization Youth Pioneer"; raised 74.712 million yuan, funded 4,942 primary and middle school students from low-income and poor families. Second, we will deepen the "youth Xing Jin" action. It provided 11,896 practical posts for students returning home during the holidays, and 5,270 Shanxi students from universities from inside and outside the province were successfully employed. Third, we will encourage young people to innovate, start businesses and create excellence based on their posts. Lead the youth to participate in the forefront of "six new" construction, in the task of "dangerous and new", select 684 outstanding young skilled talents; regularly carry out various innovation and entrepreneurship competitions. The 4th "Shanxi Youth Entrepreneurship Award" selection activity provides the youth with venture capital of 3.46 million yuan, and the 9th "Chuang Youth" Shanxi Youth Innovation and Entrepreneurship Competition attracts 1511 entries. Fourth, we will strengthen the youth volunteer action. We organized 20,787 volunteer service activities such as "neighborhood watch" and "ecological and environmental protection" for teenagers, with volunteer service credit lasting 4.807 million hours and 1.079 million participants. Heart is youth, around the youth demand continuous optimization service in Shanxi 12355 youth public service platform for epidemic prevention psychological assistance line, for students affected by the epidemic, and fight in a line of medical, police, and other staff and the masses to provide free psychological support services, point to point to send text message prompt message 2.48 million, a total of 197 online case service. This year, the Shanxi 12355 youth public service platform has accepted a total of 27,270 consultations. The Shanxi Communist Youth League did everything possible to do practical things and solve difficulties for the youth. First, we will help young people find better jobs. Youth league organizations at all levels in the province provide 4,015 social practice positions for youth people; organize youth league cadres to help low- "double first-class" college graduates find jobs. Up to now, 1,671 college youth league cadres have paired up with 2,935 graduates. Second, to help young people in difficulties. We built two "pony Library", added five "childlike innocence Harbor" institutes, conducted more than 200 "childlike innocence Harbor" project activities, providing various forms of care services for rural left-behind children and migrant workers; subsidized 7,559 students in compulsory education, and helped students from low-income families and poor families to improve their learning and living conditions. Third, we will safeguard the legitimate rights and interests of young people. A press conference was held to make an in-depth interpretation of the Youth Development Plan of Shanxi Province (2021-2025), issue the Implementation Plan of the Pilot Social Support System for the Judicial Protection of Minors, and actively promote the construction of two pilot areas of Taiyuan Jian Lawn and Changzhi Luzhou. Have the courage to self revolution, work new vitality and career new atmosphere of Shanxi provincial party committee office building for Shanxi youth movement and the communist youth league history exhibition hall, build the first by office building transformation of stereo, immersive red clock, to the provincial youth vivid show under the leadership of the party, the Shanxi communist youth league and promising image. Focusing on the fundamental direction of "political nature, advanced nature and mass nature", the Shanxi Communist Youth League insists on self-revolution. First, to grasp the organs of the regiment. Completed the change of county and rural three-level league organizations, realize the promotion of education, age reduction, the quality of the grassroots youth League committee is significantly enhanced; 23 counties (cities, districts) were selected into the league grassroots organization reform pilot, the vitality of the grassroots organization of the league continued to improve. Second, to strengthen the organizational construction of the league. Middle school strong foundation, university vitality enhancement, urban and rural regional league building, non-public enterprise league building, network organization system is constantly improved; "online Communist Youth League" let the youth within reach. Third, strict management of the youth league organization. We will standardize the systems of "three meetings, two systems and one lesson", "league member + volunteer" and "promoting excellent party membership", and focus on the daily education and training of the league members, so that the sense of honor and advanced nature of the league members will be continuously enhanced. China Youth Daily China Youth Network reporter Hu Zhizhong source: China Youth Daily

2022-06-20 Sichuan: 2022 Energy conservation Publicity Week theme volunteer service demonstration activity held Sichuan: 2022 energy conservation publicity Week theme volunteer service demonstration activity held in Chengdu Beijing June 20 (reporter Zhang Jianwei, correspondent Zhao Qinghua) June 19 morning, By the communist youth league party committee of Sichuan province, Sichuan province government affairs administration, the communist youth league Chengdu municipal party committee and other units of "I do the practical work for the masses" - "low carbon make life better" in 2022 energy-saving publicity week theme volunteer service demonstration activities in tianfu new district xinglong lake community, Taurus, jinxiu, longquanyi district hua chuan community four communities held simultaneously. About 200 people from the panda Man volunteers and representatives from the community residents attended the event. Pictures of the event site. Correspondent for figure in tianfu new district xinglong lake community, panda man volunteers led the community residents visited Chengdu first "nearly zero carbon building" office building southwest China building design and research institute co., LTD., watched the building energy saving low carbon popular science exhibition wall, environmental protection public wall, distributed photovoltaic panels, automatic lighting patio, etc., to further understand the meaning of "nearly zero carbon building", function and function, and through energy saving and carbon reduction knowledge flip wall fun games, energy saving materials assembled manual experience activities have a deeper understanding of green building. In Chengdu Gas Supply Pipeline Network Branch, Panda Man volunteers of Chengdu Gas Group led the community residents to visit the service hall, gas ball tank and dispatching digital screen, so that the public can understand the gas operation situation in Chengdu, obtain the gas service content, and improve the awareness of safety and gas conservation. At Chengdu No.4 Regenerative Water Plant, the Panda Man volunteers of Chengdu Environment Group led the community residents to visit the sewage treatment process, understand the relationship between the purified water and the public's daily life, and raise the public awareness of water saving and water protection. Pictures of the event site. In Huachuan Community, Longquanyi District, Panda Man volunteers led the community residents to visit the community "enzyme hut" and the community garden, and shared the garbage classification, enzyme production methods, as well as the process of residents' independent participation in environmental greening to revitalize the old community. The children who participated in the activity painted and made earthworm green towers, painted the imaginary green home with paintbrushes, and made earthworm towers with discarded beverage bottles and plastic tubes to provide the red scarf power for the construction of the community garden. Pictures of the event site. After the visit, panda Man volunteers and residents worked together in the Xinglong Lake, baseline River, Shahe River and Dongfeng Canal, picked up green garbage, inspected the sewage outlets, and distributed electronic environmental protection publicity materials by scanning the QR code, so as to save resources and protect the environment with practical actions. Learned, this week, the provincial youth league organizations at all levels widely "low carbon make life better" in 2022 energy saving publicity week series of volunteer service activities, cities (state), county (city, area) and relevant school youth league organizations to reduce haze, reduce plastic, emissions, resource saving, design and development of volunteer service and creative works, organize youth widely involved in the tour river tour lake, green travel, food waste, reduce emissions theme activities, encourage teenagers to actively participate in energy conservation and carbon reduction, promote the whole society to form green life new fashion. Up to now, 122 themed activities have been carried out in the province, with 1,267 participants and a service time of 6,204.6 hours.

2022-06-20 Shaanxi "walking class" years cover member youth over one million shaanxi "walking class" years cover member youth over one million people reporter recently learned from the communist youth league committee of shaanxi youth league, for deep youth thought lead responsibility field, youth shaanxi party committee actively carry out the "youth learning", the struggle of the youth of the most beautiful distinct theme, various forms of preaching propaganda activities. At present, these "walking group lessons" not only expand their influence and coverage of publicity, but also improve their brand awareness. According to preliminary statistics, more than 100 million youth league members are covered annually."Walking group class" focuses on the party's innovation theory, important historical nodes, typical deeds of youth and other aspects, and the propaganda content is closely related to the current political hot spots, and the propaganda form is constantly innovated. Since the beginning of this year, around the "welcome the party's 20 th" and "celebrating the 100th anniversary of the founding of the league" work, "walking group class" has carried out a number of small-scale, face-to-face, interactive publicity and communication activities, so that the party's theory into the youth, attract the youth, lead the youth, let the youth receive education in emotional resonance. Tuan shaanxi provincial party committee before the summary of experience, from the lecturer selection, curriculum, preaching form, business training, dynamic management, cadres on the platform, six aspects, optimal, continuous optimization selection lecturer, release about topic menu, online combined into the workshop, workshop, classroom, community, the fields interaction, continuous regular training preach personnel, open audience scoring channel, clear fixed youth corps committee team members, cadres in preaching times, ask cadres to do have food, can speak, everyone can speak, audience love to listen. In the recently launched "Welcome 20 Always follow the Party" theme education practice activities, "Youth learning" first demonstration publicity exchange activity had more than 20,000 online learning, 4 "China (Shaanxi) Youth May 4th Medal Sharing meeting" had more than 100,000 people watched the study. In the face of the impact of the COVID-19 situation, The Communist Youth League and Shaanxi Provincial Party Committee focused on innovating the form of publicity, Create high-quality products, In the form of league joining demonstration ceremony, speech under the league, visiting old sites, theme drama and "red script kill" and other activities, Guide the youth league members to inherit the red gene, Draw on the strength to move forward; Actively contact local cities, universities, enterprises, social professional and cultural companies, Together to create excellent quality, novel content, widely publicized cultural products, For example, the launch of the party history cartoon "Red Star Shine", video clips "Proud Youth", "Footsteps", "New Era is youth", micro film "Youth", "Red Arrow youth", MV "Mountains and Rivers starlight", "Have Me" and other excellent literary works, Carry out the theme activity of # 100 schools relay singing for one hundred years #, Continue to export high-quality cultural products. Source: China Youth Daily

2022-06-17 The "2020 Hope Project Always follow the Party in the 2022 Hope Project Dream Realization Action" was jointly launched by the Foundation. The 2022 Hebei Province "Project Hope Dream Realization Action" will start from June to the end of December.period, Hebei Provincial Youth League Committee and Hebei Provincial Youth Development Foundation will cooperate with the municipal implementation agencies of the project to cooperate with the media, Public welfare publicity, Advocating the concept of public welfare, Raising money through social mobilization, According to the compulsory education stage students of 1000 yuan per year, high school (secondary school) students of 2000 yuan per year, vocational college students of 3000 yuan per year, university students of 5000 yuan per year (except military students, national defense students, public funded normal university students, commissioned students) funding standard, Help primary and middle school students with poor families and excellent students to complete their studies, Priority will be given to students from poor families affected by COVID-19. The "Hope Project Dream Realization Action" has been implemented in 2004 and has been implemented for 18 consecutive years. A total of more than 50,000 students from poor families have been funded to realize their dreams, which has received support and participation from all sectors of society.

2022-06-17 The Fujian provincial party committee held Chinese youth May 4th medal sharing group Fujian provincial party committee held China youth May 4th medal sharing jianwei) from May to June, the Fujian provincial party committee successively in Fujian economic school, minjiang college and fujiang Fuzhou Fujian province "welcome 20 forever with the party forever new journey" -the Chinese youth May 4th medal sharing. Nearly 150 people attended the event, including "China Youth May 4th Medal" and "Fujian Youth May 4th Medal" winners, as well as youth league member representatives from the grassroots level. The sharing meeting was broadcast live on the Internet simultaneously, covering more than 436,000 person-times. Share the pictures of the live meeting. Members of Fujian Provincial Youth League invited 6 "Chinese Youth May 4th Medal" and "Fujian Youth May 4th Medal" to have in-depth exchanges and learn from the spirit of General Secretary Xi Jinping's important speech, and share their progress with the Party and personal advanced deeds. You Yancheng, the winner of the "China Youth May 4th Medal", executive vice President, distinguished professor and doctoral supervisor of the School of Aeronautics and Astronautics of Xiamen University, through online shared the scientific research path of integrating his personal ideals into the development of national aviation and aerospace industry and making unremitting efforts to achieve high-level self-reliance and self-improvement in science and technology. Li Dong, the winner of the "Chinese Youth May 4th Medal" and a technician in charge of the Immunology Program Institute of the Fujian Provincial Center for Disease Control and Prevention, shared his experience in Xiangyang, Hubei province and the Hong Kong Special Administrative Region to participate in the frontline epidemic prevention and control work, showing the responsibility and role of young medical workers in the new era in the fight against the epidemic. Deng Wei, winner of the "China Youth May 4th Medal", weightlifter of Fujian Weightlifting Management Center and Olympic women's weightlifting 63 kg champion, shared her inspirational story of the Olympic dream, hard training and competition amid setbacks, and finally reached the podium. Liu Zirun, the representative of the "Fujian Youth May 4th Medal Collective" and the leader of the Fujian Men's Badminton Team, told the hard journey of the development of the new China badminton industry since its establishment, and shared the story of the hard work and outstanding achievements on the court. Lin Lulu, winner of "Fujian Youth May 4th Medal" and secretary of Longxiang Village Branch, Songkou Town, Yongtai County, the Communist Party of China, shared the youth story of returning home to start his own business and taking root at the grass-roots level. From young to skilled, she constantly integrates new ideas and developing new business forms for rural development and contributes to rural revitalization. Zhang Huiling, winner of "Fujian Youth May 4th Medal" and deputy secretary general of Fuzhou Youth Affairs Social Workers Association, shared the touching story of being engaged in youth social work services, being committed to the development of youth affairs social workers, serving teenagers in difficulties, and being willing to be a "confidant". Winners also interact with the scene of young friends, inspire young friends to outstanding youth as an example, inherit and carry forward the spirit of the May fourth Movement, hard work in their jobs, sheer will in the hard struggle quality, growth work ability in practice, contribute to the new stage of development new Fujian youth strength, to meet the party's 20 victory with honors.

2022-06-16 Group Hebei provincial party committee to implement the "five project" Hebei provincial party committee to implement the "five project" group Hebei provincial party committee has drafted and issued by the "about the" welcome 20, always follow the party, new journey "theme education practice notice, to strengthen theoretical study, organize team activities, practice activities, strengthen service lead, propaganda and cultural work for activity content, implement the" five project ", build online coordination linkage, combining theory and practice of learning system, focusing on 17 specific work. League of Hebei provincial party committee will write a book, complete the new youth dialogue whole interview print work, in the province into 100 whole in industries based on the post tenacious struggle, dedication, make outstanding contributions, represented by the "Ji Qing star" industry outstanding youth and whole dialogue, listen to inculcate, inspire the youth inheritance revolutionary spirit, guide the actual work. An exhibition has been launched, together with the Communist Party Member magazine, which has collected more than 800 pictures, manuscripts and other materials, and an online league history exhibition hall has been opened on the new media platform of the Hebei Communist Youth League, which is expected to be officially launched in mid-to-late June. A series of theme publicity and education activities were carried out, including: "Welcome the 20s, Always follow the Party, forge ahead on a new journey- -Years of Youth singing the Song of the New Era" video collection activities, the theme of "100 League Members on League History" video collection and selection activities. Create a number of fine cultural works, including: production of "Youth izhuang" micro film, the launch of high-quality network cultural works- -hand-painted long scroll "Picture of Chinese Youth in the New Era", shooting and release " Struggle, China!"Youth version MV, planning and shooting" New Era Hebei Youth " video propaganda video, etc. Select trees to promote a group of new era Hebei youth model, including: 3 Hebei youth selected as "national good youth"; launch "hundred cloud speech" activities, to promote the era of the outstanding youth from all walks of life; identify 100 "digital Hebei youth model" candidates, guide the youth to participate in the development of digital economy. Source: China Youth Daily, June 16,2022, edition of the 02nd edition

2022-06-16 Correspondent Chen Kai) On June 12, the final of the "Welcome 20, Always follow the Party and forge ahead on a New Journey" was held in Guiyang. Pictures of the event site. This competition is jointly hosted by the Communist Youth League Guizhou Provincial Party Committee and China Telecom Guizhou Branch. College students from the province 20 universities in major achievements, around the party's struggle, the party led the Chinese youth movement, rural revitalization, epidemic prevention and control, through vivid interpretation, reproduce the story of youth example in different historical period, shows the Chinese youth in one hundred firm with the party's youth choice and youth elegance, for the audience brought a wonderful audio-visual feast. Pictures of the game scene. In the more than three hours of performance, the actors were fully engaged, the audience was passionate and gave continuous applause, and the struggle of generations of Chinese youth slowly unfolded through the performance, touching everyone on the scene. Stage stills. The participating play "One Hundred Years of Youth" reproduces the founding history of the Communist Youth League of China, and has artistically refined and restored the history. The stage performance is rich, and the actors are seriously involved, which is very infectious and makes people's blood boil. The participating drama "Qiannan Normal University for Nationalities" starts with the events in the fight against COVID-19 epidemic, showing the responsibility of contemporary youth in facing the test of life and death affection. The drama conflicts are distinct, and the actors vent the emotions of the whole drama to the extreme with their very infectious and impact performance. The play "Bright Moon of Moon Mountain" by Guizhou Open University, against the background of "College Students Volunteer Service in the West"., tells the story of an ordinary volunteer teacher who "illuminate the moon mountain", and finally the students they taught feed back to their hometown. The whole performance level is clear, the transition is smooth, exquisite clothing and Guizhou characteristics, has a strong artistic appeal. The simple story, because of the sincere and cordial feelings and performance, touched the hearts of the audience, and won a warm applause from the audience. After fierce competition, 3 first prizes, 7 second prizes, 10 third prizes, 5 popularity awards and 5 organization awards were awarded.

2022-06-15 Inner Mongolia to carry out the "youth learning" about communication into grassroots activities in Inner Mongolia "youth learning" about communication into grassroots activities JianWei correspondent wang Chen) on June 8 solstice 10, the Inner Mongolia autonomous region committee, Hohhot municipal party committee jointly organized to carry out a series of "youth learning" about communication into grassroots activities, common pass xi general secretary of the communist youth league of the 100th anniversary of the conference of China's important speech spirit."The teacher's teaching gave me a deeper understanding of General Secretary Xi Jinping's speech. I have become more determined to protect the people's property and ensure their happiness, and strengthened the spirit of daring to fight hard, to eat great hardships, to break new ground, and to strive for the first place."After listening to the lecture, Hohhot city fire rescue detachment special service station deputy stationmaster Li Tong so firmly said."Youth big study" publicity and exchange theme activities into the industrial and commercial Bank of China. Zhang Amy, an employee of the Inner Mongolia Branch of the Industrial and Commercial Bank of China, said, " I deeply feel that our young people in the new era not only have a broad space for development, but also shoulder the important responsibilities of The Times. In the future, I will unswervingly follow the pace of the Party, based on the post, actively struggle, with practical actions to practice the original mission."The Northern Xinjiang youth teaching group, the youth typical publicity group and the red scarf publicity group members have walked into the organ community, rural pastoral areas, schools, enterprises and other grass-roots frontline, for different fields of youth league members, hierarchical and classification of publicity."Youth study" publicity and exchange theme activities into the liaison station of league representatives. In the Yuquan District Youth League representative liaison station, the young lecturers walked into the community and sat together face to face with the young people in the community, sharing and communicating deeply. In Bakou community, Youyouban Town, rural youth learned that the pace of youth can only follow the pulse of home and country."Youth big learning" publicity and exchange theme activities into the campus. In the riverside campus of University Road Primary School, the team cadres of the primary and secondary schools in Saihan District listened to the teaching of the red scarf publicity group members of the autonomous region. In Saihan District Ethnic Primary School, the young Pioneers small backbone brush brush their hands to communicate with teachers and share their learning experience. In the next step, the Youth League Inner Mongolia Autonomous Region Committee will continue to carry out the "youth learning" publicity and exchange activities into the campus, into the grass-roots activities, guide the district youth to continue to learn, actively learn, in-depth learning, so that the spirit of the speech internalized in the heart, externalized in the line, with practical actions to meet the Party's 20th victory held.

2022-06-15 Group of Jiangxi provincial party committee of youth branch concentrated preparation seminar group youth branch concentrated preparation seminar in Beijing on June 15 (reporter Zhang Jianwei, correspondent gui-peng huang) for further study publicity and implement xi general secretary in celebrating the 100th anniversary of the founding of the communist youth league of China's important speech spirit, further develop "youth big learning" about exchange theme activities, on June 13, group of Jiangxi province party committee in Jiangxi province youth branch preparation seminar in Jiangxi agricultural university, more than 20 province youth branch members. In order to improve the level of branch members to preach, the lesson preparation activities invited the Jiangxi province radio and television association broadcasting and hosting professional committee deputy secretary-general Cheng Shan teacher field guidance, through the lecturer, the teacher comments, for each lecturer to preach anatomy sparrow type analysis comments, further enhance the actual effect of preaching."The presentation can be integrated with multiple elements or have rich forms of expression, but it must not be the host, or it will put the cart before the horse."Instructor Cheng Shan's words, let the present lecturer immersed in meditation."In the past, we always tried to attract the attention of young people, but the teacher Cheng reminded us that we should focus more on the main theme of the lecture, and the key is to speak the theory alive and thoroughly."Said Liu Lin, from Jiangxi Normal University of Science and Technology, a member of the Jiangxi Provincial Youth Teaching Group."How to make it more vivid, more vivid, and more more infectious?"" We should be good at finding 'small cuts' from 'big themes', use true and vivid stories to show the power of ideals and beliefs, and thoroughly explain the truth of institutional confidence, so that young people can truly empathize and truly identify from their hearts."Chen Yang'an, a member of Jiangxi Youth Lecture Group and from Jiangxi University of Fashion Technology, expressed his feelings in the discussion and exchange."How to make the propaganda truly accessible and close to the youth?"" Propaganda should be a dialogue with the youth. We should abandon the condescending theoretical preaching, but use the language of the youth to have empathy and stimulate their resonance, so as to better convey the mainstream values."In the intensive discussion and exchange meeting, Members of the youth teaching group, combined with their own publicity experience, Keep asking questions, Study and solve problems, In the fierce collision of ideas, share the experience, Immersion in learning, Innovative ideas are inspired out, Detailed speeches have been polished out.... After the intensive lesson preparation seminar, As the Jiangxi Provincial Committee will "Welcome the 20th Party, always follow the Party, and forge ahead on a new journey", Continue to organize members of the youth teaching group, all kinds of young models into the campus, into the community, into the production line, Focusing on the publicity and interpretation of the spirit of General Secretary Xi Jinping's important speech, Extensive "youth learning" publicity and exchange theme activities, To convey the ideological strength to the majority of young people, to answer the ideological confusion.

2022-06-15 Sichuan launched a new round of "hundred thousand" preaching in Sichuan launched a new round of "hundred thousand" preaching activities jianwei correspondent qing-hua zhao) on June 13 afternoon, Sichuan provincial party committee XuanJiangTuan new era civilization practice youth group held in Chengdu about mobilization and collective lesson preparation, officially launched a new round of "one hundred thousand" preaching activities. The picture shows the scene of the publicity mobilization meeting and the collective lesson preparation meeting. Correspondent for the picture meeting, the Sichuan Provincial Committee of the new era civilization practice youth publicity group leader, secretary of the Provincial Youth League Committee Zhang Rong made a guidance report. He said that we should combine the "two", combining the spirit of the provincial Party Congress with General Secretary Xi Jinping's important speech at the celebration of the 100th anniversary of the founding of the Congress of the Communist Youth League of China, and with learning from the important instructions of General Secretary Xi Jinping's inspection visit to Sichuan. To do "six clear", clear the new era of shu Xingchuan fundamental follow, clear over the past five years keep in mind xi general secretary entrust major achievements, clear the new era of shu Xingchuan overall planning, clear the new era of shu Xingchuan strategy traction, clear in the next five years the province's economic and social development tasks, clear to self revolutionary spirit further promote comprehensive governing party's clear requirements. At the same time, it pays attention to the systematic interpretation and authoritative interpretation of the major ideological views, major institutional arrangements and major work arrangements, to guide the young people in the province to continuously deepen their understanding and understanding, and to better unify their thoughts and actions into the important instructions and the spirit of the Party Congress. He also made a comprehensive mobilization and deployment of publicity work from three aspects: grasping the focus of publicity, clarifying the keynote guidance and focusing on key groups. The three representatives of the youth publicity group exchanged their learning experience based on their own study and work. At the meeting issued the "12th Provincial Party Congress spirit of the special publicity work plan" (hereinafter referred to as the "plan")."Plan" regulation, from early June to the National Day, will be the provincial party committee XuanJiangTuan new era civilization practice youth XuanJiangTuan members as the main body, for teenagers start "one thousand field" preaching activities, the provincial cadres at all levels, youth branch members, and youth league, union, less committee, etc, will also focus in teenagers widely preaching work, to achieve full coverage. According to the relevant person in charge of the Provincial Communist Youth League Committee, in order to expand the influence of the publicity work, will also open "Xiang Bashu" group is a new media column, and explore the form of network broadcast, online communication and interview, launch a series of theoretical publicity media products for youth groups, to form the strongest voice of online publicity. Members of the secretary of the Provincial Communist Youth League Committee, responsible members of all departments and directly affiliated institutions, and representatives of the youth group of the New Era of Civilization Practice attended the meeting in the main venue of Chengdu. The members of the youth League committee of each city (state) are mainly responsible comrades, the responsible comrades of the publicity department, and the members of the new era civilization practice youth publicity sub-group participated in the video way in the sub-venue.

2022-06-15 Fourteen departments of Fujian province joint issued to carry out the "sail plan" fourteen departments in Fujian province joint issued to develop college students internship "sail plan" jianwei) recently, the Fujian provincial party committee joint development and reform commission, Fujian provincial department, provincial education department, provincial department of fourteen departments jointly issued the "about 2022 in Fujian province college students internship" sail plan "notice" (hereinafter referred to as the "notice")."Notice" is put forward, this year in the province party and government organs, institutions, state-owned enterprises and financial institutions, all kinds of scientific research institutions and large private enterprise units for not less than 30000 quality internship positions, for the province Fujian students and college students in the province are widely released, promote not less than 15000 college students to participate in the summer internship. This activity aims to thoroughly study and implement xi general secretary about new era of new ideas, new strategy, the full implementation of provincial party committee talent work meeting deployment requirements, focusing on the provincial party committee "improve efficiency, improve efficiency, increase efficiency" action, around the "four big economy", help college students in practice deeply understand social conditions, set up the correct employment, the own career development positioning, reserve work experience, improve employment core competitiveness, and drive a batch of Fujian students outside Fujian employment, to promote the development of high quality beyond provide solid talent support. The Notice clarifies the division of responsibilities of each participating department, highlights the focus of the work at each stage, and provides scientific guidance and mechanism arrangements for the orderly promotion of the "Sailing Plan". It is reported that up to now, Fujian Province college students internship "Sailing Plan" has mobilized 2,357 employers, providing 21,863 internships, including 2,524 "digital economy" positions, 194 "Marine economy" positions, 1,665 "green economy" positions, 1,585 "cultural tourism economy" positions, attracting students to send 40,324 resumes. After the college students complete the summer internship, the employer will issue the internship appraisal, and issue the internship certificate uniformly customized by the Fujian Provincial Party Committee of the Communist Youth League. At the same time, employers are encouraged to find a number of outstanding young talents through the internship activities. If the conditions permit, both parties can appropriately extend the internship period or reach the employment intention after reaching an agreement through consultation. The Provincial Communist Youth League Committee will also identify a number of internship bases for college students in Fujian Province. If you are interested to participate in the employers and college students, you can register through the "Fujian Sailing Plan" small program.

2022-06-15 Beijing: the 26th China youth May 4th medal sharing and 2022 shougang youth model preach theme activities in Beijing: the 26th China youth May 4th medal sharing and 2022 shougang youth model preach theme activities held Beijing on June 15 (reporter Ji'an-wei zhang, correspondent, bilLAN Yang Jing) recently, sponsored by the Beijing municipal party committee, Beijing youth federation, The "Shougang Group Youth League Committee hosted the 20th Party forever" - -the 26th China Youth May 4th Medal (collective) Sharing Meeting and the 2022 Shougang Youth Model publicity theme activity was held through the network live broadcast. In the nearly 2 hours of live broadcast activities, the number of participants exceeded 16,000, and the number of thumb up people was more than 50,000. Shougang Group organized more than 1,500 youth league members to watch the whole live broadcast, which won the unanimous praise of the youth league members. Screenshot of the live broadcast event. Correspondent for the picture sharing meeting in the central League "Chinese Youth May 4th Medal" propaganda film "100 years of struggle always follow the Party" began. Zhang Qi, winner of the 26th China Youth May 4th Medal and squadron leader of the Police support detachment of Tongzhou Branch of Beijing Municipal Public Security Bureau, and Huang Zhen, deputy chief designer of the overall Design Department of the Fifth Academy of Aerospace Science and Technology Corporation, shared their youth story of moving forward. The 26th China youth May 4th medal winner (collective) six representatives combined with their own learning xi general secretary in celebrating the 100th anniversary of the founding of the communist youth league of China's important speech spirit comments and they in the games service security, community epidemic prevention and control and grassroots innovation and efficiency work, share the "with the party, listen to the party order, with the party struggle" personal course, shows the contemporary shougang youth love the motherland, burden, chicheng dedication of youth.

2022-06-15 Chongqing 29 entries for the 13th "challenge cup" Chinese college students business plan competition 29 entries for the 13th "challenge cup" Chinese college students business plan competition Wang Xinxin) on June 12,2022, "wisdom" youth "the 13 th" challenge cup " Chinese college students business plan competition division finals held in Chongqing university of post and telecommunications, a total of 29 entries for the national competition. The final was hosted by Chongqing Municipal Party Committee of the Communist Youth League, Chongqing Municipal Education Commission, Chongqing Human Resources and Social Security Bureau, Chongqing Association for Science and Technology, and Chongqing Students' Association. A total of 95 final works competed for the qualification to participate in the national competition, and 25 expert judges produced the finalists through the online evaluation. Online review is in progress. Since the launch of the competition, the Chongqing Municipal Committee, Chongqing Union, Sichuan Provincial Committee and Sichuan Province have jointly carried out a one-month "forum" talk "", "enterprise" see "", "employment" employment "," style "exhibition" "," challenge "competition" " and other more than 100 theme activities, covering more than 300,000 college students, creating a good atmosphere for scientific and technological innovation. In this competition, a total of 80 schools in the city, 18,424 works participated in the competition. It is understood that the competition set scientific and technological innovation and future industry, the revitalization of rural and agriculture and rural modernization, social governance and public services, ecological environmental protection and sustainable development, cultural creativity and regional cooperation five groups, on the basis of project business value, pay more attention to the students understand the social status quo, pay attention to social people's livelihood, solve social problems, ability and level, promote competition return to education beginner's mind."The works submitted by our team belong to the category of scientific and technological innovation and future industry. We have studied the advanced flexible sensing technology and its application for people's life and health, and developed the ultra-light, ultra-soft and ultra-sensitive smart bed sheets that can realize the auxiliary diagnosis of cardiac diseases and sudden cardiac death warning."From Chongqing University of Posts and Telecommunications automation school electrical engineering and automation major Chen Shifan said. Zhang Bingfei school of electrical engineering, Chongqing university, different metal reliable connection is one of the challenges facing manufacturing, their team relying on transmission and distribution equipment and system safety and the state key laboratory of new technology, developed the pulse discharge connection equipment, can realize copper-aluminum, aluminum, magnesium and other commonly used metal connection.

2022-06-14 Fujian Provincial Party Committee to carry out the "loyalty, post dedication" clean education theme Party day activities Jian Wei) recently, the Communist Youth League Fujian Provincial Party Committee went to Fuzhou Luzhou Chen family on the fifth floor of the family training hall to carry out the "loyalty, post dedication" clean education theme Party day activities. Li Teng, Shao Mingsong, Zhang Afeng, deputy secretary of the Provincial Communist Youth League Committee, and more than 70 party members and cadres of the government organs and directly affiliated units attended the activity. This activity around the young cadres absolutely loyal education theme, combined with the "Fujian fresh air, clean authority" clean cultural education and provincial party committee is carrying out "keep discipline, fear, bat love" special education, focus on good made authority honest education resources, education guide young cadres to predecessors martyrs as the mirror, constantly enhance political consciousness, improve political ability, inheritance Dan family precepts, carry forward the traditional virtues, with party spirit cultivation polish loyal background, take practical action to reflect loyalty consciousness. Party members learned the excellent family traditions and instructions passed down by the Chen family in Luozhou, and carefully felt the ideology and culture of Chen Baochen, Chen Ruolin and other sages of the past dynasties, which were diligent for the people, loyal and patriotic. We have said, to this activity as an opportunity to earnestly study and implement xi general secretary about pay attention to family tutor family trait construction important discourse, prison ideal faith "master switch", adhere to the absolute loyalty, adhere to the clean politics, clean power, integrity, integrity, carry forward the traditional family of the Chinese nation virtues, continuously red, edify family trait, moral sentiment, culvert good Dan, conservation authority wind is political ecology, create model organs, building clean authority, with good mental state and work results to meet the party's 20th victory.

2022-06-14 This morning, the donation ceremony of "Hubei Hope Project bosom Friend peer Student Fund" and the face to face activity was held in Wuhan University. Erken Jiang Tulhong, member of the Standing Committee of Hubei Provincial Party Committee and Minister of United Front Work Department, attended and awarded the "Outstanding Contribution Award of Hubei Hope Project" to the Provincial Tobacco Monopoly Administration (Company). At the donation ceremony, Hubei Provincial Tobacco Monopoly Administration (company) donated 10 million yuan to Hubei Hope Project. According to reports, in 2018, Hubei Provincial Tobacco Monopoly Administration (Company), together with Hubei Provincial Party Committee of the Youth League and Hubei Provincial Youth Development Foundation, jointly established the "Hubei Hope Project Scholarship Fund" to subsidize high school students, freshmen and college students from families with financial difficulties. In the past four years, the fund has invested a total of 40 million yuan to help nearly 12,000 students in need, which is the public welfare project with a single project coverage and the largest number of students since the implementation of Hubei Hope Project. In bosom friend students face to face activities, Wuhan university doctoral Zhang Liang, Hubei university undergraduate Jian prosperous, Wuhan east lake middle school students nuli, tells the story of the student fund help to study hard, actively the story, said it will remember the care, do grateful endeavour, actively participate in public welfare activities, return to society. The relevant person in charge of the Hubei Provincial Party Committee of the Communist Youth League said that efforts should be made to make the "Hubei Hope Project peer Scholarship Fund" effective, expand the influence, effectively convey the care of the Party, and provide new help and sow new hope for the young people. Source: China Youth Daily, June 14,2022, edition of the 02nd edition

2022-06-14 Shaanxi province youth safety production ShiFanGang standardization to create site advance will be held "the production safety youth first" shaanxi province youth production safety ShiFanGang standardization to create site advance will be held in xi 'an JianWei correspondent Zhang Wenhao) on June 10, sponsored by the communist youth league party committee, shaanxi province emergency management hall, iron bureau to undertake "production safety youth first" youth production safety ShiFanGang standardization create site advance will be held in xi' an. Shaanxi Provincial Secretary Xu Yongsheng attended the meeting and made a speech. Relevant leaders of Shaanxi Provincial Emergency Management Department and China Railway First Bureau attended the meeting, which was presided over by Huang Hua, deputy secretary of Shaanxi Provincial Party Committee of the Communist Youth League. Part of the Shaanxi Provincial Committee of the key contact state-owned enterprises secretary of the Youth League Committee, youth safety production demonstration post representatives attended the meeting. The participating leaders wore armbands and backpacks for the representatives of the youth safety demonstration post. Correspondent for the picture meeting, the participating leaders for the youth safety production demonstration post representative awarded the youth commando team flag, and wear the youth safety production demonstration post armband and backpack. Participants observed the work demonstration of the standardization construction, cultural construction and system construction of the youth safety production demonstration post of China Railway First Bureau, and witnessed the launch of the "Communist Youth League" shield machine in the project department of Xi'an Metro Line 8 of China Railway First Bureau. Observe the standardization construction of the youth safety production demonstration post of China Railway First Bureau. Xu Yongsheng, secretary of Shaanxi Provincial Party Committee, pointed out that the creation of youth safety production demonstration post, is an important entry point and focal point for the Communist Youth League to participate in the production safety, is the majority of young workers to reflect the responsibility, play a favorable role of the vanguard position. Youth league organizations at all levels should raise awareness, take active actions, effectively enhance the sense of responsibility and mission of youth work safety, and lead the majority of young workers to play the role of a new force, commandos, do in practice, walk in the forefront. Xu Yongsheng stressed that the youth safety production demonstration post to create pilot units to go ahead in the pilot demonstration. It is necessary to further sort out and form the detailed standards for the creation of "Qing'an Gang", improve the creation system, refine the experience and practices, and create the demonstration benchmarks that can be replicated and promoted for reference. To complete the provincial youth civilization and "youth'an post" and "number post" integration and creation of pilot work tasks, to promote the integrated development of brand work. Xu Yongsheng asked the state-owned enterprise youth League committee to create and promote the "Qing'an Gang" ahead. It is necessary to go deep into the front-line research to understand the actual role of "Qing'an Gang", find out the problems, clarify the shortcomings and weaknesses, and find practical measures to improve and improve in the comparison. To rely on the "Qingan Gang", to carry out a good "safety volunteers in action" activities. The "Communist Youth League" shield machine of the Xi'an Metro Line 8 project of China Railway First Bureau was launched. Correspondent for figure group shaanxi provincial party committee, shaanxi province emergency management hall to shaanxi province "green AnGang" collective and the young worker initiative, to take the lead to firmly establish safety consciousness, take the lead in improve safety in production skills, take the lead when young safety pacesetter, active youth safety post, drive the youth around safety practice, efforts to create a good atmosphere of "everything about safety", take practical action to help shaanxi high quality development, for the party's 20 victory to create a stable safe production environment.

2022-06-13 Beijing communist youth league: unity led the capital youth good practice "education" Beijing communist youth league: unity led the capital youth good practice "education course" theme education practice, Beijing municipal party committee adhere to the first standard to consolidate improve "three force once two security" work pattern, focusing on the city's overall situation and central task, mobilize the capital youth members and cadres actively in the epidemic prevention and control war, let the teenagers in epidemic prevention and control "education" practice the capital youth "please rest assured, power have me" youth oath.―――――――――― continues to improve the leading force, always condensed the youth under the banner of the Party to seriously carry out organized learning, strengthen the theoretical arm of young people. Organized learning and education guidelines for grass-roots youth league organizations, further promoted the "youth learning", and produced and broadcast 17 "Red Scarf Cloud Team classes", with related courses covering nearly 9 million young people. We will continue to study and implement the spirit of General Secretary Xi Jinping's important speeches among youth league organizations, Young Pioneers, Young Pioneers, Students' Federation and emerging young groups, and educate and guide young people to strengthen their confidence and faith in listening to the Party and follow the Party. Give full play to the advantages of new media matrix, do a good job in the party's innovative theory of youth interpretation. In the "Youth Centennial Heart to the Party" column was set up on the all-media platform of "Youth Beijing", releasing important activities and speeches, important theoretical articles, learning trends and graphic information, and setting up topics such as "Beijing Color Youth" and "This Moment of Youth" every day, attracting more than 120 million people online. Jointly produced and promoted more than 30 new media products such as "Born to the Light" and "A Strong Country Has I," together with the youth League district committees and the Youth League committees of various industries. Create characteristic cultural products, and strengthen the positive guidance to young people. Strive to build high quality youth thought guide products, elaborate "learning xi general secretary in celebrating the 100th anniversary of the important speech spirit" theme youth songs, make "Beijing small group take you through one hundred" video, launched "youth" theme city roaming activities and online H5, related cultural products played more than 1.3 million times. Focusing on the epidemic situation, we have set up topics such as "Youth Beijing" and "My Youth Action" on other new media platforms, with a total reading volume of more than 73 million people, thus providing a vivid "great ideological and political lesson" for young people in the hot battle against the epidemic. We will build a strong organizational force and lead young people to shoulder a strong mission in building a strong organizational system in the fight against the epidemic. Implement the secretary of the central committee about "when the party and the people need charge in front, the epidemic prevention and control front bear as" instructions, quickly set up Beijing municipal party committee to participate in scale COVID-19 outbreak emergency work leading group, formulate the capital communist youth league in scale will be coronavirus outbreak emergency work plan "and" about mobilizing volunteers in the city to participate in response will be coronavirus epidemic prevention and control work plan, establish and perfect the city, district two-level league organization linkage mechanism. Leading young people in the fight against the epidemic. Beijing after the outbreak, pku organized more than 50000 league youth and cadres, 25000 volunteers, 25000 college students, to participate in community big data sent verification, nucleic acid detection, material distribution, provide tutoring, language translation, volunteer service for more than 203000 hours, relying on the "volunteer Beijing" platform, set up epidemic prevention and control volunteer service 1845, establish enterprise youth commandos more than 2500, reserve volunteer service force nearly 300000 people. In addition, the Communist Youth League Municipal Committee also widely launched the network big V, the Youth Federation members to record videos around the material supply, policy publicity, sympathy and support and other content, the whole network broadcast more than 212 million times. Provide strong support and guarantee. The information system of "league members returning to the community for registration" was improved, which effectively promoted the 100% real-name registered volunteers of the 4,407 newly developed league members in the city. We will strengthen the support of the "Volunteer Beijing" information platform, accurately sort out the situation of volunteers, accurately grasp the volunteer forces that can be mobilized, support the community to release special epidemic prevention and control volunteer projects, and do a good job in recording the duration of volunteer service, insurance guarantee, issuing certificates and other incentive work. Effectively enhance the service force, always become the party and the youth the strongest bridge innovative working methods, to provide accurate services for young people. The Opinions of the Opinions of the Capital Communist Youth League on Carrying out the Work of "Handling Before Litigation", made full use of the analysis mechanism of 12345 citizen hotline and the three-level linkage mechanism of city, district, street and township, and with the help of big data analysis of online platforms, accurately grasp the demands of teenagers, and strive to improve the effectiveness of service. We continued to improve the precise assistance database, covering the situation of 9,850 teenagers in distress in the city, and sent epidemic prevention materials and condolence money to families in need. In view of the practical impact of the epidemic prevention and control situation on college students in the capital, the university youth league organizations were organized to establish a mechanism of "handling complaints immediately immediately" to address students' demands within 24 hours, 48 hours and 72 hours. We will focus on key areas and actively safeguard the development rights and interests of young people. We will thoroughly implement the Beijing Youth Development Plan for the 14th Five-Year Plan Period, and implement practical projects to serve young people. Relying on the mechanism of the municipal joint conference on youth Work, we will implement the working system of proposals and suggestions of the Youth Federation, actively promote the introduction of policies to serve the development of teenagers, and strive to enhance their sense of gain and happiness. According to the Opinions on the Pilot Construction of Youth Development City, the pilot work of youth development city will be carried out in municipal districts, and to explore and create practical results that can be replicated and promoted to serve the priority development of youth. Strengthen organizational construction, and constantly enhance the sense of belonging of teenagers. To implement the work responsibilities of the whole league, study in advance to strengthen the working mechanism of promoting the Communist Youth League and the Communist Youth League, take the initiative to connect with the Organization Department of the Municipal Party Committee, promote the implementation of the establishment of the league building system and mechanism, and constantly build the chain of education of the Party, the league and the team firmly. We will deepen the grass-roots mechanism, formulate the development plan for the youth social organizations in Beijing, constantly enhance the influence of the community youth gathering, vigorously enhance their sense of gain and honor, and continuously enhance the sense of belonging of the youth to the league. Source: China Youth Daily, June 13,2022, edition of page 07

2022-06-13 Henan college students "report to the grassroots" activities in 2022 the provincial college students "report to the grassroots" activities Jianwei correspondent guo-chao li) to guide and help young college students combined with practice of "education", in the social classroom education, long ability, contribution, the grassroots service, service the masses as advanced important practice carrier, give full play to the communist youth league force and commandos, recently, the Henan provincial party committee issued notice, Henan communist youth league college students "report to the grassroots" in 2022 activities. Notice stressed that to highlight the theme, focus on publicity and implement xi new era the ideas of socialism with Chinese characteristics, let return home college students by reporting to the grassroots, from the epidemic prevention and control, rural revitalization, grassroots governance and other vivid practice understand the scientific connotation of new ideas and practice, to more consciously armed with new ideas mind, to guide practice. To ensure safety, all city and county youth League committees should strictly abide by the local epidemic prevention and control requirements, pay attention to the strict management and strengthening training of the safety protection of returning college students, and strictly implement the protective measures for students on duty to ensure the health and safety of returning college students. We should adhere to the principles of localization, community and organization, take the township and street youth league organizations as the organization carrier, make full use of the system platform to carry out the mobilization, recruitment, training and management of the returning college students, organize the returning college students to participate in the community and rural grid management nearby, and cultivate and highlight the advanced nature of the league members in practice. To stimulate vitality, youth league organizations at all levels to grasp the grassroots construction and improve organizational combat effectiveness highly unified, pay attention to work achievements, explore returning college students league, volunteer service "TuanBan youth club", merit absorbing returning college students for grass-time cadres, promote the work of grassroots youth league organization construction, stimulate the grassroots youth league organizations. The notice of the notice that the work responsibility should be compacted, and the county-level youth League committee should assume the main responsibility of the activity, responsible for formulating the regional activity implementation plan, optimizing the allocation of various resources, and do a good job in the mobilization and recruitment of returning college students through the system platform. All municipal youth League committees should strengthen work guidance, coordinate the sinking of all kinds of resources to the grass-roots youth league organizations, and ensure the orderly development of service and guarantee activities. The youth League committees of all colleges and universities should do a good job in organizing and launching, and mobilize the college students in all counties (cities, districts) in the province to actively register through the platform and report to the grass-roots youth league organizations. To strengthen the incentive guarantee, the county-level youth league committee should be in various ways to participate in the activities of returning college students to give volunteer time identification, issued commemorative certificates and other spiritual incentives. The league organizations of colleges and universities should take the registration of returning college students to the grass-roots level as an important reference for the student league members to participate in the league honor, promote the excellent party membership and the backbone selection and breeding, and incorporate it into the "second class report card" and the students' comprehensive quality evaluation system, so as to create a strong atmosphere for making progress. It is necessary to timely summarize and sort out. Youth league organizations at all levels should test the establishment and improvement of the grass-roots organization system and organizational mobilization mechanism in their activities, find out the weak links in the construction of grass-roots youth league organizations, discover and train the backbone of the work, innovate the organizational form, and timely transform the work achievements into organizational and political achievements. It is reported that in recent years, to play to the practice of education group advantage, the Henan provincial party committee organization launched the "green" heart cohesion grassroots I-returning college students to the community (village) registration activities, more than 158000 returning college students, to participate in the epidemic prevention and control, flood control and disaster relief, rural revitalization of the work, became the grassroots youth league organization an important new force. In order to further break the school to the communist youth league work information barriers, play to the advantage of the county "students", let the summer home college students can more convenient to participate in the county league social practice activities, establish outside college students and hometown contact institutionalized channels, the Henan communist youth league college students "report to the grassroots" system platform.

2022-06-13 Lin Jie, reporter of China Youth Network) Recently, the 12th Congress of Guangdong Students' Federation concluded. On the same day, the "Together to the Future" youth sharing meeting was held. At the scene, Chinese professional basketball player Hu Mingxuan, Olympic champion Chen Aisen, Luo Wei, head of fish and vegetable symbiosis project in Jiangmen National Agricultural Science and Technology Park, scientific research star Yao Anzi, Zhu Anliang, rural revitalization volunteer and other young role models had face to face communication with students face to face. Hu Mingxuan tells the story of chasing his chasing basketball dream since childhood.'Work hard is a quality that everyone must have,' he said. From the beginning of 2011, when he was at a loss about basketball training in the Guangdong Junior team, to winning the championship for three consecutive seasons after 2021, he felt the power to strive for his dream. He was named the Most Valuable Player in the 2020-2021 China Men's Professional Basketball League Finals. Chen shared the importance of mentality. Although he is the Olympic champion in diving, he has also faced being returned to the provincial team, declining in form and dying on the eve of the Olympics. Faced with these difficult moments, he said his biggest insight was to be firm and down-to-earth. At the same time, adjust your mentality, to maintain the momentum of the goal to make progress. Luo Wei Te, a young man from Hong Kong, is the epitome of thousands of young entrepreneurs from Hong Kong and Macao. With his original intention of generating income for farmers, he devoted himself to the application of fish and vegetable symbiosis technology after graduating from university. Once, he suffered from the failure to grow vegetables and the results destroyed by the typhoon. In the end, the team found that the root cause of the problem was the rift between foreign technology and China's climate. When the planting techniques suitable for China were successful and high-quality vegetables grew in the vegetable sheds, he said, " What connects ideal and reality is struggle and action."Yao Anzi, a student from South China University of Technology, holds 11 SCI papers. But she admitted that her scientific research path is not plain sailing- -after more than 100 polysaccharide degradation experiments, more than 500 extraction experiments, to spend five hours a day to shave the experimental mice... in the face of many difficulties, Yao did not give up, with the support of the mentor, the school and provincial scientific research funding, finally achieved results."When I can overcome many difficulties and make one small breakthrough after another, it is not just about my own personal efforts. In addition to its own efforts, it also benefited from the school's platform, the state and Guangdong province, and more from this era."Zhu Anliang is a volunteer of the Guangdong Province University Volunteer Rural Revitalization Initiative. Graduated from Sun Yat-sen University, he resolutely decided to serve in the countryside after graduation. He introduced a little girl he had visited from a poor family, and the conversation with her immediately touched him. At this point, he is more determined to join the ranks of serving the countryside. It is reported that the 12th Congress of Guangdong Students 'Federation deliberated and passed the resolution (draft) on the Work Report of the 11th Committee of Guangdong Students' Federation and the resolution (draft) of the Constitution of Guangdong Students 'Association (Amendment), and elected the 12th Committee of Guangdong Students' Federation. From March to May this year, Guangdong Students' Federation organized the application activity of "I do practical things for the Students", which won the graduate union response of the province. 943 application projects and 30 projects won the quality service project approval award. On-site, excellent proposals and quality service projects were awarded. Photography: Luo Jinzu

2022-06-13 Hainan green enterprise association sharing will help young entrepreneurs rescue Hainan youth enterprise association sharing will help young entrepreneurs rescue Ren Mingchao) on June 10 afternoon, Hainan tax system and Hainan youth entrepreneurs association in Haikou hosted a tax policy knowledge sharing, help enterprises fully use Hainan free trade port tax policy dividend and help enterprise rescue policy measures, ease the pressure of enterprise capital, solve the specific difficulties of enterprise tax. About 50 members and business representatives listened to the sharing. Hainan Youth Enterprise Association is the link and bridge between the Hainan Provincial Party Committee of the Communist Youth League and young entrepreneurs. The relevant responsible person in charge said that this year is a crucial year for the construction of Hainan Free Trade Port, and also a key year for the operation of customs closure. In the critical period of both opportunities and challenges, the young entrepreneurs of Hainan Province, under the guidance of the Provincial Communist Youth League Committee, will give full play to the leading role of the young pioneers, and strive to guide their members to participate in the construction of the free trade port, show their strengths, contribute their youth strength and achieve their life value.

2022-06-11 Chongqing held the second assistive volunteer service project competition final Chongqing held the second assistive volunteer service project competition finalists Wang Xinxin) on June 9 in the afternoon, by the Chongqing municipal party committee, Chongqing disabled persons' federation jointly hosted "welcome 20, always follow the party, forge ahead new journey" the second Chongqing assistive volunteer service project competition final held in Yubei District. Since its launch in April, the competition has received a total of 58 projects from government organs, enterprises, public institutions, universities, and social organizations, covering 8 volunteer services, including nursing care, rehabilitation training, entrepreneurship and employment, culture and sports, education, rights and interests protection, and rural revitalization. The final drawing supply of Chongqing Municipal Youth League was conducted in the form of road show. The jury comprehensively evaluated and scored the entries from the dimensions of service objectives, service content, implementation methods and service effects. The final finally selected 2 gold awards, 3 silver awards and 5 bronze awards. It is reported that the Chongqing municipal party committee, Chongqing disabled persons' federation for disabled action "sunshine", has formed two "xin Xiaoqing" assistive youth volunteer service municipal team and 20 counties, university teams, around the disabled breathing care, universal sign language popularization, disabled family children academic counseling, intellectual and mental people community integration, rehabilitation of disabled children to carry out assistive volunteer service.

2022-06-11 Jianwei) Recently, the 6th China Youth Volunteer Service Project Competition Henan Division Competition and 2022 Henan Youth Volunteer Service Project Competition was officially launched. The competition is organized by the Henan Provincial Party Committee of the Communist Youth League, Henan Provincial Civilization Office, Henan Provincial Civil Affairs Department, Henan Provincial Department of Ecology and Environment, Henan Provincial Department of Water Resources, Henan Provincial Health Commission, Henan Provincial Disabled Persons' Federation, With the theme of "Welcome the 20th Plan, Volunteer for the New Era", Set up the project declaration, evaluation, promotion and communication, tracking and cultivation and other links, The competition covers 13 categories of rural revitalization, environmental protection, civilized practice, care for children, service for the elderly, sunshine helping the disabled, health, emergency rescue and epidemic prevention and control, community governance and neighborhood watch, water saving and water protection, cultural communication and tourism services, legal services and drug control education, and other fields; A total of 39 gold MEDALS, 50 silver awards and 60 bronze awards. It is understood that since 2014, Henan province has successfully held five provincial youth volunteer service project competition, attracted the province all kinds of youth volunteer service team, youth social organizations and social welfare forces, foster project 412, won the China youth volunteer service project competition gold medal 17, silver 66,72, has become a set of project display, communication, cultural leading, resources docking youth volunteer service integrated platform, in promoting volunteer service project, institutionalization, normalized development played an important role. In the next step, all kinds of youth volunteer service teams at all levels in Henan Province will pass the preliminary competition, provincial preliminary evaluation and provincial final evaluation, play a fierce competition, show their youth style, and welcome the 20th victory of the Party with outstanding results.

2022-06-10 Gansu "youth heart to the party to forge ahead new era" art performance held in Gansu "youth heart to the party to forge ahead new era" art performance held in Lanzhou on 10 (reporter Zhang Jianwei, correspondent Li Jing) late June 8, Gansu "youth heart to the party to forge ahead the new era" art performance held in the west of the chengguan campus in Lanzhou university gymnasium. More than 600 members of the Gansu Youth Joint Conference, some universities, enterprises and institutions, central units in Gansu, Gansu Youth Federation, and young students in Lanzhou universities watched the performance. Live pictures. The whole performance is divided into "dawn", "inheritance", "development", "attention", "set sail" five chapters, "by" dawn "," pay fire "," unforgettable 8.26 "," sing "," youth prose poetry "," touch the motherland "," always follow the party "," "" keep the heart in the place "," you and people heart to heart "," youth is sail "," classmates young "" heart " fourteen programs, 755 young students to participate in the performance. Live pictures. Through situational performance, dance, song, recitation and other artistic forms, the historic achievements of the cause of the Party and the country since the 18th National Congress of the CPC, Comprehensively review the historical context of Gansu youth movement, Fully reflect the political nature, advanced nature, mass nature, artistic quality, Reflecreflect the mission of the young generation to always follow the Party and contribute to the new era, Reviewing with the young friends on the scene, the Chinese Communist Youth League has always been in the same root with the Party, geng toward the Party, unswervingly follow the party's struggle, Under the leadership of the Chinese nation, the CPC has made a historic leap from standing up, becoming rich and becoming strong, Under the leadership of the Party, Longyuan land for a hundred years. Live pictures. Correspondent for figure in the end of the day, the audience stand up, together sing no communist party, no new China, the art performance atmosphere to a climax, fully expressed the new era of longyuan youth always listen to the party, feeling DangEn, follow the party, li strenuous, forge ahead, hard work, yong yi, to my sense of urgency, to give me whose sense of mission, struggle with the people, and the motherland forward confidence and determination.

2022-06-10 The first Sichuan university volunteer service project competition award ceremony held the first Sichuan university volunteer service project competition award ceremony held in Chengdu youth network Beijing on June 10 (reporter zhang, reporter qing-hua zhao) on June 9, group, Sichuan province and Sichuan provincial party committee education working committee jointly hosted the first Sichuan province university volunteer service project competition (hereinafter referred to as the "contest") award ceremony held in Chengdu university in the form of online combination. Mainly in charge of Sichuan Provincial Youth League Committee, comrades in charge of Education Working Committee of Sichuan Provincial Party Committee, and relevant principals of Sichuan Provincial Civilization Office and Civil Affairs Department attended the event. Representatives of some award-winning projects (works), representatives of excellent instructors, representatives of excellent organization award, and representatives of Sichuan Youth Volunteer service expert talent pool attended the activity. According to the relevant person in charge of the Sichuan Provincial Committee of the Communist Youth League, since the launch in December 2021, the competition has received a total of 836 participating projects (works) recommended by 98 universities, including 505 practical projects, 167 venture capital projects and 164 cultural product design works. Through publicity and mobilization, project submission, expert preliminary evaluation, pre-competition training, expert final evaluation and other links, a total of 54 gold award projects (works), 95 silver award projects (works), 95 bronze award projects, 96 excellent instructors, 29 excellent organizations and 1 special contribution award were selected. The competition aims to further stimulate the innovation driving force of volunteer service, use competition instead of training and competition for promotion, and promote the normal project and professional development of volunteer service in colleges and universities. Competition set practice project, venture capital projects and cultural products design three track, practice projects and venture capital project track in addition to set up "community governance", "ecological environmental protection" and other traditional volunteer service category, add "party history learning education and preach" class, cultural product design track around plane, video, animation, interactive categories, vigorously promote the "panda man" culture IP. At the same time, the university volunteer service cloud pavilion was officially unveiled at the award ceremony. Cloud pavilion set up "Sichuan youth volunteer service development process", "panda under the culture IP exhibition", "college volunteer service", "college volunteer service gold medal project" exhibition "project to build cloud docking" five plates, aims to make full use of the Internet for online volunteers to provide a permanently open to social communication display platform. In the next step, the Sichuan Provincial Committee of the Communist Youth League will continue to build a good interactive platform for volunteer service exchange and display, transform the achievements of the award-winning projects in combination with the Sichuan University volunteer service promotion plan, carry out project publicity, project promotion, project co-construction and other related work, and invite experts to follow up and cultivate.

2022-06-09 In 2022 "Hainan youth branch" training in Haikou in 2022, "Hainan youth branch" and authority about backbone ability promotion training in Haikou Beijing on June 9 (reporter Zhang Jianwei, correspondent Xu Richard) on June 7,2022, "Hainan youth branch" and authority about backbone ability promotion training in Haikou, pku secretary Chen Yu to attend the opening ceremony and speech, deputy secretary of Chen presided over the opening ceremony. It is understood that in May this year, the Hainan Provincial Party Committee of the Communist Youth League established the 2022 "Hainan Youth Lecture Group". The training course, aims to promote youth branch members and provincial party committee authority to preach backbone preaching level and ability, in order to further develop good grassroots preaching activities, speak the party's ideological strategy, party history and positive youth story lay a solid foundation, lead the broad youth active in Hainan free trade port construction, to realize the great rejuvenation of the Chinese nation the Chinese dream of unremitting struggle. Opening ceremony, group of Hainan provincial party committee secretary Chen Yu in communication with students, points out that the youth preaching work is of great significance, to further enhance the level and ability to preach, do where the youth, branch will go, to their own thought and behavior model for the youth tree benchmarking, for example, to ensure that propaganda work achieve actual effect. In 2022, more than 80 people, including members of the "Hainan Youth Lecture Group", leaders of all departments and departments of the Provincial Communist Youth League Party Committee and business backbone, attended the training. In the next step, the provincial Communist Youth League Committee will organize the members of the youth teaching group and the government youth league cadres to go deep into the majority of young people to carry out a series of theme publicity activities.

2022-06-09 Group Yunnan provincial party committee to carry out the "happy to be team" theme team day group Yunnan provincial party committee to carry out the "happy to be team" theme team day activity wei) during children, a group of children gathered in Yunnan province star torch flag, wearing bright red scarf, become a glorious young pioneers, played the pursuit of political progress "song". The picture shows the scene of the theme of "Welcome 20 to be a good member". The counselors and workers of the Young Pioneers in Yunnan Province insist on arming their minds with the spirit of General Secretary Xi Jinping's important speech and promote their work. Around the spirit of the general secretary's important speech, the young pioneers at all levels in the province rose the upsurge of learning, publicity and implementation. On May 20th, The Provincial Communist Youth League Committee held a symposium on studying and implementing the spirit of the General Secretary's important speech in Kunming University of Science and Technology, The general counselor of Dali Prefecture, on behalf of the young Pioneers counselors of the province, shared their experience in learning the spirit of the general Secretary's important speech and expressed that they would follow this as one, To ite and lead the young pioneers to listen to the Party and follow the Party; Wang Ning, Secretary of the CPC Yunnan Provincial Committee, attended the symposium and interacted with you, point out, Youth league organizations at all levels and the majority of youth league cadres, youth league members should deeply study and understand the profound connotation of General Secretary Xi Jinping's important speech, To deeply grasp the strategic position of youth work, the original mission of the Communist Youth League, and the valuable experience of the Communist Youth League's century-old journey, And the major issues of the Chinese youth movement and youth work in the new era, Draw on your wisdom and strength. On May 23, the provincial party committee, provincial less committee held in Kunming, Yunnan young pioneers study and implement xi general secretary in celebrating the 100th anniversary of the founding of the communist youth league of China's important speech spirit symposium, province, city, county and school levels, director of the young pioneers counselor and young pioneers from different angles, combining their own reality, closely around xi general secretary of the speech spirit and how to implement speech spirit of communication and speech. The picture shows in Sima Wa Village, Qingshui Township, Tengchong, Baoshan City, the counselors and teachers lead the young pioneers into the rural rich path, visit the folk exhibition hall and experience the national culture. The Yunnan Provincial Party Committee of the Communist Youth League guided the work direction for the province to carry out the extensive activities of the theme. The Provincial Communist Youth League Committee and the Provincial Youth League Working Committee organized the provincial-level centralized demonstration activity of "Welcome the 20th Plan and Strive to be a good Team Member" in Changchun Primary School, Kunming Wuhua District, Nie 'er's Alma mater. Event, red scarf narrator led party members, members, young pioneers representatives review Nieer under the influence of the May 4th movement, the marxist philosopher-siqi, the first secretary of Yunnan provincial party committee-Li Guozhu advanced figures led under the league, the story of the party, called on the children remember xi grandpa entrust, aspire to, have a dream, go good pursuit of political "life trilogy" of progress. At the joining ceremony, more than 60 children joined the Young Pioneers and became glorious young pioneers. Tang Yuan, secretary of the Provincial Communist Youth League Committee, told the young pioneers after the joining ceremony, remembering grandpa Xi Jinping's instructions, walk well in life and button the first button of life. Point out that to firm ideal faith, have an ideal life to sail; to be determined to study hard, learn scientific and cultural knowledge, temper their skills; to cultivate noble moral character, do a healthy and noble person, do a person of home and country; to exercise a strong body, strong body is the basis to contribute to the country and the people. At the same time, the provincial authority 12 department (room) and 12 schools in Kunming pair, walked into the campus, into the squadron, perform the duties of "ensemble", truly cadres down, platform, with the young pioneers "June 1" children's day at the same time closely around the general secretary in celebrating the 100th anniversary of the important speech spirit for the young pioneers and young pioneers counselors to preach. League provincial party committee, provincial committee also joint provincial women's federation, provincial education department in Kunming museum theme activities, under the guidance of the red scarf small commentator to visit the city museum "welcome 20, always follow the party, new journey" under the leadership of the yunling youth struggle in one hundred, listen to the red story, feel a smell, Li Dazhao, the communist movement pioneers, feel the communist party of China, the Chinese communist youth league history, firmly consciously inheriting red genes, be a new era of good players. The picture shows the scene of the theme of "Welcome 20 Striving to be a good Member" in Nicxiang Primary School in Shangri-La City, Diqing Prefecture. The Communist Youth League Yunnan Provincial Party Committee for various forms of theme team day activities are widely carried out. In Lijiang Huaping Girls' High School, Ms.Zhang Guimei, the model of The Times and the school counselor of the Young Pioneers of Yunnan Province, shared with the young pioneers the spirit of the important speech of the general Secretary, leading the young pioneers to enhance their sense of honor. Zhu Youyong, an academician of the Chinese Academy of Engineering, a model of The Times and a counselor of the Young Pioneers in Yunnan Province, brought a vivid and scientifically interesting activity class to the young Pioneers to love science and learn science since childhood, and serve the motherland and serve the people with what they have learned they growing up. Guo Weiyang, the Olympic champion and the school counselor of the Young Pioneers of Yunnan Province, brought a special "gift" to the children- - "Trump substitute". By telling the growth path of the champion, the young pioneers were encouraged to be not afraid of difficulties, hone their will and grow into towering trees in the future. In Simola Wa Village, Qingshui Township, Tengchong City, the Young Pioneers followed General Secretary Xi Jinping's visit to Smola Wa Village, Qingshui Township. Through the search activities, they personally felt the earth-shaking changes in the rural areas. In Nujiang longjiang township, the young pioneers counselors led the young pioneers to review xi general secretary to longjiang township people reply spirit, feeling xi general secretary of the deep care and entrust talk, the young pioneers expressed themselves will study hard, day day up, never live up to the expectations and entrust of the general secretary, for the Nujiang development construction of a new chapter, contribute to contribute in the process of a better tomorrow. Chuxiong Prefecture was held during the "61" period to welcome the party's 20th Chinese nation as one family together to build the Chinese dream " theme painting, calligraphy, photography competition. Young Pioneers at all levels in Diqing Prefecture organized to carry out a variety of cultural performances in combination with characteristic ethnic culture, and visited children in need. Dali Prefecture held a centralized demonstration activity in Yunhe No.2 Primary School of Heqing County, held a standardized entry ceremony, a large recess report performance and a demonstration theme squadron meeting of "Red scarf Listening to the Five Old Stories", and the "Hope Project 1 + 1- -Fantasy Student Aid Plan", benefiting 1,975 first-grade girls. Dehong Prefecture organized the young pioneers to seek great achievements, listen to stories of struggle, and actively carry out "red scarf narrator" activities in patriotism education bases, revolutionary memorial venues, martyrs memorial facilities, museums and other places. On May 26,27, Tang Yuan, secretary of the Communist Youth League, visited the theme of "Welcome 20", and told the majority of children; Wenshan Prefecture also carried out many young pioneers with ethnic characteristics, Zhuang, Miao, Yi, Yao and other ethnic young pioneers expressed their love for the motherland, expressed the unity and friendship among the ethnic groups. Lincang city launched the "eight one" series of activities, to organize the important speech delivered by General Secretary Xi Jinping in the 100th anniversary of the Communist Youth League of China; hold a young pioneers enrollment ceremony; find a group of examples around; collect a heartfelt message to the Party; establish a group of red scarf explanation station, conduct a caring activity; publish a letter of sympathy, and conduct a literary works competition. The picture shows the young Pioneers of Yunhe Second Primary School in Heqing County, Dali Prefecture taking the oath under the leadership of the team leader. During this year's "June 1" period, the Young Pioneers of Yunnan Province closely followed the theme of "Welcome the 20th Top Ten and Strive to be a good team member", and carried out a series of young Pioneers activities in combination with the local reality. The activity covered 13,000 young Pioneers brigades, 101,500 young Pioneers squadrons, and nearly 4 million young pioneers in the province.

2022-06-08 Shaanxi: more than 900 hope project power ankang rural revitalization of shaanxi hope project rural education funding promotion action donated more than 900 ten thousand yuan power ankang rural revitalization wei correspondent Zhang Wenhao) on June 6, guided by the shaanxi provincial party committee, shaanxi youth development foundation, group ankang municipal party committee to undertake in 2022 shaanxi hope project rural education funding promotion action donation and ankang youth power rural revitalization demonstration launch ceremony in ankang municipality hengkou demonstration area Chen jiaying primary school. Leaders of Shaanxi Provincial Communist Youth League, Shaanxi Provincial Youth Development Foundation, Ankang Municipal Party Committee, Ankang Municipal Communist Youth League, The Party Working Committee of Hengkou Demonstration Zone, and representatives of the project implementation district (county) Youth League Committee and youth social organizations jointly witnessed the launch activities. In 2022, Shaanxi Hope Project, together with a number of caring units, donated more than 9 million yuan of the project to the first batch of rural revitalization key assistance districts (counties) of Ankang City, Among them, the "Hope Project- -a classroom good Love" music classroom project donated 3 million yuan, To build 20 hope primary schools and rural primary school music classrooms in Hanbin District, Langao County, Hanyin County and Ziyang County, And funded art education, teacher training, art troupe performances; "Hope Project 1 + 1- -Fantasy Education Program" project donated 1.75 million yuan, To subsidize 1,750 originally registered family students in Ankang City, Consolidating the achievements made in poverty alleviation; Donated 1 million yuan for the smart campus project of Hedong Nine-year School in Ankang Hengkou Demonstration Zone; "Hope Project New Financial Action- -Smart Village" smart platform project donated 1 million yuan, Funding 500,000 yuan in Hanyin County and Langao County; "Project New Financial Action- -Rural Toilet Revolution" project donated 1 million yuan, Donof Hanbin District, Ziyang County toilet Revolution project 2, Each unit is $500,000; The "health protection public welfare project of" Health Plan "and health protection project, Haier Hope Primary School construction assistance project," Guanghua Love Library "donation project," Project Hope Teacher Candlelight Award " project, and Project Hope Love gift package project for left-behind children donated 1.25 million yuan. As of early June, Shaanxi Province has raised more than 20 million yuan, funded 11,320 students from originally registered families, and assisted in more than 40 Hope Primary School upgrading projects.

2022-06-08 Jiangxi Provincial Party Committee of the Youth League held the "Youth Learning" publicity and exchange activity on campus (a special event for Jiangxi Normal University). Correspondent for figure China youth network Beijing on June 8 (reporter Zhang Jianwei, correspondent Huang Guipeng huang), Jiangxi province first "youth learning" about communication into campus theme activities held in Jiangxi normal university, Jiangxi province youth branch members, Jiangxi normal university, student representatives of a total of more than 70 people attended the activity in the scene, the province youth synchronization in Jiangxi communist youth league online platform to watch, a total of 1.2 million people. The campus lecture opened the prelude to the "youth learning" lecture and exchange theme activities. In the theme presentation session, Member of Jiangxi Provincial Youth Lecture Group, Wang Yuxin, a professor at the School of Marxism of Jiangxi Normal University, titled, Sowing Youth on the Journey of National rejuvenation, It tells the magnificent course of the Chinese youth movement over the past 100 years, Leading the youth league members to firmly listen to the Party and follow the Party's ideals and beliefs; Member of Jiangxi Provincial Youth Lecture Group, Zhang Fucheng, member of the Party Committee and minister of the Publicity Department of the School of Modern Economics and Management of Jiangxi University of Finance and Economics, with the title of "The Power of Jiangxi Province in the Hundred Years of Youth League History", It tells about the spirit and value pursuit of the youth of Jiangxi Province, Guide the youth league members to actively participate in the hot practice of "six Jiangxi" construction; Collective representative of the Chinese Youth May 4th Medal, Aviation industry Hongdu Company, a young front-line worker Tang Jie to share the growth process, Encourage the youth league members to integrate the ego into the ego, Interpret the responsibility and responsibility of contemporary youth with practical actions; Member of Jiangxi Provincial Youth Lecture Group, Guan Wen, host of Jiangxi Radio and Television Station, titled "Youth, Light and the Lonely brave", It tells the ordinary story of his efforts to make life shine, Encourage the youth league members to live up to their youth, Write a hymn to youth with hard struggle. In the exchange and interaction session, the representatives of the league members put forward, " What is the best attitude for contemporary youth to contribute to the country?"" How to revitalize the old revolutionary base areas in the red land, inherit the red gene " and other questions, several young lecturers combined with their own experience and perception on the scene to answer, triggered a warm response."When we are born in the era of a strong country, we should consciously shoulder the historical mission entrusted to us by The Times, take the struggle as the most beautiful background color of our youth, and strive to run this stick that belongs to us."Said Leng Xuan, a 2020 student majoring in geography science from the School of Geography of Jiangxi Normal University, after the exchange activity. One of the characteristics of this theme lecture is to combine the history of youth sports with the vivid practice of contemporary youth, and to explain the internal relationship between the Chinese dream and the youth dream from the macro, medium and micro perspectives, so that the content of the lecture is more visible and sensible. The activity focuses on the use of interaction and communication, so that the publicity is both high and down to earth, both resonance and collision, which further triggers the profound thinking of the youth league members and ignites their enthusiasm for learning. In order to carry out in-depth publicity and exchange activities, The Jiangxi Provincial Party Committee of the Communist Youth League relies on the members of the provincial Youth Lecture group, To attract outstanding young models, previous Jiangxi Province "Micro group class" competition winners and other young representatives from all walks of life, Set up a 160-person provincial "youth learning" publicity group, Carry out small-scale and interactive publicity activities for young people in all fields, From May to December of this year, With the Youth League Jiangxi Provincial Committee of "Welcome the 20th, always follow the Party, forge ahead on a new journey", Extensive "youth learning" publicity and exchange theme activities, Promote theory preaching into communities, enterprises, schools, and rural areas, Constantly innovative forms of activities, Enrich the activity content, The publicity activities will cover every county (city, district) in the province, Let the lecture into the youth, attract the youth, lead the youth.

2022-06-07 20000 youth will be free in shaanxi 20000 youth will be free in shaanxi new vocational training wei correspondent Zhang Wenhao) recently, shaanxi party committee, shaanxi people club hall jointly issued by the about 2022 youth new vocational training work notice, clear before the end of 2022, will rely on social training institutions for free for 20000 youth new vocational training. The new vocational training focuses on all kinds of young urban and rural migrant workers aged 16 to 35 who are willing to find jobs and start businesses and need training (except those who are receiving national compulsory education). The number of training types has been increased from 15 to 25, covering Internet marketing engineers, artificial intelligence trainers, and all-media operators, etc. The course content is mainly practical courses, with class hours arranged between 48 and 72 class hours. After the training, the employment rate and training effect of the participants will be assessed, and subsidies will be given according to the training employment rate according to relevant regulations. Youth new vocational training is the implementation of the shaanxi provincial party committee, shaanxi provincial government "six stability" "six protect" task and the communist youth league central to promote college students 'employment action, is the communist youth league of shaanxi provincial party committee, shaanxi people club hall, shaanxi province federation of industry and commerce jointly issued by the jointly promote college students' employment eight measures notice " one of the key service content. In the next step, the Shaanxi Provincial Party Committee of the Communist Youth League will lead and mobilize the young people to actively participate in the training of digital skills, promote the high-quality employment of the young people, and help the high-quality economic and social development of Shaanxi province.

2022-06-06 League of Sichuan province launched cadres ability style construction special action of Sichuan province launched cadres ability style construction "four than four grasp four promote" special action wei correspondent Zhao Qinghua) recently, the Sichuan provincial party committee issued the communist youth league of Sichuan provincial party committee about "four than four grasp four promote" special action to strengthen the provincial cadres ability style construction of the implementation plan, through a series of measures, to further strengthen the ability of cadres style construction. According to the relevant person in charge of the Provincial Communist Youth League Party Committee, The special action aims to implement the four clear requirements put forward by General Secretary Xi Jinping at the conference celebrating the 100th anniversary of the founding of the Communist Youth League of China, To implement the spirit of the 12th Party Congress of Sichuan Province, Focusing on the fundamental tasks, political responsibilities, and the main work lines, Promote the individual League cadres "comparison", the overall League organization "grasp", Significantly improve the political quality, work level, work style and team image of the youth league cadres at all levels in the province, Significantly improve the ability of youth league organizations to lead and gather youth, organize and mobilize youth, and contact and serve youth, Significantly improve the overall contribution to the work of the Communist Youth League, In order to promote the governance of Shu and revitalize Sichuan in the new era to a new level, Strive to write a new chapter of the comprehensive construction of socialist modernization in Sichuan to contribute to greater youth strength. It is reported that the special action will start from June throughout the whole year, divided into three stages: learning mobilization, overall promotion, summary and promotion.

2022-06-07 Hubei launched the "Hundred Years of pursuit" theme music group class provincial tour Lei Yu) received the spiritual baptism in the music edification, and pursued the original mission in the century-old history. Recently, the Hubei Provincial Committee of the Communist Youth League launched a "century-old years of pursuit" theme music group class in Huangshi city- -to study and implement the spirit of General Secretary Xi Jinping's important speech at the celebration of the 100th anniversary of the founding of the Communist Youth League of the Communist Party of China. Zhou Senfeng, secretary of Hubei Provincial Committee of the Communist Youth League, and Wu Zhiling, Deputy Secretary of Huangshi Municipal Committee and Mayor of Huangshi attended the relevant activities. On June 2nd, Hubei "Centennial Pursuit" theme music team class and music group class were held in Huangshi Shifu Road Primary School and Huangshi No.2 Middle School respectively. The keynote speaker was Chen Li, deputy secretary of the Hubei Provincial Party Committee of the Communist Youth League. In the infectious speech of the speaker, "dislocation of time and space" "half life snow" and other teenagers catchy contemporary songs and a hundred years of history agitation collision, yun Daiying and Huang Jiguang heroic stories moved everyone to tears; the Beidou spirit inspired the power of infinite struggle; interactive question "what impressed me most story" intense discussion. In particular, the two provincial youth typical on-site communication link, the students rushed to ask questions, once again pushed the atmosphere to a climax."The first time I heard such a form of group class, it was very vivid and attractive. I hope I can do this in the future!"Said Cheng Wei, a senior one student at Huangshi No.2 Middle School."No hero from the sky, only stand up to the mortal" "through the mountains and rivers, still need to travel"..., co., LTD., the central youth information group member Yan Shuai for the enterprise young workers held xi general secretary in the celebration of the important speech spirit of special theory, analysis, simple system combing speech spirit, witty, golden sentences frequently, the truth speak deep, thoroughly, live. Site enterprise workers have said that this flexible reasoning way is both ground and penetrating. On the evening of June 2nd, the Water Lotus Theater of Hubei Normal University was greeted with thunderous applause. The music group class kicked off in the micro group class of "The Torch of Youth" told by Yan Shuai. Youth representative led the audience to open the "journey", with "newborn" "follow", "challenge", "fight" "song" five chapters, through the form of "scene interpretation + art", system learning xi general secretary of the important speech spirit, review the Hubei communist youth league under the leadership of the party unity led the broad youth explorations mission, overcome the magnificent history of progress. It is understood that the provincial tour is the Hubei provincial party committee to carry out the "grassroots observe the people solution people sorrow warm people" practice of concrete measures, the good courses to the grassroots, to the youth, the party's care warmth to the youth, in the interactive preaching survey understand the youth's thoughts and expectations. Source: China Youth Daily, June 07,2022, edition 02

2022-06-07 Sichuan: "hope project 1 + 1-magic aid plan" first grants in Sichuan: "hope project 1 + 1-magic student plan" public welfare project first grants wei correspondent Zhao Qinghua) according to the Sichuan province youth development foundation (hereinafter referred to as the "provincial youth foundation"), as the "hope project 1 + 1-magic student plan" public welfare project first grants were allocated to local youth corps committee, students around the province during the "June" this year received a special holiday gift. The picture shows the staff of youth League committees at all levels visiting the students of the "Fantasy Education Program" project. According to the relevant person in charge of the Provincial Youth Development Foundation, The "Hope Project 1 + 1- -The Magic Side Student Aid Program" charity project is supported by the China Youth Development Foundation and the Ningbo Magic Side Investment Management Co., LTD., Aim to thoroughly implement the spirit of General Secretary Xi Jinping's important speech at the conference celebrating the 100th anniversary of the founding of the Communist Youth League of China and the important message to the 30th anniversary of the implementation of Project Hope, The project is aimed to 66 poor families in former state-level poverty counties, By giving 1,000 yuan each to their children in primary school, Send them care from the party and the government and care from all sectors of society, The project plans to fund 11,550 primary school students, A total of 11.55 million yuan will be granted in grants. The picture shows the staff of youth League committees at all levels visiting the students of the "Fantasy Education Program" project. In the next step, the Provincial Youth Foundation will continue to focus on the fundamental task of educating the Party and the Party for the country, and make solid progress in the receipt and confirmation of grants, sampling inspection, and investigation, to ensure that every grant can be distributed to the students on time and in need.

2022-06-06 Gansu province "welcome 20 always with the party forge ahead new journey" Chinese youth May 4th medal sharing meeting in Gansu province "welcome 20 always with the party forge new journey" Chinese youth May 4th medal sharing meeting held in Qingyang years net Beijing on June 6 (reporter Zhang Jianwei, correspondent qing-hua zhao) recently, sponsored by the central committee, the national youth league, Gansu province, Gansu province, communist youth league Qingyang municipal party committee, Qingyang youth May 4th medal sharing meeting held in Qingyang. More than 40 people from party and government organs at all levels in Qingyang city, enterprises and institutions, members of the youth Federation, and young representatives of emerging fields listened to the scene. At the same time, more than 100,000 youth members of the province watched online through the network live broadcast. Share the pictures of the live meeting. At the photo sharing meeting of the Communist Youth League Gansu Provincial Party Committee, the participants attended the young people watched the 26th Chinese Youth May 4th Medal propaganda video "One Hundred Years of Struggle Always Follow the Party". Wang Yali, winner of the 25th China Youth May 4th Medal and chief of Nanliang Revolutionary Memorial Hall exhibition section, with "the most beautiful struggle of youth", the first national rural revitalization youth pioneer winner, president of Huanxian College Students Sheep Industry Association Ji Yongfeng to " gather the strength of youth? Under the title of Entrepreneurship Achievement Dream ", I exchanged and learned the spirit of General Secretary Xi Jinping at the celebration celebrating the 100th anniversary of the founding of the Communist Youth League of China. Based on my own work and life, I shared my personal course of working with the Party, listening to the call of the Party and fighting with the Party. At the sharing meeting, some grassroots cadres and youth representatives interacted with Wang Yali and Ji Yongfeng. Share the pictures of the live meeting. At the scene of the photo sharing meeting of Gansu Provincial Committee of the Communist Youth League, the young people attending the meeting all said that the outstanding youth representatives fully felt their youth style with ideals, ability and responsibility. In the future work and study, will take the example of advanced youth typical, learning from advanced model, set up for the motherland for the people permanent struggle firm ideal, patriotism, temper character, innovation, hard work, in the rural revitalization of the innovation, the first line, grassroots service society, take practical actions to meet the party's 20th victory.

2022-06-06 The second batch of economical organs to create work in shaanxi province will hold the second batch of economical organs to create work will hold Beijing on June 6 (reporter zhang, correspondent zhang) recently, organized by the shaanxi province government affairs service center of shaanxi province the second batch of economical organs to create work will be held in the communist youth league of shaanxi provincial party committee. Huang Hua, deputy secretary of Shaanxi Provincial Party Committee, attended the meeting, and Ji Zhiqun, director of the Energy Conservation Department of Shaanxi Provincial Government Offices Service Center, presided over the meeting. Principals of energy conservation of 43 government organs and units in Shaanxi Province visited the Shaanxi Provincial Party Committee of the Communist Youth League to observe and study, carry out experience exchange, and do a good job in the establishment of energy-saving organs in Shaanxi Province. The relevant person in charge of energy saving authority service saving work and the next step work ideas and energy consumption data, saving authority to create work evaluation index of the detailed experience, to create work task to deploy and mobilize, further create specific requirements and scoring rules, and is the shaanxi provincial party committee to create work data preparation with the participating units to share communication and discussion. Observe and learn the pictures. Correspondent for figure then, the participants in shaanxi provincial party committee, the scene visited the youth stores youth leading function and promote green office energy conservation and emissions reduction related equipment and measures, and the use of new energy charging equipment, especially the hospital green plant micro sprinkler irrigation equipment using natural water pressure irrigation, both water energy saving, and simple safety "saving authority" to create the work site. The promotion meeting aims to learn from the advanced experience of creating "energy-saving organs" of the Communist Youth League Shaanxi Provincial Party Committee, give full play to the demonstration and leading role, and further promote the standardization, refinement and information management level of creating "energy-saving organs" of public institutions in Shaanxi Province. The meeting required all party and government organs to effectively improve the sense of responsibility and urgency of the creation of the work, to seriously study and master the evaluation standards, strengthen the implementation of responsibility, establish goal-oriented, implement the content of the creation, solid and orderly promotion of the creation of energy-saving organs, to complete the requirements and goals of "energy-saving organs".

2022-06-01 Lin Jie, reporter of China Youth Network) The 13th "Challenge Cup" Guangdong College Students Business Plan Competition concluded at Guangdong University of Foreign Studies. Guangdong University of Technology and other 10 colleges and universities won the "Winning Cup" of the undergraduate group, 10 colleges and universities including Dongguan Vocational and Technical College won the "Winning Cup" of the higher vocational college group of this competition, and South China University of Technology won the "Challenge Cup" trophy with the first place in the total score. A total of 516 works from 121 universities in Guangdong province were shortlisted for the final final. A total of 131 works from 57 universities won the gold medal. With the theme of "Challenging the New Era of Entrepreneurship to the Future", the competition focuses on the complementary channels of "entrepreneurship" and "employment". Since its launch in February, the competition has received 1,763 entries from 151 universities, with more than 100,000 participants. Photo provided by the Guangdong Provincial Committee of the Communist Youth League

2022-05-31 Gansu Province youth self-care education activity launch ceremony was held "youth self-protection has you have me" Gansu Province youth self-care education activity launch ceremony was held Jianwei correspondent Li Jing) Recently, "youth self-protection has you have me" Gansu Province youth self-care education activity launch ceremony was held in Jiuquan Vocational and Technical College. Launch ceremony, Gansu province NPC standing committee office of secondary inspector, on behalf of the personnel committee on behalf of the liaison director Xie Wentao announced the launch, Jiuquan municipal committee, propaganda minister war eagle delivered a warm speech, provincial secondary inspector Yang Xiaorong is the province teenagers since the nursing education work arrangement deployment, more than 500 universities anti-drug alliance on behalf of the collective oath. The anti-drug initiative was also issued at the scene, and letters of appointment were issued to the legal and psychological counseling experts of the 12355 youth service desk, awarding a flag to the anti-drug volunteer service team. At the same time, issued the "notice on the development of 2022" Easy test preparation 12355 Walk with you "secondary school and college entrance examination decompression and summer vacation during the organization and winter vacation education activities", the province's youth self-care education and a series of activities began. The activity requires that the Communist Youth League organizations at all levels should actively provide practical and effective help to teenagers, give full play to the socialized mobilization ability of the Communist Youth League organizations, and constantly expand the field of youth self-care education activities. Youth legal psychology volunteers at all levels should be people-oriented, service first, provide a good environment for teenagers to grow up, constantly increase the integration and allocation of resources, pay attention to students' mental health, pay attention to the psychological dynamics of students in entrance examination for college and entrance examination. The majority of young people should be confident and sunshine, set up lofty ideals and beliefs, study scientific and cultural knowledge hard, and strive to become what can bear the pillars of talent. After the launch ceremony, Professor Mo Xingbang, doctor of Psychology, senior engineer of the Chinese Academy of Sciences and president of the Gansu Society of Psychological Counselors, gave lectures on mental health knowledge to the students of Jiuquan Vocational and Technical College around the mental health education of teenagers. At the same time, experts and scholars from relevant provincial units went to schools, enterprises and communities in Jiuquan to give a series of knowledge lectures on stress reduction for secondary school or college entrance examination, self-protection education, drug control and law education. This activity is a practical measure taken by the Gansu Communist Youth League to implement the important speech of General Secretary Xi Jinping at the conference celebrating the 100th anniversary of the founding of the Communist Youth League of China. It is a concrete starting point to deepen the medium-and long-term youth development plan and care for the healthy growth of young people. Through the effective implementation of the activities, it will further expand the coverage of youth self-care education, effectively do practical things, solve problems, and provide practical help for teenagers. On the day of the activity, the leaders who attended the launch ceremony went to Jinta County to investigate the protection of youth rights and interests. The research team successively went to the county court, Northeast Street Community, Jinta Town and other places to inspect the "National Youth Rights Protection Post", "Youth Home", the construction of youth legal publicity and education base, as well as the employment and entrepreneurship of young wealth leaders, and the Communist Youth League's efforts to promote rural revitalization. In recent years, the provincial party committee to implement the Gansu province long-term youth development plan (2018-202520) is suggested, focus on prevention of juvenile crime and rights and interests maintenance work, strengthen the political responsibility bear, optimize service security object, highlight care key groups, establish and improve the working mechanism, publicity and education coverage, youth healthy growth environment improvement, the communist youth league lead, organization, service enhancement, each work has made new achievements.

2022-05-31 Guangdong held the "Children's Day" Young Pioneers theme Team Day activity. China Youth network Beijing, May 31 (reporter Zhang Jianwei, correspondent Yue Qing) " to become a young pioneer feel very happy, very glorious!"On May 30, sponsored by the Guangdong provincial party committee, Guangdong province working committee, Guangzhou less committee, liwan district working committee of" welcome 20, be players "-" children "2022 in Guangdong experimental middle school li wan school second theme day activities, activities held a" join the young pioneers from primary school pioneer " new team ceremony, He Tinghao classmate is one of the young pioneers in grade. Representatives of the new young Pioneers took the oath under the team flag. The Guangdong Provincial Committee of the Youth League commended the outstanding young pioneers of Guangdong Province and the seventh session of the most beautiful Southern Guangdong youth representatives, invited the national model workers, the provincial May Day Labor medal winners and other pioneer model representatives to share their struggle feelings, for the young pioneers of the new team gave gifts."May you wear red scarves have been determined to fulfill the solemn promise of 'rest assured that the Party and the country has me'."Guangdong Experimental Middle School Party secretary, president Quan Hanyan said. Guangdong pioneer model representative to the young pioneers presented 61 gifts and speech. At the scene of the activity, 20 young pioneers representatives, under the oath of the counselors and teachers, wearing bright red scarves, vowed to "contribute to the cause of communism", and performed the program "China in the Children's Voice"."Join the Young Pioneers, I am very happy, mom and dad all praise me!"On the activity," Guangdong Red Scarf Bus School " special bus held the departure ceremony, the first grade student Deng Ziyan and the students under the leadership of the bus red scarf guide, carried out a variety of interesting activities."Guangdong red scarf bus school" is the new platform of Guangdong young pioneers social practice activities, dedicated bus to tram as the carrier, through the loop operation, with 13 line museum as the end, via Chen temple, nanyue king museum exhibition, farmers, a big square museum and Guangdong young pioneers campus practice education camp (base), let the young pioneers in practice, learning historical knowledge, listen to the struggle story, understand development achievements, feel the development of the country and take-off. At the same time, the special line bus connects with the subway and the regional conventional bus line network, providing safe and convenient bus travel supporting services for the young Pioneers to carry out a variety of off-campus education practices and search and research activities, to meet the travel needs of the young Pioneers and their parents. The Young Pioneers lined up to board with the first commemorative tickets of the "Guangdong Red Scarf Bus School"."Please get on the bus in an orderly manner."On the same day," Guangdong red scarf bus school ", in the red scarf narrator, yuexiu district of Guangzhou dongshan pei is primary school fourth grade students Chen Yiming, students have on the bus, to a big memorial hall, Guangdong electric power exhibition hall of Guangdong young pioneers campus practice education camp (base) to carry out social practice activities, learning the party history knowledge, the" double carbon " knowledge learning, experience and exploration. At the event site, the national model worker Li Zhijian, the national most beautiful bus driver, the provincial May Day Labor Medal winner Liang Yukang, the city outstanding "March 8th Red Flag bearer" Yao Yuyan, Lin Hongying, an outstanding Communist Party member, the outstanding young representative Huang Zhicong and other 6 comrades were employed as the first batch of "Guangdong Red Scarf bus school" conductor."Guangdong Red Scarf Bus School" first launch exclusive commemorative tickets. Group of Guangdong provincial party committee for group Guangdong provincial party committee relevant controller introduces, during the period of "children", Guangdong province around the unified deployment, widely integrate local resources, through the young pioneers organization linkage online forms of theme team day activities, new team ceremony, combined with advanced recognition, example lead, charity sale, solicitation show, short video works for clock, red performance, studies practice, led the province the young pioneers in the practice of education character, growth ability, realize comprehensive development, physique, with good face to meet the party's 20 victory. In addition, the Guangdong Provincial Committee of the Communist Youth League and the Guangdong Provincial Youth Working Committee will also combine the "61" activities with solving practical problems for children, and use the good mechanism of "I do practical things for the masses", to help solve practical problems. By giving full play to the rescue function of Guangdong Red Scarf Fund, the young working committees at all levels were organized to visit the young pioneers suffering from serious diseases in the province and sent condolence money and small gifts of love.

2022-05-27 Hainan Railway safety publicity into the campus Hainan Railway safety publicity into the campus Youth network reporter Ren Mingchao) On May 26, Hainan province held the "526 I love the road" railway safety publicity into the campus demonstration activity. The event was jointly hosted by the Political and Legal Committee of Hainan Provincial Committee, Hainan Provincial Committee of the Communist Youth League, the Youth League Committee of China Railway Guangzhou Bureau Group Co., LTD., Hainan Railway Road Protection Office, and each Station and Section of Hainan Railway Co., LTD. The activity aims to further enhance the brand influence of the Communist Youth League of "Dream Avenue-Ring High-speed Railway", strengthen safety protection education along the Hainan Railway, create a new form of law popularization, and a new pattern of law and law. 55 teachers, student representatives and youth railway league members are invited to participate in the activity. In Hainan Railway Museum, a national research and practice education base for primary and middle school students, teachers and students learn railway safety knowledge through knowledge popularization, guessing with prizes, reading open letters and animated video display.

2022-05-27 Lin Jie, reporter of China Youth Network) Today, 100 jurists held the 92nd lecture of Guangdong Provincial Party Committee in Guangzhou. Zhang Hongwei, dean of the School of Humanities, Jinan University, professor and doctoral supervisor of the Juvenile and Family Law Research Center, delivered a special report on the theme of "Countermeasures to prevent juvenile delinquency". More than 400 people from Guangdong Province listened to the report. Conference, Zhang Hongwei combined with the newly revised law of the prevention of juvenile crime, from its legislative purpose, revised background, significance, around the present situation of juvenile crime, the law legislation and style, the law key content interpretation, the communist youth league in the status and role, etc., further expounds the significance of preventing juvenile illegal crime work, from the practical level, the communist youth league to prevent juvenile crime work has guiding significance. Deng Yuanqiang, vice president of the Guangdong Law Society and former first-level inspector of the Provincial Political and Legal Committee, pointed out that the 100 jurists and the Guangdong Rule of Law Report are an important platform for party and government leaders at all levels in Guangdong province to learn the deployment of the central major rule of law construction, the spirit of the rule of law and legal knowledge. At the same time, we will strengthen legal education among teenagers, enhance their legal concept, improve their legal quality, and provide support and guarantee for the prevention of juvenile delinquency. The prevention and governance of juvenile crimes is an important area for the Communist Youth League to participate in social governance. All the time, the Guangdong Provincial Party Committee of the Communist Youth League has explored new ideas and new modes of prevention and governance of juvenile delinquency in combination with the new changes and new characteristics of juvenile delinquency work in Guangdong Province. The relevant person in charge of the Guangdong provincial party committee pointed out that the Guangdong communist youth league to actively develop youth rule of law publicity and education work, deepening "youth zero crime zero victim community (village)" pilot to create work, "youth QuanQuanGang" peer "partner" youth community correction work brand projects, to promote the prevention and management of juvenile illegal crime work quality and efficiency. Up to now, Guangdong province has held 1,329 reports on 100 jurists and reports on the rule of law in Southern Guangdong, with an audience of more than 2.94 million people.

2022-05-27 In 2022 Sichuan province youth culture and art performance kicks off "new era shu youth" 2022 youth culture and art performance in 2022 hot kicks off Ji'an-wei zhang correspondent qing-hua zhao) on May 27 morning, "new era shu youth" 2022 Sichuan province youth culture and art performance (hereinafter referred to as the "performance") news briefing was held in Chengdu. Learned, performance activities by the Sichuan provincial party committee, Sichuan province spiritual civilization construction office, Sichuan province education department, Sichuan province science and technology hall, culture and tourism, Sichuan province, radio and television bureau, Sichuan province concerned about the next generation working committee, Sichuan province young pioneers working committee, is the province's only one jointly sponsored by eight provincial units of youth culture and art performance. This year's performance activities set for "stage art, visual art, scientific and technological innovation, the young pioneers practice" four categories, and the stage art vocal music performance optimization upgrade into chorus performance, add drama and drone project, aims to further carry forward the excellent traditional culture, stimulate the youth spirit of innovation, guide the children to listen to the party, follow the party. According to the relevant person in charge of the Provincial Communist Youth League Committee, this year's performance registration channel will be fully opened in early June, and children aged 6-18 in all cities (prefectures) can register through the official wechat public account of "Sichuan Social Education". Relevant leaders of 8 organizers, leaders of after-school youth activities in cities (prefectures) and some representatives of young Pioneers participated in the news briefing by combining online and offline.

2022-05-26 On the morning of May 25th, Sichuan Provincial Young Entrepreneurs Association donated the forest and grassland fire fighting water truck to Xide County, Liangshan Prefecture. The ceremony was held in Xide County. Leaders of the Provincial Communist Youth League Committee, Liangshan Prefecture and Xide County, member representatives of the provincial Youth Entrepreneurs Association, and member representatives of the Xide County Emergency Rescue Team attended the donation ceremony. It is reported that according to the unified deployment of Sichuan Forest Prevention Bureau, the Provincial Communist Youth League Committee has been in charge of the contact and supervision of Dechang County and Forest and grassland Fire County of Liangshan Prefecture since April 1. In order to perform the job responsibilities, the Provincial Communist Youth League Committee set up a special work team to carry out a regular all-weather bao county contact and supervision, and adhere to the combination of supervision and service, to effectively help solve practical problems. When learning about the lack of fire water trucks in Xide County, the Provincial Communist Youth League Committee immediately organized and mobilized the members of the provincial Young Entrepreneurs Association to donate more than 900,000 yuan to purchase 5 fire water trucks, which will effectively alleviate the shortage of mobile fire fighting facilities in Xide County. According to the relevant person in charge of the provincial Communist Youth League Committee, since the bao county contact supervision work, the provincial Communist Youth League Committee team members and 20 cadres, has gone deep into the two counties and towns (streets), villages (communities) and forest card prevention point, front garrison point, fire � t sentinel and other places to carry out the work. At the same time, relying on the "volunteer" Sichuan platform, planning to launch "Sichuan province forest grassland fire prevention youth volunteer service special action", guide the city, county and township league to mobilize youth volunteers into the country, school, "two sentinel" and other key areas, close to a line to carry out the fire hazard patrol, civilization persuasion and combustible clean forest grassland fire prevention volunteer service. Up to now, a total of 3,952 emergency volunteer service teams have been set up at all levels in the province, with more than 61,500 volunteers recruited. The "Volunteer Sichuan" platform has released more than 7,000 forest and grassland fire prevention volunteer service projects, and more than 50,000 volunteers have participated with a service time of more than 300,000 hours.

2022-05-25 Capital college students 'business plan competition revitalize the country successfully held in Beijing: "green Beijing suburbs" youth in 2022 "challenge cup" capital college students' business plan competition rural revitalization special successfully held jianwei correspondent Ren Yi Yan Liang Ma Xiaoyu) "qing vibration suburbs" youth in 2022 "challenge cup" capital college students' business plan competition rural revitalization special recently ended. The competition review committee selected a total of 19 gold, 104 silver and 153 bronze works."Qing vibration suburbs" is the league Beijing municipal party committee based on Beijing "small agriculture" cities "big suburban small city" cities agriculture, combining the reality of the communist youth league and youth work, focusing on the main business, to service, cohesion, cultivating young talents as the breakthrough point, into the "rural revitalization" work the focus of the construction of brand, brand covers green selected, green, green, green love, green broadcast rural revitalization of the "green" font size series of activities."Qing vibration suburb" youth "challenge cup" capital college students business plan competition for the country revitalization of special use "list" mode, focusing on urban and rural integration, combining with Beijing rural revitalization, highlight the beautiful rural development concept of green development needs, to solve the practical problems of Beijing "three rural" development, through the form of the township list, students team, according to the corresponding entrepreneurial form and solutions, in the capital rural revitalization of the stage burden, achievements. The competition released 19 lists, attracting 597 projects from 74 universities in the city to register, with nearly 10,000 participants. Next, according to the results of the competition, combined with college students rural entrepreneurship support plan work requirements, fine implementation of the "communist youth league to promote college students' employment action", focus, focus, go all out to help college students improve social ability, to build platform for youth innovation entrepreneurship, entrepreneurship to promote employment.

2022-05-25 In order to promote the employment of college students, the Shaanxi Provincial Party Committee of the Communist Youth League recently jointly launched the relevant departments of the province to promote the "promote the employment of college students", implementing eight special measures to serve the high-quality employment of college students. It is understood that the "college students employment action" to the project to help employment, entrepreneurship with employment, training to promote employment as the key implementation direction. Among them, "Project Employment Assistance" focuses on the "Sailing Plan" public employment service based on employment guidance, career planning, and career experience; relying on the youth employment service platform of "Qin Yun Employment" and "Qin Qing Spread its wings", carries out campus employment recruitment activities based on online recruitment and live broadcasting, and carries out social practice projects such as "Western Plan Volunteers", "Returning Home", "Government internship" and "Sailing Plan"."Lead employment through entrepreneurship" focuses on the project loan, financial support and assistance from entrepreneurship mentors. For small and micro enterprises, deepen the "Qin preferential loan" project, increase the loan amount to RMB 5 million; establish the "Financial Youth Service Group", set up the "Qin Chuang" (scientific and technological innovation) and rural revitalization work class, focusing on innovation and agriculture-related enterprises; for returning students within two years, Shaanxi Youth Entrepreneurship and Employment Foundation supports 100 entrepreneurial projects with interest-free loans; play the role of Shaanxi youth entrepreneurship mentor think tank, carry out face-to-face mentor consultation, and promote the "entrepreneurial bud" support program."Promoting Employment through Training" focuses on implementing the Communist Youth League, carrying out online and offline youth entrepreneurship training in various fields; continuing to implement new vocational training for youth, and promoting the inclusion of key subsidies in vocational skills training scope. Source: China Youth Daily, May 25,2022, edition of the 02nd edition

2022-05-24 Yunnan held a symposium to study and implement the spirit of General Secretary Xi Jinping's important speech at the conference celebrating the 100th anniversary of the founding of the Communist Youth League of China. Yunnan held a study and implement xi general secretary in celebrating the 100th anniversary of the founding of the communist youth league of China's important speech spirit symposium Zhang Wenling) on May 20, Yunnan province xi general secretary in celebrating the 100th anniversary of the founding of the communist youth league of China's important speech spirit symposium and situation policy report, held in Kunming university of science and technology. Yunnan Provincial Party Secretary Wang Ning presided over the meeting and made a speech. He encouraged the youth of Yunling Mountain to contribute their youth strength in writing the Yunnan chapter of the Chinese Dream, and to welcome the victory of the 20th Congress of the Party with outstanding achievements. At the symposium, Wang Ning listened to the speeches of 10 young representatives and interacted with them. Wang ning pointed out that the provincial youth league organizations at all levels and the general cadres, youth members to study deeply to grasp the profound connotation of xi general secretary of the important speech, deeply grasp the strategic position of youth work, the communist youth league's mission, the communist youth league journey of valuable experience, and the new era of Chinese youth movement and youth work major issues, draw wisdom and strength. Wang Ning stressed that the young people should study hard, work hard, and strive to be a new force to realize the great rejuvenation of the Chinese nation. We must be firm in our ideals and convictions, grasp the right direction, and grow into a good young man in the new era with ideals, dare to take responsibility, be able to bear hardships, and be willing to struggle. We should study hard and make progress, read more books, read good books, learn theory, culture, science and skills. To be committed to hard work, to create their own wonderful life. We should actively innovate and start businesses, combine the development of Yunnan's characteristics and advantages of industries, and have the courage to participate into practice, so that innovation becomes the driving force of youth voyage, and entrepreneurship becomes the energy of youth fighting. We should have the courage to take responsibility and play a play to the role of the vanguard. We must temper our noble character, consciously practice our core socialist values, and be a person of noble moral character and a very useful person to the country and society. Wang Ning stressed that the youth league organizations at all levels in the province should earnestly implement the requirements of "adhering to educating people for the Party, consciously taking responsibility, caring for the youth, and having the courage to self-revolution", constantly enhance the leading, organizational, service and combat effectiveness, and strive to do the work of the Communist Youth League in the new era better. Colleges and universities should implement the fundamental task of cultivating people by virtue, strengthen and improve the construction of ideological and political courses, and strive to train new people who should shoulder the great responsibility of national rejuvenation. Party organizations at all levels should implement the system and mechanisms for party building and leading youth league building to create a better environment and conditions for the growth and development of young people. On the same day, Wang Ning also participated in the "Youth Marxist Training Project of the Elite Class" Youth League branch in Chenggong Campus of Kunming University of Science and Technology, and visited the key laboratory of Intelligent Control and Application of universities in Yunnan Province to understand the learning and work of young scientific and technological talents.(Lei Tongsu / Photography)

2022-05-24 China Youth Network, reporter Ren Mingchao) From May 20 to 21, the Hainan Youth League held the theme of "Strengthening the imprint into the rainforest". More than 60 people from members of the leading group of the League, all cadres of organs and subordinate units participated in the activity. The delegation from the Hainan Provincial Party Committee visited the exhibition of comprehensively deepening reform and opening up and the construction of a free trade port with Chinese characteristics, and held the flag awarding ceremony for the Wuzhishan Youth Volunteer Service Team of Hainan Tropical Rainforest National Park. In Maona Village, Shuiman Township, Wuzhishan City, we conducted a field survey on the local rural revitalization construction, gave a theme group class of "Power imprint- -The Happy footprints of Mauna Village", and reviewed the red history in the Memorial Park of Wuzhishan Revolutionary Base. League Hainan provincial party committee secretary Chen yu pointed out that in the new era of free trade port construction in Hainan, Hainan communist youth league to deepen reform, pays special attention to the youth thought lead, promote youth development friendly free trade port construction, focus on the construction and use of China (Hainan free trade port) youth dream avenue youth mobilization leading service platform, improve the communist youth league "once".

2022-05-23 Shaanxi Provincial Youth Federation held the fifth session of the 12th Chairman (expanded) meeting to study the spirit of General Secretary Xi Jinping's important speech at the celebration of the 100th anniversary of the founding of the Communist Youth League of China. Shaanxi province youth league held twelve fifth chairman (expand) meeting project learning xi general secretary in celebrating the 100th anniversary of the founding of the communist youth league of China's important speech spirit, JianWei correspondent Zhang Wenhao) on May 20, shaanxi province youth federation held twelve five chairman (expanded) meeting, special learning xi general secretary in celebrating the 100th anniversary of the founding of the communist youth league of China's important speech spirit. Xu Yongsheng, secretary of Shaanxi Provincial Committee and chairman of Shaanxi Provincial Youth Federation, presided over the meeting and delivered a speech. Vice chairman of the 12th Shaanxi Provincial Youth Federation, chairmen and secretary-general of all walks of life attended the meeting. Focusing on how to study and implement the spirit of General Secretary Xi Jinping's important speech, Xu Yongsheng, secretary of the Shaanxi Provincial Youth League Committee and chairman of the Shaanxi Provincial Youth Federation, put forward four requirements: First, we should always bear in mind the original mission and faithfully educate people for the Party. Vigorously carry forward the glorious tradition of "patriotism, unity, follow the party", further promote the "welcome the 20, always follow the party, forge ahead new journey" theme education practice, spread the voice of the party with the youth language, guide the youth of all nationalities and all walks of life in the province firmly ideal and faith, firmly support the "two established", "two maintenance"; second, to always adhere to the center, serve the overall situation of the province. Relying on the "technology", "support" youth brand work, guide the youth of all ethnic groups and all walks of life in the province in innovation-driven development, industrial optimization and upgrading, steady growth, in epidemic prevention and control, rural revitalization, risk prevention, in ecological protection, livelihood security, reform and opening up; as, three is always firmly rooted in the youth, as a good bridge. Carry out the "youth association" activities, explore the establishment of the provincial youth federation proposal working committee, organize and promote the members of the youth to strengthen the investigation of the youth group, understand and grasp the common and urgent demands, make full use of the Party Congress, NPC, CPPCC and youth organization channels, reflect and express the youth voice; fourth, to always practice strict requirements, and strengthen the self-construction. Centering on maintaining and enhancing the political nature, advanced nature, adhere to the problem-oriented, and continue to deepen the reform of the Youth Federation. We will continue to govern the Party with strict discipline, earnestly ensure strict governance, set an example of vigor and spirit, and set an example recognized by the Party and the government and satisfied by the young people. Next, shaanxi youth league will through youth league members training class, seminar, sector work conference, and other forms, in-depth study and implement xi general secretary of the important speech spirit, quickly in shaanxi youth league members learn xi general secretary of the important speech spirit, with excellent work performance to meet the party's 20th victory.

2022-05-23 Guangdong: "ideal lamp" "light of faith" into the campus of Guangdong: "ideal lamp" "light of faith" into the campus youth network Beijing on May 23 (reporter Zhang Jianwei, reporter Zhu Changbiao) recently, in 2022, "lighthouse project-Guangdong youth learning practice xi new era of socialism with Chinese characteristics action" preach communication into the campus of Guangdong first demonstration activities held in shunde vocational and technical college. The activity focused on the important speech spirit of General Secretary Xi Jinping at the celebration of the 100th anniversary of the founding of the Communist Youth League of China, and guided the majority of young people to light up the light of faith and stimulate the power of youth. More than 100 young students watched the scene, and displayed it as a quality demonstration group class resources in the whole province after recording. This year, the Youth League Committee of Shunde Vocational and Technical College won the title of the National May 4th Red Flag Youth League Committee in the national May 4th "Two Red and two Excellent" commendation. To this, shunde vocational and technical college youth corps committee secretary Liu Jinxin to "let every student have a chance to brilliant life-school mission and bear" theme, she said: "our school communist youth league to enhance the" three force once ", youth temperature, good youth story, spread youth voice, leading the youth league banner with the party, to do Renaissance pillars, yong bear power mission."The picture shows the members of the Guangdong Youth Lecture Group carrying out a theme lecture. Correspondent for figure preach activities, Guangdong youth branch members, the bay area brother ideological class guest, south China normal university of marxism professor zhang to learn xi general secretary at the celebration of the 100th anniversary of the founding of the communist youth league, as the theme of the history of the story of the revolutionary struggle, he told the school teachers and students: " based on the new centennial starting point, strive to be big, lead the youth; build great politics, care for youth; establish a great view of history, ambition."The picture shows the secretary of the Youth League Committee of Shunde Vocational and Technical College giving a theme lecture. Correspondent for figure shunde vocational and technical college student member at the event, and the students to listen to the leadership of the Chinese youth movement in one hundred, she was greatly encouraged: " as a communist youth league member, to actively party organization, with party member standard strict with themselves, study hard, determined to become a skillful craftsman, power, run the best result on the youth track."" should know and act, practice, with excellent party members predecessors as an example."Wang Yifan, a student of Shunde Vocational and Technical College, who is also feeling very passionate, believes that standing at the historical intersection of the" two centenary years ", as a young student, we should redouble our efforts to study, strive to be a pioneer, drive the students around us to make progress together, and show the style that the young students should be. It is reported that the activity plans to carry out more than 100 games, to achieve the province's 21 cities, 161 universities full coverage. The relevant person in charge of the provincial Communist Youth League Party Committee said that the "youth learning" publicity group gathered the strength of thousands of young lecturers, aiming to speak deep, thoroughly and live the latest theory of the Party through the "green language", so that more young teachers and students can understand and remember.

2022-05-23 Sichuan province youth culture and art performance activities held provincial special "new era shu youth" 2021 Sichuan province youth culture and art performance provincial special wei correspondent Zhao Qinghua) on May 21 to 22, "new era shu youth" 2021 Sichuan province youth culture and art performance provincial special (stage art) successfully held in Chengdu. According to the relevant person in charge of the organizer, the number of the provincial special children registered reached 245,868, up 18.1% compared with last year. After online registration, online preliminary evaluation and municipal performance, a total of 251 stage art programs and 575 visual art contestants were selected as provincial special performances. Activities by the Sichuan provincial party committee, Sichuan spiritual civilization construction office, Sichuan province education department, Sichuan science and technology, culture and tourism, Sichuan province, radio and television bureau, Sichuan working committee about the next generation, Sichuan province young pioneers working committee eight units, the stage art special activities through "Sichuan communist youth league" weibo and "Sichuan social education" WeChat public for the whole live, online viewers and interaction.

2022-05-13 Group Gansu provincial party committee secretary (expand) meeting project convey learning xi general secretary in the communist youth league was founded the 100th anniversary of the conference's important speech spirit group Gansu provincial party committee secretary (expand) meeting project convey learning xi general secretary in the communist youth league was founded the 100th anniversary of the conference important speech spirit on 13 (reporter Zhang Jianwei, correspondent Li Jing) on May 10 in the afternoon, The Communist Youth League Gansu Provincial Party Committee held a secretary (expanded) meeting, To convey the spirit of General Secretary Xi Jinping's important speech at the celebration of the 100th anniversary of the founding of the Communist Youth League of China, We will study and implement the guidelines. Communist Youth League Provincial Party Secretary Dong Anning presided over the meeting. Conference pointed out that xi jinping, general secretary in celebrating the 100th anniversary of the founding of the communist youth league of China's important speech, comprehensively reviewed the 100 years the communist youth league unswervingly, follow the party's youth, fully affirmed the communist youth league under the leadership of the party, unity led generations of youth to realize the great rejuvenation of the Chinese nation the Chinese dream made important contribution, profoundly illustrates the historical experience of the work of the communist youth league and youth expectations, to the contemporary new era of the communist youth league work put forward clear requirements. General Secretary Xi Jinping's important speech is full of the CPC Central Committee's cordial care for the young generation and attaches great importance to the cause of the Communist Youth League. It is highly political, ideological, strategic and guiding. The meeting stressed that thoroughly studying, publicizing and implementing the spirit of General Secretary Xi Jinping's important speech is the primary political task of the provincial Communist Youth League, Youth League, Students' League and Young Pioneers at all levels at present and in the future, and also the core content of the education and practice activities of the theme of "Celebrating the 20th National Congress, Always following the Party, and forging ahead on a new journey". Focusing on educating people for the Party, It has always become the requirement of a political school leading the ideological progress of Chinese youth, Focusing on the fundamental plan of having successors to the Party's cause, Focusing on the ideological and political guidance of the Communist Youth League, With Xi Jinping Thought on Socialism with Chinese Characteristics for a New Era, Build the ideological leading work system of "green" brand name, Thoroughly implement the "youth xinglong" education project, Constantly consolidate the "1 + 5 + N" education work system, Continue to play the role of the online Communist Youth League, To guide the majority of youth league members in the ideological baptism, to be Chinese in practice forging to constantly enhance the ambition, backbone and confidence, Let the revolution be passed on from generation to generation. Around consciously bear responsible, always become the Chinese youth permanent struggle pioneer force, further implement the requirements of the "three new high" major strategic requirements and "four" action deployment, further promote "rural youth contribution", "beautiful youth action", community youth action, "pomegranate seed members" theme practice, unity led the youth with youth power and creativity of surging spring tide, with the wisdom of youth and sweat construction happiness new Gansu, create a new situation of rich people. We must focus on the hearts of the young people and always become the strongest bridge between the Party and the young people. We must always keep our eyes down and shift our focus down, and enrich our strength and resources to the community level, so that community-level youth league organizations can play a greater role in serving the young people. We will continue to promote the implementation of the medium-and long-term youth development plan in Gansu Province, and encourage Lanzhou city, Ganzhou District, Yumen City and Linxia City to actively carry out the pilot construction of national youth development cities (counties). With difficult family children ordinary college funding projects, "group to help employment", youth innovation entrepreneurship competition, 12355 youth service platform brand work as the carrier, one thousand ways to do the practical work for youth, solving, take the initiative to youth thought, urgent youth, let the youth really feel by the party's care is around, care is in sight. To around the self revolution, has always become closely with the party in the era of advanced organization, consciously to the comprehensive governing party experience, strict in TuanZhiTuan, innovation to carry out the "three force once two guarantees" KPI index assessment work, with innovation spirit and strict wind to strengthen their own construction, in the comprehensive, high standard forging glow the communist youth league uplifting era. Meeting requirements, the province the communist youth league member, cadres to consciously understand xi general secretary of the important speech spirit, according to the important requirements of general secretary, par with the party, more consciously and firmly combined with the people's growth path, find their own gap, thorough introspection, take the lead to strengthen theoretical study, temper party spirit cultivation, lead the firm ideal faith, carry forward the country feelings, take the lead to practice mission, people, take the lead for the party takes responsible, the courage to overcome difficult, take the lead in close ties with youth, maintain a fine style of work. Meeting requirements, the provincial communist youth league organization to the above rate play a demonstration leading role, the provincial league leading organs at all levels to learn one step, learn deep layer, make full use of the secretary of the conference, theoretical study group meeting, listed project, plan, thorough communication discussion, do learn to understand, learn, learn something. We should innovate ways and methods, enhance the learning effect, organize the members of the youth teaching group to go to the grass-roots level, carry out "face to face" and "heart-to-heart" learning and publicity for the youth league members, and incorporate the spirit of the general Secretary's important speech of the general secretary into the compulsory courses of the youth league training courses at all levels. It is necessary to create a strong atmosphere for learning and implementation, rely on all kinds of media, especially the new media affiliated to the league, to carry out multi-level, all-round and three-dimensional publicity, and truly effectively convey the care and ardent expectations of General Secretary Xi Jinping to the youth league members. We should closely revolve around the "youth welcomes the 20th party", extensively carry out the mass theme publicity activities, increase the publicity of typical youth, guide the young people to better play their role of new forces and commandos in their respective fields and posts, and take concrete measures to welcome the successful convening of the 20th Party and the 14th Party Congress of Gansu Province with practical actions. Members of the provincial party secretaries of the Communist Youth League attended the meeting, and the main leaders and leaders of all departments and subordinate units attended the meeting.

2022-05-18 Gansu: condensed youth strength improve service overall contribution of Gansu communist youth league: condensed youth strength improve service overall contribution, Gansu communist youth league organization and lead the province youth widely participate in poverty engines and rural revitalization, active in epidemic prevention and control volunteer service, comprehensive ecological management and protection, pay attention to the youth national unity education in the Chinese nation community consciousness, many measures and guide the youth in Gansu will patriotism into practical action, efforts to become the national rejuvenation era, the communist youth league around the center service overall contribution rising. During his visit to Gansu province, General Secretary Xi Jinping stressed the need to deepen poverty alleviation, uphold the target, focus and standards, and resolutely conquer the last fortress of poverty. In recent years, the Gansu Provincial Committee of the Communist Youth League has thoroughly implemented six "youth poverty alleviation" actions, such as poverty aid and poverty assistance, employment assistance, entrepreneurship assistance, spiritual poverty alleviation, voluntary poverty alleviation and consumption poverty alleviation, and actively organized young people in the province to participate in the main battlefield of poverty alleviation. Gansu Communist Youth League raised 277 million yuan to fund students from poor families to complete their studies and improve the conditions of rural primary and secondary schools in poor areas; established more than 5,600 spiritual poverty alleviation teams, conducted 14,000 activities, implemented youth volunteer poverty alleviation relay plan, recruited 3,568 members, formed 31 teams to implement the "youth students" achievement plan, and conducted 15,000 volunteer poverty alleviation activities, with a total service time of 3.18 million hours. In order to effectively contribute to the development of the agricultural industry, Gansu Communist Youth League to create a series of brand activities of "youth poverty alleviation energy to help agriculture" and "rural revitalization of youth to help agriculture", Helhelp the sales of 106 million yuan of agricultural products, The "Youth to Help Agriculture" series of activities are listed as an excellent typical case of national consumption assistance to help rural revitalization in 2021; To provide designated assistance, Has helped Tianzhu County, Tongwei County, Heshui County, As the provincial direct support group leader unit of Heshui County, To organize and mobilize assistance units at all levels to implement 1,050 assistance projects, Assistance fund of 235 million yuan; besides, Also actively carry out eastern and western league organizations, Implementing 171 collaborative projects, Funds involved were 54.425 million yuan. After winning the overall victory of the battle against poverty, the Gansu Provincial Party Committee of the Communist Youth League effectively focused on the consolidation and expansion of the achievements of poverty alleviation and rural revitalization, and further promoted the "rural revitalization youth contribution" action. In 2021, he assisted 42.466 million yuan, trained 3,000 rural entrepreneurs and rich leaders, established youth volunteer service team of New Era Civilization Practice Center and relocated community volunteer service team, with over 460,000 hours, over 150,000 participants; helped more than 7,000 youth from poor families to achieve employment, and donated 21,046,900 yuan to extremely poor people. League flag in the epidemic prevention and control line flying high since the new crown outbreak, in view of the outbreak repeatedly, around Gansu province give full play to the role of the communist youth league powerforce and commandos, organization member youth charge in the front, the dedication, in the supply, community prevention, nucleic acid detection, etc., everywhere with the young volunteers. In recent years, the Communist Youth League of Gansu province has set up 7,756 youth commandos and epidemic prevention volunteer service teams in key areas, recruited more than 450,000 young volunteers of various types, and mobilized more than 18,000 returning college students to report to villages (communities), with a service time of more than 5 million hours. In order to help the Communist Youth League effectively participate in epidemic prevention and control, the Gansu Provincial Committee of the Communist Youth League successively allocated 800,000 yuan for epidemic prevention and control. At the same time, it also gave full play to the advantages of socialized mobilization, raised 10,066,900 yuan, raised and allocated 25.84 million yuan of various anti-epidemic and living materials worth, and took the lead in supporting Hubei to fight the epidemic. Over the past two years, youth league organizations at all levels in Gansu have received a total value of more than 15 million yuan, effectively helping the overall epidemic prevention and control in the province. To fight disease resistance as the reality teaching material, Gansu communist youth league also more measures and speak good Chinese disease resistance achievements, deepen the youth "four confidence" education, People's Daily online, Xinhua News Agency, China youth daily and other media have reported youth league organizations at all levels, youth involved in the epidemic prevention and control related content 900 article (times), fully demonstrate the Gansu youth disease resistance, effectively passed the youth positive energy. The construction of beautiful Gansu province, youth first is located in the northwest inland, Gansu province ecological environment is fragile, ecological protection and construction task is heavy. In recent years, the Gansu Communist Youth League has taken the "Beautiful Gansu Youth Action" as the carrier to extensively mobilize the youth forces to participate in ecological construction. The province has set up 550 green youth volunteer service teams, participated in 176,000 ecological environmental protection practices, organized 256 ecological environmental protection practices and micro-public welfare projects, and built 130 youth forests; carried out provincial demonstration activities for youth tree planting and green protection, and youth league members have planted more than 1.27 million trees. In addition, it has also successfully applied for two national key funding projects for mother River protection. Focusing on the national strategy of ecological protection and high-quality development of the Yellow River Basin, the Gansu Communist Youth League also carried out the theme activity of "7 Members", organized more than 30,000 volunteer team of "He Xiaoqing Youth", and carried out more than 6,000 activities, covering 1.02 million teenagers. At the same time, it named 28 youth ecological civilization education and practice bases in Gansu province, regularly organized youth publicity and education activities, guided young people to firmly establish the ecological civilization concept of "clear waters and green mountains are gold and silver mountains", and take the initiative to be propagandists, practitioners and leaders of ecological civilization construction. Casting prison community consciousness of the Chinese nation, deeply rooted in the hearts of Gansu province is a multi-ethnic province, in recent years, to cast up the Chinese nation community consciousness as the main line, the communist youth league actively carry out all kinds of propaganda and education, training, discussion, practice education and other activities, guide the youth cast prison community consciousness of the Chinese nation. Focusing on the Publicity Month of Ethnic Unity and Progress in Gansu Province, youth league organizations at all levels in the province have carried out 14,000 education and practice activities with the theme of "Pomegranate Seed as one family", "Ethnic unity as one family to build the Chinese Dream", and "Ethnic unity and children of all ethnic groups to the Party", covering 2.148 million young people. The Gansu Provincial Committee of the Communist Youth League organized young pioneers from ethnic minority areas in the province to participate in the "Pomegranate Seed as One Family" young Pioneers summer camp in Beijing, Tianjin and other places to continuously enhance the recognition of children of all ethnic groups to the Chinese nation and Chinese culture. In addition, it has also actively promoted the plan of youth development to Tibet and Xinjiang, employing more than 4,000 college graduates from Gansu province to Xinjiang and 600 people to Tibet, and sent 1,129 volunteers from the Western China Program to the Xinjiang Autonomous Region, the Xinjiang Corps and Tibet.

2022-05-13 The organs directly under the Sichuan Provincial Communist Youth League Committee held to study, publicize and implement the important speech spirit of General Secretary Xi Jinping at the celebration of the 100th anniversary of the founding of the Communist Youth League of China. The conference group was held by the organs directly under the Sichuan Provincial Party Committee. Study, publicize and implement the spirit of General Secretary Xi Jinping's important speech at the celebration of the 100th anniversary of the founding of the Communist Youth League of China. Meeting on May 13 (xinhua Zhang Jianwei, correspondent Zhao Qinghua) on May 12 morning, Sichuan provincial party committee directly held learning propaganda implement xi general secretary in celebrating the 100th anniversary of the founding of the communist youth league of China's important speech spirit meeting, further learning grasp xi general secretary of the important speech spirit and Sichuan provincial party committee symposium spirit, research deployment of provincial party committee organs and all fronts learning propaganda implementation measures. At the meeting, four young cadres from the organs directly under the Provincial Communist Youth League Committee respectively made exchange speeches based on their own work, around the study, publicity and implementation of the spirit of General Secretary Xi Jinping's important speech. They all said that General Secretary Xi Jinping's important speech is far-reaching, rich in connotation and inspiring, and they will closely combine with their own work and earnestly implement it. The meeting stressed that conscientiously studying, publicizing and implementing the spirit of General Secretary Xi Jinping's important speech, and thoroughly implementing the spirit of the symposium of the Provincial Party Committee are the primary political tasks of youth league organizations and youth league members at all levels in the province at present and in the future. In accordance with the deployment requirements of the Provincial Party Committee and the Central Committee of the Communist Youth League, play a good role in the youth League organizations at all levels and the youth league members in the province, and strive to promote the study, publicity and implementation of the spirit of General Secretary Xi Jinping's important speech to go deeper and practical. The meeting required that the party secretaries of the Provincial Communist Youth League Committee should learn one step first, take the important speech of General Secretary Xi Jinping as the core content of the theoretical central group of the Provincial Communist Youth League Committee, guide the departments in charge and all front fields to earnestly study study, publicity and implementation measures, and transform the learning results into political and work results. All departments and offices of the government should participate in and be extensively launched, and should study, publicize and implement the spirit of General Secretary Xi Jinping's important speech as the main content of the education and practice activities with the theme of "Welcome the 20th Party Congress, always follow the Party, and forge ahead on a new journey". Organs and directly affiliated institutions cadres to the grassroots league branch and "youth home" activities to preach propaganda, youth league, union, the young pioneers, and green enterprise association, green and other organizations to carry out various forms of learning propaganda implementation activities, league school and league training education base to learn the important speech spirit as an important content of the grassroots cadres education and training. Implement list management, define people and responsibilities, to ensure practical results, through the formulation of responsibility list, task list, supervision list, comb the implementation of key projects item by item, clear the relevant responsible departments, implementation measures, to ensure that the formation of a solid work closed loop. The party secretaries of the provincial Communist Youth League Committee, the staff of the organs and the main persons in charge of the directly affiliated institutions, the cadres (on-post training) and the volunteers of the Western Plan attended the meeting.

2022-05-13 Sichuan youth of all ethnic groups from all walks of life study and implement xi general secretary in celebrating the 100th anniversary of the founding of the communist youth league of China's important speech spirit symposium, held in Sichuan youth of all ethnic groups from all walks of life xi general secretary in celebrating the 100th anniversary of the founding of the communist youth league of China's important speech spirit symposium held in Chengdu. On May 13 (xinhua Ji 'an-wei zhang, correspondent qing-hua zhao) on May 11th afternoon, Sichuan youth of all nationalities from all walks of life to study and implement xi general secretary in celebrating the 100th anniversary of the founding of the communist youth league of China's important speech spirit symposium held in Chengdu, 12 youth respectively combined with their own study and work practice to share the learning experience. The meeting pointed out that General Secretary Xi Jinping's important speech at the celebration of the 100th anniversary of the founding of the Communist Youth League of China was full of the CPC Central Committee's cordial care for the younger generation and its great importance to the cause of the Communist Youth League, and is highly political, ideological, strategic and guiding. We should deeply understand the importance of the Party in leading the establishment, the original mission of the Communist Youth League; the goals of the new era, and the strategic tasks of the Party's youth work in the new era. The meeting stressed that learning the important speech of General Secretary Xi Jinping is to earnestly shoulder the glorious mission entrusted by the new era, with the strong faith strength, tenacious struggle spirit, firm historical confidence, to unite and lead the provincial youth league members in the era of the comprehensive construction of modern Sichuan. To focus on the leadership of the league, to consolidate and expand the youth mass foundation of the party; to promote the reform to form a more functional youth work system; to continue to pay close attention to the comprehensive strict governing the league, to establish the strict image of the new era. The meeting required that to earnestly study, publicize and implement the spirit of General Secretary Xi Jinping's important speech and thoroughly implement the spirit of the symposium of the Provincial Party Committee are the primary political task of the youth league organizations in the province at present and in the future. Provincial youth league organizations at all levels to a high degree of political consciousness, thought consciousness and action consciousness, by pays special attention to the thorough convey study discussion, continue to carry out propaganda about interpretation, step by step detailed implementation measures, strive to promote learning propaganda implement the important speech spirit go deep, go to real, quickly learning publicity implement upsurge, earnestly the xi general secretary of the care and ardent expectations into promoting the development of the provincial youth and the communist youth league career driving force. Members of the provincial Communist Youth League Committee, as well as responsible comrades of provincial organs, cities (prefectures), universities, districts and counties, members of the provincial youth League, executive chairman of the provincial Students' Federation, young Pioneers counselors, representatives of young volunteers, representatives of the provincial "two red and two excellent" representatives, scientific and technological innovation workers and other young representatives of all ethnic groups and all walks of life attended the symposium.

2022-05-16 The Shanxi Provincial Party Committee of the Communist Youth League and the Shanxi Institute of Surveying, Mapping and Geographic Information have compiled the Historical Map of the Shanxi Youth Movement and the Communist Youth League. This is the first map of the communist youth league history in Shanxi province, is to deepen the "welcome 20, always follow the party, forge ahead new journey" theme education practice, help teenagers understand the glorious course of the party leadership of Shanxi youth movement, and Shanxi communist youth league to celebrate the 100th anniversary of the founding of the communist youth league of China "listen to the party with the party youth contribution new era" one of the theme activities. Shanxi is one of the earliest provinces in China to establish local party and league organizations in Shanxi. The history of youth movement in Shanxi is an important part of the history of Chinese revolution and youth movement. Over the past 100 years, the Shanxi Communist Youth League has always kept in mind its original aspiration and mission of unswervingly following the Party and making unremitting efforts for the cause of the Party and the people, adhered to the glorious tradition of "the Party has a call and the league has an action", centering on the central tasks of the Party in different historical periods, united and led generations of young people to take the lead and act responsibly. The Historical Map of the Shanxi Youth Movement and the Communist Youth League systematically summarizes and comprehensively displays the glorious course of the Shanxi Youth Movement under the leadership of the Party, Covering 14 typical figures, 10 important places, 26 community journals and 79 events in the four historical periods of the new democratic revolution, socialist revolution and construction, reform and opening up and socialist construction, and the new era of socialism with Chinese characteristics, To guide the provincial cadres and youth league members to draw experience and wisdom from the history of Shanxi youth sports and gather the strength to forge ahead, Further firmly listen to the Party and follow the Party's ideals and beliefs, It is of important theoretical value and practical significance to actively participate in the vivid practice of promoting high-quality development in Shanxi Province.

2022-05-16 On the morning of May 10, a grand conference to celebrate the 100th anniversary of the founding of the Communist Youth League of China was held in Beijing. Jing Junhai, secretary of Jilin Provincial Party Committee, and the youth league members watched the live TV broadcast and listened carefully to the important speech of General Secretary Xi Jinping. After the conference, Jing Junhai presided over a symposium on the exchange of youth representatives, together with everyone to study and implement the spirit of General Secretary Xi Jinping's important speech. Zheng Weifeng, secretary of the Jilin Provincial Party Committee of the Communist Youth League, Yang Yongxiu, winner of the "Chinese Youth May 4th Medal", and Zhang Chaofan, the "National upward and Good Youth", made speeches successively. They combined with the actual work, talk freely about the learning experience. Jing Junhai pointed out that General Secretary Xi Jinping's speech is full of the CPC Central Committee's cordial care for the younger generation and attaches great importance to the cause of the Communist Youth League, which points out the direction and provides fundamental guidance for us to do the work of the Communist Youth League and the youth well. Standing at the new historical starting point of the Party's youth movement, the contemporary Jilin youth are ushering in a rare life opportunity of growing up and making contributions. It is hoped that the youth members of Jilin Province will always bear in mind the inculcation and entrust of General Secretary Xi Jinping, earnestly accept political training, strengthen political forging, pursue political progress, grow into a qualified Communist Party member as the goal, and contribute the youth power and creativity to the revitalization and development of Jilin. JingJunhai requirements, Jilin province youth league organizations at all levels should unswervingly adhere to the leadership of the party, unswervingly follow the development path of socialism with Chinese characteristics, adhere to the training of socialist builders and successors as a fundamental task, to consolidate and expand the ruling youth mass base as political responsibility, around the center, as the service work, strengthening leadership, organization, service, better to unite youth, organization, mobilize, to realize the second goal in one hundred, to realize the struggle of the great rejuvenation of the Chinese nation the Chinese dream. Jing Junhai pointed out that party committees at all levels (leading Party groups) in Jilin Province should do everything possible to do practical things for the youth, and take the initiative to be the confidant of young people, the enthusiasm of young people and the guide of young people. Party organizations at all levels should ensure that Party building leads to youth league building, implement Party requirements into the process of youth league building, and support youth league organizations at all levels in carrying out their work creatively. Source: China Youth Daily, May 16,2022, edition of 02

2022-05-16 Shanxi symposium to celebrate the 100th anniversary of the founding of the Communist Youth League of China was held in Taiyuan. Shanxi Provincial Party Committee Secretary Lin Wu attended the meeting and made a speech. Shang Liguang, deputy secretary of the provincial Party Committee, presided over the meeting. Vice Governor Yu Yingjie attended the meeting. Lin Wu, on behalf of the Shanxi Provincial Party Committee, first extended warm greetings and sincere greetings to the provincial youth league members, youth league organizations, youth league cadres and youth workers at all levels. He pointed out that xi jinping, general secretary in celebrating the 100th anniversary of the founding of the Chinese communist youth league conference's important speech, profoundly illustrates the historical experience of the communist youth league and the youth work, the contemporary youth expectations, to make the new era communist youth league work clear requirements, fully embodies the concern of the party leader to the youth love, attaches great importance to the communist youth league work, has a strong political, ideological content, strategic, guiding, is the new era of the party's youth work programmatic document, to seriously study, in-depth implementation. Lin Wu pointed out that Shanxi Province is one of the first provinces in China to establish local Party and league organizations. Youth League organizations at all levels unite and lead the youth league members to listen to the call and struggle with the Party, demonstrating the sincere responsibility of "the Party has the call and the league takes action". Youth league organizations at all levels in the province should strengthen ideological guidance, serve the overall situation of the center, serve the youth, strengthen their own construction, and strive to achieve political, ideological, ability, style of comprehensive and excellent. Lin Wu stressed that the province the youth to firm ideal faith, prison life course, consciously the personal development and progress and promote prosperous province, realize national rejuvenation, serve the motherland, the hometown, service people, consciously set up and practice the socialist core values, Ming virtue, social morality, yan private, correction on the road to life, go further. At the meeting, Zhang Jun, secretary of the Shanxi Provincial Party Committee of the Communist Youth League, exchanged speeches with the outstanding college students, outstanding entrepreneurial youth, outstanding young pioneers workers and the winners of the "Shanxi Youth May 4th Medal".

2022-05-12 Ningxia: the communist youth league reform "flowering" fruitful in Ningxia: the communist youth league reform "flowering" fruitful year in January, in Changsha civil affairs vocational and technical college school after 00 girl Yuan Jingyi through Ningxia ningchuan county back (in) township students village (community) deputy secretary of the league branch selection, become yongning li town feng deng village part-time deputy secretary of the youth league branch. Since participating in the work of the Communist Youth League in the village, Yuan Jingyi has completed the work of standardizing basic league affairs, identification of special teenagers, and identification of poor family teenagers. Combined with her professional social work expertise, she has formulated a detailed work plan, providing new ideas for serving the teenagers in the area."College students have many ideas and live ideas. Although they are part-time cadres, they can help teenagers in the village solve many problems."Mention Yuan Jingyi, Li Jun Town Fengdeng village youth League branch secretary Chen Yuemei repeatedly praised. Since the launch of the reform of the Communist Youth League in counties, the league organizations at all levels in Ningxia have firmly grasped the correct direction of the reform, adhered to the targeted efforts, targeted measures, effectively alleviated the blockage and difficult problems, and innovatively explored a set of new path for the reform of the Communist Youth League in counties that is in line with the reality of Ningxia. A few years ago, because of the sense of existence of the community youth league organization is not strong, the initiative is not enough, in the largest migrant relocation community in Lingwu City, migrant children have no place to go after school, no one is not tutoring questions. Since the transition of the Youth League organization in June 2021, the new team has taken the initiative to undertake the community service demonstration project of poverty alleviation relocation relocated by the Ningxia District Committee, and opened the "Red Scarf Growth Classroom", creating a platform for migrant children to grow up after class."Now, children can go here after school to do homework and play games. There are also volunteers to provide volunteer services such as schoolwork counseling, interest training, psychological counseling and family care, which effectively solve the worries and troubles of migrant families."Said Zhang Hongxia, the newly elected community youth league branch secretary. Cadres are the key to doing a good job. In the view of Lv Huibing, party secretary of the Youth League Lingwu, " matching the strong village (community) league general branch secretary, is the primary task to further enhance the vitality of the village (community) league organization."In 2021, Lingwu city to the village (community) party organization change as an opportunity to promote the city's 85 village (community) youth league branch change. Among the newly elected league branch team members, 85 are branch secretaries, with an average age of 32,4 years younger than before the transition. Except for the league branch secretary, the rest of the committee member is 277 members. After the change, the village (community) youth league branch, the village (community) two committees and youth league branch members 105,77 party members, 176 women, rich leaders 7,10 veterans, effectively broaden the coverage of youth league cadres."Grass-roots work is numerous, the league cadres often hold multiple positions, coupled with the frequent change of grass-roots staff, youth work no one to do, the league cadres are powerless phenomenon is more common."In the grass-roots work for many years, Yongning County Minning Town Communist Youth League Committee Secretary Chen � S to the grass-roots youth league cadres confused to see in the eye, urgent in the heart. After the reform of the grassroots organizations of the county Communist Youth League was started, Yongning County issued the "Yongning County to promote the reform of the county Communist Youth League grassroots organization pilot work implementation plan", Innovation put forward the "1 + 2 + X" youth league cadre allocation model, That is, a "backbone", Township (street) Party committee members are selected as the secretary of the Youth League Committee, To be responsible for planning and guiding the work of the regional Communist Youth League and young people; 2 "Auxiliary Hands", Select a part-time deputy secretary from the young cadres in the township (street), Select a full-time deputy secretary among the western Plan volunteers; Multiple "main forces", Select a number of outstanding members in the district, In the organizational system, "weave dense" the working force."The first one of the guidelines on the reform of the Communist Youth League at the county level is the 'selection mechanism of the working force of the reform'. Whether the cadre system of the Youth League is perfect and whether the team is strong and powerful directly determines the effectiveness of the reform."Said Zhang Hui, Party secretary of Tuan Yongning County. Since last year, Yongning County through the county, township (street), village (community) three-level youth league cadre team reform and construction, up to now, the county has a total of full-time youth league cadres 561 people, temporary youth league cadres 1 people, part-time youth league cadres 111 people. Seize the opportunity of reform, activate the youth work "a pool of spring water" Wuzhong city li tong district is the hometown of the national outstanding communist party member wang orchid, in recent years, group li tong district party committee make full use of the county communist youth league grass-roots organization reform pilot opportunity, vigorously inheritance "close to me, warm you" orchid volunteer service spirit, lead the youth in the social classroom education, long ability, contribution. Litong District Youth League has vigorously implemented the "practical education" project, organized the "Little Orchid Action". At present, 46 primary and secondary schools in the district have set up "little orchid" volunteer service teams, 16,000 young people become "little orchid" volunteers, often carry out love activities. In addition, relying on the Litong District Youth Volunteers Association, it has explored and implemented the working mode of the "Communist Youth League + social organizations + youth volunteers", further leading the majority of young people to be the "little orchids" in the new era."Up to now, we have issued 54 recruitment orders for volunteers in community governance, epidemic prevention and control, civilized city building, Mother River Protection, and rural revitalization, with more than 7,400 young volunteers registered for a total of 413,000 hours, serving social governance at the community level with practical actions."Said Jia Peng, president of the Litong District Youth Volunteers Association. In recent years, the creation of a national civilized city, protect the mother river ecology, care for the elderly, help orphans and disabled children... where there is a need, there are "little orchid" volunteers."They are in the charge, fearless, and strive to be the inheritors, practitioners and leaders of orchid volunteer service in the new era."Litong District Jinxing Town Golden Garden Community Party Secretary Zhang Liping said. In Jinfeng District, Yinchuan City, The Shanghai Lvyang Village Restaurant is a non-public organization with more than 80% of young employees. Three years ago, with the establishment of the youth league organization, the working atmosphere of the restaurant is getting better and better, and the employees are more motivated."After the establishment of the youth League branch, the restaurant jointly carried out various activities, which not only enriched the spare time life of the employees, but also stimulated their enthusiasm. The enthusiasm and initiative of the enterprise employees in working hard and serving the business circle have been greatly improved."The green Yang village restaurant league branch secretary Tang Liang said. In recent years, jinfeng area based on Yinchuan metropolitan area core advantage, constantly broaden the grassroots youth league organizations in the jurisdiction of three major business district coverage, has promoted business circle youth league, league, guide business circle party members, members of merchants through the way of "1 + N", forming Ningxia green Yang village restaurant youth league branch, read city business circle apartment joint 11 non-public youth league branch. The construction of youth league organization is fully rolled out, and the key is to normalize the theoretical armament and learning education of youth members in the business circle. Jinfeng district innovation to carry out the "youth heart to the party sand painting DangEn" ground large sand painting activities, "communist youth league and enterprise youth face to face" communication activities and "Yang youth youth" celebrate the 100th anniversary of the 14th youth day flash activities, the audience of more than 30000 people, form "youth" + youth "group building + dating", "group building + volunteer" "group building +" new model, committed to promote business circle group further coverage. Strengthen the top-level design, with "real" measures to "lead" in the key place to promote the reform of the county Communist Youth League, policy support is the guarantee. To strengthen the party leading, compaction grassroots party building group responsibility, in August 2021, the Ningxia district party committee revised the draft on strengthening and improving the new era party building work, condensed youth in the zone construction opinions (ning DangBan hair [2021,68), issued by the autonomous region party committee general office, for the youth league innovation at all levels, work release policy dividends. The reform of the Communist Youth League is not a matter of the Communist Youth League, but needs to be coordinated by relevant departments. To promote the national county communist youth league organization reform pilot task, according to the party building work requirements, the Ningxia district party committee coordination county into the communist youth league reform key reform task list, and jointly with the autonomous region party committee reform office held two projects, inform the national pilot task propulsion, listen to district, county party and government in charge of the leadership progress report, targeted to arrange the deployment of the next stage of key work. To promote reform measures to the ground work, Ningxia also set up by the Ningxia district party committee overall responsibility, pilot city, county two levels of youth corps committee integrated grasp the implementation, pilot county (city, area) party committee specific implementation of special working group, strengthen the reform of the county level pilot research, guidance, promote and implement, regular pilot area stagnation point research, in a line guidance to solve the problem. At the same time, the establishment of a quarterly notification, monthly supervision system, timely grasp the progress, refine the experience, interview and supervise the counties (districts) with lagging progress, effectively promote the youth league organizations at all levels to play their subjective initiative, and earnestly promote the implementation of the reform tasks. China Youth Daily China Youth Network reporter Ma Fuchun source: China Youth Daily

2022-05-12 Hubei "one hundred pursuit" theme music class premiere in Hubei "one hundred pursuit" theme music class in han premiere green network reporter LeiYu) on the evening of May 10, by the communist youth league of Hubei provincial party committee, provincial youth federation, provincial student federation, provincial less working committee, Wuhan rural commercial bank of "one hundred pursuit" theme music class premiere in Wuhan. Member of the Standing Committee of the CPC Hubei Provincial Committee, ahong, Minister of the United Front Work Committee of the Hubei Provincial Committee, Liang Weinian, deputy director of the Standing Committee of the Provincial People's Congress, Vice Governor Ning Yong, Vice Chairman of the Provincial CPPCC Zhou Xianwang attended the meeting. Music lesson in the "internationale", Hubei province "green horse project" students and the young pioneers open "journey", with "the newborn" "follow" "sheer" struggle "" song "five chapters," by "situational deduce + artistic" "across time and space dialogue" review Hubei communist youth league under the leadership of the party unity lead the youth brave mission, overcome the magnificent history of progress. Young representatives from all walks of life in the province gathered together, in this "immersive" musical epic, to pursue the original mission of the Communist Youth League unswervingly following the Party, striving for the Party and the people, deeply understand the source of vitality of the Communist Youth League, draw on its wisdom and strength, determined to strive to be the first, face the future, and make new achievements. It is understood that the Hubei provincial party committee will simultaneously launch the "one hundred" theme music tour tour, the class to the youth, leading teenagers to study and implement xi general secretary in celebrating the 100th anniversary of the founding of the communist youth league of China's important speech spirit, take practical action to meet the party's 20th victory.

2022-05-12 Inner Mongolia youth general secretary xi jinping important speech Inner Mongolia youth general secretary xi jinping important speech Shi Jia) on May 10,10, to celebrate the 100th anniversary of the founding of the communist youth league congress held, the CPC Central Committee general secretary, state President, central military commission President xi jinping attended the conference and delivered an important speech. General Secretary Xi Jinping's important speech has aroused warm response and heated discussion among the youth League members of all ethnic groups and all walks of life in the Inner Mongolia Autonomous Region. Chen Xiaodong, secretary of the Inner Mongolia District Committee of the Communist Youth League, said that Xi Jinping's important speech guided the course and inspires people, pointing out the direction for the growth of young people and providing fundamental guidance for the work of the Communist Youth League. District youth league organizations at all levels to improve the political stance, carry forward the fine tradition, earnestly the thought and action unity to xi general secretary of the important speech spirit, firmly support "two established", strengthen the consciousness of "four", "four confidence", "do" two maintenance, li strenuous, yong yi, the communist youth league construction more dynamic, more strong, with work reveal the party call, the action of loyalty and bear. Hohhot white tower airport customs youth league branch secretary Zhang Shiyu said, as the epidemic prevention and control line of customs youth, will be based on the customs post, actively into "I do the practical work for the masses", in the epidemic prevention and control, optimize the business environment, build abroad defence as, earnestly the political bear reflected in the role and check, serve the people in the actual work. Urige is a Courier in Alatan EMomo Town, New Barhu Right Banner, Hulunbuir City, Inner Mongolia, and is also the winner of the Inner Mongolia Youth May 4th Medal. He said, as a youth volunteer, will actively respond to the call of general secretary xi jinping, dare to call the sun in the new day " morale, overcome difficult, challenge, to our youth, move forward with the country, in the realization of the great rejuvenation of the Chinese nation torrent, yong forward, on the track of the youth run out of the contemporary youth's best grades. Teng Rulei, driver of the Communist Youth League locomotive locomotive of China Railway Hohhot Bureau Group Co., LTD., said, " General Secretary Xi Jinping's important speech touched me deeply. Our " Communist Youth League'locomotive group has been passed down from generation to generation, the first generation of drivers far away from home, came to the harsh natural environment of western Inner Mongolia to contribute their youth, sweat, struggle and entrepreneurship, witnessing the grassland railway from scratch. Today, our new generation of'Communist Youth League " locomotive group youth, will surely live up to the great trust of the Party and the people, shoulder the responsibility of transportation power and railway first, and win a greater victory and glory on the new era and new journey."Gao Lei, a senior welder technician at the Steel Structure Engineering Technology branch of China Second Metallurgical Group, said," As a winner of the Inner Mongolia May 4th Youth Medal, I will cherish this honor and thank the Party and the country for their attention and cultivation to the young generation. Youth by hone and brilliant, life because of struggle and sublimation. As a young skilled worker in the new era, in the future work, I will continue to do not forget the original aspiration, study hard on welding technology, and strive to improve their operation technology level, with exquisite skills and craftsmanship quality to show the new era of young skilled workers, with practical actions to meet the twentieth victory of the Party.”

2022-05-11 Guangdong member youth debate xi jinping general secretary of the important speech Guangdong youth hot xi jinping general secretary of the important speech in the youth network reporter Lin Jie) on May 10, to celebrate the 100th anniversary of the founding of the communist youth league of China congress was held, the CPC Central Committee general secretary, state President and central military commission chairman xi jinping attended the conference and delivered an important speech. The Guangdong Provincial Party Committee of the Communist Youth League organized the youth league members of the whole province to study the spirit of the speech together, and they launched a heated discussion combined with what they had learned, saying that they would "stir up the surging spring tide of national rejuvenation with the power and creativity of youth". South China University of Technology teachers and students watch the scene group a big square volunteer station master Gan Yongle said, " young people should contribute to the society!"He has been standing guard in the big memorial square of the regiment for 10 years, and saw thousands of aspiring young people full of vigor and vitality, dedicated to the party and ambition."We must bear in mind the inculcation of General Secretary Xi Jinping: be down-to-earth, realistic and pragmatic, endure hardship first and enjoy hardship later, and be willing to be a screw that will never rust."Gao Dawei, party secretary of the Shenzhen Communist Youth League, said that on the new journey, the Communist Youth League should spread its leading, organizational and service power to the grass-roots level and around the young people in Shenzhen. Do everything possible to do practical things for the youth, solve difficulties, pass the Party's care for the youth to the Shenzhen youth with practical actions, enhance the leadership and cohesion of the league through real service, let the majority of young people sincerely listen to the Party, follow the Party, and constantly consolidate and expand the young mass foundation of the Party's governance. We will work hard to promote the development of a youth-development city, a city for entrepreneurship and innovation in Shenzhen and Hong Kong, and a volunteer city. We will improve the "100" projects for young people's livelihood, and let young people judge the effect of these "100" projects for people's livelihood. Liu Jiaxin, executive chairman of the Guangdong Provincial Students 'Federation, said, "The hope of the Party and the country rests on the young people", which is a great encouragement to our young students. I have more deeply felt the general Secretary's cordial care and ardent expectations for our young generation. As a bridge and link between the majority of students, we should always adhere to from the students, to the students, solid "I do practical things for the students", take the initiative to solve the students "urgent difficult worry hope" problem, let the warmth of the party to the hearts of the students, help the students in the youth track to run out of the best results of contemporary youth. Jiang Zunguo, secretary of the Youth League Committee of Foshan University of Science and Technology, said that as cadres of universities, we should strive to be the party's loyal assistant and reliable reserve army, and constantly improve the "leading force", "organization force", "service force" and "overall contribution degree" of grassroots youth league organizations. Be a good "close friend" who can be trusted, reliable and inseparable from young people, combine "influence" with "affinity", and guide them to polish their youth background through dedication, and cultivate a sense of social responsibility in practice. League Guangzhou municipal party committee secretary He Lulu said, on the new era of new journey, the Guangzhou communist youth league will earnestly lead condensed youth, mobilize youth, contact service duties, do our best for the party training, forging when the era of national rejuvenation, unity lead the youth firmly follow the party, contributing to the new era. We will further promote exchanges and integration among young people in the Guangdong-Hong Kong-Macao Greater Bay Area, help them better solve their worries about education, employment, marriage, and child education, and further improve the environment for their growth, so that they can feel the care and warmth of the Party. Zhang Biwan, deputy director of the First Tax Office of Guangzhou Tianhe District Taxation Bureau of the State Administration of Taxation, said that as a leader leading 400 people to stick to the "National Youth Civilization" line on the front line of tax service, her team is a young pioneer who can bear hardships, fight hard and innovate."We will continue to lead the young people roots, let the youth shine in responsibility, the 'service for the people' to every detail, to ensure that the new combined tax support policy in the front line; we will continue to encourage youth unyielding, through the" youth school "and other channels, in the" youth learning " growth."Guangzhou youth volunteer association, deputy secretary general, Guangzhou association of volunteer service Liang Xiufei said, as a member of Guangdong province youth branch, will continue to tell the story of the new era, lei feng, the volunteer spirit and the party's ideological content depth fusion, through the form of preaching, condensed youth, called for youth, to join the team of volunteers, where the motherland needs, the people need place, dedicated youth strength, be a good time answer. Guangdong university of finance and economics held learning important speech symposium southern power grid Heyuan power grid co., LTD Heyuan power supply bureau youth corps committee secretary Zeng Rui said, as a cadre, will keep in mind the mission, strengthen the responsibility, unity led power supply member youth always follow the party, actively implement the new development concept, based on the new journey, power new power system and modern power supply service system construction, speed up the innovation drive and digital transformation, to build a happy harmonious beautiful Heyuan to provide strong power support. Guangzhou gold domain medical inspection center co., LTD., Guangdong province, secretary of the youth corps committee, said we use youth blood practice the responsibility of the younger generation, we use practical action to practice the "please rest assured, power have me" youth oath, always put himself and power dream China dream closely, always fulfill the responsibility of medical inspection mission closely combined. In the future, we will more firmly inherit and carry forward the spirit of "fighting, fighting and good at fighting", feel the Party, listen to and follow the Party, and strive to write a greater chapter of a healthy Chinese dream with our youthful passion. Peng Jiajia of Tantou Primary School in Nantang Town, Lufeng, Shanwei City, said that as a volunteer teacher of the Mountain Plan, we should play the role as a volunteer teacher to promote rural revitalization through education. Never forget to study hard to improve their ability cultivation, learn excellent examples, and develop targeted volunteer teaching plans based on their own major and school weak areas; guide students to establish lofty ideals and realize personal and social values; and do our best to link all resources to promote the implementation of various public welfare projects in rural schools. Wu Jiaxi, an assistant librarian of the memorial hall at the former site of the Chinese Comrade MAO Zedong hosted the Peasant Movement Institute, said, " Hot youth needs firm ideals and beliefs. From 1921 to 1949, the number of 3.7 million famous martyrs listed in the revolution led by the Chinese Communist Party has reached China. Many of the martyrs died in their 20s, practicing their ideals and beliefs with their lives. At the age of 25, I am a young narrator. My ideal is to let more people remember the stories of these heroes who seek happiness for the Chinese people and seek rejuvenation of the Chinese nation."Shen Zhigang, a lecturer at the School of Marxism at South China Normal University, said that as a contemporary youth, especially as a young scholar studying the history of the Communist Youth League and youth movement, I am deeply encouraged. In the following work, I will further explore the history of the league, deepen the research of the history of the Guangzhou youth movement, and dig out more red materials for the youth ideological and political education of the Party and the League. Youth federation of Guangdong province, Macao youth volunteers association director pei said, I will adhere to promote youth participation in volunteer service, make volunteer service become a large bay area of Guangdong and hengqin Guangdong Macao depth cooperation area youth communication and growth bridge, ji combined with Guangdong Macao C "i volunteer" system, actively promote the bay area volunteer service exchanges and cooperation. With a sense of mission to my original aspiration, I will call and unite them, cultivate them to become a solid patriotic and Macao lover, and make patriotic volunteer service become the common value of the young people in the Bay Area and even the whole country. Yang Cui'er, the executive committee and vice president of the Hong Kong Shekou Association, said that no matter how The Times change, every young man has a sense of love and patriotism and the spirit of being passed on from generation to generation. I was born and raised in Hong Kong, and participated in the mainland leader exchange activity as a university representative. Now, I have developed my own business projects and displayed my volunteer red in the Bay Area, and become the organizer of the Shenzhen-Hong Kong youth exchange platform. From "participant" to "promoter", I hope to continue to influence more young friends to show the vitality of the youth in the Bay Area, and become the "inheritors" of the power to love the country and Hong Kong.

2022-05-11 The spirit of General Secretary Xi Jinping's important speech at the celebration of the 100th anniversary of the founding of the Communist Youth League of China. The special study meeting of the Xi Central Group thoroughly studied and implemented the spirit of General Secretary Xi Jinping's important speech at the celebration of the 100th anniversary of the founding of the Communist Youth League of China. Yang Zheng, secretary of the Provincial Communist Youth League Committee, presided over the meeting and made a speech. Members of the theoretical learning center group attended the meeting, and all members of the leading group and relevant responsible comrades of departments and directly affiliated units made exchanges and discussions. Meeting that xi jinping, general secretary in celebrating the 100th anniversary of the founding of the communist youth league of China's important speech spirit for the new era of the communist youth league for the future, made new provides the fundamental follow and action guide, on the new journey to promote the communist youth league high quality provides forward power and method path, to lead the province member youth firm ideal faith, strong spiritual accomplishment, more confident, consciously listen to the party, follow the party, has great and far-reaching significance. Conference pointed out that xi jinping, the general secretary in the important speech for the communist youth league "always become a political school, pioneer power, Bridges, advanced organization" four hope "," asked youth be "five model", the cadres "four requirements", comprehensive, three-dimensional for our communist youth league organization describes a new blueprint, pointed out the direction, for the youth issued a great struggle youth, more for our cadres in one hundred journey sounded the horn. The meeting stressed that the provincial communist youth league organization to study publicity and implement xi general secretary in celebrating the 100th anniversary of the founding of the communist youth league of China's important speech spirit, as the current and future a period of Anhui communist youth league work primary political task, careful planning, careful organization, careful arrangement, quickly learning publicity to upsurge. It is necessary to make full use of the propaganda carrier position of the league, innovate the forms and means of publicity, comprehensively and deeply publicize and interpret the spirit of General Secretary Xi Jinping's important speech, carry out learning and publicity activities with their own characteristics and various forms, and create a strong atmosphere for learning, publicity and implementation. To study propaganda and implement xi general secretary in celebrating the 100th anniversary of the founding of the communist youth league of China's important speech spirit with the communist youth league each work tasks, to "celebrate 20, always follow the party, new journey" theme education practice is suggested, further implement the provincial communist youth league "3520 + action plan", li strenuous, yong yi, strengthening leading force, organization, service, celebrate the party's 20 victory with honors.

2022-05-10 Anhui Communist Youth League, Anhui Province, launched a variety of "cloud activities" Wang Haihan Wang Lei), recently, in Anhui, a attracted 1.237 million views, 130,000 likes live activities circle countless fans. This is the first stop of the "My Youth in My Hometown" live broadcast of the youth ideological and political class carried out by the Anhui Provincial Youth League and the Publicity Department of the Provincial Party Committee. In Anhui communist youth league WeChat video and Internet live platform today, along with the host and local entrepreneurial youth, tourist reception center and village history, circle farm, hand with store, entrepreneurs, transforming customs pavilion of more than ten scenes, in turn, presents rural farming, catering accommodation, studies education, electricity, wen and experience in the integration of the industrial ecological pattern. In recent days, affected by the epidemic, many offline activities have not been carried out as scheduled. Anhui Provincial Party Committee of the Communist Youth League led the youth league organizations at all levels in the province to change their thinking, with the theme of "Welcome the 20th Party, Always follow the Party, forge ahead on a new journey", and carried out various forms of ideological and political leading activities for young people by relying on the new media platform of the Communist Youth League. According to the staff of the Publicity Department of Anhui Provincial Party Committee, the first stop of the "My Youth in Hometown" live broadcast activity was selected in Maying Village, Changfeng County, Hefei City, in order to show the group image of youth struggle to help rural revitalization and enrich rural formats. In 2015, the local government launched the "Ma Ying Plan", with the mission of "building Bridges between cities and villages" for entrepreneurial youth, and the three sub-plans of "helping students", "helping farmers" and "helping the villages" as the starting point to help Ma Ying Village to transform from a backward situation with no industry to an innovative model of rural pastoral complex."Netizens in front of the camera, please guess, Ma Ying has a horse? In fact, where we are now once had no mountains, no rivers, no rivers, no industries. Can't you believe it?"" However, all this has changed dramatically because of a project called the Ma Ying Project and a group of loving volunteers."During the one-hour live broadcast, the anchor also introduced the efforts and efforts of young entrepreneurs and volunteers for rural revitalization. Netizens brush screen countless, questions and praise coexist. In addition to the intuitive and vivid live broadcast activities, the Communist Youth League Anhui Provincial Party Committee has also planned a series of new media activities."The league flag symbolizes the faith of the youth league members, and a group photo is a period of youth!"Recently, a young police officer in Hefei" posted "their feelings of participating in the" group photo ". The "group photo" he mentioned was the "I took a group photo with the youth league flag" activity of the Anhui Provincial Communist Youth League. The activity is open to the public. Young people from all walks of life stand in front of the youth flag and place a young and beautiful POSE. Young painters will make group photos into cartoon form through wonderful writing, which will be displayed on the official microblog of Anhui Communist Youth League."Creating low threshold and high quality activities makes it easier to stimulate the sense of honor and belonging of youth members, and realize ideological guidance."The above staff members introduced. In addition, the Anhui Youth League WeChat public account and B station, "league knowledge hundred answers" online answer, live broadcast, "league history cartoon words one hundred years", the organization department of the provincial Party Committee and the provincial student union in the provincial youth party (league) extensive red information collection activities, through video, pictures and other forms of works to show the youth style. In recent days, many youth league cadres and young workers in Anhui province have forwarded the original micro video titled "Youth to the Party" in their circle of friends. In this video, with more than 3.5 million views, the precious video materials in the history of youth groups in Anhui province are presented one by one, which echoes the struggle scenes of Anhui youth in various fields in the new era. The new era of college students, public security, medical care, transportation industry youth issued a character monologue and youth cry in different scenes, highlighting the image of contemporary youth forging ahead... this is the scene in the original video "How You Look" produced by the Anhui Communist Youth League. The film online platform, offline subway station large screen and other scenes repeatedly broadcast. It is the work goal of Anhui Provincial Party Committee of the Communist Youth League to closely follow their online reading and movie-watching habits and produce more video online cultural products with appearance level, connotation and thinking. Since May, the Anhui Provincial Committee of the Communist Youth League has produced dozens of youth positive energy video cultural products, MV, micro video "East is Red" and "Loud Youth Blossom" for 16 million times. More than 40 universities are organized to respond to the "one hundred years" activity initiated by the Central Committee of the Communist Youth League and the National Federation, and the video has been played 1.6 million times. At the same time, the provincial party committee to carry out the "five" one hundred cultural products show activities, through the "cartoon treasure" weibo topic interaction, "answer chapter" group knowledge ask a H5, "comic group history" hand-painted history in one hundred, "one hundred school relay" red song relay MV, "product in one hundred" media matrix linkage, guide Anhui youth wholeheartedly to the party, in the struggle. In the future, Anhui Provincial Committee of the Communist Youth League will set up Anhui Province youth learning and education platform of "Youth Welcome 20 Striving to the New Era", invite members of the youth teaching group to record a series of audio and video, and create an immersive cloud lecture hall. For a long time, Anhui Communist Youth League has paid attention to the new media matrix building work of the youth league organizations at all levels and in various fields in the province, and guided the youth league organizations in all cities, counties and districts to build online ideological leading platforms. During the May 4th Movement period, the influence of the matrix was given full play. According to statistics, the Communist Youth League organizations at all levels in Anhui released nearly one hundred posters on Weibo, including hand painting, printmaking, and more than 3 million views; Hefei, Huaibei, Maanshan used the booths of "Youth Heart to the New Era"; Tongling Municipal Party Committee produced MV; Bozhou Municipal Party Committee and Qiao District Committee plan to collect 100 youth punching points online to provide urban youth travel, sports, parent-child functions. Twenty-eight universities in Anhui province jointly recorded the video "Heartbeat Spectrum", which was watched by more than 1.6 million people on Station B. The Youth League Committee of Anhui Medical University has opened the "AHMU Sports and Health Air Lecture Hall", covering singing, dancing, folk music, Taijiquan, photography, medical care and other contents. Anhui Provincial Youth League Committee requirements, Anhui Youth League organizations at all levels to young people and children's growth characteristics and acceptance habits, targeted creation and production of excellent cultural products, provincial will select high-quality products in the form of network green evening broadcast. League of Anhui provincial party committee secretary Yang zheng said, Anhui communist youth league will continue to deepen the "welcome twenty big, always follow the party, forge ahead new journey" theme education practice, with teenagers favorite language, teenagers around vivid examples, teenagers favorite fashion elements, strengthen the theme of education practice appeal, influence and effectiveness.

2022-05-08 Tian Hongwei, reporter of Zhongqing Network) Today, the "Digital youth gather in Fuzhou" face-to-face activity for young entrepreneurs and young science and technology talents was held. More than 80 young scientists, entrepreneurs, entrepreneurs and student representatives in the field of digital economy in Fujian Province attended the event. The event is co-sponsored by the Fujian Provincial Committee of the Communist Youth League and the Fujian Provincial Association for Science and Technology. The organizer organized the young representatives to visit Fujian Hengshen Holding Group Co., LTD., Digital Fujian Cloud Computing Center, Fujian Provincial Government Affairs Cloud Platform, and Binhai New City Planning Exhibition Hall, and held a symposium on the innovation and development of digital economy. The young participants had in-depth exchanges. At the symposium, Shi Bin, vice chairman of Fujian Association for Science and Technology, encouraged young scientific and technological talents to practice the spirit of scientists in the new era, have the courage to deeply cultivate interdisciplinary fields, and strive to be the pioneers of technological innovation. Li Teng, deputy secretary of Fujian Provincial Committee of the Communist Youth League, spoke to young entrepreneurs and young scientific and technological talents to seize the opportunities of The Times and make new contributions to the construction of digital Fujian. Huang Senkun, member of the National Youth Federation and executive president of Fujian Young Entrepreneurs Association, introduced the development status of digital economy in Fujian Province at the symposium. Huang senkun hopes that young people should first realize the importance of digital economy and see the development prospects of digital economy. With the theme of "The Future, The Decade of New Hardware", Li Zhiqin introduced the past and future of chip development, the development of the industrial chain of integrated circuits, and the global IC current situation facing the four major challenges of COVID-19, geopolitical conflicts, international situation and natural disasters.

2022-05-08 Tianjin: welcome 20 childlike dream painting and calligraphy art curtain "welcome 20 forever follow the party new journey" childlike dream painting and calligraphy art curtain Hu Chunyan) recently, in Tianjin, under the guidance of the committee of Tianjin (Tianjin children's palace) joint Beijing children's palace, Shanghai youth activity center, Chongqing children's palace, Guangzhou children's palace and other units jointly organized "welcome 20 forever new journey" childlike dream painting and calligraphy art exhibition opened in Tianjin art museum, and will continue until the 15th of this month. The exhibition aims to promote the implementation of xi new era the ideas of socialism with Chinese characteristics, focusing on the party's cause successor someone the fundamental plan, from the planning to the implementation are firmly grasp the communist youth league central in the "welcome 20, always follow the party, new journey" theme education practice deployment requirements, further publicity xi general secretary of care for children, lead the children study party led the Chinese youth movement, unity leading children firm always follow the party, a new era of determination. This exhibition displays nearly 300 selected works from everywhere. During the work collection period, teachers and students overcame the impact and difficulties of the epidemic, and adopted a combination of online teaching and offline individual tutoring, aiming to broaden their ideas, exchange techniques and complete the works. Through the presentation of art works, we will review the history of the Party and the history of youth sports again, constantly integrate red elements in the education and teaching activities, and take artistic creation as a way to carry out ideological and political education for children.

2022-05-06 Sichuan: youth May 4th medal ceremony held in Chengdu, Sichuan: "one hundred struggle road youth heart to the party" art performance and youth May 4th medal ceremony held in Chengdu network Beijing on May 6 (reporter Zhang Jianwei, correspondent qing-hua zhao) on May 5, "one hundred struggle road youth heart to the party" art performance and youth May 4th medal ceremony held in Sichuan radio and television station. The art performance takes the "awakening years", "burning years", "spring tide agitation" and "the road of revival" as the main line. The art reproduces the glorious course of the Chinese youth and the struggle of the Party. Yu Lijun, member of the Standing Committee of the CPC Sichuan Provincial Committee and head of the Organization Department, Wang Fei, Deputy Director of the Standing Committee of the Sichuan Provincial People's Congress, Yang Xingping, Vice Governor of the Sichuan Provincial People's Government, and Zhao Zhenmian, Vice Chairman of the Sichuan Provincial People's Political Consultative Conference attended the event. Pictures of the event site. The Communist Youth League Sichuan Provincial Party Committee opened the picture performance in the magnificent and exciting music sound of "Towards the Renaissance", Scene performance "graduation song", poetry recitation "a hundred years of youth" shows the new democratic revolution period of the Chinese youth to strive for national independence in the revolution of the lofty aspirations; Human screen interaction "that year that rabbit those things", songs string burning "The Times of youth force" shows the socialist revolution and the construction period of the Chinese youth hard work, dedication to the motherland spirit; programs such as "Never Say Goodbye" and "Panda Man" tell the stories of Chinese youth who have devoted themselves to socialist modernization and contributed to the society since the reform and opening up. Programs such as "A Dream on the Clouds" and "Grass" show the touching stories of Chinese youth in the new era serving the overall situation, the society and the fight against the epidemic. During the period, the provincial leaders awarded the 26th "Chinese Youth May 4th Medal" Sichuan winning collective and individual awards, and the 25th "Sichuan Youth May 4th Medal" winning collective and individual representatives held a centralized display. There are also 120 youth league organizations in the province that won the title of May Fourth Red Flag Youth League Committee and Youth League Branch of Sichuan Province, and 270 comrades won the title of Excellent Communist Youth League Member and Cadre of Sichuan Province. Pictures of the event site. On the night of the event, the Sichuan Communist Youth League Committee broadcast live through Xinhua Cloud, Sichuan Observation APP, as well as the official Weibo and wechat platforms of the Sichuan Communist Youth League. According to the relevant person in charge of youth league provincial party committee, the activity is the Sichuan communist youth league in accordance with the unified deployment, to carry out "welcome 20, always follow the party, new journey" theme important carrier of education practice, the purpose is to inspire the province youth don't forget to beginner's mind, confident new journey, new era, take concrete actions to meet the party's 20 and provincial twelfth congress victory.

2022-05-06 In 2022 the commendation ceremony held in 2022 Fujian province commendation ceremony held wei) on May 5 afternoon, Fujian provincial party committee in 2022 held the awards ceremony, give full play to the youth exemplary role, motivate teenagers li strenuous, yong yi forward, to comprehensively promote the development of high quality beyond the contribution of youth strength. At the ceremony, the representatives who won the national May 4th Red Flag Youth League Committee (Youth League branch), National Excellent Communist Youth League Member, National Excellent Communist Youth League Cadre, Fujian Youth May 4th Medal, Fujian May 4th Red Flag Youth League Committee (Youth League branch), Fujian Province Excellent Communist Youth League Member, Fujian Province Excellent Communist Youth League Cadre title were awarded."National May 4th red flag youth corps committee" representative-Fujian Fuzhou bodong photoelectric technology co., LTD., from "Fujian youth May 4th medal pacesetter" representative-yongtai town Longxiang village branch secretary Lin Lulu, "Fujian youth May 4th medal pacesetter" representative-Zhangzhou second hospital infection ward head nurse Gao Yibin, "Fujian red flag group (total) branch" representative-Xiamen university graduate education pioneer league branch secretary Lin Yingjing respectively came to share. More than 40 people attended the award ceremony, including the members of the provincial Party Committee of the Communist Youth League, the national "Two Red and the Two Excellent", "the" Fujian Youth Medal "and the" Two Red and the "Fujian Excellent" representatives.

2022-05-06 On the afternoon of May 4th, the 2022 "Hubei Youth May 4th Medal" award activity was held in the Hubei Provincial Party Committee of the Communist Youth League. Event, deputy secretary of Hubei provincial party committee wang read out the central, the communist youth league about awarded the 26th Chinese youth May 4th medal decision "the provincial human resources and social security hall, the communist youth league Hubei provincial party committee, provincial youth federation about awarded 2022" Hubei youth may fourth medal "decision", the Hubei provincial party committee leadership members for the 26th "China youth May 4th medal" winner and 2022 "Hubei youth may 4th medal" individual, collective awards."Each era has the historical task of each era, and each generation of young people has the historical responsibility."Zhou Senfeng, secretary of the Hubei Provincial Party Committee, sent the delegation to the provincial Party Committee to congratulate the honored youth. He said that the "Youth May 4th Medal" is the highest honor awarded by the Communist Youth League and the Youth Federation. The typical youth who grew up in the new era lead the lead in all fields and fronts, and practice the youth oath of "please rest assured of the Party, a strong country has me" with practical actions. Hope that the honored youth remember the instructions of General Secretary Xi Jinping, vigorously promote the spirit of the May 4th, bear the historical responsibility, let the spirit of the May 4th emit more dazzling light; strive to achieve "virtue" double integrity, cultivation and integrity, integrity, and focus; play a good demonstration role, run in the youth track, to promote the high-quality development of Hubei youth positive energy. League Hubei provincial party committee, Hubei youth federation called on the provincial communist youth league, youth league organization to xi jinping new era the ideas of socialism with Chinese characteristics as guidance, unity lead the member youth to advanced model, always follow the party, new journey, in order to speed up the "built fulcrum, walk in the forefront, compose Marty natalegawa" to make new greater contributions, to meet the party's 20 victory with honors!

2022-05-06 The Inner Mongolia district party committee held special theme TuanRi activities "youth heart to the new era" Inner Mongolia theme TuanRi activities in the youth network held in the popular newspaper reporter Shi Jia) to celebrate the 100th anniversary of the founding of the communist youth league, inspire the new era of Inner Mongolia youth forever follow the faith confidence, on May 5, the Inner Mongolia district party committee hosted "youth heart to the new era" the Inner Mongolia autonomous region special theme TuanRi activities held in Hohhot. Chen Xiaodong, secretary of the Inner Mongolia District Party Committee, delivered a speech, requiring the regional Communist Youth League to earnestly study and implement the rich connotation and practical requirements of General Secretary Xi Jinping's important speech spirit during his visit to Renmin University of China, effectively improve the ideological understanding, strengthen the learning consciousness, and constantly enhance the sense of responsibility and mission to do a good job in youth work for the Party. The Communist Youth League organizations at all levels and the youth league members in the region are required to be loyal to, support, maintain, and closely follow the core, firmly support the "two establishment", firmly achieve the "two maintenance", take over the baton of history, show their youth responsibility, and make unremitting efforts to build a bright Inner Mongolia and realize the great Chinese dream. The Inner Mongolia youth representatives sang the song "Pilot". The pictures were provided by the interviewees. Three young representatives from Inner Mongolia from different fields shared their stories of struggle, showing the spirit of Chinese youth in the new era of "time is quiet without losing struggle". The scene also held a centralized ceremony to join the league, the old league member representatives for the new league member representatives issued the league seal and league member card, wearing the league emblem, the new and old league members jointly facing the league flag solemn oath. The activity also commended the representatives of the Inner Mongolia Youth May 4th Medal collective and individual winners, and the national and Inner Mongolia "two Red and two Excellent" winners.

2022-05-05 � In 2022, the 20th Congress of the Party will be held, and the Chinese Communist Youth League also marks the 100th anniversary of its founding. Today, the Party History Research Office of the CPC Shanghai Municipal Committee, the Shanghai Municipal Committee of the Communist Youth League, Shanghai University, the Youth Newspaper, and the Memorial Hall of the former site of the Central organs of the Chinese Socialist Youth League jointly released the "100 Keywords of Chinese Youth", which together draw the most beautiful youth map of Chinese youth. On December 31 last year, the above-mentioned units jointly launched a collection campaign of "100 Key words for Centennial Chinese Youth", aiming to review the history of the youth movement under the leadership of the Party and understand the Chinese youth of the Party through 100 key words. After the solicitation order was issued, it received a warm response from young people from all walks of life. Of these 100 key words officially released today, Both the May 4th Spirit, awakening, the pursuit of truth, patriotism, new youth, Yuyang Li and other youth initiatives, There are also flash youth, public welfare positive energy, burning, hardcore, China-fashion, slash youth, thumb up, very sa, have light and other contemporary youth atlas; With the spirit of unremitting self-improvement, going to Yan'an, building a new China, serving the people wholeheartedly, and voluntary reclamation, Perfect summary of the "broken defense" struggle and persistence to the pioneering era, There are also Hope projects, Volunteerism, To the west, to the grassroots, poverty alleviation, rural revitalization, The epidemic will not spread, and we will not retreat, Clear love is only for China, etc., It shows the common responsibility of Chinese young people of different times. There are the resumption of the college entrance examination, knowledge to change the fate, sent out the rush of the young people of the call of the stars and the sea; there are also pursuing the dream, the red gene from generation to generation, shoulder the responsibility of national rejuvenation, sent out the new era of the "young people" of the strong voice. These words are classic and precious. They are the true voices of Chinese youth in the history of a hundred years of struggle. A contributed the "awakening" "please rest assured, power have me" keywords such as young netizens said in the message, this activity to look back at one hundred history, for the future of the responsibility in the shoulder also more feeling: " in one hundred, the Chinese youth take on the historical task, with high passion, fighting courage, unremitting efforts to write the youth glorious chapter. Chinese youth should also seize the opportunities of The Times to make steel, enhance their own ability, and strive to be a passionate youth of the new era.”

2022-05-05 Group Yunnan provincial party committee held theme exhibition immersive experience yunling youth in one hundred struggle journey group Yunnan provincial party committee held theme exhibition immersive experience yunling youth in one hundred struggle journey newspaper correspondent reporter wen-ling zhang) today, the communist youth league of Yunnan provincial party committee "welcome twenty big, always follow the party, forge ahead the new journey" theme exhibition opened in Kunming museum. The exhibition broke the traditional exhibition of static display, through immersive interpretation and interactive, with "youth" time capsule as the main line, let the viewer in real historical atmosphere, look back on Yunnan communist youth league around the central task in different historical period, unity led generations of YunLing youth charge in the front, bear as a journey in one hundred. At the ceremony, Tang Yuan, secretary of the CPC Youth League Yunnan Provincial Party Committee, delivered a speech. The Provincial Communist Youth League Committee has established the "Yunnan Provincial Youth Ideals and Faith Education Base" in Kunming Museum. This theme exhibition is a blockbuster product of the theme education and practice activity of "Welcome 20, Always follow the Party, forge ahead on a New Journey" organized by the Provincial Communist Youth League Committee. It is composed of Yunnan Youth Business Exhibition, Art Exhibition and Calligraphy Works and "One Hundred Years of Journey" immersive interactive experience theater. Some precious historical relics were displayed in the business exhibition, such as the article "My Marxist View" article published by Li Dazhao in volume 6 of New Youth in 1918; the leaflet of "121" tragedy " sent to the public on December 9,1945; the Yunnan Communist Youth League with bronze medal. At the same time, through the application of multimedia technologies such as holographic projection, naked eye 3D and arc projection, Yunling youth has fully demonstrated their hard work, upward and good work, especially in the high-quality development of Yunnan since the 18th National Congress of the CPC. Different from the traditional exhibition, this theme exhibition has set up a "century-year journey" immersive interactive experience theater. By performing major historical events and interacting with the audience, the visitors are guided to review the century-old struggle history of Yunling youth under the leadership of the Party. According to the introduction, the theme exhibition can be integrated and split, according to the visit needs of different groups, different audiences for dynamic adjustment, in order to meet the visitors personalized experience exhibition experience. The Immersive Interactive Experience Theater will perform two performances a day from May 4. Group of Yunnan provincial party committee will be through the history exhibition, interactive exhibition, exhibition of painting and calligraphy exhibition as one of the exhibition complex, build the theme exhibition into this year the province youth ideal faith education demonstration site, theme team, designated activities and university ideological offline theme practice course, become a group green characteristics, by teenagers favorite red "clock".(Photo provided by Yunnan Provincial Party Committee) In addition, on May 3 and May 4, Yunnan Provincial Party Committee held the theme of "Welcome 20, Always Follow the Party, forge ahead on a New Journey" at the Red Army in Xundian County and Kunming Museum.(Photo provided by the Yunnan Provincial Party Committee)

2022-05-04 On the occasion of the May 4th Youth Day approaching, the results of the 2022 "Tianjin Youth May 4th Medal" were announced. The Tianjin Municipal Party Committee of the Communist Youth League decided to award the title of "Tianjin Youth May Fourth Medal" to 60 comrades including Yu � and the title of "Tianjin Youth May Fourth Medal Collective" including the Advanced Medical Materials and Innovative Devices Research Team of Hebei University of Technology. According to the introduction, the honored youth and youth collective, under the leadership of the Tianjin Municipal Committee, actively participate in the high-quality development of Tianjin, the courage to innovate and create, dare to entrepreneurship and develop, stronger than excellence, fully demonstrated the spirit and value pursuit of Tianjin youth in the new era. In 2022, the 20th Congress of the Party will be held, and the Chinese Communist Youth League will celebrate the 100th anniversary of the founding of the League. The youth in the city will be advanced typical for example, and more closely unite around the party central committee with comrade xi as the core, in-depth study and implement xi new era the ideas of socialism with Chinese characteristics, profound comprehension the decisive significance of "two established", strengthening the consciousness of "four", "four confidence", "two maintenance", determined to national rejuvenation, not young, not time, not people, on the youth track to run, strive for the best result of the contemporary youth! With practical action to welcome the party's twentieth great victory held! Tianjin youth May 4th medal winner list (a total of 60) in � (female) Tianjin nankai district people's procuratorate second prosecutors, prosecutors in Tianjin public security bureau command center three staff Malone, director of engineering of biological university of science and technology Tianjin Tianjin port container terminal co., LTD. Technology deputy manager Ma Jing (female) Tianjin public security bureau forensic team 13 team a brigade of police technology three director wang to wisdom Tianjin public security bureau ring drug security team a team of three chief wang Tianjin university of technology textile science and engineering institute researcher Wang China hydropower bureau co., LTD., Tibet branch party secretary, deputy manager Wang Lei (female) between China 3522 fittings accessories co., LTD., party-mass work minister YouHong for Tianjin aquatic research institute section chief NiuZhiqiang material green creation and manufacturing haihe river laboratory researcher;Nankai university school of chemistry institute of chemistry and engineering institute Mao Fuxin Tianjin vocational technology normal university engineering training center lecturer zhu ping blood hospital, Chinese academy of medical sciences (Chinese academy of medical sciences institute of hematology) researcher Zhu Xiaohao Tianjin ear eye Fried cake catering co., LTD., general manager of Tianjin urban construction university, deputy director of Tianjin vocational university school of tourism management teacher Sun Zhili Tianjin commercial university thermal energy and power engineering experiment teaching demonstration center, deputy director of Tianjin economic and technological development zone eight street fire rescue station political instructor sun Qian (female) to Tianjin Tianjin co., LTD. Thermal control class vice monitor sun qiang, the central radio and television station in Tianjin station reporter Rui QiSong Tianjin maritime bureau sea patrol law enforcement deputy captain wanchao li Tianjin cui lake construction engineering co., LTD., general manager li � l town deputy mayor li yuehui Tianjin xiqing district people's court jingwu court vice President li (female) Tianjin planning and natural resources south bureau deputy director li xuliang Tianjin huaneng co., LTD. Maintenance department monitor Yang Kaijun (female) The second affiliated hospital of Tianjin university of Chinese medicine head nurse Yang Pengyu Tianjin peace Gene Chinese medicine clinic co., LTD., general manager of Tianjin advanced technology research institute, deputy chief engineer He Shuang (female) Tianjin hedong district second center primary school team counselor Shen Yawen Tianjin international joint institute of biological medicine co., LTD., chairman and general manager Song Kaihai oil engineering special equipment branch welder senior technician, skills experts Zhang jianfang (female) Tianjin small station fragrant rice industry co., LTD., general manager Zhang Heng Tianjin ore biotechnology co., LTD., founder and CEO zhang Chen Na, Vice President of the Institute of Science and Technology Development, Tianjin University (female) Luo Jingshan, Vice President of the School of Journalism and Communication, Tianjin Normal University, Professor, School of Electronic Information and Optical Engineering, Nankai University; Jin Huiqiong, Deputy Director, Institute of Optoelectronic Thin Film Devices and Technology (female) Feng Hao, Lobby Manager of Tianjin Binhai Rural Commercial Bank, Associate Professor, School of Precision Instrument and Optoelectronic Engineering, Tianjin University;Tianjin fine instrument technology co., LTD., founder and chairman Zhao Xiubao (female) Tianjin first center hospital intensive care, deputy director physician Jiang Lei nuclear industrial physical and chemical engineering institute eight party committee, assistant director of the Tianjin blue sky sun technology co., LTD., senior research and development engineer He Jia (female) of nankai university finance dean Yuan Hongfei baodi district family youth volunteer service director Xu Shenyang Tianjin general assembly macro technology development co., LTD., general manager Guo Lei Build three innings group Beijing co., LTD. Tianjin branch nankai university frontier interdisciplinary center project manager Guo Yuewu yi pu photoelectric (Tianjin) co., LTD. Chairman Huang Xu its Tianjin electric power company east power supply branch, deputy director and planning technology office director and comprehensive management party branch secretary Huang Chongbiao Tianjin medical university tumor hospital senior ward chief physician Cui Meng Tianjin new steel joint special steel co., LTD. Technology center section chief Korea dove Tianjin eye hospital vitreous retinal attending physician Han Ye HaoYe technology co., LTD., founder, general manager of miss Tianjin discipline inspection commission of Tianjin, Tianjin Tianjin supervisory committee 14 review office director clerk tong handsome Tianjin south glass energy saving glass co., LTD. Chief engineer Wen Simin Tianjin medical university second hospital urology physician, deputy director of the institute of the Chinese academy of sciences researcher Xie Aihua guoxing capital company Tianjin urban planning and design research institute co., LTD., planning and design seven hospital chief planner Jane zhijun Tianjin beiyang intelligent medical engineering innovation research center director of the institute of material science and engineering Li cadres in Tianjin cadres Tianjin youth May 4th medal collective list of Hebei university of technology advanced medical materials and innovative equipment research team in 2 fourth construction engineering co., LTD., youth commandos, Tianjin jizhou fire rescue team pan mountain fire rescue station of Tianjin beichen district revenue flower volunteer service Tianjin chest hospital emergency department of Tianjin university of technology and barrier-free intelligent technology research and development team China day Chen dionitrile technology innovation team Tianjin public security bureau traffic police brigade south road brigade sea light temple group Tianjin rail transit operation group co., LTD State Grid Tianjin Electric Power Company Youth Volunteer Service Team

2022-04-30 League Hainan provincial party committee held young staff ability promotion lecture hall group Hainan provincial party committee authority in 2022 "free trade port youth said" young staff ability promotion lecture hall activity jianwei) on April 27 afternoon, Hainan provincial party committee authority in China (Hainan free trade port) youth dream avenue China youth hall held in 2022 "free trade port youth said" young staff ability promotion lecture hall of the first activity. Xu Bin, deputy secretary of Hainan Provincial Party Committee of the Youth League, attended the activity and delivered a speech. All Party members, cadres and workers of the Party branch of the Youth Development Department, and cadres and workers of other departments and subordinate units attended the activity. The lecture hall of young cadres and workers is divided into four links: "youth speaking", "interactive learning", "branch evaluation" and "secretary mentioning". In the "youth said" link, the development of youth party branch sheep Yang Lin to "commemorate the patriotic movement", tells the background of the young students, process and significance of movement, and combined with their own actual talk "one hundred years ago they and one hundred years after we" thinking; Lin Xuan to "youth said-approached the awakening of Chen Yannian", from the time background, Chen Yannian life introduction and provincial port strike three aspects, and combined with the specific work to share the service of the youth work. In the "interactive learning" section, the lecturers and the listeners have in-depth learning and communication, and answer the questions targeted. In the "party branch evaluation" link, Lin Jiachuan, secretary of the Party branch of the Youth Development Department, affirmed the wonderful teaching of the two comrades, and pointed out the highlights of the teaching and the shortcomings that need to be improved. In "secretary review" link, deputy secretary of TuanOuWei XuChangBin pointed out that we as the communist youth league cadres, will write, will speak, dare to speak is a young thought lead, unite youth an important ability, hope youth provincial authority youth staff cherish young staff ability improve lecture hall the stage, take the opportunity, in practice, continuously improve writing ability and language ability, the relevant departments to higher standards, higher requirements for good subsequent youth staff ability improve lecture hall activities.

2022-04-30 Inner Mongolia "youth express" train departure to attract youth clock in photo "youth express" train in Inner Mongolia to attract youth clock in photo (in the green newspaper network reporter Shi Jia) on April 28, to "welcome twenty big, always follow the party, forge ahead new journey" as the theme of the communist youth league "youth express" train and theme station launch ceremony held in Hohhot rail transit line 1 dam weir (airport) station. Hohhot youth punch in and take photos at the theme station. Pictures provided by respondents "youth express" train with original hand-painted and graphic design, six cars into thought leading, national unity, innovation, volunteer service, dating, group led six themes of "youth station", the party's care care to teenagers, make teenagers can afford, find, rely on the communist youth league. Today, this "youth Express line" special train officially started. As soon as the youth got on the bus, they were attracted by the colorful colors and elaborate slogans inside the carriage, and they punched in and took pictures. Zhao Jingfeng, a student from Hohhot Mongolian Primary School, said, " I was particularly surprised to visit the special train. It is full of youth, and I can learn a lot of knowledge."Inner Mongolia teenagers visit the" Youth Express Line " special train. In the green newspaper reporter Shi Jia according to the relevant controller of the Inner Mongolia district party committee, build "youth express" train and theme station, aims to create a strong atmosphere, help Hohhot to carry out the national civilized city and the national youth development city, show youth, leading youth fashion, stimulate youth creativity, promote the development of youth quality, let the city and youth closer, youth more promising in the city.

2022-04-29 Guangxi has extensively carried out youth classic cloud reading activities. China Youth Daily client news (correspondent Wang Qiuhe, China Youth Daily China Youth Network reporter Xie Yang) Youth League Guangxi District Committee, Guangxi Youth Working Committee, Guangxi Students' Federation recently launched the "Welcome 20 always follow the Party to forge ahead on a new journey" - -Guangxi youth classic cloud reading activity. Colleges and universities in Guangxi have carried out various activities to guide students to understand the glorious course and great achievements of the Party in the process of reading the Party history and classic works, and to further feel the Party's grace, listen to, and follow the Party. The Youth League Committee of Guangxi Normal University organized young students to punch in and read the classic campus landmarks such as the CPC Brief History Hall, the Duxiu Study and the Library, read the red classics, and invited all colleges (departments) to carry out the reading relay challenges. Guangxi University of Science and Technology collects reading videos of young students' party history and broadcast them on official new media platforms such as "Youth of Guangxi University of Science and Technology". Baise Vocational and Technical College has established the "Party History Classics online reading Group" to carry out online reading sharing meetings. The Guangxi District Committee of the Communist Youth League regards the study and education of Party history as a major political task, and carries out the publicity and education of Party history through cloud reading. Around the "study history theory, history credit, chong DE, history" requirements, focusing on "the study party history, enlightenment thought, do the practical work, open new bureau" goal, launched youth league organizations at all levels, drive the youth to actively participate in the party history classic reading, promote the party history learning education normalized, guide teenagers firmly listen to the party, follow the party. At the same time, transform the learning and education results into concrete practical results, and unite and lead the youth league members to forge ahead on a new journey and make contributions to a new era.

2022-04-29 Sichuan university "youth employment lecture hall" launched @ Sichuan college graduates "youth employment lecture hall" opened the network Beijing on April 29 (reporter Zhang Jianwei) " I hope that through my speech, let more college graduates choose to take root in the grass-roots, contribute to the grass-roots, grass-roots."Tang Jing, head of the Sichuan University Volunteer Service Project, told college students. On April 27th, the launching ceremony and offline demonstration and publicity activity of Sichuan University "Youth and Employment Lecture Hall" was held in Chengdu, jointly hosted by the Sichuan Provincial Party Committee, Sichuan Provincial Department of Education and Sichuan Provincial Department of Human Resources and Social Security. More than 40 people from the organizer, representatives of lecturers, student representatives and media representatives attended the event. Issue the letter of appointment to the representative of the lecturer. Group of Sichuan provincial party committee for figure activities, the organizer related responsible comrade issued for the lecturer representative, southwest jiaotong university admissions employment teacher easy � try �, Guangyuan city Jiange county grassroots civil servants zhang xuan, Sichuan province college students volunteer service western plan project director Tang Jing respectively around "employment situation and policy interpretation" "grassroots work experience and comprehension" "college students volunteer service western plan" and other topics for the offline demonstration. Wang Qingxia, a student from Chengdu University of Traditional Chinese Medicine, said, " This lecture is full of dry goods and brings me a lot of insights. I also want to sign up as a volunteer of the Western China Project to accept the test where the motherland and the people need it most, and grow up."Pictures of the event scene. Group of Sichuan provincial party committee for figure, "youth employment lecture hall" activities, from the province selected a batch of outstanding graduates and policy interpretation experts, establish provinces and universities level 3 "lecturer", focus on college graduates employment concept training, employment policy interpretation to preach training, encourage college graduates firm ideal faith, change employment concept, to remote and poor areas, border minority areas, old revolutionary base areas and grassroots line work growth. Southwest Jiaotong University recruitment and employment department teacher Yi � brave � encouraged the students to say: " To keep confidence, get ready, in the grassroots employment 'track' rushed out of their own world!"It is understood that the university youth League committee through strengthening the coordination with the school employment guidance department, actively absorb young teachers to join the publicity group, for graduates to carry out employment policy interpretation and publicity. Zhang Xuan, a grassroots civil servant in Jiange County, Guangyuan City and the "excellent first secretary" of Sichuan Province, said: " Recalling the passion of choosing to work at the grass-roots level and the experience I have gained along the way, I can firmly tell you that I do not regret choosing the grass-roots level, and I do not regret choosing the countryside."It is understood that the cities and states have also extensively explored a number of excellent performance of grass-roots selection, western plan volunteers," three support and one support " personnel to join the publicity group, combined with their own work experience and experience to carry out publicity, give full play to the leading role of peers, encourage graduates to the grass-roots achievements. Next, the province, the city, the school three levels will focus on action, let the "youth employment lecture hall" to the graduates side, and strive to receive each graduate at least one employment guidance training, widely improve the socialization ability of college students, effectively serve the employment of college students.

2022-04-29 Chongqing) On April 26, " Welcome the 20th, always follow the Party, forge ahead on the 100th celebration of the demonstration was held in Chongqing Barchuan Middle School. The activity was organized by Chongqing Municipal Party Committee of the Communist Youth League, organized by Tongliang District Party Committee, Tongliang District Education Committee and Tongliang District Youth Working Committee, and co-organized by Chongqing Barchuan Middle School. The activity was carried out in the form of "main venue + sub-venue". The main venue was located in Chongqing Bachuan Middle School, and the sub-venue was in the Memorial Square of Qiu Shaoyun Martyrs, Tongliang District of Chinese revolutionary figures. The activity is divided into three parts: bidding farewell to the young Pioneers, entering the Communist Youth League, and turning the youth to the future. In Bachuan, Chongqing, 2,774 young pioneers left the team. Facing the league flag, the 106 outstanding young pioneers solemnly swore to join the Communist Youth League of China. It is understood that the youth league organizations at all levels in Chongqing will carry out the education ceremony of joining the league around the May 4th Movement, so that the young people can accept the influence of ideals and beliefs in the solemn ceremony.

2022-04-29 Sichuan: Make the report to the provincial Party Committee. According to the provincial party committee standing committee of comprehensive governing group deployment requirements, the Sichuan provincial party committee set up a comprehensive governing group leading group, earnestly study the implementation measures, formulate the new era of comprehensive governing group implementation outline implementation plan, the formulation of 80 specific measures from five aspects, play a comprehensive governing group "combination", set up the new era of Sichuan communist youth league sealed image."Each forum sharing not only has novel forms and rich content, but also the enlightenment of thought and vision development, more feelings of cultivation and spiritual edification."Luo Hua, a young cadre, wrote down his thoughts after attending the micro-forum. Luo Hua said micro BBS, is the Sichuan provincial party committee since 2019 "10:30 waiting for you to share" authority micro BBS activities, has carried out 95, once a week, each led by one party branch secretary xi general secretary, three party members and cadres to share, leading the young cadres consciously do "two established" strong advocates and "two maintenance" firm practitioners. In recent years, the Sichuan Provincial Committee of the Communist Youth League has persisted in communicating learning and making arrangements for General Secretary Xi Jinping, as well as the decisions and arrangements of the CPC Central Committee. Every week, the Office of the Sichuan Provincial Committee of the Communist Youth League will organize the latest important speeches, important instructions and instructions, as well as the decisions and arrangements of the CPC Central Committee, and these contents will be collectively studied and implemented at the Party secretaries' meeting as the "first topic". In terms of the ideological and political guidance of young people, the Sichuan Provincial Party Committee of the Communist Youth League closely follows the development needs of young people, takes strengthening the ideological and political guidance of young people as the primary task, and carries out various forms of thematic learning activities to create an atmosphere for young people to actively learn new ideas."We must tell the young people the stories of the Party's history and the theory, line, principles and policies in the way they like them."Li Xing, a member of the Party History learning and education publicity group of Sichuan Province, and vice president of the Provincial Youth League School, said that since the establishment of the publicity group last year, every" one hundred people, one thousand people " party history learning and education publicity activity has been loved by the young people. This year, the publicity group will also carry out the theme of "Welcome the 20, always follow the Party, forge ahead on a new journey" education and practice activities. Since 2019, the Sichuan provincial party committee to city (state) comprehensive ranking, monthly report to the party committee in charge of the leadership, questioning continuous ranking youth league is mainly responsible for comrades, as a whole using "youth learning" learning "red scarf love" online theme group (team) class, attract more than 300 youth a week, more than 50000 young pioneers squadron online clock learning new ideas."We have established a working mechanism for the important instructions and instructions of the CPC Central Committee and the work requirements of the Central Committee of the Communist Youth League and the Provincial Party Committee of 'special reports, with special responsibility, special classes, and special supervision'. We adhere to weekly spot inspection, monthly inspection and quarterly supervision, so that everything has been implemented and everything has been answered."Said the head of the provincial Communist Youth League committee office. Last year, the Sichuan Provincial Party Committee of the Communist Youth League specially promoted the implementation of the medium-and long-term youth development plan into the Provincial Party Committee for supervision and filing, and led 16 departments, universities and scientific research institutions, including the Organization Department of the Provincial Party Committee, to jointly set up seven research and research groups to carry out field research and supervision in 22 pilot counties (cities and districts). At the end of March, the "Emergency Youth Volunteer Service drill for COVID-19 Prevention and Control in Sichuan Province" was held, organized by the Provincial Communist Youth League Committee, the Volunteer Department of the Chengdu Universiade Executive Committee and other units. The person in charge of the relevant department of Sichuan Provincial Committee of the Communist Youth League said that in the previous emergency volunteer service practice for epidemic prevention and control, Sichuan has gradually explored the workflow of "emergency reserve-start response-announcement-activation reserve-launch service-release response" according to the "1 + 3 + N" promotion mechanism. The "1 + 3 + N" promotion mechanism is the Sichuan Provincial Party Committee to break the administrative barriers, Explore the effective practice of connecting the work of the Communist Youth League, That is, to set up a special committee for the regiment's work, Set up three leading groups of grassroots, school, and comprehensive strict governance group, Focusing on epidemic prevention and control, earthquake relief, the development of the twin cities economic circle, and the effective connection between poverty alleviation and rural revitalization, Set up N special work classes, The province's 21 cities (states) are divided into five areas, It has formed an integrated and coordinated working pattern of connecting the members of the provincial Communist Youth League Committee, leading and cooperating by relevant departments, and flat dispatching by the secretaries of the Communist Youth League Committee in 183 counties (cities and districts). At the end of 2021, a county communist youth league organization reform pilot full coverage of pilot research guidance activities in the province, the provincial secretary of the team members in accordance with the fragmentation area scheduling mechanism, successively to their contact the pilot areas to carry out field research guidance, will work signal and pressure to the end, get through work "the last kilometer". In order to further enhance the organizational strength, Sichuan has focused on the grassroots construction of the regiment for three consecutive years, and determined one main direction every year to carry out the "100-day attack". In 2022, the "assembly" to promote the construction of urban residential communities. By the end of this year, the density of social organizations in the province will double from the end of 2018, reaching the goal of 80 league organizations for every 10,000 young people in the social field. The Sichuan Provincial Communist Youth League Committee also focuses on deepening the reform of the student Union, guiding student cadres to be "student friends" and not "student officials"; conscientiously performing the duties of leading the youth League, and constructing a ladder growth incentive system for young Pioneers. On the evening of November 27,2019, the secretaries of the Communist Youth League Committee of all cities (prefectures) and counties (cities, districts) who came to attend the training received a surprise exam on the night of their registration."The purpose of the exam is to promote learning, test the grasp of the system and deployment of the group, and truly implement the requirements of strict governance of the group, so as to focus on the main responsibility and main business of the group."The Youth League provincial committee main responsible comrade said. Since then, the provincial Communist Youth League Party Committee insisted to the youth League cadres training class before the class must test, do not notify the time in advance, do not delimit the examination question bank, do not limit the examination questions, on the spot, the real name announcement results, effectively grasp the quality of education and training of youth League cadres. In addition, the CPC Sichuan Provincial Committee organized the "practical ability competition", guide the cadres in practice; establish a talk system, the main responsible comrade of the new city (state), provincial affiliated league (work) committee secretary talk reminder, "point to point" clear task requirements, to promote the new league secretary to enter the role faster. On the cadre work style construction, actively advocate dare to grasp dare to tube, business, dare to criticism, questioning accountability, public review, reporting measures reversed transmission responsibility implementation, issued to strengthen and improve the organs directly under the league document work 7 measures, the wrong drying, reporting, aging management and a series of systems, let the city and county youth league committee "head" blush sweat. It is understood that in the past two years has interviewed the city (state) youth League committee is mainly responsible for comrade 6 times, 5 cities (state) youth League committee on the key work to promote the ineffective provincial conference review speech, two cities (state) youth League committee for a written review."Since the implementation of the league member number system, we have uniformly printed the league membership application form, 'two up and two down' to determine the league membership quota and other ways, and effectively do a good job of 'county coordination, city supplement, provincial control of the total amount', the advanced nature of the league members is significantly enhanced."The person in charge of the grassroots department of the Sichuan Provincial Youth League Committee said that Sichuan province has always controlled the number of league members and standardizing the procedures of league members as one of the important ways to maintain and enhance the advanced nature of league members. China Youth Daily China Youth network reporter Wang Shanshan, correspondent, Yue Taipeng, Xiong Xiaotian

2022-04-28 The establishment ceremony of Henan Provincial Science and Technology Innovation Working Committee of the Communist Youth League (referred to as "Henan Provincial Science and Technology Innovation Youth League Working Committee") was held in Zhengzhou today. Chen Xiangping, secretary of the Party Leadership Group and director of Henan Provincial Science and Technology Department of Henan Province, and Wang Dubo, deputy secretary of Henan Provincial Communist Youth League inaugurated the Henan Provincial Science and Technology Innovation Working Committee. More than 30 people attended the activity, including relevant personnel of Henan Provincial Science and Technology Department and Henan Provincial Youth League Party Committee, members of the first committee of Henan Provincial Science and Technology Innovation Youth League Working Committee, and the first batch of hired mentors of Henan Provincial Science and Technology Innovation Youth League Working Committee. Chen Xiangping pointed out that at present, the Henan Provincial Party Committee and the provincial government attach great importance to the work of scientific and technological innovation, and the establishment of the Provincial Science and Technology Innovation Youth League Working Committee will open a new chapter in the work of youth scientific and technological innovation in Henan Province. She encouraged the newly established provincial Science and Technology Innovation Youth League Working Committee to build a good youth home and build a brand of youth science and technology innovation. Wang Dubo hope province kechuang league committee to play a good lead as a whole, coordination and demonstration leading role, on the leading condensed kechuang youth, high in contact service kechuang youth temperature, on the organization to mobilize kechuang youth, motivate kechuang youth in the national innovation highland construction pioneer, be rushed, contribute to the modernization of Henan youth strength. At the ceremony, Chen Xiangping and Wang Dubo issued letters of appointment to Han Yifan, Zhang Haiyang and Li Gaopeng, the first mentors of Henan Provincial Science and Technology Innovation Working Committee of the Communist Youth League. The first phase of the "Create and Win the Future Scientists Face to Face" activity was held at the same time. Han Yifan, a Changjiang Scholar of the Ministry of Education and a professor of Zhengzhou University, shared the theme in the form of online and offline linkage live broadcast.

2022-04-28	Gansu: implement the revitalization of rural six action lead youth in the great practice in Gansu province: the implementation of rural revitalization of six action lead youth in the great practice jianwei) group Gansu provincial party committee thoroughly study and apply xi general secretary in the national poverty crucial summary recognition conference of the important speech spirit, xi general secretary of three rural work important discourse and in the central rural work conference's important speech spirit, Earnestly implement the series of decisions and arrangements of the Gansu Provincial Party Committee and government on consolidating and effectively linking the achievements of poverty alleviation with rural revitalization, We will help consolidate and expand the achievements of poverty alleviation and rural revitalization as an important task and battlefield to focus the central task and serve the overall situation, Closely combined with the situation of Gansu Province and the actual work of the Communist Youth League, We carried out six initiatives to make contributions to rural revitalization, Firmly grasp the fundamental task of serving, condensing and cultivating young people, We will earnestly organize and mobilize the youth league members of the province to actively participate in the great practice of all-round rural revitalization in the new era. The implementation of the "youth to help agriculture" action, effectively increase the consumption assistance work widely held "youth to help agriculture" series of activities, will help broaden the "sweet" agricultural products brand market space, increase consumption assistance as to promote the industrial revitalization of the "first chess" and "highlight", in 2021 to help consumption assistance of 42.466 million yuan. The series of activities of "Youth Helping Agriculture" have been listed as excellent typical cases of national consumption assistance and rural revitalization in 2021. The "youth to help farmers" public welfare live broadcast activities. Provincial public welfare live broadcast demonstration activities were held to vigorously promote "sweet taste" products and cultural tourism resources. The number of live viewers reached 12.82 million (the first among the online viewers of the whole network on that day). The total guided transaction volume exceeded 18.23 million yuan, and the total number of guided transactions exceeded 501,000. At the same time, it organized the local youth Internet celebrities to promote the "sweet taste" regional brands, and carried out 121 public welfare live broadcasts, with a sales volume of 5.27 million yuan. We will build a model of interconnected consumption assistance and sales. Give full play to the advantages of poverty alleviation cooperation mechanism between east and west, sell 12.3 million yuan of featured agricultural products based on Tianjin e-commerce platform and supermarket; invite 3.9 million yuan for celebrity endorsement and public welfare to sell agricultural products, and organize Gansu Youth Federation members to sell 2.246 million yuan of agricultural products on live broadcast platform; and sell 520,000 yuan by opening offline stores, entering online poverty alleviation mall, and centralized procurement of employee welfare. Formed a multi-party participation, resource sharing, effective interaction of the work force. We will implement the "Youth Revitalization" campaign, and vigorously cultivate young talents for rural revitalization. With the focus on cultivating local talents to revitalize townships, serving foreign talents for returning to their hometowns, and mobilizing social talents to the countryside, we will vigorously cultivate, select, and pool a number of young talents for rural revitalization. To build a platform for youth entrepreneurship. We will increase support for rural youth entrepreneurship, encourage 71 counties in the province to set up youth entrepreneurship alliances, and carry out entrepreneurship service activities on a regular basis. A team of youth entrepreneurship mentors in Gansu Province has been established, and 32 Chinese youth entrepreneurship mentors have been recommended, and 35 provincial youth entrepreneurship mentors have been appointed. The "Rural Revitalization Youth Helping Agriculture" Youth Innovation and Entrepreneurship Project Competition was held, 218 outstanding youth entrepreneurship projects in the province were recruited, and 15 young "entrepreneurs" were cultivated. Two provincial-level youth entrepreneurship training camps were held, and more than 1,700 young entrepreneurs were trained online and offline. We held 18 training courses for young rural leaders to get rich, training more than 1,100 people. We will develop and build a cloud platform for youth entrepreneurship and innovation incubation in Gansu Province, and promote the establishment of a provincial youth talent pool. We will strengthen the cultivation of young e-commerce talents. We will effectively increase the cohesion of rural e-commerce talents and live streaming salesmen. By holding live broadcast training camps, competitions and other activities, we will effectively build a youth employment and entrepreneurship platform, contact and serve more than 200 youth Internet celebrities, and strive to build a backbone team of live broadcast to speak for "sweet" agricultural products and beautiful countryside. The "Youth to Help Agriculture" origin public welfare live broadcast activity led 207 young anchor teams in the province to participate, and effectively improved the skills level of young e-commerce youth. Pay attention to the advanced typical tree selection. In combination with the selection activities of Gansu Youth May 4th Medal, 11 outstanding youth and teams in the field of poverty alleviation and rural revitalization were selected and commended; the typical activities of "Rural Revitalization Youth Pioneer" were widely selected to establish a clear value orientation, guide the young people to learn advanced, and encourage the youth members to make contributions. We will implement the "Youth Volunteer" campaign to promote the quality and efficiency of rural volunteer services, give full play to the organizational advantages of the Communist Youth League, organize and guide young volunteers to participate in the construction of a civilization practice center in the new era, and help improve the people's sense of gain and happiness. Carry out the "youth volunteer to the countryside" activity. Relying on the county-level youth volunteer association, 60 youth volunteer service teams of the New Era Civilization Practice Center have been set up, carrying out extensive rural volunteer service activities. The county and rural volunteer service time has exceeded 460,000 hours, and more than 150,000 young people have participated. College students "a program under which officials" social practice, practice team 2517, including 50 national team, 100 provincial team, the country, into the farmers, policy preaching, science and technology supporting agriculture, education, culture, art, love, care for left-behind children, health cleaning, draw culture wall and health census and other volunteer service more than 5000 games. Pair of immigrant relocation communities. The 2021 Provincial Rural Revitalization Youth Volunteer Service Action Commitment Conference was held in Fumin New Village, Gulang County, and 14 universities in the province were mobilized to pair up with cities and prefectures to provide normal volunteer service activities for key communities relocated. At the same time, we will build a regular, permanent and professional youth volunteer service base in Fumin New Village, Gulang County, the largest immigrant community, to promote the precise, project-oriented and branded development of volunteer services in immigrant communities. We will carry out relay programs for community-level volunteer services. We will encourage the volunteer services of the Western Western Autonomous Region Plan and the graduate education group to favor key areas and rural areas. This year, we will recruit 708 fresh graduates and graduate students to the grassroots to carry out one-to 3-year volunteer services, and the number of volunteers will remain at over 1,000. The implementation of the "Beautiful Gansu" action, lead young people to participate in ecological environmental protection practice to help build the national western ecological security barrier, improve the rural living environment, implement the "Beautiful Gansu Youth Action", guide the province's youth to actively participate in ecological environmental protection and beautiful rural practice activities. We will carry out ecological and environmental protection theme practices. Establish "the communist youth league + youth environmental protection social organizations + youth volunteers + youth commandos" of "river xiaoqing" ecological protection volunteer team 304, to carry out the tree lake green, ecological concept, environmental protection technical services, river protection, "three minus section" and other forms of ecological environmental protection volunteer activities 1796 games, the province drive 200000 teenagers to participate in. In Lanzhou city qilihe district Wei Ling township in Gansu youth ecological restoration forest farm, held "the 100th anniversary of the founding, the revitalization of the tree of hope" Gansu province youth planting green action and Gansu youth ecological restoration forest unveiling ceremony, organization cadres, youth members, youth social organization members, young volunteers, college students and enterprise, a total of more than 3000 people partial tree planting activities. Thick value of ecological and environmental protection concept. Actively strive to apply for the national "Mother River Protection" key funding projects; jointly select the second batch of provincial youth ecological civilization education and practice base with the provincial Department of Ecology and Environment and other departments, providing a platform for the provincial youth to carry out ecological civilization education experience and practice activities. Issued by the "beautiful China, I am an actor" promote citizen consciousness of ecological civilization action plan in Gansu province (2021-2025), organize the first youth ecological civilization innovation entrepreneurship competition in Gansu province, strengthen our province youth thought lead and ecological civilization values, lead the youth and the social public to participate in the ecological environment protection. We will actively participate in the improvement of the rural living environment. Youth League committees at the city and county levels organized youth league members to carry out more than 80 environmental sanitation cleaning activities in the major streets of the county and rural areas and in the urban and rural fringe areas. The province's middle school youth league organization and the young Pioneers organized to carry out the "small hand to pull the big hand to beautify the environment, I can do" more than 100 practical activities. We will implement the action of "strengthening the foundation and strengthening the foundation", effectively stimulate and enhance the vitality of grass-roots organizations, vigorously promote the standardization of rural grass-roots youth league organizations, and realize the effective coverage and role of county and rural youth organizations. We will carry out a centralized transition of village-level youth league organizations. With the help of the village "two committees" change, the village level league organization centralized election work, standardize the province's village (community) league organization election work, further strengthen the construction of grass-roots league organization, and constantly improve the grass-roots league organization "three forces once". Up to now, the province's 15,943 administrative villages, 1,460 communities in the transition have been changed, the transition rate of 100%, the province's village-level league organizations from the education, age structure has been further optimized. We will expand the coverage of community-level youth organizations. We will promote the coverage of county and county organizations, township township positions, and competent people in the work objectives of villages, and play the role of community-level youth organizations. At present, 70 county-level youth volunteer associations and 71 county-level youth entrepreneurship alliances have been established. Implementation of "consolidate" action, effectively consolidate expand poverty crucial achievements as a whole to consolidate expand poverty crucial achievements with rural revitalization effective cohesion, focus on poverty areas, teenagers practical difficulties, continue to implement the communist youth league aid trapped, employment support brand project, consolidate expand poverty crucial achievements, to effectively join rural revitalization of solid foundation, solid foundation. We will implement the "Jinsui Project" project to subsidize college enrollment for children from poor families. Relying on the brand of Project Hope student aid, we strengthened social mobilization, through targeted donation from 11 caring enterprises, promoted the application of donation small program for scanning code and micro donation, extensively mobilized youth league members to make public donations through the theme activity of "New Youth with the Long March", and raised a special student fund of 4.1 million yuan. Education, civil affairs and youth League committees at all levels were organized to compare, review and publicize the information of the admitted candidates, and allocated 46.49 million yuan to 4,971 students according to the standard. We will provide good assistance to young people with finding jobs. The provincial, municipal and county youth League committees work together to carry out special offline recruitment activities and online online recruitment activities. More than 1,500 employers were invited, providing more than 50,000 jobs, the number of 310,000, and 6,922 employment intentions. At the same time, online live employment guidance and training were carried out, providing live lectures for young people in employment needs in the province, providing services such as policy interpretation, career planning, rights protection consultation, legal aid and other services, with more than 1.2 million online viewers. The campaign of "thousands of schools" for college and technical secondary school students was implemented. Cadres at all levels in the province helped 4,374 graduates from poor families in 2021 and 2022, and helped 1,114 find jobs. It held 6 double selection meetings of "Thousands of thousands of schools", with a total of 1,100 participating enterprises, 55,684 positions and nearly 30,000 students. The 2021 "Sailing Plan" for College Students in Gansu Province, 831 students from 48 universities in the province went to 25 provincial Party and government organs, directly affiliated enterprises and institutions, and municipal (state) governments and enterprises to carry out employment internships, laying a good foundation for college students to increase their talents and find successful employment. We will provide care and service initiatives. The government issued the Implementation Plan of the Gansu Communist Youth League's Action on Care and Services for Rural Left-behind Elderly, Women and Children and People in Extreme Poverty, and carried out more than 6,600 "Hand in hand" activities, serving more than 21,000 hours, and donated living materials worth 21,046,900 million yuan. The campaign to help young people in distress, including 2,000 yuan per year, and 3,600 yuan per year. 4.18 million yuan was allocated to 2,069 young people in distress.

2022-04-28	Shaanxi communist youth league to carry out the "group" support action the communist youth league to carry out the "group" support action jianwei) in April 2022, shaanxi communist youth league further implement the "I do something for the youth", around power rural youth entrepreneurship and real economy and small micro enterprise development, give full play to organizational advantages, mobilize resources, with talent, projects, financial support, deepening including "elite green farmers" rural youth entrepreneurship support action, "Qin Qing preferential loan" project "," group " support measures. Focus on "four batch", help rural youth entrepreneurship among them, "elite color green farmers" rural youth entrepreneurship support action, with talents, projects, training, into the form of the countryside, focus on 26 rural revitalization of key support county and central direct contact rural revitalization of youth work action key county, each city selected 1 demonstration county to give key support. The campaign will focus on "cultivating a group of young entrepreneurs", Create online and offline "youth innovation classes", Carry out youth innovation training; For college graduates, 20,000 yuan for small and micro projects (within 2 years after graduation) who return to rural revitalization, promoting employment and consolidating achievements in poverty alleviation, Facing with a certain operational and development prospects, College graduates with strong financial needs (within 2 years after graduation) who return to the countryside to start-up small and micro projects (give priority to support projects that are greatly affected by the epidemic or disasters), Provide interest-free loans of no less than 20,000 yuan, Loan cycle is 1 to 3 years; At the same time, it will also provide qualified young entrepreneurs in key districts and counties, including the China Youth Entrepreneurship mentor and the "Flower Bud Program" of the Provincial Youth Entrepreneurship Mentor Association. Around "supporting a batch of green enterprises", we will organize the team to conduct special research on green enterprise development in demonstration counties; further promote "QinQing preferential loan" project, "QingLi supports agriculture and revitalization" action, provide policy support; and select a batch of regional representative and competitive characteristic products, hold production and marketing matchmaking meetings, and carry out live public welfare demonstration activities with goods. Based on the "Select a group of young Talents", the training and competition qualifications will be recommended for the outstanding young talents and quality projects discovered during the event; under the same conditions, the outstanding young talents discovered during the event will be included in the provincial rural revitalization youth talent pool as the key object of the league resources. Focusing on "supporting a number of youth innovation organizations", county youth innovation organization support projects will be implemented in 2022, with special funds will support demonstration county (district) youth entrepreneurship organizations to carry out rural revitalization youth entrepreneurship activities and guide them to carry out standardized construction. To solve the problem of rural youth entrepreneurship is the first step. How to support the sustainable development of small and micro youth entrepreneurship enterprises, including rural start-ups and a wider level of society, still needs more efforts. Under the normalization of the epidemic, the capital problem has become a key bottleneck restricting its development. Based on this, in March 2020, Shaanxi Provincial Committee of the Communist Youth League and Xi 'an Branch of the People's Bank of China jointly launched the "Qin Preferential Loan" project with four financial institutions. Up to now, the project has issued a total of 4.363 billion yuan of loans, the average loan interest rate of 4.23%, a total of 5,257 young people start small and micro businesses, greatly reducing the financing cost of enterprises. In 2022, in the face of rural revitalization and small green and enterprise development new situation, new demand, recently, the communist youth league of shaanxi provincial party committee joint people's bank of China xi' an branch, shaanxi province youth federation issued "about deepening the" Qin Qing preferential loan "project notice", is deepening the "Qin Qing preferential loan" project to make further work arrangements."Notice" clearly through the following four aspects of the in-depth promotion of the "Qin Qing preferential loan" project landing. First, to promote the project branding, From the original two-year time limit to a long-term brand work persistent implementation; Second, to strengthen financial supply, The host bank has increased from four banks to women's families, Covering state-owned banks, joint-stock commercial banks and local corporate financial institutions; Third, increase the loan amount and further reduce the loan interest rates, The loan amount was increased from RMB 3 million to no more than RMB 5 million, The loan interest rate was reduced from the original loan market quoted interest rate (LPR) with a little 120 basis points to a little 100 basis points; Fourth, the establishment of the "financial youth Service group", Made of youth business backbone of the host bank of the "Qinqing Preferential Loan" project and members of Shaanxi Financial System Youth Federation, Set up two special work classes, "Qin Chuangyuan" (scientific and technological innovation) and rural revitalization, Focus on serving science and technology innovation enterprises and agriculture-related enterprises to help rural revitalization, Providing a practical financial service model, Through the three modes of regular service, special class service and direct service, the financial projects can publicize and popularize the financial knowledge, specially promote the projects and serve the development of the real economy.

2022-04-27	Group Beijing municipal party committee to carry out the "group" project solicitation group Beijing municipal party committee to carry out the "group" project wei correspondent Jiang Qian) to celebrate the 100th anniversary of the group, strengthen the demonstration lead, promote experience, further stimulate the youth vitality, improve youth organization service youth, service ability, take concrete action to meet the party's 20 victory, the Beijing municipal party committee organization launched the "group" brand project solicitation. Starting from the end of March 2022, the solicitation will be carried out in six stages: "Recommended declaration- -organizational review- -comprehensive evaluation- -offline road show- -typical publicity- -achievement application". Focusing on summarizing the experience and practices of serving the party and government centers, contacting and serving the youth, grassroots organization construction, rural revitalization, and participating in major activities, we will summarize and incline to the projects of grass-roots youth League committees and youth League branches. After the solicitation notice was issued, the departments of each front and the youth league and district committee responded positively, organized the declaration at various levels, and collected nearly 200 brand projects. Through classified audit, comprehensively considering the innovation, influence and promotion of the project, 120 brand projects were selected. Online and online voting and offline selection stage are currently under way. In the next step, the Beijing Municipal Party Committee of the Communist Youth League will determine the list of the top 100 teams according to the selection results, and select the top 20 of the top 100 teams to carry out offline roadshow exhibition and exchange activities, and invite the previous "two red and two excellent" winners, young committee members and grass-roots representatives to elect the "top 100 teams". Excellent projects will be publicized and displayed around the May 4th Youth Day and in the second half of the year through special display boards, project compilation, network columns and other forms.

2022-04-27	Qinghai promoting youth career development made all-round progress adhere to the party tube youth principle service youth urgent needs, safeguard the rights and interests of youth development youth career development progress Jianwei correspondent a Shuyuan) group Qinghai provincial party committee adhere to xi jinping, the thought of socialism with Chinese characteristics as the guidance, To fully implement General Secretary Xi Jinping's important remarks on the work of youth and the Communist Youth League, Adhere to the principle that the Party supervises the youth, Explore the law of youth work, Optimize the environment for youth growth, Serving the urgent needs of the youth, To safeguard the rights and interests of youth development, To promote the all-round development of young people, We will gradually build a work pattern of Qinghai youth development under the leadership of the Party committee, the government's main responsibility, the coordination by the Communist Youth League, the joint management by all parties and the extensive participation of the whole society. Focusing on strengthening the ideological and political guidance of the teenagers, the ideological and moral construction level of the youth in the province has been continuously improved. Firmly grasp the fundamental task of the party education, to xi jinping, the youth of socialism with Chinese characteristics as the main line, to "learn the history of the party, strong faith, follow the party" history learning education as the gripper, deepen the "youth learning action" "red scarf love learning", "youth branch" "red scarf" tour "plan, expand the communist youth league lecture hall, lecture hall, promote the" green horse project " quality expansion, strengthen the youth ideological and political leading pertinence and effectiveness. Expand innovation of the communist youth league, youth league, union, the young pioneers organization organized education carrier, give full play to the province more than 15000 grassroots youth league branch and more than 14000 young pioneers squadron role, extensive "please party power have me" "red scarf heart to party" and other kinds of theme education practice, education leading teenagers to listen to the party, follow the party's confidence and determination, better growth into qualified builders and reliable successors to the cause of socialism with Chinese characteristics. In the past five years, the youth league organizations at all levels in the province have organized and carried out more than 70,000 various youth education and practice activities, covering more than 6 million people. We formulated and established a policy system to promote the all-round development of young people, and improved the pattern of all-round development of young people. We will accurately focus on the rights and interests of youth development, grasp the focus of youth work, strengthen the top-level design of youth work, strive to build a policy system to promote the all-round development of youth in the province, and promote the formation of a large pattern of youth development. The Qinghai province long-term youth development plan (2018-2025), establish and improve the planning and implementation of provincial joint meeting mechanism, around the plan to determine 11 key areas and 45 development measures, comprehensive coordination contact 43 made members, through special consultation, information sharing, comprehensive supervision, jointly research youth issues, coordinate the youth policy, youth projects, implement cross-sectoral youth work consultation mechanism. Through the establishment of the provincial youth development planning expert committee, the pilot construction of a youth development city, exploring the construction of a "youth meeting room", and the release of the annual statistical monitoring report of the Plan, the policy advocacy and social advocacy have been actively carried out, and the policy environment for youth priority development has been continuously optimized. At the same time, the cities and counties have been promoted to establish a joint conference mechanism for youth work under the leadership of party committees and governments, and the three-level joint conference mechanism for youth work at provinces, cities and counties, and the youth development planning system has been continuously improved. The policy system for serving the youth growth and development has been improved, and the province's youth development platform has been continuously improved. Combined with the current diversified growth and development needs of teenagers, deepen the connotation of The Times of the "Qing" brand service project, and make every effort to build a service youth work mechanism covering the whole field, all-factor participation and all-round promotion. In-depth implementation of the communist youth league to promote college students 'employment, focus on providing employment support and enhance social ability, strengthen employment guidance, strengthen the construction of level 3 youth employment trainee base, to build "home" "sail plan", "thousands" school "a program under which officials", the Yellow River basin college students innovation entrepreneurship training camp to promote college students' employment practice brand. In the past five years, the province has established a total of 667 trainee bases of provinces, prefectures and counties. Provide more than 88,000 internship and jobs such as "thousands of schools" and "Sailing Plan"; give full play to the political and social functions of the Communist Youth League, focus on "disadvantaged youth, disadvantaged youth, marginal youth and troubled youth", take "ten" search "as the main path, relying on the Communist Youth League characteristic brands such as" Five Projects "of Hope Project", "12355 Youth escort Action", and continuously improve the professional and accurate level of youth work. In the past five years, a total of 34.812 million yuan of public welfare grants have been raised, supporting 13,931 students from poor families; the whole process service system of youth innovation, entrepreneurship and excellence has been continuously improved, and a comprehensive service system of Qinghai youth entrepreneurship and employment has been built with Qinghai Youth Pioneer Park as the leader and local entrepreneurship and employment service stations as the radiation. Qinghai Youth Pioneer Park has incubated more than 1,000 enterprises and created nearly 10,000 people. We will strive to increase financial support for youth entrepreneurship and training for young entrepreneurs. Over the past five years, a total of 321.28 million yuan of loans have been issued for youth to start up businesses. More than 5.4 million yuan has been invested in training funds to train 1,471 young entrepreneurs. We will vigorously encourage young people to take responsibility as contributions, and constantly improve the mechanism for mobilizing young people to make contributions. Centering on the development strategy of "one excellent and two high schools" of the provincial Party Committee and the construction of "four places", the central work will gather the wisdom of the youth, play the role of the youth, deepen the "four major actions", serve the major strategy, and organize and mobilize the young people to make contributions and grow up in the overall situation of the service center. We will further implement the youth volunteer service initiative. Giving full play to the unique role of young volunteers in serving the western region, large-scale activities, looting, insurance and disaster relief, public service and other fields, more than 14,000 special volunteers from the western Plan, the Southern Youth Plan and grassroots youth were selected to 44 counties (cities, districts) in the province to carry out volunteer services. The number of registered young volunteers in the province has exceeded 300,000, and the total number of volunteers participating in the service has reached 1.7 million (times); and the Communist Youth League has steadily participated in ecological civilization construction. Taking the Implementation Plan of the Qinghai Provincial Committee of the Communist Youth League on Implementing the Work Plan of the Provincial Party Committee and the Provincial Government to Building "Eight New Highs", the normalization and long-term ecological environmental education for teenagers was promoted by promoting ecological civilization education into the classroom, into the team class, and the establishment of the green classroom practice school of the Communist Youth League. To build five ecological and environmental protection brand activities, such as "Protect Qinghai Lake, I am a volunteer", with a total investment of more than 40 million yuan and 31,000 mu.innovatively established 213 provincial, prefectural, county and rural level ecological volunteer service teams, carried out more than 1,500 volunteer service activities such as "River Xiaoqing Beach Action", attracting 50,000 young people; comprehensively promote the creation of youth ethnic unity and progress action. We have carried out 11,000 educational activities on young people with youth characteristics, such as the Publicity Month of "the Chinese nation is One Family and Casting the Chinese Dream", to guide young people to firmly establish the sense of community of the Chinese nation. Relying on the work brand of "youth contributes to national unity and progress", we will gradually build a working pattern of youth ethnic unity and progress with "typical guide, demonstration drive, strong points and comprehensive progress". More than 300 provincial and county three-level youth ethnic unity and progress demonstration sites and 20 temple youth homes have promoted the youth contribution action of rural revitalization, deeply implemented the Implementation Plan of the Qinghai Provincial Party Committee of the Communist Youth League, and actively contributed to the "five" revitalization. We have thoroughly carried out the "Rural Revitalization Youth Achievement Action- -Mentor Service into the Grass-roots level", fully implemented the "Rural Revitalization Youth Pioneer" training project and the "Qinghai Province College Students Youth Designers" training program, and organized and mobilized the youth league members of the province to actively participate in the rural revitalization youth contribution action. A total of more than 43 million yuan has been allocated to the Provincial Special Financial Support Plan for Youth Agriculture and Animal husbandry Entrepreneurship. Nearly a thousand college students have actively participated in the training program of "Qinghai Province College Students Youth Designers". We will continue to strengthen the protection of the rights and interests of young people, and improve the environment for their healthy growth. Strengthen the publicity and education of the rule of law for teenagers, extensively carry out a series of legal publicity and education activities of "youth and law", youth mock court, youth publicity and education activities for the rule of law in the province, and strive to create a good atmosphere of "respecting, studying, abiding by and using" for teenagers. A total of 9,530 publicity and education activities have been carried out, and 243 youth mock courts have been held. Issued by the Qinghai province on further deepening the prevention of juvenile crime work implementation opinions ", the" a appraisal three basis " as an important gripper to establish perfect the green work system, institutionalized state green work, longitudinal form provinces and counties level 3 green work system, transverse establish member unit contact coordination work system. We have further promoted the service and management of key youth groups, established 66 youth rights protection posts, established 5 youth rights protection workstations in Qinghai Province, and hired 24 experts and scholars who have long served on the frontline of youth rights protection services for the province to escort the healthy growth of teenagers in the province.

2022-04-27	Chongqing held the national rural revitalization of youth pioneer deeds sharing in Chongqing held the national rural revitalization of youth pioneer deeds sharing xin-xin wang) to widely publicized "national rural revitalization of youth pioneer" deeds, lead more aspiring youth active in rural revitalization, on April 25, "national rural revitalization of youth pioneer" (Chongqing) award ceremony and deeds sharing was held. This event is sponsored by the Chongqing Municipal Committee of the Communist Youth League and the Chongqing Agriculture and Rural Affairs Committee, organized by the Bank of Chongqing, and co-organized by the Chongqing Radio and Television Group (General Group) Social and France Channel. The event is held both online and offline ways, and the main venue is located in the headquarters of the Bank of Chongqing. Based on the story of the "National Rural Revitalization Young Pioneers" rooted in the "three farmers" and rural revitalization, they told the experience of the villagers, and working hard to actively promote the development of the industry and improve the income of farmers, which aroused the strong resonance of the audience. The "National Youth Pioneer of Rural Revitalization" was launched by the Central Committee of the Communist Youth League and the Ministry of Agriculture and Rural Affairs in July 2021, aiming to further implement General Secretary Xi Jinping's important instructions on rural talent revitalization, implement the relevant arrangements of the CPC Central Committee and The State Council, and train and select young talents for "agriculture, rural areas and farmers" through multiple channels. After candidate application, qualification examination, public evaluation, centralized publicity, naming and commendation, Chongqing Li Tao and other 9 young people in the agriculture-related field were awarded the first "National Rural Revitalization Youth Pioneer".

2022-04-26	"Entrepreneurship qilu preferred Shandong" youth talent service week activities start "entrepreneurial qilu preferred Shandong" young talent service week activities start the youth newspaper network reporter XingTing) for the implementation of the provincial party committee talent work conference spirit, further promote "talent education innovation", close service young talents to lu innovation entrepreneurship, on April 21, Shandong provincial party committee talent work leading group office, human resources and social security department, Shandong province talent demand "cloud" conference, start 2022 "entrepreneurial qilu optimization Shandong" young talent service week activities. Gong Wendong, Vice Minister of the Organization Department of the Shandong Provincial Party Committee, Zhou Chunyan, member of the Party Group and Deputy Director of the Shandong Provincial Human Resources and Social Security Department, Shang Shengbo, Deputy Secretary of the Shandong Provincial Party Committee, and Pan Jun, member of the Party Group and deputy director of the Shandong Provincial Department of Science and Technology attended the event and delivered a speech. The "cloud" press conference released a total of 13,592 employers in the province in 2022, with 86,601 talent demand, and released young talent support policies at the provincial and municipal level. Shang Shengbo, deputy secretary of Shandong Provincial Committee of the Communist Youth League, introduced the work of serving young talents and the activities of young talents in his speech. As a specific measure to help college students find employment, introduce and nurture young talents, and local enterprises resume work and production, the young Talent Service Week will continue to make efforts in the four aspects of attracting talents, using talents, gathering talents and benefiting talents, so as to achieve the "open service". Focusing on the development of employment services, we will continue to promote the cloud recruitment of "Bluebird Plan Select Shandong" and the air double selection meeting of "thousands of schools have excellent employment positions", carry out the employment work of helping students from low-income families in general colleges and universities, accurately serve the employment of young students, and help enterprises resume work and production. Around booster school cooperation, extensive links, inside and outside innovation entrepreneurial resources, guide high-end young talents and innovation elements to accelerate agglomeration docking, focusing on "top ten industry", superior disciplines, release local talent and technology demand library, universities and kechuang achievement resources supply library "two library" list, give play to the role of youth innovation entrepreneurship alliance needle, targeted to carry out two-way docking. Around the power ZhaoCaiYinCai, overall planning well-known college students to practice, perception of Shandong activities, mining enterprises and institutions practice positions, released young talents, college students "sail plan", "home", "local travel" and other activities, to attract more young talents perceive qilu, choose Shandong. Around the service hui to stay, combing the Shandong provincial party committee organization department, Shandong province, Shandong province human resources and social security department and other departments service youth science and technology talent policy, forming young talent policy package, facing 16 cities, 153 colleges and universities and 66 provincial well-known universities "green bird station" concentrated release, multi-channel promotion policy awareness. In 2022, the Shandong Communist Youth League will take the "Entrepreneurship Shandong" project as the carrier, focus on the "ten innovations", integrate and enhance the "Green Bird Project" and "Green Qilu" work brand, continue to make efforts in the aspects of young talent education, school and local integration, and strive to improve the service of talent and high quality development contribution.

2022-04-26	Shi Jia, the project sponsor of Inner Mongolia.) Recently, the "Hope Project 1 + 1- -Fantasy Education Program" project was launched in Inner Mongolia Autonomous Region. A total of 5,394 previously registered poor families in 31 counties in Inner Mongolia Autonomous Region, families with special difficulties, minimum living allowance families, and junior primary school girls with family difficulties due to serious diseases and accidents, received 1,000 yuan per person, with a total subsidy of 5.394 million yuan. It is understood that the project, through the active application of students and the recommendation of the school, through the preliminary examination of the county and district youth league organization, the final examination of the Inner Mongolia Youth Foundation, the final determination of the list of aided students, and was allocated to the aided students in May and December 2022."Fantasy Education Program" is a project to provide new impetus and sow new hope for the growth and development of rural teenagers. The effective implementation of the project in the Inner Mongolia Autonomous Region plays a positive role in further promoting the rural revitalization work of the Communist Youth League, helping rural poor students in their studies, and serving the healthy and happy growth of young people. This year marks the 100th anniversary of the founding of the Communist Youth League of China and the 32 years since the Inner Mongolia Communist Youth League implemented the Hope Project. Inner Mongolia will continue to use practical action to actively organize "welcome 20, always follow the party, new journey" theme practice education activities, focus on the responsibility of the party education mission, deepening hope project connotation, continue to intensify efforts to hope project 1 + 1, love bird student fund, tianxiang fund, sunshine young, feeling warm minority girls, interpreta dream university student projects.

2022-04-26	Beijing: "green vibration Beijing suburb" brand fully open Beijing: "green vibration Beijing suburbs" brand fully open the communist youth league power rural revitalization road jianwei correspondent Ren Yi Ma Xiaoyu Yan Liang) recently, the Beijing municipal committee of the rural revitalization of youth achievements "green vibration Beijing suburb" brand theme activities in the pinggu district of Beijing Beijing tile agricultural science and technology innovation center. The Beijing Municipal Party Committee of the Communist Youth League, the Agriculture and Industry Committee of the Beijing Municipal Party Committee, the Municipal Bureau of Agriculture and Rural Affairs (the Municipal Rural Revitalization Bureau), the Pinggu District Party Committee and the District Government, and the Beijing Rural Commercial Bank will jointly help the rural revitalization. Focus on rural revitalization and take advantage of regional advantages. Start a comprehensive strategic cooperation with China Pinggu Agricultural Zhongguancun, jointly promote the establishment of "Qingzhen Beijing suburb" live broadcast delivery base, build a platform for industry-university-research and application, and carry out live broadcast training to help farmers. We will jointly train talents, strengthen talent exchanges, and promote the selection and training activities of "good youth in every village". We will focus on organizational revitalization, consolidate the foundation of rural community-level governance, promote the construction of rural "community youth clusters", strengthen the construction of online communities, and carry out landmark service projects and activities for rural revitalization. Pay attention to horizontal union, mobilize social resources. Jointly working with the Agriculture and Industry Commission of the Beijing Municipal Party Committee, the Municipal Bureau of Agriculture and Rural Affairs (the Municipal Rural Revitalization Bureau), and the Beijing Rural Commercial Bank to create advantageous brand projects, which has been included in the relevant documents of the Beijing Municipal Party Committee and the Beijing Municipal Government. Deepen group silver cooperation, green choose the most beautiful home stay facility, green rural youth innovation entrepreneurship competition, green painted in the heart of the beautiful countryside, green love suburban youth friendship fellowship activities, green broadcast green Yun China live help farming activities and other series of brand activities, the financial policy, financial services, better build "green vibration suburbs" brand, will serve rural revitalization of the gas. Dig deep into the needs of young people and stimulate their enthusiasm. Promote the Beijing youth home stay facility entrepreneurship league, Beijing university communist youth league service rural revitalization alliance, play cohesion, cultivating young entrepreneurial talents, encourage college students to return home, contribute to the role of rural wisdom, will drive the rural tourism gear upgrades, create new hot spots, shaping new rural era, inject momentum for rural sustainable development.

2022-04-24	Jianwei Correspondent Yue Qing) On April 23, the "Guangdong, Hong Kong and Macao" jointly held by Guangdong Youth Federation and Guangdong Students' Federation was held in Guangzhou. The event was broadcast live on major online platforms, attracting 582,000 young students from Guangdong, Hong Kong and Macao. It is reported that the first plenary meeting of the 12th Committee of Guangdong Youth Federation and the 12th Congress of Guangdong Students' Federation will be held in May this year. This exchange meeting is one of the series of activities of the conference.site of activity. Group of Guangdong provincial party committee for figure in this activity of Guangdong, professor Shen Minghao first of Guangdong large bay area development trend and youth opportunities to share, he points out that a large bay area of Guangdong has location advantages, for youth provides all-round, multi-level development opportunities, called for Guangdong young students to bear the era of national rejuvenation. Professor Shen Minghao, president of the Guangdong-Hong Kong-Macao Greater Bay Area Research Institute, shared the theme of the Development Trend and Youth Opportunities of the Guangdong-Hong Kong-Macao Greater Bay Area. Four young student representatives from Guangdong, Hong Kong and Macao shared them from the perspectives of scientific and technological innovation experience, Guangdong entrepreneurship experience and social practice experience at the meeting. Wen Xiaoxiao, a young "outstanding student" from Guangdong province studying in South China University of Technology, not only led his team to win the national first prize in the "Challenge Cup" competition, and approved the key projects of the climbing plan, but also won 25 patents."Young students should have enough knowledge reserve, the fundamental curiosity, and the courage to show their youthful responsibility in the tide of The Times," she said."I am a farmer of the new era, and I am very proud."Luo Wei Te, a young Hong Kong man, shared his story of" growing vegetables " in Guangdong. After graduating from university, he took root in rural Jiangmen for five consecutive years to carry out scientific research on fish and vegetable symbiosis technology. He has the fish and vegetable symbiosis industrialization project with independent intellectual property rights, among which the 6200 square meter fish and vegetable symbiotic plant factory has been awarded the "Vegetable Production Base for Hong Kong and Macao" and "Guangdong-Hong Kong-Macao Greater Bay Area Vegetable basket Production Base". He himself has also won the Guangdong Youth May 4th Medal and National Youth Pioneer of Rural Revitalization. From "Hong Kong and Macao youth being served" to "youth serving Hong Kong and Macao" is a transformation of growth. Xue Yujun, a young student from Hong Kong who studies at Jinan University, shared her story about promoting the entrepreneurship and employment of young people from Hong Kong, Macao and Taiwan. In the "Spread the Wing Plan" special internship campaign for college students from Hong Kong, Macao and Taiwan, she served more than 1,500 young students from Guangdong, Hong Kong and Macao, including 1,451 students from Hong Kong, Macao and Taiwan, and 482 students from Hong Kong, Macao and Taiwan."I am very lucky to provide advice and help for young students working in Hong Kong and Macao from the perspective of students from Hong Kong and Macao!"She said. Four sharing guests shared and exchanged views at the event. When Lin Junle, a young student from Macao and a student at Southern Medical University, was asked why he wanted to join the national flag guard, he said, " Watching the flag bearer stand like a silver gun and feel like a meteor, which is a big reason why I joined the team. But I think the experience in the national team is a rare spiritual treasure for me."Four sharing guests jointly issued the Proposal for Young Students from the Guangdong-Hong Kong-Macao Greater Bay Area. Group of Guangdong provincial party committee for figure activities in the end, four young students representatives of Guangdong and Guangdong large bay area youth students initiative, called for Guangdong youth students challenge home feelings, brave bear the youth mission, in the new era of a large bay area of Guangdong in the wave of construction, singing style passionate bay youth main melody, be bear the national rejuvenation era.

2022-04-24	Sichuan to celebrate the 100th anniversary theme exhibition in Chengdu to celebrate the 100th anniversary theme exhibition, China youth network reporter wang xinxin) China socialist youth league Luxian branch early precious archives pictures, the important information of xiao pusheng, Sichuan first rural youth league organization director Zhao Yiman historical pictures... on April 22 afternoon, by Sichuan province, the museum of Sichuan province to celebrate the 100th anniversary theme exhibition in Chengdu. The ceremony was held right there. The theme of this exhibition is "Welcome the 20th Party and Always follow the Party". It takes the form of combining historical pictures, cultural relics, audio and video, and interactive experience, and shows the historical traces of the Sichuan Communist Youth League and youth cause in the past hundred years. The introduction of Zhao Yiman. The total area of the exhibition hall is more than 2,000 square meters, with 918 pictures and more than 1,000 physical objects displayed. The exhibition combines the characteristics of youth and integrates digital technology, and specially sets up "group history answer mini program" and "scene restoration photo area". The audience can conduct immersive interaction and communication by answering questions and taking photos. In the physical part of the exhibition, the relevant materials collected from the Sichuan Communist Youth League system and many cultural relics and physical exhibits copied from many museums, memorials and archives in the province are displayed. The exhibition will last until the end of 2022 and will feature an online panoramic display.

2022-04-23	Shanghai's 78,000 young commandos are involved in the epidemic prevention and control commandos is a glorious initiative for the Communist Youth League to shoulder the heavy burden in the socialist construction. In the face of the impact of the epidemic, the Shanghai Communist Youth League gave full play to its organizational advantages and strengthened its organization and mobilization. Through the formation of youth commandos, in the "urgent, difficult and dangerous" task and the race against the virus, the regiment flag and the youth commandos were always flying in the front line of the "fight against the epidemic" in Shanghai. According to incomplete statistics, since March, Shanghai youth league organizations at all levels have set up 2,553 youth commandos, and 78,000 youth league members have devoted themselves to the epidemic prevention and control. Take the heavy burden, charge in front is the youth commando youth footnote, for this "war epidemic", they are willing to "green" do their best."We are young people, we do not go who!"" we are young, we do not go who!"The epidemic prevention and control situation in Shanghai is grim, and many urgent tasks urgently need to be undertaken. The youth commandos expressed their determination in this simple sentence. After receiving the construction task of makeshift hospital construction, Lingang Group set up a number of youth commandos, and organized more than 100 young people to invest in the makeshift traffic construction, on-site operation and logistics support lines. The youth commandos were led by Chen Haici, secretary of the Youth League Committee of Lingang Group. The traffic construction youth commandos completed the site survey and coordinated the preliminary design drawings of the Lingang cabin within 24 hours. We contacted Pudong Electric Power Company and Lingang Water Supply and Drainage Company in the first time to complete the application for on-site temporary hydropower access, thus ensuring the "lifeline" in the process of operation. Luo Yuan and other young commando members of the design and management department of the group gave full play to their professional strengths, organized the design unit to communicate with Huashan Hospital, site operation team, sanitation, public security, fire protection and other departments, and completed the cabin design optimization work within 48 hours after the start of the project to ensure the smooth implementation of the project. The "blue zone" of the harbor cabin is the closest area to the patient. Wang Fei, member of the Youth League General Branch of Pudong New Economy Company, and the team are responsible for docking and implementing the maintenance needs inside and outside the square cabin. Nearly 200 various repair needs flock to the maintenance support team every day. A string of data can record their daily records: more than 100 phone calls a day, more than 20,000 steps, and work for nearly 20 hours a day. Jinshan makeshift Hospital is another "battlefield", with a 66-member youth volunteer service commando team managing the operation and peacekeeping service and support work, selected by the Shanghai Construction Engineering Youth League Committee from the 280 registered youth League members.cabin sign posting, bed laying, goods placement, cabin material procurement, material inventory and storage, material handling, network layout... The commandos actively assisted medical personnel to carry out nucleic acid testing on site, contributing to the timely opening of Jinshan makeshift Hospital."Disinfection" difficult "" started, come on!"" We're going to start to work, come on!"At present, Shanghai is carrying out half a month of a new round of disinfection assault work, Shanghai fire rescue team each youth commandos back on the disinfection equipment, on the Yang building, temporary isolation points, public environment for disinfection. By April 18, the Shanghai fire and rescue teams had carried out 286 disinfection duties, covering 8,265.17 square meters, covering 1,193 hours. In the community, a special "sterilization commandos" were set up. At 8 o'clock in the morning, the "disinfection commandos" have been busy in the Jiangsu Road street community, they shoulder back sprayer, holding a spray head, wearing a full set of protective equipment at each point, the residential public areas of the comprehensive elimination. In this batch of xiaosha commandos, there is a "xinhua property youth xiaosha commandos", by the new changning group xinhua property young member cadres and a group of relatively experienced property staff of temporary, after sanitizers expert pre-service professional training, in prevention area and control area, the corridor handrails, bin room, courtyard corner and other public parts for effective sanitizers. Since most of the sterilization area is the husband room, the members of the sterilization team had to carry heavy disinfection equipment and hiking at least 60 times every day. At the end of the day's disinfection work, after taking off their protective clothing, they were sweating."In the six-story house, each of us has to run 8-10 brand numbers every day, carrying more than 30 jin of disinfectant bucket running up and down, the physical consumption is very big."New Changning Group Xinhua property of Song Huiying sigh. Pharmacy is also a difficult problem during the outbreak. Order to buy medicine in Shanghai Internet hospital or online drugstore, can you get medicine? In Beicai Town, Pudong, the core area where the epidemic was first sealed down in Shanghai, a youth commando team fighting the epidemic has linked up the "drug delivery chain". The team consists of more than 100 party members and volunteers from Shanghai Chengtou and Shanghai Police School. One of the teams is responsible for the reception, sorting and distribution of medicines in local Internet hospitals. Young commandos from Shanghai Police School investigated five cadres of the neighborhood committee, who said the problem was widespread. " Due to the long community closure time, a large number of residents need to use medicine. Some medicines can not be matched near, Pudong can not match, need to go to the Puxi designated pharmacy to match."Youth commandos captain to said, they in the dispensing at the same time, using data formatting import residents dispensing demand, and adopt complex address manual review way, calculate the optimal distribution route, distribution of carding procurement process, difficulties and precautions, summarizes four process points, three experience, for the subsequent drug security task experience. Facing patients and viruses, from day to night, young medical workers always face up to danger and race against time against the virus. The Youth League Committee of Shanghai Public Health Clinical Center has set up exclusive youth commandos. The post-80s play a backbone role, and the post-90s play the main body. Since February 2020, they have been on the front line of the fight against COVID-19, with the microscope as their "scope" and the pipette as their "gun". With various chemical or physical effects, they have followed the treatment process of more than 7,000 COVID-19 patients."max" wrote "Shuguang Zhang Xing", and he became one of the first 28 people composed of doctors, nurses and hospital feeling in the Shuguang Medical Team affiliated to Shanghai University of Traditional Chinese Medicine. He supported the treatment and rescue of patients in Shanghai Public Health Clinical Center and Minhang Geriatric Medical Center. Two years ago, also on an emergency phone call, Zhang Xing immediately signed up to support Leishenshan in Wuhan. This time, he also did not hesitate. This post-80s, post-90s and 90s youth commando team practiced the original mission of medical practitioners with practical actions. With 526 youth league members gathered, the Youth League Committee of Huashan Hospital affiliated to Fudan University set up youth commandos in Lingang makeshift hospital and the designated hospitals in Baoshan Hospital respectively. In the Lingang makeshift Hospital, the commandos put into the overall planning, diagnosis and treatment standard process formulation, patient medical care, information technology support, equipment management and other work. The youth commandos of Baoshan Hospital actively connect with the region to ensure the smooth medical first aid channels for COVID-19 positive patients with acute and serious diseases, provide a green transfer channel, carry out multiple emergency operations, escort the health of patients, and practice the mission and responsibility of "serving the people and striving for a strong country". Hospital front line is full of "battlefield", put on isolation clothes, they become the "big white mother" of the newborn babies in the ward; 24-hour shift, to ensure that the COVID-19 nucleic acid test results can be reviewed and sent accurately, as soon as possible. The Youth League Committee of the Pediatric Hospital organized the youth League members to set up four youth commandos: Jinshan Public Health COVID-19 treatment, neonatal isolation ward, COVID-19 nucleic acid testing, and material support. The team members took over the children's ward in the Shanghai Public Health Clinical Center, established the "second battlefield" of pediatrics, and practiced the responsibility and responsibility of young people in the epidemic prevention and control. Nucleic acid testing, material transportation, village residence order maintenance, community epidemic prevention is one of the main battlefields of the "epidemic war". There are many tasks and heavy tasks. Every youth commando that undertakes the task is a banner of the "epidemic" line. Less than an hour after receiving the order to set up a nucleic acid sampling team in the rescue community, the Youth League Committee of the Affiliated Cancer Hospital of Fudan University gathered 30 people to form the "Fudan Cancer" community nucleic acid sampling youth commando team. After the closure, they fought on the front line of the community road and Caohejing streets. On April 4, the day of the city, they sampled 13,159 people in the community within six hours. One hundred "vegetable man" fighting in the Changning community, before the closure, Changning Youth League District Committee first time set up 103 Changning transport "vegetable" alliance youth commandos, sinking 10 streets in the district to participate in the material distribution work. Youth conductor Ge Weiyue "cross-district" registration, even the people with the car was assigned in the Zhoujiaqiao street, but also became one of the leaders of the youth commandos. The six-man team once carried nearly 30,000 materials with their bare hands. After handling practice, they also summed up their experience, " Carrying heavy boxes, 10 rows of three or four layers, light boxes can be stacked 1010, while vegetable bags can be 50 for a pile."Post-95 commando member, the Communist Youth League member Ni Jiahao also rushed to the front line, not only repeatedly moved, unloading and transporting anti-epidemic supplies, but also in the geographical west neighborhood committee to participate in the nucleic acid testing volunteer work. Sometimes after a busy day, the hands are too sour not to lift up, but to see the residents received the joy of life supplies, again bitter and tired it is worth it."If we do more, the residents can get the supplies as soon as possible. And with the cooperation of the residents, the epidemic can end a day earlier."In Pudong Wanxiang town, the town youth League committee in just a few hours quickly gathered the members of 30 youth members, they became" Baymax ", formed a special youth commando. Commando was a "propaganda detachment", of which Pan Siwen was one. Seemingly young, he is already a veteran of the epidemic. In 2020, he signed up as a college prevention volunteer and went to the crossing to guard the entrances and exits of a city. This year, when the epidemic hit, Pan Siwen responded to the call of the town youth League Committee, joined the youth commandos again, and went from street to street together with other team members to publicize the latest epidemic prevention measures, and guided the village residents to stick to the "stay at home" and "do not gather". This "war epidemic", let more people see the youth commandos not afraid not retreat the youth.

2022-04-22	Jiang Yutong reporter, correspondent Cong Yang) " We daily walk from street to street, and contact thousands of people every day, especially during the epidemic period, we also have concerns during the process of work. Now with the free COVID-19 insurance provided by the Youth League, the bottom!"After applying for the special COVID-19 insurance on the wechat mini program" Dear Youth Little Brother Online ", meituan little brother Ye Wenbin smiled on his face," Thank the Communist Youth League organization for doing practical things for us."Ye Wenbin get the special insurance, is the Zhejiang provincial party committee to carry out the Zhejiang provincial government about caring logistics practitioners, joint insurer in Zhejiang branch, provincial postal administration, provincial transportation hall, for delivery, delivery, logistics for chang industry youth launched a new service, the first to the express little brother, delivery little elder brother, truck drivers, network about car driver group 100000 new crown outbreak special insurance. Little brothers just need to log in "pro brother online" WeChat small program, after simple registration and real-name authentication can be directly received. It is reported that the coverage of this insurance service includes COVID-19, hospitalization and death for COVID-19. A one-time subsidy of 2,000 yuan for COVID-19 cases or above, 100 yuan per day for hospitalization for COVID-19 infection, and a one-time compensation of 100,000 yuan for death of COVID-19. Recently, the group of Zhejiang provincial party committee secretary He Libin to Hangzhou downtown triumph street kai @ home youth home, green green street delivery station and other sites to see Courier little brother and delivery rider, understand during the epidemic prevention and control safeguard measures, and specifically for bao chang industry youth on the insurance package, "you guarantee our express, logistics, this time for us to protect you". He Libin, secretary of Zhejiang Provincial Youth League, visited the Courier and delivery rider. Photo provided by the Communist Youth League and Zhejiang Provincial Party Committee. Since 2021, Zhejiang province, city and county level 3 youth league organization linkage, to carry out the "thousands of dry pair little brother" practice research activities, combined with "I do the practical work for the youth", cadres through a day little brother, interview, and little elder brothers to eat, work, understand their survival situation, thought dynamic, according to the little of the elder brothers' practical difficulties and practical needs, tailored. One of the "seven tips" care services is to allocate special tour fees to buy epidemic prevention and living materials for young people in the industry, focusing on providing masks, gloves, disinfectant and other protective equipment necessary for work and necessary daily items during the quarantine period. The Zhejiang Provincial Party Committee of the Communist Youth League has allocated 100,000 yuan of special tour fees in advance to purchase epidemic prevention materials and send them to his little brother. Zhejiang youth league organizations at all levels relying on the "youth", service center, the new era civilization practice site, government affairs hall, "youth civilization" unit window service position, construction of "little brother station", equipped with mobile phone charging device, raincoat rain gear, medical first aid kit, epidemic prevention materials and other facilities, to provide the little elder brother with comfort rest, life supply, warm heart care basic services and scratch first aid, heat preservation and other emergency services. Next, the Zhejiang Provincial Communist Youth League Committee will also fully promote the use of the "digital platform online", integrate various resources online to provide diversified services for the youth in the industry, actively respond to the urgent needs of the youth, and relieve the worries of the youth in the industry.

2022-04-22	Gansu communist youth league: highlight Gansu group optimization service protection Gansu communist youth league: highlight key group optimization service guarantor Ma Fuchun) in recent years, Gansu communist youth league insist service party and government center work and safeguard teenagers legitimate rights and interests, give full play to department functions, more measures and safeguard the legitimate rights and interests of teenagers, promote the healthy growth of teenagers. Youth league organizations across Gansu province have deepened volunteer services to care for left-behind children in rural areas, actively organized youth volunteer actions, and carried out actions to care for left-behind children through pairing of schools, pairing of children of migrant workers, and the "2 + 1 Peer Plan". In recent years, a total of 1,290 Communist Youth League "colorful hut" projects of caring for left-behind children have been completed, and three consecutive Gansu Province Youth Volunteer Service Project competitions have been held, and the content of "Caring for Children Volunteer Service" has been included in the competition project. We have actively carried out the work of helping young students for the poor, thoroughly implemented the "Elegant and elegant youth plan to help the poor" and the provincial government college enrollment funding for children from urban and rural subsistence allowance families for the people, so that teenagers from poor families can fully feel the warmth of the Party and the government. Since last year, actively carry out the "China maotai pillars-2021 hope project interpreta dream action large public welfare student activities", the 24th "lotus students" large public welfare activities, 2021 "my university" dream scholarship activities, "hope project 1 + 1" funding projects, etc., the annual funding of 3953 students, allocated grants 6.8324 million yuan, continue to improve support effect, to ensure the safety of student aid gold high quality and efficient distribution. We will carry out further activities to create "youth rights protection posts". In recent years, according to the national youth QuanQuanGang to create work deployment, the province each relevant system grassroots unit closely combined with their functions, around the youth rights and interests maintenance and healthy growth and work actively, enhance the sense of teenagers, happiness and security, in the protection of minors and youth rights has made outstanding contributions, emerged a batch of advanced typical. The province's 32 grass-roots units were named as the "national youth rights protection post" by the central Committee of the Communist Youth League. We carried out face-to-face activities between the Communist Youth League and NPC deputies and members of CPPCC committees. Strengthen the people's congress, CPPCC member contact with teenagers, further open youth appeal reflect channels, issued by the communist youth league to the NPC, CPPCC members face to face activities, in order to "enhance the level of adolescent mental health services" as a "the communist youth league and the people's congress, CPPCC members face to face activities" theme, the youth league organizations at all levels in the province. Up to now, the municipal and county-level plans have been collected and completed, the research work is being carried out in an orderly manner, and the work of protecting the legitimate rights and interests of minors has attracted wide attention.

2022-04-22	Jianwei correspondent Sun Bo) Recently, the 2022 Henan Province College Students Volunteer Service Western Region plan recruitment mobilization meeting was held. Wang Jiwei, deputy secretary of the Provincial Communist Youth League Committee and director of the Western Provincial Planning and Project Management Office, attended the meeting and delivered a speech. Responsible comrades of the Provincial Education Department, the Provincial Finance Department, the Provincial Department of Human Resources and Social Security, and relevant responsible comrades of the project office of 30 universities in Zhengzhou attended the meeting. The meeting stressed that the province's university project office should earnestly study and implement the important spirit of the teleconference work conference, and do a good job in the 2022 college students volunteer service plan in Henan Province. We should deepen our theoretical study, and improve our political standing. The project offices of colleges and universities should thoroughly study General Secretary Xi Jinping's important thoughts on youth work, accurately grasp the new requirements for the development of volunteer services with Chinese characteristics, and earnestly carry out all the work of the Western China Plan. To thoroughly mobilize the deployment, do a good job of recruitment and publicity. Innovative publicity methods, with "green language", "net language", short video, H5 and other young people popular content and ways of continuous publicity, dig excellent deeds, singing "to the west to the west, to the grass-roots, to the most needed place of the motherland" melody of The Times. Efforts should be intensified to ensure the completion of the recruitment task. Under the overall deployment of the local epidemic prevention and control work, the recruitment and selection work should be done in strict accordance with the principle of "three open, four necessary, five passes and six priorities", so as to ensure the successful completion of all the work of the western plan and welcome the victory of the Party with outstanding achievements. It is reported that college students' volunteer service plan in Henan for 19 years, the province selected recruit 12779 volunteers, including 8582 volunteers to Xinjiang, Tibet, Gansu, Guizhou, Qinghai, Inner Mongolia and other central and western regions, 4197 volunteers to the key support county service in the province, to promote the local economic and social development contribution youth wisdom and strength. In 2022, Henan province will continue to recruit young volunteers from western provinces (autonomous regions) and key counties in Henan province, and increase the recruitment of graduates from agriculture and forestry technology, medical nursing, social work, basic education and other majors, so as to continuously improve the effectiveness of services.

2022-04-20	A total of 19,442 new local confirmed cases and asymptomatic infections have been reported in Shanghai. On the same day, 23,286 people were discharged from Shanghai, surpassing the first number of new asymptomatic local infections on the same day. This is a major boon since the COVID-19 outbreak in Shanghai on March 1. China Youth Daily and China Youth Daily has previously opened the platform for collecting news clues about the epidemic in Shanghai. From thousands of calls every day, our reporter found that the "silent minority" life security issues who can not surf the Internet and are not good at group buying has become an important concern. Recently, the Shanghai Municipal Committee of the Communist Youth League, the Municipal Service and Protection Office, the Municipal Youth Federation and the Municipal Youth Development Foundation jointly launched the "youth war against the epidemic" youth assistance campaign for teenagers in distress, sending real help to more than 3,000 young people in distress in Shanghai. In the interview, the reporter found that this was a closed-loop chain of the whole process from finding problems, coordinating and solving problems, delivering materials and returning materials to the return visit and feedback, "breaking a way" for young people in difficulties under the epidemic. In the process of "breaking a way", Shanghai Youth Service and Protection Office is responsible for arranging the needs of teenagers in advance, checking the distribution information, and coordinating the distribution points with the Shanghai Department with the material warehouse; the members of the Youth League, the staff and Sunshine staff formed a 10 transport youth commandos, together with hundreds of grassroots cadres and youth social workers, to get through the "last kilometer" and "last 100 meters". Starting from April 10, a working group was jointly formed by the Shanghai Municipal Service and Protection Office, the relevant departments of the Municipal Communist Youth League Committee and the Municipal Youth Development Foundation to send "timely rain" to the families of minors in distress. In the last week, the day of Wang Yibo, a member of the Shanghai Youth Federation, began at 5 am. Loading, unloading and delivering, closing the packed carriages, wearing protective clothing, wearing masks and protective masks, he began the day of delivery work. The two cans of eight-treasure porridge on the car are his daily "rations". What made him so "fight" was more than 3,200 troubled teenagers in Shanghai. The epidemic has pressed the "slow traffic button" in Shanghai, but the help work of teenagers "can not slow down". Under the epidemic situation, the situation of teenagers in difficulties is complex, with factors such as cross-region and limited reception. According to the preliminary situation, the municipal Service Insurance Office confirmed that more than 3,200 families in distress in need of key assistance."There are a large number of children who lack guardianship and family difficulties, and their life problems need to be solved urgently."Liu Zhuofang, deputy director of the Shanghai Service Office, said," Some children live with their grandparents and have to be careful every month. Now the elderly can not buy group, even no mobile phone, eating has become the biggest problem, how to do?"According to the results of the service protection office, the secretariat of the Municipal Youth Federation released the needs in the loving enterprises and the member groups. Twenty-four hours later, more than 3,000 "four-in-one" love gift bags were born, including study items, daily necessities, fruits, vegetables, food, and epidemic prevention materials. With supplies is the first step, "the last kilometer" and "the last 100 meters" will defeat many heroes. Chen Feng, director of the unity and liaison Department of the Shanghai Municipal Committee, is the general dispatch of the commando transport capacity deployment. "We strive to reach the 'door' of the teenagers in difficulties, but now the transport capacity is very tight. In order to send things to the children in a short time, we have to find our own way."At this time, Wang Yibo, Wang Hongsheng and Wu Ren, members of the Municipal Youth Federation, actively registered in the group, saying that they could provide transport capacity, and could "even take the driver with the car to assist the delivery"."I happen to have a spare warehouse and manpower, and I have participated in the material deployment work before, 'when I can'."Wang Yibo said. So, the 10-man transport youth commando team began a whole week of day and night. In the commandos, two large trucks are responsible for delivery, and four private cars are responsible for delivery. According to the local epidemic prevention requirements, the gift package is also delivered in two forms: one is delivered to the teenagers, and the other is transferred by the street or the youth league district committee."This is a relay of love, sent by the commandos sent to the street town, and then contact the cadres of the district or youth affairs social workers as volunteers, and then send the gift package to the community. There are no shelves at the gate of some communities, and volunteers have to call their children's families. They can't put their things down and leave, so they have to confirm that they got them."Su Yufeng, director of the social work department of the Shanghai Service and Insurance Office, said that a few days it rained heavily in Shanghai a few days ago. In order not to let the love overnight, grassroots cadres and social workers delivered emergency delivery under the bad weather. A social worker wrote on his circle of friends: " Although I am a little cold, I hope my child will feel warm."" Extreme "transportation volunteers in the warehouse" busy residual shadow " transportation process, far more difficult than imagined."It's hard to pick it up at first place."Wang Yibo said that there is no extra manpower in the warehouse, all the materials need to load the commandos themselves," thousands of copies, we are generally 'double combat', a driver and a deputy, still in the warehouse at 10:30 PM."The workload of packing thousands of supplies is staggering. The first batch of supplies was 2,000 pieces of vegetables, each weighing about 10 jin. Wang Yibo and his teammates were too tired to pack and sort them out. On the evening of April 11, the first batch of supplies were delivered to the teenagers overnight through the Youth League Jiading District Committee. He said that if a time-lapse video camera in the Jiading warehouse, we would be "out of the busy film."All the 16 workers in the factory participated in the packaging of the gift package, including both the factory workers, the construction workers who had originally been involved in the fire control renovation, and the employees of a nearby logistics company. That night, they loaded and unloaded more than 7,500 boxes, packed 1,500 gift bags the next day, and distributed 1,000 copies by 2:00 p. m."Thank you very much for solving our urgent need."While preparing for the exam, Xiao Liu comes from a subsistence allowance family in Jinshan District. On April 14, she received a gift package from the Communist Youth League and the Shanghai Municipal Party Committee. Seeing my mother busy grabbing food every day, she wrote a letter to the Shanghai Municipal Party Committee of the Communist Youth League: " I have collected many warm stories on the Internet, in the news, and around myself in the epidemic. They encouraged me, supported me, and brought me infinite warmth."In this help work, in addition to love material support, each child's personalized needs are also tried to care for: such as online class equipment, academic counseling, psychological counseling and so on. At present, Shanghai Youth Federation has raised and distributed 27 online course learning terminal equipment; the school work Department of the Communist Youth League Municipal Committee docking "cloud classroom" service, one to one academic counseling for 32 people; the Municipal Service and Insurance Office docking professional psychological counselors, has provided psychological counseling for 8 people. Chen Jiayin, China Youth Daily, China Youth Network, reporter Wang Yejie, source: China Youth Daily, April 20,2022, edition of the 201st edition

2022-04-20	Gansu to build "force once two security" cohesion high quality development build "force once two guarantee" assessment system cohesion Gansu communist youth league high quality development correspondent Chai Jianlin) since 2022, the communist youth league of Gansu province party committee focus on "three" to "four" reform and development requirements, adhere to the party building, to celebrate building in one hundred as an opportunity to introduce the Gansu communist youth league 2022 annual "force once two security" evaluation plan, built under the leadership of the provincial party committee secretary team, various departments of professional should, promote the evaluation work pattern. To "improve organization, improve leading force, improve service, contribution" four dimensions work quality effect as the goal, strengthen the "comprehensive governing group" "comprehensively deepen reform" political guarantee, innovation to build "three force once two guarantees" evaluation system, establish "plan, execution, evaluation, notification, improve" closed-loop management linkage mechanism, task oriented more clear, target, "chess" work concept, realize the Gansu communist youth league dynamic with data, green work data analysis, work with data evaluation, evaluation results with data push "wisdom group built" new results. Innovation evaluation system to ensure the results "chapter" league provincial party committee "three force once two guarantee" evaluation plan firmly grasp the sixth plenary session of the eighteenth work deployment, closely around the 2022 league work points, strictly implement the new era comprehensive governing group implementation outline, innovation put forward "multiple evaluation subject, accurate evaluation content, online evaluation process, long-term results, promote process and results, quantitative plan and evaluation, annual evaluation and democratic evaluation integration organic link" evaluation mode. A list of 69 key tasks was formulated to provide guidance for the scientific formulation of assessment indicators and evaluation points. Evaluation system to build a "group provincial party committee secretary team as the main responsibility, organization department led execution, each department coordination" evaluation responsibility system, adhere to the problem oriented, result oriented, with quantitative indicators clear department key objectives and tasks, in science as a whole departments business practice at the same time, more reflect the division of labor collaboration, effectively promote the evaluation results to the ground. The assessment focuses on the goal of "consolidating the foundation, strengthening management and grasping innovation", and sets the indicators into 14 general indicators and 12 indicators in the field respectively. General indicators highlight the basic standardization, and reflect the standardization, completeness and timeliness of the completion of the prescribed tasks. The indicators in this field highlight the quality and efficiency of the professional management of the departments, and the method of "quantitative + qualitative" is adopted for evaluation. According to the professional nature and business scope, the 79 participants are divided into three categories of "cities, prefectures, directly affiliated universities, enterprises and institutions" for quarterly evaluation. Horizontal evaluation and classified ranking of similar league organizations are implemented to highlight the differences of the assessment objects and ensure that the assessment results are objective, fair and accurate. To implement the closed-loop management waving assessment "baton" in "assessment of" three force once two guarantees "under the framework of annual appraisal index, each led department by the OA system released key work plan and prompt, clear work objectives, indicate the assessment direction," dynamic refinement adjustment " quarterly appraisal list, assessment content more accord with the work practice, directivity more clear, promote the key key index assessment closed-loop management institutionalization, standardization and scientific. Strengthen the process supervision of daily assessment and the full application of evaluation results, all departments according to the actual work, scientific and flexible adjustment of evaluation methods, through systematic access, field visits, information access, discussion and communication, fully grasp the basic and actual work of the grassroots. The quarterly evaluation results will be taken as the basic score, and two sections of "democratic evaluation" and "additional points" will be added in the annual assessment to reflect the outstanding contribution and brand building, break the previous single assessment mode of "making the final sound" with the hammer, and improve the quality and expand the scope through the actual performance orientation. In order to ensure the fairness and justice of the evaluation process and improve the work efficiency and accuracy, the evaluation and scoring forms are specially set up for the participating evaluation departments respectively, and the function publicity nested corresponding evaluation indicators are scientifically introduced, which effectively avoids the possible deviation in the process of artificial scoring, unified scoring and scoring. At the same time, according to the provisions of the evaluation method, the evaluation results of "comprehensive index management" are released quarterly, and the quality and efficiency of the work are comprehensively evaluated from the three dimensions of basic management benchmarking, the implementation of innovation results and the work contribution. Strong support tree good incentive "vane" adhere to the "compaction base quality, expansion rushed" principle, comprehensively strengthen the results, according to the "comprehensive index management" evaluation results, quarter selected comprehensive flow benchmark 1-2, the annual evaluation "good, good, good, general" four times, to be named "good" unit, from the support funds, projects, resources, evaluation and training tilt incentive, effective director entrepreneurial enthusiasm. Through the establishment of quarterly evaluation problem rectification ledger, holding special training, on-site teaching, centralized discussion, professional guidance and other forms, to promote the exchange of experience and expand working ideas. For the league organizations that have been awarded "general" for two consecutive quarters, we will cancel the evaluation qualifications within the year, and make every effort to promote the league organizations at all levels in the province to further "strengthen the weak areas and strengthen the weak links". In the next step, the Communist Youth League Gansu Provincial Party Committee will make full use of the evaluation results of "three forces and two guarantees", timely and objectively summarize the work achievements and highlights, point out the problems and deficiencies, feedback the assessment results one by one, and implement the problem rectification. Take the lack of the work of the last quarter as the key supervision content of the next quarter, constantly promote the high-quality development of the work of the provincial youth league, constantly increase the promotion of typical experience, and explore the formation of a "Gansu model" for the work of the Communist Youth League.

2022-04-19	Jianwei) Recently, the fifth plenary session of the 12th session of the Communist Youth League Ningxia District Committee was held in Yinchuan. The meeting comprehensively summarized last year's work of the year, studied and deployed tasks for this year, and made systematic arrangements to deepen the comprehensive and strict governance of the League in the new era. Chen Yong, deputy party secretary of Ningxia Hui Autonomous Region, attended the meeting and delivered a speech. Gong Xuefei, secretary of the Ningxia District Committee, reported the work to the plenary session on behalf of the 12th Standing Committee. In his speech, Chen Yong affirmed the achievements made in the work of the regional Communist Youth League in the past year. He stressed that the Communist Youth League organizations at all levels in the region should meet the study, publicize and implement the Party's 20th as the main line, adhere to the party flag is the direction of the league flag, strengthen the political direction of the youth, strengthen the theory of the youth armed, guard the ideological position, and guide the majority of young people to enhance the political identity, ideological identity, emotional identity of the Party. We should take the youth contribution action, focus on the major strategies deployed by the CPC Central Committee and the key tasks set by the Party Committee of the Autonomous region, and organize the young people to be a new force in economic development, rural revitalization commandos, and the pioneer in volunteer service, so as to act as and make contributions in promoting the high-quality development of Ningxia. We should focus on solving the urgent problems of young people, vigorously serve their development, make great efforts to solve their difficulties, and safeguard their rights and interests. We should make all our work more proactive, more solid, and more effective by strengthening service awareness, innovating service mechanisms, and improving service capacity. To further promote the comprehensive governing group as security, stick to strong "three", "four", comprehensively strengthen the league organization, team, style construction, to ensure the reform of the communist youth league, the further development, constantly improve the attraction, cohesion and combat effectiveness of the communist youth league, take concrete action to meet the party's 20 and the 13th party congress victory. The meeting noted that, Youth league organizations at all levels in the region should thoroughly implement General Secretary Xi Jinping's important thoughts on youth work and the spirit of his important speech on inspecting Ningxia, Earnestly implement the work plans of the Autonomous Regional Party Committee and the Central Committee of the Communist Youth League, Focusing on the fundamental tasks, political responsibility and main work line of the Communist Youth League, By closely maintaining and enhancing the political nature, advanced nature and mass objectives, To meet and study to publicize and implement the Party's 20th Congress as the main line, To celebrate the 100th anniversary of the group, Fully implement the arrangements of the 13th Party Congress of the Autonomous Region, Highlight the "five focus, five improvements", Unite and lead the youth league members to contribute their youth strength to building a beautiful new Ningxia with prosperous economy, beautiful environment and beautiful people. Focus on the party's 20, carry out "welcome 20, always follow the party, new journey" theme education practice, continue to deepen youth theory, extensive ethnic youth communication activities, casting a community consciousness of the Chinese nation, to maintain youth political and ideological security, improve the effectiveness of ideological and political lead. Focus on beautiful new Ningxia construction, closely around the autonomous region party committee to determine the major tasks and the Yellow River basin ecological protection and high quality development zone construction, give full play to the youth advantages, organize the implementation of ecological construction, industrial development, rural revitalization, grassroots governance four youth contribution action, gather youth wisdom, condensed youth strength, improve the contribution of the new era. Focusing on the concerns of young people, we will further implement the medium-and long-term youth development plan, provide contact services to emerging young people groups and social organizations, care for and help them in need, deepen and expand the "I Do Practical Things for Young People" campaign, and improve the accuracy of contact services. Focus on comprehensively deepening the reform, closely around the "grass-roots youth League organization reform year", continue to deepen the reform of the county Communist Youth League, the youth League organization reform, the school and the leadership of the whole youth league, promote the implementation of reform measures, enhance the cohesion of the grass-roots youth league organizations. We will focus on comprehensively governing the League with strict discipline, strengthen the overall leadership of the Party, strictly improve the political, organizational, and institutional implementation of the League, strictly manage the discipline style of its cadres and league members, and enhance the combat effectiveness of the League. The meeting required that the youth league organizations and cadres at all levels in the district should be good at judging the problems politically and understanding the requirements of implementing the deployment, make great efforts to strengthen the political construction, and constantly improve their political judgment, political understanding and political execution. We should show the loyalty, cleanliness and responsibility of young cadres, bear in mind the organizational nature of the Communist Youth League, take out the spirit that young people should have, and show the practical state that young organizations should have. Under the guidance of the Party, we must conscientiously ensure the implementation of our work, give priority to key work such as political guidance for young people, serving the overall situation, contacting and helping them, strictly governing the Youth League, and preventing risks, and accomplish all the tasks set at the plenum to a high standard. Meeting deliberated and approved the Ningxia communist youth league in 2022 work points "," the Ningxia communist youth league about deepening the new era comprehensive governing group implementation opinions " and ten working mechanism, adjust the group of Ningxia district party committee in rural areas, enterprises, schools, the young pioneers, emerging field five special committee, discharge, co-elected the 12th committee members and standing committee. The meeting was held in the form of a video conference. More than 230 people, including members of the 12th Ningxia Youth League Committee, the municipal, county (district) youth League committee, the youth League working Committee, directly affiliated units and youth league organizations, responsible members of the central enterprises in Ningxia and youth representatives, attended the meeting.

2022-04-18	Sichuan has issued a "sprint order" just three years before the end of the medium-and long-term youth development plan, and Sichuan province has issued a "sprint order". At the fourth plenary meeting of the Joint Conference on the Implementation of the Sichuan Province Medium-and Long-term Youth Development Plan (2017-2025) (hereinafter referred to as the "Plan"), Sichuan Province launched a number of work plans and systems to ensure the completion of the targets and tasks set out in the plan. The plan will be implemented from 2017 to 2025, and more than half of the time has been implemented until now, and it has achieved phased results. For example, some target tasks in key areas are completed ahead of schedule. Data show that in 2021, the number of new labor force education years in Sichuan province has reached the planning target of 13.5 years, and the gross enrollment rate of higher education has exceeded the planning requirements of 50%, and the physical fitness rate of young people having reached the standard has exceeded the standard of 90%. However, the meeting also pointed out that there are still big shortcomings in some areas. Yu Lijun, a member of the Standing Committee of the CPC Sichuan Provincial Committee and head of the Organization Department, said that if we do not work hard, it will be difficult to achieve the planned goals. At the meeting, Yu Lijun took the surveyed unemployment rate of urban youth aged 16 to 24 as an example, and asked the relevant member units of the joint meeting to "focus on this matter", carefully study, and put forward countermeasures to be "effective and effective". Yu Lijun said, especially in youth education, youth health, youth and other social security binding indicators, the joint conference members to systematic comb, good analysis and judgment, which indicators data still gap, which guarantee policy or blank, which projects have not been implemented, must be clear. The meeting reviewed the "on further promoting the Sichuan long-term youth development plan (2017-2025) further implementation of three-year action plan (2022-2024)" (hereinafter referred to as the "three years action plan") and "Sichuan long-term youth development plan (2017-2025)" implementation joint conference annual reporting system "(hereinafter referred to as the" annual reporting system "). The three-year action plan defines the tasks in five aspects: building a more efficient youth work system, establishing a more accurate youth policy system, forming a more scientific youth work mechanism, carrying out more effective research on youth development, and creating a more friendly environment for youth development. Three years action plan, around the youth ideological and moral, education, health, employment entrepreneurship, rights and interests maintenance and other key areas, focus on 2-3 aspects a year, promote landmark youth development policy, a real "hard measures", create inclusive friendly "soft environment", solve practical problems, realize the youth development policy key breakthrough. The annual reporting system set up at the meeting requires the person in charge of the member units of the joint meeting and the convenor of the municipal (state) youth work joint meeting to report to the provincial joint meeting every year. The report includes the study and implementation of General Secretary Xi Jinping's important thoughts on youth work, the implementation of the Party's principle of managing youth, and the concept of "youth priority development first". Zhang Rong, head of the Office of the Joint Conference and secretary of the Sichuan Provincial Committee of the Communist Youth League, said that the implementation time of the plan has already been half over, and the task is urgent. The launch of the above work plan and system at this point is to mobilize, deepen and implement it again. At present, the implementation of the plan has entered from the second half of the stage, the introduction of these work plans and systems, is also to urge the city and county level party committees and governments to continue the implementation of the plan, to avoid the work gap, and to make breakthroughs in key areas.'Young people still have many things, even distress, to worry about and worry about job hunting, innovation and entrepreneurship, social integration, dating, children's education and support for the elderly, 'Mr.Yu said.' We need to formulate targeted policies and take more effective measures so that young people can feel that the Party committees and the government are helping and caring for them.'Source: China Youth Daily, April 18,2022, edition of the 02nd edition

2022-04-18	Beijing build young pioneers education service community Beijing build young pioneers education service community ning) on April 15th afternoon, Beijing municipal party committee, Beijing municipal education commission, Beijing less working committee in China aerospace science and technology group five institutes to carry out the "red scarf love capital" young pioneers off-campus characteristic practice project release activities and "red scarf school" the first lesson. Activities, 10 units became the first "red scarf love capital" Beijing young pioneers campus practice education base, including: Chinese CPPCC, China aerospace science and technology group five yuan exhibition center, China song ching ling young science and technology culture exchange center, China garden museum, the communist party of China early Beijing revolutionary activity memorial, xiangshan revolutionary memorial hall, Beijing garrison political work (national flag class), Beijing youth palace, Beijing children's palace, Beijing jade deep pool park. The aerospace spirit publicity group of the fifth Academy of Aerospace Science and Technology Corporation was appointed as a member of the "Red Scarf Lecture Group" in Beijing. The Beijing Municipal Committee of the Communist Youth League and the Beijing Municipal Youth Working Committee carried out the first lesson of "Red Scarf School-China Flying Dream" simultaneously online and offline, and 25,000 primary and secondary school squadrons watched the live broadcast. On the same day, the "Red Scarf Love the Capital" scientific practice map was also released simultaneously. The "Red Scarf Love the Capital" scientific practice map integrates the high-quality course resources of 21 popular science popularization bases, reduces tickets and provides preferential courses for young pioneers, and optimizes the guarantee mechanism for young pioneers to participate in social practice. To jointly promote the campus form "young pioneers education service community", researcher of Chinese Academy of Sciences, Chinese Academy of Sciences, aerospace science and technology group five institute chang e 3 chang'e probe system chief designer, chief designer day asked 1 Mars probe system sun Zezhou hired Beijing young pioneers outside school counselor. Source: China Youth Daily, April 18,2022, edition of the 02nd edition

2022-04-17	Jianwei correspondent Wu Jing) On April 15th, the fourth plenary (expanded) meeting of the seventh session of the Fujian Provincial Youth Working Committee was held in Fuzhou. Members of Fujian Provincial Youth Working Committee, relevant responsible comrades of the Youth League committee and education bureau of each district and Pingtan Comprehensive experimental area, secretary of the Youth League Committee of the county (city, district) youth League committee, director of the provincial primary and secondary school youth working Committee, and counselors of the young Pioneers brigade attended the meeting. Deputy secretary of the provincial Youth League Committee, provincial department of education party group members, deputy director, provincial education working committee member Li Xuan attended the meeting and speech. Meeting thoroughly implement the xi general secretary about children and young pioneers work important discourse, convey the spirit of the 11th party congress spirit, the sixth plenary session of the 18, league party 14th sixth plenary session spirit, combined with the actual work, focus on the "three, three effect", stick to the "five grasp, four focus" work layout, further promote the central committee of the communist party of China "on comprehensively strengthening the new era young pioneers work opinions" (hereinafter referred to as the "opinions") each work in place; meeting summarizes the provincial young pioneers in 2021, deployment of young pioneers work in 2022. The meeting pointed out that the young pioneers organizations at all levels in the province should systematically study a series of important instructions and requirements of General Secretary Xi Jinping and the CPC Central Committee for the work of the Young Pioneers since the 18th CPC National Congress, and further enhance the political consciousness, ideological consciousness and action consciousness of the work of the young Pioneers in the new era. We must always uphold the Party's leadership over the work of the Young Pioneers, To faithfully implement the Party's requirements into the work and construction of the Young Pioneers; We must always bear in mind the responsibilities and missions of the young pioneers in the new era, Fully understand the importance of strengthening the work of the young Pioneers from a strategic height; To accurately grasp the main line of the young pioneers' work in the new era, Starting from the standardization of building the young Pioneers 'organization, actively improving the organizational life of the young Pioneers, and strengthening the influence of the young Pioneers' culture and education, Constantly enhance the young pioneers' sense of honor and the sense of belonging to the organization; To firmly seize the important opportunity of the young pioneers in the new era, Actively strive for the work guidance and support of the local Party committees and governments, Make full use of the policies of the young Pioneers in the new era, Vigorously promote the formation of a young Pioneers working system and mechanism under the leadership of the Party committee, government support, the leadership of the Communist Youth League, league and education cooperation, and social coordination, Strive to create a good atmosphere that the whole society cares about children and attaches importance to the work of the young pioneers. The meeting required that all levels of league organizations to the political, organization, team, work with the young pioneers, strong team, with the young pioneers work planning, integrated promotion, integrated assessment, to establish the team integration work ideas, and the young pioneers into the communist Youth League "three force once" work pattern integrated deployment, strive to build a dynamic, strong organization of the new era. The meeting required that the education departments at all levels in the province should have a deep understanding of the important position of the Young Pioneers in the Party's educational cause, and give full play to the unique educational role of the young Pioneers in basic education. In accordance with the "opinions" requirements, to do a good job of the young pioneers work as a political responsibility to grasp the real. It is necessary to deepen the cooperation between the league and education, start with improving the system and mechanism, strengthen the guarantee of conditions in all aspects, and work together to promote the new development of the young Pioneers of Fujian Province in the new era. The meeting was held in the form of video, the League Longyan Municipal Committee, Cangshan District Committee, Fu'an Education Bureau responsible person made a typical speech at the meeting.

2022-04-20	With social organizations as the fulcrum to explore the "cloud classroom" with the theme of the maximum service force in Tianjin is so popular. A few days ago, the event quickly attracted hundreds of people to sign up after the official account of "Chenqing e-color" of the Beichen District Committee of Tianjin, Tianjin. This hot scene let Han � hammer core Chang Chang Jin Jin � she knows, most of these are "old fans" and "iron fans", "the children like us very much, recently have online classes at home, looking forward to having activities to play together". This is one of the youth after-school practice brand projects carried out by Tianjin Beichen District Committee relying on youth social organizations. To get through the county communist youth league organization of the reform of "the last kilometer", in recent years, the youth league organizations to develop youth social organizations as the fulcrum, to explore the group "vegetables", social organizations "cooking", evaluated integratedly village "order", teenagers "teenagers" taste ", the social from all walks of life" review " social full closed operation mode, leverage the whole society service youth. Cultivate the soil suitable for the growth of social organizations as a grass-roots labor coordinator, in the neighborhood committee as a social worker, Han � � in the first 10 years, are concerned about the people's family affairs. When she quit her stable job in the neighborhood committee and settled down to run the Xingyao Future Social Work Service Center, most of her family and friends did not understand. But she hopes to "realize her ideals while she is young", and "likes to deal with people and be willing to serve people" is her original intention to establish a youth social organization. She has been serving the community residents. She not only understands the rules and procedures of grassroots government operation, but also knows the most concerned issues of the common people and the troubles of people of different ages. Using her expertise in coordination and communication, she has launched a series of volunteer service projects to meet the needs of different residents. In the early stage of its establishment, both the site and the personnel were difficult problems. The Beichen District Youth League organization helped her coordinate about 1,000 square meters in the community party and mass service center as a fixed place for office work and activities. More importantly, through government purchase, the brand project of the social work service center was included in the "Weekend Practice Camp" series of projects of the Communist Youth League District Committee, and included into the series of "chapter competition" activities for the Young Pioneers. Nowadays, the towns and villages in the whole district can "order" according to the characteristics and preferences of the teenagers in the area, and the teenagers can "taste" the colorful activities and "feast" at home. Group beichen district party committee deputy secretary XingHongDan said, while the youth group growing diversity, personalized service demand, one side is the number of grassroots cadres is very limited, develop more and better youth social organizations, became the important way of the communist youth league contact service guide teenagers, "the development of social organizations help to promote the reform of the communist youth league, is also beneficial to extend the power of youth work". In recent years, the Beichen District Committee of the Communist Youth League makes full use of the resources and talents of the Communist Youth League to vigorously cultivate an excellent social soil suitable for the growth of professional youth work institutions. At present, it has guided the establishment of 10 youth social organizations and recruited more than 100 full-time social workers. On the one hand, relying on the "youth home" and other positions to coordinate towns, provide support for social organizations; on the other hand, jointly formulate the community social work service youth incentive system, youth affairs social work project points system, establish youth affairs social work rotation mechanism, two-way communication mechanism, formed the work force of "municipal full-time social workers + social organizations + community social workers" complement each other. Let professional people do professional things is like the seeds of fertile soil. With the support and endorsement of the youth league organizations, a number of rapidly growing young social organizations also increasingly show the unique professionalism, flexibility and youthful vitality of social forces. ChenqingQihang Social Work Service Center is the first "Qing" social work service center under the guidance of Beichen Communist Youth League. According to the idea of "independent operation of social workers and multi-party social participation", the social work service center has explored the joint working mode of "social workers + social organizations + communities + volunteers + community enterprises", becoming a comprehensive window and platform for the Communist Youth League to directly face and serve the youth. In 2017, the social organization received the first public welfare project that the youth league organization will "Pay for" - -the "Careful communication and love peer" youth crime prevention project. It is a special service for teenagers with behavioral bias or bad behavior tendency. At that time, a boy had a very nervous relationship with his parents, and was in a long-term depressed state, with poor sleep condition. His mother insisted on taking him to the activities, and the psychological counselors gave professional counseling while encouraging him to participate in various volunteer service activities. Gradually, the boy found confidence in the service of others, the sunny and confident smile on his face. After years of deep work in the prevention of juvenile delinquency, the social work service center has become the first socialized judicial and social service center for minors in Tianjin. Zhang Kun, the head of the center, said that the community where the center is located has a large floating population, and the family economic conditions of the residents vary greatly, and there are many "marginal" teenagers. Especially after the implementation of the "double reduction" policy, many migrant children do nothing on weekends, and their parents are unable to discipline them. In response to this phenomenon, Zhang Kun and his colleagues contacted the community and opened a young eagle class for these children. During the group activity at the Young Eagle class, the social worker found two problem teenagers. One of the children suffered from great psychological trauma due to the death of his mother. The social worker immediately communicated with the community. With the consent of his father, he went to the child's home for many psychological counseling to help the child slowly get out of the haze. XingHongDan xing said, in recent years, the youth league organizations actively explore social operation mode, closely combined with youth interests integrate youth social organization resources, gradually expand the service scope to the rule of law education, psychological counseling, security, quality development, etc., to the government and other forms of purchasing services to support outstanding social organization growth. In recent years, the Youth League Beichen District Committee carefully polished a number of hands, public recognition, targeted service brands, more and more people began to take the initiative to participate in volunteer service, participate in community governance. Since the outbreak of COVID-19 this year, the Beichen District Youth League organization has quickly gathered more than 4,000 young volunteers, and set up 16 youth commandos and 243 teams to participate in the epidemic prevention and control."Social organizations have played a very important role in organizing and dispatching the volunteers. Some social organizations have led the volunteers to actively contract for the various service tasks of some nucleic acid testing sites."Said Xing Hongdan. Han �, The "small public welfare experience officer" activity, founded by the � incident, encourages children to understand the great truth through personal participation in practice. For example, in the "civilized dog raising" education activity, children go into the community residents to conduct research and listen to different views; independently consult various materials, think about the relationship between man and animals, and how man and nature live in harmony, and finally show their views through research reports and debates. Safety self-care education class is not simply a simple copy to the book, young social workers designed safety knowledge "everyone to find fault" activities, as well as safety knowledge quiz and other contests, teaching and fun."A lot of kids just run over to our office."Han � onion ψ Pan Dan � and many parents have volunteered in recent years. Cultivating, developing and actively guiding youth social organizations is an important way to enrich the form of grassroots organizations of the Communist Youth League, and also a new force for the reform of the Communist Youth League. Fang Wei, minister of Tianjin Municipal Committee, said that the process of leading and helping social organizations vertically promotes the self-innovation of youth league organizations, actively updating and reforming the needs of youth groups; the growing strength of youth social organizations also extends the tentacles and strength of youth league organizations, making the service more considerate and closer to the youth. Source: China Youth Daily, April 20,2022

2022-04-20	Shanghai communist youth league launched youth support action Shanghai youth support action Jianwei correspondent Chen Jiayin) recently, the Shanghai municipal party committee, Shanghai youth service and rights and interests protection office, Shanghai youth federation, Shanghai youth development foundation jointly launched youth "youth war disease hope warm heart" support action, has let Shanghai more than 2000 troubled teenagers get real help, fully feel the party and the government for their care. Moreover, this love is still continuing to pass on... under the epidemic situation, the situation of teenagers in difficulties is complex, and there are factors such as cross-region and limited reception. The Shanghai Youth Service and Rights and Interests Protection Office has confirmed that more than 3,200 families are in need of key assistance. In order to send the love materials to the young people in distress as soon as possible, members of the Shanghai Youth Federation, staff of the Shanghai Youth Development Foundation and staff of the Sunshine Center set up a 10-person transport youth commando team. Together with hundreds of distribution commandos composed of grassroots cadres and youth affairs social workers, they worked hard to get through the "last kilometer" and "last 100 meters" of assistance. It is reported that in this help work, in addition to love material support, each child's personalized needs are also tried to care for: such as online class equipment, academic counseling, psychological counseling and so on. At present, Shanghai Youth Federation has raised and distributed 27 online course learning terminal equipment; the school work Department of the Youth League Committee connects with "cloud classroom" service, one-to-one academic counseling; Shanghai Youth Service and Rights Protection Office connects with professional psychological counselors, and has provided psychological counseling for 8 people.

2022-04-17	The 41st Sichuan-Chongqing "Campus Spring" activity cloud curtain the 41st Sichuan-Chongqing "Campus Spring" activity cloud curtain Wang Xinxin) recently, "Welcome the 20th, always follow the Party, forge ahead on a new journey" the 41st Sichuan-Chongqing "Campus Spring" cultural, art and sports activities opened. The event will run from April to June. The event was jointly organized by the Publicity Department of the CPC Chongqing Municipal Committee, the Education Working Committee of the CPC Chongqing Municipal Committee, Chongqing Municipal Education Commission, Chongqing Municipal Committee of the Communist Youth League, Sichuan Provincial Committee of the Communist Youth League, Chongqing Students 'Federation and Sichuan Students' Federation. Marathon on the cloud. According to the COVID-19 epidemic prevention and control requirements, The event opened in the "cloud", Combining online and offline products, Organization guide teachers and students small-scale, decentralized "run youth" cloud marathon activities, "youth" wisdom "university students science and technology innovation culture festival," youth heart to the party new era "university students campus singing contest," youth praise "college students painting and calligraphy contest," yue youth "," speculative youth "university students debate contest," "job" in "the college students workplace 8 hours challenge," rural revitalization Youth to help farmers "agricultural products packaging and brand logo design competition," public welfare youth "college students volunteer service project design competition," youth faith " micro group class sharing competition and closing ceremony and college students science and technology innovation exhibition and other 11 sub-activities. The 41st "Campus Spring" activity is closely held around the theme of "Welcome the 20th anniversary, always follow the Party, and forge ahead on a new journey", combined with the celebration of the 100th anniversary of the founding of the youth league. For the first time, the activity included young teachers from Sichuan and Chongqing colleges and universities, focusing on the deep integration of Sichuan and Chongqing, and invited five sub-activities from Sichuan colleges and universities to participate in the whole process. In addition, the activity around the center, the service general situation, the project design focus on rural revitalization, Chengdu-Chongqing region twins economic circle construction, science and technology innovation, party and government center, focusing on employment practice, style ability promotion of young teachers and students, focus on education evaluation and deepen the reform of college group, cultivating moral, intellectual and physical all-round development of socialist builders and successors. This year, the Sichuan and Chongqing Youth League organizations jointly launched the 2022 "Shuangcheng Young Talents Campus Pioneer" typical tree selection activity, and selected a group of outstanding individuals and teams from the teachers and students of Sichuan and Chongqing colleges and universities to show it in the closing ceremony of the 41st "Campus Spring".

2022-04-15	Jianwei correspondent Li Jing) On the morning of April 14, the Gansu Communist Youth League helped the rural revitalization work promotion meeting and the eastern and western youth league organization cooperation signing ceremony was held in Lanzhou. The meeting reviewed the 2021 Gansu, and arranged the key tasks in 2022; Nankai District, Tianjin, Qingdao, Dingxi, Gulang, and Lanzhou Jiaotong University discussed their respective work. At the promotion meeting, the Youth League Tianjin Municipal Committee and the Youth League Gansu Provincial Committee signed the 2022 Agreement through video conference cloud signing; the Youth League Gansu Provincial Committee signed the Framework Cooperation Agreement with the Provincial Postal Administration; and signed the 2022 Letter with the municipal (prefecture) Youth League Committee. Provincial party secretary Dong Anning around why to promote rural revitalization, rural revitalization of the "youth contribution" action to anchor work goal, rural revitalization of youth league, cadres, the youth, how to ensure the "rural revitalization of youth achievements" action orderly advance four aspects made a speech, points out that the youth league to be a good integration of resources "operator", cadres to be good "fighters", the youth to be good service general "force", and is "youth xinglong" education project and poor people care to support two work arrangement deployment. The meeting required that the youth league organizations at all levels in the province should consolidate and deepen the effectiveness of the Gansu Communist Youth League to help the rural revitalization brand project, and continue to implement the six actions of rural revitalization deeply. To earnestly fulfill the political responsibility of the Communist Youth League for the party, the "youth" education project- -rural youth talent cultivation action throughout the whole process of rural revitalization work; to integrate superior resources, accurately implement the "two help and two help" project (i. e., help rural youth growth, help rural social construction, help rural students study, and help rural youth entrepreneurship); focus on key groups, and develop rural elderly women and children and children; implement the project to the national and provincial key support counties. Tianjin, Shandong province youth league organization related departments responsible for comrades, provincial agriculture, rural and rural revitalization bureau, provincial postal bureau related departments responsible for comrades, provincial authority departments, units responsible for comrades, cities (state), county (area) youth corps committee, part in directly affiliated colleges and universities, enterprises and institutions are responsible for comrades, youth league overseas league committee is responsible for comrades to attend the meeting.

2022-04-19	Shaanxi Provincial Party Committee held the 2022 "Double-carbon" Camp under the background of 2022 "Double-carbon" activity) Recently, Sponsored by the Shaanxi Provincial Committee of the Communist Youth League, Shaanxi Provincial Education Department and Shaanxi Provincial Youth Federation, The "Xi 'an 2022' Double Carbon 'Background" organized by Xi' an Liangjiatan International School, Xi 'an Foreign Language School and Xi' an Second International Language School was successfully held in Xi'an Liangjiatan International School, Nearly 120,000 people participated in the activity online. Xu Yongsheng, secretary of Shaanxi Provincial Youth League Committee and chairman of Shaanxi Provincial Youth Federation, and Liu Baoping, deputy director of Shaanxi Provincial Education Department attended the event and delivered a speech online. In his speech, Xu Yongsheng pointed out that teenagers, as future builders, are the backbone of actively responding to global environmental problems and achieving the "dual-carbon" goal. He hoped that young people would strive to be the advocate of "double carbon" goal, energy conservation and emission reduction, green and low carbon "goal, and the practitioner of" double carbon " goal, dare to practice, and lead the dream of China. In his speech, Liu stressed the importance of realizing the "two-carbon" goal for building a community with a shared future for mankind. He pointed out that in the process of achieving the "dual-carbon" goal, we should not only play our subjective initiative and act actively at the technical level, but also broaden our horizons, build more communication platforms and carry out more scientific and technological exchanges and cooperation worldwide. The activity is divided into two parts: "Youth keynote speech" and "youth speech". In the first half, five experts and professors from different fields to the space power technology application in energy conservation and environmental protection, the energy transformation and zero emissions background of global future development, "intelligent Internet era energy system operation management," "double carbon" global sustainable development and "under the background of" double carbon "flood simulation and monitoring as the theme of deep sharing, jointly explore the new era background of" double carbon " goals and the new direction of global sustainable development and new measures. In the second half, from xi 'an high-tech zone experimental primary school, xi' an foreign language school, xi 'an second foreign language school, xi' an high first high school campus, xi 'an jiaotong university affiliated middle school, shaanxi normal university and xi' an liangjiatan international school of more than 50 students, combined with their own understanding, share from many aspects to practice the goal of "double carbon". It is reported that the contemporary youth, as the future builders, are bound to become the forerunner and main force in the new round of scientific and technological revolution and industrial transformation. When with different culture and educational background of young learners gathered, focus on the world proposition is closely related to human beings, intense thought collision can not only help them deepen the understanding of sustainable development goals, but also show their as world citizens sense of responsibility and the new era of bear, starts from itself, practice green development and way of life, for the construction of ecological civilization and beautiful earth, to achieve sustainable development and build human destiny community make positive contributions.

2022-04-18	Group Hainan provincial party committee held theoretical learning group learning (expand) group Hainan provincial party committee held theoretical learning group learning (expand) meeting communicate learning xi general secretary visited Hainan's important speech spirit JianWei correspondent Wu Hao) on April 15, Hainan provincial party committee held theoretical learning group learning (expand) meeting, project communicate learning xi general secretary visited Hainan's important speech spirit. Chen Yu, secretary of the Provincial Communist Youth League Committee, presided over the meeting and made a concluding speech. Members of the provincial Communist Youth League leadership, organs and subordinate units above the deputy department level (including) participated in the study, the provincial Communist Youth League team members, the main person in charge of various departments of the Communist Youth League combined with the actual work, talked about the learning experience and work planning. Conference pointed out that in the island closure operation preparations to accelerate the important moment, xi jinping, general secretary in Hainan inspection guidance, and published a lofty conception, profound thought, rich connotation of the important speech, gives Hainan new mission, put forward higher requirements, great significance, far-reaching, the communist youth league, youth league, union, the young pioneers organization to earnestly study and implement xi, general secretary of the important speech spirit. Study publicity and implement xi general secretary of the important speech spirit, the Hainan provincial party committee secretary Chen Yu delegation provincial party committee put forward five opinions: one is to study publicity and implement xi jinping, general secretary of the important speech spirit as the current and future a stage of the primary political task, quickly in the province youth league organizations at all levels of learning publicity boom. Second, we should grasp the strict political construction as the fundamental, unite and lead the provincial league cadres to set up a strict style, show a high state, make solid achievements, to meet the party's 20th victory. Third, it is necessary to further promote the deepening of the reform of the Hainan Communist Youth League to go solid and deeper, further improve the working mechanism of the implementation of the reform, strengthen supervision and supervision, step by step compaction responsibility, to ensure that the reform measures of the provincial Communist Youth League, the Youth League, the Student Federation and the Young Pioneers are implemented. Fourth, we need to deepen the construction of the "six dreams" carrier platform of China (Hainan Free Trade Port) Youth Dream Building Avenue, jointly build the concentric circle of the Chinese Dream of Hainan youth, dig into the stories behind each base point, and explain the connotation of education clearly and thoroughly. Fifth, we should have a deep understanding of the significance of carrying out the theme of "welcome the 20, always follow the Party, forge ahead on the new journey", effectively enhance the appeal and effectiveness of publicity and education, and strive to create a strong atmosphere of high spirits, unity and progress, and constantly deepen the ideological and political guidance of the youth in the province.

2022-04-17	Inner Mongolia communist youth league in Inner Mongolia to promote "green and easy loan" project communist youth league: "green and easy loan" for youth business loan 50 million yuan shi jia) on April 14, the Inner Mongolia district party committee and Hohhot golden valley rural commercial bank co., LTD., in Hohhot golden valley bank yuquan branch held "green gen easy loan" youth entrepreneurial loan service project agreement signing ceremony, for Inner Mongolia entrepreneurial youth and small micro enterprise financial loan services, and in Hohhot as the pilot, in Inner Mongolia to promote "green and easy loan" youth business loan project. The "Qingchuang Yidai" project is funded by Inner Mongolia Youth Entrepreneurship and Employment Foundation to set up risk compensation for start-up loans and provide risk compensation for Jingu Bank to issue "Qingchuang Yidai". The loan scale is enlarged by the 1:10 pool of risk compensation funds, that is, the total loan amount is 50 million yuan. This activity is a concrete measure taken by the Inner Mongolia District Committee of the Communist Youth League to help Inner Mongolia optimize the business environment, help young entrepreneurs overcome the impact of the epidemic and help the resumption of work and production by means of "public welfare + finance"."Qingchuang Yi dai" project cooperation signing ceremony site. It is understood that "Qingchuang Yidai" is divided into three types, namely, "credit loan", "guaranteed loan" and "pledge loan", and the longest loan term is not more than 2 years. Young people aged 18 to 40 in Inner Mongolia with full civil acts and small and micro enterprises in Hohhot can apply for "Qingchuang Easy Loan", with pledged personal loans up to 500,000 yuan and pledged enterprises up to 2 million yuan. Projects recommended by youth league organizations at all levels in Inner Mongolia can get further preferential treatment in terms of loan interest rate and credit line. Relevant departments will provide young people with post-loan entrepreneurship assistance, entrepreneurship mentors, entrepreneurship guidance, decision-making consultation and other services. In the next step, the Youth Inner Mongolia District Committee will continue to deepen the "Youth Innovation and Easy Loan" project, fully connect with financial institutions, and develop a variety of bank cooperation projects to meet the diversified development needs of young entrepreneurs and small and micro enterprises.

2022-04-15	Jiang Yutong, reporter of China Youth Network) On the afternoon of April 14, the fourth plenary meeting of the Zhejiang Provincial Youth Work Joint Conference was held. Huang Jianfa, deputy secretary of Zhejiang Provincial Party Committee, attended the meeting and made a speech. He stressed the need to have a deep understanding of the significance of youth and youth work, put youth development in the big pattern of common prosperity, and strive to build a "youth-development province" into a landmark achievement of a common prosperous and beautiful society. The meeting summarized the work since the third plenary meeting of the Joint Conference of Youth Work in Zhejiang Province, and deliberated the relevant documents of youth development in Zhejiang Province. The Organization Department of Zhejiang Provincial Party Committee, Zhejiang Provincial Construction Department, Zhejiang Provincial Health Commission, Hangzhou Xiaoshan District Party Committee, Ningbo Yinzhou District Party Committee, Wencheng County Party Committee and Jiashan County Party Committee made exchange speeches. Huang Jianfa stressed that to do a good job in youth development this year, to guide youth on the ideological, political and spiritual temperament, increase the young talents "education work" the policy support, around the three kechuang highland policy, create the environment, pays special attention to the Asian games, the games volunteer recruitment and management training, give full play to the positive role of youth in grassroots governance, integrate youth team strength, in the integration to promote digital reform, comprehensively deepen reform, common prosperity demonstration area major reform condensed youth together. To focus on social construction areas involving youth problems, around building youth high quality employment entrepreneurship system, crack the low youth fertility problem, promote youth physical and mental health tasks, strengthen coordination between departments, improve the policy guarantee system, improve the social support system, cultivate "successors" all-round development. We should pay attention to young people with new employment and urban migrant workers, their life, study and employment under the influence of the epidemic, and prevent poverty caused. At the same time, we should focus on providing better and fairer public education services for young people, effectively protect their legitimate rights and interests, and promote the sharing of the fruits of development. Huang stressed that Zhejiang took the lead in proposing the goal of building a "youth development province". Various departments should be around this goal, effective play to the role of the joint meeting, reshape the work process, pays special attention to the evaluation supervision, to lead a systemic reform measures, compiling the implementation of Zhejiang youth development index, promoting the construction of youth development cities and counties, promote more friendly, Zhejiang youth development, youth to Zhejiang development more bear, more promising. Wang Wenxu, vice governor of Zhejiang Province, pointed out that we should improve the responsibility implementation mechanism, according to the work tasks, pay close attention to the implementation, to ensure that the youth work in our province is effective, and continue to walk in the forefront of the country. We need to improve multi-cross coordination mechanisms, strengthen horizontal and vertical coordination, work together to do all work well in the youth field, deepen the development of a youth-development province, and promote scientific decision-making and targeted decision-making through digital reform. We need to improve the mechanism for striving for excellence, and take the opportunity of the mid-term evaluation of the medium-and long-term youth development plan as an opportunity to increase policy supply, address the pain points, difficulties, and obstacles in the process of youth development, and create more hardcore achievements that young people can feel, replicated, and promoted.

2022-04-15	Beijing: forging dare to fight to win the cadres cling to the "key minority" firm ideal faith Beijing: forging dare to win the cadres, regiment quickly implement the plenary spirit, comprehensive governing group thought consciousness and action consciousness to further enhance, path carrier more clear, work from the point breakthrough gradually to the surface, new progress has been made. Around to meet and study propaganda and implement the party's 20 this work the main line, combined to celebrate the 100th anniversary of the founding, from now on "celebrate 20, always follow the party, the new journey" column will regularly published regional units youth league to implement the spirit of the sixth plenary session of the 18, promote the typical practice of comprehensive governing group, show the fresh image of the communist youth league in the new era.―――――――――― league Beijing municipal committee thoroughly implement the spirit of the sixth plenary session of the communist youth league, seize the cadres of the comprehensive governing group of the key minority, further implement the jinping general secretary of young cadres, especially the series of important requirements, to strengthen the political ability and style construction, improve the cadre breeding work the whole chain mechanism, strictly grasp the cadre team management, strive to build loyalty clean bear high-quality professional cadres. The Beijing Municipal Party Committee seized the favorable opportunity of forging the political ability of the League cadres in the service guarantee of major activities, carefully selected forces and actively put into work to temper the political character and work style of the League cadres. In 2021, more than 320 cadres of the founding of the Communist Party of China, and more than 320 cadres of the municipal Communist Youth League system were dispatched to participate in the organization and implementation of volunteer services. In the process of serving the Beijing Winter Olympics and Paralympic Games, more than 980 cadres of the Beijing Communist Youth League system directly participated in volunteers, urban volunteers, volunteer accommodation support, gathering and evacuation of opening and closing ceremonies, experienced political experience in practice and showing their youthful responsibility. Qu Bin, deputy secretary of the Youth League Committee of Beijing Jiaotong University, was seconded to the deputy director of the Beijing Municipal Party Committee. He said, " Participating in the Winter Olympics work, I feel what is 'safe'. Our job is to show China's image to the whole world, which is an all-round experience for the cadres participating in the Winter Olympics work, so that everyone can grow up rapidly in the high intensity, fast pace and strict standard work."In the major activities to temper the league cadres, the care for the cadres is also essential. The leadership of the Beijing Municipal Party Committee of the Communist Youth League took the lead in conducting one-to-one and full-coverage heart-to-heart talks with the cadres, carrying out targeted education and guidance, and required the organization department to regularly visit the front-line youth League cadres, and send the care and care to the organization."Today, I have received a letter of sympathy and festival daily necessities from your organization. I know that you have performed very well in the Winter Olympics. Your mother and I are both proud of you. You must do well, do not live up to the expectations of the organization, and also take good care of yourself."This is a warm scene during a video between Sun Lixiang, a party cadre of the Beijing Communist Youth League, and his parents in Mudanjiang city, Heilongjiang Province, after this year's Spring Festival. As the only cadre stationed in the closed-loop management of Yanqing Winter Olympics Village (Winter Paralympic Village), Sun Lixiang came to Yanqing Winter Olympics Village on September 10,2021, serving as the deputy manager of the reception and visitor center. While doing a good job, he also wrote and directed five issues of "Sun VLOG Winter Olympic Village", immersed in the daily display of volunteers in Yanqing Winter Olympic Village and the group images of young people in the Winter Olympic Village, and won a lot of praise. In the face of the important task of COVID-19 prevention and control, youth league officials at all levels in Beijing are duty-bound to devote themselves to the frontline and act uniformly and contribute in urgent, difficult, dangerous and heavy tasks. In October 2021, a local epidemic outbreak occurred in Hongfuyuan Community, Changping District, and Yang Wen � B, Secretary of the Youth League General Branch of Changping District Landscaping Bureau, was arranged to support the prevention and control line of the community. This is a large community, with more than 110 residential buildings, and the workload is very heavy. But he said: " The moment I put on my protective suit, I felt the responsibility on my shoulder. As a league cadre, I had to stand it at the critical moment."After the outbreak in 2020, mentougou district party committee all the cadres stop back, into the epidemic prevention and control community (village) working group, district party committee and district party committee organization department office responsibilities, district party committee secretary as office director, for more than two years adhere to the mechanism, measures, team, play to the office of comprehensive coordination and overall scheduling, keep the whole community (village) prevention and control organization system in normal operation."In the past year, under the great attention and cordial care of the District Committee, the construction of the Fangshan District Youth League cadres has made new achievements. The leadership allocation rate of the Communist Youth League District Committee is 100%, the allocation rate of government cadres is 100%, and the on-post rate is 100%. I sincerely request you to give more attention to the development of the Beijing Communist Youth League and the construction of the youth League cadres in the overall work layout in 2022."This is the content of a letter to the Beijing Municipal Committee of the Fangshan District Committee at the beginning of 2022. In order to further strengthen the cooperative management of the cadres of the Communist Youth League committee of the city, the Beijing Municipal Committee of the Communist Youth League established the mechanism of "three notices, two examinations and one listening", and one of the important measures is to inform the main responsible comrades of the district Committee of the Communist Youth League cadres. In March 2020, the Fangshan District Committee received a letter from the Beijing Youth League Municipal Committee and the cadres of the Youth League Committee of Beijing 16 District, and attached great importance to the situation of the leadership rate and the cadres need to be improved, and made instructions in the first time, organized the departments to strictly implement and carefully select. In October of that year, the leading group allocation rate of the Youth League Fangshan District Committee, the allocation rate of government cadres and the post rate all reached 100%. Not only that, according to the report of the Beijing Municipal Party Committee of the Communist Youth League, the Communist Youth League Fangshan District Party Committee drew lessons from one example, comprehensively sorted out the allocation of cadres in the lower-level youth league organizations, and further strengthened the matching management."Three to two test a listen" system, in addition to early report to the district party committee is mainly responsible for comrades league cadres with situation, also includes quarter to the district party committee in charge of the leadership and organization department minister notice, targeted to the district party committee organization department, directly involved in the TuanOuWei principal adjustment, strengthen TuanOuWei cadres with results assessment, attend TuanOuWei democratic life will listen to TuanOuWei leadership members work report, etc. In 2021, taking the opportunity of the change of office, the Organization Department of the Xicheng District Party Committee selected outstanding young cadres from the central units of CNNC and the head office of ICBC to serve as part-time deputy secretaries of the Communist Youth League District Committee. They gave full play to their advantages, actively coordinated resources, and played an important role in supporting the epidemic prevention and control in the region, and serving the new employed and new business form groups such as express delivery boys and food delivery riders. Gao Jian, secretary of the Xicheng District Committee, said that the Beijing Municipal Committee and the organization department have increased communication and optimized the environment for the selection of cadres of the Xicheng District Committee. The Communist Youth League of Chaoyang District clearly requires league cadres to "not be young officials, to be youth friends". In December 2021, the report and evaluation of the street and township youth league (work) committee of the whole district was conducted in the form of the whole network live broadcast, and the young people from all walks of life watched it online in real time, truly realizing the "turning the young people from onlookers into participants". The report requires the league cadres to undraft, make open and transparent results, and face the supervision of young people from all walks of life. At the same time, members of the Youth Federation, representatives of non-public enterprises and social organizations were invited as comment guests to "promote construction through evaluation" with new angles and multi-dimensional sharing. In 2021, the Chaoyang District Committee of the Communist Youth League timely reminded and talked to the cadres with emerging and tendentious problems, with a total of 11 people, and two people became regular members. In order to avoid the "formalism" of the youth league cadres, the Chaoyang District Communist Youth League made a random telephone inspection of whether the materials were accurately distributed, whether they communicated with the youth and asked about the needs. As the person in charge of the newly established enterprise league organization, the youth League leadership of Shunyi District Committee had an in-depth talk with Sun Xu, secretary of the General branch of the Youth League of Beijing Shunyi Property Service Group Co., Ltd., before taking the post of the Communist Youth League. This made Sun Xu have a comprehensive understanding of the Communist Youth League, and had a deep understanding of the responsibilities of the Communist Youth League cadres."The direction of my efforts is more clear, and I am more confident to lead the youth league members to implement the results of the league building into the community governance and property services in 63 communities, so as to better do practical things and do good things for the residents in the area."Sun Xu said. It is one of the specific practices of the Shunyi District Committee to attach importance to the construction of the League cadres to strictly examine and carry out targeted talks with the new cadres of the League. In addition, it also includes the formulation of management measures for the selection and appointment of temporary posts and part-time cadres of the youth League district Committee, establishing a classified incentive and restraint mechanism for the youth league cadres, and actively communicating with the organization Department of the District Committee and the inspection office of the district Committee, and promoting the youth league construction and team construction into the district party building work assessment and the inspection and supervision of the district Committee. In recent years, under the leadership of the Party Committee, the Youth League Committee of Beijing Hospital Management Center has incorporated the quantitative index of "Party building leads the league building performance assessment" into the overall assessment index system of Party building, taking this as a starting point to continuously grasp the strict governance of the Youth League. Through on-site evaluation, daily assessment, comprehensive evaluation and other methods, the key work of the youth League committee of 19 directly affiliated hospitals is assessed once-twice a year, and the annual results are transformed into the overall party building performance assessment results according to a certain proportion. The effectiveness of the Beijing Hospital Management Center has formed a strong impetus around the center and serving the overall situation. The Youth League Committee of the Beijing Hospital Management Center has organized more than 150 youth commandos, and more than 8,000 people have participated in emergency volunteer services, playing a role as a new force and commandos in urgent and dangerous tasks such as epidemic prevention and control and medical treatment. This year marks the 100th anniversary of the founding of the Communist Youth League by the Party. Strict governance of the League is an eternal subject in the centenary course of the Communist Youth League and a distinct theme of the League's self-construction. The Beijing Municipal Party Committee of the Communist Youth League will strictly improve the ranks of its cadres, lead the cadres at all levels to strengthen their dedication, practice real efforts, boost their spirit, promote the comprehensive and strict governance of the Communist Youth League with stronger determination, stronger will and more sufficient tenacity, and welcome the victory of the Party's 20th Congress. China Youth Daily, China Youth Network, reporter Du Yimeng, correspondent, Gao Haina, Ma Rui, source: China Youth Daily, April 15,2022, edition of 01

2022-04-14	Jianwei correspondent Qiu Yuan) On April 13, "Hunan Communist Youth League" was online. This study power number is the important position of Hunan Communist Youth League under the strong leadership of Hunan Provincial Party Committee, the important position of the deep study and implementation of Xi Jinping Thought on Socialism with Chinese Characteristics for the New Era, the important carrier of youth work closely around the center, and the important window of youth learning and communication integrating "political nature, youth flavor, Hunan style and professionalism". Aned to play a leading role in thought, service youth healthy growth, show the new era lead the provincial communist youth league teenagers active in "building prosperous and strong democratic civilization harmonious beautiful socialist modernization new Hunan" hot practice, show in the "welcome twenty big, always follow the party, forge ahead new journey" strong atmosphere contribute the spirit of youth strength."Hunan Communist Youth League" has opened four columns: "Youth learning", "Youth learning", "Reunion power", "Song of Youth" and "Youth Hunan", adhere to the theoretical knowledge and party and government news interpretation, fully display the work of Hunan youth and the Communist Youth League work, and spread the voice of Hunan youth in an all-round way.

2022-04-14	"Guangxi youth love Guangxi" action to help to Guangxi youth employment entrepreneurship to Guangxi youth love Guangxi youth employment entrepreneurship on 12, the reporter learns from the Guangxi district party committee, for college graduates employment problem, Guangxi communist youth league in Guangxi "Guangxi youth love Guangxi" employment promotion action, through the "1 + 10" support plan, let more youth fall in love with Guangxi, to Guangxi development or stay in Guangxi employment. The "1 + 10" support action, That is, with the theme of "Guangxi youth love Guangxi", Develop 10 specific projects, Including the "return to home" social practice, internship "sail plan" and community practice plan mainly for college students, Guide college students to further understand Guangxi, understand Guangxi and love Guangxi in practice; Employment assistance plan for students from low-income families, targeted employment plan for college students staying in Guangxi, western Volunteer Service Plan, rural entrepreneurship assistance program for fresh graduates and college students within two years of graduation, Let college students find better employment and entrepreneurship in Guangxi; The "Youth Employment Port" assistance plan for urban and rural youth, the "Yan Return" employment plan for young people to return to Guangxi, Provide "doorstep" employment services for local youth, Let the youth people "leave their hometown"."Guangxi xi youth love Guangxi" also includes help youth play a bigger role in social development of "youth development friendly area to create" action, and help the youth better entrepreneurship in Guangxi the "Guangxi green ear financial services" action, etc., from employment guidance, practice, entrepreneurship training, financial support support for the development of youth to Guangxi. Source: China Youth Daily, April 14,2022, edition of the 02nd edition

2022-04-14	Correspondent Wang Qiuhe) 2022 is the transition year of Guangxi Zhuang Autonomous Region Student Federation (hereinafter referred to as Guangxi Students' Federation). At the just-concluded transition conference, Xu Jinghuan, the new president of Guangxi Students' Federation, said with emotion: " In the past five years, flowers and applause are together, and responsibility and responsibility go together. Guangxi Students' Union to serve the students wholeheartedly for the purpose, think of what the students think, urgent students urgent, solve the difficulties of the students, one want to build the student union into a students can remember, find, reliable family organization."In the past five years, the autonomous region young Marxist training project (college class) has trained 942 students. Adhering to the general principle of "selection and breeding", and striving to cultivate and convey loyal political character, strong national feelings, solid theoretical foundation, and outstanding ability and quality for the Party, we are committed to the young political backbone of Guangxi taking root in Guangxi and building a magnificent socialism with Chinese characteristics in the new era. In 2021, Guangxi young marxist training engineering university class, all the students for three days of concentrated study, learning classic marxist theory, visit the red revolution base, guide the students in learning practice enlightenment consolidate theoretical foundation, the enhancing of ideological foundation, efforts to grow into a firm marxist belief, having both ability and political integrity, all-round development of youth political backbone."Such activities deepen our mutual feelings, and also enhance our collective centripetal force."Said cadet Li Chenruibo. In 2021, TuanOuWei play organizational advantages and educational function, integration around OuZhi authority and municipal youth league resources, in Guangxi college students internship "sail plan", organize more than 439 college students to organs at all levels, township grassroots, enterprises and institutions internship, for the first time for OuZhi authority solicitation internship "sail plan" jobs, help college students set up the correct view of employment, improve employment competitiveness. Huang Yiyuan from Guangxi University for Nationalities participated in the "Sailing Plan" government affairs internship in Shuli Village, Nanpo Township, Jingxi City. She said, " Through this internship, I deeply realized the importance of grassroots organizations and saw the new challenges faced by grassroots Party organizations. I hope that more young people can pay more attention to the rural construction, especially the young party members can actively participate in the construction of their hometown, and play a vanguard and exemplary role of the party members in promoting the rural revitalization."In the" Challenge Cup " Guangxi College Students Extracurricular Academic Science and Technology Works Competition, the National College Students Entrepreneurship Competition and other brand competitions, Guangxi youth's scientific and technological innovation ability has been constantly improved. In the national "Challenge Cup" competition, a total of 3 works won the national first prize, 10 works won the national second prize, and 104 works won the national third prize."Light up dreams and volunteer service with stars."For three years, Guilin aerospace industry institute youth volunteers volunteers have three consecutive years in the summer organization youth volunteers to rural primary school volunteer service a program under rural officials practice, around the academic counseling, family company, quality development, psychological and behavioral counseling to carry out the colorful holiday volunteer service activities, in need of left-behind children and trailing children to provide help and services. In the past five years, Guangxi, the communist youth league organizations at all levels and the league youth students active responsibility, active in COVID-19 epidemic prevention work, participate in "a program under which officials" "home" and other social practice, love classroom volunteer teaching, in power poverty engines, service in the practice of rural revitalization strategy of education, long ability, contribution. Young student volunteers have become the most important volunteer force in Guangxi.

2022-04-13	Fujian "one stand two" mechanism power epidemic prevention and control of Fujian "two" mechanism power epidemic prevention and control reporter Chen Qiang) Fujian at all levels to promote "two" mechanism construction, the special committee, on behalf of the sinking line organization member youth active in COVID-19 epidemic prevention and control, participate in the temperature measurement, nucleic acid detection, personnel registration, convenient service, protection propaganda and other volunteer service. Jinjiang city is the key area of this round of epidemic in Fujian province. In the face of the severe and complex epidemic situation, the city's league representatives liaison station organized the city's league representatives at all levels to set up more than 120 youth commandos, launched more than 4,200 youth league members, as nucleic acid "collector", information "collector", epidemic prevention "waiter", knowledge "propagandist", material "porter" and other roles. Local delegation representatives have also actively contacted representatives of youth businessmen and the youth Federation to donate money and materials. So far, they have donated more than 1,800 copies of epidemic prevention and living materials to 16 units. Xiamen Siming District Committee of the League, through 25 representatives of the League representative liaison station, mobilized all units in the district to set up youth commandos and staff teams of more than 300 people to settle in 10 sealed control areas and control areas. Youth commandos assisted in setting up sealing and control enclosure, setting up temporary tents, regiment representatives assisted the community to do 24 hours on duty, point-to-point distribution of living materials, door-to-door garbage clearance, centralized disinfection and treatment, and try their best to ensure the life of residents. The liaison station of Gulou District in Fuzhou fully integrated the resources of its field and unit, set up three youth commandos totaling more than 200 people by organizing league cadres and recruiting volunteers, and assisted in the sampling work of more than 69,000 nucleic acid sampling sites. Relying on the Youth Volunteer Service special group of the Fuqing Municipal Committee, the youth volunteers for epidemic prevention and control at the grassroots level at the town (street) and village (residential) level. Li Yongsong, the representative of the Fuqing Youth League Representative Liaison Station, organized the catering service industry Association to send bread, cold drinks, mung bean soup and other comfort products to the medical staff of the nucleic acid testing base. Hanyuan Yiyuan Community is an epidemic sealed control area in Longwen District, Zhangzhou City. Under the organization of members of the special committee and representatives of the league, young volunteers and community residents quickly become "one family" through the WeChat group."On the evening of March 18, we put into the closure and control work of Hanyuan Yiyuan. And the anti-epidemic commandos soon provided their service to ensure the operation and stabilize the people."The League member of the special committee, the League Zhangzhou Municipal Party Committee Organization Department Minister Lin Ling introduced, they will mix the staff and volunteers for building group management, and then through the internal volunteers for the community material distribution, to achieve internal and external linkage, joint management and service seal control area. Huang Yulin, the representative of the youth league, is the youth League branch secretary of Cangban Community in Zhangzhou Taiwan Investment Zone. After participating in the registration and review of nucleic acid sampling personnel, he made a spare time to make short videos of fighting the epidemic to relieve the anxiety of the people in the sealed control area. CAI Jinzhao and CAI Qiming from Zhangzhou Longxi Rat Hunting Team are "father-son soldiers" who have been volunteering for a long time. They are actively engaged in the epidemic prevention and control of the epidemic after establishing contact with the regiment representatives. In the process of volunteer service, Cai Jinzhao did not pick the shift time, service positions, wearing protective clothing for four hours, his personal clothes were wet, his fingers were sweat soaked white and wrinkled. He said: " In the current epidemic, as the community epidemic prevention and control checkpoint gatekeeper, I think it is very meaningful."Source: China Youth Daily, April 13,2022, edition of the 02nd edition

2022-04-13	Shaanxi province youth volunteers planting green demonstration activities "beautiful China youth action" shaanxi youth volunteers planting trees green demonstration activities JianWei correspondent Zhang Wenhao) on April 8, sponsored by the communist youth league in shaanxi province, shaanxi province han wei engineering construction co., LTD., shaanxi province youth volunteers association to undertake "beautiful China youth action" -2022 shaanxi youth volunteers planting green demonstration activities in han wei engineering ditch water distribution hub. Members of the provincial Communist Youth League Committee, department-level cadres, as well as the provincial Communist Youth League Committee organs, the provincial Introduction Hanjiwei Engineering Construction Co., Ltd. cadres and workers, volunteer representatives of the provincial youth volunteer association to participate in the voluntary tree planting activities. Activity pictures. Correspondent for the picture tree planting site, we under the guidance of technical personnel, shovel shovel soil, carrying saplings, soil cofferdam, water irrigation, planting Qinling compound green main tree species white bark pine and ornamental tree species magnolia, begonia and other seedlings more than 300 trees. After the tree-planting activity, the volunteers adopted each sapling one by one, and named the grove the "Youth Volunteer Forest". Event, pku provincial party committee to youth league organizations at all levels, environmental protection organizations from all walks of life, youth volunteers and youth members: let us act, earnestly practice xi ecological civilization thought, thoroughly study and apply xi general secretary to visit important speech important instructions and in the capital compulsory tree planting activities of important speech spirit, starting from a tree, practice green development concept, promote the whole society to form green planting, caring natural good fashion, be practitioners of ecological civilization construction, promoters, take practical action to add green kinetic energy, for the party's 20 gift. Activity pictures. Correspondent for figure in recent years, the shaanxi provincial party committee continue to deepen the youth ecological civilization values, mobilize youth league organizations at all levels and the youth focus in the spread of the concept of green, practice green life, advocating green production, cultivating green team, green engineering construction continued hard, focus on qinling ecological protection and the Yellow River basin comprehensive management, vigorously develop the national compulsory tree planting, total organization to mobilize youth planting green more than 50000 people, planting more than 150000 plants, to promote the greening, construction of beautiful shaanxi youth strength. Next, pku provincial party committee will thoroughly study and apply xi general secretary in the capital compulsory tree planting activity's important speech spirit, closely around the ecological civilization construction overall goal task, vigorously carry out the province "beautiful China youth action" youth planting green and ecological environmental protection practice, organization guide the teenagers and social people from all walks of life practice green development concept, actively participate in the construction of ecological civilization.

2022-04-13	Hainan: youth war "epidemic", the communist youth league show ability to bear Hainan: youth war "disease", the communist youth league to play jianwei correspondent Wu Yuhua) since the new round of COVID-19 outbreak in Hainan province, Hainan provincial party committee rapid response, immediate action, timely deployment, coordination, guidance, cadres at all levels led the young volunteers into epidemic prevention and control sniper war, overall war, active war, by participating in the task under epidemic prevention actual combat, temper cadres excellent disposal ability, strengthen the cadres bear since their ability. Strengthen organizational leadership. Established with the league provincial party secretary as group leader, deputy secretary as deputy leader of the epidemic prevention and control of volunteer service leading group, organize the implementation of the provincial party committee, the provincial government and the central league on epidemic prevention and control work deployment, research and decision of the communist youth league to participate in the epidemic prevention and control volunteer work, promote the epidemic prevention and control of volunteer service work. At the same time, the Communist Youth League Hainan Provincial Committee's Emergency Plan for Epidemic Prevention and Control was revised and issued, holding a video conference on volunteer services for epidemic prevention and control, and making arrangements for the next step of the provincial Communist Youth League to help epidemic prevention and control, which set off a wave of young people in the province to actively participate in volunteer services for epidemic prevention and control. Strengthen publicity and guidance. League provincial party committee joint provincial youth league, provincial union issued to Hainan communist youth league member, youth league organizations at all levels issued the epidemic prevention and control volunteer recruitment order, at full speed to mobilize the provincial youth volunteer service force into the epidemic prevention and control, preparation, battle state, actively participate in the epidemic prevention and control related work, take concrete actions to reveal youth bear. At the same time, we will actively mobilize the new media matrix and working force of the league, and jointly create a good atmosphere for scientific prevention and rational response by timely releasing authoritative information, spreading health knowledge, and relieving panic. Vigorously publicize the outstanding deeds of advanced examples on the front line of epidemic prevention and control, and call on youth league organizations at all levels in the province to keep up with the outstanding advanced deeds and outstanding advanced young people who have emerged in the epidemic prevention and control work. We will provide voluntary services in an orderly manner. A new round of COVID-19 outbreak, pku provincial party committee fully carry forward the "party call, group action" fine tradition, quickly assembled, charge in the front, actively organize mobilization, industry system and youth commandos and youth civilization, according to the local epidemic prevention and control work requirements and grassroots actual demand, in the supply, nucleic acid detection, flow tracking, propaganda guidance, emergency services and other fields play the role of powerhouse. As of April 6, nearly 20,000 young volunteers have participated in the fight against the epidemic, and 527 youth commandos have been set up, serving more than 146,000 hours and serving more than 720,000 people. The epidemic prevention and control continues. Youth League organizations at all levels in the province will strive to improve the ability of youth League cadres to overcome difficulties, conduct risk prevention and control, organize and mobilize, actively organize young volunteers to participate in the strong and orderly emergency volunteer services in epidemic prevention and control, and build a youth defense line for epidemic prevention and control with youth volunteer services.

2022-04-13	The Guangxi Communist Youth League reporter Xie Yang) Today, "Welcome the 20th, always follow the Party, forge ahead on a new journey" the Guangxi Communist Youth League related work press conference was held. Meng Qipeng, secretary of the Communist Youth League Guangxi District Committee, said at the meeting that this year around to meet the party's 20th victory held and study to publicize and implement the spirit of the conference of the main line, Guangxi Communist Youth League in addition to high quality to complete the arrangement of the superior action, will also focus on building three characteristic brands. Meng Qipeng introduced that the Guangxi Communist Youth League will, in accordance with the deployment of the 12th Party Congress of Guangxi Zhuang Autonomous Region, build the three "common visions" of the Guangxi Communist Youth League centering on the three "common visions" of "forging the sense of Chinese national community, actively serving the construction of China and asean community with a shared future, and steadily promoting common prosperity". The first characteristic brand is to anchor the "casting the consciousness of community of the Chinese nation", and to carry out the demonstration activities of casting the community consciousness of the Chinese nation among the young people in the whole district. The second characteristic brand is to anchor "actively serve the construction of a China-Asean community of shared future", and provide more opportunities and stages for the friendly exchanges between China-Asean young people. The third characteristic brand is to anchor the "solid promotion of common prosperity", the solid development of the magnificent Guangxi youth to build new achievements action. Guangxi Communist Youth League will welcome the party's 20th victory and study, publicize and implement the spirit of the conference, the main line, To celebrate the 100th anniversary of the group, According to the unified deployment of the whole regiment, In the youth of the district, to carry out the education practice with the theme of "Welcome the 20, Always follow the Party, forge ahead on a new journey", Including playing the influence of the "Qing" brand name, Conduct theme group (team) day activities, Continue to deepen the "youth learning" action, Further implement the "young Marxist" training project, "youth teaching group" program, "Red scarf tour group" program and other publicity and education brand projects, Do a good job in the party's innovative theory of youth interpretation, Carry out the "Youth Volunteer Tour Welcome the 20" 2022 youth volunteer service series activities, promote the magnificent Guangxi youth contribution action, and so on, To guide the youth league members to make contributions in the new era and forge ahead on a new journey. At the same time, the Guangxi Communist Youth League will also hold an exhibition on the history of Guangxi Youth Sports, set up positive topics on online platforms such as the "Guangxi Communist Youth League" microblog, TikTok and B stations, and create and launch a number of cultural products popular with teenagers, such as script killing, picture album and animation, to guide them to actively learn, publicize and implement the spirit of the conference.

2022-04-08	The CPC Youth Tianjin Municipal Party Committee held the construction work meeting of the 2022 Party Building Work Conference to summarize the work in 2021 and plan the work tasks in 2022. League secretary, League system party building leading group leader Li Long attended and speech. Members of the leading group of the Communist Youth League Municipal Committee, secretaries of Party branches, secretaries of Party committees (branches), Party cadres, and discipline inspection cadres participated. The meeting conveyed the spirit of the meeting of the Standing Committee of the CPC Municipal Committee and the meeting of the Party building leading group of the CPC Municipal Committee and the spirit of the 2022 Party work and discipline inspection work meeting in 2022, and conveyed the instructions of Comrade Xiang Jun. The meeting noted that, The Party building work of the Communist Youth League Municipal Committee in 2022 should adhere to the guidance of Xi Jinping Thought on Socialism with Chinese Characteristics for a New Era, We will fully implement the guiding principles of the 19th CPC National Congress and the 19th CPC Plenary Session, To fully implement the general requirements for Party building in the new era, Following the spirit of General Secretary Xi Jinping's 79 and 72 important speeches as the fundamental guideline, Thoroughly implement the party building deployment requirements of the Municipal Party Committee and the municipal organ working Committee, Consciously use the party's century-old historical experience of the struggle, Carry forward the great spirit of party building, To meet, study, publicize and implement the Party's 20th spirit, and conscientiously study and implement the spirit of the 12th Party Congress of the city, Building a political, learning, practical and clean organization, To build the party building work to promote Tianjin youth work to a new level. The meeting stressed that efforts should be made to strengthen political leadership, highlight the welcome, study, publicity and implementation of the twentieth main line, carry out the publicity and education practice activities with the theme of "welcoming grand events, casting loyalty, strong responsibility and creating achievements", and guide Party members and cadres to consciously be the model of supporting, following and defending the core. We need to make more efforts in ideological guidance and continue to bring Xi Jinping Thought on Socialism with Chinese Characteristics for a New Era into our minds and hearts. We will continue to consolidate and expand the achievements made in the study and education of Party history, and form a regular and long-term system and mechanism, deepen the study of the Party's innovative theories, and deepen our ideological and political work. We should see tangible results on the basis of consolidating the grassroots level, and forge a contingent of young cadres who are practical and responsible. Strengthen the construction of the "four strong" party branches, further strengthen the daily education and management of party members, pay close attention to the implementation of the organizational life system, and vigorously select the tree publicity and learning advanced models. We need to make real efforts to promote the comprehensive and strict party governance, ensure a clear political ecology, deepen the construction of a clean Tianjin, lay a solid foundation of a clean culture, further strengthen the education, management, and supervision of young officials, and create an atmosphere of integrity, responsibility, and innovation. Conference pointed out that to deeply understand and grasp the "the new great party construction project has a decisive role, is the key to" four great ", to strengthen the party's construction as an important premise of all the communist youth league work and strong guarantee, understand and grasp the importance of party construction in 2022, further strengthen the quality consciousness, improve work standards, do" basic work specification, deepening, innovation window ", make" one kilometer "" the last kilometer ", the high quality requirements in the party construction process all aspects. Meeting requirements, pku party organizations at all levels should strengthen the standard consciousness of consciousness, fine consciousness, carry forward the spirit of nail real party construction, close to the actual, vigorously promotes the implementation, li strenuous, relentlessly resourceful not idle, improve the quality of the Tianjin communist youth league party, boost Tianjin economic and social development, with honors to meet the party's 20 and the 12th party congress victory!

2022-04-07	Shanghai: Young volunteer medicine delivery teams were active in the streets for the nucleic acid testing work of the "quick decision". At the same time, Pudong and Puxi of Shanghai entered the most critical sealing and control moment. However, the prevention and control of COVID-19 cannot control the daily drug needs of residents. In the green newspaper network reporter noticed that Shanghai now empty streets active with a "young volunteers delivery team", the team by Sinopharm holding Shanghai hospital management headquarters of more than 50 young senior managers, office clerk, etc., they every day wearing protective clothing, driving disinfection after private cars shuttle in the streets, send medicine for Shanghai citizens. At 3 o'clock in the morning of April 1st, it was the start time of the Shanghai Pudong and Puxi seal and control policies. On the night of March 31, when many Puxi citizens were shopping in major supermarkets, dozens of young Sinopharm volunteers went retrograde to the Hanting Hotel in Baoshan district of Shanghai. According to the Shanghai city's prevention and control requirements, they should stay in the hotel and "closed-loop control" themselves so that they can deliver drugs throughout the city the next day. The company is responsible for sending nearly 50 percent of the Internet hospital orders in Shanghai, each of which may be a "life-saving medicine" for a single resident. But earlier, the logistics and express delivery companies that were originally in charge of the delivery work were shut down due to the epidemic, with a large number of drugs stuck in hospitals and warehouses. The party committee of the company immediately called on young people, middle and senior cadres and party members to take the lead and set up this "young volunteer medicine delivery team". On the evening of March 31, Zheng Xin, an employee of the company, arrived at the closed loop hotel at 23 hours, but he and several other colleagues were asked not to check in for the nucleic acid test results on the day. A few people sat outside the hotel lobby for the night, until the next day of nucleic acid results before checking in. After checking in, and immediately went out to deliver medicine. The original work plan of the team was to complete the work in three to five days, but the prevention and control situation is constantly changing, and the team may have to be stationed for a long time. Early in the morning of April 3, the company issued a notice saying that if employees are struggling at home, they can go home with the permission of their community according to the prevention and control requirements. In the end, only three employees returned home with no one to take care of their children, and all the others chose to stay. On that day, the team temporarily received a more urgent task. The epidemic prevention materials ordered by the government should be distributed to the distribution points in various districts, streets and towns in the shortest possible time. Each point is less than 200 boxes, more than nearly 2000 boxes, sealed control under the Shanghai has not found any handling stevedores. The team is fully mobilized, the male and female staff together, from the "Courier" to the "porter". Emergencies occurred one incident after another. On the night of April 3, the party secretary of the company led a team to deliver 10,000 sets of protective suits and 5,000 N95 masks to Dachang Town, Baoshan District. At less than half an hour after returning to the station, he received a new task- -in order not to affect the streets of the day for the masses, a batch of materials should be checked overnight. In the evening, more than 20 volunteers and the party secretary went to the storage site more than 20 kilometers away to check them, and did not complete the task until about 3 am on April 4. In addition to delivering drugs, the team also sent thanks to the Shanghai people for the "Shanghai" staff. When they learned that a large number of anti-epidemic materials were sent to the warehouse, but the restaurant stopped operating and drivers, the team immediately sent 200 self-heated rice from their own rations to them. This "youth volunteer medicine delivery team" not only sent the warmth to others, but also felt the warmth passed on by all walks of life in Shanghai. After learning that Sinopharm Shanghai Hospital Management Headquarters urgently needed a closed-loop management hotel, Huazhu Hotel Group immediately prepared the Hanting Hotel with isolation experience for it, and specially configured a working team with isolation experience. According to the relevant person in charge of the company, they also obtained 800 self-heating rice coordinated by the Pudong New Area Commission of Commerce, and large drug transportation trucks coordinated by the Shanghai Municipal Commission of Transportation.�� source: China Youth Daily, April 07,2022

2022-04-07	Shanghai "cloud care class": let the love service continue online volunteer tutoring, teach English, two children said their mother recently always go out early and return late... and I said, my sister isolation for nearly a month, miss the family, have the opportunity to tell her mother 'How much I love you' every day."This is the evening of April 3, Shanghai Jiao Tong University law major sophomore Zhang Zhining posted on the circle of friends. In late march this year, the Shanghai municipal party committee opened "love cloud class", organize the university youth corps committee to recruit outstanding academic performance, communication ability of college students volunteers, one to one for COVID-19 epidemic prevention and control line personnel children and in square hospital of primary and middle school students, provide mental health guidance, inspirational education, scientific enlightenment, professional counseling and other services. Zhang Zhining is one of the volunteers. In good English, she also designed an immersive English short film reading activity for the two sisters, teaching them some common English expressions while telling fairy tales. As the head of the management center of the school's youth volunteer service team, Zhang saw backstage that more than 300 students had signed up for the volunteer activity. According to the age characteristics and interests of the children, we designed the rich "cloud companionship" content such as "How does sugar be made", "Why can N95 masks prevent the virus", bird knowledge popularization and painting skills sharing. In Zhang's opinion, the volunteer service of college students during the epidemic period is more like a kind of self-management. Volunteers "go from students to students", complete their work efficiently, and touch each other. In order to relieve the anxiety and online learning pressure of primary and middle school students in makeshift hospitals, the "Love Cloud Support Class" - -the online companionship service for primary and middle school students in Shanghai makeshift hospitals was launched simultaneously. Zhang Haiyang, a doctoral student at Shanghai Jiao Tong University School of Medicine, joined a sixth grade student at the makeshift hospital of the Expo Exhibition Center. The student's parents told Zhang Haiyang that the child was not motivated to learn English, and the experienced tutoring team focused on tutoring his English. "I hope to encourage him to contact English videos and animation, cultivate interest and develop the habit of learning English."Not only that, he also took the initiative to increase the scientific use of the eyes and other medical knowledge and popular science content. Zhang Haiyang told reporters that his most ideal career is a teacher, "doctors treat the body, teachers cultivate the mind, all have a sense of achievement."At East China Normal University, the volunteer team of "Love Cloud Support" was formerly the 2020 "Cloud Guidance" team. At that time, the team had more than 600 normal university students serving the children of medical, police, community and other frontline workers in 14 districts of Shanghai, as well as children from rural families in Xundian, Wuding, Yunnan Province and Jing'an, Jiangxi Province. This year, in addition to providing one-to-one homework tutoring, normal university students' volunteers also arranged scientific and Olympic knowledge explanation, calligraphy and other features, paying attention to daily care and companionship and mental health education. Su Fanghao, a senior physics major in the university and a member of the graduate volunteer teaching group, told reporters that he was connecting with Xiao Wang, a senior three student in a makeshift hospital at the World Expo Exhibition Center. Xiao Wang is preparing for the college entrance examination. " In order to reduce her anxiety, I made a lot of preparation before tutoring, and also tried to encourage her in a humorous tone. I can feel that she was a little disheartened from the beginning, but now her confidence has increased a lot."Said Su Fanghao. For Su Fanghao, volunteering is not unfamiliar. In his freshman year, he signed up for public welfare activities and went to primary schools every week to teach reading classes for the children of migrant workers, and attended about 10 times; In 2019, he served in Changshou Road Street as a volunteer of the "Love Summer Care Class" of the Communist Youth League Committee."After the recent outbreak of the epidemic, I got my graduation thesis in my dormitory every day. My volunteer classmates were responsible for organizing nucleic acid and cleaning up the floor garbage. Every time I saw them all excited, so I think it was very meaningful to participate in this cloud companionship volunteer service."He said. According to data from the Shanghai Municipal Party Committee, a total of 1,165 frontline children in the city have been registered on April 4, and 2,385 volunteers from eight universities, including Fudan University, Shanghai Jiao Tong University and Tongji University, have signed up to provide childcare services. Shanghai University of Finance and Economics, Shanghai University of Medicine, Jiaotong University and Shanghai Conservatory of Music, have created nearly 500 interest and health self-care courses based on their professional characteristics. March 28 solstice on April 4, "love cloud support class" -Shanghai square hospital primary and middle school students online company service has contact Zhongshan hospital, huashan hospital, benevolence hospital nine medical teams, covering the expo exhibition hall, minhang stadium, cao bao road and other seven square hospital, has been more than 40 sick primary and middle school students to carry out professional counseling services. In the future, the service will be carried out successively to help more families of primary and middle school students.�� source: China Youth Daily, April 07,2022

2022-04-06	In 2022, the "red scarf" for the people's heroes standing reporter Zhao Anqi) Today is the Qingming Festival, 24 young pioneers from Beijing Jingyuan School came to Tian' anmen Square in the morning, in the name of the red scarf, standing guard for the people's heroes. At 9 o'clock, the first class of young pioneer post members on time on duty, 6 young pioneers wearing bright red scarves, wearing white gloves, holding high the flag of the Chinese Young Pioneers, with a strong pace to the Monument to the People's Heroes. For the first time so close close to the Monument to the People's Heroes, Zhang Chenyi was very excited, " Through time and space, the heroes set up a spiritual roadmap for us, guide us in the direction forward."" These people's heroes shed their heads, shed blood in exchange for our happy life, we as the young pioneers of the new era should study hard, grow up to serve the motherland!"Fan Xinyue talked freely about her experience after standing guard. It is understood that to celebrate the party's 20, to celebrate the 100th anniversary of the founding of the communist youth league of China, further guide the capital young pioneers of revolutionary martyrs, red genes, Beijing municipal committee, tiananmen district management committee, the Beijing capital in 2022, the first guard players stand guard on April 3, the first day of the qingming holiday, a group of six, each group of rotation on guard for 15 minutes, a total of 1 hour. This year, the event will last until October 13, a total of 193 days, when there will be more than 1,000 outstanding young team members from 16 districts and Yizhuang Economic Development Zone actively close to the Youth League organization standing guard in front of the People's Heroes Monument, expressing their simple feelings of love for the Party, patriotism and socialism with practical actions. After standing guard, the team members will also visit the Chairman Mao Memorial Hall, pay their respects to Chairman Mao's legacy, continue to receive patriotism education, and continue to deepen the effect of education. It is understood that the Capital Youth Pioneer Post is an important activity with strong capital characteristics and special educational significance jointly founded by the Beijing Municipal Party Committee, Tiananmen District Management Committee and Beijing Young Working Committee in 1986. It is an important ceremony for the young Pioneers of the capital to express their nostalgia for the revolutionary martyrs. So far, the activity has attracted more than 300,000 young pioneers to participate on guard, and millions of young pioneers have been educated. It has played an important role in guiding the young pioneers from primary school pioneers and growing up, and strengthening the political enlightenment and value shaping of children in the capital. In addition, as an important part of the theme education and practice activities of "Welcome the 20th grade, always follow the Party, forge ahead on the new journey", each district has simultaneously launched the district-level, school-level youth pioneer post activities, further improving the city, district, school three-level youth pioneer post activity system. In standing good young vanguard at the same time, the district, school will also be "red scarf heart to the party" "red scarf love the capital" as the theme, organization on guard speech in the school flag-raising ceremony, participate in the theme essay activities talk about guard experience and experience, continue to cultivate children simple political emotion, further enhance the young pioneers glory, show the spirit of the young pioneers in the capital.

2022-04-12	Hainan nearly twenty thousand young volunteers fight epidemic line Hainan nearly twenty thousand young volunteers fight epidemic line "please wear a good mask" "please show your health code"... on April 5 afternoon, Haikou qiongshan district Fucheng middle school nucleic acid testing point, from Hainan provincial party committee party volunteers Wu Jiayan and volunteers partners are guiding the nearby people to orderly nucleic acid testing. Some of the volunteers involved in the fight against the epidemic were responsible for the temperature measurement, some carefully maintained the order of the scene, and some were carefully checking the health code of the people's travel code and other information. The Hainan Provincial Party Committee of the Hainan Youth League, Hainan Youth League and Hainan Students' Federation issued an initiative on April 3, calling on the members of the Communist Youth League and the youth of the province to be the first and truly become a new force and commando in the fight against the epidemic. As of April 6, nearly 20,000 young volunteers from all walks of life in Hainan province have served more than 146,000 hours in the fight against the epidemic, with 527 youth commandos and nearly 450,000 people, according to an official from the Hainan Provincial Committee of the Communist Youth League. On the morning of March 30, the Haikou Municipal Epidemic Prevention and Control Headquarters issued a notice to seal, control and control measures in some residential areas in Xiuying District. At 7 am on the same day, the TuanXiuying District Committee launched the emergency plan under the guidance of the League Haikou Municipal Committee, and launched more than 90 youth commandos to cooperate with Xiuying Street and Haixiu Street to carry out regional epidemic prevention and control. In less than a week, Haikou city and district two-level youth league organizations organized more than 1,000 youth volunteers and set up 48 youth commandos, serving 160,000 citizens. Since the outbreak, the Sanya municipal party committee formed by the cadres, youth volunteers, youth affairs social workers and social forces of "youth volunteer tiejun", to assist the major hospitals, moving station for nucleic acid detection, village (community) to carry out full nucleic acid detection, and assist in flow information registration, the new crown vaccination, sweep the health code, etc. The Sanya Municipal Youth League has launched more than 3,200 young volunteers and set up 29 youth commandos. Danzhou Danzhou municipal party committee after the outbreak to Danzhou epidemic prevention and control headquarters, organize more than 80 young volunteers, by calling confirmed cases of time with personnel telephone, detailed screening confirmation, and timely to check the information and found problems feedback to the city epidemic prevention and control headquarters, provides a strong support for the flow of work. The Wanning Municipal Party Committee guided the Dongao Town Committee and the Wanning Longyuan Property Service Co., Ltd. to distribute food, vegetables and other living materials to the people in the closure and control area. The Qionghai Youth Volunteers Association, together with the Qionghai Red Lady Rose Company Love Association, has carried out voluntary services for epidemic prevention and control in the urban farmers' markets. The League Lingshui County Party Committee and Hainan Railway Company have set up a youth commando team for epidemic prevention and control at Lingshui Railway Station, with more than 800 young volunteers and a total service time of nearly 6,500 hours. After the outbreak of the epidemic, the Communist Youth League Working Committee of Hainan Province acted quickly and extensively launched the provincial youth league departments to organize youth commandos and youth volunteer service teams to actively connect with the local youth League committees, streets and communities to help prevent and control the epidemic in Haikou. On March 29, the Youth League Committee of Hainan Provincial Higher People's Court organized more than 80 youth league members and policemen to set up youth commandos, which took the initiative to connect with the Youth League organization of Qiongshan District and went to support the nucleic acid testing work in several controlled communities. On the evening of April 4, the Youth League Committee of the General Office of Hainan Provincial Party Committee received a notice from Haikou Meilan District on the lack of some nucleic acid testing points to maintain order management personnel, and immediately organized more than 20 young volunteers to Liupo Community of Bailong Street to carry out volunteer services. The Youth League Committee of the Hainan Provincial Taxation Bureau has set up more than 500 youth commandos from the provincial tax system to participate in the epidemic prevention and control work in Haikou, Sanya, Danzhou, Dongfang, Ledong and other cities and counties. The People's Procuratorate of Hainan Province and other provincial departments and state-owned enterprises such as China Telecom Hainan Company have also set up youth commandos to reach the grassroots level in accordance with the principle of "being nearby" to help with the prevention and control of the epidemic. Source: China Youth Daily, April 12,2022

2022-04-07	Henan County Communist Youth League grass-roots organization reform pilot fourth scheduling meeting held the Henan Provincial Party Committee held the provincial county Communist Youth League grass-roots organization reform pilot fourth scheduling meeting Wei) recently, the Communist Youth League Henan Provincial Party Committee in the form of network video conference held the province county Communist Youth League grass-roots organization reform pilot fourth scheduling meeting. Relevant leaders of the Henan Provincial Youth League Committee, leaders of the provincial youth League Committee, leaders of relevant departments, and all members of the county (city, district) Youth League Committee of the pilot organization of the reform of the Communist Youth League in each county attended the meeting. Meeting on the reform pilot interim assessment, analyzes the main problems of the pilot reform, the meeting pointed out that promote the reform of the communist youth league to the county grassroots, is the implementation of xi general secretary of the important thought of youth work, is to adhere to the problem oriented and crack the key to the group of weak situation, is an important political task given by the central committee, undertake pilot reform is not "certificate", but a "writ", the "mission". All pilot counties (cities, districts) must view the pilot reform pilot work from the overall perspective of politics, enhance their sense of responsibility, sense of mission, sense of urgency and urgency, and promote the pilot work with greater determination, clearer goals and more effective measures to ensure the completion of the pilot reform pilot tasks. The meeting stressed the need to compaction compaction responsibility. Each municipal youth League committee should implement the responsibility of integrated implementation, and the pilot county (city, district) youth League committee with the same question, bow into the bureau, front command, adhere to the youth League party secretary personally grasp, in charge of the deputy secretary and the main responsibility department directly grasp, monthly regularly to the pilot county (city, district) special research and guidance, jointly promote the reform pilot work. The youth League committee of all pilot counties (cities, districts) should strengthen the main responsibility, and the party secretary of the Communist Youth League is the direct responsible person. He must work hard, take the initiative to take responsibility, overcome difficulties, boldly reform and innovate, have the courage to fight for themselves, and ensure that all measures of county reform are implemented. To fill the short board, the pilot county (city, area) to adhere to the problem oriented, in view of the existing problems of wall charts, inversion period, concentrate, concentrate, concentrate resources, focus on crucial specialist and hang cadres, building (team built) into the party appraisal difficult reform matters, to ensure that the final sprint not wrestling, not off speed, not deviation. To highlight the organizational function. The youth League committees of the pilot counties (cities, districts) should further establish and improve the organizational mobilization mechanism and improve the practical education mechanism with the "six modernization" goals, test the reform effectiveness of epidemic prevention and control, and reflect the leading, organizational and service power of the Communist Youth League to the overall situation. Strict assessment should be made. The provincial Communist Youth League Committee will evaluate the reform progress of the pilot counties (cities, districts) and local cities according to the high standards and strict requirements of the pilot reform of the county level and local cities, combined with the daily grasp, data monitoring and field research. To exercise your ability and style. Cadres of the youth League in all pilot counties (cities and districts) regard the pilot reform work as an important battlefield and a major test of improving their ability and work style, take learning from their work and learning as the only way to develop their real skills, and improve their ability to solve practical problems in the process of "urgent, difficult, dangerous and heavy". At the meeting, the responsible comrades of the youth League committee of the provincial cities and the main responsible comrades of the youth League committee of the pilot counties (city, district) reported on the progress of the reform, and the relevant comrades of the Organization Department of the Provincial Communist Youth League Committee (grass-roots Organization Construction Department) commented on the problems existing in each pilot county (city, district). It is reported that the meeting is the implementation of the provincial youth League committee to take the overall responsibility, in the county Communist Youth League grass-roots organization reform pilot work started, the fourth organization of the reform pilot scheduling meeting, to further understand the grass-roots situation, exchange reform progress, strengthen specific guidance. In the next step, the Henan Provincial Party Committee of the Communist Youth League combined with the action of the provincial Communist Youth League "strengthening ability, forging style, grasp the implementation", designated contact to help guide the reform pilot work, anchored the reform objectives and tasks, and went all out to overcome difficulties to ensure the qualified reform answer paper as scheduled.

2022-04-07	Ningxia: The Ningxia Hui Autonomous Region Committee held the 2022 Party building work conference month 7 (reporter Zhang Jianwei, correspondent Zhang Zhuo) recently, the Ningxia Hui Autonomous Region Committee held the 2022 Party building and clean government construction work conference. Autonomous region Youth League Committee secretary, secretary of the Party Committee Gong Xuefei attended and delivered a speech. Meeting convey learning xi jinping, general secretary of the central party school (national school of administration) young and middle-aged cadres training class KaiBanShi important speech spirit, the 19th session of the central commission for discipline inspection, the twelfth regional commission for discipline inspection and 2022 OuZhi authority party construction work conference spirit, summarizes the autonomous region youth corps committee in 2021 party and party to build a clean government work, arrange work in 2022, signed the party to build a clean government responsibility. The meeting pointed out that 2022 is the year of the 20th Party Congress, and the autonomous region will also hold the 13th Party Congress, which is of great significance to do a good job in party building in organs and building party conduct and clean government. To meet and study propaganda and implement the party's 20th, autonomous region 13th party congress as the main line, combined with the 100th anniversary of the party's political construction, efforts to build political, pragmatic, service-oriented, honest, civilized model organs, improve the quality of the party's construction, to promote the development of youth work high quality to provide a strong political guarantee. The meeting stressed the need to firmly uphold the political direction and resolutely defend the "two establishment". We will continue to strengthen our political study and theoretical arms, strengthen our political belief in understanding and implementing Xi Jinping Thought on Socialism with Chinese Characteristics for a New Era, and better shoulder the political responsibility of consolidating and expanding the young mass foundation of the Party's governance. We must strengthen our political responsibility and focus on the central task of Party building. Always in mind "the big country", closely around the Yellow River Basin ecological protection and high-quality development pilot zone construction, in-depth " Welcome the Party Congress? Gift 20- -strive to be the pioneer of the pilot zone construction "theme practice activities, organize the majority of the district youth to fully participate in the" welcome 20, always follow the Party, forge ahead on the new journey " theme education and practice activities, do a solid job in the establishment of model organs, party building and business integration development, serving the masses and other work. We must strictly enforce discipline and continue to create a clean and upright environment. Fully implement the party construction responsibility system, vigorously promote the construction of a clean government, form a strong force to promote the party construction; vigorously promotes the cadre style construction, study, problem oriented and work diligently, promote the formation of "grassroots to find problems, running problem work, focus on the target implementation" work situation. Members of the secretary of the Youth League Committee of the Autonomous region, leaders of the discipline inspection group of the Discipline Inspection Commission in the Federation of Trade Unions, and all party members and cadres of the organs and subordinate institutions attended the meeting.

2022-04-06	Group Heilongjiang provincial party committee to carry out the "2022 online heroes" activities of Heilongjiang communist youth league to guide the youth in qingming festival theme education memorial youth reporter Han Rong) today, the Heilongjiang provincial party committee combined with the reality of epidemic prevention and control work, relying on the network new media platform, organize the provincial youth "2022 endeavour online heroes" theme education activities, carry forward the heroic spirit of, inheriting red genes. It is understood that the Heilongjiang Provincial Party Committee of the Communist Youth League has opened an "online sacrifice to heroes" entrance on the official microblog of the Heilongjiang Communist Youth League to guide young people to pay tribute to revolutionary martyrs online, lay flowers, wipe tombstones, leave messages for memory and so on, through interaction to integrate the spirit of heroes into the blood and purify the soul. The event also carefully presented 13 city-level and (prefecture-level) -level red memorial venues in Heilongjiang Province, allowing teenagers to accept the online "immersive" spirit of baptism. Teenagers in the province actively participated, with more than 400,000 people participating in online punching interaction and more than 300,000 messages. The Heilongjiang Provincial Party Committee and Harbin Martyrs Cemetery taught the online cloud group class with the theme of "carrying forward the heroic spirit and continuing the spiritual blood". The narrator led the teenagers into the martyrs cemetery in the form of "cloud perspective", participated in the flag-raising ceremony, listened to the stories of the heroes, and recalled the eventful years. At the same time, I participated in the "today, lay flowers for the heroes" slow live broadcast activity. As of 12 noon, more than 600,000 people and 5.5 million likes. In addition, on the Tomb-sweeping Day, youth league organizations around Heilongjiang actively promoted the offline and online "Qingming Festival heroes" activities. Tuan Nenjiang Municipal Party Committee organized more than 10,000 people in 14 towns, 48 schools in the city, to carry out the theme of "youth heart to the party era" as the special theme team day of more than 700 activities; In order to create a good atmosphere of "civilized sacrifice and green Qingming Festival", Help to build a civilized city, To the city's youth "civilization sacrifice green qingming | to the city's teenagers a proposal", Advocate the city's Communist Youth League members and young people to take the lead in practicing the sacrificial concept of green memorial and civilized memory, Recalling our ancestors, martyrs and sages in a civilized and modern way, Break down from the old customs, consciously abide by social ethics.

2022-04-08	Henan) The plenary meeting of the leading group of the Henan Communist Youth League of Youth was held in Zhengzhou. Wang Qiang, deputy secretary of the Communist Youth League Provincial Party Committee, attended the meeting and made a speech. Members of the leading group attended the meeting. The meeting pointed out that the overall plan of the Henan Provincial Party Committee on comprehensively promoting rural revitalization should be fully grasped, and the tasks of effectively connecting the achievements of poverty alleviation with rural revitalization should be effectively implemented. From an overall perspective, we should fully understand the significance of carrying out youth contribution actions to help rural revitalization, effectively unify our thoughts and actions into the deployment and requirements of higher levels, focus on the main responsibilities of the Communist Youth League, and organize and mobilize young people to participate in the implementation of the rural revitalization strategy. The meeting stressed that, To be based on the reality of the Communist Youth League, To implement the "two help and two help" as the basic content, Focusing on industry, talent, culture, ecology and organizational revitalization, Focusing on rural industrial development, social construction, talent training, and young people's academic needs, entrepreneurship, and employment needs, With "the first League branch Secretary +" as the main line, Further promote the "youth to help rural living environment centralized improvement", "green man countryside" cultural revitalization action, "youth mental health education into villages (communities) action", "Dream Realization Action", We will continue to focus on the projects such as "Spring Goose Action", "Lead Goose" plan, "three to the countryside", "returning home", "New Youth OFFER Festival", "Sailing Plan" and other projects, Make positive contributions to helping rural revitalization. The meeting required that all member departments should shoulder the responsibility of steady pressure, pay close attention to the implementation of the work, and constantly expand the depth and breadth of the work. It is necessary to strengthen internal cooperation and external coordination, form the provincial Communist Youth League "a game of chess", give full play to the advantages of the organizational system, make overall use of external resource projects, and further improve the quality of work. We should innovate ideas and methods, change the style of work style, take the activity of developing the "Ability and work style construction year" as an opportunity, and steadily promote the relevant work with excellent ability and work style. To dig deep into the typical, increase publicity and guidance, adhere to the typical grasp the brand grasp the leading, focus on the typical key publicity and reports, through the formation of typical demonstration guidance, promote the work to do deep and bright to make characteristics. Meeting learning to convey the central, provincial rural work conference spirit, and the provincial government of the key work about 2022 promoting the rural revitalization of the implementation opinions, the general office of the communist youth league central "on deepening the implementation of rural revitalization of youth meritorious action work plan", the Henan communist youth league rural revitalization of youth meritorious action for the deployment, the leading group members reported the specific key matters in 2022.

2022-04-03	The Federation of Trade Unions, the Provincial Communist Youth League Committee and the Provincial Women's Federation jointly issued the Notice on the Selection and Commendation of the Seventh Moral Model in Shaanxi Province, which marked the official launch of the seventh Moral model selection and commendation activity in Shaanxi Province. According to the notice, the seventh moral models in Shaanxi province are divided into five categories: "helping others model", "brave model", "honest model", "dedicated model", "filial piety and love model", each category of about 10, the total number of no more than 50. The official candidates who are not elected as the seventh Moral Model of Shaanxi Province will be awarded the nomination award of Shaanxi Provincial Moral Model. The notice stipulates the basic conditions and classification conditions for moral models in Shaanxi Province. Citizens over the age of 18 who meet the selection criteria and work and live in Shaanxi can be recommended as candidates. Previous moral model nomination winner at or above the provincial level, selected tree from all walks of life, major model "era", "power" craftsman "model" "integrating ecology" sanqin ", and at and above the provincial level" civilized family "" good people "around" most beautiful people "" good youth "upward" model worker "" May 1 labor medal " honor winner can be recommended first. At the same time, special attention should be paid to the discovery and recommendation of advanced examples in the fight against COVID-19. Previous provincial and above moral models are no longer recommended."Notice" stressed that the seventh shaanxi province moral model selection recognition activity is divided into publicity, selection candidates, consultation, recommendation, determine the formal candidates, recognition list, publicity and grand recognition eight steps, cities (area) and provincial departments and units in accordance with the selection criteria and requirements, step by step audit, select the list of candidates, accept social supervision, widely listen. Before the National Day, the seventh moral model commendation activity was held in Shaanxi Province.

2022-04-07	The parents of the children are on the front line of fighting the epidemic. It will take a month to go home. Their children have become their biggest concern."Recently, the Shanghai Municipal Committee of the Communist Youth League, the Municipal Youth Volunteers Association and the Municipal Students 'Association provided the" love cloud care class " care service for the children of Shanghai epidemic prevention and control frontline personnel. At the same time, college student volunteers walked into the anti-epidemic families to become AIDS in education, and participants in children's growth. Yangyang, who has just finished the "Love Cloud Trust Class" course, was a little excited. He is 7 years old, just in the first grade. His father is a doctor in Renji Hospital, who is supporting the World Expo Exhibition Center makeshift hospital, and his mother is a doctor in the children's health care department of the City Children's Hospital."Today the teacher taught us to do a scientific experiment," Yang said. He was taught him to cut a flower with paper and put it in the water. As the flowers gradually get wet, the petals will slowly stretch out, "especially fun."In Shanghai, Yangyang still has many "Yunshang classmates" who have not met. As of 12:00 on April 4, a total of 1,165 frontline children in the city had registered for the nursery. Yang Fan, the mother of Yang Yang, who is engaged in research on children's development behavior, said that many of the frontline prevention and control workers are in the first stage of entering school, which is a period of need to accompany and run in. The epidemic has disrupted the pace of schools and left parents exhausted."The 'cloud support class' of college student volunteers is very timely and heart-warming. There are also special courses on weekends, and you can communicate with other children, and the children especially like them."How to make up for the temporary lack of family companionship, the answer from the volunteers is to" exchange heart for heart "."I was so lucky to get to know Miss Wu through this activity."A parent thanked Wu Fei, a volunteer from the chemistry department of China Normal University, on WeChat. After each class, she received a screen full of the sincerity and enthusiasm of college student volunteers."I listened to the class for a while today, and I feel that you spoke very well. No wonder Jianing looks forward to it every day."What moved her most was that the college student volunteers not only brought company to their children, but also brought their learning initiative. From resistance to active thinking and expecting solutions, the spiritual influence brought by volunteers is more valuable. College students grow up in the "cloud classroom", and they are also experiencing the growth."The evening was the first day when I started the 'cloud of Love company'. Two children of eight or nine years old called to my sister and asked me how old I was in the first grade."Donghua University volunteer Zuo Naixin said with a smile."When you graduate from primary school, finish the junior high school for three years, and then experience three years of high school to my age!"With that, she suddenly realized that she was exactly ten years older than them. That night, Zuo Naxin wrote in his notes: We also grew up from their age, to know the world, like the world, and finally into the world. And now we have become their brothers and sisters, so the cycle of new replacement let me feel surging, sigh that "the society teaches me- -I serve the society" two-way rush, happy in this era has a steady stream of new blood cast the ideal of youth. Wang Shengxu of Grade 19 at Donghua University and Chen Cheng of Grade 21 have completed four tutoring courses. In order to close the distance with the child, Wang Shengxu specially played a video of the frontline workers fighting against the epidemic in the first class."Do you know what mom and dad are doing?"Wang Shengxu hopes to use this way to intuitively tell the children" why their parents are not with their side ". According to the children's personality, learning situation, interests and hobbies, the volunteers also explore more targeted, individualized and warmer teaching service forms through caring and companionship, chatting and talking, so as to accompany the children's growth in addition to learning. Opening ceremony of "Huali Dream" cloud nursery class of East China University of Science and Technology. The most happy thing this week is to meet with her children in the cloud, and she most like the "sister" she called on the other end.� h's parents are both working in the fight in Shanghai, while � h and his grandmother are in Jiangsu province. So, Liu Xiuyan acted as a big sister on wechat."She shared with me all kinds of interesting things in my study and life and the things I didn't understand. As I enlightened her, I also grew up myself."Volunteer activities of the continuation of the characteristics of the" cloud "class" since March 28, "love cloud class" volunteer recruitment started, has from fudan university, Shanghai jiaotong university, tongji university, east China normal university, east China university of science and technology, Shanghai foreign studies university, donghua university, the university, Shanghai university and other eight universities of 2385 volunteers to provide hosting services. For many universities in Shanghai, the "love cloud support class" is the continuation of the volunteer activities of the university Youth League committee. It is understood that in early 2020, the new crown outbreak, "goose Dan dream" plan online tutoring volunteer team for Hubei area family staff can provide help, now 33 "goose Dan dream" volunteers to join the cloud with guidance, become the main cloud class volunteer service team, the school of international relations and public affairs of junior Li Bingxuan is one of them. She tutored one-to-one siblings, mainly improving their spoken English by reading textbooks and practicing dialogue."It's one of the few ways we can repay them with real action, and it's worth it."Mr.Li said. The Cloud companionship Volunteer Team of East China Normal University was formerly known as the "Cloud Guidance" team. The team was founded at the time of the COVID-19 outbreak in 2020. Under the guidance of the CPC Communist Youth League and the university Youth League Committee, the team has organized more than 600 normal university students to serve the children of medical, police and community workers in 14 districts of Shanghai, and children from Xundian, Wuding, Yunnan and Jingan, Jiangxi for more than 12,000 hours. The "Love cloud nursery class" novel class form is also a "circle of fans" for many people. The hosting adopts the mode of "cloud company + interest courses + self-care tutoring". Among them, cloud company mainly provides red education, physical and mental health, growth navigation, puzzle games, etc., senior students can provide academic guidance without violating the "double reduction" related regulations; interest course professors mainly provide financial education, aesthetic education, including not limited to music, dance, painting and calligraphy content; health self-care counseling mainly provides physical education, labor education guidance, including exercise, vision protection, mental health and other contents. Shanghai university of finance and economics, Shanghai jiaotong university school of medicine, Shanghai university of traditional Chinese medicine, Shanghai normal university, Shanghai academy of music, Shanghai drama academy, Shanghai sports institute, Shanghai lixin accounting finance institute, Shanghai business school, Shanghai health medical school, Shanghai school of visual arts, Shanghai league school 12 schools combined with professional characteristics, to build nearly 500 interests, health self-care courses. Among them, Shanghai University of Finance and Economics and Shanghai Institute of Physical Education provide interest courses and health self-care courses respectively."Despite the different forms, people want to express their gratitude to the medical staff in the way they can."Mr.Wang said. In addition, the Communist Youth League Committee and Renji Hospital launched the "Love Cloud Support Class" in Shanghai makeshift Hospital on March 28. As of April 4, has contact Zhongshan hospital, huashan hospital, Renji hospital, ninth people's hospital, the first people's hospital, tongji hospital, longhua hospital, Yueyang hospital, minhang district central hospital nine medical teams, covering the expo exhibition hall, minhang stadium, cao bao road, seven new international expo center, for more than 40 sick primary and middle school students to carry out professional counseling services. It is reported that the service will be launched in the city's makeshift hospitals to help more families of primary and middle school students.(China Youth Daily China Youth Network reporter Zhang Jianwei correspondent Zhang Chen)

2022-04-02	Group of Inner Mongolia autonomous region fourteen six times QuanWei (expand) meeting of Inner Mongolia autonomous region fourteen six times QuanWei (expand) meeting held Shi Jia) on March 31, the communist youth league of Inner Mongolia autonomous region fourteen six times QuanWei (expand) meeting in the form of video conference, the meeting summarizes the work in 2021, the deployment of work tasks in 2022. Inner Mongolia Autonomous Region Party Committee of the Standing Committee, Minister of the Organization Department Yang Weidong attended the meeting and made a speech. Yang Weidong said that the youth league organizations at all levels in the region should deeply contact the youth, serve them attentively, practice and implement the people-centered development thought, and promote the solution of the urgent problems of the youth group. We must continue to be problem-oriented, strengthen our awareness of problems, and continue to deepen the reform of the Communist Youth League. Conference pointed out that in 2021, the youth league organizations at all levels earnestly implement the general secretary xi about youth work the important thoughts and important speech important instructions to Inner Mongolia instructions spirit, in accordance with the autonomous region party committee and the central deployment requirements, earnestly implement lead condensed youth, organization mobilization, contact youth service youth responsibility, new progress was made in each work. The meeting stressed that in 2022, the youth league organizations at all levels to closely around to meet and learning propaganda and implement the party's 20 this main line, combined with the group to celebrate the 100th anniversary of, to "celebrate 20, always follow the party, forge ahead new journey" as the theme, completes the theory armed, team activities, practice, service lead, propaganda and cultural five aspects, to promote youth ideological and political lead targeted effectiveness. To insist on under the overall situation of thinking, action under the overall situation, widely cast up the Chinese nation community consciousness education practice, in-depth implementation of the new era of league organization promotion action, youth employment entrepreneurship service action, youth talent cohesion, rural revitalization, the youth contribution action, community youth action, youth volunteer action, strive to write a new chapter in the communist youth league cause. Meeting requirements, the youth league organizations at all levels to strengthen the goal orientation and result oriented, the achievements in the implementation of xi general secretary of a series of important requirements to test, in the big, area, in the communist youth league leading force, organization, service and overall contribution to test, to promote work results into the party's assistant and reserve comprehensive jump, into the communist youth league deepening reform quality efficiency, into the party education work and political achievements, with practical action to meet the party's 20th victory. According to the provisions of the League rules, the meeting removed, co-elected and replaced members of the 11th Committee of the Communist Youth League Inner Mongolia Autonomous Region.

2022-04-01	Shaanxi: ten thousand students benefit rural education funding promotion action in shaanxi: ten thousand students benefit rural education funding promotion action in the green newspaper reporter Sun Haihua) the reporter learns from the communist youth league of shaanxi provincial party committee, more than 10000 from shaanxi old revolutionary base areas, economic underdeveloped areas, will get from shaanxi hope project rural education funding promotion action. These funding, partly derived from the communist youth league of shaanxi provincial party committee, shaanxi hope project public welfare volunteer guidance center, shaanxi youth development foundation and China youth development foundation, Ningbo magic investment development co., LTD., the implementation of the "hope project 1 + 1-magic student plan", used to fund all 56 shaanxi poverty of the original national counties (area) 9800 primary school dilemma family girls. Another part is 1,200 primary school students in troubled families in Yan'an Old District, Shaanxi province, jointly funded by the Dongrun Public Welfare Foundation, with a total of 10.4 million yuan for student aid. As one of the important carriers of the Shaanxi Hope Project "Seven AIDS" for education- -rural education funding promotion action, "Project Hope 1 + 1- -Fantasy Education Program" aims to safeguard girls' right to education, safeguard social equity and promote social civilization. All the 56 state-level poverty-stricken counties (districts) in Shaanxi that have been lifted out of poverty receive 175 people in each county (district), with a standard of 1,000 yuan per person. Families of aided students can consult with the Youth League Committee of the county (district) where their household registration is located. The deadline for funding application is April 10. After the review, the funding will be allocated to the account of the county (district) committee where the student works in late May. In recent years, deepening shaanxi hope project "seven help once" action, to improve the service dilemma youth satisfaction as the measure, continue to hope project booster student xing education, education, fitness, science and technology, health guardian, public welfare development, emergency rescue, such as seven action, to solve the dilemma teenagers growing troubles, has become the shaanxi social organization "I do the practical work for the masses" brand practice.

2022-04-01	Tian Hongwei, Xi reporter) CAI Yuxiong is the director of the Youth Civilization Number of the Quanxiu Community Health Service Center in Quanxiu Street, Fengze District, Quanzhou, Fujian Province. After the outbreak of the epidemic, he and his teammates immediately engaged in nucleic acid sampling, specimen verification, guiding testing sites and other epidemic prevention and control work. On March 13, since the outbreak of the local epidemic in Fujian, the Fujian Provincial Committee of the Communist Youth League quickly deployed six youth actions for epidemic prevention and control, and youth commandos at all levels actively responded to the call to devote themselves to epidemic prevention and control work such as monitoring and early warning, professional medical care, logistics and distribution, and emergency duty. Quanzhou is the city with the largest number of confirmed COVID-19 cases in Fujian province, among which Fengze District is the key area of epidemic prevention in the city. Since the positive COVID-19 infection was found on the evening of March 12, the Quanxiu Street Community Health Service Center of Fengze District sent 20 nucleic acid sampling teams, 5 people participated in the flow transfer work, 6 people participated in the district disease control laboratory testing, and 9 people were stationed in the quarantine hotel for personnel health monitoring and environmental disinfection work. In the Mingguang Community of Jiangnan Street, Licheng District, Quanzhou City, the youth commandos of Jiangnan Fire and Rescue Station joined the crowd guidance, queuing guidance, body temperature measurement, material transportation, environmental elimination and other work, and assisted a total of 82,000 people in nucleic acid testing. Tong Yanqi is the captain of the youth commando team. In the youth commandos, the team members immediately submitted a "battle invitation", and the youngest team member was only 24 years old."When I chose to do the fire rescue work, I was ready to face all kinds of dangers and emergencies at any time."Young people not only appear on the front line of epidemic prevention and control, but also in the public security bureau, exit and entry border checkpoints, state Grid and other units. Youth commandos and Youth Civilization brands collectively work overtime in their responsible areas to win the battle of epidemic prevention and control. The youth commandos of 110 Intelligence Command Center of Fujian Provincial Public Security Department carry out data verification, flow tracing and close connection and expansion to accurately guide the closed-loop management of key epidemic risk personnel in the province. Youth Civilization of Fuzhou Entry-exit Border Inspection Station properly tested positive for COVID-19, ensuring "zero input, zero transmission and zero infection". The youth commandos of State Grid Putian Xianyou County Power Supply Company dynamically grasp the information of 16 key epidemic prevention and isolation sites in the county to ensure the safety of power supply. As of March 28, Fujian province had set up 1,024 youth commandos and 289 youth civilization groups, mobilizing more than 36,500 youth league members for the epidemic prevention and control work.

2022-04-07	Gansu communist youth league: extensive "welcome 20, always follow the party, forge ahead new journey" theme activities in Gansu communist youth league: extensive "welcome 20, always follow the party, forge ahead new journey" theme activists Ma Fuchun Wang hao) recently, the communist youth league of Gansu province party committee deployment "welcome 20, always follow the party, forge ahead new journey" theme education practice, the provincial youth league organizations at all levels, take concrete actions to meet the party's 20 and the fourteenth party congress victory in Gansu province. The Lanzhou Municipal Committee held the "Youth Reading Proposal On the Way to the Examination" for young literature lovers from all walks of life to read good books and discuss the classics; the " to guide the century-old youth movement history under the leadership of the Party. Various forms of groups and classes will fully mobilize the enthusiasm of teenagers to learn. In Wuwei City, Youth league organizations at all levels launched the "100 youth league classes into the grass-roots level" activity, To continuously deepen the achievements of party history learning and education, currently, More than 30 times have been carried out; Tuanding Dingxi Municipal Party Committee through online law popularization, watching warning educational films, Offline special lectures, posters distribution, theme class meetings, etc., Carry out further anti-drug publicity and education activities among teenagers, Improve the awareness of drug identification, drug prevention and drug resistance of teenagers; Tianshui city borrowed the 2022 "Young Marxist Training Project" training class, To cultivate young political backbone with loyal political character, strong feelings of family and country, solid theoretical foundation, and outstanding ability and quality; Longnan City young pioneers at all levels to carry out the Qingming festival heroes theme team meeting, watching heroes and martyrs video, online flowers, hand-copied newspaper creation, planting memorial trees, visit the historical memorial hall and other martyrs activities, Carry forward the spirit of martyrs, Cultivate patriotic feelings. At the same time, youth league organizations at all levels take the initiative to seek practical results and take practical measures, and extensively carry out voluntary services, public welfare assistance, promote youth growth and other theme practical activities. League Zhangye Municipal Party Committee to help the "one old one small one poor" masses to solve practical difficulties, Start with the small things, With the true feelings of the service; Gannan Prefecture held the "student aid, dream building, casting people" public welfare student aid conference, Grant grants to student representatives and sign a "one-to-one" funding agreement; Youth League Jiuquan Municipal Party Committee based on the "Youth Home" activity position, Organize and mobilize volunteers to take care of students after class, holidays and other periods, Help solve the problem of "students have nowhere to go, parents are difficult to care for"; Youth League Linxia State Party Committee founded the online "red scarf small class", Young Pioneers to explain the history of the Party, national conditions, national unity, civilized etiquette, young Pioneers knowledge and other content; TuanJiayuguan Municipal Party Committee aims at youth employment, Hold the Jiayuguan City 2022 "Spring Breeze Action" series of job fairs. With the "Lei Feng spirit" to gather the youth strength, the Communist Youth League Baiyin Municipal Party Committee organized the voluntary consultation, love and sympathy, voluntary haircut, mental health consultation, garbage cleaning, safe electricity publicity, Lei Feng spirit publicity, tree planting and green protection and other volunteer service activities. In Qingyang city, the CPC Communist Youth League Municipal Committee organized the activity of "Welcome the 20th Joint Youth Forest" to convey the positive energy of planting green, protecting green and loving green. League Gansu provincial party committee related responsible comrade, said around "strengthen theory armed, organize team activities, practice, strengthen service lead, do a good job of propaganda and culture" extensive theme education practice, aims to guide members and teenagers deep comprehension "two established" decisive significance, strengthen the consciousness of "four", "four" confidence firm, "two maintenance", in order to achieve the second goal in one hundred, realize the great rejuvenation of the Chinese nation the Chinese dream condensed strong youth power.

2022-04-06	The seventh plenary meeting of the 13th Shaanxi Provincial Committee of the Communist Youth League was held in Xi'an. Correspondent, Li Fengwei photo China Youth network Beijing, April 6 (reporter Zhang Jianwei, reporter Zhang Wenhao) recently, the Communist Youth League of the 13th Shaanxi Provincial Committee of the seventh plenary meeting was held in Xi'an. The meeting focused on learning the spirit of the Sixth Plenary Session of the 19th CPC Central Committee, the Sixth Plenary Session of the 18th CPC Central Committee of the Communist Youth League and the 10th Plenary Session of the 13th Shaanxi Provincial Party Committee, summarized the work in 2021, and deployed the key work in 2022. Xu Yongsheng, secretary of the Shaanxi Provincial Committee, delegation the Standing Committee of the Shaanxi Provincial Committee to report the work to the plenum and make a concluding speech. The meeting held that in 2021, under the correct leadership of the Provincial Party Committee and the Central Committee of the Communist Youth League, the work of the Shaanxi Communist Youth League has stepped up to a new level. Shaanxi 2022 the communist youth league in 2022 in accordance with the provincial party committee and the central work deployment, to meet and study propaganda and implement the party's 20 spirit of work as the main line, celebrate the 100th anniversary of related work, focus on leading condensed youth, mobilize youth, youth service basic responsibility, further build the "five" system, comprehensively deepen reform, comprehensive governing group, to improve organization, lead, service and contribution to the overall situation, unity led the members of youth, to promote the development of high quality in shaanxi province contribute youth wisdom and strength. The meeting stressed that comprehensively enforcing strict governance of the League is an inevitable requirement for the Communist Youth League to fulfill its political responsibility of educating people for the Party. Shaanxi Communist Youth League should bear in mind its duty and mission, deeply understand that the inevitable requirement of the party, is the important premise of the struggle, is the eternal theme of the cause of the Communist Youth Party, promote the overall spirit, promote the party spirit and yan spirit, " style, strengthen the confidence and determination of the method and path, remember the fundamental starting point and foothold, understand the path mechanism, and detail the standard results. Meeting deliberated and approved the "about of the communist youth league of the thirteenth session of shaanxi province special committee member adjustment of confirmation case" about promoting shaanxi new era comprehensive governing group resolution "the communist youth league in shaanxi provincial party committee to implement the <new era comprehensive governing group implementation outline> plan of action" about Duan Xiaolong comrade discharge to the provincial party committee's confirmation case "" about the fourteenth congress of the communist youth league in shaanxi province resolution ". During the meeting, also held the provincial Communist Youth League city and county two levels of the youth League committee secretary training class. League provincial party committee member, not the provincial party committee member, alternate members in shan group 18 central members, alternate members, cities (yangling demonstration area, han city), made youth league committee is mainly responsible for comrades, industry league committee is mainly responsible for comrades, league provincial party committee authority ministries (room), the directly affiliated units is mainly responsible for comrades, a total of 86 people to attend the meeting in xi'an. Members of the youth League Committee, the main responsible comrades of the internal organs of the Communist Youth League Committee and the secretaries of the youth League counties and districts in the sub-venue of each city (Yangling Demonstration area and attended the training.

2022-03-30	Hundreds respond to a single call! More than 380,000 volunteers in Guangdong have participated in the fight against the epidemic! More than 380,000 volunteers in Guangdong have participated in the fight day. Facing the severe and complex COVID-19 situation, the Guangdong Communist Youth League quickly launched the three-level emergency prevention and control response mechanism and issued recruitment orders. "Volunteer red" has become a beautiful scenery on the battlefield of epidemic prevention and control. Among these volunteers, there are enterprise white-collar workers, delivery boys, housewives, veterans, college students, etc. They stick to their posts, and the residents in the area to jointly build a "Great Wall of steel". According to the "i volunteer" system, as of March 17, since this year, 384,700 volunteers have participated in epidemic prevention and control, including 9,275 volunteer service organizations (groups), providing a total of 6.148 million hours, of which party members and league members accounted for 53.77 percent of the epidemic prevention volunteers, and young volunteers accounted for 54.2 percent."It is a sense of accomplishment to help others."Luo Zaiyan, a girl from Guizhou province who works in Shenzhen, is a post-1995 delivery rider. The community she lives in has become a control area. She led a team of more than 20 riders to join the material delivery volunteer team and contribute to the community's fight against the epidemic. Since March, an average of nearly 2,000 new volunteers have been registered in Shenzhen every day. Shenzhen Municipal Party Committee volunteer work responsible person introduced, since this Spring Festival, the city's organs, enterprises and institutions quickly carried out the "youth war epidemic ten actions", the establishment of 1339 youth volunteer commandos a total of more than 36,000 people, sinking Futian, Luohu, Nanshan, Baoan and other key areas. At the vaccination site in Shekou, Shenzhen, a Mexican girl dressed in a red vest has helped many foreigners solve their language barriers and different types of documents. Her name is Sophia. She has 3 years of volunteer service experience and can master Chinese, English and Spanish. She and a large number of foreign volunteers are as active in the epidemic prevention line. According to statistics, Shenzhen has launched 351 volunteers from Hong Kong and Macao and 203 foreign volunteers to provide diversified and international anti-epidemic services, and solidified the normal mobilization mechanism of Hong Kong and Macao volunteers and foreign volunteers. In Luohu, Shenzhen, Wu Dongmei, a volunteer, can not count how many phone calls she has made. Collect, summarize, organize, push, all information should be accurately verified, accurate investigation, fine record. For Wu Dongmei, who is racing against the virus, "when phone numbers turn into data forms and are handed over to street workers, it is the most fulfilling moment."In the interview, the reporter learned that not only the flow of work, more and more professional volunteer services are highlighted in the epidemic prevention and control. Zhu Yi, a national second-level psychological consultant, joined the "0131" temporary psychological care group of Shenzhen Bao'an District Committee to listen to the troubles of the people in the sealing and control area, ease their anxiety, and provide simple and effective self-regulation methods. The Zhuhai Municipal Party Committee of the Communist Youth League has set up a foreign language and dialect inquiry team. If any foreign or Cantonese dialect area people call for inquiries, the professional volunteers will deal with it uniformly. In recent years, there have been many obvious changes in the epidemic prevention volunteer services, and the volunteer response speed has become faster. Within half an hour, a WeChat group of 300 volunteers was set up in Haizhu District, Guangzhou; in just two hours, Shenzhen recruited 2,892 volunteers in a large scale. The person in charge of the Guangdong Volunteer Action Guidance Center said: " The organization of volunteer service is more orderly and efficient, and the grassroots league cadres are more confident."In 2021, the Guangdong Provincial Committee of the Communist Youth League established a reserve team at the provincial, municipal and county levels to produce and promote training courses on epidemic prevention and control. For example, Guangzhou, Yangjiang and other places have held hierarchical and hierarchical large-scale volunteer service training and drills for nucleic acid testing to normalize the construction of youth league members' commandos for fighting against the epidemic in various key areas. It is reported that the Guangdong Communist Youth League and the Guangdong Volunteer Federation have issued specific guidelines to fix the scientific, efficient and practical working mechanism accumulated in the practice of epidemic prevention and control. At present, 135 municipal youth League committees in Guangdong province have directly contacted and guided 11 emergency volunteer service teams. In case of an epidemic, they will immediately report to the local headquarters and be ready to fight. Source: China Youth Daily, March 30,2022, edition 02

2022-03-30	Yunnan Communist Youth League: "Bring the phoenix back to the nest" enables rural revitalization for children in the mountains to go to school."Henan Institute of Engineering civil engineering major student Xin Liang Yunan said. Xin Liang, Chongqing's hometown in Xishuangbanna Dai Autonomous Prefecture, Yunnan province, signed up to participate in the "return to hometown" social practice activity for college students organized by the Yunnan Provincial Party Committee earlier this year."This practice has strengthened my determination to return to my hometown after graduation."Xin Liang Yunan said."Yunnan sends a large number of students to universities inside and outside the province every year. They have a strong desire to participate in public welfare activities and make contributions to their hometown."Youth League Yunnan Provincial Party Committee related responsible person said. The Jinuo Mountain Township in Xishuangbanna Prefecture is the main settlement of the Jinuo people. There are no interest classes, remedial classes in the mountain village here, and adults do farm work in the fields every day."Children in the mountains are also eager to learn, but there are not too many platforms. They need the attention of youth league organizations."Jinuo Mountain ma village committee general branch secretary Li Li said. During the winter vacation, the township youth League committee recruited returning college students to set up knowledge classes on painting, epidemic prevention and safety for the children, and also led them to clean up the garbage on the village roads and classify them. The children were enthusiastic and were active in every day."Refuse to lie flat" is the new attitude for many college students returning to Yunnan in winter vacation. In order to help the implementation of the "double reduction" work and improve the quality and level of after-school services, Yunnan Provincial Committee of the Communist Youth League, Yunnan Provincial Department of Education and Yunnan Provincial Youth Working Committee launched the work of "helping the 'double reduction' " for students in compulsory education in January this year. The activity came to a climax during the winter vacation, with more than 3,500 returning college students studying in more than 100 universities across the country cooperating with youth league organizations across the province, providing services to 15,800 children. In order to do a good job, as early as before the winter vacation, the youth league organizations at all levels in Yunnan will actively plan, strive for the support of various social organizations, training institutions, writers, calligraphy, art, dance associations and other professional forces, design the content of activities, enrich the form of activities. Youth league organizations in 16 prefectures (cities) and 87 counties (cities and districts) have set up 165 service points in more than 110 communities (village groups) with the principle of "being nearby and ensuring safety", facilitating the participation of returning college students and local teenagers. Returning college students offer academic guidance, reading guidance, youth self-care and other courses for primary and middle school students; take them to visit the fire brigade, village history room, red education base, museums and other places; and carry out volunteer services such as "garbage cleaning" and "garbage sorting" in the streets, forest parks and river courses. Returning home service makes many college students feel "enhance their feelings for their hometown" and "the importance of lifelong learning". Zhang Yingyan, a senior majoring in clinical medicine at Kunming University, has "returned to her hometown" at the Zhongying Health Center in Kaiyuan City, Honghe Hani and Yi Autonomous Prefecture. For a month, she was exposed to trauma and illness unheard of."At present, township hospitals have changed a lot, the equipment is becoming more advanced; but on the other hand, the types of diseases is increasing. As a medical student, only lifelong learning can eliminate the disease with the best plan."She said. When Li Tao, a student in Hubei University, returned to his hometown in Jinping County, Honghe Prefecture, he got to learn about the green pepper planting and the e-commerce sales of agricultural products in Jinping County. At the same time, he also conducted a survey of 100 questionnaires that found that " More than 40 percent of people think farming should be encouraged and developed. In remote mountain villages in Yunnan province, planting industry is the first source of income for farmers.""This social practice shows me that the people in my hometown are full of expectations and enthusiasm for the rural revitalization, which is of great help to us to join the rural revitalization in Jinping County."He said."Only by letting young people know and love their hometown can we attract them to their hometown."Said He Jintao, deputy party secretary of Tuan Jinping County. He Jintao, also a native of Jinping County, worked from China Electrical Appliance Research Institute Co., Ltd. after graduating from Jinan University in 2013. In 2016, she quit her job in Guangzhou and returned to Jinping to become a grass-roots league cadre. Lv Junyan, a part-time deputy secretary of Qujing Qilin District Committee and a sophomore student of Qujing Normal University, has the same understanding with He Jintao. In 2019, under the guidance of the Qujing Municipal Party Committee and the Youth Qilin District Party Committee, Lu Chunyang, who often participated in the volunteer service, set up the "Dacheng Little Love" volunteer service team. Then he was still a high school student. In the past three years, the "Big City Little Love" youth volunteer service team has become the volunteer service brand of Qilin District. Although he is a college student who has not left his hometown, Lu Chunyang is very concerned about the young people returning home to start their own businesses. Luohu Village, Yuezhou Town, Qilin District, is an ancient ceramic town. With the promotion of the construction of Luohu ceramic art characteristic town, more and more young people return to their hometown to learn this ancient craft. In 2021, Lu junyang organized a team of college students to produce special publicity videos for Yuezhou, and also conducted a live broadcast with goods for Luohu Ancient Tao. At the beginning of this year, Qilin District Committee set up a cooperation platform with 56 communities and 20 direct units in 8 streets to jointly carry out the social practice of "Big City Little Love" college students "returning to their hometown". Li Yongsheng, a master's degree from the School of Government of Yunnan University, lives in Fengqing County, Lincang City. He has participated in the social practice activity of "Returning to his hometown" twice, and completed the paper "Exploration of the Model of Social Organizations in Improving Basic Education in Rural Areas". Now, Li Yongsheng, graduating graduate, has decided to return to work in Lincang. He said: " It is the practice that sowed the seeds of my return home. The "sense of belonging" is the most important point for Liu Yinchen, vice minister of the Development Rights Department of the Youth Kaiyuan Municipal Committee, when serving the returning college students. In her opinion, to let the "return home" college students take the initiative to participate in the social practice, dedication, bend over the grassroots, listen to the hearts of the people, precipitate the sincere feelings to the hometown, buried the seeds of "feedback to the hometown" after graduation, the test is the stage set by the league organization. To this end, she plans to integrate the Kaiyuan city "colorful classroom", "silver hair surfing" and "city color" volunteer service projects with the "return to home" activities, expand the participation, care about the grassroots "hot spots", help special groups, and provide accurate services for the masses."Only good activities can bring play to the intelligence and potential of college students."Liu Yinchen said. The "sense of belonging" also strengthens the emotional bond between the "returning home" college students and the Yunnan Communist Youth League. Youth League committees at all levels set up temporary youth league organizations among college students "returning to their home" to carry out theme activities, so that college students can rely on them in an organization and form a harmonious atmosphere for something to do. Local Party committees, governments and practice units also give great support to youth league organizations: providing internship positions, buying insurance for all students, providing meals and accommodation for some students, and issuing "warm heart energy packages". Although the current activity temporarily comes to an end, youth league officials from all over the country still keep in touch with college students, constantly launching the stories of "returning home" college students on the wechat official account, and building a "returning home" talent pool to the local organization department. The social practice activity of "returning to home" has also been praised by the local organizational departments, believing that this activity has become a stable channel for the grass-roots youth league organizations to contact the college students outside China, and the grass-roots youth league organizations have played a positive role in "attracting the phoenix back to the nest". China Youth Daily, March 30,2022

2022-03-29	"It is really not easy for the baby to come to this world. Thank you."Seeing the newborn child, Wan Qin, lying in the delivery room was a little excited. She was anxious just a few days ago when she was 40 weeks pregnant and pregnant. Wanqin lives in Nanchang new � jie � on March 16, long � q �, the implementation of traffic and personnel control, all personnel, vehicles only in, the local epidemic prevention and control emergency headquarters after timely open green channel, " we through the Nanchang municipal party committee, and the first affiliated hospital of Nanchang university like lake branch contacted, arranged the love vehicle escort wan qin couple admitted to hospital."Xiong Qun, deputy secretary of the Xinjian District Youth League Committee, said. On the afternoon of August 18, Wan Qin and his wife arrived at the hospital safely and went through the admission procedures with the assistance of volunteers. On March 18th, I learned that the Nanchang Municipal government would build the Xinyue Lake makeshift hospital, and the Jiangxi Branch of the China Construction Third Bureau immediately called on the frontline young builders."Let me go! I have the experience!"When Fang Qing, a young cadre who had previous experience in the construction of" Huoshenshan ", he could not resist his anxiety and took the initiative to sign up to participate. In just one hour, more than 200 youth members were assembled and rushed to the construction site. 42 youth commandos undertook urgent and dangerous tasks such as material personnel dispatch and ground paving and other tasks. We do not rest, many people 24 hours, seize the transformation progress. After the outbreak, the Jiangxi provincial party committee timely formulate the communist youth league COVID-19 epidemic prevention and control volunteer service emergency plan COVID-19 epidemic prevention and control of youth volunteer service work guidelines and the large-scale coronavirus nucleic acid detection youth volunteer service plan in Jiangxi province, etc., in orderly organization around all kinds of youth volunteers scientific and efficient participation in epidemic prevention and control volunteer service, widely issued initiative, mobilization order, recruitment, to mobilize the provincial youth league organizations at all levels and the youth volunteers to participate in the epidemic prevention and control work. As of March 23,10 points, the provincial youth league organizations, youth volunteers association formed WeChat disease resistance group of 1986 volunteers, recruit 89783 young volunteers, reserve war epidemic youth volunteers 402, mobilize 11338 volunteers to provide volunteer service against disease, order maintenance, information input, inquiry guidance, site disinfection, material transport and information MoPai statistics, space and time companion survey, etc. In the face of epidemic prevention and control, a number of "post-90 s" and "post-00 s" in Jiangxi have put on protective suits and become strong and indomitable youth forces in the "fight against the epidemic" battlefield. In March, it was rainy in the south of the Yangtze River, and the temperature difference between day and night was large. Yang Hui, wearing only one short sleeve, was busy with a protective suit and a raincoat in the cold and rainy night, but he thanked the community staff with a smile: " It is not convenient for us to wear coats, which will affect the progress of testing information input."Since the emergency outbreak in Xinjian District of Nanchang city, Yang Yanhui, as a member of the" low-light " youth volunteer service team of Nanchang Xinjian District Power Supply Company of the State Grid, has immediately devoted himself to the epidemic line to cooperate with the community to carry out nucleic acid testing work. From noon on The 16th to 11 PM on the 17th, Yang Hui participated in completing the nucleic acid detection and information collection of � 2,300 people in the long � Nuo foot. He goes to and forth from the company and �. � has not been home for three consecutive days. On March 17, Nanchang city launched the first round of large-scale nucleic acid testing. Tu Xinchen, a 22-year-old senior who was at home for epidemic prevention and control holidays, immediately applied for the volunteer service. Wang Juntao, a hoarse volunteer who insisted on the nucleic acid detection point in Tangshan Town, Qingshan Lake District, to maintain the order. During the volunteer service, he spent his unforgettable 18th birthday. A few days ago, a 29-year-old young man, from Xinjiang province, arrived to a nucleic acid testing site of the European Industrial Park in Nanchang High-tech Zone with a box of freshly baked Nang buns to deliver free lunch to medical workers and volunteers. Miradili came to Nanchang, Jiangxi province in 2013, and then took root in Nanchang and started her own business. Now it now has its own special barbecue shop. As a member of the All-China Youth Federation, he said that he should play a leading role. "This time it takes us to stand up and give back to the society."Photo source: The Communist Youth League of Jiangxi Provincial Party Committee

2022-03-28	Since the outbreak of COVID-19, the Liaoning Youth Volunteers Association has timely organized and mobilized youth volunteer service forces to enter a state of war, war preparation and response, and actively and orderly participated in the epidemic prevention and control work. The province has recruited 280 youth volunteer service organizations and more than 70,000 young volunteers. At present, more than 60,000 young volunteers fighting the fight against the epidemic. On March 15, the emergency detachment of Liaoning Provincial Youth Volunteer Service Corps went to Huashan Community, Huashan Street, Huanggu District, Shenyang city to assist medical staff to input nucleic acid testing information and assist the community to carry out epidemic prevention and control publicity, and testing 7,000 people every day. Shenyang Youth Volunteers Association recruited more than 270 epidemic prevention volunteers to assist the nucleic acid testing sites in Shenyang, and were on duty at the gate of each community. Fushun Qingyang Social Work Service Center set up an epidemic prevention and control team to prevent and control it at the checkpoint of Shenfu Avenue in Wanghua District, Fushun. They checked the vehicles and pedestrians and nucleic acid test reports together with the epidemic prevention personnel, and advised the vehicles and pedestrians who did not meet the access conditions to return in time. On March 22, Liaoning Provincial Youth League Committee and Liaoning Provincial Youth Volunteers Association openly recruited young volunteers to the whole province, expand the emergency volunteer service team (detachment) of Liaoning Provincial Youth Volunteer Service Corps; integrate social resources to give insurance to volunteer groups, and strengthen the guarantee of young volunteers for epidemic prevention and control. According to reports, young volunteers over the age of 18 will log on to the official website of the volunteer in Liaoning province (http: / / www.zyln. After downloading org) the "Volunteer Liaoning" App, click the "I want to apply" button on the home page to apply for the "Liaoning Province Registered Volunteer Certificate". When successfully applying for the certificate, the insurance function is automatically activated, and each registered volunteer can get the exclusive insurance guarantee of 328,000 yuan. Source: China Youth Daily

2022-03-26	Heilongjiang more than 4000 young volunteers power epidemic prevention and control of Heilongjiang province more than 4000 young volunteers power epidemic prevention and control moment, COVID-19 outbreak hit again, Heilongjiang province youth league organizations at all levels active war, to fight, the first blow war "disease" assembly, full speed organization mobilize youth volunteer service forces into the battle, preparation, battle state, actively and orderly participate in the epidemic prevention and control related work. As of March 20, Heilongjiang province has convened 4,471 people, including cadres of the Youth League committees at all levels, members of local volunteer service organizations, and college students, to participate in the volunteer service, escorting the province's epidemic prevention work. The Harbin Municipal Party Committee of the Communist Youth League immediately issued the Proposal to Young Volunteers in Harbin on the WeChat public platform, calling on mobilizing youth volunteer service organizations at all levels and young volunteers to take active actions, serve nearby, give full play to their strengths and fight the epidemic scientifically. Daowai District of Harbin city is the first place of this round of epidemic. After the outbreak of the epidemic, Daowai District of Tuan Harbin City immediately issued a volunteer recruitment announcement. Due to the epidemic, He Yu'ang could not return to school, so he immediately signed up for the volunteer recruitment announcement issued by the Daowai District."I hope I can contribute to my hometown when I need it."These days, and Yu Ang is busy at the forefront of the fight against the epidemic. The 20-year-old is responsible for a number of work, such as nucleic acid scanning code entry, order maintenance, isolation unit sealing and control, and garbage transfer in Northeast New Community, Daowai District, Harbin City. He is often busy with civil nucleic acid testing and active in the isolation and sealing and control site. All the cadres of the District Committee assisted the streets with more than 2000 people through the "bungalow District Communist Youth League" and "bungalow District Volunteer Service Federation" WeChat public platform, and recruited more than 300 young epidemic prevention volunteers to form the youth epidemic prevention volunteer service team and reserve epidemic prevention reserve forces. The Nangang District Committee again issued the "mobilization order" according to the arrangement of the District Committee and the District Government. Within one hour, 22 members of the Youth Federation were rushed to 10 testing points in Wanggang Town to scan the code for prevention and control work, nucleic acid sampling, and maintain the order of the nucleic acid testing site. Universities have also carried out epidemic prevention and control work. Heilongjiang university youth corps committee relying on the college youth volunteer association, formed more than 20 campus epidemic prevention propaganda volunteer service, in the school canteen, dormitory building 13 student activities intensive area to carry out a week of campus epidemic prevention propaganda volunteer service activities, remind the teachers and students actively cooperate with the epidemic prevention and control requirements, to wear masks correctly, build prevention and control barrier. According to statistics, more than 500 people participated in the volunteer service activity, with a total service time of nearly 800 hours. Students from the Discipline Inspection Department of the School of Artificial Intelligence and Robotic Engineering of Harbin Far East Institute of Technology used their spare time to carefully wipe the surface, door handle, blackboard and other details exposed in the air to the air to ensure disinfection and sterilization of every classroom. During the outbreak, Harbin Engineering University convened more than 1,000 young volunteers to cooperate with medical staff to effectively carry out nucleic acid testing order maintenance, vaccination, quarantine and food delivery, and epidemic prevention materials distribution."As a student who was been quarantined, I know the inconveniences of living in isolation in a student hotel."Shi Diko, a student at the School of Shipbuilding Engineering at Harbin Engineering University, volunteered to become a volunteer after being quarantined for epidemic prevention and control reasons. As a volunteer, he is always busy, trying his best to help his quarantined classmates solve their life difficulties.

2022-03-26	Recently, the epidemic in Gansu has been repeated again, and the prevention and control situation has become increasingly severe. The Gansu Provincial Youth League Committee and the Provincial Youth Volunteers Association have responded in time, sounding the "rallying call" for young people to join the fight against the epidemic. The Gansu Provincial Committee of the Communist Youth League immediately sent cadres to go to Zhangjijiayuan Community, Jiuquan Road Street, Chengguan District, Lanzhou City to actively assist the community in volunteer services for epidemic prevention and control. In recent days, the province's cities and state youth league organizations, youth volunteer associations also heard the news and move, active action. In Lanzhou, in response to the call of the regiment organization, a "red vest" charge in the front."Hello, are you the owner of this community? Please check it up."" Please show me your health code and take your temperature."Whenever on duty at the gate of the community, such words of Honggu District young volunteer Wang Yan to speak countless times a day. In her opinion, the prevention and control is at the grass-roots level, and the community is the focus. By performing the duty of "gatekeeper" well, the epidemic prevention and control work can be permeated into the "cells of the community". In yuzhong county, and rescue team, Gansu longyuan emergency rescue team yuzhong brigade, veterans volunteer organizations, volunteer organizations, a total of more than 100 people volunteered, in nucleic acid detection point to maintain order, the key places sanitizers, help unattended community residents move life supplies, transfer milk powder drugs, etc., got the residents' recognition and thumb up. For 18 consecutive days, volunteers from the Love Station in Chengguan District of Lanzhou city have been working continuously- -265 volunteers have participated, killing 1.496 million square meters for 170 points, providing volunteer services for 10 nucleic acid testing points, driving 62 vehicles and setting up 2 tents. During the epidemic period, the Lei Feng motorcade, composed of Lei Feng, XX, �� and �, was also busy on the front line of fighting the epidemic, picking up medical staff and transporting epidemic prevention supplies, and racing against time."In order to protect the health of the people, the volunteers are at the forefront of prevention and control."CAI Jinyan, the principal of Gansu Zhenghao Vocational Training School, decided to offer his love. On March 17, he donated 50,000 yuan worth of masks, instant noodles, protective suits and rubber gloves to the Yining Road Youth Home of the Communist Youth League. In Baiyin City, the Baiyin District Committee of the Youth League, together with volunteers, made every effort to prevent and control the epidemic at the West Railway Station. At the same time, actively docking with relevant units to raise protective equipment, daily necessities, to solve the worries of volunteers. As a local enterprise, Gansu Zhongheng Group has actively responded to the epidemic prevention and control work. By March 23, it has donated 720,000 yuan worth of epidemic prevention materials to 40 units and 179 epidemic prevention and control points in Baiyin City, and has decided to donate a makeshift hospital to Pingchuan District of Baiyin City."Although it is very hard, people will still stick to their posts and hope that the epidemic will soon retire and return to normal life."In recent days, Tian Xiaohui, a cadre of the Jingtai County Health Bureau, responded to the call of the Communist Youth League County Party Committee and fully participated in the epidemic prevention and control as a young volunteer. Twenty-one volunteers from her unit went to the front line of prevention and control to carry out services such as policy answers, information sorting and material support. Since a positive case was found in Jinta County, Jiuquan City on March 6, the youth league members of Jiuquan CDC have taken the initiative to undertake the nucleic acid testing, flow tracing and elimination of the epidemic situation of the whole city. Every morning, put on protective clothing and start to work, for several hours do not eat, drink, go to the toilet, the forehead goggles was left a deep imprint, the young people still stick to their posts, no one flinch. In grass-roots townships in Suzhou District, Jiuquan City, youth league members used loudspeakers, banners, electronic screens and other carriers to widely publicize important reminders on epidemic prevention and control, so as to further improve the people's ideological awareness of epidemic prevention and control and self-epidemic prevention and control ability. In Linxia Prefecture, the Linxia city youth volunteer service team is called upon to build a "red dam" to fight against the epidemic. The united emergency rescue team of Yongjing County has set up an emergency volunteer service team to carry out comprehensive defensive prevention and epidemic prevention work in key areas and crowded areas for epidemic prevention and control. Since March 21, the Youth League and Kangle County Party Committee and Kangle County Longren Youth Volunteers Association to recruit more than 90 young volunteers, through the "offline + online" mode, to build a solid defense line of youth. At 8:30 every morning, volunteers quickly arrived in place and went to several nucleic acid testing sites in the county to assist the community with elderly residents operate mobile phones to apply for health codes, personnel diversion, order maintenance, and epidemic prevention publicity. It is understood that at present, the Gansu province recruited youth volunteers more than 30000 people, more than 130 cadres sink grassroots, cumulative receive all kinds of epidemic prevention materials worth more than 200 ten thousand yuan, the total service for 300000 hours, and successively reply psychological calls consulting more than 100, psychological counseling live more than 20 games, with practical action to practice war "disease" have my youth oath.

2022-03-26	Guangdong: "plan" service "double hundred thousand" youth employment internship in Guangdong: "wings plan" service "double hundred thousand" youth employment interns Lin Jie) recently, 2022 "plan" Guangdong communist youth league special to promote youth employment, in 2022 Guangdong province shortage of enterprises public recruitment series of state-owned enterprises special fair held in south China university of technology. At the scene, all units played a combination of "stable employment", attracting a total of 288 state-owned enterprises and public institutions to provide 6,663 quality jobs, attracting more than 1,000 graduates on the same day. In addition to the post supply and demand docking, the job fair also has a "wings plan" workplace gas station career development consultation, college students volunteer service western (mountain) plan recruitment, youth housing security and other functional service special exhibition. On this basis, the organizer carries out career tutors for job-seeking students to "feel the pulse", guide graduates to the grass-roots practice employment, and effectively solve the housing problem for graduates and other services."This year, youth league organizations at all levels in Guangdong province plan to provide employment docking and career growth services for no less than 100,000 young people, and encourage no less than 100,000 college students to participate in practice and grassroots services."Guangdong Youth Entrepreneurship and employment Promotion Center related person in charge of the introduction, with the" spread the wings plan " as the whole, to further establish and improve the youth employment service system, improve the youth career growth mechanism. It is understood that the Guangdong provincial level will focus on the employment and career selection concept guidance, employment practice ability improvement, education and vocational skills improvement, key youth groups employment assistance, housing security and other content to launch various service projects. The municipal level will also carry out the "set up a team of mentors, carry out a career guidance activity, hold a graduate job fair, organize a skill training activity, implement a local characteristic project" and other actions. The job fair is co-hosted by the Guangdong Provincial Committee of the Communist Youth League, the Guangdong Provincial Department of Human Resources and Social Security, the State-owned Assets Supervision and Administration Commission of the Guangdong Provincial People's Government and the South China University of Technology. On the same day, the 2022 Guangdong Communist Youth League to promote youth employment special campaign also kicked off. The "Spread Your wings Plan" promotes employment through the provincial and municipal youth League committees to set up youth career mentor groups, open online consultation areas, and develop various online career guidance courses, so as to provide vocational guidance and consulting services for young students nearby. At the same time, it will timely sort out, interpret and transmit employment information, and carry out online and offline supply and demand docking activities and various social practice and skills training activities for college students, so as to concentrate on helping young people improve their career development ability and achieve higher quality and full employment. The Guangdong Provincial Party Committee and other units have implemented the western (mountainous area of volunteer service plan for Guangdong college students). In 2022, they plan to recruit about 5,000 college graduates to the grassroots frontline of Tibet, Xinjiang and other provinces and regions, and 12 cities in eastern Guangdong, Zhaoqing city and Longmen County in Huizhou city to carry out volunteer services. Among them, the Mountain Area Plan- -Guangdong College Graduates volunteer service for rural revitalization action is also included in the top ten major Guangdong provincial government practical matters for people's livelihood in 2022. On the site, in addition to all kinds of recruitment enterprise booths, there are also "youth housing security service area", for college graduates to bring all kinds of affordable housing, youth post station, social preferential rental and other housing information consultation and services. Since 2020, the Guangdong provincial party committee joint province live built hall in the country to launch the first "youth housing plan" college graduates housing security services, through the "physical security, monetary subsidies, social supplement, information construction, industry specification", promote affordable housing, talent room, youth station construction, and the relevant agencies to launch preferential housing, focus on service college graduates. At present, the youth housing information inquiry system has been launched, launching more than 9,000 units of public rental housing, talent housing and other affordable housing, helping 7,572 graduates to obtain housing security, and providing information services to more than 500,000 people.

2022-03-25	Inner Mongolia communist youth league: strengthen grassroots forces closely linked youth communist youth league: strengthen grassroots closely linked youth academy forces of college students he happy, this winter vacation in his hometown of ordos city, Inner Mongolia, jin Lin Gaole community as part-time league branch deputy secretary, as the grassroots cadres in the grassroots, go deep among the masses and youth."I used to think that grassroots work was of low value, but now my mind has changed completely. I want to go back to my hometown and do something after graduation."He said happily. This is not alone. In July 2021, the Inner Mongolia Ordos Municipal Party Committee recruited 384 Ordos native college students serving as deputy secretary of the city. The youth League members also solved the problem of "no one", which is the "dividend" brought by the reform of the Communist Youth League in Inner Mongolia county. Since the central county of the communist youth league organization reform to expand the pilot work, the Inner Mongolia district party committee adhere to the top design, key breakthrough, first try first, set up by the secretary of the leader of the work class and deputy secretary of the responsibility of the four reform team, promote 12 league city all establish work class. Through half a year's efforts, the reform measures of the grass-roots organizations of the county Communist Youth League have been implemented effectively in Inner Mongolia, and the organization force, service force and cohesion of the grass-roots youth league organizations in the pilot areas have been significantly improved. Open channels: cadres form "a pool of living water" in Inner Mongolia la shan league alshan right flag committee from diversification, universality and influence, explore the "learning" + "matching +" + "service" new path, strengthen flag county, wood township, GaZha village (community) cadres team construction, three-level linkage orderly increase grassroots work force, open grassroots cadres "choose tube" new mode, for grassroots cadres filled "oil", "strength". After the reform, the cadres of 40 Gasha villages and 9 communities of Alxa Right Banner realized their age and education, and all the league secretaries of Gacha Village (community) entered the team; the Party and government members and the cadres of counties expanded from 1 to 2 to 4 after reform, and 49 college students were selected as deputy secretaries of Gacha Youth League Branch."After the strength of grassroots league organizations, many work has changed from passive to active."Gao Jing, secretary of the League Alxa Right Banner Committee, introduced the convenience after the reform, the league secretary into the" two committees "team," we have the right to "speak", work more smoothly."Gao Jing said that there are 49 Gacha villages (communities) in Alxa Right Banner, with a permanent population of only more than 20,000 people." There are fewer Gacha people, even fewer young people, and the working force is very limited."Now every Gacha village has a college student serving as the deputy secretary of the Communist Youth League branch. These college students not only bring many good ideas, but also lead more young people to stay around the youth league organization. In addition to actively explore practice on growing strength, xingol league in Inner Mongolia under flag committee in broadening the channels of cadres selection continuously, establish effective constraints and incentive mechanism, for the grassroots cadres grow up the ladder of progress, form personnel can enter and out, can on the working pattern, grassroots cadres to form a pool of "living water". The flag youth League committee to implement the youth league cadre target responsibility system, according to the annual quantification, detailed to the month, responsibility to the person, regular publicity. The excellent Gacha village league organization secretary (community) is recommended as the local "one shoulder to shoulder" reserve candidate, and the excellent league organization secretary of Sumu township and banner direct units is recommended to the Party organization, and promoted or further used according to the procedures. Evaluation of the unqualified cadres, the League flag committee to interview to remind, rectification within a time limit, two consecutive evaluation of unqualified proposal adjustment. When recruiting the staff of the public institutions belonging to the league, the work experience of Gacha village (community) cadres should be taken as the additional conditions; and the youth affairs, youth volunteer service working years and experience should be taken as the additional conditions for the recruitment of public welfare positions. Since the implementation of the reform of the grass-roots youth league organizations in Inner Mongolia, the pilot areas have reformed the selection and appointment system, explored the socialized selection path, and tried to diversify the backbone of the grass-roots work. The working force of the youth league organizations at all levels has increased significantly, and the weak situation at the grass-roots level has been improved. In order to extensively contact the youth league members and open up the "last kilometer" of youth service, the Saihan District Committee of the Hohhot Communist Youth League has created the "double" mode of the Communist Youth League online community, and linked the youth through "online + offline" ways. Saihan District Committee of Hohhot sets up WeChat tower group in the traditional field, and youth league cadres, young backbone and volunteers settle in the WeChat tower group of "group leader-member-" seed "youth" for the supervision and management of the young cadres, publicity and mobilization, and "seed" youth people are outstanding volunteers and responsible for driving the remaining volunteers. In February this year, after the outbreak of the epidemic in Hohhot, the Saihan District Committee of the Youth League used the existing online community model to immediately issue the recruitment order for young volunteers for epidemic prevention and control, and set up a youth commando team for epidemic prevention and control in Saihan District. Nearly 3,000 young volunteers signed up to participate. During the epidemic period, the young volunteers will receive small videos of recording their work recorded by the Communist Youth League Committee of Saihan District Committee every night, and feel the care of the youth league organization. Recently, the reporter came to is located in Baotou city, Inner Mongolia lucheng building "youth" love "(" reporter note- "love" for "home", and "love neighborhood"), see learning club, mind SPA, "Kun Xiaoqing" classroom, city bookstore, web celebrity clock point five functional areas, yellow and green small pure and fresh decoration style suit young people's preferences. Young people who work in buildings can read and study here, play social games, and seek psychological counseling services. In addition, with the help of the project-oriented operation mode, Lucheng Building "Youth Love Li" has carried out a series of activities popular with young people. The Beilun District Committee of Baotou City attracts more and more building youth to join through the "Monday hour" high-frequency, fixed and characteristic activities, forming the building "youth love circle", and creating a comfortable, convenient and warm activity position for the building youth. Service youth: weave dense contact problem "a network" " agricultural technology training lack, online sales ability lack, agricultural products supply and demand information is wrong, hope the league organization to give attention and help."" I hope the League will actively strive to improve the treatment of young Pioneers counselors and help solve problems in the evaluation and employment of professional titles."" I hope the Youth League organization will increase assistance to poor students in private vocational colleges."" We hope to strengthen guidance and support for youth employment and entrepreneurship in terms of platform building, talent training, and supply and demand docking.”...... These are written on the "appeal wall" of the league representatives of Saihan District of Hohhot City. The members of the league and the league representatives claim to "take orders" according to their own actual conditions, conduct two-way communication with the youth members, actively serve the urgent needs of teenagers, and respond to everything. To solve the youth think hope urgent things, Hohhot saihan district group on behalf of the contact station to deepen close contact youth mechanism, group representatives into a line to ask, ask, ask politics in youth, for the youth around the tangible and practical work, to improve for the youth to solve problems, the ability to do the practical work and level, improve the youth satisfaction. Zhao Qian collected the wishes of students from five rural primary schools in Dengkou County, Bayannur City, Inner Mongolia. Zhao Qian and other volunteers were busy purchasing goods and helping them realize their small wishes. This is a part of the local volunteer service to "help build rural school children's palaces". Since the successful declaration of central project, group of bayinnaoer city dengkou county deputy secretary zhao jiahui chose five teachers relatively lack of rural schools, formed five youth volunteer team, using school second class, weekends, quality development, safety since protection, ideological guidance, psychological counseling and a series of theme activities, form a long-term mechanism, the care and care to teenagers, let teenagers have more feeling. It is understood that the Inner Mongolia Communist Youth League will continue to enhance the political nature, advanced nature, mass nature, effectively grasp the reform in the hand, carry the responsibility on the shoulder, enhance the political function of the league, enhance the social function, so that the grass-roots Communist Youth League really strong.

2022-03-22	Tian Hongwei, An asymptomatic infected persons and confirmed cases of COVID-19 have been found in Zhangzhou, Quanzhou and Xiamen, Fujian province since March 11. As of March 19, the Fujian Provincial Health Commission has reported a total of 636 local confirmed cases of COVID-19. After the outbreak of the epidemic, the Fujian Provincial Committee of the Communist Youth League quickly launched the recruitment and reserve work of youth volunteer service teams for epidemic prevention and control. Organize according to the needs of flow traceability, sealing control management, nucleic acid testing, convenience service, etc. Up to now, the youth league organizations at all levels in Fujian province have recruited 25,000 young volunteers and carried out 73,000 volunteer services. Youth League organizations at all levels in Fujian have also mobilized young people in the transportation, emergency support, medical and health care industries to set up youth commandos, and actively participate in the epidemic prevention and control frontline of monitoring and early warning, professional medical care, logistics and distribution, and emergency duty. Up to now, Fujian province has set up 1,456 youth commandos, with 26,000 people. On March 13, the first confirmed cases of COVID-19 were reported in Quanzhou city. On the same day, the Quanzhou Municipal Party Committee of the Communist Youth League dispatched 45 Party cadres and young volunteers to form an epidemic prevention and control team to enter Fengze New Village, the containment area, to carry out front-line epidemic prevention and control work. Xu Yue, secretary of the Youth League, deputy secretary as the leader of the responsible area. The task force is mainly responsible for sorting out the information of 975 residents in 15 buildings in the sealed and control area, guiding the residents to carry out nucleic acid testing in an orderly manner, and publicizing the knowledge of epidemic prevention and control. The Communist Youth League cadres and volunteers also need to understand the daily needs of residents, coordinate the distribution of living materials and medicines, and maintain a normal life during the quarantine period. Xu Meifeng, a youth affairs social worker, was the first member to enter Fengze New Village to participate in the epidemic prevention and control work. She started to work at around 6 am every day, starting to know the daily needs of residents and guiding residents to go downstairs for nucleic acid testing in time and in an orderly manner. Xu Meifeng told China Youth Daily that although they have set up a WeChat contact group for residents, many elderly people in the community do not use smartphones. Volunteers should specially mark such residents out, specially lead the elderly downstairs to do nucleic acid testing, communicate with the living needs of the elderly in time, and deliver the required materials to their homes. During the epidemic containment work, Xu Meifeng encountered a small episode. Mr.Yang, who lives in Fengze New Village, took his family home after the second round of nucleic acid testing and was ready to change shoes for the children. In a hurry, Mr.Yang left the key in the lock hole of the inner door, and locked the outside door when entering the door, so that both doors could not be opened inside and outside. Xu Meifeng and her colleagues came to check whether the residents have completed the nucleic acid tests and found that Mr.Yang's home situation. While reassuring the residents not to worry, they contacted the lock picker everywhere. Because Fengze New Village belongs to the sealing control area, the lock master can only remotely guide the lock. In the end, the two sides worked together to open the door. Mr.Yang also posted a poem on his wechat moments to thank the volunteers for their help. Lin Shubin, a young volunteer involved in epidemic prevention and control in Zhangzhou Taiwan Investment Zone. On March 11, an asymptomatic case of COVID-19 infection was reported in the Taiwan Investment Zone of Zhangzhou city, Fujian Province. In response to the call of the local Zhangzhou Municipal Government, led by Zhang Chenhui, Party secretary of the Communist Youth League, 8 cadres rushed to the Taiwan Investment Zone to participate in the epidemic prevention and control work. Lin Shubin is one of them. Lin Shubin recalled that residents were queuing up for full nucleic acid tests that day, and she noticed that a white-haired grandmother was also in the queue."The sun was very high, and the temperature was about twenty-eight (eight degrees Celsius)."Lin Shubin was worried that the old man would stand in line for too long, so she moved a bench to accompany the old man to finish the nucleic acid and send her home."At that time, the old woman asked me where I came from, and I told her that she came down from downtown Zhangzhou. The old woman kept saying, thank you for coming all the way to help us, thank the government and thank the country."Zhang told the China Youth Daily of China Youth Network that about 1,800 young volunteers are currently involved in the epidemic prevention and control work in the entire Taiwan business investment zone. In the two closed control areas of Cangban Farm and Jiaomei Middle School, more than 50 Party cadres and young volunteers of the Communist Youth League Municipal Committee were responsible for the daily needs of about 1,300 people during the quarantine period, and guided them to carry out nucleic acid testing in an orderly manner. Zhang has noted that in the epidemic prevention and control process, they have explored a working mode of "Party building + youth league building + community volunteers". In short, in addition to the party cadres and young volunteers who came from outside, the Communist Youth League Zhangzhou Municipal Party Committee also mobilized the volunteers in the sealed control area. The community volunteers are familiar with the local folk customs and communicate with the public more effectively, which contributes a lot to the epidemic prevention and control work. Born in 1998, Huang Yulin is the youth league branch secretary of the Cangban community. She said that the original was to set up volunteers in the control area to participate in the distribution of materials. Given the increased risk of internal and external cross-infection, volunteers were left in the containment area to serve."We will give the volunteers nucleic acid tests first every day to ensure that they are safe and then serve other residents."On March 14, Xiamen city found two people tested positive for nucleic acid tests among those who returned to Xiamen, and they were immediately transferred to Xiamen to a designated hospital for treatment. The Xiamen Municipal Party Committee of the Communist Youth League and the Communist Youth League Committee of each district and each system responded quickly to the recruitment of young volunteers to reserve candidates. So far, five recruitment groups of Xiamen youth anti-epidemic reserve volunteers have been opened. At the nucleic acid sampling point in Xingang Community, Huli District, Xiamen, a volunteer from the Huli District Taiwan compatriots Volunteer Service Alliance can be seen every 10 meters. Up to now, the alliance has 108 Taiwan compatriots volunteers. Since the establishment of the alliance in August last year, Taiwan compatriots have participated in eight volunteer services such as donating and raising anti-epidemic materials. CAI Hansheng, born in 1993, is a health officer of Houkeng Community, Jinshan Street, Huli District, Xiamen. The two natural villages of front and rear society of Houkeng Community are mostly populated by people and have dense houses, close to the municipal main road, and naturally forming 72 entrances and exits. CAI noted that the situation will increase the pressure on control during the epidemic prevention and control period. In the past few months, he and other colleagues have set up 35 closed flexible ports and kept 7 main intersections according to the actual needs and conditions of the village. This work has played a role in the current round of the outbreak stage in Xiamen. Starting from March 11, CAI and his colleagues have controlled community personnel by limiting flexible entrances and exits, and put people on duty 24 hours at the main entrances and exits. Xiamen also urgently gathered 300 nucleic acid sampling personnel in the early morning of March 16, and successively went to Quanzhou to assist in the nucleic acid testing work. The nucleic acid sampling team covers medical, nursing and technical personnel, many of whom are veterans who have supported other places for many times before. Zhuang Yuanhong, a veteran nurse in charge of the department of respiratory and critical care medicine at the Affiliated Zhongshan Hospital of Xiamen University, said, " It is in a special hurry to get the news in need of support. However, our hearts is early ready, always hope to do a part! I am Quanzhou people, in my hometown need themselves when the duty-bound, rushed to the front line."In addition, in Fuzhou, Ningde, Longyan, Sanming, Putian and pingtan comprehensive experimental area, youth league at all levels issued the epidemic prevention and control emergency reserve volunteers online recruitment, mobilize the youth in war" disease " line, set up youth commandos, scientific and orderly participation in the temperature measurement, nucleic acid testing, personnel registration, convenient service, protection propaganda and other volunteer service work.

2022-03-22	Guizhou for the national youth volunteers IP image of Guizhou for the national youth volunteers IP image Li Yajuan) recently, to further promote the "build youth volunteer service new heights" work requirements, set up a new image of young volunteers in Guizhou, Guizhou volunteers volunteer action guidance center (Guizhou youth rights protection center), Guizhou youth volunteer service foundation for the national solicitation "Guizhou youth volunteers IP image" design. It is reported that the IP image can be a young man and a woman, can also be a neutral image, or animals, plants, utensils and other material images. The image needs to reflect the palm symbol of young volunteers, image expression, youth elements and regional characteristics of Guizhou; should conform to the aesthetic trend of youth in the new era, with the atmosphere of the Times and strong regional cultural temperament. Contributors must provide the name of the IP image, the design conception and the annotation of the meaning of the expression, the three-dimensional image of the front, side and back of the IP image, and a set of expression design of the IP image (not less than 4 kinds). After the deadline for submission on April 5th, the organizer will select 5 plans from the submission and enter the re-evaluation. In the re-evaluation stage, two plans will be selected by the public network evaluation and the expert review, and in the final evaluation stage, the secretary of the Guizhou Provincial Party Committee will finally select 1 plan. The final will is RMB 40,000 yuan and the organizer issue the adopted certificate; RMB 10,000 yuan for the final evaluation, and the shortlisted certificate, RMB 5,000 Yuan and the shortlisted certificate. Source: China Youth Daily, March 22,2022, edition of the 02nd edition

2022-03-22	Recently, the sixth plenary session (expanded) session of the 15th session of the CPC Shanxi Provincial Party Committee was held in Taiyuan. Shang Liguang, deputy secretary of the Shanxi Provincial Party Committee, attended the meeting and made a speech. Shang Liguang fully affirmed the work of the Communist Youth League Shanxi Provincial Party Committee in the past year. He pointed out that the Communist Youth League is an advanced youth mass organization under the leadership of the Party. Facing the new era, being the party's assistant and the reserve army is glorious and arduous. The provincial youth league organizations at all levels and the broad masses of cadres should firmly grasp to realize the great rejuvenation of the Chinese nation of the Chinese dream of struggle era theme, to meet and study propaganda the party's twenty major as the main line, solid lead condensed youth, mobilize youth, contact youth service youth each work, resolutely shoulder the sacred mission given by the party. We should further learn and deeply understand General Secretary Xi Jinping's important discourse on youth work, understand the essence of thought, grasp the core essence, constantly improve our political judgment, political understanding and political execution, and always keep the correct work of the Communist Youth League in the right direction. We should further focus on our main responsibilities and main businesses, establish a youth-centered work orientation, closely follow the situation and tasks, innovate ideas and methods, strengthen ideological and political guidance, and organize and mobilize young people to give full play to the role of new forces and commandos in comprehensively promoting high-quality development. We should further deepen the reform of the league organs and at the grass-roots level, continue to work hard to "strengthen the three characteristics, decentralization and the four modernizations", and constantly enhance the vitality and vitality of the Communist Youth League. We must further improve our self-improvement, strengthen Party building, enhance our capabilities, build a strong contingent of cadres, and welcome the Party's 20th year victory with first-class performance. Party committees at all levels should attach great importance to the work of young people and the Communist Youth League in the new era, further strengthen their organization and leadership, improve policies and measures, solve difficult work problems, and create favorable conditions and a favorable environment for the Communist Youth League to carry out its work. Before the meeting, Shang Liguang also visited the Shanxi Youth Think Tank, the Shanxi 12355 Youth Public Service Platform and the youth League affiliated media achievements display board, and had cordial exchanges with the cadres of the Provincial Communist Youth League Party Committee. Zhang Jun, secretary of the Shanxi Provincial Party Committee of the Communist Youth League, reported to the plenary session and made a speech entitled "All-round focus of youth, high-quality development, and welcome the twentieth victory of the Party with outstanding achievements". The meeting deliberated and adopted the Implementation Opinions of the Shanxi Provincial Party Committee of the Communist Youth League on Deepening the Comprehensive and Strict Governance of the Communist Youth League and the Resolution on convening the 16th Congress of the Communist Youth League of Shanxi Province, and made arrangements for key work tasks. Source: China Youth Daily, March 22,2022, edition of the 02nd edition

2022-03-22	Bi Yufeng, an epidemic prevention volunteer who should arrive at duty at 11:30 am, arrived at the gate of Binhu Runyuan Community, Yinghuai Community, Yicheng Street, Baohe District, Hefei, half an hour earlier. She has been working for six consecutive days and is somewhat hoarse."Old man, please put on your mask, come on! Take your temperature."Every day, she repeats similar words hundreds of times a day. In recent days, COVID-19 has occurred in some cities and counties in Anhui province, increasing the pressure to prevent and control the epidemic. This weekend, in order to support and enrich the front-line prevention and control work force, the Hefei Municipal Party Committee of the Communist Youth League and the Municipal Youth Volunteers Association have arranged and mobilized the youth groups in advance. On March 19 alone, more than 500 young volunteers from different posts in Hefei rushed to the front line of epidemic prevention and control. Bi Yufeng, who works in the Comprehensive Security Department of Hefei Impression Binhu Tourism Development Co., Ltd., volunteered. That night received the news, she was assigned to the Yicheng street Yinghuai community. Early the next morning, she came to the community to report. With registration, temperature measurement and disinfection, Bi Yufeng carried out health publicity for every person in the community, reminding residents to pay attention to personal hygiene, minimize going out, and check whether the masks were worn correctly. Bi Yufeng, a retired female soldier born in 1989, is also a veteran of the volunteer service against the epidemic. As early as January 2020, Bi Yufeng voluntarily became a community purchasing volunteer without telling her family; donating medical protective materials to Wuhan Huoshenshan Hospital in the name of retired veterans. She registered as a volunteer for epidemic prevention and control, still hiding from her family."I used to be a health worker, but I can't be at the front line like a medical worker. All I can do is to guard the 'door' of the community, so as to keep the healthy defense line of the city."She said."We sent the children to our parents on the weekend and we went to the epidemic prevention work."On the evening of March 18, Yao Yuheng, a volunteer of Hefei � Street Cultural Tourism Development Co., Ltd., discussed with his wife. On the weekend morning, he arrived at the gate of Zhengding Community on time, put on a red vest, put up slogans for epidemic prevention and control propaganda, strictly observed the prevention and control duty points, checked and registered the information of people entering and leaving the community, guided the residents to show their health code and travel code, and measured the temperature in an orderly manner. In recent days, Anhui province at all levels of youth league organizations and youth league members are in action. The Anhui Provincial Committee of the Communist Youth League has decided to allocate 400,000 yuan from the provincial management league fee for the epidemic prevention and control work of grass-roots youth league organizations. At the same time, the Anhui Provincial Youth Volunteers Association issued the "Epidemic Prevention Volunteer Training Micro Lecture Hall", "Youth Volunteer Service Work Guidelines" and other graphic contents, calling for guiding the provincial youth league members to help the epidemic prevention and control in a scientific and orderly manner."My original intention is to protect the people in the quarantine point. As a party member cadre and a youth League cadre, we should be in the front of the 67 people in the isolation point."Since the early morning of March 16, Huang Yueyi, party secretary of Dangtu County, has been working hard at the isolation point. She integrated young volunteers and young entrepreneurs, with the help of the professional disinfection team, sent condolence materials to the staff of the isolation site, and wrote encouragement cards with warm words. From the three meals a day, the division of labor, to the cleaning of the isolated places, and the communication and coordination with all parties, Huang Yueywas busy. Two days before entering the quarantine site, she only slept for three hours and developed anemia due to overwork. These days, in Yixiu District, Anqing City, Yang Chengjun, secretary of the southern Anhui Company, recruited 20 young volunteers, went to the company project team overnight to deploy protective equipment, and also took volunteers to yixiu District 7 nucleic acid testing points, for the elderly with mobility difficulties to register and test. On March 17th, Wuhu Municipal Committee issued a special recruitment order for youth volunteers and volunteer service organizations. Up to now, 3,000 young volunteers serving nearly 20,000 hours; Youth members and volunteers from Tongguan District, Ian District, suburban area, Zongyang County gathered to the community. Hefei Feidong County Committee quickly arranged young volunteers to join the epidemic prevention and control line, participating in the nucleic acid testing, health screening, order maintenance, psychological counseling, publicity and guidance. Source: China Youth Daily, March 22,2022

2022-03-22	The Zhejiang Provincial Communist Youth League Committee and the Provincial Volunteer Association of the Chinese epidemic responded quickly and organized volunteers to go to the frontline of fighting the epidemic and participate in the epidemic prevention and control work, such as crossing testing, community investigation, isolation and duty, in an orderly manner. Behind the volunteers' "youth list", the Zhejiang Communist Youth League has become the most solid supporting force."On the premise of reasonable arrangement of working time, always pay attention to the psychological situation of volunteers, timely counseling. Counties (cities and districts) that experience the epidemic must open a special 12355 psychological counseling line to ensure a 24-hour online duty and keep the hotline smooth and running smoothly. While providing psychological counseling services for the masses, the mental health of the work of frontline volunteers."Youth League Zhejiang Provincial Party Committee concerned personage introduced. In view of the current epidemic situation, the Zhejiang provincial party committee issued the volunteer association at all levels against COVID-19 outbreak volunteer service emergency plan the university communist youth league response disposal epidemic volunteer service work guide "love epidemic" warm heart "prevention and control volunteers" six ", guide the youth league organizations at all levels in the normalized against COVID-19 outbreak background continue to do a good job in emergency volunteer service, set up a perfect system of the communist youth league university response outbreak volunteer service rapid response mechanism, safeguard volunteers at guard, warm heart. At the same time, in order to quickly feedback the epidemic prevention information and show the actions of volunteers, the Zhejiang Provincial Party Committee of the Communist Youth League and the "Volunteer Collection" platform jointly set up a special area for epidemic prevention and control in Zhejiang province to facilitate the registration of volunteers. An official from the Zhejiang Provincial Party Committee of the Communist Youth League said that when volunteer associations at all levels fight against COVID-19, they should abide by the four principles of unified command, local services, equal protection and strict protection. In Wenzhou, Zhejiang province, youth league members and youth volunteers from counties (cities and districts) went into parks, vegetable farms, factories and other elderly gathering areas to wear masks, not gather less, to further enhance their awareness of epidemic prevention. The Wenzhou Municipal Party Committee of the Communist Youth League innovatively launched the "isolation point help code" to collect the service needs of the personnel in the isolation point in real time, and send the humanistic care and warm heart service to the isolation personnel through the immediate response mechanism of "ordering-sending the order-receiving orders". Group Huzhou municipal party committee organization launched a "critical moment, youth list-youth global war disease mobilization order", more than 800 "lake xiaoqing" volunteers for the first time, then sink to the community, villages, park, efficient, timely and accurate docking community epidemic prevention needs, to "housekeeper" volunteer service build a home disease resistance wall. The Taizhou Municipal Party Committee of the Communist Youth League launched the "Health Savings book" point card to guide young volunteers to extensively participate in epidemic prevention and control through the form of exchanging points for small gifts, and actively guide young people to participate in guidance and consultation, epidemic prevention and control drills, community inspection and other work. According to the Zhejiang Provincial Committee, the Communist Youth League Committee and the Provincial Volunteer Association will make special certificates for the volunteers in this round of epidemic prevention; the volunteers participating in epidemic prevention and control through the "volunteer gathering" platform can also apply for exclusive volunteer service medal online, and publicize the advanced collectives, advanced individuals, typical cases and touching stories on the frontline of epidemic prevention and control. Source: China Youth Daily, March 22,2022, edition of the 02nd edition

2022-03-21	In Beijing: Since July 7, the situation of COVID-19 epidemic prevention and control in Beijing has been tightening. This round of epidemic shows the characteristics of many points and wide areas, not only a number of imported transmission chains outside Beijing, accompanied by further cases, but also many family infections and cluster infections, and the first student infection. Facing the challenge of the epidemic, youth League organizations at all levels in Beijing immediately set up a youth force to rush to the front line of the epidemic, including ordinary youth League officials, Youth League members, volunteers, social workers and security personnel... On March 7, a positive case of COVID-19 appeared in Building 7, Shuangyushu Xili, Zhongguancun Street, Haidian District. As an ordinary security officer in the street, Pang Chaofeng immediately rushed to the scene to provide services for the daily life of 229 quarantined workers from 97 households here. In the courtyard of Building 7, two temporary tents, the " home of Pang Chaofeng and several other volunteers, can be seen for more than 10 days. They transport daily supplies and garbage for home workers, having to travel 70 or 80 trips every day. Despite his poor living conditions and high risk, Pang still said confidently: " overcome the difficulties, fight the epidemic, come on!"In this round of the epidemic, the Dongcheng District Heping Li Fourth Primary School first showed two students confirmed cases. In order to make every effort to gain time for epidemic prevention and control, the Dongcheng District Communist Youth League set up a youth commando team to check the movements of key groups and collect statistical information. At present, the youth commandos have more than 80 people, and the team continues to expand.(At 3 o'clock in the morning of March 18, Dongcheng youth commandos still stick to their posts, racing against time to carry out the flow transfer work.) At the same time, under the mobilization of Dongcheng Youth League District Committee and Dongcheng District Volunteer Service Federation, all volunteer service organizations and volunteers also participate in nucleic acid testing, information statistics, epidemic prevention publicity, running errands and other voluntary services. Since March 14, he has participated in more than 2,500 person-times and contributed more than 21,000 hours of volunteer service. On March 17, the Standing Committee of the Political Bureau of the CPC Central Committee held a meeting to analyze the COVID-19 epidemic situation and make strict arrangements for the prevention and control of the epidemic. The meeting triggered a strong response among the youth league organizations at all levels in the capital and among the young people fighting the epidemic. Everyone expressed that they should highlight the true qualities of the young pioneers through practical work, build a solid defense line with their youth strength, and be a healthy "gatekeeper" of the residents. Ai Cheng, a social worker at the Yushan Meidi Community Neighborhood Committee in Shunyi District, remembers that he received the community closure control notice at 3 am on March 11. I quickly returned to work to answer questions for the residents in the closed area, solve the problem of health treasure, provide material support for medical staff and residents, install door magnetic for home observers, and check the big data dispatch system.... busy from 5:30 am to 9:30 PM every day. When asked if he had never returned home, Ai Cheng smiled and said, "No time."As a member of the Communist Youth League, Ai Cheng felt: " Persistence is victory. We used to be protected by the world, but now it is our turn to protect the world. As the young generation, we must have the courage to take responsibility, play an active role, go to the front line of prevention and control, contribute our youth strength, help the people solve their problems, and ensure the safety of our party."Li Haoze is a staff member of Qinghe Sub-district Office in Haidian District. Since January 2020, he has participated in the transfer of overseas personnel back to Beijing and the centralized isolation and transfer of medium-high-risk personnel. So far, more than 110 trains and more than 1,200 residents have been transferred.(Epidemic prevention and control personnel are transferring risk personnel.) His special experience has enabled him to rapidly grow into the backbone of the epidemic prevention and control. After the recent closure and control of Anning Huating Community in Haidian District, he relied on his experience in many emergency emergencies, assisted the medical staff to conduct nucleic acid tests for the home quarantine units where the confirmed cases living and working, and calmed the home observers. He felt that the general Secretary emphasizes scientific and precise prevention and control, and puts the importance of people first and life first. As a young man, he should be on the front line of serving the people and build a strong defense line of mass prevention and control. In Qinghe Street, there is also a "fresh and warm" volunteer service team composed of new employment groups. They use their rest time into the epidemic prevention and control in Anning Huating Community, and transfer materials to the closed residents in the buffer area of the community, and the total volunteer service time has reached more than 50 hours.(New employment group "fresh and warm" volunteer service team delivers supplies in the buffer zone.) Sf express youth league branch secretary, SF site head Sun Zhenya introduced, Courier this group every day shuttle between the big business super building community, on the one hand, to do a good job of personal protection, on the other hand, also should not forget the beginner's mind, contribute their own strength, strive to be responsible as a good youth. Also rushed in the front line of the Chaoyang District of Beijing Lianjia youth commandos. Using HOME LINK stores in the streets, community advantage, HOME LINK youth league secretary wang ying organization agent HOME LINK youth commandos volunteers, not only take the initiative to take care of residents in daily life work, also notice propaganda, maintain order, scan code registration nucleic acid sampling auxiliary work, assist medical staff to complete the sampling. She said, under the background of epidemic normalized, HOME LINK youth volunteers will continue to uphold the beginner's mind, root community, the community service, when the residents most need to rush out, stand out, play the cutting edge exemplary role, in the epidemic prevention and control work to continue to play the role of the community emergency team, to protect the life and health of the masses to make their own contribution.

2022-03-21	The employment season is good and timely rain- -The Jiangsu Communist Youth League to promote the employment of college students action series reports held one of the "send the post through train" live post recommendation activity was held in the Sina live broadcast room. During the activity, the Nanjing Municipal Party Committee of the Communist Youth League invited 8 enterprises to promote high-quality positions related to the intelligent manufacturing equipment industry online. According to statistics, the live broadcast received 530,000 interactive views online. Every March, when the new semester has just begun, the atmosphere of the graduation season has been "full" - -this is the busiest time for the Jiangsu Communist Youth League to promote the employment of college students."During graduation season, use everything you can to help them at critical moments."Said a college youth League committee teacher. To do a good job in college students' employment, the most direct way is to provide jobs. The annual job fair organized by the participation of the Communist Youth League is becoming an important channel for college students to obtain their posts. As the only public undergraduate vocational education pilot university in China, the graduates of Nanjing Polytechnic University do not worry about work. Take the class of 2021 graduates as an example, the employment rate of students is 98.24 percent, and there are 15 optional jobs per student. Talking about the reasons for the good employment performance, Ji Haibo, secretary of the Youth League Committee of the school, introduced that the school Youth League Committee actively expands the employment channels of graduates around the overall arrangement of the employment of graduates. Every semester, it cooperated with the employment guidance center to contact more than 200 enterprises and organize more than 18,000 positions."We also actively explore the secondary college sub-youth League committee combined with the professional characteristics of the college, to carry out nine live post recommendation of the 'post delivery through train' at the college level."Ji Haibo said. It is understood that in 2021, Jiangsu Communist Youth League implemented the employment assistance action of "hundreds of schools, thousands of enterprises, ten thousand posts". In total, a total of 142 "post delivery through train" live post recommendation activities were held throughout the year, raising nearly 300,000 employment, internship (internship) positions from more than 8,800 enterprises and institutions. One more position gives students one more choice. In order to raise as many positions as possible, Jiangsu Communist Youth League initiative to collect recruitment information to industrial, social, state assets and other departments, actively connect with high-tech industrial park and economic development zone, focus on integrated circuit, biomedicine, Internet of things, construction machinery and other key industries, organize job resources; Meanwhile, calls on large state-owned enterprises, advanced industries and scale private enterprises to provide quality positions, targeted recruitment. The Youth Federation and the Youth Chamber of Commerce are also one of the important sources of recruitment positions. In 2021, the Jiangsu Communist Youth League integrated the resources of the provincial, city and county level youth Federation and youth business entrepreneurs, and organized special recruitment activities for the Youth Federation and youth business enterprises. With sufficient positions, league organizations at all levels should try their best to accurately convey job information to every graduate. In Nanjing Polytechnic Vocational and Technical University, the university Youth League Committee includes the assessment of employment information transmission, explores the implementation of the league branch secretary part-time as the class employment information officer, and strives to achieve "employment information transmission without loopholes"; in Changzhou Electromechanical Vocational and Technical College, in order to ensure the accurate transmission of post information, in addition to making offline publicity atlas, it also establishes social media contact group, and transmits "point-to-point" employment information through WeChat push, campus OA system and class QQ group. At the provincial level, in recent years, Jiangsu Communist Youth League, with the help of the employment service platform of "Tuan Micro employment youth", has realized the online "access" of employment policy, post release, job application and other functions. It has always been an important part of the efforts to pay attention to low-income families of the Jiangsu Communist Youth League to promote the employment of college students. Yang � r, deputy secretary of the Communist Youth League Committee of Jiangnan University, remembers that during the 2021 graduation season, she noticed that Ding Jie (pseudonym), a normal school undergraduate in the School of Humanities. Ding Jie was born in the old area of Jiangxi Province. Her father works for work all the year round. Her mother suffers from chronic diseases and her family living conditions are difficult. In the first semester of her senior year, Ding Jie signed up for teachers to recruit in advance, but was eliminated without passing the resume screening, which made her deeply suspicious of herself. Yang � r recalled that at that time, Ding Jie frequently gave up the interview opportunity, and always avoided employment with no time to prepare for her graduation design, lack of competitiveness, recruitment distance from school, and trouble in recording videos. In order to help Ding Jie regain her confidence, Yang � r talked with her alone for four times, helped her establish the correct professional values, and comprehensively analyzed her personality characteristics according to the theory of "person and job matching", and put forward suggestions on employment and career selection. In July 2021, with the encouragement and suggestions of her teachers, Ding Jie signed up for the recruitment of special post teachers in Jiangxi Province, and successfully passed the written test and retest, and found an ideal job in her hometown. In the process of helping people with employment, students from poor families are not the only ones receiving attention. Some ethnic minority students from underdeveloped areas have also become the focus of the school Communist Youth League. At Jiangsu Shipping Vocational and Technical College, Yu Xuan, deputy secretary of the Communist Youth League Committee, found that the employment concept and living habits of Tibetan students were very different from those of mainland students. "Many Tibetan students want to return to the district, but their career choices are concentrated in civil servants and public institutions, and the employment path is very narrow."To this end, the school Youth League Committee specially held a forum for Tibetan students. On the one hand, it invited Tibetan graduates working in the mainland to share their special topics, and on the other hand, experts from the Xizang District Committee were invited to share their employment policies inside and outside the region, so as to effectively help students broaden their employment choices. It is understood that the Jiangsu Communist Youth League organized college cadres, youth League members, members of the Youth Chamber of Commerce to pair with college students to carry out "one-to-one" special guidance, in-depth understanding of demands, answer doubts, provide guidance, to help more than 8,000 graduates from low-income families to achieve employment. Psychological help do heart-warming people, connect the society do close friends " I want to graduate this year, but now the postgraduate entrance exam failure, work is difficult to find, 'test public' also don't know can take the exam, really don't know what they can do."Whenever similar words appear on the" Su Xin " App, the psychological self-help platform for college students in Jiangsu Province, peer volunteers from the general branch of the College Student Mental Health Association of Nanjing Audit University will actively contact each other and ask if they need help. In recent years, affected by the epidemic and the employment situation, the graduation season has become a period of increasing students' anxiety and psychological pressure. In order to solve this problem, Nanjing audit university based on the communist youth league set up in the school of Jiangsu province college students 'psychological crisis prevention and intervention research center, through the school mental health guidance center established college students' mental health association league, play to the power of the youth league organization and youth members, establish peer mutual assistance mechanism, through one-to-one, one-to-many employment psychological support counseling, alleviate students' employment anxiety."The employment assistance of the Communist Youth League in the school should not only be limited to the jobs, but also give full play to the characteristics of their own 'young friends', organize students to help each other, digest the anxiety, and actively face the critical period from the school into the society."Said Yang Fangmin, secretary of the Communist Youth League Committee of Nanjing Audit University. Yang Fangmin told reporters that the peer volunteers also set up a micro group class video production team, through shooting group class video, to introduce the knowledge of employment emotional adjustment, employment skills improvement and other aspects of everyone. In the employment season, the Jiangsu Communist Youth League is not only limited to the campus, but also extends the fulcrum to the society, and strives to leverage more resources. In Nanjing, the Nanjing Municipal Party Committee of the Communist Youth League actively participated in the "Ningju Plan", increased the support for graduates' entrepreneurship and employment, and comprehensively issued housing rental subsidies, employment subsidies, social insurance subsidies, entrepreneurship support and other support policies. It is understood that by January 2022, the total rental subsidies of Nanjing college graduates have been guaranteed to more than 260,000 people, and the amount of subsidies is more than 2.8 billion yuan. In Suzhou, the Suzhou municipal party committee is relying on the "Su Qinghui" youth service cloud platform, provide characteristic guidance for college graduates, employment entrepreneurship, life services, social integration package of services, and through the "youth" Chinese youth innovation entrepreneurship comprehensive service platform can assign county youth entrepreneurship organization, for more graduates entrepreneurship mentor services, online training, investment and financing docking and other comprehensive services."At present, college graduates are no longer satisfied with 'employment', but forward to 'better employment'. Therefore, the communist youth league to carry out college students' employment services, both actively to integrate resources, build online platform, actively for college graduates and unit of choose and employ persons build a bridge of efficient communication, also want to organize them to participate in social practice exercise improve social ability, adhere to provide actual support of employment and strengthen employment concept guide two-way, continue to focus on general low-income family graduates of college students employment services, to help the employment stability of Jiangsu communist youth league."Said Si Yong, party secretary of Jiangsu Province. China Youth Daily, China Youth Daily, March 21,2022

2022-03-21	Before Liu Ning, the plenary session of the 11th Committee of Guangxi Youth Federation, the 11th Congress of Guangxi Youth Federation and the 12th Congress of Guangxi Youth Federation were held in Nanning. Liu Ning, party secretary of the Guangxi Zhuang Autonomous Region and director of the Standing Committee of the Guangxi Zhuang Autonomous Region People's Congress, sent a congratulatory letter. Liu ning pointed out that in the congratulatory letter over the past five years, under the leadership of the party, under the help of the communist youth league, in the youth league and union organizations at all levels of unity, the district youth and young students remember xi general secretary entrust, new era, gratitude, new era, dedicated in rejuvenation, prosperous Guangxi hot practice, especially in poverty engines, COVID-19 epidemic prevention and control test by wind and rain, see the world, strong bones, long talent, reveals the ZhuangXiang youth patriotism and responsibility. Liu ning stressed that the youth league and union organizations at all levels to keep pace with The Times, accurately grasp the characteristics and rules of youth work, strengthen the political, advanced nature, mass, earnestly started and responsible, deepen reform and innovation, unity led the youth party, listen to the party, follow the party, further strengthen historical confidence, build a historical memory, with confidence together to the future. Party committees and governments at all levels should strengthen their leadership over youth work, care for and support the work of the Youth Federation and the Students 'Federation, create a favorable environment for young people to grow up and make contributions, gather the most active and dynamic youth forces in all aspects, and welcome the Party's 20th victory with concrete actions. Congress deliberated and approved the Guangxi youth federation of the 11th session of the tenth committee of the standing committee work report on the resolution of the Guangxi zhuang autonomous region students' federation eleventh committee work report " resolution of the committee of the Guangxi youth association of Guangxi zhuang autonomous region articles of association (amendment) resolution and other documents, elected a new youth league of Guangxi and Guangxi league leadership organization. Source: China Youth Daily

2022-03-20	Jilin: 28581 young volunteers fight against the epidemic and there is a force behind the war, 240,3685,8757,13053,28581... this is the youth epidemic prevention volunteers they come from different positions of teachers, workers, Courier, car drivers, freelancers... under the call of the Jilin Communist Youth League volunteered, quickly assembled, immediate action In the epidemic prevention and control battle annihilation war backlight speed, quenching youth, smell ling, with the flag rushed after the outbreak of the provincial youth league organizations at all levels, initiative, mobilization, recruitment to the youth immediate response through WeChat group, circle of friends, 24 hours hotline rushed in the group rushed " I am a youth, Request to fight, "My daughter has become a close contact on the fight against the epidemic, I want to stand guard for her "" unite together to overcome the epidemic "... young volunteers a loud name, a red brand in the epidemic prevention and control critical period, was" wiped " more bright in order to implement the volunteer protection measures at all levels to buy accident insurance for volunteer service and each volunteer signed volunteer agreement issued masks, protective clothing, goggles, alcohol and other necessary protective equipment in order to improve the volunteer prevention and control ability online youth prevention volunteers guide and volunteer training tutorial On March 14, at the call of the Changchun Municipal Committee of the Communist Youth League, the next team was composed of 25 people, A volunteer team of young epidemic prevention workers, with an average age of 24, quickly assembled and went to Jiutai District overnight to cooperate with nucleic acid testing. " I have never felt my responsibility so great, I must rush. "" I don't know where, No matter where you go, "" Mom and dad are all for me, I must do it well!"...... in the" last kilometer " line of defense, the volunteers dressed in protective clothing are like a faint light condensed into a powerful beam of light blooming the most dazzling light! Jilin city vocational education park isolation shelter to accept patients immediately after receiving instructions Jilin municipal party committee organize 60 youth epidemic prevention volunteers against the wind and spring snow to Jilin city vocational education park isolation shelter, on the premise of their own protection to assist staff in only 2 hours will weigh more than 2000 kg of 80 quilts, 1500 boxes, 63 waste recycling bucket carried to the designated place to complete 4 building isolation point about 1800 isolation shop bedding distribution and put a lot of volunteers' work hair And shoes were snow wet clothes were sweat in Jilin city changyi district birch skin factory town multiple village (community) is designated as the risk area changyi district party committee gathered from all walks of life youth epidemic prevention volunteers formed "epidemic prevention sanitizers youth volunteer service" they receive epidemic prevention supplies from the morning to birch skin factory town and heavy disinfectant to residential areas, markets, back streets and other key areas for repeated spraying to ensure disinfection sterilization no dead Angle to block the spread of epidemic risk " in this crisis moment these river youth Every day I was deeply touched by them "youth epidemic prevention volunteers Li Jiaxi said according to the epidemic prevention and control requirements Siping city static management and vehicle traffic control many uremia patients do hospital dialysis treatment became a problem group Siping municipal committee immediately contact lei feng's team 11 players volunteered to undertake the task" shouted many times for cities around the country today I want to cheer for hometown Siping, we must do our best to help dialysis patients contribute to the epidemic prevention and control work "... lei feng team members Has set up life for 16 dialysis patients with green channel so far the province 28581 youth epidemic prevention volunteers assembled epidemic prevention line with community screening, traffic epidemic prevention point temperature measurement, epidemic prevention propaganda, food sympathy, transportation volunteer service accumulated 62034 pioneer flag flying in the battlefield outbreak is command, prevention and control is the responsibility in the province youth league organizations at all levels called and mobilize the 462 youth commandos, youth civilization 15164 youth fully active in fire control, transportation, production and other key areas Give full play to the role of youth powerhouse and commandos Changchun fire rescue team, form a young pioneer commandos rescue Changchun exhibition center square hospital quickly develop special operational plan day and night to kill and hidden perils in build tight defense against fire protective clothing one marked "Jilin fire" "red and blue logo", became the square hospital every staff in the night sky the brightest star Jilin airport group youth commandos, received support in Jilin province medical team flightsCharter flight to ensure 12 youth commandos going the wrong way in the snow and cold, Outbreak earnestly under the double test of complete flight outbound aircraft release check deicing operations and epidemic prevention sanitizers work high standard high quality flight guarantee task Jilin port gas company youth corps committee, quickly formed youth assault team encryption inspection, leakage, designated monitoring 40 people 24 hours on duty, the city's 1800 gas well room every three days nearly 900 kilometers of pipeline patrol every day again 23499 building introduced every one long in the pipe network of nearly 400000 residents gas for Jilin city central hospital, Affiliated hospitals and other epidemic resistance line provides a safe and stable gas supply of Jilin province youth civilization, its matsubara power supply company power dispatching control center area dispatching class day and night to the work of county, plant and line timely scheduling, detailed equipment operation comprehensive power grid operation monitoring and fault and abnormal disposal work at any time due to the rain and snow freezing weather emergency guarantee the power grid stable operation and epidemic prevention important users power supply accurate help make prevention and control more temperature "sudden outbreak makes me nervous..." " recently online class, I was somewhat anxious... "" I'm in the hospital now, A little afraid... "... In order to timely relieve the anxiety during the epidemic, Jilin Province 12355 youth service desk selected 30 experienced psychological counseling counselors as psychological volunteers, continuous online psychological counseling services every day to build a bridge with the transport students Siping Municipal Party Committee prepared the unusual" meeting ceremony "elaborate" peace war epidemic hand in hand,Group and accompany with you "theme welcome card and seven color thousand paper crane" youth war epidemic peer "spring planning" group small lecture hall "" scholarly war disease transmission power "online reading salon sharing" as your hero "vlog short video contest online" group "birthday party and other characteristic activities group songara city committee opened youth service desk" heart oxygen bar " hotline to recruit 52 psychological counseling volunteers, issuing warm prompt card 1100 psychological counseling phone for isolated students to provide 24-hour online counseling services Group Baicheng municipal party committee organization young entrepreneurs to isolate students and epidemic prevention frontline staff sent "love meal" 700 warm heart pizza meihekou municipal party committee opened online psychological consultation room through the "green" listen to "disease" road "accompany you broadcast good night story audio package live street dance teaching every day with a flowers" daily sign "way to help students to reduce anxiety strong to sincerely warm heart, with true war outbreak help let love relay inheritance watch is help, waiting is spend the provincial youth development foundation for the first time" against the outbreak Hope peer " called on all walks of life to love people, public welfare organizations and love enterprises to extend a helping hand in just 3 days to raise 7.11 million yuan 23,500 sets of medical protective suits 195,000 pairs of disposable medical surgical masks 48,500 N95 masks 31,700 medical isolation masks 1. Medical gloves 4500004500 youth epidemic prevention volunteers vest worth 1.18 million yuan epidemic prevention supplies to epidemic prevention front at the same time under the call of Jilin province youth entrepreneurs association youth development promotion association, actively launched member units to donate boxing love gathered true current, trickle blood dip Baishan pine propaganda pen together to fight epidemic you choose retrograde, I choose peer communist youth league network xuan force all listed 428 group of new media account linkage as a whole propaganda provincial party committee and government epidemic prevention and control of powerful measures And epidemic prevention scientific knowledge, timely forward authoritative information, active response to social concerns, release works 476 total reading 26.1461 million open "youth war disease" action "" youth war epidemic power "" epidemic line youth image " and other eight columns through graphic, short video, posters for epidemic prevention and control of typical youth and team portrait depth and publicity form advanced learning, to be the pioneer of the young pioneers recorded disease resistance propaganda video 2325 propaganda and epidemic prevention knowledge, transmission epidemic resistance power cut network views 125,10,000 likes 55. Joint 60000 provincial youth new media association members with youth like discourse system and expression creation "home life small cartoon" Changchun chang "" war epidemic youth is backlight speed " and other original network cultural products 34 condensed the outbreak of strong force only for the passionate struggle of youth only the tenacious struggle of youth, only contribute to the people's youth will leave full, warm, lasting, regretless youth recall the new era of Jilin youth watering with faith, with interpretation mission, with struggle to write youth All the backlight fast Jilin youth salute to you!

2022-03-20	Jianwei Correspondent Lin Yucheng) Recently, the 2022 Henan Provincial University Communist Youth League Work Conference was held in Zhengzhou in the form of video conference. Wang Dubo, deputy secretary of the Communist Youth League and leader of the Communist Youth League leading group, attended the meeting and delivered a speech. Wang Jiwei, deputy secretary of the Communist Youth League and executive deputy leader of the Communist Youth League Leading Group, presided over the meeting. Members and liaison members of the leading group of the Communist Youth League, deputy secretaries of the Communist Youth League Committee of the provincial cities and Jiyuan Demonstration area, leaders of the school department, responsible members of the provincial Communist Youth League working Committee, and secretaries of the Communist Youth League Committee of the colleges and universities attended the meeting. The meeting pointed out that the provincial Communist Youth League in colleges and universities should focus on the main responsibility and main business, and enhance the sense of crisis, urgency and responsibility of the work of the Communist Youth League in colleges and universities. From the perspective of external environment, the severe and complex situation brings serious impact and challenges to the ideals, beliefs and value orientation of young college students, and the post-metaphor cultural era brings new challenges to the ideological and political education of college students. We must enhance the sense of crisis in the ideological and political guidance work of the Communist Youth League in colleges and universities in the new journey of the new era. From the internal conditions, for the xi jinping, general secretary of universities and the communist youth league to play a major role in the young students thought lead, must keep a clear mind, modest and prudent, accurate understanding in the pattern of "big politics" in the short board and insufficient, with clear positioning and self innovation courage, enhance the contribution to the party education. From their own cognition, the work of the Communist Youth League in colleges and universities still has the myth of "high level wandering", and there is the phenomenon of "high not low" in the work. We must really stand on the commanding point of ideals and beliefs to lead the thoughts of the young students' minds, establish a student-centered view, revolve around students, care for students and serve students. The meeting stressed that the Communist Youth League of colleges and universities should earnestly grasp the advantages of work and find the entry point and combination point of work. We will effectively grasp the foundation of organizational education. Based on the fact that the university Communist Youth League has a complete organizational system, it should form differentiated functions with the school teaching, moral education and other work, innovate the organizational life style of the league, and constantly enhance the ideological and political work function of receiving education in the organization. It is necessary to promote the construction of the organizational system as a whole, strengthen the linkage of schools, departments, classes and youth league organizations in the deepening of reform, strengthen the linkage of the youth league organization, the student union and the student associations, and build an organizational system under the leadership of the Party committee and led by the Communist Youth League. Focusing on the characteristic of practical education, we should fully grasp the traditional advantages, working mechanism and implementation path of the Communist Youth League in practical education in colleges and universities, and guide young students to take "great ideological and political courses" well. We should make good use of the broad track of the second classroom, coordinate the resources of the Communist Youth League inside and outside the school, optimize the linkage mechanism of the local Communist Youth League, and empower the league and learning work in colleges and universities. We should deepen the reform of the "second classroom report card" system, carry out our work with the goals and standards of political work, effectively transform the rich and colorful practical activities into educational products, and transform them into the action consciousness of young college students to listen to the Party and follow the Party. Give full play to the advantage of network education, to clear cognition the university communist youth league in the field of new media first, deep, accumulation, continue to deepen the product strategy, using green and young college students, promote the network product "supply side reform", production, supply more quality content, in subtle education function, the huge young students fans into network education the biggest increment. The meeting called on the Communist Youth League of universities to seize the opportunity to consolidate and deepen the reform of the league and study, and fully implement the strict governance of the league. To focus on the reform tasks and achieve results, We have always highlighted the internal driving force of deepening reform, To fully grasp the requirements of the new stage of development, the new development concept and the new development pattern for the youth and the Communist Youth League, Compact the main responsibility of university Youth League committee, Clarify the main objectives of the reform, Improve the work implementation mechanism, Make great efforts and resolve to solve the crises and problems caused by the external environment, internal conditions, and self-cognition, Focusing on "improving the mechanism of political education, improving the mechanism of practical education, improving the mechanism of organizational operation, and innovating the way of organizational mobilization", Further focus on the primary responsibility and main business, To identify the actions of the Communist Youth League under the great ideological and political pattern, To transform the service achievements to young college students into organizational, ideological and political achievements, To measure the effectiveness of the reform, Coallying people for the Party. We must promote the implementation of the work of the League, accurately grasp the meaning and characteristics of the comprehensive and strict governance, and accurately grasp the target and focus of the league. Cadres of the League should carry forward the fighting spirit, enhance their fighting skills, dare to grasp and manage them, and be loyal, clean and responsible. We must strictly enforce the standards for the development of league members, strengthen their awareness education and discipline requirements, establish the orientation of promoting excellent party membership, and constantly send new blood to the Party. League organizations must continue to improve their systems and rules, follow the Party Constitution and league rules as the fundamental principles, formulate, introduce, and resolutely implement them. To thoroughly implement the "strong ability, forging style, grasp the implementation of the" action, to strengthen the training as the focus to improve the ability and quality of the cadres, promote the province's colleges and universities cadres to practice investigation and research, scientific decision-making, pay close attention to the implementation of the basic skills, become an expert in their own work. We should change the style of youth league cadres at the grass-roots level, promote the focus of work downward, the work force sinking, in the process of mingwith the majority of young students, change the style of work, and constantly activate the nerve endings of the grass-roots youth league organization, smooth capillaries. We should strengthen responsibility as the fundamental to promote the implementation of the work, work hard to promote the implementation of key work, effectively promote the construction of league cadres into the implementation of key work, and fully show the new image of league cadres in the new era. Meeting learning to convey the Henan 15 second QuanWei (expand) conference spirit, on the 2021 provincial communist youth league work assessment, Luoyang normal college youth corps committee, Zhengzhou university of light industry, Xuchang college, Henan vocational and technical college, Kaifeng municipal party committee respectively for the special reporting on activities, the provincial related comrades responsible for the provincial communist youth league work points in 2022.

2022-03-20	League Xinjiang district party committee to carry out the reform of grassroots organization work view exchange league Xinjiang district party committee organization to carry out the county communist youth league organization reform view Jianwei) to further promote the reform of the county communist youth league organization, effectively promote reform work experience, improve the quality level of county communist youth league organization reform level, On February 26 and March 11, the Xinjiang Uygur Autonomous Region Committee organized the delegation of 48 people to Taizang Village, Sanbao Township, Gaochang District, Munna Community, Grape Town, European E-commerce Co., Ltd., the First Middle School, Yucai Catering Service Co., Ltd. for on-site observation and study. The observation is mainly conducted by viewing materials, experience sharing, discussion and exchange. The observation group inspected the archives in Taizang Village, Sanbao Township, Gaochang District, and Munar Community, Grape Town, on how the village (community) could implement the Party building and the league building, the standardized construction of the league organization, and the education and management of the league members. League Xinjiang District Committee for the picture view group in the grape town Munar community to view the archives. League Xinjiang Uygur District Committee for the picture observation group in Turpan Oubai E-commerce Co., Ltd. to understand the operation of the company league organization. In Turpan Oubai E-commerce Co., Ltd. and Turpan Youcai Food and Beverage Service Co., Ltd. understood the construction of the "two new" youth league organization and the employment and entrepreneurship of the youth league members in detail. The head of the enterprise introduced the operation of the company and the youth education and management of the youth league members. The observation group and the village (community) youth league organization responsible person discussion and exchange. Xinjiang District Committee for the picture observation group and village (community) league organization responsible person discussion exchange. League of Xinjiang district party committee for figure discussion exchange, the county communist youth league secretary around the county communist youth league organization reform four aspects, ten measures to carry out in-depth exchanges, we have said: " through the scene view, everyone of the county communist youth league organization reform work have intuitive understanding, inspired and encouraged, deeply realize the necessity and urgency of the reform, to the table excellent county communist youth league organization reform work saw its own shortcomings and gap, for late to promote the county communist youth league organization at the grassroots level reform work, injected the impetus.”

2022-03-20	Hunan youth education base punch card program officially launched Hunan youth education base punch card program officially launched punch card book. League of Hunan provincial party committee for China youth network Beijing on March 20 (xinhua Ji'an-wei zhang, correspondent, ChengJun Yang Liu Bingbing) to promote party history learning education normalized long-term, around the "welcome 20, always follow the party, forge ahead new journey" this theme, construction in one hundred, by "Hunan red tourism hand-painted electronic map" as the basis of "Hunan youth education base card small program" officially launched. Small program can realize the base one key navigation, scenic spot voice tour, clock in, writing travel notes, explaining words recording, one key sharing and other functions. After completing the base card, you will also get an e-certificate. Since last year, the Hunan communist youth league to vigorously promote to "study party history, strong faith, follow the party" as the theme of the youth party history learning education, relying on 107 home countries in the province, the province two levels of youth education base, launched "Hunan red tourism hand-painted electronic map" small program, extensive "flash footprint" the province youth base practice clock activities, the majority of teenagers. Tuan Chenzhou Municipal Party Committee made the "Show out your shining footprint" punch card book, Set up more than 20 clock-in and chapter collection points, More than 260,000 teenagers in the city participated; The experience of actively cultivating the bilingual "red scarf narrator" of the Miao and Han people was introduced by the 61st issue of the Central Party History Learning and Education Brief; Yiyang Municipal Committee combined with the "red trace- -I walked the road when you came" Yiyang red activity, Organize the city's youth Party members, league members and youth to take branches, families or individuals to the red education base, During the event, more than 600 punch-in videos were received, The Red Education base visits more than 5,000 people a month... The picture shows the poster of the small program of Hunan Youth Education Base. The Hunan Provincial Party Committee for the map small program will be widely promoted in the provincial Communist Youth League members, young pioneers, young people, guide the youth along the "general secretary's red footprint", "young MAO Zedong study tour", "Hunan and Jiangxi red" and other boutique lines, punch in the Sanxiang land "red landmark", and cultivate the love of the Party, the country and socialism. It is reported that you can participate in the "Flash Memory" Hunan Communist Youth League Youth Education Base clocking practice activity, interact with @ Youth Hunan Weibo topic # Flash Memory #, and share the punching footprints. So far, # Flash Memory # has been read 150 million times.

2022-03-20	Shanxi Provincial Party Committee of the 15th sixth plenary (expanded) meeting held the League of the 15th sixth plenary (expanded) meeting held month 20 (reporter Zhang Jianwei, correspondent Zhang Jinrong) March 17, the Communist Youth League of Shanxi Provincial Party Committee of the sixth plenary (expanded) meeting held in Taiyuan. Guided by Xi Jinping Thought on Socialism with Chinese Characteristics for a New Era, the meeting earnestly implemented the decisions and arrangements of the Provincial Party Committee and the relevant requirements of the Central Committee of the Communist Youth League, summarized the work, deployed the tasks, and further united and mobilized the youth league organizations at all levels and the youth league members of the province to forge ahead on a new journey and show a new style. Shang Liguang, deputy secretary of the CPC Shanxi Provincial Party Committee, attended the meeting and delivered a speech. He pointed out that the Communist Youth League is an advanced young mass organization under the leadership of the Party. Facing the new era, it is glorious and arduous to be the party's assistant and reserve army. The provincial youth league organizations at all levels and the masses of cadres should firmly grasp to realize the great rejuvenation of the Chinese nation of the Chinese dream of era theme, to meet and study propaganda and implement the party's twenty major as the main line, solid lead condensed youth, mobilize youth, youth service work, resolutely shoulder the sacred mission given by the party. We should further learn and deeply understand General Secretary Xi Jinping's important discourse on youth work, understand the essence of thought, grasp the core essence, constantly improve our political judgment, political understanding and political execution, and always keep the correct work of the Communist Youth League in the right direction. We should further focus on our main responsibilities and main businesses, establish a youth-centered work orientation, closely follow the situation and tasks, innovate ideas and methods, strengthen ideological and political guidance, and organize and mobilize young people to give full play to the role of new forces and commandos in comprehensively promoting high-quality development. We should further deepen the reform of the league organs and at the grass-roots level, continue to work hard to "strengthen the three characteristics, decentralization and the four modernizations", and constantly enhance the vitality and vitality of the Communist Youth League. We must further improve our self-improvement, strengthen Party building, enhance our capabilities, build a strong contingent of cadres, and welcome the Party's 20th year victory with first-class performance. Party committees at all levels should attach great importance to the work of young people and the Communist Youth League in the new era, further strengthen their organization and leadership, improve policies and measures, solve difficult work problems, and create favorable conditions and a favorable environment for the Communist Youth League to carry out its work. Before the meeting, Shang Liguang visited the Shanxi Youth Think Tank, the Shanxi 12355 Youth Public Service Platform and the youth League-affiliated media achievements display board, and had cordial exchanges with the cadres of the provincial Communist Youth League Party Committee. Zhang Jun, secretary of the Shanxi Provincial Party Committee of the Communist Youth League, reported to the work of the Standing Committee and made a speech entitled "All-round focus of youth, high-quality development, and welcome the twentieth great victory of the Party with outstanding achievements". The meeting deliberated and adopted the Implementation Opinions of the Shanxi Provincial Party Committee of the Communist Youth League on Deepening the Comprehensive and Strict Governance of the Communist Youth League and the Resolution on convening the 16th Congress of the Communist Youth League of Shanxi Province, and made arrangements for key work tasks.

2022-03-18	Youth representatives from all walks of life in Guangxi carried out the 2022 voluntary tree planting activity) "Welcome the 20th Youth Forest planting activity" Youth representatives from all walks of life in Guangxi voluntary tree planting activity was recently held in Gaofeng Forest Park, Nanning. On the same day of the event, the leaders of the Guangxi Zhuang Autonomous Region Party Committee and the Guangxi District Working Committee, the Guangxi District Committee of the Communist Youth League, the Forestry Bureau of the Autonomous Region and other units jointly unveiled the "Guangxi Youth Forest". In the mobilization speech, the head of the Guangxi district committee pointed out that the youth is always the new force and commandos of ecological civilization construction, the youth should keep in mind the general secretary Xi Jinping "Guangxi ecological advantage", firmly establish "Guangxi youth love Guangxi" ownership, jointly protect the Guangxi golden signboard, and proposed to the youth "to establish the concept of green development; to plant green, love green and protect the green magnificent home" three initiatives. From Guangxi in all ethnic groups from all walks of life youth represent more than 500 people participated in the "plant a concentric forest" "plant a friendship tree" activities, take concrete actions to advocate the general teenagers continue to set up "plant green, protect green, love green" green development concept, be the disseminator of ecological culture, builders of the green home, promoters of environmental protection. The activity was jointly organized by the Guangxi District Committee of the Communist Youth League, the Working Committee of Guangxi Zhuang Autonomous Region and the Forestry Bureau of the Autonomous Region.

2022-03-20	Guizhou "colorful Guizhou youth green movement" public tree planting activities in Guizhou communist youth league to carry out the "colorful Guizhou youth green movement" 2022 I for qian mountain xiushui planting tree-I participate in my happy public tree planting activities Beijing on March 20 (reporter Zhang Jianwei, correspondent Chen Kai) recently, the Guizhou provincial party committee "colorful Guizhou youth green movement" 2022 I for qian mountain xiushui planting tree-I participate in my happy public tree planting activities held in longli county crown mountain street sowing skip village lime kiln. Li Jian, deputy secretary of Guizhou Provincial Committee of the League, made a speech and announced the start of the public welfare tree planting activity. Deng Shoucheng, the second-level inspector of Guizhou Provincial Committee of the League, presided over the ceremony. More than 150 people participated in the public tree planting activity, including cadres and cadres of provincial finance Department, provincial archives, responsible comrades of Qiannan Youth League Committee, leaders of Longli County Party Committee, entrepreneurial youth, caring people and young pioneers, participating in the public tree planting activities. Pictures of the tree-planting activity. It is understood that during this year's Arbor Day, the province's league organizations at all levels of the organization to give full play to the advantages of the Communist Youth League organization and social mobilization, the organization called on the majority of teenagers throughout the province to participate in the "colorful Guizhou youth green movement" - -2022 I for the Qianshan Xiushui planting tree public welfare tree planting activities. By creating "less forest", "common forest" form, in the youth spread the concept of "green, low carbon, environmental protection", guide them a variety of trees, good trees, protect the forest, advocate everyone love green plant green protect green civilization, build green homes, youth bear practice of good social atmosphere, common power green ecological environment construction, the colorful Guizhou makeup is becoming more and more beautiful.

2022-03-17	The Shandong Communist Youth League has fought with nearly 40,000 young volunteers to build the youth fortress of epidemic prevention and control, breaking the peace of Laixi in Qingdao. Young volunteers quickly gathered together and hurried to the front line of epidemic prevention and control. Within half an hour, 12 youth commandos, more than 230 commandos were assembled. According to the regiment Laixi Municipal Party Committee statistics, as of today, the town has gathered nearly 100 youth commandos. At present, the epidemic prevention and control situation in Shandong province is complex and severe, and local outbreaks have occurred in many places. Youth league organizations and young volunteers at all levels in Shandong fully carry forward the fine tradition of "the Party has a call, the league has an action", organize the majority of young volunteers to participate in the front line of the fight against the epidemic, and write their youth responsibility with practical actions. Smell and move young volunteers quickly assembled after the outbreak, the Shandong communist youth league immediately issued the epidemic prevention and control youth volunteer service guidelines in Shandong province the communist youth league party committee about COVID-19 epidemic prevention and control initiative, the provincial youth league organizations at all levels have issued initiative, mobilization order, recruitment, mobilize the provincial youth league organizations at all levels and youth volunteers scientific and orderly to participate in the epidemic prevention and control work. On March 13, a news that the Tuan Changyi Municipal Party Committee continued to recruit young preparatory volunteers for epidemic prevention and control "flooded" local people's WeChat circle of friends. As soon as the recruitment order was issued, it quickly received a positive response from the local youth league members and volunteers. In less than an hour, volunteers reported their location and said they could charge to the front line at any time. As of March 15, more than 1,200 young volunteers have been recruited. In the province, youth volunteer service teams from all over the country have also responded to the call and immediately rushed to the frontline of fighting the epidemic, assisting street and community workers, doing order maintenance, site disinfection, bayonet duty, material distribution and other work, so as to comprehensively help the local epidemic prevention and control. According to incomplete statistics, up to now, youth league organizations at all levels in Shandong province have recruited 1,312 epidemic prevention and control commandos (reserve teams) and more than 80,000 epidemic prevention and control volunteers. At the same time, according to the needs of epidemic prevention and control, youth league organizations at all levels have made plans in advance, timely reserve the "reserve echelon" of professional volunteer services, and done a good job in volunteer training to ensure that they can fight immediately upon call. For the first time in Shandong province after the confirmed case, the entire province of the youth league organizations at all levels immediately into the emergency state, the cadres, young volunteers for the first time, working with "white", actively participate in nucleic acid sampling, order maintenance, "three yards" inspection, site sanitizers, temperature measurement, information entry, epidemic prevention propaganda, material distribution, etc. On the evening of March 4, after receiving a notice of a confirmed case, the "Walk with You" youth commandos completed an emergency gathering and rushed to five epidemic prevention sites to strictly guard them. So far, the 100 members of the commandos are divided into eight groups and have been on duty in all key areas of the city. From the time they put on protective clothing, they stay tight and work hard. Up to now, 38,800 young volunteers in the province have been fighting on the frontline of fighting the epidemic, serving more than 930,000 hours. Care a line steady do rear "guardian" at the same time, Shandong communist youth league organization continue to carry out the "dedication to devotees" volunteer service, focus on fighting in the frontline of disease resistance staff, policemen, grassroots cadres and their families to run errands to buy, family life care, such as volunteer service, to provide "NiXingZhe" strong rear security. There is such a group of caring young people, silently contributing to the fight against the rear in the epidemic. Rizhao Juxian raindrops public welfare development center organized volunteers to stick to the epidemic prevention line of Juxian traffic police brigade all police officers to send love lunch. Liu Yanzhi, a member of the Laixi Youth Federation, has set up a youth commando team to cook and deliver meals to the frontline staff in his own restaurant, and has sent hundreds of working meals to the frontline. Liu Yanzhi said: " I heard before that some front-line staff often fail to eat, and the prepared meals are cold, so we just think that everyone can drink hot soup on the front line."In Qilu, there are many such young volunteers who uphold the concept of" dedication to the devotees ", whether cooking love meals, delivering epidemic prevention materials, or helping to take care of the elderly and children, they silently contribute to relieve the frontline epidemic prevention workers' worries and protect the" home front ". Unis strength watch mutual "heart to heart" these days, Texas yucheng rural good youth alliance WeChat group often appear personnel registration, donations, material deployment information, the alliance backbone members Shandong guanghua real estate deputy general manager, Shandong food doll technology co., LTD., YuCheng Ding run new energy technology co., LTD., Yang took out more than 50000 yuan, for the prevention and control point to protective clothing, disinfectant and other materials. Youth league organizations at all levels in Shandong gave full play to their advantages and quickly collected all kinds of materials to deliver the rear love to the front line of the fight against the epidemic. The Shandong Provincial Party Committee of the Communist Youth League promptly allocated 1 million yuan to the Qingdao Municipal Party Committee for epidemic prevention and control. The Qingdao Municipal Party Committee of the Communist Youth League allocated 220,000 yuan to the West Coast New Area and Laixi City respectively for the epidemic prevention and control work. At the same time, youth league organizations at all levels in Shandong Province actively launched the youth enterprises Association, the Youth Federation and other caring enterprises on various fronts to extensively collect protective clothing, masks, disinfectants and other epidemic prevention materials to ensure grass-roots youth league organizations to carry out voluntary epidemic prevention volunteer services. The youth of Shandong always adhere to the principle of the Party flag is the league flag, uphold the spirit of volunteer service, and build a youth fortress of the prevention and control of the epidemic in Qilu with love and warmth. Correspondent Wei Hua, China Youth Daily, China Youth Network, reporter Xing Ting, Source: China Youth Daily, March 17,2022,01 edition

2022-03-17	Jiangxi Ganqing Commando: they can fight at the moment of call, the three electronic screens of the command center broadcast the ganqing commandos emergency rescue propaganda video. Zhou Jiayu, including the captain of the provincial ganqing commandos, here and the provincial flood control and drought relief headquarters video link, command, dispatch around the ganqing commandos. Here is not only the headquarters, but also a conference room, a training room. More than two months ago, the successfully registered Ganqing commandos held their inaugural meeting here. In June 2020, many places of Jiujiang and Poyang province in Jiangxi province were hit by floods. The Jiangxi Provincial Committee of the Communist Youth League issued an emergency mobilization order. Young people from government organs and public institutions, college students and state-owned enterprises in Jiangxi province gathered from all directions. Within 48 hours, more than 800 youth commandos and nearly 20,000 young people rushed to the disaster area. After the flood situation, with the team of the youth League provincial party committee cadres Gan Xiaoping began to think: how to keep a youth force, when the society needs to gather at any time, the first time to play a role? Gan Xiaoping's original vision was: " It is first of all a team of social volunteers with young people and emergency response capabilities. It is also a pivotal organization of social rescue forces, playing a pivotal role in connecting the government and social rescue forces."After receiving the support of Jiangxi Provincial Department of Water Resources, Jiangxi Provincial Department of Emergency Management, and other units, the Jiangxi Provincial Party Committee of the Communist Youth League began to prepare for the formation of this team, and learn from the management experience of the blue Sky rescue team, the Eagle rescue team and other social emergency rescue organizations, a little more clear thinking. In October 2020, Jiangxi Provincial Committee of the Communist Youth League summarized the experience of epidemic prevention and flood control, led the establishment of the first commando of Jiangxi Youth in Jiangxi Province, and formed a team according to the three-level joint construction mode of provinces, cities and counties, and 87 members were initially recruited. Over the past year or more, the volunteer team has acquired its own logo, office space, team flag, team training, clothing and organizational structure, established regulations and management system, and attracted more and more young people with emergency rescue expertise to join us. Not long ago, it welcomed its first full-time deputy secretary-general. At present, ganqing commandos have set up a total of 112 teams at the provincial, city and county levels, and registered 5,463 members of the province, with the average age of 34. Ganqing commando team follows the captain responsibility system, the core team recruitment all adopt social mobilization. Gan Xiaoping said: " As a youth-oriented social emergency rescue team initiated by the youth League committee, in order to maintain the vitality and independence of the team, more often, the youth League Committee does not help but do not extend their hands too long."Emergency rescue tasks involve mountain rescue, medical rescue, high-altitude rescue, event guarantee and other aspects. The key to keeping this team fighting power is to improve its professionalism. At the early stage of its formation, the team took the regular practical training as the core of the team management. In addition to at least two sessions of training every month, Ganqing Commando pays attention to strengthening the linkage with fire protection, blue sky rescue team and other rescue forces, and regularly carry out regular joint practical training. Jiangxi Provincial Committee of the Youth League also provides the team members through various social financing and cooperation channels. For example, through coordination, Nanchang Fire and Rescue Detachment provides more than 300 square meters for the commando headquarters; by raising social funds, Jiangxi Provincial Youth Foundation allocated 300,000 yuan to purchase equipment and customized clothing in the team. Over the past year, ganqing commandos have been present at the emergency response volunteer service sites such as Henan Flood, Poyang Lake International Bird Watching Week, Jiangxi May 4th Youth Singing Party, and the World VR Industry Conference. In the Spring Festival of 2022, nine Ganqing commandos were on duty at Nanchang Railway Station, Nanchang West Railway Station and Tengwangge scenic area for eight consecutive days for emergency preparedness, patrol and evacuation, and providing temperature detection, consultation and guidance services for passengers and tourists. The construction of Ganqing commandos is an exploration of the construction of social rescue force, and also an effective carrier to play the youth force. However, as a social rescue team led by government departments, it is faced with problems such as shortage of activity funds and insufficient treatment guarantee of full-time personnel. In addition to actively coordinating social funds and seeking support from relevant functional units, the team has established a joint meeting system to regularly contact and cooperate with the local Blue Sky rescue teams, Eagle rescue teams, Blue Leopard rescue teams, Shuguang rescue teams and other social rescue teams to jointly discuss and solve the problems together. In the future, the Jiangxi Provincial Committee of the Communist Youth League will also take the management of team members as an important category of youth management, bring it into the service objects of the Communist Youth League organization, and strive for various resource allocation and incentive measures in the fields of employment, schooling, entrepreneurship, commendation and so on. Source: China Youth Daily, March 17,2022

2022-03-16	The ninth "green miao cup" Anhui Province project capital matchmaking held the ninth "green miao cup" Anhui Province project capital matchmaking held in Hefei Beijing on March 16 (reporter Zhang Jianwei, correspondent Xu Fei Zha Wei Ting) March 12 to 13, the ninth "green miao cup" Anhui Province project capital matchmaking held in Hefei. Leaders of Anhui Provincial Party Committee, Anhui Zhongan Chuanggu Science and Technology Park, Anhui University Students Innovation and Entrepreneurship Promotion Association and other leaders, some college Youth League committee, investors and college students representatives attended the activity."Green Miao Cup" Anhui Province Project Capital Meeting is one of the important forms for the Communist Youth League organization to serve youth innovation and entrepreneurship, build a face-to-face communication platform for innovation and entrepreneurship teams and investment institutions, to help the young students in innovation and entrepreneurship practice, lead the young students to their entrepreneurial dreams, life dreams into the great practice of the construction of modern Anhui, into the comprehensive construction of a modern socialist country, celebrate the party with excellent achievements. The "Green Miao Cup" is co-sponsored by Anhui Provincial Committee, Anhui Provincial Students' Federation, Anhui Provincial Innovation Association, co-organized by Anhui Zhongan Innovation Valley Science and Technology Park, Enlightenment Star Hefei Incubation Base, Green Network Science and Technology Park, Wuhu Tongcheng Youth Pioneer Park, and supported by ape Innovation Home Enterprise service. Since the launch of the event, 290 projects have been selected for the university-level competition, and have passed the provincial preliminary examination and review. A total of 30 projects from 14 universities have been shortlisted for the final examination roadshow. After fierce competition, 10 projects including Hefei Xingmou Biotechnology Co., Ltd. won the "Entrepreneurship Gold Award"; 14 units including the University of Science and Technology of China won the "Excellent Organization Award"; and Representatives of the two investment institutions signed investment intention agreements with the project team representatives at the award ceremony. Up to now, the "Qingmiao Cup" has been held for nine consecutive years, with an intended investment of 450 million yuan and a project financing of 310 million yuan.

2022-03-14	Jianwei) Recently, the 2022 enterprise league organization and construction in Henan Province was held in Henan Province. More than 140 people from the provincial league committee, provincial enterprise working committee, provincial financial league committee, affiliated youth league organizations, provincial youth league member enterprises, provincial youth enterprise association, provincial youth association and technology association member enterprises, state-owned enterprises, non-public enterprises and industrial agglomeration areas, and provincial cities participated in the training. This training takes the form of special topic teaching, case teaching, discussion and learning, exchange and sharing, and on-site observation, and has organized and designed a rich course content. Training class, provincial government counselor zhang warehouse around Henan, the main characteristics of Henan economic development and innovation driven the development of high quality focus, provincial party committee party school, provincial league school teachers around jinping new era, the ideas of socialism with Chinese characteristics of the spirit of the 19th, group history guide and basic group, how to do the communist youth league work teaching guidance, round square group group is responsible for the comrades around non-public enterprise building experience sharing. During the training, the Henan provincial party committee compiled issued the provincial enterprise group building work guidance manual, organize students successively to build seven bureau, miss you health food co., LTD. View state-owned enterprises and non-public enterprise league organization construction, and around the enterprise group how to power service technology innovation, "everyone certificate, skills in Henan" work has carried on the discussion. At the same time, in accordance with the requirements of the comprehensive and strict governance of the group, the students were organized at the end of the training for the completion examination. It is understood that the Henan provincial party committee held the training course to implement the spirit of the fifteenth session spirit and Henan communist youth league "strong ability, forging style, grasp the implementation of the" action work deployment, through learning training, to further enhance the ability of enterprise cadres, stimulate vitality, consolidate and expand the enterprise group coverage and work coverage, improve the level of the provincial enterprise youth league organization construction.

2022-03-14	League shaanxi provincial party committee to carry out youth volunteers "learn lei feng" demonstration activities shaanxi provincial party committee to carry out youth volunteers "learn lei feng" demonstration activities Jianwei correspondent Zhang Wenhao) recently, sponsored by the communist youth league, the communist youth league xi 'an municipal party committee, to "gather youth power power high quality development" as the theme of 2022 shaanxi province youth volunteers "learn lei feng" demonstration activities in xi' an new city huaqing university city community, Weiyang District xujiawan community, Yanta community held 212 communities. The activity attracted more than 100 units from fire, traffic police, hospitals, engineering construction units, performing arts institutions, social organizations and other fronts, various fields to participate. Activity pictures. Correspondent for figure event, the youth volunteer service organizations in the community small square, basketball court and other open field for ecological civilization display, garbage classification knowledge, fire knowledge propaganda consultation, fire fighting, civilized traffic knowledge, yizhen service, legal consultation, fraud prevention education, free hydropower maintenance, compulsory haircut and other convenient and beneficial services, for the masses to provide "use" volunteer service, send warm heart service to the community masses. Activity pictures. The location of this activity is the community youth action pilot community, aiming to deepen the implementation of the community youth action by providing menu-style volunteer services through pair assistance. The Communist Youth League has built a "four-in-one" working mode for the communist Youth League participating in community governance personnel, projects, positions, and resources, leading the young people to improve their awareness of participation, strengthen practice, and increase their skills and abilities in the community, and to become an important force to assist the Party and the government to strengthen and innovate social governance. Activity pictures. Correspondent for figure according to the provincial party committee is responsible for comrade, in recent years, the shaanxi provincial party committee always put the youth volunteer action in the overall situation of economic and social development and planning, to "party and government concern, social concern, youth can" as the breakthrough point, constantly broaden the field of volunteer service, form a wide coverage, rich in content, with high professional level of volunteer service project system. This year to continue to in accordance with the group "welcome 20, always follow the party, forge ahead new journey" theme practice deployment, around the elderly assistive, community service, ecological environmental protection, large games, emergency rescue, social management, cultural construction, rural revitalization of eight special operation 30 key projects, leading the province broad members and youth volunteers extensive volunteer service activities.

2022-03-12	The second batch of youth red gene inheritance base naming ceremony held the second batch of youth red inheritance base in Zhejiang province red gene naming ceremony held in Hangzhou green newspaper reporter Jiang Yutong, correspondent Yang �) on March 11th afternoon, the communist youth league of Zhejiang provincial party committee, provincial association for the promotion of the construction of the development of old revolutionary base areas, provincial veterans affairs office jointly organized the second batch of young people in Zhejiang province red gene inheritance base naming ceremony. Naming ceremony of the second batch of youth red gene inheritance base in Zhejiang Province. Forty-three units including the Memorial Hall of the People's Liberation Army were awarded the second batch of youth red gene inheritance base. Naming the youth red gene inheritance base is an important measure for Zhejiang Province to strengthen the education of the youth party history, national history and remember the memory of the martyrs, aiming to further gather the consensus of the young people to keep the "red roots" and help common prosperity, and provide an important position for the majority of young people to receive patriotism education. Chen Tiexiong, vice chairman of the CPPCC of Zhejiang province and chairman of the provincial old revolutionary base area development and construction promotion association, pointed out at the meeting that the naming ceremony of the second batch of Zhejiang province youth red gene inheritance base is the 20th celebration of the party. Young people are an important part of the Party's cause and the backbone of the great rejuvenation of the Chinese nation. We should keep in mind the instructions of the general secretary, use the inheritance base to let the young people pass on the red gene, cultivate them into a new generation with all-round development of morality, intelligence, physique, beauty and labor, and help them aspire to realize their life ideals. Chen Tiexiong pointed out that in thought, politics, action and the party central committee with comrade xi as the core remain highly consistent, support "two established", "two" maintenance ", under the strong leadership of the provincial party committee and provincial government, persistent practice of" August strategy ", carry forward the glorious tradition of revolutionary predecessors, adhering to the revolutionary wishes, deepen reform, forge ahead, pragmatic innovation, in order to achieve the" two high level "goals and the great rejuvenation of the" Chinese dream " of the Chinese nation. The meeting read out the second batch of Zhejiang Province youth red gene inheritance base naming documents. Zhu Huamin, director of the Party History Research Office of Chun 'an County Party Committee, shared the typical practice of Chun' an County to play the role of the youth red gene inheritance base. Liu Fangjing, a member of Hangzhou Youth Theory Publicity Group and Zhejiang Red Scarf Tour Group, told the story of leading teenagers into the red Gene inheritance base. The Communist Youth League Zhejiang Provincial Party Committee, the provincial old revolutionary base area development and construction promotion association, the provincial Department of veterans affairs related leaders, 43 Zhejiang Province where the first youth red gene inheritance base in the development and construction promotion association and the main leaders of the Youth League committee, representatives of the youth publicity group attended the ceremony.

2022-03-11	Jianwei correspondent Yue Qing) Recently, the 2022 Guangdong Lei Lei Feng National Volunteer Service Action Month launch ceremony was held in Foshan. Wu Zuqing, deputy director of Guangdong Provincial Civilization Office, Li Jiaxian, deputy secretary of the Working Committee of institutions directly under Guangdong Province, Liang Junda, deputy secretary of Guangdong Provincial Communist Youth League, Ni Qian, member of the Standing Committee of Foshan Municipal Party Committee and minister of the Publicity Department and other representatives of advanced volunteer service attended the event. The picture shows the launch of the Guangdong Learning from Leifeng National Volunteer Service Action Month in 2022. Group of Guangdong provincial party committee for the event site, the main undertaking unit representative for 2021 Guangdong learn lei feng volunteer service advanced typical representative and 2021 Foshan advanced volunteer service typical representative awards, for a new batch of five-star volunteers of Guangdong province certificate, issued the 2021 Guangdong ("i volunteer" system) volunteer service development index. The 2022 Guangdong Learning from Lei Feng National Volunteer Service Action Month will take the theme of "Welcome the 20th Volunteer Achievements", On the premise of good epidemic prevention and control, Give full play to the vanguard role of Party members and league members and the exemplary role of public officials, Focusing on spreading new ideas, maintaining social stability, promoting rural revitalization, building a beautiful Guangdong, helping economic development, spreading volunteer culture and other key areas carefully planning to carry out various forms, rich content of learning from Lei Feng volunteer service activities, In strive to realize the mission and task entrusted by the General Secretary to contribute volunteer service, Write the story of Lei Feng in the new era with practical actions, To meet the party's 20th victory held with outstanding results. The picture shows the advanced model of learning from Lei Feng in Guangdong Province in 2021. Recently, 2021, the Guangdong Province learned Lei Feng volunteer service advanced model announced, selected the "most beautiful volunteer", "the best volunteer service organization", "the best", "the best volunteer service project" and "the most beautiful volunteer service community" 160. These typical volunteer long-term volunteer service activities, in the epidemic prevention and control, civilization practice, grassroots governance, rural revitalization, crisis, emergency relief, disaster relief, ecological environmental protection, caring, cultural services has made outstanding achievements, drive more people to join the volunteers, take practical actions to write a new era of lei feng's story. The information development of volunteer service is the only way to promote the high-quality development of volunteer service. Based on the data of the "i volunteer" system and the Guangdong volunteer service interaction system, the Guangdong Volunteer Federation has developed the volunteer service development index and measurement standard of the Guangdong ("i volunteer" system) around four dimensions: volunteers, volunteer service organizations (groups), volunteer service duration and the number of volunteer service activities. The index took the lead in exploring the establishment of a scientific and objective weighted index evaluation system for the development of volunteer service, to find out the actual development status of volunteer service in Guangdong, and to guide the volunteer service in various fields. The picture shows the advanced model of learning from Lei Feng in Guangdong Province in 2021. At the site of the event, Guangdong Provincial Volunteer Federation released the 2021 Guangdong ("i Volunteer" system) Volunteer Service Development Index. At present, Guangdong province has more than 14.07 million registered volunteers, and more than 132,000 volunteer service organizations and groups, with a total service time of more than 700 million hours. In terms of volunteer gender, male volunteers account for 48% and female volunteers 52%; from the age of volunteers, youth is the main force of volunteer service, 14 to 40 old volunteers account for 74.92%; from the political situation of volunteers, party members and league members account for 43.76%. By administrative division, the top cities include Guangzhou, Shenzhen, Zhuhai, Foshan, Shaoguan, Dongguan and Jiangmen in the province include Guangdong University of Foreign Studies, South China Agricultural University, Xinhua College of Sun Yat-sen University, South Medical University and South China University of Technology.

2022-03-11	Gansu: start learning lei feng youth volunteer service activities "carry forward the lei feng spirit condensed youth strength" 2022 Gansu learn lei feng youth volunteer service activities launched jianwei correspondent Li Jing) recently, the Gansu provincial party committee joint provincial civilization office, provincial department, provincial ecological environment department, provincial WeiJianWei, provincial bureau of forestry and grassland bureau, provincial disabled persons' federation, provincial Red Cross, China merchants bank Lanzhou branch of "carry forward the lei feng spirit condensed youth strength" 35 lei feng volunteer service activities launch ceremony held in Lanzhou Zhongshan bridge. Leaders of relevant units attended the launch ceremony together, and more than 500 people from the university youth volunteer service team, the provincial youth volunteer association, Lanzhou youth volunteer service team, the media and the youth volunteers from all walks of life participated in the Lei Feng activity. Pictures of the event site."You are a lighthouse, Shining on the sea before the dawn; You are the helmsman, Master the direction of the navigation. "" Love my China, Love my China... " Before the event begins, On the Zhongshan Bridge Square in Lanzhou City, A flash mob consisting of songs like "Follow the Communist Party" and "Love My China" opened the prelude of the Lei Feng month volunteer service launch ceremony, When the melody of the song plays out, People gathered spontaneously gathering towards the song, Everyone stopped to listen, Immersed in the cheerful and passionate atmosphere of the songs, In the familiar song, Young volunteers and the citizens waved the national flag together, Singing the Motherland Singing the Party, Then, the volunteers' dedicated street dance performance began, Push the atmosphere of the event site quickly to a climax. At the launch ceremony, the participating leaders presented certificates and MEDALS to the 5th Gansu Province Youth Volunteer Outstanding Individual Award, the Organization Award, the 13th China Youth Volunteer Outstanding Individual Award, the organization award winner, and the 5th National Public Entrepreneurship Competition in Gansu; China Merchants Bank Lanzhou donated "public vending machine" to 10 universities. Subsequently, the leaders jointly launched the "Carry forward Lei Feng Spirit cohesion youth strength" 35 Lei Feng volunteer service activities. Pictures of the event site. With the launch of the activity, the song of "Learning from Lei Feng" sung for 59 years sounded at the activity site, which also revealed that the Lei Feng youth volunteer service activity was fully opened. At the same time, the provincial city (state) and directly affiliated youth League committee also carried out offline activities in each sub-venue. After the ceremony, the scene sounded a familiar song: "I and my motherland, a moment can not divide, no matter where I go, all out of a hymn........." a song "I and my motherland" ignited all the enthusiasm, attracted a large number of passing people have to participate in the activity, in the square "learn from Lei Feng with volunteer" a wave of upsurge. At the event site, each volunteer service team carried out various volunteer service activities such as "voluntary blood donation", "Green Yellow River clean Beach" and "Civilized traffic guidance". The volunteers had a reasonable division of labor and actively cooperated, forming a beautiful scenery on both sides of the Yellow River. Pictures of the event site. At the site of the activity of Gansu Provincial Committee of the Communist Youth League, volunteer service activities such as healthy life, ear care, legal publicity, love free medical diagnosis, emergency rescue, epidemic prevention and control, psychological counseling, youth volunteer service stations were displayed, and "Lei Feng seeds" and various brochures were distributed to the masses. According to the citizen Mr.Wang at the event, " They have sung these songs for decades, every time we have a new experience and feeling, this time and we sing the motherland singing Lei Feng, the heart is very excited, I wish our great motherland more prosperous and strong!"Citizen Mr.Li said:" Lei Feng died is also a young man, now see so many young volunteers friends, with their own youth strength in the beautiful Gansu and dedication, the heart is very feeling, they are a living little Lei Feng ah!"Another citizen said," I hope to carry out more of these activities in the future, so that the masses can participate in them, so that the Lei Feng spirit is really integrated into the hearts of the citizens and the people's lives."In recent years, the Provincial Communist Youth League Party Committee and the Provincial Youth Volunteers Association have actively guided social organizations, caring people and young volunteers to participate in various volunteer service activities, such as social governance, rural revitalization, ecological and environmental protection, and ethnic unity. According to statistics, at present, there are 4.2 million registered young volunteers in Gansu Province, and 7,424 volunteer service teams have been set up, with a total of 63,000 volunteer service activities and a total of 40 million hours. These gathered love together constitute a strong pulse of the Lei Feng spirit in the new era of Gansu province, highlighting the vivid interpretation of the Lei Feng spirit by the majority of the majority of young volunteers."March spring breeze warm, Lei Feng spirit". In order to further enhance the influence and participation of the Lei Feng Month activities, the Provincial Communist Youth League Committee has launched the "35" Lei Feng Volunteer Service Month activities with the theme of "carrying forward the Lei Feng spirit and gathering the youth strength" from now on.

2022-03-10	Jianwei) On March 7th, the 16th and fifth plenary (expanded) meeting of the 16th Jiangxi Provincial Committee of the Communist Youth League was held in Nanchang. Wu Zhongqiong, Deputy Secretary of Jiangxi Provincial Party Committee and Secretary of Ganzhou Municipal Party Committee, attended the meeting and delivered a speech. Members of the provincial Communist Youth League Party Committee leadership attended the meeting. Conference, Wu Zhongqiong stressed that the provincial youth league organizations at all levels to xi jinping new era the ideas of socialism with Chinese characteristics as guidance, further implement xi general secretary of the important thought of youth work, to meet, study, publicity, implement the party's 20 as the main line, combined to celebrate the 100th anniversary of the party's cause after the fundamental plan, good lead condensed youth, organization to mobilize youth, contact youth, service basic responsibilities, comprehensively deepen reform, comprehensive governing group, unity led the province youth in the comprehensive construction of socialist modernization Jiangxi contribute youth strength, writing the youth chapter. Wu Zhongqiong affirmed the work of youth league organizations at all levels in the province in the past year. She stressed that the Communist Youth League was created by the Party, and the party flag refers to the league flag. We must strengthen our political orientation and continue to strengthen political guidance. We will deepen the theoretical arm of young people, vigorously carry forward the red gene, defend the ideological position, earnestly fulfill the political responsibility of consolidating and expanding the young mass base of the Party's governance, and unite the young generation closely around the Party. We must closely follow the overall situation and continue to strengthen our responsibility. Find the right work combination point, entry point, focus point, to boost the "Double No.1 Project", actively participate in social governance, vigorously advocate the new wind of civilization, unite and lead the majority of young people to actively participate in the hot practice of "six Jiangxi". To grasp the pulse of the youth, and continue to strengthen the contact service. We will fully implement the medium-and long-term development plan for young people, improve the system of having direct contact with and serving young people, strive to safeguard their rights and interests, and strive to be trusted, reliable, and inseparable from the hearts of young people. We will deepen reform and innovation and continue to strengthen self-improvement. In keeping with the goals and requirements of "strengthening the three characteristics and eliminating the four modernizations", we will earnestly strengthen Party building in government organs, continue to consolidate the foundation at the grassroots level, and strictly manage the league cadres and League members, so that the work of the Communist Youth League will radiate new vigor and energy. The conference heard the work report of the Standing Committee of the Provincial Communist Youth League Committee, the meeting conveyed the spirit of the sixth Plenary Session of the 18th Central Communist Youth League, conducted the discharge and co-election of members of the 16th Provincial Communist Youth League Committee, deliberated and approved the Implementation Plan of the Comprehensive Strict Governance of the Jiangxi Communist Youth League, and reported the work evaluation of the provincial Communist Youth League in 2021. More than 90 people attended the meeting, including members of the 16th Provincial Committee of the Communist Youth League, units directly under the CPC, provincial universities and the main heads of subordinate units.

2022-03-10	Gansu: "spring breeze action" and the fifth "youth fair" start "spring breeze action" in 2022 and Gansu communist youth league fifth "youth fair" start jianwei correspondent Li Jing) on March 6 morning, by the Gansu provincial party committee, province, people club hall, provincial education department in 2022 "spring breeze action" and Gansu communist youth league "youth fair" the fifth in Gansu province human resources market officially launched. The Communist Youth League provincial Party Committee book, the provincial Department of human resources and social security party group related responsible person attended the launch ceremony. Job fair site. Group Gansu provincial party committee for the job fair is to promote "six protect" "six stability" work, the full implementation of the long-term youth development plan in Gansu province (2018-2025), consolidate poverty crucial achievements, promote the important measures of rural revitalization strategy, and further broaden the college graduates and unemployed youth employment channels, promote the poverty areas effective way to achieve stable income. Through the youth federation, young entrepreneurs association and the caring forces from all walks of life, the job fair invited nearly 300 employment enterprises from Beijing, Shanghai, Tianjin, Guangdong, Jiangsu, Shandong, Fujian, Gansu and other provinces and cities to provide more than 10,000 jobs. Job fair site. The fifth youth recruitment fair and online recruitment activities were carried out simultaneously. The provincial recruitment special event is located in the human resources market of Gansu Province. More than 120 employers from all over the country provide more than 8,000 jobs for young people, and 14 cities (prefectures) in the province have set up sub-venues. At the same time, from March 6 to April 3, the job fair will also release online recruitment information through online employment service platforms such as Gansu Talent Network, "Gansu Youth" WeChat public account, "Tuan Employment" and other online employment platforms, and carry out various online recruitment activities such as online double selection meetings and video interviews to further expand the coverage of the activities. Job fair site. The jobs provided by the CPC Youth League Gansu Provincial Party Committee cover more than 20 industries such as wholesale (retail), construction engineering, education (training), Internet (e-commerce), involving accounting, sales, clerical, graphic design, education, new media operation, security officer and other jobs and jobs. In accordance with the epidemic prevention and control requirements, young job-seekers and college and technical secondary school students came to the recruitment site in batches and in different time periods. On the day of the activity, job seekers sent 3,075 resumes to employers through offline and online, and 476 voluntary employment agreements were initially reached offline. During the job fair, set up in the studio, invite employment mentors, lawyers and employment enterprises head through TikTok, B station webcast platform, for the province has employment demand of youth live teaching, provide policy interpretation, career planning, rights protection consulting, legal aid and other services, to watch more than 10000 people online.

2022-03-10	Zhang Wenling, the fifth plenary (expanded) meeting of the fifth plenary (expanded) meeting of the 14th Committee of the 14th Yunnan Provincial Committee of the Yunnan Provincial Committee of the Communist Youth League was held in Kunming. Shi Yugang, deputy secretary of Yunnan Provincial Committee, attended the meeting and delivered a speech. Tang Yuan, secretary of Yunnan Provincial Committee, delivered a work report to the Standing Committee of Yunnan Provincial Committee. Shi Yugang fully affirmed the work of the Standing Committee of the Yunnan Provincial Party Committee in 2021. He praised the youth league organizations at all levels, youth league cadres and youth for contributing to the high-quality leapfrog development of Yunnan in helping poverty alleviation and rural revitalization, "digital Yunnan" construction, epidemic prevention and control, and stabilizing border prevention. This meeting around the Yunnan Provincial Party Committee on the implementation of the style revolution, strengthening the efficiency of the construction of the arrangements, put forward the "change style, strong efficiency, grasp the implementation of the" target requirements, with the style revolution, efficiency revolution to promote the specific practice to improve the quality and efficiency. Meeting requirements, To "welcome the 20, always follow the party, forge ahead on the new journey" theme education and practice activities, As an important work carrier of deepening the ideological and political guidance of young people; To transform the "big task" of the Communist Youth League into a "small project" of youth work; Continue to carry out the "on the platform, down to the grassroots" work, Move the office to the grass-roots youth league organization front line, the activity position front line; Do practical things and do good things for the grass-roots level and for the youth; Promote the construction of "Yunnan Youth Development Center", Carry out projects such as "mass entrepreneurship and innovation star", "one star in one county" and "one industry in one star", Increase the cultivation of young e-commerce talents; Make good use of financial support means, We will do a good job in starting up guaranteed loans, Promote the establishment of Yunnan entrepreneurship youth database. Focus on the rural revitalization and youth contribution initiative, carry out the "Dream Road" series of activities, promote the rural public service projects; and continue to promote the implementation of the "double reduction" work measures. We will stimulate new drivers of Project Hope and do a good job in helping young people in need. The solid work reflects the responsibility of the Communist Youth League. The meeting deliberated and adopted the Resolution of the Yunnan Communist Youth League on Implementing <Yunnan Province on Promoting the Work Style Revolution and Strengthening the Efficiency Construction of Organs (Trial)>. According to the relevant provisions of the Youth League Chapter, some members and alternate members were discharged, replaced and re-elected.

2022-03-09	Guizhou, month 9 (reporter Zhang Jianwei, correspondent Chen Kai) Recently, Guizhou Province Medium-and long-term youth Development Planning joint Conference member unit liaison meeting was held in Guiyang. Liaison officers or relevant responsible comrades of 49 member units of the joint meeting attended the meeting. Provincial Committee of the Communist Youth League planning office responsible comrade presided over the meeting. Meeting to learn the long-term youth development planning implementation inter-ministerial joint meeting the fourth plenary meeting spirit, summarized in 2021 the Guizhou province long-term youth development plan (2019-2025) (hereinafter referred to as the "plan") implementation, reported the Guizhou youth development blue book writing work advance, meeting in 2022 the plan implementation points for detailed interpretation, and communicate around the key work, further improve the understanding, unified thought, condensed work together. The meeting held that the implementation of the National Medium-and long-term youth development Plan is a work that General Secretary Xi Jinping has personally cared for, guided and promoted. It is both a major political responsibility and a major historical opportunity. The implementation of the Plan is to adhere to the principle of the Party governing the youth, actively win the care and support of the party and government; the focus is to actively create a strong atmosphere of youth priority development, and provide a real sense of gain by introducing policies and implementing projects; the key is to play the role of the planning joint meeting mechanism, improve the cross-departmental and hierarchical linkage mechanism, and form a strong working force and joint management situation. The meeting pointed out that under the strong leadership and care and support of the provincial Party Committee and the provincial government, and with the joint efforts of the member units of the provincial joint conference, the implementation of the Plan in 2021 has achieved remarkable results. Building "youth friendly growth province" into the provincial government work report; last September as one of two provincial youth committee representatives, make experience exchange in the national planning pilot seminar training class; the implementation of the plan into the high quality development performance appraisal of the city (state), provincial units, one of 10 special, promote the party committee governments and functional departments to pay more attention to youth work, create youth priority development of strong atmosphere; the youth central committee in the annual work assessment of Guizhou province "plan" in the central region positive evaluation. The meeting stressed that the member units of the joint meeting should focus on the goals and tasks of the ten fields and ten projects in the Plan, strengthen the implementation of responsibilities, strengthen the "prescribed action", do the "voluntary action", and make better results. We should jointly plan and implement the "Ten Practical Things for Young People", and implement projects and introduce policies for the urgent concerns of housing, employment, entrepreneurship, marriage, love, and health, to do practical things and solve problems for young people. To speed up the construction of "youth friendly growth province", from the provide the stage of innovative entrepreneurship, growth space, development opportunity, into the integration environment and matching policy breakthrough five aspects, vigorously build "youth friendly growth province", make Guizhou more friendly to youth, youth more promising for Guizhou, youth better growth in Guizhou. The meeting also made arrangements for the recent key work of the member units of the joint conference.

2022-03-08	Hainan)'s first "juvenile police academy" was officially unveiled in Wenchang Public Security Bureau on March 6. At the ceremony, Chen Yu, secretary of the CPC Hainan Provincial Party Committee, and Long Weidong, secretary of the CPC Wenchang Municipal Party Committee, unveiled the Wenchang Youth Police School, and Long Weidong presented the flag to the juvenile police officers. Xu Chang Bin, honorary president of the youth police school, deputy secretary of the Hainan Party Committee of the Youth League Province and director of the Hainan Youth Working Committee, said in his speech that the establishment of Wenchang juvenile police school has made a demonstration and example in the province for promoting the socialization development of the young pioneers. The curriculum content of the juvenile police school should be integrated into the spirit of patriotism, collectivism and revolutionary heroism, and should enhance the patriotism and heroism of children and young pioneers through police training, public welfare classes, observation and visit, competition and competition. It is reported that wenchang "young police academy" is sponsored by wenchang communist youth league, is the exploratory mode of sunshine happy education, relying on wenchang city public security bureau, the police station, rehab, wenchang city fire rescue team, wenchang city people's court, wenchang city people's procuratorate for positions, through five entity project content operation. Starting from this month, Wenchang Youth Police School will use its spare time to carry out a series of activities such as "On duty with Wenchang police", "My Day with heroes and models", "warning education charity class", and set up "police camp small press station". During the winter and summer holidays, we will recruit students from the whole society to carry out police course training.

2022-03-08	Fujian university youth volunteer service team and community pair to build the ceremony held youth to create civilization model city, Fujian university youth volunteer service team and community pair to build the ceremony held Beijing March 8 (reporter Zhang Jianwei) March 4, On the occasion of the 59th "Learning from Lei Feng Day" and the 23rd "China Youth Volunteer Service Day", Sponsored by Fujian Provincial Committee of the Communist Youth League and Fuzhou Municipal Committee of the Communist Youth League, Youth volunteers association of Fujian province, Fuzhou youth volunteers association, Fujian province volunteers public foundation, medical volunteers association, Fuzhou Fuzhou drum tower district committee, Fujian stars youth art institute of "youth model to create civilization city" university youth volunteer service and community pairing to build the ceremony held in Fuzhou hot spring park square. League Shao Mingsong, vice secretary of Fujian provincial party committee, Fuzhou CPPCC, vice chairman, Fujian province, Fujian province, Fujian education department, Fujian municipal party committee, Fuzhou municipal party committee, Fuzhou municipal party committee, gulou, in banyan, head of the university youth corps committee, Fuzhou related county urban youth corps committee, head of the community representative, in banyan university youth volunteer service representatives and community residents, etc. Minjiang University youth volunteer service team of "Haizi accompanies growth, water protection and environmental protection line" to carry out volunteer services of river patrol and water protection in the joint construction of communities. At the scene of the activity, a children's voice singing "Listen to me to say thank you" paid tribute to the majority of selfless dedication of the volunteers, opened the prelude. Representatives of young volunteers, university youth League committee and community representatives spoke to the stage in turn, thanking and welcoming the joint construction activity to build a platform for the needs of volunteer youth in universities and communities and promoting the in-depth integration of district and schools. Fuzhou Zheng Zhanggan, vice chairman of the CPPCC affirmed the provincial communist youth league youth volunteer service tentacles to the community, innovation in college youth volunteer service and community pair building activities, leading the young volunteers to help Fuzhou civilization model city, booster Fuzhou area first-class university city construction, the construction of Fuzhou modern international city do beneficial exploration and practice. Shao Mingsong, deputy secretary of the Fujian Provincial Committee of the Communist Youth League, stressed that the district-school joint construction activity is not only a concrete measure to promote the deep integration of the district-school, but also a concrete action to implement General Secretary Xi Jinping's important thought on youth work and celebrate the 100th anniversary of the founding of the Communist Youth League. Volunteer service activities in insist, in the normalized, he hopes the volunteer service organizations to improve the long-term mechanism of volunteer service work, make the ground community project closer to the needs of the community residents, set up good service platform, to attract the public to participate in the volunteer service team, form the national participation in volunteer service, to build a harmonious society, to help Fujian volunteer service contributions to a new level of youth.site of activity. League of Fujian provincial party committee for the leaders for college youth volunteer service representatives for the flag, the first in banyan university 70 volunteer service project and Fuzhou 100 community pairing, will be around the new crown epidemic prevention and control, assistive, caring left-behind children, health care, family education, environmental protection and other fields content to carry out the normalized volunteer service activities. The site also set up a demonstration area of community youth volunteer service project in colleges and universities to explain the service content of the project to the community residents, and carry out convenient voluntary services such as free consultation, legal consultation, and people's rights and interests protection. Fujian Medical University "Intelligent Science and Technology Pension- -Sunset Red Physical and mental Function Activation" youth volunteer service team into the joint construction community to carry out volunteer services to help and care for the elderly. The university youth volunteer service team and the community pair joint construction activity is the Youth League Fujian Province Committee, Fuzhou Municipal Committee to implement the central youth volunteer "community action", Organize young volunteers to participate in volunteer services, Promote the long-term pairing service between university volunteer service organizations and surrounding communities, To guide young volunteers to play a role in key work such as striving for a civilized model city, social governance, rural revitalization, and building a center for civilized practice in the new era, Innovative measures and positive practice to continuously improve the contribution of the Communist Youth League to the overall situation, at the same time, It also provides a platform for grassroots practice and volunteer service for young students in Rong University, Open up the "last kilometer" of ideological and political classes in colleges and universities, Vigorously promote the deep integration of district and school, It has realized the benign interaction between practical education and community service. In the next step, the CPC Fujian Provincial Party Committee of the Communist Youth League and the CPC Youth Fuzhou Municipal Party Committee will further demonstrate and promote the communities and villages in Fuzhou on the basis of the first 100 pilot communities, and water the flowers of civilization with youth volunteer services.

2022-03-08	Hunan launched million youth volunteer service action Hunan launched million youth volunteer service action network reporter Hong Kewei) recently, "welcome the 20 always follow the party on a new journey" Hunan million teenagers learn from Lei Feng volunteer service action launch ceremony was held in Changsha. It is reported that the activity by the Youth League Hunan Provincial Committee, provincial Youth Federation, provincial students 'Federation, provincial young working committee sponsored, in the province's 14 cities and states synchronous launch, will welcome the victory of the 20 victory and celebrate the 100th anniversary of the important work content throughout the year. The site of the event released the "" Lei Feng Hometown Learn from Lei Feng "Million Youth Volunteer Service Action Plan" and the fifth Hunan Province "Lei Feng Cup" Volunteer Service Project Competition 120 final projects list, and announced the establishment of Hunan Youth Volunteer Service Fund. Participating leaders and guests presented flags to 15 volunteer organizations, and jointly lit up the volunteer service map of the province. Li Zhichao, the secretary of the Provincial Communist Youth League Committee, said that Hunan is the birthplace of Lei Feng's spirit, and the youth in Hunan have always been "setting an example, leading the way in Lei Feng's hometown" with the glory and responsibility of "Lei Feng" in the Lei Feng activities. Youth league organizations at all levels in the province will continue to deepen the "Hunan One million youth Learn from Lei Feng Volunteer Service Action", vigorously promote the volunteer spirit of "dedication, friendship, mutual assistance and progress", and drive and attract more young people to learn from Feng and strive to be Lei Feng.

2022-03-07	Recently, Beijing has held the work conference of the Municipal Communist Youth League in 2022, which conveyed the spirit of the meeting of the Beijing Municipal Party Committee and the Central Communist Youth League, summarized the work in 2021 and made arrangements for the key tasks in 2022. Zhang Yankun, deputy Secretary of Beijing Municipal Party Committee and deputy director of the Standing Committee of Beijing Municipal People's Congress, attended the meeting and delivered a speech. In his speech, Zhang Yankun fully affirmed the achievements made by the Beijing Communist Youth League over the past year. Conference pointed out that to continue to thoroughly study and apply the party's spirit of the sixth plenary session of the 19, deepening youth ideological and political lead, promote the spirit of the sixth plenary session of the 19th study and implement to further, to meet and study propaganda and implement the party's twenty big work, pay attention to prevent dissolve youth work political risk, maintain youth field political security and ideological security. To adhere to the service overall situation and service youth, unity led the city's youth meritorious new era, new journey, responsible in the Beijing winter Olympics, winter paralympics, contribute to the development of the new era, in solving the youth "urgent sorrow" as the initiative, to enhance youth sense, happiness, security. We must adhere to the principle of "strict", deepen the comprehensive and strict governance of the League in the spirit of self-revolution, fully understand the importance of the new requirements of comprehensive and strict governance, always adhere to the principle of comprehensive and strict governance, and earnestly promote the comprehensive and strict governance of the League in the spirit of self-revolution. The meeting called on all districts, departments and units to fully implement the principle of the Party governing the youth, strengthen the leadership over the work of the Communist Youth League, adhere to the party building leading the league building, and increase the training and care for young cadres. We should attach great importance to the implementation of the development plan for young people, and give more preference and support to funds, resources and projects, so that young people can fully feel the warmth of the Party and the country. The meeting called on the city's young people to actively participate in the hot practice of the capital's development, with practical actions to meet the victory of the Party's twenty years. Source: China Youth Daily, March 07,2022, edition 04

2022-03-07	Ren Mingchao, who started the establishment of high-speed railway volunteer service stations around Hainan) On the afternoon of March 5th, the launch ceremony of the Youth Dream Avenue- -the Communist Youth League brand building and regular volunteer service activities was held in Haikou East Railway Station. At the ceremony, Sun Chengxiong, deputy Secretary of the Party Committee of Guangzhou Railway Group, delivered a speech on the brand establishment of the Communist Youth League work of the high-speed railway around the island. Representatives of the young volunteers of Hainan Normal University made a speech, and the young volunteers attending the meeting took a collective oath. Chen Zhiyuan, deputy secretary of the Hainan Provincial Committee of the Communist Youth League, pointed out that the establishment of a volunteer service station on Dream Avenue (high-speed railway around the island) should uphold the concept of "a game of chess" of the province's youth work and coordinate and coordinate. To improve the station position, fully understand the significance of the "Dream Avenue- -Roundabout high-speed railway" brand building activities; to carefully organize, to ensure the effectiveness of the "Dream Avenue- -island high-speed railway" brand building activities; to strengthen the brand, to build the work of the Communist Youth League brand Hainan paradigm. It is reported that the activity aims to build the Youth Mobilization and Leading service Platform of China (Hainan Free Trade Port) as the hub and Hainan High-speed Railway as the carrier to build the Communist Youth League work brand of Dream Avenue-high-speed Railway around the Island, and help the construction of Hainan Free Trade Port.

2022-03-07	Xi reporter Tian Hongwei) March 5th is the 59th "Learn from Lei Feng Memorial Day", the Communist Youth League in Fujian province carried out learning from Lei Feng theme volunteer service activities. In the Hot Spring Park Square of Fuzhou, Shao Mingsong, deputy secretary of the Communist Youth League Committee of Fujian Province, and Zheng Zhanggan, vice chairman of the Fuzhou Municipal People's Political Consultative Conference, attended the ceremony of the college youth volunteer service team of "Youth and the community". At the launch ceremony, the first batch of 70 volunteer service projects in Rong University will be paired up with 100 communities in Fuzhou to carry out regular volunteer service activities in epidemic prevention and control, helping the elderly and the disabled, caring for left-behind children, medical and health care, family education, and environmental protection. We also set up a display area for community youth volunteer service projects in colleges and universities, to explain the service content of the projects to the residents, and to carry out volunteer services such as free medical consultation, legal consultation, and protection of the rights and interests of young people's rights and interests. The Xiamen Municipal Party Committee of the Communist Youth League, together with many units, invited volunteer service teams and caring enterprises of various industries to provide policy, legal, psychological and other consulting services, as well as skills training and free diagnosis and other voluntary services. In addition, 200 private enterprises in Xiamen were invited to participate in the 2022 Spring Breeze Action site of the "New Journey to the Future" job fair. We have set up a live broadcast post belt area, enterprise recruitment area for new employment forms, employment policy consulting desk, business guidance staff consulting desk, etc., to provide more than 8,000 employment positions for job seekers. Guided by the guidance of the Quanzhou Municipal Party Committee of the Communist Youth League, the theme activity of "Old Things Charity Sale to Love the World" has attracted many young people to participate in the Quanzhou Fengze New Village Legal Park. Among them, the students from Quanzhou No.2 Experimental Primary School prepared old picture books, books, small dolls, stationery, small home appliances and other items for charity sale, attracting caring people to stop. According to the person in charge, all the proceeds from the activity will be used for the "Mother Health 1 + 1 public fundraising" activity. The Sanming Municipal Committee organized a number of volunteer teams into the city special education school to send books, cups and other supplies for the students here. Students from the Xizang class of Liedong Middle School sang "Learn from Lei Feng" in sign language and danced the gesture dance of "Together for the Future". Taiwan volunteers from the School of Physical Education and Health Care of Sanming University have provided scientific fitness and health care service training for students in special schools. Gongjue Zhuoma, a student from the Xizang Class of Leidong Middle School, said that he and his classmates often receive care from caring people in the society. Today, they came to a special education school to participate in volunteer service activities, hoping to spread this love and make the love continue.

2022-03-07	The Tibet Communist Youth League organized the "Learn from Lei Feng Memorial Day" activity. The "Lei Feng people" on the plateau. These young people on the plateau paid tribute to the "Lei Feng Spirit" with their volunteer service! The Tibet communist youth league joint Lhasa youth league on March 5, the communist youth league Tibet autonomous region committee to "practice the lei feng's spirit to carry forward the new air" as the theme, the joint communist youth league committee of Lhasa, organize the mobilization of the communist youth league member, enterprises and institutions youth, western plan volunteers, returning college students to carry out the "learn lei feng anniversary" activities. At about 10 a. m., Yutuo Road was filled with the joy of the Tibetan New Year. Dozens of stalls carefully arranged by the event organizer formed a beautiful scenery in the endless crowd. The booth is not only about the popularization of national defense education, laws and regulations, garbage classification, anti-fraud and fraud prevention, civilized new style and other knowledge, but also about the convenience of consultation on financial policies, postal policies, and entrepreneurship policies. The voluntary consultation and voluntary hairdressing jointly conducted by Fukang Hospital, holy drugstore and backlight craft barber shop make Lhasa in the Spring Festival full of different warmth. At the same time, the communist youth league committee of Tibet autonomous region organized 100 communist youth league, enterprises and institutions, western plan volunteers and returning college students are divided into three groups, respectively in fukang hospital to post building, hundred trade to nokhang temple square, the autonomous region party committee propaganda department to Lhasa municipal party committee road to carry out garbage cleaning activities. Young volunteers, dressed in uniform, rolled up their sleeves, took brooms, shovels, pliers and garbage bags, and carefully cleaned up the garbage on the street. They use small shovels to clean up small advertisements, with a broom and pliers to clean up the garbage, they are not afraid of dirty bitter, sweep the road, clear dead corners, dry sweat still do not rest, full of energy, effectively do a renovation, clean, beautiful beautiful, but also the street beautiful and tidy. It is understood that the activity issued a variety of more than all kinds of publicity and education manuals more than 1200 copies, a variety of learning books 300, in addition to 70 publicity handbags, anti-drug stationery bags 40, environmental protection publicity map 20, the number of consultations reached 279 people, 28 people, cleaning the road more than 10 kilometers of time. The "Learn from Lei Feng activity" is a volunteer service activity that the Tibet Communist Youth League has upheld for a long time. The Tibet Communist Youth League actively plans and recruits volunteers to participate extensively. Through the "learn lei feng anniversary" activities, the Tibetan communist youth league and the youth volunteers to the society, youth positive energy youth contributed their own "light" and "hot" for Lhasa, take concrete actions to show the Tibetan youth civilization, carry forward the lei feng spirit in the new period, build the thick atmosphere of learning lei feng's spirit. On the morning of March 5th, the Xigaze Youth League Municipal Party Committee, together with the Municipal Youth Federation and the Tibet Tibet Hospital, went to the Pomqing Community to carry out the love free clinic activities. At the scene of the event, Tibetan medicine experts started from the concept of Tibetan medicine health, introduced the knowledge of Tibetan medicine health, and patiently and carefully consulted the masses, gave scientific guidance on how to take medicine correctly, good living habits and reasonable diet, and distributed Tibetan medicine worth more than 15,000 yuan to more than 60 residents in the community. This free clinic activity builds a convenient health consultation platform for the community residents, provides intimate services, sending care and love to the residents, and further enhancing the sense of gain and happiness. Fire safety knowledge into the community on March 5, xigaze pku municipal organization to mobilize xigaze forest fire brigade youth volunteers into the community about fire safety knowledge, demonstrate the correct use of daily fire equipment, and organize young volunteers to community residents issued fire safety knowledge leaflets, printed with fire safety knowledge stop plate, mouse pad, a total of more than 500 publicity materials, further enhance the community residents fire safety consciousness. Care for left-behind children "book" warm heart sympathy combined with xigaze municipal party committee and city do "about learning lei feng volunteer service notice" requirements, xigaze pku to mobilize the youth volunteers, raising more than 130 for less children reading books, donated to north street left-behind children, and hope to donate students through reading, writing, cultivate the children's reading interest and writing level. At the same time to north street 20 left-behind children shoes, clothing, lunch boxes, and other young volunteers and left-behind children cordial conversation, encourage them to study hard, study hard, the party and the government's care, the social from all walks of life, into learning power, with optimistic spirit to meet learning, life difficulties and challenges, cultivate noble sentiment, set up lofty aspirations, self-esteem, self-confidence, self-reliance, self-improvement, do qualified small citizens. In order to further carry forward the spirit of Lei Feng and the volunteer spirit of "dedication, friendship, mutual assistance and progress", and to carry forward the traditional virtues of the Chinese nation and spread the concept of volunteer service, on March 5th, all units in Shannan City (District) actively carried out a series of volunteer service activities to learn from Lei Feng. The picture shows volunteers cleaning up by various units in Shannan city. During the activity, volunteers from each unit took garbage bags, brooms, tongs and other tools to their respective locations according to the region. Volunteers carried forward the spirit of not being afraid of dirty and tired, and carefully cleaned up the plastic bags, beverage bottles, cigarette butts and other garbage on the streets. The picture shows the volunteer service activity site. Mei Yunliang, a volunteer, said, " The environmental cleaning campaign not only promoted the importance of environmental protection, but also promoted the traditional Chinese virtues, and further enhanced the awareness of the masses of learning and inheriting the Lei Feng spirit. Through this big cleaning activity, "" has created a clean, clean and comfortable living environment, and guided the broad masses to be the practitioners and practitioners of inheriting the Spirit of Lei Feng and carrying forward the new trend of The Times."Volunteer Xie Xiaoming said. The picture shows Shannan City organizing a series of volunteer service activities to learn from Lei Feng. Everyone said that learning from the Lei Feng spirit should start from small things, practice, integrate civilized behavior into daily life and study, consciously practice the Lei Feng spirit, strive to be a disseminator of civilization, and transfer the positive social energy to every corner. It is understood that a total of 71 units of 761 people participated in the activity. The picture shows Qiongjie County organizing volunteers to help people clean up. At the same time, all counties (districts) of Shannan City have carried out various forms of learning from Lei Feng volunteer service activities simultaneously in accordance with the unified deployment of the Municipal Civilization Office. Qiongjie County organized a volunteer service activity with the theme of "Learning from Lei Feng visits poor families to help warm their hearts", sending rice, quilts and other comfort items to the pair of families to help the pair object to clean the courtyard health. Volunteers have expressed that they should strive to practice the core socialist values and learn from Comrade Lei Feng with practical actions. The picture shows longzi County organizing volunteers for free free medical treatment for the masses. Longzi County and the resident troops carried out the "take the lead in practicing the Spirit of Lei Feng, the military and local forces jointly build warm Longzi" activity. Volunteers and troops walked into the people's homes to clean clothes, housework, indoor and courtyards for the elderly. Volunteers were also organized to carry out free clinic activities in Baekatang Village, Ridang Town, so that farmers and herdsmen can enjoy medical and health services nearby. The picture shows volunteers organizing volunteers to help people clean their clothes. Zacang County organized to Jiru Township Wulong Village to carry out learning from Lei Feng activities. Extensive theory to preach, health clinic, legal publicity, literature and art propaganda series of activities, to the masses on the civil code of the People's Republic of China, the Tibet autonomous region national unity progress model area to create regulations, etc., issued the New Year pictures calendar, hats, cups, umbrellas and other promotional materials, effectively improve the farmers and herdsmen masses of laws and regulations and the party's huimin policy awareness. Luoza County organized volunteers to provide volunteer services such as free medical treatment, free haircuts and policy explanation to the past people, distributed network security leaflets (manuals) and so on, and carried out questions and answers with prizes. Volunteers also took to the streets to clean up garbage, small advertisements and damaged slogans on both sides of the county's main road. The picture shows Qusong County carrying out volunteer service activities. Qusong County has organized a volunteer service activity with the theme of "Practicing the Spirit of Lei Feng and promoting the new wind of The Times". The young volunteers were enthusiastic and full of energy, with the spirit of "not afraid of dirty, not afraid of hardship, not afraid of tired", to clean up the flower beds, remove the "psoriasis" advertisements, and to concentrate on cleaning up the garbage around the National Unity Square. The Nyingchi Municipal Youth League Committee of Nyingchi City organized 35 young volunteers from the Western China Plan, young volunteers from the National Energy Group and the fifth branch of the Hydropower Bureau to carry out the volunteer service activity of "Practicing the Lei Feng Spirit and carrying forward the New Style of The Times". Activities take diversified project design, organize and implement the unification, personnel decentralization, volunteers will be divided into concentrated demonstration group, clean health services, civilized traffic persuasion group, propaganda group along the street, the volunteers according to the specific division of labor and task arrangement to Xiamen square, happiness village wetland park, double road community, Hong Kong road pearl river farmers market, urban traffic arteries intersection civilized traffic guide, legal policy propaganda, health cleaning and other volunteer service activities. In Xiamen Square, a centralized demonstration and publicity site was set up to publicize laws and policies, fire protection knowledge, garbage classification, the protection of minors, the prevention of juvenile crimes, and distribute water cups, umbrellas, handbags, notebooks and other daily necessities.1. Volunteers at the Pearl River Farmers' Market on Hong Kong Road have publicized and distributed legal knowledge and epidemic prevention and control materials to the street citizens through the form of mobile publicity.2. Publicize and distribute legal knowledge, epidemic prevention and control related information at the intersection of Deji Road and Bayi Street, and cooperate with the traffic police as volunteer advocates to guide the citizens and passing vehicles to consciously obey the traffic rules, yield to pedestrians, and travel in a civilized way. In the Wetland Park of Xingfu Community and Shuangyong Road streets, volunteers clean up and classify the white garbage, solid waste and other waste around the park and in the community streets.4 Cleaning garbage and classification processing prosperous city card if district on March 5, qamdo city card if district organization youth staff, community residents' committees staff and other more than 200 people of 15 volunteer service, deep card if district community streets, organize "carry forward the new era of lei feng spirit to build beautiful and harmonious new card if" lei feng anniversary theme activities. At the beginning of the New Year, all the volunteers actively went from street to street, went deep into the streets, shops, and communities to carry out volunteer publicity activities, and distributed brochures to the masses on learning about Lei Feng, work safety, ethnic unity, and minor protection. The citizens responded positively and learned all kinds of relevant knowledge in the volunteers' enthusiastic explanation. The Lei Feng Memorial Day special volunteer publicity activity distributed more than 6,000 brochures, carried forward the Lei Feng spirit and the spirit of volunteer service, to create a harmonious, positive, lively, happy, festive and peaceful festival atmosphere for the broad masses of the people. Nagqu city Naqu pku joint Naqu city fire detachment, city WeiJianWei, seni district youth corps committee, color district traffic police brigade residents during the New Year traffic safety, fire knowledge and safety education knowledge, for the cold, stomach, anti-inflammatory and other kinds of drugs more than 300 copies, toilet paper, gargle cup and other articles for daily use more than 200 copies, minor protection, fire control, traffic and other brochures more than 100 copies. The Naqu Municipal Project Office organized volunteers from the Western Project to carry out publicity on the rule of law and epidemic prevention and control knowledge in passenger stations and Zhejiang Mall, and distributed more than 200 brochures on juvenile crime prevention and drug control and AIDS prevention, and more than 200 copies of epidemic prevention masks. Youth civilization number unit combined with the actual, hanging banners or electronic screens, to "serve the people, the masses are satisfied" as the standard, implement the improvement of the style, pay close attention to the implementation of the requirements, and further highlight the youth civilization number to learn from Lei Feng atmosphere. The Youth Volunteers Association organized a voluntary blood donation activity with the theme of "Striving to be a pioneer of The Times and highlighting the volunteer feelings". On the afternoon of March 5th, Ali Youth League Local Committee contacted the Garggar County Youth League Committee to carry out the activity of "Warm sympathy to the Warm Winter" with the theme of "Practice the Lei Feng Spirit and Carry forward the New wind of The Times". First of all, Zhang Yunan, a volunteer from the Western China Project, was selected to explain self-care knowledge to 28 young students and their parents. Zhang Yunan with simple words, around the "identify danger signals away from dangerous areas", "resist network poison health online" network security education, "how to cultivate self prevention awareness" and so on three aspects for young students on the importance of nursing education, education to guide young students in their daily life to enhance self prevention awareness in learning. At the end of the event, on the occasion of the Tibetan New Year, the Ali Youth League local committee distributed 8 toys, 20 school bags and other school supplies to 28 young students. The majority of young volunteers have conveyed the positive energy of youth to the society, contributed their own "light" and "heat" to Tibet, demonstrated the civilization of Tibetan youth with practical actions, carried forward the spirit of Lei Feng in the new era, and created a strong atmosphere of learning from the spirit of Lei Feng.

2022-03-07	Zhang Wenling) In the just-ended winter vacation, the Yunnan Provincial Party Committee of the Communist Youth League launched the "double reduction" of "Tuantuan companionship and help" activity, attracting more than 3,500 returning college students to participate. They cooperated with the grassroots youth league organizations throughout the province to carry out a variety of activities, providing services for more than 15,800 children, and spent a happy holiday with them. In order to help the implementation of the "double reduction" work and improve the quality and level of after-school services, the Yunnan Provincial Committee of the Communist Youth League, Yunnan Provincial Department of Education and Yunnan Provincial Youth Working Committee launched the work of "helping the 'double reduction' " for students in compulsory education in January this year. During the winter vacation, the activity received a positive response from returning college students who studied in more than 100 universities across the country. Tens of thousands of people signed up, and finally more than 3,500 returning college students attended. They make use of their own learning and strengths, for the primary and middle school students to carry out youth self-protection, culture and science, safety education, outward bound training and other content of the courses. They also moved the classroom outdoors, and led the children to visit the fire brigade, village history room, red education base, museum and other places, to gain knowledge in the experience and practice. It is worth mentioning that, according to the principle of "territorial management, nearby", many local grass-roots youth league organizations will be "group to accompany" activities arranged in the grass-roots front line, which greatly facilitates the participation of rural college students and primary and middle school students. Xishuangbanna Jinghong City, Jinuo Mountain Township, 32 kilometers away from the township government. In winter vacation, the village no interest classes, remedial classes, home adults are farmers, busy working in the fields, returning college students to learn painting, correctly wear masks, fire escape and other safety knowledge, clean up the garbage in the village, meng mine village, returning college students to share learning experience, about "the wonderful world outside". They divide the children into homework tutoring, extracurricular reading, puzzle groups so that every child can learn. The "Tuan Tuan and group company" activity was highly evaluated by the parents. After attending the activity, the children were in a positive mood, and they finished their homework more seriously and were more willing to communicate with their parents. In particular, rural children encounter academic problems during the holidays but have no tutoring. Some children are addicted to their mobile phones and are not interested in learning, so they get some help. College students who participated in the activity also felt that they had gained more, believing that they had improved their sense of social responsibility, increased their understanding of their hometown, deepened their understanding of professional knowledge, and realized their own shortcomings in practice. The relevant person in charge of Yunnan Provincial Party Committee of the Youth League introduced that in the process of organizing activities, the grass-roots Youth League Committee made the work project, project list, grasped the time node, defined the objectives and tasks, and improved the organization and service power of the normal volunteer service and left-behind children care and service activities. By telling about the changes of hometown, excavating the traditional culture of hometown, learning the traditional crafts of hometown, and making suggestions for the development of hometown, the enthusiasm of returning college students to contribute to the development of their hometown is stimulated, and a number of talented talents have emerged, enriching the young talent pool for promoting rural revitalization.

2022-03-05	On March 3rd, the sixth plenary (expanded) meeting of the 14th Guangdong Provincial Committee of the Communist Youth League was held in Guangzhou. The meeting summarized the work of the Guangdong Communist Youth League in 2021, made arrangements for the key work in 2022, deliberated and approved the Key Work Points of the Guangdong Communist Youth League in 2022 and other documents, and co-elected the Standing Committee, member and alternate members of the Guangdong Provincial Committee of the Communist Youth League. Relevant leaders of Guangdong province attended the meeting and spoke. The meeting pointed out that we should effectively strengthen the political consciousness and mission of the youth work in Guangdong in the new era. We need to focus on the overall situation of the central task, focus on the "1 + 1 + 1 + 9" work plan, and unite and lead young people to make achievements. We must firmly establish the concept of giving priority to youth development, care for them with our heart and affection, and constantly promote their development and guide them to become talents. We should comprehensively strengthen the self-construction of the Communist Youth League to provide a strong guarantee for the youth work. The meeting stressed that the convening of the Party's 20th Congress is a major event in the political life of the Party and the country, and at the new starting point of the centenary of the league, all the work of the Guangdong Communist Youth League should be carried out around this overall situation. We should comprehensively strengthen and promote Party leadership and Party building in the Communist Youth League system to a new level. We must vigorously govern the "key minority" with a new atmosphere. We need to assume new responsibilities and make new actions in preventing and defuse political risks in the Communist Youth League and youth work. To share the party in the overall situation of the service center to do new achievements. New progress needs to be made in serving the healthy growth of young people and solving the problem of urgency and sorrow. In accordance with the relevant provisions of the Constitution of the Communist Youth League of China and the Regulations on the Election of Local Organizations of the Communist Youth League of China, the meeting confirmed the discharge of 17 full-time league cadres transferred from their posts, with 5 alternate members as members, and 2 standing members, 12 members and 2 alternate members were co-elected. After the completion of the discharge and co-election, 63 members of the 14th Provincial Communist Youth League Committee have 63 members (including 11 standing Committee members) and 2 alternate members. The conference also awarded awards to the 2021 advanced units of Guangdong Communist Youth League and the units that won the excellent research award. At the meeting, some cities and university youth League committees were arranged to exchange experience. Chen Weihao, deputy secretary general of Guangdong Provincial Party Committee, Chi Zhixiong, Secretary of Guangdong Provincial Party Committee, Liang Junda, Liu Zicai, and Zhong Zhenyu, deputy leader of the discipline inspection group of the Provincial Commission for Discipline Inspection Commission in the Provincial Federation of Trade Unions attended the meeting. Members and alternate members of the 14th Guangdong Provincial League Committee attended the meeting, including members and alternate members of the 18th Guangdong Communist Youth League, and some cities set up TV and telephone sub-venues to attend the meeting.

2022-03-02	The sixth Plenary Session of the 15th Shanghai Municipal Committee of the Communist Youth League was held. Correspondent for the picture of China youth network Beijing on March 2 (reporter Zhang Jianwei, correspondent Zhang Chen) recently, the Communist Youth League Shanghai Committee of the 15th session of the sixth plenary meeting was held. Municipal Standing Committee, Organization Department Minister Hu Wenrong attended the meeting and spoke. Municipal Party Committee Deputy Secretary-General Yan Shuang attended the meeting. Secretary of the Municipal Committee of the Municipal Standing Committee to make a work report, and a summary of the meeting. Meeting that in 2021, under the strong leadership of the municipal party committee, youth league, Shanghai earnestly study and implement the party's 19th and 19th plenary session spirit, further implement the general secretary xi about the important thoughts of youth work, unity led the city youth league organizations at all levels, earnestly started and responsible, deepen reform and innovation, maintain and enhance political nature, advanced nature, mass, improve, leading force, organization, service and the overall situation contribution, promote the work made new progress. In 2022, The Shanghai Communist Youth League should meet, study, publicize and implement the Party's 20th Congress as the main line, With the celebration of the 100th anniversary of the founding of the league and the 12th Party Congress of the city, Focusing on the fundamental plan of having successors to the Party's cause, To perform the basic duties of leading and gathering youth, organizing and mobilizing youth, and contacting and serving youth, Focus on improving the leading, organizational, service and overall contribution, deepen the reforms in an all-round way, Comprehensively and strictly governing the regiment, Constantly maintain and enhance the political nature, advanced nature, mass nature, Unite and lead the youth league members to always follow the Party and forge ahead on a new journey, To help Shanghai to create new miracles and show a new atmosphere, Take practical actions to welcome the party's 20th victory held. The meeting stressed that comprehensively enforcing strict governance of the Communist Youth League is a basic strategic measure in the Communist Youth League's work pattern of "three forces, once and two guarantees". We need to thoroughly implement the important instructions and instructions of General Secretary Xi Jinping, and earnestly implement the requirements of comprehensively and strictly governing the Party and the Shanghai Communist Youth League in the whole process and in all aspects of the work of the Shanghai Communist Youth League, so as to provide a solid organizational guarantee for better performing our duties and missions. Within the system framework of the central Committee of the Communist Youth League to comprehensively and strictly govern the league, based on the characteristics of Shanghai in the city and the reality of the Communist Youth League, we should face up to the problems, targeted rectification, take advantage of the situation, deepen the reform, and strive to promote the work of the Shanghai Communist Youth League to a comprehensive level and show a new atmosphere. The meeting called for the work of the Shanghai Communist Youth League this year to meet, study, publicize and implement the Party's 20th National Congress as the main line, consciously take the lead, set a good example, and fully demonstrate the due style of the Party's assistant and reserve army. We must firmly uphold the political soul of the Party's overall leadership, firmly fight with the Party, shoulder the political mission of educating the Party bravely, and always maintain the political sobriety of keeping up with the Party. We should accurately focus on the overall situation of the main line of work, adhere to the bottom line thinking, do a good job of positive publicity, strengthen the characteristics and highlights, strengthen the weak links, and effectively improve the contribution of the overall situation. We must strictly manage the ranks of cadres of the Communist Youth League, and in accordance with General Secretary Xi Jinping's important requirements for young officials, start from the integrity, study, work, fearless, respect, and study, comprehensively improve all abilities and abilities, and earnestly shoulder the mission and trust entrusted by the Party. The meeting deliberated and approved the Confirmation Case of the Sixth Plenary Session of the 15th Shanghai Municipal Committee of the Communist Youth League on the removal of the Shanghai Communist Youth League Committee, the Implementation Opinions of the Shanghai Municipal Party Committee on Implementing the Implementation Outline of Comprehensively Strictly Governing the Communist Youth League in the New Era, and the Key Points of the Work of the Shanghai Communist Youth League in 2022. TuanWeiWei, in Shanghai central committee, the municipal youth league organization is mainly responsible for comrades, the municipal commission for discipline inspection committee in municipal federation of trade unions organ discipline watchdog group leader, pku organs all cadres (including temporary cadres), the directly affiliated units is mainly responsible for comrades, relevant WeiBan bureau (company) group is responsible for comrades to attend, attend the meeting. In order to expand the organizational coverage and work coverage of the league, and strengthen the contact services for new business forms and new employment groups, the conference also specially invited non-public enterprises and online new economy enterprises to attend the meeting, and listen to their opinions and suggestions on the work of the Shanghai Communist Youth League.

2022-03-02	Group shaanxi provincial party committee to carry out the concentrated study research deployment group of the communist youth league shaanxi provincial party committee theory study group concentrated study deeply grasp the provincial party committee meeting on group work instructions spirit wei correspondent zhang) recently, the communist youth league shaanxi provincial party committee held theoretical study group (expand) learning meeting, further study grasp the recent provincial party committee standing committee meeting about group work instructions spirit, study the deployment to carry out the work. Members of the provincial Communist Youth League Committee, department-level cadres and departments, the main principals attended the study, deputy secretary of the Provincial Communist Youth League Committee Xu Yongsheng presided over the meeting. At the meeting, each team member around the implementation of the provincial party committee instructions requirements, combined with the actual work of exchange and discussion. Conference pointed out that the provincial party committee standing committee to listen to under work report, study under work, fully embodies the provincial party committee leadership of under work and attach importance to, the provincial communist youth league organization to study and implement the provincial party committee meeting related instructions spirit as an important opportunity, continue to strengthen the party's leadership of the communist youth league, strengthen the ideological and political lead, efforts to promote the communist youth league overall contribution. The meeting stressed that to grasp the political, highlight the publicity and implement the sixth plenary session of the 19 and the party's 20 spirit, in the important node, facing the key crowd, using "youth learning", youth branch carrier for theme activities, guide the cadres and youth members to understand "two established" profound meaning, make the new era of socialism with Chinese characteristics in the youth ear into the brain into the heart. We need to enhance our spirit, focus on helping the high-quality development of Shaanxi, strengthen the understanding of the overall situation of the party and government center, and guide and lead the youth league members to actively participate in economic and social development in key areas such as the original platform construction, rural revitalization, ecological and environmental protection, and epidemic prevention and control. We should steadily promote the reform, deepen the reform requirements, carefully organize the transition of the provincial Communist Youth League Committee, the provincial students 'Federation and the provincial youth working Committee, continue to promote the reform of the Communist Youth League, the Youth League, the Students' Federation, and the Young Pioneers to deepen the reform, and constantly consolidate and strengthen the political nature, advanced nature and mass nature. We need to improve self-improvement, pay close attention to the three main bodies of league cadres, league members, and league organizations, and comprehensively and strictly govern the Youth League, take the implementation of the medium-and long-term youth development plan as an important carrier, effectively strengthen the function of the Communist Youth League in connecting and serving the youth, and establish a good image of the Communist Youth League. The meeting stressed that the provincial communist youth league to the spirit of the sixth plenary session of the 19, xi jinping, general secretary to shan important speech important instructions spirit, the 13th session spirit and the upcoming party's 20, province 14th congress spirit propulsion, implementation, the work forward, to mobilize the youth play a bigger role in writing a new chapter of high quality development. At the meeting, we also conveyed the spirit of General Secretary Xi Jinping's speech at the 36th collective study of the Political Bureau of the CPC Central Committee, Liu Guozhong's speech at the provincial high-quality project promotion meeting and the concentrated commencement of key projects in the first quarter of 2022, and He Junke's speech at the deepening reform deployment meeting of the Central Committee of the Communist Youth League.

2022-02-28	Sichuan bright "Chongqing twins to the future" work brand the communist youth league show "Chongqing a qing twins to the future" work brand xin-xin wang) on February 25, the communist youth league of Sichuan provincial party committee, the communist youth league of Chongqing municipal party committee power Chongqing-Chongqing region shuangcheng economic circle construction of the communist youth league joint meeting for the third meeting, the meeting held in the form of video. Zhang Rong, secretary of Sichuan Province, presided over the meeting and delivered a speech. Liao Chuanjin, secretary of Chongqing City, attended the meeting and made a speech. The meeting deliberated and approved the Summary of the Work of Sichuan-Chongqing Communist Youth League to Help the Construction of Chengdu-Chongqing Shuangcheng Economic Circle in 2021, the List of Key Work of Sichuan-Chongqing Communist Youth League to Help the Construction of Chengyu Shuangcheng Economic Circle in 2022, and the Implementation Opinions of Sichuan-Chongqing Communist Youth League on Helping the Construction of Science and Technology Innovation Center with national influence. Chengdu Municipal Party Committee of the Communist Youth League, Yuzhong District Party Committee, Youth League Committee of Sichuan University and Youth League Committee of Chinese Academy of Sciences made exchanges and speeches on helping the construction of chengzhou-Chongqing Shuangcheng economic circle. The meeting decided to take "Sichuan and Chongqing family to the future" as the work brand of the Sichuan and Chongqing Communist Youth League. At the same time, the Communist Youth League of the two places will work with several units to build young scientific and technological talents exchange service stations in the Western Science City, build platforms for young scientific and technological talents and transformation of scientific research achievements, and provide precise services for young scientific and technological talents in employment and entrepreneurship, marriage, social integration, and growth incentive. Over the past two years since the construction of the Chengdu-Chongqing Shuangcheng Economic Circle, the Communist Youth League of Sichuan and Chongqing have met the other halfway to help build the Chengdu-Chongqing Shuangcheng Economic Circle, and carried out a series of work in strengthening the linkage and integration of work, promoting the exchange of cultural ideas, and serving the growth of young people. The Communist Youth League of the two places held a series of activities, such as the Youth Youth Culture Development Summit in the Shuangcheng Economic Circle, the Youth Entrepreneur Development Summit, the "Create Youth" Youth Innovation and Entrepreneurship Competition, and the "Sichuan-Chongqing River Xiaoqing" Volunteer Service Action to Protect the Yangtze River.

2022-02-28	Group Henan 15 second QuanWei (expand) meeting in Zhengzhou Henan 15 secondary QuanWei (expand) meeting held in Zhengzhou in the green newspaper network reporter zhi-Xi'an pan, intern hui-hui zhang) on February 23 to 25, the communist youth league in Henan province 15 second QuanWei (expand) meeting and Henan communist youth league "ability style construction year" activity training class held in Zhengzhou. In the group central committee, alternate members, Henan provincial party committee 15th committee, alternate members, not provincial committee or alternate members of the provincial committee (including Jiyuan demonstration area) youth corps committee is mainly responsible for comrades, some directly affiliated units is mainly responsible for comrades, part of the provincial university youth corps committee is mainly responsible for comrades, Henan provincial party committee departments and directly affiliated units responsible for comrades, a total of more than 80 people to attend the meeting. The meeting conveyed and learned the important instructions of the Standing Committee of the Henan Provincial Party Committee on the work of the masses, the spirit of the Henan Provincial Party Committee and the spirit of the Sixth Plenary Session of the 18th Communist Youth League, comprehensively summarized the work in 2021, and scientifically planned the work in 2022 by welcoming, learning, publicizing and implementing the 20th main line of the Party. Wang Dubo, deputy Secretary of the Henan Provincial Committee of the Communist Youth League, reported the work to the plenary session on behalf of the Standing Committee and summarized the meeting. The meeting stressed that to implement the work plan for 2022, we must adhere to the responsibility and practical work, and fully implement it through strong ability and forging style. The meeting deliberated and adopted the Working Rules of the Henan Provincial Committee of the Communist Youth League of China and the Resolution of the 15th Henan Provincial Committee of the Communist Youth League on Establishing a Special Committee.

2022-02-28	On February 24,25, the 14th session of the 14th session of the Communist Youth League was held in Nanning. Liu Xiaoming, deputy secretary of the Guangxi Zhuang Autonomous Region Party Committee, attended the meeting and delivered a speech. Liu Xiaoming fully affirmed the work of youth league organizations at all levels in Guangxi in 2021. He pointed out that youth league organizations at all levels in the region should focus on the overall goal of building a magnificent socialism with Chinese characteristics in a new era, and follow the goals and tasks set by the twelfth Party Congress of the autonomous region, unite and lead the young people to forge ahead and work hard, and demonstrate the role of the Party's assistant and reserve army with practical actions. We should be the "guide", "leader" and "close friend" of young people, guide them to deeply understand the decisive significance of the "two Establishment" through learning, thinking and practice, stimulate their innovation and creativity potential, promote their exchanges, and promote their participation in social governance. Focus on the "young people in Guangxi who love Guangxi", focus on key youth groups and emerging youth groups, and solve their problems. Mission organizations have become the force that young people can remember, find and rely on when they encounter difficulties. We must be a good "practitioner" of strictly governing the Youth League, strengthen the leadership of the Party, continue to deepen reform, strengthen self-improvement, and constantly enhance our organizational, leading, and service capabilities. Party committees at all levels should earnestly strengthen their leadership over the work of the youth and the Communist Youth League, strive to create favorable conditions for youth development, provide strong support for youth work, and constantly open up new prospects for youth work, so that young people can be better and more promising in Guangxi, and welcome the victory of the Party with outstanding achievements. Meeting thoroughly study and implement the party's 19 big and 19 all previous plenary session spirit, further implement the general secretary xi the important thoughts about youth work, earnestly study and implement the autonomous region twelfth party congress spirit and the sixth plenary session of the eighteenth spirit, summarized in 2021, the deployment of 2022 key work, examined and approved the "communist youth league in Guangxi district party committee about promoting the new era of comprehensive governing group implementation opinions" and other documents. Youth League Guangxi District Party Committee Secretary Meng Qipeng delegation Guangxi District Party Committee secretary team to make a work report to the plenum. Source: China Youth Daily, February 28,2022, edition of the 02nd edition

2022-02-28	The sixth plenary session of the 14th Hubei Provincial Committee of the League was held, Lei Yu) recently, The sixth plenary session of the 14th Hubei Provincial Committee of the Communist Youth League was held in Wuhan, Hubei Provincial Standing Committee of the Communist Party Committee, United Front Work Department Minister Erkenjiang Turahong attended the meeting and made a speech, Emphasizing the need to deeply study and practice General Secretary Xi Jinping's important thoughts on youth work, Closely related to meet the party's 20th and the 12th provincial Party Congress, Strengthening ideological and political guidance, Build the foundation of young people's faith, cultivate their ambition to become a strong country, and innovate the strategy of gathering young people, To guide the young people to unswervingly listen to and follow the Party, In Hubei "build the fulcrum, walk in the forefront, write a new chapter" journey to contribute to the youth strength. He pointed out that where the central task of the Party lies, the strength of the regiment should converge. To adhere to the service overall situation and service youth organic unity, effective combination, sincerely make friends with youth, patience to do the practical work for youth, support youth entrepreneurial innovation creation, actively help regional development layout implementation, strong province of science and technology construction, provincial governance modernization, for Hubei "start stabilization, compound yuan flat, continue wonderful". He called for promoting the comprehensive and strict governance of the Communist Youth League, strengthening self-construction under the guidance of the Party's political construction, guiding the cadres of the Communist Youth League to adhere to the "two establishment" and "two maintenance", enhance the ability to perform their duties, temper the excellent style of work, and build a "good, honest, practical and excellent" and clean organs. We should closely adhere to the "three characteristics" to eliminate the "four modernizations", promote the deepening of the reform of the Communist Youth League, improve the grass-roots organization system and service system of the League, and constantly enhance the cohesion and combat effectiveness of the league, so that the young people can feel that the Communist Youth League is around us. The meeting announced the 2021 Hubei Provincial advanced units of the Communist Youth League, Shiyan city, Yichang City, Xianning City, Wuhan Wuchang District, Suizhou Zengdu District, China Three Gorges Corporation Co., LTD., China Aerospace Sanjiang Group Co., LTD., Central China Normal University, the main leaders of the Youth League Committee at the meeting. Source: China Youth Daily, February 28,2022, edition of the 02nd edition

2022-02-28	Beijing Municipal Party Committee launched the work of the centenary of the advanced typical to stimulate grassroots vitality Jianwei correspondent, Li Cihang Ren Yi) to select the advanced collectives and individuals, better encourage the league organizations, league members, cadres at all levels based on their posts, solid work. Recently, the Beijing Municipal Party Committee of the Communist Youth League launched the selection and commendation of Beijing advanced organizations and advanced individuals for the 100th anniversary of the founding of the Communist Youth League. Previous Beijing "two red and two excellent" three years a review. The selection for the Beijing Municipal Committee of the Beijing Human Resources and Social Security Bureau, and the Beijing Municipal Committee agreed after the one-time special recognition. In order to ensure the continuity of the commendation work in the league, the Beijing "two red and two excellent" award classification is used. Considering the proportion of grassroots organizations, league members and cadres of the Beijing Communist Youth League, the total number of places in the selection has increased from 400 to 800, including 160 May Fourth Red Flag Youth League Committee and May Fourth Red Flag Youth League branches, and 240 outstanding Communist Youth League members and 240 outstanding Communist Youth League cadres. In this selection and commendation, the Communist Youth League District Committee can participate in the municipal May Fourth Red Flag Youth League Committee, young Pioneers counselors, youth affairs social workers can participate in the outstanding Communist Youth League cadres. In light of the actual work in Beijing, the selection and commendation of organizations and individuals who have participated in the COVID-19 prevention and control struggle, supported services for the Winter Olympics and Paralympic Games, and made outstanding achievements in county reform, and tilted to emerging areas. To implement the requirements of "the whole league focuses on the grassroots" and "comprehensively and strictly governing the league", organize the awards with the completion of the key work of the construction of grassroots organizations as the basic condition, and increase the requirements for the comprehensive evaluation of personal awards for the review and assessment. In strict recommendation, preliminary examination and review, the grass-roots unit shall declare the difference at 120%, and each sub-special committee shall conduct preliminary examination and report to the leading group of selection and commendation work for review and evaluation. The candidate list and the list of proposed commendation shall be publicized for no less than 5 working days each time.

2022-02-28	Jianwei) On February 25th, the 6th plenary (expanded) meeting of the 14th Fujian Provincial Committee of the Communist Youth League was held in Fuzhou. Meeting adhere to xi jinping, the new era of socialism with Chinese characteristics as guidance, thoroughly study and apply xi general secretary of the important thoughts of youth work and the spirit of the sixth plenary session of the 19, earnestly implement the 11th party congress of Fujian province and the sixth plenary session of the 18 work deployment, convey learning provincial party committee leadership instructions spirit, summarizes the work situation in 2021, the deployment of priorities in 2022. Xiao Huaxin, secretary of the Communist Youth League Committee, delegation the standing Committee to report to the plenary session. Conference pointed out that since the eighteenth congress, xi jinping, general secretary from the party's career, successor some strategic height, deeply grasp the party's youth work law, put forward a series of strategic, era, pioneering new argument new requirements, formed the important thought about youth work, to do the new era of Fujian communist youth league work pointed out the direction, provides the fundamental to follow. Youth league organizations at all levels in the province to earnestly to xi jinping general secretary important speech important instruction spirit as the general program always follow, adhering to the general secretary xi jinping in Fujian work created important ideas and major practice, strive to fully grasp, learn deep enlightenment, achieve mastery, to ensure that the communist youth league work moment does not deviate from the right direction. The meeting stressed that in 2022, the provincial youth league organizations at all levels to meet and study propaganda and implement the party's 20 as the main line, combined to celebrate the 100th anniversary of the provincial party committee and the central work deployment, in-depth implementation of "improve efficiency, improve efficiency, efficiency" action, earnestly, to bear, unite led the province broad member youth to comprehensively promote high quality development beyond, strive to compose a comprehensive construction of modern socialist country Fujian chapter contribution youth strength, take practical actions to meet the party's 20 victory. The meeting stressed the need to continue to deepen the ideological and political guidance of young people, to meet, study, publicize and implement the Party's 20th Congress as the main line, and carry out solid education and practice activities with the theme of "Welcome the 20th Congress, always follow the Party, and forge ahead on a new journey". We will make solid progress in the youth contribution initiative, further implement projects such as the "Challenge Cup", "Create Youth", "Young entrepreneurs visiting Fujian" and "syndicate cooperation", and do a solid job in the work of young talents such as the "Sailing Plan". We will further implement the youth exchange project between Fujian, Taiwan, Hong Kong and Macao, take the opportunity of the 20th anniversary of the Straits Youth Forum and the 10th anniversary of the Straits Youth Day to upgrade the 101 Taiwan youth entrepreneurship support plan, and deepen the "Four Hundred" exchange plan and the "Sea Tide Plan" for Fujian and Hong Kong youth, so as to help build demonstration zones for integrated development across the Taiwan Straits. We need to focus on the prominent problems of young people's "urgency, difficulties and worries", take the medium-and long-term youth development plan of Fujian Province, promote the introduction of more inclusive youth development policies, and constantly improve the professional and precise level of serving youth work. The meeting stressed that to adhere to the strict word pledge, strengthen their forging, always adhere to the overall leadership of the party, constantly improve political judgment, political understanding, political execution, unswervingly deepen the reform of the communist youth league, earnestly accomplish league organization construction, league cadre management, team management, to self revolutionary spirit further promote comprehensive governing group. The provincial cadres should earnestly implement the important requirements of General Secretary Xi Jinping, especially young cadres, earnestly achieve firm faith, absolute loyalty, hard to learn, down-to-earth, loyal to forgive, dare to do, dare to do, dare to fight, and strive to grasp the work well this year. Meeting deliberated and approved the communist youth league of Fujian provincial party committee about thoroughly study and implement the spirit of the sixth plenary session of the 19 earnestly implement the 11th party congress and the sixth plenary session of the deployment, unite the league youth to promote the development of high quality beyond strive to write a comprehensive construction of modern socialist country Fujian youth power resolution, the Fujian youth league in 2022, the communist youth league of Fujian province committee work rules, the communist youth league of Fujian province 146 plenary meeting about the provincial party committee member discharge confirmation case. During the meeting, also held the province 11th party congress spirit special guidance report. League, deputy secretary of the summary speech, provincial party committee deputy secretary Shao Mingsong, Li Wenjie, even Zhanji, Liu Anjuan, not youth league committee members of youth corps committee, provincial education league committee, provincial sasac league committee, Fujian financial league committee, league party committee departments and directly affiliated units main head to attend the meeting.

2022-02-27	On February 24,25), the seventh plenary (expanded) meeting of the seventh Hainan Provincial Committee was held in Haikou. The meeting conveyed the spirit of the Sixth Plenary Session of the 18th CPC Central Committee of the Communist Youth League, summarized the work in 2021, and deployed the key work tasks in 2022. The meeting deliberated and adopted the seventh Plenary (expanded) Meeting of the seventh Session of the Hainan Provincial Committee on the Confirmation of the discharge of the Members of the Hainan Provincial Committee (draft) and the seventh Plenary (expanded) Meeting of the seventh Session of the Communist Youth League Hainan Provincial Committee on the Adjustment of the Special Committee of the Hainan Provincial Committee of the Communist Youth League (draft). The meeting pointed out that the youth league organizations at all levels in the province should grasp the evaluation standards of the work of the Communist Youth League in the new period. Actively change the working ideas, constantly improve the communist Youth League contact youth system, serve the youth system, improve the interests of the youth system; actively build the youth League brand work, pay attention to the creativity of the grass-roots level, explore, refine and promote the good experience and good practices of the grass-roots youth work. The meeting required that the youth league organizations at all levels in the province should solve the problem of position shortage by "not owned, but used", "adapt to the youth, play a role", solve the problem of position activity, and "enter the network, use the network", to realize the position and activities where the youth are. The meeting stressed that the youth league organizations at all levels in the province should find their own unique perspective in the intervention of youth work, to act as the bridge and link between the youth and the government and society; to enhance the mass character; to enhance the attraction of the youth league organizations; to fully guide and support the young people and to mobilize the initiative of the young people. At the meeting, the main principals of 6 directly affiliated league organizations were selected to report on their work on behalf of the committee at the same level. Chen Yu, secretary of the Communist Youth League Hainan Provincial Party Committee, made a work report to the whole committee, and made a summary speech. Members and alternate members of the 18th Central Committee, members of the seventh Committee of the Hainan Provincial Committee, cadres above the deputy department level of the League, and heads of youth League organizations in 11 key parks of Hainan Free Trade Port attended the meeting.

2022-02-26	The communist youth league of Anhui province 14th eighth plenary session and deepen the comprehensive governing group will hold the communist youth league in Anhui province 14th eighth plenary session and deepen the comprehensive governing group will advance Beijing on February 26 (reporter Zhang Jianwei) on February 25, the communist youth league in Anhui province 14th eight plenary session and deepen the comprehensive governing group will be held in Hefei. The meeting followed the guidance of Xi Jinping Thought on Socialism with Chinese Characteristics for a New Era, Earnestly study and implement the guiding principles of the 19th CPC National Congress and the 19th CPC Plenary Session, To thoroughly implement General Secretary Xi Jinping's important thoughts on youth work, To convey the spirit of the provincial two sessions, the province to improve the work style for the private practical work for the enterprise excellent environment conference and the sixth plenary session of the 18th Communist Youth League Central Committee, To convey the spirit of the speech of Cheng Lihua, deputy secretary of the Provincial Party Committee, at the group organization research symposium, Deliberated and adopted the Confirmation Case of the Eighth Plenary Session of the 14th Anhui Provincial Party Committee of the Communist Youth League on the Cancellation of Members of the CPC Anhui Provincial Party Committee (draft) and the Resolution of the 14th Anhui Provincial Party Committee of the Communist Youth League on Adjusting the Establishment of the Special Committee (draft), We deliberated and studied the Key Points of the Work of the Anhui Communist Youth League in 2022 (Draft) and the Implementation Opinions of the Anhui Communist Youth League Anhui Provincial Party Committee on the Implementation of <The Implementation Outline of Comprehensively Strictly Governing the Anhui Youth League in the New Era> (Draft). Provincial Communist Youth League Party Committee related responsible comrades attended the meeting. The meeting stressed that it is necessary to deeply grasp the work situation, and effectively enhance the sense of direction, sense of mission and sense of urgency of the work of the provincial Communist Youth League. To meet the publicity and implementation of the Party's 20th plan, we will effectively improve our political position, grasp the changes in the situation facing the two overall interests, always strengthen our responsibilities, and pay attention to addressing new and major risks and challenges to ensure that risk prevention and control are in place. Focus on implementing the important requirements of strengthening "two persistence" and realizing "two bigger", deeply understand the strategic planning of modern and beautiful Anhui construction, focus on the province to build an influential "three places and one district" and other priorities, deeply plan the tactical ideas of "beautiful Anhui meritorious action"; focus on the provincial Party Committee to implement the important deployment to deepen the comprehensive strict Party governance, effectively improve the fighting ability of the provincial Communist Youth League service center. The meeting comprehensively analyzed and reviewed the implementation of the "Anhui Communist Youth League 3520 Action Plan" since last year, using data, cases and other forms, objectively evaluated the work achievements, in-depth investigation of the lack of problems. The meeting noted that, The overall thinking for our work in 2022 is to follow the guidance of Xi Jinping Thought on Socialism with Chinese Characteristics for a New Era, We will fully implement the guiding principles of the 19th CPC National Congress and the 19th CPC Plenary Session, To fully understand General Secretary Xi Jinping's important thoughts on youth work, Earnestly implement the series of important speeches and instructions made by General Secretary Xi Jinping to Anhui, Always firmly sincerely support the "two establishment", faithfully practice the "two maintenance" political consciousness, In mind "two overall interests", bear in mind "the big country", Carry forward the great spirit of party building, Highlight the party's 20th party celebration, To celebrate the 100th anniversary of the group, Fully implement the work arrangements of the Provincial Party Committee and the Communist Youth League Central Committee, Continue to thoroughly implement the "Action Plan of 3520 +" of the Provincial Communist Youth League, Unite and lead the majority of youth league members to work hard, earnestly practice not idle, Loyalty and due diligence, striving for the first place, To write a new chapter of modern and beautiful Anhui construction to contribute to the youth strength. The meeting required to focus on the heartfelt support of the "two established" and faithfully practice the "two maintenance", continue to implement the "political ability improvement action"; closely follow the party's 20 main line, continuously implement the "Red Gene Inheritance Action", highlight the construction of the comprehensive strict league work system, continue to implement the "youth organization gathering Action"; continuously implement the "Youth Development Promotion Action", enhance the work effectiveness, and continuously implement the "Beautiful Anhui Achievement Action". The meeting stressed the need to carry forward the spirit of self-revolution, promote the actual results of the comprehensive and strict governance of the league under the new situation, and provide a strong guarantee for the work of the provincial Communist Youth League and youth. The sixth Plenary session of the 19th CPC Central Committee will adhere to the self-revolution as an important historical experience of the party's centenary struggle, the provincial league organizations at all levels and the majority of league cadres should deeply study and fully understand. To further implement the important instructions of xi General Secretary and the CPC Central Committee, to the provincial party committee and the central set up political benchmarking, comprehensively carry forward the revolutionary spirit of self, set up more sealed political standards, working standards, ability standard, style standards to test the work, test their own construction, with more sealed spirit to meet the party's 20. The conference was held as a teleconference. In Hefei 14 league provincial party committee members to attend the meeting at the main venue, ShengJiWei supervisor committee in provincial federation of trade unions organ discipline watchdog group related comrades, Hefei municipal party committee team members and departments are mainly responsible for comrades, made league committee, provincial sasac league committee, mainly responsible for fat system, universities and enterprises is mainly responsible for comrades, youth league provincial party committee authority departments, directly affiliated units is mainly responsible for comrades in the main venue to attend the meeting. The provincial cities will set up sub-venues. Members of the 14th Provincial Communist Youth League Committee in Hefei will attend the meeting in the city. The main responsible members of the municipal Communist Youth League Committee and departments, and the provincial Communist Youth League Committee of universities and enterprises will attend the meeting in the municipal sub-venue in the city.

2022-02-25	"Youth Blessing See Together to the Future" was held in Fujian and Taiwan youth was held in Fuzhou. Xiao Huaxin, secretary of the Communist Youth League Committee and honorary chairman of the Provincial Youth Federation, attended the meeting and delivered a speech. Li Teng, deputy secretary of the Communist Youth League Committee, presided over the meeting. At the meeting, in Fujian and Taiwan green representatives combined with their own growth and development experience, share the experience of living in Fujian, around the enterprise development, recruitment employment, policy implementation, business support put forward suggestions, and on Taiwan green enterprises help "start" in the first quarter, promote the development of Fujian economy high quality. After listening carefully to the speeches, Xiao Huaxin said, The provincial Communist Youth League at all levels, the youth Federation organizations will further expand the exchange platform, With the Straits Youth Forum, the Straits Youth Festival and other activities as the carrier, To achieve non-stop communication, intensity and heat, Let the young people on both sides of the Taiwan Straits feel closer and feel deeper; Further improve the effectiveness of the service, Deepening the 101 Taiwan Youth Entrepreneurship Support Program, Taiwan College Students and young Social Workers in Fujian and other projects, Extensive soliciting of opinions and suggestions from Taiwan, youth and Taiwan students, To coordinate and promote the implementation of various policies to benefit Taiwan and youth; Further strengthen the incentive orientation, Expand the proportion of Taiwan and youth in the evaluation of "Fujian Youth May 4th Medal" and "Creative Youth" Innovation and Entrepreneurship Competition, Promote more Taiwan youth to join the youth enterprise associations at all levels, youth chambers of commerce and youth innovation associations, To better help Taiwan and youth to take root in Fujian and grow and develop. During the event, the participants also visited Fujian Hante Cloud Intelligent Technology Co., Ltd., Provincial Taiwan Youth Entrepreneurship Service Center, Taiwan Association Hall, and Straits Architects Home. It is reported that the 23 young people from Taiwan who attended the symposium are not only practitioners and entrepreneurs in the fields of digital economy, Marine economy, green economy and cultural and tourism economy, as well as high-tech young talents in the key areas of Fujian and Taiwan industrial cooperation, such as advanced equipment manufacturing and new materials.

2022-02-23	The 6th (expanded) Meeting of the 14th Qinghai Provincial Committee (expanded) Meeting of the 14th Qinghai Provincial Committee (expanded) meeting was held in Xining. The Qinghai Committee (expanded) meeting was held in Xining. Provincial Committee of the Standing Committee, Provincial Federation of Trade Unions Chairman Ma Jixiao attended the meeting and made a speech. Ma Jixiao fully affirmed the work of the provincial Communist Youth League in 2021 and put forward requirements for the work in 2022. He stressed that the provincial Party committee and the provincial government have always attached great importance to the work of the Communist Youth League, and the youth league organizations at all levels should fully implement the tasks and requirements with practical actions. To build ideological consensus to achieve great unity, To meet, study, publicize and implement the party's 20th and the 14th Provincial Party Congress work main line, Efforts to unite the province's youth closely around the party; Based on the service development to reflect the great role, Stick to the overall situation, Find the right job entry point, To organize and mobilize young people to make contributions in the practice of economic construction; We should focus on contact coverage to promote great alliance, Exploring the construction of a network organization system, To achieve the effective coverage of young people in all fields and at all social strata, Constantly gather together the wisdom and strength of the young people; To strengthen the self-construction and play a great function, Persistent efforts to promote the comprehensive and strict governance of the regiment, To give better full play to the political and social functions of the league, To promote the high-quality development of the work of the Communist Youth League in the new era; To promote coordinated development and build a large group, Improve the long-term mechanism for the "six Communist Party" construction, Actively carry forward, extensive and deep integration, Ensure the effective operation of the mechanism of coordination and the implementation of the collaborative work. The meeting heard the work report of the 14th Qinghai Provincial Standing Committee of the Communist Youth League and the annual work report of the cities and prefectures, discharged their duties and replaced members of the 14th Qinghai Provincial Committee of the Communist Youth League, and deliberated and approved the Implementation Plan of the Qinghai Provincial Party Committee of the Communist Youth League on Comprehensively Strictly Governing the Communist Youth League in the New Era.

2022-02-24	Fujian teenagers love eye classroom light environment improvement start red scarf light "eyes" color, Fujian youth love eye classroom light environment improvement launch ceremony held in Fuzhou JianWei correspondent Huang Peng) on February 21 morning, sponsored by the communist youth league of Fujian provincial party committee, Fujian province committee "red scarf light" eyes "-Fujian teenagers love eye classroom light environment improvement start and" for " photonic crystal eye lamp hope project donation ceremony held in Fuzhou jin an Rongbo primary school. Li Teng, deputy secretary of the Provincial Communist Youth League Committee, and relevant leaders of the provincial Youth Working Committee, provincial Hope Office and Fuzhou city and Jin'an District attended the activity. The ceremony kicked off in the young Pioneers' finger exercise "Little Eye Protection Talent". Love unit, Xiamen for the photoelectric technology co., LTD. Dr Ye Zhicheng speech, he said, the company has been to "long-term care teenagers bright eyes" for already, under the guidance of the provincial party committee, provincial less committee, the company will actively participate in the provincial classroom lighting upgrade public welfare projects, take concrete action to help protect adolescent vision health. At the ceremony, the Provincial Communist Youth League Committee and the Provincial Youth Working Committee awarded the "Youth Eye Protection Project Joint Construction Unit" and the Fuzhou Jin'an Rongbo Primary School the "Juvenile Myopia Prevention and Control Model School"; donated the photonic crystal eye protection lamp to the Provincial Youth Foundation; the Provincial Youth Working Committee issued the letter of appointment to the newly appointed red scarf counselors and young Pioneers volunteers. After the ceremony, Xue Ying, a volunteer worker of provincial Young Pioneers and deputy chief physician of ophthalmology of provincial Hospital, walked into the demonstration classroom of the photon crystal eye protection lamp and gave a lecture entitled "Protect Vision, Stay away from myopia" for the young Pioneers. The light environment improvement plan of the youth eye love and environment protection classroom is one of the important contents of the practical projects implemented by the provincial Communist Youth League Committee and the Provincial Youth Working Committee. The next stage, Fujian province will continue to play a leading role of the provincial demonstration school, combined with the "June 1" children's day, the national eye day and other important time node, continue to carry out popular science education, promote science with eye knowledge, create science for teenagers with the growth environment, correct their bad behavior with the eyes, help them develop good habits with eyes.

2022-02-24	The 10th Plenary Session of the 14th Zhejiang Provincial Party Committee held the 10th Plenary session of reporter Jiang Yutong, correspondent Yang � S) From February 21 to 22, the 10th session of the 14th Zhejiang Communist Youth League (expanded) meeting was held in Hangzhou. He Libin, secretary of the Zhejiang Provincial Party Committee of the Communist Youth League, sent a delegation to the Provincial Standing Committee to make a work report to the plenum, urging him for guarding the "red roots", helping common prosperity, and delivering a new answer of "I write youth at the window" with a new atmosphere of comprehensive and strict governance of the Communist Youth League. The Zhejiang Communist Youth League will anchor the goal of "building a transformative Communist Youth League organization", Adhere to the digital reform of the Communist Youth League led by the reform of the construction of the cadres of the comprehensive and strict governance of the deepening of the "two grasp" the main tone, Implement the "three reshaping" concept and methods of reshaping the organizational foundation, reshaping the work system and reshaping the image of the team, Promote the youth big study big publicity big practice action, "I write youth in the window" Zhejiang youth hand in hand common prosperity pioneer action, Zhejiang Communist Youth League reform full-speed race run comprehensive promotion action, deepen the province's youth league cadres style construction three-year action "four actions" task arrangement, Focus on creating more recognizable work results. Since 2021, Zhejiang Communist Youth League continues to be at the top of the league, in the annual work evaluation; undertook the eighth "Chuang Youth" China Youth Innovation and Entrepreneurship Competition (Internet Special Competition), ranked first in the overall performance; the National Medium and Long-term Youth Development Plan 5 affirmed Zhejiang practices and achieved new results in youth growth; "Digital Children's Palace" and other four scenarios were connected to the "One Account S1 Major Application" of digital reform in Zhejiang Province, and "youth help' double reduction 'volunteer service" was included in the province's major reform project of "launching the whole body" in one province in 2022.

2022-02-21	The "Outstanding Fujian College Students" symposium of the Spring Festival held by the Provincial Students' Federation was held in Industrial Bank. Chen Yanbiao, deputy director of the Organization Department of the CPC Provincial Party Committee and director of the Veteran Cadres Bureau of the CPC Provincial Party Committee, and Xiao Huaxin, secretary of the CPC Communist Youth League Party Committee attended the symposium and delivered a speech. Lu Jiajin, secretary of the CPC Provincial Committee and chairman of Industrial Bank, delivered a speech. Representatives of Fujian students from 14 universities including Peking University and Tsinghua University attended the discussion. Chen Yanbiao pointed out in his speech that the province at all levels and the Communist Youth League organizations should thoroughly study and implement the spirit of the central and the provincial Party Committee talent work conference, and further enhance the sense of responsibility, mission and urgency to do a good job of young talent work. We need to make solid progress in key work, highlight political guidance, closely follow the overall situation of our work, adhere to integrity and innovation, and constantly improve the efficiency of serving the development of young talents. We must uphold the Party's overall leadership over the work of talents and form a strong joint force in doing the work of young talents well. Xiao Huaxin summarizes the 2021 communist youth league youth talent work, and from expanding college students internship "sail plan", continue to build Taiwan youth talent landing first home, deepen the province Fujian youth students strategic tracking training, actively promote young talent innovation entrepreneurship, to optimize the growth environment of young talents and so on five aspects, made the specific deployment of the work in 2022. At the meeting, 5 student representatives and 2 representative representatives of employing enterprises exchanged and spoke, and held the awarding ceremony of Fujian Provincial Youth Talent Growth Plan Scholarship and the awarding ceremony of Fujian Provincial College Student Internship Base. On the morning of the same day, more than 40 Fujian students from "double first-class" construction universities were organized to visit enterprises, visiting well-known enterprises such as Industrial Bank, Fuyao Glass, Guanjie Electronics, BOE, Netcom Network, Zhongjian Strait and so on, to promote their in-depth exchanges and contracts with enterprises to sign employment. Provincial party committee, deputy secretary of li chaired the symposium, societe generale party committee, chairman of the supervisory committee Jiang Yunming, provincial party committee organization department talent work director Chen Zhaoxun, provincial student union, the district youth league, pingtan comprehensive experimental area youth corps committee, made authority youth league committee, provincial education committee, provincial sasac league committee, Fujian financial league committee principal and related enterprises to participate in the symposium. This "Sailing Plan introduces talents into enterprises" activity is an innovative extension based on the summer "Sailing Plan". It aims to take advantage of the important opportunity for students to return home in the winter vacation, and the youth League committees at all levels will organize enterprises to provide a number of high-quality jobs, so as to establish channels for excellent Fujian students to return to Fujian for employment. So far, 464 companies have posted 5,887 jobs, and 4,817 students have signed up.

2022-02-21	Jianwei Correspondent A Shuyuan) On February 17, the fourth Standing Committee of the 11th Qinghai Youth Federation was held in Xining. The meeting thoroughly studied, publicized and implemented the spirit of the Sixth Plenary Session of the 19th CPC Central Committee and the spirit of the tenth and 11th Plenary sessions of the 13th CPC Provincial Party Committee, reviewed and summarized the main work in 2021, arranged the work tasks for 2022, and united the young people of all ethnic groups and all walks of life in the province to contribute their youth strength to the comprehensive construction of a modern socialist country in Qinghai. The Communist Youth League of Qinghai Provincial Party Committee Secretary Dong Yuyi attended the meeting and made a speech. The meeting stressed that we should focus on welcoming, learning, publicizing and implementing the Party's 20th Congress, extensively carry out the theme of "Welcome the 20th Congress, always follow the Party, and forge ahead on a new journey", and consolidate the common ideological foundation for the united struggle of young people of all ethnic groups and all walks of life in the province. We must firmly grasp the theme of extensively uniting young people of all ethnic groups and all walks of life, pay attention to young people of ethnic minorities, emerging fields and other youth groups, and constantly extend the work radius of the youth patriotic united front in the new era. We must focus on the central work of the Party and government, take the "one excellence", "building the" five demonstration provinces "," cultivating the "four economic forms", "and" eight new heights " as the center and basis for our work, and more consciously unite and lead young people to firmly follow the Party and forge ahead on a new journey. Based on the organizational positioning of the youth patriotic united front in the new era, we should improve the organizational system of the Youth League with the Communist Youth League as the core, the Youth League as the hub, and the youth associations as the close level, constantly enhance the universality and representativeness of the Youth League, and deepen the reform of the Youth League on the new journey. Dong Yuyi, secretary of the Provincial Communist Youth League Party Committee, pointed out in his speech that the second centenary goal is beckoning to the young people in the new era, and the great task requires extensive unity and extensive unity to create great achievements. The youth federation at all levels in the province should strive on building a youth league organization with clear guidance, strong leadership, vitality, extensive representatives and obvious role, and build a team with excellent political integrity, moral character, excellent performance of duties and ability. Province youth league to give full play to the core hub in the organization system, to promote the construction of the broadest possible youth united front, active service party and government center work, actively participate in the communist youth league work pattern, strengthen the youth league team construction, improve the working mechanism, build a more open youth league work system and other aspects work to a new level, unity condensed the youth of all ethnic groups from all walks of life in the province to realize the great rejuvenation of the Chinese nation Chinese dream writing every youth splendid chapter. At the meeting, the relevant personnel matters were deliberated and approved. Solandeji, deputy secretary of the Qinghai Provincial Communist Youth League, was elected as the chairman of the Provincial Youth Federation. Qinghai Youth New Media Association was recruited as a member group, and Li Qiang, secretary general of the Provincial Youth Federation, made a work report. Leaders from all walks of life, representatives of vice-chairmen and representatives of the Standing Committee respectively reported on their work in 2021, and commended the outstanding sectors and outstanding members in performing their duties in 2021. Summer Cang Dan-zeng Jiumei, E Chongrong, Wang Honglun, Xue Ting, Long Zhoucaijia, vice chairman of the Provincial Youth Federation, attended the meeting. More than 100 members of the National Youth Federation, standing Committee members and outstanding members of the Provincial Youth Federation attended the meeting.

2022-02-20	League of Henan provincial party committee to carry out a number of "youth guard peace Spring Festival" activities of Henan provincial party committee to carry out a number of "youth guard peace Spring Festival" theme activities JianWei correspondent Wei Wenjie) during the Spring Festival, the Henan provincial party committee organization to mobilize the provincial youth members of powerforce and commandos, actively participate in the "six prevent six promote" special operation, solid theme activities, give full play to the communist youth league organization, lead, service "three force synergy" role, for the construction of a higher level of peace in Henan, the rule of law in Henan youth strength. Province disputes during the Spring Festival screening resolve special action deployment, the first convey the meeting spirit, set up a special class, selected Zhengzhou erqi district, xinan county, Xi'an county, 10 long-term youth development planning, the county communist youth league reform pilot county (city, area), 100 good work foundation the first league secretary in village deployment related work, held the provincial communist youth league video conference, a comprehensive deployment. Online, through the online youth league branch, facing the province issued the province broad youth members, the Spring Festival such initiative, mobilize to be in community service volunteer youth, physical and mental development of healthy youth, carry forward the era of healthy youth, neighborhood help enthusiastic youth, respect law-abiding civilized youth. Henan Province 12355 Service Desk has launched a series of network literacy open classes, opened 12355 self-care small classes, law popularization small classes, and local youth league organizations online to launch new media products such as winter vacation safety guidelines. Offline, more than 30,000 copies of publicity products such as yuxiaotuan Youth self-care manual, youth law popularization brochure, youth peace "ten want ten No" calendar posters, and group talk religious pocket books have been distributed to guide teenagers to improve their awareness of safety and self-care and enjoy a healthy New Year. At the same time, the Provincial Communist Youth League Committee led the establishment of a number of volunteer service teams, focusing on returning college students, and organized the COVID-19 epidemic prevention and control youth volunteer service team to build a youth volunteer service team for themed activities, to timely absorb and supplement personnel and strengthen their working strength. We have set up 3,473 youth volunteer service teams and recruited 83,684 young volunteers. A stepped psychological service team of more than 300 operators + psychological counselors + crisis interveners + psychological supervisors has been set up. Relying on the 12355 youth service desk in Henan Province, psychological counseling is online and offline for teenagers and people in need. Set up the league cadres volunteer service team, full-time and part-time cadres at all levels of the league, the first league branch secretary to take the lead to the village (community) line to participate in the relevant activities, focusing on the youth "anxious difficult worry hope" problem, through sending warmth, send knowledge, send health, send services, constantly improve the work ability of the masses, temper the style. In order to ensure the theme activities smoothly, youth league provincial party committee to establish flat work scheduling mechanism, guide the epidemic prevention and control of youth volunteer service on the basis of the local epidemic prevention and control work, on the theme activities of youth volunteer service, direct scheduling first league secretary, 12355 psychological counselors, youth affairs social workers, young entrepreneurs and other kinds of social forces, resources will sink grassroots. Spend 200,000 yuan to support and encourage grassroots league organizations to carry out theme activities to help "six prevention and six promotion"; guide league organizations at all levels to coordinate resources to provide guarantee in raising love materials and volunteer purchase of insurance. While improving the activity guarantee mechanism, the youth league organizations at all levels in the province have extensively carried out five types of volunteer services, including assisting in household investigation, mental health services, legal publicity and education, care for teenagers in need, and self-care education for teenagers. According to incomplete statistics, during the Spring Festival, the provincial youth league organizations at all levels in the rule of law propaganda more than 1300 times, covering more than 120000 people, since nursing education more than 2800 times, covering more than 120000 people, love left-behind children, caused by plague trapped teenagers difficult youth group activities more than 3000 times, covering more than 50000 people, mental health services more than 2800 times, covering nearly 60000 people, activities covering community (village) more than 23000.

2022-02-18	Qinghai Provincial Committee held a special conference on propaganda ideological and cultural work Qinghai Provincial Committee held a special meeting on propaganda ideological and cultural work Jianwei correspondent A Shuyuan) recently, the League Qinghai Provincial Committee held a special meeting on propaganda ideological and cultural work. Dong Yuyi, secretary of the Provincial Communist Youth League Committee, attended the meeting and made a speech. All the staff of the Publicity Department and information Center of the Provincial Communist Youth League Committee attended the meeting and spoke one by one. Meeting thoroughly study and apply xi general secretary of the important thought of propaganda and ideological work, earnestly study and implement the national, the provincial propaganda minister conference spirit, convey the learning league of Qinghai communist youth league about instructions spirit and 2022 propaganda and ideological work video conference spirit, 2021 national communist youth league new media summit spirit, summarizes the 2021 the provincial communist youth league propaganda, ideological and cultural work, analyzes the problems existing in the work, to completes the 2022 propaganda ideological and cultural work has put forward clear ideas and requirements. Dong Yuyi in affirming the provincial communist youth league propaganda ideological front achievements, points out that in 2022 is the party's 20, the provincial communist youth league propaganda and ideological and cultural front to jinping new era of socialism with Chinese characteristics as guidance, firmly grasp the flag, morale, new people, culture, the mission of the main line, combining the 100th anniversary of the youth ideological and political lead the task, guide the youth always follow the party, forge ahead new journey, strive to compose a comprehensive construction of a modern socialist country in Qinghai chapter. Dong Yuyi stressed that the propaganda and ideological front of the provincial youth league should adhere to the integrity and innovation, build consensus, earnestly unify the thought and action into the decision and deployment of the CPC Central Committee, the provincial Party Committee and the Central Committee of the Communist Youth League, and focus on creating new contributions in educating the Party and serving the overall situation.

2022-02-18	Beijing, February 18 (reporter Zhang Jianwei, correspondent Wu Yan of the 19th CPC Central Committee) In order to earnestly implement the spirit of the sixth Plenary session of the 19th CPC Central Committee, The Heilongjiang Provincial Party Committee of the Communist Youth League further promoted the practice of party history learning and education of "I do practical things for the youth", During the New Year's Day and the Spring Festival, Organized and mobilized the province at all levels, young volunteers to carry out the Communist Youth League to send warm "youth warm current action", For left-behind children in rural areas, express delivery youth, and college students volunteers who serve our province, To carry out academic counseling, psychological counseling, care and sympathy and other services, It has carried out 367 activities in the province, Caring for more than 21,000 people, The total amount of assistance funds is more than 2.85 million yuan. The Communist Youth League sends warm activities. Correspondent provided pictures to play an exemplary and leading role, Integrate resources to participate in the provincial Youth League committee team members led the team to go deep into the community, schools, Visited 109 left-behind children, poor households and community workers, A total assistance fund of 41,000 yuan; At the same time, we will actively integrate social resources, For 5,000 left-behind children in the province, they distributed school bags, insulated lunch boxes and other life and learning love bags, Mobilize the provincial Youth Federation to raise more than 500 love gift packages for left-behind children; Jointly with the provincial Sports Bureau to carry out two youth ice and snow fitness sports competitions, Organized 100 left-behind children to participate in free skiing teaching, snow football and other fun ice and snow sports; The provincial Communist Youth League working team sent more than 40,000 yuan worth of study and daily necessities to 60 left-behind children in Hongguang Village, And by organizing game interactions, Close up the relationship with left-behind children, Let the children feel the warm company in the winter. The Communist Youth League sends warm activities. Relevant leaders of the provincial Communist Youth League committee and the provincial Postal Administration visited the express delivery enterprises, and presented 50 "Courier boys" representatives with rice, soybean oil and other gifts worth 15,000 yuan. At the same time, the party members of the organization and the "Courier boy" worked together to sort and carry the express packages, to help them complete the day's sorting task, and to experience the courage and spirit of the grass-roots people and the frontline staff to bear hardships and overcome difficulties in the volunteer service. The Communist Youth League sends warm activities. Heilongjiang province hope project donation center, provincial youth development foundation joint 6 cities (ground) 27 youth league "youth warm hope project love" activities, called on social caring people to raise 371,900 yuan, docking 3000 love materials, more than 5700 left-behind children; in-depth "warm childlike innocence with children" public service project, organize 576 college students volunteers to provide care for more than 1000 left-behind children. The Communist Youth League sends warm activities. Correspondent for figure actively play Spring Festival work leading group member unit responsibilities, combined with the current COVID-19 epidemic prevention and control situation, to mobilize the provincial youth league organizations at all levels safe and orderly good volunteer service Spring Festival "warm winter", in the airport, railway station, passenger terminal, 418 volunteer service positions, accumulated 1216 service volunteers. Give full play to the advantages of practical education, carry out volunteer service activities. Youth league organizations at all levels in the province actively give full play to the advantages of volunteer service and practical education, organize and mobilize all institutions, enterprises, youth associations, youth volunteer organizations to carry out in-depth volunteer service care actions. Group Harbin municipal party committee joint youth league, city green enterprise association visit designated village, community workers, policemen and community poor families, for they sent to nearly 50000 yuan worth of rice flour oil sympathy items, the city youth league organizations at all levels donated sympathy supplies more than 150000 yuan, to help left-behind children, difficult students to solve the practical difficulties in life and study. League Mudanjiang municipal party committee called on the city's communist youth league organizations, city youth federation at all levels, city youth entrepreneurs association, city youth civilization collective, youth social organizations such as sympathy care activities, during the New Year's day, Spring Festival, visiting difficult youth volunteers, epidemic prevention volunteers, helping poor, community workers, etc., solve the practical difficulties in life and study, the city's communist youth league to send warm action "youth warm" 32 games. Tuan Hegang Municipal Party Committee, the city less working committee joint love enterprises for more than 60 college students volunteers and young pioneers representatives of life, learning gifts, to carry out public welfare classes, discussion exchanges, winter camp and other activities, the city carried out a total of 26 "youth warm current action", covering 786 young people, and other groups, a total of more than 118,000 yuan. Tuanhuzhong District Committee recruited 14 returning college students to pair with left-behind children to provide professional assistance, safety education and mental health activities; Mohe City, Jiagqi District and Xinlin District organized young volunteers to carry out volunteer service activities of information consultation, information verification, passenger transfer and baggage handling at the station. Yichun Municipal Committee organized Municipal Youth Federation and Municipal Youth Entrepreneurs Association to carry out public welfare viewing activities for 20 left-behind children, and distributed food, toys and stationery packages for 48 young underage children, and launched "Snow Challenge" for 1741 students and left-behind children. The Qiqihar Municipal Party Committee organized the Municipal Youth Chamber of Commerce to distribute more than 100,000 yuan worth of rice, noodles and oil to 109 families, and went into 4 poor families to send living materials and condolence money to them. The city has carried out 44 sympathy and care activities. TuanJixi Municipal Party Committee and Municipal Youth Volunteers Association held 10 online public welfare classes, and nearly 500 students in the city participated in online classroom learning. TuanJiamusi Municipal Party Committee, together with the Municipal Youth Enterprise Association, visited community workers, raised more than 80,000 yuan, and distributed 400 cotton clothes to community workers, sanitation workers and families in need and other groups. Daqing Tuan Zhaoyuan County Party Committee distributed cotton clothes, cotton hats, scarves and other warm clothes to 100 students; Daqing Datong District Committee conducted online student class activities around academic guidance, family companionship, quality expansion and other contents, and granted 80,000 yuan to 40 poor students.

2022-02-18	Beijing: "my dream flying dream" aerospace cloud camp successfully held Beijing: "my dream flying dream" aerospace cloud camp successfully held, China youth network Beijing on February 18 (reporter Zhang Jianwei, correspondent, Sun Shihao Ren Wenhui), by the Beijing municipal party committee, hope project Beijing development center, Beijing youth development foundation, Beijing aerospace museum, Beijing wisdom and education technology co., LTD., to undertake "my dream flying dream" aerospace cloud camp group special activities online. Before the activity began, the Rights and Interests Department of the Beijing Youth League Committee of the Communist Youth League publicized the youth of the whole city, and hoped that the project and the Beijing Youth Foundation communicated closely with the activity support units, to prepare for the successful holding of the activity. The picture shows watching the course. The online activity is divided into three links, In the "Curator takes you to the cloud exploration hall" stage, In the footsteps of the Korean Army curator of the Beijing Aviation and Aerospace Museum, "Into" the Beijing Aerospace Museum, Taking the course of human flight and flying dreams as clues, Visit the museum boutique exhibition items and precious physical objects, Learning aerospace science and technology knowledge and scientific principles; During the "Flying Dream of a Sheet of Paper" stage, Students follow the teacher to learn the aircraft structure and flight principles, And to put the theory into practice, Enlighten your hands with paper, Fly and go free with both hands, Fun paper plane takes off with everyone's dreams; In the "little aircraft designer" phase, Students use the common materials around them, Designed and produced a "aircraft" independently, By coloring the reinforcement and explaining the instructions, Show your own ideas boldly. In the final lian wheat interaction, hundreds of students enthusiastically showed their results, a delicate, special planes appeared in the studio. The picture shows the design work and the description. The activity attracted more than 2000 primary and middle school students to participate online, and more than 1000 students submitted videos or pictures, which won unanimous praise from parents and students. In the next step, the organizers of the Communist Youth League Beijing Municipal Party Committee, the Beijing Youth Development Foundation and other activities will organize and evaluate the works of the students who take part in the activities, and reward the students who have won the excellent works.

2022-02-16	Beijing: 12777 volunteers with "the most beautiful smile" for the games escort Beijing: 12777 city volunteers with "the most beautiful smile" for the games built wei correspondent Liu Da Yang wei Wang Xin) to ensure the snow traffic safety and convenience, since February 12, the Beijing municipal committee of Beijing volunteer service federation, widely mobilize city volunteers to participate in snow deicing volunteer service activities. To carry out the "early prevention, full preparation, careful deployment, rapid response, coordination, decisive disposal" related work requirements, guide the Beijing municipal TuanOuWei, district hub volunteer organization designated personnel responsible for the regional task, to mobilize urban volunteers to participate in the key areas, key sections of snow shovel ice work. The "Actively Participate in snow sweeping and deicing City Volunteer Service Proposal" was issued to the majority of city volunteers, and at the same time, to strengthen the reporting of snow sweeping and ice shoveling work information and typical deeds, increase the publicity of typical cases, and encourage and drive more volunteers to participate in snow clearing and ice shoveling. Priority should be given to the snow removal and ice removal work of "five districts", "four lines", "and three surrounding areas". Chaoyang District, Haidian District, Shijingshan District, Shijingshan District and Yanqing District, as the Winter Olympic venues, have also mobilized all forces to form volunteer efforts to ensure the audience to organize the evacuation of urban volunteer service work. Up to now, a total of 624 volunteer service organizations in the city have participated in snow removal, traffic guidance, urban operation and other related volunteer service activities, with a total of 12,777 urban volunteers and a service time of 26,143 hours. The 758 Winter Olympic city volunteer service stations operate in an orderly manner. The city volunteers stick to their posts without fear of the cold, and escort the Winter Olympics with the "the most beautiful smile".

2022-02-15	Ningxia authority cadres political ability and professional ability promotion project reading class group Ningxia district party committee held cadres political ability and professional ability promotion project reading class held cadres political ability and professional ability promotion project reading class, through expert teaching, communication discussion, promote cadres practice internal work, improve accomplishment, enhance ability, for this year work good article, open bureau, good step lay solid foundation. With reading class for "charge", heart cohesion drum energy, continue to pay special attention to the spirit of the sixth plenary session of the 19th, the autonomous region party committee of the 14th plenary session of the in-depth study and implementation, the quasi political direction, enhancing political loyalty, enhance political ability, strengthen the political bear, firmly defend the "two established", resolutely do "two maintenance". To pragmatic in the annual work, to further improve the political judgment, political understanding, political execution, the absolute loyalty reflected in the daunting task of political bear and action consciously, fully pays special attention to the communist youth league in the rural revitalization work, steadily advancing the reform of the communist youth league grassroots, community youth action national reform pilot projects, solid normalized epidemic prevention and control of volunteer service, comprehensively promote governing group. The reading class invited Dr. Song Pingwei, a teacher of the Party school of the autonomous region, a member of the Central Committee of the Communist Youth League and the youth teaching group of the autonomous region, to give lectures on "improving the political ability of leading cadres". Around "for the comprehensive governing party, deepening comprehensive governing group" theme, the cadres focus on learning xi general secretary in the 19th session of the central commission for discipline inspection six times the important speech spirit, comrade Chen Runer in the autonomous region commission for discipline inspection twelve six plenary meeting speech spirit, comrade He Junke in the communist youth league of the sixth plenary session of the eighteenth first plenary meeting theme report "to self revolutionary spirit deepening comprehensive governing group", etc., watched the special documentary. Eleven cadres spoke randomly, combined with the reality of learning, harvest and actual work, talked about feelings and experience, deepened understanding and built consensus in the collision of ideas, and became more firm in the belief of working with the Party and fighting with the Party. Mr.He Bao, invited from the Cadre Supervision Department of the Organization Department of the Autonomous Region Party Committee, to explain the personal report of leading cadres. Liu Shengxiang, deputy Secretary of the Party Committee of the Youth League Committee of the Autonomous Region, explained in detail how to grasp the party building work of the branch. At the same time, the government cadres have thoroughly studied and planned the work of the Ningxia Communist Youth League in 2022 in the way of party branch discussion and centralized listening of the secretary team. Four comrades took to the platform to share their experiences on "how young cadres can improve their political and professional ability". In the reading class, the Ningxia Youth Development Service Center, the newly named civilized unit of the district government, was awarded, and the advanced collective and individuals of the 2021 Ningxia District Committee were commended. Next, the autonomous region youth league committee will consciously to the comprehensive governing party, deepening comprehensive governing group, the "two established" into blood, put into action, unity, lead the youth, around the center, the service general situation, faithfully implement the central and autonomous region major policy decisions, with honors to meet the party's 20th and autonomous region thirteenth congress victory.

2022-02-14	Gansu Province 13th Committee (expanded) meeting held the 13th Committee (expanded) meeting held Fuchun) February 13, the 13th Committee (expanded) meeting held in Lanzhou, the meeting summarized the work of the provincial Communist Youth League last year, this year's work for research deployment. Gansu Provincial Party Committee Standing Committee, Executive Vice Governor Cheng Xiaobo attended the meeting and made a speech. Cheng Xiaobo, on behalf of the provincial Party committee and the provincial government, affirmed the work of the provincial Communist Youth League last year. He pointed out that this year is a year of special importance in the new era and the new journey, and the Communist Youth League organization should focus on welcoming the publicity and implementation of the Party's 20th work main line, and unite and lead the young people to create a good political and social atmosphere. Cheng Xiaobo made four suggestions on the work of the provincial Communist Youth League this year: first, to highlight the political construction, comprehensively strengthen the leadership of the Party; second, to improve the political standing, enhance the sense of responsibility and mission; three to closely follow the theme, enhance targeted effectiveness; four to adhere to strict standards, and constantly strengthen self-construction. Dong Anning, secretary of the Gansu Provincial Communist Youth League Committee, made arrangements for implementing the instructions and requirements of the Provincial Party Committee and implementing the key tasks of the Gansu Communist Youth League throughout the year. The meeting also conveyed and learned the spirit of the sixth Plenary Session of the 18th CPC Central Committee of the Communist Youth League, deliberated and passed the relevant proposals, and commended the advanced units of the work last year. Source: China Youth Daily, February 14,2022, edition of 02

2022-02-12	Our brother and grandpa live in another room. Our greatest wish is to have our own room. Now our wish has come true, with a clean and beautiful room, there are many volunteers uncles and aunts care for us."For Xiao Rong and Xiaodong, 13-year-old twins in Xianggong Street, Hedong District, Linyi City, Shandong Province, the" Hope House " is the starting point for their dreams. The "Hope Cabin" child care project, launched by the Provincial Communist Youth League Committee, was included in the first key livelihood projects of "I do practical things for the people" in 2021. Up to now, the project has raised more than 210 million yuan of public welfare funds, and 15,162 cabins have been built or under construction, and helped more than 15,000 children in difficulties, becoming a typical example of promoting the transformation and upgrading of the Hope Project nationwide. In these hope cabins, clean walls, clean floors, brand new desks... refresh the old impression of children in distress accommodation. As early as June 2020, the Provincial Communist Youth League Committee, the Provincial Youth Federation and the Provincial Youth Development Foundation jointly launched the "Hope House" child care project. For children in difficulties aged 8 to 14 who have no independent living and learning environment, relying on the original housing to create an independent learning and living space, and supporting caring volunteers to pair up to promote children's good habits and self-reliance ability. At the beginning of the project, The Shandong Communist Youth League has made great efforts to integrate social resources, By establishing a project cooperation mechanism led by the league organization and the participation of social forces, Cultivate a relatively mature construction and execution team, Establish a reliable and stable partner and a practical and feasible working mechanism: broaden the "Internet +" fundraising publicity channels, In-depth cooperation with Internet platforms such as "Tencent Charity", "Alipay Charity" and "Sina Micro Charity", Online fundraising of 118 million yuan of love funds; Explore the process reengineering of public welfare undertakings, Open up the cooperation path of "youth league organization + social organization", Building a good ecology of "foundation + social organization", Youth league organizations at all levels have established close and good cooperative relations with more than 170 social organizations in the province; During the project implementation process, Youth league organizations at all levels strive to tell cottage stories well, Pass on the positive and optimistic spirit of the assisted children, Transfer donation units, caring people selfless dedication, generosity to the poor touching good deeds."Really want to pull up a child from the poor family, built cabin alone is not enough, must effectively follow up volunteer service more effort, always adhere to the specialization, institutionalization, normalization, the volunteer follow-up service as the project in the second half, realize from 'cabin refresh' to'the growth of the spirit of color."Said Liu Tiandong, the provincial party secretary. From "campus" to "outside", from "single" student "to" comprehensive development ", from" pratt & whitney "to" more accurate "exploration and practice," hope hut "become Shandong communist youth league power poverty engines, power comprehensive well-off, but also in the" two have three security ", Shandong communist youth league deep youth demand, practical service youth growth demonstration project."House" hope "children care project will continue to expand the construction coverage, expand the scope of support object, pay more attention to the second half management, strict" hope hut "construction quality, pay special attention to the built" hope hut "maintenance, improve the level of volunteer pair support, organize assisted children summer camp, winter camp, normalized to provide family company, psychological counseling, academic counseling, feel the city" 5 + X " services. At the same time, to help the development of teenagers along the Yellow River, this year, the Shandong Communist Youth League plans to build 1,000 "hope cabins" for the nine provinces (autonomous regions) along the Yellow River. Increase support for the nine cities in our province, establish pair support mechanism for backward cities and counties to benefit more children along the Yellow River. In serving major national strategy, build 200 "hope cabins" to Xinjiang and Qinghai in 2021. Focusing on the public welfare undertakings of poverty reduction in countries along the "Belt and Road" routes, we will cooperate with the overseas institutions of central enterprises to create an overseas version of the "Hope House" and make Shandong contribution to people-to-people connectivity.

2022-02-10	Ren Mingchao, secretary of Hainan Province, On the morning of February 8th, Chen Zhiyuan, deputy Secretary of the Youth League, came to Haikou Bus Passenger Terminal and Haikou East Railway Station to visit the young volunteers of the Spring Festival travel rush on duty. It is reported that Hainan province has served 23,538 hours during the 2022 Spring Festival travel rush. Chen yu highly praised and sincerely thanked the volunteers for their hard work, and reminded them to strengthen their protection and protect their own safety. According to reports, during the 2022 Spring Festival travel rush, 18 cities and counties in Hainan province have launched a "warm Winter Action" with the theme of "youth volunteer travel, warm the New Year". By the end of January, youth league organizations at all levels in Hainan province had set up 48 service stations in train stations, passenger stations, terminals, airports and other traffic stations with frequent returnees turnover, with 3,629 volunteers participating.

2022-02-09	Jiangxi qing commandos grassroots security work in Jiangxi Jiangxi qing commandos security work during the Spring Festival, China youth network Beijing on February 9 (reporter Zhang Jianwei, correspondent Wang Weijian) during the Spring Festival, led by Jiangxi provincial party committee of Jiangxi qing commandos in Nanchang railway station, Nanchang west railway station, teng wang pavilion scenic area security work, grassroots line service masses, with practical actions to help people spend peace and peace, festival and healthy festival. Carry out the "warm winter action" service at the station. At Nanchang Railway Station and Nanchang West Railway Station, the team members are on duty and on emergency patrol inside and outside the station to deal with possible accidents at any time. At the same time, in response to the call of the Provincial Communist Youth League Committee and the Provincial Youth Volunteers Association, we actively participated in the "Warm Winter Action" during the Spring Festival travel rush, providing passengers with temperature detection, consultation and guidance and other services. Emergency preparedness near the drama viewing station. In the scenic area, team members of each place with a large flow of people guide tourists, or take emergency standby near the drama viewing station, or patrol the scenic area, to provide guarantee for the safety of tourists."The Spring Festival I am on the job, have I please rest assured!"Since the first day of the Chinese New Year, Ganqing commandos have carried out safety work in three places including the railway station for eight consecutive days.

2022-02-09	The Hunan provincial party committee held care express delivery youth "warm bee" group Hunan provincial party committee held care express take-out youth "warm bee" net Beijing on February 9 (reporter Zhang Jianwei, correspondent Peng Jiameng) recently, the Hunan provincial party committee in Changsha Meituan special site held care delivery delivery youth "warm bee action", for riders send "fu" word, send Spring Festival couplets, for 31 family consolation money difficult rider. Relevant leaders of the Provincial Youth League Committee, the Provincial Young Calligraphers Association and the Meituan Public Affairs Department attended the meeting. During the activity, Jiang Shen, a model rider of Meituan food delivery who caught the thief, shared his story, " I just did what a young man in Changsha and a retired soldier should do. Whether being a soldier or delivering food, they all serve the people. As long as people who make money with their own hands, they are worthy of respect. Since the outbreak of the "" epidemic, food delivery riders, like medical workers and grassroots cadres and other frontline workers, have been at the forefront of the fight against the epidemic, making great contributions to the fight against the epidemic and ensuring people's livelihood."Xia Binhua, deputy secretary of the Provincial Communist Youth League Committee, suggested that delivery platforms should strengthen the main responsibility of enterprises for production safety, explore more effective service paths, and safeguard the dignity and professional rights and interests of delivery riders. Youth league organizations at all levels in Hunan will also extend their working arms, innovate forms, strengthen leading and condensing young food delivery riders, and guide the riders to be new young people who cultivate their feelings of home and country and adhere to the worship of virtue and good. The activity also invited the Hunan Young Calligraphers Association to question "fu" and send Spring Festival couplets to the delivery riders. In recent years, the Communist Youth League at all levels in Hunan has regularly organized the "Express Delivery Youth Service Month" activity, specifically implemented the "7 Care" specific actions, invested a total of 4.5 million yuan in three years, carried out more than 500 sympathy activities, and helped more than 2,300 people. In the near future, the Hunan Provincial Communist Youth League Committee will also work with the Hunan Postal Administration to visit 30 couriers from poor families. In the next step, Hunan Provincial Party Committee of the Communist Youth League will continue to expand the coverage of the organization and work, establish more youth league organizations among the youth groups of express delivery industry, and select the young express delivery models among the "green" brands such as "Youth Civilization creation", "upward and Good Youth" and "Excellent Communist Youth League Member".

2022-02-08	Guangdong over thousands of college graduates volunteers in rural revitalization of a line over thousands of college graduates volunteers in rural revitalization of a line wei correspondent YueQing) in 2021, the communist youth league committee of Guangdong province, Guangdong party committee agriculture, Guangdong development and reform commission, Guangdong education department, Guangdong province, Guangdong departments, human resources social security hall, Guangdong agriculture hall launched the "Guangdong college graduates volunteer service rural revitalization action". After the recruitment announcement of the project was issued, the majority of college graduates warmly responded, a total of 7,515 people registered through the "i volunteer" system, and finally selected more than 1,000 college graduates volunteers, all as members of the work team, to participate in the rural revitalization work. Zhou Yangcheng (first from right) went to Guiling Town ecological grape manor to investigate the planting situation of featured agricultural products. After several struggles, Han Ting, the Party member volunteer, decided to give up a job in a state-owned enterprise in Guangzhou and resolutely returned to her hometown, Bachi Town, Pingyuan County, Meizhou City, to become a rural revitalization volunteer, because this was a wish buried in her heart for many years. When she was in junior high school, what Han Ting was most looking forward to was the college student volunteers to come to Bachi Town to volunteer teaching, who had brought Han Ting an endless yearning for the outside world."And now I have seen the outside world, and I want to bring what I saw and learned outside, back to my home town. The "" answered the call, duty-bound."With the dream of grassroots work and the passion of building his hometown, Zhou Yangcheng, who returned from his master's study, came to Guiling Town, Jiedong District, Jieyang City, expecting his youth in this fertile soil," to realize the value in the work, and strive to make common prosperity take the lead in Guiling."Since seeing people in difficulties since childhood, Zhou Yangcheng always expects to provide help within his capacity. Now, after finishing a visit to the marginal poor families, he said with a smile, " Now I can do something to make them happy and warm."From the volunteer pre-job training, Yunfu Youth League Municipal Committee Que Han on the frequent communication with volunteers, he said, the young volunteers strive to serve as the town to help the village team" three hands a book ", that is, small expert, small helper, small assistant and small secretary, heart with love to do little things. In the first batch of more than 1,000 volunteers of the Volunteer Service rural Revitalization Initiative, the proportion of which 73 percent had a bachelor's degree or above and 27 percent were a junior college. According to the arrangement, the volunteers' grassroots services involve rural planning and industrial development, agricultural products e-commerce promotion, agricultural technology promotion, policy and law publicity, rural culture construction, rural ecological civilization construction, rural education, rural public services, grassroots party and league organization construction and other work. Long Huiying, a native of Yao nationality, has similar wishes."We Yao girls have been taught to have a good embroidery, but this craft is gradually declining."With the desire to revitalize Yao embroidery, after graduating from university, Long Huiying chose to return to her hometown, Liannan Yao Autonomous County, Qingyuan City, to become a volunteer in Zhen Help Town. Volunteers swear the "Volunteer Rural Revitalization Action". Long Huiying's dream is to gradually promote the establishment of the Yao embroidery workshop in the village with the conditions, organize the yao embroidery craftsmen, and do regular training for the young people in the village. Now she teaches herself e-commerce operation, hoping to find opportunities to further spread and develop Yao embroidery culture through the form of e-commerce. According to the relevant person in charge of the Guangdong Provincial Committee of the Communist Youth League, the recruitment scale of the "Volunteer Service Rural Revitalization Action" in the next three years has been clear: 4,000 people in 2022,5,000 in 2023, and 5,000 in 2024. The 2022 recruitment plan for Guangdong Province has been written into the newly announced 10 practical matters concerning people's livelihood in Guangdong Province in 2022.

2022-02-08	Shanghai: Parent-child interaction "menu" allows children to gain happiness with more opportunities to communicate with their small partners and parents."Lian Anqi, a seventh-grade young pioneer of Shanghai Fengxian, took the lead to set up a mixed-age team of the building group during the winter vacation, and did two exercises at home and participated in the community micro-update. The first winter vacation after the "double reduction" came as promised. How to make the students who get rid of all kinds of missed lessons have a safe and substantial winter vacation has become one of the recent work priorities of the Communist Youth League Shanghai Municipal Party Committee and the Shanghai Municipal Youth Working Committee. The Shanghai Young Pioneers have combined with the work of the "15-minute Community Young Pioneers Happiness Circle", the Shanghai Young Pioneers have launched a series of parent-child interaction "menus", so that the young pioneers can gain happiness in the community life, professional experience, physical exercise and other activities."This year, my daughter does not have to attend remedial classes. She can play all her favorite activities. There are also young Pioneers organizations in the community, and there are many meaningful activities to participate in the door."Shanghai Putuo longevity Road street fourth grade young pioneer Wu Yiping's mother told reporters, in the past out of" do not want to let the child to lose at the starting line "idea, she" follow the crowd " to her daughter to register a training class, the child also had to study in the holidays. Now that the children have more opportunities to participate in the social practice at home, she also has a new understanding of their children. For Wu, the double reduction brings her the opportunity to show her talent and make more friends. In the past semester, she has participated in many performances and speech activities in the school and in the district. After the winter vacation began, the community became a new stage for her. Through the formation of the building group of mixed-age team, she led the students in the community to play their strengths and participate in the vaccination publicity. Wu Yiping's mother also served as a volunteer counselor, accompanied her children to participate in the community young Pioneers reconstruction corridor activities, " looking at the drawings to build bookshelves, beautify the walls, although the weather is cold, but the children are very enthusiastic, I am also very gratified."Wang Feifei, who lives in Minhang Kangcheng District, Shanghai, is the head of a public welfare organization. As the parent of the first grade child, she felt that after the" double reduction ", the children's life has returned to the source of happiness; the community young working committee also arranged a" red scarf nursery " on the roof of a residential building for the children to claim by the team."Children in the city do not know much about the growth process of vegetables. With this nursery, children have more opportunities to participate in social practice and popular science activities."After the winter vacation began, Wang Feifei also connected with the community youth working committee, with the young pioneers in the community to experience the garbage classification work. By interviewing cleaners, sanitation workers and residents, the children learned about the whole process of garbage sorting and wrote observation reports. With his spare time and career experience, Gong � r, a fifth-grade young pioneer in Yangpu, Shanghai, finally had his "own winter vacation" this year. Without having to go to cram school, he punched in the "home practice" every day: shopping daily supplies and ingredients to prepare lunch and helping his parents share the housework. In the Neighborhood Center of Jiangpu Road Street, he also participated in the community Young Pioneers parent-child picture book reading, firefighter professional experience and other activities."Under the arrangement of the neighborhood committee, the firefighters taught the children to operate the fire equipment, let them realize the difficulty of firefighters, but also learned how to deal with emergencies; the parent-child reading activities close to life, the children saw very touched."Gong � r's mother, Wang � B, felt that by participating in the community young Pioneers activities, his son had expanded his knowledge and understood a lot. In Shanghai municipal party committee, city, health emergency public welfare organizations also positive action, this winter vacation, they in "young pioneers happy circle" 15 minutes community, launched medical science classroom project, invited surgical experts for the young pioneers development stage of science operating room, explain the working principle of medical instruments, and set up the surgical instruments peeling eggs, surgical knot, let the young pioneers get "immersive" experience. Ms.Wang, a parent who took her son to the activity, thought it was a great experience. " It not only let the child know the daily work of a doctor, but also planted the seeds of ideal in his heart. He wrote in his composition, 'I want to take clinical medicine and be a doctor in the future'."In the camera, screen to find" happiness circle ", Xuhui District Lingyun Street seventh grade young pioneer Zhu Chen, this winter vacation finally have time to clock in" red scarf medal ": write Spring Festival couplets; according to the content of English textbooks; with mom and dad to play their own" invented "card games, read books... Ms.Zhu Chen's mother Chen feel," double reduction ", children less homework, parents have more time to accompany children grow up, more communication. Ms.Chen felt that improving the parent-child relationship would help ease parents 'anxiety about their children's learning performance. " On the past weekends, adults rested for his missed lessons, and time always clashed. Now the child is willing to share with us about the school and participate in activities with his community friends. We can feel his ambition in study, and encourage him to communicate more with his classmates and have a walk outside."During the winter vacation, parents can do many things together: open the" cute Shanghai "WeChat public account, into the audio area, then" cross "into the online young pioneers happiness circle, players can directly click learning" vision loss "," weight rise "," little care "," mind guardian "and other special content; parents click" cute baby parents grow camp ", can harvest" principal lecture camp "," parents "" parents good time " and other tips. These contents belong to the Shanghai Youth Working Committee and the "Parent-child Happy Time" audio class launched by Himalaya platform. The winter vacation screen is also very busy. Shanghai Youth League Shanghai Municipal Committee and Shanghai Youth Working Committee cooperated with Xiao Ying Star to launch the 2022 "Growth Carnival Screen Winter Camp" winter holiday special program, focusing on the characteristic practices and innovative experience of "15 minutes of community young Pioneers happiness circle" under the background of "double reduction", showing the happy life of the front-line young pioneers. Relying on the "Eagle Cup" Young Eagle Red scarf News commentator Challenge, Shanghai Youth Working Committee also encourages children not to be "lying flat" and consciously make a winter vacation plan to show their happy life when children stay in Shanghai during the Chinese New Year and winter vacation. So far, nearly 2,000 team members have uploaded and shared their happy times during their winter vacation. The Shanghai relevant person in charge of the Shanghai municipal committee, Shanghai less committee said, "15 minutes community young pioneers happiness circle" through the "double reduction" after the barriers between school, family and community, form inside and outside each complement, organic linkage, the young pioneers generally benefit the young pioneers social work new situation, both meet the "double reduction" policy after the young pioneers education demand, alleviate parents' education anxiety, also expand the young pioneers social growth space.�� source: China Youth Daily, February 08,2022

2022-02-04	Group of Henan provincial party committee to carry out the "youth guard peace Spring Festival" Henan provincial party committee to carry out the "youth guard peace Spring Festival" research wei) according to the province "six prevention six promote" special action requirements and Henan communist youth league "youth guard peace Spring Festival" theme activity deployment, recently, deputy secretary of Henan provincial party committee Wang Dubo to zhongmou han temple town research "youth guard peace Spring Festival" theme activities, understand home college students volunteer service work, visit condolences to the affected difficult teenagers. Wang Dubo came to the Party and Mass Service Center of Gucheng Village, Hansi Town, to learn more about the formation and role of the returning college students, and communicated with the returning college students. In the affected teenagers at home, for the children to bring rice, noodles, oil, down jacket warm package and teenagers care manual, safety education calendar, etc., during the research, with Zhengzhou municipal party committee, zhongmou county leaders about the work communication, he pointed out that youth league organizations at all levels should be in the theme activities for the breakthrough point, to organize rich and colorful rural cultural activities, focus on caring left-behind children, plague trapped teenagers difficult youth groups, to the party's care to teenagers, in the daunting show youth.it is reported, In order to thoroughly implement the spirit of the special action deployment meeting of the investigation and resolution of conflicts and disputes during the Spring Festival, The Provincial Communist Youth League Committee held a provincial video conference on January 26 to deploy the Henan Communist Youth League "youth guard the safe Spring Festival" theme activity, To organize and mobilize the youth league organizations at all levels and the general youth league members to play the role of new forces and commandos, Assist in the household investigation, To carry out mental health services, publicity and education on the rule of law, self-care education, caring for teenagers in need, and strengthen the management of league members, so far, The province has set up more than 2,560 service teams to report to the community (village) party branch, Establish a flat work scheduling mechanism, two-wing linkage mechanism, regular statistics and report mechanism, Promote the development of activities in an orderly manner.

2022-01-29	2022 Fujian and Taiwan youth words reunion symposium held in banyan "year" the 2022 Fujian youth words reunion symposium held in banyan JianWei correspondent Chen Chen) on January 26, in the three lane seven lane strait architect home, laughter, the sense of spring, young architects from both sides of the strait, teachers, doctors, social workers and entrepreneurs representatives together, to welcome the arrival of the year of the tiger. Affected by COVID-19 outbreak, this year there are many green choose to stay in Fujian New Year, to rich in Fujian Taiwan youth life, better warm heart, popularity, youth league at all levels in Fujian province, some universities combined with the actual location and green demand, visit condolences, family reunion, family dinner activities, for Taiwan to send New Year wishes, coordinate to solve practical difficulties, let green feel thick flavor and members on both sides of the warm. Youth representatives from both sides visited the exhibition of Taiwan young architects participating in Fujian Township construction, and learned about the situation of Taiwan youth actively participating in rural revitalization, flying their youth dreams and realizing their own value. At the symposium, they exchanged their experiences of study, employment, entrepreneurship, and life in Fujian, and their experience and feelings. Fujian Taiwan youth star "entrepreneurship", orange xiang travel development co., LTD., general manager of Zeng Zhiying said, as a representative of the twelfth session of the CPPCC five times, into the hall has a very sacred feeling, to promote the communication between architects on both sides of the future, to attract more Taiwan young architect team involved in Fujian township built township, for the rural revitalization of their own. Fujian youth may 4th medal winner, Fujian normal university academy of sports teacher Zhang Zhaorui said, to Fujian, deeply feel the concern of green, this year as the keynote speaker in the strait youth BBS share the story of employment entrepreneurship in Fujian, also organized a Fujian youth three basketball game, hope to help more Taiwan youth to the mainland, but also hope to be able to participate in the depth of the youth communication activities, promote the development of Fujian and Taiwan youth fusion. National Taiwan association vice CEO, Fuzhou Taiwan association, vice chairman Jane to letter, in recent years, Fuzhou Taiwan association is committed to help to Fujian employment, entrepreneurship, study of Taiwan green, "ground", promote cross-strait integration, hope that social aspects can provide more opportunities to experience local life, especially hope that universities can actively participate in promoting cross-strait youth integration, let more to banyan alone to study out of the campus, into the ground, let more youth are not only is "coming" more to "stay". After the symposium, the Secretariat of the Fujian Youth Federation distributed New Year gifts and New Year wishes to the representatives of Taiwan.

2022-01-29	Jianwei correspondent Liu Yong) On January 26, the Henan Communist Youth League "Youth guard safe Spring Festival" theme activity video conference was held in Zhengzhou. Wang Dubo, deputy secretary of the Provincial Communist Youth League Party Committee, presided over the meeting and made a speech, Wang Anlin, deputy secretary of the Communist Youth League, arranged relevant work, Wang Qiang, deputy secretary of the Provincial Communist Youth League Committee, conveyed the spirit of the special action to investigate and resolve conflicts and disputes during the Spring Festival, Wang Jiwei and Wang Haiping, deputy secretaries of the Communist Youth League Provincial Party Committee, attended the meeting, Relevant departments of the Provincial Communist Youth League Committee and directly affiliated units attended the meeting in the main venue, Main responsible comrades of the youth League committees of the provincial cities (demonstration areas), comrades in charge of safety construction, and main responsible comrades of relevant departments (units), More than 2,600 people who are in charge of the county (city, district) and township (city and sub-district) youth League committees, in accordance with the relevant requirements of the local epidemic prevention and control requirements, Join the conference through video link in each sub-venue. The meeting pointed out that the Spring Festival is a high incidence period of conflicts and disputes, and the investigation and resolution work is of great significance to creating a good environment for the people. In the process of carrying out thematic activities, all localities should strictly implement the requirements of the work plan, focus on the "six prevention and six promotion", closely adhere to the time node, extensively carry out social mobilization, set up a young volunteer team, investigate the ideological trends and practical difficulties of teenagers, and solve the problem of "urgent and anxious" according to law and regulations. At the meeting, the League Zhengzhou Party Committee, League Puyang Party Committee, League Luoyang Mengjin District Committee on the relevant work exchange speech. It is reported that in order to thoroughly implement the spirit of troubleshooting and resolving conflicts and disputes during the Spring Festival, in accordance with the relevant requirements of the "six prevention and six promotion" special action, the Provincial Communist Youth League Committee fully carried out the Henan Communist Youth League "youth to protect the safe Spring Festival" theme activities. By organizing and mobilizing youth league organizations at all levels, youth league members to play the role of fresh force and commandos, to assist in household investigation, carry out mental health services, legal publicity and education, youth self-care education, care for the youth groups in difficulties, strengthen the management of the league members and other activities.

2022-01-29	Around Yunnan group organizations to carry out the rural left-behind children care service activities in Yunnan youth league organizations to carry out the rural left-behind children care service activities Zhang Wenling) recently, around Yunnan communist youth league to village level to establish normalized work positions, gather resources and strength, continue to carry out the "childlike innocence harbor" care for rural left-behind children work project. In Qujing City, Yunnan Province, Tang Yuan, secretary of the Yunnan Provincial Committee of the Communist Youth League, and other Party cadres, youth League cadres and young volunteers, went to the Qujing Liangzi Village Group to visit the left-behind children here. At the same time, the youth league organizations in Yuxi, Wenshan, Baoshan and other places in Yunnan province organized love service teams to visit the families of left-behind children and send them books, schoolbags, pencil cases, colored pens and clothes. Some focus on "parental care" and "mobile phone addiction", let the children encourage and warm each other by playing games, telling stories, and lead the community to welcome the Winter Olympics and celebrate the Spring Festival; Some invite firefighters to the children's fire safety class, explained the use of fire extinguishers, and fire safety during the Spring Festival holiday, to help the children improve their awareness of fire prevention. The Youth Weixin County Party Committee selected six "child companion mothers" from kindergarten teachers, community women cadres and "surrogate mothers" in Longxi community to accompany the children to conduct rope skipping competitions, origami and drawing activities. With the efforts of the Lijiang Municipal Party Committee, the children's first "childlike innocence harbor" and a small library with more than 300 books were opened. Lijiang Youth Working Committee donated stationery and other supplies to the library and the children, and issued letters of appointment to "children with mother". TuanJinggu County Party Committee set up "childlike innocence harbor" service station in the county youth activity center, and organized returning college students to accompany the service station for holidays.

2022-01-25	Gansu "millions" entrepreneurial leading project power rural revitalization of Gansu province "millions" entrepreneurial talent selection activities and "rural revitalization of youth help farmers" youth innovation project competition award ceremony successfully held Beijing on January 25 (reporter Zhang Jianwei, correspondent Wei Yi) on January 23rd afternoon, by the Gansu provincial party committee, human resources and social security hall, central branch of Lanzhou "millions" entrepreneurial leading talent selection activities and "rural revitalization of the youth help farmers" youth entrepreneurial innovation project competition ceremony was successfully held in Lanzhou. It is a specific measure to implement the "Millions of Millions" entrepreneurship leading project of Gansu Province and the "Rural Revitalization Youth" youth entrepreneurship and innovation project competition to hold the "Millions of Millions" entrepreneurship leading project in Gansu Province, and an effective practice to promote the action of the "Rural Revitalization Youth Achievements" of the Communist Youth League. The successful holding of the competition has built a practical platform for serving the youth entrepreneurship and innovation, supporting the majority of young people in Gansu to make contributions to the new Gansu province, and showing the new achievements. We will encourage young people to devote themselves to entrepreneurship and innovation with the sense of opportunity for time, embrace entrepreneurship and innovation with indomitable faith, practice entrepreneurship and innovation with a down-to-earth spirit, pursue entrepreneurship and innovation with the ideal of pioneering cause, and contribute our youth to the realization of the Chinese dream of the great rejuvenation of the Chinese nation.site of activity. This competition has been through step by level selection and recommendation, provincial final selection, to the final provincial final selection, after nearly 2 months. The competition is divided into two tracks: entrepreneurship expert group and live broadcast talent group. After the preliminary registration and preliminary examination, a total of 218 participating projects (165 entrepreneurship talent group and 53 live broadcast talent group) were determined. Through the project review, there are 48 finalists (30 entrepreneurship expert groups and 18 live broadcast expert groups). Final on January 21, after fierce competition, determine the first prize 2 (entrepreneurial group and live talent group 1), second prize four (entrepreneurial group and live talent group 2), third prize 6 (entrepreneurial group and live talent group 3), each of 16 excellence (entrepreneurship group and live talent group each 8), another provincial "entrepreneurial talent" award 15. At the award ceremony, the youth entrepreneurship and innovation incubation cloud platform in Gansu Province was launched, 32 Chinese youth entrepreneurship mentors in the new phase were announced, and they were awarded appointment letters to 8 representatives on the spot. The three winners shared their entrepreneurial journey, which reflected the contemporary Longyuan youth dare to "try", "break", "fight", "create" entrepreneurial spirit, showing their "courage to take responsibility and be good at acting" spirit. Gansu provincial party committee organization department vice minister, provincial people club hall party secretary, director of li-ning zhou, Gansu province party committee secretary Dong Anning, Lanzhou central branch of the People's Bank of China party committee member, deputy governor Feng ZongJing related departments responsible for comrades and multiple industries in the province of entrepreneurial innovation entrepreneurs, investment and financing experts, participating project team representatives and news institutions media friends, investment institutions attended the award ceremony. The award ceremony was broadcast live through a new media platform, attracting more than 6.62 million viewers online.

2022-02-07	Guangdong "love together small bird's nest" holiday camp into Zhanjiang Guangdong "love together small nest" holiday camp activities into Zhanjiang wei correspondent YueQing), by the Guangdong party committee, Guangdong less working committee "love together" small bird's nest Guangdong red scarf fund care left-behind children holiday camp into Lijiang town, will be a across more than 400 kilometers of care to left-behind children, let the children in the young pioneers outside school practice warm for the winter. The Young Pioneers counselor explained the relationship between the party and the team to the children. With the melodious music of the Young Pioneers' song "We are the successors of communism" sounded, the holiday camp activities officially began. Guo Hua, deputy chief counselor of young Pioneers in Haizhu District, Guangzhou city, and deputy director of Guangdong Red Scarf Fund Management Committee, led 40 representatives of left-behind children to play an ice-breaking small game, and the children also gradually became active. Later, Ding Jinxing, director of the office of the Guangdong Football Center, led the children to have a football lesson on the pitch. Penalty kick, bouncing the ball, pass... students from the beginning did not dare to try to have to "grab the ball", laughter one after another. The children played football interactive with the football teacher. The activity also invited three science and technology teachers, including Li Zhibo, a national social sports instructor and a national referee of aviation model, to test a simulated aircraft and launch simulated rockets on site, giving left-behind children a vivid lesson."Five, four, three, two, one, launch!"The warm sun shines, Jishui Middle School on the green runway, 40 students surrounded the model, as the rocket lifted off smoothly, the playground sounded a cheer. An aviation class in a rural primary school sowed the seeds of space in the hearts of children. At the scene, the students took the rocket remote control, shouted the slogan and then pressed the button, the rocket instantly "sex" flew to the blue sky, " teacher, the rocket separation will hit people? How did the "" rocket slow down during the landing process?"The students have been asked questions. Children experience launching model airplane rockets."There is a ribbon in the rocket model that slows down the friction with the air."Science and technology teachers answered the students' questions one by one, and some students picked up a small book to write it down. At the end of the activity, the staff of the Guangdong Red Scarf Fund distributed school bags, stationery, thermos cups, air models and other gifts to the children, and sent the New Year blessing character.

2022-02-04	Guangdong "wings plan" Hong Kong, Macao and Taiwan internship students sympathy activities in Guangdong "wings plan" Hong Kong, Macao and Taiwan internship students sympathy activities held wei correspondent YueQing) recently, Guangdong overseas association, Guangdong province youth federation into the young accounting firm, sympathy "wings plan" Hong Kong, Macao and Taiwan internship college students, to intern representative shopping package and New Year wishes. More than 30 people, including Guangdong Overseas Association, Guangdong Youth Federation, Ernst & Young Accounting Firm leaders and interns from Hong Kong, Macao and Taiwan, attended the event. Send a Spring Festival gift package to interns from Hong Kong, Macao and Taiwan. Group of Guangdong provincial party committee for figure activities, Guangdong overseas association, Guangdong youth league leadership guest speech respectively, introduces the "wings plan" Hong Kong, Macao and Taiwan college students internship special action implementation, said will for Hong Kong, Macao and Taiwan youth in Guangdong learning, employment, innovation and life to create more favorable conditions, promote Guangdong Hong Kong, Macao and Taiwan youth comprehensive communication, depth blend, guide more Hong Kong, Macao and Taiwan youth in the bay area construction, into the national development. Representatives of interns from Hong Kong, Macao and Taiwan shared their internship experience, believing that the "Wings Spread Program" provided a good "window" for them to understand the cultural atmosphere of the mainland workplace, deepen their understanding of the economic and social development of the mainland, and strengthen their confidence and determination to integrate into the development of the Bay Area. Start the meeting site. Group of Guangdong provincial party committee for figure, "wings plan" Hong Kong, Macao and Taiwan college students internship special action by Guangdong provincial party committee, Guangdong federation of industry and commerce of Guangdong province, Guangdong, Guangdong federation launched in December 2020, innovation to "first clear demand, after development with post" mode, has been held three consecutive winter and summer vacation internship, attract 119 from 119 colleges and universities 1916 students signed up at home and abroad. The activity integrates nearly 4,000 high-quality internship positions in more than 1,300 institutions, and has promoted more than 1,300 people. In the return visit survey, the interns' satisfaction with the project exceeded 98%. At present, the "Wings Program" special action for college students from Hong Kong, Macao and Taiwan has become a youth internship program for Hong Kong, Macao and Taiwan with the largest number of participants, the widest coverage and the best youth reputation in China.

2022-02-07	Recently, the Henan Provincial Party Committee held a leading group of the Communist Party League held a special democratic life on Party history study and education. Wang Dubo, deputy secretary of the Provincial Communist Youth League Committee, presided over the meeting and made a concluding speech. Relevant comrades from the second circuit steering group of the provincial Discipline Inspection Committee and the provincial Commission for Discipline Inspection in the provincial Federation of Trade Unions attended the meeting for guidance. All the members of the provincial Youth League Committee attended the meeting, and responsible comrades of relevant departments attended the meeting as non-voting delegates. The meeting reported the rectification and implementation of the problems of the democratic life of the Provincial Communist Youth League Committee in 2020. Wang Dubo, deputy secretary of the Provincial Communist Youth League Committee, delegation the leadership of the provincial Party Committee of the harvest and experience of the party history learning and education, made a profound inspection and reflection, and put forward the next rectification measures. Subsequently, the team members one by one for individual examination and reflection, seriously carry out criticism and self-criticism. Next, the league will continue to pay special attention to the problem of the rectification implementation, guide the lower party organizations democratic life, persistent serious party life, the political life of a good democratic life of some effective practice applied to the daily political life, carry forward the fighting spirit, highlight the party spirit exercise, with good criticism and self-criticism weapons, improve political judgment, political understanding, political execution, to grasp the new stage of development, carry out the new development concept, build a new development pattern of political ability, bow into bureau, bear as, more brilliant for the new era of central plains make greater contribution.

2022-01-28	The Guangdong Provincial Party Committee of the youth League joined the Spring Festival. The 2022 "warm youth" caring for domestic service youth activity was held in Guangzhou. Through sympathy, communication, research and other forms, the activity understood the status quo of improving the quality and expansion of the domestic service industry, and cares about the development and current needs of the young people in the domestic service. At the scene, all the young representatives from the domestic service industry respectively told their personal stories and expressed their professional speeches and aspirations. During this period, Liang Junda, deputy secretary of the Guangdong Provincial Committee of the Communist Youth League and chairman of the Provincial Youth Federation, sent New Year wishes and condolence gift packages to the young representatives who are still serving on the front line of the festival, and presented flags to the newly established "Xiang Xiang Volunteer Service Team". According to the introduction, the "want to volunteer service team" is composed of 51 family housekeeper organization to stay in Guangzhou during the Spring Festival, will play the team around the Spring Festival, to provide public welfare housekeeping services for the elderly, left-behind children and other special groups, carry forward the traditional virtues, through the love and warmth to the society through the strength of each person. In the eyes of most people, housekeeping is doing housework and taking children, and most of the employees are middle-aged and elderly people with low education. In fact, with the explosive growth of the demand for the new homemaking market, homemaking practitioners show a trend of specialization, professionalism and youth. Professionalism and service level are the most important factors for domestic service consumers today. After 3 years of experience, Xiaoling has the biggest feeling is that the organizer is a group of very positive people. She said that the storage market prospect is good, the income is also good, the monthly income of ten thousand yuan in their team is a general level, more of the high monthly income of 40,000 yuan, is indeed a very attractive young people's sunrise industry. Approaching the Chinese New Year, the 23-year-old Xiao Ning received a lot of orders to clean air conditioning, smoke lampblack machine."My income has more than doubled," he said. "My brother has no worry about college tuition and living expenses."In April this year, from a low-income family in Maoming, he participated in the "Two Help and Two Promotion" South Guangdong Domestic Vocational Skills and Employment Training class jointly organized by Guangdong Youth Entrepreneurship and Employment Promotion Center and 51 Family Butler through the "Easy to Spread its wings" platform. After professional training and internship, he became a home appliance maintenance engineer. Xiao Yang, a post-90s student from Meizhou, Guangdong province, is the leader of a platform. After graduating from South China Agricultural University in 2018, he entered the housekeeping industry. Yang said he chose the homemaking industry when he graduated from college because he was optimistic about its salary, promotion space and development prospects."The domestic industry needs professional talents, and the future domestic helpers will have stronger market competitiveness."According to the survey, the average age of domestic service practitioners in the past was over 45 years old. In recent years, the industry has become more and more younger. In 2021, the average age of Guangdong province has dropped to 36.51 According to the 2,000 employees, 80 percent are 80 percent born in the 1990s, 60 percent were born in the 1990s, and more than 30 percent have a college degree or above. In 2022, the Guangdong communist youth league will deepen the implementation of "wings plan" Guangdong communist youth league promoting youth employment, linkage 21 provincial youth league organizations at various levels, plan for not less than 100000 youth docking employment information, career guidance and growth services, promote not less than 100000 college students to participate in practice and grassroots services, help more youth set up a positive career, achieve high quality employment.

2022-01-21	Beijing: small closed loop volunteers sympathy online activities in Beijing "green force games warm heart volunteer" volunteer series sympathy small closed loop volunteers online sympathy activities held net Beijing on January 21 (reporter zhang, reporter Shi Jijun) recently, "green force games warm heart volunteer" volunteers series of sympathy-small closed loop volunteers online sympathy activities. Li Jian, deputy secretary of the Communist Youth League and Beijing Municipal Party Committee, attended the meeting. Secretaries of the Communist Youth League Committee of Beijing Forestry University, Beijing Foreign Studies University, Renmin University of China, Communication University of China, University of Chinese Academy of Sciences, China Foreign Affairs University and Beijing City University, as well as representatives of volunteers in the small closed-loop station, attended the conference online. At the meeting, Gan Zhilin, resident manager of the main media center, and Zhang Ye, resident director of the Capital International Airport, briefly introduced the relevant situation around the work characteristics, work progress and work difficulties. Eight volunteers spoke in turn to express their determination to do a good job in the volunteer service work of the Winter Olympics. Online sympathy activity site. On the basis of the preliminary investigation of the actual needs of the volunteers, the Beijing Municipal Party Committee has specially prepared a New Year gift package for the volunteers, which will be sent to the station according to the transportation requirements of the closed-loop management materials. Group Beijing, deputy secretary of the municipal party committee delegation Beijing municipal party committee delegation has mount guard and will fight in the games the line of young teachers and students and volunteers students, and put forward three hope: hope the students can be a good example, experience yourself, the main media center and the capital international airport is the Olympics "window" and "door", you students to improve professional skills, for follow-up volunteers example. It is hoped that students can combine work and rest, abide by rules and regulations, and strictly abide by various management requirements and regulations in their work, and especially put epidemic prevention and control in the first place. I hope all the students can feel at ease and work hard. The Beijing Municipal Party Committee of the Communist Youth League, universities and residents have been doing their best to create a warm and safe working and living environment for everyone. I hope that students can unload their burden, work hard, win glory for the motherland and cheer for their youth.

2022-01-20	Inner Mongolia young pioneers to participate in special training tells the story of the party in Inner Mongolia young pioneers to participate in special training tells the party's story Shi Jia) on January 13, "the story of the party to be red scarf narrator" Inner Mongolia young pioneers winter vacation practice experience in Inner Mongolia district committee youth library, activities jointly organized by Inner Mongolia and Inner Mongolia television, from Hohhot and surrounding areas of more than 30 young pioneers participated in the first training activities. Three tutors respectively to tell the story of the party to be a new era good young "the story" the most beautiful sequel " titled special training, from the history of the party to preach content, preach, preach skills guidance for the players, and guide the players to study their own actual history in one hundred, tell the story of the party, from the party's great spirit, draw strength, firmly listen to the party, follow the party's determination. In the training, some of the young pioneers also had a wonderful lecture, showing the good spirit and preaching foundation as the red scarf narrator. Next, the team members will serve as the red scarf guide of the revolutionary tradition education and practice base of The Inner Mongolia Autonomous Region, and they will regularly publicize the stories of the Party and carry forward the revolutionary tradition. It is understood that the activity is the "red scarf heart to the party" the young pioneers theme education practice series, aims to promote the "double reduction" policy implementation, rich players winter vacation life, expand the young pioneers practice, guide the players to further understand the party's history of the party, guide the young pioneers inheritance red gene, continuously red blood, to be a new era of good youth.

2022-01-21	"New youth to help revitalize" Chinese youth shopping festival in Gansu province held the venue "new youth to help revitalize" Chinese youth shopping festival in Gansu branch event host Wang Hao) on January 18, by the communist youth league committee of Gansu province, Gansu province youth federation held the "new youth to help revitalize" Chinese youth necessities festival in Gansu venue activities in the Yellow River building in Lanzhou. With the help of the high yield and high sales of agricultural and sideline products before the Spring Festival, this activity invited members of the frontline youth Federation, who have made outstanding contributions in various fields of Gansu Province, to walk into the online broadcast room to promote all kinds of characteristic agricultural products in all cities (prefectures) and key counties (districts) for rural revitalization. On the day of the event, the number of viewers in the main Gansu branch studio reached 86,949. Through the "network power", to further enhance the influence of the "sweet taste" agricultural products. At the same time, it has given full play to the talent and intelligence advantages of the Youth Federation, led the young people to further promote the "Rural Revitalization Youth Achievements" action, and contributed its youth strength to the implementation of the rural revitalization strategy and accelerating the pace of agricultural and rural modernization. It is reported that since January 11, nine cities (states) in Gansu province have taken advantage of the opportunity of the Chinese Youth Spring Festival Shopping Festival to widely help sell "sweet" agricultural products on various live streaming platforms, with a total of more than 640,000 online viewers and a cumulative sales of more than 700,000 yuan. Follow-up activities will continue until January 24.

2022-01-28	In Fujian province young pioneers outstanding individual and collective award ceremony held in Fujian province young pioneers outstanding individual and collective award ceremony and red scarf theater art performance JianWei correspondent huang peng) recently, sponsored by the Fujian provincial party committee, Fujian province less working committee of Fujian province young pioneers outstanding individual and collective award ceremony and red scarf theater art performance held in Fujian radio and television grand theatre. League of Fujian provincial party committee, Fujian province education department, Fujian province working committee launched in August 2021, the provincial young pioneers outstanding individual and collective selection, the grassroots recommendation, expert review, assessment of the public, selected "outstanding young pioneers counselor in Fujian province" 98, "Fujian" team 100 first, "Fujian province" 50, "Fujian outstanding pioneers" squadron 49. The picture shows the representative of Fujian Provincial Excellent Young Pioneers Brigade. Li Xun, inspector of the Fujian Provincial Department of Education and director of the Provincial Youth Working Committee, Li Wenjie, deputy secretary of the Provincial Communist Youth League Committee and other leading guests attended the award ceremony to award the outstanding individuals and collectives of the Young Pioneers, and watched the Red Scarf Theater art performance. The first 100 young actors from the provincial Red scarf Art Troupe, Fuzhou Qiantang Primary School Pingnan Primary School, Xiangfeng Central Primary School, Tongpan Central Primary School, Jin 'an Rongbo Primary School and other high-level children's art groups and schools participated in the performance. Song "Red Flag fluttering". The photo art performance provided by Fujian Provincial Party Committee includes martial arts performance "Heroes from Ancient Times", melodrama "Dialogues from Time and Space Memory", "Crossing a Hundred Years of Original Aspiration", dance "Early Garden in Spring", song "If Love Has Wings", song and dance "Fly Higher", allegro "Medal Flash My Growth". Performance site. In the way of cultural programs, since 2021, the young Pioneers at all levels in the province have organized the pioneering action of "Red scarf Heart to the Party", implemented the achievements of the measures of the "double reduction" policy, and showed the spirit of the Fujian Young Pioneers from various perspectives. In the next stage, the provincial Youth Working Committee will continue to play the demonstration and leading role of the Red Scarf Theater as the young Pioneers off-school practice education position, and continue to carry out the young Pioneers art performance, work display and other comprehensive quality development activities.

2022-01-24	Henan: Cyberspace Matrix combined with the Communist Youth League to build a "city service desk", chest tightness, dizziness and eyes pain, because of the epidemic control can not go out, want to find one or two boxes of right zuo PI clone."At 9:18 am on January 13, a netizen @_LIns_o3o posted a message for help through the hashtag # Henan City service desk #.@ Henan Network information and @ Henan Communist Youth League for the first time to make contact with netizens, detailed understanding of the situation. Because the drug must be purchased by prescription, the problem was immediately transferred to the Cyberspace Office of Zhengzhou Municipal Committee for coordination and solution. At 10:12 a. m., the netizen posted a tweet telling them that the matter had been resolved. Later, he not only posted the two boxes of medicine that he got, but also shared the strategy to get quick rescue through the "Henan City Service Desk" in an emergency."Help each other", "cooperate to fight 'epidemic' ", "Henan refueling"... this is the most used comments on the topic # Henan City Service Desk # in recent days. On January 12,1.15 million fans commented: " It is rare for Henan network information system to take the initiative to launch the epidemic prevention and epidemic prevention work into a mass 'online and offline' war! The launch of 'Henan City Service Desk' provides a good answer to the question of how to play the function of government microblog to participate in social emergency governance."In order to take the online mass line, on January 10, in view of the current epidemic situation in Henan, Henan Network Information Matrix, @ Henan Communist Youth League and Weibo to set up the" Henan City service desk ", with the help of the fast Internet and convenient interaction and sharing, quickly help netizens to solve the difficulties encountered in the epidemic. The next day, all the "city service desks" of 17 provincial cities in Henan province and the Jiyuan Demonstration Zone were launched, and they were settled in the "Henan City Service Desk". Henan provincial party committee net letter, Henan province youth volunteers, two levels of functional departments, around the city, sina weibo, mutual collaboration, seamless "Henan city service desk" work including information collection, information sorting, ground processing, return feedback, etc., formed from online to offline, eventually return to online work closed-loop, efforts to do "you call, I should". With this platform, when users need to consult or seek help for COVID-19 related problems, they can bring # Henan City Service Desk # inscription to post microblog or private message @ Henan Network Information Network, and also find the entrance of the service desk in their hometown on the # Henan City Service Desk # topic page for consultation and help. In addition to responding to netizens' help, the "Henan City Service Desk" also provides communication channels for raising epidemic prevention materials and promoting positive energy against the epidemic. On January 12, the Anyang Charity Federation issued a donation proposal for epidemic prevention and control, urgently in urgent need of masks, protective suits, protective masks and other protective materials. On the morning of January 13, @ Cyberspace Anyang released an emergency help through the "Anyang City service desk". These news get "Henan city service desk" weibo matrix forward spread, then Henan daily, river money cube, southern metropolis daily media blue V also focus diffusion, related topics # Anyang need masks protective clothing #, # Henan Anyang emergency protective clothing and epidemic prevention supplies # read 32.464 million people, 21.142 million people respectively. On January 14, the shortage of local epidemic prevention materials in Anyang has eased. Jin Xuejie, chief editor of Henan Communist Youth League Weibo, said, " In order to cooperate and serve the Henan City Service Desk well, we have organized a team of 520 volunteers to communicate with netizens about the details of their needs and respond to inquiries online. At present, there are about 100 information for help collected through topics and private messages."" has a staff member responsible for collecting and recording the netizens' help information, coordinating various functional departments to effectively solve the problems of netizens."Zhao Liang, editor of Henan Cyberspace Weibo, will open a green channel, point to point with provincial and municipal functional departments; for the epidemic prevention policy consultation, volunteers will directly answer online; for general life and epidemic prevention materials needs, will contact netizens for details, and then coordinate with relevant departments to help. According to the data released by the microblog platform, as of January 17,131 government microblogs and 83 media microblogs have spoken frequently in # Henan City Service Desk, responding to the concerns of netizens and solving the needs of netizens, and more than 50 literary big V circles, such as @ Liu Haoran, @ Lang Lang, @ Zhang Zifeng, have helped to jointly spread the anti-epidemic information in Henan. The relevant person in charge of Weibo government affairs said: " 'Henan City Service Desk' is a new attempt by Weibo to actively explore new government media to improve social emergency service capabilities. We hope to work together with Henan government official Weibo and Henan Weibo users to overcome the difficulties."As of 12:00 on January 18th, the topic # Henan City Service Desk # has been read 290 million times and discussed 943,000 times; Henan City service desk responded to 16,812 consulting questions, asked 70 online for help, and coordinated with provincial cities and functional departments offline through the city service desk mechanism. China Youth Daily China Youth Network reporter Pan Zhixian source: China Youth Daily

2022-01-27	Henan province and Zhengzhou city "communist youth league and people's congress, CPPCC face to face" activities held in 2022 in Henan province and Zhengzhou "communist youth league face to face with NPC and CPPCC" activities held in Zhengzhou JianWei correspondent Liu Yong), in 2022 in Henan province and Zhengzhou "communist youth league and people's congress, CPPCC face to face" activities held in Zhengzhou. Wang Tianshun, deputy director of the Proposal Committee of the CPPCC, Wang Anlin and Wang Haiping, deputy secretary of the Provincial Communist Youth League, and leaders of the relevant committees of the Provincial people's Congress attended the event. More than 20 people, including deputies of the provincial people's Congress, members of the CPPCC, youth workers and mental health experts, attended the event. Symposium, from education, medical care, public security, colleges and universities, social work institutions, enterprises and other industries of youth representatives around the "promotion of adolescent mental health services", combined with the reality of their own work, from different angles to share the current adolescent mental health service related work, analysis to the current main problems, work opinions and Suggestions are put forward. After listening to the speeches, the NPC deputies and the CPPCC members responded to and supplemented the questions raised by the youth deputies, and put forward their respective views and views. Representatives and committee members agreed that the mental health problems of teenagers are very high social attention, to further improve the adolescent mental health service system, establish a psychological crisis intervention mechanism. After the symposium, the participants investigated the Henan Province 12355 Youth Service Center. CPPCC Wang Tianshun, deputy director of the proposal committee pointed out in his speech that youth's mental health is not only a personal problem, is also family problems, social problems, hope the CPPCC will finish related issues, strive to form a powerful proposal on the "two sessions" for service, called for the healthy growth of teenagers, to better help them solve the interests. Wang Anlin, deputy secretary of the Provincial Communist Youth League Party Committee, stressed in his speech that the healthy growth of teenagers is not only related to personal families, but also related to the future of the country. It requires multi-linkage and coordination of all sectors of society to build a interconnected mental health service mode. We need to attach great importance to and deeply understand the significance of guiding young people to participate in an orderly political manner, work together to improve the level of youth mental health services, build platforms, and continue to guide young people to interact with NPC deputies and CPPCC members. In recent years, Henan province youth league organizations at all levels around the "young talent power rural revitalization strategy" "better play to the role of youth in urban and rural community governance" themes such as concentrated activities, more than 350 listening activities, covering teenagers more than 75000 people, promote representatives and members in the provincial, city, county "two sessions" put forward the bill, 267 proposals. In the next step, the Provincial Communist Youth League Committee will rely on the provincial 12355 youth service platform to carry out mental health education on a regular basis, strengthen offline services on the platform, and actively explore new paths of youth growth services.

2022-01-26	League of Fujian provincial party committee party history learning both the practical measures cover more than 1.94 million league Fujian provincial party committee party history learning practical measures cover members more than 1.94 million qingwang trainee reporter Tian Hongwei) on January 19,2022, the communist youth league of Fujian provincial party committee secretary Xiao Huaxin in reviewing the Fujian communist youth league party history study education work, the province to carry out the theme learning 669000 games, covering more than 1.947 million members. In 2021, the Youth Party History Story Competition hosted by Fujian Provincial Committee of the Communist Youth League, 10 participating teams, in the form of music, pictures and videos, told the red boat of South Lake in Jiaxing, "a single spark can start a prairie fire", fighting the United States and aiding the DPRK, reproducing the glorious course of the centenary struggle of the Communist Party of China. Zhang Wenjing, a contestant from Fujian Normal University, said that as a youth in the new era, we should make our good voice and hope to tell the history of the Party, learn with more young students about the red story and learn the strength to move forward. The official Weibo account of the Fujian Provincial Party Committee opened the topics of "Fujian Youth Group to Learn Party History" and "Party History Online", which were read nearly 18.53 million times. The wechat official account has opened up columns and topics of "Tuan Tuan Learn the History of the Party", and pushed more than 95 related posts, with a total reading volume of more than 3 million. It has released 46 videos related to party history learning and education on short video platforms such as TikTok and Kuaishou, with more than 8.6 million cumulative views. Focusing on five themes, including strengthening ideological and political guidance for young people and deepening youth exchanges with Taiwan, Hong Kong and Macao, we conducted visits and investigations, held 19 symposiums in 19 times, and collected more than 240 suggestions. In the process of learning the history of the Party, Fujian Communist Youth League adheres to the theory and reality and carried out the activity of "I do practical things for the youth". We launched 10 projects, including the Youth Innovation and Entrepreneurship Promotion Program, raised more than 26.67 million yuan in the Hope Project, and helped 8,953 young people in need. In 2021, the Sailing plan will practice for 10,987 students, and it will provide 5,708 jobs. A total of 267 youth dating activities were held, attracting nearly 14,000 young people of the appropriate age. During the Children's Day period, 89 special group care activities were held, serving 8,286 students with disabilities. More than 800,000 new young volunteers were registered in the province, and nearly 138,000 volunteer services were carried out, covering 13.855 million hours.

2022-01-19	Youth mental health services into the village (community) listed in Henan people's livelihood practical youth mental health services into the village (community) for the first time in Henan province key people's livelihood practical newspaper youth reporter Pan Zhixian) Henan provincial party committee general office, Henan provincial government general office recently issued by the 2022 key in Henan province livelihood practical work plan, and issued a notice to local departments earnestly implement, among them, led by the communist youth league Henan provincial party committee of youth mental health services into the village (community) is listed as one of the key livelihood of Henan province in 2022. It is the first time that the youth mental health work led by the Communist Youth League has been included in the province's key livelihood matters. The mental health of adolescents is related to the future of the country and the nation. With the rapid development of economic and society, the pressure of teenagers in learning, adapting to the environment and interpersonal relationship increases, and the incidence of adolescent mental health problems shows an increasing trend. There are 10 key work matters on people's livelihood listed in Henan Province in 2022, such as vocational skills training, evaluation and evidence collection, expanding the scope of direct settlement of hospital outpatient expenses in different places, etc. The ninth item is to "carry out adolescent mental health services into villages (communities) action". The plan clearly requires strengthening adolescent mental health services, arranging no less than 1,500 youth mental health services into the village (community) activities throughout the year, realizing more than 8,000 case consultation times, and building 200 "Qingyi Home" working positions. Henan Provincial Party Committee of the Communist Youth League introduced, In order to effectively improve the psychological level of teenagers, Henan Provincial Committee of the Communist Youth League will work with the Political and Legal Committee of Henan Provincial Committee, Provincial Civil Affairs Department, Provincial Finance Department, Provincial Health Commission and other relevant units, In various online and offline forms, Organize and carry out psychological lectures, league and auxiliary activities, psychological assessment and other at least 1,500 activities of mental health services, such as entering the village (community); Set up a professional psychological counseling team, Through the hotline, Internet, face to face, case-to-case consultation services, Formation of mental health records, Regular tracking services, More than 8,000 people case consultation; Relying on the "youth home", the community Party and mass service center and other activity positions, To realize the psychological assessment, psychological counseling, psychological lectures and other functions, Equipped with related psychological service hardware, software and software equipment, Professional psychological counselors provide regular mental health services, Build 200 "green wing home" working positions. In recent years, Henan communist youth league continue to pay attention to youth mental health, to the construction of Henan province 12355 youth service desk as the gripper, to further promote the long-term youth development plan in Henan province (2019-2025) " as an opportunity to carry out a series of publicity and education, psychological counseling services, promote parents, schools, social jointly maintain and promote adolescent mental health. Group of Henan provincial party committee officials said, Henan youth league organizations at all levels will give full play to the organization mobilization and social mobilization advantage, actively integrate resources, high quality construction in Henan 12355 youth service desk, carry out various forms of mental health education services, focus on left-behind, single parent, disability, strengthen psychological counselors, volunteers and other mental health service team, promote the construction of youth mental health service system, the escort for the healthy growth of teenagers.

2022-01-21	Fujian "syndicated cooperation" power Fujian rural revitalization work will hold Fujian "syndicated cooperation" power rural revitalization work will be held in Sanming on January 21 (reporter Zhang Jianwei, correspondent Xu Jingxian) recently, the Fujian provincial party committee, Fujian silver inspect bureau in Sanming Fujian "syndicated cooperation" power rural revitalization work will advance. Fujian Provincial Youth League Committee, Fujian Banking and Insurance Regulatory Bureau Party Committee, Sanming Municipal People's Government relevant responsible comrades attended the meeting and made a speech. Nearly 150 people attended the meeting, including the youth League committee of relevant districts, the working committee of financial league, financial institutions, the leaders of relevant county (city, district) people's governments of Sanming City, the tenth batch of financial temporary league cadres and youth representatives of entrepreneurs.site of activity. Fujian provincial party committee for map Fujian provincial party committee deputy secretary li teng message the tenth batch of financial temporary cadres: to establish industrial thinking, effectively integrate resources, focus on support a number of high technical content, production scale, good market prospects of rural youth entrepreneurship projects, help implement the rural revitalization strategy, promote rural economic and social development. It is necessary to conduct in-depth investigation and research, innovate and develop a number of characteristic financial products with "syndicated cooperation", promote the innovation of rural financial services, and improve the ability and level of grassroots youth league cadres and rural young entrepreneurs to use modern financial services. We should adhere to the combination of internal and external, promote the integration, give full play to the organizational advantages of the Communist Youth League and the financial system, integrate the resources of Party and government organs, enterprises, public institutions and all sectors of society, focus on cultivating a number of typical young people getting rich, and drive more young people to start businesses and become rich. It is necessary to integrate into the reform process, give full play to our professional expertise, focus on the reform objectives of the Communist Youth League, build the brand of youth financial work, and continuously expand the popularity and influence of the "syndicated cooperation".site of activity. Group of Fujian provincial party committee for figure meeting, released in 2021, Fujian banking insurance service rural revitalization work and the key situation in 2022 work plan, to promote the "syndicated cooperation" power rural revitalization of characteristic financial products, also held the rural revitalization of cooperation agreement signing ceremony, and for the entrepreneurial youth representative credit card and policy. The Youth League Committee of CCB Fujian Branch, Zhangzhou Municipal Party Committee of the Communist Youth League and representatives of entrepreneurial youth respectively exchanged experience in financial assistance to rural revitalization. During the meeting, the 10th departure ceremony and special training for the tenth batch of temporary financial cadres of Fujian "syndicated Cooperation" were also held.site of activity. Group of Fujian provincial party committee for the figure, "syndicated cooperation" is the Fujian provincial party committee and Fujian silver protects inspect bureau in the national first work brand, since May 2010, has selected 10 batch financial institutions 661 youth cadres, by providing "financing" services, strengthen support "wisdom", "financing" platform, in power poverty engines, service rural revitalization, helping youth entrepreneurship, strengthen grassroots youth league organization has achieved good results. Up to now, a total of 15.977 billion small loans for rural entrepreneurial youth, create 1,530 "rural youth entrepreneurship financial service station", held 11,097 "send financial knowledge to the countryside" activities, training rural entrepreneurial youth more than 99000 person-time, Fujian "syndicated cooperation" work mode by the central into the communist youth league poverty alleviation practice 20 cases.

2022-01-25	Fujian "red scarf" in the garden winter vacation public hosting class in Fujian: 2022, "red scarf in the garden" winter vacation public hosting class class JianWei correspondent Huang Peng) on January 24, guided by Fujian province education department, Fujian provincial party committee, Fujian province, Fujian provincial hospital in 2022 "red scarf in the garden" public hosting class in Fuzhou garden primary school. Responsible comrades of the main and undertaking units attended the opening ceremony. Young Pioneers flash mob interactive "Drum Tower good young song". The drawing ceremony of the CPC Fujian Provincial Party Committee began with the chorus of "We are the successors of communism". The Provincial Communist Youth League Committee and the Provincial Youth Working Committee awarded the Garden Primary School "Fujian Red Scarf Pioneer Children's Palace". The Provincial Youth Working Committee has appointed a group of young doctors from Fujian Provincial Hospital as after-school counselors and volunteer workers of the Young Pioneers to provide health education courses and medical security for the cold care class and various young Pioneers public welfare care classes in the future. Fujian Youth League Committee and Fujian Youth Working Committee awarded Huayuan Primary School "Fujian Red Scarf Pioneer Children's Palace". After the ceremony provided by Fujian Provincial Party Committee, the leaders and guests also visited the red scarf class display of the cold nursery class, and experienced the colorful quality education courses, such as physical training, calligraphy practice, Chinese herbal medicine bag sachets making, handmade clay figures and so on. In the first lesson, the members of the national red scarf tour group went deep into the class, brought a wonderful and vivid party history learning education lecture for the members, the teachers with easy to understand, humorous children, to tell the story of the party history, education and guide them to feel the party, listen to the party, follow the party. Members of the National Red Scarf Tour Group conducted lectures. Since the introduction of the "double reduction" policy, the provincial Youth Working Committee has successfully held four summer care classes in Fuzhou Qiaoyuan Primary School and Jin'an Rongbo Primary School. The cold nursery class will continue heat class model, to "trust" and "tube", "learn" and "play" way, by the young pioneers school counselors, volunteer workers, "five old" personnel, TuanGan, social workers and university teachers teaching team, open party history learning education, family education, eye knowledge, eye knowledge, physical training and other more than 10 courses, to further meet the needs of children comprehensive development, let parents, players happy, to solve the trouble back at home of parents. Next, the province less committee will continue to strengthen the young pioneers social work system construction, relying on Fujian red scarf pioneer children's palace, further integrate social resources, play a leading role, using winter and summer vacation, weekends, with public welfare hosting project as the core, expand to build studies as the main body of the children practice experience, the brand, normalized public welfare activities, promote the formation of the whole society care about children, support the young pioneers work good atmosphere.

2022-01-19	Shanghai: listen to the voice of "youth" has a multilingual platform to tell the story of Chinese, community non-motor vehicle charging problem in addition to management, to Shanghai youth need a more convenient access to public service information, community housing can be transformed into a child-friendly space... the city of Shanghai, is carefully listening to the voice of youth. On January 18, the Shanghai Letters and Visits Office, together with the Shanghai Municipal Party Committee, focused on the collection of people's suggestions on the young people. A young suggestion soliciting mailbox appears in the memorial hall of the former site of the Yuyang Youth League. In the future, all young people who come here to "punch in" can send their suggestions to the mailbox, so that their "golden ideas" will turn into "golden fruit" to promote social progress. In the future, such a mailbox will also appear in all kinds of young people in Shanghai, specially collecting their "golden ideas"."At the end of 2020, since the establishment of the Communist Youth League Municipal Committee people's proposal collection workstation, the Youth Shanghai WeChat official account has collected 250 people's suggestions, of which 140 are valid suggestions."Wu Bin, deputy secretary of the Shanghai Municipal Party Committee, said that the municipal people's suggestions collection workstation is one of the contact points of the six municipal people's suggestions collection work in Shanghai, mainly responsible for collecting youth suggestions. One of the characteristics of these youth recommendations is their high quality. In 2021, the youth suggestions received many highlights. A number of insightful, warm and innovative youth suggestions, embedded in social governance, have become the highlights of Shanghai. For example, Han Lu and three students, a post-00s student from Shanghai Materials Engineering School, visited and investigated the "building readable" situation of Hengshan Road-Fuxing Road, Xuhui District, and put forward suggestions on further strengthening the information update and upgrading of the QR code of old buildings. Recommended by the Xuhui District Committee, the proposal was adopted by the Xuhui District government, and the QR code update work is under way. Roark, an international student from Tongji University, is from the Republic of Benin in south-central West Africa. During his study in China, he found that "Shanghai is really great". "You can order takeout anytime, anywhere, have coffee anywhere, where you go, and go out no matter what time in the evening."But these "ways of living a happy life" were completely unknown before he came to China."So I only knew that there were giant pandas in China."Rock said. To this end, Chen Siyao, a student at Tongji University, suggested building a multilingual demonstration platform to tell Chinese stories, with overseas students in China as the "center of the circle". In 2021, Tongji University welcomed more than 1,100 new international students from 115 countries and regions. Together with Chinese students, they jointly created the "Panda Talk- -International Students Tell Chinese Stories" project. So far, the project has shot more than 200 short videos and have been promoted through platforms such as Facebook and wechat official accounts. Overseas fans in 185 countries have received more than 170 million views so far. Ding Zixuan, a student from Guyi Primary School in Jiading District, became the guest of Li Qiang, Shanghai Party secretary, by proposing "people's suggestions". Taking a four-story tower in her community as an example, she proposed transforming the community room into a child-friendly space, and received a face-to-face reply from Li Qiang. Just like every youth center in Shanghai has a unified LOGO, logo, cultural and creative display in the whole city, the people suggest that the collection mailbox should also have its own unified visual logo and distribution of materials. Behind each mailbox is a set of standardized management process- -when to open the box to check the letter, how to submit youth suggestions, how to deal with them after receiving youth suggestions, the full set of procedures to ensure that the "Voice of Youth" has a reply."A good youth advice can improve a policy to improve some people's livelihood."Wu Bin said that today's young people do not have enough knowledge and ability. What is more important than knowledge and ability is the" responsibility "of every young people." I sincerely hope that young people can be good masters, practice the social responsibility of young people in the new era, and contribute to the better life of beautiful Shanghai."It is reported that "youth," Shanghai WeChat public number will become the future pku people's suggestion online window, Shanghai union will add people's suggestion solicitation work committee, the people's suggestion solicitation work combined with the group representative liaison station construction, build a collection, feedback and propaganda guide in the integration of the closed-loop working mechanism. The people's proposal collection will be "1 + 3 + 16 + X" entity construction layout pattern. Among them, "1" is 1 center, namely the people's proposal solicitation center, "3" is the youth League, youth Federation, and respectively; "16" is the people's suggestion solicitation stations, "X" is several points, mainly the city, youth league organization, especially the construction of youth center, and community youth council and red scarf council. Zhang Lirong, deputy director of the Shanghai Letters and Visits Office, said that the office also wants to find a new channel to communicate with the youth through the people's suggestion collection workstation. " Our government departments cannot play and sing by themselves. We should give the young people a platform to communicate with the government. Our relationship with young people is not me and you, but our relationship. The voice of the youth should be heard by the relevant authorities."Shanghai, January 18

2022-01-19	Guangdong held the Young Pioneers Off-campus Theme Team Day Demonstration Activity Guangdong held Jianwei correspondent Yue Qing) On January 16, Guangdong Provincial Youth League Committee, Guangdong Provincial Youth Working Committee jointly held the "search for electricity and light footprint continue a hundred years of salary fire" Guangdong Young Pioneers off-campus theme team day demonstration activity. Theme demonstration activity site. The organizer of the delegation of Guangdong Provincial Committee awarded the plaque of "Guangdong Young Pioneers After-school Practice Education Camp (Base)" to the five enterprise exhibition halls of Guangzhou Southern Power Grid, and included it in the extracurricular practice platform open for primary school students in the province. Ten volunteers of "Blue Charity Red Cotton" from Guangdong Guangzhou Power Supply Bureau of China Southern Power Grid were officially employed as after-school counselors of Guangdong Young Pioneers, Guangdong Province, and five Young Pioneers received the certificate of "Red Scarf Guide", issued by Guangzhou Electric Power Exhibition Hall of China Southern Power Grid. Guangdong Province Young Pioneers after-school practice education camp (base) awarding ceremony. In the activity provided by Guangdong Provincial Committee, Guangzhou Youth Working Committee and Guangzhou Electric Power Exhibition Hall jointly held a holiday practice camp. Under the leadership of external counselors, more than 30 young pioneers learned, experienced and explored the century-old electric power history, low-carbon and environmental protection. Drive the young pioneers of the province to cultivate their character in practical activities, and realize the all-round development of morality, intelligence, physique, beauty and labor. Tang Wanfeng, general counselor of Guangzhou Young Pioneers and deputy director of Guangzhou Children's Palace, said, " We should give full play to the advantages of organizing education, independent education and practical education, and do a good job in the young Pioneers' activities outside the school.""'s current screen is the documentary, which was shown at the United Nations General Assembly in 1974. It tells the story of women workers in Guangzhou Power Supply Company who went to the tower, repaired lines and fought the arc to protect the lights of thousands of homes."Under the vivid explanation of the red scarf narrator, the young pioneers intuitively understand and learn the century-old history of Guangzhou electric power development process and electric power science knowledge. The Young Pioneers are participating in practical education activities. Chen Ying, a former primary school teacher in Yuexiu District, Guangzhou city, said: " The red scarf narrator uses the discourse system of his peers to explain, which makes some complex and abstract content easier to understand. Such a practice mode is a recommended way for teenagers to spend the winter vacation under the 'double reduction' policy."For the implementation of the deployment of comprehensively strengthening the new era of the young pioneers work, build the new era of the young pioneers socialized work system, improve the level of Guangdong young pioneers socialization work, Guangdong provincial party committee, Guangdong province, Guangdong provincial party committee propaganda department, Guangdong civilization office 19 units jointly issued the about building a new era of Guangdong young pioneers socialization work system implementation opinions."Opinions" widely mobilize social resources, expand the channels of the young pioneers organization, gradually build organization coverage, mechanism specification operation, resource integration, make full use of positions, various forms, players actively participate in the new era of Guangdong young pioneers social work system, form inside and outside the school, children generally benefit from the new situation of the young pioneers work. At present, Guangdong has built nearly 400 off-campus practice education camps (bases) for the named young Pioneers, and will launch the "Guangdong Young Pioneers Off-campus Practice Education Map". During the holidays, the venue will regularly be held open to the young Pioneers organizations and the young pioneers, and will carry out colorful young Pioneers practice activities to lead the young pioneers to enrich the holiday life.

2022-01-18	The Inner Mongolia autonomous region party committee to carry out the New Year sympathy activities of Inner Mongolia district party committee "feeling warm childlike unity hand in hand national unity" New Year sympathy activities on 18 (reporter Zhang Jianwei, correspondent wang Chen) to thoroughly implement the party and the sixth plenary session of the 19th 11th autonomous region spirit, inspire the young pioneers cast the Chinese nation community consciousness, continuous care for rural pastoral children, at the beginning of the New Year, the Inner Mongolia autonomous region committee in Hohhot in 2022 "feeling warm childlike innocence national unity hand in hand" New Year sympathy activities.site of activity. At the event, more than 50 young pioneers from Juhua No.2 Primary School and Aihua Primary School in Hongsheng Industrial Park, Hohhot City, performed wonderful programs, giving New Year gifts, shaking hands and pairs, holding them tightly like pomegranate seeds.site of activity. The correspondent awarded the "Red Scarf Book Corner" to Juhua No.2 Primary School and Aihua Primary School in Hongsheng Industrial Park, Hohhot City, and donated books worth 24,000 yuan. Activities on the same day, the young pioneers came to the Olympic theme of snow world, in snow exhibition, base library ice and games knowledge, understand the spirit of the games, in the snow market, ice and snow park quality development activities and games, the players experience snow fun, enhance physique, temper will, with their own practical action to express look forward to the 2022 Beijing winter Olympics, painting and calligraphy Olympic plan, imagine the Olympic ideal.site of activity. Correspondent for the next step, pku will continue to focus on children's political enlightenment and values, around the implementation of the Inner Mongolia autonomous region on comprehensively strengthening the new era of the young pioneers work implementation opinions, to carry out colorful practice, coagulation hearts meet force service children, promote the young pioneers work to a new level.

2022-01-17	In 2022, the "Communist Youth League and deputies to the NPC and CPPCC members" activity was held opposite in Rong. Beijing, China Youth network, January 17 (reporter Zhang Jianwei, correspondent Dong Danhong) recently, 2022 Fujian Province "the Communist Youth League and deputies to the National People's Congress, CPPCC members face to face" activities held in Fuzhou. Relevant leaders of the Provincial Communist Youth League Committee, Provincial People's Congress and Provincial CPPCC, some provincial and municipal people's Congress deputies, members of the CPPCC and young representatives from related fields attended the event. At the symposium, the youth representatives put forward a series of opinions and suggestions on youth scientific and technological innovation, youth entrepreneurship and employment, young talent training, integrated development of Fujian and Taiwan youth, comprehensive promotion of rural revitalization, and youth mental health. The NPC deputies, members of the CPPCC and young deputies had face-to-face exchanges, in-depth interactive communication, and extensive exchange of views. Xiao Huaxin, secretary of the Provincial Communist Youth League Committee, pointed out in his concluding speech that the youth league organizations at all levels in the province should reflect the demands of the youth, give full play to the role of "face to face" activities, and rely on the institutionalized platform of the NPC and the CPPCC to advocate policy and social advocacy. We should strengthen the consultation mechanism, enrich the connotation of the activities, actively promote the transformation of the results of the proposals and suggestions, and constantly improve the effectiveness of the "face-to-face" activities. We need to form synergy to build a good platform and create good conditions for deputies to people's congresses and CPPCC members at all levels to participate in youth activities, so as to jointly serve the healthy growth of young people. It is reported that since the "Communist Youth League" face to NPC deputies and CPPCC members campaign in 2008, youth league organizations at all levels have used this important communication platform to achieve positive results and play an important role in representing and safeguarding the rights and interests of youth development and guiding young people's orderly political participation. In 2021, deputies to people's congresses and CPPCC members at all levels in Fujian carried out listening activities in various youth groups to carry out listening activities, actively carried out research projects, and put forward 663 NPC proposals and CPPCC proposals. In 2021, in the "face to face" theme research and evaluation of the group, a total of 5 research reports were awarded excellent, ranking the second in China.

2022-01-14	In the eyes of many parents, it has to send their children to schools and professional training institutions to be a "qualified" science and technology innovation education. In the next five years, teenagers aged 9-35 in Shanghai will be able to find suitable innovative courses and activities in the "innovation laboratory" walking a quarter of an hour away from home, and they will also become "managers" to pass on their ideas and knowledge to everyone. In order to bring the "stage" of innovation to more young people as much as possible, the Shanghai Municipal Committee of the Communist Youth League launched the "Five-year Action Plan for Shanghai Youth Development", the "14th Five-Year Plan for Shanghai Youth Development" in 2021. The action plan includes "one innovation college, two working brands, X demonstration areas" and the "youth innovation index" monitoring system, which will be comprehensively promoted through physical space transformation, innovation atmosphere creation, innovation curriculum research and development, and innovation resource integration, so that the seeds of innovation can take root and sprout in the hearts of more young people. In the community of four primary schools, she found that after the "double reduction", after school and holidays, how to settle the children, and how to let the children learn and grow in happiness, has become a great concern for parents. Lin � has gained several years of human resources work experience in �. In her opinion, after young people enter their career, their education and major will be weakened, and the ability to communicate and cooperate and solve problems is more important, which need to be cultivated from an early age. She looks forward to a place around the community that can not only take care of their children, but also let them know friends of all ages and participate in community affairs, so as to exercise their creative thinking and comprehensive quality. By participating in the creation of the social enterprise "WEable youth innovation", Lin � blowing sodium � is becoming a reality. WEable Youth Innovation was initiated by "Enpai public welfare", the first public welfare incubator in China. By holding "3-hour Extreme innovation" workshops and innovation competitions, it provides online and offline training courses and links enterprise resources to empower college students and community teenagers. What are the pain points of teenagers engaged in innovation and entrepreneurship? Lin � summarized several aspects: " Many college students are very actively involved in innovation and creation, but some of them only master professional knowledge and do not know how to transform knowledge into products or services; some think of good ideas, but have no ready-made and specific methods to achieve it; and after writing the project plan, it is difficult to implement the project due to the lack of people and resources."Recently, the Shanghai Municipal Party Committee of the Communist Youth League established the Shanghai Youth Innovation College in the Shanghai Youth League School. The Innovation College will serve as a platform and hub to gather resources, and gather the WEable and other social innovation, cultural innovation, science and technology innovation organizations, as well as the strength of various commissions and enterprises, to empower the whole Shanghai youth who are interested in innovation and creation. Pku relevant department controller introduces, innovation college to cultivate youth creativity, create force, action as the theme, will create an open, sharing, to promote youth innovation support system, by commission provide policy support, enterprises, research institutes, universities, social organizations, such as courses, experience services, town, park, provide "capillary" actual combat scene-door and unit of "innovation laboratory", carrying resources, radiation surrounding teenagers, through universal type, mixed age innovation education "the last kilometer". In Xinhua Road Street, Changning District, and Caoyang Xincun Street, Putuo District, two "Shanghai Youth Innovation Laboratory" have been launched. Xinhua road street is a typical case of Shanghai "community update", social organizations big fish community build development center (hereinafter referred to as the "big fish build") through hardware transformation, and hold public fair, art performance, theme discussion, let residents and community establish deep connection, guide teenagers to actively participate in community life and community governance. Big fish build founder he jia, said his team is exploring building "youth innovation stronghold", by forming a "innovation cooperative", "good community store", invite experienced innovation mentor together hatch ideas, lack of resources and partners "slash youth", let more youth in the community to explore career and the possibility of innovation in life. For social organizations such as big fish construction, rising costs and operating difficulties are important factors hindering them from landing their ideas, and the support of the Shanghai Municipal Party Committee will help them "help". Thanks to this, the big fish construction team in Shanghai Changning District, Shanghai Hongxian Community, Xianxia Street to build a "leisure cooperative". Its head zhang huan is a 95 youth, more than two years ago, her team cooperation with district civil defense office and other departments, on the basis of the residents demand research, the village idle shelter for many years into 36 different function independent space, open to all-age residents "manager" application, can be used for parent-child parenting, game hand, second-hand market, etc. Zhang found that young children were more interested in participating in community activities than their parents."Leisure cooperative", the sixth grade student Xiao Yuan opened "small yuan chess club", "idle" stall "and visitors play chess, the next game receive two yuan, although win less lose more, but he enjoyed it; and xiao Yuan in the same grade 3 girls master" star hall "space, visitors can spend two or three dollars to watch 20 minutes of" created " singing and dance performance. Recently, the Leisure Cooperative has opened two new technology innovation studios, one offering STEAM courses for international schools in Shanghai, which will hold regular robotics and programming classes for teenagers, and an environmental organization that will hold workshops on new ways to recycle and use plastics in Shanghai."In the context of 'double reduction', the community can provide the families with the required companionship functions and aesthetic education, physical education and science and technology innovation courses."If parents can trust the community and more young people can participate, the community building will form a virtuous circle. We expect the government to provide top-down guidance to promote young people to actively participate in community life. In a primary school in Pudong New Area, there is such a boy, described by his mother as "not loving learning", who was naughty in class and refused to cooperate with others. In 2019, his mother, with the mentality of "trusteeship", helped him sign up for the science and technology innovation characteristic love summer care class of Zhangjiang International Youth Innovation and Entrepreneurship Practice Base (hereinafter referred to as the "Mass Entrepreneurship and Innovation Base"). In a "water purification experiment class", the teacher dug out his hidden advantages: interested in doing experiments, strong hands-on ability. Under the guidance of his teacher, his team cooperation ability and confidence have been improved, and he has also become a "loyal fan" of the mass entrepreneurship and innovation base. In Pudong New Area, the Mass Entrepreneurship and Innovation Base is a platform combining high-quality research institutes, science and technology venues and top enterprise partners. It can provide a series of courses, experiences and services for teenagers on and off school, online and offline, and cultivate their innovative spirit and practical ability. Lin Andu, director of the mass innovation base, told reporters that primary and secondary schools have uneven science and technology innovation project resources, and strong schools have strong teams of teachers, and win many awards; while in weak schools, a science and technology teacher has to serve thousands of students. In order to enable children to receive inclusive science and technology innovation education, the mass entrepreneurship and innovation base provides schools with zero-foundation, science popularization courses and high-level science and technology innovation courses, and provides channels for students to participate in professional experience, social investigation and innovation competitions. For example, Lin said, there is a middle school with weak science and technology education ability near Zhangjiang Science City. The team of the mass entrepreneurship and innovation base has helped the school create AI intelligent horticulture characteristic courses suitable for students of all grades, which are very popular among children. Recently, the project was also selected as the national campus AI feature project of the China Children's Center, which will be replicated and promoted nationwide. In the Shanghai municipal party committee issued "vitality and city" under the background of five-year action plan, the pudong new area pudong building "kechuang dynamic ecosystem" plan, double gen base as an important gripper plan, is "link" more commissions, community, schools, enterprises and other main body, to benefit more primary and middle school students in the city. In the future, the pudong new area will build a batch of aims to stimulate youth innovative thinking "dream and workshop" youth center, small guest lab and other physical space, continue to hold diversified youth and BBS, green events, green and training activities, for youth kechuang "enthusiasts" to provide more interactive communication, resource sharing platform.�� Source: China Youth Daily, January 14,2022

2022-01-14	Group of Ningxia hui autonomous region committee of Ningxia youth federation held a press briefing in Ningxia Ningxia youth federation held a press briefing JianWei correspondent Zhang Zhuo) a few days ago, the Ningxia hui autonomous region committee, Ningxia youth federation held a press briefing, the Ningxia youth league transition preparations and "I do the practical work for the masses" practice situation to the news media. Li Feng, deputy secretary of the Communist Youth League Autonomous Region Committee and chairman of the Ningxia Youth Federation, presided over the meeting. Han Fan �, deputy secretary of the Communist Youth League Autonomous Region Committee, Shi Yang, some members of the team members and representatives of Ningxia Youth Federation, had discussions with reporters from more than 10 media units, such as Ningxia Branch of People's Daily, Xinhua Ningxia Branch and Ningxia Daily Press Group."Press briefing" scene. At the meeting, the correspondents provided the picture on the basic consideration of the change of the Ningxia Youth Federation, the size and composition of the committee, the recommendation conditions, standards and generation methods of the members, and the relevant requirements of the chairman, vice chairman and the Standing Committee, etc. The 10th Committee of the Ningxia Youth Federation will expire in March 2022. The 11th Committee will have about 360 people, including 15 sectors including agriculture and rural areas, social organizations and social services, press and publication and new media, Internet and information services. The selection of committee members highlights the political requirements, and the average age of around 35 at the time of the transition. Shi Yang and Bai Xizhong, vice chairmen of Ningxia Youth Federation, respectively introduced the study and education of party history, especially the practice of "I do practical things for the masses", to constantly improve the contribution of Ningxia Youth Federation to the "four promotion actions" and solve the concerns of the youth. After the release of the relevant work, the participants had in-depth exchanges with the media reporters on the establishment of a regular communication mechanism, and increased publicity and reporting on the key work of the 100th anniversary of the youth League and the Communist Youth League's participation in rural revitalization.

2022-01-14	Jiangsu: strict group strict out new atmosphere let the league organization built up, live, strong up Jiangsu: strict group strict out new atmosphere "and parents watched the movie" long jin lake "... in Suzhou Wuzhong district yingchun middle school 7 (7) squadron Zhou Guo ya" red bud " growth points manual, she recorded her process as the communist youth league member."Double product and double evaluation" points into the league is the first system of the Communist Youth League in Jiangsu province. The two points are combined with two democratic evaluations, and each young pioneer has to go through two rounds of screening before becoming a member of the Communist Youth League. Yingchun Middle School has innovatively designed three growth manuals, which can make points and evaluate the students applying for joining the league from five dimensions, including civilized quality, activity participation and volunteer service."'Double accumulation and double evaluation' can cultivate and select excellent league members, and also make the league activists and the development objects of the league members become more scientific and democratic."Li Chenjie, secretary of the school's youth League committee, found that the increased difficulty of joining the league did not hit the students' enthusiasm, but made them grow in the process of joining the league. For a long time, the Jiangsu Communist Youth League has effectively improved its political standing, set the standards and practices of comprehensively governing the Party with strict discipline, had the courage to make self-revolution, and further promoted the comprehensive and strict governance of the League to the grass-roots level and implemented. The construction of the league should be strict, and the construction of the league organization should also be standardized. The blank place and the slack place of the grass-roots level is a major chronic disease of the league construction, and the quantity and quality simultaneously to expand the coverage of the youth league organization is a good prescription. Tuan Xinyi Municipal Party Secretary Xu Rui told reporters that in the past, non-public league organizations always gathered and scattered, scattered and gathered together. Lack of activity, lack of close contact between league members and organizations are the main reasons. In recent years, the league Xinyi Municipal Committee of the industrial chain and industrial platform as the main position of the league building, focusing on improving the non-public enterprise league organization vitality and enterprise development vitality: often hold "youth lecture hall" in the enterprise park, invite the technical backbone to share professional knowledge; hold safety production knowledge competition, strengthen the red line awareness of youth... intimate service and diverse activities let the youth together, also let the league organization burst out infinite vitality. In the past five years, Jiangsu province has built 106,000 new league organizations, including 71,000 in the "two new" fields. Both the density of organizations in the social field and the total number of non-public enterprises rank first in the league. To expand the effective coverage of the league organization, the foundation is under construction, the key lies in living. As early as 2016, the Jiangsu Provincial sinking Group of the Communist Youth League Committee in Suzhou spent four months to conduct a full sample survey of the stations at the grass-roots level. In recent years, the Jiangsu Provincial Committee of the Communist Youth League has regularly carried out the "four no two straight" research and the smart league construction system, rectifying 47,000 weak and lax league organizations. In 2019, the youth League branch work list system was introduced, so that every league branch secretary can know "what the league organization does, how to do, how to evaluate". Sun Zhen, the league branch secretary of Jiawang Village, Pomegranate Street, Donghai County, showed his work list to reporters: the league organization helped a pair of factual orphan sisters to complete the "dream hut" transformation; the league members and love people started to complete the "micro wish" of troubled children... Sun Zhen felt that the work list makes his work direction more clear, down-to-earth to complete each basic item, can enhance the combat effectiveness and cohesion of the grassroots league organization. In the work list of Shen Manyi, the general branch secretary of Huangtu Village, Huangtu Town, Jiangyin City, Wuxi, there is a more sense of art.beard Town, the "web celebrity card" in Huangtu Village, gathers young artists from all over the world. They create characteristic homestays and shops to bring learning and development space to the town. Based on this, the general branch of Huangtu Village has built a youth dream space youth learning club with the theme of rural revitalization. Youth league members often sit around in the cafe, learning the history of the Party, giving league lessons, and making suggestions for local development. After the organization of the league is built and comes alive, there should be more comments and reviews. In order to strictly manage, Jiangsu Communist Youth League implements every training. The Jiangsu Provincial Party Committee of the Communist Youth League started two large-scale telephone random sampling inspections, which directly inspected the political theory and professional quality and youth satisfaction of the youth league cadres at all levels in the province. The sampling inspection results were included in the comprehensive evaluation indicators. The relevant person in charge of the Jiangsu Provincial Communist Youth League Party Committee told reporters that in 2021, a total of 39,560 youth League members and cadres were inspected in the province. Although this work is complicated, but we can listen to the aspirations of the league members, urge the league cadres to carry out the work in a standard way, so that the most grass-roots league organization is strong. Since 2017, the Jiangsu Communist Youth League has carried out the "double review and double evaluation" work of the league branch secretaries. In 2021, more than 210,000 youth League branches in Jiangsu province carried out "double review and double evaluation", and more than 10,000 grass-roots youth league (work) committee secretaries completed their review. Chu Weifeng, secretary of Dongtai Tangyang Town Committee, attended 6 times. He believes that the "double description and double evaluation" can not only enhance the sense of responsibility and honor of the youth league branch secretary, but also improve the learning initiative of the youth league members."In the past, the league members always passively watched the 'youth study', but now they are active in learning, more and more active and progressive in thinking."League cadres are the bridge between the league organization and league members, only league cadres close contact with the youth league members, league organization can be really strong and powerful. In 2020, the Jiangsu Communist Youth League began to build a joint youth service station. Every month, cadres visit villages, enterprises, schools and other grassroots units to contact the youth, carry out more than 25,000 joint youth service activities, and help 31,000 young people. The new atmosphere ideological education of the league is the first focus to ensure the advanced nature of the league members. In 2021, all 236,000 youth League branches in Jiangsu province carried out party history education, and reported college students and league members to youth league organizations in towns, streets and villages, on a regular basis. In 2019, the Youth League Committee of Changshu Institute of Technology signed a comprehensive cooperation agreement with the Changshu Municipal Party Committee of the Communist Youth League. In 2021, college students of Changshu Institute of Technology reported to the town and served as assistant to the league secretary of the Youth League and assistant to the league branch secretary of the league. They carried out more than 300 community registration projects, more than 600 practical positions, and more than 5000 college students participated in the registration work."This has opened up a new battlefield for the young people to make contributions, and also enriched the connotation of 'the whole regiment grasping colleges and universities'."Said Lu Yu, deputy secretary of the school's Communist Youth League committee. No matter in grassroots communities, or the frontline of the epidemic, there is always the Communist Youth League members. On July 21,2021, after repeated COVID-19 outbreaks in Nanjing, the Nanjing Municipal Party Committee of the Communist Youth League issued a volunteer recruitment announcement. In less than two days, nearly 6,000 young people have signed up in the city online. More than 20,000 young volunteers have served for more than 170,000 hours at hospitals and nucleic acid sampling sites. In 2021, more than 220,000 youth league members of Jiangsu province have joined the front line of epidemic prevention and control. They have demonstrated the advanced nature and leading power of the league members with practical actions, demonstrating the initial results of the Jiangsu Communist Youth League in strictly governing the youth League. Si Yong, secretary of the Jiangsu Provincial Committee of the Communist Youth League, said: " In recent years, the Jiangsu Communist Youth League has always set the requirements of comprehensively and strictly governing the Party, consciously strengthened the leadership of the Party, from the political, organizational, cadres, league members, system and other aspects of coordination, comprehensive and strict, strict out the advanced nature of the league members, the new actions of the league organization, and the good style of the league cadres."Intern, Chen Yuwei, China Youth Daily, China Youth Network reporter Li Runwen source: China Youth Daily, January 14,2022, edition of 01

2022-01-14	Chongqing through the youth theory armed "last kilometer" mainstream voice become network strong Chongqing through youth theory armed "last kilometer" and historical experience resolution spoke highly of winning the fight against COVID-19 outbreak this landmark event, difficult, we have a deep experience, such as China in the world to control the epidemic, the first to resume work and production... " a video briefing, nearly 200 people online. Recently, Ma Kai, a member of the Youth League group of Chongqing Municipal Youth League Committee and deputy secretary of the Youth League Committee of MCC CCID Group, passed on the spirit of the sixth Plenary Session of the 19th CPC Central Committee to the front-line youth online for the fact that the young employees are scattered around the project sites at home and abroad. After the closing of the sixth Plenary Session of the 19th CPC Central Committee, the Youth League Youth Lecture Group of Chongqing Municipal Party Committee focused on learning and implementing the spirit of the plenary session, and went deep into enterprises, rural areas, schools and other places to carry out publicity, setting off a wave of learning, publicizing and implementing the spirit of the sixth Plenary Session of the 19th CPC Central Committee among the youth. On the second day of the plenary session, Su Mingqi Jin, a member of the Youth Lecture Group of Chongqing Municipal Youth League and a member of the working team of Huaxi Village, Zhongyi Township, Shizhu County, Chongqing, came to Huaxi Village to share the spirit of the plenary session with the local young people. After listening to Su mingjin's speech, Ma Yuanli, the head of the local "one house" farmhouse, has more confidence in the operation of the farmhouse. She said that the industry must be managed and managed well, let the life over the more prosperous. Last year just selected the "Chongqing talents innovation entrepreneurial talents" of Chongqing medical university affiliated children's hospital, deputy director of Zhao Yao, in science and technology laboratory "science and technology innovation achievements, through subject analysis and discussion, the plenary session of the" youth element " to the youth medical science and technology workers, encourage them to be the leader of the progress of science and technology and striver. These propaganda scenes are just the epitome of the youth teaching group of Chongqing Municipal Party Committee going deep into rural areas and entering college students and emerging fields. According to the relevant person in charge of the Chongqing Municipal Committee of the Communist Youth League, since the launch of the sixth Plenary Session of the 19th CPC Central Committee, the Chongqing Youth League Youth Lecture Group has carried out more than 400 "small-scale" lectures, covering more than 26,000 young people. The Youth Lecture Group of the Chongqing Youth League Municipal Committee was established in 2019. It aims to build a "light cavalry", open up the "last kilometer" of the youth theory arm, and realize the normal and systematic interpretation of the Party's scientific theory among the youth group. This year, the Chongqing municipal party committee around building high standard, high quality youth branch team, focus on cultivating a batch of "star lecturer", build a "star team", through directional hiring, organization recommendation, personal recommendation, selection procedures, to determine the 100 lecturers, including young cadres, youth social science experts, youth science and technology innovation talents, etc. Now, as an important part of the municipal youth publicity team to study and implement the spirit of the Sixth Plenary Session of the 19th CPC Central Committee, the Youth Lecture Group of the Chongqing Youth League Municipal Committee is actively carrying out the publicity. Among them, the Internet has become this "light cavalry" to carry out another plenary session. Combined with the cognitive characteristics and learning habits of the youth, they have actively carried out online publicity, and created and launched a series of new media learning products, such as short videos and dynamic posters. Recently, Du Huichao, a member of the Youth League group of the Communist Youth League, as the leader, participated in the recording of the "Youth Learning" of the Central Youth League. He said that the use of short videos to explain the spirit of the plenum is meaningful, interesting, and more likely to become "popular". In the opinion of Meng Yuhang, a student from the School of Beauty Film of Chongqing University, the inquiry, heuristic and visual presentation have a sense of participation, experience and gain, which also gives him more motivation to learn. It is understood that participating in the "youth learning" online theme group class has become an important form for Chongqing young students to learn the "second class". More than 1.5 million young people in Chongqing have learned the spirit of the plenary session through online themed group classes called "Youth Learning" and interactive questions and answers and questionnaire surveys. Recently, Zheng Lingfeng, a member of the Youth League teaching group of Chongqing Youth Party Committee and deputy director of the Municipal Youth New Media Center, was very busy. She led the team to continuously create the spiritual-themed online cultural products of the sixth Plenary Session of the 19th CPC Central Committee. Relying on the fan image of "Little Hotpot" and "Little Hongmei" of Chongqing Communist Youth League, they created a series of products such as key words, golden sentences, cute emojis, dynamic pictures and Jiugong Ge. Through mobile phone wallpaper, chat background and other forms, these products enter the youth mobile phone, embedded in the youth life, integrated into the youth social networking, so that the mainstream voice becomes the Internet strong voice. Zheng Lingfeng said that the "Internet + propaganda" must be done, and it must be done well. Up to now, the Youth League Youth Teaching Group of Chongqing Youth League has launched more than 30 online cultural learning products at the Sixth Plenary Session of the 19th CPC Central Committee, covering more than 4 million young people. Youth League committees of districts, counties and universities have also set up teaching teams according to local conditions, create "walking classes" for young people in different fields, relying on the local unique resources, and send the spirit of the plenary session to Courier boys, online ride-hailing drivers, volunteers and other groups. The Youth Lecture Group of Shapingba District of Chongqing organized micro learning, publicity and communication with other red resources such as Gele Mountain Martyrs Cemetery, Bai Mansion and Refuse Pit. The activity of "Shamagnetic Youth Talk" attracted more than 1,000 young people to participate. Group the relevant person in charge of Chongqing municipal party committee, said the youth branch is leading the brand of youth thought, Chongqing communist youth league will continue to deepen the youth, youth, lead the youth, learning propaganda and implement the spirit of the sixth plenary session of the 19th, do "party and government identity, social recognition, youth satisfaction", let the new era of the party's ideological flag and spirit flag flying in the youth heart. Correspondent He Kai, China Youth Daily, China Youth Network reporter Wang Xinxin source: China Youth Daily

2022-01-18	Guangdong "the communist youth league and people's congress, CPPCC members face to face" held in Guangdong province "communist youth league and people's congress, CPPCC face to face" JianWei correspondent YueQing) recently, 2022 "the communist youth league and NPC, CPPCC face to face" activities in Guangzhou, Guangdong, Guangdong social construction committee, CPPCC social and legal system committee responsible comrades to attend the activities. The deputies to the National People's Congress, the deputies to the Provincial People's Congress, the members of the CPPCC, the provincial Health Commission, the provincial Department of Justice, as well as psychological counselors, lawyers, the backbone of young social organizations, teachers, students and other 30 people attended the symposium. Symposium site. Group of Guangdong provincial party committee for figure symposium, combining their respective industry direction and work, study experience, around the "build social youth mental health service mechanism" and "establish and improve the" three lost "(unemployment) youth group support system" theme, share the good experience and put forward the "strengthen the construction of local laws and regulations and professional team" "strengthen psychological service institutions, especially non-profit psychological service support" "attaches great importance to subject education training and employment problems, strengthen employment and unemployment security" and other Suggestions. At the people's congress, CPPCC members listen to speak, discussed the opinions of Suggestions, and said "face to face" activities for youth to express interests provides a good communication platform, after the opinions of the meeting will be organized, through Suggestions, proposals, proposals and other form selected at the two sessions. The meeting called for: to thoroughly study and understand General Secretary Xi Jinping's important remarks on the whole process of people's democracy, further deepen the "face to face" activities, and guide the young people to continuously enhance their awareness and ability of orderly political participation. We should give full play to the positive role of deputies to the NPC and CPPCC members in the healthy growth of youth, and mobilize more NPC deputies and CPPCC members to shout loudly for the growth and development of youth and the protection of their rights and interests. We should cooperate with relevant functional departments, psychological counselors, lawyers, social workers and other professional forces to promote the formation of a joint force for home, schools and communities to care for the healthy growth of young people. The young participants took an active part in the discussion and exchange. The "Face to face" activity is an important carrier and brand activity for the Communist Youth League to effectively perform the function of "endorsement" and institutionalize the social interest expression channels of the NPC and the CPPCC to reflect the demands of the youth. The "Guangdong Provincial Communist Youth League face to face with deputies to the NPC and CPPCC members" activity has been held for 14 consecutive years since 2008. Next, the group of Guangdong provincial party committee will take the symposium as an opportunity to strengthen the communication with the relevant units and people's congress, CPPCC members, actively cooperate with the proposal, proposal preparation, and continue to track the suggestion, proposal implementation, with concrete actions to support and guide social forces to participate in adolescent mental health and "three loss" youth group support work.

2022-01-13	Fujian People's Congress representative CPPCC members and youth discuss how to discuss how to youth discuss how to introduce talent youth network trainee reporter Tian Hongwei) introduced high-quality talents only in the countryside exercise a few years were transferred; beautiful countryside construction good, industrial development still lacks talent.... this is Lin Lulu in the Fujian Communist Youth League and NPC deputies, CPPCC members "face to face" activities. This year is the 14th year of the "face to face" activities held by the Fujian Communist Youth League and NPC deputies and CPPCC members. This year's event invited youth representatives from relevant fields of the province to participate in the work on many aspects of youth scientific and technological innovation, youth entrepreneurship and employment, rural revitalization, integrated development of youth in Fujian and Taiwan, youth mental health and so on. Among them, how to introduce and cultivate young talents has become the core issue of these work. Lin Lulu is the village branch secretary of Longxiang Village, Songkou Town, Yongtai County, Fuzhou City, and also a returning young entrepreneur. She said the rural streets are clean and the environment is better, but the problems of talent revitalization and industrial prosperity are still unsolved. She has seen many young people who are enthusiastic about participating in rural revitalization, but many have come and leave because of the lack of a long-term mechanism to take them deep in the countryside. Lin Lulu is currently leading the village to try to develop local specialties and build a communication space suitable for young people. Lin hopes the government will increase its support for rural revitalization talents in terms of policies and funds in the future. Taiwan youth Guo Yifan, the founder of the brand "Carry coffee" brand, currently has two stores in Fuzhou Sanfang and Seven lanes and Shangshanghang Historical and Cultural Scenic Spot. Guo Yifan started from the problem of recruitment difficulty of "baristas" and combined with his own research data. He hoped to cooperate with schools, trade unions and other organizations in the future to provide a new path for the development of young talents by opening coffee majors or providing vocational skills training for baristas. Also encountered on the recruitment problem and Fuzhou City CPPCC member Yin Jun. His company posted a position online that had been neglected for three months. Yin jun hopes that Fujian province will improve the market matching degree in cultivating and introducing talents in the future. LAN Haodong, an intern of Fujian Province's "Sailing Plan" in 2021, hopes that Fujian will not stick to one pattern when introducing talents. He believes that should be according to the actual needs of all walks of life in the province to identify standards, at the same time strengthen communication with provincial young talents, establish a long-term, multi-level communication mechanism as soon as possible, such as "sail plan", "good years in Fuzhou" talent activities, to further improve the quantity and quality of the work. League of Fujian provincial party committee secretary XiaoHuaxin said, the Fujian provincial party committee will closely around the youth universal interests, find the communist youth league and the youth should be for combining point, breakthrough point, key point, serious planning work ideas, for the majority of teenagers grow up to create a good environment, condensed youth strength, active in the comprehensive construction of modern socialist country in Fujian the great practice of chapter.

2022-01-11	The first batch of "Zhejiang youth craftsmen" training project selected list announced the first list of "Zhejiang youth craftsmen" training project selected list net reporter Jiang Yutong) recently, by Zhejiang human social security hall, Zhejiang communist youth league committee jointly organize the implementation of the "Zhejiang youth craftsmen" training project, released in 2021, the province a total of 1950 young skills talents for the first "Zhejiang youth craftsmen" training project. According to the Opinions on the Implementation of Zhejiang Craftsman Cultivation Project in the New Era issued by the Provincial Government of Zhejiang Province, Zhejiang Province launched the "Craftsman Selection Action" in 2021, including four levels: Zhejiang great craftsman, Zhejiang Outstanding craftsman, Zhejiang craftsman and Zhejiang Young craftsman. Among them, Zhejiang young craftsmen are jointly organized and implemented by Zhejiang Provincial Department of Human Resources and Social Security and Zhejiang Provincial Party Committee of the Communist Youth League. The selection is organized once every year. It is planned to focus on cultivating about 10,000 young craftsmen in Zhejiang by 2025. Shortalists for the first Zhejiang youth craftsman training project personnel, are a senior and above professional qualification (vocational skills level) of a line of skilled talents, mainly from Zhejiang province economic development leading industry, key industries and has a higher technical skills level of young industry workers selection, the average age of 31.2 years old, including enterprise skills talents accounted for 82.6%, women accounted for 17.2%. After the list is announced, the selected personnel will be trained for one year, and the training funds will be supported. The funds will be allocated to the units of the selected personnel, mainly for technical innovation, skill training, master guidance, team building, cooperation and exchange, etc. In recent years, according to the Zhejiang provincial government on the construction of high quality strong talent team deployment requirements, Zhejiang human social security hall, Zhejiang communist youth league focus on youth skills of personal growth and career development, continue to concentrate youth skills condensed service work, relying on the "revitalization cup" youth vocational skills competition, "young marxist training project", "Zhejiang youth craftsmen" selection, "golden blue-collar" vocational skills promotion action carrier, encourage the youth skills success, skills, high quality development construction for Zhejiang province youth strength.

2022-01-10	The second Volunteer Guangdong Public Welfare Night held in Guangzhou was held in Guangzhou, Jianwei correspondent Yue Qing) " Voluntary public welfare cause is a good thing, and Guangdong volunteer public welfare cause has developed very well."Mr.Chen Jinzhang, the" landscape master "of Lingnan School of Painting, donated his precious masterpiece" Spring Mountain and Clouds rise " to speak for the volunteer public welfare... Tonight, the Guangzhou Tower is full of love, art performances, charity sales, charity donations gathered into another beautiful scenery in the volunteer Guangdong. Volunteers show the charity sale works. On the evening of January 8th, the second Volunteer Guangdong Charity Night of "Come to Guangdong for Love together" was held in Guangzhou Tower. This activity by the Chinese youth volunteers association, the communist youth league committee guidance, Guangdong volunteers federation, Guangdong artists association, Guangdong volunteer career development foundation, attracted more than 200 caring entrepreneurs, caring people and social organizations, foundation representatives to participate in the charity sale, pledge and other activities. Chi Zhixiong, secretary of Guangdong Provincial Party Committee of the Communist Youth League, Gu Zuoyi, president of Guangdong Volunteer Federation, and Li Jin, vice president of China Artists Association and president of Guangdong Artists Association, � � activity. Leaders of the organizer issue commemorative cards to artists who support public welfare undertakings. There are many artists who love public welfare undertakings in Lingnan. They silently support this cause of serving the people with their pens, their voices and their performances. In this activity, more than 20 famous artists and social caring people have donated their favorite works of art to support voluntary public welfare undertakings. Chen Yongqiang, vice president of the Chinese Painting Society, believes that "volunteers are a kind of personality and a character serving the society". Lin Lan, president of the Guangdong Art Academy of Painting, who has donated his paintings twice in a row, said, "We should put the power of art on a big platform of public welfare and do our due public welfare responsibilities, which is our due responsibility."Literary volunteers perform their art on the scene. Group of Guangdong provincial party committee for figure in the evening, in addition to the charity sale, Guangzhou children's palace after the rain rainbow fusion troupe, the national youth league member of south China normal university music college, vice President of � retting drip mound � province youth singer shan-shan feng teacher and xinghai music college students volunteers, literary volunteers also brought to volunteer public welfare as the theme of the art performance. In 2020, the first "Volunteer Guangdong" public welfare Night activity sold 26 charity sales, and the funds raised were used for the implementation of volunteer service projects, volunteer growth and care, support for volunteer service organizations, and other needs to promote the institutionalization and normal development of volunteer services in Guangdong. All the guests took a group photo in the form of the volunteers' "heart and hand logo". This year, all the funds raised by the second Volunteer Guangdong Public Welfare Night will be used to support the development of volunteer service in the province. The Guangdong Volunteer Cause Development Foundation, in charge of the Guangdong Provincial Committee of the Communist Youth League, will be responsible for managing the fund raising and management of the event. The foundation is the first provincial-level public volunteer service foundation in China, and has funded more than 2,458 volunteer organizations to carry out volunteer service projects.

2022-01-10	The first youth in Gansu province ecological civilization innovation entrepreneurship competition successful packaged "dayu water saving cup" the first Gansu province youth ecological civilization innovation entrepreneurship competition successful packaged Wang Hao) on January 9, by the communist youth league committee of Gansu province education department, human resources and social security hall, ecological environment hall and other units "dayu water saving cup" the first Gansu province youth ecological civilization innovation entrepreneurship competition ceremony and youth ecological civilization BBS held in Lanzhou, Gansu province. After fierce competition, the organizing committee and evaluation committee in 372 entries, selected social entrepreneurship group, colleges, public project, propaganda creative group four groups first prize 7, second prize 14,21, third prize, 76, also selected 10 outstanding organization, 30 excellent teachers. The entries involve the high-quality development of the Yellow River Basin, ecological and desertification control of the Qilian Mountains, environmental health, energy conservation and emission reduction and green development, waste classification and resource regeneration, water saving and water protection, tree planting and green protection, rural revitalization and other application fields. At present, the excellent award-winning works have been compiled into a book, as a quality case of youth ecological and environmental protection projects in Gansu Province, which are centrally displayed and recommended on the official website and wechat public account. At the scene, Pan Baotian, member of the Standing Committee of Lanzhou University Party Committee and Vice President of the Yellow River Basin Green Development Institute and other experts and scholars delivered keynote speeches. Pan Baotian said that "the fifth major ecological protection and high-quality development of the Yellow River Basin", as the fifth national regional strategy, is related to the national take-off and national rejuvenation, and is a major event for governing the country. It is also a major development opportunity for Gansu, which must be firmly seized. The relevant responsible comrade of the Gansu Provincial Communist Youth League said that the Gansu Communist Youth League will establish a youth ecological and environmental protection project pool and talent pool in Gansu Province, and promote the incubation, resource docking and training and promotion of award-winning projects and institutions. Further strengthen the cooperation with financial institutions, venture capital institutions and venture capital institutions, and provide comprehensive support for the project in combination with the Gansu Youth Innovation and Entrepreneurship Fund. To help the young people contribute their youth to the construction of a new Gansu with beautiful mountains and rivers, good ecology and beautiful environment.

2022-01-10	Fujian youth branch to carry out concentrated demonstration preaching Fujian youth group Jianwei correspondent Zhou Weiliang) recently, according to the unified deployment of TuanOuWei, Fujian youth branch closely around the sixth plenary session of the 19 and the 11th party spirit, fanned out the province nine cities and pingtan comprehensive experimental area to carry out concentrated demonstration preaching activities. More than 30 provincial and municipal youth branch members into school, into the community, into the countryside, into the new era of civilization practice center, contact the party's struggle in one hundred journey, combined with the history of Fujian story, face to face, interactive preaching, to ground, steaming expression, promote the plenary and provincial party spirit in the youth into the brain into the heart, take root. Nanping lecture site."When we talk about yesterday, today and tomorrow, what will you think of? Today, I would like to talk to you about the Communist Party of China yesterday, today and tomorrow, and talk about the suffering and glory of yesterday, today's mission responsibility and the great dream of tomorrow."In Fuzhou taijiang district Hangzhou community story hall, Fuzhou Fujian youth corps committee propaganda department vice minister Lin min to "do not forget yesterday, clear conscience today, not tomorrow", around the sixth plenary session of the 19th and the 11th party congress, combined with the typical youth deeds, vivid interpretation of the communist party of China yesterday, today mission and tomorrow's great dream, called for a new era of youth to high morale, new era, yong when dreamer. In Xiamen, Fujian youth branch members, jimei university college party committee propaganda department minister associate professor Lin Weiyi "in summarizing the historical experience better future", guide the students to further study and understand the sixth plenary session of the 19 and the 11th party spirit, learn the feelings, learn confidence, learn the strength. Zhangzhou lecture site. In Zhangzhou, Chen Yi, a member of Fujian Youth Lecture Group and associate professor of Zhangzhou Municipal Party School, walked into Longwen No.1 Middle School and Longhai No.1 Middle School. He combined with his own work experience and experience in the village, and told the youth members about the "valuable Experience and Spiritual Wealth of a Hundred Years of Struggle" with vivid party history stories and vivid language. In Quanzhou, Fujian youth branch members, provincial party committee branch group "new era preacher" week into the dawn professional university, with recently popular network song "2035 to Taiwan", review the party history and cross-strait relations, interpretation "to promote melting, to promote harmony, to promote melting", looking to the youth on both sides of the landing "first home" integration development. Quanzhou propaganda site."Concentrating forces on accomplishing major tasks is a significant advantage of China's national system and national governance system, which is not only derived from the Marxist theory of proletarian dictatorship, but also derived from the traditional governance view of Chinese civilization". In Sanming, Fujian youth branch members, Fujian art vocational college of marxism dean Zheng Yuankai into sanyuan area second experimental school, detailed interpretation of the basic principle of marxism combined with the Chinese excellent traditional culture theory logic: " throughout the development history of Chinese civilization, it is not difficult to find that our nation has inherent organization and discipline, is a good at organizing the nation. From the institutional level, the reason why the Communist Party of China was able to reverse the decadence of modern China and make great achievements in the Chinese revolution and socialist modernization drive is that the secret of its success is also the characteristic of 'organization'."Putian lecture site. Group of Fujian provincial party committee for figure in Putian city li city new era civilization practice center, Fujian youth branch members, associate professor of Fujian engineering institute fan made entitled " from <resolution> read why can the communist party of China preach report, combining the reality of Fujian and youth work, youth for grassroots deep understanding, grasp and practice plenary session and the provincial party congress spirit provides powerful guidance."The resolution consists of seven parts, and the full text is more than 36,000 words. Such a magnificent masterpiece, and we have what to do with? And where should we start learning it?"In Nanping, Lin Meng, a member of Fujian Youth Lecture Group and head of the Publicity Department of Nanping Municipal Party Committee, sorted out the historical logic of the great rejuvenation of the Chinese nation. In Longyan, Li Tianli, a member of the Fujian Youth Lecture Group and the counselor of Fujian Normal University, explained the Extreme importance of Ideological Work from <Resolution> from the situation, the role, how to consolidate and occupy the position. Lin Aiqing, the member of the Fujian Youth Lecture Group, the director of the lecture group of the CPC, stressed that the new era must maintain the "four confidence" to face all difficulties and challenges without fear, and unswervingly open up new horizons and create new miracles. Ningde preaching site. League of Fujian provincial party committee for figure in Ningde, Fujian youth branch members, Fujian technical normal college youth teacher Dong Lin with a lot of rich and vivid youth stories and cases, reviewed the four historical period, four great leap forward, tells the past five years of the struggle of Fujian and the future development layout, clarify youth is an important force to promote the development of history and social progress, new era youth should learn party history, strengthen the responsibility, constantly draw strength from party history in one hundred. In Pingtan, Chen Dong, a member of the Fujian Youth Lecture Group and associate professor of the School of Marxism, Minjiang University, with the title of "The Wind and the Waves", Taking General Secretary Xi Jinping's "Pingtan complex" as the entry point, From "bear in mind what the Communist Party of China is, what it does, and what it wants to do" to " keep in mind the 'two establishment', Do 'two maintenance' "" adhere to the self-revolution, Contribution to the new era " three aspects of the spirit of the sixth Plenary Session of the 19th CPC Central Committee in a simple way, vivid and wonderful interpretation, Encourage young students to " look at the great achievements, Be a confident Chinese "," learn from science and culture, Be a new generation" Listen to the party consciously, Follow the party firmly. It is a major political task to study, publicize and implement the spirit of the sixth Plenary Session of the 19th CPC Central Committee and the 11th Session of the Provincial Party Congress. In order to effectively enhance the authority and timeliness of publicity work, Fujian Youth Teaching group selected capable forces to carry out centralized demonstration publicity. In order to tell the story of the Party history vividly, clear historical logic, thorough institutional confidence, clear political pursuit, and cultivate young people with the great spirit of party building, in addition to the necessary political quality and theoretical foundation, we also need to continuously acquire the latest and most authoritative spiritual essence. To this end, the provincial Communist Youth League Committee organized the youth teaching group members to listen to the central, the provincial Party Committee publicity group report and participate in the special training class, timely convey the central and provincial Party Committee on learning, publicizing and implementing the spirit of the sixth Plenary session of the 19th CPC Central Committee and the 11th provincial Party Congress."As a member of the youth teaching group, I should first learn one step forward and learn a deeper level. At the same time, I should do a good job in the youth, contemporary and popular interpretation of advanced theories, so that the spirit of the plenary session will enter the ears and hearts of the young people."Said Zheng Yuankai, a member of Fujian Youth Lecture Group and dean of Marxism College of Fujian Art Vocational College. The provincial Communist Youth League Committee also actively promoted the members of the young teaching group to carry out centralized learning exchanges, organize manuscript groups to polish courseware, and effectively improve the quality of publicity. Based on the basic basis of the publicity outline of the Central Committee and the Provincial Party Committee, combined with the characteristics of Fujian Communist Youth League and youth work, we organized forces to compile the publicity courseware with accurate content, popular language and infectious appeal. For example, "from the <resolution> read why the Communist Party of China can", is the three members of the Fujian youth teaching group repeatedly polished, collective completion after the use of reference for high-quality courseware. In the form of publicity, in addition to the centralized demonstration and publicity, the youth lecturers' members are also arranged to interact face to face with the grass-roots youth, and truly respond to the hot and difficult issues concerned by the youth. In addition, the Provincial Communist Youth League Committee organized members of the youth teaching group to record videos, and extensively carry out online publicity through the all-media platform of the Fujian Communist Youth League, effectively expanding the coverage of publicity. At the same time, the provincial Communist Youth League Committee deployed the youth league organizations at all levels to carry out individual self-study, special study, special training, knowledge competition and other activities, in-depth study, publicity and implementation of the spirit of the sixth Plenary Session of the 19th CPC Central Committee and the 11th Provincial Party Congress, and guide the youth league members to listen to the Party and follow the Party. Thought baptism, coagulation heart gather force a report, a thought baptism; a speech, a heart gather force. Many youth members said, Fujian youth branch on the theme of preaching the review history summarizes experience and facing the future in the struggle together, the reasoning, history, achievement, both rigorous and vivid, both highlight the key and grasp the key, to increase wisdom, enhance unity, increase confidence, enhance morale."The speech is vivid and profound," said Wu Jianzhong, a 2019 tourism major student from Wuyi Tourism and Business School, saying, " We should not forget the hardships of the past, cherish the happiness of the present, but also to remind ourselves to study hard, learn excellent skills, and be a new youth with confidence."" listened to teacher Lin Min "do not forget yesterday, clear conscience today, live up to tomorrow- -learn the spirit of the sixth Plenary session of the 19th CPC Central Committee" sharing, let me interpret the spirit of the sixth Plenary session of the 19th CPC Central Committee from the historical dimension, so that I made clear the historical positioning of the answer paper in the new era."Fuzhou Taijiang District Youth League cadre Wang Dan said. Gao Pengwei, a cadre of Putian Licheng Communist Youth League District Committee, said, " As a youth League cadre, I will carefully gather together to serve the youth, improve the effectiveness of the youth work, and strive to do a good job in the party's youth work in the new era."" The wind is good, forge ahead ". Fan Shengfei, a young cadre of Jiangkou Town government, Jianyang District, Nanping City, said, " Take the 'woodcutter' Comrade Liao Junbo as an example. Be loyal to the Party, ask for the people, self-serving, selfless dedication, strive to be a loyal, clean and responsible good cadre in the new era, and contribute to the chapter of building a modern socialist country in Fujian.”

2022-01-03	Qinghai province youth work joint meeting the third plenary meeting of youth work joint meeting in Qinghai province wei correspondent a Shuyuan) recently, Qinghai province youth work joint third plenary meeting held in Xining, communicate learning long-term youth development planning implementation work of the third plenary meeting spirit, the deployment of the long-term youth development plan in Qinghai province (2018-2025) implementation work, Qinghai provincial party committee, federation of trade unions chairman Ma Jixiao attended the meeting and speech, vice governor Yang Fengchun chaired the meeting. Ma Jixiao stressed the need to adhere to the problem-oriented approach, highlight key tasks, and vigorously promote the implementation of the plan. We need to do a good medium-term assessment, identify the weaknesses in the implementation process of the Plan, thoroughly analyze the reasons, formulate effective measures, and continue to promote its implementation. All member units should strengthen the overall linkage, constantly optimize the monitoring indicators, fully implement the working mechanism, and effectively form the overall joint force. Party committees and governments at all levels should be concerned about the growth of young people, proceed from the practical needs of the young people, plan good youth development projects and formulate good policies for youth development, and constantly create favorable conditions for their growth and development. Conference, released the 2020 annual youth development planning in Qinghai province statistical monitoring report, examined and approved the "youth development work in Qinghai province information submitted system" the youth development work reporting on activities appraisal system "of the youth work in Qinghai province joint conference key work supervision method" of the youth work in Qinghai province work rules (revised) ". Dong Yuyi, secretary of the Provincial Communist Youth League Party Committee and deputy convenor of the joint meeting, reported the implementation progress of the province's "plan" and arranged the next key work. The Provincial Bureau of Statistics made the explanation on the statistical monitoring of the "Plan in 2020", and the leaders of Xining City and Provincial Human Resources and Social Security Department exchanged speeches, and the Provincial Public Security Department, Provincial Department of Civil Affairs and Provincial Cyberspace Office exchanged written experiences. Members in charge of the liaison units of the joint meeting, the convenor of the joint meetings of the city and state, responsible leaders of the Youth League committee, and responsible leaders of all departments of the provincial Communist Youth League Committee attended the meeting.

2022-01-02	Jilin university prosperous enriching practice team to ceremony in Jilin province students prosperous and enriching practice team to ceremony Wang Peilian) recently, Jilin province party committee joint Jilin provincial party committee organization department, Jilin provincial party committee propaganda department, Jilin province education department and other department held in Changchun university of technology "I and my hometown" in 2021 Jilin province students social practice achievements and Jilin province students prosperous enriching practice team to ceremony, for Jilin province college students prosperous practice team flag, for 2021 college students "a program under which officials" summer social practice outstanding typical representative certificate. It is understood that in the summer of 2021, the Jilin Provincial Committee of the Communist Youth League built 194 social practice bases for college students, developed 8,391 practice positions, established more than 4,000 college students' practice teams, and mobilized more than 60,000 youth college league members to go throughout the province to carry out practical activities. Next, 186 teams set up by 62 universities in Jilin Province will carry out precise docking with border villages to help realize the border prosperity and enrich the people. The activity was broadcast live through the official account "Jiqing Feiyang" on the WeChat platform of Jilin Provincial Communist Youth League, and the number of likes reached 177,000. In 2021, outstanding typical representatives of college students '"three to the countryside" summer social practice activities and college students' "return to home" and "Sailing Plan" social practice students participated in the activity.

2021-12-31	Youth branch for Inner Mongolia youth preach sixth plenary session spirit youth branch for Inner Mongolia youth preach spirit sixth plenary session stone jia) for days, the Inner Mongolia district party committee organization youth branch members around Inner Mongolia, for the general member youth preach the sixth plenary session of the 19 and the 11th party congress of the Inner Mongolia autonomous region spirit. On December 16, Mo Ling, a member of the Inner Mongolia Autonomous Region Youth Lecture Group, spoke to the youth league members of North United Power Co., Ltd. in an online video form. Mo Ling took the Resolution of the CPC Central Committee on the Major Achievements and Historical Experience of the Party's Struggle in a Century as the starting point to explain the significance of the Sixth Plenary Session of the 19th CPC Central Committee and the course of the Chinese people in the choice of the CPC. Mo Ling to the North United Power members of Co., LTD. Image was provided by the respondents. On December 17, Guo Yongxing, a member of the youth teaching group, came to Jiayuan Community, Hushan Street, Jining District, Wulanchabu City, Inner Mongolia, using vivid cases, rich historical facts and detailed data to make a systematic publicity and in-depth interpretation of the spirit of the Sixth Plenary Session of the 19th CPC Central Committee. Recently, at Inner Mongolia University and Inner Mongolia Normal University, Wang Qianyu and Zhao Weiwei, two members of the Inner Mongolia Youth lecturers, brought face-to-face and interactive special lectures to students and youth League officials around the spirit of the sixth Plenary Session of the 19th CPC Central Committee and the spirit of the 11th Party Congress of Inner Mongolia Autonomous Region. In the next step, the Youth League Inner Mongolia District Committee will continue to organize the district youth lecturers members to go deep into schools, street communities, rural pastoral areas and other grass-roots frontline, for the youth of different ages, different areas of the spirit of the Party plenary session and the spirit of the Inner Mongolia Party Congress of the popularization, youth, elemental explanation.

2022-01-10	Hunan "the communist youth league and people's congress, CPPCC members face to face" activities "the communist youth league and people's congress, CPPCC members face to face" activities held in Changsha JianWei correspondent Liang eting) recently, 2022 "the communist youth league and NPC, CPPCC face to face" activities held in Changsha, and around the "promotion level of adolescent mental health service" theme held a symposium. Ye Hongzhuan, deputy director of the Standing Committee of the Provincial People's Congress, attended the meeting and delivered a speech. Li Zhichao, secretary of the Communist Youth League Committee, presided over it. Liu Jing, deputy secretary of the Communist Youth League Committee, made a keynote speech. More than 40 people attended the symposium, including provincial People's Congress Social Committee, Joint Working Committee, Provincial CPPCC Social Law Committee, Provincial Education Department, Provincial Health Department, Provincial Health Department, Provincial Health Commission, and representatives, teachers' representatives, parents' representatives and student representatives. Pictures of the event site. Correspondent for figure "face to face" activities has continued for 14 years, stick to the pulse topic presentation, around the party and government concern, social concern, youth concern, further teenagers widely listen, research, and fully negotiate and build consensus with representatives, members, form a bill, suggestion or proposal, through two channels, appeal for teenagers appeal provides the upward channel, has become an important carrier of Hunan communist youth league to participate in socialist consultative democracy. The symposium pointed out that the in-depth mental health education for teenagers is a practical and urgent task, and the NPC deputies and CPPCC members should pay close attention to it, and actively perform their duties and play a role in helping them cultivate positive, optimistic, rational and peaceful psychological qualities. Youth league organizations at all levels should deepen their "face-to-face" brand activities and continue to speak out for young people. Relevant departments under the province should perform their respective duties to jointly promote the comprehensive development of adolescent physical and mental health. Deputies to people's congresses and CPPCC members at all levels should vigorously support "face-to-face" activities, translate their demands and expectations for healthy growth into high-quality advice, provide broader policy space for their growth and development, and jointly protect their strong growth.

2021-12-31	In the park, the old factories are as old as old, integrating the Beiyang industrial style and contemporary architectural art, which is quite popular with young people. The two high-rise apartments in Linhai River have the most beautiful landscape view in the park, which is a "home" for young talents- -talent apartment. Standing in front of the sand table in the public art space of the creative park, Li Meng, secretary of the Hedong District Party Committee of Tianjin Municipality, said that the youth league organization is helping the struggling young people to make their dreams come true."We have found that a considerable number of young people are temporarily in the 'sandwich layer' of the policy, making it difficult to apply for public rental housing or shared property rights, and they are unable to buy commercial housing."Li Meng cut to the point, cut into the key problem of housing difficulties for young people. Recently, with the theme of "paying attention to the current situation of youth housing in big cities", the Tianjin Communist Youth League and the deputies of the NPC " walked into the new urban citizens and youth groups, listen to the troubles of young people about housing, and understand the actual needs of youth development. Subsequently, the cadres of the Communist Youth League introduced the investigation to the NPC deputies. This year, the group hedong district party committee for all enterprises, administrative institutions, urban new immigrants, college graduates, new social classes and other youth groups on the housing problem, the results show that the vast majority of young people are more inclined to choose to live alone, more than half of the people think that rent or mortgage accounted for 20% -30% of the monthly income has less impact on the quality of life. The reality is that house prices or rents are always too high."This is one of the important problems facing young people, such as new immigrants, enterprises and migrant workers in cities."Li Meng said. Young people also raise many expectations on how to "live in housing", such as strengthening the construction of public rental housing and low-rent housing, providing rent reduction policies, and expanding market channels for investing in youth housing projects. In addition to the high rent, renting in the process of frequent "stepping on the pit" also let many young people upset. Li Haibin, a young lawyer working in Hedong District, analyzed that the proportion of college students graduating within 5 years is low, and most rent to meet the needs of work and life; while college students who just come out of the campus usually lack experience, "such as house viewing, house checking, house collection and rent negotiation before the contract signing, but they often do not have it". As a result, once problems arise, it is difficult for college students to choose legal weapons to protect their legitimate rights and interests. One of the typical "pit" is the malicious competition and false publicity problems existing in the rental intermediary market. In the cases in which Li Haibin contacted, there are often the ownership problems and quality defects of the deliberately concealing the leased house, and then cause litigation or retaliation, "which will lead to further deepening of the contradiction". In order to solve a series of problems encountered in the process of renting houses, Tianjin municipal, district governments and youth league organizations have thought of a lot of ways."We are just long-term rental apartments tailored for urban youth."The relevant person in charge of Tianjin residential group, day live cotton three shop is the city's first built by state-owned enterprises centralized youth rent apartment, but also Tianjin talent apartment, the occupancy rate of 91%," we play a state-owned enterprises in the leading and leading role in the housing rental market, to Tianjin struggle for the development of young people first warm home ". According to statistics, most of the tenants here are currently young people under 35, of which 40% are under 28 and 45% are from 28 to 35; 52% are talents introduced through "Haihe Talents" program, 30% are temporary cadres and selected students, and 18% are municipal introduced talents. Li Meng said that eligible young talents can provide free check-in services, including double first-class universities, temporary cadres, as well as senior executives from key enterprises in the district. "More than 160 young talents live in through various forms every year," he said."In Heping District, a golden area of Tianjin, the Youth League organization has also created a "youth home" for young talents through multi-party communication and coordination. Heping District is a pilot area for the implementation of the medium-and long-term youth development plan. It is building a "youth-friendly city", attracting young talents from all over the world. Wu Xiangxiang, director of the Youth Development and Rights Protection Department of the CPC Tianjin Municipal Party Committee, said that in the "Haihe Youth Listening" practice activities carried out by the Tianjin Youth League Tianjin Municipal Party Committee, many young people reported that "the central urban rent is high and young people are difficult to stay". To this end, the Youth League organization and CCB Tianjin Branch conducted in-depth research and active planning, by revitalizing the idle assets of CCB located in the city center, transforming the old building into a youth long-rent apartment, and providing rental housing and preferential policies for college graduates, Haihe Yingcai and "New Tianjin Power". Now, relying on the comprehensive housing rental service platform "CCB Jianrong Home", the Youth League organization not only provides rental housing for young people, but also creates a characteristic "youth home youth station", building a warm home for young strivers. Here entrepreneurs harbor activities, employment and entrepreneurship policy consultation and youth reading club activities; also relying on "Jin Cai Youth" and "CCB University" free online public welfare classroom platform, provide real-time mobile learning "cloud" service, providing one-stop service for the Communist Youth League. CCB Tianjin branch relevant controller introduces, is currently working in Hebei district Fen garden for youth strivers to build exclusive rental project, set sail in binhai new district home to provide rental housing, in xiqing district for the one's deceased father grind students to build exclusive brand rental project, etc., hope young people here "rent is long live long, long live is home". Li Long, party secretary of the Communist Youth League, said that the housing problem is one of the "urgent and anxious" problems such as "new Tianjin forces" and young college graduates studying in Tianjin. Solving this problem is also an important guarantee for Tianjin to introduce, retain and gather talents in Tianjin. Youth league organizations at all levels in Tianjin reflect the demands of the youth through the "Haihe Youth Listen" and other ways, "speak" for the youth, gradually promote the solution of the "housing" problem, and "strive to help the young people to better integrate into Tianjin". Source: China Youth Daily, December 31,2021, edition of the 01st edition

2021-12-30	Beijing, Dec. 30 (Guangdong) - -Guangdong-The second plenary meeting of the implementation of medium-and long-term youth development plans in Guangdong province was held in Guangzhou. Relevant leaders of Guangdong province attended the meeting and spoke. Chen Weihao, Deputy Secretary-general of Guangdong Provincial Party Committee, Chi Zhixiong, Secretary of Guangdong Provincial Party Committee, Deputy convenor and Office Director of the Joint Conference of the Communist Youth League, Liang Junda, Deputy Secretary of Guangdong Provincial Communist Party Committee, members of the joint conference, liaison officers, representatives of specially invited units and some grass-roots implementation units attended the meeting. The meeting stressed the need to thoroughly study and implement General Secretary Xi Jinping's important thoughts on youth work, and effectively enhance the sense of responsibility and mission of doing youth work well. We need to focus on key tasks and take greater efforts and more concrete measures to make the plan more effective. Strengthen the ideological and political guidance of young people, cultivate the new people to shoulder the national rejuvenation; actively mobilize young people to serve the overall situation, support their achievements, love and love youth, effectively serve youth development; to build the "youth Bay Area" Guangdong-Hong Kong-Macao youth development Greater Bay Area, demonstrate the construction of "dynamic Guangdong" youth development province. We should strengthen the organization and leadership, establish the idea of a game of chess, continuously strengthen the overall coordination, follow-up effectiveness, pilot work, and form a joint force to promote the implementation of the planning. Meeting listened to the Guangdong long-term youth development plan (2018-2025) and the first 14 youth people's livelihood practical project implementation, Guangdong youth development statistical monitoring report, reviewed and approved the 2022 <Guangdong long-term youth development plan (2018-2025)> implementation key work "the second batch of youth livelihood practical project" Guangdong long-term youth development plan implementation work member unit work rules and other three documents, Guangdong human resources and social security department, Zhaoqing, Maoming xinyi is responsible for comrade exchange speech."Youth Bay Area vitality of Guangdong" - -Guangzhou Tower lights up for the development of youth in Southern Guangdong. On the basis of summarizing the progress of the first batch of practical projects on youth people's livelihood projects, the conference successively launched the second batch of 5 practical projects on youth people's livelihood projects. Build the "Youth Bay Area" youth development-oriented Greater Bay Area, focusing on employment, education, housing, physical and mental health, dating and other prominent issues, improve the care and support mechanism for key youth groups, make the Bay Area more friendly to the youth, ideals, talent cohesion, innovation and entrepreneurship, quality of life improvement, youth culture and other fields. We will carry out the "Youth Finance Project" to help young people from Hong Kong, Macao and Taiwan integrate into the development of the Bay Area, implement three major plans for young talents, youth partners and youth services in the Guangdong-Hong Kong-Macao Greater Bay Area, and strengthen exchanges and cooperation to serve the needs of internship, employment, innovation and entrepreneurship. Carry out the "Youth Aviation Project" to promote the return of overseas students to Guangdong, and guide overseas students to integrate into the country's development. The 2022 Guangdong College Graduates Volunteer for Rural revitalization campaign, and recruit 4,000 college graduates from inside and outside the Guangdong province to eastern and northern Guangdong to carry out a 2-3-year volunteer service for rural revitalization. Promote Guangdong youth housing affordable rental housing project construction, plans to build a batch of affordable rental housing, for new to Guangdong business employment without housing in the mainland and Hong Kong, Macao and Taiwan youth, in Guangdong business employment college graduates to provide housing, and set up youth innovation business incubator, youth career growth service station, youth home and other comprehensive service functions.

2021-12-30	Young pioneers working society in Jiangxi province, the fifth member congress of Jiangxi young pioneers working society the fifth member congress zhang correspondent Cao Zhangyue) in order to further promote the construction of scientific research and theory development, on the basis of the young pioneers working association in Jiangxi province, December 28 morning, Jiangxi young pioneers work society the fifth member congress held in Nanchang. Luo Hua, deputy secretary of the Provincial Youth League Committee and director of the Provincial Youth League Working Committee, was elected as the new president of Jiangxi Young Pioneers Working Association, and 29 members were elected as the new council members of Jiangxi Young Pioneers Working Association. More than 110 member representatives attended the meeting. The meeting deliberated and adopted the report on the work of the fourth Council. During the work of the fourth Council, Learn to earnestly study and implement General Secretary Xi Jinping's important discourse on the work of children and Young Pioneers, Remarkable achievements have been made in promoting the professional construction and theoretical research of Jiangxi Young Pioneers, Approved by the national Young Pioneers major research project and a key research project, Published the provincial young Pioneers research project of 20 key projects, 50 general topics; Combined with the situation of Jiangxi Province and the young Pioneers work characteristics to organize the young Pioneers activity course guidance book "Young Pioneers Activities"; Awarded three National Young Pioneers Master studios, Construction of 20 provincial young Pioneers counselors famous teacher studios; Promote Nanchang University to set up the second-level discipline of "Children's organization and Ideological education" under the first-level discipline of "pedagogy", It has enrolled 23 master's students; The implementation of the young Pioneers counselors professional title evaluation "double-line promotion" policy, There are 8 young Pioneers counselors in the province who were awarded the senior title of young Pioneers activities; Set up an 885-member provincial, municipal and county levels of red scarf patrol group, Carry out 2,134 lectures, It covered 1.281,500 million young Pioneers and 33,500 counselors. The meeting also decided to allow Jiangxi Young Pioneers 'Work Society to set up six professional committees, namely, the young Pioneers' theoretical research, discipline construction, organization construction, counselors, publicity and education work, and socialization work, to further deepen the discipline construction and theoretical research of the young Pioneers, and promote the scientific development of the young Pioneers' work.

2022-01-05	Ningxia District Committee held the study and implementation of the spirit of the Sixth Plenary Session of the 19th CPC Central Committee Ningxia Autonomous Region Committee held the young backbone study and implement the spirit of the sixth Plenary Session of the 19th CPC Central Committee training class and youth teaching group propaganda mobilization and deployment training class site. Correspondent for figure China youth network Beijing on January 5 (reporter Ji'an-wei zhang, correspondent Zhang Zhuo) recently, Ningxia youth backbone study and implement the spirit of the sixth plenary session of the 19th training and youth branch preach mobilization deployment will be held in Yinchuan, to lead the youth backbone learn deep enlightenment through the plenary session spirit, emphasizing teenagers preach interpretation do enough theoretical preparation. The training is carried out in the form of special guidance, group discussion, centralized lesson preparation, and annual work mutual evaluation. Liu Yu, a member of the publicity group of the Autonomous region, deputy secretary of the Party Leadership Group and president of the Ningxia Academy of Social Sciences, gave guidance on "in-depth study and implementation of the spirit of the Sixth Plenary Session of the 19th CPC Central Committee and the 14th Plenary Session of the 12th CPC Central Committee of the Autonomous Region" to help the participants further understand and grasp the spirit of the plenary session and do a good job in the youth publicity work. Excellent young representatives from municipal youth League committee, county (district) youth League committee, youth League, Students' Federation, Young Pioneers, Youth Enterprise Association and youth teaching group, made wonderful exchanges and sharing around learning the spirit and experience of the plenary session and combined with the actual work.

2021-12-30	Gansu: Inheriting red fire group in the name of youth Organization mobilizes college students to explore and spread red resources Gansu: Inheriting red fire fire in the name of youth has left many glorious footprints. The year 2021 marks the 100th anniversary of the founding of the Communist Party of China. The Gansu Provincial Committee of the Communist Youth League has extensively organized and mobilized college students in the province to actively participate in the mining, learning and dissemination of red resources, and inherit the red fire in the name of youth. In April this year, the Gansu Provincial Committee of the Communist Youth League, the Education Department of Gansu Province and the Provincial Students' Federation joined TikTok held and launched the "Red Youth Longyuan" Douyin Challenge, recording and pursuing the glorious footprints left by Chinese Communists on the land of Longyuan, in the name of youth. The challenge extensively mobilized college students from the province to focus on the theme of "Red youth dialect of Longyuan", focusing on the "four history" learning and education, combined with rural revitalization, youth achievements and hometown changes, creating novel, positive and vivid red stories. The Challenge also builds a "red classroom" on the cloud through the selection of excellent works and the coordination of media communication, providing "red textbooks" to the youth, and focusing during the May 4th Movement period, forming a wide social influence. Up to now, the "Red Youth Talk Longyuan" TikTok challenge has 2,668 video participants, with a total exposure of 40.772 million. The Gansu Provincial Committee has selected the comprehensive work views, thumb up, video content correlation and other indicators, and widely promoted in the provincial Communist Youth League system. The Communist Youth League, Gansu Provincial Party Committee, the Gansu Provincial Students' Federation also launched the "red youth light up Gansu" online answer small program. The organizer has carefully set up the red map of Gansu province, and after the respondents enter the small program, and choose the red base that they are interested in, you can learn the materials and answer the questions. When college students participate in answering the questions, they must first learn the red story of a city before they can start to answer the questions. After answering all the questions, they can light up a landmark. When all the cities are lit up, they can check the ranking."Every time I answer the question, it is a process of learning red knowledge. By answering the questions, I light up the red landmark of Longyuan land, and I can also invite your friends to participate in the answer challenge. The activity is very interesting and meaningful."Said Huang Yongwei, a student from Gansu Agricultural University who participated in the challenge. According to statistics, 217,600 young people from the province took part in the online answer challenge. According to the answers, the Gansu Provincial Committee of the Communist Youth League finally selected 12 outstanding organization awards in (city) and states, universities, enterprises and institutions, and awarded 260 outstanding individuals by means of random lottery. This year, the Gansu Provincial Committee of the Communist Youth League also extensively organized and mobilized college students in the province to participate in the red special activities of the "Challenge Cup" competition, guiding college students to feel the great strength of the red spirit of the Party by tracing the red footprints, tracing the red memories, interviewing the red characters, digging the red stories and understanding the red culture. During the activity, college students, in the form of "short video +" research reports, using sections of practical images and research reports, to express their understanding of the red spirit, the harvest of the practice process and the emotional recognition of the Party. The forms of the activities are novel and rich, with strong appeal and wide transmission power. Walking on the land of Longyuan, harvesting and growing "one inch of blood" in learning, thinking and realizing the practice, Gaotai County of Zhangye City has rich red cultural resources and red cultural memory. When Liu Xin, a student from Lanzhou University, based on the red cultural resources of Gaotai County, he traced the red stories by visiting the memorial hall of the Chinese Red Army and the red spirit, and finally shot the deeds of Dong Zhentang, commander of the fifth Red Army in the Battle of Gaotai, into video. Liu Xin said that through this practice, she learned more red stories in her hometown Gaotai, and had a deeper understanding of the heroes who made great contributions and sacrifices to her hometown. Nanliang, located in Huachi County, Qingyang City, Gansu Province, is a major historical town of the Chinese revolution. In the 1920s and 1930s, the Communists represented by Liu Zhidan, Xie Zichang and Xi Zhongxun successfully established the Shaanxi-Gansu border revolutionary base with the Southern Liang as the center. From then on, the Nanliang red flag did not fall, stood up the backbone of the Chinese revolution. Since this year, Gansu universities have extensively organized college students' practice groups to walk into Nanliang and feel the charm of the Nanliang spirit. During the summer vacation, Lanzhou University went to the Youth Practice Group of Huachi County, Qingyang City, to visit the Nanliang Revolutionary Memorial Hall to have an in-depth understanding of the development history of the Nanliang revolutionary base and the Shaanxi-Gansu revolutionary base. In the process of visiting the individual exhibition hall, the leaders of the base area's fine revolutionary style of keeping close contact with the masses and maintaining flesh and blood ties with the people made the practice team members feel the most."These outstanding representatives of the Chinese Communists have won the recognition and support of the masses, and vividly demonstrated the fundamental purpose of the Chinese Communists."Said the college students who attended the event. This summer, Jia Yongsheng, a counselor from the School of Economics and Management of Lanzhou University of Technology, walked into the Red Long March Victory Memorial Hall in Huining, Gansu province, and gave a vivid lesson on party history to teachers and students. This is one of the series of activities of "Seeking the history of the Party and telling the Red Story of Gansu" launched by the Youth League Committee of Lanzhou University of Technology. During the one-month-long preparation time, Jia Yongsheng repeatedly asked the narrator of the memorial hall for advice, and one by one, the touching stories finally became a direct touching history."As a counselor, I would like to be a spark to sow the red seeds into the hearts of more college students and light the torch of faith in their hearts."Said Jia Yongsheng."At the scene to listen to Teacher Jia told the history of the Party class, once again felt the great spirit of the Long March, the story of the small bugle is like blowing a horn on the road to the new era, inspire us to firm ideals and beliefs, brave to bear the national rejuvenation of the new people."Said Liang Xuejie, a student at Lanzhou University of Technology. China Youth Daily China Youth Network reporter Ma Fuchun source: China Youth Daily

2022-01-02	Henan province held "the communist youth league and people's congress, CPPCC members face to face" activities in 2022 in Henan province and Zhengzhou "communist youth league and NPC and CPPCC members face to face" activities held Pan Zhixian) on December 31,2021,2022, in Henan province and Zhengzhou "communist youth league and the people's congress, CPPCC members face to face" activities held in Zhengzhou. Wang Anlin and Wang Haiping, deputy secretary of Henan Provincial Party Committee, Wang Tianshun, deputy director of Henan Provincial Committee of Handling Proposals of the CPPCC, and heads of relevant special committees of Henan Provincial People's Congress attended the event. More than 20 people, including deputies of Henan Provincial People's Congress, members of the CPPCC, representatives of youth workers and mental health experts, attended the event. Symposium, from education, medical care, public security, colleges and universities, social work institutions, enterprises and other industries of youth representatives, around the "improve the level of adolescent mental health services", combined with their own work practice, from different angles to share the current adolescent mental health service related work, analysis to the current main problems, put forward work opinions and Suggestions. Henan province 12355 youth service platform head Zhang Zongwei with a large number of cases and data, he said: " in recent years, adolescent psychological counseling demand rose significantly, adolescent mental health relationship youth growth, family future and social stability, government and social departments from all walks of life should increase attention and attention, build consensus, better safeguard students 'mental health, promote their growth, avoid students' psychological crisis events."After listening to the speeches, the NPC deputies and the CPPCC members responded to and supplemented the questions raised by the young deputies, and put forward their own views and views. Henan Provincial People's Congress, provincial CPPCC responsible comrades on the symposium. Deputies to the Henan Provincial People's Congress and members of the CPPCC attending the meeting agreed that the mental health problems of teenagers are of high social attention, and the adolescent mental health service system should be further improved, and a psychological crisis intervention mechanism should be established. People have said that they will strengthen the urgent need of adolescent mental health in the "two sessions" in Henan Province. Wang said that with the rapid development of social economy and the increasing competitive pressure, the mental health problems of young students are also increasing, adding."This is not only a personal problem, but also a family problem and a social problem. I hope you will do a good job in summarizing the relevant problems, and strive to form strong proposals and proposals at the two sessions to serve the healthy growth of young people and better help them solve their interests."Wang believes that teenagers are the future and hope of the motherland, and the healthy growth of teenagers is not only related to personal families, but also related to the future of the country. It requires multi-linkage and coordination of all sectors of society to build a interconnected mental health service mode. We should attach great importance to the importance of guiding the youth in their orderly political participation, unite to enhance the youth mental health service level, and build a platform to continuously guide the positive interaction between the youth group and the NPC deputies and the CPPCC members. After the meeting, the participants visited the Henan Province Youth All Media Center and Henan Province 12355 Youth Service Center. The activity of "the Communist Youth League face to face with deputies to the People's Congress and members of the CPPCC" is an important way to guide young people to participate in political affairs in an orderly manner, an important form of activities for the League to safeguard the rights and interests of young people, and an important institutionalized arrangement for the Party's youth work well. It is understood that in recent years, the Henan Communist Youth League has been around the "young talent power rural revitalization strategy", "better play the role of youth in urban and rural community governance" and other themes to carry out concentrated activities, a total of 351 listening activities, covering more than 75,559 young people, in the provincial, city and county "two sessions" put forward relevant proposals and suggestions. Wang Anlin said that in the next step, the Henan Provincial Party Committee of the Communist Youth League will rely on the provincial 12355 youth service platform to carry out regular mental health education, strengthen the platform offline services, actively explore a new path of youth growth services, and constantly promote the youth work in Henan to a new level.

2021-12-29	Sichuan communist youth league: escort students light thought lighthouse focus big politics, big brand, big platform of Sichuan communist youth league: escort students light thought lighthouse to lead "work idea, to promote leading force as the key, stick to the group" study party history, strong faith, follow the party "deployment requirements, focusing on" big politics "," brand "" platform ", combining the actual innovation in Sichuan universities, made the positive results of the communist youth league propaganda, ideological and cultural work. Focus on the "ideological" leading college students in Sichuan communist youth league always grasp the thought leading the theme and the main line, starting from the university education law and characteristics of college students growth, guide the Sichuan college students firmly establish a consciousness of "four", "four confidence", do "two established" firmly advocates and "two maintenance" firm practitioners. Adhere to the "one game of chess" overall planning. Sichuan communist youth league organization launched Sichuan university general secretary thoroughly study and implement xi series of important instructions spirit and the spirit of the sixth plenary session of the nineteenth, widely carry out "study of party history, strong faith, follow the party" learning education, "four" propaganda and education and the provincial youth "always follow the party" learning education activities, provincial youth league, provincial league the first release practice "please rest assured, power have me" solemn promise "initiative", requires universities to class league branch, timely special learning and special organization life, to ensure that high quality to promote party history learning education in college students into the mind. Up to now, the theme education of "learning the history of the Party, strengthening faith, and following the Party" education has covered 99.62% of college students. At the same time, in order to better coordinate the province university level thought leading work, the Sichuan communist youth league in the communist youth league work leading group, the provincial school communist youth league reform steering committee and the school, on the basis of the school league organization reporting on activities system, promote the province 183 counties (city, area) education league committee, explore education league secretary of city (state) deputy secretary of the league, coagulation hearts meet force pays special attention to the Sichuan university communist youth league work. Adhere to the "integrated" learning. In order to consolidate the ideal and faith foundation of college students in Sichuan Province and improve the quality and efficiency of learning, Sichuan Communist Youth League gives full play to the organizational advantages of the Communist Youth League, and promotes the league organizations and students of colleges and universities to focus on organizational learning and carry out targeted learning at different levels and categories. Online, relying on the "youth learning", "first class" and other carriers, achieve nearly 1.5 million college students every week, all college students in the province listen to the "first class"; offline, focus on the selection of a group of outstanding university youth, join the provincial youth branch and the new era civilization practice youth branch, carry out special demonstration, headed by the party secretary. Through the establishment of the "green horse project" training class, college students backbone training class and other forms, to pay close attention to the student cadres, student party members and other key groups of learning. At important time points, experiential, interactive and immersive learning and practice activities such as theme group day and relay tracing the Long March. Adhere to the "one network" linkage. In order to promote the ideological dynamics of young students in prospective universities, the Sichuan Communist Youth League focuses on the three-dimensional linkage of "point, line and surface", and makes concerted efforts to improve the ideological and political quality of young people. Continue to strengthen the risk analysis and judgment in the ideological field of colleges and universities, establish provincial, municipal and university information direct reporting points and university information personnel team; carry out dynamic ideological research for college students, establish a dynamic evaluation mechanism of students 'thoughts, and grasp students' ideological dynamics in real time. Focusing on the major decisions and arrangements of the CPC Central Committee and the Sichuan Provincial Party Committee, the Sichuan Communist Youth League constantly explores and innovates practice carriers and brand projects, and continues to create an IP with Sichuan characteristics and deeply loved by college students. Strengthen practical education, and comprehensively improve the comprehensive quality of students. Sichuan Communist Youth League pays attention to the national education function of practice projects. By implementing the system of the "second classroom report card" in colleges and universities, carrying out the social practice of "three to the countryside" in summer vacation, the social practice is an important platform for college students to grow up and become the "Dream pursuit plan". At present, more than 1,000 "three rural practice teams" have been established to carry out themed social practice activities in more than 500 regions across the country, and help more than 90,000 college students to practice in government organs, enterprises, financial institutions, scientific research institutes and other places. Strengthen service and education, and continue to expand the growth pattern of students. Sichuan Communist Youth League pays attention to the demonstration and leading function of service projects. By carrying out a series of volunteer service activities of "youth volunteer", college students volunteer service plan, "twin Cities Young Talents Plan" and other projects, the service and education run through the whole process of college students. Up to now, 3,092 communities in 21 cities (prefectures) in the province have been organized to carry out volunteer service activities such as family care and mental health counseling. More than 41,000 graduates and graduate students from universities all over the country to participate in serving the economic and social development of Sichuan. For example, the "Gesang Flower" graduate volunteer teaching group of Southwest University for Nationalities takes root in the snowy plateau, 3,507 meters above sea level, and adopts the "volunteer + relay" designated volunteer teaching team for seven consecutive years to serve numerous students in ethnic minority areas. More than 11,000 enterprises and institutions to provide more than 530,000 jobs to serve the employment of university graduates in Sichuan and Chongqing. Strengthen care and help, carefully care for the healthy growth of students. In the practice activity of "I do something for my students", colleges and universities launched brand service projects such as "rights nest", "information through train" and "good morning students", and so on to guide the majority of students to participate in campus governance in an orderly manner, benefiting more than 100,000 people. In the employment assistance of college students from thousands of schools, we have established a pair of employment assistance mechanism for poor graduates, and recommended more than 1,300 graduates from poor families in 2020 to employers. In the "youth escort" series of activities, the provincial Public Security Department of the province, the Provincial Department of Justice and other departments cooperated to carry out the "youth escort" series of activities, escorting the growth of youth from the perspective of life safety, drug control and AIDS prevention, and prevention of telecom and network fraud. Focusing on the "big platform" to gather college students, the Sichuan Communist Youth League strives to seek the convergence point between new media and the work of the college Communist Youth League, giving full play to the function of network education, and making it an important position to educate and guide the youth, and an important means to contact and serve the youth. The ame frequency resonance, strengthen the management of new media platform in universities. Take the construction of new media matrix in universities as the top priority of the network new media work, and strive to build a new media matrix on campus with "Sichuan Students' Federation" WeChat and Weibo platforms as the core and supported by the new media platforms of universities. At present, there are 192 school-level new media accounts belonging to the youth league in the province. We will continue to standardize the operation and management of new media accounts in colleges and universities, effectively find out the base number of new media accounts in colleges and universities, regularly carry out the clean-up and rectification action of "zombie accounts", and release the information to strictly follow the process of "three trials and three schools". For example, Sichuan University has established the "Mingyuan Media" and the League and Learning New Media Alliance, strictly implemented the "review and release system, developed the new media content monitoring and management system, cleaned up" zombie accounts " and rectify low-vitality accounts. After combing, the number of new media accounts has dropped from more than 900 to 206. Content is king, the original launch of university new media products. Adhering to the use of new media means to "translate" the party and league information into the language and the picture that college students can understand, we originally launched network cultural products such as "CD Man" MG animation and "Panda Man" MV, and organized activities such as short videos of new military training style to shorten the distance with young students. The original flash mob video of "I am a CD-ROM Man" was praised and forwarded by mainstream media such as People's Daily and Learning Power, with more than 100 million page views. Positive guidance, continue to carry out network publicity and guidance. Adhere to completes the university communist youth league network hot observation, establish Sichuan university new media emergency response mechanism, in accordance with the "hierarchical response, hierarchical management, group linkage" principle, the college youth concern about social hotspot and the communist youth league related topics timely understanding, real-time analysis, in major time node and important events actively carry out positive energy propaganda guide, continuous singing network theme, build clear cyberspace. Source: China Youth Daily, December 29,2021

2021-12-28	Guangxi held "Hope Reading" achievement exhibition activity Xie Yang) Reading Yue Beautiful Swallow Hope Cloud- -Guangxi "Hope Reading" achievement exhibition and Nanning Yangmei Hope Primary School Young Pioneers theme team meeting was held recently. Su Yan, founder of the Swallow Hope Fund of the Guangxi Youth Development Foundation, encouraged students to develop good reading habits and hobbies from an early age. Liao Kunfa, the principal of Yangmei Hope Primary School, shared the school's experience in launching the "Hope Reading" public welfare project and the positive changes that the "Hope Reading" project has brought to the students. Under the guidance of the Young Pioneers counselors, the students started a "reading journey on the cloud", on the "Cloud Long March" and "Cloud View Revolution Memorial Hall" of "Swallow Hope Cloud" through learning interaction and knowledge quiz. Through AR live presentation, the students can visit the Red Army Long March Breakthrough Xiangjiang Memorial Hall remotely, and further enhance their perception of the revolutionary spirit and red culture. In April this year, the Guangxi District Committee of the Communist Youth League and the Guangxi Youth Development Foundation launched the "Hope Reading" public welfare project, which was supported by outstanding young entrepreneurs and donated 10 million yuan to the Project Hope to set up the "Swallow Hope Fund". Project to "elegant floating eight laurel reading DangEn" as the theme, by increasing reading content lead, innovation "hope reading" way, establish reading service system, highlight the educational function of the hope of the project, has been in the district 15 hope primary school, 19 "Guangxi green space" to carry out, to provide more than 80000 rural youth reading services.

2021-12-28	The 15th Congress of the Communist Youth League in Henan Province closed in Zhengzhou today. Vice Governor of Henan Province Dai Baihua attended the closing ceremony and delivered a speech. The meeting deliberated and adopted the work report of the 14th Provincial Committee of the Communist Youth League of Henan Province, elected a new committee, and defined the goals and tasks for the next stage. Dai Baihua on behalf of the Henan provincial government to the success of the conference, the newly elected Henan provincial party committee member, alternate congratulations, hope the provincial youth and youth league organizations at all levels earnestly study and implement the provincial party committee, central instructions spirit, to achieve "two ensure" grand goal, strive to compose a new era of central plains more brilliant chapter contribution youth power. Dai Baihua stressed that doing a good job in youth work in the new era is related to the transmission of the Party's cause from generation to generation and the sustainable development of the Chinese nation. The league organizations at all levels in the province should focus on the successors, strengthen the political guidance, and mobilize the youth to think and act on the initiative, to serve the young people, respond to the demands of the youth, strengthen the youth human resources, promote the youth's all-round development; to anchor the political nature, advancement and mass work, and to forge the modern Henan construction. At the first plenary meeting of the 15th Henan Provincial Committee of the Communist Youth League held this afternoon, Wang Yi was elected as secretary of the 15th Henan Provincial Committee of the Communist Youth League, and Wang Dubo, Wang Anlin, Wang Qiang, Wang Jiwei (temporary), Wang Haiping (temporary), Liang Jing (part-time) and Pei General (part-time) as deputy secretaries. Source: China Youth Daily, December 28,2021, edition of 02

2021-12-28	Anhui has the games volunteer service "standard guide" article 133 precise guidance reveals the volunteer humanistic care in Anhui has the games volunteer service "standard guide" to participate in the world manufacturing conference, Hefei college English professional students liu danlu personal feel, the local volunteer service atmosphere and organization, execution has significantly improved."The epidemic prevention situation is grim, but the volunteer service work is orderly, As an indispensable member of the conference, we get respect, care, a sense of gain, and also deeply realized the responsibility and responsibility!"Liu Danlu gave many examples- -in management, the staff measured the temperature of the volunteers and disinfected the ward rounds on time every day, which was full of security; in daily security, the competition team fully considered the volunteers' meal time, place, and even the details of clothing color. At the 2021 World Manufacturing Conference held recently, 136 young volunteers from Hefei showed their youth in the exhibition hall service, foreign language translation, logistics support, consultation and reception and other positions, which won unanimous praise from Chinese and foreign guests and staff. The Hefei Municipal Party Committee of the Communist Youth League, the leading unit of volunteer service, also specially dispatched a volunteer service support team to the conference to ensure that the volunteer epidemic prevention work was safe. Before this year's World Manufacturing Congress, the Hefei Municipal Committee of the Communist Youth League (hereinafter referred to as the "Standard") proposed and formulated by the Hefei Municipal Committee was officially issued. This is the first provincial local standard in the field of volunteer service in Anhui province, and also the first provincial local standard led by Anhui mass organizations, which is rare in the country. The total service is 8,488 hours, with 831 people working hard- -the volunteer service of the World Manufacturing Conference has delivered a wonderful "Anhui answer paper". This is inseparable from the scientific, standard, perfect policy guarantee, but also the best test of the standards. The reporter noted that the standard full text of 20 pages, divided into seven parts, a total of 133 detailed rules. Detailed regulations shall be made on the volunteer service management norms, volunteer service requirements, responsibilities and procedures of relevant parties for large-scale competitions. From the principles of volunteer recruitment, training, post, management, guarantee and incentive, to the volunteers on how to serve the disabled group, how to communicate with the media and other specific post rules, at a glance."With this 'magic weapon' guidance, each position needs several volunteers, who can accurately measure it. Before the conference, we spent a very short time to draw up a volunteer service work plan, determine the total number of volunteers, and improve the work efficiency."Said Chen Hong, deputy director of the Foreign Investment Promotion Bureau of the Anhui Provincial Department of Commerce, as the organizer of the World Manufacturing Conference. Since 2014, the Hefei Municipal Party Committee of the Communist Youth League has organized more than 20,000 young volunteers to serve large-scale competitions, and 342,000 registered youth volunteers at all levels in the city. However, the reporter learned that in many areas, the responsibilities of the competition organization, volunteer management department and volunteer service department are not clear, resulting in more rework in practical work and low communication efficiency. There are multiple departments of large-scale comprehensive exhibitions participating in them, and each department has different volunteer service needs. It is not clear about who proposes the final service needs, who evaluates, and who determines the final service needs. In addition, there is no clear policy basis for the service guarantee of the volunteers. Sometimes, the relevant departments directly issue meals. However, the volunteers are limited by the time and place, and they can not eat hot meals, and can only order takeout, and food safety cannot be guaranteed. The introduction of the standard has cracked the long-term pain point of volunteer service in Anhui Games. Since February this year, under the guidance and support of Anhui Provincial Committee, Hefei Municipal Committee organized special teams to sort out the problems exposed in previous work, visited the organizers and volunteers, learning relevant laws and regulations, drafting standards; held 7 forums to solicit opinions from the whole society, municipalities, relevant government departments, universities, volunteers and experts, and revised for 5 times."Before the standard was introduced, we often need to find similar references online for large games."Said Guo Xiaotong, the fourth-level director of the Youth Volunteer Work Department of Hefei Municipal Committee, that with the standards, the quality of volunteers can be better improved; for the volunteer management department, it can provide the operation process and management standards; and for the organizer, it can reasonably formulate the work plan and the number of volunteers in advance. It is worth mentioning that the "Anhui characteristic" of this standard lies in that it highlights the humanistic care for volunteers. Some chapters focus on standardizing the service guarantee, encouragement and recognition, summary and improvement, and give guidance on the comprehensive quality improvement of volunteers."The standard requires that after the activity, the volunteer management department should accurately evaluate the service effect of the volunteers through questionnaires, individual interviews and symposiums, and hold the volunteer experience exchange meetings in due time."Guo Xiaotong said that the standard appendix added volunteer service publicity examples, emphasizing the publicity work throughout the whole process of volunteer service," this year's World Manufacturing Congress, the Hefei Municipal Committee on the first time opened a volunteer service publicity column, through new media dissemination, tell the story of volunteers.""The standard effectively combines the work of the Communist Youth League and the characteristics of young volunteers with the urban development of Hefei. In the future, the working mechanism and other spiritual achievements of the competition, talent team, experience, methods and cultural concepts formed in the large-scale competitions will be transformed into the driving force to promote the development of local volunteer service."China Electric Science 38 researcher, large-scale competition volunteer service management standard evaluation expert Zhang Hongqi said."Volunteers need to master the history, culture, local customs of the venue of the competition. Standards are good guidelines to make volunteers more clear about their job responsibilities."Said Gu Zihan, a student from Hefei University, who has volunteered for many times."Volunteer service has become an indispensable part of the process of high-quality urban development. We take volunteer service as an important means and carrier to organize young people to participate in grass-roots community governance and realize the ideological guidance to young people."Ma Jinwei, deputy secretary of the Hefei Municipal Party Committee of the Communist Youth League, said that the standard emphasizes the adherence to the Party's leadership of volunteer service work, and giving play to the vanguard and exemplary role of party members and league members in volunteer service. The youth league organization will take the implementation of the standards as an opportunity to continuously promote the standardization, normalization and standardization of youth volunteer services, so as to better focus on the center and serve the overall situation. China Youth Daily, China Youth Network, reporter Wang Haihan, Wang Lei, correspondent Wang Cong, source: China Youth Daily, December 28,2021, edition 01

2021-12-28	Shandong Communist Youth League opened the youth. This time, the door is the standing Committee of the Shandong Communist Youth League. On December 20, a special meeting of the Standing Committee of the Shandong Provincial Committee of the Communist Youth League was held for the first time. Participants were no longer limited to 12 Standing Committee members of the Communist Youth League Committee. 18 youth representatives from the Shandong Youth Federation, Youth associations and various fields also participated in the deliberation and voting of five key work of the provincial Communist Youth League."The establishment of the meeting mechanism of the Standing Committee of the Provincial Communist Youth League Committee is not only the optimization and upgrading of the deliberation body of the Communist Youth League, but also a breakthrough in the thinking and means of administrative organs, relying on social channels to gather resources, follow social operation rules and integrate resources, so as to promote the formation of an efficient and fully functional youth work system."The Communist Youth League of Shandong Provincial Party Committee Secretary Liu Tiandong introduced. Starting with this, Shandong communist youth league to build Shandong youth development dialogue platform, innovation traditional organization, hierarchical communication mode, with the help of socialization, flattening, mass, with youth wisdom gather youth, enhance service overall contribution at the same time, make the youth have more feeling, thus deepening the reform of groups to achieve new breakthroughs. As a youth representative in various fields, Ding Yi, deputy Secretary of the Party Committee and general manager of Shandong Guoxin Culture and Tourism Group, is a new face of the meeting of the Standing Committee of the Shandong Provincial Party Committee (Expansion) of the Communist Youth League. During the one-hour meeting, he brought suggestions to increase the "craftsman spirit" when reviewing the relevant work. Based on his own research and observation, he introduced to the participants that Shandong, as a large population province, has a high total employment pressure; meanwhile, as a large industrial province, Shandong has a huge gap among young craftsmen with knowledge and skills in promoting the transformation of old and new growth drivers."According to relevant think tanks, low income and infreedom are the main reasons why young people are reluctant to enter the factory. More than 30% of the food delivery riders were once industrial workers, and the manufacturing industry was not in the industry where graduates want to find jobs most. In the long run, it will not be conducive to the cultivation of craftsmen."Ding yi thus suggested adding the" craftsman spirit " to guide more young people to move to the manufacturing sector. The Shandong Provincial Party Committee of the Communist Youth League has fully absorbed and revised and improved the 55 suggestions from 18 new faces, and included them in the five key tasks for review. According to the Rules of Procedure of the Standing Committee (expanded) Meeting (Trial), the members of the Standing Committee (expanded) meeting take the designated seats of the vice chairmen, standing committee members of the league and the leaders of the youth associations as the main topics of each Standing Committee (expanded) meeting, and some youth representatives in the relevant fields. These members are entrusted with important job responsibilities, including proposing topics for the key direction of the Standing Committee (expanded) meeting; timely making suggestions on the key work of the Provincial Communist Youth League Committee; and participating in the supervision and implementation of the key work of the Provincial Communist Youth League Committee. This means that the key work of the Communist Youth League is no longer only promoted by a few departments, but to open up ideas, relying on the strength of all walks of life, from "passive service" to "active planning". Institutional innovation reflects the reform concept of "three changes" of Shandong Communist Youth League in recent years. Work concept, adhere to the "youth oriented", deepen the normal grassroots work, improve the municipal youth league work evaluation, the voice, evaluation to youth, bold innovation operation mechanism, set up 7 key work class, innovation implementation "provincial county, city township" flat management mechanism, build the province online communist youth league comprehensive platform; work style, strictly avoid formalism, activity thinking, bonsai routine, forcing all levels to work hard. If the opening of the door is compared to the opening of the curtain, then the subsequent discussion link is a real blockbuster debut."Open the door discussion, focusing on the central work, combining the actual needs of the youth in the province, by constantly improving and standardizing the operation of the youth consultation mechanism, give full play to the industry advantages and influence of think tank experts and young representatives in various fields, and drive more young people to interact, communicate and make suggestions."Said Liu Shaohua, deputy secretary of the Shandong Provincial Communist Youth League. It is refreshing that at the Shandong Youth Development Dialogue held from December 20 to 21, the key work of the Communist Youth League in 2021 and the work report of the Shandong Youth Federation were publicly released to the youth representatives from all walks of life for the first time, and accepted the youth comments. Constantly gathering social resources, gathering young talents, gathering intellectual achievements, service projects and capital elements in various fields, Shandong Communist Youth League has changed from "fighting alone" into "fighting together". For the Shandong Communist Youth League, "coordinated combat" is first reflected in the comprehensive aggregation of brand work. In recent years, the Shandong Communist Youth League has focused on building the word "green" brand, with new projects to "drive the youth to do". Today, the word "green" the brand has realized matrix development, comprehensive coverage service youth in all fields, including: "I do the practical work for teenagers" for the service urgent sorrow, green enterprise summit as a platform to promote the development of high quality, to "rural good youth" selected culture to help the rural revitalization, to "green bird plan" support service employment entrepreneurship, to "hope hut" as the carrier to consolidate poverty crucial achievements, green committee construction affects grassroots vitality."Collaborative operation" is also reflected in the innovation of resource allocation. The Shandong Youth Development Dialogue has opened a new way for the Shandong Communist Youth League to "make friends" with the youth, that is, to take the initiative to face the society and fully leverage all sectors of society and the potential resources behind it. The "Hope House" child Care program, which is dedicated to creating an independent living space for children aged 8-14, is a typical case of promoting work through socialization. The project integrates the whole process of 170 young social organizations in the province, innovates the new model of "Internet + public welfare", raises 210 million yuan, and a total of 15,162 cabins have been built or under construction."Collaborative operation" also means adequate working force. Shandong Communist Youth League seeks helpers from the youth, condenses the young talents who are really willing to do youth work for the Party and love the cause of the Communist Youth League, and changes the service object into the working force. With the reform orientation of the Shandong Communist Youth League "focusing on the grass-roots level", now, all 1,824 towns and streets in the province have established youth working committees to deeply implement the "partnership plan" in the "two new" fields, absorbing and serving a wider range of youth groups with the help of the organizations and platforms of various new youth leaders in new fields and new industries. At the Shandong Youth Development Dialogue, with the official opening of the "Shandong Communist Youth League to serve ecological protection and high-quality development of the Yellow River Basin in 2022", covering the nine provinces (regions) along the Yellow River means that "coordinated combat" is once again endowed with new connotation. The establishment of "I want to participate" and " Shandong Youth Development think tank has further condensed the strength of the youth, and provided a good platform for Shandong to implement the general requirements in the national strategy of ecological protection and high-quality development in the Yellow River Basin."Yuan Hongying, deputy Party secretary and president of the Shandong Academy of Social Sciences, said that she now has another identity- -a special expert of the Shandong Youth Development Think Tank. The first batch of special experts consisted of 30 influential representatives in the fields of enterprises, finance, culture, education and other fields. According to the plan, the operation mode of the think tank includes seminars, research, listening to the work briefing of the Provincial Communist Youth League, participating in the evaluation of Shandong Province Medium-and Long-term Youth Development Plan (2019-2025), the expert evaluation of youth development-friendly city construction, and making suggestions and suggestions for youth development."The establishment of the think tank will bring a multi-dimensional perspective and practical guidance to the development of the Shandong Communist Youth League."Said Li Guixun, head of the United Front Work Department of the Shandong Provincial Party Committee. On the new youth development dialogue platform, the "face-to-face consultation process between the Communist Youth League, NPC deputies and CPPCC members" has been further optimized. Different from the previous face-to-face activities, this year, in the form of activities, the first time included the relevant functional departments into the face-to-face "circle of friends", asked young people to talk about appeals, departments to answer measures, the Communist Youth League proposals, representatives and members table opinions, the CPPCC push implementation, forming a closed loop of work. At the same time, the participation of NPC deputies and CPPCC members will be taken as the pre-procedure for the formulation of major decisions of the Shandong Communist Youth League, strengthen two-way communication with league cadres and young people in various fields, and further improve the effectiveness of the Communist Youth League through institutionalized channels such as the People's Congress and the CPPCC. More young people can see the direction forward from the new dialogue platform."As a young representative of scholars from Taiwan, Hong Kong, Macao and overseas, I will lead the members of the Local Youth Federation to serve the country and better promote exchanges and cooperation among young people in Shandong, Hong Kong, Macao and Taiwan."Said CAI Junsi, deputy director of Taiwan, Hong Kong, Macao special guests and overseas scholars of the Shandong Youth Federation and executive director of Hong Kong Xinhua Group. China Youth Daily China Youth Network reporter Xing Ting source: China Youth Daily

2021-12-27	Hainan training young pioneers brigade counselors (backbone) Hainan training young pioneers brigade counselor (backbone) reporter Ren Mingchao) on December 24, by the Hainan provincial party committee, Hainan province education department, Hainan province working committee jointly organized 2021 Hainan young pioneers brigade counselors (backbone) training graduation ceremony held in Haikou. It is reported that the training course lasts for four days, a total of more than 2000 young Pioneers counselors and grassroots youth cadres participated in online training. In order to optimize the training effect, this training course adopts the forms of work deployment meeting, classroom teaching, special guidance, sharing and communication, practical experience, event observation and so on. In addition to policy theory and professional knowledge, the course content also offers courses of youth psychology counseling, how to carry out adolescent drug prevention and in school education, so as to further enhance the work and professional knowledge and comprehensive quality of the young Pioneers counselors. The award ceremony of the 2021 Hainan Provincial Party History Theme Outstanding Young Pioneers Activity Class Competition was also held in Haikou on the same day.

2021-12-27	On December 25, the final of the "Micro-League Class" competition sponsored by Jiangxi Provincial Education Working Committee and Jiangxi Provincial Communist Youth League Committee was held in Nanchang. At this point, the fifth Jiangxi Provincial School Communist Youth League "micro group class" competition ended. This competition has set up four groups, including college youth league teacher group, young teacher group, college student group and secondary school youth league teacher group. Since its launch in September this year, more than 110 preliminary competitions, 18 provincial semi-finals and 4 provincial finals, and more than 300 group class works have participated in the provincial semi-finals. The competition is divided into two parts: theoretical test and group class teaching, with 20 special prizes, 26 first prizes, 32 second prizes and several third prizes produced. During the competition, the contestants used youth language, classroom teaching, speech and speech, character interview and other forms to share and deduce the "upward to good" example stories and spiritual strength. Since 2017, the Jiangxi Province "Micro-Group Class" competition has been held for five consecutive sessions, covering more than 6 million teachers and students. The "Micro-group class" competition is an important measure to strengthen the education and management of league members and implement the system of "three meetings, two systems and one lesson". Through the form of "micro group class" of "seeing the big in the small", the competition promotes the learning and applies the learning, enhances the political literacy of college group cadres, young teachers and students, and improves the systematic, scientific and standardized level of group class education. Source: China Youth Daily, December 27,2021, edition 04

2021-12-27	In Fujian province county youth social organizations in Fujian province county youth social organization training in qingwang trainee reporter Tian Hongwei) on December 22 to 24,2021 in Fujian province county league youth social organization training and "green horse project" social organization class held in the provincial school, a total of more than 70 county league, head of youth social organizations to attend. The training course, sponsored by the Fujian Provincial Party Committee of the Communist Youth League, aims to strengthen the political and value guidance of the backbone of young social organizations, discover and cultivate a number of young political backbone, promote the standardized construction and high-quality development of youth social organizations at the county level, and guide youth social organizations to participate in social governance and rural revitalization. Training with expert teaching, communication, sharing, work seminars, field teaching, around the spirit of the sixth plenary session of the 19, the new era of youth social organizations to participate in social governance innovation, youth social organization ideological position construction for teaching, and organize students to Fuzhou shoushan township nine peak village to carry out the on-site teaching.it is reported, in recent years, Fujian Communist Youth League and Youth Federation organized and implemented the "three qing, four, five guarantees" work measures, A total of 437 youth social organizations under the county youth league have been promoted, Among them, volunteer service, entrepreneurship and employment, art and sports organizations achieved 100% coverage in the province; We has set up 51 service groups, including "revitalizing agriculture through science and technology", "beautiful rural planning and construction", "industrial revitalization" and "e-commerce to help agriculture", We launched 149 activities to promote rural revitalization; Through donations, software support, teacher training and other ways to create 132 "hope cabins" in remote villages, Carry out 165 docking activities; To cultivate five national "Partnership Program" demonstration and key projects, including Ningde Fu'an Yangfan Youth Affairs Service Center, Fang Chuhong, president of Fujian Marine Ecological Civilization Promotion Association, and Guo Yan, director of Putian Sunshine Youth Affairs Service Center are two "national upward and good youth".

2021-12-25	Jiangsu Provincial College Students Micro video Creation Competition tour exhibition and award activity and the Changzhou Municipal party and League "Spark Red League" brand press conference was held in Changzhou University. The event, composed of chapters like "Youth 'Village' " Memory "," Youth Return to Home "and" Revitalization with Me ", kicked off in the opening micro video titled" Rural Revitalization in My Eyes ". In the event, the original feature films, animated short films and documentary works such as "When the Mountain Flowers are in Full bloom", "Huang Dafa: Cutting the Sky Canal to benefit the People" and "The Watchmen" will be displayed. The competition selected 10 special prize works, 14 first prizes, 22 second prizes, 31 third prizes, 15 excellent organization units, and the leaders attending the meeting awarded certificates of honor to the winning units. During the activity, Changzhou city talent and innovation policy promotion, the signing ceremony of Changzhou city and universities, and the release of the "Spark Red League" party and league joint construction brand project. The competition aims to arouse the public's attention to rural education, ecology, industry, consumption, culture and other fields from the perspective of youth through the power of images, so as to effectively consolidate and expand the achievements of poverty alleviation and rural revitalization. The competition consists of three categories: original feature films, animated short films and documentaries. Since its launch in September, it has received more than 1,000 works from more than 100 universities in Jiangsu province. The competition is sponsored by Jiangsu Provincial Party Committee, Jiangsu Provincial Student Federation and China Guanghua Science and Technology Foundation, organized by Changzhou University and Changzhou Municipal Party Committee of the Communist Youth League, and co-organized by Wujin District Party Committee of Changzhou Communist Youth League.

2021-12-23	Hubei thousands of left-behind children interpreta dream micro wish Hubei left-behind children interpreta dream micro wish report reporter LeiYu) this morning, the Hubei provincial party committee, Hubei youth foundation joint tencent charity foundation "retrace red road building fulcrum-learn the party history I do the practical work for the masses" interpreta dream left-behind children hope micro wish public welfare donation activities. Tencent Foundation donated 1 million yuan to Hubei Youth Foundation to realize micro wishes for 10,000 left-behind children. Since the implementation of the activity, more than 2,000 teams, 100,000 people to participate in the activity, lasted 100 days, a total of 10 billion steps, successfully exchanged 1 million yuan of love donations, for 10,000, left-behind children to achieve micro wishes. Hubei Youth Development Foundation awarded commemorative donation plaques and "Hubei Hope Project Co., Ltd. Charity Partner" awards to Tencent Charity Foundation and Tencent Technology (Wuhan) Co., Ltd. The participating leaders distributed the "Hope Micro-wish" gift package to the participating student representatives. After the meeting, the student representatives visited the exhibition hall of Tencent Wuhan RESEARCH and Development Center. They understood the Jingchu culture, Wuhan various index ranking and the development process of Tencent through video, time tunnel, virtual roaming scene, light carved sand table and other technological means, so as to stimulate the students' dream of becoming a strong country in science and technology. Source: China Youth Daily, December 23,2021, edition of 02

2021-12-23	Driver Hui Jiangtao was invited to the center stage to present award certificates and trophies to the Tianjin Communist Youth League. Recently, in Tianjin municipal party committee organization department, municipal party committee propaganda department, municipal party committee net letter office of Tianjin "I do the practical work for the masses" classic case final evaluation event, 100 citizens invited, on behalf of the city more than 1000 people to listen to the government departments, organs work report, a fair scores, awarded the trophy in the heart. Gold cup and silver cup is not as good as the common people's reputation."The people see and understand how much grassroots workers have done and how they have done."The organizers hope to give the right to the people, close the relationship between the party and the masses, harvest the people praise from the heart. Jiang Weiwei, who specially chose a "more formal" dress, arrived at the selection scene early, "the town informed me to score to the government, it is really surprised and happy."The first one to show on the stage is the "Haihe Qing Listening to Youth Dream Gas Station" project of Tuan Tianjin Municipal Party Committee. Watching the small figures of those left-behind children and children from poor families in the video, Jiang Weiwei has a little sour nose. She works for many such children in Dasi Town, Xiqing District, Tianjin."The young people of the Communist Youth League accompany them during the summer vacation and show them to the world. These children need such love and warmth."Said Jiang Weiwei. The on-site participation in the scoring of both the postman, security guards, hotel waiters and other grass-roots workers, but also excellent Communist party members, the most beautiful volunteers, model workers and other advanced models. Everyone is feeling the service level and fine management ability of the city."In my opinion, to truly solve the difficulties of the common people is to do practical things for the common people."Jiang Weiwei said that coming here, his biggest feeling is," no matter how small the people are, people manage!"It is a seemingly insignificant a small thing, gathered up the hearts of the people. What impressed Zhang Zhendong, an 80-year-old man who has been a volunteer commentator for 19 consecutive years since his retirement, most was the city's warm-hearted policy of extending heating for many consecutive years. In his opinion, the house to improve these several degrees, really warm the heart of a city people, "the people will see the facts around, can let him personally experience, on the score."The 20 projects that can go on the stage of the final evaluation are selected from more than 590 declaration cases of "I do practical things for the masses" in Tianjin. The service content of these projects involves all aspects of people's life. According to the relevant person in charge of the Cyberspace Office of Tianjin Municipal Party Committee, more than 3.9 million votes were received during the initial online centralized exhibition stage, and then representatives of online media and experts and scholars from all walks of life were invited to conduct mid-term reviews, and nearly 4 million valid votes were cast. The votes reflect the efforts and efforts of government departments to solve the difficulties at the grassroots and the troubles of the masses. Each project department shows the enthusiasm for serving the masses through video clips, melodrama performances, allegro and other ways. The "Jinxin Office" project selected by Tianjin Big Data Management Center promises to "do all the people". Through digital technology, they have made that the government services "one network management" and urban governance "one network management" are all integrated in the square screen at their fingertips. Today, there are 460 convenient services that can be handled through the "Jinxin Office". Standing in front of a big screen full of blue skies and white clouds, a staff member of the Tianjin Ecological Environment Bureau said: " The number of blue skies in the city is 100 days more than it was eight years ago!"The applause from the audience is the common people's sense of recognition of the ecological environment. When Jiang Tao nodded and clapped, he said: " Now that we are off the night shift, we can always see the stars in the sky, which is the happiness that money can't buy."The police officers of the Tianjin Public Security Criminal Investigation Corps used the" reunion action " to help more than 30 separated families to reunite. Behind the tears of the reunion on the screen, the public security police officers worked day and night across the mountains and rivers. One of the longest-consuming cases, the police deed and did not want to chase for 32 years. This for the common people to realize the dream of the persistent, has won a long time of applause. Ma Peng, director of the operation and management department of the Tianjin Convenience Special Line Service Center, said that this long-term persistence touched people. "To work for the people, we should continue to do it, and we should not avoid it when we encounter some difficulties.""Master, your family also has children, the elderly. You don't want them to breathe the polluted air, right?"In the melodrama, ecological environment law enforcement officers are communicating with polluters. The lead actress, Chang Wentao from the Atmospheric environment department of the Tianjin Municipal Bureau of Ecology and Environment, said: " I play my daily life."For this exhibition, she and her colleagues reviewed the work of the past few years. One of the deepest insights is that to do the things of the common people well, we must first win the hearts of the common people. After being a bus driver all his life, the No.619 bus driving back to Jiangtao has been praised as "bus first class" by the citizens. Over the years, he has been studying to meet the needs of passengers with more detailed services. He circled the scale, painted full of symbols, "directly tick the content moved me, some moved to cry; the circle is good; some will question mark, ask how to do here.""Service should go deep into the end", serving the masses is meticulous, is an important standard for Hui Jiangtao to judge the quality of service. He believes that as long as the people put forward reasonable and legitimate needs, providers should try to solve this problem, "can't say that this matter is not under my care, it will cool a person's heart."Ma Peng believes that this goodwill is indispensable to do a good job of service work. He said that there is always a long telephone line between the 12345 government service hotline and the public. "I came here today to show it to the public. We want to tell you that there are a group of young people on the telephone line, full of enthusiasm and try to do our best to serve you well."At the moment he received the certificate, each winner got a priceless praise: the most prominent position on the certificate was the handwritten signature of hundreds of citizen judges. The staff of the Communist Youth League Tianjin Municipal Party Committee held the certificate and said with emotion: " The whole page of the name is full of the trust of the people."China Youth Daily, China Youth Network reporter Hu Chunyan, source: China Youth Daily, December 23,2021, edition of the 01st edition

2021-12-27	Fujian to study the spirit of the Sixth Plenary Session of the 19th CPC Central Committee of Fujian Youth Lecture Group to study the spirit of the sixth Plenary Session of the 19th CPC Central Committee of China Youth Daily trainee reporter Tian Hongwei) " Today, I want to talk to you about the Communist Party of China yesterday, today and tomorrow. Talk about yesterday's suffering and glory, today's mission and tomorrow's great dream."This is the keynote speech delivered by Lin Min, a member of the Fujian Youth Lecture Group, in learning, preaching and implementing the spirit of the sixth Plenary Session of the 19th CPC Central Committee and the 11th Party Congress of Fujian Province. From December 23 to 24, the Fujian Provincial Committee of the Communist Youth League hosted the Fujian Youth Lecture Group on the theme of the spirit of the Sixth Plenary Session of the 19th CPC Central Committee and the 11th Fujian Provincial Party Congress were held in Pingtan Middle School affiliated to Fujian Normal University and Shanghang Road Community, Taijiang District, Fuzhou. At the lecture, Chen Dong, associate professor of the School of Marxism, Minjiang University, explained the great achievements and typical figures created by the Communist Party of China in various periods, and systematically publicized and in-depth explained the spirit of the Sixth Plenary Session of the 19th CPC Central Committee. Chen Dong encouraged the young people to fully absorb the strength of the party history, learn the excellent traditional Chinese culture and natural science knowledge, cultivate the spirit of responsibility, set up lofty ideals, and be a new youth with knowledge and ambition."The teacher explained the advanced characters deeds are very inspiring, the country's various undertakings from scratch, national strength from weak to strong, inseparable from their hard struggle, we should cherish the present life, study hard, to contribute their own strength to the development of society."Student Lin Wan Ting said after the lecture, feeling a lot. It is understood that for the teenagers to study and implement the party and the sixth plenary session of the 11th party spirit of Fujian province, Fujian communist youth league with youth branch for the gripper, take collective preparation, group grinding courseware, group to preach, into universities, into the enterprise, into the community, into the countryside, into the network to preach, a total of 20 games, covering nine cities in Fujian province and pingtan comprehensive experimental area.

2021-12-24	Henan province 15th congress will open on December 26, Henan province 15th congress will open on December 26, the youth network reporter pan zhixian intern zhang hui hui) in this afternoon of the communist youth league of Henan province congress press conference, Henan provincial party committee deputy secretary Wang Dubo, approved by Henan provincial party committee and league, the communist youth league congress in Henan province on December 26-December 27, held in Zhengzhou. The theme of the 15th Communist Youth League Congress of Henan Province is: holding high the great banner of Xi Jinping Thought on Socialism with Chinese Characteristics for a New Era, We will fully implement the guiding principles of the 19th CPC National Congress and its previous plenary sessions, Thoroughly study and implement General Secretary Xi Jinping's important thoughts on youth work and his important speeches and instructions for inspecting Henan province, Implement the spirit of the 11th provincial Party Congress, In accordance with the work plan of the 18th National Congress of the Communist Youth League and the work layout of the Communist Youth League to improve its organizational, leading, service and overall contribution in the new era, Carry forward the great spirit of party building, Inheriting a century of revolution, Keep in mind the mission of building a strong country, With determined reform and innovation, In order to ensure the high-quality construction of modern Henan and ensure the high-level realization of modern Henan, Strive to write a new era of central Plains more brilliant brilliant chapter to contribute to the youth strength. There are four main items on the agenda: to listen to the speeches of the leaders of the Henan Provincial Party Committee; to hear the speeches of the Central Committee of the Communist Youth League; to hear and deliberate the work report of the 14th Committee of the Communist Youth League; and to elect the 15th Committee of the Communist Youth League of Henan Province. According to the introduction, in addition to the specially invited representatives, attended the Communist Youth League in the 15th Congress of Henan Province, a total of 689 representatives, by the league member representatives of all fronts, the league full-time, temporary, part-time workers representatives and young Pioneers counselors representatives constitute. The generation of representatives adopts the bottom-up, up-down combination, repeated brewing, step-by-step selection method. Among the 15th Youth League Congress of Henan Province, 499 were grassroots and front-line representatives, or 72.4% of the total, 437 CPC members (including probationary members), or 63.4% of the total, 341 female representatives, or 49.5% of the total; the average age was 28.8 years old. The sources of representatives are more extensive, and the industry distribution is more comprehensive, which fully reflects the political nature, advanced nature and mass nature, and fully shows the new achievements of Henan Communist Youth League in the reform and strict governance of the Communist Youth League in recent years.

2021-12-27	Beijing: the fifth youth skills contest summary recognition and youth style show held Beijing authority the fifth youth skills contest summary recognition and youth style show Ji'an-wei zhang held correspondent, Liu Xuchun, Guo Hongbo Jiang Sheng male) recently, "youth loyalty essence struggle gift founding in one hundred" -the fifth Beijing authority youth skills contest summary recognition and youth style show successfully held in the national grand theatre lake theater. The fifth Beijing Municipal Government Agency Youth Skills Competition was hosted by the Municipal Government Working Committee and the Beijing Municipal Party Committee of the Communist Youth League from April to December 2021, With the theme of "Youth keeps loyalty and struggle to present a century to the founding of the Party", For young cadres under the age of 35 in government organs and public institutions in Beijing, Focusing on the services for the 100th anniversary of the founding of the Founding of the Party and the preparations for the Winter Olympics and Paralympics, Focusing on improving the ability of young people to learn, preach, write and make video, Set up learning ability competition, publicity ability competition, writing ability competition, film review writing ability competition, video production ability competition, at the same time, After the end of the 4 competitions, Combination of online and offline products, By organizing demonstration competitions, online display of award-winning works, series of interviews, publicity of award-winning contestants and other ways, Deepen the competition results, Expand the impact of the competition. Since the competition began, more than 20,000 young cadres from 97 units have participated in the competition. The Party leaders of the Beijing Municipal Party Committee, the Leading Party Committee of the municipal organs, the Discipline inspection and Supervision Working Committee and some municipal organs attended the event. More than 100 winners and young cadres participated in the event, and nearly 10,000 young people from the municipal organs watched the live broadcast. At the event site, through the four chapters of "learning history to realize the original aspiration", "preaching Geng blood", "film review youth" and "together to the future", in melodrama, recitation, singing and dancing and other forms, summarize the results of the skills competition, show the youth style of the organization, and award the award-winning units and individuals.

2021-12-26	Jiangxi province "green horse project" young pioneers counselors in Jiangxi province successfully held "green horse project" young pioneers counselor class successfully held) on December 19 solstice 23, the communist youth league of Jiangxi provincial party committee, Jiangxi province education department, Jiangxi province less working committee jointly organized in jinggangshan city in 2021 in Jiangxi province youth marxist training project training class (young pioneers counselor class) and the provincial young pioneers counselor backbone teacher training class. A total of 200 people from all over the province, counselors and other representatives of the school attended the training. Opening ceremony, deputy secretary of the provincial party committee, provincial less working committee director Luo Hua expressed a warm welcome to trainees, and from strengthening political lead, improve ability, promote the reform of the young pioneers three aspects for mobilization speech, hope you cherish the "green horse project" training opportunities, ideal, learning practice enlightenment, enhance the young pioneers to carry out political enlightenment and values shaping ability level, promote the new era of Jiangxi young pioneers career to achieve high quality development. Photo description: the deputy director of the provincial young working committee, the provincial young Pioneers general counselor Wen Yaoting scene demonstration teaching "meet the red scarf", practical operation demonstration of the young pioneers of the basic etiquette knowledge. It is understood that the training is the first time in Jiangxi province to the young pioneers counselor backbone teacher training into the "green horse project", and after the concentrated training, students will track training, through organizational theory study, practice, volunteer service, special research activities, efforts to build a high-quality young pioneers counselor backbone team.

2021-12-25	The 5th Jiangxi Provincial School Communist Youth League "Micro League Class" competition College League teacher group final held the 5th Jiangxi Provincial School Communist Youth League "Micro League Class" competition College League teacher group final held " competition college League teacher group final was held. The competition is divided into two parts: theoretical test and group class teaching. The 19 participating teams adopted various forms of case explanation, video display, melodrama performance and other forms to present a micro-league class around the century-old brilliant party history of the CPC and the league member consciousness education, combined with the physical and mental characteristics of the youth members. The judges scored the teaching theme, content, form and effect. Finally, the participants from Jiangxi Normal University, Jiangxi Vocational College of Foreign Studies, Jiangxi University of Finance and Economics, Jiangxi University of Traditional Chinese Medicine and Jiangxi Vocational College of Tourism and Trade won the special prize. The competition was organized by the Education Working Committee of Jiangxi Provincial Committee and Jiangxi Provincial Committee of the Communist Youth League, organized by Jiangxi University of Finance and Economics of the Communist Youth League. Teachers from 19 universities in Jiangxi Province participated in the competition, 1.79 million people from all walks of life watched the competition online, and more than 600 teachers and students from Jiangcai watched the competition.

2021-12-23	Anhui Communist Youth League held Made in China Day Anhui theme network activities Anhui Communist Youth League held Made in China Day Anhui theme network activities China Youth Daily youth network reporter Wang Haihan) " We are in the design of Hefei No.7 Middle School station to what as the theme? Which district of Hefei is the Quantum Science Center Station located in? What is the theme of the design of the research station?"This morning, as the Anhui communist youth league anchor and Hefei rail company introduction and questions on behalf of the youth league, Anhui provincial party committee propaganda department, Anhui sasac committee and Anhui province youth new media center jointly launched" made in China day "Anhui article theme network activities officially launched, the youth netizens gathered group online live platform for" branch gas " made in China in Anhui article immersive learning and experience. On the day of the live site route for Hefei rail transit line 4 in Hefei in station-quantum science center-first institute, in elaborate live location, the host detailed introduction full of experience and science and technology site design, lead young netizens perceive young students dream set sail vigor, appreciate the charm of quantum science and technology and other cutting-edge science and technology. The activity was carried out through offline live broadcast and online lottery. According to statistics, the activity was live broadcast on government affairs Weibo, wechat video number, TikTok, Today Internet live broadcast room and other platforms, with a total of 1.653 million viewers. On the same day, the event was jointly organized by Port and Shipping Group, Construction Engineering Group, Diversion Jiangjihuai Group, Salt Industry Group, Civil Aviation Airport Group, Glory Terminal Co., LTD., and Hefei Rail Transit Group Co., LTD. In recent years, the group of Anhui provincial party committee relying on "made in China day", design launched youth participation in immersive technology experience, successively into the Chinese valley, Anhui innovation pavilion and other places to carry out offline activities, with teenagers enjoy the teenagers appreciate Anhui science and technology development and manufacturing industry, deepen its confidence in Anhui and Anhui confidence, help Anhui enterprises speak good "made in China" story.

2021-12-23	Jiangxi province "revitalization cup" health service industry vocational skills competition closed in 2021 "revitalization cup" in Jiangxi province health service industry vocational skills competition closed, provincial state-owned assets supervision and administration commission, provincial federation of trade unions, provincial party committee, provincial women's federation organization, Nanchang human resources social security bureau, Nanchang qingshan lake district government in 2021 "revitalization cup" in Jiangxi province health service industry vocational skills competition finals were successfully held in Nanchang. A total of 284 contestants participated in the province. It is reported that the final adopts a combination of theoretical examination and practical operation competition, involving postpartum rehabilitation, breastfeeding, the elderly ability assessment, health care, community health, public nutrition and other aspects of the content, a comprehensive assessment of the professional quality and skill level of health care service personnel. After fierce competition, Zeng Jianfang and other 36 contestants stood out and won the first, second and third prizes. According to the relevant documents, Jiangxi Provincial Human Resources and Social Security Department will award the title of "Jiangxi Provincial Technical Expert" and corresponding profession (type) to the contestants, the title of "Jiangxi Provincial Young Post Expert" and Jiangxi Provincial Women's Federation the title of "Jiangxi Provincial Female Achievement Model". The competition aims to stimulate health care skilled talents to develop and innovate, study hard, train and select more highly skilled talents, effectively create a good social atmosphere of advocating skills and respecting the spirit of labor, and make new contributions to the construction of health care highly skilled personnel team in Jiangxi Province.

2021-12-22	On December 21, Hainan Youth League), Hainan Provincial Committee of the Communist Youth League held a special meeting to comprehensively strengthen the work of the young Pioneers in the new era. Meeting to learn the CPC Hainan provincial party committee on comprehensively strengthen the new era of the young pioneers work implementation opinions "on the construction of a new era of young pioneers socialization work system implementation opinions" and other file spirit, inform Hainan province young pioneers work, for the next stage of work deployment, and listen to Haikou, Sanya, qionghai, in higher four cities and counties representative work report. So far, the group of Hainan provincial party committee, Hainan province education department, Hainan less committee has jointly issued the measures for the implementation of Hainan "red scarf medal", and other important policy documents, provincial, city and county level less committee transition work completed, the province has established young pioneers organization 1483, accounting for 96.61%, the practical education activities 3920 games, more than 900000 people participate in. Group Hainan provincial party committee secretary Chen Yu at the meeting, Hainan young pioneers work in the future toward socialization, networking, internationalization work goal and direction, strengthen all kinds of policy docking and resource support, build perfect online, offline platform position, through the concept of "a road a dream", building dream avenue national youth "big politics" practice education base, jointly build Hainan young pioneers work characteristic brand. The opening ceremony of the 2021 Hainan Provincial Young Pioneers Brigade Counselor (backbone) Training course was also held in Haikou.

2021-12-22	Guizhou: Guide young students to "button the first button". Guizhou "Qingfeng Action Campus Plan": Guide young students to "button the first button" for a very unforgettable class. The college student, who had always thought that the words "corruption" and "corruption" were far away from him, was shocked to see the case of young people jailed for corruption. On December 13th, the packed auditorium of Guizhou Medical University saw more than 500 fresh graduates taking a specially-tailored course. This is their first clean education class before they work. Combined with the party history learning education "I do the practical work for the masses" practice, Guizhou discipline inspection provincial prison committee authority, Guizhou education department, Guizhou provincial party committee jointly launched a "breeze action campus plan" volunteer service activities, set up volunteer service, form XuanJiangZu, into the university campus, for graduating college students to preach education. The launching ceremony and the first activity of the "Qingfeng Action Campus Plan" was held in Guizhou Medical University. The members of the Qingfeng Publicity volunteer service team followed the idea of "button the first button of entry" to educate and guide students to deeply grasp the first button on the harm of corruption. Doing clean education for college students who are about to graduate is not without a target. Guizhou province commission for discipline inspection committee of the province young cadres violations done special research, data show, from the party's 19 to June this year, the Guizhou province disciplinary inspection organs after 80,90 young cadres, accounted for nearly a quarter of the same punishment, 391 people were transferred to the judicial organs, younger corruption is shocking."After some young cadres were punished, they regretted it, but it was too late."Huang Wensheng, executive deputy secretary of the Guizhou Provincial Commission for Discipline Inspection and deputy director of the Provincial Commission for Supervision, said that if these young people are not at work or just started, they will be targeted to keep the bottom line of life and take the first step in their career, maybe they will not go astray. Huang Wensheng engaged in discipline inspection work for more than 10 years, has seen all kinds of young cadres astray cases, he summarized the five outstanding reasons: some "three view" distortion, desires, pleasure, blind comparison; some lawless, take chances, for example, a provincial rural credit cooperatives cadre ke, this is a scientific research talent, but the intelligence in loopholes, illegal possession of public funds; Some people are empty, vulgar, and cannot be drawn up from bad habits. For example, Zhu, the deputy general manager of a state-owned enterprise in a county, is addicted to online games, and uses public funds to recharge games and upgrade game equipment. In addition, there are some people accidentally pulled into the water for making friends."As you gaze at the abyss, it also looks at you."Huang Wensheng borrowed the classic quotes of the philosopher Nietzsche to encourage the students," Of course, life should not always wander between sin and non-sin, nor can it only stay in the external supervision of discipline and law constraints, but also have the inner introspection, so there should be a higher moral bottom line."After listening to the lecture, Long Qizhou's dormitory held a small seminar."They are all senior brothers and sisters walking in front of us. Some of them go astray when they step into the society and make their lives fall into a doomed situation."Long Qizhou said," Life has not come over again, the workplace start is too important! The road must not go wrong, the direction must not be biased, the values of the 'master switch' can not go wrong at any time."Wu Tianwei, China Youth Daily, China Youth Network, reporter Li Yajuan, source: China Youth Daily, December 22,2021, edition of 03

2021-12-22	Hubei held 2021 "thousand green" internship employment activities in Hubei 2021 "thousand green" internship employment activities in the youth network reporter LeiYu) on December 20, Hubei province Taiwan affairs joint provincial people club hall, provincial party committee, held in 2021 in Hubei province "thousand green green" Taiwan youth internship employment special with post live activities, help Taiwan born green to Hubei development. China Railway Bridge Bureau Group Co., Ltd., Tianfeng Securities Co., Ltd. provided 515 internship jobs on Hubei Public Recruitment Network and its WeChat official account "Live with Post"; With over 1200 jobs released in this spring employment season, another 9 new companies released recruitment information and provided 327 internships, attracting more than 180,000 people online. Lai Yanjun, a Taiwanese teacher from Zhongnan University of Economics and Law, and Li Fangzhong, a young Taiwanese student from China Telecom Optical Valley Group, shared their thoughts on their employment and growth experience after graduation from a university in Hubei province online. In November 2017, Hubei province launched the plan. Over the years, we have held a number of large-scale special job fairs for offline internship and employment for young people from Taiwan, and given guidance and support to young people from Taiwan to start their own businesses in Hubei. According to statistics, up to now, more than 1,000 young people from Taiwan have practiced and employed in Hubei, more than 300 registered enterprises and teams, and 8 national and provincial entrepreneurship and employment bases for Taiwan and youth (counseling centers) and demonstration sites.

2021-12-22	Group shaanxi provincial party committee held the spirit of the sixth plenary session of the nineteenth training league shaanxi provincial party committee held the spirit of the sixth plenary session of the 19 and authority party construction, discipline inspection training on December 22 (reporter Zhang Jianwei, correspondent Zhang Wenhao), the communist youth league shaanxi provincial party committee held the party's spirit of the sixth plenary session of the 19th and authority party construction, discipline inspection training work. Training course site. Correspondent for figure training using classroom teaching, field teaching, seminars, combining examination, invited shaanxi provincial party committee party school (shaanxi administration institute) level 1 inspector professor AnRui as the party's struggle in one hundred major achievements and historical experience, study and implement the spirit of the sixth plenary session of the 19th special counseling, shaanxi provincial party committee, party secretary of the party member cadres violations case warning education, shaanxi provincial party committee party supervision guidance minister yong-jun li around authority party construction practice communication counseling. At the same time, the training class also organized students to carry out on-site teaching in Xi'an warning education base, and carried out the theoretical knowledge test that party members and cadres should know and know. Training course site. Correspondent for the training using tencent conference APP using the combination of online and offline training, organs and related directly affiliated units above place level cadres and party cadres of more than 40 people in the main venue, shaanxi youth vocational college (province) place level cadres above party cadres and more than 80 people in the venue through online video conference form took part in the training.

2021-12-21	The 2021 Jiangxi Provincial Counselor Style Exhibition activity was held in Gao 'an in Gao' an. Correspondent for the picture of China youth network Beijing on December 21 (reporter Zhang Jianwei, correspondent Cao Zhang Yue) recently, by the Communist Youth League Jiangxi Provincial Party Committee, Jiangxi Provincial Department of Education, Jiangxi Provincial Youth Working Committee sponsored by the 2021 Jiangxi Provincial Young Pioneers counselor style exhibition activities held in Gao'an city. Eleven teams of outstanding young Pioneers counselors from all districts and cities in the province participated in the activity. This style exhibition is divided into five links: professional knowledge written test, theme publicity, case explanation, young Pioneers activity counseling skills exchange, work situation processing and defense. The written test tests the professional knowledge and policy documents of the young pioneers, examines the ability of the counselors of the case explanation, discusses the skills of the counselors of the young pioneers of the young pioneers, and evaluates the comprehensive quality of the counselors by randomly selecting and solving the relevant problems of the young pioneers. Nanchang city counselor, Yao Qinhua, guided the young Pioneers to carry out the theme team class. Correspondent for figure after fierce competition, from Nanchang, Ganzhou city young pioneers counselor team won group prize, Pingxiang should Kelly, Ganzhou city Chen Haili link theme to preach prize, Nanchang Zhong Baoting, Xinyu section needle team cohesion case prize, Nanchang Yao Qinhua, Yingtan Tian Hui tutoring skills communication prize, Nanchang liu min, Ganzhou city Li Yeqing scene processing defense link. The event was held by "live + live broadcast", with a total of 5 million people watching it online.

2021-12-19	Sichuan: Members of the 19th CPC Central Committee of the Sixth Plenary Session of the 19th Sichuan Youth Federation was held in Chengdu today. The meeting required that the majority of Youth Federation members should actively carry out grassroots publicity activities, especially for the spirit of the sixth Plenary Session of the 19th CPC Central Committee. After the closing of the sixth Plenary Session of the 19th CPC Central Committee, the Sichuan Youth Federation made timely arrangements, and the members of the Sichuan Youth Federation from all walks of life carried out special study and discussion. Sichuan Youth Federation also opened an online learning column on "Learn the Plenary Session, Take the Mission" to continue to deeply interpret the spirit of the plenary session. The third standing Committee (expanded) meeting of the 14th Sichuan Provincial Youth Federation was held in Chengdu. Through the youth teaching group, members of the Sichuan Youth Federation at all levels carried out 560 publicity, exchanges and sharing on party history and the spirit of the Sixth Plenary Session of the 19th CPC Central Committee, covering more than 44,000 people. The youth league of all cities (prefectures) in Sichuan set up a youth League study group, paired up with 227 grass-roots youth league branches, and carried out various learning and exchange activities such as theme youth league days. The third (expanded) meeting of the Standing Committee of the 14th Sichuan Youth Federation required that learning, publicity and implementation of the spirit of the Sixth Plenary Session of the 19th CPC Central Committee be the most important political task at present. Relying on the youth lecture hall and other carriers, the members of the Youth Federation should actively carry out the publicity of the Youth Federation at the grass-roots level. Meeting requirements, the provincial youth federation chairman team, standing committee, contact deputy secretary general of all sectors of groups, combined with their own posts, take the lead at the grass-roots level, for the youth propaganda of the spirit of the sixth plenary session of the 19th CPC Central Committee, each year to carry out at least a year. The meeting also called for members of the Youth League to regularly contact the youth, focus on their practical problems, fully understand their ideological situation, make good use of the political resources of the Communist Youth League and the Youth League, and actively reflect their interests. Zhang Rong, secretary of the Sichuan Provincial Committee of the Youth League, said that the spirit of the sixth Plenary session of the 19th CPC Central Committee, to go deep into the grass-roots level, the front line, can be small batches, interactive, can talk about experience, cognition, the party's innovation theory clearly, clear, thoroughly, to create a strong learning atmosphere among the youth group.

2021-12-17	Guangdong province first youth calligraphy and seal cutting exhibition opened the first youth calligraphy and seal cutting exhibition opened on December 17 (reporter Zhang Jianwei, reporter YueQing) on December 16 morning, under the guidance of Guangdong provincial party committee, sponsored by the Guangdong young calligrapher association, Guangzhou young calligrapher's association, Guangdong youth calligrapher association committee and Guangdong youth calligraphy magazine to undertake the first youth calligraphy and seal cutting exhibition opened in Guangzhou dongshan hotel. Liang Junda, deputy secretary of the Provincial Communist Youth League Committee and chairman of the Provincial Youth Federation, attended the event. Guangdong Calligraphy Association, Guangdong Province, Guangdong Youth Calligraphy Association and representatives of the winning authors participated in the event, and held the award ceremony and the launch of the works at the scene. This call for papers has been actively supported by the young calligraphers in the province, and a number of fine works with traditional skills and the style of The Times have emerged. A total of 1,375 submitted works were received from all over the province. On the premise of taking into account different styles and styles, a total of 10 winning works, 20 nominated works and 229 exhibited works were selected. Award certificates to the winning authors. The organizer of the Communist Youth League said that the event was held to thoroughly study and implement the spirit of General Secretary Xi Jinping's series of important speeches on literature and art work and youth work, and vigorously promote traditional culture and enhance cultural confidence among the young people of the Communist Youth League. Constantly innovate the form of youth literature and art work, extensively contact, gather strength, fully display the creative strength of the youth calligraphy in Southern Guangdong, and promote the new situation of the development of the cause of Guangdong youth calligraphy. Guests visited the first Youth Calligraphy and seal cutting Exhibition in Guangdong Province."This batch of works are prepared in various styles, diverse styles, rooted in tradition, diverse and inclusive."After the opening ceremony, the calligraphers who attended the exhibition visited and stopped in front of the exquisite works for a long time." This is a rare exhibition of works, but also provides us with a good platform for us to communicate."It is reported that the exhibition also exhibited from the provincial calligraphy association, the provincial youth calligraphy association and the city youth calligraphy association 67 specially invited works, the exhibition will end on December 21.

2021-12-15	"Green and Beijing dream peer" emerging youth creative exhibition launched "green and Beijing dream peer" Beijing emerging youth creative exhibition launched the youth network Beijing on December 15 (reporter Zhang Jianwei, correspondent, Li Jinfeng Fu Zhen) recently, "green and Beijing dream peer" Beijing emerging youth creative exhibition and 2021 "youth" Beijing outstanding young artists exhibition opening ceremony in tongzhou zhangjiawan design town future design park. The event is organized by the Beijing Municipal Committee of the Communist Youth League and the Beijing Architectural Design and Research Institute Co., LTD., organized by the Beijing Youth Service Center and the Beijing Youth Art Development and Promotion Association, and with academic support provided by the Central Academy of Fine Arts. At the opening ceremony, the emerging young people from the fields of network literature, street dance, independent literature, national music brought their wonderful performances; the participating cultural and creative enterprises and the purchasing intention signed successfully; the participating leaders unveiled the "Dream Space" of Zhangjiawan Design Town, Tongzhou and issued the letter of appointment to the "Youth Master". Transfer of the Winter Olympics-themed art works. In the next step, the CPC will implement the CPC deployment requirements and 22 specific measures to further promote the package work plan of connecting the emerging youth, adhere to both top-level design and outstanding implementation; solve centralized demands and respond to individual needs; emphasize both ideological guidance and capacity improvement, both make the contact coverage, and strengthen the service guarantee. At the same time, through the establishment of "strong country I build a dream peer" and other work brands, with practical actions to connect and serve the majority of emerging young people. The series of activities as the party's centennial birthday and Beijing Olympics held major opportunity, based on the emerging youth talent, original works, project display opportunities and platform, guide more youth in emerging industry innovation and development, in the capital cultural development and social governance as new, the activity in the new form of "emerging youth creative exhibition", strive to build a broad stage for Beijing emerging youth groups. As a series of themed exhibition activities, the exhibition gathers and covers the young designers, young painters, hip-hop dance youth, youth musicians, network literature youth and other emerging youth groups in different professional fields.site of activity. It is reported that, "Green and Beijing dream peer" theme series of activities through creative service urban development achievements, creative service rural revitalization achievements, creative service better life display, creative services, the games cultural communication exhibition, social organizations, and park characteristic achievements display, Beijing design youth works exhibition, Beijing built courtyard "star plan" display, And to " Always follow the Party, Youth Winter Olympics " art exhibition, street-hop new scene as the theme of Beijing outstanding young artists exhibition and other 10 exhibition blocks, And supplemented by forum, salons, lectures, aesthetic education classes, hip-hop performance and other forms of activities, Three-dimensional, in-depth, vivid and complete display of the creative ecology of Beijing's emerging youth and the achievements of promoting the development of Beijing's emerging youth in recent years.

2021-12-15	Group of Heilongjiang provincial party committee to build "five one" learning system of Heilongjiang provincial party committee to build "five one" learning system to the spirit of the sixth plenary session of the 19th in youth heart "take root" in "propaganda column, Harbin normal university education science college 2018 preschool education students Zhang Yuqi was touched by a paragraph:" as a " fire join the party, I don't have to succeed in my spirit and success must have my mission, with hard backbone, wide shoulder mission, be a new long march when the era of youth."This is the Harbin first hospital nurse, aid Hubei medical team member Kan Xinxin published the feeling. In addition to the perception of the youth, the "Sixth Plenary Session of the 19th CPC Central Committee" publicity column also set up the spirit of the sixth Plenary Session of the 19th CPC Central Committee interpretation, grassroots learning interaction. Group of Heilongjiang provincial party committee secretary xu, as the theme of youth ideological and political leading work of the main line, Heilongjiang provincial party committee will be in accordance with the "comprehensive" "interactive a", "widely" said "thorough" "practice line" five aspects specific deployment, guide teenagers consciously do long-term learning, continue to follow up, efforts to create a strong atmosphere of "than learn to help super". In terms of "comprehensive learning" and "interactive answer", the Heilongjiang Provincial Party Committee of the Communist Youth League relied on the influence of the "Heilongjiang Communist Youth League" and the advantage of gathering 7 million fans, and the interactive reading volume was more than 1 million times. After the closing of the Sixth Plenary Session of the 19th CPC Central Committee, the Heilongjiang Provincial Committee of the Communist Youth League, in accordance with the work deployment of the Heilongjiang Provincial Party Committee and the CPC Central Committee, carefully issued the Notice on learning, propagating and implementing the Spirit of the Sixth Plenary Session of the 19th CPC Central Committee with the characteristics of The Times. In order to strengthen the youth interpretation and scientific expression of the spirit of the Sixth Plenary Session of the 19th CPC Central Committee, the Heilongjiang Provincial Committee of the Communist Youth League launched a special episode of "Youth Learning the Spirit of the Sixth Plenary Session of the 19th CPC Central Committee of the CPC" on the new media platform, with more than 1.2 million youth league members participating in the study. At the same time, in order to create an interactive platform for young people to love and participate, the Heilongjiang Provincial Committee of the Youth League held an online answer contest. By making a special question bank of knowledge of the Sixth Plenary Session of the 19th CPC Central Committee, relying on the "century-old Party History" online answer and battle platform, we launched one issue of 10 questions per week, deepening the young people's understanding of the major achievements and historical experience of the Party's century-old struggle. Fu Yiying, a 2019 student majoring in Chinese Language and Literature from the School of Humanities of Jiamusi University, has participated in online question answering competitions for many times. She said that in the process of answering the question contest, she not only learned to understand the spirit of the sixth Plenary session of the 19th CPC Central Committee, but also gained the fun of learning with netizens in the PK. In addition to exploring the learning methods that young people are willing to participate and easy to participate, the Heilongjiang Provincial Party Committee of the Communist Youth League also trained a group of young lecturers who "widely" and "in-depth", and spread the spirit of the Sixth Plenary Session of the 19th CPC Central Committee to the minds of young people through their strength."I saw the notice issued by the Heilongjiang Provincial Committee."Zhang Dan, deputy secretary of the Youth League Committee of Heilongjiang University, said that in order to do a good job in this lecture, she once again studied the books" A Brief History of the Communist Party of China "and" A Brief History of Socialist Development "." Only by extensive study and deeply armed with theory can we fully explore the perspective of youth and tell China story well."It is understood that the Heilongjiang Provincial Party Committee of the Communist Youth League will set up a spirit of the sixth Plenary Session of the 19th CPC Central Committee youth publicity group, through the combination of online and offline publicity. Online, the Heilongjiang Provincial Party Committee of the Communist Youth League will select lecturers to record the theme micro-group class "cloud lecture", and deliver the spirit of the sixth Plenary Session of the 19th CPC Central Committee more creatively and accurately to the youth group. Offline, the theory will be carried out to the grassroots activities, each person at least once before the end of the year. At the same time, we will also organize cadres of the Communist Youth League, members of the Youth Federation, students of the "Green Horse Project", and members of the Red Scarf Tour Group to visit the grassroots to carry out mass focus and interactive theme lectures, and accelerate the spirit of the Sixth Plenary Session of the 19th CPC Central Committee to take root and sprout in the hearts of young people in Heilongjiang. In order to study and publicize the spirit of the Sixth Plenary Session of the 19th CPC Central Committee in a simple way, and to help young people to enhance practical identification, emotional identity and rational identification. According to the media use preferences of different youth groups, the Heilongjiang Provincial Party Committee will also actively produce, promote and disseminate accurate and rich online cultural products through new media platforms and new technologies through long picture posters, audio and videos."As the Party's assistant and reserve force, the Communist Youth League should accurately focus on the fundamental task of educating people for the Party, and take learning, publicizing and implementing the spirit of the Sixth Plenary Session of the 19th CPC Central Committee as the primary political task at present and for a period to come."Xu Changyong said, as the theme of youth ideological and political leading work of the main line, Heilongjiang provincial party committee will strive to promote the party history learning education heating strength, go deep, to study the results of the spirit of the sixth plenary session of the 19th into power the revitalization of longjiang development action, let" please rest assured, power have me " youth oath become Heilongjiang youth era. China Youth Daily China Youth network trainee reporter Han Rong source: China Youth Daily

2021-12-14	The 11th Guangdong Volunteer Culture Month opened the 11th Guangdong Volunteer Culture Month Lin Jie) Recently, the 11th Guangdong Volunteer Culture Month opened. On the site, the organizer awarded the Guangdong Five Star Volunteer certificate to the volunteer service time reached 1500 hours. Ai Hexu, a foreign aid volunteer who had participated in the fight against the epidemic, Zhang Yingying, a national moral model, and Wang Zhiyong, an emergency volunteer who led the team to help the Henan flood, talked about their volunteer stories. Starting from this year, Guangzhou and Shenzhen will join hands in the volunteer service training, cultivation and exchange, the standardization system construction for volunteer service development, the integrated development of volunteer service in the Guangdong-Hong Kong-Macao Greater Bay Area, and the cultivation of Guangzhou-Shenzhen volunteer service brands. Such as deep each year will take turns to hold volunteer service exchange activities, jointly training volunteer service talents, volunteer service theory research, promote the development of a large bay area, Guangdong volunteer service fusion, promote volunteer service in building Guangdong work sharing social governance pattern play a more positive role, in order to realize the deep "shuangcheng linkage, fly wing to wing" contribution. To find out the actual development of volunteer service in Guangdong province, establish an objective evaluation mechanism, Guangdong volunteer federation based on Guangdong volunteer information management service platform ("i volunteer" system) and Guangdong volunteer service interaction system data, around volunteers, volunteer service organizations (groups), volunteer service duration, volunteer service activities, such as four dimensions, developed the Guangdong ("i volunteer" system) volunteer service development index and measurement standards. The relevant person in charge of Guangdong Volunteer Federation introduced that the data of the "i volunteer" system is strict and transparent in the collection standards of data such as volunteer identity and volunteer service duration. The index takes the lead in exploring the establishment of a scientific and objective weighted index evaluation system for the development of volunteer services, which will guide local volunteer services in various fields. Data show that as of November 30 this year, there were more than 14 million real-name registered volunteers in Guangdong province, 132,000 volunteer service organizations and groups, and 4.24 million volunteer service activities, providing more than 700 million hours. The CPC members accounted for 27 percent, and the Communist Youth League members accounted for 35 percent. The 2021 Guangdong ("i Volunteer" system) Volunteer Service Development Index is expected to be released after the New Year's Day of 2022, and the list of cities and universities will be published. During the Guangdong Public Welfare Volunteer Culture Month, all parts of Guangdong will extensively organize and mobilize volunteer service organizations and volunteer service organizations to carry out a series of distinctive and distinctive volunteer services in communities and villages. Photo is provided by the Guangdong Provincial Committee of the Communist Youth League

2021-12-14	Shandong "Hope House": On the road of charity to help common prosperity with the hand of public welfare? Since the launch of the "Hope Cabin" child care project in June 2020, more and more social forces have participated in it. The hut project has not only become a typical demonstration of national public welfare, but also become an effective exploration for children in distress to enjoy the fruits of common prosperity and development. In June 2020, Shandong Provincial Party Committee of the Communist Youth League, Shandong Youth Federation and Shandong Youth Foundation fully launched the "Hope House" child care project. The project plans to build no less than 20,000 "hope lodges" in the province in three years. After the launch of the project, Shandong Communist Youth League has continuously expanded the fundraising channels, and actively explored the new mode of "Internet + public welfare" on the basis of the combination of organization and social mobilization. During the "99 Tencent Charity Day" in 2020 and the "520 Tencent Charity Season" in 2021, both online fundraising has achieved fruitful results, and further expanded the social influence and awareness of the "Hope House". Winding along the mountain path, sleepy at night, running under the scorching sun... the province's more than 20,000 cadres, more than 24,000 volunteers, 113,500 love teams, completed another impressive feat in September this year. With the extensive participation of 1.321,100 people, 50,702,500 yuan of charity donations, it ranked first among provincial public offering institutions and the total amount of single projects of Tencent public welfare platform in China. This is the "report card" delivered at the end of the "99 Public Welfare Day" activity of "Hope House" project by Shandong Provincial Party Committee of the Communist Youth League in 2021. Looking across the province, online fundraising is in full swing, and offline activities are also warm their hearts. In the "Youth Ode to the Party" to celebrate the 100th anniversary of the founding of the Communist Party of China, more than 40 members of the Provincial Youth Federation members, young entrepreneurs donated painting and calligraphy works, a total of 3.32 million yuan, all used to support the construction of "Hope House"."It is very meaningful to see that everyone is forwarding and participating in the online fundraising activity of 'Hope House'."On September 6, Weifang Shouguang city caring citizen Yu Mei sprint to help the elderly, was awarded by Alibaba" daily positive energy special award "and won the positive energy bonus of 5,000 yuan, Yu Mei in the first time will be fully donated to the bonus to her most trusted" hope cabin ". On October 29, Ju slimming, Liu Qianchao and Li Shunhao, three post-90s employees of Weifang Port Lianhua Storage Company, Shandong Port, rescued two victims of the accident and received a 10,000 yuan positive energy bonus. The three decided to donate the prize money to the Hope Hut Child Care Program. Under the love of all walks of life, the trickle into the sea, little stars illuminate the sky. Stimulate charity power, write a common rich chapter " association as a social donation organization, has donated 'hope hut' 11, undertake the construction of 23, at the same time relying on the 'grass partner youth volunteer service,' love mother 'service brigade, form a complete set of volunteers tracking company, help troubled children realize from' cabin refresh 'to' spirit."In recent years, many social forces and individuals have joined Xin Wei, president of Rizhao Sunshine Volunteer Association to Help the Disabled, in building the" Hope House ". Every time a fund is raised, you can build a more "hope hut", and you can have a more accurate transmission of love."Hope House" not only makes it a reality for everyone to participate in public welfare, but also builds a bridge for enterprises to actively participate in charity. Located in Hedong District of Linyi City, catering enterprises First Every Group has been widely involved in social public welfare undertakings for many years. In addition to donating 240,000 yuan to contract the "hope hut" in three villages in Meijiabu Street, Hedong District, the company has also set up millions of love funds to help children in distress. In the golden autumn of October, the good news came from the distant Xigaze City in Tibet and Haibei Tibetan Autonomous Prefecture in Qinghai Province."I hope the cabin is a great help to us and our children. Gatso has his own learning space. I sincerely thank you for your support to our family."This is Xizang Anren County troubled child Jia uo's uncle from the heart of the interview. A few months ago, the nine-year-old had to help his working uncle take care of his 80-year-old grandmother. The father of Xiao Xin, a troubled child in Haiyan County, Qinghai Province, was paralyzed by an accident in 2017. The 10-year-old family dilemma has become withdrawn. He is eager to have his own small world. Now, he has become the small owner of the first "hope hut" in Haiyan County. Since this year, in order to make the teenagers in ethnic minority areas also feel the care and warmth of the Party like the children in Shandong province, the Shandong Provincial Party Committee of the Communist Youth League not only donated 2 million yuan to the two places, but also gave the "Hope House" construction standards, use management, volunteer service and other complete set of working modes. At present, the construction task of the "Hope House" in the two places has all been completed, and more than 140 children from Tibetan, Hui, Turkish, Mongolian and other ethnic groups have lived in their new rooms, truly enjoying the development fruits of common prosperity. Up to now, the Shandong Provincial Party Committee of the Communist Youth League has raised more than 210 million yuan in one and a half years, and has built 15,036 "hope cabins" under construction. More than 15,000 children in difficulties have their own independent school living space and more care from social caring people. China Youth Daily China Youth Network reporter Xing Ting source: China Youth Daily

2021-12-13	Guizhou project seminar learning the party's spirit of the sixth plenary session of the 19 Guizhou communist youth league propaganda and implement the party and the sixth plenary session of the 19 and 12 provincial plenary session spirit project seminar held Beijing December 13 (reporter Zhang Jianwei, correspondent Chen Kai) on December 8 solstice 10, Guizhou communist youth league learning propaganda and implement the party's sixth plenary session of the 19th and Guizhou provincial party committee 12th ten plenary session spirit project seminar held in Guiyang. More than 130 people from the municipal, prefectural, provincial universities, industry system, county (city, district) league organizations and all departments of the provincial Youth League Committee and directly affiliated units participated in the seminar. More than 13,000 people, including provincial, municipal, county and industrial system youth league cadres, university youth league cadres, and young volunteers, participated in the study through live video broadcasts. The special seminar is divided into two stages: individual self-study and concentrated learning. On the afternoon of December 9th, Shi Qilin, a member of the publicity group of Guizhou Provincial Party Committee and deputy secretary of the Provincial Communist Youth League Party Committee, delivered a special publicity report entitled "Always adhere to the Leadership of the Party and firmly support the" Two Establishment "and strive to write a new chapter of the high-quality development of the Provincial Communist Youth League work" for the seminar students. The Special topic seminar is on-site. Wu Zuping was specially invited, former deputy director of the Policy Research Office of the Provincial Party Committee, former counselor of the provincial people's Government, and advisory expert of the Standing Committee of the Provincial People's Congress, to have an in-depth and systematic interpretation and interpretation of the resolution adopted at the sixth Plenary session of the 19th CPC Central Committee. After listening to the two special guidance reports, the participants carried out group learning and discussion based on their own learning experience and work practice."Through the spirit training of the Sixth Plenary Session of the 19th CPC Central Committee and the 100 Plenary Session of the 12th CPC Provincial Committee, I am more deeply aware of the Party's centenary struggle and major historical achievements, and more deeply aware that a Party history is the history of generations of Chinese youth's struggle with the Party."Tuan Guiyang municipal party committee secretary PeiYingLei said, will lead the communist youth league Guiyang municipal party committee, in the direction of the direction, to the party's will, focus on Guiyang" strong capital "center work, highlight the political construction, highlight the service general situation, outstanding service ties with the masses, highlight their own management, leading the youth issued" strong capital "youth strongest, to be the" strong capital " vanguard. The Special topic seminar is on-site. Correspondent for figure Guizhou nationalities university youth corps committee, deputy secretary of hu said, as a new era of the communist youth league cadres, to fully grasp the party's sixth plenary session of the 19 theoretical connotation and practice direction, strengthen the "two established" political identity, ideological identity, emotional identity, the "two established" as the first position, first principle, first requirement and the first test, with practical action to write the communist youth league work high quality development of writing and youth chapter."We need to deeply grasp the core meaning of the spirit of the plenary sessions of the Central Committee and the Provincial Party Committees, draw on the strong strength of faith entrusted by the great achievements of the century, and be firm believers and loyal practitioners."Tuan kaili municipal party committee secretary Huang Juan said, as a new era of cadres, to strengthen the consciousness of" core ", firmly implement cultivating socialist builders and successors this fundamental task, resolutely carry era mission, yong bear the communist youth league work responsibility, closely around the" four new "main" four ", brain has ideas, under the way, in the heart has the bottom line, to promote the communist youth league work innovation situation."The Sixth Plenary Session of the 19th CPC Central Committee deeply summarized the major achievements of the Party's major achievements and historical experience in the Party's centenary struggle, and called on the whole Party, the army and the people of all ethnic groups in China not to forget yesterday's suffering and glory, be worthy of today's mission, and live up to tomorrow's great dream of prosperity. As a communist youth league cadres, we should take history as a mirror, always adhere to the leadership of the party, inheriting red gene, continuously red blood, bear the beginner's mind mission, around the center service general situation, further promote the reform of the communist youth league organizations at the grassroots level, strengthen the political function, improve social functions, contributing to realize the great rejuvenation of the Chinese nation the youth strength."Said Yang Zhengsen, Party secretary of Tuansongtao County. After the sixth Plenary Session of the 19th CPC Central Committee and the 10th Plenary Session of the 12th Guizhou Provincial Committee, Youth league organizations at all levels in the province to grasp the key points of learning, focus on the "key minority", stratified classification to carry out centralized learning, special seminars, counseling reports and other more than 170, Establish the "Guizhou Communist Youth League to study, publicize and implement the spirit of the Sixth Plenary Session of the 19th CPC Central Committee and the 10th Plenary Session of the 12th Provincial Party Committee", Organized 41 "youth lecturers", "red scarf tour group", "national unity publicity team" of 1,236 people, Go deep into the grassroots front line, go into the youth, Relying on the Red Education Base and Youth Home, Guizhou Youth Rural Revitalization Night School, and "Hope Small Classroom", 1,746 face-to-face interactive lectures were held.

2021-12-13	Beijing, Dec. 13 (China) - -Zhang Jianwei, correspondent Zhao Yun) Recently, the Xinjiang Uygur Autonomous Region Committee sent a letter of thanks to the Shanxi Provincial Committee of the Communist Youth League. The letter noted that the western Xinjiang plan project has reached a record high of 6,830 people, with 441 students enrolled, ranking the highest among the 29 recruited provinces. I sincerely thank the Shanxi Provincial Committee of the Communist Youth League for its great attention and help to the youth work in Xinjiang and the western plan over the years. I hope the Shanxi Provincial Committee of the Communist Youth League can continue to support and encourage more college graduates to serve in Xinjiang, grow in Xinjiang, stay for development and speak for Xinjiang. In recent years, under the strong leadership of the Provincial Party Committee and the Central Committee of the Communist Youth League, it has steadily promoted the youth volunteer work and carefully deployed the planned projects in the western region. In the past five years, the Western Planning and Project Office of Shanxi Province has selected and dispatched 2,857 college students from more than 60 universities in the province to the Xinjiang Uygur Autonomous Region, Xinjiang Production and Construction Corps, Tibet Autonomous Region, Inner Mongolia Autonomous Region, Shaanxi, Yunnan, Gansu and other provinces. With 296 volunteers dispatched in 2017,382 in 2018,479 in 2019,758 in 2020 and 942 in 2021, the number has increased steadily."The Western Student Volunteer Service Plan is referred to as the" Western Plan ", which is a major talent project jointly organized by the Central Committee of the Communist Youth League of the Communist Youth League, the Ministry of Education, the Ministry of Finance and the Ministry of Human Resources and Social Security. In July this year, more than 750 young volunteers from Shanxi went to Xinjiang, Tibet and other places to carry out 1-to 3-year rural education, rural construction, healthy countryside, rural social governance and grass-roots youth work services. At present, the western plan has become an important way for the Communist Youth League organization to serve the youth. Under the guidance of the Chinese Youth Volunteer Action, a large number of young people realize their own value in the process of contributing to the society and serving the people. In the practice of volunteer service, they have strengthened their understanding of the national conditions, people's conditions and social conditions, tempered their will and character, enhanced their volunteer feelings, and strengthened their ideals and beliefs. Careful organization to service western youth career development center in Shanxi Province, in recent years, the provincial party committee joint provincial education department, provincial people club hall, provincial finance department established in Shanxi Province college students volunteer service plan leading group and project management office, universities have also set up by the school party and government leadership team leader of the leading group, the recruitment provides a strong organizational guarantee for propaganda work. At the same time, the provincial Communist Youth League Committee actively coordinated the relevant departments for the expiration of the service for the volunteers in the civil service institutions to set up special recruitment positions, postgraduate entrance examination written test plus 10 points, specialized volunteers can be exempted into the adult promotion school and other preferential policies. In addition, in the annual recruitment stage, we actively coordinate the provincial TV stations, radio, newspapers and other news media to carry out extensive publicity, and mobilize the enthusiasm of volunteers to participate in the western plan. In the annual training stage, the organization unit to formulate detailed training programs, carefully design and arrange the form and content of training. Meanwhile, specially invited relevant experts and teachers for volunteers to carry out the lectures, reports, and watch the video and sports activities and other rich variety, lively teaching form, focusing on political and ideological education and volunteer service concept, volunteer service management regulations and other service work necessary professional skills, the communist youth league work, local conditions and customs and health care, emergency knowledge to carry out the training. At the same time, according to the principle of giving priority to the grass-roots positions and referring to the personal wishes of the volunteers, we actively contacted the provincial project office and assigned the volunteers to each corresponding position, so that the quantity is applicable. Where there are needs, there are volunteers, not only the western project is active with volunteers, where there is a need, there is their dedication and persistence. Shanxi university graduate volunteers for 17 years, adhere to the "hard scare, difficult pressure not collapse" service spirit and "teaching based, service for this" working principle, recruit 186 masters to Xinzhou city, Shanxi Province, Datong guangling county and shaanxi Yan'an huangling, Baise, Guangxi zhuang autonomous region Xilin County teaching service, cumulative teaching time of 600000 hours. In recent years, Shanxi Province has continued to carry out volunteer service activities, calling on the volunteers of the province to base themselves on the new era, show new actions, write the story of Lei Feng in the new era with practical actions, and push the volunteer service work to a new height in the creation of civilization. At the same time, widely mobilize the social forces from all walks of life, carry out volunteer service activities, promote volunteer service institutionalization, standardization, normalization, formed the party member volunteers, community volunteers, worker volunteers, youth volunteers, college students volunteers, female volunteers, family volunteers, elderly volunteers, the Red Cross volunteers, popular science volunteers, environmental volunteers and other kinds of volunteer service. The relevant person in charge of Shanxi Youth Cause Development Center said: " Shanxi Province has been implemented for nearly five years, and we deeply realize that 'dedication, friendship, mutual assistance and progress' is the action slogan of young volunteers engraved in the bottom of their hearts with love and action. This slogan has inspired groups of volunteers from the Western China Plan to go to the forefront of the motherland, fight side by side in the front line of the poor areas in the central and western regions, and spread the advanced and serve the grassroots. It is believed that groups of volunteers will make positive contributions to the harmonious development of the society, economy and culture in the service place with their youth and sweat, and will also write down the brilliant tomorrow of the western plan with practical actions.”

2021-12-13	Zhang Jianwei) A few days ago, "Henan Communist Youth League legal escort" Henan Communist Youth League youth legal publicity and education activity was held in Henan University of Finance and Finance. In the 2019 "Praise the Constitution" on the theme of the Youth League branch, the league members introduced the importance of the Constitution through the melodrama "If there is no Constitution", deepened the understanding of the Constitution through the knowledge contest, and praised the authority of the Constitution and the strength of the motherland through the poem reading "Praise the Constitution"."I feel that many of the contents of the Constitution are still far away from our daily life, so how should college students apply the spirit of the rule of law to their future study and life after studying the Constitution?"" If college students encounter fraud, how should they recover their losses or how to protect their legitimate rights and interests?"In the subsequent rule of law to preach, Henan province youth branch members Xie Wenjuan, Henan province people's procuratorate supervisor, Henan run Lin law firm Han Fumin, Zhengzhou city public security bureau crime investigation bureau against fraud center publicity group chief ice for the students in the spirit of the 19th, xi jinping about the rule of law thought, constitutional knowledge, and around the youth focus on labor security, personal information protection, raise awareness away from fraud trap sharing interaction. Wu Jingjing, a student from Class 3 of International Economics and Trade, 2020, Henan University of Finance and Finance, said that she hopes to use her constitutional and legal knowledge to preach to more students in the future, and also sow a legal seed in their hearts. This event is sponsored by the youth league provincial party committee, group Zhengzhou municipal party committee, Henan finance institute youth corps committee, in the constitution propaganda and education specially set up the spirit of the sixth plenary session of the 19th preach link, at the same time combined with the provincial party congress report "construction of higher rule of law in Henan" related deployment, and the youth learning life employment entrepreneurship urgent needs for legal knowledge popularization and answer. Under the arrangement of the provincial deployment, the provincial youth league organizations at all levels in the "constitution publicity week" to "qing peer legal escort" as the theme focused on the theme team, knowledge contests, the rule of law, public lectures, micro class and other forms of the rule of law publicity and education, and organize "juvenile judicial social service center", "youth zero crime zero victim community (village)" and "youth QuanQuanGang" unit (collective) teenagers provide legal aid, psychological counseling and other services.

2021-12-12	Wang Hao) On December 10th, the third plenary meeting of the Joint Conference on Youth Work in Gansu Province was held in Lanzhou. The meeting conveyed the spirit of the third plenary session of the Inter-ministerial Joint Conference on the Implementation of the National Medium-and Long-term Youth Development Plan, summarized the progress of the Medium-and Long-term Youth Development Plan in Gansu Province, and studied and deployed the key work in 2022. Sun Wei, deputy secretary of Gansu Provincial Party Committee, presided over the meeting and made a speech. Sun Wei pointed out that in recent years, the youth work in Gansu province has achieved remarkable results, and the role of youth as new forces and commandos has been further played. To improve, he said, the political stance, the youth development and youth work as a fundamental plan, xi jinping, new era of socialism with Chinese characteristics education, with the party's ideal and faith, with socialist core values, with the historical mission of the great rejuvenation of the Chinese nation, carry out the "four history" education, deeply understand the decisive significance of "two established", cultivate a large number of successors. Sun Wei stressed that we should grasp the new characteristics and new laws of youth growth, focus on the key points, take practical measures to do practical things and solve difficult problems for the youth, and constantly improve the effectiveness of the work. We need to focus on youth work, pay close attention to implementation, continue to deepen ideological and political guidance, thoroughly implement the strategy of giving priority to development, effectively safeguard development rights and interests, and constantly raise the overall level of youth work. We need to strengthen the Party's leadership over youth work, gradually improve the working pattern of "leadership of the Party committee, government responsibility, the Communist Youth League coordination, and joint management by all departments", unite and lead the young people to practice the youth pledge of "trust the Party and make the country with me", and welcome the Party's 20th victory with outstanding achievements. The meeting also called on Party committees and governments at all levels in Gansu to put youth work on the agenda and bring it into the overall arrangements for economic and social development. The member units of the joint conference should start from their respective business areas, introduce a series of landmark service policies, implement a series of inclusive and practical projects, and enhance the systematic, integrated and collaborative nature of the youth policies. The conference adopted the form of "main conference + sub-conference", and deliberated and adopted the key implementation key work of the 2022 Medium-and Long-term Development Plan for Youth in Gansu Province (2018-2025). The responsible comrades of the Gansu Provincial Committee of the Communist Youth League made relevant explanations on the Plan, and the relevant responsible comrades of the provincial Civil Affairs Department, the Provincial Health Commission, Wuwei City, Pingliang City, Chengguan District of Lanzhou City and Gaotai County of Zhangye City made exchange speeches on the implementation of the Plan.

2021-12-12	Weifang in Shandong province youth league, student union elected a new leadership of Shandong Weifang youth league, student union elected a new leadership correspondent careful in the green newspaper network reporter XingTing) on December 9-10, Weifang in Shandong province, youth federation of the ninth plenary session, Weifang student federation held the eighth congress. The conference summarized the work of the Municipal Youth Federation and the Students 'Federation in recent years, studied and determined the work tasks for the next five years, and elected the new leading organs of the Municipal Youth Federation and the Students' Federation. Shandong youth federation sent a letter to congratulations, Weifang municipal party committee, united front work department minister, deputy secretary of CPPCC party Wang Zhaohui to attend the opening ceremony and speech, Weifang, vice director of Gu, vice mayor, Weifang, Weifang CPPCC Zhao Chongfa attended the opening, Weifang federation of trade unions party vice secretary, vice President Gu Ping, Weifang municipal party committee secretary Zhao China speech respectively. In his speech, Wang Zhaohui called on the youth federations and student associations at all levels to play the role of the Party as a bridge between the youth and young students, strengthen ideological guidance, and guide the young people to constantly enhance the "four consciousness", firm the "four confidence", and resolutely achieve the "two maintenance". Party committees at all levels should stand at the strategic height of consolidating and expanding the foundation of the Party's governance, strengthen their leadership over the Youth Federation and the Students' Federation, and not only set the direction and also support them. The Communist Youth League organizations at all levels should better play their leading and guiding role to the Youth League and the students' Federation, and help them to carry out their work creatively in accordance with the law and in accordance with the articles of association. All sectors of society should care for and support for the development of youth undertakings and for the growth and progress of young people and young students. The congress deliberated and adopted the Resolution of the Plenary Session of the Ninth Committee of the Municipal Youth Federation on the Work Report of the Eighth Standing Committee and the Resolution on the Work Report of the Seventh Committee of the Municipal Students 'Federation, and elected the new leading organs of the Municipal Youth Federation and the Students' Federation. The conference also made arrangements for the work of the city's youth federation and students' federation in the next five years.

2021-12-10	Hubei long-term youth development planning joint conference in Hubei long-term youth development planning joint conference, Ken river stressed to promote youth development work deep go solid Zhu Juanjuan) "Hubei province long-term youth development planning (2017-2025)" (hereinafter referred to as the "planning") the third plenary meeting held in Wuhan on December 8, hall. The meeting deliberated on the work plan for 2022 work plan for 2022, the 2020 statistical monitoring report, and the Construction Plan of Hubei Youth Innovation Park (2021-2025). Hubei Provincial Committee of the Standing Committee, Minister of the United Front Work Department Erkenjiang Turahong attended and delivered a speech, Vice Governor Ning Yong presided over the meeting. Erkenjiang Turahong pointed out that the implementation of the youth development plan is a major deployment made by the CPC Central Committee with Comrade Xi Jinping as the core, a consistent requirement of the provincial Party committee and the provincial government for youth work, and a practical need for the healthy growth and development of young people. He stressed the need to earnestly identify the focus of the implementation of the Plan, do a solid job in the mid-term evaluation of the Plan, and improve the index monitoring mechanism of the Plan. We should attract young people, compete for young talents and tie their hearts, and explore a youth development city construction model with Hubei characteristics on a pilot basis. We should strengthen the construction of Hubei Youth Innovation Park, and strive to provide youth priority policy supply and youth-friendly service experience for young people who stay in Hubei. We should earnestly strengthen the organization and leadership, constantly strengthen coordination and linkage, continue to strengthen supervision and implementation, and promote the formation of a strong joint force for the implementation of the Plan. Ning Yong requested that the member units to timely convey the spirit of the meeting, implement the key work tasks, pay attention to the youth development city pilot and Hubei green innovation park construction and other key tasks to promote. We need to cooperate with the mid-term evaluation and annual statistical monitoring and evaluation of the Plan to jointly promote the effective implementation of the work of the Plan. Source: China Youth Daily, December 10,2021, edition 03

2021-12-09	Pan Zhixian, the spirit of the Sixth Plenary Session of the 19th CPC Central Committee), held a special lecture in Zhengzhou No.8 Middle School, Henan Province. Three and a half sentences, poetry recitation, said allegro... by the youth members of the original "the Party is the brightest star" and other programs, passionate, the sonorous sound. Henan youth branch members, Zhengzhou eight moral and rule of law group leader Wei Xinxin combined with the students 'performance, from the party's three historical resolution, the core of the sixth plenary session of the 19th and draw momentum from the party's struggle in one hundred, all the young students how to respond to the party's call on four aspects, for the students comb and preach the spirit of the sixth plenary session of the 19th. Recently, the Henan Provincial Committee of the Youth League launched the spirit of the sixth Plenary session of the 19th Central Committee of Henan Youth Lecture Group. Henan youth branch members respectively into Zhengzhou, Luoyang, Puyang, Xuchang, enterprises, villages, middle schools, colleges and universities, a small range, face to face, interactive communication, preach pay attention to questions, response to youth concerns, make "tall" plenary spirit more "ground", let the spirit in Henan youth heart."In-depth study of the spirit of the sixth plenary session of the 19th party, we can first through a theme, two established, three historic leap, four great achievements, the party's one hundred years of struggle of five historic significance, ten clear, thirteen aspects of historical achievements, 1,2,3,4,5,10,13 the seven figures to understand."On November 30th afternoon, in Nanyang Zhang Zhongjing museum, Henan youth branch members, the communist party flag county party school Zhang Tianye three young lecturer and pavilion youth walk, from Zhang Zhongjing" not "for good, when good doctor" the way, to save the people of the Chinese communist party "happiness for the Chinese people, for the rejuvenation of the Chinese nation" beginner's mind mission, speaking, simple, to the spirit of the nineteenth session of the rich connotation and the core essence of the detailed interpretation and analysis. On the same afternoon, at the ele. me delivery food delivery boy in Puyang City, there were interactive communication, music rendering, story telling and theoretical explanation, full of dry goods and positive energy. Jin Huiling, a member of Henan Youth Lecture Group and the leader of the teaching and Research Group of Hualong District Experimental Middle School, Puyang City, carefully observed the working status of the delivery boy and captured pictures of their food delivery. Before the lecture, she became the "little fans" of the delivery boy at the beginning, which quickly narrowed the distance with the young people present. Subsequently, she used the way of Lao home to enter into the growth experience of four generations around her, to see the big, from the personal growth experience to the development of the motherland, leading the young people in the emerging fields to feel the extraordinary achievements of the motherland. Young lecturers also walked into Luoyang mengjin district often bag town Ma Ling village, Henan Yellow River industrial group co., LTD., north China university of water conservancy and hydropower, 2019010 youth league branch, the Yellow River water conservancy vocational and technical college network technology 2003 class league branch, from the theoretical level, more to the party and national development achievements, youth side change, for example, profoundly convey the spirit of the sixth plenary session of the 19th, actively guide the member youth to strengthen ideological consciousness and action consciousness."Today, after listening to the teacher on the spirit of the Sixth Plenary Session of the 19th CPC Central Committee, I feel very proud to live in China in the new era."Xiao Jiayu, a student of Suntech Class 3 in Zhengzhou No.8 Middle School, said:" As a Communist Youth League member, we should unswervingly listen to the Party, follow the Party, study hard scientific and cultural knowledge, exercise a strong body, integrate the ego into the big self, and strive to be a new person worthy of the great responsibility of national rejuvenation."it is reported, After the demonstration activity starts, The Henan Provincial Party Committee of the Communist Youth League immediately organized the members of the provincial youth teaching groups at all levels, For youth league members in different fields, in different industries and at different ages, Focusing on studying and implementing the guiding principles of the Sixth Plenary Session of the 19th CPC Central Committee, Centralized and continuous face-to-face and interactive communication, To guide the youth league members of the province to study the "Resolution of the CPC Central Committee on the Major Achievements and Historical Experience of the Party's Hundred Years of Struggle" as the outline, A deep understanding of the Party's major historical conclusions, Constantly enhance the rational identification of the Party, So as to be more consciously a firm believer in and faithful practitioner of Xi Jinping Thought on Socialism with Chinese Characteristics for a New Era, Sing " Please rest assured, Strong country has me " the youth strongest voice.

2021-12-09	Chongqing Communist Youth League: transport the youth power to the grassroots line light home loquat; the city farm project invested in the village; go out to study the rural revitalization experience and industrial development ideas... this year, the party branch secretary and first secretary of the league branch of Fusheng Town, Jiangbei District, Chongqing Chen Chao is very busy, also very fulfilling. The participation of the youth force has helped Huashan Village to break through the difficulties and embark on the fast lane of development. In the process of promoting the reform of the grass-roots organizations of the county Communist Youth League, Chongqing has expanded the source channels of the work backbone of the league, paying attention to importing "youth strength" to the grass-roots level, and injecting youthful vitality into the grass-roots governance. When Chen Chao came to Huashan Village two years ago, he faced many difficult problems, such as backward infrastructure, scattered industrial development, and a lack of young and middle-aged labor force. At that time, fewer than 30 huashan people had more than 500 young people."Lack of youth strength" often makes the rural leader feel "willing but weak". Chen Chao's distress is typical in the grassroots of Chongqing. Grassroots work ultimately needs people to implement, how to solve the problem of the weak work force of the grass-roots league? In accordance with the guidance of the Central Committee of the Communist Youth League on broadening the source channels of the work backbone of the league, the league organizations of all districts (counties) in Chongqing explored the establishment of cadre management and incentive methods in line with the characteristics of the mass organizations, and tried to transfer the youth strength to the front line of work. In Chen chao jiangbei, jiangbei district party committee through the implementation of the "four hundred project", in the party and government organs, enterprises and institutions to select young cadres and workers, to carry out the "hundreds of youth", "hundreds of the" hundreds of youth into the enterprise "hundreds of youth to a line", "hundreds of youth home" the special plan of action, build a multisectoral collaboration, the regional overall cadre selection and appointment, training, pushing optimal incentive new pattern. Zeng Yu, secretary of the Jiangbei District Committee, said that after the implementation of the "400 Project", the youth commandos formed by the Jiangbei District Committee came to Huashan Village. They went to the village, face to face with the villagers, asked and solved the practical problems faced by the masses. Some young entrepreneurial teams have also come here to invest in the industrial development of Huashan Village. In Shapingba District of Chongqing, with an average age of 35, more than 50 percent of the youth population and 16 universities, the power of college students has been mobilized. On September 28, Shapingba District launched the "millions of millions" youth talent program. The plan selects 100 part-time deputy secretary of league (industry) committee or league construction instructor; the first selects 1000 grassroots youth service talents to set up youth work class, pairs with 50 qualified villages (communities), and carries out practical activities; recruits 10,000 grassroots public youth talents to establish district-level volunteer reserve talent pool. Through the "Millions and Millions" Youth Talents Plan, the youth grassroots service team of Sichuan Fine Arts Academy has paired up with Zhongliang Town, Shapingba District to establish a rural revitalization sketching base (art workshop). In addition to regular sketching and creation, they also provide help in the design and operation of villagers who are willing to develop homestays. So far, they have established one-to-one services with 12 households. Rural revitalization, elderly care services, legal aid, youth mental health... 1,000 grassroots service young excellent talents focus on the practical needs of paired villages (communities), set up professional service teams, to provide targeted help to grassroots development. Zhao Lei, part-time deputy secretary of the Youth League Committee of Zhongliang Town, Shapingba District, is one of the 100 young talents in grassroots governance. At the beginning of taking office, he spent two months running through the town together with the young cadres of the town. Zhao hopes to stay in the village after one year of service and do more for the people."Young people can do or even great things at the grassroots level."" grass-roots employment needs, college students also hope to go to the grass-roots line practice. The league organization should be a bond and build a platform to tie both sides together."Li �, secretary of the Youth League Shapingba District Party Committee, and this Dan �," We should not only supplement the youth strength to the grass-roots front line, but also drive more people to understand and pay attention to the work of the Communist Youth League."To mobilize social resources to open up the grass-roots governance" last meter " not only to mobilize the youth into the grass-roots level, some district (county) youth league organizations in Chongqing are also looking for ways to mobilize the endogenous power of the grass-roots youth, to attract local young talents for the construction of their hometown advice. Rongchang District, Chongqing, has built traditional courtyards of 10 to 20 households with "new style courtyards". Conditions of the courtyard set up a league member grid, elected a youth league member into the small courtyard council, by the village (community) cadres and to the village reported league members of the "league group", regularly to the village (community) "new wind small courtyard" to carry out environmental remediation guidance, civilization new wind dissemination and other activities."Xinfeng Courtyard" adopts the mode of "social workers + volunteers + local villagers". Through purchasing social workers to organize professional services and business training and participating by young volunteers, it drives the residents of the courtyard to participate in courtyard activities and environmental governance, helping each courtyard to set up its own volunteer service team. On weekdays, the volunteers in the small courtyard contracted for the sanitary cleaning of the public areas of the village, cleaned the courtyard for the "five guarantees" in the village, and helped the elderly to take care of the farmland during the autumn harvest. He Ping, deputy secretary of the Youth League Rongchang District Committee, introduced, relying on the "new wind small courtyard" set up the young volunteer team, can extend the volunteer activities to the doorstep of the masses, and effectively promote the grass-roots governance "the last meter". In addition to leveraging social resources, the "New Style Small courtyard" also mobilizes young people working outside to give advice for the development of their hometown. Luo Yanjie is a villager of Tong 'an Village, Anfu Street, Rongchang District. After graduating from Southwest University of Political Science and Law in 2018 and becoming a lawyer, he became the legal adviser to the "Sanchongtang Small Courtyard" in Tong' an Village. Private lending, land contracting, marriage disputes, child support... Luo Yanjie often returns to the village, helping the villagers to understand the legal knowledge based on the most asked questions in WeChat and specific legal cases. The legal lecture hall in the courtyard dam does not need multimedia equipment or list the law. The villagers either sit with small benches, stand beside them, or even sit on a motorcycle to listen to Luo Xianjie explain the legal stories around them."In the past, villagers did not give priority to solving legal channels when they encountered conflicts and disputes, and few people asked me about legal questions. Through several case explanations, we gradually realized that some problems could still be solved through lawsuits, and more villagers came to consult about legal problems."Luo Xianjie said that he is also a member of the" small courtyard ", more understand the ideas of the rural people, according to choose the people interested in the legal cases, so that everyone can listen to, but also remember."In the past, when arranging work in the village, the young cadres in the village always said that 'no � N line', 'no � N way' and" clang a possible ". Now people often say that 'find a way'."As the village league branch secretary, Chen Chao said that only by changing the thinking of the grassroots youth and activating the youth strength, can we better serve the grassroots development and promote rural revitalization. Seizing the opportunity of the reform of the Communist Youth League at the county level, 38 districts (counties) in Chongqing focused on the work force selection mechanism of the reform league, and explored a number of work plans, and the reform effect became increasingly apparent. Shapingba District, Jiangjin District and Yongchuan District have successively issued the league cadres recruitment and management related systems, Standardize the selection of youth League cadres; Jiangbei District, Dadukou District, through the special projects, Enrich the working force of the regiment; Fengjie County, Fuling District has built a talent pool, Smooth channels for selection and appointment; Rongchang District, Wanzhou District, Dianjiang County to enhance the treatment of grass-roots league cadres, Set up a special establishment to ensure the treatment of league cadres... The Organization Department of Chongqing Municipal Committee said, next step, The Chongqing Communist Youth League will further focus on key points and implement measures, Promote community-level youth league organizations to strengthen their political functions and enhance their social functions, To explore more and better experience, practices and plans for the reform of the whole regiment, Be a good "pathfinder" of reform. Intern, Ma Xiaoqing, China Youth Daily, reporter of China Youth Network, Wang Xinxin, Source: China Youth Daily, December 09,2021, edition 05

2021-12-08	Youth League Inner Mongolia District Committee held the legal industry special live with post activity Inner Mongolia "thousands of schools waiting for you" live with the post legal industry special premiere Shi Jia) recently, the youth League Inner Mongolia District Committee held "thousands of schools thousands of posts waiting for you" legal industry special live with post activity. This live broadcast selected legal related enterprises and posts, a total of 20 enterprises registered to participate in and opened the online resume delivery channel, providing more than 60 positions. It is understood that Beijing Tianchi Juntai (Hohhot) Law Firm, Inner Mongolia Dingyin Law Firm, Inner Mongolia Jianbang Law Firm, Inner Mongolia Doli Law Firm four enterprises to participate in the live recruitment. The live broadcast has received more than 20,000 clicks, and has received more than 60 resumes so far. Live invited the national 13th session of the youth league members, the Inner Mongolia autonomous region twelfth session of the youth league committee, Beijing day chi jun tai (Hohhot), Inner Mongolia, director of the town into the studio to share lawyer career planning and development experience and advice, patient answer studio audience apply for questions."Thousands of schools and posts waiting for you to come" the legal industry special live broadcast with the post activity scene. School (image provided by respondents) "thousands of jobs waiting for you" special live with post activity is the Inner Mongolia district party committee to carry out the "I do the practical work for the youth", build refined youth employment services, according to the characteristics of different industry, the different employment orientation groups for specialization, personalized, targeted special recruitment, has launched special salary 4K +, civilian, legal industry, etc.

2021-12-11	Gansu Communist Youth League Care Service Action Warm Heart Gansu Communist Youth League Care Service Action Warm Heart Zhang Jianwei) Since the launch of Gansu Province Care service Action, The Communist Youth League Gansu Provincial Party Committee earnestly implement the province's rural left-behind elderly women and children and extremely poor people care and service action and rural revitalization "post training, business competition" activity launch meeting of the important spirit, In accordance with the requirements of the Notice on Promoting Care and Services for Women and Children Left behind in Rural Areas and People in Extreme Poverty, Taking the care and service action as an important practice carrier for the Communist Youth League to help consolidate and expand the achievements of poverty alleviation and rural revitalization, and "I do practical things for the people", Combined with the responsibilities, tasks and the actual work, It formulated and issued the Implementation Plan of the Gansu Communist Youth League to provide Care and Services for Women and Children Left behind by Rural Elderly and People in Extreme Poverty, Organize and mobilize the youth league organizations at all levels in the province to closely revolve around the demands of the masses, Start with the small things, With true service, We will effectively help the elderly, one small and one poor people solve their practical difficulties. We will implement the action to help students and help the poor. Carry out the "Dream Realization university" activity. Led the implementation of the provincial government of urban and rural low family children ordinary college enrollment funding for the people practical project, with Gansu hukou and in the province in the national unified examination, and was admitted to ordinary college undergraduate, college (vocational) level 2021 urban low full security family and rural low, second security family children academic funding, admitted to undergraduate one-time subsidy of 10000 yuan, admitted to junior college (vocational college) one-time subsidy of 8000 yuan. Departments of education, civil affairs, youth League committee and other departments at all levels were centrally organized to compare, review and publicize the information of the admitted candidates, and distributed a subsidy of 46.076 million yuan to 4,958 students according to the standard. We will provide assistance to young people in difficulties. We took a number of measures to expand the financing channels for Project Hope, extensively pool social resources, and raised 24.92 million yuan to help students from poor families to complete their studies and improve the running conditions of rural primary and secondary schools. For Qingyang, Lanzhou, zhang ye, Wuwei, Dingxi, Gannan, Linxia, silver, Longnan, out 10 cities (state) of difficult family students from primary school to high school funding (pupils funding standard is 2000 yuan / person years, middle school students funding standard is 3600 yuan / person years), has issued 4.18 million yuan, funding troubled teenagers 2069. The municipal and county youth League committees were organized to make thorough statistics on the freshmen of extremely poor families in the province, and supported them based on Project Hope. They received a one-time grant of 5,000 yuan, a total of 56 students and a grant of 280,000 yuan. At the same time, 100,000 yuan was raised to build five hope cabins in the countryside. We launched a series of activities on "rural revitalization". Centralized demonstration activities: November 29 solstice 30, the Gansu provincial party committee in Qingyang city pool county, water "rural revitalization of volunteer" the communist youth league care rural left-behind elderly women and children and needy service action demonstration activities, hand in hand love enterprises to pool and water county donated worth 814,000 yuan and 1,004,000 yuan of love materials. Donated love materials to Qingyang City, built 5 "hope hut" and supporting books, with a total value of 2.99 million yuan. Carry out science and technology to campus and send health to the grass-roots level, free more than 500 people, and distribute drugs worth more than 50,000 yuan free. Form youth care service team: relying on the youth volunteer association at all levels and college students "a program under which officials" social practice team youth care service team, focus on rural left-behind elderly and the disabled to carry out the "special volunteer service" hand in hand, to carry out life care, cleaning, meals, health, census, family company, psychological counseling, act as agent buy generation care service more than 6600 times, service time more than 21000 hours. Carry out activities to care for the left-behind children in rural areas: Continue to carry out the "child companion plan" of the Communist Youth League to care for the left-behind children, and carry out the practical needs of the rural left-behind children and the children in difficulties, and conduct various forms of care activities such as love materials gift, academic guidance, self-care education, and family companionship. We will integrate volunteer resources from high-quality teachers, college students, volunteers from the Western China Program and graduate students, and set up volunteer service teams for public welfare teachers to carry out online and offline academic counseling for left-behind children in rural areas. To develop adolescent mental health assistance: to further enhance the level of 12355 youth service desk, recruitment to national secondary and third level of psychological counselors as the main body of youth volunteers 253 people, focus on the province's rural left-behind children and teenagers to provide professional and standardized mental health counseling, legal aid, case support, difficulties and other services, has provided online consulting services of more than 2450 people. At the same time, we will give full play to the advantages of the "volunteer gathering" platform, strengthen the construction of the volunteer youth legal aid and mental health services team, and carry out psychological crisis intervention and daily psychological counseling for left-behind children in rural areas. Integrate resources to help you with practical things. Donation of living materials. In view of the actual difficulties and practical needs of the people of "one old child, one small child and one poor", the Gansu Provincial Communist Youth League Party Committee has extensively mobilized and actively connected to win the attention and support from all sectors of society. Launch the youth Federation members, enterprises and caring people to lend a helping hand to donate love materials, contact 6855 body sensing lampblack machines, 1091 sets of love bags (including laundry detergent, milk powder, milk bottles, etc.), as well as love clothes, schoolbags, books and other materials, with a total value of 18.816,900 yuan. Give full play to the advantages of cooperation between the east and the west: focus on the academic difficulties of teenagers from poor families, and actively strive for the support of the organizations. The Tianjin Municipal Party Committee of the Communist Youth League launched the activity of "Books to send sunshine to light up dreams", and donated 120,000 public welfare books worth 1.8 million yuan to Baiyin City. Donation 528,000 yuan to Jingtai County to fund 155 students from poor families; Qingdao Municipal Party Committee donated 220,000 yuan to build 1 Hope Primary School in Lintao County and 90,000 yuan of computers and teaching materials to improve the hardware and software infrastructure; Jinan Municipal Party Committee raised 300,000 yuan to build 30 houses for poor families, disabled families and orphans and unattended children. Tuan Tianjin Jinnan District Committee donated 300,000 yuan "warm heart" to 200 rural children in Lingtai County, Nankai Middle School donated 30,500 yuan love clothes and materials; Tuan Hongqiao District Committee for more than 3100 left-behind children in Heshui County "micro wish" interpreta dream activities, purchase "micro wish" items of more than 50,000 yuan, donated 15,000 yuan for 3 community left-behind children holiday love care service. Youth exchange activities such as "Pomegranate Seed One Family" and "Jinlong One Family Integration Summer Camp" were held, and more than 70 teenagers from poor families in Gannan and Tianzhu Tibetan areas were organized to participate in the summer camp in Tianjin. Guide children to strengthen emotional communication in hand in hand pairing, red scarf practice experience and sharing activities, and promote healthy growth. Next, the Gansu provincial party committee will continue to further promote care service action, fine fall small work requirements, further the history of the party education achievements into "does the practical work for the masses" concrete measures, as a whole resources, funds, power to care service action, effectively improve the rural people sense of gain, happiness and security.

2021-12-09	Henan province youth: for more teenagers to send warm and hope innovation mechanism to strengthen service Henan province youth: for more youth to send warmth and hope at the same time, will also be a heavy responsibility on our shoulders, that is to love as a torch relay relay... " in September, the student Wang Xixin in Henan hope project public account background message wrote. Such a trapped student can enter the university gate to start a new journey of life, which cannot do without the protection of the hope project. In his message to the Hope Project, General Secretary Xi Jinping stressed that in the new era, the Communist Youth League should make the Hope project better, and strive to provide new assistance and sow new hope for young people. Bearing in mind the instructions of the General Secretary, Henan Youth Foundation Foundation follows the development of The Times and the changing needs of young people, dares to take responsibility and acts bravely in major emergency events such as fighting the epidemic and fighting floods, constantly innovates the social mobilization mechanism, exercises the skills of the team members, enhances the effectiveness of service, and brings warmth and hope to more young people. Fast response, wide mobilization, win two field war COVID-19 outbreak, Henan green gay quick response, elaborate preparation, extensive mobilization, efficient operation, specification in accordance with the law for fundraising activities, become the first to participate in COVID-19 epidemic prevention and control work of charity organizations, Henan is also designated four COVID-19 epidemic imports one of the recipient units. Henan youth will quickly through the Henan youth development foundation online fundraising platform, has launched a "outbreak rescue in Hubei" "care a line medical staff-hope project special action" two public offering projects, to support Hubei, Henan line to carry out the epidemic prevention and control work and care against disease frontline medical staff children and other youth groups. In mid-July 2021, many parts of Henan province were hit by heavy rain. After the flood and disaster, Henan Youth Development Foundation quickly responded to the assembly order of "to fight hard, youth charge", launched the emergency plan, and formulated the flood control and relief project plan of Henan Youth Development Foundation of "Flood relief hope". The project has been strongly supported by the China Youth Foundation and the Youth Foundation of all provinces (autonomous regions and municipalities directly under the Central government). Young people from all walks of life in Hong Kong have also enthusiastically donated money to gather strong efforts to support Henan. From July 21 to August 30, the Henan Provincial Youth Development Foundation received a total of 190 million yuan worth of donations to help the disaster areas and poor families overcome the difficulties. Through the traditional channels to carry out the fundraising at the same time, Henan youth will actively build "Internet + public welfare" service platform, to carry out the network campaign, rely on the basis of extensive coverage and strong organization mobilization advantage, the members, young pioneers, college students, league association member youth number into traffic resources, drive the province more than 600000 dedication love, received online donations more than 23 million yuan. A hard war, is an experience; a "battlefield", is a classroom. In the two tough battles of flood control and epidemic prevention, Henan Youth Development Foundation not only paid attention to the process and effect of fundraising, but also enhanced the education of young people in the loving participation through extensive mobilization, which enhanced the organization, influence and cohesion of the Communist Youth League."Put the safety and health of the majority of young people first."Whether it is the epidemic or the flood situation, this is the first criterion for all the staff of the Henan Provincial Youth Development Foundation in the face of major emergencies. During the outbreak of COVID-19, all members of the Henan Youth Foundation Foundation took the lead, acted bravely and resolutely, and stuck to their posts in a "wartime state", so that all the members participated in the war, went online all day and fully guaranteed. In flood control and disaster relief work, the Henan provincial party committee secretary front command, rushed in a line, in the most serious moment, led the cadres sink a line, ordinary party members rushed to play an exemplary role, material deployment and temporary turnover center more than 10 fixed telephone and mobile phone sound, dispatching hundreds of ten batch of supplies, for accurate docking, fast delivery, each to receive hundreds of phone calls every day, many comrades are hoarse voice, also from no complaints. In the temporary material turnover center, there is no moment of rest. The busy workload attracts many volunteers and enthusiastic passers-by, and more than 200 students are busy here every day. Zhang Haibo, a veteran member of Henan Youth Foundation Foundation, took the lead, Despite waist disease, Insist on the loading and unloading of goods, Coordinate and command until aphasia, Still insist on in the front line; Unnamed donations came in from all over the country, A loads of drinking water, thousands of boxes of fast food, And clothing, rain gear; A couple in Zhongyuan district saw that the staff and volunteers were too busy to eat, Then I cooked mung bean soup at home, cooked meals to drive more than ten kilometers to deliver... good innovation, strong service, Hope Henan Youth Foundation always pays attention to play the advantages of the league organization system, Innovate social mobilization mechanisms, Constantly convey the warmth of the Party to the youth, Spread the power of hope. During the period of epidemic prevention and control, Henan green gay with WeChat as the main online fundraising platform for project release, optimize the mechanism of the raise, form with organizational support, leading force for traction, to promote service force of hardcore advantage, drive nearly 250000 donations, form teenagers all do public good atmosphere, make hope project love mutual assistance, relay of mass public welfare activities. In "outbreak hope peer-Henan hope project love disease resistance line medical staff children, care for disease trapped youth special action", in Henan province to provincial, city, county group as the circle, with radius association at all levels, guide the youth league organizations at all levels to participate in the team "donate", layer upon layer of mobilization, comprehensive coverage, � ν � heart draw "hope engineering disease resistance of concentric circles". From Hubei to Henan, from medical supplies to humanistic care, from the community line to the scientific research front, from the timely assistance to the continuous assistance, the Henan Youth Foundation Foundation has conveyed the care and warmth of the Party organization wherever the battlefield is. After the flood situation in July this year, Henan Youth Foundation is problem-oriented and targeted assistance according to the situation to help teenagers learn and grow: for families killed on duty, school funding for teenagers affected by the disaster, school repair assistance for the damaged Hope Primary school; for the youth psychological problems, mental health education volunteer service... an innovation, is a warm. A project, it is a hope. Through these projects, the Henan Provincial Youth Development Foundation not only brings academic assistance to the affected teenagers, but also makes them feel the care and care of the Party. Source: China Youth Daily, December 09,2021

2021-12-06	The 11th Guangdong public volunteer culture month series activities held the 11th Guangdong public volunteer culture month series activities held JianWei correspondent YueQing) on December 5th afternoon, by the Guangdong provincial party committee, Guangdong volunteer federation, Guangdong youth volunteer association hosted the 11th Guangdong public volunteer culture month series activities "life, there should be volunteer" annual share in the second quarter held in Guangdong province cultural center, to volunteer public keynote speech, tells the story of volunteer Guangdong, transfer volunteer spirit. It was broadcast simultaneously online through mainstream live streaming platforms, attracting more than 6.123 million viewers. At the event, the audience listened carefully to the guests' speeches. Group of Guangdong provincial party committee for figure sharing activities, Guangdong volunteer action guidance center (Guangdong hope engineering service center) director YuYueE, Foshan pineapple rescue team director wang, Shenzhen yuyan disabled care career development center director zhang, famous ophthalmologist tao, "western plan" sun yat-sen university graduate volunteers volunteers, the first affiliated hospital of sun Yat-sen university, deputy director of wang, a professor of Peking university school of journalism and communication, from different angles to share their volunteer story. Weng Shuo, a grass-roots staff and young volunteer from Shantou Welfare Home, brought a fully open wheat for this sharing. Guangzhou landmark "small waist" for volunteers. Guangzhou Tower, Guangzhou International Media Port, Zhuhai Opera House, Dongguan CBD, Foshan, Jiangmen and other landmark buildings, the collective bright " volunteers happy holiday!"" Volunteers, thank you " light curtain. In addition, the "Volunteer Guangdong" theme carriage of Guangzhou Metro Line 1 is online, and the doors, seats, Windows and other positions show volunteer services for epidemic prevention, Spring Festival travel and warm winter action, "Jinhui Action" to help the elderly, red publicity volunteer service, subway urban traffic volunteer service and other scenes. On December 4, Huang, a college student volunteer who took the subway, said, " I am also a young volunteer. I have participated in the 'Read China' conference volunteer service and community volunteer service. I was very moved by seeing the poster and felt warm in my heart."Guangzhou Metro" volunteer Guangdong " theme carriage online. According to the "i volunteer" system, there are more than 14 million registered volunteers in the province, and 132,000 various volunteer service organizations and groups, providing a total of more than 700 million services. On average, for every 10 Cantonese people have one real-name registered volunteer. Volunteers have all played an irreplaceable role in accompanying the healthy growth of young people and children, epidemic prevention and control, ecological construction, helping the elderly and the disabled, emergency rescue, rural revitalization, and large-scale competition events."Because of you is great" and other tribute volunteer posters will continue to be publicized throughout the province for one month, three-dimensional, all-round, infiltrating dissemination of volunteer culture.

2021-12-06	On December 4th, Hubei held the "Salute Volunteers- -Volunteer Charity Creation Camp 2021" style exhibition and Hubei Youth Volunteers "12.5 International Volunteer" Day demonstration activity. Fang Qingzhong, deputy secretary of Hubei Provincial Party Committee, attended and presented gratitude to the loving enterprises. Thirty volunteer team leaders and individual volunteers from the province took the stage to tell stories about their participation in volunteer service. Their services cover more than a dozen volunteer services, including rural revitalization, helping the elderly and the disabled, care for children, ecological and environmental protection, and emergency rescue. After nearly two hours of style display, 10 teams (individuals), including Wuhan Jiangan District Snail Home Mind, Geng Hui, founder of Intellectual Disabled Service Center and Xiangyang Grass and Grass Volunteer Association, stood out and entered the "Salute to Volunteers- -Volunteer Public Welfare Creation Camp 2021" salute. The activity by the Hubei provincial party committee, Hubei province government and other units, aims to find, support and promote a batch of social response is good, great development potential of volunteer service organizations and keen and long-term volunteer service volunteers, guide, encourage and promote more social forces attention, support volunteer service, help social civilization progress. Since the event was launched in mid-October 2021, the event has received applications from 244 teams (individuals) from 17 cities and states in the province. Finally, 30 teams (individuals) were determined to participate in the "Salute to Volunteers- -Volunteer Public Welfare Creation Camp 2021" style exhibition activity. The 10 teams (individuals) shortlisted for the "tribute ceremony" will receive 40,000 yuan of volunteer service project support funds respectively, and the remaining 20 teams (individuals) will receive 15,000 to 20,000 yuan of volunteer service project support funds.

2021-12-06	Fujian youth volunteer service advanced deeds report youth volunteer dedication new era Fujian provincial party committee to carry out the "Fujian province youth volunteer service advanced deeds report" on December 6 (reporter Zhang Jianwei, correspondent Huang Yuehong) to celebrate the arrival of the 36 "international volunteer day", carry forward the "dedication, love, mutual aid, progress" volunteer service spirit, December 5 morning, group of Fujian provincial party committee, minjiang college jointly organized "Fujian province youth volunteer service advanced deeds report". Lian Zhanji, deputy Secretary of the Communist Youth League Committee, Chen Xi, Deputy Secretary of the Party Committee of Minjiang University, leaders of the Youth League Committee of Rong Provincial colleges and universities and key volunteers attended the report.at the meeting, Qian Qiaoling, an environmental protection volunteer committed to building a beautiful hometown; Zheng Caiyun, a volunteer teacher in rural education from Yushu, Qinghai Province, Lang County, Xizang Province, to her hometown; Dai Baiyan, a "good neighbor" who is enthusiastic about caring and volunteer service; On the road to fighting the epidemic, Without hesitation, brave and retrograde, With practical actions to highlight the youth responsibility of Lian Peiqi; Liu Jiali, a college student volunteer of the Western Project, "Go to the west, the grassroots and the country where the people need it most"; Ye Yuyuan, a volunteer of Fujian Province College Students Volunteer Service in Underdeveloped Areas Program who serves the grassroots to help rural revitalization; Zhong Liqing, who shows the youthful style of contemporary youth; Eight reporters, including Ruan Wenjie, the student leader of the Xiaozhi Project of Minjiang University, who publicized the river chief system, water knowledge and water environment protection, shared their advanced deeds in volunteer work in turn. Report site. At the meeting, Lian Zhanji, deputy secretary of the Communist Youth League Committee, put forward suggestions on promoting the comprehensive active and long-term development of youth volunteer service, and will focus on the construction of the youth volunteer action system, realize the institutionalized development of youth volunteer action, strengthen the demonstration of key projects, improve the youth volunteer action project system; and build the volunteer service platform to promote the professional information construction of youth volunteer service.

2021-12-01	Jiangxi Provincial Party Committee issued new measures to promote the reform of the Communist Youth League Jiangxi Provincial Party Committee issued 5 reform measures to promote the reform of the county Communist Youth League grassroots organizations Jianwei correspondent Huang Zhangyue) recently, Jiangxi Provincial Committee of the Communist Youth League issued the Notice on Further Improving the reform of the Communist Youth League in the province, Launched traction reform measures such as "appointing youth affairs workers, strengthening the management and operation of youth volunteer associations, making good use of the activity position of 'youth home', increasing the training of 'rural good" youth', and regular recruitment of college student volunteers ", It also plans to invest nearly 3 million yuan a year to support related work starting from 2022. According to the communist youth league central committee "about expanding the county communist youth league grassroots organization reform pilot guidance" file spirit, in order to further promote the reform of the communist youth league in Jiangxi province at the grassroots level, in view of the current county the communist youth league at the grassroots level organization reform difficulties, the Jiangxi provincial party committee as a whole launched five traction reform measures, promote reform work deep walk. Five traction reform measures will be carried out in 20 pilot counties for the reform of the Communist Communist Youth League this year, and will be rolled out across the province in 2022. Specifically including each county (city, district) youth League committee appointed at least one new youth affairs workers, Specializing in the league's work; Promote the full coverage of youth volunteer associations in all counties (cities, districts) of the province and allocate special funds to support the construction of the associations every year; Make good use of the "Youth Home" campaign position, allocate funds for each "youth home" every year; Actively recommend the "rural good youth" to enter the youth league organization as the youth league position, Establish the "Rural Good Youth" alliance, Carry out the training of "rural good youth" regularly; Establish a rotation mechanism for college student volunteers to carry out work in county (city, district) youth League committees, Set up a college student volunteer service team with stable source, excellent quality and efficient service. The five traction reform measures were promoted by the Provincial Communist Youth League Committee and provided policy and financial support. Through the implementation of the five reform measures, further broaden the reform work ideas, stimulate the reform power, drive the reform of the county Communist Youth League grass-roots organization tasks smoothly promoted. At present, relevant measures have been implemented in 20 pilot counties (cities, districts) for the reform of grass-roots organizations in the province. After the reform was rolled out in the province in May 2022, the provincial Communist Youth League Committee will tilt its resources downward to help the construction of grass-roots league organizations, and effectively enhance the contribution and influence of grass-roots league organizations.

2021-11-30	The 17th "revitalization of the cup" Hainan awards ceremony held in Haikou the 17th "revitalization cup" Hainan awards ceremony held in Haikou green reporter Ren Mingchao) on November 27, the 17th "revitalization cup" national youth vocational skills contest of Hainan province and Hainan province youth service skills competition ceremony held in Haikou, competition gold, silver, bronze, outstanding organization award winner was awarded. It is reported that the 17th "Revitalization Cup" National Youth Vocational Skills Competition of Hainan Province and the Hainan Provincial Youth Service Skills Competition were jointly organized by the Hainan Provincial Party Committee of the Communist Youth League, Hainan Provincial Department of Human Resources and Social Security, and Hainan Provincial Department of Education. Since the start on September 28 this year, the main competition has attracted 98 contestants from many colleges and universities in Haikou, Sanya, Chengmai and other cities and counties in Hainan province.

2021-11-27	Shandong: study propaganda and implement the party's spirit of the sixth plenary session of the 19th study publicity and implement the spirit of the sixth plenary session of the 19th Shandong youth XuanJiangTuan provincial demonstration preach XingTing) on November 26 morning, study propaganda and implement the party of the spirit of the sixth plenary session of the 19th Shandong youth XuanJiangTuan provincial demonstration to preach held in Shandong transportation institute. Liu Shaohua, deputy Secretary of Shandong Provincial Communist Youth League Committee, and Li Zhentao, deputy Secretary of the Party Committee of Shandong Jiaotong University attended the meeting. Young teachers and students of the school listened to the lecture. 1.267 million teenagers in the province watched the live broadcast through the "Youth Shandong" WeChat, Weibo and other media platforms, and had an ideological and political class with the audience. Speak in further study to promote and implement the spirit of the sixth plenary session of the 19th as the theme, "youth branch" members, youth example, youth cadres such as youth XuanJiangTuan members, youth universities, into the youth, through the "youth" to preach, guide the provincial youth in the province "three walk before" always follow, total positioning, total beacon requirements, yong set best, yong pioneer. Four members of the publicity group made speeches around the theme. Hu Tao, secretary of the Youth League Committee of Harbin Institute of Technology (Weihai), with the title of "Why We Follow the Party", guided young people to learn the history of the Party, strengthen their faith, and follow the Party from four aspects of road confidence, theoretical advantages, original aspiration for the people, and ideals and beliefs. Wang Peipei, secretary of the South District Party Committee of Qingdao City, showed the achievements of "I do practical things for young people" with the title of "Building a practical job for young people". Wu Yuanyuan, general manager of provincial "Rural good youth" and general manager of Shandong Huimin Qifa Fruit and Vegetable Co., Ltd., took the theme of "Big story" in "Little mushroom", told about his return to start his own business, and practiced his sincere heart of getting rich for his hometown and helping rural revitalization with practical actions. Xiao Dao, chairman of "The Most Beautiful Youth in Qilu in the New Era" and chairman of Shandong Beicheng Environmental Engineering Co., Ltd., will guide them to actively participate in the tide of innovation and entrepreneurship, and contribute their youth wisdom and strength to the economic and social development. It is reported that in order to better study, publicize and implement the spirit of the sixth Plenary session of the 19th CPC Central Committee, the Shandong Provincial Communist Youth League Party Committee has set up a provincial youth publicity group of 30 people and 60 municipal people. Next, the Shandong provincial party committee will give play to the role of youth XuanJiangTuan, and "I do the practical work for teenagers" to preach, into the enterprise, universities, community, rural, "two new" organization, to "youth" language interpretation of the party's latest theory achievement, tell the party's story of the good, share youth struggle, more ground propaganda good plenary session spirit, lead the league youth unswervingly listen to the party, follow the party.

2021-11-27	Vocational colleges "education" education summit held in Hainan free trade port 2021 vocational colleges "education" education summit held in free trade port in Hainan chao river bank Mongolia) on November 25, "Hainan free trade port China youth development summit and 2021 vocational colleges" education "education summit" was held in Haikou city, Hainan province. The two-day summit was hosted by China Youth Newspaper, the Communist Youth League Hainan Provincial Party Committee and the Education Department of Hainan Province, and organized by Hainan Free Trade Port New Era Youth Research Center, Hainan Youth Development Center and Hainan Youth Activity Center. More than 50 higher vocational colleges from all over the country attended the summit. Chen Yu, secretary of the Hainan Provincial Committee of the Communist Youth League, said in the opening ceremony that the Hainan Communist Youth League has built the "China (Hainan Free Trade Port) Youth Dream Avenue" and is committed to building Hainan into a national platform for youth activities and a window for world youth exchanges. Dream Avenue will provide a platform for the national youth to carry out ideological and political practical education, and polish the brand of practical education. We sincerely invite and look forward to the fact that colleges at all levels and young students at all levels, including vocational colleges, will come to Hainan to experience different ideological and political practical education. The relevant person in charge of China youth newspaper office, said the hope with the help of "China (Hainan free trade port) youth dream avenue" youth mobilization lead service platform such as carrier, discover more "education" education innovation mode and methods, jointly improve the quality and effectiveness, make youth chapter in "free trade port construction" and other national major strategy building dream, interpreta dream. At the summit, important experts were invited to give keynote reports, and the secretaries and principals of the participating universities were organized to conduct exchanges on the topics of "three comprehensive education", employment competitiveness, online ideological and political education (financial media), and the construction of "big ideological and political courses" system. After the meeting, the participants visited the 2021 "Big Ideological and Political Course" education case exhibition in vocational colleges, and inspected the big ideological and political education bases in Hainan Province, such as the Haikou Jiangdong New Area Exhibition Center, The Dream Avenue Research and Research Base, and the Youth Employment and Entrepreneurship Base. It is understood that the summit opening ceremony also held the China (Hainan free trade port) youth dream avenue youth studies practice education alliance member unit awarding ceremony, held the China (Hainan free trade port) youth dream avenue xing dream studies travel innovation workshop, "big politics" practice education base, youth studies practice education alliance opening ceremony, and free trade port in Hainan new era youth research center opening and expert employment ceremony.

2021-11-27	In Zeku County, the autonomous prefecture of the plateau, the cold winter always comes earlier. In recent days, when Tuanjie Village, Wang's hometown in Zeku County, was again hit by a cold wave, 1,017 herdsmen there were pleasantly surprised to receive a "warm current" from the shore of the Bohai Sea. The Tianjin Communist Youth League, who married them, traveled from 1,500 kilometers away and sent the supplies to every herdsman in Tuanjie Village in time."Huangnan Prefecture is a key area for the Tianjin Youth League organizations to help them work in pairs and identify their relatives. Therefore, the plight of the local villagers has become the concern of their 'relatives' in Tianjin."Group Tianjin municipal party committee secretary Li Long said, the sixth plenary session of the 19th successful closing sounded the horn in the new descend road create history, Tianjin communist youth league will continue to play to its own advantages, integrate resources from all walks of life, through one real action to pass the warmth of the party and the government, in power to consolidate expand poverty crucial achievements with rural revitalization effectively link up good step, open good bureau show Tianjin youth as. In early November, a new round of epidemic suddenly appeared in some provinces, and many places were in urgent need of emergency epidemic prevention and living supplies. The news came, the Tianjin Youth League organization immediately launched a linkage of love relay. The Communist Youth League Municipal Committee and the Municipal Youth Federation contacted to confirm the demand, and began to mobilize, and finally determined that China Tianchen Engineering Co., Ltd., Tianjin Xuhui Hengyuan Plastic Packaging Co., Ltd. and other caring enterprises to donate. Within a week, the company worked overtime to make masks, protective suits, disinfectants and other anti-epidemic materials, and urgently deployed vehicles, rapid loading... Soon, the materials loaded with the love of Tianjin people were sent to Zeku County."Thanks to the multi-level and multi-form exchanges and interactions between the youth league cadres and the local people, we can only understand what Huangnan needs in the first time."Tuan Tianjin municipal united front work department minister Yan Zengzhi said, in order to make national unity pairing activities go deep to real, Tianjin youth group set up" establish a communication channel, solve a practical difficulties, realize a micro wish, give a holiday gift, buy a consumer support products "of" five "mode," heart, hard, with love to carry out every exchanges and cooperation ". To serve the masses and the youth, we should not simply donate money or hold several activities. The service group of Tianjin Youth League is an important carrier for Tianjin Youth League to carry out the party history learning and education and practice activities of "Youth concentric Follow the Party forever", and it is an innovative measure to transform the achievements of Party history learning and education into practical work. Yan Zengzhi introduction, has been by Tianjin youth league members at all levels and experts and scholars from all walks of life "industry revitalization" "beautiful rural planning and construction" "electricity farmers" agriculture "agriculture" technology four service group, play their respective strengths, for the development of rural industry, improve infrastructure, beautify the rural environment, promote industrial upgrading of zhenhai efforts. The head of the "Industrial Revitalization" service group is Zuo Zheng, a member of the All-China Youth Federation, a member of the Tianjin Youth Federation, and a post-90s entrepreneur. At the end of September this year, he flew to the Hotan region of the Tianjin Municipal Party Committee, which was supported by Tianjin, and got a detailed understanding of the general situation of the local area, especially the actual characteristics of economy, agriculture, culture and tourism. He then donated 600,000 yuan worth of high-quality breeding cattle to the local herdsmen."I donated the first batch of breeding cattle in 2020, and now the cattle are growing very well and are well adapted to the local soil."To his delight, during his trip to Hotan this year, he traveled to a vast grass area and immediately recognized the dozens of cattle he had donated." Their size and color are very different from the local cattle, and they are easy to identify."It is understood that the local people every breed 1 breed of cattle, the annual income can reach about ten thousand yuan. This also made him more convinced of his own judgment that by donating high-quality cattle breeding, he could help to develop the local cattle industry and promote rural revitalization. He has dealt a lot with local herdsmen. " They are not well educated and do not communicate well with the outside world in Mandarin, but they raise cattle and sheep as experts. Donating high-quality breeding cattle is really teaching them how to fish."He has a clear idea in his mind: it will be a long industrial chain- -breeding, feed planting, output processing, fattening and later slaughtering, processing, etc. Subsequently, the service group of Tianjin Youth Federation members had in-depth exchanges with the local people around optimizing the layout of livestock and poultry industry and enriching the form of tourism industry, helping the local people to fully explore the ecological advantages, cultural advantages and tourism advantages, and strive to promote cooperation in deeper levels and more fields, and help the local rural revitalization. Taking root at the grass-roots level to do practical things for the common people is an important task of the youth Federation organizations at all levels in Tianjin this year. The key is to "go down" and go deep into the grass-roots level, the youth and the masses. The service group of Tianjin Youth League members is more targeted to "inquire and consult" to each recipient area, and then take targeted measures and targeted assistance."The Communist Youth League, as the party's assistant and reserve force, must play a good role as a bridge."Li Long said that the Communist Youth League, Youth League, Student League, Young Pioneers and other organizations to study, publicize and implement the spirit of the sixth Plenary Session of the 19th CPC Central Committee as the primary political task, widely gather the majestic power on the road, education and guide the youth members to unswervingly listen to the Party, follow the Party, bloom youth in the struggle. China Youth Daily, China Youth Network reporter Hu Chunyan source: China Youth Daily

2021-11-27	Youth ecological civilization education in Hubei special activities held youth ecological civilization education in Hubei special event organizers Juan-juan zhu) on November 24, sponsored by the central social liaison department, the Hubei provincial party committee, China three gorges group youth corps committee, Ministry of Water Resources of the Yangtze river youth corps committee, ecological environment Yangtze river bureau jointly undertake "beautiful China youth action" youth ecological civilization education in Hubei special activities, held in the three gorges group. Activities invited national youth zero carbon technology exhibition pilot project winners, hydropower planning and design institute international business director Jiang Hao, the 7th "mother river award" green guard award winners, Jilin province tongyu county environmental volunteer association secretary-general Wan Xiaobai, the tenth "mother river award" green team award representatives, the Yangtze river ecological environmental protection group, Hubei Yangtze river water ecological protection and restoration of youth science and technology innovation team chief four speakers, around "carbon neutral" "carbon peak" goal, beautiful rural construction, the Yangtze river basin ecological environment management, science and technology power the Yangtze river ecological protection and restoration, etc. Central social liaison department social mobilization office, deputy secretary of Hubei provincial party committee Wang Yunqing, China three gorges group work, deputy director Yin Liangrun to attend the event and speech, the Ministry of Water Resources of the Yangtze river committee, ecological environment Yangtze river bureau responsible comrades and the provincial youth environmental organizations, head of youth environmental protection volunteers representatives to participate in the activities. It is reported that the event set up the main venue in Wuhan, in Hubei province cities and states set up a sub-venue, and through the "youth Hubei" Douyu studio live broadcast, live heat 151,000. The Hubei Provincial Party Committee of the Communist Youth League will continue to carry out relevant publicity activities online and offline ways in the near future.

2021-11-26	Lei Yu, reporter of website) Today, Hubei 2021 "Baisheng Forum" provincial excellent micro League class, vitality League branch final and demonstration exhibition activities were held in Wuhan. A total of 117 universities in Hubei province participated in the event, covering tens of thousands of league branches and more than 400 league branches. After the "class (youth league branch), school, school" level by level linkage, "school, division, province" layer elected, 8 outstanding youth league cadres from 14 colleges and universities and 8 youth league branches in the province stepped on the final stage. On the platform, a famous youth cadres with youth words to promote the great party spirit, the founding chapter in the name of youth. A youth branch in a rich and vivid form of learning and publicity of their innovation and practice achievements, show the belief pursuit and practice of the youth in the new era. After the on-site competition, Li regretful, secretary of the Youth League Committee of the First Clinical College of Huazhong University of Science and Technology, won the provincial champion of "Excellent Micro League class of" Baisheng Forum ", and the Youth League branch of the Young Marxist training class of Huazhong University of Science and Technology won the provincial champion of" Baisheng Forum ". Since 2015, the Hubei provincial party committee, Hubei province union to "forum" as the carrier, through the way of young people, innovative ideological and political lead, focus on promoting the youth corps cell, in play a role of leading peer at the same time, encourage university cadres to learn, speak good xi new era the ideas of socialism with Chinese characteristics, strive to lead young students to listen to the party, follow the party. The "forum" to "theoretical study me-learn the history of the party, realize beginner's mind spectrum Marty natalegawa" as the theme, through the demonstration tour, excellent micro group class evaluation, excellent speaker and vitality league branch elected, continue to promote the new era of socialism with Chinese characteristics into the youth league branch, into the community, into the network, into the class. Source: China Youth Daily

2021-11-29	Hope project hundreds of young "nan yue" and sponsors punch in red memorial hope project hundreds of young "nan yue" and funders clock in red memorial Lin Jie) on November 26, "build hope" the 12th hope project "nan yue" open in Guangzhou, five hua, letter from Guangdong province, sealing, guang ning, even 100 rural students, in Guangzhou and support their brother and sister, uncle and aunt, have a happy weekend. This year, more than 2,000 rural students are expected to receive funding. The association is jointly sponsored by Guangdong Provincial Committee of the Communist Youth League, Guangdong Youth Federation, Guangdong Provincial Working Committee of the Young Pioneers and Guangdong Youth Development Foundation. During the event, the organizers organize caring people and the students to the site of three memorial and Guangzhou farmers movement workshop site card red cultural places, all the way to appreciate the revolutionary ideal feelings and struggle spirit, understand the Chinese communists in one years of struggle in Guangzhou, deep feeling of one hundred brilliant party history, draw upward red power. The reporter learned that this year, many "post-95" and "post-000" youth forces have emerged among the funders. Among them are young civil servants, white-collar workers in enterprises, university teachers, volunteer volunteers, and even college students. The post-1995s and post-2000s generation took over the baton and fulfilled the responsibility of the youth in the new era with practical actions. In addition to the event, this year's meeting also continued the "cloud meeting" way, so that the caring people who cannot come to the event site and 100 rural students gather in the cloud. I hope that teacher Yi Xia, a volunteer of the village teacher program, a charity sponsor, and her husband and her sick father will meet with the aided students through the "cloud will kiss". Although she is far away from Xinjiang, She still cares about students in difficulties and provides grants to four rural students. Ms.Song, the charity sponsor, has participated in the Guangdong Hope Project since 2005, directly and indirectly subsidizing more than 80 students with a amount of more than 100,000 yuan. This year is the second year for Ms.Song to attend the "Cloud Association". She hopes to establish love links for the children and encourage and support each other. It is reported that the hope Project "Guangdong Association" activity originated from "Yangcheng Association" held in Guangzhou in 1994 and "Pengcheng Association" held in Shenzhen in 2003. In 2008, the activity was upgraded to "Guangdong Association" held in 21 cities in Guangdong Province. In the past 13 years, the project has helped 34,746 poor children to complete their studies, and the funding amount is more than 53 million yuan. It has gradually developed into a brand project of Guangdong Hope Project, and has won the national 20-year National Hope Project Classic Project, Guangdong Poverty Alleviation Excellent Project and other honors.

2021-11-23	The 2021 Fujian Provincial Young Pioneers Counselors backbone Training Class was held in Fuding, Ningde. Correspondent for the picture of China youth network Beijing on November 23 (reporter Zhang Jianwei, correspondent Huang Peng) on the afternoon of November 21, by the Communist Youth League Fujian Provincial Party Committee, Fujian Provincial Department of Education, Fujian Provincial Young Working Committee held the 2021 provincial young Pioneers counselor backbone training class in Ningde Fuding opened. Provincial Youth League Committee, provincial youth working Committee, Fuding city related responsible comrades attended the event. The 5-day training course aims to further study and implement the spirit of the Sixth Plenary Session of the 19th CPC Central Committee and the spirit of the Opinions of the CPC Central Committee on Comprehensively Strengthening the Work of the Young Pioneers in the New Era, conduct in-depth discussion of the three contemporary topics of the Young Pioneers, and strengthen the professional construction of the young Pioneers counselors. Training to take special teaching, live view, experience teaching, communication, invited to the national red scarf tour, save less society, provincial youth branch units of experts teaching for students, training content theme, features, covering the spirit of the sixth plenary session of the 19th, "red scarf medal" for activities, the young pioneers ideal faith education, counselor professional growth, such as theme, will also arrange students to watch the young pioneers for experience, related content will also face "letter hand in hand" to live online provinces. At the same time, the teacher trainers of the provincial emergency rescue training center were also invited to carry out rescue safety training for all the participating counselors.2021 Provincial Young Pioneers counselor backbone training class site. During the training, we will also organize the province's young pioneers to study and implement the spirit of the sixth plenary session of the 19th CPC Central Committee, convey the spirit of the sixth plenary session of the 19th CPC Central Committee, and discuss the province's young pioneers organization to study publicity and implement the spirit of the central ideas and measures. More than 120 people from the provincial districts, counties (cities, districts) and Pingtan comprehensive experimental area, members of the provincial red scarf tour group and provincial school brigade counselors participated in the training.

2021-11-23	The Ningxia District Committee held the press conference of the Ningxia Hope Project to help the "Four Promotion Actions" project to help the "Four Promotion Actions". Conference, Ningxia district party committee deputy secretary li feng, high, Ningxia youth development foundation secretary-general Ma Bing, youth development head Chen Pingping around the "four big promotion action" implementation of immigration support, help farmers, student education, health guardian and other four projects and phased results, for "youth science and technology innovation fund service groups and implementation" "youth in rural revitalization of the carrier" a reporter asked. Li Feng released the "four major promotion actions" to help the Ningxia Hope Project, and answered questions on "what the Ningxia Communist Youth League has been doing in the rural revitalization". Gao xianghu answered questions on "how the Ningxia Hope Project will play its role in education and lead young people to help the 'four major promotion actions' ". Ma Bing answered questions on "which people the Ningxia Hope Project Youth Science and Technology Innovation Fund has been targeted at and what work has been carried out". Chen Pingping answered questions on "how project Hope will implement the concept of collaborative and opening up in the" four promotion actions "and" what public services will Hope Project Ningxia has carried out in this round of epidemic ". Since the beginning of this year, the Ningxia District Committee of the Communist Youth League has carried out community volunteer services, training and skilled personnel training of young leaders for becoming rich people. In the communities relocated from inhospitable areas, 11 social public service projects for young people have been implemented, so that migrant young people can integrate, retain, and take root. Focusing on improving the income increase of urban and rural residents, it has provided more than 5,700 employment and internship positions for college students, organized young wealth leaders and industrial development leaders to carry out industrial docking in Zhejiang and Fujian, and reached 10 cooperation projects with cooperation intentions of more than 60 million yuan, helping young people expand employment, return to their hometowns to start businesses and revitalize industries. To improve the quality of basic education, we have carried out psychological self-care of adolescence, psychological decompression for middle school or college, and software improvement in Hope Primary School, and funded 3,756 outstanding students so that they can learn to learn, become talented and report to their hometown. To improve the health of the whole people, we will provide assistance to serious diseases, scientific prevention of "small glasses" and "small fat piers", and public welfare surgery for children with hip joint difficulties, so that every young person can grow up healthily and full of hope. At the same time, it raised 5 million yuan to set up a youth science and technology innovation fund, targeting three groups of primary students, young science and technology workers, and innovative and entrepreneurial talents, including popular science education, scientific research funding, project incubation, talent reward, and achievement transformation, to comprehensively improve their scientific and technological innovation literacy. In order to promote the work effectively, on July 18, the Ningxia District Committee coordinated the party committee and the government office issued the "nine key industries", "ten projects", "four promotion actions" ten measures; on August 13, held a special deployment meeting in Yinchuan, and 10 units to introduce the implementation plan; on September 28, held in Wuzhong City, and issued the Ningxia Hope Project, defined the work direction of transformation and upgrading. At present, Ningxia Hope Project has raised more than 36.5 million yuan through the "My Ningxia" APP, all of which are used for related projects. Among them, 320,000 yuan of money was raised during the current epidemic period to provide living and protective materials for frontline volunteers.

2021-11-23	"Shaanxi" yao future hope children's palace public welfare project in shaanxi: more than 17000 left-behind children, trailing children will have "hope mentor" Sun Haihua) on November 21, "shan" yao future hope children's palace public welfare project launched in shaanxi, xi 'an, Yan' an, Shangluo three cities of 60 schools, more than 17000 rural left-behind children, city trailing personnel children will be under the guidance of "hope mentor", such as quality education promotion series of public welfare activities. The "Shaanxi" Yao Future Hope Children's Palace charity project is co-sponsored by Shaanxi Provincial Committee of the Communist Youth League, Shaanxi Youth Development Foundation, Kweichow Moutai Maotai Liquor Marketing Co., Ltd., and organized by Xi' an Youth Palace, Yan'an Youth Palace and Shangluo Youth Palace. Project aims to crack the remote left-behind children art specialty education resources, basic education is relatively weak, intergenerational education for children interest training and personality development lack of a series of problems, accelerate the high quality art education resources to remote areas, adhere to the aesthetic education, to culture, pave the way for the all-round development of rural children."The project will select 60 rural schools with relatively large numbers of left-behind children, and build a unique quality-oriented education and assistance mechanism in the field of rural revitalization."Shaanxi province hope engineering public welfare volunteer guidance center director of wu, said the project will face colleges and universities, primary and secondary schools, social education institutions, civil society to recruit 60 covering vocal music, painting, instrumental music, dance and other professional fields of" hope teacher ", to improve the level of project implementation, form pratt & whitney, public welfare education platform, to create an influential education support new brand. At the launch ceremony, Kweichow Moutai Maotai-Fragrance Liquor Marketing Co., Ltd. and Xi 'an Chengcheng Yulong Trading Co., Ltd. respectively donated RMB 1 million yuan to the "Shaanxi" Yao Future Hope Children's Palace public welfare project. Shaanxi Provincial Committee of the Communist Youth League and Shaanxi Provincial Youth Development Foundation presented the commemorative card of Shaanxi Hope Project Contribution Award in the New Era to the caring enterprises.

2021-11-22	Group of Guangdong provincial party committee convey study and implement the spirit of the sixth plenary session of the 19th group of Guangdong provincial party committee convey study and implement the spirit of the sixth plenary session of the 19 Beijing on November 22 (reporter Zhang Jianwei, correspondent YueQing) recently, the secretary of the Guangdong provincial party committee meeting, project convey learning xi general secretary at the sixth plenary session of the party's important speech spirit and the plenary session spirit, study the implementation measures. Chi Zhixiong, secretary of the Guangdong Provincial Party Committee of the Communist Youth League, presided over the meeting. Liang Jun Anda, deputy secretary, attended the meeting, and the cadres above the department level and the team members of the directly affiliated units attended the meeting. The meeting stressed that, To have a deep understanding of the great historical and practical significance of the plenary session, To unify our thinking to the major arrangements of the CPC Central Committee; Bearing in mind the glorious responsibility of the party assistants and reserve forces, Faithfully implement the specific requirements of educating people for the Party put forward in the resolution; To learn and deeply understand the rich connotation and spiritual essence of General Secretary Xi Jinping's important speech and the Resolution, Working both as a doer and as a propagandist, With vivid language to explain the spirit of the plenary session deeply and thoroughly; We should study, publicize and implement the spirit of General Secretary Xi Jinping's important speech at the Sixth Plenary Session of the 19th CPC Central Committee as the primary political task of the Guangdong Communist Youth League in the coming period, Deepening the study and education of Party history, Turn learning gains into work results, Show loyalty, cleanliness and responsibility with practical actions. Meeting requirements, Guangdong youth league organizations at all levels to study publicity and implement the plenary session spirit and thoroughly study and implement the "July 1" important speech spirit, xi jinping, general secretary of the important thought of youth work, important speech to Guangdong and instructions instructions spirit, the foothold official duty post, with real work performance to implement the plenary session spirit. We should work hard in the study, quickly introduce specific and feasible implementation plans, mobilize the youth league cadres at all levels to take the lead in learning to deeply understand the spirit of the plenary session, organize and carry out the theme team day activities, and bring the spirit of the plenary session into the teaching content of the main youth league school. Efforts should be made in publicity, implement the system requirements of the party and league class, carry out the "lighthouse project", strengthen the linkage role of the matrix of the new media platform affiliated to the league, implement the spirit of the plenary session to the grass-roots level, armed to the league members, and affect the youth. Efforts should be made to implement the history of the study and education of the youth Party and the practical activities of "I do practical things for the masses", to promote the implementation of the work of the youth development plan, strengthen the construction of the Communist Youth League in the new era, systematically plan the 100th anniversary of the founding of the League, and promote the development of the cause of the Guangdong Communist Youth League to a new level.

2021-11-19	The Qinghai Provincial Party Committee communicates the Spirit of the Sixth Plenary Session of the 19th CPC Central Committee) On November 15, the Qinghai Provincial Party Committee held an office meeting to convey the spirit of the Sixth Plenary Session of the 19th CPC Central Committee and deploy the study, publicity and implementation work. Dong Yuyi, secretary of the Provincial Communist Youth League Committee, presided over the meeting. Leading members of the Provincial Communist Youth League Committee and all departments (units) attended the meeting as non-voting delegates. Conference pointed out that the sixth plenary session of the party's 19th is in the important historical moment of the centenary of the party and the people victory in the first one hundred goal, build a well-off society in an all-round way, is toward build comprehensive socialist modernization power second one hundred goal of major historical juncture held a very important meeting, to promote the party further unified thought, unified will, unified action, unity led the Chinese people of all ethnic groups to seize the new era the new great victory of socialism with Chinese characteristics, has great practical significance and far-reaching historical significance. Plenary session of the central committee of the communist party of China on the party's struggle in one hundred major achievements and historical experience resolution, based on a comprehensive review summarizes the party's struggle in one hundred and achievements, concise put forward the historical significance and historical experience, is a continuity, has great political value, theoretical value, practical value and landmark of programmatic document. The meeting stressed that the youth league organizations at all levels in the province should study, publicize and implement the spirit of the sixth Plenary Session of the 19th CPC Central Committee as the primary political task, to study the spirit of the sixth Plenary session of the 19th CPC Central Committee as an important content of the provincial Communist Youth League Party history learning and education, quickly set off a learning upsurge. We must earnestly improve our study. Provincial Communist Youth League Party committee organs should play an exemplary role, and leading cadres should take the lead in learning, careful research, work hard to "learn the original text, understand the principle", and carefully organize the study and discussion. Provincial youth league schools should give full play to the role of the main position of education and training of youth league cadres, organize forces to study and prepare lessons for the spirit of the plenary session, and promote the spirit of the plenary session learning into training and classroom. To do a solid job in publicity. It is necessary to implement the strategy of producting the propaganda thought of the Communist Youth League in depth, rely on the new media platform, combined with the actual situation of Qinghai, focusing on the achievement education and situation education, design and launch a batch of theoretical publicity works, and carry out the publicity and discussion work innovatively, and lead the young people to learn actively and learn extensively. The spirit of the plenary session should be organically integrated with the study and publicity of General Secretary Xi Jinping's July 1 important speech, incorporated into the content of the mass publicity, and mobilize the youth league members and young pioneers to take the initiative to participate in the study and publicity, and create a good atmosphere for full participation. We need to promote our work pragmatically. To study and implement the party's spirit of the sixth plenary session of the 19th CPC, and study and implement xi general secretary about youth work important discourse and major requirements for Qinghai work, in-depth implementation of mid-and long-term youth development plan, promote the communist youth league to participate in the construction of "eight new heights", power rural revitalization of the key work to carry out the ground. Continue to deepen the reform of the communist youth league, around the "work project, survival socialization, running flat, cadres source diversification, lead the mobilization network, organization diversification" reform requirements, thinking work path, measures and carrier, strive to build an open type of the communist youth league work system, continuously enhance the political function of the organization and social function. Learn from the work experience of party building work, continue to promote the comprehensive and strict governance of the league, plan the "construction of the Communist Youth League action branch", "league construction project construction", "league member points system management" and other work, strictly promote the construction of grass-roots league organizations and league members. Systematic planning to celebrate the centennial series of activities of the founding of the league, fully show the high style of the province's youth league members, with excellent results to meet the twenty major victory of the party.

2021-11-18	From November 8 to 11, the sixth plenary session of the Central Committee of the 19th Communist Party of China was held in Beijing. Jiangxi Communist Youth League organized young people from all walks of life to study extensively through the learning conference bulletin, organizing and watching press conferences, holding special studies and discussions, and writing their learning experiences. Young people have expressed that they need to thoroughly study and implement the spirit of the Sixth Plenary Session of the 19th CPC Central Committee, respond to the Party's call, and contribute their youth to the realization of the second centenary goal and the Chinese dream of national rejuvenation. Huang Guipeng, cadre of the Publicity Department of Jiangxi Provincial Party Committee of the Communist Youth League: A country, a political party, the leadership core is very important. Only when the CPC Central Committee has a core and the whole Party has a core can the Party have strength. Since the eighteenth congress, xi jinping, general secretary led the party of the whole country people carry forward the great historical initiative, create a new era of socialism with Chinese characteristics, promote the party and the state cause made historic achievements, historic changes, in China to build a well-off society in an all-round way, become expected, deserved the core of the party, the people's leader, army commander-in-chief. The Sixth Plenary Session of the 19th CPC Central Committee stressed the "two establishment", which reflects the common aspiration of the whole Party, the army and the people of all ethnic groups, including hundreds of millions of young people. It is of decisive significance to the development of the cause of the Party and the country in the new era and to the historical process of advancing the great rejuvenation of the Chinese nation. As cadres of the Communist Youth League, we should keep the "two establishment" in mind, into the "two maintenance" ideological consciousness, political consciousness, action consciousness, and constantly enhance the "four consciousness", firm "four confidence", and achieve the "two maintenance". We need to be more keenly aware that with the CPC Central Committee with Comrade Xi Jinping at its core leading at the helm, no difficulties and obstacles can stop our progress, and no risks and challenges can stop the historical process of great rejuvenation. I will do my best to implement the specific requirements of educating people for the Party put forward in the Resolution, and do a good job in the youth work in the new era with my heart and affection. League Nanchang City Qingshan Lake District Party Committee Secretary Chen Cheng: the long river is mighty, unstoppable. On the occasion of the 100th anniversary of the founding of the CPC, the CPC Central Committee held the Sixth Plenary Session of the 19th CPC Central Committee. Its main task is to focus on comprehensively summarizing the major achievements and historical experience of the Party's centenary struggle. It is yet another mobilization meeting to unify the thinking of the whole Party, strengthen our confidence, and continue to strive for the second centenary Goal. I think this conference is a comprehensive and systematic study and deployment of the CPC Central Committee and two questions raised by the General Secretary at the July 1 celebration: to see why we succeeded in the past and how we can succeed in the future. I as a communist youth league cadre, I should thoroughly study and implement the spirit of the sixth plenary session of the 19th as a top priority for a period of time, first of all to take the lead in learning, deeply understand the conference spirit, extensive mobilization, make good use of all kinds of propaganda positions and platform, build a strong learning atmosphere, the first time the party's spirit, the party's instructions convey in place, further the youth will to build socialist modernization power, realize the great cause of the second century goal. Nanchang city teng wang pavilion tourism industrial center narrator service center director Wu Tingting wu: one hundred party history, the past one hundred, the party to the people, to history delivered an excellent answer, now, under the leadership of the general secretary in jinping as the core of the party, we set foot on the new way to achieve the second goal in one hundred. As the national youth civilization-TengWangGe narrator service center long, I know that the communist youth league is the party's assistant and reserve, according to the requirements of learning to have a good grasp of doing real organization TengWangGe youth league learn all spirit, armed with the spirit of the mind and guide the work, but also to' I do the practical work for the masses as the starting point, based on jobs, actively in tourism development, epidemic prevention and control, volunteer service work such as youth strength. Fu Xiangyang, a young employee of Aviation Industry Hongdu Company: After learning the spirit of the sixth Plenary Session of the 19th CPC Central Committee, I have strengthened my ideals and beliefs, and strengthened my steadfast work pace. It is through all the struggles, sacrifices and creations of the Chinese people united and leading by the Communist Party of China that we now have a stable life. As a front-line employee of aviation weapons and equipment production, I will always bear in mind the original mission, work hard, constantly adapt to the current new changes, stay true to the original aspiration, keep in mind the mission, and give full play to the vanguard role of party members. Ye Jianeng, young firefighter of Fang Zhimin Avenue Fire and Rescue Station in Yiyang County: The sixth Plenary Session of the 19th CPC Central Committee is a meeting of great historical significance held at an important historical juncture, shouldering the great mission of building on past achievements and forging ahead. After the brigade organized us to carry out the discussion and exchange, I deeply believe that the victory of the Sixth Plenary Session of the 19th CPC Central Committee also has a very important practical significance for promoting the fire and rescue work. As a firefighter who has joined the team for many years and changed with the team, I am deeply aware that we shoulder the mission of the team, and in the past three years, the historic changes of the team, especially the great new achievements in the system. In the future work, I will take root in the red fertile soil of Fang Zhimin's hometown, take responsibility, work solid, work hard, make new contributions to the Party and the people, and contribute to the sound and rapid development of the resident economy and society. Huang Wei, young volunteer of Qianshan County: The Sixth Plenary Session of the 19th CPC Central Committee reviewed the glorious history of the Communist Party of China, and made me understand that the mission of a generation has a generation. In the historical inheritance of the country and the nation, the young people are the inheritors and successors of The Times. As a young volunteer, we should have the responsibility of the youth. In the COVID-19 sniper battle in Qianshan County, I immediately put myself into the battle. There are a lot of people ask me, is so dangerous outside, why not rest at home, but I think, we had the honor to live under the red flag, long in the spring breeze, and my hometown is the time we need us, "stick" passed to the hand, only rushed in front, can not era, to fight the epidemic work dedicated their own strength. In the long years of my life, I will continue to hone my will, give full play to my own advantages, and contribute to the new journey of realizing the second centenary goal, and join hands to write the chapter of comprehensively building a modern socialist power in Jiangxi. Peng Xiaoliang, young teacher of Jiangxi University of Science and Technology: History is witnessing and the people are looking forward to it. The Sixth Plenary Session of the 19th CPC Central Committee comprehensively summarized the Party's major achievements and historical experience in the century of struggle. With no change, the road unchanged firm, from today to tomorrow. There was a spark of fire, and Yin thunder was heard everywhere. The Communist Party of China was born from the broken crisis of the mountains and rivers, tempered in the bloody war, stood upright in the passionate socialist construction, and grew in the tide of reform and opening up. The Party united and led the people to describe the road of rejuvenation with the aggressive spirit of "crossing mountains and building Bridges in water". As a young teacher, I am committed to educating people for the Party and the country, and write a magnificent chapter of life in the journey to realize the Chinese dream. Yin Ziyi, a young student of Jiangxi University of Science and Technology: A hundred years of intersection, planning and layout. Over the past one hundred years, the Party has delivered an outstanding answer to the people and history. Now, the Party has united and led the Chinese people on a new road to achieve the second centenary Goal. The Sixth Plenary Session of the 19th CPC Central Committee held in Beijing led us to see why we have succeeded in the past and how we can continue to succeed in the future. It also clearly pointed out the historical mission of the young generation and placed high hopes for the youth. Youth, the country and The Times are the iron triangle and the waves boosting each other. The meeting expressed ardent expectations for the young people, urging them to ride the tide of The Times and strive to realize the Chinese dream. I will try my best to learn skills, devote myself to the construction of the motherland, and live up to the expectations of the Party. Ye Lintao, League member of Nanchang Institute of Engineering: After studying the Sixth Plenary Session of the 19th CPC Central Committee, I was deeply inspired. As a youth league member, I will not forget yesterday's suffering and glory, fulfill today's mission, live up to tomorrow's great dream, and create new brilliance on the new exam on the road to the examination. Chen Xiaoyu: Over the past one hundred years, the Communist Party of China has faithfully fulfilled its original mission and united and led the Chinese people of all ethnic groups in writing an epic on the land of China. I as a grassroots people, will unite closely around the party central committee with comrade xi as the core, comprehensive in-depth implementation of the spirit of the sixth plenary session of the nineteenth, under the strong leadership of the municipal party committee and municipal government, seize the opportunity, challenges, power rural industry revitalization, good science and technology innovation, intensify product research and development, improve product quality, construction of local brand, contribute to the rural industry revitalization.

2021-11-18	Capital youth from all walks of life study and implement the spirit of the sixth plenary session of the 19th the youth from all walks of life to study and implement the spirit of the sixth plenary session of the 19th zhang correspondent LanJianhua) on November 15, Beijing capital youth representatives of all walks of life learning the spirit of the sixth plenary session of the 19 symposium and Beijing pku theoretical study group (expand) meeting, communicate study and implement the sixth plenary session of the 19th spirit. More than 40 young representatives from all walks of life in the capital from government organs, enterprises, schools, and emerging fields attended the meeting online and offline, and the 10th Steering Group of the CPC Party History Learning and Education Municipal Committee attended the meeting online. At the meeting, the youth representatives collectively watched the video of "News Broadcast" and learned the communique of the Sixth Plenary Session of the 19th CPC Central Committee. The youth representatives exchanged and shared their learning experience around studying and implementing the spirit of the Sixth Plenary Session of the 19th CPC Central Committee, combining with the experience of Party history learning and education and their own work reality. The games volunteers, Peking University students Jiang Yuxin said, by learning all spirit, realize the significance of the winter Olympics volunteer service, the winter Olympics volunteers struggle direction, realize as the historical task of the new era youth, in the upcoming Olympics, to show the new era of Chinese youth style to the world, do a piece of "burning snow". Li Qiufu, president of the Beijing Students 'Federation and a student of Tsinghua University, said that the Municipal Students' Federation will do a good job in publicity and mobilization, and take the upcoming 13th Congress of the Municipal Students' Federation as an opportunity to gather positive energy, carry forward the main theme, and create a country of youth with the self of youth."Capital universities organize teachers and students to learn the spirit of the plenary session in the first time, which has triggered a wide heated discussion among teachers and students." Qi Chenglong, secretary of the Youth League Committee of Capital Normal University, introduced the situation, saying that the university Youth League Committee will organize young teachers and students to earnestly study and understand the spirit of the plenary session, cast the soul and educate people, and do a good job in the current university youth work. From build eight bureau capital stadium Lv Yusheng, combined with the experience in the games of engineering construction said, " the development of China building gradually grow with the development of the country, the form of building also highlights the people centered development concept, in the construction of beautiful China, build a harmonious livable road, as building youth will be more powerful steps."" In the process of designing rural revitalization projects before, I felt the trust and recognition of the villagers and village cadres to the Party, felt the support of the Party and the country, and saw the earth-shaking changes in the countryside."Luan Yiyi, a young designer, said that as a new young man," our designers need to promote design to play a greater role in people's life, cultural communication, scientific and technological innovation, urban construction and rural revitalization."" although I am not a party member, but I want to actively to the party organization ", from fengtai district and righteousness outlets express little brother li said, in the ordinary post fully feel their own value, hope to do a contribution to the development of the motherland, learning the plenary session spirit, looking back at the party's journey in one hundred, inspiring, inspiring, will actively to the party organization. Wang Miaomiao, head of the orchid group of the greenhouse center of the Beijing Botanical Garden Management Office, said that the communique stressed that the CPC Central Committee with unprecedented efforts to grasp the ecological civilization construction, ecological environmental protection has undergone historic, turning and overall changes, as the young people engaged in plant work are full of vision for the future. Yu Yan, deputy secretary of the Party Committee of Shell Housing Search, introduced the work of establishing a "little brother gas station" relying on the advantages of the store, with "building a new living, always follow the party" as the core, adhere to the "party building with the group" as the support, and draw the strength of unremitting struggle from the hundred years of party history. Chaoyang public security bureau Feng Rui said, do not forget the original intention, keep in mind the duty and mission, loyal to perform their duties, dare to take responsibility, not time, not young, escort for the safe construction of Beijing and the high-quality development of the capital, to achieve the goal of the second one hundred, to realize the Chinese dream of the great rejuvenation of the Chinese nation and unremitting struggle. Zhao Siping, a young returning entrepreneur from Miyun District, expressed his honor to be in this era by sharing entrepreneurial stories and the experience of learning the spirit of the Sixth Plenary Session of the 19th CPC Central Committee through returning home, cross-border and expectation. In the near future, the Beijing Youth League Municipal Committee will continue to deeply study and implement the spirit of the plenary session, to enterprises, rural areas, organs, campuses, communities, combined with the actual work of all fronts, all departments to carry out theme activities.

2021-11-25	The Theoretical Learning Center Group of the Shanghai CPC Central Committee studied the spirit of the Sixth Plenary Session of the 19th CPC Central Committee. Zhang Jianwei, correspondent Zhang Chen) On November 22, The Theoretical Learning Center Group of Shanghai Municipal Party Committee to carry out special study and discussion, Further study and implement the spirit of the Sixth Plenary Session of the 19th CPC Central Committee, Focusing on the study of the Resolution of the CPC Central Committee on the Major Achievements and Historical Experience of the Party's Struggle in a Century " and General Secretary Xi Jinping's explanation on the Resolution, To study and put forward the Shanghai Communist Youth League to implement the learning objectives and tasks in accordance with the requirements of the Central Committee of the CPC, the Municipal Party Committee and the Central Committee of the Communist Youth League. Youth League party secretary, directly affiliated organs party secretary, chairman of the City Youth Federation on the Guanjian presided over. Municipal Commission for Discipline Inspection in the Municipal Federation of Trade Unions organs discipline inspection group deputy leader Tao Jianhua attended the meeting. The Communist Youth League municipal party committee secretary group made a keynote speech respectively. Communist Youth League part of the department responsible comrade speech. In the study and exchange, we felt that the Sixth Plenary Session of the 19th CPC Central Committee was an important meeting held at an important juncture of the Party's centenary birthday and at a critical juncture in the history of the Two Centenary Goals. The Resolution comprehensively summarizes the Party's major achievements and historical experience in a century of struggle, It deeply reveals "why we succeeded in the past and how we can continue to succeed in the future", It is a programmatic document that shines with the brilliance of the truth of Marxism and will surely go down in history, It is a political declaration for Chinese Communists in the new era to bear in mind their original mission and uphold and develop socialism with Chinese characteristics, It is a guide to action to learn from history, create the future and realize the great rejuvenation of the Chinese nation, It is also an authoritative textbook to help young people to strengthen their ideals and beliefs and inherit the red gene, To promote the whole Party to further unify its thinking, will and action, More firmly and consciously practice our original mission, To better uphold and develop socialism with Chinese characteristics in the new era, It is of great significance and has a far-reaching influence. It is believed that to study and implement the spirit of the Sixth Plenary Session of the 19th CPC Central Committee, we must learn the historical materialism and correct view of Party history, and strengthen the political consciousness of making good use of the laws of history. The Resolution focuses on the Party's practical exploration and theoretical creation over the past century, focuses on explaining the historic achievements and changes made in the cause of the Party and the country since the 18th National Congress of the CPC, and sets out clear requirements for the realization of the second centenary Goal. The CPC Central Committee with Comrade Xi Jinping at its core has made the Resolution with a strong sense of historical consciousness and historical responsibility, which enables us to learn that "taking history as a mirror and creating a future" has strong practical significance and is a mission and responsibility that we need to be more consciously and firmly fulfilled. From the "resolution" of the CPC Central Committee, from the party's one hundred struggle why can, marxism, socialism with Chinese characteristics, thus more closely combined with the party's youth work, the cause of the Chinese youth movement and the communist youth league, understanding, grasp and use the historical law, anchor goals, based on reality, completes the present, in the new era better uphold and develop socialism with Chinese characteristics, guide the youth on the new journey, vigor, along the right direction firmly forward, open up the future. We should learn to understand the decisive significance of "two establishment" and more firmly into concrete actions to achieve "two maintenance". Plenary session pointed out that the party established the core of the CPC Central Committee comrade xi, the core position of the party, establish xi jinping new era the guiding position of socialism with Chinese characteristics, reflects the whole party and army the Chinese people of all ethnic groups common wish, to the new era of the party and national career development, to promote the great rejuvenation of the Chinese nation has decisive significance. Since the eighteenth congress, our party in adhere to the party's overall leadership, comprehensive the governing party, economic construction, comprehensively deepen reform and opening up, political construction, the rule of law, cultural construction, social construction, ecological civilization, national defense and military construction, maintain national security, adhere to the "one country, two systems" and promote the reunification of the motherland, diplomatic work, has made historic achievements, historic changes, in the final analysis because establish the core of xi General Secretary of the CPC Central Committee, the core of the party, establish xi new era of the guiding position of socialism with Chinese characteristics. General Secretary Xi Jinping has led us to be self-confident, upright and innovative. We has fully demonstrated our talents and strategy as leaders of the Party and won the wholehearted support of the whole Party and the people. This is good for the Party and the country, the people and the nation. The strong core of leadership and scientific theoretical guidance are fundamental issues concerning the future and destiny of the Party and the country and the success or failure of the cause of the Party and the people. The pioneering "two establishment" at the plenary session, which is of great political and far-reaching historical significance. To more firmly safeguard xi the core of the General Secretary of the CPC Central Committee, the core of the whole party, resolutely safeguard the authority and centralized and unified leadership, insist with xi new era of socialism with Chinese characteristics armed mind, guide the work of the Shanghai communist youth league, unswervingly put the "two established" truly into "two maintenance" thought consciousness, political consciousness, action consciousness. We should learn and make good use of the historical experience of "ten persistence" to fully stimulate the strength to take the new road to the examination. The plenum highly summarized and refined ten historical experiences of fundamental and long-term significance. The "Ten Persistence" is not a temporary experience, but a valuable experience running through a hundred years and spanning time and space. It is a precious spiritual wealth of Communists and a magic weapon to success. Only by making good use of the Party's historical experience can we win the initiative, advantages and the future in the face of various risks and challenges. Unswervingly upholding the leadership of the Party is the unbroken truth revealed by the Party's century of struggle and the most fundamental guarantee for us to win new victories. The Communist Youth League is a mass organization under the direct leadership of the Party and the Party's assistant and reserve force. The Party places high trust and ardent expectations on the Communist Youth League. To learn from the Party's major achievements and historical experience of the Party's century of struggle, the first thing is to unswervingly implement the Party's leadership and fulfill our mission as the Party's assistant and reserve force. The meeting put forward that the party flag refers to is the regiment flag to the direction. In accordance with the requirements of the CPC Central Committee, the CPC Municipal Committee and the Central Committee of the Communist Youth League, with a high sense of political responsibility and mission, we must consciously integrate our thinking and actions into the important speeches of General Secretary Xi Jinping and the spirit of the plenary session, have a deep understanding of the Party's major achievements in a century, carry forward the Party's historical experience, and resolutely fulfill the duties and missions of the Communist Youth League. The meeting required that at present and for a period to come, the Shanghai Communist Youth League, Youth League, Students' League and Young Pioneers organizations should study, publicize and implement the spirit of the sixth Plenary Session of the 19th CPC Central Committee as the primary political task, and quickly rise the upsurge. To strengthen political guidance, further study propaganda and implement the party's struggle in one hundred major achievements and historical experience, strive to guide the youth league organizations at all levels and the youth learning deep enlightenment through the spirit, the cadres to take the lead to grasp the original, word for word, learn the basic content, grasp the important view, understand work requirements, always keep in mind that xi general secretary of the communist youth league, the young generation entrust talk, unswervingly listen to the party, follow the party, with "big", strengthen the consciousness of "four", firm "four confidence", "two maintenance". We must continue to encourage people to take responsibility, make good use of the major achievements and historical experience of the Party in the past century, carry out in-depth study and education of Party history, encourage all grass-roots youth league branches to take immediate action, and organize all league members to study conveniently and effectively. The spirit of the plenary session into the education and training of youth League cadres, and the thoughts and actions of the Shanghai Communist Youth League into the spirit of the plenary session. To strengthen the propaganda lead, relying on the new media platform, adhere to the focus spread, with good practical products, pays special attention to the achievement education and situation education two key, innovation in simple, tangible and effective preaching, discussion, interpretation, motivate the youth practice internal work, improve accomplishment, more active bear as, active in the people's city construction, promote urban soft power and other vivid practice, efforts to be useful, can bear the burden of pillars. To adhere to the comprehensive governing group, learn deep enlightenment through the party's struggle in one hundred major achievements and historical experience, always adhere to the party to party, comprehensive governing party, comprehensive governing group, strict political discipline and rules, fundamental task, political responsibility, work, resolutely implement the CPC Central Committee, municipal committee and the central deployment requirements on deepening the reform of the communist youth league, further enhance the organization, leading force, service and overall situation contribution, for Shanghai to create a new miracle, show new atmosphere contribution youth wisdom and strength.

2021-11-23	Zhang Jianwei and correspondent Xu Jingxian) Recently, the spiritual exchange and sharing meeting of the Sixth Plenary Session of the 19th CPC Central Committee was held in Fuzhou. At the exchange and sharing meeting, young representatives of Fujian and Macao talked about their experiences around learning and implementing the spirit of the Sixth Plenary Session of the 19th CPC Central Committee. We agree that the sixth plenary session of the 19th comprehensively summarizes the communist party of China struggle of major achievements and historical experience, the plenary session of the central committee of the party's struggle of great achievements and historical experience resolution, is the history as a mirror, create the future, realize the action guide of the great rejuvenation of the Chinese nation, including Macao compatriots, all the Chinese people to achieve the second goal in one hundred, to realize the great rejuvenation of the Chinese nation and unremitting struggle. Everyone have said that Fujian Macao youth should unswervingly implement the policy of "one country, two systems", actively integrated into the national development, seize the Fujian Macao all-round cooperation development favorable opportunity, intensify exchanges, expand cooperation areas, in a large bay area of Guangdong and Fujian comprehensive promote high quality development beyond the hot practice. League of Fujian provincial party committee secretary, honorary chairman of the youth XiaoHuaxin encourage Fujian Australia youth and youth organizations shoulder responsibility, carry forward the fine tradition, as a good bridge, from the spirit of the sixth plenary session of the 19th, continuously struggle spirit, strengthen the sense of identity and centripetal force, for the motherland development, Macao prosperity, hometown construction and Fujian Macao youth communication career to make greater contribution. Li Teng, deputy secretary of the Communist Youth League Committee, Chen Yanying, assistant of the Coordination Department of the Liaison Office of Macao, Shi Jialun, deputy of the National People's Congress and member of Macao Legislative Council, Xu Qingliang, member of Macao Fujian Youth Association, More than 40 people participated in the representatives of the event.

2021-11-17	Zhang Jianwei correspondent Zhang Jinrong) Recently, Shanxi Taiyuan Youth Palace (Taiyuan Youth Activity Center) the first youth congress and youth working committee founding conference was held. More than 80 people of the Youth League, the office of Shanxi Youth Working Committee, representatives of Taiyuan Youth Palace staff and representatives of young Pioneers attended the meeting. Taiyuan Youth Palace (Taiyuan Youth Activity Center) the first youth congress and youth working committee inaugural meeting. Correspondent for figure newly elected Taiyuan youth palace less committee director Ma Bin said in a speech, the next step, Taiyuan youth palace less committee will take youth activities as the gripper, unswervingly strengthen the political lead, focusing on political enlightenment and values shape the main business, constantly enrich the young pioneers practice education system, and improve the young pioneers organization system, closely around strengthening the young pioneers honor, sense of mission, to create a good environment for the healthy development of the young pioneers, promote Taiyuan youth palace young pioneers work to a new level, for the young pioneers outside education and activities. At the same time, taking the implementation of the "double reduction" policy as an opportunity, we will actively explore a new way of youth after-school education of "palace-school linkage", adhere to the unity of organizational education, independent education and practical education, learn pioneers to grow up to be pioneers, and make important contributions to the cause of the Party and the people from generation to generation. The Young Pioneers recited "Report on the Land of China" and "Chinese Youth". At the meeting, the representatives of the Young Pioneers recited the "Report on the Land of China" and the "Chinese Youth" expressed all the young pioneers "please trust the party strong country has me" firm confidence and determination.

2021-11-17	Seven thousand Qinghai young volunteers quickly assembled in epidemic prevention and control work seven thousand Qinghai young volunteers quickly assembled in epidemic prevention and control work Zhang Jianwei correspondent A Shuyuan) in the face of the current outbreak, Gansu provincial party committee act quickly, on October 21, research deployment of the communist youth league to participate in epidemic prevention and control work, request city, counties, advance recruitment volunteers, mobilize the provincial youth league organizations at all levels and youth, youth volunteers to actively participate in the epidemic prevention and control. On October 25, the Qinghai provincial party committee, Qinghai province youth volunteer association again to the province initiative, called for youth league organizations at all levels, all members of youth, youth volunteers, vigorously carry forward the "dedication, friendship, mutual assistance, progress" volunteer service spirit, give full play to the positive role of young volunteers in the epidemic prevention and control, scientific and orderly participation in the epidemic prevention and control work. Provincial, municipal (prefecture), county (urban) three-level youth league organizations and young volunteers quickly gathered in the fight against the epidemic, actively responded to the call, enthusiastically registered, once again wearing the "red vest", in the "epidemic" prevention and control battlefield, the plateau youth demonstrated the mission of "prevention and control of the epidemic, and volunteer" with practical actions. As of 12:00 on October 28, the league of 8 cities and prefectures and 45 counties (cities) in the province organized more than 60 teams of more than 7,000 young volunteers to form the epidemic prevention and control youth volunteer commandos, ready to participate in the epidemic prevention and control work. Haidong and Xining delegations promptly launched volunteer services for youth epidemic prevention and control, quickly organized volunteer teams for epidemic prevention and control, and participated in epidemic prevention and control in accordance with the requirements of local epidemic prevention and control headquarters. 1,400 young volunteers in Xining fought on the front line of epidemic prevention and control. The provincial Health Vocational and Technical College and the Youth Volunteer service team of State Grid Qinghai Electric Power Company volunteered to submit the letter of invitation. Members of the Provincial Youth Foundation, the Youth Federation at all levels and members of the youth enterprise associations actively participated in the epidemic prevention and control, and donated 170,000 yuan of protection funds and materials for young volunteers. In the next step, the Qinghai Provincial Party Committee of the Communist Youth League will continue to mobilize to attract more young volunteers to participate in it according to the epidemic prevention and control situation. At the same time, in accordance with the needs of epidemic prevention and control, municipal, state, county and district youth league organizations will be organized to conduct volunteer job docking, distribution and pre-job training, and volunteers will be organized to carry out volunteer services for epidemic prevention and control in an orderly, scientific and reasonable manner in accordance with the principle of "nearby".

2021-11-16	Tian Hongwei, trainee reporter of China Youth Network) " Can a friendly Rongtai basketball match better promote the cross-strait youth relations?"Recently, the idea came to the mind of Zhang Zhaorui, a teacher at the School of Sports Science of Fujian Normal University. He hopes to make the youth in Fujian and Taiwan and the mainland better understand and integrate each other through sports activities. In 2002, Zhang Zhaorui, who was still in primary school, first came to Fujian from Taiwan for the handball exchange activity. Since then, he developed a strong interest in Chinese mainland and came to Fujian to work after graduating from university. This year, the Fujian Provincial Committee of the Communist Youth League awarded Zhang the "Fujian Youth May 4th Medal", saying that he will work harder to serve the exchanges between young people on both sides and promote the better integration of the two sides. In recent years, the Fujian Provincial Committee of the Communist Youth League has earnestly implemented the important arrangements of the Central Committee and the Fujian Provincial Committee on youth exchanges among Fujian, Taiwan, Hong Kong, Macao and overseas Chinese, and continuously deepened exchanges and exchanges among key youth associations, youth industries and youth representatives. Among them, from the exchange of learning, employment, the exchange and integration of young people across the Taiwan Straits from the aspects of exchange, employment, study and entrepreneurship. In order to adapt to the new changes of the situation, overcome the impact of the epidemic, deepen the brand, and ensure the "constant, constant, many", the Fujian Provincial Committee continues to hold the Straits youth forum, Straits youth Day and Straits youth festival; coordinate the cities and related universities, continuously promote the communication with important youth associations in Taiwan; take the focus of the Taiwan youth "entrepreneurship star" selection. Up to now, a total of 21 Taiwan youth individuals and 8 Taiwan youth collective have been awarded the "Fujian Youth May 4th Medal".

2021-11-17	Beijing, Nov. 17 (reporter Zhang Jianwei, correspondent Yue Qing, Guangdong) " I would like to become a glorious volunteer. I promise: do my best, without pay, to help others, serve the society, practice the spirit of volunteerism, spread advanced culture, and contribute to social progress!"On the afternoon of November 15, Clank oath echoed in the auditorium of Qingyuan International Convention and Exhibition Center. By the Guangdong provincial party committee, Guangdong civilization, Guangdong agriculture, development and reform commission of Guangdong province, Guangdong education department, Guangdong province, Guangdong departments, Guangdong province human resources and social security hall, Guangdong agriculture hall jointly organized the 2021-2022 annual Guangdong college graduates volunteer service rural revitalization of action ceremony (hereinafter referred to as "volunteer service rural revitalization action") held in Qingyuan city. Group Guangdong provincial party committee secretary Chi Zhixiong, Guangdong province volunteer federation Gu Zuyi, Qingyuan ZhaiXuemei, deputy secretary of the municipal party committee, Guangdong agriculture and rural hall secondary inspector Song Zongyue main undertaking unit leadership, as well as Qingyuan city volunteer service rural revitalization action volunteer, vocational education city of Guangdong province college students volunteers to attend the event. The representatives of the volunteers who are about to go to the countryside spoke. The ceremony. In addition to the main venue in Qingyuan, 11 cities in the eastern and northwestern regions of Guangdong and Zhaoqing and Huizhou set up sub-venues. A total of 1,000 newly recruited volunteers in the province participated in the activities online and offline. In the event of the main venue, all the participants watched the volunteer service rural revitalization action propaganda, the new volunteers to share the service rural revitalization as a life choice of beginner's mind and vision, and combined with the spirit of the sixth plenary session of the 19 and xi general secretary of the new era of Chinese youth affectionate message, expressed the nan yue youth will their own development and the integration of national destiny ambition and enthusiasm. The scene also awarded flags for 14 city service teams. Chi Zhixiong, secretary of the Communist Youth League, awarded the flag to the temporary Youth League Committee of the "Volunteer Rural Revitalization Action". It is reported that after the recruitment announcement of the "Volunteer Service Rural Revitalization Action" was issued, a vast number of college graduates warmly responded to it, a total of 7,515 people registered through the "i volunteer" system, after examination, interview, physical examination, publicity and other links, more than 1,000 people to be admitted. Among the volunteers to be enrolled, 90.8 percent of eastern and northwestern Guangdong accounted for 90.8 percent. With the good wish of building their hometown, many volunteers participating in the "Volunteer Service Rural Revitalization Action" took this journey to serve the countryside as the origin of their dream, and also the starting point for them to inherit their original mission and shoulder the important responsibilities of The Times bravely. Long Huiying, a girl from Liannan Yao nationality in Qingyuan who graduated from Zhanjiang University of Science and Technology, has witnessed the transformation of her hometown since the implementation of the rural revitalization strategy. She resolutely quit her job and firmly joined in the volunteer service of rural revitalization action.site of activity."I want to be the last kilometer problem of logistics distribution in my hometown."Li Yihao, a master's degree in logistics engineering and management at Zhejiang University, is a native of Yunfu. She hopes to play her major to build her hometown during her volunteer service. According to the organizers, starting from November 12, the 2021 "Volunteer Service Rural Revitalization Action" will hold pre-job training for 1,000 volunteers, and organize volunteers to deeply study the spirit of the Sixth Plenary Session of the 19th CPC Central Committee. At the same time, in order to further member volunteer contact service and education management, approved by the provincial party committee study, set up volunteer service rural revitalization of volunteer service temporary youth corps committee, at the same time, Guangdong project office will also promote qualified service set up temporary party branch, further condensed the vanguard power of the volunteers.

2021-11-16	The CPC Gansu Provincial Committee conveys the spirit of the sixth Plenary Session of the 19th CPC Central Committee) On November 15, the CPC Gansu Provincial Committee held a special meeting to convey the spirit of the sixth Plenary session of the 19th CPC Central Committee. Dong Anning, secretary of the Gansu Provincial Party Committee, led the communique of the plenary session, conveyed the spirit of the relevant meeting, and arranged the provincial Communist Youth League to study, publicize and implement the spirit of the plenary session. The meeting stressed that the Gansu communist youth league organization to further enhance the consciousness of "four", "four confidence", "two maintenance", keep in mind the party's assistant and reserve glorious responsibility, the thought and action to the central plenary session spirit, will implement the spirit of the sixth plenary session of the 19 as the current and future a period of the provincial communist youth league, youth league, young pioneers, the primary political task, rapid learning boom. The meeting required that the leading organs and cadres of the League should lead and set an example; the provincial Communist Youth League, youth league, student union and young pioneers organizations should closely combine the characteristics of teenagers, innovate ways, activate the carrier, pragmatic, systematically, and promote the spirit of the "study party history, strong faith, follow the Party" learning education, guide the League members to study the resolution as the outline, further grasp the major historical conclusion of the Party history, and constantly enhance the rational recognition of the Party. It is reported that the Communist Youth League Gansu Provincial Committee will also promote the work of the Communist Youth League, adhere to the spirit of the plenary session as the guidance, unite and lead the youth league members in the rural revitalization, ecological civilization, ethnic unity, social governance and other central overall situation to contribute to the youth strength. The meeting deliberated and adopted the Gansu Communist Youth League to study, publicize and implement the spirit of the Sixth Plenary Session of the 19th CPC Central Committee and the Gansu Provincial Communist Youth League Committee to study, publicize and implement the spirit of the Sixth Plenary Session of the 19th CPC Central Committee. Members of the party secretaries and responsible comrades of the provincial departments attended the meeting as non-voting delegates.

2021-11-16	The development process of young makers with "magnetic suction" has been established in the company and established as a logistics base."The Shunhang Truck App of Li Zhiqiang's team entered Jilin Youth Entrepreneurship Park after winning the App group champion of" Innovation Youth " China Innovation and Entrepreneurship Competition. The Pioneer Park not only helped him get 20 million yuan of financing, but also provided one-to-one help in financial and legal aspects for him to connect with well-known accounting firms. At the beginning of this year, Jilin Province issued several Policies and Measures to Stimulate Talent Vitality and Support Talent Innovation and Entrepreneurship, to attract talents with more active, open and effective talent policies. Only around the center can we find the right direction, and only by serving the overall situation can we reflect the value. The Jilin Provincial Committee of the Communist Youth League is committed to creating the whole chain of employment and entrepreneurship services for the returning and returning youth, attracting the white mountains and pine water, and helping the revitalization and development of Jilin. Jilin Youth Pioneer Park, located in Changchun Jingyue High-tech Development Zone, is the only national science and technology business incubator independently built by the Communist Youth League in China. On the eve of this year's May 4th Youth Day, Jing Junhai, secretary of Jilin Provincial Party Committee, visited Jilin Youth Pioneer Park for investigation, stressing that the Communist Youth League organizations at all levels in the province should play a good role as a bridge and link, further stimulate the innovation and entrepreneurship vitality of young talents, and create superior conditions for their grow up. Jilin youth pioneer park has been upgraded to version 3.0, to park service institutionalization, project content listing, flow chart, double gen policy manual, business to template "five" form, in rent-free, bag in preferential policies, for green and enterprise to provide project financing, human talent, fiscal and tax consulting, legal aid and other nine categories of 62 services. Since the establishment of Jilin Youth Pioneer Park, 117 enterprises have enjoyed the entrepreneurial services organized by the Communist Youth League, covering e-commerce, Internet of Things, equipment manufacturing and other fields, and have 167 effective intellectual property rights. High-tech enterprises, small giant enterprises and specialized and new enterprises are emerging and have grown into emerging forces to the revitalization and development of Jilin. Jilin Youth Pioneer Park also favors resources, provides models and exports experience to all cities and states in the province, driving youth league organizations at all levels to build 37 Jilin Youth Pioneer Park (demonstration zones) with a total incubation area of 572,400 square meters, forming a green brand incubation network system covering the whole province with its own characteristics, complementary functions and standard management. In order to enable more start-ups to enjoy the same incubation policies as the incubators in the physical entrepreneurship park, the virtual service platform of "Jilin Youth Entrepreneurship Cloud" came into being, and the incubation network of "mass entrepreneurship and innovation" tends to improve, and the power of backflow is gradually demonstrated. With the continuous improvement of the whole chain service of "mass innovation incubation acceleration" organized by the Jilin Youth League Organization, more and more young entrepreneurs in first-tier cities such as Beijing, Shanghai and Hangzhou return to Kyrgyzstan with their entrepreneurial teams to start their own businesses. Wang Guancheng, the founder of "Ten Wood E-commerce", is one of them. He attracted 13 like-minded e-commerce youth from Shanghai and Hangzhou to start their own businesses together."I am willing to return to my hometown, not only because I want to see the development potential of Jilin's e-commerce field, but also because I can find a pioneer park with high standards of hardware facilities, professional services and a strong entrepreneurial atmosphere."Wang said the atmosphere will help protect start-ups to focus on expanding their business. At present, "Ten wood E-commerce" has grown into a comprehensive e-commerce enterprise integrating e-commerce operation, data management and e-commerce training. Xu Meng, secretary-general of Jilin Youth Entrepreneurship and Employment Foundation, said that the Jilin Provincial Committee of the Communist Youth League takes the pioneer Park as the carrier to carry out regular "mass entrepreneurship and innovation" activities, such as road shows, information meetings and exchange meetings, to create an atmosphere of "innovation and entrepreneurship, youth first". To accelerate the development of enterprises, Jilin Provincial Youth League Committee also established Jilin Youth E-commerce live broadcast selection Center, gathering 636 agricultural products from various cities and states of Jilin Province, and signed cooperation agreements with 117 national well-known anchors and 14 national well-known e-commerce enterprises to jointly promote the sales of Jilin characteristic products and the development of e-commerce."The Jilin Youth League organization focuses on serving the whole chain of entrepreneurs rather than providing nanny-style support."Xu Meng said that it is to build a entrepreneurship platform for enterprises, providing a complete set of youth entrepreneurship and employment service system, such as policy guarantee, capital docking and capacity improvement. Gathering talents "is" in Jilin around Jilin province talent strong province strategy, Jilin provincial party committee will combine social practice and youth employment, with the help of the communist youth league brand activities "home", and college students and universities docking, guide the youth league organizations actively coordinate local resources, gradually expand the scale of supply, improve the quality of jobs. Up to now, the youth league organizations at all levels in the province have developed 13,800 positions of various types, and selected 11,000 Jilin college students to work. Jilin Provincial Communist Youth League Committee thoroughly implemented the college students internship "sail plan", The provincial Department of Human Resources and Social Security jointly built 399 "youth employment internship bases", Development positions of 3,885, Mobilize 2,572 college students, especially those who are about to graduate, to participate in the summer employment practice; Jointly with the provincial Department of Education to jointly carry out the "thousands of schools and thousands of posts" special recruitment activities, In the first half of this year, 78 special job fairs were held, Of the 11,900 participating enterprises, Offering 279,000 jobs, 74,000 people participated; Actively organize and mobilize youth league cadres at all levels to pair up one to one with graduates from poor families to carry out targeted employment assistance work, A total of 4,850 graduates from poor families have been contacted and helped to find jobs. In order to guide the development of high-tech talents in the province, Jilin provincial committee also cooperated with the Chinese Academy of Sciences "auspicious light plan" scientific research practice, attract young and middle-aged teachers and college students to declare scientific research projects, and training in scientific research practice, optimize the policy, attract them to stay in Jilin after graduation employment; implementation of "engineer training" plan, recruit 91 universities and enterprise practice, 63 people signed an agreement for employment. The activity of "100 universities enter Baishan", which has been carried out for many years, is an important activity carried out by the Youth Development Department of the Central Communist Youth League, and an active exploration of a new mode of cooperation between the local government and the Central Committee of the Communist Youth League and universities. In recent years, nearly 5,000 young students from 425 practice teams from 100 universities nationwide have carried out practice activities in 503 villages in 47 towns and townships, injecting fresh blood and inexhaustible impetus into the revitalization and development of Baishan. In Yanbian Korean Autonomous Prefecture, the implementation of the six-year "talent introduction" project has become the brand work of the Communist Youth League. The person in charge of the school department of the Yanbian Prefecture Party Committee of the Communist Youth League introduced that, in Beijing, Shanghai, Qingdao, Seoul and other 8 areas, etc., we established the Yanbian college Students Alliance, and set up a comprehensive service exchange platform for Yanbian college students and international students. With the help of this platform, the Youth Yanbian Korean Autonomous Prefecture Committee holds a series of activities of the "talent Recovery" project every year to promote the Yanbian and returning home for employment and entrepreneurship policies. Wang Peilian, Wang Shanshan, Du Yimeng source: China Youth Daily, November 16,2021

2021-11-13	"Literature and Art Dinner" was sent to Anhui Medical University. The "Cultural Masters" and "Intangible Cultural Heritage" into campus " activities co-sponsored by the Education and Working Committee of Anhui Medical University, the Provincial Federation of Cultural Arts and the Provincial Communist Youth League Committee entered Anhui Medical University. Event, Anhui province association, artists, music, dance, association, film and television association team into the college students, combined with university characteristic community activities, hold disease resistance theme exhibition, and painting, music, dance and other volunteer tutoring activities, and opera film and television art popularization, patriotic film public screening and other activities. Anhui Intangible Cultural Heritage Research Association organized representative inheritors of national and provincial intangible cultural heritage projects such as colored pottery firing, paper paper processing and Luzhou nuclear carving to display their representative works and traditional skills, guiding students to have hands-on experience, so that teachers and students can feel the charm of intangible cultural heritage at close range. In the evening, a wonderful artistic performance was also held, and the well-known opera, song and dance, quyi, acrobatics and other artists, non-genetic inheritors and the teachers and students of Anhui Medical University jointly presented a high level of "literary feast".

2021-11-09	Tianjin: Let the grassroots youth league organization "strengthen the physical fitness" when the Communist Youth League grassroots reform to gather the youth, serve the youth, gather the youth Tianjin: Let the grassroots youth league organization "strengthen the physical fitness" work foundation. This year, the Tianjin Communist Youth League focuses on the key problems and deep-seated problems in the grass-roots construction of the league, pays close attention to the style construction of grass-roots organizations, and unblocked the "nerve endings" of the league. In Tianjin Binhai New Area, located on the coast of the Bohai Sea, the strategic emerging industries have attracted a large number of young talents to gather here. The Youth League Binhai New Area Committee has steadily promoted the Haihe "green" listening and practice activities. Research and statistics show that there are more than 15,000 non-public enterprises in the region, with more than 400,000 employees and more than 540 social organizations. With a large number of migrant young people and strong mobility, urban integration, partnership and social education have become the "short board" in youth work. In an area of more than 2,000 square kilometers, the youth league organization is facing the problem of "more false points, long lines, and incomplete coverage". Based on the actual regional situation, the Binhai New Area Committee of the Communist Youth League has explored the working method of "one main line, six starting points and three platforms", weaving a dense three-dimensional network to shorten the distance between grass-roots league organizations and youth league members. The district has established the "two new" league working committee, youth League general branch and joint league branch at the district level, street town, functional areas and village residence. Full-time and part-time league cadres at all levels have entered the "two new" league organizations to realize the refinement, standardization and informatization of service management. The city's first Internet industry youth League working committee was established not long ago. In view of the characteristic development needs of young people in 16 Internet enterprises, the Communist Youth League Working Committee took the initiative to talk with them through new media, carrying out network security vocational skills competition, party history learning and education targeted run, youth friendship and other characteristic activities, to gather the youth group. Combined with the scattered situation of the area, the high-tech zone divides 3 parks into 19 areas, each area with one liaison, who is responsible for contacting 30-35 enterprises, so as to realize the grass-roots group service management grid combining blocks, horizontal to the edge and vertical to the end. Zhang Jian, a youth liaison officer, found that some young people in the Internet industry have a common demand- -dating. Therefore, the league organization introduced a professional dating platform, and carried out a series of online and offline single youth dating activities. In order to gather the young people scattered in various parks and buildings together, the Youth League Binhai New Area Committee has also explored a new path for leading enterprises to drive the parks, industrial chains, partners, buildings and large talent agencies. The Economic Development District Youth League Committee of FAW-Volkswagen Tianjin Branch took the construction of the youth league organization as the sample to promote the upstream and downstream enterprises and human resources companies to establish the youth league organization, and also solved the problems of the transfer and management of the youth league organization relationship in the form of labor dispatch. Now, the action to promote the full coverage of emerging areas has been fully rolled out in 16 districts and counties in Tianjin. The "two new" organizations were established in transportation, Internet, ride-hailing, express delivery and catering service; the non-public enterprises in the city reached 97.5%; the social organization density in the city increased from 45.6 to 63.1 per 10,000 young people. Strengthening the party and the league, the coverage of the league organization is not extensive, the position of the league cadres is not sufficient, the effectiveness of the league cadres team training gap... in the annual party building with the league building work conference in Tianjin Beichen District, the then deputy secretary of the Beichen District Party Committee mercilessly pointed out the outstanding problems existing in the league organization. Under the audience sat the town and street party (work) committee secretary, deputy secretary, the district direct unit party organization in charge of the leadership, the village residence party organization secretary and the district at all levels of the youth league organization secretary. Xu Yan, secretary of the Beichen District, said that facing the problems can accurately solve the main contradictions and prominent problems that restrict the development of the league organization. It is closely rely on the party and the league to build a chess game, Beichen District from the regulation system, so that the work has rules to follow. Around the various problems exposed by the named grassroots organizations, by the grassroots party committee lead the rectification, actively docking the district committee office, the district committee organization department, the district committee inspection office, the communist youth League work into the district governing party main responsibility assessment, and into the performance appraisal; and give sufficient funds, namely the town and street annual work funds are not less than 50,000 yuan. At present, these prominent problems are being targeted rectified and improved, and phased progress has been made. Supported by the district party committee organization department, beichen district issued the "party with" party "push the party" and "green horse engineering training" and a series of documents, from high standard, high quality with qi village, community organizations, to strengthen the party's leadership of youth work, improve the youth league organization construction, strengthen league cadre team construction, completes key areas and optimize security mechanism 5 aspects of 13 working mechanism, promote the sustainable development of youth league. This is an epitome of the comprehensive promotion of the grassroots Party building work in Tianjin. At present, the 16 districts of the city have completed the league building work into the district-level party building assessment, performance assessment, or the implementation of the main responsibility assessment of strict party governance, and clearly assigned points, to achieve the grassroots party building more accurate and powerful. Since September, the Xiqing District Committee of the Communist Youth League has launched the "Young Talent Volunteer Plan" to explore, cultivate and absorb a large number of young volunteers with professional professional ability and comprehensive quality ability. This is a brand new attempt of regional group building work. Li Ying, party secretary of the League Xiqing District, said that how to gather a number of universities, public institutions and industrial parks to help a large number of highly educated young people find a path of development is a problem that the league organization has been thinking about. Haihe "Qing" found that many young people have enthusiasm and dedication, but can not find a platform to display their talents; at the same time, the professional professional requirements, but can not find suitable talents. Relying on the Regional Youth Working Committee, the Communist Youth League of Xiqing District has sorted out the needs of professional volunteer service talents in related fields, focusing on the central work of regional development, such as rural revitalization, investment attraction, scientific and technological innovation, and elderly care service promotion. On this basis, the youth league organization through cooperation with local universities, municipal organs, state-owned enterprises, social organizations and other targeted recruitment of young volunteers, and deepen youth league education cooperation, league cooperation, youth contact, establish a talent pool, as far as possible to provide a platform for young people to display their talents and make contributions. The stories of revitalizing regional high-quality resources, finding and retaining youth with brand activities can be seen everywhere in the youth homes throughout the city. Liu Juan, a post-85 young man, is a young parent of the Four Seasons Flower City community. In her spare time, she will go to the Youth home to tell stories to the children in the community. Like her, many of the volunteers at the Youth Home are young parents. Five years ago, the volunteers set up the "Flower City Story Club" charity club, and actively contacted the young volunteers from Tianjin University and Tianjin Normal University to join us. Today, it has become a popular "weekend children's story meeting" among residents. More and more young volunteers join in it, and the activity content is also extended to cultural exchange lectures. In order to form a long-term mechanism for regional league construction, 244 streets and towns have been set up in 16 districts and regional youth working committees, and established discussion and consultation, resource integration, work linkage and information sharing mechanisms. Li Long, party secretary of the Tianjin Youth League, said that the ultimate purpose of changing the situation of the league organizations fighting alone, linking the most extensive resources, and making the grassroots league organizations "strengthen the body", and the ultimate goal is to gather, serve and gather young people more effectively. Source: China Youth Daily, November 09,2021

2021-11-08	Zhang Wenling, the initiator of the ninth "Yunnan Youth Entrepreneurship Governor Award" selection activity.) The ninth "Yunnan Youth Entrepreneurship Governor Award" selection activity has been launched recently. Leaders of start-up enterprises or teams aged 18 to 45 in Yunnan province with industrial layout, forming a certain economic scale and influence through innovation and entrepreneurship, and assuming effective corporate social responsibility can apply for the evaluation."Yunnan Youth Entrepreneurship Governor Award" is the highest honor of youth entrepreneurship activities in Yunnan Province, and the selection activity has been successfully held for eight times. More than 20,000 young entrepreneurs have signed up to participate in the competition, with a total of 80 "Yunnan Youth Entrepreneurship Governor Award" and 146 nominated awards, exerting a wide and good influence in Yunling. The "Yunnan Youth Entrepreneurship Governor Award" was jointly organized by 17 units, including the Yunnan Provincial Party Committee of the Communist Youth League and the Provincial Department of Human Resources and Social Security. Finally, 10 people will be awarded the "Yunnan Youth Entrepreneurship Governor Award" and 20 people will be nominated. At the same time, the organizing committee will select 1-3 special prizes from the 10 main prize winners. Unlike previous years, the selection for the first time increased the final evaluation link, will take the form of project roadshow, enterprise display, field scoring, organize relevant experts and scholars, outstanding entrepreneurial youth representatives, well-known enterprises, financial institutions, venture capital institutions, etc, for the winners to show their corporate image, spread entrepreneurial ideas, promote the entrepreneurial spirit platform, from the aspects of policy, intelligence, finance for entrepreneurial enterprises continue to fu can. At the same time, will combine the actual eligible object, through the review of entrepreneurial individual (team) and enterprise policy support, accurate project support, and in the resources docking, platform building, mentor pairing, communication and learning, etc, boost its further growth development, strive to focus on building a batch of YunLing "little giant" enterprise, play "Yunnan youth entrepreneurship governor award" demonstration role.

2021-11-07	Hebei: Create good conditions to support youth growth Hebei: Create good conditions to support youth growth Youth League Provincial Party Committee pilot reform the operation mechanism of county youth work to create good conditions to support youth growth Luanzhou city invested 1.13 million yuan to build a youth entrepreneurship and innovation base- -youth entrepreneurship center, Attract 16 young small and micro enterprises to settle in for free; Dachang Hui Autonomous County has implemented the "youth-friendly" city strategy, Construction of youth parent-child park, urban youth sports park and other facilities, Supporting young talents settlement, medical education, entrepreneurship and employment and other measures; Wu'an city in the main city to build the first youth square in the local; Guangyang District of Langfang City, Qiaodong District of Zhangjiakou City and many other places have launched the "Huiqing Card"... in the past two years, Hebei province 4 medium-and long-term youth development plan national pilot, 12 provincial pilot counties (cities, districts) first trial, Adhere to the Party's philosophy that it gives priority to youth and youth development, Find out the most pressing needs of the youth, Create a good environment and conditions for young people to grow up and make contributions. We will care about the growth of young people and support their development. In order to promote the implementation of the first youth development plan in the history of New China in Hebei, the Provincial Communist Youth League Committee adheres to the pilot first, and according to the requirements of "planning, mechanism, policy, project, guarantee and implementation", reforms the operation mechanism of county youth work, and creates a sample of grass-roots youth development work in line with Hebei characteristics. Specific implementation, the league provincial party committee to guide the pilot county (city, area) in the name of the party committee, government issued the construction plan, led by the party committee, government mainly pilot work leading group, standardize the county youth work joint meeting, support pilot county (city, area) in accordance with the standard will work special youth development funds in the annual budget, promote the "party leadership, government responsibility, the communist youth league coordination, all aspects should" work pattern. In addition, give full play to the functional advantages, regularly hold the provincial joint meetings, and guide the provincial member units to give priority to the policy resources to the pilot counties (cities, districts). In the national pilot of Handan Fengfeng mining area, the medium-and long-term youth development plan, the youth development work has been put on the important agenda of the district Committee and the district government. The Standing Committee of the District Committee regularly listens to reports and conducts key supervision. Today, Cizhou Kiln youth e-commerce town helps young people to solve the problem of entrepreneurship, injecting new momentum into rural revitalization; Cizhou Kiln "mobile small classroom", covering 26,000 young people.(Correspondent Zhang Yili, reporter Xue Huijuan)

2021-11-16	Group secretary of Henan provincial party committee office (expanded) meeting to convey learning the spirit of the sixth plenary session of the 19th study propaganda implement work group secretary of Henan provincial party committee office (expanded) meeting communicate study the spirit of the sixth plenary session of the 19 study deployment propaganda implementation work zhang) on November 15, the secretary of Henan provincial party committee office (expanded) meeting, convey learning the spirit of the sixth plenary session of the 19, study deployment propaganda implementation work. Wang Yi, secretary of Henan Provincial Party Committee of the Communist Youth League, presided over the meeting and delivered a speech. Wu Hao, Wang Dubo, Yuan Hao, deputy Secretary, Wang Xin, Han Bing attended the meeting. The main responsible comrades of all departments and directly affiliated units attended the meeting. Meeting that the sixth plenary session of the party's 19th is in the important historical moment of the founding centenary of our party, the party and the people to achieve the first goal in one hundred, build a well-off society in an all-round way, is toward build comprehensive socialist modernization power of the second goal of major historical moment, held a great practical significance and far-reaching historical significance of important meeting. The Resolution of the CPC Central Committee on the Major Achievements and Historical Experience of the Party's Centennial Struggle, deliberated and adopted by the plenum, As the third historical resolution in the history of our Party, Systematically summarize the great path, great cause, and great achievements of the Party at all stages of history over the past one hundred years, Concely put forward the five historical significance of our party's century-old struggle, Deeply reveal the 10 historical experience of our Party over the past century, To solemnly declare that our Party unites and leads the Chinese people to learn from history and open up the future, The firm confidence and determination to forge ahead on the new road to achieve the second centenary Goal, Is a link between the past and the future, A programmatic document of great political value, theoretical value, practical value and milestone significance. The party established comrade xi, the core of the CPC Central Committee, the core of the party, establish xi jinping, the guiding position of the ideas of socialism with Chinese characteristics, reflects the whole party, including hundreds of millions of teenagers, the Chinese people of all ethnic groups common wish, to the new era of the historical process of the party and the national career development, to promote the great rejuvenation of the Chinese nation is of decisive significance. The meeting pointed out that we should grasp the core essence, and earnestly unify the thinking, will and action into the spirit of the Central Plenary Session. Deeply understand the significance of the central plenary session, fully understand the party deeply grasp the law of historical development, always grasp the history of the development of the party and national career initiative and mission, fully understand the party based on the present, for the future, pay attention to summarize and use historical experience of vision and foresight, from the party's one hundred struggle why we can succeed, understand how we can continue to succeed, to more firm we in the second goal in one hundred of the new exam confidence and determination. Deeply understand the major achievements of the central plenary session, learn deep enlightenment through the central committee of the communist party of China's struggle in one hundred major achievements and historical experience resolution, fully understand the history of the historical penetration, thought leading force, political mobilization force and era appeal, fully understand the decisive significance of "two established", fully understand the "ten insist" is the spirit of the party and the people to create wealth, to guide us on the new journey with indomitable struggle attitude. Deeply understand the important requirements of the central plenary session, based on the "two big situation", with "big", strengthen the consciousness of "four", "four confidence", "two maintenance", consciously with the central plenary session spirit unified thought, unified will, unified action, to achieve "two ensure" goals, strive to compose a new era of central plains more brilliant contribution of youth power. The meeting stressed that we should strengthen the original mission and shoulder the historical responsibility of stabilizing and mobilizing the province to make contributions to the new era. Carry stability for the historical mission of the party education, always remember in one hundred party history is a generation of aspiring youth with the struggle of youth history, make clear historical logic, with good reality teaching material, innovative education way, leading the provincial youth unswervingly listen to the party, follow the road of socialism with Chinese characteristics, for the party continuously when the era of national rejuvenation. Carry steady youth achievements modernization of Henan historical mission, around Henan party congress "two ensure" "top ten" strategy and message to the province youth, combined with Henan "difference" planning "perfect youth development policy system" special policy support, further enhance the consciousness of answer, know heavy load, dare to good, lead the province teenagers consciously personal ideal pursuit into the cause of the party and the country, in the realization of "two ensure" vivid practice. Carry steady construction of modern youth organization historical mission, standing next group 100th anniversary of historical node, summarizes the historical experience of the party's youth movement, promote the reform of the communist youth league, further positioning in the modernization of the national management system and management ability, always grasp the three fundamental problems, anchor enhance political, advanced, mass work direction, perform good lead condensed youth, mobilize youth, contact service youth three basic functions, grasp the new development stage, implement the new development concept, into the new development pattern, strive to forge and modern modern youth organization. The meeting called for doing a good job of overall planning and completing the political task of studying, publicizing and implementing the spirit of the sixth Plenary Session of the 19th CPC Central Committee. Overall pays special attention to the convey learning, and further implement the general secretary xi "July 1" important speech spirit, and further implement xi general secretary visited Henan important speech important instructions, and further promote party history learning education, and further implement the spirit of the 11th party congress in Henan province, through theoretical study group, five classroom forms, promote city organization and industry committee take the lead, pays special attention to the green horse project key groups, speed up the first youth research studio construction, promote the central plenary session spirit into training, into teaching materials, into the mind, further learn deep enlightenment, go deep go real. To coordinate the publicity and publicity, the new media matrix should be fully launched, adhere to the concept of mass differentiation and immersive communication, make cultural products popular with young people, improve the ideology, pertinacity and effectiveness of the products, strengthen the linkage communication, and do a good job of fission publicity and promotion. Timely to carry out offline publicity, into the campus, into enterprises, into the countryside, into communities, into the organs, into the youth home. Timely organize practical education for young people, continue to carry out good youth search, red scarf search and other key projects, achieve theory and practice. We will comprehensively implement the theme activity of "Stepping into a Brilliant New Journey and building a New Era of youth", and translate the spirit of the Plenary Session of the CPC Central Committee into practical results in doing all our work well. Strengthen the political construction of the Communist Youth League in the province, pay attention to draw spiritual power from the centenary history of the Party, to carry out a good ability and style construction year as the guarantee, to ensure that General Secretary Xi Jinping's important thoughts on youth work and the important speeches and instructions of the inspection of Henan are implemented, implemented and effective in the Henan Communist Youth League. Pay special attention to the Henan province party congress report target tasks and the 15th in Henan province league congress preparations, pays special attention to the party history learning education work under the 100th anniversary of the work, as a whole the sprint annual target tasks and planning next year, to maintain castle peak not relax persistence and line thyme half ninety awake, lead the entire province teenagers with high style and excellent performance to meet the party's 20th victory. At the meeting, arrangements were also made for the reform of the Communist Youth League at the county level, deepening the work of the Young Pioneers, making regular epidemic prevention and control, and ensuring security and stability.

2021-11-15	Fujian in key cities, colleges and universities to build liaison service point for young talents in Fujian in key cities, universities to build liaison service point for young talents xi reporter Tian Hongwei) on November 10, the Fujian provincial party committee in Fujian province in Shanghai jiaotong university, fudan university, tongji university, east China normal university four university talent workstation officially awarded. Many enterprises in Shanghai have been awarded the title of Fujian Young Talent Social Practice and Internship Base, providing high-quality internship positions for Fujian college students. These work comes from the excellent college student exchange activity of "Zhihui Fujian, Only gather in Fujian", which is jointly sponsored by the Fujian Provincial Party Committee of the Communist Youth League, The Talent Office of the Fujian Provincial Party Committee and the Industrial Bank. It is a supporting activity of the "Talent Fujian Week" in 2021. At the event, relevant departments and enterprises introduced the introduction and selection policies, employment and internship, enterprise recruitment and other contents to Fujian students. In recent years, the Fujian Provincial Committee of the Communist Youth League, relying on the Foreign Affairs Office of the Provincial government and the Working Committee of the Communist Youth League abroad, has built innovative platforms such as the Fujian Provincial Young Talent Work Liaison Office, "double first-class" university workstations and social practice base for young talents in some key cities nationwide. Through regular theme activities such as "Talent Fujian Week" and "Youth Sharing and Collection", we will promote Fujian talent introduction policies and build a liaison and service bridge for college students from outside Fujian. By organizing a series of tracking and training measures, such as visiting studies and exchange projects, social practice, scientific research project declaration, transformation of scientific and technological achievements, and innovation and entrepreneurship support, it provides support and guarantee for the growth and development of outstanding young students from outside Fujian, and attracts more young talents to find jobs and start businesses in Fujian. Up to now, the Fujian Provincial Committee of the Communist Youth League has set up liaison offices for Fujian Young Talents in Beijing and Shanghai, and set up workstations in 11 "double first-class" universities. In the future, Fujian young talent work liaison offices and university workstations will be set up in key cities including Tianjin, Wuhan, Xi'an and other key cities.

2021-11-06	"Zhejiang tide sound" online micro group class show activities start "Zhejiang tide sound" online micro group class show activities start clerk YuXinJi, in the green newspaper network reporter Jiang Yutong) on November 5, by the communist youth league party committee of Zhejiang province, Zhejiang daily newspaper group jointly organized "Zhejiang tide sound" Zhejiang youth online learning platform online micro group class show activities, in Zhejiang news client officially launched. The "Zhejiang Chaoyin" online learning platform will broadcast excellent micro-group class videos from youth league organizations and young lecturers at all levels in Zhejiang Province every day. As long as teenagers log in to the Zhejiang News client and complete the organization information input, they can demand video online and conduct independent learning. The platform also has interactive sections such as daily answers and likes for your favorite videos. New online "Zhejiang tide sound" Zhejiang youth online learning platform, actively explore the digital reform scenarios, and is developing in Zhejiang province common rich youth preach action, innovation opened "youth lecturer" platform, invited province, city, county level 3 communist youth league youth lecturer, form a new era "Zhejiang tide sound" Zhejiang youth branch matrix. At the same time, the platform opens the online application channel, and the young people who are interested in participating in the party's innovative theory propaganda can apply to join the corresponding level of youth lecturers online. The "Young Lecturers" platform will also develop functional modules such as online invitation and video on demand to realize the transformation of traditional theoretical learning from "what to have and what to listen to" to the menu of "what to listen to and what to choose", and create a new mode of online booking and offline sharing. By expanding the team of lecturers, enriching the types of courses, breaking down the hierarchical barriers, and realizing the joint construction and sharing of youth preaching teachers and courses, the "Zhejiang Chaoyin" online learning platform can truly become a service and growth platform for teenagers in the province to learn theories, expand their horizons and increase their knowledge. In the next step, the organizer will also hold the "Zhejiang Tide sound" boutique micro group class selection and competition, so that the "Zhejiang Tide sound" new era of Zhejiang youth teaching group from online to offline, so that the Party's innovative theory into thousands of households.

2021-11-05	Xing Ting) Today, the Shandong Provincial Government Information Office held a press conference to introduce the employment situation of 2021 college graduates in Shandong Province. Shang Shengbo, deputy secretary of the Shandong Provincial Party Committee of the Communist Youth League, said that since November 2019, the Shandong Provincial Party Committee and the Shandong Provincial Human Resources and Social Security Department jointly launched the "Green Bird Plan", pooling resources from cities, counties, enterprises and universities, and taking multiple measures to serve college graduates and escort their "stable employment". It is understood that the "Green Bird Plan" by building a docking bridge, accurately serve the employment of college graduates. Focusing on key enterprises, and making overall use of resources such as the Youth Federation, the Youth Enterprise Association, the "Youth Enterprise Summit" and other resources, the provincial, municipal and county level youth league organizations are working hard to explore high-quality jobs, precise promotion and matching, and send the jobs with good treatment and great potential to college graduates. At the beginning of this year, by conducting "green bird plan choose" Shandong employment service season, accurate MoPai local pillar industries, iconic enterprise demand of choose and employ persons, build enterprise and college graduates negotiate docking platform, one by one opened the provincial and 16 city special "live hall" "cloud interview", released 179000 high quality jobs, offline with post into directional recruitment, promote talent policy, for more than 4700 enterprises to attract talents, to 36700 employment intention, promote local industry demand and graduates demand "two-way docking". The "Green Bird Project" focuses on extending the service chain and building a service carrier. This summer, the Shandong Communist Youth League launched the "Green Bird Program Practice Season", attracting 21,500 prospective graduates to practice in more than 3,200 government institutions and local landmark enterprises in Shandong province. 594,000 college students participated in rural revitalization, social governance, epidemic prevention and control and other social practices at the grass-roots level. It is worth mentioning that the "green bird plan" also promote tsinghua university "rural revitalization" workstation in Weihai, Jining, establish 66 well-known universities such as Beijing university "green bird station", condensed in Shandong students, talents, more than 8500 people, normalized push employment information, talent policy, organization "perception" "home" communication activities such as 212 games, more than 8000 graduates field perception in Shandong. In addition, the "Green Bird Project" focuses on the construction of "youth post stations" by expanding employment services. Focusing on solving the problems of job-seeking college students and unemployed young people in job hunting and job docking, the "Youth Station" project in Shandong. As provincial party history learning education "I do the practical work for the masses" practice is one of the people's livelihood projects, Shandong communist youth league give full play to the advantages of social mobilization, mobilize the youth league, green enterprises to support the construction, high standard construction covering 16 city 136 counties (city, area) 228 "youth station", to provide seven days free accommodation, policy interpretation, job promotion, skills training, social integration "one-stop" service, has served more than 3800 people."Next, the provincial party committee will be in accordance with the provincial government unified deployment, around the general secretary of the three leading development requirements in our province, system to promote the blue bird plan, depth into the" entrepreneurial qilu plan "and" entrepreneurial qilu happy Shandong action, go all out to lu lu college graduates to build stage, provide services, more powerful youth development friendly city, power the new era of modern province construction."Shang Shengbo said.

2021-11-10	In Fujian province youth palace director and the young pioneers outside counselor training class in 2021, Fujian province under youth palace director and the young pioneers outside counselor training class held in taining Zhang Jianwei correspondent huang peng) on November 9, sponsored by the Fujian provincial party committee, Fujian province, league school, Fujian youth palace association and the young pioneers outside counselor training class held in taining. Youth League Provincial Committee, Youth League Sanming Party Committee, Taining County Committee, Taining County Datian Township Party Committee, Youth League Taining County Committee, provincial young working committee, provincial Youth Palace Association related responsible person attended the opening ceremony. Provincial youth palace association member list of the youth palace young pioneers workers, young pioneers counselors and other more than 100 people participated in the training. The meeting site. Correspondent for figure on KaiBanShi, provincial party committee, youth minister, provincial less working committee executive deputy director Shi Zhiyong around in-depth study and implement xi general secretary about children and young pioneers work important discourse, fully implement the opinions of the CPC central committee on strengthening the work of the new era (hereinafter referred to as the "opinion") spirit, the next stage of the province outside the young pioneers work, build a new era of Fujian young pioneers social system put forward guidance. He points out that the, The Opinions put forward clear requirements for the work of the young Pioneers in the new era and pointed out the way forward; To integrate resources to build Fujian young Pioneers social brand, Explore the formation of a joint construction mode of home, school, society and palace, Promote the organization and construction of the young Pioneers into the community, the young Pioneers 'guidance force into the community, the young Pioneers' independent activities into the community; To strengthen the construction of young Pioneers, such as the youth palace, The youth palace as an important fulcrum of the young Pioneers organization to the society, Promote the formation of a youth palace education model with Fujian characteristics; To strengthen the construction of the young Pioneers outside the school, Improve the county (city, district) after-school counselor resource database, Invite advanced figures from all fronts as off-school counselors, Provide a strong guarantee and strong support for the work of the Young Pioneers. It is reported that this training course takes the form of expert teaching, on-site teaching and group discussion, inviting after-school education teachers and industry experts to teach to the students, aiming to further improve the work level of the director of the youth palace and the young Pioneers 'external counselors, and promote the high-quality development of the young Pioneers' work outside the school. During the training, the second meeting of the eighth council of the provincial youth Palace Association will also be held.

2021-11-05	Hubei Province held green horse students symposium Hubei Province held green horse students symposium Yu) recently, Hubei Province held the "young Marxist training project" students symposium, Hubei Provincial Party Committee Standing Committee, provincial United Front Work Department Minister Er Kang Jiang Tulhong attended the meeting and speech. After listening carefully to the representatives of the students, he pointed out that the students of the "Yangma Project" love the Party and love the country, are practical and willing to contribute, which reflects the spirit and youth responsibility of the Chinese youth in the new era. He encouraged everyone to be firm in their ideals, to strengthen their belief in Marxism and communism, to strengthen believe in socialism with Chinese characteristics, and to enhance their confidence in realizing the great rejuvenation of the Chinese nation, and to be a young Marxist with ambition. To straighten the backbone, ceaseless red blood clank of the iron bone, strengthen the sonorous pride of unremitting self-improvement, forge persistent and tenacious tenacious muscles and bones, do the backbone of the young marxist. We must cultivate one's morality, be diligent in learning, be sensitive to virtue, be good at cultivating our moral nature, and be a confident young Marxist. Hubei provincial party committee, he said, attaches great importance to the young marxist training project, to take it as an important political task, outstanding for the party training and transport the youth political backbone function positioning, to build the training system, perfecting the training content, improve the management mechanism, constantly explore, the "green horse project" to do better and more solid. It is reported that Hubei province "green ma project" implementation for more than 7 years, has been divided into 8 periods of selection of more than 450 college students for centralized training, more than 160 students through the transfer (civil servants) recruitment to the grass-roots towns and party and government organs at all levels of work. According to the investigation and return visit of the Hubei Provincial Party Committee of the Communist Youth League, the qingma students have become the backbone of their work within 1 to 2 years because of their high political quality and strong adaptability, and have been highly praised. Source: China Youth Daily

2021-11-04	Human organ donation behavior in Guangdong province advocate volunteer service project start jianwei correspondent YueQing) recently, by the Guangdong provincial party committee, Guangdong civilization, Guangdong health committee, Guangdong Red Cross guidance "and I am willing to" -human organ donation behavior in Guangdong province advocate volunteer service project in Guangzhou. The project is committed to taking voluntary service as the carrier, to improve the correct understanding of human organ donation behavior from all walks of life, and to promote the development of human organ donation cause. More than 100 people, including Chi Zhixiong, secretary of Guangdong Provincial Communist Youth League, Gu Zuoyi, president of Guangdong Volunteer Federation and other unit leaders, volunteers and representatives of organ donation registration, participated in the activity."I am willing to" - -Guangdong Province human organ donation behavior advocacy volunteer service project was officially launched. Group of Guangdong provincial party committee for the figure to better promote organ donation behavior advocate volunteer service work, the first affiliated hospital of sun yat-sen university, Guangdong volunteer career development foundation decided to set up the project special fund, by the provincial volunteer enterprise development foundation invested 500000 yuan as a special fund startup funds, encourage units, enterprises, individual donated funds to develop organ donation behavior advocate volunteer service projects, for economically difficult organ donors family to provide the necessary humanitarian assistance and help. The Guangdong Volunteer Development Foundation and the First Affiliated Hospital of Sun Yat-sen University jointly set up a special fund for organ donation. The China Organ Transplant Development Foundation, together with Guangdong Volunteer Federation and Guangdong Volunteer Cause Development Foundation, also launched the recruitment of organ donation volunteers, and set up the registration entrance for human organ donation volunteers based on the "i Volunteer" system. Are willing to participate in organ donation voluntary registration, just focus on "i" volunteer "or" Guangdong "public, click on the public menu at the bottom of the number bar" volunteer "options, and then choose" organ donation voluntary registration ", can jump to small program registration, open volunteers and other masses to participate in organ donation voluntary registration channels. During the event, badminton Olympic champion Zhang Jiewen, table tennis Olympic champion Liu Shiwen, diving Olympic champion Xie Sisheng, and the host of Guangdong Radio and Television Station Li Xiao served as the ambassadors of the organ donation initiative, devoting themselves to the organ donation initiative and raising the public awareness of voluntary donation. As early as 2016, Zhang Jiemin has completed the voluntary registration of organ donation, becoming the 1,15,423rd organ donation volunteer from the China Organ Transplant Development Foundation. Guangdong is the main source of modern volunteer service in China. Up to now, more than 14.4 million volunteers have been registered through the mission of the "i-volunteer" system. Carrying out advocating voluntary services for human organ donation behavior, and vigorously publicizing and popularizing the knowledge of human organ donation, will help to increase the people's sense of identity with human organ donation behavior, and promote the social atmosphere and benign interaction of citizens' voluntary organ donation.

2021-11-03	On October 30-31, the ninth Jiangsu Academy of Juvenile Sciences was held in Wuxi Hechang Experimental Primary School. Eighty-eight young authors of outstanding scientific suggestions stood out and were appointed as the "academicians of the ninth Jiangsu Academy of Young Sciences", among which 10 young pioneers were awarded as the "Top Ten Little Academicians". On the scene, all the finalists elaborated on the topic selection background, research process, results and other contents of the scientific suggestions through the exhibition board and the display board. After the competition of expert judges' field inquiry and basic scientific knowledge test and other links, 88 young authors of excellent scientific advice finally stood out. Zhou Liang, deputy secretary of Jiangsu Provincial Party Committee of the Communist Youth League, pointed out that "small people do big things" is the glorious tradition of Jiangsu young Pioneers. As a young pioneers of Jiangsu in the new era, we should also learn and understand the grand strategy and policy of national construction. Held "small five-year plan-I for high quality development" scientific advice show and "small" review awards, is to answer xi grandpa expectations, by the example of "academician", big scientists, inheritance carry forward the "scientist spirit", determined to listen to the party, follow the party's determination, set up the "science and technology" ambition, growth from the ability of "building socialism". In may, since the "small five years plan-I plan for high quality development" scientific advice and "the ninth Jiangsu youth academy of sciences" appraisal activities launched, Jiangsu province youth around the "small five years plan-I offer for high quality development" this theme, based on the "difference" planning and 2035 vision, focus on a practical problem closely related to people production, life, actively conduct investigation and research, put forward a series of innovative value Suggestions, for the hometown of high quality development contribute their wisdom. The activity received more than 400 pieces of scientific and technological innovation works, and 88 first prizes, 110 second prizes, 155 third prizes were awarded, and 121 tutors were awarded as "Excellent tutors". After expert evaluation, 72 excellent scientific suggestion works were selected for the "academician of the ninth Jiangsu Academy of Young Sciences". The activity was organized by Jiangsu Provincial Party Committee of the Communist Youth League, Jiangsu Association for Science and Technology, Jiangsu Youth Working Committee, and organized by Wuxi Municipal Party Committee, Wuxi Association for Science and Technology, Wuxi Education Bureau and Wuxi Youth Working Committee. Party member of Jiangsu province association for science and technology, vice chairman Fang Sheng former group, deputy secretary of Jiangsu provincial party committee, deputy secretary-general cao, China shipbuilding industry corporation 702 institute "deep space station" structure group leader, national key research and development plan chief scientist, national super Wuxi center distinguished experts Zou Songming and other leading guests to attend the award ceremony.

2021-11-02	Hainan communist youth league held Hainan green farmers brand promotion communist youth league brand seminar Ren Mingchao) on October 30, Hainan provincial party committee in China (Hainan free trade port) youth dream avenue youth public welfare center held a free trade port youth agricultural products brand promotion, lasted nearly three months of "youth chapter dream future" in 2021 Hainan free trade port youth agricultural products brand building series of activities. According to the introduction, the youth agricultural products brand building series of activities as one of the special activities of talent development, has been successfully held for five consecutive years, is one of the youth League of Hainan Provincial Committee of the youth creation brand projects. As an important part of the innovation and entrepreneurship section of "China (Hainan Free Trade Port) Youth Dream Avenue", this activity will release the project information on the "Dream Avenue" APP and set up an investment and financing platform. The projects involved in the brand building are recommended by the Rural Revitalization Youth Alliance, covering 98 youth alliances and 19 Provincial Youth Wealth Leaders associations, such as the "Haicheng Wuding" Rural Revitalization Youth Alliance and the Greater Sanya Regional Youth Entrepreneurship Association. In the next step, the Hainan Provincial Party Committee of the Communist Youth League will continue to focus on the front-line areas of rural revitalization, gather and explore outstanding returning young entrepreneurs and rural youth leaders to become rich, create a number of influential agricultural products brands, and lead more young people to return to their hometowns to start businesses and stay in Qiong.

2021-11-01	Fujian communist youth league organization to carry out the "I for youth" youth needs highlight the "I for youth" activities Jianwei correspondent Xu Jingxian) in accordance with the "party history strong faith with the party" learning education requirements, the Fujian provincial party committee to "I do the practical work for youth" practice is suggested, in-depth implementation of the "implementation of youth innovation entrepreneurship promotion plan" "care key youth action" "youth mental health service activities" and other key projects, actively do the practical work for youth, solve problems. Focusing on employment and entrepreneurship, the Fujian Provincial Party Committee focused on the key links and key issues of college students' internship and employment, jointly launched the "Sailing Plan" with the provincial Education Department, the Provincial Finance Department and other six departments, raised 27,000 internship positions through multiple channels, and attracted 31,000 students to deliver more than 87,000 resumes. The Youth League Sanming Municipal Party Committee promoted the introduction of the "Ten Measures to Promote the Employment of College Graduates in Sanming City", to promote the local employment development of college graduates, through policy publicity, providing humanized housing security, giving one-time living subsidies, and giving entrepreneurial loan discount interest support measures to serve youth employment. Around the growth guide, foster education to help double worker family solve the problem of summer "care", Fujian province less working committee, youth league, for the provincial young pioneers, priority guarantee of migrant workers trailing children, single-parent families, left-behind children, family economic difficulties young pioneers, promote the provincial young pioneers organizations at all levels rely on the youth palace, red scarf, red scarf pioneer campus base after-school hosting, summer classes, summer camp and other services, covering more than 100000 people. Around care support, do the practical work for teenagers widely deployed youth affairs social work around the grassroots line, relying on the "12355" youth service desk, take "online + offline" service mode, psychological counseling, policy interpretation, social work services, employment support, for teenagers to provide psychological assistance, online course counseling, the rule of law propaganda services, service more than 1 million people. Around the unsalable products, "round" activities affected by COVID-19 outbreak cause agricultural products sales blocked, active docking Sanming tai ning (yellow peach), Zhangzhou peace (� g g), Putian Xianyou (pomelo), Longyan changting (passion fruit), Nanping pine brook (harvest festival), five special activities "round" network 5, help unsalable growers solve the urgent, sales amount of nearly 600000 yuan. Under the call of the Provincial Communist Youth League Committee, the youth commandos, youth volunteer service teams and other places actively serve the grass-roots front line. Tuan Gutian County Party Committee organized more than 40 volunteers to go to the coast of Cuiping Lake to carry out the activity of "protecting the clear water with me" to protect the mother lake, distributed environmental protection leaflets, talked about the concept of garbage classification, and enhanced the people's awareness of environmental protection. The Youth League Committee of Fuzhou University organized young volunteers to carry out the "public welfare activity of protecting Mother River and releasing the Scientific Release of animals", and sampled and tested the water quality, picked up all kinds of garbage, and carried out environmental protection publicity activities.

2021-10-30	Shaanxi Youth League Organization: Help youth find the "youth password" to social governance) How to stimulate the power of youth and guide them to integrate into the community and serve the community? Since May this year, with the continuous promotion of the "Community Youth Action" of the Communist Youth League, the ways for youth from various cities and industries to sink into communities and participate in social governance have been continuously broadened, and more and more Sanqin youth have found the "youth password" to make contributions in social governance."Former young people, hardly talk to us old people. Now, the youth volunteer service team often chat to us, to help a lot!"Said Auntie Zhang, a resident of Yanliang District, Xi'an, with more than 5,500 permanent residents. On May 14,2021, the Community Youth Action of the Communist Youth League Committee was launched throughout the province- -centering around the most urgent needs and difficulties of the community people, youth league organizations at all levels organized youth forces to explore and develop normal, project-based and scientific services. In view of the crown community population aging status, xi'an municipal party committee, yan liang district party committee and crown community MoPai contact 1895 community youth, collect youth demands, jointly launched "strong position, strong service, strong participation" construction mode, through strong community youth volunteer service, red running errands team, youth station partners and a series of projects, promote youth to become the force of community governance. Xiao Liu, who is a volunteer service member in the unit, also signed up for the community youth volunteer service team and lived a "double" volunteer life. In the community, he led the establishment of a volunteer team to help the elderly and the young, providing purchasing and other assistance services for residents living on high floors and with mobility difficulties, and becoming the "crown good youth" in the hearts of the community people."We launched the community service project collectively six action, including contact a group of community youth, form a youth volunteer service team, introduce a batch of professional social organizations, to complete a position construction, docking a batch of organizations, the purpose is to strengthen, work, sharing, draw contact common youth the largest concentric circles for community service."Crown community party branch secretary Fu Lina said. In addition, Crown Community also focuses on youth development. Through the form of "online + offline" to create "Qingqing Academy", "psychological counseling studio", "Learning Club" and other characteristic functional areas, more than 40 cultural activities have attracted more than 1,200 young people to participate in, building a solid "base" for the community youth. In order to continuously promote the community youth action, the Shaanxi Provincial Party Committee of the Communist Youth League has set up a special work leading group, and established 25 communities in the province as the first batch of pilot communities. According to the actual situation, the pilot communities have carried out the exploration and innovation of the work of the community Communist Youth League. Founded in 2011, Dongling Community of Baoji City has a service site of more than 2,000 square meters, and has also built an intelligent management platform for property service. It is an intelligent community evaluated as "sincere, kind, young and harmonious". On the basis of the original community governance grid, the youth of Dongling community are correspondingly assigned to the corresponding grid, and the community youth serving as the grid members, the community Party organizations, the youth league organizations and the residents jointly build an autonomous platform for the grass-roots people. Through the community "cloud platform", the young people will know the demands as soon as the residents are put forward, ensuring that all the major community events and the youth league members can timely grasp, actively participate in and solve them effectively. As a young employee of Dongling Group, Ma Yi (pseudonym) became a young grid member with the support of the group's Youth League Committee and the community."He was warm-hearted and could always help solve any problems."Mention the pony, the community of grandma Liu thumbs up: become a young grid member, the pony is a work into the community," is not to help the owner, is to the west home to run errands, busy not to stop. Combined with the needs of youth development, "" has mobilized youth league members to report to the district. We have formed the youth work organization system, working mode and operation mechanism with Dongling characteristics, which not only improves the sense of gain of the youth, but also greatly enhances the satisfaction and happiness of the community people."The community youth League branch secretary Yang Xue said. In August this year, Mianxian County, Hanzhong city in southern Shaanxi province, was hit by heavy rain. In the face of the sudden disaster, the youth practice group of Minan community played a prominent role."Before the holiday is 'home squatting', lying in bed all day to think about life. When I went home this summer vacation, the community was organizing a youth practice group project and wanted to do something in their free time. Unexpectedly, the worst flood disaster in a hundred years."As a community youth liaison, Niu Hongbin (pseudonym), a junior at a university in Xi'an, worked together with the dredging militia company on the front line of the disaster. In his view, the holiday was "fulfilling and meaningful". Centering on the community youth action, the Youth Mian County Party Committee launched the Youth Practice Group project, which will set up practical posts for college students returning home, providing a new path for them to give back to the community and self-exercise. This summer, the Min'an Community Youth Practice Group has received more than 20 returning college students. In the early stage, these young people were mainly engaged in epidemic prevention publicity, community care and other posts. After the rainstorm disaster, the youth practice group formed a flood control and disaster relief team with young students as the main body, actively participating in silt cleaning, material distribution, flood control knowledge publicity and other actions, becoming a new force in the disaster relief front. Under the banner of Community Youth Action, Various forms of exploration are underway across Shaanxi: in Xi'an, Youth League organizations strive to promote the full coverage of community youth social organizations, Extensive environmental renovation, respect for the elderly, children's care and other community youth volunteer service projects; In Baoji, The Youth league organizations actively connect with the colleges and universities under their jurisdiction, Guide college youth to participate in community governance with the opportunity of college students to return home and practice; In Ankang, With the government purchase of services, The assistance to key groups, employment and entrepreneurship promotion, environmental beautification and other services, Solve the practical difficulties of the relocated community residents... " Next, The Shaanxi Communist Youth League will continue to focus on the needs of the community and focus on what the youth think, Focus on exploring the project, socialized operation mode, Inject youth vitality into the community work in Shaanxi province."Said Jia Lin, deputy secretary of the Communist Youth League.

2021-11-05	Henan Province: Jianwei correspondent Guo Xiangyu) in order to further promote the pilot reform of the county Communist Youth League in Henan Province, Strengthen the provincial youth League committees to take overall responsibility, the city and county level youth league committees to implement the responsibility, To strengthen the guidance and supervision of the pilot reform of grass-roots organizations of the county Communist Youth League in the province and the construction of grass-roots league organizations in various cities, a few days ago, Henan Provincial Committee of the Communist Youth League held the pilot reform training seminar of the county Communist Youth League in the provincial youth League school, Held the province city grass-roots league organization construction work report promotion meeting and pilot county (city, district) work report promotion meeting. Wang Dubo, deputy secretary of the Communist Youth League Provincial Party Committee, attended the meeting and made a speech. The relevant responsible comrades of the Organization Department of the Provincial Communist Youth League Committee (grass-roots Organization and Construction Department), the youth League committee of the provincial cities and Jiyuan Demonstration area, the leaders of the organization department, and the 31 pilot counties (cities, districts) of the province attended the training. The meeting affirmed the progress and achievements of the pilot county (city, district) youth League committee and the youth League committee of the provincial Youth League committee of the county level since the launch of the reform of the pilot work, and made arrangements for the problems existing in the promotion of the current work and the next key work, especially the successful completion of the pilot evaluation work. The meeting pointed out that we should strengthen study and research, set the direction of reform, and deepen understanding in reform practice. To further study grasp xi jinping, general secretary of the important reform, from the new era of the party construction, the general requirements of the communist youth league development and new characteristics of contemporary youth profound understanding of why to reform, what, to what kind, firmly grasp the correct direction of reform, anchor reform goals, strive to break the "institutionalized" style and "administrative" dependence, efforts to realize the work project, survival socialization, running flat, cadres source diversification, leading mobilization network, organization diversification, promote reform truly "spirit". The meeting stressed the need to adhere to the problem-oriented approach, boldly explore and practice, and constantly innovate and make breakthroughs in the reform practice. To focus on the universality of the county grassroots league problems and weak links, accurately grasp the important principles of deepening reform and main measures, closely combining the local work reality, fully absorb the national first round of the communist youth league reform based on the comprehensive pilot experience, dare to rush dare to create, have the courage to emancipate the mind, bold reform and innovation. To deal with the universality and particularity of reform, dare to walk others without road, in practice, find rules in the exploration, constantly form new experience, deepen new understanding, contribute new solutions, earnestly through the reform of the county communist youth league organization to enhance political function, form social function, strengthening party and government feeling, youth, and the sense of personal growth. The meeting stressed the need to focus on key areas, strengthen summary and refinement, and constantly contribute wisdom to the reform practice. We should fully realize that the reform experience and cases are the concentrated embodiment of the work effect, and be good at mining and improving on the basis of the innovative reform work. While promoting the quality and efficiency of the local reform work, we should promote the overall improvement of the provincial reform work from point to point. The youth League committees of the provincial cities should join the bureau and guide the youth League committees of the pilot counties (cities, districts) to introduce a batch of reform experience that can be copied and popularized from the reform perspective, overall vision and integrated ideas, strengthen the tangible presentation of the reform results, and effectively improve the social influence of the reform work. We need to work hard on the mechanisms under construction, carefully summarize the existing achievements of reform practice, and promptly refine them into experience and institutions, so as to contribute more wisdom and strength to comprehensively advancing reform. The meeting called for strengthening the results-oriented approach, paying close attention to the implementation of the work, and constantly acting in the practice of reform. To strictly mark the table on the central the county communist youth league organizations at the grassroots level reform pilot evaluation guidance standard, further refine the reform index task, clear work progress, strict time node, inversion period, chart operations, pressure stubble, to ensure that all reforms, landing to the ground, everything work, the successful completion of the reform pilot task. In accordance with the requirements of the Central Committee of the Communist Youth League, the provincial Communist Youth League Committee will take overall responsibility, conduct a comprehensive evaluation of the progress of this round of reform pilot, supervise and promote the areas with backward work, and report the evaluation results to the party committees at the same level of the pilot counties (cities, districts). It is understood that the training using classroom teaching, practice teaching, seminars, etc, the trainees successively to the first round of the national communist youth league organization reform comprehensive pilot mou, lankao, and invited the central responsible comrades on the county communist youth league organization reform pilot evaluation guidance standard for the training guidance, the central school, provincial party school teachers respectively around the county communist youth league organization reform, study and implement the provincial party congress work report spirit for the guidance.

2021-10-30	Group Chongqing municipal party committee jointly organized the flood control and emergency volunteer service desktop deduction Chongqing municipal party committee jointly relevant departments for flood control and emergency volunteer service desktop deduction Wang Xinxin) on October 28 afternoon, Chongqing municipal committee of Chongqing civil affairs bureau, Chongqing, Chongqing emergency administration, Chongqing civil air defense office in Chongqing youth social organization growth development base "youth volunteer flood control and disaster reduction" flood control and emergency volunteer service desktop deduction. The desktop deduction simulation continuous heavy rains in Chongqing, most areas appear floods, especially the jialing river basin ocean to Shapingba water level continues to rise, Shapingba area seriously threatened by flood scene, around the disaster collection and rapid assessment, emergency response, transitional resettlement, post-disaster reconstruction content to carry out flood control and emergency volunteer services. The picture shows the desktop deduction scene. In the exercise, after simulating the flood situation, Chongqing flood control and drought relief headquarters will immediately start the emergency response, deploy emergency volunteer service related work, establish information transmission channels with the disaster league organization, inform the "Mountain City Snow Leopard" Youth Volunteer Team to start the emergency rescue plan; send the "Mountain City Snow Leopard" rescue and relief young volunteers to the front line to assist the Shapingba Youth League District Committee to carry out the work, mobilize social organizations, caring enterprises and caring people to help the disaster reconstruction. After the deduction, the relevant departments in Chongqing made a review and comment, believing that the desktop deduction was creative, rich in content, close to the reality, showing a high level of professionalism. Group the relevant person in charge of Chongqing municipal party committee volunteer work said, held the desktop deduction is mainly to find the emergency youth volunteer service work mechanism of weak link, strengthen the upper and lower linkage and horizontal coordination, strengthen the emergency youth volunteer service command is scientific, effectiveness and operability, to improve the emergency youth volunteers to emergency comprehensive disposal ability and level. The person in charge said that in the future, we will give full play to the advantages of the Communist Youth League, strengthen communication and cooperation with relevant departments, extensive link to resource support, and encourage more

2021-10-30	Qinghai: Ma Fuchun, 7,000 young volunteers involved in epidemic prevention and control) The Party has called for the group to take action. Facing the current outbreak, recently, the Qinghai provincial party committee, Qinghai province youth volunteers association again to the province initiative, called on youth league organizations at all levels, all members of youth, youth volunteers, vigorously carry forward the "dedication, love, mutual assistance, progress" volunteer service spirit, give full play to the positive role of the young volunteers in epidemic prevention and control, scientific and orderly participation in epidemic prevention and control work, to resolutely curb the current spread, win the epidemic prevention and control battle contribution youth strength. Provincial, municipal (prefecture), county (urban) three-level youth league organizations and young volunteers quickly gathered in the "epidemic" test, actively responded to the call, enthusiastically registered, once again on the "red vest", in the "epidemic" prevention and control battlefield, the plateau youth with practical actions to demonstrate the mission of "prevent and control the epidemic and volunteer with me". As of 12:00 on October 28, the youth League of 8 cities and prefectures and 45 counties (cities) in the province has organized more than 7,000 young volunteers to form the epidemic prevention and control youth volunteer commandos, ready to participate in the epidemic prevention and control work. The Haidong and Xining delegations launched volunteer services for youth in epidemic prevention and control in a timely manner, quickly organized volunteer reserve teams for epidemic prevention and control, and contributed to the epidemic prevention and control work in accordance with the requirements of local epidemic prevention and control headquarters. 1,400 young volunteers from Xining fought on the front line of epidemic prevention and control. Qinghai Provincial Health Vocational and Technical College and the youth Volunteer service team of State Grid Qinghai Electric Power Company volunteered to submit the letter of invitation, explaining the responsibility of the plateau youth in the new era with their own practical actions. Members of the Qinghai Youth Exchange Foundation, the Youth Federation at all levels, and the associations of Youth enterprises actively participated in the epidemic prevention and control, and donated 170,000 yuan of protection funds and materials for young volunteers. In the next step, the Qinghai Provincial Committee of the Communist Youth League will continue to mobilize more young volunteers to participate in it according to the epidemic prevention and control situation. At the same time, in accordance with the needs of epidemic prevention and control, municipal, state, county and district youth league organizations were organized to connect, allocate and provide pre-job training for volunteer posts, and volunteers were organized in an orderly and scientific and reasonable manner in accordance with the law, so as to jointly write the response of the youth on the plateau.

2021-11-03	Henan Communist Youth League held community youth action work promotion meeting Henan Communist Youth League held community youth action work promotion meeting Jianwei) recently, Henan Communist Youth League community youth action work promotion meeting was held in Zhengzhou. League, deputy secretary of Henan provincial party committee, provincial youth volunteer association li attended the meeting and speech, the relevant departments (units) responsible for comrades, Zhengzhou party committee, committee, Luoyang Anyang related responsible comrade, Zhengzhou county youth corps committee is responsible for comrades, Henan first community action community organizations responsible for comrades, Luoyang provincial community youth action community youth league organizations responsible for comrades, Henan service change of poverty alleviation relocation community national demonstration projects or key projects of social organizations responsible for comrades, a total of more than 60 people to attend the meeting. The meeting deeply studied the spirit of the 11th Provincial Party Congress, carefully studied the spirit of the central community youth action phased work coordination meeting, carried out exchanges and sharing, summarized the progress of the work since the community youth action work deployment meeting, and studied and defined the focus of the next work. The meeting pointed out that the spirit of the 11th Party Congress of Henan Province should be thoroughly studied and implemented. To deepen the study, fully understand. We should combine the actual work to study deeply, carefully, and integrate the learning results into the practice of guiding the work, and better serve the overall situation and serve the youth. Get into the game and act actively. We should make contributions to building a new development pattern and the historical opportunities for ecological protection and high-quality development in the Yellow River Basin, and make innovations in anchoring the "two guarantees" and implementing the "ten strategies". To gather strength, more brilliant. We should strengthen the overall leadership of the Party, bear in mind the distinctive attributes of the political organs, temper our fine style of work through deepening reform and strictly governing the Youth League, and write a more brilliant chapter in the Central Plains in the new era. During the meeting, the Henan provincial party committee combined with the central community youth action phased work coordination conference spirit, further improve the community youth action collaborative propulsion working mechanism " community youth action quantitative assessment rules, the Henan provincial party committee authority related departments report the work support measures, the Zhengzhou municipal party committee report work advance, related community youth league organizations on behalf of the work communication. It is reported that the Henan Provincial Party Committee of the Communist Youth League has thoroughly implemented the work deployment of the Central Committee of the Communist Youth League on the community youth action, conducted in-depth research, held the work deployment meeting of the community youth action, formulated the division of labor and cooperation mechanism, quantitative assessment rules, and completed the department division of labor, system implementation, work assessment and other aspects of the top-level design. For the key implementation points of the community youth action, promote the realization of full coverage of the youth home construction, coordinate the injection of youth activity resources, carry out volunteer services for flood control and epidemic prevention, "4:30 classroom", care for vulnerable teenagers, beautiful community construction and smart help for the elderly and other work projects, and complete the community work of the exploration and innovation groups. In the next step, the Provincial Communist Youth League Party Committee will carry out the community network live broadcast activities, strengthen the resource investment, stimulate the community vitality, carry out the regular supervision, and promote the community youth action to a new level.

2021-11-04	Henan Provincial Party Committee held "Youth Society School" community youth action special training class Henan Provincial Party Committee held "Youth Society School" community youth action special training class Jianwei) recently, Henan Communist Youth League "Youth Society School" community youth action special training class was held in Zhengzhou. League of Henan provincial party committee is responsible for comrade, provincial capital city youth corps committee responsible comrade, Zhengzhou first implementation of 29 community youth action community youth league organizations responsible comrade, Luoyang community youth action pilot community youth organizations responsible for comrade, participate in the change of poverty alleviation relocation community service social organizations responsible for comrade a total of more than 50 people to participate in the training. Training in-depth study xi general secretary about social construction, especially community governance important speech spirit, learning group central community youth action periodic work coordination spirit, provincial relevant responsible for comrade on the community youth action overall advancement, summarizes the community youth action since the implementation of the work progress, research clear the next work focus. The training course takes the form of concentrated study and discussion, on-site observation and learning, highlighting the study of social work majors and the practice of project design. Excellent social workers in Henan province were invited to teach "Community Youth Action Work Project and Case Analysis", excellent media propagandists in Henan province were invited to explain the ideological and political courses of community youth in the new era and carry out publicity planning, and collectively went to the urban community service center to observe the key work projects of the community. It is reported that since the central community youth action, the provincial committee attaches great importance to, strengthen the research and guidance, held special work deployment meeting, establish division of labor and cooperation mechanism, actively integrate resources, promote the construction of youth community youth home, distribute "green fashion" youth ecological education reader and green cloud classroom video CD, for community volunteers including service resources package; in the community "home" social practice activities, serve the community masses.

2021-10-29	The Communist Youth League Fujian Provincial Party Committee has launched the Red Dream Tour Series Activities) Since the Communist Youth League launched the theme activity of "Casting the Soul to Build a Dream" in May this year, all primary and secondary schools have responded positively and held a series of "Red Dream Tour Tour" related activities. Various universities in Fujian widely tell the story of the party, the revolutionary martyrs, red landmarks, red cultural relics, hero model, for outstanding young party members, red pioneer and other series of practice, at the same time "the" a program under which officials "home", members report to the community (village) and other social practice and volunteer service. The "Light Horse" red publicity group of Huaqiao University and the "Do not forget the Road from Liming Vocational University" went into the campus, community, troops and other places to carry out various forms of publicity, sharing and exchange. All middle schools carry out the "red micro classroom" activities, and organize the theme youth league day activities at the important time nodes such as "May 4 th" and "July 1". The primary schools in Fujian combined with the activities of the Young Pioneers to carry out the Fujian Young Pioneers Pioneer action of "Red Scarf Heart to the Party to be a good member in the New Era", and carried out the education of "Four History and Three Love" for the young pioneers. During the "61" period, the province's young pioneers held a centralized "red scarf heart to the party to be a good member of the new era" theme squadron meeting. The Fujian Provincial Committee of the Communist Youth League relies on the network media and the wechat public accounts of the Communist Youth League and universities. Colleges and universities will report and push the relevant activities of their own practice teams respectively, and the qualified practice teams will live broadcast or record small videos in the practice process, so that more teenagers can participate in the network cloud.

2021-10-28	Fujian held youth vocational skills competition selection competition Fujian held youth vocational skills competition selection competition youth network reporter Chen Qiang) the 17th "revitalization Cup" national youth vocational skills competition lathe project in Fujian Province selection competition recently held in Longyan city. After a fierce competition, Longyan City Technician College and overseas Chinese vocational and technical secondary school of the two players swept the top two, they will represent Fujian province to participate in the national final. According to reports, the competition is sponsored by the Communist Youth League Fujian Provincial Party Committee, Fujian Provincial Department of Human Resources and Social Security, the Communist Youth League Longyan Municipal Party Committee, Longyan City human resources and social security Bureau, Longyan Technician College undertook. With the theme of "Follow the Party to show youth style, practice skills and build new Fujian", the competition guides the young people to take the road of becoming talented with skills and serving the country with skills, and makes new and greater contributions to promoting high-quality development in all aspects and accelerating the construction of new Fujian in the new era. More than 200 contestants from 9 municipal teams in Fujian Province participated in the competition, with 1 first prize, 2 second prizes and 3 third prizes. The top three contestants will represent Fujian Province to participate in the 17th "Revitalization Cup" National Youth Vocational Skills Competition held in Hangzhou, Zhejiang Province. Communist Youth League Longyan Municipal Party Committee and Communist Youth League Sanming Municipal Party Committee won the Excellent Organization award.

2021-10-28	Jiangxi province youth branch demonstration training class held in jinggangshan more than 80 theory about "light cavalry" ready "important speech spirit, promote the party history learning education, October 20-22, the Jiangxi provincial party committee held in jinggangshan" 2021 Jiangxi province youth branch demonstration training ", from young teachers, young scholars, enterprise workers, entrepreneurial youth, college students, party and government organs at all levels and cadres, more than 80 proposed hire lecturer candidates participated in the training. The training was divided into special research groups and set up an immersive learning environment to build a platform for young lecturers to collide and communicate. Training invited China jinggangshan cadre academy professor Li Chungeng special tutoring learning xi general secretary "July 1" important speech spirit, the national youth jinggangshan revolutionary tradition education base for director of basic theory about ideological theory teaching, and Jiangxi radio and television association Cheng Shan teacher preach skills training, from how to build high-quality goods courses, how to improve oral communication and communication, etc., for the theory of armed work "light cavalry" implementation equipment upgrade."Youth Lecture Group" is a brand project that the Communist Youth League focuses on the main responsibility and main business and deepens the ideological and political guidance of the youth. Jiangxi province youth branch since its establishment in 2019, for the province selection, cultivate a batch of political stand firm, solid theoretical accomplishment, personal deeds, ability to preach excellent outstanding young lecturer, active in the party's theoretical propaganda line, with "small and fine", "small and beautiful" face to face corners to preach, promote the party's theory in the youth into the brain into the heart, provincial youth branch members have preach more than 1300 games. At the graduation ceremony, the training class organized the student representatives to make a report for the local teenagers. MAO Haofu is a "post-85 returnee". He grew up listening to the red story, gave up a high-paying job and returned to return to Jinggangshan to become a red propagandist. As a member of the youth teaching group, he innovatively carried out the way of "preaching + live broadcast" and tailored the party history learning live broadcast for young people. The so-called "the key to a well-off society depends on the fellow villagers", from "overall moderately prosperous society" to "moderately prosperous society in all respects", from "comprehensive construction" to "building a moderately prosperous society in all respects", what exactly is a moderately prosperous society? Liu Jia, a teacher from East China Jiaotong University, shows the brilliant achievements of the Party in uniting and leading the people in a simple and understandable language."To advocate heroes produces heroes, and to be heroes produces heroes in large numbers."Dr. Li Jiangbo, the image ambassador of Nanchang conscription and the School of Marxism of Nanchang Aviation University, combined with his own experience in joining the army to explain the connotation of The Times of inheritance of red gene and continuous spirit and blood with simple language. Through this training, the participants, under the guidance of on-site experts, concentrated on lesson preparation, trial lecture, discussion, and collectively created a number of high-quality demonstration courses. In the next step, Jiangxi Provincial Youth Lecture Group will combine the party history learning education and the publicity and education of "Four History", with "Qingyan Qing language" and "Internet language" with the characteristics of The Times, use Jiangxi red resources, carry out differentiated and differentiated publicity at the grass-roots level, and get through the "last kilometer" of youth theory armed work.

2021-10-27	Zhang Jianwei) On October 24, the ninth chairman (expanded) meeting of the 12th session of the Henan Provincial Youth Federation was held in Zhengzhou. Deputy Secretary of the Communist Youth League Provincial Party Committee, Provincial Youth Federation Chairman Wu Hao attended the meeting and made a speech. Wang Dubo, deputy secretary of the Communist Youth League Committee and vice chairman of the Provincial Youth Federation attended the meeting. Provincial Youth Federation vice chairman Sun Tian Tian presided over the meeting. Communist Youth League provincial second-level inspector, provincial Youth Federation secretary General Han Bing attended the meeting. Meeting to communicate learning the xi general secretary of the series of important speech spirit, the national youth league thirteenth session of the second standing committee (expand) meeting spirit, provincial party committee work conference spirit, summarizes the provincial youth federation eight chairman of the twelfth meeting, especially in winning flood control COVID-19 epidemic two war disease work situation, is the next stage deployment of key work. The meeting stressed that, We should make it our top political task to thoroughly study and implement the spirit of General Secretary Xi Jinping's important speeches, Continue to study deeply, return to the roots of the study, Effectively implement the learning results to do the work, promote the development of the cause; Centering on the general requirements of 'anchoring' two guarantees 'and implementing' ten strategies' " put forward by the provincial Party Committee, Give full play to the role of great unity and great unity, Mobilize young people to participate in key tasks such as innovative development, cultural prosperity, and rural revitalization, To contribute the youth strength to the construction of modern Henan; In accordance with the requirements of the Central Committee of the Communist Youth League, Active benchmarking table, By deepening the reform, strengthening the organizational construction, strengthening the function of endorsement, and adhering to the strict governance committee, To ensure that the reform of the Youth Federation is effective. The meeting was held in a video format. Directors of all walks of life, secretary-general and secretaries-general of municipal youth federations attended the meeting as non-voting delegates. During the meeting, some committee members shared their experiences on learning and implementing the spirit of the General Secretary's series of important speeches.

2021-10-27	Hainan free trade port youth network anchor public audition officially launched in 2021 Chinese youth dream season youth network anchor charity audition officially launched Zhang Jianwei) recently, in 2021 Chinese youth dream season free trade port youth network anchor public audition launch ceremony and Hainan free trade port youth positive energy network celebrity exchanges in China (Hainan free trade port) youth held dream avenue China youth hall. Chen Yu, secretary of the Hainan Provincial Committee of the Communist Youth League, announced the official launch of the 2021 China Youth Dream Season. At the launch ceremony, Wang Yingcheng, deputy director of the Cyberspace Office of the CPC Hainan Provincial Committee, put forward two requirements for the contestants. The first is to stick to the bottom line, consciously resist bad information, and strive to spread the positive energy of the network; the second is to play an exemplary role in the innovation and entrepreneurship practice, and help the network public welfare undertakings in Hainan Province. Li Yuan, deputy secretary of Hainan Provincial Party Committee, hopes to pave the way through the competition and smooth the communication channels for youth; second, she hopes to carry out deep guidance of network anchors to jointly build online and offline concentric circles; and third, he hopes to use the power of network example to carry forward the cultural theme of the new era. After the ceremony, the invited young positive energy network celebrities shared their experience and exchanged their interactions on the scene, discussing the development trend of anchors, the form of network positive energy communication, and the development of young anchors themselves. It is reported that the activity was jointly organized by the Hainan Provincial Committee, the Hainan Provincial Cyberspace Office of the CPC Committee and the China Youth Newspaper. Youth career development center, Hainan province youth activity center, Hainan province, Hainan nanhai network media co., LTD., aims to hold the contest, China Hainan free trade port youth dream avenue youth mobilization leading service platform, build communication platform for youth network anchor, and cultivate a group of concerned love in Hainan, youthful vigor, professional skills of the youth positive energy network anchor, for free trade port in Hainan reserves more youth network talents, better service free trade port construction. In 2021 China youth dream season free trade port youth network anchor public welfare audition as "2021 (the first) China youth dream season" one of the series of activities, attracted more than 500 from universities, enterprises and institutions, people's groups to participate in registration, the contest trill topic also synchronization online, so far the short video total playback has more than 11.1 million. Next, through the audition, semi-final and final tests of the event, the top ten contestants and the second and third place will be determined to become the star anchors of the second Hainan Free Trade Port Youth Network Anchor Public Welfare Selection Competition.

2021-10-27	Guangxi for rural revitalization of "hope head goose" special training for rural revitalization of "hope head goose" special trainers Xie Yang correspondent Yang Dongjie) sponsored by Guangxi district party committee, Nanning normal university continuing education college to undertake in 2021 high-quality youth farmers and the communist youth league rural revitalization "hope head goose" autonomous region level training class, was held in Nanning. This training class attracted 50 returning young entrepreneurs from Guangxi to participate in the study. Through expert lectures, it enabled young entrepreneurs in terms of entrepreneurial ability, entrepreneurial knowledge and entrepreneurial policies. At the opening ceremony, Rong Liwei, deputy secretary of the Guangxi District Committee of the Communist Youth League, said that young rural entrepreneurs are the leaders and pioneers of rural revitalization. They hoped that the students would increase their skills in learning, and be down-to-earth, diligent and pragmatic to increase their knowledge, character and skills. It is hoped that the students can expand their rural undertakings in practice, apply what they have learned, integrate knowledge and practice, apply their knowledge to practice, and then find a new entrepreneurial road in line with the local industry reality. It is hoped that the students will show their youth in their struggle, stand at the tide, strive to be pioneers of rural industry revitalization, and be pioneers, pioneers and devotees of rural revitalization. Since this year, the Guangxi District Committee of the Communist Youth League has provided a learning platform for young rural entrepreneurs through combining online and offline entrepreneurship training. In June this year, the group of Guangxi district party committee cooperation with China charity federation to implement the fourth phase of the rural revitalization "leader" online training plan, for more than 8000 young entrepreneurs to provide systematic, professional courses, and held in cities and counties "hope head wild goose" offline training, through the policy interpretation, training and exchange green and service activities effective service rural youth entrepreneurship.

2021-10-26	The Gansu Provincial Committee of the Communist Youth League allocated 300,000 yuan to support the Gansu Provincial Committee allocated a special fund of 300,000 yuan to support the fight against the epidemic and launched the "gather the youth strength to escort the white soldiers" special action network reporter Ma Fuchun) Today, The Gansu Provincial Party Committee of the Communist Youth League has decided to study, Emergency allocation of a special fund of 300,000 yuan, It is mainly used to support Lanzhou, Zhangye, Jiayuguan, Longnan and Tianshui, five counties with heavy COVID-19 epidemic prevention and control tasks, and counties (cities and districts) with confirmed cases; at the same time, Issued the Notice of Gansu Communist Youth League on the Special Action of "CondensYouth Strength to Escort Soldiers in White" (hereinafter referred to as the Notice), Young volunteers will be mobilized to serve the closed-loop management of medical staff, nucleic acid testing personnel, community medical and health personnel and their families in the frontline fight against the epidemic. It is understood that the special funds will be used for the epidemic prevention and control youth commandos and frontline volunteers to buy protective goods, provide life necessary and emergency transportation security, the youth league care of warm spread to fight in the epidemic prevention and control of the first line and the general youth league organizations, support and help grassroots youth league organizations to participate in the epidemic prevention and control of the war. In addition, in order to ensure the daily life needs of medical staff and their families, and reduce the worries of medical staff, the notice pointed out that young volunteers in the province will be mobilized to carry out refined and personalized volunteer service activities such as life services, care services, psychological assistance, and academic counseling.including, Set up a volunteer service Lei Feng team, Free travel services for closed-loop managed medical staff, nucleic acid testing personnel, community medical and health personnel and their families; Volunteer health management services, Establish a medical and health volunteer service team, Regularly provide health management and care services for the families of medical staff, Organize volunteers to provide accompanying patients for medical treatment, hospital escort and other services when necessary; Organize the provincial psychological counseling volunteer service team, To provide psychological counseling services for medical staff and their families; Integrate provincial high-quality teacher resources, college students, members of graduate volunteer teaching groups and volunteers of the Western Plan, To provide one-to-one " online academic counseling volunteer services for the children of medical staff. Source: China Youth Daily, October 26,2021, edition of 02

2021-10-25	Jiangxi province youth branch demonstration training class held in jinggangshan more than 80 theory about "light cavalry" ready to 2021 Jiangxi province youth branch demonstration training held in jinggangshan zhang correspondent Huang Guipeng) on October 20-22, Jiangxi provincial party committee held in jinggangshan "2021 Jiangxi province youth branch demonstration training", from the province in various fields of young teachers, young scholars, enterprise worker, entrepreneurship, youth, college students, party and government organs youth cadres and cadres more than 80 proposed to hire lecturer candidates participated in the training. Members of the youth lecturers' group attended the demonstration training course. This training is divided into special research groups, and set up an immersive learning environment, to build a platform for thinking collision, learning and communication for young lecturers. Training invited China jinggangshan cadre academy professor Li Chungeng special tutoring learning xi general secretary "July 1" important speech spirit, the national youth jinggangshan revolutionary tradition education base director for basic theory about ideological theory teaching, and Jiangxi radio and television association Cheng Shan teacher preach skills training, from how to build high-quality goods courses, how to improve oral communication and communication, etc., for the theory of armed work "light cavalry" implementation equipment upgrade. Jiangxi province youth branch since its establishment in 2019, for the province selection, cultivate a batch of political stand firm, solid theoretical accomplishment, personal deeds, ability to preach excellent outstanding young lecturer, active in the party's theory propaganda line, with "small and fine", "small and beautiful" face to face corners to preach, promote the party's theory in the youth into the brain into the heart, provincial youth branch members have preach more than 1300 games. At the graduation ceremony, the training class organized the student representatives to make a report for the local teenagers. MAO Haofu is a "post-85 overseas returnee". He grew up listening to the red story, and chose to return to Jinggangshan to become a red propagandist. As a member of the youth teaching group, he innovated the way of "propaganda + live broadcast", and tailored the party history learning live broadcast for the young people. Liu Jia, a teacher from East China Jiaotong University, shows people the brilliant achievements of the Party in uniting and leading the people in a simple and understandable language. Dr. Li Jiangbo, the image ambassador of Nanchang conscription and the School of Marxism of Nanchang Aeronautical University, combined with his own military experience, and explained the connotation of inheriting the red gene and continuing the spirit in simple language. Through this training, the participants, under the guidance of on-site experts, concentrated on lesson preparation, trial lecture, discussion, and collectively created a number of high-quality demonstration courses. In the next step, Jiangxi Provincial Youth Lecture Group will combine the party history learning education and the publicity and education of "Four History", with "Qingyan Qing language" and "Internet language" with the characteristics of The Times, use Jiangxi red resources, carry out differentiated and differentiated publicity at the grass-roots level, and get through the "last kilometer" of youth theory armed work.

2021-10-24	Reporter Hong Kefei) On the morning of October 21st, "Furong Students Rural Revitalization" young talent Forum was held in Hunan University. Began in 1998, the implementation of Hunan hope engineering brand project "lotus students" large public welfare activities, by the China youth development foundation, the communist youth league of Hunan provincial party committee, Hunan tobacco industry co., LTD., Hunan youth development foundation, is a aims to reward, funding outstanding plight college students compound large-scale public welfare activities. Liu Lianyu, Secretary of the Party Group and Deputy Director of the Standing Committee of Hunan Provincial People's Congress, Cao Jiongfang, member of the Party Group and Deputy Secretary General, Mei Feng, Deputy Secretary of the Party Committee, Vice Chairman and Secretary General of China Youth Development Foundation, Li Zhichao, Secretary of the Hunan Communist Youth League, and more than 150 young people and college students representatives from Changsha attended the event. Liu Lianyu fully affirmed the contribution of the Hunan Hope Project and the large-scale public welfare activities of "Furong Students". She proposed that college students would go into the countryside, serve and contribute to the countryside, and that the Hunan Communist Youth League would continue to focus on the educational nature of the league organization. It is understood that since the implementation of the Hunan Hope Project 32 years ago, it has raised 1.15 billion yuan, funded more than 470,000 students from poor families, and has helped build 1,759 Hope primary schools.

2021-10-22	The final of the first Guangdong College Students Network security attack and defense Competition was successfully concluded. Group of Guangdong provincial party committee for figure China youth network Beijing on October 22 (reporter Zhang Jianwei, correspondent YueQing) recently, by the group of Guangdong provincial party committee, Guangdong department of education, Guangdong province, Guangdong science and technology association, Guangdong association jointly launched the first Guangdong college students network security competition held in Guangdong technical normal university, Chinese Academy of Engineering, Guangdong technical normal university, deputy secretary of Guangdong technical normal university party secretary li and the host unit representative and final team representatives attended the closing ceremony. The first Guangdong college students network security attack and defense competition final scene. This network security attack and defense competition is committed to building a multi-dimensional communication platform in the whole field, and continuing to output practical talents. The competition adopts the combination of online and offline methods, for full-time students in universities in the province, and students participate in the form of teams. The competition adopts basic safety knowledge popularization, CTF flag winning competition, AWD attack and defense, comprehensive penetration competition and other competition systems, Complete the designated questions on the designated platform through the Internet, It is mainly divided into six categories: web security, reverse analysis, mobile security, binary vulnerability mining, exploitation and utilization, cryptography, and comprehensive miscellaneous items, A total of 255 teams were attracted to participate, After the theoretical knowledge competition, online promotion competition, provincial preliminary competition and evaluation, etc., A total of 29 teams from 16 universities were shortlisted for the final, Finally, the Sloth team of South China Normal University won the grand prize, Five teams, including the WaterDrop-master team of Sun Yat-sen University, won the first prize, Nine teams including Jinan University Xp0int teams won the second prize, Guangdong Technical Normal University gpZuZ1cT team and other 15 teams won the third prize."Young College Students and National Network Security" theme Roundtable. Group of Guangdong provincial party committee for figure contest, held the network security frontier technology peak BBS expert lectures, Chinese Academy of Engineering Shen Changxiang shared the core network space security science and technology, promote discipline construction and talent training, the theme of the report, in the subsequent round table, experts and scholars around the young college students and national network security theme communication. Guangdong young college students network security talent training alliance launch ceremony. Group of Guangdong provincial party committee for figure in Guangdong party committee, Guangdong union, pearl river kechuang center under the guidance, Jinan university, south China normal university, foreign trade university, Guangdong, Guangdong technical normal university, Guangzhou university, Huizhou college, Dongguan institute of technology and other seven cyberspace security college or professional universities, in Guangdong college students "young marxist training project" framework, jointly launched "QingMa HongKe 100" Guangdong young college students cyberspace security talent training alliance. At the event site, Guangdong Normal University of Technology, Shenzhen Qianhai Hengchang Technology Development Co., Ltd., Shenzhen Xinlun Advanced Materials Science Research Institute Co., Ltd., and Guangzhou Huizhi Communication Technology Co., Ltd., were awarded as the "Young Red Guest 100" Guangdong young college students cyberspace security talent training base.

2021-10-26	Henan "pier seedling" plan exchange office cadre symposium Henan provincial party committee held a "pier seedling" plan exchange office cadre symposium Ji'an-wei zhang) recently, Henan provincial party committee held a "pier seedling" plan exchange office cadre symposium, all the provincial party committee, organs and directly affiliated units above deputy place level cadres attended the meeting. At the meeting, the youth League provincial party committee "pier seedling seedling" plan exchange office cadres made a speech. Henan provincial party committee secretary wang yi delegation provincial party committee to congratulate them, respectively to the league provincial party committee to the grassroots five comrades gave leave work souvenirs, to the league provincial party committee authority of three comrades issued a mount guard certificate, and revisit the party oath, league oath, communicate with young cadres to share the learning experience. She pointed out that the implementation of the "pier seedling seedling" plan is an important strategic decision made by Henan Provincial Party Committee to thoroughly implement General Secretary Xi Jinping's important discussion on training and selecting outstanding young cadres, earnestly practice the Party's organizational line in the new era, proceed from the actual situation of Henan and focus on the overall situation of modern Henan construction. This plan takes up nearly a thousand key and important posts to build a stage for young cadres to exercise and grow up.

2021-10-20	Zhang Wenling, 2018,2021 "Zhenxing Cup" Yunnan Youth Vocational Skills Competition began, with 36 teams from 21 universities in the competition. The competition is jointly sponsored by the Yunnan Provincial Party Committee of the Communist Youth League, the Yunnan Provincial Department of Education of Yunnan Province, and the Department of Human Resources and Social Security of Yunnan Province, and organized by Kunming Metallurgical College, and becomes the training base of the "Revitalization Cup" National Youth Vocational Skills Competition in Yunnan Province. The theme of the competition is to "follow the Party to learn practical skills, capable skills and serve the country", setting up three competition occupations (jobs), including lathe worker, industrial robot system operator, computer programmer and computer computing designer (cloud computing platform and operation and maintenance). Among them, lathe workers and industrial robot system operators are single players, and computer programmers (cloud computing platform and operation and maintenance) are double competitions. The top three winners of the single competition and the top two winners in pairs will be selected to participate in the final of the 17th National "Zhenxing Cup" competition. Get the corresponding ranking and meet the conditions, can be promoted to the technician (senior worker) after being approved by the provincial department of human resources and social security professional qualification or vocational skill level, already have the technician (senior worker) professional qualification or vocational skill level, can be promoted to the senior technician (technician) professional qualification or vocational skill level. Those who are awarded the top 10 players of various types of work will be awarded the honorary title of "Yunnan Youth Post Expert" by the Yunnan Provincial Party Committee of the Communist Youth League. It is reported that since its holding, the competition has won high attention and recognition from schools, enterprises, the market and the society. The competition has become a high-quality platform for the majority of young skilled talents to compete, exchange and learn, and improve together. It is also a practical classroom for professional youth in the province to cultivate professional spirit and learn from professional examples.

2021-10-27	Hainan youth service skills competition English final in Hainan youth service skills competition English final net reporter Ren Mingchao) on October 24, sponsored by the Hainan provincial party committee of the 17th "revitalization cup" national youth vocational skills contest of Hainan province and Hainan youth service skills competition English final in Haikou, from Hainan province park, industry (system) foreign service window collective 9 teams about 50 players. The first team of Haikou Meilan Airport, Haikou Customs and Haikou Port Customs team and Danzhou Siyuan Middle School team won the provincial gold, silver and bronze awards in the English competition respectively. In addition, those who meet the gold medal requirements will be recommended as candidates for "Hainan Youth Post Expert". It is understood that the 17th "Zhenxing Cup" National Youth Vocational Skills Competition is a national first-class vocational skills competition. In 2021, the student group competition will be held, with the main body competition (vocational skills competition), and Hainan Province will hold provincial trials accordingly. This English competition is to strengthen the Hainan Youth Service Skills Competition held simultaneously by the Youth Collective internationalization Construction Institute of Hainan Free Trade Port.

2021-10-26	Hainan communist youth league party history youth go grassroots preaching activities in Hainan provincial party committee launched in 2021 Hainan communist youth league "learn party history, strong faith, follow the party" youth grassroots preaching zhang correspondent zhang dream) recently, sponsored by the Hainan provincial party committee in Hainan in 2021 the communist youth league "party history, strong faith, follow the party" youth walk grassroots preaching first briefing and launch ceremony in China (Hainan free trade port) youth dream avenue "China youth hall". At the meeting, Li Yuan, deputy secretary of the Provincial Communist Youth League Committee, presented ribbons to the youth'grassroots publicity representatives of the Hainan Communist Youth League in 2021. At the first lecture, three young publicity representatives carried out demonstration lectures around the theme of "learning the history of the Party, strengthening faith, and following the Party". Among them are young college teachers trained deeply by young marxists, leaders of famous auxiliary studios who devote themselves to the cause of the young Pioneers, and leaders who get rich and serve the innovation and entrepreneurship of rural youth. The delegates shared the theory of party history, youth cause practice and youth inspirational stories respectively, calling on the majority of Hainan youth to inherit and carry forward the red spirit of the revolutionary ancestors on the basis of party history study, inspire the great power of youth with the responsibility of youth, and devote themselves to the construction of Hainan Free Trade Port with more enthusiasm. Next, the youth provincial party committee will organize youth history XuanJiangTuan, provincial youth league, provincial enterprise association, provincial youth volunteer college multiple fronts youth lecturer hierarchical classification to preach team, multiple parallel, deep across the province enterprises, rural areas, schools, communities, villages and towns line units, deep into the youth, focus to carry out the party history to preach activities. According to the introduction, the campaign focused on telling the story of the Communist Party of China and the Qiongya revolution "23 years of red flag" red story, further increase the history of the Party, the history of new China, the history of reform and opening up, the history of socialist development publicity and education and the party leadership of the youth movement history knowledge popularization efforts.

2021-10-20	Hainan start youth go grassroots preaching activities in Hainan start youth go grassroots publicity activities green reporter Ren Mingchao) on October 18, sponsored by the Hainan provincial party committee in 2021 Hainan communist youth league "party history, strong faith, follow the party" youth walk grassroots preaching activities first briefing and launch ceremony in China (Hainan free trade port) youth dream avenue "China youth hall". At the meeting, Li Yuan, deputy secretary of the Hainan Provincial Committee of the Communist Youth League, presented ribbons to the youth'grassroots publicity representatives of the Hainan Communist Youth League in 2021. At the first lecture, three young publicity representatives carried out demonstration lectures around the theme of "learning the history of the Party, strengthening faith, and following the Party". Next, the Hainan provincial party committee will organize youth party history XuanJiangTuan, youth league, Hainan province, Hainan province youth volunteer college multiple fronts youth lecturer hierarchical classification to preach team, more line parallel, across Hainan province enterprises, rural areas, schools, communities, villages and towns first-line units, into the youth, focus to carry out the party history to preach activities.

2021-10-19	Henan Youth Innovation and Entrepreneurship Competition Henan Youth Innovation and Entrepreneurship Competition First and First Special Competition Network reporter Pan Zhixian) The 8th "Creative and Youth" China Youth Innovation and Entrepreneurship Competition Henan Branch Competition and "Creative and Colorful" Henan Youth Innovation and Entrepreneurship Competition was held in Zhengzhou today. With the theme of "Innovation and Entrepreneurship, Create Youth, Practical Learning and Practical Work to Follow the Party", this competition encourages and guides the young people in Henan to walk in the forefront of mass entrepreneurship and innovation, and realize the dream of innovation and entrepreneurship. The competition focuses on the 14th Five-Year Plan and the long-term goals of 2035, pointing out the direction for young people to implement the new development concept and build a new development pattern. For the first time, the competition has created four special competitions, including "Science and Technology innovation Special Project", "Rural Revitalization Special Project", "Internet Special Project" and "Social Enterprise Special Project". Since its launch in July, 1,337 participating projects have been received from the provincial cities. After the qualification examination of the national competition organizing committee and the selection of the provincial competition judging committee, 60 projects participated in the provincial final examination. After fierce competition, the final gold, silver, bronze award 6. After the final, the 60 projects can apply to settle in the Henan Provincial Youth Innovation and Entrepreneurship Service Center, giving priority to enjoying preferential business support policies and free business incubation services. The competition is jointly hosted by the Henan Provincial Committee of the Communist Youth League, the Henan Provincial Department of Human Resources and Social Security, the Provincial Department of Agriculture and Rural Affairs, the Provincial Department of Commerce, and the Provincial Rural Revitalization Bureau. Source: China Youth Daily

2021-10-19	Shandong province held a youth development friendly city construction press conference in Shandong province youth development friendly city construction press conference in Beijing on October 19 (xinhua Ji'an-wei zhang, correspondent zhang nan li bo) recently, the Shandong province government information office held a press conference, introduces the youth development friendly city construction in Shandong province. Liu Tiandong, secretary of Shandong Provincial Party Committee of the Communist Youth League, the Party Committee of the Institute of Sociology of the Chinese Academy of Social Sciences, and the Shandong Provincial Department of Human Resources and Social Security attended the press conference, and more than 30 news media attended the press conference. More than 1.8 million people watched the press conference through the live streaming platform. The press conference made a comprehensive introduction on the situation background, objectives and principles, main contents, organization and implementation of the youth development-friendly city construction in Shandong Province. In recent years, Shandong Province has formulated and implemented the Shandong Province Medium-and Long-term Youth Development Plan (2019-2025), established a joint conference mechanism for youth work, and promoted great progress in youth development. Shandong province youth work joint conference on the new era of modern province construction and youth priority development strategy, took the lead in the province within the scope to start "youth development friendly city" construction work, the purpose is to better stimulate the youth in the force of the construction of the province, at the same time promote the whole society concern, attention, care youth growth, realize the organic integration and urban development of youth development, benign interaction. The main contents of youth development-friendly cities include 10 items, including improving the quality of urban employment, mass entrepreneurship and innovation, housing security, education security, cultural service efficiency, health security, business services, ecological livability, social governance, and social participation of young people. Through this work, we will strive to build a number of "young" youth development friendly cities, so that the city more friendly to youth development, and make the young people more promising for urban development. It is reported that Shandong province is the first province to promote the construction of "youth development friendly city", the first provincial province to formulate the "youth development friendly city" construction index system and the first province to promote the construction of "youth development friendly city" simultaneously at the city and county levels. Early, the Shandong provincial party committee together with the institute of sociology, Chinese Academy of Social Sciences, in-depth research youth development friendly city subject, thorough understanding of youth groups from all walks of life in employment, entrepreneurship, housing, education, marriage and other different fields of development demand, on this basis formulated the "about youth development in Shandong province friendly city construction work guidance". As a guide, youth development friendly city construction will be city and county (city, area) two levels to promote, by the provincial youth work joint conference is responsible for the implementation, the construction main body is the province 16 cities, adhere to the principle of voluntary, construction cycle is not less than two years, control the Shandong province youth development friendly city index system (trial) for youth satisfaction evaluation. The promotion of this work will play a good role in promoting the urban development, to stimulate the urban vitality, gather talents, promote the transformation of kinetic energy, and improve the quality of the city. In recent years, to serve the youth better development, help youth better into urban construction, the Shandong provincial party committee around service old and new kinetic energy conversion and high quality development, high standard three youth entrepreneurs innovation and development international summit, building platform, aggregate resources, promote investment intention in Shandong project 220, a total investment of 217.584 billion yuan, build 1.6 square meters of green enterprise summit headquarters, set up green enterprise summit industrial park alliance, accurate service enterprises, projects, resources. Around the service youth employment, relying on the "green bird plan" for the social from all walks of life youth launched employment service season, practice, skills promotion, project recruitment season theme service, to cover 16 cities 136 counties (city, area) "youth station" 228, more measures and solve the practical problems of young talent, build heavy to love, respect with good atmosphere, let the youth of Shandong city identity, feeling, happiness more sufficient, more sustainable. Around power city to retain more young talents, upgrade from the construction of the "youth", to deepen the urban and rural volunteer service, to service emerging youth development needs, extensive urban and rural community youth into action, to build young people to stay, enjoy the urban environment, attract more youth groups in Shandong development. Next, Shandong communist youth league organizations at all levels will rely on the youth work joint conference mechanism, bring together more youth work force, through the "green enterprise summit", "green bird plan", urban and rural community youth into action such as brand work, help improve the city of youth attraction, cohesion, bearing capacity, expand youth participation in urban development, innovation, contribution, promote the development of Shandong high quality and youth all-round development benign interaction.

2021-10-18	Shanghai league school built launched 70 years "young cadres education + AI" classroom Shanghai league school built 70 years launched "young cadres education + AI" classroom on October 16th is the 70th anniversary of Shanghai league school in new "pioneer square" held "birthday", put forward the exhibition teaching combination, "young cadres education + AI" and other new teaching mode. Among them, the "combination of exhibition and teaching" mainly focuses on the integration and innovation of space business forms, establishes the situational party and league class studio and participates in the development of the red landmark situational party class, builds the "Pioneer Square", and starts the first theme education space "Communist Youth League of China and Shanghai" project. The newly completed "Pioneer Square" will become a public space integrating display, education, communication and interaction, which can tell the history and culture of the Communist Youth League to the school youth and connect with the history and culture of the North Bund of Hongkou. This is the first public space for the national provincial youth League school to show the early pioneers of the Communist Youth League, and also a milestone in the business format adjustment of the Shanghai Youth League School."Young Cadre Education + AI" is committed to enhancing the awareness and ability of young cadres in digital transformation. The project will develop a digital literacy course for youth League cadres, an artificial intelligence teaching method based on intelligent screen, an artificial intelligence laboratory for young cadres, a literacy evaluation system for young cadres, and a growth database of young cadres. In the future, the Shanghai Youth League School will establish a life-cycle training file for every young cadre. Using AI technology, the youth League school can accurately obtain the training needs of each young cadre in different stages of career development, and track and train the students throughout the whole process. The evaluation of young cadres' quality will also be justified. The young cadre quality evaluation system can evaluate the political quality and ability of cadres in key subject shifts and form a report. Young cadres will have their own growth database to support the data for the evaluation and future selection of Shanghai Qingma Project students and Shanghai young characteristic talents. It is reported that in the past 70 years ago, it has created many "national first" - -it is the first full-time team cadre college school directly recruited from fresh high school graduates, published the first professional book carrying the century-old history of local youth movement, and built the first "youth research data platform". At present, Shanghai Youth League School is building a "Shanghai Youth League School Discipline System", including four first-level disciplines, 22 second-level disciplines and General Secretary Xi Jinping's important discourse on youth work, Youth Organization, Youth Training, and several three-level research directions. Source: China Youth Daily

2021-10-18	Fujian carries out network security publicity week youth day activities Fujian carries out network security publicity week youth day activities on October 18 (reporter Zhang Jianwei, correspondent, Xu Jingxian Huang Qiuhua) on October 16, "network security publicity week-youth day" activities in Minjiang College. This youth Day is hosted by the Cyberspace Office of Fujian Provincial Committee and Fujian Provincial Committee of the Communist Youth League, and organized by the Youth League Committee of Minjiang University.site of activity, The staff gave out questionnaires to the passing students, Investigate and understand the students' understanding of network information security knowledge, And to the students participating in the answer of the "Fujian school small salary" peripheral products as a reward, Minjiang College network information speaker volunteers explained the network security knowledge to the students, Sharing the experience of network information screening, Students from CAI Jikun Conservatory of Music also combined with their own major, Adapt the network security knowledge into the allegro words, Universal network security knowledge to students with their popular allegro performance, The event site also has a TV broadcast related promotional video, The staff have also made cybersecurity knowledge posters and display boards, Call on the students to sign their names on the display board, Together agreed to become a small network security guard. It is understood that this year, under the guidance of the Fujian provincial party committee, colleges and universities to carry out various forms of network security publicity and education, using local characteristics will combine network security education and professional disciplines, enhance the participation and effectiveness of network security education activities, improve the youth network security ownership consciousness, develop youth civilization online, consciously maintain network security good quality, enhance the network security consciousness and ability.

2021-10-20	Guangdong held 2 young middle-aged middle-level cadre training class KaiBanShi Guangdong in 20212nd young middle-aged middle-level cadre training class KaiBanShi Beijing on October 20 (reporter Zhang Jianwei, correspondent YueQing) on October 18 morning, sponsored by the Guangdong provincial party committee organization department, the Guangdong provincial party committee in 2021 young middle-level cadre training class (2) KaiBanShi in Guangdong youth political college (Guangdong league), Guangdong provincial party committee, Guangdong provincial party committee organization department cadre training related responsible comrade to attend, all the students a total of more than 80 people. Liang Junda, deputy Secretary of Guangdong Provincial Committee, joined the students at the opening ceremony to be the firm believer and practitioner of Xi Jinping Thought on Socialism with Chinese Characteristics for a New Era; young cadres should stick to their original mission, cultivate the glorious tradition and fine style of the Party, strengthen the chemical practice, and share the vanguard forces of reform, development and stability in Guangdong. It is reported that this training course lasts 60 days, the students are from the provincial units and the province party and government organs, enterprises and institutions under the age of 35 years old outstanding young cadres. Training course combined with the founding theme in one hundred, the field teaching as an important form of young cadres party spirit education training, integrating red resources in the province, arranged to visit the party history learning education exhibition, the new democratic revolution and the history of reform and opening up field teaching, by strengthening the "four" education learning, further enhance the party spirit cultivation of young cadres.

2021-10-22	Gansu communist youth league to form youth commandos, volunteer service to participate in epidemic prevention and control of Gansu communist youth league formed youth commandos, volunteer service to participate in epidemic prevention and control Ji'an-wei zhang correspondent wei yi) on October 20, Gansu provincial party committee issued on strengthening the construction of epidemic prevention and control of youth commandos work notice, requirements in enterprises and institutions, urban communities, rural key areas such as epidemic prevention and control youth commandos, according to the unified requirements of the local party committee government, scientific and orderly participate in the prevention and control work. On the same day, the Provincial Communist Youth League Committee urgently set up five volunteer service teams for provincial emergency rescue, material coordination, psychological assistance, professional medical care, and information reporting. It has guided the municipal, prefectural and county districts to set up 14 municipal-level volunteer service teams and 86 county-level volunteer service teams, which are directly led by the local youth League committees and participate in epidemic prevention and control under the unified deployment of the local Party committees. Gansu Provincial Committee of the Gansu Youth League and Gansu Provincial Youth Volunteers Association issued the Volunteer Recruitment Notice of Epidemic Prevention and Control Reserve in Gansu Province, recruiting volunteers for epidemic prevention and control from the whole province, calling on youth league members, social caring people and young volunteers to actively participate in the epidemic prevention and control volunteer services. Up to now, the youth league organizations at all levels in the province have been extensively mobilized, young party members, youth league members take the initiative to participate, a total of 1171 youth commandos, 14,976 people, recruited more than 30,000 young volunteers, actively participate in various volunteer services. Social organizations, public welfare organizations and caring enterprises have responded positively, raising 270,000 masks and 16 boxes of alcohol and epidemic prevention kits to effectively support the epidemic prevention and control work.

2021-10-16	Young Pioneers at all levels in Guizhou Province organized the theme team Day demonstration activity "Please rest assured that the Party, a strong country has me". Young Pioneers at all levels in Guizhou Province organized the theme team Day demonstration activity competition, and the winning players and the winning teams awarded awards and MEDALS. Correspondent for figure China youth network Beijing on October 16 (xinhua Zhang Jianwei, correspondent Chen kai) recently, Guizhou provincial party committee, provincial education department, province less working committee held in Tongren bijiang district "please trust power have me" Guizhou young pioneers theme demonstration activities, activities to further study xi general secretary in celebrating the 10 0th anniversary of the founding of the communist party of China's important speech spirit, leading the young pioneers to inherit and carry forward the party's glorious tradition and fine style, arouse patriotic enthusiasm, strengthen the country feelings. In the activity, 9 red scarf small associations through the red story explanation, paper-cut creation, calligraphy display, art painting, sports fitness, tea art display, musical instrument performance and other ways, to fully show the new era of the young people practice "please trust the party strong country have me" clank oath. At the joining ceremony, all the members faced the national flag. the young pioneers raised their hands high over their heads to extend their lofty salute to the great and sacred motherland. Zhao Jihong, member of the Standing Committee of the Tongren Municipal Party Committee and head of the Organization Department of the Tongren Municipal Party Committee, delivered a speech on behalf of the Tongren Municipal Party Committee, and Zhang Qin, secretary of the Tongren Municipal Party Committee and director of the Tongren Municipal Youth Working Committee, announced the list of new members. Shi Qilin, deputy secretary of the provincial young pioneers, expect they have fire, firm faith, light, character, strong, hone ability, brave tide, be pentium young people, to realize the great rejuvenation of the Chinese nation of the Chinese dream, create colorful Guizhou new future ready, solid ready, blooming red scarf light; hope the provincial communist youth league, the young pioneers organization further focus on the main business, strengthen political enlightenment, deepen reform and innovation, optimize education system, promote the party's children cause high quality development. In the style display demonstration activity, the young pioneers used dance, allegro, dance poetry sitcom, recitation and other ways to express their determination to become red children, as good successors. Finally, the participating leaders presented certificates and MEDALS to the winners and the winning teams of the 2021 Provincial Young Pioneers Counselor Talent Competition. It is reported that during the "113" period, the province's young pioneers at all levels through the theme activities, practical education and theme squadron meeting and other ways, online and offline combined with "please rest assured strong country have me" team day theme activities more than 9000 times, covering more than 4 million young pioneers.

2021-10-15	Hainan youth agricultural products brand series activities Hainan youth agricultural products brand series activities Ji'an-wei zhang correspondent Wu Yuwa) by the communist youth league, Hainan province agriculture and rural areas hall, Hainan commerce, Hainan rural revitalization bureau jointly hosted the "youth chapter dream future" in 2021 Hainan free trade port youth agricultural products brand series activities the successful completion of the second stage of training promotion, training goals at the same time, 21 green farmers brand propaganda video production and design packaging upgrade work is also in development. In order to build the 21 youth agricultural products projects well this year, the organizer carefully designed and arranged a complete system of training plan, invited leaders and experts in agricultural fields, marketing fields and other industries to serve as training mentors, and conducted systematic comprehensive ability improvement training for all the project members participating in the brand building. The training according to the needs of subsequent project development, mainly in four angles and two highlights, respectively is the brand management enterprise digital transformation, the way of electricity brand entrepreneurship, all media operation perspective, from the macroeconomic perspective of Hainan agriculture four angles, experts and scholars team and field research become a bright spot. In the training and promotion stage, the experts and mentors conducted targeted face-to-face discussion and training according to the field investigation results, the content of agricultural product brand projects, and the content of brand strategy, operation combat and policy interpretation.

2021-10-19	Guangdong communist youth league held network security publicity week youth day network security youth league in 2021 youth day activities Ji'an-wei zhang correspondent YueQing) on October 16, by the Guangdong party committee net letter office, Guangdong party committee, Guangdong less committee "network security, network security for the people by the people" in 2021 network security publicity week youth day activities held in Guangzhou longkou west primary school. The activity was held in the form of a squadron meeting. The red scarf members from the sixth (3) Squadron of Longkou West Primary School, accompanied by the parents of the teachers and the guidance of the counselors, had a three-way "Miao protection class" together. Young Pioneers, teachers and parents took a "three-way class". Group of Guangdong provincial party committee for figure activities hosted by the counselors and the young pioneers, a directed by the young pioneers from the Internet surfing the family sitcom, ignited the enthusiasm of the students, children, parents and teachers three parties respectively on the field, combined with the "network security for the people, network security by the people" theme, discuss how to healthy on the Internet. On the scene, guests from different fields such as the procuratorate, young Pioneers, lawyers and the media reviewed the content of the discussion as the off-site observation group. The young pioneers on the field actively participated in the discussion and actively expressed their views. Before the start of the Guangdong Provincial Party Committee, the Provincial Communist Youth League Committee and the Provincial Youth Working Committee invited Cao Yue, the after-school counselor of the sixth (3) Squadron and the tutor of Tencent's "Miao Protection tripartite class", to carry out a questionnaire survey for parents and young Pioneers. According to the survey results, nearly 40 percent of their parents thought they were "more dependent" on the Internet, while some children said they had had minor disputes with their parents for using the Internet. During the live discussion, the children also paid close attention to their parents' online time, including some people complaining that their parents were too noisy when they used their mobile phones. The 2021 Guangdong Provincial Network Security Publicity Week Youth Day activity site."Today, our class is to ask the three parties on the field to make equal expression, listen to each other, and promote mutual understanding."Cao Yue gradually led the three parties on the field into the state, on" learning and entertainment both "," minors how to moderately on the Internet "," how to arrange an hour online time " and other issues. Watch historical videos, participate in the "Red Scarf Love Learning", and learn about the news and current affairs... During the discussion, the Young Pioneers know everything about the various uses of the Internet. At this time, the parents and teachers found that the children paid great attention to the current political news, study, history, music and other elements while going online."I had never noticed what he was looking at before. I thought I was playing a game when I picked up my phone."Some parents began to realize that in their daily life, there is really a lack of attention and guidance to their children online, and always subconsciously think that" holding the phone is just don't want to learn."After the course, the experts of the off-site observation team combined their own professional knowledge to give pertinent advice on how to develop good habits of using the Internet. The young Pioneers also opened their ideas in the "tripartite class" activity on Youth Day, "ready to go home and make a network agreement with our parents". It is reported that the 2021 Guangdong Province Network Security Publicity Week Youth Day activities in the province, the youth League committees at all levels play the advantages of media matrix communication and the advantages of the youth palace and other positions, to carry out online and offline youth Day activities. Through a series of activities, the majority of young people are guided to fully realize their own responsibilities and missions as the direct beneficiaries of network security, important promoters and technological innovators, to distinguish right from wrong, and jointly protect the network ideological security.

2021-10-14	Shanxi youth league organizations at all levels to guard home build "youth dam" Shanxi youth league organizations at all levels to guard home build "youth dam" Zhang Jianwei correspondent super shu yue) recently, Shanxi more time, range, intensity rainfall weather, affected by continuous heavy rainfall, 37 river floods in the province, highway, provincial trunk line, railway operation affected by certain. Youth league organizations at all levels in the province heard the "flood" and move, set up youth commandos, volunteer service teams, young people quickly gathered in the flood control and disaster relief line to guard their homes to build a "youth dam". The Shanxi Provincial Party Committee of the Communist Youth League immediately held an emergency meeting to deploy the provincial Communist Youth League to participate in the flood control work, requiring the youth league organizations at all levels in the province to follow the unified arrangements of the local Party committee and the government, closely monitor the changes in the flood situation, carry out risks and hidden trouble investigation, help transfer personnel and materials, and protect the lives and property safety of the people stationed in the village. To guide the Provincial Youth Development Foundation and many social organizations to jointly open the post-disaster reconstruction donation channel of "timely rain emergency rescue platform" and the post-disaster reconstruction fundraising channel of "Sanjin Civilization watch special fund", to call on the loving enterprises and enthusiastic public to help the affected areas and the masses, and to actively connect with the affected areas to ensure that the donated money can reach the affected people timely and accurately. Taiyuan Tuan Qingxu County Party Committee in the first time through the "online + offline" way, publicity, transmission of flood situation danger information, mobilize youth members to participate in flood control and disaster relief, went to mengfeng breach line, into disaster relief and rescue, cooperate with the party and government to do a good job in river repair, mass transfer and other work. The county has more than 150 grass-roots youth league organizations, more than 300 youth league members to actively and orderly participate in the emergency rescue, resettlement and reconstruction, publicity and education, psychological counseling, environmental remediation and other work. Jinzhong Jinzhong new youth university volunteers emergency rescue team organized young volunteers to go deep into the Dongzhao Township Shishan Village geological landslide point, in the collapse of the village investigation and rescue, love enterprises to provide vehicles to help changning town people transfer. A total of 78 people in 35 cave households were surveyed, and all the cave residents were moved out. Yuci District young volunteers insist on the charge in the front, the front-line fighting, in-depth farmers' homes, rivers, fields and other key areas of inspection and risk discharge. Timely evacuation of the masses, drainage of water, set up warning signs, and comprehensively build a strong defense line for flood control and disaster resistance. TuanTaigu District Committee actively organizes and mobilizes the youth league members to strengthen the investigation of dilapidated houses and villagers' agriculture, timely grasp and eliminate various potential hidden dangers, and prepare for flood control to key groups such as the lonely elderly; implement the personnel on duty, keep the information open and pull the team at any time; allocate all kinds of flood control materials in case of emergency; carefully review the implementation of the work, and contact the Party committee and government with any problems. In the face of recent continuous heavy rainfall and severe flood control situation, the Youth League Yushe County Party Committee mobilized the majority of youth league members to actively join the Yushe Youth Volunteers Association to participate in the flood control work, with day and night for the people to build a strong fortress of flood control and rescue. In the face of the sudden flood disaster, jiexiu city youth volunteers actively carry out all kinds of volunteer service, in jiexiu town, hongshan town, the youth volunteers 174 people, rescue boats 2, transfer trapped people 66 boat 460 people (including more than 40 pregnant women), transfer livestock 15 boats 105 head, transport supplies more than 50 boats, sandbags more than 800 bags. Affected by the rare and continuous heavy rainfall, floods, debris flows, mountain collapse and other natural disasters occurred in many places of Linfen, and many rivers and reservoirs in the territory approached or exceeded the warning level. Under the strong leadership of the Party committee and the government, the young people are called on to report to the Party and the Communist Youth League organization in the city nearby, and to participate in the flood control and disaster relief work in a scientific and orderly manner."I am a young volunteer. I want to contribute to the rescue and relief in my hometown."Young volunteers assisted in the transfer of people in flood control and disaster relief, patrol and drainage, drainage of water, transport supplies, emergency repair infrastructure..." Volunteer Red "and" Anxin Blue " show the responsibility and responsibility of the young generation. Up to now, the Communist Youth League and Linfen Municipal Party Committee has organized 17 rescue teams, and more than 400 young volunteers have participated in the flood control and disaster relief work. At the same time, the Youth League Linfen Municipal Party Committee actively contacted local foundations to raise materials. The first batch of 115,000 disaster relief materials arrived in Linfen on the morning of October 11, and the subsequent supplies have been delivered. Currently, the number of various materials has exceeded 130,000. The two disaster relief materials organized and coordinated by the Linfen Municipal Party Committee of the Communist Youth League were also officially put into use on October 11. Special personnel in the middle transfer warehouse were on duty 24 hours a day to receive relief materials from all walks of life and distribute materials on demand. The Yuncheng Municipal Party Committee of the Yuncheng Youth League allocated a special Youth League fee totaling 30,000 yuan to the affected Youth League committees of Xinjiang, Jishan and Hejin counties (cities) to support the Yuncheng grassroots Youth League organizations to carry out flood control, flood fighting and rescue work. At the same time, raising flashlights, water pumps, raincoats, rain shoes and other value of 150,000 yuan of scarce supplies, sent to the above three counties (city) flood control headquarters. Tuan Xinjiang County Party Committee actively collects information, take the initiative to undertake tasks, docking needs, and cooperate with the local party and government to do a good job in rescue and support work. Quickly set up flood fighting youth commandos, immediately reach the front line of flood rescue; emergency procurement and timely delivery of urgently needed materials in resettlement sites; organize young volunteers to form art teams, carry out 6 performances, provide more than 3000 youth services and serve nearly 2000 people. Tuanjishan County Party Committee organized more than 60 young volunteers to provide traffic guidance, material transportation, logistics and other work; immediately purchased flood control supplies and donated 19 batches, over 25,000 pieces, worth over 4020,000 yuan to the frontline; assisted the emergency management department to provide volunteer flood control services and delivered meals to ensure that the frontline emergency workers eat well and energetic. Tuan Hejin Municipal Party Committee organized all the staff to stick to the dam 24 hours a day, conduct careful investigation, closely monitor the changes of the flood situation, and protect the lives and property safety of the people stationed in the village. More than 30 volunteers were organized to go to the centralized resettlement site, helping to transfer more than 800 villagers, install 400 relief beds, help make beds, cleaning, disinfection, and epidemic prevention, providing a comfortable and safe environment for the villagers in the resettlement site. At the same time, a number of cultural sympathy performances were organized to help the affected people relieve their tension. The Luliang Municipal Party Committee of the Luliang Youth League immediately required the youth league organizations at all levels of the city to actively report to the party committee and the government at the same level, and take the initiative to claim and undertake the work tasks. At the same time, the organization of youth league cadres at all levels and youth social organizations to set up youth commandos to participate in various risks and disaster relief activities in a scientific, orderly and safe way, extensively mobilize social caring people and youth league members in various fields to contribute love, and actively donate money and materials for the affected areas and the affected people. The Xiaoyi Municipal Party Committee of the Youth League issued a " proposal to dedicate youth strength to the youth league members at all levels, calling on the youth league members to participate in the flood control and relief work in a scientific and orderly manner. Docking changzhi success group lian sheng coal industry, form 77 people youth rescue commandos, with a generator, 8 diesel pumps, drainage pipe, wire, cable and other professional equipment, on 11 from changzhi emergency in big filial piety town village village, drainage rescue operations, mobilize love enterprises, school young pioneers for the affected village, school donated supplies more than 31 ten thousand yuan, donated thermos, hats, textbooks, school supplies, more than 1300 meals. TuanFenyang Municipal Party Committee organized youth volunteer organizations to actively participate in personnel evacuation, drainage and rescue, dam building and other work, a total of more than 160 people helped transfer 982 affected people, carrying 60 tons of cement and other disaster materials, and donated instant noodles, ham, beef and other living materials to the affected people. Tuan Wenshui county committee to mobilize the youth social organizations to provide rescue needed machinery and equipment and love materials, and organize Wenshui County nearly 100 people to participate in the county each disaster point for flood control and disaster relief work, a total of distribution of materials, sympathy, relief, transfer of the affected people about 760 people, dredging more than 20 wading sections, clean up 3 collapsed houses. TuanShilou county joint Shilou youth volunteer association and community committee quickly established flood control youth commandos, to assist LingQuan town and management committee of the county in geological hazards, and help transfer the affected people, so far, to assist the transfer of the affected people 1600 people, to help find out the geological hazard point more than 70, and raise a value of 5000 yuan of towels, blankets and other articles for daily necessities. In order to ensure the safety and smooth flow of the main railway artery, the Youth League Committee of China Railway Taiyuan Bureau organized the youth League members to join in the flood fighting and rescue and passenger service work. Among them, all members of Taiyuan South Works Section gave up holidays and set up 11 youth commandos to comprehensively strengthen the inspection and control of key flood control sections, cutting embankment slopes and drainage facilities. More than 30 members of Yanggaonan High-speed Railway Comprehensive workshop visited day and night to ensure the safe arrival of passengers. The Youth League Committee of Taiyuan Station organized 45 young people to guide cancellation and change, organize passengers and ensure traffic safety. In the next step, youth league organizations at all levels and youth league members in the province will actively respond to the call of the provincial Party Committee and the provincial government, and cooperate with the party and government at all levels to do a good job in helping the disaster-stricken people, hidden trouble investigation, and epidemic prevention and control, so as to help speed up the restoration of production and life order in the disaster areas.

2021-10-14	On October 12, the Shanghai Young Pioneers announced the official launch of the Shanghai Young Pioneers happiness education campaign in celebrating the 72nd anniversary of the team, helping the young Pioneers happy childhood after the "double reduction". Shanghai Young Pioneers released the "15-minute Happiness Circle" activity menu, 41 primary and secondary schools and communities were awarded the "Young Pioneers Happiness Education Experimental School and Experimental Community" card, three after-school activity places, such as the Chinese Welfare Association Children's Palace, set up brigade committees, and 14 district-level after-school youth working committees were awarded. Earlier, the Central Committee of the Communist Youth League and 13 other departments jointly issued the Implementation Opinions on Building the Socialized Work System of the Young Pioneers in the New Era, which clearly strengthened the organization construction of the young Pioneers in places such as youth palaces and other off-campus activity venues. In response to the above work requirements, the Shanghai Youth Working Committee took the lead in the city to build the children's Palace, the city youth activity center and the Young Pioneers Youth Daily small press team, the future will cover the urban two-level youth palace, student activity center, youth activity center, etc. The reporter noted that this round of happiness education action launched in Shanghai is an important part of the young Pioneers socialized work system."After the young Pioneers build the team, they should also pay attention to the team building problem. Let the young Pioneers organization live in all fields."Shanghai young working committee director, the city young Pioneers general counselor Zhao Guoqiang said. The Shanghai Municipal Youth Working Committee also gave the young Pioneers a big gift- -the "15-minute Happiness Circle" activity menu. This is an innovative model built by the Shanghai Young Pioneers' Working Committee in combination with the 2035 "15-minute Life Circle" plan of Shanghai. It encourages the young working Committee at all levels to integrate regional red, education, entertainment and other practical resources, so as to provide convenience for the team members to carry out practical activities nearby. In the future, the community young working committee can "order and order" to the municipal young working committee, and the municipal young working committee will distribute the activities to the community. In the cultural activity center of Wuliqiao Street, Huangpu District, the projects such as playing basketball, learning calligraphy and learning oil painting are distributed by the Huangpu District Youth Working Committee and the Municipal Youth Working Committee, and some of which are originally created by the community. According to the newly released Shanghai Young Pioneers happiness education experiment program, both experimental schools and experimental communities have been required to "innovate the first launch activities and form a team and one product" to enrich their extracurricular life after the "double reduction". Zhao guoqiang said that the happiness education experiment program focuses on making children experience family affection, partner friendship and community warmth. In the future, Shanghai Young Working Committee will promote the development of young Pioneers happiness education to flourish throughout the city. Source: China Youth Daily

2021-10-18	Guangdong "jinwan cup" the eighth "the youth" contest successful packaged Guangdong "jinwan cup" the eighth "youth" contest successful packaged Zhang Jianwei correspondent YueQing) on October 14 to 15, by the group of Guangdong party committee, Guangdong education department, Guangdong province, Guangdong province, Guangdong agriculture, Guangdong market supervision bureau, Guangdong financial regulator, Guangdong, Guangdong, Zhuhai municipal government units hosted "jinwan cup" the eighth "the youth" Guangdong large bay area youth innovation entrepreneurship competition and communication camp in Zhuhai jinwan area successful packaged. Leaders of the organizers and support units, youth innovation mentors, and shortlisted young entrepreneurs attended the finals and the exchange camp activities, and nearly 200,000 young entrepreneurs participated in the learning and exchange activities online. The award ceremony and the Guangdong-Hong Kong-Macao Greater Bay Area Youth Innovation and Entrepreneurship Exchange Camp site. This final exchange activity was held in both online and offline, They held the Guangdong-Hong Kong-Macao Greater Bay Area Youth Innovation and Entrepreneurship Training Conference in 2021 (Guangdong-Hong Kong-Macao special event), learning, publicizing and implementing the spirit of the construction plan of the two cooperation zones, the "China Youth Innovation Board" Industry Matching Meeting, Zhuhai Jinwan District Business Environment Investigation and other activities, Invite experts and professors, senior investors, entrepreneurs and young people to talk about various incentive policies and industrial development opportunities in the new development stage, Carry out industrial docking, resource docking and landing docking activities, To study, publicize and implement the spirit of the "two cooperation zones" construction program throughout the whole process of the activity, The "Creative Youth" competition will be made into a carnival event for innovation and entrepreneurship youth in the Guangdong-Hong Kong-Macao Greater Bay Area. Chi Zhixiong, secretary of Guangdong Provincial Committee of the Communist Youth League, awarded the first prize of "Create youth" project and took a group photo."In addition to winning the honor of the competition, I also met like-minded entrepreneurial partners through 'ChuangYouth', the industrial trends and opportunities in the Bay Area, and received investors with cooperation intention, which is much more abundant than the form of simply 'comparing the competition'."Cheng Qi, an entrepreneur from Shantou, to this kind of integration of competition, communication, and resources docking of the competition process is very recognized. After five months of fierce competition, 21 cities and cities in the province held municipal competition exchange activities, Hong Kong and Macao respectively held regional trials. All localities mobilized the competition units, and a total of 6,000 youth innovation projects were excavated and contacted, including 120 from Hong Kong and Macao. The average age of the main principals of the participating projects is under 28 years old, high-level talents with doctors or above account for more than 30%, and scientific and technological innovation and rural revitalization projects account for more than 62%. Finally, a total of 48 winning projects and 10 "green farmers is superior" brand, share a total of 1.75 million bonus and Guangdong large bay area youth innovation entrepreneurship highest honor, "peace" chip "UHD image chip and system" "seconds to workplace" project won the first prize, "Chen-build the world's largest tangerine peel genuine traceability system and overseas mall" "based on plant reactor production β -nicotinamide single nucleotide" "butterfly plant factory" project won the first prize in agricultural rural group. On the "Youth" competition, young people from the Guangdong-Hong Kong-Macao Greater Bay Area competed and had in-depth exchanges. Group of Guangdong provincial party committee for the contest since 2018, every year in Hong Kong and Macao selection exchange, set up "China green gen board" Hong Kong and Macao zone, establish ground incubation service mechanism, nearly 700 services, including in 2021120 Hong Kong and Macao green gen project focus on biomedical, Internet, a new generation of artificial intelligence and other fields, its quantity and quality are significantly improved.

2021-10-15	Guangdong university lighthouse learning and theme TuanRi provincial demonstration activities in Guangdong university lighthouse learning and theme TuanRi provincial demonstration activities Ji 'an-wei zhang correspondent YueQing) on October 14, sponsored by the committee of Guangdong province, Guangdong student federation, Foshan institute of science and technology to undertake "study and implement xi general secretary' July 1'important speech spirit" lighthouse learning and "Guangdong young college students strengthen do Chinese ambition, backbone, confidence" theme TuanRi provincial demonstration activities held in Foshan college of science and technology xianxi campus student activity center. Members from Guangdong Provincial Party Committee, Guangdong Student Federation, Academy of Buddhism and other relevant units attended the activity, and more than 250 teachers and students and representatives of Class 1 of 2019 of Medical College participated in the activity. The picture shows Professor Zhang Yonggang's "lighthouse project" demonstration micro-group class. Group of Guangdong provincial party committee for figure activities, all the teachers and students watched by "lighthouse project" youth mentor, "Guangdong youth, Guangdong youth May 4th medal" winners, nan yue outstanding teachers, south China normal university, marxism college professor yong-gang zhang taught to "do ambitious new era of youth" as the theme of the "lighthouse project" demonstration micro group. Professor Zhong Xianfeng, winner of "Guangdong Youth May 4th Medal Nomination Award" and president of the Research, Development and Achievement Transformation Institute of Foshan University of Science and Technology, shared his teaching and research experience with the participants from the aspects of student training, academic achievements and personal experience with the title of "Cultivating people for the Party". The picture shows Professor Zhong Xianfeng sharing the words "Education for the Party and education for the country". At the scene, all the teachers and students studied the third season of "Youth Learning", and invited the student representatives of the school of Medicine and Law and Intellectual Property to share their experience of learning the spirit of "July 1" and their own learning experience. At the study meeting, artistic performances such as dance "Prosperity", solo "Never forget the Original aspiration", poetry recitation "Set sail to the Party Lighthouse" and solo "One Hundred Years" were also held. The picture shows the College Student Art Practice Center of the Communist Youth League Committee performing the Chinese dance in Foshan University of Science and Technology. Since the development of Party history learning and education, Guangdong Provincial Party Committee has effectively embedded party history learning and education into the chain of education work, driving the student union organizations at all levels and 5.63 million middle school students to actively carry out the learning and education of "learn Party history, strengthen faith, and follow the Party". It is reported that the Provincial Communist Youth League Committee will combine the study and implementation of the spirit of General Secretary Xi Jinping's "July 1" important speech, and extensively carry out the big discussion activity of "Guangdong young college students to enhance the ambition, backbone and confidence of being a Chinese person".

2021-10-14	Fujian to celebrate the 72nd anniversary of the youth team theme team activities held in Fujian to celebrate the 72th anniversary of the Chinese young pioneers team theme team activities Ji'an-wei zhang correspondent huang) on October 13, by the Fujian party committee, Fujian province, Fuzhou municipal party committee, Fuzhou civilization, Fuzhou education bureau, Fuzhou young committee jointly organized "red scarf heart to the party, head up" hui "growth" -Fujian young pioneers to celebrate the 72nd anniversary of the Chinese young pioneers team theme team day activities held in the first primary school affiliated to Fuzhou institute of education. Li Teng, deputy secretary of the Provincial Communist Youth League Party Committee, the provincial youth working committee and the relevant leaders of the Fuzhou Communist Youth League Municipal Party Committee, the Municipal Civilization Office and the Municipal Education Bureau attended the activity. The Young Pioneers' dedication. The ceremony began with the chorus of "We are the Successors of communism". The representatives of 17 Young Pioneers delivered a speech, the Provincial Youth League Committee and the Provincial Youth Working Committee awarded the First Primary School of Fuzhou Institute of Education "Fujian Red Scarf Pioneer Children's Palace" and "Fujian Red Scarf' Sunshine Physical Fitness Base". The Fujian Youth Palace Association and the First Affiliated Primary School presented the Fujian Red Scarf "Home School Social Palace" co-construction unit plaque. Provincial young working Committee, provincial children's Publishing House presented "Red scarf Medal medal manual". Subsequently, Fujian Provincial Youth Working Committee issued letters of appointment to 5 "red scarf outside counselors" and 5 "Young Pioneers volunteers" of the Red scarf Pioneer Children's Palace of the First Affiliated Primary School. The provincial Youth Working Committee and the provincial Children's Publishing House presented the Red Scarf Medal Competition Manual to the teachers and students of the school. At the ceremony site, we also held the Young Pioneers' entry ceremony. 226 new members joined the Chinese Young Pioneers with honor. The guests, counselors and representatives of the young Pioneers wore bright red scarves for the new members. New entry team young Pioneers call sign. Before the ceremony, the leaders and guests visited the on-site display of the intangible cultural heritage sachets, Pucheng paper cutting, and kiln white porcelain of the Young Pioneers of the First Affiliated Primary School, to learn about the intangible cultural heritage courses of the school. It is understood that the central committee of the communist party of China on comprehensively strengthen the work of the new era young pioneers opinions since issued, provincial party committee, provincial committee focus on building the young pioneers social work system, radiation driving effect, relying on the good basic advantage of hospitals, communities, schools set up Fujian red scarf pioneer children's palace, explore the combination of school, family school community children's palace of the young pioneers work new mode, help promote the "double minus" policy implementation. In the next stage, the provincial young Working Committee will continue to integrate social resources, relying on the young Pioneers counselors to open young Pioneers activity classes, carry out the young Pioneers practice experience activities and cultural and sports and art activities, promote the formation of a good atmosphere for the whole society to care for children and support the work of the young Pioneers, and comprehensively promote the healthy growth of children in the province.

2021-10-14	Jiangxi province to commemorate the 72nd anniversary of the young pioneers founding theme team held provincial demonstration activities "please rest assured, power have me" in Jiangxi province to commemorate the 72nd anniversary of the young pioneers founding theme team provincial demonstration activities held Zhang Jianwei) on October 12, "please rest assured, power have me" to commemorate the 72nd anniversary of the young pioneers founding theme team, Jiangxi provincial demonstration activities held at small red valley beach. Leaders of the Provincial Communist Youth League Committee, the Nanchang Municipal Government, the Municipal Education Bureau and the Municipal Communist Youth League Committee attended the event. Jiangxi Province to commemorate the 72nd anniversary of the youth team theme team day provincial demonstration activity site. Correspondent for the picture to enter the school, the guests visited the art exhibition under the guide of the red scarf narrator, observe the tea art performance, handmade model airplane site production, and the young pioneers together to experience the colorful campus life, and for the representatives of the young pioneers presented red books. The theme team day activity opened in the flag issuing ceremony, the new young Pioneers of the primary school attached to the South University walked onto the podium, and the old members wore red scarves for the new members, and took an oath together, "... ready, contribute to the cause of communism" neat oath echoed in the playground. Later, the guests attending the event awarded the representatives of the Young Pioneers who won the "Red Scarf Medal". The young Pioneers took the oath collectively in the activity. In the activity, the young pioneers also showed the theme team day activities: little dancers bring ballet performance "moss", pay tribute to the party's cadets bring the women's dagger exercise, men's military boxing show, show the young pioneers inherit the red gene, promote the "August 1 spirit"; red scarf narrator tells the story of "Jiangxi big hostel", led the young pioneers to find the place where the people's army was born. Students from the Nanchang Junior Military Academy show off men's military boxing. Luo Hua, deputy secretary of Jiangxi Provincial Party Committee and director of Jiangxi Provincial Working Committee, told the young Pioneers: to establish lofty ideals, ready to realize the Chinese dream of great national rejuvenation; to inherit the red gene, strive to become the narrator of revolutionary stories, revolutionary spirit successor, the disseminator of revolutionary culture; to achieve comprehensive development, learn knowledge, strong body, love labor, temper will, to add a new era of glory to the red scarf.

2021-10-14	Zhejiang held "learning party history strong faith with the party" keynote speech contest held in Zhejiang "learning party history strong faith with the party" keynote speech contest network reporter Jiang Yutong) today in Zhejiang communist youth league "learning party history strong faith with the party" theme speech contest finals, Ningbo haishu district volunteers association Sun Jiayi from the movie "long jin lake", tells the story of her help behind the martyrs search over the years. On the TikTok "Youth Zhejiang" and "Zhejiang Youth League Provincial Committee", a large number of viewers watched the finals online. Guided by the CPC Zhejiang provincial party committee propaganda department, the communist youth league Zhejiang party committee, Zhejiang provincial party committee party history and literature research, Zhejiang daily newspaper group, the communist youth league Hangzhou municipal party committee, Hangzhou normal university to undertake "learning party history strong faith follow the party" keynote speech contest finals held in Hangzhou normal university, provincial party committee propaganda department, provincial party committee, provincial party history and literature research, Zhejiang daily, provincial party history learning education tour steering group, Hangzhou normal university leadership and a total of 400 people from teachers and students. Eleven groups of 11 outstanding players from 11 cities in the province, with their own deep understanding of learning the history of the Party, competed on the same stage, and finally Zhu Dandan from Hangzhou won the grand prize of the final. Hangzhou Revolutionary Memorial Hall narrator Zhu Dandan with a brief sitcom, for everyone to reproduce the revolutionary ancestors fearless and firm calm. Liang Zhenwei, a student from Donghai School of Science and Technology of Zhejiang Ocean University, had a space-time dialogue to interpret the Power of Faith. Taizhou fire rescue detachment Qu Zibo to tell the "blue" friends in the fire water forward moving story. In addition to the keynote speech, the contestants also improvised on key entries such as "window", "red", "practical" and "digital". Shu Hyatt, a reporter from the Jindong District Financial Media Center in Jinhua City, chose "red", saying that every historical event enters the "red" of The Times, and that the young people in the new era forge ahead to realize the great rejuvenation of the Chinese nation. Zhou Di, a nurse at Huzhou Hospital of Traditional Chinese Medicine, chose the "window". In the past six months, she has received 600 or 700 people vaccinated against COVID-19 every day. She knows that she represents not only individuals, but also millions of medical staff, and everyone is a window of this era. Party history learning education, the communist youth league of Zhejiang provincial party committee earnestly implement the provincial party committee and the central decision deployment, under the guidance of the provincial party committee propaganda department, high quality to promote "learning party history strong faith with the party" youth preach, the province 11 cities more than 60000 youth XuanJiangYuan grassroots, carried out more than 20 thousand field online, covering youth member more than 600 ten thousand people. In the next stage, the majority of young speakers will also go deep into the fields, schools and factories, widely spread the Zhejiang "three places", "August 8 Strategy" a blueprint drawn to the end, "the taste of truth is very sweet" and other stories, so that the party's innovative theory "fly into ordinary people's homes". Source: China Youth Daily

2021-10-14	Inner Mongolia young pioneers show excellent textbook play to celebrate the 72nd anniversary of the Inner Mongolia young pioneers performance excellent textbook play to celebrate the 72nd anniversary of the electricity (the green newspaper reporter Shi Jia) on October 13, "please rest assured, power have me" Inner Mongolia young pioneers to celebrate the 72nd anniversary of the theme team day activities and "red scarf heart to the party" excellent textbook play (sitcom) performance held in Hohhot, Inner Mongolia. Since the party history learning education, Inner Mongolia the young pioneers organizations at all levels closely around strengthening the young pioneers honor the key goal, focusing on political enlightenment and values shaping the main business, the cast of the Chinese nation community consciousness education into party history learning education, with "red scarf heart to party" as the theme, to carry out "the party's story I tell-be red scarf narrator" "do a good thing for the people" "revolutionary martyrs inheritance red gene" "drum sound party", "red scarf" five years plan " red practice education and team activities, help the young pioneers in Inner Mongolia lay bright red background. Show the textbook drama "Shanghai, 1921". In the green newspaper network reporter Shi Jia / taken for rich party history learning education form, deepen the education results, group of Inner Mongolia district party committee, Inner Mongolia young pioneers organizations in Inner Mongolia to carry out "please rest assured power have me" in the young pioneers series of theme activities and "red scarf heart to the party" excellent textbook drama (sitcoms) solicitation, after layers of selection, expert selection, etc, finally determine eight excellent works to participate in the performance. Show the textbook drama "yellow chrysanthemum open". Pictures provided by respondents performance, "1921 Shanghai" "red rock" "forever nine" "cry" "national children" "yellow chrysanthemum open" "peak" and "boat" and other plays, through panoramic type, immersive performance, reproduce the communist party of China unity led the Chinese people of all ethnic groups unite as one, hard struggle glory years, inspire the young pioneers in Inner Mongolia from one hundred party history draw spiritual nourishment and strength, firmly listen to the party, feeling party grace, follow the party faith confidence, cast the Chinese nation community consciousness, efforts to grow to bear the rejuvenation of the era of new.

2021-10-14	Guangdong held the young pioneers commemorate the 72nd anniversary theme team day activities in Guangdong held the 72nd anniversary of the founding theme team day activities Zhang Jianwei correspondent YueQing) on October 11, sponsored by the Guangdong provincial party committee, Guangdong municipal committee, Guangzhou municipal working committee to undertake "please party assured power have me" -Guangdong young pioneers to commemorate the 72nd anniversary of the theme team day activities held in Guangzhou the second children's palace. Provincial party committee propaganda department, provincial government, provincial department, provincial brigade hall, provincial veterans affairs department, provincial sports bureau, provincial academy of sciences, provincial women's federation, provincial association for science and technology and other related bureaus, Guangzhou related departments, the first batch of Guangdong young pioneers campus practice base and the young pioneers on behalf of more than 200 people gathered, the province cities in the form of venue synchronous theme team activities. Chi Zhixiong, secretary of the Provincial Communist Youth League Committee, Luo Jicheng, deputy secretary of the Guangzhou Municipal Party Committee and honorary director of the Municipal Youth Working Committee, and the Young Pioneers jointly launched the first "Guangdong Red Scarf Culture Season". On the day, Chi Zhixiong, secretary of the Communist Youth League Committee, Luo Jicheng, deputy secretary of the Guangzhou Municipal Committee and honorary director of the Municipal Youth Working Committee jointly launched the first Guangdong "Guangdong Red Scarf Culture Season", and announced the theme logo of the activity to the society. It is reported that this activity is the first provincial "red scarf culture season" series of activities, to better display the young Pioneers culture, drive the Guangdong Province Young Pioneers organization into the society, enter the online, this activity will continue until December. Activities, the relevant bureaus for Zhongshan library of Guangdong province, Guangdong museum, Guangdong cultural center, Guangdong Olympic sports center, microbial institute of Guangdong academy of sciences, women and children activity center of Guangdong 11 first young pioneers outside practice education camp (base), less committee, Guangzhou children's palace youth palace, huangpu district wen chong street less committee, southern newspaper small press corps (Guangzhou) young pioneers brigade four pilot in Guangzhou young pioneers organization. Guangdong Young Pioneers after-school practice education camp (base) awarding ceremony. At the scene, 20 young Pioneers performed the song "Youth" and the original children's song "Dream in the Heart" in the form of Acabella and flash mob. The melodious melody and warm atmosphere infected many tourists around them. After the ceremony, the participants watched the immersive music children's drama "We are the Successors of communism" created by the Guangzhou Second Children's Palace at the Guangzhou Second Children's Palace, and led the young pioneers to recall the revolutionary spirit, review the revolutionary history and feel the red culture. The Young Pioneers' song shows "Dream in the Heart". It is understood that the Guangdong Provincial Committee and Guangdong Youth Working Committee will play the role of the first young pioneers practice education camp (base), expand practical projects and carriers, strengthen the young pioneers counselors, organize and guide the young pioneers and counselors to fully support the activities, improve the young pioneers' responsibility consciousness, innovative spirit and practical ability, and build a new mode of education."We are the Successors of Communism" is an immersive musical children's drama created by the Guangzhou Children's Palace. Li Cheng, a young pioneer of Kang Youwei Memorial Primary School, Liwan District, Guangzhou city, said, " We will actively participate in the young Pioneers' practice activities, increase knowledge, exercise will and enrich experience in another classroom and another world, and strive to grow into qualified successors and builders in the new era."The relevant person in charge of Guangdong Provincial Committee of the Youth League and Guangdong Provincial Youth Working Committee introduced that during the team construction day, relying on the" Guangdong Red Scarf "wechat public account, we innovatively launched the" I Love the Red Scarf " H5 game, which has attracted more than 1 million young pioneers to participate in the launch day.

2021-10-13	Sichuan youth life education public welfare project "package" Sichuan youth life education public welfare project "package" in the youth network reporter xin wang) on October 12, the communist youth league committee of Sichuan province, Sichuan province education department jointly organized in Sichuan province "please rest assured, power have me" theme team day activities and life education plan pilot school licensing ceremony. At the event site, the after-school counselors of the Young Pioneers from many industries led the primary school students to experience learning the self-protection and self-rescue knowledge in the immersive activity area around the fire safety, emergency first aid, traffic safety and other contents. Off-school counselors introduced fire safety knowledge to the young Pioneers. Wang Longhua / taken according to introducing, for the implementation of the Sichuan provincial party committee on strengthening adolescent mental health education and life education deployment, Sichuan provincial party committee joint of Sichuan province education department of youth league issued "about comprehensively strengthening the adolescent students' mental health education and life education implementation plan", for the province young students send 9 package, comprehensive escort teenagers healthy growth. The plan is put forward by the Sichuan provincial party committee to implement the "youth life education plan" public welfare project, the project plan in 21 cities in Sichuan province (state) of 44 schools to carry out the pilot, aims to strengthen the professional curriculum education, play parents school education function, supplement family, school, society, help the healthy growth of adolescent body and mind. The plan also includes a survey of family conditions and students' mental health in middle and secondary schools; Develop mental health education and life education theme group (team) courses; Upgrade the "12355" adolescent mental health hotline; Together with the National Health Commission and other departments and professional social organizations, Recruit, train, and certify a group of professional volunteers, Regularly go into the community to carry out disaster prevention, life first aid, anti-fraud, stay away from drugs and other life education volunteer services; Focusing on the function improvement of the Wenchuan Youth Activity Center, Open mental health education and life education, training and experience projects; Expand the functions of youth home, youth palace, children's companion home and other group activity places; Design and develop a series of short videos, animation, posters and other network cultural products, To organize and conduct research on mental health education and life education topics, Public release of related topics, Soliciting research results from the society, Provide decision-making reference.

2021-10-13	Shanxi: Youth forces gather in the slope protection rock mass of the flood fighting temple museum. Recently, most areas of Shanxi have suffered continuous heavy rainfall, causing mountain floods and geological disasters, with some rivers in danger. The reporter understands from Shanxi Province bureau of cultural relics, as of October 11,19, Shanxi cities reported the province a total of 1783 cultural relics to varying degrees of roof leakage, wall cracking collapse, foundation collapse and surrounding slope protection, wall collapse, nine museum memorial (ancient building museum) also appear small area leakage, and so on and so forth. At present, the rescue and restoration work of cultural relics in Shanxi Province is being carried out in an orderly manner. As early as more than 10 days ago, the rare continuous rainfall in the southern Jin Dynasty caused Yang Yongping's vigilance. As the person in charge of the Shanxi New Youth Emergency Rescue team, he and his team members should make a decision to prepare for duty in advance, waiting for the notice at any time, and set out for emergency rescue. Yang Yongping's feeling is not wrong. From 20:00 on October 2, Shanxi appeared a large range of heavy precipitation, the provincial meteorological bureau launched a rainstorm four emergency response the next day. It was not until 10:00 on October 7 that the fourth-level rainstorm emergency response, which lasted nearly 90 hours, was lifted. In the meantime, the province's 18 counties (cities, districts) precipitation in more than 200 mm, 51 counties (cities, districts) precipitation in between 100 mm and 200 mm, the maximum cumulative precipitation of 285.2 mm. Extreme abnormal precipitation leads to fenhe River, Qin River, Zhuozhang River and other rivers have water rise process and danger. On October 5, Yang Yongping received a call from the Xiaodian District Emergency Management Bureau of Taiyuan city, affected by several days of rain, the area of Foxconn Zhenwu Road area of vehicles were flooded, in need for rescue. The Shanxi New Youth Emergency Rescue Team completed 13 rescue operations in downtown Taiyuan on Monday. As they were about to end the rescue work, Yang Yongping learned that the Wuma River in Qingxu County, Taiyuan City had burst. The Wuma River is a regional tributary of the Fen River. After the river entered Qingxu County, due to the increased terrain and other geographical conditions, it became a hanging river, several meters higher than the surrounding flat ground, usually dry up without water. The dam of the Uma River was hit by the morning of heavy rainfall and the discharge of water from upstream reservoirs. From the afternoon of May 5, the river water volume increased rapidly, from more than 40 cubic meters per second to more than 100 cubic meters per second, and the flood began to overflow the river embankment. Five day and night, who had no time to catch a breath, Yang Yongping quickly contacted the emergency management department and applied to and rushed to the wuma River burst to participate in the rescue. On this night, 20 young emergency members who had just experienced more than 10 hours of intensive rescue rushed to the flood scene in the rain overnight. This is a established under the guidance of Shanxi league organization, professional rescue qualified volunteers, the average age of about 30 years old, some railway workers, some young entrepreneurs, some just graduated college students... on October 5 to 11, Shanxi new youth emergency rescue team in qingxu, Xiaoyi city, jiexiu, shop, jinyuan, rescue for many times, raise 500 supplies, 600,600 rescuers, rescue more than 500 people, rescue more than 5000 people, rescue travel more than 1000 kilometers. On the 11th, the reporter arrived at the Dazhai village with the rescue team, where the rain has stopped, but the lower terrain in the village can still see the deep water, people and vehicles are inconvenient, rescue workers are on the ground after the heavy rain. From October 2 to 7, Shanxi suffered the strongest autumn flood season since meteorological records. Affected by the continuous heavy rainfall, 37 rivers in the province were flooded, and expressways, national and provincial trunk lines and railways were affected to some extent. Fenhe River Xinjiang section, Wuma River Qingxu section, Ciyao River Fenyang section, Xiaoyi section and many other breach, Nantongpu line Qi County Changyuan River bridge bridge was washed down, sleepers suspended, resulting in the suspension of the train. Several days of rainfall and the impact of loess geology in Shanxi Province, Jinzhong city, Luliang city, Linfen city have occurred a number of collapses, landslides and other geological disasters, causing casualties. As of press release, the province's 76 counties (cities, districts) in 11 cities, 1.7571 million people affected, 15 deaths, missing 3 people, emergency relocation of 120,100 people, crops affected area of 3.576,900 mu, 19,500 collapsed houses, 18,200 serious damage, direct economic loss of 5.029 billion yuan. Disaster is an order, and flood control is the responsibility. Provincial youth league organizations at all levels heard the "flood" and move, quickly set up the youth commandos, volunteer service teams, the youth quickly gathered in the flood control and disaster relief front line, to guard the home to build a "youth dam". The Shanxi Provincial Party Committee of the Communist Youth League held an emergency meeting to deploy the provincial Communist Youth League to participate in the flood control work, requiring the provincial youth league organizations at all levels to follow the unified arrangements of the local Party committees and the government, closely monitor the changes in the flood situation, carry out risks and hidden trouble investigation, help transfer personnel and materials, and protect the lives and property safety of the people stationed in the village. At the same time, the Shanxi Provincial Youth Development Foundation was guided to actively connect with the donors and the affected areas to ensure that the donated money and goods can reach the affected people in a timely and accurate manner. The Qingxu County Party Committee of Taiyuan City immediately transmitted the flood situation information through "online + offline" way, mobilized youth members to participate in flood control and disaster relief, organized youth commandos to go to the front line, put into disaster relief and rescue, and cooperated with the party and government to complete river repair and mass transfer. The Luliang Municipal Party Committee of the Youth League organized youth League cadres at all levels and youth social organizations to set up youth commandos to participate in various disaster relief activities in a scientific, orderly and safe way, extensively mobilized social caring people and youth league members in various fields to contribute their love, and actively donate money and materials to the affected areas and the affected people. Since October 6, Zhang Zhexian, the 26-year-old party secretary of the Tuan Xinjiang Wan 'an County Committee, and the young volunteers have been stationed at the Wan' an Middle School resettlement site for seven days and seven nights to provide warm services to the people there. Under the call of the league organization, more than 30 youth league volunteers in the town rushed to the resettlement site."Our volunteers are young, but they do things very well. At the beginning of the evacuation days, hundreds of displaced people came here, everyone did not shout tired, day and night to service."Zhang Zhexian said that the busy in the resettlement site and the surrounding residents, everyone is very concerned about the life of the affected people in the resettlement site."Many nearby residents have sent their own cooking dishes, rice soup, and some calls to donate supplies. In fact, we have enough supplies now. Thank you for your concern."Zhang Zhexian said. After the disaster, the Yuncheng Communist Youth League Municipal Party Committee for Xinjiang, Jishan, Hejin three county (city) youth League Committee urgently allocated special league fees to support the flood rescue. TuanXinjiang County Party Committee quickly connected with the resettlement sites to continuously follow up the needs of the affected people; Tuanjishan County Party Committee recruited more than 60 volunteers to carry out volunteer services such as traffic diversion and duty; TuanHejin Municipal Party Committee actively mobilized young volunteers to patrol in key areas such as urban streets and canal dam. Zhang Zhexian told reporters that the disaster is painful, but the mutual assistance in the disaster is touching, " in times of crisis, people help each other every scene makes people feel warm, full of confidence."China Youth Daily China Youth network reporter Hu Zhizhong source: China Youth Daily

2021-10-13	October 13, the young Pioneers of China) is the founding day of the Young Pioneers of China. Today, the Shanghai Young Pioneers announced the official launch of the Shanghai Young Pioneers happiness education action at the founding of the team, releasing the "15-minute Happiness Circle" activity menu. Fifty-one primary and secondary schools and communities were awarded the "Young Pioneers Happiness Education Experimental School and Experimental Community" card, three after-school activity venues such as the Zhongfu Children's Palace set up brigade committees, and 14 district-level after-school youth working committees were awarded. Earlier, the Central Committee of the Communist Youth League and other 13 departments jointly issued the Implementation Opinions on Building the Socialized Work System of the Young Pioneers in the New Era, which clearly strengthened the construction of the organization of the young Pioneers in places such as youth palaces and other off-campus activity venues. The Shanghai Municipal Youth Working Committee took the lead in trying to build a team in the Shanghai Chinese Welfare Association Children's Palace, the Municipal Youth Activity Center, and the future team will cover the urban youth palace, student activity center and youth activity center. The reporter noted that this round of happiness education action launched in Shanghai is an important part of the young Pioneers socialized work system."After the young Pioneers build the team, they should also pay attention to the team building problem. Let the young Pioneers organization live in all fields."Shanghai young working committee director, the city young Pioneers general counselor Zhao Guoqiang said. Today, the Shanghai Municipal Youth Working Committee also gave the young Pioneers a big gift- - "15 minutes of happiness circle" activity menu. The "15-minute Happiness Circle" is that the Shanghai Young Working Committee combined with the Shanghai 2035 "15-minute life Circle" plan, based on the innovative mode of Shanghai Young Pioneers happiness education construction, encourages the young working committees at all levels to integrate regional red, education, entertainment, physical exercise and other practical resources, to provide convenience for the team members to carry out practical activities nearby. In the future, the community young working committee can "order and order" to the municipal young working committee, and the municipal young working committee will distribute the activities to the community. In the cultural activity center of Wuliqiao Street, Huangpu District, the young pioneers come to play basketball, calligraphy, oil painting, and flower arrangement, etc. These projects are distributed by the Huangpu District Young Working Committee and the Municipal Young Working Committee, some of which are created by the community. According to the newly released Shanghai Young Pioneers happiness education experiment plan, both experimental schools and experimental communities have been required to "innovate the first launch activities and form a team and one product" to enrich the young Pioneers' extracurricular life after the "double reduction". In Guanlan Primary School, established girlfriends family team, create a new model of family mutual assistance, in Hongkou Jiangwan Town street, built a rainbow weekend camp for students, with the street each citizen station platform, systematic, curriculum community activities in the spare time; Fudan junior middle school organized team members to form a number of "community micro update adjacent team" according to the community nearby, lead the building team joint activities, with practical action for "urban micro update" advice. Zhao guoqiang said that the happiness education experiment program focuses on making children experience family affection, partner friendship and community warmth. In the future, Shanghai Young Working Committee will promote the development of young Pioneers happiness education to flourish throughout the city.

2021-10-12	Heilongjiang young pioneers to carry out the 72nd anniversary of the young pioneers founding theme team day activities in Heilongjiang province to carry out the 72nd anniversary of the young pioneers founding theme team day activities (youth network trainee reporter Han Rong) today, the Heilongjiang provincial party committee, Heilongjiang province less work committee of Harbin municipal party committee, Harbin less work committee to carry out the "please party assured power have me" to celebrate the 72nd anniversary of the young pioneers team online theme team day activities. Online theme team day activities through the young pioneers joining ceremony, watch the history of the party learning education achievement video video, Heilongjiang province red scarf tour theme tour, "cloud visit" the young pioneers practice education base, led the young pioneers listen to era pioneer affectionate message and blessings, for a new era of youth party members, members of feeling spiritual strength. It is understood that this activity will continue to carry out from October 11 to October 13, TuanJiamusi Municipal Committee, Jiamusi City Young Working Committee, TuanJixi Municipal Committee, Jixi City Young Working Committee will celebrate in the next two days in turn. At the same time, in order to celebrate the 72nd anniversary of the founding of the Young Pioneers, Heilongjiang Provincial Young Working Committee, Jilin Provincial Young Working Committee and Liaoning Provincial Young Working Committee will also jointly carry out the "Please rest assured, strong country has me" new era young people online young Pioneers theme activities. In addition, the Beidahuang spirit short film "March on the Black Land" shot by the Heilongjiang Provincial Youth Working Committee will be broadcast in the live cloud team class themed "Please rest assured of the Party, the strong country has me" held during the "113 th" period of the National Youth Working Committee.

2021-10-12	Fujian "interpreta dream action" for Fujian 66000 difficult students dream refueling "dream action" 15 years for Fujian difficult students dream refueling zhang correspondent, Zheng Xiaofang Chen Fangting) " with your funding, I was able to devote myself to study, thank the warmth of the social from all walks of life, I must become excellent students, better return to my motherland in the future!"In August 2021, Fujian Provincial Hope Project Office and Fujian Provincial Youth Development Foundation received a letter from Xiao Xie, a 2021 freshman from China University of Political Science and Law. Xiao Xie is the recipient of the "2021 Fujian Province Hope Project to Realize the Dream Action". With the help of all walks of life, she can finally pursue hope with her own efforts. Xiao Xie's family is in the Sanming Mountain area, the junction of Fujian and Jiangxi provinces. There are only three people in the family. His father suffers from illness since childhood, and the action is not convenient, and the labor force mainly depends on his grandfather. She was admitted to the China University of Political Science and Law with a good score of 614. After learning about the situation of Xie's family, with the active assistance of Officer Xiao, the cadre in the village, and with the help of the local Communist Youth League organization, the Provincial Hope Project Office and the Provincial Youth Development Foundation, Xie received the "2021 Fujian Hope Project Dream Realization Action" and successfully embarked on the road of study and dream realization. Learned, "Fujian province hope project interpreta dream action" since its launch in 2006, has been for 15 consecutive years, in the provincial communist youth league organizations at all levels and the joint efforts of hope project agencies, has raised about 160 million yuan, accurate funding family difficulties college students more than 66000, has become the communist youth league of Fujian province to accurate aid, help the healthy growth of the public student brand. In June this year, "2021 hope project interpreta dream action" in Fujian province continue to carry out, focus on entering double first-class colleges of Fujian nationality difficult family students, orphans, disability, due to major family changes cause economic difficulties of college freshmen, help difficult families students raise money from home to school, help them interpreta dream university. By August 2021, the province had raised more than 11.8 million yuan to help 2,891 freshmen from poor families successfully enter college smoothly. In the process of implementing the "Fujian Province Hope Project Dream Realization Action", the local Communist Youth League organizations constantly innovate the working mechanism, strengthen the publicity and guidance, actively mobilize the attention and support of all sectors of society, and pay attention to the ideological guidance of the assisted students. Youth League Longyan Municipal Party Committee to carry out the "Hope Project Dream to realize the Action" as an important measure to deepen the "I do practical things for the masses" and "let the Party flag fly high at the grass-roots line"; Quanzhou Municipal Party Committee will "Hope Project Dream Action" and the talent harbor plan innovation integration, Carry out the "Youth Dream World Heritage Quanzhou" activity, Organize the assisted students to participate in the world heritage experience and interactive communication activities, Let the students know about their home, and love their hometown, Return to my hometown after learning; The Ningde Municipal Committee of the Youth League combined the party history learning and education with the "Hope Project Dream Action" grant distribution ceremony, Organize the assisted student representatives to visit the Mindong Light Education Base, Learn about the local red culture story; The Sanming Municipal Committee of the Youth League organically combined the "Hope Project Dream Action" and "Casting soul dream and youth to protect the young" crowdfunding project, Form a linkage with all counties (districts), Seeking various funds for students, Realize the dreams of college freshmen from poor families. Youth league organizations at all levels and hope engineering implementation agencies strictly implement the "hope project interpreta dream action" funding management procedures, adhere to accurate funding, in the beneficiary choice, capital delivery, open and transparent key link rigorous detail, ensure the funding process fair, just, through the "through train" form, directly grants to the students' bank account, ensure that grants issued 100% in place to the people, accurately and efficiently to the party and the socialist family warmth to the beneficiaries.

2021-10-12	Sichuan introduced the young pioneers socialization work three years action plan in the street (township), community widely established young work committee in the street (township), community widely established less work committee, the province all primary and secondary school each brigade equipped with 1 school counselor, promote the community party organization into the overall work arrangement... recently, the Sichuan Youth League Committee, Sichuan Province, Sichuan Young Pioneers socialization work three years action plan "(hereinafter referred to as the" plan "), the young pioneers socialization work put forward a series of plans and measures. The Plan puts forward a clear three-year plan in consolidating the organizational foundation of the young Pioneers outside the school, strengthening the construction of the counselors, and strengthening the construction of the young Pioneers."The plan" proposed, in the streets (township), communities (there are junior high schools, primary schools or young pioneers in more than 100 people), from 2021 to the end of 2023 built 500 each year, with 3 years to basically build the province's streets (township), community young working committee work pattern. Street (township), community less working committee to do what? The Plan makes it clear that according to the physical and mental characteristics and interests of the young pioneers, we should actively organize the young pioneers' activities such as red education, volunteer service, search and experience, community governance, and public welfare help. In the youth palace, student activity center, children's activity center, youth activity center (camp), science and technology museum and other places off campus, the plan also puts forward the goal of covering the construction of the committee. In terms of the construction of after-school counselors, the Plan requires that by the end of 2021, all primary and secondary schools in the province will be equipped with one counselor for each brigade and one counselor for every two squadrons. In terms of the construction of the young Pioneers 'practice and education positions, the plan proposes that by the end of 2021, the young Pioneers' practice and education base should be established at a proportion of no less than one base per 10,000 young pioneers. By the end of 2022, each base will basically have "six one" standards: a set of operation mechanism, a team of young Pioneers outside counselors, a pair of schools, a brand practice activity, an activity "card" mechanism, and a "Red Scarf Medal" characteristic chapter."Planning" requires that the city and county young working committees should timely carry out planning evaluation, the evaluation results into the work performance, strengthen the supervision and inspection of the planning implementation, widely listen to the young Pioneers, young Pioneers counselors and grass-roots young Pioneers organizations feedback and suggestions, timely improve the work, to ensure the smooth completion of the planning objectives and tasks. Source: China Youth Daily

2021-10-12	Zhejiang young pioneers to commemorate the 72th anniversary of the theme activities held the 72nd anniversary of the theme activities held the China youth daily client Hangzhou on October 11 (the youth newspaper reporter Jiang Yutong) on October 11 afternoon, the young pioneers in Zhejiang province commemorate the 72nd anniversary of the theme activities, opened in the loud team singing. More than 200 young pioneers workers and representatives of the young pioneers from all over the province gathered in Nanhu Lake, Jiaxing, where the revolutionary red boat set sail, to celebrate the young Pioneers' founding day. This activity is hosted by the Communist Youth League Zhejiang Provincial Party Committee, Zhejiang Provincial Civilization Office, Zhejiang Provincial Department of Education, Zhejiang Provincial Youth Working Committee. The activity is divided into two phases. The first stage of the theme activities by the "red boat beginner's mind does not forget" "red roots, childlike innocence guardian" "dream power in one hundred I" three chapters, through sitcoms, preaching, interviews, singing and dancing, show the Zhejiang young pioneers in the party history learning education, expressed the Zhejiang young pioneers inheritance red gene, keep red keep good roots, to be a new era of good determination. Red boat young forum theme activities, also launched the "Zhejiang" red scarf college power double reduction service action ", youth corps committee in Zhejiang, Zhejiang province by" red scarf college "established at all levels, improve the curriculum and training system, hire each front advanced figures, youth corps committee, less working committee institutionalized as a" red scarf college "counselor, launched" double reduction "service activities list, give full play to the" red scarf college "at all levels in the positive role of" double reduction " work."Red scarf retrace a road" red studies activities in the second stage organization "red scarf retrace a road" red studies activities, the guests, the young pioneers will pursue the communist party of China "a" representative in Jiaxing, in the immersive experience visit review the glorious history, feeling the party's beginner's mission, grasp the party's innovation theory, recognize the party's spirit pedigree, guide the young pioneers heartfelt love the party, love the motherland, love the people. It is reported that since this year, Zhejiang Provincial Young Working Committee around the "pay tribute to the century-old great cause, strive to be a good member in the new era" theme, deepen the development of the "red boat reflect the red heart red scarf heart to the party" party history learning and practice activities, a total of more than 4 million young pioneers participated. Through theoretical study, ritual edification, practical experience, education and guide the young pioneers from primary school pioneers, grow up to be pioneers, with practical actions to practice the "please rest assured, the strong country has me" clank oath, for the realization of the Chinese dream of the great rejuvenation of the Chinese nation always ready.(All the pictures in this article are provided by the Youth Department of Zhejiang Provincial Party Committee)

2021-10-11	Shandong: reporter Xing Ting "power youth red reading" theme activity, promote youth development friendly city construction theme seminar and "youth sunshine" youth elite symposium, "college students employment promotion action... in the first half of this year," serve youth development construction youth city " 2021 youth development friendly city (sunshine) activity season launched, through a series of concrete measures, let youth and city promote each other, grow together, common development, to create a benchmark for youth development friendly city. Rizhao's specific practice is an epitome of the implementation of the Shandong Communist Youth League of the Medium-and Long-term Youth Development Plan (2016-2025) and the Shandong Province Medium-and Long-term Youth Development Plan (2019-2025) (2016-2025) issued by the CPC Central Committee and The State Council. Since June 2019, Shandong communist youth league aimed at the "planning" target task, combining the actual situation, to serve youth development as the foothold, focus on building youth development friendly city, around strengthening youth thought lead, support youth in strong province construction, promote the development of youth education, service youth employment entrepreneurship, improve youth physical and mental quality, promote the development of youth culture, promote youth social integration, safeguard youth legitimate rights and interests of eight aspects, put forward article 22 specific measures. At present, the Youth League committees of Jinan, Zibo, Weifang, Rizhao and other cities take the lead in blowing the youth rally call, and the local municipal Party committee and municipal government have also clearly proposed to promote the construction of a youth-friendly city, and constantly improve the city's absorption, attraction and carrying capacity of outstanding youth inside and outside the province."Our youth started in Jinan" 2021 Jinan college students graduation ceremony on June 18, Shandong provincial party committee, Jinan municipal party committee secretary Sun Licheng in "our youth in Jinan" 2021 Jinan college students graduation activities, with full sincerity to nearly 200000 college graduates issued "new era new Jinan" invitation, with "talent 30" "double gen 19" "bird fly dream springs" action, etc., to promote the construction of "youth friendly city". At present, Shandong is in the critical period of "vacate the cage for the bird" and "Phoenix nirvana ��". How to realize the high-quality economic and social development of the province by promoting the construction of youth development and friendly city has become an urgent issue to be solved. Shandong communist youth league give full play to the role of the provincial joint meeting, further promote the planning landing implementation of the powerful force, clear and detailed specific departments and responsibility, in accordance with the principle of strengthening work pertinence and effectiveness, normalized in-depth study, highlight the key and difficult problems of youth development, constantly forming good experience, good practice. At the same time, strengthen the work supervision and inspection of the city and county level units of the system, providing a strong organizational guarantee for the implementation of the plan. At the same time, continue to broaden the carrier, enhance the vitality. In recent years, Shandong relying on "green summit" "green bird plan" "urban and rural community youth action" "school cooperation youth double gen action" "hope" child care project, Shandong province "skills" vocational skills competition and a series of work carrier, in the provincial level youth development friendly city index system research, promote the development of Shandong province and youth all-round development of benign interaction. The 10th Shandong Youth Innovation and Entrepreneurship Competition Award and Quancheng Youth Innovation Conference 10th Shandong Youth Innovation and Entrepreneurship Competition has attracted more than 8,600 young entrepreneurship teams and 40,000 young entrepreneurs, covering more than 200,000 youth employment and entrepreneurship. In three years, the "Good youth" selection and training program has cultivated more than 155,000 good rural young people with good moral character and strong ability, forging a youth "new force" for rural revitalization... a series of strong measures have made more and more young people truly feel the friendliness of Shandong to the youth.

2021-10-10	A series of special lectures on "Youth Learning" was held in Guangxi Academy of Agricultural Sciences. At the meeting, the leaders of the Key Laboratory of Guangxi Academy of Agricultural Sciences and the members of the rural revitalization work of the Department of Ecology and Environment of Guangxi Zhuang Autonomous Region respectively shared the stories of youth struggle to help rural revitalization with the participants through their own experience. The event was jointly organized by the Youth League Working Committee of Guangxi Zhuang Autonomous Region, the Youth League Committee of Guangxi Academy of Agricultural Sciences and the third cooperative group of the Communist Youth League. More than 200 people from the leaders of all government organs, enterprises and institutions), colleges and universities in Guangxi, and youth league members listened to the lecture.

2021-10-10	Youth gathered Shanghai red theme post office "to" teenagers of all nationalities gathered Shanghai red theme post office "to" Wang Yejie) recently, students from Shanghai health medical school in Tibet, Shanghai nanhui middle school students in Xinjiang and Shanghai huang xing school, Shanghai Nanyang junior middle school, Shanghai jinattached students gathered in Shanghai red young theme post office, express the blessing of the motherland. This activity is one of the main contents of the theme of "Red Imprint- -Celebrating National Day and Talking about The Chinese Dream". It is co-sponsored by the Cooperation and Exchange Department of the Shanghai Municipal Committee, the ninth Group of Shanghai Tibet Aid Cadres Liaison Group, and the Shajiabang Army History Research Association of the Shanghai New Fourth Army. During the activity, the narrator of the National anthem exhibition hall told the students the extraordinary story behind the national anthem "March of the Volunteers". Gu Hailou, a centenarian of the New Fourth Army, Liu Shi'an, a veteran of the Army, Zhang Zhiyong, the "most beautiful volunteer" in Shanghai, Han Binghua, a famous patriotic designer in Hong Kong, and the Qiang military wife bless the motherland and sent messages to the young people through audio postcards. It is reported that in order to further expand the coverage of youth communication between the eastern and western regions, the organizers of the event have held a new round of letter communication activities in 1,002 teenagers from 12 schools in Shanghai and the western counterpart support region in September this year. Meanwhile, 7,500 postcards sent by primary schools in Shanghai on June 1 have also received replies from the western regions. It is understood that during National Day, studies activity organizers conducted seven theme 72 hours public welfare live activities "I grow with the motherland-I and the motherland 72 change, through the video, TikTok and linkage, around the motherland change, carry forward the" love party, patriotism, love home "red theme, help children know society, return to nature, dialogue, take you into the rural school poverty after crucial rural change, into schools across the country to share" double minus " National Day holiday new harvest. In addition, 17 national outstanding teachers review their own growth process and tell their own growth stories.

2021-10-13	Shandong province "skills lu" professional skills competition final opening in Shandong province "skills" vocational skills competition in Shandong province 17th Shandong youth vocational skills competition computer programmer final opening zhang correspondent BaiChen) on October 10, sponsored by the Shandong provincial party committee, provincial people club hall, pku, city people club bureau, Jining vocational and technical college in 2021 of Shandong province "skills lu" professional skills competition-the 17th Shandong youth professional skills competition computer programmer (cloud computing platform and operations) final officially opened. Relevant leaders of Shandong Provincial Party Committee, Jining Vocational and Technical College Party Committee, Jining Vocational and Technical College, contestants, referees, media journalists and student representatives from all cities in the province participated in the activity. The picture shows the opening ceremony of the "Skills Xinglu" vocational skills competition. Correspondent for figure at the opening ceremony, Jining vocational and technical college, vice secretary of party committee, dean Li Xindou introduced the college development and educational situation, expressed a warm welcome to the event, and said it will lift the floor, to create good conditions for contestants, to provide high quality service, for the competition smoothly, successfully held, provide a strong guarantee. Associate Professor Shen Yanzeng, deputy director of the Department of information of Heze Technician College, and the Department of Electronic Information Engineering of Jining Vocational and Technical College, Teacher Liu Lu, respectively took the oath and spoke as the representatives of the judges and the contestants. League provincial youth development minister xiao-xue li announced the final opening and in his speech, pointed out that the youth vocational skills contest is an important content of "I do the practical work for the youth", the communist youth league organizations at all levels should continue to do education guidance, build stage of skills, continue to optimize service content, comprehensive build "green and qilu" work brand, guide the young generation learning ability, long ability, skills, skills of patriotism. Young people should play the role of a new force, hone their skills down-to-earth, and strive to grow into high-quality skilled personnel, skilled craftsmen and craftsmen of great countries. The picture shows the final of "Skills Xingfu" vocational skills competition. Correspondent for figure, the theme of the provincial competition theme is "history doing new work skills shine new youth", the final lasted 3 days, 53 teams from the province cities, a total of more than 200 contestants will through theoretical knowledge, practical operation competition competition, outstanding players will represent Shandong province in the 17th "revitalization of the cup" national youth vocational skills competition.

2021-10-10	The Youth Vocational College held the launching ceremony of "Jiangxi Youth Future Academic Award" - - "Jiangxi Youth Future Academic Award". At the ceremony, Dr. Zha Xiaochun, a hope project recipient of the University of California, Berkeley, donated 10 million yuan to Jiangxi Hope Project and set up the "Jiangxi Provincial Youth Future Academic Award", which set a new record of individual donations from Project Hope in recent years. Group the relevant person in charge of Jiangxi provincial party committee, "Jiangxi province youth future academic award" sponsors check xiaochun, "Jiangxi province youth future academic award" academic award jury judges Hu Gang, Zhong Hongjun, Jiangxi youth vocational college party secretary Guo Changliang, Jiangxi province youth development foundation, secretary-general MAO Hongshan jointly launched "Jiangxi province youth future academic award".

2021-10-09	The 11th "youth" Anhui youth innovation and entrepreneurship competition successfully concluded the 11th "youth" Anhui youth innovation and entrepreneurship competition successfully concluded Beijing on October 9 (reporter Zhang Jianwei, correspondent, Chen Wenjing Zha Weiting) recently, the 11th "youth" Anhui youth innovation and entrepreneurship competition finals held in Hefei. Hu Jing, deputy secretary of the Anhui Provincial Committee of the Communist Youth League, attended the competition and presented awards to the winners. Leaders of the business department of the provincial co-organizers jointly attended the event. 40 youth innovation and entrepreneurship project teams from all over the province participated in the competition. The competition by the Youth League Anhui Provincial Party Committee, Provincial Party Committee Publicity Department, provincial Cyberspace Office, Provincial Department of Economy and Information, Provincial Economy and Information Technology, Provincial Department of Human Resources and Social Security, provincial Department of Agriculture and Rural Affairs, provincial Department of Commerce, provincial Rural Revitalization Bureau, the competition is co-organized by the provincial youth entrepreneurship and employment Foundation, the provincial Youth Entrepreneurs Association, the provincial Young Entrepreneurs Association. The project involves energy conservation and environmental protection, information technology, biomedicine, bio-medicine, agricultural products processing and sales, e-commerce, Internet equipment, big data, artificial intelligence, smart city and many other fields, showing a wide range of areas, a large number of participants and great social impact. Than the technology, see the data, see the actual results. At the competition site, the innovative projects with breakthrough core technology and market prospects emerged under the passion elaboration of the players. The judges made sharp comments and witty remarks, and "independent intellectual property rights", "fill in the gap" and "breakthrough" became the high-frequency words."Create Youth" Anhui Youth Innovation and Entrepreneurship Competition has become the training ground for innovation and entrepreneurship of small, medium and micro enterprises in Anhui Province. A total of 4 first prizes, 8 second prizes, 12 third prizes and 24 excellence prizes were selected in the final of this "Innovation" Anhui Youth Innovation and Entrepreneurship Competition. The science and technology innovation group of L lactic acid separation special high-speed horizontal spiral unloading settlement centrifuge project, rural revitalization group of nine China huang jing tertiary production fusion project, Internet group ring which zha-based on 5G visual artificial intelligence education industrialization application, social enterprise group of the earth diary-natural studies and natural museum integration operation and other four projects won the first prize respectively."Youth" Anhui youth innovation entrepreneurship competition has been held for 11 consecutive years, this year's competition to "innovative entrepreneurship and youth practical party" as the theme, around the new stage of development, carry out the new development concept, build a new development pattern, actively lead the youth to implement the "difference" plan and 2035 vision struggle, efforts to stimulate Anhui youth creativity, encourage and support the youth in public entrepreneurship, peoples innovation, show the province youth in rural revitalization, beautiful construction of Anhui.

2021-10-08	Yu Jiahong has been living in Aida 88, the only medium-risk area in Harbin, for five days. At his busiest time, Yu Jiahong registered more than 300 material bills in the morning."We set up a disinfection area at the gate of the community, for the people who came to send supplies to the residents of the community to kill and goods registration, and then the special staff with carts from door to door."Yu Jiahong said that because of the sudden outbreak of the epidemic," Aida 88 " was classified as a medium-risk community, and more than 500 residents in the community have implemented closed management. These days, there are many people outside the community to send materials for the owners in the community, elimination work is more and more important. On September 21, a new novel coronavirus positive infected person was reported in Bayan County. The investigation report showed that the case involved many activity sites in Harbin. The epidemic prevention and control situation is grim and complex, and the prevention and control work has also turned into an emergency situation. The Heilongjiang Provincial Party Committee of the Communist Youth League immediately mobilized and deployed to league organizations at all levels in the province, and issued the Notice on Immediately Joining in COVID-19 Prevention and Control, calling on league organizations at all levels and youth league members to give full play to the role of new forces and commandos. After receiving the notice, more than 50 cadres and workers of the Heilongjiang Provincial Party Committee and directly affiliated units of the Communist Youth League volunteered to participate in the epidemic prevention and control work in the sinking community. After screening, 10 of them formed the "Long Xiaoqing" Youth Commando of the Heilongjiang Provincial Party Committee, and Yu Jiahong was one of them. After putting on red vests and coming to the "Aida 88" community, the volunteers immediately connected with the community, participated in the community epidemic prevention and management, and guaranteed the daily life of the quarantined residents, so as to ensure that the residents in the community had no contact with the outside world. In the face of the epidemic, everyone has s responsibility. After the Heilongjiang Provincial Party Committee of the Communist Youth League issued the Notice on Immediately Joining in COVID-19 Prevention and Control, the youth organizations of the Communist Youth League at all levels also responded and put on red vests into the fight against the epidemic. The Harbin Municipal Party Committee of the Communist Youth League has set up "youth volunteers" and "youth" commandos ", and organized 2,650 young volunteers to participate in the fight against nucleic acid testing, community duty, material transportation and other work in an orderly manner. At Harbin Normal University, more than 100 college student volunteers volunteered for more than 10 hours in the rain, providing convenience for more than 30,000 teachers and students."After receiving the notice from the teachers of the school Youth League committee to convene the students to participate in the nucleic acid testing volunteer activities, the students were very eager to sign up."Sun Hongda, chairman of the Xingzhi Youth Volunteers Association at Harbin Normal University, told reporters that more than 100 students had signed up shortly after the news of gathering the volunteers was sent out. The volunteer service lasted from 5:30 p. m. to 3:30 a. m., and the volunteers stood on their "ground" without complaint."When we got back in my dormitory, it was as if my legs were not our own, but compared with the medical staff, our hard work was nothing."Said Sun Hongda. The epidemic is an order, and prevention and control is the responsibility. Harbin after the outbreak, the communist youth league Mudanjiang municipal party committee attaches great importance to, organized 1641 young volunteers to assist community workers to carry out the returning personnel screening registration, community public code, temperature, vaccination inspection, covering the city's 118 communities and 402 CunTun, volunteer service for more than 64500 hours, volunteer service per capita 39.6 hours. Young volunteers from the Shuangyashan Municipal Party Committee of the Communist Youth League also wore red vests to join the front line of epidemic prevention and control. They have distributed leaflets on epidemic prevention and control knowledge to key street shops, market vendors and passers-by, and explained tips on epidemic prevention and control. During the event, the young volunteers handed out more than 1,400 leaflets and more than 200 masks. In order to accelerate the spread of epidemic prevention knowledge to the masses, we will accelerate the construction of a strong vaccination barrier. The Daqing Municipal Party Committee of the Communist Youth League relies on the new media matrix platform of the Communist Youth League to popularize epidemic prevention and control knowledge, and produces interesting and popular epidemic prevention videos in combination with the characteristics of teenagers. Up to now, Daqing new media platform has released more than 400 epidemic prevention and control information, with a total of more than 800,000 views. In Aihui District of Heihe City, the young volunteers will do the monotonous and mechanical work of checking the health code and registering the information carefully. Over the nine days, 47 volunteers have investigated more than 60,000 people in 15 key public places, and 1,959 people have not been screened and registered. The communist youth league qiqihar municipal party committee recently organized will be coronavirus vaccination work concentrated propaganda day activities, cadres, members at all levels of social organizations, youth volunteers represent a total of 483 people into the city 44 business super, set propaganda at the entrance, promote the importance of will be coronavirus vaccination, positive screening immune blank population, service for 1255 hours, screening out not vaccinated 735 people, for citizens to get full "security" to add a youth power. On the battlefield of epidemic prevention and control in Heilongjiang Province, young commandos and young volunteers sang the song of youth on the front line of epidemic prevention and control, integrating the volunteer service spirit of "dedication, friendship, mutual assistance and progress" into their practical actions. Wu Yan China Youth Daily China Youth network trainee reporter Han Rong source: China Youth Daily

2021-10-08	Shanxi "send training grassroots" and green horse engineering training in Shanxi "send training" and green horse engineering training Jianwei correspondent jin-rong zhang), sponsored by Shanxi provincial party committee, provincial league to undertake "learn party history, strong faith, follow the party" -Shanxi provincial party committee "send training" and green horse engineering training into the organs, universities, state-owned enterprises, united front, organizations, social organizations, focus on preach jinping general secretary "July 1" important speech spirit, more than 800 young people listened to preach. During the lecture, the lecture teacher also taught the glorious deeds of fu Jiao Wang, an old model worker around him, by learning the deeds of Comrade Jiao Yulu, an excellent Communist Party member. Wu Lei from Yaodu Commercial Bank of Linfen walked into the Linfen Youth League Municipal Committee, combined with the spirit of the Jinggangshan carrying pole, the local Eighth Route Army headquarters and the carrying pole of the headquarters of the city, systematically taught the branch of the Taihang spirit spectrum- -the carrying pole spirit. Zhang Jiangwei, from Shanxi Academy of Economic and Management, systematically taught the significance, content and methods of party history learning education for 186 students, including key party trainees, young teachers and horse students. During the training, the teachers had in-depth discussions with the youth on implementing the spirit of General Secretary Xi Jinping's important speech on July 1. Taiyuan university of technology gao hang into the information technology application innovation base in Shanxi Province, for the taihang letter and talent education alliance, genting academy of 114 youth, explained how to act in the industry, and said college students to promote the development of Shanxi letter and industry as the mission, to crucial letter and hardcore technology as the goal, based on Shanxi, deep in Shanxi, rooted in Shanxi. Zhang Li from Shanxi University walked into the School of Chemistry and Chemical Engineering, and explained to 140 "Qingma" class students, youth league cadres, teachers and student party members how to play the leading role of the national graduate model Party branch and the importance of college students to establish a correct world outlook, outlook on life and values. After listening to the lecture, the student Chu Zhipeng sighed: " The lecture context is clear, the focus is prominent, with positive energy deeds elaborated the political theory in simple terms, encourage everyone to read the original work, learn the original text, understand the principle, improve the interest in learning the Party history, and further enhance everyone's understanding of the educational significance of the Party history study.”

2021-10-05	Jianwei Correspondent Zhang Zhuo) Recently, the Ningxia Hui Autonomous Region Party Committee held a special reading class for young cadres in the whole region. The reading class lasts for 5 days, through inviting experts and scholars to give special guidance, interviewing the advanced deeds of Wang Lanhua, the winner of the National "July 1 Medal", going to the Red Education Base to observe and study, and carry out group theme discussion and other forms. KaiBanShi, all the students to review the xi general secretary "July 1" important speech spirit, concentrated study the general secretary xi in the central party school (national school of administration) young and middle-aged cadres training KaiBanShi's important speech spirit, communicate learning the autonomous region party committee standing committee "adhere to the case to promote the deepening style construction" project democratic life meeting spirit. After the opening ceremony, Song Liping, director of the Office of the Party Committee of the Autonomous Region, gave the first special lecture on "Studying and implementing the spirit of General Secretary Xi Jinping's" July 1 "" for the students. Representatives to the 18th National Congress of the Youth League, all the cadres of the organs of the Youth League committee of the autonomous region, the city, the county (district), the leaders of the youth League committee of the district, the youth league cadres and the youth representatives attended the reading class.

2021-10-02	Guangxi "youth learning" party history knowledge challenge offline contest held in 2021 in Guangxi "youth learning" party history knowledge challenge offline contest held reporter Xie Yang correspondent Li Wei Zhao Yuxin) on September 28 to 29, "learning party history, strong faith, follow the party" -2021 in Guangxi "youth learning" party history knowledge challenge offline contest held in Liuzhou city, Guangxi. The party history knowledge Challenge is divided into two parts: online competition and offline competition. The teams participating in this offline competition are 52 excellent teams selected from Guangxi cities, counties, schools, enterprises and institutions from the bottom up. Ten teams entered the final through online learning, online challenge, offline preliminary test, and the final written test answer. The final is divided into four parts: required questions, rush questions, risk choice questions, and interactive audience answers, covering the history of the Party, the history of new China, the history of reform and opening up, the development history of socialism and the history of Guangxi revolution. After fierce competition, the Youth League Committee team of Guangxi Airport Management Group won the first place, the League Liuzhou Municipal Committee team won the second place, and the League Beihai Municipal Committee Team won the third place. Fangchenggang Municipal Committee, Guangxi Art University, Nanning Vocational and Technical College, Chongzuo Preschool Normal College, Nanning Financial Service Center of Bank of Communications (District Financial Youth League Working Committee), Guigang Municipal Committee and Beijing Youth League Committee won the excellence award. The activity was sponsored by the Guangxi District Committee of the Communist Youth League, and organized by the Liuzhou Municipal Party Committee and the Youth League Committee of Liuzhou Railway Vocational and Technical College. Since this year, the Guangxi communist youth league combined with party history learning education, further implement the general secretary xi visited Guangxi's important speech spirit, "youth learning" as the gripper, build closely combined with local red resources of "guide, teaching, studies, learning, practice, the superintendent" six one learning system, the learning kung fu real, lead the thought leading work live. Since the online themed Youth League class began in 2021, Guangxi has organized more than 38 million youth league members to participate in the study, ranking first in the youth league for 20 consecutive periods, and more than 1 million youth league members have participated in the "Youth Learning" Party History Knowledge Challenge.

2021-09-30	Chongqing: Brand building of "youth lecturers". Some revolutionary ancestors joined the League first before joining the Party, does it mean that "the League was born before the Party"? During the fight against COVID-19, how did the volunteer service action with the most participants, the longest service time and the most services in the history of volunteer people in Shapingba District of Chongqing realize it? How should such a "young river chief" with no rank, no official, only obligation and no power be regarded? This year, as the members of the Chongqing Youth League Committee of the Communist Youth League went deep into schools, enterprises, communities and other places, these topics triggered thinking and discussion among the youth groups, and ignited their enthusiasm for the history of the Party and the Communist Youth League, current affairs and other hot topics. Founded in 2019, the Chongqing Youth League Municipal Committee was launched in youth lectures, covering rural areas, government organs, campuses, communities and networks to young people from all walks of life. Around the current hot topics concerned by young people, members of the teaching group can always appear in front of young people in time, or based on professional research to analyze in a simple way, or combined with their own experience to share immersive, for the majority of teenagers, and played a enlightening and leading role. The discussion on whether the league before the party was held in a lecture by CAI Fei, a member of the teaching group and doctoral supervisor of Southwest University of Political Science and Law. For this topic, which has been widely discussed, Cai Fei explained an important conclusion on the history of the Party and the history of the youth with rigorous arguments: the Communist Party of China was the founder of the Communist Youth League of China. For many young people, such discussions give them a deeper understanding of the party history and the league history. Yang Xia, a student at Chongqing Vocational College of Economics and Trade, said that she prefers this kind of guided and exploratory interactive preaching to the rigid preaching of indoctrination and preaching. At the beginning of this year, the Chongqing Municipal Party Committee of the Communist Youth League focused on building a high-quality youth lecturers' team, focusing on cultivating a group of "star lecturers" and building a "star team", and recruiting outstanding young backbone with firm political stance, high theoretical accomplishment, outstanding personal deeds and strong propaganda ability as young lecturers. After targeted selection, organizational recommendation, personal recommendation, and selection procedures, the Chongqing Municipal Party Committee of the Communist Youth League adjusted and enriched the team of young teaching groups, and redefined 100 lecturers, including party and government cadres, theoretical experts, party history experts, young teachers, media workers and other workers. Ding Yingshun, a deputy research librarian at the Chongqing Hongyan Revolutionary History Museum, is one of them. He took several months to carefully create the exclusive course, "Carry forward the Red Rock Spirit and Inherit the Red Gene". In order to make the courseware more close to teenagers' learning habits and discourse system, Ding Yingshun repeatedly polished and changed the manuscript. He asked himself to a high standard: " The details still need to be considered, and the online promotion is not fully complete."The Chongqing Municipal Party Committee of the Youth League also divided the youth lecturers into the party's innovative theory," four histories ", youth example and other research groups, and separately organized research and made courseware. According to the idea of "mature one to put one one", the Chongqing Municipal Party Committee of the Communist Youth League plans to complete 100 high-quality courseware within this year and put it into the "lecturer online classroom", "cloud children's Palace" and other platforms, to meet the needs of teenagers' extracurricular learning. Chongqing has a glorious revolutionary tradition. In order to transform the red resources into the spiritual nutrition of the majority of young people, the Chongqing Municipal Party Committee of the Communist Youth League organized the youth League committees at all levels to develop a number of high-quality courses relying on the local unique red resources, which has become the favorite "MOOC" of the Party history for the majority of young people. In Tongliang District, the local Youth League organization planned and filmed 20 "Tongliang Tuan Party History Micro Class", relying on the Qiu Shaoyun Martyrs Memorial Hall and Qiu Shaoyun's former residence. In the video, pages of cultural and historical materials and precious images explain the great spirit around them. In Shapingba, Youyang, Tongnan and other districts (counties), the local youth league organizations, relying on Bai Mansion, Refuse Pit, former residence of martyr Zhao Shiyan, have carefully shot and produced special videos of Party history and spread them on the Internet. Chongqing Youth League School focuses on Yang XX Gong, Jiang Zhuyun and other revolutionary youth, excavates the party history education materials from the perspective of youth, carries out financial media design, and creates a series of micro video courses of "Sichuan and Chongqing Youth League Qingyun". It has launched a total of 15 works with prominent themes, novel forms and well-made financial media works. Especially since July 1 this year, the youth propaganda teams that have studied and implemented the spirit of General Secretary Xi Jinping's important speech have been active in Chongqing to provide theoretical nourishment to the young people. In order to make the lecturers of the young lecturers have a sense of organization, personal identity and social honor, the Chongqing Municipal Committee of the Communist Youth League specially created the exclusive logo of the young lecturers 'group. From the notebook for lesson preparation to the flip pen for class, from the work permit to the teaching AIDS bag, the shining logo makes the image of the young lecturers' group deeply rooted in the hearts of the people. The relevant person in charge of the Communist Youth League Chongqing Municipal Party Committee said that, through "intensive cultivation", the standardized construction of the youth lecturers team, the youth lecturers into a brand leading the thought of the youth, to achieve "party and government recognition, social recognition, youth satisfaction". Correspondent He Kai, China Youth Daily, China Youth Network reporter Wang Xinxin source: China Youth Daily

2021-09-30	The 8th "Youth" Shanxi Youth Innovation and Entrepreneurship Competition successfully concluded the 8th "Youth" Shanxi Youth Innovation and Entrepreneurship Competition successfully concluded Jianwei correspondent Zhang Jinrong) thought sparks splash, creative ideas flying, youth blood filled with. In the China Shanxi Overseas Students Pioneer Park, the 8th "Create Youth" Shanxi Youth Innovation and Entrepreneurship Competition site master "cloud" set, the summit duel. Through the fierce competition in the semifinal, 72 contestants stood out from 149 works and entered the championship competition. After four hours of wonderful online road show, the dust of the awards was settled, and the first, second, third and third awards were finally decided. So far, the 8th "Create Youth" Shanxi Youth Innovation and Entrepreneurship Competition was successfully concluded. The contest lasted three months, by the Shanxi provincial party committee, provincial network letter office, provincial people club hall, provincial agriculture and rural areas, provincial rural area management committee, jointly sponsored by China Shanxi and park, science and technology innovation, rural revitalization, Internet, social enterprise four special, each special and divided into innovation group, startup group, growth group, including startup and growth group first prize subsidies of 100000 yuan, second prize subsidies 80000 yuan, third prize subsidies 60000 yuan. On the day of the final, four competitions will be opened at the same time. Each competition will have 1 provincial and two local evaluation experts from investment institutions, entrepreneurship mentors and teachers in the form of "1 + 2" as judges and scores. Each project selects 1 member with strong expression ability to conduct online defense, including 5-minute PPT presentation and 5-minute judges' questions. Since 2014, the Central Committee of the Communist Youth League, the Ministry of Education, the Ministry of Human Resources and Social Security, the China Association for Science and Technology, and the All-China Students' Federation have decided to jointly organize the "Create Youth" China Youth Innovation and Entrepreneurship Competition on the basis of the original "Challenge Cup" China College Students Business Plan Competition every two years. This year's "Innovation Youth" Shanxi Youth Innovation and Entrepreneurship Competition and "Innovation Youth" China Youth Innovation and Entrepreneurship Competition is the eighth consecutive year that the Provincial Communist Youth League Committee has held this competition jointly with relevant departments of the province."Compared with previous years, this competition is full of highlights."Competition organizing committee staff introduced, first of all, the support for entrepreneurial youth is greater. Last year changed from the commercial and industrial group, agricultural and rural group and the Internet group to four special competitions: science and technology, rural revitalization, "Internet plus" and social enterprises, the total number of awards increased from 63 to 72, and the prize money increased from 3 million to 3.52 million. The scope of awards, the number of awards and the subsidy funds reached a new high. Second, to attract a wider range of quality projects. Organize and mobilize all cities, universities, parks of comprehensive reform demonstration zones and scientific research institutions to recommend and register. At the same time, we will go deep into the innovation and entrepreneurship carrier promotion competitions of various incubators and mass innovation Spaces, and conduct policy publicity, training and guidance, road show docking and other related activities to attract more excellent projects to register for the competition. Third, the service for youth entrepreneurship measures are more detailed. Before the competition, the Provincial Communist Youth League Committee guided the Municipal Communist Youth League Committee to hold information meetings and training meetings, so as to help the participants understand the competition rules and the competition process, organize the winning project leaders to introduce the entrepreneurial experience, and encourage more young people to join the entrepreneurial practice. At the same time, we will continue to track and support the winning projects, and promote the docking of the projects with investment institutions and mass entrepreneurship and innovation carriers. The winning enterprises that meet the conditions in the final competition are recommended to be listed in Shanxi Youth Gem. Finally, the atmosphere of innovation and entrepreneurship is stronger. This year in the competition at the same time, will also hold excellent project special exchange camp activities, provide communication display platform for contestants, and recommend excellent projects to attend the central special exchange camp and comprehensive exchange activities, through the exchange camp activities speak good Shanxi communist youth league help rural revitalization, service high quality transformation development and Shanxi youth innovation entrepreneurial story, comprehensive build Shanxi youth innovation entrepreneurship thick atmosphere.

2021-09-29	League Fujian provincial party committee to implement "sail plan" to promote students to Fujian communist youth league organization implementation of "sail plan" to promote students to practice reporter Tian Hongwei) since this year, the communist youth league Fujian provincial party committee around the "six stability", implement the "six protect" task and "I do the practical work for the masses" practice deployment, focus on the key link and key problems of college students internship employment, multiple integration of resources, multi-channel raising jobs, in-depth implementation of college students' practice "sail plan". Up to now, a total of 3,217 employers have been organized to provide 27,473 internship positions, and 31,432 college students are attracted to submit 87,576 resumes, and 10,987 students are recommended for internship, covering 121 well-known universities at home and abroad, including Tsinghua University and Carnegie Mellon University. In order to ensure that all the tasks of this activity were done in a solid and deep way, the Fujian Communist Youth League strengthened the coordination and cooperation with multiple departments, and actively constructed the linkage matrix between the league inside and outside the league. At the work coordination meeting, each member unit clarified the division of responsibilities, and each district and city formulated the implementation plan according to the actual situation, and constructed the three-level linkage work pattern of province, city and county. Relying on the "smart system", this "Sailing Plan" implements the "half a month notification" of key data indicators such as the number of post solicitation, the number of applicants and the number of internships, and strengthens the whole process and integrated tracking and implementation. Broadcast publicity videos on major media platforms, sending brochures to 214 high-quality enterprises in Fujian Province, and sending letters to 80 Party and government organs, central institutions in Fujian, and central enterprises to extensively raise high-quality government affairs and enterprise internship positions. Select 50 career planners, employment guidance experts and career planners from universities and enterprises in the province, set up a team of internship mentors, and make pre-job training videos to help the interns quickly adapt to the working environment and work smoothly. This activity innovatively uses the combination of online platform and offline channels to guide Fujian students from outside the province to return to Fujian for practice. From bottom to up, an information database of excellent students from outside Fujian has been established at provincial, city and county levels, with 36,000 people. Through E-mail, SMS and other ways, point-to-point internship needs, internship policy introduction and internship post push. Relying on the wechat official account of "Fujian Communist Youth League", we develop the small program of "Sailing Plan", and divide the internship positions by region. Students can check the internship content, duration, location and subsidies online, and understand the job registration and resume delivery progress. In order to support the "Sailing Plan", this activity raised 4 million yuan and set up 2.7 million yuan outstanding intern scholarship. Launch the "Sailing Promotion Star" ranking list, encourage interns to invite more alumni of "double first-class" universities outside the province to pay attention to the "Sailing Plan" and understand the Fujian talent policy. Strive for the support of Fujian Provincial Finance Department, and make it clear that in principle, the labor remuneration of top interns should not be less than 80% of the salary standard of the probation period of the same post. Coordinate with employers to provide subsidies for interns, purchase personal insurance for accidents, and encourage qualified employers to provide accommodation. After the internship, the host unit will issue the internship certificate uniformly. The Fuzhou Municipal Party Committee of the Communist Youth League provides interns with "shared dormitories" in the university town and a transportation subsidy of up to 1,000 yuan.

2021-09-27	Yunnan: 12355 service desk let teenagers need "find" Yunnan: 12355 service desk let teenagers demand "find" the zhang) reporter from September 23 held in Kunming 12355 youth service desk area work video scheduling conference, Yunnan communist youth league vigorously promote 12355 specialization, public welfare, project construction, multiple measures, more power, 12355 overall activity, become an effective carrier to participate in grassroots social governance, in promoting the development of youth rights and interests work, has played a positive role. Li Songtao, deputy secretary of the Communist Youth League of Yunnan Provincial Party Committee, introduced, The Yunnan Communist Youth League focuses on the party and government center and takes the initiative to act as a role, Find the right job combination and focus, Combined with the characteristics of Yunnan border ethnic areas, Set up more than 1,000 people "youth teaching group", to "HuoNight School", "Chinese bilingual lecture", "Hello college students" columns and other acceptable ways to carry out theme education activities, Establishing legal publicity and education bases for youth in 129 counties in the province; More than 30 "4:30 classrooms" have been built in urban communities, urban-rural fringe areas, and relocation sites for poverty alleviation, To care for the minor children, left-behind children, migrant children and other groups of the poverty alleviation workers, Provide interest training, psychological counseling, holiday trusteeship, self-care education, urban integration, parent-child relationship adjustment and other services, Actively help the smooth completion of the province's poverty alleviation targets and tasks; In 2017, in cooperation with the People's Procuratorate of Yunnan Province in 2017, Build China's first community "one-stop juvenile protection center" in Panlong District, Kunming City, Explore the "one-stop" evidence collection for juvenile victims, Put forward the first national "double consultation system", Implement the construction concept of special judicial protection for minors; Continue to implement the "For tomorrow- -the construction project of social service system for preventing juvenile delinquent crimes", the demonstration project of juvenile rights and interests, Organize professional social workers and volunteers to participate in advance prevention, critical prevention and recidivism prevention, Establish direct stability support and service contact with key youth. In the past 5 years, it has built 74 provincial demonstration projects; developed and applied WeChat mini program "Yunqing Care-One Mobile phone Care for growth" to realize online consultation, message answering, online evaluation and telephone consultation interactive services, relying on 12355 service desk data, monitoring and analysis, form reports, timely communication and consultation with relevant departments, and provide decision-making reference. It is worth mentioning that, through the integration of resources, standardized and professional operation, 12355 in some areas of Yunnan has become a "found" and "trusted" service team when teenagers need it. For example, Kunming City 12355 has 704 experts and volunteers in the fields of psychology, law, career planning, family education and other fields, Regular cooperation with 12 social organizations and social service agencies; Select some Kunming primary and secondary schools, secondary vocational schools, universities, communities, Established the "12355 Work Site", Carry out all-round youth affairs service activities; "Yixing youth flower young Time" series of projects to provide teenagers with 12355 hotline, case counseling, "entrance examination for secondary school or college decompression", minors' mental health through train, psychological assistance series courses, family education lecture hall, As well as youth life education, sex education, school bullying prevention, safety and self-care education and other services. At the same time, the national 12355 referral mechanism was entrusted, and two cases in Guangdong and Shanghai were handled, realizing the linkage and cooperation among regional centers. Zhaotong city 12355 offline 6 judicial, psychological counseling professional team recruited 3024 young volunteers, the communist youth league in Zhaotong over 910,000 people to help people "housing, income, civilization, seedling" four actions, help to buy the starfish action charity association social services, work in Zhaotong general vocational integration middle school, focus on integration education, do not let a compulsory education stage students to drop out of school due to poverty. In addition, Lijiang 12355 online "Aunt Xiao Fang Chat Room" program, covering four counties in Lijiang District and minority teenagers, Shangri-La, Dali, Sichuan Liangshan; offline "Aunt Xiao Fang Chat Room" parent training school, the last weekend of the month; Lincang and Xishuangbanna continue to carry out "easy preparation 12355".

2021-09-26	Chongqing care for vision health public service into Tibet qamdo Chongqing care for vision health public service into Tibet qamdo wang xinxin) recently, the Chongqing municipal party committee, Chongqing, Chongqing youth union, love mic eye hospital organization care for vision health public service into class wuqi county, to carry out the "class wuqi county primary and middle school students to send light" activities. In early September this year, the Chongqing municipal party committee, Chongqing youth league to Tibet Tibet work in 2021, in the process of visiting, Chongqing youth committee, Chongqing love magh eye hospital CEO Yang Yu learned, because of traffic inconvenience, lack of eye medical services, and lack of professional mirror institutions, part of primary and middle school students myopia can't get timely correction, learning and life affected. A student is trying on his glasses. In view of this situation, Yang Yu and the Chongqing Municipal Committee of the Communist Youth League and the Chongqing Youth Federation actively prepared for the vision and health public service activities after returning to Chongqing. In the Tibet autonomous region prosperous municipal party committee, class wuqi county people's government, Chongqing ninth batch of aid wuqi cadre talent task force support, care for vision health public service in wuqi county 15 schools to carry out the light environment monitoring, myopia prevention and control of science, vision screening and love medical mirror such as eye health support services, vision screening of primary and middle school students more than 5200. SYangji (pseudonym) of Wuqi County Middle School is the student with the highest degree of myopia found in this activity, 900 degrees right eye and 1000 degrees left eye, only 14 years old. If not timely mirror correction and medical intervention, she will face the risk of blindness fundus diseases caused by high myopia. The 10 (pseudonym) of Wuqi No.1 Primary School was not only a refractive error, but also diagnosed with endotropia and amblyopia in his left eye."Although we have missed the best period of correction and treatment, we still need to be scientifically corrected as soon as possible for the children's future eye health and study quality of life."Yang Heping, director of the optometry department of Chongqing Ermag Eye Hospital, who participated in this activity, explained the examination results to the head teacher of the school, and put forward corresponding suggestions for correction. During the activity, the public service team also completed the mirror data collection, evaluation and frame fitting selection for nearly 500 primary and secondary school students with abnormal vision and meet the conditions of myopia correction. After the glasses processing and quality inspection are completed, these love glasses will be sent to the students.

2021-09-28	Shandong province rural revitalization youth innovation and entrepreneurship exchange held in Rizhao Shandong province rural revitalization youth innovation and entrepreneurship exchange held in Rizhao China youth network in Beijing, September 28 (reporter Zhang Jianwei, correspondent phase) chang-hui, Shandong province rural revitalization youth innovation and entrepreneurship exchange launched in Wulian pine lake. The Communist Youth League of Shandong Provincial Party Committee, Rizhao Municipal Party Committee and other relevant responsible comrades attended the event. At the meeting, Rizhao introduced the industries and supporting policies related to rural revitalization, and communicated and connected the projects with nearly 20 entrepreneurs face to face. Everyone said that Rizhao is rich in resources, has distinctive industrial characteristics, and has strong policy support. It is very suitable for investment and development in high-quality agricultural products, agricultural industry chain, and life and health industry. We will continue to pay attention to Rizhao, further explore cooperation, and achieve win-win results. During the period of sunshine, nearly 200 entrepreneurs representatives, winning projects, provincial rural good youth guests, will also field view sun city manor, incense in cloud valley soup club, egret bay art town, 95 agricultural science and technology industrial park and vibration bearing agricultural company, to carry out the keynote speech, interview sharing, project docking and other activities.

2021-09-24	Guangdong will be coronavirus nucleic acid testing volunteer service training drills in Guangdong will be coronavirus nucleic acid testing volunteer service training drills JianWei correspondent YueQing) recently, group, Guangdong provincial party committee, Guangzhou municipal party committee, Guangzhou volunteer action guidance center, Guangzhou youth league school (Guangzhou volunteer college), Guangzhou youth volunteer association, Guangzhou Guangzhou volunteer service foundation of Guangdong and Guangzhou hierarchical stratification will be coronavirus nucleic acid testing volunteer service training and drills held in Guangzhou youth league school. The heads of volunteer services for epidemic prevention and control at the city, county (city, district) and township (street) levels of Guangdong Province and the backbone of volunteers participated in the training by watching live online videos, and the representatives of the youth epidemic prevention and emergency volunteer reserve teams (commandos) at all levels and of all types in Guangzhou participated in the on-site training. Online live streaming has accumulated nearly 40,000 views. Large-scale Novel Coronavirus nucleic acid testing volunteer service training and drill in Guangdong Province and Guangzhou City. The training course of Guangdong Provincial Committee is mainly divided into two parts: theoretical training and nucleic acid testing combat drill. In the theoretical training course in the morning, the organizer organized the students to carefully study the spirit of General Secretary Xi Jinping's important speech on July 1st, and invited the professional medical staff from the First Affiliated Hospital of Guangzhou Medical University to teach personal protection knowledge to the young volunteers. In addition, representatives of the Youth League Committee of Haizhu District of Guangzhou were invited to share the experience of establishing a reserve team for emergency epidemic prevention in Haizhu District. In the afternoon of the same day, the volunteers practiced the whole process of nucleic acid testing and epidemic prevention work according to the division of different positions. They successively carry out order maintenance, admission temperature measurement, waiting diversion, marking registration, testing and guidance, emergency logistics and other related services. Volunteers cooperated closely according to the scheduled plan and completed all the tasks of the drill in an orderly and efficient manner. After the drill, the training lecturers made repeated comments on all links of the drill process, and put forward specific suggestions for the improvement of the problems arising in the drill process, so as to achieve the expected purpose of leading the demonstration, training the team and the running-in mechanism. Large-scale Novel Coronavirus nucleic acid testing volunteer service training and drill in Guangdong Province and Guangzhou City. Zeng Lifang, the backbone of the Yuexiu District Epidemic Prevention and Emergency Volunteer Reserve Team, said, "This training paid special attention to practical operation. Combined with our previous experience in epidemic prevention and control work, we detailed the working mechanism, work process and job responsibilities, which played an important role in guiding our work."The relevant person in charge of the organizer, said the main purpose of the training is through clear nucleic acid detection and vaccination volunteer service workflow, translation work at any time, further enhance the youth volunteers epidemic prevention and control emergency disposal ability, to ensure that large-scale nucleic acid detection, vaccination volunteer service scientific arrangement, smooth and orderly, fast and efficient, safeguard volunteers before, on-the-job and post security protection closed-loop, to help epidemic prevention and control work.

2021-09-24	Jiangxi: create ideal space for the youth targeting youth needs, people hope, society, Jiangxi: for the youth to create ideal space youth liu, looking forward to the youth bring "welfare": skills training, financial policy, financial support... this is the league Jiangxi provincial party committee to serve "Jiangxi rural good youth" a series of measures. A few years ago, he graduated with a master's degree, and returned to his hometown, Jinshi Township, Suichuan County, Jiangxi Province, to raise pigeons, and became known as the famous "prince of raising pigeons". Now participating in the selection of "Jiangxi Province Rural Good Youth" has made him more determined to take root in rural areas and devote himself to rural revitalization. Party history learning education, Jiangxi provincial party committee according to the "party needs, social concern, the communist youth league for" requirements, called for the provincial youth league organizations at all levels and youth members actively carry out "I do the practical work for the masses" and "I do something for youth" activities, targeting youth needs, people hope, society, launched ten class huimin convenience, service youth work, involving talent support training, entrepreneurship, employment training, dating, poverty alleviation, etc. Related projects not only meet the prominent needs of the young people, focus on handling the urgent expectations of the youth, but also identify the social access incision, and give full play to the majority of young people to do practical work for the masses. Let the ideal take root in the countryside earlier this year, according to Jiangxi provincial party committee according to "about the implementation of the" new era of Jiangxi province rural good youth "selected culture plan opinion requirements, specially issued" about the 2021 "new era of Jiangxi province rural good youth" candidate recommended notice, began to do good youth selected tree work. The selection and training program of "Jiangxi Province Rural Good Youth" provides support and impetus for youth development in terms of capital, technology, training and platform experience. Through the three-level division of labor of "provincial demonstration training, key training of cities and counties, and full coverage training of towns", the first batch of selection attracted nearly 3,000 employed people from outside the province to return to their hometowns for employment and start businesses. Currently, Jiangxi Provincial Committee leads the establishment of the provincial "Young Marxist Training Project" rural class, establishes the "Good Youth" alliance, builds a platform for learning and exchange and resource sharing, organizes 3000 "good youth" to attend higher vocational colleges and improve their skills, and negotiwith Jiangxi CCB and CMB exclusive financial products to relieve the funding difficulties... and a series of initiatives to encourage the rural youth. As one of the important starting points of "I do something for the youth", the selection and training of "Jiangxi Province Rural Good Youth in the New Era" is not only the process of selection and training policy publicity, organization and mobilization of young people, but also further stimulates the awareness of grass-roots organizations to grasp the talent work, creating a good atmosphere for rural Party organizations to pay attention to the training of young talents. More and more grassroots cadres realize that the use of existing policies to introduce, train and retain more young talents, and to invigorate the "pool of spring water" of rural development with the "living water" of talents is rural development."Thanks to such a 'good youth' selection and training policy, the concept of the village people began to gradually change. The idea of 'young people return to the village and their development is worthless' is not only among the young people, but also no longer the mainstream among the majority of villagers. More and more young people are willing to devote themselves to the rural work. This is not only to bring fresh water to rural development, but also to remove the psychological burden for young people to return to their hometown for development, and to create a friendly atmosphere to return home for entrepreneurship."Jiang Honghong, branch secretary of Yanqian Village, Jiaxuan Township, Yanshan County, Shangrao City, compared the selection of" Jiangxi Poyang Rural Good Youth in the New Era "to a rain to quench his thirst. In the" good youth "selection, he found several good seedlings, which solved the distress of" no village organization successor " that had troubled him for many years."Hello, what business do you want to deal with? You can first come here to check "" Hello, please take the number to queue up here, convenient for business as soon as possible "... in the office hall of Yongxiu County, Jiujiang City, Jiangxi Province, the volunteers of the youth volunteer service station warmly guide the people to come to handle affairs. Volunteers master the operation methods such as all-in-one query machine, self-service form filling machine, intelligent queuing and calling system, and help to guide the masses to handle various businesses, which has won unanimous praise. The service station also set up a special youth civilization demonstration post and league member demonstration post, unified production of post card, so that young volunteers show their identity and responsibilities. The volunteer service of Yongxiu County Government Affairs Service Center is an epitome of the Jiangxi Provincial Committee of the Communist Youth League to carry out the practical activity of "I do practical things for the masses", and also one of the important projects to carry out "I do something for the youth". League of Jiangxi provincial party committee will "I do something for the youth" as an important focus of party history learning education, aiming at youth to and social needs, the member youth conduct "I do the practical work for the masses" and Jiangxi provincial government to build "policy optimal, lowest cost, best service, the fastest" "four" business environment, in the provincial government affairs service hall establish youth volunteer service station. By building a youth service platform, we will guide young people to participate in the provincial reform of "delegating power, delegating control and service", solve the problems such as the shortage of personnel and inconvenient people to handle affairs, and help Jiangxi to build a first-class province with national satisfaction of government service. At present, 107 youth volunteer service centers have been established in the service halls of various government affairs service centers in Jiangxi Province, which have organized more than 7,000 young volunteers to serve at the posts, serving more than 850,000 people for a total of 60,000 hours."Under the arrangement of the youth League organization, I got this internship opportunity, which changed my understanding of my work from 'hearsay' to 'osmosis'."Guo Qingqing, an undergraduate from Jiangxi Normal University, participated in the government and enterprise internship of college students '" Sailing Plan " sponsored by the Jiangxi Provincial Committee of the Communist Youth League and was assigned to the Legislative Working Committee of the Standing Committee of the Provincial People's Congress. She said that the nearly one-month internship has fulfilled her strong desire to connect with the society as soon as possible and calmed her employment anxiety. This summer vacation, Jiangxi Provincial Committee of the Communist Youth League launched the 2021 "Sailing Plan" in Jiangxi Province. 256 college students went to provincial organs, enterprises and institutions for one month, and provided internship positions for 2,712 college students in municipal and county units and enterprises. As a combination project of the Communist Youth League "I do something for the youth" and the youth "I do practical things for the masses", the "Sailing Plan" not only provides high-quality internship opportunities for the young people, but also brings a new wind to the government and enterprise units."These young people are highly motivated, comprehensive quality, can bear hardships, can contribute, strong ability, highly evaluated by relevant internship units. They bring a wind of youth to the organs and units, so that everyone eliminates some stereotypes of the post-90s and post-00s."Youth League Jiangxi Provincial Party Committee school department minister Li Lengsheng introduced. In the next step, the "Sailing Plan" of college students will extend the internship time based on the actual situation of the interns, promote the communication among the internship students by holding symposiums and holding group activities, improve the effectiveness, and further integrate the expectations of the young people into the social needs. Huang Zhangyue, China Youth Daily reporter Chen Youth Network source: China Youth Daily

2021-09-30	Capital young pioneers "spark youth" group to carry out the "double minus" work requirements to build campus practice brand capital young pioneers "spark youth" open TuanJianwei correspondent PeiBeibing) recently, the Beijing municipal party committee, Beijing municipal committee innovation of the young pioneers campus practice education brand-the capital "spark youth" group activities were officially held. Nearly 100 young pioneers from 20 schools in 9 districts participated in the two-day and one-night camp practice through organizational recommendation and social recruitment. The Young Pioneers jointly held the Young Pioneers ceremony, and became the first members of the capital "Spark Youth Group" through learning and exchange activities such as military experience, outdoor camping, safe self-protection, and collective sharing. Capital "spark youth league" is the implementation of the new era of the young pioneers work measures of specific measures, aiming at "double minus" parents and the children of the actual needs, actively find the education reform under the background of the young pioneers work growth point and the foothold, to the "after class" and "outside", improve the young pioneers education and after-school service effect, lead the young pioneers inheritance red gene, enhance honor and organization sense of belonging, realize all-round development. Highlight the red inheritance, focus on political guidance. In the design of the activity, we will unswervingly put the political enlightenment and value shaping in the first place, and guide the young pioneers to "listen to the Party and follow the party". Based on the physical and mental development characteristics of the young pioneers, military skills training projects are designed. By telling the revolutionary history and simulating the battlefield scenes, immersive and experiential party history learning and revolutionary education are conducted, so as to light up the ideals, beliefs and patriotism in the hearts of the young pioneers. Strengthen the organization of education, enhance the sense of honor education. In the opening and graduation ceremony serious young pioneers ceremony, highlight the red scarf, team, flag, team logo education significance, adhere to the young pioneers organization education, independent education, practice education, the young pioneers activity performance in the school "red scarf medal" for reference conditions, guide the young pioneers in the collective, practice to enhance the sense of honor and organizational sense of belonging. Expand the practice carrier, practical service growth. Mining "spark youth league" brand connotation, expand practice project and the carrier, focusing on red inheritance education, national defense military education, security since the education, science and technology art innovation, outdoor exercise, Chinese traditional culture, the capital development achievement, the games ice and snow experience eight themes, fusion military training, camp life, urban tread, career education, public volunteer, subject learning carrier, let children grow in the overall development of the capital. Capital "spark youth league" holiday practice will be in the National Day and other statutory holidays, weekend double holidays and normalized during winter and summer vacation, and further integrate the capital advantage resources, give full play to the youth practice position, gradually form the city, district, street township three young pioneers education and service system, create "double reduction" under the background of primary and middle school students aspire to, character, practice skills of "youth".

2021-09-28	College students in Shanxi Province "home" social practice successful MSC 2021 "youth xing Jin" college students "home" summer social practice successful MSC JianWei correspondent, Zhang Jinrong Guo Li) in September is the season of harvest, with the opening of the new semester, Shanxi Province in 2021 "youth xing Jin" college students "home" summer social practice complete packaged. In 11,448 practical posts, 5,883 Shanxi students from universities from inside and outside the province were successfully employed, 117 temporary youth league branches and practice alliances were established, and 407 various forums and other theme activities were held."Return to home" students spread their wisdom and sweat in the land of Sanjin, in practice, "receive education, long talent, make contributions", and handed over a qualified youth answer paper. Since 2018, the Shanxi Provincial Party Committee of the Communist Youth League has thoroughly implemented the strategy of strengthening the province with talents, implemented the work requirements of the Central Committee of the Youth League of the Communist Youth League, and launched the summer social practice activity of "Youth Prosperity" for college students connecting young students. Through continuous improvement and accumulation, Formed the working principle of "due to post, according to post, two-way selection, territorial management, nearby", Through the hierarchical management of the provincial and county levels, Unified collaboration, Establish a summer social practice special registration and recruitment system, Realize college students' practice will and practice units "two-way choice"; Through conducting targeted pre-job training, Unified issuance of identification as a student professional practice, comprehensive evaluation and "second class" transcript identification of the verification materials, Unified purchase of life insurance and other measures for students, To ensure the efficient and orderly activities; By organizing college students to visit and investigate in Party and government organs, local transformation projects and large enterprises, Feel the change of your hometown, Feel the achievements of the transformation, We will improve the willingness of college students to return to their hometowns to start businesses and find jobs. Over the past four years, a total of 16,000 students from Shanxi Province have returned to their hometowns to conduct practice in poverty alleviation, rural revitalization, changing customs, livelihood services, and social governance. Youth League organizations at all levels have encouraged young students to provide more than 10,000 volunteer services for COVID-19 prevention and control. Through practical experience, the young students in the province feel the strong atmosphere of seeking transformation and seeking talents in their hometown."Youth Xing Jin" has only a starting point, no end. Next, the provincial party committee will continue to further implement the general secretary xi "July 1" important speech spirit, according to the history of the party in the education "I do the practical work for the masses" work requirements, according to the provincial party committee and provincial government on the implementation plan of deepening provincial cooperation requirements, focus on power college students practice base construction, youth league organizations at all levels to assist the party talent department, make cooperation universities to jin students contact, service and long-term contact work. At the same time, the outstanding students found in the practice and training will be taken as the key contact and training objects, included into the service scope of youth league organizations at all levels, attract young students and talents to work in Shanxi, and effectively serve the provincial Party Committee to strengthen the provincial strategy with talents.

2021-09-23	Sun Haihua) the fifth Shaanxi Provincial Youth Public Welfare Project Competition is being held, inviting the public to vote for 100 youth public welfare projects. By the communist youth league, shaanxi youth development foundation of shaanxi provincial committee, shaanxi net letter office units jointly organized the fifth shaanxi youth public welfare project competition, for shaanxi communist youth league organizations at all levels, civil affairs department registered social organizations, college youth league and student management student associations and keen on public welfare enterprises and institutions, solicitation, selection, cultivate a batch of outstanding public welfare projects, service youth development power shaanxi charity healthy development. Since its launch in May this year, the competition has attracted wide attention and active participation from all walks of life, and has received a total of 401 applications for public welfare projects. According to the project category statistics, including rural revitalization and rural basic education class accounted for 19%, community governance and public service accounted for 9.2%, care for rural left-behind children and migrant workers trailing children accounted for 17.5%), epidemic prevention and control and emergency relief accounted for 3.5%, also including network civilization and civilization practice, legal donations and drug AIDS prevention, ecological environment and water saving water protection, culture and technology communication and tourism services, sunshine assistive and service for the old and so on category content. After a preliminary evaluation by experts, 100 projects entered the exhibition and voting process. From 0:00 on September 22 to 24:00 on October 26, the selected projects will be displayed and voted on online. According to the online voting results, the competition will select 10 categories, a total of 50 excellent projects for defense and evaluation, and finally select 20 gold awards and 30 silver award projects, and the competition sharing meeting will be held to release the awards of the project, inform excellent organization organizations, and organize the exhibition of winning projects. Please click on the "Public Welfare Competition" in the lower left corner of the wechat official account of Shaanxi Provincial Youth Development Foundation (website https: / / ebke5g.wx.lppoll.com/act/vote?act=94602)。Voting rules require: each wechat ID can vote 10 times a day and vote for the same project per day.

2021-09-23	Recently, reporter Lei Yu), the 2021 "Creative Youth" Hubei Youth Innovation and Entrepreneurship Competition award ceremony and the "Yijing Jingen" city cluster youth innovation and entrepreneurship exchange meeting was held in Yichang. Since its launch in July this year, the competition has collected 264 projects from the science and technology innovation group and rural revitalization group, combining both online and offline, and held preliminary, semi-final and final competitions successively. After fierce competition, 18 outstanding projects, such as the industrialization of high-end LED light source and the integrated development of the citrus industry chain, stood out, including 4 gold awards, 6 silver awards and 8 bronze awards. At the meeting, the Yichang, Jingzhou, Jingmen and Enshi youth league groups organized and established the "Yijing Jingen" city cluster Youth Innovation and Entrepreneurship Alliance to deepen the agglomeration effect of youth innovation and entrepreneurship and build a youth vitality and innovation community. The Yichang Municipal Party Committee of the Communist Youth League explored the launch of the "Youth Innovation Farm" project, released the first batch of 20 winning projects, provided financial support in accordance with the way of "small funding, awards instead of subsidies", integrated resources, and optimized the rural innovation and entrepreneurship environment. The activity also held the "Yijing Jingen" urban agglomeration youth innovation and entrepreneurship exchange meeting, and organized the heads of the municipal youth league organizations and entrepreneurial youth to observe the city innovation and entrepreneurship base and the "green excellence products" exhibition. Qin Longping, the person in charge of Yichang Meng Guoguo E-commerce Co., Ltd., shared his 10-year entrepreneurship and hard work from his senior year. The company was recognized as Hubei Province e-commerce demonstration enterprise by the Provincial Department of Commerce in 2020."At the most difficult time, we won the runner-up and the prize money of 180,000 yuan in the business plan competition organized by the Provincial Communist Youth League Committee, which gave us the opportunity and courage."Leaders of relevant departments directly under Hubei Province, leaders of youth league organizations of various cities and states, well-known entrepreneurs, and representatives of young entrepreneurs participated in the activity.

2021-09-23	Shandong Provincial Party Committee of the Communist Youth League and Central Conservatory of Music signed a cooperation agreement in Beijing. China Youth League Shandong Provincial Committee for Beijing, September 23 (reporter Zhang Jianwei, correspondent Xiang Changhui) recently, the Communist Youth League Shandong Provincial Committee and the Central Conservatory of Music signed a cooperation agreement in Beijing. Liu Tiandong, secretary of the Provincial Communist Youth League Party Committee, Yu Feng, president of the Central Conservatory of Music, Yu Hongmei, vice president of Jinan City, and Wang Guiying, vice mayor of Jinan City attended the signing ceremony. President Yu Feng, on behalf of the Central Conservatory of Music, extended a warm welcome to the CPC Communist Youth League Provincial Party Committee and the Jinan Municipal Government, and introduced the educational concept, educational characteristics, cutting-edge research and the teaching achievements made in recent years. Shandong culture, he says, profound, young talents, Jinan is music, sea right city, college will give full play to the talent advantage, with good teaching resources, constantly strengthen communication with the provincial party committee, Jinan, around talent training, course research and development, teachers training, positions construction for further cooperation, set up the central music academy of Chinese excellent traditional culture heritage base in Shandong practice center, support the city 2, provinces and cities youth palace units bold exploration, first try, made new achievements in the whole process, new breakthrough in teaching education. Liu Tiandong's delegation to the Provincial Party Committee thanked the Central Conservatory of Music for its continuous care and support. He said that Shandong is a province with a large population, culture and education, and the Central Conservatory of Music is the highest institution in music education research and talent training in China. The cooperation signing is of great significance and will strongly promote the development of youth art education in Shandong. League provincial party committee as a bridge of cooperation, will give full play to the Shandong humanities, location, resources advantage, fully support the central academy of music teachers and students in Shandong to carry out all kinds of music art practice, performance, communication activities, provide comprehensive, high quality service guarantee, and broaden the cooperation areas, jointly promote the central music center in Jinan art institute, promote the development of Shandong music education and cultural undertakings. After signing the contract, the provincial Communist Youth League Committee investigated and studied the relevant teaching work of the Central Conservatory of Music.

2021-09-22	Hainan communist youth league research free trade port youth agricultural products brand building Hainan communist youth league research free trade port youth agricultural products brand builder Ren Mingchao) by Hainan provincial party committee jointly sponsored the "youth glorious chapter dream future" in 2021 Hainan free trade port youth agricultural products brand building series activities ended, screening a batch of planting, breeding, rural tourism, technology promotion, a total of 21 youth agricultural products projects. The research activity invited four experts from the fields of Hainan Academy of Agricultural Sciences in planting, processing, storage and preservation, e-commerce and other fields, to carry out a 6-day in-depth research. Next, the youth agricultural products brand activities project team will be around 21 youth agricultural products brand, brand marketing strategy, brand VI image design, promotional production, entrepreneurship training and a series of activities based on improving brand image, cultivate a group of understand agriculture, love the countryside, can entrepreneurial excellent rural youth team and talents.

2021-09-26	Chongqing) On September 24, the Communist Youth League, Chongqing Finance Bureau and the People's Bank of China jointly held the rural revitalization youth loan project launch meeting and policy briefing, and announced the official launch of rural revitalization youth loan financial products. Rural revitalization of the youth loan by Chongqing municipal party committee, municipal finance bureau, the people's bank of Chongqing business management joint related banking institutions and Chongqing agricultural financing guarantee group co., LTD., mainly for rural revitalization, agriculture and rural modernization development of youth to provide credit support, aims to promote the implementation of the "rural revitalization" youth meritorious action, help rural talent revitalization, industry revitalization. The loan objects of the project mainly includes two categories, one is the agriculture-related entrepreneurship young individuals under the age of 45, and the other is the agriculture-related business entities with the main responsible person or legal representative under the age of 45. The interest rate of the loan project shall not exceed 5%, and the maximum loan amount shall reach 3 million yuan. Have loan demand and meet the requirements of loan application of rural revitalization of industry development youth leaders or agricultural business subject, can be through the Yangtze river Chongqing financing online platform for application, the relevant financial institutions and Chongqing agricultural financing guarantee group co., LTD., after reviewing the project, to meet the requirements for loans. The relevant person in charge of the Communist Youth League Chongqing Municipal Party Committee said that the product will help to solve the financing problems of insufficient mortgage, insufficient guarantee, poor information and high cost.

2021-09-22	In 2021 Guangxi youth volunteer service public entrepreneurship competition final held in 2021 Guangxi youth volunteer service public entrepreneurship competition final held Xie Yang intern QinFangPing) sponsored by Guangxi district party committee, Guangxi volunteer guidance center, Guangxi youth volunteer association to undertake 2021 Guangxi youth volunteer service public entrepreneurship competition final was held in Nanning, Guangxi, 30 entrepreneurial projects from cities, industries. The "translation" water project from Guangxi Normal University for Nationalities won the gold medal; the "aviation craftsmanship", the 21st century, Guangxi University Graduate Volunteer Team; 5 teams from Guilin Normal College and Hezhou Love Home Association won the bronze award, and 19 teams from Nanning Normal University won the excellence award. Since its launch in July this year, the competition has been widely soliciting volunteer service projects from all departments in Guangxi, and as many as 101 projects were shortlisted in the final round of the whole region alone. In the final, after the qualification examination, semi-final selection and media publicity, the 30 projects shortlisted for the final all reflected the distinctive characteristics of the theme of "Volunteer heart to 'benefit' to the Party and create the future".

2021-09-19	The communist youth league of Fujian provincial party committee issued epidemic prevention and control initiative, the communist youth league Fujian provincial party committee issued epidemic prevention and control in network trainee reporter Tian Hongwei) today, the communist youth league of Fujian provincial party committee issued the provincial cadres and youth league initiative, initiative provincial cadres, youth members to carry out the epidemic prevention and control deployment requirements, stand up, the charge in the front, solid epidemic prevention and control work. The "Proposal" proposed that all youth league organizations at all levels should actively carry out the youth war "epidemic" commando action. In accordance with the unified deployment of the local Party committee, youth league members were organized to actively report to communities (villages), quickly set up "epidemic" youth war commandos, and set up "vanguard posts" for league members " in epidemic prevention and control. In Xiamen, Putian and Quanzhou, the Communist Youth League should form more than 30 youth commandos, and more than 10 in other districts. According to the actual needs of epidemic prevention and control, conduct youth volunteer service operations, recruitment, reserve professional medical services, convenience services, order maintenance, logistics, etc. We should adhere to the "safety first", strictly implement the protective measures for the on-duty volunteers, so that the protective measures are not in place, and do not work if the protection training is not qualified. The Communist Youth League of Xiamen, Putian and Quanzhou should set up youth volunteer service teams with more than 3,000 people, and other districts and cities with more than 500 people. At the same time, local youth league organizations at all levels should carry out youth care and help actions. With online activities such as "Warm childlike innocence and Care for left-behind children" and "Colorful Classroom" as the carrier, we will help home primary and middle school students better adapt to the home learning environment. Special volunteer services called "you and me behind" have been launched to solve problems for frontline prevention and control workers. A volunteer service team of psychological care and youth assistance has been set up, to timely understand and respond to the psychological confusion and needs of teenagers, and to provide public welfare psychological counseling and counseling services. College youth league organizations should actively help college students to solve the difficulties in their study and life. Launch youth federations at all levels to carry out youth care activities in Fujian and Taiwan. Actively mobilize the youth civilization number collective and the industry youth League committee to visit the frontline police, community workers, express workers and other young workers who stick to their posts during the "Mid-Autumn Festival" and "National Day"; and deeply carry out the youth network civilization action. Strengthen online publicity, timely and accurate release of authoritative information, take the initiative to voice, positive guidance. According to the proposal, youth league officials and youth league members should take the lead in strictly abiding by the provisions of epidemic prevention and control, enhance self-protection awareness, develop good living habits, timely vaccination, and cooperate with nucleic acid testing. Do not go to medium-high risk areas, reduce unnecessary travel, do not gather, no dinner. We will guide relatives and friends around us to correctly understand and treat the epidemic, do not spread, spread or believe rumors, and maintain social stability and stability.

2021-09-18	Guangdong communist youth league propaganda and ideological and cultural work will be Guangdong provincial party committee of Guangdong communist youth league propaganda and ideological and cultural work will build wei correspondent YueQing) on September 15, Guangdong communist youth league propaganda and ideological and cultural work, analysis summarizes the new era of the communist youth league propaganda, ideological and cultural work planning (2019-2023) " since the implementation of the work, the deployment of the next stage of work, the provincial youth league organizations at all levels of propaganda work front related comrades through network video live. The meeting summarized and analyzed the propaganda, ideological and cultural work of the Guangdong Communist Youth League since the Third Plenary Session of the 18th CPC Central Committee of the Communist Youth League. First, there were new achievements in serving the "three-force coordination", new breakthroughs in the layout of the "lighthouse project", and third, new achievements in building the "Great Wall of Thought". Wu Yiting, deputy secretary of Guangdong Provincial Party Committee, stressed the need to accurately grasp the focus and requirements of the ideological and cultural propaganda work of the Communist Youth League under the new situation. We need to light up the "beacon" of studying, publicizing and implementing Xi Jinping Thought on Socialism with Chinese Characteristics for a New Era, bring Xi Jinping Thought on Socialism with Chinese Characteristics for a New Era into the thoughts, emotions, and hearts of young people, and guide them to form practical, rational, and emotional identity. It is necessary to comprehensively build the integrated ideological and political guidance work system of the Guangdong Communist Youth League, and realize the political effect of the Party educating the people, consolidating and expanding the young mass foundation of the Party's governance. We should continue to promote the strategy of producing propaganda ideology and culture, and adhere to the unity of high-quality content and fashion forms. We should build the main position of propaganda ideological and cultural work and consolidate the main battlefield of Internet propaganda struggle. We should grasp the opportunity of "double reduction" policy and promote the construction of an all-round education platform combining online publicity and offline practice. Meeting to communicate learning the group of the Guangdong provincial party committee standing committee study and implement xi general secretary of the "July 1" important speech spirit project reading class spirit, the "lighthouse project-Guangdong youth learning practice xi new era the ideas of socialism with Chinese characteristics action" propulsion has carried on the periodic summary, read the communist youth league Internet propaganda, ideological and cultural work related requirements. The main leaders of Guangzhou Youth League, Zhongshan Committee, Qingyuan Youth League and Lingnan Normal University made speeches on promoting the theme of ideological and cultural productization strategy, promoting the online theme of "youth learning", the promotion of propaganda action, and the ideological and political guidance of the Communist Youth League in colleges and universities.

2021-09-17	The first Chinese youth dream season launched in Beijing in 2021 (the first) Chinese youth dream season start and "millions of youth see free trade port" series of activities cloud conference held in Beijing JianWei correspondent Wu Yuhua) to implement the national strategic deployment, for the free trade port construction and development gather more youth strength, further enhance the influence of free trade port in Hainan, Hainan provincial party committee joint about national youth organizations, media units, think-tank and some provincial youth corps committee, youth organizations, etc., jointly build "China (Hainan free trade port) youth dream avenue" youth mobilization leading service platform. On September 15th, the launch ceremony of the 2021 (the first) China Youth Dream Season and the cloud press conference of the "One million Youth Watch the Free Trade Port" series of activities was held in Beijing. The China Youth Dream Season is jointly organized by China Youth Daily, Hainan Provincial Party Committee of the Communist Youth League, Shenzhen Reform Office of Hainan Provincial Party Committee (Free Trade Port Working Committee Office) and Hainan Provincial Department of Tourism, Culture, Radio, Television and Sports, With the theme of "Youth, a magnificent chapter, building dreams for the future", It aims to build a "China (Hainan Free Trade Port) Youth Dream Avenue" youth mobilization and leading service platform, Gathering national youth organizations and youth innovation and entrepreneurship resources, Give full play to the mobilization advantages and leading service function of youth organizations, To attract national and even international youth to Qiong for employment and entrepreneurship, Provide high-quality, efficient, targeted growth and career development services, Inject new momentum into the construction of Hainan Free trade Port. In 2021 (the first) Chinese youth dream season to "dream avenue millions of youth see free trade port" theme activities, "Hainan free trade port China youth development high-end summit" and build "dream avenue China youth hall" three major activities as the main content of the time, in the next three months, will be held a series of youth dream activities, for "youth seed" cultivating employment development "fertile soil", and by sharing and spreading free trade port construction results, radiation influence national and global youth. Cloud conference synchronous launch "Hainan free trade port China youth cloud living room", will launch "dream big discussion" activities, launched "dream weavers free trade port" series of micro video, etc., promote various fields of youth and youth organization collaboration, full-service youth growth, gradually expand the free trade port youth circle of friends, accurate service free trade port high quality development.

2021-09-16	Fujian province set up the first Chinese horseshoe crab science education point in Fujian "protect beautiful coastline we act together" clean beach public welfare activities to set up the province's first Chinese horseshoe crab science education point JianWei correspondent Pan Jiangbin) recently, Fujian province green ecological service line deep pair service pond east village "protect beautiful coastline we act together" net beach public welfare activities, guide the local resident beach protection and coastal biological volunteer service, set up the first Chinese horseshoe crab protection mission point in Fujian province. Zhang Yongjun, deputy director of Committee of Population, Resources and Environment of Fujian CPPCC attended the event and delivered a speech. Some members of the Fujian Youth Federation Ecological Service Group took a group photo under the blue sky. In the activity provided by Fujian Provincial Party Committee, the leaders and guests jointly unveiled the publicity board of "The first Chinese Limulus Protection Science Selection and Education Center in Fujian Province", awarded the flag to "Jinjiang Jinjing Town Protection Beach and Coastal Biology Volunteer Service Team", and issued the initiative of "cherish the earth and protect the ocean" to the whole society. The Ecological Service Group of Fujian Youth Federation, Tangdong Village, Jinjing Town, Jinjiang City, Beijing Jingshi (Quanzhou) Law Firm, and Fujian Marine Ecological Civilization Promotion Association respectively signed contracts with eco-environmental protection and perennial public welfare legal advisers, jointly providing resources, intellectual support and legal guarantee for the ecological civilization construction of Tangdong Village. Xu Yunli, member of the All-China Youth Federation, vice chairman of Fujian Youth Federation, public welfare ambassador of Fujian Marine Ecological Civilization Promotion Association and member of the Youth Federation Service Group, donated clean beach tools to the volunteer service team on behalf of the service group. The volunteers went to clean the beach and released 5,000 Chinese horseshoe crabs, a national second-class protected animal. Fujian Youth Federation ecological service group and the pair of the village signed a support agreement. Group of Fujian provincial party committee for figure the event sponsored by the China Marine development foundation, activities with national clean beach public welfare activities as the carrier, in the clean beach, establish green league service unity to support mechanism, guide the local volunteer service organizations, set up animal science mission, organization science proliferation discharge activities, will actively promote pond east village coastal beach environment and ecological resources sustainable protection, help rural green development. The event also invited Li Yuhong from Huaqiao University, Professor Cai izhe from Xiamen University and other experts to local schools to give public lectures such as "Understanding Wetland and Protecting Home" for teachers and students. The ecological service group led the volunteer service team to carry out the beach-clearing activities. Group of Fujian provincial party committee for figure, Fujian youth ecological service by the national youth league, Fujian province association for the promotion of Marine ecological civilization, the national youth league, Fujian youth league, vice President, Fujian normal university, Chinese women's volleyball team former world champion Xu Yunli, the national youth league, Beijing capital (Quanzhou) lawyer Yang Jiawen, and Fujian, city, county level 3, a total of 15 related youth members in the field.

2021-09-22	Group Guangdong provincial party committee held study and implement xi general secretary "July 1" important speech spirit special reading class group Guangdong provincial party committee held study and implement xi general secretary "July 1" important speech spirit special reading class JianWei correspondent YueQing) recently, the Guangdong provincial party committee standing committee in Guangdong league school (Guangdong youth political college) held study and implement xi general secretary "July 1" important speech spirit special reading class, from around the province, industry more than 80 cadres attended the meeting. The meeting site. Everyone agreed that in-depth study of General Secretary Xi Jinping's important speech on July 1 is the primary political task of the Guangdong Communist Youth League at present and in the future, and also the core content of the education of youth Party history in Guangdong in the next stage. We need to deeply study and understand the rich connotation and significance of the spirit of General Secretary Xi Jinping's important speech on July 1, and further enhance our sense of political responsibility and historical mission in doing youth work well for the Party. Chi Zhixiong, secretary of the Guangdong Provincial Party Committee of the Communist Youth League, stressed that we should learn and make good use of the Marxist thinking methods and working methods contained in the spirit of General Secretary Xi Jinping's July 1 important speech, adhere to the comprehensive, dialectical and historical thinking, and correctly grasp the work of Guangdong youth and the Communist Youth League in the new era. We must clearly uphold the overall leadership of the Party, have a deep grasp of the "three-force coordination" of the Communist Youth League, enhance the overall contribution of the Communist Youth League, and improve the youth work system in the new era. We need to thoroughly implement the guiding principles of General Secretary Xi Jinping's important July 1 speech closely in the light of actual conditions, and promote the high-quality development of the work of the youth and the Communist Youth League in the new era. To focus on educating the party, to strive to cultivate the new people of the party rejuvenation, to share the party, to improve the effectiveness of serving the party, to gather the party, to expand the young mass foundation of the party, and to fully implement the requirements, to forge a loyal, competent, competent, courageous and exemplary cadres, to promote the high-quality development of the youth and the Communist Youth League in the new era. During the study period, the participants conducted group study and discussion around the important speech spirit of "July 1", and visited the first provincial-level Party history learning and education exhibition for teenagers- - "Stay true to the original aspiration and Keep in mind the Mission" Guangdong Youth Party History Learning and Education Exhibition.

2021-09-15	Sichuan documents to promote volunteer institutionalization normalized Sichuan file to promote volunteer institutionalization normalized optimal annual volunteer service time must be more than 20 hours students volunteer service time into the "second class" transcript, eligible star youth volunteers into trustworthy joint incentive object, governments at all levels will youth volunteer service work funds into the fiscal year budget at the same level... recently, Sichuan Province issued the Notice on Further Deepening the Reform of Youth Volunteer Service and Promoting the Institutionalization and Normalization of Volunteer Service (hereinafter referred to as the Notice), A series of "welfare" for young volunteers and policy "gift packages" to support youth volunteer service work."Notice" by the communist youth league of Sichuan province, Sichuan provincial party committee organization department, Sichuan civilization, development and reform commission, education department, department, departments, people club hall, transportation hall, culture and tourism hall, Chengdu branch of the People's Bank of China, around the growth incentive, campus incentives, public service incentives, credit incentives, financial support, platform support, project foster seven aspects, introduced many policies. According to the notice, the volunteer service situation should be taken as an important reference for the selection and evaluation of cadres. Candidates who participate in the "May 4th Medal", "Two Red and Two Excellent" and "Outstanding Youth Volunteers" of the Communist Youth League system will participate in the volunteer service for no less than 20 hours last year. For college students, the notice pointed out that student party members and league members should not serve for less than 15 hours annually. The annual volunteer service time of young college students will be more than 20 hours, as the necessary condition for applying for the A-level comprehensive quality certificate, evaluating the best, and promoting the best to join the league. The length of student volunteer service is taken as an important indicator of comprehensive quality evaluation, included in the "second class" transcript, and loaded into the student's school status files. In terms of credit incentives, "notice" is put forward, to promote youth volunteer service incentives and social credit system construction, promote that departments (units) will be eligible star youth volunteers into the "credit system red list", encourage banking financial institutions, financing guarantee institutions, platform company for star youth volunteers in related business to provide corresponding convenience. The notice also requires governments at all levels to include funds for youth volunteer services into the fiscal year budgets at the same level to ensure the normal development of youth volunteer services. We will support local governments in optimizing government purchase of services and increase support for voluntary services. In recent years, Sichuan has continuously deepened the reform of youth volunteer service and promoted the institutionalization and normalization of volunteer service. In 2019, the General Office of the CPC Sichuan Provincial Committee and the General Office of the Sichuan Provincial People's Government issued the Notice on Further Promoting the institutionalization and Normalization of Volunteer Service, and the Sixth Departments of Sichuan Province issued the Pilot Plan for the Reform of the Sichuan Youth Volunteer Service System. Zhang Rong, secretary of the Sichuan Provincial Committee of the Communist Youth League, said that the notice aims to further promote the implementation of various incentive policies, effectively play the practical role of volunteer service in education, and is also a concrete measure for the party history learning and education to do practical things for the young people. Source: China Youth Daily

2021-09-15	Hubei Communist Youth League: Community "small plan" leversocial "big resources" to build a community youth volunteer service team that can not go. A group of children aged 3-9 will wait for the arrival of volunteers of Hubei Business and Trade College. Since July last year, Fang Cheng, a young teacher from the school, has led a professional public welfare organization, " Wuhan Hongshan District Youth Reading Activity Center, to the community every week to help children with their homework and hold hand-drawn classes and reading groups. At the same time, the team provides many professional services, such as helping the elderly and physical exercise guidance. At the height of the COVID-19 outbreak last year, Fang Cheng picked up medical staff for half a month, and voluntarily collected and transported 6,000 jin of radishes for the staff in the sealed control. In Hubei province, tens of millions of young volunteers like Fang Cheng have stepped forward to participate in the community sealing and control, supermarket purchasing, psychological counseling and other services, contributing to the community's fight against the epidemic. As the epidemic prevention and control turned to normal, volunteers returned to work one after another, "find when you need, use it up". The "chronic disease" of scattered and poor community youth volunteer service in the past has emerged again. After the big test, how to consolidate the achievements of community youth volunteer service during the epidemic prevention and control period, and help normalize community volunteer service, has become a new issue for the Hubei Communist Youth League to serve urban community governance. Since June 2020, the Hubei Provincial Party Committee of the Communist Youth League has selected 206 pilot communities in the province to explore the implementation of the "community plan" for youth volunteer service. By means of joint construction, financial support, professional guidance, project incubation and other ways, each community is promoted to establish a "Qingbrand" volunteer service team, create a number of volunteer service brand projects close to the needs of residents and regular operation, and improve the professional and institutionalized level of community volunteer service. Regular voluntary services will be integrated into the "nerve endings" and "community plans" of community governance, focusing on epidemic prevention and control, community convenience services, mental health, and protection of rights and interests. In the initial stage, the Hubei Provincial Party Committee of the Communist Youth League guided the pilot units to explore around the team building, project implementation, cultural cultivation, mechanism norms and other aspects, and formulate work guidance manuals."Where do people come from" as young volunteers is one of the difficulties faced by local communities to carry out volunteer services. The "Community Plan" thus breaks the ice and advocates on cultivating local youth volunteer service forces in the community. During the epidemic period, Hubei province fully implemented the "double registration" system for Party organizations and Party members and cadres in government organs, enterprises and public institutions, and more than 1.04 million Party members and cadres in the province participated in the work of sinking communities."Community plan" takes advantage of "Party cadres sinking community" activities to launch a group of Party members youth volunteers in the community; meanwhile, introduce the surrounding university volunteer team, leading by the community league branch secretary, cultivating a normal youth volunteer service team composed of "grassroots cadres + Party members youth volunteers". In Hongguang Community, Fancheng District, Xiangyang City, some residents are free to come to the neighborhood committee after work. Community party committee and the area of 16 "double registration" party members after consultation, set up the party and the party post station, organized young party members volunteers to take turns on duty, launched the "7:30 post" service. From 7: to 7:30 every night, the young party members take turns to be on duty, collect residents to do things; the next morning by the community branch and the community contracted to the neighborhood committee, industry committee and property "three parties" to solve the problem. Once the service was launched, it was widely popular. In the Second Road Community, Xiling District, Yichang City, there are more than 300 double-worker families within the jurisdiction, and the parents usually have limited time to supervise their children. The community launched school resources within its jurisdiction, recruit to 10 professional teacher volunteers, through the communist youth league to recruit 16 college students volunteers, form a teacher volunteer team, for double worker family children after school and holiday hosting services, including academic counseling, interest training, art healing, etc., directly benefit nearly 3000 people. In Nanyuan Community, Qingshan District, Wuhan, there are 107 people over 80 years old. Most of the party members of Nanyuan community are employees of Baowu Group. From the community has discovered a group of young party members with expertise to carry out pipeline maintenance, home appliance repair and other services. Pilot communities will explore the self- "hematopoietic" mechanism to mobilize social volunteer service forces to participate in the "community plan", and help excellent volunteer service projects are incubated in the community for implementation. The Hubei Provincial Youth League Committee, together with the Propaganda Department and other units, has held volunteer service community special competition and "Dream Community- -Volunteer Charity Creation Camp"; and jointly launched the "Colorful Community" project, selected 60 volunteer service teams, college volunteer organizations, social workers, youth volunteer associations and community volunteer service teams in 60 communities, providing 10,000 yuan for each project. The college student volunteer service team led by Fang Cheng and the Wuhan Beigang community, where the team serves, are the beneficiaries. Fang Cheng admitted that there are many volunteer service activities for college students on campus, but due to the lack of funds and other factors, most of them are relatively scattered and lack of professionalism. They are often "where they want to do it" and "organize them when they have time", which is far from the real needs of the service objects. Since last summer vacation, with the support of the "Community Plan" of the Hubei Provincial Committee of the Communist Youth League, the team has participated in professional training, upgraded the scattered volunteer services to professional public welfare institutions, and received a 50,000 yuan special support fund of "Dream Community- -Volunteer Public Welfare Creation Camp 20,020" to provide regular services to the Beigang community. The continuous cost of the community normalized volunteer service is high, and the financing channels are limited. Under the promotion of the "community plan", the pilot community has explored the self- "hematopoietic" mechanism. In Zhangwan District of Shiyan City, Sunshine Community donated 2 million yuan through joint real estate enterprises to establish a community public welfare fund, which provided guarantee for the implementation of volunteer service projects and team incentive, and carried out ten volunteer services such as environmental protection and cultural associations. In Nanhu Street, Wuhan city, the "small and beautiful" community fund has formed a special youth volunteer service. Through the Double Ninth Festival and community Lantern Festival, mobilize the community and surrounding businesses to raise resources, developed the "small courtyard" ecological base income recycling project, "for my tomorrow" and other services for the elderly continue to carry out smoothly. Some young volunteers have gradually found the right service direction at the community grass-roots level, and promoted community governance in the volunteer service projects. Yuntian Community, Wansong Street, Jianghan District, Wuhan city, was one of the first pilot units, with more than 3,000 households. Community league branch secretary Wang Tianhong introduced that last August, the community through the "demand research meeting" learned that the area of more school-age children, parents have a greater demand for parenting knowledge and parent-child activities. At the same time, among the young residents in the community, more highly educated and high income, many are good at photography, painting, English tutoring. Parents working in education institutions, self-taught himself a lot of parenting knowledge, also obtained the relevant qualifications, community party trained him for volunteer service projects, building platform launched "the raise", collect young parents, service willingness, together formed a "giraffe parent-child alliance", has carried out the "treasure hunt" "natural science classroom" "parents" classroom "English corner" and other activities, has attracted more than 400 families to participate in. To consolidate the "community plan" pilot work, the Hubei provincial party committee, provincial volunteer association, provincial youth development foundation launched in March 2021 youth volunteer service "community plan" competition, provincial, city, county level linkage format, launched 571 city communities in the province, invested nearly one million yuan reward funds, promote the communist youth league and youth volunteer service organizations into the grassroots social governance."Volunteer services are weak in the streets and lanes, but it affects thousands of families and concerns people's livelihood. The 'community plan' is a beneficial exploration of the Hubei Communist Youth League to serve urban community governance and innovate the normal volunteer service mechanism after the epidemic test."Zhou Senfeng, secretary of the Hubei Provincial Communist Youth League, said that the Hubei Communist Youth League will continue to leverage social" big resources "through community" small plans ", cultivate local youth volunteer service forces, improve the community volunteer service system, and help grassroots social governance for a long time. Hu Lin China Youth Daily China Youth Network reporter Zhu Juanjuan Lei Yu source: China Youth Daily

2021-09-14	The communist youth league of Fujian red dream tour "red classroom" Fujian red dream tour "red classroom" activities xi reporter Tian Hongwei) to further carry out red education, do focus on university, middle school, primary school youth groups, Fujian provincial party committee in summer resources in colleges and universities, let the students into primary and secondary schools to open "red micro classroom", the red teaching and preaching activities, education to guide the province all the teenagers of love party, patriotism, love socialism, let the red gene, revolution. The "Light cavalry" propaganda group of Huaqiao University went into the classroom of primary and secondary schools, and brought the party history theme of "Making youth China with youth" to the primary school students. For nearly 600 middle school students, the publicity group combined with the knowledge of history and geography course knowledge, supplemented by animation micro video, to make the theoretical problems story, character, scene, to create a theoretical height and interesting party history class. The "Xinglin Child Walk" volunteer service team of Fujian University of Traditional Chinese Medicine has carried out 35 special courses of traditional Chinese medicine culture, red history, civilized etiquette, environmental protection, mental health, and national geography in Wuzhai Primary School, Shipbuilding Farm Town, Zhangzhou City. The practice team composed of the Department of Nursing of Xiamen Medical College went to Guanghe Middle School in Gansu Province to hold a special sharing meeting for the senior three students with the theme of "Returning to discuss the students in Xiamen". The practice team of Fujian Chuanzheng Transportation Vocational College went to Nanchuan Village, Malien Township, Xiji County, Ningxia, to preach the story of the Party history to the local villagers, and learned the dance performances and sang classic red songs with the villagers.

2021-09-14	Wang Xinxin, reporter of China Youth Daily) On September 10th, the Sichuan Transportation system league building brand activity "Traffic Youth Talk" was held in Chengdu. Leaders of the Youth League Committee of the Sichuan Provincial Transportation Department and the Youth League Committee of the Shudao Group visited the Sichuan Provincial Youth Volunteer Service Center and the Sichuan Provincial Youth New Media Center, and had a heated discussion on "how to do the Party's youth work well in the new Era". The activity is jointly hosted by the Youth League Committee of Sichuan Provincial Transportation Department and the Youth League Committee of Shu Road Group. On the same day, the participants went to the Sichuan Provincial Committee and Sichuan Chengyu Expressway Co., Ltd. for exchange and study. In the Sichuan Provincial Youth New Media Center, the delegates learned about the exploration of the group's new media to build an accessible and trustworthy youth circle of friends. At the Sichuan Provincial Youth Volunteer Service Center, delegates learned about the development of volunteer work since the launch of the pilot reform of the youth volunteer service system in December 2019. At the theme salon site, the participants discussed the topics such as party building and youth league building, how to connect youth work and rural revitalization, and the grass-roots construction of the Communist Youth League. The activity has inspired many youth league cadres. The person in charge of the youth League organization of Sichuan Highway Engineering Consulting and Supervision Co., Ltd. suggested that the youth can help them realize their own value to the collective and the society through volunteer service. Yang Quan, secretary of the Youth League General Branch of the Department of Architectural Engineering of Sichuan Communications Vocational and Technical College, believes that in the field of volunteer service, the way of youth service to the society should not be limited to communities, schools, especially college students, who can make some contributions to the industry. It is reported that the traffic youth said is the Sichuan transportation system building youth brand, series of salon activities by inviting industry youth models on the growth story, show a batch of amiable, respectable, credible, learn youth example, guide the transportation industry youth be "patriotic, inspirational, realistic, practice" new era.

2021-09-13	Jiangsu communist youth league: activate the grassroots reform new expand youth circle of friends and resource circle of Jiangsu communist youth league: activate the grass-roots reform new, he thinks, " this round of county communist youth league reform measures is very real, the key is the grassroots 'four' problem, help us expand the youth circle of friends and resource circle, change out of the youth league momentum."In the past three months, the Standing Committee of the Haian Municipal Party Committee studied the reform of the municipal Communist Youth League grassroots organizations, the main leaders of the Municipal Party Committee listened to the work report of the Communist Youth League twice, the Municipal Party Committee in charge of the leadership to promote the reform work for four times, and set up the reform leading group as the leader of the main leaders of the Municipal Party Committee. The Haian Municipal Party Committee Office, the Organization Department of the CPC Municipal Party Committee and the CPC Municipal Inspection Office issued three documents on the reform of the Communist Youth League, and the Municipal Communist Youth League established the Municipal Youth Affairs Service Center, which doubled the work funds of the Communist Youth League, and selected the cadres of the Communist Youth League in a larger scope. These changes all come from this round of county communist Youth League grass-roots organization reform work. In June this year, 25 counties (cities and districts) in Jiangsu province, including Haian, were designated as the pilot reform of the primary organizations of the county Communist Youth League."Now the provincial youth League party committee leads us to do, the municipal Party committee supports us to do, the grass-roots youth league cadres strive to do, our work confidence and confidence is more sufficient!"Xu Li said proudly. One by one analysis accurate pulse to the train of thought " grass-roots county reform what? Where to change? How to change? In fact, we did not know very well at the beginning, but through several rounds of precise guidance from the provincial and municipal youth League committees, our reform path became more and more clear."Said Wu Zeyu, party secretary of Tuan Jiangyin. From June to August this year, like Wu Zeyu pilot county (city, area) communist youth league secretary participated in the Jiangsu provincial party committee organized the county communist youth league reform seminar, training and hall, focus on the reform of the communist youth league "three race" research, respectively around the county league, youth league members, cadres "three" in-depth analysis. Zhang Yingchun, deputy secretary of the Jiangsu Provincial Committee of the Youth League, said, " The reform should first find out the weak links and identify the right target. The league, district and youth conditions are different places. We focus on the 'three where' to plan the reform to make the reform more targeted and effective, and make the political and social functions of the county league more prominent."" We collected the flow and distribution data of youth groups in the whole region to draw the 'heat map' of youth services, and the reform is more targeted."Li Jing � t, secretary of Xiangcheng District Party Committee of Suzhou City, was deeply touched. Combined with the precise positioning of" heat map ", the Communist Youth League District Committee has built 4 youth homes, 8 youth V + stores and 68 non-public youth league organizations, helping regional development in the integration of industry and city, social governance, cultural inheritance and other aspects. The Jiangsu Provincial Party Committee of the Communist Youth League has deeply realized that the establishment of a long-term mechanism is the key to this round of reform of the county Communist Youth League, and has guided all localities to strive to actively achieve the "three inclusion" of the party building and team building mechanism in the fields of party building assessment, party committee inspection, education evaluation and education supervision. Yangzhong city will be the party building with the league construction, team construction into the municipal party committee inspection content, issued the list of key work inspection list, put forward three aspects of 10 specific requirements, to provide a strong political guarantee for the reform work. Chen Jian, party secretary of the Communist Youth League, sighed with emotion: " The precise guidance of the superior youth League committee has optimized the policy environment for the reform. All the reform measures are progressing smoothly, and the city's youth League cadres and officers are more energetic."For strong guidance, efficient reform, Jiangsu provincial party committee established the provincial county communist youth league organization reform at the grassroots level the work leading group, the central four dimensions 10 aspects reform measures for 39 specific reform tasks, establish provincial, city, county level linkage mechanism, close communication with local party committee, direct guidance 25 pilot counties (city, area). A reform plan, Xu Li, secretary of the Communist Youth League Haian Municipal Party Committee, and the Provincial Communist Youth League Committee and the Municipal Communist Youth League Committee discussed back and forth 10 times."It was the first time I saw the reform of the Communist Youth League involving so many dimensions. In the process of revising the plan, I felt full of motivation, because it is really to really promote the role of grass-roots league organizations and inject vitality into the grassroots."On August 18, the haan communist youth league grassroots organization reform plan officially issued, the haan committee seize the reform opportunity, promote district town (street) full-time cadres with 100%, hang, part-time cadres with rate rose 200%, and set up the city youth affairs service center, pku work funds from the original 14-35 years of permanent youth of 1.5 yuan / person to 3 yuan / person."This joint youth service station activity, I took my son to the pair of 'little relatives' Yao Yuhao's dream cabin, played basketball together, they have become good friends, good partners."League Xinyi City Party Committee member, Xinyi City Youth Chamber of Commerce President Song Wensheng said. The work of the United Youth Service Station has been integrated into the daily life of the youth league representatives, committee members and cadres of the Communist Youth League at all levels in Jiangsu. Through the "January One Theme", the province has carried out centralized services with 12 themes, and the youth League cadres at all levels have carried out a total of 11,000 activities and helped more than 25,000 young people. This summer, the Youth League Jiangyan District Committee relied on 16 "youth homes" to carry out "youth charity summer care classes", serving 355 young people. This year, 932 such "love summer care classes" were opened in Jiangsu province, including 100 provincial demonstration classes and helping 35,000 young people together. Focusing on the "double reduction policy" and focusing on how to run classes, the Provincial Communist Youth League Party Committee also jointly formulated the guidance manual, and hired a third party and parents to jointly supervise and guide them to ensure the standardized operation of summer care classes. In the face of major and difficult problems in this round of reform, Jiangsu Provincial Youth League made careful plans and actively strive for various policy breakthroughs. At the provincial level, we fully contact with the CPC Organization Department, CPC Inspection Office, CPC Reform Office and other departments to introduce "policy packages" covering the allocation, use, management, incentive and restraint of the pilot league cadres, and actively promote the establishment of reform leading groups to form fundamental and long-term institutional achievements. The lack of work force of the youth League is a "difficulty" that has long troubled the development of grass-roots youth League organizations, and it is also the top priority of the "problem" of the reform. In the view of the Communist Youth League Nanjing Municipal Party Committee, the reform is first to ensure that the force is in place. To this end, the Nanjing Municipal Party Committee of the Communist Youth League spends 2 million yuan financially funded to purchase youth social work services every year. At present, the city has established a 100-full-time youth affairs social workers team, covering all towns and streets. At the same time, the implementation of the community (village) Communist Youth League work assistant project, raised supporting special funds of 520,000 yuan. Group 10 county in accordance with the provincial party committee organization department and youth league provincial party committee on the province of the township, village (community) youth league transition work notice requirements, seize the transition opportunity, held the county village (community) youth league transition deployment advance, the county village (community) "two committees" young members of the youth league secretary reached 100%. By the end of August, 95 percent of the township and village (community) youth league organizations in Jiangsu province had been replaced, and more than 80 percent of the "two committees" were also youth league secretaries. The average number of members of each village (community) youth league was close to five."We won the honor of the National May Fourth Red Flag Youth League Committee this year, which cannot be separated from the role of the 'three orders, four degrees and five feedback' mechanism."Said Wang Zhibin, who was just appointed as the temporary deputy secretary of the Yangzhong Youth League Municipal Party Committee. Around the League cadres work energy is not focused and other problems, Yang Zhong clear league cadre post full-time special post, the term is not less than two years. Through the three lists of "basic league affairs, key projects, characteristics and highlights", the term and annual objectives are clearly defined, and the evaluation and feedback is conducted through the overall contribution, work completion, youth satisfaction, etc., and a "military order" is issued for the part-time deputy secretaries like Wang Zhibin, forming a management closed-loop to promote the promotion of youth League cadres. In the repeated struggle against the new round of epidemic, the Nanjing Jiangning District Committee really tested the effectiveness of the reform. Through the establishment of district-level backbone group, sub-district (district direct) contact group, community (school) service group at three levels, and 515 wechat tower groups, more than 3,000 youth league members were quickly recruited to join the front line. In this round of epidemic prevention and control battle, Jiangsu has mobilized more than 223,000 youth league members to report to villages (communities), and organized 4,138 youth commandos and 64,000 youth commandos to show the responsibility of youth on the frontline of medical and health care, urban and rural joint prevention and control, traffic control, and material support. League of Jiangsu provincial party committee secretary SiYong said: " Jiangsu communist youth league will strengthen the political nature, advanced, mass throughout the county of the communist youth league organization reform the whole process, resolutely implement the reform task, the reform signal to the end, the power resources to the grass-roots level, let the county communist youth league really strong, make society more perception, youth have more feeling."China Youth Daily, China Youth Network reporter Li Runwen, correspondent, Yang Yisheng source: China Youth Daily

2021-09-12	Jiangsu: the new development of public welfare jobs for urban and rural employment difficulties in Jiangsu: new development 7000 public welfare jobs for urban and rural employment difficulties in rhyme green newspaper network reporter li runwen) on September 10, Jiangsu province people's government held "I do the practical work for the masses" the third press conference, conference focused on college students, migrant workers, flexible employment personnel stable employment of the people's livelihood. Among them, this year's Jiangsu provincial government work report clearly proposed that 7,000 new public welfare jobs to relocate urban and rural people with employment difficulties, as an annual people's livelihood. Jiangsu province has innovated the post development mode of "assigning posts according to demand and setting up posts according to people". From January to August, Jiangsu province has developed 9,589 various public welfare positions, and employed 5,134 people with difficulty in finding jobs. Zhang Hongwei, a member of the Party Leadership group and deputy director of the Human Resources and Social Security Department of Jiangsu Province, said, " Public welfare post is a measure to help people who have difficulty finding jobs. By formulating measures for the development and management of public welfare posts, we will strive to improve the quality of posts and the matching degree of resettlement."It is reported that Jiangsu expanded the scope of unemployment insurance protection, to receive the unemployed who are still unemployed, do not meet the conditions of receiving unemployment insurance benefits of the unemployed, unemployment subsidies. From January to August, the province paid 4.385 billion yuan of unemployment insurance benefits, benefiting 539,900 people, and the per capita unemployment insurance benefits level was 1,653 yuan; in the first half of this year, the unemployment benefits were 1.334 billion yuan, benefiting 421,200 people. From January to August, the province helped 192,600 urban and rural people with employment difficulties to find jobs. Jiangsu is a major employment province for migrant workers, with a total of more than 23 million migrant workers. In the face of regular epidemic prevention and control, Jiangsu has vigorously implemented efforts to stabilize enterprises, carried out special actions to stabilize posts and retain work, and promote return to employment. Organized the special action of "sending warmth and stabilizing to in the Spring Festival", guided foreign employees to stay in the city for the Spring Festival and return home at the wrong peak. The province issued 927 million yuan of subsidies, held 2,793 job fairs, and provided 2.8098 million jobs. We will ensure the point-to-point services for rural migrant workers returning to work, carry out a special campaign to "jointly fight the epidemic and help find employment", and deliver 112,100 workers directly to enterprises. At the same time, we will promote human resources and social security departments to help enrich the people and strengthen the villages, and continue to promote the rural revitalization strategy. We will carry out a special campaign to improve vocational skills and a five-year training campaign for rural migrant workers returning to their hometowns to start businesses. We will adopt a "through train" to rural areas and online training to improve their ability to find jobs and start businesses. By the end of August, Jiangsu Province had conducted 397,800 rural workers, 238,400 new-generation migrant workers, 34,900 farmers' entrepreneurship training, and 30,300,4.214 billion guaranteed loans. Zhang Hongwei introduced that Jiangsu is located in the developed region of the east, with developed industries and market segments, and the rapid development of platform economy and digital economy, which is the "fertile soil" for the growth of new employment forms. At present, the province's flexible employment personnel have reached 11 million. We will vigorously expand new space for flexible employment by taking measures to encourage the development of business forms, improve security policies, and provide convenient services. At present, Jiangsu province employs more than 4 million employees in express delivery, food delivery, online ride-hailing and e-commerce platforms.

2021-09-09	Hainan hope project grants for 620 students in Hainan hope project interpreta dream action 11 long-term fund grants 620 students funded wei correspondent Wu Yuhua) on the morning of September 8,2021 hope project of Hainan province power rural revitalization interpreta dream action 1 a long-term fund grants ceremony held in Haikou, Hainan provincial party committee and other organizers leadership, 11 long-term fund donation unit representatives to attend the ceremony. At the ceremony, the leaders attending the event gave 5,000 yuan in grants to each of the 620 student representatives funded by the 111 long-term funds. As of September 7, the 2021 Hainan Hope Project to help realize rural revitalization dream action has received positive response and enthusiastic participation from all sectors of society. The province has raised 19.09 million yuan to support 2,818 college students from low-income families. Hainan Hope Project Dream Realization action has been implemented for 19 consecutive years, raising 292 million yuan, helping 56,295 students realize their dream of college, from 2008 to more than 10 million yuan, has become an important carrier of Hainan Communist Youth League to serve young growth and the most famous poverty alleviation project brand in our province.

2021-09-09	The fifth in Anhui province middle school students career simulation challenge (final) held youth dream, not youth in the fifth middle school students career simulation challenge (final) successfully held Beijing on September 9 (reporter Zhang Jianwei, correspondent, ya-dong liu check WeiTing), sponsored by Anhui province, Hefei one and provincial mental health association youth career development education committee to undertake the fifth Anhui middle school students career simulation challenge (final) successfully held online. Leaders from the school departments of the Provincial Communist Youth League Committee, the Provincial Academy of Education and Science, the Provincial Mental Health Association, the Hefei Education Bureau, the Hefei No.1 Middle School and other units were invited to attend the online opening ceremony. The competition lasted for 7 months and was divided into two stages: preliminary competition and final competition. The instructor training meeting was organized in the early stage of the competition preparation, and nearly 100 teachers participated. The competition attracted 170 students from 44 schools in 13 cities in the province to participate. After the preliminary evaluation, a total of 60 students entered the final. The final part adopts online competition, and the contestants and judges are randomly divided online to ensure the fairness, justice and openness of the competition. After strict evaluation by the jury, 7 students including Chang Yutong from Hefei No.1 Middle School won the best contestant, best production, best planning and Best Display Award; 10 students including Yang Jingwei from Feidong No.1 Middle School won the group grand prize, 19 students from Huaibei Experimental High School, 26 students including Wu Wenhan from Tongling No.3 Middle School won the second prize; 115 students including Wang Yanxi from Tongling No.1 Middle School won the group No.1 Middle School; 23 schools including Susong Middle School won the Best Organization Award. The provincial vocational simulation Challenge for middle school students is a practical activity to cultivate young students' comprehensive ability, such as communication ability, innovation ability and coordination ability. It is an effective carrier for the interaction and integration of the first classroom and the second classroom of school talent education and training. As a key brand competition for middle school students organized by the Provincial Communist Youth League and the Student Union, this competition has been successfully held for five sessions so far. The influence of the competition is constantly improving. It has become an important stage for middle school students in the province to correctly understand themselves, scientifically plan their life, and be brave in career exploration.

2021-09-08	Hubei open up the county communist youth league grassroots organization reform "new battlefield" communist youth league grassroots reform party committee to promote provincial and county level three linkage Hubei open up the county communist youth league grassroots organization reform "new battlefield" green net reporter Lei Yu) " dare to rush dare to try, dare to think dare to do, the league county committee became a few things all dare not think before."Li Yangbing, party secretary of the Communist Youth League, Jingzhou City, Hubei Province, was excited about the reform. In just 1 month, Youth Jiangling County Party Committee was approved to establish a public institution directly under the Youth League Party Committee, Three additional business cadres, Completely change the weak situation of the full-time backbone force; In the youth concentrated express delivery, e-commerce, hotel industry and other fields of the youth League working committee, Contact with the social field of youth has a new grasp... this year, the central Committee of the Communist Youth League grass-roots organization reform expansion pilot work mobilization and deployment meeting was held, The Hubei Provincial Communist Youth League Party Committee officially launched the second round of pilot reform, In the province's 17 cities (states) of 26 counties (cities, districts) opened up a "new battlefield" of grass-roots organization reform, To jointly win the support of the Party committees, Expand horizontal collaboration, Form the provincial, city and county "three-level linkage" work situation. In November 2019, Hubei Qianjiang, dae both in the central county communist youth league organization reform in the first round of pilot, in the party building system implementation, cadres choose mechanism innovation, organization innovation �, a new way, a strong basic youth league network gradually clear, also "weave" out of the grassroots league youth cohesion, centripetal force, pilot task completion rate of 100%, laid the foundation for Hubei to expand the pilot. Since the reform pilot project, Hubei Provincial Party Committee attaches great importance to, high-level promotion. In July this year, the provincial party committee reform office issued by the reform on accelerating the county communist youth league organization pilot work notice, the pilot work added in the annual reform work project and parameter, and timely jointly with the provincial party committee organization supervision inspection, report work progress, related evaluation will serve as an important basis for the annual reform assessment, institutional and institutional "hard constraints", for the county communist youth league organization reform pilot work into momentum. The reform is based on the grass-roots level and oriented to the front line. It does not mean that the reform is only a matter at the grass-roots level, but needs to be promoted by the youth league organizations at all levels to form synergy. The Hubei Provincial Party Committee of the Communist Youth League has explored the mode of "three-level secretaries working at the grass-roots level", and issued a special "work manual" to clarify the work boundary, list work lists, clarify work responsibilities, and clarify the work path of the reform of grass-roots organizations at the provincial, municipal and county levels. Hubei Provincial Party Committee established leading group contact bao reform key units, clear each team member contact 3-5 cities and municipal reform pilot counties (cities, districts), the pilot reform as a key issue; strong liaison for pilot counties (cities, districts), improve the working mechanism of weekly analysis, double weekly, monthly scheduling, and improve the provincial working force system to promote reform. The municipal youth League committee gives full play to the advantages of the territory, mainly focusing on the responsible comrades and coordinating with those in charge, gathering the advantageous working strength and guaranteeing the resources to the reform counties and cities. The county-level youth League committee focused on the reform and innovation of 10 measures in four aspects, including the selection of league cadres, the league organization setting and operation, the education and management of league members, and the leadership guarantee. Pilot counties (cities, districts) combined with the local conditions, to explore the characteristic path. Wuchang District, Wuhan organization, team cooperation, practice cooperation "three cooperation", establish league "two-way office system", set up 21 people education league working committee, create a studio, implement "big moral education" to promote the integration of education; Xiangyang Fan District innovation grassroots youth league assessment system, set up KPI index, monthly follow up, quarterly report, annual evaluation, strengthen the league cadres perform office assessment."Take the initiative to promote the level, as a good grassroots reform of the 'propellant' 'catalyst' 'lubricant' ", since the implementation of the pilot work, the Communist Youth League Hubei Provincial Party Committee Secretary Zhou Senfeng put forward that the reform work to "the main leadership grasp, the main energy grasp", according to half a year time inverted schedule, wall chart operations, pressure stubble to advance. Up to now, 100 percent of the 26 counties (cities, districts), responsible members of party committees have listened to reports on the pilot work, and 69 percent of the standing committees of county-level Party committees or secretaries of the reform have deliberated on the reform plan. The Communist Youth League's "reform and reform" has been played. Source: China Youth Daily

2021-09-08	Shandong "Hope House" project 99 Charity Day online fundraising is fully launched to integrate feelings, wisdom and strength of Shandong school Communist Youth League with "a cabin" to convey "human love". China youth network Beijing, September 8 (reporter Zhang Jianwei, correspondent Zhang Kangning) on September 7 to September 9 at 24 hours, Shandong "hope cabin" project 99 public welfare day online fundraising was fully launched. Based on the organization foundation of Project Hope, the "Project Hope House" integrates social resources, extends the service carrier, serves the grass-roots level and helps children as the purpose, innovates the practice, and provides the "Qilu Plan" for the Project Hope to serve the society. The project mainly provides "love assistance" for children aged 8 to 14. Through the planning, design and renovation of their original houses, combined with necessary desk furniture and school supplies, it can improve the children's living environment and create a good learning atmosphere. Weifang University carried out the "Hope House" theme Youth League Day activity. After the comprehensive promotion, the "Hope House" has become an important position for the Shandong Communist Youth League to integrate into the overall situation of the party and the government, participate in the local construction, eliminate the "intergenerational transmission" with the "education and poverty alleviation", and help the rural revitalization. Data show that since the launch of the project, more than 165 million yuan of public welfare funds have been raised, 12,739 cabins have been built or under construction, 24,776 volunteers, benefiting more than 10,000 children, and have been awarded the "Top 50 Poverty Alleviation Cases of Social Organizations in China", which has become a typical case of promoting the transformation and upgrading of the Hope Project in China. Based on the solid experience and fruitful achievements of social practice, the Communist Youth League of Shandong School has widely publicized the "Hope House" project, and mobilized all the young students, practice teams, alumni and alumni enterprises to show their love."Nest" don't forget to "warm nest", pay more attention to solid "hope hut" in the second half, organize the provincial college students around "hope hut" pair support, academic counseling, psychological counseling, and other volunteer service activities, help realize from "cabin refresh" to "refresh" spirit, pass good "hope hut" value, do a good job in practice education, promote the comprehensive growth of young students.

2021-09-07	The first class of correspondent Yue Qing) " What does the new drugs look like?"" What are the dangers of drugs? "" How do teenagers prevent new drugs?"Recently, the first lesson of the 2021 autumn anti-drug group class premiered on the online platform, sponsored by the Guangdong Provincial Drug Control Office, Guangdong Provincial Youth League Committee, Guangdong Provincial Department of Education, Guangdong Provincial Department of Human Resources and Social Security, and organized by the Guangdong Provincial Juvenile Crime Prevention Research Society. Zhong Nanshan, Du Feng, Su Bingtian and other big names sent messages to middle school students in the province through video, calling on young people to cherish their lives, stay away from drugs, and enhance their awareness and ability to resist and prevent drugs. The anti-drug themed group class was broadcast live through the official account of "Guangdong Communist Youth League", "Guangdong Anti-drug", "Guangdong Public Security" new media matrix and other platforms, and nearly 9.4 million middle school students from more than 5,000 schools watched it online simultaneously. Guangdong Experimental Middle School organized students and league members to watch the first lesson of the 2021 anti-drug group class together. Correspondent for figure activities around "what is a new drug" "where easy to appear new drugs" "how to prevent new drugs" "accidentally infected with new drugs how to save themselves", through the scene deduction, video display, experts, Guangdong drug cartoon image "yue tiger" popular science multidimensional to teenagers propaganda drug knowledge, let teenagers in the immersive classroom deeply realize the harm of drugs, to improve the awareness and ability to prevent drugs. Scene interpretation: drug dealers lure teenagers to take drugs. The activity also invited Nadila, a police officer from the Anti-drug Guidance Department of Guangzhou Public Security Bureau, to lead the students to dismantle the common "toxic tactics" of drug dealers to lure teenagers to take drugs one by one, teach the skills of "open tactics", and educate and guide teenagers to resist the temptation of drugs and stay away from drugs. At the end of the activity, Quan Hanyi, the principal of Guangdong Experimental Middle School, told the teenagers to always bear in mind the life concept of "healthy life, green and non-toxic", have lofty ideals, and be the youth of The Times. Zhaoqing Industrial and Trade School organized student league members to watch the first lesson of the 2021 anti-drug group class. Correspondent for the figure, the activity aims to focus on the school main position, student main groups, targeted ground to young students to carry out drug prevention education, expanding drug propaganda influence and coverage, enhance the ability of young students to prevent and resist new drugs, as the escort for the majority of young students healthy growth. The video will be posted and replayed on official accounts such as "Guangdong Communist Youth League" and "Guangdong Anti-Drug Control", and all youth members can watch it online at any time.

2021-09-07	Jiangxi "a program under which officials" volunteers in hot practice refinement success "volunteers" a program under which officials in hot practice refinement success wei correspondent wang � h) this summer, Jiangxi college student volunteers "a program under which officials" summer social practice launched more than 284000 students actively participate in, form 3107 practice team, young students in social classroom education, long ability, contribution, with practical action to practice "please rest assured, power have me" youth oath. Ceaseless blood, launch the red engine of coagulation soul gathering gas " inheritance of red gene, ceaseless spirit blood."The province actively launched more than 15,000 teachers and students to form 1137 party history study and practice groups, innovative forms, rich connotation, promote the party history study education to go deep, to the heart, to the real, for the party history study education" heating up ". Nanchang university more than 100 practice team relying on the path of "faith" party history, fanned out around the province hot theory, take the initiative to become the party history education "seeder", "propaganda", humanities college Hu Bangning teacher felt "red fresh party history story, characters, let us more firm faith in socialism with Chinese characteristics, more continuously spirit blood, completes the founding spirit of learners, propagandists and practitioners". Jiangxi agricultural university "red gene" practice group to revolutionary history memorial facilities, site and patriotism education base to visit learning, with the youth perspective story, with new media means of jinggangshan spirit and Soviet spirit, in the form of popular red culture, let the red history "live", let the red education "fire". Nanchang engineering institute "painters picture scroll painting in one hundred, vigorous renewed sail spectrum Marty natalegawa" social practice group 28 teachers and students to Yingtan Zou Taofen hometown picked up the brush, with a picture of bright color, complete composition, prominent theme of hundred picture scroll depicts the communist party of China the world, 12 chapters, one hundred party history leap present on the wall, the new era of young students love party, power and patriotism. To inject the development vitality of rural revitalization into the fields and empower rural development. This year, Jiangxi province continues to mobilize the majority of young students to go deep into the countryside, from the fields to the minds of the people, and use their professional strengths to inject youthful vitality into the rural development. The practice group of "Communication and Flying Dream" in the School of Humanities of Jiangxi University of Finance and Economics used the 4:30 class of Qingbo Home School to carry out accompanying education activities, offering courses of calligraphy, traditional paper cutting and Putonghua promotion, which solved the problem of some children staying behind in summer vacation. With the strong support of the school library and the college, we have collected and purchased a large number of basic book resources. A total of more than 600 copies were donated to Qingbo Family School, Yuan Yu Civil Public Library and Bailiang Middle School. Jinggangshan university college of life science practice group into the jinggangshan old revolutionary base areas to visit sampling, field picking, environmental research, technology to the countryside and other activities, for the local lily industry form scale effect and brand effect, and use the new media platform for "goods" live, in the promotion of tourism and effectively alleviate the agricultural unsalable problem. Jiangxi Institute of Applied Science and Technology, Jiangxi Township team combined with professional characteristics, give full play to the advantages of the discipline, using professional building instruments and equipment to carry out field measurement and planning, design ecological routes according to the local ecological planning needs, adjust measures to local conditions, on the basis of protecting the original buildings and respecting the wishes of residents, reasonable layout, optimization and transformation."Ethnic unity is one family and work together to build the Chinese dream."While going deep into the villages of ethnic minorities in Jiangxi province, the practice group also organized seven provincial key teams to Xinjiang and Tibet to deepen exchanges and cooperation with teenagers in Xinjiang and Tibet, and help college students strive to be practitioners of ethnic unity and progress. Jiangxi normal university into the Tibet Shannan beauty county middle school to carry out the safety knowledge small lecture hall, quality development activities, science and technology enlightenment education, promote mandarin and other activities, also use the cloud to lead the students to visit the bayi uprising memorial and other red attractions, accept red education, inheritance red genes, strengthen the patriotic feelings, guide the students determined to do when the rejuvenation of the new era."I want to have a desk" "want to see the flag with parents"... Nanchang aviation university practice team in addition to open red music class, popular aviation knowledge, discussion research, visit local people, but also for local students to carry out "light micro wish" solicitation, back to school by the Nanchang aviation university youth league branch claim pair, for minority children. Wang Xiuxiu, a member of the practice group, said, " This practice will combine small campus classes with large social classes, hoping to learn professional knowledge well, and devote himself to the border construction in the future, so that the youth can shine in the most needed places of the motherland."" Communist Party hard for the nation, the Communist Party he dedicated to save China "," Jiangxi Normal University of Science and Technology "pomegranate seed" practice team into the Xinjiang International Bazaar to hold a flash mob activity, singing the youth hymn to the party and the motherland. The team members also invited Huo Jiajia, deputy secretary of the Youth League Committee of Xinjiang Production and Construction Corps, to give you a vivid ideological and political lesson. The spirit of the corps from the "earth" was deeply rooted in the soil and planted the seeds of integration into the self in the hearts of the practice team members. During the summer vacation, Henan was hit by extreme heavy rainfall and caused floods. Zeng Lifang, the leader of the rescue team and a student from East China Jiaotong University, led the team members to the front line overnight to transfer and rescue the trapped people. Liu Bingbing, a student of the former Shandong Armed Police Corps, rode a motorcycle from Changsha and arrived in the disaster area. He said, " As a veteran and a young college student, I will not forget your duty and rush to the front line of flood control and rescue, where there is a need."This year, the Jiangxi Provincial Committee of the Communist Youth League also organized more than 2,500 college students from 105 universities to participate in full-time activities in communities (villages) for daily work. Big bones adhere to close contact with the masses, based on the demand of the masses, solve the masses, combined with professional knowledge to carry out policy preaching, assist in epidemic prevention and control, technology promotion and free clinic activities; at the same time as "one yuan donation" public welfare angel to help the development of public welfare undertakings, so far launched more than 95,000 participation, raise more than 125,000 yuan.

2021-09-06	Shanghai communist youth league: people from the grassroots to grassroots project to break the administrative style to remove the Shanghai communist youth league: people from the grassroots to project to the grassroots about the "difference" period to deepen the reform of important instructions spirit, promote the reform of the communist youth league extends to the county, meet the requirements of the party central committee, in June 2021 launched the county communist youth league grassroots organization reform to expand pilot work, 31 provinces (autonomous regions and municipalities directly under the central government) of 621 county youth corps committee into the pilot. In order to strengthen the typical guidance and demonstration drive, our newspaper has specially opened a column on "When the grassroots reform of the Communist Youth League is ongoing" to exchange reform practices, show the progress of reform, build consensus on reform, and stimulate the impetus of reform. The spring breeze of the reform of the Communist Youth League in---------------County is warming the hearts of more grassroots youth in Shanghai. Some newly established "two new" organizations, get the resources directly sent by the district youth League committee, can also "order" request the superior youth league organization to send activities; some backbone youth who actively participate in and organize youth work, become part-time, temporary, professional cadres; some street town youth League cadres because of the basic manpower and material resources, more energetic. Earlier, the General Office of the Central Committee of the Communist Youth League issued a notice on determining the pilot areas of grassroots organization reform of the Communist Youth League at the county level, and the 16 districts in Shanghai also became part of the pilot areas of 621 counties (cities, districts and flags) nationwide. Reporters from the China Youth Network of China Youth Daily noted that Shanghai is the first pilot area of mass and league reform in China. On this basis, the reform of the county Communist Youth League has also produced a lot of "hardcore" experience."The 16 districts in Shanghai are promoting reform at the same time, striving to diversify grassroots organizations, socialized survival, project work, flat operation and diversified backbone, so as to truly break the organizational style of work and remove administrative dependence."Shanghai Guanjian, party secretary of the Shanghai Communist Youth League, said that the ultimate goal of the Shanghai Communist Youth League reform in the Shanghai county level is to strengthen the political function of the youth league at the grass-roots level and promote the formation of social functions. In early September, when the effectiveness of the "double reduction" policy gradually emerged in Shanghai. Shanghai is one of the pilot cities of the Ministry of Education's "double reduction" policy. After the "double reduction", the number of primary and secondary school students has become less homework, and less training courses on disciplines. How children play on weekends and how to have meaningful games have become the top priority for young parents. In Pudong, Shanghai, teenagers in the community are playing with science and technology experiment devices. They are applying for the "Youth STEAM Science Course" in the "Youth Caravan" project through the platform of the Youth Pudong District Committee. They find the science of "seeing is not believing" at home and understand the mystery of science and technology in life. The "Youth Caravan" is led by the Pudong District Committee of the Communist Youth League, which provides the community teenagers with diversified services such as learning and training, parent-child education, physical fitness, cultural entertainment, anti-fraud and fraud prevention. The project provides more than 10,000 point and order services for 1,3006 "doorstep" youth centers in the district, covering 500,000 people. Sub-district and township youth League committees can order on the platform according to their actual conditions, and the district Youth League Committee can deliver the activities to the points on time and on demand through the public service of youth League members, volunteer services of commissions, offices and bureaus, and the purchase of services by social organizations."Youth STEAM Science Course" is a youth science exploration project purchased by the Youth Pudong District Committee from the Shanghai Youth Development Center in Shanghai. In each activity, the youth social organization will take a variety of small items in life as a lecture props, so that the children can understand the scientific knowledge in life."Almost all streets and villages are very welcome, and demand exceeds supply, relying on grabbing orders."The center director-general Du Liang told reporters that the center also participated in the Youth League Pudong District Committee of the love summer care class, youth club salon and other directly distributed to the grass-roots projects. In Shanghai's current popular international Qiantan community, a "new space" located on the fifth to eighth floors of high-end office buildings is being built. Qiantan community is known as the second Lujiazui in Shanghai, where high-end shopping malls, office buildings, international hospitals and international schools gather. According to preliminary statistics, 68,000 people are currently living here, of which 66,000 are all young people and about 1,000 are foreigners, which is expected to increase significantly in the future. Every noon from Monday to Friday, yoga, fighting and dance classes in the "Tuan Youth Relations New Space" are in short supply. Tang Jia, deputy secretary of the league Sanlin Town Committee, introduced that according to the international characteristics here, the league organization has launched a "membership system" - -young people can participate in volunteer activities such as garbage classification, COVID-19 vaccination, and epidemic prevention and control to get points, and apply for noon characteristic activities with the points. Young people who participate in volunteer activities can also enjoy the service of "youth league organizations to help them with their children". " They are volunteers, and we organize go classes and piano classes to help them take care of their children."Recently, the league organization has also delivered Chinese cultural characteristics and other services, such as dragon and lion dance and zongzi making, directly to foreign companies working in Qiantan community. Who "does practical things" at the grass-roots level is the key issue. In fact, behind the league-run projects that become "hot goods" in Shanghai is the core of the reform of the county Communist Youth League at the grassroots level- -people come from the grass-roots level, the project is sent to the grass-roots level, and the system closed-loop design is in place. Take Pudong New Area as an example. From the part-time deputy secretary of the Communist Youth League District Committee to the full-time deputy secretary of the grass-roots streets, there are all "young backbone" from the front line. For example, Tang Jia, deputy secretary of the Sanlin Town Committee, was just a volunteer actively participating in the league's activities. Born in 1992, she began working in the Sanlin Town City Operation Office in 2016. Later, she attended the May 4th theme rally, worked as a volunteer, and worked as a volunteer at the youth center. In 2018, when Shanghai launched a nationwide pilot mass reform, she became a part-time deputy secretary of the Youth League Sanlin Town Committee."At that time, there was a secretary, a social worker, and two social workers to prevent juvenile delinquency. We suddenly went to three part-time deputy secretaries, which was nearly half stronger."Tang Jia said that after she and two other part-time deputy secretaries" arrived ", the work strength of the Tuan Sanlin Town Committee was suddenly enriched. They helped to promote the Sanlin Old Street Youth Center to become one of the first 5A level youth centers in Shanghai, helped to receive all over the youth league organizations to visit and study, and later also helped to start a love summer care class. In April this year, Tang Jia, who has been a part-time deputy secretary of the Communist Youth League Committee for two and a half years, was officially appointed as the deputy secretary of the full-time Communist Youth League Committee of Sanlin Town. Reporter noted that the county communist youth league reform guidance of the first is the "reform work force selection mechanism", broaden the group of work backbone source channels, not stick to one pattern strict selection politically strong, enterprising young talents into the work backbone team, explore to meet the characteristics of cadres management and incentive way. Now in Shanghai, there are many cases of "young backbone becoming full-time group cadres" like Tang Jia. The youth League district committees at all levels in Shanghai regard the training of young backbone as a major event. Shanghai yangpu district launched "community youth pioneer training plan", from colleges and universities, community youth leaders focus, training, has three batches of 81 youth leaders become "youth pioneer", including 11 people into the industry authority, 424 from law, engineering, finance, social work and other areas of youth community governance "think tank". Liu Weiwei, an associate professor of urban planning and management at the University of Shanghai for Science and Technology, is the deputy director of the industry committee of Shengshi Yulong wan Community. He also trained two young post-80s people to participate in community governance through the Youth Pioneer Program. Jinshan District, located in the outer suburb of the southwest of Shanghai, has long been some problems, such as the single source of the league cadres, the relatively weak working force of the grass-roots league work, and the insufficient reserve force of the league work. For this, jinshan launches the youth league cadre talent pool and jinshan nationality college students on the "golden seed" plan. In 2021, the "Golden Seed" plan received 633 Jinshan students from 107 universities, and finally 316 students participated in the temporary employment practice, and were included in the "Golden Seed" talent pool. Currently, 24 "Golden Seed" college students have enriched their strength in the league work. Team, resources, system can not have the front-line "young backbone" to join, the league activities want to really become the grass-roots demand, the "practical project" in the eyes of the youth, but also need the support of funds and system."People, money and institutions are indispensable."Lin Tingjun, secretary of the Youth League Pudong District Committee, told reporters that in addition to appointing young backbone, Pudong is also increasing the funding to the grassroots, last year the Youth League District Committee to support the grassroots funds accounted for 57 percent of the total annual project funds. The new economic organization represented by Ding Dong Vegetable Shopping Company has also received various "benefits" from the Communist Youth League District Committee. This Spring Festival, all over the country called for the "local New Year". The Pudong District Committee of the Communist Youth League sent gift packages to the delivery boys left behind in Shanghai. Later, according to the needs of young people in enterprises, it contacted the principals of famous schools in Pudong to give lectures for young parents to relieve anxiety. The company's front-line employees and young backbone also get the opportunity to join the Youth League and participate in the "Youth Talent Camp" training respectively. Since 2019, the Communist Youth League District Committee has provided more than 100 themed education, learning, life services, and social welfare services for non-public enterprise league organizations. This year, the Communist Youth League District Committee also provides league construction toolkit and funds for each new non-public league organization. It is reported that the principle of fund allocation adopted by the Communist Youth League Pudong District Committee is "district-level compensation, grass-roots level, social funding, a point". This low-cost, sustainable operation mode directly helps the grass-roots youth league organizations to gather popularity, enhance feelings, and has won the reputation and praise of the young people in the area. Lin Tingjun introduced that Pudong adopts the method of "money follows the project". Grass-roots youth league organizations and youth social organizations can undertake projects from the Communist Youth League district Committee and get financial support. Behind each sum of money, the equivalent is the responsibility to complete the project. The Communist Youth League District Committee evaluates the implementation of each project every year. The evaluation mainly includes three aspects: the first, the third-party social organization evaluates the implementation of the project; the second, whether the task is completed with good quality through the youth interview and the annual work assessment; the third, solicits the evaluation of the project implementation. Take the "youth caravan" project as an example, the street town raised demand, social organizations or commission bureau volunteer organizations to receive orders, and the Communist Youth League District Committee set up a docking platform. This "platform" is also called the order system, which should give feedback to the course quality after enjoying the project service."Popular activities continue to do, unwelcome ones to change."The Communist Youth League district committee can also grasp the preferences of the grassroots youth. In Xuhui District, this socialized institutional arrangement is also used vividly. The Communist Youth League District Committee recruited and established the "Xuhui Young People's City Practice Group" through socialized registration, including six teams, including red commentator, professional experience officer, "Water Drop" volunteers and entrepreneurial partners. These practice groups all have their own "social circle layer". They plan popularity gathering activities online, organize interactive punching tasks offline, strengthen the identity of the members of the practice group through political guidance, practice exercise and ritual education, and establish a virtuous cycle mechanism of online and offline bidirectional diversion. In addition, the practice group also works with regional units and colleges and universities in Shanghai to build a cooperation platform for the youth practice group to make contributions and provide education, recommend outstanding youth to the Xuhui District Talent Reserve Plan, and constantly stimulate the social vitality of innovation, entrepreneurship and creation. China Youth Daily China Youth Network reporter Wang Yejie source: China Youth Daily

2021-09-08	League Hainan provincial party committee does the practical work to service youth see pragmatic group Hainan provincial party committee does the practical work to service youth see pragmatic wei reporter WuYuwa) group Hainan provincial party committee further promote party history learning education go deep, focusing on the party and government needs and teenagers hope precision, around the "youth power rural revitalization, youth power free trade port construction, service youth growth" and other areas, promote "I do the practical work for the masses" practice. Since the launch of the activity, in strict accordance with the work promotion schedule and task chart, compaction and compaction work responsibility, work together to promote the implementation of various practical results. Planning work has a "height" organization to create the "Hainan free trade port countries build dream avenue" youth comprehensive service platform, service teenagers growth, organization drainage national and international youth and its organizations in Hainan, power Hainan free trade port construction, studies through train and island studies train 28 shifts (period), about 1360 people to attend. Service youth have "strength" to carry out the youth power rural revitalization action, the implementation of create "rural revitalization of youth league", cultivating local youth talents township, service youth talents home, mobilize social youth talents to the countryside, service rural development, the rural revitalization of the youth league as the communist youth league of the important auxiliary force and youth power rural revitalization of the "assembly station". So far, the province's 18 cities and counties (excluding three sand) to pilot model have set up 60 rural revitalization of youth league, absorbing outstanding youth 805 people, determine the work project 56,53 activities, covering 5861 people, make young people grassroots agricultural and rural development injected new vitality, create a strong clerkstarts entrepreneurial atmosphere. Contact youth have "speed" to carry out the service youth talent action, create youth (talent) dream station, the province 16 build dream station has been online operation, using "online and offline" mode, to Joan employment entrepreneurship youth free one-stop temporary accommodation, employment guidance, policy consultation, social integration services, build free trade port in Hainan service young talents "service window". At present, the registered users have reached 903 in the post station system, and 666 people have applied for the Dream Building Youth Card. They have provided free accommodation services for 129 young people, and provided low-price preferential accommodation services for 35 young people. Guarantee youth "deep" service youth growth action, prepare to carry out "sea green edge" friendship activities, to solve the problem of single youth marriage; hold 2021 "easy preparation 12,355" stress psychological counseling lectures 65, covering more than 56,000 teenagers and their parents; carry out " youth self-care? Summer safety " youth self-care education activities, the province has carried out more than 60 self-care education lectures, covering more than 26,000 teenagers. Volunteer warm "temperature" to expand the Hope Project work, implement "Project Hope Dream Action", this year has raised 11.28 million yuan, "Project Hope Vocational Education Funding Program" action, has raised 2 million yuan for 40 migrant children with free secondary vocational education; youth "micro wish" work, has light the micro wish for 502 children from low-income families.

2021-09-04	Fujian communist youth league held new media network training class Fujian communist youth league held new media network training network reporter Chen qiang) Fujian communist youth league new media network training class was recently held online, from all over Fujian province, Tibet, Xinjiang, Ningxia cadres and the new media staff more than 170 people to participate in a three-day training. According to the introduction, this training class aims to further improve the Fujian Communist Youth League propaganda front and counterpart assistance to ethnic areas of the Communist Youth League and youth network propaganda backbone team ability. Around the party history study education and xi general secretary of the "July 1" important speech spirit and the communist youth league network new media work theory and practice, short video creation and communication courses, the central propaganda department cadres, the central youth branch members, Fujian youth branch members, Fuzhou university professor and B station, TikTok, the relevant person in charge of the new media platform for the teaching of the students. The training content includes theoretical learning, business promotion and practical operation training. Wu Simin, a cadre of Xinjiang Mulei County Party Committee who participated in the training, said that through the training, he realized that the work of the Communist Youth League should use new ideas and new methods, make full use of Bilibili, TikTok, Kuaishou and other emerging media to carry out the work, and play the exemplary and vanguard role of the Communist Youth League members in the new media trend.

2021-09-04	Henan Communist Youth League with Chinese painting on a flood relief aesthetic education ideological and political class Henan Communist Youth League with Chinese painting on a flood relief aesthetic education ideological and political course Pan Zhixian, intern Yang Lu) " See this Chinese painting, my heart has a high resonance, can not help but think of one emergency moment in the flood relief."On the afternoon of September 1, in the Communist Youth League Henan Provincial Party Committee organs held the stone Chinese painting" flood relief praise youth " work donation ceremony, the delegates, Henan Provincial Youth Volunteer Association Yuqing Qiming Emergency rescue Youth volunteer service team leader Li Zhipeng was filled with emotion. This group of Chinese painting works, "Flood Relief and Praise Youth", was created by Professor Shi Ping, former deputy Party Secretary of North China University of Water Resources and Hydropower and dean of Art and Design, president of Henan School Art Education Association. The work contains 12 pictures, 12 scenes, the youth commandos before departure solemn flag ceremony, young volunteers carry sandbags to block the flood, transfer to escort the masses, the young pioneers to help carry rescue supplies... from different angles to describe the majority of youth in the frontline of dangerous scenes, depicting a vivid and moving moments of youth. Talking about the reason for the creation, Shi Ping said that this summer, many places in Henan province suffered severe floods, all sectors of society quickly formed a flood relief army, among which, the majority of young people is particularly prominent, young volunteers quickly gathered, youth commandos rushed to fight, build an indestructible youth dam."As an old educator, I was moved by the behavior of the youth members and felt proud of them! As an artist, it is also my mission and responsibility to shape their image and carry forward their spirit with my paintbrush!"Stone said, from the perspective of art creation, a picture no matter how big size, it is difficult to show the youth flood relief this grand theme, and using several works of picture" cloth combination " composition form, painted 12 pieces, each work from different sides show a scene, concentrated together fully highlight the theme of the flood relief praise youth. Since the outbreak of the massive flood in Zhengzhou, the Yuqing Qiming Emergency Rescue Youth Volunteer Service Team immediately responded to the call of the Henan Provincial Party Committee, and the first batch of 94 elite soldiers immediately rushed to the front line of rescue to carry out deep water rescue and urban search and rescue. After the "double-line battle" of flood control and epidemic prevention began, 17 young professional rescue volunteers were immediately dispatched to six boats to fight in Xingyang, Hebi, Xinxiang and other worst-hit areas for three consecutive days, and the area of epidemic prevention and elimination exceeded 40,000 square meters in Zhengzhou."We are just a microcosm of the young volunteers. In the flood prevention and epidemic prevention test, the touching youth stories emerge in an endless stream, and the dedication of the retrograde charge inspires the morale. This volunteer spirit can be displayed, condensed, sublimated, inherited and carried forward in Professor Shi Ping's works."Li Zhipeng said that in the future, he will stick to the original aspiration, unswervingly, help the epidemic prevention and control, serve the resumption of work, and do a solid job in post-disaster reconstruction. Henan youth branch members xiao-jing zhang said, every picture has a meaning, whether "rescue Henan" rescue team, love supplies, or "rescue moment" youth initiative, rushed to flood rescue line, all embodies the "the more difficult the more forward" Chinese power, the spirit of China and China's efficiency!"Youth commandos", "Young Pioneers in action" and other pictures, but also the youth of Henan youth responsibility, show incisively and vividly. Zhang Xiaojing said: " As a teacher of ideological and political courses in colleges and universities, I understand from these works that students should follow more ideological work well. We should guide students to understand the position of the people, consolidate the foundation of confidence, and hone their youth responsibility in the big war test!"At the donation ceremony, Wang Yi, secretary of the Henan Provincial Party Committee, delegation the Provincial Party Committee to accept the donation and issued the donation certificate to Shiping. Shi pin said that in the future, it will continue to pay close attention to the work of the Communist Youth League and the youth group, continue to create more literary and artistic works recording the responsibility of young people, vigorously promote the core socialist values, and spread positive social energy. Wang Dubo, deputy secretary of the Henan Provincial Party Committee of the Communist Youth League, believes that Shi Ping's works are both sincere and solid, with the dual functions of aesthetic education and moral education, and it is a timely and vivid model of aesthetic education and ideological and political course textbooks. It is understood that during the opening season, the Henan provincial party committee will put the flood relief praise youth works fully into the communist youth league member, young pioneers "first lesson", and Henan province "the disaster when the teaching material, grow with the motherland" theme education activities fully integrated, let the teenagers in the vivid aesthetic education contains the spiritual connotation. The relevant person in charge of Henan provincial party committee, said the next step, the provincial party committee and Henan province federation, provincial artists association, based on The Times, find the topic selection, deepen cooperation, hand in hand to create more young happy work, in aesthetic education, to culture to further guide the province broad league youth active bear, shoulder the responsibility of modernization in Henan era, to compose a new era of more brilliant chapter in the youth.

2021-09-03	Ningxia district party building with league building work deployment will be held in Yinchuan Ningxia party building with league building work deployment will be held in Yinchuan wei correspondent Zhang Zhuo) recently, Ningxia party building work deployment will be held in Yinchuan, deputy secretary of TuanOuWei Li Feng chaired the meeting and speech, district, city and county organization department, reform, civil affairs and the communist youth league system related responsible comrade more than 180 people to attend the meeting. The meeting put forward work requirements for the three national pilot reforms of the Communist Youth League undertaken by Ningxia, and made arrangements for the party building of the youth league and the construction of grass-roots youth League organizations. The meeting pointed out that to promote the county communist youth League grass-roots organization reform, the implementation of medium-and long-term youth development plan, community youth action implementation of the three national youth pilot work, to carry forward the spirit of reform, to "please the party rest assured, strong country" lofty ambition, be a good vanguard of reform. We should seize the reform opportunities, deeply understand that the hard-won bold reform opportunities in Ningxia have come by, cherish the opportunities, make good use of the opportunities and create experience. We need to fulfill the responsibilities of reform, take measures in light of reality and local conditions, know, clarify, and fulfill our responsibilities, and work hard to ensure the completion of the pilot tasks. The meeting required that to win the battle of grassroots organization construction, to focus on the "three areas", highlight the basic, source, strategic position of the school field, consolidate the standardized construction of the traditional field, expand the organization coverage of emerging areas; to anchor the "three paths", promote the Communist Youth League organization data modeling more accurate, more comprehensive coverage, organization vitality; to maintain the "three concentration", unswervingly loyal to the party, practice the strict style of concentration, adhere to the determination of the people's feelings. At the meeting, the relevant departments of the district Committee of the Communist Youth League gave special guidance on the construction of the league, the implementation of the youth development plan, the work of the Young Pioneers and the united Front and social liaison work of the Communist Youth League.

2021-09-02	Group of Qinghai provincial party committee learning "July 1" important speech spirit reading class the communist youth league of Qinghai provincial party committee held learning xi general secretary "July 1" important speech spirit special reading class wei correspondent Han Xiao) on August 30, the communist youth league of Qinghai province 14 six standing committee (expand) meeting and study and implement general secretary xi "July 1" important speech spirit special reading class, from across the province, industry more than 50 cadres attended the meeting. At the meeting, the youth league provincial party committee secretary Dong Yuyi around the communist youth league learning propaganda to implement the general secretary xi "July 1" important speech spirit, Qinghai provincial party committee of the thirteenth tenth plenary session spirit made a special guidance report, from the grasp of the new era, learning new experience, anchor new goals, practice the new mission of four aspects were discussed. Emphasizing that the Communist Youth League should deeply grasp the characteristics of the new era, Grasp the characteristics of the network, organizational differentiation and appeal diversification of youth survival in the new era, Understand the differentiated, personalized, three-dimensional and diverse demands of youth, Adjust the ways of serving the youth from the supply side; To learn from the experience of the "nine Must", Find out the combination point and entry point that the Communist Youth League can do good deeds, To integrate the work of the Communist Youth League into the overall cause of the Party and the country; To anchor the second centenary goal, Clarify the direction of the work of the Communist Youth League, Enhance the awareness of initiative, anticipation, and danger, Prepare for thought, ability, and work in advance; We must fulfill the political mission of training new people worthy of the era of national rejuvenation, Strengthen the problem-oriented, Centering on the center and serving the overall situation, Constantly improve the Communist Youth League's "three forces once". Conference discussed the communist youth league of Qinghai provincial party committee to implement the xi general secretary of the "July 1" important speech spirit implementation plan (draft) "the communist youth league of Qinghai provincial party committee on the implementation of the provincial government to build" eight new heights "work deployment implementation opinions (draft)" and "the communist youth league of Qinghai provincial party committee power rural revitalization work implementation opinion (draft)". After the meeting, it will be revised and improved according to the opinions and suggestions solicited, and issued in the near future.

2021-09-03	Fujian communist youth league new media network training class held in Fujian communist youth league new media network training class wei correspondent Zheng Yanling) recently, the Fujian communist youth league new media network training class held online, from all over Fujian province, Tibet, Xinjiang, Ningxia cadres and the new media staff more than 170 people to participate in a three-day training. This training course aims to further improve the ability of the Fujian Communist Youth League propaganda front and the Communist Youth League and youth network propaganda backbone team in ethnic areas. Invited the central propaganda department cadres, central youth branch member, Fujian youth branch member, Fuzhou university school of marxism, the relevant person in charge of the party history learning education and xi general secretary "July 1" important speech spirit, the communist youth league network new media work theory and practice, short video creation and communication courses for training, training includes theoretical study, business promotion and field training. In the practical operation training, the students focused on strengthening the network new media work of the Communist Youth League, exchanging advanced work experience of each unit, facing the main problems, and the opinions and suggestions on improving the work, combined with the party history learning and education and the spirit of General Secretary Xi Jinping's "July 1" important speech, group new media product planning and production.

2021-09-06	Henan held flood control and disaster relief reconstruction and epidemic prevention and control work inventory will Henan provincial party committee held "do the practical work" the provincial communist youth league power flood control and disaster reconstruction and epidemic prevention and control work inventory will wei correspondent Liu Yong) recently, the Henan provincial party committee held "do the practical work" the provincial communist youth league power flood control and disaster relief, reconstruction and COVID-19 epidemic prevention and control work inventory, sum up experience, to the situation, and the deployment of the next step of work. Henan Provincial Committee of the Communist Youth League, Provincial Commission for Discipline Inspection in the provincial Federation of Trade Unions discipline inspection teams, departments and directly affiliated units attended the meeting. The meeting stressed the need to follow the mass line in helping with flood control and disaster relief, post-disaster reconstruction and epidemic prevention and control work. The provincial communist youth league to thoroughly implement xi general secretary of flood control and disaster relief and epidemic prevention and control work important instructions, according to the post-disaster meeting in Henan province recovery and reconstruction work leading group work deployment, implement the Henan communist youth league power province post-disaster reconstruction and return to school and return to work and production work guide "the province school the communist youth league, young pioneers organization help" safe school, safe " work guide, the results of the results, do the practical work to guide the youth solid power on flood control and disaster relief, post-disaster reconstruction and epidemic prevention and control work. The meeting required that learning history be practiced in the result direction. To build the "four beams and eight pillars" of the work in the next five years, Further clarify the working principles, ideas, overall layout, path and methods, working motivation and mechanism guarantee for the work of the Provincial Communist Youth League to adhere to; All fronts should be built on the basis of the preliminary work, Further focusing on seven important aspects, Continue to use discussion and exchange, field visits, online consultation and other ways, To carry out research in various local units and various key youth groups; Further publicize General Secretary Xi Jinping's important remarks on the work of the youth and the Communist Youth League, Vigorously publicize the work and achievements of the Communist Youth League in serving the overall situation and serving the youth in recent years, To guide the youth of the province to enhance their political expression and political participation in the form of caring about the league congress, Jointly to promote the province's youth and the Communist Youth League cause of high-quality development, suggestions and suggestions.

2021-09-06	Group of Jiangxi province party committee in the province to prevent drowning special action group of Jiangxi province party committee to carry out the "youth protect summer safety" drowning special action wei correspondent Zhou Ying) in order to further improve the youth drowning awareness and protect self-help ability, maximum prevent and reduce the occurrence of juvenile drowning accidents, ensure the life safety of minors, recently, group of Jiangxi province party committee in the province to carry out the "youth protect summer safety" drowning special action. The picture shows a special campaign to prevent drowning. Correspondent for pictures to do practical publicity and education. Carry out the drowning prevention safety knowledge into the campus activities. Using online curriculum mode, for the province's primary and middle school students and their parents, focusing on rural left-behind children and children in difficulties, to carry out the youth safety education small classroom "anti-drowning" publicity activities, covering more than 1.5 million teenagers, effectively enhance the safety awareness of teenagers. Expand publicity channels. Make anti-drowning propaganda posters, comprehensive use of radio, television, newspapers, group affiliated WeChat, Weibo, TikTok and other media channels, widely carry out anti-drowning self-protection education, popularize anti-drowning safety knowledge, teach anti-drowning self-mutual rescue skills, so that "cherish life, prevent drowning" to form a social consensus. Carry out anti-drowning publicity and education into the community, into the rural activities. Using youth homes and "double zero" communities to create pilot villages (communities), young social organizations, young volunteers and youth affairs social workers were mobilized to carry out more than 800 anti-drowning education activities in communities, villages and families, covering more than 700,000 teenagers. The picture shows a special campaign to prevent drowning. Correspondent for pictures to do real volunteer service. Carry out swimming resistance volunteer service. We mobilized villages, schools, enterprises and other forces to set up 334 volunteer service teams for preventing drowning prevention and swimming resistance, carried out volunteer services such as patrols and wild swimming dissuasion in key time periods, comprehensively investigate rivers, ponds and other places with potential safety risks, set up safety warning signs, and worked together to weave a tight drowning safety net. Give full play to the strength of college students. The drowning prevention work will be combined with the "summer" and "return to home" college students social practice activities, mobilize college and technical secondary school students to participate in summer volunteer activities, drowning prevention swimming volunteer services, safety and other publicity work, to help local prevention and control safety risks and ensure the safety of teenagers. At the same time, the swimming resistance volunteer service is also combined with the river Xiaoqing activities, the volunteers in the river patrol to dissuade teenagers from swimming, popularize the knowledge of drowning prevention, at the same time, pick up the river bank garbage, clean up the river, to further create a clean and tidy living environment. The picture shows a special campaign to prevent drowning. Correspondent for pictures to do real rural left-behind children to help. Do real rural left-behind children to prevent drowning safety education. Focus on rural left-behind children, to carry out a series of drowning prevention publicity and education. For example, post, hang a batch of drowning prevention safety propaganda and education slogans and banners, watch a drowning prevention safety education feature film, to participate in the Jiangxi Province youth safety education small class "anti-drowning" publicity activities. The Provincial Communist Youth League Committee also listed "strengthening the rule of law and self-protection education for left-behind children in the 'childlike innocence harbor' " as the project of "I do practical things for the masses" in the party history learning and education, effectively solving the problems that the rule of law and self-protection ability of rural left-behind children need to be strengthened. Enrich the summer life of rural left-behind children. Organize activities such as baby village, summer summer camp, summer care services and so on to enrich the summer life of left-behind children. In the province 11 "childlike harbor" to carry out love in the summer "five one" activities, namely a drowning safety education, a reading challenge, a summer psychological adjustment training, a "wish" warm activities and a "happy sports" carnival activities more than 160 games, covering more than 600 people, for left-behind children to provide a colorful summer life.

2021-09-01	Group of Shandong provincial party committee held study and implement general secretary xi "July 1" important speech spirit preach report group Shandong provincial party committee held study and implement general secretary xi "July 1" important speech spirit preach report wei), the communist youth league Shandong provincial party committee held study and implement xi general secretary xi "July 1" important speech spirit preach report, Shandong youth school party committee, the relevant person in charge of the Shandong provincial party committee to attend the meeting. Shandong province party secretary and executive vice President MAO jintao focus on study and implement general secretary xi "July 1" important speech spirit, combining the reality of the communist youth league work, from grasp the practical significance, historical significance and theoretical significance, grasp the new ideas, new ideas and new requirements, pays special attention to the speech spirit to study and implement the three aspects. Everyone agreed that through this report, we further deepened the understanding and grasp of the spirit of General Secretary Xi Jinping's important speech on July 1. The next step is to improve our political position, to unify our thoughts and actions into the spirit of Xi Jinping's important speech on July 1; to strengthen the party building, to strengthen the responsibility, to build the branch stronger, to grasp the organizational life stricter, to do better work; and to transform the results, and to promote the work effectively.

2021-09-01	The young pioneers of Fujian province in Fuzhou to the first lesson "champion into the campus to carry forward the Olympic spirit" in Fuzhou began wei correspondent Huang Peng) on September 1 morning, sponsored by the communist youth league of Fujian provincial party committee, Fujian Fujian young pioneers first lesson in Fuzhou jin rongbo elementary school, courses to "champion into the campus to carry forward the Olympic spirit" as the theme. Li Teng, deputy secretary of the Communist Youth League Committee, and relevant responsible comrades from Jin'an District of Fuzhou attended the activity. Event, preparing for the 14th national games unable to attend Huang Dongping, Lu Yunxiu, Li Wenwen, Li Fabin four Fujian Tokyo Olympic champion in the form of video for the young pioneers school blessing, encourage the players firm ideal faith, set up the feelings, inheritance and carry forward the good Olympic spirit, and extend to study and life, good study, strong, ready to contribute to realize the great rejuvenation of the Chinese nation the Chinese dream. At the event, Fujian Youth Palace Association signed a strategic cooperation agreement of "double reduction" with Fuzhou Jin'an District; Fujian Provincial Youth Working Committee awarded "Fujian Red scarf Broadcasting Station" to Fujian Traffic Broadcasting Station (FM100.7), and appointed three anchors of Fujian Traffic Broadcasting Station (FM100.7) as Fujian Young Pioneers. After the activity, Mr.Chen Si, a young rookie of Fujian meteorology and an expert of the provincial climate Center, brought a unique meteorological science class to the young Pioneers with the title of "Meteorological Talent on the sports field". It is understood that in Fujian province red scarf theater, red scarf radio station, is the provincial party committee, less committee to carry out the policy of "double reduction", the practice of "I do the practical work for the masses" concrete measures, aims to further strengthen youth thought lead, for the provincial young pioneers to spread culture, cultivate aesthetic education, edify sentiment, show talent, mutual learning art stage and activities. The next stage, Fujian less committee will integrate social resources, the red scarf theater in Fujian province to carry out art performances, works and other comprehensive quality development activities, relying on the red scarf in Fujian province red scarf pulpit, promote various forces to join the red scarf culture brand construction, build the whole society good atmosphere of care, support the young pioneers.

2021-08-30	Group Hainan provincial party committee to carry out social practice for free trade port construction reserve talent Hainan provincial party committee to carry out the "home" social practice for free trade port construction reserve young talent JianWei correspondent Wu Yuhua) as Hainan free trade port construction, to do a good job of young talent reserve, Hainan provincial party committee from the youth practical demand, to "China (Hainan free trade port) youth dream weavers avenue" as the hub, for the social college students provide entrepreneurial employment, internship trainee quality service. This summer, the provincial Communist Youth League Committee for the province's 24 counties (districts) to carry out college students "return to home" social practice activities, guide and help young students to establish the grassroots rural world is broad, can also achieve the concept of employment, after graduation actively to the grass-roots talent, in the implementation of the rural revitalization strategy. Do a good job of top-level design, multi-channel publicity launch. Adhere to the "education, long talent, contribution" tenet, around government practice, enterprise practice, volunteer service and so on seven plates, in county (city, area), play the advantage of "students" of the county youth corps committee, using home resources to create conditions, make security, take the form of offline, online, build students and home normalized practice of bridge, efforts to guide the students returning home employment entrepreneurship. In accordance with the principles of independent application, voluntary registration, two-way selection, and selective selection, the returning college students are arranged to the county units, towns, and enterprises to carry out social practice. According to statistics, since the launch of the activity, it has received tens of thousands of college students' attention and consultation, and 2,046 college students from Hainan have participated in the activity. We will highlight the education in ideals and beliefs, and strengthen ideological and political guidance. The book "xi jinping and college students friends" as a social practice guide and vivid teaching material, strengthen party history, new China, reform and opening up, socialist development education, through the form of social practice, combined with "I do something for the youth", help and guide college students fully feel home change, remember the party's struggle, strengthen the service people, return home sense of responsibility sense of mission. At the same time, in the activities to carry out the "six" project, namely mining a batch of practical jobs, with a system, held a pre-service meeting, set up a temporary league branch, held a policy briefing, choose a batch of typical practice, promote "home" social practice effectively, let it become the communist youth league practice new growth point of education work. Make a good summary afterwards, and constantly improve the quality of activities. Adhere to the event summary system, actively participate in the central released "home" series of online activities, completes the "home" practice of excellent typical publicity and work case promotion, students to participate in "home" social practice as an outstanding party member, outstanding member, the May 4th medal, good youth for good youth. Further expand the impact coverage of activities, improve the organizational working mechanism, and form practical work results. At the same time, the provincial Party Committee of the Communist Youth League planned in advance, did a good job of top-level design, constantly improved the system, and planned in advance to start the social practice activities of "returning to home" during the winter vacation.

2021-08-30	The Qinghai Provincial Party Committee four measures to build College Students 'Employment Service System Four measures to build College Students' Employment Service System Jianwei Correspondent A Shuyuan) The Communist Youth League Qinghai Provincial Party Committee in strict accordance with the provincial Party Committee, the provincial government and the Communist Youth League Central Committee on the overall deployment of college students' employment work, To serve the employment of college students as an important entry point to convey the party's concern for college students and enhance the youth's sense of gain, To mark the youth needs, Focus on the key links of job hunting and employment, Through four measures to build up the "training, refining, innovation, help" parallel complementary service college students employment work system, Encourage and support college graduates to find flexible employment through multiple channels, Effectively improve the service ability of the youth league organizations to the youth. With "training" as the first one, improve the employability and pay attention to the cultivation of students' employment concept. Encourage guide school youth league organization to carry out multi-level, multi-angle, high quality related student employment activities, organize the university youth league joint school is a department to carry out employment guidance lectures, simulated recruitment, workplace experience, pre-service training, employment experience exchange activities, actively guide college graduates to correct understanding of employment situation, set up the concept of merit, career everywhere. Pay attention to the improvement of students' examination-taking level. The third-party professional training institutions are entrusted to provide free public examination training on theoretical learning, interview guidance, and practical training, and to strengthen the training and guidance on the problems and weak links existing in the public service examination of the past graduates, so as to help candidates improve the exam-taking level and help college students improve their employability. Pay attention to students' ability to improve. Strengthen the deep integration of "employment and major", rely on provincial brand competitions such as "Challenge Cup", "Create youth" and "Bank of Communications Cup", strengthen students 'participation in "science and technology innovation Competition" and "Employment and entrepreneurship Competition", form a provincial and school-level linkage activity mode, and improve the competitiveness of college students' employment and entrepreneurship. Focus on "refining", focus on education and enrich social practice. We have actively carried out the social practice of "three to the countryside" and "returning to home". Every year, more than 2,000 young students are organized at the provincial, university and department levels at three levels to carry out social practice activities in the fields, rural pastoral areas and ethnic minority areas, and guide and help students to take the "employment class" that combines good with reality. Strengthen voluntary services. Every year, more than 700 college students are organized to visit towns and streets to help them understand the national and social conditions and conditions, establish a correct view of career selection, accumulate work experience and improve their employability. In 2021,774 college students were selected to engage in volunteer service work. Strengthen practice and practical training. We will continue to implement the "Sailing Plan" for college students, integrate the resources of party and government organs and enterprises at all levels, and provide nearly 200 internship positions for college students every year to cultivate students' personal ability in different positions. Innovative evaluation system. We will actively explore the implementation of the "second classroom report card" system of the Communist Youth League, deeply explore its practical education function, gradually establish a diversified evaluation system for college students, promote the reference basis for the selection and appointment of social employers, and improve the employment level of serving students. To "gen", optimize business service adhere to the "horse" and "send", by building the provincial youth entrepreneurship employment service system, efforts to build the youth business incubation, training, capital, publicity, electricity, accelerate and so on six big platform, optimize employment entrepreneurship of college students group security, groundwork for college graduates employment entrepreneurship. Business incubation services have been continuously upgraded. A total of 1,014 enterprises inside and outside the provincial Pioneer Park, 128 successfully incubated and graduated enterprises, supported more than 1,018 young entrepreneurs, and directly promoted nearly 10,000 employment people. Start-up financial services continued to expand. Jointly with Provincial Agriculture and Animal Husbandry Credit Guarantee Co., Ltd. launched the "Qinghai Youth Financial Support Plan for Agriculture and Animal Husbandry Entrepreneurship", helping young entrepreneurs in agriculture and animal husbandry industry solve the problem of financing difficulties and expensive financing through the special plan, with a total of 126.98 million yuan of loans (of which 6.9 million yuan was issued in the first half of 2021). We deepened the "Youth Entrepreneurship Poverty Alleviation Action", cooperated with the Provincial Poverty Alleviation and Development Bureau, and issued loans of 303 million yuan. The development of the "Youth Innovation and China Bank Gang" youth entrepreneurship small guarantee loan project. Effective coverage of entrepreneurship training. A total of more than 150 youth innovation mentors have been recruited, providing entrepreneurship training, project guidance, resource docking and other public welfare services for nearly 2,000 hours. It has provided more than 60 mentors at the grassroots level, guided and helped 2,548 young entrepreneurs, and successively conducted 52 activities such as youth entrepreneurship incubator management and operation talent training, youth entrepreneurship course public welfare lecture hall, and youth entrepreneurship elite training, covering more than 11,700 young people. Based on the basic function of the Communist Youth League in organizing and serving the youth, we will weave a dense "service network" to help college graduates find employment, so as to lay the foundation for the employment and entrepreneurship of college graduates. Focus on employment training. Together with the Provincial Department of Human Resources and Social Security, we will continue to carry out practical skills training for employment and entrepreneurship in the form of the youth league organizations at all levels, the human resources and social security departments to designate special training institutions, and the government bears the training costs. We will provide employment assistance. To clarify the object of assistance, find out the employment intention, provide employment guidance, recommend employment information, pay attention to employment progress, continuous tracking services as the content, the league cadres and difficult graduates pair, help measures to accurately achieve the head, solid mass work methods to solve students' employment difficulties. Grasp the position docking. We have carried out the "thousands of schools and thousands of posts" employment assistance campaign. Over the past five years, nine "double-line" job fairs have been held, providing 68,977 jobs, and providing 1,185 students with successfully employed. Focus on platform construction. We will focus on establishing 667 employment internship bases in provinces and counties in small and medium-sized enterprises and non-public units, providing more than 8,200 internship positions for college graduates every year, and encourage and guide college students to face the grassroots level and work in small and medium-sized enterprises and non-public units.

2021-08-30	The Jiangxi Provincial Red Scarf Public Welfare Center, a non-profit social public welfare organization under the Jiangxi Provincial Party Committee of the Communist Youth League, was inaugurated on August 28. In the next three years, the center plans to serve the healthy growth of more than 30,000 young Pioneers, help the young Pioneers counselors education and teaching practice of more than 3,000 people, and strive for social love money and goods to help the center of more than 10 million public welfare projects. The first council held on the same day deliberated and adopted the election method, the articles of association, the development plan and other relevant documents, and 34 management systems such as personnel and finance. The center by expanding and integrating social resources, for the young pioneers to provide funds, projects, site, etc to support, has established a "red scarf love angel" system, the plan through the implementation of "red scarf story" "red scarf lesson" "red scarf dream fund" "red scarf public BBS" and other public welfare projects, help the healthy growth of the young pioneers, funding the young pioneers counselor education practice, promote the development of Jiangxi young pioneers. Source: China Youth Daily

2021-08-30	More than 5,000 poor students won the theme activity of "Realizing the University" and the grant ceremony was held in Lanzhou, Gansu Province, on August 27. Sun Wei, deputy secretary of the CPC Gansu Provincial Party Committee, and Zhang Shizhen, vice governor of Gansu Province, attended the ceremony and gave academic grants to 16 subsidized students' representatives respectively. Jiang Xiaoli, secretary of the Gansu Provincial Party Committee of the Communist Youth League, presented the plaque of "Loving Enterprises of the Provincial Party Committee and the Provincial Government in 2021" to 8 representatives of caring entrepreneurs. In order to effectively help students from poor families to successfully complete their higher education, the Gansu Provincial Party Committee and provincial government have included the college entrance aid for children of urban and rural low-income families into the practical project in 2021. The student financial aid work is led by the Gansu Provincial Committee of the Communist Youth League and jointly implemented with the Department of Education, the Department of Civil Affairs and the Department of Finance of Gansu Province. This year in Gansu Province, more than 5,000 urban families with full subsistence allowance and rural first and second class subsistence allowance families' children were admitted to ordinary colleges and universities, all of whom were included in the scope of financial aid. Among them, the one-time 2021 freshmen receive a one-time subsidy of 10,000 yuan, and the 2021 freshmen admitted by the ordinary college (vocational college) receive a one-time subsidy of 8,000 yuan. At the ceremony, the participants watched the propaganda video of the provincial government for the people. Four representatives of the subsidized students told about their study experiences of overcoming difficulties and inspiring success. Two previously funded youth representatives shared their growing experiences. Source: China Youth Daily

2021-08-30	Fujian by the end of the qualified "two new" organization promote building work in Fujian before the end of qualified "two new" organization to promote building work reporter Tian Hongwei) recently, the Fujian provincial party committee "two new" committee agreed, the communist youth league of Fujian provincial party committee issued a notice, in the province to carry out the "two new" group building crucial action, strive to promote before the end of 2021 to meet the conditions of the "two new" organization coverage. The "Two new" organizations are short for the new economic organizations and the new social organizations. New economic organizations refer to the non-state-owned economic organizations, such as private enterprises, foreign-invested enterprises, Hong Kong, Macao and Taiwan, private science and technology enterprises, individual businesses and mixed-owned economic organizations; the new social organizations and private non-enterprise units. Notice requirements, by the county youth League committee is responsible for, to the township (street) youth League committee, to the area of non-public enterprises and social organizations, to visit, "two new" organization league members and youth registration, the establishment of provincial, municipal and county level ledger. We will strengthen the group construction of express delivery logistics enterprises, and continue to strengthen the organizational coverage of emerging young groups such as express delivery boys. For the "two new" organizations that have no league members but have more than 10 young employees under the age of 35, the work of the league should be covered by establishing interest groups, youth social organizations, and through the selection of league building instructors. The "two new" organizations are more developed city and county youth League committees should actively explore the establishment of the "two new" organizations youth League working committee. Notice also requires that the youth corps committee at all levels to choose well with strong "two new" organization head, timely training, improve the system of "three will be two system a lesson", focus on the "three one" activities, namely "held a founding meeting, held a party history learning education activities, to carry out a meritorious action", let the group "live". With the youth innovation competition, youth innovation loan, youth civilization, youth volunteers, youth post experts and other activities as the starting point, give full play to the role of the youth league members as a new force, widely carry out colorful learning exchanges, dating and voluntary public welfare activities, to promote the growth and development of the youth league members.

2021-08-30	Southern area of Hainan province youth entrepreneurship employment theme BBS held south of Hainan province youth entrepreneurship employment "check point, broken problem, promoting development" theme BBS successfully held JianWei correspondent WuYuWa), hosted by the league committee of Hainan province southern area youth entrepreneurship employment "check point, broken problem, promoting development" theme BBS held in Sanya cliff bay technology city, from Sanya, wanning, five mountains, ledong, lingshui, baoting six cities and county youth corps committee, deputy secretary, director and entrepreneurial youth on behalf of about 45 people to participate in the event. This BBS is the pku provincial party committee to implement the general secretary xi the important thoughts about youth work, according to the communist youth league central about <about strengthening the communist youth league to promote the construction of youth entrepreneurship employment service system implementation opinion> and the notice of the CPC Hainan provincial party committee office issued by "about" check blocking point, broken problem, promote development "activity implementation plan" notice ", launched a" I do the practical work for the youth " the specific work. In order to further effectively solve the problems and difficulties in the process of youth entrepreneurship and development in the southern area of Hainan Province, promote the docking of various market resources, guide young people to gather strength and grow together, and promote the sustainable development of young enterprises. The BBS invited digital China (China) digital agriculture rural institute Zhou Qiuping, Hainan bo yu financial group experts, accounting, vice President of the Chinese and foreign humanities exchange institute, Hainan tropical ocean institute, Hainan transportation logistics association lu xiaoli respectively bring the county digital agriculture architecture and development strategy, the enterprise fiscal and tax policy interpretation, the modern logistics technology and future development of the keynote speech. From the perspective of digital agriculture, enterprise fiscal and tax preferential policies and future logistics development, we can empower the business thinking of young entrepreneurs. At the same time, we invited Bank of China, Bank of Hainan, Hainan SF Express Co., Ltd., Sanya Sutai Express Co., Ltd., LTD. (YTO Sanya Branch), and Hainan Boyu Financial Group to promote the project, expanding the channels to solve the problems of financing difficulties, expensive logistics and complicated taxation for young entrepreneurs. On August 26, the participants visited the Sanya cliff state bay city of science and technology exhibition hall, under the introduction of the narrator, learn about the park overall planning, further deepen the Sanya cliff state bay science and technology city will be built into a national deep sea science and technology innovation center, "south numerous silicon valley" and production city depth fusion site, promote scientific and technological innovation and industrial development, occupy the future high-tech industry commanding heights, in order to promote the free trade port construction with strong momentum of understanding. Then, he went to Sanya Meishan Revolutionary History Museum to visit the red education base. In Meishan revolution offspring under the guidance of the narrator, the participants listened to the Meishan revolution record, glorious battle course, visit the museum of various red exhibits and historical data, watch the Meishan revolution witness about revolutionary history, feel the revolutionary ancestors fought, open up red land, not afraid of sacrifice, indomitable heroism. League provincial party committee will take the BBS activities as an opportunity to the big Sanya area youth entrepreneurship alliance, on the basis of continue to expand the southern area of youth, the power of Hainan southern area youth development, promote the southern cities and counties youth entrepreneurial resources connectivity, formed under the condition of marketization of youth entrepreneurship work effective mechanism, promote all kinds of entrepreneurial service resources and effective docking of young entrepreneurs, further promote the rural revitalization strategy and the implementation of the free trade port in Hainan action.

2021-08-30	Guangxi: Let the young generation take over the baton of faith and pass over the circle of friends of Bagui youth. As the former first secretary of Baini Village, Leye County, Baise City, Guangxi Zhuang Autonomous Region, her 30-year-old life is always fixed on the road of poverty alleviation. Recently, 100 Guangxi Huang Wenxiu style good youth in batches went deep into the youth league members throughout the district, walked on the platform to preach Wenxiu deeds, in-depth grass-roots discussions and exchanges, into the youth to answer questions. On July 1,2019, General Secretary Xi Jinping made important instructions on Comrade Huang Wenxiu's advanced deeds. In July 2020, the Guangxi District Committee of the Communist Youth League launched the "Youth for the country" - -striving to be a Huang Wenxiu style good youth theme activity. For more than 1 years, youth league organizations at all levels in Guangxi around "Wuxi, innovative entrepreneurship, study progress, poverty" four categories, from the roots service people of civilian hero layer selected a batch of trees, stand positive energy youth, through example selection tree, sharing, communication discussion, quickly in the earth do eight laurel wen-xiu huang type good youth learning boom, for the construction of the new era of socialism with Chinese characteristics in Guangxi injected strong spiritual power."The sea breeze is not enough, the boat song is not central, rooted in the fishing port, the heart without regret. This is my 19 years stationed on the North Overseas Sand Island clang oath."On July 9, the passengers fire rescue detachment sea city brigade sea squadron equipment technician, Guangxi youth branch member li came to Guilin electronic university of science and technology Beihai campus, to leave holiday more than 300 youth members share wen-xiu huang deeds, and how to learn from the cable, wash deck, step by step for the fire boat pilot youth story. After the sharing meeting, Li Hongliang was surrounded by youth members who still wanted more to communicate about "how to practice the spirit of Huang Wenxiu in study, life and life" and "what to do as college students in the" inner "and" lying flat "in the new era". After the sharing, Fan Yifan, a student from Beihai Campus of Guilin University of Electronic Science and Technology, said excitedly, " Li Hongliang risked his life to protect people's lives and property against the fire until it was put out, showing his excellent qualities of responsibility and dedication like Huang Wenxiu. As a youth league member in the new era, I will also strive to become a new person of The Times with ideals and responsibility like them."On the morning of July 29, in the realization of the whole poverty alleviation huanjiang Maonan autonomous county, Chen Shuang village, golden rice fields, Huang Wenxiu gave his precious life for poverty alleviation story by Guangxi Jinxiu yao autonomous county town liu duan village villagers, Huang Wenxiu type good youth Wang Yungui to the fields, to the youth side. As a master in Dayao Mountain, Wang Yungui not only shared her understanding of the spirit of Wenxiu, but also told her story about how to bring Yaoshan delicacies to the country through live streaming to help poverty alleviation and rural revitalization. Eye-opening skills with goods, inspiring local goods sales, so that the young people to learn Huang Wenxiu spirit at the same time, listen to the more absorbed."Today's lecture, let me further learn to understand the spirit of Wenxiu, enhance my understanding that rural revitalization should be youth first. After graduation from university, I will also return to my hometown to contribute my youth to the construction of a magnificent Guangxi."Huanjiang County youth League member, Jilin Engineering Technology Normal University student Wei Yangqian said. Since June this year, the Guangxi Huang Wenxiu Good Youth Sharing Group has visited 100 youth sharing meetings and exchange seminars in Baise, Hechi and Chongzuo, including 14 cities, schools and communities, enterprises and factories. For aid to Hubei disease resistance and delay the wedding after 90 party members huang qijing, write the paper in the tea garden of science and technology correspondent, building Guangxi university first polymer lithium ion battery production line of young teachers Lai Feiyan with qing yan qing language about advanced deeds of Huang Wenxiu, share their own growth experience, preach the party's struggle in one hundred, and the new era of the party's innovation theory. Huang Wenxiu-style Good youth Sharing group not only shares with young people face to face offline, but also makes Huang Wenxiu spirit active on the mobile phones and in front of the youth members through new media platforms such as online video numbers. According to statistics, up to now, the direct audience of the sharing group has reached 11,000, and more than 250,000 people have watched it on new media platforms such as Weibo, wechat video account and TikTok 20. Through careful planning, precise positioning, the new media products are promoted by "Huang Wenxiu Style Good Youth" in an all-round way to the online youth groups. On May 25 this year, the story of Liuzhou Liubei Liubei fire rescue station stationmaster He Bo boarded on the gold medal young lecturer said online column. As soon as the video was released, the thrilling scenes of the rescue, the moment of the fire, the III degree burns, and the dedication spirit touched many young people who watched in front of the screen. Soon the video became a popular product for Huang Wenxiu good youth online. The relevant person in charge of the Guangxi District Committee of the Communist Youth League said that in the next step, the youth league organizations in Guangxi will take the party history learning and education as an opportunity to continuously promote the "I do something for the youth" through the theme of youth league day and youth league classes, and lead more young people to learn the glorious course of the Party and practice the spirit of Huang Wenxiu. Online, Huang Wenxiu type good youth struggle will also be VLOG short video, animation, cloud class, such as youth popular form, continue on the new media platform, comprehensive incentive youth to comrade Huang Wenxiu as an example, don't forget to beginner's mind, keep in mind that the mission, have the courage to bear, dedicated, the youth struggle into the cause of the party and the people, not time, not young, walk the long march of the new era of the long road. China Youth Daily, China Youth Network, reporter Xie Yang, correspondent, Liao Zongyuan, Li Wei, source: China Youth Daily (August 30,2021, edition 04)

2021-08-29	Shaanxi: hope project to help 60 freshmen university dream shaanxi: hope project to help 60 new university dream hai-hua sun) in 2021 shaanxi hope project interpreta dream action in hand one of the series of activities- "xi my university" dream scholarship ceremony held in xi'an, 60 from shaanxi, low-income family university freshmen received 5000 yuan respectively. By the communist youth league of shaanxi provincial party committee, shaanxi province hope project public volunteer guidance center, shaanxi youth development foundation joint Guizhou maotai distillery (group) liquor co., LTD., Guizhou youth development foundation of the project, in shaanxi landing three years donated 900000 yuan, to help 180 students interpreta dream university. It is also understood that since the Shaanxi Hope Project Dream Realization Action was launched in 2005, it has raised more than 200 million yuan to help 76,961 poor students in Shaanxi province realize their dream of college education. At the same time, it provides more than 13,950 charity education positions for poor students, which has become an important platform and brand project for the Shaanxi Communist Youth League to serve the growth of young people in difficulties.

2021-08-29	Zhejiang held a new era youth craftsmen speak in Zhejiang held a new era youth craftsmen speak activities (in the green newspaper network reporter Ji'an-ping li) today, in Zhejiang Shaoxing ke bridge green gathered into technology co., LTD. Workshop temporary platform, Shaoxing secondary professional school training instructor Zheng Ke inspirational speech, favored by the young worker. The "I Write Youth at the Window- -Zhejiang Young Craftsans in the New Era" was held in the company, jointly sponsored by the Zhejiang Provincial Party Committee and Zhejiang Human Resources and Social Security Department, and organized by the Communist Youth League Shaoxing Municipal Party Committee and Shaoxing Human Resources and Social Security Bureau and Shaoxing Federation of Trade Unions. Sheng Le, deputy secretary of the Provincial Communist Youth League Committee, Wang Jigang, deputy secretary of the Party Leadership Group and deputy director of the Standing Committee of the Shaoxing Municipal People's Congress, as well as representatives of young craftsmen and young workers from Zhejiang province attended the event. Five young craftsmen born in the 1990s in the province told about their growth process and their insights of ingenuity, youth and dreams. From the ingenuity of the students, the ingenuity of the competition, to the work, they bring the craftsman culture into the team culture, consciously resonate with the dream of a strong country, and become the youth example for the majority of young people to learn from. Zhejiang Provincial Party Committee of the relevant person in charge said that in recent years, the Youth League Shaoxing Municipal Party Committee to the youth skills training action as the whole, strive to create a whole chain of young workers training system, for the province's young skills training to explore a good experience. On this basis, the province will face the entire province youth skills, looking for a group of outstanding Zhejiang youth craftsmen, by sharing their personal youth struggle story, achievements, further show the youth youth, condensed the development of high quality youth power construction common prosperity demonstration area of youth consensus, guide the youth go skills, skills.
