# Supplementary material for: Serving organization goals by organizational information dissemination: An empirical study from the Communist Youth League of China
Source: PLoS One. 2023 Jan 20;18(1):e0280221. doi: 10.1371/journal.pone.0280221 (PMC9858461; doi:10.1371/journal.pone.0280221)
Supplement: S1 Data — (ZIP) [file pone.0280221.s001.zip › Supporting Data/Version of Chinese to English/School Committee(English).docx]

2022-06-29 Xiamen university for graduate student on "before the last party class" Xiamen university for graduate student on "before the last party class" Yang Baoguang) on June 23 afternoon, in Xiamen lujiang road waters "figure" party history education theme ship, "red sail pilot, dream mountain" Xiamen university the 24th graduate student on "before the last party class" before the learning education activities. Activities, Xiamen university, deputy secretary of party committee Xu Jingang JiaoJiaoTuan players watched the "red sail" immersive performance, and to "absorb Minning spiritual nutrients, inspire the force of youth forge ahead", for the upcoming to Ningxia haiyuan county, longde county, pengyang county and yongning county to carry out a year of teaching of 22 players teaching before the last party class. Xu Jinguang briefly reviewed the glorious course of Minning poverty alleviation cooperation. He said that in the past 26 years, Minning has been thousands of miles apart from mountains and seas, fighting poverty together, which has not only become a model of poverty alleviation cooperation between the east and the west of China, but also formed an inspiring "Minning spirit". Xiamen university graduate volunteers as "MinNing counterpart cooperation for poverty alleviation" aid ning one of the important members of the group, to learn comprehension MinNing spirit, and draw spiritual nutrients and wisdom, in poverty engines and rural revitalization as a whole, with practical action to reveal Xiamen youth bear and as a, make graduate university youth volunteer service society, take root roots in western grassroots a banner, become Xiamen youth practice of socialist core values of vanguard and vanguard. Xu Jinguang put forward three hopes for the graduate volunteer teaching group: first, to be the fertile soil of education; second, to be the disseminator of the red gene; and third, to be the practitioner of Minning spirit. At the activity, Xu Jinguang awarded the team flag to the 24th Graduate Student Support Group, Li Feng, director of the Student Work Department and Zeng Zheng, secretary of the Youth League Committee of the university, awarded the certificate of completion to the students of the special seminar of "ma Project" Graduate Student Support Group. Participants also celebrated a collective "political birthday" together and reviewed their party membership vows."As a Xiamen university students, I will inherit Mr Chen countries feelings, based on the three feet platform for teaching to teach the children, efforts to teach to students 'four lead, to teaching action for' MinNing collaboration ', for Ningxia rural revitalization career contribute their own strength."Mao Yanting, head of the volunteer teaching group, made a commitment to practice after listening to the party class. It is reported that since 1999, as the first "Chinese youth volunteers for poverty alleviation relay graduate student team project" team, Xiamen university 323 graduate student team adhering to the "unyielding, perfect" school motto spirit, the "west solid" by the United Nations food development agency as "one is not suitable for human survival area" the pursuit of ideal, selfless dedication, experience, relay education for poverty alleviation for 23 years, participate in and witnessed the "ground not long grass" dry beach ", has become xi jinping general secretary said" golden beach " of historic changes. In 2020, Xiamen University Graduate Volunteer Support Group, as one of the "Minning Cooperation and Aid Ning Group", was awarded the title of "Model of The Times" by the Propaganda Department of the CPC Central Committee.(Photo: Xiamen University Youth League Committee)

2022-06-24 Southwest jiaotong university teachers and students to malkang earthquake psychological counseling in southwest jiaotong university teachers and students to markang earthquake psychological counseling youth reporter Wang Xinxin) June 21 solstice 23, southwest jiaotong university of Sichuan malkang 6.0 earthquake mental health counseling social practice, successively to Aba malkang second middle school, the fourth primary school, songgang town central primary school, ZhuoKeJi town central school, for nearly 1000 from the affected families and the epicenter of primary and middle school students to carry out psychological counseling activities. Malkang after the earthquake, southwest jiaotong university youth corps committee according to the needs of the malkang disaster area, formed composed of psychology professional graduate and graduate volunteers volunteers of mental health counseling social practice team, southwest jiaotong university applied psychology institute dean professor ning Weiwei as expert service experts in Sichuan province, as a teacher and went to malkang psychological counseling, psychological reconstruction after the earthquake. Recently, members of the social practice team of Southwest Jiaotong University are carrying out league auxiliary activities. Southwest jiaotong university for figure in the three-day malkang psychological counseling activities, southwest jiaotong university teachers and students through seminar, group counseling, psychological games and one to one answer psychological confusion, help the children with scientific attitude correct understanding of disaster, eliminate the fear after the disaster, with a positive attitude to adapt to the new learning and living environment."I will definitely face the temporary difficulties with a positive, sunny and grateful attitude."Tashi Nam, a sixth-grade student from Cadeng Township Central Primary School, said she will study hard to be admitted to a good university and build her hometown more beautiful in the future. Recently, Professor Ning Weiwei, dean of the Institute of Applied Psychology of Southwest Jiaotong University, held a group support activity. Southwest jiaotong university for southwest jiaotong university psychology graduate student Zhu Junnan, QiYunFang said, remote poor areas of education resources, especially mental health education is still weak, as a college students, to apply the professional knowledge to social life, contribute to ethnic areas of primary and middle school students' mental health education. Southwest jiaotong university, deputy party secretary of ai-hua Yang said that the school to form a professional social practice team to malkang post-earthquake psychological counseling activities, is to guide the young students to the motherland and the people most need by education, contribution, long ability, to participate in cultivating family feelings, growth ability, in learning practice enlightenment firm ideal faith, efforts to grow as the revival of new era.

2022-06-23 Guangzhou university of traditional Chinese medicine to carry out Hong Kong, Macao and Taiwan students national education activities of Guangzhou university of traditional Chinese medicine in Hong Kong, Macao and Taiwan students national education activities Lin Jie correspondent Liao Xiaojian) on June 21, Guangzhou university of traditional Chinese medicine held national education series of activities summary, at the same time to get "youth gift 20 power have my new journey" photo exhibition of Hong Kong, Macao and Taiwan overseas Chinese and international students. Ren Tiejian, Vice Minister of the Guangdong Liaison Department of the Liaison Office of the Central Government in Australia, presented the award to the winners. Guangzhou university of Chinese medicine for figure to celebrate the 25th anniversary of Hong Kong's return to the motherland, Macao 23rd anniversary of the return to the motherland, to meet the party's 20 victory, Guangzhou university of Chinese medicine held a "youth gift 20 power have my new journey" as the theme of the photography and video production contest, essay contest, the best dormitory competition, disease resistance students building long comparison, outstanding graduates comparison and other national conditions education activities. Party secretary of the university of Chinese medicine in Guangzhou zhang pointed out that the school adhere to the party education, yucai, organization of Hong Kong, Macao and Taiwan students to participate in military training, red studies, rural revitalization research, disease resistance volunteer service activities, hope the students grasp the development opportunities of a large bay area, Guangdong, will put their ego into the motherland, time with the motherland, common destiny. Jin Wenjie, deputy director of the Guangdong Provincial Bureau of Traditional Chinese Medicine, said the CPC Central Committee has placed TCM more prominent in the prevention and control of COVID-19 and upgraded the development of TCM as a national strategy. In the face of new opportunities and challenges, as a young generation of traditional Chinese medicine people, students should invest the greatest energy, learn basic skills, practice real skills, and serve the country and the people."Youth tribute to the 20 great powers have my new journey" photography exhibition award scene. At Guangzhou University of Traditional Chinese Medicine, 2022 outstanding graduates from Hong Kong, Macao, Taiwan and international students presented graduation souvenirs to the university. Li Ka-hong, from Hong Kong said that after returning to Hong Kong after graduation, he will strive to shine for the cause of TCM, and always be a firm patriot and contribute to the prosperity and stability of Hong Kong. Lin Leshi, from Macao, said that it is a great honor to be a witness and dreamer of this beautiful era. Wang Shanqing, a master student from Taiwan, said that he will continue to pursue excellence and strive to be an elite of The Times. Ren Tiejian, Vice Minister of the Guangdong Liaison Department of the Liaison Office of the Central Government in Australia, presented the award to the winners. It is reported that Guangzhou University of Traditional Chinese Medicine has begun to recruit overseas Chinese students from Hong Kong, Macao and Taiwan since the 1980s, and has trained more than 6,000 outstanding senior TCM talents for Hong Kong, Macao and Taiwan. At present, there are 1226 students from Hong Kong, Macao and Taiwan. Since 2019, the university has made use of the excellent traditional culture of traditional Chinese medicine and the excellent red resources, inside and outside the province, to carry out a rich and colorful national conditions education activities of "Chinese Love and China Travel" for overseas Chinese students from Hong Kong, Macao and Taiwan, and constantly enhance the national awareness, patriotism and sense of belonging of overseas Chinese compatriots from Hong Kong, Macao and Taiwan to the motherland.

2022-06-14 Guizhou Qiannan college of science and technology spring large campus job fair successfully ended Guizhou Qiannan college of science and technology in the spring of 2022 large campus recruitment successfully ended jianwei correspondent, Chen Yingmei YanTing YanChun) to implement the promotion of college graduates employment policy decisions, recently, "dream south Ming achievement future" and 2022 Guizhou Qiannan college spring large campus job fair held in the football field. Relevant leaders of Guizhou Qiannan Institute of Science and Technology attended the job fair. The picture shows the job fair site. A total of 100 enterprises participated in the job fair, providing more than 200 jobs, attracting more than 2,000 people, and delivering more than 4,000 resumes. The offline job fair was crowded with people. Students held carefully prepared resumes, walked in front of the booth of each enterprise, understood the recruitment position information and employment requirements of each enterprise, and selected the enterprises they were interested in to fill in the application form and conduct an interview. The picture shows the students being interviewed by the employers."Excellence is the most basic thing that a job seeker should have."The person in charge of Guizhou Provincial Wine Group Co., LTD., said that the company is more inclined to attract positive, multi-faceted development of talents."In the process of job hunting, I pay more attention to the environmental facilities, salary, benefits, and development prospects of employers, and I prefer to choose positions that match my major."Wang Jiabin of the Planning Department 182 suggested that the younger students should get more skill certificates, participate in more social practice, accumulate practical experience and cultivate practical ability. Looking for opportunities is more important than waiting and seeing there. The graduates establish the concept of "making achievements everywhere", always base themselves on their own duties with a high sense of responsibility and mission in any position, work diligently and conscientiously, and show the good spirit of the students of the School of Science and Technology. Zhao Ping, vice president of Guizhou Qiannan University of Science and Technology, said that the college will do a good job in graduate employment by adopting departmental incentive policies, strengthening departmental employment guidance, employment policy publicity, and expanding employment channels. In addition, the recruitment activity site through the establishment of policy publicity points and Nanming employment small program, public number, TikTok number of the QR code screen, for graduates to answer the employment and entrepreneurship subsidies, social security payment, talent settlement, employment rights and interests protection, housing security and other policies. The picture shows the relevant staff of the conscription office of the Huishui County Armed Forces Department answering the conscription policy for the students. Guizhou Qiannan college of science and technology for the figure, the job fair by the CPC nanming district party committee talent work leading group office, Guiyang nanming district human resources and social security bureau, Guizhou Qiannan institute of science and technology (former college of science and technology), Guiyang nanming district employment and vocational skills development center, Guizhou radio and television public channel "the people employment" group. The job fair is mainly aimed at all fresh graduates of 2022, in the form of on-site resume delivery, face-to-face negotiation, and two-way selection. Enterprises and job seekers can sign the employment intention on the spot in the signing area.

2022-06-06 Anhui Agricultural University launched the fifth "Maker Salon" of 2022) Recently, the fifth phase of the "Maker Time" series of activities "Maker Salon" was held in the road show hall of College Student Maker Space Zone A. Cheng Rui, a 2020 graduate of resource utilization and plant protection major, general manager of Luan Keydar Biotechnology Co., LTD., and gold medal winner of the 7th China International "Internet +" College Students' Innovation and Entrepreneurship Competition, made a wonderful special sharing with the theme of "How to participate in innovation and entrepreneurship activities". The event was carried out in the form of a live broadcast of the Tencent Conference. More than 300 entrepreneurial teams who have settled in the Maker Space and students interested in innovation and entrepreneurship have participated. Based on his own experience in innovation and entrepreneurship practice and competition, Cheng Rui told the participants how to participate in innovation and entrepreneurship activities during the school period from the aspects of project creative source, core competitiveness building and improving the details of the competition works. He suggested that people actively participate in social practice, pay attention to social needs, select practical research and market demand; encourage students to actively participate in laboratory projects, apply for major innovation projects, and actively integrate into teachers and research teams, so as to continuously improve the core competitiveness of entrepreneurial projects; Entrepreneurial team members should adhere to professional work, fine management, efficient attitude, and improve team cooperation ability. Finally, Cheng Rui to "do not envy others' success, to respect the labor behind it" to encourage the students to be down-to-earth and work hard to realize their entrepreneurial dream. In the interactive session, Cheng Rui answered the questions raised by the students in detail and gave reasonable suggestions. The activity aims to build a platform for college students to exchange experience in innovation and entrepreneurship competitions, and lay a foundation for the continuous improvement of the innovation and entrepreneurship establishment rate of Anhui Agricultural University college students and the new breakthroughs in the achievements of the innovation and entrepreneurship Competition.

2022-05-31 Jiangsu university to carry out the twenty theme education practice in Jiangsu university to carry out the "always follow the party forever forge ahead new journey" theme education practice JianWei correspondent, Ding Yijuan wang hope) " the first time to participate in the immersive theme TuanRi activities, through punching in red landmark, complete the answer task, really let me benefit a lot."Said Jiang Qian, a student at Jiangsu University. Recently, the Youth League Committee of Jiangsu University organized the spirit of the Youth League Day activity at the celebration of the 100th anniversary of the founding of the Communist Youth League of China. More than 600 youth members from different colleges participated. Activities to clock in the form of "green history on the map", set up the red building, Guangzhou east garden eight red landmark clock point, participate in the students to complete the corresponding answer, puzzles, music tasks can finish clock, through immersive learning the glorious course of the youth movement, strengthen the youth members always follow the party's faith confidence. The picture shows the theme of "Youth Heart contributes to the Party in the New Era". Correspondent for the picture. In the previous "Welcome the 20th Party and Always follow the Party" competition of ideological and political and skills of Jiangsu University, 38 league cadres and young teachers from various colleges displayed. The picture shows the ideological and political competition of professional and temporary youth league cadres and young teachers in Jiangsu University. Correspondent for the picture. Cadres and young teachers around the "welcome twenty big" theme, combining the reality of the communist youth league work education class, to preach about the content also combining the time characteristics of characteristics, youth, etc., the party's scientific theory in youth language and way of interpretation, strive to speak the truth deep, through, live, inspire the youth in realizing the great rejuvenation of the Chinese nation Chinese dream on the new journey. The picture shows the full-time Youth League cadres of Jiangsu University studying and implementing the spirit of the important speech delivered by General Secretary Xi Jinping at the celebration of the 100th anniversary of the founding of the Communist Youth League of China. Correspondent for the picture. Jiangsu University also held a symposium for full-time youth league cadres to study and implement the spirit of General Secretary Xi Jinping's important speech at the celebration of the 100th anniversary of the founding of the Communist Youth League of China. The theme education practice aims to form the whole school "welcome twenty big, always follow the party, forge ahead new journey" rich atmosphere, leading the cadres and youth members to further study and apply xi general secretary in celebrating the 100th anniversary of the founding of the Chinese communist youth league conference's important speech spirit, motivated youth li strenuous, self-motivated, to meet the party's 20 victory with honors.

2022-05-30 Wuhan university of science and technology: hand-painted creative video "number" Wuhan university of science and technology: hand-painted creative video "number" history, tribute built one hundred JianWei correspondent zhang) recently, Wuhan university of science and technology youth corps committee public released a series of video works "the" number " said one hundred history, to present the 100th anniversary of the founding of the communist youth league of China. This series of video works, led by the Youth League Committee of the school, was jointly produced by dozens of students of the seventh "Youth Horse Project" talent leading flight, some students of painting major and youth Media Center. The school green horse class students CAI Zhengge, video series of ten, they selected the "54 (1919.5.4 May 4th movement)", "1957 (China new democratic youth league congress)", "1988 (song)" group has special significance of historical history, from the digital, combined with hand-drawn form and audio commentary, made into video, show the development of the Chinese communist youth league in one hundred years. The picture shows the Qingma class students discussing the production details. Since January this year, the students of qingma class have been divided into six groups, including painting, editing, dubbing and video, respectively responsible for picture hand-drawing, data collection, script writing, audio recording, post-editing and other work."Despite the difficulties, everyone thought the work was very meaningful and everyone was very serious."When Yu Zijie, who had never tried dubbing, recorded the third audio issue, she recorded the first version on March 27 and completed the final speed editing on April 4, recording 27 versions of the recording. The picture shows the students drawing it by hand."This year is a hundred years of the founding of the group. In such a special period, we are all very happy to use our ability to do something meaningful. The process of painting is also a process of learning."Nearly one-third of Ma Xiaoyu's class, a student in the 2001 painting class, volunteered to participate in the hand-painted painting work."We hope to take a new perspective, more in line with the aesthetic concept of the Communist Youth League of China, from the perspective of the youth, from the light to the deep."The school, deputy secretary of the Youth League Committee, the seventh green horse class teacher Ye Xianwei introduced.

2022-05-27 Hebei Youth Management Cadre Institute Launch Week 2 year Science and Technology Week launch ceremony. This science and technology activity week arranges artificial intelligence experience hall, VR experience, science and technology exhibition. In the artificial intelligence experience hall, intelligent service robot, humanoid intelligent robot, six-foot bionic robot, driverless car, VR-Touch touch all-in-one touch machine, 3D printer, etc., make people feel the charm of intelligent technology. In the VR experience hall, we watch popular science videos and visit the patriotism education base. In the science and technology exhibition area, we will experience intelligent voice, intelligent programming, automatic ticket purchase, SAAS education platform, China Postal Savings UMCloud project, etc. Tech Week will last until May 28. During this period, Hebei Youth Management Cadre Institute will hold a class meeting themed "Popular science Knowledge on campus" and a science and technology film exhibition for all students to broadcast high-quality science and technology documentaries and science fiction films, popularize scientific knowledge, promote the scientific spirit, present scientific life, and improve their scientific literacy.

2022-06-29 Weifang vocational college held "green horse project" training graduation and "a program under which officials" departure ceremony Weifang vocational college held the eighth "young marxist training project" training graduation ceremony and 2022 summer "a program under which officials" social practice ceremony wei correspondent Liu Jia Zhao Shu warm) recently, Weifang vocational college eighth "young marxist training project" training graduation ceremony and 2022 summer "a program under which officials" social practice ceremony in binhai campus qingze Lou lecture hall, kuiwen campus teachers and students to attend online ceremony. More than 300 people attended the activity, including the head of the Student work Office of the college Youth League Committee, the teachers of the youth League general branch of the secondary college, the instructor of the "three to the countryside" social practice team and all the students of the eighth "Young Marxist Training Project" training class. The activity is presided over by the head of the student Work Office of the Communist Youth League Committee. Pictures of the event site. The conference kicked off with the solemn national anthem. At the meeting, the person in charge of the Student work Office of the Youth League Committee of the college read out the decision of the "Top ten Students" and "Excellent students" commendation of the eighth "Young Marxist Training Project", and awarded the certificate of completion to the eighth "Young Marxist Training Project" training course, and awarded the award to the "Top ten students" and "Excellent students". The eighth "Qingma Class" training started on May 4th, lasted for 6 weeks, and organized a total of 11 lectures. After examination, 306 students successfully completed, among which 10 students were rated as "Top ten students", and 60 students were rated as "Excellent students". Pictures of the event site. At the launch ceremony of the social practice activities, the Student work Office of the Youth League Committee praised the outstanding social practice team of "three to the countryside" in 2021, and arranged the organization and publicity work of the "three to the countryside" social practice activities in 2022. The "Three to the countryside" social practice team instructor representatives and student representatives promise to go all out to successfully complete the scheduled practical tasks, and show their style, contribute wisdom and contribute strength at the grass-roots level. The head of the student work office of the Communist Youth League Committee awarded the flag to the social practice team. This summer "a program under which officials" social practice to "welcome twenty forever with the party forever new journey" as the theme, the innovation implementation "six combination" work mode, guide the young students will practice thought into practical action, in the social classroom education, long ability, contribution, take concrete actions to gift in one hundred, to meet the party's twenty big victory, constantly create a new situation of practice education.

2022-06-28 Jianwei Correspondent Wu Libo) On June 26, the social practice team of "Tongxin Zhen Township" of the School of Marxism of Xihua University held a special lecture in Lion Village, Langchi Street, Yingshan County, Nanchong City, Sichuan Province. The social practice team of "Tongxin Zhen Township", all members of the Lion Village Village Committee, some representatives of party members and villagers attended the seminar. Presentation picture. At the seminar, Du Jing, director of the School of Marxism, Xihua University, introduced the main purpose of the seminar and the connotation of the summer social practice activities of "three to the countryside". Subsequently, Yan Yuanshuang, a graduate student of the "social practice team of Tongxin Zhen Township", gave a lecture on the theme of "Scientific and technological talents revitalize the countryside", highlighting the key role of science and technology and talents in the rural revitalization strategy, and the important role of these two elements in promoting the modernization of rural governance system and governance capacity. Peng Zi � Z gave a lecture on the theme of "establishing clean family style, cultivating clean and clean family wind", listed several typical positive cases of ancient family style construction, pointed out that family style tutoring is the most precious wealth, and called on everyone to take the lead in building good family style. Xu Zhiying took the title of "new development of rural education, promote common prosperity", clarify the connotation of common prosperity and the significance of realizing common prosperity, and put forward opinions and suggestions on how to promote the development of rural education and achieve common prosperity. Presentation picture. Correspondent for figure to preach, Yin Dezhi, vice President of the school of marxism and field personnel interaction, stressed the technology talents for the key of rural revitalization, the necessity of leading the construction of good Dan, and efforts to achieve common prosperity, called on participants to practice xi general secretary to visit important instructions spirit and the 12th party congress of Sichuan province spirit, take practical action to meet the party's 20th victory.

2022-05-26 China university of geology (Wuhan) held red special class China university of geology (Wuhan) held the first phase of "youth heart to party new era" red may special group class on 26 (reporter Zhang Jianwei, correspondent, Chen Wenting Gong Weihang) to further study and implement general secretary xi in celebrating the 100th anniversary of the founding of the communist youth league of China's important speech spirit, guide the youth league to strengthen political theory study, improve political theory accomplishment, recently, China university of geology (Wuhan) organization for the first phase of "youth heart to party new era" red special group. Ma Jinxiang, an assistant researcher of China Youth and Youth Research Center and a doctor of political science from Beijing Normal University, was invited to give a special lecture. Nearly 100 students of the "Qingma Project" of the university participated in the online study. Team lesson screenshots. Correspondent for figure Dr Ma Jinxiang to "one hundred dream: the party leads the Chinese youth movement in 100", through detailed data, vivid cases, from the formation of the Chinese youth group, the generation of the Chinese youth movement and the party leads the Chinese youth movement in different historical period performance further introduces the party leads the Chinese youth movement in 100 years. Teaching finally to one hundred journey of four historical experience, put forward four hope to the communist youth league, the communist youth league member put forward five models, put forward four requirements, the cadres put forward six requirements five aspects, in-depth interpretation of the xi general secretary in celebrating the 100th anniversary of the founding of the communist youth league of China's important speech spirit. Chinese youth have always been the vanguard force in realizing the great rejuvenation of the Chinese nation. Chinese youth in the new era should turn the Chinese dream of the great rejuvenation into reality with an unremitting spirit and a stagnant attitude. China university of geology (Wuhan) engineering college youth corps committee secretary of wen said, group is rich in content, simple, through learning further deeply understand the party led the Chinese youth movement in one hundred and experience, youth league should first demonstration, in the form of young students to guide young students to strengthen party history, youth history and study, lead the young students struggle the youth into the great rejuvenation of the Chinese nation in the new journey. It is understood that to meet the party's 20 victory, to celebrate the 100th anniversary of the founding of the communist youth league of China, China university of geology (Wuhan) invited many domestic famous experts and scholars to build "youth heart to the new era" red may series boutique class, build red may learning atmosphere, continue to strengthen the youth theory, guide the youth from the struggle of one hundred journey, on the track of youth forward, run out of the best achievement of the contemporary youth.

2022-05-25 Anhui university of technology to carry out the grass group theme group activities Anhui university of technology to carry out the grass group group activities in youth network wang haihan) " I will be in teaching service about ecological civilization, environmental protection knowledge and other activities, in the grassroots education work at the same time, to help children build up ecological environmental protection consciousness."Yao Jinfang, a member of the 24th Graduate Student Teaching Group of Anhui University of Technology, sat on the grass and shared her experience with her friends. Anhui University of Technology recently held a themed league day activity. Zhao Yinghong, a post-90s "the most beautiful counselor", and her students sat on the grass of Xuejiawa, Ma 'Anshan City to carry out grass reading activities, to learn the spirit of General Secretary Xi Jinping's important speech at the 100th anniversary of the founding of the Communist Youth League of China. Lu Lin, Party Secretary of Anhui University of Technology, Li Bo, Deputy Secretary of the Communist Youth League, Zu Ming, Deputy Secretary of the Party Committee of Anhui University of Technology, leaders of the Communist Youth League and Maanshan Municipal Committee of the Communist Youth League attended the event. More than 40 college students shared and exchanged views around keywords such as "commitment" and "bridge" responsibility ". At the scene, each student personally folded a thousand paper crane, wrote the blessing to the motherland, put the thousand paper cranes in the transparent glass bottle, the students also gave the bottle had a nice name- "youth dream bottle". Wang Yingying, a grade 18 student majoring in automation, School of Electrical and Information Engineering of Anhui University of Technology, was recommended to study for a master's degree in Tongji University, with a research direction of medical micro-nano robot. Many people around her were curious about her decision to choose this direction of research."I have seen countless medical workers and science and technology workers engaged in the epidemic, and my blood has been ignited. I want to use my next life to contribute to the development of the medical cause of the motherland."She said. Participants also listened to the Ma'Anshan Youth Federation vice chairman, municipal Party School associate professor Chen Qunxiang to the detailed introduction of the target."This unique form, rich content of the youth league day activity makes me feel very excited, this is the real youth ideological and political class held in the motherland."Fany, a 20th master student from the School of Chemistry and Chemical Engineering, said that as a young man in the new era, she will inherit the red gene and let her youth blossom where the motherland and the people need it most.

2022-06-16 Shandong university to build "everyone volunteers" brand, has registered more than 50000 volunteers in Shandong university to build "everyone volunteers" brand, has registered more than 50000 volunteers WengXiangdong youth network reporter XingTing) in recent years, Shandong university around khalid ents fundamental task, adhere to the "quality, strong quality, wide coverage" work ideas, vigorously carry out the youth volunteer service work. With the focus of the whole chain layout as the carrier, the all-round participation as the focus, and the goal of full-staff training as the goal, the school scientifically and orderly leads the whole school youth to be dedicated, virtuous and virtuous, and constantly creates three programs of "everyone volunteers". From the perspective of system, system and team layout, Shandong University has gradually consolidated the "four beams and eight pillars" of the development of volunteer service. The university has issued 8 institutional documents, including the Management Measures for Young Volunteers of Shandong University, the Detailed Rules for the Management of Student Volunteer Service Lengths of Shandong University, and the Measures for the Management of Volunteer Service Projects of Shandong University, to promote the institutionalization of volunteer service. At the same time, relying on the "Shandong university students expand training plan comprehensive management system" open volunteer service sector, realize voluntary service information management, form "school-college-league branch (volunteer service)" three-level linkage, and establish "youth volunteer federation council meeting", "public community alliance" "volunteer league" and other volunteer service organizations, strengthen the construction of the volunteer service team system. Grasp the important education nodes such as Lei Feng Day, through the political consciousness of Lei Feng spirit to love the Party and the country and the noble sentiment of "serving the people", constantly cultivate the simple feelings of young people who know history, love the Party and the country, and promote the normalization and long-term education of the study of Party history. In order to ensure that the supply of volunteer services is "more solid" and "more sufficient", Shandong University continues to promote the construction of volunteer service projects around multiple levels. In the campus, Shandong University Youth Volunteers Federation has long carried out the "Star Wish Action" harmonious campus construction plan, and established more than 10 long-term special volunteer service teams, such as campus security volunteer service team and library collection sorting volunteer service team. During the 120th anniversary of Shandong University, 1,941 volunteers of the university spent more than 20,000 hours and submitted satisfactory answers to the 120th anniversary. Outside the university, Shandong University insists on promoting the community service action, covering all kinds of places in the district, establishing more than 200 volunteer service bases, and integrating the discipline characteristics into the people's livelihood services. Among them, "Minxiao Xinfeng" community historical and cultural construction activities, "Spring City Ancient Rhyme" - -the traditional culture inheritance public welfare project, "Medicine fei" - -focus on the health of residents' medication and other projects are widely loved by the community residents. Since its launch, the Service Community Action has been awarded several national awards, such as the National Service Community Demonstration Project and the Bronze Award of the Fifth China Youth Volunteer Service Project Competition. The school's 30 volunteer teaching teams bring thousands of young students to the "three regions and three prefectures" areas every year to help promote rural revitalization. For 24 consecutive graduate volunteer teaching groups, 400 members serve more than 30 schools in 10 provinces, benefiting more than 60,000 children; Chunhui Society of Shandong University docking in Ningxia, Henan, Shanxi and other volunteer teaching places, launched education support activities for many years. Nowadays, Shandong University has formed a volunteer service and education system with full-staff training, hierarchical training and high-quality cultivation. The school jointly offers special compulsory courses for volunteer service, summer courses for volunteer service, and gives young volunteer backbone training courses with "practice +", "project +", "ideological and political +" and other contents. Inspired by the concept of "everyone is volunteers", Shandong University has emerged a number of volunteer service "willing people" with the dedication of home and country. Zhang Muzi, a master from 2021, joined the Chinese Bone marrow Bank, becoming the 108th donor in Jinan and the 15th donor from Shandong University. Yu Xuze, an undergraduate from 2021, participated in the translation of letters from foreign friends in the Foreign Liaison Department of the Organizing Committee, contributing to the Olympic event with rigorous and meticulous style. Recently, the domestic epidemic situation is severe and complex, Shandong University reserves more than 5600 volunteers in Jinan 6 campus, fully ensure the orderly epidemic prevention and control work in school; actively undertake the provincial volunteer service "cloud classroom", the epidemic prevention and control youth volunteer care knowledge and emergency disposal seminar, related practice is promoted nationwide. In addition, according to the actual needs of the minor children of faculty and medical staff and college students, Shandong University has organized a special volunteer service of "Dedication for devotees", and set up a special team of more than 400 volunteers to provide academic guidance, spiritual companionship and interest training for more than 200 recipients. At present, the school has registered more than 50,000 volunteers, with a total volunteer time of more than 1.44 million hours.

2022-06-15 Guizhou university the first guofeng culture festival and tea culture promotion activities commendation conference held in Guizhou university the first wind culture festival and tea culture promotion commendation conference held jianwei correspondent, Liu Yanchun Chen Yunxia) to commend advanced, build a good atmosphere of learning and inheriting Chinese excellent traditional culture, promoting "three education" work effect, constantly deepen the "five education and" rich connotation, on June 11, Guizhou university the first wind culture festival and tea culture promotion activities commendation conference success. The chairman of the Guizhou Provincial Party Committee, the president of the Guizhou Students' Federation, the relevant personnel of the school Youth League Committee and the representatives of the student associations gathered together to share the cultural feast. Photos of the event site. At the commendation conference, the guests awarded awards to honorary teams and individuals such as "Best Cultural Inheritance Society", "Best Organization Association" and "Best Innovation Society". After the commendation, the first Guizhou National Style Culture Festival and Tea Culture Promotion performance of Guizhou University, "Meet with national Style, blooming youth style", officially began. The Chinese martial arts, brought by Guizhou University Wushu Association and Boxing Association, let us appreciate the spirit and spirit of the Chinese nation; Hanfu song of Guizhou University shows the beauty of Chinese traditional dress; Miao Studies youth group of Guizhou University, Guizhou University Shui Culture Research Association, Guizhou University Dong Culture Association and Guizhou University Minority Culture Association "Exploration, Fashion", fully shows the charm of Guizhou national culture. Photos of the event site. Correspondent for the next step, Guizhou university youth corps committee will take the "wind culture festival" as the starting point, around the school center work and key projects, continue to build "wind culture street" "wind show" "wind creative competition" and other brand activities, encourage your youth fully explore the traditional culture resources, innovative form, to show the new era of Chinese youth, to better serve the present people's cultural life.

2022-06-10 Jiangxi youth vocational college to carry out the mental health theme class meeting focus from the "heart" Jiangxi youth vocational college to carry out the mental health theme class meeting JianWei correspondent Cheng Hong Liu Xin) to guide students to maintain a positive life state, cultivate students good psychological quality, promote mental health knowledge, recently, Jiangxi youth vocational college organization classes to carry out the mental health theme class meeting. The picture shows a mental health-themed class meeting. Correspondent for figure college counselors through the form of online, to reflect the college students' common interpersonal communication, emotional regulation, love psychology, stress management, life safety education and other mental health problems, take psychological knowledge preaching, actual case analysis, collective discussion and sharing, and the students in a spiritual collision class meeting. The picture shows an online mental health-themed class meeting. In the next step, the school will continue to focus on the fundamental task of moral education, in the mental health education of college students, starting from the "heart", adhere to the combination of heart education and moral education, to create a "depth, temperature, strength" psychological education work mechanism.

2022-06-10 Anhui agricultural university youth volunteers to carry out the "green classroom" activities in Anhui agricultural university youth volunteers to carry out the "green classroom" youth corps committee for China youth network Beijing on June 10 (reporter Ji'an-wei zhang) on June 7 afternoon, Anhui agricultural university youth volunteers to amber street "youth home" to carry out the school "agriculture" characteristics of volunteer service brand "green classroom" activities. Zhu Yueyue, a lecturer of the "Green Classroom" service group of the Youth Volunteers Federation of Anhui Agricultural University, told the children about the climate characteristics and biodiversity of the tropical rainforest in the Beiyuan Village Community Party and mass service center, saying, " What is a tropical rainforest?"As the entry point, attract children's attention and trigger thinking, and then lead to the theme of the course, through the interval, by introducing the latitude and dimensions of the tropical rainforest, as well as its unique climate characteristics, gradually uncover the" mysterious veil " of the tropical rainforest, so that children have a preliminary understanding of the tropical rainforest. Finally, with the help of group pictures and short videos, the children were further introduced to some of the rare animals and plants in the tropical rain forest, and explained the importance and necessity of protecting the tropical rain forest. The "green classroom" activity popularized the relevant knowledge of the community children, helped the children establish environmental protection awareness, and also fully exercised the social responsibility and comprehensive ability of the young volunteers of Anhui Agricultural University.

2022-06-09 Guangxi art institute held "our festival Dragon Boat Festival" theme activities Guangxi art institute held "our festival Dragon Boat Festival" theme activities Beijing on June 9 (reporter Zhang Jianwei, correspondent, Wang Qiuhe lu hui) to celebrate the party's 20, inheritance and carry forward the excellent traditional culture of the Chinese nation, recently, Guangxi art institute in south lake campus and acacia lake campus to carry out "our festival Dragon Boat Festival" theme activities. Students participated in the prize-winning knowledge quiz activity. On the day of the activity, the teaching units of the participating students dressed in faucet sets, wearing wooden shoes, racing against time to the zongzi making point, began to make zongzi, do sachets. After the task is completed, each team brought zongzi and sachets back to the starting point. Finally, the judges rated the first, second and third prizes according to the appearance and number of zongzi and sachets of each team. Traditional ethnic culture display activities. During the activity, the scene also prepared a prize-knowledge quiz activity, the students in the game at the same time to understand the traditional cultural knowledge of the Dragon Boat Festival. Then, in the south lake campus to carry out the "dancing youth gift 20" -south lake dance night and in acacia lake campus "reed dragon boat gift 20" -han culture community display activities to the festival atmosphere to a climax, the students in watching the traditional dance performance, hanfu culture show feel the thick festival atmosphere and unique charm.

2022-06-07 Shandong Weifang vocational college held college graduates employment entrepreneurship guidance Weifang vocational college held "bird plan kite in Weifang" college graduates employment entrepreneurship guidance into campus activity figure China youth network Beijing on June 7 (reporter Zhang Jianwei, correspondent Zhao Shu warm) recently, Shandong Weifang vocational college held in binhai campus "bird plan kite is in Weifang" college graduates employment entrepreneurship guidance activities, college department heads, student representatives, a total of more than 200 people to participate in the activities. Zhou Fajia, deputy secretary of Weifang Municipal Party Committee of the Communist Youth League, awarded the industry-University-Research Transformation Liaison Service Center, and the Youth League Committee of the college and Binhai Party and Mass Work Department exchanged university-local cooperation agreements. The picture shows the scene of the employment and entrepreneurship policy report. Su Ming, chief of college and Technical Secondary School Graduates Service Section of Weifang Public Employment and Talent Service Center, made a report on the employment and entrepreneurship policy, interpreted the employment and entrepreneurship policy of Weifang city in detail, and analyzed the employment situation in the special period of epidemic prevention and control. And for the students concerned about the employment procedures, employment internship, grassroots employment, entrepreneurship subsidies and other procedures were answered one by one. Graduate employment and entrepreneurship guidance activity site. This activity is a specific measure to thoroughly implement the "2022 Shandong Provincial College Graduates Employment and Entrepreneurship Policy Publicity Month Activity Plan" of the 2022 College issued by the Shandong Provincial Human Resources and Social Security Department and the 2022 Employment Promotion Week series activities of the College. The content of the activity is not only full and rich, but also vivid, which promotes the students to fully understand the employment situation, understand the employment situation of employment and entrepreneurship policies, establish the correct employment concept, and cultivate the spirit of innovation and entrepreneurship.

2022-06-02 Sichuan liberal arts college held "welcome twenty big youth heart to the party" large youth song held in Sichuan liberal arts college "welcome twenty big youth heart to the party" large youth song China youth network Beijing on June 2 (reporter Zhang Jianwei, correspondent qing-hua zhao) on May 31, Sichuan liberal arts and sciences college in lotus lake campus track and field held large youth song. The song will be divided into four chapters: "Ode to Party grace", "hymn of time", "Song of youth" and "Dream song". The picture shows ten thousand people singing "Ode to the Motherland" together. According to the relevant person in charge of the school Youth League Committee, this song party is the largest number of participants in the school, with more than 600 teachers and students from 14 colleges, and more than 4,000 people from 15 colleges and industrial and Commercial Bank of China and other units to sing. In order to provide students with a better viewing experience, the song club is presented in the form of an open-air stage. The whole stage is designed in the shape of "The Phoenix spreads its wings", the school emblem of Sichuan University of Arts and Sciences, meaning that the phoenix is about to fly. The picture shows a group photo of the cast and crew. The club also broadcast live on the official B station of Sichuan University of Arts and Sciences, the official TikTok of Sichuan University of Arts and Sciences, the video number of Sichuan University of Arts and Sciences and other platforms.

2022-05-24 China academy youth corps committee to carry out the biodiversity protection action China academy youth corps committee to carry out the biodiversity protection actors Ren Mingchao) on May 22 is the international biodiversity day, the same day, the Chinese academy youth corps committee organization Hainan tropical rain forest national park biodiversity protection volunteers, to "build a common future for all life" as the theme, to the public about science rainforest biodiversity knowledge. As the highest academic institution of comprehensive tropical agricultural scientific research in China, the Chinese Academy of Thermal Sciences has a team of young scientific and technological personnel to investigate, study and popularize the biodiversity of tropical rainforest all the year round. Dozens of unique new species of sedaceae, Pepaceae, Orchaceae, Hainan medicinal plants, and new species of large fungi have been discovered, and thousands of special popular science activities have been carried out. In order to better protect the biodiversity of Hainan Rainforest National Park, the Youth League Committee of the Chinese Academy of Thermal Sciences recently organized a group of outstanding young scientific and technological personnel to form a volunteer team of biodiversity conservation of Hainan Rainforest National Park, named "Rainforest Youth". In the next step, the Youth League Committee of the Chinese Academy of Thermal will organize more young volunteers to tell the story of Hainan to the world.

2022-05-24 Guizhou Qiannan college of science and technology for disaster prevention and mitigation safety knowledge lectures in Guizhou Qiannan institute of science and technology for disaster prevention and mitigation safety knowledge lecture wei correspondent, xue-mei zhang, Yang Fangxue Chen Yingmei) to enhance the safety of staff awareness, improve the ability of emergency response to disasters, on May 18 afternoon, Guizhou Qiannan institute of science and technology "reduce disaster risk protect better home" disaster prevention and mitigation safety knowledge theme lecture held in sunshine building 110 liberal lecture hall, college teachers and students represent a total of more than 250 people to attend the lecture. The picture shows the lecture site. Correspondent for figure lecture, Guizhou Qiannan institute of science and technology, vice minister XiaoMing through PPT and field demonstration, combining the earthquake, flood, debris flow, landslides, typhoon, lightning, storm, dust, fire eight signs of eight common natural disasters, disasters and the self-rescue communal safety knowledge for the teachers and students, and led the teachers and students to the disaster response manual systematically study, let the teachers and students feel benefit a lot. The picture shows the lecture site."Safety is the premise of the healthy growth of every student, to strengthen the risk and disaster resistance and self-rescue and mutual rescue ability of publicity and education is the link we can not ignore."Du Fengxiang, executive vice president of Guizhou Qiannan University of Science and Technology, said that the university teachers and students should effectively improve the awareness of disaster prevention and mitigation, emergency avoidance skills and self-rescue and mutual rescue ability, and jointly build a safe, stable and harmonious campus.

2022-05-23 Henan Kaifeng institute of science and technology media red classic reading contest held in Henan Kaifeng institute of science and technology media red classic reading contest held China youth network Beijing on May 23 (reporter Ji'an-wei zhang) recently, Henan Kaifeng institute of science and technology media auditorium, undertaken by the school of economics "welcome 20 forever go with the party ahead new journey" red classic reading contest held the final. School of communication "the dawn of the May 4 th", the school of medicine inherited the ambition of five or four hundred, youth reading predecessors voice, school of economics fire team "commemorate 129, we when self-improvement", tells the story of the establishment of the communist youth league of China, called for contemporary youth cherish happy life, shoulder the mission, in the experience in bear, in responsible growth. Pictures of the event site. Henan Kaifeng University of Science and Technology Media for the reform vanguard of Tu Business School brought " Reform and opening up, The China Miracle, It shows the great changes brought about by China's brilliant reform and opening up strategy to China's development; College of Art has my "youth" best team " youth volunteer line, Building the Chinese Dream together, School of Humanities for spring to return to wild geese back to the team "for spring back to wild geese", The struggle of the school of Economics is youth, youth to the party team brought " youth heart to the party, Welcome 20 shows that the young generation has ideals, capable, responsible, To the motherland most needs the place dedication of youth style; The school of Physical Education, the gravel ice tour team brought the " double Olympic appointment, The Gravel Ice Journey shows the sports spirit of striving and striving... Pictures of the event site. Henan Kaifeng University of Science and Technology Media for the final scene was solemn and passionate. The performance of the participating teams led everyone to look back at the centennial history of the Communist Youth League of China, showing the youth style of the youth in the new era.

2022-05-23 Guizhou Qiannan college of science and technology the fifth "green cup" environmental creative competition final Guizhou Qiannan college of science and technology the fifth "green cup" environmental creative competition final jianwei correspondent, hongyan Yang Caifen) recently, Guizhou Qiannan college of science and technology "protect green mountains to build green academy" the fifth "green cup" creative environmental design competition ended. The picture shows the participating team representatives explaining the works. In the final scene of the picture, the contestants of the poster design group and the handmade group explained the works from the theme, core design concept, constituent elements and creation process through the combination of PPT explanation and physical display. The picture shows the students' works displayed. The field judges will comprehensively score the performance of the contestants according to five aspects: the theme, structure, color, creative concept and the explanation effect of the team design. One team each won the first prize, second prize, third prize and the highest popularity award, two excellence award teams, and the first prize, second prize, third prize, excellence award and the highest popularity award teams in the poster production group. The picture shows the students' works displayed."When the artworks made of waste items appear in front of me, they are just like the magic given by the producer, and I feel the magic of turning waste into treasure."Zeng Chunyang, a finance major from Guizhou Qiannan University of Science and Technology, said that through the careful production of creative environmental protection manual works, he can more deeply feel the significance of environmental protection. The picture shows a group photo. It is reported that this activity is a total of more than 100 teams to register for the competition, after the preliminary, semi-final, semi-final three stages of screening, a total of 11 groups of teams entered the final. Each work of the handmade team is made of discarded wood boards, paper shells, plastic straws, plastic bottles and plastic bags.

2022-05-20 Guizhou university first "challenge cup" Chinese college students 'business plan competition training camp in Guizhou university first "challenge cup" Chinese college students' business plan competition training camp figure China youth network Beijing on May 20 (reporter Zhang Jianwei, correspondent, Liu Yanchun GouMengmeng) on May 18, the 13th "challenge cup" Chinese college students' business plan competition training camp (phase 1) held in xishan theater. More than 300 secretaries of the Youth League Committee of Guizhou University colleges, "Challenge Cup" project instructors and representatives of participating students attended the event. Pictures of the event site. Correspondent Yao Yi Hu Shuikun for figure this training camp keynote speaker Yi Yongxiang to "challenge cup" competition analysis as the theme, around what is the "challenge cup", preparation points, material preparation and roadshow points, and combined with case preparation details, remind the team pay attention to the topic selection, candidates, grinding, also with the scene students features of gold medal project, how to prepare, how to better show project to interact. Pictures of the event site. Correspondent Yao Yi Hu Shuikun for the provincial competition team students said that the training camp benefited them a lot, but also felt more, the future will better improve the entries according to the knowledge learned, strive to achieve good results. In the next step, the Youth League Committee of Guizhou University will continue to organize various forms of "Challenge Cup" competition and training, do a good job in cultivating the competition projects, enhance students' awareness of scientific and technological innovation, and enhance their innovation and entrepreneurship ability.

2022-05-20 Changzhou institute of technology held faith public class and May 4th commendation conference Changzhou institute of technology held faith public class and May 4th commendation conference Yang Baoguang) in today's Tianjin university zhang memorial room, have a signed more than 100 years ago did not take the diploma, it wrote: students zhang let wujin county, Jiangsu province, now 23, in our law law examination pass, grant graduation."This paper in today have a very high value of the graduation certificate, at that time the society is an official office pass, why not be taken away?"Dr. Gao Xuan, a young teacher from the School of Marxism, Changzhou Institute of Technology, asked the students."Because Zhang Tailei said to his classmates, 'I won't be a lawyer in Shanghai after graduation. Everyone is responsible for the fate of his country. Only by taking the road of the October Revolution can we save China '. For this reason, he changed his name to 'Tai Lei', 'willing to turn into the old world of the thunder', dispel the haze, and transform the old society."Gao Xuan explained. Recently, Changzhou Institute of Technology held the "hold high the league flag with the party to forge ahead to the future" faith open class and May 4th commendation conference. During the activity, Gao Xuan brought the students a "Zhang Tai Lei: Seek happiness in the future forever" micro group class. He introduced the passionate youth in Changzhou from five aspects, including enterprising search, brave leadership, centennial league history, study and wealth, and heroic struggle, running on the road of saving the country and fighting for the people, and played a unique and important role in the creation and development of the Communist Party of China and the Communist Youth League. Dr. Xu Yiwei, a young teacher of the Normal College of the university, also sang the original song "Lei", praising comrade Zhang Tai Lei's sacrifice to the revolution, reminding and inspiring the youth of the school, as college students in the new era, should work hard to serve the motherland. During the activity, Bi Shida, a graduate of the 2020 College of the Western Program, and Tong Lin, a graduate of the School of Humanities of the Northern Jiangsu Program, also shared their volunteer service experience in Xinjiang Corps and the grassroots level in northern Jiangsu online. Jiangsu province in 2022 school eugenics XuanDiaoSheng 18 trade a Xu Tianyu, Changzhou wan help star charging technology co., LTD., data analyst 18 statistics Li Jinzhou and university of Chinese Academy of Sciences 2022 master 18 energy two Chen Lin jointly bring faith public class "the struggle of the youth is the most beautiful", to the students about their dream, dream, dream journey. Cao Yuping, Party Secretary of Changzhou Institute of Technology, sent the blessings of the festival on behalf of the youth league members. He led the students to review the past 100 years under the leadership of the CPC, generations of young people, forge ahead, united struggle, hard work and dedication. At the end of the activity, the participating leaders presented awards to the representatives who were awarded the 2021 "Top Ten Youth", "May Fourth Red Flag Youth League Committee", "May Fourth Red Flag Youth League Branch", "Excellent Communist Youth League Cadres" and "Excellent Communist Youth League Member". The conference ended successfully with the loud song of the Communist Youth League of China.

2022-05-19 Guizhou Qiannan institute of science and technology held "prevent telecom fraud" knowledge lecture Guizhou Qiannan institute of science and technology held "prevent telecom fraud" knowledge lecture Jianwei correspondent, Huang Mengyao YanChun) "what is the common form of telecom fraud, telecom fraud, how to identify telecom fraud..." recently, Guizhou Qiannan institute of science and technology invited Huishui County public security bureau Lianjiang police station deputy director Tan Fengqun for college teachers and students to carry out "prevent telecom fraud" safety education lecture."Telecom fraud prevention" lecture site. Correspondent for the picture " students do not trust the unknown object and suspicious information, do not be greedy for small profits and by the temptation of illegal text messages, do not call the strange phone number in the text message, do not remit money to strangers, do not disclose personal information, especially bank card information."Lecture, Tan Fengqun combined with their own experience and actual case to the teachers and students on fraud commonly used fraud, and told teachers and students improve telecom fraud awareness, strengthen the protection of personal information, and guide the teachers and students to install national fraud center APP, called on students to the friends, classmates and relatives propaganda to prevent telecom fraud knowledge, strive to play the role of" education a student, drive a family ". Huishui County Public Security Bureau Lianjiang police station deputy director Tan Fengqun explained the "prevention of telecom fraud" knowledge. Correspondent for the picture tourism management class 191 of Zhai Yadi was deeply inspired by the lecture, " in the future life, I will not have the pie in heaven psychology, try not to do not greedy, do not trust, do not disclose, consciously do a good job of 'anti-fraud propagandist', the anti-fraud knowledge to the classmates, relatives, friends around the publicity."Lecture in the end, Guizhou Qiannan college of science and technology resident police RanMaoFeng through the form of case interpretation, around the campus security, broken card, campus bullying, fire safety for college students on a" rule of law ", in order to further improve the safety awareness of teachers and students and self-protection ability, building a safe and harmonious campus laid a solid foundation.

2022-05-18 West farmers big group organization to build "five education" comprehensive education new pattern west agriculture big group organization to create "five education" comprehensive education new pattern green network reporter Huang Bo) recently, the northwest agriculture and forestry university of science and technology youth league organization students held "high flag with the party, stride by dream offer youth" youth help dream running activities, guide students to exercise strong body, temper firm will. This is an important part of the education work carried out by the Youth League Committee of XAgricultural University in order to create a new pattern of comprehensive education and take "five education" as the starting point- -adhere to the sports people and strengthen the young people. The so-called "five education" is a new systematic education project summarized by the Communist Youth League Committee of the work requirements of the new era and the new characteristics of young students, including the theory of the youth, and the practice as the foundation of the youth tree. To this end, the university youth corps committee refinement and organized a series of corresponding activities, such as the red culture corridor, southeast kiln experiment station platform as the main position for young students patriotic, love party, love the school strong feelings, with the Chinese excellent traditional culture, red culture infiltration student mind, to the campus culture construction lead young students firm cultural confidence, etc."Culture Box" is an entrepreneurial project led by college students created by the Youth League Committee of West Agricultural University for one year. It aims to build an education platform for innovation and entrepreneurship, and provide students with systematic entrepreneurial guidance from entrepreneurship "germination" to "incubation and growth" and finally "fighting the market". At present, the Youth League Committee of the university has built 5 platforms for student innovation and entrepreneurship, incubated more than 140 student enterprises, and selected typical maker models such as "Nongsang Maker", "Jiang Yiliang and" Tu Pioneer " Hao Yifei. The Youth League Committee of Western Agricultural University has also steadily promoted the "systematic, project-based and curriculum" of social practice, established a base of labor and farming and reading education practice, taken the lead in opening public elective courses of labor education in China, and compiled labor curriculum textbooks. Li Guolong, secretary of the Communist Youth League Committee of the school, said, " The various youth league organizations of the school will continue to take the 'five education' as the starting point to create a new pattern of comprehensive education.”

2022-05-17 Shandong Jianzhu University: Let labor become the best habit for students to become a sense of participation and identity, and then become their habit? At Shandong Jianzhu University, Labor education is no longer a straight-faced "old gentleman", More like the "friends" accompanied with students day and day: it is the various volunteer service "little vest" shuttling on campus, It is a practical classroom living in flowers and trees, It is a group of students explaining the beauty of campus architectural culture... The word "Lao" is embedded in volunteer service, Since the temporary closed management of Shandong Jianzhu University campus in March this year, Many "colorful vests" appear on the campus, The Orange color of the School of Thermal Engineering, The Academy of Art in Red, School of Computer Science & Technology and the Blue, The School of Foreign Languages in Green... Together with the second opening of winter jasmine, magnolia and pink cherry, Make up the different scenery line of this spring. Since the outbreak of the epidemic prevention and control battle, the Youth League Committee of Shandong Jianzhu University has actively explored a new mode of volunteer service with "labor" as the core, combined labor education with volunteer service, integrated the strength of public welfare associations and school-level volunteer service teams, and built a two-level volunteer service system to boost the implementation of labor education. Since the launch of the activity, more than 5,000 young volunteers from various colleges, wearing vests representing the color of the college, have changed their jobs according to the volunteer work schedule formulated by region and date, and undertook all the daily volunteer service work required throughout the school. Nucleic acid detection point daily flow of thousands of people, the weather, but the same is the student volunteers again and again "please keep a meter distance, wear a mask, show the reservation code", intimate tips, and in the restaurant persuasion interval queuing and scattered dining, in the supermarket playground and other crowded places remind wearing masks to help maintain order of busy figure. In addition to the necessary life materials, the temporary closed management of the campus is also shipped into many "love gifts". T-shirts, milk, masks and hand sanitizer... are a "gift package" for students. Student volunteers waited early at the school gate and carried boxes of love materials to the dormitory building. At 2 PM, the sun was still a little hot. The sweat of the volunteers ran down their cheeks to the cardboard boxes they were carrying, but they were not afraid of fatigue and distributed them one by one. This university regards labor education as an important part of the education project, and gives love and feedback to the payers, so that the concept of labor glory is truly deeply rooted in the hearts of the people. On Labor Day, May 1, the teachers and students at the university received a special gift- -two round boiled eggs. Since 7 am, the school has delivered more than 30,000 eggs in three canteens to comfort the teachers and staff who have contributed to the epidemic prevention and control work."Today is the working people's festival, the restaurant staff not only did not rest, but also got up early to cook for us eggs, how can we be indifferent, always have to do what we can."Some of the students who received the" Love eggs " volunteered to help the restaurant staff clean up and recycle the tableware after the event. Canteen here has "love egg" labor feeding, teaching building there there is social practice labor mobilization. On Labor Day, the teachers and students of the School of Foreign Languages established two themed social practice teams, "Beautiful China" and "Promoting Rural Revitalization" of Shandong Jianzhu University. They integrated labor education through the foreign language talent training system, and combined with "foreign language education" and "curriculum ideological and political education" to further expand the education breadth of "labor + professional practice". Walking in the campus of Shandong Jianzhu University, careful attention will find that the semester responsible for campus sanitation and beautification staff seems to change a group of new people, their average age is only 20 years old, energetic, energetic, waving a big broom to carry the long water pipe effortless. But it seems that some are not very professional, the broom a careless did not take steady on the foot, carry the water pipe but did not control the direction. They are not high work efficiency, the work process, but throughout the energy, work results are commendable: the scattered garbage in the grass clean, watering the green area, help clear the silt of the snow lake, moving on the shared bikes on the road to the roadside... this is the students involved in campus labor, they are also involved in the practical course. Shandong Jianzhu University organically combines labor education with general education, professional education, and innovation and entrepreneurship education, and offers a labor education compulsory course, "Labor and life", consisting of 8 class hours of theoretical education and 24 class hours of labor practice. In response to the call of the school, the colleges have moved the labor education classroom according to the "safety and civilization responsibility area" divided by the school, moved the classroom out of the classroom, and moved to the public facilities and flowers and trees on the campus."Before, I often saw people busy beside the green belt. I felt that the work of watering, watering and cutting branches was not complicated. After I did it myself, I realized that the seemingly simple labor was not easy."Chen Liyang, a student who participated in the course, said," One experience, several hard work, and multiple insights, the work is very difficult, and the workers are very glorious. ". The school also further implemented the "school-wide cleaning" work every Thursday afternoon, distributing garbage bags and 84 disinfectant to the dormitories, and encouraging the school counselors and head teachers to join the workforce, and the teachers and students work together to create a clean and comfortable living environment. The college has also carried out various forms of civilized dormitory creation activities, "the most bright and clean dormitory", "the most green dormitory", "the most warm and friendly dormitory" have appeared, fully mobilize the enthusiasm of young students. In the campus of this university, there are several famous "old stars": the railway architecture pavilion with yellow walls and red tiles, the primitive and natural Daiyue One Residence pavilion, and the map land deed pavilion with over 100 years... they are the shining "name cards" of the school, transmitting a different campus architectural culture to the outside."Behind the scenes", these "old stars" are introduced by a campus cultural landscape volunteer explanation group composed of student volunteers from various colleges. With enthusiasm for these old buildings and love for the campus landscape, the members of the group are on duty in each pavilion every week on rest days, telling the cultural stories of these old buildings to the students, teachers and visitors to the pavilion. Before the campus was closed to the public management, they undertook the reception work of more than 1,000 people per academic year. Their professional interpretation and patient question-answering have brought an enduring fame to these old buildings."The exhibition hall is both our friend and our 'home'. Those days of explaining the work here have already tied us closely together with us here. We know all their stories, and they carry many of the stories that we work with our partners and understand architectural culture with our friends."The narrator Yan Lexin classmate said. In order to combine the practicality of labor education with the self-restraint function of aesthetic education, Shandong Jianzhu University also carried out the theme of "labor education + aesthetic education" education activities, leading the students to carry out green planting and green protection activities. In the opinion of Wang Peng, secretary of the Youth League Committee of Shandong Jianzhu University, it is the original aspiration and mission to carry out labor education to train builders and successors with both integrity and ability, and also the starting point and goal of further implementation of labor education in the future. China Youth Daily China Youth Network reporter Xing Ting correspondent Yang Jing Zhang Zhiwen source: China Youth Daily

2022-05-17 Southeast university held a "youth heart to the party, the new era" theme TuanRi activities and 2022 may 4th commendation congress southeast university held "youth heart to the party, the new era" theme TuanRi activities and 2022 may commendation congress wei correspondent, NingJunKang QuGang hang add) recently, the southeast university of Nanjing, Jiangsu province "youth heart to the party, the new era" theme TuanRi activities and 2022 may 4th commendation congress held in jiulonghu campus lecture hall. School party secretary, member of Chinese Academy of Sciences, President Huang Ru, all the school leadership, the party committee, the relevant functional departments is responsible for the comrades, the college party committee is responsible for the activities, all the comrades, award-winning young teachers and students, the college youth corps committee is responsible for the comrades and some outstanding members on behalf of the scene. Activity pictures. Before the start of the conference, all watched the video "The progressive Southeast University Communist Youth League", reviewing the struggle of the Southeast University Communist Youth League over the years. Ren Yanjun, representative of innovation and Entrepreneurship youth and doctoral student of School of Mechanical Engineering, Wang Min, Top Ten Young Volunteers of Jiangsu Province and 2021 master student of School of Foreign Languages, and Hao Menglong, winner of Jiangsu Province "Youth May 4th Medal" and professor of School of Energy and Environment, delivered a wonderful speech. The scene poem and painting "Talents serve the Country" brought by the college student art troupe, leading all teachers and students to feel the national rejuvenation and the frontier of science and technology. The 15th "Jiangsu Youth May 4th Medal Collective", Network Communication and Security Youth Research Team, 2020-2021 national "University Vitality Youth League Branch", 2017 top Innovative Youth League Branch of medical College for style display. Southeast university red mei an XuanJiangTuan members bring under the leadership of the party of the southeast university youth movement in one hundred to share the theme, the "red mei an" online exhibition hall, aims to further use good red resources, continuously red blood, guide the youth in intuitive, convenient, diverse way to see exhibition, listen to lectures, enlightenment beginner's mind, words in the future. The choir of the College Student Art Troupe first sang the original chorus "Original Heart According to Mei'an", singing the strongest voice of youth in the new era. At the award ceremony, Xing Jihong, deputy secretary of the Party Committee of the University, read out the decision to commend the 2021 "Two Red and Two Excellent" and the May Fourth Medal, Youth. The participating leaders presented awards to the advanced collectives and individuals. Principal Huang Ru presented awards to the winners of the "National Flag Youth League Branch" and the "National Flag Youth League Branch (nomination)", and awarded the flag of the "National Flag Youth League Branch" of Jiulong Lake Campus in 2021. It is reported that the Youth League Committee of Southeast University closely around the comprehensive reform of "three complete education", innovation to promote the "casting soul", "rock" and "good knowledge" three major projects, with platform construction, content innovation as the focus of the reform and improvement of the "four schools", to improve the leading force, organization, service force and the overall contribution of the Dongda Communist Youth League.

2022-05-14 Northern University for Nationalities leads the young people to study the history of the Youth League to explain and visit the theme of "the Communist Youth League of the Communist Youth League", leading the young people to study the history of the history of the Youth League together. Since the exhibition of the Communist Youth League in May, Gong Xuefei, Secretary of the Communist Youth League, Han Fan, Deputy Secretary of the Communist Youth League Ningxia District � [and Wang Hongwei, deputy Secretary of the Education Working Committee of Ningxia Hui Autonomous Region, visited the "Centennial Communist Youth League" of Northern University for Nationalities and gave full recognition. Up to now, the Northern University for Nationalities has organized the class and youth league branch to visit intensively through the second class system, and more than 5,000 students have visited the exhibition. It plans to cover more than 20,000 young students by the end of June. Before the theme group history exhibition, in order to make group history exhibition better play the education effect, northern university for nationalities party committee proposed to cast the Chinese nation community consciousness as the main line, relying on the group history of the party of the history of Chinese youth movement, at the same time to make good use of the office space, clever design tour route, set the group history exhibition group history education propaganda display center, long-term for school teachers and students and surrounding teachers and students, social league organization exhibition, give full play to the group history education display center service school and social function. The Northern University for Nationalities organized more than 50 youth league cadres and key students to form a preparatory working group for the youth league history exhibition, Through reading Excerpts of Xi Jinping's Discussion on the Work of Youth and the Communist Youth League and Xi Jinping and College Students, Thoroughly study General Secretary Xi Jinping's important thoughts on youth work; Through the discussion of the History of the Communist Youth League of China, Photo of the History of the Youth League- -A Documentary of the Growth of the Communist Youth League of China, and Chinese Youth in the New Era, Deeply understand the glorious course of the Party leading the Chinese youth movement; By studying the Biography of Ethnic Minority People in the History of the Communist Party of China, Constantly understand that the great rejuvenation of the Chinese nation is for the people of all ethnic groups under the leadership of the Party, A great feat of bloody struggle and united struggle together. Through the collective studies, the group history exhibition is divided into five parts, the first four parts show the youth league in the new democratic revolution, socialist revolution and construction period, reform and opening up and socialist modernization construction in the new period, the new era of socialism with Chinese characteristics always with the party concentric, with the party struggle, always contribute youth, establish important meritorious historical facts. The fifth part mainly shows that the Ningxia Communist Youth League has always been under the strong leadership of the Communist Party of China, united and led the young people in all periods, adhered to the truth, made patriotic contributions, wrote touching poems of national unity on the land of the fortress, and created the historical facts worthy of the brilliant achievements of the May 4th ancestors. More than 100 students from the publicity group of "Pomegranate Seed" of Northern University for Nationalities, during the preparation of the theme group history exhibition, jointly discussed how to make the history of the group more accurate and appealing; on the other hand, how to reflect the main line of forging the sense of community of the Chinese nation. Everyone around the previous congress of the league and all previous reform of the party concern, during the May 4th movement and the emergence of patriotic youth representatives, the party's national policy, the red army long march touching story, millions of youth in the western construction and other content repeatedly discussed, strive to explain the more comprehensive, accurate and vivid.

2022-05-10 Five schools seec LianXue build exchange activities to share the games spirit five schools seec LianXue build exchange activities to share the games spirit xin wang) recently, by tsinghua university, Beijing university of aeronautics and astronautics school of energy and power engineering, Tianjin university precision school of instrument and optoelectronic engineering, university of electronic science and technology, southwest jiaotong university school of information science and technology jointly launched the "flag lead the games, youth cohesion to the future" learn build communication activities held online. The event, organized by the Party and Youth League branch where the five schools have similar disciplines, aims to inspire young students by exchanging the spirit of the Winter Olympics. Five universities have set up a number of sub-venues, with more than 1,000 people online and offline participating. Beijing university of energy and power engineering school 2020 master NiTianxuan, Beijing university of Aeronautics and aerospace energy and power engineering college 2019 undergraduate NiShu, tsinghua university automation department 2020 doctoral guo, tsinghua university automation department 2020 master NiuJiaHe five Beijing Olympics, winter paralympic games volunteers, With the title of "We are Beyond the spotlight", "To the vast and Snow the Chinese Dream", "Standing the Bird's Nest Technical troubleshooting", "Snowflakes flying on Xiaohaituo Mountain", About volunteering during the Beijing Winter Olympic and Paralympic Games, In particular, the application of the team's innovation results in the Winter Olympics volunteer service. They said that the volunteer service experience of the Winter Olympics is a valuable wealth of life, and they encourage them to study hard and contribute to building a modern socialist country. Tianjin university precision instrument and optoelectronic engineering college 2018 undergraduate pan guangtong, southwest jiaotong university school of information science and technology 2018 undergraduate Zhao Pan, electronic technology university automation engineering college 2020 master Xu Xinjie, respectively to the concerted resistance to disease ambitious youth bear "one hundred Beijing intelligent new face power games jiaotong university bear" "persist Beijing games spirit power national" double carbon "strategy" as the theme, combined with the school and the professional, share the learning experience. They have expressed their determination to fulfill their mission in the major national strategies and social needs from the perspectives of fighting the epidemic, the strategy to strengthen the transportation country and the "dual-carbon" strategy.

2022-05-09 Changshu Institute of Technology refined "faith open class" into "provincial quality demonstration class 1 year" faith open class "provincial demonstration class selection results" notice, determined 40 "faith open class" as the provincial quality demonstration class. The "Letter of Faith" submitted by the School of Foreign Languages of Changshu Institute of Technology was selected into the provincial quality demonstration class."Faith open course" is a work project that unremittingly arms the youth with the latest achievements of adapting Marxism, guides the practice of youth league and learning, and promotes the work of ideological guidance. The open class was conducted both online and offline, with a total audience of more than 30,000 people. In recent 4 years, the school "classic translation station" learning club to develop provincial "faith open class" 2 times, 8 times, more than 50 times, 800 times, the league branch, organized the campus display 2 times, theory study more than 30 times, social practice more than 50 times, the total number of educated students; the school held the provincial "faith open class" twice awarded Jiangsu Province "faith open class" quality demonstration class. The school of foreign languages will continue to explore the "party + professional" "education + professional" financing education, to "classic translation" learning club as the platform, the combination of party construction, education, professional education, through expanding "faith public class" form, improve the affinity and pertinence of talent training and targeted, let students in different education courses set up the lofty faith and correct life values.

2022-04-29 Hefei normal college was founded to welcome the party's 20 young students XuanJiangTuan Hefei normal college was established to meet and learn to implement the party's 20 young students XuanJiangTuan JianWei) recently, Hefei normal college in Anhui province to meet and learn to implement the party's 20 youth students XuanJiangTuan founding meeting in jinxiu campus line know building, tencent meeting. The leaders of the publicity Department of the Party Committee, the Youth League Committee of the college, all the staff of the school and 30 students from the party history education students attended the meeting. The meeting was presided over by Zong Biao, the temporary deputy secretary of the school Youth League committee. The meeting read out the "notice on the establishment of welcome, study, publicize and implement the party's 20 young students publicity group". Lv Zihan, the head of the publicity group, made a speech, saying that he should strengthen the theoretical study, strictly abide by the work discipline, unite and cooperate sincerely, and carry out the publicity work with high quality and high quantity in accordance with the requirements. The meeting also issued letters of appointment to the members of the young student publicity group. Party committee propaganda department vice minister zhao is introduced and study propaganda and implement the party's 20 youth students XuanJiangTuan background and significance, points out that young students XuanJiangTuan into students preach is learning propaganda and implement the party's 20 spirit, is to guide the teachers and students to study since the eighteenth big original thought, transformative practice, breakthrough and the important carrier of landmark achievements. He asked the students of the publicity group to firmly correct the political direction, strengthen the study of political theory, conduct in-depth discussions, prepare lessons carefully, preach confidently, follow the characteristics and rules of young students' growth, and successfully complete the publicity task with the words that the students can understand and listen to. After the founding meeting, Chen Jinkun, a member of the Anhui Provincial University Student publicity Group, the "Excellent lecturer", and the 2020 discipline teaching (mathematics) graduate student of the School of Mathematics and Statistics, gave the first concentrated training for the members of the publicity group. It is understood that according to the "welcome 20, always follow the party, forge ahead new journey" theme education practice and "youth gift 20, power have my new journey" to meet the learning propaganda party's 20 theme publicity and education activities, the party committee propaganda department, school youth corps committee for floor students to meet and implement the party's 20 youth students XuanJiangTuan members. After individual application, college recommendation, publicity and trial lecture, and interview and selection, 30 students from 15 colleges were finally identified as members of the publicity group of Party history learning and education students, including 1 master graduate student and 29 undergraduate students. They will closely follow the welcome and study publicity implementation of the party's 20 this main line for the whole school students to carry out a series of special publicity.

2022-04-25 North China Electric Power University Baoding Campus held "waste into treasure" handmade competition Jianwei correspondent, Zhang Yifan Ma Caieng) recently, North China Electric Power University Baoding campus held "waste into treasure" handmade competition exhibition. Young college students use waste newspapers, waste electronic components, plastic food boxes, express boxes, dry branches and flowers and other materials to design and produce a variety of environmental protection handmade works, convey the concept of environmental protection, with action to help the "double carbon goal", so that waste goods find "spring". Pictures of some works. In order to pay their respect to the staff fighting on the front line of the COVID-19 epidemic and their hope for the early end of the epidemic, college students made all kinds of handicrafts with the theme of fighting the epidemic. The little blue flower team used scratch paper, old clothes, plastic bottles and other materials to make healthy hydrangea and small daisies meaning happiness to pay tribute to the workers fighting the epidemic. Pictures of some works. Online shopping has become an essential part of the life of college students' pictures, and a large number of express packaging boxes have also become a source of garbage that can not be ignored on campus. The contestants supplemented these delivery boxes with paper cards, dried flowers and other materials to bring a "new look" to the delivery boxes. Flying Youth Team made four express boxes of the same size into four small and simple desktop classification garbage bins, in the "turning waste into treasure" while showing the idea of garbage classification, and advocating the implementation of garbage classification. The BFRP101 team used the combination of express box, dry flowers, dead branches and handmade paper cutting, and made the paper extraction and potted handmade products with the creative idea of "wonderful paper and potted flowers", realizing the unity of ornamental and practical value. Pictures of some works. Correspondent for pictures to protect the earth, with professional knowledge to help the "dual-carbon target" participating students also combined with professional characteristics and made a variety of professional appliances. The creative youth team used the discarded cans and water bottles in daily life to create a simple and environmentally friendly filtration and power generation system, so that students can understand the working principle of the clean and environmentally friendly power generation system. Use flexible solar panels and some discarded paper panels to make small electric fans with easy to carry, low carbon and environmental protection characteristics. Huadian scavenger team uses simple drive systems, vacuum cleaners and paper boxes to make handheld automatic vacuum cleaners.... the students integrate the "double carbon" goal into their works and use their expertise to protect the environment. More than 90 pieces of all kinds were exhibited in the handmade competition exhibition, which attracted more than 400 students to stop to watch and write down their feelings on the activity message board. It is reported that the school Youth League Committee will focus on the "dual-carbon" goal, combined with the actual work, continue to carry out a variety of online and offline activities. Cherish the resources, the garbage "turn waste into treasure"

2022-04-22 Guizhou university welcome Guizhou university theme demonstration preach Guizhou university "welcome 20, always follow the party, new journey" theme demonstration demonstration held in Beijing on April 22 (reporter Zhang Jianwei, correspondent, Liu Yanchun coke) recently, Guizhou university "welcome 20, always follow the party, new journey" theme demonstration preaching activities successfully in the school of marxism. Information, marxism Chinese professional 2020 graduate student Kevin Yang, ideological and political education professional 2020 graduate students ever made, Chinese modern history basic problem research professional grade 2020 students, with youth perspective for the youth brought a "words the communist youth league history write a new chapter" under the title of vivid preaching. The lecture is skillfully integrated into interactive questions and answers, reading letters, watching videos and other popular forms, telling the centennial history of the youth league, let the youth league members stay in the red story environment, feel the red spirit, and inspire the youth league members to develop themselves in the new era. For the history, the group song is a kind of inheritance, and for the future, it is a kind of responsibility, with a distinct mark of The Times. Lecturer Luojie uses red music to carry out patriotism education, through the awakening of the nation from the May 4th torch, to the youth lighting the future with their lives, and then to the magnificent cause to inspire the past and the future, so that the youth members are immersed in understanding the "Glory"! The historical background and creation story of the Communist Youth League of China feel the glorious course of the history of the League. Combined with their own majors, the students deeply explored the red story and told the historical contributions of the Communist Youth League uniting and leading the young generation in the new democratic revolution period, the socialist revolution and construction period, the new period of reform and opening up and the socialist modernization drive, and the new era of socialism with Chinese characteristics. To preach and study the glorious deeds of the revolutionary martyrs and heroes in their hometown, and to seek the national spiritual strength by reviewing the stories of their ancestors. Let the youth members read in the progress of The Times thought inspired, the revolutionary martyrs for national independence, national self-improvement and set up the lofty ideals, feel the lofty faith, great integrity and revolutionary pride of the ancestors. It is reported that the publicity of the Youth League history is an innovative measure for the Communist Youth League of Guizhou University to study, publicize and implement Xi Jinping Thought on Socialism with Chinese Characteristics for a New Era, and constantly strengthen and promote the youth theoretical armed work. It is an important carrier for the continuous and in-depth study and education of the Party history. Since march this year, the school of marxism, Guizhou university from the ideological and political education, marxism, marxism, basic principles of Chinese modern history research and other professional elected a batch of political stand firm, high theoretical accomplishment, preach ability of excellent member representative, strive to build a based on youth perspective, focus on youth work, response to youth concerns, covering youth groups of high-quality "student branch" team.

2022-04-20 Jiangsu university youth corps committee held youth league branch secretary of the game in Jiangsu university held "welcome 20, always follow the party, new journey" theme micro league game China youth network Beijing on April 20 (reporter Zhang Jianwei, correspondent, Ding Yijuan liu) recently, Jiangsu university youth corps committee held "welcome 20, always follow the party, new journey" youth league branch secretary micro league class game, from the school of finance and economics students on the stage about the story of "two brothers" Chen Yannian, Chen Qiao. The picture shows the youth league branch secretary micro-youth league class competition venue. A total of 60 youth league branch secretaries from 29 colleges signed up to participate in the competition. After the preliminary evaluation organized by the school youth League committee, 28 youth league branch secretaries were shortlisted for the final and displayed. In the display, the league branch secretary around the "welcome twenty big" theme, focusing on a major event, a great spirit, a great man show the party's one hundred history, the new era of historical achievements and no great changes in one hundred, combined with the characteristics of time, youth, etc., with value leading oriented, actively carry forward the positive energy, spread the good voice.

2022-04-20 National agriculture and forestry universities on the games of agriculture and forestry universities on the games of agriculture students joint XuanJiangTuan starts Yang Baoguang) on April 15, the national universities Beijing 2022 Olympics and winter paralympics agriculture students joint XuanJiangTuan founding ceremony and the first preaching report will be held in China agricultural university. This activity was initiated by the National Communist Youth League Work Alliance of Higher Agriculture and Forestry Universities. More than 900 teachers and students from Beijing Agricultural University, Beijing Forestry University, Beijing Agricultural University and Hebei Agricultural University participated in the Beijing 2022 Winter Olympic and Paralympic Games, according to the report. In order to fully explore the advanced models and moving deeds emerging in the process of service guarantee, let the teachers and students of higher agriculture and forestry colleges and universities across the country understand the story behind the volunteer service, and jointly improve the ideological and political courses of the Winter Olympics, the National Communist Youth League Work Alliance of Higher Agriculture and Forestry Colleges established a joint publicity group for agricultural students. From mid-April to the end of July, the delegation will conduct various forms of publicity and exchanges at universities according to the needs of the alliance and the epidemic situation. It is understood that before this, the four publicity group source colleges and universities have been respectively for the publicity group members to carry out the training work, and through the competition, lesson preparation, trial lecture and other ways, to promote the publicity work landing effective. So far, four XuanJiangTuan source university has been widely in the school preaching activities, through into the classroom, into the class, into the branch, combined with various forms such as online to preach series of more than 20 games, at the same time through the docking Beijing district youth corps committee designated teaching service, build primary and secondary schools to preach activities, widely praised by inside and outside the school. Launch ceremony, China agricultural university youth corps committee secretary Fan Danian read "about the establishment of the national agriculture and forestry colleges in Beijing 2022 Olympics and winter paralympics" agricultural students joint XuanJiangTuan "decision", the communist youth league central youth volunteer action guidance center director Shi Xinming for XuanJiangTuan university representative flag, China agricultural university, deputy secretary of the party committee Wang Yong for XuanJiangTuan representatives. Fan Chenhui, deputy Secretary of the Youth League Committee of China Agricultural University, Gan Zhilin, deputy Secretary of the Youth League Committee of Beijing Forestry University, Chen Yong, Secretary of the Youth League Committee of Beijing Agricultural University, and Qiao Mengtao, Secretary of the Youth League Committee of Hebei Agricultural University, introduced the services of the Beijing Winter Olympics and Paralympic Games and the preparation of the publicity group respectively. Li Guolong, secretary of the Youth League Committee of Northwest A & F University on behalf of the alliance, Li Jincai, Secretary of the Youth League Committee of Northeast Forestry University, and Peng Xiaochuan, Secretary of the Youth League Committee of Huazhong Agricultural University, exchanged speeches online. After the launch ceremony of China Agricultural University, 7 members of the publicity group gave their first lecture. Li Mohan, a volunteer from the National Sleigh Center, told about where I stand is China's national feelings; Leon told the youth story of serving the Winter Olympics event at home; Liu Wei, the volunteer manager of the main media center, told about how to do a good job in the Winter Olympics media press release services in the global media spotlight; Volunteer Du anna told how she invented the QR code to solve the travel problem of foreign journalists; Wang Zhentao, a volunteer at the Capital International Airport, talked about his transformation into Baymax, Stand well "the first post", Keep the "first pass of the first door" personal perception; Zhang Zhiying, a volunteer from the Winter Olympics Village, talked about what athletes think and what athletes need, Touching experience of actively coping with various risks and challenges; Guo Xiaojun, a city volunteer, is proud of witnessing the glory of the motherland. The lecture content won the applause of the audience. It is understood that more than 6,400 representatives of the Communist Youth League of the Beijing Winter Olympics and Paralympic Games, and the representatives participated in the activity online and offline. In 2016, the National Communist Youth League Work Alliance of Higher Agriculture and Forestry Universities was established, which has played an active role in promoting ideological guidance, practical education and youth work exchanges among various universities.

2022-04-15 Zhengzhou university youth corps committee to carry out the "cloud games" campus sports activities in Zhengzhou university to carry out a number of activities for epidemic prevention and control youth power build wei correspondent Liu YingXie Yutong) in order to encourage students actively exercise during COVID-19 outbreak, enhance immunity and resistance, develop good exercise habits, a few days ago, Zhengzhou university youth corps committee to "offline free movement, online upload comparison" a variety of ways to carry out the "cloud sports" campus sports activities. The activities included eight events, including one minute push-ups, one minute Bobby jump, one minute rope skipping, one minute sit-up, one minute shot basketball, one minute football, one minute mat volleyball, and standing long jump. Participants recorded and uploaded their own sports videos, and the staff assessed the awards online. There are a large number of participants in the activity, on the playground, dormitory, square, there are students sports everywhere. Not only that, the school youth corps committee also adopts new media means, actively promote epidemic prevention policies and knowledge, to carry out online "academic salon" "singer contest" public lecture " and other activities, not only enrich the students' campus life, more help the young students to relieve stress, keep positive attitude and uplifting spirit, actively cooperate with the school epidemic prevention and control measures, in the outbreak work. The Communist Youth League of Zhengzhou University uses campus media platforms such as Zhengzhou University Youth Assembly and Houde Assistance Sister, Online continuous publicity of disease prevention and control knowledge, relevant laws and policies, life and epidemic prevention knowledge, To improve the ideological understanding of teachers and students, Enhance the civilized quality of teachers and students, self-protection awareness, prevention and control awareness, at the same time, Give full play to the advantages of new media platforms, Republish the relevant epidemic prevention information and the prevention and control results, Timely publicize the relevant requirements and work arrangements for epidemic prevention and control, Accurate guidance; Conduct a series of reports, Explore the epidemic prevention stories of youth league members, The typical cases of young volunteers taking the initiative, brave the vanguard, and actively participating in the front line of epidemic prevention and control, Positive guide to all the teachers and students to strengthen confidence, Scientific prevention and control. In order to enrich the campus activities and help students to maintain optimistic and physical and mental health under the normal epidemic prevention and control situation, the school Youth League Committee actively explored the activity mode, integrated the platform resources, and organized a series of online characteristic activities that students enjoyed. Invite media celebrities to hold a youth innovation lecture hall, Improve the students' media literacy; Organize academic salons, Invite senior professional professors and outstanding seniors to exchange you literature, innovation, scientific research knowledge and experience, Serve young students to grow up; Holding the Challenge Cup Competition public lecture, Provide competition guidance and experience sharing for the participating students; United Law School held an online 315 equity system legal knowledge training lecture, Popularize legal knowledge, Help students to better safeguard their own rights and interests; Holding public lectures on youth career Support programs, To answer questions for young students, Help young students to plan their career direction and solve their employment problems; Host an online campus Singer Contest, Build a platform for students to show their talents; Through the recruitment of online volunteer teachers in the "Cloud Classroom", Encourage young students to practice their responsibility of The Times. At the important moment of the prevention and control of the epidemic on campus, the majority of young volunteers participated in the school epidemic prevention work, and the youth league members took the initiative to apply for nucleic acid registration, order maintenance and other epidemic prevention volunteer actions, and assisted the school in the epidemic prevention and control work until late into the night. Whenever and wherever, young volunteers of Zhengzhou University: return home in winter vacation, volunteers actively report to the community, into the epidemic prevention and control work without return; return to school, actively assist the school persuasion, evacuation, help students back to school; the school epidemic situation is increasingly severe, all nucleic acid crowd, volunteers actively assist in testing, maintain order, all day.

2022-04-11 Guangxi normal university students with youth sowing green hope Guangxi normal university students with youth sowing green hope China youth network Beijing on April 11 (reporter Zhang Jianwei, correspondent, Liang Ziyan Cui Yidan) "grass is ecological xing", under the concept of ecological civilization construction, recently, the Guangxi normal university youth corps committee organized the "welcome 20, planting forest" voluntary tree planting activities, through green planting, ecological protection system of green campus construction project, in response to the call of afforestation cause, with youth to sow the seeds of beautiful China. More than 70 young teachers and students from different colleges gathered together to shovel soil, cofferdam and irrigation to create green."After you really do it, I will feel that afforestation is not as simple as imagined. There is a lot of attention only to planting a small bamboo plant. Let me realize that building a beautiful China really needs everyone's unremitting efforts."Said Bin Lu, president of the School of Arts / School of Journalism and Communication Branch of Guangxi Normal University, when participating in the tree-planting activity. The Youth League Committee of the school has carried out "green adoption activities for the future". Through online and offline publicity, it calls on teachers and students to pay attention to the environment, encourage young students to observe and understand the characteristics of plants in the campus by writing, shooting, drawing and other forms; and guide teachers and students to care for the environment. For a long time, Guangxi Normal University has formed a campus project construction system represented by associations, organizations and volunteer service teams in terms of green planting and ecological protection. The environmental protection association of Guangxi normal university, Guangxi normal university youth volunteer association as the leading role, drive multiple organizations, associations to participate in the "China water week" theme activities, "air pollution knowledge science" "environmental protection knowledge competition" recycling-drifting bottle " activities and so on a number of activities, has successfully led tens of thousands of young students to participate in the practical activities of the green campus construction. In 2019, the Youth Volunteers Association of Guangxi Normal University built the "River Guard" project, which focuses on all branches of the Lijiang River Basin, and the project is committed to protecting local rivers with the core of " protection, culture, popularization and integration) as the core. More than 23,000 volunteers have participated so far. Really let the students out of the limitations of ecological cognition, into the outdoor, focus on the environment, innovative ecological development. While building a civilized campus, it also contributes a youth strength to building a beautiful China with always green mountains and long green water. The Youth League Committee of Guangxi Normal University makes full use of the second classroom to guide young teachers and students to pay attention to ecological civilization. Through the series of activities of the forum, young teachers and students are guided to understand the ecological situation and solutions, and multiple professional problem classes such as "global warming" and "environmental pollution around" are set up, providing opportunities for all teachers and students to reserve ecological knowledge and cultivate civilized ecological habits. In rich and diverse forms, the concept of ecological civilization is rooted in the hearts of every young student. It has successively cultivated the "common green forest" and "green horse forest" in the campus green area combining ideological culture and ecological civilization. Huang Li, who majored in ideological and political education at the School of Marxism, said, " Every time I pass by the'Qinglin, I will feel the real concept of clear water and green mountains passed on to me, but at the same time, it represents the new force of our young Marxists.”

2022-04-11 Liaoning university of science and technology held "war" epidemic "youth said" theme information of Liaoning university of science and technology held "war" epidemic "youth said" theme speaker Wang Chen) "you are shining the liao university campus colorful light, dribs and drabs, let me unforgettable..." recently, Liaoning university of science and technology youth league committee held "war" epidemic "youth said" theme information in school. Liu Xiaowen, a member of the New Era college students 'theory publicity group, delivered a lecture on the theme of "The epidemic can't steal our youth", which told the touching story of school leaders, teachers, dormitory administrators, canteen staff and student volunteers actively participating in the front line of the fight against the epidemic, presenting the daily life during the closure of Sunshine Music School. Li Fupeng and Li Kaijie, the captain of the youth commando team, respectively told the touching story of the 24-hour escort with the injured foot roommates as a "personal nanny" and the story of the school volunteers. Sun Xiaohua, deputy secretary of the university Party Committee, said that the university's epidemic prevention and control work has entered a critical period, which is a "big test" that cannot be lost. Liao ke adults know heavy load, face up to the difficulties, to show loyalty, dedication and responsibility, for the school to build a protective wall of life and health. The lecture showed the students' rational cognition of the epidemic, their sunny attitude, their feelings of gratitude and their positive spirit.

2022-03-17 Service college students 'employment "five one" service college students' employment "five one" guidance, practice, difficult support, result orientation in five aspects of employment work deployment, from the employment concept, skills, practice three dimensions, focusing on the main business, to the youth demand, build service college students' employment "five one" work system.1. Focusing on highlighting value guidance and building the core value system of employment values for guiding students, the school and college youth league organizations organize both "guidance and supervision" and "docking and contact", and encourage students to take root in the grass-roots employment and entrepreneurship. Carry out "grade customized" employment view micro group class activities to understand, experience, match and achieve occupations, Improve students' development initiative, adaptability and possibility; Open "like young Xi Jinping without regret youth" " youth learning, Employment Dialectic, Learn to Do Employment Guidance with Comrade Xi Jinping, and Students, Please accept your 14th five-year work guide " and other employment ideological and political courses, More than 50,000 online and offline viewers; Carry out the "Western Plan", "Northern Jiangsu Plan", conscription information conference, Nearly 100 graduates have signed up for grassroots programs, Twelve outstanding graduates went to Xinjiang, Tibet, Ningxia and other western regions to carry out volunteer services for one and three years through the "Western Plan" and "Northern Jiangsu Plan" assessment.2. Highlight employment guidance, optimize the employability improvement system, focus on improving students 'employability, combine the employability improvement curriculum with campus cultural activities, and continue to empower and enhance students' employment. More than 500 lectures on "going ahead" employability promotion, Help students to enhance the core competitiveness of employment; To carry out the employment policy interpretation activity of "Jiangsu University Vocational Studio", Career guidance for students; Holding the "Career Future- -mock interview Contest", Provide students with close to real job hunting experience; Establish college student innovation club, innovation leader training camp, entrepreneurship community and other student associations, Launch the "Entrepreneurship class" activities, Invite guests from Suzhou Tiangong Incubator and Global Entrepreneurship Week Campus Center (GCC) with rich experience in operating entrepreneurial enterprises, Provide experience sharing for students who are starting a business or have a business intention in the future.3. Highlight the practice and training, improve the employment practice system to focus on strengthening students' practice and training, based on the big data analysis of graduation direction, to help students to create more internship opportunities. Promote school-enterprise construction, build youth employment and entrepreneurship internship bases in 40 high-quality enterprises, combine the "returning home" social practice, implement the "community registration" system, provide more than 500 government practice internships, create an atmosphere for college students, actively organize students to participate in various high-level innovation and entrepreneurship competitions, promote entrepreneurship through project training, and accelerate entrepreneurship through project incubation.4. Focusing on targeted assistance to graduates with difficulties, we will customize a series of career guidance, and actively recommend employers to them to help students find jobs. Organize 100 full-time and part-time league cadres and 311 graduates of 2021 employment difficulties pair, implement the support process ledger record system; to "online + offline" mode, relying on "micro employment" service youth employment platform, actively collect job demand list, raise 13,478 jobs, actively encourage graduates online resume, hold 21 recruitment activities, "jobs" Jiangsu college students "live" activity 2, invite more than 270 high-quality enterprises to participate. The graduation rate of 2021 was 93.39 percent.5. Highlight the effect evaluation, establish the employment feedback system to focus on the effect evaluation, establish a three-party contact feedback mechanism between the university youth League committee, students and employers, to help students find high-quality employment. Joint recruitment and employment Department of Soochow University, take the initiative to visit the human resources departments of 100 key enterprises, understand the job requirements, sort out job information, and promote supply and demand docking; conduct graduate employment return work, continuously track graduate employment situation, serve student needs, and provide detailed and complete reference opinions for employment guidance. Yu Qianchi, Secretary of the Communist Youth League Committee, Soochow University

2022-03-16 Donghua university youth corps committee: social practice reveal youth bear Donghua university youth corps committee: social practice reveal youth bear wei correspondent Jiang Hao Luo Mei) recently, the university youth corps committee to "please rest assured, power have me" as the theme, the above good national conditions, education as the goal, around the party history learning education, rural revitalization, the Beijing games and volunteer service project, organization guide the whole school member youth feelings in social practice, take practical actions to interpret youth bear. Deepen the party history study and education, ceaselessly red blood. Xiao Juanhong, a 2020 information management and information system major of the School of Science, came to the Yudu River to visit the monument of the Red Army Long March. In the large-scale sculpture "Crossing the Yudu River at Night", the scene of "Uncle Zeng donated longevity materials to build a pontoon bridge" deeply attracted her."The history of the Party is a spiritual treasure house, which has let me absorb the spiritual motivation to forge ahead. I was deeply moved by the deep story of the army and the people in the Soviet area, and I also felt the high revolutionary morale and brave revolutionary spirit of the army and the people in the Soviet area."Xiao Juanhong said. Zhang Nijie, the grade 20 English major of Fuzhou Normal University, walked into the Memorial hall of Pingxiang Anyuan Road Mining Workers' Movement to review the history of the older generation of revolutionaries leading the workers on the revolutionary road in Anyuan. Her heart was strongly shocked. " This is the first independent leadership of the Chinese Communist Party of China and a completely successful strike struggle, which is a feat in the history of the Chinese workers' movement. I sincerely pay my high respect to the revolutionary martyrs."Focus on the Beijing Winter Olympics, leading the sports fashion. Ma Cheng, the 2020 surveying and mapping engineering major of the School of Surveying and Mapping Engineering, was deeply encouraged by the Winter Olympics athletes to challenge the snow mountain summit. He made full use of his professional knowledge, using satellite images and mountaineering track to make mountaineering plans, paid close attention to temperature, precipitation, wind speed and wind direction and other meteorological information, and conducted targeted physical and technical training. At 14:50 on January 22, he successfully climbed the peak of Mount 5072 in Sichuan province, cheering for the Winter Olympics in his own way."The spirit of the Winter Olympics has infected every Chinese people, as college students, we should learn from the Winter Olympic athletes are not afraid of hard, tenacious struggle spirit, walk their own life."Said Ma Cheng. Chen Zhijie, a 2021 applied chemistry major from the College of Chemical Materials, made full use of the winter vacation to learn the knowledge of the Beijing Winter Olympics. Chen Zhijie won the first prize in the 2022 Winter Olympics."In this way, I want to influence and drive the people around me to popularize the knowledge of the Winter Olympics, understand ice and snow sports, and understand the Winter Olympics culture, so as to participate in ice and snow sports."Said Chen Zhijie. We will examine social and public conditions and help promote rural revitalization. Li Jun of the School of Water Environment and his partners formed a research team to carry out the research on "the current status and future outlook of biomass energy utilization in rural areas under the background of 'carbon peak and carbon neutrality' ". Through face-to-face interview and questionnaire survey, to understand the development and utilization of biomass energy in rural areas, and to put forward suggestions for the optimization and upgrading of biomass energy development and utilization in rural areas. Qiu Chenye, who majored in Data Science and Big Data Technology at the Institute of Information Technology, signed up as an intern in the Data Development Center of Honghe Prefecture, Yunnan Province. He used his professional knowledge to participate in the development of basic databases with his professional knowledge."Through the internship, I realized that the deep integration of big data with agriculture and rural areas can inject new impetus into rural revitalization. In the future study, we should be more down-to-earth, constantly improve our professional knowledge and skills, and contribute to the rural revitalization construction of my hometown."College of science level 19 statistics professional Fu Ziyue in xijiang county" childlike innocence harbor " left-behind children's home to carry out the franco-prussian propaganda, she used plain language and the case guide children how to prevent danger, resist infringement, how to be a law-abiding good student, let the left-behind children to enhance the ability to distinguish between right and wrong, improve the self-protection consciousness. Carry out civilized practice and promote the spirit of volunteerism. College 2019 Ma Yunhui in hometown, Xinjiang wusu signed up for the "red scarf small class" activities, in the local new era civilization practice center to carry out the socialist core values education, to children in detail the glorious history of red scarf, the young pioneers knowledge, and vividly tells the story of "bright emblem" of the Xinjiang uncle, also through painting, reading, fun games education guide children set up ambition. To control college LeiSenHua back to home yugan county actively participate in the county bus station to carry out the "warm winter action" volunteer service, for the Spring Festival passengers to provide guide consultation, order maintenance, nucleic acid testing registration convenient and beneficial services, " three days of volunteer service is very full, very happy, let me learn to communicate with people, care for others, also let me more deeply understand the connotation of the volunteer spirit."It is reported that on the premise of strictly observing the COVID-19 prevention and control policies, more than 10,000 students participated in the COVID-19 prevention and control policy. They give full play to their academic characteristics and professional specialties, temper their will, strengthen their character and increase their talents in practice, and practice the youth pledge of "please rest assured of the Party and strengthen the country with me".

2022-03-15 Anhui Medical University held young volunteers public health skills training Anhui Medical University held young volunteers public health skills training network reporter Wang Haihan Wang Lei) " layer upon layer off the protective clothing, again and again disinfection, seemingly simple, in fact, the steps are very complex. When I put on my protective suit, it was already hot. During the epidemic, countless medical workers had to wear it for several hours, which is admirable."Said Sun Tao, a 2021 medical imaging major at the First Clinical Medical College of Anhui Medical University, after experiencing wearing and taking off protective clothing. On March 13, Anhui Medical University held a special public health volunteer skills training class on "Epidemic prevention and Control in Southeast School". The 13 medical staff from the second affiliated hospitals and the fourth Affiliated hospitals of the university brought professional skills training to the 540 volunteers and the class health committee members from each college. The main teacher explained the seven-step washing technique, throat swab collection method, wearing and taking off protective clothing and other details. After watching the demonstration, the volunteers stepped onto the platform to practice, and the teachers commented."The students had a lot of participation, and some students volunteered to practice repeatedly. I found that we all realized the difficulty of epidemic prevention and control, and the frontline medical workers fighting the epidemic expressed more admiration."The school's fourth affiliated hospital head nurse Zhang Yan regrets. Cheng Ying, a student from the University Youth Volunteers Federation, said, " I have participated in the community epidemic prevention and control work many times during the holidays. In the past, I had to do some information registration work. After training, I not only learned how to collect throat swabs, but also learned how to wear and take off my protective clothing correctly. The two words "" "science" and "meticulous" are the deepest impression of Dong Fangyan, a nursing major in grade 2021."When throat swabs are collected, a casual accidental touch and an unscientific wipe will affect the accuracy of the test results. Hundreds of repetitions, all to test the patience of workers. Not to mention the efficiency, the comfort of the recipient, and so on. Salute to the medical workers!"The training is sponsored by the Party Committee of Science and Work Department, the Party Committee of Research and Work Department, the Youth League Committee. The video is produced by Cheng Qi and Tu Huaxin

2022-03-15 Guizhou Qiannan college of science and technology held student photography, painting exhibition Guizhou Qiannan college of science and technology held student photography, painting exhibition China youth network Beijing on March 15 (reporter Zhang Jianwei, correspondent YanChun) to enrich campus cultural activities, active campus art atmosphere, a few days ago, Guizhou Qiannan college of science and technology held student photography, painting exhibition, exquisite exhibition attracted the masses of teachers and students to stop to visit. The picture shows the students recording their favorite works with their cameras. The correspondent offered the picture " the faces of the painting, the details of the food color sketch and the different composition of the photography work... it amazed me that a small picture, a small picture, is like the perfect picture of the upcoming centenary of our group."As a member of the Communist Youth League, Huang Mengyao students felt that after watching the exhibition under the leadership of the Communist Youth League of China, can sit in a clean classroom class, walking in the beautiful campus, frolicking is a happy thing. It is reported that the student art exhibition is divided into two parts: photography exhibition and painting exhibition, a total of 81 works are exhibited, including 51 student photography works, 30 student paintings.

2022-03-14 Shaanxi energy vocational and technical college to learn from lei feng civilization practice volunteer service activities in shaanxi energy vocational and technical college to carry out "learn lei feng tree behavior" civilized practice volunteer service activity Beijing on March 14 (reporter Zhang Jianwei, correspondent often lu), initiated by shaanxi energy vocational and technical college youth corps committee, coal and chemical industry college to undertake, to "learn from lei feng tree behavior" as the theme of volunteer service activities in Xianyang weigarden community. The school 50 young volunteers, Xianyang Wisdom enjoy billion environmental protection propagandists and Wenyuan community staff to participate in the activity. Mobile phone repair service. On the same day, the volunteers gathered together and went to Wenlin Community and Xiyan Railway Community respectively, and began to arrange the activity site. The activity set up community free clinic, garbage classification, mobile phone maintenance, photography and other convenient services. Garbage classification knowledge publicity. Volunteers measure free blood pressure, massage and moxibustion, provide health consultation services; publicize epidemic prevention knowledge and garbage classification knowledge in the community; help the elderly to clean up and repair their mobile phones, and teach them to use WeChat, TikTok and other software. The students of the photography group used the photos to record the beautiful moments and freeze everyone's happy smile. Play chess with the old man. Volunteer Yuan Hao is our school chess good lover, accompanied an old man to play three sets of chess, the result of three sets are lost."Uncle's chess age is older than I am. I am still proud of my defeat."Yuan Hao said that he would come to the community to accompany the elderly to play chess on weekends. At the same time, the volunteers also provided household services, under the leadership of the Wenyuan community staff, came to the home of the elderly, measure their blood pressure, chat, to help the elderly clean up, tidy up the room. Since the Central Committee of the Communist Youth League launched the community Youth action, the Youth League Committee of the College has played a role as a bridge and built a social practice cooperation unit with Wenyuan Community. This activity, taking the "Learn from Lei Feng" activity month as an opportunity, aims to strengthen the communication between the youth and the community through medical treatment, environmental protection and convenient services into the community, and further promote the implementation of the community youth tour.

2022-03-14 Southwest University of Finance and Economics students return home winter vacation social practice to sell local products to overseas Southwest University of Finance and Economics students return home winter vacation social practice to sell local products to overseas youth network reporter Wang Xinxin) in the past winter vacation, Southwestern University of Finance and Economics junior Li Yu Chen had a busy and full. In response to the call of the local youth league organization, he gave full play to his professional expertise, and devoted himself to the social practice of "returning to his hometown" in the form of opening a shop and starting a business, and sold the local products of his hometown overseas. For a while, his story was widely told in his hometown. On the day when he left home and returned to school, he also rushed to the "Rongxian Rural Agricultural and sideline Products Business Department" opened in Xuyang Town, Rongxian County, Sichuan Province, checking the turnover of 1029.5 yuan in the morning, and repeatedly told the "bartender" to guard the shop, pay close attention to the sales information and timely delivery. Li Yu Chen shop sales of products. Jiang Bing for the picture in this business department, with Li Yu Chen winter vacation business practice story, but also has his use of the "international business" knowledge to help his hometown rural revitalization feelings. Li Yu Chen is a student of international Business School of Southwestern University of Finance and Economics, he responded to the Communist Youth League college students "return to home" social practice call, immediately devoted himself to the "return to home" business practice activities in early January."We will consolidate our achievements in poverty alleviation and help rural revitalization."Li Yu Chen in such ideas, opened the winter vacation entrepreneurship practice. On the first day after the holiday, he made a business plan to manage the second day, he thought of the summer "three to the countryside" chili sauce social practice in 2020, and carried out market research and analysis. On the third day, he walked into the 30,000 mu pepper planting base in Ledu Town, Rongxian County, went into the greenhouse to understand the varieties, quality, production and sales, and then to the pepper sauce production factory in Shau Kizui Village, Lide Town, rolled up his sleeves to experience fried pepper, broken pepper, making pepper sauce, packaging and handling, and became a worker. Li Yu Chen in the pepper seedlings to understand the planting situation. After the inspection, he took out more than 10 years of his parents gave him a scholarship and lucky money, rental shops, business license, tax registration, food small business shop (food sales) record certificate, became a self-employed. At the same time, registered Taobao store, adopt the combination of online and offline mode of Rongxian local specialty "Shau Kei mouth chili sauce". Offline open business department, individual customer retail, supermarket wholesale, door-to-door delivery, he is busy from morning to night; online sales, registered Taobao store, TikTok shop, micro store, QQ shop, he personally. In order to do a good job in product promotion, he also specially invited the local web celebrity team, into the production workshop live broadcast with goods, shooting video online publicity. On the same day of the live broadcast, I received orders one after another, and opened the shop for one month, selling more than 2,000 bottles in total, and achieving a turnover of more than 30,000 yuan. Li Yu Chen is in the production workshop. Jiang Bing for figure Li Yu Chen said, online sales to sell the hometown specialty to the north, Guangzhou, also sold to the UK, France, the United States, Japan, Malaysia Chinese circle, let exotic Chinese taste home, Chinese flavor, also let the overseas Chinese understand the "rong county", is very helpful to promote home popularity."We never thought that the chili sauce produced in poor villages could still be sold abroad, so that overseas Chinese can eat a 'happy flavor'."Lide town Party Secretary Deng Rongzhong quite regrets. Li Yu Chen said, more than a month of entrepreneurial practice, there are three gains: one is the use of "international business" expertise and communication platform, "Shau Kui chili sauce" in the village is exported to all over the country, the second is to open the physical business department and online sales stores, accumulated operation and management knowledge; the third is through the experience of entrepreneurs."In the future, I will continue to operate, manage them remotely during my school years, and return to my hometown to manage my shops during holidays, so as to further expand my sales channels and contribute to the rural revitalization."Li Yu Chen said.

2022-03-12 Anhui Engineering University, reporter Wang Haihan) More than 2,000 young entrepreneurs have settled in Yin Lingang Art Creative Town, which has become the practice base of Anhui Engineering University. The town is located in � b District, Wuhu Bay, Anhui province, with an old workshop transformed into an art workshop. Art candidates can practice sketching here, and the relevant departments and colleges of Anhui Engineering University's entrepreneurship project roadshow, e-commerce forum, maker salon and training camp, policy publicity and other activities are also carried out regularly here. Recently, Anhui University of Technology is working with Yingang Art Innovation Town on the construction and acceptance of the "three complete education" practice education base, to understand the spatial layout, characteristic industrial agglomeration, innovation and entrepreneurship of the town. In October 2021, the School of Humanities of Anhui Engineering University signed a cooperation agreement with the town operation unit to jointly build a graduate practice education base. Liu Jun, president of the School of Humanities, said that the two sides should step up cooperation in cultural governance, rural governance and cultural development to provide talent support for comprehensively improving industry-university-research cooperation. At the end of last year, Anhui University of Technology conducted exchanges and discussions on the comprehensive reform pilot of "three comprehensive education", professional practice base construction, innovation and entrepreneurship base construction, quality expansion base construction, and art practice base construction. Su Guohong, deputy party secretary of the university, said, social practice activities should be combined with professional learning, employment and entrepreneurship to improve the "second classroom" of school-local cooperation."Relying on the advantages and practical experience of Yin Lingang Town, we will optimize the curriculum system of practical education, and make efforts in the standards, teachers, courses, achievements and projects of practical education."She said that in recent years, the school has promoted the establishment of art and design, architecture, food, chemistry, textile and other majors in Yin-Lingang Creative Town, allowing students to indirectly participate in rural revitalization and urban construction."The school holds more than 10 activities such as Party school training, research, green horse training and quality development activities in the Lingang Town Practice Base every year. In the past two years, a group of graduates have chosen to stay in Wuhu and small towns to work and start businesses."Su Guohong also suggested that we should explore the town education resources, create practical courses such as innovation, entrepreneurship and rural revitalization, promote the planning and suggestions of various functional departments to take root, build the education brand, and improve the talent training and evaluation system of the practice base.

2022-03-10 Winter vacation can assign, practice spring your university power home development winter vacation can practice spring your university can help home development JianWei correspondent, Liu Yanchun, Song Hongqing Gou Mengmeng) to meet the arrival of the arrival of the centenary of the founding of the communist youth league, Guizhou university youth corps committee carefully organized the 2022 "return home" winter vacation social practice, lead your big youth promote social skills in practice, active in home construction, bear youth, draw strength. Looking for red resources to consolidate the history of the party learning education achievements in order to further consolidate the history of the party history learning education achievements, 2022 winter vacation social practice, your university teachers and students use hometown red resources, online linkage, organization to retrace red footprints, interview red people, connect red culture and other activities, practice continuously red blood youth oath. The "Red Dream" went through the field visit, study, preach, local red culture and century-old veterans of the Red March. The team visited the Long March to complete the biographies, research reports and research videos. The picture shows the practitioners visiting the veterans of the Anti-Japanese War. Correspondent for figure youth power the games volunteer service dedication youth power during the winter vacation when the Beijing Olympics, Guizhou university youth corps committee relying on a new media platform actively build the games atmosphere, popularize knowledge of the games, organize teachers and students to participate in the "welcome the games, I play" activities, guide students to feel the games culture, to participate in the games volunteer service. Wang Yashu from the School of Public Management passed the assessment to become a volunteer of the Winter Olympics City in Zhangjiakou Competition area. In the 20-day volunteer service, she played the song of youth dedication with enthusiasm, kindness and patience, and interpreted the social responsibility of the youth in the new era with practical actions. In order to consolidate the achievements of COVID-19 prevention and control and build a strong safety barrier for COVID-19 vaccine, your students devoted himself to the epidemic prevention work in your hometown communities, bus stations, sub-district offices and other places, writing the responsibility and responsibility of young people in the new era. The day before the city-wide nucleic acid screening in Tianjin, Wang Wei � u, an active member of the Party, submitted an application for volunteer service. Carrying supplies, maintaining order, answering questions, Wang Wei � u devoted himself. Zhang Yilian joined the epidemic prevention and control volunteers to register information and take their temperature at the bus station, and assist medical staff to do nucleic acid tests. The picture shows the photos of volunteer services for epidemic prevention and control. The social practice of "returning to my hometown" is deeply practiced in 2022. Through colorful practical activities, the majority of young people can deeply feel the new changes in their hometown, better understand their hometown, and then love it and build their hometown. The picture shows the activity of writing and sending Spring Festival couplets. Yangming College Yuan Shuang Shuang participated in the "Spring Festival couplets" activities, the love for hometown, dating kindness, into blessings to thousands of families; after the students said, "the rural river pollution control is profound responsibility, I will have an opportunity to contribute to the hometown river pollution control from what I have learned".

2022-03-08 The Research Branch Group of Nanjing University of Posts and Telecommunications opens the "first lesson of the new semester".) To deeply learn and apply Xi Jinping Thought on Socialism with Chinese Characteristics for a New Era, Closely around the fundamental task of cultivating moral education, Help to improve the education system of moral, intellectual, physical, aesthetic and labor comprehensive training, Promote the healthy growth and comprehensive development of students, At the beginning of spring 2022, Nanjing university of post and telecommunications 23rd graduate teaching group members respectively in the Xinjiang production and construction corps 8 division Shihezi city 29 middle school, Qinghai province, Guizhou Tongren tujia autonomous county along the river plate field town junior middle school and Sichuan Dazhu County second middle school attentively built a bacon cast soul, "the first lesson" theme education practice. The 23rd Research Corps, corps branch of Nanjing University of Posts and Telecommunications in Shihezi City 29 Middle School to carry out the theme of "young strong China strong" young pioneers, leading the young pioneers to study the history of the Party, cultivate patriotism. At the team meeting, the members of the corps detachment first led the students to review the oath of joining the team and sing the team song of the Chinese Young Pioneers, thus enhancing the determination of the young Pioneers to stay true to their original aspiration and follow the Party and strive to be the successors of communism. And use the video and courseware to lead the students to review the history of the young pioneers, understand the nature of the young pioneers, purpose and logo of the young pioneers, combining the young pioneers correct salute, standard red scarf wearing and party history knowledge quiz, free speech, guide all the young pioneers learn advanced example, practice the socialist core values, efforts to become the builders and successors of the cause of socialism with Chinese characteristics. Carry forward the lei feng's spirit, a fascinating volunteer civilization education in Qinghai team will the seeds of lei feng spirit is rooted in the students' heart, put the lei feng spirit broadcast in the west of the motherland, establish Qinghai province national middle school "youth" volunteer service community, in the "first lesson" played song, revisit beginner's mind, solemn oath, with a "hand" badge writing dedication of youth commitment."Make a drop of water in the sea", "do a screw that will never rust", the vivid volunteer civilization education leads the majority of the Communist Youth League members and young volunteers to do a good thing for the class, do a good thing for the school, do a good thing for the society, with full of struggle attitude, let the spirit of Lei Feng from generation to generation. With a live "Winter Olympics" material, A unique ideological and political education Guizhou detachment effectively activated the spirit of the Winter Olympics education elements, Deeply excavating the valuable educational value in the spirit of the Winter Olympics, The combination of ideological and political education, Promote the spirit of sports culture, In the "first lesson" through reviewing the wonderful, concentrated propaganda, free sharing and other forms to guide students to learn the Olympic knowledge, exchange athlete stories, In a way that students are willing to accept, lively, and happy, The "fighting spirit", "Chinese confidence", "openness and inclusiveness" and other Winter Olympic spirit imprint firmly engraved in the hearts of students, Let the students in the new era to feel and truly practice the spirit of the Beijing power of the Winter Olympics, Join hands for the future in the new spring semester. The Sichuan team of an interesting mental health education, focusing on mental health education, has carried out a "first lesson" with temperature, depth and strength, to help students "heart to the sunshine". Classroom on the basis of adolescent psychological needs and personality characteristics, around "know yourself, find yourself, believe in yourself" three dimensions, lead students feel infinite potential, draw confidence strength in example story, in the classmate evaluation to face their own shortcomings, digging in the self cognition, to "love education" to help students build a "protective wall" of mental health. A full of personality "highlight card", a heartfelt "I am really good", the students in the classroom correctly understand themselves, brave embrace confidence, with a positive attitude and full state to meet the challenge of the new semester. Li Li forge ahead spectrum new chapter, dream sail set sail again. The new semester of the 23rd Volunteer Teaching Group of Graduate Students of Nanjing University of Posts and Telecommunications starts with the "first lesson", encouraging itself with the spirit of "trust reaching the world, unremitting self-improvement", helping students, casting dreams and educating people, serving the rural revitalization, and welcoming the 20th victory of the Party with outstanding achievements.

2022-03-07 Liaocheng university: "5 minutes micro education courses" open learn lei feng anniversary Liaocheng university: "5 minutes micro education courses" open learn lei feng anniversary XingTing) on March 4, the 59th "learn lei feng anniversary" and the 23rd "Chinese youth volunteer service day" approaching, Liaocheng university school of marxism firmly grasp the classroom main channel, with "lei feng spirit and volunteer service" as the theme of education activities, elaborate "5 minutes micro ideological courses". In class, under the guidance of the teacher, the students sing "Learn from Lei Feng", enjoy Lei Feng's dedication, listen to the youth volunteer service deeds of Liaocheng University, stimulate the sense of pride and responsibility; fill in the "Carry forward Lei Feng spirit, practice volunteer service" personal wish card, solemnly write down their recent volunteer service plan, to promote the dedication to take root. After class, college students said, " The short five or six-minute class made us feel that the anniversary of Learning from Lei Feng and the China Youth Volunteer Service Day are more ceremonial, and it also made us like this micro-ideological and political class."" In the heated discussion of what we have learned from Lei Feng, we really felt the significance of Lei Feng's spirit in the new era, and ignited the enthusiasm for volunteer service."" In order to expand the micro class, we also recorded a video to promote the Lei Feng spirit, leading you to appreciate Lei Feng's style, and discuss how to practice the Lei Feng spirit in the new era. At present, it has cooperated with the school Youth League Committee to launch multiple platforms, which can not only serve as an effective carrier of ideological and political education for students, but also radiate a wider audience."Teacher Liu Linlin said. In 2022, Liaocheng university to implement the major festival anniversary ideological and political education calendar, including 36 major festival anniversary, as an educational, leading and cultural value carrier, school departments, college linkage, as an opportunity and the carrier to promote ideological and political education, strengthen the college students' ideological understanding of socialist core values and emotional identity, strive to realize the political guidance and value shaping of college students.

2022-03-03 Strengthen tracking guidance and extending student employment service after graduation Strengthen tracking guidance and leading the process of extending student employment service after graduation ", forming a working pattern of full employment and promoting employment. In 2021, the initial employment rate of school graduates was 96.25%, and the year-end employment rate was 98.9%, ranking among the top in Jiangsu Province. From the "face-to-face help" universal service to the "point-to-point help" refined service upgrade. School youth league for two consecutive years and Wuxi municipal party committee jointly held "thousands of enterprises" "post through train" activities, Jiangsu, panasonic energy and other well-known enterprises to participate in live JianGang, the scene for school graduates employment internship jobs 264, and more than 170 enterprises cooperation to participate in online recruitment, for tin job graduates provides more than 8000 jobs, better meet the needs of the graduates to jobs. From pre-graduation employment promotion to post-graduation tracking guidance extension. The school of 13 full-time and part-time youth league cadres pair of employment difficulties of 33 students. Through career planning guidance and employment information recommendation, the employment problems were solved in a regular, targeted and relay manner. Among them, the employment rate of the graduates in 2021 reached 100%. Teacher career guidance and peer lead and driving force. The school has carried out employment guidance and service activities such as "Le" career guidance month, career planning competition, employment core ability training, "100-Day Sprint" employment promotion action and post-graduation tracking service. At the same time, it also gave full play to the driving effect of peers, organized a series of lectures of outstanding alumni "Alma mater Appointment" and a series of sharing meetings of "Growth back from internship", and guided the graduates to actively join the army in response to the call of the state through the National flag Guard and the Olive Green Military Association. In 2021,157 students signed up to join the army. 45 students entered the barracks after physical examination and political examination. The Communist Youth League of the school regards helping the transformation of innovation and entrepreneurship projects as a stone to improve young cadres to solve practical problems. First, relying on the running characteristics of the school and professional advantages to explore and cultivate competition projects, strive for technical support and industry recognition; second, increase the cultivation and assistance of youth entrepreneurship projects and alumni enterprises highlighting professional advantages, and strive for the transformation of resources and landing platform. Since 2019, the university has won 37 awards in innovation and entrepreneurship competitions at or above the provincial level. The youth League Committee and the relevant departments of the university have provided entrepreneurship venues, technical teacher assistance, business mentor configuration, product and project promotion services, alumni enterprise application, e-commerce platform promotion and other measures. Zheng Yueyuan, Deputy Secretary of the Youth League Committee of Wuxi Vocational and Technical College, Source: China Youth Daily, March 03,2022 edition, edition 05

2022-03-03 Create "1234" College students employment assistance project to build "1234" college students employment assistance project joint construction, innovative employment guidance methods and carrier construction, broaden the channels for multiple employment measures, focus on the implementation of "1234" college students employment assistance project. To build an online employment cloud service platform. Through cloud service platforms such as "Tuan Tuan Micro Employment", extensive contacts between employers and job-seeking students have been established to make the employment information smooth and efficient and provide an information platform for students to seek jobs and employment. In the fall of 2021, more than 3,500 job information through online platforms. Build two major carriers. Every year, Changzhou University holds a special live job recommendation activity for college students in Jiangsu Province, which provides employment services for graduates, especially graduates from poor families, through the combination of online live job recommendation and offline on-site recruitment. In the autumn of 2021,12 post recommendation activities were held, and the offline venue brought together more than 600 well-known enterprises inside and outside the province, bringing more than 6,700 jobs. Online live broadcast venue, a number of high-quality enterprises were invited to live broadcast lectures on the online platform for college students in the province, and some students have reached a preliminary employment intention with the recruitment enterprises. Focus on three types of key student groups to help. Through MoPai inputting tent card in our school, the basic situation of ethnic minorities and disabled students, to carry out targeted support measures, established by inputting tent card unemployed college students cadres one-on-one-on-one pair support mechanism, college two levels of 28 cadres docking 22 graduates, strengthen personalized employment guidance, real-time tracking of their job employment situation. We will expand the four types of employment channels. Based on the characteristics of the integration of petroleum and petrochemical, industry and education in our school, we vigorously promote the policy publicity of western plan and northern Jiangsu Plan, encourage the majority of young people to go to the grass-roots level and increase their talents. By encouraging entrepreneurial college students to carry out special actions to promote employment, more college students to get employment smoothly. In 2021,7 students from northern Jiangsu and 8 from western China and 40 college students. Gu Xianpeng, Deputy Secretary of the Youth League Committee of Changzhou University, is available from China Youth Daily

2022-03-03 Since 2020, the education case of Jiangsu Institute of Technology helping college students find jobs and become talented people has been listed on the national media platform twice. One is "People's Daily" to " graduate Su Haichao looking for a job, no problem! The story of Su Haichao, a 2020 graduate, looking for a job, is about his failure in the postgraduate entrance examination and the most difficult employment season under the influence of the epidemic in 2020, and finally found his favorite job through many efforts. The Inner Mongolia boy, who majored in communication engineering, has participated in many discipline competitions at school, and was determined to become a compound talent with both technology and management. However, when he failed in the postgraduate entrance examination, Hai Chao found that he had missed many opportunities for campus autumn recruitment and civil service examination. Meanwhile, affected by the epidemic, the demand for spring recruitment positions was reduced, and the employment channels were narrowed, so he felt under great pressure to find a job. Fortunately, our youth League cadres, employment instructors, counselors, professional mentors and class teachers collectively work together to care about the job hunting problems of college students with employment difficulties, providing them with interview guidance, push employment information, relieve psychological pressure and so on. After many efforts, Su Haichao was employed by a number of enterprises at the same time, and he finally chose a professional counterpart company connected by the school Youth League Committee- -Su Xiuwen Electric Energy. In just over a year, Su Haichao has grown into a legal person of a subsidiary company of Xiuwen Electric Power, and can independently lead the team to complete the research and development, manufacturing and sales of the equipment. Under the drive of Su Haichao, a total of 15 college students who have worked in the Student Union have entered Suwen Electric Power for employment, and their working ability and comprehensive quality are highly recognized by the enterprise. Second, the case of Xiao Dingmeng, a 2021 graduate of our school, who registered for the "Western Plan" to participate in the grassroots construction in the report of the "Guangming Daily" client titled "If I Want to know the motherland, I must first go to the front line.". Xiao Dingmeng is the president of the student Union of our school. She is a young political backbone who grew up in the league and school work. Due to some reasons, she failed to apply for her preferred employment position successfully, and she was once very upset. But because he has some experience and ability of participation in grassroots social governance, as the school student union President, province, the "green horse" students, participated in "central" a program under which officials "summer social practice group project, participated in hometown epidemic prevention and control volunteer service and won recognition, the school encouraged him to try to prepare to sign up" western plan " volunteer service projects, and give theory and interview guidance, finally XiaoDing Meng selected, currently in Guangyuan city, Sichuan province zhaohua tiger jump town people's government work. Due to his accumulated experience of youth league school work during the school period, he was selected by the local youth league organization to start to work in the youth league. What impressed us deeply was that when he first started working, he participated in the local establishment of the Lizhou District College Students Union and sent the conference photos to the teachers of the school Youth League Committee. He said: whenever he participated in these work as a cadre, will think of the teacher of the Youth League to accompany the student Union to carry out the situation. This is a story from the Communist Youth League to the Communist Youth League. The student cadres trained by the Communist Youth League returned to the Communist Youth League team, and eventually grew into a young post-00s league cadre, inheriting the cause of the Communist Youth League. Changzhou University focuses on the implementation of the "1234" college student employment assistance project by strengthening the organization and leadership, strengthening the employment work team construction, innovating the employment guidance methods and carrier construction, and broadening the channels for diversified employment. To build an online employment cloud service platform. Through cloud service platforms such as "Tuan Tuan Micro Employment", extensive contacts between employers and job-seeking students have been established to make the employment information smooth and efficient and provide an information platform for students to seek jobs and employment. In the fall of 2021, more than 3,500 job information through online platforms. Build two major carriers. Every year, Changzhou University holds a special live job recommendation activity for college students in Jiangsu Province, which provides employment services for graduates, especially graduates from poor families, through the combination of online live job recommendation and offline on-site recruitment. In the autumn of 2021,12 post recommendation activities were held, and the offline venue brought together more than 600 well-known enterprises inside and outside the province, bringing more than 6,700 jobs. Online live broadcast venue, a number of high-quality enterprises were invited to live broadcast lectures on the online platform for college students in the province, and some students have reached a preliminary employment intention with the recruitment enterprises. Focus on three types of key student groups to help. Through MoPai inputting tent card in our school, ethnic minorities and the basic situation of the disabled students, to carry out targeted support measures, established by inputting tent card unemployed college students cadres one-on-one pair support mechanism, college two levels of 28 cadres docking 22 graduates, strengthen personalized employment guidance, real-time tracking their job employment situation. We will expand the four types of employment channels. Based on the characteristics of the integration of petroleum and petrochemical, industry and education in our school, we vigorously promote the policy publicity of western plan and northern Jiangsu Plan, encourage the majority of young people to go to the grass-roots level and increase their talents. By encouraging entrepreneurial college students to carry out special actions to promote employment, more college students to get employment smoothly. In 2021,7 students from northern Jiangsu and 8 from western China and 40 college students. Wuxi Vocational and Technical College, Deputy Secretary of the Youth League Committee of Wuxi Vocational and Technical College, has fully implemented the "top job process" of employment work, forming a working pattern of all staff grasping employment and promoting employment. In 2021, the initial employment rate of school graduates was 96.25%, and the year-end employment rate was 98.9%, ranking among the top in Jiangsu Province. From the "face-to-face help" universal service to the "point-to-point help" refined service upgrade. School youth league for two consecutive years and Wuxi municipal party committee jointly held "thousands of enterprises" "post through train" activities, Jiangsu, panasonic energy and other well-known enterprises to participate in live JianGang, the scene for school graduates employment internship jobs 264, and more than 170 enterprises cooperation to participate in online recruitment, for tin job graduates provides more than 8000 jobs, better meet the needs of the graduates to jobs. From pre-graduation employment promotion to post-graduation tracking guidance extension. The school of 13 full-time and part-time youth league cadres pair of employment difficulties of 33 students. Through career planning guidance and employment information recommendation, the employment problems were solved in a regular, targeted and relay manner. Among them, the employment rate of the graduates in 2021 reached 100%. Teacher career guidance and peer lead and driving force. The school has carried out employment guidance and service activities such as "Le" career guidance month, career planning competition, employment core ability training, "100-Day Sprint" employment promotion action and post-graduation tracking service. At the same time, it also gave full play to the driving effect of peers, organized a series of lectures of outstanding alumni "Alma mater Appointment" and a series of sharing meetings of "Growth back from internship", and guided the graduates to actively join the army in response to the call of the state through the National flag Guard and the Olive Green Military Association. In 2021,157 students signed up to join the army. 45 students entered the barracks after physical examination and political examination. The Communist Youth League of the school regards helping the transformation of innovation and entrepreneurship projects as a stone to improve young cadres to solve practical problems. First, relying on the running characteristics of the school and professional advantages to explore and cultivate competition projects, strive for technical support and industry recognition; second, increase the cultivation and assistance of youth entrepreneurship projects and alumni enterprises highlighting professional advantages, and strive for the transformation of resources and landing platform. Since 2019, the university has won 37 awards in innovation and entrepreneurship competitions at or above the provincial level. The youth League Committee and the relevant departments of the university have provided entrepreneurship venues, technical teacher assistance, business mentor configuration, product and project promotion services, alumni enterprise application, e-commerce platform promotion and other measures.―――――――――― "a branch of a famous" help students 'employment, Changzhou mechanical and electrical vocational and technical college youth league secretary since 2019, our school in every new league branch implementation "a branch" school hand in hand, combining youth pilot engineering, build double main sports mechanism, by the enterprise human resources manager as a part-time teacher in charge, responsible for students' professional planning, professional quality and career culture lead, training, enterprise technical director is responsible for the professional knowledge and technical skills points and education. Organize each new youth league branch to carry out the professional education, experience the career situation, and feel the corporate culture. Changzhou Bo Rui Electric Power and other 28 enterprises have come into the class to carry out the theme group day activities, to help students to adjust to themselves as soon as possible, clear the development path, and do a good job of career planning. The school Youth League Committee has created a "one student and one special project" vocational skills upgrading project for special students. Starting from 2021, special skills training for students from poor families, recommended part-time jobs and vocational skills upgrading training for students from poor families and point-to-point services. In the form of the work, the Communist Youth League of the school insists on the combination of "please come in" and "going out".one side, Regularly invite enterprise experts, skilled craftsmen, successful alumni and so on to the school, Combined with corporate culture, craftsman spirit, innovation and entrepreneurship and other themes to carry out the "electromechanical forum", at the same time, Establish cooperation with local well-known enterprises, Encourage enterprises to set up order classes and enterprise scholarships in the campus; on the other hand, Organizing students to enter enterprises as an important part of professional education, career planning and employment and entrepreneurship education for students, Regularly organize school students into zhongtian Iron and Steel, CRRC Group, Hengli Hydraulic and other well-known enterprises, Develop career experience, Feel the corporate culture, The university Communist Youth League adheres to the combination of "professional learning" and "social practice". As an engineering higher vocational college with distinctive intelligent equipment manufacturing majors, the Youth League Committee encourages students to carry out the social practice of returning to their hometown in combination with their majors, and gives key support to excellent projects with a high degree of integration with their majors through the project approval and evaluation. For example, organize e-commerce students to carry out "live help farmers" activities, help farmers do agricultural products online sales, build agricultural products characteristic pavilion, promote rural e-commerce standardization construction, brand development; to "rooted in fertile soil, write youth" theme, guide agricultural machinery students to actively participate in rural revitalization, deep into rural fields, popularize plant protection UAV knowledge, maintain agricultural machinery, experience new agricultural machinery development... the Communist Youth League adhere to the "universal service" and "accurate help" combination. One is the joint school-enterprise cooperation office, in May and November to invite quality cooperative enterprises to all students to carry out "school enterprises" live recommendation activities, offline help to practice campus students to implement the post, online to help has not implement jobs or willing to change work unit students secondary employment; the second is to carry out full-time cadres pairing support action, implement the "life case" responsibility mechanism, to document tent card family economic difficulties graduates, especially A students, disabled graduates, priority recommend jobs, let students more gain through accurate employment services. China Youth Daily, China Youth Daily, March 03,2022

2022-03-03 Ningbo institute of finance and economics: when the Asian games volunteers "green lotus" Ningbo institute of finance and economics: when the Asian games volunteers "green lotus" Jiang Yutong correspondent hai-feng zhang) on February 28 solstice on March 3, Ningbo institute of finance Asian games volunteers recruited into the second round of selection, enter the interview stage of 346 teachers and students from nine colleges, including English, Japanese, Korean and other languages. Ningbo University of Finance and Economics Hangzhou Asian Games volunteer interview and selection site. Not long ago, the young volunteers of Ningbo University of Finance and Economics successfully completed the volunteer service guarantee work of the 14th National Games Sailing Competition (Ningbo Competition Division). The Youth League Committee of the college carefully planned the volunteer recruitment and publicity activities of Hangzhou Asian Games, and actively mobilized all the teachers and students to participate in the Asian Games volunteer service work of Asian Games, endurance "Little green fan", and strive to be "Little lotus". Wu Zhenyu, a sophomore senior student from Ningbo University of Finance and Economics, once participated in the military parade celebrating the 70th anniversary of the founding of the People's Republic of China in 2019."There is a saying in the army that 'discharge does not fade'. If I can be successfully selected, I will definitely complete the volunteer service work in accordance with the high standards and high requirements of the mission in the army."Miao Xiaoxuan, a junior student, has accumulated 530 hours of volunteer service time at school. Liu Beni, a junior majoring in business administration, hopes to use her dancing skills to show the youth of Chinese college students to the world on behalf of the school. It is reported that all the Hangzhou Asian Games volunteer interview and selection will be completed by March 10, after the selection will participate in the general training, and the recruitment will be completed by the end of June. Before the post in September, venue training and post drills will be conducted to ensure that volunteers serve the competition at a professional and standardized level.

2022-02-24 Hometown: "3 D" class: "3 D" class Jianwei correspondent Wang Chong Yi Song Chen Zhaoxuan) " I have to cover with my hand, or the glue will solidify."Zhang Sichen, from the School of Economics and Management of East China University of Technology, posted anti-fraud posters outside more than minus 20 degrees Celsius. During the holiday, more than 200 youth members dedicated silently in the streets, towns, transportation centers and civilized practice centers, feeling the vivid group class outside the campus. Xiao Yong, a student of 2019, returned home in the winter vacation and conducted disinfection work at a quarantine hotel in Ganzhou. Carrying 18 kilograms of disinfectant and wearing heavy protective clothing, he struggled to lift his hand, and the goggles were often covered with fog. Even so, he still sticks to it and does a good job. "Moving forward" is his working attitude on the front line of epidemic prevention."I cook a meal for my parents", "Focus on the Beijing Winter Olympics, leading the healthy fashion"... a series of "down to earth" "classroom homework" has aroused the enthusiasm of the youth league members of the college. Hu Huixuan, of the class of 2021, plays in in sports every morning. From fine housework to popularizing various knowledge for the community residents, her winter vacation life is full and meaningful."The General Pavilion is located in Xingguo County, Jiangxi Province. The pavilion reproduces the heroic deeds of the founding generals in the form of pictures, physical objects and characters."Said Wang Junpeng, a student of grade 2021. This is the college youth League committee of the winter vacation homework "highlight", red books, red movies, field studies, watching the movie "Changjin Lake", reading books "red rock", online "cloud tour" jinggangshan... through a variety of online and offline practice study tour, the majority of youth members actively complete the "red homework", feeling the red spirit, continuously red blood."Through completing these 'homework', I feel the parents are not easy, but also realized the strength of the motherland, more deeply realized that it is the blood and tears of the ancestors in exchange for today's peaceful life."During the winter vacation, under the guidance of the college Youth League Committee and the Student Union, our 'second class' became more colorful, and these 'courses' were very memorable."East China university of technology, economics and management youth corps committee in compliance with the provisions of COVID-19 epidemic prevention and control regulations, formulated the member youth winter vacation to participate in volunteer service work guide, encourage guide college youth return home, does the practical work, extensive outbreak zone spreading, township community labor practice, Spring Festival warm winter action, red knowledge, etc., play the advantages of the communist youth league organization mobilization and practice education, with the book," grassroots "" practice "," and "masses" for the youth "serve people, contribute country" vivid lesson.

2022-02-24 Xi 'an jiaotong university: with youth "light" light village hope xi' an jiaotong university: with youth "light" light village hope jianwei correspondent, Cui Yike, Zhou Chunyan, Liu Yifei Huang Bin) Xi'an jiaotong university in every winter and summer vacation held "light" public online volunteer teaching activities has been successfully completed 3 periods. Volunteers from many universities across the country have become "low light", providing personalized learning guidance and diversified public courses for rural students, turning knowledge into energy, and illuminating people far away with science and technology. The third phase of "Micro Light" volunteer teaching follows the training method of "one-to-one question answering" and public courses. In view of the students' weaknesses, the volunteers provide one-to-one tutoring to the students, and maintain two-way communication with the students and their parents in the process, to ensure that the volunteer teaching achieves practical results. Li Xiaojuan, a volunteer, said, " I will run in with my students to enhance their relationship, so that the subsequent answering questions or student psychological counseling can go more smoothly."Through exploration and communication, the volunteers and the students gradually formed a mutual adaptation mode of sharing and communication. Student Ping Xiangqi said when mentioning one-to-one q & A:" Online class freedom is relatively high, I can ask questions at any time, I feel I can learn a lot."In addition to" one-to-one answering questions ", the online volunteer teaching also offers a variety of public courses for students and parents, including university introduction, English phonetic standards, space knowledge science, media literacy, 100,000 medical lessons, and pioneer party members in the fight against COVID-19. Yao Qianshen, a student, said, " I invited my classmates to participate in this activity. Their comments are all good. We all hope to volunteer here in the future."Wang Fengjuan, a student, wrote in a letter to volunteer Ma Lei:" I didn't dare to speak English before. These days, I gradually let up under the leadership of my teacher. Teachers gave targeted questions I didn't understand in school, which helped me a lot."" I know that there is a gap between urban and rural educational resources, but this volunteer teaching has exposed the gap to me for the first time. Although poverty alleviation has been achieved, rural revitalization still has a long way to go."Li Yijing, a volunteer, sighed after 16 days of volunteer teaching. In addition to Li Yijing, many volunteers also actively shared their feelings. Bai Xiaoyu said, " During the epidemic period, I could not teach around my classmates. Online volunteer teaching has greatly weakened the infreedom of the region and time, allowing me to help my students in need. Volunteer teaching will not stop, and my love will not be cut back. At present, "" Micro Light " volunteer teaching has been successfully held for three periods, recruiting more than 500 volunteers from more than 30 universities at home and abroad, and supporting more than 500 rural students in nearly 20 provinces in China. The second issue formed an academic paper, trying to comprehensively analyze the cloud volunteer teaching of college students from the perspective of communication science, and put forward the optimization path. In the third phase, the education situation of the students' villages and towns was understood in the form of interviews, and three research reports were formed from different professional perspectives to summarize the problems and experience.

2022-02-24 Jiangnan university: facing new forms, chain employment support for new forms, chain employment support set up the correct employment values, on the macro level, promote the career and employment guidance required course content into the youth corps cell theme lesson education, set up jiangnan university student career development association, through entrepreneurs counselors, experts, outstanding alumni seminar, etc., continue to strengthen the employment situation education, change the students' employment concept. In 2021, the school will carry out 8 various employment value leading activities, with a total of nearly 5,000 students participating. We also hosted the 2021 Jiangsu College Students Volunteer Service Plan in the Western Region and the Northern Jiangsu Plan (Jiangnan University Station), singing the theme of going to the west, going to the grass-roots level and going to the most needed places of the motherland. In terms of "micro" for individual, strengthen the ideological education, psychological counseling, employment guidance, classification help students do career planning, and help difficult students set up the correct view of employment and career, guide them to the grassroots and production the first line of employment, and through psychological counseling, mental health services, timely channel their employment problems caused by anxiety and pessimism. Second, we will improve the system of employment practice. We strengthen the students' practice, social practice as the carrier to help students experience the workplace, expand youth employment internship trainee base, established with Wuxi lakeside pku district party committee district school youth talent alliance, organize green horse engineering students into the grassroots community street with post trainee, in order to help students through social practice to achieve seamless docking with the workplace. Adhere to the original mission of "educating people for the party, cultivating talents for the country", actively organize the national college graduates "three one help" plan and the central "college students volunteer service western plan" and other projects, guide and encourage more youth employment aspirations and the national needs of frequent resonance, to the most need of the motherland. Among the graduates of 2021, the total number of national and local grassroots employment projects, such as selected, drafted, "three branches and one support", western Plan and Northern Jiangsu Plan, increased by 22.1% year on year. Third, we will improve the employment service system. It is clear that each full-time and part-time youth league cadre will pair up with no less than 3 graduates in difficulty, and input the "group league micro employment" assistance small program. The success rate of 69 graduates in 2021 is 100%, and 105 graduates in 2022 have been paired up and provided follow-up assistance. At the same time, we use SMS, telephone, QQ, WeChat and other ways, and make full use of social resources, and actively provide employment opportunities. Up to now, the university has held 9 online and offline job fairs for 2022 graduates, with more than 1,000 recruitment units, including 65 top 500 enterprises, providing 38,211 positions and more than 3,000 students participating. Yang � r, Deputy Secretary of the Youth League Committee of Jiangnan University Source: China Youth Daily

2022-02-24 Nanjing agricultural university: cadres employment support success rate 100% cadres employment support 100% employment ability promotion activities, build online employment channels, improve students' employment ability, efforts to provide students with all-round, warm heart, effective employment services, help students to achieve higher quality more full employment. We will cultivate students' awareness of employment and entrepreneurship, and guide young people to devote themselves to rural revitalization. Carry out agriculture-related brand activities for all teachers and students, focusing on skills improvement, entrepreneurship training, space incubation, etc., to create a strong campus atmosphere for employment and entrepreneurship on campus, guide youth league members to actively participate in employment and entrepreneurship internship and agricultural and rural practice, and improve the enthusiasm of young people for rural development. Opening of special training camps related to agriculture, Organize students to systematically learn agricultural development theory and practical skills, Using the training method of centralized training, thematic discussion and practical visit, Provide students with opportunities to teach tutors, share experience, practice experience and exchange investigation, In 2021, a total of 28 special activities on agricultural employment and entrepreneurship, More than 5,000 teachers and students were attracted to participate in the activity, Continue to cultivate students' "agriculture, rural areas and farmers" feelings; Implementing special plans such as "helping rural revitalization", Organize and carry out the special action of "one hundred villages and one hundred lines" for social practice, Establish 323 practice teams, Through "visiting the surrounding areas", "returning to home" and other forms to further promote the "100 villages and 100 lines" action, Practice footprint across the country's 28 provinces (autonomous regions, municipalities) in nearly 1,100 rural communities of more than 6,100 households, Carry out more than 400 times of theoretical popularization and propaganda; Set up the Shuo and Bo technology service group, Regularly organize professional students to carry out a series of practical activities, such as household research, field visits, interviews with village cadres, and planning preparation, in majiang, the corresponding poverty alleviation units of the school, Suggestions for the implementation of the local rural revitalization strategy, More than 10 research reports and video pictures and other practical results have been formed. We will build diversified employment assistance platforms to strengthen students' practice ability. Deepen the implementation of the "hundreds of schools, thousands of enterprises and thousands of posts" employment assistance action for college students, Hold 2 "post delivery through train" live post recommendation activities, A total of 78 companies were invited to participate, Raising 1,834 jobs, The number of students interviewed was 1,094, More than 7,000 students can watch live streaming online, Send out more than 2,000 resumes online and offline, Effectively guide enterprises and students to achieve accurate docking and two-way selection; To guide full-time youth league cadres to help graduates with employment difficulties, The proportion of employment assistance and youth league cadres reached 100%, the proportion of youth League cadres logging in to help small programs reached 100%, and the success rate of assistance reached 100%, Do a solid job of filing card work, Precise help to help poor students find employment; Implement the "new farming Elite" employment internship program, A total of 3,788 employment internship positions from 590 agriculture-related enterprises in the province, Posts cover 17 categories of agricultural technology, management, service, sales and assistant, Actively provide young people interested in agricultural employment and entrepreneurship with agricultural products production, sales, logistics, e-commerce, after-sales, 7-15 days of employment internship opportunities, Attracting more than 1,500 young people to participate in the internship, Effectively guide youth league members to devote themselves to rural revitalization. We will actively select advanced models of employment, and strengthen services for employment and entrepreneurship. Continue to optimize the employment and entrepreneurship platform services, create functional departments, colleges, experts, industry experts, improve relevant support policies, accurate docking industry-university and research achievements, help students to achieve high quality entrepreneurship; deepen employment advanced typical publicity activities, online "era new farmers" special column, offline organization "maker face to face" sharing meeting, vigorously select and promote employment and entrepreneurship advanced typical, play an example to drive students to establish correct view of employment and positive entrepreneurship, guide students to combine professional knowledge and times mission, encourage more students to grassroots employment entrepreneurship. Tan Zhi, Secretary of the Youth League Committee of Nanjing Agricultural University, is � S. Source: China Youth Daily

2022-02-24 Nanjing Audit University: do practical things with heart to do practical things "I do practical things for the youth", "with heart" to do a good job of college students employment assistance. Help college students' employment concept education and employment skills improvement. At present, there is a slow employment, slow employment, no employment mentality among college graduates. Relying on the forms of "faith open class" and the second class series of activities, the Communist Youth League invited experts and scholars, outstanding alumni and outstanding graduates to talk with students about planning and employment, leading students to establish correct employment values and buckle the "first button" of employment concept. At the same time, students' employment skills are improved through career planning competition, resume making competition, employment experience sharing and elite training camp. Help college students' employment psychological adjustment and guidance. With the intensification of employment competition, employment anxiety among college students is becoming more and more common, and the process of career selection is also a comprehensive test of various psychological quality and overall quality of college students. South of the communist youth league based on set up in the campus of Jiangsu province college students psychological crisis prevention and intervention research center, through the school mental health guidance center established college students mental health association league, through the "one to one" "one-to-many" employment psychological support counseling, employment psychological adjustment micro group class alleviate students employment anxiety, set up a good employment mentality. Help college students to expand their employment resources. First, to assist the employment center of the school to do everything possible to expand resources and strengthen the supply, actively carry out the connection between schools, schools and enterprises, carry out the job fair of "hundreds of schools, thousands of enterprises and thousands of posts", help college students to incubate business projects, and recommend the volunteers of the "Western Plan" and "Northern Jiangsu Plan" through targeted cooperation with the local Youth League committee. It also provides young students with more internship, employment internship opportunities, to improve the ability, and to help more young teachers and cadres to help students find employment; third, to increase the "Western Project", "Northern Jiangsu Plan" and other basic service projects, national audit talent demand and other projects, actively lead students to go to the grassroots, the local employment. Yang Fangmin, Secretary of the Youth League Committee of Nanjing Audit University, Source: China Youth Daily

2022-02-17 Nanjing University of Aeronautics "China Southern Airlines") The total employment rate of 2021 graduates is 94.3%, ranking among the top in Jiangsu Province. As the organization of the Communist Youth League, we have been thinking about how to work hard on employment help, which can not only help young students solve the pain points and difficult problems in the growth, improve the service ability of the league, but also serve the "double first-class" construction center task, serve the overall situation of discipline construction and development, and improve the contribution of the league. First of all, what can the Communist Youth League do in the employment assistance? At the beginning, we had questions, exactly what role could we play? To this end, we have specially carried out special research and discussion. We found that in the post-outbreak era, for young college students, both employment and entrepreneurship, are facing great difficulties, these difficulties are not only the epidemic impact to economic development, more important is difficult, employment psychological pressure, students don't know what to choose a career, don't know what direction of life should develop in the future. Such a situation has inspired us, the main responsibility and main business of the Communist Youth League is ideological guidance, so the Communist Youth League to do employment assistance, we must focus on the value of employment values to lead this key issue. China southern airlines was born in the war, born for aviation, with aviation and long, always do not forget to "cast jack for the country, for the party education ying only" mission, cultivate the "beidou", "chang e" and C919 big aircraft and a number of national aerospace major model engineering chief designer, flowing in the blood "aviation patriotic" red gene. From the employment situation of students of China Southern Airlines in recent years, the employment rate is not our most worrying problem, our employment goal is to send more graduates to aviation, aerospace, civil aviation and other units, leading students to continue the red gene, pass the blue dream, and better serve the development of the motherland three aviation cause. Therefore, the employment assistance of the Communist Youth League of China Southern Airlines is not only done for young students, but also, we hope to encourage young students to integrate their personal ideals into the development of the motherland and the nation through the guidance of employment values. According to the characteristics of contemporary young students, we selected the students like short video way, the stage and microphone to students, focus on building "group when the anchor employment I" online brand activities, through the "six" action, namely, each college, send a student, research an industry, select an enterprise, interview an alumni, record a video, to live with goods, aiming at students fragmented time, with 3 minutes to understand the basic situation of the enterprise, use of choose and employ persons demand, development prospects, etc. At present, we have invited to the "mountain eagle" chief designer Hu Jianxing aviation industry your fly, "AG600" chief designer huang Lingintroduced aviation industry fly south China, "chang 3,4", "day 1" chief designer Sun Zezhou introduces space five courtyard, etc., have broadcast 33 video, a single article highest reading of 48000, accumulated interaction of more than 300000, by the campus young students and campus unit of choose and employ persons. We have also launched special videos on resume production and English job interview skills, which will not only guide employment values, but also provide guidance on employment skills. Wang Jing, Secretary of the Youth League Committee of Nanjing University of Aeronautics and Astronautics

2022-02-17 Nanjing China's national strategy and national economic construction needs, formerly known as Nanjing University of Meteorological, is known as "the cradle of meteorological talents". The university attaches great importance to the employment of college students, and in 2020, it launched six quality improvement projects to serve their employment. As the organization of the Communist Youth League of the university, we do a good job in helping college students from three aspects around the six quality improvement projects. First, take the initiative to carry out the "100 schools, thousands of enterprises and thousands of posts" college students employment assistance activities. In March 2021, under the guidance and support of Jiangsu Provincial Party Committee and Nanjing Municipal Party Committee of the Communist Youth League, the university undertook the first launch ceremony of the "Through train" of college students in Jiangsu Province, and launched more than 500 enterprises with more than 6,000 positions for on-site recruitment. Second, collaborative linkage, actively assist the student affairs office and colleges to find well-known enterprises to establish employment bases. At present, it has signed employment base agreements with nearly 1,000 large enterprises such as Tencent and Huawei. The Communist Youth League of the university has also jointly carried out more than 30 special online and offline recruitment activities with the Student Affairs Office and various colleges. The school's Communist Youth League also helps to improve students' employability from two aspects. First, the College Students' Career Development Association has been established to carry out employment improvement through self-management, self-education and self-service. During the year, more than 20 activities were carried out, including employment situation analysis, administrative ability training, employability training, postgraduate entrance examination experience sharing, and public examination compilation training, to guide students to establish a correct concept of employment. Second, actively mobilize students to make contributions to the grassroots level, guide graduates to take root through the western plan and northern Jiangsu plan; guide studying graduate students to participate in the graduate student volunteer teaching group; organize students to participate in the college student employment internship plan. Shen Weifeng, Secretary of the Youth League Committee of Nanjing University of Information Technology, Source: China Youth Daily, February 17,2022, edition 07

2022-01-28 Jiangsu university about practice team to carry out the personal information protection dissemination activities of Jiangsu university about practice team to carry out the franco-prussian personal information protection activities map China youth network Beijing on January 28 (reporter Zhang Jianwei, correspondent Sun Jie) since November 1,2021, the personal information protection law of the People's Republic of China, the protection of personal information has been valued and guarantee. Recently, the personal information protection publicity practice team of Jiangsu University walked into the Zhenjiang Charming City Primary School and the Wuli community construction site of Zhenjiang City to carry out the personal information protection law popularization activities. The picture shows the volunteers introducing the cases of information leakage to the students. Correspondent for figure activities, volunteers use leaflets, display board to the students and workers introduced the personal information protection law, at the same time combined with the often encountered in daily life personal information leakage, network authority setting, express single leak personal information, telephone fraud, etc., the possible consequences of personal information leakage is introduced and explain the personal information leakage and encounter fraud response, also to the participants presented the personal information protection law. The picture shows volunteers distributing promotional materials to migrant workers. In this law popularization activity, the college student team went to the grass-roots level to publicize the law, while enriching their own legal knowledge, enhanced the legal awareness of primary and middle school students and migrant workers, and established the concept of the rule of law to protect personal information.

2022-02-10 Shandong university (Qingdao) life rainbow support agriculture teaching research association to carry out the winter vacation online teaching activities of Shandong university (Qingdao) life rainbow support agriculture teaching research association for winter vacation online teaching activities JianWei correspondent Zhang Yang liu nan), Shandong university (Qingdao) life rainbow agriculture support teaching research association with the help of tencent meeting, in the form of online teaching, for Heze city in Shandong province peony district zhou Lou elementary school grade one to six students opened the winter vacation online teaching project. Previously, the team in charge of "Life Rainbow" contacted with Shandong Heze Zhoulou Primary School and Heze No.1 Middle School and the principals of primary and middle schools about online teaching in winter vacation, and obtained the permission and support from the principals of the two schools. The weeks elementary school floor all students winter vacation online teaching activities, a total of two weeks, nearly 90 students participated in the "cloud teaching" online teaching activities, teaching content according to the grade division, covering Chinese, mathematics, English, and quality development courses, in tutoring students test subjects and help them to broaden their horizons, rich knowledge reserves.

2022-02-08 The research team of Ocean University of China carried out the winter vacation social practice activity, Beijing, February 8 (reporter Zhang Jianwei) In order to deeply understand and inherit the spirit of the new Long March, Let the young people of the new era fight bravely on the new Long March of realizing the Chinese dream of national rejuvenation, The School of Management, Ocean University of China held the winter vacation social practice activities, "Hai University'tracing the red footprint, Inheriting the Spirit of the Long March'" The research team went to the Red Army Long March through Beichuan County, Sichuan Province to carry out research, By searching the red footprints, Enter the party members and carry out volunteer services. Led by Wang Zhiyi, the head of the local cultural station, the team members visited the Red Army Long March Memorial Hall, the Red Army Forest of steles, the Martyrs' Cemetery and the Qianfoshan Campaign Memorial. Team members visit the Martyrs' Cemetery. Ocean university of China research group for figure team members, head of the local cultural construction webmaster wang and has 55 years probationary party member comrade Liu Rongfang conducted the interview, in the interview process, the team members learned to the 1950s, 60s life hardships and the difficulty of reading, the whole study lei feng spirit, lead by example, hard work for education. During the practice, the team members learned that because of the remote location of Yuli town, lack of access to transportation, There are still many deficiencies in the construction and publicity of the local red education base, the Red Army Long March Memorial Hall for the education of teenagers and the inheritance of the Long March spirit with local characteristics, In response to these problems, the team proposed to promote the local red gene construction and education through Internet methods such as publishing push and cloud visit, In addition, because Yuli has the special cultural background of Dayu's birthplace and the Qiang ethnic group gathering place, The team members proposed that they could combine the Dayu culture and the spirit of the Long March to form the unique Long March spiritual connotation of Yuli Town, While enriching the red spirit, Develop tourism to help with the local economic development. After two days of practice and study, the team members said that they would carry forward the long March spirit of the revolutionary ancestors who are not afraid of hardship and invincible, and called on the majority of young friends in the new era to approach the revolutionary site, understand the revolutionary spirit, and strive to be a new youth with faith, responsibility and achievements.

2022-01-28 Liaocheng University School of Life Science held anti-fraud propaganda activities Liaocheng University School of Life Science held "improve prevention awareness and strengthen self-protection" activity Wei) Winter vacation is coming, all kinds of network fraud are showing a high incidence trend, especially for the youth groups. In order to increase the publicity of young groups to prevent fraud, recently, Liaocheng University School of Life Science "prevention in the heart, fraud difficult to line" service team in Heze City, Shandong Province, Heilongjiang Province, Yichun City, Changshu City, Jiangsu Province and other places to hold "improve the awareness of prevention, strengthen self-protection" activities. Volunteers collect common fraud methods, typical cases and measures to prevent fraud in advance. On the day of the activity, volunteers came to the community with large flow of people to look for teenagers for anti-fraud publicity, broadcast and watch anti-fraud knowledge publicity video together, with animation and actual life situation as the content to deepen teenagers' understanding and understanding of telecom network anti-fraud. Video watching in the process, volunteers synchronization for teenagers, summarize fraud including communication tools fraud, network dating fraud, network game fraud, winning fraud, etc., teach teenagers to identify network fraud, told teenagers to pay attention to personal information security, don't use the parents mobile phone network payment, he have to verify with parents.

2022-01-24 North China electric power university Baoding campus to carry out the "green horse project" training of Baoding campus of north China electric power university youth corps committee more forms to carry out the "green horse project" training JianWei correspondent Zhang Jian Zhang Yifan Gao KaiYing) recently, Baoding campus of north China electric power university youth corps committee by organizing special training, sharing experience, watching documentaries and quality development training and other forms to carry out the "green horse project" training in 2021. Guo Xiaofeng, deputy Secretary of the Party Committee of the University, Zhang Jinhui, former Deputy Secretary of the Party Committee and vice president of the university, and Qi Xiuqiang, associate professor of the School of Marxism, respectively carried out special theoretical teaching and training. The sixth plenary session of the 19th CPC Central Committee was held on November 8 to 11 in Beijing. Students of "Yangma Project" of North China Electric Power University first studied the spirit of the plenary session. The students have talked about the great achievements of the Communist Party of China in the past century, the concise historical experience and the personal progress and development direction, combined with their own learning and life reality. To carry forward the spirit of Korean, guide the youth firm ideal faith, continuously red blood, practice good skills, explorations era, in strictly abide by the relevant provisions of the school epidemic prevention and control, Baoding campus "green horse project" training conducted red theme watching activities, watch the theme documentary "1950 they are young". Invite professional quality development teachers to carry out team quality development activities for ma students. Students constantly sum up the experience, find ways to break the game, in the harvest of victory while fully experience the red culture and love the party patriotic spirit. It is reported that the centralized training lasted for more than 2 months, from the centralized theoretical study, practice and practice exercise, daily tracking and training of the three sections of planning. More than 200 students from 2021 freshmen, league activists and backbone of major school-level associations attended the training.

2022-01-19 Teachers and students sent us the blessings, window decorations and dolls of the Spring Festival. We fully feel the strong flavor of the New Year and the deep friendship, and we are warm in our hearts."Recently, Hefei City Shushan District Sanli'an street Meishan Road community Ge uncle said. On January 14, Anhui medical university youth corps committee organization youth volunteer federation carried out the "with warm, welcome Year" warm winter action, aims to further carry forward and practice the socialist core values, give full play to the role of volunteer service activities education, further promote the party history learning education "I do the practical work for the masses" practice go deep go real."As the Spring Festival was approaching, I failed to receive the blessing from my teachers and students. As a staff member of the canteen, I was very moved. I think the harmonious relationship between the school logistics and the students is the best New Year gift for me."School canteen staff master Chen said."In this cold winter, we send New Year's greetings to the canteen staff who work hard every day, thank them for preparing delicious meals for us. It is their considerate service that makes our campus life better. We went to the community to send blessings to the community residents, they repeatedly thanked them, very happy about our arrival, our small move, warm the residents of the community, I felt the pride of serving as a volunteer. Volunteer when you have time, and volunteer for you when you have problems. Volunteering, we are always on the way."Said Xiao Wenxuan, a student volunteer from the Youth Volunteer Federation of Anhui Medical University. The school Youth Volunteer Federation practices the spirit of volunteer service with practical actions, and further promotes the practical activities of "I do practical things for the masses" in the vivid volunteer service activities.

2022-01-28 I am very proud of serving the volunteer service jointly organized by the Youth Committee and making a small contribution to Qingzhen."Said Pu Qing, a student from Guizhou Industrial Vocational and Technical College. Qingzhen City of Guizhou Province gives full play to the advantages of Guizhou (Qingzhen) Vocational Education City, strives to build a volunteer service brand of "Nest Project" with vocational education characteristics, and actively provides a platform for urban vocational education youth to participate in social practice, community governance and rural revitalization. Through party building with league building and party members with league members, Qingzhen city has signed cooperation agreements to transform the service advantages of local Party committees into "strong talents" advantages and comprehensively promote the construction of a youth-friendly city. Since the launch of the "Wild Goose Nest Plan" volunteer service, more than 900 young volunteers have been recruited, more than 130 civilized etiquette persuasion and community volunteer services have been organized, and 60 periods of "double reduction" care and companionship have been carried out. Source: China Youth Daily, January 28,2022, edition of the 02nd edition

2022-01-25 Jiangsu university volunteers to carry out fun party history small classroom practice in Jiangsu university volunteers to carry out fun party history small classroom practice China youth network Beijing on January 25 (reporter Zhang Jianwei, reporter correspondent Wang Yaodong) recently, Jiangsu university law school volunteers came to Zhenjiang run business primary school, launched the party history fun classroom activities. With the theme of "Staying true to the original aspiration and undertaking the mission, with red hearts to the Party", combined with PPT and videos, the volunteers told the glorious course of the Communist Party of China in lively and vivid teaching methods. The picture shows the students showing the made party history button painting. The interesting party history class also set up the party history manual works creation link, under the guidance of the volunteers, the children group completed the button painting in the form of the party history theme creation works, a "the party in my heart", "my heart to the party" show on the paper, more printed in the heart.

2022-01-25 North China electric power university Baoding campus "4S" work system improve games volunteer training north China electric power university Baoding campus building "4S" work system promote games volunteer training effect Jianwei) since September 2019 was identified as the "Beijing 2022 Olympics and winter paralympics" Zhangjiakou division uniform and registration branch and accommodation volunteer source universities, north China electric power university Baoding campus pay attention to form innovation and enhance effectiveness, build the "thought, type, real, potential" (4S) training system, improve volunteer training effect. Learning experience sharing meeting. The school Youth League committee for the first "S": grasp the "thinking". To lead the overall training system, set up temporary party branches and league branches, give play to the role of vanguard party members and league members, hold many theme party days and league day activities, strengthen volunteers' political awareness, overall situation awareness and political stance. The second "S": identify the "formula". North China Electric Power University provides a combination of online independent learning and offline communication learning, and both online learning theory and offline learning practice, with comprehensive training for volunteers. At the same time, make full use of the IKM learning platform online, and offline to carry out daily learning progress punching in in groups, forming a learning atmosphere of "without a whip from the hard hoof". The Winter Olympics volunteers' launch ceremony. The school youth League committee for the third "S": fall in the "real". Experts from related fields were invited to give the reserve volunteers special training on medical emergency aid, assistance for the disabled, epidemic prevention skills, etiquette and language expression. Volunteers were sent to Zhangjiakou, Beijing and other places to participate in backbone exchange camps, field survey and other training and studies. Establish a physical exercise mechanism, and encourage volunteers to carry out long-term running and other physical exercise through activities such as punching in and prize competition. Organize the Winter Olympic Games volunteers to provide volunteer services for various sports events such as Baoding Marathon, school autumn track and field games, school snow and ice games, and school sports festival, so as to promote success through practical work and promote improvement through practice. The fourth "S": integrate into the "potential". The Youth League Committee of Baoding Campus of North China Electric Power University has integrated into the general trend of the national Winter Olympics, integrated the training into the major nodes, and cooperated and interacted with other universities and enterprises. On the occasion of the first-year countdown to the Beijing Winter Olympics, we took the lead in holding a one-week special training activity in Hebei Province of the uniform and registration Branch in Chongli Galaxy Ski Resort. Strengthen exchanges and cooperation with enterprises, and provide each volunteer with cotton clothes, pants, shoes, scarves, suitcases, charge banks, thermos cups and other daily necessities. All the Winter Olympics volunteers took a group photo. After more than a year of training and assessment, a total of 46 teachers and students from the Baoding campus of North China Electric Power University became official volunteers for the Beijing Winter Olympic Games and Paralympic Games. At present, the first batch of 29 volunteers from the uniform and Registration Branch entered the venue on November 22,2021, responsible for the reception and translation, document production management, uniform distribution, personnel management and other work, and have officially carried out the volunteer service work, and the other volunteers went to Zhangjiakou to report for service. As the volunteer Shi Zhekai said: " I will certainly live up to the expectations of the school, win glory for Huadian, add luster to the Winter Olympics, and contribute my own strength to the motherland.”

2022-01-16 Anhui university of science and technology: guide students to find the right direction in the innovation practice of Anhui university of science and technology: guide students in the practice of innovation to find the direction wang haihan) from freshman to participate in "green horse project" training courses, interest in innovation research and development, to now more than two years have passed, innovation entrepreneurship has become Anhui university of science and technology of civil construction college junior Li Shenqing study, part of life."If you choose to take this path, you have to work more hard than others."At that time, the project instructor Chen Xiaozhu and senior Shen Zejun told him so. Traditional central air conditioning has industrial pain points and technical problems such as excessive energy consumption, unstable system state and condensate treatment defects. This is the direction that Li Shenleqing chose for innovation. He and his team have investigated a number of large public places, using the fresh air volume and water pump speed segment control, and developed a new central air conditioning energy saving system. Last year, the project won the first prize of the "Challenge Cup" College Students' Extracurricular Academic Science and Technology Works Competition in Anhui Province, and was shortlisted for the final final of the national competition, and also won the first prize of the national Youth Zero-carbon Science and Technology Project (creativity) collection. Like Li Shenleqing has a large number of young students with innovative thinking in Anhui University of Science and Technology. Anhui University of Science and Technology is the only one in Anhui province to be selected as the "National Innovation and Entrepreneurship Typical Experience University", and "one of the first national demonstration universities for deepening innovation and entrepreneurship education reform", with a number of national and provincial scientific research platforms. For a long time, under the leadership of the university Party Committee, the university Youth League Committee and other departments, relying on various competitions, pay attention to the cultivation of young students' innovation and entrepreneurship genes, and serve the growth of students in an all-round way. In the past five years, the university has won more than 30 national first prizes in the A-class discipline competitions, such as the "Challenge Cup Competition" and the "National College Students Mathematical Modeling Competition". In the past three years, 373 national and national innovation and entrepreneurship training programs for college students and 746 provincial programs have been approved."As an indispensable production and living equipment, the central air conditioning's effect of energy saving and consumption reduction has a far-reaching impact on alleviating the pressure of power supply and demand shortage."Li Shenliqing introduced, the project started from scratch, focusing on the central air conditioning energy saving and HVAC system, and then improving the wind system and water system. With a preliminary idea, several people began to build a model, first to study the 3D printing modeling technology, and then try to print the PVC pipeline, water pump materials. People often stay in the laboratory, looking up information and professional papers. During the research and development period, Li Shenleqing's winter and summer vacation is always "put later" than others. In half a year, the team explored to create an air conditioning system model. Li Shenleqing introduced that he is currently training the team of freshmen, to the central air adjustment house energy saving innovation direction force. Liu Haofeng, a post-2000s student majoring in computer science and technology, and Liu Jiale, who majored in automation, are roommates and entrepreneurial partners. In their freshman year, the two participated in science and technology innovation club activities and had the idea of making intelligent hardware interdisciplinary. In the second semester of the freshman year, a five-member research and development team was formed. They consider to build a comprehensive machine carrier, using the professional knowledge of learning control, artificial intelligence and computer inside and outside the class. In early 2020, the school term was postponed due to the outbreak. The team focused on research at home, held meetings online, reported the progress of their respective direction, and focused on the next plan. During that time, the team figured out the knowledge of robot control algorithm and other knowledge. In September 2020, it quickly implemented the design and processing of mechanical structure, robot control algorithm and circuit system integration, visual perception and navigation and other technologies. In June 2021, the project won the first prize of the "Challenge Cup" provincial Competition, and also won the incubation fund of 1 million yuan."At least participated in more than 20 simulation reports and defense."Liu Haofeng clearly remembers that the project has grown through several rounds of grinding. During each preparation for the innovation competition, the school Youth League Committee will invite experts and teams to help them, from the design of operation scenarios, polishing the significance of the project, and guiding and improving the project book. In the first semester of their junior year, the teachers of the Youth League committee of the college suggested that they participate in the challenge cup and "collide" with the masters."When preparing for the school competition, the work was not yet mature, and the text and video materials submitted did not fully show the potential of our work. The teacher also spared no effort to often help revise the work declaration until the early morning."Liu Haofeng recalled. At a defense meeting, an expert said: " What is the core competitiveness of your robots? I don't think simple ground movement is not enough. In the coal mine search and rescue, robots will be complex environment, look through the wire fence, you can consider improving your project."Yang Wei, a 24-year-old graduate student majoring in safety science and engineering, also felt deeply about the school's warm help. He and Wu Xueqiang, who both spent time in Anhui University of Science and Technology, jointly developed the colloidal foam intelligent fire fighting system, trying to solve the shortcomings of the traditional fire fighting technology, such as low fire fighting efficiency, narrow range of use and lack of intelligence. Since 2019, the team has been constantly solving problems through research, manufacturing equipment and competition. Yang Wei said that when he first entered the school, people did not know much about the Challenge Cup, and thought it was "taking something to participate in the competition". However, by writing the application form, setting up the project project, and then participating in the competition, we felt that the competition was a huge and systematic process, and we had to do every little thing in great detail."Not only do we gain personal growth, we also know how to work together. After the competition, everyone even write the paper, format, binding, specification will not go wrong."The school has always attached great importance to the" Challenge Cup " project construction. The school Youth League Committee took the lead to hold the training meeting, inspection meeting, promotion meeting of the entries, several rounds of defense, from the pulse of the declaration, PPT, video, physical model and other links. At present, the university has 149 innovation and entrepreneurship instructors, and employs 185 alumni entrepreneurs, successful entrepreneurs, venture investors and experts and scholars as part-time innovation and entrepreneurship mentors. At the same time, the school Youth League Committee sets up innovation and entrepreneurship student associations and clubs, including 12 academic science and technology associations, such as Robot Association, Model airplane and Science and Technology Practice Association, 3D Innovation Design Association and Business Management and Enterprise Innovation Association, and two innovative and entrepreneurship associations; and carries out "Science and technology Innovation Festival" and "Anli Youth said" entrepreneurship simulation training activities to create a good ecological environment."Save a drop of water, save a kilowatt hour of electricity, and jointly protect our only home. Do all the children remember it?"This is the school" colorful holiday " social practice group in Huainan City Datong District Kongdian township central primary school popular science class. Volunteers used elaborate promotional videos and cartoon cartoons to show primary school students the importance of low-carbon and environmental protection."If the Challenge Cup and other competitions are a high-level test and hone of students' innovation and entrepreneurship ability, then the usual science practice education can let more students know that mastery, feedback theoretical growth, find the right learning direction, so that the concept of innovation and entrepreneurship to the greatest extent."Cheng Xiuxia, secretary of the Youth League Committee of Anhui University of Science and Technology, introduced that every summer" three to the countryside "social practice activities, the school will set up more than 50 teams to go to the grass-roots level, robot science popularization performance," Internet + " science and technology supporting agriculture, ecological environment protection, water quality testing and other popular science practice. Suntai Village of Maoji Experimental Area in Huainan City suffers from eutrophication pollution in aquaculture waters, which makes the villagers difficult. In July 2021, the volunteer service team of the University Student Green Association took samples and analyzed the contaminated water sources respectively, and found that the problem was found in a full set of automatic water treatment equipment. Volunteers quickly came up with a solution, made and developed "public welfare bags" for removing phosphorus and heavy metals combined with their professional knowledge, and provided them to the villagers free of charge. In recent years, the school Youth League Committee adheres to the concept of "popular science publicity is as important as scientific and technological innovation", and gives full play to the strength of the community to publicize the achievements of scientific and technological innovation to primary and middle school students and the public. At the end of 2020, the school's "big hand holding small hand" of science popularization into the campus and college student science popularization publicity and education base was listed. College Student Robot Association, College Student Association of Model airplane and other associations joined hand in hand with primary and middle school students to strengthen the sharing of popular science resources and the creation of scientific and technological works. Guo Yongcun, secretary of the Party Committee of Anhui University of Science and Technology, said that the university has always attached great importance to innovation and entrepreneurship education and practice. Students actively participate in various scientific and technological innovation competitions and achieve excellent results, which is the result of the university's further promotion of the reform of innovative and entrepreneurial talent training mode, and also a demonstration of the school's running level and comprehensive strength. He pointed out that the school will continue to improve the quality of talent training as the core, and focus on building a new mechanism of innovative talent training, cultivate young students' "small in the school", "big in the province" and "dedication to the country" feelings, and guide them to contribute their youth strength to the national scientific and technological innovation and development.(The video was shot and produced by Wang Haihan, a reporter of China Youth Network of China Youth Daily)

2022-01-16 Northwestern polytechnical university with practical action to practice "please rest assured, power have me" oath "please rest assured, power have me" northwest polytechnical university with practical action to practice "please rest assured, power have me" oath jianwei correspondent, Ma Tingting Yang province � � " I should be in the motherland need our young generation firmly stand out, to make their own contributions to society, realize their own value."This is in the face of the lens of a student of the true feelings, but also thousands of university youth charge horn. Facing COVID-19 epidemic prevention and control situation, under the strong leadership of the school party committee, in the superior youth league, under the guidance of the communist youth league immediately condensed "leading force, organization, service", launched the league organizations at all levels, leading the backbone of the charge, led the university son condensed the communist youth league strong fortress, with practical action to practice "please rest assured, power have me" clank oath, contribute to win the epidemic prevention and control of annihilation youth strength. In the face of the epidemic, no one is a bystander. The Communist Youth League of Northwestern Polytechnical University responded quickly and issued "a Written Proposal to All Students", "A Letter to All Epidemic Volunteers" and " Assembly Order to the whole university! Youth League members, act "and other proposals, called on all the young students to participate in the epidemic prevention and control work in the school; combined with the" Notice on the theme of "fighting the epidemic, youth gathering and brave" "," the series of Youth League branch activities collection order! The document requires that the Youth League branch should be mobilized to carry out colorful organizational life; to issue the Notice on Developing Special Social Practice Activities to Fight the Epidemic, Notice on Soliciting Publicity Materials for Epidemic Prevention and Control on campus, and List of College Epidemic Fighting Activities to enrich the campus cultural life. After the volunteer recruitment announcement was issued, the young people of the school heard the order. In just 30 minutes, more than 300 students volunteered. Up to now, 4,745 emergency volunteers and more than 2,000 building food delivery volunteers have been stored. After the work task was issued, the young volunteers have said, "I can" and "we beg us to go to war". In the process of fighting the epidemic, the youth of each groups became the most beautiful volunteers. School continue to strengthen the top-level design, overall planning "school volunteer service department + volunteer service team leader + various service volunteers" three-level linkage mechanism, led the Chang'an campus, friendship campus emergency volunteers QQ group, arrange students to participate in epidemic prevention and control volunteer service, as of January 10,8754 people to assist total nucleic acid, research, material handling, dormitory delivery volunteer service, total service time 32000 hours, complete nucleic acid detection 14 rounds of 350000 people, for dormitory meals 28 rounds 592000, carrying nearly one hundred tons of supplies. The epidemic is merciless, and public welfare activities light the torch of hope."A hundred years of struggle begins a new journey, Online presentation on the fight of the Future" Gathering strong spiritual efforts to fight the epidemic, The studio popularity value hit a new high, More than 15,000 yuan; "Meet in Chang'an Flower" New Year charity cloud concert, It took us 163 minutes to present a special "New Year gift", With 1,951 bullet screens, For a "cure" for the anxiety in the epidemic; "Between outbreaks, Warm feeling of the world " college volunteer service community special exchange and sharing meeting, More than 120 students from 10 volunteer public welfare associations of "Wuhan University-Northwestern Polytechnical University" have exchanged views and shared their experiences in the cloud; Student associations such as Sunshine Project Youth Volunteer Service Team, Xingxing Environmental Protection Volunteer Association, Student D Design Association and so on have contributed their youth strength to epidemic prevention and control with their own characteristics. In the battle against the epidemic, every adult worker is a hero. The publicity backbone of the school Youth League Committee and the members of the student photography society recorded the touching with the nib and captured the moment with the lens, leaving the touching drops of the most beautiful volunteers and epidemic prevention workers in the epidemic prevention and control. During the period, editing complete volunteer service work briefing 12 issues, complete all kinds of epidemic prevention and control of push 73, war epidemic related work including the news broadcast of the shaanxi daily and the People's Daily client media reported more than 30 times, make the image of nTU youth volunteers more vivid display in front of the people of the whole country. Volunteers "epidemic" is bound to rush ahead, warm service silently follow up in the rear. School in order to further standardize the west university student volunteer service recruitment process, simplify information sorting, school youth corps committee joint NPU-Devhub developers community student team speed developed university emergency volunteer service WeChat small program, divided into the latest notice, material demand, dining statistics, volunteer service, information summary, psychological counseling and so on seven plates, greatly improve the volunteer recruitment, registration and information statistical screening efficiency, convenient volunteers to use. The number of visits exceeded 50,000, related media special reports. In order to further reduce the risk of epidemic spread, reduce the waiting time, students, students, graduate students with the help of QQ small niu platform dormitory building nucleic acid testing schedule, starting from nucleic acid testing time calculation, with 15 minutes for a cycle, for students update Chang 'an, friendship campus nucleic acid detection queue, solve the problem of students' concerns. In addition, the Cultural and Sports Center of the School Youth League Committee and the School Student Union took ten days to carefully create the students' original music MV "Against the Wind", which was officially released on the whole network on December 30."In the fight against the epidemic, our young students have continued to grow up mature, responsible and willing to contribute."Said a volunteer after the work. With practical actions, they have fulfilled the pledge of "please ask the Party to rest assured that the strong country has me", and interpreted the responsibility of the youth of ICBC with sweat.

2022-01-14 Shanghai Jiao Tong University Youth League Committee of Shanghai Jiao Tong University: Do a good job of the transformation of the transformation of the Youth League Committee: Do a good job of the transformation position, inheritance, reform and innovation, take the youth as the focus of the core, adhere to the content operation, with the favorite voice of young people, do a good job of value guidance. In the early days of reform and opening up, social thought repeatedly, Shanghai jiaotong university teachers and students are eager to morale, is thought, Shanghai jiaotong university youth corps committee to "enlighten themselves, friends" for the purpose founded the friends, deep original text, reflect students ecological, spread the campus information, advocating fashion, has been 36 years, never stopped more. With the rapid development of the Internet, how to keep up with The Times and lead the cohesion of youth has become an important topic for the Youth League Committee of Shanghai Jiao Tong University. Since 2014, the Youth League Committee of the school has added a new media operation track, opened the wechat public account "Youyou sjtu", and continuously expand the new media position, and seize the advantages of print media and new media, operating and running in tandem. The Youth League Committee of Shanghai Jiao Tong University positioned the construction of new media platform, focusing on timely content, directly hit the scene, chase hot spots, create hot spots; paper media platform is more professional, with "deep" generation "new", with profound thinking and resonance of young students, and bimonthly into major time node distribution, ritual reading. At the same time, the school youth League committee retained the friends of the editorial department, set up "new media editorial department", "visualization center" and "affairs center", cultivate all technical expertise; with new technology packaging content, for different student groups, using new media directional precision information push, "campus new" as the starting point, using the convenience of new media, the fastest follow up. In combination with the needs of college students for typical in-depth reports of students in various fields such as annual figures and national scholarship, the Youth League Committee of the university has set up columns such as graduate volunteer teaching group in the print media, forming a good peer incentive effect. General Secretary Xi Jinping has stressed that for the news media, content innovation, form innovation and means innovation are all important, but content innovation is fundamental. Shanghai jiaotong university youth corps committee always adhere to the content operation as the core, with youth focus, formed the "patriotism education, strengthen the country feelings" "encourage practice education, youth confessions motherland" lead "outstanding example, set up the spiritual coordinates" "strongest practical dry, campus life" and a series of rich ideological connotation, youth love to listen to see the series of products. The Youth League Committee of Shanghai Jiao Tong University has successively launched themed pictures, cartoons, videos and innovative form products, such as "Start", "My Youth", "and the Hand of Colorful Clouds", which constantly arouse emotional resonance with heartfelt, heart-warming and sincere original stories. In the face of the network fragmentation, emotional information, the school from the perspective of ideological education topic, with paper media carrying depth comments, with new media platform for the first time, from "passive" to "proactive", guide students to more objective and dialectical view, turbidity Yang qing, and always adhere to the print media and new media spread double operation mode, experienced the transition of touch, continue to win the young students wide attention in cyberspace. In the ranking of new media platform, "Youyou" has been the first place in the list for a long time and is widely loved by young people of Jiaotong University. At the same time, the new media work of the Communist Youth League of Jiaotong University has also realized the education, and the team has cultivated a group of excellent talents with comprehensive quality. Source: China Youth Daily, January 14,2022

2022-01-13 Jiangsu university school of teacher education graduate member into the campus study party history activities in one hundred struggle continuously red gene blood in Jiangsu university school of teacher education graduate student member into the campus study party history practice JianWei correspondent Cao Dong Zhang HaoChen) at the beginning of the New Year, Jiangsu university school of teacher education graduate students came to Zhenjiang China road primary school, with more than 100 third grade three young pioneers "members and players in one hundred struggle, hand hand continuously red gene blood" practice. The practice team was composed of 10 normal graduate students. All who gave full play to their professional expertise, carefully prepared and actively participated, and planned patriotic poetry recitation, sharing party history stories and watching the video of the Anti-Japanese War. The picture shows the practice team members hosting the activity. Correspondent for the picture to recite patriotic poetry, members and members of the hundred years of struggle "poetry recitation" link, practice team members and the young pioneers read patriotic poetry articles together, leading the children to eulogize the Chinese nation to realize the great rejuvenation of the Chinese dream struggle, feel the spirit of patriotism."Many members of our team are subject teaching Chinese professional, everyone of the Chinese teaching material patriotic poetry is very understand, also mastered certain recitation skills, hope that through our recitation of" the Chinese youth ", can let the children feel as the Chinese young sense of responsibility and love for the national great rivers. We also felt the power of the new generation from the children's recitation, and we and them are the hope of the country's future."Said team leader Zhang Haochen. The picture shows the activity link of "poetry recitation". Correspondent for the picture to tell the story of revolutionary history, big hand holding small hand ceaselessly red gene blood."Share the story" link, practice team members led the children know the flag, emblem, learning group related knowledge, encourage the children to study hard, the future efforts to join the Chinese communist youth league, with loud internationale, participate in the activities and the young pioneers waving the national flag, pursue the glorious footprints of revolutionary predecessors. The picture shows the team members sharing their New Year's wishes with the children. Finally, the children shared their New Year wishes with the members, such as "hope the epidemic will end soon", "hope the family health" and the "hope that every test can test a hundred"... the team members as "big brothers and sisters" to answer the children in life and study confusion. The picture shows both the team members and the children. Correspondent for figure after the activity, practice team members share their practice comprehension, subject teaching Chinese professional 2020 graduate students Sun Wenjing touched: " before we learned to and more than 100 third grade children, thought the order will be chaotic, but they entered, like an orderly little soldiers, under the guidance of the teacher, the children lined up to their seats. Several children read the beginning of Chairman MAO's poems were shocked, surging emotion and proper pause stress, can not help but sigh at the excellence of the younger generation, which is also the expected result of the revolutionary ancestors.”

2022-01-05 Jiangxi youth vocational college held the second course ideological teaching competition Jiangxi youth vocational college held the second course ideological teaching competition on 5 (reporter Zhang Jianwei, correspondent Zeng Chenxi) for the implementation of the school "three education" work requirements, promote the course ideological construction, improve teachers' teaching ability, Jiangxi youth vocational college held the second course ideological teaching competition final. After the preliminary recommendation of each department, a total of ten course teams entered the final. In the competition, all the participating teams dug deep into the ideological and political elements through the field lecture and micro-class teaching links, combined with the course characteristics, and organically integrated the ideological and political education into the classroom teaching. One first prize, two second prizes and four third prizes were awarded. Through this competition, the teaching ideas are broadened, the curriculum of ideological and political construction consciousness is strengthened, the professional courses and ideological and political theories are run together, and the education pattern of all staff, whole process and all-round of the school is constructed.

2022-01-05 Guizhou university research group to carry out micro wish student activities Guizhou university research group to carry out micro wish student activities in Beijing on January 5 (reporter zhang, correspondent liu) "thank your big brothers and sisters, this is the best New Year gift" "good, I always want to have a basketball" "wow, I want to read books", the children's laughter and thanks over the school, more added a bit of the joy of the New Year. Photos of the event site. Correspondent for the figure recently, Guizhou university youth corps committee secretary Liu Kui, deputy secretary Yang Xiaochun, ting-ting wang respectively led to Guizhou university graduate FuJiaoTuan service to danzhai xingren middle school and zhenfeng second middle school to carry out the "research have love, wish shine" micro wish student donation activities, for xingren middle school and zhenfeng second middle school left-behind children to your big teachers and students of the New Year, and visit is the 23 session of graduate education members. Received New Year gifts for the kids."When I grow up, I also want to do meaningful things, return to the society, and warm more people". The children's messages deeply touched everyone who participated in the activity."Wish is passing on, love is continuing, and I think this is the meaning of the micro-wish public welfare activities," said Wang Na, a student from the Activity Development Center of Guizhou University. Received New Year gifts for the kids. Correspondent for the figure, the micro wish student donation activities have been successfully held four periods, the micro wish student activities is in Guizhou university youth league, danzhai county, under the guidance of the county, xingren middle school and zhenfeng the second middle school school support help, by Guizhou university research league joint Guizhou university student union and student activities development center. After a week of wish claim recruitment, more than 1,800 expensive universities participated, raising more than 1,700 books and 289 younger brothers and sisters wish gifts.

2022-01-12 Shaanxi energy vocational and technical college held a "challenge cup" field heats review shaanxi energy vocational and technical college organized 2022 "challenge cup" field heats review Jianwei correspondent Wang ruo) recently, shaanxi energy vocational and technical college organization online in 2022 "challenge cup" field heats three rounds of review, the review invited to professional innovation entrepreneurship research of three provincial experts to enter the field heats project review, the relevant personnel and field preliminary selected 50 project team report personnel to participate in the meeting. The defense of this competition takes the form of "online". The whole defense adopts the inspection room to check the identity. The process is open and transparent. The defense students use online display PPT to realize the defense sharing. In this competition defense, the contestants combined the packaging of agricultural and sideline products in the field of rural revitalization, the transformation of new achievements with intangible cultural heritage and traditional culture, the integration of scientific and technological innovation and the "one black and one white" professional characteristics of the school, and investigated the current needs around them to introduce the introduction of the project. Each project defense students combined with PPT report, work topic based on professional characteristics, stick to the pulse of The Times, contact the social livelihood of the people's livelihood, review after the report, the judges teacher pointed out the advantages and disadvantages of each project team, and for how to optimize the PPT put forward some valuable Suggestions, but also on the interpretation of the project, display skills and improvement direction to give guidance. After the defense evaluation, 30 teams will be selected to enter the university-level final, and the selected excellent projects will be set up.

2022-01-06 Wang Haihan, reporter of China Youth Network) In order to thoroughly implement the "double reduction" policy and promote the integration of universities, primary and secondary schools, Anhui University of Technology and Maanshan Experimental Middle School regularly carry out after-school delayed volunteer services. Under the organization and mobilization of the school Youth League Committee, college students volunteers will go to Maanshan Experimental Middle School every Monday to Friday at noon and afternoon to give special courses. The life track of ancient poets, the development course of architectural history, and the clever memorizing method of English words, these rich and interesting courses are much welcomed by the students of Maanshan Experimental Middle School. At the same time, the volunteers combined their professional knowledge and junior high school students' learning interest points, to carry out interactive teaching in humorous language, and the classroom atmosphere was warm. Student volunteers from the School of Foreign Languages also assist middle school teachers to organize students to carry out group-based personalized tutoring, and improve students' interest in English learning through questions and answers and dialogue. Nowadays, in Ma 'Anshan Experimental Middle School, parents pay more attention to "what courses their children can learn", "whether they can broaden their children's horizons through after-school services" and other issues. More and more parents encourage their children to participate in after-school activities to develop their interest in learning. In the early stage, each college student volunteer went through declaration, multi-level screening, management and training to strictly ensure the quality of after-school services. Volunteers prepared different characteristic service contents to teach students in the form of classes."Rich after-school delayed activities can also allow students and volunteers to grow up together."In charge of the Youth League committee of Anhui University of Technology said that the volunteers strive to transform the" 2 hours of after-school service "into" 2 hours of quality improvement ", so that the" double reduction " can be implemented in detail. In the future, volunteers will strengthen after-school tutoring, community activities and other work innovations to help the high-quality development of education in Ma'Anshan city.

2022-01-05 Chaohu College: From December 6 to December 30,2021,28 members of the "Party History Learning and Education Student Publicity Group" went to various colleges and students to organize 30 party history publicity activities. They told the glorious history, great achievements and historical experience of the Communist Party of China around 20 themes, such as "The May 4th Movement to Arouse the People", "A Great Gathering," groundbreaking "," Red Separation "," Catching Fire "," National Revitalization, The Happiness of the People ", and" Focus on strength to do great things "."Rich in content and deeply inspired!"Is the feeling of the school of Machinery class 2020 material control class league branch secretary Wu Hao after listening to the lecture. He said that in his future study and work, he will make down-to-earth contributions to serving students and schools. In 2021, under the guidance of the Publicity Department of the Party Committee of the University, the Youth League Committee of Chaohu University selected and set up a student publicity group for the whole school, and specially invited teachers Xiang Zexiong and Xia Mingqun from the School of Marxism to serve as the training instructors of the publicity group. Under the encouragement and guidance of the instructor, the 28 members worked hard to "prepare lessons", closely related to the theme of publicity, selected red stories, red views, red heroes and other "dry material materials", and made the spiritual essence, historical significance, and the value of The Times were refined to form the "vernacular" speech that students like. Up to now, there have been more than 60 lectures, with more than 5,000 listeners. Some of the class youth league branches of the school also invited the members of the publicity group to the class for the theme youth league group class, to participate in the youth league day activities, and to interact with the youth league members."I benefited a lot from every live lecture. As a college student speaker, we had to consult a lot of materials to help the students establish their feelings of home and country."Said Cai Jinglei, a member of the publicity group. Zhou Yu, a teacher from the school's Youth League Committee, said, " We will continue to contribute our youth strength to the school's high-quality party history learning and education with high standards and high quality, so that young students can learn nutrients from the study of Party history.”

2022-01-04 Guizhou Qiannan Institute of Science and Technology winter vacation social practice launched Guizhou Qiannan Institute of Science and Technology held 2022 "new era new journey new youth xing hometown" winter vacation social practice launch ceremony di Tu China youth network Beijing on January 4 (reporter Zhang Jianwei, correspondent, Song Huixin Yan Chun) recently, Guizhou Qiannan buyi and miao autonomous prefecture hui county "chunhui rural revitalization of a think-tank" committee, college students chunhui service was established and a think-tank, experts, chunhui messenger salute and Guizhou Qiannan institute of science and technology in 2022 "new era new journey new youth xing hometown" winter vacation social practice launch ceremony held in the college of science and technology, complex building 211 lecture hall. For the "Chunhui rural revitalization think tank" experts issued a letter of appointment. Correspondent Yang Di for the picture group Huishui County Party Committee, College of Science and Technology Party Committee related officials attended the ceremony, "Chunhui Rural Revitalization think tank" experts, Chunhui emissary, teachers and student representatives, a total of more than 400 people attended the ceremony. At the scene of the event, we issued letters of appointment to the experts of "Chunhui Rural Revitalization Think tank" and Chunhui emissary, and awarded licenses to the expert committee of "Chunhui Rural Revitalization Think Tank" and Huishui County College Students Chunhui Service Club. For the Chunhui emissary issued the letter of appointment. Correspondent Yang di for figure hui shui county "chunhui rural revitalization think tank" expert representative, Guizhou Tianjin zhi accord culture development co., LTD. General manager Ran Guangjin made a speech, she said, as long as perseverance, meager strength, a bit of love will eventually converge into a surging jianghai, called on everyone to adhere to the spread of chunhui love, spread human love. For Huishui County "Chunhui rural revitalization think tank" expert committee awarded. Correspondent Yang Di for the picture of Huishui County college students Chunhui service club president, college of science and technology Youth League committee teacher Yuan Tiansen spoke. He promised to use the existing resources of the college to establish a service position of Chunhui Action, ensure the high-standard and standardized construction of Chunhui Society, and strive to build the Chunhui service project of "Chunhui Action- -I practice together with my hometown". For Huishui County college students Chunhui service club card. Correspondent Yang Di for the picture Zhang Yaofang awarded the flag for Guizhou 2022 Qiannan University of Science and Technology winter vacation social practice team and made a summary speech. He pointed out that the Chunhui Action is not only a hope project that focuses on people's livelihood, education and students, but also an important heart-warming project that reflects our love, warms our hearts and builds our confidence. It is hoped that all the students of CAS will take the winter vacation of 2022 social practice activities as an opportunity, take "family affection, nostalgia and friendship" as the link, carry forward the Chinese civilization, feed their native relatives, jointly promote the economic and cultural development of their hometown, and promote social harmony and progress.

2021-12-28 Beijing, Dec. 28 (Xinhua) - -China Youth Network- -Cultivate the spirit of volunteering and gather the strength of youth. Recently, Nanjing University of Posts and Telecommunications "Orange Wind Set Post Sichuan" winter charity sale was successfully held. In 2010, the "Winter Charity Sale" first appeared on the South Post campus, where volunteers raised money to sell apples to fund leukemia patients. In the following ten years, the charity sale took root in NanPost and passed down from generation to generation. The year 2021 is another year for the "Winter Charity Sale" to accompany students of South Post and warm volunteer teaching children. On the 100th anniversary of the founding of the Communist Party of China, the teachers and students of South Post cherish the strong country, love the Party, practice the volunteer line, and help the dream of studying with the heart of volunteer, which warms the whole winter. On the morning of December 9th, the winter charity sale of "Orange Wind Set sail, Love Sichuan" kicked off in the postal campus. Outfield activity layout. As the organizer and planner of the winter charity sale of "Orange Wind Set sail, Love Post to Sichuan", the volunteers of the South Post Youth Annals Union have endowed them with different meanings for oranges."Sincere heart" to celebrate the 100th anniversary of the founding, "royal post orange" show grapefruit deeply love for south post, "orange like brocade" blessing grapefruit final refueling, academic success, "research orange" wish grind prepare for the grapefruit ashore, "orange to your heart" feedback warm our people... "orange" full, grapefruit in giving love at the same time, also sent out a good wishes. The winter charity sale of "Orange Wind Set sail to Sichuan" lasts from 8 a. m. to 9:30 p. m. The volunteers stick to their posts, with publicity, on-site guidance and form registration... "Red Vest" are active in every corner of the South Post campus. The enthusiastic, patient and careful service attitude ensures the orderly and efficient promotion of the outfield activities, and also builds a bridge between the students of Nanjing University of Posts and Telecommunications and Dazhu County No.2 Middle School in Sichuan Province. The outfield activity is over, and the volunteer service is not over. The pomelo volunteers dressed in red vests delivered oranges and love to the hands of more than 4,000 teachers and students of Nanyou, becoming a beautiful scenery in the winter campus of Nanyou. Post-stage packaging and distribution. Nanjing university of post and telecommunications youth corps committee for figure, "orange wind set sail post chuan" winter charity sale as south post youth long-term public welfare project "pomelo plan" important part, has funded Tibet, Guizhou, Sichuan students, in 2018 in south post graduate student volunteers in Sichuan province second middle school special set up "green scholarship", cumulative scholarship more than 40000 yuan, to reward teaching and excellent, both ability and political integrity, motivate bamboo students forever youth vitality, chang huai grateful heart, down-to-earth struggle, perseverance. To help the growth of western students and promote the rural revitalization of volunteer teaching areas. Qingzhi Scholarship award site. Nanjing University of Posts and Telecommunications Youth League Committee for the picture years because of the youth clearly went to go and more beautiful, the world because of the youth stand up forward and more magnificent. The annual winter charity sale of "Orange Wind Sailing Love Post in Sichuan" not only tightened the common bond between South Post and Sichuan Dazhu No.2 Middle School, but also wrote a vivid footnote for volunteer service. Volunteer in the heart, dedication in the line, "grapefruit people" has been on the road.

2021-12-28 Jiangsu universities: The online Communist Youth League is "within reach" 6,92,089 grassroots youth league branches, and the number of college youth league members in universities has reached 2.2584 million. In recent years, the communist youth league in Jiangsu province always adhere to the "strengthening political attribute, to adapt to the characteristics of youth, the media ecology" work concept, expanding new media work platform position, positive innovation condensation lead the way of service youth, strive to build popular new media products, political firm, dynamic, within reach "the communist youth league" construction, effective. Outstanding khalid ents, build a bacon cast soul network position around the fundamental task of khalid ents in colleges and universities, combined with the party history study education and learning propaganda and implement the theme of the spirit of the sixth plenary session of the 19th, Jiangsu university communist youth league make full use of new media platform for the broad masses of the young college students to carry out the ideological and political leading work, guide the youth members more unswervingly listen to the party, follow the party. Jiangsu Communist Youth League extensively conducts the "youth learning". On the one hand, it strengthens the organization and mobilization, specially issues work guidelines, and launches the "Work Ten methods"; on the other hand, it conducts special network activities on how to promote the "youth learning" in college freshman league members. The participation rate of most colleges and universities in the province is above 70%, and one third is over 100%. The total participation rate and the number of students remain in the forefront of the whole league. Since the end of 2018, Jiangsu communist youth league in the province normalized "faith class" online class, including "new ideas public class" public class "accomplishment" "dream class", etc., preferred ideological experts, cadres, young teachers, outstanding alumni, youth model for the speaker, in the form of young students happy good faith class, let the ideal faith in the fine fall small implementation of spring breeze rain, into the brain into the heart. Jiangsu Communist Youth League selects high-quality courses from the demonstration courses declared by colleges and universities every year for processing and improvement, and shows them in online classes. At present, there are nearly 100 online high-quality demonstration courses. Starting from January 2021, Jiangsu Communist Youth League has planned and launched a series of micro videos titled "14th Five-Year Plan on My Learning Experience" around the "14th Five-Year Plan". Selected young teachers and students from colleges and universities in the province, started with "small cuts" and "small stories", review the history and tell the future in the language of youth. The column was all collected by the national platform "Youth to the Party" and displayed on the home page, with more than 120 million clicks. The Jiangsu Communist Youth League pays attention to transforming the party history learning and education into interesting online and offline activities in a way that the young people are interested in, so as to achieve the best education effect. In 2021, around the centenary of the founding, the network theme campaign of "Dream youth follow the Party" script "Sin", collected 85 script works, including 7 performances; the "red classic growth" network singing campaign, two of which played video works were praised and recommended by the Internet; "Praise write on the earth- -online marathon and red landmark" campaign, attracting 3.26 million participants, a total mileage of 140 million kilometers. In the network world, young people are divided into "circles" and groups and circles with "interest" to build different "small" circles in different cyberspace. At present, the Jiangsu university communist youth league new media account a total of 4918, including WeChat public, weibo, QQ space, TikTok, B station and other nearly 20 college students often gathered in cyberspace, through optimal content construction, let high quality works in the "broken" spread youth consensus, youth voice, spread the youth positive energy. Aiming at the Communist Youth League of various audio and video platforms represented by "TikTok", Jiangsu Universities takes the initiative to occupy the network position, and seize the "fragmented" time of college students with the positive energy of audio and video content reflecting the core values, so that more high-quality content can be spread among young students. A short video produced by Jiangsu Communist Youth League "I finally controlled tears in the dormitory with Nanjing University of Aeronautics and Astronautics, Nanjing University of Technology, Nanjing Police College, but tears burst in the subway..." was praised by millions of people after it was released at Station B. The Jiangsu Communist Youth League also launched the Listen at Station B on the National Memorial Day for the Nanjing Massacre victims on December 13! The Message: The original video. The video, shot and produced by new media backbone from Nanjing Normal University and Nanjing University of Media and Communication, was viewed 800,000 times on the National Memorial Day, with a full screen of "Forget the national humiliation, revitalize China" and resonated with all the young people in the station. Jiangsu Communist Youth League provides practical and effective online services, gives full play to its organizational advantages, coordinates resources for college students, builds an online comprehensive service platform, and vividly presents the second classroom activities and the online platform of student organizations. Students choose to participate in the activities independently, and the platform automatically records the credits of participating in the activities, and accurately records the real-time situation of each student participating in the second class activities in the form of transcripts. At the same time, Jiangsu Communist Youth League also established the data application center of the second class to provide accurate guidance for the work, promote the application of the second class report card, and improve students' sense of gain. In the past two years, in the face of the impact of the COVID-19 epidemic on the employment of college students, the Jiangsu Communist Youth League has timely shifted its employment services from offline to online services, and organized various localities to actively carry out the "post delivery through train" live post recommendation campaign to ensure the continuous line of employment services. We adhered to standardized deployment, formulated normative implementation plans, and promoted 144 live post recommendation and cloud relay sessions. Since last year, relying on online and offline work carriers, we have raised more than 530,000 jobs for college graduates in the province, and helped 23,040 college students to achieve employment intentions. The new media work of the Communist Youth League in colleges and universities needs the support of the offline real teams. Most universities in Jiangsu Province have established the operation and maintenance teams with more than 10 people to carry out the new media work. In the future, the Jiangsu communist youth league will further implement the university new media work conference spirit, earnestly learn from brother provinces (autonomous regions, municipalities), good practice, good experience, further play to the role of Jiangsu communist youth league media center this year, continue to cooperate with Nanjing normal university and other key universities, constantly promote the communist youth league in Jiangsu university new media work to achieve new development, achieve greater results. Source: China Youth Daily, December 28,2021

2021-12-24 Shaanxi energy vocational and technical college to carry out the epidemic prevention and control warm heart activity shaanxi energy vocational and technical college to carry out the "warm friendship warm warm warm love" epidemic prevention and control activity Ji'an-wei zhang correspondent xiao-hong Chen) recently, shaanxi energy vocational and technical college youth corps committee to carry out the "warm friendship warm warm love" epidemic prevention and control warm heart activities, for teachers and students "a bag of masks, two lollipops, three warm words" "warm heart three sets". In the campus roadside benches, garden trunk, playground football rack, stair handrails, classroom aisle and other positions there are "small gift" prepared "warm heart three sets" for everyone. Students, teachers, dormitory administrator aunts and canteen aunts who received the "warm heart three-piece set" all said that they would set an example to lead people around them to do a good job in epidemic prevention. The heart-warming activity aims to encourage teachers and students to understand and support the school's epidemic prevention requirements of the school, actively cooperate with the school's "school closure" management, pay attention to the mental health of teachers and students during the epidemic period, and provide warmth to the staff who stick to their posts and students who review carefully during the epidemic period.

2021-12-22 Anhui Medical University set up a youth publicity group to promote the spirit of the plenary session into the youth heart test, we delivered the 'China's answer ', is the result of the CPC Central Committee decisive decision, calm response, adhere to the people first."Recently, Dong Yongpeng, the winner of" The National Excellent Communist Youth League "and" the most Beautiful Family in Fighting the Epidemic ", combined with the couple's experience in Wuhan, told about the understanding of the spirit of the sixth Plenary Session of the 19th CPC Central Committee, which deeply infected the audience. On the same day, Anhui Medical University held the appointment ceremony and interactive publicity activity of "youth group". Members of the publicity group interacted with the audience face to face, and promoted the spirit of the plenary session into the heart of the youth with a strong "youth flavor"."We need to see from the century-old struggle of the Communist Party of China why we have succeeded in the past and how we can continue to succeed in the future. The original mind is easy to win, but always difficult to keep. Only with history as a mirror can we create the future."Chen Xin, a member of the youth publicity group, said to the audience that he joined the army in 2019 and has now returned to the nursing major. He will not forget his original intention to serve the country in medicine, and should protect the health of the people just like guarding the national flag. In order to pass the voice of the voice of contemporary college students' struggle, bear, the school selected 16 outstanding youth representatives from the grassroots youth league organizations, through the establishment of a vigorous outstanding young teachers and students theory propaganda team, with "green language" to do a good job in the spirit of the sixth plenary session of the 19th CPC interpretation, set off a new round of preaching upsurge. At the lecture, the audience listened attentively and wrote notes from time to time. In order to test the early learning results, the scene also carried out a knowledge contest activities, the audience enthusiastically raised their hands to answer."This seminar gave me a more comprehensive and clear understanding of the spirit of the Sixth Plenary Session of the 19th CPC Central Committee. As the young generation, we should make clear our responsibilities and burdens, integrate our thoughts and actions into the spirit of the plenary session, implement them into action, implement into our study, and escort the health of the people."Said Zhen Ziyi, a 2020 medical laboratory student at the university's First Clinical Medical School. The lecture used a combination of online live broadcast and offline lecture, watching 1,000 people online.

2021-12-16 North China electric power university: labor education "play out" new pattern of north China electric power university: labor education "play out" new pattern for figure China youth network Beijing on December 16 (reporter Zhang Jianwei, reporter Zhang Jian Zhang Yifan) recently, north China electric power university Baoding campus youth corps committee organization theme manual lantern show, school youth corps committee combined with work practice, strengthen planning design, through the rich and colorful theme activities, continue to guide students to establish correct labor consciousness, form the correct labor concept, improve practical ability. The manual theme lanterns creation display competition to "carry forward the labor spirit inheritance excellent culture" as the theme, breakthrough past only limited in the proposition of labor education design, follow the era theme, designed to celebrate the founding in one hundred, green low carbon environmental protection, youth power games, celebrate New Year's day 2022 four themes, collecting students made 250 lanterns. The lanterns are made with newspapers, colored cardboard papers, cartons and other materials. Combined with the study and education of the Party history, the students made the landmark events in the century-old history in the form of carving or painting, showing the lanterns and also showing the brilliant achievements of the Party and the deep blessing of the young students to the Party. This year is the second year of the North China Electric Power University handmade lantern creation and exhibition competition. The original intention is to let the students use their brains, frequent hands, enrich the extracurricular cultural life. Nowadays, lantern creation has become an important carrier for the school to carry out labor education, besides the traditional manual labor of cleaning campuses, planting trees and weeding.

2021-12-17 Xiamen University: Youth micro lecture let the spirit of the sixth Plenary Session of the CPC "voice" into the hearts of the people Yang Baoguang) " Resolution gathers great wisdom, review the glorious history of the Party; two establish a clear direction, ten adhere to the good position!"" The 'big' of the country is to protect the people's sense of security, gain and happiness!"" A Red Army well reflects the unchanging original aspiration and mission of Chinese Communists for a hundred years!"...... recently, Xiamen university party committee party school 85 the party's basic knowledge learning class ushered in a batch of" 95 "after" after 00 "youth lecturer, they to" beginner's mind as rock, power generation have me " as the theme, into the storytelling, allegro, magic, short video, pipa playing, such as the form of youth happy, for the audience brought a party class feast. From Tan Kah Kee's trip to Yan' an in 1940, to the happiness code on the road to prosperity; from the twists and turns of Qian Xuesen back to China, to the confidence and strength of the "great power"; from the help and cooperation of Minning, to the youth where the party and the people are most flower... 8 young student lecturers, or telling their stories, telling the story, or refining the profound theory, using the grounded, warm language, the spirit of the sixth Plenary session into a vivid story, allowing teachers and students to sit down, willing to listen, and use."Would you have the courage to come if you knew the life you have to face?"Che Xiaoxuan, from the law school, quoted the movie line first with such a question. Then, she led everyone through a hundred years of time and space, back to the birth of the Communist Party of China, by telling the story of revolutionary martyr Fang Zhimin and combat hero Zhang Fuqing, show the spiritual quality of the Communist party "advocate hero will produce a hero, hero to hero...." Xu Wei from the school of Economics micro propaganda "each such you and I, are a hero", with a "grassland hero little sisters" pipa performance opening, let everyone fresh and fresh. Shang Zhaoyan, wearing a long lab coat at the School of Public Affairs, played allegro and played magic on the platform, explaining the back story of "China's hope is in Yan' an" in the form of storytelling. Zhu Xueqing, School of Architecture and Civil Engineering, brought a micro lecture, "Chinese Bridges Map the centenary History of the Party", which tells how Chinese bridge builders have made the impossible possible in the past hundred years. Yu Zhewei, from the School of Pharmacy, a member of the 21st Graduate Student Support Group at Xiamen University, taught the theme of "A Story to Join the Party". In just over 10 minutes, he shared the changes brought by the CPC Central Committee's targeted poverty alleviation policy to the western region with his own experience and experience as a volunteer of poverty alleviation based on education. The theme of the micro lecture was distinct and rich in content. The speaker on the stage was eloquent, and the audience listened attentively."The teacher group's lecture speaks to my heart! As an active member of the Party, I will firmly listen to the Party and follow its ideals and beliefs, color my youth with struggle, and follow the 'baton' of the new era."After listening to the micro party class lecture, Zheng Shaoqing from the School of Humanities was deeply touched."Youth theory propaganda should realize precise drip irrigation from flood irrigation. Only by linking from small angles, selecting people around to participate in, and promoting the normal development, can the attraction, appeal, pertinence and effectiveness of the theory propaganda be effectively improved."Said Liang Zhenwei, deputy secretary of the Communist Youth League Committee of Xiamen University. It is understood that the Xiamen University firefly Spark Youth Lecture Group was established in May 2021, and composed of 46 young students. Starting from December 11, the lecturers will carry out tour lectures through the branch, the apartment, the associations and the network, further expand the influence and radiation of the lectures, and promote the spirit of the Sixth Plenary Session to take root and sprout in the hearts of young students.

2021-12-16 Wuxi process vocational and technical college: three-dimensional building ideological education system of Wuxi process vocational and technical college: three-dimensional building ideological education system "always follow the party, the new era" as the theme, to "study party history, enlightenment thought, does the practical work, open new bureau" as guidance, based on online, campus, conduct red courses, red art education, red social practice, three-dimensional build red education system, make the red education "blossom everywhere" on campus. All the teachers and students of the school have firm ideals and beliefs in the learning and experience, forge good character in the practice and experience, and write patriotism, patriotism and dream of becoming a strong country with their youth. The school gives full play to the advantages of "curriculum ideological and political development", and consciously organically integrates red resources such as Party history into professional teaching, skills training and other links. In the ideological and political seminar of the "Red Gene Casting Soul Education" course held this year, the representatives of professional teachers shared how they took their needs, showed their strengths, and worked together to do a good job in the big article of "Party history + curriculum ideological and political education". Mr.Li, a teacher from the School of Environmental Art and Design, has created a new teaching model with a "red ideological and political flavor". Miss li in originally pure technical course added the "red factor", to "design to retrace the long march road" as the carrier to teach, through the long march starting point of postcards, Guizhou Zunyi long march important location of Logo, climbing snow grass animation snow, the old red army photo repair, long march theme movie posters copy production full of long march elements of course training content, let the students in every real project case from easy to difficult, understand the use of software, deepen the skills. When designing and improving the project in the footsteps of the teacher, the students not only have not only the satisfaction of improving their own skills, but also the pride in the great spirit of party building. The students also connected all the cases into a Long March route, which became the "Long March journey" in the new era in the course. They explored the Long March Road online and deeply learned the spirit of the Long March. The school takes ideological and political courses as an important position to carry out ideological and political education, and adopts the idea of taking professional courses as the carrier, mastering skills as the goal, and taking ideological and political elements and red stories as the medium to carry out curriculum teaching, paying attention to silent education and endless aftertaste. Especially since this year, the school has taken the party history learning and education as an opportunity to guide teachers to integrate red ideological and political elements through and into the whole process of education and teaching, to improve students' moral quality from multiple angles and in an all-round way, and to cultivate patriotic feelings. In order to find the best fit point of knowledge transmission, teachers will spend more time learning the knowledge of party history, understand the story of party history, and think and study how to organically integrate red elements into professional courses. For teachers and students, this is undoubtedly a "two-way growth" process. While subtly influencing students, professional course teachers have also experienced ideological growth, and their moral awareness and ability have been significantly improved. Open the "Tao Run Hundred Years of Works", you can see the works full of red elements designed or created by the school teachers. Since this year, the school relying on art professional resources and talent advantage, throughout the scope of the "thousand people thousand art salute in one hundred" artistic creation activities, guide the teachers and students on the basis of thorough study of the history of the party, using professional director, the red elements and artistic creation organic fusion, actively reflect the great journey of art in one hundred, a "red tide" on campus. Teachers and students use purple sand pot, pottery, sculpture, clothing, painting, calligraphy and other artistic forms to express their sincere feelings of love for the Party and the motherland. At the same time, the school also extends the activity of "Centennial Respect of Respect" to the countryside, and sets up a special creation working group of "Tao Run Centennial Respect", to carry out cultural creativity, artistic design, academic guidance and other work, guide Yixing rural purple sand artists to create fine red art, and pay tribute to the centennial anniversary of the Party. Exhibition, teachers and villagers cooperation of the red army soul teapot attracted many attention, work creation inspiration from yuan blue and white porcelain "general", lid imitate the red army cap modelling, using asymmetric tendon capsule design, the overall appearance of the red army soldiers, let the viewer in feeling the teapot traditional charm at the same time, more feel the beauty of red poetry and the nobility of the revolutionary spirit. The teachers and students of the school feel the great strength of thought from the centennial history of the Party, draw the strength to forge ahead, and learn the history of the Party, understand the thought, strengthen the faith, and follow the party in the unique red art creation. Social practice shows the mission. For the social practice team of "revitalizing green Tea Culture", more than ten teachers and students spent a different summer vacation. In Jinzhai, Lu'an City, Anhui Province, they went deep into the homes of tea farmers, used professional knowledge for cultural and creative design, live delivery for tea farmers, and played a distinctive "tea culture, tea brand" on the Internet, enabling rural revitalization with practical actions. Through the sales and promotion activities of featured agricultural products and cultural products, students can help farmers with science and technology, promote employment with entrepreneurship, and describe and tell the stories of China's beautiful countryside from the perspective of college students. In addition, many students also actively contributed to the epidemic prevention and control. With the purpose of "serving the society, helping others, improving themselves and promoting new trends", they went deep into various streets and communities, participated in the epidemic prevention and control work in their hometown, and contributed to the epidemic prevention and control. Members of the practice group said that as the youth volunteers in the new era, they should forge ahead and make the "youth" force, so that the youth power can shine in social practice, practice the mission of youth with practical actions, and shine the light of youth. Since the education of the history of the party, the school adhere to the combination of self-study and league branch collective learning, organize youth members to actively participate in a series of online theme class, with the party's struggle history, the party inspirational story, the party achievements, leading the youth members to love the party patriotic ideological foundation; dig into the typical characters and typical deeds, strengthen the advanced typical demonstration effect, the formation of the "good" good atmosphere. At the same time, teachers and students integrate the red spirit into life, and carry out a series of activities in various forms and rich connotations centering on ten themes, such as "traditional culture inheritance", "education care and service" and "enabling rural revitalization". The relevant person in charge of the school said that the school always takes practical education as a key link of education and teaching, strives to create a high-quality "walking classroom", promotes the majority of young people to deeply inherit the red gene, and practice their mission with practical actions. Source: China Youth Daily

2021-12-15 Nanchang University held the final of the red Film Dubbing Competition of the Student Culture and Art Festival. After selection, 15 teams from different colleges entered the final. In the final, the contestants voiced the classic red films such as "Revolutionary", "Big River", "Railway Guerrillas" and "Founding Ceremony". In the end, Xin Tian Group from the School of Journalism and Communication of the university won the first prize in the professional group, and Zhang Xiaolei Group from the School of Journalism and Communication and Qian Yulu Group from the School of Basic Medicine of the university won the first prize in the non-professional group. The competition is sponsored by the Youth League Committee of Nanchang University, the Art Education Teaching Department of Nanchang University and the Student Union, and organized by the Youth League Committee and the Student Union of the School of Journalism and Communication.

2021-12-13 Guizhou Qiannan institute of science and technology held party history knowledge contest in Guizhou Qiannan institute of science and technology "lead the growth red light youth" knowledge contest Beijing on December 13 (reporter Zhang Jianwei, correspondent YanChun) recently, Guizhou Qiannan college of science and technology "lead the growth red light youth" knowledge contest heats, finals held in the complex building 211 lecture hall. The competition is divided into three stages: preliminary, semi-final and final. A total of 32 teams will participate in the preliminary competition. The picture shows the contestant answering questions in the knowledge contest. After the competition, a total of 8 teams entered the final round. The semi-final is divided into four types of questions: group answer, limited time answer, situational answer and risk question. It mainly revolves around the youth study, party history, new China history, reform and opening up history, socialist development history and other related contents of the contestants. Finally, four teams were selected to enter the final. The picture shows the contestant answering questions in the knowledge contest. The final set of individual required questions, rush to answer questions, additional questions, the scene of each team players knowledge reserve, strain ability, team spirit and psychological quality of the wonderful competition won the audience bursts of applause. After fierce competition, "Riding the Fire Phoenix Team" won the first prize, "Party Affairs Pioneer Team" won the second prize, "Youth Learning Team", "Star Dream team" won the third prize, "Go forward team", "Spark prairie fire team", "Build dream team" and "Red Star Flash team" won the award of excellence.

2021-12-12 Nankai students to the national college league branch endorsement hometown products nankai students to the national college league branch endorsement home products Li Mingwei) on December 9, nankai university history college 20 undergraduate class 1 league branch held a "rooted long mu, to know" online theme TuanRi, youth league branch secretary Song Jinke initiative to the national college league branch, collect 100 youth league branch to join the power rural revitalization of action."Eighty-six years ago today, that group of patriotic students of about our age shouted,'Long live the Chinese nation!'' Struggle for the freedom of the motherland '! Today, we commemorate the 129th patriotic Movement in this way, which is to draw spiritual strength from the four histories ', inherit the red gene, and continue the important responsibility of The Times."Song Jinke said. During the "Double 11" period, the youth league branch held the theme of "Live with goods Double 11, across the mountains and rivers", promoting the most characteristic apples, potatoes, brushes, lanterns and other characteristic products in Zhuanglang County, Gansu Province, so that many students can feel the sense of gain in the process of helping farmers with goods."Students hope to help more places and more farmers to promote characteristic products, and pay attention to agriculture, rural areas and farmers, and serve the responsibility of contemporary youth. Therefore, we launched the name of the youth League branch to collect 100 youth League branches from universities across the country to jointly contribute a youth strength to the national strategy of rural revitalization."Said Ma Chao, secretary of the Communist Youth League Committee of the School of History. The initiative calls on the youth league members to pay attention to the "A Bite of hometown", in the form of micro video to promote hometown products, introduce local conditions and customs; on the basis of fully understand the hometown food, characteristics, history and cultural heritage, relying on the public welfare platform to help farmers to expand sales channels; at the same time, the youth league members to respect and love the hometown in daily conversation, let their words and deeds become the name card of the hometown. The initiative was released simultaneously on the official Weibo account of Nankai University and the official account of Nankai History History. Hao Chenyan, secretary of the Youth League branch of Xi'an Jiaotong University Student Micro Publicity Group (Dream Society), expressed his willingness to participate in it and gather youth strength for the national strategy of rural revitalization. In this activity, the students independently planned the theme exhibition of "Taking root in Longchuan mu, Learning from the past- -Rural Revitalization Timeline" was carried out simultaneously online and offline, and the students drew a "time sample" of poverty alleviation. In Nankai Boling Class, students gave full play to their advantages of being keen on academic exploration and held four reading seminars to deepen their understanding of rural revitalization. Nankai university youth corps committee secretary He Wenxia said, guide the students in rural revitalization, do the knowledge on the Chinese earth, is not only the "know China service China" academic tradition, and contemporary college students bear "love Chinese rejuvenation" era of responsibility, is to commemorate the "129" movement, carry forward the spirit of patriotism of nankai expression.

2021-12-11 Guizhou Qiannan college of science and technology theme lang song game ended he party in one hundred birthday continuously red blood Guizhou Qiannan college of science and technology theme lang song game ended Beijing on December 11 (reporter Zhang Jianwei, correspondent, Huang Mengyao YanChun) on December 6, Guizhou Qiannan college of science and technology "he party birthday in one hundred continuously red blood" theme lang song game final concluded."One Hundred Years, Heaven Yao China", "Dear Mother- -The Communist Party of China", "Clear Love is Only for China", "One Hundred Years of Youth"... In the final scene, the contestants combined with the background music, PPT and their own understanding and perception of the recitation content, led the present teachers and students to review the glorious course of the Party. The picture shows the contestants reciting affectionately."I seem to have a dream, I stood confused at the intersection of an era set sail, watching people hurried by me, some in suits and shoes, some ragged, they are so different, but so similar, it seems to be 1921...." contestant Luo Xiaobei read affectionately expressed their love and praise for the motherland, brought the audience an auditory feast, won bursts of applause. The picture shows the contestants reciting affectionately. The judges of the contestants were comprehensively scored for their performance in four aspects: pronunciation, language skills, stage performance, and appearance. After fierce competition, Luo Xiaobi from Class 208 Chinese Language and Literature won the first prize, Liu Wen from Class 211 won the second prize, Liu Peng from Class 211 won the third prize, Wang Yihan, Zhang Simiao, Yuan Bin � D from Xiang Yihan from Class 211, Zhu Jinhao and Lu Lu from Class 208 Chinese Language and Literature won the excellence prize. It is reported that the competition lasted for 10 days, divided into the preliminary competition, the final two stages, a total of 25 contestants to participate, after layers of competition, 12 contestants entered the final.

2021-12-11 Tsinghua university held inheritance "129" spirit relay activities at tsinghua university "inheritance" 129, new one hundred "theme relay running activities China youth network Beijing on December 11 (reporter Zhang Jianwei, reporter Xu Hui Chen Tianyi)" inheritance "129, new one hundred" theme relay activities recently held in tsinghua university, more than 500 teachers and students staff with running historical spirit, express patriotism. The participating teams will warm up before the match. This year's relay running site also set up a special exhibition, to show the glorious history of the party history and the outstanding figures, before the start of the activity, the athletes came to the auditorium early to visit the special exhibition, detailed narration and exquisite production also attracted visitors to stop. On December 5th, students, counselors, staff, middle-and long-distance running sports teams and others formed nearly 50 teams to participate in the activity. Former deputy secretary of the school party committee wang, director of the sports department liu, soil water college wang, deputy party secretary of social academy, RongKe, deputy party secretary of law school, dynamic Chen ping student group leader zhang Yang, college party committee joint student working group leader kai-feng wang, sports jun-hua wang department guests to attend the event. Students of each great time. In the morning, nearly 50 teams gathered to sing the school song in front of the auditorium. In order to let the students remember the history and feel the changes of The Times, this long-distance commemoration has carefully designed the track stick. The activity named the location of the important events in the "129" movement, such as Xizhimen, West Fourth, Xinhua Gate, Tianqiao and other places, and set up the corresponding exhibition areas according to the general spatial distribution of each region. At the same time, each of the first five sticks restores a specific scene in the 129th movement, and the last five sticks integrate elements into the centenary of the founding of the Party, the 110th anniversary of the Revolution of 1911, the Winter Olympics and the 110th anniversary of the anniversary of the university. Students of each great time. This long-distance running activity gives each stroke a profound meaning, aiming to deeply integrate the spirit of Tsinghua University with the great era, and the process of long-distance running and the process of national historical development are closely combined, so that teachers and students can better review the stirring history in the long-distance running, and pay tribute to the spirit of The Times. After the competition, the teachers and students stopped in front of the display board to take photos. Every teacher, students and children present gave full play to their best state. In addition to the ranking, what is more important is everyone's perception and inheritance of the spirit of "129 th".

2021-12-09 Southwest People Research Branch Group: watering with youth sweat "Gesang flower" Wang Xinxin with youth sweat, can still bloom strong in high altitude, cold areas, is a symbol of love and auspicious flowers. This is also the name of the graduate Student Support group of Southwest University for Nationalities. Since 2015, the "Gesang Flower" graduate volunteer teaching group of Southwest University for Nationalities has sent 30 volunteers from all ethnic groups to Hongyuan County, Aba Tibetan and Qiang Autonomous Prefecture, Sichuan Province, about 3,500 meters above sea level. On October 21st, the "Gesang Flower" graduate volunteer teaching group of Southwest University for Nationalities held a reading sharing meeting themed group day activity in Hongyuan County Middle School. Due to the lack of local teacher resources, the members of the "Gesang Hua" research branch group hold several positions. For example, the first volunteer of Zuo Yaozhou students, at the same time to give students on the first math, politics, two politics, computer, politics and two politics and other courses. Get up at six o'clock every day to prepare lessons, teach, organize activities, and correct homework. From grade one to grade three, after three volunteer relay sessions, three Tibetan students, Geng Gadolinium Wo, Ye Xima and Chen Yufang from Class 2, Grade 2015 of Hongyuan County Middle School, achieved excellent results in the 2018 high school entrance examination, and were pushed to the Middle School affiliated to Minzu University of China. In the 2019 high school entrance examination, three students of Park Jinglin, head of the third "Gesang Flower" Research Group, applied for the High School Affiliated to University of Central Civil University, standing out among more than 8,000 students in Aba Prefecture, ranking first in Hongyuan County, Heishui County and Songpan Prefecture respectively. In the unified testing of 2020-2021, the Chinese discipline of Class 1 of class 2020 taught by Zheng Zudie of the fifth "Gesang Flower" research group and the political discipline of class 1 of class 2019 taught by Gu Yunqiu all won the first place in Hongyuan County. In addition to daily teaching, the members of the "Gesang Flower" research and branch group of Southwest University for Nationalities also actively cooperate with various channels such as the school and the society to improve the local basic education environment. With the support of all walks of life, the school has built two "only wish" computer laboratories, installed the love of drinking water purifier and other equipment, and set up the plateau "love library". In addition, caring people set up 294,000 yuan of "care Hongyuan Tibetan qiang youth grants" and "Gesang flower" scholarship, help Hongyuan more than 20 orphans, excellent students 100, "only wish" if the poor grants helped 30 if the primary and middle school students... in 6 years, the local accumulated donations and awards, grants of more than 2 million yuan. Relying on special group class, class meeting and poetry recitation and sharing meeting, southwest University of Nationalities "Gesang Flower" publicity and education activities with the theme of "five identities", national unity and socialist core values. On the occasion of the 70th anniversary of the founding of the People's Republic of China, the "Gesang Flower" research group led the students to the Rganqiao Wetland of Wach Town to carry out the theme of "Youth Heart to the Party, Building the New Era". By retracing the Long March of the Red Army, reviewing the oath of joining the League and listening to the Long March of the Red Army, they stimulated the students on the snowy plateau to love the Party and their patriotism. At the same time, the "Gesang Flower" research branch group also actively explores the mode of "teachers and students order- -volunteers receive orders". First of all, through the offline "ordering", students learned the students' needs for standardized pinyin spelling, and then cooperated with the Chinese and Chinese bilingual volunteer service group of Southwest University for Nationalities, set up a "little pomegranate" broadcast room, using Internet technology to carry out lectures and launch a series of online micro courses.

2021-12-08 Chongqing University will combine the best, education and promotion to let the students take the "first kilometer" of active applicants to join the Party!"Cui Xinyue immediately called her parents to tell them the good news. On this year's May 4th Youth Day, the 2020-level Youth League branch of Chongqing University Academy of Management Sciences held a conference to discuss and recommend a group of outstanding league members as active party members, and Cui Xinyue is one of them. On the road to becoming a glorious Communist party member, Cui Xinyue took a solid step of "the first kilometer". For a long time, Chongqing University attaches great importance to the work of "Party building and league building", and constantly deepens and improves the Communist Youth League to join the Party. The Party Committee of the University has successively issued the Implementation Rules for the Education and Management of Party Member Development and Party Members of Chongqing University and the Implementation Plan for the Reform of the Communist Youth League of Chongqing University, which has clearly incorporated the promotion of the Communist Youth League into the Party member development plan of the University. On this basis, the Party Committee of the University issued the Implementation Rules of Chongqing University on the Work of Recommended Excellent League Members as Active Members of Party membership last year, further refining the relevant work requirements and procedures, and actively promoting the effective implementation of the excellent work. Wang Xu, deputy secretary of Chongqing university party committee, said: " we always adhere to the fundamental task of khalid ents, practice for party education, yucai's mission, give full play to the role of the communist youth league as the party's assistant and reserve, strengthen the political lead of the communist youth league member, combining selection, education, optimization, training, guide, recommend comprehensive development of excellent member to become the party activists, actively send fresh blood to the party organization."At the beginning of enrollment, ushered in my" highlight "moment" to promote the party is a systematic project, from the students entered the school to participate in the military training, the party enlightenment education began, from the moral, intellectual, physical, aesthetic and labor for students to describe a red plan, this is also a roadmap for students to grow up."The school youth League committee in each level of freshmen military training division will set up a temporary youth corps committee, really a good" entrance pass ", the youth league branch built on the" company ". At the important node of the centenary of the founding of the Party this year, the theme of "Please trust the Party, strengthen the country with me" is more deeply rooted in the hearts of the people, and it will lead the freshmen to actively draw close to the Party organization through various activities, so that the short-term achievements of military training and education can play a long-term role. During the 20 days of military training, it has become a habit of freshmen of Chongqing University to participate in the online Youth League committee and the online themed Youth League class of "Youth University Learning". Through learning to consolidate and understand and absorb, many students have become the "masters" of party history knowledge. Among them, Huang Yaobiao, a new student from law and journalism categories, signed up for the speech contest of "Inheriting the Red Gene, Striving to be a New Person of The Times" held by the temporary Youth League Committee and won the second prize, which made him gain confidence and growth, and encouraged him to get more close to the Party organization actively. Let the "guarantee of supply" gradually change into "active promotion", so that students have goals, direction, motivation. The Youth League Committee of Chongqing University explored the implementation of the "star evaluation" system for student league members in 2018, and asked students to make clear how to become "five-star league members" and "Sunshine League members" from the five aspects of morality, intelligence, physique, beauty, beauty and labor, and then give priority to obtain the excellent qualification. The school committee, said: "the clear, complete, rich 'training plan as fit each student to the party' customs clearance secrets, in learning and practice to enjoy the happiness of jin upgrade, more important is the students 'personal comprehensive quality, in the school members created' than learn to help super thick atmosphere, the students will be earlier, more in one after another belong to their" highlight " moment."In addition to studying political theory, Chongqing University also pays special attention to practice in the training of league members. The Youth League Committee of the university further promotes the "tree sound" action of innovation and entrepreneurship, the volunteer service opening state action and the social practice furnace action, so as to provide precise supply and realize accurate education for cultivating excellent league members. At the beginning of 2020, in the face of the sudden outbreak of COVID-19, the outstanding performance of the young generation is gratifying and touching."Seeing so many young party members write down the invitation letters to go to the front line, so many young party members stick to the community (village) bayonet guard, I also want to do something like them."Wang Ge, a student of Public Management, made up his mind. During his stay at home, Wang Ge has been participating in the "guarding the rear" charity volunteer service organized by the Youth League Committee of the school, attending more than 150 hours and tutoring more than 10 medical children. After the opening of the school, the Youth League Committee of the school carried out the political guidance and absorption of advanced youth in the fight against the epidemic, adhered to the standards, ensured the quality, effectively completed the work of more than 50 students, including Wang Ge, and effectively enhanced the training, service and guidance of excellent league members in special periods. After getting the push form stamped by the league organization, Wang Ge said with a smile: " This is the 'privilege' of a party member, when the party and the people need it, we must rush in front!"Wang Ge counselor, grade of party branch secretary BaoHan said:" more and more student members are more yearning for the communist party member's cutting edge exemplary role, in ordinary times can see out, the critical moment to stand out, crisis clear out, thanks to the youth league push optimal platform, let us to carry out students' ideological and political work has a more powerful gripper."In providing multiple practice platform at the same time, Chongqing university pays more attention to follow-up tracking training, forming process assessment and overall evaluation of dynamic management mechanism, gradually promote the optimal practice education scale, form system, paradigm, and will meet the standard of optimal youth members one by one identified establish optimal object list library, to ensure that qualified youth members can be recommended in a timely manner. Whether the league member is excellent or not, the branch has the final say to do a good job in promoting the league organization, design the "push" system procedures and "excellent" evaluation standards, and implement the work is the top priority. To this end, the school did three aspects: one is the full implementation of youth league branch "benchmarking project", in accordance with the "contact an excellent party member, held a volunteer service activities, read a red classic, held a demonstration TuanRi activities, make a branch wind collection" "five" evaluation criteria, polished youth corps cell card, enhance the branch work scientific and credibility. Second, the Youth League Committee of the university and the Party organization of the college coordinate, so that the Youth League Committee and the Youth League branch of the college work under the leadership of the college Party Committee, not only pay attention to the evaluation process, but also pay attention to the evaluation results, so as to promote the fairness and justice of the evaluation of the branch. Third, to realize the "school-school-class" multi-level, all-round supervision and guidance, through public defense, network publicity, video display and other forms, effectively improve the transparency and influence of the work. Multiple measures to solve the problem of "league branch good or not good, league members excellent not good, league cadres do the same" problem, to ensure that the excellent work let students receive education, branch add vitality, the party flag add luster. School each secondary college party committee strictly implement the main responsibility, strengthen the college party leadership and supervision, will push the party, the reform of the communist youth league as an important content of college party construction, at the same time the completion of the annual push optimal work and college youth corps committee, youth league branch and its head of the annual assessment, assessment awards link the system into effect. The person in charge of the school Youth League committee said: " In the past two years, the school has achieved 'two 100%' of student party members, that is, students under the age of 28 to join the Party, 100% from the league members, 100% through the whole process and each link of training, investigation and recommendation of the league branch."The Communist Youth League is the Party's assistant and reserve force, and it should train qualified builders and reliable successors to the cause of socialism with Chinese characteristics for the Party."Training" is first of all political training, "qualified" and "reliable" the key is political qualified and reliable. Chongqing university youth corps committee push optimal to join the party and strengthen the construction of advanced member efforts to grasp, highlight political standards, strengthen education management, promote role, has achieved periodic results, since 2021 has recommended 2366 members become party activists, help them to walk good down-to-earth the party "the first kilometer", efforts to grow into be loyal, absolutely reliable, critical moment can charge for the cause of the party youth political backbone. China Youth Daily China Youth network reporter Jin Zhuo correspondent Bao Han source: China Youth Daily

2021-12-07 Yancheng Normal University: Polish the practice education background color with red, and deduce the party history story in the form of "script killing", the scene has become popular in the campus."Immersive experience" and "cross-time dialogue" allow the participating young students to "enter the play" quickly, and the college student Chen Jiawei is even immersed in it. Because of these stories, it is what he participated in the "search for the New Fourth Army veterans" practice regiment income. This year, Chen Jiawei followed the "search for the New Fourth Army veterans" practice group, in Yancheng to search for 87 New Fourth Army veterans, the production of the "pursue the iron army footprint continuously red century" historical documentary and more than 10 thousand words of research report, with words and images to make this party history memory in reality "resurrection"."The number of surviving veterans of the New Fourth Army is decreasing year by year, and it is urgent to preserve this historical data."Said Chen Jiawei. In 2016, Yancheng Normal University set up a practice group of "Searching for veterans of the New Fourth Army", and nearly 200 college students participated, collected more than 200 hours of audio and video, and collated more than 400,000 words of text materials. At the school, there are 16 such red practice regiments. College students walked into memorial halls, martyrs' cemeteries and historical sites to "punch in" in more than 100 red bases in Yancheng to share their experiences online."Yancheng's rich red resources are' living teaching materials' for schools to conduct practical education."The school vice principal Zhang Guihua introduced. Yancheng, located in northern Jiangsu province, was once the location of the New Fourth Army and also the center of forging the "iron army spirit". According to statistics, there are 248 red heritage sites in Yancheng, and 128 towns and villages named after martyrs. Wulie Town, the hometown of Cao Kefan, is one of the 128 towns and villages. Last summer vacation, Cao Kefan and more than 200 college students visited the towns and villages named after the martyrs. They traveled more than 3,000 kilometers, distributed more than 2,000 questionnaires, interviewed more than 200 people, and wrote relevant research reports. Yancheng Normal University also actively explores the "same-frequency resonance" between social practice and local development. In July, Hu Xinyi, a college student, led the villagers to read the classic passage from the Red Rock in a book building in a rural library in Zhangdian Village, Longgang Town, Yancheng City. The college students' social practice group of "Helping Rural Libraries for Rural Revitalization" has visited 2,221 rural libraries in Yancheng, "bringing the red culture to the rural libraries and making the rural libraries become a gas station for party history learning and education". In October this year, the 25th batch of college students went to Xinjiang, and 20 college students went to primary and secondary schools and vocational schools in Yili, Qapqal, Hami and other places for practice and volunteer teaching. Since 2009, the university has sent 25 groups of 1,426 internship and volunteer teaching college students and 45 instructors to Xinjiang."This is the vivid embodiment and strong proof of the school to cast the soul of the red culture."Dai Binrong, party secretary of the university, said," In the process of deepening practice education, the university revitalized red resources and improves the effectiveness of education; inherits the red gene and cultivates red successors."Ding Xuankai, China Youth Daily, China Youth Network, reporter Li Chao, source: China Youth Daily, December 07,2021, edition 02

2021-12-03 Guangxi normal university held in 2021 cadre quality ability contest of Guangxi normal university in 2021 cadre quality ability contest network Beijing on December 3 (reporter Shi Weiqiang, correspondent Wang QiuHe) on November 29, sponsored by the youth corps committee of Guangxi normal university, school of politics and public administration to undertake the Guangxi normal university in 2021 in yanshan campus successfully ended. It is reported that the competition lasted for one month, divided into two links of college (department) preliminary competition and university-level final, taking the theoretical knowledge test and micro-group class teaching two forms. Focus on the theoretical knowledge level, comprehensive coordination ability, language expression ability and other aspects of the competition.

2021-12-03 School of Science and Technology, University of China) has launched the rural revitalization action plan and the "one class, one village" action plan. At the launching ceremony, the participating leaders presented to flag the "Student Rural Revitalization Volunteer Team" of Nanchang Jiaotong University and inaugurated the Rural Revitalization Research Institute. The relevant person in charge of Nanchang Transportation University awarded the "Rural Revitalization Practice Education Base" to Jing'an County, Yichun City. Jingan County Party Committee and county government issued the letter of appointment of county-level rural revitalization consultant to the relevant responsible person of Nanchang Transportation University. Nanchang Jiaotong University and the Jing'an County government signed a cooperation framework agreement to help rural revitalization. Ma Zhiwu, deputy director of the Standing Committee of Jiangxi Provincial People's Congress, Sun Zhonghua, president of China Cooperative Economics Association and former chief agronomist of the Ministry of Agriculture, and Guo Jiezhong, deputy Secretary of the Education Working Committee of Jiangxi Provincial Party Committee and director of the Provincial Department of Education attended the event.

2021-12-03 Southeast university: optimize the whole chain "push" education new results southeast university: optimize the "push" the whole chain education new results passed by the sixth plenary session of the 19th of the central committee of the party's struggle in one hundred major achievements and historical experience resolution, points out that the development of the cause of the party and the people need generations of the communist party of China in the struggle. We must constantly recruit advanced elements from all sectors, especially outstanding young people, into the Party. The Communist Youth League is the Party's assistant and reserve army. Strengthening the political guidance to the Communist Youth League members, actively sending fresh blood to the Party organizations and "optimizing" are the political responsibilities entrusted to the Communist Youth League by the Party and a key measure for the Communist Youth League to implement General Secretary Xi Jinping's important thoughts on youth work. Since the issuance of the Implementation Measures for the Communist Youth League (Trial), youth league organizations at all levels have carried out practical exploration. This newspaper now offers a special column, publishing a batch of good experience and good practices from the grass-roots level. Since---------------submitted the application for party membership to the School of Public Health of Southeast University, has felt the standardization and effectiveness of the "promotion" process of the university in the whole chain of the university. Under the systematic training arrangement and one-to-one contact person training, Guzanurinur received regular guidance and training while becoming an active member of the Party. In recent years, Southeast University has continuously improved the work of "optimization", and issued several Measures for Deepening the Reform of the Communist Youth League of Southeast University. Taking the opportunity of participating in the pilot work of "optimization" of the Central League, the pilot reform of "optimization" has been taken as an important measure to deepen the reform of the Communist Youth League. The innovation and effectiveness of the "promotion" work and the satisfaction of teachers and students continue to rise, and the development quality of student party members has steadily improved."Colleges and universities are the main position for recommending outstanding youth league members to join the Party. It is an important part of the work of 'promoting excellence' and actively sending new blood to the Party organization, which is an important embodiment of the colleges and universities of the Communist Youth League to play the political function of the Party's education."Said Zheng Jiamao, deputy Party secretary of Southeast University. From the source of the southeast university school of transportation students in the whole chain of "push" work guidance, has a clear, complete, rich personalized training program, through all kinds of "micro classroom", "cloud lecture" and a variety of forms of theme education activities, Cao irui in the process of joining the party more sense of growth. These mechanisms will improve the working mechanism for the training and education of active applicants, realize the double improvement of the quantity and quality of the school "promoting excellence", further strengthen the foundation for the growth of young students, and create a new pattern of education for the Party."Since the launch of the pilot reform of 'promoting excellence', all departments of the university have taken classified measures based on the basic work requirements, promoted them in an orderly manner, strengthened the Party building and the youth league building, and highlighted the training and education of active Party applicants. Under the strong leadership of the party organization of the department, the youth League organization actively promotes the establishment of positions, courses and projects for training active applicants, and constructs a scientific, reasonable and guaranteed 'excellent' work system with quality and quantity."Southeast University Youth League Committee director Yang Wenxie introduced. The construction of various kinds of positions, courses and projects provides support for the scientific and standardized party membership work of the league members, and truly makes the advanced league members ideologically refined and theoretically improved in the process of party membership, and completes the "entrance pass" for the development of the party members. In 2020 and 2021, the school will resolutely implement the two "100%" of student party members, fully achieve the students under the age of 28 to join the Party, 100% from the league members development, 100% through the standardized "promotion" procedures and the whole process training link. Mechanism innovation escorts "excellent" escort accurate "excellent" and "excellent" mechanism is the premise of "excellent" work. Southeast University has constantly improved the working mechanism of the key party members to contact the youth league branch of the class, and continued to strengthen the guidance of the "promotion of excellence" and other work. In view of the large number of ethnic minorities and students from poor families, Wu Lingyao, the secretary of the Party Committee of the School of Public Health of the school, regularly conducted talks and research with the youth League branch, and accurately formulated the "promotion" development plan in line with the actual situation of students."In the School of Public Health, as soon as the freshmen enter the school, the teachers of the college will go deep into the youth league branches to introduce the glorious history and great cause of the Party and stimulate their enthusiasm to join the Party."Gulzaninur said," The college has arranged one-to-one contact people to conduct regular tracking and training, to solve the weak links in the training process."In the past two years, the overall application rate of students from the School of Public Health has increased significantly, and students with different characteristics have received precise guidance in the process of" promotion ", and the training quality of active applicants has been steadily improved."The Party committee of the college strictly implements the main responsibility, strengthens the leadership and supervision of the Party organization of the college, brings the overall work of 'excellence' into the party building assessment point of the college, and brings the standardization of 'optimization' into the assessment and evaluation system of the league organization of the college. According to the academic performance, comprehensive evaluation and mass base, earnestly formulate the evaluation indicators of excellent league members, and strictly do a good job of 'promoting excellent' work."Said Qian Yijun, deputy party secretary of the School of Energy and Environment. At the same time, Southeast University focuses on the construction of grass-roots League cadres, regularly organizes the study of relevant system documents, helps the League cadres to get familiar with and master the policies and requirements of the "optimization" work, constantly analyze the characteristics of students in the new era, standardize the "promotion" procedures, strengthen the training process, and improve the level of "optimization" work. How to implement the work of "promotion" work, how to make students have a more sense of gain in the process of "promotion", and how to improve the effectiveness of the training of active applicants, has become a question of Tang Dawei, secretary of the Graduate Party branch of the School of Information Science and Engineering students of the university."In our student dormitory community, laboratory public space, the college carries out party building and youth league building, so that we can effectively use the fragmented time to study."Tang Dawei introduced. Tang's college has also established a relatively fixed position for active applicants to study, activity and discuss. Here, students can exchange and discuss and share their learning experience. In the past year, the study and education of party history has become the distinct theme of these learning positions, which has greatly promoted the students' study and cognition of party history. It is understood that the "excellence" pilot work of Southeast University through more than a year of creation, effectively strengthen the guidance and training before the "excellence", focus on the process of "optimization" guidance and training, normal continuation of the promotion of the "excellence" education and training, so that the "excellence" before, middle and after the three links of organic linkage. In the work of "optimization", each department comprehensively inspects the participation of youth league members, faith open class and the "Qingma Project", and implements the implementation of the "1 + 10 + 20 + X" cultivation rules, that is, participate in no less than one week; participate in no less than 10 thematic education activities carried out by the Party and league, and obtain no less than 20 hours of volunteer service. In addition, relying on the characteristic projects in the party building work and the brand activities in the work of the Youth League Committee, each department carries out the characteristic cultivation links for the active party members. Lin Qiong, secretary of the Youth League Committee of the School of Economics and Management, said, " It is the implementation of these detailed rules that promote the theoretical level, practical ability and dedication of active applicants in the training process, have a deeper understanding of the history and fine traditions of the Party, and continuously deepen their feelings for the Party."Yang Wenxie said that the establishment of the whole chain of" promotion "work system, improve the training program of active applicants, to standardize the system and optimize the mechanism to strictly do a good job of" optimization "work, is the main measures of Southeast University to further promote the reform of" promotion " work. The Youth League Committee of the school will further strengthen the political guidance to the league members, perform the important political responsibility of "promoting the Communist Youth League entrusted by the Party", continuously absorb the outstanding youth into the Party, and constantly highlight the political function of the Communist Youth League organization as the Party to train and transport the backbone of the youth. China Youth Daily China Youth Network reporter Li Runwen, correspondent, Ning Junkang source: China Youth Daily

2021-12-02 Jiangsu University: "Three-dimensional" classroom makes the party history education have more temperature and more depth to educate you. When you grow up, I hope you don't forget that your mother died for her country! After my mother dies, my child will continue to fight for her mother!"Not long ago, in the Youth League Committee of Jiangsu University held the" study the history of the Party, strong faith, follow the Party " theme micro youth league class competition scene, Jiao Xinyi from the medical school students told the story of the female Communist Party Zhao Yiman wrote home to his son before the heroic death. Since the launch of the party history learning and education, the Youth League committee of the school has responded positively. Nearly 1,000 league cadres and youth members of the school have taken to the platform to tell about the profound connotation of the revolutionary spiritual spectrum formed by the Chinese Communists in different periods. At the same time, they also continue to innovate the content and form of the party history learning and education, and steadily promote the party history learning and education into the brain into the heart, go deep and solid."Walking" party history class, to learn "to learn from comrade Zhao Yafu, breathe with the common people destiny, tao heart ask changes in warm, do enthusiastic service to the people, selfless dedication to society..." in jurong city Zhao Yafu deeds hall, Jiangsu university school of marxism graduate Shi Haijiang as a representative, took the bright red party flag read "resolution". In recent years, the Youth League Committee of Jiangsu University, closely relying on the red resources of Zhenjiang, has organized the youth league members to revisit the red route, punching in the seven "red landmarks" of Zhenjiang city in turn, and connecting the "walking party class points" in various forms. In addition, relying on the "faith open class", league class and other work carriers, they promote the system of league cadres on the platform and promote the ordinary youth league members; organize the 100 faith open class demonstration class of "celebrate the centenary of the Party" and sing the main theme of loving the Party to tell the Party and deepen the history of the Party."Immersive" art class, to the exhibition to promote learning " villagers! If we give up resistance and let the Japanese invade our land, won't we become deserters! I must stay and fight against the Japanese army!"This is the fourth Jiangsu University undergraduate youth League branch style display competition, the school of Management manpower 2002 youth League branch performance scene. This competition closely around the party history learning and education, firmly grasp the class league branch, by showing the league branch members to retrace the red footprints, tell the red stories, enrich the form of learning and education. Not long ago, the university held a report performance of the 2021 Jiangsu University College Students Art Troupe in detail. The host told the party history stories, which was integrated with the program background of the performance, so that the college students could receive an "immersive" party history education. The performance is based on the important events in the history of the Party as the context. More than 300 teachers and students from the college student art troupe performed the revolutionary course and struggle epic of the Party in various songs, dances and other artistic forms."Our proposal of installing charging piles for electric vehicles in the dormitory area at the student congress has really been implemented."Said Zhu Yeting, a student at the School of Finance and Economics at Jiangsu University. Since the launch of party history learning education, the school youth League committee carefully planned to implement the "I do the practical work for students" practice, students through open online "rights mailbox", collect students study, life and other problems and feedback; held "315" rights knowledge exchange, to enhance students' awareness and ability to safeguard their legitimate rights and interests. Cadres at all levels of the school take field visits, concentrated discussion, questionnaire survey as the main forms, widely listen to students 'demands, collect students' opinions, do a good job of students 'urgent and anxious practical things, and constantly enhance students' sense of gain and happiness. In addition, the Youth League Committee of the school and the Zhenjiang Municipal Party Committee of the Communist Youth League jointly carried out the theme practice activity of "Party members learn Party History hand in hand" in Zhenjiang city, giving full play to the role of college students' Party and league members in the study and education of Party history, so that the red gene and revolutionary fire are passed on from generation to generation. Jiangsu university youth corps committee secretary wang said, will continue to innovate party history learning education form, education guide the youth to understand the party's glorious history, feeling the party's beginner's mind mission, grasp the party's innovation theory, recognize the party's spirit pedigree, inheriting the party's red gene, to ensure that the youth party history learning education has both "depth" more "temperature". China Youth Daily, December 02,2021

2021-12-02 "World AIDS Day" Correspondent Jianwei, Liu Yanchun, Li Xinjie) At noon on December 1st, Guizhou University 2021 "World AIDS Day" publicity and education activity was held in Yawen Building Square. More than 3,000 people attended the meeting of the Party committee, relevant functional departments and representatives of teachers and students of the college. Photos of the event site. At the beginning of the activity, all the staff carefully watched the anti-AIDS cartoon theme exhibition, from the communication channels, prevention and control knowledge of AIDS. Linghu Caitao, deputy secretary of the Party Committee of the university, read out the activity declaration to all the teachers and students: improve personal protection, build a strong "safety wall" to prevent AIDS; care for AIDS patients, gather the "salary power" of AIDS prevention; youth help red ribbon, strive to be the "willing person" to end AIDS. Hold up life with love, pour the future with love, care for the truth with respect, and show the dignity with understanding. Let us join hands to resolutely fight against AIDS and COVID-19, isolate the virus from the campus, and jointly build a beautiful, harmonious and healthy campus. Photos of the event site. The student representative Yao Liangliang issued an activity initiative: to understand AIDS, improve the awareness of "AIDS prevention", hand in hand to resist, eliminate the "AIDS ambiguity" psychology; actively publicize, and jointly build a "zero AIDS" society. The new generation of young people, bearing new responsibilities and missions, should actively participate in the AIDS prevention and control work, jointly build and share, advance to the "zero AIDS" society, and create a healthy environment for the whole society to jointly prevent AIDS. Photos of the event site. At the scene of the activity, the college students preached on the AIDS prevention theme, the medical staff and the volunteers distributed the AIDS prevention publicity materials and protective equipment to the teachers and students, and the students actively participated in the AIDS prevention knowledge questions and signed on the AIDS prevention signature wall. It is reported that recently, Guizhou University Red Cross, Youth Volunteers Federation and other student organizations also held anti-AIDS knowledge contest, anti-AIDS knowledge lectures, anti-AIDS movie watching party, anti-AIDS cartoon contest and other activities.

2021-12-01 Nanjing Industrial Vocational University Youth League Committee of multiple measures to promote the study and education of Party history Nanjing Industrial Vocational University Youth League Committee to promote the study of Party history education around history knowledge, history credit, history Chongde, history practice, combined with the work and the actual youth of the Communist Youth League, multiple measures to promote the party history study and education. The school youth corps committee relying on youth learning club and youth learning club red line, through the red attractions "clock" movie "follow the history of the party", to carry out the "study the party history, strong faith, follow the party" party history learning education project youth league branch conference, "feeling great course, condensed strength" party history learning education project organization life, "youth to the party, struggle power" series theme TuanRi activities such as more than 500 grassroots youth league branch activities. At the same time, the school youth corps committee also carried out the "party history youth" youth speech contest, "faith public class" cadres ideological skills tournament, the excellent class works through the network form, within the scope of the party history learning education expert report, heroic deeds lecture, party history learning education reading 12, secondary college to carry out party history learning education project more than 50 games, branch more than 500 games.

2021-12-01 Shaanxi energy vocational and technical college social practice base of shaanxi energy vocational and technical college garden community and community to build social practice base opening ceremony JianWei correspondent li) to achieve universities and community in the harmonious development goal, enhance college students service community and experience ability, recently, shaanxi energy vocational and technical college and Xianyang garden community in office building on the fourth floor Wen Lin lecture hall "emotional support to promote harmonious community to build spectrum Marty natalegawa" social practice base opening ceremony. Representatives of the two parties shall sign a cooperation agreement. Ren Xiangming, deputy secretary of the Party Committee of the College, delivered a speech on behalf of the school and put forward the requirements for the joint construction of the social practice base, requiring highlighting the guidance, influence and integration of the social practice base activities, to ensure the safety of social practice activities and enhance the timeliness of social practice activities. The construction of social practice base should be regarded as an important starting point and education platform to strengthen the ideological and political education, patriotism education and labor education in the new era, so as to play the positive role of practical education. College volunteer team teachers on behalf of the teacher, volunteer student representative ji first statement, to the leaders, guests and all the teachers and students, volunteer service will play the role of the bridge between school and social practice base, to practical action to lead the youth to participate in the social practice activities, exercise ability, growth ability. Xiao Bin, the first secretary of Wenyuan Community in Xianyang City, signed a cooperation agreement with Feng Chao, the secretary of the School Youth League Committee, and the Party committee of the college, Xianyang Municipal Party Committee, Xianyang Wenyuan Community, and the Party Working Committee of Weicheng District Office jointly unveiled the social practice base. Social practice base opening site. Correspondent for figure finally, group Xianyang, deputy secretary of Gao Jun speech and pointed out that colleges and community to build a harmonious community is both optimize education environment, the need to achieve the training goal, is the needs of community construction and development, colleges and universities and community, can the resource sharing, complementary advantages, improve together, requires the youth in the community construction base to carry out colorful, various forms of practice activities, cherish exercise opportunity, work attitude, strict with oneself, the community, serve the masses, combine their learning income and practical work effectively, in practice by wind and rain, make contribution, long talent.

2021-12-06 Jiangsu university college students backbone training class the third graduation and the fourth opening ceremony held in Jiangsu university college students backbone training class the third graduation and the fourth opening ceremony held jianwei correspondent liu Dan) recently, Jiangsu university college students backbone training class the third graduation and the fourth opening ceremony held in the conference center of the first lecture hall. Relevant persons from the Party Committee, Working Committee of Jiangsu University were invited to attend the ceremony, presided over by Wang Haijun, Secretary of the Youth League Committee of Jiangsu University. More than 130 teachers and students attended the leaders of the secondary league organizations, representatives of the third students and the fourth college student backbone training class. All the leaders and guests presented certificates to the representatives of the third "Outstanding Students". Correspondent for figure Jiangsu university deputy party secretary, doctoral supervisor professor hong-bo li on behalf of the school party committee to congratulate all the students and attend the ceremony for the fourth students representative flag, officially open a new phase field "green horse project" global work, youth corps committee secretary of hai-jun wang on the third phase and the fourth phase of admissions, party committee minister Yang Daojian read "about the third phase of Jiangsu university college students backbone training" outstanding students "decision". All the leading guests presented the book to the fourth session of the students. Xu Jialu, the representative of the fourth phase of the students, spoke, calling on everyone to "firm ideals and beliefs, keep in mind the original mission", "always keep sober, the key test", "always maintain confidence and gratitude, live up to the time of The Times". The ceremony is the Jiangsu university youth corps committee to further implement the "green horse project" work important link, with the help of ceremony education infection, vividness, through flag, recognition, books, initiative, further guide the "green horse students" firm ideal faith, in the theoretical study, red education, practice exercise improve political character, cultivate the country feelings, improve ability and quality.

2021-11-28 Zhejiang University emergency epidemic prevention (China Youth Daily reporter Jiang Yutong) " Teacher, teacher, we are volunteers! Teacher, what need we to do! Teacher "", I have collected it, I want to be a volunteer!"" Our college only 100 (volunteers) places, two minutes to report full, do not grab the report."On the night of November 25, groups of volunteers arrived at the nucleic acid collection site under the lights of the Zijingang campus of Zhejiang University. Liu Bo, a teacher from the school's research and work department, said that the help of the volunteers ensured the orderly on-site registration."The feet were numb when the stall was closed with cold, but my heart was always moved by the warmth."On the afternoon of the same day, the zijingang Campus of Zhejiang University was found. According to the order of the provincial and municipal Epidemic prevention and Control Leading group Office, the zijingang Campus of Zhejiang University was closed for management. The school immediately launched the emergency plan for epidemic prevention and control, transferred teaching activities online, quickly organized nucleic acid testing for all staff, and placed those identified as close contact or subclose contact under isolation and observation. All teachers, students and staff suspended all gathering activities and outdoor exchange activities. The Youth League Committee of Zhejiang University organized 484 volunteers within one hour to conduct collective training and mobilization in the small theater. The training content was the nucleic acid testing and sampling process and the use method of the special App for information input. At the training site, the volunteers wore their face masks. In the evening, the volunteers went to several points in zijingang, Yuquan, Xixi and other campuses to conduct information collection and order guidance for volunteer services. The latest group of volunteers stayed until 4 a. m. On this night, all the street lights were on on the Zijin Port campus of Zhejiang University. The "Can if stars" lights that were only celebrated at the school anniversary and New Year's Eve lit up this unforgettable night when the medical staff, volunteers and all the teachers and students worked together. At 7 am on November 26,107 volunteers took up again to provide services at several points in zijingang, Yuquan, Xixi and other campuses. In the afternoon, the second batch of 230 volunteers conducted intensive training and participated in the later work. On the morning of November 26, Zhejiang Provincial Committee of the Communist Youth League and Zhejiang Volunteers Association immediately sent 200 red vests, 2,500 gloves, 681 disinfectant boxes and 500 masks to Zhejiang University to assist in the campus fight against the epidemic.

2021-11-26 Your big youth take you to use VR party history immersive! Your big youth take you with VR party history of youth network Beijing on November 26 (reporter Zhang Jianwei, correspondent, liu yanchun, Song Hongqing Yang Yuqing) in Guizhou university college students cultural exchange park, students from the school of computer, using VR technology by modeling the picture of the scene, let your university "through" to decades ago, immersive feel the extraordinary history. In 2021 is the centenary of the founding of the communist party of China, Guizhou university innovation learning form, solid party history learning education, with the support of the school, the college of computer science and technology students combining professional expertise, form a VR team, develop VR party history learning platform, lead the youth immersion, sharing, interactive learning party history in one hundred, inheriting red genes. VR Party history learning platform photos. The platform provided by correspondents is divided into two sections: the century-old party history corridor and the party history reappearance and interaction. Corridor of party history, with the new democratic revolution period, socialist revolution and construction period, reform and opening up and the new era of socialism with Chinese modernization, and present the new history of the party in the past, allowing learners to clearly understand and study of the party history, the party history period. Schematic diagram of using the VR Party history learning platform. The correspondent provided " using VR technology experiential learning, will greatly increase the students' interest in learning, better lead the majority of young people to firmly study the history of the Party, feel the Party, the faith to follow the party, let the red gene from generation to generation."Team member Wang Qi said. It is reported that the project won the silver prize in the 7th "Internet +" College Students' Innovation and Entrepreneurship Competition "Red Dream Tour" track in Guizhou Province. At present, we have cooperated with some enterprises, and we will further optimize the system to bring quality content output and realistic experience to learners.

2021-11-25 Changchun University of Science and Technology: Professional assessment allows counselors to visit poor students. Counselors run psychological workshops. Rural volunteer teaching is an important experience for many college students. For Hu Ruiyang, a student from the School of Physics of Changchun University of Science and Technology, she gained more than 20 days of rural volunteer teaching in the sophomore summer vacation and grew a lot. Different from most students who spontaneously organize to volunteer teaching, the activity is accompanied by college counselors to ensure their accommodation and safety, and guide them to teach volunteer teaching. Hu Ruiyang said that from the early preparation to the finishing arrangement, the college counselor Yu Changming all devoted a summer vacation time to the students participating in the volunteer teaching. For the counselors, this is not only a sentimental effort, but also an active work practice in the atmosphere where the school counselors are more active than learning and catching up. In the past two years, Changchun University of Science and Technology, known as the "cradle of Chinese optical talents", has explored the reform of the counselor work assessment system. This exploration is specifically undertaken by the Student Engineering Department and the Youth League Committee of the university. The new assessment mechanism has broadened the channel for counselors to evaluate and choose the first, built a platform to show their expertise, and made college counselors clearer about their future career development. In September this year, Changchun University of Science and Technology announced the assessment results of the 2020 counselors. 41 of the 83 full-time counselors were rated as excellent in the special assessment, and 8 students won the title of Advanced Worker in Student Work. With the core idea of building a versatile counselor, Changchun University of Science and Technology tries to divide the assessment into special assessment and comprehensive assessment. In the special assessment, for the main responsibilities and content of counselor work, set ideological lead and capacity building, student management, financial management, mental health education, the communist youth league, network ideological politics, employment and entrepreneurship seven special, require counselors to declare at least 1 a year, and the special work assessment results as the annual results. Only the counselors who are excellent in the special assessment are qualified to participate in the comprehensive assessment. After passing the inspection of morality, ability, diligence, performance, honesty and other aspects, they can be rated as advanced workers in student work, and enjoy a variety of excellent opportunities and related subsidies. Who is the subject of evaluating counselor work? As the focus of the reform of counselor work assessment, this problem has been repeatedly discussed in the school special meetings for several times. Wang Chao, minister of the student work department, said that in order to ensure the scientific and fairness of the assessment, the school held more than 20 meetings on the reform of counselors for repeated discussion. After the formation of each plan, it will be tried out in a small range to evaluate the assessment effect, and then further improve it. Students are the direct service of counselors, and have the best say; the leaders and colleagues of the college are also witnesses and supervisors of the work of counselors; Students believe that the evaluation should not only have subjective evaluation, but also pay the support of objective materials; the counselors worry about whether human evaluation is too much. Considering the above considerations, Changchun University of Science and Technology refines the investigation indicators and procedures around different evaluation subjects, requires the counselors to submit objective performance materials according to the indicators, issue electronic questionnaires to students to evaluate the work of the counselors, and organizes the heads of the college and the head of the student department to give subjective scores, and evaluate the guidance performance. Sun Ji, the counselor of the School of Computer Science and Technology, was awarded the special excellence in network ideological and political work in the assessment. The network ideological and political field is Sun Ji's specialty. In recent years, less efforts have been made in this aspect. However, because other achievements are not outstanding enough, the previous assessment results are difficult to be related with excellence. It is understood that in previous years, there is only one evaluation channel for counselors' assessment, and the excellent quota is very limited. Many counselors, who have expertise in some businesses, but are not outstanding in their ability in other businesses, show their weakness in the assessment, which affects the work enthusiasm of the counselors."The special assessment has made many young and practical counselors stand out."Wang Chao bluntly, previous assessment by the college leadership and school functional departments to counselors, marking the lack of understanding of counselors work, thus leads to working fixed number of years, good interpersonal relationship counselor can often be good, and a short fixed number of years, college leadership is not familiar with, get good probability is very small. Yu Changming, a physics counselor, is the big winner in the 2020 counselor assessment of Changchun University of Science and Technology. He applied for four special projects: ideological guidance and capacity building, student management, network ideology and politics, and the work of the Communist Youth League, all of which were awarded excellent, and won the title of "Student Advanced Worker". But in the past, despite his working as a counselor for years, Yu Changming, who kept a low profile, did not often attract the attention of people around him. Hu Ruiyang and his classmates can often see Yu Changming still busy in the office during work hours or on weekends. She said: " Teacher Yu deserves the award."" good mechanisms can activate a pool of spring water."Yang Yuxin, Party secretary of Changchun University of Science and Technology, said that making good use of the baton of assessment is a key measure to promote the high-quality development of the university's student work. The counselors are more motivated and have stronger organizational strength, cohesion and combat effectiveness."We feel the most directly."Said An Mengyu, a senior student at the School of Optoelectronic Engineering. This year, An Mengyu was recommended to major in photoelectric engineering at Tsinghua University. During college, he often participated in scientific and technological innovation and entrepreneurship competitions, and won many national and provincial gold MEDALS. Good results can not be achieved without the guidance of professional teachers. At the same time, the resource docking provided by counselors and the guarantee of the competition link are also essential. In Changchun University of Science and Technology, counselors are not only a simple provider of opportunities for students to participate in competitions, but also need to build a resource platform for students to participate in competitions. Large-scale competitions often require multiple departments to cooperate, which requires counselors to coordinate and integrate work. Liang Xue, a national outstanding Youth League cadre and secretary of the Youth League Committee of Changchun University of Science and Technology, introduced that before the assessment reform, the counselors did these work more out of enthusiasm and feelings, and was not sustainable. Now this work has become an assessment index in the special employment and entrepreneurship work of the counselors, ensuring that the counselors can serve the students more attentively and professionally."Counselors give full play to their respective expertise and design more forms of student activities."Said Xiang Tianjun, a junior law school junior. In the past two years, the counselors organized them to participate in many social practices, and entered the communities and villages to carry out legal publicity. The counselors will ask the students to focus on the introduction of the Civil Code, how to deal with homestead disputes and other concerns for rural residents."In the past, social practice needed us to find opportunities on our own. Now, counselors contact and lead teams, and people are more targeted and fruitful when participating in social practice."Xiang Tiansong said. In the 2020 annual assessment, Liu Xiangfei, a counselor at the School of Computer Science and Technology, whose student management expertise attracted the attention of many counselors. Students like to talk to him, and often talk to him about postgraduate entrance examination, job hunting and future development. Recently, Zheng Meiling, who is preparing for the postgraduate entrance examination, hesitated to apply for the two universities. One afternoon, near work, she saw counselor Liu Xiangfei in the office and went for advice. Talking for an hour, Liu Xiangfei helped her analyze the pros and cons, Zheng Meiling suddenly enlightened, had the final decision."As long as there is a question to find Mr.Liu, he can not eat, not off work, also must put you talk about all doubts."Zheng Meiling said with a smile. Zhang Renkai, a junior student, is the deputy secretary of the Youth League Committee of the School of Chemistry and Environmental Engineering. In his opinion, after the work of the Communist Youth League became a special assessment of counselors, the counselors were more targeted in leading student cadres to carry out the work of the Communist Youth League. In the past, it was not clear what the deputy secretary of the Youth League committee should do, Zhang said. Now the tutor will inform the work in detail and give guidance. Liang Xue introduced that, combined with the needs of the reform of the Communist Youth League in colleges and universities, the special assessment of the work of the Communist Youth League of counselors involves 7 first-level indicators, and each first-level indicator is also set with multiple second-level indicators. How to carry out the work and how to do it better are all reflected in the assessment indicators."Students will focus on the guidance and services they need."Liang xue said that the professional growth of counselors makes students become direct beneficiaries. In recent years, Changchun University of Science and Technology has achieved a new breakthrough in student training. Students from Changchun University of Science and Technology have been winning money and silver in various competitions, such as "Challenge Cup", "Internet +", "National Volunteer Service Competition" and "College Students' Art Performance", with many great achievements."Every counselor who is excellent in the special work assessment is an example for other counselors in various business development directions."Liu Xinran, a counselor who has just joined the school of Liberal Arts for two years, said that now I not only know which direction, how to do it, and to what extent, but also know who to learn from, how and what level to learn. In the first job, Liu Xinran has a lot of jobs do not know how to start, and can not always ask the college leaders. Now, she consulted the relevant counselors according to the list of each special assessment. This year, Liu Xinran took a graduate class and didn't know how to approach the students because he had no experience. After consulting with excellent counselors in student management, Liu Xinran was relieved. As for many students of Chinese and art majors who choose to take the civil service and postgraduate entrance examination because of poor employment, Liu Xinran also consulted with other excellent counselors and taught students in accordance with their aptitude to guide them to find timely employment."The assessment orientation determines the direction of our efforts. Counselors in the new era must be specialized and versatile to promote their career development and better adapt to the new situation and new requirements of student work in the new era."Said Yang Guang, deputy party secretary of Changchun University of Science and Technology and leader of the counselor work assessment leading group."The assessment criteria are very scientific and very convincing."Said the counselors interviewed. At the same time, in the view of many counselors, compared with the honor, the comprehensive recognition from the college leaders, colleagues and students is the biggest harvest brought by the assessment, which makes them feel the value of hard work. Happily, under the reasonable assessment mechanism, the work enthusiasm and professionalism of the counselor team were fully stimulated. In 2020, the number of counselors in Changchun University of Science and Technology, the number of papers published and the number of individual awards all increased by 100% year-on-year. National outstanding youth League cadres, outstanding youth of Jilin Province, Jilin Province counselor of the year and other role models have emerged. At present, the counselors of Changchun University of Science and Technology are giving full play to their different specialties and make more efforts in their own positions to promote the high-quality education of the university. China Youth Daily China Youth network reporter Wang Peilian, correspondent Wang Chao source: China Youth Daily

2021-11-23 Recently, under the leadership of the Youth League Committee of Shanghai Jiao Tong University, all the students of Shanghai Jiao Tong University have carried out a special education on labor education. To Marine ocean and architectural engineering institute, mechanical and power engineering college, material science and engineering, environmental science and engineering, agriculture and biology, kaiyuan law school, design, wisdom energy innovation institute, the school youth corps committee organized nearly 2000 students, successively to songjiang town zhu village, fengxian town road village, minhang district xu bridge town bright village, chongming district xing town Pan Shi village, minhang district wujing town spark village and songjiang district town Zhang Ze four village to carry out "harvest in the autumn" series of labor practice. After listening carefully to the action points of cutting rice, we put on gloves, feet stand separately, bent down, grasp two or three bundles of rice left hand, the right hand with a sickle cut down, the whole set of action at one go. Then the cut rice is tied up, manually beating and threshing in the grain bucket, the grain grains fly everywhere, and everyone is in the "grain rain". Near noon, everyone began to prepare lunch, cutting firewood, making a fire, picking vegetables, washing vegetables and cooking. Some students use local materials, pick up dry wood, digging pits, baked sweet potatoes, and some students into the farmers home, making dumplings under the guidance of the villagers. The "Harvest in the Golden Autumn" series of labor classes is one of the latest measures of Shanghai Jiao Tong University to comprehensively strengthen the labor education of college students in the new era. The school explored the joint mode of labor practice base of school-school, school and enterprise joint. For the first time, it launched the harvest theme labor skills class such as "rice fragrance", and put the second class labor education course to the fields. School relying on the subject characteristics of agriculture and biology college, pilot built the "1 + 3" labor education mode-with a fine labor education education courses as the core, with productive agricultural labor, service practice labor, creative kechuang labor three forms of labor education as the carrier, the labor education into class, form the integration of the first classroom and the second classroom labor education mode. Previously, Shanghai Jiao Tong University students have also taken courses such as "Entering the Mystic Tomato World" and "Grape and Wine Culture".

2021-11-23 Wang Lei, correspondent Zhao Junlong) On November 20th, the 12th Congress of Anhui Agricultural University was held in the auditorium of the university. Li Bo, deputy secretary of the Anhui Provincial Committee of the Anhui Provincial Committee, congratulated on the victory of the conference. He pointed out that to actively integrate into the service youth, will all work consciously into the school khalid ents work, fully integrated into the school talent training global, wholeheartedly service youth as the starting point and the foothold of all work, fully trust youth, enthusiastic concern youth, strict with youth, give full play to the party contact the youth bridge and link, lead the youth thought, service youth growth. Jiang Chun, secretary of the Party Committee of Anhui Agricultural University, encouraged all the youth league members and students of the university to inherit the spirit of "Dabie Mountain Road" and strive to grow into a new person of The Times who can be useful and can shoulder heavy responsibilities. He pointed out that the school youth league organizations at all levels should pay attention to their own construction, and effectively enhance the ability to serve the growth of young people. In innovative work methods, in building education brand, strength on the governing group, build a batch of AnNong characteristics, AnNong style and agriculture temperament of original cultural high-quality goods, set up the clerkstarts fresh vital qi, build than learn to catch up with good sweet atmosphere to drive the youth to carry forward the patriotic struggle spirit, a new era. The conference listened to, deliberated and approved the work report made by Lin Xudong on behalf of "Remember the history, keep the original aspiration, follow the Party with the Youth League banner held high, and strive to write a new chapter of youth on behalf of the 11th Youth League Committee of the university". The congress elected 23 members of the 12th school Youth League Committee. After the closing ceremony, the first meeting of the 12th Committee of the Communist Youth League of Anhui Agricultural University was held, and the standing member, secretary and deputy secretary of the 12th Communist Youth League Committee were elected.

2021-11-19 Guizhou Qiannan college the second student congress ended Guizhou Qiannan college (Guizhou university college) the second student congress ended Beijing on November 19 (reporter Zhang Jianwei, correspondent, Yang Fangxue Chen Yingmei) recently, Guizhou Qiannan college (Guizhou university college) the closing ceremony of the online and offline, the conference is divided into the venue and venue, a total of more than 340 teachers and students to attend the conference, college of science and technology youth corps committee teachers Liu Xuechen report to the conference election results."All student cadres should have the consciousness and determination of 'new thinking every year, a new spirit, new breakthroughs every year, and a new image every year'. With the courage of innovation, fighting spirit and pragmatic style, we should make our school of science and technology built into a better and stronger place."School of Science and Technology Party Committee propaganda Department minister, secretary of the Youth League Committee Zhang Yaozhi hope that the newly elected student cadres must learn from the experience and lessons, further improve themselves, seriously learn scientific and cultural knowledge, and constantly improve the ideological and cultural quality, really play an exemplary role in college students. It is reported that at the election conference, the participants after full consideration to cast a solemn and sacred vote, after the vote counting personnel statistics determined, elected the new presidium members of the Student Union of the College of Science and Technology. Finally, the second student congress of Guizhou Qiannan University of Science and Technology (College of Guizhou University) ended with the song of the Chinese Communist Youth League.

2021-11-18 Nankai University: heart and love hard for students to solve the garbage classification game, stimulate the interest of students at the same time to achieve the purpose of garbage classification propaganda "" We plan to move the curling field and equipment to the scene, to facilitate the students to experience the current ice and snow sports courses "... some time ago, in Nankai University Bali campus sea ice building, contact the first monthly work held as scheduled, the school to better serve students brainstorming. In recent years, the Student Union and Graduate Association of Nankai University have found the right organizational positioning, actively explored the reform path by problem-oriented, actively carried out the theme activity of "I Do something for Students", and solved problems for students with heart, affection and efforts. At the end of last year, Nankai University innovatively set up a working group on connecting the youth and service coordination (hereinafter referred to as the "Working Group"). This is a working group led by the university Youth League Committee, including 17 university-level functional departments, such as the academic work Department of the Party Committee, the Party Committee, the security Department, the Sports Department, the library, and the catering service center, as well as student representatives. It is worth mentioning that the mechanism pays attention to "two-way service". While effectively responding to the needs of students, it helps students to understand the work of various departments, establish a sense of gratitude, implements the concept of service management and education, and promotes the implementation of "three complete education". How much power can such a working group exert? Small to the canteen night operation in short supply, to the list of youth services, are within the scope of the working group. Through practice and exploration, this working mechanism has gradually become mature. Not only pay attention to the vital interests of students' daily study and life, but also look to the future and pay attention to the long-term development. In April this year, in two nankai university in 2021 the communist youth league "hai he river green" listen "practice and nankai university" difference "development plan outline comments at the meeting, the presidium of the student union, graduate students will rely on the" haihe river green listen "practice platform for" I do something for students " read the work. According to the preliminary investigation and professional characteristics, the representatives of the student Union and graduate committee of each college put forward suggestions on the Outline of Nankai University (Draft), covering many aspects of promoting the integrated development of disciplines, improving the support system for innovation and entrepreneurship, and strengthening the protection of rights and interests. In the first year of the difference, the Student Union and Graduate Association of Nankai University organized student representatives to provide nearly 10 constructive opinions for the preparation of the class of 2021 and the 41st University Leaders Reception Day, making suggestions for double first-class construction development and forming more than 20 proposals successively."From the fat-reducing meal launched in the canteen to the landmark of the campus during the graduation season, I have increasingly heard and learned about the changes brought by the student Union organization to the campus of Nankai University. Starting from the needs of their students, they take warm measures. Such a student organization makes people feel warm."Said Shi Xinqi, a 2018 undergraduate student at the School of Physical Sciences. It is a required course for the Student Union and graduate Association of Nankai University. Since this year, the student Union and the graduate association have organized more than 10 collective theoretical studies to study the speeches and messages of Xi Jinping, General Secretary, in the first time, firm their ideals and beliefs, and strengthen the theoretical arm. Many members of the Student Union and graduate Association signed up for the "Qingma Project" and the training camp of the Youth League School (Huangpu Youth League School) and were successfully selected. A number of key members participated in social practice activities such as theory publicity. In Nankai University, a three-level linkage mechanism of "school-college-class" has been gradually formed. Through the organization of the student union and the construction of the student representative team, the linkage pattern of "school president discussion, college president and class support" has been established. The relevant person in charge of the Youth League Committee of Nankai University introduced that through the two student proposal competition, the standardized and scientific level of student proposals was improved, and the work quality and ability of student representatives were improved. Student representatives use the proposal to solve the "problem", to warm the service "heart", to promote the construction of "decentralized problems of direct contact, centralized problems to form proposals" contact service working mechanism, so that "I do something for students" to further focus on the actual needs of students. In September this year, nankai university students to explore the implementation of the "cut jade plan" new growth training camp, through theoretical study, business training, research and activities founded module, focusing on the students growth fundamental demand, practice of "service students, dedication to nankai" tenet, in improve the students' comprehensive quality, improve the quality of school talent training play a positive role. Source: China Youth Daily, November 18,2021

2021-11-18 Jiangsu university youth corps committee organization to carry out the "faith" class demonstration class Jiangsu university youth corps committee organization "please party power have my jiang youth said" 2021 "faith class" demonstration class China youth network Beijing on November 18 (reporter Zhang Jianwei) recently, Jiangsu university youth corps committee in the conference center first lecture hall organized "please party power have my river youth said" Jiangsu university "faith class" demonstration class in 2021. Jiangsu university closes working committee, youth corps committee, part of the secondary league organization, head of the fourth college students backbone training class all the students, school two levels (youth learning club), 2021 freshman league branch secretary, college students and student community representatives of about 240 people to attend, activities live through the network platform, more than 200 new league secretary online participation. The demonstration class invited to Jiangsu grassroots cadres ideological skills tournament first, second prize winner, Zhenjiang party history learning education XuanJiangTuan members, Zhenjiang "youth XuanJiangTuan" members, youth education teachers and Jiangsu university support Nanjing outbreak student volunteers, around the "power faith, bear, power spirit, power youth" four chapters, carry out the party history learning education, participate in the epidemic prevention and control theme "faith public class". In the "first chapter of the Strong Country Faith", Yue Zhiqiang, a teacher from the School of Marxism of Jiangsu University, shared the theme of "<the Youth Code in the Awakening Age>". At the beginning, Teacher Yue said to all the students, " What is youth?"Ask, triggered the whole audience to think. Combined with historical data, film and television work fragments, Teacher Yue told in detail the lovely youth group portrait in the party's revolution and construction cause, and defined the "youth code" as faith, responsibility, struggle and original aspiration, profound and simple, arouse the resonance of students, effectively stimulate the enthusiasm of young people to love the Party and their patriotism. In the "second chapter of taking responsibility as a Strong country", Liu Yunxi, a graduate student of the School of Pharmacy of Jiangsu University, a student of the fourth backbone training class for college students, and a volunteer to fight the epidemic in Nanjing, wrote, " If faith has color, it must be China red!"" To share the personal experience and vivid cases of the volunteer work of nucleic acid collection in Nanjing this summer vacation, and to tell the personal stories and personal journey of fighting the epidemic to the teachers and students present. In the "Third Chapter of the Spirit of Strong Power", the participants first watched the video clip of the Zhou Enlai documentary "He Then". After, Jiangsu university energy and power engineering school of undergraduates, Jiangsu grassroots cadres ideological skills tournament first prize winner guo chenlong classmates do "for the world heart" theme sharing, elaborated the profound connotation of zhou enlai spirit and era significance, and focus on the national poverty crucial model Zhao Yafu and Jiangsu university to carry out scientific research crucial story, looking for the inheritance of the zhou enlai spirit and continuation. In the "the fourth chapter power youth", graduate school of marxism, Zhenjiang "youth XuanJiangTuan" member carina li classmates do the struggle, sacrifice, creation: one hundred Chinese youth movement spirit map theme sharing, from xi general secretary in celebrating the 100th anniversary of the founding of the communist party of China conference speech, lead us to review the different times of Chinese youth movement spirit background, profound, thought-provoking. After the sharing session, Li Wenqing, deputy director of the Working Committee of Jiangsu University, and Cheng Shan, deputy secretary of the Youth League Committee, respectively issued letters of appointment to the teachers and students. The "faith and open class" through the online and offline linkage, "speaking, performing, singing, broadcast" interactive form, for the two student backbone and youth league members of the "customized" party history publicity.

2021-11-16 Nanjing university of technology: polish the "second class" signboard Nanjing university of technology: polish "second class" signboard green reporter li chao) recently, is looking for a job of Nanjing university of technology school of economics and management human resource management senior hu proud "heart very confident", because hands have a "3 years" special transcript-in her mobile phone "pocket university" (PU) App, 286 practice hours, 867 points, this is her university to participate in the "second class" harvest perfect answer. Since 2013, the school has been exploring a quantitative assessment of college students' "second class" hours. As long as college students install a PU, they can know all the activity information of the "second class" of scientific and technological innovation, culture and art, and practical services in the first time. After participating in any activity, as long as you scan the QR code of the activity through the mobile phone, the background can record the name of the activity you participated in, and record the corresponding class hours. Hu Ao Ran, 22, is very keen on softball culture festivals. She knew nothing about softball before attending college. The first time she saw her classmates training in softball, she was very envious but not confident. With the encouragement of Zhang Qian, secretary of the college Youth League committee, she signed up. The school women's softball team players taught her hand in hand. She learned that the school has a deep sports culture, and the Softball Culture Festival has been held for 15 years. This year, the school's Jiangsu women's softball team also represented Jiangsu province in the National Games and won three consecutive championships. In addition to the rich cultural and sports projects, Hu Ao Ran also participated in the "Think + Thinking Loud" free forum, which cultivates college students' critical thinking ability, "almost one session has more people than another". The Olympic spirit, the story of the fight against the epidemic, the 100th anniversary of the founding of the Party... all the "major events" were held by the students. Now, originally a little stage fright, Hu Ao ran, but also because of attending the forum has become talkative. On several occasions, she stood on the podium and facing a hundred people. In order to integrate the first class and the second class, the school has issued a number of relevant institutional documents in recent years, and the social practice credits are coordinated by the school Youth League Committee, and they have entered the students' first class transcript. In addition, there are also capped rules for the practice hours, in case that students are too enthusiastic about participating in the second class and ignore the first class learning. After years of exploration, the school has established a clear achievement oriented "fashion south" and "line" innovation "climbing" "art" four "the second classroom" practice education curriculum system, all the boutique courses "have a complete training plan, professional teachers, personalized evaluation mechanism, have obvious educational effect". At present, more than 8,500 related courses are carried out throughout the year. In November this year, Nanjing Gaoxin Experimental Primary School in Jiangbei New District welcomed special guests, volunteers from the School of Environmental Science and Engineering of Nanjing University of Technology. In the experimental class on the day of the activity, Yao Huan-di, a volunteer, added activated carbon to the muddy and smelly water body, and witnessed the "miracle" together with the children: the water quality gradually became clear, " This is the simplest way to improve the water quality- -adsorption."" My second class score is already full marks."In the past four years, Yao Huandi has participated in more than 100 volunteer service activities, lasting 5,305 hours. Now, as a "volunteer talent", he has signed up for a graduate student volunteer teaching group, hoping to contribute his youth to the most needed places in the western part of China."There are 1,754 public service projects in China alone this year!"Xiong Jian, a junior majoring in civil engineering at the university, said that he is the student head of the quantitative assessment platform for the second class hours."In 2019, there were 1,088 sports and art activities, and in 2020, the number of sports and art activities due to the epidemic dropped to 802."To Xiong Jian's surprise, ideological and political activities" rose rather than decreased ". In 2020 alone, the school held 7,529 ideological and political activities, accounting for more than 60 percent of the total number of second classroom activities, a record high."Political leadership is stronger, and league organization synergy is more effective."Wang chao, secretary of the Communist Youth League Committee of the school, said, the survey found that in 2017, the school carried out 5,646 campus cultural activities, including 395 students 3 points (full score 5 points); in 2020,11,827 campus cultural activities, including 237 student evaluation less than 3 points."Satisfaction with points below 3 has dropped from 7 percent to 2 percent."In addition, the" second classroom " also makes the school's quality education more accurate. In 2020, the school won the national college students summer social practice advanced unit, "challenge cup" Chinese college students business plan competition gold medal, outstanding organization, China youth volunteer service project competition gold medal, the national college students art performance and other honors, it is closely related to the vigorous development of the college students' second class. In 2017, the country's first second classroom data application center for college students was settled in the university. Today, the school is paying more and more attention to the big data analysis of youth development. For example, from the perspective of education and teaching reform from the perspective of "second classroom", use the platform big data to improve the curriculum evaluation and feedback mechanism and the system of the party and government decision-making reference system. In addition, the analysis and research on the quality of talent comprehensive quality training, the quality of the second classroom practice education project, students' personal career tendency and ability structure, so as to provide more comprehensive reference information for youth development. Wang Yuchao said that on the basis of the comprehensive coverage of the ideological and political education work in the current "second classroom", the school strengthens the ideological content in various "second classroom" activities, actively creates the league branch work transcript, provides the whole process record for the daily education of the league members, and makes the golden signboard of the "second classroom" brighter.

2021-11-14 The completion ceremony of the fourth "Green Horse Project" training course was held by Sichuan Sanhe Vocational College. Correspondent for the picture of China youth network Beijing on November 14 (reporter Zhang Jianwei, correspondent Gong Na) recently, Sichuan Sanhe Vocational College of the fourth "green horse project" college student backbone training class graduation ceremony was held in the academic lecture hall. Leaders and teachers of the university Party Committee, the School of Preschool Education, the Ideological and Political and Basic Teaching Department, and the University Youth League Committee attended the ceremony, etc. 185 students attended the ceremony. At the ceremony, Gong Na, deputy secretary of the school Youth League Committee, made a summary of the fourth "Yangma Project" training class. Guo Wenyu, secretary of the school Youth League Committee, read out the commendation decision, and the leaders attending the meeting awarded certificates of honor to the outstanding students. Zhang Jun, the representative of the students, said in his speech that the training has strengthened his belief in Marxism and paid more attention to the combination of theoretical study and social practice. Youth is fearless, take the lead, this is not only a slogan, but also a spirit. As a member of the Qingma class, to establish a firm service consciousness, set an example, to apply the knowledge and skills to practice; to explore and innovate, to serve the students as the fundamental purpose, and to contribute to the construction and development of the school. Ye Changlun, party secretary of the university Party Committee, made a speech entitled "Stick to the faith", I hope the majority of young college students will take Marxism as a bright background of life, Continue to study and practice Marxism; To draw the spiritual strength from the history of the Party, To learn history, history credit, history Chongde, history practice; Pay attention to the unity of knowledge and action, Combine theory with practice, Constantly summarizing the working methods, Improve the working ability and level, To achieve the moral and technical double repair, To contribute our own wisdom and strength to the great rejuvenation of the Chinese nation, A strong country has me; Keep in close contact with your classmates, Abandabandon the tendency of "young officials", Firm the consciousness of "youth friends" in the depths of thought, Make direct contact, direct service and direct guidance of youth become the action consciousness and normal work of youth league cadres, Improve service awareness. The ceremony ended with the enthusiastic singing of all the students. The training lasted for 6 months and was rich in content. Through special lectures, group discussions, watching videos and practical research activities, we carried out ideological and political education, party history learning education, professional knowledge training and quality development training. Through the training, all the participants can quickly grow into the excellent backbone of college students, optimize the talent training, promote the league and learning work, and serve the development of the school.

2021-11-12	Recently, 118 college students from Shanghai Ocean University held a themed party day at the 4th China International Import Expo. It is reported that the school set up the CIIE "small leaf" temporary party branch, the party branch in the sixth plenary meeting of the 19th CPC Central Committee held, around the "great spirit of the Party in the CIIE volunteer work practice" to carry out the theme of study and exchange. Han Wei, a volunteer and party member of the Expo, shared his learning experience of learning the precious spirit of "serving the people wholeheartedly" of the general secretary around the spirit of "dedication". He hoped that he could shoulder heavy responsibilities in the state of "no me", and live up to his PhD and classmates. It is reported that Han and Wei had previously been a flood relief volunteer. He called on students to actively participate in volunteer service and realize the value of youth through dedication. Yan Yu, the secretary of the temporary Party branch, is a member of the "one hundred hundred lectures" group of Shanghai Ocean University. She once entered her class and entered the Chongming community to preach. This time, she shared her understanding of the "pioneering spirit" based on her volunteer work experience. She said that she firmly believed that under the concept of "building an open world economy", the CIIE will be done better and better. Wang Hongzhou, party secretary of Shanghai Ocean University, looked at the high spirits of the students and recalled his story of attending the Shanghai World Expo more than 10 years ago. In 2010, Wang Hongzhou, as a teacher of the university education system, participated in the opening ceremony, work preparation and closing ceremony of the Shanghai World Expo. He hoped that the students will work dedicated and dedicated in the Expo, feel the confidence and pride in China's development, and hoped that they could turn the brief experience into their own life motivation and bring the spirit of the Expo to their personal study and life.

2021-11-11	The 11th cultural and creative universities across the Taiwan straits BBS concluded the 11th cultural and creative universities across the Taiwan straits BBS concluded the youth network Beijing on November 10 (reporter Zhang Jianwei, correspondent Yang Heng horse access) on November 9 afternoon, the 11th universities across the strait culture and creative BBS concluded in overseas Chinese university. Wu Jianping, president of Huaqiao University, and Ma Ruicheng, Chief Vice President of Taiwan China Youth Innovation Network Association attended and delivered a speech. More than 300 students from 34 universities on both sides of the Taiwan Straits participated online and offline. On behalf of the school, Wu Jianping expressed his gratitude to the young talents and hardworking staff from both sides of the Taiwan Straits who attended the forum, congratulated on the success of the forum, and affirmed the achievements of the forum since its establishment. He hoped that young students from both sides of the Taiwan Straits would continue to strengthen exchanges, enhance consensus and sublimate friendship in the process of collision of thinking and emotional exchanges, jointly promote the "family of both sides of the Taiwan Straits as one family", and contribute wisdom and strength to the realization of the Chinese dream of national rejuvenation. Ma ruicheng said that the more difficult the conditions are, the more difficult they will hone the ability of young people. He hoped that young people on both sides of the Straits would bear in mind their obligations and missions and maintain eager exchanges regardless of the road obstacles. The guests presented the certificates to the winning students. At the closing ceremony, the students of Huada also performed the dance "Hometown Love" to show the consistent history and culture of Fujian and Taiwan. Video Review "Fujian and Taiwan Rim" leads you to review the unforgettable moments of the preparation of the forum, and jointly appreciate the works carefully created by the participating teams. Zhang Qiaoyun from Zhongyuan University and Liu Jiaming from Wuhan University delivered their acceptance speeches, thanking Huaqiao University for creating for them for cross-Straits youth exchange opportunities, and expressed their expectations for next year's forum. Other players also said they had gained a lot. Huang Yuqin, a member of Kaohsiung, Taiwan, said, " This participation is a different experience for me. In addition to having a deeper understanding of Chinese culture and history, I also realized that the relationship between the two sides is actually very close. I hope I can have more opportunities to communicate with you in the future!”。Xie Zhaoye, a student from Huaqiao University, said that during the lecture and study tour, we deeply understood the complex about the "five relationships" between the two sides. In the subsequent video shooting process, they also felt the cultural integration between the two sides and realized the mutual dependence between southern Fujian and Taiwan. It is reported that the forum is hosted by Huaqiao University and Taiwan China Youth Innovation Network Association. Thirty-three students from 19 universities including Taiwan Tsinghua University and Chenggong University and 115 mainland students formed 25 teams to participate in the short video competition of "Cross-strait college students". The "Menglian Strait" Cultural and Creative Works Design Competition has received 278 mainland cultural and creative works, and 51 mainland cultural and creative works from 5 universities including Zhongyuan University and Yuanzhi University in Taiwan. Over the past 11 years, the Forum has been committed to youth cooperation and exchange, creative convergence and emotional interweaving, promoting college students to show their talents and enhance friendship in practice, and promoting exchanges and cooperation between universities on both sides. With the attention and efforts of universities and all sectors of society on both sides, it has become an important platform for creative exchanges in the creative field on both sides.

2021-11-08	The fourth International Expo of Shanghai Jiao Tong University was held in Shanghai, bringing the spirit of Jinbo to remote mountainous areas. The reporter noted that some of the nearly 5,000 volunteers organized by the Youth League committee of 40 universities in Shanghai are themselves members of the graduate volunteer teaching group. These special volunteers have brought the volunteer service spirit of the "little leaves" to the most remote mountainous areas in China. This year, 105 "little leaves" from Shanghai Jiao Tong University were devoted to the service guarantee work during the Expo. They come from 22 colleges, including volunteers, sports and academic talents, and 19 members of the 24th graduate volunteer teaching team of the university. At an exchange meeting, this group of "little leaves" found that people in the central and western regions had little understanding of the Expo, and their understanding of the culture and the significance of the Expo was seriously insufficient. So this research group and the last research group hit it off. The members of the research and support team participating in the CIIE prepared courseware and video materials, and the members of the last service place brought the Expo culture out of Shanghai, so that the fourth CIIE was "dry" in the classroom in the central and western regions. At present, in Shanghai jiaotong university counterpart of ordos city, Yunnan, Yunnan Yibin city, Sichuan, Guangxi Liuzhou four service 10 medium and small schools, have carried out nearly ten vivid theme "into the bo class", through the "small leaves" sharing, radio station news broadcast, group learning form, help the children in the heart will be "Shanghai import expo" a pen outline, and with colorful color. In addition, the publicity of the research team also visited the local people, who visited 30 or 40 local educators, parents of students 'parents and people from nearby villages and towns, introduced them to the Expo and publicized the country's high-level opening-up measures. After Yang Zile, a student from NaringXili Primary School in Ejin Horo, Ejin Horuo Banner, Ordos City, listened to Jia Zengguang's class introduction, I don't enjoy enough, Use all the free practice time of the information technology class to get more information about the Expo; Liang Yiman, vice president of the Student Union of Guangxi Sanjiang Middle School, Attended in the teacher Wang Xingyu's lecture into bo spirit class said, "I really want to have the opportunity to experience'Make the Chinese market a world market, a shared market, To inject more positive energy into the international community "; Song Hujun, principal of Inner Mongolia Bulian Primary School in Inner Mongolia, noticed the spirit of volunteer service conveyed by members of the research team, He specially came to the volunteer teacher's office to consult humbly.

2021-11-18	Jiangsu University Communist Youth League Energy Alliance was founded in Soochow University, Li Chao) On November 14, initiated by Soochow University, Southeast University, China University of Mining and Technology and other universities responded positively, Jiangsu University Communist Youth League Energy Alliance was established. Since the innovative implementation of the Communist Youth League Cadre Energy Increase Plan in 2018, the Communist Youth League Committee of Soochow University has held monthly regular working meetings of the secretaries of the Communist Youth League Committee, issued guiding documents and compilation of theoretical learning materials, and carried out extensive learning and exchange activities, and actively selected outstanding backbone to participate in various temporary post training. We will strengthen the commendation and incentive for outstanding community-level youth League cadres, and promote innovation in youth league building. In recent years, the Youth League Committee of Soochow University has continued to deepen the League cadre enhancement plan, and innovatively implemented the League enhancement plan. Qingma Project, faith open class and other political literacy project, summer social practice and volunteer service, focuses on youth innovation ability training, holds special guidance to stimulate youth innovation potential; and comprehensive literacy improvement project relies on the rich campus culture. Alliance established meeting, China university of mining, Jiangsu university of science and technology, Jiangsu normal university, Suzhou university of science and technology, Suzhou economic and trade vocational and technology college, Suzhou arts and other colleges and universities respectively around enhancing between the communist youth league, pay attention to cadres and youth members can assign and promote the development of the communist youth league communication and discuss high quality.

2021-11-06	Nankai students introduce live streaming to rural revitalization Youth Daily reporter Hu Chunyan correspondent Song Jinke) " Students, do you want to taste the potatoes just out of the pot? Face and sweet potatoes, come and be welcome!"" Students to taste the apples produced by Damei Zhuanglang, crisp and sweet!"At noon on November 5th, Nankai University Jinnan campus in front of the liberal arts canteen" changed into " the popular broadcast room, attracted many students to stop. It turned out that a unique theme group day activity was under way here. This is a series of activities, "Live streaming with goods on Double 11, with True Love across the mountains and rivers", launched by the students of the School of History, Nankai University, in order to help the rural revitalization of Zhuanglang, Gansu Province. Today's theme group day is the Zhuanglang special product tasting party, the most representative local potatoes and apples have become the "appetizer" for students."The apple is so sweet, I want to scan the code to pay attention to the studio, to buy a box of Zhuanglang apples for my family..." to taste the food here, there to brush ink. I saw a member of the Youth League branch holding a senior writing brush produced by Zhuang Lang, writing on the rice paper, waving a "rural revitalization, help Zhuang Lang" calligraphy works. Next to the students of a doggerel to speak vigorously, like Tianjin allegro: " Yu points Jiuzhou place Yong Jiang, Zhuang Lang domain in Long Pingliang. Hills and gully customs, Liupan mountain extended pale yellow."The lively rhythm and interesting content tell the history of Zhuanglang, the story of Nankai and Zhuanglang, as well as the future of rural revitalization and industrial development, which has attracted bursts of applause. Immediately, the knowledge contest began, the questions are all designed around the "Nankai and Zhuanglang", "rural revitalization strategy" and other themes, answer more than four questions can get a lovely succulent small potted plants as prizes. With questions and answers, more and more teachers and students have more understanding of Zhuang Lang, thousands of miles away, and have more expectations for the future of rural revitalization there."In the unprecedented poverty to be completed, the Chinese people, including countless young people with 'insist qingshan not relax' strength, 'dare to teach the sun in the new day' drive, 'not break loulan not' resilience forging formed the strength of the poverty crucial spirit, we witness is the vast youth love, more witness is with mountains build the Chinese dream of China! Ma Jinxia from the "Shi in Nankai" publicity group was also invited to participate in the youth League Day activities to preach the great spirit of poverty alleviation. Yu Jiayi, a member of the Youth League branch, was deeply touched that his hometown is a relatively remote place. When he came to Nankai University to study in Nankai University, he thought he should work more hard to learn his skills and contribute to the rural revitalization. Helping Zhuang Lang is a glorious political task entrusted to Nankai University by the CPC Central Committee and The State Council. Since the end of 2012, when the designated assistance to Zhuanglang County, the University Party Committee has always adhered to the spirit of General Secretary Xi Jinping's series of important speeches as the key point, and carried out the designated assistance work with a high sense of responsibility and a lofty sense of mission. In 2020, Zhuanglang County was officially approved by the people's government of Gansu Province to withdraw from the poverty-stricken county list. The school of History of the school actively explores new ways to help Zhuang Lang, and plans a series of activities in the form of party branch and youth League branch linkage. It is understood that on the occasion of the Singles' Day, the first Party branch of the School of History in 2020 will also rely on the e-commerce platform "reduced pure direct purchase" provided by Nankai alumni to live broadcast the goods. Related person in charge said that in order to ensure the effect of the live broadcast, the activity first conducted online internal testing and offline product promotion through the theme group day, and the official live broadcast will be launched on the 9th, with the enthusiasm and wisdom of college students for the rural revitalization.

2021-11-05	Shantou university held "rewalk the long march road" project sharing Shantou university held "rewalk the long march road" project sharing wei correspondent Huang Jiaman) recently, in the 85th anniversary of the red army long march victory, Shantou university held "lighthouse learning" and "rewalk the long march road" project sharing, share "rewalk the long march road" party history learning education the members see, hear, think, think, think, understand. Shantou University "Lighthouse Learning Meeting" and "revisiting the Long March Road" project sharing meeting was successfully held in the lecture hall of the library. Group of Guangdong provincial party committee for the graph to promote party history learning education deep real, let the red gene, revolution generations, Shantou university, engineering, youth corps committee on October 1 solstice 7 organized 20 teachers and students in ababa Tibetan and qiang autonomous prefecture of Sichuan province to carry out the "retrace long road" party history learning education, the red army "LuHua meeting" site, the red army offspring, and walk over the long march of the third, is the most akxia snow mountain, let the teachers and students in practice "immersion" learning the red army pioneers, not afraid to sacrifice the long march spirit."Rewalk the Long March" party history learning education project group members over the Yakxia Snow mountain dumb mouth. The activity of "I hope the students can take the history of the Party for their growth as the rich spirit of nourishment, in the process of following the red footprints, and have a deep experience of the hard-won happy life" began in the speech of Zhou Zhensong, deputy secretary of the Party Committee of the university."The rejourney of the Long March is the road of pilgrimage, roots and adults," said Dr. Ma Yanchao from the School of Marxism during a special lecture on "Light up the lighthouse". Dr. Ma Yanchao had previously held a heroic lesson in front of the 4,200-meter-elevation martyr's tomb, and concluded that all the people who struggle tirelessly for the freedom and liberation of the people are all heroes. Ideological and political courses were held at the tomb of the Red Army martyrs at 4,200 meters above sea level."The Red Army often surprised me to break the limits of human beings by faith. Today, I have also experienced the great role of spiritual power, with a firm belief, I will be able to create miracles."Wang Weijing shared it after the project introduction. The project in the process of preparation, carried out the summer physical training (total sports clock distance of 2259 km), long march and outdoor professional knowledge learning, although well prepared, but in high altitude hypoxia, low temperature environment, there are some students appear headache, swelling, and even a player had to retreat. It is conceivable that the Red Army soldiers relied more on the support of a belief, which is the long March spirit and lighthouse in the eyes of the youth in the new era. The project team members struggled forward in the rain."I hope you can learn the spirit of the Red Army further," Su Lang Pengchu expressed his hope to the students. The first stop of the project arrived at the ruins of the Luhua Conference, listening to the guardian of the site, the Communist Party member Su Lang Pengchu introduced the revolutionary history of the Red Army in Heishui County, and the local Red Army descendants back of the same year Zhu De commander-in-chief dug wild vegetables through a section of the mountain road."In the future, I will certainly practice this spirit," Zhuang Peican students in the highest altitude of the Red Army martyrs tomb choked up to express his feelings. After crossing the pass, the team members came to the highest tomb of the Red Army martyrs. All the team members took off their felt hats for a minute in silence and bowed three times to the tomb. Then, they stood straight, with fists, reviewing the oath of joining the regiment.

2021-11-05	Anhui normal university volunteers open "smartphone school" wang haihan, correspondent, Zhang Yuanyuan Gu Jing) "I can now through WeChat and daughter video, taobao shopping, book train tickets, also learned to homemade music album, the class and the elderly set up their own WeChat public account..." after a few weeks of study, 80 years old Anhui normal University retired teacher ji can skillfully use smart phones. As one of the key university-level key projects of "I Do Practical Things for Teachers and Students" of Anhui Normal University, the "Sunset e Road" volunteer service team of the School of Computer and Information went into the University for the Elderly and set up smart phone schools to help the elderly learn smartphone operation. As early as April 2018,15 college student volunteers from the School of Computer Science and Information formed the "Sunset e Road" practice team to love and respect the elderly. In November 2018, under the guidance of the school Youth League Committee, the "Sunset e Road" volunteer service team was established, with more than 100 college student volunteers who joined it successively. Volunteers use summer social practice and weekends to teach lessons for the elderly. The project adopts the "1 + 3 + N" personnel setting, that is, 1 community deputy team leader, 3 group leaders and a number of student volunteers in the designated service community. After the course, volunteers will set up regular consolidation classes for after-school tutoring for the elderly's weak memory and software updates. In order to have a good class, the volunteers understand the information of the community and the elderly in advance, and develop personalized plans; set up teaching groups, compile the tutorial, simulated teaching, form a complete teaching materials, through the "hand in hand", "face to face" and "key to key" service. During the lecture, a volunteer on the stage gave lectures through PPT and blackboard writing, and more than ten volunteers under the audience answered questions to the elderly in the form of "one-to-one" tutoring. In terms of course setting, there are both basic courses such as alarm clock setting and mobile phone photography, and also software use methods such as wechat use and song download, as well as advanced operations such as appointment registration and mobile payment. In class division, volunteers provide "order-based" services, and the elderly can choose basic and improved classes according to their own conditions."Before each lecture, many elderly people arrived at the classroom half an hour earlier and took notes while listening. This rigorous and serious attitude infected us."Yang Liya, a student volunteer, said that helping the elderly learn smartphones is not only a one-way teaching of knowledge and skills, but also the life experience and learning habits. The "Sunset e Road" volunteer service team insists on taking Wuhu city as the center, radiating more left-behind elderly people in the old revolutionary base areas. At present, classes have been held in 24 communities in 4 provinces and 10 cities, serving more than 8,000 elderly people.

2021-11-04	Harbin commercial university held to deepen the party history learning education results on Harbin commercial university held "green horse project" student backbone to deepen the party history learning education results on the reporter Han Rong) recently, Harbin commercial university held a "green horse project" student backbone to deepen the party history learning education and learning the xi and college students friends results on. On online, in the form of "report + interview", including video playback, interview, live interaction, around the young students rooted grass-roots, committed to learning work, participate in volunteer service, in innovative entrepreneurship, pursue the red footprint of vivid practice, shows the school "green horse project" student backbone learning "xi and college students friends" book learning results. Among the key student representatives of the "Qingma Project" who participated in the briefing were the volunteers of the "College Student Western Volunteer Service Plan" who contribute to the west, take root at the grass-roots level and serve the society, members of the graduate student teaching group of "revitalizing border areas and enriching the people", and representatives of the "student backbone group of the" Qingma Project ". Sun Xianmin, secretary of the university Party Committee, and the young students listened to the results briefing together. After the meeting, Sun Xianmin said that recommending young students of the school to read and study "Xi Jinping and College Students Friends" is to hope that students can bear in mind the inculcation and ardent expectations of General Secretary Xi Jinping, and strive to grow into socialist builders and successors with all-round development of morality, intelligence, physical fitness, beauty and labor. The relevant person in charge of Harbin University of Commerce said that the school will take this briefing as an opportunity to further lead the majority of young students to learn the party history of this "compulsory course", strengthen the love of the Party, the country and socialism, so that the youth for the motherland, the nation, the people and the human unremitting struggle.

2021-11-05	Guizhou university research group "secretary President special award" solid education poverty alleviation active in rural revitalization of Guizhou university research team "secretary President special award" on November 5 (reporter Zhang Jianwei, correspondent, Liu Yanchun, Song Hongqing, Xu Xinran Li Yuhan) in recognition of Guizhou university graduate education solid education for poverty alleviation, active in the rural revitalization. Recently, Guizhou University awarded the "Secretary and President" of the 19th, 20th and 21th Graduate Volunteer Teaching Group "Special Award". In the subsequent "secretary of the principal special award" YanZhiTuan recognition team volunteer service work exchange meeting, the 20th, 21st YanZhiTuan members, said "secretary of the principal special award" is given in school education teaching, scientific research, management services and poverty engines work outstanding performance, make special contributions to the school of teachers and students issued by the lofty honor, honored as a member of the award. It is hard to join the land of the motherland, to be a teaching dream builder, a pioneer of poverty alleviation and education, which is the most beautiful memory of my youth. Members of the 20th and 21st sessions of the research delegation delivered a speech. Members of the 24th Research Group sent their best wishes to the seniors and said that they would strive to continue the youth baton of the research Group, inject great strength into rural revitalization with hard work, and hand over the new generation of your adults to stick to the original aspiration and brave the era of the mission. It is understood that the 19th, 20th, 21st graduate teaching group members, since August 2017 for a long time in zhenfeng LuRong township junior middle school, danzhai xingren middle school poor rural education such as solid education line, poverty alleviation, continue to carry out public welfare student, pass good poverty engines and rural revitalization effectively cohesion baton, with youth practical action to practice the "mingde to good, erudite relentlessly resourceful" school motto spirit.

2021-11-05	Wenzhou Medical University launched the "mobile party history hall" the original study of party history this school has a unique trick!...... A "Mobile Party History Museum" in Wenzhou Medical University allows young students to learn the original knowledge of Party history without leaving the school. Where to learn the original party history? How to realize the immersive study of party history? How to realize the history of the Party? Recently, in Wenzhou Medical University, these problems have been solved! In order to create a new carrier of "full coverage", "no closing" and "effective" of the red research of Party history, Wenzhou Medical University innovates ideas and transforms "teachers and students going out" into "Party history hall coming in", and launched "floating Party History hall", so that teachers and students can study the history of the Party and understand their thoughts without leaving their homes. In the first phase, Wen Medical University reached in-depth cooperation with an exhibition hall of the Communist Party of Zhejiang Province, introduced the original party history display design, and created a reduced version of "an Exhibition hall of the CPC Zhejiang Province" in the public space of the teaching building, so that students can meet the party history hall and enter the Party history hall on the way to class and study, and receive party history education. A major provincial exhibition hall is located in Fengwo Town, Pingyang County, Wenzhou City. It used to be that it took four hours to go to a large exhibition hall in the provincial level. Now I can and the branch students at any time to the "mobile Party history hall" to carry out the league day activities. Hong Xiaochang, secretary of the Youth League Committee of Wen Medical University, said: In fact, this is already the 2.0 version of the "Mobile Party History Museum" of Wen Medical University. Version 1.0 is to make the knowledge of the party history into a display board, in the campus flow, so that students can use the fragmented time to understand the history of the Party. Version 2.0 we hope to bring teachers and students an immersive experience of party historical environment education. The current campus "Mobile Party History Museum" not only ensures the "original formula" of the Party history Museum, but also lets the Party history Museum walk into the campus and approaches the teachers and students, which is more conducive to promoting the "full coverage" of the teachers and students in the red study of the Party history. So, in addition to the exhibition hall please into the school "mobile party history hall" also "not proofing"! On the one hand, the school has introduced the acoustic explanation audio and VR mini program of the exhibition hall. Students can scan the QR code to listen to the original commentary and watch the field Party history hall on the cloud. At the same time, under the training of "One major Zhejiang Provincial Exhibition Hall of the Communist Party of China", the "Party History Youth explanation Team" of Wenzhou Medical University was jointly established, which is open to teachers and students, and created a youth explanation team that "is rooted in the campus" and "can not take away". Cheng Jinguo, Deputy Secretary of the Party Committee of Wen Medical University, the "Mobile Party History Museum" is an important platform for the university to innovate the party history education carrier and promote the full coverage of the Party history education. The next step will be to cooperate with more Party history museums to break the shackles of time and space, and let the teachers and students of Wen Medical University walk into the "Mobile Party History Museum" on the campus to accept the baptism of the revolutionary spirit.

2021-10-29	The communist youth league work association communication symposium in Jiangxi vocational college communist youth league work association communication symposium in Jiangxi industry and trade Shi Weiqiang correspondent, Zou Fanyu Sun Danqing) in order to further promote the work of vocational colleges, strengthen the communication between colleges and universities, recently, Jiangxi communist youth league work association of vocational colleges communication symposium held in Jiangxi industrial trade vocational and technical college. It is reported that the exchange forum a total of Jiangxi tourism business and other colleges and 18 universities to attend. Liu Guohong, member of the Party Committee and vice president of Jiangxi Industrial and Trade Vocational and Technical College, and Zhang Lei, chairman of the Association and secretary of the Youth League Committee of Jiangxi Tourism and Trade Vocational College, delivered a speech and speech. Wang Xing, secretary of the Youth League Committee of Jiangxi Industrial and Trade Vocational and Technical College, shared and exchanged views on the work priorities, brands, characteristics and highlights of the college in recent years. Conference, the college cadres on how to broaden the effective way of new era student member training education management, how to effectively crack group building and business work "two skin" problem, "challenge cup" "revitalization cup" competition experience and incentive mechanism, "second classroom" management advanced experience, aesthetic education work advanced experience and higher vocational colleges departments two levels of the communist youth league work management method is discussed.

2021-10-28	Nanjing Vocational College of Science and Technology: Make contributions in social practice, Nanjing Vocational College of Science and Technology: grow ability to make contributions in social practice, forge ahead in the new era "summer social practice theme, carry out the" one hundred years cup " college students summer social practice activities. Key team around 100 study the history of the party, the theory to preach, national observation, rural revitalization project, in the social classroom education, long talent, contribution, help to consolidate poverty crucial achievements, in rural revitalization strategy, in the practice of middle school party history, strong faith, follow the party, the patriotism, the patriotic ambition, patriotic line consciously into the new era dream journey. The "white dove" volunteer practice team came to a primary school in Tianchang, Anhui province to open the social practice activity of "deep ploughing into the party history and aesthetic education field, red seeds sprinkle childlike innocence". Volunteers donated books and stationery for the small students, built "love library", to the poor students donated grants; "four history education" as the main line, from the aesthetic education, labor practice, "hand in hand with party history", "long march story" "into the warrior life" labor experience activities, red education seeds into the students labor education, aesthetic education, red gene, build ideal and faith. Volunteer Li said, seeing the children's eager eyes, my classmates and I should pass on the faith of the volunteers, warm the children, and serve the society. In the face of the outbreak, the school of nearly 400 volunteers positive response, practice "please rest assured, power have me" youth oath, carry out "a line have me, team out" action, active in epidemic prevention and control, the sampling detection, sealing area service, epidemic prevention propaganda, order maintenance play the role of youth powerforce and commandos, full service general situation, jointly build epidemic prevention and control security lines, fully promote hardship, not tired, brave burden spirit, show youth in the epidemic prevention and control, with action and love interpretation volunteer spirit, to resolutely win the epidemic prevention and control war contribution youth strength. Teacher party member Teacher Zhu first time came to the community to report, "I am a party member, I must go up". Put on the protective suit, Ms.Zhu entered the first round of nucleic acid testing volunteer service. After the completion of the test, she began to cooperate with the community. Then, she continued to put on the second round and the third round... Party activist Zhang has been rotating in the community."As a college student, I should stand up even more."She said. Every red education base is a vivid history education classroom."The red memories of unforgettable memories not only contain the spiritual code of 'where we come from', but also mark the spiritual roadmap of 'where we go'. Every red mark has deepened our understanding of the party's centennial glorious course."Said one volunteer. With "e-commerce" professional background volunteers teachers and students as "postman", they went to wujiang, Lianyungang, manzhouli and other places, visit characteristic food, characteristic process and intangible elements, using e-commerce professional knowledge, through the network, carry forward the local characteristics, service home economic development. In the summer vacation, the "white dove" volunteer practice team transformed the "faith" into "action", and had a colorful "big ideological and political lesson" combined with the reality. Through practice, teachers and students learn to think, understand and gain, in practice to cultivate the feelings of home and country, enhance their mission, and make contributions to their ability."Social practice is the tangible carrier of practical education, is a powerful measure to comprehensively improve the comprehensive quality of college students, through the summer social practice activities, practice group members out of school, contact with society, understand the national conditions, theory and practice combined, consolidate the party history learning and education achievements."Said the head of the relevant school department. In the future, the school will continue to strengthen the construction of practice education system, innovative practice form, expand coverage, the campus small classroom with social big classroom, education guide more students into social practice, in the service to the people, realize the value of life, in education in social practice, long ability, contribution, yong do when the era of new people.(Data and information source: Nanjing Vocational College of Science and Technology) October 28,2021, edition, 07

2021-11-03	Guizhou Qiannan college of science and technology held campus rainbow run Guizhou college of science and technology (Guizhou university college of science and technology) held "new life run" campus rainbow running activities on November 3 (reporter Zhang Jianwei, correspondent, Chen Yingmei YanChun), Guizhou Qiannan technology college (Guizhou university college of science and technology) "new life run" campus rainbow run held in college football field. More than 700 students participated. The activity kicked off in the "Dance string Burning" brought by the College Youth Volunteers Association. Event photo. Correspondent for figure this game has red, orange, yellow, green, green, blue, purple seven sites and each site has a team game, contestants need to complete the "opposite" and "hands and feet" "hula hoop" game task set to return to the venue to complete the final puzzle, if any mistakes in the process of the game, will be the staff "rainbow powder" punishment. Event photo."This is a different activity, brought me a lot of happiness, although very tired, but also 'siege', but more meaningful than lying in the dormitory playing mobile phone play."After completing the race, her hair and clothes were in different colors, but she was still enthusiastic. Event photo. It is reported that the campus rainbow running life aims to convey the concept of healthy life, enrich the campus cultural life of college students. In the early stage of the activity, the college adopted online QQ, wechat promotion + offline publicity for team recruitment, which expanded the scope of publicity and the coverage of students, and broke the traditional single on-site recruitment mode.

2021-10-28	Anhui engineering university to establish the first off-campus labor education practice base of Anhui engineering university to establish the first off-campus labor education practice base Ji'an-wei zhang correspondent, Guo Guangchun check WeiTing) recently, Anhui engineering university art college party committee, Anhui engineering university, the party committee, the party branch and Anhui Wuhu Sanshan street party construction cooperation agreement, jointly develop party history learning education, the first off-campus labor education practice base, the wall painting power rural civilization volunteer service. Su Guohong, deputy Secretary of the University Party Committee, attended the event and spoke at the signing ceremony, She points out that the, The school and Wuhu city have both made common development and progress for a long time, Today's cooperation is a vivid practice of the school's "1 + 3" Party building model of "promoting Party building through cooperation and promoting development through Party building", The university secondary party committee, grass-roots party branch signed with the local party construction cooperation and joint construction agreement, We should leverage the advantages of both sides, Through the development of joint construction, Building a working system of resource sharing, organization and co-construction, and complementary advantages, Strive to make the school scientific research results to serve the local governance and development, Let the local volunteer service and social practice carrier service schools to implement the fundamental task of moral education, Gradually explore joint construction mechanisms that can achieve long-term adherence, produce results and achieve mutual benefit, Form a replicable model for demonstration and promotion. Art college party committee, the school youth league, the party branch and sanshan street party working signed the grass-roots party construction cooperation agreement, clear the "joint party spirit education, thematic party day activities, joint volunteer public welfare activities, employment village (house) environment image design consultant, establish summer" a program under which officials "social practice base, set up the labor education practice base" and other five to build content. The two sides came to the Sanshan Revolutionary Martyrs Cemetery to jointly carry out the party history learning and education, laid flower baskets to the martyrs buried here, and collectively reviewed the party membership oath. The two sides came to Changba Village, Sanshan Street, to view the wall painting with the theme of filial piety and love for the elderly and relatives, agricultural harvest and living environment. Su Guohong and Zhang Xinfeng, secretary of the Party Working Committee of Sanshan Street, jointly inaugurated the "Labor Education Practice Base of Anhui University of Engineering and Qingma Engineering Practice Education Base", and issued the letter of appointment to the labor education practice tutors after-campus and on-campus respectively. The students of the tenth "Qingma Project" college students backbone training class labor practice group listened to the explanation. Anhui engineering university labor education practice base, "green horse project" practice education base is one of the key content of the cooperation, provided by Sanshan street working committee, office of the area within the jurisdiction, after leveling delivery of Anhui engineering university, specially used in schools to carry out the labor education practice, it is also to carry out the school labor education plan an exploration attempt. Next, the Academic Affairs Office, the Student Affairs Office and the school Youth League Committee will specially set up labor education practice courses around the theme of labor education, and lead the teachers and students to carry out crop planting practice experience activities in the base.

2021-10-22	Guizhou Qiannan college of science and technology red narrator contest (Guizhou university college of science and technology) red narrator contest on October 22 (reporter Zhang Jianwei, correspondent, Yang Caifen, Chen Qinghong, wanjia billion Chen Yingmei) recently, Guizhou Qiannan college of science and technology (Guizhou university college of science and technology) "red spark light qian your earth" red narrator contest in complex building 211, college relevant functional departments, the department responsible for teachers and students represent a total of more than 300 people to participate in the activities. The competition is divided into two parts: personal presentation and story explanation. The 4 teachers and 11 students respectively explained the red story of the Red Army's Long March, the memory of a city, the victory of the cause of communism through video, singing, music, dancing and other forms. The judges scored the speech content, skills, image and effect. Finally, Xu Lei won the first prize, Tang Chuanchun won the second prize, He Kejian won the third prize, Cui Mengting won the second prize and Luo Xuelian won the third prize. Business administration of class 211 Liao Xinyi classmate understanding of Guizhou and more, " I came to school from other provinces to Guizhou, not familiar with Guizhou, but through the interpretation of the players, I felt the Guizhou deep red, also learned some of the history of Guizhou, have the opportunity to go to Anshun must visit comrade Wang Ruofei's former residence.”

2021-10-21	Northern University of Nationalities held the "Students of All ethnic groups" activity of "Students from All ethnic groups See the Beautiful New Ningxia". More than 100 students from all ethnic groups participated in the activity. Activities, the students visited research in Ningxia minning town, Ningxia hui autonomous region stone chateau, Ningxia museum, white beach, the Yellow River in Ningxia Wuzhong city section, Shizuishan mouth industrial site park, "the national ecological culture village" longquan village, xinghai Hunan domain wetland, Shizuishan dry school museum, Wu Zhongjin garden community, ningdong energy and chemical industry base, six panshan red army long march memorial and fort monument, etc.

2021-10-18	Sichuan culture media vocational college held a national poverty alleviation day special action Sichuan culture media vocational college held national poverty alleviation day special action zhang jianwei) on October 17, is the eighth national poverty alleviation, is the 29th international poverty eradication day, before winter comes, Sichuan culture media vocational college special call for floor teachers and students to warm clothing fundraising, the fundraising collected nearly 70 boxes, a total of more than 2000 pieces of cold supplies, has been sent to shiqu county must gong horse township. In the past three years, Shiqu has been adhering to the concept of "there is no small matter on the road of poverty alleviation, and the poverty alleviation work should be specific", deeply cultivating education and poverty alleviation, and paying attention to the growth of children in Shiqu. This charity fundraising activity was also personally deployed and coordinated by the leaders of the college themselves. When the cold winter was coming, the party and government leaders of the college had participated in the process of the activity initiative and call for action, taking the lead in fundraising, assisting in sorting out and making the packaging car. During the one week of centralized fundraising, the school Youth League Committee and the Student Affairs Office organized student cadres to collect point-to-point clothes, and the Party branch of the Department of Art Education and Performance also organized teachers and students majoring in character image design to repair and transform the clothes collected. To actively cooperate with the strategy of rejuvenating the country, consolidate the crucial poverty achievements, continuous security stone canal children education resources, college cooperation with provincial radio and television network company "rural cloud classroom", so far, this year "cloud classroom" teaching 15 times, a total of 30 hours, and combines the founding in one hundred, recitation, art, dance, music, etc.

2021-10-10	In the first "Cloud Games" Putian University held the first "Cloud Games". Due to the epidemic, the school's offline sports meeting this year was held online. According to Lin Chengshan, a member of the presidium of the Student Union of the university, the "cloud Games" was jointly organized by the school Youth League Committee and the Student Union to promote the physical and mental health of college students during the epidemic prevention and control period. The event was launched on the eve of National Day and received a positive response from students, and many people signed up for each project. The new events of the first "Cloud Games" mainly include three categories: self-presentation, physical fitness events and team events, among which the self-presentation category includes Baduanjin, Mazu Gong, Taijiquan and cheerleading. Traditional physical items include: push-ups (boys), sit-ups (girls), open and off (boys), and plank (girls). During the competition, the participants shoot themselves indoors and upload the videos independently. Finally, the competition is evaluated according to the completion degree."Yang Shuqing, Huang Miaojuan and Dong Hong", have a self-presentation team project called "Don't Get Angry aerobics," which has attracted a lot of attention. Yang shuqing said that they often do such relaxed sports in their spare time. " The simple movements and cheerful music can not only relax, but also exercise."Chen Sifan, one of the leaders of the event, said:" The 'Cloud Games' is loved by the students, which shows that the concept of the national movement has become popular among the young students, and it is no longer the previous paper talk. I hope more students can participate in sports, fall in love with sports, and stick to sports.”

2021-10-08	Southwest jiaotong university student backbone to Yan 'an to carry out the party history study education southwest jiaotong university student backbone to carry out the party history study education jianwei correspondent, He FuXin li) for further study general secretary xi "July 1" important speech spirit, vivid to carry out "party history, strong faith, follow the party" learning education, southwest jiaotong university student backbone in the Chinese revolution Yan' an "please rest assured, power have me" party history education, comprehension Yan'an spirit, ceaseless red blood. The party history study education content includes the theme party class, visit study, real scene teaching and other forms. Students will recall the revolutionary years of the Communist Party of China in Yan 'an University, the Yan' an Revolutionary Memorial Hall, and the former site of the Zaoyuan Revolution in Yan 'an, and understand the great Yan' an spirit. During the study period, the students planned and shot a series of micro-group class videos with the theme of "Inheriting the Yan'an Spirit" based on their own insights. After returning to school, they went deep into student organizations and youth League branches to conduct lectures, leading the students to learn the history of the Party, strengthen their faith and follow the Party. Southwest jiaotong university youth corps committee secretary zhang junqi said that the school in the process of promoting the reform of the student union organization exploration established by the student union (graduate school) party member student backbone of temporary party branch, the to Yan'an theme education practice, aims to guide the students from the extraordinary course of the communist party of China deeply feel the truth of marxism power and practical power.

2021-09-30	Xi 'an aviation institute "little Qin treasure" power 14 games volunteer service work xi' an aviation institute "little Qin treasure" power 14 games volunteer service work 163 volunteers and 52 city volunteers service in outdoor rock climbing project competition organization, information technology, audience service, administrative reception and so on 10 offices and xi'an several traffic arteries. The rock climbing competition was held in the outdoor sports rock climbing venue in Yanliang District, Xi'an city, Shaanxi Province. During the competition, 163 competition volunteers served the work tasks of 27 positions in 10 offices, including the competition organization, audience service and media broadcasting, and the total volunteer service duration reached 6,520 hours. Volunteers conduct daily inspection of the equipment room in the equipment support room of the competition organization office, the audience service office of the rock climbing competition project, and the volunteer services, such as information consultation, civilized guide and cultural display. Fifty-three volunteers provided route guidance, information consultation and emergency assistance, and assistance for special groups to citizens and tourists in Datang West City, Bell and Drum Tower, Yanliang District and other places, with a total volunteer service duration of more than 3,120 hours. In order to ensure the smooth progress of the test competition volunteer service work, the volunteers received more than 10 comprehensive pre-job training in terms of basic skills, service skills, etiquette norms, action norms, and post skills. In addition, according to the epidemic prevention and control requirements of the Climbing Competition Committee of the 14th National Games, 112 volunteers conducted closed and centralized control. The university has formulated the Management Regulations on the Closed Period of the Volunteers in the 14th National Games of Xi 'an University of Aviation, and established the temporary youth league branch of the volunteers in the closed management Zone of Xi' an University of Aviation, to ensure the study, life, mental and physical health of the volunteers during the quarantine period.

2021-10-08	Guizhou university college of science and technology held the third counselor quality ability contest of Guizhou university college of science and technology held the third counselor quality ability contest for China youth network Beijing on October 8 (reporter Zhang Jianwei, correspondent, Liao Xinyi, Yang Fangxue, Xu Rong lei Chen Yingmei), the third session of Guizhou university college counselor quality ability contest in complex 211 multi-function hall. The competition is divided into four parts: basic knowledge test, case analysis, heart-to-heart talk and theory lecture. Fifty-three counselors participated, and more than 300 students watched it. The picture shows the talk session of the counselor quality and ability contest."Student work is the top priority of the counselor's work. The counselor's work should be student-centered and serve the students wholeheartedly."College student work department minister Xu Man commented to."It is easy to get, but teachers are hard to find. Counselors should strengthen the theoretical knowledge reserve, enrich their own experience, and influence and move people with a deeper and comprehensive personality charm."Fan Wei, secretary of the Party Committee of the college, affirmed the wonderful performance of the contestants. He hoped that all the counselors could comprehensively improve their professional skills and work level, deepen their ideological height, make concerted efforts to promote the education development of the college, and cultivate reliable socialist builders and successors for the country. The picture shows a group photo of the college competition. It is reported that the competition is organized by the student work department of the college, aiming to conduct an all-round investigation of the counselors' ideological and political quality, language expression ability, problem handling ability, and communication and coordination ability. In the end, Zhang Lilin won the first prize; Mo Ting of Law and Public Administration, Xu Lei won the second prize, Luo Ting, and Xie Huili won the third prize.

2021-09-30	Nanchang University held "young scientists into the campus" theme day activities "young scientists into the campus" theme day activities into the high school affiliated to Nanchang university, popularize scientific knowledge for teenagers; young scientists play a role model, strive to cultivate the spirit of scientific exploration. At the beginning of the semester, Dr. Li Wei of Software School gave a keynote lecture on "3 D point cloud vision driven AI Application"; Dr. Lin Deyu of Software School gave a lecture on the theme of "integrated communication and interconnection of everything". During the activity, the Youth League Committee of the School of Software of Nanchang University also carried out the theme signature activity of "Please trust the Party to have me" in the high school of the High School affiliated to Nanchang University. Junior and senior high school students experience university courses in the theme of youth league day activities, master advanced scientific concepts, inspire wisdom, and anchor their positions more accurately, so as to inspire faith, gain inspiration, draw strength, and write a song of progress with youth. The activity was hosted by the Youth League Committee of Nanchang University and jointly organized by the Youth League Committee of the Software College of Nanchang University and the Youth League Committee of the affiliated Middle School.

2021-09-30	Binzhou medical school: learning practice enlightenment in the "field" Binzhou medical school: learning practice enlightenment in the "field" Chinese students political XuanJiangTuan in the vast field, record example around example, carry forward the great founding spirit, practice practitioners benevolence... 1050 college students, 405 preach, preaching team, all over the country, 412 grassroots party members typical, carry out various forms of 548, the audience of more than 42,000 people."When the village party secretary recommended Chen Baoguo to me, I was suddenly stunned. Isn't this the ordinary old grandfather in the village? Walk into his cabin, see a pile of red certificates, MEDALS, just know that the face is a veteran of many meritorious service, veteran party members."Chen Xinyu, a lecturer of the Shi Jian Pioneers who lives in Pingyi County, Linyi city, Shandong Province, wrote in her interview record:" He has been an engineer for decades and experienced life for 56 years. He has built countless roads and caves, and still has scars on his legs and hands. But he was very calmly said, 'endure hardship is to do good for our people ah, as a Communist party member, the car does not fall just push, as long as there is a breath, the heart will have to hold the people'."It is understood that during the summer vacation, similar publicity and interview records have accumulated nearly 600,000 words. Cao Xishan, the instructor of the publicity group, said that in the centenary of the Party, General Secretary Xi Jinping brilliantly summarized the great spirit of building the Party in 32 words in his important speech on July 1. In order to better inherit this precious spiritual wealth, the school launched thousands of college political publicity activities at the beginning of the summer vacation, encouraging students to "break out of the ivory tower, record the example around, and tell the spirit of building the Party with abundant field perception". The students were enthusiastic and divided into 405 teams to sink into communities, factories and villages. Soon, more than 400 vivid examples emerged, including veterans of the war, grass-roots cadres, community workers, and people's teachers. Veteran Zhang Zizhen with a strong sunshine accent told about the scene of the battle of crossing the River, "I didn't know the fear back then, If I just want to follow the party, Crossing the Yangtze River, Free the whole of China "; Retired doctor Mei Zhiying faces a male patient who failed to defecate smoothly for three weeks, Without hesitation, he leaned over to relieve the patient with his hands, "This is the daily routine of a doctor, in emergency circumstances, I only remember myself as being a party member, A doctor. "; Police Wang Xuantao faced the suspect kidnapped hostage with grenades, Resolutely rushed up to grab the explosive opener, "Such case must come up, To protect people's lives and safety, Is the duty of the police "... the extraordinary move behind the ordinary, Great love bosom in simplicity, Constantly stirring the hearts of the students. Mei Jingchen said that the interview with party members is like a folk treasure hunt, which left me a precious red memory, worth a lifetime of treasure. Chen Xinyu and other students immediately wrote the application form for joining the Party after the interview... How to light the star torch of the field of fire, sparks, with the power of propaganda, gathered into the torch to light up the grass-roots fields? Binzhou Medical College focuses on innovating the publicity mode, opening up two offline and online positions, highlighting the scene, story and colloquial expression, and making the theory publicity become interesting, sentient and meaningful. Drawing on the "dry goods" accumulated in the early interviews, each team developed a new way, and launched a series of "flavor meals" that the grass-roots people like. Youth to the party propaganda team in Binzhou City, Shandong Province, according to the characteristics of primary school students, specially made for them a group of party history stories, a string of red classic songs, the children listening while singing, singing and dancing to feel the glorious course of the Party. The Red Culture publicity team took more than 30 local villagers into the Party and mass service center, which not only reviewed the centennial history of the Party, but also told about the changing history of the towns and villages. The great historic leap of "standing up and getting rich" became "what happened at home", which aroused the resonance and applause of the villagers from time to time.231 The flood fighting team is very down-to-earth. They go from village to village, into the elders home to sit on the kang Lao; meet student children, gathered to the courtyard to chat. While teaching villagers to watch computer videos to learn about the state affairs in Henan, he explained the knowledge of drought and flood control according to the local weather. The old people looked at the young soldiers fighting in the war of tears, while the children asked how to donate their pocket money to Henan. On the Internet, young college students take out their own skills and unique skills, the video propaganda.307 Beauty girl rainbow team invited the interviewed party members directly into the studio, while the interview while feeling, while preaching while editing, the original taste at one go. Team 288 will preach into the red memorial site, from the Dezhou Revolutionary Martyrs Cemetery to the Huaiyu Zhou Temple, from Jiao Yulu's former residence to the Menglianggu Campaign Memorial Hall, the members are divided into several places, and interact between the air, so that the soldiers, heroes, great men and role models of different times together "gather together" to preach the stage, together to interpret the password of the great party founding spirit. Up to now, the college student current political publicity group has carried out more than 500 propaganda lectures, and uploaded 405 propaganda videos on the B station, lasting more than 2,500 minutes. Because of the strong local flavor, story flavor and emotional taste, the audience is called the era of "tide products" from the field."To realize the Chinese dream and create a better life for all the people, every one of us should continue to work hard and make hard work!"After finishing the propaganda report entitled" the red spirit spread forever ", the happy planet propaganda team of 3 members are still can not calm," always feel to do something!"Before the end of the summer vacation, the three college students majoring in hearing and speech rehabilitation and pharmacy, respectively, submitted more than 2,000 words of practice research reports. Under the theme of "Rural Revitalization and Listening to China", the report investigated the hearing status and the popularity of hearing AIDS in rural villagers, providing first-hand information for the prevention and rehabilitation of hearing impairment in rural areas. Wei zhiwei said that the test results were not optimistic, and the middle-aged and elderly people in rural areas had serious hearing loss. What is even more regrettable is that these losses could have been avoided by preventing deafness from high noise and accidental injuries. The next time, the team members will focus on teaching the villagers to do ear exercises, popularize the knowledge of preventing deafness and wearing headphones. As an excellent team of summer "social practice activities", the current political publicity group always adheres to the whole line of "learning, speaking and doing". This summer publicity, the school further advocated the publicity group to do a good job of record report, publicity report and practice report, combined with the director of medical students, to do practical things for the masses. Li Juanjuan, who lives in Bincheng District, Binzhou City, held a "1 + 1" publicity activity in the local middle school. On the one hand, she carried out a pioneer story exchange meeting for party members, and on the other hand, she popularized health first aid and epidemic prevention and control knowledge to the students. She led everyone to practice earnestly and gathered a number of "popular science fans". Zhao Haibei, a member of the publicity group who lives in Zhaoyuan, Yantai, took the initiative to claim the epidemic prevention task at the critical moment when the Delta epidemic affected the local area. He braved the scorching heat to help collect information at the monitoring points and stopped for 10 consecutive hours."A strong country has me, focus on temper", according to the head of the propaganda department of the university, the recent school will put these 00 college students in the field of learning and practice, further "back to the furnace" refining, refined a number of excellent propaganda talents and propaganda quality, in the school to carry out the tour, and into the teachers' ideological and political classroom. China Youth Daily, China Youth Network reporter Xing Ting source: China Youth Daily

2021-09-17	Foshan institute of science and technology the Red Cross first aid training activities Foshan institute of science and technology the Red Cross "world day" the first aid first aid training activities China youth network Beijing on September 17 (reporter Zhang Jianwei, correspondent Xiong Hang) in order to better spread first aid knowledge and concept, first aid knowledge and skills, enhance communal consciousness, should Guangdong Red Cross and Foshan Red Cross initiative, Foshan institute of science and technology the Red Cross in xianxi campus, jiangwan campus held first aid training, training theme is "first aid hero" do school, community, the school more than two hundred students to participate in the training. In his speech, Jiang Zunguo, secretary of the Youth League Committee of the school and the head of the Red Cross Office of the school, first introduced the "World First Aid Day" and the basic situation of the Red Cross Society of Foshan University of Science and Technology, calling on the students to actively learn and publicize first aid knowledge, master more first aid skills, and help more people in need. In jiangwan campus training meeting, Foshan college of science and technology medical school teacher Zhu Fan teacher introduces the basic situation of the Red Cross organization, explained the common first aid knowledge and skills, including saving prime time, how to call emergency phone, syncope, respiratory blockage, burns and other emergency what first aid methods, etc. At the training meeting of Xianxi Campus, Wang Ling, the emergency nurse of the Fifth People's Hospital of Nanhai District, explained the basic knowledge of first aid, and demonstrated various first aid skills such as risk escape, cardiopulmonary resuscitation and trauma rescue to the students."Assessment the environment, assess consciousness, shouted for help, flat limbs, judge breathing, chest compressions, open airway, artificial respiration..." training site, the two lecturers carefully explained for the students of cardiopulmonary resuscitation, heim rick first aid and drowning, heat stroke and other common life accident injury prevention and on-site emergency treatment measures.

2021-09-17	Anhui Engineering University of Technology held a special meeting on strengthening and improving the style of the Communist Youth League Student Union to strengthen and improve the style of work Jianwei Correspondent, Sun Linyao Zha Weiting) recently, Anhui University of Engineering held a special conference on the Communist Youth League Student Union to strengthen and improve the style of work in the academic lecture hall. The whole school has a full-time and part-time league cadre, 2021-2022 student organization backbone, student club backbone to attend the meeting. The backbone of the International College of Engineers attended the conference online. Su Guohong, deputy secretary of the Party Committee of the university, took the important speech on July 1, To struggle to practice the "please party rest assured that the strong country has me" youth oath "," practice true knowledge, Practice is long and true talent, Strive to be the pillars of the talent " as the theme, To study the spirit of General Secretary Xi Jinping's important speech on July 1 and the important speech at the opening session of the Training class for young and Middle-aged Cadres of the CPC Central Committee (National Academy of Administration) in the autumn semester of 2021, It is hoped that the backbone of the school will strengthen the study of party history, study practice, strengthen and improve the style of work, Promote student organizations to better play their leading role as Bridges and services, Build an ideal, care about students, fresh and sunshine student organization image. The head of the Youth League Committee of the university read out the list of the backbone of the new university student organization, the Regulations of the Communist Youth League Student Union of Anhui Engineering University on further strengthening and improving the style construction, from the "three hearts" and "three forces" students, and deployed the new students in 2021. Student representatives of the school Youth League Committee, school student Union, and school League Association propose to strengthen and improve the work style, urging all students to establish lofty ideals and beliefs, learn knowledge, practice, and improve hard quality, promote the standard construction, promote volunteerism, and promote the purpose of service, accept supervision, and lead the work style.

2021-09-16	Shandong university will college students 'social practice and party history learning education with frequency resonance of Shandong university: guide college students' social practice and party history learning education with frequency resonance XingTing) this summer vacation, Shandong university to carry out the "party history, strong faith, follow the party" 2020-2021 students summer social practice, a school 50 departments unit of nearly 17000 students to participate in various forms of 1690 teams, social practice with full social practice to guide the young students with practical action to promote party history learning education landing effect. During the activity, the college topics in eight directions covered many fields of returning to help agriculture, current political analysis, supporting teaching and poverty alleviation, and epidemic fighting service, and simultaneously opened five key topics, including "Party History Learning and Education Youth Publicity Group", "Serving Shandong Mountain University Program" master and doctoral Service Group, and "Green Bird Project" post internship. Not only that, Shandong university also dig deep, make full use of red resources, active docking hero mountain Jinan battle memorial, liberation pavilion Jinan liberation memorial red education base, establish effective contact mechanism, guide young students to carry out research practice, theory, the new era youth responsibility bear research activities, promote the party history learning education precision to the ground. At the same time, we will deepen the cooperation between schools and enterprises, and cooperate with China Railway, Shandong Provincial Taxation Bureau, Tuantuan Zibo Municipal Party Committee and other relevant units to realize complementary advantages, resource sharing, and help young people grow up. Shandong university also set up the history of the party learning education "youth XuanJiangTuan", for the school formed 101 "youth XuanJiangTuan" team, actively carry out the "one hundred days team hundred about one hundred" activities, from 50 departments of more than 1000 people involved, covering the medical disciplines, doctoral period, each team accumulated practice activities more than 80 times. In addition, based on the "Green Bird Plan", it anchored the "urgent, difficult and anxious" problem of youth employment and job selection, and appointed more than 40 liaison officers of the "Green Bird Plan" college of Shandong University to effectively follow up the employment, entrepreneurship and practice of the college students' "Green Bird Plan", and combine the party history learning and education with youth development. In the process of guiding practice, Shandong University pays attention to innovative forms and excavates new drivers of party history learning and education through volunteer service. Fixed-point contact of marxism institute, school of philosophy and social development, international education institute and other related college, accurate guide related professional volunteer service force depth participation, to form a "one hundred elegance, youth set sail" youth volunteer XuanJiangTuan, "green wing has a dream," history "ambition" volunteer service six special team, reserve nearly hundreds of volunteers, in party history learning education practice. In addition, to volunteer service as the gripper, form a "hope hut mountain bear" social practice special team 23, with the history of the party learning education new kinetic energy booster "hope hut" children care project deep walk fine, for children do the practical work, solving problems, in the history of the party story children interpretation at the same time, to promote party history learning education into the brain, into the heart, into action.

2021-09-15	Anqing normal university after 00 students "retrace long march road" practice activities Wang Haihan, correspondent, Zhou Ziqi Li YanChunhui) from Anqing, Anhui city, Nanchang, Ruijin, yu, finally arrived in Guizhou Zunyi, seven days... this summer vacation, Anqing normal university foreign languages college "retrace long march road" practice group 6 after 00 students along the red footprint feeling ancestors spirit. In the thick and old sheepskin coat, the mildew and broken small rattan basket, and the marching pot with guns, in the central Long March departure memorial hall, the players are attracted by the objects. Zhang Zilong, the team leader, took out and took a picture with his mobile phone."Many soldiers were as young as us and joined the Long March without hesitation. I admire this spirit."Zhang Zilong said. With damp watermarks on the walls, moss on the steps, and small wooden doors, the bumpy ground, two long wooden benches and thin wooden board beds are where Chairman MAO lived in the revolutionary struggle in Ruijin, Jiangxi province. According to statistics, in the seven days, the six students, under the guidance of their teachers, visited and punched in 45 old revolutionary sites, battle sites, memorial halls and red scenic spots in the three provinces and four cities."My grandfather is a veteran. He often tells me the story of the Red Army climbing the snow-capped mountains and crossing the grass."" Just as 'the Red Army is not afraid of an expedition and is only idle', we should also carry forward the spirit of hard struggle."Du Jianxing, a member of the team, said that along the way, we helped each other to overcome the hot weather and other difficulties. At the end of each day, the members of the practice group will also make the graphic and text materials of the red scenic spots with the students' harvest feelings and share them on the new media platform. In the end, they took 813 photos of red scenic spots and wrote about nearly 10,000 words. Photo taken by Li Yanchun and Li Yilin

2021-09-10	Ningxia national vocational and technical college to carry out the first class theme TuanRi activities Ningxia national vocational and technical college youth corps committee to carry out the first lesson "please rest assured, power have me" theme TuanRi activities for China youth network Beijing on September 10 (reporter Zhang Jianwei) recently, Ningxia national vocational and technical college youth corps committee carried out the first class "please rest assured, power have me" theme TuanRi activities. Gao Xiaolian, a member of the "Wuzhong Youth Power" Lecture " group and a student of the Education Department at Ningxia Vocational and Technical College for Nationalities, made a speech on the spirit of General Secretary Xi Jinping's important speech on July 1. During the discussion and exchange session, the young people of Ningxia Vocational and Technical College for Nationalities have made speeches. They said that the youth of China, the youth oath, as the youth of the new era in the construction of a modern socialist country, to achieve the great rejuvenation of the Chinese nation in the new journey, shoulder the heavy responsibility, indomitable, run the baton of history.

2021-09-10	Hainan tropical ocean institute summer social practice out of the "Hainan rocks" Hainan tropical ocean institute summer social practice out of the "Hainan rocks" JianWei correspondent Zhou Yutong) according to the central league, Hainan provincial party committee and other departments about 2021 summer college students "a program under which officials" social practice related file spirit, summer, Hainan tropical ocean institute youth corps committee organization practice team 112, participating in 653 students, in various cities and counties to carry out the summer practice activities in 2021."Tell the party history story and spread the red gene" - -college students party history education publicity practice team to preach the theme. Correspondent for figure college around the "one hundred is youth" theme social practice, "youth power free trade port" theme social practice, "rooted in China, help rural revitalization" -college students "home" theme social practice, "youth dream beautiful China" theme social practice, "youth heart to the party, dream new journey" social practice brand project five practice theme of party history learning preach, free trade port construction, rural revitalization, volunteer service, environmental protection and other activities. Huang Huiqiong (2 from left), the inheritor of Li costume, communicated with the practice team about the production and sales of Li Jin products at the institute. This social practice is in the centenary of the founding of the Party, and it is also the first anniversary of the release of the Hainan Free Trade Port Overall Plan for the Construction of Hainan Free Trade Port. Highlight Hainan features become the summer social practice content, party history education practice team, "Hainan free trade port into the construction of a large bay area research", "one hundred light red gene search" and "island Marine culture and social governance" research group and school secondary college combined with professional characteristics, formed different forms of practice team to carry out the "a program under which officials" social practice, with practical action has achieved fruitful results.

2021-09-09	Students of Suzhou university of science and technology ideological education leads the student community development of Suzhou university of science and technology ideological education leads the student community development in the youth newspaper reporter Li Runwen) recently, Suzhou university of science and technology construction association youth league branch secretary Xi Zhihua participated in the Suzhou university of science and technology "community spring sound" red classic series of community activities, and share their feelings online. Since the middle of May, the Youth League Committee of Suzhou University of Science and Technology has selected 30 club activities for inheriting the red classics, including the classic selection and reading, thought exchange, red song singing, drama performance, knowledge competition, poetry writing, etc. Building association club "spring Gusu, red painting" see ", with college students painted Suzhou" flowering "party building billboard and surrounding landscape, learning excellent landscape construction design as the main content; south wind literature club", to the history of the essay competition ", from the perspective of college students about the party building history, lofty ideals and the future development of the party; guitar club" red new youth playing ", playing red songs as the main content, combining the college students favorite art form with the red culture. In addition, the club activities also focus on highlighting the political, ideological and contemporary nature. During the summer vacation, the Young Marxist Association set up the "Qingma Party History Publicity Group" to study and prepare lessons online around Xi Jinping's "July 1" speech, and walked into the celebrity community in Cangzhou, Hebei. Baichuan Knowledge Society held an online "faith open class", leading the community students to review the glorious history of the Party, and read the full text of Xi Jinping's "July 1" speech to think about the historical proposition of national rejuvenation. Wang Rongqing, deputy secretary of the Party Committee of Suzhou University of Science and Technology, said, " In the background of comprehensive education, the university takes the important component of the second classroom- -community activities as the fresh carrier and path of ideological and political education, and actively explores the integration of ideological and political elements into community activities. The strong organizational guarantee of the Communist Youth League, the rich implementation carrier of community activities and the sufficient participation and coverage of community students have all laid a solid foundation for the development of ideological and political education in the associations.”

2021-09-16	Foshan University of Science and Technology More than 10,000 students have visited more than 10,000 students to carry out social practice) In order to deeply study, publicize and implement Xi Jinping Thought on Socialism with Chinese Characteristics for a New Era, Education, grow ability in the social classroom, make contributions, In the observation and practice, learn the party history, strong faith, follow the party, In the Summer season of 2021, Foshan University of Science and Technology has established 50 university-level key teams, As well as the school-level key teams and a number of class, youth league branch as the unit of the team, With the principle of "student declaration, college integration and school overall planning", Set up social practice teams from different levels, such as schools, colleges and grassroots youth League branches, Covering the whole school youth League branch, In total, more than 10,000 students went to various places to carry out " social practice activities. The countryside team members for pre-departure training. The Party Committee of Foshan University of Science and Technology attaches great importance to it, and orderly organizes and mobilizes the Youth League Committee of the school to extensively publicize the "social practice activities to the countryside" through online and offline publicity sessions and new media, so that all teachers and students can fully realize the significance of social practice activities of college students. In social practice preparation, the school youth corps committee carefully planned many offline briefing, from a program under which officials practice content, purpose, significance and overall process is introduced, and detailed the field key team project process, declaration and knot requirements, propaganda manuscript writing, video production, etc., also introduces the social practice and the voice of "Guangdong youth" platform combining way. At the same time, we also invited the outstanding team representatives of the three to the rural areas in previous years to share their experience and enhance the students' overall understanding of the social practice of "three to the countryside". The Sanxiang team discussed the development path of rural characteristic industries in Lianzhang Village. The whole school has set up a total of 25 lighthouse practice teams and 25 key practice teams, and at the same time, it has won more than 30 national and provincial key project approval teams. Each a program under which officials team combined with their own professional characteristics, around the party history study, rural revitalization, theory, national observation, national unity, new era civilization six directions, xi jinping, the ideas of socialism with Chinese characteristics and the "July 1" important speech spirit, the history to the countryside, rural revitalization, "two help two promote", pushing activities, COVID-19 epidemic prevention and control about service, love medical care and other social practice. The practice location is throughout Foshan, Zhanjiang, Shaoguan, Meizhou, Qingyuan, Yangjiang and other more than ten prefecture-level cities, across Hunan, Guangxi, Guizhou, Sichuan and other provinces. The activity covers left-behind children, teenagers, rural people and other groups, with a total of more than 44,000 people through online and offline services and research objects. The team members conduct party history education in the countryside. Foshan institute of science and technology for figure learning history don't forget to beginner's mind, red gene fire party history learning education propaganda is an important theme of this year, the practice team, relying on the university science and technology culture health talents, through the collective preparation, project group, door propaganda, field classroom, online preaching, linkage practice residents, middle school youth members, young pioneers, the old revolutionary base areas village visits, senior sympathy, red culture, youth micro group teaching and recording various forms of party history learning education campaign, promote the party history knowledge and red culture spread in local residents."The light of Buddha xiao" team in practice to carry out the "learning history keep beginner's mind, red fire" project series of micro group class teaching and recording, micro group class by the team young students as the speaker, vivid language collocation vivid field historical material, several red culture micro group class for team members and accompanying local primary and middle school students by education and encouragement. In addition, the team led local teenagers to sing patriotic songs on the site of the revolution through several teaching classes.

2021-09-08	Beijing, September 8 (Xinhua) - -On September 4th, Qiannan University of Science and Technology in Guizhou province welcomed the first batch of freshmen of grade 2021. In order to do a good job of comprehensive orientation, ensure the freshmen smoothly, youth corps committee students, and the department under the guidance of school leadership of the orientation work for meticulous and thoughtful arrangement deployment, in the city depot and set up the orientation, the car shuttle freshmen and parents, near the campus also affixed with striking signs, and set up the campus in the campus gate profile panel introduces the basic situation of the college. In order to prevent and control the COVID-19 epidemic, a body temperature check, health code, travel code check and luggage disinfection are provided at the school gate. In addition, considering the weather reasons, there are also a number of drinking water points and summer places set up in the welcome site, to prevent students and fellow parents in the middle of the school emergency. Volunteers and party members in the school wear red armbands, and students from the student Union and various departments also wear uniform clothes, which is convenient for everyone to quickly identify, and can timely and accurately help when they encounter problems. Freshmen enter the campus site. After the freshmen enter the campus, handle the report procedures in turn according to the report flow chart of the freshmen at the entrance of the school playground, the process recommendation on the ban app, and the procedure of the school official account. With the help of other teachers and staff, the freshmen will handle the luggage storage, class report, handling the league organization relationship transfer, payment, receiving the military training uniforms and other matters according to the order of the process table."Classmate, do you need any help?" " Hello classmate, please come here."The welcome staff warmly welcomed the new students, reduce the burden for them, led them to a process. A sweating but smiling staff member said, " When I came here, thanks to the help of my last senior senior, I reduced a lot of trouble. Now I am also very happy to help my new junior junior."In addition, the school publicity department offers a live broadcast to understand the reporting process and reporting office, and to answer their questions. At the same time, it also provides ways for parents due to epidemic prevention and control.

2021-09-07	University of Science and Technology Beijing has completed the welcome work for more than 7,000 freshmen of 2021) Recently, the Student Union and graduate Association of University of Science and Technology Beijing organized more than 500 volunteers to successfully complete the welcome work of 2021, for more than 7,000 new "shell" sail escort."This is my third time to participate in the orientation, which has not only witnessed my own growth and transformation, but also tested the 'background color' and 'kinetic energy' of the whole student Union organization to do practical things for students," said Azhhati, executive chairman of the Student Union. It is understood that in order to ensure the new safety, efficient and thoughtful, under the guidance of the department and youth corps committee, north university student union organization to start orientation work in July, from early planning, supplies, volunteer recruitment, station spots, to the final service, the student union staff and all the volunteers are continuous efforts. According to the new standards and new changes, due to the epidemic prevention and control, parents can only watch their children go to the school alone to complete the registration. In order to reassure parents and students, the volunteers left to pick up their buses at 6 am, and all the volunteers were on duty at 7 o'clock. The orientation around the campus set up luggage register, reception, information office and service desk of different nature, volunteers, in accordance with the "guarantee one to one, more for one" service principle to guide new incoming, and help new carrying luggage, store luggage, guide the directions, photos, etc., provide school basic situation, guide the canteen campus card circle machine full dimension of volunteer service, enthusiasm, meticulous help freshmen complete the registration. Beijing university of science and technology youth corps committee secretary Wang Li said, orientation is the student union organization practice service tenet, especially in the student union organization reform "thin", in the form of special recruiting volunteers to participate in the practical work for teachers and students, can be formed, dedicated to pay "magnetic effect", realize the practice of education and service linkage, expand the leading coverage of young students.

2021-09-06	Xidian University promotes the high-quality connotative development of social practice. Xidian University Youth League Committee, Beijing, September 6 (reporter Zhang Jianwei, correspondent Yin Peng, Yuan Xiangyu) In 2021 summer vacation, The Youth League Committee of Xidian University focuses on the theme of "the century of the founding of the Party and the ninth rank of XD", Give full play to the practical characteristics of "peer teachers and students, research collaboration, interdisciplinary collaboration, and dual-college linkage", Organize and carry out social practice activities in the countryside in summer vacation, To guide the majority of young students to take root in China to understand the national conditions, In practice, feeling the great new achievements of the Party in leading the people in building the motherland, In the observation and practice, learn the party history, strong faith, follow the party, In the social classroom education, long ability, make contributions. The summer social practice, invited 38 college party and government leadership, 349 education teachers, professional teachers involved in guidance, 74 college students backbone training members as practice assistant, organized 1916 practice team, 9479 xd youth across the country, into the countryside, grassroots, on the premise of strict COVID-19 epidemic prevention and control work, actively involved in service national development, social governance and the people's livelihood construction learning practice. The year 2021 marks the 100th anniversary of the founding of the Communist Party of China, the first year of the 14th Five-Year Plan, and the beginning of a new journey to comprehensively build a modern socialist country, The Communist Youth League of the school grasps its function positioning in the pattern of "great ideological and political education" and "three complete education", Focusing on the five practical sections of party history study, theory publicity, national conditions observation, ethnic unity, and rural revitalization, Set up the " One Hundred Years of Understanding journey, Inheriting the red gene "," Taking root in grassroots research, Seek common development of people's livelihood. "" Write the mission, Youth dedication home, "" live up to the glory of The Times, Committed to serving the country with science and technology "and" revisiting the original aspiration, Root search nine rank west electricity " special practice theme, Create a practical education classroom combining online and offline, in class and after class, inside and after school. The picture shows Xidian University improving the second class service power with its quality and ability. The Youth League Committee of Xidian University provides the guidance of Party building, and promotes the original aspiration to the study and education of Party history. According to the "ego into the big self, youth to the motherland" the history of the party learning education practice requirements, each college to establish social practice team temporary youth corps cell, all team set up temporary party class study group, in the local materials for at least one party building activities, improve the occasion to mobilize the party, party members, probationary party members can further review the party oath party day activities. In 106 red revolution memorial sites, including the site of the CPC National Congress, the former site of the New Youth Editorial Department, the Xiangjiang Campaign Memorial Hall, the Xiangshan Revolutionary Memorial Hall, the Chahar Martyrs' Cemetery, and the Memorial Hall of the Northwest Bureau of the CPC Central Committee, Relying on the school "hundred years of red soul" Internet + competition project team to produce the "Communist Party of China" knowledge map 100 meters long scroll, Practice team members combined with the practice of red resources to carry out a series of activities of "one-day narrator", In the process of telling the story of the party history, we should have a deeper understanding of why the CPC "can", why Marxism "does", why socialism is "good" with Chinese characteristics and other basic principles are "good", Effectively activate the sense of responsibility and mission of contemporary college students. Stage coverage, to expand the quality and ability to improve the second classroom service ability. Follow comprehensive ability to improve, deep learning practice, multidimensional value dissemination implementation of ideas, detailed school combing before, middle, late work, around to solve the "travel difficult", "less tube" "no refining" three aspects, through online teaching, practice base construction, organization report, improve the depth and breadth of young students to participate in social practice. We will improve the multi-level resource supply system and fully mobilize the joint efforts of practical education. In 2021, which coincides with the 90th anniversary of Xidian University, the university specially set up a special practice action of "revisiting the road of the original aspiration and searching Nine rank West Electric", with "revisiting the road of moving the school again" as the main line to pursue the road of red radio wave education. The practice teams went to the practical areas closely related to the construction and development of XD, represented by Ruijin, Changting, Fujian, Guangxi, Zunyi, Guizhou, Yan 'an, Shaanxi, Yulu, Hebei, Zhangjiakou, Hebei, and Xi' an, Shaanxi, to carry out practical activities, retracing the 90 years of wind and rain road and reviewing the 90 years of love. Participate in the teachers and students, combining with the specific situation of the practice place, determine the practice theme, carry out practical activities, realize the unremitting pursuit of always forging ahead in the forefront of The Times, and jointly compile the characteristic school history with space as the context.

2021-09-02	Anhui Medical University set up a summer social practice special scholarship from the visit to the first site of the Communist Party of China and the Red boat of the South Lake, all the way to review the history of the Party, ok?"" We can also print some medical science knowledge brochures, while exploring the development and changes of the old revolutionary base areas, but also can play a professional expertise, to make some contributions to the local residents... "This is the Anhui Medical University," seek a root search and build a dream, build a good doctor " social practice team members before this summer brainstorming scene. In recent years, with the goal of "great ideological and political courses" in Anhui Medical University, we will build a diversified evaluation system, stimulate students' internal motivation, and build a new mechanism of practical education in line with the law of education development and talent growth. It is worth mentioning that this year, the school specialized in the establishment of summer social practice team scholarship, in order to achieve good social benefits and educational effect of the school, school two level social practice team reward 5,000 yuan or 10,000 yuan special bonus, excellent teams and individuals can also get advanced social practice and other exclusive honors."The establishment of the scholarship is an important incentive measure for practical education in schools. The bonus follows the principle of 'award instead of compensation'. The purpose is to encourage students to enhance their sense of social responsibility and guide them to learn real knowledge, find real problems, carry out real research and put forward real opinions in the 'walking classroom'."A staff member of the school Youth League Committee introduced that the scholarship is reviewed once a year and issued around the end of September every year. It must all be used for the reasonable expenses incurred by the team students to carry out social practice activities in the current year. Since 2018, Anhui medical university to explore the implementation of the Anhui medical university, the comprehensive quality evaluation method, innovative labor education into the evaluation index, and develop the Anhui medical university students social responsibility education training plan for the Anhui medical university "second classroom transcript" system implementation plan (try out) system, combining quantitative evaluation and qualitative evaluation, process evaluation and result evaluation, combining solid evaluation and democratic appraisal. The annual summer "three to the countryside" social practice activity is an important link in the multiple evaluation system. In addition, Anhui medical university has been focused on talent training goals, dig deep into the second classroom education value, consciously into the ideal faith, home country feelings, moral quality and the spirit of the rule of law, set up, including innovative entrepreneurship, style, volunteer service, social practice, group organization work, the second classroom practice education platform, provide clear practice path for students to grow up. Students can get corresponding points for participating in southeast School, Qingma Project, college student backbone training main class training experience, participating in the theme league day class meeting, situation and policy, and other ideological activities, visiting the patriotic education base, safe, harmonious and civilized dormitory evaluation, etc. The platform will be evaluated by scientific quantitative standards. Counselors and teachers can also log in to the system to learn about the students and guide them. According to the introduction, the school's second classroom activity form is more flexible and diverse, by time, space restrictions. This kind of "fit" learning makes students play their subjectivity, creative practice more calm and firm. This summer, with the theme of "Always follow the Party to forge ahead in the new Era", Anhui Medical University has set up 46 key teams and 2,306 young students to join the grassroots practice of party history learning and education, theoretical publicity, national conditions observation, rural revitalization, ethnic unity, serving the people and other contents. Xia Ziwei, a counselor at the First Clinical Medical College, said, " The biggest difference this year is that more than half of the teams use 'red' as the practice keyword."Gu Jiashan, Party Secretary of the Party Committee of Anhui Medical University, said that the university will actively promote the construction of a practical education community featuring the government, society and schools in accordance with the requirements of curriculum goals, project mode, socialization methods and brand strategy, and persistently stimulate students' initiative and drive. China Youth Daily China Youth network reporter Wang Haihan, correspondent Wang � F � F Ding Yi source: China Youth Daily

2021-09-02	Xiamen University "fireflies spark youth branch" into the Guizhou Miao village Xiamen University "fireflies spark youth branch" into the Guizhou Miao village party history story how to "fly into the ordinary people" home listen to my reason... " July 17 evening, in the Qiandongnan village of Guizhou Province, the villagers shaking the fan, sitting under the big banyan tree in the village. A group of Xiamen University students dressed in red T-shirts stood among the villagers, holding a civil code and speaking with catchy rhymes to more than 20 villagers. From July 16 to 22, nine members of the "Spark Youth Lecture Group" of Xiamen University went to Bao Village and Changtan Village, Laotun Township, Taijiang County, Qiandongnan Miao and Dong Autonomous Prefecture, Guizhou Province, to launch a one-week party history publicity practice activity. How to make the century-year party history story "fly into the ordinary people's homes"? This is a question that the lecturers have been thinking about. In order to make the lecture more "local" and "umami", the team members contacted the local college students in Miao Village in advance, collected the topics of the local people through them, and claimed the publicity topic according to their professional expertise. Players of the "strange", in the preaching form integrated music, opera, allegro, magic, dance and other fashion elements, set up the "bench classroom", "field song", "red horn" characteristic preaching platform, in plain language to let the villagers understand, remember, greatly enhance the party history about the appeal and appeal."The students of Xiamen University spoke very interestingly. I listened carefully to the story of the Luo Yangcai martyr just told by the young man in the form of storytelling. I want to tell my grandson back."Yang from the long beach village after listening to the team members of the speech, thumbs up."Sing a folk song to the party, I compare the party to the mother; the mother only gave birth to my body, the glory of the party according to my heart...." After dinner, the "loudspeaker" of the service village sounded a beautiful melody. Team members Li Yifan and Xiao Zhonghong became "red announcers", bringing the village an "art Party class" through the village radio. In preaching the history of the party, the players also combine "I do the practical work for the masses" activities, the huimin policy interpretation, legal knowledge popularization, miao village architectural culture, garbage classification science into the bench classroom content, and actively participate in the manual embroidery, mushroom picking, garbage cleaning, cultural wall painting labor practice, let the party history about and the life, leisure, daily focus on organic integration, truly ground, run hearts. Founded in May 2021, was founded in May 2021, composed of 46 young students from Xiamen University. Based on the perspective of the youth, the teaching group closely adheres to the theme of the party history learning and education, and delivers the propaganda full of the flavor of The Times to the masses with the "green words and green language". At present, the lecture group has carried out more than 100 lectures both inside and outside the school."The young lecturers should be good at 'watching people and serving dishes'. Only when they are in line with the 'appetite' of the common people can they really go deep and solid."Said Liang Zhenwei, deputy secretary of the Communist Youth League Committee of Xiamen University. China Youth Daily, China Youth Network reporter Yang Baoguang source: China Youth Daily

2021-09-02	Shaanxi energy vocational and technical college based on the "three heart" good school first lesson shaanxi energy vocational and technical college based on the "three heart" good "first lesson" wei correspondent Zhao Lingjuan) on August 30 is shaanxi energy vocational and technical college of the new semester, at the beginning of the new semester, the school based on "heart, heart, sweet" measures and "first lesson" series of activities, a solid completes the new semester student education management. Good humanistic care class, be a good "close friend". The school principals will check on the teaching situation, teaching facilities and logistics support. Put forward solutions to the problems found in the inspection, and further strengthen the teaching management and students' daily management work. Good ideal and faith class, inject "heart booster". The School of Marxism, combined with the characteristics of the discipline, takes the "spirit of the important speech of July 1" as the content of the "first lesson of the semester", and vividly tells the story of the Communist Party of China seeking happiness for the people, rejuvenation for the nation and great harmony for the world. Good discipline rules class, pull tight the "heart rope". On August 29, the department arrange the class teacher in charge into class new semester first class meeting, fully grasp the student situation, around COVID-19 epidemic prevention and control, student management, teaching services, logistics considerations detailed arrangement, education and guide students to strictly abide by the provisions of the school on epidemic prevention and control and the management of student management "home", with good learning and living habits growth progress.

2021-09-01	Nanjing university of post and telecommunications: research red base, spread red spirit of Nanjing university of post and telecommunications: research red base, spread red spirit enlightenment spirit of huaihai campaign, review the history of the communist party of China plowed foundation, feeling big party firm beginner's mind mission, recently, Nanjing university of post and telecommunications school of communications and information engineering and huaihai campaign martyrs monument administration college students social practice base and signing ceremony was held in huai tower administration. Wen Liang, director of the Education Exchange Department of Huaita Administration Bureau, Mr.Wen Youlong, a teacher from the School of Communication and Information Engineering of Nanjing University of Posts and Telecommunications, and all members of the red special social practice group of "Dream Building Red Brigade Youth Post me" attended the signing and opening ceremony. Mr.Wen Youlong first introduced the School of Communication and Information Engineering of Nanjing University of Posts and Telecommunications and the red special social practice group of "Dream Building Red Tour, Youth Post Me", and explained the specific situation of the team project in detail. This summer, the practice group will take the "historical implication of the spirit of the Huaihai Campaign and the value of The Times" as the main line, with patriotic education bases, traditional villages, township enterprises, community streets, schools, nursing homes and wetland parks, to carry out social practice activities. Inherit the red gene, based on the development of The Times, transform the "spirit of Huaihai Campaign" into a spiritual pillar leading high-quality development, deeply explore the educational significance of red resources, actively explore the spiritual connotation of red culture, and give full play to the leading role of revolutionary spirit. Director Wen Liang welcomed the "red special social practice team of" Dream Red Brigade, Youth Post Me " to the Huaihai Campaign Memorial Hall to carry out practical research and visit and study. Through the research of the red base, students can further understand the red culture, feel the great spirit of red, receive education in social practice, and firm their ideals and beliefs. Director Wen Liang hopes that the students can draw wisdom and strength from the spirit of the Huaihai Campaign, in the future study and work, the integration of practice, knowledge and action, enhance their mission, and accumulate strong power for the voyage of youth. Huai tower administration bureau of education exchange Wen Liang do important speech activities, huai tower administration bureau of education exchange Wen Liang and Nanjing university of post and telecommunications institute of communications and information engineering "dream red brigade youth post me" red special social practice group guidance teacher WenYouLong signed "youth employment entrepreneurship trainee base" cooperation agreement, and jointly for college students' social practice base. After the ceremony, the teachers and students of the practice group paid their respects to the martyrs of the Huaihai Campaign, remembered the revolutionary martyrs, and visited the Huaihai Campaign Memorial Hall under the guidance of the narrator. The Huaihai Campaign shows the party's strategic vision and command ability, as well as the military and civilian integration and deep feeling. Through the college student social practice base signing and construction and memorial hall visit and study, students deeply realized the Communist Party of China through the vicissitudes of life, across the difficult years, as well as with the blood of watering youth pride, with life to carry the banner of victory promising responsibility. At the same time, the inheritance of red gene into social practice education, and the true learning, true understanding, true trust, and true use in the party history learning education will help students to guide them to button the first button in the "jointing and pregnancy period" of life, and play a good "combination of boxing" of ideological guidance. Students have expressed that they should strengthen their faith, stimulate their confidence and motivation, stay true to their original aspiration and keep their mission in mind. Teachers and students of the practice regiment took a group photo in front of the Martyrs' memorial Tower of the Huaihai Campaign

2021-08-31	Recently, the teachers and students of the "Mountain and Sea Meet" practice team of Xiamen Marine Vocational and Technical College held a Marine summer camp in Datian County, Fujian Province. The summer camp through the party history study, popular science lectures, VR boat driving and other activities, let the primary school students in Junxi Town, Datian County spent a different summer vacation. Volunteers led small campers to learn the history of red Party culture at the site of the "Second Jimei Learning Village". There are more than 70 revolutionary sites in Datian County, among which the old site of "The Second Jimei Learning Village" is a national site of the Anti-Japanese War, and also a provincial patriotism education base and a party history education and learning visit site. The "Mountain and Sea Meet" practice team is here to carry out on-site teaching of red Party history for small campers, and to carry out "cloud learning" of party history education with the help of live network broadcast for college students. The practice team also led the young campers to review Chen Kah Kee's anti-Japanese speech in Datian "A branch has leaves, a country has a home", and used VR modeling technology to move the Jiageng Memorial Hall in Xiamen- -Aoyuan to the scene, so that the young campers could more deeply understand the spirit of Kah Kee and the red culture of Jimei Learning Village. Volunteers take small campers to experience the VR underwater world. In order to make the young campers who have been living in the mountains have a more intuitive experience of the ocean, the "Mountain and Sea Meet" practice team introduced VR technology and designed the underwater world and sailing driving experience module. Lin Xinyuan, a small campers who had just come down from the experience machine, shared with her friends in surprise: " It was amazing. When the waves came over, I felt that I had really become the captain and was sailing to the sea."Su Jinhang, another small campers, said:" When I just put on VR glasses to swim under the sea, I seemed to touch the clownfish."The young campers actively answered questions in the Marine environmental protection class. The practice team introduced an anatomical video of a seabird, letting the young campers understand the current severe situation of Marine pollution, and guiding them to think about how to protect the ocean through daily small things. The practice team donated the ocean knowledge corner for Junxi Central Primary School. Tang Zhongmin, deputy Secretary of the Party Committee of Xiamen Ocean Vocational and Technical College, and his delegation also held the signing ceremony and donation ceremony of the rural school children's Palace in Junxi Central Primary School. The college donated Marine animal models, Marine specimens and Marine books to Junxi Central Primary School. The practice team designed Marine knowledge corners for the children. Xu Chenchen, deputy secretary of the Youth League Committee of Xiamen Ocean Vocational and Technical College, said that in the future, the school will conduct more in-depth cooperation with Datian Working Committee and the Party Committee of the Communist Youth League, to realize the common meeting of mountains and sea, "old, young and young", and jointly promote the construction of Shanhai to build a social practice base for college students.

2021-08-30	Nanjing university of post and telecommunications: in social practice with youth interpretation home countries as Nanjing university of post and telecommunications: in the summer social practice with youth interpretation as Sichuan correspondent Qin Changjian) this summer, Nanjing university of post and telecommunications youth corps committee to "always follow the party forge ahead new era" as the theme, around the party history study, theory to preach, rural revitalization, national observation, love school and hometown enterprise research project, form a summer social practice team, including field key team 32, the provincial key team 15. Each team above good "a biggest professional education practice class" and "the biggest national conditions and social situation ideological and political class" as the goal, in the social classroom education, long ability, make contributions, in the observation and practice of the party history, strong faith, follow the party, cultivate patriotism, strong country, serve the country. Nanjing University of Posts and Telecommunications summer social practice skimming. Correspondent for the picture to learn a hundred years of party history, ceaseless red blood. On the centenary of its founding, The "Heart Language wish" practice group of the School of Society and Population of Nanjing University of Posts and Telecommunications, and the Red Ying Team practice group of the School of the Internet of Things visited the Memorial Hall of the Victims of the Nanjing Massacre by Japanese invaders, the Victory Memorial Hall of Crossing the River and other red landmarks, Reviewing the extraordinary years of the revolution, Explore the connotation of red culture, In the memory of the martyrs to draw the strength, inspire the will to serve the country; "Pomelo Red Travel Love, Post dream sweet rain walk " practice group went to Kaishan Island to interview the time model Wang Shihua, Listen to the 32 years' history of defending the island with the "life mission", Inheriting patriotism and dedication, Promote the sense of responsibility; The School of Geography and Bioinformatics, through a combination of online and offline methods, Visit and investigate 30 red theme memorial halls in Nanjing, Detailed the the in detail with videos and photographs, Combining geographic information expertise to make "panoramic VR", "map story", "visit vlog", Reusing the GIS technology, With the dynamic map and the interactive system as the carrier, Space visualization display of the practice results, Realize the red culture education on the map, Tell the red story well; School of Automation, School of Artificial Intelligence "mail I Jinling" practice group walked into Nanjing Maqun Street, Hold the " Listen to the original mind story, Feeling the mission feelings " special activities, Visit the winner of the "50 Years of Glory in the Party", Listen to them, " Once joining the Party, Life for the Party. " The red story, Inherit and carry forward the distinctive character of loyalty, selflessness and progress of the older generation, The interview transcript will also be prepared, Show respect to the predecessors. Nanjing University of Posts and Telecommunications summer social practice skimming. To gather the strength of youth and help rural revitalization. During the summer vacation, more than 20 students from the School of Media and Arts came to Nantong, Jiangsu province to carry out the "Centennial Dream" wall painting creation activity. Students play professional expertise, painting, for the village monotonous wall with "new clothes", colorful color add vitality; "grapefruit youth" practice group into Jiangsu Suqian, with the theme of the founding "hand-painted beautiful countryside" wall painting activities, draw a good vision, salute the centennial journey, let the cultural wall become "red scenery" along the way. In the process of practice, teachers and students feel the happiness of contributing to their youth and helping the rural construction, enhance the volunteer spirit of serving the society, deepen the connection between the majority of young people and the countryside, and make contributions to the publicity of red culture and the construction of a beautiful Jiangsu province. Nanjing University of Posts and Telecommunications summer social practice skimming. Correspondent for picture knowledge to light up childlike innocence, youth dream dream in the future. In visiting and practice, The volunteer service group of "The School of Communication and Information Engineering" went to Lilou Primary School, Laohekou City, Xiangyang, Hubei Province, Carry out the "return to home" practice activities, Volunteers around the childlike innocence to sing to the motherland, science and technology to change life, interesting English, the Chinese dream of space dream, garbage classification civilized life, traditional culture, mental health and other topics, Focus on creating 20 quality classes, Accompany the children through a full and unforgettable "colorful holiday"; School of Foreign Languages " Colorful Holiday, Childlike innocence to the Party " volunteer teaching group for Hubei Enshi County Gaoping Town Mingde Primary School to carry out cloud red teaching activities, Focusing on the centenary of the Communist Party, Develop a red education list, Create the red quality courses, By singing red songs, drawing red pictures, reciting red books and other teaching forms, Integrating red into thematic education courses, For students on the "compulsory course" of thick planting national feelings, Injection of the red gene, Polish the youth background color; The "Pomelo Youth" red practice group of the Institute of Applied Technology went to Liulpeng Village, Suqian, Jiangsu province to publicize party history and care for left-behind children, Raise 500 books to build a "popular science home", Combination of popular science and educational activities, Show the youth style in practice. Nanjing University of Posts and Telecommunications summer social practice skimming. Correspondent for the picture deep grass-roots line, talk about the development of The Times. Feel the spirit of The Times, Listen to the voice of The Times, School of Communication and Information Engineering, " Dream Building Red Brigade, The " practice team went to Xuzhou, Jiangsu, Huaibei, Anhui and Shangqiu, Henan, In-depth patriotism education bases, traditional villages, manufacturing enterprises, communities and streets, Return to home to see the changes, go back to the old place to see the new face, walk into the front line to see the development, Feel the pulse of China's development in the new era; The School of Automation, the School of Artificial Intelligence "mail I Jinling" practice group and Nanjing Maqun Street jointly held the special "College students see Maqun" practice activity, Twenty-seven temporary college students by visiting street social innovation demonstration sites, Understand the overview of street governance innovation, Understanding the power of grassroots innovation. Nanjing University of Posts and Telecommunications summer social practice skimming. Join COVID-19 prevention and control, shine with youthful responsibility. Since Nanjing lukou international airport cluster outbreak, Nanjing university of post and telecommunications nearly 500 pomelo volunteers positive response, in epidemic prevention and control of volunteer service, at a critical moment "stand out, top up, the charge in the front", report to the school, report to the community, complete information submitted, full nucleic acid detection, policy mobilization, answering, build mutual protection net, gather unity is strength, � ν � heart positive energy, reveal youth bear. It is reported that the social practice is the bridge of effective communication between the youth and the society, can let the students in the social "melting pot" influenced by education, long ability, make contributions, buckle the first button of life. This summer, the students went out of the campus, into the countryside, into the enterprises, into the grassroots, and on the front line of the fight against the epidemic. Centering on the historical background and social needs, they gave full play to their academic characteristics and professional expertise, and practiced the youth oath of "Please rest assured of the Party, the strong country has me".

2021-08-28	Anhui Medical University set up a summer social practice special scholarship Wang Haihan, correspondent Wang � F � F Ding Yi) " This year is the 100th anniversary of the founding of the Communist Party of China, let's start from the site of the first Congress of the Communist Party of China, all the way to review the history of the Party, ok?"" we can also print some medical science knowledge brochures, in the development of the old revolutionary base areas, but also can play a professional expertise, to make contributions to the local residents......"This is this summer, Anhui Medical University" roots and dream " social practice team members brainstorming scene. Ding Xiangsheng, the instructor of the team and a teacher of the School of Marxism, said, " Last semester, when I talked about the first Congress of the Communist Party of China, I wanted to 'move' part of the course content to the historical place and integrate theoretical education into personal practice. This year, this idea was put into practice."In recent years, Anhui Medical University above the good practical education" big ideological and political course " as the goal, to build a diversified evaluation system, smooth and effective education path, stimulate students' internal motivation, and to build a new practical education mechanism in line with the law of education development and the law of talent growth. At the same time, the school specially studies the establishment of summer social practice team scholarship, to the school and school social practice team reward 5,000 yuan, 000 yuan of social practice teams or 10,000 yuan special bonus, excellent teams and individuals can also get exclusive honors such as advanced social practice. On August 26, the university passed the scholarship related rules."The establishment of the scholarship is an important incentive measure for the school's practical education, and is an important component of the school's social practice incentive and evaluation system and the student support system. According to the principle of 'reward instead of compensation', the purpose of the bonus is to encourage students to enhance their sense of social responsibility, and guide them to learn real knowledge, find real problems, carry out real research and put forward real opinions in the'walking classroom "."A staff member of the Youth League committee of the school introduced that the scholarship is reviewed once a year and issued around the end of September every year, which must be used for the team students to carry out social practice activities in the current year. Since 2018, Anhui medical university to explore the implementation of the Anhui medical university, morality, physique and labor comprehensive quality evaluation method, innovative labor education into the evaluation index, and develop the Anhui medical university college students social responsibility education training plan for the Anhui medical university "second classroom transcript" system implementation plan (try out) system, combining quantitative evaluation and qualitative evaluation, process evaluation and result evaluation, combining solid evaluation and democratic appraisal. The annual summer "three to the countryside" social practice activity is an important link in the multiple evaluation system. This year, the school has 46 key teams, 2,306 young students into the grassroots practice. Before the activity began, the school youth corps committee also specially issued the "Anhui medical university college students summer social practice work regulations (trial)" notice, emphasize the principle of social practice, tasks and objectives, practice team to deal with the school actual practice and local demand, brand inheritance and innovation, key group and full participation. For a long time, Anhui medical university focus on talent training goals, dig deep into the second classroom education value, consciously into the ideal faith, the country feelings, moral quality and spirit of the rule of law, etc, including innovative entrepreneurship, style, volunteer service, social practice, group organization work, the second classroom practice education platform, provide clear practice path for students to grow up. Students can get corresponding points by participating in southeast School, Qingma Project, college student backbone training main class training experience, participating in the theme league day class meeting, situation and policy and other ideological activities experience, visiting patriotism education base, safe, harmonious and civilized dormitory evaluation, etc. The platform will be evaluated by scientific quantitative standards. Counselors and teachers can also log in the system to learn about the students and guide them. Liu Xiaoyu, a student, said, " I really like to participate in the 'Class 2' activities, not just to get points, but to gain a different understanding and experience. Some classroom knowledge points, become easy to understand in practice. By improving the talent training program and evaluation system, "" extends the understanding chain of 'practice, understanding and re-practice', and constructs a new education cycle of classroom teaching, school activities and social practice."Gu Jiashan, Party Secretary of the Party Committee of Anhui Medical University, said that the university will actively promote the construction of a practical education community featuring the government, society and schools in accordance with the requirements of curriculum goals, project mode, socialization methods and brand strategy, and persistently stimulate students' initiative and drive.

2021-08-26	The State Grid Zhoushan Power Supply Company held the launching ceremony of the "Youth Achievement Alliance". Correspondent for the picture of China youth network Beijing on August 26 (reporter Li Chuan correspondent Ma Ni) on August 24, the State grid Zhoushan power supply company in the Dinghai power supply branch held the "youth contribution alliance" establishment ceremony."Alliance" youth alliance "around the company production safety, power grid construction, marketing services, operation and management, the party construction center, set up production, development, marketing, data, scientific research, skills, party construction, propaganda, art, 10 branches, to alliance, through positions, factory, matters, problem solution, break the professional barriers, open business communication, forging young talents in learning and practice, expand the comprehensive ability of youth in the field of various professional, for the construction of national grid strategy landing demonstration window" island demonstration " boat electric youth power. On August 24, State Grid Zhoushan Power Supply Company held the establishment ceremony of "Youth Achievement Alliance". Correspondent for the establishment ceremony, Zhoushan power supply company discipline inspection commission secretary, chairman of the trade union Wang Guang issued a letter of appointment for the head of the alliance. Zhoushan power supply company Party secretary, deputy general manager Huang Jiong for the alliance unveiled and delivered a speech. Huang Jiong said that the youth should be ambitious, constantly "mind me", practice "please rest assured, power me" youth oath; to be good at innovation, always "struggle", innovation breakthrough "promoter", "leader"; to settle down, do "I", focus on the "tower spirit" to perform the job responsibilities. On August 24, State Grid Zhoushan Power Supply Company held the establishment ceremony of "Youth Achievement Alliance". Correspondent for figure understands, "youth construction alliance" is Zhoushan power supply company focus with Chinese characteristics international leading energy Internet enterprise strategic goal innovation consciousness, the implementation of the SGC construction with new energy as the main body of the new power system construction important measures, is also Zhoushan power supply company to the practice of "stand-alone, bear dedication, scale new heights" tower spirit, guide the youth post work, stimulate youth potential and vitality platform. The establishment of "Youth Achievement Alliance" is another innovative exploration of the Youth League Committee of Zhoushan Power Supply Company in gathering youth, serving youth and leading youth work. Next, the company youth corps committee will by promoting the alliance established and subsequent professional alliance activities, encourage the youth in the bank is better than words, achievements in bear dedication, in the pursuit of excellence, build "island demonstration", the construction of a new power system provincial demonstration area contribution youth strength.

2021-08-18	South post students: let the youth in the colorful practice colorful glorious south post students: let youth in colorful practice brilliant correspondent Qin Changjian) this summer vacation, Nanjing university of post and telecommunications students around the founding theme in one hundred, on the basis of epidemic prevention and control work, to the country take an active part in summer social practice, deep study party history, rooted in a line, service society, make positive contributions to home development, glow youth brilliance in practice."Hundred years of journey road, the old place to find the original heart" red special social practice group visited the site of the Hongjing Revolution. In the summer practice, the "century-old journey road, the old place to find the original heart of the original" red special social practice group visited the Hongjing revolutionary site group, Yeping revolutionary site group, Ruijin Revolutionary Martyrs Memorial Hall and the Yunxi Mountain Long March site. Practice group members in the photos and text in revolutionary predecessors, under the ancient trees and house revolutionary spirit, discovered in cultural relics and nicks, determined to learn from predecessors beginner's mind does not change the spirit, self-motivated quality, pioneering style of determination, also determined in the pursuit of the party's ideal, to realize the great rejuvenation of the Chinese nation on the road of faith."Dream red brigade, youth mail me" red special social practice group into the Huaihai Battle Ran Zhuangwei Battle Memorial Hall. Correspondent for figure "dream red brigade, youth mail me" red special social practice to "the spirit of the huaihai campaign historical implication and era value" research as the main line, based on patriotism education base, traditional villages and social practice, came to Xuzhou if huai hai campaign ground ZhuangWei battle memorial and Xuzhou national defense education park, and the huaihai campaign martyrs monument administration to build college students' social practice base, inheriting red spirit in practice. The volunteer service team of "Qingxin Dream" took a group photo with the teachers and students of Lilou Primary School. Correspondent for figure focus grassroots power rural revitalization in Xiangyang city, Hubei province, "green heart build dream line mail I" volunteer service team in li Lou elementary school launched the "colorful holiday" summer camp activities, volunteers from thought lead, civilized life, health, physical and mental science and technology innovation, happy learning, artistic accomplishment, cultural inheritance, build the boutique class, help the children have a full and unforgettable "colorful holiday". While accompanying the children to grow up and bringing them wonderful courses and academic guidance, every volunteer is also making continuous progress and improvement. He has honed himself in practice, practicing the volunteer service spirit of "dedication, friendship, mutual assistance and progress", and bringing warmth to more people."Dream red brigade, youth mail me" red special social practice group visited Xugong automobile enterprise exhibition hall and product exhibition hall. Correspondent for figure deep enterprise comprehension spirit in Xuzhou, "dream red brigade, youth mail me" red special social practice group into the xugong automobile manufacturing co., LTD., visit Xuzhou construction machinery group co., LTD., feel the enterprise culture, understand the machinery manufacturing process and enterprise personnel management and with enterprise science and technology and entrepreneurship symposium, on college students employment and social needs to ask questions. During the visit and study, the members of the practice group realized Xugong's core values of "taking on great responsibility, walking the road and becoming great tools", and personally felt the development and change of China's equipment manufacturing industry."A hundred years of journey road, the old place to find the original heart" red special social practice group and the elderly exchange. In Ruijin, Jiangxi province, the "red special social practice group, the century-old journey, the old place" came to Ruijin Shazhouba Nursing Home to carry out the volunteer service activity of "respecting the elderly". In the nursing home, the members communicate with the elderly, clean up for the elderly, take photos and print life photos for the elderly, and inherit and carry forward the traditional virtues of the Chinese nation."Dream Red Brigade, Youth Post me" red special social practice group members visited Xuzhou "real thumbnail". In Xuzhou, the red special social practice group of "Building a Dream Red Brigade, Youth Post me" went to Xuzhou Urban Planning Museum to visit and study. The staff of the exhibition hall led the teachers and students of the practice group to review the urban development history of Xuzhou together, described the future development plan of the city for the members of the practice group, and introduced the future development plan of Xuzhou in industrial economy, reform and innovation, urban construction, rural revitalization, cultural construction, ecological and environmental protection, people's livelihood security, social governance and other aspects. It is reported that the social practice is a bridge of effective communication between the youth and the society, which can let the students receive education, grow their talents and make contributions in the social "melting pot".

2021-08-17	Morning Correspondent Yang Xu) In the summer vacation of 2021, students of Donghua University went deep into the Red Revolutionary base to carry out the "self-study course of Party History". Pay attention to combine professional, do practical things in the party history study education. It is understood that the Textile College of Donghua University has led the organization of "Belt and Road" National College Students Textile Aid to Xinjiang Practice Group for 10 consecutive years to help the development of Xinjiang textile industry with "professional yarn". Students investigate the cotton field situation of Xinjiang. The School of Clothing and Art and Design of Donghua University went to Shanghai to visit Fengjing Ancient Town, Jinshan District, Shanghai to carry out field investigation and rural design, so that the "beauty of design" can truly enter the countryside and help the rural revitalization. Students from the School of Mechanical Engineering use the current popular AR technology means popular with young people to build an "online + offline" immersive learning platform for red cultural sites. Students of the School of Foreign Languages gave full play to their advantages of language discipline, organized professional teachers and students to carry out volunteer education and red culture publicity in Xinjiang, and built the "Guanghua Silk Road Library" in Changji, and the "Kashgar Guanghua Silk Road Library" and "Party History Silk Road Library" in Kashgar, so as to help improve the local education and teaching level in Xinjiang.

2021-08-12	Chengdu university of information engineering in the social practice of comprehension "meteorological" Chengdu university of information engineering: social practice comprehension "weather" xin-xin wang) recently, in 2021 the national college student volunteers summer culture of science and technology health "a program under which officials" social practice "the first city Yibin line" special activities in Yibin city, Sichuan province. The "Sichuan Red Practice Team" of Chengdu University of Information Engineering participated in the activity, and college students devoted themselves to rural revitalization with practical actions. The teachers and students of Sichuan Red Practice Team visited the representative enterprises in Yibin, including Wuliangye Group, Kaiyi Automobile Smart Factory, Lingzhi Valley Technology, etc., to understand the economic development of Yibin, and feel the livable environment of Yibin in the Yangtze River and the charm of the city as the "first city of the Yangtze River". Junlian County, Yibin City is a famous black tea producing area, with the "hometown of Sichuan Red". The Youth League Committee of Chengdu University of Information Engineering has set up a "rural revitalization" practice base in Xunsi Town, Junlian County. College students have carried out a social practice activity with the theme of "Exploring the beauty of the Sichuan Red River, helping the rural revitalization" in Yinxing Village, Xunsi Town, the "Rural Revitalization Demonstration Zone". In order to have a deep understanding of the local tea industry, the teachers and students visited the village secretary of Yinxing Village and the old tea makers, and learned about the history of tea planting in Yinxing Village, the development of the tea industry and the bottlenecks encountered. In the local well-known tea enterprises and the smart tea garden, the teachers and students learned the tea picking technology, visited the local smart tea garden and the "pig-marsh-tea" planting and breeding cycle mode, and felt the transformation and upgrading of the traditional tea area in the comparison. In the Junlian County Meteorological Bureau, the teachers and students visited the National Meteorological Observation Station to learn more about the weather modification facilities and the operation mechanism of the emergency early warning system. In practice, the volunteers combined with the school's meteorological characteristics and professional background, publicized the knowledge of meteorological disaster prevention and reduction in Xunsi Village and other places, explained the meteorological knowledge to the local children, and popularized the causes and scientific countermeasures of natural disasters such as rainstorm, flood and landslide. Xu Qiucheng, a college student volunteer, said, " In This practice, we have deeply understood the significance of comprehensively promoting rural revitalization, and also realized the importance of professional knowledge we have learned. We have strengthened the confidence and determination to study hard, study hard, and devote themselves to rural revitalization in the future.”

2021-08-12	Qingdao University: At the beginning of the summer vacation..., the children of the summer vacation, Langgan Village, Yingwustan Township, Kashgar District, Xinjiang Uygur Autonomous Region welcomed a group of friends coming from afar- -Qingdao University. The cloud volunteer teaching service team composed of Qingdao University Youth Volunteers Federation has 20 members from 9 different colleges, including Normal College, School of Literature, Journalism and Communication, and School of Fine Arts. All of them have passed the professional volunteer teaching training in the early stage and have outstanding comprehensive ability. From the coastal city of Qingdao to Kashgar, Xinjiang, "we dragged the heavy suitcase in a hurry, in the middle of the narrow sleeper suitcase support desk, in the driving car to discuss the planning of the teaching arrangement..." The volunteers passed 25 stations on the train, after a long 38 hours and 28 minutes, to arrive at the destination. From being in front of the screen to face to face, Zhang Yao, a student at the School of Arts, felt that the problem seemed more complicated. For example, her Sinology class has set up morning reading, because many of the children are Uyghurs, and Mandarin is not very standard. Although there is no big obstacle in communication, the problems in the recitation process are very prominent. The children's voice is very loud, but the pronunciation is not standard. After the senior, who volunteered together, raised the question, Zhang sent the clips she recorded in class to several teachers for support."Although I am a teacher of my children, I am still a student who can find my problems from my children and learn from my predecessors."Zhang Yao likes helping each other. The problems found and information collected in offline volunteer service can timely guide the improvement of online education, which is exactly what Xu Guohui, vice president and secretary general of Qingdao University Youth Volunteers Federation and founder of QiliCloud Volunteer Teaching, expects. Xu Guohui introduced: " This trip to Xinjiang for volunteer teaching is the first offline practice and exploration of the cloud volunteer teaching project."To do a good job in online volunteer services, offline field visits are indispensable. He believes that only by really getting close to the students can we understand their real needs and solve the real problems for students. Kashgar, Xinjiang, is a city with the counterpart assistance of Shandong Province. It is particularly important to carry out online volunteer services in Xinjiang, and to understand the local educational needs and local customs in advance. The 15 days of Xinjiang kashgar teaching, Xu Guohui led the volunteer service team also "beans to melon", through communication with Shandong Xinjiang headquarters, both sides reached intention, the future Qingdao university volunteers can not only in kashgar more schools to carry out the "one to one" online service, can also use the summer vacation, in offline teaching to carry out these schools."As soon as the novel mode of online volunteer teaching appeared, it made me sign up to join QiliCloud Volunteer teaching without hesitation."Li Yahui is a master's degree in piano performance. Before participating in cloud volunteer teaching, she once taught online music courses and accumulated about 2,000 lessons of experience. She still did not dare to relax in treating cloud volunteer teaching courses. After determining the topic and carefully preparing the teaching plan, she will be familiar with PPT again and again and simulate the class before class."These children are less lacking and need art education, and their thirst for knowledge is even stronger."Unvolunteering, pandemic or not, and students and volunteers can choose both ways across the country. Such a cloud volunteer teaching model not only makes full use of everyone's fragmented time, but also expands the scope of choice. Kong Yiming, from the School of Economics, is an English volunteer who has spent 60 hours online on the QiliCloud volunteer teaching platform. He still remembers his caution and neglect in his first class. In the dormitory, he carefully asked his child about the learning progress and led him to learn slowly from English numbers. Never familiar with online English teaching, to gradually becoming popular with children, Kong Yiming felt that they made him have more respect for the profession of teaching. In the service platform of cloud volunteer teaching, the volunteers' strengths and characteristics are open to the registered students like the "menu". The assisted students can "order" their volunteers to provide services according to their needs in various aspects. In addition, the cloud volunteer teaching platform includes not only disciplinary and non-disciplinary volunteer services such as language, voice voice, body and beauty, as well as social assistance services such as family companionship, medical guidance and psychological counseling. The composition of volunteers is also very diverse, including college teachers and students, professional volunteers such as medical staff, as well as social organizations."During the service process, the volunteers will encounter all kinds of large and small situations, but they will have the same goal."Kong Yiming said, no matter how the study and teaching process is, we want to build the most solid knowledge foundation for our children- -just to help their children fly and make everyone feel at ease."Volunteers can only need one mobile phone to provide volunteer services for friends in the western region without geographical and space restrictions. The 30-minute service time can make college students become a stage to dedicate their love."Xu Guohui introduced that since its launch in June 2021, a total of 286 registered volunteers, 387 students have received assistance, providing volunteer service for nearly 20,000 hours. In order to encourage volunteers to serve better, volunteers can get the volunteer service certificate issued by Shandong Youth Volunteers Association for every 60 credit hours (30 hours). According to the background data, the volunteers have accumulated 94 hours of online service."The development of the hai cloud volunteer teaching makes the distance between the children in the east and the western mountainous areas can attend the music class carefully prepared by the volunteers of Qingdao University through the big screen."Xiangchang Primary School, Sishui County, Shandong Province, is one of the volunteer schools. Yan Tingpeng, the principal, felt that the participation of the volunteers not only broadened the horizon of the students in the mountainous areas, but also made them get infected and encouraged in the contact with the volunteers. Different from the traditional volunteer teaching during cold and summer periods, cloud volunteer teaching is conducted simultaneously online and offline between the middle line and winter and summer vacations. Moreover, only the best among the best with outstanding online performance can have the opportunity to participate in offline volunteer teaching services."Provide continuous attention and help to the aided students, both online and offline ways."Ma Lingang, secretary of the Youth League Committee of Qingdao University, expects that college student volunteers can take the hai cloud volunteer teaching as an opportunity to help them explore the form of volunteer service in the post-epidemic era. China Youth Daily China Youth Network reporter Du Yimeng source: China Youth Daily

2021-08-11	Beijing jiaotong university practice group into Hebei tunnel memorial hall for heroes footprint Beijing jiaotong university practice group into Hebei tunnel memorial hall for heroes footprint China youth network Beijing on August 11 (reporter Zhang Jianwei, correspondent wen-bin lu) Ranzhuang tunnel warfare site is an important war site in the Anti-Japanese War, is also one of the national hundred revolutionary tradition education base. In order to understand the heroic deeds of the revolutionary ancestors, inspire patriotism, encourage themselves to love the Party and the country, and contribute to the people, the "Hot Blood Youth Practice Group" of the School of Economics and Management of Peking Jiaotong University recently walked into the Ranzhuang Tunnel Memorial Hall in Hebei province to find the footprints of heroes and tell revolutionary stories. The practice group visited the exhibition areas of "The Fall of central Hebei", "national difficulties", "and" turn the tide ", and went deep into the" underground Great Wall " to feel the difficult environment of the anti-Japanese struggle of Ranzhuang revolutionary ancestors at that time. After visiting the Tunnel War Memorial Hall, the practice group visited the former residence of martyr Wang Xia, the pioneer of tunnel warfare in Ranzhuang, and had the honor to visit martyr Wang Xia's grandson and daughter-in-law. According to Wang Xia's grandson daughter-in-law, Wang Xia got rid of several raids of Japanese enemies in the anti-Japanese struggle, and transferred tens of thousands of information, making great contributions to the victory of the anti-Japanese struggle in central Hebei. It is reported that the practice group is composed of 11 party members, among which 5 are graduate part-time counselors, 4 are graduate party branch secretaries, and 2 are the party branch branches. This activity is a preliminary exploration of Beijing Jiaotong University in the three rural activities and the party branch theme education activities combined, will provide experience for the development of the party branch activities.

2021-08-11	Students of Foshan University of Science and Technology held the flag awarding ceremony of "three social practice in the countryside". Correspondent for figure China youth network Beijing on August 11 (reporter Zhang Jianwei, correspondent Jiang Zunguo) summer, Foshan college of science and technology around "always follow the party, forge ahead the new era" theme for college students volunteer summer culture science and technology health "a program" social practice, the school formed school key team, level key team and teachers and students team to multiple cities, counties and villages to carry out social practice activities. The picture shows a member of the "Zhuoyue Culture and Medical Team" interviewing Li Mugang, a hero of the Anti-Japanese War (party member). The undergraduate Party branch of the Department of Medical Laboratory of Foshan University of Science and Technology and the "Zhuoyue Cultural and Medical Team" jointly held a series of Party history learning meetings in the Double � � � Department of Yangchun City, Yangjiang City. During the practice, the team members interviewed the heroes and veteran party members of the Anti-Japanese War, led the local villagers to visit the Party history learning gallery and the heart pavilion between the Party and the masses, and told the villagers the stories of the Party doing practical things for the people. The picture shows the Red Boat, a wood carving celebrating the centenary of the founding of the Party. After members of the "Chaoshan Research Team" searched for intangible cultural heritage in Huilai County, Jieyang City, Guangdong Province for intangible cultural heritage- - "Chaoshan wood carving", they had a deeper understanding of the cultural inheritance. Through the interview and investigation of Mr.Chen Huanhui, the inheritor of the intangible cultural heritage, the team members learned more about the characteristics, history and development status of Chaoshan wood carving, the carving form of wood carving and the carving steps of Chaoshan wood carving. The picture shows members of the Xinchao Culture team visiting the ecological greenhouse of Qiaochen Village. Foshan Institute of Science and Technology Xinchao Culture team in Shantou Bridge Village, through the village cadres interview, careful investigation of the industrial development of the village, a comprehensive understanding of the Bridge Village, through the professional study to help the masses to solve practical problems. In the summer of 2021, Foshan University of Science and Technology set up 50 university-level key teams, more than 300 university-level key teams, and more than 4,000 teachers and students went to many cities, counties and villages inside and outside the province to carry out social practice activities from the aspects of party history study, theory publicity, national conditions observation, rural revitalization and other aspects.

2021-08-10	The social practice group of Zhejiang University students works together with the rural students to "color their hometown" to emphasize the matters needing attention when painting. Zhejiang university in Anhui Fuyang slide town central primary school social practice for China youth network Beijing on August 10 (reporter Zhang Jianwei, correspondent, Xu Chuyang Deng Rui) recently, Zhejiang university students three rural association in Anhui Fuyang slip town central primary school summer social practice practice team organized an outdoor painting activities, under the guidance of the practice team, the students on the flat canvas on the ground with imagination, with brushes and paint painted home. At the end of the activity, the students of each group held up the creation results and took an aerial photo. All the teachers participated in the theme class meeting, and jointly guided the students to paint the theme of "coloring their hometown" and stimulate the students' creative ideas. In the class meeting, each class teacher introduced the relevant arrangement of outdoor painting activities to the students, and grouped the students."Their paintings are filled with innocence and childlike interest. As I watch them paint them carefully with their brushes, I can really feel their emotions," said Wang Yu, the head teacher of the class group. It is reported that this outdoor ground painting activity is an expansion activity in the summer camp, aiming to cultivate students 'group cooperation ability and artistic creation ability, and cultivate students' "love their hometown, love the motherland" emotion.

2021-08-08	Yangzhou University students use stamps to tell the red spirit practice group and carry out 100 places card project. The practice group chose stamps as the carrier of red culture, and relying on the scene, micro video recording. Practice group members from the beginning began to action, in the early stage of the collection stamps, determine the process of clock location, they went to the red stamps, and through various channels in the network collection search for related materials, borrow stamps to select representative, historic red stamps, contact the corresponding red stamps brigade base as a team clock in. Up to now, the practice team has reached 6,000 kilometers, and the footsteps of the team members are spread throughout most of China, including Beijing, Tianjin, Zunyi, Jinggangshan and many other red cultural tourism bases. Team officials said that all the micro videos shot in the later stage will be released regularly on the mainstream video platforms.

2021-08-08	The Youth League Committee carried out the summer social practice activities with the theme of "Attention to the elderly, people with the heart of youth", "Love to the elderly". The team members have visited the retired party member teachers of the school and some 50 + old party members in Changzhou city, listening to the progress of the predecessors, and absorbing the strength of the young people. Visit and learned the "Party flag flying dragon city- -Changzhou to celebrate the 100th anniversary of the founding of the Communist Party of China" theme exhibition. In addition, we also cooperated with Changzhou Sanjing Street Zijiang Community, Tian'an Community, Yonghong Street Xianghe River Huating Community, to carry out smart phone operation popular science lectures. In the next step, the Youth League Committee of the school will take this as a starting point to carry out regular and continuous volunteer services to help the elderly, and create an atmosphere of "the elderly are supported, supported, happy and safe".

2021-08-07	Sichuan liberal arts college party history study practice group revisit long march Sichuan liberal arts college party history study practice group revisit long march spirit revisit youth newspaper reporter Wang Xinxin) visit Zhu Mao old residence, retrace the red army path, hiking over jinshan, research old revolutionary base areas green development... this summer, Sichuan liberal arts college "study party history, strong faith, follow the party" party history study practice group teachers and students by retrace long march, revisit a memorable long march history. In a week of practice, the practice group from Sichuan Dazhou, Chengdu to baoxing, all the way north to bilateral two estuary town, visited the red army long march over clip jinshan memorial hall, MAO zedong zhu long march, clip jinshan red army trail, clip jinshan five turn, David bridge, David Lama temple, Mao achievement site, two mouth meeting memorial hall, solid bridge and saddle bridge and Yingxiu epicenter earthquake site memorial hall. Teachers and students climbed over Jiajinshan. In the snow-capped mountain that the Central Red Army once crossed, Jiajinshan, teachers and students experienced the final sprint before the Red Army reached the summit. They climbed over the Long March, about 4,000 meters above sea level, with steep mountains and misty clouds overhead. The cold wind in their ears made people still feel the chill in the summer. Some students have serious altitude sickness, but they still insist on climbing. We helped each other and spent nearly two hours on the Long March and experienced the difficulties of the Long March. While pursuing the footsteps of the Red Army, the members of the regiment also communicated with the local residents. In Dawei Town, Xiaojin County, Aba Tibetan and Qiang Autonomous Prefecture, they visited the Jinshan Rose Garden, a local model of rural revitalization industry, and interviewed two senior local party members to tell their stories about the history of the party. Members of the practice group interviewed local senior party members. Chen Wanghui, the "rose sister" of Jinshan Rose Garden for the map of Sichuan University of Arts and Sciences, took the lead in establishing Qingduoxiang Rose Planting Professional Cooperative and Jiajinshan Qingduoxiang Wild Resources Company. It adopted the green industry development mode of "company + cooperative + farmer + base" to promote the revitalization and development of rural areas of Maoshui Village, Xiaojin County through combining production and tourism. When listening to the stories of the old party members, Dawa, a member of the practice group, remembered what the old man said: " The essence of the Party is to serve the people. We should not think that we are superior to others, but we should be modest and humble."Dawa said that now some people think that money is becoming more and more important, and the old man's story tells me that something that can never be measured by money, like spirit, like faith. The day before the return trip, the teachers and students of the delegation also visited the ruins of Yingxiu Xuankou Middle School and the Memorial Hall of Yingxiu Town in Wenchuan County, Tibetan and Qiang Autonomous Prefecture, Aba, to learn about the May 12 Wenchuan earthquake, earthquake relief and post-disaster reconstruction, and presented flowers to remember the victims.

2021-08-07	Anhui university of technology "hand in hand" XuanJiangTuan into the grassroots Anhui university of technology "hand in hand" XuanJiangTuan into the grassroots of the party history reporter wang haihan) recently, according to the Yangtze river delta region universities "red jiangshan red road youth power youth line" activity theme, Anhui university of technology "hand in hand" XuanJiangTuan into the ma'Anshan city streets, rural practice. According to the characteristics of the audience of primary and middle school students, the team members carefully prepared the content and way of preaching. In Liyuan Community, Taoyuan Street, Ma'Anshan City, the team members carried out the activities of "One hundred years of Party history", "Party history knowledge you answer" and "Red song and dragon singing", telling the story from the May Fourth Movement to "South Chen and North Li, meet for the founding of the Party", and led the children to review the founding process of the Communist Party of China. In Maanshan library, the team members and the local "youth volunteer group" children to learn "young MAO Zedong circle" and other red stories, read the red letters from revolutionary martyrs; the team also together youth volunteers came to the village, to the children youth party history learning cartoons, telling the story behind the songs such as "No Communist Party, no New China". The publicity team of "Learning Party History hand in hand" is also the key team of Anhui University of Technology and the School of "Materials Science and Engineering". This year, the Youth League Committee of the school innovatively created a column entitled "Learning Party History in Minutes". Teachers and students shoot and record micro videos and micro audio lectures of Party history, and regularly broadcast them on the online platform."In this activity, we took the online learning results into the grass-roots level, and primary and middle school students together in the practice of middle school party history."Said team instructor Zhao Yinghong.

2021-08-06	Hefei Normal University: 2,340 "little gentlemen" participating in the "Xingzhi School" Wang Haihan, correspondent Cai Hailong) " I understand the whole process of the development and growth of the New Fourth Army, we should remember the red years of burning passion."Recently, in Jing County, Anhui Province, Hefei Normal University, a network and new media professional student, Ren Zhengwei, visited the New Fourth Army military and political headquarters and other former sites. He also has another identity-the school Xingzhi school "little gentleman" (the school social practice volunteer nickname). Since 2012, the Youth League Committee of Hefei Normal University has created a brand activity of "Xingzhi School". During the holidays, the "little gentlemen" went to the rural grassroots, offering public welfare schools for left-behind children, the children of migrant workers and other groups, such as classrooms, communities and offices. This year, 2,340 students from Hefei Normal University participated in the Xingzhi School activities. In addition to teaching at the grass-roots level, the school has added the theme of learning Party history to the activity menu of Xingzhi School. The "Little gentlemen" carried out activities such as revisiting red footprints, redrawing red memories, singing red songs and reviewing red films, and then told the local children what they have learned about party history."Students, when we just entered the garden, a big stone written on the two big characters, what to read?'revere'! We should respect the revolutionary heroes, who will exchange their lives for our happy life today."At the end of July, when the" little gentlemen " from Xingzhi School in Zhubei Town, Qingyang County, led the left-behind children to visit the memorial tower of revolutionary martyrs, Shi Juan, a student from Liu Mingchuan College of the school, explained. In the Yunhua community of Hefei city, the "little gentlemen" brought video cameras and tripods to organize the primary school students in the community to shoot red movies together. Chen Xi, a Chinese language normal major at the university, said: " The young students are interested in performing the roles in the film, and they have a deeper understanding of the image of a revolutionary hero."" sings a folk song to the Party.... " On July 26, neat songs echoed in the classroom of Liuzhu Primary School in Gaogong Town, Guoyang County, Anhui Province. She said: " Singing can improve children's confidence, and they will hum the songs we teach after class. After returning home, I still sing it to my grandparents.”

2021-08-05	The newly revised Law of the People's Republic of China on the Protection of Minors (hereinafter referred to as the "Uninsured Law") has just been implemented on January 1. Hu Xinyue and her classmates brought the latest information about school bullying to three kindergartens in Jiangning district, Nanjing city. In the kindergarten, they brought the teachers special lectures on the prevention of school bullying, hoping to "promote the stable development of the kindergarten education and teaching, and escort the growth of the children with the law". Hu Xinyue is a college student volunteer of the Law School of Southeast University. Since the establishment of the publicity group in 2017, this volunteer team of "student autonomy" and "college self-help" has been paying attention to the legal education and physical and mental health of minors. Through various characteristic activities to promote the law popularization, it have promoted the law popularization and abiding by the law. For students, hands-on practice often detects practical problems better than in class. In the process of popularizing the law, the volunteer team found that many current ways of popularizing the law do not adapt to the actual needs, among which the way of education of popularizing the law for minors is more obvious."The autonomy of volunteer service organizations is weak", "the responsibility system has not yet been implemented", "the role of the legal teaching group is not obvious", "the assessment mechanism is not sound"... Gradually, many problems were exposed to everyone. To this end, the members of the publicity group began to try to innovate the mechanism of college students' associations to participate in the legal education of minors. In the summer of 2018, the team cooperated with the Beautiful Village Law Popularization Service Center of Jiangning District of Nanjing, and began to walk into various communities in Jiangning District of Nanjing to conduct practical research on law popularization. In this process, they listened to the actual difficulties of the local people, and then carried out in-depth legal publicity. In 2019, the team visited Hebei province and Shandong Province to investigate the "Rule of Law Publicity in 100 Schools". They combined with the local actual situation to "suit the case", the theoretical knowledge applied to practice, for the rights and interests of minors protection and for the local grass-roots legal services to advise. During the epidemic period in 2020, offline activities cannot be carried out, so the team volunteers contacted the town Central Primary School and Sunjiawan Primary School in Hubei Province to broadcast online legal teaching. Qin Jiayin, a law student at Southeast University, said that in the summer social practice, they had 5-8 "in-class" questions after each law popularization course. The test found that the audience could only answer the knowledge points explained, but still did not understand the legal principles behind it."Only offline teaching is a way to popularize the law, which is obviously insufficient, and the legal knowledge received by the audience is scattered and unsystematic". In 2021, the team began to think on the transformation of practical experience into theoretical support. They summed up the "lubricant", "warm warming" and "freshener" methods, and explored the multi-subject cooperation mechanism and "wechat-Weibo-micro-video" three-dimensional publicity network. In order to solve the problem of poor linkage of the current social mechanism of the law, the law, the team believes that college student associations should play the role of "lubricant" and use professional knowledge to seek "government-university research base-college student associations-social organization-service point" in five dimensions of law popularization, dimension and audience viscosity. They set up a teaching group for college students, relying on the "Jiangsu Youth Work Research Base (Prevention of Juvenile Crimes)" of Southeast University Law School and Jiangning District Beautiful Village Law Popularization Service Center, to regularly carry out regular volunteer training meetings to convey the spirit of dedication. In the aspect of legal education for minors, they improve the combination of legal cultural products and pursue diversification. By comparing the stage characteristics of the legal education of minors, especially aiming at the particularity of the juvenile groups such as left-behind children and troubled teenagers, the hierarchical classification is strengthened to enhance the sense of participation in the interactive experience of minors. They focus on the special groups of minors, and provide targeted heart-warming and legal education for the minors in the special schools. For left-behind children who lack the company of their parents, teenagers who lack legal awareness and family education, and are sensitive and lonely, add psychological counseling, future development planning and other targeted content on the basis of popularizing the law, so that children can gain more warmth in the activities of popularizing the law. The team will also take the innovation of legal popularization and cultural products as the entry point, B station, TikTok, wechat public account trinity, through the production of short videos, writing small articles, play the role of "freshener" of cultural products. China Youth Daily, China Youth Network reporter Yang Baoguang source: China Youth Daily

2021-08-04	Hefei university of technology "a program under which officials" team retrace the reclamation of Hefei university of technology "team" summer "reclamation" social practice key team- "explore corps spirit, continuously red history gene" practice to the Xinjiang production and construction corps, through field visits, retrace army reclamation, interview dialogue reclamation practice form, mining the outstanding children construction of Xinjiang, defend the frontier, and practice the spirit of the era value. In army reclamation culture museum branch of the Xinjiang production and construction corps reclamation frontier history exhibition, the team through song into Xinjiang, hard work, passion burning, the role of the "three teams" and other four exhibition chapter understand the historical development of the Xinjiang production and construction corps, especially the people's liberation army in the desolate wilderness on the Nanniwan spirit, one hand gun, pick, unprecedented army reclamation in human history.

2021-08-03	Volunteers from Nanjing University of Posts and Telecommunications are volunteers to help with epidemic prevention and control. China Youth Network, Beijing, August 3 (reporter Li Chuan, correspondent Qin Changjian) The epidemic is an order, and prevention and control is the responsibility! Since Nanjing lukou international airport cluster COVID-19 outbreak, from full nucleic acid detection point to Nanjing health information center, from the epidemic prevention and control propaganda to the hotline, from information data collection to sampling order maintenance, from community material distribution to management site sanitizers, Nanjing university of post and telecommunications nearly 300 pomelo volunteers responded, play youth volunteer dedication, at a critical moment, up, charge in front, report to the school, report to the community, in epidemic prevention and control of volunteer service. The picture shows pomelo volunteers from Nanjing University of Posts and Telecommunications on the front line of the epidemic. It is reported that more than 60 youth league members of Nanjing University of Posts and Telecommunications took the initiative to sign up as fixed volunteers to take turns, becoming the school's full nucleic acid testing work, and helping the school's epidemic prevention and control work. The picture shows pomelo volunteers from Nanjing University of Posts and Telecommunications on the front line of the epidemic. More than 70 youth league members of the School of Applied Technology took the initiative to register, To Nanjing International Exhibition Center, Suojin Village No.1 Primary School, Mount Wutai Mountain Gymnasium and other nucleic acid testing sampling sites; Jiang Jiaojiao, a student from the School of Communication and Information Engineering, attended the hotline consultation service of Nanjing Health Information Center, Clearly and clearly interpret the epidemic prevention and control policies to the public; Yang Ke, a student at the School of Automation and Artificial Intelligence, became a community volunteer, Put on a red vest, Responsible for checking the health code and the temperature measurement of the incoming and outgoing personnel; Wang Yuxuan, a student from Modern Institute of Post, registered as a nucleic acid testing volunteer in Meiyuan Xincun Street, Xuanwu District, Nanjing, Over 7 hours a day, Do a good job to maintain order, handling materials, site disinfection and other work... The picture shows pomelo volunteers from Nanjing University of Posts and Telecommunications on the front line of the epidemic."In the most critical moment, go to where it is most needed."In the battle against the epidemic, many of the same green pomelo volunteers volunteered to go to various districts, streets and communities in Nanjing to support the epidemic prevention and control, and to build a" line of youth defense ".

2021-08-02	Guizhou university college of science and technology practice team in service country college of technology practice team in service country increase ability long ability contribution jianwei correspondent Zhang Yaogift) in recent days, Guizhou university college of science and technology "institute inspirational line Chinese dream" summer social practice service into huishui water town dragon village, practice group rural revitalization practice team, theory about practice, volunteer practice team seven teams around the party history study, rural revitalization, theory, volunteer, into the masses, into the countryside, in the service of rural social practice by education, long ability, make contribution. Service of rural development, help rural revitalization of " due to the shelf life of edible fungi is short, even after picking immediately sent to cold storage, there will be some edible fungus sales due to various reasons, so we are chicken and duck breeding near the base, to the excess mushrooms as feed, the development of the second industry, this is the benefits of national poverty alleviation industry to us."Said Ms.Duan, the head of the base. The picture shows the practice team members packaging bacteria sticks. Under the guidance of the person in charge of the edible fungus planting base of Dalong Village, Yali Water Town, the practice team members observed the fungus sticks closely, learned the packaging fungus sticks and mushroom planting methods, and helped the villagers to put the 500 fungus sticks neatly in the shed according to the requirements."Mushroom with corn cobs, straw, wheat and other materials as the main nutrients, planting the best temperature in 21° -22°, a greenhouse can produce 1,000 stick mushrooms a day."Liu Wenting, a member of the practice team, said that only by visiting the field for research and understanding the development of poverty alleviation industry projects, can he effectively help the local villagers with practical actions, which is his biggest harvest as a member of the volunteer service group in the countryside. Theory into the countryside, civilized practice to help development practice team 20 members from door to door, and the villagers "pendulum", distributing posters, brochures, broadcast video and other ground form, and the villagers together, with concise language, video to the masses "July 1 spirit", "cherish life, stay away from drugs" and other theoretical knowledge, carefully to the villagers on COVID-19 epidemic prevention and control measures, the essence of the rural revitalization policy. The picture shows the members of the practice team popularizing the epidemic prevention knowledge in the villagers' homes and asking them about their vaccination situation."On July 1st, I got up early in the morning to watch General Secretary Xi Jinping's speech. I was very proud to grow up in China and grow up in China."In the process of preaching the spirit of General Secretary Xi Jinping's important speech at the celebration of the 100th anniversary of the founding of the Communist Party of China, a villager of Dalong village excitedly said."This theoretical publicity activity is mainly to help the villagers deepen their understanding of the party history, epidemic prevention and control measures, and their understanding and application of current affairs and policies, to help the rural development; we also hope to train the team members' solidarity, language expression and their ability to deal with and solve problems in practice."Instructor Yuan Tiansen said."'bike', everyone read with me, 'bike' " English class teacher Li Jiayu is teaching students simple English words. This year is the 15th year of the summer volunteer teaching activity held by the School of Science and Technology of Guizhou University. This love volunteer teaching activity was carried out in Heai Primary School, Dalong Village, Yahui Town. A total of calligraphy, English, handicraft, vocal music and other 7 interest classes, six courses a day. In class, the volunteer teachers of each class taught the students the basic knowledge of sketch, handmade skills, song singing methods, the difference between horizontal and vertical in calligraphy and other contents. The picture shows the volunteer teacher giving one-to-one tutoring to the children. Correspondent for figure before classes, volunteer teaching practice team members to dragon village with group, Yang see group of the villagers group door to door, face to face, with parents admissions propaganda, guide the parents lead children to service point, fill in the enrollment form, let the children according to their interests choose to participate in the course. The practice group visited 157 households, and 122 primary school students signed up to participate. After a day of teaching tasks, the volunteer teachers walked together to send the children to their homes safely."After a day of volunteer teaching activities, I deeply realized that the difficulty of teaching is not easy, but when I saw their innocent smiles and soft voices, you will melt together."Said Peng Kaili, a member of the practice group.

2021-07-31	Anhui Vocational College of Finance and Trade, the inheritor of the intangible cultural heritage) In recent days, the practice team of Anhui Vocational College of Finance and Trade has gone to Dangtu County, Anhui province to carry out the social practice activity of "three to the countryside" to pursue the red memory and inherit the intangible cultural heritage. Closely related to the unified theme of this year's Yangtze River Delta Communist Youth League organized "Red Jiangshan Red Road Youth Power Youth Tour", the first stop of the team came to Dangtu Museum. Photos of the People's Navy, military kettle, the New Fourth Army... precious revolutionary relics and documents displayed here."Behind every old object is a touching story, who pursues the red memory during the visit and taught the history of the Party."Team instructor Zhang Gaoyang said. In order to further explore the relevant culture and knowledge of Dangtu folk songs- -the national intangible cultural heritage, the practice team has gone to many towns and communities on the basis of collecting data to understand the local inheritance and development of Dangtu folk songs. Students have learned that in recent years, local artists and artists have created innovative ideas, creating red songs such as "The People's Mountains are Long", "Always Follow the Communist Party" and folk songs such as "Sing the Green Sea and the Golden Wave" and "Beat the Wheat Song" filled with new words."In recent years, schools for the elderly in the village have collected tunes from folk songs, compiled their own contents to teach and sing, and arranged them into artistic programs to promote traditional culture."Taibai town garden village party branch secretary Wang Xiaoyun introduced. During the activity, the practice team also taught local pupils to read Chinese characters, read ancient poems and sing folk songs. Dai Xiaoqiong, the instructor of the team, said that while investigating the intangible cultural heritage, the young students will carry out volunteer services on the spot, sing Dangtu folk songs, and do practical things for the masses.

2021-07-30	Wang Xinxin) From only sing Chinese songs to spell lyrics and sing Chinese. On July 18 to 27, the China West Normal University team held a 10-day volunteer service in Litang County, Ganzi Tibetan Autonomous Prefecture, Sichuan Province, bringing a series of special Mandarin courses to local Tibetan children. During the ten days, nine college students from various colleges and majors of China West Normal University offered courses and activities, such as learning Mandarin, stories about Party history, singing red songs, and red classics in art works, and presented a Xinhua Dictionary to each of the more than 80 local Tibetan children. Zerathasi, who is studying in the sixth grade, is a famous local little singer, but due to his poor Mandarin foundation, he can only hum many Chinese songs with melodies. After nearly 10 days of learning Mandarin, Xiao He has been able to read pinyin and sing songs such as "Love My China" in Chinese. During the volunteer service, Wang Fei, the team instructor and national Mandarin level tester, also gave public lectures on oral communication and Mandarin learning for the staff of the Communist Youth League Litang County Party Committee and other units, and guided the Litang Mandarin Language Association to carry out the Mandarin test and promotion work.

2021-07-30	Guizhou university college of science and technology students listen to the red story red gene Guizhou university college of science and technology to carry out "listen to the red story, red gene" practice JianWei correspondent, Chen Jiayu JiaoCheng Li Gen Yang ZhongCanwei) recently, Guizhou university college of science and technology "institute � q inspirational line � q Chinese dream" summer social service team pursue red gene practice in elegant water town dragon village to "listen to the red story, red gene" as the theme of the practice. The 24 members of the practice team explored the local red resources, and found the old party member grandpa of "Glory in the Party for 50 years" in Dalong Village, and grandpa Yang Yingsong, a retired hero who had participated in the self-defense and counterattack war of Vietnam. The team members of the practice took them to the home of the two old people to listen to the old party members and revolutionary veterans of "glory in the Party", feel the original mission and gain the strength forward. The picture shows the practice team members sitting around the old party member petrochemical state grandpa listening to the red story. Talking about the experience in the party, petrochemical state grandpa is very excited, and proud of being a Communist party member."At that time, the family conditions were very difficult, and my parents were also very old, but I still wanted to put the great cause of the country and the Party first, listen to the Party, and follow the Party. Without the Communist Party, there would be no new China."Grandpa Shi said then took out his" glory in the Party 50 years " medal of honor, encouraged the team members to study hard, day day up, in the future to do the second centenary goal of the pillars of the talent, for the realization of a modern socialist power and struggle, live up to this great era. The picture shows retired veteran Yang Yingsong sharing his combat experience with the practice team members. Correspondent for figure to participate in Vietnam self-defense counterattack Yang Yingsong grandpa tells the story of his experience in 1980 in Guangxi border forces to join the army, as a retired hero, he regrets: " as conscripts recruits to participate in the war against Vietnam counterattack, conditions is very hard, many comrades are gone, they sacrifice themselves for the cause of the party and the country, the great spirit is worth future generations to learn. Today's better life is the great achievement of the Party seeking happiness for the people and doing practical things. We must believe that only peace can develop and the country can prosper."This listen to the old party member to tell a story, practice team member Li Dengyou has a deep feeling: when we see the stone grandpa stooped waist to the village under the tree, I can imagine this once tall body experienced how much wind and rain and hardships. When the stone grandpa said, 'Without the Communist Party, no us, without the Communist Party, no new China,' when I eyes tears in his story, I feel the greatness of the Party, the glory of the Party, the Party has been for our welfare, do practical things.

2021-07-29	Guizhou university college of science and technology to prevent fraud knowledge preaching about Guizhou university college of science and technology to promote fraud knowledge jianwei correspondent JiaoCheng Liu Yuping, Zhong Canwei Chen Jiayu) late July 25, Guizhou university college of science and technology "science feeling inspirational line Chinese dream" summer social practice service theory about practice team in dragon village rock foot group to prevent fraud knowledge preaching. Instructor Yuan Tiansen led the 38 members of the practice team to the villagers' homes to conduct door-to-door visits and lectures. The picture shows the practice team members watching the anti-fraud movies together with the villagers. Correspondent for figure in addition, the practice team in love elementary school building to deduce fraud skits, play theme movies, distributing posters for dragon village rock foot group villagers present all kinds of fraud, such as fake health insurance, social security, entertainment, high salary recruitment fraud cases, in order to improve the villagers' fraud awareness."This anti-fraud theory propaganda I explained a lot of places that they do not understand, in the process of preaching to exercise my language expression ability, I think this is my biggest harvest."Chen Jiayu said, a member of the practice team, that in life and study, people should also improve their own awareness of anti-fraud in life and study, as much as possible to help people around them to prevent fraud. The anti-fraud theory propaganda, on the one hand, to promote the anti-fraud series of knowledge deeply popular, deepen the villagers' understanding and understanding of fraud means; on the other hand, also let the practice team members have a broader and profound understanding of fraud prevention, in the future study and life will be practice and theory combined, do a good job of volunteer service.

2021-07-28	Shanxi Datong University carries out summer Social Practice to help Rural Revitalization " practice team carries out summer social practice in Guangling County, Datong City. The practice team went to Changshibao Village and Jianxi Village respectively to visit the rural revitalization industries, learn folk culture, understand how the local people get rid of poverty, implement the work layout of the "rural revitalization" strategy and make their achievements, and review the heroic stories of the revolutionary martyrs. Subsequently, the practice team visited the Yanbei Spark Party history education exhibition hall, visited the "glory in the Party for 50 years" veteran Party members, the Party members faced the party flag, reviewed the oath of joining the Party. The practice team in Xiadu Central Primary School, answered questions for children, carried out volunteer services such as knowledge supplement and psychological counseling.

2021-08-01	The elderly can enjoy high-quality medical services at home. It is really convenient and warm the heart. Thank the Party and the government for always taking us to heart."Living in Zunyi City Huichuan District Gaoping Street Xuetang bao community uncle Wang regrets. It is understood that uncle wang mouth activity is the street focus "I do the practical work for the masses" practice of "love in the community, feeling warm the old man heart" health free physical examination activity, the activity mainly for jurisdiction over 65 years old, free physical examination, physical examination project includes blood pressure, blood sugar, blood lipids, electrocardiogram, B ultrasound, etc., timely understanding of the elderly health, let the elderly fully enjoy the basic public health services the huimin policy. The activity first started in Gaoping Street Xuetang bao community. Early in the morning of the same day, more than 500 elderly people successively went to the community health service center to participate in the physical examination. Nearby, the volunteers patiently explained the physical examination process and precautions for the elderly, and guided them to complete all the physical examination items."The street will continue to integrate service resources from all walks of life, and successively carry out free health physical examination activities in all villages (communities) under the jurisdiction, to constantly improve the sense of gain and happiness of the elderly."Gaoping street responsible person introduction. Recently, the health free physical examination activities into the Gaoping Street Renjiang Village. The General Party Branch of Renjiang Village and the Youth League Committee of the Affiliated Hospital of Zunyi Medical University organized the medical and health science popularization knowledge and free clinic service activity of "I do practical things for the masses, popularize health science and warm the people". The activity invited experts and doctors from the department of respiratory medicine, neurology, cardiology, cardiology, thoracic surgery and spine surgery of the Affiliated Hospital of Zunyi Medical University to Renjiang Village to provide free medical treatment for senior party members, veterans and the masses, and popularize health knowledge for the medication and health management of multi-system diseases such as hypertension, diabetes and emphysema for the elderly."Old age, all kinds of small problems have come to us. Learning some health knowledge is conducive to our health."Old party member Deng Juxian side carefully recorded the doctor told about the content, while told reporters. At the end of the activity, the Youth League Committee of the Affiliated Hospital of Zunyi Medical University also distributed emergency medical aid kits with cotton swabs, alcohol, band-aids, iodine and other common items. According to the relevant person in charge of Gaoping street, next, the street will continue to carry out all kinds of activities to benefit the people, and effectively transform the party history learning and education results into the power to serve the people, do good things for the broad masses, do practical things.(Zhou Jingmei, Zhou Li)

2021-07-27	Fuzhou University: Learning the History of the Party on the Road of social practice. In Shuiwei Village, Jianning County, there are many and more complete dwellings and Red Army living sites, among which the Red Army hospital, Red Army bank and Red Army factory have completely restored the living atmosphere of the rest of the Red Army and the military and civilian situation with the local residents. While visiting these sites, the social practice team members began to record micro videos, and introduced the stories of some characters happening here, and the red stories and the history of the army and the people, through the short videos popular with college students, which made the revolutionary site of Shuibei Village really alive. For example, in the process of making earth grenades in the Arsenal, the gunpowder is mainly filled by the local villagers, and the village women always carry grass ropes and repair straw sandals for the Red Army soldiers anytime and anywhere. In Xiang 'an mansion village, minhou pukou village, building ning water tail village, players interview many whole, some of them have glorious in the party more than 60 years, have a plenty of the old secretary in the village, have a plenty of troops to join the party has been home in agricultural production after the first line, some teach in the village for more than 40 years, the players feel from them to the older generation of communist party member beginner's mind and mission. Jianning is an important area of the "Young International Division" activities, around this revolutionary history, the team members play their professional advantages, waving the youth brush, in the She primary school with wall painting to tell the "Young International Division" little hero story, let the red thought rooted in teenagers, the red gene from generation to generation.

2021-07-26	The 28 colleges and universities launched green long march Yellow River research practice 28 colleges jointly launched youth green long march Yellow River research practice du river bank Mongolia) on July 23, "beautiful China youth action" -2021 youth green long march Yellow River research practice ceremony held in Beijing forestry university, using the way of webcast online synchronization. The Yellow River basin research practice by Beijing forestry university, Chinese Academy of Sciences, Chinese Academy of Sciences, Lanzhou university, Qinghai university, Henan agricultural university and other 27 universities, the national protection of mother river action leading group office, the national forestry and grassland propaganda center, the national youth ecological civilization education center, ideological and political work innovation and development center, Beijing forestry university, the Yellow River basin ecological protection and high quality development institute to support. The event will focus on "green scientific research" and "red search", focusing on ecological protection and rural revitalization in the Yellow River Basin, and strengthen the study of party history education in the search for red stories."Since 2007, north forest play young students in the construction of ecological civilization, spread green culture, building a harmonious society leading, demonstration role, joint 50 universities jointly launched the national youth green long march activities, accumulated more than 30000 college students involved, affect hundreds of thousands of people, has achieved a wide range of social influence."President of Beijing forestry university, the Beijing forestry university of the Yellow River ecological protection and high quality development institute, said, this year's research practice into, active docking, active service in the Yellow River ecological protection and high quality development major national strategy, with higher stance, more real measures, shoulder the Yellow River basin ecological protection and high quality development, for the party and the country to cultivate more support for the Yellow River ecological protection and high quality in the field of development of the future talents. Wang Zhen, deputy director of the Publicity Center of the National Forestry and Grassland Administration, and Ai Shuigen, deputy director of the Social Liaison Department of the Central Social Liaison Department of the Communist Youth League, jointly awarded the flag to the Youth Green Long March Yellow River Basin Scientific Research and Practice Group. Wang Lina, representative of teachers and deputy secretary of the Youth League Committee of Lanzhou University, Wang Heng, representative of teachers and Party Secretary of Ordos Vocational College of Ecology and Environment, and Wu Tong, representative of the Yellow River Basin Scientific Research Practice Group, spoke respectively. Yin Weilun, academician of the Chinese Academy of Engineering and former president of Beijing Forestry University, gave a special guidance report entitled "Thoughts on ecosystem protection and High-quality Development of the Yellow River Basin" to the teachers and students online, which kicked off this year's scientific research practice. It is reported that up to now, 28 colleges and universities have set up 497 teams, and more than 5,000 people have gone to 9 provinces and autonomous regions in the Yellow River Basin to carry out practical scientific research activities through the combination of green scientific research and red search. Since 1984, Beijing Forestry University has successively launched the "green consulting", "green bridge", "green Bridge", "green Long March", "A4210" and other well-known environmental protection practice activities for college students in the capital and even the whole country. Every year, the school sets up more than 300 social practice teams to go to all over the country to guide the youth league members to temper their skills in practice. In 2021, with the theme of "Always follow the Party", Beijing Forestry University will set up seven special projects, including youth to the Party, green Long March and rural revitalization, with a total number of more than 3,600 participants. Among them, 106 teams of 650 people went to nine provinces and autonomous regions along the Yellow River Basin to carry out scientific research and practice activities.

2021-07-26	Yangzhou Vocational University: Li Chao, reporter of China Youth Daily) " The panda house is a one-way route, please go according to the signs on the ground."Under the scorching sun, the two volunteers still stood upright and greeted each tourist with enthusiasm and smile. They are Zhang Yuxin and Liu Yunan, volunteers from Yangzhou Vocational University, and the responsible area is the most popular panda house. The 2021 International Horticultural Expo opened in Yangzhou on April 8. As the university with the largest number of volunteers, Yangzhou Vocational University has sent 500 volunteers to serve here for two months since late May. During the service period, the volunteers successfully completed the volunteer service work of each post with full mental outlook and excellent professional quality, which was fully affirmed and highly evaluated by the organizing committee and tourists from all over the world. The scorching sun in early July scorched the park, and the panda house was more enthusiastic than the temperature of that day. Before the morning period of work is over, Zhang Yuxin's voice has been slightly hoarse, and their original white faces were baked a piece of red marks, the neck and arms also appeared a clear black and white dividing line, sweat soaked the clothes. In addition to guiding the visitors, they kept giving customized small fans to the park, hoping to bring a touch of coolness to the tourists. After the large crowd receded, only one old man took the children to watch the panda. The child was deeply attracted by the charming panda, regardless of the drops of sweat flowing down. At the moment, did not guide the task of Zhang Yuxin picked up a small fan behind the old man and children fan. The cool tourist looked at them sweating profusely and gave them a thumbs-up. When the head of the volunteer department of the Expo sent the video to the school, Zhang Yuxin said that this is what young volunteers should do! Guidance, security check, answer questions... In the World Horticultural Expo Park, the volunteer service work of all positions is being carried out in an orderly manner, and the volunteers of Yangzhou Vocational University have also been proficient, providing warm and professional services for the tourists. Wei Qi, from the School of Foreign Languages of the university, is a member of the publicity group of "Red Gene Party History of Yangzhou". In Jiaxing Garden, where she serves, it is a popular red culture punching place. This also provides Wei Qi with an excellent opportunity to publicize the knowledge of party history. Every day, facing many citizens and tourists who came to feel the revolutionary spirit, Wei Qi took the initiative to introduce the party history of Yangzhou Party to them after doing her own work well. Volunteers from the tourism management major of the School of Tourism give full play to their professional expertise, get familiar with and accurately recite the display content of the service park in advance, and strive to explain the more comprehensive and profound design connotation and traditional cultural background of the horticultural landscape to the citizens and tourists. In the process of explaining again and again, they also deeply felt the extensive and profound traditional culture of the Chinese nation, and their pride arises spontaneously."Although the volunteer service period is only two months, our preparatory work will start in the second half of 2020, with early planning, more consideration and quick solution to ensure the service quality of the volunteers in an all-round way."Wang Miao, deputy secretary of the school's youth League committee, said that the school has purchased rechargeable desk lamps to ensure the orderly development of volunteer service work on the premise of ensuring that students can complete their academic tasks. From that day on, we prepared iced mung bean soup for volunteers every day, so that volunteers could feel the attention and care of the volunteers as soon as they got off the bus. The school also organized a three-day volunteer training from March 17 to 19. Through the training, the volunteers had a detailed understanding of the history of the Expo, the overview of the Yangzhou Expo, how to provide quality services, and how to deal with complaints."No matter which position you are in, we will meet the challenges with the best appearance and highlight the responsibility and responsibility of the young people in the new era!"Xu Jinru, deputy secretary of the Party Committee and vice president of the University, said," It is both a great honor and an experience to undertake the volunteer task. As the successors of the socialist cause, young students should have the courage to undertake the responsibility and mission entrusted by the new era and show their passionate youth in the process of volunteer service.”

2021-07-26	Shaanxi energy vocational and technical college practice in Tongchuan summer social practice in shaanxi energy vocational and technical college practice "Ming founding in one hundred, social practice" summer social practice member for China youth network Beijing on July 26 (reporter zhang, reporter NiGuoBin) recently, shaanxi energy vocational and technical college "era" practice in Tongchuan with the theme of "Ming founding in one hundred, show social practice true knowledge" summer social practice. During the activity, the practice group traveled to 2 districts and 3 towns in Tongchuan, traveled a total of more than 400 kilometers, visited 3 red revolutionary education bases, visited 2 villages and 1 community, and carried out nearly 20 special education and practice activities successively. The activity was widely praised by the practice units and the residents, signed the summer social practice base agreement 3, held the summer social practice base awarding ceremony 3, received 7 letters of thanks, including 3 units, 4 individuals, received 1 banner. The picture shows the members of the practice group giving a free medical treatment for the villagers of Mengjiangyuan Village. The correspondent practice group successively came to Zhaojin Red Revolutionary Base, Chuankou Community, Wangyi District, Mengjiangyuan Village, Mazui Village, Yaozhou District and other places, and went deep into the fields and communities to carry out party history publicity, health science popularization, free clinic service, interview and research and other activities. On the basis of completing the practical activities, Li Yunnan, a national outstanding Communist Party member and Party branch secretary of Mazzui Village, Du Tianxiang, the "advanced individual of grassroots theory propaganda" of the Propaganda Department of the CPC Central Committee in 2018, and Zheng Xiaojun, a rich expert of Mazzui Village, were interviewed to further review the glorious history of poverty alleviation.

2021-07-24	A doctoral service team composed of more than 20 doctoral students on Sichuan-Tibet Railway construction was established in Southwest Jiaotong University. It is reported that this is the first doctoral service team in China to serve the construction of the Sichuan-Tibet railway, which went to Tibet for the first time on July 22. The first members of the doctor service group from multiple disciplines, professional, including geological engineering, surveying and mapping science and technology, civil engineering, bridge and tunnel engineering, mechanical engineering, electrical engineering, environmental engineering, rail transit information and control, transportation, logistics engineering, high wet environmental medicine, Chinese language and literature, marxist theory, etc. Southwest Jiaotong University set up a Sichuan-Tibet Railway construction doctoral service group. Southwest jiaotong university for figure southwest jiaotong university youth corps committee secretary jun-qi zhang is introduced, the doctor service will be normalized into the Sichuan-tibet railway construction related unit research, according to the demand to provide targeted scientific research, technological innovation and humanistic services, at the same time to strengthen the spirit of "two road" and the Qinghai-tibet railway spirit study, research, preaching, comprehensive power Sichuan-tibet railway construction. It is reported that the doctoral service team has gone to Tibet on July 22 to carry out the first practical service. They will visit relevant design and construction units, visit experts in related fields, and carry out research and exchange, on-site investigation and theoretical publicity. The construction of the Sichuan-Tibet railway is a major strategic deployment made by the CPC Central Committee and The State Council based on the overall situation and with a long-term view. It is a century strategic project for China to build itself into a strong transportation country. The Sichuan-Tibet Railway has complex topographic geology and climatic conditions and fragile ecological environment, which is called the most difficult super project in the history of human railway construction. Southwest jiaotong university school of earth science and environmental engineering doctoral supervisor Zhao Xiaoyan said, Sichuan-tibet railway across the first and second ladder in our country, cross the transverse mountain, site mountain canyon, geological disasters is very developed, building difficulty is rare, the construction of Sichuan-tibet railway is not only our national strength, but also the highland of scientific research innovation and talent training. Southwest Jiaotong University is the birthplace of higher education in civil engineering and transportation engineering in China. It has a complete discipline system and comprehensive advantages in the field of rail transit in China. It is located at the starting point of Sichuan-Tibet Railway, and has unique conditions for scientific research and engineering practice services. Shen Huoming, deputy secretary of the Party Committee of Southwest Jiaotong University, said that the establishment of the doctoral service team is intended to give full play to its advantages in disciplines and talents in the field of rail transit, and lead young students to make contributions to the construction of the Sichuan-Tibet railway.

2021-07-24	The volunteer service team of Fuzhou Vocational and Technical College carried out the activity team went to Wushi Town, Zixi County, Fuzhou City to carry out the "three to the countryside" activity. In the Wushi Town Central Kindergarten, the volunteers helped the children to establish a firm awareness of saving food through the theme of "advocating conservation and anti-waste". After class, the children reflect on whether they have any waste phenomenon by practicing the "CD Action", and promise to be the practitioner, model and disseminator of thrift. In order to let the children intuitively understand the knowledge of environmental protection, the volunteers help the children to build environmental awareness through the form of "acting, speaking, painting, doing" and other forms. Volunteers and children made clothes from discarded colored cards and did an environmental runway show. Children wear their own designed "new clothes", enjoying the results and fun of "turning waste into treasure". Volunteers also guided the children to express their most true feelings for the Party and the motherland through collage, gratitude classes, party history education classes and other forms.

2021-07-23	Southwest jiaotong university graduates party members pay a "special dues '" southwest jiaotong university graduates party members pay a "special dues' " Wang Xinxin this graduation season, southwest jiaotong university school of electrical engineering undergraduate course 2017 five party branch jointly organized a special DangRi activities-through the sale raised a special "dues '" to the college party committee, all the party members to end their college career. At the recent graduation ceremony of the class of 2021 students of the School of Electrical Engineering of Southwest Jiaotong University, the total "special party fee" of 1,557 yuan was officially paid to the Party Committee of the School of Electrical Engineering. A person in charge of the Party committee of the School of Electrical Engineering of Southwest Jiaotong University said that the "special party fee" explains the love of the Party, carries the gratitude of the party organization, and is a affectionate confession from the students on the 100th anniversary of the founding of the Communist Party of China. The idea of raising "special party fees" through the charity sale stems from an earlier "mentoring" activity launched by the college. Zhang Yi, a counselor at the School of Electrical Engineering at Southwest Jiaotong University, said that starting from 2020, graduate party members will collect their books in the college affairs center for free use from lower-grade students. However, from the affairs center feedback, the work was not as effective as originally thought. Zhan Xiangwen, the party branch secretary, found that not many junior students knew or responded, resulting in a low utilization rate of books. After consultation, the party branch decided that the graduate party members retained the form of old books unchanged, but the circulation method was changed to through flea markets, idle fish, campus vendors and other channels for secondary sales, the sales income as a "special party fee". This idea has been echoed by many graduates of 2021, including party members and classmates, and has also served as a joint party day activity for the five Party branches of the college at the 2017 undergraduate level. Through the education lectures of each party branch and the publicity of the class qq group, the senior students of the School of Electrical Engineering left the old books, and set up the old things donation points at the entrance of the boys 'dormitory and the girls' dormitory, collecting books, clothes and other items that could not be taken away but need to be dealt with after graduation. Wang Chuang, the secretary of one of the party branches, recalled the sorting of the items, and said that everyone was very motivated, and that the donated items and books were almost piled up. The graduate party members also set up a book flea market in the campus living area, mainly for the lower grade students in the school of electrical engineering, at a lower price, "almost half sell, half give away."The charity sale lasted for two days. The first day was a stall in the school park, and on the second day, it sold the remaining items through a recycler. On the day of the charity sale, the party branch informed the junior students in the college to start the charity sale around 8 p. m. Wang chuang said that as student party members and volunteers of the charity sale, they did not expect the students to respond so well at the beginning of the activity. In addition to the lower grades in the college, students from other colleges came over to participate."It felt very good to see the sales amount increase gradually and slowly exceed my expectations."Zhan Xiangcheng said that he was in charge of collecting the money on the day of the charity sale. At first, he expected to earn 300 to 400 yuan, but he raised more than 800 yuan in more than two hours, raising a total of 1,557 yuan in two days. Wang Chuang said that the charity sale allowed the students to choose the books they needed from the large number of professional books, which not only reduced the cost of buying new books, but also realized the second use of resources, killing two birds with one stone. Wang chuang said that when he gave the party fee to the Party committee, he had a sense of responsibility as a student party member."On the graduation, through such a form to contribute their own strength, but also left a good memory to the youth."

2021-07-23	Chen Qiang, reporter of China Youth Daily) " Wow! Look at this peach, red and white, so beautiful ah! These are we just picked from the tree, very fresh, eat sweet extraordinary oh! Worthy of the gutian Baifeng peach, love love!"Recently, the" Beautiful Village "special social practice team of the School of Culture and Communication of Putian University came to Changling Village, Gutian County, Fujian Province to carry out the live broadcast activity of" Strong Love in the Peach Garden, Integrating media to promote innovation " to help rural revitalization. It is understood that every Changling village family planting peach, currently there are 7000 mu of peach trees, lotus plum 5000 mu. At the invitation of Changling Village College Students' Innovation and Entrepreneurship Base, 8 teachers and students of Putian College came to conduct a three-day social practice activity to help the peach industry of the village become famous and expand its brand influence. In temporary studio-long ridge village rural cultural center, they set up live equipment to introduce thou farmland specialty peach, lotus plum and color and color, taste and picking, then on the way to pick peach led the audience to visit the qing dynasty star national treasure residence, national treasure park, sweet cemetery, etc., by introducing the local beautiful ecological environment, simple local custom folk way, the depth experience of promoting propaganda."The 80-minute live broadcast 'lead tour' allows teachers and students to enter the countryside, combine the professional knowledge of news and advertising, have an in-depth understanding of the implementation effect of the rural revitalization strategy, and contribute to the rural revitalization of Gutian County."Said LAN Yu, secretary of the Youth League Committee of the School of Culture and Communication of Putian University.

2021-07-23	Fujian college students summer "a program under which officials" into a line of community correction college students summer "a program under which officials" into the community correction line can Li Jiapeng) to assist the community correction work smoothly, on July 10, Fujian agriculture and forestry university to pingtan comprehensive experimental area "national conditions" summer social practice team in pingtan judicial office community correction center, held by "growth, communication" as the theme of the briefing, briefing is divided into two in the afternoon, area two area of more than two hundred community correction object attended. The members of the practice team took the inspirational stories of community correction as the entry point, and admonished the three types of community correction objects of different ages, with typical transformation cases, to actively face the correction and move into a new life. In the process, the practice team members play the professional relevance, targeted psychological counseling to the community correction objects, and eliminate and reduce the potential conflict and vigilance of the community correction objects. On this basis, the members of the practice team stressed the necessity of abiding by discipline and law, and encouraged the community correction objects to actively learn laws and regulations and epidemic prevention knowledge, give full play to their own subjective initiative, and rebuild their life order. Xiao Li (pseudonym), a community correction subject who was suspended for drunk driving, said: " The lecture made us feel the good intentions of the correction center. We can think calmly and rationally about our mistakes and feel a lot. People only have calm reflection, will have unexpected harvest."" social work is a core course of my major. Such a practical course in advance is helpful for me to better master the theoretical knowledge and apply what I have learned. At the same time, this activity also encourages us to pay attention to the special group of social correction objects, and to integrate the personal value into the career needs of the society."Su Xufei, a member of the practice team at Fujian Agriculture and Forestry University in 2019, concluded.

2021-07-22	Hebei University of Science and Technology 67 social practice teams of Hebei to help rural revitalization " special practice activities, set up 67 practice teams to help rural revitalization. From July 3 to 12, the "Light of Science and Technology" research team went to Zanhuang County, Luancheng County, Pingshan County and Shijiazhuang Youth Palace to carry out popular science publicity activities. Nearly 300 teenagers felt the colorful and charm of science and technology in the activities. From 3D printing, three-view drawing tips, the programming secrets behind the music fountain, to the significance behind garbage classification and the handmade production of the "intangible cultural heritage" hairy monkey, the public welfare class adopts an open learning mode to guide teenagers to understand the world and think about the world from a scientific perspective. From July 6 to 18, the star research team combined social practice with subject research, and went to Wen'an County, Langfang City to investigate the treatment of pesticide packaging waste, to help improve the agricultural and rural ecological environment. The practice team traveled to more than a dozen villages, such as Ji Village, Mazhuang Village and Zhao Village, and popularized the harm of improper treatment of pesticide packaging waste and rural ecological environment through field visits and questionnaire surveys, and collected data and villagers' attitudes and opinions on the treatment of pesticide packaging waste.67 Social practice team fanned out in the Midwest, ethnic minority areas and less developed areas of the country, through investigation, research, insight into the current rural development, combined with professional knowledge to solve practical problems, at the same time to collect and excavate the rural revitalization of social governance, volunteer service, cultural inheritance, innovation in the fields of youth representatives, typical demonstration in the youth. Wu Jing, secretary of the Youth League Committee of the School of Mechanical Engineering at Hebei University of Science and Technology, who has led teams to guide students 'social practice for six consecutive years, said the social practice aims to improve college students' ability to adapt to society and solve practical problems with what they have learned, and enhance their sense of responsibility and mission.

2021-07-22	Students of Nanchang University have carried out the summer practice of "remembering the development of Party and historical medicine" in Gansu, Qinghai and Jiangxi provinces respectively. In Gansu province, the practice team of "remembering the Development of the Party Long March" Victory Memorial Hall, to understand the difficult process of the Long March of the Red Army, Party members face the party flag, review the party membership oath; listen to the revolutionary martyrs "remember the education of revolutionary tradition to enhance the humanistic quality of college students in the New Era" theme lecture, and talk around the spirit of the Long March. Subsequently, the practice team went to Lanzhou No.5 Hospital to learn the rules based on the specifications of clinical medication in the hospital medication, shared the report on the application of microneedle patch in the drug vehicle system, and provided volunteer services such as blood pressure, blood glucose measurement and oral examination for the community residents. In Qinghai, the team members visited the local rural revitalization; participated in the publicity and education activities on animal and plant protection, and had a preliminary understanding of the ecological resources, the daily work of the managers and the monitoring of researchers using modern science and technology. In addition, the team members also visited and practiced the green space ecosystem, combined with professional knowledge, focusing on understanding the local vegetation species, the latest grassland ecosystem related scientific research results, common medicinal plants and their effects. In Jiangxi province, the practice team of "Pharmaceutical Jiangxi Journey" went to Ruijin, the capital, to study in the Yeping Revolutionary site group and the site of the Second Soviet University.

2021-07-22	Jiangsu university students to Yancheng rural to carry out the franco-prussian and practice Jiangsu university students to Yancheng rural to carry out the franco-prussian and practice figure China youth network Beijing on July 22 (reporter Zhang Jianwei, correspondent Ding Wenwen) to guide the young college students in the social classroom education, long ability, contribution, contribute to help rural revitalization of youth strength, recently, the law school of Jiangsu university team into Jiangsu Yancheng constant north village, launched a series of the franco-prussian and social practice activities. We see villagers browsing the Rural Revitalization Promotion Law. Correspondent for figure in the local farmers' home, team members to the villagers presented including "civil code" rural revitalization promotion law ", legal text and brochures, to the villagers popularized the civil code real right and marriage and family related legal knowledge, such as real right for the protection of the interests of the villagers, homestead inheritance problems, etc., to help the villagers to solve the specific legal problems encountered in life. The picture shows the enterprise staff consulting the students about legal issues. Correspondent for figure in constant north village original hot spring resort hotel, team members asked the hotel managers about company profile, legal needs, land contract management and sales contract disputes, etc, for enterprise staff consulting private lending, team members also from the loan contract limitation of limitations, litigation, preservation solutions, and prompt employees to improve legal awareness. The picture shows the students explaining the leaflets to the villagers. In the rural fields, the team members addressed some legal problems troubling farmers, such as the relevant matters related to the conclusion of supply and marketing contracts, combined with the display boards and leaflets, and made farmers understand how to use the law to avoid risks to safeguard their legitimate rights and interests. This practice activity of popularizing the law and benefiting farmers not only improved students 'ability to use professional knowledge to solve specific practical problems, but also strengthened students' efforts to learn legal knowledge and contribute to the comprehensive promotion of the rule of law.

2021-07-22	Jiangxi University of Finance and Economics to build party history learning education "red learning circle" project system operation youth league branch to promote- -Jiangxi University of Finance and Economics to build party history learning education "red learning circle" will collision out what kind of sparks? In April this year, the original scene stage play "Jiangshan Love" was premiered in the auditorium of Jiaoqiao Campus of Jiangxi University of Finance and Economics. The play is interspersed with important historical events such as the Sanwan adaptation, and the 20-year-old "we" and the 20-year-old "they" go across time and space to have a youth dialogue. After visiting the red exhibition hall, 100 young students from the two youth League branches of the "Spark" and "Liaoyuan" of Jiangxi University of Finance and Economics went to the Jinggangshan National Youth Revolutionary Traditional Education Base to carry out the red day reading activities. In this red museum without walls, young students stop at pictures, objects and stories; pursue the footprints of revolutionary martyrs in experiential teaching on marching, weaving straw sandals and homemade Red Army meals. Study up the mountain, go down the mountain into the play. Through the red search, Jiangcai students wrote, directed and performed it by themselves to welcome the centenary birthday of the Party with this well-made red stage play. Counselor Xie Er Ali was deeply touched: " The historical reproduction of the melodrama and the wonderful interpretation of the stage play make the party history learning and education from the scripted to the immersive. In Jinggangshan, I and my classmates visited the historical relics of the world; under Jinggangshan, I and my classmates jointly perform the vivid lesson of party history learning and education."Since this year, the Youth League Committee of Jiangxi University of Finance and Economics to the project system operation as the starting point, has launched the" Jiangshan sentiment "scene stage play," speech under the national flag " theme group day and a number of party history learning and education activities, the circle of numerous fans. Project system operation to promote the party history learning education deep go real as early as 2018, the school youth corps committee to explore in the school to establish project work system, and eventually at the school level planning set up thought lead, the second classroom, grassroots, volunteer service, aesthetic education, innovation practice, six project team, attract secondary college teachers, student backbone within the framework of the project team of the specific operation of a work. Xia Xianfeng, secretary of the Youth League Committee of Jiangxi University of Finance and Economics, introduced that after the establishment of the project working mechanism, some of the special league affairs of the university was subcontracted to the grass-roots league organizations through the "unveiling of the list" in the form of the project system, which strengthened the linkage between the university and the integration of resources. According to the Party History Learning and Education Activity Program of the Youth League Committee of Jiangxi University of Finance and Economics, the University Youth League Committee has customized 13 characteristic Party history learning and education activities for all the youth league members of the school, and six of them are operated in the form of project system. The history of the party learning education, Jiangxi rich youth corps committee continue to play to the project system operation organization advantages, to build to build six project team, cultivating dozens of characteristic projects, build hundreds of youth corps branch, cultivate tens of thousands of backbone, driving students as the basic framework and implementation path of the "millions" project work system, live party history story, with live red resources, innovative education form. Speech, study Tours, movie viewing, reading groups, red cultural creation, stage plays... How to transform the quantity improvement of party history learning and education activities into quality improvement? The school Youth League committee adheres to the problem-oriented, held many student symposiums, let the young students talk about the feelings and experience of participating in the party history learning and education."Only when students become the main body of the history of the Party, can they truly draw strength from the history of the Party!"A youth representative said at the symposium. Jiang CAI youth is not only the main body of learning, but also has become the main force of speaking. Under the national flag, a party history learning and education activity called "one hundred hundred hundred days" is continuing. The speaker and organizer of the activity are the representatives of the league members from the grassroots youth league branch. The Youth League Committee of Jiangxi University of Finance and Economics has integrated "Party History Every Day" into the ideological and political brand of "National Flag Rising Every Day" for 31 years, organizing 100 youth League branches to carry out the theme of "Speech under the National Flag", and 100 graduate party history lecturers have made 100 party history lectures under the national flag."At 7 o'clock in the morning, the five-star red flag rose slowly in the passionate national anthem, which is the most beautiful pattern on campus in the morning. Learning the history of the Party is the best way to open up a new day."Finance and taxation 2019 class 1 league branch secretary Fang Zhinan's experience is that under the national flag of the party history propaganda, a sense of ceremony more affinity. In Jiangxi University of Finance and Economics, the class league branch, community league branch, student union league branch, big bone class league branch and other grass-roots youth league branches undoubtedly play a main role in the party history learning and education, which comes from the school youth League committee "all work to the branch" and "resources supporting to the branch" work concept. In March this year, the school started the party history learning and education "100 excellent youth League branch" application, set up 100 youth League branches, and supporting a series of resource support measures. The youth League branch seriously carry out the party history learning education theme youth league day, and actively participate in the school youth League committee party history learning education "youth learning", "youth said", "youth line" three modules of the series of activities, you can apply for a project to become the party history learning education "100 excellent youth League branch"."The threshold is not high, many opportunities to declare conditions attracted many league branches to take the initiative to uncover the registration."Said Ye Chenghao, head of the student union. Up to now, the whole school has 100 youth league branch formally approved. The establishment of "100 excellent youth League branch", let the party history study education direct "organization end". In order to further deepen the learning results, the Youth League Committee of Jiangxi University of Finance and Economics will select 30 of the 100 "excellent Youth League branches" to further establish "ma Project Student Project" practice projects and give financial support, so that the Youth League branch will burst out more vitality. In order to explore the new model of party history learning and education theme, the school Youth League Committee introduced the "Party History Footprint: Four History Learning and Education Sandtable Course", and held the first demonstration teaching on April 13. This course simulates the "four histories" historical events of the party history footprint, etc. According to the real historical background of the "four histories", the "four histories" is studied by means of situation simulation and sand table deduction. The teachers and students also went to the ruins group of Fujian, Zhejiang and Jiangxi Soviet Area in Shangrao Hengfeng County, Hengfeng Revolutionary Martyrs Memorial Hall and Fang Zhimin Memorial Hall and other places. Through the restoration and display of the venues, they personally realized the great practice of planning economic construction, arranging and commanding battles in these simple adobe rooms. And in Hengfeng County, opened the red cultural creation special training class. Jiangxi is the cradle of the Chinese revolution and the place where the military flag was raised. This red land has nurtured the Spirit of Jinggangshan, the Soviet area and the Long March spirit, and carries the original mission of the Chinese Communists. Relying on the red resources around it, the Youth League Committee of Jiangxi University of Finance and Economics opened up the "second classroom" according to local conditions, organized youth league members to explain on site and experience the "classes" outside Jinggangshan, Yiyang and Geyuan, and carried out more than 20 immersive practical teaching activities."One, two, or even ten or 20 activities have become the campus burst, which does not mean that the party history learning and education has a large enough coverage and sustainability. The establishment of a working mechanism can ensure that this work is carried out regularly, so that students can make a difference in the broad extracurricular space and network space."In the process of guiding students to carry out the party history learning and education activities, Liao Chunyan, deputy secretary of the school Youth League Committee, gradually thought deeply on the project system work system. In order to guarantee the quality of one hundred party history XuanJiangTuan, marxist institute organization teachers and students wrote "my heart to the party-red land 100 classic party history story, establish" source check, collective preparation "quality assurance system and" self education, to promote learning " education mechanism, implements the party history learning education and discipline construction, the depth of the talent training fusion."Come to Jiangcai, you must go to the Kerui cultural museum to get a card."The renovated four-story building, which gathers the university history hall, the alumni hall and China's first tax ticket museum, is now a" fixed classroom " for a series of party history learning and education activities. The school gives full play to the role of the new era civilization practice center in the museum, simplifies the borrowing and approval process, and provides convenience for students to use the site to carry out distinctive and diversified special activities such as special guidance, Party history publicity and volunteer service. Project system operation does not represent the operation of the project team. In the party history learning and education series activities, Jiangxi finance youth league committee temper "inner brain", "party history footprint" party history learning education situation simulation sand table course demonstration class teaching, let teachers and students to learn more spiritual strength; introduce "outside brain", invite experts and scholars responsible for the characteristic project approval, project review, put forward professional suggestions for the project system, steadily promote the implementation, management, assessment and acceptance of each characteristic project. The school Youth League Committee also provides specific guidance for the operation of the project system through the preparation of work standardization manuals. During the study and education of Party history, it has compiled and completed many working manuals such as the youth League daily guidance manual on the theme of "Speech under the National Flag". China Youth Daily China Youth Network reporter Chen Zhuoqiong, correspondent Fu Quan source: China Youth Daily

2021-07-21	Summer practice team of Henan university of technology to carry out the red research activities of Henan university of technology three rural practice field to expand practice middle school understanding jianwei correspondent Zhou Beibei) to guide the young students out of the campus, to the social feeling red culture, red spirit, good combining theory and reality "education", in the social classroom education, long ability, contribution. The School of Civil Engineering (Architecture) of Henan University of Technology has sent a number of social practice teams to various places to carry out the red spirit search, research and publicity activities. The picture shows Wang Maogui, the veteran commentator of the current site of HuaShanzhai, explaining the Long March process of the 25th Red Army. The site of the Huashanzhai Conference in Guangshan County, Xinyang City, is the source where the 25th Red Army held a meeting and decided to implement the strategic transfer. Huashanzhai is the beginning of the arduous Long March of the 25th Red Army, and also an important turning point for its future and destiny. During the practice, members of the "Xingguang" practice team interviewed Wang Maogui, an 85-year-old anti-American aid to the DPRK and the current volunteer commentator of the Flower Shanzhai Site. In the interview, the old man said, " Without the Flower Shanzhai Conference, there would be no Long March of the 25th Red Army."When asked why he has been staying here for more than 40 years, the old man said," I insist on being a volunteer commentator here, because I just want to let more younger generations understand the red spirit of the revolutionary martyrs, and let the public know that the better life has not come easily."The picture shows the" starlight " practice team members reading the oath. China Youth Network correspondent Yuhang photo "starlight" practice team members also jointly visited the flower shanzhai conference site- -the 25th Red Army military command post."Here is very shabby", which is the first impression of the team members of the site, and it is in this shabby earthen embryo house, the 25th Red Army opened the prelude to the great Long March. The practice members said, " Under the leadership of the Communist Party of China, the 25th Red Army overcame numerous difficulties, and countless revolutionary martyrs shed their blood and became the first team to successfully reach northern Shaanxi. This arduous history needs every citizen to carry forward and inherit. The picture shows the practice team doing the experiential research assistant work and publicity work. The Huaihai Campaign is the most important battle of the People's Liberation Army, with the largest number of enemies, the greatest political influence and the most complex war style. The "Qingma" community "original aspiration" Party building studio of Civil Engineering (Architecture) College went to the Huaihai Campaign Memorial Hall in Xuzhou City, Jiangsu Province to carry out experiential research. On the basis of the preliminary docking, the practice team members serve as the research assistants in the museum, respectively responsible for the organization of a part of the activities in the museum and the guidance of the research tour team. To memorial to carry out the research activities of the youth team under the guidance of practice team field visit around annihilation Huang Wei corps, battlefield rest, battle victory, four parts of content, after experiential studies, studies tutor, assistant and studies swim youth with "for the people" huaihai campaign spirit theme scene party class.

2021-07-21	Group photo of Southwest Jiaotong University Graduate Basic Education Group relay Help Qin'an Basic Education Association. Correspondent for figure China youth network Beijing on July 21 (xinhua Ji'an-wei zhang, correspondent Zhu Hui) recently, southwest jiaotong university qinan education support work symposium held in Gansu province, southwest jiaotong university, 22,23 graduate volunteers volunteers transition, relay in qinan to carry out a one-year basic education teaching volunteer service work, and more measures in the designated help work power qinan education career development. According to reports, Southwest Jiaotong University will start to send volunteers from graduate volunteer teaching groups to Qin'an County Qiaonan Middle School and Xingguo Middle School to provide one-year volunteer teaching services in 2020. Over the past year, the first batch of six volunteers have adhered to their original aspiration of volunteer teaching and poverty alleviation, actively participated in grassroots education and teaching, and tried their best to teach and solve the children. Teaching for poverty alleviation, practice education, leading the fashion, they complete the basic education teaching work at the same time, also give full play to their respective advantages, in the teaching school guidance to establish student organizations, guide students kechuang practice, rich and colorful campus cultural activities and "dream dilemma students for one", "the" warm winter action "," teaching to the countryside "," read a book " book donation and other public welfare activities, they take concrete actions to drive volunteer spirit relay, but also in the children heart sow the seeds of love. During the discussion and exchange, the leaders of Qin 'an County Education Bureau, County Youth League Committee, Qiaonan Middle School, Xingguo Middle School and other units expressed high recognition of the contributions and achievements made by the volunteers of Southwest Jiaotong University in their dedication to the basic education cause of Qin' an with heart and affection. At the meeting, Zhang Junqi, secretary of the Youth League Committee of Southwest Jiaotong University, handed the flag to the graduate teaching group and pointed out in his speech that As an outstanding representative of the contemporary youth, I hope the volunteers of the new graduate volunteer group: to firm their ideals and beliefs, Integrate their own ego into the motherland, the people's ego of the ego, Better to realize the value of life; To make unremitting struggle, Teach every lesson carefully, correct every homework well, organize every activity well, Give full play to their strengths to do a good job of volunteer teaching; Take on the responsibility of The Times, Cherish this great era and shoulder the mission entrusted by The Times, Experience in responsibility, grow in responsibility, With practical actions to contribute to the development of Qin'an education cause of the youth of Jiaotong University. At the meeting, also held the "youth growth" youth summer camp flag awarding ceremony. It is reported that the summer camp, funded by the Sichuan Provincial Science and Technology Poverty Alleviation Foundation, supported 30 students in Qin'an County to go to Chengdu for a one-week summer camp from July 24 to 30.

2021-07-21	Guizhou University College of Science and Technology in 2021 "Three to the Rural" summer social practice service group to Jianwei, Guizhou University correspondent Jiao Cheng Chen Jiayu Yang Changming) " Three to the countryside, we go! Please rest assured that a strong country has me!"With the loud slogan shouted by the members of the practice group, on the morning of July 19, the 2021" Three to the countryside " summer social practice service group of Guizhou University College officially went to Heai Primary School in Dalong Village, Yaishui Town, Huishui County, Qiannan Prefecture to carry out social practice activities."College students should use their own enthusiasm, intelligence, knowledge and skills to serve the society and the grass-roots level, temper themselves, and contribute their youth strength where the people need it most."Fan Wei, secretary of the Party Committee of the School of Science and Technology of Guizhou University, urged the delegation to educate the teachers and students of the delegation, strengthen the history of the Party and follow the Party in a broader world. Before arriving at Heai Primary School, the summer social practice group of College of Science and Technology of Guizhou University arrived at the People's Government of Yasha Town to hold a discussion and exchange meeting. Huishui county water town party committee member, chairman of the National People's Congress dawn, town party committee member, deputy mayor Yang Shengpeng, Luo Yingfen and related departments, head of the water town "four big card", population composition, traffic conditions, covering area, make a brief introduction, make practice members have a preliminary understanding of elegant water town, have a certain understanding of industrial resource chain. It is reported that the seven teams of the "Three to the countryside" summer social practice service group of the College of Science and Technology of Guizhou University will carry out a 10-day volunteer service activities in the aspects of party history study, rural revitalization, theory publicity and volunteer teaching.

2021-07-19	College students of Jinzhong Institute of Information Technology feel the rural charm of the new era. China Youth Network, Beijing, July 19 (reporter Zhang Jianwei, correspondent Liang Jing and Feng Jiahui), in order to understand the rural construction, Exploring the road of rural revitalization and Party building and development, Strive to achieve the great goal of "science and technology, culture, health" to the countryside, An investigation into rural development in the new era, On July 10th-16th, Shanxi Jinzhong Institute of Information Jicheng Academy new youth village research and observation group into Shuixiu Village, Jinzhong City, Shanxi Province, Launched a seven-day summer vacation "three to the countryside" trip, Explore the road of rural revitalization of the ancient city of Jin merchant culture, Feel the rural charm of the new era under the national prosperity and development. It is reported that the New Youth Research and Observation Team of Jicheng Academy is composed of 15 volunteers. Through the century-old party building process, it focuses on rural revitalization and explores the current situation of rural development in the new era. Understand the development process and current situation of Shuixiu Village by visiting the net red wall and chess competition; by communicating with party members, village cadres and villagers, listen to the struggle meaning behind village revitalization; design creative graffiti, give sachets and DIY, and contribute to village construction with their professional knowledge."Through this practice activity, I not only have a deeper understanding of the development process of Shuixiu Village, but also have a deep understanding of the significance of the activity of college students going to the countryside."Zhang Yuyan, a member of the research team, said that she will continue to take an active part in social practice activities, explore the mysteries of the development of The Times, and use her professional knowledge to contribute to the society.

2021-07-19	Shaanxi Industrial Vocational and Technical College took multiple measures to promote the party history learning and education of the Communist Youth League members, go deep, heart and practical members and guidance report. Shaanxi industrial vocational and technical college for figure China youth network Beijing on July 19 (reporter Zhang Jianwei, correspondent Zhao Shuangjun) to promote the youth study party history, strong faith, follow the party, shaanxi industrial vocational and technical college closely combining the reality of the school, adhere to the five education, the overall planning and classification implementation, do online and offline propulsion, organic collection of learning and practice, innovative learning education form, promote the party history learning education go deep. Classified implementation, Deepening the three-dimensional inheritance of the red gene combined with the actual situation of the school Communist Youth League, The Youth League Committee of the school formulated the implementation Plan of "Learning Party History, Strengthening Faith, and Following the Party", Clear target requirements, schedule arrangement and key tasks; Taking the establishment and cultivation of the first batch of youth league building demonstration universities in Shaanxi Province as an opportunity, Establish the three-level youth league organization linkage mechanism of school, school and class, Held "learn the party history strong faith to follow the Party" learning education mobilization and guidance report, Early mobilization and early deployment of learning and education, Plan in advance, continue to promote, and implement it well; Relying on the second classroom report card network platform, Through the " youth learning, three meetings, two systems and one lesson, the league evaluation first incentive, social practice, volunteer service and other carriers, Explore a good way to study party history, Combine the study and education of party history with the promotion of business work organically, To ensure that the two levels of the league organization prescribed action "gesture" clear ", Choose the optional action "clear direction". The picture shows winning the first prize in the national "Challenge Cup" red special final. In addition, the school adheres to the "five integration", takes multiple measures at the same time, implements classified implementation, and integrates the party history learning and education into the "ideological guidance, campus culture, team construction, key work, publicity and education". Relying on the statistics of "Smart League Building" system, as of July, more than 530 leagues in the school have carried out the learning and education activities of "Learning the Party and follow the Party". The online participation rate of "Youth Learning" has exceeded 75%, ranking among the top higher vocational colleges in Shaanxi Province. School also through organizing the whole school youth league branch to carry out the "youth to the party power" "learning party history strong faith with the party" "always follow the party, set sail new journey" theme TuanRi activities, "red film history in one hundred" theme practice, "have revolutionary historical monument, noble revolutionary spirit" tomb-sweeping day activities, let the youth members in the activity thick plant love party, patriotic feelings, inheriting red gene, draw spiritual strength. Hold brand cultural activities such as "green rhyme, praise the party grace" grass music festival, "dancing the new era youth offer the party grace" campus dance competition, so that students in the artistic practice subtly understand the power of faith, ceaseless red blood. The implementation of the system of "league cadres on the platform" was implemented, held the training of league cadres' ability improvement and league cadres quality expansion activities, docking the provincial competition, held the "100 league cadres on the history of the Party" quality and ability competition, 116 teachers and students of the league cadres actively participated. Relying on the Challenge Cup "National College Students extra-curricular Academic Science and Technology Works Competition, young students were organized to retrace the red footprints, trace the red memories, interview the red characters, dig the red stories, and understand the red culture, and won the first prize in the red special final of the National" Challenge Cup " class competition. The publicity matrix of "network, micro, screen and end" was constructed. The new media of the Youth League Committee opened the columns of "Youth study of the History of the Party" and "Qingxin Radio Reading Red Letters", and produced more than 10 youth League cadres telling the history of the Party, so that the "tide of Party history learning and education" rose ". The picture shows "Always follow the party to forge ahead in the new era" party history knowledge final. Shaanxi industrial vocational and technical college for figure features, singing always follow the party theme the secondary college league using "red originality" educational resources, online linkage, with "immersive" "experiential" and "interactive", widely in young students content colorful, valuable, positive, healthy, lively and effective publicity and education and practical activities. Among them, the Youth League General Branch of the School of Mechanical Engineering carried out the keynote speech contest of "Learning the glorious spirit, gathering the strength of youth" to remember the red history, recall the extraordinary years, and help students to "button the first button of life". The General Branch of the Youth League of the School of Electrical Engineering carried out the party history knowledge competition of "Always follow the Party and forge ahead in the new Era". Through knowledge contest, party history telling, expert comments and other ways, it created a good atmosphere of striving to be the first and catching up. The School of Aeronautical Engineering has carried out a series of activities of "100 years of the Founding, generation to generation" to interview 100 outstanding teachers and party members. By listening to the original aspiration of old party members to join the Party, the red gene is implanted into the blood of students. Trade and circulation league branch of the college held the micro marathon, "celebrate the red gene", through the miniature "long march" track, let the students in running for red memory, feeling the great long march spirit....... the picture is "non-toxic youth, healthy life" anti-drug publicity and education activities. With the practical activity of "I do practical things for the students" as the starting point, the satisfaction and sense of gain of the youth league members are taken as an important yardstick to evaluate the effectiveness of the party history learning and education of the Communist Youth League. Hold youth league members symposium, listen to the aspirations of the youth league members, focus on the general needs of the youth and the practical difficulties of youth, establish a list of practical projects, do practical things, do good things for the youth league members. We will deepen the implementation of the "second class transcript" system, investigate the problems in the second class transcript system, add the "six hundred" education action module, revise the identification standards of the second class transcript hours, achieve more scientific and comprehensive record certification, more optimized and convenient operation process, and help young students grow up in an all-round way. Focusing on the needs of the problem, we carried out anti-drug publicity and education activities of "non-drug youth and healthy life" to improve students' awareness of identifying, resisting and anti-virus drugs.

2021-07-21	Shandong taishan college of science and technology college students: trace mountain village red light Shandong taishan college students: trace mountain village red year network correspondent Song Kai China youth network Beijing on July 21 (reporter Zhang Jianwei, reporter Chen Chuang Zhang Weixu) Shandong taishan college of science and technology "salute 1332" party history study practice on July 17, came to Jinan Laiwu district cattle spring town pigeon building village to take root base, go deep among the masses of a program under which officials social practice, common red footprint, inheriting red gene. With the theme of the team, "Party history learning and education", To record "the" earth-shaking new changes in the old site of Shandong Provincial Working Committee " as the main line, Through the form of "I tell you the history of the Party" to show the red story of the base pavilion; Red Movie in Geflou Village Square, Reviewing great films, Understanding the revolutionary spirit; Interview with senior party members, front-line workers of base construction, principals of venues and heads of the village committee, Listen to the changes in the new countryside under the influence of base construction from different perspectives; The whole process of shooting "smile under the Party flag" as tribute to the centenary of the Communist Party of China; Visit the grass-roots people and support the "I do practical things for the masses" activities, Help the villagers to renovate the fields, pick up firewood, repair the field paths, Providing practical services, At the same time, distribute and explain the party history party and government brochures, questionnaire survey to understand the impact of the construction of the old site of the provincial working Committee of Gelou village. Shandong Taishan University of Science and Technology "pay tribute to 1332" party history study and practice group in the observation and practice of a new understanding of social practice, the team members said that they would listen to the teachings of the revolutionary predecessors, learning and thoughts into practice, the party's story widely and continuously told to more people.

2021-07-18	In 2021, Guizhou University for Nationalities will go in the summer vacation, gathering its youth and gratitude. On July 15th, 2021, the launching ceremony of the summer culture, science, technology and health activities of "three to the countryside" was held in the lecture hall of the library of Guizhou University Town Campus. Chu Guangrong, Secretary of the University Party Committee, Zhang Pengcheng and Yang Shengguo, Deputy Secretary of the University Party Committee, and Ren Jie, member of the University Party Committee, Minister of the Publicity Department and the United Front Work Department attended the meeting. The main principals of the school office, the student work department and the school Youth League committee, the main principals of the party committee of each school, the leaders of the college Youth League committee, the instructor of the social practice team and the representatives of more than 100 members of the practice team attended the meeting. Li Jianling, deputy secretary of the school Youth League Committee, presided over the ceremony.site of activity. Party Secretary of the University Party Committee Chu Guangrong awarded the flag. Zhang Pengcheng, on behalf of the school Party committee and the administration, encouraged the teachers and students who will go to the grassroots to participate in social practice: learn the party history, strengthen faith, follow the party; learn vision and skills in social practice; promote unity, warm hearts and make contributions in social practice. Hope that the majority of young students carry forward the spirit of bearing hardships and standing hard work, give play to the advantages of knowledge and skills, full of enthusiasm, dedicated service, do good things, do the practical work, truly do their own exercise, the masses benefit, in the hot social practice to serve the people's mission, earnestly learn the history of the party, strong faith, follow the party. Hu Bin, deputy Secretary of the Youth League Committee of the University, made a report on the preparatory work of the social practice activities of the summer culture, science, technology and health activities of Guizhou University for Nationalities in 2021.take a group photo to mark the occasion. At the ceremony, representatives of the national and provincial teams made a statement.(Article / Photo: Pan Xiaolu)

2021-07-17	The founding ceremony of the Capital University Science and Technology Volunteer Service Corps was held in Beihang University. Beijing, July 17 (reporter Li Chuan correspondent Ding Ruiyun) On July 12, the establishment ceremony of the Capital University Science and Technology Volunteer Service Corps and the 2021 Capital College "youth serving the country" student social practice ceremony in Beijing University was held in Beijing University of Aeronautics and Astronautics. Party member of China association for Science and Technology, office secretary of Wang progressive, Beijing party secretary Li Junhui, China association for science and technology popularization vice minister Liao Hong, party member of Beijing association for science and technology, vice chairman Chen Weicheng, vice secretary of Beijing municipal party committee of li, Beijing university party secretary cheng bo guests, Beijing municipal party committee, Beijing association for science and technology and representatives of Beijing university teachers and students of more than 300 people to attend the event. The activity was presided over by Li Jian, deputy secretary of the Communist Youth League. Before the event, Cao Shumin, Secretary of the Party Committee of Beihang University, met with all the guests and talked on volunteer service, social practice and science and technology innovation education. At the event, participants watched the video of the Capital University Science and Technology Volunteer Service Team. Participants jointly launched the establishment of the Capital University Science and Technology Volunteer Service Corps. China youth network correspondent Diegret for figure Cheng Bo said that the capital university science and technology volunteer service team will be committed to gather the capital university science and technology volunteer service resources, to "green and Beijing" for traction, into the campus, into the community, into the countryside, into the grassroots, to serve the people, with innovation to create contribution country, help the people such as youth scientific quality improvement. At the same time, Beihang University, as the secretariat unit of the corps, will fulfill its duties, do a good job in service guarantee, jointly promote the sustainable development of the corps with various universities, and make the social practice activities of the youth in colleges and universities in science and excellent. Li Junhui said in his speech, set up the capital university science and technology volunteer service team, is to further deepen the "green and Beijing" work of important measures, aims to promote scientific and technological innovation and "youth service country" social practice brand project of organic integration, realize kechuang education and practice education in common, youth success and youth achievements together, talent training and service society. At the same time, he also raised three hopes to the students, first, deeply feel the magnificent history of the Party, and further strengthen the party and love the country, through social practice, further stimulate the mission of The Times, and through social practice, the comprehensive image of Chinese youth, further help the Chinese and foreign people. Wang progressive in his speech, pointed out that college students science and technology volunteers is an important part of science and technology volunteers, today in China science and technology volunteer service corps launched the capital university volunteer service corps, aims to further coordinate university science and technology volunteer service resources, for rural revitalization and science and technology power gathering youth power."First, we should promote the scientific spirit and popularize scientific and technological knowledge. Second, we should tell scientific stories and establish scientific ideals. Third, we should use the achievements of science and technological innovation to help rural revitalization."Wang said that college students' science and technology volunteers have good scientific literacy, sincere feelings of home and country, and a strong responsibility. I hope that students can better realize the value of life in the science and technology volunteer service, and be worthy of the new task of national rejuvenation. Wang progressive and Li Junhui presented flags to representatives of the Capital University science and Technology Service team and the "Youth Service Country" social practice team from 20 universities including Peking University and Tsinghua University. China youth network correspondent, Diegret for figure from renmin university of China science and technology volunteer service, Beijing normal university, the youth games have my practice, Beijing university of Aeronautics and Astronautics inheritance flame of the three students representatives speak respectively, introduces the basic situation of their schools, team, and on how to better carry out the volunteer service of science and technology for the outlook. Student representatives of the Capital University Science and Technology Volunteer Service Corps took the oath. They said that they will abide by the constitution of the corps, fulfill the obligations of science and technology volunteers, carry forward the volunteer service spirit of "dedication, friendship, mutual assistance and progress", and strive to realize the Chinese dream of the great rejuvenation of the Chinese nation. The activity ended successfully with the loud singing of "No Communist Party, no New China". Before the ceremony, Li Junhui and his delegation also investigated the construction and development of the Communist Youth League of Beihang University and the innovation and entrepreneurship of students. It is reported that the capital university science and technology volunteer service team from Beijing university, tsinghua university, Beijing university of aeronautics and astronautics and other universities more than 20 teams, they will through conducting volunteer service of science and technology, help solve the remote areas, poor mountainous areas and old revolutionary base areas popular science work imbalance, insufficient problems, for rural revitalization and science and technology power construction in the youth of colleges and universities.

2021-07-21	Henan university of technology and the huaihai campaign memorial to practice education base of Henan university of technology and the huaihai campaign martyrs memorial tower administration to build students party history learning education base jianwei correspondent Zhou Beibei), Henan university of technology and the huaihai campaign martyrs monument administration to build the party history learning education base signing and opening ceremony was held in Xuzhou. Leading Party members of Huaita Administration Bureau, Education Exchange Department, Publicity Department of the Party Committee of Henan University of Technology, relevant Party Committee of the College of Civil Engineering (Architecture) and all practical members of the "original aspiration" Party building studio attended the signing ceremony. Civil engineering (construction) college deputy party secretary wang di pointed out that the school, the school will party history learning education and students social practice organic integration, organize youth marxist community members to revolutionary museum, red education base, patriotism education base to carry out social practice, really make social practice into a solid theoretical study, touch the soul of political baptism, coagulation hearts meet the spirit of refinement "ideological classroom", hope to practice team long ability in practice, long ability, long knowledge. Zhou Yongjie, member of the Party group and deputy director of the Huaita Administration Bureau, introduced the basic situation of the Huaihai Campaign Memorial Hall and the development process of the park in recent years. He said that the joint construction of the school is only the beginning, and he hoped to carry out in-depth cooperation in the protection of red buildings and ideological and political drama performances. After the speech, the two sides signed the school-construction agreement and jointly inaugurated the party history learning and education base. The base is the first party history learning and education base set up by Henan University of Technology, and also the first practical education base jointly built by universities in Henan Province and the Huaihai Campaign Memorial Hall.

2021-07-17	Nantong college students 'social practice help "dream house" fly out of the golden phoenix Nantong college students' social practice power "dream house" fly out of the golden phoenix green net reporter li chao) on July 13, Jiangsu Nantong communist youth league "dream transformation" + "care plan" heart yue classroom " class and Nantong university social practice base opening ceremony held in Nantong tongzhou district xingren town. On the same day, the "Jianghai Xinyue" Dream child care service station of Nantong University was inaugurated. Since October 2020, the Jiangsu Provincial Party Committee of the Communist Youth League has launched the "Dream Transformation +" care plan, focusing on nearly 10,000 virtually homeless children in the province, a key youth group in distress. Held in March this year in Jiangsu province "dream hut" centralized construction propulsion meeting, Nantong university "jianghai heart yue" team as the first university in the province team selected "dream transformation +" care plan, the scene released a "heart yue classroom" care services, online fusion heart yue small classroom, heart yue summer camp and other activities. In the past four months, the "Jianghai Xinyue" team of Nantong University has recruited more than 30 volunteer service teams. The steps of teachers and students have been spread across 48 old revolutionary base areas across the country, giving deep and multi-dimensional care and help to no supported children in the old revolutionary base areas, which has received good social response. Nantong university party committee member, vice President hou fully affirmed the Nantong university "jianghai heart yue" team "dream +" summer social practice of early results, think this is the school actively implement the general secretary xi "July 1" important speech spirit and important discourse about education of concrete action, is to implement the important task of the "khalid ents" important measures, is to carry out the party history learning education vivid practice."We must bear in mind the general Secretary's instructions and encouragement to the young people, amplify the effect of social practice, and ensure that the practical results are truly reflected in doing practical things for the people.'Jianghai Xinyue' enabling 'dream cabin' can fly out of the golden phoenix, and can make due contributions and efforts to the development of the whole society."The relevant person in charge of Nantong municipal party committee said, Nantong university in tongzhou district set up social practice base and service station, is the guarantee of deepening the" school cooperation "innovative achievements, for the city to consolidate" dream + "" 1 + 3 + 6 "care service system," dream + " the second half of the love article provides a reference, can promote typical experience. Up to now, the League Nantong Municipal Party Committee has raised 3.86 million yuan online by relying on the Su Qingyi fundraising platform, and pledged 95 "dream cabins" offline. All 207 "dream cabins" in Nantong have been completed, and 465 "factual orphans" in the city have all implemented the "one person, one gear" under the "1 + 3 + 6" care and service network of youth league cadres at the city, county and township levels, and conduct one-to-one pairing to promote volunteer service projects.

2021-07-16	Anhui university of Chinese medicine youth teachers and students to send health knowledge to Anhui university of Chinese medicine youth teachers and students to send health knowledge to the grassroots wang haihan) recently, Anhui university of Chinese medicine "a silver needle a originality" summer social practice team came to Hefei new high-tech zone, community, acupuncture, acupuncture, massage, cupping, ear and the demonstration professor, first aid technology demonstration professor, common acupressure professor, and bring Chinese medicine health sachets, medicinal diet experience, traditional Chinese medicine health tea experience. At the same time, the volunteers specially prepared health knowledge brochures to publicize the symptoms of cervical spondylosis, periarthritis of shoulder, lumbar disc herniation and other common diseases, prevention, rehabilitation and exercise. After the popularization of health free diagnosis and first aid knowledge, the volunteers also brought a carefully rehearsed fitness Qigong wuqinxi, which led the residents to perform action decomposition exercises together. For the people with mobility difficulties, the volunteers, under the leadership of the modian community staff and the community new home public welfare organization, went into the people's homes to carry out diagnosis and treatment, giving drugs and sachets for epidemic prevention and health preservation."We provide meticulous diagnosis and treatment services for the people, and integrate traditional TCM health care knowledge into people's daily life."Team leader Li Yifan regrets."We have also specially prepared the heat and summer heat flower tea, dietary diet."Qi Yajun, the team led the teacher, the initiator of the free clinic activity and the most beautiful volunteer in China, said that the volunteer service has been carried out for many years, providing more opportunities and platforms for young students to perceive the grassroots, apply what they have learned, and integrate knowledge and practice. In order to normalize, standardize and institutionalize voluntary services, on July 14, the School of Acupuncture and Massage of Anhui University of Traditional Chinese Medicine and the Modian Community Management Committee jointly built a social practice base for Anhui University of Traditional Chinese Medicine college students.

2021-07-20	Guizhou university: promote both youth exchanges spread red spirit blood Guizhou university: promote both youth exchanges spread red spirit blood-2021 Macao college students to qian red culture learning experience activities officially opened China youth network Beijing on July 20 (reporter Zhang Jianwei, correspondent, yan-chun liu, Song Hongqing Liang Qian) on the morning of July 17, Jointly organized by Guizhou University and The University of Macao, the 2021 Macao college students' red culture learning and experience activity and the gift of Macao return- -the opening ceremony of "Come sweet from Hard" was held in the Mian School of Culture Academy, Guizhou Province, Hong Kong and Macao Affairs Office, The Hong Kong, Macao and Taiwan Office of Guizhou University, the University Youth League Committee, 16 student representatives from the University of Macao, and more than 60 students from Guizhou University attended the meeting. University of Macao student representative Lin Weiqi expressed the university of Macao students of the red culture learning experience activities, hope that through this activity can learn and experience the red army long march spirit, through the establishment and continuation of culture and art, to build the development of the national youth bridge, together to carry forward and inherit the great spirit of the Chinese nation. Zhang Peiqi, the student representative of Guizhou University, said that he hoped that through the red culture learning and experience trip, we could strengthen the exchanges and cooperation between the youth of the two places, take drama performances as a bridge to enable the young people of the two places to blend together because of art, and take the opportunity of red Guizhou to review the great history of the Party and appreciate the glorious course of the Party. The four students of Guizhou University introduced the situation of Guizhou province from the geographical location, natural resources, cultural characteristics, red tourism and other aspects, which increased the understanding and understanding of the students from the University of Macau. The picture shows the student representatives of the two schools giving each commemorative gifts. After the opening ceremony, students from the University of Macau and student volunteers from Guizhou University visited the Cultural Academy together and carried out ice-breaking exchange activities. In the afternoon, students from the University of Macau visited the University History Hall and library of Guizhou University to further understand the historical evolution, faculty strength, academic contributions and foreign exchanges of Guizhou University. As the opening ceremony, 2021 Macao college students to qian red culture learning experience activities and Macao return gift- "sweet" national tour activities officially launched, it is understood that in the following activities, the university of Macao students will perform in Guizhou university small theater "sweet" drama, to Zunyi conference site, Gou dam meeting site visit red attractions, depth experience of red culture.

2021-07-15	Guizhou university, 159 teams more than 2700 teachers and students in rural revitalization of Guizhou university 159 team more than 2700 teachers and students in rural revitalization youth network Beijing on July 15 (reporter Zhang Jianwei, correspondent, Liu Yanchun Song Hongqing) on July 14,2021 summer "a program under which officials" social practice ceremony in Guizhou university, school related functional departments, summer 2021 social practice led teachers and practice team representatives attended the ceremony. Li Jianjun, secretary of the Party Committee of Guizhou University, awarded the national key team project approval certificate to the colleges and units awarded by the national key team project, and awarded flags to the summer social practice team representatives in 2021. It is reported that in the summer vacation of 2021, Guizhou University will set up 159 social practice teams in the "three to the countryside" social practice activities, covering more than 2,700 teachers and students of undergraduate, master's and doctoral students, as well as teachers and professors. Among them, there are 9 national key teams, 53 national special plan teams, 42 provincial special plan teams and 55 university-level special plan teams. The picture shows the departure ceremony scene. In this year's preparation work, the teachers and students of Guizhou University closely combined with their professional characteristics and advantages, closely around the party history study and education strategy, and formed a red, specialized and accurate rural revitalization strategy. Among them, the rural revitalization practice team represented by the "Challenge Cup" national competition team, the "ego into the big me, the youth to the motherland" to celebrate the first time, and the "Star Spiritual Angel Service Team" and "Funding Ambassador" in cooperation with the external resources, and the Youth League Committee of Guiyang City and Huaxi District to form the "Civilized Tourism Publicity Practice Team". After the launch ceremony, more than 2,700 teachers and students from 159 teams of Guizhou University will go to the motherland to devote themselves to rural revitalization.

2021-07-15	Jinzhong Institute of Information college students immersive experience rural representative representative election Jinzhong Institute of Information college students immersive experience rural representative election China Youth network Beijing July 15 (reporter Zhang Jianwei, correspondent, Wang Yingbo Xie Yuqian) July 13, During the 2021 national summer culture, science, technology and health "three to the countryside" social practice activity, With the theme of "Reform and Deepening Reform", Seriously study the unique "socialist rural collective property rights system with Chinese characteristics" in Xiaoyi Village, Xiaoyi City, Deep excavation " farmers citizens shareholders new people enjoy golden profits, Rent and salary shares more gold to benefit the people " behind the profound connotation. The election of shareholders is directly related to the construction of grass-roots economic organizations and social stability in Luyi Village. After visiting the village history development wall of Luyi Village and reviewing the development process of property rights system reform, the team members were deeply curious, which simulated the election meeting of Xiaoyi Village in Xiaoyi City. Players in familiar with the relevant process, turned into a "villagers", came to the simulation, in the entrance to sign in and receive votes, shareholder election, by the "director" read the specific rules, the family representatives introduce their family situation, check by the "supervisor", people election, announced the election results and issued the shareholders, then shareholders representative election, sing the vote, vote, monitor and the results of the public. Finally, the shareholders raised their hands to express their trust and expectations for their representatives. The immersive experience of "simulating and restoring the election of shareholders 'representatives of village-level organizations" makes the contemporary young college students have a better understanding of the reform of China's rural politics and economic system, and be firm in their ideals and beliefs, and serve to witness the reform and development of China's rural revitalization.

2021-07-15	The Central China Normal University has promoted the study of Party history.) Since March this year, The central China Normal University graduate student volunteer teaching group has carried out the "seven one" Party history learning education, That is, by "reading a book on Party history", "watching a film on Party history", "singing a classic red song", "listening to a special lecture", "visiting a red scenic spot", "paying tribute to a revolutionary martyrs" and "participating in a volunteer service", Truly achieve "learning history and understanding, learning history to increase credit, learning history to worship virtue, learning history to practice", We will effectively promote the study of party history and education to be sincere, deep, and solid. The central China Normal University Graduate Support Group of Supporting Education has called on all members of the group to read the original work, learn the original text and understand the principle. At the same time, we also strive to innovate the learning way, enrich the learning carrier, through watching red movies, singing classic red songs and other ways to let the party history story into the eyes, ears, brain, into the heart, really learn to think, thinking, understanding, understanding. In May, the Xinjiang Branch of the 22nd Research and Branch Group of Central China Normal University organized the "Seven One" sharing meeting on Party history learning and education. At the meeting, the players are early reading party history book a brief history of the communist party of China exchange, captain Gao Ruikuo share: " we should consciously adhere to the latest achievements of marxism, the scientific theory into understanding the world, transform the world's powerful material force, with fresh rich Chinese practice to promote the continuous development of marxism, gradually realize the great rejuvenation of the Chinese nation."At the sharing meeting, the team members reviewed some clips of the party history drama TV series" The Age of Awakening ", and sang the classic red song" Without the Communist Party, No New China " together. In Heshan Middle School of Yuqing County, Service County, the Guizhou Branch set up a "learning" study club with the history of the Party as the main task, leading local young students to learn the history of the Party and understand their ideas, and to train a group of advanced and firm middle school students. The study club actively participated in the county "youth home" related activities, focusing on telling the stories of party members, carried out party history publicity activities in the sinking community, and assumed the responsibility of party history learners and disseminators. The 23rd research group to take the combination of online and offline, called on everyone to read the party history works and held a reading sharing meeting, the members actively exchange their experience in the party history reading, share the party history story to guide the members to feel the great spirit of the party in various historical periods. In addition, the members of the research branch group also carried out the activity of singing classic red songs together, and 27 members sang "singing a folk song to the Party" together in their hometown. Learning the party history story is a "required course" for the members of the research group. In order to further promote the party history learning and education, it is necessary to explore the red resources of the volunteer teaching places and schools, and create a good "second classroom" of party history learning and education. To this end, the members of the service places consciously went to the local red education venues to visit and study, on a good and vivid party history "experience class". Members of the Xinjiang detachment went to visit the Museum of Bortala Mongolian Autonomous Prefecture to see the cultural relics. They felt the patriotism of the troops to the army and the establishment of the autonomous region. The team members were not only attracted by the long history presented in the museum, but also moved by the pioneering spirit of the reclamation generation, determined to contribute their contribution to the national unity and social development of Xinjiang. The members of the Guizhou detachment visited the Breakthrough Wujiang River Memorial Park and the site of the Zunyi Conference successively, and reviewed the great feat of the Red Army in breaking through the Wujiang River and the great turning point at the Zunyi Conference. While fully excavating the red resources everywhere, the research team of Central China Normal University actively invited well-known experts and teachers from the school and in the volunteer teaching places to organize several special lectures on party history, bringing a great "research class" of party history study and education to the members. Li Xiaoping, secretary of the General Party Branch of Bole Senior High School, gave a special party history lesson to the members of the Xinjiang detachment. She combined with the specific examples of the development and growth of the Communist Party of China, let the team members see the Communist Party of China dare to adhere to the truth, the spirit of self-innovation. The members of Guizhou Branch actively integrate party history education into daily teaching and bring party history learning education to the classroom. When serving as an ideological and political teacher, captain Yu Yifei prepared his lessons carefully and brought vivid ideological and political lessons to the students, explaining the history of the Party to the students and why the Communist Party of China could. In the theme group class of the volunteer teaching place, the team members introduced the outstanding alumni of Central China Normal University, the Chinese proletarian revolutionary, one of the leaders of the early youth movement of the Communist Party of China, and led the young students to have "patriotism, reported national conditions, strong ambition" through the story of the party history figures. Out of the school, the players also went deep into the community and yuqing county change of poverty alleviation relocation product good community to build "China erudite hall" at 4:30 class, to carry out "big hand holding hand, childlike innocence to the party", "concentric direction, young painting dream" theme education activities, in the form of popular, sow the red seeds in the hearts of the children. The Yunnan Branch participated in the youth League Day activity of "Party building, Youth League building, Learning and Growth together" held by the local Communist Youth League County Party Committee, listened to the speech of Li Xiaojuan, secretary of the Communist Youth League County Party Committee, and learned the special report made by Tang Xiaobo, director of the Party School Office of the Mouding County Party Committee. In this study class, the team members really learned the history of the Party, read the Party constitution, improve the party spirit, improve their own party spirit cultivation, strengthen the political literacy. Relying on the resources of the school, the 23rd Research Branch Group invited well-known experts and scholars to give special lectures for its members. Zhao Lingyun, secretary of the Party Committee of Central China Normal University, sent a message to the members of the research group in the party history learning and education activity and the launching ceremony of the 23rd Research Group: " This year coincides with the centenary birthday of the Party, and the students marching at this time has a symbolic significance and a special mission."When talking about the expectations of the members of the research branch group, he proposed that everyone could do a good job of spreading the red culture and the creator of the red spirit in the volunteer education area. Good recall the ancestors "moral education class", remember the struggle process "learning history Chongde, learning history practice", the party history study must be engraved in the heart, practice in the line. In order to ensure that the party history learning and education really came into the mind, the central China Normal University graduate volunteer teaching group carried out activities to commemorate the martyrs around the Qingming Festival, and worked together to thank them and recall the "moral education class" of the revolutionary ancestors. During the Qingming Festival, the Xinjiang Corps detachment went to the Bortala Memorial Park to remember the martyr Xu Yanchao. In the solemn atmosphere, all the staff in front of the martyrs monument to the martyrs to pay high respect and infinite grief. At the school, the Bole team learned the glorious history of the Party and the deeds of the revolutionary ancestors by watching the video "Eleven Books to Witness the Original Heart and Mission", reviewing the oath of joining the Party, and observing it in silence and reciting it. During the Qingming Festival, the members of the Guizhou Team organized the youth league members of the service school to carry out the activity of "learning the History of the Party and offering sacrifices to heroes" in the Yuqing County Revolutionary Martyrs Cemetery, leading the youth league members to pass on the revolutionary spirit from generation to generation. This year marks the 90th anniversary of the sacrifice of yun Daiying, a famous revolutionary alumnus of Central China Normal University. During the Qingming Festival, the members of the 23rd research branch group participated in the central China Normal University "remembering the martyrs to pay tribute for the fire inheritance" young students to honor the revolutionary martyrs yun Daiying ceremony. The members of the research branch group remembered the revolutionary martyrs, inherited the legacy of the ancestors, from the glorious deeds of comrade Yun Daiying drew infinite strength. It is essential to take the "practice lesson" of volunteer service, practice the study and education of the Party history, and serve the people. Only by implementing the achievements of the study and education of Party history can we constantly ask our original aspiration, constantly adhere to our mission, and truly achieve our original aspiration as a rock and the mission on our shoulder. The research and branch group of Central China Normal University has integrated the party history learning and education into the volunteer service, giving new significance to the work of each team. On May 23, members of the Xinjiang Bole Branch of the research group went to the fourth Congress of the Bortala Mongolian Autonomous Prefecture of the Chinese Young Pioneers to carry out volunteer service. The conference strongly stimulated the simple feelings of the young Pioneers to the Party and the socialist motherland, and the members of the Bole team contributed themselves to the strength of the young volunteers to the complete success of the conference. Also in May, in the fifth volunteer public welfare project of "Building Dream China University and Walking with Love" launched by Guizhou Branch, the team members organized the young students of the service school to visit Wuhan Central China Normal University. The visiting delegation came to the Wuhan Revolution Museum to experience the red culture of the heroic city of Wuhan. At the end of March, the members of the 23rd Research Branch Group carried out a volunteer service activity of "Wash all the lead in China for You" to clean up the statue of our ancestors and inherit the spirit of Chinese teachers. Twenty-seven members went to yun Daiying, Light Weiwei, Tao Xingzhi and other ancestors of the statues, carefully wipe away the dust for the ancestors, through this way to express their gratitude and thoughts to the revolutionary ancestors. In mid-June, in central China normal university to celebrate the 100th anniversary of the founding and 2021 graduation party, the 23rd research team members to prepare and participate in the poem "let the youth bloom in the struggle of brilliant flower", "to the grassroots, to the west, to the motherland most need place" "with a long time, do a lifelong unforgettable" "to youth of me, dedication country, keep in mind the entrust, not mission", a sonorous oath, show research team members aspire to western education career dedication youth beginner's mind and determination.

2021-07-14	Hunan University of Science and Technology "Three to the countryside" practice group in-depth carried out "I do practical things for the masses" activity Zhao Zhiying Beijing, July 14 (reporter Zhang Jianwei, correspondent, Cao Wenxing, Zhao Zhiying, Liu Zixuan) July 11, Hunan University of Science and Technology summer "three to the countryside" practice group went deep into various areas of Yongfa Village to carry out the "I do practical things for the masses" series of activities, In a colorful form, in a kind and warm way, Send knowledge of culture, science and technology, and safety to the masses, Promote the summer "three to the countryside" practice activities to carry out in depth. In order to improve the safety consciousness of the villagers and children, volunteers into primary and secondary schools, cultural square population gathering point to promote drowning safety knowledge, broadcast drowning project education propaganda, lead and teach the villagers square dancing, play red movie series of activities, heart hard to solve the villagers 'difficulties, the troubles of the masses, enhance the people's sense of security. The picture shows the electrical appliance obligation maintenance. China youth network correspondent zhi-ying zhao for figure activities on the same day, "a program under which officials" volunteers into the home inspection and repair circuit aging, in the village committee pendulum "voluntary maintenance" activities, free to help villagers repair fans, board, rice cooker, induction cooker and other small household appliances, with professional knowledge practice "I do the practical work for the masses" mission and bear, to let the masses in the "home" enjoy huimin service. Next, Hunan university of science and technology summer "a program under which officials" practice group will continue to carry out the "I do the practical work for the masses" practice, practice "culture, science and technology, health" to the countryside responsibility mission, strive to do the practical work, open a new bureau, carry out local custom civilization, agricultural products packaging design, left-behind children support and other practical activities.

2021-07-13	Henan Agricultural University social practice service team to carry out anti-drowning publicity activities Henan Agricultural University School of Animal Medicine practice team to carry out the "anti-drowning" publicity activities Jianwei correspondent Lu Yilin) recently, Henan Agricultural University social practice service team in Hebi city to carry out the "anti-drowning" safety education special activities. Team members took to the streets to help the residents enhance the safety awareness of drowning, master the knowledge and skills of drowning, and improve the ability of self-rescue and mutual rescue. During the lecture, the team members introduced the safety knowledge of drowning prevention to the public through the form of questionnaire survey, introduced the principle of "six short time" in detail, invited local children to deeply understand the drowning incident, and sounded the alarm for them. At the same time, the team members also broadcast homemade promotional videos to the general public. During the lecture, the students expressed their views and feelings in an orderly manner. Team members suggested that citizens should go to regular swimming places. Children under 15 children should be accompanied by their parents to guide citizens to cherish life, grow up healthily, prevent drowning and care for their families. After video publicity and members' explanation, the team members distributed anti-drowning leaflets and anti-drowning safety knowledge questionnaires to the residents, and hung banners and slogans in dangerous areas and more obvious areas to carry out anti-drowning publicity and education in the research area, calling on everyone to improve their awareness of prevention and cherish life. Finally, the members of the publicity group organized the public to conduct the "drowning alarm bell often ringing, safety education in mind" signature activity, and the participating citizens have solemnly signed their names on the propaganda banner. Volunteers issued publicity pages and warned the drowning accidents in other areas, calling on parents to earnestly fulfill their responsibility of guardianship while reminding their children to consciously stay away from dangerous waters, and work together to do a good job in the safety education of students.

2021-07-12	Chizhou college: let young students in "between" appreciate the party history of Chizhou college: let the young students in "between" appreciate the party history in one hundred reporter wang haihan) recently, Anhui Chizhou college held to "witness glorious history stamps show one hundred glory" as the theme of stamp exhibition, let young teachers and students learn the history of the party in the stamp exhibition story."Square inch, inclusive the world; small pieces of paper, read a hundred years of wind and rain. Many stamps have never been seen in our generation, and such exhibitions are very interesting and instructive."Said Chen Yu, a 2019 real estate development and management student at Chizhou University. The event lasted for four days, with 15 boxes of nearly 500 stamps on display. From the "epoch-making" when the red boat, a big site, to the "founding ceremony" of tian'anmen, China table, fireworks, to "sail" in the Renaissance high iron, daxing airport, stamps condensed the great achievements of the communist party of China in one hundred, recorded the party unity led the Chinese people the extraordinary years of arduous struggle. The activity was jointly organized by the Publicity Department of the Party Committee of the university, the university Trade Union (retired work office), the university Youth League Committee, and the Chizhou branch of China Post Group Co., LTD.

2021-07-15	Tsinghua Academy of Navigation jointly submitted a series of low-carbon College and Fudan University of Microelectronics to carry out a series of characteristic Party history learning and education theme activities Jianwei) Recently, 192 Party Branch of School of Aeronautics and Astronautics, Tsinghua University, 201 Party Branch of Tsinghua University, Temporary Party Branch of Tsinghua University and Practice Branch of COMAC Test Flight Center, We jointly carried out a series of characteristic party history learning and education theme activities with the 2019 Student Party Branch of the Sino-British International College of Shanghai Jiao Tong University, the Party Branch of the 2020 Graduate students and the 2020 Handan Party Branch of the School of Microelectronics of Fudan University. This activity carried out the secretary of the party committee (party) party class, teachers and students chorus a song, went to Shanghai to the communist party of China a site and visit the memorial site of the communist party, party development communication, three party history project reading sharing, to Jiaxing in a big cruise and visit the south lake revolution memorial hall, young alumni symposium and other series of activities. Mr.Cao Bingyang, secretary of the Party Committee of Tsinghua Aviation Institute, first took the lead in telling the first party class with the theme of "Start a prairie fire, stay true to the original aspiration". Through the founding of the communist party of China and in the great revolution, in the land revolution war in rural road surrounded the city, in the history of the history of the party, to show the communist party of China from the turmoil to led the national people won the final victory of the great course, at the same time, introduces the history of tsinghua navigation courtyard and navigation courtyard in the unremitting efforts, called on the young comrades to enhance responsibility consciousness, work enthusiasm, scientific research work hard. Mr.Wang Anhua, deputy Secretary of the Party Committee of Fudan University of Microelectronics, gave a lecture with the theme of " 'core' fire research and serving the country". Mr.Wang first told about the magnificent history of the Communist Party of China since the reform and opening up, and introduced the Fudan Institute of Microelectronics in the torrent of reform and opening up, seize the opportunity of The Times, facing the key technology fields, overcome one difficulty after another, and constantly create new brilliant. Mr.Wang encouraged everyone to take history as a mirror, further enhance their ideals and beliefs through learning the history of the Party, and constantly strive for the great rejuvenation of the realization of the Chinese nation. Mr.Xu Min, deputy secretary of the General Party Branch of the Low Carbon College, brought the third course of a series of party lessons with the theme of "three new and one high, bravely bear the mission". Mr.Xu introduced the basic situation and important contents of the previous Party congresses and the achievements of the CPC in leading the people since the founding of new China, and talked about the mission of universities in the new era. He urged everyone to be committed to independent innovation, study hard, make unremitting efforts, and make their own contributions in their respective professional fields.

2021-07-14	The Party Committee of the School of Science and Technology of Guizhou University held a material donation ceremony to carry out the Party building and poverty alleviation activities. Jiang Bayi Primary School held in horseshoe Town, Bozhou District, Zunyi City. Correspondent for figure China youth network Beijing on July 14 (reporter Ji'an-wei zhang, correspondent, Song Huixin Yang di) recently, Guizhou university college of science and technology party committee to zhunyi sowing state area horseshoe town of party construction for poverty alleviation activities and held a symposium, for horseshoe town center school students brought bags, pencil case, football and other love materials 520 pieces (a). Fan Wei, Party Secretary of Guizhou University School of Science and Technology, Pan Xin, Deputy Secretary of Party Committee, Zuo Zhangquan, Deputy Secretary of Party Committee, Zhao Ping, Vice President, Yang Shoudong, Party Secretary of Horseshoe Town, Yang Feng, Secretary of Commission for Discipline Inspection attended the meeting, and heads of relevant departments of both sides attended the meeting. The meeting was presided over by Yang Feng."You send love and kindness to the horseshoe, which is an honor of our horseshoe education, and it is a blessing for our village children."Luo Shuangyu, president of Horseshoe Town Central School, said that we must remember the deep feeling of the School of Science and Technology of Guizhou University, and repay this love with the action to revitalize rural education."The college will continue to give full play to its advantages, gather strength and integrate resources to make due contributions to the further development of education in Horseshoe Town."Fan Wei, Party Secretary of Guizhou University School of Science and Technology, summed up a speech. He said that the School of Science and Technology of Guizhou University will actively advocate young college students to think of the source when drinking water, devote themselves to the grassroots level, feed their hometown, help Guizhou develop, and strive to be "new people of The Times". It is reported that the School of Science and Technology of Guizhou University has sent 9 teachers and more than 200 college student volunteers to carry out assistance activities based on the summer social practice activity of "The Institute of Science and Technology Inspired for the Chinese Dream" for five consecutive years. Among them, more than 1,270 primary school students participated in the volunteer teaching, more than 24,000 copies of health, drug control and legal publicity materials were distributed, and more than 40,000 yuan of love materials were donated to help horseshoe Town win the battle of targeted poverty alleviation.

2021-07-08	Hefei University of Technology: An undergraduate graduate has received his own "second class report card". The report card is an important part of the school's talent training system, and also a "hard indicator" for students to graduate. Since 2016, the first batch of the communist youth league "second classroom transcript" pilot unit, the school continues to promote the second classroom and its curriculum, systematization, standardization construction, constantly highlight the second classroom in the important role in the talent training system, formed the first classroom and the second classroom depth fusion, classroom extracurricular efficient collaborative education mechanism, let the students really realize the intelligence and physique of all-round development."The device we have developed can simulate a foreign body embedded between the comb tooth board and the pedal surface, Trigger escalator / sidewalk stop, Completed the reliability test of the comb tooth panel safety protection... " Recently, The results of the 2021, the 17th "Challenge Cup" National College Students Extracurricular Academic Science and Technology Works Competition of Hefei University of Technology were officially announced, Huo Jide, a 2018 undergraduate, and his students independently designed "an escalator comb tooth board safety protection detection device", which won the special prize, Another 99 entries were also awarded, The school will give all the winning students stars in the "Science and Technology Innovation" module according to the second class. In recent years, Hefei University of Technology has deeply implemented the trinity education and teaching integration system of "moral education, ability-oriented, innovation and entrepreneurship", and incorporated the standard requirements of the second class into the talent training program and undergraduate graduation standards, striving to achieve the simultaneous education of "morality, intelligence, physical education, beauty and labor". In the second classroom modular education system, set up "ideological learning" "scientific and technological innovation" "sports fitness" three compulsory modules and "public service" "social practice" "entrepreneurial activities" and other six elective modules, each module is refined the training objectives, project content and evaluation criteria, all grades using the "five-star" evaluation, 1 star is qualified, the highest score is 5 stars. According to the goal of the second classroom education, all the relevant units of the school constantly enrich the supply of various module projects, activities and content, to meet the development needs of students' second classroom development to the greatest extent. According to statistics, in 2020, the second classroom of the school released 2,338 projects, with nearly 300,000 students participating. The university clearly stipulates that, starting from 2019 undergraduates, all compulsory modules and at least one elective module in the second class can only meet the standards. The transcripts will be handed out by the university and put into the graduate files."In the context of deepening the reform of education evaluation in the new era, the school focuses on the core and key of talent training, constructs a scientific second classroom education mode, and guides students to improve their comprehensive quality in the combination of theory with practice and achieve all-round development."Liang �, president of Hefei University of Technology, �, a mirror of the growth of students." Without filling in the form, and running no errands, in the activity released by the system, click 'I want to register' to generate a task form, and the results can be found on the fastest day at the end of the activity."Huang Juntao, a 2017 undergraduate, has signed up for several second class projects through the system. In Qinxinhu Community, Hefei city, he and his classmates have long carried out volunteer services such as visiting the lonely elderly and senior Party members, and tutoring primary and middle school students for a total time of nearly 200 hours, which are automatically converted into the corresponding stars in the system. The school took the lead in independent research and development based on PC and mobile terminal "second classroom transcript" information management system, set project release, project audit, student registration, achievement import, the public and student evaluation function, to fully meet the teachers in the second classroom work and students to participate in the second class project personalized needs. In the system, the "Second classroom Teaching Plan" can automatically generate menu lists, and teachers can publish items according to the modules. Students can check in real time in the WeChat small program, and quickly complete the registration with their fingers. Through objective recording, scientific evaluation, grading certification, students' experience and achievements in the second class are comprehensively recorded and evaluated. Now, open the school's "second class report card big Data Center" interface, all the important data of all the students at a glance. Based on the advanced Internet and big data technology, the center has the total number of projects, participants, winners, volunteer service duration monitoring in real-time, the quality project quality, project goal, the college students average star, student interest distribution function of wisdom, fully implement the second classroom project implementation quality, educational results and the scientific evaluation of supply and demand dynamic, to dynamically adjust the course project system, promote the project perfect provides a strong support."The system record and evaluation of the second class is like a mirror, helping us to develop habits, take the initiative to mark the nine modules, strengthen the strong and weak points in a planned way, and consciously improve the comprehensive quality through the exercise of each module."Huang said."High-speed entrance and exit parking charging is not only time-consuming and laborious, but also brings hidden security risks. We have developed a complete dynamic data network platform charging system to realize the functions of non-parking charging and real-time navigation."During the school period, the school 2020 graduate Shen Jiaying and her students independently developed the" Virtual Toll Station Design based on Dynamic Service Level in the Internet Environment " stood out in the school traffic technology competition. At the same time, she has also shown outstanding performance in cultural and sports activities, public service and social practice, and has won several national and ministerial awards. When she entered the graduate job market in 2020, Shen's second class transcripts her confidence. Now, in Huawei Technology Co., LTD., she is not only a good job in her own work, but also the literary and artistic backbone of the unit, and the comprehensive ability can be reflected."Through the second class transcript, we can systematically grasp the development situation and ability level of students outside the classroom, so as to clearly understand the comprehensive quality of graduates."Ma Jun, deputy director of the human resources department of China National Machinery Industry Group Co., LTD., said that the group company recruits the graduates of NTU every year, and the second class report card more intuitively reflects the comprehensive quality of the graduates, which is conducive to improving the matching degree of the recruitment work. According to statistics, in recent years, employers satisfied more than 95% of graduates and graduates with the employment of the university. The number of recruitment enterprises has increased from 2910 in 2015 to 5258 in 2020. The contract salary of undergraduates has increased by 53% and the contract salary of graduate students has increased by 45%. This well-designed "second class report card" not only provides a reference for employers to select and employ people, but also becomes a key evaluation index for students' comprehensive quality evaluation, award evaluation, promotion of excellent Party membership, and exemption of graduate students. Today, the impact of the school's second class transcript has expanded beyond the campus. Not long ago, the school published the domestic first department about the second classroom education of high-quality teaching materials, with simple language and detailed rich examples of the relevant experience of the second classroom practice system, with strong policy, theoretical and practical to promote the second classroom education provides theoretical support and practical guidance. The school has also established the magazine "Second Classroom Education Research and Practice" to timely and comprehensively report the vivid practice and achievements of the second classroom in the school, and build a dynamic and academic exchange platform for the construction of "second classroom report card"."Through the construction of the 'second classroom report card', the school has continued to deepen the reform of the talent training system, and integrated moral education into ideological and moral education, cultural and knowledge education, and social practice education."Yu Qijun, Party secretary of Hefei University of Technology, said that the university will further promote the integration of the two classrooms, constantly improve the quality and level of talent training, and train socialist builders and successors who develop moral, intellectual, physical, aesthetic and labor for comprehensively building a modern socialist country. China Youth Daily, China Youth Network reporter Wang Lei, Wang Haihan, correspondent, Liu Hongping, Zhou Hui source: China Youth Daily

2021-07-09	Henan agricultural university animal medical school to carry out along the yellow social practice of Henan agricultural university animal medical school practice team along the yellow social practice wei correspondent Lu Yilin) on July 6, Henan agricultural university animal medical school student team, using a program under which officials "summer social practice, went to the Yellow River garden area along the yellow social practice, to" search the Yellow River culture, protect the Yellow River ecology " as the theme, from the details, to make their own contribution to defend the Yellow River. The picture shows the team members learning the history of the Yellow River. Correspondent for figure water pollution prevention and control work is an important content of the construction of ecological civilization, Henan agricultural university animal medical school student team innovation to carry out the "river xiaoqing" power the Yellow River ecological protection project, "small" power the Yellow river basin central urban garbage classification project, "river small enterprise" young entrepreneurs in the Yellow River basin. The picture shows the team members posing at the scraping scene. By visiting the "1938 scraping place" and picking up garbage publicity garbage classification and treatment, the team members remember that as the new era of youth, should be the former benchmark, strive to be a model for the younger generation. It is a long process to develop the practice of good garbage classification that consciously protect the environment around us. As long as we continue to do it, we call on more people to participate in it and return the Yellow River to be "clear".

2021-07-12	Party members and cadres of the School of Science and Technology of Science and Technology of Guizhou University carry out righteousness education activities for party history learning and education activities. Correspondent for figure China youth network Beijing on July 12 (xinhua Zhang Jianwei, correspondent, Song Huixin Yang Di) in order to further implement the party history learning education and "remember the earnest entrust, loyalty clean bear, celebrate the founding in one hundred" project education requirements, July 8 afternoon, Guizhou university college of science and technology party secretary Fan Wei led the leading cadres, teachers and students to the national patriotism education demonstration base conference site to carry out the party history learning education activities. In Gou dam conference site, we walk experience MAO zedong, zhou enlai walked, carefully visit Gou dam conference revolutionary history memorial hall, Gou dam conference site, MAO zedong once lived houses, by watching museum historical pictures, physical exhibition, text materials, listen to the explanation to understand the Gou dam conference in the significance of the history of the Chinese revolution, sentimental revolutionary achievements, deeply understand the MAO zedong and other older proletarian revolutionaries adhere to the truth, dare to bear, be loyal communist ideal and faith. In front of the Gouba meeting site, all the party members wore the party emblem, raised their right hands and faced the party flag, and reviewed the oath of joining the Party under the leadership of Fan Wei, the party secretary of the college. We will experience the magnificent course of the Chinese revolution and draw on the wisdom and strength to move forward."Through this study and education of party history, I have a deeper understanding of the difficult process of establishing the red regime under the leadership of the Communist Party of China, and further deepened my understanding of the great historical significance of the Gouba Conference."Participating in the activity of the teacher party member Yuan Tiansen said, in the future work will consciously inherit and carry forward the revolutionary tradition, inherit the red gene, the communist" I will not live up to the people " lofty feelings into the work, love and dedication, willing to contribute, to make positive contributions to the high-quality development of the college.

2021-07-08	Zhejiang University: Correspondent Ye Sheng � B) " 14 years ago, I was still a doctoral student. General Secretary Xi came to our laboratory to see the demonstration and asked me what I was professional and how long I had been doing this project. During the conversation, he also smiled and encouraged us to say that the research on the brain-computer interface is very interdisciplinary, very cutting-edge, and very meaningful. I hope to continue to study deeply in the future and realize the transformation of the results as soon as possible."In the" four history " education education public class, Zhejiang university higher institute researcher zhang teacher for young students brought this special course, from the perspective of the witness, for the students tells the story of 2007 xi in Zhejiang university, and his more than ten years to engaged in interdisciplinary research experience to encourage young students. Youth reading, youth flash mob, youth lead learning... The Youth League Committee of Zhejiang University to carry out the "four history" learning education, education throughout the whole process of moral education, highlight the taste of youth, promote the "four history" education into the brain into the heart, see the effect, stimulate young students to know the history of patriotic enthusiasm, draw the strength from the history. The young man said, " This is the 'treasure of the town college' of the Agricultural College of Zhejiang University, collected in Yishan, Guangxi, on the road to the west of Zhejiang University. During the Anti-Japanese War, the old president of Zhejiang University led the teachers and students to move to the west. The journey was far away and everything could be thrown away, but the precious specimens could not be thrown away, so he followed Zhejiang University back to Hangzhou, which has been well preserved."Yu Fengzhe, a sophomore at the College of Agriculture, tells the story of the treasure of the town college. This is an episode of the "My Subject has Stories" video series at Zhejiang University. This series of videos dig into the short stories in the discipline development history of the university: the first computer in Zhejiang Province, ZD-1, the first university botanical garden in China, the structure competition held for more than ten years... reflects the progressive story of generations of people in Zhejiang University, let the discipline story in history glow with new vitality, become an inexhaustible driving force to promote the development of the discipline. Bo Zheng, the planner of the event and the secretary of the Youth League Committee of Zhejiang University, said, " We must tell these unknown touching stories well, so that the contemporary young people of Zhejiang University can understand the past, never forget the original aspiration, and turn the touching and touching into the spiritual power to make achievements."The Youth League Committee of Zhejiang University also launched the theme activity of" Recalling the History of Youth Sports of Zhejiang University ", which participated in teachers and students to consult millions of words of information, condensed into 12 sets of small stories and shot into short videos, to pay tribute to the young seniors in the history of Zhejiang University."Independent learning attention is not easy to concentrate," and "I hope my partner will supervise me to get up early every day"... Through the research, the students will summarize the students' more concentrated learning needs: privacy, convenience, and improve efficiency. The student Union has launched the "cloud study room" and formulated the study room learning convention: advocating locking the entertainment APP and improving the learning concentration. Meanwhile also launched the "early rise supervision group", let the students play the leading role, each other supervision, encourage each other."I hadn't been very good at using the resources around me, and I didn't know who I could learn from. With the cloud study room, I asked questions in the group, and many enthusiastic students helped to answer them."The student barely passed his English course a semester ago to more than 90 points. Take the lead in learning the history of the Party, create a good style of study, enrich cultural and sports activities............ these have been written into the student union "I do practical things for the students" work task list, one by one promotion, fixed point implementation, so that the party history education to understand the thought, do practical things extension. Do practical things, not only for the students around you. Zhejiang university student association-students grassroots work service association for work XuanDiaoSheng alumni launched a "yi" plan, invited expert teachers in the form of "online + offline" initial training, on-the-job training training chain training services, covering theoretical accomplishment, document writing, management ability, and other aspects, the first launched more than 20 courses."Figure" to learn the history of the party: walk the heart more new youth to the party, associations to learn. A red cultural market with student associations as the main body was held on the campus. Ren Shaobo, secretary of the Party Committee of the university, completed the paper-cut work- -a red boat- -together with the students from the fingertip traditional handicraft club. Dozens of student associations give full play to the special power in the learning and education of the "four histories" in the form of knowledge questions and answers, cultural experience, popular science publicity and volunteer services. With the deepening of party history learning education, the pace of learning extends from the school to outside the school. The student backbone of the school Youth League Committee recalls the revolutionary history in Yizhou, Guangxi, School young marxists (student backbone) training college organized students to come to Nanjing Yuhuatai to pay respects, The student Union went to Wuhan to understand the great spirit of fighting COVID-19... This summer, Nearly 10,000 students and nearly 1,000 teams of Zhejiang University will set foot on the red line carefully selected by more than 100 schools, The special practice of "Party History Youth Walk" is to " walk a red line, Seeking a veteran party member, Shooting a red video, Do a practical thing for the masses, Conduct a party history propaganda, Organize a note on Party history, Form a research report "" seven one " red series of activities as the main body. In order to make the research practice more scientific, thorough, the school opened "cognition and practice" first general class elective courses, the 16 class national policy theory teaching and 48 class research practice, lead young students to learn, walk, while watching, while doing, the "walk of the party history classroom" walk deeper, more walk more solid.

2021-07-08	The summer social practice team of Northwestern Polytechnical University went to the revolutionary site to learn the red spirit special training class to take a group photo. China youth network correspondent Cheng Jiao jiao for figure China youth network Beijing on July 8 (reporter Zhang Jianwei, correspondent, Zhang Zhiming, Zhang Quankui Zhang Ying) on July 5 afternoon, northwest polytechnical university "don't forget to road, remember the party history" summer social practice to ruijin, Jiangxi province the second national congress site and red jing scenic spot to visit the study and research. The practice team members increase their knowledge of party history through the visit of the old site and the study of relevant knowledge. At the same time, they issue questionnaires to investigate the understanding degree and attention of all kinds of visitors on party history knowledge, so as to make the research results more data, rigorous and efficient. During the visit and learning process, the practice team members distributed questionnaires in groups for research. Through communication with the questionnaire fill-bearers, they understood the learning degree of party history, and learned the ways and methods of learning party history in different industries, which provided theoretical and reference significance for the subsequent party history learning and education. Members of the practice team had an in-depth exchange and study with the special training class of "Learning Party History and Strengthening Party Spirit" by the young backbone of the Aviation Industry Helicopter Institute and the summer social practice team of Nankai University Law School. They listened to the introduction of the revolutionary site and carried out the party history learning education in an all-round way. In Hongjing scenic spot, the practice members of Northwest Polytechnical University and the social practice members of Nankai University discussed the study of party history. In the visit and study of the old site of the Red Revolution, the practice team members measured the road of the revolutionary martyrs with their footsteps. Experience the history of the Party in practice and inherit the spirit of the Party.

2021-07-06	Nanjing Xiaozhuang University School of Environmental Science in the red Li Lane to carry out summer social practice activities correspondent Bao Ruiqiong Beijing, July 6 (reporter Li Chuan correspondent Bao Ruiqiong) to continue the red blood, inherit the revolutionary spirit, Firm in the ideals and beliefs of young college students, On July 2nd, School of Environmental Sciences, Nanjing Xiaozhuang University, " Pay tribute to a hundred years of glory, Youth to practice the original aspiration- -to study the history of the Party, Find the red trace " research group of 10 people, Under the leadership of Wang Xiaolei, deputy Party Secretary of the School of Environmental Sciences, Go to the red Li Lane to carry out the " study of the history of the Party, Find the red trace " research activities. The research group visited the command center of the New Fourth Army and the red relics group of the Anti-Japanese War, known as the "Little Yan'an in South Jiangsu", reviewed the history of the Anti-Japanese War of the New Fourth Army in Nanjing area, and inherited and carried forward the spirit of the Anti-Japanese War. Nanjing Xiaozhuang University School of Environmental Science "study the history of the Party, looking for the red trace" research group research practice in the red Li Xiang. China youth network correspondent BaoRuiqiong for remember history, candle in the future, under the guidance of the narrator, the delegation has visited the Anti-Japanese War collection, the new fourth army honest education, Chen yi, in revolutionary martyrs fighting pictures and some relics, while recalling the arduous years, revisit the revolutionary course, listen to learning li lane battle story and glorious deeds, feel the older generation of proletarian revolutionaries great patriotism and indomitable fighting spirit. On the scene, facing the bright red party flag, under the leadership of the old party members, the members of the research group collective oath, review the oath of joining the Party. It is reported that this survey is a vivid school of environmental science to guide young students to learn the revolutionary spirit of the history of the Party. Study a hundred years of the history of the party, draw the strength to forge ahead, through this research, the students have said to adhere to the original aspiration, brave mission, with my youth, my struggle, and strive to help the party to continue to write a new historical chapter of the new era.

2021-07-05	Fujian: Jimei University carries out the youth red song flash mob activity, and develops the double first-class " youth red song flash mob activity. Shen Canhuang, secretary of the Party Committee of the university, and Hong Wenjian, deputy Party secretary of the university, celebrated the centenary of the founding of the Party. Shen Canhuang and his youth league members sang together the classic songs such as "No Communist Party, No New China" and "Sing a Folk Song to the Party". After the chorus, all the teachers and students present shouted in unison "Ode to the party, forge ahead double first-class". At the scene of the event, Shen Canhuang message of youth members, hope everyone continue Luo Ming, Luo Yang, YanLiBin set big predecessors left red footprints, red genes, keep in mind the youth mission, full of the heart of the party and the people, further firm ideal faith, have feelings for the people, enhance ability, have the courage to bear as. Shen Canhuang also on behalf of the school on the "July 1" birthday member youth representatives presented "brief history of the communist party of China" of the history of China communist party " record the party's history of red books as a birthday gift, encourage students to study the struggle course of the communist party of China and fine tradition, draw strength from the history of the party in one hundred.

2021-07-05	Yangzhou university school of marxism deeply plant "the flower of ideological education" Yangzhou university school of marxism deeply plant "the flower of ideological education" graduate party branch 15 party members to the national era model, the national moral model, the most beautiful striver Qu Jianwu teacher letter, to express his love for education, education education, stick to education dream admiration, and determined to take his example, deep plant ideological "three inches platform", being a good young students grow lead. On June 28, Qu Jianwu wrote back to the branch, and sent out messages and expectations to the students as a senior party member and guidance counselor. Received Qu Jianwu's reply, the party branch immediately organized all the party members to hold to "learn from the model of The Times to draw the power of the example" as the theme of the learning exchange meeting. The branch of 15 doctoral student party members have joined the college students red theory publicity group. This year, the XuanJiangTuan to "tell one hundred party history story condensed youth strength" as the theme, combining with theory to preach, works tour, creating design, sitcom deduce the "door" preaching way, active in enterprises and institutions, villages, communities, schools and other campus inside and outside, to spread the party's theory of campus "light cavalry", reveal the era of young students.

2021-07-03	Anhui Agricultural University, the name of a revolutionary martyrs, shining a golden light; a century of important events, the history of the party, combined into a towering tree; a beam of lights, illuminate the party century of brilliant history... a full of creative posters, the party century of the history of important events, revolutionary martyrs, with an artistic way. Recently, students of Anhui Agricultural University used "art + Party building" to create 100 distinctive works to present a gift to the centenary of the Party. The poster has been welcomed by young students since it was exhibited inside and outside the school. Sang Yihan, a student majoring in tea culture and trade at the school, said, " It is very creative and very shocking. The posters reproduce the great struggle of the Party in the way of art, which is very vivid."Yin Shi, the planner of the poster exhibition and the secretary of the Teaching staff Party branch of the School of Light Textile Engineering and Art, said that this year is the 100th birthday of the Party. As a teacher and party member, he has always wanted to present a gift to the Party in a different way."Later, I mentioned this matter to my classmates, but I didn't expect to get a positive response. We find the combination point of course learning and party history learning and education."" This is a very solemn thing, not only to design a sense of art, but also to rigorously restore the history of the Party."In two months, Yin Shi led a team of students to study the history of the Party carefully while conducting artistic design. Soon, a series of works have a preliminary appearance. Zhang Qi, a member of the creative team, felt deeply about the creative process."It is a good polishing and discussion, the connotation of the work, this is a very rare party history education class.”

2021-07-02	Guizhou university college of science and technology let the party history learning education more "heart" go "new" party history learning education "new" more "heart" Guizhou university college of red flag relay contest wei correspondent Zhang Yaoji) recently, Guizhou university college of science and technology held in the football field "academy youth hearts to the struggle in one hundred youth" red flag relay race. More than 460 people from 60 student teams and 2 volunteer teaching teams participated in the competition. The picture shows the competitors running on the competition field. Correspondent for the activity set up "red boat station", "spark" station "," great transition "," to victory station "," reform and opening up " five levels, in the process of competition, each player holding flag and task to the corresponding levels to write down the answer to the corresponding party history questions, and the flag and task single pass to the next relay player, group of six players run 3 km schedule is to complete the race. Party history questions are correct, the total time is reduced by five seconds, otherwise add five seconds, to determine the winning team according to the final time. The picture shows the contestants answering the questions at the level. After nearly four hours of seesaw war, the "wind-chasing youth Team" of the Engineering department won the first prize in the student group, the "self-surpassing team" of the literature department won the second prize in the student group, and the "whew and sex team" of the Business department won the third prize in the student group. The picture shows a group photo of the winning team and the staff."The first time to participate in this form of relay run, I think it is very novel!"Wang Shengming said he also learned a lot of party history in the event."Sports competition is an essential sport in campus cultural life. It can not only enhance students 'physical fitness, but also cultivate students' unremitting self-improvement and indomitable will. A qualified college student should be able to climb new heights both in his study and in the sports field."Du Fengxiang, executive vice president of the college, said that the Red Flag relay race will combine sports and party history learning and education, and promote the party history learning and education into the hearts of students in the form of education and fun.

2021-07-02	Shaanxi energy vocational and technical college held party history class micro video game shaanxi energy vocational and technical college held "hundreds of party" league micro video game Wei correspondent li peng) recently, shaanxi energy vocational and technical college youth corps committee held "hundreds of party history" group micro video game, the event by the league of coal and chemical industry college, the school seven league branch. The group class competition is divided into two stages: preliminary and final. The preliminary competition (selection) was arranged by the league general branch and the league branch according to the actual situation, and organized the students to extensively carry out the micro video competition with the theme of "learning the history of the Party and following the Party". Each league always completed the preliminary competition as scheduled according to the requirements, and finally selected 105 videos to participate in the final. After strict and fair selection, this activity selected: 3 first prize, second prize 6,10 third prize, 10 excellence award, 2 excellent organization award 2.

2021-06-30	Guizhou university college of science and technology to love station volunteer service activities volunteer service warm heart love pass tender Guizhou university college love station volunteer service activities JianWei correspondent Song Huixin) recently, Guizhou university college of science and technology in dormitory Taoyuan 1, Taoyuan 2, Taoyuan 6 dormitory building set up three post love service point, organize volunteers to help graduates carry luggage and receive donated idle goods, the graduates exclamation " too convenient and happy. When "" graduated, he found that he had accumulated a lot of things in his four years of college life, but there were also many memories that could not be taken away, such as these professional books. I hope the predestined people can cherish them."Chen Xin, a journalism graduate of 2021, said that I am very grateful to the students who helped me carry my luggage. I hope they can keep awake in the future study, do what they should do at every stage, always keep a bright and kind heart, and try to catch what they want."Name", "class", "dormitory address", "handling time", "donation goods"... in each registration form of luggage handling and goods donation, there are 20 graduates who need luggage handling information, volunteers will not only provide "door-to-door" service, but also orderly go into the dormitory or on the campus road to help graduates send luggage to the designated place, but also transport the graduates donated idle goods to the service point. According to incomplete statistics, within one day, we helped nearly 200 graduates to carry their luggage and collected nearly 1,000 donated unused goods."Sister, this is too heavy, let me come", "thank you", "hard", "I help you to mention, senior"... a warm words, between graduates and volunteers. Behind the warm-hearted action is the active organization and planning of the Youth League Committee of the college, which is the rotation and unremitting efforts of more than 150 volunteers composed of the College Youth Volunteers Association, the College Student Union and other groups. The service time lasts for 10 hours in a day. Most of the first-year students participated in the volunteer activity, including Song Qian from English Class 203, who said, " I feel very happy to help seniors do some warm memories on campus."Pay attention to publicity, before the online and offline love post volunteer service activities, the Youth League Committee of the college hung banners, posters, offline points, posted tweets on the wechat public account and other online and offline forms, to ensure the full coverage of graduates."Although the students graduated, but the heart of his Alma mater is always together with them" college, deputy secretary of party committee ZuoZhangQuan stressed at the meeting, to do the practical work for graduates, intensify propaganda, through online multiple channels, let the graduates understand the college organized volunteers to help carry luggage, let them can feel the love of his Alma mater."I noticed the news on the WeChat official account. I saw the service point downstairs on the same day I moved the luggage and immediately registered the information."Hu Dan, a 2021 journalism graduate, said the college's actions warmed her heart's heart."Books", "the little fan", "the notebook", "the guitar"... As the "flea market" will sell the idle items in a few days, the student cadres of the College Youth Volunteers Association are working overtime to check and register the items."The proceeds from the sale of unused items will be donated to the Guizhou Red Cross in the name of graduates who donated them, and the unsold items will be classified and donated to special education schools or other institutions in need."Zhang Yaogong, director of the Publicity Department of the College Party Committee and secretary of the Youth League Committee, said, since the development of the party history learning education, the college continues to deepen the work of serving teachers and students, and at the same time will do practical work to the outside of the school, truly serve the society and the masses.

2021-06-30	Xidian University has launched a column titled "Youth Lead Learning" to tell hero stories to young people to tell you hero stories ". Beijing, June 30 (reporter Zhang Jianwei, correspondent Chen Haitao) in order to celebrate the 100th anniversary of the founding of the Communist Party of China, Leading the youth league members to cultivate their love for the Party, patriotism and socialism, Under the guidance of the Communist Youth League Committee of Xidian University, The Youth League Committee of the School of Physics and Optoelectronic Engineering of the school closely focuses on the learning history, the learning history of increasing credit, Focus on the "hero" theme, Make good use of the "textbook" of heroic deeds and heroic spirit, Relying on the official wechat of "Xidian and Light Institute" and "Himalaya FM" radio station, we launched the "Youth Lead Learning" program, Educate and guide young people to talk with "heroes", Stimulate the responsibility of young students.100 Issues. Xidian university youth corps committee for figure since March this year, xi'an university of electronic science and technology opened party history learning education project "youth led-the hero story to you", 100 young students as "party history lead", dig deep hero models typical deeds 100, through the form of "green voice praise" dialogue hero, good hero story, share learning comprehension, recording audio in school media and "Himalayan FM" radio online program 100, well received by the teachers and students. At present, "youth led-to tell the hero story to you" 100 online columns will be updated, not only including li dazhao, Xie Zichang, zhi-dan liu revolutionary martyrs, and lu xun, nier, Xi'an, as well as Bethune, Edno, Gregore garage shen international fighters, the hero story is to adhere to the "khalid ents" the most vivid textbooks. The picture shows the "youth Lead Studies" audio album. Xi'an university of electronic science and technology youth corps committee for figure college youth corps committee opened "youth led" online column 100 period, from material collection, manuscript writing, audio recording to poster design, tweet propaganda all completed by the group graduate student backbone team, at the same time, the college will be "youth led" recorded audio album, give "led youth" collection, hope the youth have the courage to issue "youth voice", transfer "youth positive energy".it is reported, To carry out a solid party history learning and education activities, Constantly consolidate independent learning and concentrated discussion of "prescribed actions", Enrich and expand practical study research, party member pioneer forum, teachers and students theory propaganda, red film screening, party history books flow and other "optional action"; Adhere to the problem-oriented, Do practical things for the masses, Implementing the "Young Teachers' Teaching Ability Training Salary Plan", Carry out "laboratory tour", "light by electricity", "professor publicity group" and other activities to help the categories of undergraduate majors, Together with the teaching staff Party branch of Chengnan School in Pucheng County, we cooperated to carry out campus cultural activities such as "ability improvement project", "Model star" character selection and "Voice of Spring" speech contest.

2021-06-25	China academy youth league committee held party history learning education exchange meeting Chinese academy youth league committee held "party history, strong faith, follow the party" learning education exchange meeting in Beijing, June 25 (reporter Zhang Jianwei, correspondent Zhang Shaoshuai) recently, the Chinese academy youth league committee held "learning party history, strong faith, follow the party" learning education exchange meeting. Zhu Anhong, secretary of the Communist Youth League Committee of the college, made a theme youth league class entitled "Learning the history of the Party, strengthening faith, and following the Party" at the exchange meeting. More than 70 youth league members attended the meeting. The picture shows visiting the Hainan Provincial Museum. At the photo exchange meeting, Zhu Anhong, secretary of the college Youth League Committee, brought a vivid and wonderful league class to all the youth league members of the college. He summed up and refined the "ten spirits", will be the party's one hundred years of struggle course. He hoped that the youth members of the college should work hard to "learn the history of the Party", further enhance the urgency of consciousness from learning, work hard on "strong faith", further find the focus from learning, "follow the Party", and further enhance the contribution of focus from learning. It is understood that the Chinese hot academy youth corps committee within the scope of full caliber party history courtyard history learning, each unit selected youth representatives, to the school exhibition hall, science and technology expo park site teaching, at the same time theme essay contest, the party history knowledge test, the party history learning education practice, in the floor continue to build a love party, patriotism, love, love the thick atmosphere.

2021-06-24	Beijing, June 24 (reporter Zhang Jianwei, correspondent Gao Shimin) recently, Jiangxi University of Science and Technology party history learning education music party history class was held in the Hongqi campus new gymnasium. This theme music party history class is the special "Long March Song", sponsored by Jiangxi University of Science and Technology, organized by Jiangxi University of Science and Technology Party History Learning and Education Leading Group Office, the Youth League Committee, the School of Marxism, specially invited Jiangxi Yudu County Changzheng Source Chorus and Jiangxi University of Science and Technology octagonal Voice Chorus to perform on the same stage. More than 3,000 people attended the lecture on the party members, teachers, student party members, active party members, youth league members and new party members in 2021. The picture shows the music party history class oath scene."I volunteer to join the Communist Party of China, uphold the Party's program, abide by the Party Constitution, fulfill the obligations of a Party member, implement the Party's decisions, strictly observe Party discipline, keep conservative secrets, be loyal to the Party, work actively, fight for communism all my life, be ready to sacrifice everything for the Party and the people, and never betray the Party."The music party history class, also held Jiangxi University of Science and Technology in 2021 new party members collective oath ceremony and the old party members review the party oath activity, the first half of the school absorbed 1631 probationary party members and the leadership of the old Party members secretary of the university Yang Bin, solemnly took the oath to the party flag. The picture shows the music party history class scene. The class was taught by the ideological and political teachers, the dance team of the college student art troupe, the students of the School of Economics and Management, and the choir singing the "Song of the Long March" and other rich forms, presenting the historical story of the Party behind the "Song of the Long March" by combining speaking, performing and singing.

2021-06-29	The School of Innovation and Entrepreneurship of Huaiyin Institute of Technology carried out the celebration of the centenary of the founding of the Party of the spirit of the history of the Party " to celebrate the celebration of the centenary of the founding of the Party of the Party. China youth network correspondent Yu Chunhong for the picture China youth network Beijing June 29 (reporter Li Chuan correspondent Yu Chunhong) June 27, Jiangsu Huaiyin Institute of Technology innovation and entrepreneurship college directly under the party branch of the "red base to learn the party history Enlai spirit forever" to celebrate the founding of the century theme party day activities. Representatives of all party members and active student members of the college participated in the activity. In the former residence of Zhou Enlai, the representatives of young teachers Sun Qianna and Yang Liuqing were respectively titled "Zhou Enlai:" Zhou Enlai: Diligence and thrift, fair and honest "and" The Story of Zhou Enlai cherishing talents ", telling the story of Premier Zhou Enlai's thirst for talents and thrift. Sun Shuhao and Li Ranran, representatives of the active members of the students, recited "This Man" on the spot, praising Comrade Zhou Enlai's noble moral character, lofty spirit and great style. Huaiyin institute of technology innovation entrepreneurship dean zhi-guo zhao said, premier zhou enlai's glorious deeds and great character is the party history learning education is the most vivid teaching material, hope the students keep in mind "reading for the rise of China" school motto, set up the correct life ideal and broad goal, study hard, dare to climb, ambitious, the unity, in order to realize the great rejuvenation of the Chinese nation of the Chinese dream. On June 27th, the School of Innovation and Entrepreneurship of Jiangsu Huaiyin Institute of Technology held a party day activity with the theme of "celebrating the centenary of the Party". In the exhibition hall of "People's Premier Zhou Enlai", all the staff visited the physical objects and video materials of Premier Zhou Enlai in different times, and reviewed Premier Zhou Enlai's glorious life of striving for happiness for the Chinese people, seeking rejuvenation for the Chinese nation, and striving for the cause of human progress. Liu Yi, secretary of the Party branch directly under the School of Innovation and Entrepreneurship, called on everyone to earnestly study Premier Zhou Enlai's loyal spirit, pragmatic spirit, innovative spirit, strict discipline and the spirit of continuous learning, so as to work harder in their respective posts and make new contributions worthy of the current era. It is reported that recently, innovative entrepreneurship college through various forms of party history learning education activities, has read to books, organized the study of zhou enlai six outstanding model essay contest, watching "with" four "ideological courses", relay to read party history, school "zhou enlai class" and Hebei normal university software institute "Deng Yingchao class" construction activities and other series of activities.

2021-07-01	Shaanxi Energy Vocational and Technical College held the first Red Cross Emergency rescue Skills Competition. Correspondent for figure China youth network Beijing on July 1 (xinhua Ji'an-wei zhang, correspondent Hu Yuanyuan) recently, shaanxi energy vocational and technical college the first Red Cross emergency rescue skills competition held in training building sixth floor lecture hall, eight teams from each college 32 players, competitive, eventually nursing school team to the group score first won the "excellent organization award". The competition is divided into three parts: CPR, trauma dressing and speech contest. In cardiopulmonary resuscitation, trauma bandage practice operation link, three players play at the same time, increased the competitive competition, each round contest the judges play test the operation results, check the hemostasis, bandage, fixed first aid operation points, and give guidance and comments, let the contestants through the nervous game to learn more practical knowledge. The Red Cross emergency rescue skills competition, for the students set up a display platform, students learn in the competition, improve in the game, the emergency rescue knowledge and skills have a more comprehensive understanding and master, improve the Red Cross volunteers emergency rescue skills, also strongly promote and popularize the Red Cross concept and emergency rescue knowledge, shows the "people learn first aid, first aid for all" game theme, let more people know about first aid, familiar with first aid, participate in first aid.

2021-06-30	Shaanxi energy vocational and technical college green horse students into Ann Wu Qing class site in red base inheritance red gene shaanxi energy vocational and technical college students into the sixth site on June 30 (reporter Zhang Jianwei, correspondent Yao Na) on June 28, shaanxi energy vocational and technical college "young marxist training project" training some students representatives, under the guidance of the teacher to JingYang AnWu Town Wu Qing class site visit study, launched a "inheriting our fine tradition, create a new situation" theme activities. The students visited the existing class headquarters, the Wu Manor, the Academic Affairs Office, the May Moon Building and the gathering site successively. Under the leadership of the teacher, through listening to the explanation, field visits and other forms of a detailed understanding of the history of An Wu Qing training class, remembering the older generation of proletarian revolutionaries to carry out revolutionary education activities in An Wu. In the youth training class, the youth people reviewed the oath of joining the league, traced their original aspiration and encouraged the future. After the visit and study, qingma students exchanged and discussed at the old site. The picture shows the activity site. This theme activity, on the one hand, let the students remember the glorious mission of the party, inherit the revolutionary tradition, carry forward the youth cadres pursuit of ideal, the pursuit of light spirit, resist aggression, serve the motherland patriotism, self-reliance, hard work and courage, not afraid of sacrifice dedication; on the other hand, to the students higher requirements, namely firm ideal and faith, give full play to the cutting edge role, and contribute for the construction and development of the motherland wisdom and strength.

2021-06-30	Shaanxi energy vocational and technical college held party history recitation contest final shaanxi energy vocational and technical college "party history, party, follow the party" recitation contest final JianWei correspondent Wang ruo) recently, sponsored by shaanxi energy vocational and technical college party committee propaganda department, school union, youth corps committee to undertake "party history, know, follow the party" the school teachers and students recitation contest final held in chrysanthemum garden college students activity center. Leaders from the organization Department, the Publicity Department, the University Party Committee, the Youth League Committee, the General Party Branch of the School of Economics and Management, the General Party Branch of the School of Continuing Education, the School of Humanities and Education, and the Trade Union office served as the judges of this competition. The 13 staff teams selected by the grass-roots trade unions and the 14 student teams selected by the youth League general branch of the secondary colleges will participate in the final. The contestants full of spirit, impassioned to read the "youth confessions motherland" "across one hundred commitment" "no communist party, there is no new China" "the five men" "always follow the party" "ode to China" and other red poetry, deeply remembering the feats of the older generation of proletarian revolutionaries, enthusiasm eulogize the socialism with Chinese characteristics, celebrate the era theme, praise the happy new life. The recitation of classic red chapters in this competition is not only a kind of memory, a kind of inheritance, but also a feeling of a passion, absorbing a kind of strength to inspire people to love the Party, the motherland and their hometown.

2021-06-29	Let the red faith rooted in the hearts of students capital medical university to create "red matrix" promote party history learning education let red faith rooted in the hearts of students to ask what you want, paper ship bright candle burning "... capital medical university campus often echoed with classic red poetry reading, some students changed WeChat signature to" do not do net red do party red ". Nowadays, to be a "party" red college student in the new era has become a new fashion among students of Capital Medical University. Recently, Capital Medical University carried out the theme of "reading the poems of leaders and exploring the red history", setting off a upsurge of "learning the history of the Party, strengthen faith and follow the Party" among teachers and students. The school designs four links: "speaking" poetry about history and Ming history, "tasting" poetry, "reading" poetry, and practicing "competition" poetry, guiding medical students to read red poetry and explore red history, inspiring them to think deeply and practice their mission and responsibility. Hu Wenliang, secretary of the Party Committee of the University, gave special training for more than 1,000 Party members and students with the theme of "Deputy Secretary of the Party Committee of the University, and" Exploring the red history "."Online Online, online, The school held the "Reading the leaders' poems to explore the red History" red poetry reading month activity, Push one or two leader poems every day, Interpretation of the distribution legend, From the time background, poetry connotation and thoughts and feelings of in-depth interpretation and analysis, Has been pushed for 100 consecutive issues; Establish an online competition platform, The Launch of the "One Daily Race" event, Set up five types of questions: leader poetry memory, leader poetry analysis, red history exploration, classic knowledge consolidation, red resource exploration, There were 12,000 people participated in the "daily competition" answer link; Create the "Top 100 Red Poetry Competition", By filling in the blanks of poems and identifying the nine grids, Fully stimulate students' enthusiasm for party history learning."In the process of the history of the party learning education, the school focus on history li today, learning important goals, will read poetry, example guide, practice education means organic into the history of the party, improve the learning education targeted and leading force, efforts to guide the medical students to further enhance the love party, the party, follow the party's spirit of faith."Said Liu Fang, deputy Party secretary of the Capital Medical University. Wu Yifan, a student of the basic medical school of Capital Medical University, wrote in his experience: From the red poetry, I feel the heart of the older generation of proletarian revolutionaries of concern and love for the people. This feeling for the people is the original aspiration and mission of medical students in the new era. Capital Medical University also invited Zhao Guoguang, president of Xuanwu Hospital, Zhang Shutian, president of Affiliated Beijing Friendship Hospital, Wei Wenbin, vice president of Affiliated Beijing Tongren Hospital and other clinical medicine "big names" to participate. Their intensive study of the history of the Party and the red poetry makes the students who just enter the medical gate feel the spirit of The Times of the red poetry, and makes the red faith take root in the hearts of the medical students. With live "party history textbooks" build a medical youth faith in addition to read the leader poetry, the capital university of medical sciences combined with the characteristics of medical students and demand, launched youth lecture hall and "always follow the party" group backbone party history learning series of activities, invite mei, TaoYong outstanding medical predecessors for the students about "medical faith, to the light" story, promote the party history learning education."As a party member, there is no retirement age for working for the Party; as a doctor, there is no retirement age for relieving patients."Lu Xinmei, winner of the" Xiude " Outstanding Alumni Award, grade 63 alumnus and former vice president of Jiaxian People's Hospital, Yulin City, Shaanxi Province, told the teachers and students of Capital Medical University about her more than 50 years of youth and perseverance on the medical road. From Shaohua to Baitou, she took root in northern Shaanxi to relieve the pain of patients, while actively promoting the development of Jiaxian People's Hospital. For more than 50 years, she has always fulfilled two commitments: she promised to her Alma mater to "obey the distribution of the motherland and go to the hardest place"; and she promised to the people of Jia County to "serve the people of Jia County for 50 years". Tao Yong, a young doctor, shared his views on the 20-year medical practice from three stages: "technology", "art" and "principles" of medical practice, and interacted with the students on the scene to explore the true meaning of the medical practice."Miss Lu's struggle let me deeply understand what is' the great from the ordinary, the ordinary makes the great ', to extend the highest respect to her!"" In Dr. Tao Yong, I see my yearning for my future medical career."Out of the lecture hall, the students felt a lot. Capital Medical University has also held a series of activities to celebrate the centenary of the Party, such as "Top Hundred Red Poetry Competition" and "Singing songs to the Party", to further broaden the path of students' Party history learning and set off a wave of teachers and students learning Party history. At 7 am, Yan Jiayu, a 2020 doctoral student at Beijing Children's Hospital affiliated to Capital Medical University, rescued a child. After the 24-hour hospital general duty, he rushed to the school without a rest to study this year's summer social practice work plan with Dong Li, a doctor of ophthalmology major in the affiliated Beijing Tongren Hospital. They are planning to recruit health volunteer service teams to Hebei and Shaanxi provinces to pair up with doctors from local village clinics. Through online interaction and joint free medical consultations during holidays, they will improve the medical service level of village doctors and open up the "last kilometer" of villagers' health services."After participating in the study and education of party history, we have more truly realized the responsibility given to us by the building of a healthy China. As a medical youth, we should transform the love for the Party and the people into the practical action of 'serving the people's health '."Yan Jiayu said that it is the fifth time that he has set up a health volunteer service team to participate in the summer social practice of Capital Medical University. For more than ten years, Capital Medical University has been setting up a health volunteer service team every summer. The team members include more than 1,000 medical experts, graduate students and outstanding student cadres carefully selected from 21 clinical medical schools. They went to economically and healthily underdeveloped areas to carry out free medical services and voluntary health education services, and provided nearly 100,000 medical services such as health examination and disease screening. China Youth Daily China Youth Network reporter Wang Lulu source: China Youth Daily

2021-06-29	Guizhou university student union held "secretary of the President and I face to face" activities of Guizhou university student union held "secretary of the principal and I face to face" activities Beijing on June 29 (reporter Zhang Jianwei, correspondent, Liu Yanchun hong-qing song) to promote party history learning education, further open student feedback channels, listen to youth, Guizhou university student union held "I do something for my classmates" "secretary of the President of the face to face with me" activities. Principals of relevant departments of the school and more than 20 relevant units, and representatives of students of the school and college student Union attended the meeting. Conference, the student union, graduate student members, the province against COVID-19 outbreak of outstanding volunteers, the top ten college students, youth kechuang mentor, "Dr village head" representative, graduate student volunteers members, a total of 12 teachers and students have as "celebrate one hundred practice youth bear" theme speech, to share their own experience, talk about the learning experience. Each college student union representatives on the current students concerned about the problems encountered in study and life free speech, such as "about Guizhou university set up the whole school online electricity channel proposal", "on improving the east (former north) bathroom environment and 10 accommodation environment", "about the construction of library seat reservation query system", "about improving the dormitory lights out power proposal" and other proposals. Secretary, the principal and the students cordial communication, listen to the students reflect the problem and asked "the head of the school units to student-oriented, to solve the problem of students, not only to reply hundred, but also solve hundred", head of the participating units said will fully research demonstration and put forward solutions, earnestly the students' study and life security. The picture shows the activity site. The President Song Bao'an affirmed the speeches and proposals of all the deputies. He pointed out that "everything for the students, everything for the students" is the fundamental starting point, the foothold of your college students work, each unit must value and earnestly implement the students 'reasonable demands, carry forward the spirit of "I do the practical work for the populace", the students do student heart, improve students' satisfaction, happiness. The symposium ended successfully with the chorus of "No Communist Party, no New China". Zhou Haodong, a student of the Student Union, said that through the activity of "Secretary and principal and me face to face", the student Union has built a close bridge between the party and government of the school and the majority of students, further unblocked the feedback channels for students, and safeguarded the legitimate rights and interests of students. It is understood that "I do something for students" practice, the students focus on service students general demand, select the service thought growth, practice, helping difficult students, safeguard legitimate rights and interests, power overall development, harmonious campus construction of specific practical projects, timely promote the implementation, the party history learning education and student union reform, let the party history learning education achievements into service the real practice of college students.

2021-06-23	Southwest jiaotong university launched the original song "new youth" southwest jiaotong university youth league committee launched "jiaotong university" new youth "theme tribute work founding century correspondent Zhu Wei)" you ask me / new youth's original mission / I said the new era / youth heart towards the flag / struggle power / the best period / shoulder / strive to be a better self... "recently, a affectionate, and full of youthful vitality of positive energy original song" new jiaotong university ", sounded and sung in southwest jiaotong university campus, by the majority of young college students popular and love.it is reported, According to the deployment and arrangement of the whole regiment of Southwest Jiaotong University, Solidly carry out the "study of the Party, strong faith, follow the Party" members of the theme of learning education and practice activities, To further promote Xi Jinping Thought on Socialism with Chinese Characteristics for a New Era into party branches, league classes, associations, the Internet, and minds, Carry out the "youth" learning campaign, Promote the "youth teacher group" to conduct in-depth grassroots and social publicity, Deepen the construction of a "youth media" financial media center, "Jiaotong University YouSi" was selected as the first batch of ideological and political universities, Carefully planned and launched the book "Learning One Hundred Years of Party History", Be the youth of The Times "," Study a hundred years of Party history, Emphaslearning methods "" glorious past, Anti-the United States and Aid to Korea engineering team "," Love the Party, patriotism, love the school "," in the patriotic struggle ceaseless May 4th spirit " and other series of theme micro group classes, As well as the original micro film "Light up the Faith with the Ceremony", "The New Youth of Jiaotong University" music MV, "Youth struggles to the Party and power- -Southwest Jiaotong University Youth says in the New Era" books and other series of theme works, Show the youth and struggle, responsibility and responsibility, original aspiration and mission of young college students in an all-round and three-dimensional way in the new era. Zhang Junqi, secretary of the Communist Youth League Committee of Southwest Jiaotong University, said that the university Communist Youth League launched a series of theme works, aimed at leading the majority of young people to establish the value orientation of being wise and striving for success, consciously integrate the personal ideal pursuit into the new journey of comprehensively building a modern socialist country, and youth to the Party and strive for a strong country.

2021-06-23	The fifth in Anhui province university students keynote speech contest final successfully held in Hefei college "learning party history strong faith with the party" the fifth in Anhui province university students keynote speech contest final successfully held in Hefei college jianwei correspondent, Su Weigang check wei ting) recently, "learning party history strong faith with the party" the fifth in Anhui province university students keynote speech contest final was successfully held in Hefei college. More than 160 people from the Publicity Department, the Anhui Provincial Party Committee, the Anhui Provincial Party Committee, the Provincial Youth League Party Committee, Hefei University, and teachers and students of Hefei University attended the activity. The picture shows the final scene. Correspondent feeds final according to the undergraduate course group, higher vocational group respectively, 16 contestants adopt the way of impromptu speech, affectionate praise the great course in one hundred, revisit the brilliant one hundred party history, express patriotic love party, feeling the party's beginner's mind mission, shows the contemporary college students positive, progressive spirit. After fierce competition, Shang Ziru from South Anhui Medical College, Bai Tianyu from Anhui Radio, Film and Television Vocational and Technical College won the special prize, 6 contestants including Zhang Yuanxiang from Hefei College won the first prize, and 8 contestants including Wu Xina from Anhui Medical University won the second prize. The activity site was also awarded the excellent teacher guidance award and the excellent organization award. Since its launch, this speech contest has won wide attention and participation of college students in the province. Competition aims to encourage the young students continue to strengthen party history learning education, from the one hundred inspire faith, inspired, draw strength, further enhance the consciousness of "four", "four" confidence "," two maintenance ", inherit red genes, set up for the motherland permanent struggle for the people, chicheng dedication of firm faith, strive to do reasonable credit, chong DE practice.

2021-06-22	Wenzhou Medical University innovative Party history education launched menu party history micro course in the vivid story of the party's spiritual monument of Wenzhou Medical University innovation Party history education launched menu party history micro course Jianwei correspondent Li Lujing) " the original Party history course can also be so, we all listen to."This semester, Wenzhou Medical University is active in such a group of people, carefully made a menu-style party history micro class, using the break time to tell the students about the original aspiration and mission of the Chinese Communists. They are the teachers and students of the "809000" lecture group. Party history class how to let the students "listen to enter, forget"?"indoctrination theory education, students often not easy to accept. To let the young people to 'cook', with the perspective of youth, youth thinking, youth practice, youth innovation, tailor the new era party history of the taste of the youth of Wen Medical University, to tell the party history story 'interesting', 'all love to listen to' and 'really believe'."Said Cheng Jinguo, deputy secretary of the university Party Committee. Lin Xuanru, a student from the Second Clinical Medical College, found the answer from the "walking red classroom". The story of Haixia excavated and recorded in the study is taken as a fresh teaching material for the study and education of the party history, and more students can understand and practice the Haixia spirit of the new era through the publicity. And Lin Xuanru, nursing school "big Chen island reclamation spirit" red studies of Wang Mengru, Alberta college "package to the prairie fire club" red studies of Chen Jiayi, flag guard "search flag guard inheritance iron soul" red studies SheHui students, also choose to speak good in Zhejiang local party history story, the "walking red classroom" practice found touching story and profound comprehension into a vivid ideological micro lesson, bring back to the campus to the students. They jointly launched the sea-a new era of "mulan" call "the pearl of the east China sea big Chen island, written" reclamation heart "", "under the flag of youth" "Wenzhou modern gene code: start a prairie fire spirit start a prairie fire" excellent education micro courses, let learning thought practice enlightenment organic connection, continue to promote the party history learning education "live" "deep". Huang Junting, a student from the First Clinical Medical College (School of Information and Engineering), chose to tell the shining stories of the teachers and students on the campus well. She condensed her personal experience into the 8-minute ideological and political micro course of "Go To Great Love, Be a Life Ferry Man", and told the young people of Wen Medical University with the shining words and deeds of the predecessors of Wen Medical University and their own personal feelings. Liu Meihua teacher north latitude 35 degrees of medical- -the power of youth and bear, Wang Yuqin teacher "grape hall, light up your eyes", Chen Zhengteacher "" green pepper "eyes" mutual care ", Du Xinyi classmates" played a new era of youth ", the youth of Xie Huating classmates," enough youth "all choose" hardcore ", in the school history, in" volunteer service warm medical phenomenon "," in the "green and university dream forward", in the "university youth example power", about the struggle story, with people around you, with moving people around. It is reported that since January this year, the school started the establishment of Wen Medical University 809000 publicity group, the first batch of recruitment of 37 after 80,90,00 after the youth publicity team, with the youth power to innovate the momentum of the party history publicity. Members of the lecture group adopted a lively and popular form to "ignite" the class during the break. Up to now, they have preached more than 300 times around the themes of "Party history education", "red classic", "development achievement", "youth growth", "service and dedication", reaching more than 1.5 people.

2021-06-21	Drama "the communist declaration" in shaanxi xi 'an jiaotong university XianZi hall "tribute one hundred does not forget youth pledge" -drama "communist declaration" in shaanxi xi' an jiaotong university XianZi hall Ann jiaotong university youth corps committee for Beijing on June 21 (reporter Zhang Jianwei, correspondent BiXiaonan) recently, by the shaanxi provincial party committee propaganda department, shaanxi party committee education committee, shaanxi party committee, the communist youth league jointly sponsored "tribute founding one hundred does not forget youth pledge" -drama "the communist declaration" into university rehearsal learning activities in xi'an jiaotong university XianZi hall. Xu Yongsheng, deputy secretary of the Communist Youth League, more than 1,400 people from the university Party Committee, trade union, Research and Work Department, Academic Affairs Office, Student Affairs Office, Security Department, Party committee, Youth League Committee and other departments, representatives of relevant units in Shaanxi Province, Xi'an, teachers, students and party members watched and studied the drama party class. The event was broadcast live simultaneously, with more than 15,000 people online. The picture shows the drama "The Communist Manifesto" being staged at Xi'an Jiaotong University. The drama is professionally guided by Xi 'an Chinese Opera Theatre Co., Ltd., Xi' an Performing Arts Group, and performed by the Student Drama Troupe of Xi'an Jiaotong University. The play made full use of the stage art of drama, successfully shaped the 1920s, a large number of communist revolutionaries who love light, would rather die than surrender, firm belief under the guidance of the spirit of the Communist Manifesto, to explore the truth, the pursuit of ideals and faith stories. The picture shows the drama "The Communist Manifesto" being staged at Xi'an Jiaotong University. The drama "The Communist Manifesto" tells the story of the female Communist Party member Lin Yufei was arrested with the reactionary military and police, as well as the fate of the businessman Kuang Zhaohe's family. The whole performance was energetic, and the cast, crew and the teachers and students present were full of passion. The actors' wonderful scenes not only received the whole thunderous applause, but also brought a strong spiritual shock to the audience. Teachers and students have said that the drama "The Communist Manifesto" not only let everyone review the history of the Party, but also made everyone have a deeper understanding and understanding of the belief in Marxism. Wenzhi academy wisdom energy and intelligent manufacturing class 023 Lv Mingyang said excitedly: " when kuang shouted out 'choose new' at that moment, really make my heart, the power of faith is infinite, as a new era of college students, we should be firm in the communism belief, in the hard study and practical struggle for the great rejuvenation of the Chinese nation."The picture shows the whole party members comrade collective solemn oath. Xi 'an jiaotong university youth corps committee for "I volunteer to join the communist party of China, uphold the party's platform, abide by the constitution of the party, fulfill party member obligations, implement party decisions, strictly observe party discipline...." at the end of the performance of the stars, the party members comrade collective solemnly raised his right hand, clenched his right fist, review the party oath, feel the original heart and mission of the communist party of China, firm their ideal and faith. The performance is the first batch of universities in shaanxi province to undertake the communist declaration performance MSC, and xi 'an jiaotong university party history learning education activities is an important link, to promote school party history learning education activities and carry out khalid ents fundamental task has a positive role, can help teachers and students in the fresh stage performance review the party's history, the revolutionary martyrs, firm ideal faith, inheriting red genes, deeply understand youth responsibility in the new era and historical mission.

2021-06-21	The 17th Campus Top Ten Singer Final of Guizhou University College of Science and Technology, Correspondent of Jianwei, Zhong Shiyun, Xiao Yan, Yan Chun) Sing the youth dream, as a tribute to the centennial birthday. In order to flourish the campus culture, strengthen the construction of campus spiritual civilization, and provide a stage for students to show themselves, the 17th Campus Top Ten Singer Final and Pepsi Best Voice was held in the 211 lecture hall, sponsored by the College of Science and Technology Committee of the Communist Youth League and jointly organized by the Student Union and the student art troupe. More than 400 lifelong members of China Guqin Association, members of Guizhou Musicians Association, Party Committee, Youth League Committee and sponsors of College of Science and Technology, Guizhou University, teachers and students attended the activity. The picture shows the contestant's style. Correspondent for the competition scene, the contestants on the stage to show their talent, the whole competition process atmosphere is intense, applause and cheers, various styles of music "I eat fried chicken" in the people's square, "the love of nature", "red rose"... again and again the competition atmosphere to the climax, perfect interpretation of the "singing youth dream, tribute centennial birthday" theme. The picture shows the contestant's style. The judges of this competition scored the 20 contestants from four aspects: singing skills, intonation and rhythm, stage performance and original works. Finally, after a fierce competition, music performance 191 class Zhao Xiujie students won the first competition, music performance of 191 class Yang Yanhong students won the second competition, music performance of class 181 ocean students won the competition third place, music performance 201 class Liu Limei students won the competition fourth place, at the same time, the four students will be recommended to Guiyang for the best semi-final. The picture shows the contestant's style. Correspondent for figure won the first Zhao Xiujie students feel on the stage singing is a great thing, ranking is not so important, she said, " I really love the stage, to I want to sing songs to more and more people hear enough, all the contestants are best, everyone is great, can get the first place, and luck has a lot to do, thank you for all affirmation and applause, I will continue to work hard, for my love of things."It is reported that the competition lasted a total of ten days, more than 200 contestants signed up for the competition, after layers of selection, the last 20 contestants entered the final, the top ten for the competition top ten singers winners.

2021-06-17	In the class of "Party History learning and Education publicity" by participating in the publicity of the hundred years of Party history, the publicity teacher was selected by the students by "ordering". On the afternoon of May 31, the young Pioneers participated in the "Archives Open Day" activity of the provincial Archives, focusing on visiting the provincial archives "History will always remember" and "Gansu Memory" archives exhibition and the archives inspection center. At the beginning of the semester in March, how to do a good job in "course ideological and political affairs" has become a topic often discussed by teachers and students of Cuiying College of Lanzhou University, which is an important case creation activity for Cuiying College to promote the study and education of Party history. The activity takes all students as the main body, encourages students to explore the party history education elements in professional courses from their own perspective and integrate them into ideological and political teaching cases, guide students to deeply read the party history, learn, think and practice, understand and worship virtue, inherit the red gene, and cultivate the feelings of home and country. In "semiconductor physics" class, for chip and related industries including lithography machine, photoresist is restricting the development of our industrial industry technology, 2017 physics extraction English class students Yao Maheng tells the story of SMIC founder Mr Chang struggle for China chip, all the students listen very carefully, finally he said "I when so also"."Our ideological and political courses take students as the starting point and rely on the knowledge points of professional courses. While learning professional knowledge, students can collect and sort out relevant party history stories, scientists' spirit, core values and advanced concepts, so as to deeply integrate party history education, ideological and political education with professional curriculum education."Said Shang Sheng, deputy secretary of the Party general Party branch of Cuiying College. At present, The College has collected more than 230 cases of "course ideological and political affairs" submitted by students majoring in mathematics, physics, chemistry, biology, literature, history, philosophy and other majors."Broad bean stalk, low and low, bear soybean iron body.... "An emotional and melodious" Soybean Ballad " led the students of Lanshi Technical School through that arduous revolutionary era. On the same day, Liu Ting, a member of the Lanzhou City youth teaching group and a commentator of the Gansu Provincial Museum, reviewed the vigorous revolutionary stories in Gansu province, with "Red Gansu: Towards 1949" as the title."There is no platform, no PPT projection equipment, every desk, every chair and every microphone, and the preaching begins. This form is very good, and there is no sense of distance with the students face to face, and the preaching is more down-to-earth."Said Luo Wei, a young teacher at Lanshi Technical School. Students can independently list, choose the teachers and topics; the party history learning and education and the practice of socialist core values, strengthen the ideological and moral construction of minors, throughout the whole education and teaching process... Recently, through the "button classroom", "childlike innocence to the party" and other theme activities, enrich the history of party learning and education form and content, to create a popular party history learning and education activities, let the party history into the hearts of teenagers."The moment I took over the flag of the 'red day study' practice team, I felt a glorious mission. On the occasion of the 100th anniversary of the founding of the Party and the 85th anniversary of the victory of the Long March of the Red Army, our college student practice team should carry out the 'red day study' practice activities, experience the red culture, understand the revolutionary spirit, and take the long March in the new era."Said Li Wenyuan, head of the college student practice team of Gansu Normal University for Nationalities. Gansu Normal University for Nationalities has recently launched an education activity with the theme of "Taking the Long March by Learning the Party History Online" with the theme of "Inheriting the red gene and taking the Long March in the new Era". Six universities along the Long March participated in the Long March, initiated by the initiative of Yan'an University. During the activity, teachers and students will sort out and show the historical stories of the Red Army when the Long March passed through its region in cyberspace. Recently, Lanzhou College of Resources and Environment launched the red film "Guild Wars Doujiashan" has attracted the attention of all teachers and students."Our campus is adjacent to the battlefield of the Lanzhou Campaign during the War of Liberation. By exploring the red resources around us, the school has launched red-themed film dramas such as micro films like Guild Wars Doujiashan and Example Five, which makes us learn the history of the Party more vivid, interesting and easier to understand."Said Sun Yinghao, a student at Lanzhou University of the Environment. Through cohesion "online" party history learning education mode, Lanzhou institute launched red speech "than", online learning "know" party history, drama film "product" party history, various forms of "set", TikTok WeChat "brush" party history, practice education "enlightenment" party history, party history learning to realize the complete coverage of the campus. Recently, under the organization of the Youth League Committee of Lanzhou University of Technology, the party history publicity group composed of 13 young people with good expression ability, strong appeal and excellent quality went into the league branch of the class and had a "dialogue about youth" with the youth members, bringing a wonderful party history publicity to the students. When talking about General Deng Baoshan, who devoted his life to the defense of the motherland, and Gao Yongxiang, the Anti-Japanese War hero who emerged from the land of Lingtai, Gansu Province, the students attentively read the glorious deeds of these great ancestors, and felt excited. The relevant person in charge of Lanzhou University of Technology said that with the publicity group into a league branch, the party's hundred years of history emerged in front of us again and again, infiltrating the hearts of college students.(Pictures of this edition are provided by Gansu Provincial Committee) China Youth Daily China Youth Network reporter Ma Fuchun source: China Youth Daily

2021-06-17	Zhou Wenjiang, a student of grade 17 of the "Baymax Studio" at Hubei University, was once depressed. He has participated in employment recruitment many times, but due to a long job and postgraduate entrance examination hesitation, lack of employment preparation, has not found a job near graduation. To this end, the big white studio guided Zhou Wenjiang to change his employment concept. After fully understanding his employment intention, it accurately selected the recruitment enterprises and provided three suitable units, and also guided their interview skills. Before graduation, Zhou Wenjiang signed an employment agreement with a Guangzhou pharmaceutical and foreign trade company."Getting students to timely grasp the effective employment information is the key to achieving employment. To provide students with employment difficulties with the job information they really need to achieve efficient employment."Said Liang Hao, a teacher at the Big White Studio. Founded in 2017, Baymax Studio is teachers of the Engineering Office of the School of Chemistry and Chemical Engineering. It specializes in solving the difficulties of students in all aspects. Since its establishment, it has helped more than 100 students with economic difficulties, academic difficulties and employment difficulties. In the employment assistance, Baymax Studio implements the relevant requirements of graduate employment guidance and service, and guides the career planning of students of all grades in the college by layers and classification. Zhu Liyun, a pharmaceutical engineering student at the college, comes from rural Gansu province. Her father died early and her family had financial difficulties. She suffered a psychological crisis at school and it was difficult to continue her studies. To this end, Baymax Studio tailored a set of assistance plan for her, recommended her to apply for work-study positions, and get paid for part-time jobs in her spare time, so as to relieve the economic pressure and study at ease. In view of Zhu Liyun's psychological problems, Baymax Studio also formulated an emotional counseling plan for her and established a good communication and interaction mechanism. After a period of guidance, Zhu Liyun's mood stabilized, and her study and life gradually got on the right track. White studio since the beginning of the new entrance for their career planning, organize freshmen in college students' career planning competition, career planning courses, around personal self-awareness, target career cognition, target career orientation and planning, etc., let the students clear the basic composition and method of career planning. In a comprehensive grasp of graduate employment progress and employment psychological dynamic, on the basis of white studio for family economic difficulties, disability, one's deceased father grind difficulties, employment enthusiasm is not strong, such as students adhere to the principle of "one cannot be less", pay attention to humanistic care and psychological counseling, after fully understand their employment intention, targeted to recommend jobs to them. In the graduation season of 2020, Liu Chang, a 2016 student at the college, narrowly missed his favorite school in the postgraduate entrance exam. After communicating with his family and counselors, he decided to take the postgraduate entrance exam in "World War II". Since then, Baymax Studio has not only provided him with a scientific research assistant position in the college to relieve his financial pressure, but also contacted the postgraduate entrance examination school and his tutor to explain the skills of the second interview to him. This year, with his own efforts and the help of the college, Liu Chang successfully took the postgraduate entrance examination. Hubei, Hubei university college deputy party secretary of liu said, white studio combined with the characteristics of students 'learning, hierarchical classification guidance, personalized employment guidance activities, guide students to set up the correct career idea, enhance their employment ability and employment consciousness, both to promote graduates more fully, high quality employment, and improve students' employment quality and employment satisfaction. In order to increase innovative measures, Baymax Studio also focuses on "Party branch +, graduating class counselor +, employed graduates +, class committee +, alumni +, class +" to promote employment, and mobilize professional teachers, counselors, class teachers and alumni to integrate employment information and gather employment assistance forces. Gan ��, secretary of the Youth League Committee of Hubei University, said that in order to highlight accurate assistance and expand employment channels, the school Youth League Committee not only invited employment guidance and career planning lecturers to teach online and build a solid foundation for employment education, but also invited entrepreneurship mentors and alumni entrepreneurs from inside and outside the university to hold discussion salons to guide students to choose a reasonable career and correct employment. Li Chuan source: China Youth Daily

2021-06-16	Huainan joint university to carry out the history of the party theme education practice of Huainan joint university youth corps committee to carry out the party "childlike innocence to the history of the party theme education practice JianWei correspondent Jiang Bo check WeiTing) recently, the Huainan joint university youth corps committee organization school trainee student cadres in the trainee kindergarten to carry out the" childlike innocence to the party " party history theme education practice. During the activity, the trainee learn dry in the youth corps committee staff and under the guidance of professional teachers, through the story of revolutionary hero, chorus red songs, draw red picture scroll and other forms of patriotism, collectivism and national spirit education, the children acceptable way to guide them to understand the communist party of China in one hundred glorious course and struggle spirit, guide them to listen to the party, follow the party's determination, to the seeds of love the party patriotic socialism in the children's heart.

2021-06-22	Foshan college of science and technology to carry out the world blood donor day series activities of the campus of Foshan college of science and technology to carry out the world blood donor day series of campus blood donation activities for China youth network Beijing on June 22 (reporter Zhang Jianwei, correspondent, Jiang Zunguo Luo Yanni), Foshan college of science and technology to carry out the world blood donor day series of campus blood donation activities. Zhong Feijian, deputy secretary of the school party committee, took the lead to participate in love blood donation, the majority of teachers and students see the blood donation initiative, actively registered blood donation, with practical action of blood to fight the epidemic, the three campuses 592 blood donation, 183,300 ml, including: May 19 to 21269 anxi campus 88,700 ml; on May 26 riverside campus 26 people donated 8200 ml; June 16 to 17 Jiangwan campus 297 people donated 86400 ml. Under the mobilization and demonstration of the school leaders, the majority of young teachers and students actively participated in the blood donation activities. According to the requirements of epidemic prevention and control, they wore masks, did not gather, waited one meter apart, and queued up for blood donation in an orderly manner. During the whole activity, the majority of students and volunteers were not afraid of the heat, and guided the teachers and students to fill in forms, measure blood pressure, blood test, blood drawing, queuing, orderly management, and distributed food, mineral water, masks, souvenirs to the teachers and students.

2021-06-16	The Youth League Committee of China Academy of Thermal organized the youth league members to carry out the party lessons to study and educate the Party history of the Party. Correspondent for the picture of China Youth network Beijing, June 16 (reporter Zhang Jianwei, correspondent Zhang Shaoshuai) a few days ago, the Chinese Academy of Thermal Science Youth League Committee innovation of the party history learning organization form, high standard, full caliber to carry out "learning the party history of the school history dedication youth intelligence struggle hot science cause" series of activities. Wang Qinghuang, president of the Chinese Academy of Thermal, and Cui Pengwei, secretary of the Party Leadership Group of the Academy attended the event and gave a vivid and profound party lesson to the youth members of the Academy, encouraging the youth of Rehe to remember their original mission, dare to take responsibility, and actively contribute their talents to the cause of tropical agricultural science and technology innovation. Chinese Academy of Thermal Sciences Youth League Committee "circuit Party class" site. Correspondent for figure according to the Chinese hot academy youth league secretary Zhu Anhong, school youth league will be party history learning education practice, knowledge contest, theme essay, discussion communication series of activities, the party history learning education do deep, guide the youth members based on post achievements, for the implementation of the "difference" plan, achieve 2035 vision contribution youth strength, to celebrate the 100th anniversary of the founding with honors.

2021-06-12	Guizhou university the third campus dubbing contest held successfully in Guizhou university "to the voice of youth, praise in one hundred" the third campus dubbing contest successfully held in Beijing on June 12 (reporter Zhang Jianwei, correspondent, yan-chun liu, Song Hongqing shu qi), Guizhou university "to the voice of youth, praise in one hundred" the third campus dubbing contest was held successfully held. More than 300 teachers and students representatives of Guizhou University attended the meeting. There are nearly 200 students who love dubbing to signed up for the competition. After preliminary evaluation, 15 teams entered the final. In the final scene, the contestants showed themselves heartily and fully enjoyed the game. In the process of the competition also inserted "learn the history of the Party, celebrate a hundred years" knowledge answer and "wechat on the wall" two small games. The picture shows the dubbing contest scene. Correspondent for figure, in order to continue to promote party history learning education, Guizhou university communist youth league held four history essay contest, poster cartoon contest, short video contest, three market poetry, four history knowledge contest, calligraphy competition, and actively organize "youth branch", "college students XuanJiangTuan" hundred community to preach, hundreds to preach into branch preaching activities, promoting party history learning education and special education work take root in the youth.

2021-06-18	Hainan software vocational and technical college "five up" to promote the party history study deep solid Hainan software vocational and technical college communist youth league system through "five up" to promote party history learning deep construction correspondent Zhou Yutong) independent learning live, organization strong up, teachers busy, learning atmosphere, do practical work... since the development of party history learning education, Hainan software vocational and technical college communist youth league system through the "five up", further promote the party history study deep solid, has achieved very good learning education effect. The picture shows the students studying the party history independently in the dormitory. In the party history learning education, the school Youth League committee launched on the public account "Party history learning every day learning" column. Organize all students to participate in the series of online theme group classes of "Youth Learning and the History of the Party together". Guide students to carry out party history study in the party history study column of yi class and school library websites. Through the introduction of the "menu type" party history learning content, multiple measures to realize the students' independent study of the party history, guide students to independently study the party history learning alive. The picture shows the majority of students participating in the party history reading class study. With the branch general meeting as the main form, the study of the Party history and the "three meetings, two systems, one system and one lesson" and other daily organizational system of the Communist Youth League combined, carefully organized reading classes, sharing meetings, special guidance, multiple measures to carry out the party history study and education. Give full play to the advantages of organizational learning, ensure that each league branch carries out at least more than four party history study meetings throughout the year, and all the members of the league will carry out one special organizational meeting with the league branch as the unit, so that the study of Party history is more organized. The picture shows Liu Mingpeng, deputy secretary of the university Party Committee, awarding the letter of appointment to the teaching group. Relying on the "Young Marxist training project" training class, the school Youth League Committee set up the special guidance on Party history and the Party history learning education theme group class, to share the learning experience of Party history. Members of the teaching group taught the party history to the young students in the Youth League committee of the secondary college. Through the "busy teaching group", to build a professional team, deepen the study of the important party history content, and guide the students to continue the red blood, bear the responsibility of The Times. The picture shows the "Red Inheritance all the way with you" calligraphy competition. The picture shows the campus marathon of ". Correspondent for figure held series of cultural activities, learning atmosphere of the communist youth league system relying on the student union, student associations and secondary college youth corps committee, held the "one hundred party history reading China" classic reading contest, "I learn the party history" fun activities, "one hundred people" campus marathon, "learn party history, strong faith, follow the party" red song chorus, at the end of each month "unplugged" red song concert, "red inheritance" calligraphy contest and other campus cultural activities, make learning atmosphere thick. Young teachers and students of China were also organized to record various forms of "experiential" Party history learning and education, such as videos of "Singing a Folk Song to the Party" and "We are all dreamers", so that students can deeply grasp why the Communist Party of China can, why Marxism works, and why socialism with Chinese characteristics is good. The picture shows the teachers and students providing English explanation and guarantee services for the Boao Annual Conference. The picture shows the volunteers ensuring the COVID-19 vaccination. Correspondent for the picture to carry out "I do something for the youth" theme practice activities-do practical things do up. This year marks the 100th anniversary of the founding of the Communist Party of China. The ultimate goal of the whole Party in conducting education on Party history is to do practical things for the people. To this end, the Communist Youth League system of Hainan Software Vocational and Technical College has carried out the theme of "I do something for the youth" practice activities, so that it can do practical things for the students. In April, the Youth League Committee of the school organized minor students to visit and study at the Juvenile Legal Education Base of Qionghai People's Procuratorate. A 130 volunteer service team composed of five English teachers and 125 student volunteers was dispatched to provide English explanation and volunteer services for foreign guests visiting five beautiful villages in Qionghai. In May, 100 volunteers were organized to ensure the vaccination of all teachers and students, and 150 outstanding volunteers excellently completed the service and guarantee work of "The Red Detachment of Women 2021 Qionghai 10,000 People Hiking Activity". Volunteers went into the Haiguifang Community to carry out community computer maintenance and network maintenance volunteer activities, and the "Wu Xiaozhi" science and technology service team went to Qionghai No.1 Primary School and Qionghai Experimental Primary School to carry out the "Science and Technology into the School" activities. In June, I carried out practical activities such as "I do practical things for the youth, get free mineral water" and "I do practical things for the youth, drug prevention education". In July, we will coordinate social practice and volunteer services such as "going to the countryside", "going back to our hometown to fight the epidemic", and the league members reporting to communities (villages). Through practical activities, students do enhance their sense of gain and happiness, so that students know history and love the party, know history and patriotism in practice.

2021-06-17	Hainan Economic and Trade Vocational College School of International Trade to carry out Party History Learning Education School of International Trade "six studies" to promote college students youth League members Party history learning education to do deep go practical correspondent Zhou Yutong) Since the development of Party history learning education, The General Branch of the School of International Trade of Hainan Vocational College of Economics and Trade combined with the actual work of the school, The first time to set up the general branch of the Communist Youth League party history learning and education leading group, Formulate the implementation plan for party history study and education, To compile the study and education manual of "Learning the history of the Party, strengthening faith, and following the Party", Held the whole learning and education mobilization meeting, Adhere to the "six studies" linkage, We will promote the study of party history for college students to be deeper and deeper. The picture shows the Youth League General Branch of the School of International Trade of Hainan Vocational College of Economics and Trade carrying out party history learning and education. Correspondent for picture cadres to set an example "learn". All the cadres of the Communist Youth League General Branch of the college set an example to "lead learning", insist on learning first, give full play to the leading role of the cadres of the Communist Youth League, learn to use the independently compiled Study Manual, consolidate the online positions such as the "International Trade Pioneer" wechat public platform, and turn paper materials into audiobooks. Around the party history study and education and semester work plan, through the regular convening of the league work deployment meeting, the implementation of the monthly work list system, strengthen work exchanges, further unified thinking. Excellent communist youth league cadres, Hainan province, party history learning education XuanJiangTuan members take the lead on the party, college league launched and joint two college synchronous "learn the history of the party, strong faith, follow the party" learning education XuanJiangHui, through the province optimal TuanGan DangTuanTang, up for good youth advanced deeds sharing, youth member youth talk experience lead the thoughts of youth, expand the influence of the youth league, a total of more than 83000 people to attend. The picture shows the Youth League General Branch of the School of International Trade of Hainan Vocational College of Economics and Trade carrying out party history learning and education. Correspondent for the picture of the student organization to seriously fulfill the responsibility of the "school inspector". Clarify the task list of party history learning and education, drive the party history learning and education in the form of activities, layer upon layer compaction class league branch secretary, organization committee members and other grass-roots league cadres, so as to ensure that the direction does not deviate, the prescribed action does not go out of shape. The general branch of the Youth League of the college has included the learning and education assessment into the May 4th evaluation, the recommendation of excellent League members for party membership, and the development of the league members, and further "supervised the school" through quantitative indicators and integral evaluation. Pay attention to the advantages of student organization and self-management, strengthen the online popularization of "youth learning" through competition ranking and weekly notification, and strictly control the progress of learning. The participation rate of "youth learning" of the college league members has reached 100% for 14 consecutive weeks. The picture shows the Youth League General Branch of the School of International Trade of Hainan Vocational College of Economics and Trade carrying out party history learning and education. Correspondent for the picture youth league branch innovative method "study". Innovate ways and methods, guide college student league members to draw wisdom and strength from learning the history of the Party, and constantly transform the learning results into learning motivation. According to the actual work of the Communist Youth League, organize the study activities, branch committee, league member evaluation meeting and so on; strictly implement the collective study system every Tuesday afternoon, carry out special study and discussion; conduct practical education activities of "study party history, strong faith and follow the Party" on the theme league day; and make good use of "Youth Study" and "International Trade Pioneer" WeChat public platform to deepen the party history league class into the class, class and dormitory. A total of 1,571 students from 38 youth league branches in the school have carried out special studies around four themes: the new democratic revolution, socialist revolution and construction, reform and opening up, and the new era of socialism with Chinese characteristics, to further enhance the vitality of the grass-roots youth league branches. The general branch of the youth League takes the party history learning education as an important index to select the annual advanced youth League branch and outstanding Communist Youth League members. The picture shows the Youth League General Branch of the School of International Trade of Hainan Vocational College of Economics and Trade carrying out party history learning and education. Correspondent for the picture of the youth league members interactive exchange "than learning". The general branch of the Youth League of the college held a symposium on the youth of the college students' league members, around the theme of how to make a good start with the youth of the study and present a hundred anniversary of achievements ". The youth members respectively exchanged the ideas of "comparing, learning and catching up" on the Communist Youth League "three forces once", talked about their experience and put forward suggestions, laying a good foundation for the development of party history learning and education. At the same time, according to the learning characteristics of college students 'youth league members, the general branch of the Youth League of the college has developed the online brush question bank and online competition system of party history knowledge, and the fragmented learning of college students' youth league members is highly popular. Up to now, a total of 5,616 people have participated in the party history knowledge contest of the college, and all the students of the college have participated for 2.4 times, with the penetration rate reaching 100%. The second classroom reform explores "promoting learning". The general branch of the college youth League Jiao gathered the needs of the youth league members, and made good use of the "second classroom report card system", so that the party history learning and education into the hearts of young college students. Further combine professional education and party history learning education, through the special study, building the party centennial professional etiquette exercise contest, the party history micro group contest, red viewing activities, playing "the sun, chairman MAO's closest" red song chorus and a series of campus cultural activities, in the tide of historical change of college students member youth to ride the positive energy. Since the beginning of the party history learning and education, the college student Union, Youth Volunteers Association, E-commerce Association and other student organizations have carried out more than 130 red-themed second classroom activities, and 5,850 people have participated. The picture shows the Youth League General Branch of the School of International Trade of Hainan Vocational College of Economics and Trade carrying out party history learning and education. Correspondent for the immersive red landmark "practice learning". Integrate the red resources of the whole province, clock in the red landmarks, carry out on-site teaching, and inherit the red gene. College set up "youth backbone" red practice education center, college league led the youth successively to Hainan liberation park, wuzhrevolutionary base memorial park, baisha uprising site, Sanya xisha battle martyrs cemetery, wanning six ridge revolutionary base to carry out red "practice", member youth immersive appreciate eventful years, revisit the oath, to the examine yourself, looking for theoretical nourishment, accept thought baptism. Organize teachers and students to experience the great spirit of the "Red flag of the 23 years of the Qiongya Revolution", carry out the theme group day activities at the former site of the Lingshui Soviet Government, and experience the difficulty of preserving the first red regime in Qiongya. The majority of youth league members through time and space, touch the history, in the pursuit of the glorious course of the party was infected by the education, with practical actions to seriously practice the party flag is the direction of the league flag.

2021-06-10	Hainan university of the first community sports festival campus hiking activities successfully held in Hainan university "celebrate the founding in one hundred, strong youth body" the first community sports festival campus hiking activities held on June 10 (reporter Zhang Jianwei, correspondent Zhou Yutong) on June 6, Hainan university "celebrate the founding in one hundred, strong youth body" the first community sports festival campus hiking activities was successfully held in haidian campus. Zhu Rongping, director of the Security Department of Hainan University, Chen Weidong, secretary of the General Party Branch of the School of Physical Education, Gong Yanfeng, deputy secretary of the Youth League Committee, Li Changyu, deputy director of the Student Affairs Department, and Cui Jianbing, deputy department-level counselor attended the event. The event was jointly organized by the Youth League Committee of Hainan University and the School of Physical Education. Nearly 300 teachers and students participated in the event. The picture shows the activity site. At the beginning of the activity, all the teachers and students of Hainan University watched the video "Learn the Party History with the General Secretary", reviewed the glorious course of the Party, praised the great achievements of the Party, strengthened the determination to follow the Party forever, and further stimulated the love of patriotism and the Party. The picture shows the activity site. Secretary Chen Weidong delivered a speech on behalf of the organizer, emphasizing that college students should attach great importance to physical exercise, actively participate in physical exercise, cultivate people with sports wisdom, and cast their soul with strong body. He hoped to take this sports festival as an opportunity, the whole school will further form a strong atmosphere of loving sports and promoting the spirit of sports, and transform the indomitable fighting spirit contained in sports activities into a strong driving force for the university to build a domestic first-class university and a world-class discipline. The picture shows the activity site. The roller skating Association and the martial arts Association will bring a warm-up performance for this activity. Hiking in Hainan university source school (north) as the starting point, along the main road around the campus, about 3.5 km, the road has five clock and 10 one hundred party history event publicity column, volunteers for teachers and students on the party history events, innovative party history learning education and sports fitness activities, combining the history of the party forward into the sea of teachers and students never stop steps, reveal the Hainan university party history learning education has been "on the road".

2021-06-10	Heilongjiang university held party history learning education about Heilongjiang university party history learning education to preach) Heilongjiang university recently held theme learning education youth students XuanJiangTuan preach report, four party history learning education XuanJiangTuan representative around the "transmission times strong voice, power poverty engines, persistent mission glory, practice disease resistance spirit" share growth experience, vivid interpretation of the new era of youth bear. Gong Jiaxin, from the Heilongjiang Radio and Television Host Competition, one of the top 10 students, said, " As a college student, I have entered the team and the league oath. Now when my heart goes to the Party, I open the oath of joining the Party, sonorous words dusty historical figures and vivid stories. The black land raised me, the story of the Northeast Resistance Union inspired me to take root in the fertile soil of Longjiang, tell the youth story, issued a black strong voice."Wang Danyang, a student on the road to poverty alleviation, said," Since 2015, I have been to remote mountainous areas in Guizhou and Chongqing four times to send education to the countryside, and twice to Luobei County and Hongtuya Town of Heilongjiang Province to help farmers out of poverty. From the northeast to the southwest, from the field to the three feet platform, I always hold the party and the people's one cavity sincere struggle in the front line."Retired college students soldiers Zhang Guowei classmates said:" xi jinping, general secretary of the new college students to nankai university, let me encouraged, determined to write, the youth dream, revolutionary ideal, the country feelings always shoulder on the youth, will 'revolutionary reason above the day' mission to bear as a lifelong faith and pursuit."The national COVID-19 outbreak youth volunteer service advanced individual, Heilongjiang province prosperous graduate students Zhao Zixu classmates said:" I am proud of the volunteers, it will be my firefly fire into the flame of disease resistance, my patriotic side faith rooted in mohe permafrost, I volunteer to youth bloom in the motherland and the people need most need!”

2021-06-09	Zhengzhou university: let the party history education "real" Zhengzhou university: let the party history education "real" relatives deeply attached heroic, with life write love to the people and loyalty to the party.... "at the end of April, Zhengzhou university" one hundred years I tell " activity launch ceremony, information management school student Wang Yige tells the story of anti-japanese hero Zhao Yiman, present teachers and students in tears. After the ceremony, 38 outstanding students went to different departments to tell the students the knowledge of party history and red stories from different aspects from the perspective of the students. Wu Hongyang, deputy secretary of the Party Committee of the university, said that "One Hundred years of Party history" is an important starting point for the party history learning and education of the university. With the language and form closer to the youth, it effectively promotes the party history learning and education to achieve remarkable results among students. The event is just a small microcosm of Zhengzhou University's party history learning and education since March. In recent months, Zhengzhou University attaches great importance to the study and education of party history for students, which has ignited the enthusiasm of all the teachers and students and set off one upsurge of party history learning and education after another. The school has held with a party history class, thousands of class party history study reading class, "one hundred emerged today, beginner's mind mission bear revival" party history drama contest, "the world expand international vision, learning history LiGeng power bear" simulation diplomats style contest tribute founding series of activities in one hundred, further broaden the students' party history learning education path. Song Zhonghui, secretary of the Communist Party Committee of Zhengzhou University, who "teaches more than 70,000 students", gave the lecture with the topic of "100,000 grand Party people first". He encouraged young students to combine their personal growth with national development and national rejuvenation, and strive to forge themselves into new people with firm ideals and beliefs who can shoulder the great responsibility of national rejuvenation. Each department has also carried out its own distinctive party history learning and education in combination with its own reality. The School of Journalism and Communication held "New Youth Talk" with the theme of "Xiaokang Talk" and "My Red Memory", Sixty young students told the stories of the Communist Party of China leading the people in fighting against poverty and the stories of revolutionary history; The School of Materials Science and Engineering has invited experts and scholars to " aim high and consistent, How to find the 'big science' from the'small problems "," Carry out theoretical micro-class for students; School of Water Conservancy Science and Engineering invited Jiang Peng, chief engineer of FAST, to give a special report; Law school organized the third party branch of undergraduate students development objects into Zhengzhou Erqi Memorial Hall and Erqi Memorial Hall, Reviewing the revolutionary passion years in the strong atmosphere of Chinese workers' revolutionary spirit... not only call on students to participate extensively, The Student Work Department of the Party Committee of Zhengzhou University also organized the "one hundred Party History publicity activities for 100 counselors", One hundred and eighty-two full-time and part-time counselors met students face to face, With the theme of "More than 100 Years of theory and Practice of the Communist Party of China", It covers 60,000 students. In addition to promoting the study and education of Party history into the mind, the Student Work Department of the Party Committee of Zhengzhou University also closely focused on the theme of "doing practical things for teachers and students" to carry out solid practical activities. Zhengzhou University has always put its students in the most important position. Improve the student evaluation system, The participation in labor education and aesthetic education into the students' comprehensive quality evaluation method, Formulate an evaluation system that can reflect the concept of scientific talent and promote the comprehensive development of students' morality, intelligence, physique, beauty and labor; Care for students with financial difficulties, Carry out party history learning, education and perception sharing activities among students from poor families, Let the students in the school warm care in the school firm ideal faith; To provide round-trip travel fee subsidies for poor students from registered families who participate in the second examination of postgraduate entrance examination, Help the poor students to smooth the second test; Print and printing "Students' Ideological and Political Work Theory Study Pleaf", Launched the party history story, "one hundred pictures, one hundred exhibition", "theoretical study" column, It is convenient for teachers and students to learn anytime and anywhere, and they can read the authoritative articles and materials of party history learning and education everywhere. Liu Chao, director of the Student Engineering Department of Zhengzhou University Party Committee, said that in the next step, we will continue to focus on "I do practical things for the youth", guide students to firm their ideals and beliefs, inherit the spirit of struggle, and strive to meet the needs of students and do practical things for students, so that the education of Party history can blossom among young students. China Youth Daily China Youth Network trainee reporter Wang Lulu source: China Youth Daily

2021-06-11	Jiangsu: Huaiyin Institute of Technology Innovation and Entrepreneurship College of Huaiyin Institute of Technology held a promotion meeting of the centenary years of the founding of the Party to create a brilliant " Party history learning and education promotion meeting. China youth network correspondent Yu Chunhong for figure China youth network Beijing on June 11 (reporter Li Chuan correspondent Yu Chunhong) to celebrate the party's birthday in one hundred, inspire the teachers and students firm ideal faith, brave era responsibility, further promote the party history learning education go deep, go real, heart, on June 9, Jiangsu huaiyin institute of technology innovation entrepreneurship institute held "have one hundred years to see today brilliant" party history learning education advance. At the beginning of the conference, Zhang Linlin, associate professor of Marxism School of Huaiyin Institute of Technology, delivered a speech. She to "draw entrepreneurial wisdom from the party history in one hundred years", from "strong team theory, team ideal faith, team organization ability is strong, team mass base, team self revolutionary consciousness" five dimensions, simple to the teachers and students to present the communist party of China from small to large, from weak to strong, from a boat to wei ship struggle and the great innovative entrepreneurial spirit. In interactive link, huaiyin institute of technology closes working committee member Zhang Naizhong, young teachers represent Yang Liuqing, and young students represent Kim, Sun Shuhong exchanges, delegates said, to constantly learn from the party history learning education, to draw the wisdom from the history of the party and power, transform their own development power, overcome difficulties, solve problems, constantly explore, striving, let the party history learning is permeated with youth. Advance meeting, huaiyin institute of innovation entrepreneurship dean Zhao Zhiguo reviewed the struggle of the communist party of China in one hundred, reviewed the development of the college since May 2019, and called on the partial party activists to do history Ming, credit, chong DE, history, set up the correct party history, strengthen the confidence and strength, firm marxist belief and broad ideal of communism, and the unremitting struggle. Innovation entrepreneurship college directly under the party branch secretary liu chaired the meeting, its from the perspective of the theoretical innovation, system innovation and practice innovation, the history of the communist party of China is a fresh innovation history, and encourage college teachers and students party members from party history learning education inspire faith, nutrition, red gene, sheer beginner's mind mission, founding with excellent achievements in one hundred years. Members of the Party History Learning and Education Supervision Group of Huaiyin Institute of Technology, all party members of the Party branch directly under the School of Innovation and Entrepreneurship, and active members of students' Party membership attended the meeting.

2021-06-11	Shandong University Youth League Committee has extensively carried out the "I Do something for the Youth" theme education action, reporter Xing Ting, "Know the students 'temperature, relieve the students' worries" - -in the campus of Shandong University, heart-warming stories are happening every day. The Youth League Committee of Shandong University has transformed the effect of party history learning and education into the internal driving force of "I do practical things for the masses", and has extensively carried out the education action with the theme of "I do something for the youth" among the youth league members. The Youth League Committee of Shandong University has highly unified the development of serving students and helped the construction of the school, focused on the shortcomings and universal demands of campus governance that are closely related to students, and taken "I do something for the youth" as an important work carrier of Party history learning and education."Right Xiaoyi studio" in this semester for the whole school undergraduate and graduate students to collect student development rights and interests for 693, through the support of the school functional departments to effectively solve the problem for 683, sports field installed lighting, library open leisure room, additional self-service printer... students' appeal from paper to reality. Relying on the campus "Changyishan University" proposal competition, the Student Union of Shandong University has extensively understood the needs of students, found and solved the practical problems existing in the campus, and received a total of 168 valid proposals. In addition, the "See Mountain University" student apartment design competition, 315 Equity Activity Week, "Canteen Recipes I Will Set" food improvement activities and seminars between teachers and students in each campus to build a bridge and link, and give full play to the positive role of young students in protecting the rights and interests of teachers and students and promoting the high-quality development of the campus. With volunteer service to lead the study and history to practice, to practice the spirit of Lei Feng against the Shandong University Party Committee on the party history learning and education arrangements, gather the potential energy of the Party building work, Shandong University Youth League Committee gives full play to the advantages of the Communist Youth League organization, position advantages, and combine the party history learning and education with solving practical problems for the masses."The hope hut" in Shandong university child care project relying on Shandong province youth gay, tencent public platform launched "hope house in May charity month" activities, the college launched "a picture read hope hut" "hope hut tender story" young students popular organization form, so far, the school has raised more than 40000 yuan, form a "team" 416. At the same time, the united Zibo municipal party committee held "learn the history of the party, strong faith, follow the party" Shandong university to zi "four history" red theme education and "green bird plan" of Shandong university school docking professional exploration special activities, the system has carried out a series of various forms, rich content activities, with the ideological pattern of the history of the party learning education new scene can assign "green bird plan" in-depth implementation. In addition, the school also pays attention to guiding young people to use voluntary actions to promote the party history learning and education into the mind and heart. At present, 18,000 volunteers have participated in the project and launched more than 130 volunteer service projects, benefiting more than 60,000 teachers and students. The "Action Plan of" I Do Good for Schools "Leifeng Month Theme Education Practice" was launched, and more than 1,000 volunteer public welfare posts were set up in dormitory areas, living areas, teaching and research areas and other areas, and more than 30 regular and specialized volunteer service teams were set up around environmental elimination, gate duty, and traffic guidance. Enrich the cultural life with campus activities, and help them grow into the Youth League Committee of Shandong University and various colleges to build rich practice carriers for students to help students increase their talents and integrate into the society. Through holding the second Shandong University Aesthetic Education Culture Festival, the school Youth League Committee and the School of Art jointly led the units of the aesthetic education work committee to hold the aesthetic education culture festival series of activities, chorus culture and art festival, elegant art into the campus, the campus top ten singer competition and other activities to improve the young students' artistic aesthetic and humanistic quality. The School of Control Science and Engineering carried out the party history learning education of "studying hard, learning history and making clear ambition". Under the strong inspiration of the "learning party history", the party members went into more than 50 old revolutionary base areas and visited more than 20 beautiful villages. The College of Basic Medicine carried out the visit to the second funeral home in Jinan, leading the students through the road of life and experiencing the whole state of life. The Student Astronomy Fans Association held a lecture on "Appreciation and Shooting of Starfield Photography Works", which made students from all aspects of Starfield photography understand the craft of gathering stars and enjoy the visual feast of light drawing. In practice, the Youth League Committee of Shandong University pays attention to the exemplary role of student party members, youth League cadres and outstanding League members in the league branch, and helps the members of the branch to solve some difficulties and troubles. On the one hand, we carry out employment assistance, organize the graduation grade branch to set up an employment experience sharing group, go deep into the employment guidance and experience sharing in the lower grade youth league branch, and fully rely on the "Youth Bird Plan" organized and implemented by the school Youth League Committee to carry out various employment and entrepreneurship services. At the same time, each league branch actively organizes and carries out various skills training according to professional characteristics and practical needs, especially students, provide accurate preparation support and timely carry out psychological knowledge popularization. In addition, social practice or practice service groups are set up for the summer social practice and practice work of each grade branch for help and guidance, and assist teams or individuals with insufficient ability to do a good job in information sorting and selecting internship positions. Each youth league branch extensively carries out the national fitness campaign, guiding the branch members to walk out of the dormitory, walk down the network, to the playground, forming a good atmosphere for sports.

2021-06-09	Hainan university youth corps committee to carry out the party history special theme TuanRi Hainan university youth corps committee to carry out the "study the party history, strong faith, follow the party" special theme TuanRi activities on June 9 (reporter Zhang Jianwei, correspondent Zhou Yutong) recently, Hainan university youth league committee youth league representatives into the mother mountain revolutionary base memorial park and China (Hainan) the south China sea museum to carry out the "study party history, strong faith, follow the party" special theme TuanRi activities. This activity was presided over by Gao Qiao, secretary of the Youth League branch of "Qingma Project" of Hainan University. Full-time league league members of the university and more than 40 student league member backbone representatives participated in the activity. The picture shows the activity site. In the memorial park of Mu Ruishan Revolutionary Base area, we have held a series of theme activities, such as remembering the revolutionary martyrs ceremony, on-site teaching, the old curator told the theme group class, and revisiting the experience teaching of the Red Army Road. The youth league members, dressed in Red Army uniforms and holding flowers in order, held a ceremony in front of the revolutionary martyrs' bronze statues of the main leaders of the Qiongya Revolution, Wang Wenming and Feng Baiju, and conducted on-site teaching in the Qiongya Revolutionary History Exhibition Hall. At the China (Hainan) South China Sea Museum, young teachers and students have an in-depth understanding of the cultural history, natural ecology, cultural heritage protection of the South China Sea, as well as the economic and trade exchanges, cultural exchanges and cooperation between China and neighboring countries in the South China Sea and countries and regions along the "Belt and Road" route.

2021-06-07	Xd Communist Youth League customized the party history learning education menu for young students to highlight the "group flavor", taste, and interesting Xidian University took multiple measures to make the party history learning education go new and more walk Xinwei correspondent Li Rongshuai) In order to deepen the party history learning education, The Youth League Committee of Xidian University combined with the red culture and education resources, Grasp the ideological characteristics and growth law of contemporary young students, Adhere to the five education simultaneously, Highlight the "group flavor", Highlight the characteristics of the double courtyard, Meet the "taste", Give Play to the vitality of the branch, Full of "fun", Customized for young students to take the "new" more walk the "heart" party history learning education menu, Lead young students to learn the history of the Party, strengthen their faith, and follow the Party. Adhere to the five education and highlight the "league flavor" - -let the party history learning and education "tide" up to create a "youth learning" online league learning brand. In order to strengthen the theoretical study, the school Youth League Committee offers an online micro-league class called "Youth Great Learning", which is published every week, with about 16,000 people participating in each study. The book "College Students Write Party History Stories", which was edited by the Xi'an Party History Research Office, and will be published before July 1. We have built a financial media bookcase, recorded and published physical books on audio party history learning. Up to now, we have pushed 58 relevant articles and videos, with a total reading volume of more than 1 million times. Deepen the "Young Pioneer Forum" model publicity brand. School youth corps committee joint institute of space science and technology, invited famous master key research areas in China, Chinese Academy of Sciences, China aerospace science and technology corporation committee director Bao WeiMin, member of Chinese Academy of Sciences, air force university of engineering professor Li Yinghong, Chinese Academy of Sciences, Nanjing university professor Wu Peiheng to school "space", online live watch more than 100000 people. Create a quality development class demonstration theme group day. The school youth League committee organized the "past 90 years, but now return to the science and technology road", "code name: Red radio wave" theme youth league day activities. The activity combines radio direction finding and campus cross-country in the form of "script killing", referring to the Red Army's Long March route and the route of moving the school, assembling the telegraph and sending ciphertext by obtaining the corresponding parts and information. The activity integrates red education into the experience of science and technology and cross-country sports, symbolizing the glorious history of running schools on the Long March Road and starting a business together with the Party, allowing students to review the red history of XD in the activity, and feel the spirit of running XD on the Long March Road in the interlace of reality and history. Set up party history learning art, ideological and political courses. With the symphony chorus concert of "Song of the" Long March "jointly performed by the college student choir and the symphony Orchestra as the main body, the Youth League Committee and the Art Education Center jointly produced the" Long March "Song" aesthetic education open class and launched the platform of "Learning in the West Electricity". Ten teachers involved in recording course a total of 120 minutes, course innovative design built the party history school history music history background and red spirit, appreciate tasting long march live song concert video, online answer the combination of course model, let the students by listening to red music masterpieces, learning party history, improve artistic accomplishment, firm ideal faith, strengthen the character cultivation, enhance quality ability. Up to now, online courses have selected 22,400 courses and received 126,000 page views. Create the "Red Dream Building" cloud volunteer teaching double-teacher classroom. Launched the "Cloud" classroom volunteer service project, combined with "one college, one product, one town, one school", and built 248 volunteers of the Red Dream science and innovation cabin. Organize students to conduct red dream building, science and technology empowerment and art support courses, establish an integrated volunteer education system of ideological and political courses in primary and secondary schools with XD characteristics, and strive to achieve the new goals and requirements of ideological and political teaching materials, teaching integration, team integration and resource exchange. Highlight double courtyard characteristics conform to the "taste" -let the party history learning education "move" up communication engineering college combining labor education practice and party history study, successively to pucheng longchi town farmers melon industrial park and pucheng county Yaotown Yan Jia village honeysuckle industrial park for college students labor education practice base, and hired Bai Runyu, Dong jianping, generation, Wang Chunyan a line of labor workers for college outside labor tutor. Let the students really grow up from the labor practice, in the labor education to adhere to the original aspiration. The Youth League Committee of the School of Electronic Engineering organized 30 students to appear on the CCTV stage and participate in the special program "Flowers in May" of the May 4th Youth Day, guided by the Publicity Department of the CPC Central Committee, the Ministry of Education and the Central Committee of the Communist Youth League, vividly performing the military program "Military Rites" and paying tribute to the great motherland with dance. College of computer science and technology youth corps committee online "party history every day" activity 29, making "party history story" column 17, nearly one thousand people, college 2017 undergraduate party qi wei through Beijing through the Ministry of Education, state language committee, the central radio and television reception desk jointly carried out the national college students party history knowledge contest conference program recording, shaanxi only six students, party qi wei total ranked first in shaanxi. The Youth League Committee of the School of Mechanical and Electrical Engineering held the first "Mechanical and Electrical Talents List" award ceremony around the history, history, history, and history, to educate and guide the youth league members to talk with the "example" and stimulate the responsibility of young students. The Youth League Committee of the School of Physics and Optoelectronic Engineering focuses on the theme of "hero", and makes good use of the "textbook" of heroic deeds and heroic spirit. 100 youth league members "talked" with 100 heroes, and launched the activity of "Tell you Hero Stories- -Youth Lead Learning". Humanities college youth corps committee organization rehearsal of chorus music "together" "sing a folk song to the party to participate in the" the red boat south lake "2021 hundred national chorus performance and the 18th south lake chorus festival, won the university silver, red boat award, through the song to" good communist party, socialism, reform and opening up, the great motherland, people of all ethnic groups good " the theme of the campus. The Youth League Committee of the Microelectronics Institute and the microelectronics Institute organized the "scientific and technological innovation discussion" activity, and organized the students of the Red Chaoyang class to carry out the "Party history and" realize the History of the Party " series of revolutionary holy places. Sun Lirui, secretary of the Youth League Committee of the college, learned more than 1w students. The Youth League Committee of the School of Life Science and Technology organized the third seminar of "Learning history, Doing New Era and the roundtable forum of" Serving at the Grassroots, Youth Responsibility ". Activities invited Tongchuan city yao zhou district according to gold town, deputy secretary of the party committee, shaanxi entrepreneurship mentor association director, shaanxi MBA association executive chairman zhao feng, xd 2021 XuanDiaoSheng Zhao Xue, the 22nd graduate students Wang Kaikai for students to "western plan" example, let the students have a deeper understanding of grassroots work, also buried a dedicated society, grass-roots, the seeds of serving the people heart and soul. The Youth League Committee of the School of Space Science and Technology held a series of student activities of "Space Culture Festival", recorded the MV video of Ode to the Party for the founding of the Party, and released it on the Learning Power, China Youth Daily, China Education Daily and other platforms, with a total broadcast of more than 50w times. Together with the school Youth League Committee, entrepreneurship alumni Zhang Wang, science and technology innovation mentor Zhang Wenbo, he went to rural campuses and old revolutionary base areas to carry out the theme practice activities of "Half radio radio inheritance of youth party history learning and education". Network and information security youth league committee through reading, learning, learning, "read poetry, learn one hundred party history" online column, nearly one thousand people participate, and reproduced by the Ministry of Education leadership, organize students to participate in the "red boat ode south lake" 2021 and the eighteenth south chorus festival, won the university group bronze award (non-professional departments best result); selected 2019 master Xie Yuhang to Beijing to the Ministry of Education with the theme of "red cultural relics, youth" video "micro party class", shaanxi only 7 students were selected. Artificial intelligence college youth corps committee to undertake the school fourteen badminton venues volunteer service work as an opportunity to hold "intelligent star" autonomous car game, the selection of excellent projects, combined with professional characteristic research and development intelligent volunteer service robot and intelligent volunteer service auxiliary equipment, science and technology innovation and volunteer service, the concept of "science and technology power patriotism" rooted in young students heart. Lilac 2 academy league committee, economics and management youth corps committee, mathematics and statistics institute, advanced materials and nano technology college youth league committee, foreign languages institute youth league, haitang 8 college league committee also respectively through "mental health festival theme party" "custom" hand-painted masks "" campus graffiti contest "" foreign dubbing contest "" kite culture festival "and other activities to inspire students with their own creative expression of" in one hundred " blessing. The Youth League Working Committee of Haitang No.7 Academy organized the "Xiao Qi Publicity Group" to carry out simultaneous online and offline publicity. Up to now, 7 sessions have been successfully held, covering more than 10,000 students inside and outside the school. The Youth League Working Committee of Dingxiang No.1 Academy has opened an online column entitled "My Story of joining the Party", inviting a student party member to share the story of joining the Party every week. A series of offline activities of "Inheriting Party history research together, Serving the country through science and technology", and organized students to go to military research institutes to create an "immersive" party history learning class. The Youth League Working Committee of Haitang No.9 Academy organized students to create "party history small story pocket books", and spread the party history and school history stories in a way popular with students. The Youth League Working Committee of Zhuyuan No.1 Academy opened a column titled "Red Letters to describe the History of the Party", where students learn and tell touching heroic stories in the history of the Party through the ideological and political ways of their friends. The Youth League Working Committee of Zhuyuan No.3 Academy organized the online party history study of "reread the glorious history", and the offline party history practice activity of "revisiting the road and practicing the example", so that students can understand the history of the Party, understand the history of the Party, and inherit the vanguard spirit of party members in the history of the Party " in their daily study and life. Give full play to the vitality of the branch is full of "fun" - -let the party history study education "live" up the class youth league branch adhere to the party history education and practice study closely combined, To guide the league members to bear in mind their original mission in learning, thinking and practice, Take the initiative to act in the unity of knowledge and action, Extensive reading of red family home, singing red songs, watching red movies, red classics into the campus, telling the party history stories and other red education theme activities, To guide the young people to know history, love the Party, know history and love the country, Vigorously promote the red culture and inherit the red gene, Ceaseless communist spirit blood; Visit the old revolutionary sites, revolutionary exhibition halls and memorial halls, The "Cloud Tour" red Pavilion, Adapting red plays, creating red works, Feel the hardships, and great changes, and brilliant achievements in the unity of knowledge and action. The Youth League Working Committee of student associations carried out the theme activity of "one hundred groups wish a hundred years", Support all kinds of student associations to carry out the theme education of eulogizing the founding of the Party in combination with their own characteristics, Stimulate the enthusiasm of student associations to study party history, Create a warm atmosphere for student associations to celebrate the founding of the Party: the youth league branch of volunteer public welfare student associations to carry out the excellent community branch cloud exhibition activity of "youth excitation tribute anniversary", Show team information in pictures and videos, Volunteer style and volunteer service situation, Let the students realize the excellent volunteer service teams through the school, Learn about the enthusiastic volunteer culture of the school; The youth league branch of sports and sports student associations actively prepares for the national college student art performance, They won the national first prize of instrumental music group (band) and the national first prize of vocal music group (chorus) respectively. During Chengdu performance, college students symphony orchestra branch, chorus branch also carried out the history of the party, feeling, follow the history of the party, the party study practice theme TuanRi activities, respectively to 12 bridge martyrs tomb, autumn road death memorial in revolutionary monument and martyrs, listen to the revolutionary heroes, learning and carry forward the revolutionary martyrs take the bull by the horns, not afraid of sacrifice heroism. The youth League branch of science and technology innovation student associations uses AI technology to repair the image quality and color of the old images, which solves the problems of traditional and historical images, including photos and videos, which can repair difficulties, long time and high cost. The team was also invited to repair the old films for the Xi'an Film Studio, Up to now, 30 films have been restored; To the former site of the third Bureau of the Central Military Commission in Yan'an, Innovative use of MR holographic reproduction technology, A panoramic image of the former site of the Third Bureau of the Central Military Commission in Yan'an, Through computer technology to achieve all-round interactive real-time viewing, Really recreate each pavilion with each exhibit, Through field visits, field shooting and other means to strengthen the learning results, To learn to think, to think to promote action; According to the theme of "Youth Learning", the youth League branch of online media student associations launched the online answering activity of "Learning the History of the Party, Strengthening faith, and following the Party", Online wechat platform "point exchange" module, Open the "integral mall" function; Launch of the GEEK junior series "blind box" and the school building series badges based on the special year of its 90th anniversary, And GEEK junior "Colorful Summer" fruit series paper bags and the 90th anniversary series T-shirts. Use the way of aggregated news communication, to further shorten the distance with the youth, stimulate the enthusiasm for learning, and improve the learning effect."XD Youth" financial media was selected into the national "first batch of key Ideological and political public accounts construction List" jointly selected by the Publicity Department of the CPC Central Committee, the Central Cyberspace Administration, the Ministry of Education and the Central Committee of the Communist Youth League, and was selected as the top 30 national Excellent University media in 2020, and is the only platform for universities in Shaanxi province. In addition, the school Youth League Committee party branch around the learning requirements, play a good learning education "combined fist". Recorded the "centennial red Boat radio wave original heart" series of cloud lecture demonstration micro party lessons. Tell the history of the Party and the history of the school through MOOCs, From a different perspective of history, To provide students with willing to listen, actively listen to the party class learning experience, Up to now, it has launched "a hundred years has never changed- -a reply across time and space", " Listen to the wind hero is unknown, "," Cut the smoke of smoke "," One Hundred Years of Red Boat phase and nine rank radio spectrum chapter "," My Red Revolutionary object "," Practice the Red Boat Spirit, "Inheriting red Gene" and other course content, The total number of attention on the whole platform has exceeded 100,000, Among them, Wang Jiayue, a full-time cadre of the school Youth League Committee, participated in the national "Hundred Years of Red Boat Youth" publicity contest and won the national third prize. Continue to launch the "morning lecture" activity, So far, 12 issues have been conducted, To Shanghai, Jiangxi, Yan'an, Pucheng to carry out the theme Party day, Through immersive practice, science and technology innovation transformation, and two-way education, Forming the idea closed loop of "practice-understanding-re-practice-re-understanding", Go to Jiangxi Ruijin Xiaobo ancestral hall live rehearsal and recording recitation drama "never disappear radio wave", With the history as the background, Tell heroic stories, Pass on the ideological and political connotation with practical actions; Centering on the theme of "I do practical things for the masses", Develop a task list, To solve the problem of insufficient venues for student associations, Relying on information means, Build a smart data platform, Online with 32 student businesses, Optimize the approval process, Reduce the complicated procedures, Building a public space for student activities, Add 15 open reservation club activity rooms; Create a model wisdom activity room, We will realize a pilot project of intelligent integration of dual activity space, multiple networks and multiple terminals.

2021-06-04	Xi'an University of Architecture and Technology "one hundred youth speak for a hundred years" Party history publicity group walked into the Affiliated Primary School of CCB. Correspondent for figure China youth network Beijing on June 4 (reporter Zhang Jianwei, correspondent KeSuJuan) to help children to learn party history, firmly listen to the party, follow the party's determination, steadily push forward "study party history, strong faith, follow the party" learning education, recently, xi' an university of science and technology youth corps committee organization "hundred youth in one hundred" XuanJiangTuan teachers and students representatives to build large affiliated primary school, for children on a unique party history education class. The members of the party history publicity group went deep into the class, combined with their own experience and understanding, to the primary school students about the glorious course of the Communist Party, deeply touching, vivid and interesting teaching content let the children applause constantly. In order to do a good job in the party history publicity, close the distance with the children, improve the children's understanding of the party history content, the school Youth League Committee specially selected some suitable for students from the course of the party history publicity group, interactive, ornamental publicity content. Xi'an architecture university of science and technology "one hundred youth in one hundred" the history of the party XuanJiangTuan school by marxist youth teachers, staff cadres, student party members and xi new era the ideas of socialism with Chinese characteristics youth study club members of youth representatives, is the school to carry out the "study party history, strong faith, follow the party" the important form of learning education activities. XuanJiangTuan in the form of "micro", for the student party branch, youth league branch, the collective, through the theme party class, party day activities, theme, class, TuanRi activities, hierarchical classification to promote knowledge of party history and the party story as the main content of preaching activities, gradually realize a complete coverage of students, at the same time in the study practice guide the school teenagers inheritance red gene, condensed youth to the party, the struggle power of youth.

2021-06-10	Wuxi lixin higher vocational and technical schools with "red rite" confessions to the motherland Wuxi lixin higher vocational and technical school with "red rite" confessions to the motherland on June 10 (xinhua Zhang Jianwei) recently, lixin "read red classic sung red melody" and 2019 grade 18 adult rite ceremony held in the rainbow in the school theater. Also attending the ceremony were parent representatives of grade 2019 and nearly 800 students. The picture shows the activity site. School leaders and parents' representatives to the 19th grade student representatives awarded the "Constitution", in the "reading of the red" classics "chapter, 10 classes respectively recited the" July hymn "," Ode to the Motherland "," Lovely China " and other works. Eleven classes sang songs such as "Me and My Motherland" and "Loyalty to the Country". The picture shows the activity site. A hundred years of party history to illuminate the future road, young students should be top gun. The activity to young students 18 adult ceremony as an opportunity to sing the era theme on campus, acura the party's glorious performance, inspire young students firmly follow the party's ideal faith, called on young students to inherit the May 4th tradition, carry forward the great national spirit of patriotism as the core, do an ideal, ability, bear the era of youth.

2021-06-07	Hainan university "one hundred" activities and si forum held in Hainan university "party history, strong faith, follow the party" of "one hundred" and si forum held in Danzhou campus in Beijing June 7 (reporter Zhang Jianwei, correspondent Zhou Yutong), sponsored by the communist youth league committee of Hainan university, Hainan university student association federation student association management center (Danzhou campus) to undertake "study party history, strong faith, follow the party" learning education "one hundred" activities and si forum 16-Su Dongpo Danzhou, in Danzhou campus of Hainan university smoothly. In this forum, Teacher Haibin, professor of the School of Humanities and Communication, Hainan University, vice president of Hainan Suzhou Research Association and deputy secretary general of Hainan Lvqiong Literature and Art Association, was invited to give a special lecture on "Hun Han Light, become a family- -Su Shi's article achievements in Hainan". The lecture was presided over by Mr.Xu Teng from the School of Ecology and Environment, and was attended by nearly 200 students from various colleges in Danzhou Campus. By analyzing Su Shi's article description of Hainan's mountains, flowers, fruits, birds and animals, and delicacies..., Su Dongpo explained the three major achievements in Danzhou: people's livelihood, articles, and personality. After three years of exile, Su Shi changed his course in Danzhou, broke down bad habits, studied agriculture and built water conservancy. At the same time, he also talked with Hainan scholars and spread the culture in the Central Plains, making Danzhou the cultural and education center of all over Hainan Island at that time. Haibin pointed out that the development of Hainan customs and the spread of Qiongya culture are all closely related to Su Shi, so the people of Hainan are still remembering Su Shi, commemorating Su Shi, and thank Su Shi for his great contributions to the development of Hainan.

2021-06-10	Jiangsu university youth corps committee to build "immersive" literary party class innovation booster party history learning education in Jiangsu university youth corps committee to build "immersive" literary party class innovation booster party history learning education deeply built wei) for solid party history learning education, Jiangsu university youth corps committee make full use of aesthetic work characteristics, the party history learning education and organic integration of stage art, with art form of party history, show the party's thought, create "immersive" literary party class. Recently, "The 100-year Heart of Youth Dream to the Party Dream of a New Journey" - -2021 Jiangsu University College Students Art Troupe Report performance was held in the small auditorium of the school department. Principal, doctoral supervisor xiao-hong yan, deputy secretary of the party committee, doctoral supervisor hong-bo li, the party committee united front work department minister Yang Jingjiang, related functional departments and the party, head of the department, college, directly under the party secretary and deputy party secretary of learning in charge of the work, secretary of the youth corps committee and the youth members on behalf of more than 700 teachers and students watched the performance. The performance to important events in the history of the party, divided into "one hundred wind and rain epoch-making", "one hundred struggle, unyielding", "one hundred journey dream" three chapters, more than 300 teachers and students from the college students to songs, dance, instrumental music, recitation, martial arts, drama performances, emotional deduce the party's revolutionary course and struggle epic, fully demonstrates the spirit of Jiangsu university youth vigor, take the initiative to bear to carry forward and inherit the revolutionary spirit of determination and faith. In the whole performance, the host with the story of the history of the party, and performance era background fusion, set artistic, ornamental, educational, not only brought the audience a shocking audio-visual feast, also let them in the classic red melody, scenes touching scene performance, accepted an "immersive" the history of the party education. It is reported that Jiangsu university youth corps committee will continue to innovate the party history of education form, education guide member youth understand the party's glorious history, feeling the party's mission, grasp the party's innovation theory, recognize the party's spirit pedigree, inheriting the party's red gene, to ensure that the youth party history learning education has both "depth" more "temperature".

2021-06-03	Wuxi commercial vocational and technical college launched party history learning characteristic class Wuxi commercial vocational and technical college: skills display stage into party history characteristic class China youth network Beijing on June 3 (reporter Zhang Jianwei, correspondent force Xie Qiang) recently, is located in Wuxi district qiao street Wuxi commercial vocational and technical college campus, sponsored by the college of tourism management, "home countries the world" vocational skills teaching exhibition is in progress. Display activities combined with the history of the party learning education, the one hundred history, red spirit into students skills, vocational skills performance and professional teaching achievements, innovative party history expression form, in the vocational education students skills at the same time, guide students to strengthen the love party patriotic feelings, motivate students to go skills success, skills. More than 3,000 people visited the scene. Party history elements embedded skills display, let the party history learning education visible, touch display activities with "love tourism, love life" wonderful tourism exhibition, "flowers praise, founding in one hundred" flower art exhibition, "carry forward the spirit of craftsmen, build skills power" pastry craft exhibition, "skills make life better" cooking craft exhibition, and other areas, the scene is lively, wonderful. The vivid telling of the local red story, praise the founding of the hundred years of creative flower arrangement works, engraved with the flag and the map of China exquisite food carving works, meaning the founding of the hundred years of special drinks, the layout of the red boat and the emblem of the theme... the activity site, a full of party history elements and highlight professional characteristics of skills works and skills display is very "eyeball". These works are carefully conceived, designed and produced by the teachers and students of the School of Tourism Management of the university. They tell the story of the history of the Party through their skill works, pay tribute to the century of the founding of the Party, and pass on the power of China. During the performance, the teachers and students also explained the story of the party history behind the works to the teachers, students and guests, leading the audience to approach a period of hardships and historical moments, so as to show their skills as the carrier to make the party history more concrete and vivid. The integration of knowledge and practice inherits the red gene, and makes the skills and serving the country become a new fashion display activities, and the characteristic achievements of the reform and innovation development of the school and the training of vocational education talents are also quite bright spots. In the "Better Life" exhibition area "," innovative carving "and" molecular cuisine "booth combined traditional skills with modern technology, the" gold mystery "booth reproduced the" gold mystery ", opened in the school" secret "of the school with high training standard, gold medal, deep teaching of industry and education, and" intelligent cooking " booth, with the exquisite skills and exquisite pastry innovative works, showing integrated and innovative professional development ideas. During the preparation period of skills display, in order to better present the works, the students formed a team under the leadership of professional teachers to discuss the materials, content and forms of the works, and carried out a targeted study of party history knowledge. The skills results show activities fully shows the Wuxi business school vocational education reform and innovation and development and students' professional skills level, embodies the students patriotic love party, strenuous enterprising spirit, also created a strong atmosphere of party history learning education, is the school party history learning education innovation, rich form of a beneficial attempt, to promote the party history learning results, harvest, promote the development of the party history study education high quality has a positive role.

2021-06-09	The Communist Youth League of Haikou University of Economics relies on the "second classroom" education position to help the party history learning and education into credits. Beijing, June 9 (reporter Zhang Jianwei, correspondent Zhou Yutong) recently, The Youth League Committee of Haikou Jiangdong New District Administration Bureau and the Youth League Committee of Haikou Economics Institute jointly held the "Launch of the Communist Youth League Party History Learning and Education Association and the Signing Ceremony of the Normal Volunteer Service Base" in the Haikou Jiangdong New Area Exhibition Center, This signing is an effective continuation of the "Volunteer Classic" brand of Haikou University of Economics, Is to build a platform position for professional volunteer service work, It is an important measure to further promote the youth league members to study the history of the Party and deepen it deeply. In "learning party history, strong faith, follow the party" learning education, Haikou economics institute youth corps committee to "the second classroom transcript" for traction, strengthen quality development "four modules, four positions, a set of credits" function, the young students learn party history and organic integration of quality education, for students to participate in online school party history, the film review, theme TuanRi, preaching, speech, knowledge contest, do the practical work, special training of party history learning education activities that quality development credits. It has effectively promoted the credit system and quantifiable system of party history learning and education, improved the participation consciousness and effectiveness of young students, and effectively promoted the orientation and coverage of the "second classroom report card" system. Thought leading position: promote the party history learning education increase gravity this year Haikou institute of economics "green horse project" practice class theme is "study party history, does the practical work, strict style", training students 230 people, opening ceremony invited professor jia party history, vice President Sun Zhi asked students: "combine the party history and strong faith, increase ability, practice, learn, letter, use, to guide their own thoughts, study, work and life"."Hainan Youth May 4 medal" winner Wu Xiaoying, "Hainan moral model" Lu Weiqing, "national outstanding Communist Youth League member" Mou Xiaohan and other young students participated in the recording of "one hundred youth model tell one hundred years of party history" short video, with the typical around for the party history narrator, in the "sea youth" video number; "sound party history 100" in the "sea youth" WeChat public account has launched nearly 40 issues, different voice transmission party history story. Volunteer service position: make the party history learning education cohesion in Haikou institute of economics "group for youth" project review meeting, "4:30 preschool class", "photo", "English through train" support activities, "elegant post" tea break project between teachers and students, "phoenix flowers have you have me-old recycling" such as 17 "do the practical work volunteer service project" stand out, the project covers the academic support, employment support, children, dormitory mutual assistance, quality improvement, and other aspects. In June, the "Volunteer Night" volunteer service project exhibition activity held in June will take the study of the history of the Party and doing practical things as the main line, and stimulate the volunteer awareness and responsibility of young college students by telling volunteer stories and interpreting volunteer experiences. Innovative practice position: make the party history learning education add power this year's "colorful club, tribute in one hundred" community carnival activities, is one of Haikou economic institute party history education activities, in the form of community performance, faith, youth, dream three chapters, club members write from the song of the flag to the direction, the drama out of the book history, melodrama war and peace, song and dance "young" 12 programs singing the main melody. The Youth League Committee of Haikou Institute of Economics held practical activities with the theme of party history education watching movies, expanding the quality of league and students. At the same time, the youth League committees of each colleges made great efforts to carry out the theme activities of "learning the history of the Party, strengthening skills" to describe colorful jiangdong, "young students enter the barracks, the spirit of the May 4th always follow", "learn the history of the Party, do practical things" to create the off-campus practice base, to promote the continuous "heating up" of the learning and education of Party history. Cultural and sports activities position: let the party history learning and education vitality before the May 4th, "tribute to the founding of the Party, the youth of the country" speech contest online and offline simultaneously, 12 contestants from different angles and fields, told the advanced deeds of outstanding Communists."Love the Party does not need a reason", "again to the Xin Chou year, has changed the world", "a hundred years of great cause, a true heart" and other stories have caused the scene of more than 1,000 teachers and students, more than 30,000 online young people resonance. The theme activity of "Watching the Centennial History of the Party" is the national flag raising theme activity carefully planned by Huadu Film and Television College of Haikou University of Economics. Acting major students reproduce the classic film "Dadu River" from the perspective of telling people. Youth league organizations at all levels spread red voices and red energy through unique cultural and sports activities such as knowledge contests, keynote speeches and fun sports meetings, organically integrate learning the history of the Party with daily activities and quality development, and realize the "zero distance" docking between theme learning and extracurricular activities. The "four modules and four positions" of the quality expansion project reflects the orientation and connotation of quality education in the school. "One set of credits" is a specific measure to bring quality education into credits and into the talent training program. On the basis of the overall planning of the school, the youth league organizations at all levels have created their own characteristic highlights, and constantly promote the innovation and heart of the characteristic activities to fully meet the learning needs of students of different types, different majors and different interests. The party history education will be integrated into the overall section of quality education, and the strength, depth and effect of student participation are evaluated with credits, so as to further gather the resultant force and enhance the influence and effectiveness of the party history learning education.

2021-06-02	In Qinghai province, the space alliance founding meeting in Qinghai university in Qinghai province, the space alliance formally established wei correspondent A Shuyuan), by Qinghai province education department, the communist youth league committee in Qinghai province, the Chinese university space alliance guidance, Qinghai university launched and undertake the "the space of the alliance in Qinghai province" founding meeting held in Qinghai university. Wu Xiaobao, convenor of the Presidium of China University Maker Space Alliance and Deputy Secretary of the Party Committee of Zhejiang University, Dong Yuyi, Secretary of the Qinghai Provincial Committee of the Communist Youth League, Mei Yan, Deputy Director of the Qinghai Provincial Education Department, Yu Hongxian, Party Secretary of Qinghai University, Ye Minsheng, Deputy Secretary of the Communist Youth League, Guo Wengang, Director of the Student Engineering Department of Zhejiang University Party Committee and Dean of the School of Innovation and Entrepreneurship, Liang Sishu, Deputy Director of the Office of the School of Innovation and Entrepreneurship, Zhejiang University, Jiang Nan, Deputy Secretary-general of China University Maker Space Alliance, Yao Jinxin, the "Big Dipper" innovation and entrepreneurship mentor and partner of Pance Capital, attended the meeting, Leaders from 12 universities, including Qinghai University, Qinghai Normal University, Qinghai University for Nationalities, and nearly 150 innovation and entrepreneurship educators attended the conference. Standing Committee of Qinghai University Party Committee and Vice President Ren Yanming presided over the conference. Yu Hongxian, secretary of the Party Committee of Qinghai University, said that Qinghai University will take the establishment of the alliance as an opportunity to work with other universities and universities to uphold the concept of "interconnection, interaction, joint construction and sharing", and provide various services such as resource sharing, cooperation and exchange for the construction, operation and management of mass innovation space of universities in the province. It will help college students to improve the level of innovation and entrepreneurship education for college students, provide low-cost, convenient, all-factor and open innovation and entrepreneurship services for college teachers and students and entrepreneurs, and contribute to promoting the transformation of scientific and technological achievements in colleges and universities, the training of college students' entrepreneurial talents, and promoting the in-depth development of innovation and entrepreneurship in the province. At the conference, Mei Yan, deputy director of the Education Department of Qinghai Province, congratulated on the establishment of the Qinghai University Mass Maker Space Alliance. He pointed out that the establishment of the alliance is of great significance and far-reaching influence for promoting the comprehensive promotion of the mass innovation space in universities, optimizing the innovation team and entrepreneurship team in the province, and creating a good innovation and entrepreneurship ecology. It is hoped that the alliance will recognize the construction positioning, strengthen integration and cooperation, focus on mass entrepreneurship and innovation activities, pay attention to the evaluation first, uphold the original aspiration, give full play to the organizational advantages, strengthen the communication and cooperation between colleges and universities, integrate innovation and entrepreneurship resources, and gather new forces for the development of mass entrepreneurship and innovation education and mass innovation space resources in Qinghai Province. Dong Yuyi, secretary of the Provincial Communist Youth League Committee, said in his speech that the establishment of the Qinghai University Mass Innovation Space Alliance will help universities to achieve the development of higher quality, faster speed and better efficiency by cooperating with all universities and all sectors of society in the province, and will surely help the innovation and entrepreneurship work of college students in Qinghai Province to a new level. It is hoped that the alliance can strive to make breakthroughs in resource integration, activity coordination, mechanism innovation and overall service. Chinese university, the presidium of the space alliance convener, Zhejiang university, deputy secretary of the party committee Wu Xiao support in Qinghai province, the gen space alliance founded congratulations, hope the gen space alliance actively play the role of regional alliance, interconnected interaction, sharing, give full play to the gen space in Qinghai province on the role of service double gen, stimulate vitality. At the conference, Wu Xiaobao, convenor of the presidium of China University Mass Maker Space Alliance and deputy secretary of the Party Committee of Zhejiang University, presented a flag to Qinghai Province Mass Innovation Space Alliance, and Ren Yanming, chairman of Qinghai Province University Mass Maker Space Alliance, received the flag on behalf of the Alliance. Over the same time, the Chinese university space alliance "red dream double innovation long march activities" and the 17th "challenge cup" Qinghai province college students extracurricular academic science and technology competition finals held in Qinghai university, for Qinghai university teachers and students, students provide patriotism practice, innovative entrepreneurship, double gen event experience such as abundant lecture resources, let the students fully feel the feast of science and technology and the unique charm of youth event.

2021-06-02	Wuxi city vocational and technical college promoting "I do the practical work for the masses" Wuxi city vocational and technical college "four focus" promoting "I do the practical work for the masses" wei correspondent Jiang Wenting) "I do the practical work for the masses" practice is an important content of the history of the party to study education, is also an effective way to further contact and service between teachers and students of the masses. Since the development of party history learning and education, Wuxi City Vocational and Technical College, located in Qianqiao Street, Huishan District, Wuxi city, has focused on the "urgent difficulties and sad hope" of teachers and students, strengthened the weak links, grasp the implementation, do practical things, and further promoted the achievements of party history learning and education to take root and bear fruitful results. Focus on the infrastructure, Improving the Educational Environment Schools insist on ensuring and improving people's livelihood through development, Members of the leading group of the university Party Committee went deep into the front line to fully carry out research, To extensively solicit opinions from teachers and students by holding symposiums and issuing forms for soliciting opinions, Grasp the needs of the growth and development of teachers and students, Providing targeted services, Enhance the supply capacity, Actively help to solve the reasonable demands of teachers and students in their work and learning, Focus on promoting campus infrastructure construction and improvement projects, Through the renovation of office buildings, smart classrooms, and libraries, Expand the psychological center, the art center for college students and other ways, Constantly improve the staff office environment and students' learning and living conditions, Effectively enhance the sense of security, happiness and satisfaction of teachers and students.? The picture shows a modern campus. The picture shows the transformation of the smart classroom. Focusing on social needs, correspondents carry out volunteer services to extensively mobilize teachers and students to carry out volunteer services inside and outside the campus. Nearly 100 volunteers participated in the school's vaccination site service, providing guidance, reception, q & A, observation and other services for the vaccination teachers and students, so as to facilitate the orderly progress of the vaccination work. During the vaccination, in order to thank the medical staff, the volunteers sent 50 hand-stacked nurse caps and milk to the medical staff helped the community to inspect nearly 2000 people in over 500 households, organized teachers and student volunteers to maintain the traffic order around the school during rush hour, to facilitate the congested traffic, and remind them to wear masks to escort teachers and students. Pictures show volunteers participating in vaccination services. The picture shows the message to the medical staff. Focus on the field, In response to the call of the national rural vitalization strategy, Give full play to the functions of higher vocational colleges to integrate industry and education and serve the society, Wuxi City Vocational and Technical College relies on its industrial college- -Wuxi Rural Electricity Business School, For the vast number of farmers and rural workers have opened a rich content of e-commerce to help farmers micro courses, It mainly includes the new farmers' e-commerce live broadcast road to prosperity, the commonly used agricultural products live broadcast platform, product interpretation and live broadcast script, e-commerce live broadcast community construction, short video marketing of agricultural products, product packaging should be the side- -agricultural products delivery and packaging, smart agriculture good helper RFID, etc., Learning through online courses, Help farmers and rural workers to quickly start the e-commerce operation, Help with rural revitalization; Set up a scarecrow rural e-commerce team, Relying on two student entrepreneurship teams and one online operation platform, we will help three farms and five cooperative companies to realize live delivery and help farmers. The picture shows the e-commerce helping agriculture micro class. The correspondent shows the activities to help farmers. Focus on the students' eagerness, It is currently in the peak employment season, The school attaches great importance to the internship and employment of graduates, Holding more than 10 special online and offline special job fairs, A total of more than 100 employers participated, Provide more than 5,000 jobs, More than 6,000 students have participated; Employment assistance, Organize teachers to connect with poor students, Help students to make clear the self-positioning of employment, Guide students to conduct job market analysis, Learn the interview skills; Held "I do practical things for the masses" symposium special students, Soliciting students' opinions and suggestions on learning and growth, campus life, internship and employment, And open the "Secretary reception day", Invite students into the secondary College Secretary's office, Communicate with the secretary face to face; In the "smart logistics" service platform online online repair and follow-up evaluation function, Integrate the campus one-card system into the "smart logistics" service platform, Realize an account to walk the whole process, Reasonable repair repair process combined with efficient maintenance quality, Greatly improve the service efficiency and the satisfaction of teachers and students. The picture shows the job fair site. With the spirit of "serving the people, I serve the children", the school organically integrates the party history learning and education with the practical work, caring for and serving the teachers and students, and bringing visible and tangible changes to the teachers and students.

2021-06-01	Carey college to carry out the love eye protection volunteer service activities Carey college youth volunteer federation to carry out the "attentively care," eyes "color" view "" volunteer service activities wei correspondent Cao Tingting) on May 25 to 27, Carey college youth volunteers federation organized volunteers to assist 21 southeast love eye hospital in may 4th square "attentively care," eyes "color" view "volunteers" volunteer service activities. The event was led by Li Jingfeng, vice chairman of the University Youth Federation. The picture shows the activity site. In order to ensure the smooth progress of the volunteer service work, the volunteers helped the doctors to carry the materials to the designated place before the activity began, and assigned various tasks, standing on the post and waiting for the arrival of the students. After the activity began, the volunteers devoted themselves to the volunteer service activity. Volunteers carefully filled in their personal information for the students who came to check their eyesight, and guided them to wait in line for the medical staff to check their eyesight. In addition, the medical staff shared with the volunteers the causes of myopia, the matters needing attention of daily eyes and other related knowledge. The volunteers listened patiently, so as to solve the doubts for the students who came to consult. Volunteers also told students who came to check their eyesight to scan the code to follow the "Qiandongnan Aier Ophthalmology" public account can receive small gifts. The picture shows the activity site. Through this volunteer service activity, more students learned about the relevant knowledge of myopia prevention and control, and enhanced the students 'awareness of eye love and eye care. The volunteers brought the eye love knowledge to the students and also passed on their love, reflecting the noble quality of the volunteers' dedication to others.

2021-05-31	The Communist Youth League of Hainan Normal University has set off a new upsurge of Party history learning and education through a variety of ways.) Extensive mobilize the school young teachers and students, From the "five dimensions", respectively, Through a variety of ways to set off a new upsurge of party history learning and education, By "telling" red history, "singing" red songs, "displaying" red works, "seeking" red footprints, and "passing" red genes, To guide the majority of young teachers and students to learn the history of the Party, Strong faith, Keep to your original mind, Listen to the party, Follow the party, Effectively achieved the "five outstanding". The picture shows the youth of Hainan Normal University reviewing their vows of joining the Youth League. Correspondent for the picture of "plastic soul", highlight the ideological and political guidance adhere to focus on the main responsibility and main business, to carry out various forms of ideal and faith education, guide the youth league members to cultivate love for the Party, patriotism, love for socialism emotion, let the red gene and revolution passed on from generation to generation. Relying on the "three meetings and two systems", "Qingma Project", the special study and training; study the history of the Party, speak the history, understand the history of the Party, for the youth members; "grounded, take the spirit", "heart" in the provincial history hall, hold more than 2000 youth members to review the league oath, and organize the youth league members to raise the national flag, and hold the newspaper competition."Innovation", highlight the combination of online and offline combination, adhere online and offline combination, actively build a financial media platform, carry forward the main theme, and spread positive energy. Using the "Communist Youth Little Sea Lion" youth financial media Center, with the help of Weibo, wechat, TikTok, QQ space and other means, to show and publicize the great process of the Chinese nation on the great road of realizing the great rejuvenation, to drive the majority of young students to understand the glorious history of the Party, and enhance their feelings and trust for the Party. Closely related to the education and study of Party history, Set up the party history study column (small class); Production and launch of "General Secretary Xi Jinping in the hot discussion of the spirit of the important speech during the visit of Tsinghua University", "Qingming Festival heroes cast the Chinese soul" and other forms of rich party history learning theme propaganda cultural products; "Hundred baton relay" continues the original mission, One hundred young teachers and students relay to do the party history leader, By recording the video, Passing the power of faith, Inheriting the red spirit; Actively mobilize students to participate in the "National College Students' Party History Knowledge Competition Conference"; "Youth big study together to learn the party history of the Party" network theme group class. Hold micro group class competition, online and offline league entry ceremony, plastic model, model, commendation, carry out the May 4th outstanding typical commendation conference, cooperate with Xinhua News Agency to carry out the "Xinhua News Agency into the campus" series of activities, relying on the Himalayan FM platform, carry out "Yue reading", "youth", "Love the Party" three theme activities. Hainan Normal University celebrates the 100th anniversary of the Founding of the Founding of the Communist Party of China and the annual award ceremony of the Communist Youth League. Correspondent for the picture "rooted", highlight cultural infiltration and conservation adhere to the concept of cultural people, actively build the practice of socialist core values as the main line, to improve the cultural heritage and comprehensive quality of the goal of elegant and inspiring campus culture brand of young college students. Relying on the "May 4 th", "the 100th anniversary of the founding of the" and other important time node, held "learning party history, strong faith, listen to the party, follow the party" college students culture and community culture festival, widely throughout the dance, singing, drama, painting, calligraphy, photography, debate, micro film, reading sharing, academic salon 24 favorite activities among students."Hundred red film show" activities, "the party in my heart" sing red song contest, "to celebrate the 100th anniversary of the founding" theme essay activities, "youth know the party history of the party" history knowledge contest, "sing a folk song to the party" flash, the history of the party learning reading sharing activities, in the form of happy to carry out the history of the party, the party education. The picture shows "Learning the history of the Party, strengthening faith, and following the Party" - -the special practice activity of student backbone patriotism in Hainan Normal University. Adhere to the practice orientation of practice orientation, sharpen the will, temper the character, increase the ability, constantly enhance the feelings with the Party, strengthen the responsibility of the youth, and cast the personal growth into the construction of the new era and the tide of Hainan Free trade Port. Using Hainan red resources, the memory of revolutionary martyrs, red base to visit experience, red landmark activities, organizing cadres and youth members to qiongya of the communist party a large site, red detachment of honor, qiongya red army yunlong adapted site, mountain revolutionary base memorial park, Hainan annals pavilion revolutionary sites and memorial venues pavilion to visit, carry out the special theory lecture into the volunteer service (social practice). Through the means of theme publicity, data dissemination, video broadcast, service for the people, art performance, social practice and typical excavation, college students are guided to consciously undertake the important responsibility of party history study and publicity. The picture shows the Communist Youth League of Hainan Normal University "Learn the History of the Party, strong faith, follow the Party" learning and education- -the Youth League Committee Secretary (Party) Youth League class activity. Correspondent for the map "empower", highlight the long-term normal construction adhere to strengthen the safeguard measures, highlight the focus of work, around the party history learning education project list ideas, measures, grasp the implementation, in the funds, personnel, site and other aspects to provide conditions. Explore the establishment of a long-term management mechanism, improve various rules and regulations, into the key work assessment of the Communist Youth League, into the "Qingma Project" training system, into the normal ideological and political education of students, strengthen the process supervision, and track the progress of the follow-up projects at any time. Relying on the "three meetings, two systems and one lesson", the party history study and education results will be reported and displayed comprehensively, and each college is displayed online, offline competition, and the whole school competes. The menu-style learning mode is designed, and students independently choose learning projects according to their own reality, combined with the second class credit management, to encourage students to normalize the study of party history. Relying on the forms of publicity meetings, exchange meetings, symposiums and other forms, the participating students are organized to tell their learning experiences and practical stories, and share the contemporary young students' deep feelings and firm beliefs for the Party, so as to inspire more young students.

2021-05-29	Anhui Normal University: Youth League study reform enhances student happiness Wang Haihan Wang Lei) " The class youth league branch launched the 'youth three walk' theme youth league day activities, the group leader took us out of the dormitory, to the playground, let me feel the different vitality of the league members!"Recently, Lu Chenxin, a student majoring in Chinese Language and Literature in 2020 at Anhui Normal University, had a deeper understanding of the functions and content of the activity through participating in the activity. In the past five years, Anhui Normal University has made clear the roadmap and timetable of the league and school reform, and various measures have been steadily promoted. Student unions at all levels focus on the actual needs of students, regularly collect rights and interests demands, sort out and analyze them, and submit them to the school, and put forward constructive plans for the work of the school. Since last year, the student Union has contacted with the city bus group in view of the problem of difficult travel during holidays, and opened the campus direct bus line to Wuhu Railway Station to escort students to travel during holidays. In the process of league and learning reform, the school focuses on building the three-level student union organization linkage pattern of "the school meeting main discussion, the main president of the school meeting and the class support". The student Union of the university formulates the development plan, resource integration and work plan for the student activities of the whole university; it also guides the class Committee to mobilize and publicize the school and the college student Union. In addition, the Youth League Committee of the school has designed and developed the "I Star" second classroom information system, and has jointly designed more than 300 second classroom activities, such as mental health education month, animation and digital art competition, and student invention patent competition. The responsible comrade of the school Youth League Committee introduced that since 2015, the requirements for joining the league have been improved, but the advanced nature and sense of honor have come up, and the number of students who actively apply for joining the League has increased year by year.

2021-05-28	Hainan normal university "five action" power "I do the practical work for the youth" "very 4 + 1": Hainan normal university communist youth league relying on the "five action" power "I do the practical work for the youth" JianWei correspondent Wu Yuhua) since the party history learning education, the Hainan normal university communist youth league will party history learning education and service for young teachers and students, adhere to the "open the door" problem, focusing on youth expectations, precise selected key, earnestly "does the practical work for the youth". Closely build and rely on the "4 + 1" platform, vigorously implement the "five major actions", among which four actions are for the general young teachers and students of the whole school, and one action is for serving the social development. Focus on solving the most direct, the most realistic problems of teachers and students, real efforts, constantly improve the sense of gain and happiness of young teachers and students, and promote the theme of education to go deep and practical. Refine the service form, and transform the learning effect into the work momentum of "doing practical things for the youth". Ideological "plastic soul" action: focus on the main responsibility and main business, focus on solving the problem of ideological guidance, ideological internalization. Relying on the league organizations at all levels, carry out thousands of themed league day activities; actively promote the construction of star grassroots league organization, organize the majority of young teachers and students to visit the red Revolution base experience, will (Party) league class to the "heart" of college students. Establish the party history learning education youth publicity group, through the youth popular theme activities; actively carry out "dialogue" with the faith series activities, through reading sharing meeting (exchange meeting), continue to grasp the youth theory study; strengthen the "Communist Sea lion" youth media platform construction, build "new media service center on the fingertips", promote the main theme, and spread positive energy. Mass entrepreneurship and innovation "dream building" action: carefully set up the innovation and entrepreneurship practice group, Actively organize the "Innovation and Entrepreneurship Practice Week" activities, Relying on the "Creative Youth" and the "Internet +" College Students' Innovation and Entrepreneurship Competition, Instead of playing games for training, Promote learning through competition, Effectively enhance the innovation and entrepreneurship ability of college students; Holding academic science and technology festival and "Challenge Cup" extracurricular academic science and technology works competition; Students are encouraged to actively sign up for the recruitment of the Midwest Project Volunteers, To guide graduates to make contributions to the grassroots level, to the central and western regions, and to the backward areas; Based on the characteristics of school teacher education, Heart about students' career development, Organize the teacher skills Competition, Build the activity brand of "Sailing Plan" to cultivate college students' professional ability, Do a good job in employment and entrepreneurship guidance; Deepening the supply-side reform of students' growth and talents, Relying on the comprehensive service platform of Guoxing Building Dream Avenue, Release announcements on innovation and entrepreneurship projects. Cultural "infiltration" action: combined with the party history learning education, develop the menu of campus cultural activities, carry out "campus culture festival", "community culture festival" and other colorful campus cultural activities; relying on the quality community creation project, vigorously support innovation and entrepreneurship, cultural education human student community development, relying on the national university science and technology park, "the creative space" and other bases to provide practice places for student innovation and entrepreneurship, to provide comprehensive support in project cultivation, policy consultation, team incubation, achievement display. Hold the "The Party in my Heart" singing contest, innovative ways, make the party history learning and education vivid; relying on the "Communist Youth film garden" to carry out a hundred red classic film exhibition, through the red dream tour cultural experience, hold the party history knowledge contest and other ways, set off a new upsurge of party history learning and education among young teachers and students. Youth "escort" action: continuously deepen the organizational reform of the Communist Youth League organization and the Student Union (graduate Association), Effectively enhance the leading force, organizational, service and contribution of the league organization; Actively respond to the concerns of young teachers and students, By setting up a special line for safeguarding students' rights and interests, regularly visiting student dormitories (classes), holding a "school leaders meeting", and establishing an exclusive mailbox for the campus service of the student Union, Safeguard the legitimate rights and interests of young teachers and students, To Solve their practical difficulties; Carry out a civilized campus, Civilized dormitory, Civilized classroom, Civilized canteen creation activities, Help the construction of campus civilization; By accelerating the construction of the college student activity center in Guilin Yang Campus, Focus on the mental health education of teachers and students, Implement full-time and part-time youth league cadres to track, contact and help students with employment difficulties, Strive to build a "youth home". Social "empowerment" action: actively build a platform for serving social development, extensively carry out "three to the countryside" social practice activities and professional, regular and long-term volunteer service activities, and guide teachers and students to serve rural revitalization, basic education, and the construction of Hainan Free Trade Port. Vigorously implement the service basic education "aesthetic education and foreign education" promotion plan, open Lingshui education interest special class, establish special volunteer teaching activities in Town and Hope Primary School, Lingshui County, actively serve the construction of Hainan education reform and innovation pilot zone, start and organize college students to set up free trade port foreign language promotion volunteer service team, accelerate the construction of Hainan free trade port, improve the foreign language level; set up garbage classification volunteer service team, popularize garbage classification knowledge, enhance the awareness of garbage classification in the island.

2021-05-28	Guizhou university "hundred preach into the branch" activities began Guizhou university "hundred preach into the branch" theme preach taken China youth network Beijing on May 28 (reporter Zhang Jianwei, correspondent, Liu Yanchun Song Hongqing) to promote the "study party history, strong faith, follow the party" party history learning education and "remember the earnest entrust, loyal clean bear, happy in one hundred" special education, guide the youth to study party history, enlightenment thought, do the practical work, on May 26, Guizhou university "hundred preach into branch" activities officially began. In the first session of the activity of "100 lectures into the Branch" of Guizhou University, hundreds of league members from more than 20 youth League branches such as Electrical Engineering 2003 Youth League Branch, Civil Engineering 2002 Youth League Branch and English 195 Youth League Branch listened and learned together in five venues. Guizhou university youth branch, university students XuanJiangTuan members around the "glorious course", "the light of faith", "beautiful China", "youth bear", "your big power" five projects, focus on the party history in one hundred years, to carry out face to face, interactive preaching communication, the party's theory clear, understand, let teenagers understand, remember and can use. The picture shows the scene of the lecture activity. It is reported that the publicity group of Guizhou University college students has participated in more than 100 lectures, focusing on the young people to lead the young people. In early 2021, in order to further enhance the propaganda strength, strengthen the party history learning education, Guizhou university open youth XuanJiangTuan recruitment, after three rounds of selection, early training, campus training, selected 26 excellent moral character, positive, good at expressing young teachers, formed the "Guizhou university youth branch". In addition to the activities of "100 lectures into the branch", the youth teaching group and college students' publicity group of Guizhou University will also carry out the theme publicity activities such as "100 associations to welcome lectures" and "100 lectures into the network".

2021-05-26	Zhejiang university of technology to carry out the rural revitalization of ideological courses theme TuanRi youth to the party booming Zhejiang university of technology to carry out rural revitalization of ideological courses theme TuanRi activities on May 26 (reporter Zhang Jianwei, correspondent, xiao tiger wang caiyu) recently, Zhejiang university of technology joint youth corps committee, the service center for the "rice field ideological courses" party history learning education project activities and theme TuanRi activities. Praise the party's grace, talk freely about the achievements of rural development. Students from the wheat field class of the college and some of the league backbone visited the rural culture auditorium under the leadership of the Party branch secretary of Sanxi Village. In the auditorium, I communicated with the local villagers carefully, listened to the stories of their hometown on the road to wealth, and felt the outstanding achievements achieved in the rural construction under the leadership of the Party. Students helped the children's homework carefully in the cultural auditorium, and learned the party history stories, local folk customs, and village rules and regulations together with the children. Afterwards, the students went to "Fenglin pharyngeal Spring" to listen to the long history story of Sanxi Village and appreciate the achievements of local characteristic culture development according to local conditions. Look at the present moment, enjoy the beautiful life in the countryside. On the day of the activity, the students walked into the Sanxi Village Youth Army (Fuyang) national defense education and training camp. Zhu Tao, the chief executive of the training camp, introduced the history of the Fuyang boat to the students in the memorial hall, and led them to visit the retired Fuyang boat. The picture shows the students experiencing the process of harvesting food in the wheat field. Correspondent to show the future, draw a blueprint for rural revitalization. The rice field is fragrant with wheat, and the breeze is blowing slowly in the mountain stream. After visiting the military education base, the students came to the wheat field in Sanxi Village to pick up the sickle and bent down to experience the process of harvesting food. At the last stop of the ideological and political course, the blueberry growers and the Party branch secretary of Dale Village told you their entrepreneurial stories of organically combining green industry development with rural revitalization. The students really felt the importance of promoting rural innovation and entrepreneurship in the new era, and truly felt the vitality and vitality of China's rural development. Fang Xiaohu, secretary of the Youth League committee of the college, said: " Through the 'ideological and political course in the rice field', the students not only experienced labor education in the countryside, but also received red education and traditional virtues, feeling the close distance by the development of China's countryside."Wu Jiamin, a student, said," After the holiday 'Challenge Cup' red special social practice and the ideological and political course study, I sincerely feel proud of the vigorous development of the country and the countryside.”

2021-05-26	Yunnan agricultural university held to celebrate the 100th anniversary of the creative painting contest held in Yunnan agricultural university to celebrate the 100th anniversary of the creative painting contest the youth network Beijing on May 26 (reporter Ji 'an-wei zhang) by Yunnan agricultural university school youth league, food science and technology institute, the school learning practice Yan' an spirit group food school team to undertake "the party shine in my heart, one hundred meters picture scroll to the party" creative painting contest lasted 7 days, ended. By 20 college from the school 32 teams to "the May 4th movement, the founding of the communist party of China, the founding of new China, reform and opening up, Hong Kong and Macao, towards a new era, disease resistance, poverty engines" as the theme, with red course, great achievements, character, times new appearance as the main content, the painting creation. After preliminary conception and screening, shortlisted works creation, exhibition, audience voting, judges and teachers, a total of 1 first prize, 3 second prizes, 6 third prizes and 22 excellence awards were selected. The picture shows the activity site. On the scene of the competition provided by the Youth League Committee of Yunnan Agricultural University, the three teams won the first prize, the school of Construction team, food school team and tea team won the second prize, the wave team, patriot team and star team won the third prize, and the rest of the participating teams won the excellence award. The picture shows the activity site. Yunnan agricultural university youth corps committee for figure school deputy party secretary LeiJianyi speech pointed out that the creative painting contest through the activity let the students further understand the significance of four history learning education, both revisit the history, and can learn from the glorious revolutionary tradition, firm ideal faith, better inheritance red gene, and carry forward the revolutionary spirit in the inheritance, let the red gene culture in inheritance and carry forward the more enduring.

2021-05-24	Beijing sports university "four" as the main line to carry out the history of the party learning Beijing sports university youth corps committee to carry out the party history learning education in youth online reporter Jiang Jibao) Beijing sports university youth corps committee recently with "four" as the main line for the history of the party learning education, for the theme of young teachers and students, various forms of "four" theme TuanRi activities, to celebrate the 100th anniversary of hundreds of kilometers personal clock relay and the second "civilized spirit savage body" weekend sports events."Four one" is around a history, a section of the centenary history of the Communist Party of China; select a specific major event or character story in the centenary history of the party, and deeply think, feel the selected facts, know the history of the party, patriotism, discuss, and personal study, work, life experience, political education, actively guide the youth members to seriously study the centenary history of the party, enhance ideals and beliefs, firmly follow the party.

2021-05-21	Morning) In order to further encourage and lead the majority of young students, carry forward the great national spirit with patriotism as the core, and create an atmosphere of learning from the Party history. Recently, the Communication Engineering College of Liaoning Railway Vocational and Technical College and the Railway Locomotive College Student Union jointly entered the Liaoshen Campaign Memorial Hall to hold the theme of "commemorating the 100th anniversary of the founding of the Party, commemorating the 102nd anniversary of the May 4th Movement, reviewing the oath of joining the League and singing the songs". Jinzhou medical university joining ceremony to the history of the party learning education and promote grassroots youth league branch vitality combined to ensure that the basic youth league branch party history learning education quality, enhance the basic youth league branch vitality, innovation of grassroots youth league branch work mode, school youth corps committee to explore in the party history learning education field culture, practice, learning activities to recruit grassroots youth league branch to participate in. On April 22, the eve of the "World Reading Day", the two-level students organized the "Youth Learning" theoretical learning center group to launch the "Party history of the celebration of the centenary of the founding of the Communist Party of China and the" Youth Learning " action. Especially, the youth League branch of Class 74, Class 2020 of the School of Humanities and Management and the newly developed league members in 2020 are specially invited to participate in learning the classic books of party history, and carry out the new league members' joining ceremony and the activity of all the league members to review the oath of joining the league. In order to commemorate the 102nd anniversary of the May 4th Movement and cultivate the students' patriotism, the School of Software specially held this "Youth Poetry Recitation Meeting". The students performed the scene of the year, which set off a boom. The volunteer service team of Panjin Vocational and Technical College came to Shaling Town, Panshan County, to carry out the red education and practice activities, to guide the students to draw the strength from the glorious history of the Party, firmly love the country and the Party, and bravely bear the mission of The Times. The volunteer service team came to the Shaling Battle Martyrs Cemetery to pay respects to the Shaling Battle monument, campaign overview wall and martyrs list wall, expressed their respect and memory for the revolutionary martyrs, and held a collective oath of joining the League. Walking into the education base, the teachers and students reviewed the development process of the party branch, and listened carefully to the older generation of revolutionaries and martyrs actively involved in the revolution.

2021-05-21	Beijing, May 21 (reporter Zhang Jianwei, correspondent Zhang Hao) " What to do if the Internet in the classroom is not good?"" Who has the black lines on the projection screen?"Recently, in the platform of Shanghai International Studies University Songjiang, Hongkou two campus nearly 300 classrooms, there is a" repair QR code ", teachers and students can scan the wechat code for the first time to report the repair, but also can check the maintenance progress at any time, teachers and students sigh," too convenient!"The picture shows the school library new books can be borrowing and compiling."In previous years, although we can recommend the school library to buy their favorite books, but the purchase and shelf also has to wait for a period of time, this year directly in the library site can register and take away the unsealed new books, too lucky!"The school first-grade graduate student Xiao Liang said happily. And located in the campus large flow of the student party building league building service center, this year has participated in the school at all levels of the party organized nearly 80 branch activities, for more than 200 branches to provide red movie, league class, red search, dream plan, party and league consultation and other services. Since this year, the school combined with the history of the party learning education carried out "I do the practical work for the masses" "teachers and students micro wish" activities, organize party members and cadres close to the teachers and students, into the teaching line, listen to the teachers and students wish, understand the actual demand, has carried out a series of practical projects for the masses, introduced a group of unreal hard recruit benefit the teachers and students. Technology enables teachers and students to achieve micro wish "keyboard", "clock" clock "," desk and chair "," floor tile "... in the repair small program page, more than 20 kinds of equipment in the classroom are described one by one, repair as long as click on the fault option, choose the repair classroom number, leave their name contact information can easily complete the repair. Since the launch of the platform, nearly 30 maintenance orders have been processed, which have won praise from teachers and students. Behind the convenient, is the office, information technology center, logistics management departments to get through data plugging point difficult cooperation and unremitting efforts, during the period, they repeatedly into college and department research, understand the actual needs of teachers and students, and technical team development team repeatedly debugging, optimize operation, improve the user experience, strive to make service more fine, more efficient, more intimate. The launch of the maintenance small program is another effort to take the party history learning and education as an opportunity, to promote the digital construction of the campus with the needs of teachers and students, to eliminate data islands, and to promote data integration. In addition, to solve the problem of teachers and students losing campus card, information technology center also launched the function of "pick up information" in the campus card self-service system, help teachers and students to find campus card; open seat reservation system for teachers and students to read; help students online retest, 20 departments, 48 classrooms, 947 candidates, 147,140 times, about 474 hours. The school continues to promote the construction of campus informatization, comprehensively improve the school's governance ability and service level, solve practical problems for teachers and students, and provide high-quality and convenient services for teachers and students, so that teachers and students can fully feel the care, and improve the happiness index of study, work and life in school. In order to better meet the reading needs of teachers and students, the school library also recently held an on-site borrowing book fair entitled "You read, I will pay". School selected domestic publishing house of the latest published books, is given priority to with high quality humanities social science academic works, involving language, literature, economy, history, art, philosophy, politics, and other disciplines, a total of more than 800 copies, display in the library multilingual reading hall, unlike previous years, the book fair readers can register directly at the scene, the first time to read the favorite books."You can pick up these new books and take them away, and we will return them in a month. Please bring the book to the service desk to register!"The site staff led the increasing number of onlookers, teachers and students. And borrow the students holding the selected new book smiling, chatting at the service desk side- - " borrow the new book also give small dolls, the school this activity is too intimate!"The picture shows reading books and magazines in the canteen or self-study. Correspondent for figure practice service of teachers and students, environmental education concept, the library also to the school restaurant, add books in the dining environment of teachers and students, build scholarly campus culture atmosphere, build broader learning reading space, communication site to listen to teachers and students, continue to improve library management services, improve reading research environment construction, create warm quiet elegant elegant atmosphere, build cultural education, environmental education position. The student party branch wants to organize a variety of activities, but can not find the resources or extend the explanation? The newly established student Party building and league building center provides party secretaries at all levels of the school, giving young students many opportunities for social practice and national conditions. By opening the door to run good contact points, the center enables the grass-roots branches, realizes the sharing of learning materials, financial media materials and social resources, and provides space for the branch secretaries to carry out salons and exchanges. The red film project provided by the center has served 56 branches, covering 14 departments, and served more than 1,100 people. From undergraduate to master's to doctoral branch, from the youth League branch to the Party branch and even the party Youth League committee, more and more people have come to the center to book the movies."Founding", "I and my hometown", "one home", "awakening s" and so on more than 20 movies, TV series for screening, for the "awakening s", "win" two films, also for the whole school branch after class lectures, let the students have more harvest, really become a learning education "guide", "branch secretary of the" family ", school and social cooperation education" connection ", student party members with ask and answer" close friend ". In fact, the school continues to "do practical things" to outside the school. The German language department of the school provides the service base for student party members- -Songjiang Huaqiao Primary School students to compile the Sino-German historical and cultural popular science book "Sino-German Those People" to apply what they have learned to help cultivate students' world vision and patriotic feelings. The book abandons obscure professional vocabulary and complex data charts, and tells illustrated stories about Chinese and foreign characters, encouraging students to compare and think across cultures. The picture shows organizing young students to communicate their reading needs with the library. In addition, the university participated in the "University Library Service Innovation Western Activities", through the donation of books to Xinjiang, Yunnan and other places, to promote the development of western school libraries, and donated professional and targeted Russian books to the first Russian undergraduate graduates of Karamay Campus of China University of Petroleum (Beijing), "I do practical things for teachers and students". In combination with the party history learning and education, the school enables teachers and students to meet the international needs of Shanghai's fine management through professional foreign language services, and effectively solves the communication problems for the smooth vaccination of foreigners during the regular epidemic prevention and control stage. Since March 2021 started in Shanghai foreigners new crown vaccine booking vaccination, Shanghai foreign studies university to undertake vaccination site translation task, set up the communication bridge between foreigners and field medical personnel, has sent volunteers 145 people to yangpu, pudong, xuhui, jing'an, minhang five municipal districts, a total of 13 vaccination provide English translation services. Volunteers commute an average of more than 2500 kilometers, work more than 600 hours a week, in addition, the school English and French, Russian, Spanish, German, Japanese, Korean six small languages online volunteers, by telephone for putuo district two vaccination provide online multilingual translation services, since the team formed on standby for more than 150 hours. Insist from set out actually, based on job functions and job characteristics, vary, advance planning, thinking, actively, Shanghai foreign studies university is planning through "face to face", "I" action, "problem", "send" warm "," offer "," pioneer "and" volunteer service "action" seven " action, attentively with love to do the practical work for teachers and students, do good deeds, problems.

2021-05-20	Nanchang Aeronautical University helped the "Red scarf narrator" trainers into Hongling School, gave the party history class to more than 80 student representatives, and carried out relevant training for the "little narrator" of the school. Speaking is divided into "here, the party eternal", "military keep beginner's mind", "blood soaked women's clothes, salt ode military and civilian feelings" and "do not forget to road, challenge forward" four chapters, respectively in sitcoms, speech, recitation and other different forms of performance, led the teachers and students to listen to the story of the party history, feeling one hundred elegance. According to the relevant person in charge of Nanchang aviation university youth corps committee, the activity from the school 11th big bone class students selected excellent backbone, formed by them into "small xuan" member youth branch team to surrounding primary school to carry out the party history story and "red scarf narrator" training, lead and help pupils and "red scarf narrator" draw nutrients from the revolutionary spirit, with their own words and deeds practice and inheritance red gene.

2021-05-27	Jiangsu information vocational technology college to let the party history learning education heart walk more "new" Jiangsu province of information vocational technology institute: be party history "a" people let party history learning education heart walk more "new" member for China youth network Beijing on May 27 (reporter zhang, correspondent, Jiang San Gao Xiulan) Jiangsu information vocational technology college youth corps committee "party history strong faith with the party" party history learning education, Not only focus on special learning, special training, online and offline knowledge competition, Also make full use of all kinds of red resources inside and outside the school, Guide youth members to learn history, history credit, history Chongde, history practice, Effectively promote the party history study and education to go "new" go "heart" more go "deep". The picture shows holding the offline "Learning Party History together" challenge. Recently, the Youth League Committee of Jiangsu Information Vocational and Technology College carried out the offline "learning Party History together" challenge in the dormitory road, setting off a new upsurge of party history knowledge education and learning. The activity site attracted many teachers and students to come, we just need to scan the QR code on the exhibition frame to learn the knowledge of the party history, through the online answer platform of the Central Committee of the Communist Youth League for two duel, the winning party to the next round of competition, will eventually produce the day champion, week champion and month champion. Song Jingyan, who won the Japanese championship, was fascinated by the Party History Knowledge Challenge. " The topic is not difficult, but the scope is a little wide. Through the challenge, I feel that I still have a lot to learn."The weekly online Party history knowledge competition has also attracted many students to participate in it, which has formed a learning atmosphere of" comparing with learning and catching up with learning ". The youth league members who participated in the answer were fascinated with the online answer. " I only answered 10 questions a week, which is not enough."The online challenge is not only accuracy, but also speed. Liu Jiankang said that the weekly online Party history Knowledge Challenge set off a small climax in learning the history of the Party in their youth league branch. People actively participated in the answer and forwarded the circle of friends for more people to participate in the answer. The picture shows the youth league members learning the "Red Boat Spirit" in the school "League Member Consciousness Theme Education Hall". Correspondent to dig the red resources inside and outside the school, let the party history learning education go "heart" " overview of the spiritual spectrum of the Communist Party of China, the red boat spirit, Jinggangshan spirit, Xibaipo spirit, the spirit of space spirit... a precious spiritual wealth, like a torch, illuminate the journey of the struggle of the Communists."Su Yongcheng, a member of the Jinsong lecture group, told the profound connotation of the" Chinese Spirit "to the youth league members in the" League Member Consciousness Theme Education Hall ". In one month, he and other members of the publicity group have received nearly 2,000 visitors from the youth League branch. In the red position of more than 320 square meters, the "red elements", "ideological and political elements" and "school elements" are fully integrated, and the education area, exhibition area, conference area are set up. Through the "reality classroom", review the oath of joining the league and joining the Party, and pursue the historical footprints. Organize 100 "post-00" lecturers, let the party history learning education go "deep" by the school cadres composed of the "one hundred hundred hundred" after the official lecture, through the wechat platform every day released "one hundred hundred hundred" party history story video, let the young generation ceaseless spirit blood, gather the strength of the youth. Every "post-00 s" speaker is a believer of the Party. He takes advantage of new media platforms and short video communication to create a youth party history course, let the closest students to guide, and let the party history stories infiltrate the hearts of college students. The picture shows holding a seminar on the theme of "Staying true to the original aspiration and remembering the Mission". The Youth League Committee also held a red gene theme of "Stay true to the original aspiration, keep in mind the mission", Jinsong publicity group members on the scene about the glorious deeds of revolutionary heroes, "history" both "material", red story let the present teachers and students unknowingly tears. Ding Peijian said, " In the past, when I told the history of the Party, I always felt that history was a little far away from us. It was not until they heard the revolutionary story at the scene that they felt the real shock.”

2021-05-19	Anhui medical university: let the students understand the medical skills perception spirit of war disease of Anhui medical university: let the students understand the medical skills perception war disease spirit youth network Beijing on May 19 (reporter Zhang Jianwei, correspondent Liu Wencai) " after training, I learned the specific steps of protective clothing, and the throat swab and nasopharyngeal swab collection method. When I put on protective clothing and goggles, I was deeply aware of the responsibility and responsibility of medical students. I will always be ready to stand up when I need me!"Said Wang Dongxu, a 2020 preventive medicine student at the School of Public Health of Anhui Medical University. Recently, the epidemic response and prevention and control work in Anhui province is progressing in an orderly manner. The school has actively set up a reserve volunteer team for nucleic acid testing and sampling. In order to further strengthen the emergency response capacity of the public health special volunteers, the youth League committee of the school held a special youth volunteer training meeting on the "Southeast School Epidemic prevention and control" on the morning of May 16. The training session will be jointly organized by the Fourth Affiliated Hospital, the School of Nursing, the First Clinical Medical College, the Second Clinical Medical College, the School of Public Health and the Management Committee of the South Campus. The picture shows the teacher and the professor wearing and taking off his goggles. Correspondent for the picture "we should scientific operation, do pollution-free, wear hats, masks, boots, goggles......" activity, five experienced the epidemic line (four had supported Hubei) medical staff present, the main teacher patiently explain, demonstration teacher synchronous accurate demonstration, teach students seven steps wash techniques, wearing protective clothing and other skills. After watching the teachers' demonstration operation, 100 young volunteers were freely divided into five groups for practice. The young volunteers collected secretions with throat swabs, then immersed the swab head into the cell preservation solution, breaking the tail sample, and the teacher guided the side. After the training, all medical staff and volunteers signed on the protective clothing and left each other as a souvenir. The picture shows the teacher and the professor wearing and taking off his protective clothing. Correspondent for the picture " off hand sleeve, off goggles, off boot cover, off protective clothing... simple and repeated steps, every step can not go wrong."Fan Lin, a 2018 clinical medicine student at Anhui Medical University, said that through the training, he felt the rigorous, pragmatic and selfless dedication spirit of medical workers. Tu Mengwei, a clinical medical student of the grade of 2018, said that in just three hours, she had realized the difficulty of medical staff in preventing and controlling the epidemic."Just trying on protective clothing has left us sweating and our goggles are foggy, so you can imagine how much willpower it takes for medical workers to work for hours in this way."The picture is a signed group photo of nostalgia. Wang Lina, a nursing student of grade 2019, said, " When I put on the isolation clothes under the guidance of my teachers, I personally realized the sense of mission and the courage of the frontline medical staff to overcome difficulties."" volunteers were interested in collecting throat swabs, wearing and taking off protective clothing, and took them seriously in practice. Proud of their more and more skilled operation, the training is worth it!"Said He Chunlin, a medical worker from the Fourth Affiliated Hospital. In the next step, the Youth League Committee of the school will also combine the professional characteristics of medical students, strengthen the training of volunteer service ability, regularly carry out the volunteer service activities of learning from Lei Feng, and guide the students to fulfill their social responsibilities.

2021-05-19	During the special activity, President Dou Xiankang and the volunteer college students provided tour guides to the medical team members who returned to Han to appreciate the cherry blossoms. What happens when integrating vivid anti-epidemic stories into ideological and political teaching? Chen Wu, a teacher at the School of Marxism at Wuhan University, still remembers choking up several times when he voiced the "instant sight" of the ideological and political finance course. This program, known as the "Wu University version", is deeply loved by students, and also let the post-00s generation truly realize the connotation of the great anti-epidemic spirit."Not born fearless, but to choose bravery."Ding Zhaoying, a student from Wuhan University, lamented that through the short films, pictures and vivid narration, Wuhan had a better understanding of the fight against the epidemic. During the COVID-19 outbreak last year, the battle of Wuhan and Hubei touched the hearts of people of the whole country. The great anti-epidemic spirit of "life first, the whole nation, sacrifice life, respecting science and sharing destiny" bred in this invisible battlefield has become a thick and vivid ideological and political textbook for millions of college students in the province. Central China normal university using the school for many years of ideological brand "good China story" theme education activities, with "disease resistance letter" as the theme, using virtual time and space special effects, combined with letters, diaries, reading, songs and dialogue story screening, on the screen, teachers and students, alumni tells a touching story to disease resistance, shows the Chinese people unity is strength, become cassia mountain a vivid ideological lesson. The Publicity Department of the Party Committee of Wuhan Institute of Technology edited the story record, which included 141 teachers, students and staff as the university's ideological and political course textbooks, and ideological and political teachers have integrated the stories into relevant courses. The story of the war "epidemic" happened into the textbook, credible and learned, straight to the heart. The letters came from the affiliated hospitals of the university, and the faces of the nurses pressed under masks were as clear as yesterday... Wang � Z, a student from the School of Chemical Engineering of Wuhan University of Science and Technology, said, "I thought the theory in the textbook was far away from me, but now the vivid images have become the best interpretation of the theory."In March this year, the Hubei Provincial Department of Education opened the Hubei provincial-level undergraduate quality education elective course "the Great Anti-epidemic spirit" officially opened. Tao Hong, secretary of the Party Leadership Group and director of the Hubei Provincial Department of Education, said at the first activity that the college students in the province sharing the lesson of "Great Anti-epidemic Spirit" is a new measure to carry forward the great anti-epidemic spirit and a new carrier to strengthen the learning and education of Party history. Gather education resultant force, guide students to grow up. The first lesson of "The Great Anti-epidemic Spirit" was delivered by Professor Shen Zhuanghai, the chief expert of the Key textbook compilation Group of the Central Marxist Theory Research and Construction Project. It took the form of "offline teaching + synchronous live broadcast". More than 25,000 college students from universities in the province participated in the course selection online in real time. The course aroused a warm response among the students, with 59,000 likes. Shen Jiao, China Youth Daily, China Youth Network, reporter Lei Yu, source: China Youth Daily

2021-05-21	Liaoning universities organize Party and League knowledge competition activities Liaoning universities organize Party and League knowledge competition activities morning) in order to strengthen students' patriotism education. Recently, the student Union of Liaoning Railway Vocational and Technical College held the "Qingyun Cup" red speech contest in the lecture hall on the fifth floor of the college student activity center. Chaoyang Normal College to carry out the "study the history of the Party, strong faith, follow the Party" - -the first Party history knowledge competition of Chaoyang Normal College. This competition inspects the study of party history of the school youth league members, deepens the understanding of the party's theory, line and principles, creates an atmosphere of party history learning and education, and promotes the in-depth development of party history education. The students expressed that they would continue to learn the knowledge of the party history, bear in mind the original mission, and write the song of youth in the new era with the struggle. Dalian art institute around "four history" organization "read China" activities competition, "in one hundred, writing brilliant" party history learning education painting and calligraphy exhibition, party history "hundred" essay "" one hundred posters "," video "" story "and" T-shirt "series of activities," history "as direction, don't forget to beginner's mind with the party" watching "history" four history "theme activities," history, "theme reading clock" four history " activities, encourage students to understand the history of the party, learning in the theme activities. In order to welcome the 100th anniversary of the founding of the Communist Party of China, Liaoning Vocational University of Technology carries forward the revolutionary tradition, carries forward the spirit of patriotism, inherits the red gene, and encourages and guides the youth league members of the university to firmly follow the Party's ideals and beliefs. The school Youth League Committee held a speech contest on "Carry forward the May 4th Spirit and Bear the Youth Mission". Shenyang pharmaceutical university to celebrate the 100th anniversary of the founding, the 90th anniversary, inheriting red gene, carry forward the red spirit, in-depth study xi general secretary in party history learning education mobilization meeting important speech spirit, in April, the school youth corps committee based on knowledge learning, in the form of network contest and offline competition, in the school "study party history, strong faith, follow the party" -Shenyang pharmaceutical university "have eventful years, exhibition Shenyang medicine style" party history school history knowledge contest. We will continue to deepen the education of loving the Party and the country, extensively carry out the education of ideals and beliefs, and draw nourishment from the history of Shenyao Red School, so that the love of the Party, the country and the school has become the firm faith, spiritual strength and conscious action of the majority of students. The preliminary contest has been completed, and a total of 4983 people from the school participated in the activity.

2021-05-19	The original musical "Faith Youth" of Dalian University of Arts premiered "Cloud Theater Party Class" Youth ". With the help of "Internet +" and "Cloud Theater", the advantage of "Faith Youth" realizes the visual presentation, interactive communication and immersive experience of party lessons, and takes the remote "zero distance" party lessons. The play was performed in the Grand Theater of Dalian Development Zone, and played online through the official WeChat video platform of the Liaoning Provincial Committee of the Communist Youth League. The teachers and students of more than 100 universities in Liaoning province watched the play with party lessons and received party history education. Through the form of "attending party lessons, learning heroes and acting heroes", the drama shapes and shows the typical image of excellent Communists in different times, and guides the young people to learn the history of the Party, strengthen their faith and understand their original aspiration. Centering on the theme of the play, the school carried out various activities, such as creation field collection, party history education seminar, "cloud visit" red education base, to innovate the learning methods of party lessons and expand the position of party history learning and education.

2021-05-18	The 11th "Challenge Cup" Yunnan College Students Academic Science and Technology Festival ended on May 15. Twenty-two works won the provincial special prize, and 50 works won the provincial first prize. Yunnan will recommend 32 works to participate in the 17th "Challenge Cup" National College Students Extracurricular Academic Science and Technology Festival. The competition received 600 works from 56 universities in Yunnan province, and the number of participating universities and the number of entries all reached a record high. The works involve the development strategy of serving the country and Yunnan province, including natural science, academic papers, philosophy and social sciences, social survey and academic papers, scientific and technological inventions, etc. It is reported that since the first "Challenge Cup" Yunnan Provincial College Students extracurricular Academic Science and Technology Festival was held in 2001, more than 600,000 young students have participated in it. The competition has played a positive role in promoting the growth of young and innovative talents, deepening quality education in colleges and universities, and promoting economic and social development. During the competition, the Yunnan Provincial Committee of the Communist Youth League invited Huang Xiaojun, vice president of Yunnan Academy of Social Sciences, to give a lecture on the study of Party history, Dr. Xu Gang of Kunming Branch of Chinese Academy of Sciences and some of the judges to discuss the science and technology innovation forum for the use of the expert judges as "think tank" to provide support and help for academic innovation and symposium of the Secretary of the Youth League Committee of Yunnan Province, and discussed the reform of the "Challenge Cup" competition system.

2021-05-17	Guizhou university college of science and technology "community style" community culture festival opening of Guizhou university college of science and technology "founding history, bright culture month" community culture festival opened Beijing on May 17 (reporter Zhang Jianwei, correspondent, Chen Yingmei YanChun) to celebrate the founding of the communist party of China in one hundred birthday, urge students to work, create rich campus culture atmosphere, recently, Guizhou university college of science and technology held a "founding history in one hundred, bright culture month" student community culture festival opening ceremony. More than 1,100 people, secretary of the Party Committee Publicity Department and Secretary of the Youth League Committee of the School of Science and Technology, Guizhou University, teachers of the Party Committee and representatives of various student groups attended the activity. Acg dance performance "Blossom With Dreams". At the opening ceremony, the Social Federation and Art Department, Huaiyin Hanfu Association, Guitar Association, ACG Animation Association and other organizations and associations respectively showed their distinctive style in the form of songs and dances, which were highly praised by the audience. Teachers and student staff took a group photo. It is reported that the community culture festival is sponsored by the Communist Youth League Committee of Guizhou University School of Science and Technology Committee, 6 student organizations jointly organized, a total of 8 theme activities, 27 associations to participate in, the activities from May 7,2021 to May 27,2021, to participate in the above activities will reach more than 4,000 students.

2021-05-10	Morning correspondent, Liu Yanchun, Song Hongqing) On May 7th, the activity of Guizhou University, " learning the history of the Party of China, was successfully held. Li Jianjun, Party Secretary of Guizhou University, and Linghu Caitao, Deputy Secretary of Guizhou University attended the event and delivered a speech.it is reported, Since the Central Committee of the Communist Youth League launched the theme education activity of "Learn the Party and Follow the Party", Guizhou University responded positively, Online leverage comes from "micro-media", Juli "financial media", Open "Tuan Tuan accompany you to learn the history of the Party", "Party History micro theater", "Party history micro classroom" and other network boutique, In the form popular with young people, To guide young people to study the history of the Party, strengthen their faith, and follow the Party; Offline activities are held in the four history essay contest, poster cartoon contest, short video contest, three market poetry, four history knowledge contest, teachers and students red song competition, calligraphy competition and other activities, And organized the "youth lecture group", "college students publicity group" to carry out 100 associations to welcome publicity, 100 branches to welcome propaganda, 100 propaganda into the network and other publicity activities, Let the party history learning and education online and offline "linkage" up, We will make solid progress in the study and education of Party history and education on special topics, taking root among young people.

2021-05-10	Zhang Wenling) Recently, under the guidance of the Youth League Committee of Yunnan University, the Student Union of Yunnan University recruited all the readers of party history stories, which attracted the attention of students, and hundreds of students participated in the selection of readers. Up to now, the audio column of "Reading Party History Example" has launched 12 party history stories, example stories and red stories read by students, which have been viewed nearly 4,000 times. Yunnan University is a university with a glorious revolutionary tradition. a large number of revolutionary martyrs emerged in the period of revolutionary construction. Mr.Wen Yiduo once delivered his "last speech" at the Zhi Hall of Yunnan University regardless of his personal safety. Recently, the student art troupe of Yunnan University performed the stage play "Mr.Wen in the Heart" in front of Zhi Gong Hall, transforming the red resources into a vivid textbook to learn the history of the Party and the history of the school well. The second season of Zhu Dan, deputy secretary of the Yunnan Provincial Committee of the Communist Youth League and associate professor of Yunnan University, presided over the first director of Yunnan University, launched the column "The Story of the Party", which has been produced for 7 issues at present. School also play to the advantages of various disciplines, history and archives institute host "one hundred burr line" series of academic lectures, school of art and design, the history of the party study education and aesthetic education, literary creation, curriculum education, launched the original party history theme illustration "youth beginner's mind inheritance", has updated 10, received wide attention by the society.

2021-05-09	Anhui Medical College: Young students "punch in" red landmark learning party history Wang Haihan, correspondent Jiang Nan Hu Anran) recently, Anhui Medical College "learning history increase credit learning history practice" series of theme activities launched. It is reported that the school organizations at all levels into the party history learning education volunteer service system, evaluation system and security system, form the party history learning education "college students branch", into the grassroots community speak party history; create "volunteer + professional + thought politics + X" practice education platform, normalized "love to help old" volunteer public welfare activities and preach party history story. At the launch meeting, 26 red education bases were lit up on large-screen maps. In the early stage, the school has selected 26 red landmarks across the country, including revolutionary memorial halls, conference sites, battle sites, martyrs' cemetery and other places. In a series of themed activities, the Party and the Youth League organization of the school will combine "Party building +", "Three parties" to the countryside "and" volunteer service " and other activities to lead teachers and students to learn the relevant knowledge of 26 red education bases online and offline through reading groups, PPT explanation, field punching, video cultural product production and other ways. At the same time, the school will also carry out a series of activities of "100 teachers" and "learning history and practice". Teachers, party members and volunteers will carry out personalized guidance and precise assistance in view of the students' ideological, economic, psychological, study, life and other difficulties or problems, so as to continuously enhance the sense of gain and happiness of teachers and students. Dong Yajie, secretary of the Party Committee of the University, and Zheng Weichao, deputy Secretary of the Party Committee and president of the University attended the launch meeting.

2021-05-07	) More than 300 young students from Shanghai University have spent an unforgettable May 4th Youth Day on May 4th in Shengqiang Film and Television Base in Shanghai. They participated in the promotion and shooting of the film "1921", which tells the story of the first Chinese Communists to shoulder the responsibility of saving the nation and make the Chinese society take on a new look. It is reported that this is an important part of the "May fourth" series of theme education practice activities and students' party history learning education series activities of Shanghai University. Before the shooting, the participating students reviewed the background and experience of the May Fourth Movement in 1919 through special lectures, theme seminars, historical materials study and visiting red venues, and studied the history of the founding period of the Communist Party of China carefully. In the film, the progressive youth who play the May 4th Movement are. The students put on student clothes and student hats, and listen to the speech of "Li Dazhao" to promote Marxism and inspire the youth to unite and save the nation. Students held high banners, raised their arms and shouted "Return to Shandong, punish national thieves."Students study the history of the Party in an "immersive" way, experience it from a first-person perspective, and enter the life and spiritual world of the young people a hundred years ago."With my classmates, I shouted the slogan of the youth 100 years for national independence 100 years ago, and read Mr.Li Dazhao's" Youth " with my classmates. Deeply feel that the beautiful life now, is the blood of countless revolutionary martyrs for."Out of the shooting base, mechanical and electrical institute of engineering and automation, 2020 graduate said, oneself to learn professional knowledge, scientific research, efforts to crack the" neck "problem, truly" I with youth, create the youth family, youth, youth nation, youth of human beings, youth of the earth of youth, the universe of youth "."This immersive party class made me deeply realize that the rise and fall of the country is responsible. The May 4th Youth led the Chinese people to fight bloody battles to save national danger and safeguard national dignity. This patriotism not only encouraged the Chinese people at that time, but also encouraged us who played them today."Said Song Haoxiang, a 2020 master student at the School of Science of Shanghai University. According to introducing, this year Shanghai university innovation education form, method, carrier, earnestly students party history learning education, through hundreds of teachers and students qi ode "the communist declaration", join "1921" one hundred reading activity with recite revolutionary youth speech, interview hundreds of party members, salute hundreds of heroes activities, graduate students "hundred +" action plan, "hundreds of party history" "graduate red Tuan" tour, graduate students "one hundred singing" youth song, "I do for classmates" activities, the party history of learning into daily learning education, and the party history learning education results into action and actual effect.

2021-05-07	Changzhou Institute of Technology "Chang Xiaoyan" college students theory lecture "Chang Xiaoyan" college students theory lecture lecture green network reporter Li Chao) recently, Changzhou Institute of Technology "one hundred years of party history youth said" college students publicity competition and "Chang Xiaoyan" college students theory lecture lecture launch ceremony was held in Changzhou Institute of Technology. On the eve of the May 4th youth day, in the group to carry out "learn the history of the party, strong faith, follow the party" learning education, "Chang Xiaoyan" theory of college students XuanJiangTuan around one hundred party history theme, fully tap Changzhou red resources, local red gene, with the voice of youth why the communist party of China why "can", marxism "line" and why "good" socialism with Chinese characteristics. The university students theory XuanJiangTuan began in 2017, the early form of college students summer social practice and volunteer service preaching activities, once in the new north, bell tower area of multiple communities to preach hundred games, won "excellent volunteer service team in Jiangsu province" "the fourth Jiangsu volunteer service exhibition gold medal project" and other honors. At the end of December 2020, Changzhou engineering institute of marxism and youth corps committee, joint new north new era civilization practice guidance center, open the "nest" recruitment, after two rounds of selection, early training, campus training, scenario, selected 60 moral good, positive, expressive, enthusiastic public welfare members, formed the "Chang XiaoYan" college students theory XuanJiangTuan. In order to test the learning effect and publicity level of the members of the publicity group, create a strong learning atmosphere of party history in the campus, and accumulate experience for the following community publicity, the first publicity of "Chang Xiaoyan" in 2021 was held through the competition at the launch ceremony. After the preliminary, preliminary, a total of 10 contestants were selected for the final, preaching the content of both women do not let men, brave female warrior Shi Liang; also like a thunder, in the revolution woke up the world one of the three outstanding Changzhou Zhang Dalei and other Changzhou revolutionary historical figures, and Guixiang street "alley premier" Xu Qiaozhen and other characters around the story. The contestants fully explored the red resources of Changzhou, inherited the local red genes, and told the stories of the party history around them with the voice of youth. Sun Junqiao, the first prize winner of the lecture competition, said that it was through the combination of theory and practice that he had a deeper understanding and understanding of the content to be preached, and he was full of expectation and awe for this activity from the bottom of his heart. After the launch ceremony, "Chang Xiaoyan" will use their spare time to go to the grass-roots level of the Xinbei District for nearly 80 lectures, sending the magnificent history of the CPC and its rich history to the grass-roots villages and communities.

2021-05-06	Southwest jiaotong university youth corps committee overall promote league history learning education southwest jiaotong university youth league committee promote league party history learning education correspondent li na zhang) southwest jiaotong university youth corps committee thoroughly implement the xi general secretary in party history study education mobilization meeting speech spirit, according to the overall deployment of the central party history learning education, give full play to the advantages of the communist youth league, with good organization life, theme education, cultural activities, social practice, promote youth league education study the history into the brain into mind, guide the young students learn history, history, history chong DE, learning history. Do a good job of top-level design, sort out the main line of party history study work. In accordance with the decision and deployment of the Central Committee of the Communist Youth League and the Party Committee of the University on the learning and education of Party history, the Implementation Plan of the Committee of Southwest Jiaotong University of the Communist Youth League on the Learning, Education and Practice Activities with the Theme of "Learning Party History, Strengthening Faith, and Following the Party" was formulated. On the one hand, it highlights the project traction, creates 17 characteristic activities of theme learning and education practice, and defines the key work of theme education practice every month according to the time line. On the other hand, the development of party history learning and education of grass-roots youth league organizations should be included in the key content of the work assessment of the school Communist Youth League, and the platform of "smart youth league building" should be played to supervise the learning and education. Highlight the theme, enrich the connotation of party history study. With the general meeting of party branch and league courses as the main form, all league branches are required to carry out at least more than four party history learning meetings throughout the year, and create the quality online courses such as "Youth teaching group" party history series of theme league courses, so as to realize the full coverage of online and offline learning and education for young students. We organized various special activities in the study of "Youth, the Light of Faith", "Stay true to the original aspiration and follow the Party", college students' "transportation public welfare" volunteer service, and the national flag raising ceremony commemorating the 102th anniversary of the May 4th Movement. Production school cloud day activities H5, widely during the May 4th "learn the history of the party, strong faith, follow the party" theme cloud day activities, 100000 + people visit the site to carry on the cloud flag-raising, party league lessons, example learning, joining ceremony activities, in the activities to grasp the party's innovation theory, experience the party's spiritual pedigree, inheriting the party's red gene. Strengthen the demonstration and guidance, build the party history study positive incentive. We will strengthen Party building and youth league building, carry out a project to guide Party members, and encourage Party branches and youth league branches to jointly study and educate Party history. The study of party history education is the main course of the "young marxist training project" training course. It is clear that all youth league cadres and youth league members should participate in no less than 8 credit hours of intensive study and training with political theory as the main content for no less than 8 credit hours per semester. Members of the publicity group of Sichuan Provincial Party Committee and other experts and professors were invited to offer special group courses on party history learning. The establishment of "specially called the league branch", "youth civilization", "youth pioneer post", to commend the education and learning outstanding performance of the secondary league organization secretary, grassroots league branch secretary, forming a positive incentive, stimulate the spirit of struggle.

2021-05-06	Changzhou Textile and Garment Vocational and Technical College launched eight special actions to lead the youth study party Shi Qing network reporter Li Chao) recently, The Youth League Committee of Changzhou Textile and Garment Vocational and Technical College focuses on the main line of leading the youth to study the party history, Give full play to the organizational function of the Communist Youth League of "three forces once" condensed youth, Stimulate the active role of the student Union in leading and serving students, Through the three main positions of "thematic learning, red quality improvement, and organizational life", Launch of "youth growth action, youth dry competition action, youth service action, youth speech propaganda action, green heart action, green party action, green horse practice action, youth creation centenary action, green travel card action" 8 special actions, Leading more than 10,000 youth league members of the school to learn the party history, strengthen faith, and follow the party. According to the introduction, the school through the main position of the party into the new league member development, league membership, student cadre selection training, outstanding league members (league cadres) selection training; with the history of the party, the "four history" into the "youth marxist training project", youth league class, faith open class platform; a batch of books such as a brief history of the mainstream reading of youth league organization life and personal growth. A few days ago, "youth to the party struggle power" theme youth day activities in Changzhou martyrs cemetery, the school youth members read out the league oath, through a across time and space "confession", to the martyrs made the Chinese dream of youth oath, opened the "green heart to the party action" curtain."Youth generation face to face" "green heart symposium" "green listening mailbox" a series of practical measures for the youth gradually launched, "youth service action" in the youth league cadres at all levels of the Youth League, student union members to collect and solve nearly 100 problems around the students, "I do practical things for the youth" has become the core of everyone's work. In addition, " Do you look at China? One hundred in the party of China "" youth learning "," around spinning group "such as online learning," I give the youth to the party "speech," dream youth with the party "script solicitation," thumb up in one hundred "and exhibition of the second classroom content successively launched" green and one hundred action ", including" spinning group micro class | one hundred party history read every day " has been released 69. It is understood that the school's "red theme film and television works" has been released in the school 10 times, participating in more than 4,000 students, "online marathon card red landmark" activity has entered museums, memorial halls, red education bases and other places. Through rich learning and practical activities, let the young students learn history Ming Ming, study history increase credit, study history Chongde, study history practice, so that the "young people should love the country, inspire faith, inspire inspiration, draw strength, and constantly strengthen the 'four self-confidence' " youth training goals can be realized.

2021-05-05	University of Shanghai university of technology hundreds of teacher cadres into the youth league branch of Shanghai hundreds of teachers cadres into the youth league branch newspaper reporter wang ye jie) today, Shanghai university of technology organization 100 teachers and cadres into 100 youth league branch, zero distance, face to face to share with young students beginner's mind and struggle, build set of "experiential, sharing, interactive" as the integration of the new mode, let the party history learning education "sink", "live". Ke Qinfei, president of Shanghai University of Applied Technology, told the students with "heaven, earth and people" about the great achievements of "opening the world", "changing the earth", "earth-shaking" and "earth-shaking". He encouraged the young students to bear in mind General Secretary Xi's earnest entrust, cherish "the great people of the country", establish great ambition, achieve great virtue, become great talents, and shoulder the great responsibility, and strive to become a new person worthy of the important responsibility of national rejuvenation, so that their youth can bloom brilliantly in the struggle."May 4 th" this year, Shanghai application technology school leaders, theoretical experts, party members and cadres, outstanding young teachers represent 100 teachers actively into the youth members, through common experience, scene interaction, listen to the youth story, talk about youth, youth dream, launched a teachers and students heart communication, thought resonance "journey" of youth. Mao Xiangdong, vice president of Shanghai University of Applied Technology, walked into the Youth League branch of the School of Economics and Management, and started the youth party class from the "Age of Awakening". Huang Yan, winner of the National Women's Ice Hockey Championship, shared her story of the branch as an excellent member of her youth struggle. Branch members shouted "the struggling youth is the brightest", and wrote down the youth blessing. The young people of the special education class of the School of Art and Design "moved" the event to the national flag. Zhao Yin, the "Star of Self-improvement of Chinese College Students", led the students to review the history of the May 4th Movement. The students "sang" the national anthem in sign language, remembering the pioneers of the May 4th Movement and expressing their patriotic feelings. It is reported that the first senior Party school training class of Shanghai University of Applied Technology was also opened recently, upgrading the party class to "interactive" and "immersive" experience exchange class, professional "technology" enabling party class, and tribute to the red education base.

2021-05-03	Party history learning education into the network Guizhou University Communist Youth League so do Party history learning education into the network Guizhou University Communist Youth League so fly correspondent, Liu Yanchun Song Hongqing) learn history Ming Li, learn history to increase credit, learn history Chongde, learn history to practice. To thoroughly study and apply xi new era the ideas of socialism with Chinese characteristics, the spirit of the fifth plenary session of the 19 and xi general secretary in party history learning education mobilization meeting's important speech spirit, Guizhou university youth corps committee with "your youth media center", close to youth demand, youth communication characteristics, seize youth accept psychology, from "micro media", deepen online combination, create diverse network, in the form of youth, guide the youth party history, strong faith, follow the party, strengthen the party history study into the network effect. We should persist in thinking, thinking, doing, and understanding in learning, and continue to push the study and education of Party history deeper and achieve practical results. Take advantage of "micro media", focus on "financial media". The output terminal of "Guizhou University Youth Financial Media Center" of the Youth League Committee of Guizhou University has formed a complete structure of "4 + 1 + 1" so far, 4 is the official website of "Online Communist Youth League", wechat, Weibo and the Voice of Youth Network platform, 1 is the cloud broadcast room of "Your University Youth" and 1 "Party Building + Youth League Building". Close to the youth demand, find the youth communication characteristics, Guizhou university youth corps committee website, official micro, official bo opened "red stream", "celebrate in one hundred" "one hundred read" the history of the party study theme column, group magazine your youth planning theme in one hundred issue, "party + group building" studio for party history education live, recording about, from "micro media", "media", really make party history learning education online "spread", to ensure the party history learning education of high quality, full coverage. Create network fine works, enrich the form of publicity. To catch the youth eyeball, "let faith strengthen the heart of the youth," your big youth media center "constantly enrich preaching form, launched" group "party history with the party history", "theater", "," party history micro class "series of network boutique, with students happy animation, sitcom, sand, posters, cartoons, allegro, short video network preaching form, really make party history learning education" live " up, effectively lead youth learning in one hundred party history, inheriting red gene, ceaseless red blood. Combine online and offline activities to enhance the publicity effect. Promoting online propaganda position construction at the same time, Guizhou university communist youth league attaches great importance to party history education offline, held four history essay contest, poster cartoon contest, short video contest, three market poetry, four knowledge contest, teachers and students, and actively form "youth branch", "college students XuanJiangTuan" hundred community to preach, hundred branch to preach, really make the party history learning education online "linkage" up, promoting the party history learning education and special education work takes root in the youth. Rely on the youth, lead the youth. When youth prospers, the country prospers. When youth is strong, the country is strong. Youth is the hope and future of the country, and the Party history is a fertile ground for the youth to absorb nutrition. The learning and education of the Party history must rely closely on the youth to truly lead the youth. The Communist Youth League of Guizhou University gives full play to the role of "youth teaching group" and "college student publicity group" to tell the wonderful stories on the party's centenary journey to all the youth of the university. During the winter vacation of 2021, the publicity group of Guizhou University held a "theme cloud publicity activity to tell the red history of the founding of the Party" (12 issues), which brought the red stories all over the country to all the teachers and students through video publicity, and achieved very good results. It is reported that Guizhou University "focus on the inheritance of the great spirit forged by the CPC in the long struggle;" party history micro theater "in melodrama, including sketch, crosstalk, harmonica, guide students to learn party history knowledge;" party history micro class " in animation, cartoons, posters to show the glorious journey of China. In the next step, "Guiyang Youth Financial Media Center" will launch a new series of programs- - "youth broadcast room", in the form of special interviews, to deeply publicize the significance of the history of the Party learning and education, and to interview the advanced examples emerging in the extraordinary struggle and the economic and social development of our province.

2021-05-01	In Linchuan No.2 Middle School, the 100th anniversary of the founding of the Party was held in Linchuan No.2 Middle School, and the tenth anniversary of the founding of the Party theme youth League Day and the May 4th commendation conference was held. China Youth Network correspondent Beijing May 1 (reporter Qin Liang correspondent Yu Hui) review the brilliant struggle of the Party history, pay tribute to a hundred years of trials and hardships. On April 29, Fuzhou, Jiangxi province linchuan 2, linchuan 2 experimental school "learning party strong faith with the party" to celebrate the 100th anniversary of the founding of the communist party of China theme TuanRi and May 4th commendation conference, including school leaders, young teachers and member student representatives, more than 900 people gathered, talk about youth story, celebrate the youth festival. Three young teachers told their own stories of struggle emotionally. China Youth Network correspondent Yu Hua hui for the picture scene, we watched the founding of the centenary theme MV "youth", once again feel the Communist Party of China led the Chinese people to practice the original mission, create a brilliant future magnificent epic. Hu Wei, teacher ethics model of Jiangxi Province and winner of Fuzhou May Day Labor Medal, Zhou Qingyun, outstanding teacher and provincial backbone teacher, and Xie Dongping, master student and outstanding head teacher of Southwest University, explained their struggle course and explained the responsibility and responsibility of young teachers in the new era. The girls' chorus "I Love China" sang loud and clear, singing the surging passion of the youth of the new era to celebrate the 100th anniversary of the founding of the Party. The poem recitation "Youth China" is inspiring, eulogizing the great China of youth, and expressing the love for the motherland. Conference read the linchuan 2,2 experimental school 2020 may 4th commendation decision ", to won the" outstanding "member" outstanding communist youth league cadres "" most beautiful students "and" may 4th red flag league branch " winning individual and the collective awards, thanks to the winning youth league organizations and individuals for the school development, the construction made outstanding contributions. In the past year, the youth league organizations at all levels of the school have closely focused on the work of the school education center, and have made new achievements in organizational construction, ideological guidance, social practice, volunteer service, campus culture and other aspects. The cadres and members of the Communist Youth League have worked hard, studied hard, practiced bravely and were willing to play an exemplary role and contribute their youth to the battle against COVID-19. Clank oath, boxing boxing original intention to focus on the mission."I volunteer to join the Communist Youth League of China, firmly support the leadership of the Communist Party of China..." at the conference, all the young teachers and students facing the league flag, clenched the right fist, with a loud, sincere emotion for a solemn oath. Young teachers and league members of the students, facing the league flag, solemnly swear. China Youth network correspondent Yu will for the picture

2021-05-09	Zhang Wenling) Recently, under the guidance of the Youth League Committee of Yunnan University, the Student Union of Yunnan University recruited all the readers of party history stories, which attracted the attention of students, and hundreds of students participated in the selection of readers. Up to now, the audio column of "Reading Party History Example" has launched 12 party history stories, example stories and red stories read by students, which have been viewed nearly 4,000 times.

2021-05-01	National college youth XuanJiangTuan in nankai university launched the national youth XuanJiangTuan in nankai university start youth network reporter Hu Chunyan correspondent Luo Fan) on April 29, to "inheritance May 4 pay fire carry forward the spirit of nankai salute founding in one hundred" as the theme of the national college youth XuanJiangTuan launch held in nankai university. Teachers and students of the youth publicity groups from Fudan University, Xi'an Jiaotong University, Lanzhou University, Hunan University, Tianjin University and Tianjin Polytechnic University gathered together to welcome the May 4th Movement."Become patriotic history in nankai" XuanJiangTuan Zhang Mingyue on behalf of nankai university, Beijing university, fudan university, xi 'an jiaotong university, Lanzhou university, Hunan university and so on six xi jinping general secretary of caring youth XuanJiangTuan, the youth to the national college students initiative: " in the future when the party's important theoretical innovation, important historical moment, we will preach as the carrier, through online, offline and other forms, together issued the patriotic voice of the contemporary young students."The meeting also established the Tianjin University Youth Publicity Alliance, and the microblog online publicity relay activity jointly launched by the publicity group of the six schools also began. Nankai university school youth corps committee secretary He Wenxia said that the founding of one hundred, in the face of the new era of new mission, youth theory preaching work should keep pace with The Times, change with each passing day, with each passing day, let more young people put the party's innovation theory to young people, let more young people education baptism, let everyone in "learning, thought, practice, enlightenment" education, "really read the communist party of China" can ", marxism why" line ", why" good ", grow to bear the era of national rejuvenation.

2021-04-30	Reporter Hu Chunyan, correspondent Luo Fan) Today, a launch meeting of the national college youth publicity group association with the theme of "honoring the founding of the Party" was held in Nankai University. Representatives of teachers and students of youth publicity groups from 20 universities from different provinces and cities, including Fudan University and Xi'an Jiaotong University, gathered together to tell us about the century-year history of the Party and express the patriotism of young students. Lin Xiaochun, the representative of the "Qingma" publicity group of Lanzhou University, told the story of revolutionary heroes deeply. The representative of the doctoral student group of the Fudan University expounded the great part of the spirit of the Long March. Han Xueze, a member of the Communist Youth Information Group of Nankai University, introduced passionately about how to help the North Korea was won under the correct decision of the Party, the brave battle of the volunteers and the full support of the people of the whole country."Become patriotic history in nankai" XuanJiangTuan Zhang Mingyue on behalf of nankai university, Beijing university, fudan university, xi 'an jiaotong university, Lanzhou university and Hunan university six university youth XuanJiangTuan, to the national college youth students initiative: in the future whenever the party's important theoretical innovation, important historical moment, we will preach as the carrier, through online, offline and other forms, such as the patriotic voice of contemporary young students together. In addition, a microblog online lecture relay activity jointly launched by the publicity group of the six universities also began, and Nankai University opened the first stop of the joint lecture on Weibo. Starting tomorrow, Xi'an Jiaotong University, Hunan University, Lanzhou University, Fudan University and Peking University will preach online.

2021-04-29	East China jiaotong university to carry out the "always follow the party" red singing contest in east China jiaotong university "always follow the party" situational red singing contest morning correspondent Guo Huiying) to celebrate the 100th anniversary of the founding of the communist party of China, to meet the 50th anniversary of the founding, will learn "four history" khalid ents throughout the school process, inspire teachers and students love party patriotic, east China jiaotong university combined with party history learning education to carry out the red song relay. Since March, the school youth League committee specific deployment implementation, the college youth League committee, class league branch, student club temporary league branch and other grass-roots league branch "point" positive response, in the pepper campus sang exciting red songs. Campus, the red song singing, "red day school" type red songs out of the campus, into the patriotic education base. The choir of East China Jiaotong University took an active part in the "New Journey of Youth to the Party" - -young people from all walks of life in Jiangxi province to celebrate the 100th anniversary of the founding of the Communist Party of China. East China jiaotong university synchronization "dialogue across time and space" original poetry contest, "heartfelt feeling" across time and space dialogue speech contest, "youth" one hundred glorious history member backbone class and "time listen to the party" party history knowledge contest series activities and "I advise" campus proposal contest, "I with youth classes" second class classes and "I help students grow" four pepper success promotion plan member youth "joy participation" series of activities, to carry out the "do the practical work, open new bureau", into the "difference" new journey. The one hundred party history red song covering east China jiaotong university campus more than 20000 youth, in the youth generation interested in online song relay way, focusing on the story behind the red song, with stage scene drama series present vivid party history education class, attract the youth member spontaneous participation in party history learning education, guide the youth member history, history credit, history chong DE, history, comprehension in the party history story young people's beginner's mind, bear and mission.

2021-04-26	Zunyi medical university school youth corps committee held league branch secretary class contest Zunyi medical university youth league held "youth league branch secretary class struggle power" morning) to promote the history of the party learning education and league system "party history, strong faith, follow the party" learning education, guide the youth in ideological and political theory learning, on April 23, Zunyi medical university held in moral lecture hall "youth to the party power" youth league branch secretary lecture class final. The final is in the passionate " glorious ah! The Chinese Communist Youth League " group song song opened the curtain. Under the early organization and planning, the youth League committee of each department vigorously publicized and extensively mobilized it, and recommended one person to enter the school-level final on the basis of the preliminary competition. In the final, 15 contestants from each department gave lectures through classroom teaching, case explanation, typical story sharing, character interview and other methods. In the next step, the Youth League Committee of Zunyi Medical University will continue to take the opportunity of "learning the history of the Party, strengthening faith, and following the Party" to further strengthen the ideological and political guidance, focus on the main responsibilities and main business, guide the youth league members to firmly uphold their ideals and beliefs, take the initiative, and celebrate the 100th anniversary of the founding of the Party with outstanding achievements.

2021-04-26	Beijing university student union: do responsive "campus harbor" I do something for students in Beijing university student union: do responsive "campus harbor", for a long time, Beijing university students to wholeheartedly service students, adhere to from the students, to build service student union brand, do yes, responsive student service organization. Academic service has always been one of the key contents of student service in the student Union. The student union of the school extensively collects students' needs and difficulties in academic development, strengthens the learning exchange and mutual assistance between students, and guides students to promote each other and grow together, so as to create a good growth environment for everyone to grow up. In a survey, the students found that students were in need for academic guidance in the mid-term and final season, and hoped to get help from excellent students in the same major. To this end, the students will actively play a role of service, linkage part of the department youth association, at the end of the autumn semester preparation organization students online counseling activities, for the epidemic prevention and control can not participate in offline course learning students to conduct online one-to-one, one-to-many course counseling, and encourage them to communicate to learn each other's culture and language of the motherland. For a long time, many students hope that there is a place to read aloud. Since 2016, the "Beida Tree Cave" has appeared many times hoping to build a reading classroom stickers. Later, the library opened a "reading pavilion", but the number was small, can not meet everyone's needs. In the 2019-2020 "Proposal Research Competition", some participating groups put forward "proposals on the addition and improvement of public reading areas on campus". After the learning of the rights proposal department of the student Union began to implement the relevant needs. After many negotiations, the school successfully opened 5 classrooms as reading space in April this year. After hearing the news, the students have thumb up. As a responsible and warm backbone team of students, the Student Union of Peking University is good at discovering all the details around it. It always actively participates in student assistance activities and volunteer activities, and provides services for students in its daily life. On the evening of March 9th, the Rights proposal Department of the Student Union received a report from students that "there is a small stone gap between the parking lot and the road. cyclists are easy to slide into and fall, and some students have been injured". The relevant person in charge of the student Union went to the relevant area that night, and the investigation found that there was indeed a large range of road damage. The next day, the student union reported the situation to the campus service center. The center has repaired the pavement within 3 days. In the future, Beijing university students will continue to implement the reform requirements, actively contact grassroots departments, timely collect opinions to the students, Suggestions and activity feedback, improve service quality, strengthen the students' sense of belonging, sense of responsibility, sense of honor, is committed to build the students to rely on and trust the "campus harbor". China Youth Daily, reporter of China Youth Network, Jin Zhuo, source: China Youth Daily

2021-04-23	Tianjin university of traditional Chinese medicine youth corps committee held party history learning education reading class Tianjin university of traditional Chinese medicine youth corps committee held "history with the party faith" party history education project reading class arrangement, to guide the general youth history Ming, history credit, chong DE, history, let red gene, revolution, Tianjin university of traditional Chinese medicine youth corps committee recently held a "history faith with the party" party history learning education project reading class activities. Reading class, Tianjin university of traditional Chinese medicine youth corps committee is responsible for the comrade led a brief history of the communist party of China, and lead you to the reading, hope the grassroots cadres should consciously put the "study party history strong faith with the party" theme learning education deepening, constantly lead all the member youth learning enthusiasm, initiative, enhancing youth thought foundation, draw from the party history in one hundred don't forget to beginner's mind, keep in mind that the mission, the spirit of struggle forever. Reading class cadres have said, in the birthday of the communist party of China in the history of the party study education project reading class, passionate everyone love party patriotism, lead everyone to understand a more deeply why the communist party of China from the "small red boat" to the new era of "wei ship", why one hundred course difficult twists and turns, ups and downs, in the wind and rain suffering.

2021-04-23	The "Zhou Enlai Class" of Huaiyin Institute of Technology and the "Deng Yingchao Class" of Hebei Normal University jointly held the cloud "sharing and exchange activity of" Learning the Party History, inherit the spirit of great men, and Be a youth in the New Era ". China youth network correspondent Sun QiNa, Lv Xiaofeng Qin Qi for figure China youth network Beijing on April 23 (reporter Li Chuan correspondent, Sun QiNa, Lv Xiaofeng Qin Qi) on April 21, huaiyin institute of technology and Hebei normal university software college teachers and students jointly held "party history, the great spirit, do the new era youth" cloud sharing communication activities, in front of the big screen, the students from two colleges together through the network. On April 21st, the "Zhou Enlai Class" of Huaiyin Institute of Technology and the teachers and students of the "Deng Yingchao Class" of Hebei Normal University jointly held a cloud sharing and exchange activity of "Learning the history of the Party for a hundred years, bearing the spirit of great men, and being a youth of the New Era". Huaiyin Institute of Technology is located in Huai'an city, Jiangsu Province, the hometown of Premier Zhou Enlai. School adhering to the spirit of zhou enlai education characteristics, since 2006 "zhou enlai class" to create activities, has more than 1000 classes to create activities, more than 90 classes was named field "zhou enlai class", 21 class was named Huai'an "zhou enlai class", two classes were named "zhou enlai class" in Jiangsu province. Hebei Normal University is located in Shijiazhuang city, Hebei Province. Mr.Deng Yingchao is the 10th grade student of Zhili No.1 Girls' Normal School, the predecessor of Hebei Normal University. Mr.Deng Yingchao has little ambition and outstanding talent. During his study, he issued a sonorous oath of "cheer up his spirit and seek the progress of the country". In recent years, the school has taken the star class of the excellent style of study and the "Deng Yingchao Class" evaluation activities as the carrier to strengthen the construction of the style of study and lead a good educational atmosphere for students to grow up. The Student Affairs Office has carried out the "Deng Yingchao Class" evaluation activity. In the past 11 years, more than 2,000 classes have participated in the activity, and more than 40 "Deng Yingchao Class" have been evaluated. Whether it is "Zhou Enlai Class" or "Deng Yingchao Class", they are the honors looked forward to by the students of both sides. The striving process has effectively improved the students' initiative and enthusiasm of students in learning, promoted the construction of the style of study, and effectively driven the construction of the style of study of the whole school class. On April 21st, the "Zhou Enlai Class" of Huaiyin Institute of Technology and the teachers and students of the "Deng Yingchao Class" of Hebei Normal University jointly held a cloud sharing and exchange activity of "Learning the history of the Party for a hundred years, bearing the spirit of great men, and being a youth of the New Era". In the cloud joint construction activity, "Zhou Enlai Class" of Xiangyu College of Huaiyin Institute of Technology and "Deng Yingchao Class" of Software College of Hebei Normal University respectively exchanged views on the construction of class study style, ideological and political education, scientific and innovation competition and other aspects. The two classes jointly held the "Deng Yingchao class", "Zhou Enlai class" class pairing and joint construction ceremony, and agreed to help each other in the next step, and gradually build a multi-dimensional learning, whole-process education, excellent study style, condensed strength of the excellent class, in the vast journey of growth and strength, to the light. On April 21st, the "Zhou Enlai Class" of Huaiyin Institute of Technology and the teachers and students of the "Deng Yingchao Class" of Hebei Normal University jointly held a cloud sharing and exchange activity of "Learning the history of the Party for a hundred years, bearing the spirit of great men, and being a youth of the New Era". China Youth Network correspondent Sun Qiina, Lv Xiaofeng Qin Qi for the two college student work leaders, counselors and teachers, presidium members of the student Union and "Zhou Enlai class", "Deng Yingchao class" students participated in the activity together.

2021-04-21	Qingdao University of Technology held community party history learning education special training meeting Qingdao University of Technology held community party history learning education special training meeting reporter Xing Ting) recently, Qingdao University of Technology held community party history learning education special training meeting. School student affairs office, youth corps committee, security department, college and other community business guidance unit is responsible for comrade, marxist college theory XuanJiangTuan members and related teachers, school of humanities and foreign languages, social work teaching and research section all party member teachers, China university of petroleum (east China) challenge cup national gold medal team representative, the challenge cup school team and more than 100 student club backbone to participate in the activities. According to the introduction, the training meeting aims to strengthen the backbone of the community party history learning education, from the study of the party history firm ideals and beliefs, learn from the work experience, further promote the standardization of the community construction, improve the level of the community work, enhance the effectiveness of the association thought guidance. Liu Shiying, director of the Social Work Teaching and Research Section of the School of Humanities and Foreign Languages, was invited to do the training of "Standardized Development of Volunteer Service from the Perspective of Social Work". China university of petroleum (east China) challenge cup national gold medal, "star road sail-make autism youth accurate employment model" project winner zhang Shared team through volunteer service focus on autism group, after in-depth investigation and research, finally created the autism youth precision employment model of entrepreneurship experience, and from the volunteer service project topic selection, team formation, publicity to share the practical experience. The training meeting also invited Dr. Liu Jinde and Dr. Ma Yanyan from the theory publicity group of Qingdao University of Technology to give two vivid party history education courses to all the backbone of the association. This theory lecture is also the second phase of the "Centennial Party History Lecture Series" of Qingdao University of Technology. Before the training meeting, the relevant responsible comrades of the Student Affairs Office, the Youth League Committee, the security Department, the colleges and other community business guidance units issued letters of appointment for the volunteer public welfare associations and 5 new thought leading associations, and the allocation of instructors provided effective help for the standardized development of the associations. Next, the student associations will take the 15th Student Association Culture Festival of "Stay true to the original aspiration and follow the Party, the community color is colorful and gather youth" as the platform, give full play to the educational function of the student associations, and unite and lead the majority of young students to inherit the red gene and fine revolutionary tradition in the rich and colorful community cultural activities.

2021-04-21	Anhui University of Science and Technology "Youth Forest" activity guides youth members to fall in love with labor Youth network reporter Wang Haihan) Recently, the 2021 "Youth Forest" launch ceremony in the university fengyang and Longhu two campuses at the same time. The school Youth League committee divided the green plant area into 36 responsibility areas according to the terrain and region, and encouraged the grass-roots youth league branch to take the initiative to claim it and the youth league members to participate in the maintenance labor. The youth League branch is responsible for the daily management and maintenance of their respective areas, including assisting the school greening department and professionals in planting seedlings, weeding, watering, fertilization, pruning, leaf cleaning, debris removal, garbage cleaning and other work."Youth Forest" is the position and carrier of the Youth League Committee of Anhui University of Science and Technology, based on the fundamental task of cultivating people by virtue, strengthening the concept of practical education, and carrying out labor education. It has been carried out for 10 years."Every year before the event starts, the registration is relatively popular."Wu Han, a teacher of the school Youth League Committee, said," This work has formed a good tradition, with high student recognition and participation."This year's activity took the form of online and offline declaration, branch joint declaration, which attracted the active participation of 109 league branches, and the number of declared league branches accounted for nearly one-third of the total number of league branches in the school. During the activity, Zhang Qiankun, secretary of the 193 Youth League Branch of the Mechanical Design, Manufacturing and Automation major of the School of Mechanical Engineering, hung the "Youth Forest" sign printed with the youth League branch information on a willow tree in the responsibility area. He said: " Working in the beautiful woods, the students are very relaxed and happy."It is worth mentioning that the work is launched once a year and dynamically adjusted according to the effect of the activities. Since the construction of "Youth Forest" was launched in 2012, with the formation of the "one school and two districts" running pattern of the school, the responsibility areas have been increasing, and 223 youth League branches have participated in the activities, covering more than 7,000 students. In order to give full play to the educational role of "youth forest" and improve the construction effect of construction, the youth volunteer association of the school carried out weekly evaluation of each responsibility area during the claim period, timely feedback existing problems, regularly published the evaluation results, as the basis and support for the selection of school-level "advanced youth League branch" and "advanced collective of young volunteers".

2021-04-19	Chaohu University Communist Youth League to carry out party History learning theme education activities Chaohu University Communist Youth League to carry out party history learning theme education activities morning correspondent Yang Feihu) since the party history learning education development, The Communist Youth League of Chaohu University responded quickly, In accordance with the unified deployment of the Central Committee of the Communist Youth League, the Provincial Communist Youth League Committee and the University Party Committee, Earnestly implement the spirit of the Communist Youth League to "study the history of the Party, strengthen faith, follow the Party learning and education", Carrying out rich content and various forms of theme education activities, To deepen and strengthen the study and education of party history, Lead the majority of youth members to learn history, history credit, history Chongde, history practice, In the vivid practice of joining the great cause of a strong country, we our youthful responsibility and greater achievements. Mobilization deployment, strengthen the leading chaohu college school youth corps committee will party history learning education as the communist youth league focus this year, stick to the political task, issued by the " about promoting the school youth league organizations at all levels, student organizations to carry out the party history learning education work tips, formulate the party history learning education schedule, comprehensive deployment, system planning, in the group quickly party history learning education boom. Since March, the school youth League committee to strengthen the political theory study, special branch theory study meeting, league work meeting, organize the school communist Youth League system to participate in the communist Youth League "learn party history, strong faith, follow the party" learning education mobilization video conference, the party history learning education to mobilize, redeployment; the college league branch detailed work plan, regular league learning cadre theoretical study, the superior league organization deployment arrangement, learning tasks, work requirements transfer to each league branch and each member. In order to deepen the study and education of the history of the Party, The university Youth League Committee established the Chaohu University "Xi Jinping and College Students Friends" reading class, Hold the "youth heart to the party to build the new era" to learn the history of the Party, strong faith, follow the party to walk the college students keynote speech contest, the 15th girls festival "inspirational youth exhibition" red story I will talk about the keynote speech contest, The university student Union organized the theme of "learning the history of the Party, strong faith, follow the Party", Tangshan Youth Media Center carried out the theme of "Learning Xi Jinping and College Students Friends", Through wechat, QQ and other online platforms, extensive publicity and mobilization, More than 500 students actively participated in the essay solicitation contest. In addition, the colleges have helped the party history education go deeper and deeper, and carried out the morning reading activities with the theme of "reading the Party history, keeping the original aspiration and undertaking the mission", the School of Business Administration and the School of Electronic Engineering have carried out the Tomb Sweeping Day martyrs cemetery memorial activities to remember and commemorate the martyrs, and carry forward and inherit the national spirit. Various forms, and strive to make effective use of the voice of Tang Qing wechat, QQ and other new media platforms, to build the main position of propaganda. On the "Tang Qing of the Voice" platform, open up the study of the Party history column, detailed theoretical study, theme education, typical selection tree, launched the party history of today, the Party history daily reading, major historical events of the Party and other articles, widely publicize the party history study and education. Use the group magazine "Tangshan Youth", open up a print media column. In the school magazine published the news, comments, insights and other articles about the centennial history of the party; the general branch of the league publicity matrix, to make learning products popular with young students, taking the opportunity of the centenary of the party, deepen the "one school, one town, one product" action, the volunteer service brand more and more bright. Chaohu college of the communist youth league will combine May 1, May 4th, July 1, National Day and other important nodes continue to celebrate the 100th anniversary of the theme publicity and education practice, normalized party history learning education, unity led the school member youth for the new stage of modern Anhui construction and characteristic local applied high level university construction contribution youth strength, with honors to the 100th anniversary of the founding of the communist party of China!

2021-04-17	Jiangsu Nantong Open University held the China Chinese Service Day theme activity garden jointly held the "Huayun Chasing far, youth first" China Chinese Service Day theme activity. At the activity, folk experts of Nantong Museum and members of Hanfu Society of Nantong Open University learned the development of Hanfu from Qin and Han to Ming and Qing Dynasties. Then the students dressed to show the beauty of Hanfu and experience the fun of ancient rhyme.

2021-04-17	On the land of Xingnan Lake, watching so many party members solemnly swear beside the red boat, Wei Ailing from Beijing University of Technology has some regrets. This is the first time to pay close respects to the South Lake red boat, the first time to listen to the spirit of the red boat in detail."Learn from the Communist Party's pioneering and pioneering spirit."She said. It is this initiative that has brought the Party from its birth to the present century of its founding. As a youth of the new era, it is more important to study and serve the country, study hard, innovate and start, and serve the society. Wei Ailing is a member of the "Zhen CAI Practical Studies" practice group of Beijing Institute of Technology. Not long ago, she and the students of the practice group went to Hangzhou from Beijing to set foot on the practice journey of the base, and began a new round of professional learning combining the industry-university-research practice. Before starting the practice, they specially went to Nanhu to carry out a special party history education activity to feel the original aspiration of the Party. Before that, they had been practicing for seven years."Material with practice, this is the original aspiration and mission of the Practice Group of Beijing University of Technology". Practice group was founded in 2014, the team actively practice xi jinping, general secretary of the Chinese root, understand the national conditions, growth wisdom ability in the innovative entrepreneurship, committed to biomedical related materials industrialization research and scientific and technological innovation application, the students' social practice, professional learning, innovation and entrepreneurship closely with national demand."In recent years, we have been paying special attention to the ideological and political education for our students, hoping to help them correctly understand their original intention of choosing a scientific research path, and let them know what path they will take in the future."Said Chen Yu, the instructor of the practice group. The members of the practice group have a deep experience of the original intention of this scientific research. From the inspection of raw materials in the field to the sterile production in the factory workshop, from more than 100 attempts in the laboratory to the stable performance of the pilot scale amplification, from the public lectures in rural schools to the online science popularization of thousands of people, the students of the practice group wrote their papers on the earth. During this period, many students from the beginning of the confusion to later gradually find the direction of scientific research to serve the country. Yang Juying, a 2015 undergraduate from the School of Materials at Beijing Beijing Institute of Technology, began scientific research training during her undergraduate period. On this road, she experienced many failures and hesitated to insist. After joining the "Zhen Xincai Real Learning" practice group, under the guidance of teachers, the influence of scientific research atmosphere and the contact with practice, she understood the industry field and innovative characteristics involved in professional learning, achieved outstanding research results, and felt the value of her scientific research to the society. During her undergraduate years, she published one SCI paper in the top journal, won the Xu Teli Scholarship, the highest honor of Beijing University of Technology, and won the national scholarship for four times. She gave up the opportunity to study doctoral outside the university and stay in our university. She hopes to continue to take the road of scientific research, integrate knowledge and practice, and adhere to her original aspiration of serving the country through scientific research. Wang Weizhe, a 2014 undergraduate from the School of Materials, Beijing Institute of Technology, is the leader of the practice team of "Zhen Material Practical Learning" in 2019. This road of practice has brought him too much harvest. Through social practice, he strengthened his original aspiration of scientific research, plunged into the research field of national defense materials, and made breakthroughs. He published two SCI papers, and won the 2019 Internet + Red Brigade Race National Competition Silver Award. He is the leader of the 2019 Communist Youth League Central Three Rural Excellent Practice Group, and the leader of the youth model team of Beijing University of Technology. In the seven years since the establishment of the "Zhen Xincai Real Learning" practice group, the team members have gone to 11 cities in Beijing, Guangdong, Zhejiang and other places, and set up 5 practice bases, covering more than 10,000 kilometers. In 2020, the team overcame the adverse impact of the epidemic and went to the Poverty Alleviation Base in Zhanjiang, Guangdong Province and the pilot base in Hangzhou, Zhejiang Province. On the one hand, they carried out technological poverty alleviation, and on the other hand, it promoted the rapid transformation of scientific and technological achievements. In Zhanjiang, the team made use of the local high-quality patchouli resources and its excellent antibacterial properties to develop the high-performance antibacterial hemostasis dressing of the essential oil to drive the local industry out of poverty. By 2020, a total of 548 people had been helped through poverty, with an increase of 3.313 million yuan. It is expected that by 2022, a total of 2,143 people will be helped, with an increase of 12 million yuan. The team members, in the cooperative pilot-scale amplification base- -Hangzhou Chemical Industry Research Institute, carried out key technologies for the industrialization and transformation of antibacterial hemostatic dressings based on patchouli essential oil, anti-mosquito gel and other new products, and accelerated the registration process of medical devices for antibacterial hemostatic dressings. Combined with the outbreak special situation, the team also actively explore the practice of "online + offline" new mode, organization "natural materials achievement better future" live science class, across Beijing, Hangzhou, Zhanjiang three cities, linkage more than a dozen experts and scholars, covering from tropical crops to Marine materials and other more than 20 kinds of natural materials, formaldehyde purification materials, hemostasis microball and so on more than 30 research results. During this practice trip in Hangzhou, the team members developed a new antibacterial mosquito repellent hydrogel around the integration of 3D printing technology and hydrogel, and printed out their own "mosquito repellent artifact". In the future, the team will continue to follow up the product testing and market research work, and promote the development of industry, university and research.

2021-04-15	Jiangsu University Youth League Committee held a micro Youth League class competition "Learn the history of the Party, strong faith, follow the Party" theme morning correspondent, Wang Haijun, Zhang Yongqiang, Ding Lijuan) " Mother does not need thousands of words to educate you, with the implementation to educate you. When you grow up, I hope you don't forget that your mother died for her country! After my mother dies, my child will continue to fight for her mother!"On April 12, in the Youth League committee of the Jiangsu University held the" study the history of the Party, strengthen the faith, follow the Party " theme micro league class competition scene, Jiao Xinyi from the medical school told the story of the female Communist Zhao Yiman wrote to her son before the heroic death. It is understood that in order to celebrate the 100th anniversary of the founding of the Communist Party of China, the Youth League Committee of Jiangsu University held the "Learn the history of the Party, strengthen faith, follow the Party" theme micro-league class competition in the first lecture hall and the third lecture hall of the Conference Center. After the preliminary competition of the college and the examination by the university Youth League Committee, 23 league branch secretaries were shortlisted and displayed. In micro class display, the league secretary around the "party history, strong faith, follow the party" theme, focusing on the red boat spirit, yuhua district heroic spirit, small spirit, poverty crucial spirit, disease resistance spirit in different periods of the communist party of China formed the connotation of the revolutionary spirit spectrum, combined with time characteristics, youth characteristics, etc., to value guide, carry forward the positive energy, spread the good voice. The theme micro group class competition aims to promote learning, class, education guide the youth members understand the glorious history of the party, feeling the party's beginner's mind mission, grasp the party's innovation theory, recognize the party's spiritual pedigree, inheriting the party's red gene, cultivate patriotic love party, truly learn history, history, history chong DE, learning history.

2021-04-15	Anqing Normal University Youth League Committee: Anqing Normal University Youth League Committee: Guide young students to spread Huangmei Opera Culture at night still hear people laughing, after all the world is more happy "... Recently, 746 students of Huangmei Opera Art Education Club of Anqing Normal University received the examination of Huangmei Opera teaching repertoire. On November 8th last year, Anqing Normal University established the Huangmei Opera Art Education Club (hereinafter referred to as "the Club"), which selected 34 students with strong comprehensive quality of Huangmei Opera as the guidance teachers, and organized 25 shifts to teach, sing and teach performances of Huangmei Opera for the whole school. Among them, each teaching class teaches a play through 7 courses, and the students are intensively assessed intensively in the last course. In order to ensure the singing quality and level of each teaching student, the school Youth League Committee invited wang Li, a national first-class actor, to train the "young teachers" in their singing skills and teaching methods. Huang Jianhao, the head of the Huangmei Opera Art Education Club of the school and the major of Huangmei Opera Performance, has been thinking about the selection of plays, the compilation of school-based teaching materials, personnel arrangement and achievement display since the opening of the club."I hope that more young college students will participate in the team of learning Huangmei Opera, so that the art of Huangmei Opera can be passed on from generation to generation."He said. Lu Mengjuan, a 2019 student majoring in Huangmei Opera performance at the school, is also one of the teaching instructors of the Huangmei Opera. She and her partner are responsible for the play "Husband and Wife Return their Home". Lu Mengjuan admitted that before the class opened, in addition to taking professional courses, she also practiced two extra hours of repertoire every day. In the process of teaching, singing and acting, she was also growing up."Lack of teaching experience, I didn't know how to start at first."After some discussion, she and her partner summed up a set of teaching methods," first to introduce the development process of Huangmei Opera, point out the learning methods and essentials, then play the video of famous artists singing, introduce the plot and characters, and finally 'dig out the details' word by word, to help the students better integrate into the situation."Lift up the orchid finger, straight up, turn around, step... in addition to singing songs, the instructors also teach opera movements."Many students have not been exposed to Huangmei Opera before, and they have many problems in their pronunciation, expression and action. We choose one boy and one girl to sing duet in each class, pointing out their concentrated and typical mistakes, and the students also give feedback to each other."Lu Mengjuan said. The school journalism major 2019 (2) class Ma Shuai is a theater fan, he said: " in the past we learn opera, we can only 'babble ah ah' sing a lively, intonation also grasp is not good, now there are professional teachers to sing word by word, the opportunity is rare!"In the last course, the Huangmei Opera Art Education Club will evaluate the students, who will sing a solo on the stage in turn, and the instructor will score the comprehensive performance according to the tones, facial expressions and movements. Yan Yu, from Class 2020 (2) of Chinese Language and Literature major, performed well in the assessment. A few months ago, she was a Huangmei opera "little white". When she learned that the school had opened a club, she immediately took her three roommates to sign up together. After class, they correct each other's pronunciation mistakes. In the process of learning, he learned about the art of Huangmei Opera before and behind the stage and the historical inheritance, and made many friends. In recent years, the school has opened various types of art clubs to teach students in art skills. Chen Dong, secretary of the school Youth League Committee, said that "everyone understands Huangmei Opera and everyone can sing Huangmei Opera" is also an important part of the school's public art education. The school is located in Anqing City, the hometown of Huangmei Opera. It should make full use of its resource advantages to guide young students to spread and inherit the excellent traditional culture. China Youth Daily China Youth Network reporter Wang Haihan Wang Lei correspondent, Wei Minghui, Wang Ruotong source: China Youth Daily

2021-04-13	Shanghai Academy of Music creates "walking ideological class" Shanghai Academy of Music creates "walking ideological class" China Youth Daily (April 13,2021 edition 01) "Honghu red guard", "white hair girl"... "red coral" these red classic opera created 60 or 70 years ago, recently become the work of generations of teachers and students of Shanghai Academy of Music. In order to let young teachers and students learn the history of the Party and present a tribute to the centenary of the founding of the Party, Shanghai Conservatory of Music launched a series of red creative programs this year, organically integrating music professional education and ideological and political education, to create a "music ideological and political education" education model. On April 10th, the national opera selection concert of "Classic Opera Collection of English", carefully planned by the National Opera Department of Shanghai Conservatory of Music, was premiered at the Shanghai Music Opera House. More than ten classic red songs, such as "Honghu Water, Wave and Wave", "Ten Miles of Snow" and "Ode to Coral", were presented to the audience with a brand new look. In order to accurately reflect the connotation of the song and achieve the perfect integration of sound and feeling, the teachers and students held high enthusiasm during the rehearsal. Chang Liuzhu, a tenor singer and professor of the vocal music opera department, aged 65 years of party age, was invited to participate in the vocal music guidance and performance of the concert. Chang Liuzhu told reporters that he was moved by the department's attention to the concert. When polishing vocal music skills and emotional expression, teachers and students are strict with themselves and give opinions to each other."For example, I gave advice to the teacher who played Yang Bailao. I don't have to be very excited and energetic when singing Ten miles of Snow and Snow, because Yang Bailao was old and cold and hungry. He should be weak at the beginning and show his old image."When he was young, Chang Liuzhu had worked in Tibet for 20 years, and was the collector and first singer of the famous song" On the Golden Hill of Beijing ". He learned folk songs from the Tibetan people, and conveyed the feelings for the Party and the PLA to the public through his own creation and singing. After returning to the Shanghai Conservatory of Music to teach in 1980, he trained a young generation of outstanding singers such as RouAmuti, Zhao Yong and Danzeng. At this concert, he led all the teachers to sing "The revolutionaries are always Young". He said: " Revolutionary vigor and revolutionary spirit are the same for people of all ages. The party has cultivated me and given me a lot of honors. I hope to make more contributions to the party's education cause in my lifetime."Professor Fang Qiong, director of the department of Vocal Opera, sang the opera" Ode to Coral " in the concert. Through the singing of the fisherman's sister, the oppressed public hoped for liberation. Fang Qiong said that in daily teaching, singing art teaching is often closely combined with the party history learning and education. For example, when young teachers take their students to rehearse the song "Embroidery the Red Flag" in the opera "Sister Jiang," the performance is often superficial before the singer has read "Red Rock" well. At this time, Fang Qiong will tell the students about that history."We will tell the children to travel into the cruel surroundings of Refuse Pit. Sister Jiang heard the news of the founding of new China, although they were in prison and could not see the success of the revolution, they were still full of faith in communism and deep feeling for the Communist Party."Fang Qiong said that after understanding the" hard-won "red flag" thoughts and feelings conveyed by the song, the students would naturally put their faces on the red flag on their chest, and enter a state of empathy. Recently, the teachers and students of the Department of vocal music Opera also stepped up the arrangement of the new era version of the "Long March Song". It is not easy for young college students to sing this group of highly professional and magnificent songs well in a short period of time. In order to perform this group of songs as perfectly as possible, the whole department of teachers use their spare time to participate in the tutoring, the department also invited the "Long March Set Song" lyricist, the founding general Xiao Hua's daughter Xiao Xia to guide. Fang Qiong said that the process of rehearsing the "Long March Song" was a vivid ideological and political lesson."Young people in peacetime today may not feel the difficulties of the Long March, but many of the soldiers involved in the Long March are younger than our students. After learning this period of history, the students can have faith, feel that singing a good song is not a big difficulty, and we can completely overcome it."Li Linzi, a 2017 student of ethnic vocal music, participated in the chorus of" Honghu Water Wave " in the concert. She said the song was chosen from the opera "The Honghu Red Guards," which tells the story of Han Ying leading the Honghu Red Guards in fighting hard against the enemy in the 1930s to defend the red regime in the revolutionary base of western Hunan and Hubei."Honghu Water Waves" shows the daily life and labor scenes of the people of Honghu Lake, as well as the people of Honghu know that a happy life is hard-won, and cherish the "paradise" life, support and gratitude for the Party leadership. Li Linzi said that as a student from Hubei province, she has heard the song from her grandmother since she was a child, and often plays by the lake. She can experience the local life of fishing sails, light boats, rice fragrance, wild ducks and peace in the song. She said: " This rehearsal and performance gave me a deeper understanding of the great contribution the Communist Party has made to the people. It is precisely because of the correct leadership of the Party and the hard struggle with us that we can have a rich and beautiful life today."Honghu Water Waves" carries the difficult years and good memories of the older generation in our hometown. I feel very honored to take this opportunity to learn the classics and inherit the feelings for the Party. I especially hope that our performance can move more people."Liu Wang, deputy secretary of the General Party branch of the vocal music and opera department of the Shanghai Conservatory of Music, said that for young students today, the red spirit cannot be forgotten. Although the times and fields are different, the responsibility and sense of mission are the same."Therefore, we make full use of the advantages and characteristics of the discipline, inherit the red culture with our songs, review the historical memories, grasp the pulse of The Times, stay true to our original mission, tell China's story well, and cultivate our patriotism."As one of the first pilot departments of ideological and political courses of the school, the Department of Vocal Music and Opera covers the ideological and political courses in all professional courses, which is reflected in the evaluation of teaching management. According to introducing, this year the Shanghai music institute will launch seven play project to celebrate the 100th anniversary of the founding of the communist party of China, including the opera "Kangding love song", opera "sentinel under the neon lights", symphony chorus suite "longhua soul", symphony suite "song", musical "loyalty" five large original works, and opera film "He Luting" and the new era version of the long march song " classic tour. These projects will not only be "walking ideological and political classes" in schools, but also make full use of new media, short videos and other forms to carry out party history learning and education in old revolutionary base areas, major national projects, and the forefront of reform and opening up, and benefit the whole society. On April 13,2021, Version 01

2021-04-12	Foshan College of Science and Technology party and history into campus and love for the disabled performance held "a hundred years of struggle road sailing new journey" Foshan College of Science and Technology Party and history into campus and love to help the disabled inspirational art performance held China youth network Beijing on April 12 (reporter Zhang Jianwei, correspondent Xiong Hang)) recently, "One Hundred Years of Struggle" Party History Campus and 2021 Foshan Love inspirational Art Performance was held in anxi Campus of Foshan University of Science and Technology, This activity is not only one of the series of activities of "Party History into the campus", It is also an important part of the school's "learn party history and follow the Party" lighthouse learning meeting and campus sports and art festival, This activity invited the Beijing Voice of the Soul Disabled Persons' Art Troupe to bring a wonderful audio-visual feast. The event was hosted by Foshan College of Science and Technology, Foshan Disabled Persons' Federation and Foshan Municipal Committee of the Communist Youth League, and jointly organized by the Communist Youth League Committee of Foshan College of Science and Technology, Guangdong Lions Club and Foshan Volunteer Action Service Center. Relevant leaders of the University Party Committee, the Municipal Disabled Persons' Federation and the Municipal Communist Youth League Municipal Committee attended the activity. The Publicity Department of the University Party Committee, the University Youth League Committee and 200 disabled representatives and students from the five districts of Foshan attended the activity. The picture shows the activity site. Foshan college of science and technology youth corps committee for figure on the literature and art performance, Beijing the voice of the disabled art troupe scene deduce the large dunhuang dance "thousand hand guanyin", "Sichuan opera face", acrobatics "drill ring", "flourishing" classic show, the performance, fully embodies the high artistic attainments and love life, the spirit of the disabled. Founded in 1992, the Beijing Soul Voice Disabled Persons' Art Troupe has performed nearly 10,000 performances at home and abroad, with more than 200 performers, among which 80% of the actors are disabled people with artistic expertise. The picture shows the activity site. Photo provided by the Youth League Committee of Foshan University of Science and Technology, The school actively pays attention to the development of the cause of the disabled, In close cooperation with the Municipal Disabled Persons' Federation and the Municipal Communist Youth League Municipal Committee, Jointly develop a number of related volunteer services, In the field of sports for the disabled, In cooperation with the Municipal Disabled Persons' Federation since 2013, To undertake the training of Guangdong Province deaf men's basketball team and Foshan City deaf men's basketball team, The school shall appoint the head coach and provide the training site, So far, more than 20 provincial team players have been trained, The team won the champion of the deaf men's basketball competition at the 10th (2019) National Disabled Games and the third place in the 9th (2015) same event. The joint performance activities in the city five district nearly 200 disabled people watch the performance, the school youth volunteer association organization completes the performance of volunteer service, recruited hundreds of school youth volunteers, from all aspects to watch the audience and attend the actors to provide warm and thoughtful service, and after the performance escorted the disabled representatives visited the campus, to the student dining hall, etc., for the escort of the activity.

2021-04-10	Northeast agricultural university held the communist youth league party history learning education launch ceremony at northeast agricultural university held the communist youth league "learn party history, strong faith, follow the party" learning education launch ceremony the cloud) on April 7 afternoon, the northeast agricultural university communist youth league "learn party history, strong faith, follow the party" learning education launch ceremony and theme TuanRi activities held in new art building modern education center live hall. Pang Yuhong, deputy secretary of the University Party Committee, and other heads of relevant departments attended the launch ceremony. In addition, more than 7,000 people, including representatives of the party history learning and education youth lecturers' group and 196 youth league branches of grade 2020, attended the main venue and 48 sub-venues of the whole school. Study the party history education activity site. Combined with the actual work of education in Northeast Agricultural University, Formulated the Plan for establishing a Youth Lecture Group for Party History Learning and Education of Northeast Agricultural University, Carefully selected and trained 100 outstanding young students with a firm political stand and those who are good at spreading the Party's theories and propositions to set up the "Party History Learning and Education Youth Publicity Group", The content of the lecture centered on six themes: "Great Journey", "Four History Education", "Beautiful China", "spiritual pedigree", "role models" and "Dongnong Power", Give full play to the role of demonstration, leading, radiation and driving, Into the branch, into the forum, into the network, into the classroom, Carry out publicity activities among all the students. At the same time, the Youth League Committee of the school specially hired 6 professional teachers including Zhong Haiyan from the School of Marxism as the instructors of the "Party History Learning and Education Youth Publicity Group". At the scene of the ceremony, the members of the "Party History Learning and Education Youth Publicity Group" delivered a publicity report on behalf of Sun Yuxin and Zhao Jiawei, telling the story of the Party, the story of revolution and the story of heroes. At the launch ceremony, Pang Yuhong said that the young students to carry out the history of the Party, strengthen faith, follow the Party " is an important deployment made by the Party committee based on the nature of the Communist Youth League and functions of the Communist Youth League, and put forward three requirements for the school to carry out the history of the Party. First, we must keep in mind our original mission and firmly uphold our ideals and convictions. Second, we should improve the ideological position, to find the right point of focus and foothold. Third, we need to deepen innovation-driven development and demonstrate our responsibility in our youth. The school Youth League committee should combine its own reality, give full play to its work advantages, promote the in-depth development of learning and education with "strict style of study" and "practical style", and show the vigorous strength and mission of dongnong youth in the new era. Study the party history education activities sub-venue. Northeast agricultural university for the next step, the communist youth league of northeast agricultural university of Ming will be closely around the history, history, chong DE, history, closely combined with the communist youth league work and youth members, "youth learning" "youth" party history education youth "classroom" "party history" boutique projects and "I do something for youth" theme TuanRi activities, social practice and volunteer service activities, etc., the party history learning education into education process, constantly improve the party history learning education height, depth, breadth, validity. Take practical actions to promote the construction of the world-class discipline and high-level university, and contribute to the comprehensive construction of the socialist modern new Longjiang River.

2021-04-09	Qingyuan city technician institute solid history learning education party history nourish the new youth technology support power dream Qingyuan city technician institute solid to carry out the history of the party learning education wei correspondent YueQing) Qingyuan city, Guangdong province technician institute give full play to the cadres advanced exemplary role, many measures simultaneously, combining with the new era of mechanic education demand steadily promote the party history learning education, guide the teachers and students to carry out the history. Do a good job of party history study mobilization, into the goal of technical training. Party history learning education mobilization meeting and party history learning education series launch ceremony, invited group Qingyuan municipal party committee, straight, municipal people club bureau, city commission for discipline inspection supervisor committee in seventh team responsible comrade to attend, college leadership members, middle-level cadres, all party members, ideological and political teachers, some students on behalf of nearly 500 people attended the activity, called for incentive floor teachers and students focus on theoretical study, comprehensive deployment of party history learning education series activities, formulate implementation measures list. Issued by the party history learning documents, through conducting party history learning education to fully open the construction of socialist modernization mechanic education new journey to lay a solid foundation, promoting school-enterprise cooperation, skills competition, "cantonese chef", "Guangdong mechanic", "nan yue domestic" and so on the key work, the successful completion of the college in 2021 top ten key tasks, to ensure that the party history learning education achieved solid results. Firmly grasp the history of the party learning education opportunity, vigorously carry forward the spirit of labor model, labor spirit, spirit, motivate young students to walk skills become skills patriotic road, cultivate more skilled personnel and powers, the implementation of the "study the party history, enlightenment thought, does the practical work, open new bureau", lead the youth set up the correct values, buried the post of the ideal faith. Set up a party history lead study team, and carry out party history publicity activities. The party committee members of the college quickly formed the Red Flag publicity team, and 10 Party branch secretaries formed the torch publicity team, and spent 10 weeks to train 100 outstanding youth league cadres to form the Red Star publicity team, forming 117 party history leaders echelon. At present, the college party secretary, dean Gao Xiangqin as a red flag preach team captain, to "revisit Qingyuan red history power hometown revitalization of development" for teachers and students speak good party history learning education in the first lesson, the torch xuan team to revisit the May 4th movement, the autumn harvest uprising development and historical significance, the next 100 "little red star" will return to class, in the youth, in the form of happy speak good "four history" story. Enrich the forms of educational activities and deepen the results of party history study. College youth corps committee under the guidance of the college party committee to carry out the "follow the film history of the heart said to the party" to celebrate the founding of one hundred classic film theme education activities, weekly regular red classic film, the students consciously record feelings and understanding of the party's movie, by the teacher in charge in weekly class meeting class organize communication sharing activities. In order to train party members and league members to strive to be Party history leaders, the college has organized a series of themed activities celebrating the 100th anniversary of the founding of the Communist Party of China, such as the red classic Party history story reading contest, essay contest, Party history knowledge contest, red song chorus contest, and theme art performance.

2021-04-09	Correspondent Liu Ye) 2021 marks the 100th anniversary of the founding of the Communist Party of China. At the intersection of the two hundred years, Xinhua College of Ningxia University took the opportunity of the founding of the Party to carry out party history education for the whole Party. In mid-to-late March, the Youth League Committee of the college and the General Youth League Branch of all grades successively carried out a series of activities with the theme of "Inheriting the Spirit of Lei Feng and Celebrating the Centennial of the Founding of the Party", and the fourth reading salon activity of "Learning the History of the Party, Strengthening Faith and Following the Party". Through the theme TuanRi activities, campus volunteer service, civilized dormitory selection, theme essay, speech contest and other online rich and colorful forms, actively explore around lei feng story, extensive learning lei feng spirit, further strengthen the young students volunteer service consciousness, guide the youth to do the new era of lei feng's spirit inheritors and practitioners. With the theme of "Learn from Lei Feng spirit, Set an example image", the salon aims to guide the youth members to understand the glorious history of the Party, feel the original mission of the Party, and guide the students to more deeply understand and learn from Lei Feng spirit at the same time, consciously take Comrade Lei Feng as an example, and be a new person with faith and responsibility. On April 2nd, the Department of Grammar and Foreign Languages of the College held a "Party history learning, education, remembering the revolutionary martyrs and patriotism education activity" in the Laoheqiao Martyrs' Cemetery in Wuzhong City. Representatives of teachers and students observed silence to the revolutionary martyrs, presented flowers and paid respects to the monument. Lu Chao, a teacher of the Marxist teaching and research department of the college, gave a micro party history class entitled "Remember the martyrs, pass the spirit, increase faith, set faith, worship virtue and practice the time". He hoped that the students would not forget the way, know what to do, and cherish today's happy life.

2021-04-05	Southwest University of Science and Technology: Red paper-cut according to the original history of the Chinese folk art, borrowing paper-cut, a Chinese folk art, with a number of elaborate paper-cut art works to praise the Communist Party of China, with different forms of creation to pay tribute to the glorious history of the Party. With the theme of "red paper cutting according to the original aspiration, combining knowledge and practice with learning the history of the Party", the activity integrates the excellent traditional Chinese culture and modern science and technology for creative composition, absorption, reference of painting, calligraphy, carving and other technological techniques for artistic creation. The teachers and students who participated in the activity completed the paper-cut works with red stories as original materials, such as "True to Learn the Red Moon" and "Clean corrupted". Among them, the biggest work "Women follow the Party to forge ahead on a new journey" (1.5x0.5 meters) under the cooperation of six teachers, in 2 days, completed on the afternoon of the 30th. Gu Song, curator of the university library, said that the paper-cut works are integrated into major party history events and blessings to the motherland, which also let teachers and students review the magnificent glorious course of the Communist Party of China once again, deepen their understanding of the Party while feeling the traditional cultural atmosphere, and love the Party and patriotism in their hearts. In addition, paper cutting also integrates "China aircraft carrier", "Tianwen 1", "5G", "high-speed rail" and other elements, to further feel the changing scientific and technological strength of the motherland in the party history learning and education.

2021-04-06	Xi 'an University of Architecture and Technology to carry out the "study the Party history sacrifice heroes" activities Xi' an University of Architecture and Technology to carry out the "youth to the Party struggle power-study the Party history, sacrifice heroes" theme group day activities month 6 (reporter Zhang Jianwei, correspondent Ke Sujuan) late spring Qingming, cautious. On April 4, Xi 'an University of Architecture and Technology organized key students to xi' an Martyrs Cemetery to carry out the "youth to the Party struggle for power- -learn the history of the Party, sacrifice heroes" theme league day activities.site of activity. The national flag guard of Xi'an University of Architectural Science and Technology entered the flag, the flag-bearers and student representatives presented flower baskets to the revolutionary martyrs, and the students observed a collective silence. Undergraduate Li Sixuan classmates, graduate students Zheng Shuchang students on behalf of the students to the martyrs, they initiative xi'an university son to firm ideal faith, study the patriotism of revolutionary martyrs, inherit the glorious tradition of revolutionary martyrs, forge ahead, do loyal to the motherland loyal to the people, willing to bear as a new era of youth.site of activity. Subsequently, the student representatives of the heroic deeds of Du Bincheng, Cao iru and other martyrs, exchange experience, with on-site teaching to let the party history education in the hearts of students.

2021-04-03	Shandong university: to volunteer service lead youth learning history of Shandong university: to volunteer service lead youth learning LiXingZhe XingTing) recently, Shandong university guide youth to actively participate in "I prepare for the school", "I for the school daily service" "I for the school employment power" "I for the school epidemic prevention unattended", "I for school dormitory culture endorsement" five aspects of volunteer service activities, promote Shandong university teachers and students medical staff based on lei feng spirit to practice the socialist core values, live with party history learning education resources, with practical actions to promote party history learning education into the brain, into the heart. At present, 18,000 volunteers have participated in the project and launched more than 130 volunteer service projects, benefiting more than 60,000 teachers and students. Shandong university around xi general secretary in the party history learning education mobilization meeting's important speech spirit, combined with the CPC Central Committee on the history of the whole party learning education notice series decision decisions, according to the group on the group "about the party history, strong faith, follow the party" learning education notice "related work requirements, control Shandong university party committee about party history learning education arrangement, aggregation party lead TuanJian work potential energy, with the help of learning lei feng spirit, give full play to the communist youth league organization advantage, position, the first time launched the" I do good for school " lei feng theme education practice action plan."I do good for the school" Lei Feng month theme education practice activities include: through the perception of Lei Feng spirit to explore the spiritual core of the party history learning education, While participating in the "search for the new Era of Lei Feng stories", carrying out online "cloud" volunteer, organizing "volunteer feeling" sharing activities, Carry out a series of activities with the theme of "Promoting the Spirit of Lei Feng and promoting the study of Party history", Create a three-point linkage pattern of "learning from Lei Feng + theme youth League day + Party history learning and education", Dig deep into the red educational resources of fresh textbooks, Create a "red heart and love" volunteer service cause; With the youth League branch as the traction to promote "I do good for the school" to do enough red article, "School, school, league" three levels of youth linkage, In the youth League committee of more than 50 colleges in one school and three places, we established a volunteer service working group of "I do good for the school", Drive the whole school 1,000 youth league branches to carry out casual public welfare. At the same time, in accordance with the requirements of the activity, in the practice of Lei Feng spirit to build a strong party history learning education campus position. More than 1,000 volunteer public welfare posts have been set up in dormitories, living areas, teaching and research areas. The young people of the school have participated in three categories of volunteer service activities: "I prepare for the school", "I do daily services for the school" and "I do a good job in dormitory culture endorsement for the school". In addition, the activity requires to seize the key minority to help the party history learning education brand development. Party backbone, outstanding TuanGan actively play the cutting edge exemplary role, to carry out "I do employment power for the school" "I for the school epidemic prevention unattended" volunteer service brand project, such as student employment, do job promotion, information release, data sorting plate, such as public employment workshop 7, can promote students full employment, around environment sanitizers, door on duty, traffic to form a normalized, specialized volunteer service team, build "party history, love dedication" positive atmosphere.

2021-04-01	In terms of rural primary schools, the university life is rich and colorful, but the most unforgettable time is the volunteer teaching time in Xiewang Primary School, Sanbing Town, Chaohu City. In her junior year, although she is busy with school, she always returns to the village primary school whenever she has the opportunity to look at those lovely faces and make up a few lessons for the children. Xiewang Primary School has been established for 70 years, and nearly half of the nearly 200 students are left-behind children. Since 2007, volunteers from Chaohu College have been teaching in the village small relay for 15 years. Every summer, volunteers will bring popular science, psychological counseling and academic assistance to the lovely children. Xu Panpan, who majored in Chinese Language and Literature at the university, volunteered to be a volunteer volunteer in June 2018, before the summer vacation of her freshman year. In June, it is the school final exam review stage. After class, Xu Panpan and his classmates first went to the library to study for themselves, and then found a classroom to discuss the volunteer teaching courses."We combined our own major, several people simulate students, several people try to speak, and prepare a variety of courses."She recalled. She remembered that during the first visit to Xiwang Primary School in July, the children ran to the school gate to meet them, jumping and jumping. There were only ceiling fans in the classroom, and she was sweating very hot as she introduced herself on the platform. At this time, a little boy silently handed over the small electric fan from home, these details moved Xu Panpan heart. In the experimental class, few people spoke, and the children were very focused. Their favorite is the egg ups and downs experiment, after class, all around the platform to ask the principle of the experiment. Peng Wei, a student majoring in mechanical design, manufacturing and automation of the university, has participated in the volunteer teaching for two consecutive years."Through volunteer teaching and interaction, I feel that the children know a sense of responsibility and learn to manage themselves. At the end of the volunteer teaching, the monitor also specially invited me to visit her home."Peng Wei served as a psychological committee member in the university class. When he volunteered, he took the initiative to teach his children psychological knowledge. In the last two classes of his first year, he gave the children a special assignment: asking everyone to go home and create a painting with their parents or grandparents. As a result, many children hand in the homework, are painted like their parents. A quiet girl painted a tile-roofed house, behind which were the mountains. In the picture, the girl is standing in front of the house, with her parents and grandparents beside her. It turned out that she had been a left-behind child since childhood, and her parents only went home for a few days during the Spring Festival. The girl said to Peng Wei, " The happiest day of my year, except for the Chinese New Year, is the day when you come to class."This has made Peng Wei feel that volunteer teaching is meaningful and has strengthened his determination to continue to volunteer teaching in the second year. Before the second year, Peng Wei specially explained the details of getting along with his children, which is a tradition of volunteer teaching volunteers at the school. At the end of volunteer teaching, volunteers share their experiences and methods. Recently, Chaohu College was listed as the "Tangshan Love School" in Xiewang Primary School. In 2010, Chaohu College established the first "Tangshan Love School" in Chengbei Primary School. Up to now, the school has set up 23 volunteer teaching sites in and around Chaohu city, creating a number of volunteer service brands. In recent years, Chaohu City has actively promoted the volunteer service action of "school-land co-construction". Chaohu University students have extended their volunteer service to all fields of regional economic and social development in combination with their professional directors and the actual needs of the towns and townships."Volunteer service also provides a platform for college students to practice social practice, cultivate team spirit and increase their talents. Generations of volunteer volunteers share experience and inherit the spirit, making the school's volunteer service brand brighter and brighter."Said Zheng Ling, secretary of the Communist Youth League Committee of Chaohu University. China Youth Daily China Youth Network reporter Wang Haihan Wang Lei correspondent Guo Chao source: China Youth Daily

2021-04-01	Shihezi university held the communist youth league party history learning education launch ceremony Shihezi university held the communist youth league "learning party history, strong faith, follow the party" learning education launch ceremony) recently, Shihezi university held the communist youth league "learning party history, strong faith, follow the party" learning education launch ceremony, the party committee, vice President Ye Hanchun to attend the meeting and make a speech. More than 600 people from the student work Department of the Party Committee, the Communist Youth League Committee, the leaders of each college and the cadres attended the meeting online and offline. Conference pointed out that in the whole school league organization to carry out the party history learning education is the school communist youth league in a period of major political task, standing in the important historical node in one hundred, how to use the way of "young people" ignite the enthusiasm of young students party history learning, to young students speak good "past story", is the communist youth league cadres and student workers "basic skills", is a good baton, motivate newcomers "required course". The meeting stressed that the whole school youth league and school organizations should thoroughly study and implement the spirit of General Secretary Xi Jinping's important speech at the mobilization conference of Party history study and education, and carry out the Party history study and education with high standards and high quality. First, we should improve the political standing and deeply understand the significance of the study and education of Party history, highlight the goal orientation, accurately grasp the political direction of the organization, and strengthen the organization and leadership to promote the study and education of Party history. Guan Xin, secretary of the Communist Youth League Committee of the school, learned the speech spirit of the speech made by He Junke, the first secretary of the Secretariat of the Central Committee of the Communist Youth League, in the mobilization meeting of "Learning the history of the Party, strengthening the faith, and following the Party", and made specific arrangements for the study and education of "learning the history of the Party, strengthening the faith, and following the Party" in the whole school. The Youth league organizations at all levels of Shihezi University will organize all the youth league members of the university to thoroughly study and implement the spirit of General Secretary Xi Jinping's important speech at the Party history learning and education mobilization conference, To implement the relevant deployment requirements of the university Party Committee, Centering on learning, education, life and practical activities, Extensive development of youth learning, "party history micro classroom", "youth to learn the history of the Party" quality project cultivation, "I do something for the youth" theme youth league day activities, social practice special activities, volunteer service civilization action, "I love my motherland" education practice activities, party history learning education report performance, Integrate party history learning and education into the whole process of education, Constantly improve the height, depth, breadth and validity of the league organization to carry out party history learning education, Guide the youth members to further strengthen their ideals and beliefs, To learn history, history credit, history Chongde, history practice, A tribute to the 100th anniversary of the founding of the Party with outstanding achievements.

2021-04-01	Henan Agricultural University: The College student volunteer service team helps with rural revitalization, and it can also improve its oral English ability. Teacher, will you still come here again?"Xiao Li, a student leader of the Houma Class School of Foreign Language Studies of Henan Agricultural University, said at the Central Primary School, Taiqian County, Puyang City, Henan Province, on March 5. Chen Yanli replied with a smile, " Of course! After we can meet every week, think the teacher can also often video!"It was a conversation between the child and the teacher after the volunteer service. On March 4, on the eve of the 58th "Lei Feng Memorial Day" and the 22nd China Youth Volunteer Service Day, Henan Agricultural University "Lei Feng" youth volunteer service team went to Taiqian County to carry out the "Lei Lei Feng" rural volunteer service activity. Li Chengwu, deputy secretary of the Party Committee of the university, encouraged the teachers and students: " The Lei Feng spirit never goes out of date. The young people should study hard, actively inherit and carry forward well. The school encourages the young teachers and students of agriculture, rural areas and farmers to apply what they learn to practical practice, giving full play to the Lei Feng spirit in the volunteer service for rural revitalization, and in the socialist modernization drive."Chen Yanli said," The tuition fee for an English teaching class on the market is tens or even hundreds of yuan, and many families in the village can not afford it. When we see the big eyes eager for knowledge, we feel that we have a burden, and we are determined to study more seriously, and strive to improve the quality of teaching, to bring better education to the children. We will carry out long-term fixed-point oral communication services online and offline to guide the children in the village to learn oral English pronunciation. It will also be expanded to more schools and serve more students."Teachers and students of Henan Agricultural University also came to Lihu Village, Malou Town, Taiqian County, to carry out the theme practice activity of" Let the light of civilization illuminate the new journey ". They explained and distributed the Regulations on Promoting Civilized Behavior in Henan Province to the local people to strengthen the daily cultivation of the civilization. Zhang Yunfan, the instructor of the Qingma Society of Henan Agricultural University and the young teacher of the School of Marxism, explained the history and feelings of the Party for the villagers. From "to sacrifice more ambition, dare to teach the sun in new day" ambition, talking to "only the communist party can save China, only socialism can save China" simple truth, again from the party's one hundred glorious achievements, carry forward the country feelings have Dang, keep in mind the mission development, zhang yunfan talk deeply attracted the villagers. The teachers and students of the Department of Sociology of Grammar College of the university give full play to their professional characteristics to carry out professional service activities for the empty nesters in Lihu Village. Social workers lead the elderly to learn "pat exercises", physical exercise, activities of their hands, wrists, elbows, shoulders, waist, etc."Paiexercise" action is small, less energy consumption, long-term adherence can play a role in slowing the physical function of the elderly. With the help of such study groups, the social workers hope to benefit the physical and mental health of the elderly, and to enhance their understanding and feelings through interactive learning. The school rural revitalization of graduate volunteer service in SunZhuang modern agriculture demonstration park research, agricultural college PhD Lv Weize said: " I to research this time, think my lotus root experiment can be used here, the village large river empty idle is also a waste, development of lotus root economy will help farmers income, I would like to do a good job of support for them."Food science and technology college graduate student silver peak said, he can contribute in food processing, the laboratory accumulation of various deep processing technology applied to the local agricultural products," on the one hand can improve its economic value, increase people's income, on the other hand, can also solve the problem of some unsalable agricultural products preservation and storage."Zhao Zhen, a graduate student at the school of Economics and Management, has some views on Taiqian county's rural economic planning. He said: " Building a new socialist countryside is a major measure to strengthen the work of agriculture, rural areas and farmers and promote the improvement of the rural social economy. I am willing to contribute to the rural economic construction with my professional knowledge and combining with the innovative ideas of the new era."Wei Wei, secretary of the Youth League Committee of Henan Agricultural University, said:" The Youth League Committee will establish a long-term mechanism for rural revitalization. This pilot activity in front of the stage is a good start. In the future, we will provide local youth volunteer services for rural revitalization projects, so as to build a platform for young people to display their talents and actively solve practical problems for local governments."According to Wei Wei, after the activity, the school will be set up in Henan agricultural university rural revitalization of youth volunteer service center, and established in the front county of Henan agricultural university college students volunteer service base, aiming to meet the actual needs of the masses, around poverty engines and rural revitalization, etc., cultivate and practice the new era civilization practice volunteer service. China Youth Daily China Youth Network reporter Pan Zhixian source: China Youth Daily

2021-03-29	Nanjing xiaozhuang students made yuhua district martyrs cemetery worship of Nanjing xiaozhuang students made yuhua district martyrs cemetery worship heroes net reporter li runwen) on March 26 morning, the communist youth league Nanjing xiaozhuang college committee in Nanjing Yuhuatai martyrs cemetery administration, Nanjing xiaozhuang college affiliated primary school and jinling remit school, in Nanjing yuhua district martyrs cemetery to carry out the "party history sing faith-inheriting red gene gift in one hundred" theme education. Yuhua heroes zhao liangzhang was born in 1921 in liuhe a poor family, 18 years old he resolutely, enter oneself for an examination, the kuomintang air force flight school, and then unwilling to the kuomintang negative anti-Japanese atmosphere, published the if I sacrifice for the truth, I can't put down the gun songs, encourage our compatriots actively anti-Japanese, save the nation. Then resolutely left the Kuomintang air force, into the Communist Party of China underground party, was arrested by the Kuomintang."After my uncle was arrested, in his dark and scary cell, he read English reciting words and composed music every morning. He also comforted his fellow inmates, 'We should use the happy time and send away the dark time'."At the scene, Zhao Liangzhang's niece Xu Caiping emotionally described the story of the martyrs to the students, encouraging the successors of the new era of socialism to be more vigorous. Wen Huibin, former director of the Nanjing Yuhuatai Martyrs Memorial Hall and director of the Cultural Relics Museum, vividly told the touching story of Yuan Zitong and other "Xiaozhuang Ten Heroes" with firm faith and lofty fearlessness. In 1928, Xiaozhuang Normal University, the predecessor of Nanjing Xiaozhuang University, established the Communist Party and the Communist Youth League organizations to lead students to carry out revolutionary activities. In 1930, Shi Jun, Ye Gang, 10 Party members, � ⒐�, Xie Wei, Yuan Zitong, Yao Ailan, Shen Yunlou, Hu Shangzhi, Tang Zao and Ma Mingju, were arrested successively, and died in Yuhuatai from August to October of the same year. The average age of the 10 martyrs was less than 20, with the youngest, Yuan Zitong, aged 16. The story of the "Ten Heroes" is passed down from generation to generation in Xiaozhuang College, and the red culture is deeply rooted in people's hearts. Xiaozhuang College in 2006 established the Yuhuatai Martyrs Cemetery volunteer explanation team, every Saturday, Sunday, the explanation team members in the Yuhuatai Martyrs Memorial Hall to do a voluntary explanation, the volunteer explanation team also set up a "little raindrops" red culture performance practice group, to spread the spirit of martyrs to the society. The scene, by the Yuhuatai martyrs memorial hall to explain the team members of the original sitcom "Xiaozhuang ten heroes" as if to bring everyone back to that bloody revolutionary era."Through the explanation and play, I have a deeper understanding of the deeds of the Yuhua heroes."Jin Jinliang, a student from the School of Tourism and Social Management of Xiaozhuang University, said that the rehearsal of touching works of art is one of the best ways to pay tribute to the heroes. At the end of the activity, the audience sang the theme song "Faith" of the centenary of the founding of the Party and the song "If I die for the truth" created by Yuhua hero Zhao Liangzhang. The passionate songs echoed in the cemetery for a long time, passing the "red scarf", the party league member students' deep respect for the revolutionary martyrs and the most affectionate blessing for the 100th anniversary of the founding of the Communist Party of China.

2021-04-06	Nanjing university of post and telecommunications "youth volunteer, dedication" difference "public carnival activities in Nanjing university of post and telecommunications" youth volunteer, dedication "difference" public carnival activities for China youth network Beijing on April 6 (reporter Li Chuan correspondent Qin Changjian) recently, Nanjing university of post and telecommunications organization "youth volunteer, dedication" difference "public carnival activities, vigorously carry forward the" dedication, love, mutual assistance, progress " volunteer service spirit, guide the south post students do volunteer spirit practitioners, volunteer culture disseminator, volunteer organizers.site of activity. China youth network correspondent Qin Changjian for figure event, Nanjing university of post and telecommunications 24 volunteer service community, more than 3600 young volunteers gathered in Xianlin campus ding xin avenue, through the "volunteer experience" "project roadshow" "sign" volunteer agreement "form, show the south post students volunteer and youth, and with the graduate student corps Xinjiang corps team" light micro wish, power micro dream " activities, light up for 244 children in the western region. This public welfare carnival is one of the series of practice activities of "four talk, four understanding and eight practice" for the party history learning and education of "youth to the Party, struggle and strong country" of the South Post. Activities before the school youth corps committee organization graduate volunteers, youth volunteers to participate in "study the history of the party full network project training", through "study party history review glorious history" "praise party revisit party oath" "strong party spirit consolidate party history knowledge", "remember the youth mission" module of learning, further understand the party in the struggle practice of glorious tradition, fine style and revolutionary spirit, learn from the glorious history of rich nourishing and wisdom strength. With the achievements of the study of party history, we will guide the young volunteers to strengthen their ideals and beliefs in learning, stimulate the strength of forging ahead, and demonstrate the responsibility of the youth. Party History knowledge competition. Cong Longtao, a staff member of the Youth Volunteers Federation of Nanjing University of Posts and Telecommunications, said that the charity carnival has created a strong volunteer atmosphere, leading more students to understand the rich daily activities of volunteers, join the ranks of young volunteers, and actively assume social responsibilities. Hao Manjia, a young volunteer, said that through the special training of learning the history of the Party network, I deeply realized the difficulty of the Communist Party of China to experience the vicissitudes of life and overcome the difficult years, as well as the promising responsibility to irrigate the youth pride with blood and carry the banner of victory with life. As a young man in the new era, we should pass on the red gene, carry forward the great love of society, and jointly create a new chapter of volunteer service in the new era. Tao Linhua, a member of the 23rd Research Group, said that the history of the Party's struggle has profound educational significance for the young generation. As a member of the graduate volunteer group, we should learn the history of the Party, strengthen faith, follow the Party, and do a good job as the inheritor and speaker of the history of the Party in the volunteer teaching.

2021-03-30	Anhui Engineering University Youth League Committee carried out the "holiday ideological and political" activity to pay tribute to the "holiday ideological and political" anniversary of the party founder Wang Haihan and Wang Lei) " In the conversation, I really feel the deep feelings of the grassroots people to the Party. As college students in the new era, we should study hard, seize the opportunity, and take over the baton of rural revitalization."Xu Wan, a post-2000 student majoring in administration at Anhui Engineering University, wrote in his research diary. In the past two months, Xu and nine friends have traveled through 26 villages in Anhui province to collect and produce 100 rural development slogans, research diaries and questionnaires on rural economy, rural education and rural customs. At the beginning of 2021, the Youth League Committee of Anhui Engineering University carried out an education activity with the theme of "paying tribute to the century of elegance, youth and continuous struggle", presenting a tribute to the centenary of the Party with "holiday ideological and political affairs". On the premise of complying with the epidemic prevention and control regulations, the school's propaganda, ideological and political, and academic departments and counselors guided the class league branches and student associations to carry out activities nearby to ensure the coverage and quality of the activities. In the end, the students submitted 2,813 research works, such as pictures and videos, which were also displayed through new media platforms such as WeChat Moments and TikTok."The village has taken on a new look. With a cultural park, a library and a big stage for the villagers, the lives of the villagers are improving day by day."Xu Wan walked into his hometown, Dingyuan County, Liuhu Li Village, a straight and spacious asphalt road to let him intuitively feel the changes in his hometown. He and his teammates visited 10 village groups in Chilingkou Town and Yangling Town of Qimen County, to learn about the drying, frying and drying techniques of Qimen red tea, a local specialty."Black tea is the pride of our Qimen people and the symbol of the tea culture in our hometown. Thank the party, thanks to such a good era, let the black tea brand really go out. ". Zhu Jinlong, a post-2000 student from the School of Textile and Garment of Anhui Engineering University, visited the red scenic spot in Yuexi County deep in the Dabie Mountain. He and his classmates recorded the local villagers' red memories, made short videos, and preached to their students after they returned to school. Eighteen students, including Shi Xinru and Zhu Xin, majoring in advertising at the School of Art, also spontaneously set up a "good people" search group. In the Chinese good man, 90 years old veteran Dai Qing home, students learned that in 1947,16 years old Dai Qing revolution, joined the Communist Party of China, has participated in the huaihai campaign, cross the river campaign... after retirement, he walked into primary and secondary schools to carry out red preach more than 570, every year out 80% of the salary to subsidize, reward poor students, a total of 600,000 yuan."A mountain figure, a bell-like loud sound, not a deep gully on his face, but still a youth flame in his eyes. For more than four hours, the old man told about the extraordinary years of his life, from the suffering of childhood to the experience of the Anti-Japanese War, and then to the kindness after retirement, he gave us a big lesson in life, but also let me more understand the Communist Party of China through 100 years of great."Zhu Xin said. The group also interviewed 12 good examples, including Miao Huanqin, a "good Chinese man" who donated tens of thousands of yuan to help 108 students realize their dreams, and Wu Chuanyun, a good man from Anhui province who stepped forward to save the mother and son. The interviews have been compiled into a book. Since March, the Communist Youth League Committee of the school has organized students to exchange and share the harvest activities, making the down-to-earth "holiday ideological and political affairs" become an important carrier of party history learning.

2021-03-29	Shaanxi university of science and technology: meet lake learning comprehension one hundred youth students meet lake learning comprehension one hundred history of Shaanxi university of science and technology to carry out the "party history, strong faith, follow the party" demonstration theme, China youth network Beijing on March 29 (reporter Zhang Jianwei, correspondent xue-jing wang) early spring afternoon, university lake sparkling, pleasant scenery. Recently, to further promote young students party history learning education, arouse the enthusiasm of grassroots youth league branch study party history, the youth corps committee of shaanxi university of science and technology and material science and engineering college party committee, under the careful guidance of shaanxi province college league building model branch-material branch 181 youth league branch, joint material 208 youth league branch and graduate youth league branch, invited shaanxi provincial youth development research center director Xue Hongping together into the "lake hall", held "study party history, strong faith, follow the party" series of youth study party history demonstration theme TuanRi. Wang Junmin, the second-level inspector of the Communist Youth League Committee, and Yan Bo, the minister of the Publicity Department participated in the whole process and gave on-site guidance. Youth study party history demonstration theme youth League day scene. Xue Hongping explained the centenary development course of the Party in a simple way, and affirmed the positive contribution of the Shaanxi Youth Movement in the centenary history of the centenary founding of the Party, especially during the War of Resistance against Japanese Aggression. A period of touching history, an emotional story has brought the students' thoughts to the hard years and vicissitudes of life, which has caused the resonance of the students present and bursts of applause. During the exchange session, Xue Hongping guided the youth league members to draw spiritual strength from the history of the Party, strengthen the practice in the grass-roots work, and serve and lead the majority of young students based on the class situation, school situation and national conditions. Youth study party history demonstration theme youth League day scene. Correspondent for the various forms of learning the history of the Party, the school teachers and students through reading the original work, learn the original text in the party history learning education first step. The secretaries of the Youth League Committee and the youth league members of the college shared their insights through reading books such as The Brief History of the Communist Party of China and Xi Jinping and College Students' Friends, and were determined to learn the history of the Party well, tell the story of China well, and be a good youth in the new era. School youth corps committee secretary li peng on demonstration theme, the review, stressed that the party called, group have action, the school communist youth league in accordance with the deployment requirements of the school party committee and the superior league organization, adhere to the learning education as the basic unit, the self-improvement throughout the whole process of learning education, practice, unity led the general youth league banner with the party. Youth study party history demonstration theme youth League day scene. Correspondent for figure recent material college youth league branch positive action, party history, feeling DangEn, listen to the party, follow the party, to "lake lecture hall" as the learning platform, to tell stories, poetry reading, sitcom, spontaneously organized unique party history learning education, the party history education from the classroom moved to the lake, offline online synchronization, through expert teaching, stories, peers share, integrating party history, national conditions, beauty, subtly prompted young students understand party history, feeling, enhance love, do history, history, chong DE, history.

2021-03-25	Shanxi Jinzhong information institute of teachers and students to carry out the party history learning education seminar Jinzhong information institute of Shanxi Province teachers and students to carry out the party history learning education seminar wei strong correspondent, Guo Jiawei Liu Fan) in order to further guide the masses of party members and cadres and teachers and students in the one hundred party history development wisdom and strength, recently, Jinzhong information institute in Shanxi Province held in school wu ma river theatre party history learning education seminar. This seminar, the school party committee propaganda department invited Shanxi provincial party committee party school visiting professor, Shanxi national defense education committee distinguished XuanJiangYuan, Shanxi university, Jinzhong municipal party committee party school visiting professor Mr Jiang Yong into the campus, for teachers and students to "learn deep enlightenment through the fine tradition of the communist party China practice xi new era of socialism with Chinese characteristics" of the theme of the school all education teachers, green horse engineering students, field student cadres, student party members, a total of more than 600 people to listen. Mr Jiang Yong from the perspective of a whole, around the "communist party of China and fine tradition background, the older generation of proletarian revolutionaries founded developed the party's fine tradition, the new era to how to practice the fine tradition of the communist party of China" these three aspects, told the red history to letter school teachers and students passed the revolutionary spirit and patriotic feelings. After the theme lecture, Mr Jiang Yong into the school party activity room, among young students, and talk about their party beginner's mind, convey the communist party in the difficult years take the bull by the horns, firm belief of revolutionary spirit and generations of Chinese, strive to seek the development of the struggle.

2021-04-02	Jiangsu university spring "garden" tea and tea banquet design contest, product culture, Jiangsu university walking aesthetic education classroom wei correspondent, wang) in order to further enrich the campus cultural life, carry forward and inherit the Chinese excellent traditional culture, recently, Jiangsu university "garden spring" tea and tea banquet design contest held in garden, for the school teachers and students brought a unique "walking aesthetic education class". At the beginning of the activity, the song dynasty tea not genetic bearing Song LianKe teacher with gentle tone, beautiful attitude, cordial interaction, tells the origin and the development of some tea, introduces some tea traditional skills and tea spirit, demonstrated the tea use method and standard tea process, the teachers and students to listen with relish, feel the essence and charm of tea culture. Along with the melodious music melody, the scene of 12 masters to "tea ten" technique in their tea, tea, tea smoke curl, tea linkage with tea of the thought and background, and revealed between the tea, the teachers and students walk between the tea and main communication tea theme and design concept, tea, enlightenment tea ceremony, feel the thick of Chinese history and cultural tea mellow. Students of the College Student Tea Art Club said that this "walking aesthetic education class" has closely combined theory with practice, and through the combination of aesthetics and cultural concepts, more college students have accepted the baptism of tea culture in an elegant atmosphere. This activity shows the charm of Chinese tea culture to the teachers and students, edify the sentiment with the tea ceremony, and spread the concept of healthy life. In the future, Jiangsu university public art education center of the Chinese excellent traditional culture education as the basis of school aesthetic education bacon cast soul, continue in the traditional culture and art refining, transformation, fusion, let the young students in aesthetic education practice for beauty and enjoy, learning and inheriting the Chinese excellent traditional culture, and promote the school aesthetic education work orderly and vigorous development.

2021-03-25	"Walking aesthetic education class" of Jiangsu university power "33 class" Jiangsu university "walking aesthetic education class" power "3:30 class" jianwei correspondent, navy-jun wang chengshan wang) on March 24 afternoon, Jiangsu university "walking aesthetic education class" held in the affiliated kindergarten, bring children rich "3:30 class". At the beginning of the activity, Yuan Hui teacher flood militia, firefighters, farmers uncle, doctors, police, teachers image pictures as an example leads to the aesthetic education classroom theme "you smile really good", let the children see in the ordinary post silently stick to smiling face, accompanied by cheerful piano melody, college students art troupe bi � h and Liu Shuwei students live "you laugh really good" dance teaching, make the children in music rhythm and dance learning enjoy beauty and joy while subtly accept patriotism, collectivism, socialism education. Later, four students from Rosenkang, Chen Kangle, Ma Yuntian and Xie Kaiyun from the College Student Art Troupe performed bamboo flute and pipa folk music. The children beat up with the music. The children were in high spirits and the scene was filled with laughter. Through this activity, the students of the art troupe enriched their practical experience in aesthetic education, improved their humanistic quality, and conveyed their love and knowledge to the children, combining education in the practice of aesthetic education activities. The warm public service of aesthetic education was highly praised by schools, students and parents. It is understood that Jiangsu university public art education center will continue to follow the aesthetic characteristics, actively practice aesthetic education infiltration plan of action, heart, with love, hard to help solve "3 o" problem, efforts to create temperature, quality and distinctive aesthetic education classroom, the seeds of Chinese excellent traditional culture into every child's heart.

2021-03-25	Beijing Institute of Technology to develop party history learning education "union association" activities Beijing institute of Technology "always follow the party, forge ahead new journey" theme day activities li tian) "sing a folk song to the party, I put the party than mother..." on March 23, Beijing Institute of Technology in shougang ski jump venues held "always follow the party, forge ahead new journey" theme day activities. This party history learning and education "joint students association lecture" activity, fully opened the Beijing Institute of Technology and Shougang ski jump platform winter games "in-depth cooperation". Beijing winter Olympics organizing committee, vice minister of media operation Han Qiang, volunteers vice minister xiu-feng zhang, shougang ski jump venue secretary general Zhao Hui, 1992 Barcelona paralympic champion, Beijing model of the year, Beijing games XuanJiangTuan members Liu Yukun, vice party secretary of Beijing institute of technology, school teachers and students and winter volunteer candidates, media broadcast trainees in the party backbone of more than 100 people to participate in the activities. A special ideological and political lesson was held. In the study of party history, Cui Jianxia, professor of Marxism at Beijing Institute of Technology, vividly explained why the Communist Party of China "can", "Marxism" did "," why socialism with Chinese characteristics is "good", and guided students to deeply understand the importance of learning the "four histories" well. Ren Wei, director of the Recruitment and Management Office of the Volunteer Department of the OCOG, called on all the reserve volunteers to carry forward the spirit of volunteering by telling about the origin, development and value of the Olympic volunteers to contribute to the success of the Olympic Games. Professor Dong Liquan, the innovation team of Beijing University, told the story of Beijing University, focusing on conquering a number of key technologies to provide core technical support for the Winter Olympics in the fields of scientific training, intelligent creation and intelligent competition watching. Zhang Erjia, a teacher of ideological and political theory course of Beijing Institute of Technology, with the title of "Learning the Party history and building a hundred years of sports power", told the great transformation of the Party leading the Chinese people from "the sick man of East Asia" to "the lion of the East", from a sports power to a sports power. Liu shared his inspirational experience of overcoming physical obstacles, training hard, winning the Paralympic title, breaking world records, and his belief in serving China's Olympic career after retirement. In the theme group day activities, the countdown to the ideological and political education practice base of Beijing Polytechnic University students and the Shougang Ski jumping venue for the Beijing Winter Olympic Games were officially inaugurated. All the teachers and students sang "I love you in China" together, all the party members reviewed the oath of joining the Party, and the representatives of each youth league branch had a style display, and jointly issued a call to be a new person of The Times. According to the introduction, This year, Beijing Institute of Technology is for all students, " Always follow the Party, A new journey "and" bear the responsibility of rejuvenation, Be a New Person of The Times " theme education activities (phase 4), Will "lift a flag-xi jinping, the new era of socialism with Chinese characteristics, tree a belief-marxism belief, take a road, the road of socialism with Chinese characteristics, called a name, the new era, round a dream-the great rejuvenation of the Chinese nation the Chinese dream" of the "five one" work objectives throughout, Deepen and continue the theme of "three together and four together" education model, Adhere to the "learning, telling, tree selection, practice, deepening" five links pressure stubble advance. To celebrate the 100th anniversary of the founding of the communist party of China, party history learning education as the theme of school ideological and political education work, the education to guide students to love the party, patriotism, love school as the main line, throughout the year to "learn", "sing", "speak", "do" as the main way of party history learning education activities, guide the youth firm faith, faith and confidence, with thought consciously lead action consciously.

2021-03-24	Beijing, March 24 (Xinhua) - - " Brothers and sisters, what does your school look like?"The mountain in front of him blocked out the children's eyes. Every summer vacation, when the "Heart to Heart" volunteer teaching summer camp, the children asked. Bus after bus between schools and villages connects the left-behind children's yearning for the outside world with the hearts of the volunteers."The child has a dream in his eyes, and we see the twinkling stars shining in the child's heart."Said a member of the Warm Club. Founded in 2014, the "Warm Sunshine Society" student club of the School of Finance of Jiangxi University of Finance and Economics, formerly known as the "Sunshine Relay" service team of Jiangcai Finance College. The members have worked hard to irrigate the children's dream of reading, and they have been serving the left-behind children in rural areas for more than ten years. Qiu Kailiang, the sixth president of Nuanyang Club, said, " I have been dazzled by college, but only Yang Yang has got my heart deep."The warm sun team has gathered a group of interesting and kind souls. Although the conditions of volunteer teaching are difficult, they can always gain friendship in the cooperation and string up a good memories in the service. The laughter in the teaching building, the chatter on the platform, the morning light sprinkled on the face, are all warm sun. Someone once asked a member of the warm club: " What is the most unforgettable thing during your volunteer teaching period?"He replied," What impressed me most was that when I came to jiao Primary School in Ganzhou City, Jiangxi Province, I found a building called Warm Building. The principal said that it was precisely because of our summer camp publicity that their primary school received a lot of social attention, and caring people donated money to build such a building. In order to thank the Yangguang club, the principal specially named it the Yangguang Building."The appearance of Nuanyang Building is undoubtedly the affirmation of Nuanyang Club's insistence on volunteer teaching for many years, and also inspires the original mission of Nuanyang people to have good classes and do a good job in volunteer teaching. It is reported that up to now, the volunteer teaching activities cover poor villages in Jiangxi, Sichuan, Guangxi, Guizhou and other provinces and cities, serving more than 2,000 rural children, donated more than 30,000 yuan, and more than 200 volunteers. Education is like, the future will be like."The moss flowers are as small as rice, full of books."Members of the warm sun club said, although we are like" moss flowers ", but also willing to open all over the motherland, with the sound of lang books, with education of children a smooth future.

2021-03-26	Jiangsu university "party history, strong faith, follow the party" learning education meeting at Jiangsu university communist youth league "party history, strong faith, follow the party" learning education meeting jianwei correspondent, hai-jun wang, Zhang Yongqiang Ding Yijuan) on March 25, Jiangsu university in the third conference hall held the communist youth league "study party history, strong faith, follow the party" learning education rally, the Jiangsu university league organizations at all levels to carry out the "party history, strong faith, follow the party" learning education centralized mobilization and comprehensive deployment. The leaders of the secondary youth league organization, all the staff of the school youth League committee, the presidium members of the university-level student organization, and the representatives of the youth league branch secretary of each college attended the meeting. The meeting pointed out that we should improve our political standing and fully understand the basic considerations of the Communist Youth League to carry out learning and education. All the youth league members should deeply understand the essence, clear practical requirements, to learn the history of the Party this "compulsory course" to consolidate the ideological foundation, calibration the direction of action, understand and grasp the basic consideration of this deployment from the source. Carrying out study and education is a major responsibility to highlight the role of the Party's assistant and reserve army, an important part of strengthening the ideological construction of the Communist Youth League, and a major opportunity to improve the ideological and political quality of the league members. The meeting stressed that we should guide our good work and closely follow the objectives and tasks of learning and education. This study and education, in essence, is a political education for the school Communist Youth League, and it is a political refinement for the school Communist Youth League to maintain and enhance its political nature, advanced nature and mass nature. To ensure that the study and education objectives are focused and focused, we must always tighten the political string, adhere to the political direction, highlight the political attribute, play the political function, stand on the political position, learn the glorious history of the Party and understand the original mission of the Party; learn the historical experience of the Party; learn the spiritual pedigree and inherit its red gene. Meeting requirements, to pay close attention to the implementation, effectively strengthen the overall coordination of the party history study education. Youth league organizations at all levels must pay special attention to the whole, strengthen coordination, the learning education really grasp comprehensive, grasp, through the whole year, to daily, focus on "special learning education, special training exchange, special organization, life, special practice", closely combined with "speak, exhibition, game, line", to ensure learning education in-depth development, obtains the actual effect. Jiangsu university league organizations at all levels will take the meeting as an opportunity to carry out the "party history, strong faith, follow the party" learning education, seize the party history, the basic point, "strong faith", "the master switch" to "follow the party" the overall goal, in order to further stimulate the new era, meritorious "difference" ambition, follow the party to achieve the second goal in one hundred achievements, for the school "double" create and high level university construction contribution youth strength, celebrates the 100th anniversary with honors.

2021-03-24	Central China Normal University) March 19, Central China Normal University announced the formal establishment of the "Party Students History publicity group", to explore the party history publicity for students, tell the centenary of the story of the Party. The university has 34 members of the party history publicity group, composed of graduate students majoring in Party history and members of the Student Study Association for Xi Jinping Thought on Socialism with Chinese Characteristics for a New Era. The publicity group will take micro publicity, micro video, micro party class and other ways, go into the red education base as the narrator, focus on introducing 100 party members' stories, telling 10 kinds of great spirit, create a stage play performance, promote the party history learning and education into the masses, the grass-roots level, and the hearts of the people. At the same time, the school presented four required reading books on the History of the Communist Party of China, Xi Jinping Thought on Socialism with Chinese Characteristics for a New Era, A Brief History of the Communist Party of China, MAO Zedong, Deng Xiaoping, Jiang Zemin and Hu Jintao's Discussion on the History of the Communist Party of China.

2021-03-24	Beijing, March 24 (China Youth Network) - -Playing guitar, doing handwork, playing basketball... in Chilowa community, Nanchang City, Jiangxi Province, every Wednesday interest class is the "favorite" of children. The Financial Youth Association of Jiangxi University of Finance and Economics connects with the community neighborhood committee to create a long-term volunteer service project with the theme of "youth and childlike innocence". It holds interest classes in the community every Wednesday afternoon to meet the needs of children. Volunteers guide the children to draw. Interest class is mainly divided into three sections: knowledge class, sports class and art class. Knowledge class is mainly to popularize some extracurricular knowledge for children and broaden their scope of knowledge, such as the teaching of garbage classification and the historical popularization of the National Memorial Day. In the PE class, the volunteers took the children to exercise through fun games, combining teaching and physical fitness, which not only ensures the fun of the class but also makes the children strengthen their health. In the art class, volunteers teach the children handmade paintings, music and dances, such as leaf stickers and paper cutting, to cultivate their practical and learning ability. The seeds of interest have been quietly sprouted in the hearts of the children. The volunteers will keep insisting and keep cultivating, looking forward to this seed to grow into a towering tree of love and dream. Volunteers took photos with the children. Photo provided by the Youth League Committee of the School of Finance, Jiangxi University of Finance and Economics

2021-03-27	Shaanxi University of Science and Technology: Young students are busy again in the volunteer service. Over the past eight consecutive years, more than 4,000 volunteers from the school have provided high-quality volunteer services for the annual Qingming Festival Xuanyuan Yellow Emperor Ceremony with high enthusiasm and high standard quality."Eight years, 0 error, 0 error, 0 complaint..." behind this number, is the epitome of the volunteer spirit of young students of Shaanxi University of Science and Technology from generation to generation. With the promotion of volunteer service activities, the spirit of selfless volunteer service has taken root in Shaanxi University of Science and Technology, and a volunteer service team that responds quickly and can fight hard battles has grown up in hone. In the years of summary, the volunteer service work of Shaanxi University of Science and Technology has formed a set of perfect management system. To promote voluntary activities to carry out normalized, service specialization, brand project, the school not only introduced the youth volunteer management regulations, volunteer incentive management plan, volunteer service emergency report handling mechanism, system, also continue to improve the volunteer line before training program, with qingming symbol of the Yellow Emperor ceremony project of volunteer service activities set up the characteristic brand. The combination of volunteer service with the disciplines and majors of the university and serving the social and economic development of western China, Shaanxi University of Science and Technology is a new type of volunteer service road explored. The Yuanmeng public welfare team has won many awards in the volunteer service project competitions at all levels. In 2013, only after the drinking of a cup of local water cellar, the source dream charity team members who went to northwest rural areas to conduct social practice suffered from diarrhea and vomiting. Such water, where the villagers but drink for a lifetime. The touched team members began to investigate on foot, analyzed the water samples taken back to the laboratory, and found that the fluoride content in the water was too high. Relying on the strength of the school's national characteristic major- -inorganic non-metallic materials engineering, ceramic materials green manufacturing and new functional application innovation team, the team proposed a plan to improve the water quality, and developed two kinds of water purification equipment: nano zinc oxide ceramsite and new activated carbon. In the past eight years, the team has led more than 20,000 college students to participate in volunteer services, directly installed more than 15,000 water purifiers, and indirectly driven the production of more than 30,000 units, benefiting 200,000 people, covering nearly 30 counties and districts in the Shaanxi-Gansu-Ningxia region. As a university in western China, most of its students come from Shaanxi province and the western regions. When organizing and carrying out volunteer services, the school pays attention to guiding students to actively participate in western China and serve their hometown. The Mabian volunteer teaching team took a group photo with the local students."Along the winding mountain road, sometimes accelerate up, sometimes slow down........... sitting in the car, I have not dare to look down the mountain."This is the 22nd graduate volunteer group member Wang Wanni on the home visit scene. In Mabian Yi Autonomous County, Sichuan Province, where the volunteer teaching group is taught, members of the volunteer teaching group will visit the countryside every weekend to do their part to control dropout and ensure school attendance in the Yi nationality area. In the face of the unsalable of local agricultural products, members of the volunteer group also become e-commerce anchors, bring goods online. Due to its outstanding achievements, it has won the 2020 County Poverty Alleviation Dedication Award issued by the Mabian County Party Committee and the county Government. The volunteer team of "Qinling Guardian" conducted research under the guidance of Professor Guo Junkang. In front of a tailings in the eastern section of the Qinling Mountains, members of the volunteer team were shocked by the slag piled up seven or eight stories high, with a stench and pungent smell... "The tailings contain a lot of heavy metals, which can cause serious pollution after precipitation."Team members are determined to fight to protect the Qinling Mountains. Under the guidance of Professor Guo Junkang, the team members went deep into several key mining areas to carry out soil pollution research. The final Investigation Report on the Current Situation of Soil Pollution and Restoration in Qinling Mountains won the 2019 Excellent Research Project Award of Qinling Ecological Scientific Research by Shaanxi Provincial Committee of the Communist Youth League. Wei Yaoyao is on the front line of epidemic prevention and control."When I stand on the front line of epidemic prevention and control, I think of the teachers and students who also stick to it, which increases my courage and motivation."Wei Yaoyao, a student awarded the title of advanced Individual by the Central Committee of the Communist Youth League and the China Youth Volunteers Association, told reporters. In Shaanxi University of Science and Technology, the spirit of "everyone volunteers" has become a common trend."Learn from Lei Feng's good example, be loyal to the revolution and be loyal to the party..." Walking in the teaching building of Shaanxi University of Science and Technology, the ear came a loud song. In the classroom, the league day activity with the theme of "Volunteer Learning from Lei Feng" is under way. After taking the volunteer oath, another group of students will go to the nursing home to start a new day's volunteer service...

2021-03-24	Wuhan sports institute to learn the party history theme Shi Ming draw strength from the history of the party learning Wuhan sports institute to carry out "learn the history of the party, enlightenment life, follow the party, round dream" theme preach jianwei correspondent Han Xiaoyi) " seize the youth, exercise ability, work hard to realize the Chinese dream, realize their brilliant in the process of struggle life!"Although the lecture has been over, but MAO Lao's words are still lingering in the ears of the martial arts students. On March 20th, Wuhan Institute of Physical Education invited MAO Lancheng, the leader of the Air Force, to report the theme of "Learning the history of the Party, understanding life, following the Party, and realizing the dream of the Party" in the small theater of the art museum. This lecture adopts two online and offline forms, covering all the youth league members of the school. This time to the martial arts propaganda, MAO Lao said that the school learning atmosphere is more strong, this implementation of the party's educational policy activities for young people, and the students under the guidance of Marxist thought presented the vigorous spirit expressed appreciation. As an "old friend" of martial arts youth, he told his profound friendship with the Party for more than 50 years with personal experience. He hoped that the new generation of young people would forget their original aspiration and keep in mind their mission in peace, so that the red spirit could be passed on from generation to generation."Cultivate thought, noble soul; temper personality, ideal lofty; hone character, high will; exercise ability, excellent ability."Is MAO Lao's sincere advice to the students present here. He told his students about his tortuous experience of joining the Party. When he was a teenager, he began to learn Marxism, and strengthened his belief in working for the Party and serving the people. For decades, he has been constantly striving and practicing his original aspiration with actions. At the same time, he called on the youth in the new era to adhere to the combination of learning people and literature, learning history and theory, learning theory, learning speech and practice, to apply theory to practice and match words and deeds. Mao old saying let every youth member present is deeply encouraged, the eyes are full of admiration and admiration for Mao Lao. After the lecture, the students rushed to ask questions to MAO Lao, through the exchange of everyone had a more profound understanding of the spirit of the Party. Each generation has its own journey, and each generation has its own mission. With his own experience, Mao told the arduous course of the older generation of party members in the last century, so that the majority of youth league members have a deep understanding of the arduous journey of unity, united struggle of the Communist Party of China.

2021-03-23	The Chengdu University "Green Orange Station" of Chengdu University and the "Green Orange Station" organized by the University Youth League Committee held a publicity activity on garbage classification in the villagers' activity room of Shijia Village, Xinshi Street, Jianyang City, Chengdu City, Sichuan Province. Shijia Village, Xinshi Street, Jianyang City, is the matching support point of the "Orange Station" rural revitalization service group. The "Orange Station" released the upcoming project in Jianyang City on its wechat official account. Students from Chengdu University can voluntarily register online according to different projects and combine their own majors, specialties and interests. According to the relevant person in charge of Chengdu university, the school set up "green orange station" rural revitalization service, on the one hand, hope to play the teachers and students team social research ability, help promote rural revitalization and accelerate the modernization of agriculture and rural areas, on the other hand also hope to let the teachers and students in practice, broad vision, education, long ability, contribution. For the party history learning education achievements, teaching practice, college students 'innovative entrepreneurship, college students' social practice applied to the specific work project, help poverty engines and rural revitalization of effective cohesion, "green orange station" rural revitalization service will be to JianYang "small scale, frequency, normalized" service practice, "and planning" celebrate one hundred good poverty alleviation story "cohesion", runoff comprehensive well-off "" focus on industry, help rural revitalization "" to meet the green universiade volunteer service country " and other four specific projects.

2021-03-22	Zunyi medical university to carry out the history of the party learning education theme TuanRi activities Zunyi medical university to carry out the "keep in mind the earnest entrust loyalty clean bear celebrate the founding in one hundred" theme TuanRi activities JianWei correspondent Chen Kai) to thoroughly study and apply xi general secretary in the history of the party learning education mobilization meeting's important speech spirit and inspect Guizhou important speech spirit, To guide young students to firmly uphold their ideals and beliefs, Enhance the consciousness of learning party history, recently, The Youth League Committee of Zunyi Medical University organized more than 100 cadres of the student Union and student associations to visit Hongjun Mountain and the theme of "Remember, loyalty, clean, responsibility and Welcome the centenary of the Founding of the Party". Fu Yanong, vice minister of the Publicity Department of the university Party Committee, participated in learning and education as the keynote teacher of the party class. In the Red Army Mountain, the league cadres carried out a contest on party history knowledge, reviewed the course of new China from standing up, getting rich to becoming strong, and remember and felt the great achievements of the Party. In front of the Red Army martyrs' monument, the cadres paid a moment of silence to pay their highest respects. Fu led the league cadres to pay respects to the Red Army martyrs relief, martyrs list wall, Deng Ping's tomb, the Red Army grave, and carried out special party lessons, focusing on the historical background, process and significance of the Red Army Long March, and guided the cadres to remember and learn from the martyrs' spirit of not afraid of hardship, dare to sacrifice and dare to win. At the site of the Zunyi Conference, the cadres visited the Zunyi Conference Memorial Hall. By browsing the cultural relics materials, watching historical videos and listening to the red stories, they reviewed the difficult course of the Long March of the Red Army, accepted the profound baptism of thought, and deeply understood the spirit of the Zunyi Conference. In the theme TuanRi activities, the school youth corps committee called on all cadres to around "keep in mind the earnest entrust clean loyalty bear celebrate the founding in one hundred" theme, inheriting red gene, continuous spirit blood, the personal ideal into Guizhou high quality development, unswervingly listen to the party, follow the party, yong do bear the national rejuvenation of the new era.

2021-03-22	Shandong University and China Railway) On March 18, Shandong University and China Railway held the signing ceremony of the youth development joint construction agreement in Jinan to deepen the cooperation between enterprises and universities and accelerate the pace of joint construction. Jing Guobin, Vice Minister of Human Resources Department of China Railway Corporation, Li Haiming, Vice Minister of Science, Technology and Innovation Department, and Wang Junsong, Deputy Secretary of the Party Committee of Shandong University attended the signing ceremony. Yang Fei, secretary of the Youth League Committee of China Railway, and Zhang Xi, Secretary of the Youth League Committee of Shandong University signed the agreement on youth development on behalf of both sides. According to the agreement, the two sides will build a "youth federation", in promoting talent recruitment, student practice, scientific research, "green horse project", mentor, youth fellowship, volunteer service and other areas such as further cooperation, youth federation, youth office, youth office is located in Shandong university, civil engineering and water conservancy institute and qilu traffic college youth corps committee. The launch ceremony also released the "4 + N" Youth Cooperation Plan for 2021. Under the plan, this year, China Railway will offer more than 1,000 internships for college students, General Shan has publicly recruited and selected a group of students to practice in China Railway; Jointly carry out a youth education study of party history theme education activity; Set up a college student volunteer teaching group, To primary and secondary schools in Guidong County to carry out volunteer teaching in Hunan Province; Planning a face-to-face event for young alumni, Gathering the young alumni of Shandong University working in various fields of the enterprise, Back to his Alma mater to carry out the "pair of help" action; Implementing the "vanguard action" of enterprise-university scientific research cooperation, Focusing on tackling major scientific research projects, Establish a mechanism for joint declaration and joint efforts, Establish N application projects, To provide a platform and financial support for youth scientific and technological innovation. Wang Junsong said that the signing of China Railway Shandong University will expand the social practice, volunteer service, internship and employment channels, and the scientific research content will be more closely integrated with the actual needs of frontier production, key technology fields, enterprise talents and development needs, and promote the further development of industry-university-research integration. Jing Guobin said that in the future, China Railway will deeply connect with the relevant institutes of Shandong University, and jointly establish a mechanism for talent training and expert resource sharing. Hai-ming li, for the implementation of the new development pattern "traffic power, railway first" strategic requirements, China railway will and Shandong university around the difficult engineering construction, adhere to the strategic guidance and innovation drive, in tunnel construction, underground engineering and technological innovation cohesion, joint research problems, realize the core technology breakthrough. During the discussion and exchange, representatives of both sides focused on the actual needs of schools and enterprises, focusing on the promotion of major projects, "bottleneck" technology breakthroughs, college students' practice, job hunting and employment, and enterprise innovation and development. Leaders of relevant departments of China Railway, China Railway 10th Bureau, China Railway Tunnel Bureau, China Railway Tunnel Bureau, China Railway Construction Engineering, China Railway Industry, Shandong University Service Shandong Office, Employment and Entrepreneurship Guidance Center, University Youth League Committee, School of Civil Engineering and Water Conservancy, Qilu Institute of Transportation, and other relevant officials attended the activity.

2021-03-16	Nantong University: Reporter Li Chao) On the campus of Nantong University, there is a red propaganda team of young people. Unlike other propaganda teams who publicize red culture and red thought with their mouths, this propaganda team painted red thought and red culture on the wall. When it was founded in 2015, the team was a Yixing painter, with only four or five people. The surrounding communities have the need of wall painting needs, and they carry out activities according to their needs, covering all aspects such as humanistic customs and socialist core values publicity. Later, they gradually realized that each wall painting should have its unique meaning, with propaganda effect."Wall painting itself is an intuitive form of expression. We can use this form to tell red stories and publicize the latest policies, and convey current affairs to the rural grass-roots level with vivid pictures, not only beautifying the rural ecological environment, but also spreading Chinese red culture."Said team instructor Ye Xinxin. In 2018, they officially established the project, which established the theme of using wall painting to convey the red stories and the red spirit to the grass-roots level. They set up a social practice team, painted buckets on their backs, put on volunteer clothes printed with the project logo, and went to various places for red wall painting activities. With their simple and sincere enthusiasm, more and more students were encouraged to join the team. From the initial shortage of manpower, gradually to the clear division of design and drawing, and then to the cooperation between multiple colleges, the team formed a team of nearly 1,000 people, and the wall drawing is countless. Five years of unremitting pursuit, day and night of hard painting, more than 30 thank-you note.... Nantong university art institute "red originality-grass-roots patriotism communication pioneer" project team has entered 7 provinces, design red theme works more than 760, carry out "a painting double speak" micro classroom 1000 times, participation in more than 2800 people, the area of more than 15000 square meters, affect the number of more than 80 thousand. The drawing of wall painting is not as simple as imagined. The walls of the community are of different sizes, not only to join the concept of the project, but also to meet the requirements of the community. The design draft of each wall painting has become a difficult problem in drawing. Many team members were extremely nervous when they first participated in the wall painting activity, and the current leader of the team, Li Yuan Guan of Art Normal Class 184, was no exception. In the heat of more than 30 degrees, she was bitten out by mosquitoes in the corridor of the community, but she enthusiastically insisted on painting a whole wall painting."I remember once it was very hot when an aunt held her child in the shade and watched us draw all afternoon."This almost became the motivation to support Li Yuan overflow to persist. In 2018, Li Yuan Yu began to participate in wall painting activities. By drawing again and again, she, who was introverted and sensitive in the past, slowly accepted the outside world. From a little girl who dare not speak on the stage to become the head of the project. These college students grew up together with the "red ingenuity". Although it is a public welfare wall painting, it is occasionally not understood. Because some communities of the elderly in the majority, for the wall painting does not understand. In the process of wall painting, an old grandfather who lived nearby mistakenly thought that a group of young people were scribbling, destroying the community environment, so strongly stop."You group of college students are indeed contributing to the society."After learning about the truth, grandpa apologized to them and affirmed what they did. In the wall painting, children and residents will constantly come to watch and cheer, which makes the member Li Chengyin think that they are not doing surface projects, but can really mobilize the residents to participate in it and understand the red story and spirit they want to express together, which is more meaningful than the painting itself. In the team's "art lights up the childlike innocence" rural art volunteer teaching activity, a letter of thanks from Feng Guoqiang, a village boy, deeply touched everyone in the team. The letter wrote: " My brothers and sisters painted a beautiful Tiananmen Square. I grew up like my brothers and sisters."On the signature, the boy printed his red fingerprints. In the past five years, the Red Craftsmanship project team has been spread to all the urban areas of Jiangsu Province, and has radiated to more than 40 rural areas in five provinces, including Heilongjiang, Shanxi, Shandong, Henan and Hebei. In 2020, the epidemic was rampant, making the team unable to conduct offline activities. They became "little fighters against the epidemic", turned to online, supported online art teaching, made video micro classes, established wall painting materials library, and created more than 800 anti-epidemic works, more than 30 anti-epidemic songs and more than 20 anti-epidemic videos. At the end of last year, the project team also won the silver medal in the fifth China Youth Volunteer Service Project Competition. They take root in the villages and communities, carry forward the Chinese red culture in practice with their paintbrushes and paints, and truly convey the spirit of love for the Party and patriotism to the hearts of the grass-roots people.

2021-03-17	On March 15, the opening class of the reading class of Minzu University of China was held. Zhang Jingze, party secretary of Minzu University of China, presided over the study meeting. Zhang Jingze led the important speech of General Secretary Xi Jinping at the Party history study and education mobilization conference, and combined with the "On the History of the Communist Party of China" from the study of the Party history, what to learn from the Party history, how to study the history of the Party learning experience."The establishment of the College for Nationalities is a pioneering work made by the Communist Party of China to solve ethnic problems. Before, there has been no ethnic college in the world before."Zhang Jingze pointed out that since the establishment of the Communist Party of China of the College for Nationalities in Yan'an in 1941, the policy, philosophy and tasks are the same line, and ethnic higher education has gone through 80 years. Guo Guangsheng, deputy secretary of the Party Committee and president of the University, read the guide "Continue to draw strength from the Yan'an Spirit" and elaborated the learning experience. He said that Minzu University of China was born during the War of Resistance against Japanese Aggression, and was born because of the Party and stood for the Party. To study the history of the Party is to draw strength from the Yan 'an spirit, integrate the Yan' an spirit into all aspects of the school running, promote the construction of "double first-class", and promote the high-quality development of the school."The Yan'an Spirit is our background color, our foundation, our development and the soul of our power."Ma Wenxi, executive deputy secretary of the university Party Committee and secretary of the university Discipline Inspection Commission, talked about his own experience around the general secretary's profound discussion on the" Yan' an spirit ". Zou Jizhong, deputy secretary of the Party Committee of the University, said when talking about the learning experience that thought is strength, and the power of Marxism lies in profoundly changing China. The century-old Party history makes us firmly believe that Marxism is a powerful ideological weapon, which must be upheld, followed and well used. Li Jiyong, deputy secretary of the Party Committee and vice president of the university, talked about the understanding of understanding the thinking, grasping the general trend and opening a new bureau. The reading class also went to the school history exhibition for on-site teaching. Reading class all the students follow the history and culture institute professor Cang Ming, from Yan'an institute of nationalities chairman MAO zedong handwritten inscription "unity", to the beginning of the party and the country to determine the "three main tasks", to xi comrade inspection pointed out that "to serve national development strategy, national unity and progress, minority talent training play a bigger role", through a vivid images, frames of precious photos, revisit the school 80 years of struggle. After the visit, the students deeply felt, the construction of central university for nationalities in 80 years, is the communist party of China in China founded great higher education model practice of 80 years, is also the school practice the education thought of the communist party of China, inherit and carry forward the Yan'an red gene education concept to explore practice for 80 years.

2021-03-12	East China university of science and technology "beautiful youth river volunteer service" established professional professor when long river, China youth river with professional "escort" green net reporter wang yejie) recently, generations of east China technology people engraved campus memory of youth river has a professional "escort" - "beautiful youth river volunteer service" was formally established, and appointed professional professor and graduate students as a long river, with professional service ecological civilization construction. The establishment of the "beautiful youth river volunteer service" is the Shanghai xuhui district new era civilization practice team, consists of 10 ring college graduate students and undergraduates, volunteer service by ecological environment monitoring, microbial water treatment, environmental toxicology assessment, dynamic simulation members of different research direction, is a professional counterpart, interdisciplinary and dynamic volunteer service team. They will carry out a series of activities around the real-time monitoring of the water quality of the Qingchun River, sensing technology research and development and intelligent inspection, ecological restoration technology, environmental science popularization and publicity and other aspects. Employed as a teacher long long professor, is not only the party secretary of the college, also national environmental protection chemical process environmental risk assessment and control key laboratory, deputy director of Shanghai environmental protection environmental standards and risk management key laboratory director of academic position, for many years has been committed to environmental standards and risk management in the field of ecological environmental protection work. Li Tao, vice president of East China University of Science and Technology, said that the university will further promote professional teachers and professional students to participate in the "Beautiful China, I am an actor" activity, to provide strong support and help for the management and maintenance of the Youth River, and help the Youth River to maintain its youth forever.

2021-03-16	China university of petroleum (Beijing) teachers and students learn "xi and students friends" China university of petroleum (Beijing) teachers and students learn "xi and college students friends" wei correspondent Wang RuJiaoLing Ying) recently, China university of petroleum (Beijing) youth corps committee organized the teachers and students to carry out the "learn" xi "timely-read" xi and college students' friends " activities. Through the combination of online and offline methods, this activity creates a closed-loop learning mode of "content guide + self-study punching in + experience exchange". Activities based on group organization online platform to carry out the "" Shi "YanShi said" and "Shi Qing" two columns, "" Shi "YanShi said" column invited 25 party and government department workers, scientific research teachers and college full-time cadres "Shi" guide team, they combined with work characteristics and their own experience, concise book 25 interview record core thoughts, help students more targeted tapping the original meaning, improve reading interest; The column of " 'Shi' Yan Qing Tan" collects reading experiences from all the students, and regularly selects some excellent manuscripts for display. By the 10th guide study, more than 460 high-quality contributions have been received. In the reading activities, the qingma class of the school has played the role of a "pioneer" in mutual peer learning, and has organized two online special reading sharing meetings of "Xi Jinping and College Students Friends". Invite karamay campus grade 2020 graduates around "let youth bloom in the motherland most needed place" theme, share communication xi general secretary of the book repeatedly encouraged college students to go to the grassroots line to accept practice exercise, "to give books knowledge, to get knowledge 'dry", only combined with the practice, to put the' moisture " out."Invited Wang Caizhong, the outstanding student of the last class, combined with the university experience and work experience, about the importance of the class to college students and the path to improve their comprehensive quality through the class platform, focusing on how science and engineering students read and learn Marxist theory books. All the colleges actively respond to the call of "learning", design carefully combined with the characteristics of the college, and put the reading activities into practice and learn deeply. At present, more than 400 youth league branches and student organizations to participate in reading activities, the school, the league branch as the basic unit, party members and activists as the lead, student associations and the "Quartz plan" backbone active participation, create the radiation learning mode, create students actively participate in and extensive learning atmosphere; school of safety and ocean engineering committee for counselors and student organization backbone bought xi and students friends books, directly mail home, to ensure the timeliness and effectiveness of learning, and organize counselors guide, students backbone lead learning, all the members to study. Next, the school youth corps committee will continue to lead the group to carry out theoretical learning activities, to "stone said" online learning as the main line, set up "teacher said green" offline sharing platform, the learning propaganda xi jinping and college students friends and promote "three education" comprehensive reform work, efforts to form propaganda force, improve the quality of ideological and political work, xi further promote the ideas of socialism with Chinese characteristics in school young students into the brain into the heart, take root.

2021-03-15	Jiangsu University "walking aesthetic education class-special orchestra" held in Jiangsu university "walking aesthetic education class-special orchestra" held JianWei correspondent Wang Xue) to enrich campus cultural activities, improve students' cognitive beauty, appreciate beauty, feeling beauty ability, Jiangsu university public art education center on March 14 in garden "walking aesthetic education class-special orchestra" activities. In March, the River plum Garden, flowers, leaves willow spring. The ear side of the birds sing warbler warbler, the body side song water flow wine cup, more accompanied by bamboo flute, erhu, guzheng, dulcimer and other traditional Musical Instruments. At the beginning of the activity, the dulcimer performance, brought by Liu Wei and Zhang Hao � h, created a jubilant opening atmosphere and ignited the enthusiasm of the audience. The tracks of Jasmine Flower, Grain in Ear, Gusu Journey, Butterfly Lovers, War Typhoon, Summer of Jjujiro, Pipa Language, Small Town ballad, War Horse Pentium, and Jiangnan Spring Festival show the beauty of traditional Musical Instruments to the audience. It is reported that in addition to the melodious music, this activity also invited the folk orchestra instructor Jin Lianghui, to spread the relevant knowledge of Chinese folk music, introduce the earliest Chinese Musical Instruments, musical instrument material classification, ancient court music, folk music, playing, and teach the basic performance knowledge of guzheng on the spot. Ms.Jin hopes that students can cherish the opportunities and platforms to recognize, appreciate and feel beauty, stimulate their youth, cultivate their sentiment and shape a beautiful soul in the colorful campus activities. Zhao Yaqin, a member of the Hanfu Club, said excitedly, " I was the first time to learn the guzheng performance and feel the beauty of the Chinese national rites and music. I am very happy and have a special sense of gain."The school folk band was founded in 2005 and won the honorary title of Jiangsu Provincial College Student Art Troupe in 2008. With the purpose of "carrying forward national culture and improving students' quality", the folk orchestra has formed a young, lively, beautiful and elegant performance style, and created a number of excellent classic songs, which have won many achievements in the Art Performance instrumental Music Competition of Jiangsu Province. It is such an excellent team that brings a scene audio-visual feast to all the teachers and students. It is reported, since the Jiangsu university on strengthening the new era of aesthetic education work implementation rules, Jiangsu university public art education center regularly held theme positive, rich in content, various forms of campus culture and art activities, deputy secretary of school, said the center will continue to carry out "walking aesthetic class" series of activities, for all students, vigorously develop extracurricular practice activities, create elegant, aesthetic, vibrant campus culture.

2021-03-11	Sichuan day a college volunteers help mianzhu wen brigade industry development [new youth see new hometown] Sichuan day a college youth volunteers help mianzhu wen brigade industry development jianwei) recently, Sichuan day a college more than 30 student volunteers into mianzhu qingping town, as a volunteer guide service the town to carry out the cultural tourism industry development."The unique temperature of Yanjing Village and the distinctive architectural features attract many tourists to spend the summer here."Liao Xueqin, a major in 2019 in Qiang costumes, enthusiastically explained the history of Qingping town, the reconstruction and the history of the panda house, and made a tour guide to the tourists. In addition to serving as on-site tour guides, the volunteers also undertook the reception work. Flight attendant professional students presented qiang red and a rose to express festival blessings, let everyone feel warm; two students wearing 4 meters high, weighing 30 jin mecha equipment, posing cool shape with tourists; the rest of the volunteers assigned in the small train, tour center and giant panda national park science publicity and education center, with love for tourists...... Warm scenes staged in Qingping town, a wipe of volunteer red became the most beautiful scenery of the day."Guiding students to dare to take responsibility and cultivate the consciousness of serving society is an important aspect of strengthening the ideological and political education of college students."Wang Qiuhong, secretary of the General Party Branch of the Department of Humanities and Art Design, said that the volunteer service is an extension of the premise of cooperation between the government and schools. The department fully combines professional advantages, actively strengthens the cooperation with Qingping Town, on the one hand, to help the development of regional cultural tourism industry. On the other hand, students should be guided to practice the Spirit of Lei Feng with practical actions and integrate volunteer service into their daily life.

2021-03-10	Nanjing Xiaozhuang University: Innovate the new mode of Party history learning to make the Party history "live" around him. Sichuan correspondent Chen Zilai) Recently, the Party history learning and education mobilization conference was held in Beijing. In order to better study the history of the Party, praise the grace of the Party and follow the Party, the School of Environmental Science of Nanjing Xiaozhuang University has focused on creating the column "Xiao" Talk of the History of the Party, "Ring" said the red trace ", by telling the people and things in the red ruins of the local and surrounding areas in Nanjing. According to Nanjing xiaozhuang institute of environmental science college party controller introduces, combined with the characteristics of the academy geography related professional, "xiao" party history, "red," ring "relying on" youth ring " WeChat public, each push a 4 to 7 minutes of voice story, with the Nanjing red relics of historical changes, characters, the present situation, etc. In the scope of red site, the column follows that the school serves Nanjing, using the party history around it: the "founding of China and the Great Revolution", "Agrarian Revolutionary War", "National Resistance War" and "National Liberation War", the column group tries to grasp the nodes to make the party history interact through time and space and reality to make the party history "live". On March 8, the International Women's Day, the column group pushed three martyrs Zhou Zhengming, Yao Ailan, Ying Boheng, the tomb of DongMartyrs, focusing on the heroic deeds of Yao Ailan, feel the unyielding and strong of women. On March 15, "Anniversary Day", the column group plans to push "Xiaozhuang Ten Heroes Monument", let Xiaozhuang red gene from generation to generation, on April 5, Qingming Festival, plans to push "Yuhua Martyrs Cemetery", let the heroic spirit to illuminate life; on May 21, it plans to push the love story in the camp (liaison station of Nanjing Underground Municipal Committee), let the love of youth can be sublimated, etc. Due to the novelty of form, the readability of content and the touch of material, the column has been widely praised by all the teachers and students since the column was pushed on March 1st. In order to build this brand project, the General Party Branch of the School of Environmental Science of Nanjing Xiaozhuang University has conducted full deliberation and research. With the help of Nanjing local Chronicles, Tourism Bureau and other units, 100 Nanjing red relics have been basically drafted. As of March 9, the program has been pushed for nine issues, and plans to complete the 100 red relics push by July 1 this year, as a gift to the 100th anniversary of the founding of the Communist Party of China. Later, Nanjing xiaozhuang institute of environmental science party will also be around the 100 red relics to carry out special party class training, social practice, visiting research series of party history learning activities, further promote the red site and the depth of the construction of professional science, with red culture education into the classroom, into the teaching material, continuously expand the depth and breadth of the brand column.

2021-03-10	Shaanxi energy vocational and technical college launched "celebrate the founding of one hundred to meet the whole event Yang volunteer air" series action shaanxi energy vocational and technical college launched "celebrate the founding to meet the whole event Yang volunteer air" series of action JianWei correspondent Chen Jie) to inherit and carry forward the lei feng's spirit, carry forward the "dedication, love, mutual aid, progress" volunteer spirit, in early March, shaanxi energy vocational and technical college youth corps committee guidance dream volunteer service officially launched the 2021 annual "celebrate the founding to meet the whole event Yang volunteer air" theme series action. Teachers and thousands of youth members signed their names on a banner reading "Celebrating the founding of the Party and welcoming the whole Games". The volunteers carried out a cleaning activity of "Make the environment around them beautiful", and cleaned up the garbage on the green belt of Wenlin Road. During the activity, Gao Bo, deputy secretary of the Youth League Xianyang Municipal Party Committee, and his delegation also came to the site of the Learning from Lei Feng activity, and awarded the banner of "Xianyang City Youth Volunteer Service Team" to the young volunteers. Volunteer service is an important symbol of social civilization and progress, and a vivid practice of carrying forward the new trend of The Times. This year, the school youth League committee to the school youth league members, hope we actively carry forward the lei Feng spirit, cohesion, enterprising, effectively implement the lei Feng spirit in learning and life, hope that young volunteers, learning example "advocate", encourage themselves to grow; do brave "practitioners", actively undertake volunteer service, do the spirit of "relay", vigorously promote the lei Feng spirit, "dedication, friendship, mutual aid, progress" volunteer spirit, for the 100th anniversary of the founding of the Communist Party of China, writing a new era, new youth lei Feng story.

2021-03-10	Jiangxi youth vocational college to carry out the party history learning education activities of Jiangxi youth vocational college the first lesson: revolutionary descendants speak the party history of Jiangxi youth vocational college "revolutionary descendants speak the party history beginner's mind" the first class activities, invited the son of founding general Zhu Xinchun, the daughter of the Zhao � F Zhao Jingya to school Zhu Liangcai and Zhao � F general heart to the story of the party, the hard struggle. Zhu Xinchun told everyone about Zhu Liangcai's experience of joining the Party, finding the Party and following the Party, and told you about the story of Zhu Liangcai's struggle for the Party, the people and the country all his life. Zhao Jingya shared the story of Zhao � F's career and growth from a young student to a founding general. For a long time, Jiangxi Youth Vocational College has created the education characteristics of league-affiliated colleges. By excavating red resources, innovating the way of ideological and political education work in schools, and integrating the red tradition and red genes into the school ideological and political education with different teaching forms, different presentation methods and different perspectives. In the next step, the school will systematically carry out the study of party history and education, combine the study of party history with summarizing the experience, observing the reality and promoting the work, truly transform the learning effect into the work effect, constantly enhance the pertinence and effectiveness of the school ideological and political education work, and realize the purpose of learning from the present and educating people. This activity is not only the first lesson of the spring semester of the school, but also one of the series of party history learning and education activities. On the same day, nearly 8,000 teachers and students participated in the study together through one main venue, 70 sub-venues and an online live broadcast platform.

2021-03-09	Changshu institute of technology practice to learn lei feng volunteer service activities changshu institute of technology practice to learn lei feng volunteer service activities 22 Chinese youth volunteers, changshu institute of technology in Jiangsu province "" not "love, spark transfer" volunteer service practice did not love public welfare together into the taixing yao wang primary school, for 40 children to learn lei feng theme education class. In the section of "Lei Feng story moisten the childlike innocence", the volunteers learned to sing the song "Learn from Lei Feng", and let the children draw the "Lei Feng" in their minds, so that the spirit of Lei Feng can take root and sprout in their young hearts."The 'not' love, spark pass" volunteer service practice since 2018, annual summer and winter vacation in taixing city to carry out left-behind children care action, for taixing city boundary village, as village, south lake village, 15 villages, more than 600 children, teaching more than 120 section, and taixing city of minors protection association, not love public together to focus on the local left-behind children, help them grow up healthy and happy.

2021-03-08	Henan Agricultural University: College student youth volunteer service team helps the rural revitalization drama, but also can improve the oral English ability. Teacher, will you still come here again?"Said Xiao Li, a student of Henan Agricultural University, head of the volunteer service team of Henan Agricultural University, at the Central Primary School in Taiqian County, Puyang City, Henan Province, on March 5. Chen Yanli replied with a smile, " Of course! After we can meet every week, think the teacher can also often video!"It was a conversation between the child and the teacher after the volunteer service. To guide the masses of young students inherit lei feng good quality, practice of volunteer service spirit, better service to rural revitalization, the new era, on March 4, the 58th "learn lei feng anniversary" and 22 China youth volunteer service day approaching, Henan agricultural university "learn lei feng" youth volunteer service to front county to carry out the "learn lei feng" service rural volunteer activities. Li Chengwu, deputy secretary of the Party Committee of the university, encouraged the teachers and students: " The Lei Feng spirit never goes out of date. The young people should study hard, actively inherit and carry forward well. The school encourages the young teachers and students of agriculture, rural areas and farmers to apply what they learn to practical practice, giving full play to the Lei Feng spirit in the volunteer service for rural revitalization, and in the socialist modernization drive."Chen Yanli said:" on the market an English teaching class are tens of yuan or even hundreds of yuan, many families in the village can not afford, when we see the pair of eager knowledge big eyes, feel they have a burden, determined to study more seriously, and strive to improve the quality of teaching to bring better education for the children. We will carry out long-term fixed-point oral communication services online and offline to guide the children in the village to learn oral English pronunciation. It will also be expanded to more schools and serve more students."Teachers and students of Henan Agricultural University also came to Lihu Village, Malou Town, Taiqian County, to carry out the theme practice activity of" Let the light of civilization illuminate the new journey ". They explained and distributed the Regulations on Promoting Civilized Behavior in Henan Province to the local people to strengthen the daily cultivation of the civilization. Zhang Yunfan, the instructor of the Qingma Society of Henan Agricultural University and the young teacher of the School of Marxism, explained the history and feelings of the Party for the villagers. From "to sacrifice more ambition, dare to teach the sun in new day" ambition, talking to "only the communist party can save China, only socialism can save China" simple truth, again from the party's one hundred glorious achievements, carry forward the country feelings have Dang, keep in mind the mission development, zhang yunfan talk deeply attracted the villagers. The teachers and students of the Department of Sociology of Grammar College of the university give full play to their professional characteristics to carry out professional service activities for the empty nesters in Lihu Village. Social workers lead the elderly to learn pat pat exercises, physical exercise, activity of the elderly hands, wrists, elbows, shoulders, waist, etc."Paiexercise" action is small, less energy consumption, long-term adherence can play a role in slowing the physical function of the elderly. With the help of such study groups, the social workers hope to benefit the physical and mental health of the elderly, and to enhance their understanding and feelings through interactive learning. The school rural revitalization of graduate volunteer service in SunZhuang modern agriculture demonstration park research, agricultural college PhD Lv Weize said: " I to research this time, think my lotus root experiment can be used here, the village large river empty idle is also a waste, development of lotus root economy will help farmers income, I would like to do a good job of support for them."Food science and technology college graduate student silver peak said, he can contribute in food processing, can be the laboratory years accumulation of various deep processing technology applied to the local agricultural products," on the one hand, can improve its economic value, increase the income of the people, on the other hand, can also solve the problem of some unsalable agricultural products preservation and storage."Zhao Zhen, a graduate student at the school of Economics and Management, has some views on Taiqian county's rural economic planning. He said: " Building a new socialist countryside is a major measure to strengthen the work of agriculture, rural areas and farmers and promote the improvement of the rural social economy. I am willing to contribute to the rural economic construction with my professional knowledge and combining with the innovative ideas of the new era."Wei Wei, secretary of the Youth League Committee of Henan Agricultural University, said:" The Youth League Committee will establish a long-term mechanism for rural revitalization. This pilot activity in front of the stage is a good start. In the future, we will connect the rural revitalization project, so as to build a platform for young people to display their talents and actively solve practical problems for local governments."According to Wei Wei, after the activity, the school will set up Henan agricultural university rural revitalization of youth volunteer service center, and in front county Henan agricultural university college students volunteer service base, to meet the actual needs of the masses, around poverty engines and rural revitalization, etc., cultivate and practice the new era civilization practice volunteer service, motivate young students learn lei feng spirit, be contemporary lei feng, firm ideal faith, keep in mind the mission, in the volunteer work, take concrete actions to write a new era of lei feng story, contribute to the service rural revitalization of youth.

2021-03-08	East China University of Science and Technology: The "junk bank" is the "stored value" financing institution of the "green city" account. At East China University of Science and Technology, there is a "junk bank". After 10 years of environmental protection inheritance, this environmental protection practice and science popularization volunteer service project named "Garbage Bank" has accumulated a valuable "wealth" - -recycling more than 12,000 jin of garbage, reducing carbon emissions by 28.81 tons, organizing 187 practical activities, and affecting more than 34,000 teachers and students. The "green seed" planted in the hearts of Huali young volunteers 10 years ago has also grown into a green shade of the campus today. The "Garbage Bank" project was initially "cold eye" also encountered. Neither the small interaction nor the small game of garbage classification can stimulate residents' enthusiasm for garbage classification."Keep doing your own thing well, regardless of what others say or think."Shao Wenli, a member of the Youth Volunteer Association of Huali Capital and Environment College, always uses this sentence to encourage herself and her friends when she encounters setbacks.500,800,1000.............. more and more students join in the environmental volunteering campaign."Garbage banks", which are constantly injecting positive energy, are "storing value" for "green city" accounts. From 2010 to 2020, from the original advocacy of garbage sorting to the current technology for environmental protection, team members also expanded from Institute to Mobile College. From the initial mechanical design and automation research to the research direction of garbage classification, to the final development of a "full sense of technology" intelligent trash cans. Team members insist on enhancing the practical effectiveness through scientific and technological breakthroughs, so that the scientific and technological achievements can benefit more people."During the epidemic, online discussions almost every day. After school, whenever I have time, I will find local governments to study and discuss offline."Said Wu Longyan, the project director of the Mobile College."The moment the garbage falls off your fingertips, then free your hands. Ma Xinling, the instructor of the Smart trash can project, is introducing a new era ICE (It Causes Effects.) Intelligent garbage can, which uses the garbage type identification algorithm, solves the problem of low accuracy and high cost of a single machine vision identification, and solves the problem of difficult garbage classification and high classification cost from the source. It is reported that students from Ma Xinling's project team have published 4 core journal papers and obtained 3 national patents. At present, the "garbage bank" volunteer service project has been carried out for 10 years, more than 1800 volunteers, organized 52 community garbage classification science publicity, 141 stalls, covering more than 10 streets in Shanghai, more than 13000 people, 165 classes in 3 primary schools, more than 2000 students; published 232 popular science articles online, people can understand the meaning of garbage classification anytime and anywhere, help environmental action."We will cooperate with more communities and primary and secondary schools to regularly publicize environmental protection knowledge to residents, and answer the environmental problems encountered in residents' life and environmental protection related rules and regulations with professional knowledge."Shao Wenli said.

2021-03-06	Correspondent, Luo Fusheng, Yuan Zhou Miqi and Wu Weize) Young students of Hainan University are brave in epidemic prevention and control, and have returned to their hometown with volunteer services. Winter vacation in 2021, Hainan university league youth actively participate in hometown epidemic prevention and control, active in the outbreak against the "battlefield", their war "disease" footprint has been deep into Hainan, Anhui, Fujian, Shandong, Henan, Yunnan, Ningxia, shaanxi, Inner Mongolia, Zhejiang, Heilongjiang, Xinjiang, Chongqing and other national more than 20 provinces (cities, autonomous regions) community and rural grassroots line, with practical action to carry forward the epidemic positive energy, with youth bear power the epidemic prevention and control war without smoke. Wang Zihao, a student from Hainan University, participated in the epidemic prevention and control work in his hometown. Correspondent, Luo Fusheng for figure zihao students is Hainan university tropical crop college 2018 agricultural resources and environment professional undergraduate students, home in huimin county, Shandong province, is a part-time cadres of Hainan university, in early 2020 had volunteered in the forefront of epidemic prevention and control, to assist the community to bayonet check, temperature measurement, information registration, personnel verification and other work. He also assisted the Communist Youth League County Party Committee to organize the first youth work joint construction committee in Huimin County, and recruited more than 80 young volunteers to participate in the epidemic prevention and control work. During the winter vacation, Wang Zihao joined the volunteer team in his hometown again, reported to the community and participated in the epidemic prevention and control work, and actively cooperated with the community to make all kinds of statistics and reports during the epidemic prevention and control period. Wang Zihao volunteered to participate in the "war" for two consecutive years, explaining the responsibility and responsibility as a youth league member. Shu Wei, a graduate student from Hainan University, participated in the epidemic prevention and control work in his hometown. Shu Wei, who lives in Jixi County, Anhui Province, is a 2019 master's student in Ecology from the School of Ecology and Environment of Hainan University. On January 29,2020, during a critical period of national epidemic prevention and control, As a graduate party member, volunteered to join the front line of Yunxi County to complete the distribution of 800 masks and 200 bottles of medical alcohol every day at the epidemic prevention headquarters office. In February 2021, Shu wei once again responded to the call of the Communist Youth League County Party Committee, and became an epidemic prevention and control guide, who took the initiative to work in the most difficult places. The epidemic is an order, and prevention and control is a responsibility. The Youth League Committee of Hainan University encourages and guides the young people to actively participate in the joint prevention and control of the epidemic on the premise of their own safety protection, so that the youth can shine at the forefront of the epidemic and the league flag can fly at the forefront of the battle against the epidemic.

2021-03-05	Chaohu College volunteer service culture month launched relay volunteer teaching village 15 years Chaohu College volunteer service culture month launched relay volunteer teaching village 15 years China Youth Daily youth network reporter Wang Haihan) this afternoon, 2021 Chaohu College volunteer service culture month launch ceremony and Xiewang Primary School "Tangshan Love School" opening ceremony was held. Chaohu College student volunteers have been volunteer teaching in the village small relay for 15 years."Rural revitalization of rural beauty, to achieve agricultural modernization......." the activity began, Chaohu City Light Primary School red scarf new era civilization practice publicity team brought the theme of publicity. Six young team members presented the touching stories and style of young people fighting the epidemic and fighting floods. Chaohu University leading teachers and volunteer representatives donated books and stationery to Xiewang Primary School; Chaohu Municipal Party Committee presented the schoolbag and stationery and other micro wish gifts to the children. Chaohu University has been attached to Xie Wang Primary School since 2007. Every summer, a group of enthusiastic big friends and a group of lovely children will gather on the campus, where college students will carry out volunteer service activities such as science popularization, outdoor development, psychological counseling and academic assistance. Huang Chuang, principal of Xiwang Primary School, said that nearly half of the nearly 200 students at the school are left-behind children. Many children usually do not talk much, very introverted, eager to contact with the outside world."The arrival of college student volunteers has enriched the school curriculum and made the children develop a positive and optimistic character."Hu Xile, a student from Xie Wang Primary School, said," Thanks to the volunteer brothers and sisters for their meticulous care. Although my parents are not around, we can learn and entertain in the school children's home. We will use the time to prove that we will not live up to your help and expectations."Xu Panpan, a junior student of Chinese language and literature of the" Tangshan Love School ", said that during the journey, he has gained good memories and learned valuable experience in carrying out activities."Before the volunteer teaching, more than a dozen of our team members took time to discuss the students' curriculum, combined with their own major, prepared a variety of curriculum arrangements, took turns to give trial lectures, and worked together. I also understand so much more about the importance of teamwork. Before the end of "" teaching, I promised the children and would come back to see them. I will also bring more humanities and scientific knowledge to them."She said. It is worth mentioning that, as early as 2010, Chaohu College established the first "Tangshan Love School" in Chengbei Primary School, which also became a volunteer service project of the school's youth League committee. In 2007, Chaohu University also built a "Communist Youth Library" in Xiewang Primary School. Up to now, Chaohu University has set up 23 "Tangshan Love School" volunteer teaching centers in the township primary and secondary schools around Chaohu City, and is committed to building the "Tangshan Love School" into a typical volunteer service brand among similar colleges and universities in Anhui Province. The school every year to lei feng month, international volunteer day anniversary as an opportunity to actively organize college students volunteers into the community, into the countryside to carry out love student, cultural propaganda and other kinds of volunteer service and social practice activities, founded the "Tangshan love school", pupils environmental education, and other brand activities, establish volunteer service base more than 50. In recent years, Chaohu City has actively promoted the volunteer service action of "one school and one school, one town, one product '". Volunteers of Chaohu University extend their volunteer service to all fields of regional economic and social development in combination with their professional directors and the actual needs of all towns and townships in Chaohu City."Volunteer service brings knowledge and joy to children, and also provides a platform for college students to practice social practice. In communication, they contact the society, understand the team spirit, increase their talents, develop love, and feed their own growth."Ruan Aimin, deputy secretary of the Party Committee of Chaohu University, said that generations of volunteers have combed through their experiences, shared their experience and inherited the spirit, making the brand of volunteer service brighter and brighter.

2021-03-04	Guangdong ocean university practice team to retrace the red road inheritance revolutionary spirit of Guangdong ocean university Guangzhou city red practice team practice inheritance revolutionary spirit wei correspondent zeng) in order to further explore the precious wealth of red culture, carry forward the three revolutionary struggle spirit of the communist party of China, recently, Guangdong ocean university suangzhou city red practice team to Guangzhou yuexiu district shirt the communist party three conference memorial to visit learning and questionnaire survey, deeply understand the spirit of the revolutionary martyrs. On the day of the activity, a total of 205 valid questionnaires were obtained, with an efficiency of up to 82%. According to the survey results, more than 90 percent of the investigators are interested in the red culture, especially the historical buildings and representatives related to the three major CPC meetings. In addition, more than 85% of the investigators conduct video learning to acquire red culture information; about 70% of the investigators browse the newspapers and magazines to enrich their knowledge and improve their political consciousness; about 50% understand the development history of the CPC through classroom learning, so as to strengthen the lofty faith of CPC leadership and promote the spirit of revolutionary culture. In the following time, the team members will continue to pay attention to the red culture, especially the historical heritage and cultural connotation contained in the three major Communist Party of China, and carry forward the revolutionary struggle spirit of the Communist Party of China ancestors team. To stay true to our original aspiration, we must always learn from our heart to brocade and contribute to inheriting the gene of the red culture and helping to realize the Chinese dream of national rejuvenation.

2021-03-02	Wang Haihan, a youth league member of Anhui Engineering University who returned to his hometown to carry out a red education practitioner.) " We took the opportunity of social practice back to our hometown to visit the red education base nearby and share our experience online."During the winter vacation, more than 30 students from Anhui Engineering University formed a red gene research team to carry out the online and offline red special activity of" Investigate the Red Exhibition Hall, Inheriting the revolutionary gene ", to feel the red spirit and present the century of the founding of the Party. During the activity, Xu Wei, a member of the presidium of the student Union of the university, led 9 members of the student Union to conduct interviews and research in the red memorial hall in their hometown. Through the excavation of the revolutionary history of their hometown, the students put forward suggestions and suggestions on the development of red scenic spots and the promotion of red culture. Grade 2020 students before winter vacation invited 10 different professional students formed the "red gene" research team, successively in jinzhai red army square, crossing the river battle general committee site, red 28 army military and political site, the new fourth army headquarters memorial, martyrs memorial in the south of Anhui incident, huai hai campaign memorial and fengyang Xiaogang village visit, to carry out the party history learning education. Some memorial halls were closed due to epidemic prevention and control. Zhou Shiyang and other 10 students from the second classroom operation center of the school formed the "Online Red Pavilion" research team. On the basis of extensive access, they collected and selected 10 national and 20 local online red pavilions for centralized research and analysis. Zhou Shiyang said: " With the development of information technology, many online red pavilions now make full use of VR technology and other modern technology means to effectively enhance the interaction of learning and education, and further enhance our experience of red culture."During the survey, the students also conducted online questionnaires and offline interviews, covering students from many universities in Hefei, Wuhu and Maanshan in Anhui province. Among them, nearly 90 percent of the respondents said they knew the red culture and red base in their hometown.

2021-03-02	The key support project "College Students' Bilingual Volunteer Service helps the communication and integration of all ethnic groups" has successfully completed the implementation of the project. According to the relevant person in charge of the southwest university for nationalities youth league, Chinese bilingual volunteer service around casting the Chinese nation community consciousness of the main line, combined with volunteer service, professional practice and popular science education to ethnic minority areas and communities, to "normalized, scale, specialization, specialization" as the construction goal, to carry out the bilingual volunteer service, promote all ethnic exchanges. The project was awarded "the 8th Sichuan Provincial Youth Excellent Volunteer Service Project". In the face of the sudden outbreak of COVID-19, the members of the bilingual volunteer service group actively participated in the community (village) grass-roots youth service volunteer team, and made use of their own language advantages to carry out scientific epidemic prevention knowledge publicity."We offer bilingual services that teach older people from ethnic minority areas how to wear face masks correctly."According to Gao Gang, president of the university Youth Volunteers Association, in the community of Airport Road from the university, the proportion of residents and the elderly from ethnic minority areas is relatively high. While providing them with bilingual services for the elderly, college student volunteers also organized publicity activities for epidemic prevention and control knowledge throughout the year. During the outbreak, at the gate of Chengdu 363 Hospital, volunteers from the "Loving Medical Care and Public Health Service Group" organized by Southwest University for Nationalities helped residents from ethnic minority areas use their health codes, take their body temperature, and provide bilingual guidance services. Ji Jie, president of the Red Cross Society of Southwest University for Nationalities, said, " Most of our activities have always been in the school. When we walk out of school and practice the spirit of 'humanity, fraternity and dedication', I feel very proud with practical actions."According to the introduction, the service group insisted on the combination of volunteer service and thought infiltration, the business school" red heart dream "bilingual volunteer service team to Aba Tibetan and Qiang Autonomous Prefecture, Sichuan Province, organized a class meeting around" casting the awareness of community of the Chinese nation "; the Chinese bilingual service group regularly to Chengdu wash bridge community to provide pair assistance, provide text translation, psychological comfort and legal aid services. The bilingual service group also combines professional knowledge with Chinese bilingual science popularization to help fight poverty alleviation in ethnic minority areas. In the summer vacation of 2020, the bilingual science popularization and summer social practice team of the School of Animal Husbandry and Veterinary Medicine of Southwest University for Nationalities went to Hongyuan County for the third consecutive year to carry out a bilingual science popularization and practice activity with the theme of "sending scientific breeding technology to pastoral areas, disease prevention and control and helping poverty alleviation"."Local yak breeds have poor production performance, serious group degradation, and the low reproduction rate of the traditional breeding mode, and the high mortality rate of calves in winter."Let Gengzhou is a herdsman in Rangkou Village, Rangkou Township, Hongyuan County. How to solve these problems in yak breeding is directly related to the livelihood of his family."The teachers and students of Southwest University for Nationalities came to us and brought yak heat us technology and artificial insemination technology in the same period, which improved the breeding efficiency and calving rate. The survival rate of calves increased a lot, the number and quality of fattening of yaks increased, and our income also increased."Let the more state say. Peng Zhongli, chief expert of yak Industry Service Group 8 of Sichuan Science and Technology Poverty Alleviation Tour and professor of Animal Husbandry and Veterinary Medicine of Southwest University for Nationalities, including 10 teachers and 12 Tibetan-Chinese bilingual students, Deep into the plateau pastoral areas, A number of special bilingual popular science lectures have been held, such as "yak scientific breeding technology", "application of knowledge and technology of calf diarrhea disease prevention and control" and "practical technology of wohuan oat planting", More than 500 volumes of Tibetan and Chinese bilingual popular science brochures, such as "Application of Knowledge and Technology for diarrhea and Disease Prevention and Control in Calves" and "Healthy and Efficient Yak Breeding Technology", have been issued, More than 2,000 copies of milk powder, feed premix and commonly used veterinary drugs and other practical materials, Field investigation visited Maiwa Township Dakunima yak breeding factory and other breeding cooperatives and industrial parks, A comprehensive understanding of the development of the local animal husbandry industry, And targeted to give herdsmen and grass-roots animal husbandry staff guidance and suggestions."We conducted short-term fattening according to the method provided by the teacher, and the fattening effect was obvious."Said Wu Qiuwuyou, a local yak farmer."In the next step, we will strengthen the construction of service groups and volunteer service work, and promote the upgrading of volunteer service capacity in the context of the normalization of epidemic prevention and control."Fang Xiaowu, secretary of the Youth League Committee of Southwest University for Nationalities, said," The university will rely on the 'volunteer Sichuan' and the 'second classroom transcript' platform to promote the informatization and standardized certification and management of volunteer service, and strengthen the construction in the aspects of identifying the service time and obtaining extracurricular practice points.”

2021-02-25	Yu chapter normal college youth students in "home" epidemic prevention service yu chapter normal college youth students in "home" epidemic prevention and control volunteer service should school youth corps committee initiative, according to the requirements of the superior youth league organization, combined with school winter vacation social practice, will participate in the nearby "home" epidemic prevention and control of volunteer service activities. Gong Yue, who lives in Chongyi County, Ganzhou City, is a 419 environmental engineering student majoring in the Department of Ecology and Environment of Yuzhang Normal University. She is actively enrolled in the front line of epidemic prevention and control. She visited the area she was responsible for every day to publicize the epidemic prevention and control policies, epidemic prevention measures, put up posters to the residents, and got familiar with the flow of people among the 76 households in the area. Zhu Jie, a student of Class 418, Chinese Language and Literature (1), College of Culture and Tourism, lives in Xuanzhou District, Xuancheng City, Anhui Province. She volunteered to provide volunteer services on the front line of epidemic prevention and control. Every day, the migrant workers and students inquired by phone, and made detailed records of their work and study place, holiday time, whether they have plans to return home, how to return home, etc., and told them to go to the designated hospital for nucleic acid testing 24 hours after returning home. Jian Yiran, who lives in Yushui District, Xinyu City, is a targeted normal university student in Class 518 (8), Primary Education College. This is her second time to participate in the epidemic prevention and control volunteer service activities. She is responsible for two places with large traffic: Jiyang Community and Xinyu non-staple food wholesale market. Arriving at duty at 8:30 every am, responsible for carrying masks, disinfectant, alcohol and other materials, urging residents and businesses to scan the code to measure the temperature and check whether they are wearing masks. Chapter normal college this winter vacation social practice mainly with online team and individual dispersed practice, which participate in volunteer service, in home construction, power the epidemic prevention and control of chapter normal college students have hundreds, they use practical action to meet the 100th anniversary of the party, realize self-worth in volunteer service, writing wonderful life chapter.

2021-02-24	Anyang Normal University: Implement the spirit of General Secretary Xi Jinping's important speech at the Symposium on Ecological Protection and High-quality Development of the Yellow River Basin, According to the work requirements of Henan Provincial Youth League Committee of the Communist Youth League to "return to home for college students" social practice activities along the Yellow River, On the basis of strictly following the local epidemic prevention and control, Carefully organized and made solid progress, Set up the "River Xiaoqing" to help the Yellow River Basin ecological protection practice group, the "River Xiaowen" in the Yellow River Basin intangible cultural heritage youth research, exchange and practice group, Leading young students to focus on the ecological quality of the Yellow River, Help to promote ecological protection in the Yellow River basin, Tell the "Yellow River story" well, Keep the cultural heritage well, To contribute to the ecological protection and high-quality development of the Yellow River Basin. Practice activities, Ann students wrote professional research report of the Yellow River ecological environment problem present situation analysis and countermeasures, interview record three, feeling nine, recording short video and environmental protection class six sets, edit the Henan Yellow River basin intangible project propaganda series, the original product new media article product back home along the yellow social practice, a figure read ~ Ann students do so, harvest rich practice, got the Ministry of Education Chinese college students online, xiaoxiang morning paper, image network, Henan higher education and other media reports. Protect speak good "Yellow River story" keep good cultural heritage from education college, school of politics and public administration, literature, economics, 35 live in the Yellow River home Ann students, to tell "Yellow River story", keep good cultural heritage, continue the historical context as the theme, form a "river small" intangible cultural heritage of the Yellow River youth studies exchange practice group. Focus on hometown intangible cultural projects, in the form of online search, research, compiled the Jiyuan Yellow River boatman song, Xinxiang YueJiaQuan, Luoyang helo drum, front county dahong boxing, Chen jiagou taijiquan, Puyang northeast zhuang acrobatics, kaiyang more than 60 palace lantern intangible cultural heritage project text, pictures and audio and video data, edit the Henan Yellow River intangible project propaganda series ", speak" the Yellow River story " in practice communication, firm cultural confidence, for the Yellow River basin intangible cultural heritage contribution. Students from the School of Resources, Environment and Tourism, the School of Chemistry and Chemical Engineering, and the School of Media focus on the ecological quality of the Yellow River, Set up the "River Xiaoqing" to help the Yellow River basin ecological protection practice group, Fully combined with their own professional, On the premise of complying with local epidemic prevention and control requirements, In Henan Zhoukou, Zhengzhou, Sanmenxia, Xinxiang, Jiyuan, Puyang, Kaifeng, Shanxi Changzhi and other cities along the Yellow River through the questionnaire survey, field research to understand the current situation of the Yellow River and the problems faced; Through one-to-one river protection propaganda, to littering tourists and the masses to persuade; Make water resources flash mob video, environmental protection micro class and other forms to publicize the knowledge of protecting the Yellow River, And let the nearby residents realize the importance of protecting the ecological environment of the Yellow River basin. The the mountains, turn the ravines, the Yellow River from ancient times, to the Chinese earth painted a powerful stroke. The members of the "River Xiaoqing" practice team answered their understanding of the Yellow River, their feelings of investigation and investigation, and what we should do to protect the Yellow River in the novel form of short videos. The micro class "The Development and Governance of the Yellow River" focuses on introducing the causes of the ecological and environmental problems in the Yellow River basin and the prominent development or prevention problems in different river sections, so as to arouse people's attention to the protection of the Yellow River. Micro class "Protect water resources, start from saving water" micro class introduced the current situation of water resources in China and Henan Province, the causes of water resources shortage and how to protect water resources to improve the utilization rate of water, enhance the importance of protecting our mother river water resources. The micro class "Protect Mother River Day" introduced the origin and the content of the festival activities, calling on more young people to join the Mother River protection action. Micro class "Protect Mother River, Start from Garbage Classification" micro class introduced the impact of garbage on the river ecological environment, the common types of garbage and how to do the garbage classification, calling on everyone to consciously do a good job in garbage classification and reduce water pollution. Original practice along the yellow cartoon draw youth style from the academy of fine arts students, will return home along the yellow social practice situation, in the form of diffuse cartoon creation, vividly depicts the practice players conduct water quality monitoring, civilized persuasion, river patrol river, recording "environmental cloud classroom", cultural search, intangible research and studies volunteer image, shows a "home" along the yellow social practice beautiful youth picture scroll. In the social practice of "returning to home" along the Yellow River, the young students bear in mind their entrust, carry the mission of protecting the Yellow River with the power of youth, sing the Yellow River, and measure the magnificent mother of the Yellow River with their youth footprints. Let us clench our fingers into a fist, let the flowers of youth bloom on both sides of the Yellow River, sing the most beautiful Yellow River chorus!

2021-02-23	Ten thousand students of Guangxi Normal University sang the theme of the era of Jianwei, Xiong Wenkai, Gui Xiao, Huang Huaqiu) " Come! Let me teach you to sing a red song!'Without the Communist Party, there would be no new China, the Communist Party worked hard for the nation, the Communist Party he dedicated to save China.....' " loud singing, smile filled with the faces of young students, the theme of The Times lingering in everyone's heart. To commemorate the 100th anniversary of the founding of the communist party of China, let the new era of the party's singing in China, Guangxi normal university youth league committee held in 2021 winter vacation during "the party's song" red song activities, called on the students to people around sing classic red songs, tell the party history story, and through the "cloud recording" sung video, tribute the anniversary of the founding. Up to now, the activity has covered more than 20 provinces and autonomous regions across the country, with tens of thousands of students participating in it. Listen to the stories of our ancestors and praise the glorious course."Reviewing that period of history, I truly feel the hellfire experienced by the Communists. From childhood to adulthood, inspired by my grandparents, I always remind myself that I can not forget the suffering once, but to face any difficulties and obstacles with more firm faith."Yang Ying, a teacher at the Conservatory of Music, said of the founding of the Party. Foreign languages institute 2020 foreign language and literature professional Xu Yipeng in Sichuan hometown to learn from a veteran and sing red song "no communist party there is no new China" and "the east is red", listen to veterans to the party's exclusive memory, feel the older generation of the communist party member hard struggle good quality, " I hope you can do a like a communist party member can bear hardships, understand the struggle."Xu Yipeng was encouraged by hearing what the veterans would say."When I sang" No New China, No Communist Party, " although her voice was a little hoarse, I could still see the twinkling tears shining in her eyes. Grandma said that the red song sounded, the heart will have faith, life will have the strength. I think the youth of the new era should also feel the passionate and burning years of their ancestors in the red songs, so as to encourage us to ride the wind and waves and continue to move forward."Said Feng Lu, who majored in Chinese international education in the School of Journalism and Communication, School of Arts. In the activity, the majority of students learn to sing red songs from the older generation of comrades, listen to the story of the Party, feel the glorious course, let the party spirit cultivation in the song improve, let the ideal and faith in the story more firm. Singing great river north and south, singing series spiritual activities, the students through chorus with their family, using the network platform cloud attachment, singing in hometown, express their patriotic love party pure heart, inheriting the excellent quality of the Chinese nation and red spirit, actively embrace the new era, new era, glow more brilliant brilliance in dedication. The singing videos recorded by the student organizations and associations of the school are also wonderful, showing the youthful vitality of the students. The chorus of Guangxi Normal University, vocal band and Acabella Team performed the red songs in different singing methods, bringing an auditory feast. Foreign friends from the International School of Culture and Education sang the complete song "No Communist Party, no New China" in Chinese to express their sincere wishes for the 100th anniversary of the founding of the Communist Party. In the singing activity, the students not only learned from the predecessors, but also passed on the great spirit to the younger generation through the singing. Sports and health college 2020 sports training professional Tang Li students said after attending the activity: "I teach at home seven-year-old brother learned to sing" no communist party, no new China, " I myself by the red song fighting spirit of the infection, hope oneself in the future to constantly learn from the elder party members, improve, but also can drive the people around progress together, their youth into the development of the construction of the motherland."Long Yi, a major in ideological and political education at the School of Marxism, said," I stood in the Nanchang Uprising Memorial Hall and was beside the young pioneers singing "No Communist Party, no New China.""He said that in the activity of his heart excited, as a new era of new youth, more to learn from excellent Chinese Communist Party members, dare to be the first, willing to contribute, with practical actions to drive the surrounding people around, with the strength of youth for the construction of the motherland. It is reported that the Youth League Committee of Guangxi Normal University attaches great importance to the "red song singing activity of the Party singing", carefully planned and implemented, gave full play to the appeal and influence of the school Youth League Committee, and led the ideological construction and promotion of the youth League members. In the activity, the students to learn from predecessors, inheriting the revolutionary spirit fire, to the junior, lit the torch era spirit, the activity form rich, including chorus, a landmark, landmark, dialect series of activities, and through video record song and story, the school will sing many youth sung "story" made into tribute of the "big story" in one hundred, all-round, multi-level show normal university member youth youth.

2021-02-15	Learning propaganda civil code of Jiangsu university youth learning in action to promote civil code of Jiangsu university youth Jianwei correspondent in action, Shi Xiangyu Shao Feng) under the new situation, Jiangsu university law school "civil code day" practice team online, follow the principle of convenient nearby, in their hometown (Jiangsu Suzhou, Lianyungang, Yangzhou, Taizhou, etc) to carry out the civil code propaganda practice, through offline research, online preaching to make the civil code to the masses, into the masses in the heart. This year is the first year for the formal implementation of the Civil Code, and the Civil Code is closely related to the lives of the people. During this practice, the team members learned more about professional knowledge, and promoted the people to learn the Civil Code, which helped to build a good atmosphere for the people to know and abide by the law, study and protect the law. Through online questionnaires, offline interview research analysis, practice team mastered the ordinary people for the understanding of the civil code and learning requirements, team members accordingly to make the approach to the civil code, open the civil code series video and published in WeChat public, weibo, B station, TikTok platform, with the help of new media platform to further promote legal knowledge, improve legal awareness, strengthen the rule of law publicity and education. Team members conduct a questionnaire survey in the community. The correspondent practice team released the "Into the Civil Code" series push with the help of wechat public account. Correspondent for the photo team members to record the "Open the Civil Code" series of videos. Correspondent for figure the series of video selection in the civil code about residence, personal information protection, divorce cooling-off period, will inheritance and other provisions are closely related to residents 'life, through the way of combining with typical cases to interpret the relevant provisions of the civil code, strive to professional legal language speak simple, easy to understand, into the residents' life. In order to further improve the publicity, the practice team, on the premise of epidemic protection, publicized the civil Code in local communities, middle schools and village enterprises, and issued publicity brochures of the civil Code to the community elderly, middle school students and enterprise employees, urging residents to protect their rights and abide by the law. A flyer made by the team members. Members of the team distributed leaflets to the community residents. Members of the team preach about the civil code at local middle schools. Correspondent for the picture

2021-02-11	Wang Yejie, a temporary Youth League branch) This winter vacation, nearly 400 youth league members of Donghua University colleges chose to stay in school. In order to enrich the life of the youth, the Youth League Committee of the school set up a temporary league branch for the students, online and offline, the menu activities are constantly surprised, so that the youth members warm around, warm in their hearts. In the past few days, the temporary youth League branch of the school students has carried out activities such as library surprise sympathy, chef teaching you to make dumplings, a greeting, a bottle of yogurt, a cultural creation, a plate of dumplings..., a small move, let the warmth in the school members pass. Ren Chutong, a student from the School of Computer Science, said after receiving the surprise yogurt from the library, " Suddenly there is a feeling of home. Mom and dad, please rest assured that I am very good for the Chinese New Year in Donghua!"It is reported that the youth members also gave the warmth to the canteen master who accompanied you for the New Year, and sent warm blessings to the members of the logistics teachers who have been warm heart for the league branch. In addition, the Youth League Committee of Donghua University has launched various online and offline learning packages in combination with the learning needs of the remaining students. Library offline learning punch card, online reading notes, cloud self-study and other activities, to meet the needs of all kinds of learning circles, to help the school students to make good teachers and helpful friends in learning, and to develop learning habits in the persistence. The school Youth League Committee also focused on the ideological guidance, launched the "Ten Winter Holiday Lesson", "Xi Jinping and College Students Friends" reading exchange to guide the students to learn new knowledge and broaden their new horizons. In order to encourage the students to insist on physical exercise during the holidays, the school Youth League Committee set up an online sports WeChat group among the school students. Students meet in the group, walked off the Internet, walked out of the dormitory, and walked to the playground together.

2021-02-15	Chinese academy of tropical agricultural sciences held member youth special learning exchange will strengthen the national strategic science and technology support leading hot area rural revitalization of the Chinese academy of tropical agriculture held youth special learning exchange Jianwei correspondent Zhang Shaoshuai) recently, the Chinese academy of tropical agricultural sciences youth corps committee to "strengthen the national strategic strength of science and technology support leading hot area rural revitalization" as the theme of special learning exchange, called on floor youth remember the mission, clear main direction, enterprising, bear, strengthen the national strategy of science and technology strength, support leading hot rural revitalization. College Youth League Committee Secretary Zhu Anhong attended the meeting and made a speech. At the learning and exchange meeting, Secretary Zhu Anhong led the spirit of the Fifth Plenary Session of the 19th CPC Central Committee, the Central Economic Work Conference, the Central Rural Work Conference and the Director Conference of the National Department of Agriculture and Rural Affairs, A comprehensive interpretation of the college's 2021 work report, He stressed that, As the backbone of tropical agricultural science and technology innovation of the college, To firmly establish the lofty ideals, Firm in the political conviction, Guided by Xi Jinping Thought on Socialism with Chinese Characteristics for a New Era, Unswervingly implement the new development philosophy, Building a new pattern of development, We will give full play to our youthful talents in building a world-class tropical agricultural science and technology innovation center, comprehensively improving the supply capacity of science and technology, supporting and leading rural revitalization in hot areas, and accelerating agricultural and rural modernization. More than 90 youth League cadres, outstanding young representatives and scientific research backbone of the institute actively spoke on the theme of "how the hot science youth play the role of 'leading goose' in their work, make contributions in strengthening the national strategic scientific and technological strength and supporting the rural revitalization of the hot area". It is reported that in the next step, the Youth League Committee of the Chinese Academy of Thermal Sciences will focus on the two strategic destinations of China hot area and the world hot area, play the vanguard and exemplary role of the youth members of the Academy, shoulder the responsibility of the national strategic scientific and technological force, and provide scientific and technological and talent support for the rural revitalization of the hot area.

2021-02-07	Guangdong communist youth league to carry out winter vacation college students sympathy activities Guangdong communist youth league in winter vacation college students sympathy activities on 7 (reporter Wang Dongwei) on February 3 morning, the Spring Festival is approaching, in response to the Chinese New Year initiative, let college students spend a warm and peaceful holiday, sponsored by the Guangdong provincial party committee, Guangdong federation "youth warm" "warm winter" winter vacation college students sympathy activities in Jinan university. Chi Zhixiong, Secretary of the Guangdong Provincial Communist Youth League Committee, and Wu Yiting, Deputy Secretary of the Communist Youth League, visited the college students and the youth League members through discussions and exchanges, and sent New Year greetings and holiday greetings to them."Although my home is thousands of miles away, but the teacher's company and love let me feel very happy and warm!"Said Tashi Doga, a student from Nyingchi, Xizang Province."I plan to enrich myself during my stay."Although Liang Huiting, a Hong Kong, China student, is the first time for the holiday abroad, she is full of expectations for the holiday planning. She hopes to seize the development opportunities given to young people in the Guangdong-Hong Kong-Macao Greater Bay Area. Zak, a representative from Congo), said: " I felt the warmth of going home at school. The epidemic has been brought under control in China, and I feel that China is very severe!"At the symposium, representatives of students from medium-high risk areas, students from Hong Kong, Macao, Taiwan, ethnic minorities, foreign students and representatives from Guangdong and Hong Kong Association spoke freely and shared their study and life experience and winter vacation plan in Guangdong. The responsible comrade of the Party Committee of Jinan University introduced the experience measures of the university in launching a series of experience courses of excellent traditional Chinese culture during the winter vacation, and the experience of delivering love and sending warmth to the students with a number of measures and multi-care."Each of you is amazing!"Chi Zhixiong asked details about students learning life and holiday arrangements, for positive response to initiative, choose" New Year " to stay in the students thumb up, to stick to work, care for school students teachers, and encourage the young students to study professional knowledge, actively participate in social practice, improve comprehensive quality, temper excellent skills, the personal struggle into the construction of national rejuvenation and socialist modernization power, with honors for the party's birthday in one hundred. It is reported, "youth feeling warm" activities has continued for ten years, is the Guangdong communist youth league at the end of youth people's livelihood practical public welfare brand project, through the travel volunteer service "warm winter action", poor students "nan yue will kiss" and other series of activities, attentively with affection for college students, migrant youth, poor youth, grassroots line young workers, emerging young teenagers to solve problems in production and living, pass the warm care of the party and the government. Since its inception in 2011, it has received the attention and support of young entrepreneurs, outstanding young people and other enthusiastic people from all walks of life. On average, it raises nearly 10 million funds and materials every year, carries out more than 3,000 care activities, invites more than 150,000 volunteers, and directly visits more than 300,000 young people in difficulties. In December, 2020, Guangdong Provincial Party Committee of the Communist Youth League, Guangdong Federation of Industry and Commerce, Guangdong Youth Federation, Guangdong Youth Federation, Guangdong Students' Federation and other units jointly launched the "Spread the wings plan" special action for college students from Hong Kong, Macao and Taiwan, The first winter vacation internship action adopts the mode of "find out the needs first, and then develop the posts later", Attracted 754 students from Hong Kong, Macao and Taiwan from 20 universities such as Sun Yat-sen University, South China University of Technology, Jinan University, Xiamen University and so on to register, Integrating more than 300 high-quality positions, such as top 500 Chinese Enterprises, Top 500 Guangdong enterprises, "unicorn" enterprises, industry leading enterprises, party and government organs and public institutions, Involving finance, technology, medicine, e-commerce, media and other fields. This winter vacation, some students from Hong Kong, Macao and Taiwan stayed in school for the Spring Festival due to the epidemic, and organized an internship in Guangdong, providing a new choice for students from Hong Kong, Macao and Taiwan to stay in Guangdong for the Spring Festival, and helping them spend a full and meaningful holiday.

2021-02-07	Du Lili, Dong Yue) " In the face of the epidemic, respond to the call of the Party, become a volunteer for epidemic prevention and control, and take good protection of my hometown, now it is the happiest thing for me."This is the voice of the volunteers returning home to fight the epidemic. New crown outbreak is still spreading around the world, domestic sporadic sporadic cases and local outbreak risk still exists, the Spring Festival, the current epidemic prevention and control is still in a critical period, Jiangsu university law school of member youth positive response "outbreak together, together to overcome difficulties" spirit, to participate in hometown epidemic prevention and control, active in the outbreak against the "battlefield".(1) Help nucleic acid testing to protect residents' health In order to strengthen the epidemic prevention and control network, To fully ensure the life safety and physical health of the community residents, For the precise "mine clearance" of epidemic prevention and control, On January 29th, The seventh round of nucleic acid testing was carried out for all, Du iley, a returning volunteer, voluntarily participated in the nucleic acid test, Assist grid workers to guide residents to install and register and use the "first teacher health line" mini program at the testing site, Help the elderly and special people to print health codes, Improve the detection efficiency, At the same time, to guide the residents to orderly test, keep a distance, Assist to complete the testing of residents of Jinlu Xuefu Community, Xingfu Road Street, The nucleic acid testing task was successfully completed. Volunteer Dooleley instructs residents to use the "One Division Health Walk" mini program to register their personal information. Correspondent for volunteers to help residents into the area for inspection. Correspondent for figure (2) fill residents information to carry out accurate epidemic prevention community is an important line of epidemic prevention and control, in view of the current epidemic prevention and control situation in Shanghai, to cooperate with sheshan community in epidemic prevention and control work, Jiangsu university law school volunteers dong yue actively participate in Chen Fang residents' committees outside MoPai registration volunteer work, contribute to hometown epidemic prevention and control. In addition to completing the information registration, it also actively publicized epidemic prevention, popularized scientific knowledge of epidemic prevention and control, and guided residents in scientific epidemic prevention. Faced with the heavy work, she said, "Although I am tired every day, I thought that there are many veteran party members in the volunteers, going from home to home and insisting on the frontline of the fight against the epidemic. As a probationary party member, I should learn from them, and make a contribution to the epidemic prevention work."Volunteers will classify and sort out the information of migrants returning to Shanghai to fill in and register in an orderly manner. In the critical period of epidemic prevention and control, the period of "leaving the Chinese New Year" has become the contribution of migrant workers to the prevention and control of the epidemic, at the sacrifice of reunion, only for peace. But many online rumors followed, and Du Lili, a law school volunteer at Jiangsu University, posted a tweet titled "Clear up the Internet and win-win fight against the epidemic" on his community public account. In order to popularize legal knowledge and traditional culture to more teenagers and children, the winter vacation activity of "big hands holding small hands, the rule of law warm winter walk" in the community cultural center, combined with their major to contribute their own strength to the community public service, and advocate a good atmosphere of the rule of law and civilization. Volunteers told teenagers about the glorious history of the 359th Brigade and encouraged students to study hard. The virus is heartless and the volunteer has love. Volunteers of Jiangsu University Law School interpret their volunteer responsibility with persistence and faith, protect their hometown with warmth and action, contribute to winning the battle against the epidemic, and make a touch of "volunteer red" highlight on the land of their hometown.

2021-02-07	Nanjing forestry university students to Wuxi village economic research in Nanjing forestry university students to Wuxi village economic research activities China youth network Beijing on February 7 (reporter Wang Dongwei, correspondent, Feng Qianqian XiaoXiao) overall planning of rural economic construction, political construction, cultural construction, social construction, ecological civilization construction and the party's construction, promote rural industry, talent, culture, ecology, organization, is the inevitable requirement of comprehensive construction of a modern socialist country. At the Central Conference on Rural Work, General Secretary Xi Jinping stressed that solving problems related to agriculture, rural areas and farmers should be the top priority in the work of the whole Party, mobilize the whole Party and society to promote rural revitalization, and make high-quality and efficient agriculture, rural areas livable and working, and farmers rich and prosperous. In 2021 is the centenary of the founding, Nanjing forestry university "Chinese village economy", teachers and students, with village economic change as the research theme, on January 30 to Wuxi 11 village to carry out field research, take practical actions to practice "focus on agriculture, care about rural, care for farmers", track the village economic changes, depicting a new picture of rural development in one hundred. Nanjing Forestry University "China Village Economy" teachers and students research group visited learning Yanqiao "one package of three changes" memorial hall. In the 1930s, 1950s, 1980s and 1990s, the Institute of Economics of the Chinese Academy of Social Sciences carried out the rural economic tracking survey of 11 villages in Wuxi for four times, and analyzed and studied its economic development and institutional evolution."The nation wants to revive, The countryside will be revitalized. ", Based on the content and results of the "Chinese Village Economy" of the Nanjing Forestry University, Once again into Liucang community, Xinan Community, Yudong Village, Zhuangqiao community, Jingxi Village, Linong community, Taihu community, Wutang community, Ma'an Village, Hongsheng Village, Qianjin community and other 11 villages, communities, Through field visits, discussion and interviews, literature review and other channels, To understand the rural construction and basic economic conditions of the 11 administrative villages and communities in the new era, From the dimensions of rural natural geographical conditions, population and assets, party building, public income and expenditure, public infrastructure, villagers' income, village enterprises and other dimensions, Outline the development track of the beautiful countryside in Wuxi, Listen to the story of the "four histories" of rural construction. Students from the research group filmed and recorded the last demolition site in Liucang community. In the investigation process of Wutang Community (formerly Wudang Village), Xuelang Street, Binhu District, Wuxi City, the research group and the secretary and director of Wutong Community discussed the historical development and current situation of Wutang Community. At present, most of tang community still maintains its original rural style, and uses sour plum, peach and bayberry as the main cash crops. In response to the governance policy and environmental protection requirements of Taihu Lake, the farmland in the community has not carried out intensive farming, avoiding the rural non-point source pollution caused by excessive fertilization, and promoting the protection of the water quality of Taihu Lake. Through logistics, the community will ensure the timely sales of fruits and vegetables in the community. In the future, it will use cold chain technology to expand the sales radius and carry out activities such as fruit and vegetable picking culture festival and online fruit and vegetable promotion meeting to promote the development of local agricultural economy. During the interview, the students of the research group deeply realized that farmland also played an important role in ecological protection. By developing ecological agriculture, reducing farmland pollution, and unifying the layout of "fields, water, forests and roads", they helped to improve the environmental governance capacity and internal impetus of the Taihu Lake Basin. The teachers and students of the research group went into the private land of the villagers in the Taihu community. Correspondent for figure nib record village history, footsteps witness change on January 30, morning, the team teachers and students a line came to Wuxi huishan district weir bridge street liu cang community (original liu lane village), this day the community completed the community last 18 households resettlement work, and this day also witnessed the liu cang community last 18 villagers to the residents. Research group students in interview with "old village book", also witnessed the change and development of liu village (now Liu Cang community): village (community) has more and more people involved in the construction, now have an average age of more than 60 years old volunteer team, they provide services for the village (community), also played a huge role in the process; in the development of the village (community), although now demolition live upstairs, using the characteristics of familiar with each other, let "something to discuss" into the corridor, encourage building things, yourself, forming residential building "micro autonomy". On January 30, this day, Nanjing forestry university "Chinese village economy" research group of teachers and students and students and community residents, looking at the blackboard with the community development blueprint, liu cang at the foot of just removed the red brick, nib records liu xiang village "the last day", also witnessed the liu xiang village completely to liu cang community of this important moment. Research group of students in taihu community natural village demolition site. Correspondent for figure party to lead multiple work, grid management data can assign "our community currently has a service center, residents committee, stock economic cooperatives and community workstation", in the research group students asked to the community management service organization, the agriculture, director of the community, this sentence is deeply printed in the research group students. As early as 2010, Linong community was removed from the original Li rural village construction, and is currently a mixed community. Through the interview, the students learned that the four organizations in the Linong community performed their duties in the community party building, public services, collective economy, community affairs and other aspects, to jointly promote the healthy and orderly development of the community. In February 1, in the process of research visit, the students also met the school graduated back to home in the construction of senior, through the "90" senior understand the present situation of community development, the students understand the community through party construction culture, with grid as the fulcrum, realize the full coverage of the grid, and in household platform to community "three public" residents in the home. After the survey, the students sighed: "Community governance is completely different from their original understanding in both the mode and technology application. It is really 'small community, big society, small grid, big responsibility'.". The students of the research group investigated and visited the enterprises in the community. Correspondent for pictures to promote rural construction and improve rural governance. At present, China is in the historical convergence period of the "two Centenary Goals". The research group of teachers and students of "China Village Economy" of Nanjing Forestry University revisited the century-old research road, perceived the transformation of "village-community" with their steps, and recorded the century-old changes of the countryside with their persistence. Through the research, the students deeply realized that every farmland is buried with the story of the country, every village is engraved with the memory of the country, and every farmer has witnessed the century-old journey of China's rural development. To build a modern socialist country in all respects and realize the great rejuvenation of the Chinese nation, the most arduous and arduous task remains in the rural areas, and it still has the most extensive and profound foundation. As a new era of young students, Nanjing forestry university students with the research opportunity, positive response to return home to do rural research, feel rural development, rural social situation, people, take concrete actions to integrate the ideal pursuit of the party and national cause, for the party, for the motherland, make more contributions to the people, for the rural revitalization of youth!

2021-02-04	Ningbo University: The "Ideological and political Micro recess" of Ningbo University: For the time-fragmented ideological and political education exploration course "If Love, Please true love"."The micro-class break refreshes us, not only because it is menu-style, but also because it is tailored to our fragmented time."This semester, the students of Ningbo University have the same expectation during the break- -" ideological and political micro break "."Ideological and political Micro recess" is an exploration of ideological and political education, carried out by the Youth League Committee of Ningbo University. It mainly designs the fragmented time of college students, and plays the effect of natural embedding of ideological and political education and silent education. It is reported that all colleges of the university carry out micro recess activities through on-site teaching and video teaching, with micro classes covering five topics, including current politics, ideal beliefs, youth growth, scientific research and innovation, and public welfare dedication. A total of 21 classes, each class lasts 5 to 8 minutes. By the end of this semester, a total of 377 micro-recess activities have been carried out, covering more than 8,320 people."Love a country, is to help her become better, if love, please true love. After receiving the invitation, Chen Jiawen, the keynote speaker of "Ideological and Political Micro Break", carefully polished every step from collecting materials, preparing speeches to making videos. Her "Ideological and Political Micro-recess" course "If You Love, Please Love, True Love" has become the "most popular" course in the course menu."What do the students like? What do the students like to listen to?"This is a problem that Chen Jiawen often considers. Her understanding of the "ideological and political micro class break" is, " to pull the lofty theory into students 'lives, to show the charm of ideological and political courses in the new era to students with the most lively language and vivid cases, and to actively adapt to the new characteristics of young students' learning and life through innovative forms."Fang Jun, secretary of the clinical 193 Youth League branch of the medical school, was one of the first" listeners "of Chen Jiawen's" ideological and political micro-break ". When the bell rang, the students were not scattered, but sat in the classroom quietly watching the micro class video, " not only will not feel tired, but like relaxing, like in the 'live version' short video, the original patriotism, national conditions, strong country ambition so close to our life. Another benefit of the "" ideological and political micro break "is that there is little need for" organization ", which greatly improves the efficiency and effect of ideological and political education."In the past, the class had to specially organize the theoretical learning activities, or it spent a lot of energy, otherwise a considerable number of students would be late or ask for leave."Said Han Xinyi, secretary of the youth League branch of English Class 2, Grade 2019, School of Foreign Languages. It is worth mentioning that the "ideological and political micro-class break" of Ning University makes outstanding college students sing the "leading role", and two-thirds of the courses are taught by outstanding college students. During the break, the students became as "ideological and political teachers"."In the past, most of the students used Weibo to play their mobile phones during classes. The keynote speakers of the 'ideological and Political Micro recess' were all our teachers and students. The content was vivid and interesting, which pulled all the students back from their mobile phones."Business school 2017 accounting class youth league branch secretary Zhang Yuqi told reporters. Law school level 2017 law students Long Xingkang is one of them, he chose to participate during the jinggangshan revolutionary base learning experience as lecture material, to "diary Jinggang, thousands of red earth find beginner's mind" as the theme, will be in the process of jinggangshan learning diary content and their thoughts, to convey to the students the fine style of the older generation of revolutionaries."I recall again the spirit of hard struggle I learned when learning to weave straw sandals, and reviewed the membership oath under the portrait of Marx and Engels in the Octagon Tower. In the preparation and telling of the 'ideological and political micro recess', my heart was once again baptized."Yang Yu, a 2020 graduate student of Marxist theory at the School of Marxism, chose the spirit of the Fifth Plenary Session of the 19th CPC Central Committee as the theme of" Ideological and political micro recess "."Contemporary college students are the main force to realize the great rejuvenation of the Chinese nation, and it is essential to deeply understand the spirit of the plenum. I want to help students understand more about the national policies and policies through the 'ideological and political micro-break', and set up lofty ideals and beliefs in the study of political theory."Zhang Xiaotian, a 2018 undergraduate majoring in computer science and technology at the School of Information Science and Engineering, was very impressed by the" ideological and political micro break " brought by Rehan, a student of the graduate volunteer teaching group."From the northwest of China to Ningbo, Zhejiang province, and to become a volunteer teacher in southwest Guizhou province, her dedication and tenacity frequently moved the students to tears in the sharing."It is reported that the" ideological and political micro class break " is further exploring the implementation of the tutorial system.3-4 students combined with the professional characteristics, jointly polished a single ideological and political micro-class, equipped with an ideological and political mentor, to form a theoretical learning team. The school Li Baogeng, dean of marxism, " 'ideological micro recess should also gradually realize ideological courses and courses' two legs walk, and the organic integration of various disciplines of professional knowledge system, lead the students online, deepen the understanding of the great cause of socialism with Chinese characteristics, consciously participate in ideological courses and courses with the practice of education."" mobile Internet is profoundly changing the way of information acquisition. Time fragmentation has become one of the important characteristics of college students' daily life. Ideological and political education in colleges and universities should be changed according to things, into the time and new according to the situation, which can enter the brain into the heart into action. Ningbo University has carried out the exploration of 'ideological and political micro class break', which is precisely hoping to strive for the reuse of fragmented time and the innovation of ideological and political course mode through the menu mode of ideological and political education, and further enhance the ideological and political education."Said Tong Xiaohui, deputy Party secretary of Ningbo University. China Youth Daily, China Youth Network reporter Yang Baoguang, correspondent, Zheng Junpeng, Bao Lili source: China Youth Daily

2021-02-02	Xi 'an graduate student volunteers activities in 2020 micro wish completed xi' an graduate student volunteers in 2020 micro wish activities completed the special team, xi'an and Hanzhong county has become a family, since accurate docking, preaching, light wish, with, deep Yang county education career, sowing with love and hope. Time came to a warm winter in 2020, xi'an construction university of science and technology 22nd and 23rd graduate volunteers held the sixth "feeling warm winter light micro wish" in wild goose pagoda and thatched cottage campus held at the same time, from the county four junior high school hard motivated, excellent by inputting tent card students 520 micro wish, attracted the attention of the teachers and students."I want a pen", "I want a document", "I want a badminton racket", "I want a thermos cup"... the children touched the hearts of every member of the research branch group, but also touched the hearts of the teachers and students of CCB. And every micro wish gift collection activities, the collection scene scene crowded, warm atmosphere, just one day, micro wish was built by teachers and students and the community claimed, we all from the heart that: "round children a dream, spread a hope". Due to the epidemic, the gifts collected could not be delivered to Yangxian County by the school teachers and students. In order not to let the children wait or make the gifts late, the school took the mail method to deliver the warmth in winter to the children deep in the mountains of Yangxian County on time. When the 520 gifts filled with hope and blessings crossed the Qinling Mountains and delivered them to the students, they carefully opened the gifts, and their frozen red faces were filled with happy smiles. These gifts are either a cotton-padded clothes to resist the cold, or a long-awaited tool book, or exercise equipment... The school has always attached great importance to and supported the work of the graduate volunteer group, and the school leaders often care about the volunteer group and the local children. Su Sanqing, secretary of the Party Committee of the school, learned that Lu Yongkang, Class 83 of Huahuiguan Junior Middle School wanted a "Red Star Shine in China", so he bought a book and wrote a book on the title page of the book: " Learn the revolutionary history of trees, establish ideals and beliefs, stick to the original mission, and work hard to lose his ambition."Principal Liu Xiaojun for Huaishu iguan Junior Middle School Class 83 He Jiaying sent a child eager for a long time of" History ", and said:" read history wise, learn from the past ". Different gifts, full of strength and encouragement words, contain the ardent hope of teachers, stimulate children's desire and pursuit for knowledge, progress, and the future, and inspire children to constantly forge ahead to realize the dream of success and serving the country. A cumulative drop of nectar, although insignificant, can make flowers bloom, with a book, a pen, a thermos cup... to connect two strangers together, a hope and encouragement, a dream and hard work. Over the years, xi'an architecture university of science and technology in the social output pillars of the talent at the same time, never forget the trend of national rejuvenation in its own mission and bear, build big students never forget the Alma mater, with a pure heart, one after another arrived in the mountains, let their youth shine there. With sincere service and selfless dedication, the members of xi 'an University of Architecture and Technology show the youth style of contemporary college students thinking about the motherland, serving the people, lofty and striving for success. They bring new knowledge, new ideas and new ideas to the students, sow the seeds of dreams, and look forward to the children's set sail. It is reported that in 2012, Xi'an University of Architecture and Technology began to implement the China Youth Volunteer Poverty Alleviation Relay Program graduate volunteer teaching group project. In the past eight years, Xi'an University of Architectural Science and Technology has selected 62 members of the graduate volunteer teaching group to Yangxian County to provide a one-year volunteer teaching service, covering tens of thousands of local students."Warm Winter and Light up Micro-wish activity" is a characteristic activity of Xi 'an University of Architecture and Technology. Teachers and students of Xi' an University of Architecture and Technology collect personal wishes from poor families in volunteer teaching service places and help them realize their wishes. Since the launch of this activity six years ago, a total of 3,120 micro wishes were collected, received more than 3,600 gifts, a total value of more than 300,000 yuan, to help 4,000 poor students to realize their micro wishes. This year, we donated more than 520 students from four schools, including books, cotton clothes, gloves, 600 items, including stationery, stationery, sports goods and clarinet, worth more than 50,000 yuan, realizing the children's micro wishes.

2021-02-01	Teachers and students of Xi Jinping and Peking Union University read it together to understand the meaning of 'applying what you learn to use', Only by actively applying the knowledge learned in practice, "" As a normal university student, I will keep in mind the General Secretary's message, "I've been considering working in the West after graduation, This book strengthened my determination to "...... At the very beginning of this winter vacation, The Youth League Committee of Beijing Union University organized all the students to read the activity of "Xi Jinping and the Friends of College Students" with the book of "Growing up in the footsteps of the General Secretary". In just one week since the beginning of the event, the official account of the Youth League Committee has received nearly 1,000 messages from students."This book is easy to understand, close to the reality of students, students through reading a clear growth path, stand firm on the people, feel the leadership style. This is a vivid youth textbook."Said He Kankan, secretary of the Communist Youth League Committee of Beijing Union University. According to him, in order to improve the pertinence and effectiveness of reading, the school youth corps committee organization 25 full-time and part-time cadres for students guide, from the story background, key content, growth enlightenment three aspects of the book 25 chapters one by one analysis, make the students on the basis of comprehensive grasp the book content, focusing on the characteristics of students, targeted to carry out the ideological and political lead. The school business college youth corps committee secretary deer to the students guide is the fifth chapter xi secretary in the evening with our social practice group discussion, she listed during disease resistance to volunteer service students model advanced deeds, let the students deeply understand book xi general secretary said "only to the grassroots, to practice, to the people, can really know how to play, how to learn knowledge to contribute to the society" meaning. Lu Ran said: " Educators receive educated first. In the process of preparing the guide, I not only improved the ability of ideological and political education, but also received a spiritual and ideological baptism."It is understood that the activity has been formally included in the school's" ideological and political theory class comprehensive practice " course, will continue to carry out. Beijing united university, deputy secretary of the party committee Zhao Feng said: " in the new stage of development, the school to carry out 'grow along the footsteps of the general secretary' series of activities, combining daily ideological and ideological courses, courses, form a synergistic effect, for the implementation of khalid ents fundamental task, cultivating all-round development of socialist builders and successors play a positive role.”

2021-01-31	Anhui university of finance and economics law school joint Bengbu women's federation symposium law school of Bengbu university of finance and economics federation symposium DongWei correspondent, Ma Chi Liang Wang JingGu beautiful) to actively play the advantages of Anhui university of finance and economics law school, using professional knowledge, promote the protection of minors, on January 11,2021 afternoon, under the guidance of the teacher, composed of our students "Daisy" project team came to Bengbu, Anhui province women association, joint city women's federation held minors care project implementation symposium. Jin Mei, party secretary and chairman of the Women's Federation, Shen Hui, Ou Lin, minister of the Rights and Interests Department, and others attended the symposium. The members of the "Daisy" team have conducted full practical research in Anhui province, and built a "comprehensive protection system for the victims of sexual assault cases", integrating the five aspects of law, medical treatment, psychology, economy and information. Before the symposium, a detailed implementation plan and exchange plan were made to protect the victims of sexual assault and publicize self-protection knowledge. When the symposium officially began, Jin Mei, president of the Women's Federation, first explained the basic situation such as the population composition of Bengbu city, expressing the importance of the protection of minors' rights and interests and the necessity of cooperation and docking with the Law School of Anhui University of Finance and Economics. Subsequently, Wei Zhen, Ma Chiliang and other students of the Daisy project spoke in turn, and made a detailed introduction on the preliminary research, overall structure and specific service content of the project successively. In this process, the participants of the Women's Federation showed strong interest, and put forward the corresponding questions, the team members actively answered, both sides specifically analyzed the potential danger sources threatening the safety of minors, the rights and interests have not been fully protected and other issues, the discussion atmosphere was warm. Finally, Associate Professor Jinyu of the college further anticipated the cooperation prospect between the university and Bengbu Women's Federation, and the chairman of Bengbu Women's Federation made a summary statement. Through this symposium, the college and Bengbu Women's Federation reached an important consensus on improving the security mechanism for minors in Bengbu, improving the legal concept of the masses, and establishing a long-term school cooperation. The connection between the school and Bengbu Women's Federation, which is conducive to establishing a joint platform for women and children's rights protection from the horizontal to the border and vertical to the end. At the same time, the symposium let the security financial law students more than an opportunity to exercise themselves, knowledge into strength. Encourage all students to actively use what they have learned to contribute legal strength to the protection of women and children!

2021-01-31	Dong Wei) The Fifth Plenary Session of the 19th CPC Central Committee proposed the initiative to promote green development and promote the harmonious coexistence between man and nature. In order to effectively understand and publicize the spirit of the plenary session, the Pengcheng 5G Spirit Team walked on the Shenzhen Bay Corridor on January 25 to observe and record the water quality of the park, the grassland environment, the number and distance of garbage cans, and the environmental protection tips. The ecological environment construction of the park has achieved remarkable results, and it is a resort for citizens to relax their leisure activities. In order to go deep into the hearts of the people, the reporter communicated and interviewed with the public to understand their satisfaction with the current ecological construction. After a series of interviews, it is found that the people are very satisfied with the achievements of environmental construction around them, and highly praise the government's governance capacity. In addition, two volunteers from Shenzhen Blue Marine Environmental Protection Association were interviewed. Through interviewing them, I learned that there were once many citizens blindly following the trend, which caused some harm to the red water gulls, the Marine environment and the human beings themselves. In order to better maintain the environment of the park, they preach in the park and remind the public not to feed red water gulls. Interview Shenzhen citizens to understand the achievements of ecological civilization construction. Correspondent for the picture of Pengcheng 5G spirit team team to take a group photo. Follow the staff for the picture

2021-01-31	Qufu normal university social practice: wisdom farmers painting qufu normal university social practice: explore farmers painting wisdom on January 31 (reporter Wang Dongwei, correspondent Cao Min Xu Linhua) on January 17,2021 afternoon, "fire" practice team ride to Joe beauty school visit farmers painting artist Qiao Nuo teacher, practice players, in turn, asked Joe teacher about the farmers painting creation and innovation, Joe teacher on the status of farmers, prospects to practice players, to discuss the farmers painting way of sustainable development."In the old days, Wang thanked the hall before Yan and flew into the homes of ordinary people's homes", expressing the feelings of the poets of the Tang Dynasty on the vicissitudes of life and the changes of the world. Everything is constantly developing, and so is art? Art comes from life. Since ancient times, the objects of appreciation and creation of art have been constantly popularized and moved down, moving down from the literati to the poor, and truly enjoy the refined and vulgar, so is peasant painting. Farmers painting originated from painting, developed after the founding of new China, to the 80s gradually formed a strong recognizable sunshine style, with line flat coating as the main technique of expression, depicts the sunshine local farming, fishermen life and other folk customs, life scene, express the efforts and pursuit of happiness, convey the truth. In 2020, China has achieved a well-off society in an all-round way, and in the stage of social development of large machine production. Why do we need to create farmers' paintings? The practice members learned that it is true that today's society is constantly developing and progressing, but peasant painting is not stuck in their own. Many peasant painters are also trying to adapt to contemporary society and coordinate with modern civilization in their creation. In modern society, the existence of peasant paintings is reasonable, and modern people use this way to remember the previous farming civilization, to spread, to remember this civilization, and to retain the history. Just as "National Treasure", "If a National Treasure Can Talk" and other programs, they are all for more people to understand Chinese history and remember Chinese history, which is the wealth of the Chinese people, and the same is true of the creation of peasant paintings. Farmer's painting. The teacher Qiao introduced that the creation of peasant painting is quite exquisite, and every step is required to the extreme. Art comes from life, and so is farmer painting. Therefore, the creation of farmer painting first needs to understand the relevant historical background according to its theme and the atmosphere of the whole painting, so as to make it consistent with the theme. Secondly, we need to draw the first draft. When drawing the first draft, we need to consider trivial issues such as the beautiful composition, whether the picture is coordinated, whether the overall layout is reasonable, which to write, or how to arrange the long and vertical bars and other issues. If inspiration arises, the first draft will be completed quickly, but in most cases, it requires repeated revision, coloring after revising the final draft, and keeping improving in every step to achieve the ideal effect. Tracing the source to the road to see the innovation era in progress, culture should develop."Pass on from generation to generation" practice team members learned from Teacher Qiao that in order to make farmer painting innovative development, we first need to clarify the most fundamental characteristics of farmer painting. Farmer painting is a kind of painting with rich colors, which gives people a strong visual impact and shows the colorful life of farmers. However, for farmer painting, people always have a misunderstanding: people think that farmer painting is the painting of farmer life created by farmers. However, through the interview with Mr.Qiao, the "generation to generation" practice team made clear the definition of farmer painting, and have a better understanding of farmer painting. Farmer paintings can be "fishermen's paintings" or "labor paintings" of migrant workers.... Farmer paintings are just a name, but the works it shows can reveal a career, a group of youth, a spirit of life. Among them, Teacher Qiao introduced to the practice members of a winning work in the national competition, using innovative modern elements, effectively explained the spirit of farmer painting and the character environment is not limited.sector. Promoting sustainable development in inheritance and innovation, improving popularity and expanding the scope of communication are an indispensable part in the process of developing cultural economy such as farmer painting. People's thoughts change with the development of The Times, and they begin to attach importance to local culture, and their acceptance of peasant paintings is also gradually improving. What cultural communicators need to do is to let people have contact with peasant paintings, and then receive the cultural connotation of the cultural works. Teacher Qiao, as a cultural communicator, is trying to make more people contact farmer painting, understand farmer painting and like farmer painting through his own efforts."Farmer painting +" has always been on the road. Through the interview, the "generation to generation" practice team members learned that Teacher Qiao combined farmer paintings with small items in daily life, so that they were decorated on silk scarves, fans, tea boxes, pillows and other gifts and souvenirs, adding features to ordinary objects, so that more people can understand and appreciate farmer paintings. Let the farmer painting no longer become the wall framed decorations, but into thousands of homes "life". At the same time, the yan product industry of farmers 'painting is also developing gradually, for example, embroidery, picture album, desk calendar, bookmarks, ashtray and other products with farmers' painting as patterns have been developed, forming industrial characteristics and driving the development of local economy. With the rise of the Internet and TikTok short videos, more and more local culture is known by people. Talking about the innovation of farmers' painting + short video, Mr.Qiao told the practice team members that they had always had the idea in this aspect, but it was limited to the cooperation of a special team. Next, farmer painting will keep up with the pace of The Times, inherit from history, melt into The Times, inheritance and development.

2021-01-26	Hebei Normal University 2020 level of education doctoral war "epidemic" documentary Jianwei correspondent Wang Guanghua) There is such a collective, which has written an ordinary and passionate collective declaration with persistence. They unite, help each other like the family, they coincide, generously pay special class fees, waiting for unified arrangements to fight the epidemic; the overall situation, they check the whole body discipline, they hear the order, silently warm you and me; they are confident and optimistic with confidence. They are brothers and sisters from the School of Education of Hebei Normal University in 2020, in four provinces and seven cities. During the epidemic period, class monitor Ren Yuhong took the lead in the volunteer work. As teachers' apartment testing coordinator, she put the work energy and enthusiasm play, three times nucleic acid detection, each coordination of 468 households of more than 700 people, from testing information confirmed to ten people group, from testing humanistic care to orderly progress, from testing information input to the results query, comprehensive organization and coordination, children no one to care, is a day, the wind war "disease" the most beautiful mother and daughter group, she laughed " as long as it is the party to my work, I am serious."She carefully completed the nucleic acid testing task in an orderly and loving manner. Li Yanling as a party of the communist party members, party members, active in community volunteer service, in yuhua district the beautiful phoenix green community, under the arrangement and scheduling, door to door MoPai check unit personnel, three times during the nucleic acid detection, are actively maintain nucleic acid detection order, seriously do a good job in community epidemic protection and volunteer service. She patiently answers questions, carefully checked household information, and was eager to solve problems for neighbors. During the epidemic period, the Qinhuangdao Municipal Bureau of Education and the Qinhuangdao Municipal Mental Health Association set up the "Qinhuangdao Comprehensive School Section Psychological Protection Working Group" to create a series of micro-lectures on psychological prevention and control. In order to make the city's students, parents and teachers make good physical and mental adjustment, Song Yue played his professional expertise to actively participate in it, and became a bosom sister to help people in difficulties during the epidemic to actively fight the "epidemic" and live an optimistic life. Li Qian actively assist the community staff service community, is responsible for the prevention and control area into the personnel registration, vehicle certificate verification and door post sanitizers work, Xingtai particularly cold this winter, the temperature is as low as minus 16℃, hand red cold numbness, she ignored, still stick to his post, until stand every shift. Wang Guanghua is a foreign student from Heilongjiang Province. She has deeply felt the enthusiasm and generosity of Shijiazhuang people in the past six months. Due to the epidemic, in order to repay the city, she joined the volunteers and worked with the angels in white to provide testing services for the elderly with mobility difficulties. The 2020 education doctor plays a variety of social roles, mostly as front-line teachers and department managers. During the epidemic period, they stick to their own work and stand well in each class. Chen Na took the lead in the epidemic, and needed to cross half the city to lead the work. Because the public transportation was suspended, she had to ride a bicycle for 10 kilometers. Every duty is more like a training to temper her perseverance. The winter is lonely and the streets are empty, while her woman is weak but enthusiastic. In the past two years, Liu Yi has been working in a temporary post, training not only in his work ability, but also in exercising his responsibility. After learning of the outbreak in Shijiazhuang, he took the initiative to isolate himself and worked online during the quarantine period. Zheng Jianhui and Wang Dongpo were both in charge of the student management work in the unit. During the epidemic period, the students went home on holiday, and the epidemic prevention work was extremely difficult. They became the "cousin" brother and the "cousin" brother, and fought side by side with the counselors to count the students' information and data every day. Care for stranded students and help them overcome fear; buy them daily necessities to meet closure. Through video conference, we also publicized the fight against the epidemic, called on everyone to actively participate in volunteer activities and fulfill the responsibility of contemporary youth. Zhang Wan is a primary school art teacher. At home in isolation due to the epidemic, in order to enrich the children's daily life, she guides her children online to use comprehensive materials to create art, so as to enrich their perceptual experience and inspire and express their emotions. Yuan Xiaoxiao, as a primary school teacher, knows the importance of labor education, she stressed that labor can exercise the body, promote good quality, a child who love labor, the heart is full, is full of enthusiasm for life. She encouraged the children to work at home to "play", let the students find the fun of family labor, to develop a good habit of loving labor. During the epidemic period, it is the business of doctoral students. If you have a dream, there is the direction of struggle. Time can not be wasted, the sun and the moon dare not neglect, the 2020 level of education doctors, during the home isolation to warm fight "epidemic", with endurance learning. Under the leadership of the monitor Ren Yuhong, the whole class was enthusiastic about scientific research. It held online academic salons every week, conducted WeChat group exchanges every day, made vacation learning plans, and shared the phased learning harvest. As a member of the doctoral publicity group, Ren Yuhong participated in the good book recommendation activities, gave full play to her professional advantages, and improved their ability to read foreign literature. Li Yanling adhered to her original academic aspiration, interacted with her tutor every week on their learning information, and urged everyone to read the literature every day to prepare for the topic selection and writing of the paper. Cao Feifei suggested that you carry out online reading activities, recommend you to read a book every week, spur each other, move forward hand in hand. Chen Xiaole will share her learning materials with her tutor, and share and provide video resources for academic lectures of the School of Education of Peking University. Although Hui Weihua is in his hometown of Shaanxi province, he is interested in the class. She provides you with relevant materials for the opening of the education doctoral thesis of Shaanxi Normal University for you to study and discuss. Guo Yuan sorted out the electronic materials, more than 100 documents, more than 50 education books, and formed a learning community with everyone, to make progress together.

2021-01-23	Hebei University of Science and Technology provides assistance services with temporary assistance funds and psychological counseling services to students who stay in schools or travel in transit cities. After the outbreak of the epidemic, the school immediately investigated the stranded situation of the students, determined the responsible person in charge of Baolian, and provided proper resettlement and provided one-to-one assistance to the stranded students through effective means such as WeChat and telephone. In order to reduce the financial pressure on students during the stay, the school gave a temporary subsidy of 1,000 yuan to 64 students from poor families in Shijiazhuang and Xingtai to ensure the normal life of students during the stay. In addition, the school's mental health center has also opened three mental health consultation hotlines to provide psychological help and support for teachers and students, especially stranded students, and guide them to do a good job in psychological protection, enhance psychological resilience and coping ability, so as to better devote themselves to study, life and work.

2021-01-22	Shaanxi normal university students with good streamline the golden key shan university students reform: with good to streamline the golden key institution setup, control scale, change of working methods, is the university students adhere to the principle of streamlining, continuous reform of the key measures, also strive to improve the students work efficiency, expand the effective coverage, solve the students work "28 phenomenon" the golden key. A series of practical explorations of the student Union of Shaanxi Normal University highlight the effectiveness of the reform."There were hundreds of students before, but now there are only dozens. What if you want to hold a big event?"Some student union members expressed concern. But Tao Lujiang, a member of the presidium of the Student Union in 2018, said, " This may be a good opportunity for the student Union to transform its functions and realize from passive to active."In a student backbone growth salon, the school student union members around the streamlining and functional transformation of a heated discussion. Since the launch of the reform in 2017, Shaanxi Normal University College Students Association has strictly followed the principle of "setting according to demand, reasonable optimization, streamlining and efficiency", and gradually implemented the "streamlining and slimming" plan. The number of members of the student Union has been reduced from over 800 to 24 now, and the number of working departments has been reduced from eight to six."When there are enough staff, you can 'rotate'. If the people reduce the 'rotation' in difficulty, we have to try to rely on the students and 'turn' around them."Said Li Yangyuan, a member of the presidium of the student Union in 2019. In order to achieve this goal, Shaanxi Normal University College Students will establish a special work preparatory group in major activities or work according to the idea of "small organization, big participation", and recruit volunteers in the form of projects, so that students can participate in the whole process of work design, decision-making, implementation and evaluation of the Student Union. The 2020 campus health run is a successful practice of this operation mechanism. Within a few hours after the volunteer recruitment notice was issued, more than 1,300 volunteers received active registration, and the activity notice was viewed more than 10,000 times."To participate in the whole process of the student Union activities as a volunteer, I have a deeper understanding of the student Union, and also have a new understanding of the positioning of 'campus dream team and heart-warming navigator'."Some student volunteers said after the event."After streamlining the personnel, what we consider most is how to improve the service efficiency and accuracy, and the research needs have become the premise of the work."Zhang Junhui, head of the rights and interests department of the university student Union, told reporters that since the launch of the reform, the student union has insisted on special research and asked questions for young students. In addition to understanding the needs of students through the "germination plan", but also a "questionnaire survey + feedback" system was established, which invited students to carry out multi-dimensional evaluation of their awareness, participation and satisfaction online and offline."How about college students' online study life at home? What are the other difficulties there are?"During the COVID-19 epidemic prevention and control period, a special survey questionnaire on college students' study and life carefully designed by the student Union was issued, and we received feedback from 4,888 students from 21 colleges within five days. In the "most needed help right now", the top three questions are "course tutoring (61.37%)", "paper writing tutoring (19.05%)" and "psychological counseling (17.31%)"."Since everyone needs course tutoring most, students should take the first action."Said Haramuji, head of the ethnic minority student working committee of the university Student Union. Such a decision, and the positioning of the reform of the student union confirms- -really beneficial to the students to do enough "addition", to go through the formalities, to do a good job of "subtraction". One hundred and one hundred and sixty-five students from 19 colleges and universities covering eight disciplines were soon active in the one-to-one precision assistance of online volunteer services. In order to better listen to the students, the school students held with functional departments face to face symposium, student proposal contest activities, and in the college student union, class committee set up rights and interests, rights, form the "school-school-class" three-level linkage feedback chain, strive to achieve students demands "every echo, everything has landed". Before this, the widespread problems of complex institutions and personnel, and the uneven quality and ability of cadres, which not only greatly restricted the service effect, but also affected the overall image of the student union. In this regard, Shaanxi Normal University students will "entrance" good students. In 2020, the comprehensive academic evaluation of all the staff of the student Union will reach the top 30% of their major. Not only that, but also launched the student backbone training program, through theoretical learning, ability expansion, practical experience and other training to improve the quality of student cadres. The student Union has also established a review system to regularly summarize the reflection and self-improvement. In the implementation of the "image building" project of the student backbone, the activities such as the "student official" issue discussion and the student proposal collection contest were launched to effectively improve the work style and external image of the student backbone.

2021-01-17	Warm winter fourth stick | cotton hat warm winter, love surviving warm winter fourth stick | cotton hat warm winter, love surviving winter wei correspondent Zhou Lei XiaoYuan Ting, Dong Shuai Wang Zhuhui) this winter, evergreen two small children not only have a new teaching teacher company, and previous teaching teacher read them, be love care through the cold winter. Pan Zheng, a member of the 21st Graduate Student Support Group of Hefei University of Technology, once served at Changqing No.2 Primary School in Danzhai County, Qiandongnan Prefecture, Guizhou Province. During the service period, I actively contacted caring people from all walks of life to carry out poverty alleviation and education. In the winter of 2019, I contacted the School of Architecture and Art of Hefei University of Technology to raise more than 1,500 pieces of warm winter clothes, benefiting all the students in the university. In July 2020, Pan returned to his Alma mater to start his graduate career and worked at the Graduate Institute. When carrying out night running activities, Pan Zheng combined his own volunteer teaching experience, and actively proposed to combine night running activities with public welfare, effectively connect physical fitness with public welfare activities, and give sports more profound significance."Benefit from Health, Walk for Love" takes the sum of the accumulated steps on the date of the activity into the specific number of donations to buy warm winter clothes and be donated to Evergreen No.2 Primary School. In the winter of 2020, Pan purchased more than 40 cotton hats for students in Changqing Second Primary School. The power is small, the love and long. With a year is not a long time, do a life of unforgettable things! For him, although the year of volunteer teaching was over, he never forgot the children of evergreen Second Primary School, actively asking him what he was missing and what he needed. Reviewing a year of volunteer teaching experience, Pan Zheng said: " For me, volunteer teaching is a fruitful year, out of the university campus, to the grassroots education, participate in the teaching front line. This year, I learned to deal with leaders, colleagues, students and other different groups, carefully complete the school assigned work, and most students. This year, an unforgettable year, witnessed too many students families, feel too many students, so I feel to do what to do for the children."Service volunteer teaching for eight years, care and challenge" Guizhou " line! This care will always pass on the writing, to give children infinite hope and future!site of activity.

2021-01-15	Jianwei correspondent Wang Xuehan Shi) Recently, the 10th "Star Cup" College Students' After-school Academic Science and Technology Works Competition final defense review meeting of Jiangsu University was successfully held. Each participating team held a 10-minute project display in each defense venue according to the groups. School teachers in various professional fields served as the judges of the competition, and guided and communicated with each project. The atmosphere of the defense site was warm. Jiangsu University "Star Cup" series of school competitions is divided into science and technology works competition and business plan competition, which is a powerful measure for Jiangsu University to select excellent project works and prepare for the "Challenge Cup" series of competitions. Since 2001, the school held "star cup" series of competition, "technology" "entrepreneurship" has been held ten sessions respectively, inspired the students love science, science, with scientific enthusiasm and initiative, guide and cultivate the students 'scientific spirit and scientific attitude, in showing the students' outstanding achievements at the same time, also created a good campus atmosphere of the science and technology culture. The 10th "Star Cup" College Students Extracurricular Academic Science and Technology Works Competition is a university-level selection competition held to prepare for the 17th "Challenge Cup" National College Students Extracurricular Academic Science and Technology Works Competition organized by Sichuan University in 2021. Since the publication of the school competition in March 2020, the college youth League committee and the student science and technology association have been widely publicized and mobilized, and students have enthusiastically participated. After the preliminary competition of the college, the youth League committee of the university, the final final was held through the team public defense and expert evaluation.

2021-01-12	Anhui wenda information engineering institute to help students safe home Anhui wenda information engineering institute to carry out the "group send you home" activities cut contact, safeguard students from the school to the station "the last kilometer" health security, on January 8 solstice 9, Anhui wenda information engineering institute in Hefei third bus company to carry out the "public group group send you home" volunteer service activities. During the event, from 9:00 to 17:00 every day, the bus company runs directly from the school to the Hefei Railway Station and the high-speed railway South station, and the two routes each have one shift per hour. More than 1,560 people signed up online for the ride. At the event, more than 50 young volunteers provided services and help to students who came waiting for the bus to help students go home safely. It is the work function and attribute of the Communist Youth League to serve the youth and to safeguard and realize its legitimate rights and interests. The youth League Committee of the school will continue to practice the working concept of "student-oriented", and strive to build a cadre team of youth league and students with quality, temperature and feelings, and become the "close friend" of the youth league members of the school.

2021-01-11	The first lecture of the young scientists theory publicity group of Zhejiang University of Technology began! The establishment ceremony of the publicity group and the first lecture of "Qingsay Qinglisten" was held in the library of Pingfeng Campus. CAI Yuanqiang, secretary of the Party Committee, Chen Jie, deputy secretary of the university, Zheng Yi, head of the Theory Department of the Publicity Department of the Provincial Party Committee, Ni Jiakai, cadre of the theory Department, Lu Jian, deputy director of Zhejiang Bureau and director reporter of Guangming Daily, and Liu Wen, reporter of Zhejiang Bureau of Economic Daily attended the event. Leaders of the publicity Department, the Student Engineering Department, the school Youth League committee, the scientific Research Institute, the Talent Office, the School of Management, The School of Marxism, the Jianxing College and other relevant functional departments, members of the theory publicity group of young scientists, representatives of new young teachers, propagandists of each college, and representatives of teachers and students attended the activity. CAI Yuanqiang and Zheng Yi jointly unveiled the letter of appointment for the "Young scientists" members of the group. The advanced deeds of two academicians Shen Yinchu and Zheng Yuguo and their scientific research team were deeply excited and encouraged by the teachers and students present.▲ "explore the unknown passion solution" theme about materials school associate professor Liu Yujing, environmental professor, chemical school professor Yang Yunfang, institute of information doctoral Peng Chunxiang, management researcher wu bao, institute of mechanical professor Tan Dapeng successively to "develop innovative thinking, climbing the scientific highland", "carbon neutral and green development", "computational chemistry: take you into the molecular world" "using artificial intelligence to explore the mystery of protein structure" "crack" double chain risk "password, protect enterprise financial security", "the weight" wisdom "pioneer" to preach, and interact with the students. Young scientists from their own scientific research direction and growth experience, to the present teachers and students about the country of science and technology, innovation power vivid practice, fully demonstrate the young scientists of Zhejiang university of technology, in-depth study and implement xi new era the ideas of socialism with Chinese characteristics, in innovation driven development, self-reliance and bear as in the construction of "important window". Yang Yansheng, a member of the lecture group and a young doctoral teacher of the School of Marxism, presided over the lecture.▲ Liu Yujing: Develop innovative thinking, ▲ Zhang Sihan: Carbon neutral and green development ▲ Yang Yunfang: Computational chemistry: Take you into the molecular world ▲ Peng Chunxiang: Using Artificial intelligence to explore the mystery of protein structure ▲ Wu Bao: crack the "double-chain risk" password, Protect the enterprise financial security ▲ Tan Dapeng: National weight "wisdom" pioneer ▲ Yang Yansheng: Host ▲ exchange and interaction Zheng Yi commented on the first lecture, He affirmed the significance of the province's first young scientists, The lecture very well reflects the characteristics of young people speaking to young people, scientists speaking about scientific theories, doing well and saying well, For the Zhejiang youth theory propaganda work has injected the new force. He hoped that the publicity group would aim at the construction direction of "three science and technology innovation highlands" in Zhejiang, and serve the construction of "important window" according to the requirements of "the country needs, Zhejiang can do, the masses hope and the future" proposed by the provincial Party Committee. It is reported that the " young scientists theory publicity group is composed of 53 outstanding young scientists, young scholars, young teachers and doctoral students in various professional fields, with an average age of less than 34 years old. They will through the theory of micro preach, micro party class, education, based on professional areas, contact innovation practice, docking national needs, for the masses of young teachers and students and the service object, preach the party's innovation theory and science frontier, promote "curriculum education" and "education", for the comprehensive construction of modern socialist country relay struggle, bear the national rejuvenation.

2021-01-08	Sichuan culture media vocational college teachers with tribute works crucial Sichuan culture media vocational college teachers with tribute poverty crucial wei correspondent Yu Xingcai) recently, Sichuan federation held 2020 annual hundred "optimal project" conference, including art, drama, folk art, photography, calligraphy, dance, quyi, music and so on 12 categories, a total of 100 outstanding original literary works, Sichuan culture media vocational institute of art design department Zhang Pan teacher of Chinese painting works "full horsepower to help poverty", and get the federation of Sichuan province creation funding support. The annual hundred "promotion project" is a key brand project specially organized by the Sichuan Federation of Literary and Art to summarize and display the achievements of the "six action plans" implemented by the literary and art circles, building the plateau peak, expanding the literary and art Sichuan army, and strengthening the creative strength. In the press conference of 100 "Promotion Projects" in 2020 held by Sichuan Provincial Federation of Literary and Art Affairs, 100 outstanding original literary works selected by 100 "promotion projects" in 2020 were commended. In 2020 hundred "push project" officially launched on October 14,2020, get the city state federation, provincial literary association and the provincial relevant literary units support, strictly abide by the principle of openness, fairness, justice, declaration, review, review, final, the public, released six procedures, implement "launch excellent works, launch talent" double push standard, good choice, best, best, not thick masters, not thin, round, recommended, from 552 qualified declaration, finally selected 100 excellent original literary works.

2021-01-06	Anhui normal university "four warm winter action" funding poor Anhui normal university warm 4721 poor students heart Wang Lei correspondent Yang Mullong) since December 2020, Anhui normal university relying on the wisdom politics big data platform, organized with a nutrition improvement plan, a air conditioning electricity subsidies, a series of growing support as the main content of the "four warm winter action", to 4721 poor students issued all kinds of funding of 3.3772 million yuan, hierarchical classification funding full coverage. Nutrition improvement plan, for example, the school first through the platform for more than twenty thousand students in consumption data tracking, identify to sign up for postgraduate entrance examination and work-study poor students, reference to Wuhu low standard (630 yuan / month) and students compared with last month's consumption decline, a total of 64 screened economic difficult students for low consumption in the month. Subsequently, the platform charged the living subsidies into their campus card in time, so that the poor students taking the postgraduate entrance examination can supplement their nutrition in the sprint stage, so that the poor students participating in the work-study program can suspend their part-time jobs and review at ease. Anhui normal university is the Anhui province tube service integration wisdom education platform construction of the first pilot universities, since July 2020, the school initially built with school characteristics of the wisdom education data platform, the integration of eight departments of 11 application system, the development of wisdom funding, psychological counseling, community management, academic management, safety management of five modules. Xiang Jiachun, director of the student financial aid management center at the university's Student Affairs Office, said the platform counted students who eat at least twice a day and have a significantly low consumption level to support the actual situation of students."In the past, most of the funded students followed the list, which had pain points such as insufficient accuracy and no dynamic adjustment. Now, in addition to using the platform, the staff will randomly select the assisted students every month to verify the situation to verify whether the data model is accurate."In recent days, the platform has also distributed winter air conditioning electricity subsidies to particularly poor students, warm clothes to orphans and students from registered poor families, and online inspirational courses to poor students.

2021-01-03	Xinyang normal college recruiting preach Xinyang normal college recruitment preach reporter dong-wei wang) to fully mobilize the enthusiasm of Xinyang normal college physics electronic college students join the army, promote the modernization of national defense and military, late December 29, the school teaching recruiting preach on the second floor, the school 2020 level all students to participate in the activity. The activity was given by Liu Dong of Grade 2018. Activities began, liu through draft propaganda show hard training, striding, dignified soldiers image, pointed out that college students enlisted is an important measure to promote the progress and development of national army construction, is an effective policy to implement the concept of national defense education, the new era of youth should actively respond to the call of the party and the state, improve their comprehensive quality, volunteer to participate in the national defense army construction. He with carefully prepared video, PPT pictures for "college students conscription need to know" the topic from "the relevant provisions of the conscription" conscription "need conditions" "men and women conscription process" "soldier ten benefits" four aspects, introduces the conscription related legal documents, deepen the students' understanding of college students to join the army. Liu Dong pointed out that in order to encourage college students to actively join the army, the state approved the adjustment of conscription to twice a year, two retirement. He stressed that the conscription process can be roughly divided into four basic steps: registration, on-site confirmation, pre-recruitment management, and political physical examination. Those who join the army should meet the requirements of moderate age, physical health, and good and correct political attitude. Liu Dong combined with political hot spots and military examples of The Times to show the image of the iron soldiers, and around the study, work, life and other aspects of the significance of college students enlisted, further called on students to actively join the army. Recruitment work, he said, is to consolidate national defense and strong army hope project, is the basic project of military modernization construction, college students join the army can not only accumulate a rich life experience, create perseverance, bear hardships and stand hard work, self-reliance of good quality, and can also enjoy the government to provide employment opportunities, subsidies tuition, living expenses, hope that young students actively sign up for the army, consciously as the revitalization of the era, be socialist successors, contribute to our national defense cause youth strength. It is reported that the school has always attached great importance to the national defense construction education and propaganda work. The preaching activities to respond to the party and national conscription, implement the school's national defense construction work, encourage all students active enlistment, students to further understand the army related information, promote the youth from the perspective of growth, deep understanding of the party and the country to create a better life for the people and soldiers defend sacrifice, enhance the consciousness of its contribution to the army construction, is of great significance to build a harmonious and stable campus environment.(Niu Wenjuan, Yang Meng, Zhang Luyao, Sun Xiaoyu / Feng Yufei, Su Roqiong / Photo)

2021-01-13	Jiangsu university volunteers to carry out the spirit of the constitution to preach Jiangsu university volunteers to carry out the spirit of the constitution to preach China youth network Beijing on January 13 (reporter Zhang Jianwei, correspondent Ding Wenwen) on January 10, Jiangsu university law school volunteers came to Zhenjiang Jingkou middle school to carry out the "gender equality education into the classroom" constitutional spirit propaganda activities. The volunteers introduced the meaning of the Constitution, the basic content of the Constitution, the effectiveness of the Constitution, and the status of the Constitution in China's legal system through vivid teaching and explanations. It focuses on the establishment and development of the basic system of gender equality in China, explains the relationship between building a moderately prosperous society in all respects and women's all-round development, emphasizes the important role of gender equality as a basic state policy, and puts forward the way to improve the protection of women's rights and interests. Finally, the volunteers had an in-depth interaction and communication with the students on the topics of gender equality, the right to education and campus violence. Volunteers presented awards to the winning students. After the lecture, the volunteers also participated in the award ceremony of the "Gender Equality, Youth First" essay contest of Zhenjiang Jingkou Middle School, which awarded the awards to the winning students and took a group photo. In response to the theme of The Times, the lecture combines law popularization activities with volunteer service, bringing constitutional knowledge into the campus, forming a legal education atmosphere of law study, usage and law-abiding. Volunteers also exercised their ability to solve problems in the process of teaching, and contributed their due strength to vigorously promote the spirit of the Constitution.

2021-01-02	The Youth League Committee of the School of Philosophy of Nankai University took multiple measures to promote the grassroots youth League building meeting as scheduled. The work evaluation of the league branch secretary is one of the many grass-roots league building work carried out by the Youth League Committee of the philosophy Institute to further strengthen the management and education of the league members, enhance the organization and guiding force of the league, and thoroughly implement the policy of "party building leads the league building". This semester, the Youth League Committee of the School of Philosophy focuses on promoting the personality development of each branch, emphasizing the importance of organizational construction for the branch, advocating the way of multiple participation, multiple measures, and all-round promotion of the construction of the grass-roots youth League organization. Report on work and review, and promote the "five good" branch construction. In the league branch secretary work review meeting, each branch league branch secretary review the semester league organization work and make a summary. Adhering to the principle of openness and transparency, the seven members of the Youth League Committee of the college and other members of the Youth League branch of the class evaluated and advised the youth League branch secretaries with the construction requirements of the "five good Youth League Branch" as the evaluation standard. The report will effectively strengthen the work exchange between the league branches, and share the work experience, which is conducive to promote the continuous improvement of the quality of the league members and the active development of the league organization construction; the league branch of the philosophy institute is generally developing towards the goal of "five good" branch with high momentum. Grassroots supervision, problem-oriented, one by one discussion. In order to promote the self-improvement and improvement of the work of each branch, and better promote the construction of "excellence" of mass organizations, the Youth League Committee of the School of Philosophy also carried out the supervision of the class branch branch one by one, combining with the implementation of the branch in depth, and actively exploring the new development path of the grass-roots supervision requirements. Mr.Liu Weishuang, secretary of the Youth League Committee of the School of Philosophy, Mr.Tian Jiajia, deputy Secretary of the Youth League Committee, and other members of the Youth League Committee supervised the work of the six branches under the school, put forward targeted suggestions on the problems existing in the work of each branch, and guided the branch to consciously carry out introspection and self-examination. At the same time, the committee members encourage each branch to foster strengths and circumvent weaknesses, give full play to the spirit of innovation, promote the "class and league integration" construction, constantly stimulate the vitality of the branch, enhance the cohesion of the class and league organization, so as to better lead the youth and serve the students. Contact and follow-up, establish the committee member-branch "1 + 1" system. In order to urge the construction of the Youth League branch, the members of the Youth League Committee of the Institute of Philosophy will take the initiative to play a guiding role. Each committee member will contact and follow up at least one grass-roots Youth League branch, and establish the "1 + 1" system of the committee member-branch. In terms of the league organization and activities, to learn together with the league members; to communicate with the branch, to adjust the working method of the branch; and to guide the branch accurately in the daily work of the league. The "1 + 1" system enables the college Communist Youth League Committee and the Communist Youth League branch to work closely together, laying a good foundation for mutual cooperation in the future work. It is understood that in promoting the overall construction of grassroots youth league organizations, philosophy school youth corps committee is brewing, pilot systematic, institutionalization of "party building" implementation plan, in order to achieve multiple measures simultaneously, multiple participation, strengthen the youth corps committee and branch, the connection between party members and league members, to promote the branch of grassroots league building work, comprehensive, multi-level, pragmatic play to the role of the good group.(Correspondent Hu Xiaofan)

2020-12-29	Panzhihua College launched the Qingma Class Student Reading Club, Clean Culture and Education Practice Base held the Qingma Class Students' reading Club and Panda clean culture volunteer service activity launch ceremony. The reading club was held in the form of the "Qingma Project" class students' clean story competition. Students stick to the theme, combined with the patriotic national spirit, three line spirit and the new era struggle spirit, etc., in turn explain the origin of the wind garden and culture on the wall of honest story, from 1927, the birth of the first party discipline commission, to the eve of the founding of new China MAO zedong led the Chinese communist party of China "Beijing descend", when it comes to the revolutionary martyrs with blood writing "prison eight", and based on the actual study work life, express oneself on how to set up honest consciousness, be self-discipline climbing adult views and views. After the reading, the launching ceremony of Panda Clean culture volunteer service activity was held. Chen Bin, the chief of the political Department group of Panzhihua Forest Fire Detachment, was specially invited to share the heroic deeds of Liangshan Prefecture in fire fighting and explain the relevant knowledge of forest fire prevention, and presented souvenirs to the students of qingma Class."Qingfeng Yuan" is the practice base of clean culture education of "Qingma Project" of Panzhihua College, and also an important position to tell the stories of clean government, climbing and Panzhihua. The activity to further enrich the school clean culture carrier, cultivate college students' awareness of honesty and self-discipline, deeply promote the construction of school clean culture, guide young college students to tell good stories of clean culture, publicize clean culture, listen to the party to follow the party, do self-discipline climbing adults.

2020-12-28	Yantai University's original dance drama "Mine War" and the 2021 New Year concert were staged in Yantai Jiaodong Theater in China Youth Daily. According to the introduction, this is the school inheritance of jiaodong red culture, and strive to create a "hot blood Jiaodong red education" series of activities. The performance is divided into the first half, the first half is the original dance drama "Mine War". By the teachers and students of the School of Music and Dance, Yantai University. Through various music and dance expression forms, the play vividly shows the familiar story of the war of land mines, and affectionately eulogizes the brave patriotism of Jiaodong children. It is reported that the creation of the drama has been highly valued and strongly supported by Haiyang City, and it has allocated special funds to subsidize and provided rich historical materials and story clues. The creative team went to the relevant red Education Base in Haiyang for many times, looking for creative inspiration and collecting a large number of related historical materials, folk stories and literary and artistic works. In order to ensure the artistic effect of the dance drama, Yantai University also invited senior Chinese dance artist Zhang Yinsong, famous Chinese choreographer Tian Lu, Beijing Dance Academy, Haiyang Yangko non-genetic inheritors to participate in the creation, to extract the fresh characters in the Haiyang mine war by artistic means. In the second half, the teachers and students of the School of Music and Dance of Yantai University presented a New Year's concert full of patriotism to the audience through various musical expression forms, such as orchestral symphony, girl chorus, piano solo, drum ensemble, male and female voice solo and multiple singing, and pipa Ruan ensemble. In recent years, Yantai university strive to build "blood jiaodong red education" series of activities, is through college students' information, red music, original red ballet performances and other forms, approached the teachers and students, out of the campus, to society, increasingly become Yantai university polished khalid ents background, strengthen the patriotism, inheritance jiaodong red gene, service civilized city to create an important brand.

2020-12-28	The Party branch of the Party Day activity with the theme of "Carry forward the great Spirit of resisting the United States and aiding the DPRK, and strive to be a promising youth in the New Era". Student party members learned the great spirit of resisting the United States and aiding the DPRK by having a dialogue with the veterans, visiting the martyrs' cemetery and watching patriotic films. On the morning of the same day, the student party members visited Xinlian Village, Hangzhou Town, Yangzhou Ecological Science and Technology New City, and visited Tang Cunfu, a veteran soldier of resisting the United States and aiding Korea. Tang Cunfu, 88, joined the army at 87. This year, the CPC Central Committee, The State Council and the Central Military Commission awarded Tang Cunfu the "70th anniversary of the Chinese People's Volunteers fighting against the United States and Aid North Korea" medal. The second stop of the party day activity, the student party members walked into the Shaobo defense war martyrs cemetery to visit and study, all the party members in front of the memorial for a silence ceremony, in order to express their deep memory of the revolutionary martyrs. Facing the bright red party flag, the student party members raised their right hands and reviewed their party membership oath. In the afternoon, all the party members of the branch watched the educational film "Diamond Chuan" with the theme of aiding North Korea, recalling the extraordinary years of fighting anti-American aggression and aid to North Korea, and paying tribute to "the most lovely people". After watching the film, the party members launched the signature activity of "the mountains and rivers are safe, the heroes are immortal, remember the history, we should be self-improvement". Finally, all the party members focused on learning the spirit of General Secretary Xi Jinping's important speech at the conference commemorating the 70th anniversary of the Chinese People's Volunteers to resist the United States and aid North Korea. Forty-eight Party members of the first undergraduate Party branch of the School of Information Engineering of Yangzhou University attended the party day activity.

2020-12-28	Jiangsu Agriculture and Forestry Vocational and Technical College held a new Tour Festival to show their youth charm. " The 2020 Association New Tour Festival activities. This activity is divided into two sections: booth exhibition and stage display. In the booth display, the poetic agricultural and forestry painting and calligraphy, mellow and strong fruit wine fragrance, full of sports atmosphere of the three-color ball arena and other clubs are unique, each show their own characteristics. In the stage show the youthful performance, the strong performance of Qingfeng Taekwondo Club, and shows the strength of T-MAX Roller Skating Club. Finally, according to the 5 expert judges and 20 public judges, the college Youth League Committee selected 10 best booth design awards. The purpose of this festival aims to show students the rich campus cultural life, enhance the ability of the club to hold activities, enhance the communication between the club, and provide a good platform for the development of the club. Jiangsu Agriculture and Forestry Vocational and Technical College currently has 52 student associations, including 2 ideological and political categories, 13 academic science and technology categories, 12 innovation and entrepreneurship categories, 13 culture and sports categories, 10 voluntary public welfare categories, and 2 self-discipline and mutual assistance categories. Among them, the total number of freshman and sophomore students was 8,227, and a total of 10,860 people participated in community activities. The relevant principals of the party committee of the college, the leaders of the student Affairs Office, the Youth League Committee and the secondary college (campus) participated on the site. In the activity, 52 university-level student associations were displayed, and nearly 2,000 students participated in the activity.

2020-12-23	Tianjin University of Traditional Chinese Medicine carries out voluntary service activities in Ximigu Village, Jinnan District, carries out voluntary voluntary service activities, and delivers TCM medical services to the villagers' doorsteps. On the site of the free clinic, 5 TCM experts provided TCM pulse diagnosis and face diagnosis to the villagers who came to consult, and put forward practical treatment suggestions according to the villagers' diseases, explained the knowledge of TCM conditioning and preventive health care, and helped everyone to develop a healthy lifestyle and living habits. Student volunteers will conduct medical guidance according to the villagers' physical conditions, measure their blood pressure, publicize their safety and health care knowledge, and provide convenient and high-quality medical services for the villagers. At the same time, the students also went deep into the fields to remove weeds for crops, in the process of labor to cultivate the spirit of hard work, let them feel the "labor is the most glorious, the most noble, labor is the greatest, the most beautiful" truth. More than 40 experts from Tianjin University of Traditional Chinese Medicine, teachers of the Youth League Committee, counselors of Traditional Chinese Medicine and student volunteers attended the activity.

2020-12-17	Northwest University of Agriculture and Forestry Science and Technology: let the students really become the master of the school construction and development of the student Union reform in the Northwest University of Agriculture and Forestry Science and Technology: let the students really become the master of the school construction and development of the knowledge is further enhanced, the work style has had a great change. The implementation of work operation mechanism flat management, the main student students should not only overall work, but also directly responsible for the special work, to ensure that everyone does his duty, everything is simple and efficient.--------------- " since the student union organization reform, I personally feel pure and fresh, not so many departments, so many we don't know the student union members to arrange some unknown so activities, and my most profound feeling is now the student union elder brother sisters are good cow oh, particularly high efficiency, also can put the learning well, is really very bad."Northwest A & F University School of Horticulture 2019 horticulture major Liu Jiani said with a worship face. In October 2019, a wechat official account of the Youth League Committee of Northwest A & F University caused a great stir, with comments from teachers and students."These people are so hard, excellent students!"" How did you win the national scholarship for two years in a row!"" Among the dozens of people, more than 80 percent have won the honorary title of 'Outstanding Student Leader'."" So many party members ah!"" God-like existence, they are all my research 'subject'!"...... This is the eve of the third academic (research) congress of the university, and the teachers and students have pushed the candidate introduction of the new student Union and graduate student association committee. Northwest agriculture and forestry university of science and technology earnestly implement the communist youth league central committee, the Ministry of Education, the national association of relevant documents spirit, in strict accordance with the new standards and new requirements hiring student union staff, through research a baseline, mobilization training, innovation restructuring, select a batch of political qualified, excellent, with wholeheartedly for the students service consciousness and ability of student backbone."I've considered a lot since I took office. I know that many people have doubts about our new group of key students. I think that some of us are nerds, not enthusiastic and passionate, and maybe their academic performance is ok, but their ability in organization, coordination and communication is too weak. Personally, I think we just do the duty of students, study good to be qualified to talk about other good, organize students will have confidence. Fortunately, the teacher gave us a 'reassuring pill', encouraged us to say that a person's learning ability is the most important, learning can learn in front, other aspects can learn, not the local teacher can teach us. In a short month, the school secretary, the principal have met with us kindly, this is a deep care for us, we have no reason to disappoint the trust of the teachers. Therefore, we should maintain a strong learning force, strive to be the teacher rest assured, let the students satisfied with the 'vanguard'."Zhang Shuo, the executive president of the Student Union, said with emotion. This is a "service group" with brightness, " a bathroom that can't drink coffee is not a good gym! Sinon did it!"" Xi Nong is now 'net red restaurant'!"" Community unimpeded direct drinking water "" net red laundry room, wait and see!"...... read more than 100,000 WeChat push, fully shows the quality of life and love of xong college students. Guo Dongliang, a cadre of the student Union, was particularly pleased to see the students excitedly post photos, dinner and fitness on the "We Media". As a student representative, he participated in the investigation and demonstration of many campus projects. He was very clear how the service projects that satisfied the students blossomed. In recent months, he and the friends of the student union deep into the students, collect students in study and life needs and opinions, and timely reflect to the relevant departments of the school, at the same time objectively feedback to the opinions of the students, under the premise of mutual understanding, slowly, the bath environment improved, dining style improved, dormitory intelligent laundry service project promoted... in the student union students enthusiastic, west campus gradually become "other people's' web celebrity'campus", students also affectionately called "web celebrity manufacturing little expert". After the reform, the student union organized the students is further enhanced, and the work style has been greatly changed. The implementation of work operation mechanism flat management, the main student students should not only overall work, but also directly responsible for the special work, to ensure that everyone does his duty, everything is simple and efficient. In the campus management work, the classroom occupies a seat problem has been a difficult to chew "bone", the student opinion is very big. Now the work is directly responsible for the executive chairman of the student union, with the help of the teacher, the executive chairman actively contact academic affairs office, student affairs, logistics, school (department,) to discuss countermeasures, by dividing responsibility area, hanging work card, the student union staff shift on duty, regular report, purchase temporary database, as far as possible to create a comfortable and equal learning environment. Not long ago, Ximeng issued the "Political Theory Study and Collective Activity System of Northwest A & F University" to innovatively carry out students' ideological and political education work. This work can be widely carried out, and the organization of the student union at all levels contributes greatly. Wang Chenling, head of the study department, said: " Political learning is a means to maintain combat effectiveness. The student union has a standardized organization and a sound system, which is easy to convey the voice of the Party and the requirements of the school to every student. Up to now, the student union at the two levels have jointly discussed the implementation of the system for 4 times, paying attention to the phased efforts to avoid being anticlimactic."In the face of the new face of the student union organization after reform, water building college 2018 civil engineering peng-fei gao students said," students will no longer set up chairman, vice President, minister, vice minister, etc, now junior students are often zhang (student executive chairman) senior how to, is who (graduate executive chairman) senior sister how how, students the big family significantly less tough and official, more harmony and popularity. In addition, the school formulated the "Northwest A and Forestry University Student Union (Graduate Association) Student Cadres Management Assessment Method", for the strengthening of the style construction and standardized management of the student Union staff is a very good constraint."This is a broad" heart-to-heart bridge "." To safeguard the legitimate rights and interests of students and to convey the reasonable demands of students is our job responsibilities. Only sitting on the same bench, can close the distance between heart and heart, hand in hand to solve each proposal, can let you and me live together in the 'happy West Nong'!"Said Xue Fang, head of the rights department of the student Union. In order to guide the majority of students to be deeply involved in campus governance, the West Agricultural Student Union has explored the formation of a three-dimensional rights and interests work structure of "petition mailbox", "political campus" and "proposal moment". In various forms such as high-quality activities, video exhibition and broadcast, students can realize the work goal of freely expressing their demands, working face to face with functional departments, and enhancing the voice of campus governance. Since may this year, the school student union successively with school logistics management, food service center, station management center, school union, the community service project, "kitchen king tournament", labor education platform construction, National Day memorial activities such as work exchange views, form student demands reflect, department explanation convey interactive platform, reduce the probability of students have misunderstanding, improve the effectiveness of the department work propaganda, achieve win-win results. Among the students' opinions, there is no lack of major problems such as "nearby factory waste gas pollution, affecting the physical and mental health of teachers and students". Li Guolong, secretary of the Communist Youth League Committee of the school, said in an interview: " Every 'proposal' of the students represents a sincere expectation. My colleagues and I were deeply moved by their love for the school and their intention to honor the school. Through the active exploration of mechanisms such as 'political campus' and so on, our students 'ability to participate in and investigate and research is constantly improving. Students' proposals have done in-depth investigation and research, which can accurately grasp the root cause of the problem, and many places have touched the dead corners and blind spots in school management. Students can stand in the height of the school development and personal development to examine the problem, sincerely put forward a heavy suggestion for the school, which is the trust and expectation of the school and teachers."On October 1,2019, the whole country solemnly celebrated the 70th anniversary of the founding of new China. The school held a staff singing contest to offer blessings to the motherland, and the whole campus was immersed in a happy festive atmosphere."There are fat sea, tea and snacks, go to replenish some energy!"" The students served them very carefully."...... in different areas of the performance site, several pieces of" Northwest A & F University Staff Singing Competition gas Station "propaganda board is eye-catching, which also wrote" confession teachers, confession school, love the motherland "," we love teachers, we love the school, we love the motherland "," teachers refueling, school refueling, China refueling!"" Charming teacher, charming China!"And other propaganda language, this is the West agricultural student Union organization spontaneously in the National Day important moment to the teacher, to the school, to the motherland affectionate confession. For two whole days, the student union staff and volunteers worked tirelessly to serve the 30 teams, handed the uncomfortable teachers a cup of tea, gently helped the older teachers off the stage, help the team evacuation channel... the youth figure of the students infected many teachers and students, through TikTok, friends circle forward their busy moving moments."Expand the brand publicity effect of the student Union, and appear at the node of major events" is a new working idea formed after the organizational reform of the student Union. On Teachers' Day, National Day, examination season, and learning the reply spirit of General Secretary Xi Jinping to the secretaries, principals and experts and professors, the West Agricultural Student Union organized an active voice and clear attitude, and clearly guided the students on the road of learning agriculture, loving agriculture, strengthening agriculture and developing agriculture. The vibrant student union has sown the seeds of dedication, responsibility and love in the campus and the hearts of xstudents. Since the 85th anniversary of the establishment of Northwest A & F University, it has shouldered the historical mission of "developing the northwest China, developing agriculture and learning". The organization of the Student Union has played an important role in promoting the development of disciplines and talent training. As Li Xingwang, party secretary of the university, said, " I be a political leader, an example in study, an example in study, and a representative in work and life. Call on the majority of students to play the spirit of ownership, to participate in the activities of the school, including management and governance and other work, so that the students really become the master of the school construction and development."China Youth Daily, China Youth Network reporter Sun Haihua source: China Youth Daily

2020-12-16	Li Chuan, correspondent Qin Changjian) In 2010, volunteers from Nanjing University of Posts and Telecommunications first raised money through an apple charity sale to fund leukemia patients. In the following ten years, the "winter charity sale" took root and sprouted in the postal campus. Every year for international volunteers, the South Post Youth Volunteers Federation participates in the charity sale as planners, volunteers and buyers, and makes their own contribution to the charity sale. In 2018, the crisp apple sweet like fire in the taste buds of the students, warm the snow winter; in 2019, the sweet orange to the students of the lamp, lit up the innocent smiling face; in 2020, winter charity volunteers continue to set sail for love, convey warmth."Gardenia" means evergreen, but also has the meaning of youth forever. In order to encourage students to study hard and make progress, and promote the all-round development of moral, intellectual, physical, aesthetic and labor, the Graduate Teaching Group of Nanjing University of Posts and Telecommunications began to prepare for the "Qingzhi Scholarship" based on the "pomelo Plan", and was officially established in the volunteer teaching area in 2018. The scholarship comes from the "charity sale" for the whole school, so as to encourage the subsidized students to be grateful and youthful and vitality forever, and create a better future with full enthusiasm, comprehensive ability and excellent quality. On December 3rd this year, the winter charity sale of "' Orange" Love Post in Sichuan "was opened, helping to help raise the fourth" Qingzhi Scholarship ". As the protagonist of the winter charity sale, the oranges were inspired by the volunteers: "Royal Post Orange" shows the students 'deep love for South Post; "Academic' Orange '" means academic success and continuous progress; "Help Orange" for the campus with youth positive energy... South Post students offer love, and harvest "orange" times warm blessing. After two days of charity sale, we waited from 8 a. m. to 9 PM. The volunteers did their best and the postman was sincere. In winter, the South Post campus becomes particularly warm because of the students' love gathering. As the main members of the winter charity sale, the volunteers in red vests are the most lovely people. They delivered oranges in a hurry, to interpret the volunteer dedication " with practical actions. They show the vitality of youth under the warm sunshine of winter, the red vest for this winter painted a gorgeous, added a warmth, lit a passion. On the road of volunteering, the students hold hand in hand, adhere to walking with the motherland, contribute to the society, and write the story of Lei Feng in the new era with their youth dreams and practical actions. From generation to generation, never stop.

2020-12-16	Jiangsu Normal University CD clocking activity leads the new "food" and the youth CD clocking leads the new "food" is still moving. In the early stage of the activity, the school Youth Volunteers Association carried out online multi-channel publicity of this activity through wechat push, QQ activity talk, Weibo topics and other ways. On campus, young volunteers set up service stations to carry out food saving publicity to teachers and students, recommend CD card action. In each school canteen, young volunteers put up posters, set up table stickers, and put the publicity videos made by the school Youth Association on the electronic screen, calling on students to love the food festival in the form of singing and dancing in the canteen. The CD punching action was responded by the youth of Su Shi, and more and more young people bring their own meal boxes and take an appropriate amount of meals to reduce waste. Through communicating with the canteen staff, the volunteers of Jiangsu Normal University Youth Association learned that the food waste phenomenon was greatly reduced after the CD-card punching activity. Meanwhile, more and more students chose to eat in the canteen to reduce the use of takeaway disposable tableware. The Youth Association of Jiangsu Normal University also distributed questionnaires to the participants of the school. More than 1,000 questionnaires were received, and all the students recognized the CD action. Nearly 90% of the participants developed more environmentally friendly dining habits. Relying on the technology platform of the third School CD Action Challenge of the Central Committee of the Communist Youth League, the school logistics Group and the Student Affairs Office provide venues, materials, personnel and policy for the support of this activity. By the end of the event, a total of 6,238 people from Jiangsu Normal University had participated, punching in more than 222,000 times and saving about 8.437 tons of grain. In the third National CD-ROM Card Challenge, it ranked the 16th in the national universities and won the "Excellent Organization Award".

2020-12-10	The provincial Blood Center has jointly carried out the activity of "blood donation without compensation to help the fight against the epidemic". For a long time, east China jiaotong university institute of technology attaches great importance to unpaid blood donation public welfare undertakings, and Jiangxi province blood center to build "college students unpaid blood donation love education base", the school around khalid ents center task, attaches great importance to students' moral education, constantly explore new mode of moral education practice, among them, with volunteer service as the carrier of moral education practice, become the second class of moral education practice, forming "voluntary blood donation without glory" school spirit tradition. The first week of the school Youth League Committee is the knowledge popularization week of blood donation every year. Through the production of documentaries, wechat push, blood knowledge lectures and volunteer speeches, volunteer services, blood donation and other public welfare undertakings are constantly popularized to every teacher and student. According to statistics, during the five-day blood donation activity, a total of 1,283 teachers and students of the Institute of Technology of East China Jiaotong University participated in the blood donation of 388,300 ml, which has broken the record of single blood donation in universities in Jiangxi Province for three consecutive years.

2020-12-10	Northeastern University held the 18th Poetry and prose recitation Contest 8th "wonderful pen flow sound" poetry and prose recitation Contest. The competition began in the collective recitation of "Young China said". The competition is divided into three chapters: "Home and country", "inheritance" and "Dream building", consisting of 10 groups of recitation works, including "Unforgettable Memory" and "Youth China". Zhao Ji, president of Northeastern University, said that Northeastern University held a poetry and prose recitation contest commemorating the "129 th" movement to remember the history and pay tribute to the ancestors. The activity has become a campus cultural activity brand that can carry forward the glorious tradition of Dongda, highlight the cultural spirit of the university, and convey the positive energy of youth. Since 2003, the "Wonderful Brush Flow Sound" Poetry and Prose recitation Contest has been successfully held for 18 sessions. More than 6,000 original literary works were collected, and more than 20,000 teachers and students participated in the activity.

2020-12-10	Nanchang Institute of Technology: the principle course "play out a new pattern" will investigate the luggage, there will be a common item- -Marxist-Marxist-Leninist classic original work."Take a few books with you" is a soft system established by the teachers of the Marxism School of Nanchang Institute of Engineering in recent years. Always do not forget to read the original work, on the original work, line the original work, this activity has long been extended from the class to the class, and from the young teachers' internal conscious discussion and learning development to drive the young students to participate together. Even in the unusual online teaching process, the original teaching series of activities have never stopped. This is a young teaching team that has been established less than 3 years ago, mainly by the post-80s young teachers. When it comes to carry out the original teaching series of activities, the principle of teaching and research section director Wang Chengde said: " all the marxist theory original classic can be understood as a certain social life reflected in people's mind, are marxist classic writers according to' they are people's life thought material to create precious wealth, we must' chew 'inherit all the excellent original ideological heritage, as our young teachers and students' from here and now people's life to create new spiritual wealth."In his opinion, in the new era, China's youth are undergoing great changes unseen in a century. Influenced by the value diversification, the international community is also paying more and more attention to the Chinese youth. How to better meet the ideological needs of young people to settle down, how to more confidently transform the classic ideas creatively, how to improve the level of school young students' Marxist theory and practice, the original work reading course teaching activities, is undoubtedly a crucial part of the countermeasures."In a word, young teachers and students need to read the original work, and the original work also needs to be rooted in the real life of young teachers and students."At Nanchang Institute of Engineering, every Tuesday and Saturday are the fixed days for teachers and students to meet with the principle course respectively. Every Tuesday morning, the teachers read the classics; every Saturday, the students of the principle course participate in the original reading."Teachers read first, and students read again, creating an atmosphere in which everyone can participate and benefit."Said Wang Chengde. During the epidemic prevention and control period, this agreement was never interrupted. People moved reading the classics and learning the original works from the daily conference room to the online learning group. Teachers and students were very supportive of this activity. In Nanchang Institute of Engineering, the original work series of teaching activities is not a random phonograph, but a teaching activity carefully created by the young teachers and students with flesh and blood, emotions, love, hate, dreams and inner struggles. Teachers and students read around Marx's "Political Economy Criticism", Engels' "Persia and China", Lenin's "On the Straight Road", Li Dazhao's "My Marxist View", MAO Zedong's "Hunan Peasant Movement Investigation Report", "Xi Jinping: The Governance of China" and other classics one by one. In the activity, we combined with the new achievements of socialism with Chinese characteristics in the new era, each express their opinions, although the debate but ideas collision very happy, finally the original reading activity process will form the original theory briefing, reserved for the reference of teaching and research, further internalized in the heart, become the conduct orientation of our study and life. Talking about the whole design process and value of this activity, Wang Chengde said: " Initially, teachers used collective lesson preparation in a small range of attempt, and then by summarizing the specific experience of tutoring and answering questions, combined with the needs and ideas, brainstorming summarized step by step. Now it has penetrated into the specific links of course teaching, practical teaching tasks. Some students also use the weekend time to participate in the course of collective reading activities, from the point to the surface, from the surface to the body, the original teaching series of activities can be benign operation. After all, he is inevitably limited by thinking when reading, and discussion can collide with new sparks of ideas."During the epidemic prevention and control period, although there was no face-to-face" principle " courses between teachers and students, the reading and discussion of the classic original works continued in another way. Before each class, the teacher will take a classic original text as a classroom introduction, and then let the students talk about their own feelings and life experiences in combination with the classics. When each student puts his or her own joys and sorrows into the pen, the teacher will give appropriate comments to ensure that the students' ideas can get timely feedback. The original boring class was connected by the small stories told by the students. The students not only exercised their language expression ability, but also listened to the already familiar "strange heart journey" of the students. The best part is that people can answer the questions and find out "what role the classic book plays in your daily life". To the surprise of Hong Yan, the 18th e-commerce class of Nanchang Institute of Technology, "the students who did not speak actively in class before all confided their feelings.". In fact, as early as last semester, Wang Chengde organized a discussion class entitled "The Historical process of human struggle against the epidemic" in combination with the question "The people are the creators of history". From Azheimer, Parkinson's son, China and so on, we have found that human history is a "history of the fight against the epidemic", but at that time, we did not expect the "theory according to the reality" so fast, the course has just ended, the novel coronavirus will sweep the world. However, at this time, the students have had a deeper understanding of the human epidemic, and they have a more peaceful and calm conscious attitude. Yin Jingjing, the first prize winner in the 2019 Jiangxi Provincial Youth Ideological and Political Course Teaching Competition and the top 10 young teacher of Nanchang Institute of Technology in 2020, said, " Classics are not boring, but fresh. What we have experienced now, in fact, most of the first people have already experienced, reading the classics can not necessarily let us recite how many sentences of the classics, but at least can let us stand on a higher level to look at the overall situation."In Nanchang Institute of Engineering, many young teachers are using their own good way to guide young students to read the original work. Wang Wei's online practice teaching emphasizes the collective communication between students. Bian Guiping pays more attention to the connection between traditional culture and Marxist theory, while Yu Houwei emphasizes that the original work should be combined with "the original thought". Hu Xian, also in the lesson preparation group, also has a different experience of reading the classics. She believes that it is better to teach them how to fish, so before the course begins, she found some electronic versions of the classic original works to share them in the teaching group. She hoped students to feel the charm of the classics by reading through the classics, and asked each student to choose their favorite content and record a short video of the recitation."Instead of using blunt language to instill the classics, it is better to let the students personally feel in the classics. Through reciting, writing feelings and other ways, let the students further deepen their understanding of the classics."In Hu Xian's opinion, the process of recitation is the process of students' close contact with the classics. Through a period of online principle course practice teaching, teachers found that students have gradually mastered the basic knowledge and basic principles of marxist learning essentials, sections of the classic original thought is infiltrating into the online classroom, copies of the introduction to the basic principles of marxist course practice task book is orderly completed. Students' understanding of principles courses is also quietly changing.

2020-12-08	China university of science and technology: with the pride of the youth with the movement of youth communication college 2018 undergraduate students Wang Sichen reading the book xi and college students friends, I seem to listen to the general secretary of the xi entrust talk to college students, really feel by xi general secretary and college students' intimacy, deeply agree with the general secretary said "born at its time, responsibility". Especially in the 17th interview, Zhao Nan, as one of the interview objects, was interviewed by China Youth Daily. In the interview, he recalled the scene of General Secretary Xi Jinping's inspection on July 17,2013. On the same day, the general secretary was about to leave UCAS and made an impromptu speech to the graduate students who came to see him off. He said to the students present: "we are in a great era, with great goals, it can be said that was born at the right time, great responsibility...." after reading this paragraph, I can not help but emerge the figure of Chinese youth this year: COVID-19 outbreak, Chinese youth white armour, retrograde forward, build the youth defense against the epidemic. I realize that the contemporary youth only put the heart of the life in the motherland, only the personal ideal into the national development, can not youth, not only to write articles published in the motherland, but also to write articles in the youth, in the crisis, in the motherland needs, stand in the tide, can bloom youth!"Looking for suffering" is the quality of life, but also a student, the school of Shipping and Ocean Engineering 2017 undergraduate students, Ye Tao carefully read "Comrade Xi Jinping advocates young people to" looking for suffering "- -Xi Jinping and College students friends (3)" this article, I was touched a lot. I want to talk about my own ideas based on my growing up experience in college. First, young people should "seek hardships" in practice. Last year, I in Lincang Linxiang District No.1 help practice, those young students with firm faith in adversity, their thirst for cultural knowledge, poor reality, gratitude to the country, gave me a life lesson; I went to Hebei Saihanba to carry out "beautiful China" research and practice, in the forest farm, I know several years of "Lin second generation" couple, their dedication let me understand that behind the "beautiful China" is countless green workers... second, youth in the grassroots, in the motherland most need. This summer vacation, I went to the Tanggu Street Working Committee of Binhai New Area, Tianjin, for a one-month government affairs internship. I really understand the saying "the most solid force of the Party's work is at the grass-roots level". Whether it is the scorching sun or the wind and rain, from fighting the epidemic and building a civilized city, to finding lost property and cleaning up for the elderly, the figures of grassroots workers are always where the people need it most. It is because of the many "seeking hardships" experience that let me understand the responsibility of young people. Therefore, I chose to become a member of the 23rd research group of the school. Next year, I will go to the most needed place of the motherland to continue to shine, and I will internalize the spirit of "seeking hardships" in my heart and externalize it in my actions, and contribute my youth strength to the cause of the party and the country. Looking back a hundred years ago, young students burst out their passion for the sake of the new democracy and pursued advanced development for the sake of the new China. A hundred years later, our nation is moving towards a great rejuvenation, and this one hundred years has also witnessed the change of our young students. We are the main force in achieving the great two Centenary Goals. Just as General Secretary Xi said, contemporary college students cherish their time, closely link their study and growth with the cause of the Party and the country, and with the needs of society and the people, pave the way with youth, and extend their ideals. I think there is a lot we can do under the great changes unseen in a century. As young people, we can find the needs of the people and the country in the era, both at the forefront of scientific research and in rural revitalization. The young generation of college students are the pillars of the future! After reading "Xi Jinping and College Students' Friends", I am more convinced that the future of the motherland needs the efforts and dedication of our young college students.

2020-12-07	China university of petroleum (east China): scientific and technological achievements, the university has held five innovation and entrepreneurship annual conference for students for micro defect intelligent detector, combustible ice efficient mining technology... on December 5, a total of 116 innovation and entrepreneurship projects, 12 outstanding achievements display, 15 colleges (center) innovation and entrepreneurship education exhibition in China university (east China) the fifth "wisdom youth create the future" innovation and entrepreneurship annual opening ceremony, more than 800 people exhibition, feel the charm of science and technology innovation and youth entrepreneurial passion. China University of Petroleum (East China) has held the College Student innovation and Entrepreneurship Annual Conference since 2012, which has now held its fifth session."Industrial equipment will also be 'sick' like people, equipment failure will even cause serious accidents, at this time, we need our industrial equipment intelligent doctors to help."Custom industrial equipment fault analysis and prediction platform project booth is crowded with interested audience. Talking about the advantages and research significance of the project, Liu Juan, 2017 Internet of Things Engineering major, School of Computer Science and Technology, said: " We have developed a variety of fault prediction and analysis technologies, such as 'looking, hearing, asking and cutting', and applied different fault types according to the data characteristics of different enterprises. At the same time, our platform is more accurate and predicts faster than other products of the same type."It is understood that the relevant results of this project have been implemented in Haier, Huabei Oilfield, State Grid and other enterprises and played practical results."Gas and humidity-sensitive sensors are widely used, but China's long-term product supply is dependent on imports. After eight years of technical breakthroughs, the team has developed more than 60 kinds of gas-and humidity-sensitive sensor chips with multiple advantages, achieving the same performance as foreign leading enterprises."Meng Weiqi, a 2017 undergraduate majoring in electrical engineering and automation of New Energy College, is the co-founder of Qingcore Technology. He introduced the achievements of the team to the audience." The products have been applied and tested in the three fields of biomedicine, industrial testing and movement monitoring, to achieve the effect of saving enterprise costs and reducing environmental pollution."Zhang solstice, a professor at the School of Control Science and Engineering, is the instructor of the project, providing the correct guidance and strong support to the team. In the process of promoting the project, Meng Weiqi's team always encountered technical bottlenecks. Zhang solstice's patient and meticulous explanation always made them feel like "another village with a bright future". The bionic flexible mechanical claw, which can rotate in various directions and realize the variant adaptation, breaks through the limitation of the existing technology; the larger load turbojet UAV that can independently avoid obstacles and plan the route, and realize the large-scale remote sensing data collection... the colorful achievement display fully demonstrates the scientific and technological innovation ability of college students. Zheng Shengjun, a 2020 student from the School of Economics and Management, said: " From high-tech bionic drones and face recognition to APP research and development, and intangible heritage protection. Each project has its own characteristics and innovations, which let us open our eyes."According to the introduction, the university's college students' innovation and entrepreneurship annual meeting aims to gather the strength of all the teachers and students, give full play to the superior resources, to build an innovation and entrepreneurship exchange platform for students, and focus on displaying the college students' innovation and entrepreneurship projects and achievements. With the theme of "Zhihui Youth Create the Future", this annual conference has a multi-module activities, including innovation and entrepreneurship achievements exhibition, academic exchange meeting, innovation and academic forum, innovation and entrepreneurship salon, expert report meeting and so on.

2020-12-07	Correspondent Jianwei, Liu Yanchun, Zeng Zhongxian, Yang Yiran) Recently, "Xi Jinping and College Students Friends" study symposium was held in the Culture Academy, East Campus of Guizhou University. More than 100 people from the heads of various functional departments and colleges, as well as the university student Union, graduate student association and qingma Engineering training class attended the event. Before the symposium, Li Jianjun, secretary of the Party Committee of Guizhou University, and Song Baoan, president of Guizhou University and academician of the Chinese Academy of Engineering, distributed learning books called "Xi Jinping and College Students" to outstanding representatives of the "Guishan Elite" College Student Backbone Training Class of Guizhou University. At the symposium, the student representatives shared their learning experiences on learning "Xi Jinping and College Students' Friends". Zhong Dayong, Chairman of the Graduate Student Association: After reading the book " Xi Jinping and College Students' Friends, I have a clearer understanding of the word youth. Only the youth of struggle and struggle has vigorous vitality, and only the youth created by practice with both legs is worth recalling. Yang Chenxi, president of the Student Union of Guizhou University: General Secretary Xi Jinping has told us that "young people have to 'suffer' ". "Only when they become one and twisted with the masses, and they have a sense of social responsibility can they gain insights."The book has repeatedly mentioned that encouraging young college students to go to practice and work with the people, only in this way can they better realize themselves. We should keep in mind entrust, their youth and the construction of the motherland, have the courage to take root at the grass-roots level, constantly temper themselves, study, practice, with the youth of me, I struggle, for the construction of the motherland, really let the youth in the party and the people most need the place bloom gorgeous flowers. Zhou Jiayi, deputy to the 27th National Congress of the All-China Students 'Federation: General Secretary Xi Jinping's encouragement and affirmation to the young people have also made me think, how does our generation stand out at the tide and give back to the great era? General Secretary Xi Jinping's words let me find the answer: "bright ambition" and "pragmatism". Volunteer is the original aspiration, and pragmatism is the mission. I will strive to be synchronized with the motherland, and resonate with The Times of your big youth, to achieve the realization of the Chinese dream of the great rejuvenation of the Chinese nation to contribute to the youth strength! Shi Bohan, outstanding student representative of Qingma Engineering Training Class: As a student leader, I often encounter difficulties such as my classmates' incomprehension and their conflict with learning when assisting teachers in my daily organization and management work. General Secretary Xi Jinping's teachings have given me inspiration. He said, " A journey of a thousand miles begins with a single step. To do the possible and possible things, we must avoid' urgent'and do not rush for success."Indeed, when I transform my perspective and put the seemingly tedious work into the overall situation of organizing and connecting the youth, these small things that I may do and can do are that I can make a small contribution to the contemporary youth with my own strength. Wu Mingshuo, the outstanding student representative of the Qingma Engineering Training course and the 26th executive president of the All-China Students' Association, said that "Young people should suffer for themselves" has always inspired me. I am a child who grew up in coastal provinces and cities, and when I came to Guizhou, I was determined by a word: I "want to go to the grass-roots level, to the countryside, and to the place where the motherland needs me most"! In the past three years, I have actively responded to the call of the school Youth League Committee and the student Union, and led the team to participate in the social practice activities of "three to the countryside" for three times. Experience tells me that only when we really go down to the grass-roots level, can we personally feel the profound changes in the countryside, deeply understand the lovely nature of the frontline staff in poverty alleviation, and truly feel the pride brought to us by the strength of the motherland.

2020-12-07	Hebei agricultural university students: to compose youth, with sweat tribute the motherland to compose youth with sweat university horticulture college freshman Guo Yu after reading the xi and college students friends after the book, I remember the most profound sentence is "through the opportunities behind the surface, find a solution to the problem, to really write a research report". As an undergraduate of Agricultural University of Hebei, I will bear in mind General Secretary Xi Jinping's instructions to young people, learn from senior Cao Binghai- -solid, solid steps, solid foundation, face the future, and move forward steadily. Unity, the future can be agricultural university of Hebei university college of land and resources freshman student mei-ping liu xi general secretary invited Hebei agricultural university positive definite cadastral students summer home make social research, encourage the youth to learn the theory knowledge to real life, and points out that learning to learn endless, all things learned is useful. Learning at the same time to find ways to apply, in the application, and then used for learning. In the international competition in the 21st century, talents occupy an increasingly important position, and the real talents are able to apply theoretical knowledge to practice. Professor Li Baoguo of our school has been rooted in the Taihang Mountain for 35 years, writing his theoretical knowledge on the Taihang Mountain, benefiting the local people and encouraging thousands of students. As the successors of the new era, we should strive to integrate knowledge and action, integrate our own strength into the rejuvenation of the Chinese nation, be down-to-earth and have a promising future. In the book "Xi Jinping and College Students 'Friends" written by Zhang Yi, a freshman at the School of Economics and Management of Hebei Agricultural University, General Secretary Xi's kindness and enthusiasm for young people are always unforgettable for me. In 1983, Xi Jinping, then party secretary of Zhengding County, invited them to eat meat steamed buns after mobilizing and instructing students in conducting rural research at Hebei Agricultural University. On the way to the hotel, he chatted very well with everyone; during the dinner, he is also have no shelf, warm hello to everyone, careful care of every student. In this interview, General Secretary Xi Jinping's kindness made me very touched, " some small I Caozhou county officials, a leaf always close feelings."General Secretary Xi Jinping has set an example, cares about the people, is close to the people, serves the people with true feelings, and moves the people with their hearts. He vividly interprets the eight characters of" close to the people, love the people, for the people, respect the people ". For college students, they are full of vigor and hope, hard work and energy. It is inevitable that they will be confused when they establish their life goals. After reading the book Xi Jinping and College Students, I have a clear answer in mind. If we contemporary college students want to keep up with the tide of The Times, we must cultivate strategic vision, scientific spirit, and develop tough character. In particular, the process of seeking knowledge in universities should be down-to-earth, and pursue knowledge and academics unremittingly, and integrate their personal destiny into the future blueprint of the motherland. Looking back, Li Meihui, a freshman student of Animal Science and Technology College from generation to generation, Hebei Agricultural University, Looking back, the spirit is a good beginning. Xi Jinping, then party secretary of Zhengding County, invited Cao Binghai and other college students to do rural research to guide them to study in practice and apply it in learning. This way of combining theory with practice is exactly what we college students should learn from. Go forward, the spirit is passed down from generation to generation. As a new generation of college students, we should inherit the enterprising spirit of seeking truth and charging into the battle, in the future, flying dreams, write youth, do the pioneers of the forefront of The Times.

2020-12-03	Nanjing Institute of Information Vocational and Technology carries out campus tea party activity to point tea and campus tea party " activity. At the scene, the historical introduction of tea ordering in the Song Dynasty and the beautiful tea art performance attracted many students to stop. Many students sat in front of the tea stage to try their skills, and read the tea music recorded in CAI Xiang's "Tea Record" of the Song Dynasty that "the cup is cold, the tea is heavy, the tea leaves scattered, the soup is more porridge noodles gather". They personally felt the elegant interest of the Song Dynasty with the tea needle as the pen and outlined a pair of "water painting" with the tea noodles as the painting paper. Nanjing college of information vocational technology adhere to the traditional culture into textbooks, into the classroom, into the campus activities, adhere to the overall design and classification, content optimization and form innovation principle of unity, relying on the "south letter green tea tea art club" "qixia dragon dance club" "the sea history culture club" and other traditional culture community to carry out rich and colorful campus cultural activities. The school has also set up a number of labor skills studios, such as "Nanxin Green Tea" and "Qixia Dragon Dance", and is equipped with professional instructors to carry out traditional culture courses and skills training for students. At the same time, the school also pays attention to leading students to go out, bringing traditional culture into the community, and radiating to the surrounding areas. The school tea culture community will traditional tea culture into volunteer service activities, and Nanjing xuanwu district lock gold five village help pension center build community old man "tea" tea "tea art community class", let students in the community in the volunteer service shoulder the responsibility of excellent traditional culture inheritance, constantly deepen the understanding of the importance of Chinese excellent traditional culture, dig into the value connotation of Chinese excellent traditional culture, further strengthen cultural confidence.

2020-12-03	Yunnan university student union: singing service students "best" Yunnan university students: singing service students "best" school mascot doll clothes welcome new students, when he took off the doll clothes, clothes are wet, this let me really feel the change of the student union, also saw the reform of the student union and results, they are really in service for the students."In 2020, freshmen enter the school, a student who participated in the orientation work said with emotion. Since 2017, Yunnan university students according to the instructions and requirements of the central, the national union, actively promote the reform of the student union, highlight basic system innovation and organization vitality, expanding work effective coverage, service students growth, service teaching and research center work, service first-class university construction ability to improve, formed the leading force fully reveal, increasingly strong organization, service, increasing cohesion of vitality, artistically vivid student union organization construction new situation."I will actively practice the core socialist values, stay true to my original aspiration, break through the difficulties on the academic road, and add luster to the construction of double first-class!"" I will get up early and run for five days a week, study hard and be self-disciplined, and strive to become a first-class student matching with a first-class university!"" I want to use the most beautiful verses to describe the most beautiful Yunnan University in my heart, with my own poetic and painting to express my gratitude to the double flow of clouds and blessing!"" I want to pick up every piece of garbage I see on campus, and protect the school environment starts from me!"In the theme collection activity of" One hundred Little Things I Can Do for 'Double First-class' "organized by the Student Union of Yunnan University, students actively participated in and contributed articles, and made suggestions for the construction of" double first-class ", starting from the little things around them to help the construction of" double first-class ". For a long time, the Student Union of Yunnan University has thoroughly studied and implemented Xi Jinping Thought on Socialism with Chinese Characteristics for a New Era, adhered to the education of ideals and beliefs as the core, and united and led the students to listen to the Party and follow the Party. Explore the student union political study and ideological and political student community construction depth fusion, set up "xi new era, the ideas of socialism with Chinese characteristics" learning club, student union executive chairman President, student union members voluntarily join, and for the masses of students recruit members, regular organization to carry out the political study, improve the students' political literacy. Build "ideological, thought, thinking" in the integration of the "YunQing thought hui" ideological independent learning plan, with "the second classroom transcript system" as the carrier, through the colorful, various forms of activities to organize the students learn related political theory, conference spirit, stimulate the enthusiasm of students independent ideological learning, learning situation by "news broadcast" reported. Innovate the carrier of ideological and political education, develop the form of ideological guidance, and enrich the content of value guidance activities. Through lectures, meetings, salons, debate competitions, knowledge competitions, new media and other forms, a series of ideological and political guidance theme education in line with the growth law of students and with Yunda characteristics has been widely carried out among students. The national flag-raising ceremony, the "Learning and doing" ideological and political salon, and the theme practice activities of "Staying true to the original aspiration and following the Party" were deeply loved by the students. Sing good service growth "aria" in the morning sun sprinkled on the track and field, but also sprinkled on the track constantly running on the students. At 7 o'clock in the morning, Wang from the School of Economics came to Nanyuan Athletics on time to participate in the activity of "I make contributions to the" Double First-Class "" organized by the Student Union. She said: " so I always waste the best time in the morning one day in the sleep late, less than class time never get up, but since this activity, I have the early motivation and enthusiasm, not only exercise the body, also can morning reading with classmates, let me more self-discipline, also let me more positive and energetic every day."Since the student Union," I make contributions to the'Double First-class "morning running morning reading yue night running" has won the support and welcome of students, covering more than 10,000 people. The activity effectively encourages the students to "walk down from the network, walk out of the dormitory, and go to the playground", develop good living habits, and promote the construction of the style of study. Based on the expansion and improvement of students' quality, the Student Union of Yunnan University takes many measures to serve their growth. Based on students' own learning duties, closely adhering to the main line of study style construction, we carry out a series of activities with the theme of "I contribute to" Double First-class class "", and organize learning activities such as "library card", Donglu forum, foreign language corner, freshmen debate competition, "reading hour", no mobile phone class and so on. Focus on campus culture construction, promote aesthetic grade, and CCTV "readers" joint "humanities east lu warm cloud" CCTV reading pavilion into Yunnan university activities, "summer light cloud goodbye" graduation season party, "we together" Mid-Autumn festival party, top ten singers, Chinese college students music festival, elegant art into the campus and a series of students happy boutique cultural activities, for the students to show themselves. Carry forward the sportsmanship spirit of the university, strengthen the physical quality training, organize and carry out physical exercise activities such as happy moving night running, tug-of-war competition, three-person basketball match, seven-person football match, three-walk CTI youth pleasure running, Central mini marathon and other sports activities. During the epidemic period, the first "Cloud Moving Youth" online sports meeting, creative sports video competition, AI physical fitness project competition and endurance sports competition were organized to encourage students to get off the Internet, go out of the dormitory and go to the playground, study hard and develop in an all-round way. Cultivate the sense of responsibility, strengthen volunteer public service, organize and carry out public welfare volunteer activities such as "Traffic Volunteers", "Volunteer Tutor", "Peking University and Taiwan Dayun University" student social service plan charity sale, and create a positive, dedicated and responsible campus cultural atmosphere. In just the end of the "one day with me-logistics early experience" activities, a participation experience the canteen table students said: " experience the canteen aunts today's work let me feel a lot, deeply realized their work hard, every bit of garbage on the table, are bent down with a towel clean, at the end of the day I have acid back pain, can think how hard their daily work. I strongly call on everyone to clean up the residue on the table after the canteen meal, so that the aunts less bend over once."In the evening, Li and his roommates from the School of Liberal Arts were walking in the campus. The street lamp stretched out their backs and reflected them on the steps at the door of the Qinglai Hall. Xiao Li said, "Before, the street lights on campus were too old, which caused some inconvenience to students. We reflected the situation through the online'School Voice 1 + 100" activity organized by the student Union. Soon after, the school logistics department replaced the light bulbs in time to realize the lights."Walking in the campus of the cloud, everywhere every road smooth and smooth, campus bus coming and going, carrying students shuttle in the campus, campus cinema, cafes, student activities, provide rich choice for students extracurricular leisure; campus network two speed up, campus WiFi full coverage, these are the result of the student union rights service work."Wow, I can actually talk to the school leaders face to face ah!"Campus cafe door, in the first phase of the" sharing time "youth meeting room xiao Yang classmate regrets said," just not only listen to the school leaders share him from middle school to university, work experience, and his interests, youth dream, successful experience, etc., also listen to the senior about his Yunnan gonglongjiang township teaching during the experience, and graduate subject research confusion and engaged in part-time counselor work harvest, let I learned a lot of knowledge from books to school, such an activity is particularly meaningful!"Yunnan University Student Union gives full play to the role of a bridge, seriously pay attention to the construction of the rights and interests service system for young students, launched the online and offline smooth mechanism, to realize the" online problems, offline solutions; online questions, offline answers; online confusion, offline communication ". Adhere to the problem-oriented, around the students concerned about things, things around, need things, targeted to supply services. To carry out the "two" love across campus transfer object service, student recommendations proposal contest, raise charity for sick students, graduates "cloud graduation photo", "preferential tickets into the campus" and airport bus free shuttle activities, regularly check, the school canteen, supermarket, apartment, to carry out the menu solicitation, held "classmate review conference". In order to help freshmen understand college life, the Student Union produces "Freshmen Treasure Book" and "Admission Guide" every year to introduce the school situation from the five aspects of "food, clothing, housing, transportation and study" and respond to the concerns of freshmen. It has become an important brand for new students to adapt to college life and understand the history of the school. Innovation and brand construction, and strive to build a "whole-hearted rights and interests" service brand. In the official account of the Student Union, the section of "Students online- -small rights masters" is opened to open the channels of rights protection and create a cartoon image of "rights Jun", so that students can "remember and remember". Offline "1 + 1" service is carried out. One member of the student Union contacts one college to provide service to the college on the reform of the student union, daily work liaison, rights and interests protection, optimize the linkage between the school and strengthen communication. How to make the original 570 students slim Union is a major problem for the student Union. A secretary of the Student Union of Yunnan University said with emotion: " At the beginning of the reform, we all thought it was just a 'little adjustment' and a 'small fight'. Unexpectedly, the students really reformed one by one in strict accordance with the requirements. The streamlining of the personnel was also unswervingly implemented. The members who did not meet the requirements left the student Union without exception. The number of members in the student union was reduced to 60, and even the presidium was reduced from 12 to 5. Now the student union staff team has become an excellent character and learning, the students are convinced, everyone recognized, with the combat effectiveness of the team."Since 2018, Yunnan university party committee has issued the implementation of the Yunnan university student union organization reform implementation plan" "about promoting the Yunnan university student union (graduate student) several opinions", strengthen the leadership of the student union work, deepening the reform of the student union organization, perseverance, for a long time to work, our actual effect. Through the reform, the Student Union of Yunnan University strives to do a good job of "addition and subtraction". First, we will take solid steps to reduce the scale, implement reform requirements, and streamline the personnel of government institutions. The second is to do a good job in the service "addition", strengthen the function positioning, adhere to the purpose of serving students wholeheartedly, set up a new rights and interests department, promote the function of contacting and serving students and safeguarding students' rights and interests to play more fully, truly sing the "strongest voice" of serving students. China Youth Daily China Youth Network reporter Du Yimeng source: China Youth Daily

2020-12-03	Guizhou university: hand in hand to epidemic prevention AIDS bear health responsibility of Guizhou university: epidemic prevention AIDS bear health responsibility jianwei correspondent, Liu Yanchun, Huang HuaLei Zhou Xia) on December 1, by Guizhou university party committee propaganda department, party committee student work department (place), party graduate work, school union, the school youth corps committee and the school hospital, the Red Cross community in 2020 "world AIDS day" publicity and education activities held in Guizhou university Xi'an is building lecture hall. More than 360 people attended the heads of the university Party committee, relevant functional departments and representatives of teachers and students of the college. At the beginning of the activity, all the staff carefully watched the public welfare publicity video of AIDS prevention and learned about AIDS from the aspects of communication channels and prevention and control knowledge. Linghu Caitao, deputy secretary of the Party Committee of the university, read out the activity declaration to all the teachers and students: attach great importance to it, Enhance the awareness of AIDS prevention, The campus has become a new battleground for the AIDS AIDS, AIDS prevention work has a long way to go; "Three complete" AIDS prevention, Full coverage, AIDS prevention and control is an overall, long-term work, All the departments of the school actively create a good publicity atmosphere, Strive to put the AIDS prevention of the safety education work to cover the whole school every teacher and student, "Prevent AIDS and everyone participates, Everyone benefits without AIDS. "; conceptual knowledge, Prevent AIDS from love, AIDS should be viewed positively and correctly, Give more care and help to AIDS patients, Strive to eliminate the misunderstanding and discrimination existing in the society. Ran Shishi, the student representative, issued the activity initiative: study hard, improve the awareness of prevention, actively publicize, create a healthy environment; care for others, and work together to prevent AIDS. It is the duty and duty of contemporary college students to do a good job in AIDS prevention and control, publicize AIDS prevention and control awareness and pay attention to AIDS patients. At the activity site outside the Yawen Building of the West Campus, the medical staff and AIDS prevention volunteers distributed AIDS prevention publicity materials and protective equipment to the teachers and students. The students actively participated in the questions and answers on AIDS prevention knowledge and signed their names on the AIDS prevention signature wall. At the same time, the activity was live broadcast by the Publicity Department of the Youth League Committee. The majority of teachers and students of AIDS actively signed the signature through the live broadcast, expressing the determination of teachers and students to wear red ribbon together to prevent AIDS. It is reported that in the near future, the Red Cross, Youth Volunteers Federation and other student organizations will also carry out a series of activities such as AIDS prevention education watching movies, AIDS prevention treasure hunting contest, online AIDS prevention knowledge answers. AIDS prevention publicity and education will also become an important part of the theme group organization life evaluation of Guizhou University in December.

2020-12-03	The community activities of Kangda College of Nanjing Medical University bloom in a different style. The 28 student associations have narrowed the distance with the young students through the community culture display, entertainment performance, network live broadcast, learning and punch card and other forms that are popular with the students. The Youth League Committee of Kangda College of Nanjing Medical University strives on building a practical education system with the growth and development of medical students, student associations as the platform and various forms of characteristic club activities as the starting point. Through innovative means, rich forms, the ideological work of youth is fully combined with the characteristics of youth, so that the "second classroom" and the "first class" can go hand in hand, and jointly help young students to develop in an all-round way. Among them, the Rehabilitation Society and the AIDS Prevention Society allow students to give full play to the professional advantages of medical students, apply what they have learned, and broaden the width and dimension of the "second classroom" with the professional knowledge learned in the "first classroom". With theoretical learning as the theme, the "Port Kong Kangda" youth learning club held the first "Youth Learning" knowledge competition clocking activity. More than 3,000 teachers and students participated in the event.

2020-12-03	The final of the second Learning Talent Challenge was held in the second Anhui University of Engineering Challenge. Since the launch of the activity in mid-November, more than 8,000 students from the school have participated in the event, covering more than 500 class youth league branches. On the basis of the comprehensive and continuous "youth learning" network theme group class, the competition takes the "four history" learning and education as the breakthrough point, the general Secretary Xi Jinping's important thoughts on youth work, the spirit of the fifth Plenary Session of the 19th CPC Central Committee, the "Party Constitution", "League Chapter" and other knowledge content throughout it. The competition working group composed of the host department guides the student backbone to form nearly 1,000 competition question banks on the basis of the early study to promote the independent learning of young people. During the competition, the organizer clarified 16 different themes, and the outstanding League branch secretary of the League branch of the "Theme League Day" activity recorded the micro-league class and displayed the "Four histories" around the final and related knowledge, so as to enhance the effect and appeal of the scene. In addition, this activity adheres to the unity of knowledge, fun and challenge, using the favorite H5 online answer form of young students, to effectively mobilize the enthusiasm of students to participate. The preliminary contest was entered into the answer system through the WeChat official account of the school Youth League Committee, and the youth league branch of each class organized and mobilized the youth league members to participate in the online answer. Participants will complete the answer challenge within a limited time and enter the final round. In the semifinal and final, the event also organized the audience to answer questions online. More than 300 teachers and students observed and participated in the simultaneous competition activities.

2020-12-02	Yancheng Normal University: It dissolved the "salt of ideological and political affairs" in the heart-to-heart talk ceremony, and 10 senior "student workers" received the letter of appointment of "heart-to-heart talk work supervisor" from the school leaders."Heart-to-heart talk is the 'diamond' for counselors to carry out ideological and political education. Only with the talk to the degree of heart-to-heart talk with depth, strength and temperature, can counselors truly become good teachers and helpful friends of students."Said Gao Juan, deputy secretary of the School of Law and Politics, who was the first supervisor of counselors. Since 2016, the school has held by the counselor talk to talk project work salon, assembly talk talk work case, organize new and old counselor pair mentoring, online project training, talk talk work experience summary communication, hierarchical classification for counselor "charging", to do a new era of college students value lead and emotional guidance work to lay a solid foundation."As ideological and political educators, we should be good at using the method of heart-to-heart talk, to approach the students, to understand the students, and then to understand the students, to better achieve the purpose of education."Said Zhang Guihua, vice president of the school. Previously, the school a student Xiao Ya was pyramid selling organization "cheat" to Guangxi. Xiao Ya did not come to the first day of class, the counselor, He teacher found that the situation was abnormal. When learning that Xiaoya did not have relatives and friends in Guangxi, He teacher realized that Xiaoya may have been cheated. In the following several days, Teacher He and his colleagues kept in touch with Xiaoya, patiently explained the illegality and danger of pyramid selling organizations to her, and actively contacted their parents. With the joint efforts of all parties, Xiao Ya finally returned to school. Not long ago, Teacher He received a good news from Xiaoya- -she has been successfully admitted to the teachers of Henan Province, to become a middle school biology teacher. Xiaoya still retains the original he teacher he and her online chat records."I chat the 15 pages of 115 will remain forever. Miss He is the person I am most grateful to, but also my example, I will become a good teacher who cares about her students in the future."Said Xiao Ya. In the school, there are many other cases like Xiaoya."Teacher, the shadow of the failure in the college entrance examination made me lose my enthusiasm for college life."" Teacher, the number of infections published online is becoming more and more, and I am a little afraid."" Teacher, we have several people coming back from Wuhan, will I be infected?"...... nowadays, "Eight talks" has become a required course for school counselors: freshmen within a month, Important time nodes, important events, special student groups and active interview students must talk about, Students who are rewarded or criticized and disciplined must talk, Students with poor academic performance and facing academic early warning must talk about it, Students facing dormitory conflicts, love, interpersonal communication problems must talk about it, Students with family financial difficulties or natural disasters or emergencies must talk... the school also has a more clear rule on heart-to-heart talk to counselors: not less than one hour every working day, Not less than 8 hours per week; The new counselor will be supervised for the first 3 talks... at the work experience exchange meeting held by the school, The counselors talked about the ideas, skills, results, insights and experiences."How to have heart-to-heart talks with your students? With norms and supervision, I have a direction and a method."Said this year's new counselor, Miss CAI."Good ideological and political work should be like the salt, but not just eat the salt. The best way is to dissolve the salt into all kinds of foods and absorb it naturally. We should dissolve the salt of thinking and politics in heart-to-heart talks, and educate people with heart and affection."Dai Binrong, party secretary of the university, said," Young students have a broad vision, public personality and pursue independence. counselors need to accurately grasp the characteristics of students, use the network platform, seize the opportunity to talk, strengthen humanistic care, and be students '' bosom friends 'and' guide '.”

2020-12-01	Fujian Quanzhou vocational and technical university: secretary of the party committee into the student community about Fujian Quanzhou vocational and technical university: secretary of the party committee into the student community about college activity room, party secretary Fu Zhixiong representative for more than 20 students about the significance of the fifth plenary session of the 19th, principles, main objectives, key tasks and vision, etc. In order to thoroughly study and implement the spirit of the fifth Plenary Session of the 19th CPC Central Committee, Quanzhou Vocational and Technical University launched a wave of learning and publicity through special learning, special lectures, publicity columns, knowledge contests and other forms, and at the same time set up a publicity group, batch into the college (student community) publicity. Grade 2019 students party activists, Chen Jiannan said, as a college student, should take the initiative to assume the responsibility of contemporary youth, good inheritance "jinjiang experience" love spell, dare to win, dare to create spirit, struggle, practice skills, become high quality skilled personnel, to serve the society, dedication to the motherland, the people, let the youth in the great rejuvenation of the Chinese nation and power journey shine. Hong Peiling, a student from Class 2 of Business Administration, Grade 2019, said that the Fifth Plenary Session of the 19th CPC Central Committee clarified the guiding ideology of economic and social development during the 14th Five-Year Plan period, and put forward the important principles that must be followed. " For young people, the national strategic layout points out the direction for individuals to display their talents and wisdom and realize their life value. As long as the realistic needs of the country as their own direction of struggle, young people will have much to do and do."Quanzhou Vocational and Technical University actively explore the education mode, innovation and implementation of the" college + college "double school system student management mode, the college party branch, the league branch, the party activity room, the implementation of the" party into the academy "," party building extended to the post internship unit ", strengthen the party spirit cultivation of teachers and students, for the party education, for the country.

2020-12-01	The fourth youth league congress of Anhui University of Technology and the fourth student congress of Anhui University of Technology were opened in the College Student Activity Center of Xiushan Campus. Liu Xinyue, party secretary of the university, attended the meeting and made a speech. He hoped that the work of the university should clearly adhere to the leadership of the Party, inspire the youth, unite the youth, and lead the youth, write the song of youth into the youth into the motherland, and enhance the development of the new era, and to enhance the combat effectiveness and educational achievements for the construction of a high-level university in the new era. After the opening ceremony, the first two generations held a plenary meeting."Two generations will" representatives to listen to Wang Jing respectively titled "firmly grasp the" three force once "is committed to promote" six big projects ", for the new era of high level university construction gathering youth strength, writing youth chapter" and yun zhengxi titled " flying youth dream singing times strong to write a new chapter of Anhui university of technology student union work work report. Leaders of Anhui Provincial Committee, Youth League Maanshan Municipal Committee and other relevant departments, secretaries of the colleges, deputy secretaries in charge of student work attended the opening ceremony of the conference. More than 450 delegates from the fourth Congress of the Communist Youth League Anhui University of Technology and the fourth student Congress of Anhui University of Technology attended the opening ceremony.

2020-11-27	Nantong Open University held a live-scene teaching themed league day school, and carried out the theme league day activity of "Learning the History of the Party, Inheriting the Red Gene". Cave School is located in Xingfu Street, Chongchuan District, Nantong City. It restores the classic red scene. The window lattice below the round circle, red pepper, bamboo hats, horse lanterns, and simple wooden tables and chairs reflect the red spirit everywhere and reproduce the classic class of Yan'an and Xibaipo cave dwellings. The Nantong Open University innovated the "Qingma Project" student backbone training method, and replaced the fixed class with the "immersive" real scene class, so that the youth members could personally feel the spirit of cave dwelling and draw the strength of progress. Through the red corridor "Yan'an Cave Memory of Youth", cave school micro-regiment class, reviewing the oath of joining the League, the students reviewed the extraordinary years of the revolutionary War and the glorious course of the Communist Party of China, and learned the ideological essence of the CPC from the 19th to the 19th National Congress of the CPC. Ideological teachers around the party history of the "yan'an cave dialogue" this learning, the general secretary xi about "marxist party to seize power is not easy, consolidate power is not easy; as long as the marxist ruling party, the socialist country will not be a big problem, we can jump out of the historical cycle rate of" prosperity, death "."Important judgment, clarify the communist party members always maintain advanced nature and purity. The relevant person in charge of the Youth League Committee of Nantong Open University said that this is a spiritual journey to stay true to the original mission, inherit the fine tradition, and be firm their ideals and beliefs. The young students will deeply study the "four histories", strengthen the "four confidence", and contribute their youth to the realization of the Chinese dream of the great rejuvenation of the Chinese nation.

2020-11-26	China University of Mining and Technology organized and carried out the 2020 League Cadre Training Project " 2020 League Cadre training class was held in the Jinghu Lecture Hall of the university. The relevant person in charge of the Youth League committee of the school gave a lecture on the theme of "the Communist Youth League work in the new Era", combining theory with practice. The head of each department took the league work, ideological guidance, campus culture construction, innovation and entrepreneurship competition, volunteer service and social practice as the breakthrough point. The education training activities mainly take the form of theoretical training, according to the combination of theoretical training and experience, organization training and self education, concentrated training and the principle of long-term training, guide the student cadres to take the lead consciously play the role of the communist youth league as the party's reserve army, completes the league branch work at the grassroots level, unity led youth active to realize "the great rejuvenation of the Chinese nation Chinese dream" in practice. Deputy student secretary of the Youth League Committee (Youth League General Branch) of each college of China University of Mining and Technology, and all the secretaries of the Youth League branch of grade 2020 attended the training.

2020-11-24	Guizhou university: love, to one's deceased father grind on the road you Guizhou university: love, to the one's deceased father grind road you Ji 'an-wei zhang, correspondent, liu, Liu Xiaoqian PuXiaoYan) when light snow, temperature, distance 2020 is only a month, Guizhou university library, escape building one's deceased father grind classroom is early filled with nervous test of students, they or writing or bow meditation, completely into the sprint state, ready to best into the examination room. At this critical moment, in order to encourage and convey the warmth and care of all the teachers and students, on the morning of November 22, the representatives of the Youth League Committee of Guizhou University with the care of all the teachers and students."Senior sister / senior, this box of milk and this bag of cookies for you, I wish your dream come true!"," senior / senior, this package of candy for you, the struggle of youth is the most beautiful, you are my example "," senior / senior, this is the blessing of all junior junior, to dream as a horse, live up to the time "... a low gentle blessing and a sincere smiling face temporarily broke the peace of the library. In order not to disturb the students preparing for the exam, the staff delivered the love breakfast to them as quickly as possible, and quickly left the classroom."Stars do not ask the traveler, time pays off", "I wish the seniors, research road sparkling", "never forget, there will be echoes", " postgraduate students come on!"... Outside the classroom, blessing wall, a horizontal one vertical, full of students to the postgraduate students 'heartfelt blessing and encouragement; a stroke, pass is the whole campus to the postgraduate students' deep care and support."Great, I have come", " the 2020 postgraduate entrance examination people are here, I will succeed, will go ashore!!!"," When the loneliness swept in, go to the library to study, enrich the mind with knowledge!"Also, the postgraduate entrance examination students also left their own firm and expectations on the blessing wall, encouraging the younger students to forge ahead. A senior sister in the corridor of the problem said to the staff: although the postgraduate entrance examination will have countless difficulties, but in this process also often harvest moved. Like today suddenly received love breakfast, very simple, but also very warm, this a care and only 9 degrees of the weather formed the obvious contrast, originally, preparation for we are not fighting alone, schools and students also pay attention to us, support us, as a one's deceased father grind, to try to the students do good example. Li Xin, a staff member, said, " I learned a lot from this activity. I was deeply moved by the serious investment and persistence of the seniors, and made me feel that a lot of preparation was worth it. I felt very happy to do something for them at the critical moment of their study."In order to thoroughly implement the construction of the style of study, vigorously support the postgraduate entrance examination students, cultivate the students' heart of gratitude, and encourage the students to work hard, the school Youth League Committee organized this activity. Is to let the postgraduate entrance examination students know that they are not alone on the road, they have our whole Guizhou University behind them! We pass on our love in the early winter, I hope it can be as warm and brilliant as the sunshine of postgraduate entrance examination students. I wish all the postgraduate entrance examination people to successfully go ashore! School youth League committee presided over the work of deputy secretary Liu Kui talked about.

2020-11-23	Liaoning petrochemical university volunteers spent two months for the community painting wall praised Liaoning petrochemical university volunteers spent two months for the community painting wall praise Ji'an-wei zhang correspondent, Yang Qingzhou Cao Jiangtao) a few days ago, the 20 days of teachers and students and zhang tao teacher late careful pen, a more than 70 square meters of wall painting in Fushun flower district new steel community. Wall painting will be an ordinary old house decorated with vitality, beautiful round beauty huan. Miao Yue, secretary of the Xingang Community Party Committee, said: " From today on, it will become a stronghold for the community residents to have a daily rest and exchange, because it is the most beautiful here."In the winter morning, with the demolition of the last scaffolding on the west wall of Building 10 of Xingang Community, a vivid and beautiful picture of" Neighborhood Harmony Map " appeared in front of the public. This is the masterpiece of more than ten teachers and students and party members and volunteers for more than half a month. Volunteers from Liaoning Petrochemical University took a group photo before completing the wall painting. At the end of September this year, Cao Jiangtao, deputy of Fushun Wanghua District People's Congress and dean of the School of Information and Control Engineering of Liaoning Petrochemical University, was entrusted by the contact unit, Xingang Community, to ask the teachers and students of the college to beautify the environment. On October 9th, the college formally set up a wall painting volunteer group, instructing Teacher Zhang Tao with rich experience as the person in charge. Mr.Zhang and the student leaders Hu Kaiwen and Kou Chenhe visited the site for many times to survey, repeatedly modified the plan and debug the software according to the community requirements and opinions, and calculated all kinds of needs. Because it is the outer wall, plus the recent winter, the painting work is facing many difficulties. In particular, the highest point is 5 meters, is in the air work, there are safety problems, Mr.Zhang repeatedly improve the plan, efforts to reduce the work to zero risk. At the same time, because it is a rough quartz sand wall, the painting materials are extremely picky, for teachers and students to calculate the materials, instruments with a lot of problems. Wall drawing site. On October 23rd, after full preparation and repeated on-site drills, the teachers and students finally started work. Just in time with the sudden drop in temperature during that period, adding a lot of "natural" disasters to the painting work. Especially in the first process on the clay base, because the low temperature must be mixed with barrels after hard daub, pure heavy manual labor, boys take turns, the girls grasped the nettle; after the coarse sandpaper grinding level, brush two latex paint is also not easy, drawing outline, calculate and revise the picture content cumbersome, after two background coloring, picture color, fine molding pure test will. In order to ensure safety, Mr.Zhang set a strict "group rules" - -girls can only be in the scaffolding a layer of homework, the second floor by the calm and careful and through safety training boys responsible for, three layers of all processes he a person. Wall drawing site. When asked about teacher Zhang Tao and his students's hard work, the students said, " We are very happy to use what we have learned to actually add color to the harmonious community."Teacher Zhang replied:" First, the students' ability and will are exercised; and then to see such a beautiful picture, we are very tired but worth it. We can come back in the future.”

2020-11-24	Nanjing Agricultural University: "CD" action can punch out koi prize Nanjing Agricultural University CD punch out koi koi prize, but also as an image ambassador for CD action endorsement? Recently, Nanjing Agricultural University "beautiful" food "light, refuse to waste" youth CD action theme practice activity is in full swing in the campus, the profound educational significance into the rich theme activities, "CD action" has gradually become a common trend in the campus. For the students, the kitchen has been "idle free". The school organized and led the students into the kitchen, participated in the kitchen work, how the food came from, and where the leftovers went.... the students' curious questions were answered here. Through the experience of the kitchen post, the students deeply understand the "a porridge and a meal when the hard-won" truth. In order to further guide the teachers and students will save food love food consciousness to action, and develop good habits, the school organization carried out the "CD action" 21 days clock card, for all teachers and students set card, complete the specified task, can participate in the "CD koi" lottery, thousands of yuan including learning necessities koi package attracted more than 3000 teachers and students stick to finish 21 days. Good habits should not only from their own form, more to drive the students around together practice, to give full play to the typical leading role, south farmers also specially held the CD action image ambassador selection activities, let the students choose their favorite image ambassador, together for the "CD action" endorsement, called on the school youth thrift, on-demand purchase, resist waste. For agricultural students university, "CD" should be deeply rooted in the people. A grain of rice from sowing to harvest, need to consume seven jin or four liang of water, through seedling raising, transplanting, weeding and fertilization, irrigation, harvesting, drying, peeling, packaging, transportation, sales, cooking and other links, can be served on our dinner table, these are closely related to the students' major. It is reported that Nanjing Agricultural University "beautiful" food "light, refuse to waste" theme league day activities, theme works collection and other series of activities are also carried out on campus, the league branch responded positively, to carry out research and visits, CD vlog shooting and other activities.

2020-11-19	Jiangsu Vocational and Technical College of Architecture integrates ritual archery culture into moral etiquette cultivation to watch the "Huaihai Battle Documentary", and carry out the "Xuzhou Archer in Han Painting" exhibition activity, to promote the integration of ritual archery culture, the two Han Dynasty culture and Xuzhou local culture. After the research on the archery program, the school integrated moral, intellectual, physical, aesthetic and labor education into it. Cultivate the students 'morality and accomplishment, combine the moral education with practice; expand the knowledge and integrate the school intellectual education; practice the etiquette and practice to achieve the sports effect, use the bones, bronze, Chinese portrait stone, calligraphy and painting, mural, painting and other works of art, cultivate the students' ability and improve the labor education. By opening relevant courses, setting up student associations, launching relevant scientific research, holding academic seminars, competitions, performances and other ritual and launching activities, we can explore its educational value and explore its educational mode. Since 2014, the school has begun to explore the culture of ritual shooting, combining professional characteristics, cultural traditions, training goals, student characteristics, student demands, growth rules, combined with the needs of contemporary education, combining the culture of ritual with the cultivation of conduct and etiquette of students, so as to contribute to the training of high-quality technical and skilled talents.

2020-11-19	The final of the third National College Students' Ship Energy and Power Innovation Competition was concluded in Jiangsu University of Science and Technology. After deciding 10 grand prizes, 20 first prizes and 30 second prizes, 12 universities including Shanghai Jiao Tong University and Dalian Institute of Technology won the Excellent Organization Award. The entries will focus on the online video display and physical display of new energy, multi-water multi-functional underwater cleaning robot, ultra-low emission ship device for solar energy applications, hull cleaning, seawater desalination, ship-efficient and energy-efficient composite energy storage system, etc. These works show the results of the combination of professional skills learning and practical application of college students. The competition aims to guide and encourage colleges and universities to actively adapt to the requirements of the new era of ship development and innovation, enhance the practical ability and innovation ability, and closely follow the research and practice of scientific and technological innovation of ship energy and power. The competition is co-sponsored by China Society of Shipbuilding Engineering, Jiangsu Association of Science and Technology, Ship Turbine Academic Committee of China Association of Shipbuilding Engineering, Jiangsu Society of Shipbuilding Engineering, Jiangsu Internal Combustion Engine Society and Jiangsu University of Science and Technology. Due to the impact of the epidemic, the final of the competition was held through online defense, and the skill competition was suspended for one time. The competition attracted more than 20100 works from more than 20 ship energy power universities, and finally from Shanghai Jiao Tong University, Dalian University of Technology, China University of Mining and Technology and Harbin Institute of Technology (Weihai) and other 12 universities to enter the final, a total of more than 100 students to participate in the online final.

2020-11-18	Hebei University of Science and Technology holds the intangible cultural Heritage dynamic exhibition from its intangible cultural heritage performance and exhibition projects, bringing a cultural feast to all the teachers and students. At the event, Jingxing flower performance, Ergui wrestling, Chen Tai Chi, diabolo, Changshan war drum, Weifang kite, straw carving, Wuqiang New Year pictures and other intangible cultural heritage projects to feast the audience's eyes and attracted many students to participate in it. Intangible Cultural Heritage dynamic exhibition is one of the series of activities of Hebei University of Science and Technology, which is the organic combination of aesthetic education and intangible cultural heritage inheritance of Hebei University of Science and Technology. Since 2005 to carry out intangible culture into campus activities, Hebei university of science and technology to combining social practice, research, etc, rich "intangible" cultural heritage form at the same time, through many intangible special performances, "enjoy the cherry blossom, intangible" display of Chinese traditional culture, realize the dynamic intangible heritage in contemporary college students, has obtained the good education effect and social response.

2020-11-18	Gansu University of Political Science and Law on November 14 and 1) held its first student congress and the first graduate congress. Ma Jiandong, party secretary of the university, and Vice president Tang Weiyao attended the meeting and delivered a speech. Ma Jiandong, on behalf of the university Party Committee, congratulated on holding the conference. He pointed out that all the representatives and all the young students of the university should take this conference as an opportunity to move forward firmly under the guidance of Xi Jinping Thought on Socialism with Chinese Characteristics for a New Era, and strive to grow into qualified builders and reliable successors of the cause of socialism with Chinese characteristics with an all-round development of morality, intelligence, physique, beauty and labor. Students' organizations at all levels should strengthen political guidance, firm ideals and beliefs, focus on functional orientation, deepen service connotation, improve work ability, play the role of example; remember the original mission, write the youth chapter, uphold the rule of law, practice social responsibility, be the youth of national rejuvenation, and contribute youth wisdom and strength to realize the Chinese dream of the great rejuvenation of the Chinese nation. Executive Chairman of Gansu Provincial Students' Federation and representatives of Lanzhou University Student Union delivered speeches respectively. The meeting adopted the articles of association and the work report, and elected the first student committee, the graduate student committee, the presidium of the student Union and the graduate student association. More than 350 people attended the meeting, including the relevant leaders of the Gansu Provincial Youth League Committee, the presidium members of the graduate Association of Lanzhou Brothers University, the heads of the youth League committee, student representatives and representatives of graduate students.

2020-11-15	Students from Sichuan Vocational College of Culture and Media met with "Golden Panda". Wang Dongwei) " Where to sign in?"" Enter the door ahead and turn left, there is a sign at the'Golden Panda " forum in the hall, and there will be a special reception staff to serve you."From uniform clothing to makeup requirements, from form etiquette to activity process... Sichuan Vocational College of Culture and Media Aviation department organized unified training, simulation exercise, and finally selected 46 flight attendant professional students to participate in November 11 to November 12 in Chengdu Jinjiang Hotel" Golden Panda " international communication Summit forum, responsible for the reception task of the activity. During this reception mission, 46 students were assigned to the airport reception group, CCO reception, check-in, and logistics groups. On the first day of the forum, guests from all over the country gathered in Chengdu. In order to make the guests feel full of enthusiasm, the students from the airport reception group waited early and enthusiastically provided guidance and consultation services for the guests going to the forum. The students of the CCO reception group provided the guests with the whole process, so that the guests could feel the better service. The students would strictly abide by the epidemic prevention and control requirements. Even through the masks, they would still smile and treat people with courtesy. The students from the check-in group helped the guests to get the guest card and the forum guide accurately, checked in smoothly, and carefully checked the identity information of the guests and the epidemic prevention measures. In order to ensure the smooth progress of the activity, the students of the logistics team upheld the service principle of "wherever I need me, I will go there" to escort the students. A high specification BBS reception not only reflects the organizers of Sichuan culture media vocational college student accomplishment and professional ability, but also provides the students to guests around the world show quality professionalism and etiquette service platform, the outstanding performance of the students won the organizers and the guests consistent high praise!

2020-11-15	Wang Dongwei, correspondent Yan Chun) From November 9 to 13,2020, the "Blood donation into the campus" activity held by the College of Science and Technology of Guizhou University was successfully concluded! A total of 1056 teachers and students donated 256800 ml. The event was sponsored by the Committee of College of Science and Technology of Guizhou University of the Communist Youth League, organized by the Pioneer Volunteer Service Team of College of Science and Technology of Guizhou University, and co-organized by Guizhou Blood Center. At the activity site, under the guidance of the volunteers, the teachers and students who came to donate blood lined up orderly, rolled up their sleeves, and actively cooperated with the staff of the blood center and the volunteers for information registration, temperature measurement, blood drawing and testing, intravenous blood collection, blood donation certificate, etc. Xiong Ruping, a pioneer volunteer service team, once again signed up for this year's voluntary blood donation activity, which is his third blood donation."The main purpose of participating in voluntary blood donation activities is to help people in need, increase the source of blood and relieve the blood tension."Xiong Ruping said that as long as he meets his blood donation requirements, he will go to the voluntary blood donation."Moderate amount of blood donation will stimulate the hematopoietic system and enhance the human body's hematopoietic capacity, and the proportion of fresh blood cells is significantly higher than that of people who have not donated blood."Dr. Lin Chengjun, a doctor of Guizhou Blood Center, said that blood donation will reduce the blood viscosity and have a positive preventive effect on reducing the incidence of cardiovascular and cerebrovascular diseases."The blood is limited, the love is infinite."The voluntary blood donation activity was held, which fully mobilized the enthusiasm and initiative of teachers and students, reflected the selfless dedication spirit of teachers and students, and contributed to the cause of voluntary blood donation.

2020-11-12	Suzhou University Youth League Committee and Suzhou Suzhou University Youth League Committee and Suzhou Xukou Town jointly Build Volunteer Service Base " signing ceremony was held in Suzhou Xukou Town Tibetan South School. During the activity, Yu Qianchi, secretary of the Youth League Committee of Soochow University, and Xu Xia, president of South Tibetan School, jointly signed the Agreement on Joint Construction of Volunteer Service Base. Zhang Jiawei, vice president of Normal College of Soochow University, and Shao Hongjun, executive president of South Tibetan School, jointly unveiled the school base of Volunteer Service of Soochow University. Xu Xia, the principal of South Tibetan School, introduced the school overview, development history and teaching achievements in recent years. In the coming year, the members of Soochow University Graduate Volunteer Teaching Group will carry out the "Colorful 4:33 Learning Classroom" project around academic counseling, family companionship, quality expansion, self-care education, psychological counseling and other contents, which is committed to promoting students to "know learning", "understand learning" and "enjoy learning". Heads of Soochow University, Suzhou Education Bureau and other relevant departments, graduate volunteers of Suzhou University and student representatives from South Tibetan School attended the event.

2020-11-11	Soochow University held the 2020 "Faith Open Class" university-level Demonstration Course Committee to carry out the university-level demonstration course of "Youth tribute to the motherland, Strengthen the System confidence" - - "Faith Open Class" of Soochow University. Students watched the video to review the reasons and great historical significance of the war to resist the United States and aid North Korea, and fully studied the important speech delivered by General Secretary Xi Jinping at the conference commemorating the 70th anniversary of the Chinese People's Volunteers to resist the United States and aid North Korea abroad. The British model Shi Guangli recalled his personal experience of fighting against the enemy aircraft on the Korean battlefield, and gave you an open theme faith class with deep feeling. In order to better inherit the spirit of resisting the United States and aiding the DPRK, and educate future generations to never forget to praise the Party's grace, Soochow University hired Comrade Shi Guangli as the special tutor of Soochow University Faith open class. The students of the Party branch of the School of Electronic Information gave full play to their professional expertise, and made an electronic embroidery with the theme of "defending the country" composed of 381 LED lights for this faith open class. The number of the LED lights is the same as the number of the bullet holes on a battle flag that had experienced the Battle of Shangganling, so as to pay tribute to the revolutionary ancestors and carry forward the great spirit of resisting the United States and aiding Korea. The electronic embroidery was lit up by Comrade Shi Guangli himself. Shi Guangli, the model of Resistance against the United States and Aid North Korea, Colonel Zhang Huijun, director of Suzhou Fifth Cadre Institute of Jiangsu Military Region, school leaders, students of the 2020, members of the 23rd Research Group, representatives of student party members of the School of Electronic Information, and representatives of freshmen of 2020 attended the activity.

2020-11-07	Heilongjiang University Youth Volunteers: Win Warm Youth Correspondent Dong Shukai) In early winter, two or two snowflakes fly in Harbin. Adhering to the concept of "pursuing dreams and having the courage to take responsibility", the young volunteers of Heilongjiang University embody the passionate responsibility of Chinese youth with their professional and warm-hearted volunteer services. Warm: Starting from a text message, " Welcome to the beautiful ice city of Harbin, I hope that during the 7th 'Create Youth' Games, our service will leave a good impression on you, and the competition volunteers will welcome you at the exit later!"As soon as the flight landed, the guests received a text message from the volunteers. Su Hang and Chen Lu from Heilongjiang University are responsible for the travel reminder work of the competition. As travel reminders, they carefully studied the whole process and schedule of the competition, and prepared 28 SMS reminder templates in advance. At the same time, in order to prevent emergencies, they have arranged various emergency plans in advance, including weather changes and changes of meeting time and place, to escort the smooth progress of the conference. And this warmth, along with the whole process of the competition. From the reception of the first day to the daily meeting schedule reminder, to the final arrangement of the delivery vehicle, the volunteers put themselves in the shoes of the guests and made warm housekeepers for the guests. In the five days, they contacted 169 guests and sent 2,362 messages. From the first reminder text message in the morning to the last good night text message in the evening, the warm and thoughtful service has won the unanimous praise of the participants. In Harbin in early winter, the temperature often falls below zero. On the first floor of the Hongqi International Convention Center, the black "red vests" left a deep impression on the participants. Song Weibin and his friends in the newspaper everywhere position close to the gate, the temperature is particularly cold. Volunteers' frozen red hands, the frosty fog on the glasses pieces....... But their faces were always bright."We should use the most spirited face of young volunteers, provide the most warm service, show the style of black youth!"Every day to stand the last shift post, do a good job of the registration of each participant, meticulous and serious, no omission. For Cui and 35 other etiquette volunteers, smiling service is not just on the face. According to the plan, they provide etiquette services for more than 20 conferences and events of the "Creative Youth" China Youth Innovation and Entrepreneurship Competition, responsible for guiding the participants to sit, maintaining the order of the venue, participating in the venue consultation, and providing etiquette services. The etiquette of big events should not be careless, and every detail must be considered thoroughly. Cui Xiangcheng and his friends received two days of pre-job training, from body movements to language norms, warm, polite, atmospheric and dignified. From the airport, from the railway station to the convention center...... Always bright and warm smile, always impeccable attitude, always like the spring breeze service attitude, these are the most true portrayal of the black big youth volunteers. When a guest from all over the world came to the ice city of Harbin, they were greeted by the most thoughtful guidance and service of the "Create youth" volunteers of Heilongjiang University, which was the vivid interpretation of "volunteer service, Black University first". The organization position of this competition is composed of computer-related professional volunteers from Heilongjiang University, who are mainly responsible for the pre-competition equipment debugging, assisting the judges in the competition, and organizing the cloud contestants to connect with the site. They apply their professional theoretical knowledge to practice and provide technical support for the competition with practical actions. In the mobile affairs group, Wang Yuexin and other four volunteers are responsible for the logistics work of the team. As the reserve members of the whole team, they do not have a fixed position, but always according to the needs of the activity, at any time, to provide a more complete guarantee for the competition, leaving a dedication, friendship, mutual assistance, progress figure. And for the volunteers Mou Yuguang, Feng Weidong, the volunteers of the vehicle dispatching team, whether in Harbin Taiping International Airport, Harbin Railway Station or Harbin West Railway Station, they are constantly running to ensure the smooth travel of the guests......"Which university do you come from?"" We are all from Heilongjiang University."" was a volunteer for the last two days."" Your volunteer service is really very good!"" we Wenzhou there more than 20 degrees, off the plane here minus 4,5 degrees, I came in at the door of the boy directly put his thick cover on my body, very warm."" Our purpose is to let you feel the warmth of Heilongjiang province. The "" really felt it."it is reported, To thoroughly study and implement Xi Jinping Thought on Socialism with Chinese Characteristics for a New Era, To implement the national plan for mass entrepreneurship and innovation, Deepening the "Create Young Chinese Youth Entrepreneurship Action", Carry forward the hard work and pioneering spirit of innovation and entrepreneurship in the whole society, To guide young people to devote themselves to innovation and entrepreneurship practice, Co-sponsored by the Central Committee of the Communist Youth League, the Ministry of Human Resources and Social Security, the Ministry of Agriculture and Rural Affairs, the Ministry of Commerce, the Poverty Alleviation Office of The State Council, the People's Government of Heilongjiang Province and the People's Government of Jiangxi Province, The 7th "Creative Youth" China Youth Innovation and Entrepreneurship Competition and 2020 China Youth Innovation and Entrepreneurship Exchange Conference organized by the CPC Heilongjiang Provincial Party Committee was held in Harbin Hongqi International Conference Center from November 3 to November 6. The Heilongjiang University Committee of the Communist Youth League has selected nearly 150 volunteers to serve Harbin Taiping International Airport, Harbin West Railway Station, Harbin Railway Station and Harbin Hongqi International Convention Center. Volunteer service involves more than 10 positions, such as vehicle scheduling, guest guidance, reception, reception affairs, guest contact, itinerary reminder, venue etiquette, activity organization, and mobile affairs.

2020-11-07	A girl with different youth sounds but equally excellent has went to Shandong University, Peking University, Fudan University and Shanghai University of Science and Technology respectively. At the right time, they are both sensitive and eager to learn, but also dare to expand the boundaries of their ability, and constantly explore the growth path beyond the GPA. With his heavy love for Jinan and his Alma mater, Ma Xiaobo will continue to attend graduate school at Shandong University in the next three years. She said that if she introduces herself in only one identity, she prefers to call herself a youth volunteer than a national scholarship winner. The Shandong University Committee of the Communist Youth League is committed to effectively organizing and guiding the volunteer service of young students, establishing a complete volunteer service mechanism, resource sharing, and the linkage of the whole school. In the campus culture of "everyone strives to be a volunteer", Ma Xiaobo, whether the early activity planning or later field layout of the booth, every step: to residents popular drug safety, rehabilitation training with children with cerebral palsy and autism... Ma opei to Fudan University prefers to stay in the laboratory, reagents and instruments always provide a comfortable and secure sense of certainty. It is with the support of the "undergraduate tutorial system" and his own dedication to scientific research and overcoming difficulties that Ma Xiaopei completed the national innovation project alone. Promoting innovation and promoting learning through competition is an important measure in the practical education system of the Shandong University Communist Youth League centered on innovation and entrepreneurship. From the declaration materials, operation experiments, report writing, the conclusion of defense, application for reimbursement. In Ma opei: " All the experience is worth it."The graduate dormitory seems to be" proud ", in fact, everyone has gone through twists and turns, the graduate Peking University Pan Yue is no exception. For five months, she was constantly self-affirming and self-suspicious. At first, people around her gained an offer, and anxiety followed- -despite her 4.66 GPA, second in her major, and having published a paper as the first author. But free and easy, she laughed that she had "two talent, seven diligence and one lucky". Just as when I first published a paper, after a long review, but received a notice of rejection. It was the experience of manuscript rejection and the ups and downs that honed her writing ability and academic research patience, and she also wanted to contribute to the field of pharmacy. There are many small "contrasts" in dormitory 2321. For example, Ran Yuting, the only southern girl, is a loyal lover of suspense and legal programs and the president of the "Cool Action" club. Shandong University has more than 100 student associations. Under the guidance of the association guidance center, it not only realizes the collision of multiple cultures, but also provides a stage for students to show themselves. Ran Yuting is good at playing badminton, and she keeps doing sports after her study. Under her guidance, others in the dormitory also joined the team, and gradually became a long-term member of the sports club."Dare to challenge and make breakthroughs" is not only Ran Yuting's sports label, but also her academic declaration. Her first place in her major led to her, but she chose to cross her major from pharmacy to biology. She said she liked a more challenging environment. Compared with her roommates, Meng Xiaolin's performance is not in the top, once became a "marginal person of graduate research" she is even uncertain whether she can get the graduate research quota. However, with the encouragement of her roommate and the college teacher, she decided to sign up for the summer camp to try it out, and finally successfully got the place to go to the school. When sending messages to her junior students, Meng Xiaolin is full of emotion, "seize the moment, everyone will have infinite possibilities."

2020-11-05	Henan University: Heart and soul, "School Hui" and you accompany the Student Union of South University. Behind the recognition of "school Hui" by "He Baoer" is the effectiveness brought by the students' insistence on serving students as its own responsibility, optimizing rights and interests services, reform and strengthening its own construction.--------------- "Songyue green, great river, Central Plains culture, long..." Henan University has experienced 108 years since its establishment in 1912. Several vicissitudes of life, now the campus is popular is the "school jun"."School Jun" is the kind name of "He Bao" of He University to the WeChat platform created by the Student Union of Henan University. Behind the recognition of "school Hui" by "He Baoer" is the effectiveness brought by the students' insistence on serving students as its own responsibility, optimizing rights and interests services, reform and strengthening its own construction. The Student Union of Henan University adheres to the goal of "gathering students, serving the school, being a good bridge and strictly governing the society", and takes rooted students, relying on students and representing students to comprehensively deepen the reform. The student Union of Henan University sets the fundamental requirements of serving students, optimizes the organization setting, simplifies the staff, continuously improves the operation mechanism, and promotes the standardized supervision and management. Continue to "slim down and keep healthy", according to the actual situation to streamline the number of personnel, the school student union to reduce the number of ministers by 23%, the number of staff by 30%, and the implementation of all the staff assessment and withdrawal system. The student union adheres to the self-examination of "the blade is inward", and gradually strengthens the "slimming" of the institutional level, the "expansion" of the work coverage, and the "warm service for the students". At the same time, the students adhere to the center of gravity down, power, sinking, through the "school seec project" "departments" "school students joint meeting" "school students members cross", form a led, multiple linkage working mode, school will coordinate school straight class, give full play to the main role of departments and class, mobilize the power of the students work, constantly improve the leading and service of the students' level and ability. The student Union constantly improves the volunteer service mechanism, and promotes the majority of students to participate in the volunteer service. We will promote the establishment of a mechanism for " small institutions and large participation to break the closed loop. As long as students have the spirit of dedication and service willingness, they can become volunteers of the student Union to serve students, or participate in it through project service. This from the mechanism to provide students to serve each other, set up the students mutual service open sharing platform, in the service "tower card students" (because the river is located next to the famous "tower", so the river university is called "tower card student" -reporter note) on the road, leaving a string of powerful footprints. With you, "the whole heart rights and interests" feeling warm "river treasure son" " 'the school will be jun', our dormitory heating is broken, the winter sleep is very cold ah!"" 'school jun', the east room on the third floor of the reading room is broken, the light is a little dark."" 'school jun', Jinming campus complex building toilet has a fault, when to repair?"As long as you receive the problems reported by the students," School Jun " will organize investigation on the same day, report the communication to the students that night, and continue to follow up the maintenance and treatment. Timely communication, real-time follow-up, "school jun" for "river treasure" to protect warmth, escort. In the spring semester of 2019,138 logistics problems were received and 109 were solved; 568 were lost and 393 were recovered; immediately responding to students' hot concerns, 15 "Heda Express" were issued and 3 canteen inspections. The problem that "HeBao" is concerned about is the focus of "school Jun" work. Return to the purpose of serving students, to focus on the main responsibility and main business."School Jun" is committed to providing efficient services, reforming and improving service quality, and systematically establishing a network matrix of rights and interests protection. Now, in addition to the official platform of Weibo and wechat official account, there are more "wechat group and QQ" rights and interests service group "established by the rights department of the Student Union, which cover the leaders and monitor of each college, and the" school leaders " in the group publish lost and found marks, and collect life problems. The student union has set up a "opinion storage box", which is distributed in apartment buildings, teaching buildings and canteen and other places. The students will regularly collect it to understand and reflect students' demands. The school student Union has become the bridge and link between the student food service center, dormitory management center, classroom management center and other school functional departments, and closely contact with students, to realize the substantive solution of rights and interests problems."Student union to return to the 'main business', in the final analysis is to let the students in the 'thing' temper, in the 'thing' improvement, improve in the 'thing' growth, so that the student union can better play the role of organizing and contacting students, serving students and leading students."The school Youth League Committee secretary Sha Sha said. With the help of the school's "second classroom report card" platform, in recent years, the students will carry out activities around seven sectors, including ideological growth, practical practice, voluntary public welfare, innovation and entrepreneurship, to give full play to the value of the second classroom education and improve the effectiveness of the second classroom education. They actively build a "face-to-face" platform at different levels between schools and students, teachers and students, and students and students, walk into the hearts of students, listen to students' aspirations, solve practical problems, improve self-construction, and truly practice the working concept of "wholeheartedly, service for you". Constantly close the psychological distance between the school and the students, so that the majority of the students understand the work of the student Union, so that the student union and the students always stand on the same front. The Student Union of Henan University serves students' spiritual and cultural needs, carries forward traditional culture and advocates new styles of civilization. Activities as the carrier, aiming to promote the comprehensive development of students' moral, intelligence, physical, beauty and labor."River big cup" football, winter cross-country, "thought the rhyme debate river wind" campus debate, dormitory culture festival, "the most beautiful river in your heart" photography competition, "school history, love his Alma mater" theme knowledge contest, and founded in 1985, has been successfully held 35 campus singer contest, etc., the campus culture, the open inclusive, advancing with The Times, spread the campus culture positive energy, advocating healthy lifestyle, won the affirmation of successive students, become a river adults common good youth memory. China Youth Daily, China Youth network trainee reporter Yang Baoguang

2020-11-03	Nantong Vocational College of Science and Technology started the "Four History" education activity of The Times "- -learning the" four History " education activity launch ceremony was held. At the launch ceremony, Hu student of Nantong Vocational College of Science and Technology participating in the 70th anniversary of the founding of the People's Republic of China parade shared the youth story of "I and my motherland"; teacher Ye Xin, a national defense education expert from Hohai University, was invited to do the theme class entitled "the war that month-from the modern national defense construction". Nantong Municipal Party Committee of the Communist Youth League, Nantong Vocational Science and Technology College, and more than 500 student cadres, party members, active members of the Communist Youth League and youth representatives attended the activity.

2020-11-02	This paper describes the story of Ninglang Yi Autonomous County, Lijiang City, Yunnan Province, which has overcome the difficulties and strengthened its weak links on the road to poverty alleviation. Ninglang Yi Autonomous County in Lijiang, Yunnan province, commonly known as "Xiaoliang Mountain", was once been difficult to travel and even harder to develop the industry. Since the start of the battle against poverty, Ninglang County has made every effort to overcome the difficulties and difficulties, lifting more than 77,000 people out of poverty. Since the beginning of this year, Ninglang County has concentrated human, financial and material resources to support the frontline "battle" to ensure high-quality poverty alleviation as scheduled. The graduate volunteer teaching group of Harbin Institute of Technology has been supporting teaching in Ninglang Yi Autonomous County, Yunnan Province for 10 years. During the volunteer teaching service, the social love in Ninglang is dedicated to the healthy growth of children in poor areas in western China. The graduate volunteer teaching group, together with social caring enterprises, foundations, Harbin teachers, students, alumni, as well as many caring people, works hand in hand to send the funding and batches of materials to each student in need of help. The university research team has covered 15 townships, 2 high schools, 13 junior high schools and more than 40 primary schools in Hongqi Township, Cuiyu Township and Xinyingpan Township in Ninglang County, directly benefiting more than 7,000 students. The work of student education has been highly recognized and frequently praised by Yunnan Provincial Youth League Committee, Lijiang Municipal Youth League Committee and leaders at all levels in Ninglang. The "Soma Fragrance, Love Full Liangshan" brand student aid activity is planned and implemented by the graduate student teaching group of HIT, which integrates social love and contributes resources to encourage poor children in Ninglang mountainous area to realize their dream of studying. The activity encourages students from primary, middle and senior high schools who are excellent in character and learning and have difficulty in living in life to work hard, get out of the mountains and give back to the society. Since its establishment, it has been widely supported and recognized from all walks of life. So far, it has raised more than 780,000 yuan to help nearly 500 poor children in nine schools to complete their studies. Over the years, 10 children from Ninglang No.1 Middle School and Ninglang Ethnic Middle School in Ninglang Yi Autonomous County have also received a "Soma Fragrance, Love Full of Liangshan" scholarship for a total of 6,000 yuan. The launch of the project not only solves the children's life difficulties to a certain extent, but also makes the children feel the warmth of the society, and stimulates the students' enthusiasm for learning. In addition, the Harbin Institute of Technology also launched a special fund of "Love", to set up annual theme projects and implement them. We have completed the "Love Health Plan", "Lighting Plan", "Sports Shoes", "Love Plan", "Love Stationery Plan" and "Backpack Plan", and the "fitness Plan" is under progress. The project aims to distribute school supplies to children in poor mountain village primary schools in Ninglang Yi Autonomous County, Yunnan Province, and help them grow up healthy and happy. The special fund has not only been greatly assisted by the Tiandi Youyou Love Foundation, but also raised a large number of love materials through the online platform. The graduate teaching group integrates and distributes the materials to the children who need them most. Perhaps a schoolbag, a pair of running shoes, or even a pencil case may inspire the children to continue to study, and decorate their youth even more colorful. Heart has faith, can go far. In 2020, Harbin Institute of Technology has sent 320 volunteers for 18 sessions to the west to spend their youth on the platform."All the motherland needs, is my hometown."Facing the new century, whether it is rooted in the western grassroots volunteer teaching, or devoted to scientific research to serve the country in the future, the volunteer teachers will firmly ideal and faith, practice the original mission in the hard work, live up to the glorious tradition of Harbin Institute of Technology, let the flower of youth bloom where the motherland most needs!

2020-10-30	Jiangsu: Huaiyin Institute of Technology held the youth innovation ability strengthening training theme activity: Creativity, innovation and Creation " youth innovation ability strengthening training theme activity. Beijing, October 30 (reporter Li Chuan correspondent, Wang Xiaohui, Chen Juan) " Innovation is the soul of a nation's progress, is the inexhaustible driving force for a country's prosperity, but also the deepest national endowment of the Chinese nation."In order to train college students 'innovative thinking, cultivate college students' innovative spirit, enhance college students' innovative ability, and fully implement the fundamental task of moral education, On October 27, Jiangsu Huaiyin Institute of Technology held the" Dialogue: Creative Innovation and Creation " youth innovation ability strengthening training theme activity. Wu Jianhua, deputy secretary of the Party Committee, vice president of Huaiyin Institute of Technology and deputy director of Huaiyin University Working Committee attended the event and delivered a speech. Wu Jianhua said that the activity is conducive to guiding young students to fully understand the situation of world scientific and technological innovation and the great significance of the national innovation-driven strategy, is conducive to stimulate and strengthen the young students 'awareness of innovation, but also conducive to the cultivation and training of young students' innovation ability. The theme activity entered the first part in watching the video of CCTV "Dialogue" program "Innovation Born under Change". The teachers and students shared the typical examples and wonderful views of Zhou Yuxian, chairman of China Building Materials Group, Jiao Chengyao, chairman of Zhengzhou Coal Mine Machinery Group, and Huang Li, chairman of Wuhan Gaode Infrared Co., LTD."Innovation is the soul and internal gene of the spirit of enterprise" "innovation is the first driving force leading China's development" "grasping innovation is to grasp development, seeking innovation is to seek the future" and such views and discussions left a deep impression on the teachers and students present. In the expert report link, huaiyin institute of technology, mechanical manufacturing and automation professional doctor, master tutor professor gao rong with their own study and work experience, put forward to the college students let the ideal faith flag young, lead the development of innovative spirit, with innovative entrepreneurial service social advice, message college students set up a good state of mind, keep healthy spirit of the body, the pursuit of excellence. Professor Dong Yun, dean of Huaiyin School of Construction Engineering, doctor of Civil Engineering and academic leader, started from a college student majoring in architecture, and shared his experience of being "specialized", "refined", management innovation, and persistent spirit and perseverance. On October 27th, Jiangsu Huaiyin Institute of Technology held the "Dialogue: Creative Innovation and Creation" youth innovation ability strengthening training theme activity. China youth network correspondent xiao-hui wang Chen Juan for figure in the leadership comments and interaction between teachers and students, huaiyin institute of technology, executive director of working Yue, closes working committee member Wang Wenke speech respectively, hope that young teachers and students to bear the national rejuvenation mission, establish innovation value orientation, strengthen professional knowledge base, expand practical ability training. Young teachers and students participating in the activity actively participated in the interaction. Bai Xiaoying, representative of young teachers, and Ding Junwen, student representative, shared the harvest of participating in the theme activities. They believe that this activity is an activity to stimulate ideals and aspirations and patriotic feelings, an activity to inspire innovative thinking and innovative spirit, and an activity to improve the comprehensive quality and practical ability. They said that they should bear in mind their instructions and teachings, lay a good foundation, increase their talents, serve the people and serve the motherland. Innovation is the open weapon; innovation is the hope of the future. Without innovation, there would be no national prosperity and strength. Without innovation, there will be no great rejuvenation and a better future of the Chinese nation. Similarly, without innovation, there will be no high-quality development of Huaiyin Institute of Technology and the bright future of young students. More than 200 representatives of retired leaders, representatives of young teachers and students from college of Mechanical Materials, School of Automation and Academy of Sciences attended the activity.

2020-10-29	Ningbo University: "Great Aesthetic Education Mode" makes ideological and political education moisten things silently."The practice of 'great aesthetic education mode' in Ningbo University has always been committed to making all students enjoy the opportunity to accept aesthetic education. Through the cognition, experience, feeling, appreciation and creation of beauty, every student shapes the concept, sentiment, taste, style and accomplishment of beauty."Said Tu Chunfei, party secretary of the School of Music of Ningbo University."Joining the chorus is the most unforgettable experience in my college life."Dai Dongjun, a medical school graduate of Ningbo University, joined the school choir in his sophomore year. In the choir, he learned a lot of musical skills, and won many honors such as the Zhejiang Provincial Grand Prize in the chorus competition."Many famous scientists have profound artistic literacy, and aesthetic education can promote professional study and research. I am a good example."Dai Dongjun entered Zhejiang University with excellent undergraduate studies, and also led the choir of Run Run Shaw Hospital affiliated to Zhejiang University School of Medicine to achieve the best results in history. Music has become a hobby that Dai Dongjun can never give up in his whole life. Dai Dongjun started his "music journey" through an elective course. After years of exploration, Ningbo University has formed a "core + characteristic" aesthetic education curriculum teaching system, realizing the "full coverage" of students in the school."Core" is the core limited elective courses, such as "Introduction to Art", "Music Appreciation", "Art Appreciation", "Film and Television Appreciation", which require all students to take at least one elective course; "Characteristic" is an open online and mixed online and offline elective courses. Dai Dongjun took three courses in the school, and entered the school choir through the course study, which has greatly improved his aesthetic appreciation and humanistic quality. The school has also introduced the "teaching team" system and promoted the integrated talent training mode of "performance, learning and innovation". Now, it has built 2 national high-quality courses and 2 provincial high-quality courses. Related courses have been launched on five domestic MOOCs platforms and a learning power, with more than 100 online and offline universities that have selected courses, and the number of courses selected has exceeded 100,000. Aesthetic education practice "ground gas" course learning no time no interest? It doesn't matter, there is also the aesthetic education practice waiting for you. Ningbo University has set up 40 aesthetic education practice projects such as college Students art Festival and College students chorus Festival. Students can get 0.5-2 quality credits. If participating in competitions and winning awards, they can get up to 8 innovation credits."I remember the first time I moved props to the drama, with only one iron frame, but I pinched it for two hours, and every time I moved it to my heart, I was worried that I was wrong and the whole play was ruined."Xiao Yu (pseudonym), a student from Ningbo University, joined the club, and was just responsible for carrying props. After a period of study, she got a very small role. She felt" very excited that she was imagining the day of the show."Rehearse all day on weekends, eating is takeout, nap on the bench, so hot weather that people "feel difficult to breathe". However, these "bitter", in everyone's eyes have become "happy", "sometimes we will read the lines on the road, just like occupying here, I think- -this is what youth should look like". Baihua Lin Drama Club of Ningbo University is one of the top ten societies in Zhejiang Province. It has won the Best Drama Award and Organization Award of Zhejiang College Students Drama Network Competition, and is one of the key aesthetic education practice platforms built by the school. The down-to-earth aesthetic education practice has not only attracted a large number of excellent students, but also achieved excellent explicit results. At the closing ceremony of the fifth National College Students Art Performance, Ningbo University Chorus conquered all the audience with a song "Yang Pass Song". The National College Student Art Show is held every three years, which is the highest standard, largest and most influential art event for college students in China. In the fifth national college students' art performance, Ningbo university chorus in the province first national performance, and in the history of Zhejiang province for the first national big art exhibition professional group choir into the live performance (only 11), finally with almost perfect performance won the first prize in vocal music b group, and as a vocal music group one of the only four choir at the closing ceremony. After the closing ceremony of the party, then deputy secretary of education committee, deputy director of education department Bao Xuejun specially visited the closing ceremony of Ningbo university teachers and students, "the performance is an opportunity, the key to strengthen the usual art education, to build the campus art atmosphere to go, represented by Ningbo university, the public art education in Zhejiang province bigger and stronger"."Lei, people struggle, hundred death for the country; the declaration lang lang, but out of my mouth, always in my heart!"In June 2020, Ningbo University Red Art Light Riding Team walked into Beilun Xiapu Street to participate in the first event when I was a party lecture speaker in Zhejiang. 270,000 party members in the province participated in the viewing. Zhu Chaoyin, director of the Xiapu Sub-district Office of Beilun District, Ningbo City, said with deep feeling: " As the people of the whole country are united in fighting the epidemic, reviewing the lyrics of the Declaration of the Original Morning and the Morning Start is more powerful."《 Morning Start Manifesto is a musical work created by the Red Literary Light Riding Team of Ningbo University, which tells the story of two generations guarding their original intention. This work has been on the stage in Ningbo University for five consecutive times, and has also entered the South Lake in Jiaxing, military barracks, old revolutionary base areas, rural auditorium and other places for the local people, with a total audience of more than 2 million people."We live in this great age, and we cannot live up to it. We need to resonate among teachers and students, and find the common background of The Times."Said Liang Qing, a professor at Ningbo University and the producer of the musical. Ningbo university red light team to carry forward and spread the red classic literature and research university "aesthetic education" as the main body, now has the national art fund project 2,2 original musical, ten original songs, music skits 1,4 academic papers, performances in Beijing, Shanghai, Yan'an, Zunyi, Ganzhou, Hangzhou, Jiaxing, Ningbo, including the national and Ningbo spiritual civilization construction "five one project award", a number of industry awards. In the past twenty years of exploration and practice, Ningbo University has built a linkage mechanism from top-level design to grass-roots level implementation, and gradually formed an aesthetic education culture with both hard power and soft power. Campus environment is the hard power of aesthetic education culture. Under the school aesthetic education work committee, Ningbo university built a large number of aesthetic education base, built Thanksgiving culture garden, ancient ceramics museum, Hu Xiaoping art center, 1986 and dream garden 10 campus aesthetic education base, and the government, enterprises to build "Ningbo help" museum, Ningbo art museum and more than 30 off-campus aesthetic education base. The school also focuses on improving the landscape of aesthetic education, with the overall planning of building names, road names and lake names, forming many public art works such as Wang Yangming bronze statue, Bao Yugang sculpture and many other works, and integrating the rich gratitude culture into the construction of aesthetic education landscape, so that every tree and grass on the campus can become the carrier of aesthetic education, forming an infiltrating aesthetic education environment. Soft power is invisible, but even more important. Aesthetic education in colleges and universities needs "building" and more needs "master". School implemented the double hundred aesthetic education teacher plan, cultivate hundreds of campus aesthetic education backbone teachers, outside hundreds of aesthetic education teacher, the existing executive chairman of China singer association, vice chairman of the Chinese artists association Pan Gongkai school teachers 56, and famous singer hu, the American national independent college of art and design alliance chairman of Tony Jones outside aesthetic education teacher 49 people. At the same time, the school has established aesthetic education studios of music, drama and art with famous teachers as the core, and now it has established 30 studios such as Xu Zhongyou Studio and Liang Qing Musical Studio. Under the framework of "building" and "master", Ningbo University takes associations and brands as the main starting point, making the infiltrating campus cultural atmosphere of infiltrating aesthetic education penetrate into the students. The school implements the construction plan of "high-quality associations" for aesthetic education, guides the student associations of aesthetic education by different levels and categories, and sets up a high-level art association team with the themes of instrumental music, chorus, dance, drama, painting and calligraphy, etc. Now, it has built 50 aesthetic education associations, such as choir, Baihua Lin dialect Drama Association and Ballroom Dance Association. At the same time, the implementation of aesthetic education brand activities cultivation plan, relying on 25 colleges to promote the construction of "one school, one product". Now, it has formed 45 school-level aesthetic education brand activities such as "New New SHOW", "Original wind" and "Ning Good Voice", as well as 10 school-level brand activities such as the top ten singers, drama festival and chorus festival. The aesthetic education culture with both hard power and soft power is going more and more out of the campus to the world. In 2019, Wang Lei, the head of the Conservatory of Music of Ningbo University, led a delegation of 7 people to the UAE to participate in the 11-day 15th World Year of Music Peace activity and the visit performance of "Listen to China", which is the largest and longest tour of Chinese folk music into the UAE universities so far. China Youth Daily China Youth Network trainee reporter Yang Baoguang, correspondent, Zhang Zhiping, Zheng Junpeng source: China Youth Daily

2020-10-28	Anhui medical university for seven consecutive years to build "everyone speech" group brand Anhui medical university for seven consecutive years to build "everyone speech" group brand Wang Lei correspondent Cheng Yan Li-juan Mao) was recently held in Anhui medical university "my Chinese dream-don't forget to beginner's mind, keep in mind that the mission of the" everyone speech final, the school 47 undergraduate and graduate students compete, about the understanding of "beginner's mind mission". Since the launch of the preliminary contest in October 2019, more than 4,000 freshmen have stood on the platform, from the perspective of historical changes, scientific and technological progress, medical development and other examples, to express their firm faith in loving the Party and the country, the courage to create and the good character of dedication with language and art. Since 2013, the school has carried out the "My Chinese Dream" speech activity for freshmen for seven consecutive years, creating a thought-leading brand for everyone, with more than 30,000 students participating. Every year, the speech activities use the way of promotion round by round for theoretical self-study, teachers, speech learning, practice deep learning, etc., to ensure that every freshman has the opportunity to take the stage.

2020-10-28	Zhang Jianwei, correspondent Liu Yong) Recently, the 2020 Qingma Open Class Demonstration class of Jiangsu University was held in the school auditorium. Wang Haijun, secretary of the Youth League Committee of the university, attended the event. More than 400 people participated in the event, including the leaders of the secondary league organizations, the youth League branch secretary of grade 2020, all the students of the third big bone class and the students of the Elite College. The event was broadcast live through the online platform and watched by nearly 10,000 people. This "Qingma Open Class" in the form of keynote speech, teachers and students style display and other forms, During the keynote speech session, School of Marxism teacher Yue Zhiqiang made "patriotism is the background color of youth" theme to share, Encourage young people in the new era to carry forward patriotism; Zhao Yanyan, an advanced individual in COVID-19 prevention and control in the national health system, shared the theme of "We are One Family", Tell touching stories during the epidemic, Highlight the mission of the young people of major countries; Li Guowen, a retired professor at the School of Food Science, wrote a difficult history of building a school, A constant love of school feelings " theme sharing, Reviewing the hard course of the early days of the school, Encourage students to have ambition and have a career; Wang Ting, graduate student of the School of Management, Zhang Shim, leader of the 21st Graduate Student Support Group, and Hong Siming, alumni of Vehicle Engineering Grade 00, also shared themes to encourage students to set up lofty ideals, dare to challenge. In the exhibition section, the teachers and students of the school of Literature brought the original poem titled "The Dream of the Agricultural Machinery Man", and the content is inspiring. Subsequently, the tutor appointment ceremony of "Faith Open Course" of Jiangsu University was held. Wang Haijun, secretary of the Youth League Committee of the university, presented the letter of appointment to the six keynote speakers.

2020-10-22	Recently, 60 brand-new bicycles have been sent to the village department of Fanzhuang Village, Shijian Town, Wuwei City, Anhui Province. Fan Qiyun, a local student, said happily after getting the bike, " I finally got my first bike. I used to rush to school before dawn, but now I can recite more books at home."On the same day, the Youth League Committee of Anhui Engineering University cooperated with the Wuhu Municipal Party Committee to guide the school Xiaoxiao service team to hold a donation activity in Fanzhuang Village and donate 60 charity bicycles to poor students. The bikes were given to poor villagers and students. Xu Dezhong, party secretary of Fanzhuang Village, praised: " The quality is very good and everyone liked them. The villagers and students who got the bikes before said they were very strong."At the donation site, some students immediately rode around the village after getting the car, very excited. Zhou Yuhang, the head of the volunteer service team, said, " The happy smiling faces of the children are the most satisfactory answer paper we have received. The volunteer service team will continue to bring happiness to more children."Since April 2019, the volunteer service team has donated a total of 210 public welfare bicycles to Yahu Village, �� -qiao District, Suzhou City, and Fanzhuang Village, Shijian Town, Wuwei City. Where did these cars come from? Tao Ye, secretary of the Youth League Committee of the School of Materials Science and Engineering of Anhui Engineering University, said that the abandoned bicycles on campus not only affects the beauty of the campus, but also hinders the campus traffic, which has become a campus "pain point". The volunteer service team gave full play to the advantages of the related disciplines of the School of Mechanical Engineering and the School of Materials Science and Engineering, and reassembled and reconstructed the abandoned bicycle on campus by relying on the bicycle disassembly competition, and the abandoned bicycle has become a new charity bicycle. Tao Ye said that the donation of public welfare bicycles to the poor areas in need not only solves the pollution problem of waste bicycles in schools, but also improves the students' professional practical ability, and also realizes the charity donation for public welfare. Founded in March 2019, the Volunteer team is committed to exploring the new development direction of campus bicycle management by solving the problem of used bicycles on campus. It has grown from more than 10 people to more than 140 core members and more than 500 participating members. After one and a half years, the team comprehensively cleaned up the main waste bicycles and battery cars on the campus. After all the bicycles were modified and donated to poor areas, all the waste vehicles that could not be reused were scrapped for sale, a total of 28,810 yuan. The money was remitted into the school financial account as a donation for poverty alleviation love. In the next step, the volunteer service team will go out of school and cooperate with the Youth League committee of other universities in Wuhu city to discuss the recycling plan of waste and uninhabited bicycles on campus; cooperate with bike-sharing related companies to provide maintenance services for them, and jointly negotiate for public bike donation activities.

2020-10-22	Changchun normal university: "volunteers 108 will" support love relay Changchun normal university: "volunteers 108 will" support love relay music, art and other art classes, also established painting, guzheng, electronic organ, hulusi and other interest groups, as far as possible to provide children to experience art, enrich their extracurricular life. In---------------, a three-month volunteer teaching experience, made Liu Haitao, a student of Changchun Normal University, decided to become a special post teacher, rooted in a rural primary school in a foreign land. Not only that, he also influenced the former classmate, now the lover Liu Mingyue also joined the team of rural full-time art teachers. Liu haitao's hometown is in Tangshan, Hebei province, as the only child. As his parents get older and need more company, Liu struggled for a long time when he chose to go from college. In the end, he chose to return to the Hongxing Township Central Primary School, where he once volunteered to teach, as a special post teacher, as a full-time music teacher."When the volunteer teaching was parted, my students' tears fell to my heart."This friendship makes Liu Haitao difficult to give up. Hongxing Township Central Primary School is located in Yushu City, Jilin Province. Changchun Normal University, the person in charge of the Youth League committee, Yushu City rural art education has been relatively weak, the city's 43 rural middle schools, primary schools do not have a full-time art teacher, slightly better conditions of rural schools at most only one part-time music or art teacher. Five years ago, when Liu Haitao first came as a volunteer teacher in Hongxing Township Central Primary School, he had to bring a small speaker every time he took a music class. He found that the local children were very short of music theory, and it was not easy to teach them music or learn the rhythm. In the past five years, the Qingsong Volunteer Teaching Group of Changchun Normal University has recruited 10 groups of 108 volunteers to 13 middle and primary schools in Yushu City for art volunteer teaching. Different from the traditional short-term volunteer teaching, each group of literary and art volunteer teaching volunteers are stationed in rural schools for at least three months in the form of graduation practice. In 2016, Liu Haitao, a junior, became a member of the Qingsong Volunteer Teaching Group. Due to the limited quota, the selected students are excellent in character and learning and willing to bear hardships and contribute. The volunteers not only opened art classes such as music and art in rural schools, but also set up interest groups such as painting, guzheng, electronic organ and hulusi, trying to provide children with opportunities to experience art and enrich their after-school life. Last year, Zhao Jiayi, a junior at the Conservatory of Music, quickly signed up when he learned that the Qingsong volunteer teaching group began recruiting again. He and his teammates left their comfortable city life and came to the remote countryside, where dirt roads were cut off in every bad weather. Zhao's volunteer school is also Hongxing Township Central Primary School, which is responsible for teaching music lessons for third to sixth grade students. A few years ago, Hongxing Township Primary School applied for government funding and bought a lot of Musical Instruments, but no teachers were always idle. During the volunteer teaching period, Zhao Jiayi cooperated with Liu Haitao to set up the school's pipe band, to teach the children to learn the saxophone, clarinet, trumpet and trombone and other musical instruments. When Zhao Jiayi finished his volunteer teaching, the wind band was already good. The children changed from being completely unfamiliar to Musical Instruments and ignorant of music scores to playing three pieces. Today, the band often participates in briefing and competes at the local children's palace."With nearly a semester of stay, we can really understand a rural school and the students there, and find the most suitable teaching method."Zhang Fangcao, a 2016 student at the Academy of Fine Arts, said that this is an effect that short-term volunteer teaching cannot achieve. Zhang Fangcao has been a volunteer teacher in the Eighth Town Central Primary School for more than three months, serving as an art teacher, teaching students to draw stick figures. When she first entered the classroom, Zhang Fangcao saw the children all sitting straight and staring at her. Such a scene, Zhang Fangcao felt "very sacred", from that moment on, she was determined to have a good class, not to live up to the expectations of the children. As a volunteer teacher, Zhang Fangcao gets up at 5:30 every day, goes to the office to prepare lessons after washing, and goes to bed at 9:30 PM. Changing her habit of staying up late, she found that her health was much better and she did not like to lose her hair. Now, Changchun Normal University's volunteer teaching service covers calligraphy, art, music, dance and other courses for rural schools. More than 30,000 rural students have benefited from the 108 volunteers. Not only do volunteers volunteer to teach literature and art in rural areas, but Changchun Normal University also regularly invites the children from these rural schools to visit the university campus and perform in the concert hall of the volunteer teachers to feel the university culture. In the view of these volunteers stationed in rural schools, the long-term volunteer teaching for college students can not only effectively change the backward teaching level in rural schools, but also make college students know more about and pay more attention to rural education. In the eyes of Qingsong literature and art volunteer teaching volunteers, Liu Haitao and Liu Mingyue are their role models. "The flower of youth blooms in the place where the country needs it most". For Liu Haitao and his wife Liu Mingyue, working and living in rural schools, not too much with vigour and vitality, but more can see the children eager to learn art eyes and their love for music every day. This made the couple think that their original choice was right. Now, as every classroom in Hongxing Township Central Primary School is equipped with multimedia equipment, Mr.Liu has added a lot of audio-visual content to the music class. Being good at playing ocarina and hulusi, Liu Haitao also set up the ocarina and hulusi club at the school. This year, due to the COVID-19 epidemic, Changchun Normal University suspended its offline art teaching activities in rural schools. In order to allow rural children to continuously learn art courses, volunteers from Changchun Normal University recorded a series of music and art teaching videos. On this year's June 1 Day, the volunteers also wrote many handwritten letters for the students, who recalled their good time during their volunteer teaching, and sent holiday blessings and encouragement to the children. After the beginning of the semester, the volunteers began to try to return to rural schools again. Considering the epidemic prevention and control factors, the volunteers can only go once a month. Unable to go face to face, the volunteers recorded the weekly art courses respectively and sent them to the rural schools. Through the online courses, the love relay of the art volunteer teaching was continued continuously. China Youth Daily China Youth Network reporter Wang Peilian, intern, Yu Xishui source: China Youth Daily

2020-10-20	Yangzhou Vocational University: "CD action" leads the saving of "CD food action" leads the saving of "new food action" constant read material difficulties."At the beginning of the semester, Yangzhou Vocational University multi-pronged to resolutely stop food waste. In order to encourage teachers and students to "CD action", the logistics department arranged the staff to send gifts to each "CD" teachers and students, to create a strong campus atmosphere of thrift and respect for labor."CD action, thrifty raise morality", "civilized dining, respect for the fruits of labor"... the public welfare signs advocating "CD action" are placed in the prominent position of Yangzhou Vocational University canteen, each canteen, advocating thrifty banners can be seen everywhere, small meals sell hot."Before the students returned to school, we posted a conservation proposal at the gate of the canteen to create an atmosphere of shame and pride on campus."Zheng Zhi, director of the food and beverage service center of the logistics management Office of Yangzhou Vocational University, said that as students return to school, the number of teachers and students in the canteen has gradually increased. In the process of cooking, the staff reminded the teachers and students to buy meals according to their own situation. The canteen also launched a special reward package, " we randomly distributed small gifts, including two yuan meal fee vouchers, stationery, milk, fruit, etc., although the amount is not much, but the 'CD' awards, encourage everyone to cultivate the good habit of saving food."Zheng Zhi said. Wang Hui, a canteen staff member, who was the distributor of the small gifts, and said that teachers and students would suggest a "CD" whenever they had leftovers. Every time she gave out the prizes, she felt very happy. As a farmer's child, her parents' hard work makes her know that everything is hard. The small dishes introduced in the school canteen also let students who want to lose weight find a new way. For Yin xuehan, a meal used to cost about 10 yuan for a meal, but now she can eat full for 6 yuan. At the same time, the Logistics Management Office of the school is committed to optimizing management and improving service quality. On the one hand, it timely connects with the academic affairs department, grasps students 'meal peak hours, understands and improves students' favorite food varieties through questionnaire survey and data analysis; on the other hand, it introduces high-quality catering companies through bidding, strengthens supervision and assessment, and improves service quality and satisfaction of teachers and students. Nowadays, less fried, more fried, batch fried, rice quantified according to demand... diversified personality service has become the "standard" of Yangzhou Vocational canteen, which not only ensures the quality and taste of dishes, but also avoids waste phenomenon. The purpose of the "CD action" is not more teachers and students, no more canteen, and no more kitchen. It is expected that through two months, students will develop the good habit of cherishing food, practicing strict economy and opposing waste. Zhou Zhong, deputy secretary of the general Party branch of the logistics management Office, said that 200 small gifts are currently delivered for a limited time every day. Zhou Sheng, Party secretary of Yangzhou Vocational University, said that thrift is a traditional virtue of the Chinese nation, and the university has been actively implementing the concept of hard work and thrift. In the process of promoting the construction of energy-saving campus, "practicing strict economy and opposing waste" has gradually become the action consensus of school teachers and students. Hua Jun Liang Xiaomei, China Youth Daily China Youth network reporter Li Chao

2020-10-20	Dalian University of Nationalities participated in the "Dalian Liupanshui Golden Autumn Consumption Festival" to provide volunteer services for the whole activity. The 20 Guizhou college students gave full play to the advantages of bilingual volunteer service, and introduced the characteristic products to support the agricultural and sideline products, tea and wine in Liupanshui and Tongren. According to the introduction, poverty alleviation through consumption is an important way for social forces to participate in poverty alleviation. By consuming products and services from poor areas and poor people, it helps the poor people to increase their income and get themselves out of poverty. The school attaches great importance to this work. The Communist Youth League of the school conducts extensive publicity and mobilization among the bilingual volunteer service group volunteers of college students, takes the initiative to meet the work needs with the organizer, and selects college students who are familiar with the cultural environment of both places and have volunteer service experience to participate in the activities with high-quality service. Xiao Zhiyun, a volunteer from Liupanshui, Guizhou province, said, " I am very proud to speak for the products of my hometown in Dalian and contribute to the cooperation between the east and the west and the national poverty alleviation.”

2020-10-13	Sichuan Culture and Media Vocational College: More highlights: Leave a good first impression Zhang Jianwei correspondent Hao Yuemin) Just after the double festival, Sichuan Culture and Media Vocational College ushered in the 2020 autumn unified recruitment freshmen. With the successful experience of single recruitment work, the college leadership and relevant departments have made unified preparations to make the autumn recruitment work with highlights and characteristics on the basis of following the epidemic prevention and control requirements. On the one hand, it should be different from the single enrollment. On the other hand, it can feel the professional education and colorful college life. In chongzhou campus autumn recruit welcome site, one side is according to the department of professional division, to help students easily complete professional registration, realize that the same professional senior seniors and teachers; the other side is according to the source of students, so that each site becomes a small "hometown station", new to inevitably unfamiliar, but meet their fellow or even classmates will make people warm. The simultaneous professional reception points of Mianzhu campus will show their professional results, including exquisite and meticulous handicrafts and magnificent ink Chinese painting. Freshmen and their parents can form a preliminary understanding of their professional learning through the explanation of students' works and site volunteers. In addition, the Communist Youth League Committee of the college also organized student volunteers and student associations to perform song and dance flash mob performances, attracting many freshmen and their parents to stop to watch and applaud. The orientation work can not only reflect the overall management and work level of the school, but also be related to the first impression of freshmen on the school. The orientation site with frequent highlights and many fun will undoubtedly become the first good memory of freshmen and literature. Luo Qin, the executive director of the college, also said that the freshmen came from different provinces and cities. The strict epidemic prevention and control and the unknown of the new life would inevitably make the children a little nervous. They would create a cheerful and lively welcoming atmosphere with their warm work appearance, so that the freshmen could feel the warmth of the message in this late autumn.

2020-10-12	The Xuanhuai College of Tianjin University explores the innovation and entrepreneurship education mode to design the future performances in the classroom. The Xuanhuai College of Tianjin University, as the "explorer" of the reform and development of innovation and entrepreneurship education in Chinese universities, recently announced that it will launch a "new engineering business plan". According to the introduction, the plan will build a cross-college and interdisciplinary project team platform, inviting all outstanding students with undergraduate, master and doctoral degrees to participate together, create scientific and technological innovative products, and improve the ability to solve practical problems in the project practice."Can you imagine swimming in the pink ocean and picking up a wisp of memory from the childhood swing? With the rapid development of The Times, more and more people are in the state of psychological sub-health, while in Mr. With the help of right, each of us can have a unique AI psychologist... "This is the first professional core course of IDBE Project 1- -the roadshow of the closing project". The former chief designer of the Nokia Research Institute was also in the audience. Different from the traditional theoretical teaching and homework reporting, this unique course has a whole new set of curriculum content and teaching system, with neither fixed textbooks or selected homework questions. What students need to do is to predict and design products that may emerge in the next 5-20 years. Making the classroom full of imagination is an important way to explore the effective mode of innovation and entrepreneurship education in recent years. In 2015, Tianjin University established the School of Innovation and Entrepreneurship, named after Sheng Xuanhuai, the founder of the university, aiming to become an "experimental area" for the reform and development of innovation and entrepreneurship education in Chinese universities. As the "test field" of new engineering education at Tianjin University, Xuanhuai College began to offer a double degree in business Administration (innovation and entrepreneurship) in 2015, recruiting students from all the sophomores of Tianjin University. So far, 182 students have received double degrees. In 2018, the college launched the first batch of national new engineering research and practice projects of the Ministry of Education- -the Qiushi Talent Class, drawing on the innovative educational practice of the world's top universities such as Stanford University and the Massachusetts Institute of Technology. The first-class universities at home and abroad jointly participated in the construction, and the graduates were awarded double bachelor's degrees. In the class here, students need to independently learn the knowledge needed in the process of the project, including 3D printing technology, video collection and editing, sensor original application, product full-case design, etc. In order to help students' unrestrained dreams come true, it has also set up a team of innovation and entrepreneurship teachers from foreign universities, domestic universities and enterprises. Under the guidance of teachers and business mentors, each student needs to complete the research and product prototype model production, writing including project work report, industry analysis report, English business plan and product design instructions, the project report, finally in different professional teachers and entrepreneurs in various fields of the review, multimedia and video product display, complete the group project final roadshow. What is more interesting is that students can also teach the "Lego play seriously" innovative experience in the manual learning laboratory, make 3D printing models in the prototype laboratory, enter the small and medium-sized enterprises to communicate face to face with the founders, and participate in the IDBE LAB summer camp concentrated project system learning. The relevant person in charge of Xuanhuai College said that he hoped to cultivate innovative and entrepreneurial leading talents with design thinking, innovative consciousness and entrepreneurial ability through different teaching modes."In the classroom, I can perceive the technological changes taking place in the world and understand what the world's advanced companies are really like."Zhuang Wenqian, the first student of the Qiushi Talent Class, believes that what she has gained is not only knowledge here, but also a state of continuous innovation. China Youth Daily, China Youth network reporter Hu Chunyan

2020-10-12	The Student Union of Dalian Maritime University: Building "Pengyou Circle" is a famous higher navigation university in China, known as "the cradle of navigator". Since the deepening of the reform, the Student Union of Dalian Maritime University has tried to build a campus "Peng-friendly circle" to serve students to grow up and guide them to consciously integrate their personal ideals into the responsibility of striving for the Chinese dream."Before mentioning theoretical study, the first reaction was boring and boring. Since the school student union launched the 'Pengyou' series, everyone rushed the theory class, for fear of falling once."Said Zhang Shihao, a student at the School of Marine Electrical Engineering."Yukun Chengpeng" is the training concept that Dalian Maritime University has always been adhering to, and "Peng" has thus become the symbol of outstanding students in the university. The "Pengyou" series is a red theoretical brand popular with young people for the students. This series of activities was studied and launched together by Liu Kexin, a presidium member of the university Student Union, and other members of the student Union. According to the different needs of the students, they carried out a series of activities, such as "Professor afternoon tea", "You Penglai", "Penglai", "Pengyou Hui" and "Pengyou said". In the process of practice, the "Pengyou" series of activities have won the students' recognition and love with their flexible and diverse forms and rich and vivid content. Students will investigate the form and content that students like in advance, and carry out activities in different groups. At the same time, after each activity, it will be timely adjusted according to the opinions and suggestions of the students, and constantly improve. In the eyes of the students, "having a strong sense of participation" is one of the main advantages of the "Pengyou" series of activities. It can not only participate on site, but also record it, allowing students who are unable to participate online. Liu Kexin said: " In the way of the students, to timely convey the voice and propositions of the Party, to serve the growth of students, guide the students consciously personal ideals into the joint struggle of the Party and the people, is the important responsibility and mission of our student Union staff."In the past two years," You Peng to "reading salon activity has been held 58, appreciate more than 60 related works, online and offline participants accumulated more than 6000," professor afternoon tea "activity also held 28, online and offline participants nearly 3000," Peng you said "," Peng peer " also held nearly 20 times. In addition to conveying the voice of the Party, the school student Union also pays attention to listening to and sorting out the general needs and practical difficulties of the students, and gives feedback to the relevant departments of the school in the first time. For example, with the great help of the school logistics group, the functional modules of "bath appointment", "logistics online warranty", "equity questionnaire", "tuition inquiry" and "empty class table query" of the school student Union have been successively put online. Students' problems in daily life can be left at home and directly reported to relevant departments through the mobile wechat client. The Rights and Interests Department of the Student Union will also timely follow up and assist in giving feedback, truly realizing the transformation from simple voice transmission in the past to two-way communication. In addition, the rights and interests department of the Student Union will also regularly organize activities such as "school leaders face to face", "functional department exchange meeting" and other activities, and the "Pengyou circle" is getting better and better. Can there be no president or vice president in the school student association? Dalian Maritime University gave a positive answer. The student union of the school standardized the organizational framework, innovatively began to explore the operation mode of "presidium + working department", and implemented the rotating system. The presidium of the Student Union is collectively responsible for the major matters of the Student Union. There is no chairman or vice chairman, but only the executive chairman, and the presidium is responsible for convening meetings and carrying out daily work. Wang Song, a member of the presidium of the university student Union, said: " Since the implementation of the rotating system, our presidium has formed a working mode of combining collective leadership and individual division of labor and responsibility."On this basis, the school made it clear that the college student union is the grassroots organization of the university-level student union, and accept the guidance of the university-level student union. This institutional arrangement strengthens the working linkage mechanism of the two-level student union, and the student union of the college gives full play to the advantages of being close to the majority of students to undertake specific work projects for the whole school students under the guidance of the school-level student union."On the one hand, we take the initiative to communicate and report our work with the student union, and on the other hand, we insist on independently solving problems within the scope of our responsibilities. We and the school student union began to truly exchange intelligence, mutual support, mutual supervision and mutual supervision, which not only enables the students to have more understanding of the student union affairs, but also enhances the students' sense of participation."Shipping Economics and management school student Union executive chairman Xia Mengsi said. In November 2018, the 27th student congress of the school was held, and the permanent representative conference system was formally implemented. As a permanent organization when the student congress is not in session, the congress recommends 22 permanent representatives elected by it to form the permanent congress, and has set up an office to supervise the rights and interests department of the Student Union, which is responsible for the collection and sorting of proposals. During the not in session, permanent representatives visited students, focused on campus problems, reflected students' demands, and solved them in the form of proposals. On this basis, the rights and interests department of the Student Union specifically classified the proposal content, and submitted it to the relevant departments of the school in the form of work suggestions and opinions to follow up the handling progress. After that, the school's proposal handling opinions will be summarized and fed back to the proposal representative, and the implementation situation will be submitted to the permanent Congress office, which will report to all the representatives."In the past, the approval of student club activity venues was a 'big old problem', students need to take the list to find the college, teaching facilities management center, the security department, the school youth League committee and other departments, each department and work in different places, often run a day, now as soon as the online submission is done."Said a student from a club at the school. With the support of the school youth corps committee, the students actively coordinate communication school departments, develop and constantly optimize the student community activity application network platform, the platform implements from activity declaration, site application, publicity, teachers and unit audit to the relevant functional departments of the whole process of network, students never leave home can complete the approval process, greatly improve the ability of the management and supervision of student community activities. China Youth Daily, China Youth network trainee reporter Yang Baoguang

2020-10-12	Huazhong Agricultural University Research Group takes students to play "science" with peaks with steep cliffs and clouds. From the beautiful Enshi to the smooth Wuhan, one teacher and three volunteers traveled 531 kilometers across the forest sea, and brought 12 children to the place where the dream of science began. Recently, the 7th rural children's city tour of "Big hands holding small hands, smile at the world" held by Huazhong Agricultural University " public welfare project was launched in Wuhan. Twelve mountain village students from Mofeng Central Primary School, Guandian Town, Jianshi County, Enshi County, Hubei Province, spent five days of National Day holiday at Wuhan Huazhong Agricultural University and the Science and Technology Museum, led by volunteers from the Huazhong Agricultural University Research Group."Read 10,000 books and travel 10,000 miles."Compared with the textbook knowledge, this scientific journey has a model, can touch, more intuitive, more specific, children can use their eyes, with their hands, with the heart to close to feel the infinite charm of science. The first stop of their trip is the insect laboratory of Huazhong Agricultural University. In the popular science class "Different insect world" of the School of Plant Science and Technology, Teacher Zhou Xingmiao went straight into the theme and guided the children to learn insect knowledge with questions."Why learn and learn about insects?"Under Zhou Xingmiao's explanation, we understand that the insect world has its own system, has been alive in the world for hundreds of millions of years. At the end of the class, Zhou Xingmiao gave each child a specimen of insects. The children sometimes held the specimen in both hands, and sometimes held the specimen tightly in their chest and began to jump excitedly. In the microbiology class at the School of Life Science and Technology, the children went into the micro world. Teacher Chen Dasong explained the existence of microbes to the children through easy-to-understand examples, and then introduced the use of the microscope, and the children also experienced observing the microbes with the microscope personally."That's amazing, too!"The students communicated with each other excitedly. Tan Chao said: " Ha ha, I still have ice blue crystals in my saliva!"Liu Botao, a fifth-grader, wrote in his diary:" I was very nervous and didn't know how to use a microscope. Fortunately, I had big brothers and sisters who taught me carefully. Originally the bacteria are invisible to the naked eye, I actually have so many microorganisms on my hands."In the food fun experience camp activities, the students from the School of Food Science and Technology explained to the children the quality of snacks and the selection method. After the explanation, under the guidance of the members of the Association of Food Science and Technology, the children began to make egg tarts by themselves. This is a special DIY food trip for the children, each student became a "little pastry chef", ready to enjoy the happy and beautiful "food" light."Wow, so sweet!"" You see, the golden egg tarts are so good-looking!”。Before the egg tart came out, the children had already stood on tiptoe, through the oven glass, close up to see the flapping egg tart liquid inside. In the Aquatic Animal Heritage and Breeding Function Laboratory of the Fisheries College, the children got to know about aquatic life and learned about ecological knowledge through creating the aquatic scenery by themselves. The stones crashed into the fish tank, and all the water plants were fixed under the water, and three or two pairs of small fish shuttled freely among them.... the children's eyes were full of expectation."When I first came to Wuhan, what impressed me about the children who just came out from the mountains most was their formality. As the natural science journey activities began, the children's changes happen quietly. They began to actively interact with their teachers, ask questions, and communicate with their classmates."Lead the teacher, the 16th BenYu volunteer service team volunteer Yuan Shihan said."In the process of landscape creation experience, hands can not only strengthen children's cognition, but also touch children's thinking, to understand 'what is, why, how to do', let them be inspired is the original intention of organizing this activity."In the virtual simulation laboratory of the College of Horticulture and Forestry, the children take turns to experience the process of the virtual simulation experiment. Combined with virtual simulation and artificial control techniques, Teacher Xu Liqing from the College of Horticulture and Forestry explained to the children the knowledge about seedling planting, grafting and fruit picking cultivation."Why can the big car go on the side?"Seeing the machinery, the boys excitedly surrounded them all up. At the electromechanical engineering training center, the children saw the two locomotive models flexibly driving on a simulated ridge road without any operation, and wondered, " Why didn't it hit an obstacle?"" How did it find the right route?"A student of the School of Technology explained:" These two model cars use ultrasonic sensing to detect the surrounding obstacles and identify weeds and crops with infrared light. The special structure of the wheels ensures that it can travel freely in the field."The children nodded and responded loudly when they heard what they know."Can the machinery go up the stairs?climb a wall? Or do you fly up? Can the car get smaller? What if the weeding is stuck?......" children associate with a lot of problems. Mei fourth grade Shaokang excitedly to lead the teacher, the 16th Ben Yu volunteer service team volunteer Deng Shasha said: " my favorite TV program is 'I love to invent', see these machines very happy ah, later I also want to like the two brothers invented mechanical car."She actively encouraged him to study hard and strive to realize his scientific dream in the future. The children also visited workshops and agricultural work cars, and experienced laser cutting technology up close in the workshop. After the visit, everyone was given a laser cutting technology made of a famous brand engraved with their own name and the school emblem of Huazhong Agricultural University. In the life exhibition hall of Wuhan Science and Technology Museum, the children know the secrets of the human organs and the magic of the life system; enjoy the essence of the universe in the corridor of the Milky Way; before the clear blue water clock... The children understand the rules of all things and feel the magic of natural power. At the science and technology museum, children experience the telescope "'All people are enrolled, the world will return to farmers. Zhang Zhidong, founder of Hubei Agricultural School and governor of Huguang, wrote the couplet at the opening ceremony, expressing his high hopes for education and the revitalization of agriculture."From this pair of couplet, the children came to visit the" School History Exhibition Hall " special exhibition, through the graphic display board and a piece of the vicissitudes of life, to understand the 122 years of extraordinary years and development achievements of the school. Xu Benyu introduced Xu Benyu's volunteer teaching deeds and campus volunteer activities, also attracted the children's attention. Everyone said that they all knew the story of Teacher Xu Benyu "moving China", and they also knew that there are many volunteer teachers in their primary school. Yuan Shihan, a volunteer of the research branch group, guided the children to think: " The teachers have gained growth and progress in serving others and contributing to the society, and found the direction of youth and life goals. So where are your goals and directions?"In the laboratory children experience virtual simulation scene research branch group volunteer T-shirt behind there is such a line of words:" with a year of short time, do a lifetime unforgettable thing."" 'frequently read force ploughing, self-talent' eight words, has been imperceptibly into our blood. Once ignorant young people, after four years of ploughing, are doing a thing of their own talent."Said Xue Jingrui, leader of the 16th graduate student volunteer service team."I hope the natural science activities of 'big hands holding small hands and smiling at the world' will help more children. One day, little science lovers will become scientists. They can use their knowledge to help other children who also love science."In the school history hall message book, Xue Jingrui message:" in the anniversary of the children to revisit the school history hall, emotion, read the responsibility, the talent figure self-improvement, we have been on the road.... "the children listen to huazhong agricultural university campus planning and construction" hand holding hand, smile to see the world " city activity has been the seventh. The activity takes natural science as the theme, which helps the children to further learn the basic knowledge of natural science, improve their natural science literacy, and cultivate their interest in learning science, the spirit of scientific exploration and the awareness of nature protection. It is reported that Huazhong Agricultural University is building a natural science science project that will benefit from students in mountain villages, and will establish more natural science laboratories to broaden the boundaries of students' natural science knowledge.

2020-10-12	The Northwest Minimally Invasive Camp was held in Xi 'an Jiaotong University in Xi' an Jiaotong University on the afternoon of October 11, Twenty-six entrepreneurial teams from 15 schools, including Xi'an Jiaotong University, Chongqing University and Southwest Petroleum University, have gathered at the Jiaotong University Innovation Port, After the project roadshow display and defense, The "Speed Map" project from Xi'an Jiaotong University won the gold medal, Xi'an Huize Power Technology Co., LTD., "Carbon Cable Future" project won the silver award, Five projects of "patron saint of Pipeline safety production", "Apple Red Red" craft industry to help farmers, Jetta Technology, BICOCS Technology and "Duzhongkang" won the bronze award. This project roadshow is part of the 2020 Guangfa Securities College Students Micro Entrepreneurship Camp in 2020. The purpose is to activate the atmosphere of innovation and entrepreneurship in colleges and universities, serve the rural revitalization strategy, encourage college students to return to their hometowns to start their own businesses, and build a roadshow platform for venture capital institutions to negotiate with young entrepreneurs. At the following project summary meeting, Zheng Qinghua, member of the Standing Committee of the Party Committee and vice President of Xi 'an Jiaotong University, Dong Shi, Deputy editor-in-chief of China Youth Newspaper and Director General of KAB National Promotion Office, He Ying, deputy general manager of Guangfa Securities Xi' an Branch of Guangfa Securities and other guests attended the summary meeting and issued certificates. Zheng Qinghua said at the summary meeting that innovation and entrepreneurship is a feature of the current era, and it also brings more opportunities for young people today. He hoped that college students would integrate what they have learned with innovation needs, integrate innovation models and innovative industries, find and solve problems in entrepreneurial practice, better serve human life and world civilization, and at the same time obtain a more wonderful and meaningful life. Speed up innovation, accelerate the transformation of development mode is the trend of The Times, concern and support for youth is the responsibility of the whole society, China youth newspaper officials said, college students micro entrepreneurship since its launch in 2015, continue to pay attention to young students of small micro enterprises to support and cultivation, hope young students with their intelligence, with the help of Internet + technology, face to face with social problems, starting from the micro, open their entrepreneurial dream. He Ying said that Guangfa Securities will give full play to its advantages and directly connect with the angel and venture capital platform for young entrepreneurs, and will work with universities across the country to actively promote the activities of college students' micro entrepreneurship action. In the three-day northwest minimally invasive camp, the organizers invited enlightenment star venture zhao mei, managing director, guangfa qian and investment co., LTD., senior investment manager Shi Tuanwei, garlic technology executive CEO JingFeng and xi'an jiaotong university professor Yang Jianjun, professor at Jilin university Wang Wencheng more than 10 experts, investors, entrepreneurial youth and campers face to face communication training. The minimally invasive camp entries covers high-tech, rural revitalization, biotechnology, etc, the organizers specially set up "innovators", "focus on new industries" two cloud lecture hall, at the same time business model canvas training, from the core resources, key business, customer groups, value services such as 9 module grinding project, and through the roadshow show let campers and investors "recruit" on the scene. The northwest minimally invasive camp by xi 'an jiaotong university, KAB national promotion office, guangfa securities social public foundation, xi' an jiaotong university and center, the communist youth league of Xi'an jiaotong university committee to undertake specific, got the guangfa securities co., LTD., China youth newspaper unit support, and in the China youth daily client and official weibo, green and headlines client live, premiered the cumulative viewers of 481000.

2020-10-10	Wuhan university of science and technology school youth corps committee held the flag into the primary school youth corps committee held the flag into the primary school flag-raising activities weave seven flag class members came to Jilin street primary school, to participate in "' celebrate the Mid-Autumn festival, celebrate the birthday-Jilin street primary school jiangnan spring city campus to celebrate the 71st anniversary of the founding" activities, a flag-raising ceremony, preach the national flag culture. In the passionate and majestic national anthem, the bright five-star red flag fluttering in the wind, the audience solemn. More than 1,000 teachers and students from grade 1 to grade 4 of Jilin Street Primary School participated in this activity. Jilin Street primary school third and fourth grade students representatives to bring "praise the motherland" poetry praise. Students of the national flag class and all the teachers and students of Jilin Street Primary School sang "Me and My Motherland". Members of the national flag class also preach the national flag culture and promote patriotism for the students. Tell the design of the flag, the iser of the flag, the symbol of the flag, the meaning and steps of the flag-raising ceremony. And in the outdoor for the children to explain the military posture and queue salute and training, encourage the children to understand the national flag, strive to be a qualified flag-bearer.

2020-10-10	China University of Petroleum (East China): "Fingertip Charity" moistens the "Hope Hut" sprouts and grows in the University of Petroleum."Wenwen (pseudonym), a 12-year-old girl from Xiamali Village, Yangxin County, Binzhou City, Shandong Province, has said goodbye to her old room full of clutter and has a clean and warm" hope hut ". China University of Petroleum (East China) tailored for her "hope cabin" not only clean floor, white wall, more beautiful desk, comfortable cot, fashionable wardrobe. Wenwen had lost her father since she was young, and her mother had remarried. She had always lived with her elderly grandmother, and she did not even have a decent piece of furniture in her room."Hope cabin" gives Wenwen a warm space for her study and life. College students from China University of Petroleum (East China) also established a long-term care plan with Wenwen, and helped her with homework online through "cabin Cloud Classroom" to solve her growing troubles."They are my bosom sisters. They also said that they took me to Qingdao to play in the winter and summer vacation. I will study hard and grow into people like them in the future. In Yangxin County, Binzhou, Shandong Province, 12 other children like Wenwen also have a "Hope Hut"."Hope hut" is a child care project jointly initiated by Shandong Provincial Committee of the Communist Youth League and Shandong Youth Federation. China University of Petroleum (East China) actively carried out the work. The first batch invested more than 70,000 yuan to build 12 "hope cabins", bringing true feelings and love to 12 families. In the new semester, the Youth Volunteers Association of China University of Petroleum (East China) held a love purchase activity of "building a hut and holding up the hope" on the campus, and raised 7,589 yuan in two days."People are very enthusiastic about public welfare undertakings, and it has become a campus fashion to show love and do good deeds together."Qi Shunxin, president of the association, said," Pay itself is also a harvest, and dedication of love is the sublimation of the soul. During the "" 99 Charity Day ", the teachers and students of China University of Petroleum (East China) set up a" love team "and launched the" Shida Hope House " project, raising 74,630 yuan."The moment the children walked into the Hope House, I saw the light in their eyes and their yearning for a better life."After Man Cong, an alumnus of China University of Petroleum (East China) and the general manager of Qingdao Shijiazhuang Chemical Co., Ltd., he not only donated 70,000 yuan to visit the poor children there, but also acted as a propagandist and actively contacted other alumni to participate in the charity donation."Fingertip public Welfare" and "Everyone Public Welfare" are "blooming everywhere" on the campus and alumni of China University of Petroleum (East China). The "Hope hut", like the seeds of hope, are sprouting and growing under the love of teachers, students and alumni. In recent years, China University of Petroleum (East China) attaches great importance to cultivating the spirit of volunteer service of college students, and strives to make volunteer service become the "spiritual coordinate" of college students, guiding students to take the initiative to understand the social conditions, care about the society, and serve the society. Under the guidance of the spirit of volunteer service, the teachers, students and alumni of China University of Petroleum (East China) actively participate in the epidemic prevention and control, poverty alleviation, rural revitalization and other work, so that their youth can shine in love and dedication."We will strive to make 'Hope House' a powerful starting point to serve the overall situation of poverty alleviation, a model project to care for children's growth, and a public welfare brand to gather social love. In the activity, we also attach great importance to the cultivation of students' feelings of volunteer service, and play a good role in educating and guiding students."Zhang Zhan, secretary of the Communist Youth League Committee of China University of Petroleum (East China), said," In the future, we will earnestly build, manage and use the 'Hope House' well, make distinctive features, and strive to make every child grow up healthily. Each 'hope hut' will be a seed of hope and a moving story."

2020-10-10	The original stage play "Dream Youth" of Dalian Art Academy was premiered in the Dalian Development Zone Grand Theater. The drama is hosted by the Liaoning Provincial Party Committee and the Dalian Municipal Communist Youth League. Play using the stage form of the musical, will fight the new crown outbreak occurred in Dalian institute of art campus reality, truth, the truth, the reality, is a for inheriting youth and love spirit and create new ideological drama, but also shows the Dalian institute of art school for 20 years in the field of ideological and political education. The whole play is divided into four acts, using the documentary style. Symphony, chorus, recitation, musical, dance and other artistic forms reflect each other, reflecting the exquisite multiple time and space, multi-situation, multi-point performance collocation; lighting, stage design, costumes, props and other extreme rendering, showing the convergence of stage art and stage technology. Ideological and political courses can be taught not only in class, but also on stage. Over the years, Dalian University of the Arts has been educating young students on their ideals and beliefs in the form of bringing ideological and political courses to the stage, and cultivating the patriotism of their teachers and students.

2020-10-02	Fudan University youth league committee launched the declaration of communist party inheritance activities wang yejie) "I am the spirit of the spirit Cai Mingyu, I am the declaration spirit inheritor Zhou Yicheng..." to commemorate 100 years ago fudan university President Chen wang dao translated the first Chinese communist declaration, the spirit of fudan university young teachers and students recently snap, learn, with the lens clock around red scenic spots, record the new changes at home, deep study practice general secretary xi reply spirit, do declaration spirit inheritor. On the eve of July 1, General Secretary Xi Jinping wrote back to all members of the volunteer service team of the Communist Manifesto Hall of Fudan University, encouraging the party members to strengthen their ideals and beliefs in learning, thinking and practicing, and practice their original mission in working hard and promising. Fudan university then study and implement general secretary important reply spirit of upsurge, the school youth corps committee launched "learning practice fudan people, the declaration of spiritual inheritance-inheritance with" youth image contest, encourage fudan youth in the compliance of the epidemic prevention and control requirements, with learning activities at any time, attracted the fudan youth teachers and students enthusiasm to participate in. It is reported that the competition is divided into two units: images and short video, including three themes: "red gene, declaration spirit", "" four history "education" and "well-off life". Young teachers and students learn at any time during the visit and record process. Their footprints throughout the communist manifesto exhibition hall, the communist party site, Guizhou Zunyi conference site, the central plains liberation memorial, Shanghai four warehouse anti-japanese memorial and other famous red historical buildings, in the glorious home, yuhang dream town, Ningxia west solid Yang Ling village has special significance also left their figure, fudan students practice original mission in social life, in the unity of active bear as.

2020-09-28	Its university held "green horse project" lecturer training camp activities its university held "green horse project" lecturer training camp project "training class demonstration training" four confidence out "lecturer training camp activities, marks the" green horse thought enjoy hui "series of activities" trilogy " (classic theory in, four confidence out, strategy execution dry) packaged. Participants in the training camp are divided into 10 groups, which select different themes for publicity and display, innovate and make breakthroughs in the form and content of publicity, and form demonstration experience. Students integrated use of speech, recitation, sitcoms, dialogue, and other forms, innovation using case introduction, multimedia interaction, simulation method, such as the understanding of "four confidence" comprehension into young people easy to accept, popular "green language", the profound historical law, majestic struggle, touching youth story and continuous cultural heritage, vivid presentation. After on-site voting, 1 first prize, 5 second prize and 4 third prizes were selected.
